# Synthesis of 4- and 5-membered fluorine-stabilised semi-saturated heterocycles via alkylidene carbenes

**DOI:** 10.1038/s41467-026-77771-y

**Published:** 2026-09-28

**Authors:** E. Harvey Randle, Alexi T. Sedikides, Julien Lefranc, Alastair J. J. Lennox

**Affiliations:** 1 School of Chemistry, University of Bristol, Bristol, UK; 2 Merck KGaA, Darmstadt, Germany

**Keywords:** Synthetic chemistry methodology, Catalytic mechanisms, Reaction mechanisms

## Abstract

Heterocycles are ubiquitous in biologically active molecules and central to pharmaceutical design. Their diverse reactivity and three-dimensional architectures make them essential scaffolds for tuning non-covalent interactions with biological targets. Saturated and unsaturated heterocycles are well established and widely exploited, but semi-saturated variants are rare, limited by synthetic inaccessibility and oxidative instability. Here we present a general strategy for synthesising fluorinated semi-saturated four- and five-membered nitrogen and oxygen heterocycles via alkylidene carbene intermediates, and show through kinetic, voltammetry and metabolism studies that fluorine is key to stabilising these underexplored motifs. The method affords oxidatively stable products with diastereoselectivity across diverse substitution patterns and functional groups. These scaffolds can be selectively derivatised to yield saturated, unsaturated, and semi-saturated fluorinated analogues to provide broader access to pharmaceutically-relevant chemical space.

## Abstract (web summary)

The authors present a general strategy for synthesising fluorinated semi-saturated four- and five-membered nitrogen and oxygen heterocycles via alkylidene carbene intermediates, and show through kinetic, voltammetry and metabolism studies that fluorine is key to stabilising these underexplored motifs.

## Introduction

**Fig. 1:**
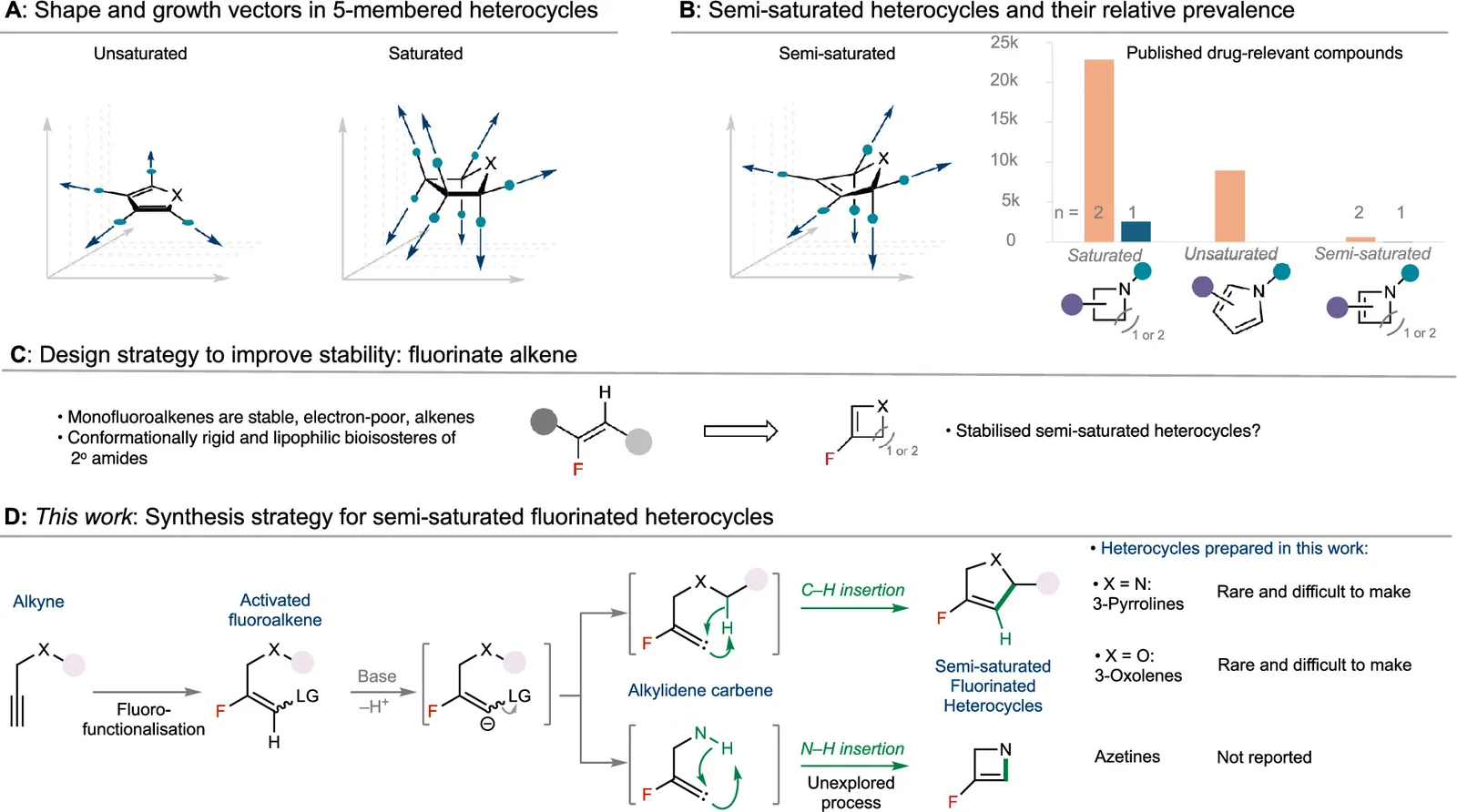
An introduction to semi-saturated heterocycles and fluorinated semi-saturated heterocycles. **A** Shape and growth vectors in saturated and unsaturated heterocycles. **B** Semi-saturated heterocycles with their relative presence in drug molecules; see SI for methodology. **C** Monofluoroalkenes as potentially more stable semi-saturated heterocycles, and **D** strategy for synthesis of fluorinated semi-saturated heterocycles.

Heterocycles are abundant in naturally occurring molecules and form essential components of all known forms of life^1,2^. This prevalence in nature is one reason why over 90% of FDA-approved small molecule drugs contain a heterocycle^3,4^, over 80% of which are nitrogen-based^5^. These heterocycles fall into two broad categories, unsaturated aromatic heterocycles and saturated heterocycles. While unsaturated heterocycles give rise to flat, rigid structures with growth vectors extending in 2-dimensions, the *sp*^3^ carbon centres of saturated heterocycles provide more flexibility and 3-dimensional character (Fig. 1A). This difference plays a crucial role in drug design by providing a means to vary the shape of a molecule, and the direction and orientation of functional groups in space, which directly influences the intrinsic potency and selectivity of a drug^6–9^.

Semi-saturated heterocycles that contain a double bond and the remaining carbons in the ring are *sp*^3^ hybridised (heterocycles containing carbonyl groups, such as α,β-unsaturated lactams, are therefore not included in this definition) constitute a third category. These semi-saturated heterocycles combine spatial properties from both the 3-dimensional saturated and the 2-dimensional unsaturated heterocycles (Fig. 1B). However, they are rare, both in biologically relevant molecules^10–14^, as well as more generally in the field of synthetic organic chemistry. This scarcity is compounded by their sensitivity to oxidation^15^, and their challenging synthesis, particularly for the four-membered azetine. We hypothesised that if semi-saturated heterocycles could be made more stable and their synthesis was facile, their unique shape and growth vectors should provide tunability in drug discovery that is not currently available^8,16^.

To increase the stability of semi-saturated heterocycles, we envisaged the removal of electron density from the alkene would reduce its propensity to oxidise and aromatise. Due to the strong sigma electron-withdrawing effect of fluorine, monofluoroalkenes are relatively inert compared to non-fluorinated alkenes^17,18^. These alkenes, which are lipophilic bioisosteres of secondary amides (Fig. 1C)^19^, are much less susceptible to oxidation and enzymatic metabolism^18,20^. Stabilising the alkene with fluorine should attenuate aromatisation while retaining the spatial characteristics of semi-saturated heterocycles^21^.

Published examples of characterised semi-saturated fluorinated heterocycles are very rare, with no examples of fluorinated four-membered azetines reported. For the five-membered 3-pyrrolines, only a single method is reported for their synthesis, but without control of diastereoselectivity, which requires specifically substituted allenes as starting materials, thereby limiting the substitution patterns potentially available^22^. Multi-step syntheses and ring-closing metathesis are two other strategies that have each been used to produce single examples of fluorinated oxolenes and pyrrolines, respectively^23–30^ (see Supplementary Tables 10 and 11 for comparisons). In the case of metathesis, only a 2-methyl substituent on the fluorinated pyrroline has been successfully demonstrated^27–30^. Hence, the development of a general and accessible method to semi-saturated fluorinated oxygen and nitrogen heterocycles would provide new building blocks for drug development^31^.

We envisaged that the fluoro-functionalisation of readily accessible heteroatom-containing alkynes could lead to an activated fluoroalkene, which, upon deprotonation, will eliminate a leaving group to give a β-fluoro-alkylidene carbene intermediate (Fig. 1D). A new route to fluorinated five-membered heterocycles should be accessible as this class of carbenes undergo intramolecular 1,5-C–H insertion reactions^32–45^. However, this reactivity has not been demonstrated for smaller four-membered rings. Hence, we envisaged that an X–H insertion may be feasible, which is a mechanism that possesses minimal precedent and has yet to be exploited in synthesis^46^. Here, we demonstrate the successful realisation of this strategy to produce diverse five-membered fluorinated heterocycles with the introduction of diastereoselectivity, and an unexplored mode of reactivity whereby four-membered rings can be formed through insertion of the carbene into N–H bonds, affording fluorinated azetines from simple alkynes.

## Results

**Fig. 2:**
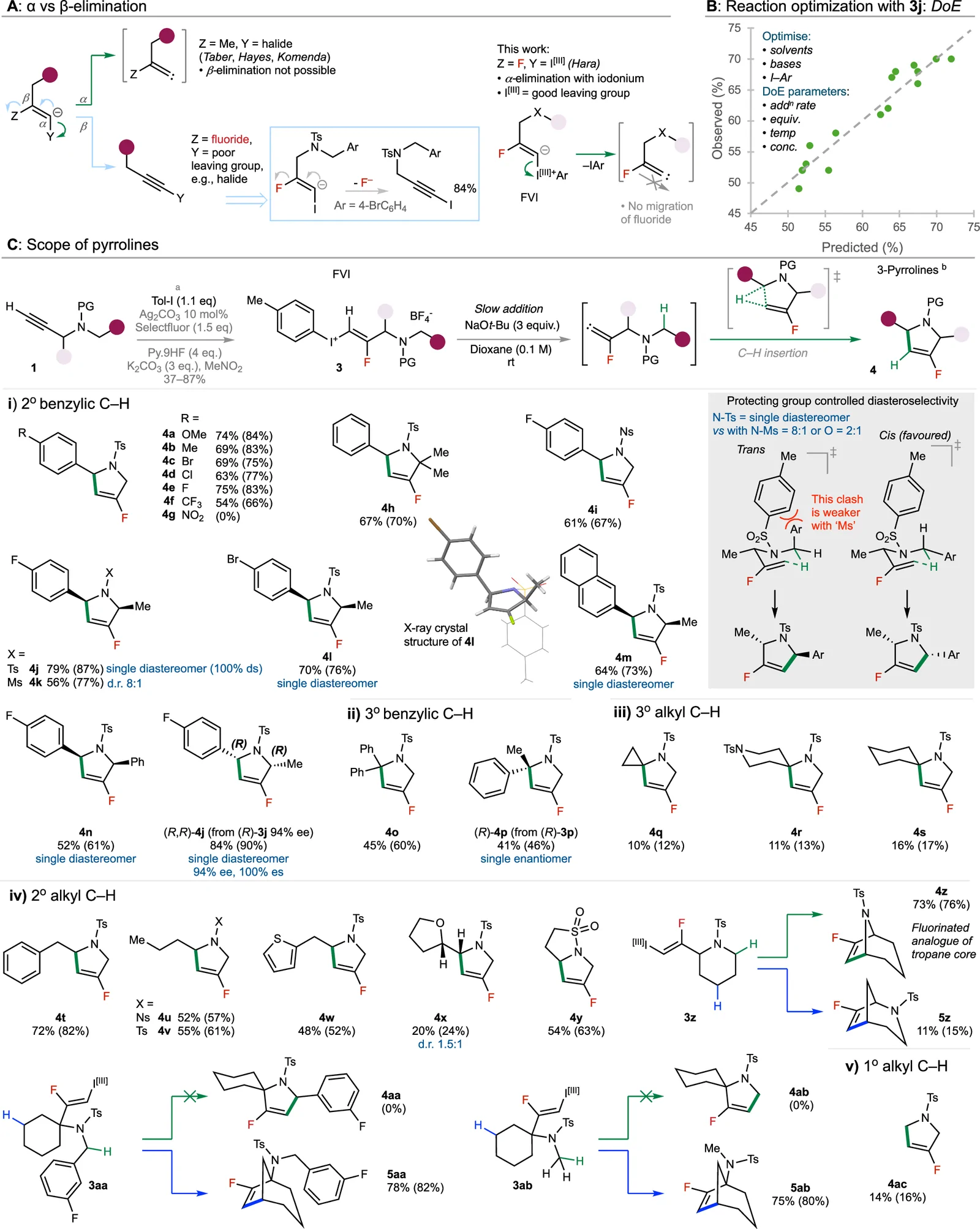
Strategy, optimisation and substrate scope for the formation of fluorinated pyrrolines **4**. **A** Design strategy for generating the alkylidene carbenes. **B** Design of experiments (DoE) reaction optimisation. **C** Substrate scope of pyrrolines showing insertion of carbene into: i secondary benzylic C–H bonds, ii tertiary benzylic C–H bonds, iii tertiary alkyl C–H bonds, iv secondary alkyl C–H bonds, e primary alkyl C–H bond. a: conditions adapted from reference^49^; b: isolated yields of product from **3**, with NMR yields in parentheses. CCSD (**4l**) = 2485038.

The generation of non-fluorinated alkylidene carbenes using halides as leaving groups has been studied by several groups, including Taber, Hayes and Komenda (Fig. 2A). The substrates tested in these studies each contain a methyl group in the β-position (*Z* = Me), meaning β-elimination to form an alkyne cannot occur. Replacing the methyl group with fluorine creates that opportunity, as β-elimination of fluoride is more favourable than α-elimination of a bromide or iodide^47^. We demonstrated this issue to be the case, as deprotonation of an iodo-fluoroalkene revealed the corresponding iodoalkyne that results from β-fluoride elimination (Fig. 2A). However, with the much stronger iodonium (I^[III]^) leaving group, such ylide intermediates can undergo the desired α-elimination, as first demonstrated by Hara in the synthesis of a simple carbocycle^37,38^. In addition, fluoride migratory aptitude is so poor that competing rearrangement to an alkyne is unlikely to occur^47^, which is not the case for non-fluorinated alkylidene carbenes^37,44,47,48^, (Fig. 2A). Hence, we employed β-fluorovinyl iodonium (FVI) salts^49,50^ as a strategy to generate these underexplored and difficult-to-access fluorinated semi-saturated heterocycles (Fig. 2A).

The synthesis of 3-fluoro-3-pyrrolines (**4**) was studied first using β-fluorovinyl iodonium (FVI) salts that were formed from unactivated alkynes in a single, silver-catalysed step^49^. Optimisation of solvent and base with **3e** as the model substrate revealed 1,4-dioxane and sodium *tert*-butoxide as a suitable combination (see SI, Supplementary Tables 1–3). A wide range of conditions were then surveyed with **3j** as the model substrate for the remaining variables, where a full-factorial design of experiments (DoE) approach was used to scan the multi-dimensional chemical space for both yield and diastereoselectivity (Fig. 2B and SI, Supplementary Table 4)^51^. This enabled reaction optimisation to be carried out in relatively few experiments and determined which variables were significant. While complete diastereoselectivity was observed under all conditions tested, optimal yields of product and minimal yields of side-products were achieved with the slow addition of FVI to the basic solution. The reaction outcomes were found to be unaffected by air and moisture, and the DoE optimisation showed the reaction to not be temperature sensitive.

With an optimised set of conditions for the synthesis of semi-saturated fluorinated pyrrolines (**4**), the generality of the substrate scope was explored (Fig. 2C). Carbene insertion into secondary benzylic C–H bonds gave moderate to excellent yields of pyrrolines **4a**–**f** containing differentially substituted rings (Fig. 2C-i), except *p*-NO_2_ (**4g**) that was unsuccessful. Insertion into more acidic C-H sites such as this proved difficult, as is shown by a comparison of yield against the Hammett parameter (Supplementary Fig. 9) and our attempted C-H insertions α to sulfur, nitrile, or carbonyl groups (Supplementary Fig. 10). Protection of nitrogen with either a tosyl or nosyl group (**4e** vs **4i**) worked similarly. The reaction proceeded with complete *cis*-diastereoselectivity for 2,5-disubstituted substrates bearing a tosyl protecting group (**4j, 4l**–**n**). Formation of the *trans*-product should be attenuated due to steric clash between the tosyl group and the substituents on the ring in the 6-membered transition state; the tosyl group occupies one side of the ring, forcing both substituents onto the opposite side, which leads to the excellent selectivity observed (inset Fig. 2C-i)^36^. Consistent with this, the diastereoselectivity dropped when the smaller methanesulfonyl protecting group in **4k** was used in place of the tosyl group. Insertion into a tertiary benzylic C–H bond worked to form **4o**-**p** (Fig. 2C-ii). Complete enantiospecificity was observed in pyrrolines **4j** and **4p** when starting from alkynes containing an enantiomerically pure stereo-centre.

Insertion of the carbene into a tertiary methine C–H bond was tolerated to form valuable spirocycles **4q**–**4s** (Fig. 2C-iii). Insertion into secondary methylene C–H bonds successfully gave 2-alkyl pyrrolines (**4t**–**x)** or fused heterocycle **4y** (Fig. 2C-iv). With a choice of C–H bonds to insert into that can each form five-membered rings, products were observed that depend on their relative reactivity and accessibility. For example, **3z** formed pyrroline **4z** in preference to the bridged cyclopentene **5z**, indicating greater reactivity of the C–H bond alpha to nitrogen. However, a similar C–H bond, now also benzylic, was not involved in the reaction of **3aa**, as the carbene preferred to form the alternative five-membered carbocyclic compound **5aa**. In this case, the tosyl group blocks access to the benzylic C–H bond due to the strong steric influence of the cyclohexane ring. This reactivity was mirrored in substrate **3ab** that contains a primary methyl C–H bond to form **5ab** in preference to **4ab**. Interestingly, insertion into a methyl C–H bond was indeed observed when no choice was provided (Fig. 2C-v), as substrate **4ac** was cleanly formed, albeit in low yield.

**Fig. 3:**
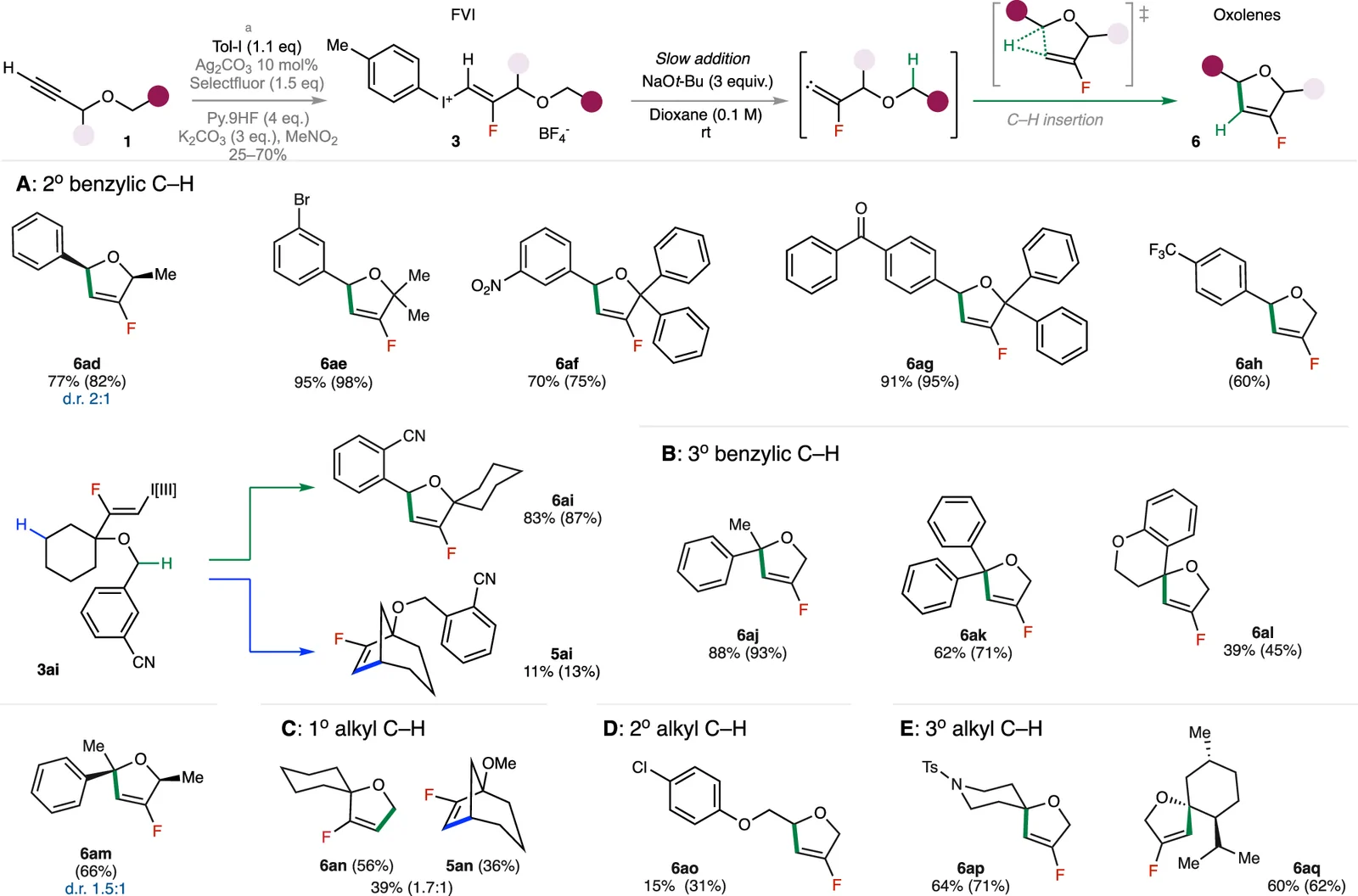
Substrate scope for the formation of fluorinated oxolenes 6. Isolated yields of product from 3, with NMR yields in parentheses. **A** insertion into secondary benzylic C–H bonds; **B** insertion into tertiary benzylic C–H bonds; **C** insertion into primary alkyl C–H bond; **D** insertion into secondary alkyl C–H bond, and **E** insertion into tertiary alkyl C–H bonds. a: conditions adapted from ref. ^49^.

Using this methodology, a second semi-saturated heterocycle could be obtained by replacing the nitrogen atom with oxygen to produce fluorinated 3-oxolenes (**6**) (Fig. 3). This heterocycle is also a rare motif but is present in a nucleotide reverse transcriptase inhibitor, which takes several steps to prepare, for treating HIV infection^52–54^. Insertion into secondary benzylic C–H bonds gave excellent yields of oxolenes with gem-dialkyl or -diaryl substitution **6ad–ah** (Fig. 3A). Substitution in the 2- and 5-positions yielded the *cis* product as the major diastereoisomer (**6ad**). The diastereoselectivity is lower than the pyrrolines (**4**), as steric clash between the lone pairs on oxygen and the substituent should be lower than with the protected nitrogen in the 6-membered transition state (see inset, Fig. 2Ci), hence, the discrepancy between the conformers is lower. When given a choice of secondary C–H bonds in **3ai**, the benzylic C–H bond was preferred to form **6ai**, which is in direct contrast to that observed with amine **3aa**, demonstrating the increased relative accessibility due to the smaller size of oxygen than NTs. Carbene insertion into tertiary benzylic C–H bonds successfully produced the corresponding oxolenes **6aj**–**am** (Fig. 3B), as well as primary, secondary and tertiary C–H bonds producing oxolenes **6an**–**6aq** (Fig. 3C–E). Successful insertion into the primary C–H bond of the methoxy group demonstrates a viable strategy for switching the position of fluorine relative to the substituents, cf. **6an** vs **6ap**.

**Fig. 4:**
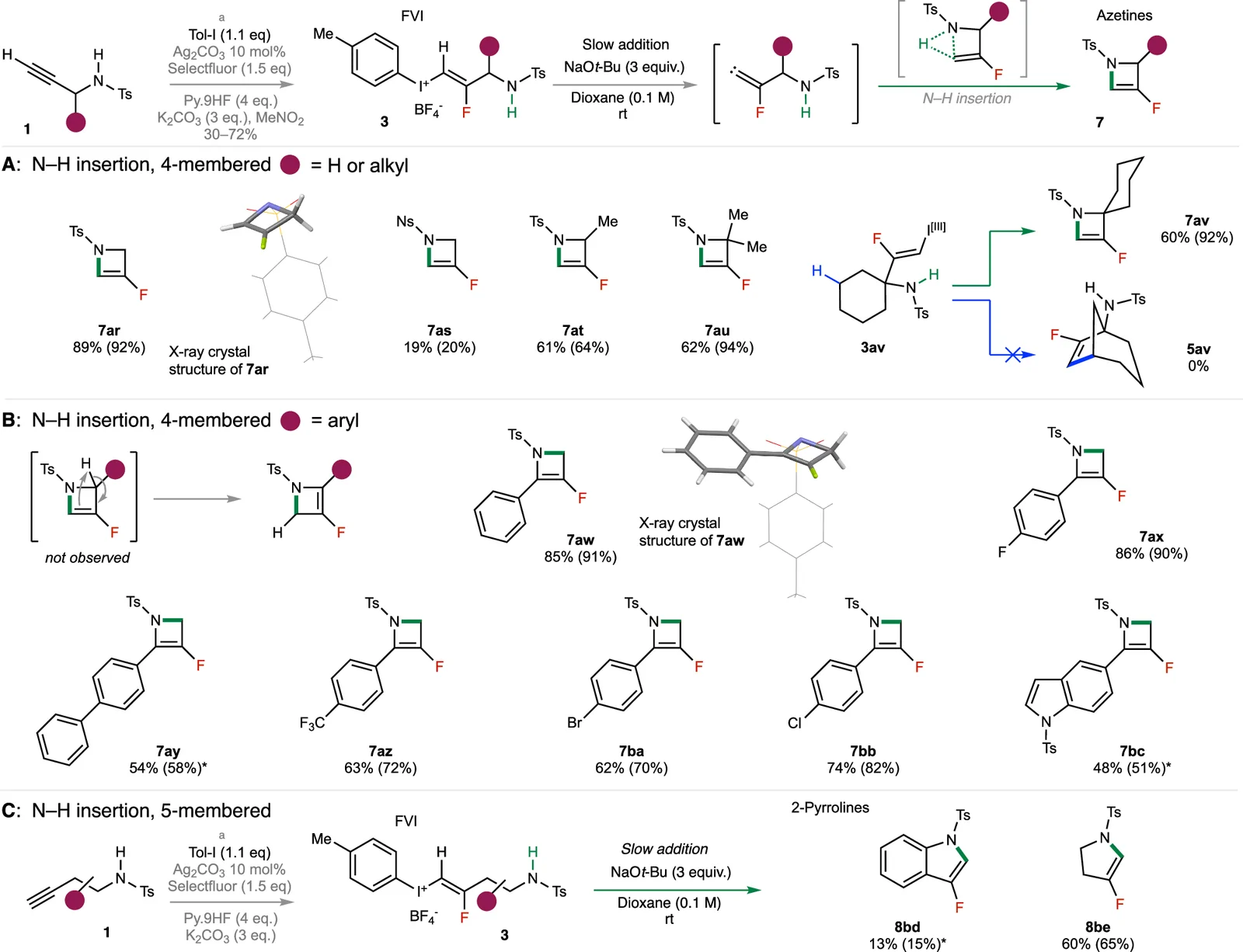
Substrate scope for the formation of fluorinated azetines and 2-pyrroline. Isolated yields of product from **3**, with NMR yields in parentheses. **A** H or alkyl-substituted propargylic amines; **B** aryl substituted propargylic amines, which undergo an allylic proton shift; **C** 2-pyrrolines formed from homopropargylic amine. a: conditions adapted from ref. ^49^. *Yield over 2 steps from alkyne. CCSD (**7ar**) = 2485040; (**7aw**) = 2486256.

Four-membered semi-saturated heterocycles constitute another rare family of heterocycle. While azetines have gained some traction recently, through reports of new synthetic methods^55–59^ and an exploration of their biological utility^60^, no methods are reported for the synthesis of fluorinated azetines. To produce four-membered heterocycles using our approach, insertion into a heteroatom–H bond must occur, Fig. 1D. When alcohols were employed for O–H insertion, products were observed from the electrocyclic ring opening of the evidently unstable, four-membered oxetenes (see Supplementary Fig. 5 for mechanism and Supplementary Fig. 10 for unsuccessful oxetene substrates). However, insertion into the corresponding N–H bond of a sulfonamide gave 3-fluoro-2-azetine heterocycles **7** (Fig. 4A) (Dimerised perfluorinated azetes have previously been formed, see Chambers et al.^61^).

The azetines formed by our method (Fig. 4A), when unsubstituted (**7ar**–**as**) or mono-substituted (**7at**), were found to be stable at room temperature, with the β-fluorine atom evidently curbing the reactivity of the enamine. The ^1^*J*_(C-F)_ coupling constants measured in these 3-fluoroazetines (330 Hz) are among the largest recorded (Perfluorocyclobutenes have slightly higher ^1^*J*_(C-F)_ coupling constants, see Zhang et al.^62^), and the C–F bond is one of the shortest C(*sp*^2^)–F bonds known (1.32 Å) (see SI, Supplementary Table 5), indicating uniquely high s-character in the hybridised orbital. Where both N–H and C–H reactions were possible in **3av**, the 1,4-N–H insertion was observed to occur with complete selectivity over the 1,5-C–H insertion, affording azetine **7av** in high yield. The two geminal disubstituted azetines **7au** and **7av** were formed in excellent yields. While products from a slow thermal electrocyclic ring-opening were observed in these two products when stored at room temperature, alkene hydrogenation produces useful and stable azetidines, vide infra. If the azetine contained an aryl substituent, a spontaneous allylic-proton shift occurred to afford styrene-type azetines (**7aw**–**7bc**), which could be readily purified on silica and were all stable at room temperature (Fig. 4B).

Formation of five-membered rings through N–H insertion successfully produced 2-pyrroline and indole products, **8bd** and **8be** (Fig. 4C); hence, access to both 3-fluoro-3-pyrrolines (**4**) (Fig. 2C) and 3-fluoro-2-pyrrolines (**8**) (Fig. 4C) can be accessed through this strategy. While 2-fluoro-2-pyrrolines can be accessed^63,64^, there are no facile routes reported for generating these 3-fluoro-2-pyrrolines, with only 3 single isolated examples reported^65–67^. 2-Pyrroline **8be** was found to be stable at room temperature, further demonstrating the important role that fluorine plays to stabilise an otherwise highly reactive enamine.

**Fig. 5:**
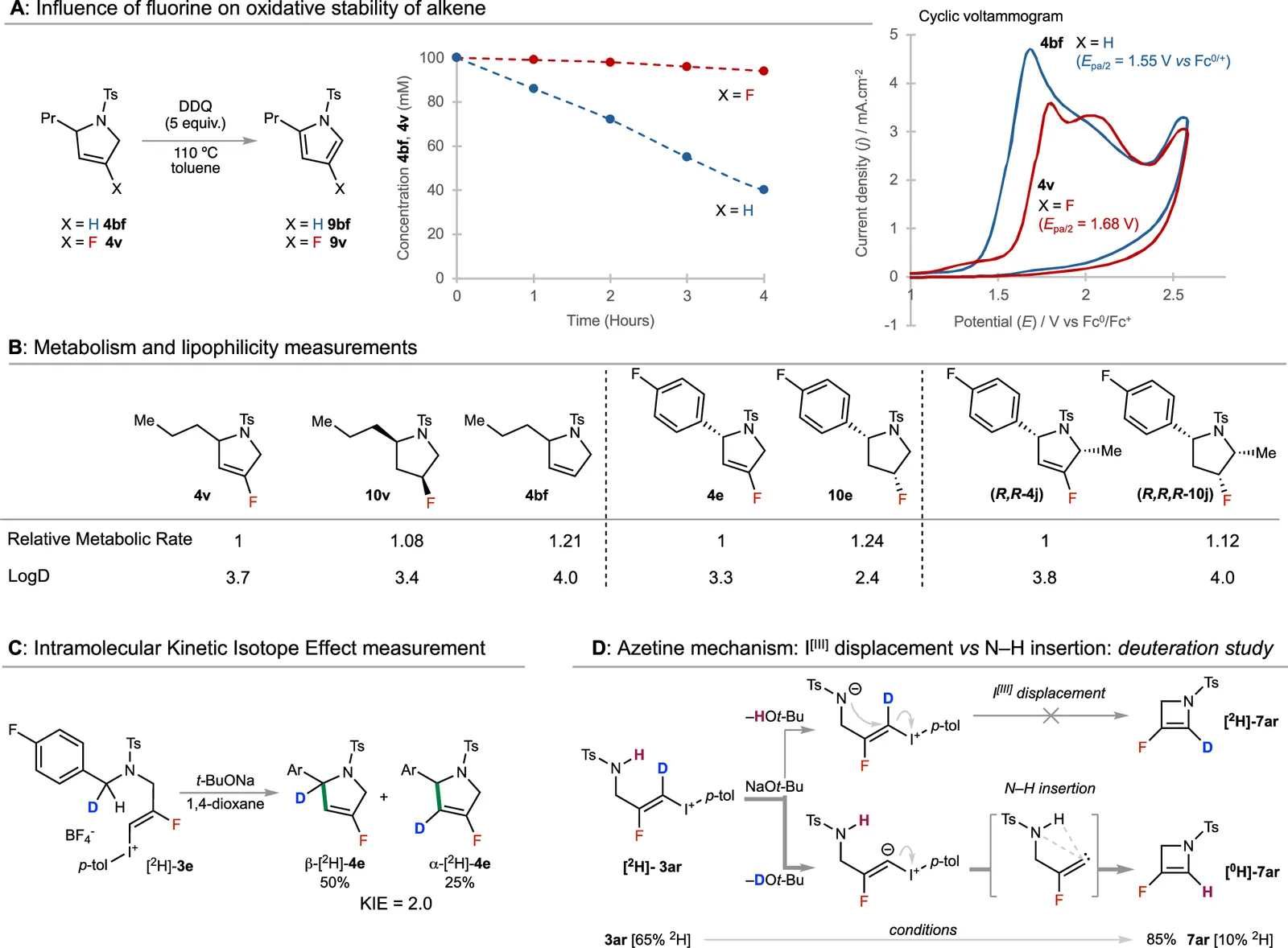
Properties of semi-saturated fluorinated nitrogen heterocycles and mechanism of pyrroline **4** and azetine **7** formation. **A** Influence of fluorine on oxidative stability of alkene. CV conditions: pyrroline (5 mM), TBAPF_6_ (0.1 M), MeCN, 100 mV s^−1^. **B** LogD (pH 7.4) and relative human liver microsomal metabolic stability assays of fluorinated azetines compared to fluorinated pyrrolidines and non-fluorinated pyrrolines. **C** Intramolecular KIE reveals two-fold preference for insertion into C–H over C–D bond. **D** Deuteration of **FVI 3ar** leads to azetine with loss of deuterium, indicating N–H insertion pathway.

The introduction of fluorine can enable access to unique and privileged scaffolds. With fluorine central to our design strategy (Fig. 1C), we wanted to assess the effect it has on the oxidative sensitivity of the alkenes. Hence, the rate of oxidation for non-fluorinated pyrroline (**4bf**), which was the only one commercially available to us, was compared to the fluorinated analogue **4v** prepared herein (Fig. 5A). We found that under the highly forcing conditions of 110 °C with 5 equiv. DDQ, fluorinated pyrroline **4v** was left untouched while non-fluorinated **4bf** reacted rapidly. Cyclic voltammetry revealed a 130 mV difference in the oxidation potential between the fluorinated and non-fluorinated pyrrolines (Fig. 5A), providing further quantification of the influence of fluorine.

Metabolism data recorded from human liver microsomal (HLM) assays revealed that the semi-saturated fluorinated pyrroline (**4**) was metabolised more slowly, and therefore is more stable, than the corresponding pyrrolidine (**10**) or non-fluorinated pyrroline **4bf** (Fig. 5B, Supplementary Tables 8 and 9). Lipophilicity LogD measurements also demonstrated either a slight increase or were comparable to the corresponding pyrrolidine. These studies highlight the tunability of properties that can be provided to heterocycles when fluorinating the alkene.

Mechanistic studies were conducted to elucidate details on the reactivity of the carbene and the C–H insertion. Molecular orbital calculations (DFT) confirmed the β-fluoroalkylidene carbene is an electrophilic singlet carbene, with orbital hybridisation that is formally filled sp_1.1_ and empty sp_11_. This electrophilicity is exemplified by the greater reaction efficiency observed with more electron-rich C–H bonds, cf. **4a** (84%, R = OMe) vs **4f** (66%, R = CF_3_) (Fig. 2C-i). An intramolecular KIE of 2.0 was measured from the reaction of monodeuterated **3e** (Fig. 5C), in which insertion into the weaker C–H bond was preferred. This KIE reflects an expected energy barrier increase of 1.7 kcal/mol for insertion into a C–D bond over a C–H bond.

For the formation of 3-fluoroazetines (**7**), we considered two possible mechanisms: (1) N-H deprotonation to form a tosyl amide and then displacement of the I^[III]^, and (2) **FVI** deprotonation, carbene formation and N–H insertion (Fig. 5D). While the pK_a_ of the **FVI** proton is lower by ca. 5 units than the TsN–H, preliminary DFT studies could not establish a clear preference for carbene N–H insertion. Hence, deuterated **3ar** was prepared (Fig. 5E), which should either maintain deuterium incorporation if undergoing the I^[III]^ displacement pathway, or lose deuterium if proceeding via the carbene N–H insertion pathway. Under the cyclisation conditions, deuterium incorporation decreased from 65 to 10% (Fig. 5D), thereby in support of carbene insertion into the N–H bond. Control studies (see SI, Supplementary Fig. 7 for details) showed that competing N–H/D exchange from the liberated *t*-BuOD meant that some deuterium was retained in the azetine product via insertion of the carbene into a newly formed N–D bond.

**Fig. 6:**
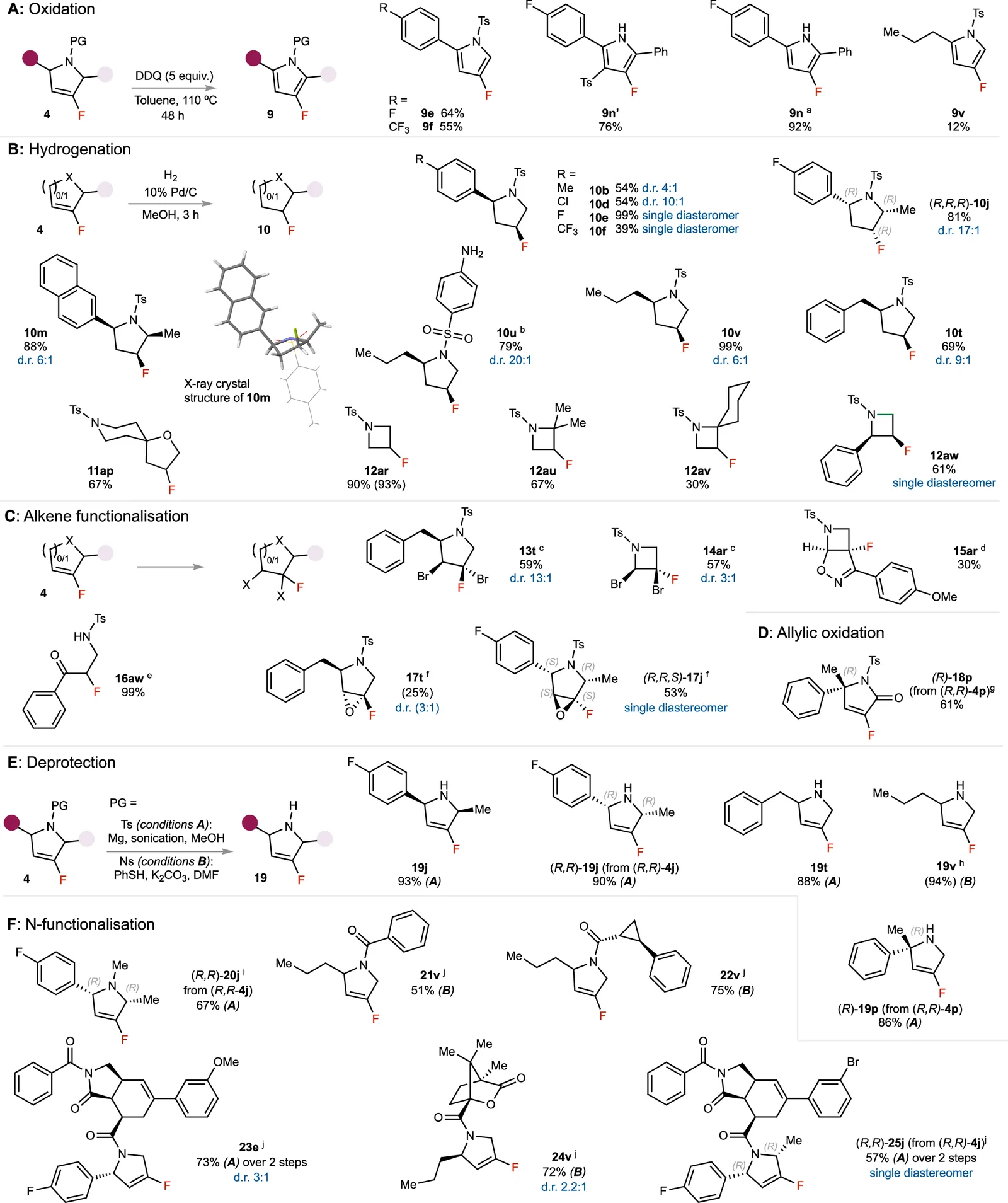
Derivatisations of semi-saturated fluorinated heterocycles. Reactions conducted on 0.2 mmol unless otherwise specified. Isolated yields shown, with NMR yields in parentheses. **A** Oxidation to pyrroles. **B** Hydrogenation to pyrrolidines and azetidines. **C** Functionalisation of the alkene. **D** Allylic oxidation. **E** Deprotection of nitrogen. **F** Functionalisation of nitrogen. a NaOtBu (6 equiv.) in DCM (0.1 M) air, 40 °C, 16 h. b Reduction of the nosyl group also occurred to give the amine. c NBu_4_Br_3_ (20 equiv.), DMF, 70 °C, 24 h. d Oxime, NCS in DMF at 0 °C, 6 h, added to azetine, NEt_3_ in *t*-amyl alcohol, 70 °C, 18 h. e H_2_O (2 equiv.), CDCl_3_ (0.2 M), 50 °C, 2 h. f *m*-CPBA (20 equiv.), CHCl_3_, 45 °C, 24 h. g CrO_3_ (20 equiv.), DCM (25 mM), pyridine (140 equiv.), reflux, 21 h. h Could not be isolated due to volatility. i MeI (1.5 equiv.), K_2_CO_3_ (3 equiv.) in MeCN (0.04 M), 65 °C, 18 h). j Either acyl chloride (1.5 equiv.), Et_3_N (3 equiv.), DMF (50 mM), rt; 18 h, or HBTU (1.5 equiv.), DIPEA (5 equiv.), carboxylic acid (1 equiv.), rt, 18 h. CCSD (**10 m**) = 2485039.

Although relatively stable alkenes, investigations into their derivatisation were conducted to assess their broader utility as building blocks towards other heterocycles. Oxidation to the corresponding 3-fluoropyrroles (**9**) could be achieved for substrates with aryl groups (**9e,** **f,** **n**) when heated to 110 °C for 48 h with 5 equivalents of DDQ in good to excellent yields (Fig. 6A). However, alkyl-substituted pyrroline **9v** was found to be more resistant to oxidation, and even under these forcing conditions gave only a poor yield of the corresponding pyrrole, further demonstrating the profound influence of fluorine on the alkene reactivity.

Hydrogenation of 3-fluoropyrrolines using H_2_ and Pd/C delivered the corresponding fluorinated pyrrolidines (**10**) in good to excellent yields (Fig. 6B). The diastereoselectivity of this reaction was excellent, with the hydrogen adding to the least hindered face. Hence, excellent stereoselectivity on up to 4 stereo-centres can be readily generated on these highly pharmaceutically relevant heterocycles. Spirocyclic oxolene **6ap** was hydrogenated to the corresponding oxolane (**11ap**) in high yield (Fig. 6B). Hydrogenation of the fluorinated azetines produced azetidines (**12ar,** **au,** **av,** **aw**) in moderate to excellent yields. The less stable gem-disubstituted azetines **7au** and **7av** transformed to stable fluorinated azetidines. Complete diastereoselectivity was observed in the hydrogenation of arylated azetine **7aw**.

The fluoroalkene was functionalised via dibromination (**13t,** **14ar**), cycloaddition with a 1,3-dipole (**15ar**), hydration (**16aw**) and epoxidation (**17t**–**17j**), with good to excellent diastereoselectivity and complete enantiospecificity (Fig. 6C). Allylic oxidation led to lactam **18p** with complete enantiospecificity, yielding pharmaceutically relevant pyrrolinones^68–71^ (Fig. 6D).

Deprotection of either the tosyl or nosyl group in pyrrolines (**4**) was facile under reducing or nucleophilic conditions, respectively (Fig. 6E), leading to the corresponding unprotected pyrrolines **19j,** **t,** **v,** **p**, that were stable and could be isolated with no loss in diastereo- or enantio-enrichment. The unprotected heterocycles were functionalised at nitrogen to form tertiary amines (**20j**) or amides (**21v,** **22v,** **23e,** **24v,** **25j**) in good to excellent yields (Fig. 6F), hence demonstrating the potential utility in drug design and development.

In conclusion, we have developed a general strategy for the efficient synthesis of semi-saturated four- and five-membered oxygen- and nitrogen-containing fluorinated heterocycles. These motifs were previously very rare and difficult to access in the case of fluorinated pyrrolines (**4**) and oxolenes (**6**), and unknown in the case of fluorinated azetines (**7**), which we formed from an N–H insertion pathway. Fluorination of the alkene stabilises these otherwise elusive classes, as demonstrated by kinetics, CV and metabolism studies, which enables the synthesis of structurally diverse scaffolds—including bridged and spirocyclic frameworks—with high diastereoselectivity and broad opportunities for derivatisation. By rendering stable fluorinated pyrrolines, oxolenes and azetines readily accessible from simple starting materials, this work opens new directions in both synthetic methodology and the design of next-generation therapeutics.

## Methods

Synthesis of FVI (3) from alkynes: an HDPE vial was charged with *p*-iodotoluene (2.2 mmol, 1.1 equiv.), Selectfluor (3 mmol, 1.5 equiv.), Ag_2_CO_3_ (0.2 mmol, 10 mol%), K_2_CO_3_ (6 mmol, 3 equiv.) and MeNO_2_ (12 mL). The mixture was cooled to 0 °C, 9HF. Pyridine was added (1.88 mL, 72 mmol, 36 HF equiv.), and the reaction mixture was stirred vigorously at 0 °C for 10 min. The relevant alkyne (2 mmol, 1 equiv.) was then dissolved in 2 mL MeNO_2_ and added dropwise to the HDPE vial. The vial was warmed to RT and stirred for 18 h. Upon completion, the reaction was diluted with H_2_O (100 mL) and dimethyl carbonate (50 mL). The organic layer was washed with H_2_O (4 × 100 mL) and a 2 M aqueous solution of NaBF_4_ (100 mL), and the aqueous washes were quenched with excess sat. aq. NaHCO_3_. The organic layer was then filtered and concentrated in vacuo to afford the crude fluorovinyl iodonium salt (FVI), which was triturated using Et_2_O with sonication to give the pure FVI.

Synthesis of semi-saturated fluorinated heterocycle: a Schlenk tube was taken into a glove box under N_2_ and charged with NaO*t*Bu (0.6 mmol, 3 equiv.). After removal from the glove box, dry 1,4-dioxane (1 mL) was added and stirred until dissolved. In a separate vial, the relevant FVI (0.2 mmol, 1 equiv.) was dissolved in 1,4-dioxane (1 mL). With the NaO*t*Bu stirring vigorously, the FVI solution was added to it dropwise over 4 min (0.05 mmol/min). The reaction was stirred for 2 h under air at RT, monitoring reaction progress by TLC. Upon completion, the reaction mixture was diluted with Et_2_O (30 mL) and washed with H_2_O (2 × 20 mL). The organic layer was dried over MgSO_4_, filtered, and concentrated *in vacuo* to afford the crude product, which was then purified by column chromatography.

## Supplementary information

Supplementary Information

Transparent Peer Review File

## Data Availability

The data that underpin the findings reported in this study can be found in the Supplementary Information file, which is available free of charge, and includes experimental and computational procedures, characterisation data for known and new compounds and spectra for new compounds. All data are available from the corresponding author upon request.
