# Supplementary material for: Synthesis of 4- and 5-membered fluorine-stabilised semi-saturated heterocycles via alkylidene carbenes
Source: Nat Commun. 2026 Sep 28;17:11200. doi: 10.1038/s41467-026-77771-y (PMC13714270; doi:10.1038/s41467-026-77771-y)
Supplement: Supplementary file 1 [file 41467_2026_77771_MOESM1_ESM.pdf]

Supplementary Information

Synthesis of 4- and 5-Membered Fluorine-Stabilised Semi-Saturated Heterocycles via Alkylidene Carbenes

E. Harvey Randle[a], Alexi T. Sedikides[a], Julien Lefranc[b], Alastair J. J. Lennox[a*]

[a]*School of Chemistry, University of Bristol, Cantock's Close, Bristol, BS8 1TS, UK.*

[b]*Merck KGaA, Frankfurter Str. 250, 64293 Darmstadt, Germany.*

**a.lennox@bristol.ac.uk*

Table of Contents

1.1. General experimental details **4**

1.2. General Non-Experimental details **6**

2. General procedures **7**

3. Optimisation Tables **13**

3.1. Discrete variable optimisation 13

3.2. Continuous variable optimisation (DoE) 15

4. Miscellaneous Experiments **16**

4.1. Variable Temperature NMR **16**

4.2. Oxidation Kinetics **17**

4.3. KIE study **18**

4.4. Oxetene Intermediate **25**

4.5. Use of less powerful leaving groups **26**

4.6. Deuteration of the FVI **27**

4.7. HOMO/LUMO of the carbene **37**

4.8. Bond Length / J-value analysis **38**

4.9. Plot of yield against Hammett parameter **38**

4.10. Long-term storage stability **40**

4.11. Acid and base stability **46**

5. Failed Substrates **47**

6. Biological Data **48**

6.1. Metabolic stability 48

6.2. Blood plasma stability 51

7. Methodology Benchmarking **53**

7.1. 3-Pyrrolines 53

7.2. 3-Oxolenes 54

8. Characterisation data **55**

8.1. Alkynes (1) 55

8.2. Fluorovinyl Iodonium Salts (FVIs) (3) 121

8.3. 3-Pyrrolines (4) 184

8.4. Other Cycles (5) 215

8.5. Oxolenes (6) 219

8.6. Azetines (7) 233

8.7. 2-Pyrrolines (8) 247

8.8. Pyrroles (9) 249

8.9. Pyrrolidines (10) 255

8.10. Oxolanes (11) 266

8.11. Azetidines (12) ... 267
8.12. Dibromides (13-14) ... 272
8.13. Cycloadditions (15) ... 274
8.14. Hydrations (16) ... 275
8.15. Epoxides (17) ... 276
8.16. Pyrrolinones (18) ... 278
8.17. Deprotections (19-25) ... 279
8.18. Pre-alkyne intermediates (26) ... 296
8.19. Miscellaneous (27-29) ... 317
9. NMR spectra for Novel Compounds ... 320
9.1. Alkynes (1) ... 320
9.2. FVIs (3) ... 373
9.3. 3-Pyrrolines (4) ... 495
9.4. Other Cycles (5) ... 547
9.5. Oxolenes (6) ... 555
9.6. Azetines (7) ... 581
9.7. 2-Pyrrolines (8) ... 610
9.8. Pyrroles (9) ... 612
9.9. Pyrrolidines (10) ... 622
9.10. Oxolanes (11) ... 645
9.11. Azetidines (12) ... 647
9.12. Dibromides (13-14) ... 657
9.13. Cycloadditions (15) ... 661
9.14. Hydrations (16) ... 663
9.15. Epoxides (17) ... 665
9.16. Pyrrolinones (18) ... 667
9.17. Deprotected Pyrrolines (19-25) ... 669
9.18. Pre-alkyne intermediates (26) ... 691
9.19. Miscellaneous (27-28) ... 695
10. Crystal Data ... 698
10.1. Pyrroline 4l (CCDC Deposition Number 2485038) ... 698
10.2. Pyrrolidine 10m (CCDC Deposition Number 2485039) ... 704
10.3. Azetine 7ar (CCDC Deposition Number 2485040) ... 710
10.4. Azetine 7aw (CCDC Deposition Number 2486256) ... 714
11. Supplementary References ... 717

1.1. General experimental details

Techniques

Unless otherwise stated, all manipulations were performed under standard atmospheric conditions. Manipulations involving air and moisture sensitive materials were conducted employing standard Schlenk-line and glovebox techniques, using vacuum lines attached to a double manifold with greaseless J. Youngs valves equipped with an oil pump (0.1 mmHg) under an atmosphere of dry nitrogen. All glassware was dried using a blowtorch, then allowed to cool under vacuum at 0.1 mmHg. The removal of solvents *in vacuo* was achieved using a Büchi rotary evaporator (bath temperatures up to 40 °C) at a pressure of 15 mmHg (diaphragm pump), or at 0.1 mmHg (oil pump) on a vacuum line at room temperature. The addition of liquids, with the exception of HF solutions, were added using a Gilson PIPETMAN p20.

Solvents

THF (tetrahydrofuran), DCM, CH_3CN and Et_2O was dried using an Anhydrous Engineering alumina column drying system situated in the University of Bristol's chemistry department. All solvents were collected using Strauss flasks using a gastight J. Youngs valve and stored over activated 4 Å molecular sieves under N_2. 1,4-Dioxane was purchased from Acros organics with molecular sieves pre-added in an acro-sealed bottle. Nitromethane, DMF, and deuterated solvents for NMR analysis were purchased from Sigma Aldrich and used without further purification.

Chromatography

Column chromatography was performed using silica gel (230-400 mesh, 60 Å pore size) or using a Biotage Selekt with Sfär Silica D Duo cartridges. Thin layer chromatography was performed using aluminium-backed F_{254} silica plates purchased from Sigma Aldrich. Visualisation was achieved by UV fluorescence, iodine vapours, potassium permanganate, or phosphomolybdic acid stains.

Reagents

Chemicals were purchased from Sigma Aldrich, Acros, Fluorochem, TCI, Fisher Scientific, Alfa Aesar, Manchester Organics, or Lancaster Chemicals and used as received unless otherwise stated.

Analysis

NMR spectra were recorded on Bruker Nano 400, Bruker Advance III HD 500 cryo, Jeol ECZ400-var, Jeol ECS400, Jeol ECZ400, Varian 600 cryo and Prodigy 500 spectrometers, recorded at 298 K unless stated otherwise. Chemical shifts (δ) are quoted in parts per million (ppm), referenced to the residual solvent peak (^{1}H and ^{13}C NMR only). Signals are described as singlets (s), doublets (d), triplets (t) and multiplets (m), or combinations thereof. Coupling constants (*J*) are quoted to the nearest 0.1 Hz. NMR shifts for novel compounds have been assigned with the use of the appropriate 2D NMR experiments, such as COSY, HSQC and HMBC. Mass spectra were recorded by the University of Bristol Mass Spectrometry Service, using a Thermo Scientific Orbitrap Elite spectrometer. Infrared spectra were recorded using a Perkin Elmer Spectrum Two FTIR spectrometer. High Resolution Mass Spectrometry was recorded on QExactive (GC-Orbitrap), Orbitrap Elite (LCOrbitrap) or Synapt G2S (IMS-Q-TOF) instruments using electron ionisation (EI), electrospray ionization (ESI), and nanospray techniques. Samples were submitted neat. Optical rotations were measured on a ADP450 polarimeter from Bellingham + Stanley Ltd. Enantiomeric ratios were determined by HPLC on an Agilent 1100 series or Agilent Technologies 1260 Infinity instrument with UV detection at 230, 280 and 254 nm.

Warning: Use of HF reagents

The hazards of hydrogen fluoride solutions are well categorised and can be viewed in this SDS (https://www.sigmaaldrich.com/catalog/product/aldrich/184225). Therefore, personal protection is of utmost importance. It is advised to wear two pairs of nitrile gloves when handling, and if the gloves come into contact with HF, they are removed immediately, the area affected washed thoroughly with Hexafluorine solution™ (http://www.medicalcare.se/diphoterine-and-hexafluorine), calcium gluconate gel is applied to the area and medical attention is sought. Hexafluorine solution™ and calcium gluconate gel must be kept nearby in case of contact with any HF containing solution.

1.2. General Non-Experimental details

Data for Figure 1B was generated from a substructure search in June 2025 on Reaxys. The results for each heterocycle were limited to those published in the following medicinally chemistry relevant journals:

J. Med. Chem.

Bioorg. Med. Chem. Lett.

Eur. J. Med. Chem.

Bioorg. Med. Chem.

Front. Pharmacology

The numbers found in each journal were then totalled and plotted.

2. General procedures

General procedure I (Tosylation)

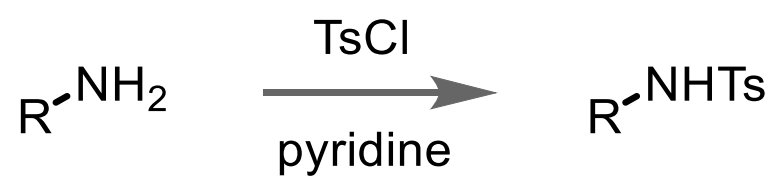

26

A Schlenk tube was charged with tosyl chloride (6 mmol, 1.2 equiv.) and purged with N_2. Pyridine (10 mL) was then added, and the tube was cooled to 0 °C. The relevant amine (5 mmol, 1 equiv.) was then added dropwise to the tube via needle (added as a solution in 1 mL pyridine if solid) and stirred at 0 °C for 2 h. The tube was then warmed to RT and stirred for a further 18 h, monitoring reaction progress by TLC. Upon completion, the reaction mixture was diluted with EtOAc (30 mL) and washed with aqueous 1 M $HCl_{(aq)}$ (2 × 30 mL). The organic layer was dried over $MgSO_4$, filtered, and concentrated *in vacuo* to afford the crude product.

General procedure II (Propargylation)

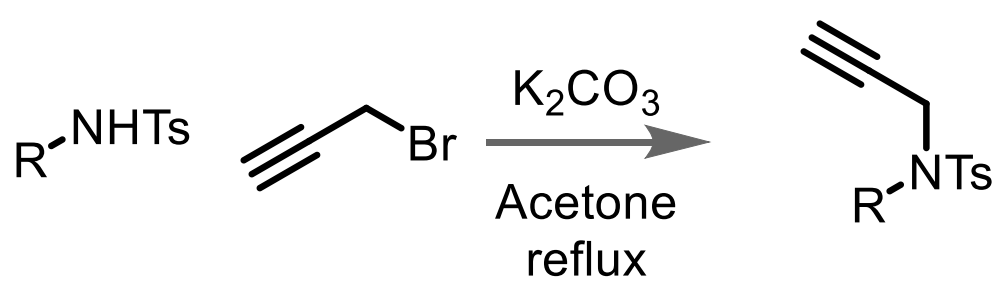

26 **1**

To a round-bottom flask containing the relevant tosyl amine (5 mmol, 1 equiv.) was added acetone (15 mL), K_2CO_3 (15 mmol, 3 equiv.), and propargyl bromide (80% w/w in toluene, 0.645 mL, 6 mmol, 1.2 equiv.). The mixture was heated to reflux and stirred for 18 h, monitoring reaction progress by TLC. Upon completion, the reaction mixture was concentrated *in vacuo*, redissolved in H_2O (30 mL) and extracted with DCM (3 × 20 mL). The organic fractions were combined and dried over $MgSO_4$, filtered, and concentrated *in vacuo* to afford the crude product.

General procedure II-A (other electrophiles)

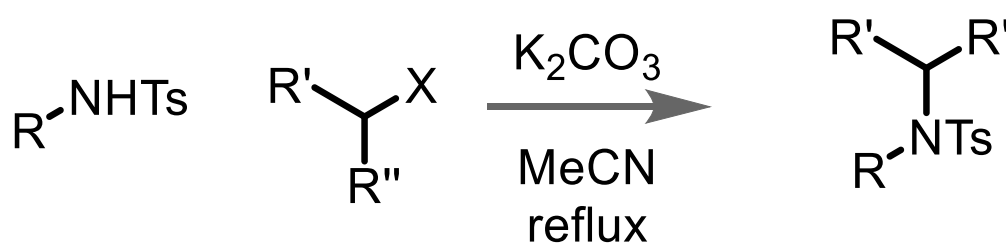

26 **1**

To a round-bottom flask containing the relevant *p*-toluenesulfonamide (5 mmol, 1 equiv.) was added MeCN (15 mL), K_2CO_3 (15 mmol, 3 equiv.), and the relevant electrophile, (7.5 mmol, 1.5 equiv.). The mixture was heated to reflux and stirred for 18 h, monitoring reaction progress by TLC. Upon completion, the reaction mixture was concentrated *in vacuo*, redissolved in H_2O (30 mL) and extracted

with DCM (3 × 20 mL). The organic fractions were combined and dried over $MgSO_4$, filtered, and concentrated *in vacuo* to afford the crude product.

General procedure III (Fluorovinyl iodonium salt (FVI) synthesis - from original FVI paper).[1]

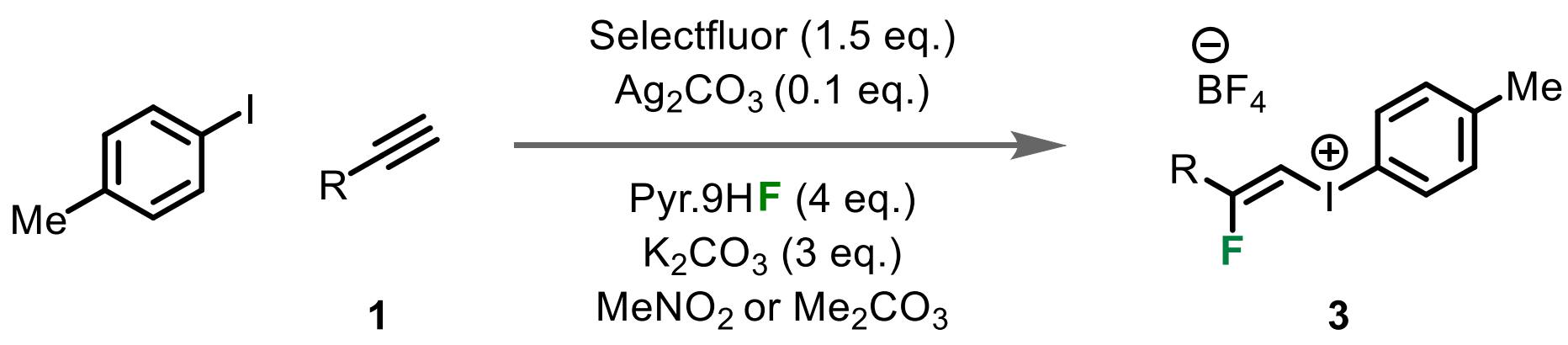

An HDPE vial was charged with *p*-iodotoluene (2.2 mmol, 1.1 equiv.), Selectfluor (3 mmol, 1.5 equiv.), Ag_2CO_3 (0.2 mmol, 10 mol%), K_2CO_3 (6 mmol, 3 equiv.), and $MeNO_2$ (12 mL). The mixture was cooled to 0 °C, 9HF.Pyridine was added (1.88 mL, 72 mmol, 36 HF equiv.), and the reaction mixture was stirred vigorously at 0 °C for 10 minutes. The relevant alkyne (2 mmol, 1 equiv.) was then dissolved in 2 mL $MeNO_2$ and added dropwise to the HDPE vial. The vial was warmed to RT and stirred for 18 h. Upon completion, the reaction was diluted with H_2O (100 mL) and dimethyl carbonate (50 mL). The organic layer was washed with H_2O (4 × 100 mL) and a 2 M aqueous solution of $NaBF_4$ (100 mL) and the aqueous washes were quenched with excess sat. aq. $NaHCO_3$. The organic layer was then filtered and concentrated *in vacuo* to afford the crude fluorovinyl iodonium salt (FVI), which was triturated using Et_2O with sonication to give the pure FVI.

General procedure III-A (FVI synthesis – stoichiometric silver, adapted from original FVI paper).[1]

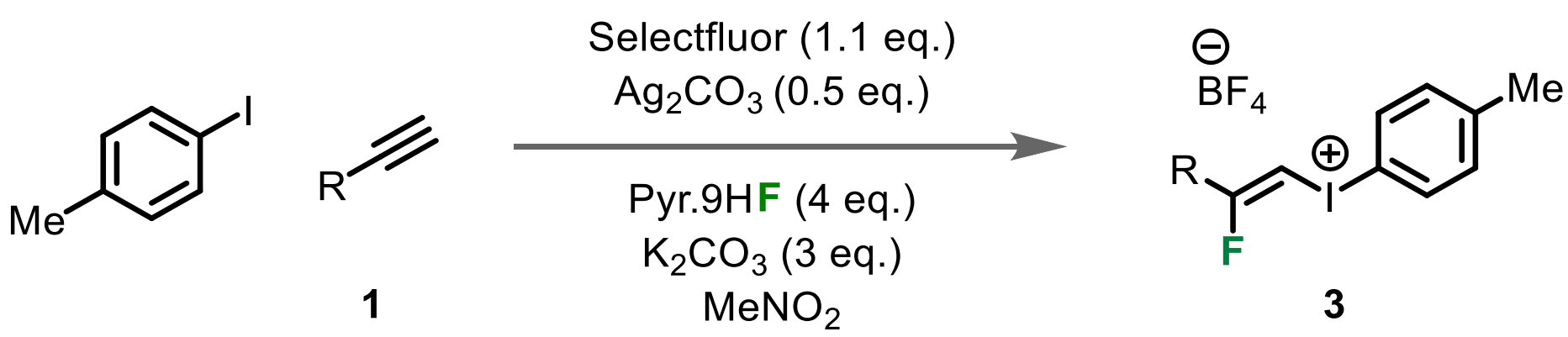

An HDPE vial was charged with *p*-iodotoluene (2.2 mmol, 1.1 equiv.), Selectfluor (2.2 mmol, 1.1 equiv.), Ag_2CO_3 (1.0 mmol, 50 mol%), K_2CO_3 (6 mmol, 3 equiv.), and $MeNO_2$ (12 mL). The mixture was cooled to 0 °C, 9HF.Pyridine was added (1.88 mL, 72 mmol, 36 HF equiv.), and the reaction mixture was stirred vigorously at 0 °C for 30 minutes. The relevant alkyne (2 mmol, 1 equiv.) was then dissolved in 2 mL $MeNO_2$ and added dropwise to the HDPE vial. The vial was allowed to warm slowly to RT for 18 h with stirring. Upon completion, the reaction was diluted with H_2O (100 mL) and Me_2CO_3 (50 mL). The organic layer was washed with H_2O (4 × 100 mL) and a 2 M aqueous solution of $NaBF_4$ (100 mL) and the aqueous washes were quenched with excess sat. aq. $NaHCO_3$. The organic layer was then filtered and concentrated *in vacuo* to afford the crude FVI, which was triturated using Et_2O with sonication to give the pure FVI.

General procedure IV (Cyclisation)

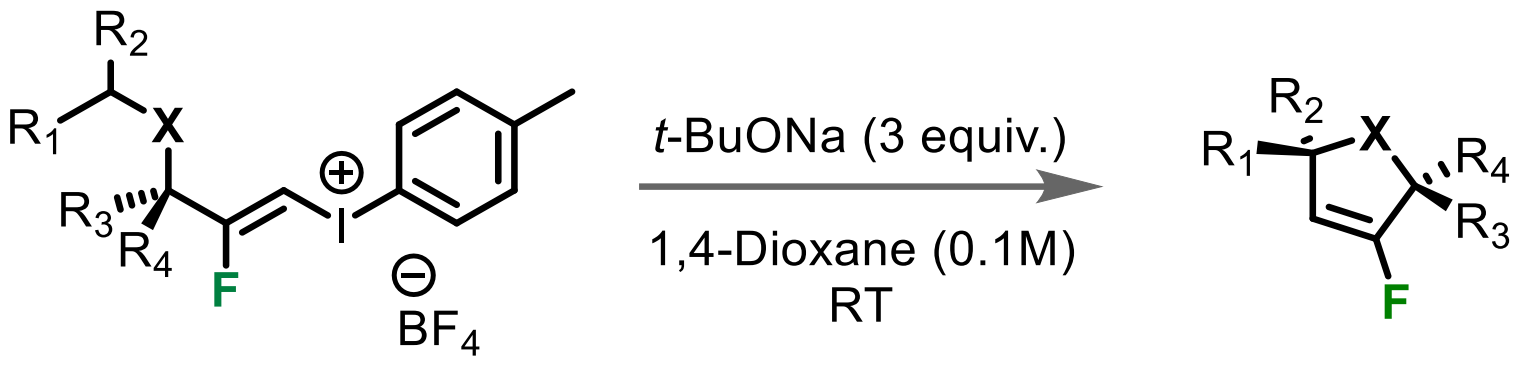

3 **X** = NTs, NNs, NMs, O **4, 6, 7**

A Schlenk tube was taken into a glove box under N_2 and charged with NaO*t*Bu (0.6 mmol, 3 equiv.). After removal from the glove box, dry 1,4-dioxane (1 mL) was added and stirred until dissolved. In a separate vial, the relevant FVI (0.2 mmol, 1 equiv.) was dissolved in 1 mL 1,4-dioxane. With the NaO*t*Bu stirring vigorously, the FVI solution was added to it dropwise over 4 minutes (0.05 mmol/min). The reaction was stirred for 2 h under air at RT, monitoring reaction progress by TLC. Upon completion, the reaction mixture was diluted with Et_2O (30 mL) and washed with H_2O (2 × 20 mL). The organic layer was dried over $MgSO_4$, filtered, and concentrated *in vacuo* to afford the crude product, which was then purified by flash column chromatography (conditions specified individually).

General procedure V (Oxidation when R = aryl)

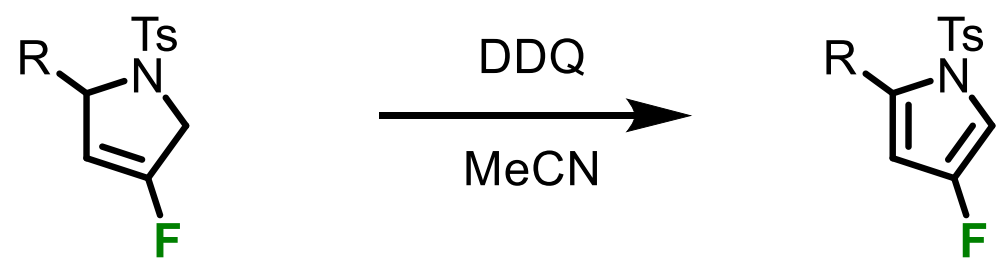

4 **9**

To a pressure tube containing the relevant heterocycle (0.2 mmol, 1 equiv.) in dry MeCN (2 mL) was added DDQ (1.0 mmol. 5 equiv.). The pressure tube was sealed and heated to reflux for 48 h. Reaction progress was monitored via TLC. Upon completion, the reaction mixture was concentrated *in vacuo* and purified by flash column chromatography to afford the pure product.

General procedure V-A (Oxidation when R = alkyl)

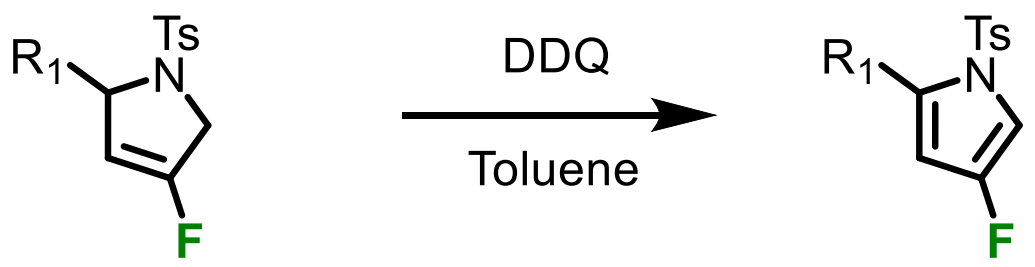

4v **9v**

To a pressure tube containing the relevant heterocycle (0.2 mmol, 1 equiv.) in dry toluene (2 mL) was added DDQ (1.0 mmol. 5 equiv.). The pressure tube was sealed and heated to reflux for 72 h. Reaction progress was monitored via TLC. Upon completion, the reaction mixture was concentrated *in vacuo* and purified by flash column chromatography to afford the pure product.

General procedure VI (Hydrogenation)

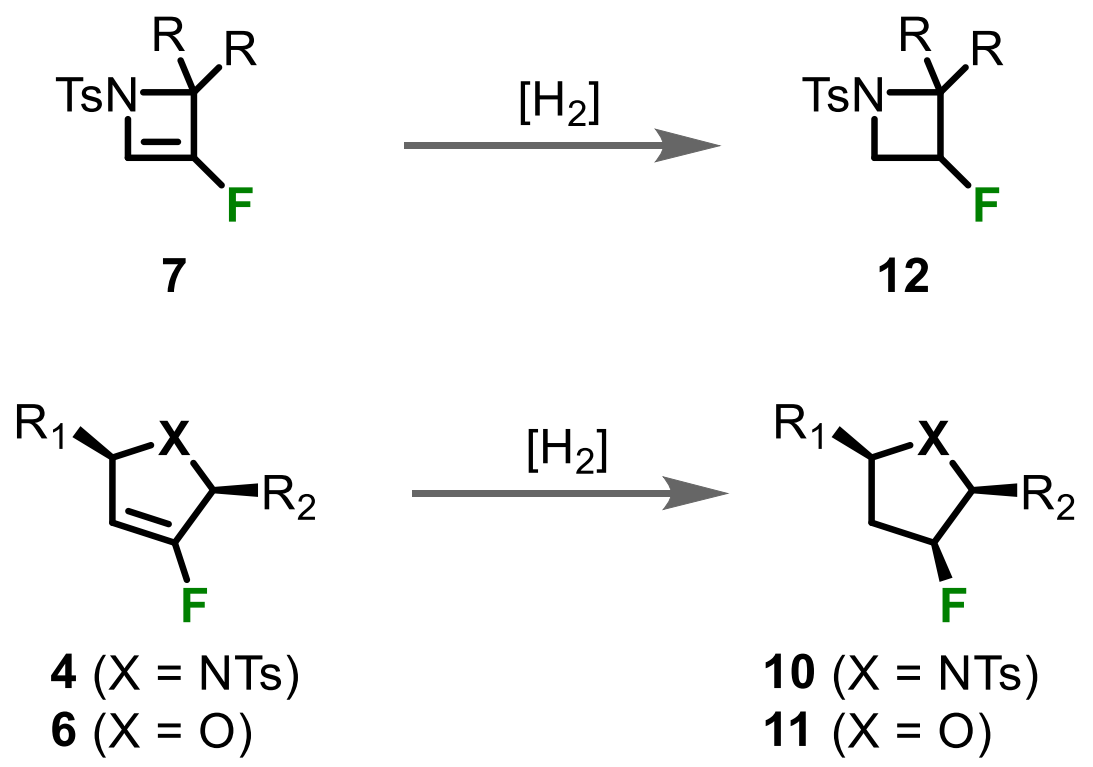

A 10 mL RBF containing palladium on carbon (10% wt., 0.02 mmol, 10 mol%) was sealed with a rubber septum and purged with N_2. A solution of the relevant heterocycle (0.2 mmol, 1 equiv.) in MeOH (4 mL) was added via needle, and N_2 was bubbled through the reaction mixture for 10 minutes. H_2 was then bubbled through the reaction mixture for 3 h using a balloon, and then the outlet needle was removed, leaving the headspace under a small positive pressure of H_2. The reaction mixture was stirred for 18 h, monitoring progress via TLC. Upon completion, the H_2 balloon was replaced with an N_2 balloon and bubbled for 10 mins. The reaction mixture was then filtered through celite and concentrated *in vacuo* to afford the crude product.

General procedure VII (Tosyl deprotection)

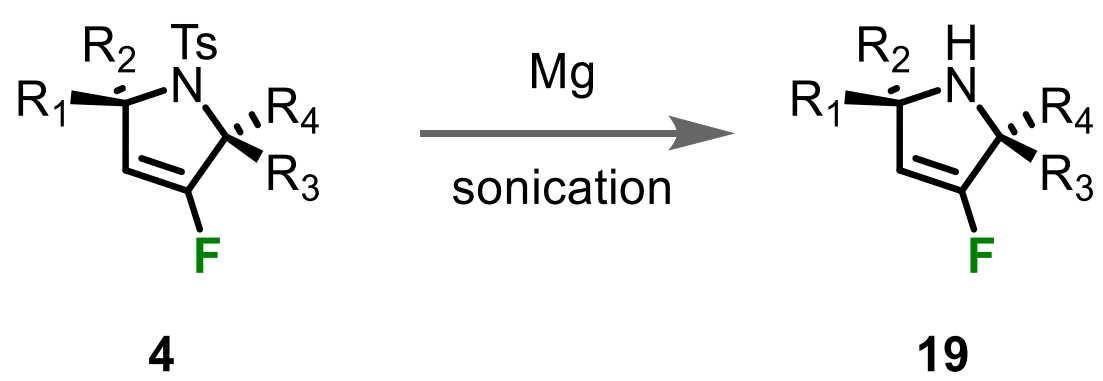

A Schlenk tube containing a magnetic stirrer bar was taken into a glove box, purged with N_2, and charged with Mg powder (10 mmol, 50 equiv.). The tube was then sealed with a rubber septum and removed from the glove box. A solution of the relevant tosylated heterocycle (0.2 mmol, 1 equiv.) in MeOH (6 mL) was added via needle and a near-empty N_2 balloon was added to the tube to contain any evolved H_2. The tube was then sonicated for 1 h, shaking the tube every 5 minutes for the first 15 minutes. Reaction progress was monitored via TLC of aliquots taken using a needle. Upon completion, the reaction mixture was poured over EtOAc (30 mL) with vigorous stirring to prevent formation of a gel. The solution was then filtered through celite and washed through with EtOAc. The organic solution

was then washed with H_2O (2 × 20 mL), dried over $MgSO_4$, filtered, and concentrated *in vacuo* to afford the crude product.

General procedure VII-A (Nosyl deprotection)

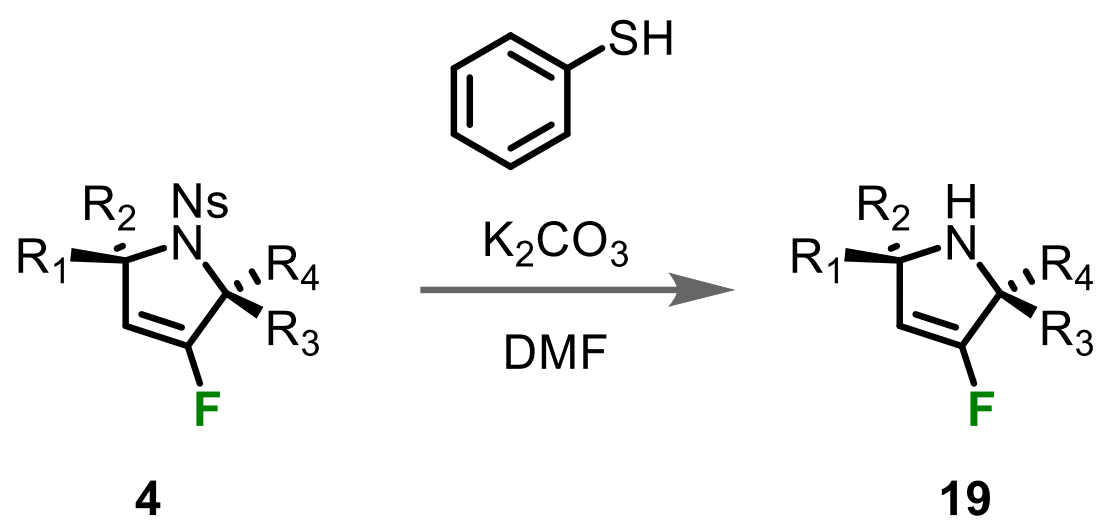

To a solution of the relevant nosylated heterocycle (0.2 mmol, 1 equiv.) in DMF (2 mL, 0.1 M) was added K_2CO_3 (0.8 mmol, 4 equiv.), followed by thiophenol (0.3 mmol, 1.5 equiv.). The reaction mixture was then stirred at RT for 4 h, monitoring reaction progress via TLC. Crude products from this deprotection were carried forward to the reprotection step.

General procedure VIII (1-phenylpropargylation)

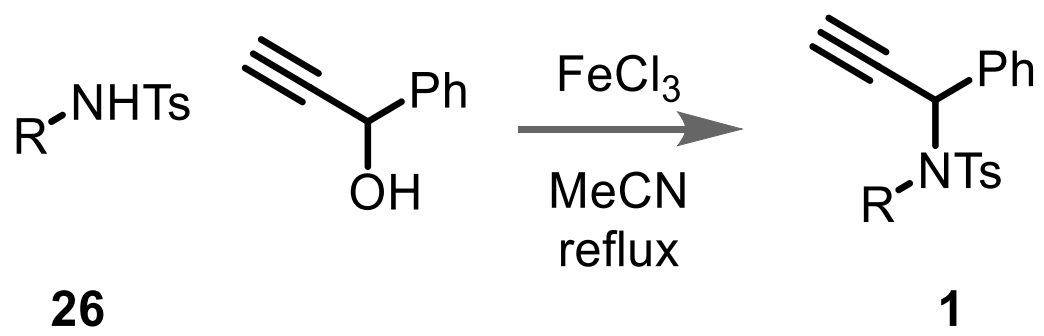

A 25 mL RBF was charged with 1-phenylprop-2-yn-1-ol (3 mmol, 1 equiv.), the relevant tosylated amine (9 mmol, 3 equiv.), and MeCN (6 mL). $FeCl_3$ (0.6 mmol, 20 mol%) was then added and the mixture was stirred at 60 °C for 18 h, monitoring progress by TLC. Upon completion, the reaction mixture was concentrated *in vacuo* and purified by flash column chromatography to afford the pure product.

General procedure IX (Etherification)

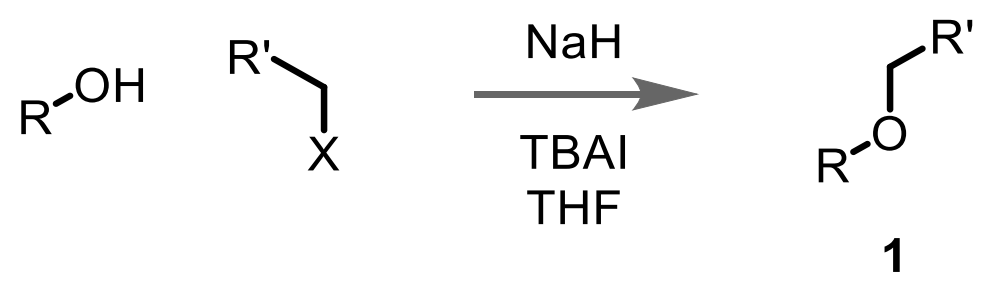

To a solution of the relevant alcohol (2 mmol, 1 equiv.) in dry THF (10 mL) was added NaH (60% dispersion in mineral oil, 2.2 mmol, 1.1 equiv.). The mixture was stirred at RT for 1 h before adding the relevant electrophile (2 mmol, 1 equiv.) and TBAI (0.1 mmol, 5 mol%). The mixture was then stirred for a further 18 h, monitoring progress by TLC. Upon completion, the reaction mixture was diluted with

Et_2O (20 mL), washed with H_2O (2 × 30 mL), dried over $MgSO_4$, filtered, and concentrated *in vacuo* to afford the crude product.

General procedure X (Addition of lithium trimethylsilyl acetylide to aldehydes and imines)

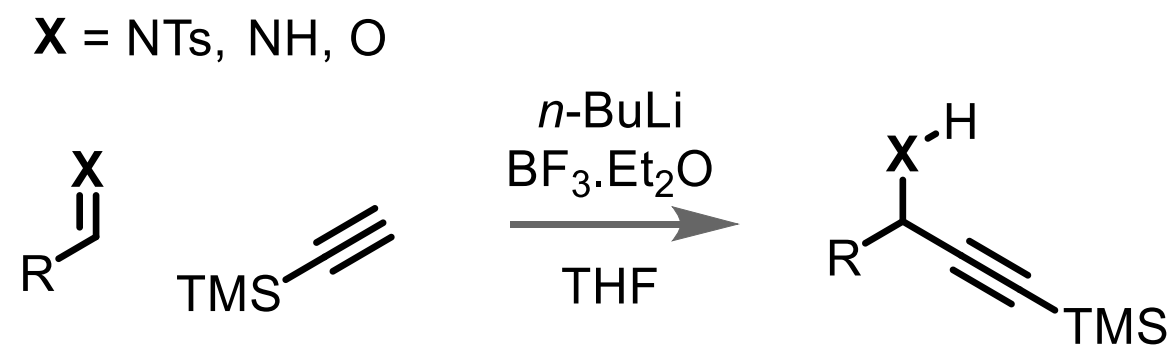

26 1

A Schlenk tube containing ethynyltrimethylsilane (29.3 mmol, 4.05 mL) in anhydrous THF (19.5 mL) was cooled to −78 °C in a dry ice / acetone bath. *n*-BuLi (2.5 M in hexanes, 23.4 mmol, 9.36 mL) was then added dropwise and stirred at −78 °C for 1 h. The relevant imine or aldehyde (19.5 mmol) and $BF_3.Et_2O$ (48.75 mmol, 6.92 mL) were then added dropwise and stirred for 30 minutes, after which the solution was allowed to warm to RT and stirred overnight. Reaction progress was monitored via TLC. Upon completion, the reaction mixture was then quenched with sat. aq. $NaHCO_3$ (100 mL) and extracted with EtOAc (3 × 50 mL). The organic fractions were combined, dried over $MgSO_4$, filtered, and concentrated *in vacuo* to afford the crude product.

3. Optimisation Tables

3.1. Discrete variable optimisation

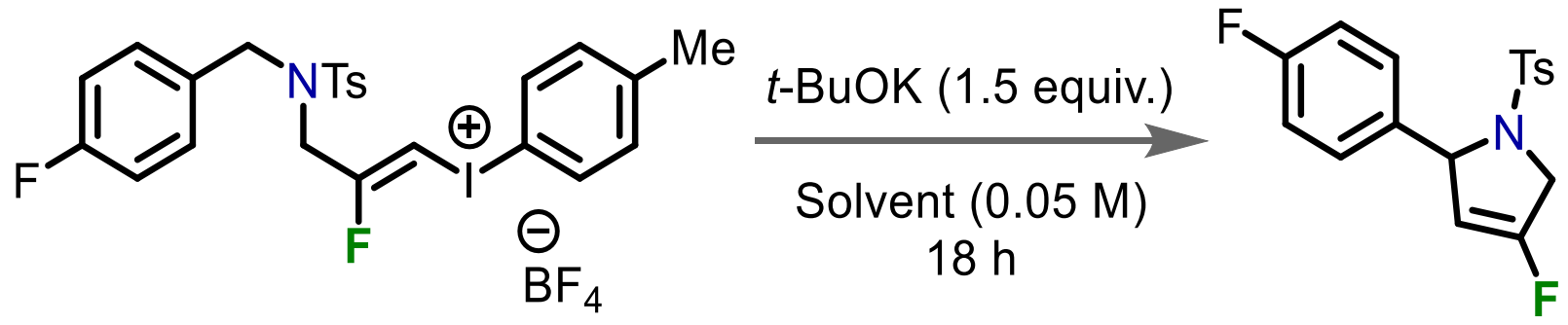

Entry	Solvent	Atmosphere	Temperature	RSM %	Yield %
**1**	dry THF	N_2	RT	0	29
**2**	dry THF	N_2	0 °C	0	28
**3**	wet THF	Air	RT	0	30
**4**	dry DCM	Air	RT	0	14
**5**	wet DCM	Air	RT	0	11
**6**	Acetonitrile	Air	RT	15	11
**7**	Dimethyl Carbonate	Air	RT	5	1
**8**	Propylene Carbonate	Air	RT	0	0
**9***	4:1 Et_2O/THF	Air	RT	0	37
**10**	Glyme	Air	RT	0	18
**11**	NMP	Air	RT	0	5

Supplementary Table 1: Air and moisture sensitivity and solvent screening. Both fluorovinyl iodonium salt (FVI) and base were dissolved in equal amounts of solvent (0.05 M) and the solution of base added dropwise to the solution of FVI. Reactions carried out on 0.05 mmol scale. *optimal result carried forward to base screening. Data show reaction not to be sensitive to air or moisture.

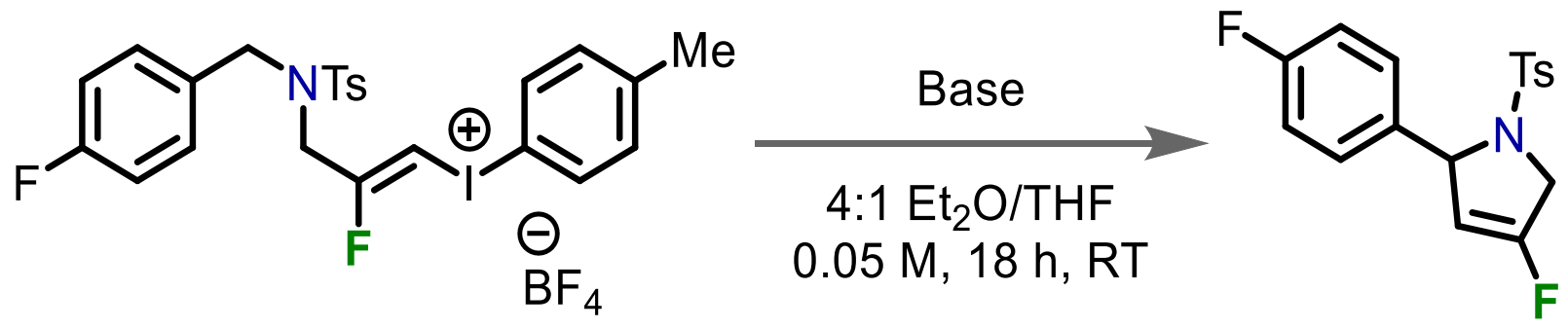

Entry	Base	Base equiv.	RSM %	Yield %
**1**	K_2CO_3	1.5	61	2
**2**	2,6-lutidine	1.5	93	0
**3**	DBU	1.5	0	0
**4**	TBAF	1.5	0	1
**5**	Et_3N	1.5	0	0
**6**	MeONa	1.5	0	6
**7**	*t*-BuOK	1.5	0	37
**8**	*t*-BuONa	1.5	0	56
**9**	*t*-BuOLi	1.5	0	55
**10**	*t*-BuONa	2	0	60
**11**	*t*-BuONa	2.5	0	71
**12***	*t*-BuONa	3	0	72

Supplementary Table 2: Base screening. Procedure unchanged from **Supplementary Table 1**. Reactions carried out on 0.05 mmol scale. *optimal result carried forward to second solvent screen.

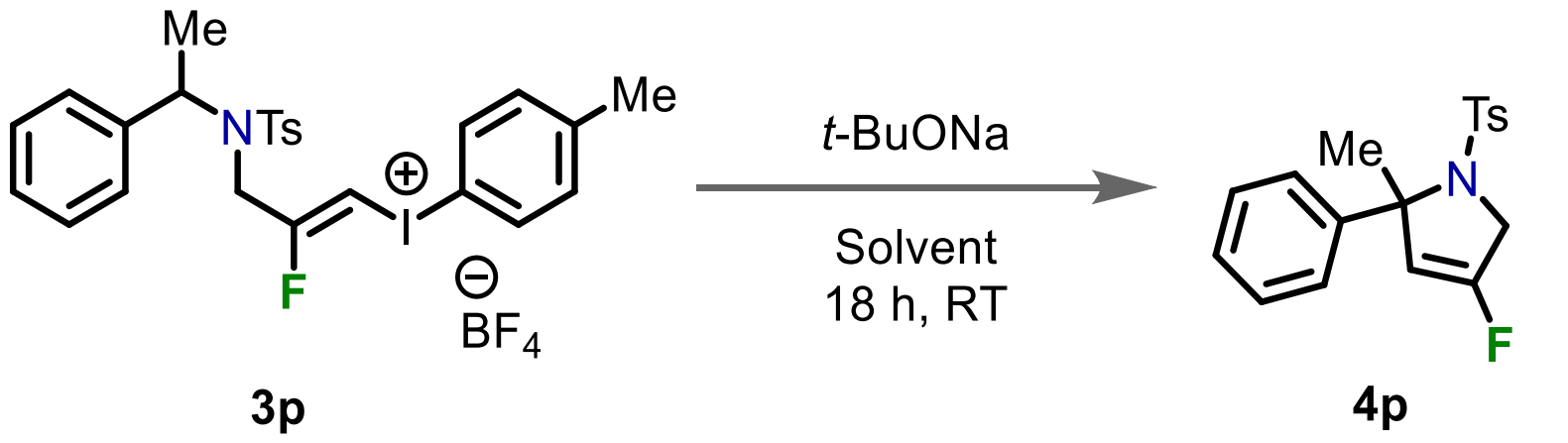

Entry	Solvent	Modification	Base equiv.	Conc. (M)	Yield %
**1**	1:1 Et_2O/THF	-	2.5	0.01	34
**2**	4:1 Et_2O/THF	-	2.5	0.01	29
**3**	THF	-	2.5	0.01	25
**4**	Et_2O	-	2.5	0.01	7
**5**	TBME	-	2.5	0.01	2
**6***	1,4-Dioxane	-	2.5	0.01	36
**7**	4:1 Et_2O/THF	2.5 equiv. 15-crown-5	2.5	0.01	6
**8**	4:1 Et_2O/DCM	-	2.5	0.01	8
**9**	1,4-Dioxane	-	1.2	0.01	26
**10**	1,4-Dioxane	-	1.5	0.01	26
**11**	1,4-Dioxane	-	2.0	0.01	29
**12**	1,4-Dioxane	-	2.5	0.0017	35
**13**	1,4-Dioxane	Base added over 16 h	2.5	0.0017	15
**repeat**					14

Supplementary Table 3: 2nd solvent screen. *optimal result carried forward to DoE optimisation of continuous variables.

3.2. Continuous variable optimisation (DoE)

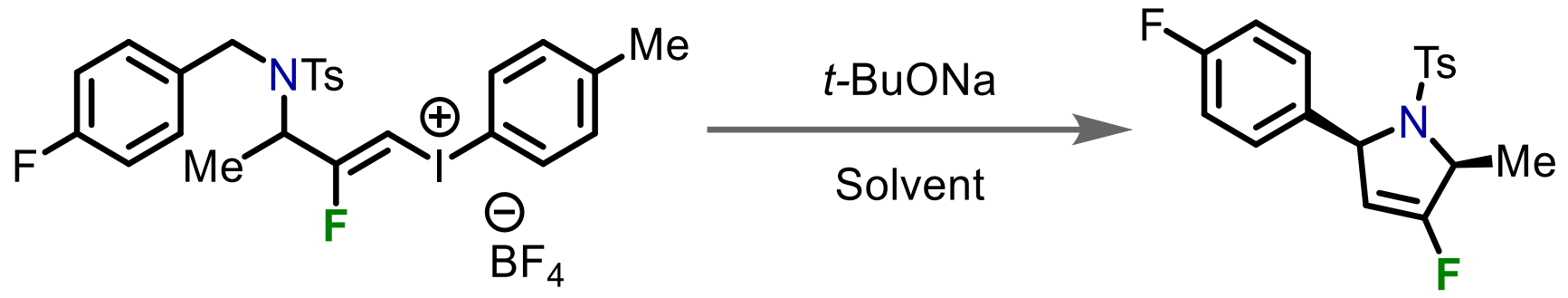

3j **4j**

Entry	Solvent	Conc. [mM]	Base equiv.	Addition rate (mmol/min)	Temp. (°C)	Time (h)	Yield %
**1**	1,4-Dioxane	10	2.05	0.1	RT	2	69
**2**	1,4-Dioxane	10	2.05	0.1	RT	2	68
**3**	1,4-Dioxane	10	2.05	0.1	RT	2	70
						4	70
						16	70
**4**	1,4-Dioxane	1	3.0	1	80	2	61
**5**	1,4-Dioxane	1	3.0	0.01	80	2	52
**6**	1,4-Dioxane	100	3.0	1	80	2	67
**7**	1,4-Dioxane	100	3.0	0.01	80	2	52
**8**	1,4-Dioxane	100	1.1	1	80	2	70
**9**	1,4-Dioxane	100	1.1	0.01	80	2	56
**10**[a]	1,4-Dioxane	1	1.1	1	80	2	48
**11**	1,4-Dioxane	1	1.1	0.01	80	2	49
**12**	4:1 Et_2O/THF	1	3.0	1	-40	2	62
**13**	4:1 Et_2O/THF	1	3.0	0.01	-40	2	53
**14**	4:1 Et_2O/THF	100	3.0	1	-40	2	68
**15**[a]	4:1 Et_2O/THF	100	3.0	0.01	-40	2	66
**16**	4:1 Et_2O/THF	100	1.1	1	-40	2	66
**17**	4:1 Et_2O/THF	100	1.1	0.01	-40	2	52
**18**	4:1 Et_2O/THF	1	1.1	1	-40	2	58
**19**[a]	4:1 Et_2O/THF	1	1.1	0.01	-40	2	61
**20**[b]	1,4-Dioxane	10	3.0	0.01	RT	2	84
**21**[b]	1,4-Dioxane	100	2.0	1	RT	2	68
**22**[b]*	1,4-Dioxane	100	2.0	0.05	RT	2	87
**23**[b]	1,4-Dioxane	100	2.0	0.001	RT	2	87

Supplementary Table 4: DoE optimisation of continuous variables. Base added dropwise to FVI as standard. [a] Anomalous result, omitted from model. [b] FVI added dropwise to base. *Optimal conditions used for substrate scope.

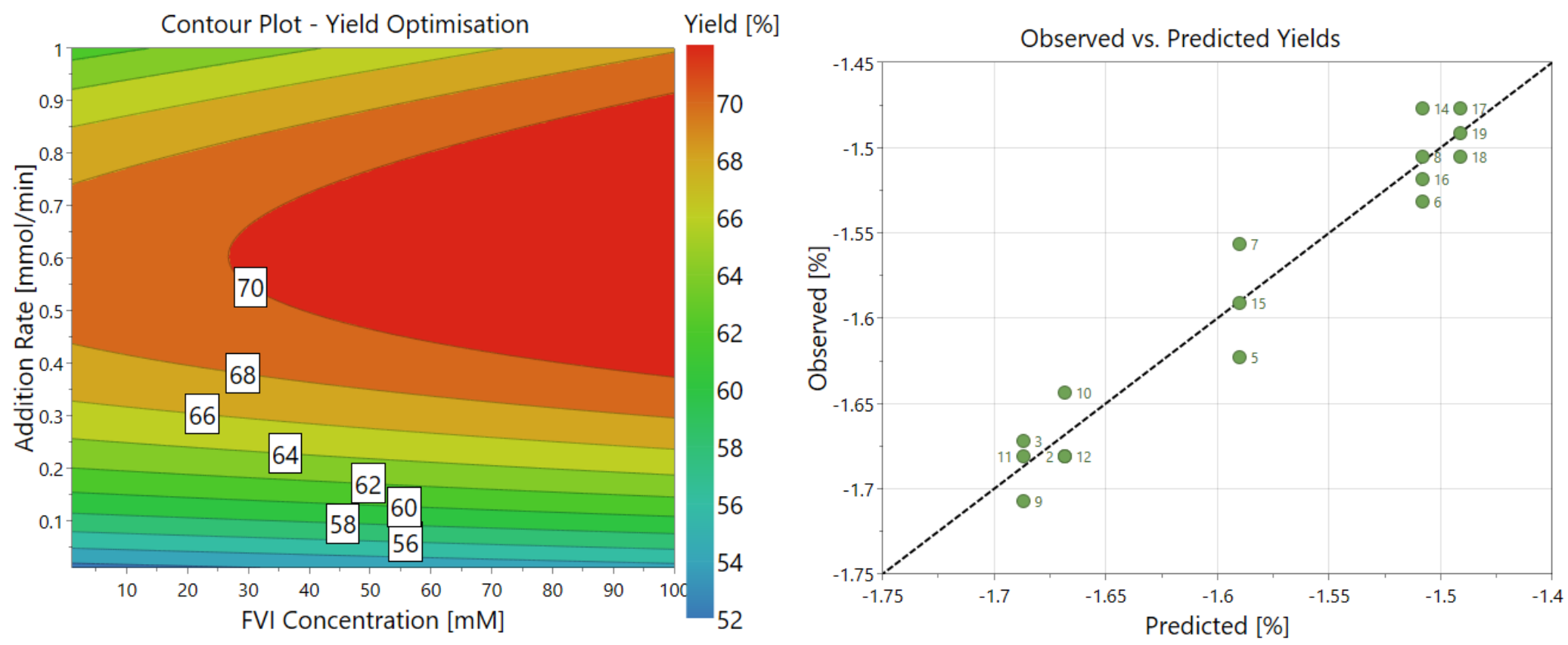

Supplementary Figure 1: Yield contour plot and observed vs predicted yields from MODDE optimisation predictive model (Full factorial design). R^2 = 0.946, Q^2 = 0.885, model validity = 0.669, reproducibility = 0.973.

4. Miscellaneous Experiments

4.1. Variable Temperature NMR

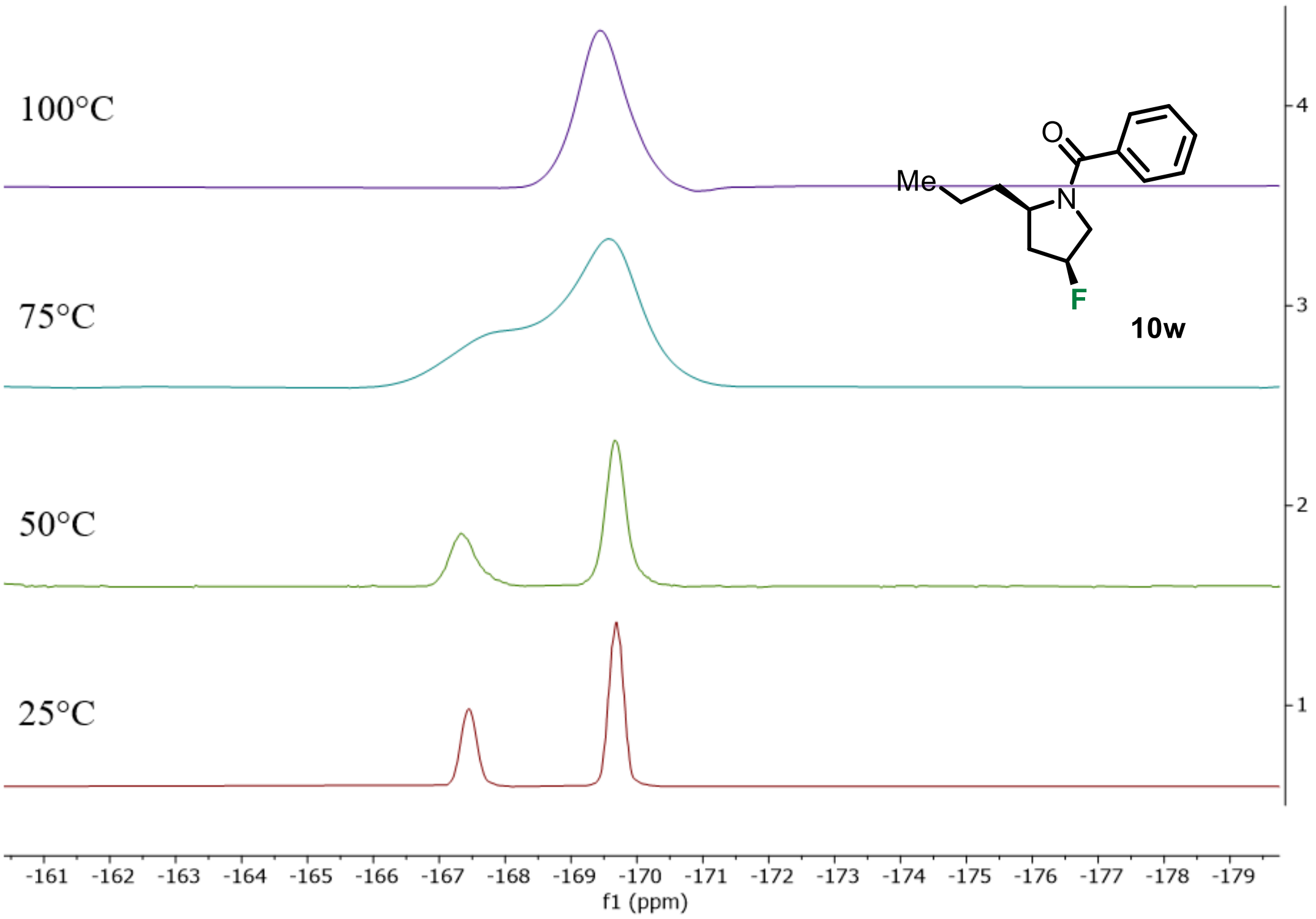

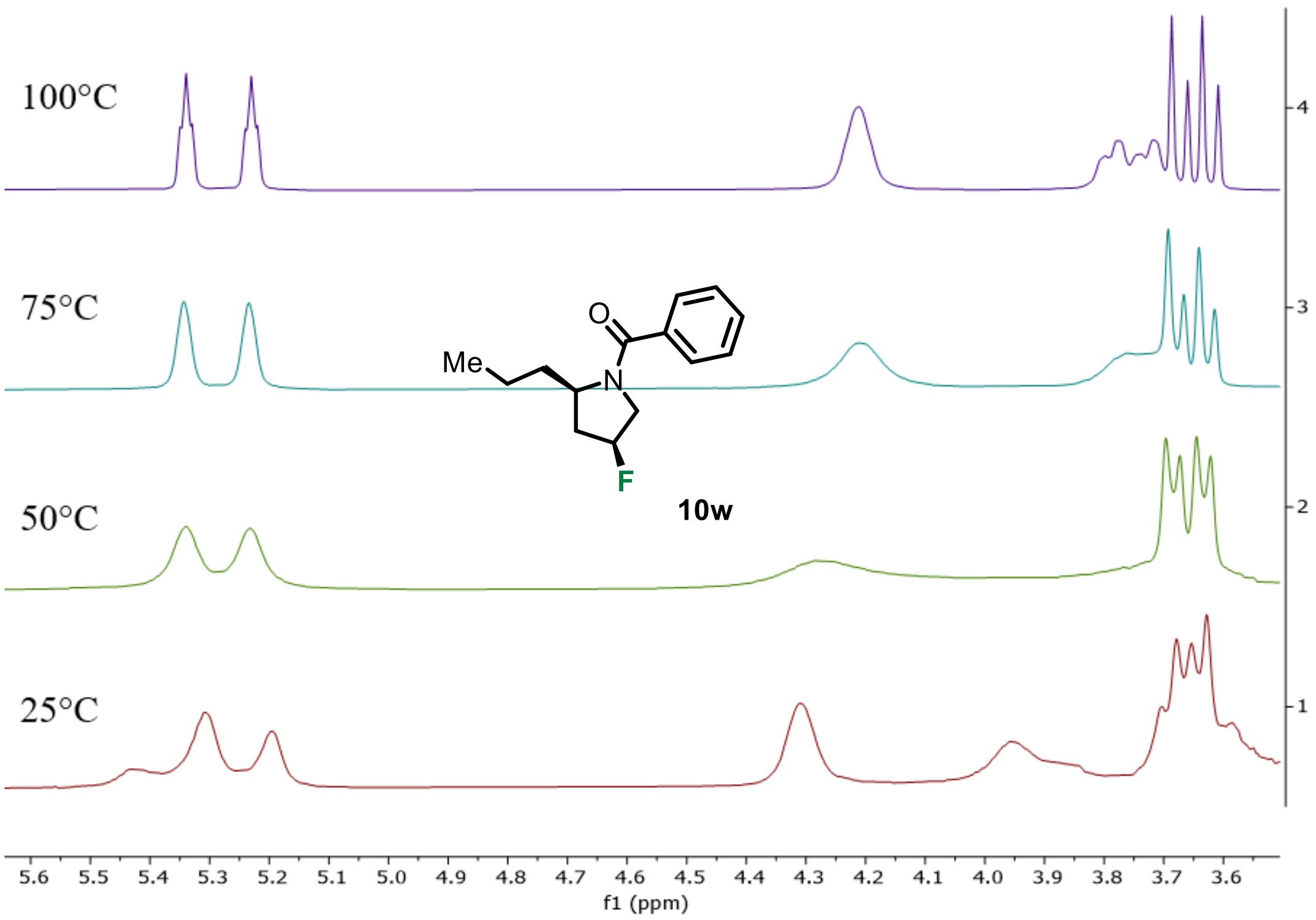

Supplementary Figure 2: Variable temperature NMR spectra for pyrrolidine **10w** (Top: ^{19}F. Bottom: ^{1}H). Spectra are taken at 25°C, 50°C, 75°C, and 100°C. The two amide rotamers are shown to coalesce into a single signal.

4.2. Oxidation Kinetics

The graph below shows the relative rates of oxidation by DDQ of a non-fluorinated pyrroline **4bf** and a fluorinated pyrroline **4v** to exemplify the stabilising effect of fluorine on oxidisable alkenes.

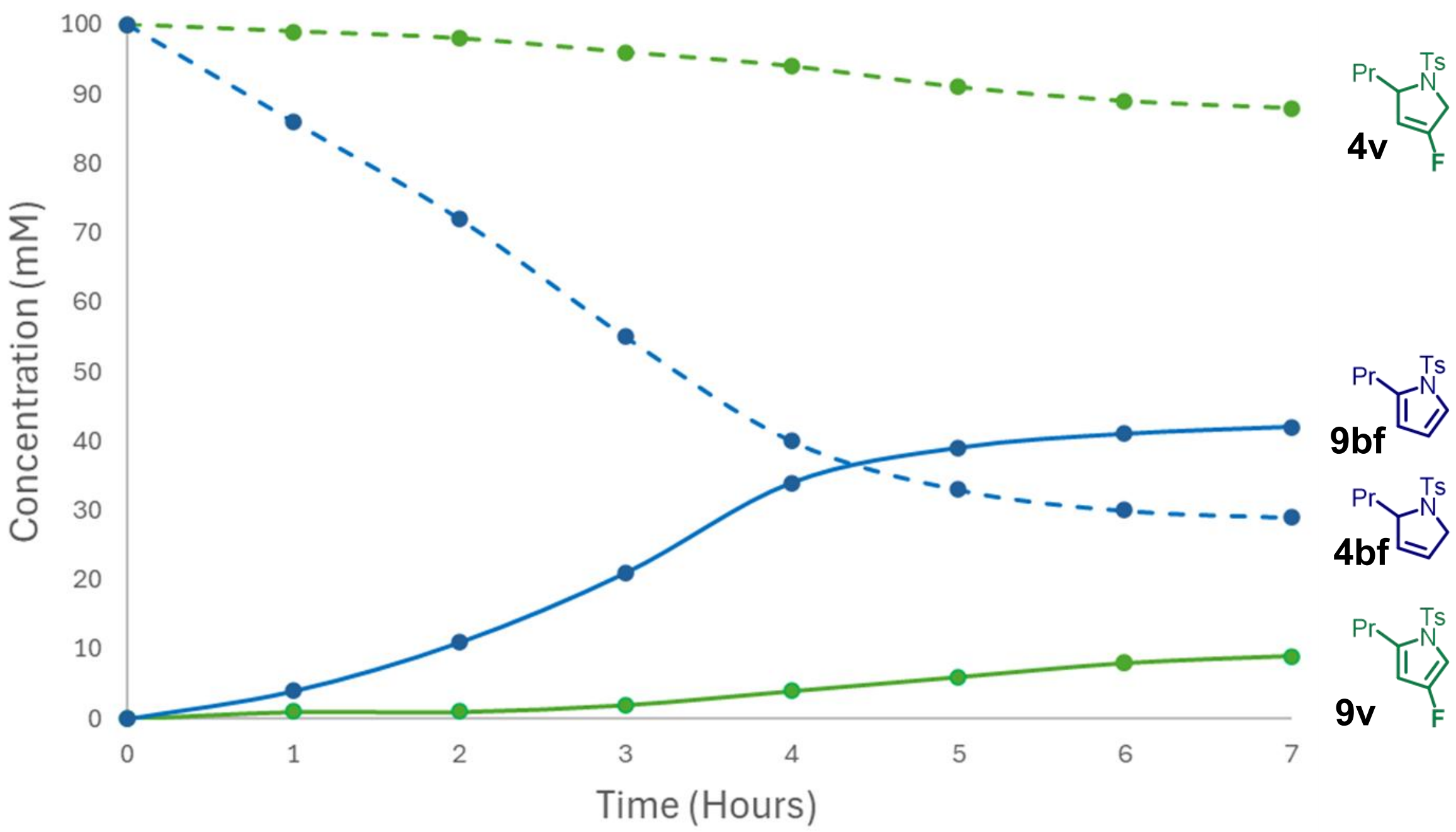

Supplementary Figure 3: Relative rates of dehydrogenative oxidation of the fluorinated *n*-propyl pyrroline substrate **4v** to the pyrrole **9v**, and the non-fluorinated pyrroline **4bf** to pyrrole **9bf**. Concentration of each compound at each time point is shown on the *y*-axis, and time points are shown on the *x*-axis. Conditions: 5 equiv. DDQ, 110°C, 0.1 M, Toluene. Concentrations were monitored via NMR sampling, using 1,3,5-trimethoxybenzene (^{1}H) and 4,4'-difluorobiphenyl (^{19}F) as NMR standards.

4.3. KIE study

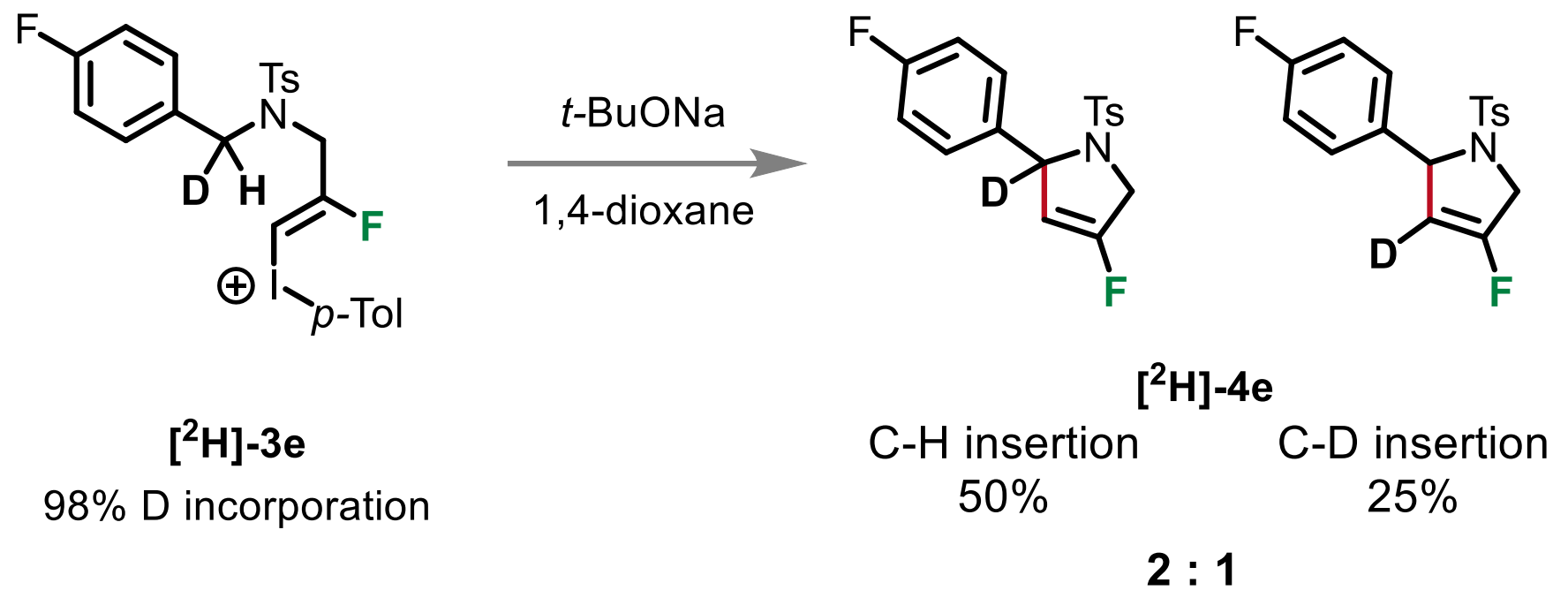

Supplementary Figure 4: Cyclisation of monodeuterated fluorovinyl iodonium salt (FVI) **[^{2}H]-3e** to pyrroline **[^{2}H]-4e** according to **General procedure IV**. NMR yields were measured from the crude reaction mixture, showing a kinetic isotope effect of 2.0. Products were isolated as an inseparable mixture, and deuterium incorporation was measured via NMR. See next pages for characterisation data of deuterated FVI and pyrrolines.

(Z)-(2-fluoro-3-((N-((4-fluorophenyl)methyl-d)-4-methylphenyl)sulfonamido)prop-1-en-1-yl)(p-tolyl)iodonium tetrafluoroborate **([²H]-3e)**

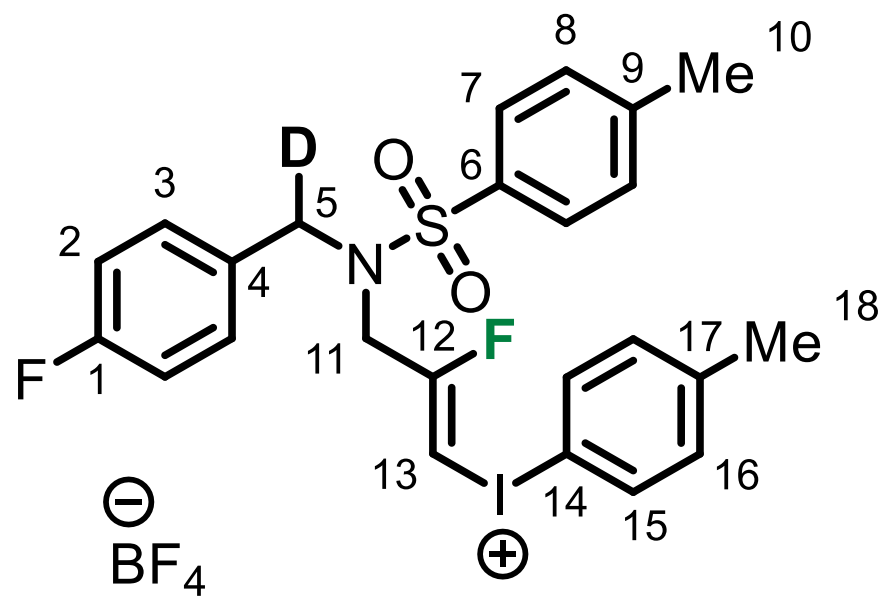

Produced according to **General procedure III** from alkyne **[²H]-1e** to afford the title compound as a white solid (896 mg, 70%, 98% D incorporation).

^{1}H NMR (500 MHz, Acetone-*D₆*) δ 7.99 (app. dt, *J* = 8.5, 1.9 Hz, 2H, C15-H), 7.80 (app. dt, *J* = 8.4, 1.8 Hz, 2H, C7-H), 7.44 (d, *J* = 8.4 Hz, 2H, C8-H), 7.42 (d, *J* = 8.5 Hz, 2H, C16-H), 7.33 – 7.26 (m, 2H, C3-H), 7.00 – 6.95 (m, 2H, C2-H), 6.97 (dt, *J* = 33.8, 1.0 Hz, 1H, C13-H), 4.35 (d, *J* = 12.2 Hz, 2H, C11-H), 4.32 (s, 1H, C5-H), 2.46 (s, 3H, C18-H), 2.45 (s, 3H, C10-H).

^{13}C NMR (126 MHz, Acetone-*D₆*) δ 168.0 (d, *J* = 278.7 Hz, C12), 163.2 (d, *J* = 244.7 Hz, C1), 145.1 (C17), 144.8 (C9), 136.8 (C6), 136.5 (C15), 133.8 (C16), 132.5 (d, *J* = 3.1 Hz, C4), 131.6 (d, *J* = 8.3 Hz, C3), 130.9 (C8), 128.3 (C7), 116.1 (d, *J* = 21.6 Hz, C2), 109.7 (C14), 80.7 (d, *J* = 19.1 Hz, C13), 52.2 (t (1:1:1), *J* = 21.2 Hz, C5), 48.0 (d, *J* = 30.3 Hz, C11), 21.5 (C18), 21.3 (C10).

^{19}F NMR (376 MHz, Acetone-*D₆*) δ −75.88 (dt, *J* = 33.7, 12.2 Hz, 1F, C12-F), −115.11 (tt, *J* = 9.4, 5.4 Hz, 1F, C1-F), −150.07 (d, *J* = 20.4 Hz, 4F, BF_4).

HRMS (+ESI) calcd: $[M]^+$ ($C_{24}H_{22}DF_2INO_2S$) 555.0520, measured 555.0498 (3.96 ppm error).

IR (neat) v_{max}/cm^{-1}: 3096, 2925, 1696, 1653, 1601, 1509, 1342, 1222, 1158, 1091, 1059, 996, 802, 664, 547.

***4-fluoro-2-(4-fluorophenyl)-1-tosyl-2,5-dihydro-1H-pyrrole-2-d (major), 4-fluoro-2-(4-fluorophenyl)-1-tosyl-2,5-dihydro-1H-pyrrole-3-d (minor)* ([2H]-4e)**

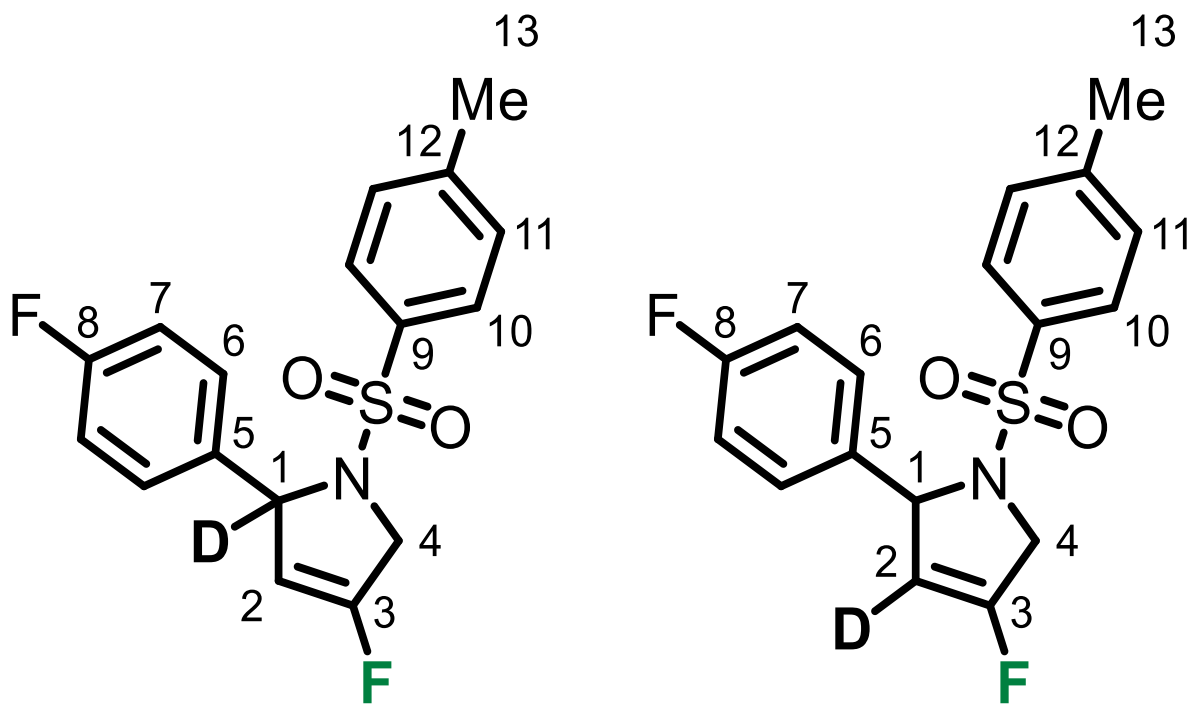

Prepared according to **General procedure IV** from **[2H]-3e** (75% NMR) and purified by flash column chromatography (2-20% EtOAc in pentane) to afford the title compound as a colourless oil (43.7 mg, 65%).

Products were obtained as an inseparable mixture (ratio 1:0.50).

^{1}H NMR (500 MHz, CDCl$_3$) Major regioisomer δ 7.49 (d, *J* = 8.2 Hz, 2H, C10-H), 7.27 – 7.23 (m, 2H, C6-H), 7.22 (d, *J* = 8.2 Hz, 2H, C11-H), 7.00 – 6.94 (m, 2H, C7-H), 5.05 (dt, *J* = 3.3, 1.6 Hz, 1H, C2-H), 4.33 – 4.26 (m, 1H, C4-H), 4.25 – 4.18 (m, 1H, C4-H), 2.40 (s, 3H, C13-H). **Minor regioisomer** δ 7.49 (d, *J* = 8.2 Hz, 2H, C10-H), 7.27 – 7.23 (m, 2H, C6-H), 7.22 (d, *J* = 8.2 Hz, 2H, C11-H), 7.00 – 6.94 (m, 2H, C7-H), 5.46 (td, *J* = 5.3, 2.4 Hz, 1H, C1-H), 4.33 – 4.26 (m, 1H, C4-H), 4.25 – 4.18 (m, 1H, C4-H), 2.40 (s, 3H, C13-H).

^{13}C NMR (126 MHz, CDCl$_3$) Major regioisomer δ 162.7 (d, *J* = 246.9 Hz, C8), 155.1 (d, *J* = 278.3 Hz, C3), 143.9 (C12), 135.9 (dd, *J* = 3.2, 2.0 Hz, C5), 135.1 (C9), 129.8 (C11), 129.2 (d, *J* = 8.3 Hz, C6), 127.4 (C10), 115.5 (d, *J* = 21.7 Hz, C7), 103.9 (d, *J* = 8.2 Hz, C2), 65.5 (1:1:1 td, *J* = 22.4, 7.3 Hz, C1), 49.8 (d, *J* = 30.7 Hz, C4), 21.6 (C13). **Minor regioisomer** δ 162.7 (d, *J* = 246.9 Hz, C8), 154.9 (d, *J* = 278.2 Hz, C3), 143.9 (C12), 136.0 (dd, *J* = 3.2, 2.0 Hz, C5), 135.1 (C9), 129.8 (C11), 129.2 (d, *J* = 8.3 Hz, C6), 127.4 (C10), 115.5 (d, *J* = 21.7 Hz, C7), 103.8 (1:1:1 td, *J* = 27.5, 8.1 Hz, C2), 65.8 (d, *J* = 7.1 Hz, C1), 49.9 (d, *J* = 30.7 Hz, C4), 21.6 (C13).

^{19}F NMR (376 MHz, CDCl$_3$) Major regioisomer δ −113.78 (tq, *J* = 8.8, 4.7 Hz, 1F, C8-F), −128.00 (t, *J* = 4.3 Hz, 1F, C3-F). **Minor regioisomer** δ −113.78 (tq, *J* = 8.8, 4.7 Hz, 1F, C8-F), −128.33 (dt, *J* = 5.3, 4.3 Hz, 1F, C3-F).

HRMS (+EI) calcd. $[M]^+$ ($C_{17}H_{14}DNO_2SF_2$) 336.0849, measured 336.0846 (0.89 ppm error).

IR (neat) v_{max}/cm^{-1}: 2924, 2871, 1696, 1676, 1604, 1508, 1348, 1160, 1094, 814, 673, 581, 569, 545.

(Z)-(2-fluoro-3-((N-((4-fluorophenyl)methyl-d)-4-methylphenyl)sulfonamido)prop-1-en-1-yl)(p-tolyl)iodonium tetrafluoroborate **([2H]-3e)**

^{1}H NMR (500 MHz, Acetone-D_6)

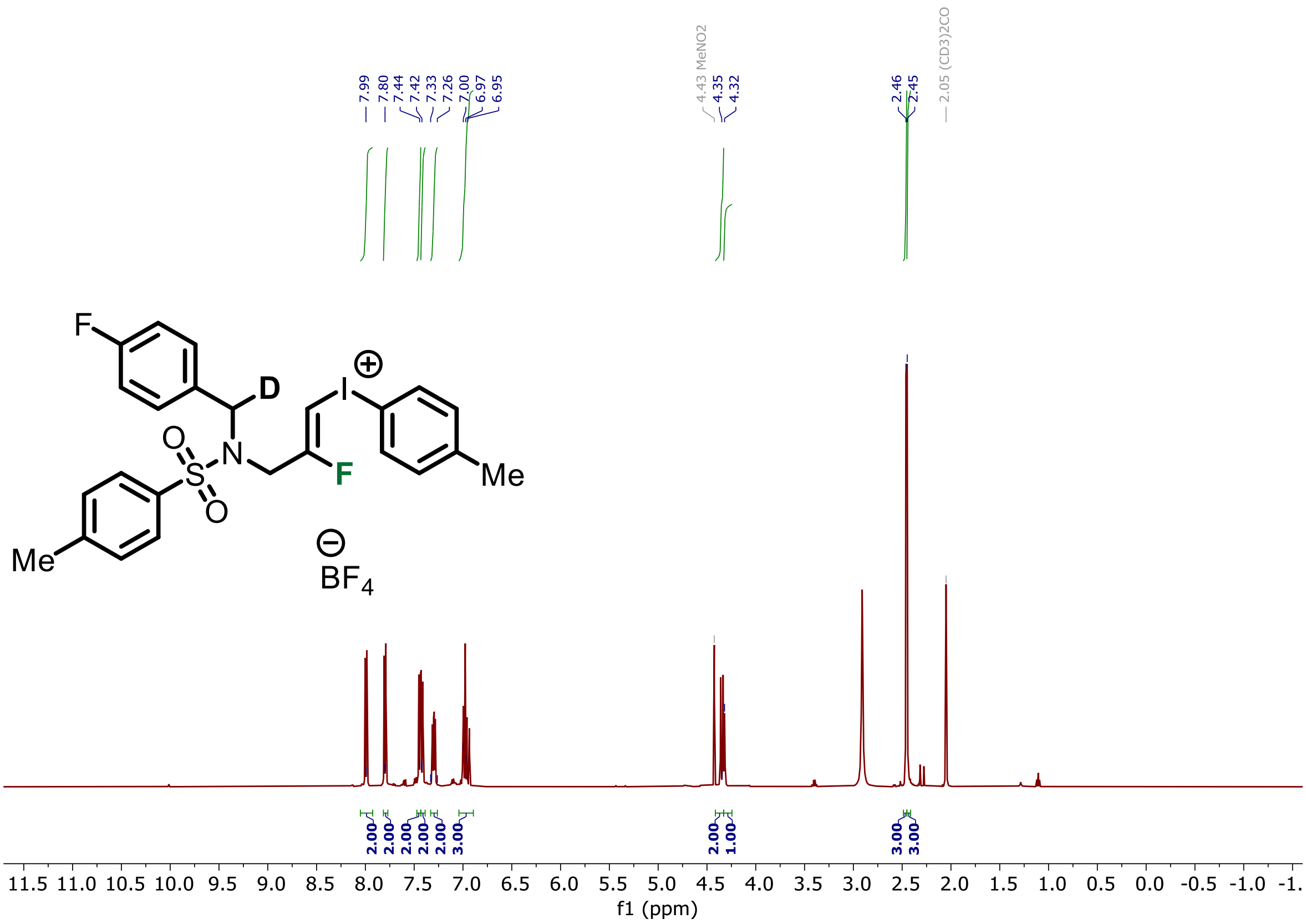

^{13}C NMR (126 MHz, Acetone-D_6)

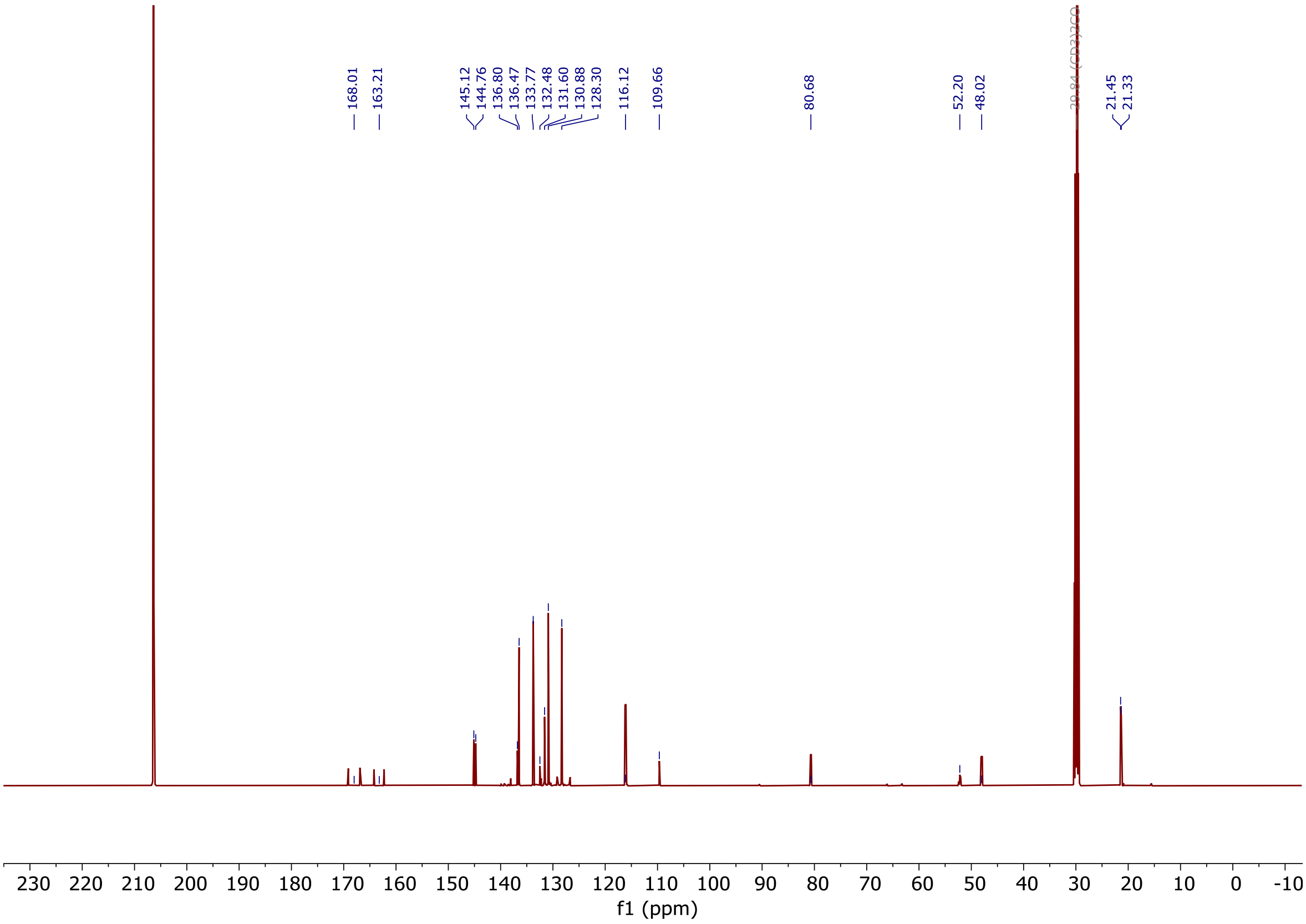

^{19}F NMR (376 MHz, Acetone-D_6)

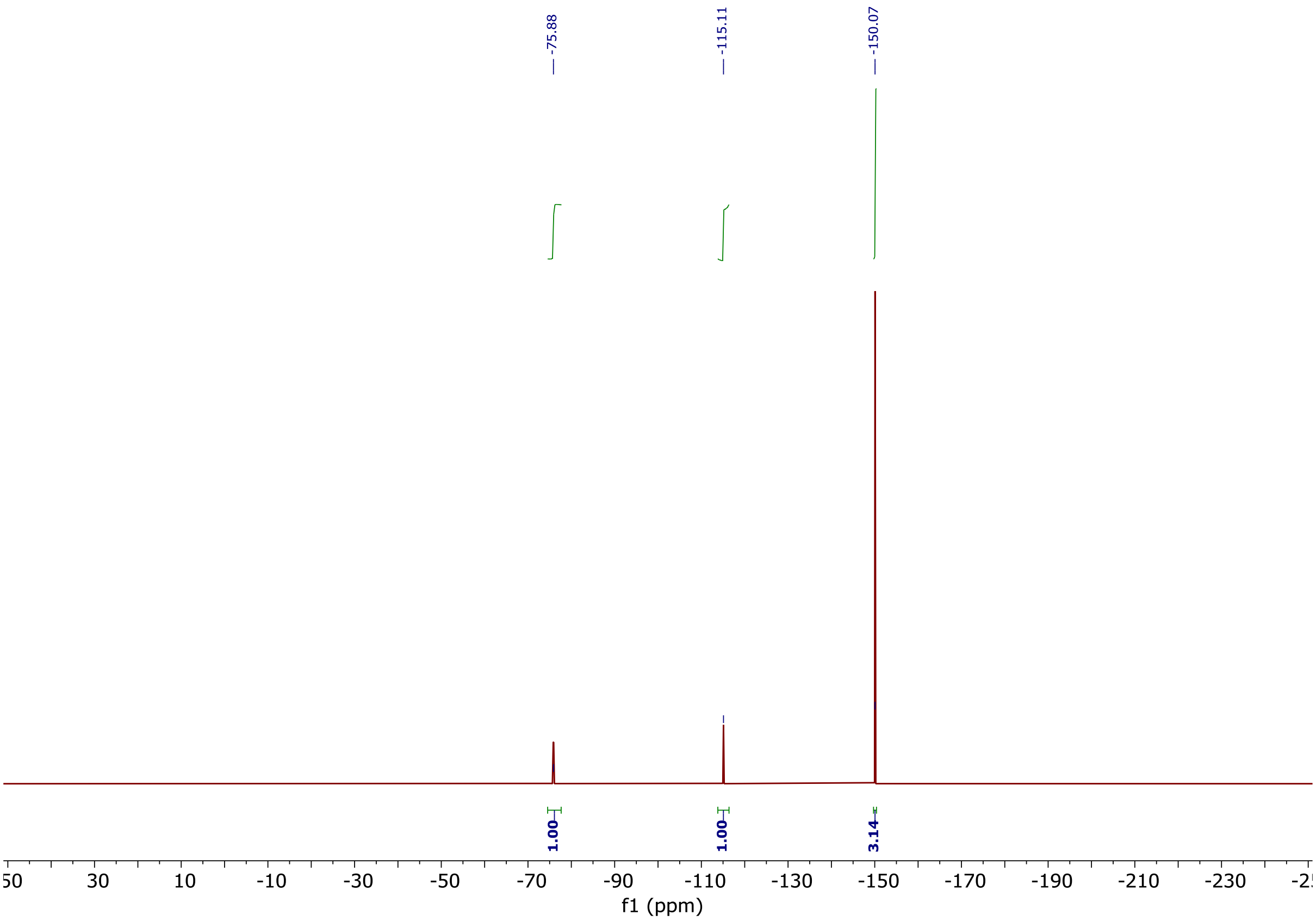

4-fluoro-2-(4-fluorophenyl)-1-tosyl-2,5-dihydro-1H-pyrrole-2-d (major), 4-fluoro-2-(4-fluorophenyl)-1-tosyl-2,5-dihydro-1H-pyrrole-3-d (minor) ([^{2}H]-4e)

^{1}H NMR (500 MHz, CDCl$_3$)

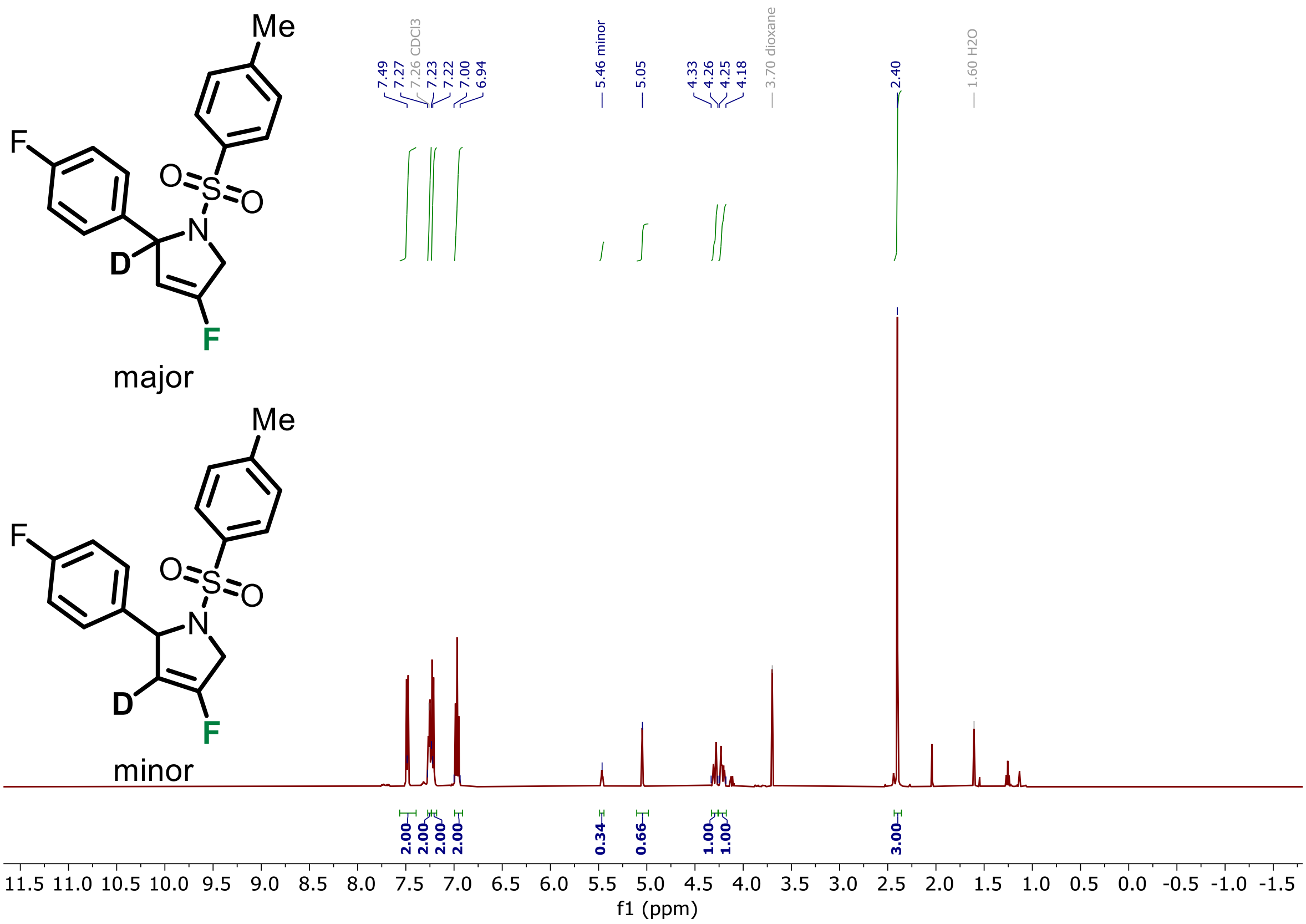

^{13}C NMR (126 MHz, $CDCl_3$)

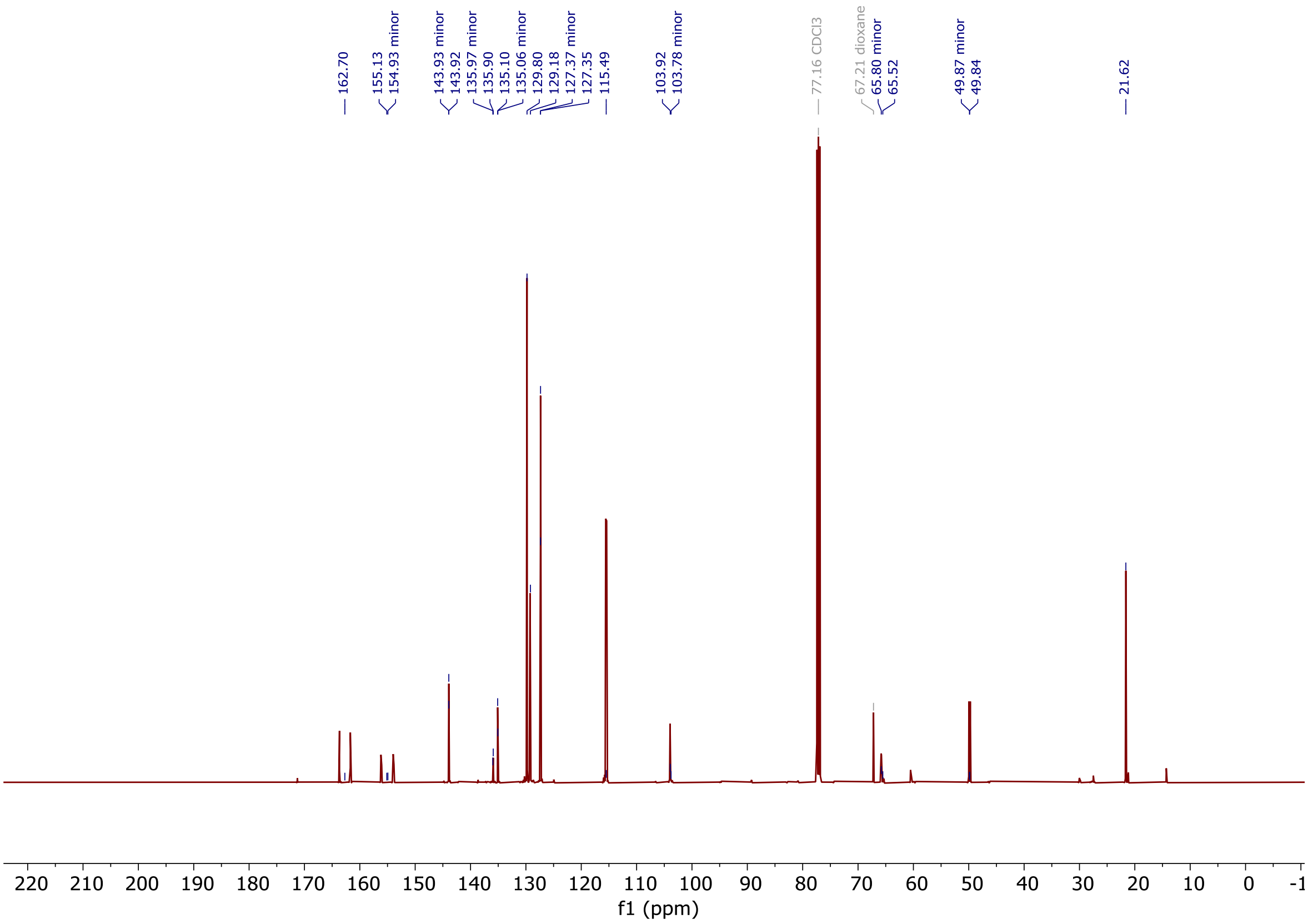

^{19}F NMR (376 MHz, $CDCl_3$)

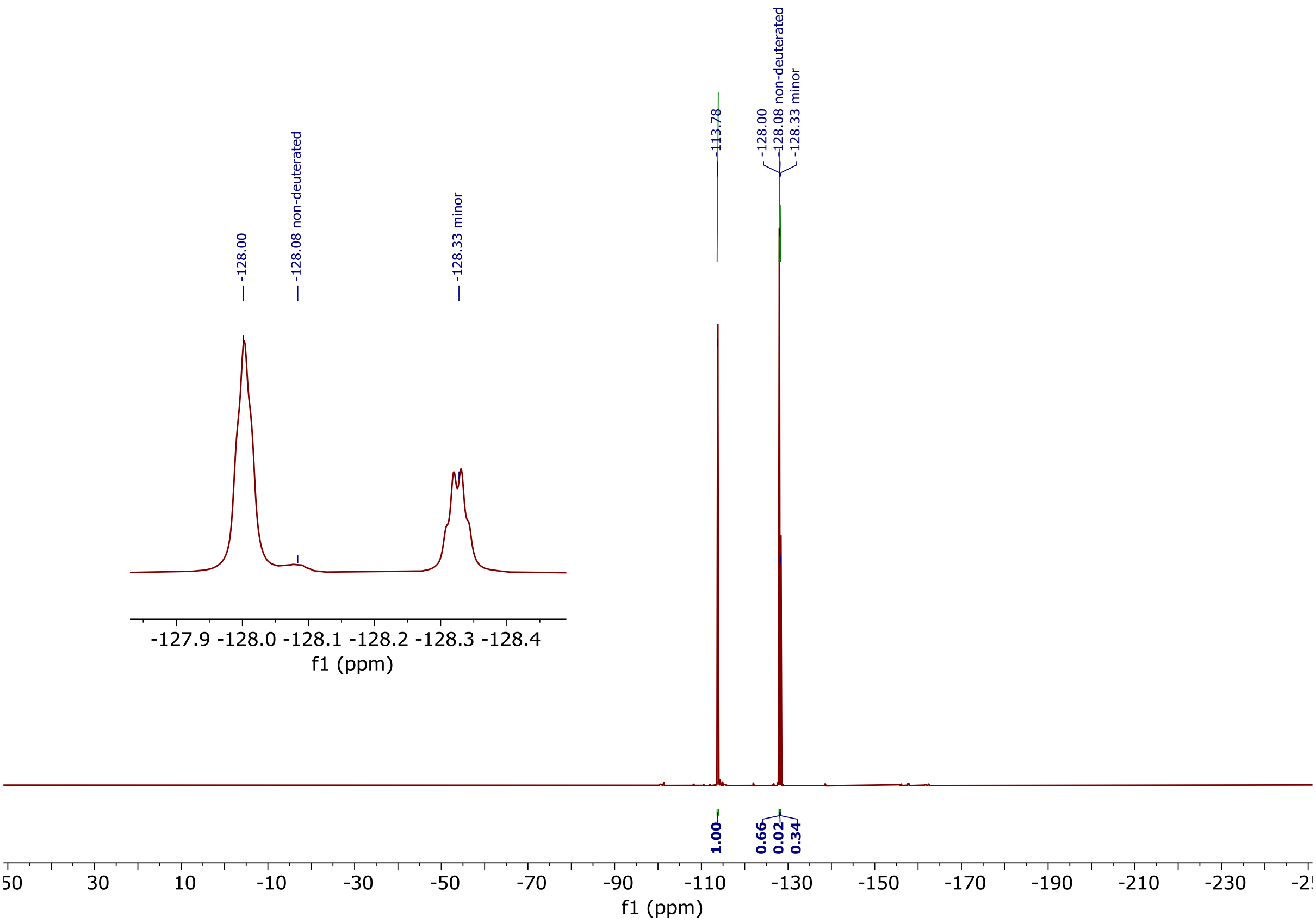

4.4. Oxetene Intermediate

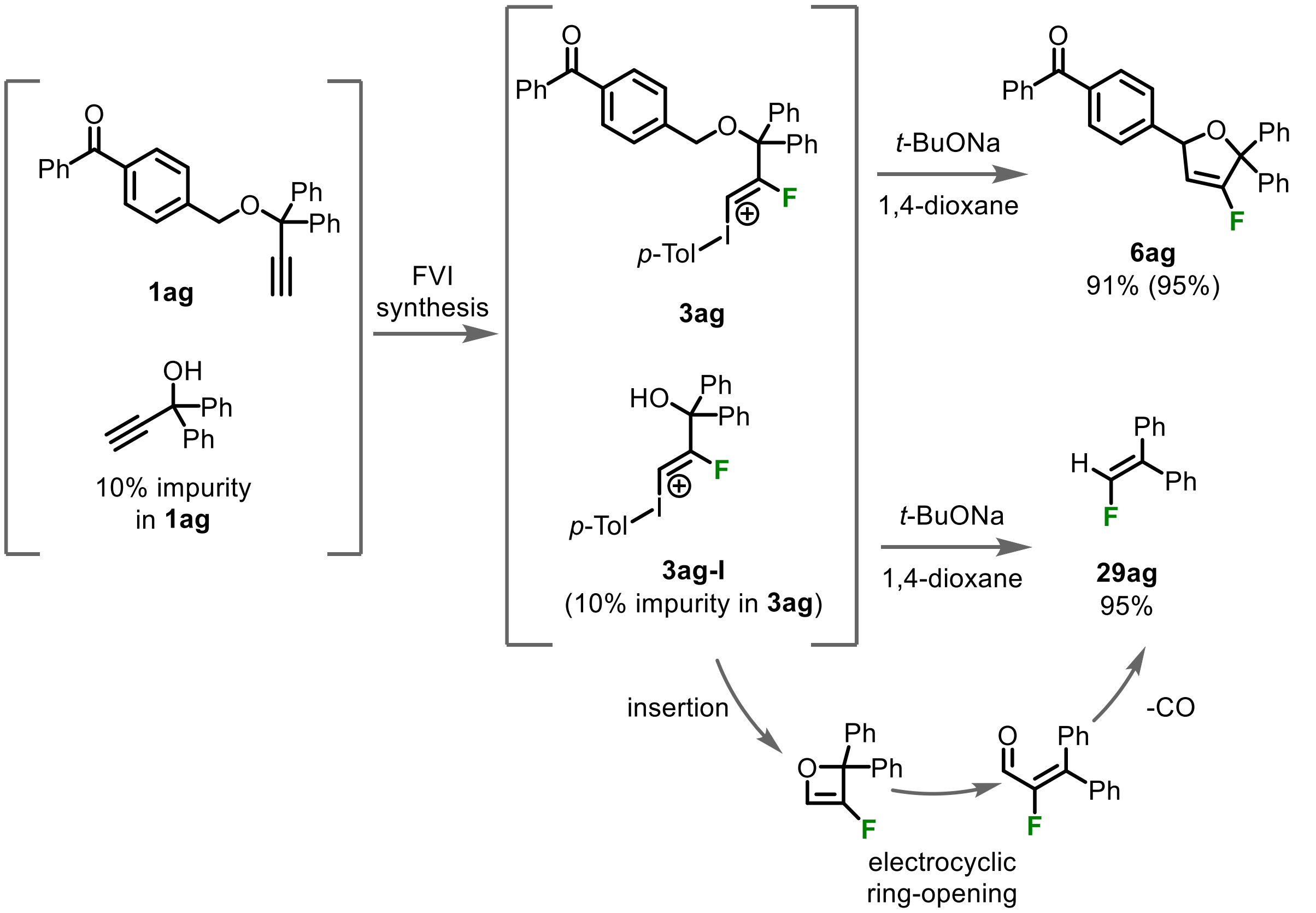

Supplementary Figure 5: Formation of alkene **29ag** during the synthesis of oxolene **6ag**.

10% of 1,1-diphenylprop-2-yn-1-ol was present in alkyne **1ag**. These were both converted into FVIs without separation, and the final post-cyclisation products **6ag** and **29ag** were separated by flash column chromatography. The formation of **29ag** in 95% yield provides evidence for an oxetene intermediate formed via O-H insertion (**Supplementary Figure 5**). Yields were determined by ^{19}F NMR using 4,4'-difluorobiphenyl as an internal standard. Yield of **29ag** was calculated relative to the quantity of **3ag-I** present in the starting material.

4.5. Use of less powerful leaving groups

Iodine was used in place of the usual tolyl iodonium, demonstrating why the iodonium hyper-nucleofuge is needed; iodine is too poor a leaving group to allow α-elimination of the iodane, and only β-elimination of fluoride was observed, forming 1-iodoalkyne **28c**, as well as de-iodination of the resulting iodoalkyne to form the terminal alkyne, **1c** (**Supplementary Figure 6**). It is apparent from this that use of weaker leaving groups than the ones used in this paper, such as iodide used by Taber and Hayes,[2–5] is incompatible with fluorinated carbenes.

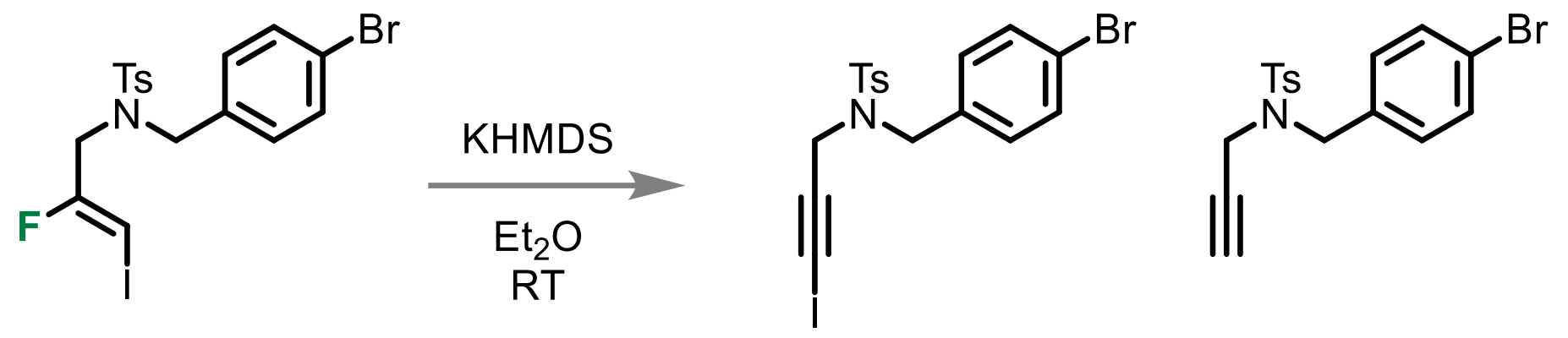

27c **28c** **1c**

Entry	KHMDS equiv.	Time (h)	% **28c**	% **1c**	% RSM
**1**	2	2	19	31	45
**2**	2	4	22	40	32
**3**	3	16	2	84	0

Supplementary Figure 6: Product distribution with use of an iodide leaving group. Conditions derived from Taber and Hayes' work.[2–5]

4.6. Deuteration of the FVI

Two alternative mechanisms were proposed for the N-H insertion: either carbene formation followed by insertion into the N-H bond, or by deprotonation of the sulfonamide followed by vinylic nucleophilic substitution displacing the aryl iodide. To investigate this, the position α to iodine was deuterated (**Supplementary Figure 7a**). If the deuterium was lost on cyclisation, the deprotonation occurs α to iodine, forming the carbene. If the deuterium is retained, the sulfonamide is deprotonated, and the reaction proceeds via vinylic substitution. On deprotonation of the sulfonamide, it is also feasible that the sulfonamide could then deprotonate the position α to iodine,

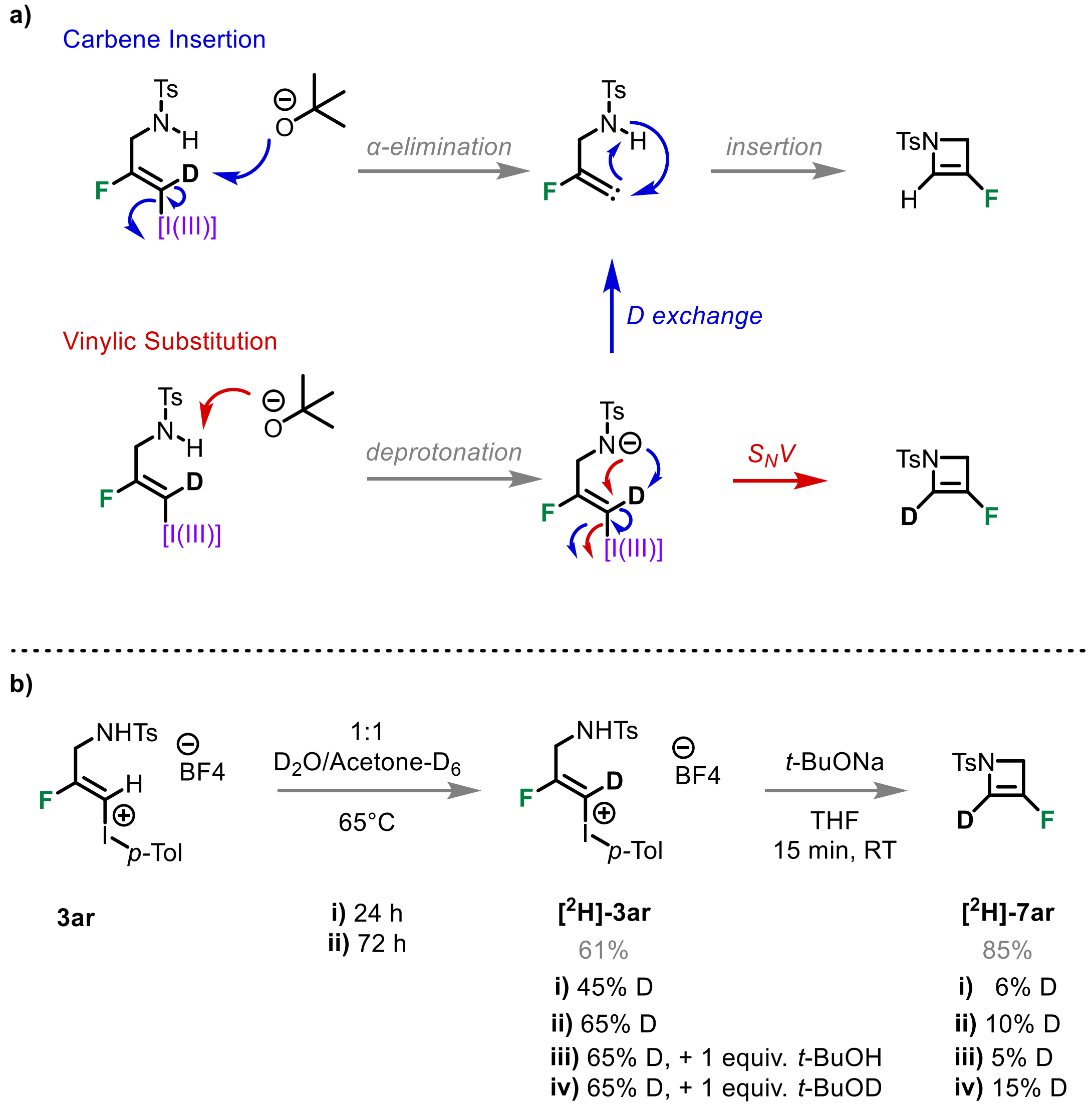

Supplementary Figure 7: a) Alternative mechanisms of azetine formation. **b)** Deuteration of the proton α to iodine in FVI **3ar** and subsequent cyclisation. **i)** Cyclisation of FVI with 45% D incorporation, resulting in 6% D incorporation in the heterocycle (87% decrease). **ii)** Cyclisation of FVI with 65% D incorporation, resulting in 10% D incorporation in the heterocycle (85% decrease). **iii)** Cyclisation of FVI with 65% D incorporation, with reaction spiked with 1 equivalent of *tert*-butyl alcohol, resulting in 5% D incorporation in the heterocycle (92% decrease). **iv)** Cyclisation of FVI with 65% D incorporation, with reaction spiked with 1 equivalent of O-deuterated *tert*-butyl alcohol, resulting in 15% D incorporation in the heterocycle (77% decrease). Deuterium incorporation was measured by NMR.

leading to a deuterated product through the carbene mechanism. Since it has been shown that the pK_a of the α-iodo proton (10-13) is much lower than that of a sulfonamide (16-17) through the base optimisation (**Supplementary Table 2**), this exchange will only go in this direction. The results of these tests are shown in **Supplementary Figure 7b**. Since the cyclisation results in a ~86% reduction in D incorporation, we conclude that the major mechanism is carbene formation, and the remaining 14% proceeds via either N-H deprotonation followed by D exchange with either the FVI or *tert*-butyl alcohol byproduct and then a carbene insertion, or via S_NV. To investigate this, the reaction was spiked with 1 equivalent of either *t*-BuOH or *t*-BuOD. The result was a 5% increase in product deuterium incorporation from the deuterated alcohol, and a 5% decrease from the non-deuterated alcohol. We conclude from this that deuterium incorporation in the product is primarily a result of exchange between the sulfonamide and the *t*-BuOD byproduct.

See next pages for characterisation data of deuterated FVIs and azetines.

(Z)-(2-fluoro-3-((4-methylphenyl)sulfonamido)prop-1-en-1-yl-1-d)(p-tolyl)iodonium tetrafluoroborate **([2H]-3as)**

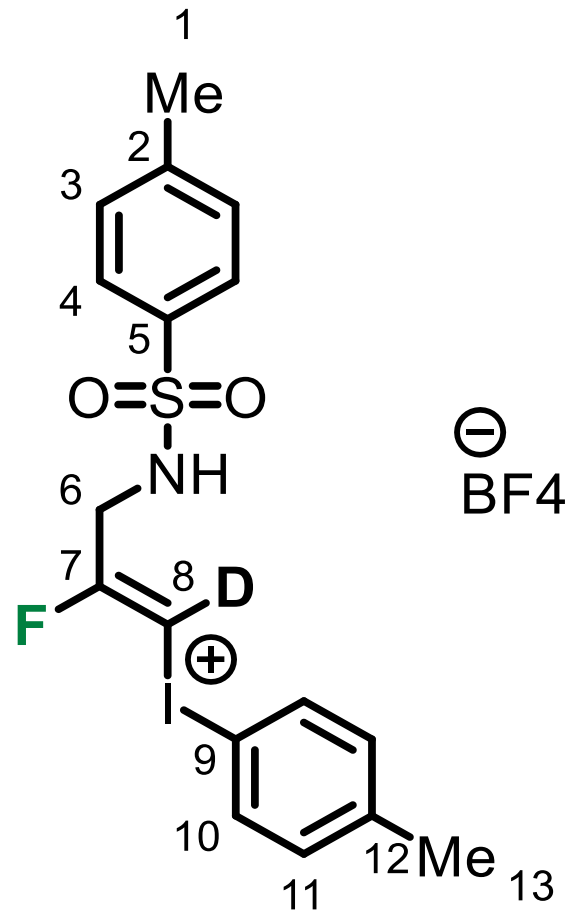

Alkyne **3ar** (0.2 mmol, 1 equiv.) in a 1:1 mixture of D_2O and acetone-d_6 (2 mL, 0.1 M) was heated to 65 °C in a pressure tube for 24 h (45% D incorporation) or 72 h (65% D incorporation). The reaction mixture was then extracted with DCM (2 × 5 mL) and the organic layer was dried over $MgSO_4$ and concentrated *in vacuo* to afford the title compound without further purification as a yellow solid (65.6 mg, 61%).

2 samples were obtained, with 45% and 65% D incorporation respectively.

^{1}H NMR (500 MHz, Acetone-*D₆*) δ 8.07 (d, *J* = 8.2 Hz, 2H, C10-H), 7.74 (d, *J* = 8.2 Hz, 2H, C4-H), 7.43 (d, *J* = 8.2 Hz, 2H, C11-H), 7.37 (d, *J* = 8.2 Hz, 2H, C3-H), 7.22 (t, *J* = 6.4 Hz, 1H, C6-NH), 7.08 (d, *J* = 33.6 Hz, 0.35-0.55H, C8-H), 4.12 (dd, *J* = 10.1, 6.2 Hz, 2H, C6-H), 2.44 (s, 3H, C13-H), 2.40z (s, 3H, C1-H).

^{13}C NMR (126 MHz, Acetone-D_6) Protonated δ 169.6 (d, *J* = 277.1 Hz, C7), 144.9 (C12), 144.6 (C2), 138.3 (C5), 136.5 (C10), 133.9 (C11), 130.7 (C3), 127.8 (C4), 109.7 (C9), 79.5 (d, *J* = 18.9 Hz, C8), 43.4 (d, *J* = 32.7 Hz, C6), 21.4 (C13), 21.3 (C1). **Deuterated** δ 169.3 (d, *J* = 276.7 Hz, C7), 144.6 (C12), 144.5 (C2), 138.2 (C5), 136.3 (C10), 133.7 (C11), 130.6 (C3), 127.8 (C4), 109.6 (C9), 79.3 (1:1:1 td, *J* = 28.7, 18.9 Hz), 43.3 (d, *J* = 32.2 Hz, C6), 21.4 (C13), 21.3 (C1).

^{19}F NMR (376 MHz, Acetone-D_6) Protonated δ −76.38 (dt, *J* = 33.6, 10.0 Hz, 1F, C7-F), −149.91 (d, *J* = 20.2 Hz, 4F, BF_4). **Deuterated** δ −76.82 (tt, *J* = 9.9, 5.0 Hz, 1F, C7-F), −149.91 (d, *J* = 20.2 Hz, 4F, BF_4).

^{2}H NMR (77 MHz, No Solvent) δ 7.13 (d, *J* = 5.0 Hz, 1H, C8-D).

HRMS (+ESI) calcd: $[M]^+$ ($C_{17}H_{17}DFINO_2S$) 447.0144, measured 447.0127 (3.80 ppm error).

IR (neat) v_{max}/cm^{-1}: 3290, 2925, 1737, 1647, 1597, 1436, 1328, 1158, 1090, 815, 664, 550.

3-fluoro-1-tosyl-1,2-dihydroazete-4-d **([²H]-7ar)**

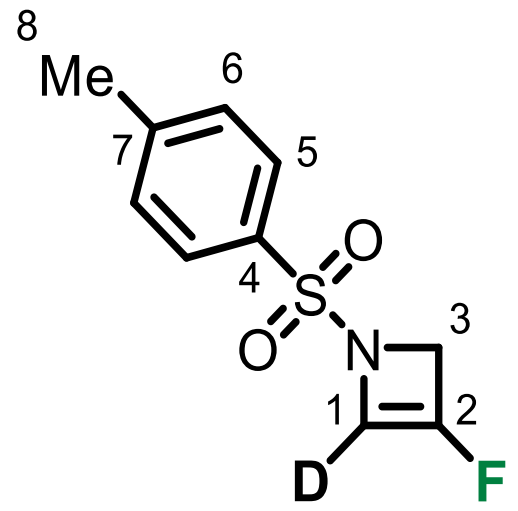

Prepared according to **General procedure IV** from **[²H]-3ar** (85% NMR) and purified by flash column chromatography (2-20% EtOAc in pentane) to afford the title compound as a colourless oil (18.3 mg, 80%).

2 samples were obtained, with 10% and 6% D incorporation, prepared from samples of **[²H]-3ar** with 65% and 45% D incorporation respectively.

^{1}H NMR (500 MHz, $CDCl_3$) δ 7.72 (d, *J* = 8.2 Hz, 2H, C5-H), 7.36 (d, *J* = 8.2 Hz, 2H, C6-H), 5.80 (d, *J* = 8.6 Hz, 0.90-0.95H, C1-H), 4.20 (d, *J* = 5.0 Hz, 2H, C3-H), 2.46 (s, 3H, C8-H).

^{13}C NMR (126 MHz, $CDCl_3$) Protonated δ 145.5 (d, *J* = 328.2 Hz, C2), 145.1 (C7), 129.8 (C6), 129.6 (C4), 129.2 (C5), 117.7 (d, *J* = 5.0 Hz, C1), 60.6 (d, *J* = 20.9 Hz, C3), 21.8 (C8). **Deuterated** δ 145.3 (d, *J* = 328.4 Hz, C2), 145.2 (C7), 130.2 (C6), 129.3 (C4), 128.4 (C5), 117.7 (1:1:1 td, *J* = 28.4, 5.1 Hz, C1), 60.6 (d, *J* = 21.0 Hz, C3), 21.2 (C8).

^{19}F NMR (376 MHz, $CDCl_3$) Protonated δ −115.22 (dt, *J* = 9.2, 5.1 Hz, 1F, C2-F) **Deuterated** δ −115.46 (t, *J* = 5.1 Hz, 1F, C2-F).

^{2}H NMR (77 MHz, No Solvent) δ 5.81 (s, 1H, C1-D).

HRMS (+ESI) calcd: $[M]^+$ ($C_{10}H_9DFNO_2S$) 228.0474, measured 228.0484 (4.39 ppm error).

IR (neat) v_{max}/cm^{-1}: 3126, 2956, 1737, 1670, 1596, 1347, 1303, 1163, 723, 676, 600, 555.

(Z)-(2-fluoro-3-((4-methylphenyl)sulfonamido)prop-1-en-1-yl-1-d)(p-tolyl)iodonium tetrafluoroborate **([2H]-3as)**

1H NMR (500 MHz, Acetone-*D6*) 45% D incorporation

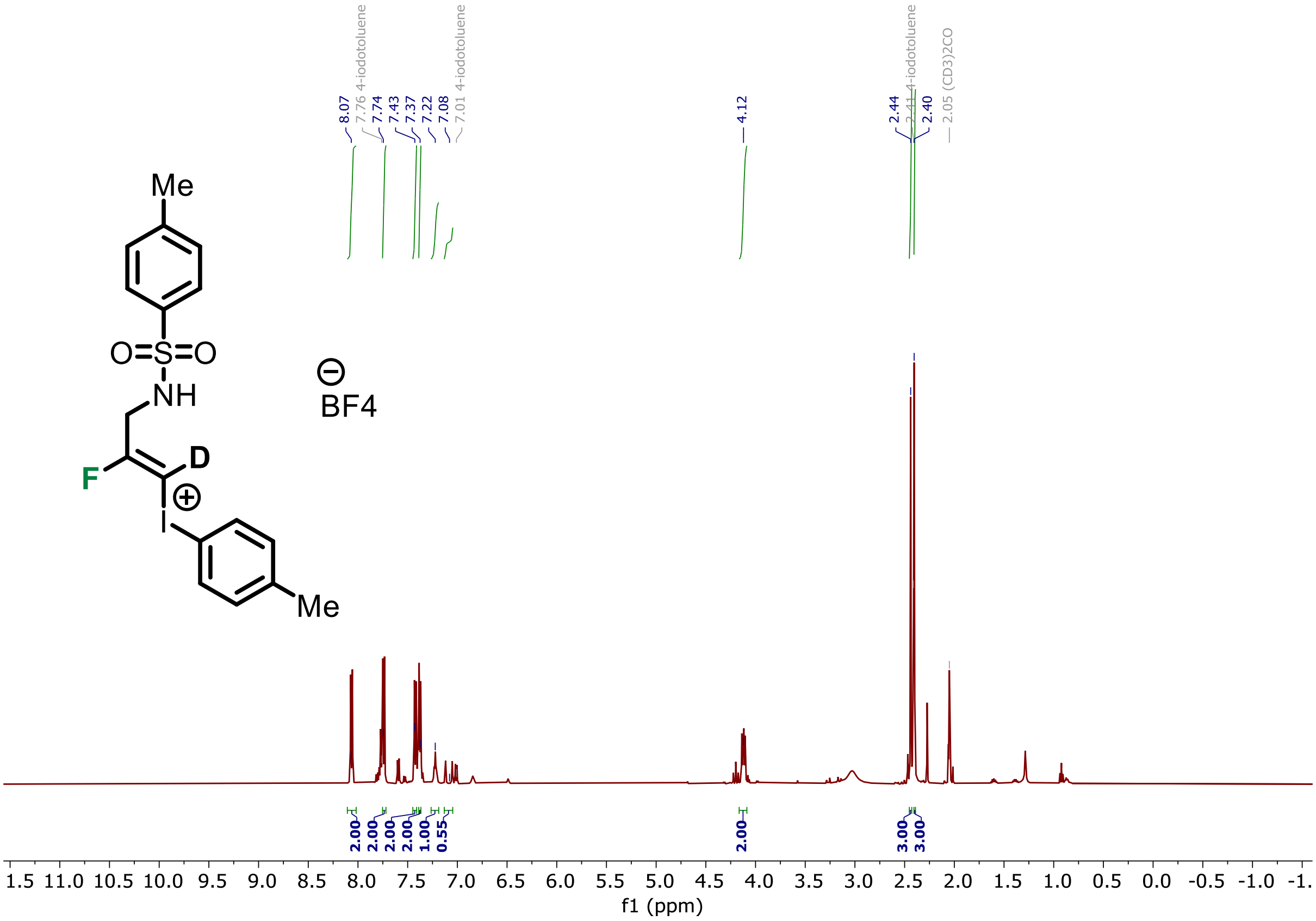

^{13}C NMR (126 MHz, Acetone-D_6)

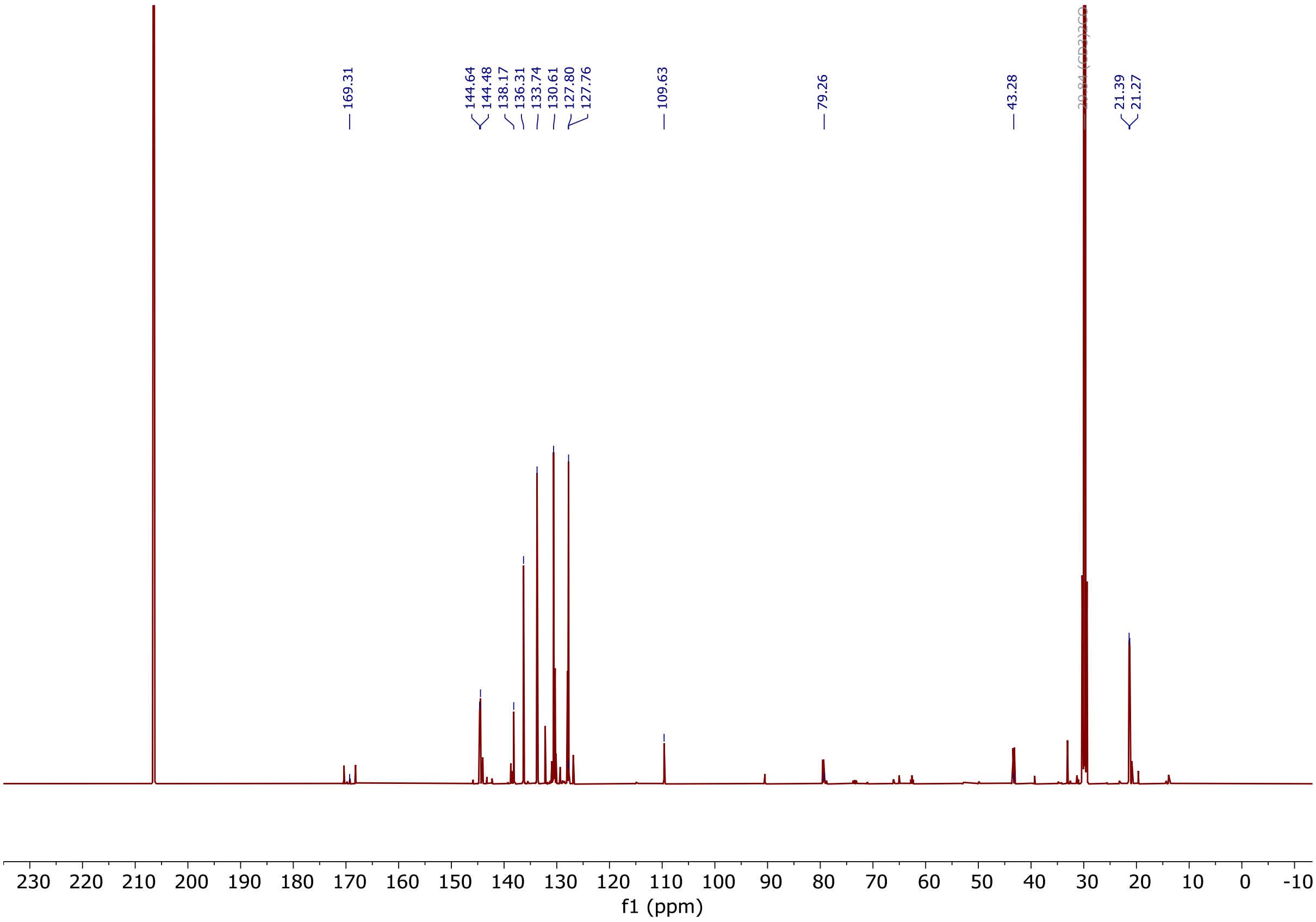

^{19}F NMR (376 MHz, Acetone-D_6) 45% D incorporation

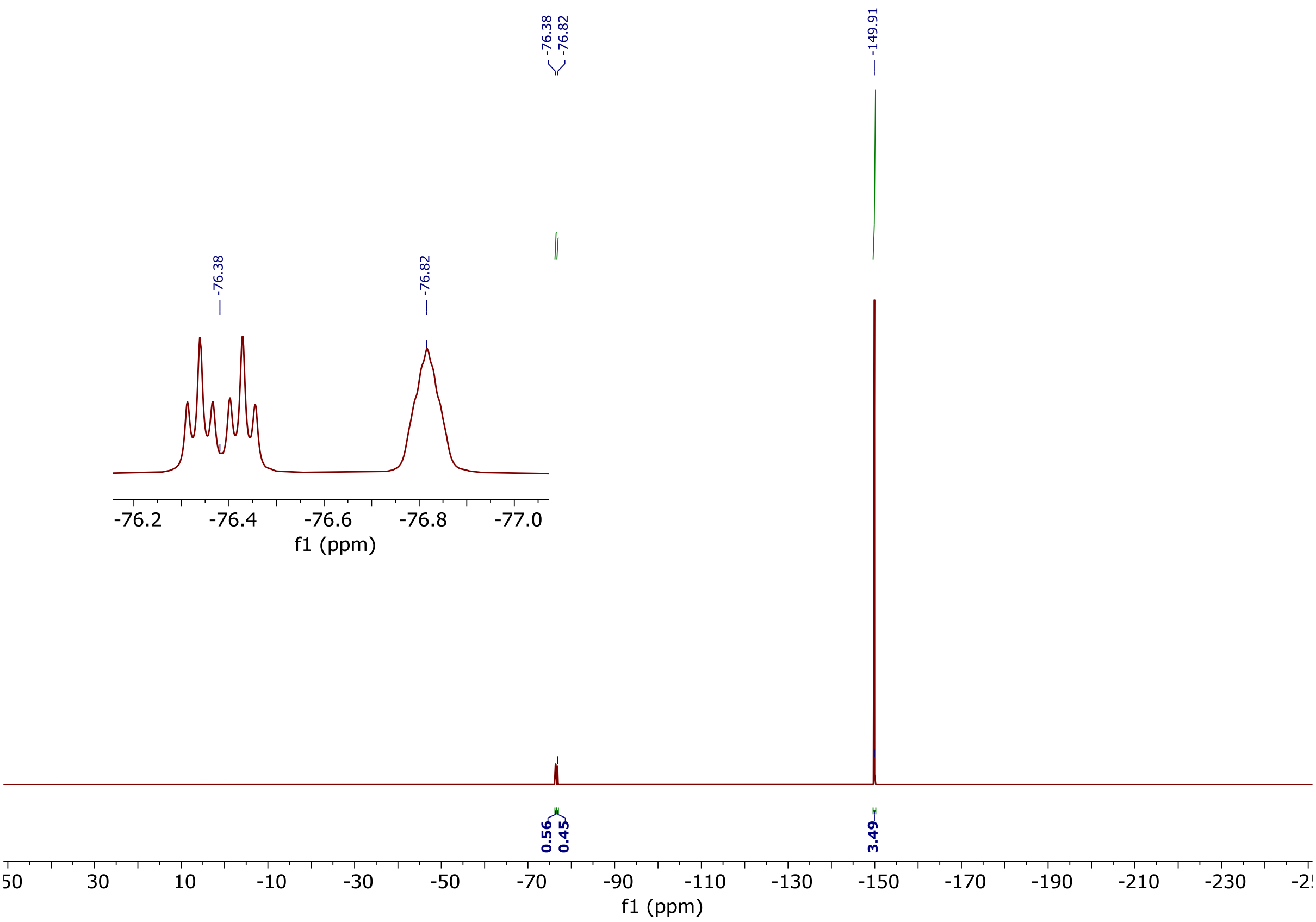

^{19}F NMR (376 MHz, Acetone-*D*₆) 65% D incorporation

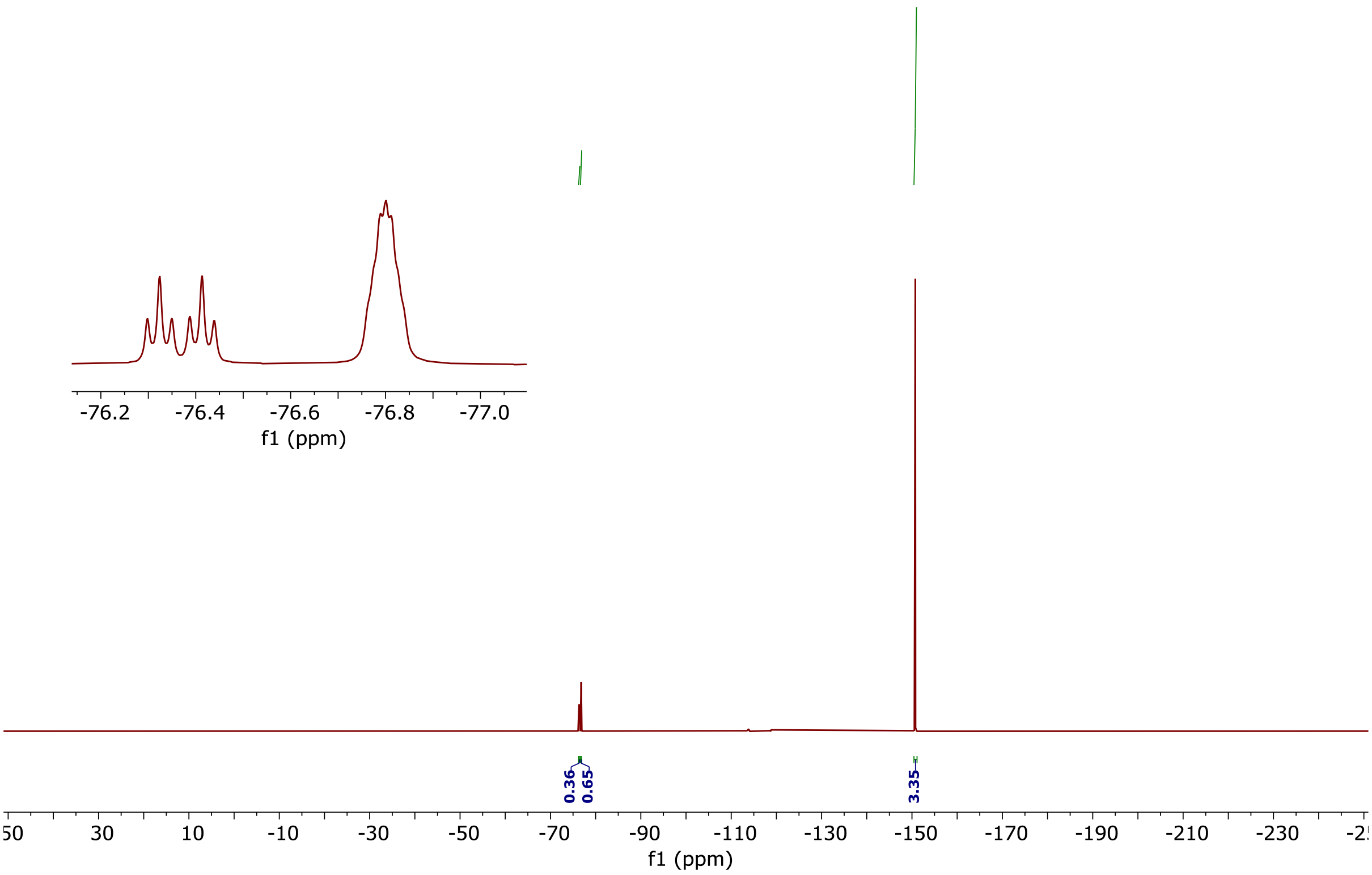

^{2}H NMR (77 MHz, No Solvent)

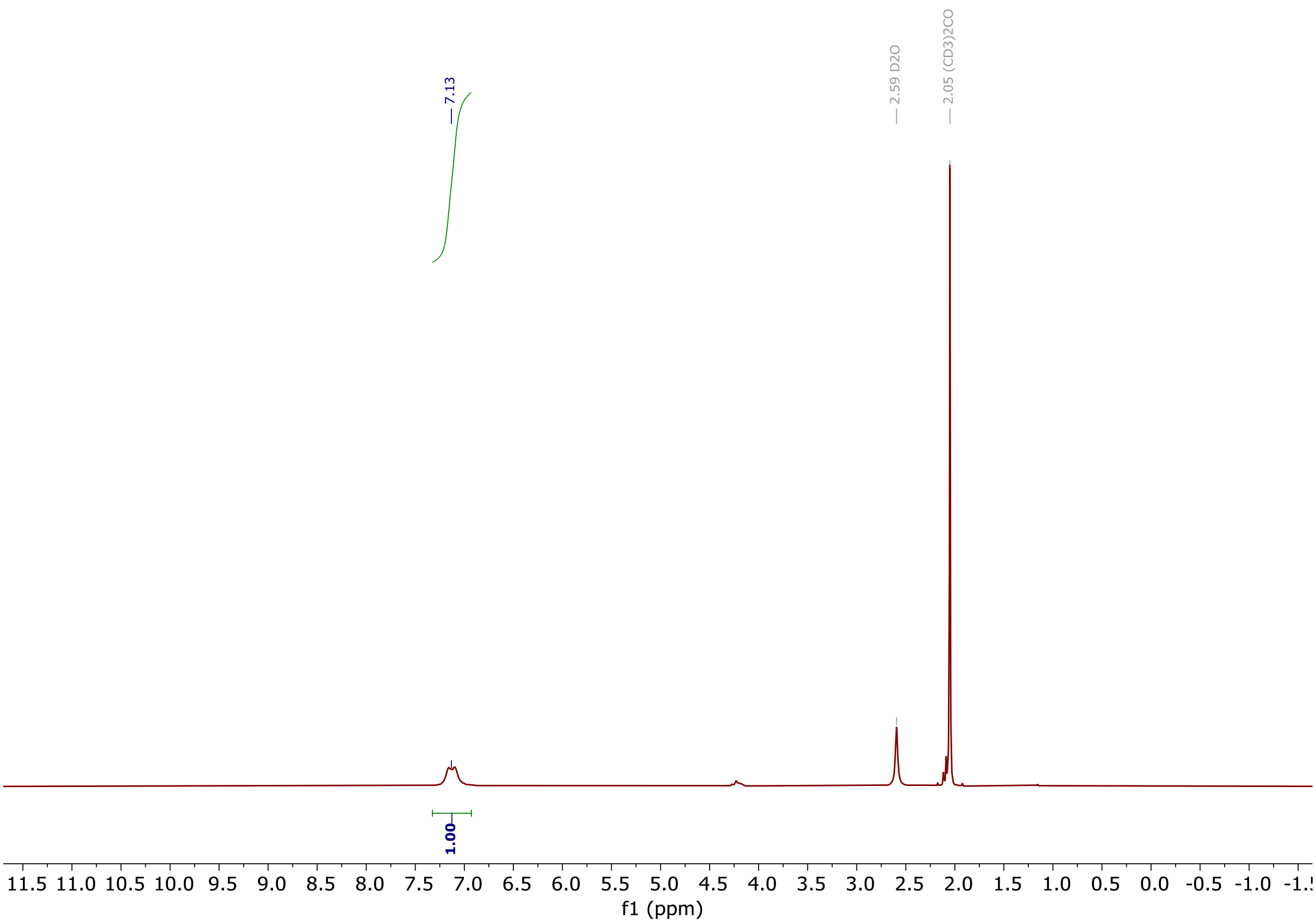

3-fluoro-1-tosyl-1,2-dihydroazete-4-d **([²H]-7ar)**

^{1}H NMR (500 MHz, CDCl$_3$) 10% D incorporation

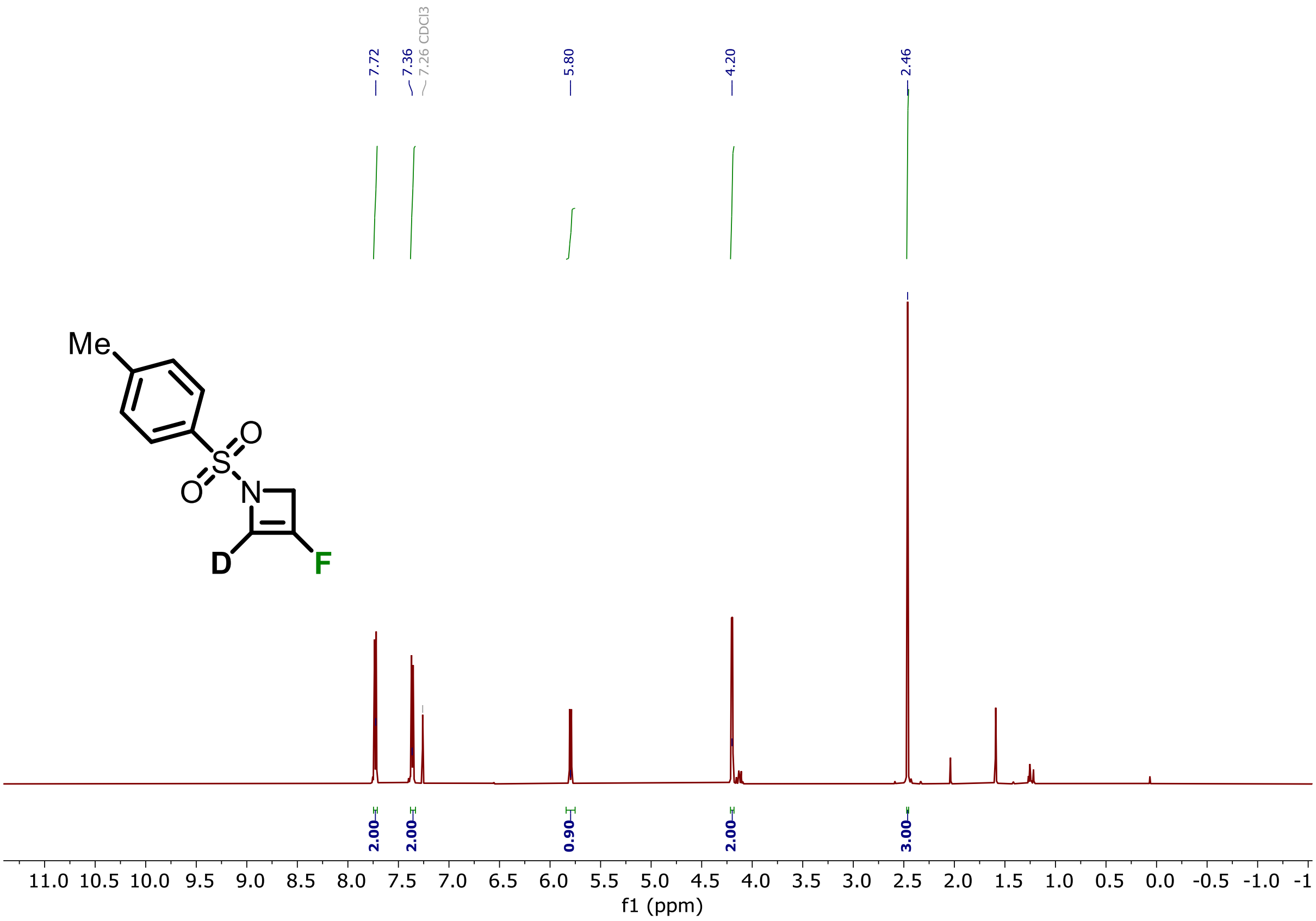

^{13}C NMR (126 MHz, CDCl$_3$)

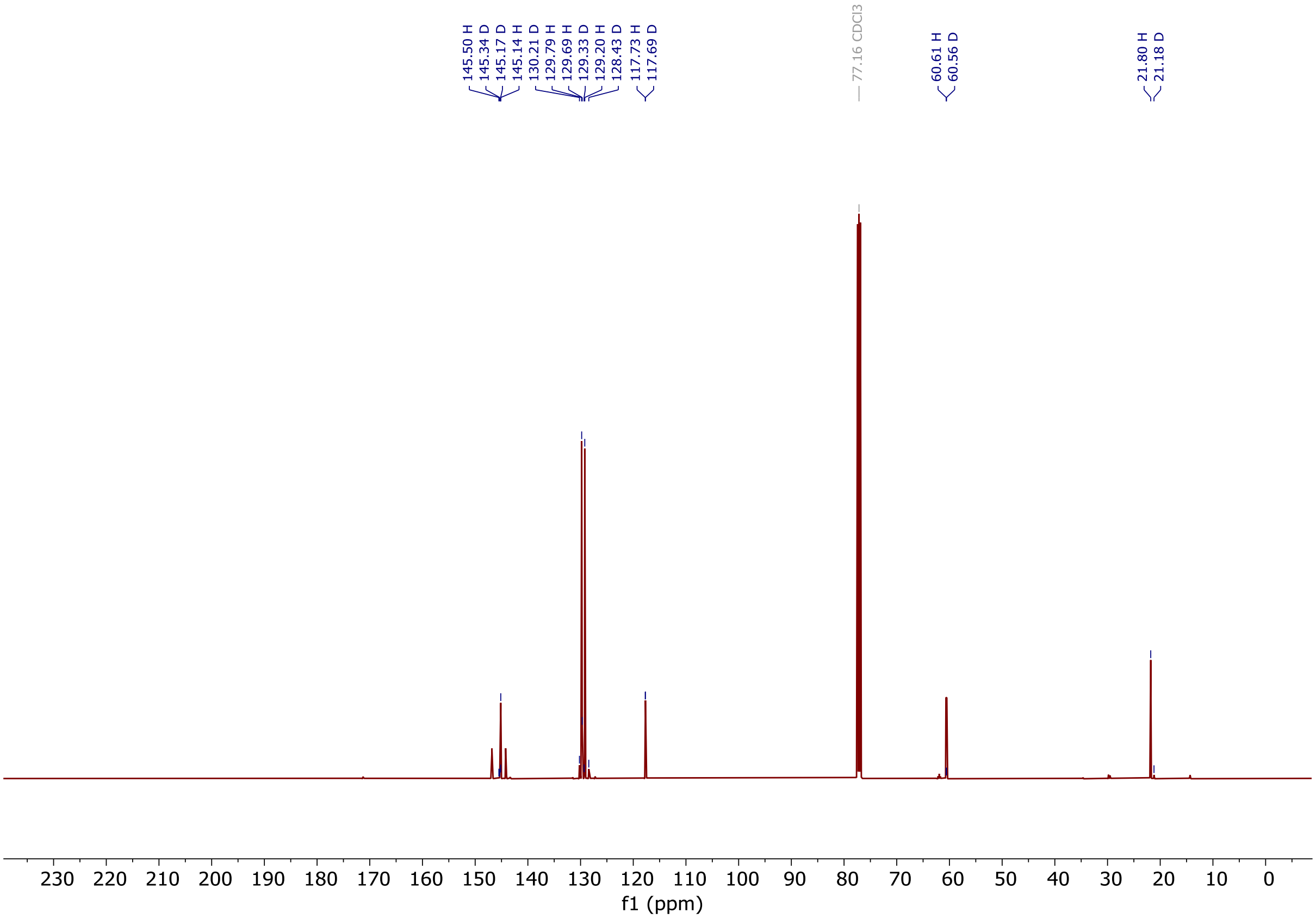

^{19}F NMR (376 MHz, CDCl$_3$) 10% D incorporation

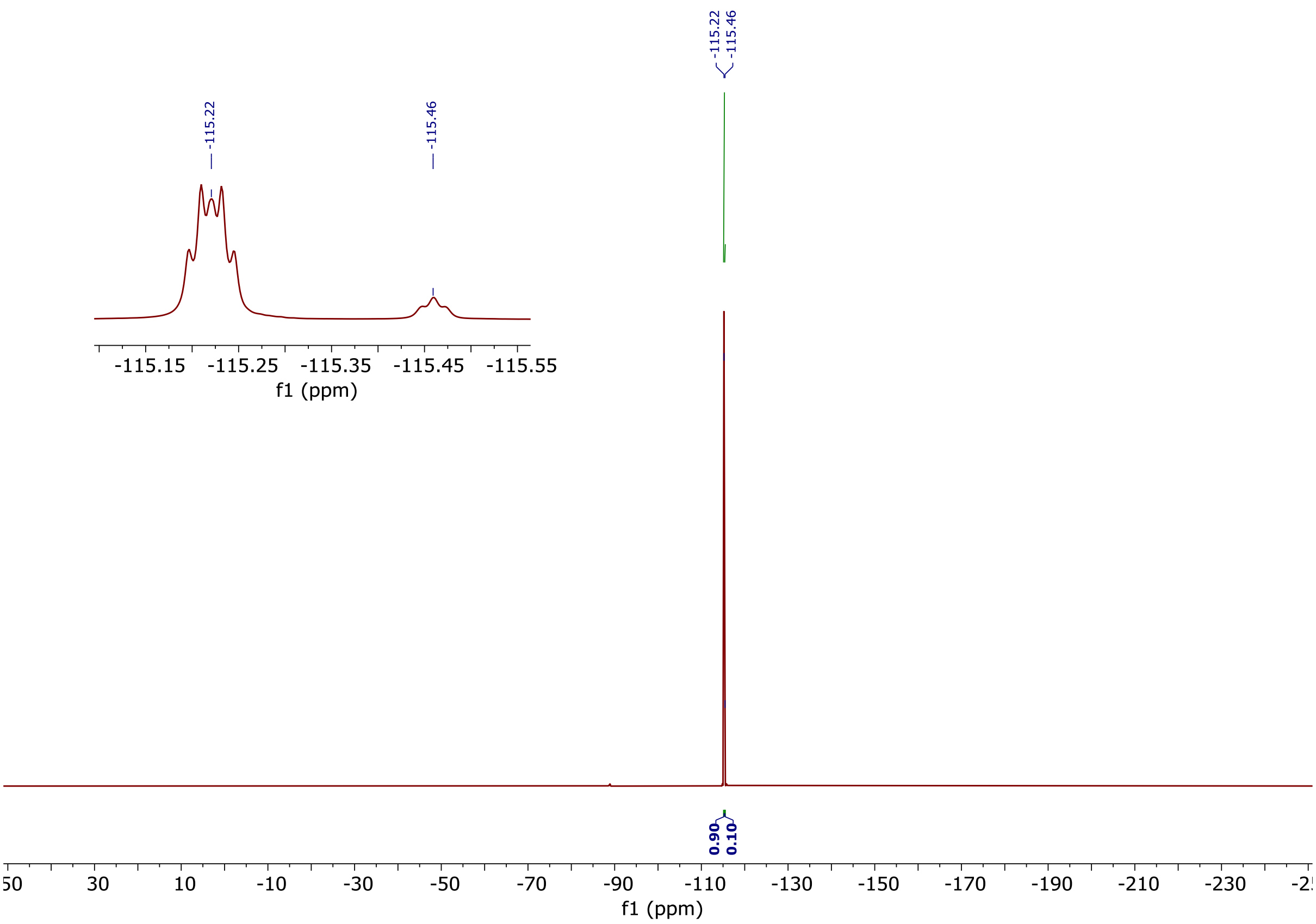

^{19}F NMR (376 MHz, $CDCl_3$) 5% D incorporation

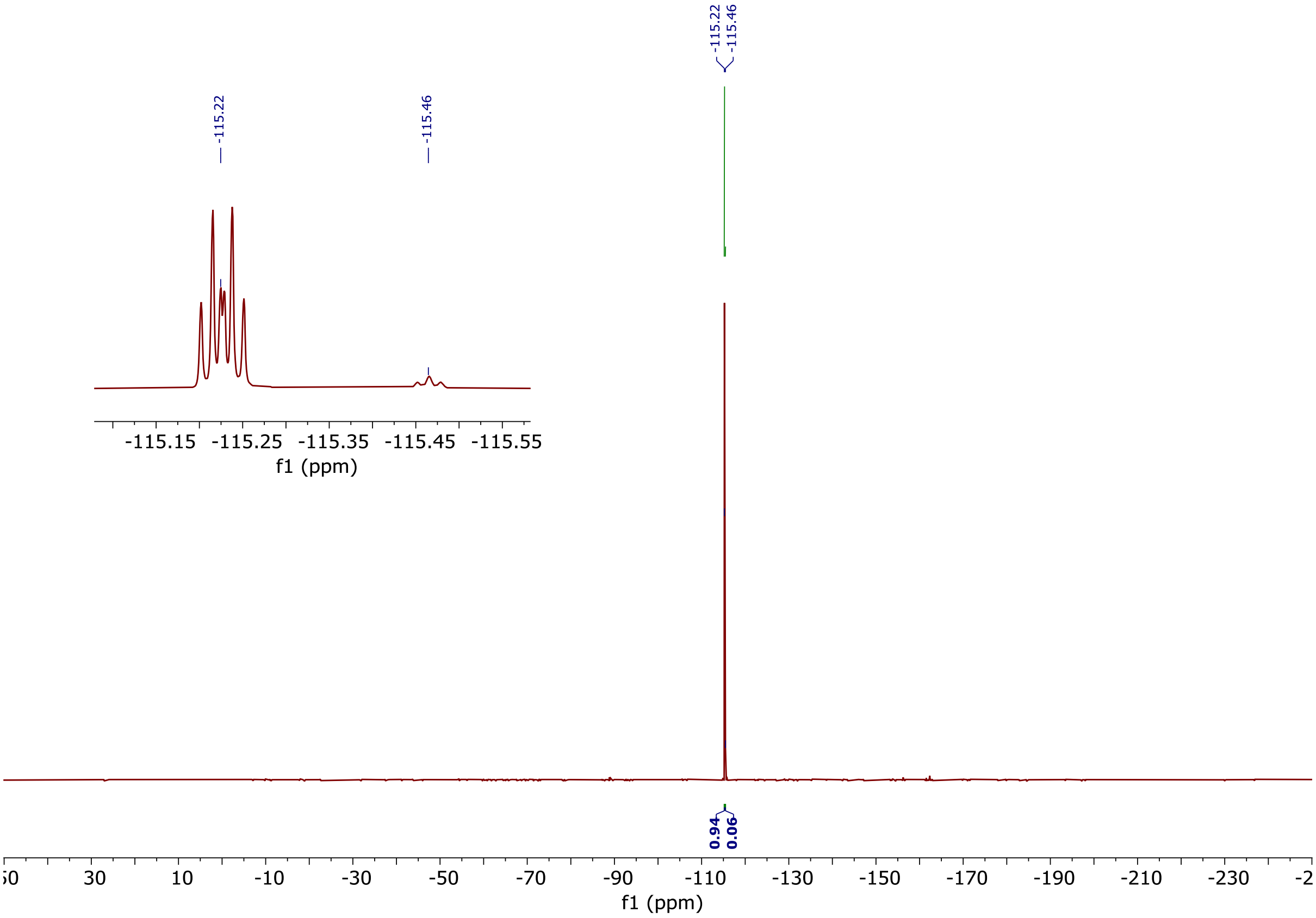

^{2}H NMR (77 MHz, No Solvent)

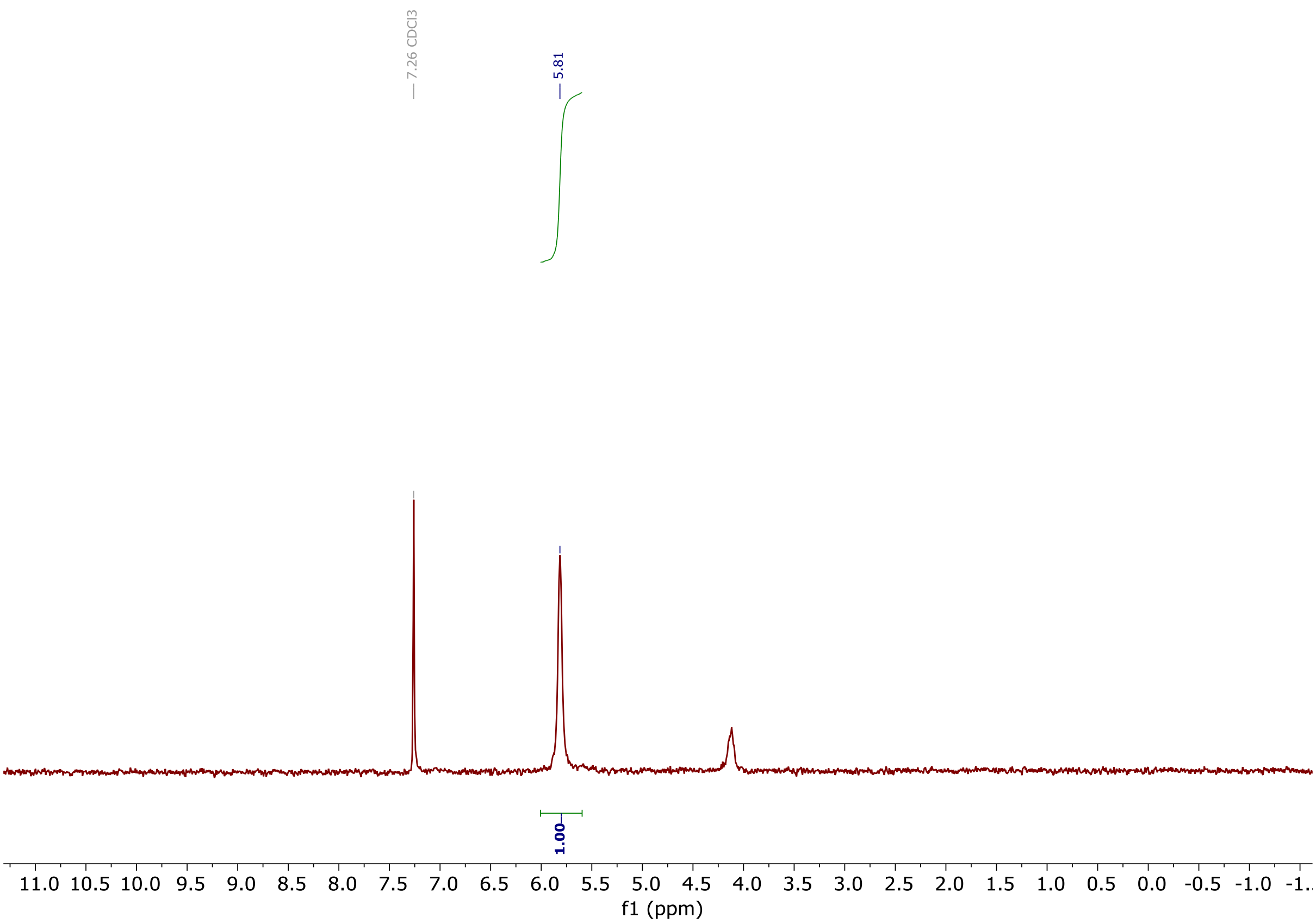

4.7. HOMO/LUMO of the carbene

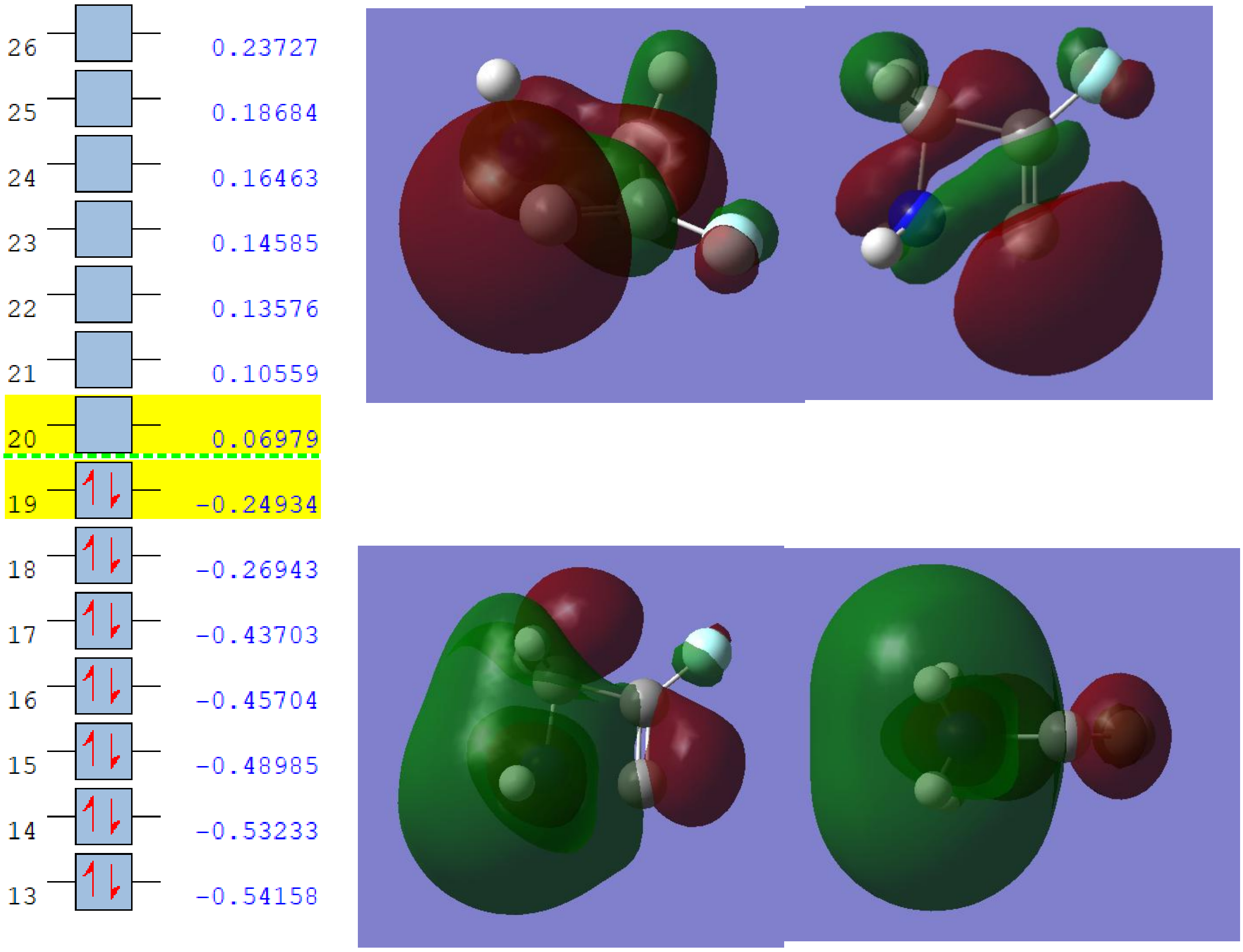

Supplementary Figure 8: Top) The HOMO of the carbene (*sp* lone pair) **Bottom)** The LUMO of the carbene (empty *p*-orbital). Shown on an unsubstituted azetine-forming carbene, with the protecting group omitted for simplicity. **Left)** MO diagram of the carbene.

4.8. Bond Length / J-value analysis

Entry (reference)	Compound	Structure	C-F ^1J (Hz)	C-F bond length (Å)
**1** ([6])	(*S*)-2-(fluoromethyl)-pyrrolidinium chloride	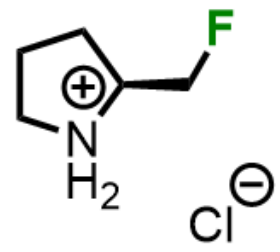	176.7	1.40
**2**	**10m**	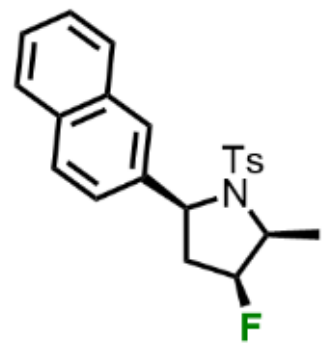	187.3	1.41
**3**	**4l**	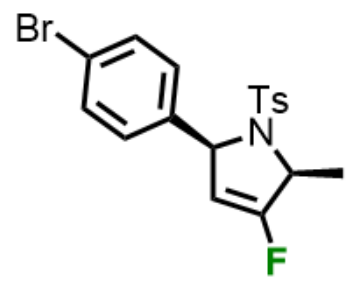	280.4	1.35
**4** ([7])	difluoroethyne	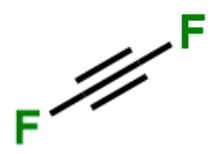	287.3	unknown
**5** ([8])	fluorotrinitromethane	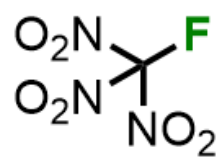	333.4	1.31
**6**	**7ar**	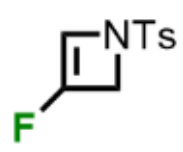	328.2	1.32
**7**	**7aw**	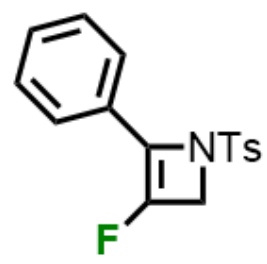	332.5	1.33
**8**	**7az***	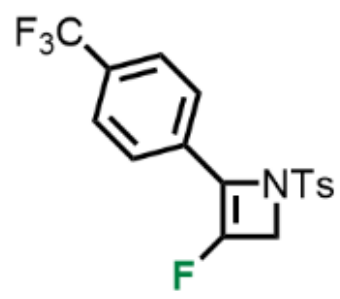	336.2	unknown
**9** ([9])	perfluorocyclobutene	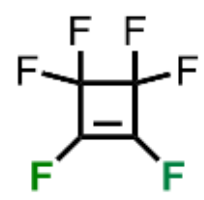	338.9	1.32
**10** ([10])	1-chloro-2,3,3-trifluorocyclobut-1-ene	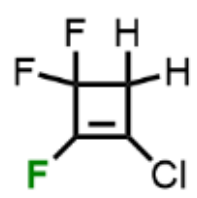	346.7	unknown

Supplementary Table 5: C-F ^1J values and bond lengths of various C-F bonds. Arranged in order of increasing ^1J value, ending with 1-chloro-2,3,3-trifluorocyclobut-1-ene, the compound with the largest known C-F coupling constant. Bond lengths taken from X-ray crystal structures. *largest J-value in this paper.

4.9. Plot of yield against Hammett parameter

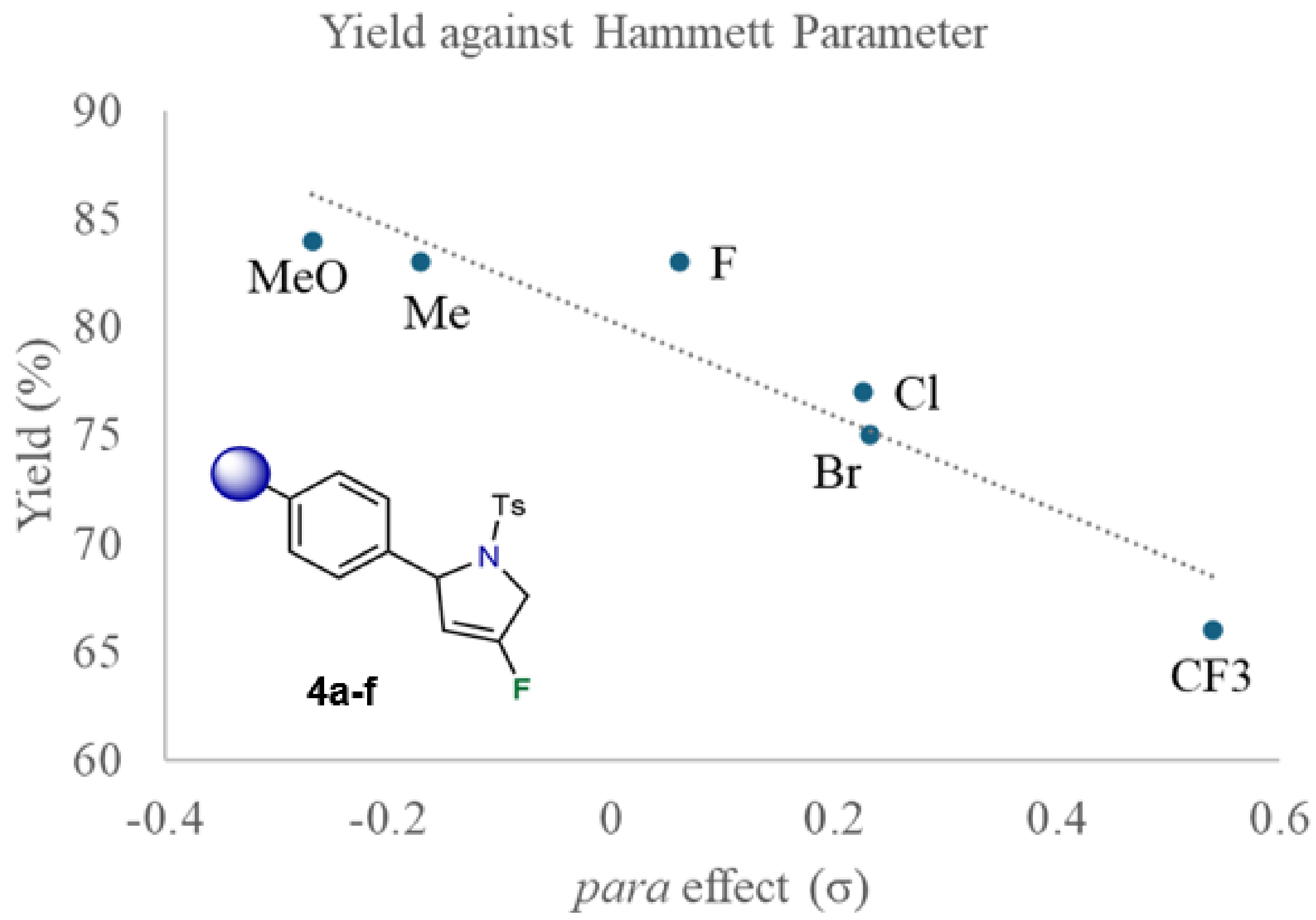

Supplementary Figure 9: A graph of yield against Hammett parameters of *para*-substituents on an aromatic ring adjacent to the insertion site (**4a-f**). Results show lower yields for more electron-withdrawing substituents. *p*-NO_2 did not cyclise at all. All reactions were run according to **general procedure IV** for 2 h.

This graph shows a significant negative correlation between yield and Hammett parameter, with more electron-withdrawing substituents affording lower yields. Literature shows alkylidene carbenes to already be electrophilic, and fluorination of this carbene will cause it to be even more so. This is consistent with these data, as a more electrophilic carbene will react faster with electron-rich C-H bonds and give higher yields.

4.10. Long-term storage stability at room temperature under air

Structure	Compound no.	Synthesised	Tested	Result	$t_{1/2}$
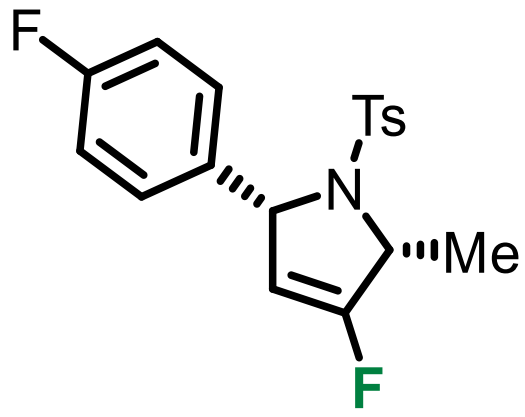	**(*R,R*)-4j**	21/06/2024	16/06/2026 (2 years)	Unchanged	>200 years*
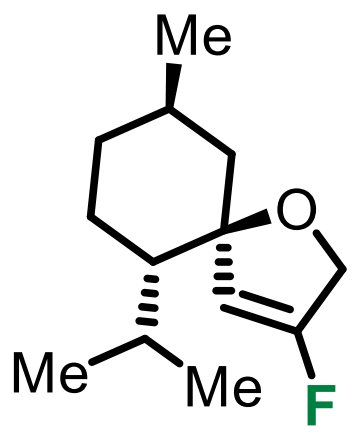	**6aq**	26/04/2024	16/06/2026 (2 years 2 months)	58% **6aq**<br>42% degraded	2.8 years
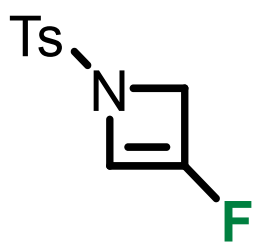	**7ar**	14/05/2025	16/06/2026 (13 months)	48% **7ar**<br>23% ring-opened<br>29% degraded	12.3 months
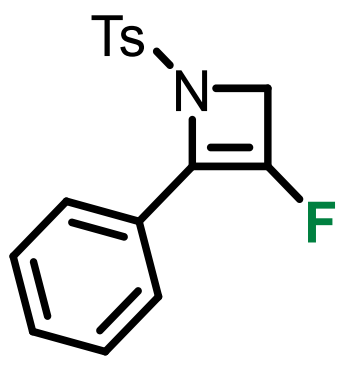	**7aw**	13/12/2024	16/06/2026 (1 year 7 months)	96% **16aw**<br>4% degraded	n/a

Supplementary Table 6: Effects of long-term storage of various substrate classes under air at RT. Representative examples from 4 substrate classes (pyrroline, oxolene, alkyl azetine, aryl azetine) were chosen based on sample availability. Results were quantified by NMR analysis. *Based on 1% margin of error.

Pyrroline **(*R,R*)-4j** showed no signs of degradation after 2 years of storage under air at RT. Oxolene **6aq** showed 58% remaining material after 2 years and 2 months under air at RT (half-life 2.8 years). Azetine **7ar** showed 48% remaining material after 13 months under air at RT (half-life 12.3 months). To prevent thermal electrocyclic ring-opening, azetines should be stored in a freezer. Aryl azetine **7aw** showed complete conversion to hydrated open chain product **16aw** after 1 year 7 months under air at RT. To prevent this hydration, this type of azetine should be stored in a sealed container under dry conditions. See below for NMR spectra of samples after the specified amount of time in storage. See section S8 for NMR spectra of the newly synthesised compounds.

NMR data (Long-term storage stability)

(2R,5R)-3-fluoro-5-(4-fluorophenyl)-2-methyl-1-tosyl-2,5-dihydro-1H-pyrrole **((R,R)-4j)**

2 years storage under air, RT

^{1}H NMR (500 MHz, $CDCl_3$)

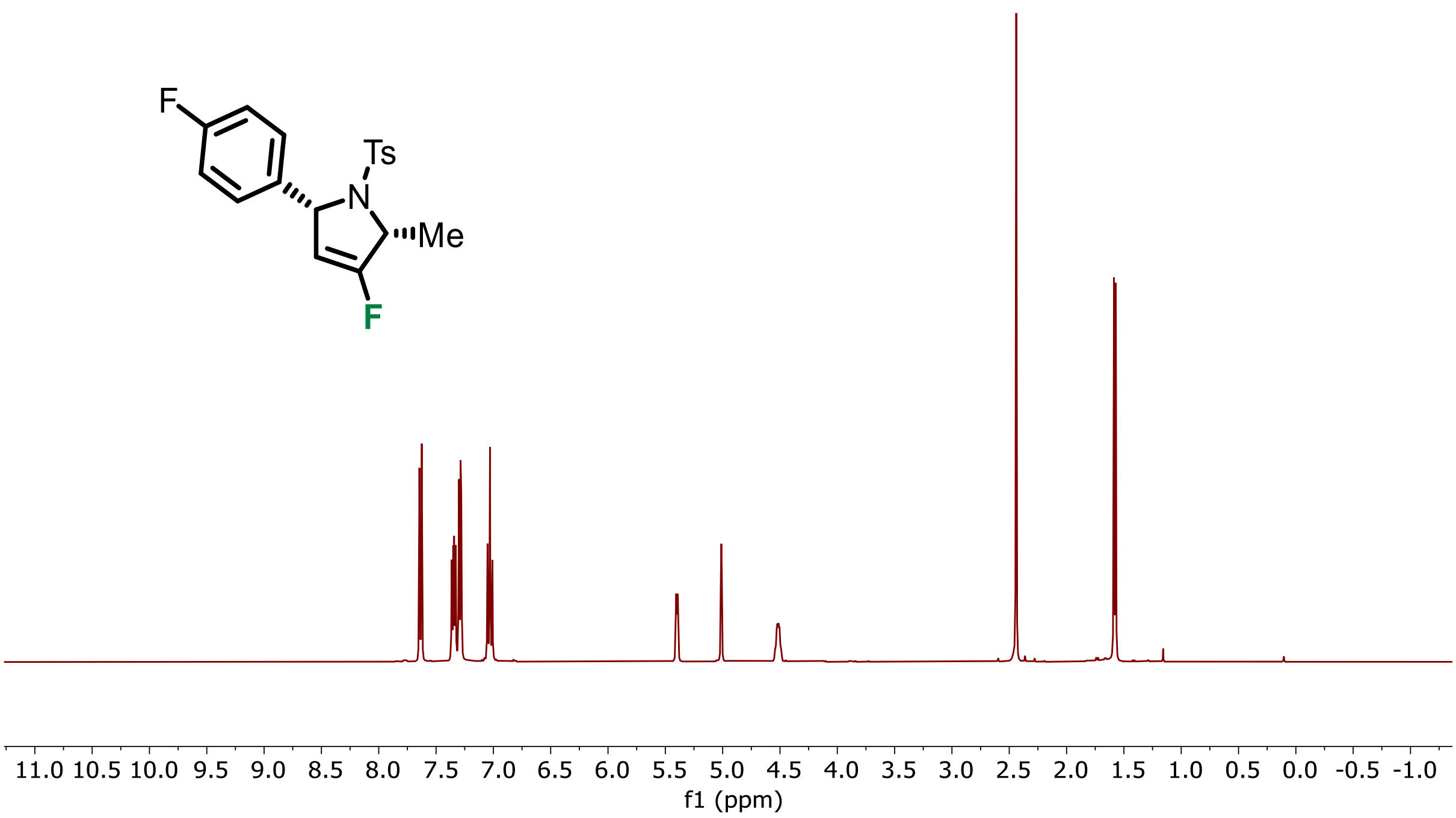

^{19}F NMR (376 MHz, $CDCl_3$)

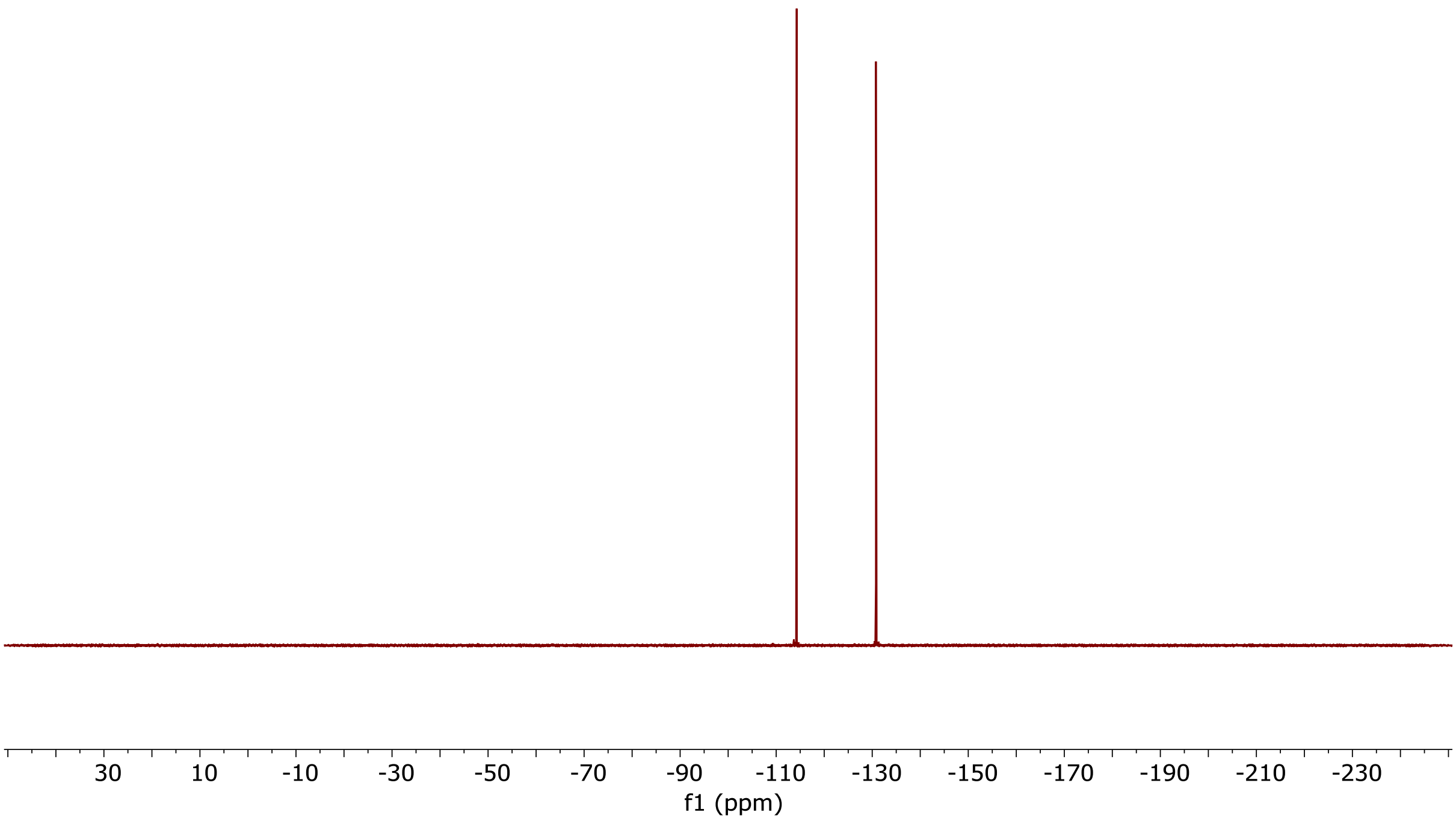

***(5R,6S,9R)-3-fluoro-6-isopropyl-9-methyl-1-oxaspiro[4.5]dec-3-ene* (6aq)** 26 months under air, RT

^{1}H NMR (500 MHz, $CDCl_3$)

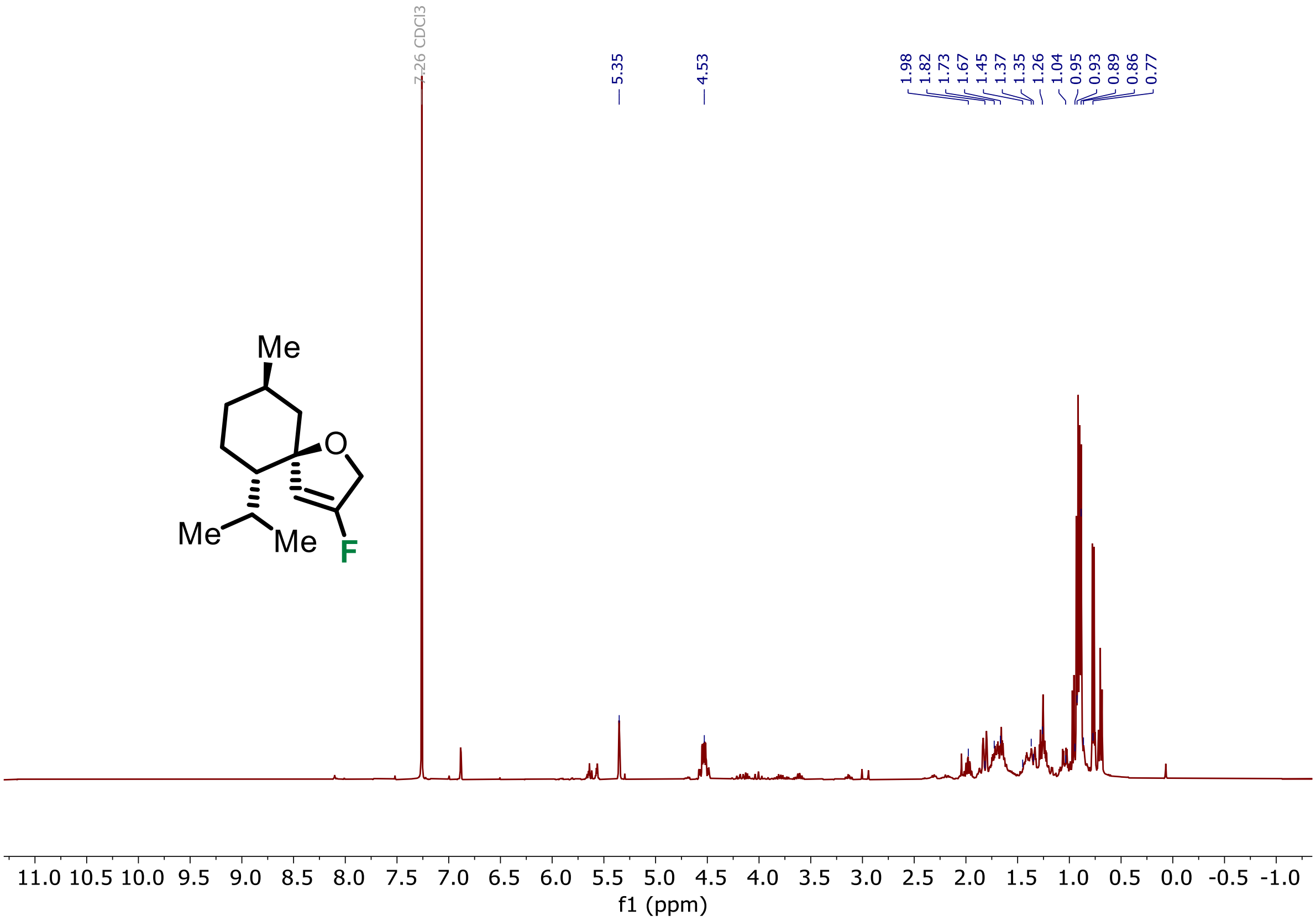

^{19}F NMR (376 MHz, $CDCl_3$)

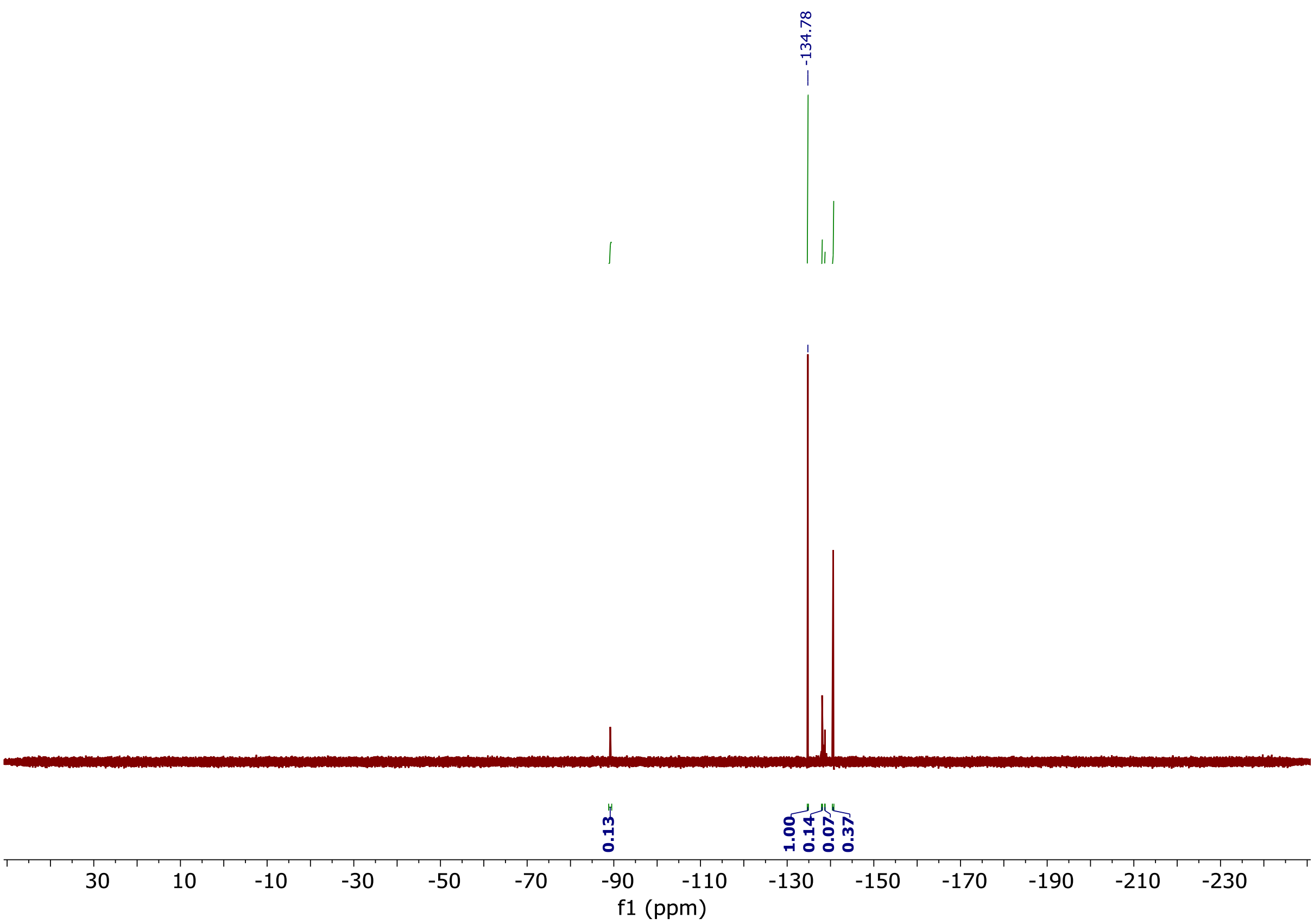

3-fluoro-1-tosyl-1,2-dihydroazete **(7ar)** 13 months under air, RT

¹H NMR (500 MHz, CDCl₃)

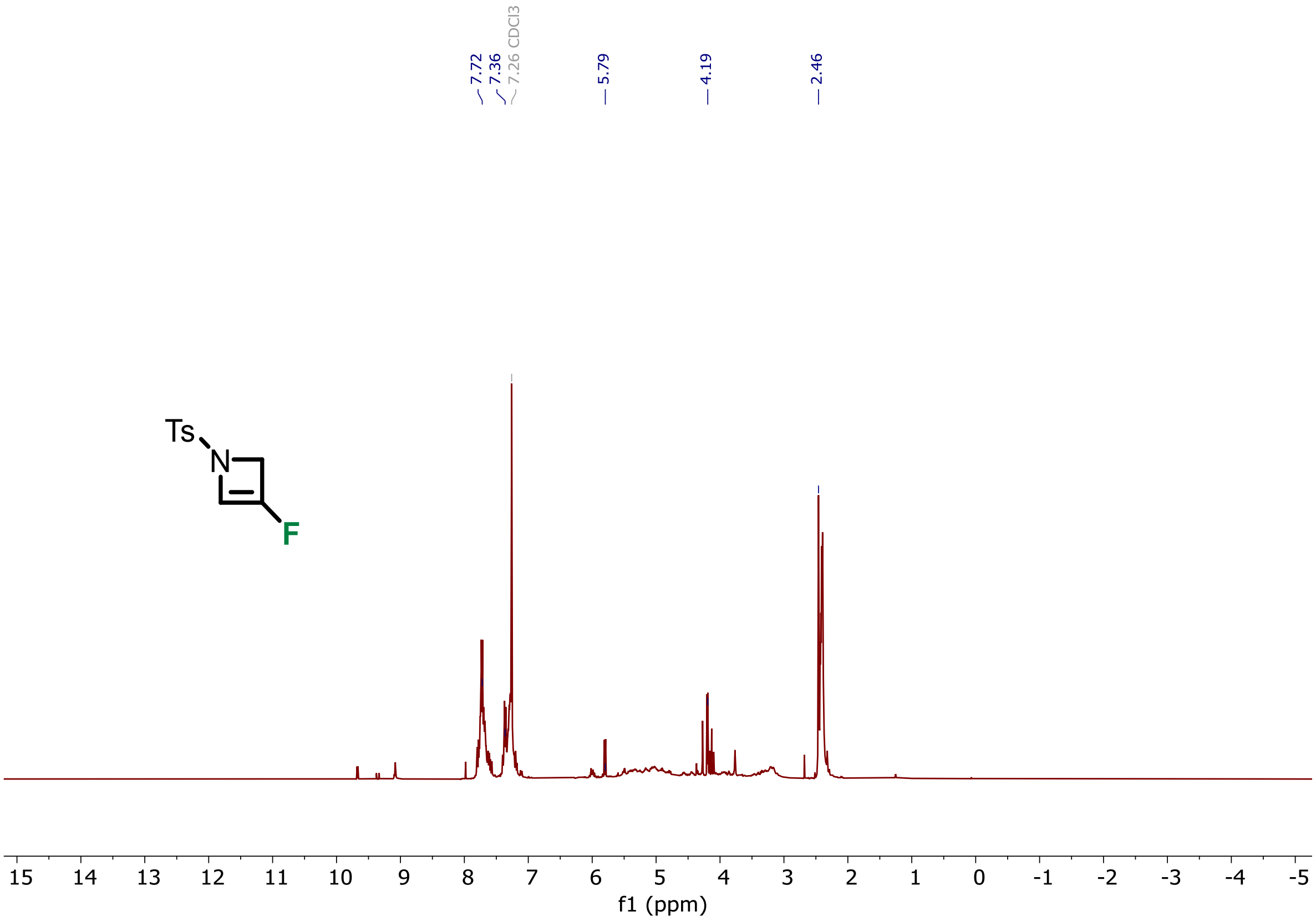

¹⁹F NMR (376 MHz, CDCl₃)

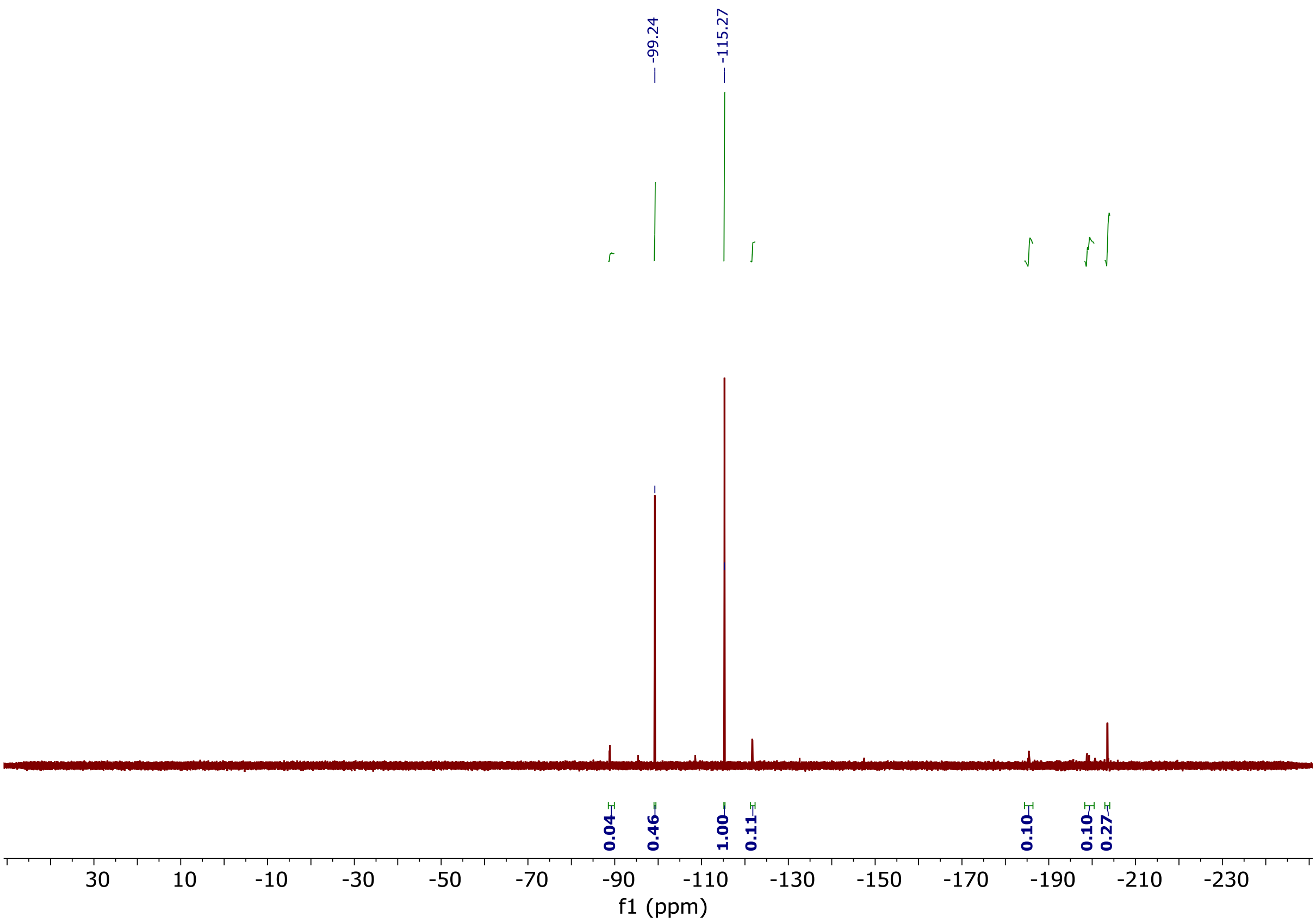

***3-fluoro-4-phenyl-1-tosyl-1,2-dihydroazete* (7aw)** 19 months under air, RT.

^{1}H NMR (500 MHz, CDCl$_3$)

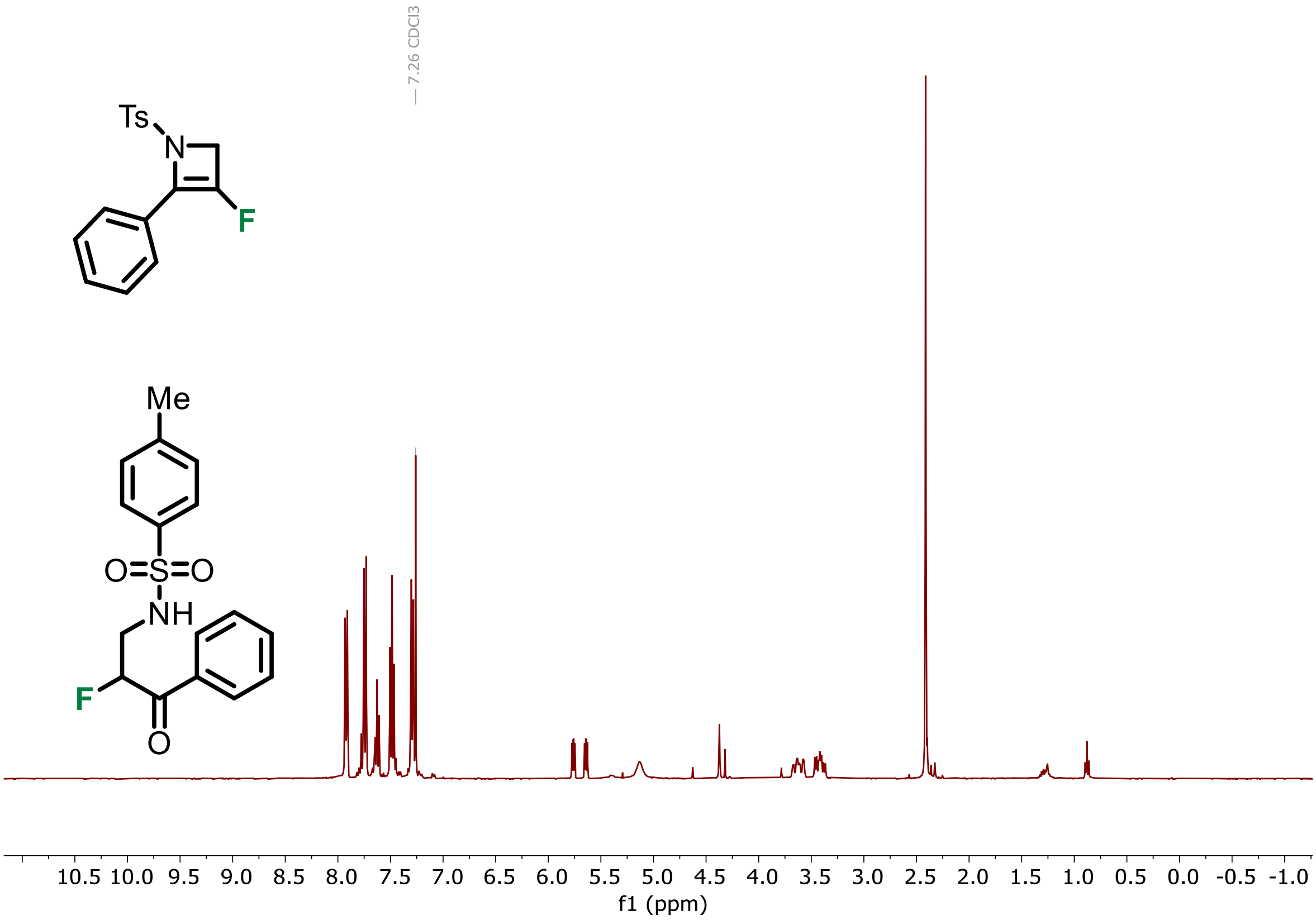

^{19}F NMR (376 MHz, CDCl$_3$)

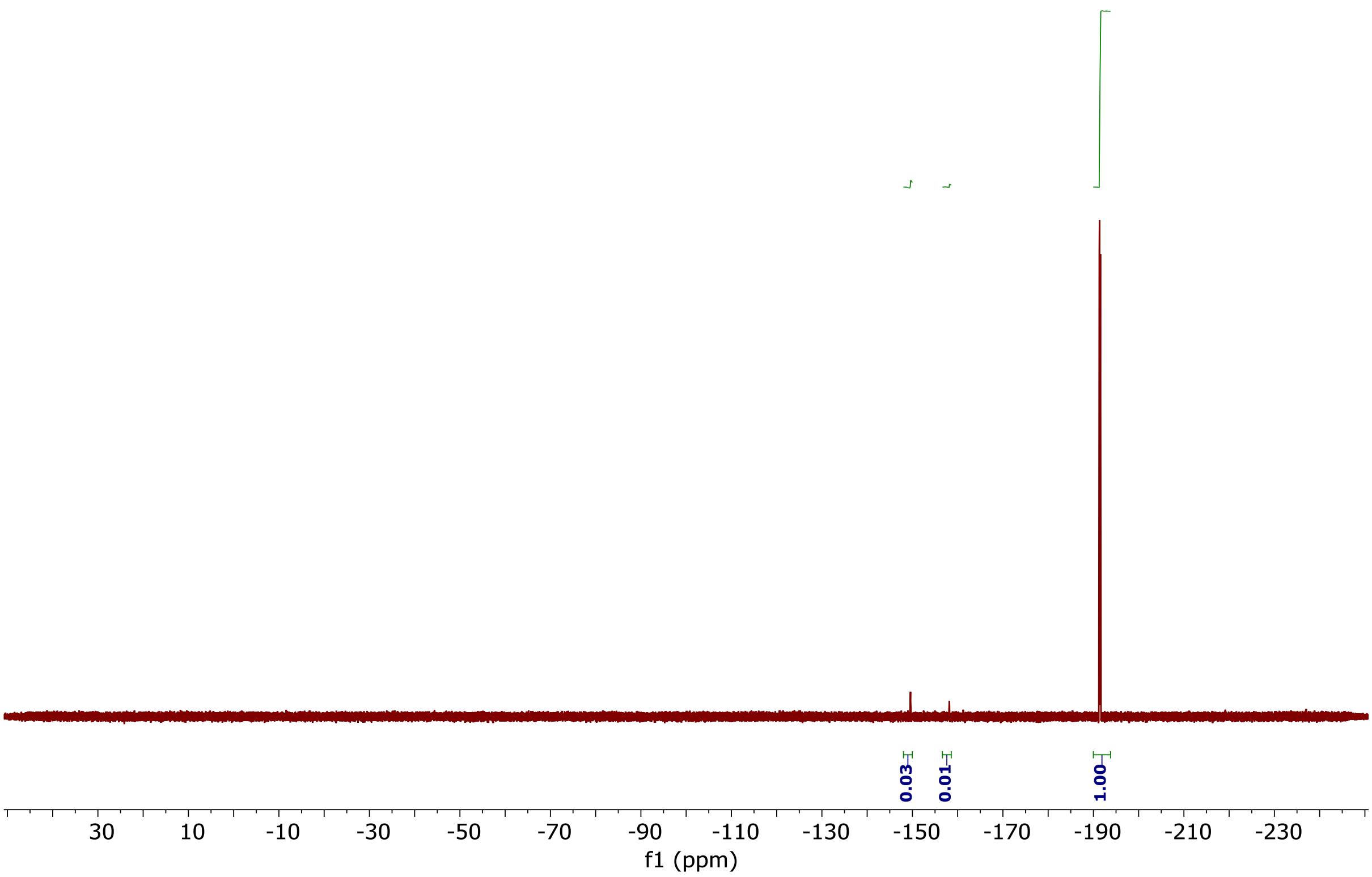

4.11. Acid and base stability

A pyrroline, an oxolene, and an azetine were subjected to acidic, basic, and neutral conditions in aqueous media for extended periods of time, and the remaining quantity of material recorded. Solutions were neutralised with either sat. aq. Na_2CO_3 or 2M aq. HCl as required.

Structure	Compound no.	pH 0 (24 h) % remaining	pH 14 (24 h) % remaining	pH 7 (48 h) % remaining
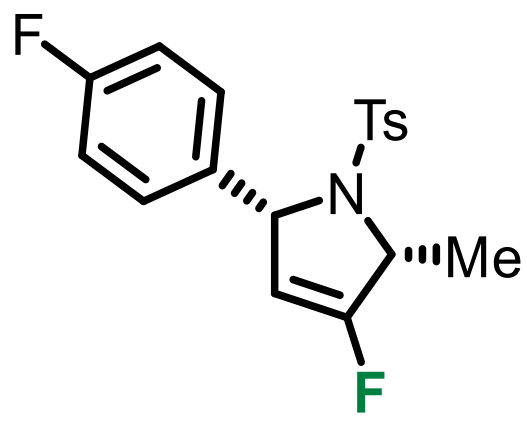	**(*R*,*R*)-4j**	100	100	100
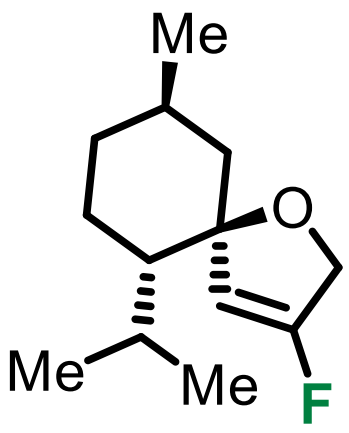	**6aq**	44	51	86
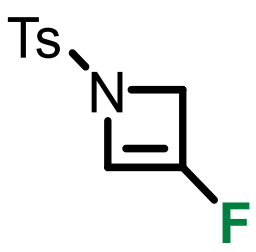	**7ar**	91	66	95

Supplementary Table 7: Remaining quantity of a selected example from each product family after stirring in either acid or base for 24 h, and stability in aqueous media over 48 h. Solutions were made up in 2:1 $MeCN/H_2O$, and made up to the required pH using HCl (37% w/w aqueous) for the acidic solutions, and DBU for the basic solutions. Substrate concentration was 0.1M. After neutralising, results were measured by ^{19}F NMR using 4,4'-difluorobiphenyl as an internal standard.

5. Failed Substrates

5.1. Failed to cyclise

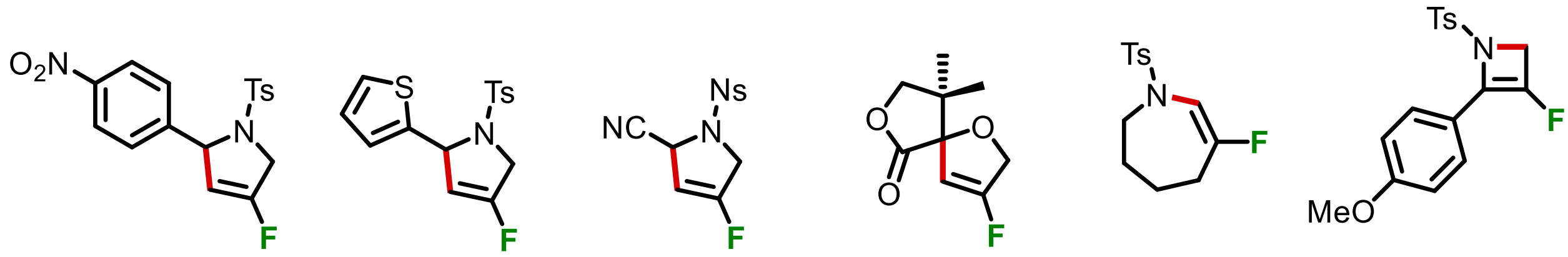

from **3bg** from **3bh** from **3bi** from **3bj** from **3bk** from **3bl**

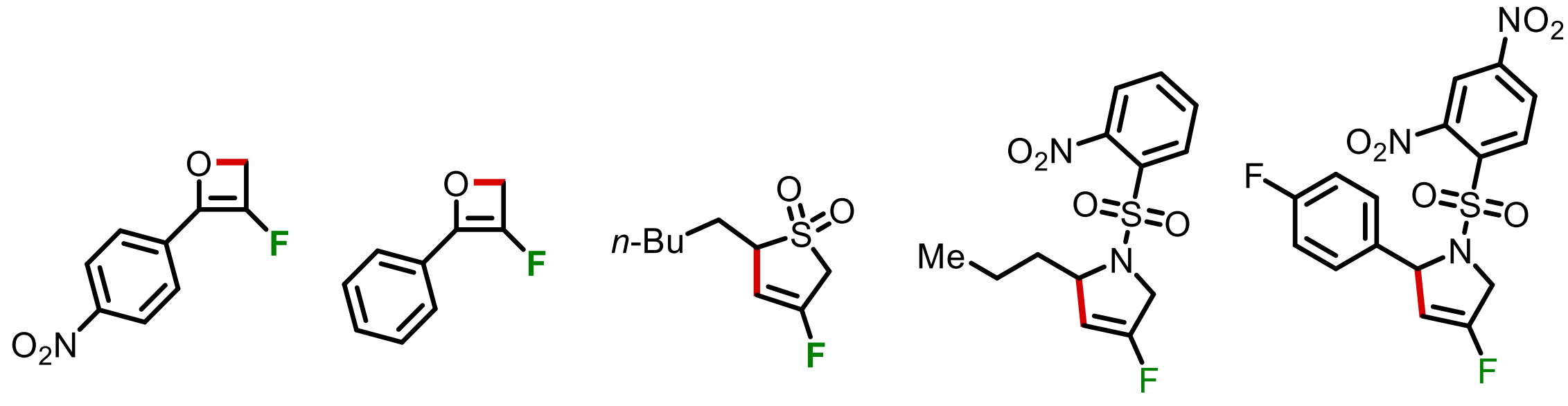

from **3bm** from **3bn** from **3bo** from **3bp** from **3bq**

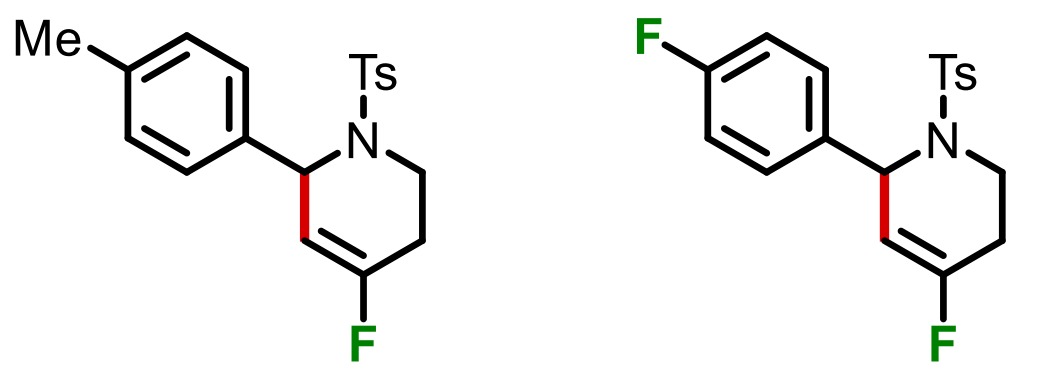

from **3br** from **3bs**

Supplementary Figure 10: Cycles that could not be synthesised from the parent FVI. Bond not formed highlighted in red. For functionalities not tolerated by the FVI synthesis reaction, see the original Lennox FVI synthesis paper.[1]

6. Biological Data

6.1. Metabolic stability

Structure	Compound number	Log D (pH 7.4)	Human CL int (µl/min/mg prot.)	Human clearance half-life $t_{1/2}$ (h)	**Relative rate of metabolism**
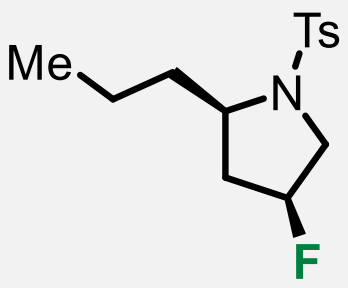	**10v**	3.4	238.0	0.10	**1.000**
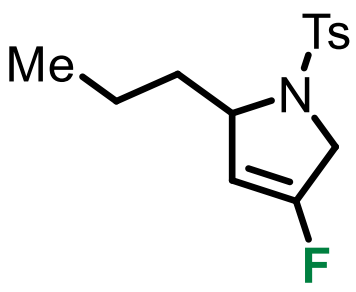	**4v**	3.7	221.0	0.11	**0.929**
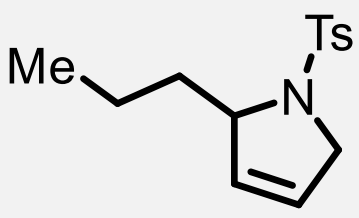	**4bf**	4.0	268.0	0.09	**1.126**
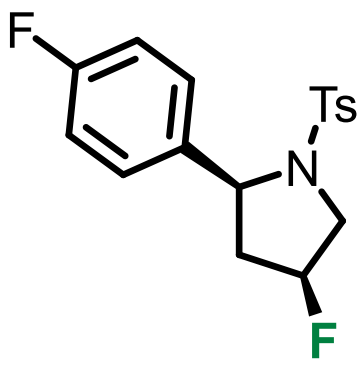	**10e**	2.4	265.0	0.09	**1.000**
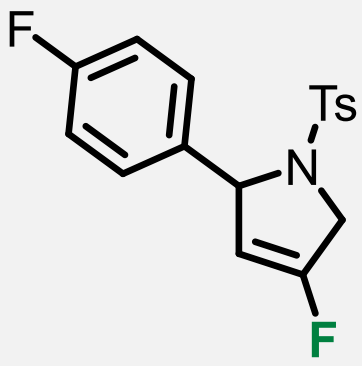	**4e**	3.3	213.0	0.11	**0.804**
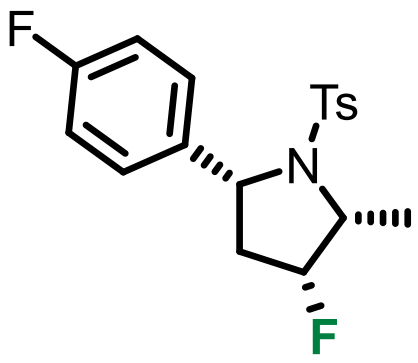	**(*R*,*R*,*R*)-10j**	4.0	282.0	0.08	**1.000**
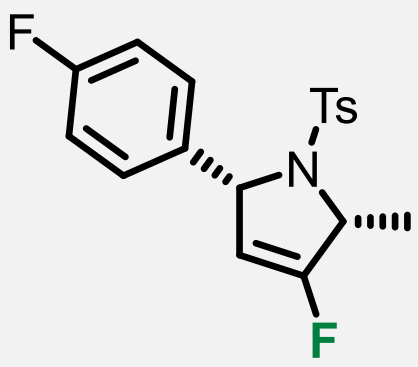	**(*R*,*R*)-4j**	3.8	252.0	0.09	**0.894**

Supplementary Table 8: Human enzyme metabolic stability data (Human Liver Microsomal, HLM) showing the differences in stability between fluorinated pyrrolidine, fluorinated pyrroline, and non-fluorinated pyrroline. Relative rate is given relative to the relevant pyrrolidine in each matched set. Measurements were conducted at Merck KGaA.

Measurement methods for metabolism data:

LogD:

LogD (distribution coefficient) is a shake flask method measuring a compound's distribution between two immiscible solvents typically octanol and water. The compound's varying solubility in each phase, dictated by its polarity, leads to different concentration levels, which are quantified through HPLC analysis.

For the sample 8µL of the 10mM compound solution in DMSO are pipetted into one safe-lock tube. 200µL of Octanol saturated Phosphate Buffer 20mM pH 7.4 and 200µL of Phosphate Buffer pH 7.4 saturated Octanol is added to this. Afterwards the tube is placed on the overhead rotator to invert for 60 minutes. After centrifugation for 2 min at 1000 rpm, both the upper phase and the buffer-phase (lower phase) taken with an insulin jab through the bottom of the safe-lock are analysed. Dilution Octanol-phase can be diluted with Methanol (dilution factor = 10) if required.

Chromatographic conditions: Column Waters XBridge Column C8 3.5µm (Waters Cat No 186003053); Column temperature 30°C; Autosampler Room temperature (approx. 25°C); Injection volume 10 µL (HPLC) / 3µL (UPLC); Wavelength 220 nm, 254 nm, 290 nm using DAD detector. Eluant A: Place 630mg Ammonium formiate in a 2000mL volumetric flask, add 4mL Formic acid and bring up to volume with Ultrapure Water; Eluant B: 50 mL Eluant A + 950 mL Acetonitrile.

HPLC Gradient:

Time	Eluent A (%)	Eluent B (%)	Flow (ml/min)
0.0	95	5	2.0
4.0	0	100	2.0
4.1	95	2	2.0
6.0	95	5	2.0

HLM: See literature procedure.[11]

Microsomal stability was assessed on a liquid handling platform (Tecan Freedom EVO-2 200 MCA384) using pooled mixed genders HLM (HLM UltraPool 150 Corning catalog no. 452117, lot no. 38291). Compounds (1 μM final) were preincubated with microsomes (final 0.5 mg/mL) for 5 minutes at 37 °C in 50 mM phosphate buffer at pH 7.4 or 7.0, followed by the start of the incubation by the addition of cofactor NADPH (final 1.5 mM). The final DMSO concentration did not exceed

0.2% (v/v). Incubations were carried out in singlicate. Aliquots were taken at time points 0, 5, 10, 20, and 30 minutes. Additionally, 2 control samples (0 and 30 minutes) lacking the addition of NADPH were included to flag compounds with nonmicrosomal metabolism. After incubation, samples were quenched by adding sample aliquots from 4 different compound incubations to the 2-fold volume of acetonitrile for bioanalytical cocktailing designed to avoid analytical interference. After centrifugation, supernatants were analyzed by a ultra-high performance liquid chromatography coupled to tandem mass spectrometry (UHPLC-MS/MS).

The data were subjected to postacquisition analysis by the software Analyst (version 1.7.2), and the percentage of compound remaining (defined as the ratio of peak area at a given time point and that at 0-minute time point) was plotted against incubation time. The first-order elimination rate constant (k_{el}) was derived by nonlinear regression using the software Biorails version 6.0 (The Edge Software Consultancy) and CL_{int} values (expressed in μL/min/mg) obtained by normalizing k_{el} with the microsomal concentration.

6.2. Blood plasma stability

Structure	Compound number	Human plasma $t_{1/2}$ (h)	Mouse plasma $t_{1/2}$ (h)	Rat plasma $t_{1/2}$ (h)	Phosphate buffer $t_{1/2}$ (h)	Simulated gastric fluid $t_{1/2}$ (h)
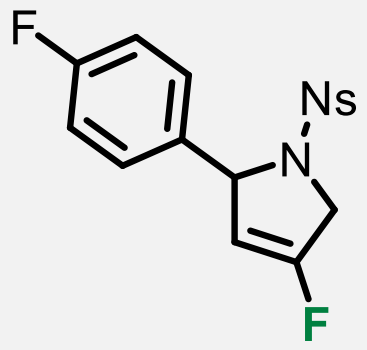	**4i**	>9.60	9.36	>9.60	>9.60	>9.60

Supplementary Table 9: Stability of a fluorinated pyrroline in blood plasma, phosphate buffer, and simulated gastric fluid. Plasma and phosphate buffer are at pH 7.4, simulated gastric fluid at pH 1.2. Measurements were conducted at Merck KGaA.

Measurement methods:

Procedure:

The incubation was done on a Hamilton ML STAR pipetting robot.

Compounds were available in a 10 mM DMSO solution.

Compounds were mixed in a cocktail with up to 9 compounds and a verification standard.

5 different matrices were used for the process: Simulated Gastric fluid, phosphate buffer, human plasma, mouse plasma and rat plasma.

The 3 plasma types were set to a pH of 7.4 using 5% 1M phosphate buffer.

The incubation period lased for 4 hours at 37°C while shaking. Samples were generated for 4h, 2h, 1h 0h and 4h total workup.

Precipitation was achieved using acetonitrile. An internal standard was added during the precipitation step. The final dilution was done using water.

Calibration and blank samples were also processed by the robot.

Measurement:

The quantitative determination was performed using an LC-MS/MS assay.

One calibration and one blank sample per matrix were prepared.

Measurements were performed on a UPLC.

Tuning data for all compounds was generated beforehand.

Detection was performed on a Tandem MS using multiple reaction monitoring (MRM) for MS/MS detection of the compounds.

The concentrations of the compounds in all timepoints and calibration standards were calculated by interpolation of the peak area ratio of the analyte/internal standard versus the ratio of their nominal concentration into the regression line obtained from the calibration standards through zero.

Equipment:

Waters H-Class UPLC,

Sciex Triple QTrap API 5500 Mass Spectrometer

Hamilton ML STAR

Chemicals Used: Water Milli-Q

Formic Acid (FAC) Art. 1.00264.0100

Acetonitrile (ACN) Art. 34998

Dimethyl sulfoxide (DMSO) Art. 1.01900.0500

di-Potassium hydrogen phosphate-trihydrate Art. 1.05099

Sodium chloride Art.1.06404

Hydrochloric acid 25% Art.1.00316

Pepsin Art. P7000

Human plasma was sourced from internal donors

CD1 mouse plasma and wistar rat plasma bought from CliniScience

7. Methodology Benchmarking

Existing methods of synthesis for 3-fluoro-3-pyrrolines and 3-fluoro-3-oxolenes are benchmarked against this work in the tables below. No table has been included for fluorinated azetines are there are currently no methods for their synthesis other than this work. Ring-closing metathesis (RCM) work on pyrrolines is by Rutjes,[12] Haufe,[13] Inoue,[14] and Brown,[15] and in the case of oxolenes is only by Inoue. Brown's attempt to synthesise a 3-fluoro-3-pyrroline was unsuccessful, and Haufe's products were pyrrolinones rather than strictly pyrrolines. Stated step count is given from simple non-fluorinated or commercially available starting materials. For more details on limitations of this work's functional group compatibility, see section S5 (failed cyclisation substrates) and previous work from our group for limitations in the FVI synthesis step.[1]

7.1. 3-Pyrrolines

Metric	**Liu *et al.***	**RCM**	**This work**
Step count	2	2-4	2
Isolated yield	28-92%	19-86%	10-79% (see manuscript)
Diastereoselectivity	1:1 (none)	n/a	>100:1 *cis*
Substitution Patterns	Broad substitution patterns, but 4-position must be ester or aryl	Limited to 1 methyl group in 2- or 4-position	Broad (see manuscript), but no substitution at 4-position
Functional Group Tolerance	Limited to esters, sulfonates, and aryl halides	Simple alkyl only	Broad (see manuscript) but no groups highly sensitive to oxidants
Synthetic Scalability	Only tested on 0.1 mmol scale (<100 mg)	20.2 mg scale	Tested on multi-gram scale

Supplementary Table 10: Benchmarking of existing methods of synthesis for 3-fluoro-3-pyrrolines against this work. Methods shown are ring-closing metathesis (RCM), and a general method from allenes by Liu *et al.*[16]

7.2. 3-Oxolenes

Metric	**Chu *et al.***	**Lequeux *et al.***	**RCM**	**This work**
Step count	6	4 steps from 3-fluorooxetane	2	2
Isolated yield	75-80%	75%	74%	15-95% (see manuscript)
Diastereoselectivity	1.3:1 *trans*	n/a	n/a	2:1 *cis*
Substitution Patterns	Single substitution pattern, small variations at 1 position	1 example, 1 position	1 example, 1 position	Broad (see manuscript), but no substitution at 4-position
Functional Group Tolerance	Only alcohol and ether groups shown	Only alcohol shown	Alkyl/aryl only	Broad (see manuscript) but no groups highly sensitive to oxidants
Synthetic Scalability	Up to 280 mg scale	30 mg scale	14.2 mg scale	Tested on multi-gram scale

Supplementary Table 11: Benchmarking of existing methods of synthesis for 3-fluoro-3-oxolenes against this work. Methods shown are a multi-step synthesis used by Chu *et al.* in the synthesis of nucleotide bioisosteres,[17–19] a single example by Lequeux *et al.*,[20] and ring-closing metathesis (RCM).

8. Characterisation data

8.1. Alkynes (1)

***N-(4-methoxybenzyl)-4-methyl-N-(prop-2-yn-1-yl)benzenesulfonamide* (1a)**

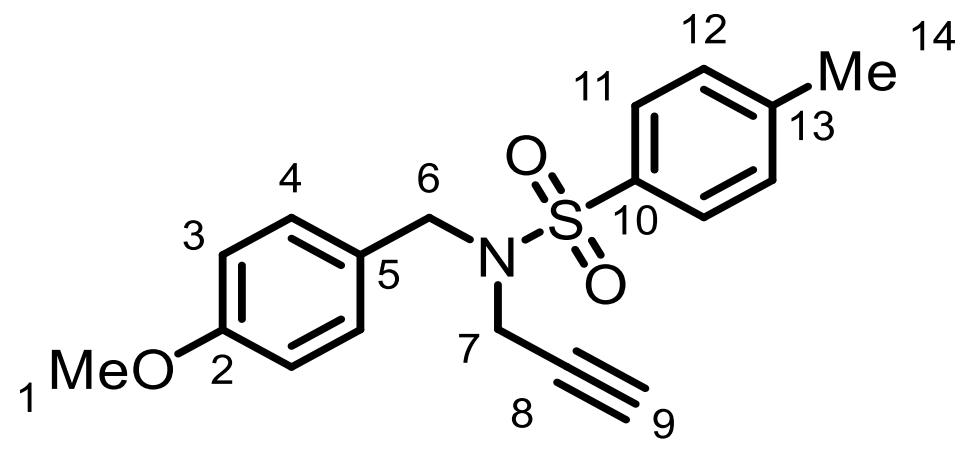

Prepared according to **General procedure II** from sulfonamide **26a**, affording the title compound without further purification as a yellow oil (1.36 g, 83% over 2 steps).

^{1}H NMR (400 MHz, CDCl$_3$) δ 7.79 (d, *J* = 8.3 Hz, 2H, C11-H), 7.32 (t, *J* = 8.1 Hz, 2H, C12-H), 7.29 – 7.26 (m, 2H, C4-H), 6.85 (d, *J* = 8.6 Hz, 2H, C3-H), 4.29 (s, 2H, C6-H), 3.93 (d, *J* = 2.4 Hz, 2H, C7-H), 3.80 (s, 3H, C1-H), 2.44 (s, 3H, C14-H), 2.00 (t, *J* = 2.4 Hz, 1H, C9-H).

^{13}C NMR (126 MHz, CDCl$_3$) δ 159.6 (C2), 143.7 (C13), 136.2 (C10), 130.3 (C4), 129.6 (C12), 128.0 (C11), 126.9 (C5), 114.2 (C3), 76.5 (C8), 74.1 (C9), 55.4 (C1), 49.3 (C6), 35.4 (C7), 21.7 (C14).

HRMS (ESI+) calcd: $[M+Na]^+$ ($C_{18}H_{19}NO_3S$) 352.0978, measured 352.0962 (4.54 ppm error).

IR (neat) v_{max}/cm^{-1}: 3286, 3015, 2970, 1738, 1611, 1512, 1441, 1365, 1352, 1229, 1216, 1160, 542, 527.

R_f 0.45 (20% EtOAc in pentane).

4-methyl-*N*-(4-methylbenzyl)-*N*-(prop-2-yn-1-yl)benzenesulfonamide (1b)

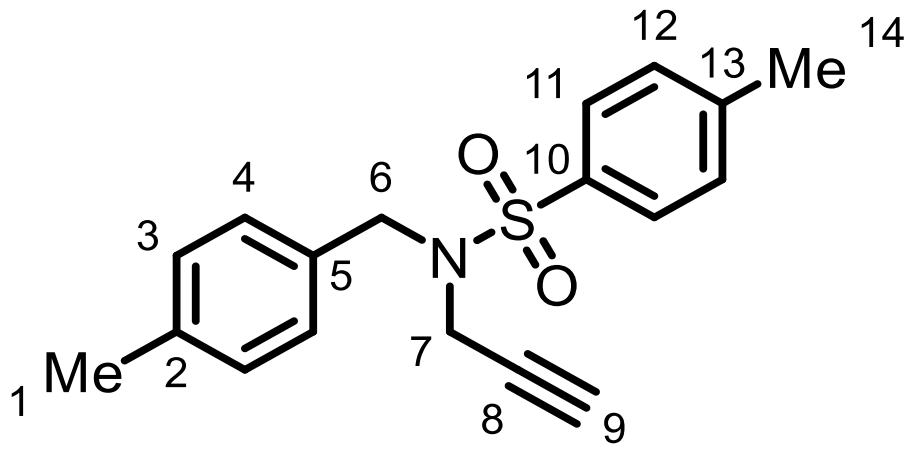

4-methyl benzylamine was tosylated according to **General procedure I**. The crude product was then propargylated according to **General procedure II**. The crude product was purified by flash column chromatography (2-20% EtOAc in pentane) to afford the title compound as an orange solid (1.02 g, 81%).

^{1}H NMR (400 MHz, $CDCl_3$) δ 7.80 (d, *J* = 8.2 Hz, 2H, C11-H), 7.32 (d, *J* = 7.9 Hz, 2H, C4-H), 7.24 (d, *J* = 8.2 Hz, 2H, C12-H), 7.14 (d, *J* = 7.9 Hz, 2H, C3-H), 4.31 (s, 2H, C6-H), 3.94 (d, *J* = 2.4 Hz, 2H, C7-H), 2.44 (s, 3H, C14-H), 2.34 (s, 3H, C1-H), 2.00 (t, *J* = 2.4 Hz, 1H, C9-H).

^{13}C NMR (101 MHz, $CDCl_3$) δ 143.6 (C13), 137.9 (C2), 136.0 (C10), 131.8 (C12), 129.5 (C5), 129.4 (C3), 128.8 (C4), 127.9 (C11), 77.4 (C8), 74.1 (C9), 49.5 (C6), 35.4 (C7), 21.6 (C1), 21.2 (C14).

IR (neat) v_{max}/cm^{-1}: 3287, 2931, 1703, 1603, 1504, 1451, 1366, 1323, 1303, 1222, 1151, 1102, 1091, 1015, 1001.

Characterisation data matches that previously reported.[21]

***N*-(4-bromobenzyl)-4-methyl-*N*-(prop-2-yn-1-yl)benzenesulfonamide (1c)**

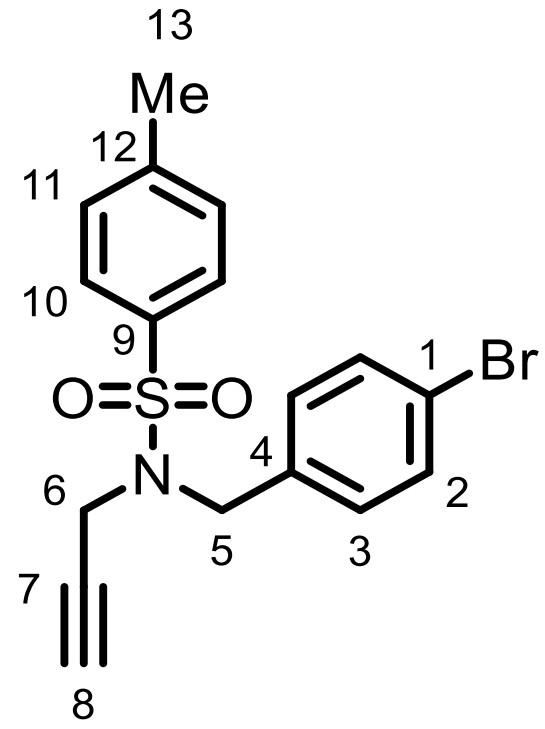

4-Bromobenzylamine was protected according to **General procedure I**. The crude product was then propargylated according to **General procedure II** to afford the title compound as a yellow oil (1.10 g, 58% over 2 steps).

^{1}H NMR (400 MHz, $CDCl_3$) δ 7.78 (app. dt, *J* = 8.4, 1.7 Hz, 2H, C10-H), 7.47 (app. dt, *J* = 8.2, 1.7 Hz, 2H, C2-H), 7.33 (d, *J* = 8.5 Hz, 2H, C11-H), 7.24 (app. dt, *J* = 8.4, 1.9 Hz, 2H, C3-H), 4.30 (s, 2H, C5-H), 3.94 (d, *J* = 2.5 Hz, 2H, C6-H), 2.45 (s, 3H, C13-H), 2.02 (t, *J* = 2.5 Hz, 1H, C8-H).

^{13}C NMR (101 MHz, $CDCl_3$) δ 143.9 (C12), 136.0 (C9), 134.2 (C4), 132.0 (C2), 130.6 (C3), 129.7 (C11), 128.0 (C10), 122.3 (C1), 76.2 (C7), 74.4 (C8), 49.4 (C5), 35.8 (C6), 21.7 (C13).

Characterisation data matches that previously reported.[22]

***N*-(4-chlorobenzyl)-4-methyl-*N*-(prop-2-yn-1-yl)benzenesulfonamide (1d)**

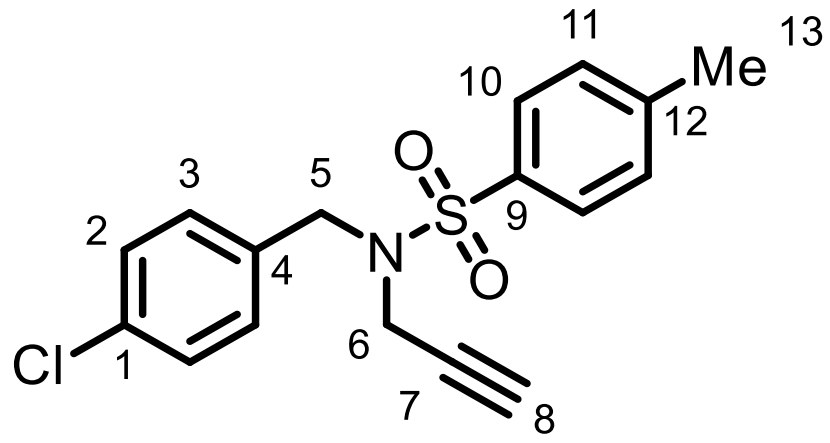

Prepared according to **General procedure II** from sulfonamide **26d**, affording the title compound without further purification as a yellow oil (1.39 g, 83% over 2 steps).

^{1}H NMR (400 MHz, CDCl$_3$) δ 7.78 (app. dt, *J* = 8.2, 2.0 Hz, 2H, C10-H), 7.36 – 7.29 (m, 6H, C2-H, C3-H, C11-H), 4.32 (s, 2H, C5-H), 3.94 (d, *J* = 2.5 Hz, 2H, C6-H), 2.45 (s, 3H, C13-H), 2.01 (t, *J* = 2.5 Hz, 1H, C8-H).

^{13}C NMR (126 MHz, CDCl$_3$) δ 143.9 (C12), 136.0 (C1), 134.2 (C9), 133.6 (C4), 130.3 (C11), 129.7 (C2), 129.0 (C3), 128.0 (C10), 76.2 (C7), 74.4 (C8), 49.3 (C5), 35.8 (C6), 21.7 (C13).

HRMS (ESI+) calcd: $[M+H]^+$ ($C_{17}H_{16}ClNO_2S$) 334.0663, measured 334.0652 (3.29 ppm error).

IR (neat) v_{max}/cm^{-1}: 3456, 3294, 3027, 2970, 2119, 1738, 1597, 1491, 1365, 1350, 1228, 1216, 1160, 1091, 900, 660, 564, 542.

R*f* 0.56 (20% EtOAc in pentane).

***N*-(4-fluorobenzyl)-4-methyl-*N*-(prop-2-yn-1-yl)benzenesulfonamide** (**1e**)

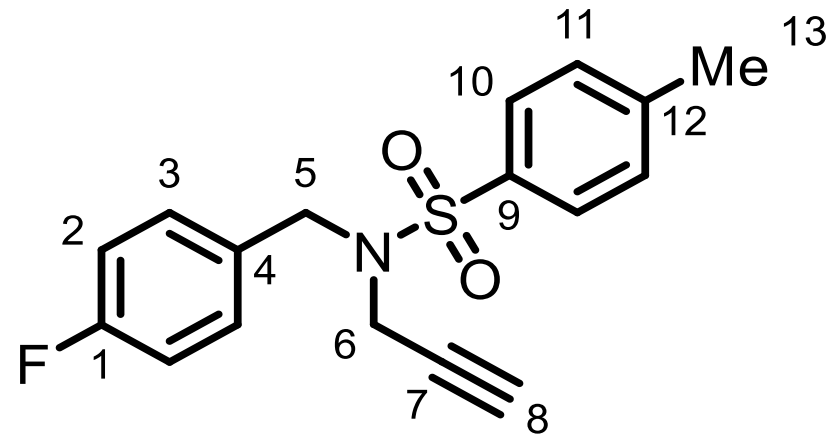

Prepared according to **General procedure II** from sulfonamide **26e**, affording the title compound without further purification as a yellow oil (1.08 g, 68% over 2 steps).

^{1}H NMR (400 MHz, CDCl$_3$) δ 7.78 (app. dt, *J* = 8.4, 2.0 Hz, 2H, C10-H), 7.36 – 7.32 (m, 2H, C3-H), 7.33 (d, *J* = 8.4 Hz, 2H, C11-H), 7.03 (tt, *J* = 8.6, 3.1 Hz, 2H, C2-H), 4.32 (s, 2H, C5-H), 3.94 (d, *J* = 2.5 Hz, 2H, C6-H), 2.45 (s, 3H, C13-H), 2.02 (td, *J* = 2.5, 1.0 Hz, 1H, C8-H).

^{13}C NMR (151 MHz, CDCl$_3$) δ 162.6 (d, *J* = 246.9 Hz), 143.7, 135.9, 130.7 (d, *J* = 3.3 Hz), 130.5 (d, *J* = 8.4 Hz), 129.6, 127.8, 115.6 (d, *J* = 21.3 Hz), 76.1, 74.2, 49.1, 35.5, 21.6.

^{19}F NMR (376 MHz, CDCl$_3$) δ −113.84 (td, *J* = 8.7, 4.5 Hz, C1-F).

HRMS (EI+) calcd: $[M-H]^+$ ($C_{17}H_{16}FNO_2S$) 316.0802, measured 316.0801 (0.32 ppm error).

IR (neat) v_{max}/cm^{-1}: 3295, 2924, 1604, 1509, 1446, 1348, 1224, 1163, 1091, 900, 815, 750, 660.

R*f* 0.63 (20% EtOAc in pentane).

***N-((4-fluorophenyl)methyl-d)-4-methyl-N-(prop-2-yn-1-yl)benzenesulfonamide* ([2H]-1e)**

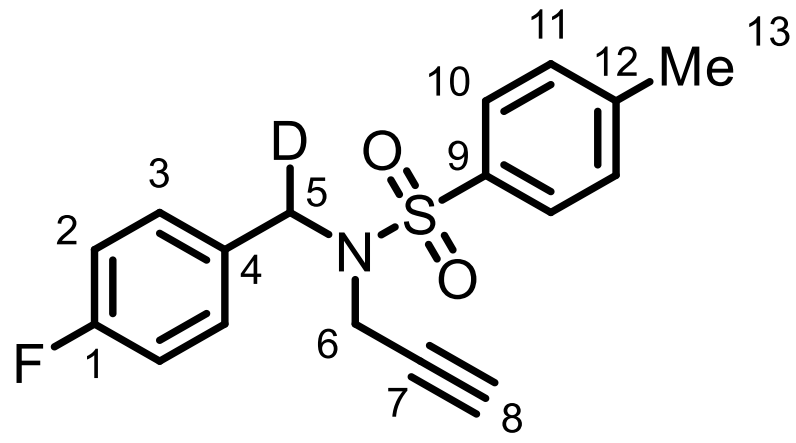

[2H]-26e was tosylated according to **General procedure I** to afford the title compound without further purification as a yellow solid (1.21 g, 81%, 98% D incorporation).

^{1}H NMR (500 MHz, CDCl$_3$) δ 7.79 (app. dt, *J* = 8.3, 1.7 Hz, 2H, C10-H), 7.37 – 7.30 (m, 4H, C11-H, C3-H), 7.07 – 6.99 (m, 2H, C2-H), 4.30 (s, 1H, C5-H), 3.94 (d, *J* = 2.4 Hz, 2H, C6-H), 2.45 (s, 3H, C13-H), 2.02 (t, *J* = 2.5 Hz, 1H, C8-H).

^{13}C NMR (126 MHz, CDCl$_3$) δ 162.8 (d, *J* = 246.7 Hz, C1-H), 143.9 (C12), 136.1 (C9), 130.7 (d, *J* = 2.5 Hz, C4), 130.7 (d, *J* = 8.3 Hz, C3), 129.7 (C11), 128.0 (C10), 115.8 (d, *J* = 21.6 Hz, C2), 76.3 (C7), 74.3 (d, *J* = 1.9 Hz, C8), 48.9 (t (1:1:1), *J* = 21.3 Hz, C5), 35.6 (C6), 21.7 (C13).

^{19}F NMR (376 MHz, CDCl$_3$) δ −113.83 (tt, *J* = 8.5, 5.3 Hz, 1F, C1-F).

HRMS (+ESI) calcd: $[M+H]^+$ ($C_{17}H_{15}DFNO_2S$) 319.1021, measured 319.1017 (1.25 ppm error).

IR (neat) v_{max}/cm^{-1}: 3290, 2923, 1728, 1601, 1508, 1347, 1221, 1159, 1093, 813, 731, 657, 570, 541.

***4-methyl-N-(prop-2-yn-1-yl)-N-(4-(trifluoromethyl)benzyl)benzenesulfonamide* (1f)**

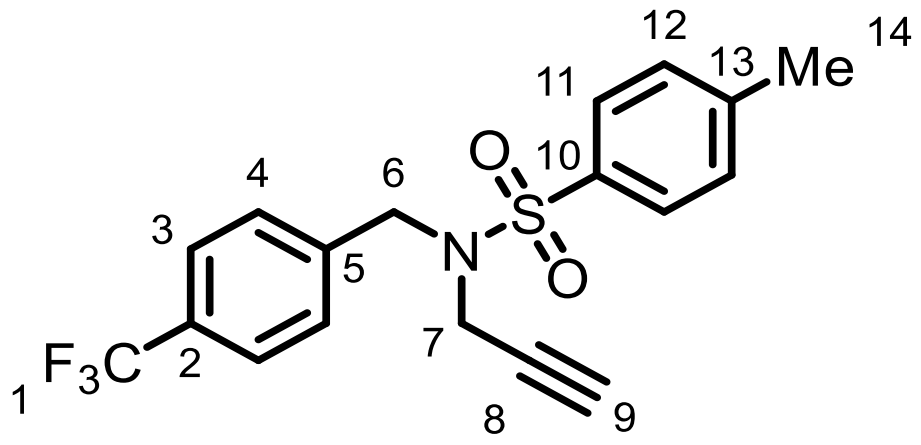

Prepared according to **General procedure II** from sulfonamide **26f**, affording the title compound without further purification as a yellow oil (1.03 g, 56% over 2 steps).

^{1}H NMR (400 MHz, CDCl$_3$) δ 7.79 (app. dt, *J* = 8.3, 1.7 Hz, 2H, C11-H), 7.60 (dd, *J* = 7.7, 1.1 Hz, 2H, C3-H), 7.49 (d, *J* = 8.1 Hz, 2H, C12-H), 7.34 (d, *J* = 8.0 Hz, 2H, C4-H), 4.41 (s, 2H, C6-H), 3.96 (d, *J* = 2.5 Hz, 2H, C7-H), 2.45 (s, 3H, C14-H), 2.03 (t, *J* = 2.5 Hz, 1H, C9-H).

^{13}C NMR (151 MHz, CDCl$_3$) δ 144.1 (C13), 139.4 (C10), 135.9 (C5), 130.6 (q, *J* = 32.4 Hz, C2), 129.8 (C4), 129.1 (C12), 128.0 (C11), 125.8 (q, *J* = 3.8 Hz, C3), 124.1 (q, *J* = 272.3 Hz, C1), 76.1 (C8), 74.6 (C9), 49.6 (C6), 36.1 (C7), 21.7 (C14).

^{19}F NMR (376 MHz, CDCl$_3$) δ −62.48 (C1-F).

HRMS (ESI+) calcd: $[M+H]^+$ ($C_{18}H_{16}F_3NO_2S$) 368.0927, measured 368.0938 (2.99 ppm error).

IR (neat) v_{max}/cm^{-1}: 3297, 2925, 1737, 1619, 1597, 1420, 1349, 1324, 1161, 1124, 1092, 1066, 1018, 903, 815, 660.

R*f* 0.66 (20% EtOAc in pentane).

4-methyl-N-(4-nitrobenzyl)-N-(prop-2-yn-1-yl)benzenesulfonamide **(1g)**

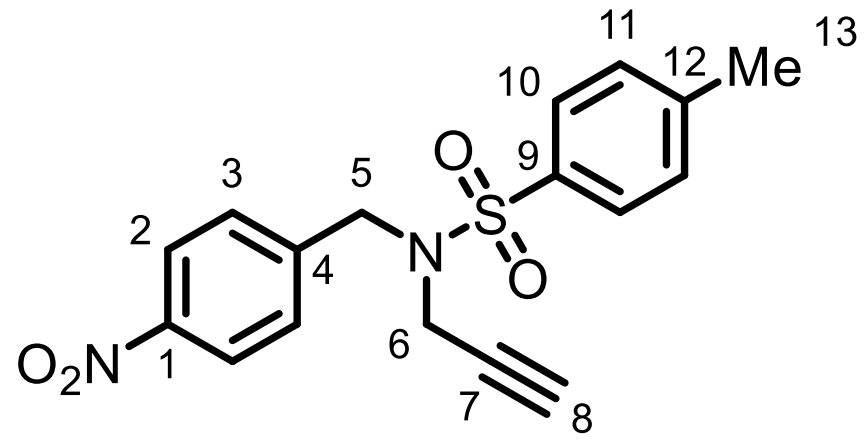

4-nitrobenzylamine was tosylated according to **General procedure I**. The crude product was then propargylated according to **General procedure II**. The crude product was purified by flash column chromatography (2-20% EtOAc in pentane) to afford the title compound as an orange solid (520 mg, 17%).

^{1}H NMR (400 MHz, $CDCl_3$) δ 8.21 (d, *J* = 8.3 Hz, 2H, C2-H), 7.79 (d, *J* = 8.0 Hz, 2H, C10-H), 7.56 (d, *J* = 8.4 Hz, 2H, C11-H), 7.35 (d, *J* = 8.0 Hz, 2H, C3-H), 4.45 (s, 2H, C5-H), 3.98 (d, *J* = 2.5 Hz, 2H, C6-H), 2.45 (s, 3H, C13-H), 2.05 (t, *J* = 2.5 Hz, 1H, C8-H).

^{13}C NMR (101 MHz, $CDCl_3$) δ 147.9 (C1), 144.2 (C12), 142.8 (C4), 135.5 (C9), 129.8 (C2), 129.4 (C11), 127.8 (C12), 124.0 (C3), 77.4 (C7), 74.7 (C8), 49.4 (C5), 36.3 (C6), 21.7 (C13).

HRMS (+ESI) calcd. $[M+H]^+$ ($C_{17}H_{16}N_2O_4S$) 345.0904, measured 345.0916 (3.48 ppm error).

IR (neat) v_{max}/cm^{-1}: 3293, 3115, 3082, 2930, 2856, 1716, 1597, 1516, 1345, 1157, 1090, 1013.

R_f 0.51 (20% EtOAc in pentane).

***N-benzyl-4-methyl-N-(2-methylbut-3-yn-2-yl)benzenesulfonamide* (1h)**

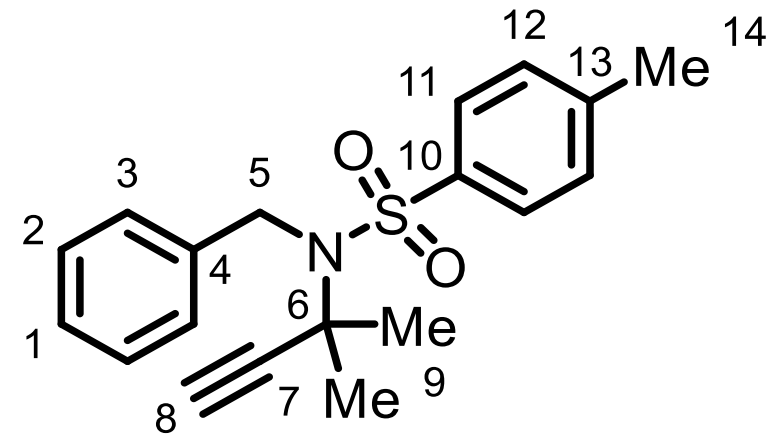

Prepared according to **General procedure II-A** from sulfonamide **1av** and benzyl bromide, with TBAI (5 mol %) added. The crude product was purified by recrystallisation from Et_2O to afford the title compound as a white solid (742 mg, 45%).

^{1}H NMR (500 MHz, $CDCl_3$) δ 7.66 (d, *J* = 8.3 Hz, 2H, C11-H), 7.48 (d, *J* = 8.1 Hz, 2H, C12-H), 7.33 (t, *J* = 7.7 Hz, 2H, C2-H), 7.27 – 7.21 (m, 3H, C1-H, C3-H), 4.82 (s, 2H, C5-H), 2.40 (s, 3H, C14-H), 2.26 (s, 1H, C8-H), 1.61 (s, 6H, C9-H).

^{13}C NMR (126 MHz, $CDCl_3$) δ 143.2 (C13), 139.6 (C4), 139.5 (C10), 129.4 (C12), 128.4 (C2), 127.7 (C11), 127.6 (C3), 127.2 (C1), 86.3 (C7), 72.3 (C8), 57.1 (C6), 52.2 (C5), 31.1 (C9), 21.6 (C14).

HRMS (+EI) calcd: $[M\text{-}Me]^+$ ($C_{19}H_{21}NO_2S$) 312.1053, measured 312.1047.

IR (neat) v_{max}/cm^{-1}: 3279, 2988, 2943, 1737, 1598, 1495, 1454, 1364, 1334, 1149, 1090, 1029, 703, 573, 542.

R_f 0.62 (20% EtOAc in pentane).

***N*-(4-fluorobenzyl)-4-nitro-N-(prop-2-yn-1-yl)benzenesulfonamide (1i)**

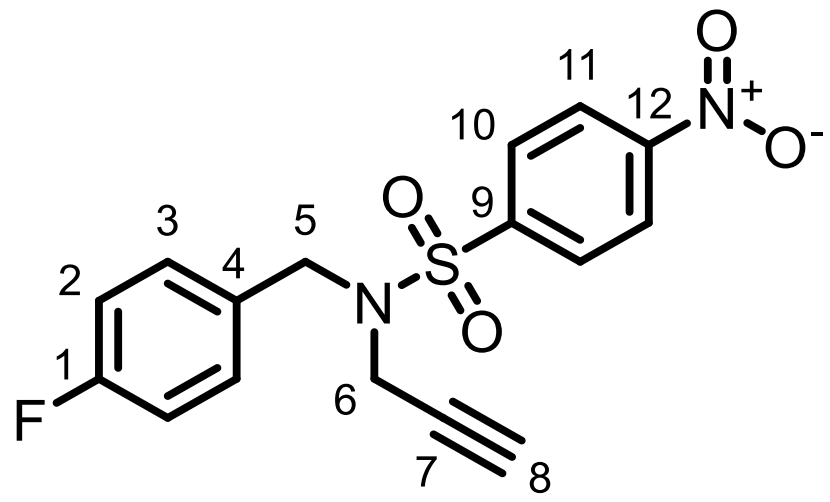

4-fluorobenzylamine was protected according to **General procedure I** using 4-nitrobenzenesulfonyl chloride instead of *p*-toluenesulfonyl chloride. The nosyl amine was then propargylated according to **General procedure II** to afford the title compound as a yellow solid (3.27 g, 94% over 2 steps).

^{1}H NMR (500 MHz, CDCl$_3$) δ 8.39 (app. dt, *J* = 9.0, 2.3 Hz, 2H, C11-H), 8.10 (app. dt, *J* = 8.9, 2.1 Hz, 2H, C10-H), 7.39 – 7.30 (m, 2H, C3-H), 7.06 (tt, *J* = 8.5, 2.1 Hz, 2H, C2-H), 4.37 (s, 2H, C5-H), 3.99 (d, *J* = 2.5 Hz, 2H, C6-H), 2.04 (t, *J* = 2.5 Hz, 1H, C8-H).

^{13}C NMR (126 MHz, CDCl$_3$) δ 163.0 (d, *J* = 247.6 Hz, C1), 150.4 (C12), 144.9 (C9), 130.7 (d, *J* = 8.3 Hz, C3), 129.8 (d, *J* = 3.2 Hz, C4), 129.2 (C11), 124.4 (C10), 116.1 (d, *J* = 21.7 Hz, C2), 75.6 (C7), 75.0 (C8), 49.5 (C5), 35.7 (C6).

^{19}F NMR (376 MHz, CDCl$_3$) δ −112.99 (tt, *J* = 8.5, 5.3 Hz, 1F, C1-F).

Characterisation data matches that previously reported.[1]

***N-(but-3-yn-2-yl)-N-(4-fluorobenzyl)-4-methylbenzenesulfonamide* (1j)**

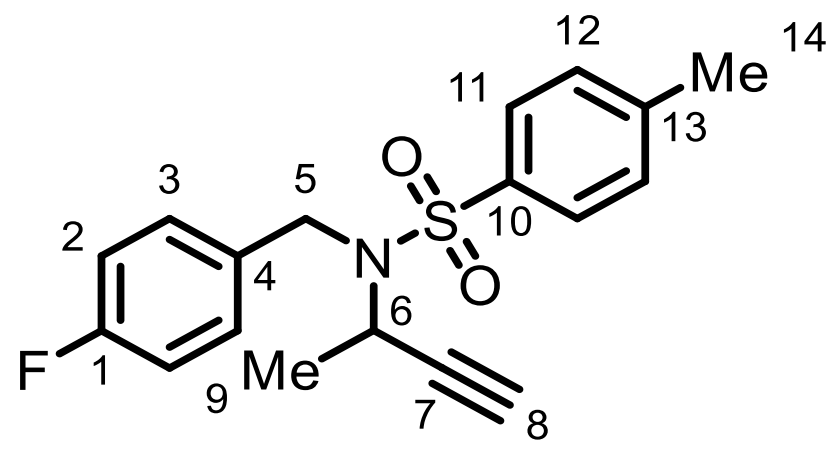

Prepared according to **General procedure II-A** from sulfonamide **26e** and purified by flash column chromatography (2-20% EtOAc in pentane) to afford the title compound as an orange oil (655 mg, 40%).

^{1}H NMR (400 MHz, $CDCl_3$) δ 7.72 (app. dt, *J* = 8.3, 2.0 Hz, 2H, C11-H), 7.43 – 7.38 (m, 2H, C3-H), 7.30 (d, *J* = 8.5 Hz, 2H, C12-H), 6.99 (tt, *J* = 8.7, 2.1 Hz, 2H, C2-H), 4.96 (qd, *J* = 7.1, 2.2 Hz, 1H, C6-H), 4.62 (d, *J* = 15.9 Hz, 1H, C5-H), 4.17 (d, *J* = 15.8 Hz, 1H, C5-H), 2.43 (s, 3H, C14-H), 2.17 (d, *J* = 2.2 Hz, 1H, C8-H), 1.16 (d, *J* = 7.1 Hz, 3H, C9-H).

^{13}C NMR (126 MHz, $CDCl_3$) δ 162.4 (d, *J* = 245.7 Hz, C2), 143.7 (C13), 136.3 (C10), 134.1 (d, *J* = 3.2 Hz, C4), 129.8 (d, *J* = 8.0 Hz, C3), 129.7 (C12), 127.7 (C11), 115.4 (d, *J* = 21.6 Hz, C2), 81.0 (C7), 74.1 (C8), 47.9 (C5), 46.7 (C6), 23.2 (C9), 21.7 (C14).

^{19}F NMR (471 MHz, $CDCl_3$) δ −115.3 (td, *J* = 8.7, 4.4 Hz).

HRMS (EI+) calcd: $[M-Me]^{+}$ ($C_{18}H_{18}NO_2SF$) 316.0802, measured 316.0798 (1.27 ppm error).

IR (neat) v_{max}/cm^{-1}: 3296, 2970, 2936, 1737, 1604, 1509, 1447, 1339, 1223, 1156, 1092, 835, 660, 583, 546.

R_f 0.58 (20% EtOAc in pentane).

(*R*)-N-(but-3-yn-2-yl)-N-(4-fluorobenzyl)-4-methylbenzenesulfonamide (*R*)-1j

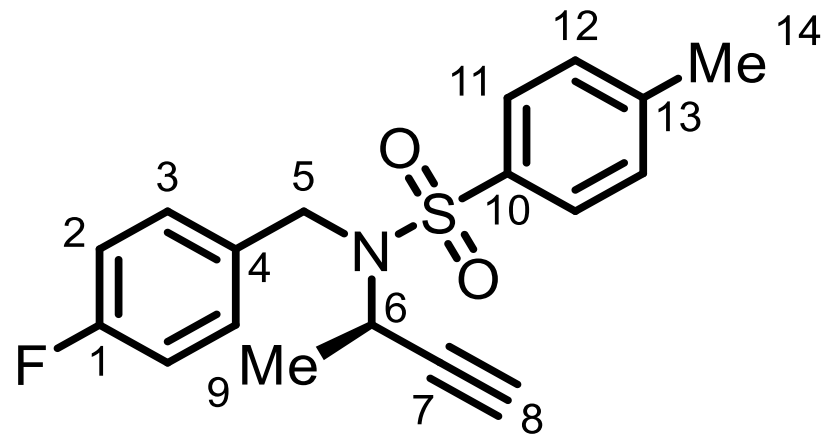

Prepared according to **General procedure II-A** from sulfonamide **26e** using (S)-(-)-3-butyn-2-ol and purified by flash column chromatography (2-20% EtOAc in pentane) to afford the title compound as an orange oil (655 mg, 40%).

^{1}H NMR (400 MHz, CDCl$_3$) δ 7.72 (dt, *J* = 8.3, 2.0 Hz, 2H, C11-H), 7.43 – 7.38 (m, 2H, C3-H), 7.30 (d, *J* = 8.5 Hz, 2H, C12-H), 6.99 (tt, *J* = 8.7, 2.1 Hz, 2H, C2-H), 4.96 (qd, *J* = 7.1, 2.2 Hz, 1H, C6-H), 4.62 (d, *J* = 15.9 Hz, 1H, C5-H), 4.17 (d, *J* = 15.8 Hz, 1H, C5-H), 2.43 (s, 3H, C14-H), 2.17 (d, *J* = 2.2 Hz, 1H, C8-H), 1.16 (d, *J* = 7.1 Hz, 3H, C9-H).

^{13}C NMR (126 MHz, CDCl$_3$) δ 162.4 (d, *J* = 245.7 Hz, C2), 143.7 (C13), 136.3 (C10), 134.1 (d, *J* = 3.2 Hz, C4), 129.8 (d, *J* = 8.0 Hz, C3), 129.7 (C12), 127.7 (C11), 115.4 (d, *J* = 21.6 Hz, C2), 81.0 (C7), 74.1 (C8), 47.9 (C5), 46.7 (C6), 23.2 (C9), 21.7 (C14).

^{19}F NMR (471 MHz, CDCl$_3$) δ -115.3 (td, *J* = 8.7, 4.4 Hz).

HRMS (EI+) calcd: $[M\text{-}Me]^+$ ($C_{18}H_{18}NO_2SF$) 316.0802, measured 316.0798 (1.27 ppm error).

IR (neat) v_{max}/cm^{-1}: 3296, 2970, 2936, 1737, 1604, 1509, 1447, 1339, 1223, 1156, 1092, 835, 660, 583, 546.

R$_f$ 0.58 (20% EtOAc in pentane).

***N*-(but-3-yn-2-yl)-*N*-(4-fluorobenzyl)methanesulfonamide** (**1k**)

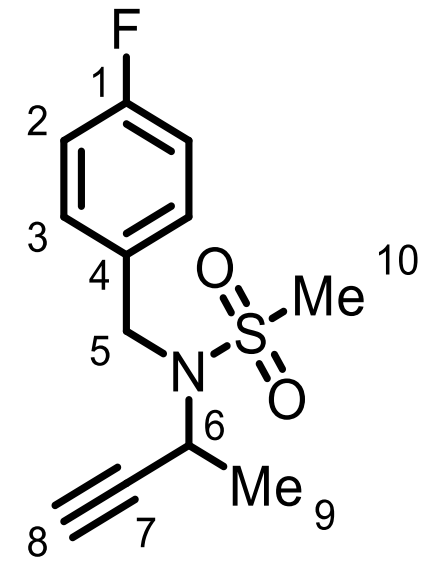

4-fluorobenzylamine (5 mmol) was alkylated with mesylate **26j** according to **General procedure II-A**. The crude product was mesylated according to **General procedure I** using methanesulfonyl chloride instead of *p*-toluenesulfonyl chloride to afford the crude product, which was purified by flash column chromatography to afford the title compound as a white crystalline solid (486 mg, 38%).

^{1}H NMR (500 MHz, $CDCl_3$) δ 7.47 – 7.38 (m, 2H, C3-H), 7.06 – 6.98 (m, 2H, C2-H), 4.85 (qdd, *J* = 7.1, 2.3, 0.8 Hz, 1H, C6-H), 4.59 (d, *J* = 15.9 Hz, 1H, C5-H), 4.32 (d, *J* = 15.9 Hz, 1H, C5-H), 2.92 (s, 3H, C10-H), 2.51 (d, *J* = 2.3 Hz, 1H, C8-H), 1.21 (d, *J* = 7.1 Hz, 3H, C9-H).

^{13}C NMR (126 MHz, $CDCl_3$) δ 162.4 (d, *J* = 246.0 Hz, C1), 133.9 (d, *J* = 3.1 Hz, C4), 129.7 (d, *J* = 8.0 Hz, C3), 115.5 (d, *J* = 21.5 Hz, C2), 81.7 (C7), 74.3 (C8), 47.8 (C5), 46.5 (C6), 38.1 (C10), 22.6 (C9).

^{19}F NMR (471 MHz, $CDCl_3$) δ −115.00 (ttd, *J* = 8.6, 5.3, 1.4 Hz, 1F, C1-F).

HRMS (+EI) calcd. $[M\text{-}Me]^+$ ($C_{12}H_{14}NO_2FS$) 240.0489, measured 240.0487 (0.83 ppm error).

IR (neat) v_{max}/cm^{-1}: 3279, 2970, 1738, 1605, 1510, 1336, 1218, 1146, 963, 834, 516.

R_f 0.46 (20% EtOAc in pentane).

***N-(4-bromobenzyl)-N-(but-3-yn-2-yl)-4-methylbenzenesulfonamide* (1l)**

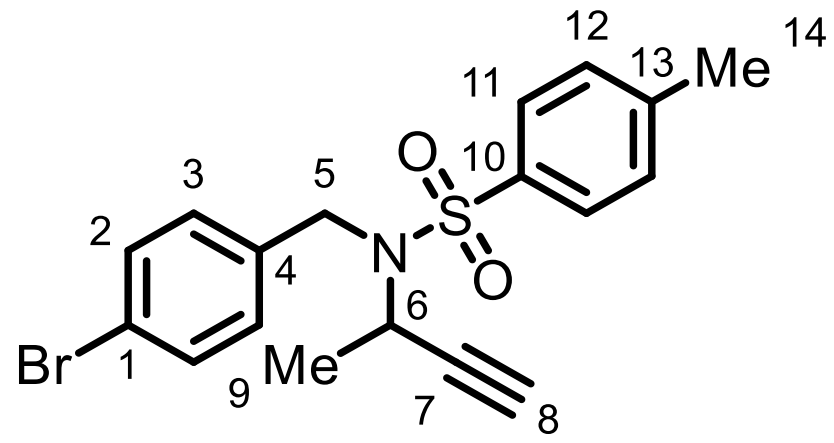

Prepared according to **General procedure II-A** from sulfonamide **1at** and 4-bromobenzyl bromide. The crude product was purified by flash column chromatography (2-20% EtOAc in pentane) to afford the title compound as a white solid (1.12 g, 69%).

^{1}H NMR (400 MHz, CDCl$_3$) δ 7.72 – 7.69 (m, 2H, C11-H), 7.42 (app. dt, J = 8.4, 1.9 Hz, 2H, C2-H), 7.35 – 7.26 (m, 4H, C3-H, C12-H), 4.94 (qd, J = 7.1, 2.3 Hz, 1H, C6-H), 4.57 (d, J = 16.0 Hz, 1H, C5-H), 4.13 (d, J = 16.0 Hz, 1H, C5-H), 2.42 (s, 3H, C14-H), 2.15 (d, J = 2.3 Hz, 1H, C8-H), 1.15 (d, J = 7.1 Hz, 3H, C9-H).

^{13}C NMR (126 MHz, CDCl$_3$) δ 143.8 (C10), 137.6 (C13), 131.7 (C4), 131.6 (C12), 129.8 (C2), 129.7 (C3), 127.8 (C11), 121.5 (C1), 81.0 (C7), 74.1 (C8), 47.9 (C5), 46.8 (C6), 23.2 (C9), 21.7 (C14).

HRMS (ESI+) calcd: $[M+H]^+$ ($C_{18}H_{18}BrNO_2S$) 392.0314, measured 392.0320 (1.53 ppm error).

IR (neat) v_{max}/cm^{-1}: 3294, 2970, 1738, 1487, 1441, 1365, 1228, 1216, 1156, 1091, 660, 543.

R*f* 0.58 (20% EtOAc in pentane).

N-(but-3-yn-2-yl)-4-methyl-N-(naphthalen-2-ylmethyl)benzenesulfonamide **(1m)**

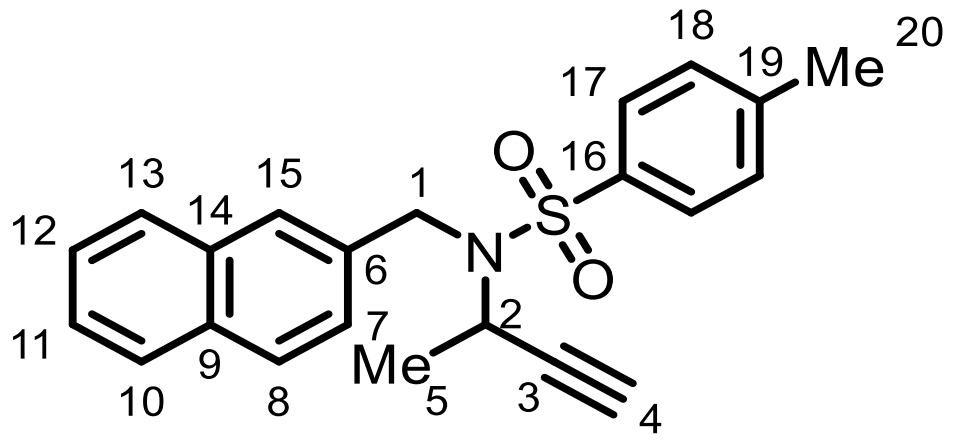

Prepared according to **General procedure II-A** from sulfonamide **1at** and 2-(bromomethyl)naphthalene. The crude product was purified by flash column chromatography (2-20% EtOAc in pentane) to afford the title compound as a white solid (1.02 g, 81%).

^{1}H NMR (500 MHz, $CDCl_3$) δ 7.84 – 7.80 (m, 3H, C8-H, C10-H, C13-H), 7.80 (s, 1H, C15-H), 7.76 (app. dt, J = 8.4, 1.7 Hz, 2H, C17-H), 7.61 (dd, J = 8.4, 1.8 Hz, 1H, C7-H), 7.49 – 7.45 (m, 2H, C11-H, C12-H), 7.30 (d, J = 8.4 Hz, 2H, C18-H), 5.00 (qd, J = 7.0, 2.2 Hz, 1H, C2-H), 4.84 (d, J = 15.9 Hz, 1H, C1-H), 4.36 (d, J = 15.9 Hz, 1H, C1-H), 2.43 (s, 3H, C20-H), 2.20 (d, J = 2.3 Hz, 1H, C4-H), 1.15 (d, J = 7.2 Hz, 3H, C5-H).

^{13}C NMR (126 MHz, $CDCl_3$) δ 143.7 (C19), 136.4 (C6), 135.9 (C16), 133.4 (C14), 133.0 (C9), 129.7 (C18), 128.3 (C13), 128.0 (C10), 127.8 (C8), 127.8 (C17), 126.9 (C15), 126.2 (C7), 126.2 (C12), 126.0 (C11), 81.1 (C3), 74.1 (C4), 48.8 (C1), 46.9 (C2), 23.2 (C5), 21.7 (C20).

HRMS (Nanospray) calcd: $[M+H]^+$ ($C_{22}H_{22}NO_2S$) 364.1366, measured 364.1363 (0.82 ppm error).

IR (neat) v_{max}/cm^{-1}: 3456, 3286, 3016, 2946, 2970, 1738, 1598, 1441, 1365, 1228, 1216, 1206, 1161, 661, 583.

R_f 0.64 (20% EtOAc in pentane).

***N-(4-fluorobenzyl)-4-methyl-N-(1-phenylprop-2-yn-1-yl)benzenesulfonamide* (1n)**

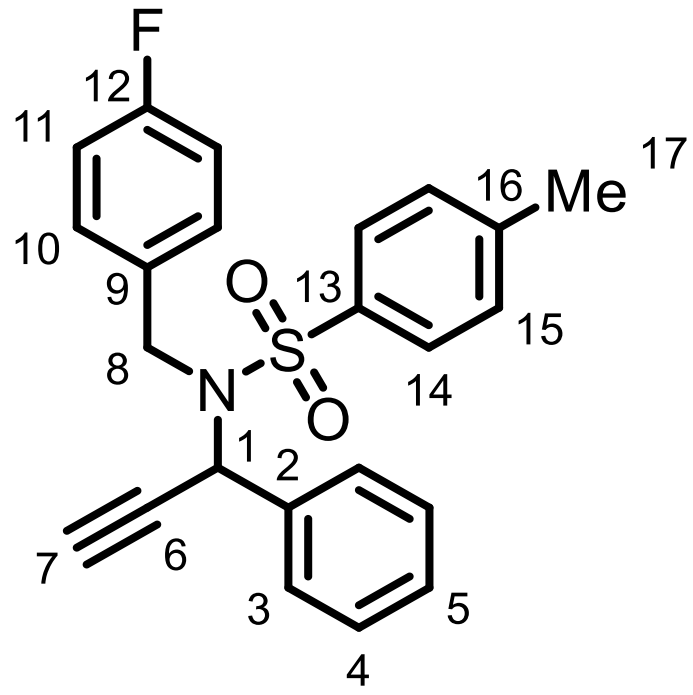

Prepared according to **General procedure VIII** from sulfonamide **26e** and purified by flash column chromatography (2-20% EtOAc in pentane) and washed with 2M $NaHSO_{3(aq)}$ (3 × 20 mL), affording the title compound as a white solid (782 mg, 26%).

^{1}H NMR (500 MHz, $CDCl_3$) δ 7.77 (app. dt, *J* = 8.1, 1.8 Hz, 2H, C14-H), 7.42 – 7.38 (m, 2H, C4-H), 7.33 (d, *J* = 8.1 Hz, 2H, C15-H), 7.17 – 7.12 (m, 3H, C3-H, C5-H), 6.94 – 6.89 (m, 2H, C10-H), 6.65 (tt, *J* = 8.7, 3.2 Hz, 2H, C11-H), 6.17 (d, *J* = 2.5 Hz, 1H, C1-H), 4.48 (d, *J* = 15.3 Hz, 1H, C8-H), 4.10 (d, *J* = 15.3 Hz, 1H, C8-H), 2.46 (s, 3H, C17-H), 2.45 (d, *J* = 2.5 Hz, 1H, C7-H).

^{13}C NMR (126 MHz, $CDCl_3$) δ 161.9 (d, *J* = 245.2 Hz, C12), 143.8 (C13), 136.3 (C16), 135.6 (C2), 132.2 (d, *J* = 3.2 Hz, C9), 130.4 (d, *J* = 8.1 Hz, C10), 129.7 (C15), 128.2 (C5), 128.2 (C3), 128.2 (C4), 127.9 (C14), 114.5 (d, *J* = 21.4 Hz, C11), 78.0 (C6), 77.3 (C7), 53.8 (C1), 48.4 (C8), 21.7 (C17).

^{19}F NMR (471 MHz, $CDCl_3$) δ −115.9 (tt, *J* = 8.7, 4.4 Hz).

HRMS (+ESI) calcd: $[M+H]^+$ ($C_{23}H_{20}FNO_2S$) 394.1272, measured 394.1258 (3.55 ppm error).

IR (neat) v_{max}/cm^{-1}: 3455, 3295, 3064, 3030, 2970, 2927, 2119, 1738, 1676, 1602, 1509, 1351, 1218, 1162, 1090, 901, 664, 547.

R_f 0.55 (10% EtOAc in pentane).

***N*-benzhydryl-4-methyl-N-(prop-2-yn-1-yl)benzenesulfonamide (1o)**

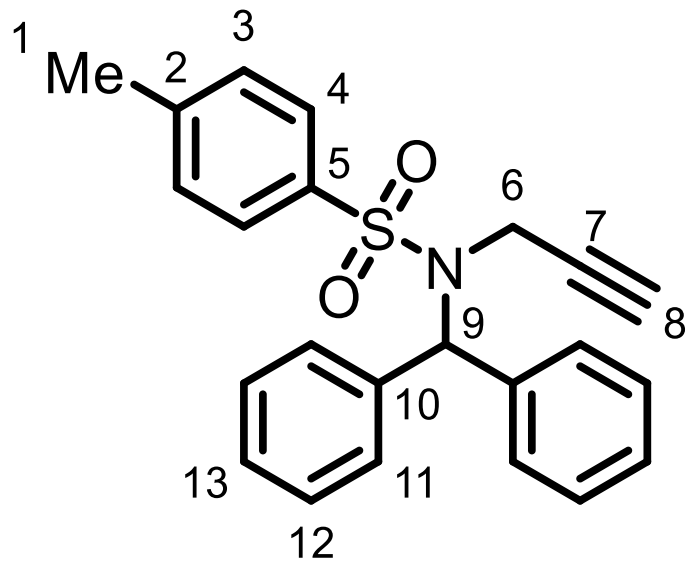

Prepared according to **General procedure II** from **ATS006** and purified by flash column chromatography (0 -50% EtOAc in pentane) to afford the title compound as a white solid (1.46g, 39%).

^{1}H NMR (500 MHz, CDCl$_3$): δ 7.72 – 7.65 (m, 2H, C4-H), 7.30 – 7.23 (m, C12-H, 6H, C13-H), 7.22 – 7.19 (m, 2H, C3-H), 7.16 – 7.08 (m, 4H, C10-H), 6.35 (s, 1H, C9-H), 4.05 (d, *J* = 2.5 Hz, 2H, C6-H), 2.41 (s, 3H, C1-H), 1.92 (t, *J* = 2.5 Hz, 1H, C8-H).

^{13}C NMR (126 MHz, CDCl$_3$): δ 143.5 (C2), 138.4 (C10), 137.7 (C5), 129.4 (C3), 129.2 (C11), 128.4 (C12), 127.9 (C13), 127.8 (C4), 79.1 (C7), 72.4 (C8), 65.3 (C9), 35.2 (C6), 21.7 (C1).

Characterisation data match that previously reported.[23]

***rac*-4-methyl-*N*-(1-phenylethyl)-*N*-(prop-2-yn-1-yl)benzenesulfonamide** (**1p**)

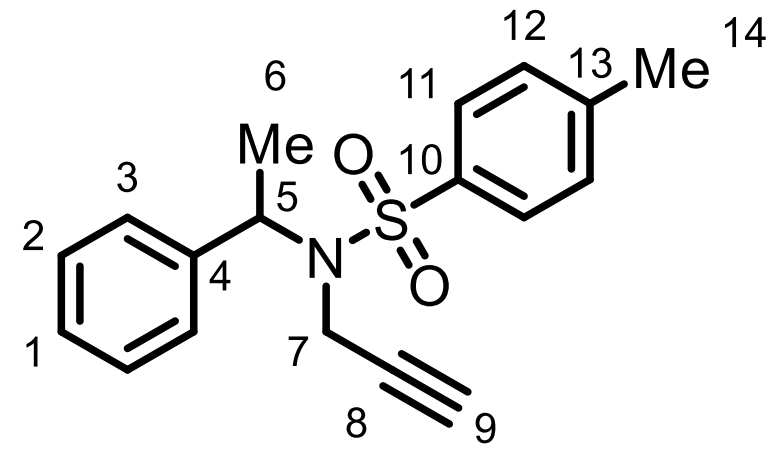

Prepared according to **General procedure II** from sulfonamide **26p**, affording the title compound without further purification as a yellow oil (1.25 g, 80% over 2 steps).

^{1}H NMR (400 MHz, $CDCl_3$) δ 7.87 (app. dt, *J* = 8.4, 1.8 Hz, 2H, C11-H), 7.35 – 7.27 (m, 7H, C1-H, C2-H, C3-H, C12-H), 5.23 (q, *J* = 7.1 Hz, 1H, C5-H), 4.19 (dd, *J* = 18.6, 2.5 Hz, 1H, C7-H), 3.54 (dd, *J* = 18.6, 2.5 Hz, 1H, C7-H), 2.45 (s, 3H, C14-H), 2.08 (t, *J* = 2.5 Hz, 1H, C9-H), 1.51 (d, *J* = 7.1 Hz, 3H, C6-H).

^{13}C NMR (126 MHz, $CDCl_3$) δ 143.5 (C4), 139.5 (C10), 138.1 (C13), 129.6 (C11), 128.6 (C2), 128.0 (C1), 127.8 (C3), 127.7 (C12), 80.2 (C8), 72.6 (C9), 56.1 (C5), 32.7 (C7), 21.7 (C14), 17.1 (C6).

HRMS (ESI+) calcd: $[M+Na]^+$ ($C_{18}H_{19}NO_2S$) 336.1029, measured 336.1042 (3.87 ppm error).

IR (neat) v_{max}/cm^{-1}: 3285, 2980, 1737, 1598, 1495, 1451, 1332, 1162, 1091, 881, 699, 660, 556.

R*f* 0.70 (20% EtOAc in pentane).

(S)-4-methyl-N-(1-phenylethyl)-N-(prop-2-yn-1-yl)benzenesulfonamide **((*S*)-1p)**

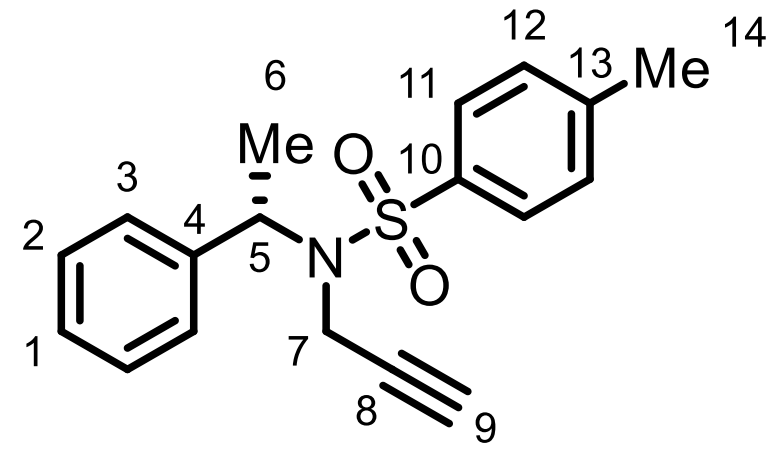

Prepared according to **General procedure II** from sulfonamide **(*S*)-26p**, affording the title compound without further purification as a yellow oil (1.16 g, 74% over 2 steps).

^{1}H NMR (400 MHz, $CDCl_3$) δ 7.87 (app. dt, *J* = 8.4, 1.8 Hz, 2H, C11-H), 7.35 – 7.27 (m, 7H, C1-H, C2-H, C3-H, C12-H), 5.23 (q, *J* = 7.1 Hz, 1H, C5-H), 4.19 (dd, *J* = 18.6, 2.5 Hz, 1H, C7-H), 3.54 (dd, *J* = 18.6, 2.5 Hz, 1H, C7-H), 2.45 (s, 3H, C14-H), 2.08 (t, *J* = 2.5 Hz, 1H, C9-H), 1.51 (d, *J* = 7.1 Hz, 3H, C6-H).

^{13}C NMR (126 MHz, $CDCl_3$) δ 143.5 (C4), 139.5 (C10), 138.1 (C13), 129.6 (C11), 128.6 (C2), 128.0 (C1), 127.8 (C3), 127.7 (C12), 80.2 (C8), 72.6 (C9), 56.1 (C5), 32.7 (C7), 21.7 (C14), 17.1 (C6).

HRMS (ESI+) calcd: $[M+Na]^+$ ($C_{18}H_{19}NO_2S$) 336.1029, measured 336.1042 (3.87 ppm error).

IR (neat) v_{max}/cm^{-1}: 3285, 2980, 1737, 1598, 1495, 1451, 1332, 1162, 1091, 881, 699, 660, 556.

R_f 0.72 (20% EtOAc in pentane).

***4-methyl-N-(prop-2-yn-1-yl)-N-(1-tosylpiperidin-4-yl)benzenesulfonamide* (1r)**

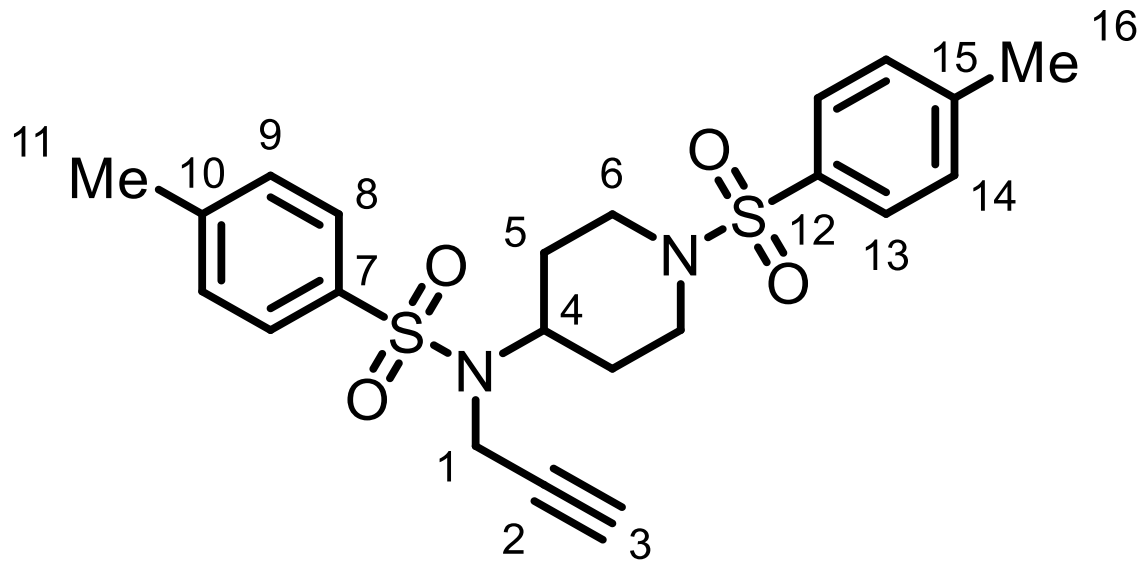

4-aminopiperidine was tosylated according to **General procedure I**, and the crude product was propargylated according to **General procedure II** to afford the title compound as a yellow oil (1.64 g, 73% over 2 steps).

^{1}H NMR (400 MHz, $CDCl_3$) δ 7.71 (app. dt, *J* = 8.2, 1.8 Hz, 2H, C8-H), 7.62 (dt, *J* = 8.2, 1.9 Hz, 2H, C13-H), 7.33 (d, *J* = 8.2 Hz, 2H, C9-H), 7.25 (d, *J* = 8.2 Hz, 2H, C14-H), 4.06 (d, *J* = 2.5 Hz, 2H, C1-H), 3.83 (dp, *J* = 11.9, 2.2 Hz, 2H, C6-H), 3.60 (tt, *J* = 12.1, 3.9 Hz, 1H, C4-H), 2.45 (s, 3H, C11-H), 2.40 (s, 3H, C16-H), 2.22 (td, *J* = 12.2, 2.4 Hz, 2H, C6-H), 2.16 (t, *J* = 2.6 Hz, 1H, C3-H), 1.94 (qd, *J* = 12.4, 4.2 Hz, 2H, C5-H), 1.67 (ddd, *J* = 12.4, 4.3, 2.1 Hz, 2H, C5-H).

^{13}C NMR (101 MHz, $CDCl_3$) δ 143.9 (C10), 143.7 (C15), 137.7 (C7), 133.1 (C12), 129.9 (C9), 129.7 (C14), 127.8 (C8), 127.4 (C13), 79.8 (C2), 73.1 (C3), 55.3 (C4), 46.2 (C6), 32.5 (C1), 29.8 (C5), 21.7 (C11), 21.7 (C16).

HRMS (+ESI) calcd: $[M+H]^+$ ($C_{22}H_{26}N_2O_4S_2$) 447.1425, measured 447.1407 (4.03 ppm error).

IR (neat) v_{max}/cm^{-1}: 3273, 2932, 2851, 1723, 1597, 1334, 1154, 1091, 726, 663, 580, 547.

R_f 0.14 (20% EtOAc in pentane).

***N*-cyclohexyl-4-methyl-*N*-(prop-2-yn-1-yl)benzenesulfonamide (1s)**

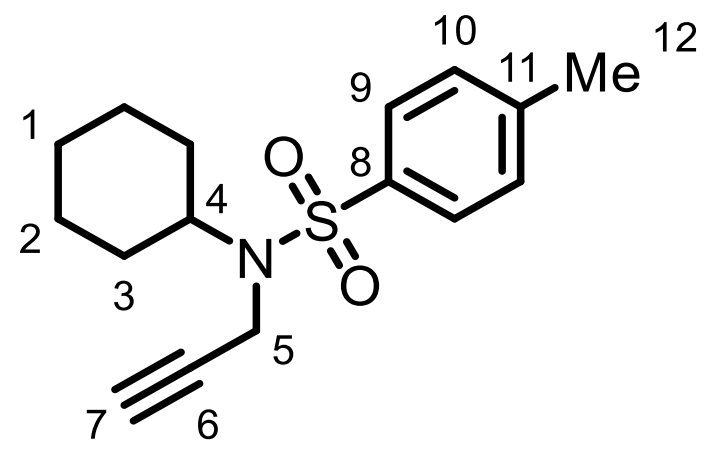

Prepared from sulfonamide **26s** according to **General procedure II**, affording the title compound as a white solid (770 mg, 53% over 2 steps).

^{1}H NMR (400 MHz, $CDCl_3$) δ 7.79 (d, *J* = 8.1 Hz, 2H, C9-H), 7.27 (d, *J* = 8.1 Hz, 2H, C10-H), 4.09 (t, *J* = 2.5 Hz, 2H), 3.67 (tt, J = 11.2, 3.4 Hz, 1H, C5-H), 2.42 (s, 3H, C12-H), 2.14 (t, *J* = 2.5 Hz 2H, C7-H), 1.75 (d, *J* = 12.8 Hz, 2H, C3-H), 1.68 (d, *J* = 12.3 Hz, 2H, C2-H), 1.64 – 1.55 (m, 1H, C1-H), 1.49 (qd, *J* = 12.8, 2.3 Hz, 2H, C3-H), 1.34 – 1.15 (m, 2H, C2-H), 1.04 (q, *J* = 13.1 Hz, 1H, C1-H).

^{13}C NMR (126 MHz, $CDCl_3$) δ 143.2 (C11), 138.3 (C8), 129.5 (C10), 127.5 (C9), 80.7 (C6), 72.2 (C7), 58.1 (C4), 32.3 (C3), 31.5 (C2), 26.2 (C1), 21.7 (C12).

Characterisation data matches that previously reported.[24]

4-methyl-*N*-phenethyl-*N*-(prop-2-yn-1-yl)benzenesulfonamide (1t)

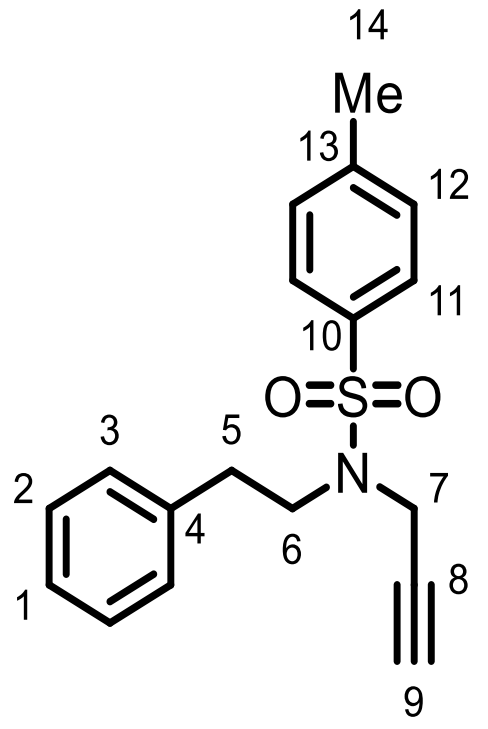

Prepared according to **General procedure II** from sulfonamide **26t**, affording the title compound without further purification as a yellow oil (1.42 g, 91% over 2 steps)

^{1}H NMR (400 MHz, $CDCl_3$) δ 7.71 (d, J = 8.1 Hz, 2H, C11-H), 7.35 – 7.21 (m, 5H, C1-H, C2-H, C12-H), 7.21 (dt, J = 6.6, 2.3 Hz, 2H, C3-H), 4.10 (d, J = 2.5 Hz, 2H, C7-H), 3.47 – 3.38 (m, 2H, C6-H), 2.95 – 2.87 (m, 2H, C5-H), 2.41 (s, 3H, C14-H), 2.06 (dt, J = 3.5, 1.7 Hz, 1H, C9-H).

^{13}C NMR (151 MHz, $CDCl_3$) δ 143.6 (C13), 138.4 (C4), 136.0 (C10), 129.6 (C12), 129.0 (C3), 128.7 (C2), 127.8 (C11), 126.8 (C1), 76.8 (C8), 73.9 (C9), 48.1 (C6), 37.0 (C7), 34.9 (C5), 21.7 (C14).

Characterisation data matches that previously reported.[25]

***N*-butyl-4-nitro-*N*-(prop-2-yn-1-yl)benzenesulfonamide** **(1u)**

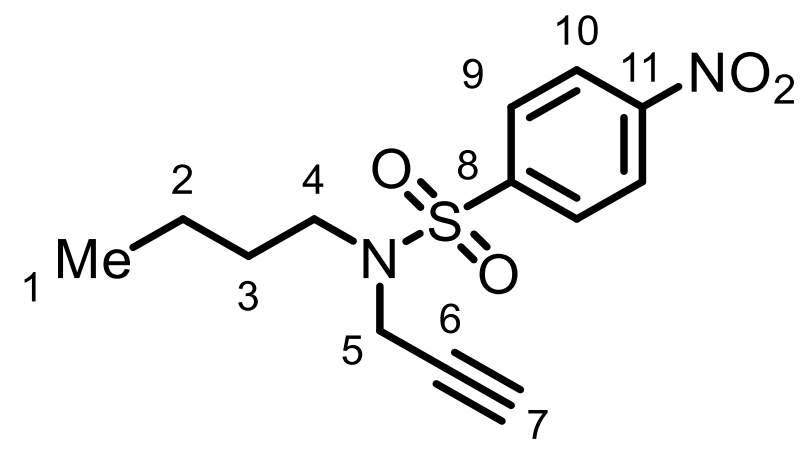

n-Butylamine was protected according to **General procedure I** using 4-nitrobenzenesulfonyl chloride instead of TsCl. The crude product was then propargylated according to **General procedure II** to afford the title compound as a yellow oil (1.75 g, 47% over 2 steps).

^{1}H NMR (500 MHz, $CDCl_3$) δ 8.35 (app. dt, *J* = 8.9, 2.0 Hz, 2H, C10-H), 8.04 (app. dt, *J* = 8.9, 2.0 Hz, 2H, C9-H), 4.19 (d, *J* = 2.5 Hz, 2H, C5-H), 3.23 (t, *J* = 7.3 Hz, 2H, C4-H), 2.01 (t, *J* = 2.5 Hz, 1H, C7-H), 1.62 – 1.53 (m, 2H, C3-H), 1.37 (h, *J* = 7.4 Hz, 2H, C2-H), 0.95 (t, *J* = 7.4 Hz, 3H, C1-H).

^{13}C NMR (126 MHz, $CDCl_3$) δ 150.2 (C11), 145.0 (C8), 129.1 (C9), 124.2 (C10), 75.9 (C6), 74.4 (C7), 46.3 (C4), 36.2 (C5), 29.5 (C3), 19.8 (C2), 13.7 (C1).

HRMS (+EI) calcd. $[M-H]^+$ ($C_{13}H_{16}N_2O_4S$) 295.0747, measured 295.0741 (2.03 ppm error).

IR (neat) v_{max}/cm^{-1}: 3285, 3106, 2960, 2934, 2872, 1739, 1606, 1528, 1348, 1310, 1161, 1088, 854, 740, 614.

R_f 0.69 (20% EtOAc in pentane).

***N-butyl-4-methyl-N-(prop-2-yn-1-yl)benzenesulfonamide* (1v)**

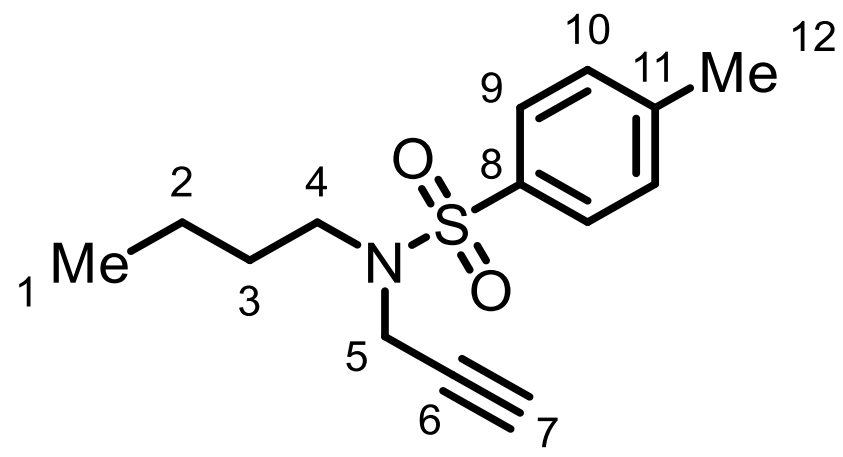

n-Butylamine was tosylated according to **General procedure I**. The crude product was then propargylated according to **General procedure II** to afford the title compound as a yellow oil (1.68 g, 57% over 2 steps).

^{1}H NMR (500 MHz, $CDCl_3$) δ 7.72 (d, *J* = 8.1 Hz, 2H, C9-H), 7.28 (d, *J* = 8.1 Hz, 2H, C10-H), 4.13 (d, *J* = 2.5 Hz, 2H, C5-H), 3.22 – 3.15 (m, 2H, C4-H), 2.42 (s, 3H, C12-H), 2.00 (t, *J* = 2.4 Hz, 1H, C7-H), 1.55 (tdd, *J* = 7.4, 6.8, 4.3 Hz, 2H, C3-H), 1.35 (h, *J* = 7.4 Hz, 2H, C2-H), 0.92 (t, *J* = 7.4 Hz, 3H, C1-H).

^{13}C NMR (126 MHz, $CDCl_3$) δ 143.50 (C11), 136.14 (C8), 129.56 (C10), 127.85 (C9), 76.81 (C6), 73.69 (C7), 46.13 (C4), 36.24 (C5), 29.65 (C3), 21.68 (C12), 19.89 (C2), 13.80 (C1).

HRMS (+ESI) calcd. $[M+H]^+$ ($C_{14}H_{19}NO_2S$) 266.1209, measured 266.1206 (1.13 ppm error).

IR (neat) v_{max}/cm^{-1}: 3278, 2959, 2932, 2872, 1740, 1598, 1454, 1345, 1157, 1090, 1039, 814, 657, 544.

R*f* 0.71 (20% EtOAc in pentane).

***4-methyl-N-(prop-2-yn-1-yl)-N-(2-(thiophen-2-yl)ethyl)benzenesulfonamide* (1w)**

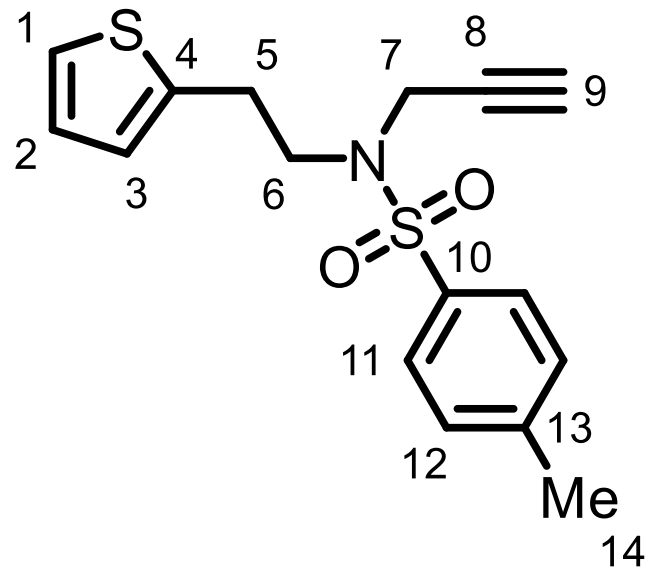

Prepared according to **General procedure II** from sulfonamide **26w**, affording the title compound as a yellow oil (1.38 g, 86% over 2 steps).

^{1}H NMR (500 MHz, CDCl$_3$) δ 7.72 (d, *J* = 8.5 Hz, 2H, C11-H), 7.28 (d, *J* = 8.5 Hz, 2H, C12-H), 7.15 (dd, *J* = 5.1, 1.1 Hz, 1H, C1-H), 6.93 (dd, *J* = 5.1, 3.5 Hz, 1H, C2-H), 6.86 (dd, *J* = 3.5, 1.1 Hz, 1H, C3-H), 4.10 (d, *J* = 2.5 Hz, 2H, C7-H), 3.50 – 3.43 (m, 2H, C6-H), 3.17 – 3.10 (m, 2H, C5-H), 2.42 (s, 3H, C14-H), 2.07 (t, *J* = 2.5 Hz, 1H, C9-H).

^{13}C NMR (126 MHz, CDCl$_3$) δ 143.76 (C13), 140.41 (C4), 135.95 (C10), 129.68 (C12), 127.82 (C11), 127.15 (C2), 125.68 (C3), 124.15 (C1), 76.79 (C8), 74.01 (C9), 48.27 (C6), 37.27 (C7), 29.18 (C5), 21.68 (C14).

HRMS (+ESI) calcd: $[M+Na]^+$ ($C_{16}H_{17}NO_2S_2$) 342.0593, measured 342.0579 (3.60 ppm error).

IR (neat) v_{max}/cm^{-1}: 3283, 2924, 1738, 1597, 1347, 1159, 1092, 815, 700, 658, 546.

R_f 0.48 (20% EtOAc in pentane).

***4-methyl-N-(prop-2-yn-1-yl)-N-((tetrahydrofuran-2-yl)methyl)benzenesulfonamide* (1x)**

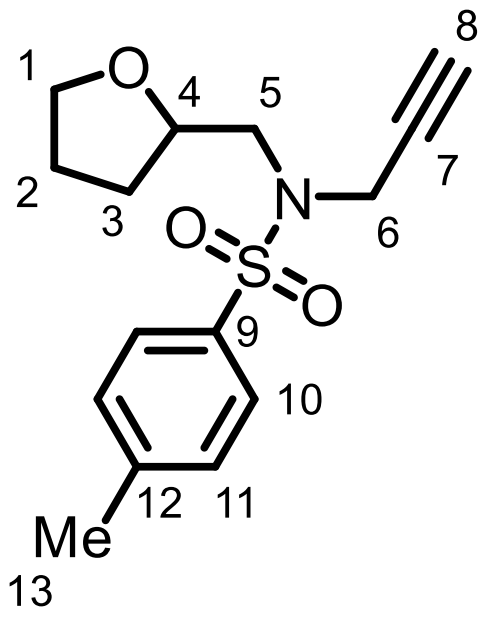

Prepared according to **General procedure II** from sulfonamide **26x**, affording the title compound without further purification as a yellow oil (1.18 g, 80% over 2 steps).

^{1}H NMR (500 MHz, $CDCl_3$) δ 7.73 (app. dt, *J* = 8.4, 1.8 Hz, 2H, C10-H), 7.30 – 7.27 (m, 2H, C11-H), 4.29 (t, *J* = 2.1 Hz, 2H, C6-H), 4.11 (qd, *J* = 6.9, 4.1 Hz, 1H, C4-H), 3.85 (dt, *J* = 8.4, 6.8 Hz, 1H, C1-H), 3.74 (td, *J* = 8.0, 6.2 Hz, 1H, C1-H), 3.36 (dd, *J* = 14.2, 4.2 Hz, 1H, C5-H), 3.20 (dd, *J* = 14.2, 6.7 Hz, 1H, C5-H), 2.42 (s, 3H, C13-H), 2.04 – 1.96 (m, 1H, C3-H), 1.99 (t, *J* = 2.4 Hz, 1H, C8-H), 1.94 – 1.82 (m, 2H, C2-H), 1.72 (ddt, *J* = 12.1, 8.6, 7.2 Hz, 1H, C3-H).

^{13}C NMR (126 MHz, $CDCl_3$) δ 143.6 (C12), 136.3 (C9), 129.6 (C11), 127.9 (C10), 78.5 (C4), 77.3 (C7), 73.8 (C8), 68.4 (C1), 49.6 (C5), 38.1 (C6), 29.1 (C3), 25.6 (C2), 21.7 (C13).

HRMS (ESI+) calcd: $[M+H]^+$ ($C_{15}H_{19}NO_3S$) 294.1158, measured 294.1145 (4.76 ppm error).

IR (neat) v_{max}/cm^{-1}: 3456, 3277, 3016, 2970, 2947, 1738, 1435, 1365, 1353, 1228, 1216, 1205, 1160, 1090, 527.

R_f 0.30 (20% EtOAc in pentane).

***2-(prop-2-yn-1-yl)isothiazolidine 1,1-dioxide* (1y)**

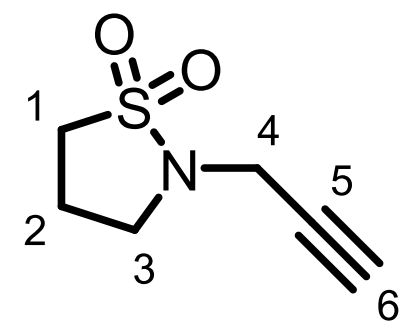

Prepared from isothiazolidine-1,1-dioxide according to **General procedure II** without further purification to afford the title compound as a yellow oil (577 mg, 82%).

^{1}H NMR (500 MHz, $CDCl_3$) δ 3.87 (d, *J* = 2.5 Hz, 2H, C4-H), 3.41 (t, *J* = 6.8 Hz, 2H, C1-H), 3.14 (t, *J* = 8.3 Hz, 2H, C3-H), 2.40 (dt, *J* = 8.3, 6.8 Hz, 2H, C2-H), 2.33 (t, *J* = 2.5 Hz, 1H).

^{13}C NMR (126 MHz, $CDCl_3$) δ 77.2 (C5), 73.9 (C6), 46.6 (C1), 46.0 (C3), 34.9 (C4), 18.9 (C2).

HRMS (+EI) calcd: $[M-H]^+$ ($C_6H_9NO_2S$) 158.0270, measured 158.0270 (1.27 ppm error).

IR (neat) v_{max}/cm^{-1}: 3270, 2924, 1739, 1426, 1302, 1137, 1061, 748, 675, 596.

R_f 0.69 (20% EtOAc in pentane).

***N-(1-ethynylcyclohexyl)-N-(3-fluorobenzyl)-4-methylbenzenesulfonamide* (1aa)**

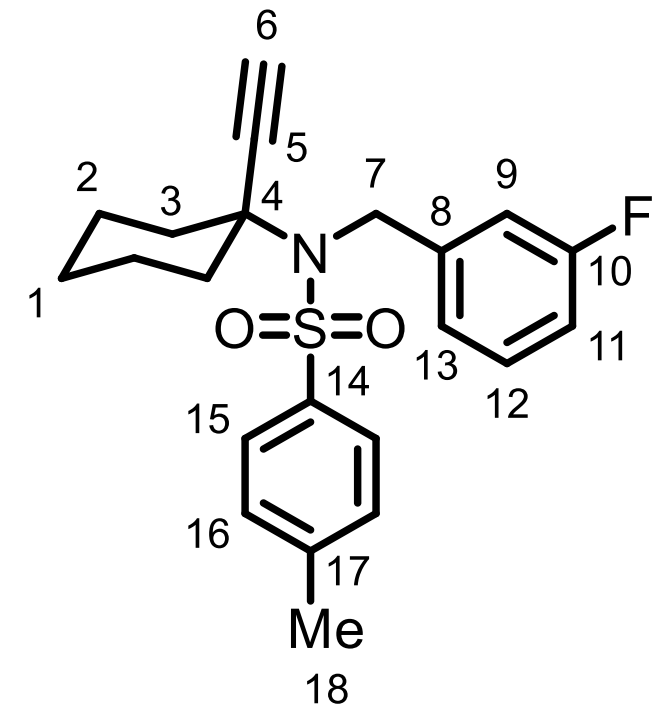

Prepared from sulfonamide **1au** and 3-fluorobenzyl bromide according to **General procedure II-A** using acetone instead of MeCN. The title compound was afforded without further purification as a pale yellow solid (419 mg, 98%).

^{1}H NMR (500 MHz, CDCl$_3$) δ 7.70 (d, *J* = 8.6 Hz, 2H, C15-H), 7.31 (dt, *J* = 7.7, 2.0 Hz, 1H, C13-H), 7.26 (d, *J* = 8.6 Hz, 2H, C16-H), 7.23 – 7.18 (m, 1H, C12-H), 7.00 (td, *J* = 8.2, 2.4 Hz, 1H, C9-H), 6.93 (tt, *J* = 8.1, 1.8 Hz, 1H, C11-H), 4.81 (s, 2H), 2.42 (s, 3H), 2.29 (s, 1H), 1.99 (d, *J* = 11.4 Hz, 2H), 1.86 (td, *J* = 12.5, 3.7 Hz, 2H), 1.66 – 1.43 (m, 6H), 1.08 (ddt, *J* = 13.8, 9.0, 4.7 Hz, 1H).

^{13}C NMR (126 MHz, CDCl$_3$) δ 163.0 (d, *J* = 245.5 Hz, C10), 143.3 (C17), 142.9 (d, *J* = 7.0 Hz, C8), 139.2 (C14), 129.9 (d, *J* = 8.3 Hz, C12), 129.5 (C16), 127.7 (C15), 122.9 (d, *J* = 2.8 Hz, C13), 114.4 (d, *J* = 22.3 Hz, C9), 113.9 (d, *J* = 21.2 Hz, C11), 83.3 (C5), 75.6 (C6), 63.1 (C4), 51.5 (d, *J* = 2.0 Hz, C7), 38.0 (C3), 24.7 (C1), 23.6 (C2), 21.7 (C18).

^{19}F NMR (376 MHz, CDCl$_3$) δ −113.11 (ddd, *J* = 10.1, 8.7, 5.7 Hz, 1F, C10-F).

HRMS (+ESI) calcd: $[M+Na]^+$ ($C_{22}H_{24}FNO_2S$) 408.1404, measured 408.1389 (3.68 ppm error).

IR (neat) v_{max}/cm^{-1}: 3292, 2939, 1739, 1364, 1216, 1168, 546.

***N-(1-ethynylcyclohexyl)-N,4-dimethylbenzenesulfonamide* (1ab)**

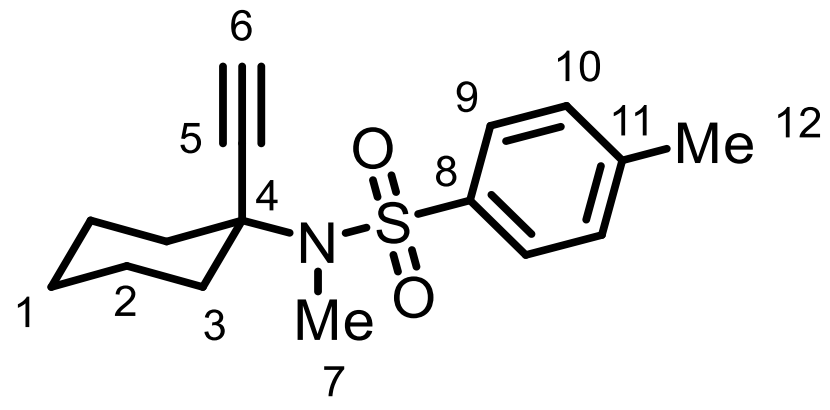

An RBF containing sulfonamide **1au** (2.96 mmol, 1.10 g), 4-methoxybenzyl bromide (3.96 mmol, 0.583 mL), K_2CO_3 (11.9 mmol, 1.64 g), TBAI (0.8 mmol, 295 mg), and acetone (20 mL) was heated to reflux for 18 h, monitoring reaction progress by TLC. On completion, the reaction mixture was concentrated *in vacuo*.

^{1}H NMR (500 MHz, $CDCl_3$) δ 7.72 (app. dt, J = 8.1, 2.0 Hz, 2H, C9-H), 7.27 (d, J = 8.1 Hz, 2H, C10-H), 3.07 (s, 3H, C7-H), 2.41 (s, 3H, C12-H), 2.24 (s, 1H, C6-H), 2.16 – 2.07 (m, 2H, C3-H), 1.92 (td, J = 12.2, 3.7 Hz, 2H, C3-H), 1.74 – 1.64 (m, 2H, C2-H), 1.63 – 1.51 (m, 3H, C1-H, C2-H), 1.22 – 1.13 (m, 1H, C1-H).

^{13}C NMR (126 MHz, $CDCl_3$) δ 143.1 (C11), 138.7 (C8), 129.4 (C10), 127.6 (C9), 83.1 (C5), 74.8 (C6), 61.0 (C4), 37.3 (C3), 33.7 (C7), 25.0 (C1), 23.4 (C2), 21.7 (C12).

HRMS (+ESI) calcd: $[M+H]^+$ ($C_{16}H_{21}NO_2S$) 292.1366, measured 292.1363 (1.03 ppm error).

IR (neat) v_{max}/cm^{-1}: 3272, 2942, 2864, 1740, 1454, 1335, 1217, 1157, 932, 671, 549.

R_f 0.63 (20% EtOAc in pentane).

***N*,4-dimethyl-*N*-(prop-2-yn-1-yl)benzenesulfonamide (1ac)**

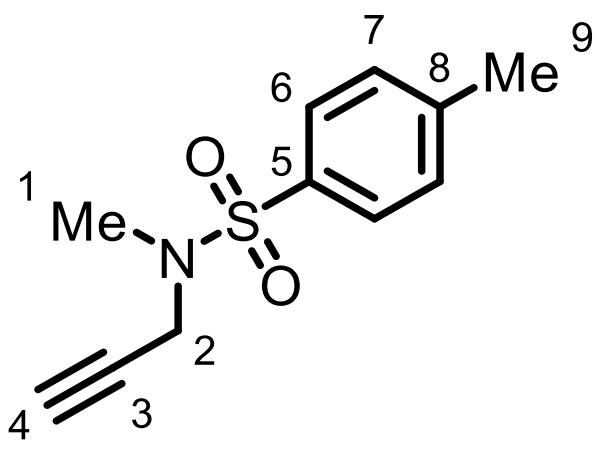

Prepared from sulfonamide **26ac** according to **General procedure II**, affording the title compound as a white solid (699 mg, 63% over 2 steps).

^{1}H NMR (500 MHz, CDCl$_3$) δ 7.71 (app. dt, *J* = 8.1, 1.9 Hz, 2H, C6-H), 7.31 (d, *J* = 8.1 Hz, 2H, C7-H), 4.02 (d, *J* = 2.5 Hz, 2H, C2-H), 2.82 (s, 3H, C1-H), 2.43 (s, 3H, C9-H), 2.08 (t, *J* = 2.5 Hz, 1H, C4-H).

^{13}C NMR (126 MHz, CDCl$_3$) δ 143.8 (C8), 134.3 (C5), 129.7 (C7), 128.1 (C6), 76.5 (C3), 74.1 (C4), 39.9 (C2), 34.4 (C1), 21.7 (C9).

Characterisation data matches that previously reported.[26]

((but-3-yn-2-yloxy)methyl)benzene (1ad)

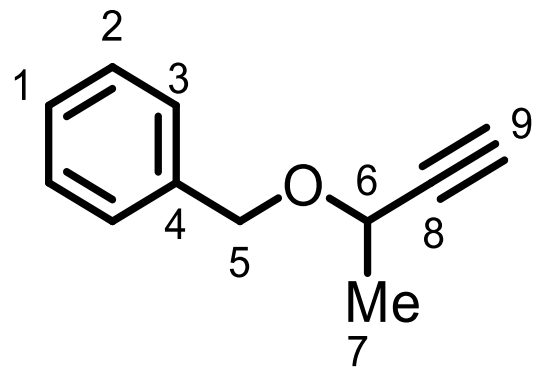

Prepared according to **General procedure IX** from but-3-yn-2-ol (2 equiv.), benzyl bromide (1 equiv.), and TBAI (20 mol%), affording the title compound without further purification as a colourless oil (1.19 g, 37% yield).

^{1}H NMR (500 MHz, $CDCl_3$) δ 7.41 – 7.32 (m, 4H, C2-H, C3-H), 7.32 – 7.27 (m, 1H, C1-H), 4.81 (d, *J* = 11.6 Hz, 1H, C5-H), 4.52 (d, *J* = 11.6 Hz, 1H, C5-H), 4.22 (qd, *J* = 6.6, 2.0 Hz, 1H, C6-H), 2.47 (1H, d, *J* = 2.0 Hz, 1H, C9-H), 1.49 (3H, d, *J* = 6.6 Hz, C7-H).

^{13}C NMR (126 MHz, $CDCl_3$) δ 137.9 (C4), 128.6 (C3), 128.2 (C1), 127.9 (C2), 83.8 (C8), 73.3 (C9), 70.6 (C5), 64.3 (C6), 22.2 (C7).

Characterisation data match that previously reported.[27]

***1-bromo-3-(((2-methylbut-3-yn-2-yl)oxy)methyl)benzene* (1ae)**

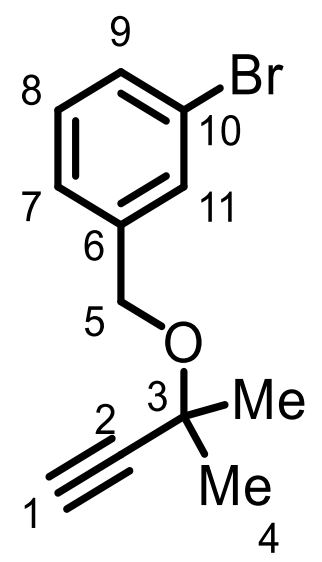

Prepared according to **General procedure IX** from 2-methyl-3-butyn-2-ol and 3-bromobenzyl bromide, affording the title compound without further purification as a colourless oil (613 mg, 81%).

^{1}H NMR (500 MHz, $CDCl_3$) δ 7.53 (t, *J* = 1.9 Hz, 1H, C11-H), 7.39 (dt, *J* = 7.9, 1.4 Hz, 1H, C9-H), 7.28 (dq, *J* = 7.7, 1.0 Hz, 1H, C7-H), 7.20 (t, *J* = 7.8 Hz, 1H, C8-H), 4.61 (s, 2H, C5-H), 2.48 (s, 1H, C1-H), 1.55 (s, 6H, C4-H).

^{13}C NMR (126 MHz, $CDCl_3$) δ 141.4 (C6), 130.8 (C11), 130.6 (C9), 130.0 (C8), 126.3 (C7), 122.6 (C10), 85.9 (C2), 72.7 (C1), 70.8 (C3), 65.8 (C5), 28.9 (C4).

HRMS (+EI) calcd: $[M-H]^+$ ($C_{12}H_{13}OBr$) 251.0066, measured 251.0062 (1.59 ppm error).

IR (neat) v_{max}/cm^{-1}: 3296, 2984, 2926, 1737, 1597, 1571, 1466, 1427, 1378, 1227, 1156, 1069, 776, 669.

R_f 0.85 (20% EtOAc in pentane).

***(1-((3-nitrobenzyl)oxy)prop-2-yne-1,1-diyl)dibenzene* (1af)**

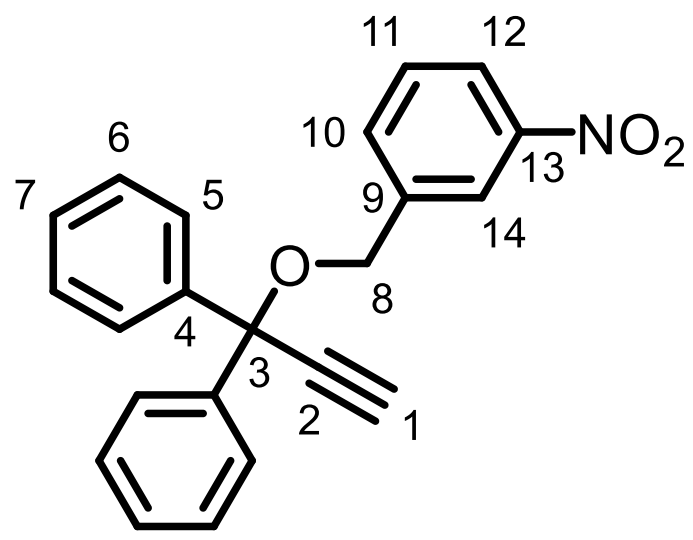

Prepared from 1,1-diphenylpropyn-1-ol and 3-nitrobenzyl bromide according to **General procedure IX** to afford the title compound without further purification as a white solid (1.01 g, 98%).

^{1}H NMR (500 MHz, $CDCl_3$) δ 8.25 (t, *J* = 2.0 Hz, 1H, C14-H), 8.15 (dt, *J* = 8.4, 1.6 Hz, 1H, C12-H), 7.73 (d, *J* = 7.6 Hz, 1H, C10-H), 7.64 – 7.57 (m, 4H, C6-H), 7.52 (t, *J* = 7.9 Hz, 1H, C11-H), 7.38 – 7.32 (m, 4H, C5-H), 7.32 – 7.27 (m, 2H, C7-H), 4.65 (s, 2H, C8-H), 2.98 (s, 1H, C1-H).

^{13}C NMR (126 MHz, $CDCl_3$) δ 148.4 (C13), 142.7 (C4), 140.8 (C9), 133.6 (C10), 129.4 (C11), 128.5 (C5), 128.2 (C7), 126.8 (C6), 122.6 (C14), 122.5 (C12), 83.0 (C2), 80.8 (C3), 78.5 (C1), 66.0 (C8).

HRMS (+Nanospray) calcd. $[M+Na]^+$ ($C_{22}H_{17}NO_3$) 366.1101, measured 3.66.1110 (2.46 ppm error).

IR (neat) v_{max}/cm^{-1}: 3286, 3062, 2925, 2112, 1739, 1528, 1449, 1349, 1092, 1061, 698.

R_f 0.69 (20% EtOAc in pentane).

***(4-(((1,1-diphenylprop-2-yn-1-yl)oxy)methyl)phenyl)(phenyl)methanone* (1ag)**

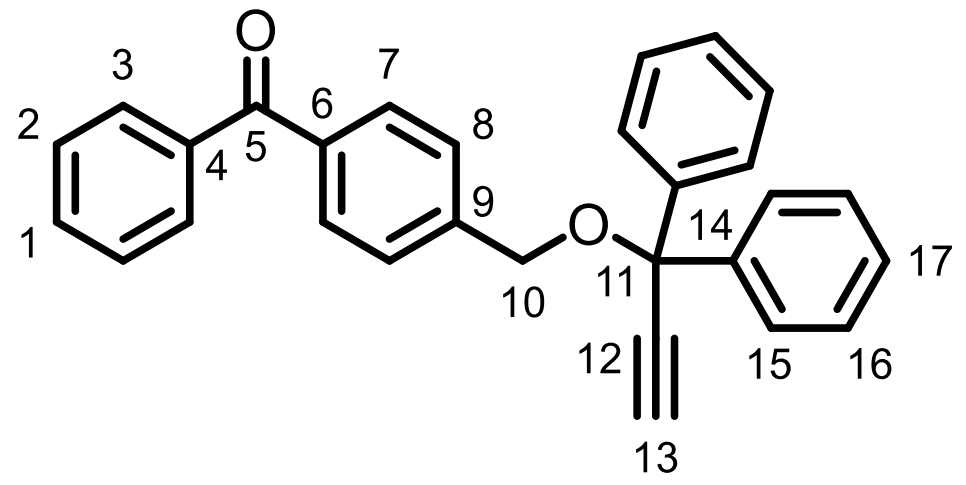

Prepared according to **General procedure IX** from 1,1-diphenylprop-2-yn-1-ol and 4-(bromomethyl)benzophenone, affording the title compound without further purification as a white solid (708 mg, 88%)

^{1}H NMR (400 MHz, $CDCl_3$) δ 7.83 – 7.79 (m, 4H, C3-H, C7-H), 7.65 – 7.56 (m, 5H, C1-H, C2-H, C8-H), 7.54 – 7.46 (m, 4H, C15-H), 7.38 – 7.27 (m, 6H, C16-H, C17-H), 4.65 (s, 2H, C10-H), 2.97 (s, 1H, C13-H).

^{13}C NMR (126 MHz, $CDCl_3$) δ 196.6 (C5), 143.6 (C9), 143.0 (C14), 137.9 (C6), 136.7 (C4), 132.5 (C1), 130.3 (C7), 130.2 (C3), 128.5 (C15), 128.4 (C17), 128.1 (C8), 127.2 (C2), 126.8 (C16), 83.2 (C12), 80.7 (C11), 78.3 (C13), 66.5 (C10).

HRMS (+ESI) calcd: $[M+H]^+$ ($C_{29}H_{22}O_2$) 403.1693, measured 403.1709 (3.97 ppm error).

IR (neat) v_{max}/cm^{-1}: 3286, 2924, 1739, 1656, 1609, 1448, 1278, 1071, 699, 657.

R_f 0.71 (10% EtOAc in pentane).

2-(((1-ethynylcyclohexyl)oxy)methyl)benzonitrile **(1ai)**

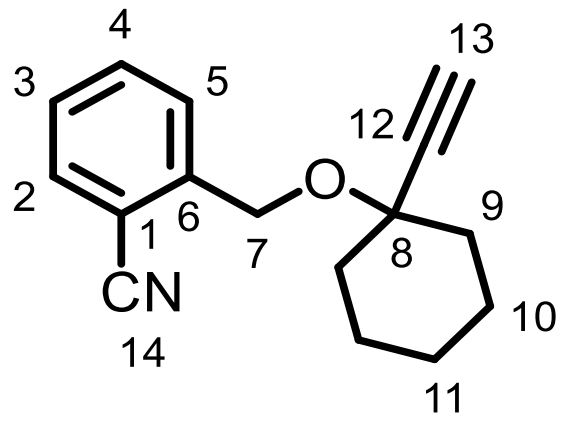

Prepared according to **General procedure IX** from 1-ethynylcyclohexanol and 2-cyanobenzyl bromide, affording the title compound without further purification as a colourless oil (579 mg, 48%).

^{1}H NMR (400 MHz, $CDCl_3$) δ 7.68 – 7.62 (m, 2H, C2-H, C4-H), 7.59 (td, J = 7.7, 1.3 Hz, 1H, C3-H), 7.36 (t, J = 7.6 Hz, 1H, C5-H), 4.85 (s, 2H, C7-H), 2.55 (d, J = 1.0 Hz, 1H, C13-H), 2.06 – 1.95 (m, 2H, C9-H), 1.80 – 1.66 (m, 4H, C9-H, C10-H), 1.65 – 1.48 (m, 3H, C10-H, C11-H), 1.40 – 1.28 (m, 1H, C11-H).

^{13}C NMR (101 MHz, $CDCl_3$) δ 142.9 (C6), 133.0 (C4), 132.8 (C2), 128.9 (C3), 127.8 (C5), 117.6 (C14), 111.3 (C1), 84.8 (C12), 74.7 (C13), 74.6 (C8), 63.2 (C7), 37.2 (C9), 25.5 (C11), 22.8 (C10).

HRMS (+EI) calcd: $[M-H]^+$ ($C_{16}H_{17}NO$) 238.1224, measured 238.1226 (0.84 ppm error).

IR (neat) v_{max}/cm^{-1}: 3295, 2935, 2858, 2225, 1727, 1449, 1109, 1076, 726, 656.

R_f 0.76 (20% EtOAc in pentane).

(1-(prop-2-yn-1-yloxy)ethyl)benzene (1aj)

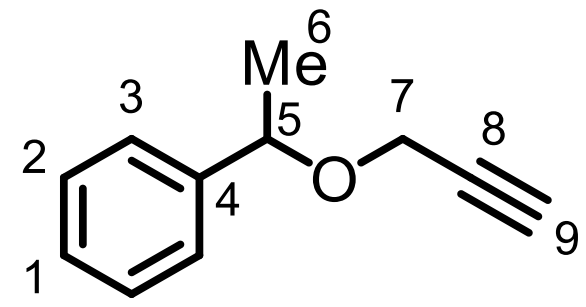

Prepared according to **General procedure IX** from 1-phenylethanol and purified by flash column chromatography (0-20% EtOAc in pentane) to afford the title compound as a yellow oil (2.88 g, 45%).

^{1}H NMR (400 MHz, CDCl$_3$) δ 7.43 – 7.09 (m, 5H, C1-3-H), 4.64 (q, *J* = 6.5 Hz, 1H, C5-H), 4.06 (ddd, *J* = 15.8, 2.5, 1.0 Hz, 1H, C7-H), 3.85 (ddd, *J* = 15.7, 2.4, 0.9 Hz, 1H, C7-H), 2.39 (td, *J* = 2.4, 0.9 Hz. 1H, C9-H), 1.46 (dd, *J* = 6.5, 1.0 Hz, 3H, C6-H).

^{13}C NMR (101 MHz, CDCl$_3$) δ 142.5 (C4), 128.7 (C2), 128.0 (C1), 126.6 (C3), 80.1 (C5), 76.8 (C8), 74.2 (C9), 55.6 (C7), 23.9 (C6).

Characterisation data match that previously reported.[28]

((prop-2-yn-1-yloxy)methylene)dibenzene (1ak)

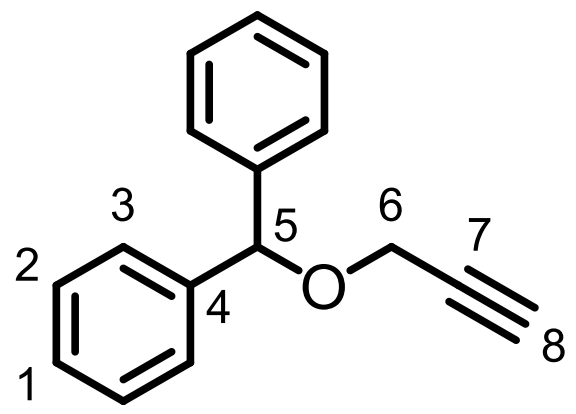

Prepared according to **General procedure IX**, using diphenylmethanol (7.37 g, 40 mmol,1 equiv.), sodium hydride (2.4 g, 60% in mineral oil, 60 mmol, 1.5 equiv.), propargyl bromide (5.7 mL, 60 mmol, 1.5 equiv.), and TBAI (2.95 g, 8 mmol, 0.2 equiv.). Purified by flash column chromatography (0-30% Et_2O in pentane) to afford the title compound as a colourless solid (4.2 g, 47%).

^{1}H NMR (400 MHz, $CDCl_3$) δ 7.42 – 7.32 (m, 8H, C2-H, C3-H), 7.32 – 7.25 (m, 2H, C1-H), 5.70 (s, 1H, C5-H), 4.18 (d, J = 2.4 Hz, 2H, C6-H), 2.48 (t, J = 2.4 Hz, 1H, C8-H).

^{13}C NMR (101 MHz, $CDCl_3$) δ 141.3 (C4), 128.6 (C2), 127.8 (C1), 127.4 (C3), 81.8 (C5), 79.9 (C7), 74.7 (C8), 55.9 (C6).

Characterisation data match that previously reported.[29]

4-(prop-2-yn-1-yloxy)chromane (1al)

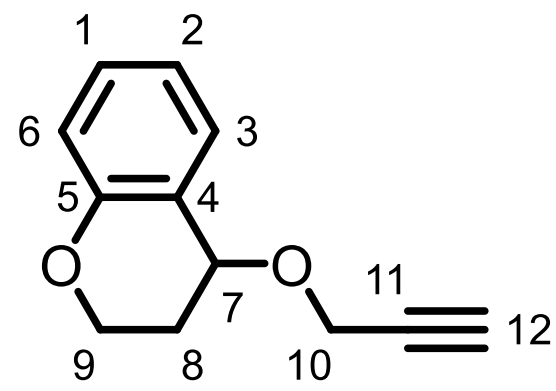

Prepared according to **General procedure IX** from 4-chromanol and purified by flash column chromatography (0-20% EtOAc in pentane) to afford the title compound as a yellow oil (800 mg, 76%).

^{1}H NMR (400 MHz, $CDCl_3$) δ 7.29 (dd, *J* = 7.6, 1.7 Hz, 1H, C3-H), 7.22 (ddd, *J* = 8.3, 7.3, 1.7 Hz, 1H, C1-H), 6.90 (td, *J* = 7.4, 1.2 Hz, 1H, C2-H), 6.85 (dd, *J* = 8.2, 1.2 Hz, 1H, C6-H), 4.68 (t, *J* = 3.5 Hz, 1H, C7-H), 4.34 – 4.19 (m, 4H, C9-H, C10-H), 2.51 (t, *J* = 2.4 Hz, 1H, C12-H), 2.21 – 1.98 (m, 2H, C8-H).

^{13}C NMR (101 MHz, $CDCl_3$) δ 155.1 (C5), 131.0 (C3), 130.1 (C1), 120.7 (C4), 120.1 (C2), 117.2 (C6), 80.2 (C11), 74.8 (C12), 68.6 (C7), 62.1 (C9), 55.1 (C10), 27.7 (C8).

Characterisation data match that previously reported.[28]

***(1-(but-3-yn-2-yloxy)ethyl)benzene* (1am)**

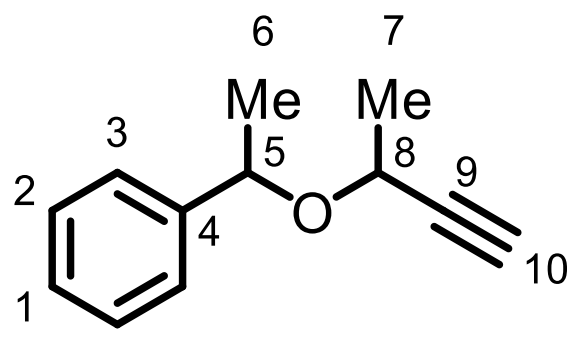

Prepared from 3-butyn-2-ol and 1-(bromoethyl)benzene according to **General procedure IX**. The crude product was purified by flash column chromatography (0-10% EtOAc in pentane) to afford the title compound as a colourless oil (113 mg, 22%).

Isolated as a mixture of diastereomers (1.17:1).

^{1}H NMR (400 MHz, $CDCl_3$) Major diastereomer δ 7.41 – 7.27 (m, 5H, C1-H, C2-H, C3-H), 4.81 (q, *J* = 6.4 Hz, 1H, C5-H), 3.90 (qd, *J* = 6.6, 2.0 Hz, 1H, C8), 2.41 (d, *J* = 2.0 Hz, 1H, C10-H), 1.47 (d, *J* = 6.4 Hz, 3H, C6-H), 1.40 (d, *J* = 6.6 Hz, 3H, C7-H). **Minor diastereomer** δ 7.41 – 7.27 (m, 5H, C1-H, C2-H, C3-H), 4.74 (q, *J* = 6.4 Hz, 1H, C5-H), 4.28 (qd, *J* = 6.5, 2.1 Hz, 1H, C8-H), 2.35 (d, *J* = 2.1 Hz, 1H, C10-H), 1.46 (d, *J* = 6.5 Hz, 6H, C6-H, C7-H).

^{13}C NMR (126 MHz, $CDCl_3$) Major diastereomer δ 143.2 (C4), 128.7 (C3), 127.8 (C1), 126.6 (C2), 84.3 (C9), 75.6 (C5), 72.6 (C10), 62.2 (C8), 24.5 (C6), 22.4 (C7). **Minor diastereomer** δ 143.8 (C4), 128.5 (C3), 127.5 (C1), 126.4 (C2), 84.4 (C9), 75.3 (C5), 72.5 (C10), 62.7 (C8), 22.7 (C6), 22.0 (C7).

HRMS (+EI) calcd: $[M-H]^+$ ($C_{12}H_{14}O$) 173.0961, measured 173.0958 (1.73 ppm error).

IR (neat) v_{max}/cm^{-1}: 3293, 3029, 2980, 2933, 2874, 1740, 1492, 1452, 1371, 1097, 1049, 760, 700, 631.

R_f 0.88 (20% EtOAc in pentane).

1-ethynyl-1-methoxycyclohexane (1an)

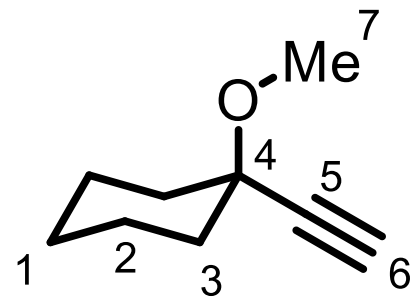

Prepared from 1-ethynylcyclohexanol according to **General procedure IX** using iodomethane instead of propargyl bromide. The title compound was afforded without further purification as a colourless oil (638 mg, 92%).

^{1}H NMR (500 MHz, $CDCl_3$) δ 3.37 (s, 3H, C7-H), 2.46 (s, 1H, C6-H), 1.94 – 1.81 (m, 2H, C3-H), 1.71 – 1.46 (m, 7H, C3-H, C2-H, C1-H), 1.39 – 1.18 (m, 1H, C1-H).

^{13}C NMR (126 MHz, $CDCl_3$) δ 85.1 (C5), 73.9 (C6), 73.8 (C4), 50.8 (C7), 36.7 (C3), 25.5 (C1), 22.8 (C2).

Characterisation data match that previously reported.[30]

1-chloro-4-(2-(prop-2-yn-1-yloxy)ethoxy)benzene **(1ao)**

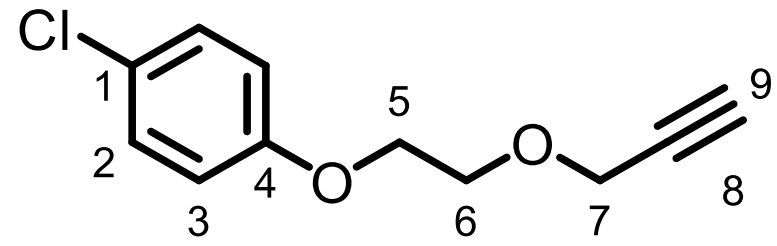

Prepared from 2-(4-chlorophenoxy)ethanol according to **General procedure IX** without further purification to afford the title compound as a yellow oil (633 mg, 99%).

^{1}H NMR (500 MHz, $CDCl_3$) δ 7.23 (d, *J* = 9.0 Hz, 2H, C2-H), 6.85 (d, *J* = 9.0 Hz, 2H, C3-H), 4.26 (d, *J* = 2.4 Hz, 2H, C7-H), 4.14 – 4.09 (m, 2H, C5-H), 3.91 – 3.87 (m, 2H, C6-H), 2.46 (t, *J* = 2.4 Hz, 1H, C9-H).

^{13}C NMR (126 MHz, $CDCl_3$) δ 157.4 (C4), 129.4 (C2), 126.0 (C1), 116.1 (C3), 79.5 (C8), 75.0 (C9), 68.2 (C5), 67.6 (C6), 58.7 (C7).

HRMS (+EI) calcd: $[M]^+$ ($C_{11}H_{11}ClO_2$) 210.0441, measured 210.0442 (0.48 ppm error).

IR (neat) v_{max}/cm^{-1}: 3296, 2928, 1737, 1597, 1491, 1243, 1104, 1094, 1035, 823, 667, 638.

4-(prop-2-yn-1-yloxy)-1-tosylpiperidine **(1ap)**

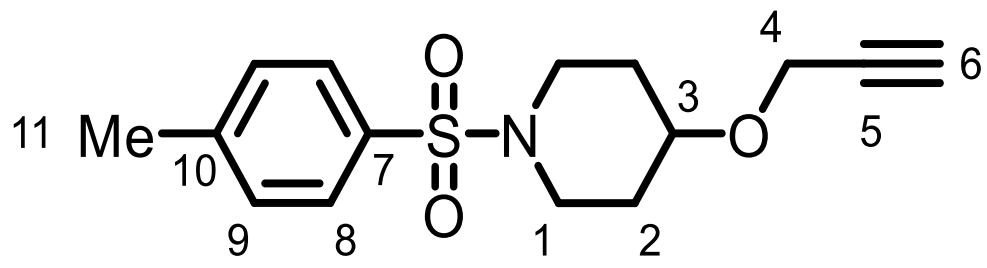

Prepared from alcohol **26ao** and propargyl bromide (80% w/w in toluene) according to **General procedure IX**, affording the title compound without further purification as a colourless oil (777mg, 99%).

^{1}H NMR (500 MHz, CDCl$_3$) δ 7.64 (app. dt, *J* = 8.2, 1.9 Hz, 2H, C8-H), 7.32 (d, *J* = 8.2 Hz, 2H, C9-H), 4.10 (d, *J* = 2.4 Hz, 2H, C4-H), 3.61 (tt, *J* = 7.0, 3.5 Hz, 1H, C3-H), 3.20 (ddd, *J* = 11.7, 7.9, 3.6 Hz, 2H, C1-H), 2.93 (ddd, *J* = 11.7, 7.6, 3.7 Hz, 2H, C1-H), 2.43 (s, 3H, C11-H), 2.37 (t, *J* = 2.4 Hz, 1H, C6-H), 1.91 (dddd, *J* = 11.7, 7.6, 7.0, 3.7, 3.5 Hz, 2H), 1.74 (dddd, *J* = 11.7, 7.9, 7.0, 3.6, 3.5 Hz, 2H).

^{13}C NMR (126 MHz, CDCl$_3$) δ 143.6 (C10), 133.4 (C7), 129.8 (C9), 127.8 (C8), 79.9 (C5), 74.4 (C6), 71.9 (C3), 55.4 (C4), 43.3 (C1), 30.1 (C2), 21.7 (C11).

HRMS (+EI) calcd: $[M-H]^+$ ($C_{15}H_{19}NO_3S$) 292.1002, measured 292.0999 (1.03 ppm error).

IR (neat) v_{max}/cm^{-1}: 3272, 2937, 2859, 1739, 1348, 1229, 1216, 1164, 1089, 1049, 935, 726, 548.

R*f* 0.34 (20% EtOAc in pentane).

4-methyl-*N*-(prop-2-yn-1-yl)benzenesulfonamide (1ar)

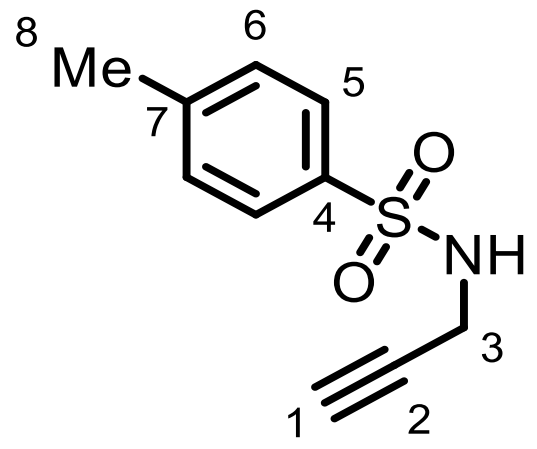

Prepared according to **General procedure I** from propargylamine, affording the title compound without further purification as a yellow solid (994 mg, 95%).

^{1}H NMR (500 MHz, CD_3CN) δ 7.80 – 7.60 (m, 2H, C5-H), 7.38 (d, *J* = 8.1 Hz, 2H, C6-H), 5.86 (d, *J* = 6.3 Hz, 1H, C3-NH), 3.72 (dd, *J* = 6.3, 2.5 Hz, 2H, C3-H), 2.42 (s, 3H, C8-H), 2.34 (t, *J* = 2.5 Hz, 1H, C1-H).

^{13}C NMR (126 MHz, CD_3CN) δ 144.9 (C7), 138.2 (C4), 130.6 (C6), 128.1 (C5), 79.7 (C2), 73.5 (C1), 33.2 (C3), 21.5 (C8).

Characterisation data matches that previously reported.[31]

4-nitro-*N*-(prop-2-yn-1-yl)benzenesulfonamide (1as)

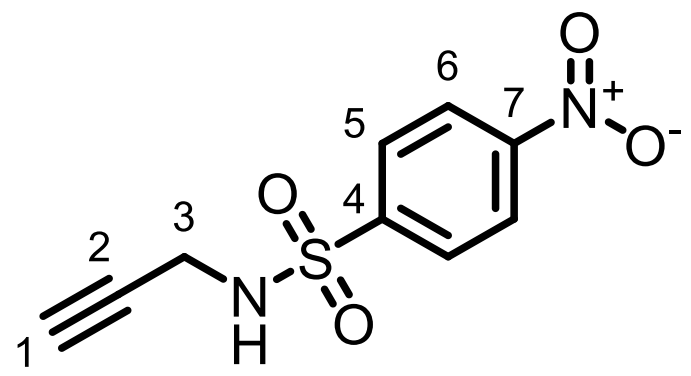

Prepared from propargylamine according to **General procedure I** using 4-nitrobenzenesulfonyl chloride instead of TsCl without further purification to afford the title compound as a yellow solid (855 mg, 71%).

^{1}H NMR (400 MHz, MeOD) δ 8.40 (app. dt, J = 8.7, 2.0 Hz, 2H, C6-H), 8.11 (app. dt, J = 8.8, 2.0 Hz, 2H, C5-H), 3.86 (d, J = 2.5 Hz, 2H, C3-H), 2.45 (t, J = 2.5 Hz, 1H, C1-H).

^{13}C NMR (126 MHz, MeOD) δ 151.6 (C7), 148.0 (C4), 129.8 (C5), 125.2 (C6), 79.2 (C2), 74.0 (C1), 33.1 (C3).

Characterisation data matches that previously reported.[32]

***N*-(but-3-yn-2-yl)-4-methylbenzenesulfonamide (1at)**

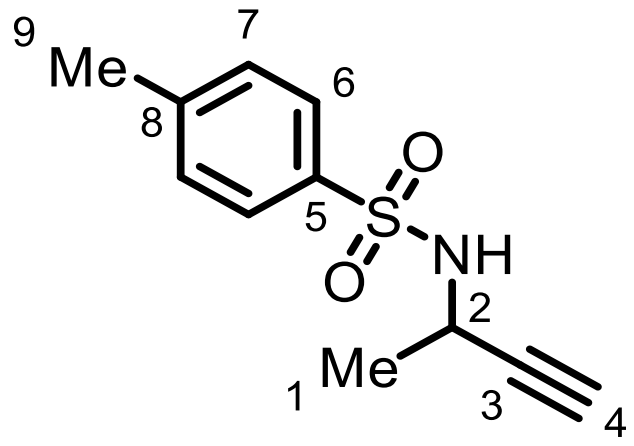

Prepared according to **General procedure II-A** from *p*-toluenesulfonamide and mesylate **26j**. The title compound was produced as a white solid (926 mg, 69%).

^{1}H NMR (500 MHz, $CDCl_3$) δ 7.79 – 7.75 (m, 2H, C6-H), 7.34 – 7.28 (m, 2H, C7-H), 4.70 – 4.53 (m, 1H, C2-NH), 4.18 (dqd, *J* = 9.1, 6.9, 2.3 Hz, 1H, C2-H), 2.08 (d, *J* = 2.3 Hz, 1H, C4-H), 1.42 (d, *J* = 6.9 Hz, 3H, C1-H).

^{13}C NMR (126 MHz, $CDCl_3$) δ 143.8 (C8), 137.4 (C5), 129.7 (C7), 127.5 (C6), 82.9 (C3), 72.0 (C4), 41.3 (C2), 23.6 (C1), 21.7 (C9).

Characterisation data matches that previously reported.[33]

4-methyl-*N*-(2-methylbut-3-yn-2-yl)benzenesulfonamide (1au)

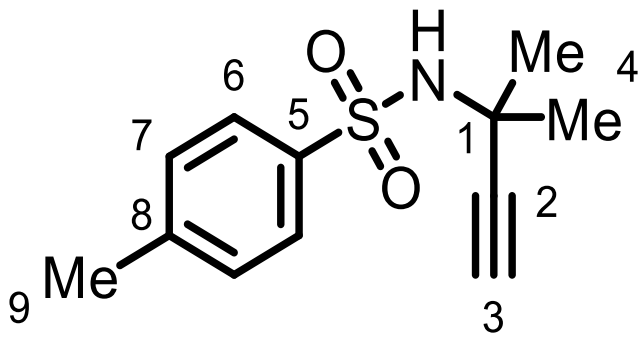

Prepared according to **General procedure I** from 2-methyl-3-butyn-2-amine. Crude product was used without further purification.

^{1}H NMR (500 MHz, CDCl$_3$) δ 7.79 (app. dt, *J* = 8.2, 1.9 Hz, 2H, C6-H), 7.30 – 7.27 (m, 2H, C7-H), 4.71 (s, 1H, C1-NH), 2.42 (s, 3H, C9-H), 2.10 (s, 1H, C3-H), 1.55 (s, 6H, C4-H).

^{13}C NMR (126 MHz, CDCl$_3$) δ 143.4 (C8), 138.9 (C5), 129.4 (C7), 127.8 (C6), 85.6 (C2), 71.3 (C3), 50.2 (C1), 30.8 (C4), 21.7 (C9).

Characterisation data matches that previously reported.[34]

***N*-(1-ethynylcyclohexyl)-4-methylbenzenesulfonamide (1av)**

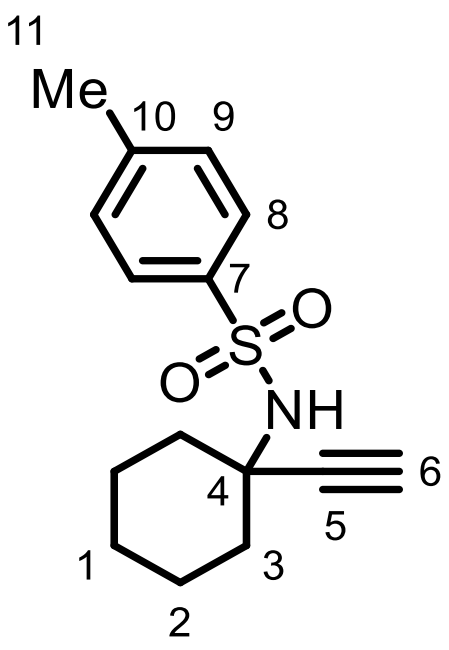

Prepared from 1-ethynylcyclohexylamine according to **General procedure I** without further purification to afford the title compound as a white solid (1.38 g, 99%).

^{1}H NMR (500 MHz, CDCl$_3$) δ 7.79 (app. dt, *J* = 8.4, 1.7 Hz, 2H, C8-H), 7.27 (d, *J* = 8.4 Hz, 2H, C9-H), 4.60 (s, 1H, C4-NH), 2.42 (s, 3H, C11-H), 2.14 (s, 1H, C6-H), 2.07 – 1.96 (m, 2H, C3-H), 1.71 – 1.46 (m, 7H, C3-H, C2-H, C1-H), 1.28 – 1.10 (m, 1H, C1-H).

^{13}C NMR (126 MHz, CDCl$_3$) δ 143.3 (C10), 139.3 (C7), 129.3 (C9), 127.8 (C8), 84.0 (C5), 73.9 (C6), 54.6 (C4), 38.8 (C3), 25.0 (C1), 22.5 (C2), 21.7 (C11).

Characterisation data matches that previously reported.[35]

4-methyl-*N*-(1-phenylprop-2-yn-1-yl)benzenesulfonamide (1aw)

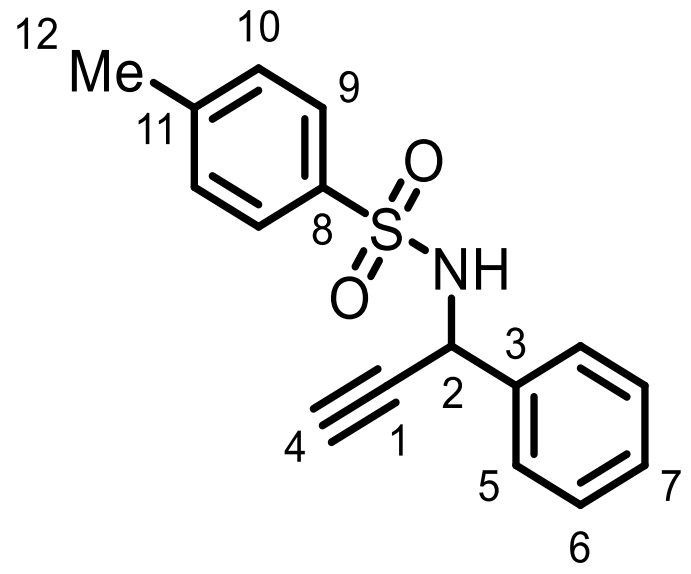

Prepared according to **General procedure VIII** using *p*-toluenesulfonamide, affording the title compound as a white solid (530 mg, 62%)

^{1}H NMR (500 MHz, CDCl$_3$) δ 7.77 (d, *J* = 8.5 Hz, 2H, C9-H), 7.48 – 7.43 (m, 2H, C6-H), 7.34 – 7.27 (m, 5H, C5-H, C7-H, C10-H), 5.33 (dd, *J* = 8.7, 2.4 Hz, 1H, C2-H), 4.81 (d, *J* = 8.8 Hz, 1H, C2-NH), 2.44 (s, 3H, C12-H), 2.33 (d, *J* = 2.4 Hz, 1H, C4-H).

^{13}C NMR (126 MHz, CDCl$_3$) δ 143.8 (C11), 137.4 (C8), 137.1 (C3), 129.7 (C10), 128.9 (C5), 128.7 (C7), 127.6 (C9), 127.3 (C6), 80.6 (C1), 74.9 (C4), 49.1 (C2), 21.7 (C12).

Characterisation data matches that previously reported.[36]

***N-(1-(4-fluorophenyl)-3-(trimethylsilyl)prop-2-yn-1-yl)-4-methylbenzenesulfonamide* (1ax)**

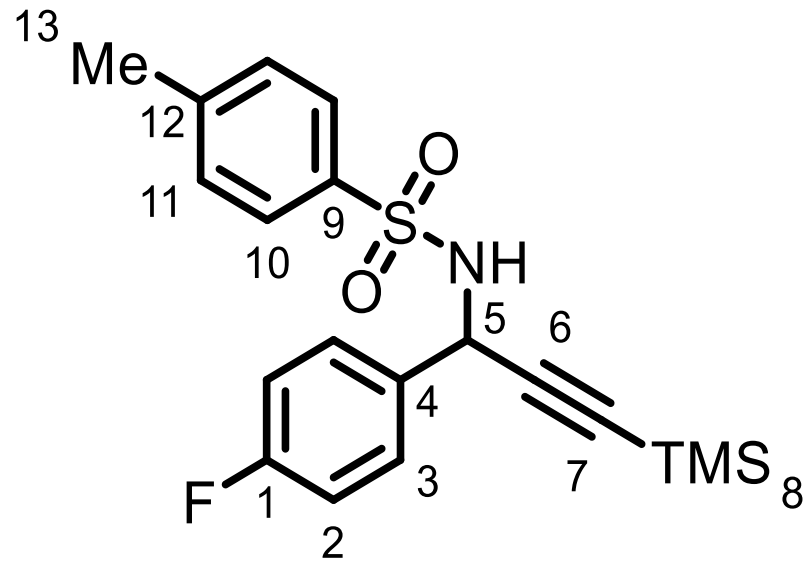

Prepared from imine **26ax** according to **General procedure I4**, affording the title compound without further purification as a blue solid (590mg, 97%).

^{1}H NMR (500 MHz, CDCl$_3$) δ 7.77 (app. dt, *J* = 8.3, 1.9 Hz, 2H, C10-H), 7.49 – 7.44 (m, 2H, C3-H), 7.30 (d, *J* = 8.3 Hz, 2H, C11-H), 7.01 (tt, *J* = 8.6, 2.1 Hz, 2H, C2-H), 5.31 (dd, *J* = 9.1, 0.9 Hz, 1H, C5-H), 4.80 (d, *J* = 9.1 Hz, 1H, C5-NH), 2.44 (s, 3H, C13-H), 0.04 (s, 9H, C8-H).

^{13}C NMR (126 MHz, CDCl$_3$) δ 162.8 (d, *J* = 247.5 Hz, C1), 143.7 (C12), 137.5 (C9), 133.3 (d, *J* = 3.2 Hz, C4), 129.7 (C11), 129.3 (d, *J* = 8.4 Hz, C3), 127.6 (C10), 115.6 (d, *J* = 21.8 Hz, C2), 101.3 (C6), 92.1 (C7), 49.2 (C5), 21.7 (C13), -0.3 (C8).

^{19}F NMR (376 MHz, CDCl$_3$) δ −113.57 (tt, *J* = 8.6, 5.3 Hz, 1F, C1-F).

HRMS (+ESI) calcd: $[M+Na]^+$ ($C_{19}H_{22}FNO_2SSi$) 398.1017, measured 398.1014 (0.75 ppm error).

IR (neat) v_{max}/cm^{-1}: 3264, 2960, 1726, 1603, 1507, 1331, 1161, 845, 570.

***N-(1-([1,1'-biphenyl]-4-yl)prop-2-yn-1-yl)-4-methylbenzenesulfonamide* (1ay)**

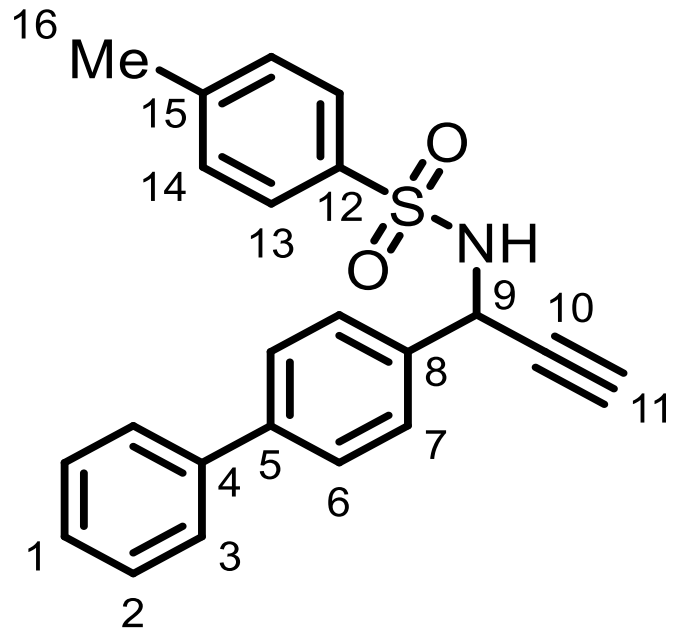

4-Biphenylcarboxaldheyde was converted into the corresponding propargyl alcohol according to **General procedure I4**. The crude product was dissolved in MeOH, and K_2CO_3 (15 mmol, 3equiv.) was added, and the mixture was stirred for 2 h. The reaction mixture was then concentrated *in vacuo,* redissolved in water and extracted with diethyl ether (3 × 30 mL). The organic fractions were dried over $MgSO_4$, filtered, and concentrated *in vacuo* to afford the desilylated alkyne. The crude product was then converted into the tosyl amine according to **General procedure VIII** using *p*-toluenesulfonamide. The crude product was purified by flash column chromatography (2-20% EtOAc in pentane) to afford the title compound as a white solid (137 mg, 8% over 3 steps).

^{1}H NMR (500 MHz, $CDCl_3$) δ 7.78 (d, *J* = 8.1 Hz, 2H, C13-H), 7.58 – 7.51 (m, 6H, C3-H, C6-H, C7-H), 7.44 (dd, *J* = 8.2, 6.9 Hz, 2H, C2-H), 7.39 – 7.34 (m, 1H, C1-H), 7.28 (d, *J* = 8.1 Hz, 2H, C14-H), 5.37 (dd, *J* = 8.7, 2.4 Hz, 1H, C9-H), 5.15 (d, *J* = 8.7 Hz, 1H, C9-NH), 2.42 (s, 3H, C16-H), 2.36 (d, *J* = 2.3 Hz, 1H, C11-H).

^{13}C NMR (126 MHz, $CDCl_3$) δ 143.7 (C15), 141.6 (C5), 140.5 (C4), 137.4 (C12), 136.1 (C8), 129.6 (C14), 128.9 (C2), 127.8 (C7), 127.7 (C1), 127.6 (C6), 127.5 (C13), 127.2 (C3), 80.5 (C10), 74.9 (C11), 48.9 (C9), 21.7 (C16).

HRMS (+ESI) calcd: $[M+H]^+$ ($C_{22}H_{19}NO_2S$) 362.1209, measured 362.1192 (4.69 ppm error).

IR (neat) v_{max}/cm^{-1}: 3270, 3030, 1738, 1665, 1599, 1486, 1328, 1157, 1092, 813, 762, 668, 578.

***4-methyl-N-(1-(4-(trifluoromethyl)phenyl)-3-(trimethylsilyl)prop-2-yn-1-yl)benzenesulfonamide* (1az)**

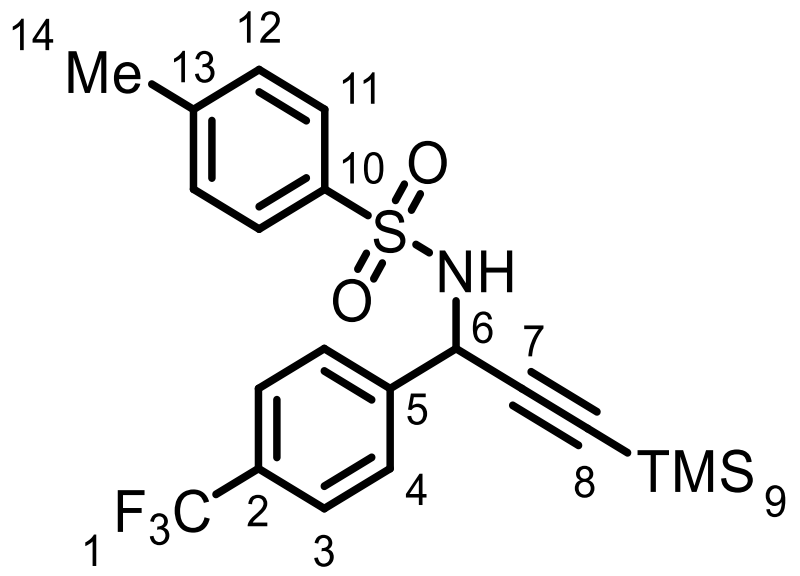

4-trifluoromethyl benzaldehyde was converted to the benzylic alcohol according to **General procedure X**, the crude product of which was then converted to the corresponding sulfonamide according to **General procedure VIII**. The crude product was then purified by flash column chromatography to afford the title compound as a yellow solid (943 mg, 63% over 2 steps).

^{1}H NMR (500 MHz, CDCl$_3$) δ 7.74 (app. dt, J = 8.3, 1.9 Hz, 2H, C11-H), 7.63 – 7.55 (m, 4H, C3-H, C4-H), 7.30 – 7.27 (m, 2H, C12-H), 5.36 (d, J = 9.1 Hz, 1H, C6-H), 4.94 (d, J = 9.1 Hz, 1H, C6-NH), 2.43 (s, 3H, C14-H), 0.05 (s, 9H, C9-H).

^{13}C NMR (126 MHz, CDCl$_3$) δ 143.9 (C13), 141.3 (q, J = 1.4 Hz, C5), 137.3 (C10), 130.7 (q, J = 32.5 Hz, C2), 129.8 (C12), 127.9 (C4), 127.6 (C11), 125.7 (q, J = 3.8 Hz, C3), 124.1 (q, J = 272.3 Hz, C1), 100.7 (C7), 92.6 (C8), 49.5 (C6), 21.7 (C14), -0.4 (C9).

^{19}F NMR (376 MHz, CDCl$_3$) δ −62.53 (s, 3F, C1-F).

HRMS (+ESI) calcd: $[M+H]^+$ ($C_{20}H_{22}F_3NO_2SSi$) 426.1165, measured 426.1182 (3.99 ppm error).

IR (neat) v_{max}/cm^{-1}: 3261, 2962, 1740, 1419, 1325, 1162, 1067, 845, 761, 647.

***N*-(1-(4-bromophenyl)-3-(trimethylsilyl)prop-2-yn-1-yl)-4-methylbenzenesulfonamide** (1ba)

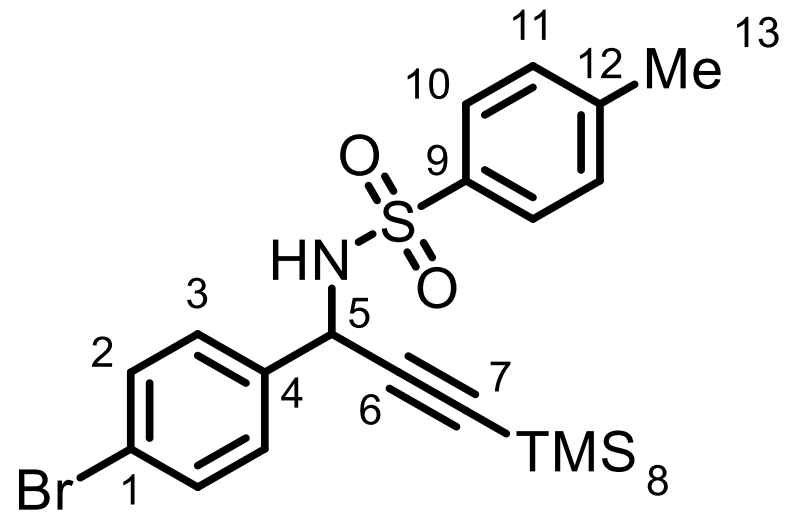

4-bromobenzaldehyde was converted to the corresponding secondary alcohol according to **General procedure X**. The crude product was then taken forward without further purification and converted to the tosyl amine according to **General procedure VIII**. The crude product was purified by flash-column chromatography (0 to 60% Et_2O in pentane), affording the title compound as a white solid (312 mg, 7% over two steps).

^{1}H NMR (500 MHz, $CDCl_3$): δ 7.76 (d, *J* = 8.2 Hz, 2H, C10-H), 7.45 (d, *J* = 8.1 Hz, 2H, C2-H), 7.36 (d, *J* = 8.2 Hz, 2H, C3-H), 7.30 (d, *J* = 8.1 Hz, 2H, C11-H), 5.28 (d, *J* = 9.1 Hz, 1H, C5-H), 4.79 (d, *J* = 9.1 Hz, 1H, C5-NH), 2.44 (s, 3H, C13-H), 0.04 (s, 9H, C8-H).

^{13}C NMR (126 MHz, $CDCl_3$): δ 143.8 (C12), 137.4 (C1), 136.5 (C4), 131.9 (C2), 129.8 (C11), 129.2 (C3), 127.6 (C9), 122.7 (C1), 101.0 (C6), 92.3 (C7), 49.3 (C5), 21.7 (C13), -0.3 (C8).

HRMS (+Nano) calcd: $[M+Na]^+$ ($C_{19}H_{22}NO_2SBrNaSi$) 458.0222, measured 458.0220 (0.4 ppm difference).

IR (neat) ν_{max}/cm^{-1}: 3242, 2940, 2923, 2859, 1487, 1439, 1327, 1158, 1007, 846, 668, 570.

***N*-(1-(4-chlorophenyl)-3-(trimethylsilyl)prop-2-yn-1-yl)-4-methylbenzenesulfonamide** (1bb)

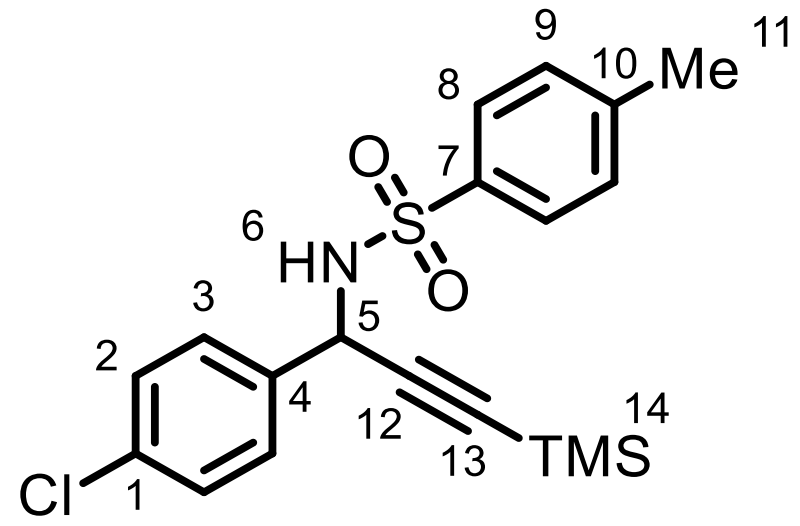

Prepared according **General procedure X** using the corresponding imine formed *in situ* from *p*-toluenesulfonamide and 4-chlorobenzaldehyde. The crude product was purified by flash column chromatography (0 to 60% EtOAc in pentane) to afford the title compound as a white solid (207 mg, 18%).

^{1}H NMR (500 MHz, CDCl$_3$) δ 7.76 (d, *J* = 8.1 Hz, 2H, C8-H), 7.42 (d, *J* = 8.1 Hz, 2H, m, C3-H), 7.32 – 7.27 (4H, m, C2-H, C9-H), 5.30 (1H, d, *J* = 9.1 Hz, C5-H), 4.81 (1H, d, *J* = 9.1 Hz, C6-H), 2.44 (3H, s, C11-H), 0.04 (9H, s, C14-H).

^{13}C NMR (126 MHz, CDCl$_3$) δ 143.8 (C10), 137.4 (C7), 136.0 (C4), 134.5 (C1), 129.8 (C9), 128.91 (C2), 128.86 (C3), 127.6 (C8), 101.1 (C12), 92.2 (C13), 49.3 (C5), 21.7 (C11), -0.3 (C14).

HRMS (+Nano) calcd: $[M+Na]^+$ ($C_{19}H_{22}NO_2SClNaSi$) 414.0727, measured 414.0719 (0.4 ppm error).

IR (neat) ν_{max}/cm^{-1} : 3244, 2961, 2180, 1911, 1685, 1596, 1490, 1430, 1410, 1328, 1306, 1291, 1245, 1185, 1159, 1119, 1089, 1062, 1008, 946, 916, 846, 823, 809, 765, 717, 705, 674, 657, 609, 570, 555, 544, 508, 499, 483, 468, 443, 428, 409.

***4-methyl-N-(1-(1-tosyl-1H-indol-5-yl)-3-(trimethylsilyl)prop-2-yn-1-yl)benzenesulfonamide* (1bc)**

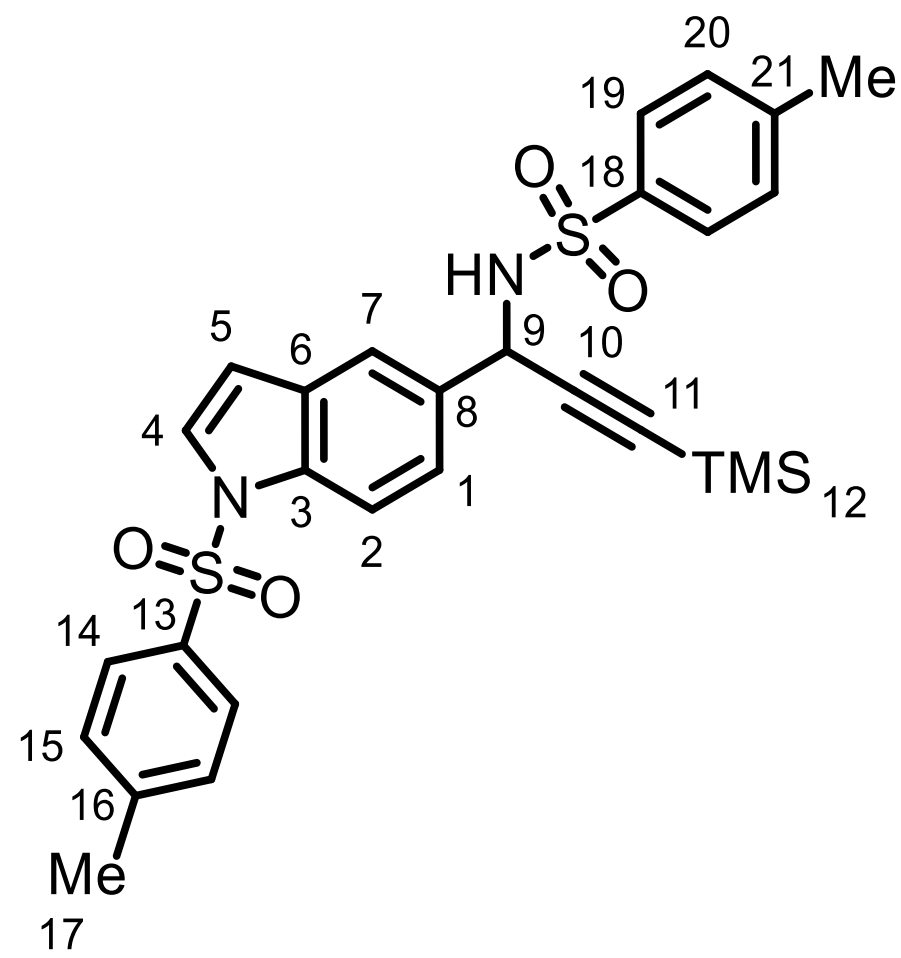

Sodium hydride (1.1 equiv., 6.6 mmol) was added to a solution of indole-5-carboxaldehyde (1 equiv., 6 mmol) in THF (12 mL) at 0°C and stirred for 2 h. The mixture was allowed to warm to RT and stirred for a further 18 h. The reaction was then quenched with sat. aq. $NaHCO_3$, diluted with EtOAc (50 mL), and washed with water (2 × 30 mL). The organic layer was dried over $MgSO_4$, filtered, and concentrated *in vacuo* to afford the tosylated indole. The crude product was redissolved in DCM (10 mL) with *p*-toluenesulfonamide (1.5 equiv., 6 mmol), pyrrolidine (0.5 equiv., 3 mmol), and 4Å molecular sieves (10 g). The mixture was heated to 60°C in a pressure tube for 24 h. The mixture was then filtered and concentrated *in vacuo* to afford the corresponding tosyl imine. The imine was alkylated according to **General procedure I4** and purified by flash column chromatography (2-30% EtOAc in pentane) to afford the title compound as a white solid (885 mg, 27% over 3 steps).

Compound contains 25 mol% 1-tosyl-1*H*-indole-5-carbaldehyde impurity.

^{1}H NMR (500 MHz, $CDCl_3$) δ 7.92 (d, *J* = 8.7 Hz, 1H, C2-H), 7.77 – 7.72 (m, 4H, C14-H, C19-H), 7.62 (d, *J* = 1.8 Hz, 1H, C7-H), 7.57 (d, *J* = 3.7 Hz, 1H, C4-H), 7.42 (dd, *J* = 8.6, 1.9 Hz, 1H, C1-H), 7.25 – 7.20 (m, 4H, C15-H, C20-H), 6.61 (dd, *J* = 3.7, 0.8 Hz, 1H, C5-H), 5.39 (d, *J* = 8.9 Hz, 1H, C9-H), 4.82 (d, *J* = 8.9 Hz, 1H, C9-NH), 2.43 (s, 3H, C17-H), 2.40 (s, 3H, C22-H), 0.03 (s, 9H, C12-H).

^{13}C NMR (126 MHz, $CDCl_3$) δ 143.6 (C16), 143.4 (C21), 135.3 (C13), 134.7 (C18), 134.1 (C6), 132.6 (C8), 130.2 (C3), 130.1 (C15), 129.6 (C20), 127.7 (C14), 127.2 (C4), 127.0 (C19), 124.1 (C1), 120.4 (C7), 113.8 (d, *J* = 2.5 Hz, C2), 109.2 (C5), 101.7 (C10), 91.8 (C11), 49.8 (C9), 21.7 (C17), 21.7 (C22), -0.3 (C12).

HRMS (+Nano) calcd: $[M+Na]^+$ ($C_{28}H_{30}N_2O_4S_2Si$) 573.1308, measured 573.1321 (2.27 ppm error).

IR (neat) v_{max}/cm^{-1}: 3269, 2960, 2175, 1738, 1695, 1458, 1372, 1334, 1172, 1160, 1123, 1093, 846, 813, 667, 583, 540.

***N*-(2-ethynylphenyl)-4-methylbenzenesulfonamide (1bd)**

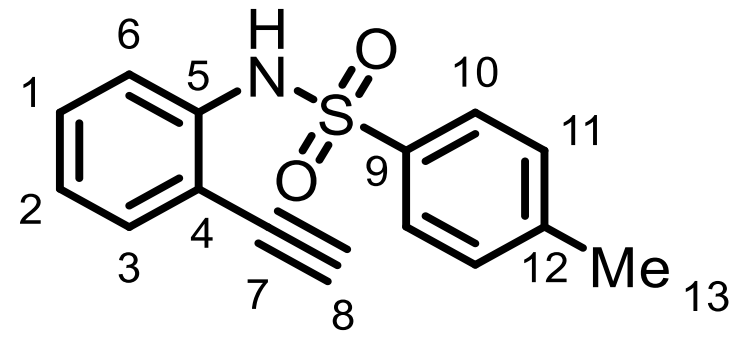

Prepared from 2-ethynyl aniline according to **General procedure I** without further purification to afford the title compound as a white solid (796 mg, 98%).

^{1}H NMR (500 MHz, $CDCl_3$) δ 7.69 (d, *J* = 8.0 Hz, 2H, C10-H), 7.59 (d, *J* = 7.7 Hz, 1H, C6-H), 7.34 (dd, *J* = 7.7, 1.3 Hz, 1H, C3-H), 7.29 (td, *J* = 7.7, 1.3 Hz, 1H, C1-H), 7.24 – 7.19 (m, 3H, C11-H, C5-NH), 7.01 (tt, *J* = 7.7, 1.3 Hz, 1H, C2-H), 3.37 (s, 1H, C8-H), 2.37 (s, 3H, C13-H).

^{13}C NMR (126 MHz, $CDCl_3$) δ 144.3 (C12), 138.6 (C5), 136.1 (C9), 132.6 (C3), 130.3 (C1), 129.8 (C11), 127.5 (C10), 124.3 (C2), 119.4 (C6), 112.8 (C4), 84.5 (C8), 78.7 (C7), 21.7 (C13).

Characterisation data matches that previously reported.[37]

***N*-(but-3-yn-1-yl)-4-methylbenzenesulfonamide (1be)**

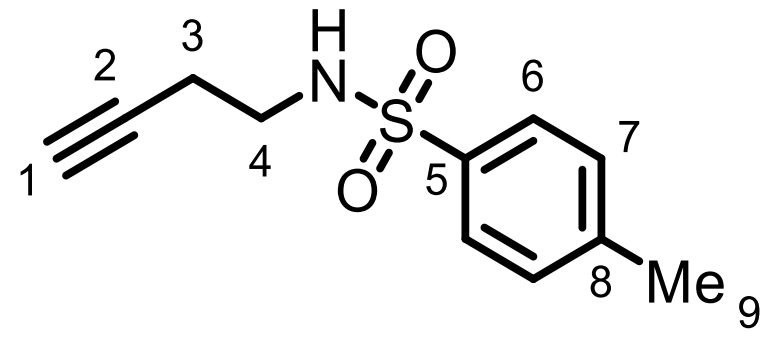

But-1-yn-4-mesylate was synthesised according to **General procedure II-A** and carried forward without further purification.

The title compound was prepared from but-1-yn-4-mesylate and *p*-toluenesulfonamide according to **General procedure II-A**. The crude product was purified by flash column chromatography (2-30% EtOAc in pentane) to afford the title compound as a white solid (244 mg, 36%).

^{1}H NMR (400 MHz, $CDCl_3$) δ 7.76 (d, *J* = 8.3 Hz, 2H, C6-H), 7.32 (d, *J* = 8.3 Hz, 2H, C7-H), 4.71 (s, 1H, C4-NH), 3.12 (dt, *J* = 6.5, 6.4 Hz, 2H, C4-H), 2.43 (s, 3H, C9-H), 2.35 (td, *J* = 6.4, 2.6 Hz, 2H, C3-H), 2.01 (t, *J* = 2.6 Hz, 1H, C1-H).

^{13}C NMR (101 MHz, $CDCl_3$) δ 143.8 (C8), 137.1 (C5), 129.9 (C7), 127.2 (C6), 80.4 (C2), 71.1 (C1), 41.7 (C4), 21.7 (C9), 19.9 (C3).

Characterisation data matches that previously reported.[38]

4-methyl-*N*-(prop-2-yn-1-yl)-*N*-(thiophen-2-ylmethyl)benzenesulfonamide (1bh)

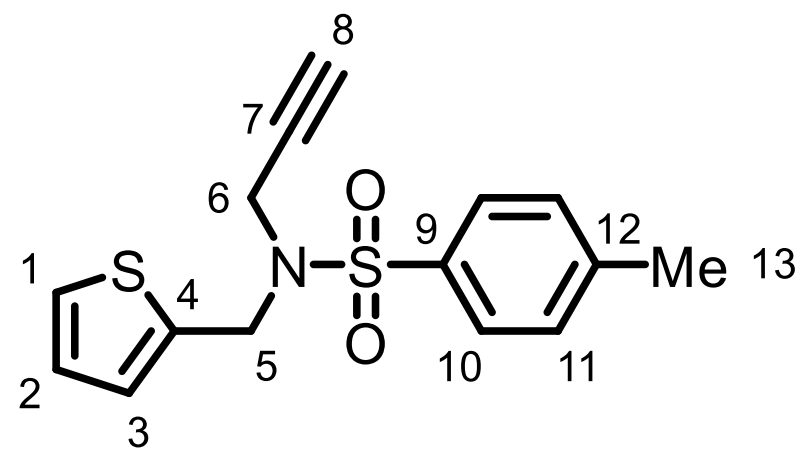

Prepared according to **General procedure II** from sulfonamide **26bh**, affording the title compound without further purification as a yellow oil (1.15 g, 75% over 2 steps)

^{1}H NMR (500 MHz, CDCl$_3$) δ 7.77 (app. dt, *J* = 8.2, 1.8 Hz, 2H, C10-H), 7.32 (d, *J* = 8.2 Hz, 2H, C11-H), 7.28 (dd, *J* = 5.1, 1.2 Hz, 1H, C1-H), 7.01 (dd, *J* = 3.5, 1.1 Hz, 1H, C3-H), 6.94 (dd, *J* = 5.1, 3.5 Hz, 1H, C2-H), 4.58 (s, 2H, C5-H), 4.03 (d, *J* = 2.5 Hz, 2H, C6-H), 2.44 (s, 3H, C13-H), 2.05 (t, *J* = 2.5 Hz, 1H, C8-H).

^{13}C NMR (126 MHz, CDCl$_3$) δ 143.8 (C12), 137.5 (C4), 135.9 (C9), 129.6 (C11), 128.0 (C2), 127.8 (C10), 126.8 (C3), 126.5 (C1), 76.3 (C7), 74.2 (C8), 44.5 (C5), 35.5 (C6), 21.6 (C13).

Characterisation data matches that previously reported.[39]

***N-(cyanomethyl)-4-nitro-N-(prop-2-yn-1-yl)benzenesulfonamide* (1bi)**

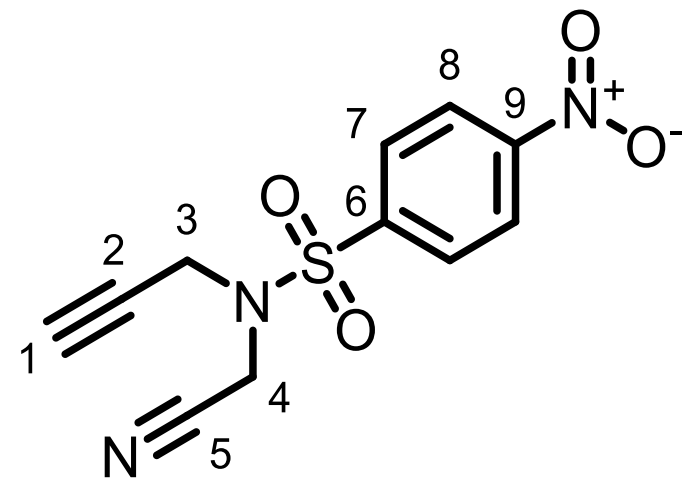

Prepared from sulfonamide **1as** and bromoacetonitrile according to **General procedure II-A** without further purification to afford the title compound as an orange solid (355 mg, 36%).

^{1}H NMR (500 MHz, CDCl$_3$) δ 8.43 (app. dt, *J* = 9.0, 2.1 Hz, 2H, C8-H), 8.07 (app. dt, *J* = 9.0, 2.1 Hz, 2H, C7-H), 4.38 (s, 2H, C4-H), 4.24 (dd, *J* = 2.5 Hz, 2H, C3-H), 2.32 (t, *J* = 2.5 Hz, 1H, C1-H).

^{13}C NMR (126 MHz, CDCl$_3$) δ 151.0 (C9), 143.0 (C6), 129.3 (C7), 124.8 (C8), 113.0 (C5), 76.6 (C1), 74.3 (C2), 38.1 (C3), 35.3 (C4).

HRMS (+MALDI) calcd: $[M+H]^+$ ($C_{11}H_9N_3O_4S$) 280.0387, measured 280.0390 (1.07 ppm error).

IR (neat) v_{max}/cm^{-1}: 3285, 2982, 1739, 1607, 1531, 1351, 1168, 1094, 889, 856, 766, 743, 620, 562.

R*f* 0.24 (20% EtOAc in pentane).

(R)-4,4-dimethyl-3-(prop-2-yn-1-yloxy)dihydrofuran-2(3H)-one **(1bj)**

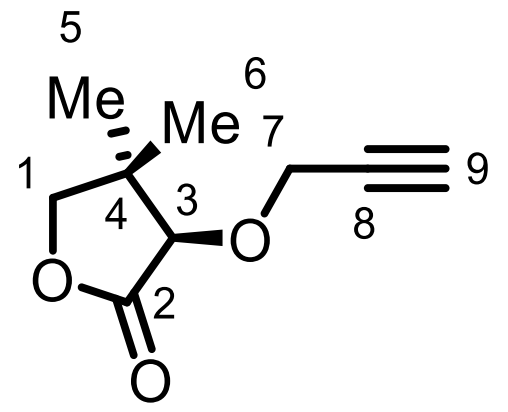

Prepared according to **General procedure IX** from *D*-(−)-pantolactone using DMF as the solvent instead of THF. To remove the DMF, the crude mixture was dissolved in Et_2O (50 mL) and washed with 2M $LiCl_{(aq)}$ (3 × 50 mL). The organic layer was dried over $MgSO_4$ and concentrated *in vacuo* to afford the title compound without further purification as a yellow oil (452 mg, 90%).

^{1}H NMR (500 MHz, $CDCl_3$) δ 4.59 (dd, *J* = 16.1, 2.4 Hz, 1H, C7-H), 4.51 (dd, *J* = 16.1, 2.4 Hz, 1H, C7-H), 4.04 (s, 1H, C3-H), 4.00 (d, *J* = 8.8 Hz, 1H, C1-H), 3.93 (d, *J* = 8.8 Hz, 1H, C1-H), 2.48 (t, *J* = 2.4 Hz, 1H, C9-H), 1.21 (s, 3H, C6-H), 1.09 (s, 3H, C5-H).

^{13}C NMR (126 MHz, $CDCl_3$) δ 175.2 (C2), 79.6 (C3), 79.0 (C8), 76.5 (C9), 75.6 (C1), 58.0 (C7), 40.3 (C4), 23.1 (C6), 19.4 (C5).

HRMS (+EI) calcd. $[M\text{-}Me]^+$ ($C_8H_{12}O_3$) 153. 0546, measured 153.0543 (1.96 ppm error).

IR (neat) v_{max}/cm^{-1}: 3285, 2968, 2933, 2875, 2118, 1784, 1465, 1371, 1230, 1116, 1006, 681.

R_f 0.44 (20% EtOAc in pentane).

***N*-(hex-5-yn-1-yl)-4-methylbenzenesulfonamide (1bk)**

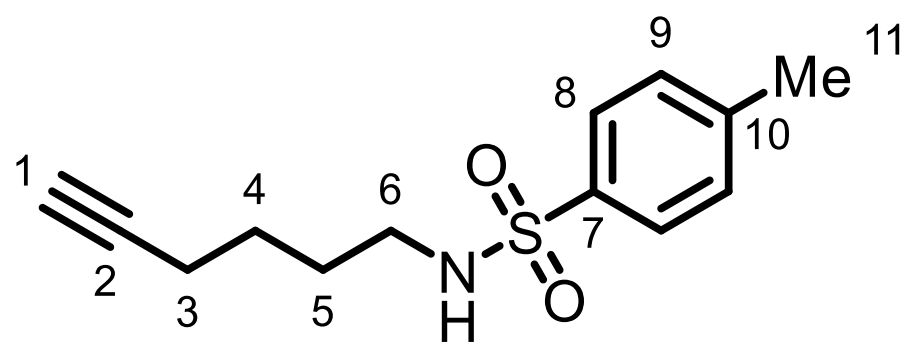

To a solution of *p*-toluenesulfonyl chloride (1.2 equiv., 7.2 mmol) and triethylamine (1.5 equiv., 9 mmol) in DCM at 0°C was added 5-hexyn-1-ol (1 equiv., 6 mmol) dropwise. The reaction was stirred at 0°C for 2 h, warmed to RT, and stirred for a further 18 h. The mixture was then diluted with water (50 mL) and extracted with DCM (2 × 20 mL). The organic layers were combined, dried over $MgSO_4$, filtered, and concentrated *in vacuo*. The crude alkyl tosylate was then redissolved in MeCN (10 mL), to which was added *p*-toluenesulfonamide (2 equiv., 12 mmol) and K_2CO_3 (3 equiv., 18 mmol). The mixture was refluxed for 48 h, after which the solvent was removed *in vacuo* and the residue was diluted with water (50 mL) and extracted with DCM (2 × 20 mL). The organic fractions were combined, dried over $MgSO_4$ and concentrated *in vacuo*. The crude product was purified by flash column chromatography (2-30% EtOAc in pentane) to afford the title compound as a white solid (1.02 g, 68% over 2 steps).

^{1}H NMR (500 MHz, $CDCl_3$) δ 7.74 (app. dt, *J* = 8.3, 1.7 Hz, 2H, C8-H), 7.31 (d, *J* = 7.9 Hz, 2H, C9-H), 4.54 (t, *J* = 6.3 Hz, 1H, C6-NH), 2.96 (q, *J* = 6.3 Hz, 2H, C6-H), 2.43 (s, 3H, C11-H), 2.15 (td, *J* = 6.7, 2.6 Hz, 2H, C3-H), 1.92 (t, *J* = 2.6 Hz, 1H, C1-H), 1.64 – 1.46 (m, 4H, C4-H, C5-H).

^{13}C NMR (126 MHz, $CDCl_3$) δ 143.6 (C10), 137.0 (C7), 129.9 (C9), 127.2 (C8), 83.8 (C2), 69.0 (C1), 42.8 (C6), 28.7 (C5), 25.4 (C4), 21.7 (C11), 18.0 (C3).

Characterisation data matches that previously reported.[40]

1-(4-nitrophenyl)-3-(trimethylsilyl)prop-2-yn-1-ol (1bm)

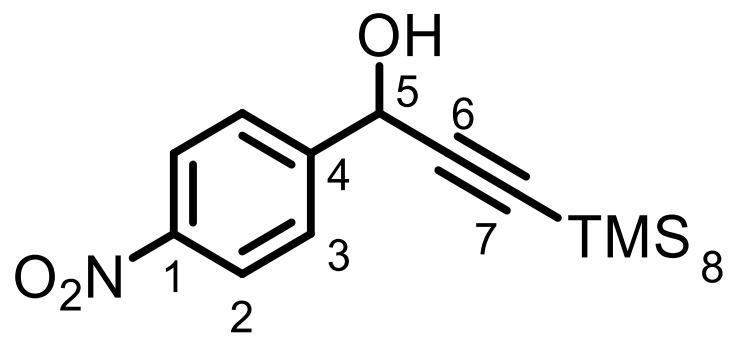

Prepared from 4-nitrobenzaldehyde according to **General procedure I4** without further purification to afford the title compound as a colourless oil (1.48 g, 99%).

^{1}H NMR (500 MHz, CDCl$_3$) δ 8.24 (dt, *J* = 8.8, 2.0 Hz, 2H, C2-H), 7.72 (dt, *J* = 8.8, 2.0 Hz, 2H, C3-H), 5.57 – 5.53 (m, 1H, C5-H), 2.43 (br s, 1H, C5-OH), 0.21 (s, 9H, C8-H).

^{13}C NMR (126 MHz, CDCl$_3$) δ 147.9 (C1), 147.2 (C4), 127.6 (C3), 123.9 (C2), 103.6 (C6), 93.2 (C7), 64.1 (C5), -0.2 (C8).

Characterisation data matches that previously reported.[41]

1-(prop-2-yn-1-ylsulfonyl)hexane (1bo)

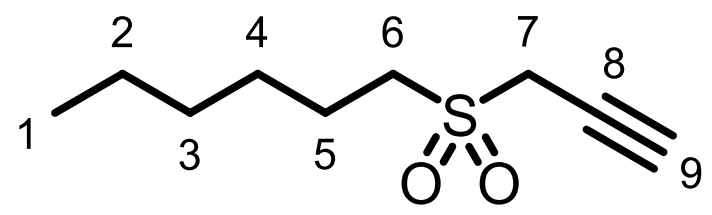

Sulfide **26bo** (2.5 mmol, 1 equiv.) was dissolved in DCM (10 mL, 0.25M) and cooled to 0°C. *m*CPBA (7.5 mmol, 3 equiv.) was then added portionwise and the reaction was allowed to warm to RT and stirred for 18 h. Upon completion the reaction was diluted with diethyl ether (50 mL) and washed with sat. aq. $NaHCO_3$ (3 × 50 mL). The organic layer was dried over $MgSO_4$, filtered, and concentrated *in vacuo* to afford the title compound without further purification as a white solid (464 mg, 90%).

^{1}H NMR (500 MHz, $CDCl_3$) δ 3.83 (d, *J* = 2.7 Hz, 2H, C7-H), 3.23 – 3.16 (m, 2H, C6-H), 2.51 (t, *J* = 2.7 Hz, 1H, C9-H), 1.92 – 1.82 (m, 2H, C5-H), 1.52 – 1.42 (m, 2H, C4-H), 1.36 – 1.30 (m, 4H, C3-H, C2-H), 0.94 – 0.85 (m, 3H, C1-H).

^{13}C NMR (126 MHz, $CDCl_3$) δ 76.5 (C9), 71.8 (C8), 51.5 (C6), 45.0 (C7), 31.3 (C3), 28.2 (C4), 22.4 (C2), 22.0 (C5), 14.1 (C1).

Characterisation data matches that previously reported.[42]

***N-butyl-2-nitro-N-(prop-2-yn-1-yl)benzenesulfonamide* (1bp)**

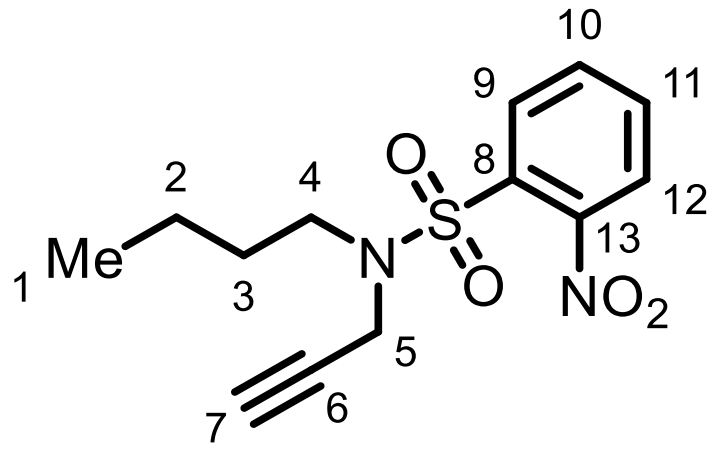

n-Butylamine was protected according to **General procedure I** using 2-nitrobenzenesulfonyl chloride instead of TsCl. The crude product was then propargylated according to **General procedure II** to afford the title compound as a yellow oil (1.77 g, 60% over 2 steps).

^{1}H NMR (500 MHz, CDCl$_3$) δ 8.04 (ddd, J = 8.0, 1.8, 1.8 Hz, 1H, C11-H), 7.73 – 7.65 (m, 2H, C10-H, C11-H), 7.65 – 7.61 (m, 1H, C12-H), 4.20 (d, J = 2.4 Hz, 2H, C5-H), 3.40 (t, J = 7.7 Hz, 2H, C4-H), 2.16 (t, J = 2.4 Hz, 1H, C7-H), 1.57 (tt, J = 7.7, 7.3 Hz, 2H, C3-H), 1.32 (h, J = 7.3 Hz, 2H, C2-H), 0.91 (t, J = 7.3 Hz, 3H, C1-H).

^{13}C NMR (126 MHz, CDCl$_3$) δ 148.4 (C13), 133.7 (C10), 133.0 (C8), 131.7 (C11), 131.0 (C9), 124.3 (C12), 77.0 (C6), 73.8 (C7), 46.7 (C4), 36.3 (C5), 29.5 (C3), 19.8 (C2), 13.8 (C1).

HRMS (+EI) calcd. $[M-H]^+$ ($C_{13}H_{16}N_2O_4S$) 295.0747, measured 295.0741 (2.03 ppm error).

IR (neat) v_{max}/cm^{-1}: 3289, 3096, 2960, 2934, 2874, 1731, 1543, 1358, 1162, 1128, 1042, 907, 769.

R$_f$ 0.31 (20% EtOAc in pentane).

***N-(4-fluorobenzyl)-2,4-dinitro-N-(prop-2-yn-1-yl)benzenesulfonamide* (1bq)**

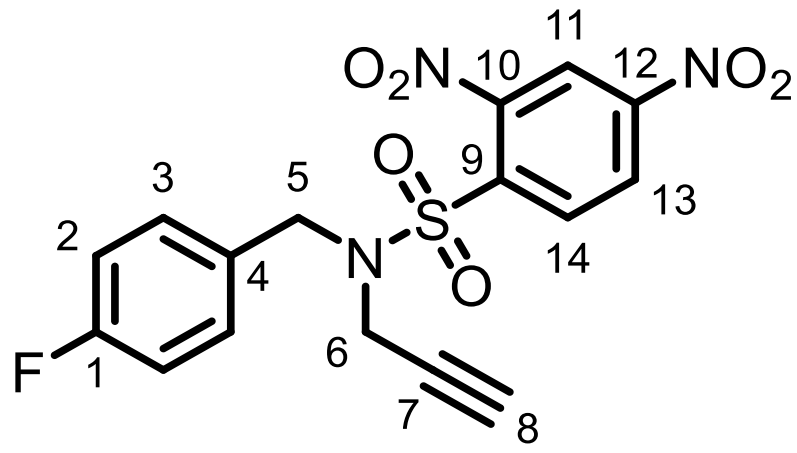

Prepared from sulfonamide **26bq** according to **General procedure II** without further purification to afford the title compound as a yellow solid (847 mg, 60%).

^{1}H NMR (500 MHz, CDCl$_3$) δ 8.54 – 8.45 (m, 2H, C11-H, C13-H), 8.29 (d, J = 9.3 Hz, 1H, C14-H), 7.38 – 7.30 (m, 2H, C3-H), 7.10 – 7.02 (m, 2H, C2-H), 4.58 (s, 2H, C5-H), 4.03 (d, J = 2.5 Hz, 2H, C6-H), 2.21 (t, J = 2.5 Hz, 1H, C8-H).

^{13}C NMR (126 MHz, CDCl$_3$) δ 163.0 (d, J = 248.0 Hz, C1), 150.0 (C10), 148.6 (C12), 138.4 (C9), 133.0 (C14), 130.8 (d, J = 8.3 Hz, C3), 129.7 (d, J = 3.2 Hz, C4), 126.2 (C13), 119.9 (C11), 116.1 (d, J = 21.6 Hz, C2), 76.0 (C7), 75.0 (C8), 50.2 (C5), 36.1 (C6).

^{19}F NMR (376 MHz, CDCl$_3$) δ −112.72 (tt, J = 9.1, 5.2 Hz).

HRMS (+MALDI) calcd: $[M+Na]^+$ ($C_{16}H_{12}N_3O_6SF$) 416.0323, measured 416.0327 (0.96 ppm error).

IR (neat) v_{max}/cm^{-1}: 3292, 3103, 1604, 1552, 1539, 1509, 1350, 1223, 1166, 1107, 904, 833, 762.

***N*-(but-3-yn-1-yl)-4-methyl-*N*-(4-methylbenzyl)benzenesulfonamide** (**1br**)

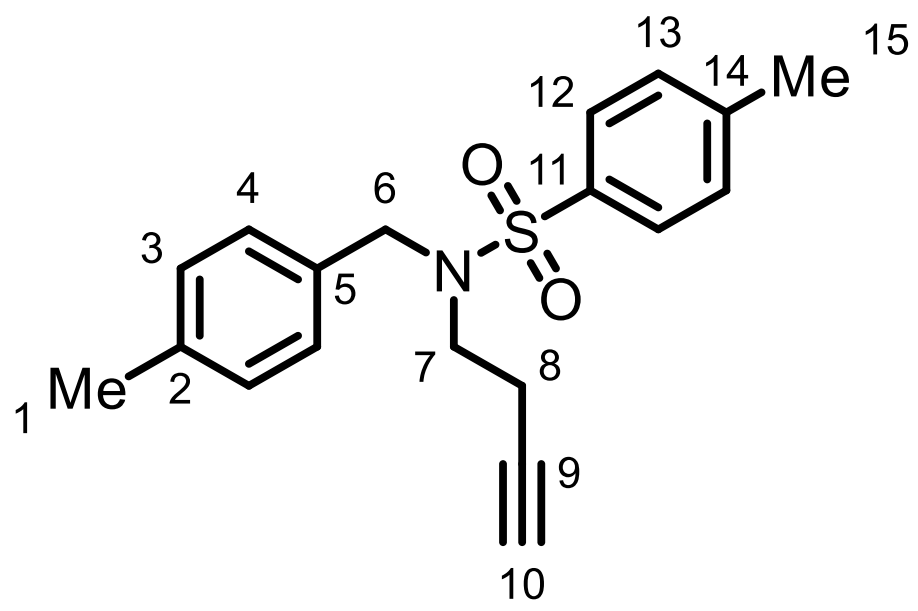

4-methyl benzylamine was tosylated according to **General procedure I**. The crude product was then alkylated with homopropargyl bromide according to **General procedure II**. The crude product was purified by flash column chromatography (2-20% EtOAc in pentane) to afford the title compound as a white solid (0.81 g, 49%).

^{1}H NMR (400 MHz, CDCl$_3$) δ 7.74 (d, J = 8.2 Hz, 2H, C12-H), 7.33 (d, J = 8.0 Hz, 2H, C4-H), 7.14 (q, J = 8.0 Hz, 4H, C3-H, C13-H), 4.32 (s, 2H, C6-H), 3.34 – 3.14 (m, 2H, C7-H), 2.45 (s, 3H, C15-H), 2.33 (s, 3H, C1-H), 2.26 – 2.19 (m, 2H, C8-H), 1.89 (t, J = 2.7 Hz, 1H, C10-H).

^{13}C NMR (101 MHz, CDCl$_3$) δ 143.5 (C14), 137.8, (C2) 136.8 (C11), 132.9 (C5), 129.8 (C4), 129.4 (C13), 128.4 (C3), 127.2 (C12), 81.0 (C9), 70.1 (C10), 52.3 (C6), 46.6 (C7), 21.6 (C8), 21.2 (C15), 19.1 (C1).

HRMS (+ESI) calcd. $[M+H]^+$ ($C_{19}H_{21}NO_2S$) 328.1352, measured 328.1366 (4.27 ppm error).

IR (neat) v_{max}/cm^{-1}: 3260, 2924, 1698, 1596, 1512, 1494, 1458, 1356, 1345, 1325, 1268, 1151, 1130, 1090, 1017, 979.

***N*-(but-3-yn-1-yl)-4-methyl-*N*-(4-fluorobenzyl)benzenesulfonamide** (**1bs**)

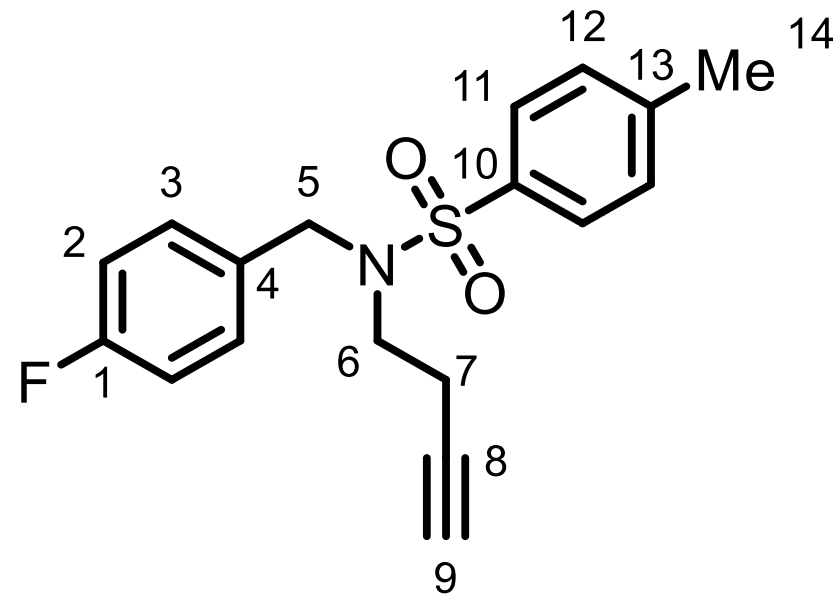

4-fluoro benzylamine was tosylated according to **General procedure I**. The crude product was then alkylated with homopropargyl bromide according to **General procedure II**. The crude product was purified by flash column chromatography (2-20% EtOAc in pentane) to afford the title compound as a white solid (723 mg, 44%).

^{1}H NMR (400 MHz, $CDCl_3$) δ 7.73 (d, *J* = 8.0 Hz, 2H, C11-H), 7.33 (d, *J* = 8.0 Hz, 2H, C12-H), 7.30 – 7.22 (m, 2H, C3-H), 7.01 (t, *J* = 8.6 Hz, 2H, C2-H), 4.33 (s, 2H, C5-H), 3.38 – 3.15 (m, 2H, C6-H), 2.44 (s, 3H, C14-H), 2.27 – 2.19 (m, 2H, C7-H), 1.89 (t, *J* = 2.7 Hz, 1H, C9-H).

^{13}C NMR (101 MHz, $CDCl_3$) δ 162.6 (d, *J* = 246.5 Hz, C1), 143.7 (C13), 136.8 (C10), 132.0 (d, *J* = 3.2 Hz, C4), 130.1 (d, *J* = 8.2 Hz, C3), 130.0 (C12), 127.3 (C11), 115.7 (d, *J* = 21.5 Hz, C2), 80.9 (C8), 70.3 (C9), 52.0 (C5), 46.8 (C6), 21.7 (C14), 19.2 (C7).

^{19}F NMR (376 MHz,$CDCl_3$) δ −114.08 (tt, *J* = 8.7, 5.3 Hz, 1F, C1-F).

HRMS (+ESI) calcd. $[M+Na]^+$ ($C_{18}H_{18}FNO_2S$) 354.0934, measured 354.0932 (0.56 ppm error).

IR (neat) v_{max}/cm^{-1}: 3291, 2991, 2919, 2868, 1596, 1577, 1514, 1427, 1401, 1325, 1163, 1090, 1053, 1018.

8.2. Fluorovinyl Iodonium Salts (FVIs) (3)

(Z)-(2-fluoro-3-((N-(4-methoxybenzyl)-4-methylphenyl)sulfonamido)prop-1-en-1-yl)(p-tolyl)iodonium tetrafluoroborate **(3a)**

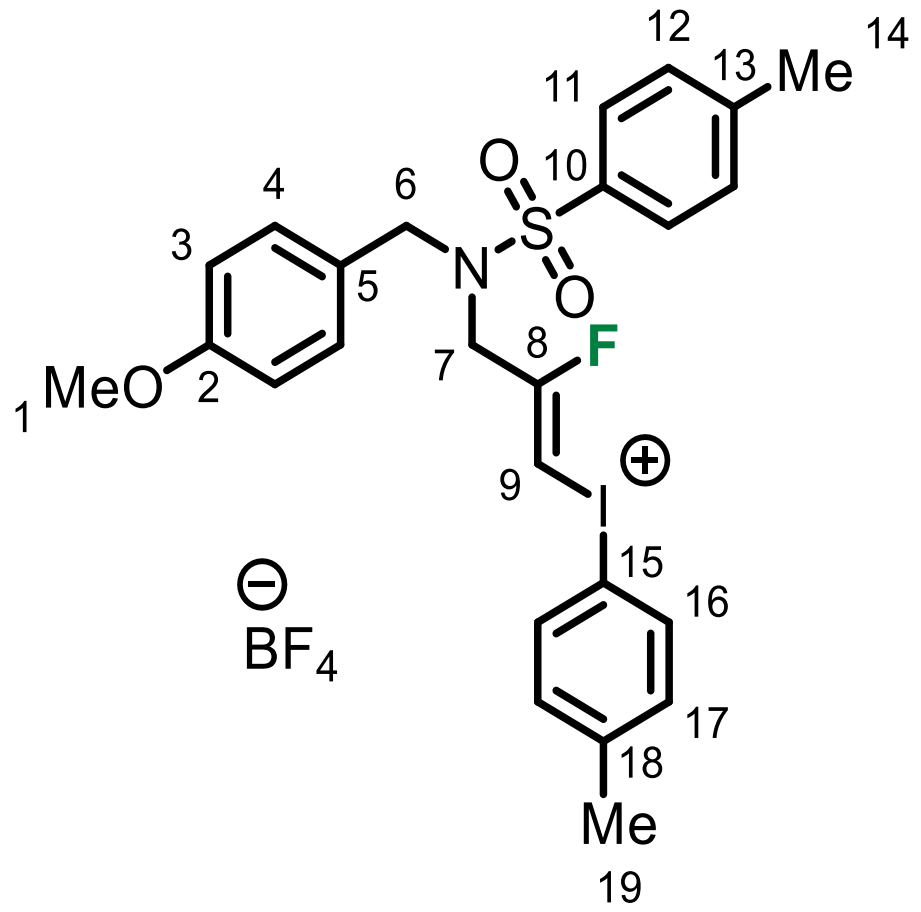

Produced according to **General procedure III** from alkyne **1a** to afford the title compound as a white solid (0.993 g, 76%).

^{1}H NMR (500 MHz, Acetone-*D$_6$*) δ 8.05 – 7.98 (m, 2H, C16-H), 7.80 (d, *J* = 8.0 Hz, 2H, C11-H), 7.48 – 7.42 (m, 4H, C4-H, C12-H), 7.15 (d, *J* = 8.2 Hz, 2H, C17-H), 6.96 (d, *J* = 34.0 Hz, 1H, C9-H), 6.79 (d, *J* = 8.3 Hz, 2H, C3-H), 4.30 (d, *J* = 11.1 Hz, 2H, C7-H), 4.27 (s, 2H, C6-H), 3.78 (s, 3H, C1-H), 2.47 (s, 3H, C19-H), 2.46 (s, 3H, C14-H).

^{13}C NMR (126 MHz, Acetone-*D$_6$*) δ 168.7 (d, *J* = 278.8 Hz, C8), 160.6 (C2), 145.1 (C18), 144.9 (C13), 136.8 (C10), 136.4 (C16), 133.9 (C12), 131.0 (C17), 130.9 (C4), 128.4 (C11), 127.8 (C5), 114.8 (C3), 109.6 (C15), 80.1 (d, *J* = 19.2 Hz, C9), 55.6 (C1), 52.8 (C6), 47.7 (d, *J* = 31.4 Hz, C7), 21.5 (C19), 21.4 (C14).

^{19}F NMR (376 MHz, Acetone-*D$_6$*) δ −75.37 (d, *J* = 34.0 Hz, 1F, C8-F), −150.66 (4F, BF_4).

HRMS (+ESI) calcd: $[M]^+$ ($C_{25}H_{26}FINO_3S$) 566.0657, measured 566.0638 (3.36 ppm error).

IR (neat) v_{max}/cm^{-1}: 3096, 2929, 1651, 1513, 1249, 1159, 1090, 1061, 1029, 803, 663, 548.

***(Z)-(2-fluoro-3-((4-methyl-N-(4-methylbenzyl)phenyl)sulfonamido)prop-1-en-1-yl)(p-tolyl)iodonium tetrafluoroborate* (3b)**

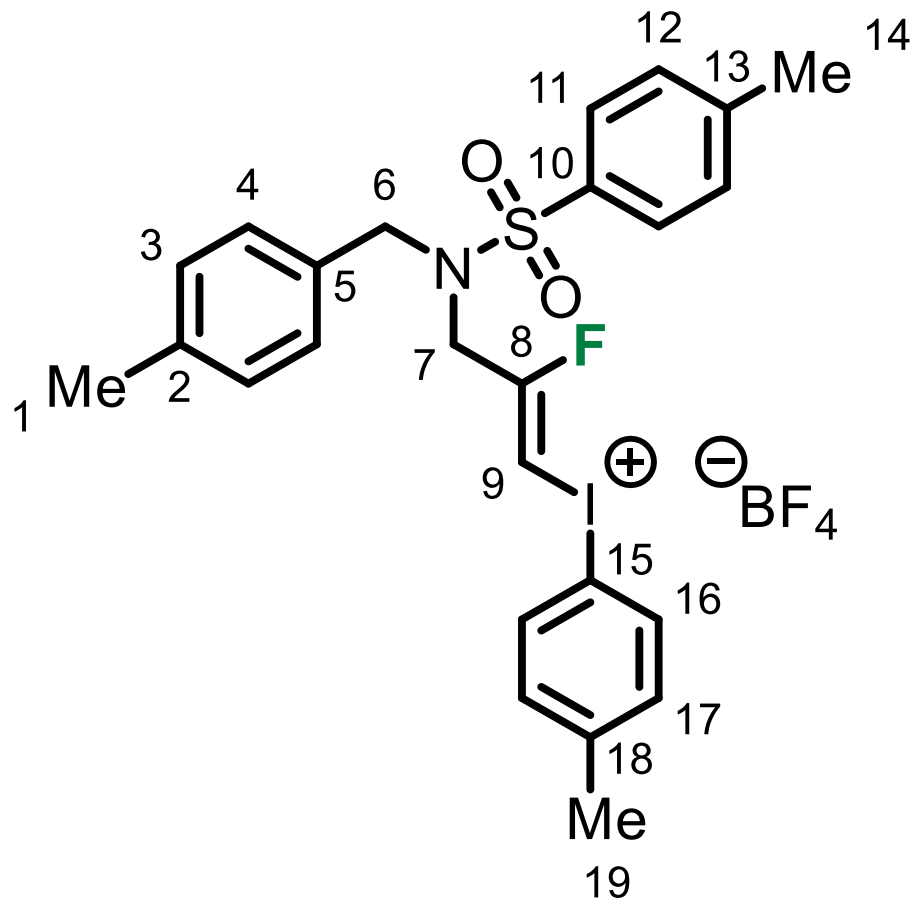

Prepared from alkyne **1b** via **General procedure III** to afford the title compound as a brown solid (1.604 g, 78%).

^{1}H NMR (400 MHz, Acetone-*D$_6$*) δ 8.02 (d, *J* = 8.4 Hz, 2H, C16-H), 7.80 (d, *J* = 8.4 Hz, 2H, C11-H), 7.45 (m, 4H, C17-H, C12-H), 7.11 (d, *J* = 8.2 Hz, 2H, C4-H), 7.06 (d, *J* = 8.0 Hz, 2H, C3-H), 6.96 (d, *J* = 33.5 Hz, 1H, C9-H), 4.31 (d, *J* = 12.0, 2H, C7-H), 4.30 (s, 2H, C6-H), 2.46 (s, 3H, C19-H), 2.45 (s, 3H, C14-H), 2.27 (s, 3H, C1-H).

^{13}C NMR (101 MHz, Acetone-*D$_6$*) δ 167.5 (d, *J* = 279.3 Hz, C8), 144.2 (C18), 144.0 (C13), 137.8 (C2), 136.1 (C10), 135.7 (C16), 133.0 (C17), 132.3 (C5), 130.0 (C12), 129.3 (C3), 128.8 (C4), 127.5 (C11), 108.7 (C15), 79.4 (d, *J* = 19.2 Hz, C9), 52.0 (C6), 46.8 (d, *J* = 30.3 Hz, C7-H), 20.7 (C19), 20.5 (C14), 20.4 (C1).

^{19}F NMR (376 MHz, Acetone-*D$_6$*) δ −75.27 (dt, *J* = 33.5, 12.1 Hz, 1F, C8-F), −150.57 (s, 4F, BF_4).

HRMS (+ESI) calcd. $[M]^+$ ($C_{25}H_{26}FINO_2S$) 550.0707, measured 550.0692 (2.73 ppm error).

IR (neat) v_{max}/cm^{-1}: 3096, 2923, 1651, 1597, 1478, 1448, 1333, 1289, 1156, 1088, 1053, 994.

(Z)-(3-((N-(4-bromobenzyl)-4-methylphenyl)sulfonamido)-2-fluoroprop-1-en-1-yl)(p-tolyl)iodonium tetrafluoroborate **(3c)**

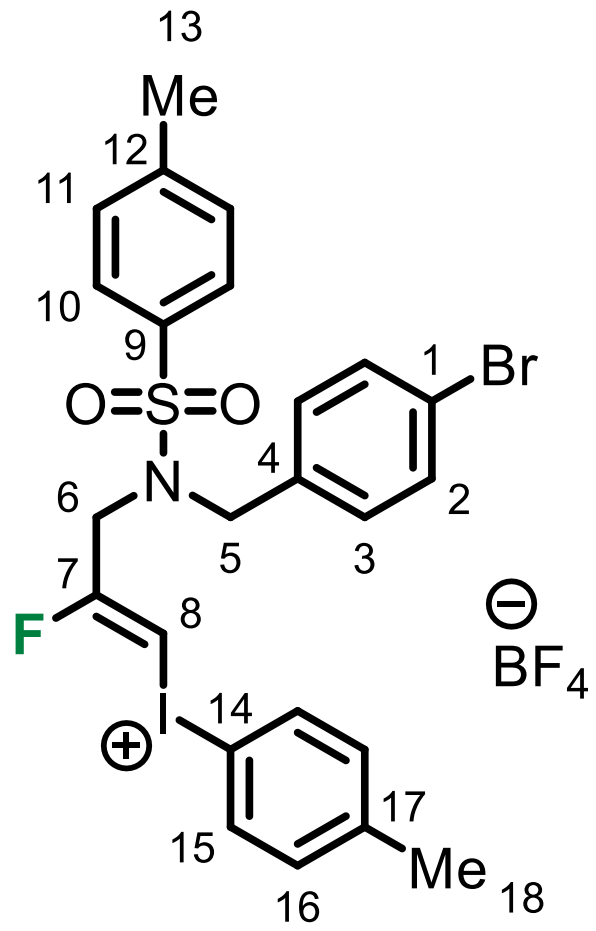

Produced according to **General procedure III** from alkyne **1c** to afford the title compound as an orange gel (1.00g, 71%).

^{1}H NMR (500 MHz, Acetone-*D$_6$*) δ 8.02 (d, *J* = 8.0 Hz, 2H, C15-H), 7.80 (d, *J* = 8.0 Hz, 2H, C10-H), 7.45 (d, *J* = 8.0 Hz, 2H, C16-H), 7.45 (d, *J* = 8.0 Hz, 2H, C11-H), 7.41 (d, *J* = 8.0 Hz, 2H, C2-H), 7.22 (d, *J* = 8.0 Hz, 2H, C3-H), 7.04 (d, *J* = 33.3 Hz, 1H, C8-H), 4.38 (d, *J* = 12.2 Hz, 2H, C6-H), 4.34 (s, 2H, C5-H), 2.48 (s, 3H, C18-H), 2.46 (s, 3H, C13-H).

^{13}C NMR (126 MHz, Acetone-*D$_6$*) δ 168.1 (d, *J* = 279.0 Hz, C7), 145.2 (C17), 144.9 (C12), 136.7 (C9), 136.5 (C15), 136.0 (C4), 133.9 (C16), 132.5 (C2), 131.5 (C11), 130.9 (C3), 128.4 (C10), 122.4 (C1), 109.6 (C14), 80.7 (d, *J* = 19.0 Hz, C8), 52.7 (C5), 48.3 (d, *J* = 30.1 Hz, C6), 21.5 (C18), 21.4 (C13).

^{19}F NMR (377 MHz, Acetone-*D$_6$*) δ −75.57 (dt, *J* = 33.4, 12.1 Hz, 1F, C7-F), −150.59 – −150.71 (m, 4F, BF_4).

HRMS (+ESI) calcd: $[M]^+$ ($C_{24}H_{23}BrFINO_2S$) 613.9656, measured 613.9652 (0.65 ppm error).

IR (neat) v_{max}/cm^{-1}: 3097, 1738, 1652, 1488, 1338, 1157, 1088, 1057, 1010, 799, 663, 545.

(Z)-(3-((N-(4-chlorobenzyl)-4-methylphenyl)sulfonamido)-2-fluoroprop-1-en-1-yl)(p-tolyl)iodonium tetrafluoroborate **(3d)**

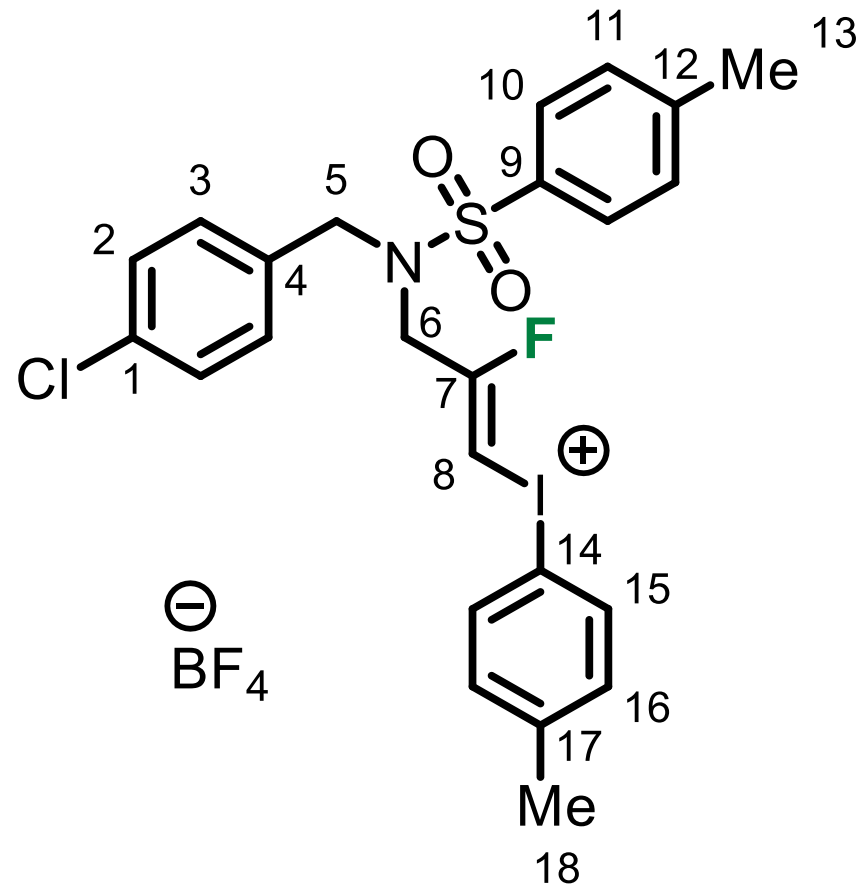

Produced according to **General procedure III** from alkyne **1d** to afford the title compound as a white solid (1.10 g, 84%).

^{1}H NMR (400 MHz, Acetone-*D$_6$*) δ 8.03 (app. dt, *J* = 8.4, 1.6 Hz, 2H, C15-H), 7.81 (app. dt, *J* = 8.3, 1.9 Hz, 2H, C10-H), 7.46 (d, *J* = 8.4 Hz, 2H, C16-H), 7.46 (d, *J* = 8.3 Hz, 2H, C11-H), 7.28 – 7.26 (m, 4H, C2-H, C3-H), 7.05 (dt, *J* = 33.5, 1.1 Hz, 1H, C8-H), 4.38 (dd, *J* = 12.2, 1.1 Hz, 2H, C6-H), 4.35 (s, 2H, C5-H), 2.48 (s, 3H, C18-H), 2.46 (s, 3H, C13-H).

^{13}C NMR (126 MHz, Acetone-*D$_6$*) δ 168.1 (d, *J* = 279.0 Hz, C7), 145.2 (C9), 144.9 (C17), 136.7 (C12), 136.5 (C15), 135.5 (C1), 134.1 (C4), 133.9 (C11), 131.2 (C2), 130.9 (C16), 129.5 (C3), 128.3 (C10), 109.4 (C14), 80.6 (d, *J* = 19.1 Hz, C8), 52.6 (C5), 48.3 (d, *J* = 30.0 Hz, C6), 21.5 (C18), 21.4 (C13).

^{19}F NMR (471 MHz, Acetone-*D$_6$*) δ −75.6 (dt, *J* = 33.5, 12.2 Hz, 1F, C7-F), −150.1 – −150.3 (m, 4F, BF_4).

HRMS (+ESI) calcd: $[M]^+$ ($C_{24}H_{23}ClFINO_2S$) 570.0161, measured 570.0146 (2.63 ppm error).

IR (neat) v_{max}/cm^{-1}: 3098, 1738, 1653, 1491, 1340, 1159, 1090, 1063, 1015, 802, 766, 568, 547.

(Z)-(2-fluoro-3-((N-(4-fluorobenzyl)-4-methylphenyl)sulfonamido)prop-1-en-1-yl)(p-tolyl)iodonium tetrafluoroborate **(3e)**

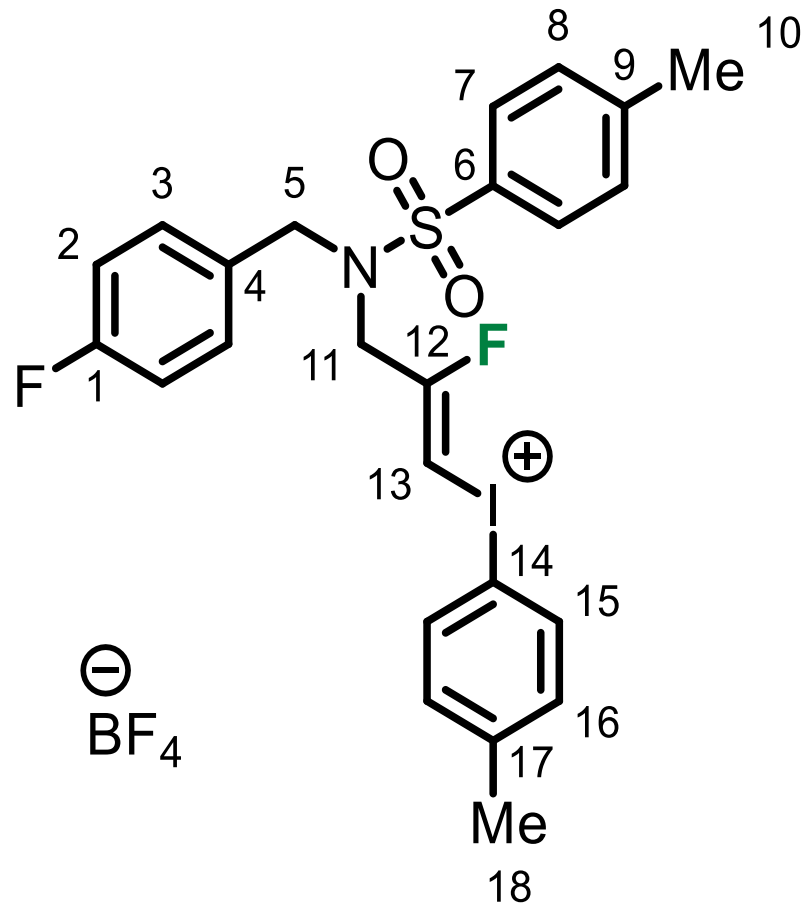

Produced according to **General procedure III** from alkyne **1e** to afford the title compound as a yellow solid (1.98 g, 82%).

^{1}H NMR (500 MHz, Acetone-*D*$_6$) δ 8.02 (app. dt, *J* = 8.5, 1.8 Hz, 2H, C15-H), 7.81 (app. dt, *J* = 8.4, 2.2 Hz, 2H, C7-H), 7.47 – 7.45 (m, 2H, C16-H), 7.45 – 7.43 (m, 2H, C8-H), 7.30 (ddd, *J* = 8.8, 5.3, 2.7 Hz, 2H, C3-H), 7.00 (dt, *J* = 33.5, 1.1 Hz, 1H, C13-H), 6.99 – 6.94 (m, 2H, C2-H), 4.36 (dd, *J* = 11.5, 1.1 Hz, 2H, C11-H), 4.34 (s, 2H, C5-H), 2.48 (s, 3H, C18-H), 2.46 (s, 3H, C10-H).

^{13}C NMR (126 MHz, Acetone-*D*$_6$) δ 168.4 (d, *J* = 279.0 Hz, C12), 163.3 (d, *J* = 244.8 Hz, C1), 145.2 (C17), 145.0 (C9), 136.7 (C6), 136.6 (C15), 133.9 (C8), 132.5 (d, *J* = 3.1 Hz, C4), 131.6 (d, *J* = 8.4 Hz, C3), 130.9 (C16), 128.4 (C7), 116.2 (d, *J* = 21.7 Hz, C2), 109.5 (C14), 80.4 (d, *J* = 19.1 Hz, C13), 52.7 (C5), 48.2 (d, *J* = 30.9 Hz, C11), 21.5 (C18), 21.4 (C10).

^{19}F NMR (376 MHz, Acetone-*D*$_6$) δ −75.42 (dt, *J* = 33.5, 11.5 Hz, C12-F), −115.04 (td, *J* = 8.8, 4.5 Hz, C1-F), -150.66 (BF_4).

HRMS (ESI+) calcd: $[M]^+$ ($C_{24}H_{23}F_2INO_2S$) 554.0457, measured 554.0431 (4.69 ppm error).

IR (neat) v_{max}/cm^{-1}: 3097, 1652, 1509, 1337, 1223, 1159, 1090, 802, 548, 521.

(Z)-(2-fluoro-3-((4-methyl-N-(4-(trifluoromethyl)benzyl)phenyl)sulfonamido)prop-1-en-1-yl)(p-tolyl)iodonium tetrafluoroborate **(3f)**

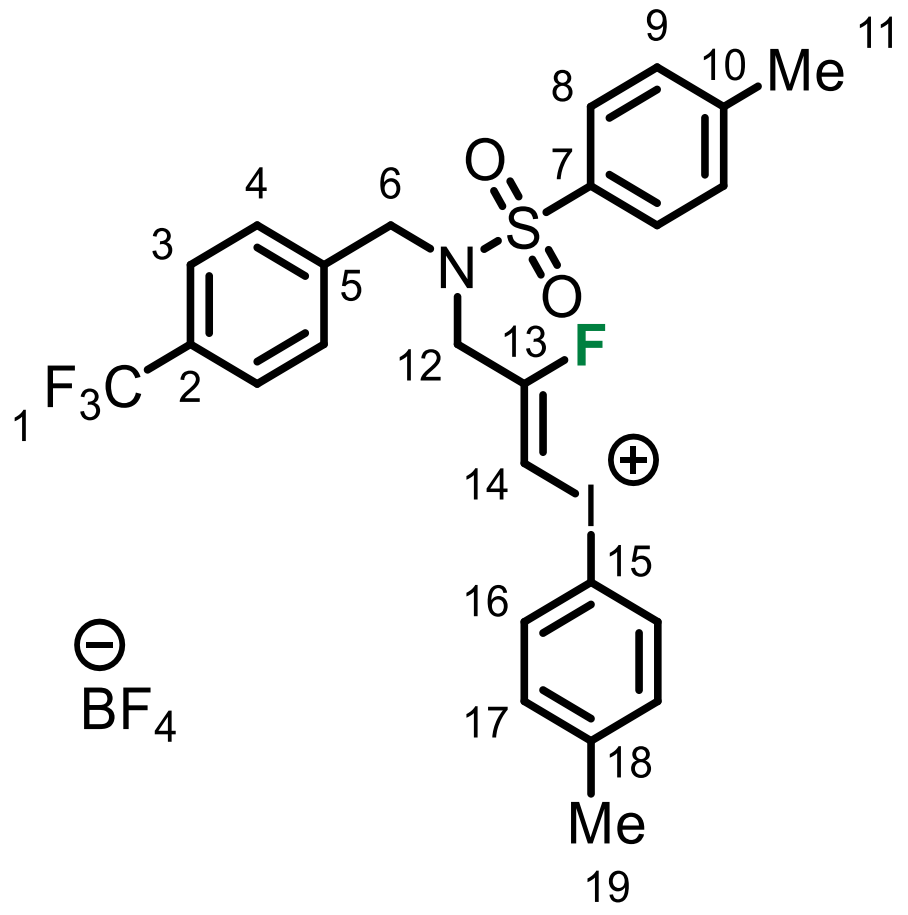

Produced according to **General procedure III** from alkyne **1f** to afford the title compound as a yellow solid (1.32 g, 68%).

^{1}H NMR (400 MHz, Acetone-D_6) δ 8.04 (d, J = 8.5 Hz, 2H, C16-H), 7.81 (app. dt, J = 8.4, 1.8 Hz, 2H, C8-H), 7.61 (d, J = 8.1 Hz, 2H, C3-H), 7.49 (d, J = 8.0 Hz, 2H, C9-H), 7.45 – 7.42 (m, 4H, C4-H, C17-H), 7.10 (d, J = 33.3 Hz, 1H, C14-H), 4.49 (s, 2H, C6-H), 4.43 (d, J = 13.1 Hz, 2H, C12-H), 2.46 (s, 3H, C19-H), 2.45 (s, 3H, C11-H).

^{13}C NMR (151 MHz, Acetone-D_6) δ 167.9 (d, J = 279.3 Hz, C13), 145.3 (C18), 144.9 (C5), 141.5 (C7), 136.7 (C10), 136.5 (C16), 133.9 (C4), 130.9 (C17), 130.3 (q, J = 31.8 Hz, C2), 129.9 (C9), 128.4 (C8), 126.3 (q, J = 3.9 Hz, C3), 125.3 (q, J = 271.7 Hz, C1), 109.5 (C15), 80.9 (d, J = 18.8 Hz, C14), 52.6 (C6), 48.5 (d, J = 29.2 Hz, C12), 21.5 (C19), 21.3 (C11).

^{19}F NMR (376 MHz, Acetone-D_6) δ −62.81 (d, J = 2.6 Hz, C1-F), −75.26 – −75.58 (m, C13-F), −150.78 – −151.07 (m, BF_4).

HRMS (ESI+) calcd: $[M]^+$ ($C_{25}H_{23}F_4INO_2S$) 604.0425, measured 604.0405 (3.31 ppm error).

IR (neat) v_{max}/cm^{-1}: 3622, 3099, 2927, 1739, 1653, 1325, 1160, 1115, 1090, 1066, 1018, 802.

***(Z)-(2-fluoro-3-((4-methyl-N-(4-nitrobenzyl)phenyl)sulfonamido)prop-1-en-1-yl)(p-tolyl)iodonium tetrafluoroborate* (3g)**

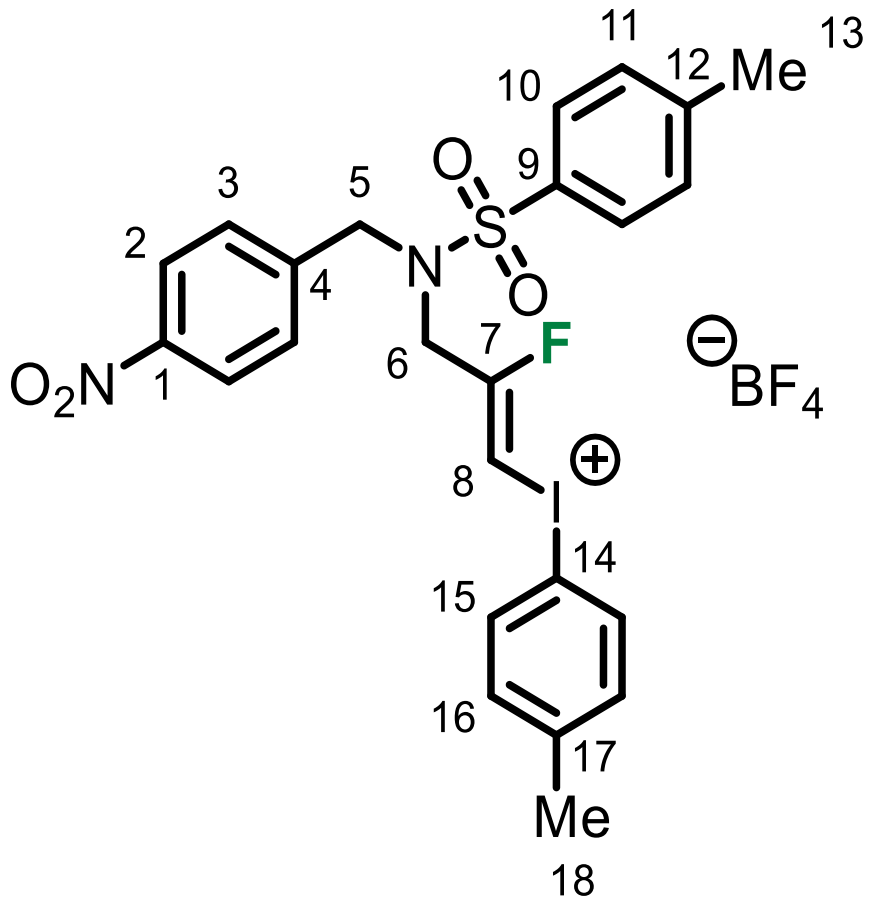

Prepared from alkyne **1g** via **General procedure III** to afford the title compound as a brown solid (0.841 g, 83%).

^{1}H NMR (400 MHz, Acetone-*D$_6$*) δ 8.08 (d, *J* = 8.5 Hz, 2H, C2-H), 7.98 (dd, *J* = 8.4, 1.6 Hz, 2H, C15-H), 7.82 (d, *J* = 8.1 Hz, 2H, C10-H), 7.57 (d, *J* = 8.5 Hz, 2H, C16-H), 7.45 (d, *J* = 8.0 Hz, 2H, C11-H), 7.41 – 7.34 (m, 2H, C3-H), 7.05 (d, *J* = 33.4 Hz, 1H, C8-H), 4.52 (s, 2H, C5-H), 4.47 (d, *J* = 13.2 Hz, 2H, C6-H), 2.44 (s, 6H, C18-H, C13-H).

^{13}C NMR (101 MHz, Acetone-*D$_6$*) δ 167.0 (d, *J* = 276.5 Hz, C7), 147.5 (C1), 144.6 (C17), 144.1 (C12), 144.0 (C4), 135.7 (C9), 135.6 (C15), 133.0 (C2), 130.2 (C16), 129.5 (C11), 127.6 (C10), 123.6 (C3), 108.5 (C14), 80.0 (d, *J* = 19.0 Hz, C8), 52.0 (C5), 48.3 (d, *J* = 29.5 Hz, C6), 20.7 (C18), 20.5 (C13).

^{19}F NMR (376 MHz, Acetone-*D$_6$*) δ −75.54 (dt, *J* = 32.8, 13.1 Hz, 1F, C7-F), −149.74 (s, 4F, BF_4).

HRMS (+ESI) $[M]^+$ calcd. ($C_{24}H_{23}FIN_2O_4S$) 581.0378, measured 581.0402 (4.13 ppm error).

IR (neat) v_{max}/cm^{-1}: 3094, 1642, 1597, 1521, 1483, 1455, 1404, 1345, 1328, 1309, 1242, 1205, 1186, 1156, 1056, 1017, 995.

***(Z)-(3-((N-benzyl-4-methylphenyl)sulfonamido)-2-fluoro-3-methylbut-1-en-1-yl)(p-tolyl)iodonium tetrafluoroborate* (3h)**

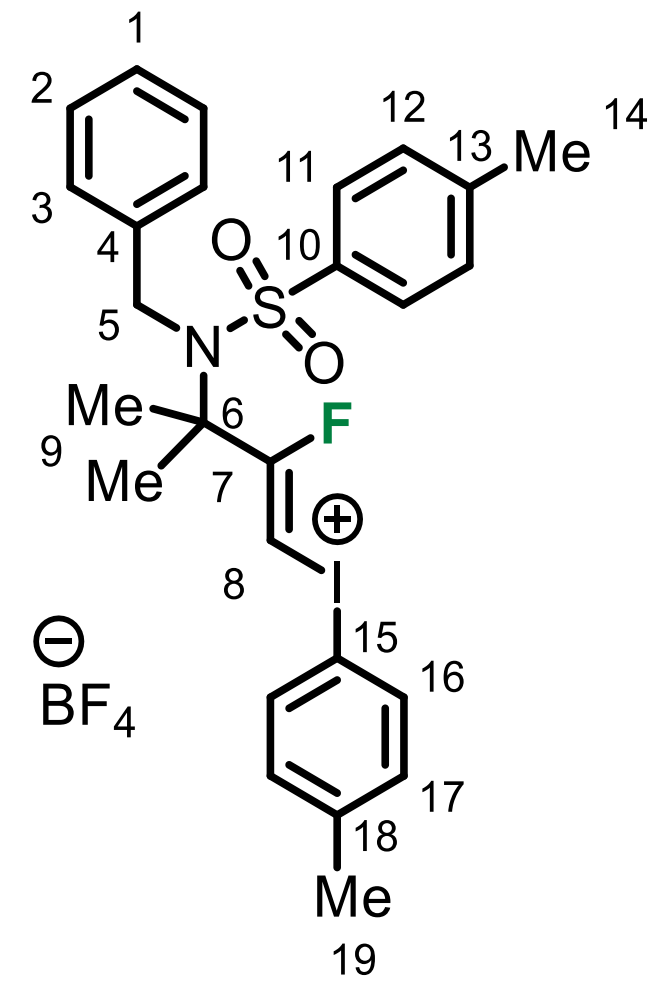

Produced according to **General procedure III** from alkyne **1h** to afford the title compound as a white solid (1.15 g, 78%).

^{1}H NMR (500 MHz, Acetone-*D$_6$*) δ 8.13 (app. dt, *J* = 8.4, 2.0 Hz, 2H, C16-H), 7.72 (app. dt, *J* = 8.2, 1.7 Hz, 2H, C11-H), 7.45 (d, *J* = 8.4 Hz, 2H, C17-H), 7.40 (dt, *J* = 8.2, 1.7 Hz, 2H, C12-H), 7.32 – 7.17 (m, 6H, C1-H, C2-H, C3-H, C8-H), 4.65 (s, 2H, C5-H), 2.43 (s, 3H, C19-H), 2.42 (s, 3H, C14-H), 1.50 (s, 6H, C9-H).

^{13}C NMR (126 MHz, Acetone-*D$_6$*) δ 174.9 (d, *J* = 280.8 Hz, C7), 145.0 (C18), 144.7 (C13), 140.1 (C4), 139.1 (C10), 136.5 (C16), 133.8 (C17), 130.7 (C12), 129.2 (C2), 128.6 (C3), 127.9 (C1), 127.6 (C11), 109.3 (C15), 78.3 (d, *J* = 20.1 Hz, C8), 63.4 (d, *J* = 21.9 Hz, C6), 50.0 (C5), 26.2 (C9), 21.4 (C19), 21.3 (C14).

^{19}F NMR (471 MHz, Acetone) δ −75.07 (ddd, *J* = 34.6, 17.1, 7.0 Hz, 1F, C7-F), −150.19 – −150.74 (m, 4F, BF_4).

HRMS (+ESI) calcd: $[M]^+$ ($C_{26}H_{28}FINO_2S$) 564.0864, measured 564.0843 (3.37 ppm error).

IR (neat) v_{max}/cm^{-1}: 3119, 1739, 1636, 1476, 1334, 1150, 1058, 996, 865, 803, 729, 675, 544.

***(Z)-(2-fluoro-3-((N-(4-fluorobenzyl)-4-nitrophenyl)sulfonamido)prop-1-en-1-yl)(p-tolyl)iodonium tetrafluoroborate* (3i)**

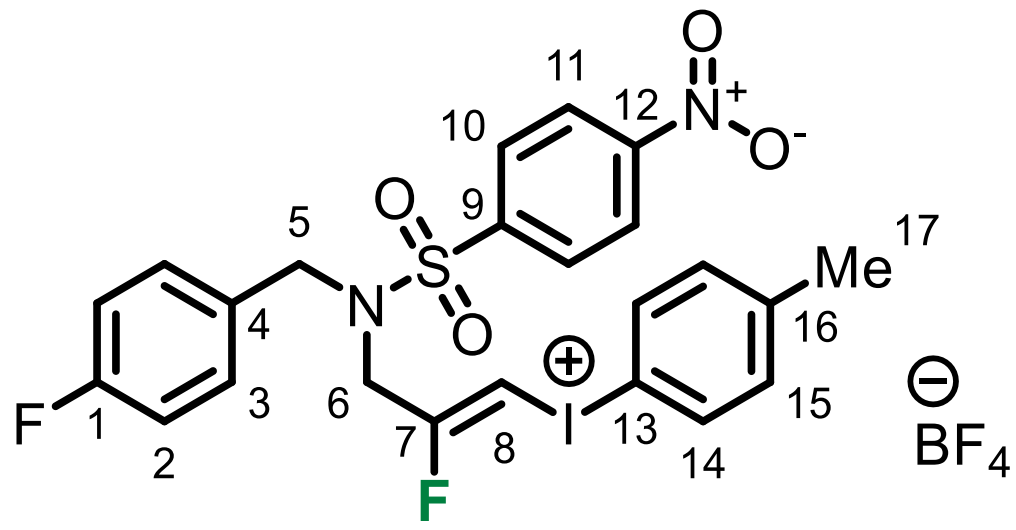

Produced according to **General procedure III** from alkyne **1i** to afford the title compound as an orange gel (4.74 g, 75%).

^{1}H NMR (500 MHz, Acetone-*D*$_6$) δ 8.43 (app. dt, *J* = 8.9, 2.5 Hz, 2H, C11-H), 8.21 (app. dt, *J* = 8.9, 2.0 Hz, 2H, C10-H), 8.03 (app. dt, *J* = 8.6, 1.6 Hz, 2H, C14-H), 7.46 – 7.41 (m, 2H, C15-H), 7.35 – 7.30 (m, 2H, C3-H), 7.06 (d, *J* = 33.7 Hz, 1H, C8-H), 7.01 (tt, *J* = 8.8, 2.3 Hz, 2H, C2-H), 4.51 (d, *J* = 12.8 Hz, 2H, C6-H), 4.48 (s, 2H, C5-H), 2.47 (s, 3H, C17-H).

^{13}C NMR (126 MHz, Acetone-*D*$_6$) δ 167.4 (d, *J* = 278.7 Hz, C7), 163.4 (d, *J* = 245.1 Hz, C1), 151.4 (C12), 145.4 (C9), 145.0 (C16), 136.6 (C14), 133.9 (C15), 132.0 (d, *J* = 3.2 Hz, C5), 131.7 (d, *J* = 8.4 Hz, C3), 129.8 (C10), 125.5 (C11), 116.3 (d, *J* = 21.7 Hz, C2), 109.5 (C13), 81.0 (d, *J* = 18.9 Hz, C8), 52.4 (C5), 48.0 (d, *J* = 29.9 Hz, C6), 21.3 (C17).

^{19}F NMR (376 MHz, Acetone-*D*$_6$) δ −75.85 (dt, *J* = 33.5, 12.7 Hz, 1F, C7-F), −114.84 (qd, *J* = 7.0, 4.3 Hz, 1F, C1-F), −149.97 – −150.27 (m, 4F, BF_4).

HRMS (+ESI) calcd: $[M]^+$ ($C_{23}H_{20}F_2IN_2O_4S$) 585.0151, measured 585.0151 (0.00 ppm error).

IR (neat) v_{max}/cm^{-1}: 3558, 3105, 2970, 1737, 1530, 1509, 1350, 1225, 1218, 1164, 1089, 1060, 1011, 782.

***(Z)-(2-fluoro-3-((N-(4-fluorobenzyl)-4-methylphenyl)sulfonamido)but-1-en-1-yl)(p-tolyl)iodonium tetrafluoroborate* (3j)**

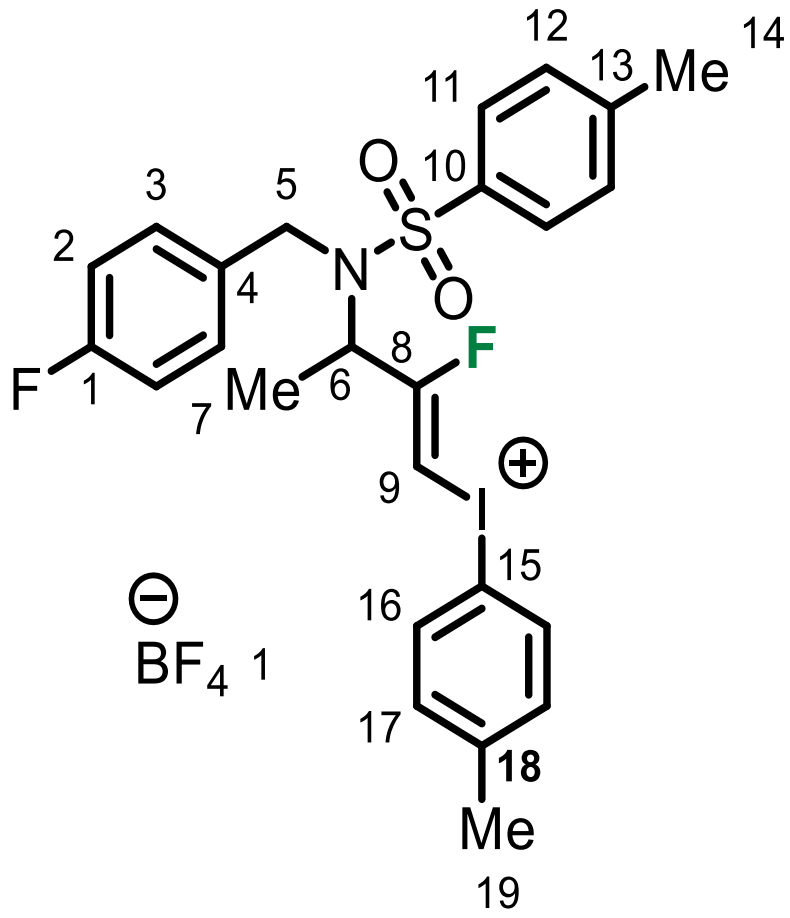

Produced according to **General procedure III** from alkyne **1j** to afford the title compound as a white solid (1.13 g, 87%).

^{1}H NMR (400 MHz, Acetone-*D*$_6$) δ 8.02 (app. dt, *J* = 8.7, 2.0 Hz, 2H, C16-H), 7.75 (app. dt, *J* = 8.4, 1.4 Hz, 2H, C11-H), 7.43 (app. t, *J* = 8.9 Hz, 4H, C12-H, C17-H), 7.34 – 7.27 (m, 2H, C3-H), 7.10 (dd, *J* = 34.2, 1.2 Hz, 1H, C9-H), 7.00 (tt, *J* = 8.8, 2.2 Hz, 2H, C2-H), 5.15 (dq, *J* = 7.2, 7.2 Hz, 1H, C6-H), 4.42 (d, *J* = 16.0 Hz, 1H, C5-H), 4.30 (d, *J* = 16.0 Hz, 1H, C5-H), 2.46 (s, 3H, C19-H), 2.45 (s, 3H, C14-H), 1.28 (d, *J* = 7.0 Hz, 3H, C7-H).

^{13}C NMR (126 MHz, Acetone-*D*$_6$) δ 170.2 (d, *J* = 279.6 Hz, C8), 163.1 (d, *J* = 244.3 Hz, C1), 145.0 (C18), 144.9 (C13), 138.3 (C10), 136.7 (C16), 134.2 (d, *J* = 3.0 Hz, C4), 133.9 (C17), 131.1 (d, *J* = 8.2 Hz, C3), 130.7 (C12), 128.1 (C11), 115.9 (d, *J* = 21.7 Hz, C2), 109.3 (C15), 81.4 (d, *J* = 20.1 Hz, C9), 54.5 (d, *J* = 24.6 Hz, C6), 48.3 (C5), 21.5 (C19), 21.3 (C14), 16.0 (d, *J* = 4.7 Hz, C7).

^{19}F NMR (376 MHz, Acetone-*D*$_6$) δ −72.55 (dd, *J* = 34.3, 7.8 Hz, C8-F), −115.85 (tt, *J* = 9.1, 5.5 Hz, C1-F), -150.86 (ddt, *J* = 20.8, 10.6, 5.7 Hz, BF_4).

HRMS (ESI+) calcd: $[M]^+$ ($C_{25}H_{25}F_2INO_2S$) 568.0613, measured 568.0602 (1.94 ppm error).

IR (neat) v_{max}/cm^{-1}: 3547, 3107, 2969, 1738, 1509, 1339, 1223, 1159, 1088, 1057, 995, 840, 666, 550, 478.

(*R,Z*)-(2-fluoro-3-((N-(4-fluorobenzyl)-4-methylphenyl)sulfonamido)but-1-en-1-yl)(p-tolyl)iodonium tetrafluoroborate ((*R*)-3j)

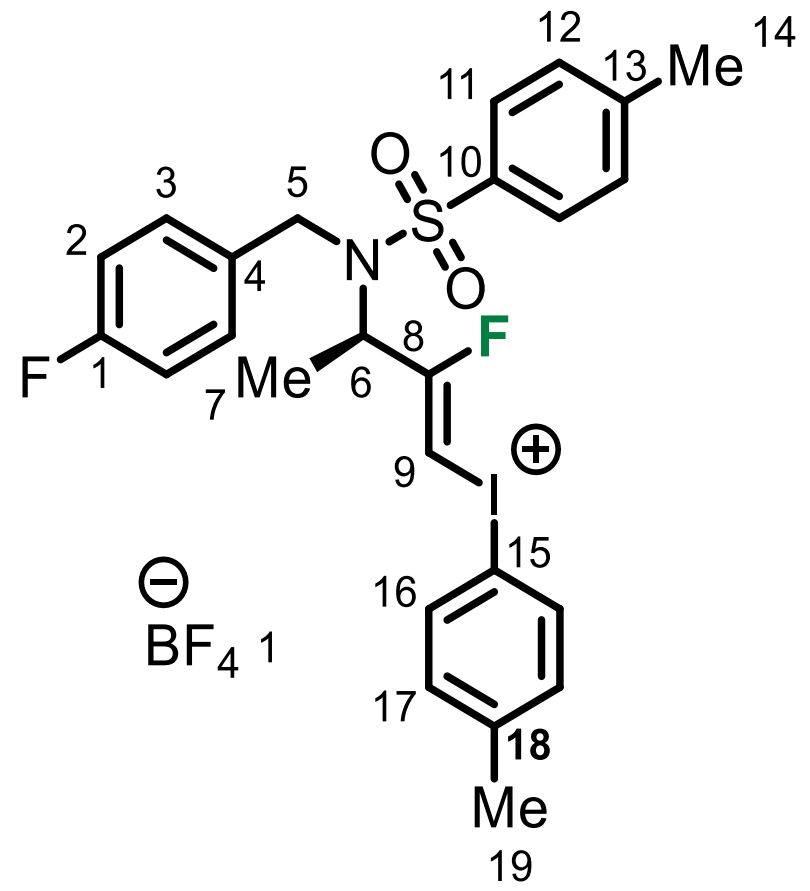

Produced according to **General procedure III** from alkyne **(*R*)-1j** to afford the title compound as a white solid (1.13 g, 87%).

^{1}H NMR (400 MHz, Acetone-*D*$_6$) δ 8.02 (dt, *J* = 8.7, 2.0 Hz, 2H, C16-H), 7.75 (dt, *J* = 8.4, 1.4 Hz, 2H, C11-H), 7.43 (apparent t, *J* = 8.9 Hz, 4H, C12-H, C17-H), 7.34 – 7.27 (m, 2H, C3-H), 7.10 (dd, *J* = 34.2, 1.2 Hz, 1H, C9-H), 7.00 (tt, *J* = 8.8, 2.2 Hz, 2H, C2-H), 5.15 (dq, *J* = 7.2, 7.2 Hz, 1H, C6-H), 4.42 (d, *J* = 16.0 Hz, 1H, C5-H), 4.30 (d, *J* = 16.0 Hz, 1H, C5-H), 2.46 (s, 3H, C19-H), 2.45 (s, 3H, C14-H), 1.28 (d, *J* = 7.0 Hz, 3H, C7-H).

^{13}C NMR (126 MHz, Acetone-*D*$_6$) δ 170.2 (d, *J* = 279.6 Hz, C8), 163.1 (d, *J* = 244.3 Hz, C1), 145.0 (C18), 144.9 (C13), 138.3 (C10), 136.7 (C16), 134.2 (d, *J* = 3.0 Hz, C4), 133.9 (C17), 131.1 (d, *J* = 8.2 Hz, C3), 130.7 (C12), 128.1 (C11), 115.9 (d, *J* = 21.7 Hz, C2), 109.3 (C15), 81.4 (d, *J* = 20.1 Hz, C9), 54.5 (d, *J* = 24.6 Hz, C6), 48.3 (C5), 21.5 (C19), 21.3 (C14), 16.0 (d, *J* = 4.7 Hz, C7).

^{19}F NMR (376 MHz, Acetone-*D*$_6$) δ -72.55 (dd, *J* = 34.3, 7.8 Hz, C8-F), -115.85 (tt, *J* = 9.1, 5.5 Hz, C1-F), -150.86 (ddt, *J* = 20.8, 10.6, 5.7 Hz, BF_4).

HRMS (ESI+) calcd: $[M]^+$ ($C_{25}H_{25}F_2INO_2S$) 568.0613, measured 568.0602 (1.94 ppm error).

IR (neat) v_{max}/cm^{-1}: 3547, 3107, 2969, 1738, 1509, 1339, 1223, 1159, 1088, 1057, 995, 840, 666, 550, 478.

***(Z)-(2-fluoro-3-(N-(4-fluorobenzyl)methylsulfonamido)but-1-en-1-yl)(p-tolyl)iodonium tetrafluoroborate* (3k)**

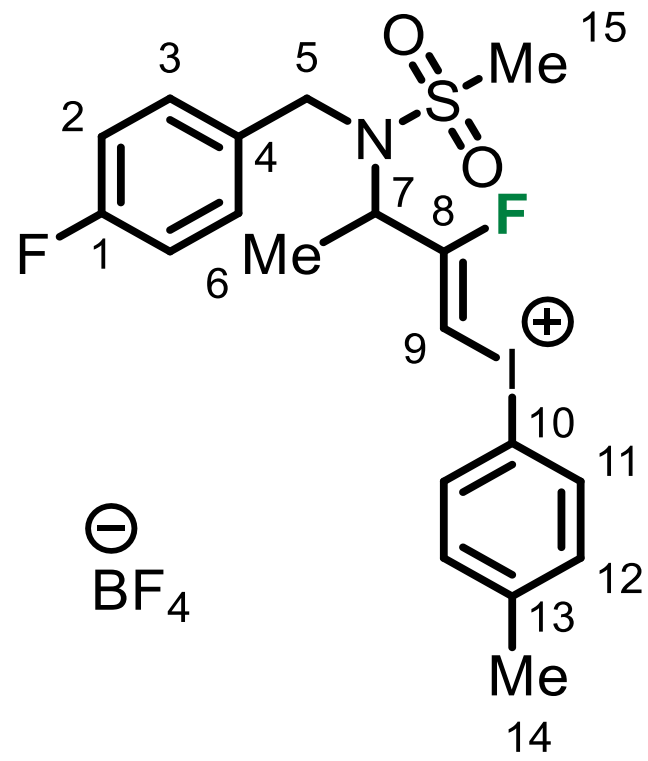

Produced according to **General procedure III** from alkyne **1k** to afford the title compound as a white solid (436 mg, 48%).

^{1}H NMR (500 MHz, Acetone-*D$_6$*) δ 8.09 (app. dt, *J* = 8.4, 1.8 Hz, 2H, C11-H), 7.45 (d, *J* = 8.4 Hz, 2H, C12-H), 7.43 – 7.36 (m, 2H, C3-H), 7.21 (dd, *J* = 34.6, 1.2 Hz, 1H, C9-H), 7.09 – 6.99 (m, 2H, C2-H), 5.09 – 5.00 (m, 1H, C7-H), 4.52 (d, *J* = 16.1 Hz, 1H, C5-H), 4.31 (d, *J* = 16.1 Hz, 1H, C5-H), 2.97 (s, 3H, C15-H), 2.46 (s, 3H, C14-H), 1.42 (d, *J* = 7.1 Hz, 3H, C6-H).

^{13}C NMR (126 MHz, Acetone-*D$_6$*) δ 170.5 (d, *J* = 278.8 Hz, C8), 163.1 (d, *J* = 244.1 Hz, C1), 145.0 (C13), 136.7 (C11), 134.6 (d, *J* = 3.2 Hz, C4), 133.9 (C12), 131.1 (d, *J* = 8.3 Hz, C3), 116.0 (d, *J* = 21.7 Hz, C2), 109.5 (C10), 81.5 (d, *J* = 20.1 Hz, C9), 54.6 (d, *J* = 24.9 Hz, C7), 48.5 (C15), 40.2 (d, *J* = 2.1 Hz, C5), 21.3 (C14), 16.7 (d, *J* = 4.4 Hz, C6).

^{19}F NMR (471 MHz, Acetone-*D$_6$*) δ −73.25 (dd, *J* = 34.6, 7.9 Hz, 1F, C8-F), −116.12 (ddd, *J* = 14.4, 9.1, 5.4 Hz, 1F, C1-F), −150.36 (d, *J* = 25.0 Hz, 4F, BF_4).

HRMS (+ESI) calcd: $[M]^+$ ($C_{19}H_{21}FINO_2S$) 492.0300, measured 492.0293 (1.42 ppm error).

IR (neat) v_{max}/cm^{-1}: 3106, 2938, 1730, 1644, 1604, 1510, 1330, 1222, 1147, 1058, 996, 966, 840, 521.

(Z)-(3-((N-(4-bromobenzyl)-4-methylphenyl)sulfonamido)-2-fluorobut-1-en-1-yl)(p-tolyl)iodonium tetrafluoroborate **(3l)**

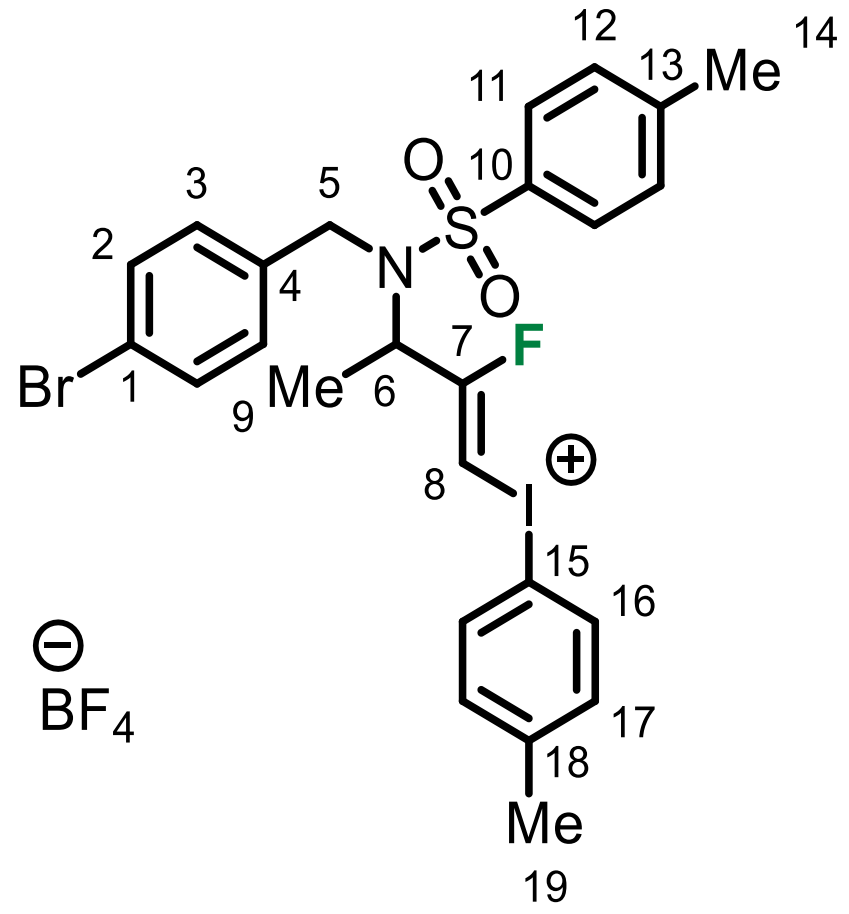

Produced according to **General procedure III** from alkyne **1l** to afford the title compound as a white solid (1.46 g, 72%).

^{1}H NMR (500 MHz, Acetone-*D$_6$*) δ 8.04 (app. dt, *J* = 8.5, 2.8 Hz, 2H, C16), 7.75 (app. dt, *J* = 8.4, 1.8 Hz, 2H, C11-H), 7.47 (d, *J* = 8.3 Hz, 2H, C17-H), 7.45 – 7.42 (m, 2H, C12-H), 7.38 (dt, *J* = 8.6, 1.7 Hz, 2H, C2-H), 7.28 – 7.21 (m, 2H, C3-H), 7.15 (dt, *J* = 34.3, 1.0 Hz, 1H, C8-H), 5.23 – 5.13 (m, 1H, C6-H), 4.44 (d, *J* = 16.3 Hz, 1H, C5-H), 4.31 (d, *J* = 16.3 Hz, 1H, C5-H), 2.49 (s, 3H, C19-H), 2.47 (s, 3H, C19-H), 1.30 (d, *J* = 7.0 Hz, 3H, C9-H).

^{13}C NMR (151 MHz, Acetone-*D$_6$*) δ 170.2 (d, *J* = 279.7 Hz, C7), 145.1 (C18), 145.0 (C13), 138.1 (C10), 137.7 (C4), 136.7 (C16), 134.0 (C17), 132.2 (C2), 132.1 (C3), 130.8 (C12), 128.2 (C11), 121.9 (C2), 109.3 (C15), 81.4 (d, *J* = 20.3 Hz, C8), 54.6 (d, *J* = 24.8 Hz, C6), 48.4 (C5), 21.5 (C19), 21.4 (C14), 16.0 (d, *J* = 4.7 Hz, C9).

^{19}F NMR (471 MHz, Acetone-*D$_6$*) δ −72.8 (dd, *J* = 34.2, 8.3 Hz, 1F, C7-F), −150.8 – −151.0 (m, 4F, BF_4).

HRMS (ESI+) calcd: [M+] ($C_{25}H_{25}BrFINO_2S$) 627.9813, measured 627.9788 (3.98 ppm error).

IR (neat) v_{max}/cm^{-1}: 2970, 1738, 1487, 1365, 1228, 1216, 1157, 1089, 1070, 1011, 547.

***(Z)*-(2-fluoro-3-((4-methyl-N-(naphthalen-2-ylmethyl)phenyl)sulfonamido)but-1-en-1-yl)(p-tolyl)iodonium tetrafluoroborate* (3m)**

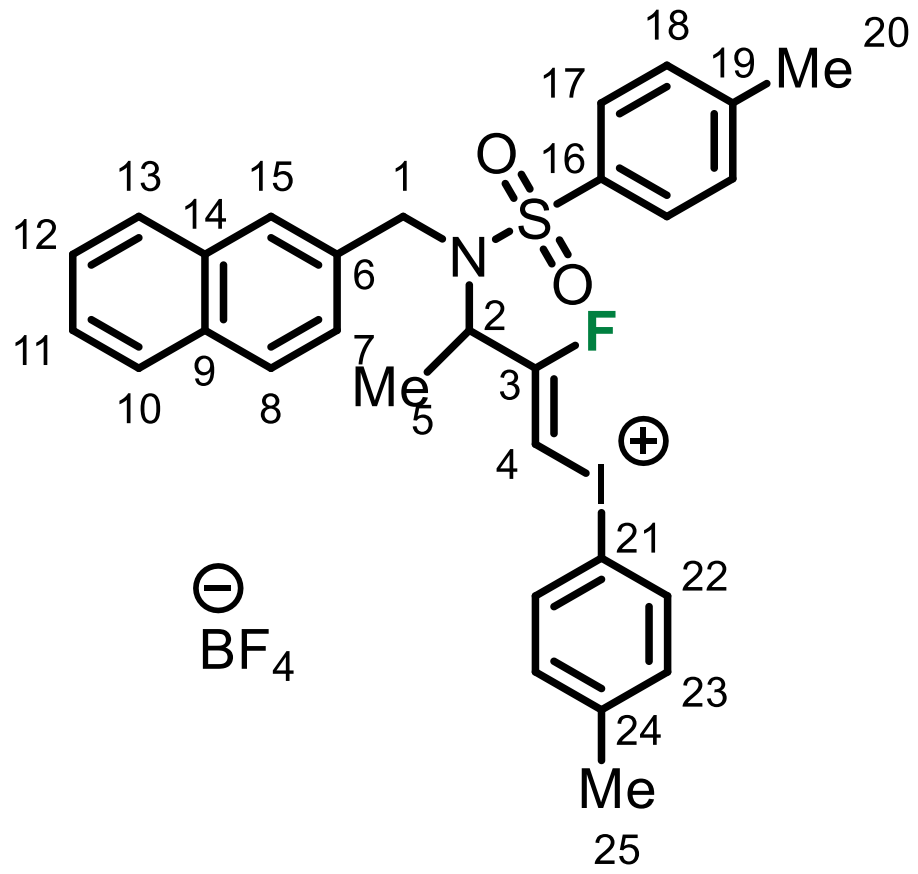

Produced according to **General procedure III** from alkyne **1m** to afford the title compound as a white solid (1.39 g, 69%).

^{1}H NMR (500 MHz, Acetone-*D_6*) δ 7.95 – 7.90 (m, 1H, C13-H), 7.93 – 7.87 (m, 2H, C22-H), 7.84 (d, *J* = 8.5 Hz, 1H, C8-H), 7.83 – 7.77 (m, 2H, C10-H, C17-H), 7.74 (d, *J* = 1.9 Hz, 1H, C15-H), 7.55 (dt, *J* = 6.2, 3.4 Hz, 2H, C11-H, C12-H), 7.48 (dd, *J* = 8.6, 1.9 Hz, 1H, C7-H), 7.43 (d, *J* = 8.2 Hz, 2H, C18-H), 7.34 (d, *J* = 8.3 Hz, 2H, C23-H), 7.08 (dd, *J* = 34.3, 1.2 Hz, 1H, C4-H), 5.20 (dqd, *J* =7.3, 7.1, 1.3 Hz, 1H, C2-H), 4.63 (d, *J* = 16.0 Hz, 1H, C1-H), 4.52 (d, *J* = 15.9 Hz, 1H, C1-H), 2.45 (s, 3H, C25-H), 2.43 (s, 3H, C20-H), 1.33 (d, *J* = 7.1 Hz, 3H, C5-H).

^{13}C NMR (126 MHz, Acetone-*D_6*) δ 169.4 (d, *J* = 280.1 Hz, C3), 149.6 (C19), 144.0 (C24), 137.5 (C16), 135.6 (C22), 134.7 (C14), 133.2 (C9), 133.0 (C23), 131.4 (C6), 129.9 (C18), 128.1 (C8), 127.9 (C13), 127.7 (C10), 127.3 (C17), 127.2 (C15), 126.3 (C12), 126.2 (C11), 126.1 (C7), 108.4 (C21), 80.3 (d, *J* = 20.2 Hz, C4), 53.7 (d, *J* = 24.7 Hz, C2), 48.5 (C1), 20.6 (C25), 20.4 (C20), 15.1 (d, *J* = 4.6 Hz, C5).

^{19}F NMR (471 MHz, Acetone-*D_6*) δ −72.28 (dd, *J* = 34.3, 7.3 Hz, 1F, C3-F), −150.53 – −150.80 (m, 4F, BF_4).

HRMS (+ESI) calcd: $[M]^+$ ($C_{29}H_{28}FINO_2S$) 600.0864, measured 600.0849 (3.36 ppm error).

IR (neat) v_{max}/cm^{-1}: 3100, 2040, 1736, 1340, 1157, 1068, 817, 665, 549, 477.

(Z)-(2-fluoro-3-((N-(4-fluorobenzyl)-4-methylphenyl)sulfonamido)-3-phenylprop-1-en-1-yl)(p-tolyl)iodonium tetrafluoroborate **(3n)**

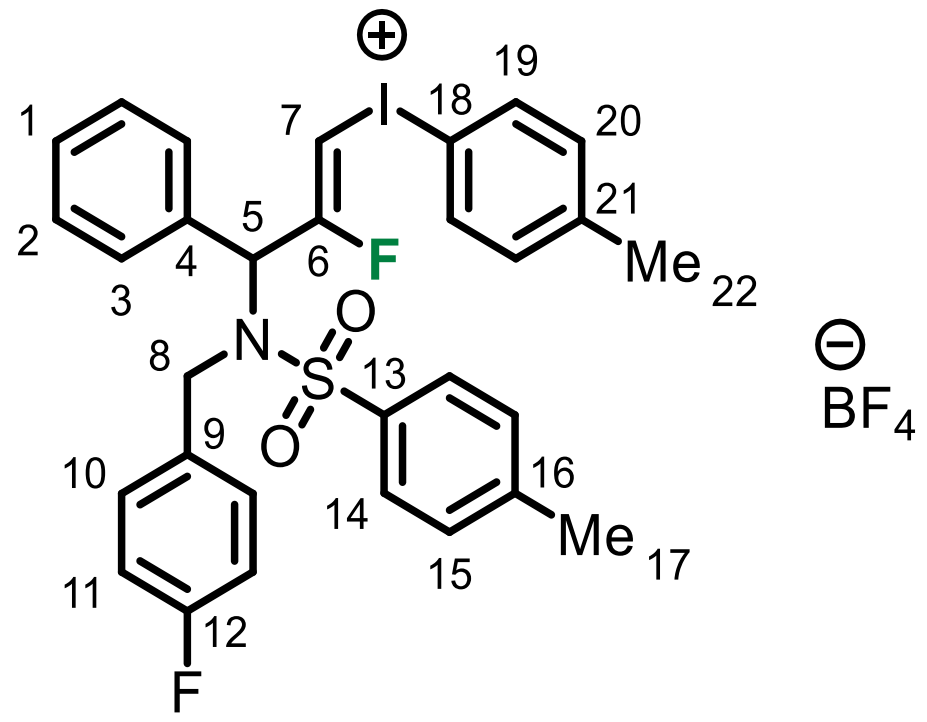

Produced according to **General procedure III** from alkyne **1n** to afford the title compound as a white solid (498 mg, 69%).

^{1}H NMR (500 MHz, Acetone-*D_6*) δ 8.08 (app. dt, *J* = 8.4, 1.6 Hz, 2H, C19-H), 7.66 (app. dt, *J* = 8.2, 1.5 Hz, 2H, C14-H), 7.48 (d, *J* = 8.4 Hz, 2H, C20-H), 7.36 (t, *J* = 7.3 Hz, 1H, C1-H), 7.34 (d, *J* = 8.2 Hz, 2H, C15-H), 7.29 (dd, *J* = 8.5, 5.0 Hz, 2H, C3-H), 7.19 (d, *J* = 33.7 Hz, 1H, C7-H), 7.07 – 7.01 (m, 2H, C2-H), 7.01 – 6.96 (m, 2H, C10-H), 6.85 (dt, *J* = 8.8, 2.4 Hz, 2H, C11-H), 6.22 (d, *J* = 9.2 Hz, 1H, C5-H), 4.50 (d, *J* = 16.0 Hz, 1H, C8-H), 4.21 (dd, *J* = 15.9, 4.8 Hz, 1H, C8-H), 2.50 (s, 3H, C22-H), 2.43 (s, 3H, C17-H).

^{13}C NMR (126 MHz, Acetone-*D_6*) δ 169.5 (d, *J* = 281.1 Hz, C6), 162.9 (d, *J* = 244.4 Hz, C12), 145.2 (C16), 145.0 (C21), 137.8 (C13), 136.6 (C19), 133.9 (C20), 133.6 (d, *J* = 1.9 Hz, C4), 133.5 (d, *J* = 2.0 Hz, C9), 131.2 (d, *J* = 8.3 Hz, C10), 130.7 (C15), 130.3 (C1), 130.1 (C3), 129.8 (C2), 128.2 (C14), 115.6 (d, *J* = 21.7 Hz, C11), 109.8 (C18), 83.2 (d, *J* = 19.7 Hz, C7), 63.0 (d, *J* = 29.4 Hz, C5), 49.9 (C8), 21.5 (C22), 21.4 (C17).

^{19}F NMR (471 MHz, Acetone-*D_6*) δ −73.47 (ddt, *J* = 33.7, 9.2, 5.0 Hz, 1F, C6-F), −116.07 (dtd, *J* = 14.2, 8.8, 5.1 Hz, 1F, C12-F), −150.23 – −150.84 (m, 4F, BF_4).

HRMS (+ESI) calcd: $[M]^+$ ($C_{30}H_{27}F_2INO_2S$) 630.0770, measured 630.0743 (4.29 ppm error).

IR (neat) v_{max}/cm^{-1}: 2970, 1739, 1507, 1365, 1217, 1157, 1089, 544.

***(Z)-(3-((N-benzhydryl-4-methylphenyl)sulfonamido)-2-fluoroprop-1-en-1-yl)(p-tolyl)iodonium tetrafluoroborate* (3o)**

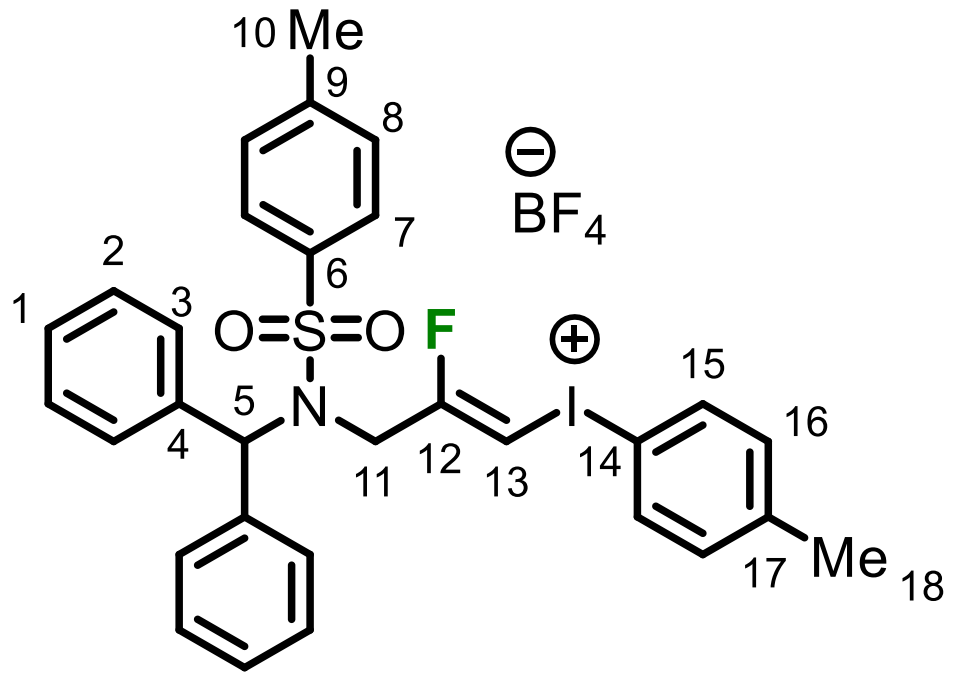

Prepared from **ATS007** according to **General procedure III** using 20 mol% Ag_2CO_3, affording the title compound without further purification as a white solid (1.75 g, 50% yield).

^{1}H NMR (500 MHz, Acetone-*D$_6$*) δ 7.99 – 7.94 (2H, m, C15-H), 7.71 – 7.65 (2H, m, C7-H), 7.46 (2H, dd, *J* = 8.8, 0.7 Hz, C16-H), 7.33 – 7.25 (2H, m, C8-H), 7.20 – 7.12 (6H, m, C1-H, C2-H), 7.01 (4H, dd, *J* = 7.2, 1.7 Hz, C3-H), 6.57 (1H, dt, *J* = 33.4, 1.1 Hz, C13), 6.40 (1H, s, C13-H), 4.57 (2H, d, *J* = 9.6, 1.1 Hz, C11-H), 2.49 (3H, s, C18-H), 2.40 (3H, s, C10-H).

^{13}C NMR (126 MHz, Acetone-*D$_6$*) δ 169.2 (d, *J* = 279.1 Hz, C12), 145.1 (C9), 144.9 (C17), 139.0 (C4), 137.3 (C4), 136.7 (C15), 133.9 (C16), 130.6 (C8), 129.7 (C3), 129.2 (C2), 128.7 (C1), 128.5 (C7), 109.5 (C14), 78.8 (d, *J* = 19.7 Hz, C13), 66.1 (C5), 46.1 (d, *J* = 31.7 Hz, C11), 21.4 (C10), 21.4 (C18).

^{19}F NMR (376 MHz, Acetone-*D$_6$*) δ −74.05 (1F, dt, *J* = 33.6, 8.9 Hz, C12-F), −151.13 – −151.19 (m, 4F, BF_4).

HRMS (+EI) calcd: $[M]^+$ ($C_{30}H_{28}FINO_2S$) 612.0864, measured 612.0843 (3.10 ppm error).

IR (neat) ν_{max}/cm^{-1}: 3107, 2929, 1643, 1598, 1494, 1454, 1340, 1188, 1163, 1145, 1038, 995, 898, 974, 803, 747, 699, 676, 656, 603, 576, 543, 522, 507, 478.

***(Z)-(2-fluoro-3-((4-methyl-N-(1-phenylethyl)phenyl)sulfonamido)prop-1-en-1-yl)(p-tolyl)iodonium tetrafluoroborate* (3p)**

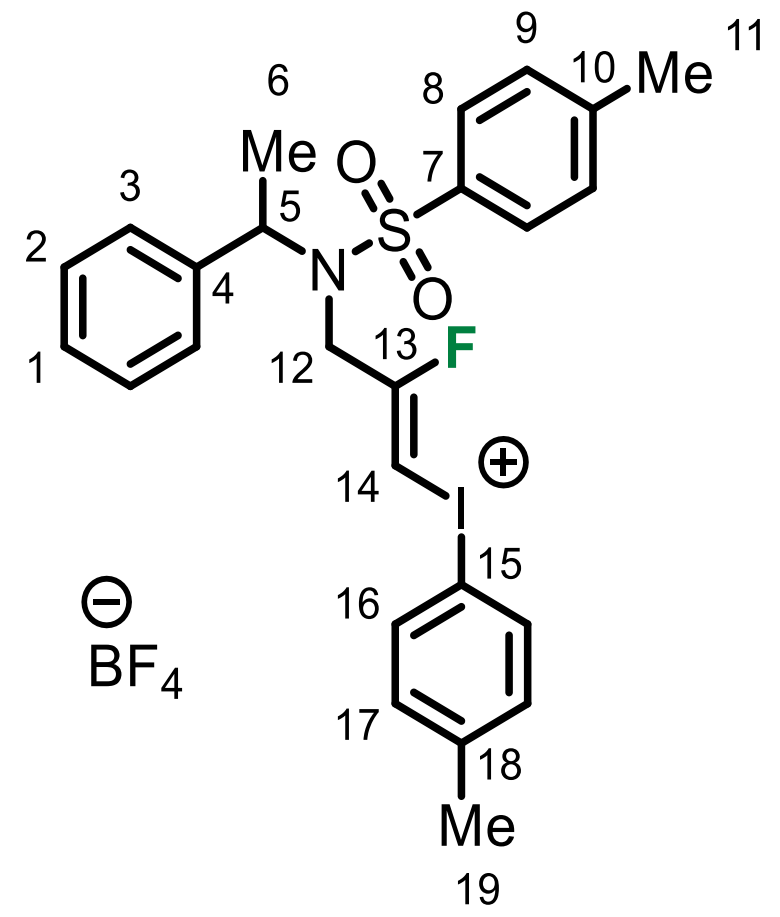

Produced according to **General procedure III** from alkyne **1p** to afford the title compound as a white solid (2.16 g, 85%).

^{1}H NMR (400 MHz, Acetone-*D*$_6$) δ 8.01 (app. dt, *J* = 8.4, 1.8 Hz, 2H, C16-H), 7.82 (app. dt, *J* = 8.4, 2.1 Hz, 2H, C8-H), 7.48 – 7.46 (m, 2H, C9-H), 7.46 – 7.42 (m, 2H, C2-H), 7.21 – 7.17 (m, 5H, C1-H, C3-H, C17-H), 6.77 (dt, *J* = 33.5, 1.1 Hz, 1H, C14-H), 5.18 (q, *J* = 7.1 Hz, 1H, C5-H), 4.39 (ddd, *J* = 18.0, 10.0, 1.1 Hz, 1H, C12-H), 4.24 (ddd, *J* = 18.0, 11.0, 1.1 Hz, 1H, C12-H), 2.48 (s, 3H, C19-H), 2.46 (s, 3H, C11-H), 1.30 (d, *J* = 7.1 Hz, 3H, C6-H).

^{13}C NMR (101 MHz, Acetone-*D*$_6$) δ 169.8 (d, *J* = 278.9 Hz, C13), 145.2 (C18), 144.9 (C10), 139.8 (C4), 138.0 (C7), 136.6 (C16), 133.8 (C9), 130.9 (C2), 129.3 (C17), 128.9 (C2), 128.5 (C3), 128.3 (C8), 109.5 (d, *J* = 0.9 Hz, C15), 79.0 (d, *J* = 19.7 Hz, C14), 56.9 (C5), 43.7 (d, *J* = 31.2 Hz, C12), 21.5 (C19), 21.3 (C11), 16.5 (C6).

^{19}F NMR (377 MHz, Acetone-*D*$_6$) δ −74.34 (dt, *J* = 33.5, 10.0 Hz, 1F, C13-F), −150.76 (d, *J* = 20.5 Hz, 4F, BF_4).

HRMS (ESI+) calcd: $[M]^+$ ($C_{25}H_{26}FINO_2S$) 550.0707, measured 550.0687 (3.64 ppm error).

IR (neat) v_{max}/cm^{-1}: 2970, 1738, 1648, 1452, 1365, 1216, 1154, 1089, 1054, 883, 664, 558.

(S,Z)-(2-fluoro-3-((4-methyl-N-(1-phenylethyl)phenyl)sulfonamido)prop-1-en-1-yl)(p-tolyl)iodonium tetrafluoroborate **((*S*)-3p)**

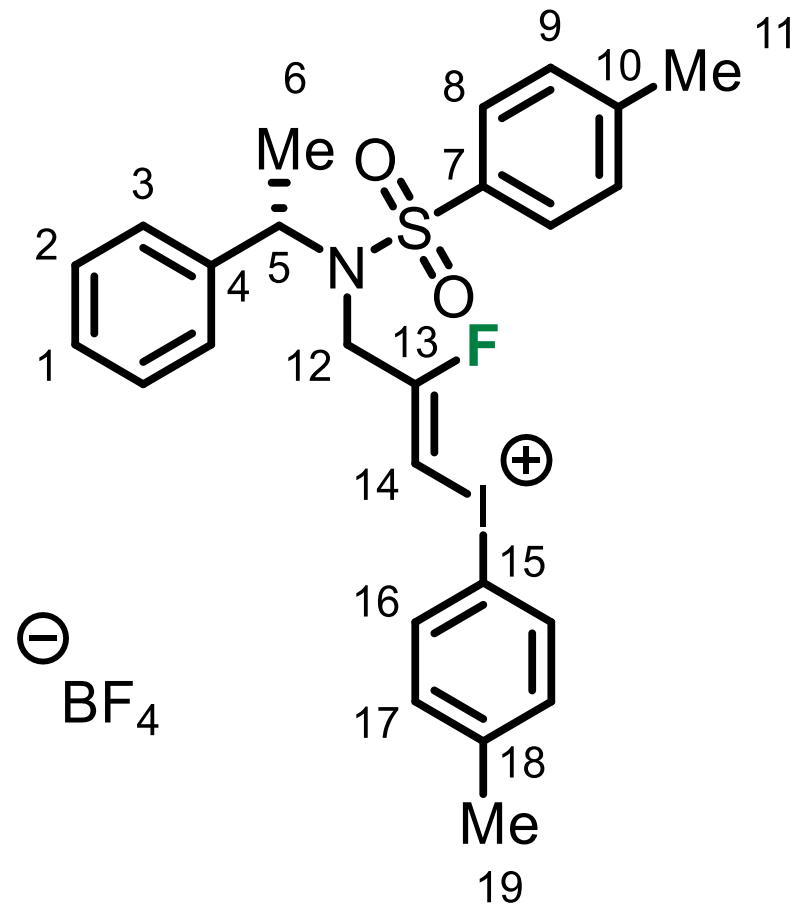

Produced according to **General procedure III** from alkyne **(*S*)-3p** to afford the title compound as a white solid (1.24 g, 65%).

^{1}H NMR (400 MHz, Acetone-*D*$_6$) δ 8.01 (app. dt, *J* = 8.4, 1.8 Hz, 2H, C16-H), 7.82 (app. dt, *J* = 8.4, 2.1 Hz, 2H, C8-H), 7.48 – 7.46 (m, 2H, C9-H), 7.46 – 7.42 (m, 2H, C2-H), 7.21 – 7.17 (m, 5H, C1-H, C3-H, C17-H), 6.77 (dt, *J* = 33.5, 1.1 Hz, 1H, C14-H), 5.18 (q, *J* = 7.1 Hz, 1H, C5-H), 4.39 (ddd, *J* = 18.0, 10.0, 1.1 Hz, 1H, C12-H), 4.24 (ddd, *J* = 18.0, 11.0, 1.1 Hz, 1H, C12-H), 2.48 (s, 3H, C19-H), 2.46 (s, 3H, C11-H), 1.30 (d, *J* = 7.1 Hz, 3H, C6-H).

^{13}C NMR (101 MHz, Acetone-*D*$_6$) δ 169.8 (d, *J* = 278.9 Hz, C13), 145.2 (C18), 144.9 (C10), 139.8 (C4), 138.0 (C7), 136.6 (C16), 133.9 (C9), 130.9 (C2), 129.3 (C17), 128.9 (C2), 128.5 (C3), 128.3 (C8), 109.5 (d, *J* = 0.9 Hz, C15), 79.0 (d, *J* = 19.7 Hz, C14), 56.9 (C5), 43.7 (d, *J* = 31.2 Hz, C12), 21.5 (C19), 21.3 (C11), 16.5 (C6).

^{19}F NMR (377 MHz, Acetone-*D*$_6$) δ −74.34 (dt, *J* = 33.5, 10.0 Hz, C13-F), −150.76 (d, *J* = 20.5 Hz, BF_4).

HRMS (ESI+) calcd: $[M]^+$ ($C_{25}H_{26}FINO_2S$) 550.0707, measured 550.0687 (3.64 ppm error).

IR (neat) v_{max}/cm^{-1}: 2970, 1738, 1648, 1452, 1365, 1216, 1154, 1089, 1054, 883, 664, 558.

***(Z)-(2-fluoro-3-((4-methyl-N-(1-tosylpiperidin-4-yl)phenyl)sulfonamido)prop-1-en-1-yl)(p-tolyl)iodonium tetrafluoroborate* (3r)**

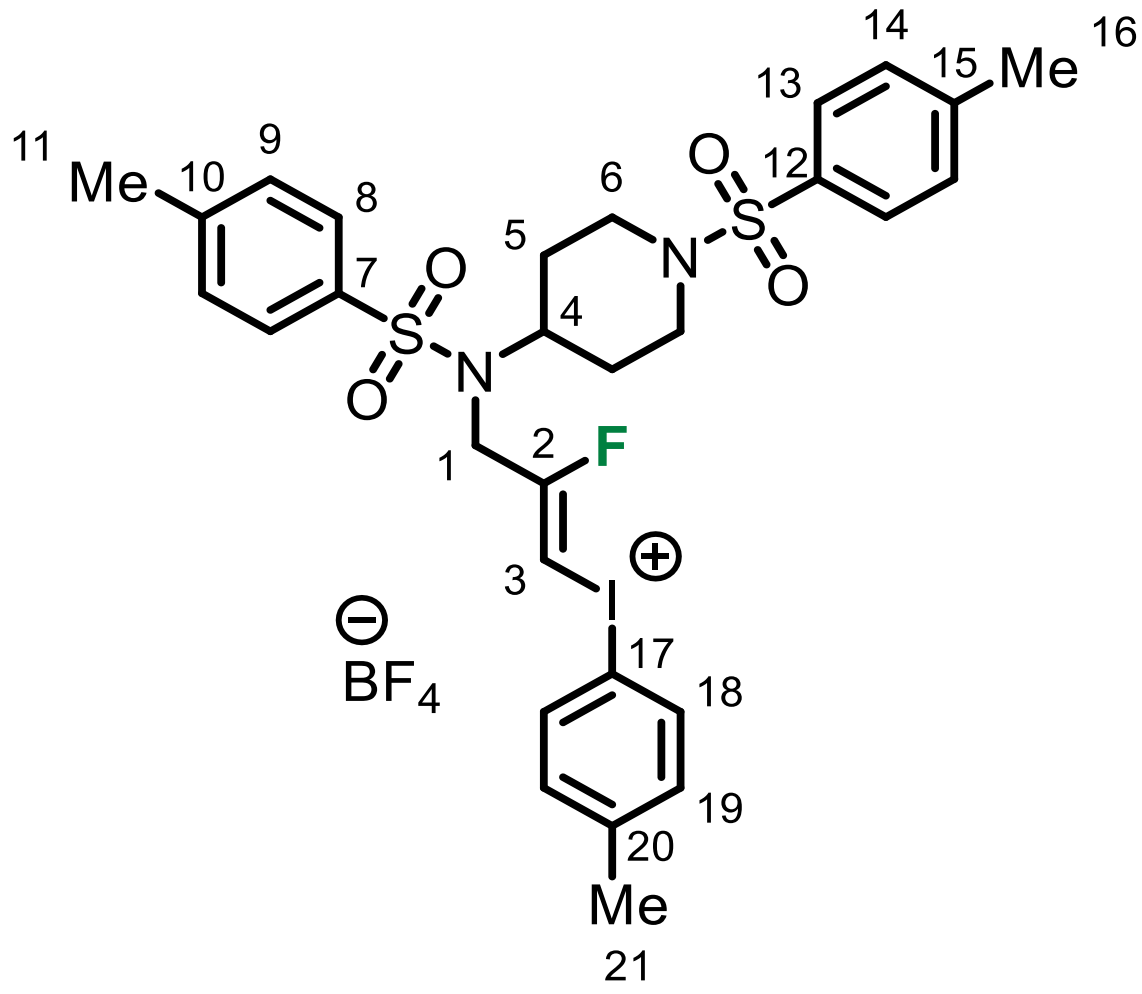

Produced according to **General procedure III** from alkyne **1r** to afford the title compound as a white solid (2.30 g, 82%).

^{1}H NMR (400 MHz, Acetone-*D_6*) δ 8.15 (d, *J* = 8.6 Hz, 2H, C18-H), 7.73 (d, *J* = 8.2 Hz, 2H, C8-H), 7.61 (d, *J* = 8.2 Hz, 2H, C13-H), 7.47 (d, *J* = 8.6 Hz, 2H, C19-H), 7.43 (d, *J* = 8.2 Hz, 2H, C9-H), 7.37 (d, *J* = 8.2 Hz, 2H, C14-H), 7.23 (d, *J* = 33.5 Hz, 1H, C3-H), 4.51 (d, *J* = 10.1 Hz, 2H, C1-H), 3.70 (tt, *J* = 12.1, 3.8 Hz, 1H, C4-H), 3.61 (dp, *J* = 12.1, 2.3 Hz, 2H, C6-H), 2.47 (s, 3H, C21-H), 2.43 (s, 3H, C11-H), 2.40 (s, 3H, C16-H), 2.23 (td, *J* = 12.1, 2.5 Hz, 2H, C6-H), 1.59 (qd, *J* = 12.3, 4.2 Hz, 2H, C5-H), 1.48 – 1.39 (m, 2H, C5-H).

^{13}C NMR (126 MHz, Acetone-*D_6*) δ 170.0 (d, *J* = 278.5 Hz, C2), 145.0 (C20), 144.9 (C10), 144.6 (C15), 138.1 (C7), 136.5 (C18), 134.2 (C12), 133.8 (C19), 130.9 (C9), 130.6 (C14), 128.5 (C8), 128.0 (C13), 109.8 (C17), 79.9 (d, *J* = 19.2 Hz, C3), 56.1 (C4), 46.5 (C6), 43.7 (d, *J* = 30.7 Hz, C1), 29.9 (C5), 21.4 (C11), 21.4 (C16), 21.3 (C21).

^{19}F NMR (376 MHz, Acetone-*D_6*) δ −75.23 (dt, *J* = 33.5, 10.2 Hz, 1F, C2-F), −150.13 – −150.36 (m, 4F, BF_4).

HRMS (+ESI) calcd: $[M]^+$ ($C_{29}H_{33}FIN_2O_4S_2$) 683.0905, measured 683.0882 (3.37 ppm error).

IR (neat) ν_{max}/cm^{-1}: 3096, 2930, 2853, 1695, 1653, 1334, 1155, 10889, 1056, 1016, 803, 727, 594, 548.

***(Z)-(3-((N-cyclohexyl-4-methylphenyl)sulfonamido)-2-fluoroprop-1-en-1-yl)(p-tolyl)iodonium tetrafluoroborate* (3s)**

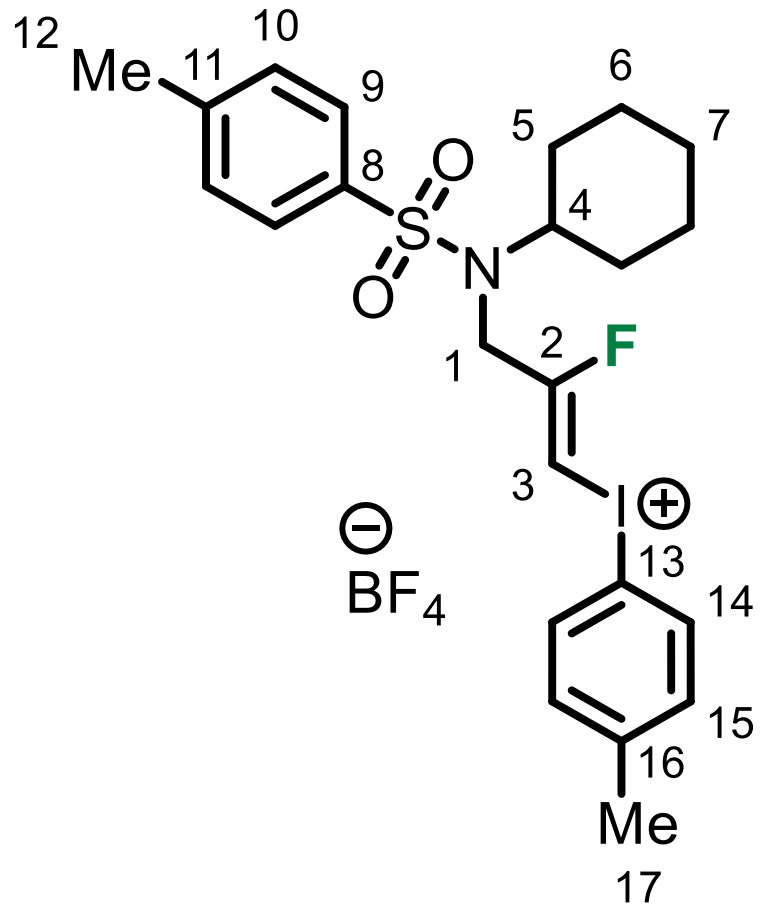

Produced according to **General procedure III** from alkyne **1s** to afford the title compound as a white solid (756 mg, 47%).

^{1}H NMR (500 MHz, Acetone-*D*$_6$) δ 8.14 (app. dt, *J* = 8.5, 1.8 Hz, 2H, C14-H), 7.75 (app. dt, *J* = 8.4, 1.8 Hz, 2H, C9-H), 7.45 (d, *J* = 8.5 Hz, 2H, C15-H), 7.41 (d, *J* = 8.4 Hz, 2H, C10-H), 7.20 (d, *J* = 33.5 Hz, 1H, C3-H), 4.47 (dd, *J* = 10.2, 1.1 Hz, 2H, C1-H), 3.62 (tt, *J* = 11.7, 3.9 Hz, 1H, C4), 2.45 (s, 3H, C17-H), 2.42 (s, 3H, C12-H), 1.59 (d, *J* = 11.7 Hz, 2H, C5-H), 1.48 (d, *J* = 14.1 Hz, 1H, C7-H), 1.40 – 1.13 (m, 6H, C5-H, C6-H), 0.92 (qt, *J* = 14.3, 4.7 Hz, 1H, C7-H).a

^{13}C NMR (126 MHz, Acetone-*D*$_6$) δ 170.5 (d, *J* = 278.9 Hz, C2), 144.8 (C16), 144.7 (C11), 138.5 (C8), 136.5 (C14), 133.7 (C15), 130.8 (C10), 127.9 (C9), 109.8 (C13), 79.7 (d, *J* = 19.2 Hz, C3), 58.9 (C4), 43.6 (d, *J* = 31.0 Hz, C1), 31.4 (C5), 26.5 (C6), 25.5 (C7), 21.4 (C17), 21.3 (C12).

^{19}F NMR (376 MHz, Acetone-*D*$_6$) δ −75.05 (dt, *J* = 33.9, 10.5 Hz, 1F, C2-F), −150.14 – −150.40 (m, 4F, BF_4).

HRMS (+ESI) calcd: $[M]^+$ ($C_{23}H_{28}FINO_2S$) 528.0864, measured 528.0855 (1.70 ppm error).

IR (neat) v_{max}/cm^{-1}: 3099, 2934, 2858, 1652, 1332, 1306, 1154, 1052, 1089, 805, 664, 584, 548.

***(Z)-(2-fluoro-3-((4-methyl-N-phenethylphenyl)sulfonamido)prop-1-en-1-yl)(p-tolyl)iodonium tetrafluoroborate* (3t)**

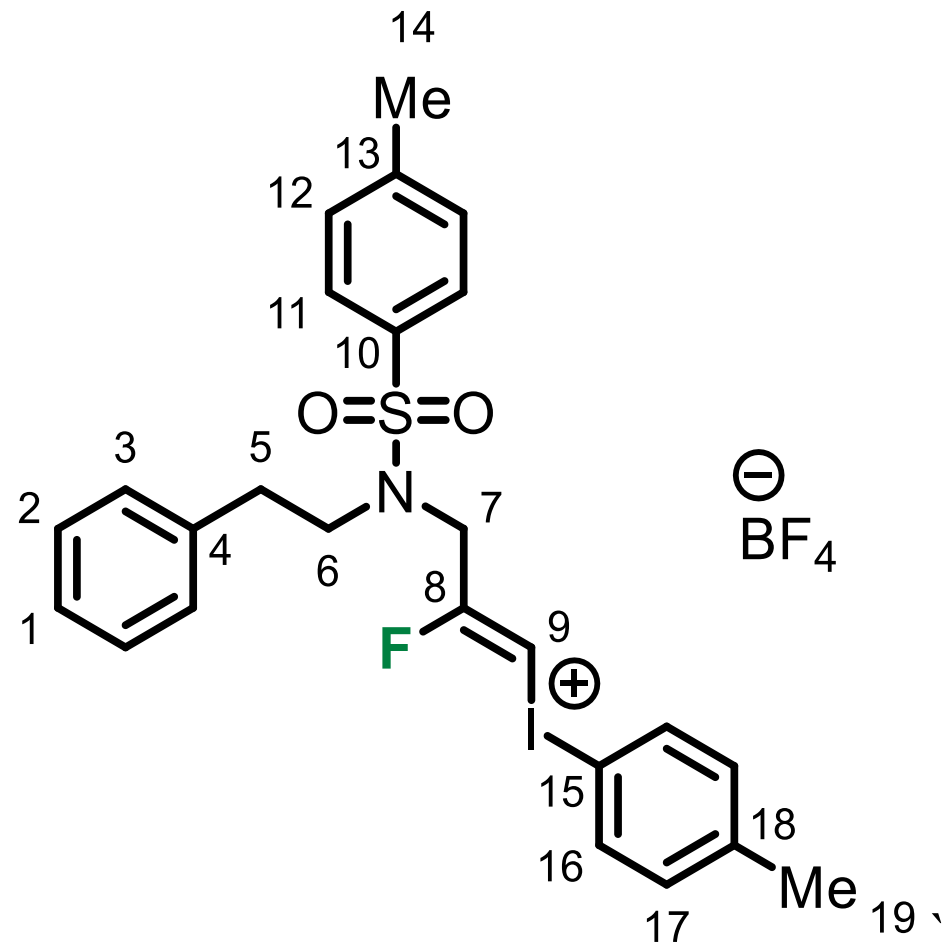

Produced according to **General procedure III** from alkyne **1t** to afford the title compound as a white solid (1.32 g, 69%).

^{1}H NMR (400 MHz, Acetone-*D*$_6$) δ 8.14 (d, *J* = 8.4 Hz, 2H, C16-H), 7.76 (d, *J* = 8.1 Hz, 2H, C11-H), 7.45 – 7.37 (m, 4H, C12-H, C17-H), 7.25 (dt, *J* = 27.3, 1.2 Hz, 1H, C9-H), 7.32 – 7.15 (m, 3H, C1-H, C2-H), 7.09 (d, *J* = 7.0 Hz, 2H, C3-H), 4.50 (d, *J* = 12.1 Hz, 2H, C7-H), 3.38 – 3.30 (m, 2H, C6-H), 2.77 – 2.69 (m, 2H, C5-H), 2.42 (s, 3H, C19-H), 2.39 (d, *J* = 1.7 Hz, 3H, C14-H).

^{13}C NMR (151 MHz, Acetone-*D*$_6$) δ 168.7 (d, *J* = 279.2 Hz, C8), 145.0 (C18), 144.9 (C13), 139.0 (C4), 137.1 (C10), 136.6 (C16), 133.9 (C17), 130.8 (C12), 129.6 (C2), 129.4 (C3), 128.2 (C11), 127.4 (C1), 109.8 (C15), 80.7 (d, *J* = 19.1 Hz, C9), 51.4 (C6), 48.7 (d, *J* = 29.8 Hz, C7), 35.6 (C5), 21.4 (C19), 21.3 (C14).

^{19}F NMR (376 MHz, Acetone-*D*$_6$) δ −75.79 (dt, *J* = 26.6, 13.2 Hz, 1F, C8-F), −150.19 (d, *J* = 13.5 Hz, 4F, BF_4).

HRMS (ESI+) calcd: $[M]^+$ ($C_{25}H_{26}FINO_2S$) 550.0707, measured 550.0690 (3.09 ppm error).

IR (neat) v_{max}/cm^{-1}: 3096, 2969, 1738, 1653, 1597, 1454, 1346, 1158, 1093, 1061, 995, 756, 548.

***(Z)-(3-((N-butyl-4-nitrophenyl)sulfonamido)-2-fluoroprop-1-en-1-yl)(p-tolyl)iodonium tetrafluoroborate* (3u)**

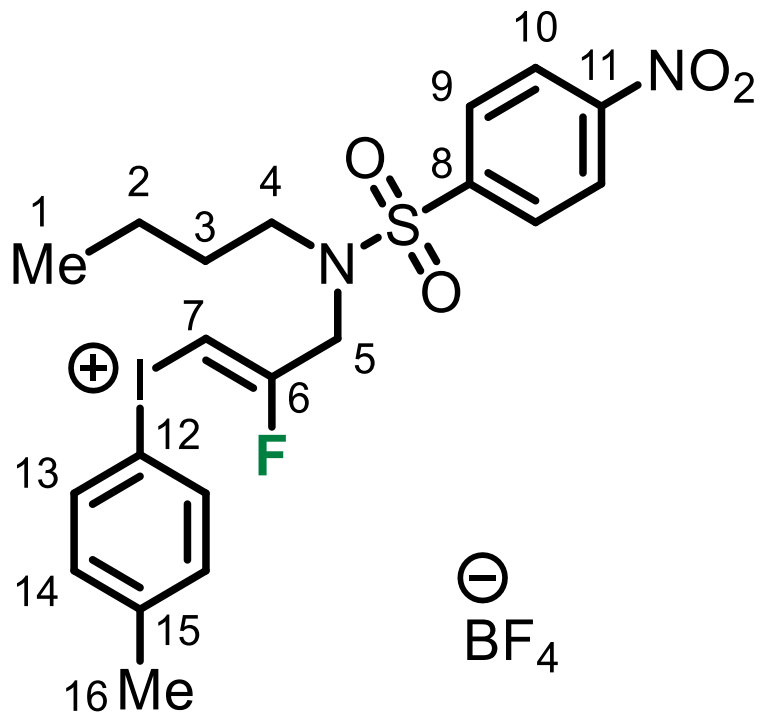

Produced according to **General procedure III** from alkyne **1u** to afford the title compound as a white solid (2.62 g, 74%).

^{1}H NMR (500 MHz, Acetone-D_6) δ 8.42 (app. dt, J = 9.0, 2.0 Hz, 2H, C10-H), 8.17 (app. dt, J = 8.8, 2.0 Hz, 2H, C9-H), 8.12 (dt, J = 8.4, 1.8 Hz, 2H, C13-H), 7.44 (d, J = 8.4 Hz, 2H, C14-H), 7.25 (dt, J = 33.6, 0.9 Hz, 1H, C7-H), 4.54 (dd, J = 13.1, 0.9 Hz, 2H, C5-H), 3.28 – 3.19 (m, 2H, C4-H), 2.45 (s, 3H, C16-H), 1.46 (tt, J = 7.7, 6.5 Hz, 2H, C3-H), 1.19 (h, J = 7.4 Hz, 2H, C2-H), 0.79 (t, J = 7.4 Hz, 3H, C1-H).

^{13}C NMR (126 MHz, Acetone-D_6) δ 168.1 (d, J = 278.8 Hz, C6), 151.3 (C11), 145.5 (C8), 144.9 (C15), 136.6 (C13), 133.8 (C14), 129.7 (C9), 125.4 (C10), 109.7 (C12), 81.0 (d, J = 18.8 Hz, C7), 49.8 (C4), 48.2 (d, J = 29.2 Hz, C5), 30.9 (C3), 21.3 (C16), 20.2 (C2), 13.8 (C1).

^{19}F NMR (376 MHz, Acetone-D_6) δ −76.06 (dt, J = 33.6, 13.2 Hz, 1F, C6-F), −149.87 – −149.93 (m, 4F, BF_4).

HRMS (+ESI) calcd: $[M]^+$ ($C_{20}H_{23}FIN_2O_4S$) 533.0402, measured 533.0379 (4.31 ppm error).

IR (neat) v_{max}/cm^{-1}: 3104, 2963, 2873, 1739, 1529, 1350, 1311, 1162, 1087, 1053, 1010, 996, 722, 603, 520.

(Z)-(3-((N-butyl-4-methylphenyl)sulfonamido)-2-fluoroprop-1-en-1-yl)(p-tolyl)iodonium tetrafluoroborate **(3v)**

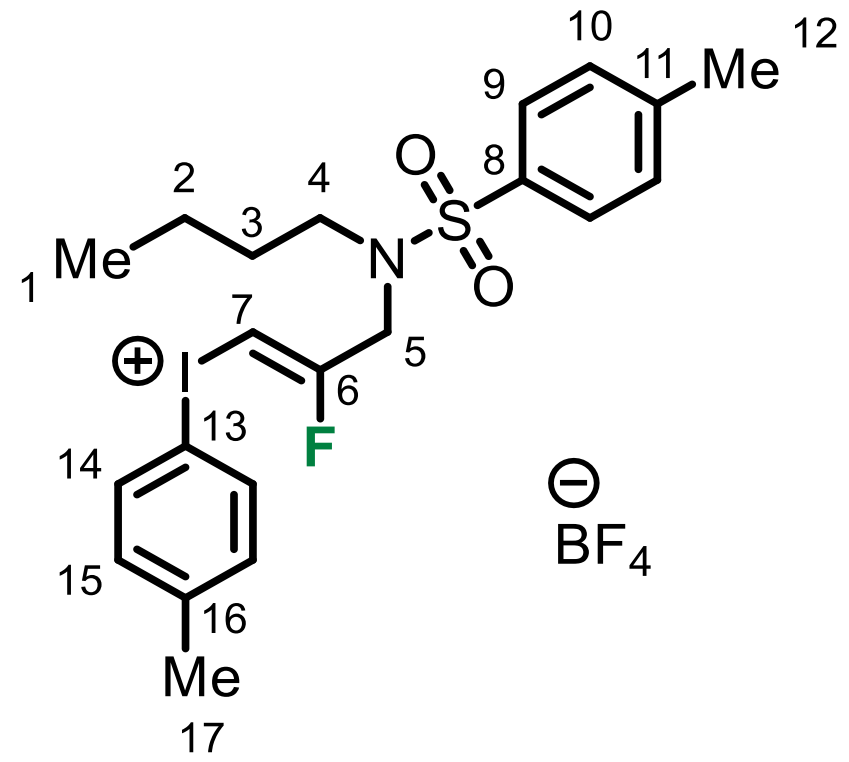

Produced according to **General procedure III** from alkyne **1v** to afford the title compound as a white solid (2.88 g, 68%).

^{1}H NMR (500 MHz, Acetone-*D$_6$*) δ 8.12 (app. dt, *J* = 8.4, 1.8 Hz, 2H, C14-H), 7.74 (app. dt, *J* = 8.2, 1.6 Hz, 2H, C9-H), 7.44 (d, *J* = 8.4 Hz, 2H, C15-H), 7.42 (d, *J* = 8.2 Hz, 2H, C10-H), 7.20 (dt, *J* = 33.5, 1.0 Hz, 1H, C7-H), 4.40 (dd, *J* = 12.3, 1.0 Hz, 2H, C5-H), 3.14 – 3.08 (m, 2H, C4-H), 2.45 (s, 3H, C17-H), 2.43 (s, 3H, C12-H), 1.46 – 1.36 (m, 2H, C3-H), 1.17 (h, *J* = 7.4 Hz, 2H, C2-H), 0.77 (t, *J* = 7.4 Hz, 3H, C1-H).

^{13}C NMR (126 MHz, Acetone-*D$_6$*) δ 168.9 (d, *J* = 278.9 Hz, C6), 144.8 (C16), 144.7 (C11), 137.0 (C8), 136.5 (C14), 133.8 (C15), 130.8 (C10), 128.2 (C9), 109.9 (d, *J* = 3.0 Hz, C13), 80.6 (d, *J* = 19.0 Hz, C7), 49.9 (C4), 48.5 (d, *J* = 29.9 Hz, C5), 31.0 (C3), 21.4 (C17), 21.3 (C12), 20.2 (C2), 13.8 (C1).

^{19}F NMR (376 MHz, Acetone-*D$_6$*) δ −76.07 (dt, *J* = 33.7, 12.3 Hz, 1F, C6-F), −150.07 (d, *J* = 20.3 Hz, 4F, BF_4).

HRMS (+ESI) calcd. $[M]^+$ ($C_{21}H_{26}FINO_2S$) 502.0707, measured 502.0686 (4.18 ppm error).

IR (neat) v_{max}/cm^{-1}: 3098, 2962, 2873, 1740, 1653, 1453, 1342, 1157, 1088, 1051, 996, 916, 802, 664, 547.

***(Z)-(2-fluoro-3-((4-methyl-N-(2-(thiophen-2-yl)ethyl)phenyl)sulfonamido)prop-1-en-1-yl)(p-tolyl)iodonium tetrafluoroborate* (3w)**

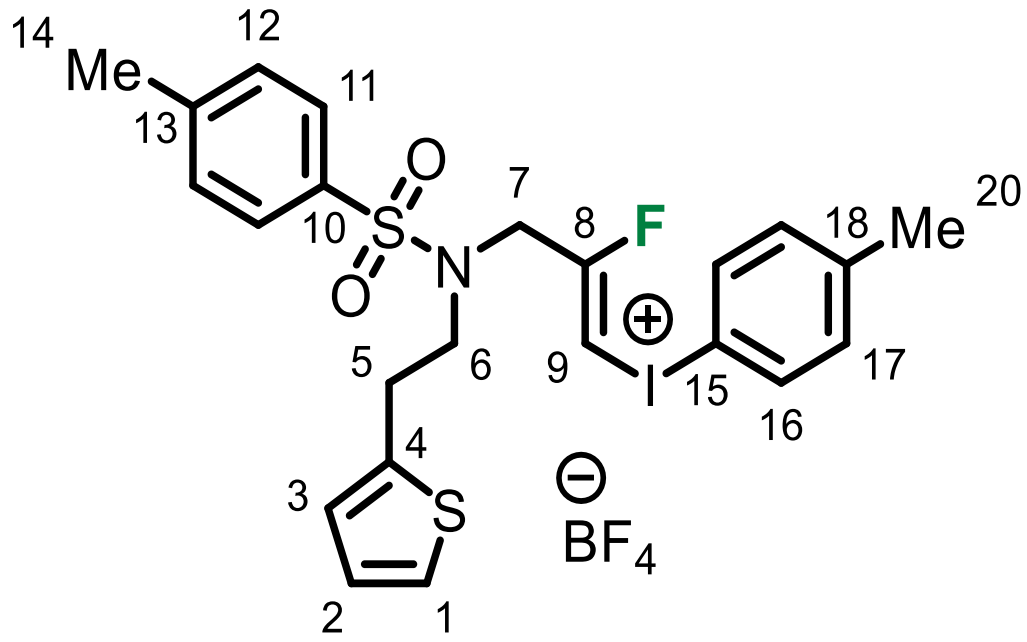

Produced according to **General procedure III** from alkyne **1w** to afford the title compound as a white solid (635 mg, 49%).

^{1}H NMR (500 MHz, Acetone-*D*$_6$) δ 8.13 (d, *J* = 8.4 Hz, 2H, C16-H), 7.76 (d, *J* = 9.1 Hz, 2H, C11-H), 7.45 – 7.40 (m, 4H, C12-H, C17-H), 7.26 (dd, *J* = 5.1, 1.2 Hz, 1H, C1-H), 7.24 (dt, *J* = 33.5, 1.0 Hz, 1H, C9-H), 6.92 (dd, *J* = 5.1, 3.5 Hz, 1H, C2-H), 6.78 (dd, *J* = 3.4, 1.2 Hz, 1H, C3-H), 4.50 (d, *J* = 11.5 Hz, 2H, C7-H), 3.41 (dd, *J* = 9.6, 6.5 Hz, 2H, C6-H), 2.98 (dd, *J* = 9.6, 6.5 Hz, 2H, C5-H), 2.43 (s, 3H, C19-H), 2.42 (s, 3H, C14-H).

^{13}C NMR (126 MHz, Acetone-*D*$_6$) δ 168.9 (d, *J* = 278.9 Hz, C8), 145.1 (C18), 145.0 (C13), 140.8 (C4), 136.8 (C10), 136.6 (C16), 133.9 (C12), 130.9 (C17), 128.2 (C11), 127.9 (C2), 126.5 (C3), 125.0 (C1), 109.8 (C15), 80.6 (d, *J* = 19.1 Hz, C9), 51.6 (C6), 48.9 (d, *J* = 30.5 Hz, C7), 30.1 (C5), 21.4 (C19), 21.3 (C14).

^{19}F NMR (471 MHz, Acetone-*D*$_6$) δ −75.72 (dt, *J* = 33.6, 11.5 Hz, 1F, C8-F), −150.88 (d, *J* = 25.3 Hz, 4F, BF_4).

HRMS (+ESI) calcd: $[M]^+$ ($C_{23}H_{24}FINO_2S_2$) 556.0272, measured 556.0252 (3.60 ppm error).

IR (neat) v_{max}/cm^{-1}: 2970, 1739, 1363, 1216, 1159, 1090, 548.

***(Z)-(2-fluoro-3-((4-methyl-N-((tetrahydrofuran-2-yl)methyl)phenyl)sulfonamido)prop-1-en-1-yl)(p-tolyl)iodonium tetrafluoroborate* (3x)**

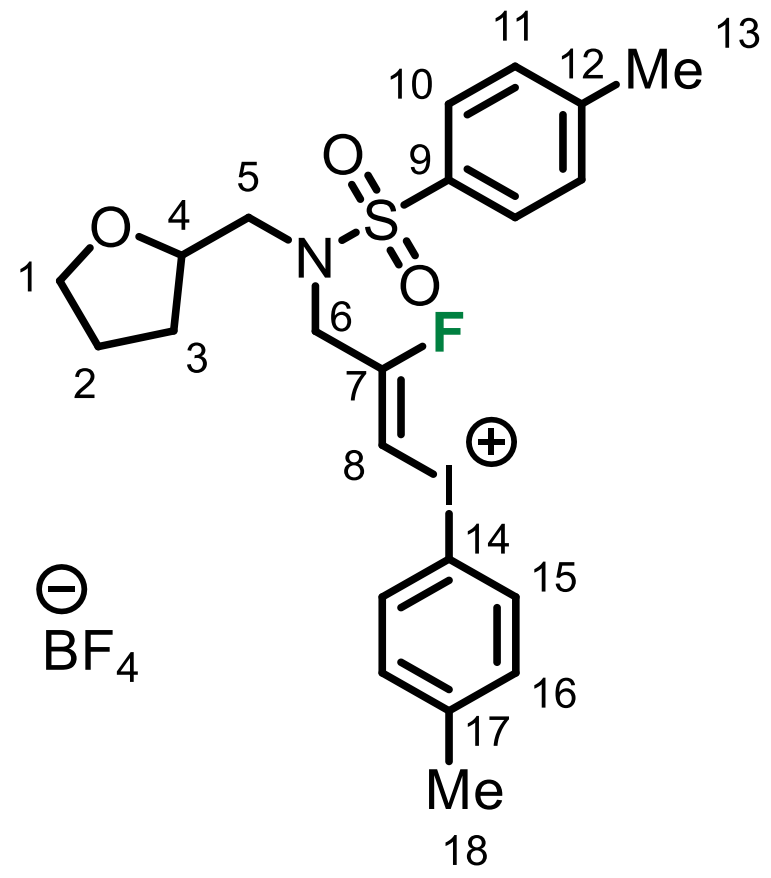

Produced according to **General procedure III** from alkyne **1x** to afford the title compound as a white solid (460 mg, 37%).

^{1}H NMR (400 MHz, Acetone-*D*$_6$) δ 8.13 (d, *J* = 8.3 Hz, 2H, C15-H), 7.75 (d, *J* = 8.2 Hz, 2H, C10-H), 7.47 (d, *J* = 8.3 Hz, 2H, C16-H), 7.45 – 7.41 (m, 2H, C11-H), 7.19 (d, *J* = 33.7 Hz, 1H, C8-H), 4.58 – 4.37 (m, 2H, C6-H), 3.88 (ddt, *J* = 8.2, 5.1, 3.2 Hz, 1H, C4-H), 3.63 (q, *J* = 7.2 Hz, 1H, C1-H), 3.49 (q, *J* = 7.2 Hz, 1H, C1-H), 3.41 (dd, *J* = 14.5, 3.2 Hz, 1H, C5-H), 3.02 (dd, *J* = 14.5, 8.2 Hz, 1H, C5-H), 2.46 (s, 3H, C18-H), 2.43 (s, 3H, C13-H), 1.95 – 1.83 (m, 1H, C3-H), 1.82 – 1.65 (m, 2H, C2-H), 1.54 – 1.40 (m, 1H, C3-H).

^{13}C NMR (126 MHz, Acetone-*D*$_6$) δ 168.8 (d, *J* = 278.3 Hz, C7), 144.1 (C17), 143.9 (C12), 135.9 (C9), 135.6 (C15), 132.9 (C16), 129.9 (C11), 127.4 (C10), 109.0 (C14), 79.0 (d, *J* = 18.9 Hz, C8), 77.6 (C4), 67.6 (C1), 52.9 (C5), 48.6 (d, *J* = 32.7 Hz, C6), 29.4 (C3), 25.0 (C2), 20.5 (C18), 20.4 (C13).

^{19}F NMR (471 MHz, Acetone-*D*$_6$) δ −75.57 (dt, *J* = 33.7, 8.9 Hz), −150.96 – −151.12 (m).

HRMS (+ESI) calcd: $[M]^+$ ($C_{22}H_{26}FINO_3S$) 530.0657, measured 530.0635 (3.96 ppm error).

IR (neat) v_{max}/cm^{-1}: 3092, 2970, 2871, 1737, 1350, 1216, 1161, 1062, 804, 663, 548.

***(Z)-(3-(1,1-dioxidoisothiazolidin-2-yl)-2-fluoroprop-1-en-1-yl)(p-tolyl)iodonium tetrafluoroborate* (3y)**

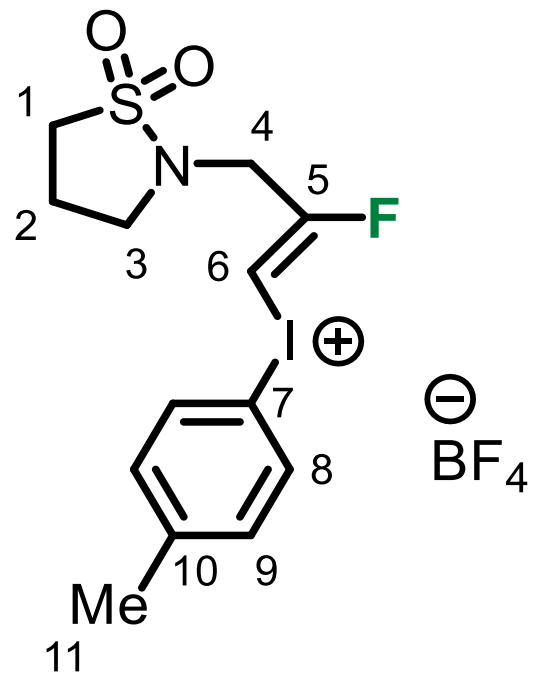

Produced according to **General procedure III** from alkyne **1y** to afford the title compound as a white solid (86.0 mg, 6%).

^{1}H NMR (500 MHz, Acetone-D_6) δ 8.13 (app. dt, *J* = 8.5, 1.9 Hz, 2H, C8-H), 7.45 (d, *J* = 8.5 Hz, 2H, C9-H), 7.23 (dt, *J* = 33.6, 1.0 Hz, 1H, C6-H), 4.22 (dd, *J* = 10.5, 1.1 Hz, 2H, C4-H), 3.40 (t, *J* = 6.7 Hz, 2H, C1-H), 3.19 (dd, *J* = 7.9, 7.1 Hz, 2H, C3-H), 2.45 (s, 3H, C11-H), 2.42 – 2.34 (m, 2H, C2-H).

^{13}C NMR (126 MHz, Acetone-D_6) δ 168.8 (d, *J* = 278.1 Hz, C5), 144.9 (C10), 136.5 (C8), 133.9 (C9), 109.8 (C7), 80.3 (d, *J* = 18.6 Hz, C6), 48.8 (C1), 46.9 (C3), 46.0 (d, *J* = 32.0 Hz, C4), 21.3 (C11), 19.9 (C2).

^{19}F NMR (376 MHz, Acetone-D_6) δ −75.53 (dq, *J* = 32.9, 10.6 Hz, 1F, C5-F), −150.70 – −150.92 (m, 4F, BF_4).

HRMS (+ESI) calcd: $[M]^+$ ($C_{13}H_{16}FINO_2S$) 395.9925, measured 395.9908 (4.29 ppm error).

IR (neat) v_{max}/cm^{-1}: 3097, 1655, 1480, 1301, 1203, 1139, 1061, 803, 162, 478.

***(Z)-(2-fluoro-2-(1-tosylpiperidin-2-yl)vinyl)(p-tolyl)iodonium tetrafluoroborate* (3z)**

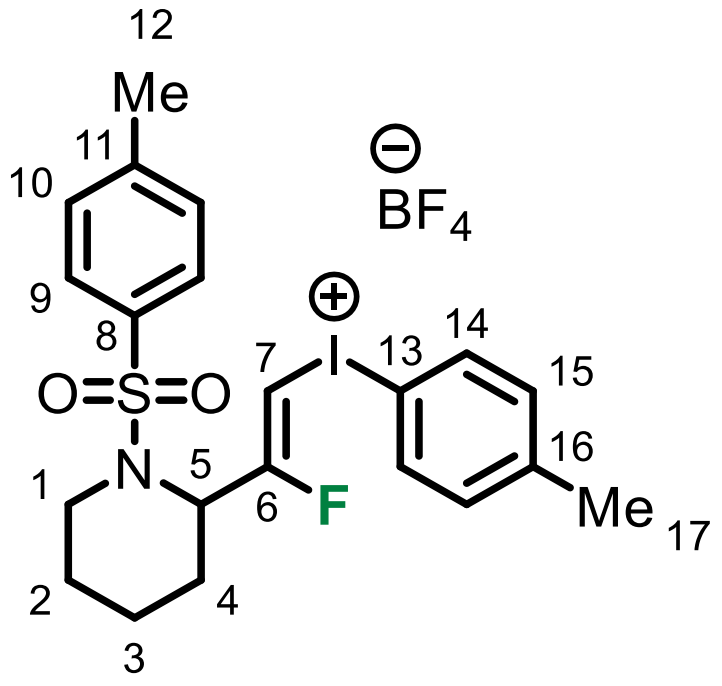

2-ethynylpiperidine was tosylated according to **General procedure I**, and the crude product was carried forward according to **General procedure III** to afford the title compound as a white solid (675 mg, 58%).

^{1}H NMR (400 MHz, Acetone-*D*$_6$) δ 8.13 (app. dt, *J* = 8.5, 2.0 Hz, 2H, C14-H), 7.75 (app. dt, *J* = 8.2, 1.8 Hz, 2H, C9-H), 7.46 (d, *J* = 8.5 Hz, 2H, C15-H), 7.39 (d, *J* = 8.2 Hz, 2H, C10-H), 7.17 (dd, *J* = 34.5, 1.5 Hz, 1H, C7-H), 5.17 (dd, *J* = 5.0, 4.5 Hz, 1H, C5-H), 3.67 (dt, *J* = 14.5, 3.6 Hz, 1H, C1-H), 3.11 (ddd, *J* = 14.5, 12.1, 3.0 Hz, 1H, C1-H), 2.46 (s, 3H, C17-H), 2.42 (s, 3H, C12-H), 2.03 – 1.94 (m, 1H, C4-H), 1.64 – 1.45 (m, 3H, C2-H, C3-H, C4-H), 1.37 (qt, *J* = 13.4, 4.0 Hz, 1H, C2-H), 1.24 – 1.12 (m, 1H, C3-H).

^{13}C NMR (126 MHz, Acetone-*D*$_6$) δ 170.2 (d, *J* = 282.5 Hz, C6), 144.8 (C16), 144.8 (C11), 138.3 (C8), 136.5 (C14), 133.8 (C15), 130.8 (C10), 128.0 (C9), 109.8 (C13), 81.3 (d, *J* = 20.0 Hz, C7), 54.3 (d, *J* = 28.3 Hz, C5), 43.2 (C1), 26.8 (C4), 24.5 (C3), 21.4 (C17), 21.3 (C12), 20.0 (C2).

^{19}F NMR (376 MHz, Acetone-*D*$_6$) δ −75.41 (dd, *J* = 34.5, 5.0 Hz, 1F, C6-F), −150.23 (d, *J* = 20.4 Hz, 4F, BF_4).

HRMS (+ESI) calcd: $[M]^+$ ($C_{21}H_{24}FINO_2S$) 500.0551, measured 500.0533 (3.60 ppm error).

IR (neat) ν_{max}/cm^{-1}: 3103, 2945, 2867, 1767, 1641, 1451, 1337, 1156, 1090, 1052, 996, 950, 802, 667, 547.

(Z)-(2-fluoro-2-(1-((N-(3-fluorobenzyl)-4-methylphenyl)sulfonamido)cyclohexyl)vinyl)(p-tolyl)iodonium tetrafluoroborate **(3aa)**

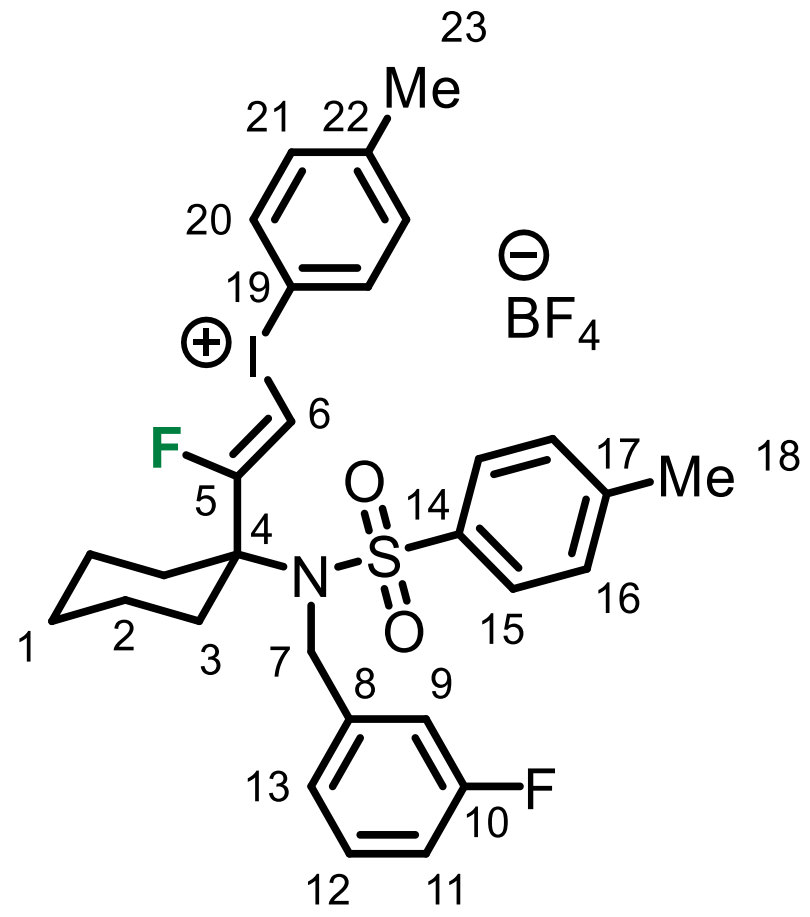

Produced according to **General procedure III** from alkyne **1aa** to afford the title compound as a white solid (453 mg, 58%).

^{1}H NMR (500 MHz, Acetone-*D*$_6$) δ 8.17 (app. dt, *J* = 8.4, 2.0 Hz, 2H, C20-H), 7.66 (app. dt, *J* = 8.3, 1.9 Hz, 2H, C15-H), 7.47 (d, *J* = 8.4 Hz, 2H, C21-H), 7.38 (d, *J* = 8.3 Hz, 2H, C16-H), 7.36 (d, *J* = 36.1 Hz, 1H, C6-H), 7.35 – 7.28 (m, 1H, C12-H), 7.14 (d, *J* = 7.7 Hz, 1H, C13-H), 7.08 (dt, *J* = 10.6, 2.1 Hz, 1H, C9-H), 6.99 (td, *J* = 8.5, 2.6 Hz, 1H, C11-H), 4.73 (s, 2H, C7-H), 2.44 (s, 3H, C23-H), 2.42 (s, 3H, C18-H), 2.19 (d, *J* = 13.5 Hz, 2H, C3-H), 1.88 (tt, *J* = 13.6, 3.9 Hz, 2H, C3-H), 1.58 – 1.42 (m, 3H, C1-H, C2-H), 1.27 (q, *J* = 13.0 Hz, 2H, C2-H), 1.19 – 1.09 (m, 1H, C1-H).

^{13}C NMR (126 MHz, Acetone-*D*$_6$) δ 169.3 (d, *J* = 280.5 Hz, C5), 162.8 (d, *J* = 243.8 Hz, C10), 144.0 (C17), 144.0 (C22), 142.8 (d, *J* = 7.2 Hz, C8), 138.9 (C14), 136.1 (C20), 132.9 (C21), 130.1 (d, *J* = 8.3 Hz, C12), 129.8 (C16), 127.5 (C15), 122.6 (d, *J* = 2.7 Hz, C13), 113.7 (d, *J* = 21.3 Hz, C11), 113.6 (d, *J* = 22.5 Hz, C9), 108.6 (C19), 83.3 (d, *J* = 21.7 Hz, C6), 67.0 (d, *J* = 22.2 Hz, C4), 49.0 (d, *J* = 2.1 Hz, C7), 33.2 (d, *J* = 3.5 Hz, C3), 24.3 (C1), 22.5 (C2), 20.5 (C23), 20.5 (C18).

^{19}F NMR (376 MHz, Acetone-*D*$_6$) δ −75.46 (d, *J* = 35.7 Hz, 1F, C5-F), −114.43 (td, *J* = 9.5, 5.9 Hz, 1F, C10-F), −150.46 (d, *J* = 20.4 Hz, 4F, BF_4).

HRMS (+ESI) calcd: $[M]^+$ ($C_{29}H_{31}F_2INO_2S$) 622.1083, measured 622.1062 (3.38 ppm error).

IR (neat) v_{max}/cm^{-1}: 3107, 2941, 2866, 1692, 1591, 1450, 1333, 1156, 1087, 1059, 799, 548.

***(Z)-(2-(1-((N,4-dimethylphenyl)sulfonamido)cyclohexyl)-2-fluorovinyl)(p-tolyl)iodonium tetrafluoroborate* (3ab)**

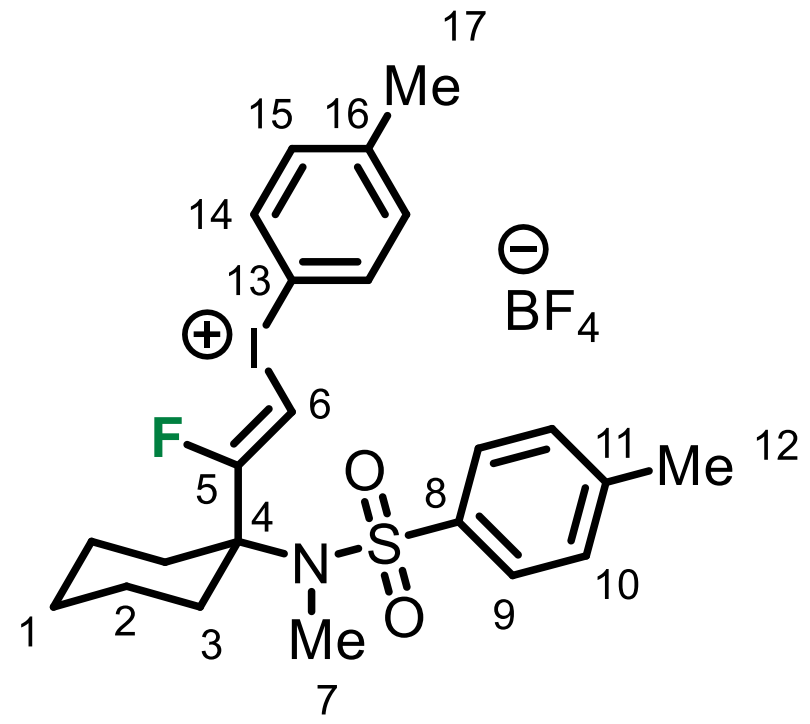

Produced according to **General procedure III** from alkyne **1ab** to afford the title compound as a white solid (757 mg, 77%).

^{1}H NMR (500 MHz, Acetone-*D*$_6$) δ 8.12 (app. dt, *J* = 8.4, 1.8 Hz, 2H, C14-H), 7.67 (app. dt, *J* = 8.2, 2.3 Hz, 2H, C9-H), 7.43 (d, *J* = 8.4 Hz, 2H, C15-H), 7.40 (d, *J* = 8.2 Hz, 2H, C10-H), 7.32 (d, *J* = 35.8 Hz, 1H, C6-H), 2.89 (s, 3H, C7-H), 2.43 (s, 6H, C12-H, C17-H), 2.17 (t, *J* = 5.6 Hz, 4H, C3-H), 1.62 – 1.26 (m, 6H, C1-H, C2-H).

^{13}C NMR (126 MHz, Acetone-*D*$_6$) δ 172.5 (d, *J* = 282.2 Hz, C5), 144.7 (C16), 144.6 (C11), 139.3 (C8), 136.6 (C14), 133.8 (C15), 130.6 (C10), 128.1 (C9), 109.6 (d, *J* = 1.0 Hz, C13), 82.0 (d, *J* = 21.6 Hz, C6), 65.7 (d, *J* = 22.3 Hz, C4), 34.0 (d, *J* = 2.7 Hz, C3), 33.0 (d, *J* = 1.5 Hz, C7), 25.5 (C1), 23.1 (C2), 21.4 (C17), 21.3 (C12).

^{19}F NMR (376 MHz, Acetone-*D*$_6$) δ −76.27 (d, *J* = 35.6 Hz, 1F, C5-F), −150.40 – −150.62 (m, 4F, BF_4).

HRMS (+ESI) calcd: $[M]^+$ ($C_{23}H_{28}FINO_2S$) 528.0864, measured 528.0841 (4.36 ppm error).

IR (neat) v_{max}/cm^{-1}: 3110, 2938, 2864, 1690, 1632, 1452, 1328, 1154, 1064, 803, 668, 549.

(Z)-(3-((N,4-dimethylphenyl)sulfonamido)-2-fluoroprop-1-en-1-yl)(p-tolyl)iodonium tetrafluoroborate **(3ac)**

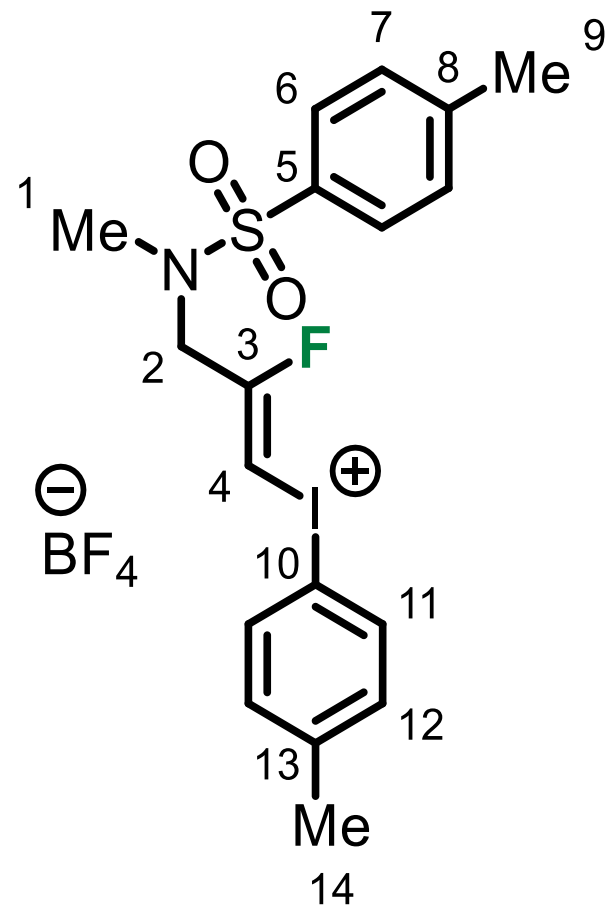

Produced according to **General procedure III** from alkyne **1ac** to afford the title compound as a white solid (810 mg, 47%).

^{1}H NMR (500 MHz, Acetone-*D$_6$*) δ 8.11 (d, *J* = 8.5 Hz, 2H, C11-H), 7.72 (d, *J* = 8.4 Hz, 2H, C6-H), 7.43 (d, *J* = 8.5 Hz, 2H, C12-H), 7.43 (d, *J* = 8.4 Hz, 2H, C7-H), 7.20 (d, *J* = 33.4 Hz, 1H, C4-H), 4.29 (d, *J* = 12.9 Hz, 2H, C2-H), 2.75 (s, 3H, C1-H), 2.44 (s, 3H, C14-H), 2.42 (s, 3H, C9-H).

^{13}C NMR (126 MHz, Acetone-*D$_6$*) δ 167.7 (d, *J* = 278.7 Hz, C3), 144.9 (C13), 144.7 (C8), 136.5 (C11), 135.1 (C5), 133.8 (C12), 130.8 (C7), 128.3 (C6), 109.7 (C10), 81.0 (d, *J* = 18.8 Hz, C4), 50.4 (d, *J* = 29.6 Hz, C2), 35.7 (C1), 21.4 (C14), 21.3 (C9).

^{19}F NMR (471 MHz, Acetone-*D$_6$*) δ −76.66 (dt, *J* = 33.5, 12.9 Hz, 1F, C3-F), −149.63 – −149.73 (m, 4F, BF_4).

HRMS (+ESI) calcd: $[M]^+$ ($C_{18}H_{20}FINO_2S$) 460.0238, measured 460.0227 (2.39 ppm error).

IR (neat) ν_{max}/cm^{-1}: 3100, 1694, 1478, 1338, 1160, 1086, 1055, 1018, 1007, 996, 923, 802, 758, 665, 549.

***(Z)-(3-(benzyloxy)-2-fluorobut-1-en-1-yl)(p-tolyl)iodonium tetrafluoroborate* (3ad)**

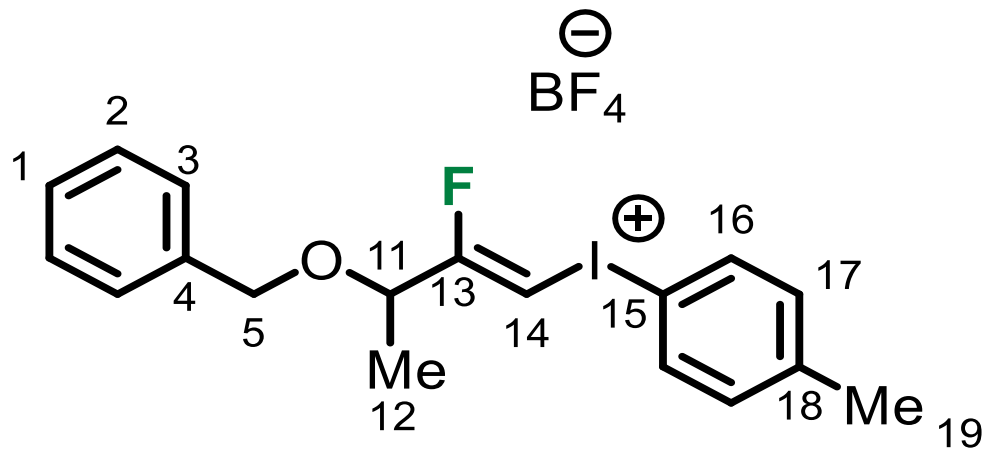

Prepared from alkyne **1ad** according to **General procedure III** with 20 mol% Ag_2CO_3, affording the title compound as an orange viscous oil (1.16 g, 48% yield).

^{1}H NMR (500 MHz, Acetone-*D*$_6$) δ 8.15 (2H, d, *J* = 8.4 Hz, C16), 7.46 (2H, d, *J* = 8.2 Hz, C17), 7.38 – 7.26 (5H, m, C1-H, C2-H, C3-H), 7.27 (1H, d, *J* = 34.3 Hz, C14-H), 4.54 (2H, d, *J* = 4.9 Hz, C5-H), 4.58 – 4.50 (1H, m, C12-H), 2.45 (3H, s, C19-H), 1.41 (3H, d, *J* = 6.5 Hz, C12-H).

^{13}C NMR (126 MHz, Acetone-*D*$_6$) δ 171.8 (d, *J* = 282.5 Hz, C13), 144.9 (C18), 138.5 (C4), 136.6 (C16), 133.9 (C17), 129.2 (C2), 128.7 (C1), 128.6 (C3), 109.8 (C15), 80.0 (d, *J* = 18.9 Hz, C14), 73.4 (d, *J* = 27.9 Hz, C11), 72.0 (C5), 21.3 (C19), 18.7 (C12).

^{19}F NMR (471 MHz, Acetone-*D*$_6$) δ −84.29 (dd, *J* = 34.3, 14.5 Hz, 1F, C13-F), −150.51 – −150.57 (m, 4F, BF_4).

HRMS (+ESI) calcd: $[M]^+$ ($C_{18}H_{19}OFI$) 397.0459, measured 397.0455 (1.01 ppm error).

IR (neat) ν_{max}/cm^{-1}: 3535, 3091, 3034, 2990, 2959, 2876, 1749, 1639, 1583, 1477, 1454, 1397, 1374, 1341, 1271, 1209, 1188, 1177, 1032, 995, 915, 835, 804, 783, 748, 718, 699, 643, 595, 575, 521, 480, 434.

***(Z)-(3-((3-bromobenzyl)oxy)-2-fluoro-3-methylbut-1-en-1-yl)(p-tolyl)iodonium tetrafluoroborate* (3ae)**

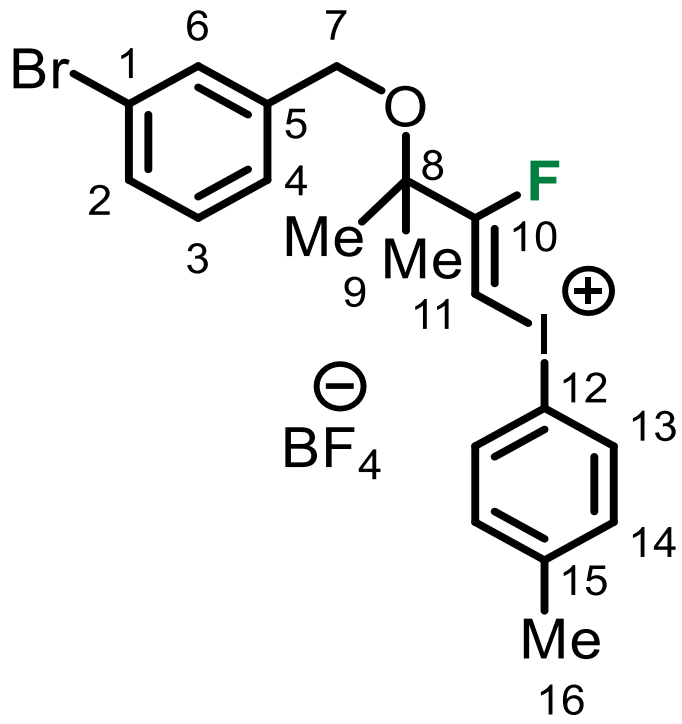

Produced according to **General procedure III** from alkyne **1ae** to afford the title compound as a white solid (685 mg, 49%).

^{1}H NMR (400 MHz, Acetone-*D*$_6$) δ 8.17 – 8.12 (m, 2H, C13-H), 7.46 (app. dt, *J* = 7.6, 1.6 Hz, 1H, C2-H), 7.43 (s, 1H, C6-H), 7.42 (d, *J* = 6.0 Hz, 2H, C14-H), 7.35 (d, *J* = 34.6 Hz, 1H, C11-H), 7.28 (d, *J* = 7.6 Hz, 1H, C3-H), 7.25 (dt, *J* = 7.6, 1.6 Hz, 1H, C4-H), 4.41 (s, 2H, C7-H), 2.42 (s, 3H, C16-H), 1.55 (d, *J* = 1.3 Hz, 6H, C9-H).

^{13}C NMR (126 MHz, Acetone-*D*$_6$) δ 172.2 (d, *J* = 282.3 Hz, C10), 143.9 (C15), 141.0 (C5), 135.7 (C13), 132.9 (C14), 130.4 (C6), 130.2 (C2), 130.0 (C3), 126.2 (C4), 121.8 (C1), 108.9 (C12), 80.4 (d, *J* = 20.0 Hz, C11), 76.2 (d, *J* = 24.6 Hz, C8), 64.7 (C7), 23.4 (C9), 20.4 (C16).

^{19}F NMR (471 MHz, Acetone-*D*$_6$) δ −80.93 (d, *J* = 34.6, 1F, C10-F), −150.07 – −150.29 (m, 4F, BF_4).

HRMS (+ESI) calcd: $[M]^+$ ($C_{19}H_{20}BrFIO$) 488.9721, measured 488.9702 (3.89 ppm error).

IR (neat) v_{max}/cm^{-1}: 3117, 2989, 1736, 1636, 1476, 1388, 1273, 1165, 1058, 996, 800, 780, 478.

***(Z)-(2-fluoro-3-((3-nitrobenzyl)oxy)-3,3-diphenylprop-1-en-1-yl)(p-tolyl)iodonium tetrafluoroborate* (3af)**

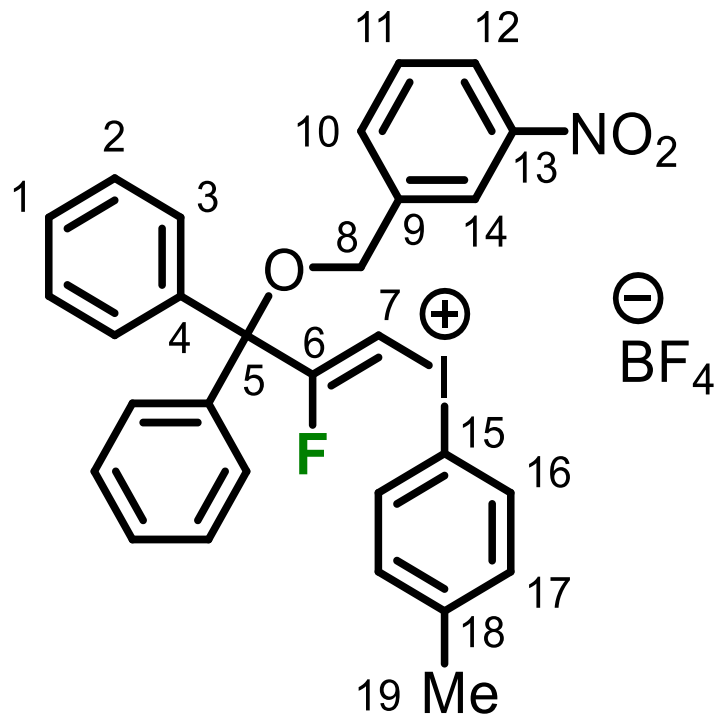

Produced according to **General procedure III** from alkyne **1af** to afford the title compound as a white solid (487 mg, 25%).

^{1}H NMR (400 MHz, Acetone-*D_6*) δ 8.14 (app. dt, J = 8.0, 1.5 Hz, 1H, C12-H), 8.09 (t, J = 1.9 Hz, 1H, C14-H), 8.05 (d, J = 8.3 Hz, 2H, C16-H), 7.76 (d, J = 7.8 Hz, 1H, C10-H), 7.66 (d, J = 33.8 Hz, 1H, C7-H), 7.58 – 7.52 (m, 1H, C11-H), 7.49 – 7.43 (m, 8H, C2-H, C3-H), 7.42 (d, J = 8.3 Hz, 2H, C17-H), 7.40 – 7.31 (m, 2H, C1-H), 4.50 (s, 2H, C8-H), 2.45 (s, 3H, C19-H).

^{13}C NMR (126 MHz, Acetone-*D_6*) δ 170.8 (d, J = 278.3 Hz, C6), 149.2 (C13), 144.9 (C18), 140.9 (C9), 138.1 (C4), 136.4 (C16), 134.3 (C10), 133.8 (C17), 130.2 (C1), 129.6 (C2), 129.5 (d, J = 0.7 Hz, C3), 127.3 (C11), 123.3 (C12), 122.5 (C14), 110.1 (C15), 86.7 (d, J = 26.9 Hz, C7), 83.2 (d, J = 19.0 Hz, C5), 66.5 (d, J = 1.6 Hz, C8), 21.3 (C19).

^{19}F NMR (376 MHz, Acetone-*D_6*) δ −75.09 (d, J = 33.8 Hz, 1F, C6-F), −150.42 – −150.69 (m, 4F, BF_4).

HRMS (+ESI) calcd. $[M]^+$ ($C_{29}H_{24}FINO_3$) 580.0779, measured 580.0762 (2.93 ppm error).

IR (neat) v_{max}/cm^{-1}: 2969, 1738, 1638, 1528, 1448, 1350, 1216, 1064, 802, 702, 477.

(Z)-(3-((4-benzoylbenzyl)oxy)-2-fluoro-3,3-diphenylprop-1-en-1-yl)(p-tolyl)iodonium tetrafluoroborate **(3ag)**

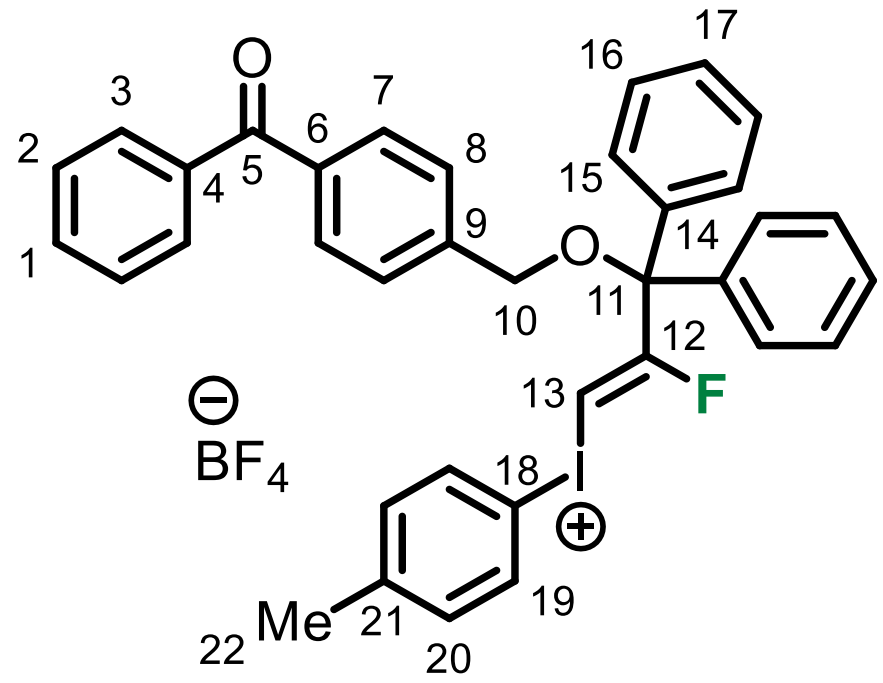

Produced according to **General procedure III** from alkyne **1ag** to afford the title compound as a white solid (897 mg, 70%).

^{1}H NMR (500 MHz, Acetone-*D*$_6$) δ 8.06 (d, *J* = 8.3 Hz, 2H, C19-H), 7.79 – 7.75 (m, 2H, C3-H), 7.75 – 7.70 (m, 2H, C7), 7.68 – 7.64 (m, 1H, C1-H), 7.64 (d, *J* = 34.0 Hz, 1H, C13-H), 7.56 (t, *J* = 7.5 Hz, 2H, C2-H), 7.49 (d, *J* = 7.1 Hz, 2H, C8-H), 7.51 – 7.41 (m, 8H, C15-H, C16-H), 7.42 (d, *J* = 8.3 Hz, 2H, C20-H), 7.39 – 7.34 (m, 2H, C17-H), 4.42 (s, 2H, C10-H), 2.45 (s, 3H, C22-H).

^{13}C NMR (126 MHz, Acetone-*D*$_6$) δ 196.1 (C5), 171.0 (d, *J* = 278.4 Hz, C12), 144.9 (C21), 143.1 (C9), 138.5 (C4), 138.1 (d, *J* = 1.2 Hz, C14), 137.8 (C6), 136.4 (C19), 133.8 (C20), 130.7 (C3), 130.5 (C7), 130.1 (C17), 129.5 (C16), 129.5 (C15), 129.3 (C2), 129.2 (C1), 127.8 (C8), 110.0 (C18), 86.6 (d, *J* = 26.6 Hz, C11), 83.1 (d, *J* = 19.1 Hz, C13), 67.0 (d, *J* = 1.5 Hz, C10), 21.3 (C22).

^{19}F NMR (376 MHz, Acetone-*D*$_6$) δ −75.00 (d, *J* = 34.0 Hz, 1F, C12-F), −150.47 (d, *J* = 27.2 Hz, 4F, BF_4).

HRMS (+ESI) calcd: $[M]^+$ ($C_{36}H_{29}FIO_2$) 639.1191, measured 639.1167 (3.76 ppm error).

IR (neat) v_{max}/cm^{-1}: 2970, 1739, 1644, 1445, 1368, 1228, 1216, 1066, 702, 527.

***(Z)-(2-(1-((2-cyanobenzyl)oxy)cyclohexyl)-2-fluorovinyl)(p-tolyl)iodonium tetrafluoroborate* (3ai)**

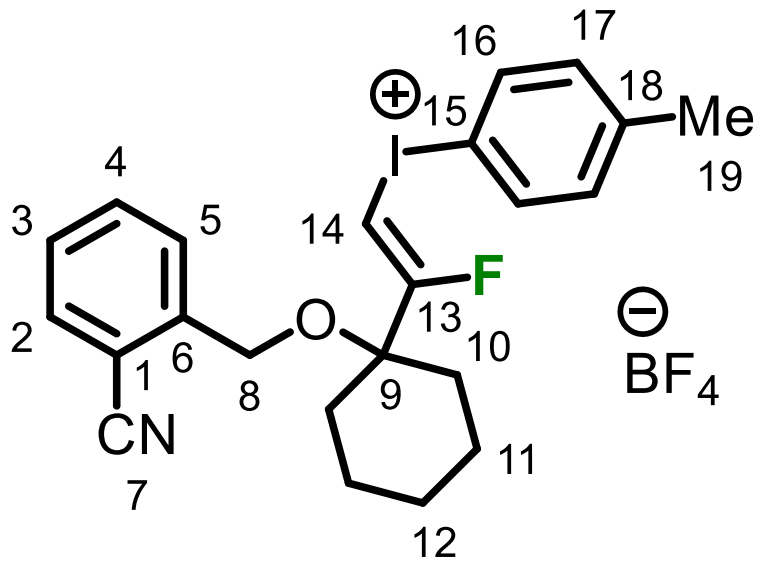

Produced according to **General procedure III** from alkyne **1ai** to afford the title compound as a white solid (833 mg, 61%).

^{1}H NMR (400 MHz, Acetone-*D$_6$*) δ 8.19 – 8.11 (m, 2H, C16-H), 7.78 (dt, *J* = 7.4, 1.1 Hz, 1H, C2-H), 7.70 (td, *J* = 7.7, 1.4 Hz, 1H, C4-H), 7.58 – 7.49 (m, 2H, C3-H, C5-H), 7.43 – 7.40 (m, 2H, C17-H), 7.39 (d, *J* = 35.3 Hz, 1H, C14-H), 4.51 (s, 2H, C8-H), 2.42 (s, 3H, C19-H), 2.13 – 2.03 (m, 2H, C10-H), 1.81 (ddd, *J* = 13.7, 11.3, 3.4 Hz, 2H, C10-H), 1.71 (ddt, *J* = 24.4, 10.5, 3.4 Hz, 2H, C11-H), 1.64 – 1.50 (m, 2H, C11-H, C12-H), 1.35 (ddd, *J* = 11.0, 8.2, 5.0 Hz, 1H, C12-H).

^{13}C NMR (101 MHz, Acetone-*D$_6$*) δ 172.0 (d, *J* = 283.4 Hz, C13), 144.8 (C18), 141.9 (C6), 136.7 (C16), 134.1 (C4), 133.9 (C17), 133.7 (C2), 130.2 (C3), 129.5 (C5), 117.9 (C7), 112.4 (C1), 109.9 (C15), 82.8 (d, *J* = 20.7 Hz, C14), 78.7 (d, *J* = 22.9 Hz, C9), 63.8 (C8), 32.5 (d, *J* = 2.0 Hz, C10), 25.6 (C11), 21.9 (C19), 21.3 (C12).

^{19}F NMR (376 MHz, Acetone-*D$_6$*) δ −81.74 (d, *J* = 35.2 Hz, 1F, C13-F), −149.78 – −149.91 (m, 4F, BF_4).

HRMS (+ESI) calcd: $[M]^+$ ($C_{23}H_{24}FINO$) 476.0881, measured 476.0871 (2.10 ppm error).

IR (neat) v_{max}/cm^{-1}: 3111, 2938, 2862, 2226, 1727, 1691, 1633, 1450, 1281, 1049, 1019, 995, 764, 479.

***(Z)-(2-fluoro-3-(1-phenylethoxy)prop-1-en-1-yl)(p-tolyl)iodonium tetrafluoroborate* (3aj)**

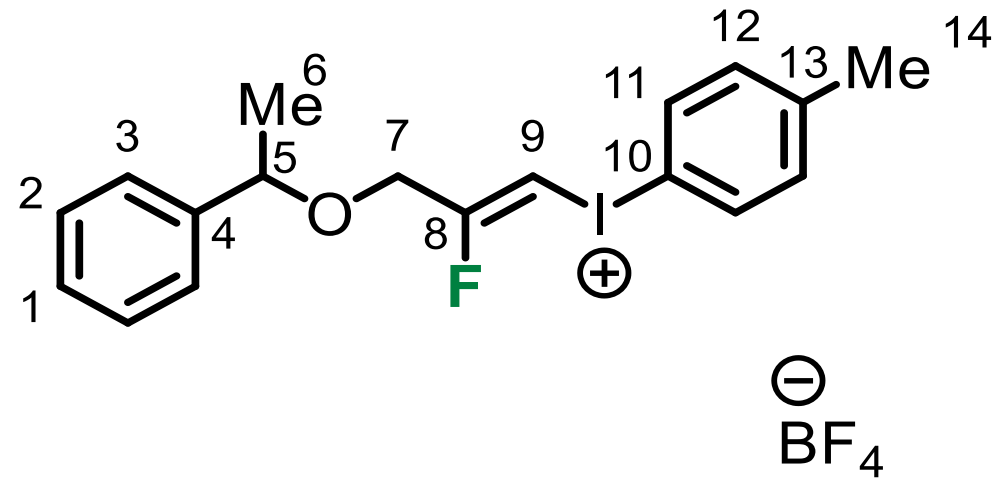

Prepared according to **General procedure III** from alkyne **1aj** with 20 mol% Ag_2CO_3, affording the title compound as a viscous brown oil (625 mg, 26%).

^{1}H NMR (600 MHz, Acetone-*D*$_6$) δ 8.12 (d, *J* = 8.0 Hz, 2H, C11-H), 7.44 (d, *J* = 8.0 Hz, 2H, C12-H), 7.36 – 7.26 (m, 5H, C1,2,3-H), 7.17 (dt, *J* = 34.2, 1.1 Hz, 1H, C9-H), 4.61 (q, *J* = 6.4 Hz, 1H, C5-H), 4.33 – 4.29 (m, 2H, C7-H), 2.45 (s, 3H, C14-H), 1.41 (d, *J* = 6.4 Hz, 3H, C6-H).

^{13}C NMR (151 MHz, Acetone-*D*$_6$) δ 169.8 (d, *J* = 276.9 Hz, C8), 144.8 (C13), 143.2 (C4), 136.6 (C11), 133.8 (C12), 129.4 (C2), 128.8 (C1), 127.1 (C3), 109.7 (C10), 79.5 (d, *J* = 18.1 Hz, C9), 79.3 (C5), 65.4 (d, *J* = 31.7 Hz, C7), 23.8 (C6), 21.3 (C14).

^{19}F NMR (471 MHz, Acetone-*D*$_6$) δ −84.29 (dd, *J* = 34.3, 14.5 Hz, 1F, C8-F), −150.51 - −150.57 (4F, BF_4).

HRMS (+ESI) calc: $[M]^+$ ($C_{18}H_{19}FIO$) 397.0459, measured 397.0446 (3.27 ppm error).

IR (neat) ν_{max}/cm^{-1} : 3069, 2989, 2667, 2551, 2091, 1916, 1686, 1602, 1583, 1567, 1484, 1452, 1421, 1393, 1321, 1272, 1179, 1091, 1069, 1055, 1026, 1007, 933, 849, 830, 801, 753, 708, 683, 667, 643, 542, 541.

***(Z)-(3-(benzhydryloxy)-2-fluoroprop-1-en-1-yl)(p-tolyl)iodonium tetrafluoroborate* (3ak)**

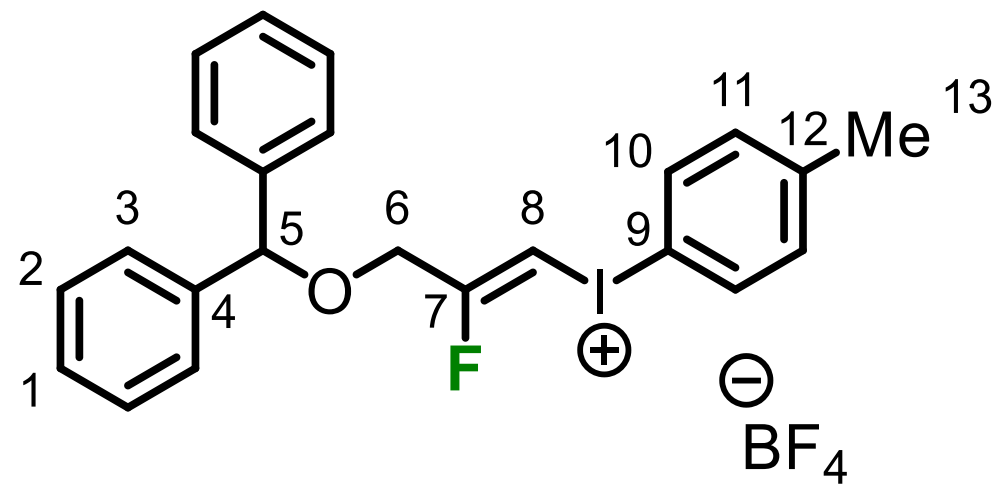

Prepared according to **General procedure III** from alkyne **1ak** with 20 mol% Ag_2CO_3, affording the title compound as a viscous dark-brown oil (1.04 g, 38%).

^{1}H NMR (500 MHz, $CDCl_3$) δ 7.88 – 7.84 (m, 2H, C10-H), 7.34 – 7.30 (m, 8H, C2-H, C3-H), 7.28 – 7.23 (m, 4H, C1-H, C11-H), 6.71 (d, J = 32.8 Hz, 1H, C8-H), 5.49 (s, 1H, C5-H), 4.31 (d, J = 6.9 Hz, 2H, C6-H), 2.41 (s, 3H, C13-H).

^{13}C NMR (126 MHz, $CDCl_3$) δ 169.5 (d, J = 279.4 Hz, C7), 144.4 (C12), 140.5 (C4), 135.8 (C10), 133.4 (C11), 128.8 (C2), 128.2 (C1), 127.2 (C3), 107.3 (C9), 84.4 (C5), 76.8 (d, J = 18.0 Hz, C8) 65.1 (d, J = 33.1 Hz, C6), 21.6 (C13).

^{19}F NMR (376 MHz, $CDCl_3$) δ −76.52 (dt, J = 32.9, 7.1 Hz, 1F, C7-F), −146.43 – −146.49 (4F, BF_4).

HRMS (+ESI) calcd: $[M]^+$ ($C_{23}H_{21}FIO$) 459.0616, measured 459.0597 (4.14 ppm error).

IR (neat) ν_{max}/cm^{-1} : 2923, 2852, 1672, 1597, 1495, 1447, 1402, 1358, 1244, 1164, 1108, 1092, 1031, 1015, 1001, 936, 840, 816, 788, 767, 706, 692, 679, 662, 650, 586, 549.

(Z)-(3-(chroman-4-yloxy)-2-fluoroprop-1-en-1-yl)(p-tolyl)iodonium tetrafluoroborate **(3al)**

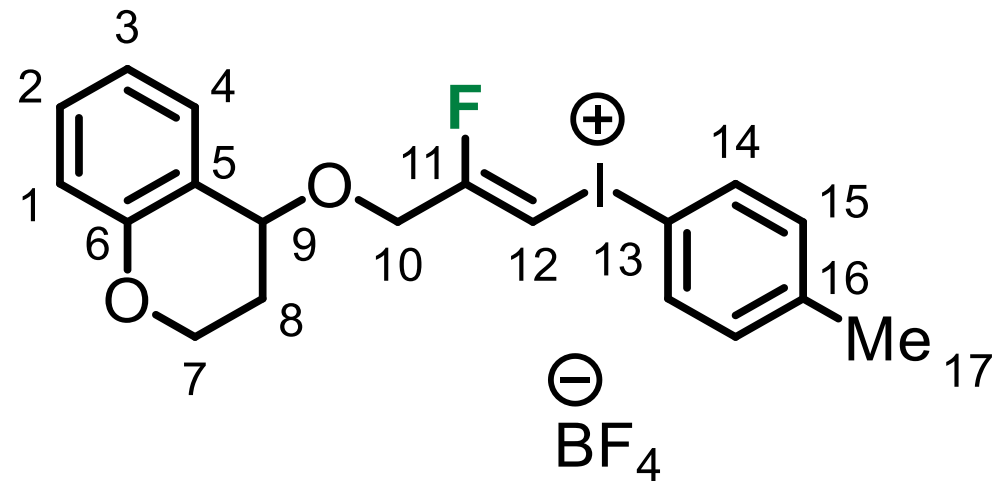

Prepared from alkyne **1al** according to **General procedure III** with 20 mol% Ag_2CO_3 (10% NMR).

Crude product carried forward to insertion step.

^{19}F NMR (376 MHz, $CDCl_3$) δ −76.86 (dt, *J* = 33.0, 7.7 Hz, 1F, C11-F), −146.59 – −146.65 (4F, BF_4)

HRMS (+ESI) calcd: $[M]^+$ ($C_{19}H_{19}FIO_2$) 425.0408, measured 425.0399 (2.12 ppm difference).

(Z)-(2-fluoro-3-(1-phenylethoxy)but-1-en-1-yl)(p-tolyl)iodonium tetrafluoroborate **(3am)**

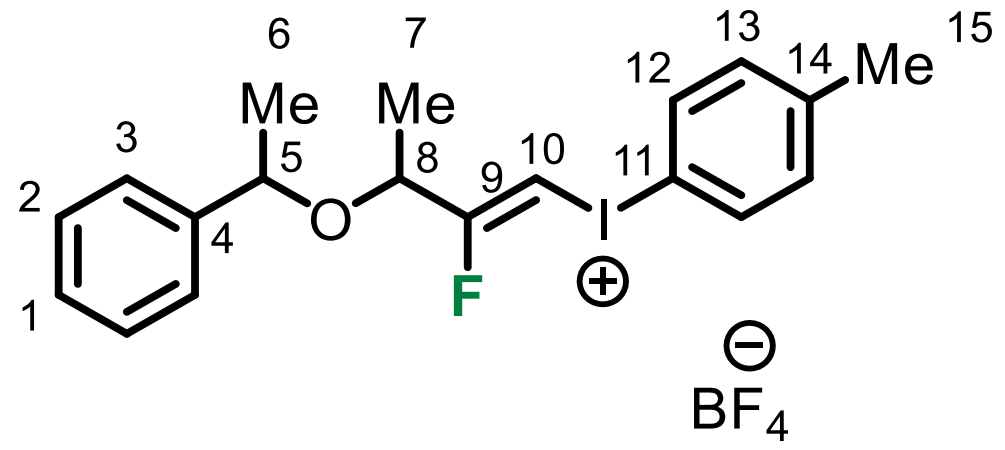

Produced according to **General procedure III** from **1am** to afford the title compound as a white solid (6.0 mg, 2%).

Isolated as a mixture of diastereomers (1.17:1).

^{1}H NMR (500 MHz, Acetone-*D$_6$*) Major diastereomer δ 8.16 (app. dt, *J* = 8.6, 1.8 Hz, 2H, C12-H), 7.49 (d, *J* = 8.6 Hz, 2H, C13-H), 7.34 (app. dt, *J* = 6.9, 1.4 Hz, 2H, C3-H), 7.32 – 7.24 (m, 3H, C1-H, C2-H), 7.22 (d, *J* = 34.1 Hz, 1H, C10-H), 4.53 (q, *J* = 6.5 Hz, 1H, C5-H), 4.19 (dq, *J* = 14.6, 6.6 Hz, 1H, C8-H), 2.47 (s, 3H, C15-H), 1.34 (d, *J* = 6.4 Hz, 3H, C6-H), 1.28 (d, *J* = 6.6 Hz, 3H, C7-H). **Minor diastereomer** δ 8.05 (dt, *J* = 8.5, 1.9 Hz, 2H, C12-H), 7.45 (d, *J* = 8.5 Hz, 2H, C13-H), 7.32 – 7.24 (m, 5H, C1-H, C2-H, C3-H), 7.12 (dd, *J* = 34.2, 0.9 Hz, 1H, C9-H), 4.67 (q, *J* = 6.4 Hz, 1H, C5-H), 4.49 – 4.39 (m, 1H, C8-H), 2.47 (s, 3H, C15-H), 1.41 (dd, *J* = 6.5, 0.6 Hz, 3H, C7-H), 1.40 (d, *J* = 6.4 Hz, 3H, C6-H).

^{13}C NMR (126 MHz, Acetone-*D$_6$*) Major diastereomer δ 172.1 (d, *J* = 283.5 Hz, C9), 144.9 (C14), 143.5 (C4), 136.5 (C12), 133.9 (C13), 129.5 (C3), 128.8 (C1), 127.1 (C2), 109.9 (d, *J* = 0.9 Hz, C11), 79.9 (d, *J* = 18.8 Hz, C10), 77.6 (C5), 71.4 (d, *J* = 27.5 Hz, C8), 24.4 (C6), 21.3 (C15), 19.3 (C7). **Minor diastereomer** δ 172.6 (d, *J* = 280.6 Hz, C9), 144.9 (C14), 143.7 (C4), 136.5 (C12), 133.9 (C13), 129.3 (C3), 128.6 (C1), 127.0 (C2), 109.6 (C11), 79.1 (d, *J* = 18.8 Hz, C10), 77.5 (C5), 71.5 (d, *J* = 28.9 Hz, C8), 23.6 (C6), 21.3 (C15), 18.6 (C7).

^{19}F NMR (376 MHz, Acetone-*D$_6$*) Major diastereomer δ −84.17 (dd, *J* = 34.2, 14.6 Hz, 1F, C9-F), −150.6 (d, *J* = 20.2 Hz, 4F, BF_4). **Minor diastereomer** δ −82.56 (dd, *J* = 34.2, 11.5 Hz, 1F, C9-F), −150.6 (d, *J* = 20.2 Hz, 4F, BF_4).

HRMS (+ESI) calcd. $[M]^+$ ($C_{19}H_{21}FIO$) 411.0616, measured 411.0597 (4.62 ppm error).

IR (neat) ν_{max}/cm^{-1}: 2970, 1740, 1368, 1216, 1228, 1075, 527.

(Z)-(2-fluoro-2-(1-methoxycyclohexyl)vinyl)(p-tolyl)iodonium tetrafluoroborate **(3an)**

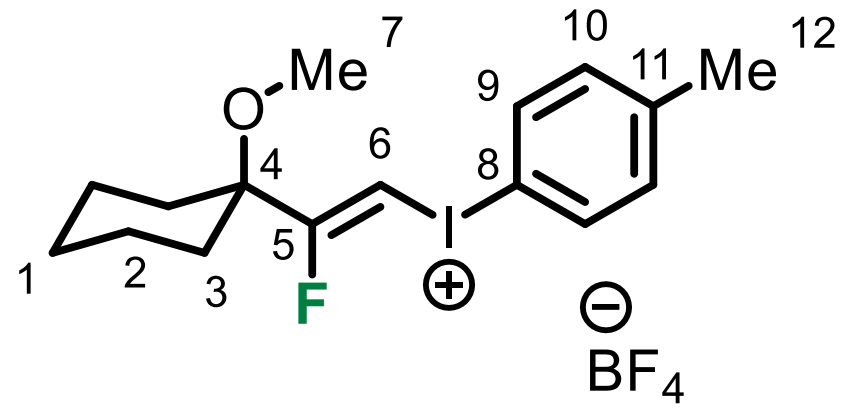

Produced according to **General procedure III** from alkyne **1an** to afford the title compound as an orange gel (313 mg, 34%).

^{1}H NMR (400 MHz, Acetone-*D*$_6$) δ 8.10 (d, *J* = 8.5 Hz, 2H, C9-H), 7.42 (d, *J* = 8.5 Hz, 2H, C10-H), 7.19 (d, *J* = 35.4 Hz, 1H, C6-H), 3.09 (s, 3H, C7-H), 2.42 (s, 3H, C12-H), 1.88 (dt, *J* = 13.6, 3.8 Hz, 2H, C3-H), 1.69 – 1.59 (m, 2H, C3-H), 1.59 – 1.41 (m, 5H, C2-H, C1-H), 1.32 – 1.20 (m, 1H, C1-H).

^{13}C NMR (126 MHz, Acetone-*D*$_6$) δ 172.7 (d, *J* = 284.4 Hz, C5), 144.6 (C11), 136.8 – 135.9 (m, C9), 133.7 (C10), 109.8 (C8), 81.9 (d, *J* = 20.5 Hz, C6), 77.9 (d, *J* = 22.5 Hz, C4), 50.8 (C7), 32.0 (d, *J* = 1.8 Hz, C3), 25.6 (C1), 21.7 (C2), 21.3 (C12).

^{19}F NMR (376 MHz, Acetone-*D*$_6$) δ −82.25 (d, *J* = 35.4 Hz, 1F, C5-F), −149.07 – −149.22 (m, 4F, BF_4).

HRMS (+ESI) calcd: $[M]^+$ ($C_{16}H_{21}FIO$) 375.0616, measured 375.0602 (3.73 ppm error).

IR (neat) v_{max}/cm^{-1}: 3100, 2929, 1654, 1491, 1244, 1090, 1054, 827, 800, 667, 477.

(Z)-(3-(2-(4-chlorophenoxy)ethoxy)-2-fluoroprop-1-en-1-yl)(p-tolyl)iodonium tetrafluoroborate **(3ao)**

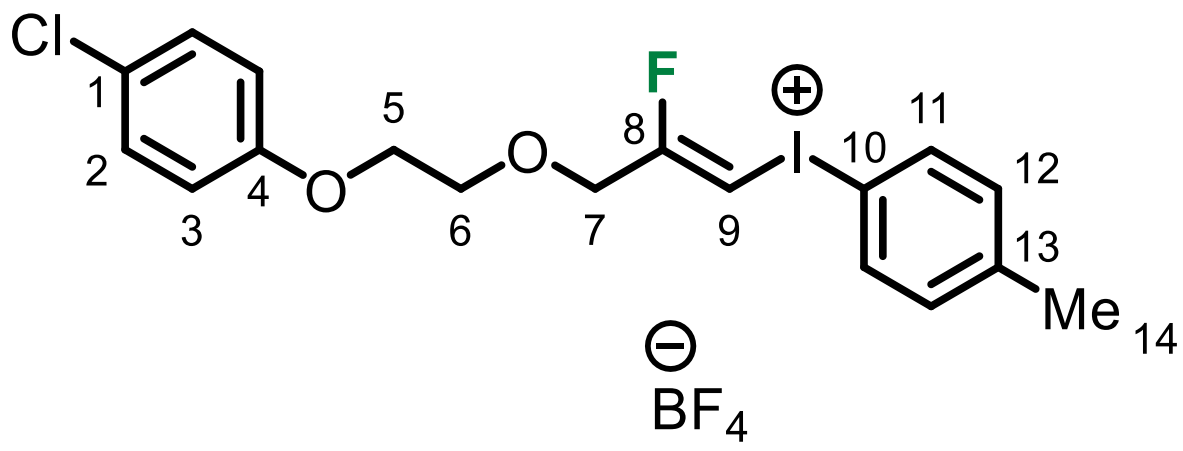

Produced according to **General procedure III** from alkyne **1ao** to afford the title compound as an orange gel (882 mg, 55%).

^{1}H NMR (500 MHz, Acetone-*D*$_6$) δ 8.13 (d, *J* = 8.2 Hz, 2H, C11-H), 7.43 (d, *J* = 8.2 Hz, 2H, C12-H), 7.28 (d, *J* = 9.1 Hz, 2H, C2-H), 7.20 (dt, *J* = 34.2, 1.1 Hz, 1H, C9-H), 6.94 (d, *J* = 9.1 Hz, 2H, C3-H), 4.58 (d, *J* = 9.3 Hz, 2H, C7-H), 4.17 – 4.14 (m, 2H, C5-H), 3.94 – 3.90 (m, 2H, C6-H), 2.44 (s, 3H, C14-H).

^{13}C NMR (126 MHz, Acetone-*D*$_6$) δ 169.6 (d, *J* = 277.8 Hz, C8), 158.5 (C4), 144.9 (C13), 136.7 – 136.5 (m, C11), 133.8 (C12), 130.1 (C2), 126.0 (C1), 117.0 (C3), 109.7 (C10), 79.6 (d, *J* = 18.1 Hz, C9), 70.6 (C5), 68.4 (C6), 67.9 (d, *J* = 31.9 Hz, C7), 21.3 (C14).

^{19}F NMR (376 MHz, Acetone-*D*$_6$) δ −80.22 (dt, *J* = 34.4, 8.1 Hz, 1F, C8-F), −150.20 – −150.54 (m, 4F, BF_4).

HRMS (+ESI) calcd: $[M]^+$ ($C_{18}H_{18}ClFIO_2$) 447.0019, measured 447.0010 (2.01 ppm error).

IR (neat) ν_{max}/cm^{-1}: 3100, 2929, 1654, 1491, 1244, 1090, 1054, 827, 800, 667, 477.

***(Z)-(2-fluoro-3-((1-tosylpiperidin-4-yl)oxy)prop-1-en-1-yl)(p-tolyl)iodonium tetrafluoroborate* (3ap)**

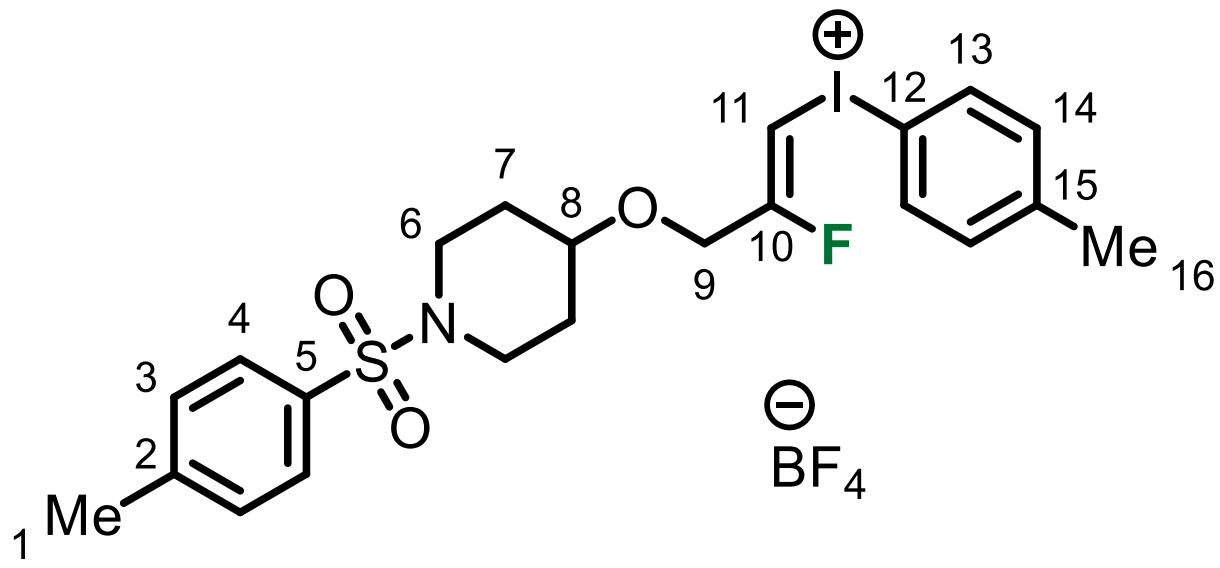

Produced according to **General procedure III** from alkyne **1ap** to afford the title compound as a white solid (737 mg, 42%).

^{1}H NMR (400 MHz, Acetone-*D*$_6$) δ 8.10 (app. dt, *J* = 8.5, 2.0 Hz, 2H, C13-H), 7.64 (d, *J* = 8.1 Hz, 2H, C4-H), 7.45 (d, *J* = 8.5 Hz, 2H, C14-H), 7.42 (d, *J* = 8.1 Hz, 2H, C3-H), 7.13 (dt, *J* = 34.2, 1.2 Hz, 1H, C11-H), 4.46 (dd, *J* = 10.2, 1.2 Hz, 2H, C9-H), 3.53 (tt, *J* = 7.6, 3.6 Hz, 1H, C8-H), 3.20 (ddd, *J* = 11.6, 7.1, 3.8 Hz, 2H, C6-H), 2.75 (ddd, *J* = 11.6, 8.4, 3.6 Hz, 2H, C6-H), 2.44 (s, 3H, C16-H), 2.44 (s, 3H, C1-H), 1.89 (dddd, *J* = 14.1, 7.6, 7.1, 3.6 Hz, 2H, C7-H), 1.60 (dddd, *J* = 14.1, 8.4, 3.8, 3.6 Hz, 2H, C7-H).

^{13}C NMR (126 MHz, Acetone-*D*$_6$) δ 169.9 (d, *J* = 277.7 Hz, C10), 144.9 (C15), 144.5 (C2), 138.1 (C5), 136.6 (C13), 133.8 (C14), 130.6 (C3), 128.5 (C4), 109.7 (C12), 79.7 (d, *J* = 18.2 Hz, C11), 75.0 (C8), 65.2 (d, *J* = 31.4 Hz, C9), 44.1 (C6), 31.0 (C7), 21.4 (C16), 21.3 (C1).

^{19}F NMR (376 MHz, Acetone-*D*$_6$) δ −79.55 (dt, *J* = 34.2, 10.2 Hz, 1F, C10-F), −150.37 (dd, *J* = 21.8, 16.5 Hz, 4F, BF_4).

HRMS (+ESI) calcd: $[M]^+$ ($C_{22}H_{26}FINO_3S$) 530.0657, measured 530.0637 (3.77 ppm error).

IR (neat) v_{max}/cm^{-1}: 3510, 3098, 2931, 1733, 1655, 1452, 1332, 1161, 1089, 1052, 936, 727, 548.

***(Z)-(2-fluoro-3-((4-methylphenyl)sulfonamido)prop-1-en-1-yl)(p-tolyl)iodonium tetrafluoroborate* (3ar)**

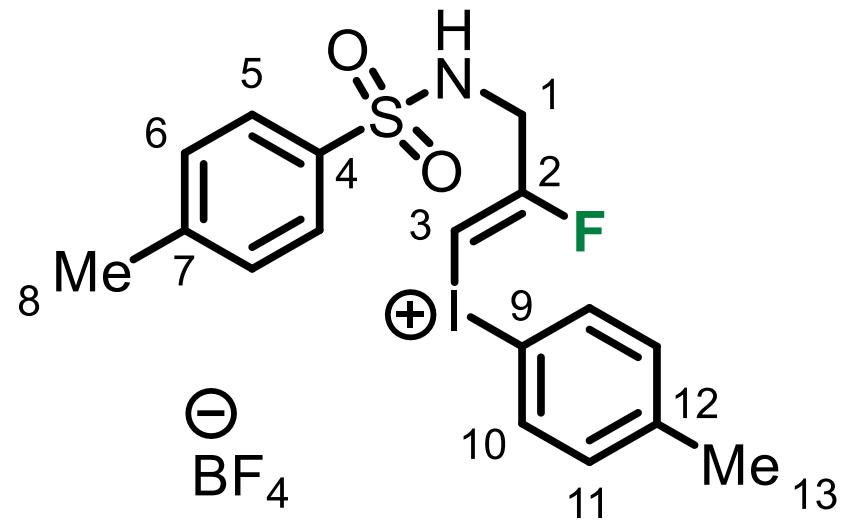

Propargylamine was tosylated according to General procedure I, and then converted into an FVI according to **General procedure III** to afford the title compound as a white solid (1.58 g, 34%).

^{1}H NMR (500 MHz, Acetone-*D_6*) δ 8.10 (app. dt, *J* = 8.3, 1.9 Hz, 2H, C10-H), 7.74 (app. dt, *J* = 8.4, 1.8 Hz, 2H, C5-H), 7.49 – 7.43 (m, 2H, C11-H), 7.40 – 7.37 (m, 2H, C6-H), 7.25 (t, *J* = 6.4 Hz, 1H, C1-NH), 7.15 (d, *J* = 33.6 Hz, 1H, C3-H), 4.15 (ddd, *J* = 9.6, 6.4, 1.1 Hz, 2H, C1-H), 2.46 (s, 3H, C13-H), 2.42 (s, 3H, C8-H).

^{13}C NMR (126 MHz, Acetone-*D_6*) δ 169.6 (d, *J* = 277.1 Hz, C2), 144.9 (C12), 144.6 (C7), 138.3 (C4), 136.5 (C10), 133.9 (C11), 130.7 (C6), 127.8 (C5), 109.7 (C9), 79.5 (d, *J* = 18.9 Hz, C3), 43.4 (d, *J* = 32.7 Hz, C1), 21.4 (C13), 21.3 (C8).

^{19}F NMR (376 MHz, Acetone-*D_6*) δ −76.27 (dt, *J* = 33.4, 9.5 Hz, 1F, C2-F), −150.71 – −151.09 (m, 4F, BF_4).

HRMS (+ESI) calcd: $[M]^+$ ($C_{17}H_{18}FINO_2S$) 446.0081, measured 446.0063 (4.04 ppm error).

IR (neat) v_{max}/cm^{-1}: 3296, 2970, 1739, 1660, 1437, 1366, 1216, 1160, 1087, 805, 660, 549.

***(Z)-(2-fluoro-3-((4-methylphenyl)sulfonamido)prop-1-en-1-yl)(phenyl)iodonium tetrafluoroborate* (3ar')**

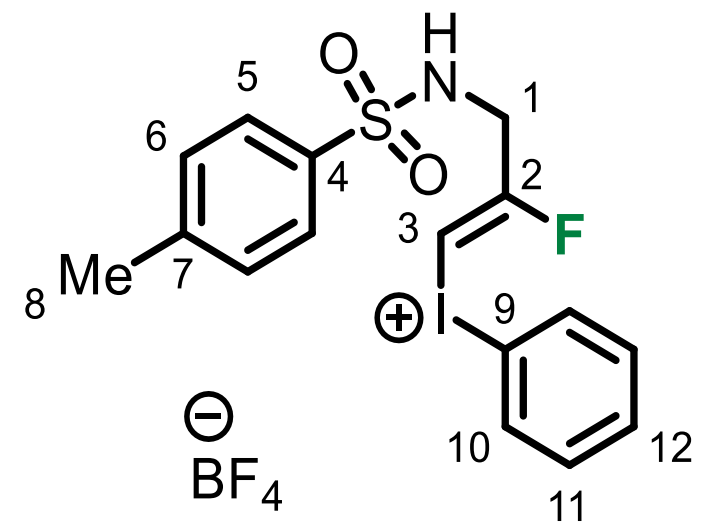

Propargylamine was tosylated according to General procedure I, and then converted into an FVI according to **General procedure III** using iodobenzene instead of iodotoluene to afford the title compound as a white solid (262 mg, 22%).

^{1}H NMR (500 MHz, Acetone-*D$_6$*) δ 8.27 – 8.20 (m, 2H, C10-H), 7.83 – 7.78 (m, 1H, C12-H), 7.74 (dt, *J* = 8.3, 1.8 Hz, 2H, C5-H), 7.67 – 7.61 (m, 2H, C11-H), 7.41 – 7.35 (m, 2H, C6-H), 7.25 (t, *J* = 6.3 Hz, 1H, C1-NH), 7.19 (dt, *J* = 33.5, 1.1 Hz, 1H, C3-H), 4.16 (ddd, *J* = 9.7, 6.4, 1.1 Hz, 2H, C1-H), 2.41 (s, 3H, C8-H).

^{13}C NMR (126 MHz, Acetone-*D$_6$*) δ 169.8 (d, *J* = 277.6 Hz, C3), 144.6 (C7), 138.2 (C4), 136.4 (C10), 133.7 (C12), 133.2 (C11), 130.7 (C6), 127.8 (C5), 113.5 (C9), 79.3 (d, *J* = 18.9 Hz, C3), 43.4 (d, *J* = 32.6 Hz, C1), 21.4 (C8).

^{19}F NMR (376 MHz, Acetone-*D$_6$*) δ −75.80 (dt, *J* = 33.6, 9.7 Hz, 1F, C2-F), −150.71 – −150.84 (m, 4F, BF_4).

HRMS (+ESI) calcd: $[M]^+$ ($C_{16}H_{16}FINO_2S$) 431.9925, measured 431.9907 (4.17 ppm error).

IR (neat) v_{max}/cm^{-1}: 3295, 2970, 1739, 1656, 1445, 1362, 1216, 1159, 1081, 987, 816, 739, 671, 550.

***(Z)-(2-fluoro-3-((4-nitrophenyl)sulfonamido)prop-1-en-1-yl)(p-tolyl)iodonium tetrafluoroborate* (3as)**

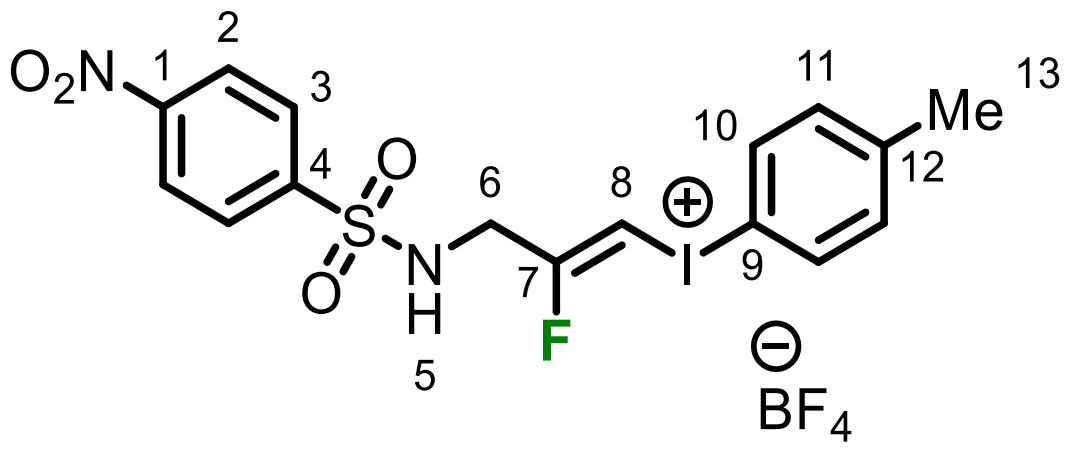

Prepared from alkyne **1as** according to **General procedure III** with 20 mol% Ag_2CO_3, affording the title compound as a brown viscous oil (1.30 g, 46% yield).

^{1}H NMR (500 MHz, Acetone-*D_6*) δ 8.39 (d, *J* = 8.2 Hz, 2H, C2-H), 8.14 (d, *J* = 8.2 Hz, 2H, C3-H), 8.07 (d, *J* = 8.1 Hz, 2H, C10-H), 7.69 (1H, t, *J* = 6.4 Hz, N5-H), 7.43 (d, *J* = 8.1 Hz, 2H, C11-H), 7.15 (1H, d, *J* = 33.7 Hz, C8-H), 4.30 (2H, ddd, *J* = 10.5, 6.3, 1.0 Hz, C6-H), 2.45 (3H, s, C13-H).

^{13}C NMR (126 MHz, Acetone-*D_6*) δ 168.8 (d, *J* = 277.0 Hz, C7), 151.2 (C1), 146.8 (C4), 144.9 (C12), 136.5 (C10), 133.8 (C11), 129.3 (C3), 125.4 (C2), 109.7 (C9), 79.9 (d, *J* = 18.9 Hz, C8), 43.3 (d, *J* = 32.2 Hz, C6), 21.3 (C13).

^{19}F NMR (376 MHz, Acetone-*D_6*) δ −76.59 (dt, *J* = 33.7, 10.6 Hz, 1F, C7-F), −150.38 – −150.43 (m, 4F, BF_4).

HRMS (+ESI) calcd: $[M]^+$ ($C_{16}H_{15}FIN_2O_4S$) 476.9776, measured 476.9756 (4.19 ppm error).

IR (neat) ν_{max}/cm^{-1}: 3288, 3107, 2159, 2024, 1655, 1607, 1527, 1479, 1433, 1403, 1348, 1311, 1248, 1213, 1163, 1088, 1055, 1009, 995, 855, 832, 801, 736, 684.

***(Z)-(2-fluoro-3-((4-methylphenyl)sulfonamido)but-1-en-1-yl)(p-tolyl)iodonium tetrafluoroborate* (3at)**

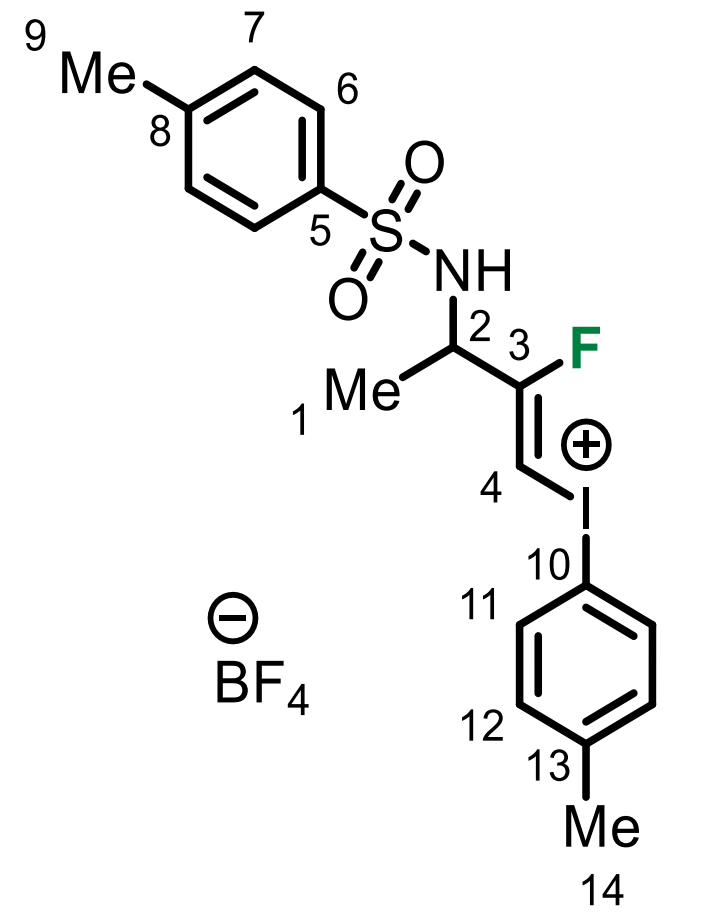

Produced according to **General procedure III** from alkyne **1at** to afford the title compound as a white solid (109 mg, 10%).

^{1}H NMR (400 MHz, Acetone-*D*$_6$) δ 8.06 (d, *J* = 8.3 Hz, 2H, C11-H), 7.70 (d, *J* = 8.0 Hz, 2H, C6-H), 7.44 (d, *J* = 8.3 Hz, 2H, C12-H), 7.35 (d, *J* = 8.0 Hz, 2H, C7-H), 7.15 (d, *J* = 33.8 Hz, 1H, C4-H), 4.42 (dq, *J* = 12.0, 6.8 Hz, 1H, C2-H), 2.44 (s, 3H, C14-H), 2.39 (s, 3H, C9-H), 1.23 (d, *J* = 6.8 Hz, 3H, C1-H).

^{13}C NMR (126 MHz, Acetone-*D*$_6$) δ 172.2 (d, *J* = 280.2 Hz, C3), 144.9 (C13), 144.6 (C8), 138.1 (C5), 136.4 (C11), 133.9 (C12), 130.7 (C7), 127.8 (C6), 109.6 (C10), 79.3 (d, *J* = 19.9 Hz, C4), 50.8 (d, *J* = 29.0 Hz, C2), 21.4 (C14), 21.3 (C9), 18.5 (C1).

^{19}F NMR (376 MHz, Acetone-*D*$_6$) δ −81.31 (dd, *J* = 33.8, 13.2 Hz, 1F, C3-F), −151.09 (d, *J* = 18.7 Hz, 4F, BF_4).

HRMS (+ESI) calcd: $[M]^+$ ($C_{18}H_{20}FINO_2S$) 460.0238, measured 460.0230 (1.74 ppm error).

IR (neat) v_{max}/cm^{-1}: 3294, 3101, 1656, 1597, 1441, 1331, 1159, 1072, 994, 809, 669, 550, 476.

(Z)-(2-fluoro-2-(1-((4-methylphenyl)sulfonamido)cyclohexyl)vinyl)(p-tolyl)iodonium tetrafluoroborate **(3av)**

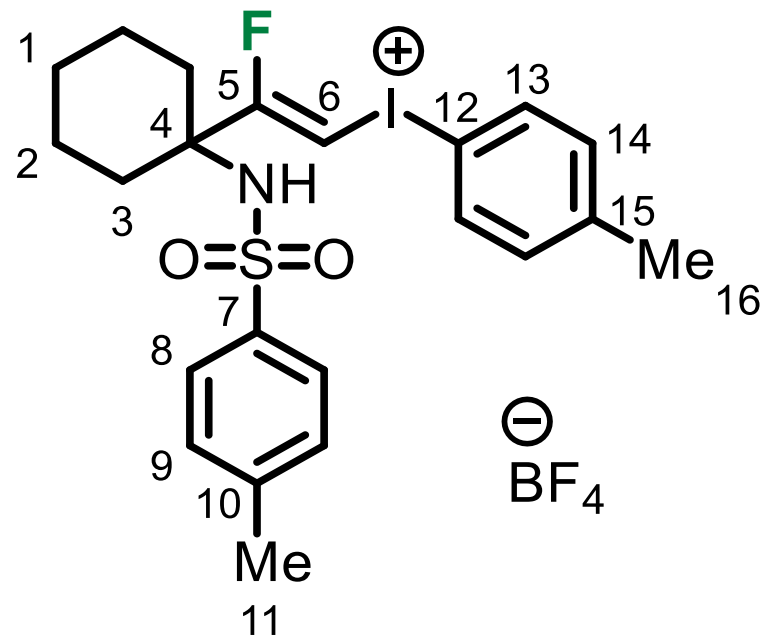

Produced according to **General procedure III** from alkyne **1av** to afford the title compound as a white solid (2.17 g, 72%).

^{1}H NMR (500 MHz, Acetone-*D$_6$*) δ 8.09 (app. dt, *J* = 8.5, 2.6 Hz, 2H, C13-H), 7.72 (app. dt, *J* = 8.3, 1.9 Hz, 2H, C8-H), 7.46 (d, *J* = 8.5 Hz, 2H, C14-H), 7.40 (d, *J* = 8.3 Hz, 2H, C9-H), 7.15 (d, *J* = 35.1 Hz, 1H, C6-H), 6.97 (s, 1H, C4-NH), 2.92 – 2.81 (m, 2H, C3-H), 2.45 (s, 3H, C16-H), 2.43 (s, 3H, C11-H), 1.79 – 1.69 (m, 2H, C3-H), 1.47 – 1.32 (m, 5H, C1-H, C2-H), 1.29 – 1.18 (m, 1H, C1-H).

^{13}C NMR (126 MHz, Acetone-*D$_6$*) δ 173.6 (d, *J* = 281.3 Hz, C5), 144.6 (d, *J* = 3.3 Hz, C15), 144.3 (C10), 140.9 (d, *J* = 2.4 Hz, C7), 136.3 (C13), 133.8 (C14), 130.4 (C9), 127.8 (d, *J* = 1.5 Hz, C8), 109.4 (C12), 79.9 (d, *J* = 21.7 Hz, C6), 60.5 (d, *J* = 22.5 Hz, C5), 33.9 (d, *J* = 1.6 Hz, C3), 25.4 (C1), 21.5 (C2), 21.4 (C16), 21.3 (C11).

^{19}F NMR (377 MHz, Acetone-*D$_6$*) δ −78.51 (d, *J* = 35.2 Hz, 1F, C5-F), −150.52 (s, 4F, BF_4).

HRMS (+ESI) calcd: $[M]^+$ ($C_{22}H_{26}FINO_2S$) 514.0707, measured 514.0687 (3.89 ppm error).

IR (neat) v_{max}/cm^{-1}: 3276, 3109, 2938, 2864, 1691, 1633, 1450, 1327, 1154, 1091, 1057, 1019, 801, 668, 550.

***(Z)-(2-fluoro-3-methyl-3-((4-methylphenyl)sulfonamido)but-1-en-1-yl)(p-tolyl)iodonium tetrafluoroborate* (3au)**

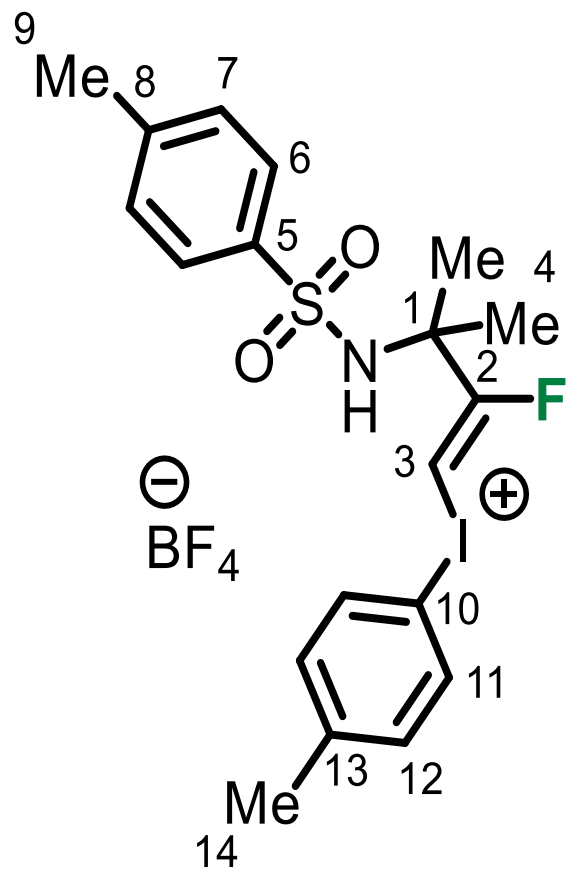

Produced according to **General procedure III** from alkyne **1au** to afford the title compound as a white solid (672 mg, 60%).

^{1}H NMR (400 MHz, Acetone-*D*$_6$) δ 8.10 (d, *J* = 8.8 Hz, 2H, C11-H), 7.72 (d, *J* = 8.3 Hz, 2H, C6-H), 7.46 (d, *J* = 8.1 Hz, 2H, C12-H), 7.39 (d, *J* = 8.0 Hz, 2H, C7-H), 7.22 (s, 1H, C1-NH), 7.21 (d, *J* = 34.1 Hz, 1H, C3-H), 2.46 (s, 3H, C14-H), 2.42 (s, 3H, C9-H), 1.46 (s, 6H, C4-H).

^{13}C NMR (126 MHz, Acetone-*D*$_6$) δ 174.2 (d, *J* = 280.4 Hz, C2), 144.7 (C8), 144.2 (C13), 141.3 (C5), 136.3 (C11), 133.8 (C12), 130.5 (C7), 127.6 (C6), 109.4 (C10), 79.1 (d, *J* = 21.3 Hz, C3), 57.5 (d, *J* = 24.6 Hz, C1), 26.0 (C4), 21.4 (C14), 21.3 (C9).

^{19}F NMR (376 MHz, Acetone-*D*$_6$) δ −78.10 (d, *J* = 34.1 Hz, 1F, C2-F), −149.91 – −151.47 (m, 4F, BF_4).

HRMS (+ESI) calcd: $[M]^+$ ($C_{19}H_{22}FINO_2S$) 474.0394, measured 474.0372 (3.36 ppm error).

IR (neat) v_{max}/cm^{-1}: 3284, 3113, 1739, 1638, 1328, 1151, 1069, 997, 801, 662, 546.

***(Z)-(2-fluoro-3-((4-methylphenyl)sulfonamido)-3-phenylprop-1-en-1-yl)(p-tolyl)iodonium tetrafluoroborate* (3aw)**

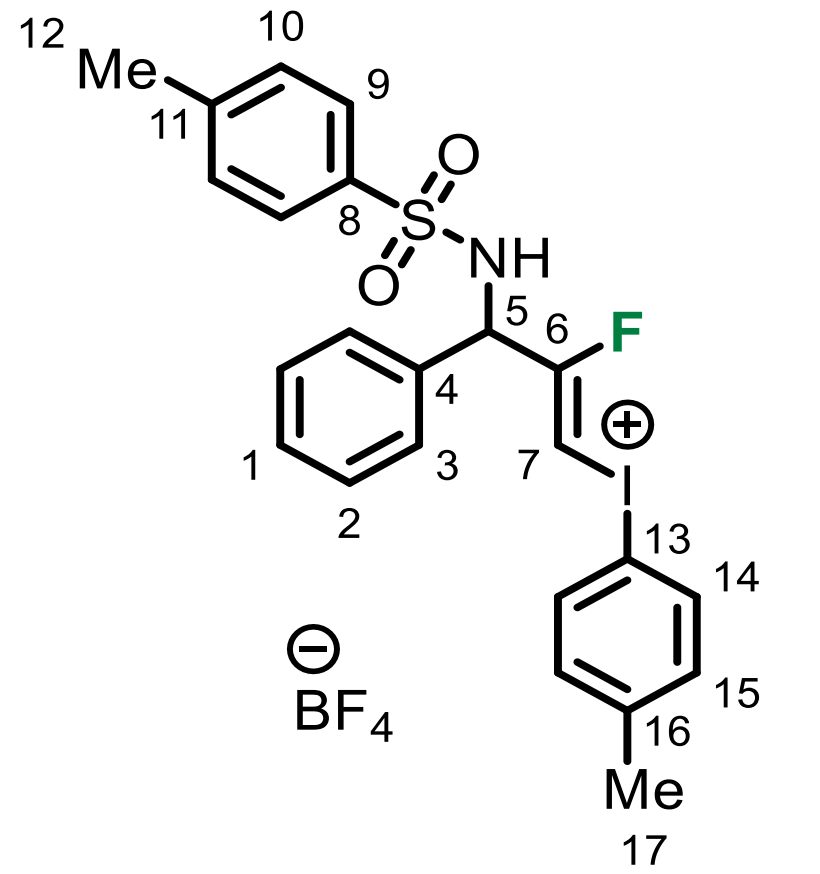

Produced according to **General procedure III-A** from alkyne **1aw** to afford the title compound as a white solid (1.896 g, 76%).

^{1}H NMR (500 MHz, Acetone-*D*$_6$) δ 8.09 (app. dt, *J* = 8.6, 2.0 Hz, 2H, C14-H), 7.69 (d, *J* = 7.9 Hz, 1H, C5-NH), 7.67 (dt, *J* = 8.3, 1.8 Hz, 2H, C9-H), 7.50 – 7.43 (m, 2H, C15-H), 7.37 (dd, *J* = 33.5, 0.9 Hz, 1H, C7-H), 7.34 – 7.25 (m, 5H, C1-H, C3-H, C10-H), 7.22 – 7.16 (m, 2H, C2-H), 5.57 (dd, *J* = 11.7, 7.9 Hz, 1H, C5-H), 2.48 (s, 3H, C17-H), 2.38 (s, 3H, C12-H).

^{13}C NMR (126 MHz, Acetone-*D*$_6$) δ 170.2 (d, *J* = 278.8 Hz, C6), 145.0 (C16), 144.6 (C11), 138.7 (C8), 136.5 (C14), 133.9 (C15), 130.5 (C10), 129.9 (C1), 129.8 (C3), 128.3 (C2), 127.8 (C9), 109.8 (C13), 81.0 (d, *J* = 19.5 Hz, C7), 58.6 (d, *J* = 30.1 Hz, C5), 21.4 (C17), 21.3 (C12).

^{19}F NMR (471 MHz, Acetone-*D*$_6$) δ −78.47 (dd, *J* = 33.4, 11.6 Hz, 1F, C6-F), −151.35 (d, *J* = 25.1 Hz, 4F, BF_4).

HRMS (+ESI) calcd: $[M]^+$ ($C_{23}H_{22}FINO_2S$) 522.0394, measured 522.0389 (0.96 ppm error).

IR (neat) v_{max}/cm^{-1}: 3270, 2970, 1739, 1441, 1366, 1229, 1217, 1160, 1092, 1068.

(Z)-(2-fluoro-3-(4-fluorophenyl)-3-((4-methylphenyl)sulfonamido)prop-1-en-1-yl)(p-tolyl)iodonium tetrafluoroborate **(3ax)**

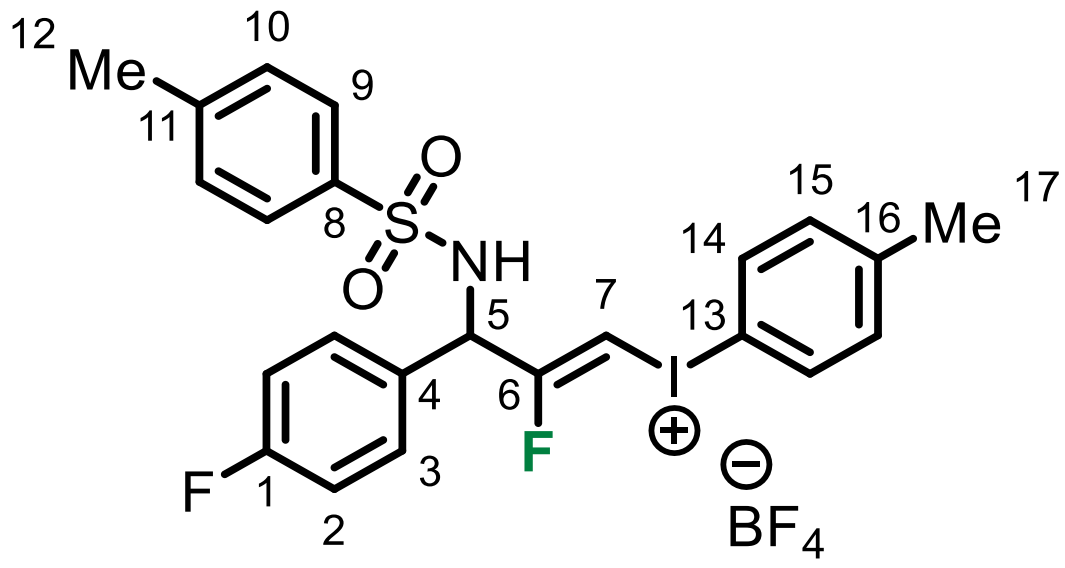

Produced according to **General procedure III-A** from alkyne **1ax** to afford the title compound as a white solid (283 mg, 30%).

^{1}H NMR (500 MHz, Acetone-*D$_6$*) δ 8.07 (app. dt, *J* = 8.4, 1.7 Hz, 2H, C14-H), 7.73 (d, *J* = 8.2 Hz, 1H, C5-NH), 7.66 (dt, *J* = 8.3, 1.9 Hz, 2H, C9-H), 7.46 (d, *J* = 8.4 Hz, 2H, C15-H), 7.34 (dd, *J* = 33.4, 0.9 Hz, 1H, C7), 7.29 – 7.25 (m, 4H, C3-H, C10-H), 7.04 (dt, *J* = 8.7, 2.3 Hz, 2H, C2-H), 5.62 (dd, *J* = 11.5, 8.1 Hz, 1H, C5-H), 2.47 (s, 3H, C17-H), 2.37 (s, 3H, C12-H).

^{13}C NMR (126 MHz, Acetone-*D$_6$*) δ 168.8 (d, *J* = 278.2 Hz, C6), 162.8 (d, *J* = 246.7 Hz, C1), 144.0 (C16), 143.7 (C11), 137.8 (C8), 135.6 (C14), 133.0 (C15), 131.1 (dd, *J* = 3.2, 1.1 Hz, C4) 129.7 (d, *J* = 8.6 Hz, C3), 129.6 (C10), 126.9 (C9), 115.6 (d, *J* = 22.0 Hz, C2), 109.0 (C13), 80.4 (d, *J* = 19.4 Hz, C7), 57.0 (d, *J* = 30.3 Hz, C5), 20.5 (C17), 20.4 (C12).

^{19}F NMR (376 MHz, Acetone-*D$_6$*) δ −78.84 (dd, *J* = 33.4, 11.5 Hz, 1F, C6-F), −113.92 (tt, *J* = 8.7, 5.0 Hz, 1F, C1-F), −150.39 (d, *J* = 20.3 Hz, 4F, BF_4).

HRMS (+ESI) calcd: $[M]^+$ ($C_{23}H_{21}F_2INO_2S$) 540.0300, measured 540.0278 (4.07 ppm error).

IR (neat) ν_{max}/cm^{-1}: 3274, 3093, 2962, 1730, 1600, 1510, 1334, 1230, 1160, 1074, 800, 667, 574, 549.

(Z)-(2-fluoro-3-((4-methylphenyl)sulfonamido)-3-(4-(trifluoromethyl)phenyl)prop-1-en-1-yl)(p-tolyl)iodonium tetrafluoroborate **(3az)**

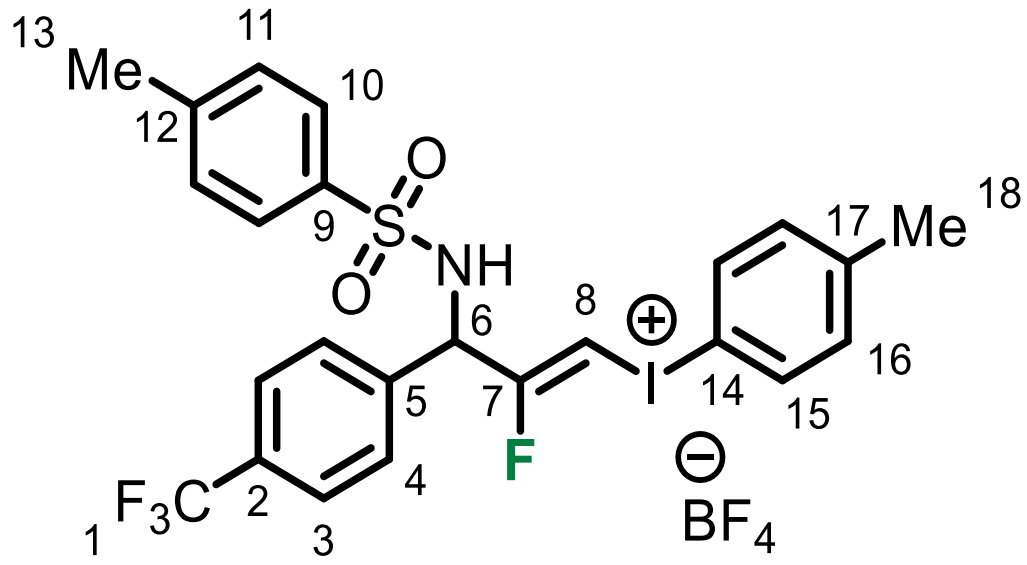

Produced according to **General procedure III-A** from alkyne **1az** to afford the title compound as a white solid (455 mg, 30%).

Contains 22% w/w unknown impurity.

^{1}H NMR (400 MHz, Acetone-*D*$_6$) δ 8.08 (app. dt, *J* = 8.3, 2.2 Hz, 2H, C15-H), 7.87 (d, *J* = 8.6 Hz, 1H, C6-NH), 7.67 – 7.58 (m, 4H, C3-H, C10-H), 7.49 (d, *J* = 8.2 Hz, 2H, C4-H), 7.45 (d, *J* = 8.3 Hz, 2H, C16-H), 7.38 (d, *J* = 33.5 Hz, 1H, C8-H), 7.24 (d, *J* = 8.1 Hz, 2H, C11-H), 5.79 (dd, *J* = 12.4, 8.6 Hz, 1H, C6-H), 2.47 (s, 3H, C18-H), 2.35 (s, 3H, C13-H).

^{13}C NMR (126 MHz, Acetone-*D*$_6$) δ 168.8 (d, *J* = 278.4 Hz, C7), 150.2 (C5), 144.9 (C17), 144.6 (C12), 138.6 (C9), 136.6 (C15), 133.9 (C16), 131.2 (q, *J* = 32.5 Hz, C2), 130.5 (C11), 129.3 (C4), 127.8 (C10), 126.6 (q, *J* = 3.8 Hz, C3), 124.9 (q, *J* = 271.6 Hz, C1), 109.7 (C14), 81.7 (d, *J* = 19.1 Hz, C8), 58.1 (d, *J* = 29.8 Hz, C6), 21.4 (C18), 21.3 (C13).

^{19}F NMR (376 MHz, Acetone-*D*$_6$) δ −63.17 (s, 3F, C1-F), −79.22 (dd, *J* = 33.5, 12.4 Hz, 1F, C7-F), −150.45 – −150.50 (m, 4F, BF_4).

HRMS (+ESI) calcd: $[M]^+$ ($C_{24}H_{21}F_4INO_2S$) 590.0268, measured 590.0286 (3.05 ppm error).

IR (neat) ν_{max}/cm^{-1}: 3271, 3092, 1738, 1683, 1632, 1325, 1162, 1122, 1066, 1017, 996, 801, 668, 546.

***(Z)-(3-(4-bromophenyl)-2-fluoro-3-((4-methylphenyl)sulfonamido)prop-1-en-1-yl)(p-tolyl)iodonium tetrafluoroborate* (3ba)**

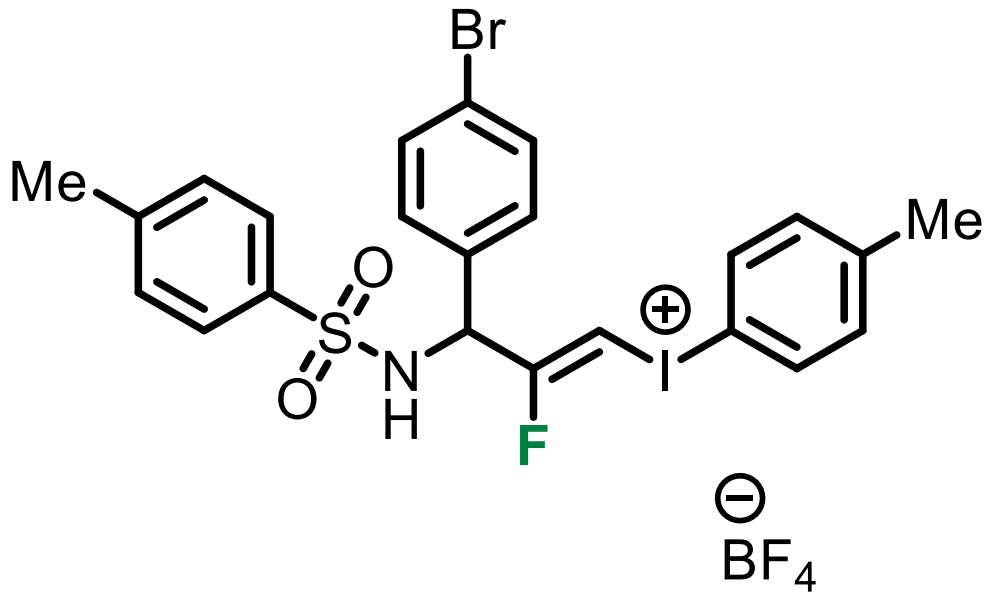

Prepared from alkyne **1ba** according to **General procedure III**. (33% NMR yield). The crude material was carried forward to the cyclisation step to form azetine **7ba**.

Identified by characteristic peaks in ^{19}F NMR:

^{19}F NMR (376 MHz, Acetone-*D6*): δ −78.91 (dd, *J* = 33.4, 11.7 Hz, 1F, C-F), −150.67 – −150.73 (4F, BF_4).

HRMS (+ESI) calcd: $[M]^+$ ($C_{23}H_{21}NO_2SBrF$) 599.9500, measured 599.9484 (2.67 ppm difference).

***(Z)-(3-(4-chlorophenyl)-2-fluoro-3-((4-methylphenyl)sulfonamido)prop-1-en-1-yl)(p-tolyl)iodonium tetrafluoroborate* (3bb)**

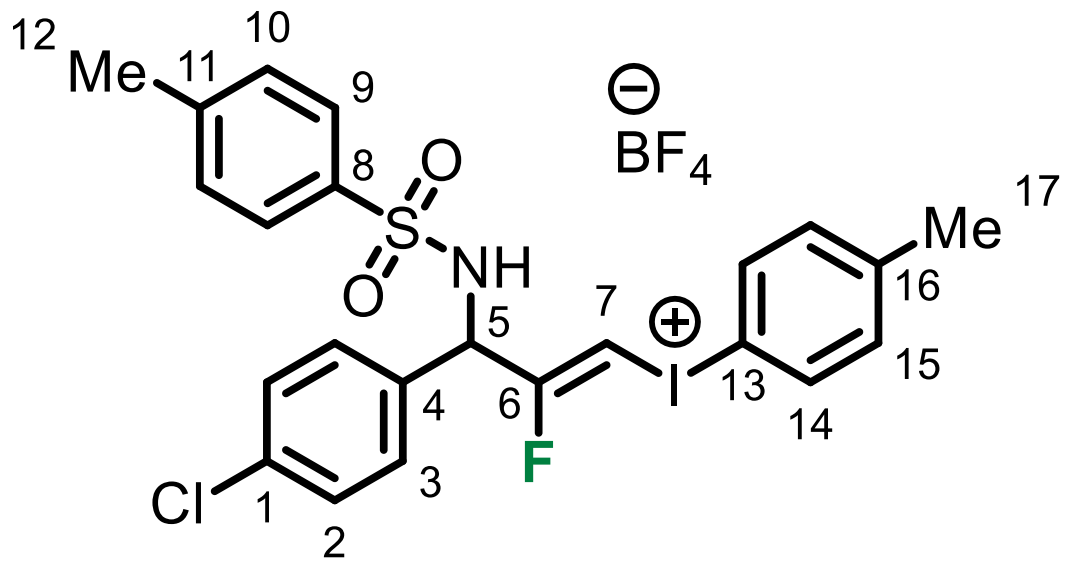

Produced according to **General procedure III-A** from alkyne **1bb** to afford the title compound as a brown solid (54.5 mg, 16%).

^{1}H NMR (500 MHz, Acetone-*D$_6$*) δ 8.04 (app. dt, J = 8.5, 1.9 Hz, 2H, C14-H), 7.75 (d, J = 8.5 Hz, 1H, C5-NH), 7.65 (app. dt, J = 8.5, 1.9 Hz, 2H, C9-H), 7.44 – 7.41 (m, 2H, C15-H), 7.30 (dd, J = 33.3, 0.9 Hz, 2H, C7-H), 7.31 – 7.24 (m, 6H, C2-H, C3-H, C10-H), 5.63 (dd, J = 11.8, 8.4 Hz, 1H, C5-H), 2.46 (s, 3H, C17-H), 2.37 (s, 3H, C12-H).

^{13}C NMR (126 MHz, Acetone-*D$_6$*) δ 169.1 (d, J = 278.2 Hz, C6), 144.7 (C16), 144.5 (C11), 138.7 (C8), 136.4 (C14), 135.2 (C1), 134.8 (C4), 133.7 (C15), 130.5 (C3), 130.1 (C2), 129.7 (C10), 127.8 (C9), 109.9 (C13), 81.5 (d, J = 19.3 Hz, C7), 57.9 (d, J = 30.0 Hz, C5), 21.4 (C17), 21.3 (C12).

^{19}F NMR (376 MHz, Acetone-*D$_6$*) δ −79.15 (dd, J = 33.6, 11.9 Hz, 1F, C6-F), −149.89 (d[1:3], 4F, BF_4).

HRMS (+ESI) calcd: $[M]^+$ ($C_{23}H_{21}FINO_2SCl$) 556.0005, measured 555.9991 (2.52 ppm error).

IR (neat) v_{max}/cm^{-1}: 3463, 3276, 2970, 1739, 1653, 1596, 1491, 1368, 1216, 1159, 1091, 814, 669, 546.

***(Z)*-(2-fluoro-4-((4-methylphenyl)sulfonamido)but-1-en-1-yl)(p-tolyl)iodonium tetrafluoroborate* (3be)**

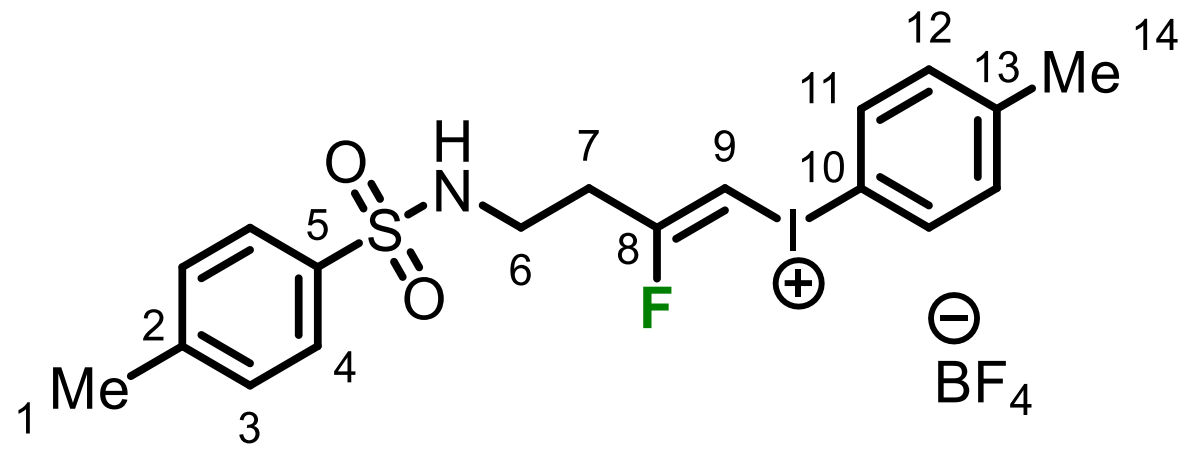

Produced according to **General procedure III** from alkyne **1be** to afford the title compound as a white solid (22.4 mg, 5%).

^{1}H NMR (600 MHz, Acetone-*D$_6$*) δ 8.10 (app. dt, *J* = 8.6, 1.9 Hz, 2H, C11-H), 7.73 (app. dt, *J* = 8.2, 1.8 Hz, 2H, C4-H), 7.44 (d, *J* = 8.6 Hz, 2H, C12-H), 7.40 (d, *J* = 8.2 Hz, 2H, C3-H), 6.96 (d, *J* = 34.4 Hz, 1H, C9-H), 6.67 (t, *J* = 6.3 Hz, 1H, C6-NH), 3.18 (q, *J* = 6.4 Hz, 2H, C6-H), 2.94 (dt, *J* = 17.6, 6.4 Hz, 2H, C7-NH), 2.44 (s, 3H, C14-H), 2.42 (s, 3H, C1-H).

^{13}C NMR (151 MHz, Acetone-*D$_6$*) δ 171.0 (d, *J* = 276.1 Hz, C8), 144.6 (C13), 144.2 (C2), 138.4 (C5), 136.2 (C11), 133.8 (C12), 130.6 (C3), 127.8 (C4), 109.8 (C10), 78.7 (d, *J* = 20.8 Hz, C9), 40.0 (d, *J* = 1.2 Hz, C6), 33.3 (d, *J* = 24.3 Hz, C7), 21.4 (C14), 21.3 (C1).

^{19}F NMR (376 MHz, Acetone-*D$_6$*) δ −69.11 (dt, *J* = 34.7, 17.6 Hz, 1F, C8-F), −150.75 – −150.87 (m, 4F, BF_4).

HRMS (+ESI) calcd: $[M]^+$ ($C_{18}H_{20}FINO_2S$) 460.0238, measured 460.0227 (2.39 ppm error).

IR (neat) v_{max}/cm^{-1}: 2927, 2856, 1634, 1451, 1159, 1061, 765.

***(Z)-(3-((N-(cyanomethyl)-4-nitrophenyl)sulfonamido)-2-fluoroprop-1-en-1-yl)(p-tolyl)iodonium tetrafluoroborate* (3bi)**

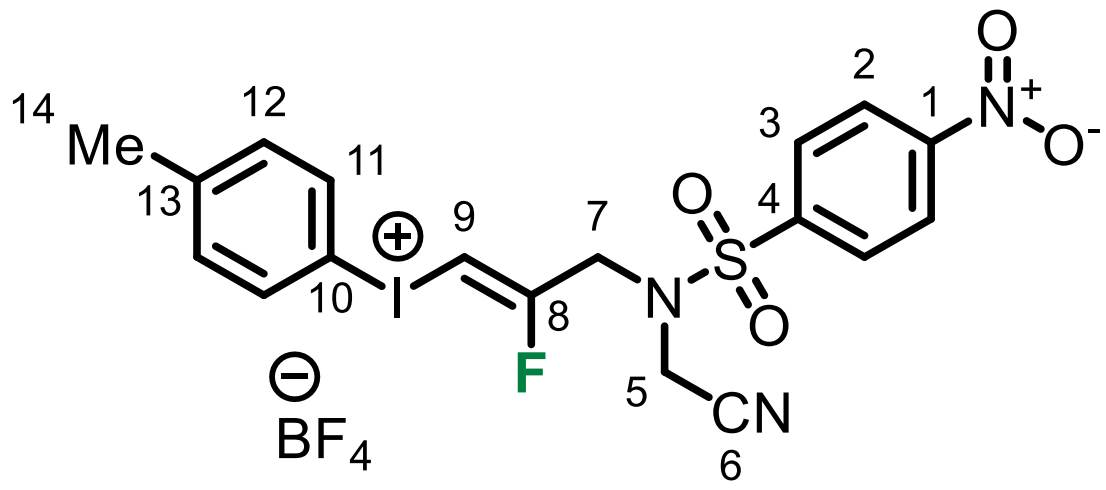

Produced according to **General procedure III** from alkyne **1bi** to afford the title compound as a white solid (413 mg, 54%).

^{1}H NMR (400 MHz, Acetone-*D*$_6$) δ 8.47 (app. dt, *J* = 8.7, 1.9 Hz, 2H, C2-H), 8.26 (app. dt, *J* = 8.7, 2.0 Hz, 2H, C3-H), 8.14 (app. dt, *J* = 8.3, 2.2 Hz, 2H, C11-H), 7.45 (d, *J* = 8.3 Hz, 2H, C12-H), 7.39 (d, *J* = 33.6 Hz, 1H, C9-H), 4.71 (d, *J* = 12.7 Hz, 2H, C7-H), 4.61 (s, 2H, C5-H), 2.46 (s, 3H, C14-H).

^{13}C NMR (126 MHz, Acetone-*D*$_6$) δ 165.4 (d, *J* = 278.0 Hz, C8), 151.0 (C1), 144.2 (C13), 143.0 (C4), 135.8 (C11), 133.0 (C12), 129.3 (C3), 124.8 (C2), 114.4 (C6), 108.6 (C10), 80.9 (d, *J* = 18.7 Hz, C9), 48.0 (d, *J* = 30.0 Hz, C7), 36.6 (C5), 20.4 (C14).

^{19}F NMR (376 MHz, Acetone-*D*$_6$) δ −77.02 (dt, *J* = 33.6, 12.7 Hz), −150.64 – −150.82 (m).

HRMS (+ESI) calcd: $[M]^+$ ($C_{18}H_{16}FIN_3O_4S$) 515.9885, measured 515.9876 (1.74 ppm error).

IR (neat) v_{max}/cm^{-1}: 3105, 2962, 1731, 1656, 1531, 1351, 1313, 1169, 1091, 856, 771, 744, 608.

***(R,Z)*-(3-((4,4-dimethyl-2-oxotetrahydrofuran-3-yl)oxy)-2-fluoroprop-1-en-1-yl)(p-tolyl)iodonium tetrafluoroborate** (3bj)**

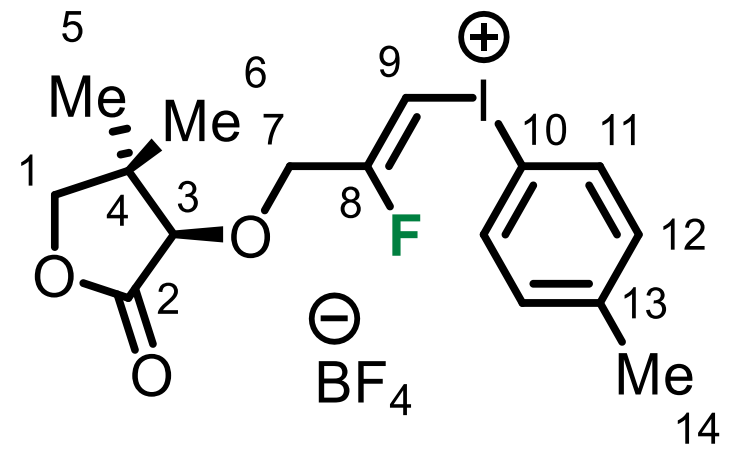

Produced according to **General procedure III** from alkyne **1bj** to afford the title compound as a white solid (228 mg, 21%).

^{1}H NMR (500 MHz, Acetone-*D_6*) δ 8.15 (d, *J* = 8.6 Hz, 2H, C11-H), 7.46 (d, *J* = 8.3 Hz, 2H, C12-H), 7.28 (dt, *J* = 34.0, 1.1 Hz, 1H, C9-H), 4.91 (ddd, *J* = 15.1, 9.0, 1.1 Hz, 1H, C7-H), 4.78 (ddd, *J* = 15.1, 10.8, 1.0 Hz, 1H, C7-H), 4.20 (s, 1H, C3-H), 4.00 (s, 2H, C7-H, C1-H), 2.45 (s, 3H, C14-H), 1.16 (s, 3H, C6-H), 1.03 (s, 3H, C5-H).

^{13}C NMR (126 MHz, Acetone-*D_6*) δ 175.2 (C2), 168.8 (d, *J* = 276.5 Hz, C8), 145.0 (C13), 136.7 (C11), 133.9 (C12), 109.7 (C15), 83.0 (C3), 80.3 (d, *J* = 18.1 Hz, C9), 76.4 (C1), 67.7 (d, *J* = 32.3 Hz, C7), 41.1 (C4), 22.7 (C6), 21.3 (C14), 19.5 (C5).

^{19}F NMR (471 MHz, Acetone-*D_6*) δ −79.95 (ddd, *J* = 34.1, 10.8, 9.0 Hz, 1F, C8-F), −150.76 (d, *J* = 25.2 Hz, 4F, BF_4).

HRMS (+ESI) calcd: $[M]^+$ ($C_{16}H_{19}FIO_3$) 405.0357, measured 405.0351 (1.48 ppm error).

IR (neat) v_{max}/cm^{-1}: 3508, 3099, 2968, 1766, 1657, 1473, 1275, 1105, 1060, 1007, 995, 803, 521, 477.

***(Z)-(2-fluoro-6-((4-methylphenyl)sulfonamido)hex-1-en-1-yl)(p-tolyl)iodonium tetrafluoroborate* (3bk)**

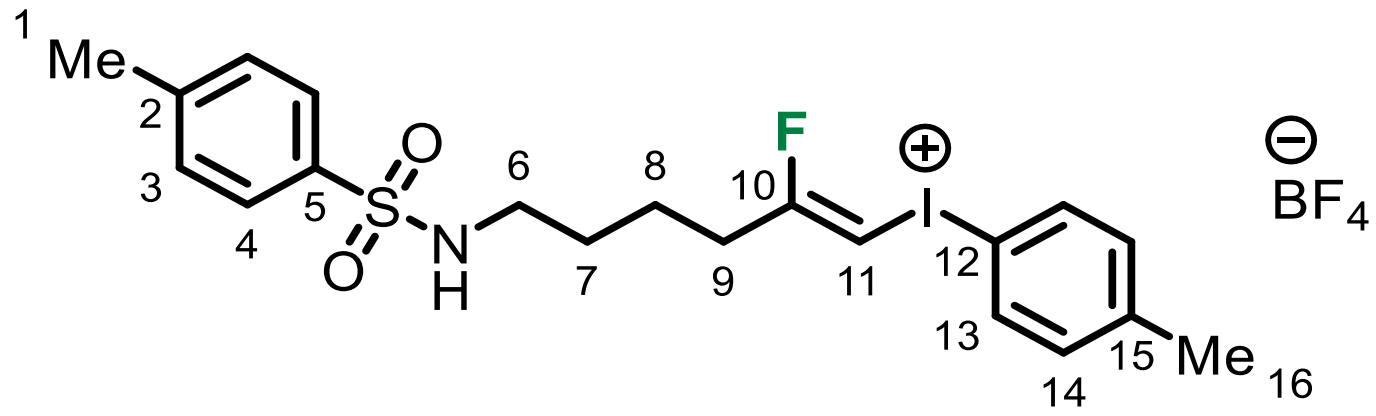

Produced according to **General procedure III** from alkyne **1bk** to afford the title compound as a brown solid (1.05 g, 61%).

^{1}H NMR (500 MHz, Acetone-*D$_6$*) δ 8.09 (app. dt, *J* = 8.5, 1.8 Hz, 2H, C13-H), 7.71 (app. dt, *J* = 8.2, 1.9 Hz, 2H, C4-H), 7.46 – 7.41 (m, 2H, C14-H), 7.41 – 7.37 (m, 2H, C3-H), 6.94 (dt, *J* = 34.4, 0.9 Hz, 1H, C11-H), 6.38 (t, *J* = 6.6 Hz, 1H, C6-NH), 2.87 (q, *J* = 6.6 Hz, 2H, C6-H), 2.71 (dt, *J* = 17.7, 7.3 Hz, 2H, C9-H), 2.44 (s, 3H, C16-H), 2.41 (s, 3H, C1-H), 1.68 – 1.61 (m, 2H, C7-H), 1.58 – 1.49 (m, 2H, C8-H).

^{13}C NMR (126 MHz, Acetone- *D$_6$*) δ 173.6 (d, *J* = 276.7 Hz, C10), 144.7 (C15), 143.8 (C2), 139.0 (C5), 136.3 (C13), 133.8 (C14), 130.4 (C3), 127.7 (C4), 109.9 (C12), 77.4 (d, *J* = 21.2 Hz, C11), 43.1 (C6), 31.8 (d, *J* = 24.2 Hz, C9), 29.2 (C7), 23.4 (d, *J* = 1.3 Hz, C8), 21.4 (C16), 21.3 (C1).

^{19}F NMR (377 MHz, Acetone-*D$_6$*) δ −67.61 (dt, *J* = 35.1, 17.9 Hz, 1F, C10-F), −150.63 – −150.74 (m, 4F, BF_4).

HRMS (+ESI) calcd: $[M-HF]^+$ ($C_{20}H_{23}INO_2S$) 468.0489, measured 468.0476 (2.78 ppm error).

IR (neat) v_{max}/cm^{-1}: 3283, 3100, 2939, 1738, 1643, 1428, 1324, 1156, 1077, 815, 664, 551, 478.

(Z)-(2-fluoro-3-hydroxy-3-(4-nitrophenyl)prop-1-en-1-yl)(p-tolyl)iodonium tetrafluoroborate **(3bm)**

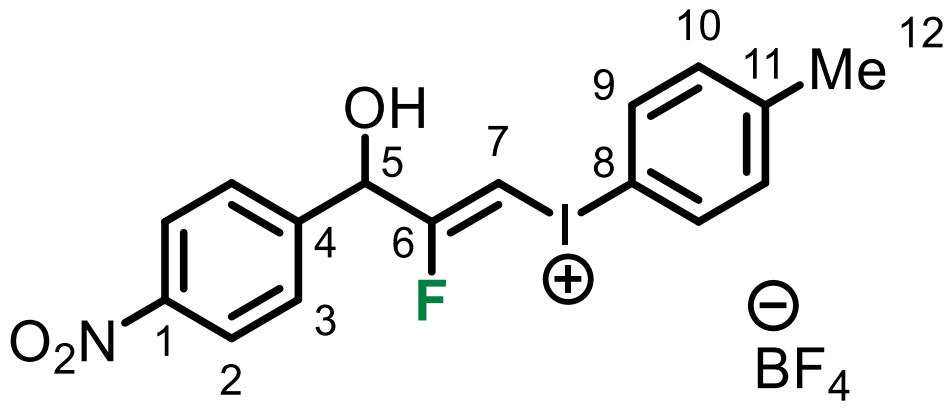

Produced according to **General procedure III-A** from alkyne **1bm** to afford the title compound as a white solid (264 mg, 12%).

^{1}H NMR (500 MHz, Acetone-*D₆*) δ 8.26 (app. dt, *J* = 8.9, 2.0 Hz, 2H, C2-H), 8.10 (app. dt, *J* = 8.5, 1.9 Hz, 2H, C9-H), 7.76 (app. dt, *J* = 8.9, 2.0 Hz, 2H, C3-H), 7.45 – 7.42 (m, 2H, C10-H), 7.42 (dd, *J* = 33.8, 0.9 Hz, 1H, C7-H), 6.12 (d, *J* = 5.0 Hz, 1H, C5-OH), 5.94 (dd, *J* = 9.5, 5.0 Hz, 1H, C5-H), 2.44 (s, 3H, C12-H).

^{13}C NMR (126 MHz, Acetone-D_6) δ 171.6 (d, *J* = 279.3 Hz, C6), 149.1 (C1), 146.3 (d, *J* = 1.2 Hz, C4), 144.9 (C11), 136.5 (C9), 133.8 (C10), 129.0 (C3), 124.5 (C2), 109.7 (C8), 80.1 (d, *J* = 18.2 Hz, C5), 71.2 (d, *J* = 30.0 Hz, C7), 21.3 (C12).

^{19}F NMR (376 MHz, Acetone-D_6) δ −82.10 (dd, *J* = 33.8, 9.5 Hz, 1F, C6-F), −150.05 – −150.17 (m, 4F, BF_4).

HRMS (+ESI) calcd: $[M]^+$ ($C_{16}H_{16}FINO_3$) 413.9997, measured 414.0001 (0.97 ppm error).

IR (neat) v_{max}/cm^{-1}: 3492, 3109, 1728, 1649, 1519, 1347, 1054, 1014, 995, 855, 801, 718, 447.

***(Z)-(2-fluoro-3-hydroxy-3-phenylprop-1-en-1-yl)(p-tolyl)iodonium tetrafluoroborate* (3bn)**

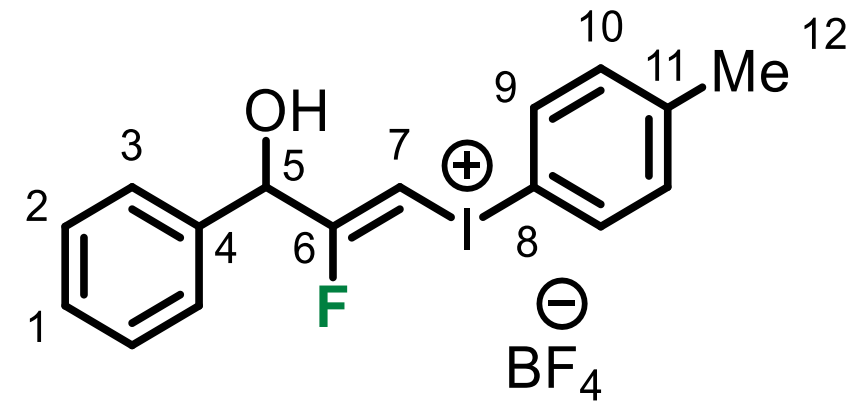

Produced according to **General procedure III-A** from 1-phenylprop-2-yn-1-ol to afford the title compound as a white solid (30 mg, 2%).

^{1}H NMR (400 MHz, Acetone-D_6) δ 8.08 (app. dt, *J* = 8.5, 1.9 Hz, 2H, C9-H), 7.47 – 7.36 (m, 7H, C1-H, C2-H, C3-H, C10-H), 7.31 (d, *J* = 33.9 Hz, 1H, C7-H), 5.67 (d, *J* = 7.6 Hz, 1H, C5-H), 2.44 (s, 3H, C12-H), 2.42 – 2.37 (m, 1H, C5-OH).

^{13}C NMR (126 MHz, Acetone-D_6) δ 172.3 (d, *J* = 278.6 Hz, C6), 143.8 (C11), 138.5 (d, *J* = 1.2 Hz, C4), 135.5 (C9), 132.9 (C10), 128.8 (C1), 128.6 (C3), 127.0 (C2), 109.0 (C8), 78.1 (d, *J* = 18.5 Hz, C7), 71.4 (d, *J* = 30.7 Hz, C5), 20.4 (C12).

^{19}F NMR (376 MHz, Acetone-D_6) δ −80.54 (dd, *J* = 33.9, 7.6 Hz, 1F, C6-F), −150.18 (d, *J* = 20.3 Hz, 4F, BF_4).

HRMS (+ESI) calcd. $[M]^+$ ($C_{16}H_{15}FIO$) 369.0146, measured 369.0135 (2.98 ppm error).

IR (neat) v_{max}/cm^{-1}: 3506, 3102, 1735, 1648, 1454, 1156, 1062, 703.

***(Z)-(3-((N-butyl-2-nitrophenyl)sulfonamido)-2-fluoroprop-1-en-1-yl)(p-tolyl)iodonium tetrafluoroborate* (3bp)**

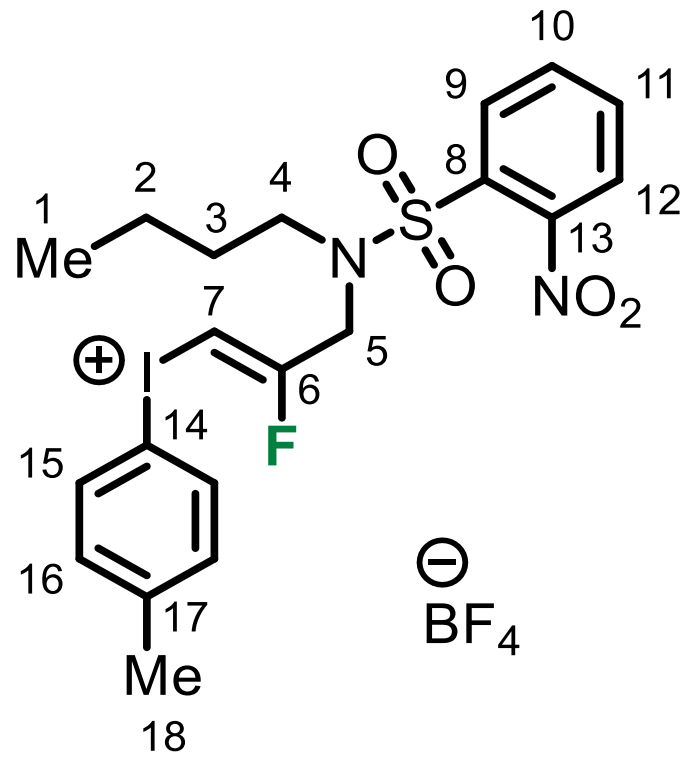

Produced according to **General procedure III** from alkyne **1bp** to afford the title compound as a white solid (1.75 g, 47%).

^{1}H NMR (500 MHz, Acetone-*D$_6$*) δ 8.15 – 8.11 (m, 1H, C11-H), 8.12 (d, *J* = 8.5 Hz, 2H, C15-H), 7.94 (ddd, *J* = 9.9, 6.4, 1.4 Hz, 1H, C10-H), 7.91 – 7.85 (m, 2H, C9-H, C12-H), 7.45 (d, *J* = 8.2 Hz, 2H, C16-H), 7.24 (dt, *J* = 33.5, 1.0 Hz, 1H, C7-H), 4.62 (dd, *J* = 12.8, 1.0 Hz, 2H, C5-H), 3.33 (dd, *J* = 8.5, 6.9 Hz, 2H, C4-H), 2.45 (s, 3H, C18-H), 1.51 – 1.41 (m, 2H, C3-H), 1.16 (h, *J* = 7.4 Hz, 2H, C2-H), 0.77 (t, *J* = 7.4 Hz, 3H, C1-H).

^{13}C NMR (126 MHz, Acetone-*D$_6$*) δ 167.9 (d, *J* = 279.1 Hz, C6), 149.0 (C13), 144.9 (C17), 136.6 (C15), 135.7 (C10), 133.8 (C16), 133.4 (C9), 132.8 (C8), 131.6 (C11), 125.4 (C12), 109.8 (C14), 81.1 (d, *J* = 18.9 Hz, C7-H), 49.2 (C4), 47.8 (d, *J* = 29.5 Hz, C5), 30.6 (C3), 21.3 (C18), 20.1 (C2), 13.8 (C1).

^{19}F NMR (376 MHz, Acetone-*D$_6$*) δ −76.34 (dt, *J* = 33.6, 12.9 Hz, 1F, C6-F), −150.11 (d, *J* = 20.3 Hz, 4F, BF_4).

HRMS (+ESI) calcd. $[M]^+$ ($C_{20}H_{23}FIN_2O_4S$) 533.0402, measured 533.0387 (2.81 ppm error).

IR (neat) v_{max}/cm^{-1}: 3098, 2962, 2874, 1738, 1690, 1653, 1542, 1359, 1162, 1053, 995, 773, 577.

***(Z)-(2-fluoro-3-((N-(4-fluorobenzyl)-2,4-dinitrophenyl)sulfonamido)prop-1-en-1-yl)(p-tolyl)iodonium tetrafluoroborate* (3bq)**

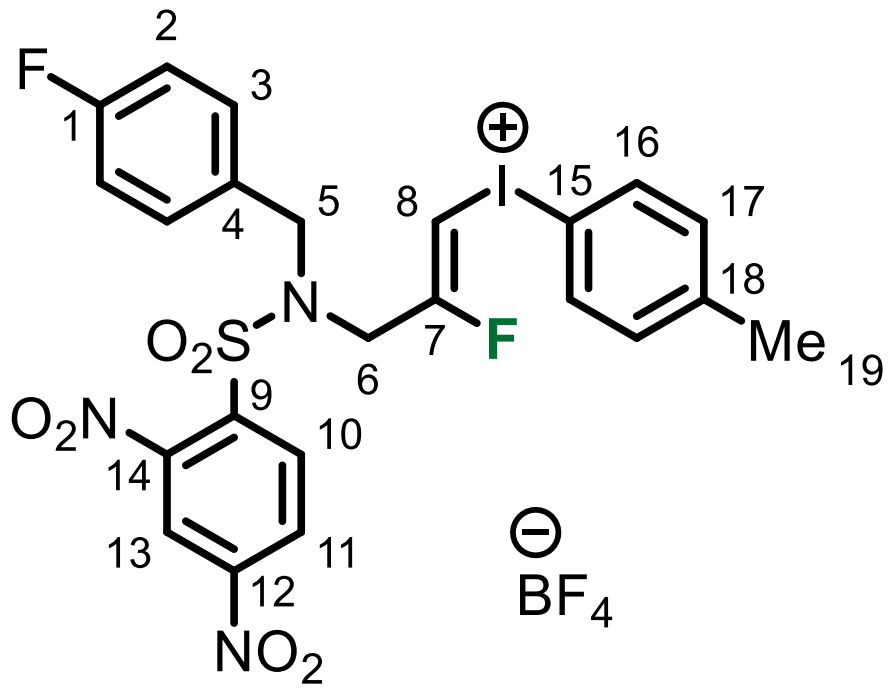

Produced according to **General procedure III** from **1bq** to afford the title compound as a white solid (1.02 g, 66%).

^{1}H NMR (500 MHz, Acetone-*D$_6$*) δ 8.80 (d, *J* = 2.3 Hz, 1H, C13-H), 8.63 (dd, *J* = 8.7, 2.3 Hz, 1H, C11-H), 8.45 (d, *J* = 8.7 Hz, 1H, C10-H), 8.05 (dt, *J* = 8.3, 1.8 Hz, 2H, C16-H), 7.45 (d, *J* = 8.3 Hz, 2H, C17-H), 7.35 – 7.28 (m, 2H, C3-H), 7.09 (dt, *J* = 33.6, 1.0 Hz, 1H, C8-H), 7.04 – 6.98 (m, 2H, C2-H), 4.64 (s, 2H, C5-H), 4.61 (dd, *J* = 12.6, 1.0 Hz, 2H, C6-H), 2.47 (s, 3H, C19-H).

^{13}C NMR (126 MHz, Acetone-*D$_6$*) δ 166.6 (d, *J* = 278.5 Hz, C7), 163.5 (d, *J* = 245.5 Hz, C1), 151.4 (C14), 148.9 (C12), 145.1 (C18), 138.1 (C9), 136.7 (C16), 133.9 (C17), 133.7 (C10), 131.7 (d, *J* = 8.4 Hz, C3), 131.5 (d, *J* = 3.2 Hz, C4), 128.0 (C11), 121.1 (C13), 116.5 (d, *J* = 21.8 Hz, C2), 109.6 (C15), 81.6 (d, *J* = 18.9 Hz, C8), 52.3 (C5), 47.7 (d, *J* = 29.5 Hz, C6), 21.4 (C19).

^{19}F NMR (376 MHz, Acetone-*D$_6$*) δ −76.42 (dt, *J* = 33.6, 12.6 Hz, 1F, C7-F), −114.54 (tt, *J* = 9.4, 5.3 Hz, 1F, C1-F), −150.30 – −150.52 (m, 4F, BF_4).

HRMS (+ESI) calcd: $[M]^+$ ($C_{23}H_{19}F_2IN_3O_6S$) 630.0002, measured 629.9971 (4.92 ppm error).

IR (neat) v_{max}/cm^{-1}: 3102, 1719, 1604, 1539, 1509, 1351, 1225, 1165, 1067, 912, 834, 783, 734.

(Z)-(2-fluoro-4-((4-methyl-N-(4-methylbenzyl)phenyl)sulfonamido)but-1-en-1-yl)(p-tolyl)iodonium tetrafluoroborate **(3br)**

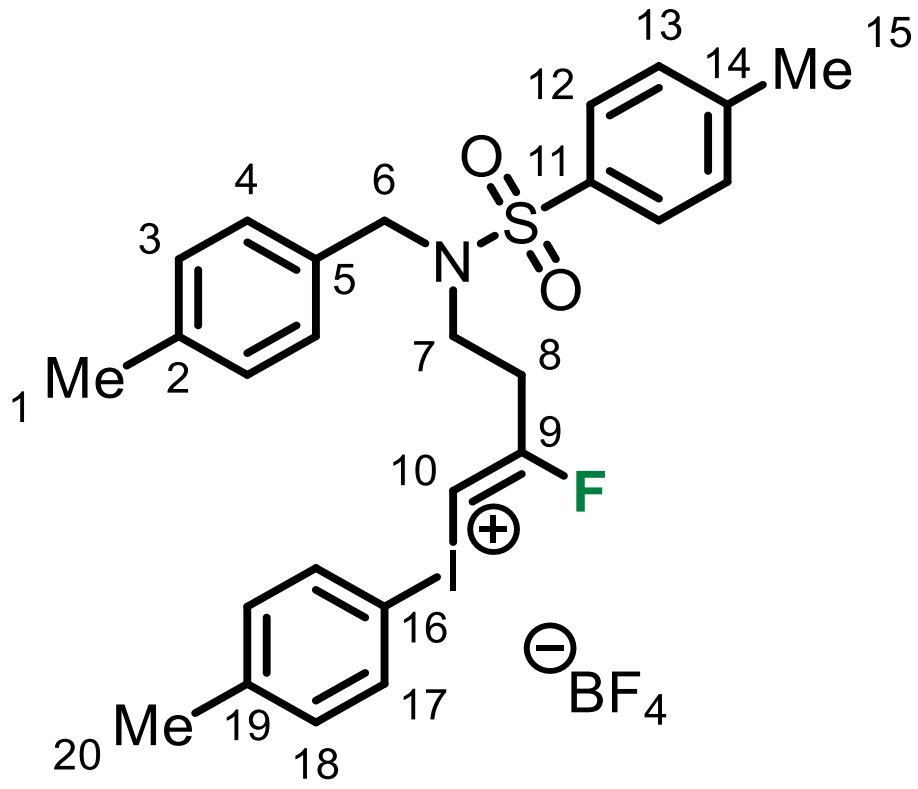

Prepared from alkyne **1br** via **General procedure III** to afford the title compound as a white solid (1.182 g, 85%).

^{1}H NMR (400 MHz, Acetone-*D_6*) δ 8.08 (d, *J* = 8.4 Hz, 2H, C17-H), 7.78 (d, *J* = 8.3 Hz, 2H, C12-H), 7.52 – 7.36 (m, 4H, C13-H, C18-H), 7.17 (d, *J* = 8.1 Hz, 2H, C4-H), 7.10 (d, *J* = 8.1 Hz, 2H, C3-H), 6.61 (d, *J* = 34.2 Hz, 1H, C10-H), 4.27 (s, 2H, C6-H), 3.42 (t, *J* = 6.7 Hz, 2H, C7-H), 2.71 (dt, *J* = 18.5, 6.7 Hz, 2H, C8-H), 2.44 (s, 6H, C20-H, C15-H), 2.29 (s, 3H, C1-H).

^{13}C NMR (101 MHz,-Acetone-*D_6*) δ 169.8 (d, *J* = 276.5 Hz, C9), 143.9 (C19), 143.8 (C14), 137.6 (C11), 136.5 (C2), 135.6 (C17), 133.8 (C5), 133.0 (C18), 130.0 (C13), 129.3 (C4), 128.7 (C12), 127.3 (C3), 108.8 (C16), 77.5 (d, *J* = 20.4 Hz, C10), 52.5 (C6), 45.0 (C7), 31.8 (d, *J* = 24.3 Hz, C8), 20.6 (C20), 20.5 (C15), 20.3 (C1).

^{19}F NMR (376 MHz, Acetone-*D_6*) δ −68.32 (dt, *J* = 34.2, 18.4 Hz, 1F, C9-F), −150.21 (s, 4F, BF_4).

HRMS (+ESI) calcd. $[M]^+$ ($C_{26}H_{28}FINO_2S$) 564.0864, measured 564.0847 (3.01 ppm error).

IR (neat) v_{max}/cm^{-1}: 3097, 2923, 1697, 1648, 1597, 1514, 1478, 1451, 1334, 1250, 1155, 1053, 1033, 995.

***(Z)-(2-fluoro-4-((N-(4-fluorobenzyl)-4-methylphenyl)sulfonamido)but-1-en-1-yl)(p-tolyl)iodonium tetrafluoroborate* (3bs)**

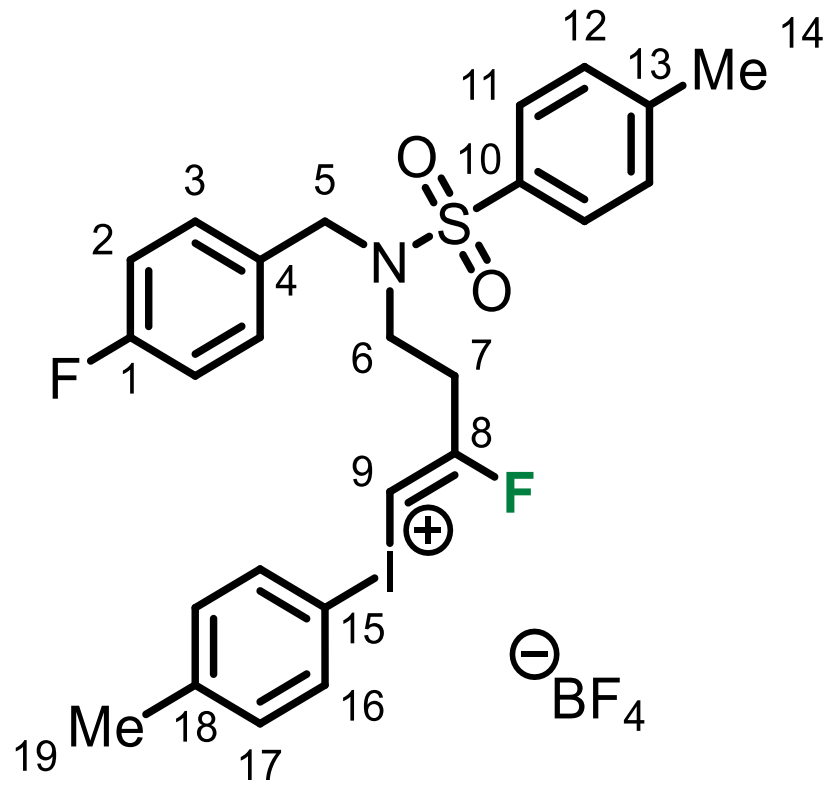

Prepared from alkyne **3bs** via **General procedure III** to afford the title compound as a purple solid (0.293 g, 23%).

^{1}H NMR (400 MHz, Acetone-D_6) δ 8.10 (d, *J* = 8.3 Hz, 2H, C16-H), 7.79 (d, *J* = 8.3 Hz, 2H, C11-H), 7.49 – 7.44 (m, 4H, C12-H, C17-H), 7.36 (m, 2H, C3-H), 7.06 (t, *J* = 8.8 Hz, 2H, C2-H), 6.73 (d, *J* = 34.3 Hz, 1H, C9-H), 4.34 (s, 2H, C5-H), 3.47 (t, *J* = 6.7 Hz, 2H, C6-H), 2.77 (dt, *J* = 18.2, 6.7 Hz, 2H, C7-H), 2.46 (s, 6H, C14-H, C19-H).

^{13}C NMR (126 MHz, Acetone-D_6) δ 169.7 (d, *J* = 276.3 Hz, C8), 162.3 (d, *J* = 244.5 Hz, C1), 143.9 (C18), 143.8 (C13), 136.4 (C10), 135.6 (C16), 133.0 (d, *J* = 3.1 Hz, C4), 133.0 (C17), 130.5 (d, *J* = 8.2 Hz, C3), 130.0 (C12), 127.3 (C11), 115.3 (d, *J* = 21.6 Hz, C2), 108.7 (C15), 77.5 (d, *J* = 20.8 Hz, C9), 51.8 (C5), 45.1 (C6), 31.7 (d, *J* = 24.4 Hz, C7), 20.6 (C19), 20.4 (C14).

^{19}F NMR (376 MHz, Acetone-D_6) δ −68.33 (dt, *J* = 35.4, 18.3 Hz, 1F, C8-F), −115.59 – -115.90 (m, 1F, C1-F), -150.77 (s, 4F, BF_4).

HRMS (+ESI) calcd. $[M]^+$ ($C_{25}H_{25}F_2INO_2S$) 568.0613, measured 568.0596 (2.99 ppm error).

IR (neat) v_{max}/cm^{-1}: 3094, 2926, 1696, 1648, 1597, 1518, 1480, 1452, 1333, 1252, 1050, 1033, 994.

8.3. 3-Pyrrolines (4)

***4-fluoro-2-(4-methoxyphenyl)-1-tosyl-2,5-dihydro-1H-pyrrole* (4a)**

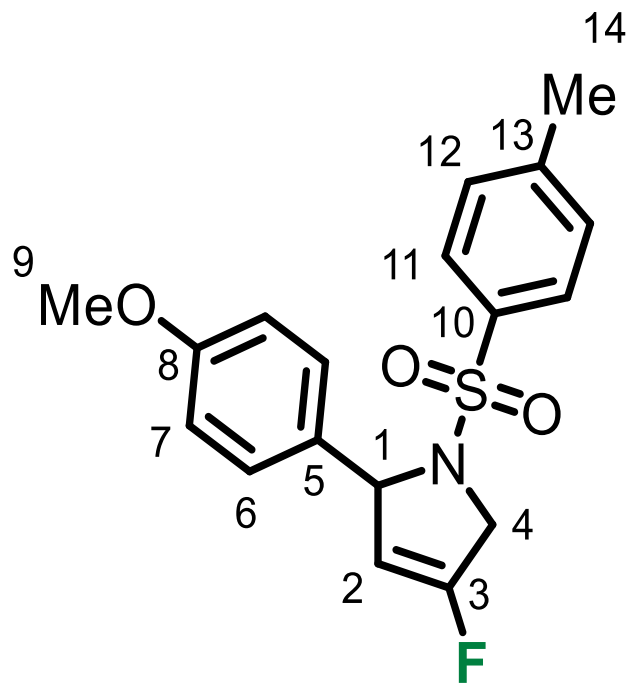

Prepared according to **General procedure IV** from **3a** (84% NMR) and purified by flash column chromatography (2-25% EtOAc in pentane) to afford the title compound as a colourless oil (51.4 mg, 74%).

^{1}H NMR (500 MHz, $CDCl_3$) δ 7.47 (app. dt, *J* = 8.4, 1.8 Hz, 2H, C11-H), 7.23 – 7.16 (m, 4H, C12-H, C6-H, C12-H), 6.81 (dt, *J* = 8.8, 2.1 Hz, 2H, C7-H), 5.45 (tq, *J* = 4.7, 2.3 Hz, 1H, C1-H), 5.05 (ddt, *J* = 2.1, 2.0, 1.9 Hz, 1H, C2-H), 4.33 – 4.15 (m, 2H, C4-H), 3.80 (s, 3H, C9-H), 2.39 (s, 3H, C14-H).

^{13}C NMR (126 MHz, $CDCl_3$) δ 159.7 (C8), 154.8 (d, *J* = 277.7 Hz, C3), 143.6 (C10), 135.3 (C13), 132.1 (d, *J* = 1.9 Hz, C5), 129.7 (C12), 128.8 (C6), 127.4 (C11), 114.0 (C7), 104.2 (d, *J* = 7.6 Hz, C2), 66.1 (d, *J* = 7.2 Hz, C1), 49.8 (d, *J* = 30.8 Hz, C4), 21.6 (C14).

^{19}F NMR (471 MHz, $CDCl_3$) δ −128.88 (q, *J* = 4.6 Hz, C3-F).

HRMS (EI+) calcd: $[M]^+$ ($C_{18}H_{18}NO_3SF$) 347.0986, measured 347.0983 (0.86 ppm error).

IR (neat) v_{max}/cm^{-1}: 3284, 2927, 1756, 1598, 1513, 1341, 1305, 1250, 1159, 1091, 1081, 814, 664, 548.

R_f 0.19 (20% EtOAc in pentane).

4-fluoro-2-(p-tolyl)-1-tosyl-2,5-dihydro-1H-pyrrole **(4b)**

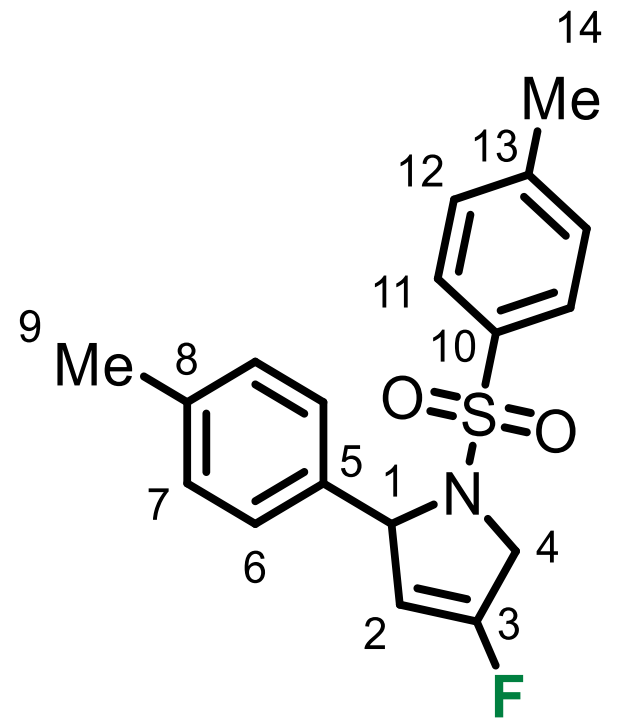

Prepared according to **General procedure IV** from **3b** (83% NMR) and purified by flash column chromatography (2-20% EtOAc in pentane) to afford the title compound as a yellow oil (45.7 mg, 69%).

^{1}H NMR (400 MHz, CDCl$_3$) δ 7.50 (app. dt, *J* = 8.2, 1.7 Hz, 2H, C11-H), 7.21 (d, *J* = 8.2 Hz, 2H, C12-H), 7.18 (d, *J* = 8.0 Hz, 2H, C6-H), 7.10 (d, *J* = 8.0 Hz, 2H, C7-H), 5.44 (tt, *J* = 5.0, 2.3 Hz, 1H, C1-H), 5.05 (ddt, *J* = 2.3, 2.0, 1.9 Hz, 1H), 4.35 – 4.16 (m, 2H, C4-H), 2.40 (s, 3H, C14-H), 2.34 (s, 3H, C9-H).

^{13}C NMR (126 MHz, CDCl$_3$) δ 154.8 (d, *J* = 277.7 Hz, C3-F), 143.7 (C13), 138.1 (C10), 137.2 (d, *J* = 2.0 Hz, C5), 135.2 (C8), 129.7 (C12), 129.3 (C7), 127.5 (C11), 127.4 (C6), 104.3 (d, *J* = 7.8 Hz, C2), 66.4 (d, *J* = 7.1 Hz, C1), 49.9 (d, *J* = 30.8 Hz, C4), 21.6 (C14), 21.3 (C9).

^{19}F NMR (376 MHz, CDCl$_3$) δ −128.81 – −128.85 (m, 1F, C3-F).

HRMS (EI+) calcd: $[M\text{-}H]^+$ ($C_{18}H_{18}NO_2SF$) 330.0959, measured 330.0955 (1.21 ppm error).

IR (neat) v_{max}/cm^{-1}: 3280, 2923, 1760, 1598, 1338, 1182, 1158, 1090, 1068, 909, 812, 729, 663, 547.

R$_f$ 0.22 (20% EtOAc in pentane).

2-(4-bromophenyl)-4-fluoro-1-tosyl-2,5-dihydro-1H-pyrrole **(4c)**

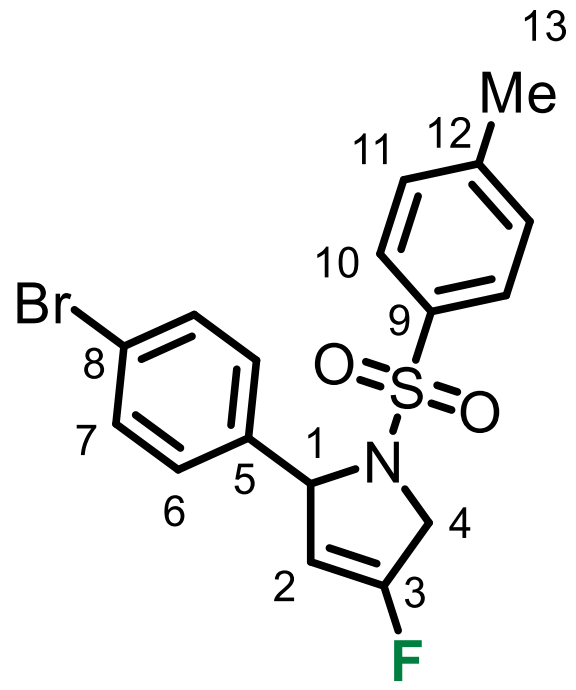

Prepared according to **General procedure IV** from **3c** (75% NMR) and purified by flash column chromatography (2-20% EtOAc in pentane) to afford the title compound as a colourless oil (54.4 mg, 69%).

^{1}H NMR (500 MHz, CDCl$_3$) δ 7.49 (app. dt, J = 8.3, 1.8 Hz, 2H, C10-H), 7.40 (app. dt, J = 8.5, 1.9 Hz, 2H, C7-H), 7.25 – 7.21 (m, 2H, C11-H), 7.15 (ddd, J = 8.5, 1.9 Hz, 2H, C6-H), 5.43 (dddd, J =5.7, 2.2, 2.1, 1.8 Hz, 1H, C1-H), 5.04 (ddd, J = 2.1, 2.0, 1.9 Hz, 1H, C2-H), 4.30 (dddd, J = 13.6, 4.4, 2.2, 2.0 Hz, 1H, C4-H), 4.23 (dddd, J = 13.6, 5.7, 4.5, 1.9 Hz, 1H, C4-H), 2.41 (s, 3H, C13-H).

^{13}C NMR (126 MHz, CDCl$_3$) δ 155.2 (d, J = 278.7 Hz, C3), 144.0 (C12), 139.2 (d, J = 1.9 Hz, C5), 135.0 (C9), 131.8 (C7), 129.8 (C11), 129.1 (C6), 127.4 (C10), 122.3 (C8), 103.8 (d, J = 8.4 Hz, C2), 66.0 (d, J = 7.1 Hz, C1), 50.0 (d, J = 30.6 Hz, C4), 21.7 (C13).

^{19}F NMR (376 MHz, CDCl$_3$) δ −127.69 (ddd, J = 4.5, 4.4, 1.8 Hz, 1F, C3-F).

HRMS (+EI) calcd. $[M]^+$ ($C_{17}H_{15}NO_2SFBr$) 394.9985, measured 394.9980 (1.27 ppm error).

IR (neat) v_{max}/cm^{-1}: 3104, 2922, 2869, 1695, 1596, 1487, 1371, 1349, 1160, 1094, 1069, 1010, 814, 670, 581, 569, 544.

***2-(4-chlorophenyl)-4-fluoro-1-tosyl-2,5-dihydro-1H-pyrrole* (4d)**

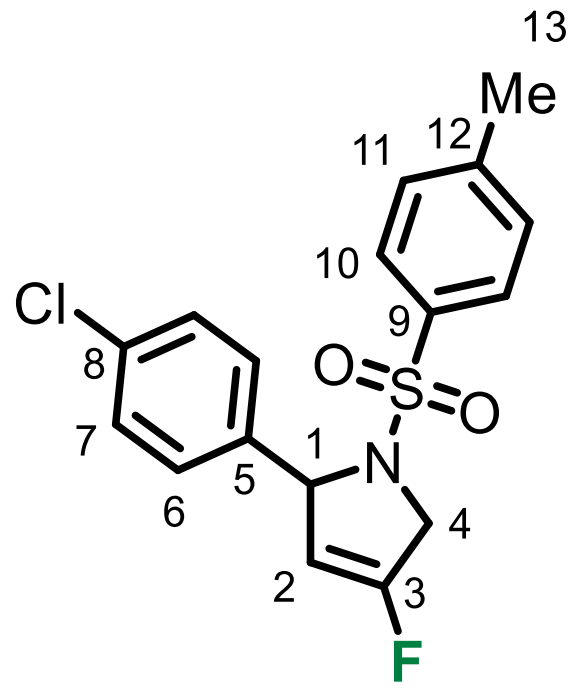

Prepared according to **General procedure IV** from **3d** (77% NMR) and purified by flash column chromatography (2-20% EtOAc in pentane) to afford the title compound as a colourless oil (44.3 mg, 63%).

^{1}H NMR (500 MHz, CDCl$_3$) δ 7.47 (app. dt, *J* = 8.4, 1.7 Hz, 2H, C10-H), 7.25 – 7.16 (m, 6H, C6-H, C7-H, C11-H), 5.41 (tt, *J* = 4.9, 2.2 Hz, 1H, C1-H), 5.02 (ddt, *J* = 2.2, 2.0, 1.9 Hz, 1H, C2-H), 4.31 – 4.14 (m, 2H, C4-H), 2.38 (s, 3H, C13-H).

^{13}C NMR (126 MHz, CDCl$_3$) δ 155.1 (d, *J* = 278.4 Hz, C3), 144.0 (C12), 138.7 (d, *J* = 2.0 Hz, C5), 134.9 (C8), 134.2 (C9), 129.8 (C11), 128.8 (C7), 128.8 (C6), 127.4 (C10), 103.9 (d, *J* = 8.3 Hz, C2), 65.9 (d, *J* = 7.1 Hz, C1), 49.9 (d, *J* = 30.7 Hz, C4), 21.6 (C13).

^{19}F NMR (471 MHz, CDCl$_3$) δ −127.89 (q, *J* = 4.5 Hz, C3-F).

HRMS (EI+) calcd: $[M]^+$ ($C_{17}H_{15}NO_2SFCl$) 351.0981, measured 351.0486 (1.42 ppm error).

IR (neat) v_{max}/cm^{-1}: 2870, 1697, 1596, 1490, 1371, 1350, 1161, 1091, 1015, 815, 674, 582, 571, 545.

R_f 0.59 (20% EtOAc in pentane).

***4-fluoro-2-(4-fluorophenyl)-1-tosyl-2,5-dihydro-1H-pyrrole* (4e)**

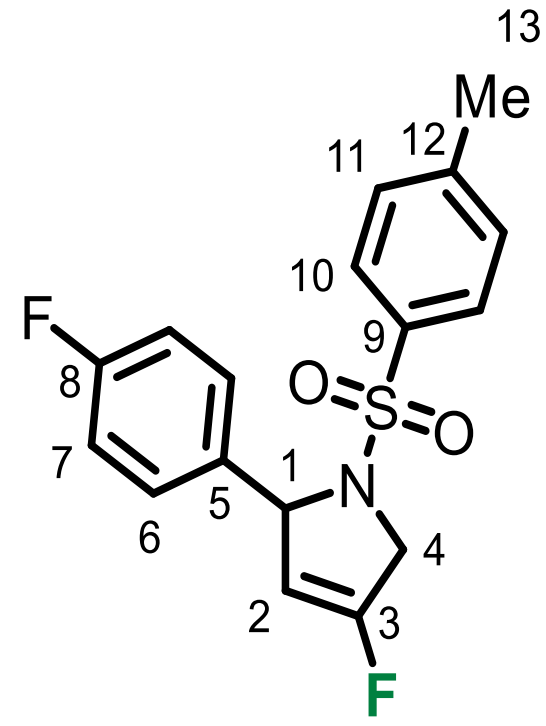

Prepared according to **General procedure IV** from **3e** (83% NMR) and purified by flash column chromatography (2-20% EtOAc in pentane) to afford the title compound as a colourless oil (50.3 mg, 75%).

^{1}H NMR (500 MHz, $CDCl_3$) δ 7.48 (app. dt, *J* = 8.3, 2.1 Hz, 2H, C10-H), 7.28 – 7.24 (m, 2H, C11-H), 7.22 (dd, *J* = 8.6, 0.7 Hz, 2H, C6-H), 6.97 (tt, *J* = 8.7, 2.1 Hz, 2H, C7-H), 5.47 (ddd, J = 8.0, 4.9, 2.3 Hz, 1H, C1-H), 5.05 (ddt, J = 2.3, 1.8, 1.6 Hz, 1H, C2-H), 4.33 – 4.17 (m, 2H, C4-H), 2.40 (s, 3H, C13-H).

^{13}C NMR (126 MHz, $CDCl_3$) δ 162.7 (d, J = 246.9 Hz, C8), 155.1 (d, J = 278.3 Hz, C3), 143.9 (C12), 136.0 (dd, J = 3.2, 2.0 Hz, C5), 135.1 (C9), 129.8 (C11), 129.2 (d, J = 8.3 Hz, C6), 127.4 (C10), 115.5 (d, J = 21.7 Hz, C7), 104.0 (d, J = 8.1 Hz, C2), 65.9 (d, J = 7.1 Hz, C1), 49.9 (d, J = 30.7 Hz, C4), 21.65 (C13).

^{19}F NMR (376 MHz, $CDCl_3$) δ −113.77 (ddd, J = 13.8, 8.7, 5.3 Hz, C8-F), −128.08 (dt, J = 4.9, 4.8 Hz, C3-F).

HRMS (+ESI) calcd: $[M+H]^+$ ($C_{17}H_{15}F_2NO_2S$) 336.0864, measured 336.0864 (0.00 ppm error).

IR (neat) $\tilde{v}_{max}$/cm^{-1}: 3277, 2925, 1696, 1600, 1509, 1344, 1225, 1159, 1094, 1057, 837, 815, 676, 582, 574, 549.

R_f 0.73 (50% EtOAc in cyclohexane).

***4-fluoro-1-tosyl-2-(4-(trifluoromethyl)phenyl)-2,5-dihydro-1H-pyrrole* (4f)**

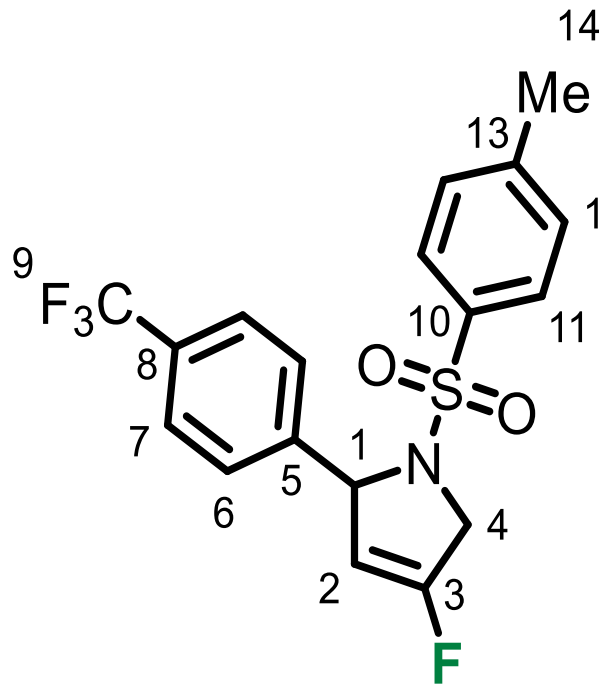

Prepared according to **General procedure IV** from **3f** (66% NMR) and purified by flash column chromatography (2-30% EtOAc in pentane) to afford the title compound as a colourless oil (41.6 mg, 54%).

^{1}H NMR (600 MHz, CDCl$_3$) δ 7.53 (d, *J* = 8.1 Hz, 2H, C7-H), 7.48 (d, *J* = 8.3 Hz, 2H, C11-H), 7.38 (d, *J* = 8.1 Hz, 2H, C6-H), 7.21 (d, *J* = 8.3 Hz, 2H, C12-H), 5.51 (tt, *J* = 5.0, 2.3 Hz, 1H, C1-H), 5.07 (p, *J* = 1.9 Hz, 1H, C2-H), 4.37 – 4.25 (m, 2H, C4-H), 2.40 (s, 3H, C14-H).

^{13}C NMR (151 MHz, CDCl$_3$) δ 155.2 (d, *J* = 279.1 Hz, C3), 144.0 (C13), 143.9 (C5), 134.8 (C10), 130.4 (q, *J* = 32.5 Hz, C8), 129.7 (C12), 127.6 (C6), 127.3 (C11), 125.5 (q, *J* = 3.7 Hz, C7), 124.0 (q, *J* = 272.2 Hz, C9), 103.6 (d, *J* = 8.6 Hz, C2), 65.9 (d, *J* = 7.1 Hz, C1), 49.9 (d, *J* = 30.7 Hz, C4), 21.5, (C14).

^{19}F NMR (376 MHz, CDCl$_3$) δ −62.49 (s, 3F, C9-F), −127.27 (q, *J* = 4.4 Hz, 1F, C3-F).

HRMS (EI) calcd: $[M-H]^+$ ($C_{11}H_8OF_4$) 231.0428, measured 231.0424 (1.73 ppm error).

IR (neat) v_{max}/cm^{-1}: 2927, 2872, 1696, 1620, 1597, 1350, 1323, 1159, 1122, 1109, 1095, 1066, 1017, 815, 677, 662, 582, 545.

R_f 0.35 (10% EtOAc in pentane)

***3-fluoro-2,2-dimethyl-5-phenyl-1-tosyl-2,5-dihydro-1H-pyrrole* (4h)**

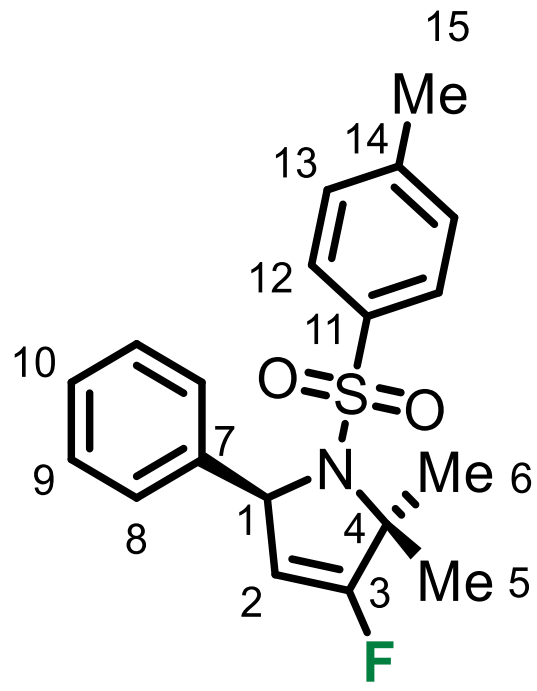

Prepared according to **General procedure IV** from **3h** (70% NMR) and purified by flash column chromatography (2-20% EtOAc in pentane) to afford the title compound as a colourless oil (46.3 mg, 67%).

^{1}H NMR (400 MHz, CDCl$_3$) δ 7.29 (app. dt, *J* = 8.2, 1.8 Hz, 2H, C12-H), 7.19 – 7.11 (m, 5H, C8-H, C9-H, C10-H), 6.98 (t, *J* = 8.2 Hz, 2H, C13-H), 5.44 (dd, *J* = 5.6, 2.1 Hz, 1H, C1-H), 4.93 (t, *J* = 1.8 Hz, 1H, C2-H), 2.30 (s, 3H, C15-H), 1.76 (s, 3H, C5-H), 1.75 (s, 3H, C6-H).

^{13}C NMR (101 MHz, CDCl$_3$) δ 160.5 (d, *J* = 280.0 Hz, C3), 142.7 (C14), 139.4 (d, *J* = 2.1 Hz, C7), 138.7 (C11), 129.0 (C13), 128.4 (C9), 128.0 (C8), 128.0 (C10), 127.5 (C12), 100.8 (d, *J* = 8.6 Hz, C2), 66.5 (d, *J* = 26.3 Hz, C4), 65.4 (d, *J* = 7.3 Hz, C1), 26.6 (d, *J* = 2.2 Hz, C5), 26.0 (d, *J* = 2.1 Hz, C6), 21.5 (C15).

^{19}F NMR (376 MHz, CDCl$_3$) δ −135.78 (dd, *J* = 5.7, 1.5 Hz, 1F, C3-F).

HRMS (+EI) calcd: $[M\text{-}Me]^+$ ($C_{19}H_{20}NO_2FS$) 330.0959, measured 330.0951 (2.42 ppm error).

IR (neat) v_{max}/cm^{-1}: 2978, 1702, 1599, 1494, 1456, 1339, 1147, 1092, 1067, 1047, 813, 697, 676, 570, 545.

R*f* 0.73 (20% EtOAc in pentane).

***4-fluoro-2-(4-fluorophenyl)-1-((4-nitrophenyl)sulfonyl)-2,5-dihydro-1H-pyrrole* (4i)**

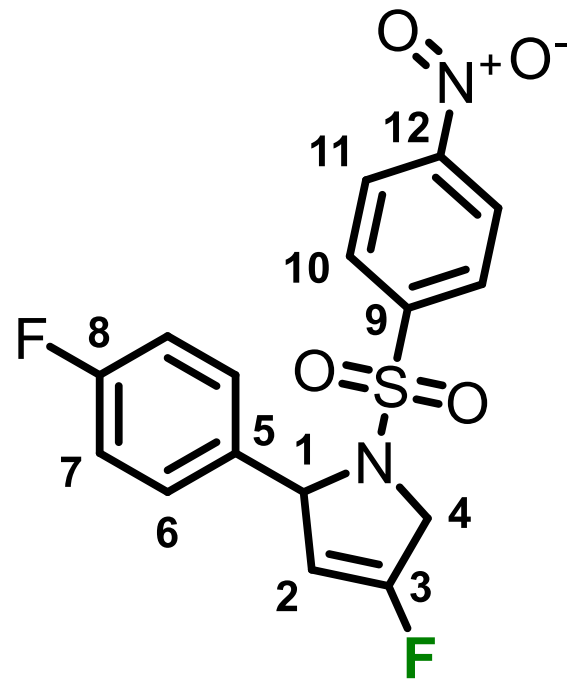

Prepared according to **General procedure IV** from **3i** (67% NMR) and purified by flash column chromatography (2-20% EtOAc in pentane) to afford the title compound as a colourless oil (44.7 mg, 61%).

^{1}H NMR (500 MHz, CDCl$_3$) δ 8.18 (app. dt, *J* = 8.9, 2.0 Hz, 2H, C11-H), 7.60 (app. dt, *J* = 8.9, 2.0 Hz, 2H, C10-H), 7.23 – 7.15 (m, 2H, C6-H), 7.00 – 6.91 (m, 2H, C7-H), 5.60 (dddd, *J* = 4.7, 4.6, 2.3, 2.1 Hz, 1H, C1-H), 5.13 (ddd, *J* = 2.3, 2.1, 1.9 Hz, 1H, C2-H), 4.44 (dddd, *J* = 13.3, 4.0, 2.3, 2.1 Hz, 1H, C4-H), 4.23 (dddd, *J* = 13.3, 8.0, 4.6, 1.9 Hz, 1H, C4-H).

^{13}C NMR (126 MHz, CDCl$_3$) δ 163.0 (d, *J* = 248.7 Hz, C8), 154.7 (d, *J* = 278.4 Hz, C3), 150.1 (C12), 144.6 (C9), 134.7 (dd, *J* = 2.7, 2.3 Hz, C5), 129.7 (d, *J* = 8.3 Hz, C6), 128.2 (C11), 124.2 (C10), 115.7 (d, *J* = 21.6 Hz, C7), 103.9 (d, *J* = 8.1 Hz, C2), 66.2 (d, *J* = 6.6 Hz, C1), 49.8 (d, *J* = 31.4 Hz, C4).

^{19}F NMR (376 MHz, CDCl$_3$) δ −112.32 (tt, *J* = 8.4, 5.3 Hz), −127.34 (ddd, *J* = 8.0, 4.7, 4.0 Hz).

HRMS (+ESI) calcd: $[M-H]^+$ ($C_{16}H_{12}N_2O_4SF_2$) 365.0402, measured 365.0396 (1.64 ppm error).

IR (neat) v_{max}/cm^{-1}: 3109, 2860, 1696, 1604, 1529, 1350, 1167, 1096, 854, 738, 612, 568, 460.

***cis-3-fluoro-5-(4-fluorophenyl)-2-methyl-1-tosyl-2,5-dihydro-1H-pyrrole* (4j)**

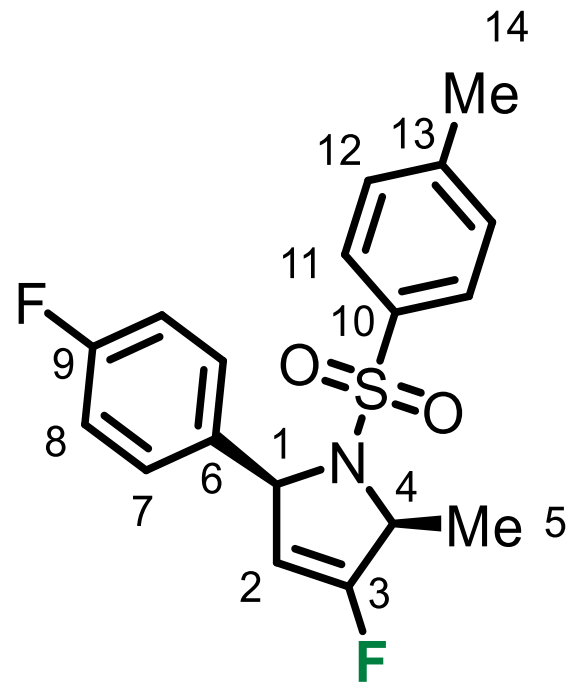

Prepared according to **General procedure IV** from **3j** (87% NMR) and purified by flash column chromatography (2-20% EtOAc in pentane) to afford the title compound as a colourless oil (55.2 mg, 79%).

^{1}H NMR (400 MHz, CDCl$_3$) δ 7.60 (app. dt, *J* = 8.4, 1.8 Hz, 2H, C11-H), 7.35 – 7.29 (m, 2H, C7-H), 7.28 – 7.24 (m, 2H, C12-H), 7.00 (tt, *J* = 8.8, 3.0 Hz, 2H, C8-H), 5.37 (ddd, *J* = 5.8, 2.1, 1.9 Hz, 1H, C1-H), 4.98 (ddd, *J* = 2.1, 1.5, 1.2 Hz, 1H, C2-H), 4.48 (qddd, *J* = 6.4, 4.1, 1.9, 1.5 Hz, 1H, C4-H), 2.41 (s, 3H, C14-H), 1.55 (d, *J* = 6.4 Hz, 3H, C5-H).

^{13}C NMR (126 MHz, CDCl$_3$) δ 162.6 (d, *J* = 246.8 Hz, C9), 158.3 (d, *J* = 280.3 Hz, C3), 144.1 (C13), 136.6 (d, *J* = 2.5 Hz, C6), 135.0 (C10), 129.9 (C12), 129.1 (d, *J* = 8.3 Hz, C7), 127.7 (C11), 115.5 (d, *J* = 21.5 Hz, C8), 102.2 (d, *J* = 8.9 Hz, C2), 65.2 (d, *J* = 7.7 Hz, C1), 57.7 (d, *J* = 27.9 Hz, C4), 21.7 (C14), 21.0 (d, *J* = 3.3 Hz, C5).

^{19}F NMR (376 MHz, CDCl$_3$) δ −114.14 (tt, *J* = 8.8, 5.1 Hz, C9-F), −130.66 (ddd, *J* = 5.8, 4.1, 1.2 Hz).

HRMS (EI+) calcd: $[M+H]^+$ ($C_{18}H_{17}NO_2SF_2$) 349.0943, measured 349.0942 (0.29 ppm error).

IR (neat) v_{max}/cm^{-1}: 2979, 2931, 1698, 1604, 1508, 1352, 1226, 1164, 1094, 835, 816, 680, 578, 569, 554.

R_f 0.59 (20% EtOAc in pentane).

(2R,5R)-3-fluoro-5-(4-fluorophenyl)-2-methyl-1-tosyl-2,5-dihydro-1H-pyrrole **((*R*,*R*)-4j)**

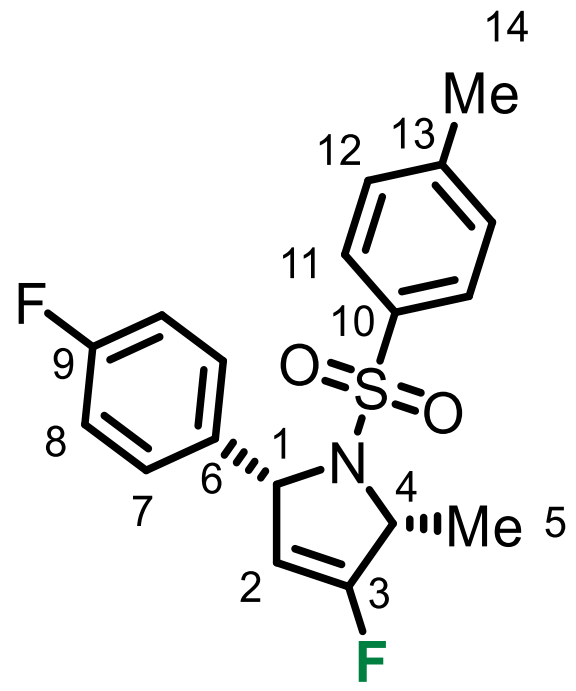

Prepared according to **General procedure IV** from **(*R*)-3j** (90% NMR) and purified by flash column chromatography (2-20% EtOAc in pentane) to afford the title compound as a colourless oil (1.81 g, 84%).

Enantiomeric excess determined by chiral HPLC (94% ee).

^{1}H NMR (500 MHz, CDCl$_3$) δ 7.60 (app. dt, *J* = 8.4, 1.8 Hz, 2H, C11-H), 7.35 – 7.29 (m, 2H, C7-H), 7.28 – 7.24 (m, 2H, C12-H), 7.00 (tt, *J* = 8.8, 3.0 Hz, 2H, C8-H), 5.37 (ddd, *J* = 5.8, 2.1, 1.9 Hz, 1H, C1-H), 4.98 (ddd, *J* = 2.1, 1.5, 1.2 Hz, 1H, C2-H), 4.48 (qddd, *J* = 6.4, 4.1, 1.9, 1.5 Hz, 1H, C4-H), 2.41 (s, 3H, C14-H), 1.55 (d, *J* = 6.4 Hz, 3H, C5-H).

^{13}C NMR (126 MHz, CDCl$_3$) δ 162.6 (d, *J* = 246.8 Hz, C9), 158.3 (d, *J* = 280.3 Hz, C3), 144.1 (C13), 136.6 (d, *J* = 2.5 Hz, C6), 135.0 (C10), 129.9 (C12), 129.1 (d, *J* = 8.3 Hz, C7), 127.7 (C11), 115.5 (d, *J* = 21.5 Hz, C8), 102.2 (d, *J* = 8.9 Hz, C2), 65.2 (d, *J* = 7.7 Hz, C1), 57.7 (d, *J* = 27.9 Hz, C4), 21.7 (C14), 21.0 (d, *J* = 3.3 Hz, C5).

^{19}F NMR (376 MHz, CDCl$_3$) δ −114.14 (tt, *J* = 8.8, 5.1 Hz, C9-F), −130.66 (ddd, *J* = 5.8, 4.1, 1.2 Hz).

HRMS (EI+) calcd: $[M+H]^+$ ($C_{18}H_{17}NO_2SF_2$) 349.0943, measured 349.0942 (0.29 ppm error).

HPLC Chiral Regis Whelk O1 250 × 4.6 mm, 5 μm, hexane:IPA = 90:10, flow = 1.00 mL/min, λ = 230 nm, tR = 25.9, 27.6 min. Enantiomeric ratio: 96.8:3.2 (ee = 94%).

IR (neat) ν_{max}/cm^{-1}: 2979, 2931, 1698, 1604, 1508, 1352, 1226, 1164, 1094, 835, 816, 680, 578, 569, 554.

$[\alpha]_D^{24}$ = +98° (c 1.00 in CHCl$_3$).

R*f* 0.59 (20% EtOAc in pentane).

Chiral HPLC chromatogram of **HR057**

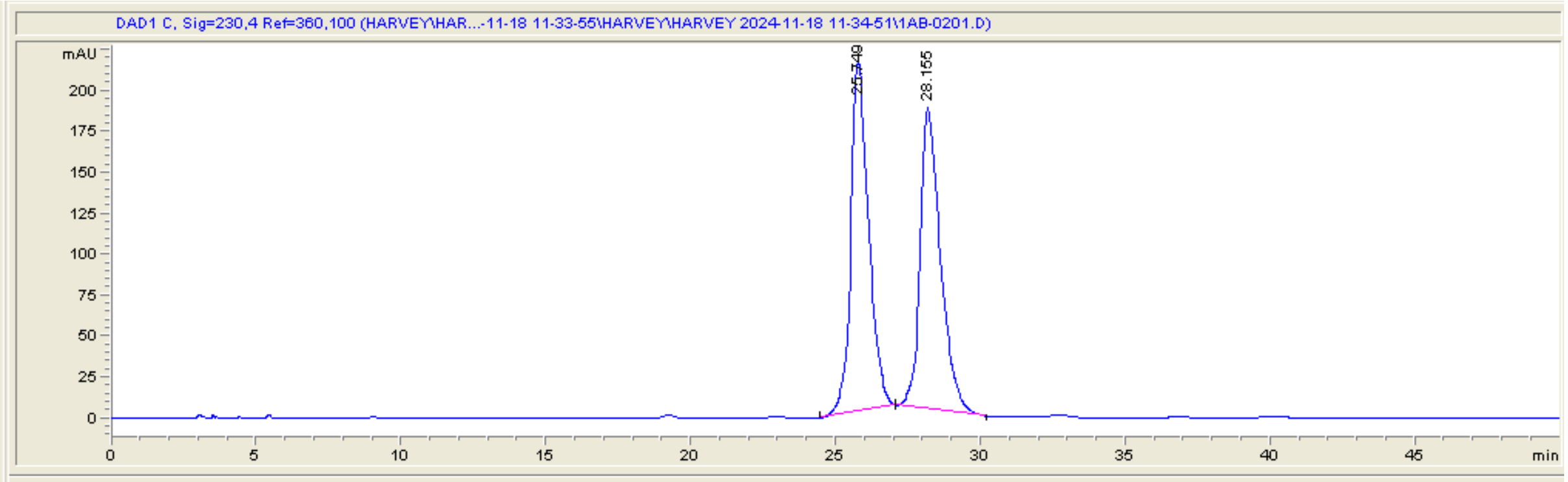

Chiral HPLC chromatogram of **HR057R**

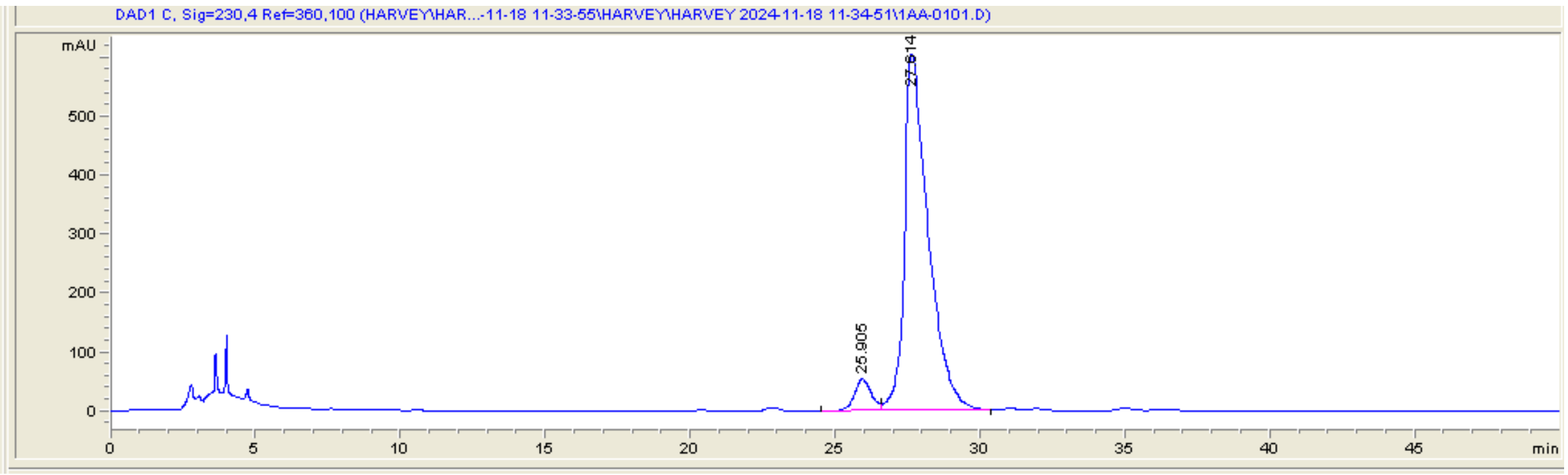

***cis*-3-fluoro-5-(4-fluorophenyl)-2-methyl-1-(methylsulfonyl)-2,5-dihydro-1*H*-pyrrole (4k)**

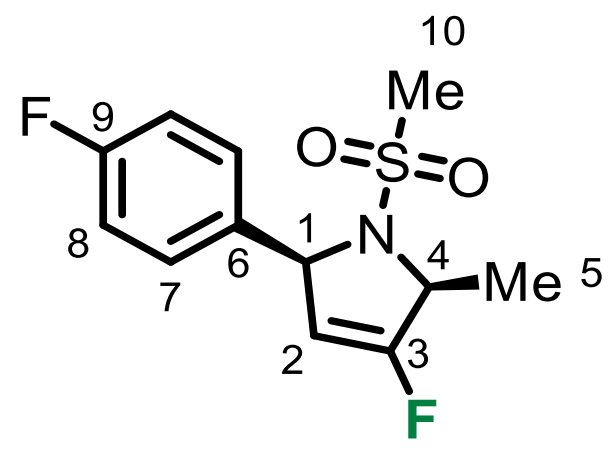

Prepared according to **General procedure IV** from **3k** (77% NMR) and purified by flash column chromatography (2-20% EtOAc in pentane) to afford the title compound as a colourless oil (30.6 mg, 56%).

Produced as a mixture of diastereomers (*d.r.* 1: 0.13). Only the *cis*-diastereomer was isolated.

^{1}H NMR (500 MHz, CDCl$_3$) δ 7.43 – 7.33 (m, 2H, C7-H), 7.12 – 7.00 (m, 2H, C8-H), 5.47 (dt, *J* = 5.6, 2.2 Hz, 1H, C1-H), 5.12 (dt, *J* = 2.5, 1.4 Hz, 1H, C2-H), 4.67 (qdt, *J* = 6.3, 3.3, 1.7 Hz, 1H, C4-H), 2.57 (s, 3H, C10-H), 1.56 (d, *J* = 6.4 Hz, 3H, C5-H).

^{13}C NMR (126 MHz, CDCl$_3$) δ 162.8 (d, *J* = 247.6 Hz, C9), 158.4 (d, *J* = 279.8 Hz, C3), 135.9 (dd, *J* = 1.9, 1.9 Hz, C6), 129.4 (d, *J* = 8.3 Hz, C7), 115.9 (d, *J* = 21.8 Hz, C8), 102.0 (d, *J* = 8.9 Hz, C2), 64.7 (d, *J* = 7.5 Hz, C1), 57.4 (d, *J* = 28.5 Hz, C4), 40.5 (C10), 20.5 (d, *J* = 3.3 Hz, C5).

^{19}F NMR (471 MHz, CDCl$_3$) δ −113.23 (tt, *J* = 8.5, 5.3 Hz, 1F, C9-F), −130.67 (dd, *J* = 5.6, 3.3 Hz, 1F, C3-F).

HRMS (+EI) calcd: [M-Me]$^+$ ($C_{12}H_{13}NO_2SF_2$) 258.0395, measured 258.0390 (1.93 ppm error).

IR (neat) ν_{max}/cm^{-1}: 2939, 1697, 1605, 1508, 1339, 1225, 1152, 1097, 1075, 834, 751, 548, 535, 518.

R$_f$ 0.22 (20% EtOAc in pentane).

***cis-5-(4-bromophenyl)-3-fluoro-2-methyl-1-tosyl-2,5-dihydro-1H-pyrrole* (4l)**

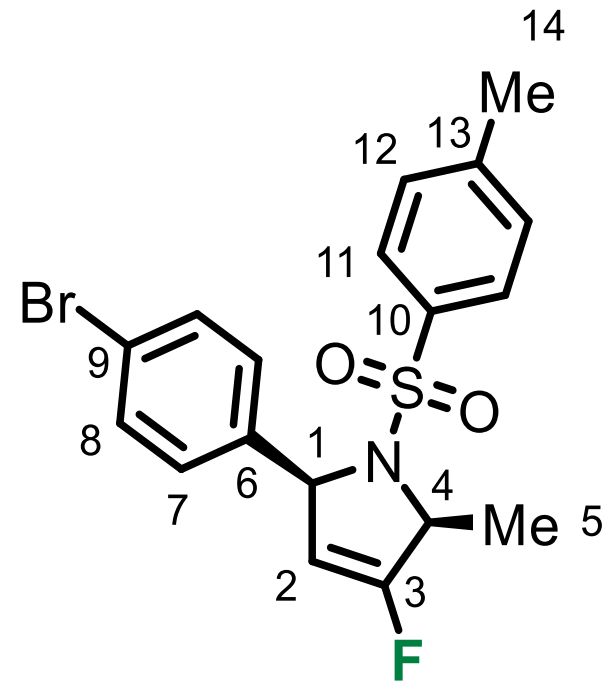

Prepared according to **General procedure IV** from **3l** (76% NMR) and purified by flash column chromatography (2-25% EtOAc in pentane) to afford the title compound as a colourless oil (57.4 mg, 70%).

^{1}H NMR (500 MHz, CDCl$_3$) δ 7.61 (app. dt, *J* = 8.3, 1.8 Hz, 2H, C11-H), 7.45 (dt, *J* = 8.4, 1.9 Hz, 2H, C8-H), 7.28 – 7.26 (m, 2H, C12-H), 7.22 (dt, *J* = 8.4, 1.9 Hz, 2H, C7-H), 5.33 (dt, *J* = 5.6, 1.9 Hz, 1H, C1-H), 4.98 (p, *J* = 1.1 Hz, 1H, C2-H), 4.49 (qdt, *J* = 6.4, 3.3, 1.9 Hz, 1H, C4-H), 2.43 (s, 3H, C14-H), 1.55 (d, *J* = 6.4 Hz, 3H, C5-H).

^{13}C NMR (126 MHz, CDCl$_3$) δ 158.3 (d, *J* = 280.4 Hz, C3), 144.1 (C10), 139.7 (C13), 134.7 (C6), 131.7 (C8), 129.8 (C12), 128.9 (C7), 127.6 (C11), 122.1 (C9), 101.8 (d, *J* = 9.1 Hz, C2), 65.1 (d, *J* = 7.6 Hz, C1), 57.6 (d, *J* = 27.8 Hz, C4), 21.6 (C14), 20.9 (d, *J* = 3.1 Hz, C5).

^{19}F NMR (471 MHz, CDCl$_3$) δ −130.39 (dd, *J* = 5.6, 3.3 Hz).

HRMS (EI+) calcd: $[M]^+$ ($C_{18}H_{17}NO_2SFBr$) 409.0142, measured 409.0142 (0.00 ppm error).

IR (neat) ν_{max}/cm^{-1}: 2978, 2931, 1727, 1698, 1596, 1487, 1351, 1163, 1093, 1073, 1010, 814, 674, 578, 564, 548.

R_f 0.66 (20% EtOAc in pentane).

***cis*-3-fluoro-2-methyl-5-(naphthalen-2-yl)-1-tosyl-2,5-dihydro-1H-pyrrole** (**4m**)

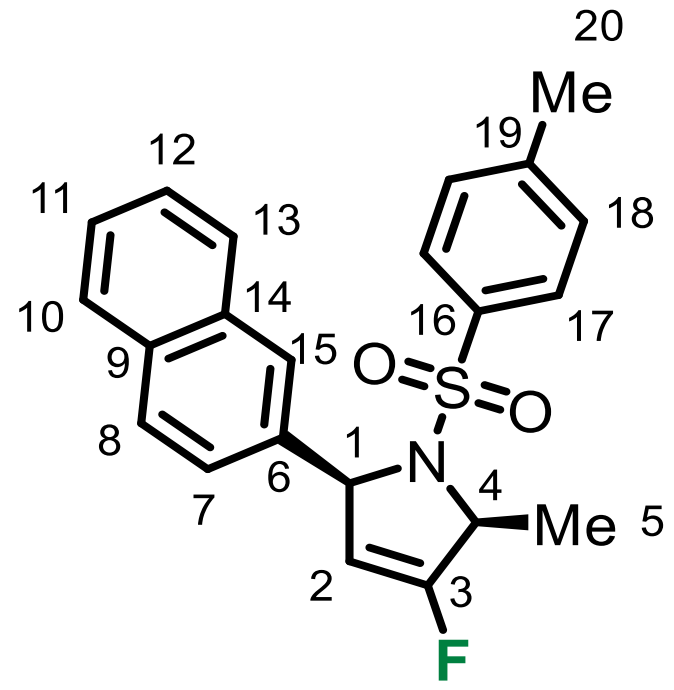

Prepared according to **General procedure IV** from **3m** (73% NMR) and purified by flash column chromatography (2-20% EtOAc in pentane) to afford the title compound as a colourless oil (48.8 mg, 64%).

^{1}H NMR (500 MHz, CDCl$_3$) δ 7.83 – 7.78 (m, 3H, C8-H, C10-H, C13-H), 7.74 (d, *J* = 1.8 Hz, 1H, C15-H), 7.60 (dt, *J* = 8.1, 1.6 Hz, 2H, C17-H), 7.49 – 7.46 (m, 2H, C11-H, C12-H), 7.44 (dd, *J* = 8.4, 1.8 Hz, 1H, C7-H), 7.17 (d, *J* = 8.1 Hz, 2H, C18-H), 5.56 (ddd, *J* = 5.7, 3.2, 2.2 Hz, 1H, C1-H), 5.08 (ddd, *J* = 2.2, 1.2, 0.9 Hz, 1H, C2-H), 4.59 (qddd, *J* = 6.4, 6.1, 3.2, 1.2 Hz, 1H, C4-H), 2.33 (s, 3H, C20-H), 1.63 (d, *J* = 6.4 Hz, 3H, C5-H).

^{13}C NMR (126 MHz, CDCl$_3$) δ 158.2 (d, *J* = 280.0 Hz, C3), 143.9 (C19), 137.8 (d, *J* = 1.5 Hz, C6), 135.2 (C16), 133.3 (C8), 129.8 (C18), 128.6 (C13), 128.4 (C14), 128.2 (C10), 127.8 (C17), 127.4 (C9), 126.5 (C15), 126.4 (C12), 126.3 (C11), 124.8 (C7), 102.4 (d, *J* = 8.8 Hz, C2), 66.1 (d, *J* = 7.5 Hz, C1), 57.8 (d, *J* = 28.1 Hz, C4), 21.6 (C20), 21.0 (d, *J* = 3.1 Hz, C5).

^{19}F NMR (471 MHz, CDCl$_3$) δ −130.97 (dd, *J* = 5.7, 3.2, 0.9 Hz).

HRMS (+EI) calcd: $[M]^+$ ($C_{22}H_{20}NO_2SF$) 381.1193, measured 381.1190 (0.78 ppm error).

IR (neat) v_{max}/cm^{-1}: 3056, 2927, 1725, 1697, 1598, 1493, 1351, 1163, 1163, 1093, 815, 685, 659, 577, 553.

R*f* 0.53 (20% EtOAc in pentane).

***cis*-3-fluoro-5-(4-fluorophenyl)-2-phenyl-1-tosyl-2,5-dihydro-1*H*-pyrrole** (**4n**)

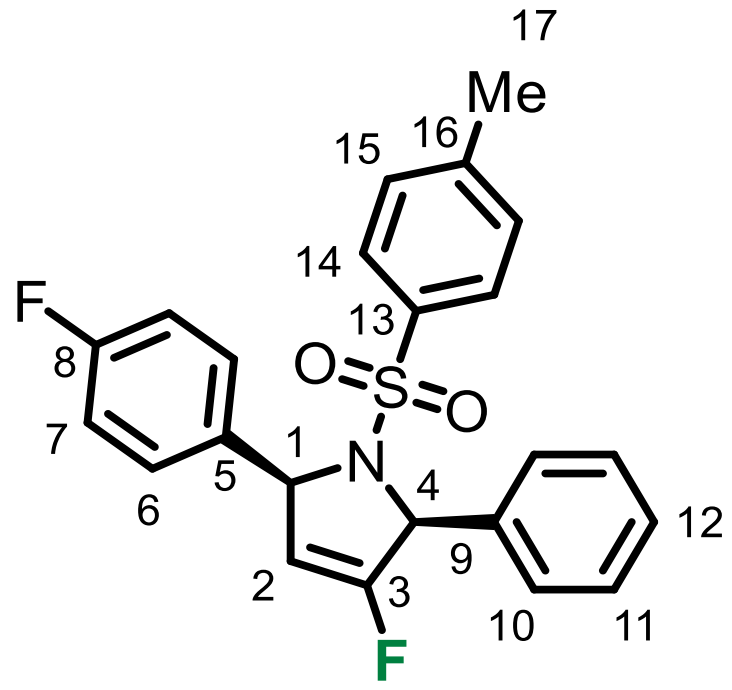

Prepared according to **General procedure IV** from **3n** (61% NMR) and purified by flash column chromatography (2-20% EtOAc in pentane) to afford the title compound as a white solid (42.8 mg, 52%).

^{1}H NMR (500 MHz, $CDCl_3$) δ 7.43 – 7.38 (m, 2H, C6-H), 7.37 (app. dt, *J* = 7.2, 3.2 Hz, 2H, C11-H), 7.35 – 7.32 (m, 3H, C10-H, C12-H), 7.26 (dt, *J* = 8.3, 1.8 Hz, 2H, C14-H), 7.07 (d, *J* = 8.3 Hz, 2H, C15-H), 7.01 (tt, *J* = 8.7, 2.1 Hz, 2H, C7-H), 5.67 (ddd, *J* = 6.8, 2.4, 2.1 Hz, 1H, C1-H), 5.59 (dd, *J* = 3.0, 2.4 Hz, 1H, C4-H), 5.21 (dd, *J* = 2.1, 2.1 Hz, 1H, C2-H), 2.34 (s, 3H).

^{13}C NMR (126 MHz, $CDCl_3$) δ 162.8 (d, *J* = 247.4 Hz, C8), 156.3 (d, *J* = 279.9 Hz, C3), 143.8 (C16), 136.8 (d, *J* = 3.1 Hz, C5), 135.8 (C13), 129.8 (d, *J* = 8.3 Hz, C6), 129.5 (C15), 128.8 (C12), 128.7 (C9), 128.7 (C10), 128.1 (C11), 127.5 (C14), 115.5 (d, *J* = 21.7 Hz, C7), 103.0 (d, *J* = 9.1 Hz, C2), 65.2 (d, *J* = 27.1 Hz, C4), 64.7 (d, *J* = 7.2 Hz, C1), 21.6 (C17).

^{19}F NMR (471 MHz, $CDCl_3$) δ −113.79 (tt, *J* = 8.5, 5.3 Hz), −127.65 (ddd, *J* = 6.8, 3.0, 2.1 Hz).

NOESY: Strong cross peak between C6-H and C10-H

HRMS (+ESI) calcd: $[M+H]^+$ ($C_{23}H_{19}F_2NO_2S$) 412.1177, measured 412.1189 (2.91 ppm error).

IR (neat) v_{max}/cm^{-1}: 2927, 1698, 1598, 1509, 1349, 1162, 1092, 816, 665, 578.

R_f 0.56 (20% EtOAc in pentane).

***4-fluoro-2,2-diphenyl-1-tosyl-2,5-dihydro-1H-pyrrole* (4o)**

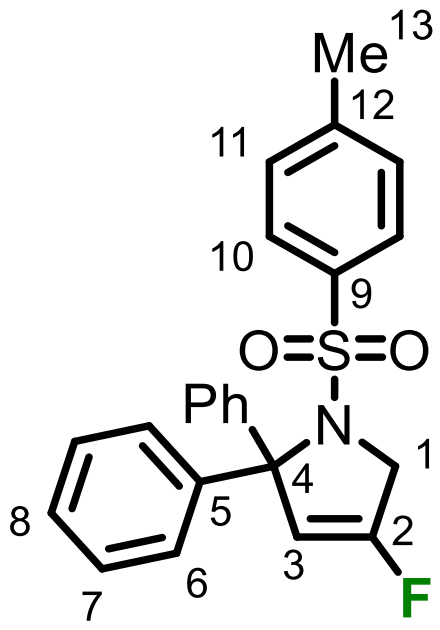

Prepared according to **General procedure IV** from **3o** (60% NMR) and purified by flash column chromatography (0 – 50% Et_2O in pentane) to afford the title compound as a colourless oil (18 mg, 45%).

^{1}H NMR (500 MHz, CDCl$_3$) δ 7.45 – 7.37 (m, 4H, C6-H), 7.35 – 7.29 (m, 6H, C8-H), 6.95 (d, *J* = 8.2 Hz, 2H, C10-H), 6.73 (d, *J* = 8.2 Hz, 2H, C11-H), 5.24 (q, *J* = 1.8 Hz, 1H, C3-H), 4.37 (dd, *J* = 3.8, 1.8 Hz, 2H, C1-H), 2.32 (s, 3H, C13-H).

^{13}C NMR (126 MHz, CDCl$_3$) δ 153.6 (d, *J* = 275.7 Hz, C2), 142.9 (C12), 141.5 (C5), 136.3 (C9), 129.1 (C10, C6), 128.1 (C7), 127.8 (C8), 127.1 (C11), 111.1 (d, *J* = 6.4 Hz, C1), 79.2 (d, *J* = 6.3 Hz, C4), 50.5 (d, *J* = 31.2 Hz, C3), 21.5 (C13).

^{19}F NMR (471 MHz, CDCl$_3$) δ −131.73 (1F, q, *J* = 3.9 Hz, C2-F).

HRMS (+EI) calcd: $[M]^+$ ($C_{23}H_{20}FNO_2S$) 394.1272, measured 394.1268 (1.01 ppm error).

IR (neat) ν_{max}/cm^{-1}: 3060, 3025, 2925, 2863, 1702, 1598, 1493, 1446, 1378, 1348, 1306, 1290, 1267, 1209, 1156, 1132, 1095, 1038, 1016, 925, 907, 817, 764, 732, 699, 666, 652, 632, 600, 581, 546, 522, 489, 455.

4-fluoro-2-methyl-2-phenyl-1-tosyl-2,5-dihydro-1H-pyrrole **(4p)**

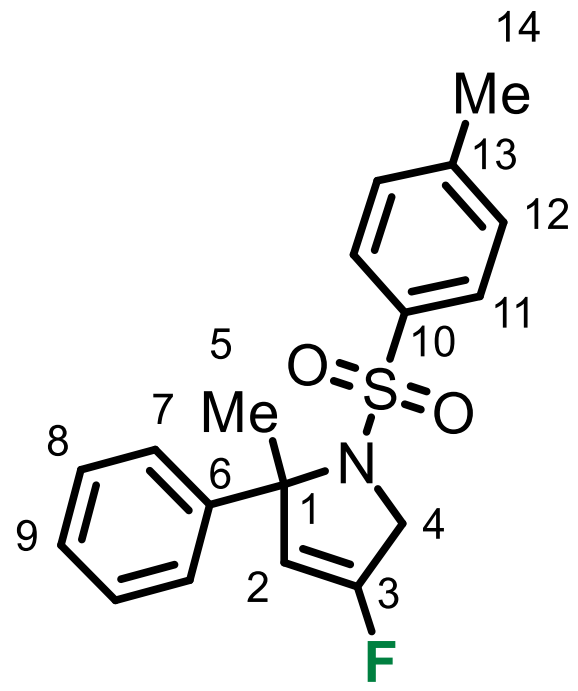

Prepared according to **General procedure IV** from **3p** (44% NMR) and purified by flash column chromatography (2-20% EtOAc in pentane) to afford the title compound as a yellow oil (26.5 mg, 40%).

^{1}H NMR (500 MHz, CDCl$_3$) δ 7.33 – 7.27 (m, 2H, C8-H,), 7.25 – 7.18 (m, 3H, C7-H, C9-H), 7.09 (d, *J* = 8.4 Hz, 2H, C11-H), 7.03 (d, *J* = 8.4 Hz, 2H, C12-H), 5.01 (q, *J* = 2.0 Hz, 1H, C2-H), 4.28 (dddd, *J* = 84.7, 12.8, 3.9, 1.8 Hz, 2H, C4-H), 2.35 (s, 3H, C14-H), 2.10 (s, 3H, C5-H).

^{13}C NMR (151 MHz, CDCl$_3$) δ 153.7 (d, *J* = 275.0 Hz, C3), 142.9 (C13), 141.5 (C6), 136.7 (C10), 129.2 (C12), 128.3 (C8), 127.8 (C9), 127.3 (C7), 127.1 (C11), 110.8 (d, *J* = 5.7 Hz, C2), 72.0 (d, *J* = 6.7 Hz, C1), 50.2 (d, *J* = 31.7 Hz, C4), 26.4 (d, *J* = 2.2 Hz, C5), 21.6 (C14).

^{19}F NMR (376 MHz, CDCl$_3$) δ −131.93 (s, 1F, C3-F).

HRMS (+ESI) calcd: $[M+H]^+$ ($C_{18}H_{18}FNO_2S$) 332.1115, measured 332.1114 (0.30 ppm error).

IR (neat) v_{max}/cm^{-1}: 2936, 1700, 1598, 1371, 1343, 1156, 1093, 1052, 814, 765, 694, 660, 578, 541.

R*f* 0.56 (10% EtOAc in pentane).

(R)*-4-fluoro-2-methyl-2-phenyl-1-tosyl-2,5-dihydro-1H-pyrrole** ((R***)-**4p**)

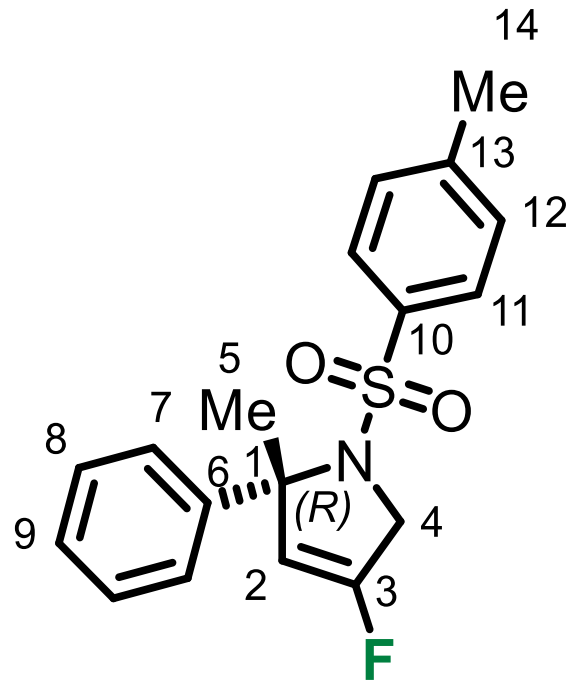

Prepared according to **General procedure IV** from **(*R*)-3p** (46% NMR) and purified by flash column chromatography (2-20% EtOAc in pentane) to afford the title compound as a yellow oil (27.2 mg, 41%).

Spectra in accordance with **4p**

^{1}H NMR (500 MHz, $CDCl_3$) δ 7.33 – 7.27 (m, 2H, C8-H,), 7.25 – 7.18 (m, 3H, C7-H, C9-H), 7.09 (d, *J* = 8.4 Hz, 2H, C11-H), 7.03 (d, *J* = 8.4 Hz, 2H, C12-H), 5.01 (q, *J* = 2.0 Hz, 1H, C2-H), 4.28 (dddd, *J* = 84.7, 12.8, 3.9, 1.8 Hz, 2H, C4-H), 2.35 (s, 3H, C14-H), 2.10 (s, 3H, C5-H).

^{13}C NMR (151 MHz, $CDCl_3$) δ 153.7 (d, *J* = 275.0 Hz, C3), 142.9 (C13), 141.5 (C6), 136.7 (C10), 129.2 (C12), 128.3 (C8), 127.8 (C9), 127.3 (C7), 127.1 (C11), 110.8 (d, *J* = 5.7 Hz, C2), 72.0 (d, *J* = 6.7 Hz, C1), 50.2 (d, *J* = 31.7 Hz, C4), 26.4 (d, *J* = 2.2 Hz, C5), 21.6 (C14).

^{19}F NMR (376 MHz, $CDCl_3$) δ −131.93 (s, 1F, C3-F).

HRMS (+ESI) calcd: $[M+H]^+$ ($C_{18}H_{18}FNO_2S$) 332.1115, measured 332.1114 (0.30 ppm error).

IR (neat) v_{max}/cm^{-1}: 2936, 1700, 1598, 1371, 1343, 1156, 1093, 1052, 814, 765, 694, 660, 578, 541.

$[\alpha]_D^{24}$ = +24° (c 1.00 in $CHCl_3$).

R_f 0.56 (10% EtOAc in pentane).

***6-fluoro-4-tosyl-4-azaspiro[2.4]hept-6-ene* (4q)**

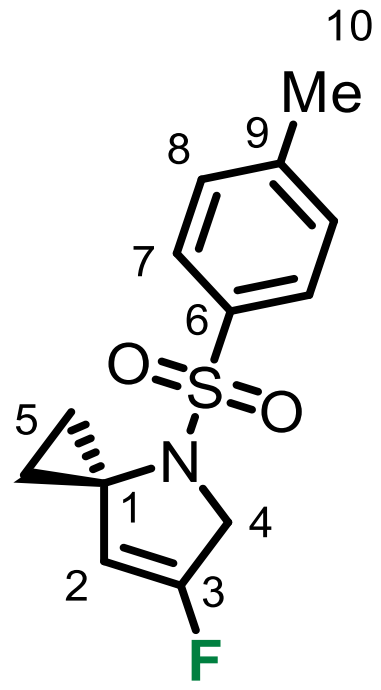

Prepared according to **General procedure IV** from (*Z*)-(3-((*N*-cyclopropyl-4-methylphenyl)sulfonamido)-2-fluoroprop-1-en-1-yl)(p-tolyl)iodonium tetrafluoroborate (in-house chemical) (12% NMR) and purified by flash column chromatography (2-20% EtOAc in pentane) to afford the title compound as a colourless oil (5.35 mg, 10%).

^{1}H NMR (500 MHz, $CDCl_3$) δ 7.75 – 7.70 (m, 2H, C7-H), 7.32 (dd, *J* = 8.1, 4.4 Hz, 2H, C8-H), 4.63 (q, *J* = 1.6 Hz, 1H, C2-H), 4.33 (dd, *J* = 4.2, 1.8 Hz, 2H, C4-H), 2.44 (s, 3H, C10-H), 1.74 – 1.65 (m, 2H, C5-H), 0.69 – 0.64 (m, 2H, C5-H).

^{13}C NMR (126 MHz, $CDCl_3$) δ 152.5 (d, *J* = 274.3 Hz, C3), 143.8 (C9), 136.1 (C6), 130.0 (C8), 127.3 (C7), 106.4 (d, *J* = 9.3 Hz, C2), 51.5 (d, *J* = 30.7 Hz, C4), 48.9 (d, *J* = 9.5 Hz, C1), 21.7 (C10), 10.5 (d, *J* = 1.4 Hz, C5).

^{19}F NMR (471 MHz, $CDCl_3$) δ −130.28 (t, *J* = 4.2 Hz, 1F, C3-F).

HRMS (+ESI) calcd. $[M+H]^+$ ($C_{13}H_{14}FNO_2S$) 268.0802, measured 268.0810 (2.98 ppm error).

IR (neat) v_{max}/cm^{-1}: 2926, 1725, 1697, 1597, 1339, 1160, 1094, 814, 585, 550.

R_f 0.57 (20% EtOAc in pentane).

***3-fluoro-1,8-ditosyl-1,8-diazaspiro[4.5]dec-3-ene* (4r)**

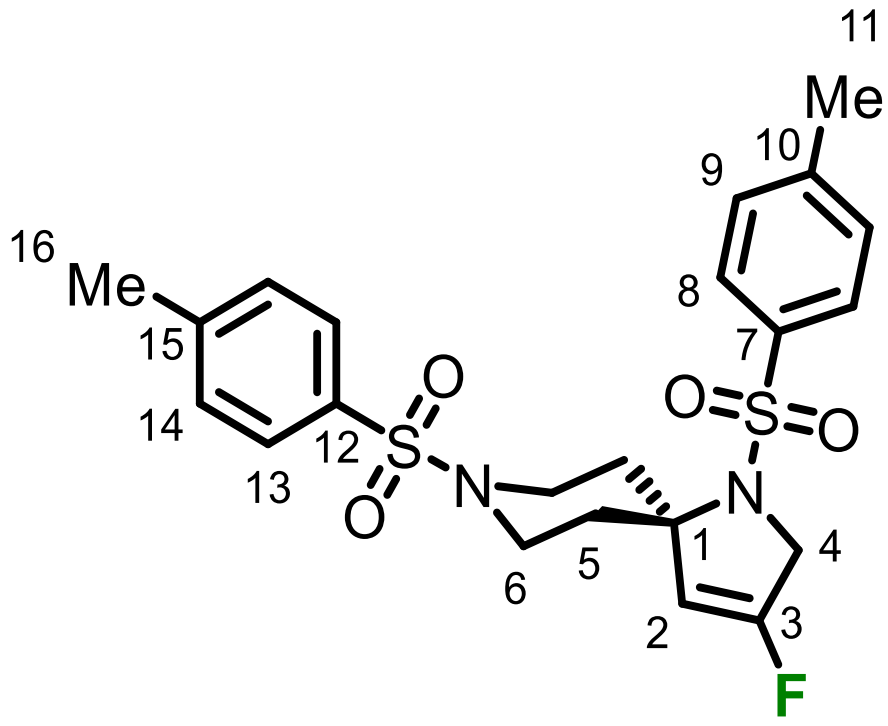

Prepared according to **General procedure IV** from **3r** (13% NMR) and purified by flash column chromatography (2-20% EtOAc in pentane) to afford the title compound as a colourless oil (10.2 mg, 11%).

^{1}H NMR (500 MHz, $CDCl_3$) δ 7.75 – 7.68 (m, 2H, C8-H), 7.67 – 7.56 (m, 2H, C13-H), 7.36 – 7.28 (m, 4H, C9-H, C14-H), 5.21 (t, *J* = 2.5 Hz, 1H, C2-H), 4.14 – 4.06 (m, 2H, C4-H), 3.83 (qd, *J* = 12.2, 6.2 Hz, 2H, C6-H), 2.89 – 2.79 (m, 2H, C5-H), 2.44 (s, 3H, C16-H), 2.43 (s, 3H, C11-H), 2.28 (tt, *J* = 12.2, 2.9 Hz, 2H, C6-H), 1.63 (m, 2H, C5-H).

^{13}C NMR (126 MHz, $CDCl_3$) δ 155.2 (d, *J* = 276.8 Hz, C3), 143.9 (C10), 143.8 (C15), 137.6 (C7), 133.5 (C12), 129.9 (C9), 129.9 (C14), 127.8 (C8), 127.4 (C13), 103.4 (d, *J* = 6.7 Hz, C2), 71.0 (d, *J* = 5.8 Hz, C1), 45.6 (d, *J* = 21.4 Hz, C4), 44.4 (C6), 36.2 (C5), 21.7 (C11), 21.7 (C16).

^{19}F NMR (376 MHz, $CDCl_3$) δ −127.88 (dd, *J* = 6.6, 2.8 Hz, 1F, C3-F).

HRMS (+ESI) calcd: $[M+H]^+$ ($C_{22}H_{25}N_2O_4S_2F$) 465.1313, measured 465.1321 (1.72 ppm error).

IR (neat) v_{max}/cm^{-1}: 2927, 2852, 1727, 1335, 1162, 1093, 1008, 727, 548.

3-fluoro-1-tosyl-1-azaspiro[4.5]dec-3-ene **(4s)**

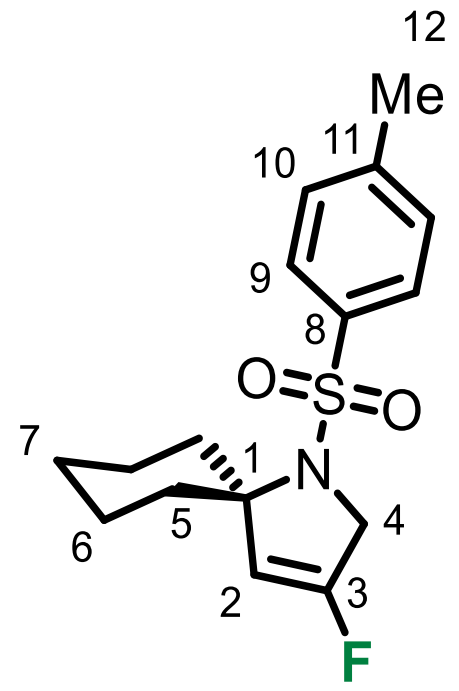

Prepared according to **General procedure IV** from **3s** (17% NMR) and purified by flash column chromatography (2-20% EtOAc in pentane) to afford the title compound as a colourless oil (9.90 mg, 16%).

^{1}H NMR (500 MHz, $CDCl_3$) δ 7.75 (app. dt, J = 8.1, 1.8 Hz, 2H, C9-H), 7.29 (d, J = 8.1 Hz, 2H, C10-H), 5.50 (dt, J = 3.4, 1.9 Hz, 1H, C2-H), 4.10 (dd, J = 4.0, 1.9 Hz, 2H, C4-H), 2.48 (ddd, J = 12.5, 9.9, 2.4 Hz, 2H, C5-H), 2.42 (s, 3H, C12-H), 1.79 – 1.63 (m, 5H, C5-H, C6-H, C7-H), 1.30 – 1.18 (m, 3H, C6-H, C7-H).

^{13}C NMR (126 MHz, $CDCl_3$) δ 153.8 (d, J = 274.1 Hz, C3), 143.3 (C11), 138.1 (C8), 129.7 (C10), 127.4 (C9), 105.7 (d, J = 5.6 Hz, C2), 74.5 (d, J = 5.6 Hz, C1), 50.0 (d, J = 32.3 Hz, C4), 37.7 (d, J = 2.2 Hz, C5), 25.1 (C7), 24.6 (C6), 21.6 (C12).

^{19}F NMR (376 MHz, $CDCl_3$) δ −130.84 (td, J = 4.0, 3.4 Hz).

HRMS (+EI) calcd. $[M]^+$ ($C_{16}H_{20}NO_2SF$) 309.1193, measured 309.1190 (0.97 ppm error).

IR (neat) v_{max}/cm^{-1}: 2930, 2862, 1703, 1455, 1378, 1362, 1335, 1157, 1095, 815, 661, 586, 545.

R_f 0.70 (20% EtOAc in pentane).

***2-benzyl-4-fluoro-1-tosyl-2,5-dihydro-1H-pyrrole* (4t)**

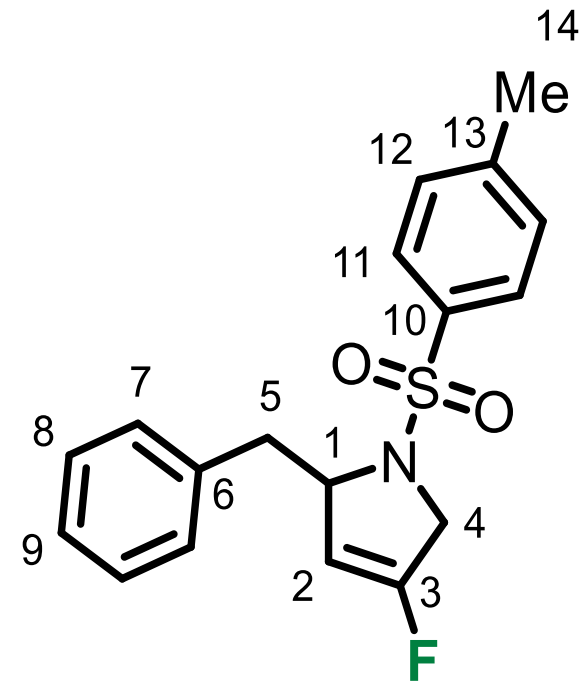

Prepared according to **General procedure IV** from **3t** (82% NMR) and purified by flash column chromatography (2-20% EtOAc in pentane) to afford the title compound as a yellow oil (47.7 mg, 72%).

^{1}H NMR (400 MHz, $CDCl_3$) δ 7.75 (app. dt, *J* = 8.3, 1.7 Hz, 2H, C11-H), 7.34 (d, *J* = 8.9 Hz, 2H, C12-H), 7.29 (m, 2H, C8-H), 7.26 – 7.20 (m, 3H, C7-H, C9-H), 4.86 (p, *J* = 2.0 Hz, 1H, C2-H), 4.59 (dtp, *J* = 7.7, 4.9, 2.4 Hz, 1H, C1-H), 4.02 – 3.87 (m, 2H, C4-H), 3.30 (ddd, *J* = 13.2, 3.6, 1.9 Hz, 1H, C5-H), 2.96 (ddd, *J* = 13.2, 8.7, 1.4 Hz, 1H, C5-H), 2.44 (s, 3H, C14-H).

^{13}C NMR (126 MHz, $CDCl_3$) δ 154.7 (d, *J* = 277.6 Hz, C3), 144.2 (C13), 136.9 (C6), 134.2 (C10), 130.1 (C12), 130.0 (C7), 128.5 (C8), 127.6 (C11), 126.8 (C9), 102.3 (d, *J* = 8.3 Hz, C2), 65.2 (d, *J* = 6.7 Hz, C1), 50.2 (d, *J* = 31.1 Hz, C4), 43.6 (d, *J* = 1.9 Hz, C5), 21.7 (C14).

^{19}F NMR (376 MHz, $CDCl_3$) δ −127.46 (d, *J* = 2.0 Hz, C3-F).

HRMS (EI+) calcd: $[M]^+$ ($C_{18}H_{16}NO_2FS$) 329.0880, measured 329.0876 (1.22 ppm error).

IR (neat) v_{max}/cm^{-1}: 2924, 2869, 1697, 1597, 1494, 1371, 1346, 1159, 1094, 815, 702, 679, 668, 660, 579, 549.

R*f* 0.50 (10% EtOAc in pentane)

***4-fluoro-1-((4-nitrophenyl)sulfonyl)-2-propyl-2,5-dihydro-1H-pyrrole* (4u)**

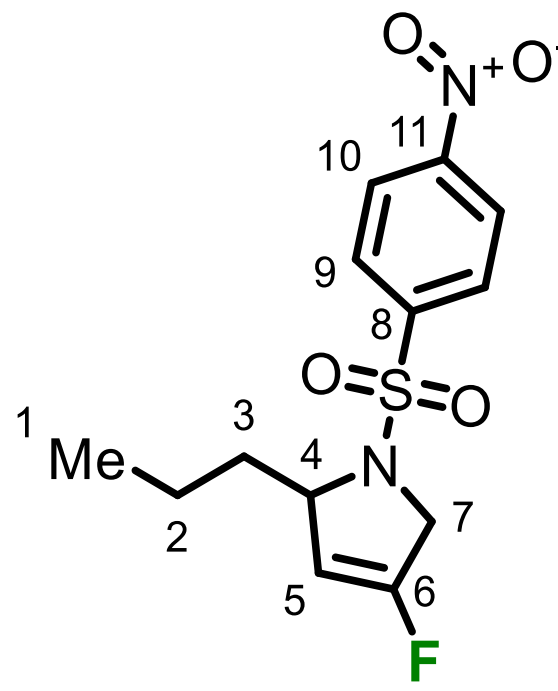

Prepared according to **General procedure IV** from **3u** (57% NMR) and purified by flash column chromatography (2-20% EtOAc in pentane) to afford the title compound as a colourless oil (662.0 mg, 52%).

^{1}H NMR (500 MHz, $CDCl_3$) δ 8.40 (app. dt, J = 8.8, 1.9 Hz, 2H, C10-H), 8.02 (app. dt, J = 8.8, 1.9 Hz, 2H, C9-H), 5.02 (ddd, J = 2.0, 1.9, 1.8 Hz, 1H, C5-H), 4.47 (ddddd, J = 9.4, 5.2, 2.7, 2.0, 1.9 Hz, 1H), 4.16 (dddd, J = 14.1, 5.0, 5.2, 2.0 Hz, 1H, C7-H), 4.06 (dddd, J = 14.1, 3.7, 2.0, 1.8 Hz, 1H, C7-H), 1.88 – 1.68 (m, 2H, C3-H), 1.33 (tq, J = 7.6, 7.5 Hz, 2H, C2-H), 0.93 (t, J = 7.5 Hz, 3H, C1-H).

^{13}C NMR (126 MHz, $CDCl_3$) δ 154.1 (d, J = 277.4 Hz, C6), 150.4 (C11), 143.4 (C8), 128.7 (C9), 124.7 (C10), 102.9 (d, J = 7.8 Hz, C5), 64.4 (d, J = 6.3 Hz, C4), 50.2 (d, J = 31.5 Hz, C7), 38.6 (d, J = 1.9 Hz, C3), 17.8 (C2), 14.0 (C1).

^{19}F NMR (376 MHz, $CDCl_3$) δ −127.59 (ddd, J = 5.0, 3.7, 1.9 Hz, 1F, C6-F).

HRMS (+EI) calcd. $[M-H]^+$ ($C_{14}H_{15}N_2O_4SF$) 313.0653, measured 313.0651 (0.64 ppm error).

IR (neat) v_{max}/cm^{-1}: 3107, 2961, 2874, 1698, 1531, 1350, 1166, 1093, 855, 736, 617, 568, 463.

4-fluoro-2-propyl-1-tosyl-2,5-dihydro-1H-pyrrole **(4v)**

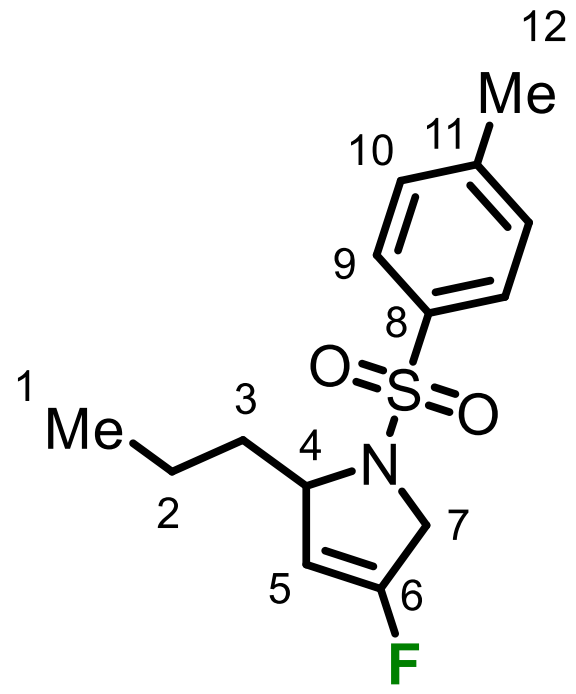

Prepared according to **General procedure IV** from **3v** (61% NMR) and purified by flash column chromatography (2-20% EtOAc in pentane) to afford the title compound as a colourless oil (763 mg, 55%).

^{1}H NMR (400 MHz, $CDCl_3$) δ 7.71 (app. dt, *J* = 8.1, 1.9 Hz, 2H, C9-H), 7.33 (d, *J* = 8.1 Hz, 2H, C10-H), 4.96 (p, *J* = 2.0 Hz, 1H, C5-H), 4.42 (tddt, *J* = 7.2, 4.9, 2.4, 2.0 Hz, 1H, C4-H), 4.09 (dtd, *J* = 14.1, 4.9, 1.9 Hz, 1H, C7-H), 4.03 (ddt, *J* = 14.1, 3.9, 2.0 Hz, 1H, C7-H), 2.43 (s, 3H, C12-H), 1.87 – 1.66 (m, 2H, C3-H), 1.42 – 1.25 (m, 2H, C2-H), 0.93 (t, *J* = 7.4 Hz, 3H, C1-H).

^{13}C NMR (126 MHz, $CDCl_3$) δ 154.4 (d, *J* = 276.8 Hz, C6), 144.0 (C11), 134.4 (C8), 130.0 (C10), 127.6 (C9), 102.8 (d, *J* = 7.6 Hz, C5), 64.0 (d, *J* = 6.6 Hz, C4), 50.2 (d, *J* = 31.2 Hz, C7), 38.8 (d, *J* = 2.0 Hz, C3), 21.7 (C12), 17.9 (C2), 14.1 (C1).

^{19}F NMR (471 MHz, $CDCl_3$) δ −128.59 (ddd, *J* = 4.9, 3.9, 2.4 Hz, 1F, C6-F).

HRMS (+ESI) calcd. $[M+H]^+$ ($C_{14}H_{18}FNO_2S$) 284.1115, measured 284.1116 (0.35 ppm error).

IR (neat) v_{max}/cm^{-1}: 2960, 2873, 1698, 1597, 1458, 1347, 1161, 1093, 815, 669, 580, 548.

***4-fluoro-2-(thiophen-2-ylmethyl)-1-tosyl-2,5-dihydro-1H-pyrrole* (4w)**

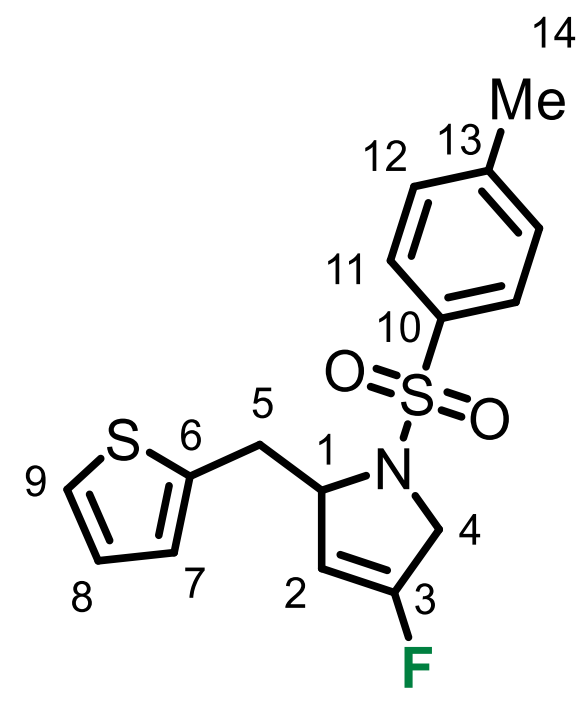

Prepared according to **General procedure IV** from **3w** (52% NMR) and purified by flash column chromatography (2-20% EtOAc in pentane) to afford the title compound as a colourless oil (32.4 mg, 48%).

^{1}H NMR (500 MHz, CDCl$_3$) δ 7.75 (dt, *J* = 8.4, 1.7 Hz, 2H, C11-H), 7.38 – 7.33 (m, 2H, C12-H), 7.17 (dd, *J* = 5.2, 1.1 Hz, 1H, C9-H), 6.94 (dd, *J* = 5.2, 3.4 Hz, 1H, C8-H), 6.86 (dd, *J* = 3.4, 1.1 Hz, 1H, C7-H), 4.93 (p, *J* = 1.9 Hz, 1H, C2-H), 4.66 – 4.57 (m, 1H, C1-H), 4.00 (tt, *J* = 4.3, 1.4 Hz, 2H, C4-H), 3.42 (dd, *J* = 14.6, 3.2 Hz, 1H, C5-H), 3.29 (ddd, *J* = 14.6, 7.7, 1.5 Hz, 1H, C5-H), 2.44 (s, 3H, C14-H).

^{13}C NMR (126 MHz, CDCl$_3$) δ 155.0 (d, *J* = 277.8 Hz, C3), 144.3 (C13), 138.4 (C6), 134.0 (C10), 130.2 (C12), 127.7 (C11), 126.9 (C7), 126.9 (C8), 124.7 (C9), 102.2 (d, *J* = 8.4 Hz, C2), 64.7 (d, *J* = 6.8 Hz, C1), 50.4 (d, *J* = 30.9 Hz, C4), 37.3 (d, *J* = 2.0 Hz, C5), 21.7 (C14).

^{19}F NMR (376 MHz, CDCl$_3$) δ −126.64 (qd, *J* = 4.3, 1.9 Hz, 1F, C3-F).

HRMS (+ESI) calcd: $[M+H]^+$ ($C_{16}H_{16}FNO_2S_2$) 338.0679, measured 338.0688 (3.25 ppm error).

IR (neat) v_{max}/cm^{-1}: 2923, 2869, 1697, 1597, 1345, 1159, 1093, 815, 710, 696, 664, 580, 549.

R_f 0.53 (20% EtOAc in pentane).

***4-fluoro-2-(tetrahydrofuran-2-yl)-1-tosyl-2,5-dihydro-1H-pyrrole* (4x)**

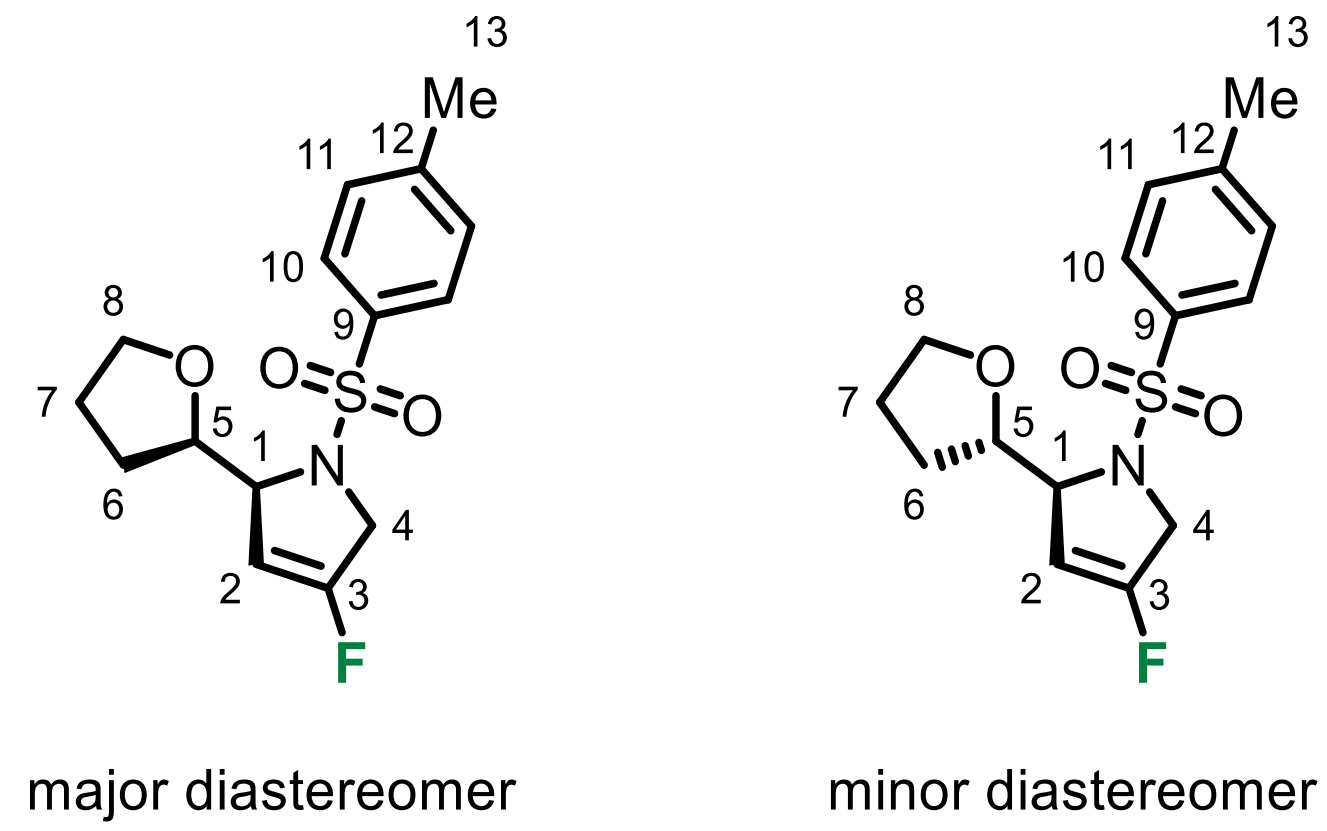

Prepared according to **General procedure IV** from **3x** (24% NMR) and purified by flash column chromatography (2-20% EtOAc in pentane) to afford the title compound as a yellow oil (12.5 mg, 20%).

Isolated as a mixture of diastereomers (1.4:1).

^{1}H NMR (500 MHz, $CDCl_3$) Major diastereomer δ 7.71 (d, *J* = 8.4 Hz, 2H, C10-H), 7.32 (d, *J* = 8.4 Hz, 2H, C11-H), 5.04 (p, *J* = 1.9 Hz, 1H, C2-H), 4.40 (dddd, *J* = 4.9, 4.9, 2.3, 1.4 Hz, 1H, C1-H), 4.15 – 3.99 (m, 3H, C4-H, C5-H), 3.85 – 3.73 (m, 2H, C8-H), 2.43 (s, 3H, C13-H), 2.11 – 2.04 (m, 1H, C7-H), 1.93 – 1.85 (m, 2H, C6-H), 1.82 – 1.74 (m, 1H, C7-H). **Minor diastereomer** δ 7.72 (d, *J* = 8.4 Hz, 2H, C10-H), 7.33 (d, *J* = 8.4 Hz, 2H, C11-H), 4.93 (p, *J* = 1.9 Hz, 1H, C2-H), 4.58 (tdd, *J* = 5.3, 4.2, 2.1 Hz, 1H, C1-H), 4.35 (tdd, *J* = 6.8, 4.2, 2.4 Hz, 1H, C5-H), 4.15 – 3.99 (m, 2H, C4-H), 3.85 – 3.73 (m, 2H, C8-H), 2.43 (s, 3H, C13-H) 1.98 (dtd, *J* = 11.5, 7.3, 5.1 Hz, 1H, C7-H), 1.94 – 1.86 (m, 1H, C7-H), 1.86 – 1.79 (m, 2H, C6-H).

^{13}C NMR (126 MHz, $CDCl_3$) Major diastereomer δ 156.3 (d, *J* = 278.4 Hz, C3), 144.1 (C12), 134.4 (C9), 130.0 (C11), 127.7 (C10), 100.4 (d, *J* = 9.3 Hz, C2), 81.9 (d, *J* = 2.0 Hz, C5), 68.9 (C8), 67.1 (d, *J* = 6.8 Hz, C1), 50.4 (d, *J* = 30.6 Hz, C4), 28.6 (C6), 25.9 (C7), 21.7 (C13). **Minor diastereomer** δ 156.0 (d, *J* = 278.0 Hz, C3), 144.3 (C12), 133.5 (C9), 130.1 (C11), 127.8 (C10), 99.8 (d, *J* = 8.6 Hz, C2), 80.5 (C5), 69.1 (C8), 65.6 (d, *J* = 6.7 Hz, C1), 50.8 (d, *J* = 31.1 Hz, C4), 26.1 (C6), 25.8 (C7), 21.7 (C13).

^{19}F NMR (471 MHz, $CDCl_3$) Major diastereomer δ −126.74 (s, 1F, C3-F). **Minor Diastereomer** δ −126.08 (s, 1F, C3-F),

HRMS (Nanospray) calcd: $[M+H]^+$ ($C_{15}H_{18}FNO_3S$) 312.1064, measured 312.1078 (4.49 ppm error).

IR (neat) v_{max}/cm^{-1}: 2927, 2873, 1696, 1347, 1159, 1092, 1065, 815, 664, 582, 547.

R_f 0.31 (20% EtOAc in pentane).

***5-fluoro-2,3,3a,6-tetrahydropyrrolo[1,2-b]isothiazole 1,1-dioxide* (4y)**

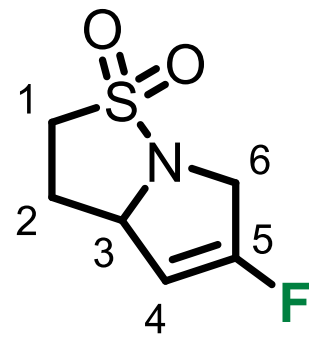

Prepared according to **General procedure IV** from **3y** (63% NMR) and purified by flash column chromatography (2-20% EtOAc in pentane) to afford the title compound as a colourless oil (19.1 mg, 54%).

^{1}H NMR (400 MHz, $CDCl_3$) δ 5.07 (td, *J* = 1.9, 1.6 Hz, 1H, C4-H), 4.75 (dddddd, *J* = 7.5, 3.7, 3.0, 2.8, 1.6, 1.4 Hz, 1H, C3-H), 4.42 (dddd, *J* = 15.4, 4.5, 3.7, 1.9 Hz, 1H, C6-H), 4.00 (dddd, *J* = 15.4, 5.2, 2.8, 1.9 Hz, 1H, C6-H), 3.23 (ddd, *J* = 12.2, 6.5, 3.3 Hz, 1H, C1-H), 2.82 (ddd, *J* = 12.2, 12.0, 7.6 Hz, 1H, C1-H), 2.65 (dddd, *J* = 12.2, 12.0, 7.5, 6.5 Hz, 1H, C2-H), 2.26 (dddd, *J* = 12.2, 7.6, 3.3, 3.0 Hz, 1H, C2-H).

^{13}C NMR (126 MHz, $CDCl_3$) δ 157.7 (d, *J* = 281.7 Hz, C5), 101.6 (d, *J* = 10.0 Hz, C4), 63.9 (d, *J* = 8.2 Hz, C3), 50.8 (d, *J* = 29.4 Hz, C6), 44.4 (C1), 27.6 (d, *J* = 2.5 Hz, C2).

^{19}F NMR (376 MHz, $CDCl_3$) δ −126.84 (ddd, *J* = 5.2, 4.5, 1.4 Hz).

HRMS (+EI) calcd: $[M]^+$ ($C_6H_8NO_2SF$) 177.0254, measured 177.0251 (1.69 ppm error).

IR (neat) v_{max}/cm^{-1}: 2955, 1693, 1330, 1165, 1148, 1098, 739, 553.

(syn)-6-fluoro-8-tosyl-8-azabicyclo[3.2.1]oct-6-ene **(4z)**

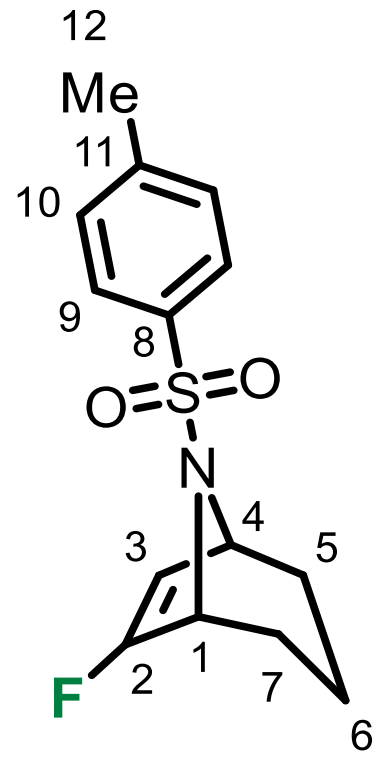

Prepared according to **General procedure IV** from **3z** (76% NMR) and purified by flash column chromatography (2-20% EtOAc in pentane) to afford the title compound as a colourless oil (41.1 mg, 73%).

Produced as a mixture with regioisomer **5z** and separated by flash column chromatography.

^{1}H NMR (500 MHz,CDCl$_3$) δ 7.61 (app. dt, J = 8.2, 1.8 Hz, 2H, C9-H), 7.27 (d, J = 8.2 Hz, 2H, C10-H), 4.78 (d, J = 2.6 Hz, 1H, C3-H), 4.41 (dddd, J = 5.2, 2.7, 2.6, 2.3 Hz, 1H, C4-H), 4.22 (dt, J = 2.7, 2.6 Hz, 1H, C1-H), 2.42 (s, 3H, C12-H), 2.00 – 1.84 (m, 2H, C5-H, C7-H), 1.82 – 1.61 (m, 3H, C5-H, C6-H), 1.51 – 1.38 (m, 1H, C7-H).

^{13}C NMR (126 MHz, CDCl$_3$) δ 160.8 (d, J = 294.0 Hz, C2), 143.8 (C11), 135.3 (C8), 129.8 (C10), 127.7 (C9), 101.7 (d, J = 7.5 Hz, C3), 59.5 (d, J = 8.8 Hz, C4), 58.8 (d, J = 26.9 Hz, C1), 25.9 (d, J = 2.7 Hz, C5), 24.7 (d, J = 3.2 Hz, C7), 21.7 (C12), 16.7 (C6).

^{19}F NMR (376 MHz, CDCl$_3$) δ −129.86 (ddd, J = 2.7, 2.3, 2.2 Hz).

HRMS (+EI) calcd: $[M]^+$ ($C_{14}H_{16}NO_2SF$) 281.0880, measured 281.0877 (1.07 ppm error).

IR (neat) v_{max}/cm^{-1}: 2938, 1668, 1342, 1302, 1161, 1095, 843, 694, 580, 545.

3-fluoro-1-tosyl-2,5-dihydro-1H-pyrrole **(4ac)**

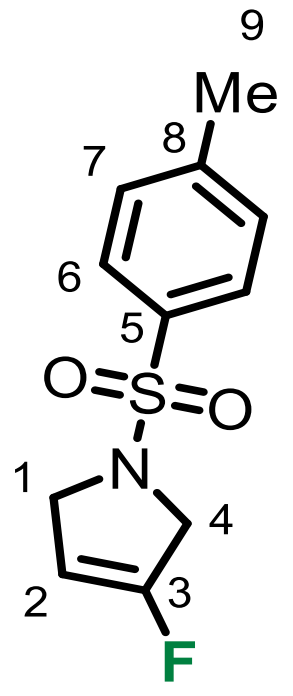

Prepared according to **General procedure IV** from **3ac** (16% NMR) and purified by flash column chromatography (2-20% EtOAc in pentane) to afford the title compound as a colourless oil (6.76 mg, 14%).

^{1}H NMR (500 MHz, $CDCl_3$) δ 7.74 – 7.70 (m, 2H, C6-H), 7.35 (d, *J* = 8.0 Hz, 2H, C7-H), 5.01 (d, *J* = 3.5 Hz, 1H, C2-H), 4.09 – 4.06 (m, 4H, C1-H, C4-H), 2.44 (s, 3H, C9-H).

^{13}C NMR (126 MHz, $CDCl_3$) δ 154.5 (d, *J* = 275.4 Hz, C3), 144.1 (C8), 133.8 (C5), 130.1 (C7), 127.6 (C6), 98.6 (d, *J* = 9.8 Hz, C2), 51.0 (d, *J* = 6.8 Hz, C1), 49.5 (d, *J* = 31.5 Hz, C4), 21.7 (C9).

^{19}F NMR (377 MHz, $CDCl_3$) δ −126.92 – −127.01 (m, 1F, C3-F).

HRMS (+EI) calcd. $[M]^+$ ($C_{11}H_{12}NO_2SF$) 241.0567, measured 241.0567 (0.00 ppm error).

IR (neat) v_{max}/cm^{-1}: 2925, 2865, 1725, 1697, 1370, 1348, 1162, 1097, 814, 670, 582, 548.

R_f 0.49 (20% EtOAc in pentane).

2-propyl-1-tosyl-2,5-dihydro-1*H*-pyrrole (4bf)

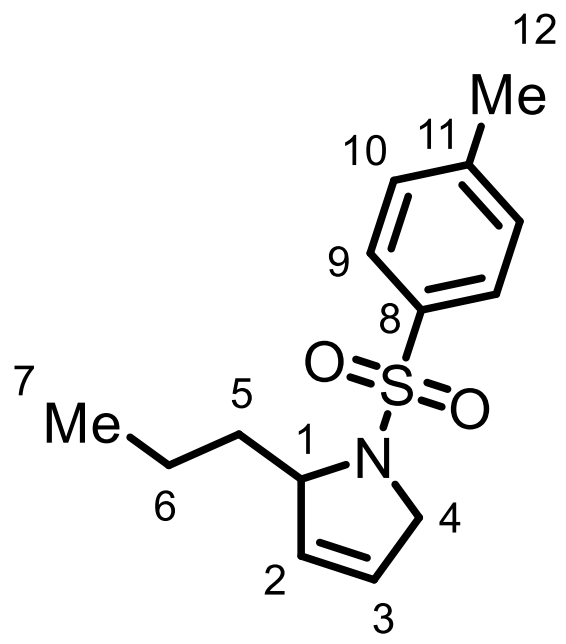

Prepared according to **General procedure II** from 2-propyl-2,5-dihydro-1*H*-pyrrole without further purification to afford the title compound as a pale brown solid (1.21 g, 91%).

^{1}H NMR (500 MHz, CDCl$_3$) δ 7.70 (app. dt, *J* = 8.4, 1.9 Hz, 2H, C9-H), 7.32 – 7.27 (m, 2H, C10-H), 5.62 – 5.54 (m, 2H, C2-H, C3-H), 4.50 – 4.42 (m, 1H, C1-H), 4.14 – 4.06 (m, 2H, C4-H), 2.41 (s, 3H, C12-H), 1.83 – 1.65 (m, 2H), 1.45 – 1.22 (m, 2H), 0.93 (t, *J* = 7.4 Hz, 3H).

^{13}C NMR (126 MHz, CDCl$_3$) δ 143.4 (C11), 135.1 (C8), 130.0 (C2), 129.8 (C10), 127.5 (C9), 124.7 (C3), 67.4 (C1), 55.7 (C4), 38.5 (C5), 21.7 (C12), 18.0 (C6), 14.2 (C7).

Characterisation data matches that previously reported.[43]

8.4. Other Cycles (5)

***(syn)-7-fluoro-2-tosyl-2-azabicyclo[3.2.1]oct-6-ene* (5z)**

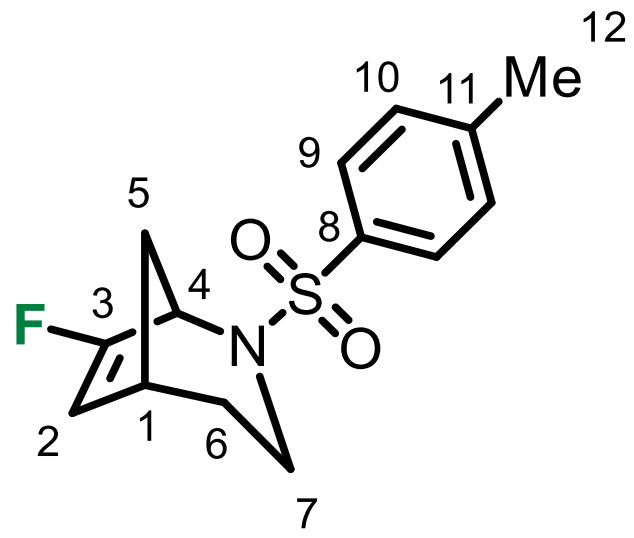

Prepared according to **General procedure IV** from **3z** (15% NMR) and purified by flash column chromatography (2-20% EtOAc in pentane) to afford the title compound as a colourless oil (6.2 mg, 11%).

Contains 44 mol% of major regioisomer **4z.**

^{1}H NMR (500 MHz, CDCl$_3$) δ 7.68 (d, *J* = 8.2 Hz, 2H, C9-H), 7.28 (d, *J* = 8.2 Hz, 2H, C10-H), 5.22 (d, *J* = 3.3 Hz, 1H, C2-H), 4.57 (d, *J* = 4.8 Hz, 1H, C4-H), 3.72 (dd, *J* = 12.0, 6.6 Hz, 1H, C7-H), 2.94 (td, *J* = 12.0, 5.2 Hz, 1H, C7-H), 2.63 – 2.58 (m, 1H, C1-H), 2.42 (s, 3H, C12-H), 2.19 – 2.14 (m, 1H, C5-H), 2.00 – 1.90 (m, 1H, C6-H), 1.69 – 1.67 (m, 1H, C5-H), 1.55 – 1.52 (m, 1H, C6-H).

^{13}C NMR (126 MHz, CDCl$_3$) δ 158.4 (d, *J* = 294.9 Hz, C3), 143.4 (C11), 135.6 (C8), 129.6 (C10), 127.8 (C9), 108.4 (d, *J* = 4.9 Hz, C2), 58.0 (d, *J* = 23.4 Hz, C4), 42.9 (d, *J* = 6.8 Hz, C5), 40.3 (C7), 34.3 (d, *J* = 6.9 Hz, C1), 27.3 (d, *J* = 3.8 Hz, C6), 21.7 (C12).

^{19}F NMR (377 MHz, CDCl$_3$) δ −124.08 (dd, *J* = 4.8, 2.7 Hz).

HRMS (+EI) calcd: $[M]^+$ ($C_{14}H_{16}NO_2SF$) 281.0880, measured 281.0878 (0.71 ppm error).

IR (neat) v_{max}/cm^{-1}: 2937, 1668, 1665, 1339, 1160, 1095, 939, 729, 695, 664, 581, 546.

***N*-(3-fluorobenzyl)-*N*-((syn)-7-fluorobicyclo[3.2.1]oct-6-en-1-yl)-4-methylbenzenesulfonamide* **(5aa)**

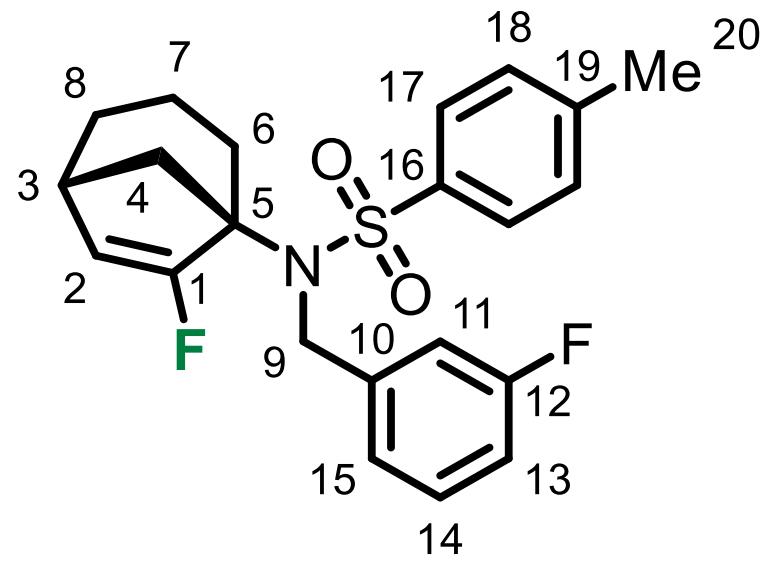

Prepared according to **General procedure IV** from **3aa** (82% NMR) and purified by flash column chromatography (2-20% EtOAc in pentane) to afford the title compound as a colourless oil (62.9 mg, 78%).

^{1}H NMR (500 MHz, $CDCl_3$) δ 7.67 (app. dt, *J* = 8.3, 1.9 Hz, 2H, C17-H), 7.23 (dd, *J* = 8.6, 3.2 Hz, 1H, C14-H), 7.23 (d, *J* = 8.1 Hz, 2H, C18-H), 7.15 (d, *J* = 7.8 Hz, 1H, C15-H), 7.10 (dt, *J* = 10.0, 2.1 Hz, 1H, C11-H), 6.89 (td, *J* = 8.4, 2.3 Hz, 1H, C13-H), 4.99 (dt, *J* = 3.0, 1.4 Hz, 1H, C2-H), 4.68 (d, *J* = 17.3 Hz, 1H, C9-H), 4.60 (d, *J* = 17.3 Hz, 1H, C9-H), 2.52 (dp, *J* = 6.3, 3.1 Hz, 1H, C3-H), 2.46 (ddd, *J* = 9.4, 5.7, 3.2 Hz, 1H, C4-H), 2.38 (s, 3H, C20-H), 1.86 (td, *J* = 12.4, 6.0 Hz, 1H, C6-H), 1.79 (dddd, *J* = 12.4, 6.5, 3.2, 1.6 Hz, 1H, C6-H), 1.64 – 1.55 (m, 2H, C7-H), 1.54 – 1.43 (m, 1H, C4-H), 1.25 (dt, *J* = 9.4, 3.1 Hz, 2H, C8-H).

^{13}C NMR (126 MHz, $CDCl_3$) δ 163.0 (d, *J* = 245.6 Hz, C12), 159.6 (d, *J* = 293.1 Hz, C1), 143.3 (C19), 142.3 (d, *J* = 7.1 Hz, C10), 139.6 (C16), 129.8 (d, *J* = 8.2 Hz, C14), 129.6 (C18), 127.3 (C17), 122.6 (d, *J* = 2.8 Hz, C15), 114.1 (d, *J* = 22.3 Hz, C11), 113.9 (d, *J* = 21.2 Hz, C13), 104.5 (d, *J* = 5.1 Hz, C2), 69.3 (d, *J* = 19.0 Hz, C5), 50.5 (t, *J* = 2.1 Hz, C9), 47.9 (d, *J* = 4.0 Hz, C4), 33.7 (d, *J* = 7.8 Hz, C3), 29.2 (d, *J* = 3.3 Hz, C6), 24.5 (d, *J* = 3.5 Hz, C8), 21.6 (C20), 20.2 (C7).

^{19}F NMR (376 MHz, $CDCl_3$) δ −113.05 – −113.14 (m, 1F, C12-F), −127.95 (dd, *J* = 6.3, 3.0 Hz, 1F, C1-F).

HRMS (+ESI) calcd: $[M+H]^+$ ($C_{22}H_{23}F_2NO_2S$) 404.1490, measured 404.1479 (2.72 ppm error).

IR (neat) v_{max}/cm^{-1}: 2939, 2862, 1739, 1661, 1616, 1591, 1450, 1331, 1155, 1090, 922, 1090, 922, 809, 660, 581, 548.

***N-((syn)-7-fluorobicyclo[3.2.1]oct-6-en-1-yl)-N,4-dimethylbenzenesulfonamide* (5ab)**

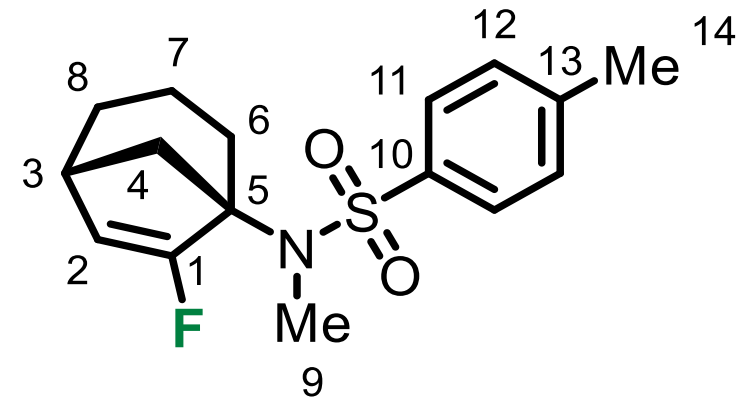

Prepared according to **General procedure IV** from **3ab** (80% NMR) and purified by flash column chromatography (2-20% EtOAc in pentane) to afford the title compound as a colourless oil (46.4 mg, 75%).

^{1}H NMR (500 MHz, CDCl$_3$) δ 7.74 (app. dt, *J* = 8.2, 2.0 Hz, 2H, C11-H), 7.29 (d, *J* = 8.2 Hz, 2H, C12-H), 5.05 (dt, *J* = 2.9, 1.4 Hz, 1H, C2-H), 2.96 (d, *J* = 1.3 Hz, 3H, C9-H), 2.61 (dp, *J* = 6.3, 3.2 Hz, 1H, C3-H), 2.43 (s, 3H, C14-H), 2.46 – 2.38 (m, 1H, C4-H), 1.99 (td, *J* = 12.4, 6.1 Hz, 1H, C6-H), 1.83 (dddd, *J* = 12.4, 6.3, 3.1, 1.5 Hz, 1H, C6-H), 1.73 – 1.64 (m, 1H, C7-H), 1.62 – 1.53 (m, 1H, C7-H), 1.49 (dd, *J* = 9.9, 3.7 Hz, 1H, C4-H), 1.36 – 1.30 (m, 2H, C8-H).

^{13}C NMR (126 MHz, CDCl$_3$) δ 159.6 (d, *J* = 293.2 Hz, C1), 143.2 (C13), 139.2 (C10), 129.6 (C12), 127.2 (C11), 104.2 (d, *J* = 4.9 Hz, C2), 68.4 (d, *J* = 18.9 Hz, C5), 45.1 (d, *J* = 4.0 Hz, C4), 33.8 (d, *J* = 7.9 Hz, C3), 33.1 (d, *J* = 3.2 Hz, C9), 28.7 (d, *J* = 3.4 Hz, C6), 24.8 (d, *J* = 3.6 Hz, C8), 21.6 (C14), 20.4 (C7).

^{19}F NMR (376 MHz, CDCl$_3$) δ −129.67 (dd, *J* = 6.3, 2.9 Hz, 1F, C1-F).

HRMS (+ESI) calcd: $[M+H]^+$ ($C_{16}H_{20}FNO_2S$) 310.1272, measured 310.1262 (3.22 ppm error).

IR (neat) v_{max}/cm^{-1}: 2938, 2864, 1738, 1661, 1454, 1328, 1305, 1156, 1088, 813, 685, 660, 583, 550.

2-(((cis-7-fluorobicyclo[3.2.1]oct-6-en-1-yl)oxy)methyl)benzonitrile **(5ai)**

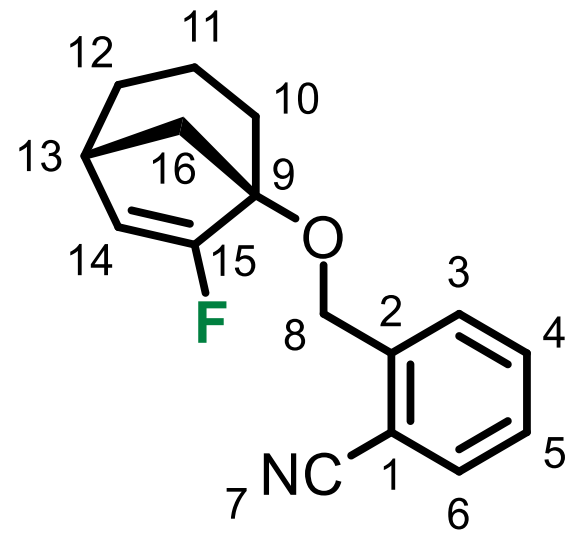

Formed as a side-product in the synthesis of oxolene **6ai** (13% NMR) and purified by flash column chromatography (0-10% EtOAc in pentane) to afford the title compound as a colourless oil (5.66 mg, 11%).

^{1}H NMR (400 MHz, CDCl$_3$) δ 7.72 – 7.61 (m, 2H, C4-H, C6-H), 7.59 (td, *J* = 7.7, 1.4 Hz, 1H, C5-H), 7.36 (td, *J* = 7.6, 1.3 Hz, 1H, C3-H), 5.14 (dd, *J* = 3.2, 1.5 Hz, 1H, C14-H), 4.85 (d, *J* = 12.5 Hz, 1H, C8-H), 4.65 (d, *J* = 12.5 Hz, 1H, C8-H), 2.72 (dp, *J* = 6.3, 3.2 Hz, 1H, C13-H), 2.50 (dt, *J* = 9.2, 4.2 Hz, 1H, C16-H), 1.90 – 1.84 (m, 1H, C11-H), 1.79 – 1.64 (m, 3H, C10-H, C11-H), 1.44 (dd, *J* = 9.2, 4.0 Hz, 1H, C16-H), 1.42 – 1.36 (m, 2H, C12-H).

^{13}C NMR (101 MHz, CDCl$_3$) δ 160.6 (d, *J* = 295.3 Hz, C15-H), 142.7 (C2), 133.0 (C4), 132.7 (C6), 128.8 (C5), 127.9 (C3), 117.5 (C7), 111.0 (C1), 105.4 (d, *J* = 3.7 Hz, C14), 83.3 (d, *J* = 20.0 Hz, C9), 63.3 (C8), 45.6 (d, *J* = 4.8 Hz, C16), 33.6 (d, *J* = 8.0 Hz, C13), 29.5 (d, *J* = 3.6 Hz, C10), 24.8 (d, *J* = 3.8 Hz, C12), 20.1 (C11).

^{19}F NMR (376 MHz, CDCl$_3$) δ −132.80 (dd, *J* = 6.3, 1.5 Hz).

HRMS (+EI) calcd: $[M]^+$ ($C_{16}H_{16}NOF$) 257.1210, measured 257.1207 (1.17 ppm error).

IR (neat) v_{max}/cm^{-1}: 2941, 2862, 2225, 1737, 1658, 1452, 1340, 1216, 1134, 1075, 763, 706.

8.5. Oxolenes (6)

***3-fluoro-2-methyl-5-phenyl-2,5-dihydrofuran* (6ad)**

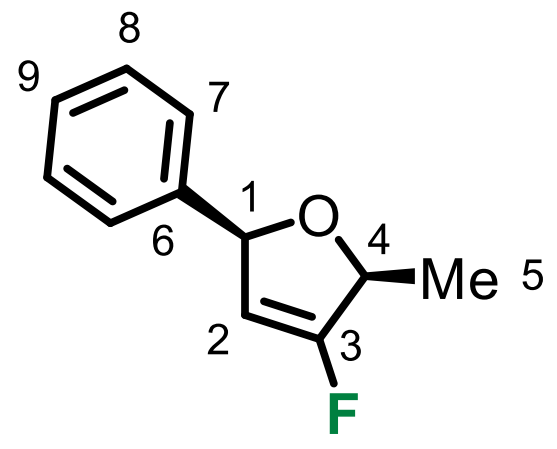

Major diastereomer

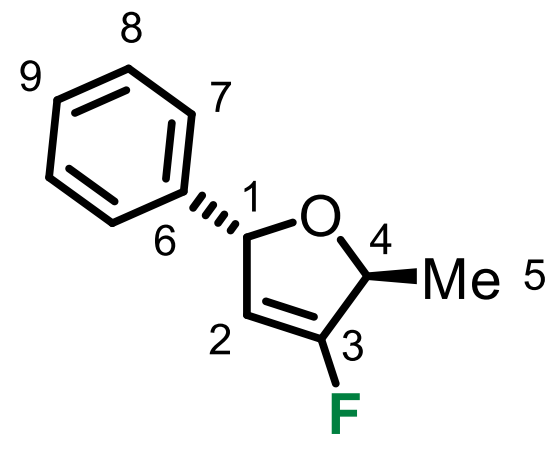

Minor diastereomer

Prepared according to **General procedure IV** from **3ad** and purified by flash column chromatography to afford the title compound as a colourless oil (27.4 mg, 77%).

Isolated as a mixture of diastereomers (1.87:1).

^{1}H NMR (500 MHz, $CDCl_3$) Major diastereomer δ 7.39 – 7.34 (m, 4H, C7-H, C8-H), 7.33 – 7.28 (m, 1H, C9-H), 5.72 (ddd, *J* = 5.2, 3.9, 1.8 Hz, 1H, C1-H), 5.24 (t, *J* = 1.8 Hz, 1H, C2-H), 4.91 – 4.84 (m, 1H, C4-H), 1.46 (d, *J* = 6.4 Hz, 3H, C5-H). **Minor diastereomer** δ 7.39 – 7.34 (m, 4H, C7-H, C8-H), 7.33 – 7.28 (m, 1H, C9-H), 5.77 (ddd, *J* = 5.8, 4.2, 1.8 Hz, 1H, C1-H), 5.27 (t, *J* = 1.8 Hz, 1H, C2-H), 5.00 – 4.93 (m, 1H, C4-H), 1.43 (d, *J* = 6.3 Hz, 3H, C5-H).

^{13}C NMR (126 MHz, $CDCl_3$) Major diastereomer δ 161.3 (d, *J* = 282.7 Hz, C3), 141.6 (C6), 128.7 (C7), 128.4 (C9), 126.9 (C8), 102.3 (d, *J* = 8.3 Hz, C2), 83.2 (d, *J* = 11.9 Hz, C1), 75.1 (d, *J* = 26.1 Hz, C4), 20.5 (d, *J* = 3.1 Hz, C5). **Minor diastereomer** δ 161.5 (d, *J* = 282.9 Hz, C3), 141.6 (C6), 128.8 (C7), 128.4 (C9), 126.6 (C8), 102.0 (d, *J* = 8.4 Hz, C2), 83.0 (d, *J* = 11.9 Hz, C1), 75.1 (d, *J* = 26.6 Hz, C4), 19.7 (d, *J* = 3.3 Hz, C5).

^{19}F NMR (376 MHz, $CDCl_3$) Major diastereomer δ −137.56 (t, *J* = 5.2 Hz, 1F, C3-F). **Minor diastereomer** −137.67 (t, *J* = 5.8 Hz, 1F, C3-F).

HRMS (+EI) calcd: $[M-H]^+$ ($C_{11}H_{11}OF$) 177.0710, measured 177.0706 (2.26 ppm error).

IR (neat) v_{max}/cm^{-1}: 3398, 3064, 2932, 1701, 1604, 1591, 1564, 1450, 1356, 1235, 1052, 774, 690.

***5-(3-bromophenyl)-3-fluoro-2,2-dimethyl-2,5-dihydrofuran* (6ae)**

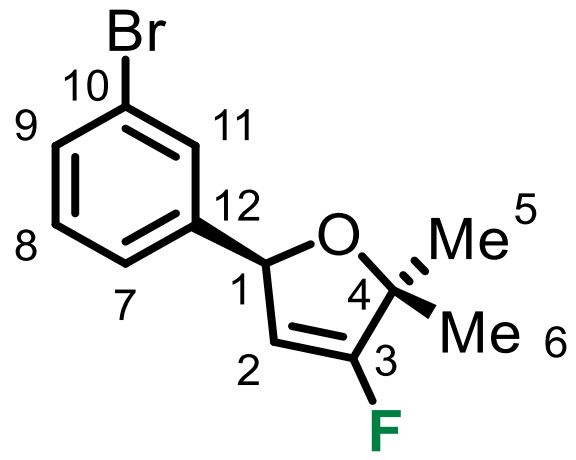

Prepared according to **General procedure IV** from **3ae** (98% NMR) and purified by flash column chromatography (2-20% EtOAc in pentane) to afford the title compound as a colourless oil (80.5 mg, 95%).

Contains 36% w/w 4-iodotoluene; could not be purified further as compound is unstable on silica.

^{1}H NMR (400 MHz, CDCl$_3$) δ 7.49 (t, *J* = 1.8 Hz, 1H, C11-H), 7.43 (dt, *J* = 7.6, 1.7 Hz, 1H, C9-H), 7.28 (dt, *J* = 7.9, 1.7 Hz, 1H, C7-H), 7.23 (t, *J* = 7.7 Hz, 1H, C8-H), 5.66 (dd, *J* = 4.7, 1.6 Hz, 1H, C1-H), 5.15 (d, *J* = 1.6 Hz, 1H, C2-H), 1.48 (s, 3H, C5-H), 1.46 (s, 3H, C6-H).

^{13}C NMR (126 MHz, CDCl$_3$) δ 164.1 (d, *J* = 285.8 Hz, C3), 144.1 (d, *J* = 2.2 Hz, C12), 131.3 (C11), 130.3 (C9), 129.9 (C8), 125.4 (C7), 122.8 (C10), 100.2 (d, *J* = 9.4 Hz, C2), 81.1 (d, *J* = 24.8 Hz, C4), 80.4 (d, *J* = 12.1 Hz, C1), 26.8 (d, *J* = 3.2 Hz, C6), 25.8 (d, *J* = 3.5 Hz, C5).

^{19}F NMR (376 MHz, CDCl$_3$) δ −137.89 (d, *J* = 4.7 Hz).

HRMS (+EI) calcd: $[M]^+$ ($C_{11}H_{11}OFBr$) 268.9972, measured 268.9968 (1.49 ppm error).

IR (neat) ν_{max}/cm^{-1}: 3089, 3070, 2978, 2930, 1689, 1602, 1581, 1556, 1474, 1360, 1170, 1050, 786, 700.

R*f* 0.42 (20% EtOAc in pentane).

***3-fluoro-5-(3-nitrophenyl)-2,2-diphenyl-2,5-dihydrofuran* (6af)**

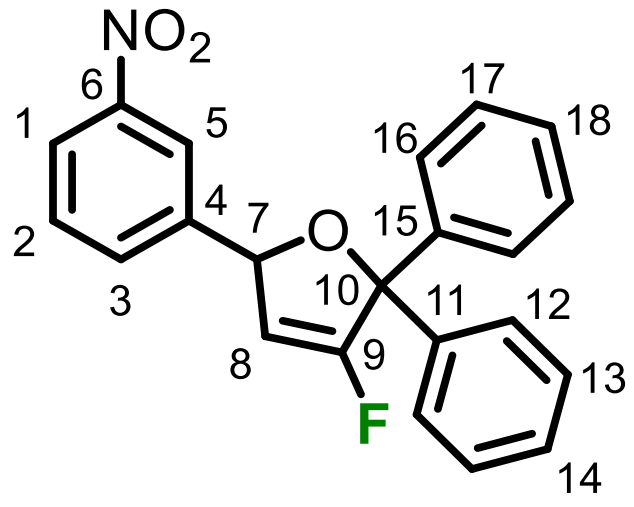

Prepared according to **General procedure IV** from **3af** (75% NMR) and purified by flash column chromatography (0-15% EtOAc in pentane) to afford the title compound as a colourless oil (50.6 mg, 70%).

^{1}H NMR (500 MHz, CDCl$_3$) δ 8.80 (t, J = 2.0 Hz, 1H, C5-H), 8.44 (ddd, J = 8.2, 2.3, 1.1 Hz, 1H, C1-H), 8.25 (ddd, J = 7.8, 1.7, 1.1 Hz, 1H, C3-H), 8.15 (dq, J = 8.1, 1.3 Hz, 1H, C2-H), 7.58 – 7.45 (m, 4H, C12-H, C16-H), 7.42 – 7.33 (m, 6H, C13-H, C14-H, C17-H, C18-H), 6.00 (dd, J = 4.7, 1.7 Hz, 1H, C7-H), 5.55 (d, J = 1.7 Hz, 1H, C8-H).

^{13}C NMR (126 MHz, CDCl$_3$) δ 160.9 (d, J = 288.1 Hz, C9), 148.5 (C6), 142.9 (d, J = 2.2 Hz, C4), 141.8 (d, J = 2.8 Hz, C15), 141.8 (d, J = 2.7 Hz, C11), 133.2 (C2), 129.7 (C3), 128.7 (C13), 128.5 (C17), 128.4 (C18), 128.3 (C14), 127.3 (C1), 126.8 (C16), 126.6 (C12), 122.3 (C5), 103.5 (d, J = 10.0 Hz, C8), 88.6 (d, J = 20.8 Hz, C10), 81.2 (d, J = 11.7 Hz, C7).

^{19}F NMR (471 MHz, CDCl$_3$) δ −129.24 (d, J = 4.8 Hz, 1F, C9-F).

HRMS (+EI) calcd: $[M]^+$ ($C_{22}H_{16}NO_3F$) 361.1105, measured 361.1109 (1.11 ppm error).

IR (neat) v_{max}/cm^{-1}: 3074, 1698, 1528, 1346, 1169, 1056, 907, 761, 732, 695, 599.

***(4-(4-fluoro-5,5-diphenyl-2,5-dihydrofuran-2-yl)phenyl)(phenyl)methanone* (6ag)**

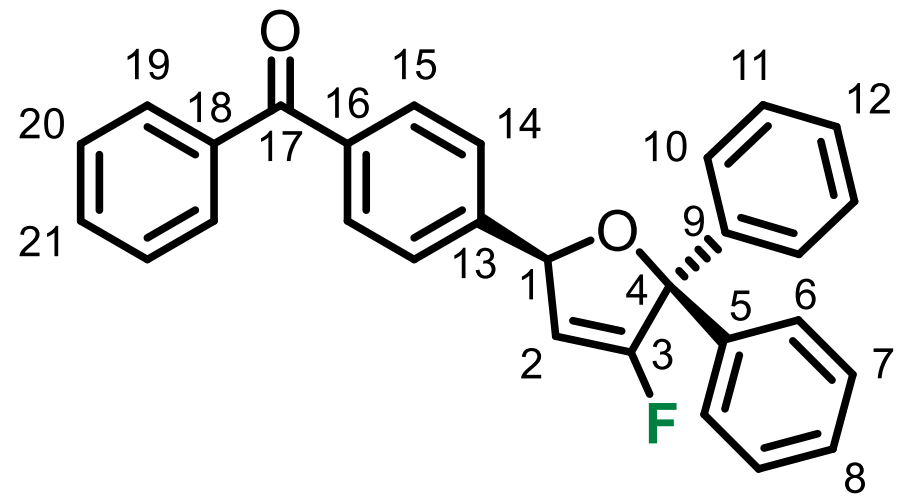

Prepared according to **General procedure IV** from **3ag** (95% NMR) and purified by flash column chromatography (2-20% EtOAc in pentane) to afford the title compound as a white solid (76.5 mg, 91%).

^{1}H NMR (500 MHz, CDCl$_3$) δ 7.79 (tt, *J* = 7.1, 1.4 Hz, 4H, C15-H, C19-H), 7.59 (tt, *J* = 7.3, 1.1 Hz, 1H, C21-H), 7.52 – 7.45 (m, 4H, C14-H, C20-H), 7.44 – 7.39 (m, 6H, C6-H, C8-H, C10-H, C12-H), 7.39 – 7.32 (m, 4H, C7-H, C11-H), 5.99 (dd, *J* = 4.8, 1.7 Hz, 1H, C1-H), 5.55 (d, *J* = 1.7 Hz, 1H, C2-H).

^{13}C NMR (126 MHz, CDCl$_3$) δ 196.4 (C17), 160.5 (d, *J* = 287.4 Hz, C3), 145.1 (d, *J* = 2.0 Hz, C13), 142.1 (d, *J* = 3.2 Hz, C5), 142.0 (d, *J* = 3.5 Hz, C9), 137.7 (C16), 137.6 (C18), 132.6 (C21), 130.5 (C15), 130.1 (C19), 128.5 (C14), 128.4 (C11), 128.4 (C7), 128.2 (C12), 128.2 (C8), 127.4 (C10), 127.1 (C6), 126.7 (C20), 104.0 (d, *J* = 9.5 Hz, C2), 88.4 (d, *J* = 20.7 Hz, C4), 81.6 (d, *J* = 11.4 Hz, C1).

^{19}F NMR (471 MHz, CDCl$_3$) δ −130.31 (d, *J* = 4.8 Hz, 1F, C3-F).

HRMS (+ESI) calcd: $[M+H]^+$ ($C_{29}H_{21}FO_2$) 421.1598, measured 421.1594 (0.98 ppm error).

IR (neat) ν_{max}/cm^{-1}: 3059, 1692, 1657, 1596, 1447, 1315, 1276, 1031, 937, 759, 698.

R$_f$ 0.56 (20% EtOAc in pentane).

***4-fluoro-2-(4-(trifluoromethyl)phenyl)-2,5-dihydrofuran* (6ah)**

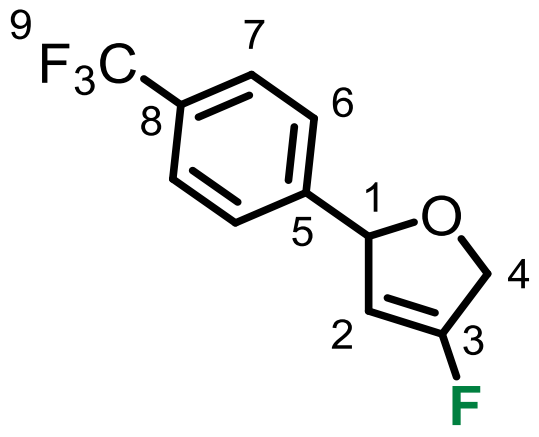

Prepared according to **General procedure IV** from **3ah**. Product not isolated. ^{19}F NMR yields calculated from crude products versus C_6F_6 internal standard (60%).

Identified by characteristic ^{19}F peaks:

^{19}F NMR (376 MHz, $CDCl_3$) δ −62.45 (s, 3F, C9-F), −135.27 (dt, J = 5.6, 4.7 Hz, C3-F).

HRMS (+EI) calcd: $[M-H]^+$ ($C_{11}H_8F_4O$) 231.0428, measured 231.0424 (1.73 ppm error).

IR (neat) v_{max}/cm^{-1}: 2923, 1783, 1695, 1413, 1325, 1169, 1131, 1068, 1017, 417.

R_f 0.31 (20% EtOAc in pentane).

2-(4-fluoro-1-oxaspiro[4.5]dec-3-en-2-yl)benzonitrile **(6ai)**

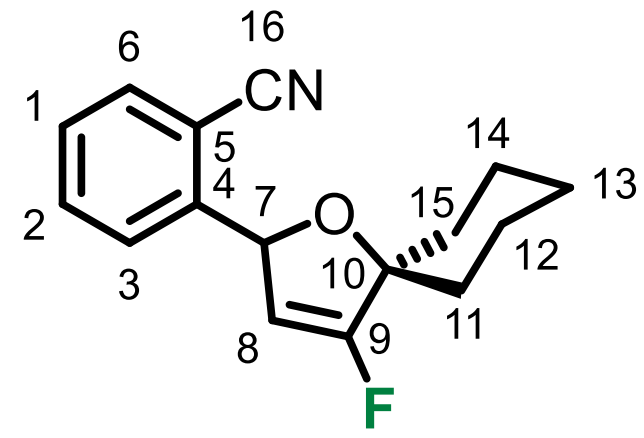

Prepared according to **General procedure IV** from **3ai** (87% NMR) and purified by flash column chromatography (0-10% EtOAc in pentane) to afford the title compound as a colourless oil (42.7 mg, 83%).

^{1}H NMR (400 MHz, CDCl$_3$) δ 7.68 – 7.59 (m, 3H, C1-H, C2-H, C6-H), 7.38 (td, *J* = 7.6, 1.6 Hz, 1H, C3-H), 6.08 (dd, *J* = 4.7, 1.7 Hz, 1H, C7-H), 5.29 (d, *J* = 1.7 Hz, 1H, C8-H), 1.88 – 1.60 (m, 9H, C11-H, C12-H, C13-H, C14-H, C15-H), 1.34 – 1.22 (m, 1H, C13-H).

^{13}C NMR (101 MHz, CDCl$_3$) δ 164.6 (d, *J* = 285.9 Hz, C9), 146.0 (d, *J* = 2.4 Hz, C4), 133.4 (C2), 132.8 (C6), 128.3 (C3), 127.1 (C1), 117.4 (C16), 110.2 (C5), 100.1 (d, *J* = 10.1 Hz, C8), 82.6 (d, *J* = 22.9 Hz, C10), 78.6 (d, *J* = 12.3 Hz, C7), 35.1 (d, *J* = 3.2 Hz, C11), 34.2 (d, *J* = 3.4 Hz, C15), 25.0 (C13), 22.1 (C12), 21.8 (C14).

^{19}F NMR (377 MHz, CDCl$_3$) δ −136.44 (s, 1F, C9-F).

HRMS (+EI) calcd: $[M]^+$ ($C_{16}H_{16}NOF$) 257.1210, measured 257.1209 (0.39 ppm error).

IR (neat) v_{max}/cm^{-1}: 2934, 2859, 2224, 1691, 1596, 1448, 1326, 1086, 1050, 1036, 977, 949, 842, 767.

***4-fluoro-2-methyl-2-phenyl-2,5-dihydrofuran* (6aj)**

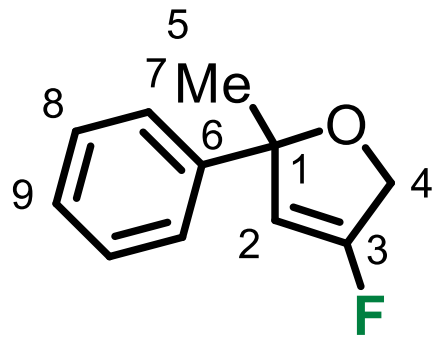

Prepared according to **General procedure IV** from (*Z*)-(2-fluoro-3-(1-phenylethoxy)prop-1-en-1-yl)(p-tolyl)iodonium tetrafluoroborate (in-house chemical) (93% NMR) and purified by flash column chromatography (0-10% EtOAc in pentane) to afford the title compound as a colourless oil (31.4 mg, 88%).

^{1}H NMR (400 MHz, $CDCl_3$) δ 7.42 – 7.32 (m, 4H, C7-H, C8-H), 7.29 – 7.24 (m, 1H, C9-H), 5.47 (td, *J* = 2.1, 0.7 Hz, 1H, C2-H), 4.68 (ddd, *J* = 12.1, 4.6, 2.0 Hz, 1H, C4-H), 4.62 (ddd, *J* = 12.1, 4.9, 2.1 Hz, 1H, C4-H), 1.69 (d, *J* = 1.4 Hz, 3H, C5-H).

^{13}C NMR (101 MHz, $CDCl_3$) δ 157.0 (d, *J* = 281.2 Hz, C3), 146.1 (d, *J* = 1.9 Hz, C6), 128.5 (C7), 127.3 (C9), 124.7 (C8), 107.2 (d, *J* = 6.3 Hz, C2), 88.5 (d, *J* = 11.1 Hz, C1), 68.0 (d, *J* = 28.5 Hz, C4), 29.3 (d, *J* = 2.0 Hz, C5).

^{19}F NMR (376 MHz, $CDCl_3$) δ −137.18 (td, *J* = 4.8, 1.5 Hz, 1F, C3-F).

HRMS (+EI) calcd: $[M\text{-}Me]^+$ ($C_{11}H_{11}OF$) 163.0554, measured 163.0550 (2.45 ppm error).

IR (neat) v_{max}/cm^{-1}: 3057, 3027, 1702, 1589, 1570, 1445, 1366, 1277, 1217, 1156, 1051, 766, 697.

***4-fluoro-2,2-diphenyl-2,5-dihydrofuran* (6ak)**

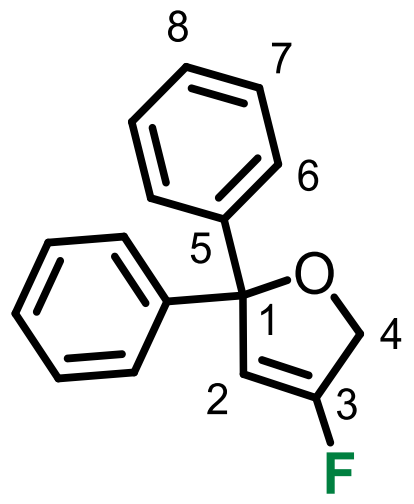

Prepared according to **General procedure IV** from (*Z*)-(3-(benzhydryloxy)-2-fluoroprop-1-en-1-yl)(p-tolyl)iodonium tetrafluoroborate (in-house chemical) (71% NMR) and purified by flash column chromatography (0-10% EtOAc in pentane) to afford the title compound as a colourless oil (29.8 mg, 62%).

^{1}H NMR (400 MHz, $CDCl_3$) δ 7.39 – 7.22 (m, 10H, C6-H, C7-H, C8-H), 5.77 (td, *J* = 2.1, 1.2 Hz, 1H, C2-H), 4.72 (dd, *J* = 4.7, 2.1 Hz, 2H, C4-H).

^{13}C NMR (101 MHz, $CDCl_3$) δ 157.9 (d, *J* = 282.2 Hz, C3), 145.0 (d, *J* = 1.9 Hz, C5), 128.4 (C7), 127.6 (C8), 126.4 (C6), 106.3 (d, *J* = 7.6 Hz, C2), 92.1 (d, *J* = 11.1 Hz, C1), 68.2 (d, *J* = 28.2 Hz, C4).

^{19}F NMR (377 MHz, $CDCl_3$) δ −135.73 (td, *J* = 4.6, 1.2 Hz, 1F, C3-F).

HRMS (+EI) calcd: $[M]^+$ ($C_{16}H_{13}OF$) 240.0945, measured 240.0942 (1.25 ppm error).

IR (neat) v_{max}/cm^{-1}: 3057, 3027, 1702, 1589, 1570, 1445, 1366, 1277, 1217, 1156, 1051, 766, 697.

***4'-fluoro-5'H-spiro[chromane-4,2'-furan]* (6al)**

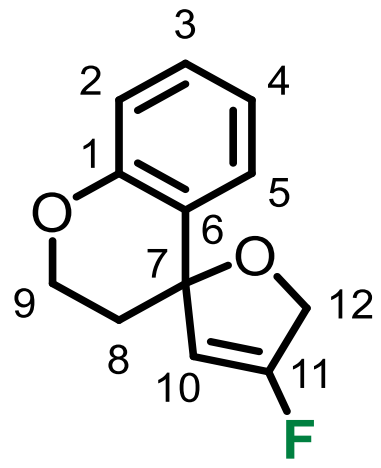

Alkyne **1ap** was converted to the FVI **3al** according to **General procedure III**, the crude product of which was cyclised according to **General procedure IV** and purified by flash column chromatography (0 to 40% Et_2O in pentane) to afford the title compound as a colourless oil (8 mg, 39% yield).

^{1}H NMR (500 MHz, $CDCl_3$) δ 7.35 (dd, J = 7.8, 1.7 Hz, 1H, C5-H), 7.20 (ddd, J = 8.3, 7.2, 1.7 Hz, 1H, C3-H), 6.93 (ddd, J = 7.8, 7.2, 1.2 Hz, 1H, C4-H), 6.84 (dd, J = 8.3, 1.2 Hz, 1H, C2-H), 5.23 (t, J = 2.1 Hz, 1H, C10-H), 4.69 (ddd, J = 12.4, 4.7, 2.1 Hz, 1H, C12-H), 4.61 (ddd, J = 12.4, 4.7, 2.1 Hz, 1H, C12-H), 4.40 – 4.21 (m, 2H, C9-H), 2.15 (dd, J = 8.9, 4.6 Hz, 2H, C8-H).

^{13}C NMR (126 MHz, $CDCl_3$) δ 158.7 (d, J = 282.4 Hz, C11), 154.6 (C1), 130.2 (C3), 128.6 (C5), 124.6 (d, J = 1.9 Hz, C6), 120.9 (C4), 117.4 (C2), 105.6 (d, J = 7.1 Hz, C10), 82.5 (d, J = 12.0 Hz, C7), 67.5 (d, J = 27.9 Hz, C12), 63.0 (C9), 35.6 (d, J = 2.3 Hz, C8).

^{19}F NMR (376 MHz, $CDCl_3$) δ −133.71 (1F, t, J = 4.7 Hz, C11-F).

HRMS (+EI) calcd: $[M]^+$ ($C_{12}H_{11}FO_2$) 206.0738, measured 206.0734 (1.94 ppm error).

IR (neat) ν_{max}/cm^{-1}: 2955, 2878, 1693, 1607, 1582, 1489, 1452, 1377, 1307, 1268, 1253, 1224, 1116, 1050, 1016, 933, 909, 825, 755.

***4-fluoro-2,5-dimethyl-2-phenyl-2,5-dihydrofuran* (6am)**

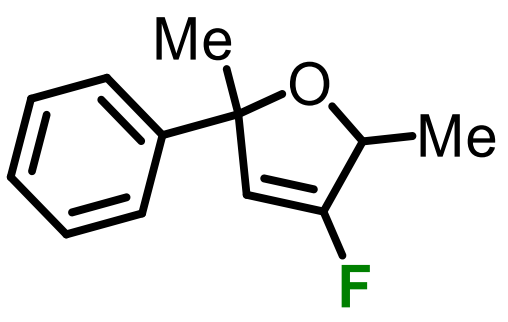

Prepared according to **General procedure IV** from **3am**. Product not isolated. ^{19}F NMR yields calculated from crude products versus C_6F_6 internal standard (66%, d.r. 1.35:1).

Identified by characteristic ^{19}F peaks:

^{19}F NMR (376 MHz, No Solvent) Major diastereomer δ -140.7 (d, *J* = 4.8 Hz, 1F), **Minor diastereomer** δ -140.9 (d, *J* = 4.6 Hz, 1F).

4-fluoro-1-oxaspiro[4.5]dec-3-ene (Major regioisomer) **(6an)**
(syn)-7-fluoro-1-methoxybicyclo[3.2.1]oct-6-ene (Minor regioisomer) **(5an)**

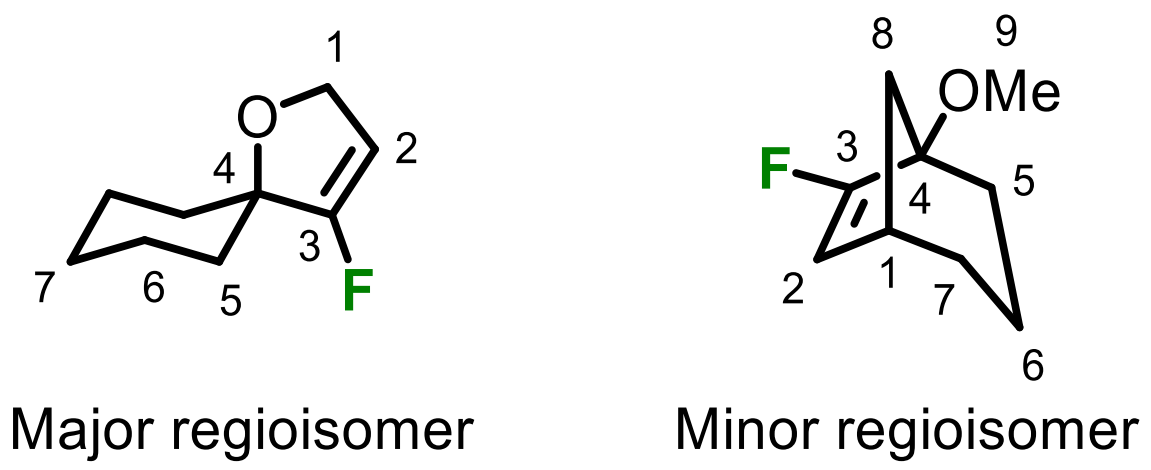

Prepared according to **General procedure IV** from **3an** as a mixture of regioisomers (r.r. 1.60:1, 56%, 36% NMR yield) without further purification to afford the title compounds as a colourless oil (55.8 mg, 39% combined yield).

Compounds were characterised as a mixture with the 4-iodotoluene byproduct as they are unstable on silica. The mass of iodotoluene (75.5 mg) has been accounted for in the isolated yield.

^{1}H NMR (500 MHz, CDCl$_3$) Major regioisomer δ 5.09 (t, *J* = 1.8 Hz, 1H, C2-H), 4.52 (dd, *J* = 5.2, 1.8 Hz, 2H, C1-H), 1.82 – 1.46 (m, 10H, C5-H, C6-H, C7-H). **Minor regioisomer** δ 5.08 (q, *J* = 1.5 Hz, 1H, C2-H), 3.31 (d, *J* = 0.9 Hz, 3H, C9-H), 2.67 (tq, *J* = 5.8, 3.1 Hz, 1H, C1-H), 2.45 – 2.38 (m, 2H, C5-H, C7-H), 1.82 – 1.46 (m, 4H, C5-H, C7-H, C8-H), 1.39 – 1.33 (m, 2H, C6-H).

^{13}C NMR (126 MHz, CDCl$_3$) Major regioisomer δ 163.3 (d, *J* = 281.8 Hz, C3), 96.8 (d, *J* = 10.2 Hz, C2), 81.2 (d, *J* = 23.5 Hz, C4), 68.4 (d, *J* = 12.1 Hz, C1), 34.1 (d, *J* = 3.4 Hz, C5), 27.6 (C7), 22.1 (C6). **Minor regioisomer** δ 161.1 (d, *J* = 295.9 Hz, C3), 104.9 (d, *J* = 3.7 Hz, C2), 82.5 (d, *J* = 19.8 Hz, C4), 51.1 (d, *J* = 1.3 Hz, C9), 44.5 (d, *J* = 5.0 Hz, C8), 33.4 (d, *J* = 8.2 Hz, C1), 29.3 (d, *J* = 3.6 Hz, C5), 24.9 (d, *J* = 3.8 Hz, C7), 20.0 (C6).

^{19}F NMR (376 MHz, CDCl$_3$) Major regioisomer δ −138.81 (s, 1F, C3-F). **Minor regioisomer** δ −133.07 (t, *J* = 4.1 Hz, C3-F).

HRMS (+EI) calcd: $[M+H]^+$ ($C_9H_{13}OF$) 157.1023, measured 157.1021 (1.27 ppm error).

IR (neat) v_{max}/cm^{-1}: 2925, 2852, 1689, 1483, 1449, 1261, 1081, 1059, 1007, 797, 474.

***2-((4-chlorophenoxy)methyl)-4-fluoro-2,5-dihydrofuran* (6ao)**

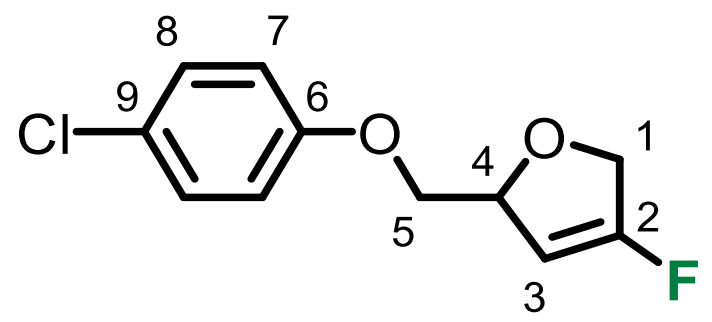

Prepared according to **General procedure IV** from **3ao** (31% NMR yield) and purified by flash column chromatography to afford the title compound as a colourless oil (6.9 mg, 15%).

^{1}H NMR (500 MHz, CDCl$_3$) δ 7.23 (d, *J* = 9.0 Hz, 2H), 6.84 (d, *J* = 8.9 Hz, 2H), 5.25 (q, *J* = 1.8 Hz, 1H), 5.14 (dddd, *J* = 9.7, 5.6, 3.9, 1.5 Hz, 1H), 4.62 – 4.49 (m, 2H), 4.04 – 3.93 (m, 3H).

^{13}C NMR (126 MHz, CDCl$_3$) δ 158.9 (d, *J* = 281.3 Hz, C2), 157.4 (C6), 129.5 (C8), 126.1 (C9), 116.1 (C7), 99.5 (d, *J* = 9.3 Hz, C3), 81.9 (d, *J* = 11.1 Hz, C4), 71.1 (d, *J* = 3.0 Hz, C5), 68.6 (d, *J* = 28.6 Hz, C1).

^{19}F NMR (471 MHz, CDCl$_3$) δ −133.39 (q, *J* = 4.7 Hz, 1F, C2-F).

HRMS (+EI) calcd: $[M]^+$ ($C_{11}H_{10}O_2FCl$) 228.0348, measured 228.0344 (1.75 ppm error).

IR (neat) v_{max}/cm^{-1}: 2923, 2858, 1696, 1516, 1492, 1246, 1091, 824.

***3-fluoro-8-tosyl-1-oxa-8-azaspiro[4.5]dec-3-ene* (6ap)**

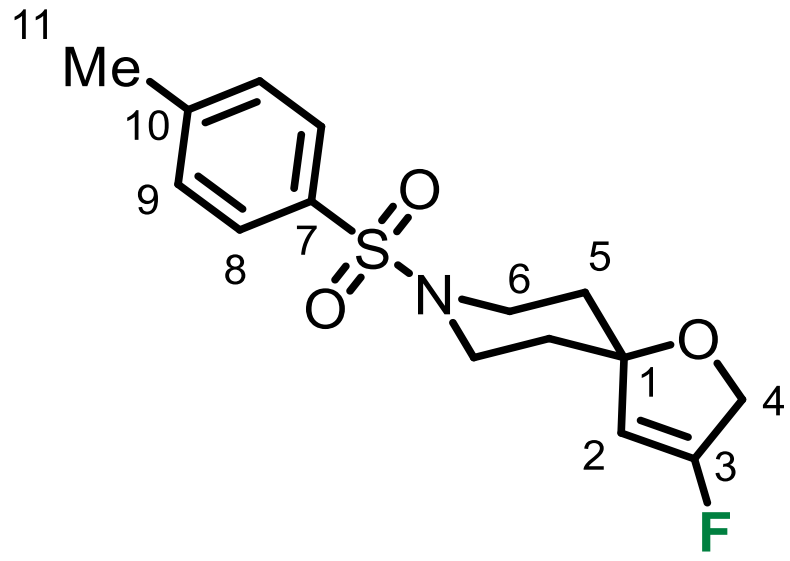

Prepared according to **General procedure IV** from **3ap** (71% NMR) and purified by flash column chromatography (0-20% EtOAc in pentane) to afford the title compound as a colourless oil (39.9 mg, 64%).

^{1}H NMR (500 MHz, CDCl$_3$) δ 7.63 (app. dt, J = 8.2, 2.1 Hz, 2H, C8-H), 7.31 (d, J = 8.2 Hz, 2H, C9-H), 5.08 (t, J = 2.1 Hz, 1H, C2-H), 4.41 (dd, J = 4.8, 2.1 Hz, 2H, C4-H), 3.50 (dt, J = 11.8, 4.5 Hz, 2H, C6-H, equatorial), 2.70 (td, J = 11.5, 3.2 Hz, 2H, C6-H, axial), 2.42 (s, 3H, C11-H), 1.83 (td, J = 11.1, 4.5 Hz, 2H, C5-H, equatorial), 1.75 (dt, J = 11.1, 3.2 Hz, 2H, C5-H, axial).

^{13}C NMR (126 MHz, CDCl$_3$) δ 157.7 (d, J = 282.0 Hz), 143.6, 133.3, 129.8, 127.8, 105.8 (d, J = 6.5 Hz), 83.8 (d, J = 10.9 Hz), 67.3 (d, J = 28.6 Hz), 42.8, 36.2 (d, J = 2.3 Hz), 21.6.

^{19}F NMR (376 MHz, CDCl$_3$) δ −134.79 (t, J = 4.8 Hz, 1F, C3-F).

HRMS (+EI) calcd: $[M]^+$ ($C_{15}H_{18}NO_3SF$) 311.0986, measured 311.0981 (1.61 ppm error).

IR (neat) v_{max}/cm^{-1}: 3103, 2934, 2861, 1791, 1693, 1345, 1351, 1327, 1164, 1063, 1051, 889, 724, 548.

(5R,6S,9R)-3-fluoro-6-isopropyl-9-methyl-1-oxaspiro[4.5]dec-3-ene (menthol derivative) **(6aq)**

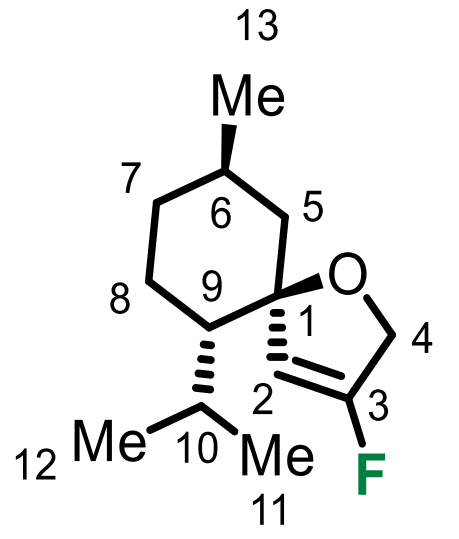

Prepared according to **General procedure IV** from (Z)-(2-fluoro-3-(*O*-menthyl)prop-1-en-1-yl)(p-tolyl)iodonium tetrafluoroborate (in-house chemical) (62% NMR) and purified by flash column chromatography (0-10% EtOAc in pentane) to afford the title compound as a colourless oil (25.5 mg, 60%).

^{1}H NMR (500 MHz, $CDCl_3$) δ 5.35 (q, *J* = 1.8 Hz, 1H, C2-H), 4.53 (qdd, *J* = 12.0, 4.9, 2.1 Hz, 2H, C4-H), 1.98 (heptd, *J* = 7.0, 2.3 Hz, 1H, C10-H), 1.82 (dt, *J* = 12.2, 2.8 Hz, 1H, C5-H), 1.73 (dp, *J* = 12.6, 3.2 Hz, 1H, C7-H), 1.67 (dq, *J* = 13.1, 3.6 Hz, 1H, C8-H), 1.45 – 1.37 (m, 1H, C6-H), 1.35 (dtd, *J* = 13.1, 3.6, 2.3 Hz, 1H, C9-H), 1.26 (td, *J* = 12.8, 3.6 Hz, 1H, C5-H), 1.04 (qd, *J* = 13.1, 3.6 Hz, 1H, C8-H), 0.93 (d, *J* = 7.0 Hz, 3H, C12-H), 0.95 – 0.86 (m, 1H, C7-H), 0.89 (d, *J* = 6.4 Hz, 3H, C13-H), 0.77 (d, *J* = 7.0 Hz, 3H, C11-H).

^{13}C NMR (126 MHz, $CDCl_3$) δ 156.3 (d, *J* = 279.8 Hz, C3), 103.4 (d, *J* = 5.7 Hz, C2), 92.1 (d, *J* = 9.5 Hz, C1), 67.8 (d, *J* = 28.8 Hz, C4), 52.3 (C9), 50.2 (d, *J* = 2.2 Hz, C5), 35.0 (C7), 31.5 (C6), 26.0 (C10), 24.4 (C8), 23.7 (C12), 22.4 (C13), 18.8 (C11).

^{19}F NMR (471 MHz, $CDCl_3$) δ −134.78 (p, *J* = 4.4 Hz, 1F, C3-F).

HRMS (+EI) calcd: $[M]^+$ ($C_{13}H_{21}OF$) 212.1571, measured 212.1568 (1.41 ppm error).

IR (neat) v_{max}/cm^{-1}: 2953, 2923, 2868, 2849, 1728, 1692, 1455, 1375, 1255, 1067, 933, 828.

$[\alpha]_D^{24}$ = -4° (c 1.00 in $CHCl_3$).

R_f 0.64 (10% EtOAc in pentane).

8.6. Azetines (7)

3-fluoro-1-tosyl-1,2-dihydroazete **(7ar)**

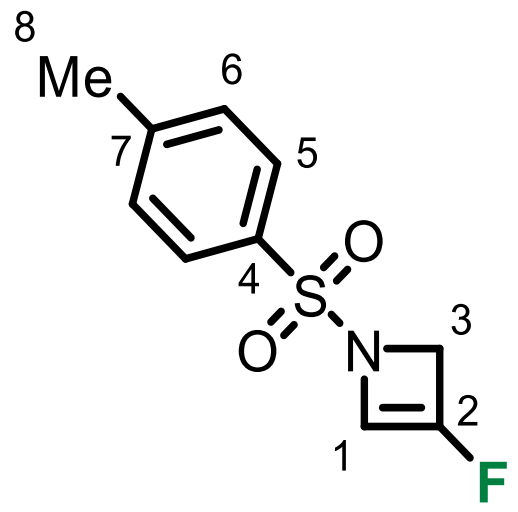

Prepared according to **General procedure IV** (scaled up 1410%) from **3ar** (92% NMR) and purified by flash column chromatography (2-20% EtOAc in pentane) to afford the title compound as a colourless oil (570 mg, 89%).

^{1}H NMR (500 MHz, CDCl$_3$) δ 7.72 (d, J = 8.2 Hz, 2H, C5-H), 7.36 (d, J = 8.2 Hz, 2H, C6-H), 5.79 (d, J = 8.6 Hz, 1H, C1-H), 4.19 (d, J = 5.0 Hz, 2H, C3-H), 2.45 (s, 3H, C8-H).

^{13}C NMR (126 MHz, CDCl$_3$) δ 145.5 (d, J = 328.2 Hz, C2), 145.1 (C7), 129.8 (C6), 129.6 (C4), 129.2 (C5), 117.7 (d, J = 5.0 Hz, C1), 60.6 (d, J = 20.9 Hz, C3), 21.8 (C8).

^{19}F NMR (471 MHz, CDCl$_3$) δ −115.22 (dt, J = 8.6, 5.0 Hz, 1F, C2-F).

HRMS (+EI) calcd: $[M]^+$ ($C_{10}H_{10}NO_2FS$) 227.0411, measured 227.0408 (1.32 ppm error).

IR (neat) v_{max}/cm^{-1}: 3127, 1667, 1595, 1345, 1302, 1161, 1087, 985, 814, 722, 673, 598, 553, 520.

R_f 0.46 (20% EtOAc in pentane).

***3-fluoro-1-((4-nitrophenyl)sulfonyl)-1,2-dihydroazete* (7as)**

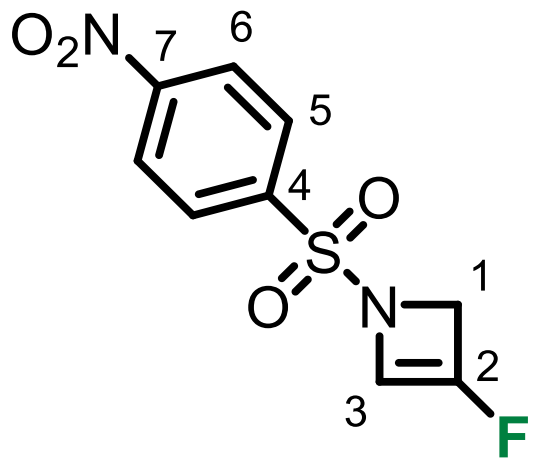

Prepared according to **General procedure IV** from **3as** (20% NMR) and purified by flash column chromatography (0 to 50% Et_2O in pentane) to afford the title compound as a yellow solid (5 mg, 19%).

^{1}H NMR (500 MHz, $CDCl_3$): δ 8.43 (d, *J* = 8.1 Hz, 2H, C6-H), 8.06 (d, *J* = 8.1 Hz, 2H, C5-H), 5.88 (dt, *J* = 8.3, 0.7 Hz, 1H, C3-H), 4.28 (2H, dd, *J* = 5.0, 0.7 Hz, C1-H).

^{13}C NMR (126 MHz, $CDCl_3$): δ 151.0 (C7), 145.8 (d, *J* = 328.8 Hz, C2), 138.7 (C4), 130.4 (C5), 124.4 (C6), 117.5 (d, *J* = 5.8 Hz, C2), 60.9 (d, *J* = 21.4 Hz, C1).

^{19}F NMR (471 MHz, $CDCl_3$): δ −113.86 (dt, *J* = 8.7, 5.3 Hz, 1F, C2-F).

HRMS (-MALDI) calcd: $[M]^-$ ($C_9H_8N_2O_4$) 258.0116, measured 258.0114 (0.76 ppm error).

IR (neat) ν_{max}/cm^{-1} : 3112, 2926, 2865, 1671, 1606, 1529, 1438, 1402, 1350, 1307, 1246, 1171, 1109, 1085, 1034, 1013, 987, 966, 856, 800, 750, 740, 684, 641, 578, 520, 465, 411.

***3-fluoro-2-methyl-1-tosyl-1,2-dihydroazete* (7at)**

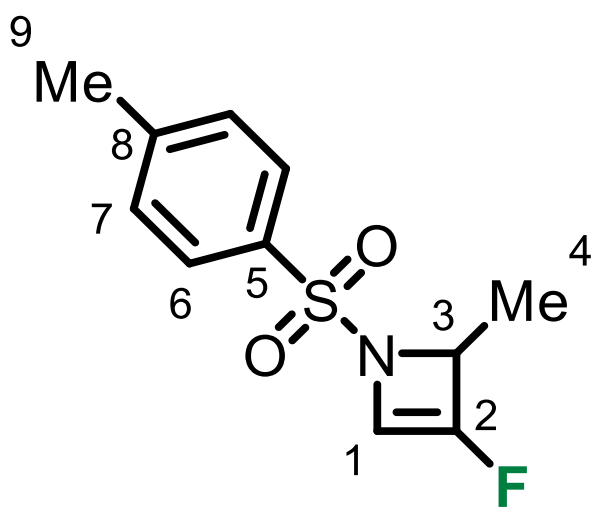

Prepared according to **General procedure IV** from **3at** (64% NMR) and purified by flash column chromatography (2-20% EtOAc in pentane) to afford the title compound as a colourless oil (29.4 mg, 61%).

^{1}H NMR (400 MHz, CDCl$_3$) δ 7.73 (d, *J* = 8.0 Hz, 2H, C6-H), 7.35 (d, *J* = 8.0 Hz, 2H, C7-H), 5.74 (d, *J* = 8.5 Hz, 1H, C1-H), 4.13 (td, *J* = 6.7, 5.0 Hz, 1H, C3-H), 2.46 (s, 3H, C9-H), 1.53 (d, *J* = 6.7 Hz, 3H, C4-H).

^{13}C NMR (126 MHz, CDCl$_3$) δ 149.9 (d, *J* = 330.8 Hz, C2), 144.9 (C8), 130.7 (C5), 129.7 (C7), 129.0 (C6), 114.9 (d, *J* = 4.8 Hz, C1), 70.4 (d, *J* = 21.6 Hz, C3), 21.8 (C9), 15.4 (d, *J* = 3.6 Hz, C4).

^{19}F NMR (376 MHz, CDCl$_3$) δ −119.76 (dd, *J* = 8.5, 5.0 Hz, 1F, C2-F).

HRMS (+ESI) calcd: $[M+H]^+$ ($C_{11}H_{12}FNO_2S$) 242.0646, measured 242.0642 (1.65 ppm error).

IR (neat) v_{max}/cm^{-1}: 3067, 2924, 1660, 1597, 1322, 1306, 1157, 1088, 875, 784, 690, 511.

R*f* 0.30 (20% EtOAc in pentane).

***3-fluoro-1-tosyl-1-azaspiro[3.5]non-2-ene* (7av)**

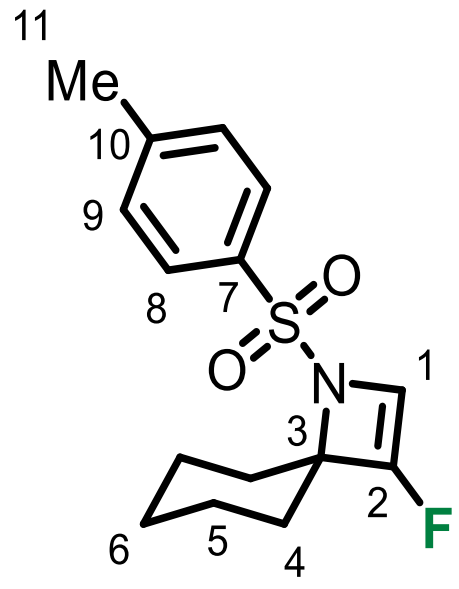

Prepared according to **General procedure IV** from **3av** (92% NMR) and purified by flash column chromatography (2-30% Et_2O in pentane) to afford the title compound as a colourless oil (31.7 mg, 50.3 mg, 60%).

Compound is unstable ($t_{1/2}$ = 43 min at RT). Spectra show product as a mixture with electrocyclic ring-opening product **HR228D** and the aldehyde resulting from its hydrolysis.

^{1}H NMR (500 MHz, $CDCl_3$) δ 7.71 (app. dt, J = 8.3, 1.9 Hz, 2H, C8-H), 7.30 (d, J = 8.3 Hz, 2H, C9-H), 5.82 (d, J = 7.9 Hz, 1H, C1-H), 2.43 (s, 3H, C11-H), 2.09 – 1.98 (m, 2H, C4-H), 1.86 – 1.77 (m, 4H, C4-H, C5-H), 1.66 – 1.57 (m, 2H, C5-H), 1.47 – 1.35 (m, 1H, C6-H), 1.29 – 1.21 (m, 1H, C6-H).

^{13}C NMR (126 MHz, $CDCl_3$) δ 154.9 (d, J = 332.1 Hz, C2), 144.1 (C10), 136.0 (C7), 129.8 (C9), 128.1 (C8), 112.4 (d, J = 6.6 Hz, C1), 86.3 (d, J = 19.5 Hz, C3), 33.3 (d, J = 3.5 Hz, C4), 24.8 (C6), 24.4 (C5), 21.7 (C11).

^{19}F NMR (471 MHz, $CDCl_3$) δ −121.13 (d, J = 7.9 Hz, 1F, C2-F).

HRMS (+EI) calcd: $[M]^+$ ($C_{15}H_{18}NO_2SF$) 295.1037, measured 295.1035 (0.68 ppm error).

IR (neat) v_{max}/cm^{-1}: 3264, 2933, 2860, 1737, 1715, 1681, 1447, 1333, 1157, 1091, 910, 812, 730, 662, 555, 539.

(E)-N-(2-cyclohexylidene-2-fluoroethylidene)-4-methylbenzenesulfonamide **(7av')**

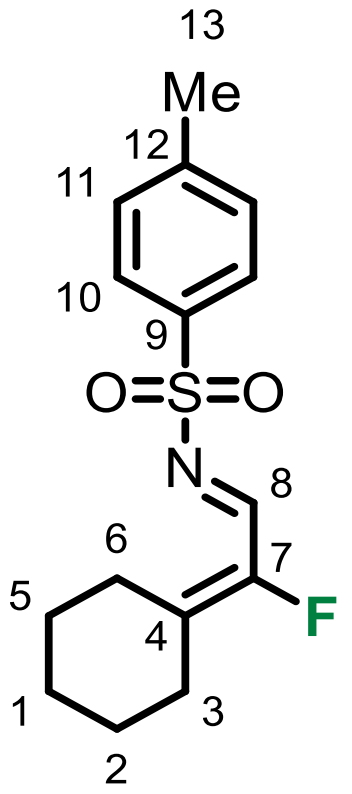

Prepared by thermal electrocyclic ring opening of **7av** by leaving at RT for 18 h.

Spectra show product as a mixture with the aldehyde resulting from imine hydrolysis.

^{1}H NMR (500 MHz, CDCl$_3$) δ 8.90 (d, *J* = 20.0 Hz, 1H, C8-H), 7.85 (dt, *J* = 8.4, 1.8 Hz, 2H, C10-H), 7.32 (d, *J* = 8.1 Hz, 2H, C11-H), 2.55 – 2.45 (m, 4H, C3-H, C6-H), 2.42 (s, 3H, C13-H), 1.76 – 1.60 (m, 6H, C1-H, C2-H, C5-H).

^{13}C NMR (126 MHz, CDCl$_3$) δ 157.0 (d, *J* = 20.0 Hz, C8), 148.2 (d, *J* = 12.2 Hz, C4), 147.0 (d, *J* = 242.8 Hz, C7), 144.8 (C12), 135.2 (C9), 129.9 (C11), 128.2 (C10), 28.8 (d, *J* = 2.8 Hz, C6), 28.3 (d, *J* = 7.2 Hz, C3), 28.1 (d, *J* = 2.1 Hz, C2), 27.3 (d, *J* = 1.3 Hz, C5), 25.9 (C1), 21.8 (C13).

^{19}F NMR (376 MHz, CDCl$_3$) δ −129.91 (d, *J* = 20.1 Hz, 1F, C5-F).

HRMS (+EI) calcd: $[M]^+$ ($C_{15}H_{18}NO_2SF$) 295.1037, measured 295.1035 (0.68 ppm error).

IR (neat) v_{max}/cm^{-1}: 3264, 2933, 2860, 1737, 1715, 1681, 1447, 1333, 1157, 1091, 910, 812, 730, 662, 555, 539.

***3-fluoro-2,2-dimethyl-1-tosyl-1,2-dihydroazete* (7au)**

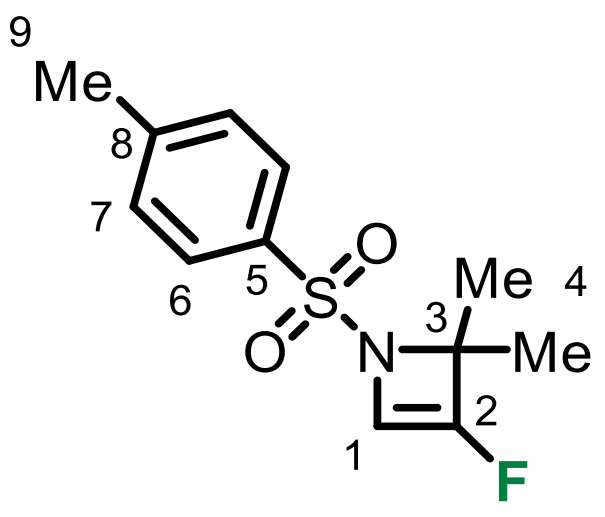

Prepared according to **General procedure IV** from **3au** (94% NMR) and purified by flash column chromatography (2-30% EtOAc in pentane) to afford the title compound as a colourless oil (31.7 mg, 50.3 mg, 62%).

Compound is unstable ($t_{1/2}$ = 76 min at RT). Spectra show product as a mixture with electrocyclic ring-opening product **7av'**

^{1}H NMR (400 MHz, $CDCl_3$) δ 7.72 (app. dt, *J* = 8.2, 1.8 Hz, 2H, C6-H), 7.33 – 7.27 (d, *J* = 8.2 Hz, 2H, C7-H), 5.76 (d, *J* = 8.1 Hz, 1H, C1-H), 2.43 (s, 3H, C9-H), 1.51 (s, 6H, C4-H).

^{13}C NMR (126 MHz, $CDCl_3$) δ 152.9 (d, *J* = 330.7 Hz, C2), 144.3 (C8), 135.7 (C5), 129.8 (C7), 128.2 (C6), 112.0 (d, *J* = 6.0 Hz, C1), 82.2 (d, *J* = 22.6 Hz, C3), 22.4 (d, *J* = 3.5 Hz, C4).

^{19}F NMR (376 MHz, $CDCl_3$) δ −127.89 (d, *J* = 8.1 Hz, 1F, C2-F).

HRMS (+EI) calcd: $[M]^+$ ($C_{12}H_{14}NO_2FS$) 255.0724, measured 255.0720 (1.57 ppm error).

IR (neat) v_{max}/cm^{-1}: 3127, 1667, 1595, 1345, 1302, 1161, 1087, 985, 814, 722, 673, 598, 553, 520.

R*f* 0.59 (10% EtOAc in pentane).

***(E)-N-(2-fluoro-3-methylbut-2-en-1-ylidene)-4-methylbenzenesulfonamide* (7au')**

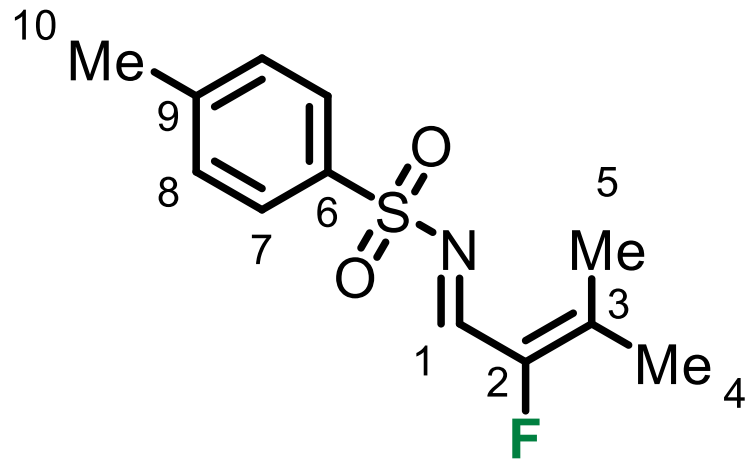

Prepared by thermal electrocyclic ring opening of **7au** by leaving at RT for 24 h (31.7 mg, 100%).

^{1}H NMR (500 MHz, CDCl$_3$) δ 8.86 (d, J = 19.8 Hz, 1H, C1-H), 7.85 (d, J = 8.1 Hz, 2H, C7-H), 7.33 (d, J = 8.1 Hz, 2H, C8-H), 2.43 (s, 3H, C10-H), 2.10 (d, J = 3.0 Hz, 3H, C4-H), 2.00 (d, J = 3.4 Hz, 3H, C3-H).

^{13}C NMR (126 MHz, CDCl$_3$) δ 157.5 (d, J = 19.8 Hz, C1), 149.6 (d, J = 242.3 Hz, C2), 144.9 (C9), 140.5 (d, J = 14.6 Hz, C3), 135.1 (C6), 129.9 (C8), 128.3 (C7), 21.8 (C10), 18.9 (d, J = 7.3 Hz, C5), 18.5 (d, J = 2.8 Hz, C4).

^{19}F NMR (376 MHz, CDCl$_3$) δ −126.29 (dqq, J = 19.8, 7.3, 2.8 Hz).

HRMS (+EI) calcd: $[M]^+$ ($C_{12}H_{14}NO_2FS$) 255.0724, measured 255.0720 (1.57 ppm error).

IR (neat) v_{max}/cm^{-1}: 3359, 3270, 2923, 1649, 1596, 1575, 1326, 1291, 1157, 1088, 852, 814, 755, 669, 555, 540.

R_f 0.30 (20% EtOAc in pentane).

***3-fluoro-4-phenyl-1-tosyl-1,2-dihydroazete* (7aw)**

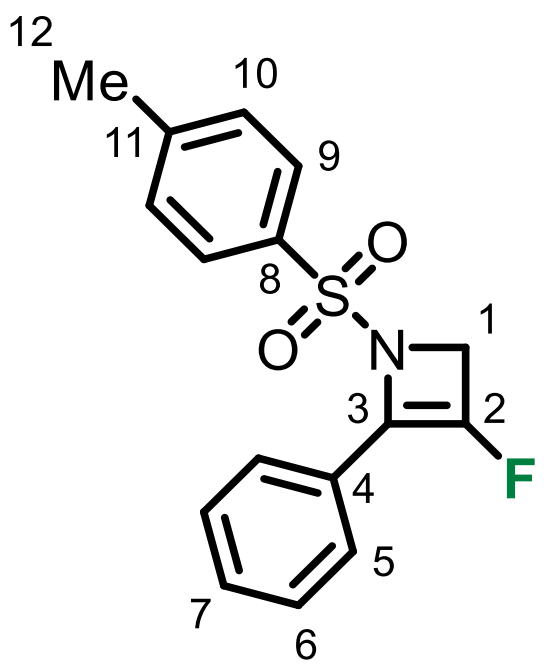

Prepared according to **General procedure IV** from **3aw** (91% NMR) and purified by flash column chromatography (2-20% EtOAc in pentane) to afford the title compound as a colourless oil (51.6 mg, 85%).

^{1}H NMR (500 MHz, CDCl$_3$) δ 7.61 – 7.57 (m, 2H, C5-H), 7.44 (d, *J* = 8.0 Hz, 2H, C9-H), 7.42 – 7.38 (m, 2H, C6-H), 7.38 – 7.34 (m, 1H, C7-H), 7.23 (d, *J* = 8.0 Hz, 2H, C10-H), 4.29 (d, *J* = 5.6 Hz, 2H, C1-H), 2.41 (s, 3H, C12-H).

^{13}C NMR (126 MHz, CDCl$_3$) δ 145.0 (C11), 140.4 (d, *J* = 332.5 Hz, C2), 130.9 (d, *J* = 2.6 Hz, C4), 129.6 (C10), 129.3 (C8), 129.2 (C9), 129.2 (C7), 128.7 (C6), 127.3 (d, *J* = 4.9 Hz, C3), 126.9 (d, *J* = 3.6 Hz, C5), 59.7 (d, *J* = 20.1 Hz, C1), 21.8 (C12).

^{19}F NMR (471 MHz, CDCl$_3$) δ −119.43 (t, *J* = 5.6 Hz, 1F, C2-F).

HRMS (+EI) calcd. $[M]^+$ ($C_{16}H_{14}FNO_2S$) 303.0724, measured 303.0719 (1.65 ppm error).

IR (neat) v_{max}/cm^{-1}: 3286, 1703, 1597, 1448, 1353, 1165, 1088, 815, 707, 693, 681, 594, 548.

R_f 0.57 (20% EtOAc in pentane).

***3-fluoro-4-(4-fluorophenyl)-1-tosyl-1,2-dihydroazete* (7ax)**

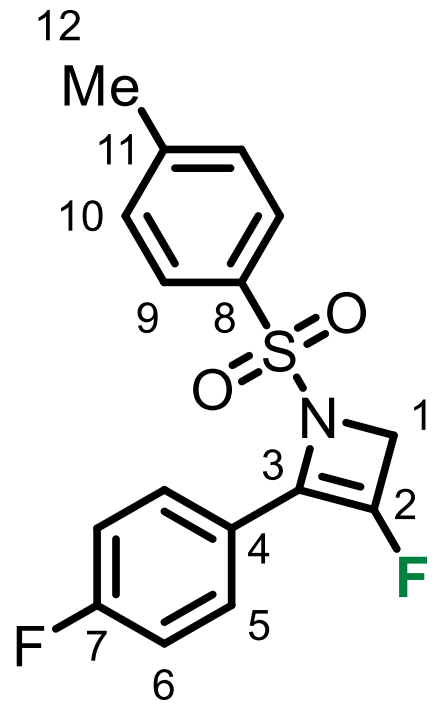

Prepared according to **General procedure IV** from **3ax** (90% NMR) and purified by flash column chromatography (2-20% EtOAc in pentane) to afford the title compound as a white solid (55.2 mg, 86%).

^{1}H NMR (500 MHz, CDCl$_3$) δ 7.65 – 7.57 (m, 2H, C5-H), 7.45 (dt, *J* = 8.3, 1.7 Hz, 2H, C9-H), 7.26 (d, *J* = 8.3 Hz, 2H, C10-H), 7.11 (tt, *J* = 8.5, 2.2 Hz, 2H, C6-H), 4.31 (d, *J* = 5.5 Hz, 2H, C1-H), 2.43 (s, 3H, C12-H).

^{13}C NMR (126 MHz, CDCl$_3$) δ 162.9 (dd, *J* = 250.4, 2.1 Hz, C7), 145.1 (C11), 140.0 (dd, *J* = 332.1, 2.3 Hz, C2), 129.9 (d, *J* = 2.5 Hz, C3), 129.7 (C10), 129.3 (C8), 129.2 (C9), 129.0 (dd, *J* = 8.4, 3.5 Hz, C5), 123.5 (dd, *J* = 5.2, 3.4 Hz, C4), 115.9 (dd, *J* = 22.0, 1.0 Hz, C6), 59.7 (d, *J* = 19.8 Hz, C1), 21.8 (C1).

^{19}F NMR (376 MHz, CDCl$_3$) δ −110.1 (ttd, *J* = 8.5, 5.3, 2.3 Hz, 1F, C7-F), −120.0 (td, *J* = 5.5, 2.3 Hz, 1F, C2-F).

HRMS (+ESI) calcd: $[M+H]^+$ ($C_{16}H_{13}F_2NO_2S$) 322.0708, measured 322.0702 (1.86 ppm error).

IR (neat) ν_{max}/cm^{-1}: 3284, 2969, 1738, 1692, 1597, 1508, 1331, 1232, 1158, 1091, 844, 814, 663, 550, 534.

4-([1,1'-biphenyl]-4-yl)-3-fluoro-1-tosyl-1,2-dihydroazete **(7ay)**

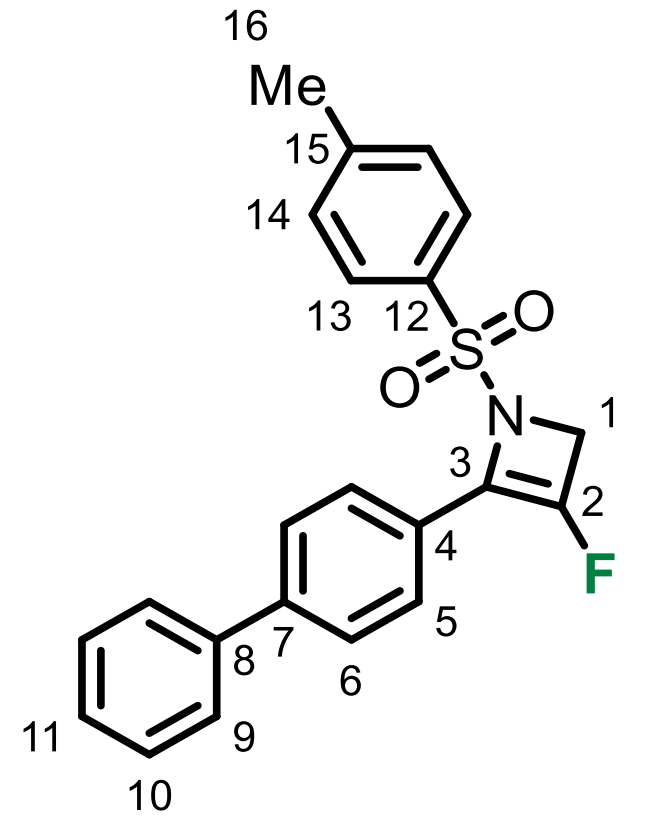

Alkyne **1ay** was converted into the corresponding FVI salt according to **General procedure III**. The crude product was then cyclised according to **General procedure IV** (58% NMR) and purified by flash column chromatography (2-20% EtOAc in pentane) to afford the title compound as a yellow oil (65.1 mg, 54% over 2 steps).

^{1}H NMR (500 MHz, CDCl$_3$) δ 7.71 – 7.65 (m, 4H, C5-H, C6-H), 7.63 (d, *J* = 7.9 Hz, 2H, C9-H), 7.51 (dt, *J* = 8.3, 1.6 Hz, 2H, C13-H), 7.47 (t, *J* = 7.9 Hz, 2H, C10-H), 7.41 – 7.36 (m, 1H, C11-H), 7.27 (d, *J* = 8.3 Hz, 2H, C14-H), 4.34 (d, *J* = 5.7 Hz, 2H, C1-H), 2.44 (s, 3H, C16-H).

^{13}C NMR (126 MHz, CDCl$_3$) δ 144.9 (C15), 141.8 (C7), 140.3 (d, *J* = 332.6 Hz, C2), 140.2 (C8), 130.6 (d, *J* = 2.3 Hz, C4), 129.8 (C12), 129.5 (C14), 129.2 (C13), 128.9 (C6), 127.8 (C11), 127.2 (C9), 127.2 (C5), 127.1 (C10), 126.0 (d, *J* = 5.0 Hz, C3), 59.7 (d, *J* = 19.7 Hz, C1), 21.7 (C16).

^{19}F NMR (376 MHz, CDCl$_3$) δ −118.9 (t, *J* = 5.7 Hz, 1F, C2-F).

HRMS (+ESI) calcd: $[M+H]^+$ ($C_{22}H_{18}FNO_2S$) 380.1115, measured 380.1131 (4.21 ppm error).

IR (neat) v_{max}/cm^{-1}: 3279, 2962, 1739, 1602, 1408, 1366, 1228, 1216, 1161, 1092, 814, 767, 697, 549.

3-fluoro-1-tosyl-4-(4-(trifluoromethyl)phenyl)-1,2-dihydroazete **(7az)**

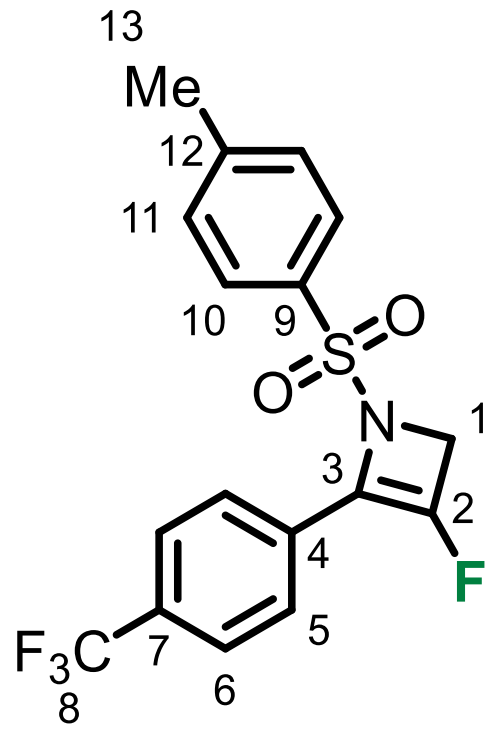

Prepared according to **General procedure IV** from **3az** (72% NMR) and purified by flash column chromatography (2-20% EtOAc in pentane) to afford the title compound as a white solid (46.8 mg, 63%).

^{1}H NMR (500 MHz, $CDCl_3$) δ 7.77 – 7.65 (m, 4H, C5-H, C6-H), 7.46 (d, *J* = 8.1 Hz, 2H, C10-H), 7.28 (d, *J* = 8.4 Hz, 2H, C11-H), 4.35 (d, *J* = 5.4 Hz, 2H, C1-H), 2.44 (s, 3H, C13-H).

^{13}C NMR (126 MHz, $CDCl_3$) δ 145.3 (C12), 142.1 (d, *J* = 336.2 Hz, C2), 130.9 (qd, *J* = 32.7, 1.7 Hz, C7), 130.5 (dq, *J* = 5.2, 1.8 Hz, C4), 129.8 (C11), 129.6 (d, *J* = 2.0 Hz, C3), 129.2 (C10), 129.1 (C9), 127.1 (d, *J* = 3.7 Hz, C5), 125.7 (qd, *J* = 3.7, 1.0 Hz, C6), 123.9 (q, *J* = 272.3 Hz, C8), 59.9 (d, *J* = 19.3 Hz, C1), 21.8 (C13).

^{19}F NMR (376 MHz, $CDCl_3$) δ −62.73 (s, 3F, C8-F), −115.29 (t, *J* = 5.4 Hz, 1F, C2-F).

HRMS (+ESI) calcd: $[M+H]^+$ ($C_{17}H_{13}F_4NO_2S$) 372.0676, measured 372.0684 (2.15 ppm error).

IR (neat) v_{max}/cm^{-1}: 3283, 2928, 1829, 1737, 1704, 1412, 1323, 1159, 1127, 1091, 1066, 1016, 814, 667, 549.

4-(4-bromophenyl)-3-fluoro-1-tosyl-1,2-dihydroazete **(7ba)**

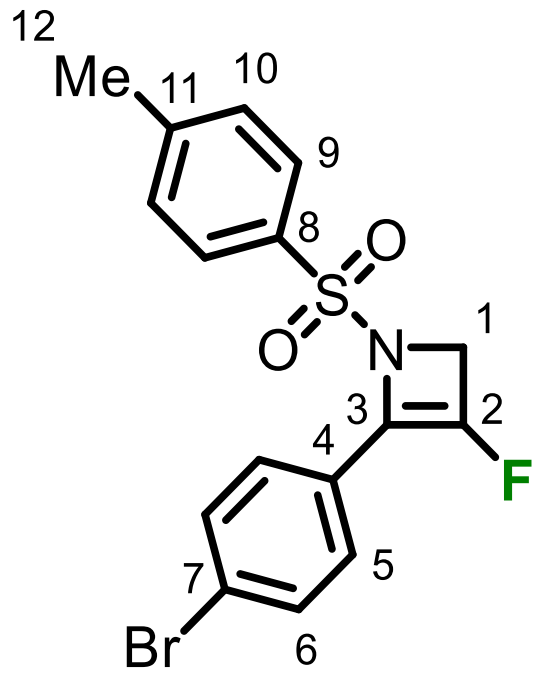

Prepared according to **General procedure IV** from **3ba** (70% NMR) and purified by flash column chromatography (2-20% EtOAc in pentane) to afford the title compound as a white solid (46.8 mg, 62%).

^{1}H NMR (400 MHz, CDCl$_3$) δ 7.56 (d, *J* = 8.5 Hz, 2H, C5-H), 7.49 (d, *J* = 8.5 Hz, 2H, C6-H), 7.46 (d, *J* = 8.2 Hz, 2H, C9-H), 7.27 (d, *J* = 7.7 Hz, 2H, C10-H), 4.29 (d, *J* = 5.6 Hz, 2H, C1-H), 2.44 (s, 3H, C12-H).

^{13}C NMR (126 MHz, CDCl$_3$) δ 145.2 (C11), 140.7 (d, *J* = 333.8 Hz, C2), 132.0 (d, *J* = 1.1 Hz, C5), 130.0 (d, *J* = 2.4 Hz, C8), 129.7 (C10), 129.2 (C9), 128.4 (d, *J* = 3.6 Hz, C5), 126.1 (d, *J* = 5.1 Hz, C3), 123.5 (d, *J* = 2.2 Hz, C7), 59.8 (d, *J* = 19.6 Hz, C1), 21.8 (C12).

^{19}F NMR (376 MHz, CDCl$_3$) δ −117.7 (t, *J* = 5.5 Hz, 1F, C2-F).

HRMS (+EI) calcd: [M]$^+$ ($C_{16}H_{13}NO_2FBrS$) 380.9829, measured 380.9819 (2.62 ppm error).

IR (neat) v_{max}/cm^{-1}: 3283, 2920, 2849, 1737, 1693, 1584, 1399, 1325, 1159, 1092, 1071, 813, 729, 661, 549.

***4-(4-chlorophenyl)-3-fluoro-1-tosyl-1,2-dihydroazete* (7bb)**

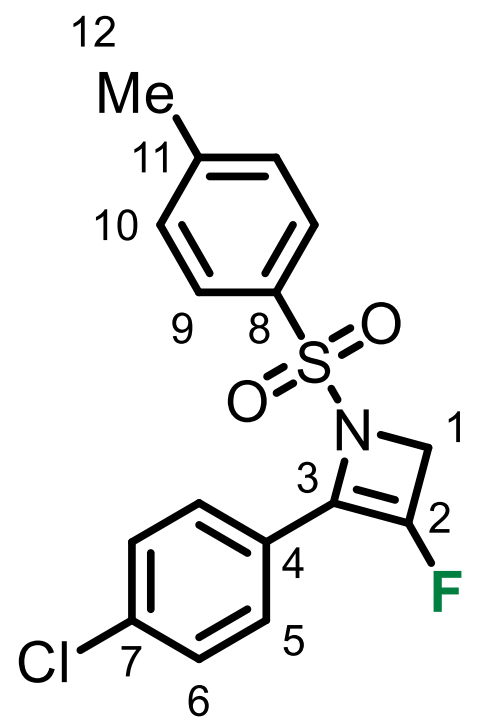

Prepared according to **General procedure IV** from **3bb** (82% NMR) and purified by flash column chromatography (2-20% EtOAc in pentane) to afford the title compound as a white solid (21.2 mg, 74%).

^{1}H NMR (500 MHz, CDCl$_3$) δ 7.56 (app. dt, *J* = 8.6, 2.1 Hz, 2H, C6-H), 7.46 (dt, *J* = 8.2, 1.9 Hz, 2H, C9-H), 7.40 (dt, *J* = 8.6, 2.1 Hz, 2H, C5-H), 7.27 (d, *J* = 8.2 Hz, 2H, C10-H), 4.31 (d, *J* = 5.8 Hz, 2H, C1-H), 2.44 (s, 3H, C12-H).

^{13}C NMR (126 MHz, CDCl$_3$) δ 145.2 (C11), 140.6 (d, *J* = 333.7 Hz, C2), 135.2 (d, *J* = 2.2 Hz, C4), 129.9 (C7), 129.9 (C8), 129.7 (C10), 129.2 (C9), 129.1 (d, *J* = 1.0 Hz, C6), 128.2 (d, *J* = 3.7 Hz, C5), 125.7 (d, *J* = 5.1 Hz, C3), 59.8 (d, *J* = 19.7 Hz, C1), 21.8 (C12).

^{19}F NMR (376 MHz, CDCl$_3$) δ −118.02 (t, *J* = 5.8 Hz, 1F, C2-F).

HRMS (+ESI) calcd: $[M]^+$ ($C_{16}H_{13}FNO_2SCl$) 337.0331, measured 337.0334 (0.89 ppm error).

IR (neat) v_{max}/cm^{-1}: 3280, 2969, 2924, 1737, 1692, 1589, 1329, 1217, 1158, 1090, 814, 663, 550.

***5-(3-fluoro-1-tosyl-1,4-dihydroazet-2-yl)-1-tosyl-1H-indole* (7bc)**

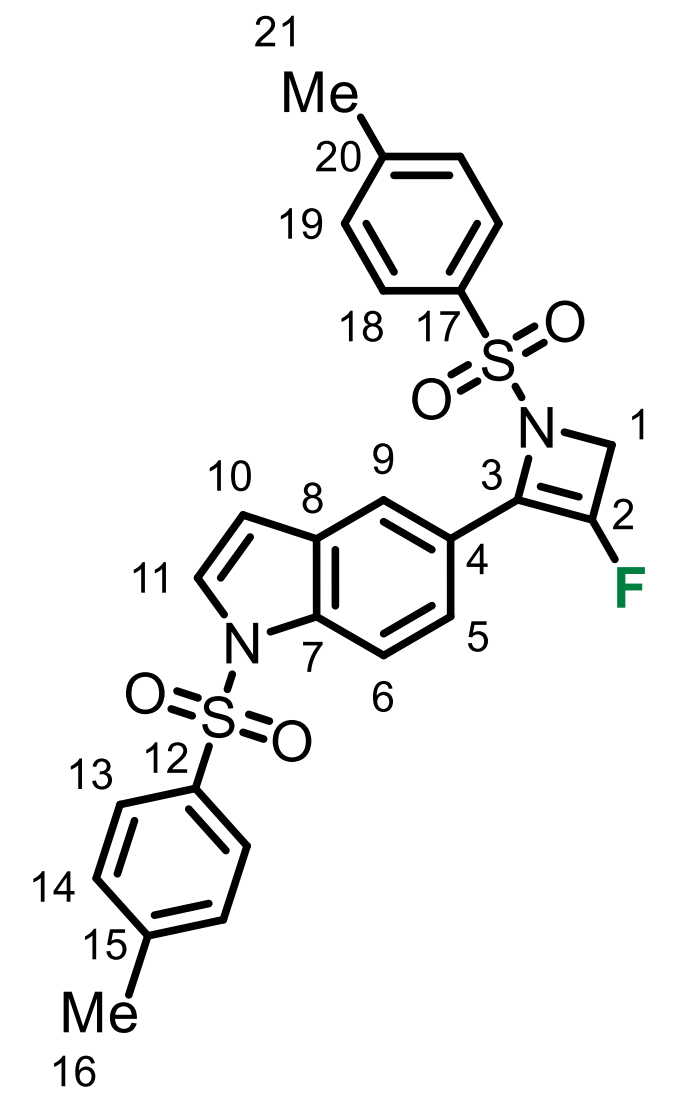

Alkyne **1bc** was converted into the corresponding FVI salt according to **General procedure III**. The crude product was then cyclised according to **General procedure IV** (51% NMR) and purified by flash column chromatography (2-20% EtOAc in pentane) to afford the title compound as a yellow oil (97.6 mg, 48% over 2 steps).

^{1}H NMR (500 MHz, CDCl$_3$) δ 8.01 (d, *J* = 8.6 Hz, 1H, C6-H), 7.86 (d, *J* = 1.6 Hz, 1H, C9-H), 7.79 (dt, *J* = 8.5, 2.1 Hz, 2H, C13-H), 7.61 (d, *J* = 3.7 Hz, 1H, C11-H), 7.50 (dd, *J* = 8.6, 1.7 Hz, 1H, C5-H), 7.43 (dt, *J* = 8.4, 1.9 Hz, 2H, C18-H), 7.26 (d, *J* = 8.5 Hz, 2H, C14-H), 7.22 (d, *J* = 8.4 Hz, 2H, C19-H), 6.70 (dd, *J* = 3.6, 0.8 Hz, 1H, C10-H), 4.31 (d, *J* = 5.4 Hz, 2H, C1-H), 2.41 (s, 3H, C21-H), 2.36 (s, 3H, C16-H).

^{13}C NMR (126 MHz, CDCl$_3$) δ 145.4 (C15), 145.0 (C20), 139.8 (d, *J* = 331.4 Hz, C2), 135.3 (C12), 134.8 (d, *J* = 1.4 Hz, C8), 130.9 (d, *J* = 2.4 Hz, C3), 130.8 (C7), 130.2 (C14), 129.6 (C19), 129.3 (C17), 129.2 (C18), 127.4 (C11), 127.0 (C13), 123.4 (d, *J* = 3.3 Hz, C5), 122.6 (d, *J* = 5.0 Hz, C4), 120.2 (d, *J* = 3.7 Hz, C9), 113.8 (C6), 109.4 (C10), 59.6 (d, *J* = 20.0 Hz, C1), 21.8 (C16), 21.7 (C21).

^{19}F NMR (377 MHz, CDCl$_3$) δ −120.64 (td, *J* = 5.5, 1.9 Hz, 1F, C2-F).

HRMS (+ESI) calcd: $[M+H]^+$ ($C_{25}H_{21}FN_2O_4S_2$) 497.1000, measured 497.0996 (0.80 ppm error).

IR (neat) v_{max}/cm^{-1}: 3291, 3144, 2924, 2855, 2257, 1737, 1687, 1600, 1440, 1372, 1333, 1189, 1161, 1128, 1092, 993, 909, 813, 730, 667, 582, 550, 538.

8.7. 2-Pyrrolines (8)

***4-fluoro-1-tosyl-2,3-dihydro-1H-pyrrole* (8be)**

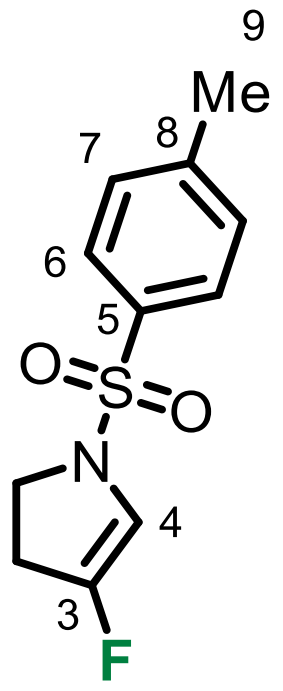

Prepared according to **General procedure IV** from **3be** (65% NMR) and purified by flash column chromatography (2-20% EtOAc in pentane) to afford the title compound as a colourless oil (28.9 mg, 60%).

^{1}H NMR (400 MHz, $CDCl_3$) δ 7.67 (app. dt, J = 8.0, 1.7 Hz, 2H, C6-H), 7.35 (d, J = 8.0 Hz, 2H, C7-H), 6.17 (dt, J = 3.1, 2.0 Hz, 1H, C4-H), 3.58 (t, J = 9.0 Hz, 2H, C1-H), 2.55 (tdd, J = 9.1, 3.4, 2.0 Hz, 2H, C2-H), 2.45 (s, 3H, C9-H).

^{13}C NMR (151 MHz, $CDCl_3$) δ 154.1 (d, J = 271.7 Hz, C3), 144.4 (C8), 132.0 (C5), 129.9 (C7), 128.2 (C6), 111.2 (d, J = 29.5 Hz, C4), 46.4 (d, J = 6.7 Hz, C1), 28.0 (d, J = 20.2 Hz, C2), 21.8 (C9).

^{19}F NMR (376 MHz, $CDCl_3$) δ −147.97 (ddd, J = 5.8, 3.4, 3.1 Hz, 1F, C3-F).

HRMS (+EI) calcd: $[M]^+$ ($C_{11}H_{12}NO_2SF$) 241.0567, measured 241.0563 (1.66 ppm error).

IR (neat) v_{max}/cm^{-1}: 2929, 2862, 1353, 1322, 1164, 1091, 815, 666, 589, 54

***3-fluoro-1-tosyl-1H-indole* (8bd)**

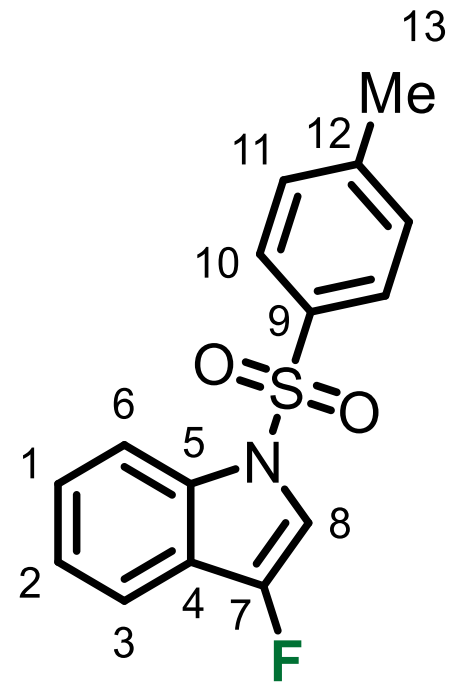

Prepared from alkyne **1bd** according to **General procedure III** (15% NMR) and purified by flash column chromatography to afford the title compound as a colourless oil (110 mg, 13%).

^{1}H NMR (500 MHz, CDCl$_3$) δ 8.02 (ddt, *J* = 8.4, 1.9, 0.9 Hz, 1H, C3-H), 7.93 – 7.86 (m, 1H, C1-H), 7.90 (d, *J* = 8.2 Hz, 2H, C10-H), 7.52 (dt, *J* = 7.8, 1.1 Hz, 1H, C6-H), 7.37 (ddd, *J* = 8.5, 7.2, 1.3 Hz, 1H, C2-H), 7.32 (d, *J* = 3.0 Hz, 1H, C8-H), 7.21 (d, *J* = 8.2 Hz, 2H, C11-H), 2.34 (s, 3H, C13-H).

^{13}C NMR (126 MHz, CDCl$_3$) δ 149.1 (d, *J* = 255.5 Hz, C7), 145.3 (C12), 134.8 (C9), 132.9 (d, *J* = 5.2 Hz, C5), 130.4 (C1), 130.0 (C11), 128.6 (C2), 127.0 (C10), 121.7 (d, *J* = 19.0 Hz, C4), 117.8 (d, *J* = 2.6 Hz, C3), 114.3 (d, *J* = 1.6 Hz, C6), 108.9 (d, *J* = 28.7 Hz, C8), 21.7 (C13).

^{19}F NMR (376 MHz, CDCl$_3$) δ −165.4 (dd, *J* = 3.0, 1.9 Hz, 1F, C7-F).

Characterisation data matches that previously reported.[44]

8.8. Pyrroles (9)

***4-fluoro-2-(4-fluorophenyl)-1-tosyl-1H-pyrrole* (9e)**

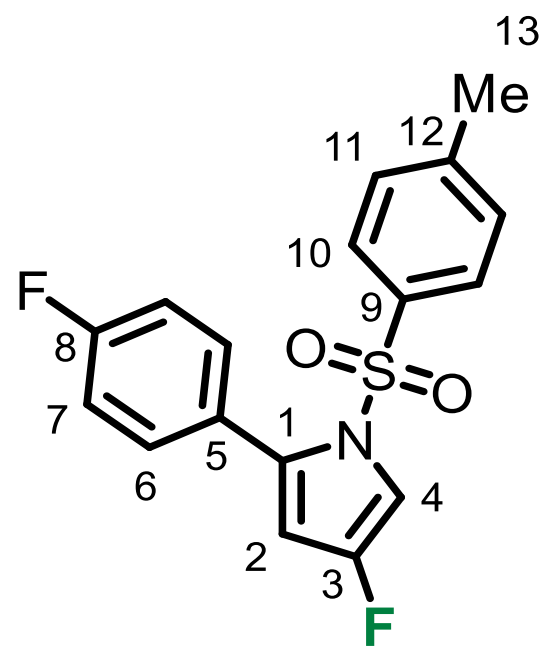

Prepared according to **General procedure V** from pyrroline **4e** and purified by flash column chromatography (2-30% EtOAc in pentane) to afford the title compound as a yellow oil (42.7 mg, 64%)

^{1}H NMR (600 MHz, CDCl$_3$) δ 7.23 (d, *J* = 8.2 Hz, 2H, C10-H), 7.22 – 7.18 (m, 2H, C6-H), 7.16 (dd, *J* = 3.2, 2.1 Hz, 1H, C4-H), 7.14 (d, *J* = 8.2 Hz, 2H, C11-H), 7.01 (t, *J* = 8.6 Hz, 2H, C7-H), 6.00 (d, *J* = 2.1 Hz, 1H, C2-H), 2.38 (s, 3H, C13-H).

^{13}C NMR (151 MHz, CDCl$_3$) δ 163.2 (d, *J* = 248.9 Hz, C8), 153.0 (d, *J* = 250.5 Hz, C3), 145.3 (C12), 135.2 (C9), 133.7 (d, *J* = 5.5 Hz, C1), 132.8 (d, *J* = 8.5 Hz, C6), 129.7 (C11), 127.2 (C10), 126.8 (d, *J* = 3.7 Hz, C5), 114.7 (d, *J* = 21.7 Hz, C7), 107.1 (d, *J* = 20.0 Hz, C2), 106.9 (d, *J* = 10.5 Hz, C4), 21.8 (C13).

^{19}F NMR (376 MHz, CDCl$_3$) δ −112.16 (ddd, *J* = 13.9, 8.6, 5.4 Hz, 1F, C8-F), −158.96 (d, *J* = 3.2 Hz, 1F, C3-F).

HRMS (+ESI) calcd: $[M+H]^+$ ($C_{17}H_{13}F_2NO_2S$) 334.0708, measured 334.0712 (1.20 ppm error).

IR (neat) v_{max}/cm^{-1}: 3237, 2925, 1715, 1683, 1597, 1508, 1450, 1237, 1164, 1087, 844, 660, 547.

R_f 0.34 (20% EtOAc in pentane).

***4-fluoro-1-tosyl-2-(4-(trifluoromethyl)phenyl)-1H-pyrrole* (9f)**

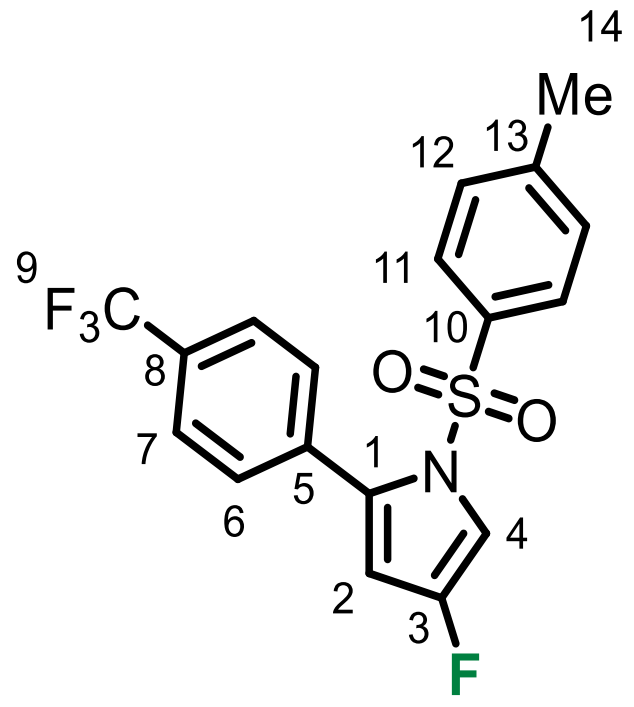

Prepared according to **General procedure V** from pyrroline **4f** and purified by flash column chromatography (2-30% EtOAc in pentane) to afford the title compound as a yellow oil (42.2 mg, 55%)

^{1}H NMR (500 MHz, CDCl$_3$) δ 7.59 (d, J = 8.0 Hz, 2H, C7-H), 7.38 (d, J = 8.0 Hz, 2H, C6-H), 7.24 (d, J = 8.4 Hz, 2H, C11-H), 7.20 (dd, J = 3.4, 2.0 Hz, 1H, C4-H), 7.15 (d, J = 8.1 Hz, 2H, C12-H), 6.08 (d, J = 2.1 Hz, 1H, C2-H), 2.38 (s, 3H, C14-H).

^{13}C NMR (126 MHz, CDCl$_3$) δ 153.1 (d, J = 251.2 Hz, C3), 145.6 (C13), 135.0 (C11), 134.4 (C5), 133.3 (d, J = 5.6 Hz, C1), 131.1 (C6), 130.8 (q, J = 32.6 Hz, C8), 129.8 (C12), 127.2 (C11), 124.6 (q, J = 3.8 Hz, C7), 124.1 (q, J = 274.1 Hz, C9), 108.0 (d, J = 11.9 Hz, C2), 107.8 (d, J = 3.1 Hz, C4), 21.8 (C14).

^{19}F NMR (471 MHz, CDCl$_3$) δ −62.66 (s, 3F, C9-F), −158.42 (dd, J = 3.4, 2.1 Hz, 1F, C3-F).

HRMS (+EI) calcd: $[M]^+$ ($C_{18}H_{13}NO_2SF_4$) 383.0598, measured 383.0592 (1.57 ppm error).

IR (neat) v_{max}/cm^{-1}: 3268, 2926, 1724, 1597, 1324, 1167, 1129, 1067, 844, 664, 547.

R_f 0.06 (20% EtOAc in pentane).

***3-fluoro-5-(4-fluorophenyl)-2-phenyl-1H-pyrrole* (9n)**

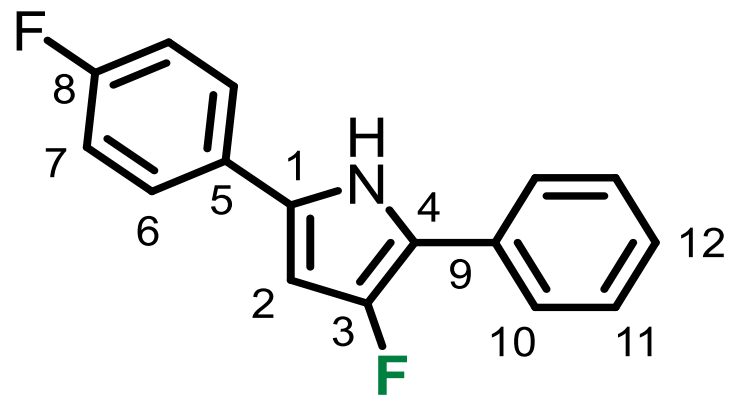

To a solution of pyrroline **4n** (0.1 mmol, 1 equiv.) in DCM (1 mL) in a pressure tube was added sodium *tert*-butoxide (0.6 mmol, 6 equiv.). The solution was heated to reflux for 16 h. The reaction mixture was then diluted with water (10 mL) and extracted with DCM (2 * 10 mL). The organic fractions were dried over $MgSO_4$, filtered, and concentrated *in vacuo* to afford the crude product as a white solid (23.5 mg, 92%).

^{1}H NMR (600 MHz, $CDCl_3$) δ 8.00 (s, 1H, C1-NH), 7.56 (d, *J* = 7.8 Hz, 2H, C10-H), 7.46 – 7.43 (m, 2H, C6-H), 7.42 (dd, *J* = 7.8, 7.4 Hz, 2H, C11-H), 7.23 (t, *J* = 7.4 Hz, 1H, C12-H), 7.10 (tt, *J* = 8.6, 2.0 Hz, 2H, C7-H), 6.31 (d, *J* = 3.1 Hz, 1H, C2-H).

^{13}C NMR (151 MHz, $CDCl_3$) δ 162.1 (d, *J* = 247.0 Hz, C8), 150.4 (d, *J* = 245.1 Hz, C3), 130.3 (d, *J* = 4.5 Hz, C9), 129.2 (C11), 128.5 (apparent t, *J* = 2.5 Hz, C5), 128.1 (d, *J* = 7.2 Hz, C1), 126.4 (C12), 125.7 (d, *J* = 7.8 Hz, C6), 124.1 (d, *J* = 4.8 Hz, C10), 116.2 (d, *J* = 21.8 Hz, C7), 115.7 (d, *J* = 20.0 Hz, C4), 96.8 (d, *J* = 17.3 Hz, C2).

^{19}F NMR (471 MHz, $CDCl_3$) δ −114.78 (tt, *J* = 8.4, 5.1 Hz, 1F, C8-F), −160.07 (d, *J* = 3.1 Hz, 1F, C3-F).

HRMS (+ESI) calcd: $[M]^+$ ($C_{16}H_{11}F_2N$) 255.0854, measured 255.0856 (0.78 ppm error).

IR (neat) v_{max}/cm^{-1}: 3331, 3069, 2924, 1724, 1599, 1506, 1449, 1233, 1158, 1100, 842, 698.

R_f 0.61 (20% EtOAc in pentane).

3-fluoro-5-(4-fluorophenyl)-2-phenyl-4-tosyl-1H-pyrrole **(9n')**

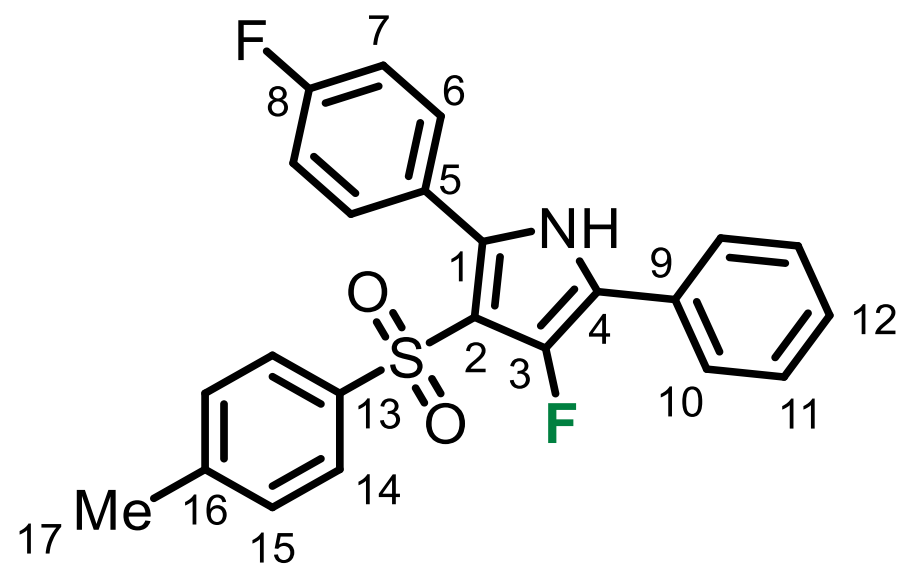

Prepared according to **General procedure V** from pyrroline **4n** and purified by flash column chromatography (2-30% EtOAc in pentane) to afford the title compound as a yellow solid (62.2 mg, 76%).

^{1}H NMR (500 MHz, $CDCl_3$) δ 8.32 (br s, 1H), 7.71 (d, *J* = 8.1 Hz, 2H), 7.56 – 7.50 (m, 2H), 7.49 (d, *J* = 7.5 Hz, 2H), 7.40 (t, *J* = 7.7 Hz, 2H), 7.31 – 7.27 (m, 1H), 7.24 (d, *J* = 8.1 Hz, 3H), 7.12 (t, *J* = 8.6 Hz, 2H), 2.39 (s, 3H).

^{13}C NMR (126 MHz, $CDCl_3$) δ 163.6 (d, *J* = 250.2 Hz, C8), 145.1 (d, *J* = 253.9 Hz, C3), 144.0 (C16), 140.4 (C13), 132.1 (d, *J* = 8.4 Hz, C6), 130.9 (d, *J* = 2.3 Hz, C1), 129.7 (C15), 129.3 (C11), 128.4 (d, *J* = 4.6 Hz, C9), 127.7 (C12), 127.1 (C14), 126.1 (d, *J* = 3.3 Hz, C5), 124.7 (d, *J* = 4.6 Hz, C10), 115.9 (d, *J* = 18.6 Hz, C4), 115.6 (d, *J* = 21.9 Hz, C7), 112.5 (d, *J* = 11.8 Hz, C2), 21.7 (C17).

^{19}F NMR (471 MHz, $CDCl_3$) δ −110.86 (tt, *J* = 8.6, 5.3 Hz, 1F, C7-F), −157.38 (d, *J* = 2.7 Hz, 1F, C3-F).

HRMS (+ESI) calcd: $[M+H]^+$ ($C_{23}H_{17}F_2NO_2S$) 410.1021, measured 410.1009 (2.93 ppm error).

IR (neat) v_{max}/cm^{-1}: 3291, 2927, 1828, 1710, 1597, 1494, 1444, 1323, 1230, 1144, 1088, 948, 662, 547.

R_f 0.25 (20% EtOAc in pentane).

***4-fluoro-2-propyl-1-tosyl-1H-pyrrole* (9v)**

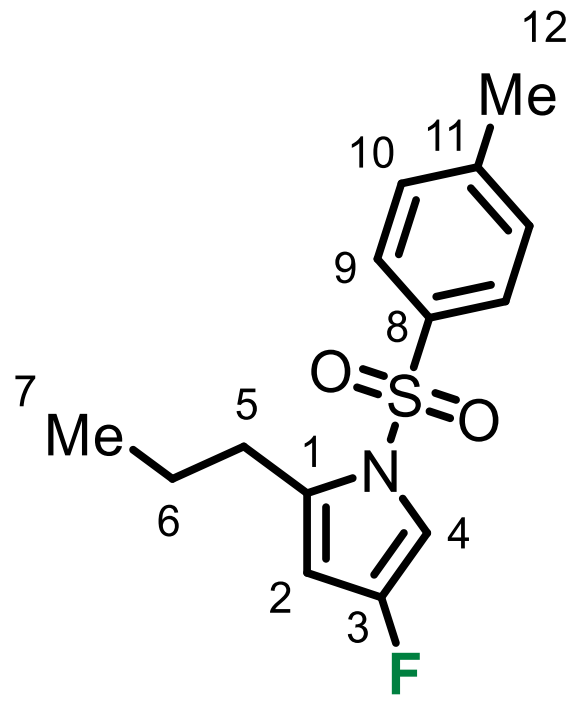

Prepared from pyrroline **4v** according to **General procedure V-A** (23% NMR yield). The crude product was purified by flash column chromatography (0-10% EtOAc in pentane) to afford the title compound as a colourless oil (14.6 mg, 12%).

Could not be fully isolated; contains 16 mol% 4-methylbenzophenone.

^{1}H NMR (500 MHz, CDCl$_3$) δ 7.65 (app. dt, *J* = 8.4, 1.8 Hz, 2H, C9-H), 7.30 (d, *J* = 8.4 Hz, 2H, C10-H), 7.00 (dd, *J* = 3.3, 2.3 Hz, 1H, C4-H), 5.85 (dt, *J* = 2.3, 1.0 Hz, 1H, C2-H), 2.63 – 2.59 (m, 2H, C5-H), 2.42 (s, 3H, C12-H), 1.55 (h, *J* = 7.4 Hz, 2H, C6-H), 0.92 (t, *J* = 7.4 Hz, 3H, C7-H).

^{13}C NMR (126 MHz, CDCl$_3$) δ 152.9 (d, *J* = 248.1 Hz, C3), 145.2 (C11), 136.2 (C8), 134.8 (d, *J* = 4.6 Hz, C1), 130.2 (C10), 126.9 (C9), 104.9 (d, *J* = 29.9 Hz, C4), 103.3 (d, *J* = 19.7 Hz, C2), 29.5 (d, *J* = 1.1 Hz, C5), 22.0 (C6), 21.8 (C12), 13.9 (C7).

^{19}F NMR (376 MHz, CDCl$_3$) δ −160.38 (d, *J* = 3.3 Hz, 1F, C3-F).

HRMS (+ESI) calcd: $[M+H]^+$ ($C_{14}H_{16}FNO_2S$) 282.0958, measured 282.0959 (0.35 ppm error).

IR (neat) v_{max}/cm^{-1}: 3268, 2926, 1747, 1661, 1597, 1449, 1367, 1264, 1189, 1171, 1089, 813, 665, 560, 545.

2-propyl-1-tosyl-1*H*-pyrrole (9bf)

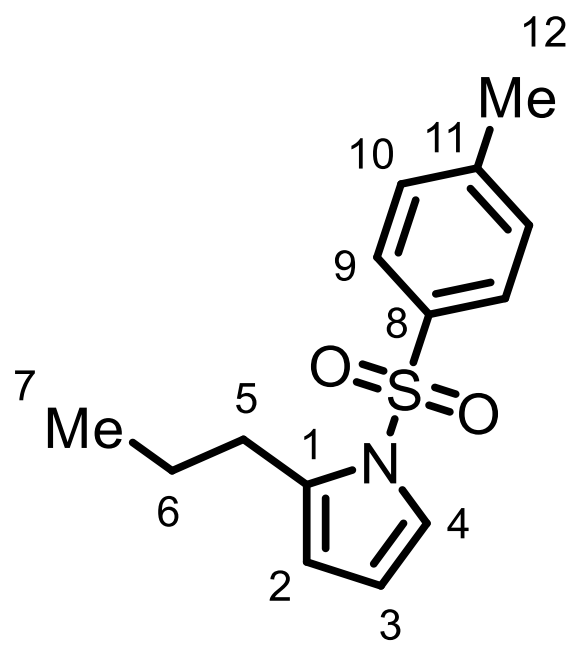

An RBF was charged with pyrroline **4bf** (0.434 mmol, 115 mg), DDQ (0.868 mmol, 197 mg), and toluene (10 mL). The solution was heated to reflux for 24 hours. The reaction mixture was then concentrated *in vacuo* and purified by flash column chromatography (2-20% EtOAc in pentane) to afford the title compound as a colourless oil (40.0 mg, 35%).

^{1}H NMR (500 MHz, $CDCl_3$) δ 7.64 (app. dt, *J* = 8.5, 1.9 Hz, 2H, C9-H), 7.30 – 7.26 (m, 3H, C4-H, C10-H), 6.19 (t, *J* = 3.3 Hz, 1H, C3-H), 5.98 (ddd, *J* = 3.3, 1.2, 1.1 Hz, 1H, C2-H), 2.63 (td, *J* = 7.7, 1.1 Hz, 2H, C5-H), 2.40 (s, 3H, C12-H), 1.58 (h, *J* = 7.4 Hz, 2H, C6-H), 0.92 (t, *J* = 7.4 Hz, 3H, C7-H).

^{13}C NMR (126 MHz, $CDCl_3$) δ 144.8 (C11), 136.7 (C8), 135.9 (C1), 130.1 (C10), 126.8 (C9), 122.3 (C4), 111.9 (C2), 111.4 (C3), 29.3 (C5), 22.0 (C6), 21.7 (C12), 14.0 (C7).

Characterisation data matches that previously reported.[45]

8.9. Pyrrolidines (10)

4-fluoro-2-(p-tolyl)-1-tosylpyrrolidine **(10b)**

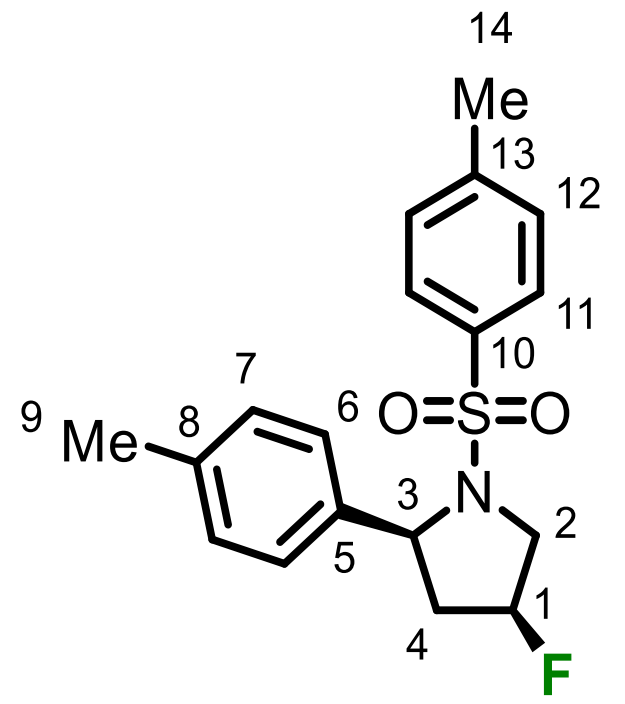

Prepared from pyrroline **4b** according to **General procedure VI** and purified by flash column chromatography (0 - 50% Et_2O in pentane) to afford the title compound as a white solid (18 mg, 54%).

Isolated as a mixture of diastereomers (4.3:1).

^{1}H NMR (500 MHz, $CDCl_3$) Major diastereomer δ 7.60 (d, *J* = 8.0 Hz, 2H, C11-H), 7.30 – 7.19 (m, 4H, C6-H, C12-H), 7.08 (d, *J* = 7.9 Hz, 2H, C7-H), 5.13 (dddd, *J* = 53.3, 4.6, 3.2, 1.5 Hz, 1H, C1-H), 4.85 (dd, *J* = 9.6, 3.0 Hz, 1H, C3-H), 3.88 (ddd, *J* = 21.6, 12.2, 1.5 Hz, 1H, C2-H), 3.61 (ddd, *J* = 31.8, 12.2, 4.5 Hz, 1H, C2-H), 2.41 – 2.15 (m, 2H, C4-H), 2.42 (s, 3H, C14-H), 2.32 (s, 3H, C9-H). **Minor diastereomer** δ 7.63 (d, *J* = 8.0 Hz, 2H, C11-H), 7.30 – 7.19 (m, 4H, C6-H, C12-H), 7.13 (d, *J* = 7.9 Hz, 2H, C7-H), 5.08 (dddd, *J* = 53.3, 4.6, 3.2, 1.5 Hz, 1H, C1-H), 4.65 (dd, *J* = 10.0, 6.8 Hz, 1H, C3-H, 4.00 – 3.93 (m, 1H, C2-H), 3.79 (dd, *J* = 13.5, 3.2 Hz, 1H, C2-H), 2.42 (s, 3H, C14-H), 2.34 (s, 3H, C9-H). 2.41 – 2.15 (m, 1H, C4-H), 1.99 (dddd, *J* = 40.5, 14.1, 10.1, 3.6 Hz, 1H, C4-H).

^{13}C NMR (126 MHz, $CDCl_3$) Major diastereomer δ 143.8 (C13), 139.0 (C5), 137.0 (C8), 134.8 (C10), 129.7 (C12), 129.1 (C7), 127.7 (C11), 126.7 (d, *J* = 2.2 Hz, C6), 92.1 (d, *J* = 180.6 Hz, C1), 62.2 (C3), 55.5 (d, *J* = 24.1 Hz, C2), 41.7 (d, *J* = 20.6 Hz, C4), 21.7 (C14), 21.2 (C9). **Minor diastereomer** δ 143.6 (C13), 138.6 (C5), 137.5 (C8), 134.5 (C10), 129.5 (C12), 129.4 (C7), 128.0 (C11), 126.4 (C6), 91.3 (d, *J* = 179.3 Hz, C1), 62.5 (C3), 56.2 (d, *J* = 22.6 Hz, C2), 44.1 (d, *J* = 21.0 Hz, C4), 21.7 (C14), 21.3 (C9).

^{19}F NMR (471 MHz, $CDCl_3$) Major diastereomer δ −172.10 (dddt, *J* = 53.6, 36.4, 32.0, 21.7 Hz, 1F, C1-F). **Minor diastereomer** δ −177.08 (dtdd, *J* = 52.3, 40.5, 24.1, 17.3 Hz, 1F, C1-F).

HRMS (+EI) calcd: $[M]^+$ ($C_{11}H_{13}NO_2SF$) 242.0646, measured 242.0642 (1.65 ppm error).

IR (neat) ν_{max}/cm^{-1}: 2950, 1728, 1597, 1515, 1494, 1455, 1432, 1345, 1306, 1290, 1249, 1185, 1159, 1092, 1029, 1016, 962, 898, 853, 814, 730, 708, 666, 603, 548, 504, 436, 414.

***2-(4-chlorophenyl)-4-fluoro-1-tosylpyrrolidine* (10d)**

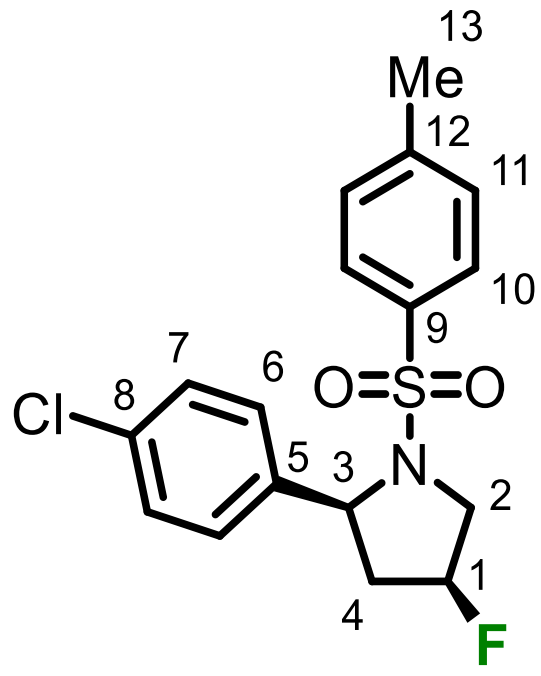

Synthesised from pyrroline **4d** according to **General procedure VI** and purified by flash column chromatography (0 to 50% Et_2O in pentane) to afford the title compound as a white solid (19 mg, 54%).

Isolated as a mixture of diastereomers (9.1:1)

^{1}H NMR (500 MHz, CDCl$_3$) δ 7.60 (d, *J* = 8.0 Hz, 2H, C10-H), 7.29 – 7.21 (m, 6H, C6-H, C7-H, C10-H), 5.16 (dt, *J* = 53.1, 4.5 Hz, 1H C1-H), 4.87 (dd, *J* = 9.8, 2.6 Hz, 1H, C3-H), 3.95 – 3.83 (m, 1H, C2-H), 3.60 (ddd, *J* = 33.1, 12.2, 4.3 Hz, 1H, C2-H), 2.42 (s, 3H, C13-H), 2.40 – 2.26 (m, 1H, C4-H), 2.26 – 2.16 (m, 1H, C4-H).

^{13}C NMR (126 MHz, CDCl$_3$) δ 144.1 (C12), 140.6 (C5), 134.7 (C9), 133.2 (C8), 129.8 (C11), 128.6 (C7), 128.24 (C6), 127.7 (C10), 92.2 (d, *J* = 180.5 Hz, C1), 61.7 (C3), 55.6 (d, *J* = 23.6 Hz, C2), 41.6 (d, *J* = 20.7 Hz, C4), 21.7 (C13).

^{19}F NMR (471 MHz, CDCl$_3$) Major diastereomer δ −171.88 (dddt, *J* = 54.1, 38.1, 33.2, 21.2 Hz, 1F, C1-F). **Minor diastereomer** δ −177.26 (dtdd, *J* = 52.3, 40.3, 24.0, 16.8 Hz, 1F, C1-F).

HRMS (+EI) calcd: $[M+Na]^+$ ($C_{17}H_{17}ClFNO_2S$) 376.0545, measured 376.0554 (2.66 ppm error).

IR (neat) ν_{max}/cm^{-1}: 2957, 2926, 2863, 1598, 1492, 1458, 1348, 1305, 1162, 1091, 1029, 1014, 962, 764, 854, 817, 709, 668, 601, 551, 511.

***cis*-4-fluoro-2-(4-fluorophenyl)-1-tosylpyrrolidine (10e)**

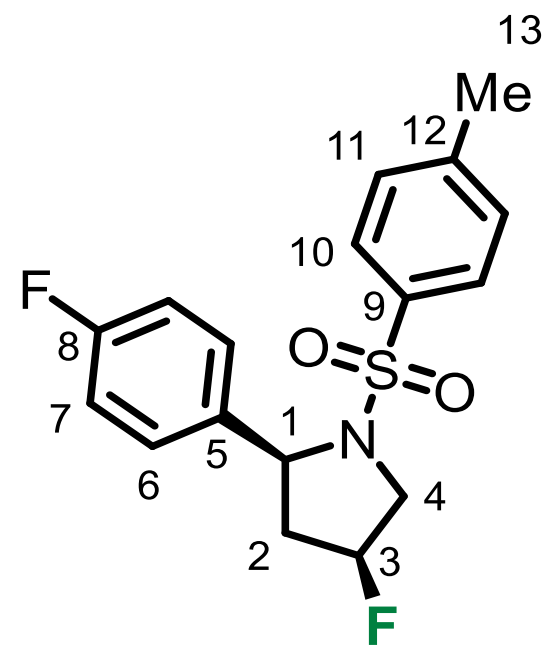

Produced according to **General procedure VI** from pyrroline **4e** and purified by flash column chromatography (2-30% EtOAc in pentane) to afford the title compound as a white solid (66.8 mg, 99%)

^{1}H NMR (500 MHz, $CDCl_3$) δ 7.59 (d, J = 8.4 Hz, 2H, C10-H), 7.32 – 7.27 (m, 2H, C6-H), 7.26 (d, J = 8.4 Hz, 2H, C11-H), 6.95 (tt, J = 8.7, 2.2 Hz, 2H, C7-H), 5.16 (dtt, J = 53.0, 4.6, 1.2 Hz, 1H, C3-H), 4.88 (dd, J = 9.8, 2.7 Hz, 1H, C1-H), 3.90 (ddd, J = 21.4, 12.2, 2.0 Hz, 1H, C4-H), 3.60 (ddd, J = 33.0, 12.2, 4.6 Hz, 1H, C4-H), 2.42 (s, 3H, C13-H), 2.41 – 2.29 (m, 1H, C2-H), 2.22 (dd, J = 20.7, 15.4 Hz, 1H, C2-H).

^{13}C NMR (151 MHz, $CDCl_3$) δ 162.2 (d, J = 245.6 Hz, C8), 144.0 (C12), 137.8 (d, J = 3.1 Hz, C5), 134.8 (C9), 129.8 (C11), 128.5 (dd, J = 8.3, 2.6 Hz, C6), 127.7 (C10), 115.3 (d, J = 21.6 Hz, C7), 92.2 (d, J = 180.5 Hz, C3), 61.7 (C1), 55.6 (d, J = 23.7 Hz, C4), 41.7 (d, J = 20.8 Hz, C2), 21.7 (C13).

^{19}F NMR (376 MHz, $CDCl_3$) δ −115.53 (tt, J = 9.1, 5.4 Hz, C8-F), −171.84 (dddt, J = 54.1, 37.9, 33.0, 21.4 Hz, C3-F).

HRMS (+ESI) calcd: $[M+H]^+$ ($C_{17}H_{17}F_2NO_2S$) 338.1021, measured 338.1015 (1.20 ppm error).

IR (neat) v_{max}/cm^{-1}: 2926, 1601, 1509, 1347, 1222, 1162, 1093, 1029, 814, 666, 603, 552.

R*f* 0.21 (20% EtOAc in pentane).

***4-fluoro-1-tosyl-2-(4-(trifluoromethyl)phenyl)pyrrolidine* (10f)**

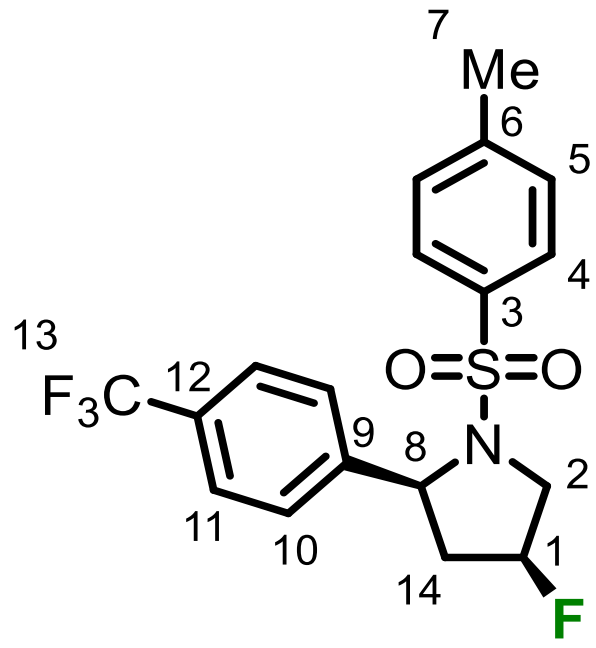

Synthesised from pyrroline **4f** according to **General procedure VI** and purified by flash column chromatography (0 to 50% Et_2O in pentane) to afford the title compound as a white solid (15 mg, 39%, single diastereomer).

^{1}H NMR (500 MHz, $CDCl_3$) δ 7.60 (d, *J* = 8.1 Hz, 2H, C4-H), 7.51 (d, *J* = 8.1 Hz, 2H, C11-H), 7.43 (d, *J* = 8.1 Hz, 2H, C10-H), 7.25 (d, *J* = 8.1 Hz, 2H, C5-H), 5.18 (dt, *J* = 52.9, 4.4 Hz, 1H, C1-H), 4.95 (dd, *J* = 10.0, 2.5 Hz, 1H, C8-H), 3.94 (dd, *J* = 21.4, 12.3 Hz, 1H, C2-H), 3.61 (ddd, *J* = 33.6, 12.2, 4.2 Hz, 1H, C2-H), 2.46 – 2.31 (m, 1H, C14-H), 2.41 (s, 3H, C7-H), 2.30 – 2.19 (m, 1H, C14-H).

^{13}C NMR (126 MHz, $CDCl_3$) δ 146.1 (q, *J* = 1.5 Hz, C9), 144.2 (C6), 134.6 (C3), 129.9 (C5), 129.6 (q, *J* = 32.4 Hz, C12), 127.7 (C4), 127.2 (d, *J* = 2.6 Hz, C10), 125.4 (q, *J* = 3.8 Hz, C11), 124.3 (q, *J* = 271.9 Hz, C13), 92.2 (d, *J* = 180.7 Hz, C1), 61.8 (C8), 55.7 (d, *J* = 23.7 Hz, C2), 41.6 (d, *J* = 20.8 Hz, C14), 21.6 (C7).

^{19}F NMR (376 MHz, $CDCl_3$) δ −62.38 (s, 3F, C1-F), −171.62 – −172.12 (m, 1F, C13-F).

HRMS (+EI) calcd: $[M]^+$ ($C_{18}H_{17}NO_2SF_4$) 387.0911, measured 387.0906 (1.29 ppm error).

IR (neat) ν_{max}/cm^{-1}: 2926, 2861, 1727, 1598, 1494, 1466, 1433, 1353, 1330, 1306, 1290, 1222, 1168, 1153, 1109, 1090, 1073, 1050, 1017, 1000, 981, 961, 939, 901, 817, 801, 749, 724, 655, 647, 590, 549, 508, 473.

(2R,3R,5R)-3-fluoro-5-(4-fluorophenyl)-2-methyl-1-tosylpyrrolidine **((*R,R,R*)-10j)**

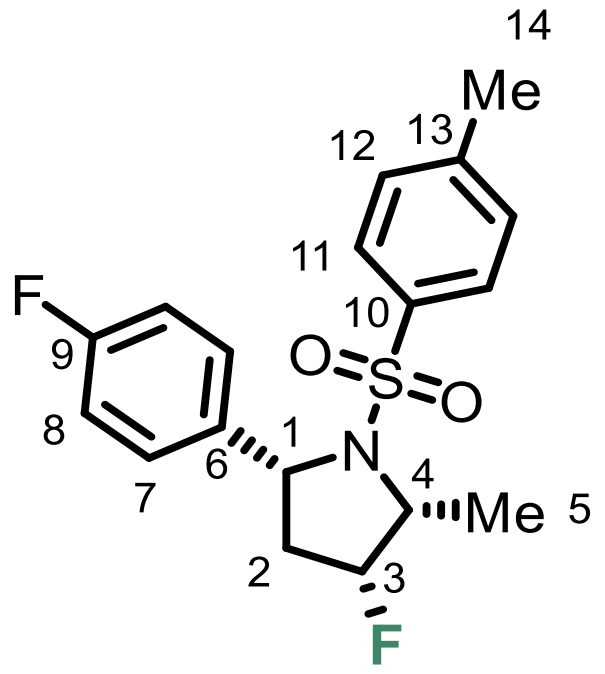

Produced according to **General procedure VI** from pyrroline **(*R,R*)-4j** (94% NMR) as a mixture of C3 diastereomers (*d.r.* 17:1) and purified by flash column chromatography (0-20% EtOAc in pentane) to afford the title compound (single diastereomer) as a white solid (142 mg, 81%).

^{1}H NMR (500 MHz, CDCl$_3$) δ 7.64 (d, *J* = 8.1 Hz, 2H, C11-H), 7.33 (dd, *J* = 8.6, 5.3 Hz, 2H, C7-H), 7.28 (d, *J* = 8.1 Hz, 2H, C12-H), 6.98 (t, *J* = 8.6 Hz, 2H, C8-H), 4.94 (dd, *J* = 8.8, 3.7 Hz, 1H, C1-H), 4.84 (dtd, *J* = 53.1, 4.8, 3.3 Hz, 1H, C3-H), 3.92 (dqd, *J* = 18.2, 6.6, 4.8 Hz, 1H, C4-H), 2.42 (s, 3H, C5-H), 2.26 – 2.06 (m, 2H, C2-H), 1.52 (dd, *J* = 6.6, 3.3 Hz, 3H, C5-H).

^{13}C NMR (126 MHz, CDCl$_3$) δ 162.1 (d, *J* = 245.3 Hz, C9), 144.0 (C13), 138.1 (d, *J* = 3.1 Hz, C6), 135.0 (C10), 129.9 (C12), 128.1 (dd, *J* = 8.1, 2.0 Hz, C7), 127.7 (C11), 115.2 (d, *J* = 21.5 Hz, C8), 93.8 (d, *J* = 186.7 Hz, C3), 62.1 (d, *J* = 1.5 Hz, C1), 60.8 (d, *J* = 21.1 Hz, C4), 39.6 (d, *J* = 20.0 Hz, C2), 21.7 (C14), 16.0 (d, *J* = 9.7 Hz, C5).

^{19}F NMR (376 MHz, CDCl$_3$) δ −115.90 (tt, *J* = 9.0, 5.3 Hz, 1F, C9-F), −189.26 (ddt, *J* = 53.1, 35.6, 18.2 Hz, 1F, C3-F).

$[\alpha]_D^{24}$ = +52° (c 1.00 in $CHCl_3$).

HRMS (+EI) calcd. $[M]^+$ ($C_{18}H_{19}NO_2SF_2$) 351.1099, measured 351.1095 (1.14 ppm error).

IR (neat) v_{max}/cm^{-1}: 2986, 2936, 1601, 1509, 1346, 1223, 1161, 1093, 1048, 666, 616, 552.

***cis-3-fluoro-2-methyl-5-(naphthalen-2-yl)-1-tosylpyrrolidine* (10m)**

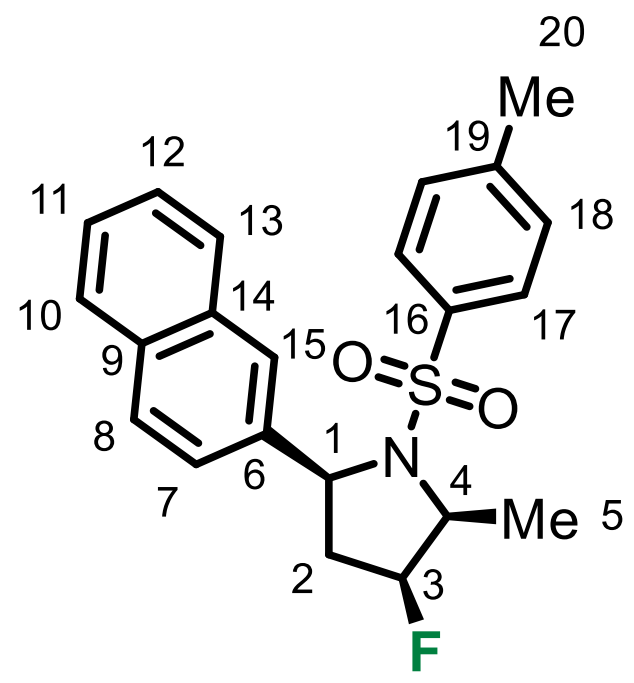

Produced according to **General procedure VI** from pyrroline **4m** as a mixture of C3 diastereomers (*d.r.* 5.6:1) and purified by flash column chromatography (2-30% EtOAc in pentane) to afford the title compound as a white solid (67.5 mg, 88%) containing both *syn* and *anti* diastereomers. The mixture was then purified again by flash column chromatography (0-30% EtOAc in pentane) over a slower gradient to afford the title compound (single diastereomer) as a white solid (53.7 mg, 70%).

^{1}H NMR (500 MHz, $CDCl_3$) Major diastereomer δ 7.83 – 7.76 (m, 4H, C8-H, C10-H, C13-H, C15-H), 7.66 (d, *J* = 8.2 Hz, 2H, C17-H), 7.50 – 7.41 (m, 3H, C7-H, C11-H, C12-H), 7.23 (d, *J* = 8.2 Hz, 2H, C18-H), 5.12 (dd, *J* = 8.8, 4.0 Hz, 1H, C1-H), 4.88 (dtd, *J* = 53.2, 5.1, 3.6 Hz, 1H, C3-H), 4.09 – 3.98 (dqd, *J* = 18.3, 6.6, 5.1 Hz, 1H, C4-H), 2.37 (s, 3H, C20-H), 2.38 – 2.19 (m, 2H, C2-H), 1.58 (dd, *J* = 6.6, 3.1 Hz, 3H, C5-H). **Minor diastereomer** δ 7.83 – 7.76 (m, 3H, C8-H, C10-H, C13-H), 7.74 (d, *J* = 1.7 Hz, 1H, C15-H), 7.68 – 7.63 (m, 2H, C17-H), 7.49 – 7.41 (m, 3H, C7-H, C11-H, C12-H), 7.25 – 7.21 (m, 2H, C18-H), 4.95 – 4.81 (m, 1H, C1-H), 4.75 (dd, *J* = 51.6, 3.0 Hz, 1H, C3-H), 4.30 (dqd, *J* = 23.8, 7.0, 1.2 Hz, 1H, C4-H), 2.38 (s, 3H, C20-H), 2.21 – 2.02 (m, 2H, C2-H), 1.53 (d, *J* = 7.0 Hz, 3H, C5-H).

^{13}C NMR (126 MHz, $CDCl_3$) Major diastereomer δ 143.9 (C19), 139.5 (C6), 135.3 (C16), 133.3 (C14), 132.8 (C9), 129.8 (C18), 128.3 (C13), 128.2 (C10), 127.7 (C17), 127.7 (C8), 126.2 (C12), 125.9 (C11), 125.4 (d, *J* = 1.1 Hz, C15), 124.5 (d, *J* = 2.0 Hz, C7), 93.5 (d, *J* = 187.3 Hz, C3), 62.7 (d, *J* = 1.9 Hz, C1), 60.6 (d, *J* = 21.3 Hz, C4), 39.6 (d, *J* = 19.9 Hz, C2), 21.6 (C20), 16.1 (d, *J* = 9.7 Hz, C5). **Minor diastereomer** δ 143.7 (C19), 138.9 (C6), 134.5 (C16), 133.4 (C14), 133.1 (C9), 129.5 (C18), 128.7 (C13), 128.1 (C10), 128.0 (C17), 127.9 (C8), 126.3 (C12), 126.0 (C11), 125.5 (C15), 124.2 (C7), 96.0 (d, *J* = 179.9 Hz, C3), 63.9 (d, *J* = 22.1 Hz, C4), 63.6 (C1), 41.8 (d, *J* = 20.8 Hz, C2), 21.7 (C20), 21.0 (d, *J* = 10.8 Hz, C5).

^{19}F NMR (471 MHz, $CDCl_3$) Major diastereomer δ −189.53 (ddddq, *J* = 53.2, 30.1, 18.3, 18.3, 3.1 Hz 1F, C3-F). **Minor diastereomer** δ −173.48 (dddd, *J* = 51.6, 43.4, 23.8, 16.3 Hz).

HRMS (+ESI) calcd: $[M+H]^+$ ($C_{22}H_{22}FNO_2S$) 384.1428, measured 384.1410 (4.69 ppm error).

IR (neat) $\tilde{\nu}_{max}$/cm^{-1}: 3056, 2984, 2933, 1598, 1508, 1344, 1161, 1093, 1049, 948, 909, 817, 735, 668, 613, 551.

R_f 0.14 (10% EtOAc in pentane).

***2-benzyl-4-fluoro-1-tosylpyrrolidine* (10t)**

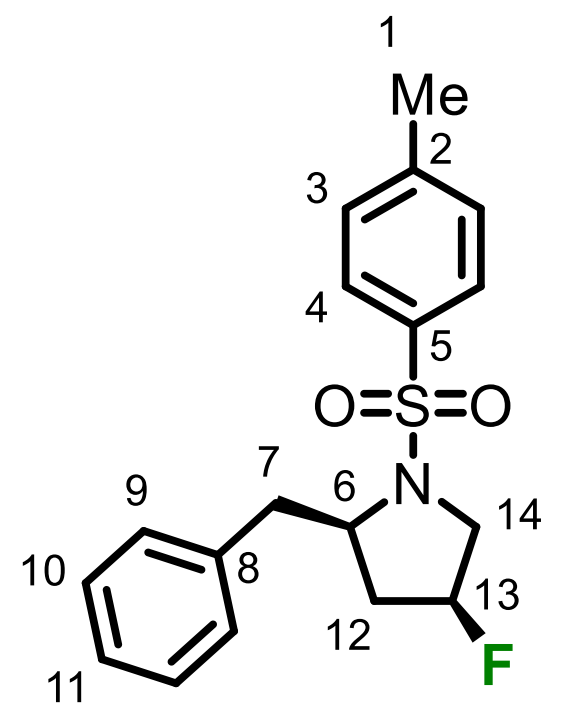

Prepared from pyrroline **4t** according to **General procedure VI** and purified by flash column chromatography (0 to 50% Et_2O in pentane) to afford the title compound as a colourless oil (23.0 mg, 69%).

^{1}H NMR (500 MHz, $CDCl_3$) δ 7.75 (d, J = 8.0 Hz, 2H, C4-H), 7.38 – 7.28 (m, 4H, C3-H, C9-H), 7.25 – 7.21 (m, 3H, C10-H, C11-H), 5.08 (dddd, J = 53.1, 4.4, 3.3, 1.1 Hz, 1H, C13-H), 4.04 (dddd, J = 11.2, 8.9, 4.3, 1.4 Hz, 1H, C6-H), 3.78 (dddd, J = 22.2, 12.4, 1.8, 1.0 Hz, 1H, C14-H), 3.43 (ddd, J = 33.7, 12.2, 4.2 Hz, 1H, C14-H), 3.39 – 3.32 (m, 1H, C7-H), 2.85 (ddd, J = 13.0, 11.2, 1.7 Hz, 1H, C7-H), 2.42 (s, 3H, C1-H), 2.21 – 1.90 (m, 1H, C12-H), 1.56 – 1.40 (m, 1H, C12-H).

^{13}C NMR (126 MHz, $CDCl_3$) δ 143.9 (C2), 138.4 (C8), 134.8 (C5), 130.0 (C4), 129.8 (C10), 128.7 (C9), 127.6 (C4), 126.6 (C11), 93.0 (d, J = 179.0 Hz, C13), 61.5 (C6), 55.0 (d, J = 24.4 Hz, C14), 42.7 (d, J = 2.4 Hz, C7), 35.4 (d, J = 19.9 Hz, C12), 21.7 (C1).

^{19}F NMR (376 MHz, $CDCl_3$) Major diastereomer δ −171.34 – −172.10 (m, 1F, C13-F), **Minor diastereomer** δ −177.32 (dtdd, J = 52.4, 39.8, 22.1, 18.4 Hz, 1F, C13-F).

HRMS (+ESI) calcd: $[M+Na]^+$ ($C_{18}H_{20}NO_2SF$) 356.1091, measured 356.1092 (0.28 ppm error).

IR (neat) ν_{max}/cm^{-1}: 2964, 2933, 2874, 2837, 1612, 1586, 1511, 1495, 1459, 1334, 1304, 1245, 1210, 1173, 1154, 1107, 1089, 1031, 1009, 945, 920, 886, 814, 771, 736, 698, 654, 583, 561, 547.

***cis*-4-((4-fluoro-2-propylpyrrolidin-1-yl)sulfonyl)aniline** **(10u)**

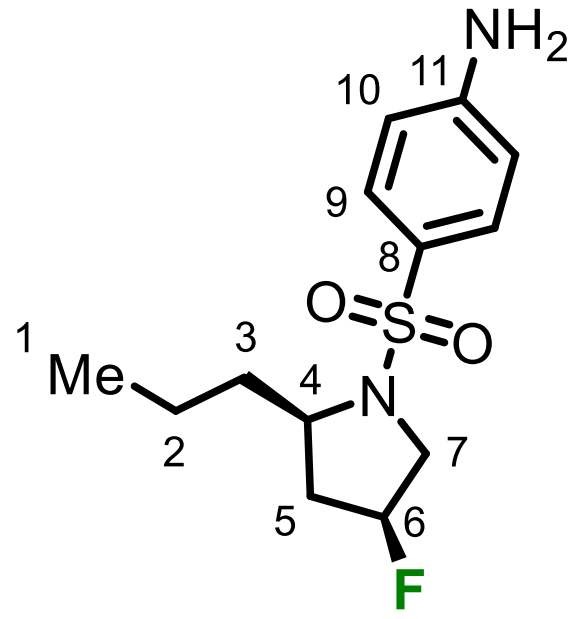

Produced according to **General procedure VI** from pyrroline **4u** (79% NMR) as a mixture of diastereomers (1:0.05) and purified by flash column chromatography (2-30% EtOAc in pentane) to afford the title compound as a white solid (41.8 mg, 73%).

^{1}H NMR (500 MHz, $CDCl_3$) δ 7.59 (app. dt, *J* = 8.6, 1.7 Hz, 2H, C9-H), 6.68 (app. dt, *J* = 8.7, 1.9 Hz, 2H, C10-H), 5.02 (dtt, *J* = 53.6, 4.9, 1.4 Hz, 1H, C6-H), 4.17 (s, 2H, C11-NH), 3.75 (tdd, *J* = 8.9, 5.6, 1.8 Hz, 1H, C4-H), 3.61 (ddt, *J* = 22.6, 12.7, 1.5 Hz, 1H, C7-H), 3.42 (ddd, *J* = 31.7, 12.8, 4.8 Hz, 1H, C7-H), 1.95 (ddq, *J* = 21.0, 14.5, 1.6 Hz, 1H, C5-H), 1.85 (ddt, *J* = 12.4, 9.7, 6.2 Hz, 1H, C3-H), 1.71 (dddd, *J* = 39.5, 14.5, 8.9, 4.9 Hz, 1H, C5-H), 1.63 – 1.52 (m, 1H, C3-H), 1.41 – 1.30 (m, 2H, C2-H), 0.93 (t, *J* = 7.3 Hz, 3H, C1-H).

^{13}C NMR (126 MHz, $CDCl_3$) δ 150.7 (C11), 129.7 (C9), 126.1 (C8), 114.3 (C10), 92.9 (d, *J* = 179.0 Hz, C6), 59.8 (C4), 54.6 (d, *J* = 24.9 Hz, C7), 38.5 (d, *J* = 1.8 Hz, C3), 36.8 (d, *J* = 20.0 Hz, C5), 19.8 (C2), 14.0 (C1).

^{19}F NMR (376 MHz, $CDCl_3$) δ −170.95 (ddddd, *J* = 53.6, 39.5, 31.7, 22.6, 21.0 Hz, 1F, C6-F).

HRMS (+ESI) calcd. $[M+H]^+$ ($C_{13}H_{19}FN_2O_2S$) 287.1224, measured 287.1212 (4.18 ppm error).

IR (neat) v_{max}/cm^{-1}: 3474, 3376, 3246, 2959, 2931, 2871, 1627, 1594, 1502, 1338, 1317, 1150, 1091, 1027, 832, 679, 601, 551.

***cis-4-fluoro-2-propyl-1-tosylpyrrolidine* (10v)**

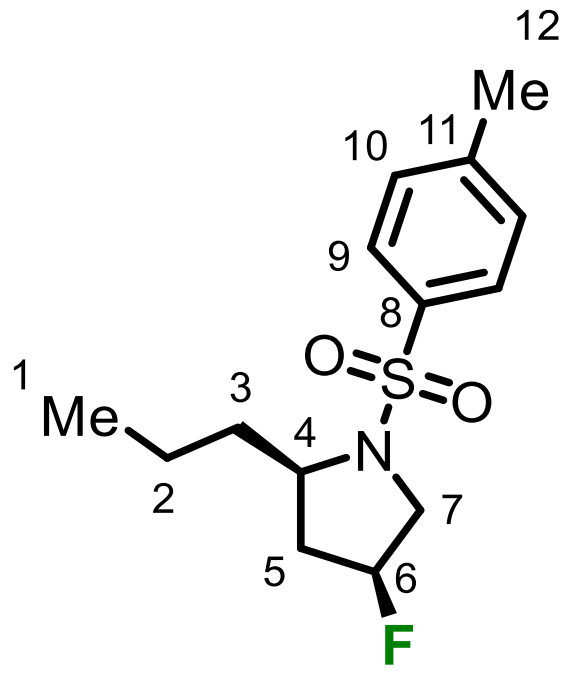

Produced according to **General procedure VI** from pyrroline **4v** as a mixture of diastereomers (1:0.18) without further purification to afford the title compound as a white solid (56.5 mg, 99%).

^{1}H NMR (500 MHz, CDCl$_3$) Major diastereomer δ 7.71 (d, J = 8.1 Hz, 2H, C9-H), 7.31 (d, J = 7.7 Hz, 2H, C10-H), 5.02 (dtt, J = 53.7, 4.9, 1.4 Hz, 1H, C6-H), 3.80 (tdd, J = 9.0, 5.5, 1.4 Hz, 1H, C4-H), 3.65 (ddt, J = 22.6, 12.7, 1.4 Hz, 1H, C7-H), 3.44 (ddd, J = 31.6, 12.7, 4.9 Hz, 1H, C7-H), 2.43 (s, 3H, C12-H), 1.97 (ddq, J = 20.6, 14.6, 1.4 Hz, 1H, C5-H), 1.91 – 1.83 (m, 1H, C3-H), 1.75 – 1.62 (m, 1H, C5-H), 1.62 – 1.53 (m, 1H, C3-H), 1.41 – 1.31 (m, 2H, C2-H), 0.94 (t, J = 7.4 Hz, 3H, C1-H). **Minor diastereomer** δ 7.71 (d, J = 7.8 Hz, 2H, C9-H), 7.30 (d, J = 8.3 Hz, 2H, C10-H), 4.98 (dt, J = 52.7, 3.7 Hz, 1H, C6-H), 3.76 (ddd, J = 23.9, 13.9, 2.3 Hz, 1H, C7-H), 3.68 – 3.62 (m, 1H, C4-H), 3.55 (ddd, J = 38.9, 13.9, 3.3 Hz, 1H, C7-H), 2.42 (s, 3H, C12-H), 2.27 – 2.15 (m, 1H, C5-H), 2.14 – 2.04 (m, 1H, C5-H), 1.64 – 1.50 (m, 2H, C3-H), 1.40 – 1.31 (m, 2H, C2-H), 0.95 (t, J = 7.4 Hz, C1-H).

^{13}C NMR (126 MHz, CDCl$_3$) Major diastereomer δ 143.8 (C11), 134.9 (C8), 129.9 (C10), 127.6 (C9), 92.8 (d, J = 179.3 Hz, C6), 60.0 (C4), 54.7 (d, J = 25.1 Hz, C7), 38.4 (d, J = 2.0 Hz, C3), 36.8 (d, J = 20.0 Hz, C5), 21.7 (C12), 19.8 (C2), 14.0 (C1). **Minor diastereomer** δ 143.6 (C11), 134.6 (C8), 129.6 (C10), 127.9 (C9), 91.5 (d, J = 178.7 Hz, C6), 59.1 (C4), 55.6 (d, J = 22.4 Hz, C7), 39.0 (d, J = 21.3 Hz, C5), 38.3 (C3), 21.7 (C12), 18.8 (C2), 14.2 (C1).

^{19}F NMR (376 MHz, CDCl$_3$) Major diastereomer δ −170.67 – −171.22 (m, 1F, C6-F). **Minor diastereomer** δ −176.43 – −177.02 (m, 1F, C6-F).

HRMS (+ESI) calcd. $[M+H]^+$ ($C_{14}H_{20}FNO_2S$) 286.1272, measured 286.1269 (1.05 ppm error).

IR (neat) v_{max}/cm^{-1}: 2959, 2932, 2372, 1598, 1457, 1432, 1343, 1159, 1091, 1029, 815, 665, 600, 550.

***cis*-4-fluoro-2-propylpyrrolidin-1-yl)(phenyl)methanone (10w)**

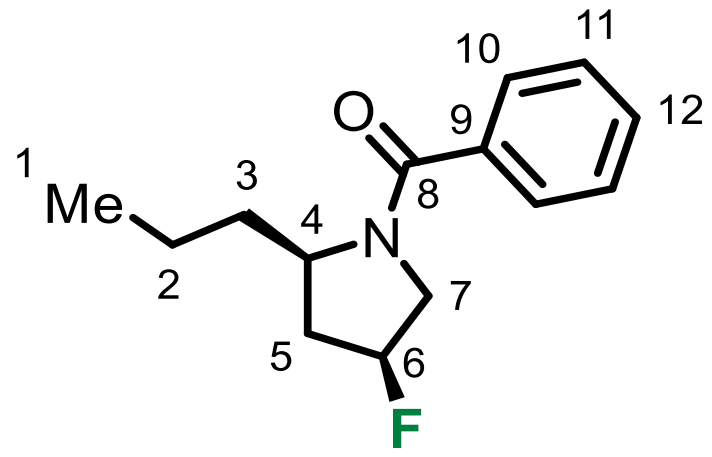

Produced according to **General procedure VI** from pyrroline **21v** and purified by flash column chromatography (0-20% EtOAc in pentane) to afford the title compound (single diastereomer) as a white solid (30.1 mg, 64%).

NMR shows a mixture of amide rotamers **(1:0.50)** at RT, which coalesce when heated to 100°C in DMSO-*D_6*.

Significant line-broadening in RT spectra due to slow amide rotation.

^{1}H NMR (500 MHz, $CDCl_3$) Major rotamer δ 7.49 – 7.42 (m, 2H, C10-H), 7.42 – 7.35 (m, 3H, C11-H, C12-H), 5.16 (d, *J* = 53.9 Hz, 1H, C6-H), 4.51 (s, 1H, C4-H), 3.98 – 3.50 (m, 2H, C7-H), 2.25 – 2.06 (m, 2H, C5-H), 1.66 – 1.52 (m, 2H, C3-H), 1.41 (s, 2H, C2-H), 0.97 (s, 3H, C3-H). **Minor rotamer** δ 7.49 – 7.42 (m, 2H, C10-H), 7.42 – 7.35 (m, 3H, C11-H, C12-H), 5.30 (d, *J* = 53.2 Hz, 1H, C6-H), 4.04 (s, 1H, C4-H), 3.98 – 3.50 (m, 2H, C7-H), 2.25 – 2.06 (m, 2H, C5-H), 1.99 (s, 1H, C3-H), 1.79 (s, 1H, C3-H), 1.29 – 1.20 (m, 2H, C2-H), 0.63 (s, 3H, C1-H).

^{13}C NMR (126 MHz, $CDCl_3$) Major rotamer δ 170.2 (C8), 137.1 (C9), 130.0 (C12), 128.5 (C11), 127.1 (C10), 92.9 (d, *J* = 178.2 Hz, C6), 56.5 (C4), 55.6 (d, *J* = 25.8 Hz, C7), 36.2 (C3), 35.8 (d, *J* = 18.9 Hz, C5), 19.8 (C2), 14.1 (C1). **Minor rotamer** δ 170.2 (C8), 137.1 (C9), 129.8 (C12), 128.5 (C11), 126.7 (C10), 92.3 (d, *J* = 174.6 Hz, C6), 58.5 (C4), 52.8 (d, *J* = 24.8 Hz, C7), 36.7 (C3), 35.8 (d, *J* = 18.9 Hz, C5), 19.5 (C2), 13.5 (C1).

^{19}F NMR (376 MHz, $CDCl_3$) Major rotamer δ −170.46 (dp, *J* = 53.9, 27.1 Hz, 1F, C6-F). **Minor rotamer** δ −167.25 (dp, *J* = 53.2, 32.7 Hz, 1F, C6-F),

[100°C] ^{1}H NMR (500 MHz, DMSO-*D_6*) δ 7.46 – 7.42 (m, 5H, C10-H, C11-H, C12-H), 5.28 (dt, *J* = 54.4, 4.9 Hz, 1H, C6-H), 4.21 (s, 1H, C4-H), 3.76 (dd, *J* = 29.3, 13.3 Hz, 1H, C7-H), 3.65 (dd, *J* = 25.7, 13.4 Hz, 1H, C7-H), 2.26 (dddd, *J* = 38.6, 14.3, 8.5, 5.1 Hz, 1H, C5-H), 2.02 (ddd, *J* = 21.5, 14.3, 1.9 Hz, 1H, C5-H), 1.67 (s, 1H, C3-H), 1.57 – 1.47 (m, 1H, C3-H), 1.38 – 1.12 (m, 2H, C2-H), 0.81 (t, *J* = 7.7 Hz, 3H, C1-H).

[100°C] ^{19}F NMR (470 MHz, DMSO-*D_6*) δ −169.47 (1F, C6-F).

HRMS (+ESI) calcd. $[M+H]^+$ ($C_{14}H_{20}FNO$) 236.1445, measured 236.1439 (2.54 ppm error).

IR (neat) v_{max}/cm^{-1}: 3062, 2960, 2932, 2872, 1697, 1601, 1575, 1449, 1418, 1362, 1256, 1215, 1175, 713, 702.

8.10. Oxolanes (11)

***3-fluoro-8-tosyl-1-oxa-8-azaspiro[4.5]decane* (11ap)**

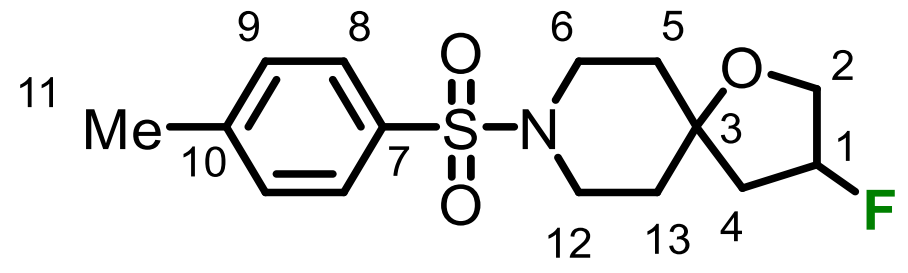

Prepared from oxolene **6ap** according to **General procedure VI** and purified by flash column chromatography (0 to 40% Et_2O in pentane) to afford the title compound as a white solid (21 mg, 67%).

^{1}H NMR (500 MHz, $CDCl_3$) δ 7.64 (d, J = 8.0 Hz, 2H, C8-H), 7.31 (d, J = 8.0 Hz, 2H, C9-H), 5.20 (dm, J = 54.7 Hz, 1H, C1-H), 4.00 (dddd, J = 23.5, 11.4, 1.7, 0.9 Hz, 1H, C2-H), 3.84 (ddd, J = 36.0, 11.4, 3.6 Hz, 1H, C2-H), 3.49 (dt, J = 11.6, 4.2 Hz, 2H, C6-H, C12-H), 2.71 (tt, J = 11.5, 2.4 Hz, 2H, C6-H, C12-H) 2.43 (s, 3H, C11-H), 2.08 (dddd, J = 25.1, 14.7, 1.7, 0.9 Hz, 1H, C4-H), 1.95 (dt, J = 13.8, 3.1 Hz, 1H, C5-H, C13-H), 1.93 – 1.82 (m, 1H, C4-H), 1.78 (ddd, J = 13.6, 11.6, 4.6 Hz, 2H, C5-H, C13-H), 1.67 – 1.59 (m, 1H, C5-H, C13-H).

^{13}C NMR (126 MHz, $CDCl_3$) δ 143.6 (C10), 133.4 (C7), 129.8 (C9), 127.8 (C8), 94.5 (d, J = 178.0 Hz, C1-H), 79.4 (C3), 72.5 (d, J = 23.5 Hz, C2), 44.7 (d, J = 20.5 Hz, C4), 43.5 (C6), 43.4 (C12), 36.1 (d, J = 1.6 Hz, C5), 35.8 (d, J = 1.2 Hz, C13), 21.7 (C11).

^{19}F NMR (376 MHz, $CDCl_3$) δ −171.33 (dtt, J = 54.7, 36.6, 24.3 Hz, 1F, C1-F).

HRMS (+EI) calcd: $[M]^+$ ($C_{15}H_{20}NO_3SF$) 313.1142, measured 313.1141 (0.32 ppm error).

IR (neat) ν_{max}/cm^{-1}: 2925, 2856, 1741, 1598, 1492, 1462, 1348, 1244, 1163, 1091, 1029, 1014, 962, 817, 765, 709, 668.

8.11. Azetidines (12)

***3-fluoro-1-tosylazetidine* (12ar)**

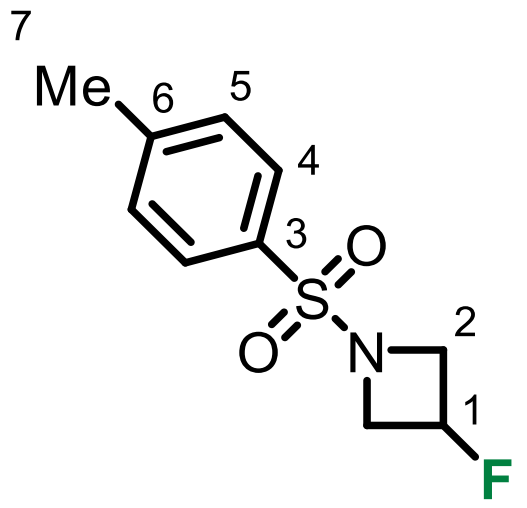

Prepared from azetine **7ar** according to **General procedure VI** and purified by flash column chromatography (0 to 40% Et_2O in pentane) to afford the title compound as a white solid (19 mg, 87%).

^{1}H NMR (500 MHz, $CDCl_3$) δ 7.74 (d, *J* = 8.1 Hz, 2H, C4-H), 7.39 (d, *J* = 8.1 Hz, 2H, C5-H), 5.10 (dtt, *J* = 56.6, 6.1, 4.4 Hz, 1H, C1-H), 4.07 (dddd, *J* = 16.8, 8.9, 6.1, 1.6 Hz, 2H, C2-H), 3.82 (dddd, *J* = 23.4, 9.0, 4.4, 1.6 Hz, 2H, C2-H), 2.47 (s, 3H, C7-H).

^{13}C NMR (126 MHz, $CDCl_3$) δ 144.7 (C6), 131.3 (C3), 130.1 (C5), 128.5 (C4), 80.1 (d, *J* = 208.2 Hz, C1), 58.2 (d, *J* = 25.2 Hz, C2), 21.8 (C7).

^{19}F NMR (376 MHz, $CDCl_3$) δ −179.62 (dtt, *J* = 57.0, 23.4, 16.9 Hz, 1F, C1-F).

HRMS (+EI) calcd: $[M]^+$ ($C_{10}H_{12}O_2NSF$) 229.0567, measured 229.0568 (0.44 ppm error).

IR (neat) ν_{max}/cm^{-1}: 2937, 1597, 1449, 1345, 1161, 1134, 1089, 1006, 893, 817, 751, 709, 667, 559, 550, 504, 447.

***3-fluoro-1-tosyl-1-azaspiro[3.5]nonane* (12av)**

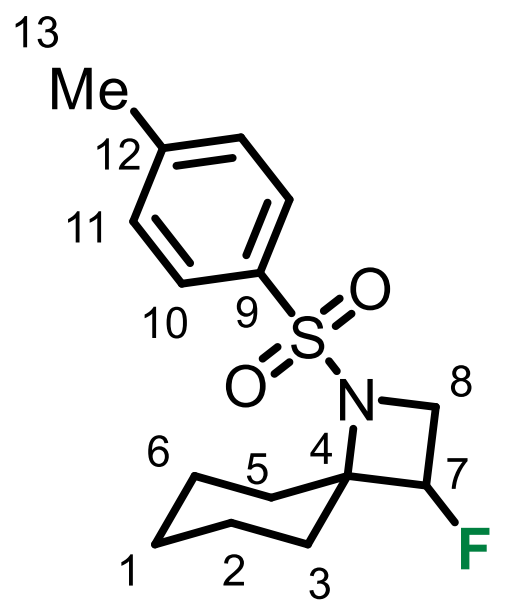

Azetine **7av** was synthesised according to **General procedure IV** from **3av** (0.2 mmol). After 10 minutes, the reaction mixture was concentrated *in vacuo* and redissolved in dry MeOH (1 mL), and hydrogenated according to **General procedure VI** to afford the crude product (35% NMR). The crude product was purified by flash column chromatography (2-10% EtOAc in pentane) to afford the title compound as a white solid (18 mg, 30%).

Product could not be fully isolated; 2 samples were obtained, as a mixture with 30 mol% 4-methylanisole and with 54 mol% of linear product **12au'**. Proton spectra are included for both samples.

^{1}H NMR (500 MHz, $CDCl_3$) δ 7.71 (app. dt, J = 8.4, 1.8 Hz, 2H, C10-H), 7.30 (d, J = 8.4 Hz, 2H, C11-H), 4.84 (ddd, J = 56.7, 6.2, 3.6 Hz, 1H, C7-H), 4.03 (ddd, J = 18.8, 9.6, 6.2 Hz, 1H, C8-H), 3.75 (ddd, J = 24.6, 9.6, 3.6 Hz, 1H, C8-H), 2.43 (s, 3H, C13-H), 1.92 – 1.66 (m, 5H, C1-H, C2-H, C3-H, C5-H, C6-H, equatorial), 1.41 – 1.12 (m, 5H, C1-H, C2-H, C3-H, C5-H, C6-H, axial).

^{13}C NMR (126 MHz, $CDCl_3$) δ 143.5 (C12), 136.9 (C9), 129.6 (C11), 127.6 (C10), 88.3 (d, J = 213.0 Hz, C7), 78.8 (d, J = 21.3 Hz, C4), 53.3 (d, J = 27.1 Hz, C8), 35.1 (d, J = 2.9 Hz, C3), 30.0 (d, J = 7.5 Hz, C5), 24.5 (C6), 23.8 (d, J = 2.1 Hz, C1), 22.8 (C2), 21.6 (C13).

^{19}F NMR (376 MHz, $CDCl_3$) δ −191.01 (ddd, J = 56.7, 24.6, 18.8 Hz, 1F, C5-F).

HRMS (+EI) calcd: $[M]^+$ ($C_{15}H_{20}NO_2SF$) 297.1193, measured 297.1188 (1.68 ppm error).

IR (neat) v_{max}/cm^{-1}: 3290, 2935, 2860, 1738, 1598, 1453, 1332, 1156, 1092, 994, 815, 716, 664, 594, 548.

(ring-opened) (E)-N-(2-cyclohexyl-2-fluorovinyl)-4-methylbenzenesulfonamide **(12av')**

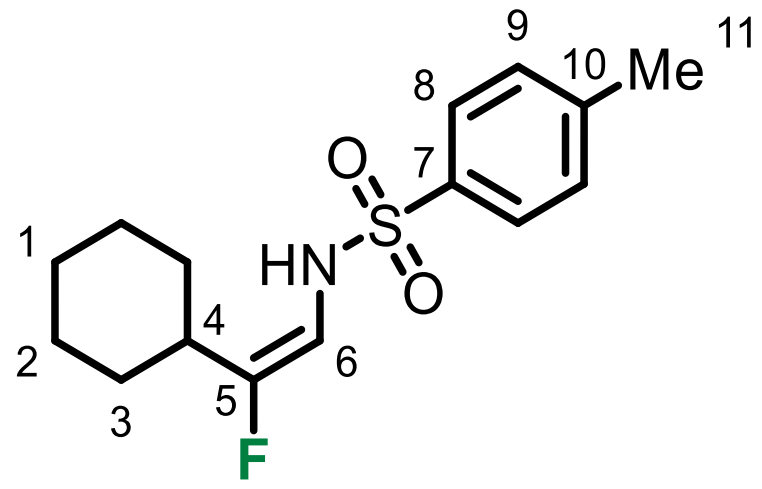

Formed as a side-product in the synthesis of **12av** (50% NMR) from hydrogenation of electrocyclic ring-opening product **7av'**. The crude product was purified by flash column chromatography (2-10% EtOAc in pentane) to afford the title compound as a white solid (26 mg, 44%).

^{1}H NMR (500 MHz, CDCl$_3$) δ 7.72 (app. dt, *J* = 8.3, 2.4 Hz, 2H, C8-H), 7.31 (d, *J* = 8.3 Hz, 2H, C9-H), 6.05 (d, *J* = 10.7 Hz, 1H, C6-NH), 5.55 (dd, *J* = 26.6, 10.7 Hz, 1H, C6-H), 2.43 (s, 3H, C11-H), 2.04 (dt, *J* = 14.6, 8.3 Hz, 1H, C4-H), 1.76 – 1.70 (m, 4H, C2-H, C3-H), 1.68 – 1.62 (m, 1H, C1-H) 1.30 – 1.03 (m, 5H, C1-H, C2-H, C3-H).

^{13}C NMR (126 MHz, CDCl$_3$) δ 154.9 (d, *J* = 251.3 Hz, C5), 144.0 (C10), 136.7 (C7), 129.9 (C9), 127.0 (C8), 102.1 (d, *J* = 12.8 Hz, C6), 38.7 (d, *J* = 21.9 Hz, C4), 29.5 (d, *J* = 2.3 Hz, C3), 25.9 (C1), 25.7 (C2), 21.7 (C11).

^{19}F NMR (376 MHz, CDCl$_3$) δ −126.77 (dd, *J* = 26.6, 14.6 Hz, 1F, C5-F).

HRMS (+EI) calcd: $[M]^+$ ($C_{15}H_{20}NO_2SF$) 297.1193, measured 297.1189 (1.35 ppm error).

IR (neat) v_{max}/cm^{-1}: 3265, 2932, 2858, 1728, 1450, 1330, 1160, 1094, 901, 813, 666, 556, 540.

***3-fluoro-2,2-dimethyl-1-tosylazetidine* (12au)**

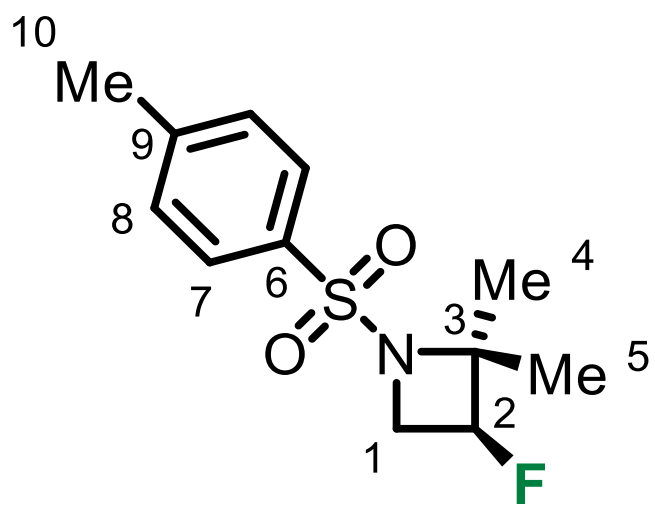

Azetine **7au** was synthesised according to **General procedure IV** from **3au** (0.3 mmol). After 10 minutes, the reaction mixture was concentrated *in vacuo* and redissolved in dry MeOH (6 mL), and hydrogenated according to **General procedure VI** to afford the crude product (81% NMR). The crude product was purified by flash column chromatography (2-20% EtOAc in pentane) to afford the title compound as a white solid (51.7 mg, 67%).

^{1}H NMR (500 MHz, $CDCl_3$) δ 7.70 (app. dt, *J* = 8.4, 1.9 Hz, 2H, C7-H), 7.30 (dq, *J* = 8.3, 2.1 Hz, 2H, C8-H), 4.76 (ddd, *J* = 57.3, 6.3, 4.4 Hz, 1H, C2-H), 3.96 (ddd, *J* = 15.4, 8.9, 6.3 Hz, 1H, C1-H), 3.75 (ddd, *J* = 22.1, 8.9, 4.4 Hz, 1H, C1-H), 2.42 (s, 3H, C10-H), 1.51 (d, *J* = 0.7 Hz, 3H, C4-H), 1.41 (d, *J* = 3.4 Hz, 3H, C5-H).

^{13}C NMR (126 MHz, $CDCl_3$) δ 143.7 (C9), 136.6 (C6), 129.7 (C8), 127.7 (C7), 88.1 (d, *J* = 214.4 Hz, C2), 75.8 (d, *J* = 22.8 Hz, C3), 53.5 (d, *J* = 26.5 Hz, C1), 26.5 (d, *J* = 2.4 Hz, C4), 21.6, 19.4 (d, *J* = 10.5 Hz, C5).

^{19}F NMR (376 MHz, $CDCl_3$) δ −190.65 (dddq, *J* = 57.3, 22.1, 15.4, 3.4 Hz).

HRMS (+EI) calcd. $[M]^+$ ($C_{12}H_{16}NO_2SF$) 257.0880, measured 257.0877 (1.17 ppm error).

IR (neat) v_{max}/cm^{-1}: 3289, 2978, 2934, 2877, 1739, 1598, 1460, 1337, 1304, 1154, 1122, 1088, 815, 709, 687, 663, 654, 609, 550.

***cis-3-fluoro-2-phenyl-1-tosylazetidine* (12aw)**

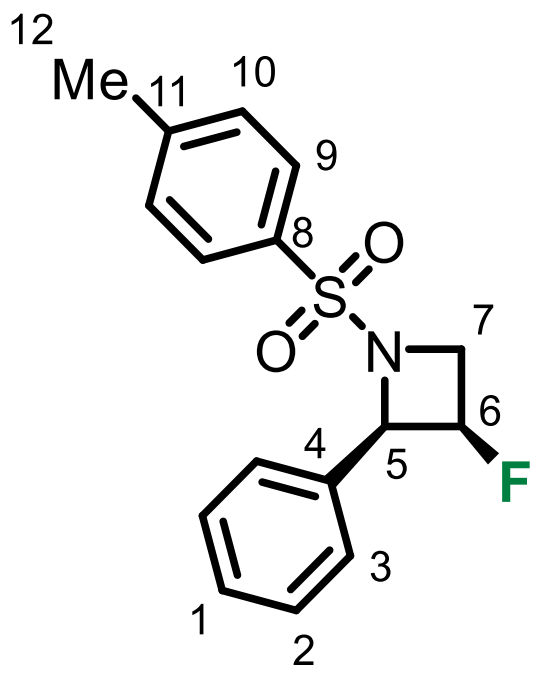

Prepared according to **General procedure VI** from azetine **7aw** to afford the crude product (76% NMR). The crude product was purified by flash column chromatography (2-10% EtOAc in pentane) and recrystallised from DCM/pentane to afford the title compound as a white solid (18.6 mg, 61%).

^{1}H NMR (500 MHz, $CDCl_3$) δ 7.71 (app. dt, *J* = 8.3, 1.5 Hz, 2H, C9-H), 7.35 – 7.21 (m, 5H, C1-H, C2-H, C3-H), 7.15 (d, *J* = 8.3 Hz, 2H, C10-H), 4.71 (ddd, *J* = 48.3, 7.0, 3.1 Hz, 1H, C6-H), 4.70 (d, *J* = 6.6 Hz, 1H, C5-H), 3.23 (dddd, *J* = 26.7, 13.9, 3.1 Hz, 1H, C7-H), 3.08 (ddd, *J* = 18.9, 13.9, 7.0 Hz, 1H, C7-H), 2.44 (s, 3H, C12-H).

^{13}C NMR (126 MHz, $CDCl_3$) δ 143.9 (C9), 136.8 (C8), 135.7 (d, *J* = 5.7 Hz, C4), 130.0 (C3), 129.4 (C10), 128.8 (C2), 127.2 (C9), 127.2 (C1), 92.8 (d, *J* = 173.7 Hz, C6), 46.3 (d, *J* = 21.1 Hz, C5), 38.7 (d, *J* = 20.9 Hz, C7), 21.7 (C12).

^{19}F NMR (376 MHz, $CDCl_3$) δ −184.30 (dddd, *J* = 48.3, 26.7, 18.9, 6.6 Hz, 1F, C6-F).

HRMS (+ESI) calcd: $[M+Na]^+$ ($C_{16}H_{26}NO_2SF$) 328.0778, measured 328.0770 (2.44 ppm error).

IR (neat) v_{max}/cm^{-1}: 3284, 3029, 2929, 1740, 1598, 1496, 1454, 1327, 1160, 1093, 814, 747, 701, 665, 550.

8.12. Dibromides (13-14)

***2-benzyl-3,4-dibromo-4-fluoro-1-tosylpyrrolidine* (13t)**

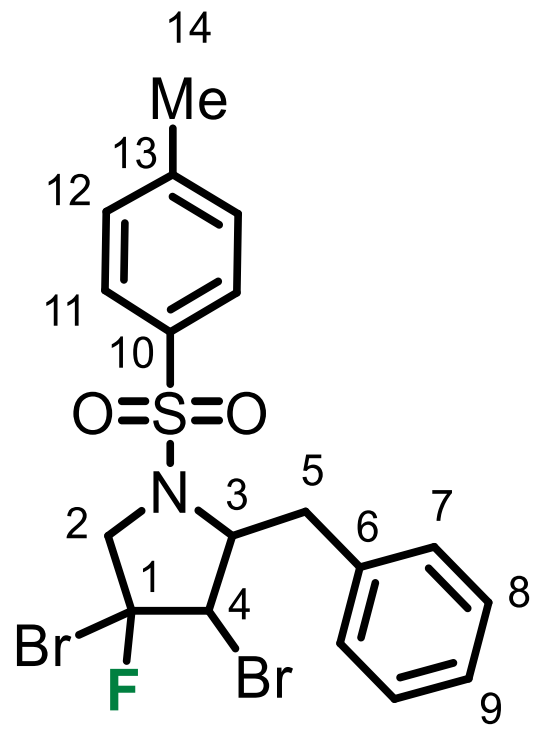

To a vial equipped with a stirrer bar, was added pyrroline **4t** (0.1 mmol, 1 equiv.) and $TBABr_3$ (2 mmol, 20 equivalents) and DMF (1 mL). The mixture was stirred at 70 °C for 24 hours, cooled to RT, concentrated *in vacuo* and purified by flash-column chromatography (0 – 50% Et_2O in pentane) to afford the title compound as a white solid (29 mg, 59%).

^{1}H NMR (500 MHz, $CDCl_3$) δ 7.77 – 7.71 (m, 2H, C11-H), 7.41 – 7.27 (m, 7H, C12-H, C7-H, C8-H, C9-H), 4.14 (dd, *J* = 15.4, 13.8 Hz, 1H, C2-H), 3.90 – 3.79 (m, 2H, C3-H, C4-H), 3.72 – 3.64 (m, 1H, C5-H), 3.45 (dd, *J* = 32.8, 13.8 Hz, 1H, C2-H), 3.05 (dd, *J* = 14.4, 2.3 Hz, 1H, C5-H), 2.45 (s, 3H, C14-H).

^{13}C NMR (126 MHz, $CDCl_3$) δ 144.8 (C13), 134.6 (C6), 132.9 (C10), 130.7 (C7), 130.1 (C12), 128.9 (C9), 128.0 (C11), 127.6 (C8), 99.7 (d, *J* = 264.8 Hz, C1), 65.8 (d, *J* = 0.9 Hz, C3), 60.0 (d, *J* = 22.9 Hz, C2), 54.8 (d, *J* = 19.9 Hz, C4), 36.3 (C5), 21.8 (C14).

^{19}F NMR (377 MHz, $CDCl_3$) δ −113.86 – −114.18 (1F, m, C1-F).

HRMS (+Nano) calcd: $[M+Na]^+$ ($C_{18}H_{18}NO_2SBr_2FNa$) 511.9301, measured 511.9307 (1.17 ppm error).

IR (neat) ν_{max}/cm^{-1}: 3066, 3027, 2926, 2178, 1621, 1598, 1495, 1455, 1401, 1352, 1306, 1267, 1245, 1217, 1168, 1157, 1089, 1025, 950, 879, 839, 814, 758, 717, 702, 660.

2,3-dibromo-3-fluoro-1-tosylazetidine **(14ar)**

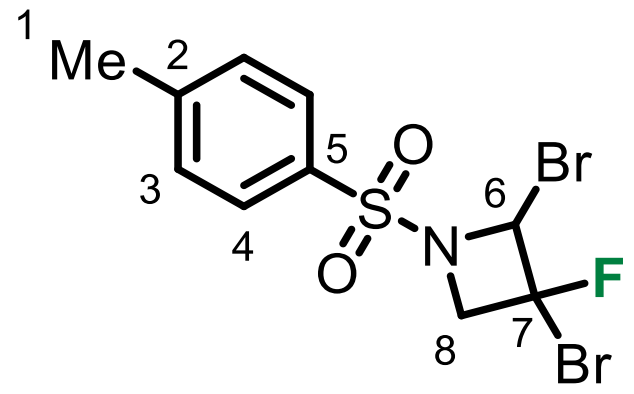

To a vial equipped with a stirrer bar, was added azetine **7ar** (0.1 mmol, 1 equiv.) and $TBABr_3$ (2 mmol, 20 equivalents) and DMF (1 mL). The mixture was stirred at 70 °C for 24 hours, cooled to RT, concentrated *in vacuo* and purified by flash-column chromatography (0 – 50% Et_2O in pentane) to afford the title compound as a colourless oil (22 mg, 57%).

Isolated as a mixture of diastereomers (2.6:1).

^{1}H NMR (500 MHz, $CDCl_3$) Major diastereomer δ 7.81 – 7.75 (m, 2H, C4-H), 7.40 – 7.36 (m, 2H, C3-H), 6.41 (dd, J = 5.9, 0.6 Hz, 1H, C6-H), 4.60 – 4.49 (m, 2H, C8-H), 2.46 (d, J = 1.8 Hz, 3H, C1-H). **Minor diastereomer** δ 7.81 – 7.75 (m, 2H, C4-H), 7.40 – 7.36 (m, 2H, C3-H), 6.21 (dd, J = 16.5, 1.1 Hz, 1H, C6-H), 4.60 – 4.49 (m, 1H, C8-H) 4.33 (ddd, J = 12.9, 9.4, 1.0 Hz, 2H, C8-H), 2.47 (d, J = 1.8 Hz, 3H, C1-H).

^{13}C NMR (126 MHz, $CDCl_3$) Major diastereomer δ 145.4 (C5), 133.5 (C5), 130.1 (C3), 128.0 (C4), 87.8 (d, J = 298.0 Hz, C7), 75.3 (d, J = 24.6 Hz, C6), 66.4 (d, J = 27.9 Hz, C8), 21.74 (C1). **Minor diastereomer** δ 145.6 (C5), 133.0 (C5), 128.2 (C4), 97.1 (d, J = 290.2 Hz, C7), 72.8 (d, J = 34.3 Hz, C6), 65.0 (d, J = 24.9 Hz, C8), 21.76 (C1).

^{19}F NMR (471 MHz, $CDCl_3$): Major diastereomer δ −104.96 (td, J = 12.1, 5.8 Hz, 1F, C7-F). **Minor diastereomer** δ −96.04 (td, J = 16.7, 12.9 Hz, 1F, C7-F).

HRMS (+Nano) calcd: $[M+Na]^+$ ($C_{10}H_{10}NO_2NaSBr_2F$) 407.8681, measured 407.8677 (1.0 ppm error).

IR (neat) ν_{max}/cm^{-1}: 3276, 1597, 1495, 1445, 1332, 1037, 1186, 1160, 1122, 1090, 921, 814, 705, 668, 555, 523.

8.13. Cycloadditions (15)

***5-fluoro-4-phenyl-7-tosyl-2-oxa-3,7-diazabicyclo[3.2.0]hept-3-ene* (15ar)**

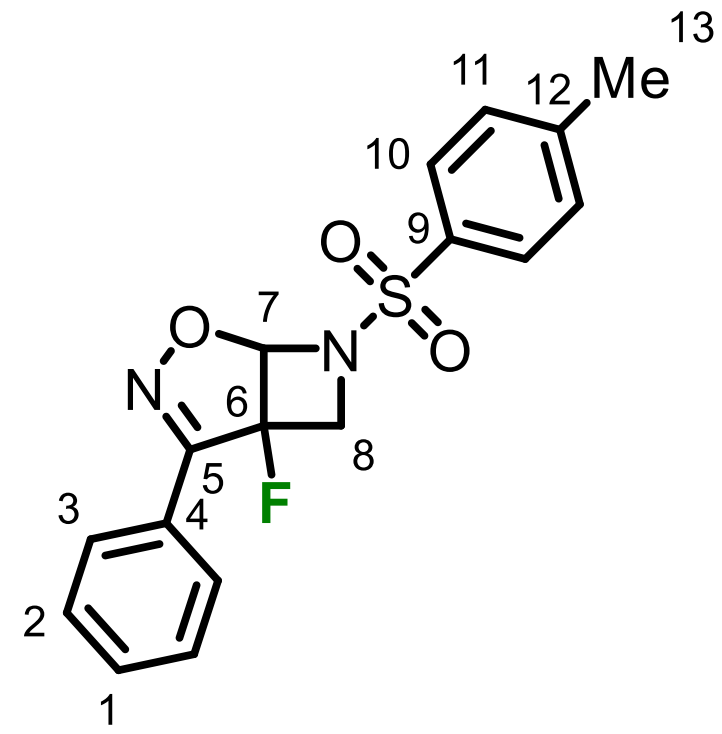

A stock solution of the *N*-hydroxyimidoyl chloride was prepared as follows. To a glass vial equipped with a stirrer bar was added benzaldehyde oxime (1 mmol) and DMF (1 mL). The solution was cooled to 0°C , and with NCS (1 mmol) was added. The mixture was stirred for 6 hours. A vial containing a stirrer bar and azetine **7ar** (0.05 mmol, 1 equiv.) in *t*-amyl alcohol (0.9 mL) was added a 100 uL aliquot of the *N*-hydroxyimidoyl chloride stock solution (0.1 mmol, 2 equiv.). While heating the fluoroalkene solution to 70°C, a separate solution of triethylamine (0.1 mmol, 2 equiv.) in *t*-amyl alcohol (1 mL) was added to the azetine solution by syringe pump over an hour. After stirring at this temperature for 18 hours in total, the reaction mixture was concentrated *in vacuo* and subjected to flash-column chromatography (2-20% EtOAc in pentane) to afford the title compound as a colourless oil (4.2 mg, 12%).

30% NMR yield. Product contains 12% **7ar** starting material.

^{1}H NMR (500 MHz, CDCl$_3$) δ 7.77 (d, *J* = 8.1 Hz, 2H, C10-H), 7.72 – 7.68 (m, 2H, C3-H), 7.52 – 7.46 (m, 1H, C1-H), 7.46 – 7.40 (m, 2H, C2-H), 7.38 – 7.31 (m, 2H, C11-H), 6.47 (d, *J* = 9.9 Hz, 1H, C7-H), 4.43 (dd, *J* = 12.1, 8.2 Hz, 1H, C8-H), 4.24 – 4.17 (m, 1H, C8-H), 2.44 (s, 3H, C13-H).

^{13}C NMR (126 MHz, CDCl$_3$) δ 156.0 (d, *J* = 18.9 Hz, C5), 144.9 (C12), 134.6 (C9), 131.6 (C1), 130.0 (C11), 129.3 (C2), 127.6 (C10), 127.1 (d, *J* = 1.2 Hz, C3), 125.1 (d, *J* = 1.5 Hz, C1), 98.6 (d, *J* = 245.5 Hz, C6), 96.4 (d, *J* = 24.0 Hz, C7), 55.3 (d, *J* = 31.0 Hz, C8), 21.7 (C13).

^{19}F NMR (376 MHz, CDCl$_3$) δ −158.61 (dddt, *J* = 11.4, 9.2, 7.4, 1.6 Hz, 1F, C6-F).

HRMS (+ESI) calcd: $[M+Na]^+$ ($C_{17}H_{15}FN_2O_3S$) 369.0680, measured: 369.0694 (3.79 ppm error).

IR (neat) ν_{max}/cm^{-1}: 2925, 2856, 1707, 1598, 1443, 1353, 1308, 1242, 1092, 1018, 842, 815, 765, 692.

8.14. Hydrations (16)

N-(2-fluoro-3-oxo-3-phenylpropyl)-4-methylbenzenesulfonamide **(16aw)**

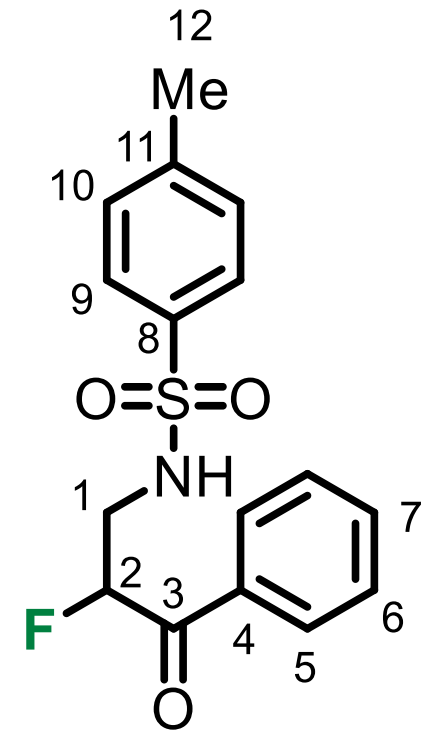

H_2O (0.2 mmol, 2 equiv.) was added to a solution of azetine **7aw** (0.1 mmol, 1 equiv.) in $CDCl_3$ (1 mL) and heated to 50 °C for 2 h. The reaction mixture was concentrated *in vacuo* to afford the title compound as a yellow oil (31.8 mg, 99%).

^{1}H NMR (500 MHz, $CDCl_3$) δ 7.92 (d, *J* = 8.1 Hz, 2H, C5-H), 7.75 (d, *J* = 8.4 Hz, 2H, C9-H), 7.63 (tt, *J* = 7.6, 1.3 Hz, 1H, C7-H), 7.49 (t, *J* = 7.6 Hz, 2H, C6-H), 7.29 (d, *J* = 8.1 Hz, 2H, C10-H), 5.70 (ddd, *J* = 48.1, 7.0, 4.2 Hz, 1H, C2-H), 5.11 (br s, 1H, C1-NH), 3.62 (ddd, *J* = 24.1, 14.5, 4.1 Hz, 1H, C1-H), 3.42 (ddd, *J* = 18.3, 14.5, 7.0 Hz, 1H, C1-H), 2.42 (s, 3H, C12-H).

^{13}C NMR (126 MHz, $CDCl_3$) δ 194.3 (d, *J* = 18.9 Hz, C3) 144.0 (C11), 136.8 (C8), 134.6 (C7), 133.9 (d, *J* = 1.1 Hz, C4), 130.0 (C10), 129.2 (d, *J* = 3.5 Hz, C5), 129.0 (C6), 127.2 (C9), 91.1 (d, *J* = 184.9 Hz, C2), 44.3 (d, *J* = 23.5 Hz, C1), 21.7 (C12).

^{19}F NMR (471 MHz, $CDCl_3$) δ −191.39 (ddd, *J* = 48.1, 24.1, 18.3 Hz, 1F, C2-F).

HRMS (+ESI) calcd. $[M+Na]^+$ ($C_{16}H_{16}FNO_3S$) 344.0727, measured 344.0723 (1.45 ppm error).

IR (neat) v_{max}/cm^{-1}: 3282, 3064, 2924, 1692, 1597, 1449, 1330, 1158, 1091, 814, 698, 663, 550.

R_f 0.12 (20% EtOAc in pentane).

8.15. Epoxides (17)

(1S,2R,4S,5S)-1-fluoro-4-(4-fluorophenyl)-2-methyl-3-tosyl-6-oxa-3-azabicyclo[3.1.0]hexane
((*S*,*R*,*S*,*S*)-17j)

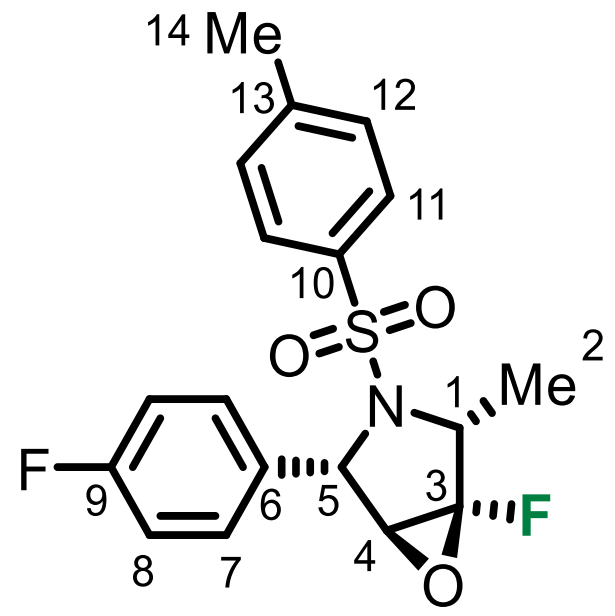

To a vial equipped with a stirrer bar, was added pyrroline **(*R*,*R*)-4j** (0.1 mmol, 1 equiv.) and *m*-CPBA (2 mmol, 20 equiv.) and chloroform (2 mL). The mixture was stirred at 45°C for 24 hours. The mixture was then diluted with DCM (20 mL), washed with 10% sodium carbonate (2 × 20 mL), dried over $MgSO_4$ and concentrated *in vacuo* to afford the crude product. This was then purified by flash-column chromatography to afford the title compound as a white solid (19 mg, 52%).

^{1}H NMR (400 MHz, CDCl$_3$) δ 7.67 (d, *J* = 8.1 Hz, 2H, C11-H), 7.44 (dd, *J* = 8.5, 5.2 Hz, 2H, C8-H), 7.34 (d, *J* = 8.1 Hz, 2H, C12-H), 7.11 (t, *J* = 8.6 Hz, 2H, C7-H), 4.70 (d, *J* = 6.4 Hz, C4-H, 1H), 4.20 (qd, *J* = 6.9, 2.8 Hz, 1H, C1-H), 3.90 (d, *J* = 2.8 Hz, 1H, C5-H), 2.45 (s, 3H, C14-H), 1.58 (dd, *J* = 6.9, 1.5 Hz, 3H, C2-H).

^{13}C NMR (126 MHz, CDCl$_3$) δ 162.8 (d, *J* = 247.7 Hz, C8), 144.4 (C13), 134.5 (C10), 132.3 (d, *J* = 3.1 Hz, C6), 130.0 (C12), 128.7 (d, *J* = 8.3 Hz, C7), 127.9 (C11), 116.1 (d, *J* = 21.7 Hz, C8), 100.0 (d, *J* = 289.3 Hz, C3), 62.7 (d, *J* = 14.5 Hz, C4), 62.6 (d, *J* = 4.1 Hz, C5), 55.4 (d, *J* = 30.7 Hz, C1), 21.8 (C14), 18.5 (C2).

^{19}F NMR (376 MHz, CDCl$_3$) δ −113.41 (dt, *J* = 8.5, 3.7 Hz, 1F, C9-F), −163.13 (s, 1F, C3-F).

HRMS (Nano) calcd: $[M+Na]^+$ ($C_{18}H_{17}NO_3SNaF_2$) 388.0788, measured 388.0795 (1.8 ppm error).

IR (neat) ν_{max}/cm^{-1}: 2923, 2851, 1731, 1604, 1510, 1452, 1410, 1380, 1325, 1307, 1224, 1191, 1165, 1125, 1093, 1073, 1012, 698, 944, 883, 852, 840, 815, 802, 788, 730, 707, 670, 652, 599, 570, 550, 537, 496, 475, 429, 419.

***(syn)-4-benzyl-1-fluoro-3-tosyl-6-oxa-3-azabicyclo[3.1.0]hexane* (17t)**

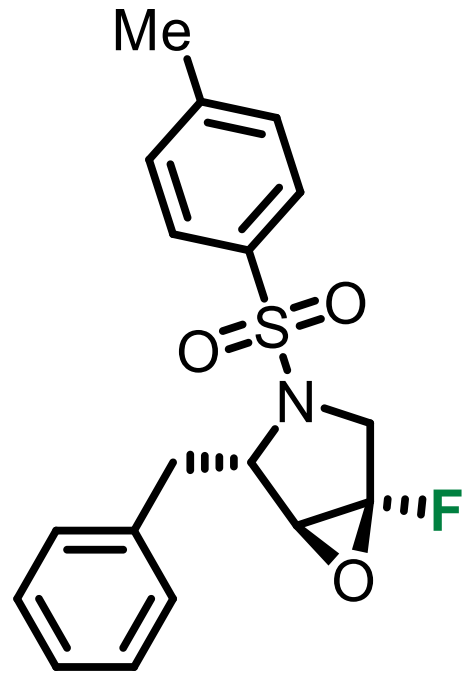

To a vial equipped with a stirrer bar, was added **HR057R** (0.1 mmol, 1 equiv.) and *m*-CPBA (2 mmol, 20 equiv.) and chloroform (2 mL). The mixture was stirred at 45°C for 24 hours. The product could not be isolated (25% NMR yield).

Identified by characteristic peak in ^{19}F NMR:

^{19}F NMR (283 MHz, No Solvent) δ -163.00 (s, 1F).

8.16. Pyrrolinones (18)

***3-fluoro-5-methyl-5-phenyl-1-tosyl-1,5-dihydro-2H-pyrrol-2-one* ((*R*)-18p)**

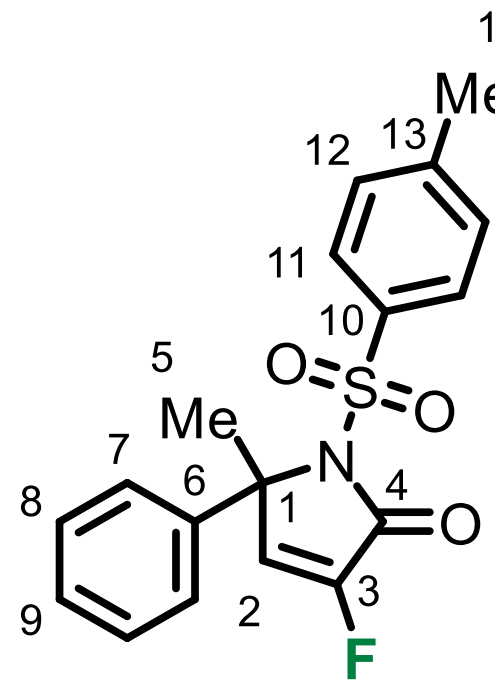

A round bottom flask was charged with CrO_3 (1 mmol, 20 equiv.), purged with N_2, and cooled to 0°C. DCM (2 mL) and pyridine (7 mmol, 140 equiv.) were then added, and the mixture was stirred for 10 minutes at 0°C. The mixture was then warmed to RT and stirred for a further 30 minutes. Pyrroline **4p** (0.05 mmol, 1 equiv.) in DCM (1 mL) was then added dropwise over 5 minutes. The reaction mixture was then heated to reflux for 21 h. On completion, an NMR yield was taken (66%) and the reaction mixture was filtered through celite and concentrated *in vacuo* to afford the crude product, which was purified by flash column chromatography to afford the title compound as a yellow oil (10.5 mg, 61%).

^{1}H NMR (500 MHz, $CDCl_3$) δ 7.44 (app. dt, *J* = 8.4, 1.8 Hz, 2H, C11-H), 7.40 – 7.31 (m, 3H, C7-H, C9-H), 7.29 – 7.26 (m, 2H, C8-H), 7.15 (d, *J* = 8.4 Hz, 2H, C12-H), 6.35 (s, 1H, C2-H), 2.39 (s, 3H, C14-H), 2.26 (d, *J* = 1.2 Hz, 3H, C5-H).

^{13}C NMR (126 MHz, $CDCl_3$) δ 161.0 (d, *J* = 32.1 Hz, C4), 148.6 (d, *J* = 279.7 Hz, C3), 145.4 (C13), 136.0 (d, *J* = 2.1 Hz, C6), 135.7 (C10), 129.4 (d, *J* = 2.9 Hz, C2), 129.3 (C12), 128.9 (C7), 128.9 (C9), 128.6 (C11), 126.7 (C8), 67.0 (d, *J* = 4.1 Hz, C1), 23.3 (d, *J* = 2.5 Hz, C5), 21.8 (C14).

^{19}F NMR (376 MHz, $CDCl_3$) δ −141.4 (s, 1F, C3-F).

HRMS (+ESI) calcd: $[M+H]^+$ ($C_{18}H_{16}FNO_3S$) 346.0908, measured 346.0898 (2.89 ppm error).

IR (neat) v_{max}/cm^{-1}: 3095, 2970, 1740, 1678, 1597, 1448, 1365, 1197, 1186, 1172, 1129, 1088, 669, 575.

8.17. Deprotections (19-25)

***2-benzyl-4-fluoro-2,5-dihydro-1H-pyrrole* (19t)**

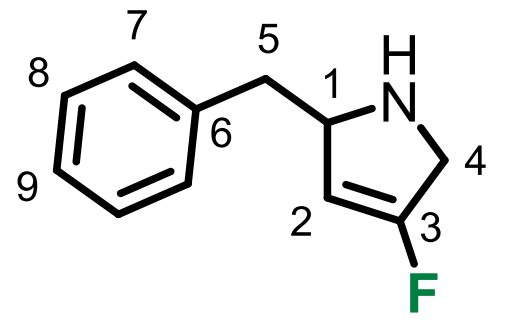

Produced according to **General procedure VII** from pyrroline **4t** without further purification to afford the title compound as a white solid (31.2 mg, 88%)

^{1}H NMR (500 MHz, CDCl$_3$) δ 7.32 – 7.27 (m, 2H, C8-H), 7.24 – 7.21 (m, 1H, C9-H), 7.21 – 7.17 (m, 2H, C7-H), 5.08 (ddt, *J* = 4.5, 2.1, 0.6 Hz, 1H, C2-H), 4.23 (tddd, *J* = 8.6, 8.6, 4.4, 2.0 Hz, 1H, C1-H), 3.72 (dddd, *J* = 4.5, 4.4, 2.2, 2.1 Hz, 2H, C4-H), 2.85 – 2.73 (m, 2H, C5-H), 1.94 (br s, 1H, C1-NH).

^{13}C NMR (126 MHz, CDCl$_3$) δ 160.4 (d, *J* = 279.4 Hz, C3), 138.6 (C6), 129.4 (C7), 128.6 (C8), 126.5 (C9), 104.1 (d, *J* = 8.2 Hz, C2), 63.0 (d, *J* = 9.4 Hz, C1), 48.7 (d, *J* = 26.5 Hz, C4), 44.3 (d, *J* = 2.3 Hz, C5).

^{19}F NMR (376 MHz, CDCl$_3$) δ −129.78 (q, *J* = 4.5 Hz, C3-F).

HRMS (EI+) calcd: $[M+H]^+$ ($C_{11}H_{12}NF$) 178.1027, measured 178.1024 (1.68 ppm error).

IR (neat) v_{max}/cm^{-1}: 3028, 2924, 2853, 1691, 1603, 1494, 1454, 1200, 1092, 814, 750, 700.

R$_f$ 0.10 (20% EtOAc in pentane).

cis-3-fluoro-5-(4-fluorophenyl)-2-methyl-2,5-dihydro-1H-pyrrole **(19j)**

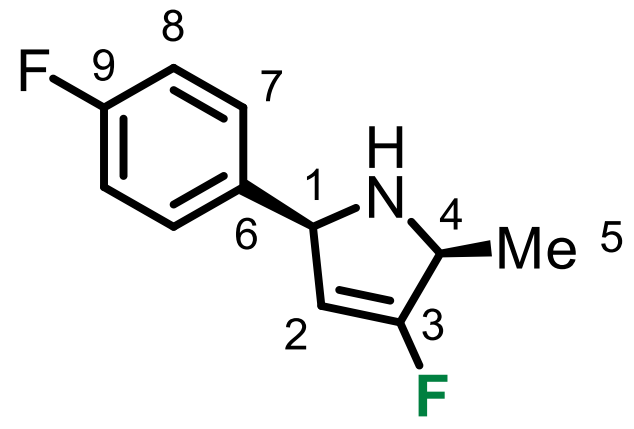

Produced according to **General procedure VII** from pyrroline **4j** without further purification to afford the title compound as a white solid (36.3 mg, 93%).

^{1}H NMR (500 MHz, CDCl$_3$) δ 7.36 – 7.27 (m, 2H, C7-H), 7.02 (tt, *J* = 8.6, 2.2 Hz, 2H, C8-H), 5.11 (dd, *J* = 2.1, 2.0 Hz, 1H, C2-H), 4.97 (ddd, *J* = 5.4, 3.8, 1.8 Hz, 1H, C1-H), 4.13 (qddd, *J* = 6.5, 6.1, 4.0, 2.0 Hz, 1H, C4-H), 1.97 (s, 1H, C1-NH), 1.32 (d, *J* = 6.5 Hz, 3H, C5-H).

^{13}C NMR (126 MHz, CDCl$_3$) δ 164.0 (d, *J* = 282.1 Hz, C3), 162.4 (d, *J* = 245.5 Hz, C9), 140.25 (dd, *J* = 3.1, 1.6 Hz, C6), 128.8 (d, *J* = 8.0 Hz, C7), 115.5 (d, *J* = 21.4 Hz, C8), 103.8 (d, *J* = 9.1 Hz, C2), 62.6 (d, *J* = 10.7 Hz, C1-H), 55.2 (d, *J* = 24.1 Hz, C4), 21.2 (d, *J* = 2.9 Hz, C5).

^{19}F NMR (376 MHz, CDCl$_3$) δ −115.11 (tt, *J* = 8.7, 5.4 Hz), −133.06 (t, *J* = 4.7 Hz).

HRMS (ESI+) cald: $[M+H]^+$ ($C_{11}H_{11}F_2N$) 196.0932, measured 196.0937 (2.55 ppm error).

IR (neat) v_{max}/cm^{-1}: 3994, 2907, 2674, 2493, 1698, 1605, 1511, 1434, 1227, 1214, 1181, 1160, 834, 618, 549, 531, 415.

R*f* 0.21 (20% EtOAc in pentane).

(2R,5R)-3-fluoro-5-(4-fluorophenyl)-2-methyl-2,5-dihydro-1H-pyrrole **((*R*,*R*)-19j)**

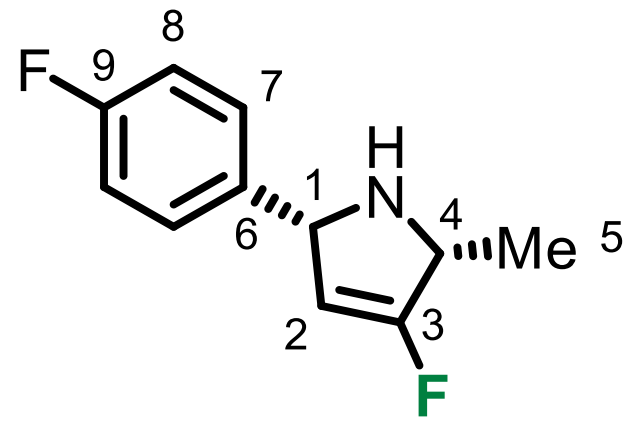

Produced according to **General procedure VII** from pyrroline **(*R*,*R*)-4j** without further purification to afford the title compound as a white solid (34.7 mg, 89%).

Characterisation data matches **4j**.

^{1}H NMR (500 MHz, $CDCl_3$) δ 7.36 – 7.27 (m, 2H, C7-H), 7.02 (tt, *J* = 8.6, 2.2 Hz, 2H, C8-H), 5.11 (dd, *J* = 2.1, 2.0 Hz, 1H, C2-H), 4.97 (ddd, *J* = 5.4, 3.8, 1.8 Hz, 1H, C1-H), 4.13 (qddd, *J* = 6.5, 6.1, 4.0, 2.0 Hz, 1H, C4-H), 1.97 (s, 1H, C1-NH), 1.32 (d, *J* = 6.5 Hz, 3H, C5-H).

^{13}C NMR (126 MHz, $CDCl_3$) δ 164.0 (d, *J* = 282.1 Hz, C3), 162.4 (d, *J* = 245.5 Hz, C9), 140.25 (dd, *J* = 3.1, 1.6 Hz, C6), 128.8 (d, *J* = 8.0 Hz, C7), 115.5 (d, *J* = 21.4 Hz, C8), 103.8 (d, *J* = 9.1 Hz, C2), 62.6 (d, *J* = 10.7 Hz, C1-H), 55.2 (d, *J* = 24.1 Hz, C4), 21.2 (d, *J* = 2.9 Hz, C5).

^{19}F NMR (376 MHz, $CDCl_3$) δ −115.11 (tt, *J* = 8.7, 5.4 Hz), −133.06 (t, *J* = 4.7 Hz).

HRMS (ESI+) cald: $[M+H]^+$ ($C_{11}H_{11}F_2N$) 196.0932, measured 196.0937 (2.55 ppm error).

IR (neat) v_{max}/cm^{-1}: 3994, 2907, 2674, 2493, 1698, 1605, 1511, 1434, 1227, 1214, 1181, 1160, 834, 618, 549, 531, 415.

R*f* 0.21 (20% EtOAc in pentane).

(R)-4-fluoro-2-methyl-2-phenyl-2,5-dihydro-1H-pyrrole **((*R*)-19p)**

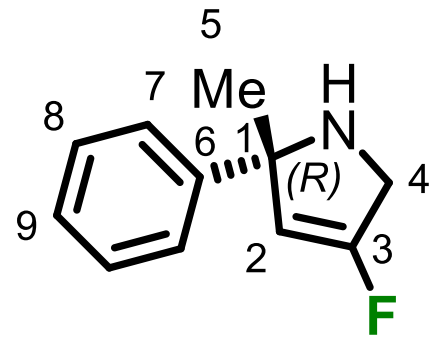

Produced according to **General procedure VII** from **(*R*)-4p** without further purification to afford the title compound as a white solid (30.5 mg, 86%).

^{1}H NMR (400 MHz, CDCl$_3$) δ 7.48 – 7.41 (m, 2H, C7-H), 7.38 – 7.30 (m, 2H, C8-H), 7.27 – 7.23 (m, 1H, C9-H), 5.42 (td, *J* = 2.0, 0.8 Hz, 1H, C2-H), 3.81 (qdd, *J* = 14.3, 4.1, 2.0 Hz, 2H, C4-H), 2.03 (s, 1H, C4-NH), 1.62 (d, *J* = 1.4 Hz, 3H, C5-H).

^{13}C NMR (101 MHz, CDCl$_3$) δ 160.0 (d, *J* = 281.4 Hz, C3), 147.8 (d, *J* = 1.9 Hz, C6), 128.6 (C7), 127.0 (C9), 125.3 (C8), 109.5 (d, *J* = 7.6 Hz, C2), 67.5 (d, *J* = 10.4 Hz, C1), 48.6 (d, *J* = 25.5 Hz, C4), 30.1 (d, *J* = 2.3 Hz, C5).

^{19}F NMR (376 MHz, CDCl$_3$) δ −131.26 – −131.30 (m, 1F, C3-F).

HRMS (+ESI) calcd: $[M+H]^+$ ($C_{11}H_{12}NF$) 178.1027, measured 178.1023 (2.25 ppm error).

IR (neat) v_{max}/cm^{-1}: 3019, 2970, 2938, 3320, 1429, 1365, 1229, 1216, 524.

***4-fluoro-2-propyl-2,5-dihydro-1H-pyrrole* (19v)**

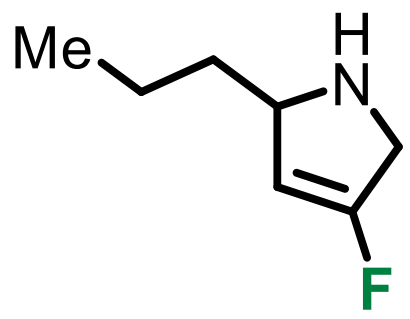

Prepared from pyrroline **4v** according to **General procedure VII-A**.

Product not isolated due to volatility. ^{19}F NMR yields calculated from crude products versus C_6F_6 internal standard (93%).

Identified by characteristic ^{19}F peak:

^{19}F NMR (376 MHz, No Solvent) δ −128.27 (s, 1F).

***(2R,5R)-3-fluoro-5-(4-fluorophenyl)-1,2-dimethyl-2,5-dihydro-1H-pyrrole* ((*R*,*R*)-20j)**

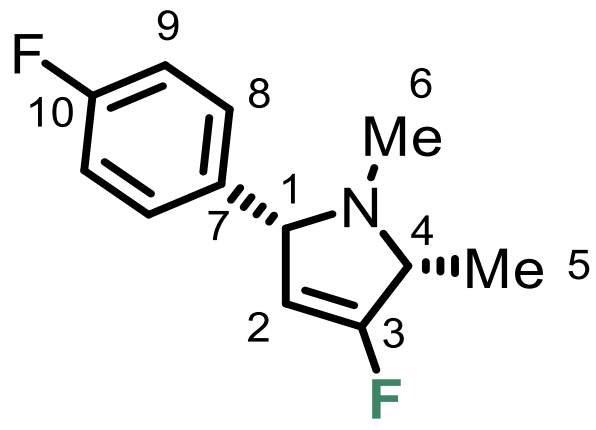

Pyrroline **(*R,R*)-4j** was deprotected according to **General procedure VII**, and the crude product was redissolved in MeCN (5 mL, 0.04M). K_2CO_3 (83 mg, 3 equiv.) and MeI (19 µL, 1.5 equiv.) were then added, and the solution was heated to 65°C for 18 h. On completion, the reaction mixture was diluted with water (50 mL) and washed with DCM (2 × 20 mL). The organic fractions were combined, dried over $MgSO_4$, and concentrated *in vacuo*. The residue was then purified by flash column chromatography to afford the title compound as a colourless oil (28.0 mg, 67%).

^{1}H NMR (500 MHz, $CDCl_3$) δ 7.34 – 7.28 (m, 2H, C8-H), 7.01 (tt, *J* = 8.5, 2.2 Hz, 2H, C9-H), 4.94 (dd, *J* = 2.1, 1.8 Hz, 1H, C2-H), 4.20 (dddq, *J* = 6.4, 4.8, 1.8, 1.4 Hz, 1H, C1-H), 3.54 (dqdd, *J* = 8.4, 6.3, 4.8, 2.1 Hz, 1H, C4-H), 2.34 (d, *J* = 1.4 Hz, 3H, C6-H), 1.29 (d, *J* = 6.3 Hz, 3H).

^{13}C NMR (126 MHz, $CDCl_3$) δ 162.5 (d, *J* = 245.0 Hz, C10), 161.6 (d, *J* = 280.9 Hz, C3), 138.7 (d, *J* = 2.6 Hz, C7), 129.4 (d, *J* = 8.1 Hz, C8), 115.3 (d, *J* = 21.4 Hz, C9), 103.4 (d, *J* = 6.7 Hz, C2), 70.8 (d, *J* = 9.8 Hz, C1), 62.2 (d, *J* = 23.3 Hz, C4), 38.2 (C6), 18.5 (d, *J* = 2.8 Hz, C5).

^{19}F NMR (471 MHz, $CDCl_3$) δ −115.55 (ddd, *J* = 14.2, 8.9, 5.5 Hz, 1F, C10-F), −131.57 (dd, *J* = 8.4, 6.4 Hz, 1F, C3-F).

HRMS (+ESI) calcd. $[M+H]^+$ ($C_{12}H_{13}F_2N$) 210.1087, measured 210.1089 (0.95 ppm error).

IR (neat) v_{max}/cm^{-1}: 3011, 1743, 1598, 1365, 1285, 1228, 1216, 1205, 1164, 528, 516.

(2R,3R,5R)-3-fluoro-5-(4-fluorophenyl)-1,2-dimethylpyrrolidine **((*R*,*R*,*R*)-20l)**

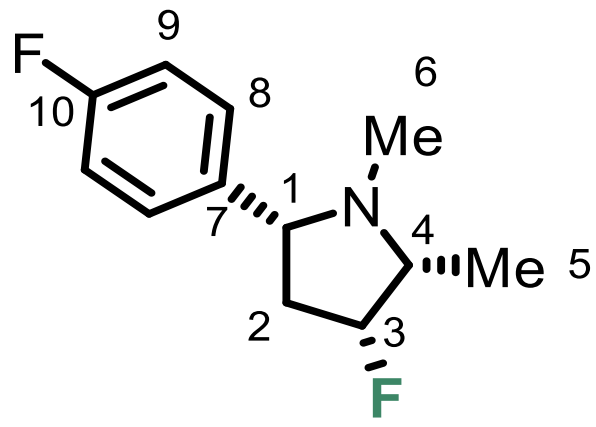

Pyrrolidine **(*R*,*R*,*R*)-10j** was deprotected according to **General procedure VII** (0.15 mmol), and the crude product was redissolved in MeCN (5 mL, 0.04M). K_2CO_3 (63 mg, 3 equiv.) and MeI (15 µL, 1.5 equiv.) were then added, and the solution was heated to 65°C for 18 h. On completion, the reaction mixture was diluted with water (50 mL) and washed with DCM (2 × 20 mL). The organic fractions were combined, dried over $MgSO_4$, and concentrated *in vacuo*. The residue was then purified by flash column chromatography to afford the title compound as a colourless oil (18.7 mg, 59%).

^{1}H NMR (500 MHz, $CDCl_3$) δ 7.38 – 7.30 (m, 2H, C8-H), 7.01 (tt, *J* = 8.8, 2.1 Hz, 2H, C9-H), 4.91 (dddd, *J* = 55.3, 8.4, 4.3, 2.0 Hz, 1H, C3-H), 3.08 (td, *J* = 8.4, 1.9 Hz, 1H, C1-H), 2.57 (dddd, *J* = 25.4, 15.1, 8.5, 6.8 Hz, 1H, C2-H), 2.32 (dqd, *J* = 26.2, 6.5, 4.3 Hz, 1H, C4-H), 2.08 (s, 3H, C6-H), 1.89 (dddd, *J* = 35.3, 15.0, 8.4, 1.9 Hz, 1H, C2-H), 1.26 (dd, *J* = 6.5, 2.0 Hz, 3H, C5-H).

^{13}C NMR (126 MHz, $CDCl_3$) δ 162.3 (d, *J* = 244.8 Hz, C10), 138.6 (d, *J* = 3.1 Hz, C7), 129.4 (d, *J* = 8.0 Hz, C8), 115.4 (d, *J* = 21.2 Hz, C9), 94.1 (d, *J* = 181.6 Hz, C3), 70.0 (C1), 66.3 (d, *J* = 19.6 Hz, C4), 41.6 (d, *J* = 22.9 Hz, C2), 38.2 (C6), 12.6 (d, *J* = 9.2 Hz, C5).

^{19}F NMR (376 MHz, $CDCl_3$) δ −115.59 (tt, *J* = 8.8, 5.5 Hz, 1F, C10-F), −182.41 (dddd, *J* = 55.3, 35.3, 26.2, 25.4 Hz).

HRMS (+ESI) calcd. $[M+H]^+$ ($C_{12}H_{15}F_2N$) 212.1245, measured 212.1239 (2.83 ppm error).

IR (neat) v_{max}/cm^{-1}: 2917, 2848, 1604, 1509, 1273, 1156, 1050, 972, 833, 793.

(4-fluoro-2-propyl-2,5-dihydro-1H-pyrrol-1-yl)(phenyl)methanone **(21u)**

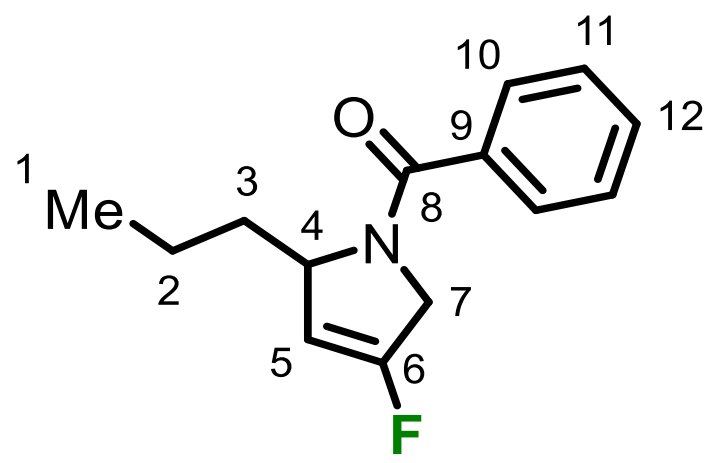

4u (0.72 mmol, 227 mg) was denosylated according to **General procedure VII-A**, after which Et_3N (0.301 mL, 2.16 mmol) and benzoyl chloride (0.249 mL, 2.16 mmol) were added, and the reaction mixture was stirred at RT for a further 18 h. Et_2O (30 mL) was then added, and the solution was washed with sat. aq. $NaHCO_3$ (3 × 20 mL). The organic layer was dried over $MgSO_4$, filtered, and concentrated *in vacuo*. The residue was purified by flash column chromatography (0-30% EtOAc in pentane) to afford the title compound as a colourless oil (85.7 mg, 51%).

NMR shows a mixture of amide rotamers **(1:0.35)**.

^{1}H NMR (500 MHz, $CDCl_3$) Major rotamer δ 7.53 – 7.47 (m, 2H, C10-H), 7.46 – 7.36 (m, 3H, C11-H, C12-H), 5.20 (t, *J* = 2.2 Hz, 1H, C5-H), 5.09 – 4.99 (m, 1H, C4-H), 4.38 – 4.28 (m, 1H, C7-H), 3.98 (d, *J* = 13.8 Hz, 1H, C7-H), 1.90 – 1.70 (m, 2H, C3-H), 1.48 – 1.29 (m, 2H, C2-H), 0.96 (t, *J* = 7.4 Hz, 3H, C1-H), **Minor rotamer** δ 7.46 – 7.36 (m, 5H, C10-H, C11-H, C12-H), 5.09 – 4.99 (m, 1H, C4-H), 4.68 (s, 1H, C5-H), 4.42 (d, *J* = 16.1 Hz, 1H, C7-H), 4.38 – 4.28 (m, 1H, C7-H), 1.21 – 1.02 (m, 4H, C2-H, C3-H), 0.66 (t, *J* = 7.1 Hz, 1H, C1-H).

^{13}C NMR (126 MHz, $CDCl_3$) Major rotamer δ 170.3 (C8), 154.2 (d, *J* = 275.0 Hz, C6), 136.4 (C9), 130.4 (C12), 128.6 (C10), 127.1 (C11), 102.9 (d, *J* = 5.5 Hz, C5), 61.0 (d, *J* = 5.6 Hz, C4), 51.7 (d, *J* = 32.0 Hz, C7), 36.0 (d, *J* = 2.2 Hz, C3), 18.1 (C2), 14.2 (C1). **Minor rotamer** δ 169.9 (C8), 155.9 (d, *J* = 274.1 Hz, C6), 136.2 (C9), 130.0 (C12), 128.7 (C10), 126.5 (C11), 102.3 (d, *J* = 7.1 Hz, C5), 62.0 (d, *J* = 5.8 Hz, C4), 49.1 (d, *J* = 32.0 Hz, C7), 36.8 (C3), 16.9 (C2), 13.7 (C1).

^{19}F NMR (376 MHz, $CDCl_3$) Major rotamer δ −129.23 (1F, C6-F). **Minor rotamer** δ −128.83 (1F, C6-F).

HRMS (+ESI) calcd. $[M+H]^+$ ($C_{14}H_{16}FNO$) 234.1289, measured 234.1279 (4.27 ppm error).

IR (neat) v_{max}/cm^{-1}: 3062, 2960, 2932, 2872, 1697, 1601, 1575, 1449, 1418, 1362, 1256, 1215, 1175, 713, 702.

phenyl(2-propyl-2,5-dihydro-1H-pyrrol-1-yl)methanone **(21bf)**

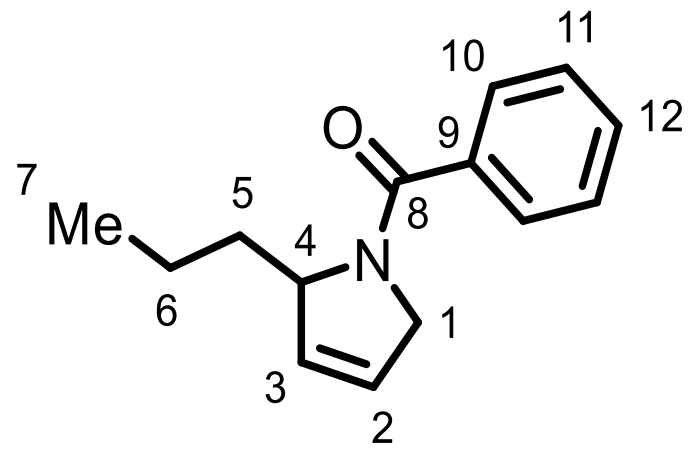

To a solution of 2-propyl-2,5-dihydro-1*H*-pyrrole (444 mg, 3.99 mmol) and triethylamine (0.84 mL, 5.99 mmol) in DCM (8 mL) at 0°C was added benzoyl chloride (0.421 mL, 3.63 mL). The reaction mixture was allowed to warm to RT and stirred for 18 h. The reaction was then diluted with H_2O (50 mL) and extracted with DCM (2 × 30 mL). The organic fractions were combined, dried over $MgSO_4$ and concentrated *in vacuo* to afford the crude product, which was purified by flash column chromatography (2-40% EtOAc in pentane) to afford the title compound as a colourless oil (704 mg, 82%).

^{1}H NMR (500 MHz, $CDCl_3$) Major rotamer δ 7.54 – 7.50 (m, 2H, C10-H), 7.42- 7.37 (m, 3H, C11-H, C12-H), 5.86 (dq, *J* = 6.7, 2.2 Hz, 1H, C3-H), 5.70 (ddt, *J* = 6.2, 2.2, 1.9 Hz, 1H, C2-H), 5.09 (dtd, *J* = 6.7, 4.3, 1.8 Hz, 1H, C4-H), 4.28 (dddd, *J* = 15.0, 6.2, 2.2, 1.9 Hz, 1H, C1-H), 4.02 (ddd, *J* = 15.0, 1.9, 1.8 Hz, 1H, C1-H), 1.90 – 1.74 (m, 2H, C5-H), 1.45 – 1.34 (m, 2H, C6-H), 0.97 (t, *J* = 7.4 Hz, 3H, C7-H). **Minor rotamer** δ 7.49 – 7.45 (m, 2H, C10-H), 7.42 – 7.37 (m, 3H, C11-H, C12-H), 5.94 – 5.88 (m, 1H, C3-H), 5.75 – 5.69 (m, 1H, C2-H), 4.70 (br s, 1H, C4-H), 4.53 – 4.45 (m, 1H, C1-H), 4.35 – 4.30 (m, 1H, C1-H), 1.24 – 1.02 (m, 4H, C5-H, C6-H) 0.67 (t, *J* = 7.0 Hz, 3H, C7-H).

^{13}C NMR (126 MHz, $CDCl_3$) Major rotamer δ 170.2 (C8), 137.3 (C9), 130.8 (C3), 130.0 (C12), 128.4 (C11), 127.3 (C10), 124.4 (C2), 64.4 (C4), 56.7 (C1), 35.6 (C5), 18.2 (C6), 14.4 (C7). **Minor rotamer** δ 169.8 (C8), 137.4 (C9), 129.9 (C3), 129.7 (C12), 128.6 (C11), 126.5 (C10), 125.3 (C2), 64.9 (C4), 54.1 (C1), 36.6 (C5), 16.9 (C6), 13.9 (C7).

HRMS (+ESI) calcd: $[M]^+$ ($C_{14}H_{17}NO$) 216.1383, measured 216.1375 (3.70 ppm error).

IR (neat) ν_{max}/cm^{-1}: 3061, 2957, 2931, 2869, 1637, 1615, 1602, 1576, 1446, 1403, 1324, 795, 699, 666.

R_f 0.35 (20% EtOAc in pentane).

(4-fluoro-2-propyl-2,5-dihydro-1H-pyrrol-1-yl)(trans-2-phenylcyclopropyl)methanone **(22v)**

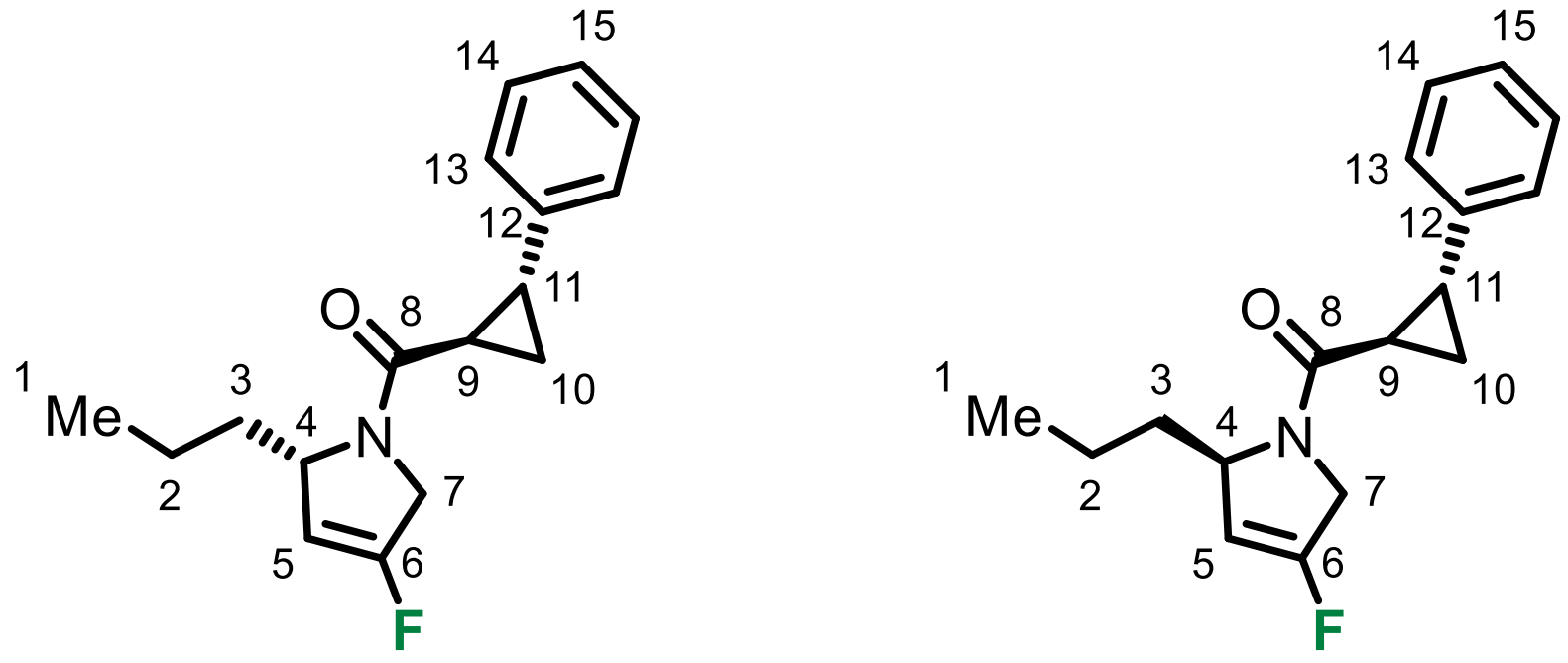

major diastereomer minor diastereomer

Pyrroline **4u** (0.2 mmol, 63 mg)was denosylated according to **General procedure VII-A**, after which Et_3N (84 µL, 0.6 mmol) and trans-2-phenyl-1-cyclopropanecarbonyl chloride (54 mg, 0.3 mmol) were added, and the reaction mixture was stirred at RT for a further 18 h. Et_2O (30 mL) was then added, and the solution was washed with sat. aq. $NaHCO_3$ (3 × 20 mL). The organic layer was dried over $MgSO_4$, filtered, and concentrated *in vacuo*. The residue was purified by flash column chromatography (0-30% EtOAc in pentane) to afford the title compound as a colourless oil (41.0 mg, 75%).

Isolated as a mixture of C4 diastereomers, d.r. = 1.1:1.

NMR shows each diastereomer as a mixture of rotamers. *Z:E* ratio = 1.7:1 (major diastereomer), 1.3:1 (minor diastereomer).

^{1}H NMR (500 MHz, $CDCl_3$) Major diastereomer δ 7.31 – 7.27 (m, 2H, C14-H), 7.23 – 7.18 (m, 1H, C15-H), 7.15 – 7.08 (m, 2H, C13-H), 5.19 (h, *J* = 2.2 Hz, 1H, C5-H), 4.78 (ddq, *J* = 10.3, 5.0, 2.2 Hz, 1H, C4-H), 4.48 – 4.30 (m, 2H, C7-H), 2.64 (dddd, *J* = 15.0, 9.1, 6.3, 4.1 Hz, 1H, C11-H), 1.90 – 1.78 (m, 1H, C9-H), 1.78 – 1.48 (m, 3H, C3-H, C10-H), 1.39 – 1.15 (m, 3H, C2-H, C10-H), 0.93 (t, *J* = 7.4 Hz, 3H, C1-H, minor rotamer), 0.92 (t, *J* = 7.3 Hz, 3H, C1-H, major rotamer). **Minor diastereomer** δ 7.31 – 7.27 (m, 2H, C14-H), 7.23 – 7.18 (m, 1H, C15-H), 7.15 – 7.08 (m, 2H, C13-H), 5.13 (dp, *J* = 4.3, 2.1 Hz, 1H, C5-H), 4.74 – 4.66 (m, 1H, C4-H), 4.47 – 4.30 (m, 1H, C7-H), 4.13 (ddtd, *J* = 15.6, 12.9, 4.9, 2.0 Hz, 1H, C7-H), 2.47 – 2.40 (m, 1H, C11-H), 1.90 – 1.78 (m, 2H, C9-H), 1.78 – 1.48 (m, 3H, C3-H, C10-H), 1.39 – 1.15 (m, 3H, C2-H, C10-H), 0.95 (t, *J* = 7.5 Hz, 3H, C1-H, major rotamer), 0.67 (t, *J* = 7.4 Hz, 1H, C1-H, minor rotamer).

^{13}C NMR (126 MHz, $CDCl_3$) Major diastereomer, major rotamer δ 170.6 (C8), 154.1 (d, *J* = 274.2 Hz, C6), 140.9 (C12), 128.6 (C14), 126.5 (C15), 126.2 (C13), 103.3 (d, *J* = 5.2 Hz, C5), 61.2 (d, *J* = 5.6 Hz, C4), 49.5 (d, *J* = 32.0 Hz, C7), 35.8 (d, *J* = 4.5 Hz, C3), 25.4 (C11), 24.6 (C9), 18.0 (C10), 16.8 (C2), 14.1 (C1). **Major diastereomer, minor rotamer** δ 170.5 (C8), 154.1 (d, *J* = 274.2 Hz, C6), 140.8 (C12), 128.6 (C14), 126.5 (C15), 126.0 (C13), 103.3 (d, *J* = 5.2 Hz, C5), 61.2 (d, *J* = 5.7 Hz, C4), 49.5 (d, *J* = 32.0 Hz, C7), 35.8 (C3), 26.6 (C11), 24.5 (d, *J* = 1.5 Hz, C9), 17.6 (C10), 15.6 (C2),

14.1 (C1). **Minor diastereomer, major rotamer** δ 170.9 (C8), 156.5 (d, *J* = 274.5 Hz, C6), 140.8 (C12), 128.6 (C14), 126.5 (C15), 126.4 (C13), 102.2 (d, *J* = 7.4 Hz, C5), 60.9 (d, *J* = 6.2 Hz, C4), 48.8 (d, *J* = 31.9 Hz, C7), 38.5 (C3), 25.8 (C11), 23.7 (d, *J* = 1.4 Hz, C9), 18.0 (C10), 17.0 (C2), 14.2 (C1). **Minor diastereomer, minor rotamer** δ 170.6 (C8), 156.4 (d, *J* = 274.2 Hz, C6), 140.6 (C12), 128.7 (C14), 126.5 (C15), 126.2 (C13), 102.2 (d, *J* = 7.3 Hz, C5), 61.1 (d, *J* = 6.2 Hz, C4), 48.9 (d, *J* = 31.9 Hz, C7), 38.5 (C3), 26.1 (C11), 24.7 (C9), 17.5 (C10), 16.2 (C2), 13.8 (C1).

^{19}F NMR (376 MHz, $CDCl_3$) Major diastereomer, major rotamer δ −129.24 (s, 1F, C6-F) **Major diastereomer, minor rotamer** δ −129.18 (s, 1F, C6-F) **Minor diastereomer, major rotamer** δ −128.68 (s, 1F, C6-F) **Minor diastereomer, minor rotamer** δ −128.85 (s, 1F, C6-F).

HRMS (+EI) calcd: $[M]^+$ ($C_{17}H_{20}FNO$) 273.1523, measured 273.1519 (1.46 ppm error).

IR (neat) v_{max}/cm^{-1}: 2960, 2871, 1790, 1740, 1698, 1639, 1444, 1365, 1229, 1216, 755, 697.

(3aS,7R,7aS)-2-benzoyl-7-(4-fluoro-2-(4-fluorophenyl)-2,5-dihydro-1H-pyrrole-1-carbonyl)-5-(3-methoxyphenyl)-2,3,3a,6,7,7a-hexahydro-1H-isoindol-1-one **(23e)**

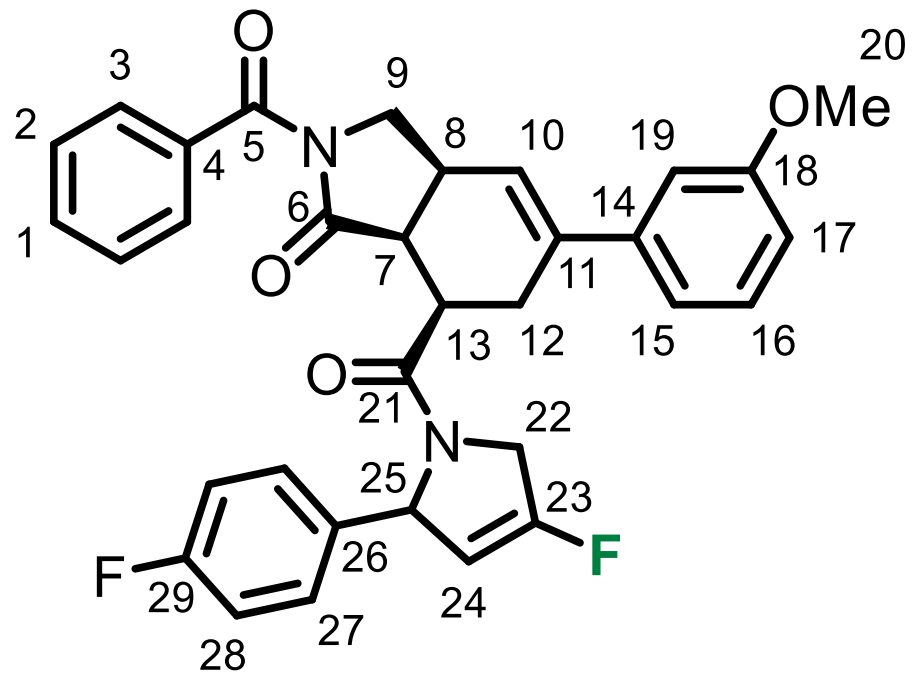

Pyrroline **4e** (0.2 mmol, 1 equiv.) was deprotected according to **General procedure VII**. The crude product was then added to a solution of DIPEA (1 mmol, 5 equiv.), and (3a*S*,4*R*,7a*S*)-2-benzoyl-6-(3-methoxyphenyl)-3-oxo-2,3,3a,4,5,7a-hexahydro-1*H*-isoindole-4-carboxylic acid (0.2 mmol, 1 equiv.) in DCM (2 mL) at 0°C. HBTU (0.3 mmol, 1.5 equiv.) was then added, and the reaction mixture was warmed to RT and stirred for 18 h. On completion, the reaction was diluted with water (30 mL) and extracted with DCM (2 × 20 mL). The organic fractions were combined, dried over $MgSO_4$, and concentrated *in vacuo* to afford the title compound as a colourless oil (81.0 mg, 73% over 2 steps).

Product NMR shows a complex mixture of 8 isomers; 2 C25-diastereomers, each showing 4 amide rotamers (C5, C21). Identified by characteristic peaks from the parent pyrroline **4e** and carboxylic acid. Isomeric ratio = 10:7:45:1:17:7:26:19.

Contains 13 mol% tetramethyl urea by-product.

^{1}H NMR (500 MHz, $CDCl_3$) δ 7.64 – 7.11 (m, 8H, C1-H, C2-H, C3-H, C16-H, C27-H), 7.08 – 6.49 (m, 5H, C15-H, C17-H, C19-H, C28-H), 6.15 – 5.90 (m, 1H, C10-H), 5.72 – 5.45 (m, 1H, C25-H), 5.26 – 5.03 (m, 1H, C24-H), 4.69 – 4.29 (m, 2H, C22-H), 4.22 – 3.86 (m, 2H, C9-H), 3.84 – 3.78 (m, 3H, C20-H), 3.51 – 3.12 (m, 2H, C12-H), 3.07 – 2.88 (m, 1H, C8-H), 2.78 – 2.39 (m, 1H, C7-H), 2.34 – 1.65 (m, 1H, C13-H).

^{13}C NMR (126 MHz, $CDCl_3$) δ 175.0 – 168.2 (m, C5, C6, C21), 166.3 – 160.9 (m, C29), 159.9 – 159.7 (m, C18), 157.6 – 151.9 (m, C23), 144.0 – 141.3 (m, C14), 138.9 – 135.5 (m, C4, C26), 135.1 – 131.5 (m, C11, C16), 130.2 – 127.1 (m, C1, C2, C3, C27), 125.3 – 122.6 (m, C10), 120.3 – 114.7 (m, C28), 113.7 – 109.2 (m, C15, C17H, C19), 105.1 – 103.8 (m, C24), 65.2 – 62.8 (m, C25), 55.5 – 55.2 (m, C20), 51.3 – 50.3 (m, C22), 49.8 – 48.7 (m, C8), 45.2 – 42.7 (m, C9), 38.4 – 32.6 (m, C12), 32.4 – 28.4 (m, C13), 27.6 – 24.7 (m, C7).

^{19}F NMR (376 MHz, $CDCl_3$) [C29-F signals] δ −111.84 (s, 0.22F), −111.94 (tt, *J* = 8.4, 5.2 Hz, 0.16F), −112.40 (tt, *J* = 8.4, 5.0 Hz, 1.00F), −113.27 (tt, *J* = 8.4, 5.0 Hz, 0.02F), −114.29 (tt, *J* = 8.6, 5.2 Hz, 0.38F), −114.58 (tt, *J* = 8.6, 5.2 Hz, 0.15F), −114.95 (s, 0.58F), −115.02 (tt, *J* = 8.4, 5.0 Hz,

0.42F). **[C23-F signals]** δ −128.53 (q, *J* = 4.4 Hz, 0.38F), −128.67 (q, *J* = 4.4 Hz, 0.16F), −128.98 (s, 1.00F), −129.25 (q, *J* = 4.2 Hz, 0.15F), −129.49 (q, *J* = 3.1 Hz, 0.58F), −129.59 (s, 0.02F), −129.69 (s, 0.22F), −129.92 (dd, *J* = 8.2, 3.6 Hz, 0.42F).

HRMS (+Nano) calcd. $[M+Na]^+$ ($C_{33}H_{28}F_2N_2O_4$) 577.1915, measured 577.1910 (0.87 ppm error).

IR (neat) v_{max}/cm^{-1}: 3443, 3348, 3198, 2936, 1739, 1698, 1667, 1603, 1578, 1508, 1447, 1428, 1365, 1319, 1291, 1219, 1172, 838, 781, 699.

(1R,4S)-1-((R)-4-fluoro-2-propyl-2,5-dihydro-1H-pyrrole-1-carbonyl)-4,7,7-trimethyl-2-oxabicyclo[2.2.1]heptan-3-one **(24v)**

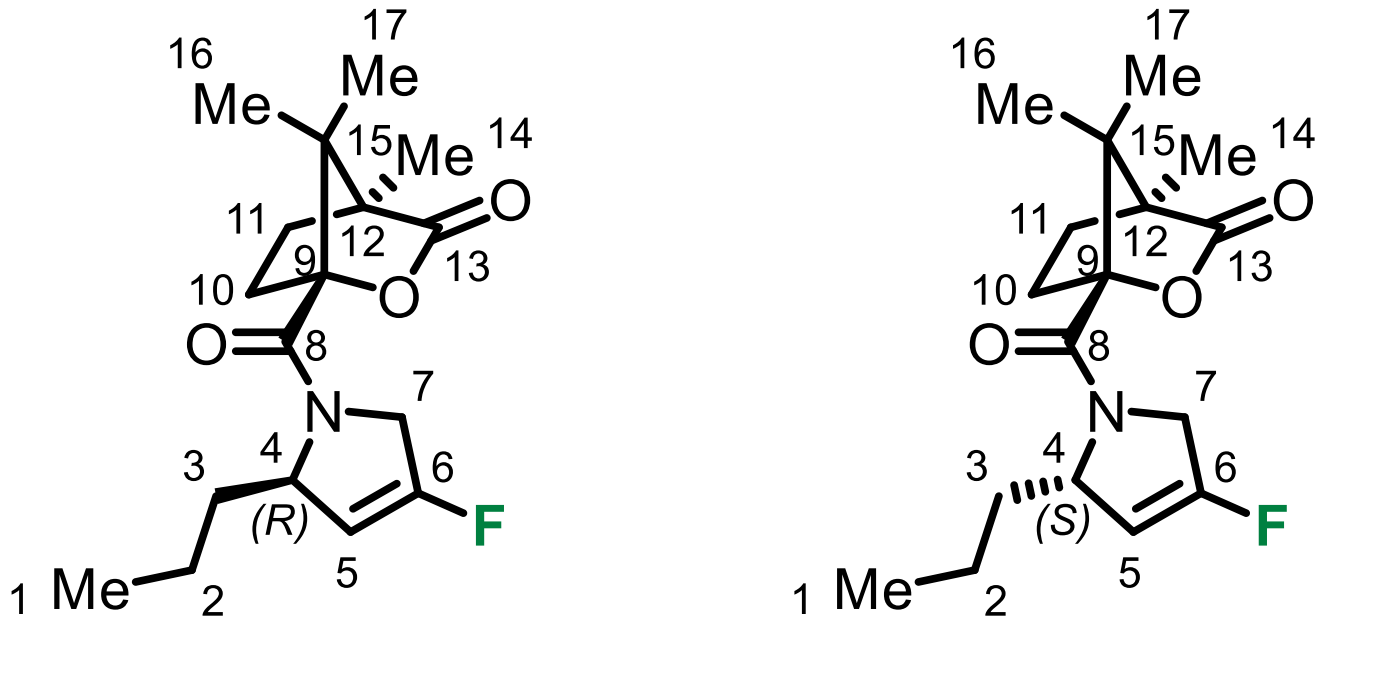

Pyrroline **4u** (0.2 mmol, 63 mg) was denosylated according to **General procedure VII-A**, after which Et_3N (84 µL, 0.6 mmol) and (1*S*)-(-)-camphanic chloride (65 mg, 0.3 mmol) were added, and the reaction mixture was stirred at RT for a further 18 h. Et_2O (30 mL) was then added, and the solution was washed with sat. aq. $NaHCO_3$ (3 × 20 mL). The organic layer was dried over $MgSO_4$, filtered, and concentrated *in vacuo*. The residue was purified by flash column chromatography (0-30% EtOAc in pentane) to afford the title compound as a colourless oil (44.6 mg, 72%).

Isolated as a mixture of C4 diastereomers, d.r. = 2.2:1 *R:S*.

NMR shows each diastereomer as a mixture of rotamers. *Z:E* ratio = 30:1 (major diastereomer), 5:1 (minor diastereomer).

^{1}H NMR (500 MHz, $CDCl_3$) Major diastereomer δ 5.15 (p, *J* = 2.1 Hz, 1H, C5-H), 4.93 – 4.83 (m, 1H, C4-H), 4.61 – 4.50 (m, 1H, C7-H), 4.50 – 4.42 (m, 1H, C7-H), 2.41 (ddd, *J* = 13.0, 10.6, 4.3 Hz, 1H, C10-H), 2.02 – 1.88 (m, 2H, C10-H, C11-H), 1.84 – 1.74 (m, 1H, C11-H), 1.70 – 1.55 (m, 2H, C3-H), 1.38 – 1.20 (m, 2H, C2-H), 1.16 (s, 3H, C16-H), 1.10 (s, 3H, C14-H), 0.96 (s, 3H, C17-H), 0.91 (t, *J* = 7.4 Hz, 3H, C1-H). **Minor diastereomer** δ 5.15 (p, *J* = 2.1 Hz, 1H, C5-H), 4.93 – 4.83 (m, 1H, C4-H), 4.61 – 4.50 (m, 1H, C7-H), 4.50 – 4.42 (m, 1H, C7-H), 4.30 (dd, *J* = 16.1, 3.3 Hz, 1H, C7-H, minor rotamer), 4.24 – 4.17 (m, 1H, C7-H, minor rotamer), 2.55 – 2.47 (m, 1H, C10-H), 2.02 – 1.88 (m, 3H, C10-H, C11-H), 1.84 – 1.74 (m, 1H, C11-H), 1.70 – 1.55 (m, 2H, C3-H), 1.38 – 1.20 (m, 2H, C2-H), 1.12 (s, 3H, C16-H), 1.10 (s, 3H, C14-H), 0.99 (s, 3H, C17-H), 0.91 (t, *J* = 7.4 Hz, 3H, C1-H).

^{13}C NMR (126 MHz, $CDCl_3$) Major diastereomer δ 178.7 (C13), 166.1 (C8), 154.8 (d, *J* = 274.4 Hz, C6), 101.4 (d, *J* = 5.5 Hz, C5), 92.7 (C9), 62.5 (d, *J* = 5.5 Hz, C4), 55.6 (C12), 54.2 (C15), 50.4 (d, *J* = 32.9 Hz, C7), 35.8 (d, *J* = 2.2 Hz, C3), 30.6 (C10), 29.3 (C11), 18.1 (C2), 17.7 (C16), 16.6 (C17), 14.2 (C1), 9.7 (C14). **Minor diastereomer** δ 178.6 (C13), 165.3 (C8), 154.8 (d, *J* = 274.2 Hz, C6), 101.5 (d, *J* = 5.4 Hz, C5), 93.1 (C9), 62.8 (d, *J* = 5.5 Hz, C4), 55.0 (C12), 54.4 (C15), 50.0 (d, *J* = 33.0 Hz, C7), 35.2 (d, *J* = 2.3 Hz, C3), 32.0 (C10), 29.0 (C11), 17.9 (C2), 17.6 (C16), 17.1 (C17), 14.2 (C1), 9.7 (C14).

^{19}F NMR (376 MHz, $CDCl_3$) Major diastereomer δ −128.62 (s, 1F, C6-F, major rotamer), −131.28 (s, 1F, C6-F, minor rotamer). **Minor diastereomer** δ −128.46 (s, 1F, C6-F, major rotamer), −131.44 (s, 1F, C6-F, minor rotamer).

HRMS (+ESI) calcd: $[M+H]^+$ ($C_{17}H_{24}FNO_3$) 310.1813, measured 310.1823 (3.22 ppm error).

IR (neat) v_{max}/cm^{-1}: 2963, 2875, 1789, 1702, 1638, 1428, 1368, 1166, 1015, 924.

***(3aS,7R,7aS)-2-benzoyl-5-(3-bromophenyl)-7-((2R,5R)-3-fluoro-5-(4-fluorophenyl)-2-methyl-2,5-dihydro-1H-pyrrole-1-carbonyl)-2,3,3a,6,7,7a-hexahydro-1H-isoindol-1-one* ((*R,R*)-25j)**

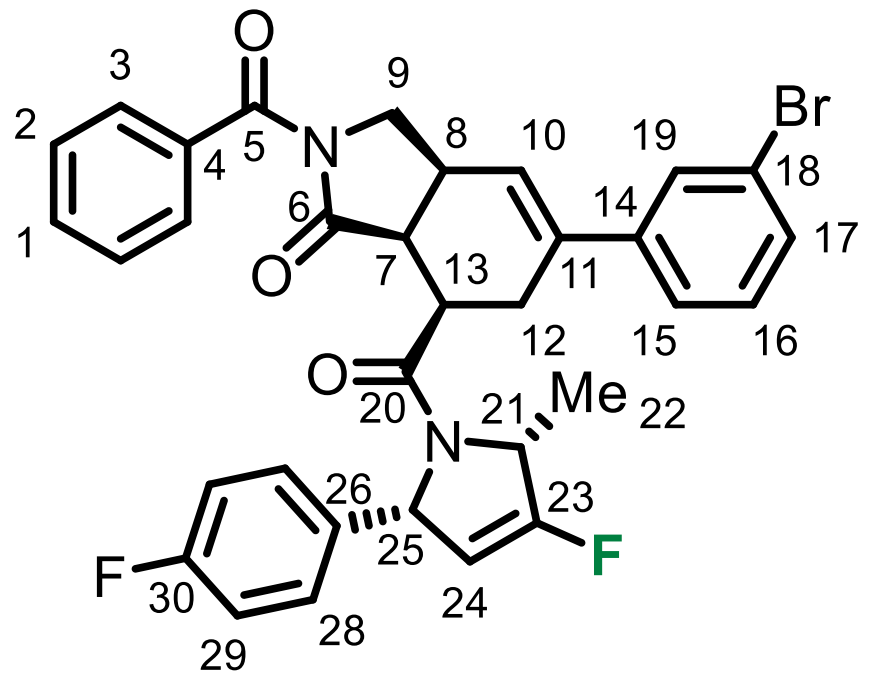

Pyrroline **(*R,R*)-4j** (0.2 mmol, 1 equiv.) was deprotected according to **General procedure VII**. The crude product was then added to a solution of DIPEA (1 mmol, 5 equiv.), and (3a*S*,4*R*,7a*S*)-2-benzoyl-6-(3-bromophenyl)-3-oxo-2,3,3a,4,5,7a-hexahydro-1*H*-isoindole-4-carboxylic acid (0.2 mmol, 1 equiv.) in DCM (2 mL) at 0°C. HBTU (0.3 mmol, 1.5 equiv.) was then added, and the reaction mixture was warmed to RT and stirred for 18 h. On completion, the reaction was diluted with water (30 mL) and extracted with DCM (2 × 20 mL). The organic fractions were combined, dried over $MgSO_4$, and concentrated *in vacuo* to afford the title compound as a colourless oil (70.3 mg, 57% over 2 steps).

Product NMR shows a mixture of 4 amide rotamers (C5, C21). Identified by characteristic peaks from the parent pyrroline **(*R,R*)-4j** and carboxylic acid. Isomeric ratio (**R1**:**R2**:**R3**:**R4**) = 25:11:1:14

^{1}H NMR (500 MHz, $CDCl_3$) δ 7.61 – 7.19 (m, 11H, C1-H, C2-H, C3-H, C15-H, C16-H, C17-H, C19-H, C28-H), 7.15 – 6.98 (m, 2H, C29-H), 6.05 – 5.92 (m, 1H, C10-H), 5.53 (s, 1H, C25-H), 5.18 – 5.01 (m, 1H, C24-H), 4.99 – 4.89 (m, 1H, C21-H), 4.16 – 4.07 (m, 1H, C7-H), 3.74 – 3.70 (m, 1H, C13-H), 3.35 – 3.19 (m, 1H, C9-H), 3.04 – 2.94 (m, 1H, C9-H), 2.93 (s, 1H, C8-H), 2.72 (s, 1H, C12-H), 2.57 – 2.45 (m, 1H, C12-H), 1.56 (d, J = 6.4 Hz, 3H, C22-H).

^{13}C NMR (126 MHz, $CDCl_3$) δ 174.7 – 170.2 (m, C5, C6, C20), 164.0 (d, J = 282.4 Hz, C23, **R4**), 162.8 (d, J = 250.3 Hz, C30, **R1**, **R2**), 162.4 (d, J = 245.4 Hz, **R4**), 159.6 (d, J = 278.2 Hz, **R1**, **R2**), 143.8 – 142.4 (m, C4), 140.6 – 133.4 (m, C14, C16, C26), 132.8 – 127.4 (m, C1, C2, C3, C11, C28), 125.9 – 122.1 (m, C10, C15, C17, C18, C19), 116.6 (d, J = 21.6 Hz, C29, **R1**), 115.6 (d, J = 21.3 Hz, C29, **R4**), 115.5 (d, J = 21.4 Hz, C29, **R2**), 115.0 (d, J = 21.2 Hz, C29, **R3**), 103.8 (d, J = 9.0 Hz, C24, **R4**), 103.5 (d, J = 9.4 Hz, **R3**), 102.2 (d, J = 8.0 Hz, **R1, R2**), 65.2 – 64.8 (m, C25, **R1**), 62.6 (d, J = 11.0 Hz, C25, **R4**), 62.4 (d, J = 8.0 Hz, C25, **R3**), 60.5 (C25, **R2**), 56.1 (d, J = 28.4 Hz, C21, **R1**), 55.5 (d, J = 26.6 Hz, C21, **R2**), 55.2 (d, J = 24.3 Hz, C21, **R4**), 54.9 (d, J = 24.4 Hz, C21, **R3**), 52.8 – 49.5 (m, C9), 46.0 – 42.6 (m, C7), 40.5 – 37.1 (m, C13), 33.8 – 30.4 (m, C8), 30.1 – 24.7 (m, C12), 21.2 (d, J = 3.0 Hz, C22, **R4**), 20.5 (d, J = 3.3 Hz, C22, **R3**), 18.6 (C22, **R1**), 14.3 (C22, **R2**).

^{19}F NMR (471 MHz, $CDCl_3$) [C30 signals] δ −112.18 – −112.32 (m, 1.00F, **R1**), −114.69 (s, 0.45F, **R2**), −115.16 (ddd, *J* = 14.2, 8.8, 5.4 Hz, 0.04F, **R3**), −115.24 (ddd, *J* = 14.2, 8.7, 5.4 Hz, 0.55F, **R4**). **[C23 signals]** δ −130.91 (s, 0.45F, **R2**), −132.63 (s, 1.00F, **R1**), −133.19 (t, *J* = 4.5 Hz, 0.55F, **R4**), −133.33 (t, *J* = 4.1 Hz, 0.04F, **R3**).

HRMS (+ESI) calcd: $[M+H]^+$ ($C_{33}H_{27}BrF_2N_2O_3$) 617.1246, measured 617.1265 (4.34 ppm error).

IR (neat) v_{max}/cm^{-1}: 3356, 2970, 2930, 1740, 1665, 1508, 1356, 1305, 1318, 1257, 1219, 1155, 840, 781, 695, 658, 548.

8.18. Pre-alkyne intermediates (26)

N-(4-methoxybenzyl)-4-methylbenzenesulfonamide (26a)

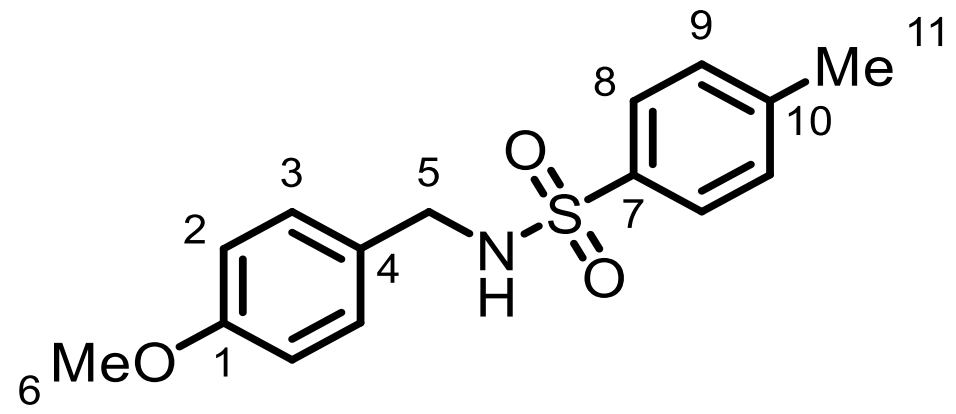

Prepared from 4-methoxybenzylamine according to **General procedure I**. Crude product was used without further purification.

^{1}H NMR (400 MHz, $CDCl_3$) δ 7.76 (d, *J* = 8.1 Hz, 2H, C8-H), 7.31 (d, *J* = 8.6 Hz, 2H, C3-H), 7.11 (d, *J* = 8.1 Hz, 2H, C9-H), 6.81 (d, *J* = 8.6 Hz, 2H, C2-H), 4.50 (s, 1H, C5-NH), 4.06 (d, *J* = 6.1 Hz, 2H, C5-H), 3.78 (s, 3H, C6-H), 2.44 (s, 3H, C11-H).

^{13}C NMR (126 MHz, $CDCl_3$) δ 159.5 (C1), 143.6 (C10), 137.0 (C7), 129.9 (C9), 129.4 (C3), 128.4 (C4), 127.4 (C8), 114.2 (C2), 55.4 (C6), 47.0 (C5), 21.7 (C11).

Characterisation data matches that previously reported.[46]

***N*-(4-chlorobenzyl)-4-methylbenzenesulfonamide (26d)**

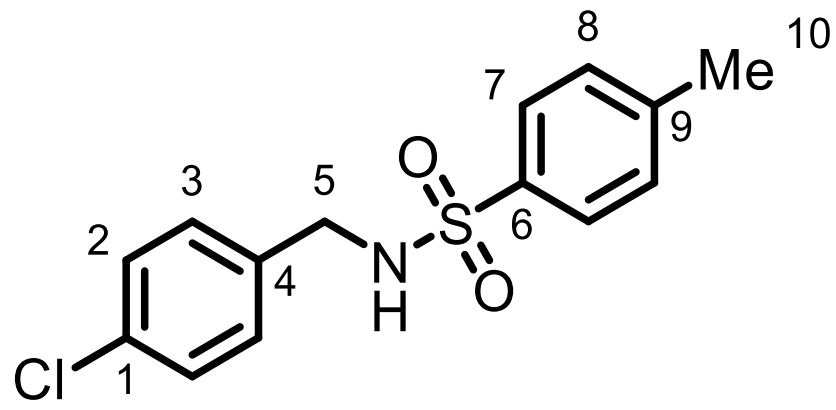

Prepared from 4-chlorobenzylamine according to **General procedure I**. Crude product was used without further purification.

^{1}H NMR (400 MHz, CDCl$_3$) δ 7.74 (d, *J* = 8.1 Hz, 2H, C7-H), 7.31 (d, *J* = 8.1 Hz, 2H, C8-H), 7.27 – 7.22 (m, 2H, C2-H), 7.14 (d, *J* = 8.4 Hz, 2H, C3-H), 4.64 (d, *J* = 7.3 Hz, 1H, C5-NH), 4.10 (d, *J* = 6.3 Hz, 2H, C5-H), 2.44 (s, 3H, C10-H).

^{13}C NMR (126 MHz, CDCl$_3$) δ 143.9 (C9), 136.9 (C1), 135.0 (C4), 134.0 (C6), 129.9 (C8), 129.4 (C2), 129.0 (C3), 127.3 (C7), 46.7 (C5), 21.7 (C10).

Characterisation data matches that previously reported.[47]

***N*-(4-fluorobenzyl)-4-methylbenzenesulfonamide (26e)**

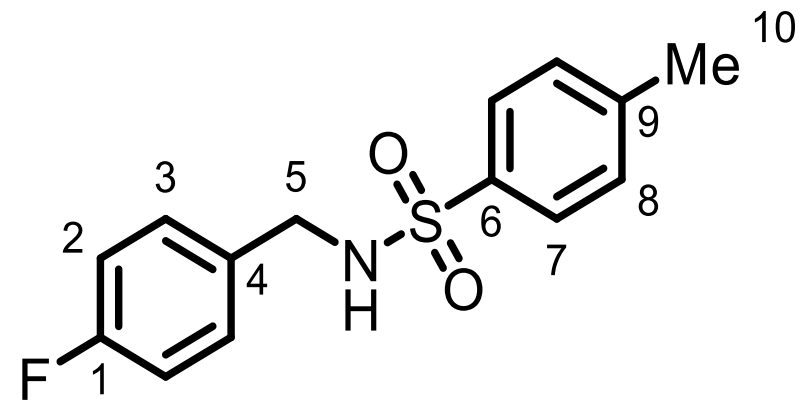

Prepared from 4-fluorobenzylamine according to **General procedure I**. Crude product was used without further purification.

^{1}H NMR (400 MHz, CDCl$_3$) δ 7.75 (d, *J* = 8.2 Hz, 2H, C7-H), 7.32 (d, *J* = 8.2 Hz, 2H, C8-H), 7.22 – 7.13 (m, 2H, C2-H), 6.97 (tt, *J* = 8.9, 2.9 Hz, 2H, C3-H), 4.55 (s, 1H, C5-NH), 4.10 (d, *J* = 6.2 Hz, 2H, C5-H), 2.44 (s, 3H, C10-H).

^{13}C NMR (151 MHz, CDCl$_3$) δ 162.6 (d, *J* = 246.5 Hz, C1), 143.8 (C9), 137.0 (C6), 132.2 (d, *J* = 3.3 Hz, C4), 129.9 (C7), 129.8 (d, *J* = 8.1 Hz, C3), 127.3 (C8), 115.8 (d, *J* = 21.7 Hz, C2), 46.8 (C5), 21.7 (C10).

^{19}F NMR (376 MHz, CDCl$_3$) δ −114.03 (tt, *J* = 8.8, 5.3 Hz, C1-F).

Characterisation data matches that previously reported.[46]

N-((4-fluorophenyl)methyl-d)prop-2-yn-1-amine **([^{2}H]-26e)**

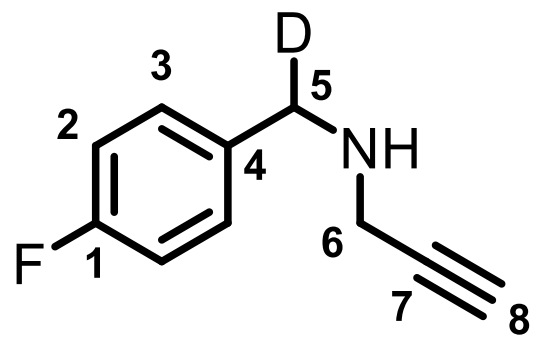

An RBF containing 4-fluorobenzaldehyde (5 mmol, 0.535 mL), propargylamine (6 mmol, 0.385 mL), and MeOH (25 mL) was cooled to 0 °C and stirred for 30 minutes. $NaBD_4$ (7.5 mmol, 314 mg) was then added in portions and stirred for 3 h at 0 °C, then warmed to RT and stirred overnight. The reaction was then quenched with sat. aq. $NaHCO_3$ (50 mL), then diluted with water (100 mL), and extracted with DCM (3 × 30 mL). The organic fractions were combined and dried over $MgSO_4$, filtered, and concentrated *in vacuo* to afford the title compound without further purification as a colourless oil (772 mg, 94%).

^{1}H NMR (500 MHz, $CDCl_3$) δ 7.35 – 7.28 (m, 2H, C2-H), 7.05 – 6.96 (m, 2H, C3-H), 3.83 (t, *J* = 1.9 Hz, 1H, C5-H), 3.41 (d, *J* = 2.5 Hz, 2H, C6-H), 2.26 (t, *J* = 2.4 Hz, 1H, C8-H), 1.44 (s, 1H, C5-NH).

^{13}C NMR (126 MHz, $CDCl_3$) δ 162.2 (d, *J* = 244.9 Hz, C1), 135.2 (d, *J* = 3.2 Hz, C4), 130.1 (d, *J* = 8.0 Hz, C3), 115.4 (d, *J* = 21.2 Hz, C2), 82.1 (C7), 71.8 (C8), 51.2 (t (1:1:1), *J* = 20.5 Hz, C5), 37.3 (C6).

^{19}F NMR (376 MHz, $CDCl_3$) δ −115.61 (tt, *J* = 8.6, 5.6 Hz, 1F, C1-F).

HRMS (+ESI) calcd: $[M+H]^+$ ($C_{10}H_9DFN$) 165.0933, measured 165.0927 (3.63 ppm error).

IR (neat) v_{max}/cm^{-1}: 3299, 2916, 2832, 1726, 1604, 1509, 1450, 1219, 1156, 1108, 818, 647.

4-methyl-*N*-(4-(trifluoromethyl)benzyl)benzenesulfonamide (26f)

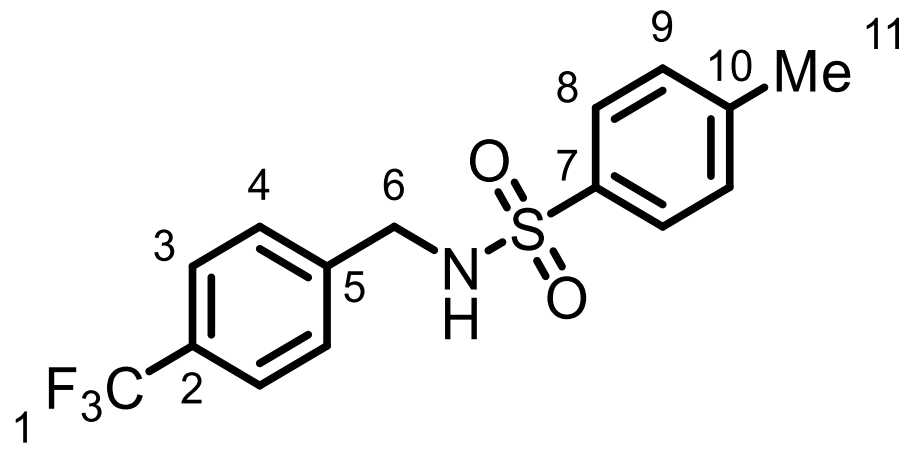

Prepared from 4-(trifluoromethyl)benzylamine according to **General procedure I**. Crude product was used without further purification.

^{1}H NMR (400 MHz, $CDCl_3$) δ 7.73 (d, *J* = 8.1 Hz, 2H, C8-H), 7.53 (d, *J* = 8.0 Hz, 2H, C3-H), 7.33 (d, *J* = 8.1 Hz, 2H, C4-H), 7.30 (d, *J* = 8.0 Hz, 2H, C9-H), 4.67 (d, *J* = 6.5 Hz, 1H, C6-NH), 4.21 (d, *J* = 6.4 Hz, 2H, C6-H), 2.43 (s, 3H, C11-H).

^{13}C NMR (151 MHz, $CDCl_3$) δ 144.0 (C5), 140.5 (C7), 136.9 (C10), 130.3 (q, *J* = 32.1 Hz, C2), 130.0 (C4), 128.2 (C9), 127.3 (C8), 125.8 (q, *J* = 3.8 Hz, C3), 124.1 (q, *J* = 270.8 Hz, C1), 46.9 (C6), 21.7 (C11).

^{19}F NMR (376 MHz, $CDCl_3$) δ −62.53 (C1-F).

Characterisation data matches that previously reported.[48]

pent-4-yn-2-yl methanesulfonate (26h)

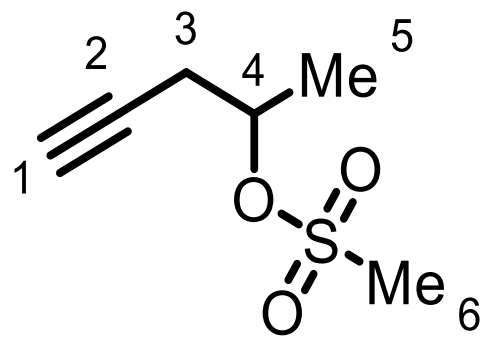

4-pentyn-2-ol was mesylated according to **General procedure I1A** without further purification to afford the title compound as a thin orange oil (1.40 g, 86%).

^{1}H NMR (500 MHz, CDCl$_3$) δ 4.88 (sextet, *J* = 6.2 Hz, 1H, C4-H), 3.05 (s, 3H, C6-H), 2.63 (ddd, *J* = 17.0, 6.2, 2.7 Hz, 1H, C3-H), 2.57 (ddd, *J* = 17.0, 6.2, 2.7 Hz, 1H, C3-H), 2.08 (t, *J* = 2.7 Hz, 1H, C1-H), 1.51 (d, *J* = 6.2 Hz, 3H, C5-H).

^{13}C NMR (126 MHz, CDCl$_3$) δ 78.8 (C2), 76.9 (C4), 71.6 (C1), 38.8 (C6), 26.8 (C3), 20.7 (C5).

Characterisation data matches that previously reported.[49]

but-3-yn-2-yl methanesulfonate (26j)

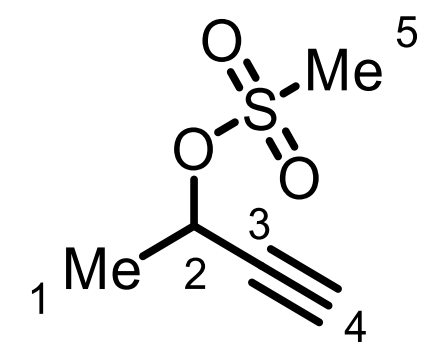

A solution of but-3-yn-2-ol (10 mmol, 1equiv.) in DCM (8 mL) was cooled to 0 °C, and Et_3N (15 mmol, 1.5 equiv.) was added. A solution of methanesulfonyl chloride (11 mmol, 1.1 equiv.) in DCM (2 mL) was then added dropwise and stirred at 0 °C for 2 h. The reaction was then warmed to RT and stirred for a further 18 h. The reaction mixture was diluted with H_2O (20 mL) and extracted with DCM (3 × 20 mL). The organic fractions were combined, dried over $MgSO_4$, filtered, and concentrated *in vacuo* to afford the product as an orange liquid (1.304 g, 88%)

^{1}H NMR (500 MHz, CDCl$_3$) δ 5.29 (qd, *J* = 6.8, 2.1 Hz, 1H, C2-H), 3.12 (s, 3H, C5-H), 2.70 (d, *J* = 2.0 Hz, 1H, C1-H), 1.66 (d, *J* = 6.8 Hz, 3H, C1-H).

^{13}C NMR (126 MHz, CDCl$_3$) δ 80.3 (C3), 76.4 (C4), 67.6 (C2), 39.3 (C5), 22.6 (C1).

Characterisation data matches that previously reported.[33]

***N*-benzhydryl-4-methylbenzenesulfonamide (26o)**

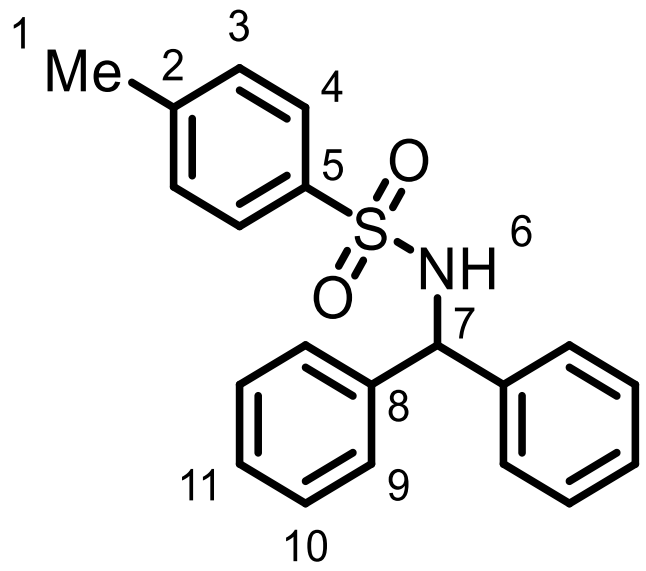

Prepared according to **General procedure I** using benzhydrylamine (50 mmol, 1 equiv.), affording the title compound without further purification as a light yellow solid (7.6 g, 45%).

^{1}H NMR (500 MHz, CDCl$_3$) δ 7.56 (d, *J* = 8.3 Hz, 2H, C4-H), 7.25 – 7.18 (m, 6H, C10-H), 7.14 (d, *J* = 8.3 Hz, 2H, C3-H), 7.11 – 7.08 (m, 4H, C10-H, C11-H), 5.57 (d, *J* = 7.0 Hz, 1H, C7-H), 5.02 (d, *J* = 7.0 Hz, 1H, C6-H), 2.38 (s, 3H, C1-H).

^{13}C NMR (126 MHz, CDCl$_3$) δ 143.4 (C2), 140.7 (C8), 137.5 (C5), 129.5 (C3), 128.7 (C10), 127.8 (C11), 127.5 (C9), 127.4 (C4), 61.5 (C7), 21.6 (C1).

Characterisation data match that previously reported.[50]

***rac*-4-methyl-*N*-(1-phenylethyl)benzenesulfonamide (26p)**

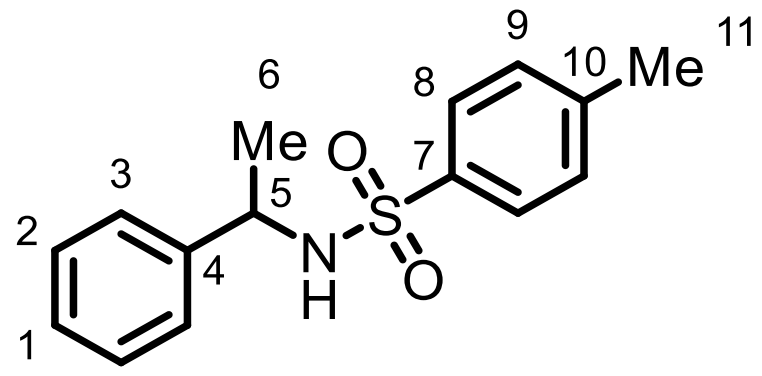

Prepared from α-methylbenzylamine according to **General procedure I**. Crude product was used without further purification.

^{1}H NMR (400 MHz, $CDCl_3$) δ 7.61 (app. dt, *J* = 8.3, 2.2 Hz, 2H, C8-H), 7.24 – 7.15 (m, 5H, C1-H, C2-H, C9-H), 7.14 – 7.05 (m, 2H, C3-H), 4.82 (s, 1H, C5-NH), 4.46 (p, *J* = 6.9 Hz, 1H, C5-H), 2.38 (s, 3H, C11-H), 1.43 (d, *J* = 6.9 Hz, 3H, C6-H).

^{13}C NMR (101 MHz, $CDCl_3$) δ 143.3 (C4), 142.1 (C7), 137.7 (C10), 129.6 (C2), 128.7 (C9), 127.6 (C1), 127.2 (C8), 126.2 (C3), 53.7 (C5), 23.7 (C6), 21.6 (C11).

Characterisation data matches that previously reported.[51]

***(S)*-4-methyl-*N*-(1-phenylethyl)benzenesulfonamide ((*S*)-26p)**

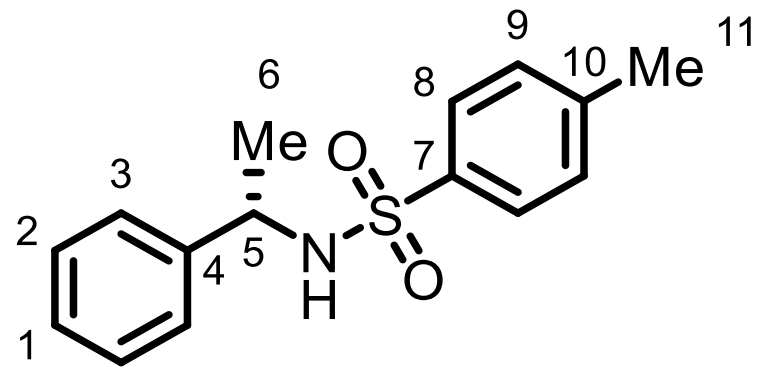

Prepared from (S)-(−)-α-methylbenzylamine according to **General procedure I**. Crude product was used without further purification.

^{1}H NMR (400 MHz, CDCl$_3$) δ 7.61 (app. dt, *J* = 8.3, 2.2 Hz, 2H, C8-H), 7.24 – 7.15 (m, 5H, C1-H, C2-H, C9-H), 7.14 – 7.05 (m, 2H, C3-H), 4.82 (s, 1H, C5-NH), 4.46 (p, *J* = 6.9 Hz, 1H, C5-H), 2.38 (s, 3H, C11-H), 1.43 (d, *J* = 6.9 Hz, 3H, C6-H).

^{13}C NMR (101 MHz, CDCl$_3$) δ 143.3 (C4), 142.1 (C7), 137.7 (C10), 129.6 (C2), 128.7 (C9), 127.6 (C1), 127.2 (C8), 126.2 (C3), 53.7 (C5), 23.7 (C6), 21.6 (C11).

Characterisation data matches that previously reported.[51]

***N*-cyclohexyl-4-methylbenzenesulfonamide (26s)**

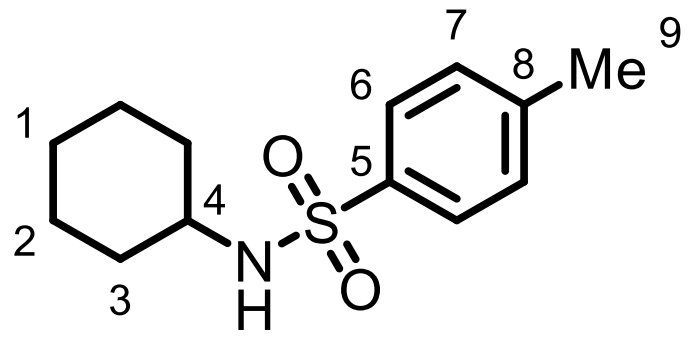

Prepared from cyclohexylamine according to **General procedure I**. Crude product was used without further purification.

^{1}H NMR (500 MHz, $CDCl_3$) δ 7.76 (app. dt, *J* = 8.1, 1.7 Hz, 2H, C6-H), 7.29 (d, *J* = 8.1 Hz, 2H, C7-H), 4.41 (br d, *J* = 7.6 Hz, 1H, C4-NH), 3.13 (dddd, *J* = 14.0, 10.1, 7.6, 3.9 Hz, 1H, C4-H), 1.79 – 1.70 (m, 2H, C3-H), 1.67 – 1.60 (m, 2H, C2-H), 1.51 (ddd, *J* = 12.7, 5.6, 3.5 Hz, 1H, C1-H), 1.25 – 1.07 (m, 5H, C1-H, C2-H, C3-H).

^{13}C NMR (126 MHz, $CDCl_3$) δ 143.3 (C8), 138.6 (C5), 129.8 (C7), 127.1 (C6), 52.7 (C4), 34.1 (C3), 25.3 (C1), 24.8 (C2), 21.7 (C9).

Characterisation data matches that previously reported.[52]

4-methyl-*N*-phenethylbenzenesulfonamide (26t)

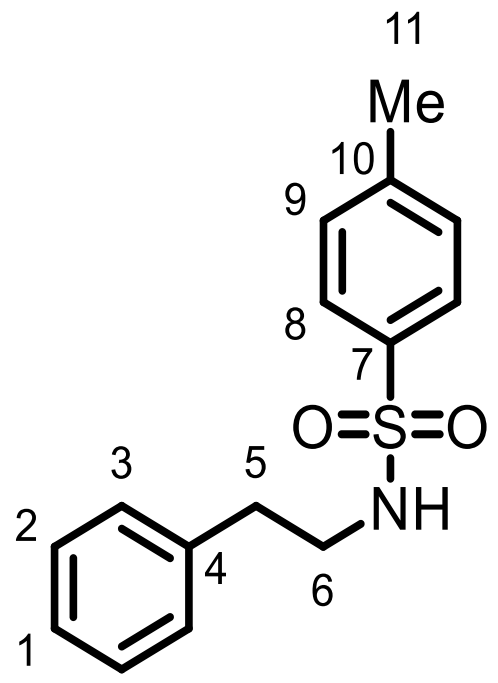

Prepared from phenethylamine according to **General procedure I**. Crude product was used without further purification.

^{1}H NMR (400 MHz, $CDCl_3$): δ 7.69 (app. dt, *J* = 8.3, 1.8 Hz, 2H, C8-H), 7.30 – 7.28 (m, 2H, C9-H), 7.28 – 7.19 (m, 3H, C1-H, C3-H), 7.11 – 7.04 (m, 2H, C2-H), 4.42 – 4.25 (m, 1H, C6-NH), 3.22 (qd, *J* = 6.9, 1.6 Hz, 2H, C6-H), 2.76 (t, *J* = 6.9 Hz, 2H, C5-H), 2.43 (s, 3H, C11-H).

^{13}C NMR (151 MHz, $CDCl_3$): δ 143.6 (C10), 137.8 (C4), 137.1 (C7), 129.9 (C9), 128.9 (C2), 128.9 (C3), 127.2 (C8), 127.0 (C1), 44.3 (C6), 35.9 (C5), 21.7 (C11).

Characterisation data matches that previously reported.[48]

4-methyl-*N*-(2-(thiophen-2-yl)ethyl)benzenesulfonamide (26w)

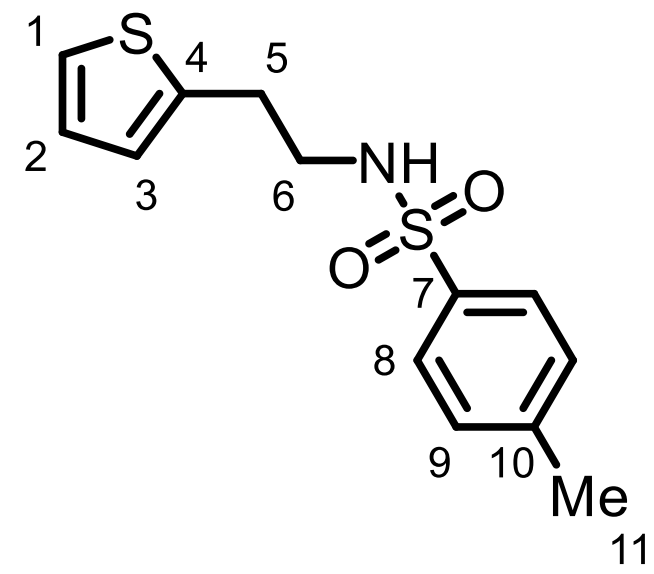

Prepared from 2-thiopheneethylamine according to **General procedure I**. Crude product was used without further purification.

^{1}H NMR (500 MHz, CDCl$_3$) δ 7.72 (d, J = 8.6 Hz, 2H, C8-H), 7.30 (d, J = 8.0 Hz, 2H, C9-H), 7.15 (dd, J = 5.1, 1.1 Hz, 1H, C1-H), 6.91 (dd, J = 5.1, 3.5 Hz, 1H, C2-H), 6.76 (dd, J = 3.5, 1.1 Hz, 1H, C3-H), 4.54 (br s, 1H, C6-NH), 3.23 (q, J = 6.6 Hz, 2H, C6-H), 2.97 (t, J = 6.7 Hz, 2H, C5-H), 2.43 (s, 3H, C11-H).

^{13}C NMR (126 MHz, CDCl$_3$) δ 143.7 (C10), 140.0 (C4), 137.0 (C7), 129.9 (C9), 127.3 (C2), 127.2 (C8), 126.0 (C3), 124.5 (C1), 44.5 (C6), 30.2 (C5), 21.7 (C11).

Characterisation data matches that previously reported.[53]

4-methyl-N-((tetrahydrofuran-2-yl)methyl)benzenesulfonamide (26x)

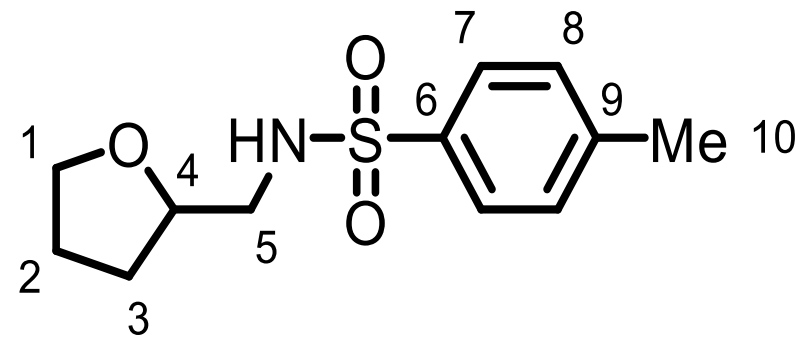

Prepared from 2-(aminomethyl)tetrahydrofuran according to **General procedure I**. Crude product was used without further purification.

^{1}H NMR (400 MHz, $CDCl_3$) δ 7.73 (app. dt, J = 8.3, 1.7 Hz, 2H, C7-H), 7.34 – 7.28 (m, 2H, C8-H), 4.72 (s, 1H, C5-NH), 3.93 (qd, J = 6.8, 3.6 Hz, 1H, C4-H), 3.77 (dt, J = 8.4, 6.5 Hz, 1H, C5-H), 3.69 (dt, J = 8.3, 6.7 Hz, 1H, C5-H), 3.11 (ddd, J = 12.4, 6.9, 3.6 Hz, 1H, C1-H), 2.89 (ddd, J = 12.3, 6.5, 5.5 Hz, 1H, C1-H), 2.43 (s, 3H, C10-H), 1.99 – 1.89 (m, 1H, C3-H), 1.89 – 1.81 (m, 2H, C2-H), 1.67 – 1.56 (m, 1H, C3-H).

^{13}C NMR (126 MHz, $CDCl_3$) δ 143.7 (C9), 139.3 (C6), 130.4 (C8), 127.8 (C7), 78.2 (C4), 68.5 (C1), 47.9 (C5), 29.3 (C3), 26.2 (C2), 21.4 (C10).

Characterisation data matches that previously reported.[54]

***N*,4-dimethylbenzenesulfonamide (26ac)**

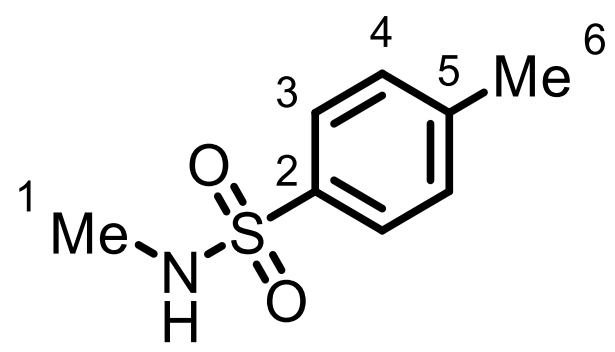

Prepared from methylamine hydrochloride according to **General procedure I**. Crude product was used without further purification.

^{1}H NMR (500 MHz, $CDCl_3$) δ 7.75 (d, J = 7.9 Hz, 2H, C3-H), 7.32 (d, J = 7.9 Hz, 2H, C4-H), 4.31 (br s, 1H, C1-NH), 2.65 (d, J = 4.1 Hz, 3H, C1-H), 2.43 (s, 3H, C6-H).

^{13}C NMR (126 MHz, $CDCl_3$) δ 143.7 (C5), 136.0 (C2), 129.9 (C4), 127.5 (C3), 29.5 (C1), 21.7 (C6).

Characterisation data matches that previously reported.[55]

1-tosylpiperidin-4-ol (26ao)

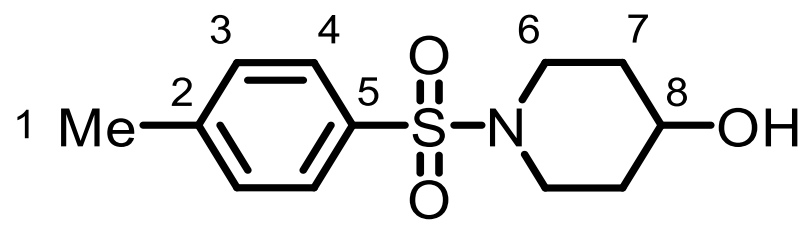

Prepared from 4-hydroxypiperidine according to **General procedure I** without further purification to afford the title compound as a colourless oil (765 mg, 60%).

^{1}H NMR (400 MHz, $CDCl_3$) δ 7.64 (d, *J* = 7.9 Hz, 2H, C4-H), 7.32 (d, *J* = 7.9 Hz, 2H, C3-H), 3.75 (tt, *J* = 7.6, 3.5 Hz, 1H, C8-H), 3.30 (ddd, *J* = 11.4, 7.3, 3.8 Hz, 2H, C6-H, equatorial), 2.89 – 2.78 (m, 2H, C6-H, axial), 2.43 (s, 3H, C1-H, 2.01 – 1.86 (m, 2H, C7-H, equatorial), 1.65 (dtd, *J* = 12.0, 7.9, 3.5 Hz, 2H, C7-H, axial), 1.47 (s, 1H, C8-OH).

^{13}C NMR (126 MHz, $CDCl_3$) δ 143.7 (C2), 133.3 (C5), 129.8 (C3), 127.8 (C4), 66.1 (C8), 43.3 (C6), 33.4 (C7), 21.7 (C1).

Characterisation data matches that previously reported.[56]

(*E*)-*N*-(4-fluorobenzylidene)-4-methylbenzenesulfonamide (26ax)

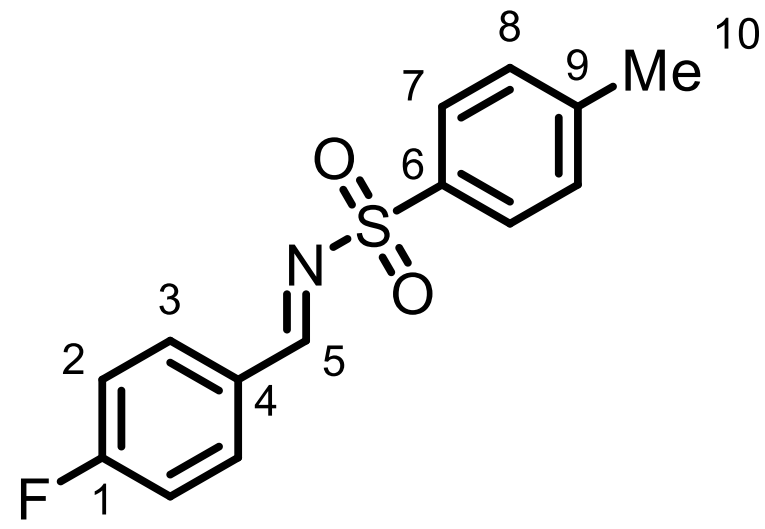

A pressure tube was charged with 4-fluorobenzaldehyde (3.3 mmol, 1.1 equiv.), pyrrolidine (0.3 mmol, 0.1 equiv.), *p*-toluene sulfonamide (3 mmol, 1 equiv.), activated 4Å molecular sieves (1.0 g), and DCM (10 mL). The reaction mixture was then heated to 60 °C for 24 h. The reaction was then cooled to RT, filtered, and concentrated *in vacuo* to afford the crude product, which was purified by flash column chromatography to afford the title compound as a white solid (328 mg, 40%).

^{1}H NMR (500 MHz, $CDCl_3$) δ 9.00 (s, 1H, C5-H), 7.99 – 7.92 (m, 2H, C3-H), 7.89 (app. dt, *J* = 8.4, 1.9 Hz, 2H, C7-H), 7.35 (d, *J* = 8.4 Hz, 2H, C8-H), 7.17 (tt, *J* = 8.5, 2.9 Hz, 2H, C2-H), 2.44 (s, 3H, C10-H).

^{13}C NMR (126 MHz, $CDCl_3$) δ 168.7 (C5), 167.0 (d, *J* = 258.4 Hz, C1), 144.8 (C9), 135.2 (C6), 133.9 (d, *J* = 9.8 Hz, C3), 130.0 (C8), 128.9 (d, *J* = 2.9 Hz, C4), 128.2 (C7), 116.8 (d, *J* = 22.3 Hz, C2), 21.8 (C10).

Characterisation data matches that previously reported.[57]

1-(4-(trifluoromethyl)phenyl)-3-(trimethylsilyl)prop-2-yn-1-ol (26az)

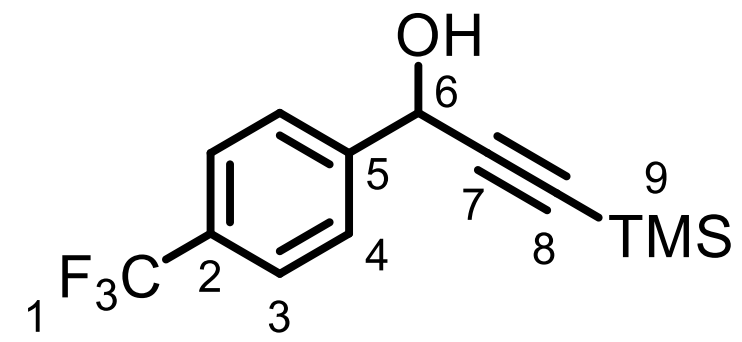

Prepared from 4-(trifluoromethyl)benzaldehyde according to **General procedure I4** without further purification to afford the title compound as a colourless oil (1.02 g, 85%).

^{1}H NMR (500 MHz, $CDCl_3$) δ 7.70 – 7.60 (m, 4H, C3-H, C4-H), 5.51 (s, 1H, C6-H), 2.31 (s, 1H, C6-OH), 0.21 (s, 9H, C9-H).

^{13}C NMR (126 MHz, $CDCl_3$) δ 144.2 (q, *J* = 1.3 Hz, C5), 130.6 (q, *J* = 32.4 Hz, C2), 127.1 (C4), 125.7 (q, *J* = 3.7 Hz, C3), 124.2 (q, *J* = 272.1 Hz, C1), 104.2 (C7), 92.6 (C8), 64.5 (C6), -0.1 (C9).

^{19}F NMR (376 MHz, $CDCl_3$) δ −62.46 (s, 3F, C1-F).

Characterisation data matches that previously reported.[58]

4-methyl-*N*-(thiophen-2-ylmethyl)benzenesulfonamide (26bh)

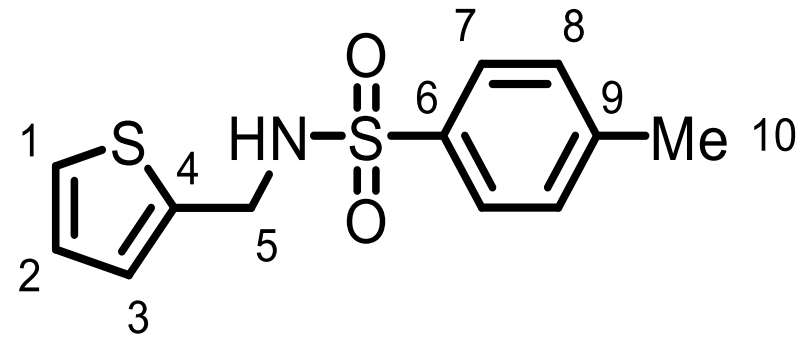

Prepared from 2-(aminomethyl)thiophene according to **General procedure I**. Crude product was used without further purification.

^{1}H NMR (400 MHz, $CDCl_3$) δ 7.79 – 7.71 (m, 2H, C7-H), 7.30 (d, *J* = 7.9 Hz, 2H, C8-H), 7.19 (dd, *J* = 5.0, 1.3 Hz, 1H, C1-H), 6.88 (dd, *J* = 5.0, 3.0 Hz, 1H, C2-H), 6.85 (dd, *J* = 3.0, 1.3 Hz, 1H, C3-H), 4.59 (d, *J* = 6.1 Hz, 1H, C5-NH), 4.33 (dd, *J* = 6.1, 0.8 Hz, 2H, C5-H), 2.43 (s, 3H, C10-H).

^{13}C NMR (126 MHz, $CDCl_3$) δ 143.8 (C9), 139.0 (C4), 136.9 (C6), 129.9 (C8), 127.4 (C7), 127.1 (C2), 126.7 (C3), 126.0 (C1), 42.3 (C5), 21.7 (C10).

Characterisation data matches that previously reported.[59]

hexyl(prop-2-yn-1-yl)sulfane (26bo)

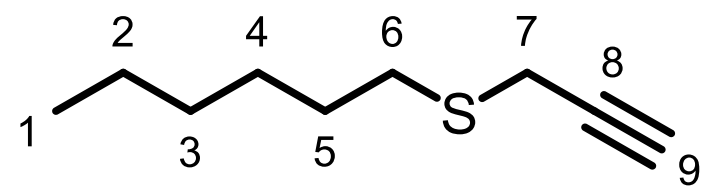

n-Hexyl mercaptan was propargylated according to **General procedure II** without further purification to afford the title compound as a yellow oil (840 mg, 100%).

^{1}H NMR (500 MHz, $CDCl_3$) δ 3.24 (d, *J* = 2.6 Hz, 2H, C7-H), 2.70 – 2.66 (m, 2H, C6-H), 2.22 (t, *J* = 2.6 Hz, 1H, C9-H), 1.65 – 1.58 (m, 2H, C5-H), 1.44 – 1.36 (m, 2H, C4-H), 1.33 – 1.25 (m, 4H, C3-H, C2-H), 0.91 – 0.86 (m, 3H, C1-H).

^{13}C NMR (126 MHz, $CDCl_3$) δ 80.3 (C8), 70.9 (C9), 31.8 (C6), 31.5 (C3), 29.0 (C4), 28.6 (C5), 22.7 (C2), 19.3 (C7), 14.2 (C1).

Characterisation data matches that previously reported.[42]

N-(4-fluorobenzyl)-2,4-dinitrobenzenesulfonamide **(26bq)**

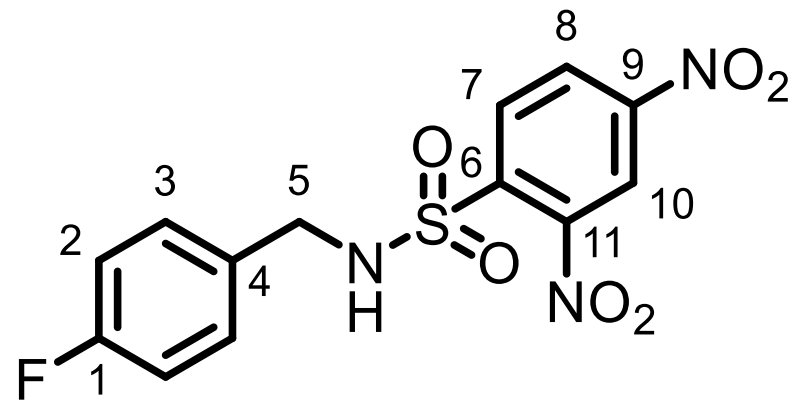

4-fluorobenzylamine was protected according to **General procedure I** using 2,4-dinitrobenzenesulfonyl chloride instead of TsCl, affording the title compound without further purification as a yellow solid (1.26 g, 71%).

^{1}H NMR (500 MHz, Acetone-*D$_6$*) δ 8.74 (d, *J* = 2.2 Hz, 1H, C10-H), 8.56 (dd, *J* = 8.7, 2.2 Hz, 1H, C8-H), 8.22 (d, *J* = 8.7 Hz, 1H, C7-H), 7.67 (t, *J* = 5.8 Hz, 1H, C5-NH), 7.41 – 7.31 (m, 2H, C3-H), 7.05 – 6.94 (m, 2H, C2-H), 4.44 (d, *J* = 6.3 Hz, 2H, C5-H).

^{13}C NMR (126 MHz, Acetone-*D$_6$*) δ 162.2 (d, *J* = 244.4 Hz, C1), 149.9 (C11), 147.9 (C9), 139.1 (C6), 133.2 (d, *J* = 3.2 Hz, C4), 132.3 (C7), 130.0 (d, *J* = 8.3 Hz, C3), 126.9 (C8), 120.2 (C10), 115.0 (d, *J* = 21.6 Hz, C2), 46.4 (C5).

^{19}F NMR (376 MHz, Acetone-*D$_6$*) δ −116.24 (tt, *J* = 9.2, 5.4 Hz, 1F, C1-F).

HRMS (-MALDI) calcd. $[M]^-$ ($C_{13}H_{10}N_3O_6SF$) 354.0202, measured 354.0207 (1.41 ppm error).

IR (neat) v_{max}/cm^{-1}: 3351, 3105, 1738, 1604, 1537, 1509, 1349, 1218, 1168, 1102, 833, 746.

8.19. Miscellaneous (27-29)

***(Z)-N-(4-bromobenzyl)-N-(2-fluoro-3-iodoallyl)-4-methylbenzenesulfonamide* (27c)**

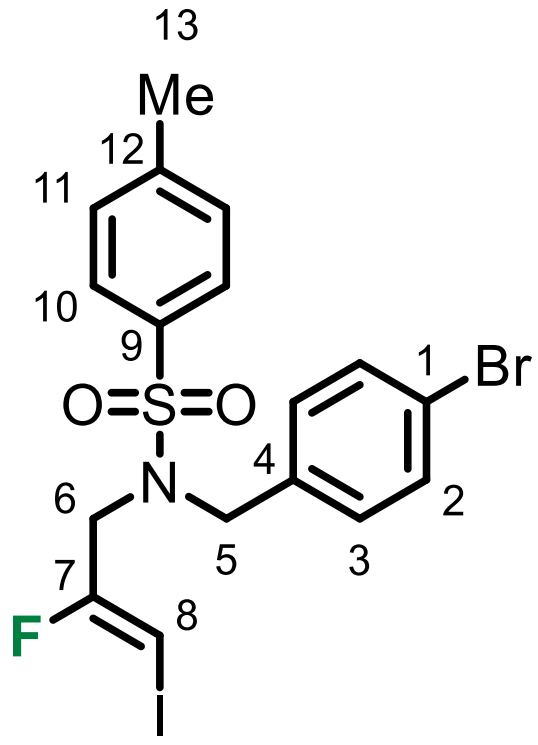

An RBF containing FVI **3c** (0.2 mmol, 1 equiv.) and TBAI (0.3 mmol, 1.5 equiv.) in MeCN (2 mL, 0.1 M) was heated to 70°C with stirring for 16 h. The reaction mixture was then diluted with diethyl ether (30 mL) and washed with water (2 × 20 mL). The organic layer was then dried over $MgSO_4$, filtered, and concentrated *in vacuo* to afford the crude product, which was then purified by flash column chromatography to afford the title compound as a white solid (81.1 mg, 77%).

^{1}H NMR (500 MHz, $CDCl_3$) δ 7.71 (dt, *J* = 8.1, 1.8 Hz, 2H, C10-H), 7.46 (dt, *J* = 8.4, 1.8 Hz, 2H, C2-H), 7.33 (d, *J* = 8.1 Hz, 2H, C11-H), 7.15 (dt, *J* = 8.4, 2.0 Hz, 2H, C3-H), 5.39 (d, *J* = 33.5 Hz, 1H, C8-H), 4.32 (s, 2H, C5-H), 3.95 (d, *J* = 16.0 Hz, 2H, C6-H), 2.45 (s, 3H, C13-H).

^{13}C NMR (126 MHz, $CDCl_3$) δ 160.5 (d, *J* = 264.9 Hz, C7), 144.0 (C12), 136.7 (C9), 134.3 (C4), 132.0 (C3), 130.3 (C2), 129.9 (C11), 127.4 (C10), 122.4 (C1), 57.7 (d, *J* = 24.1 Hz, C8), 50.7 (d, *J* = 1.3 Hz, C5), 46.6 (d, *J* = 29.0 Hz, C6), 21.7 (C13).

^{19}F NMR (376 MHz, $CDCl_3$) δ -86.18 (dt, *J* = 32.8, 16.0 Hz, 1F, C7-F).

HRMS (+Nano) calcd: $[M+H]^+$ ($C_{17}H_{16}BrFINO_2S$) 523.9187, measured 523.9193 (1.15 ppm error).

IR (neat) v_{max}/cm^{-1}: 3087, 2922, 1657, 1596, 1487, 1336, 1157, 1090, 912, 813, 665, 561, 545.

***N*-(4-bromobenzyl)-*N*-(3-iodoprop-2-yn-1-yl)-4-methylbenzenesulfonamide (28c)**

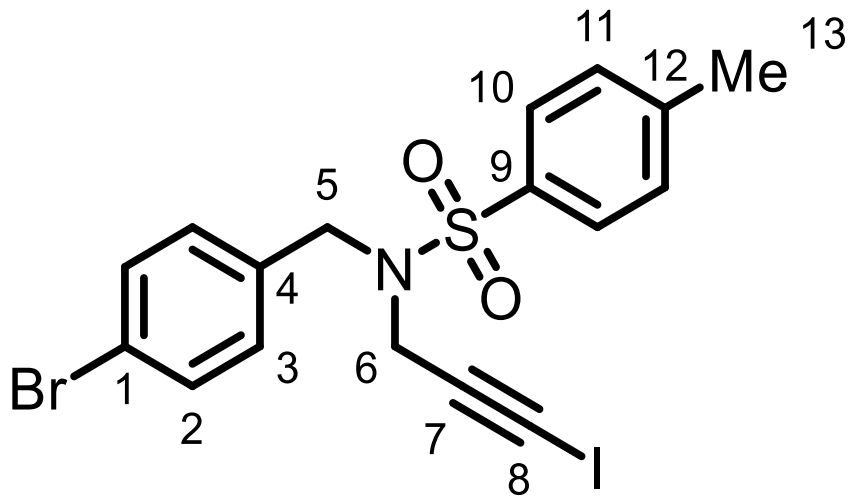

A Schlenk tube was purged with N_2 and charged with iodofluoroalkyne **27c** (0.08 mmol, 1 equiv.) in Et_2O (1 mL). A solution of KHMDS (0.16 mmol, 2 equiv.) in Et_2O (1 mL) was then added dropwise at RT via a needle. The reaction mixture was then stirred for 2 h, and an NMR yield was taken (22% NMR). The reaction mixture was then concentrated *in vacuo* and purified by flash column chromatography (2-20% EtOAc in pentane) to afford the title compound as a white solid (8.1 mg, 20%).

^{1}H NMR (500 MHz, $CDCl_3$) δ 7.76 (d, *J* = 8.3 Hz, 2H, C10-H), 7.47 (d, *J* = 8.3 Hz, 2H, C2-H), 7.36 (d, *J* = 8.3 Hz, 2H, C11-H), 7.22 (d, *J* = 8.3 Hz, 2H, C3-H), 4.26 (s, 2H, C5-H), 4.03 (s, 2H, C6-H), 2.46 (s, 3H, C13-H).

^{13}C NMR (126 MHz, $CDCl_3$) δ 144.0 (C12), 135.4 (C4), 134.0 (C9), 132.0 (C11), 130.6 (C10), 129.8 (C2), 128.0 (C3), 122.4 (C1), 86.5 (C7), 49.9 (C5), 37.8 (C6), 21.8 (C13), 2.3 (C8).

HRMS (+ESI) calcd: $[M+H]^+$ ($C_{17}H_{15}BrINO_2S$) 503.9124, measured 503.9108 (3.18 ppm error).

IR (neat) v_{max}/cm^{-1}: 2969, 1738, 1487, 1346, 1159, 1090, 1069, 1012, 903, 748, 662, 560, 542.

(2-fluoroethene-1,1-diyl)dibenzene (29ag)

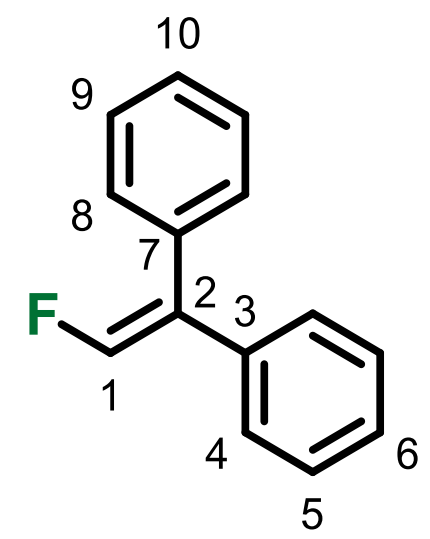

Produced as a side-product during the synthesis of oxolene **6ag** (4.8 mg, 12%).

^{1}H NMR (500 MHz, $CDCl_3$) δ 7.40 – 7.28 (m, 8H, C4-H, C5-H, C8-H, C9-H), 7.26 – 7.22 (m, 2H, C6-H, C10-H), 6.97 (d, *J* = 83.4 Hz, 1H, C1-H).

^{13}C NMR (126 MHz, $CDCl_3$) δ 145.9 (d, *J* = 268.5 Hz, C1), 137.1 (d, *J* = 7.9 Hz, C3), 135.3 (C2), 129.9 (d, *J* = 4.2 Hz, C4), 128.8 (d, *J* = 3.1 Hz, C8), 128.6 (C5), 128.4 (C9), 127.9 (C6), 127.9 (C10), 126.4 (d, *J* = 5.7 Hz, C2).

^{19}F NMR (471 MHz, $CDCl_3$) δ -128.1 (d, *J* = 83.4 Hz, 1F, C1-F).

Characterisation data match that previously reported.[60]

9. NMR spectra for Novel Compounds

9.1. Alkynes (1)

***N-(4-methoxybenzyl)-4-methyl-N-(prop-2-yn-1-yl)benzenesulfonamide* (1a)**

1H NMR (400 MHz, $CDCl_3$)

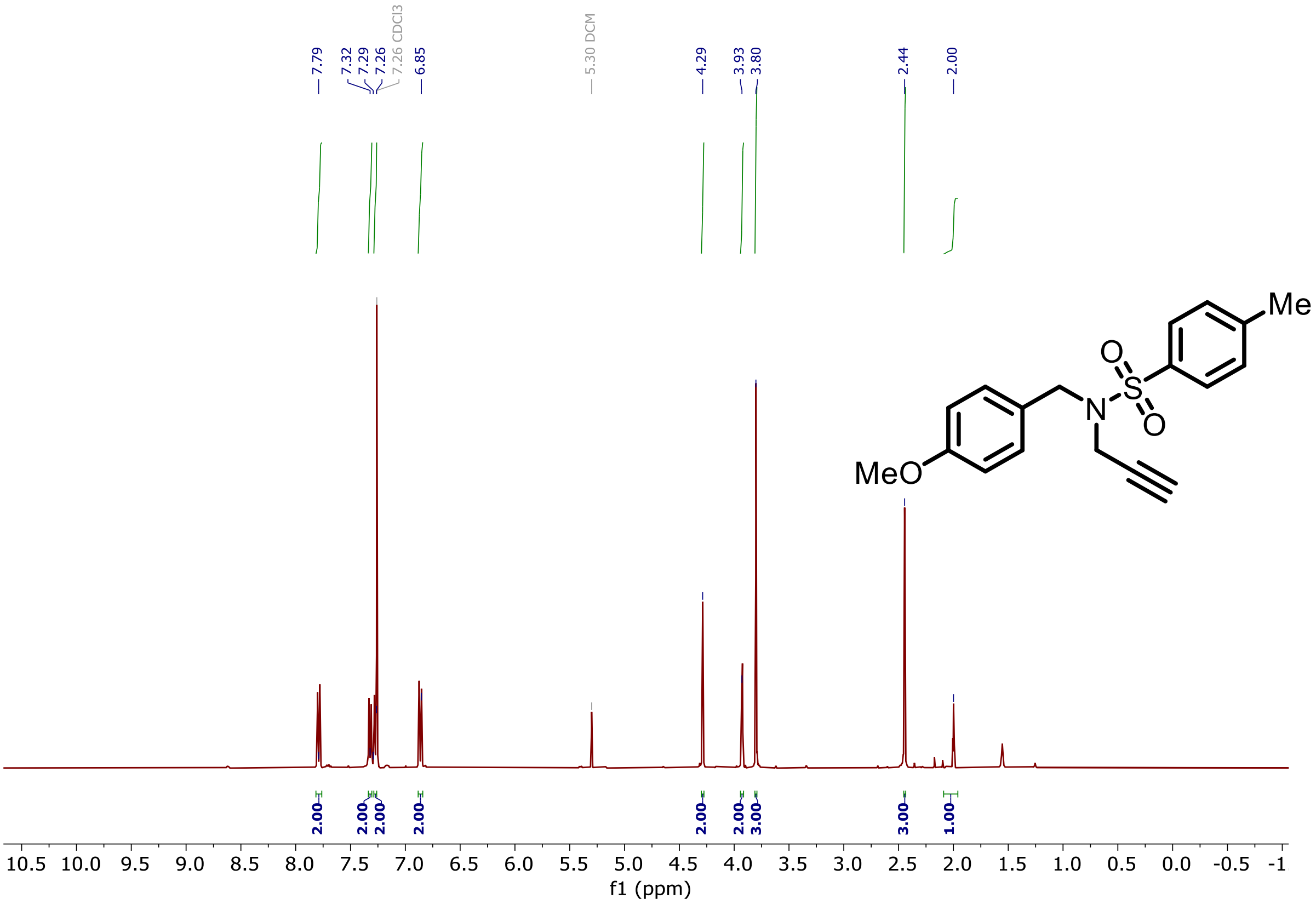

^{13}C NMR (126 MHz, $CDCl_3$)

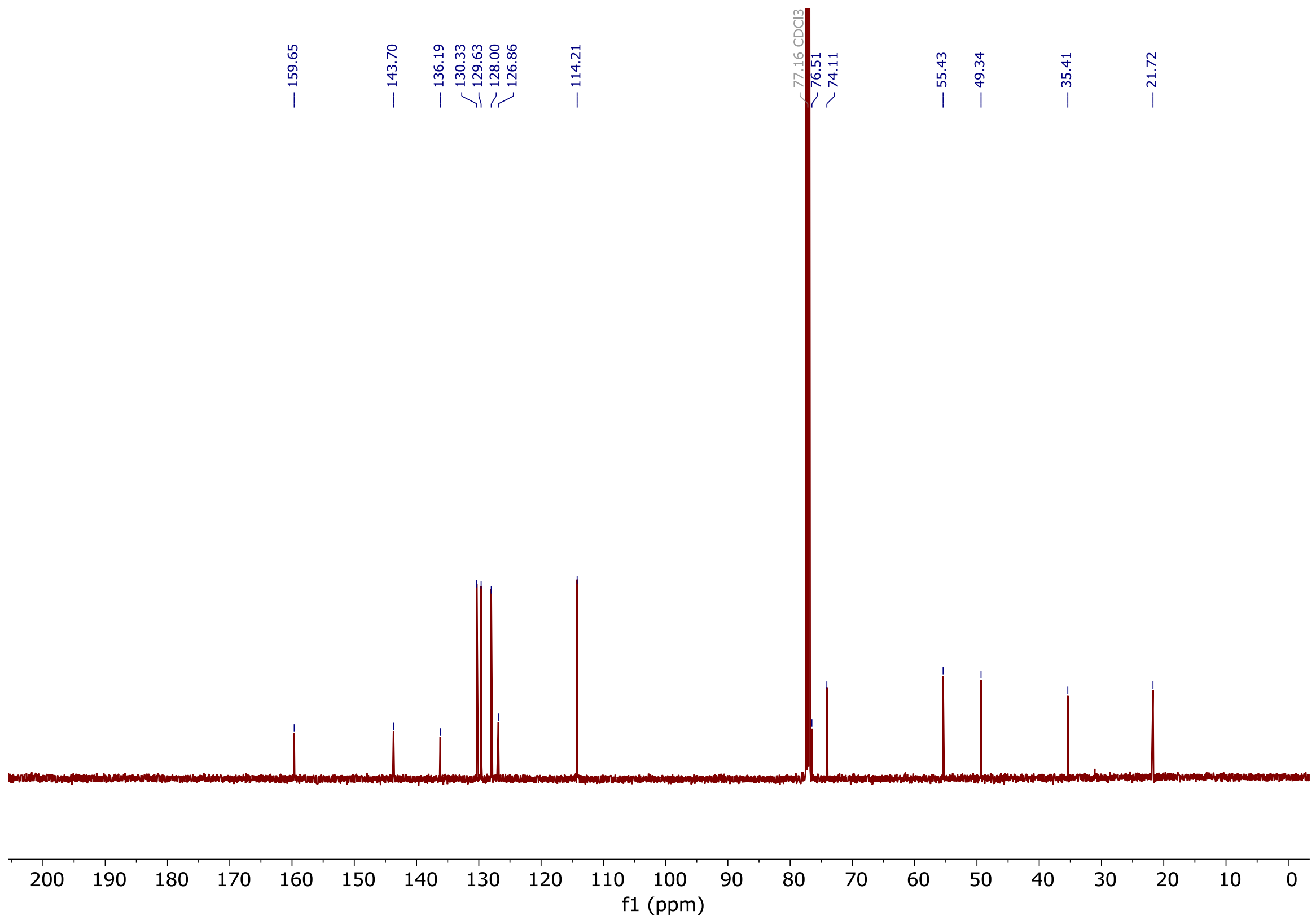

N-(4-chlorobenzyl)-4-methyl-N-(prop-2-yn-1-yl)benzenesulfonamide **(1d)**

^{1}H NMR (400 MHz, $CDCl_3$)

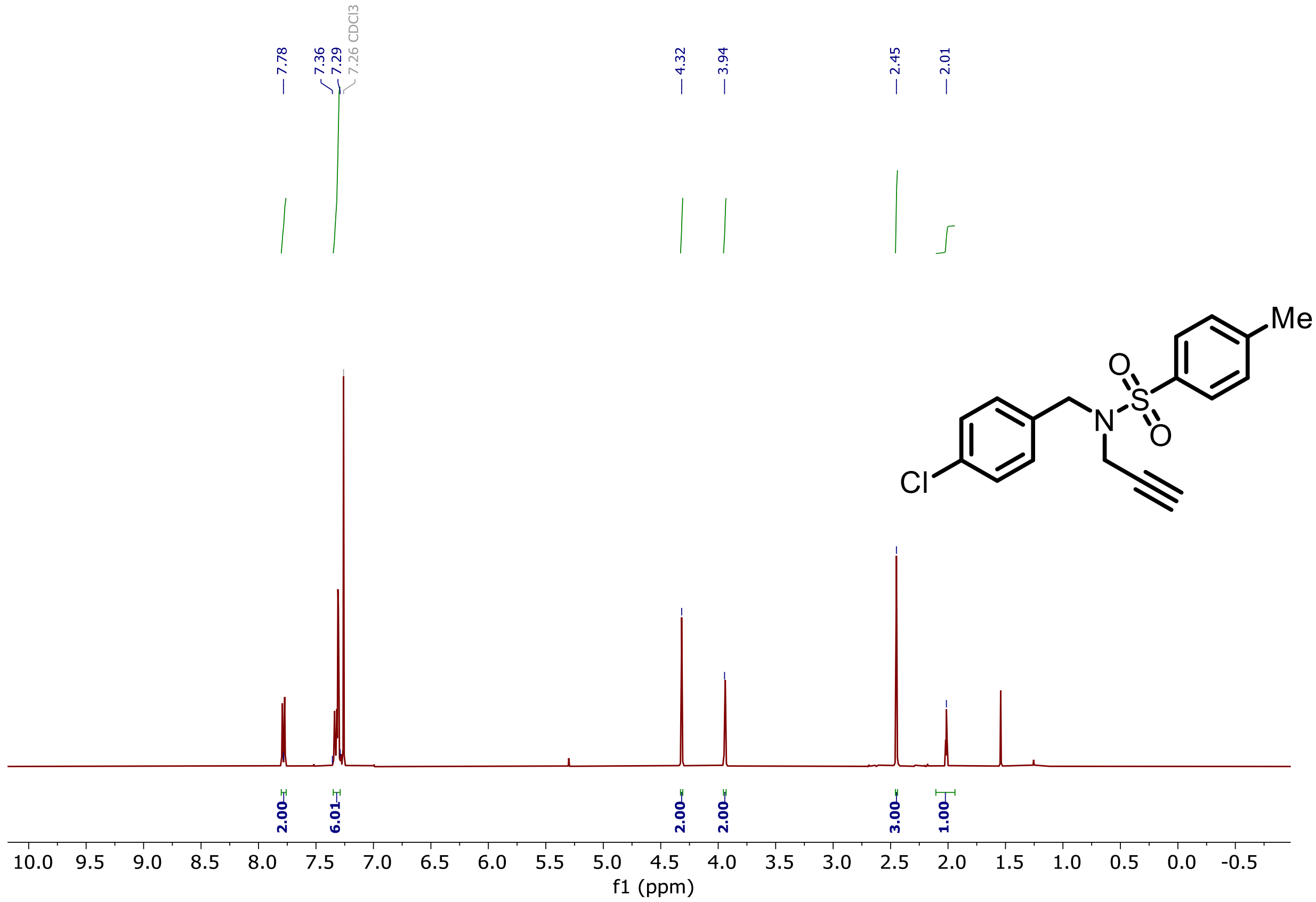

^{13}C NMR (126 MHz, $CDCl_3$)

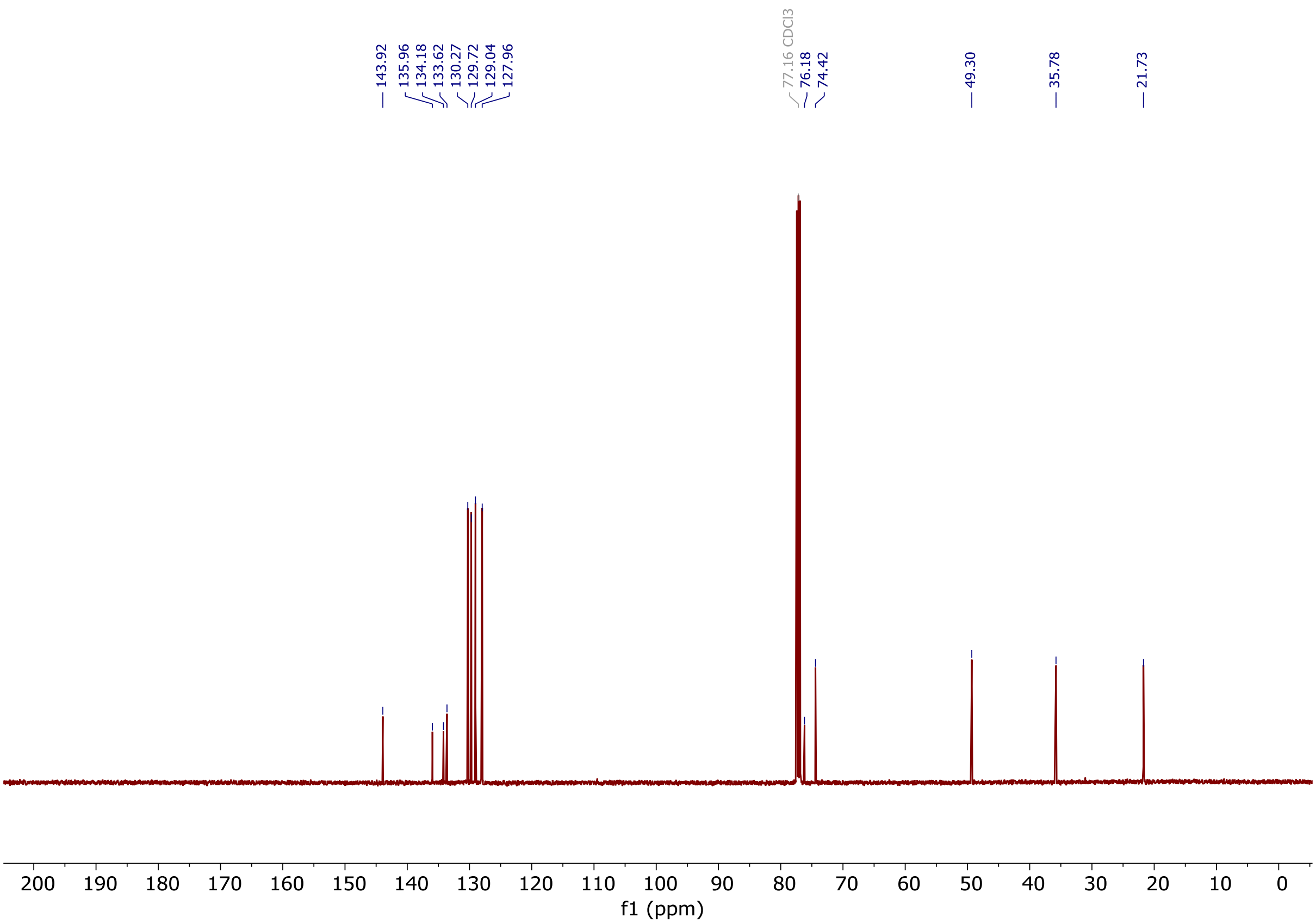

N-(4-fluorobenzyl)-4-methyl-N-(prop-2-yn-1-yl)benzenesulfonamide **(1e)**
^{1}H NMR (400MHz, CDCl$_3$)

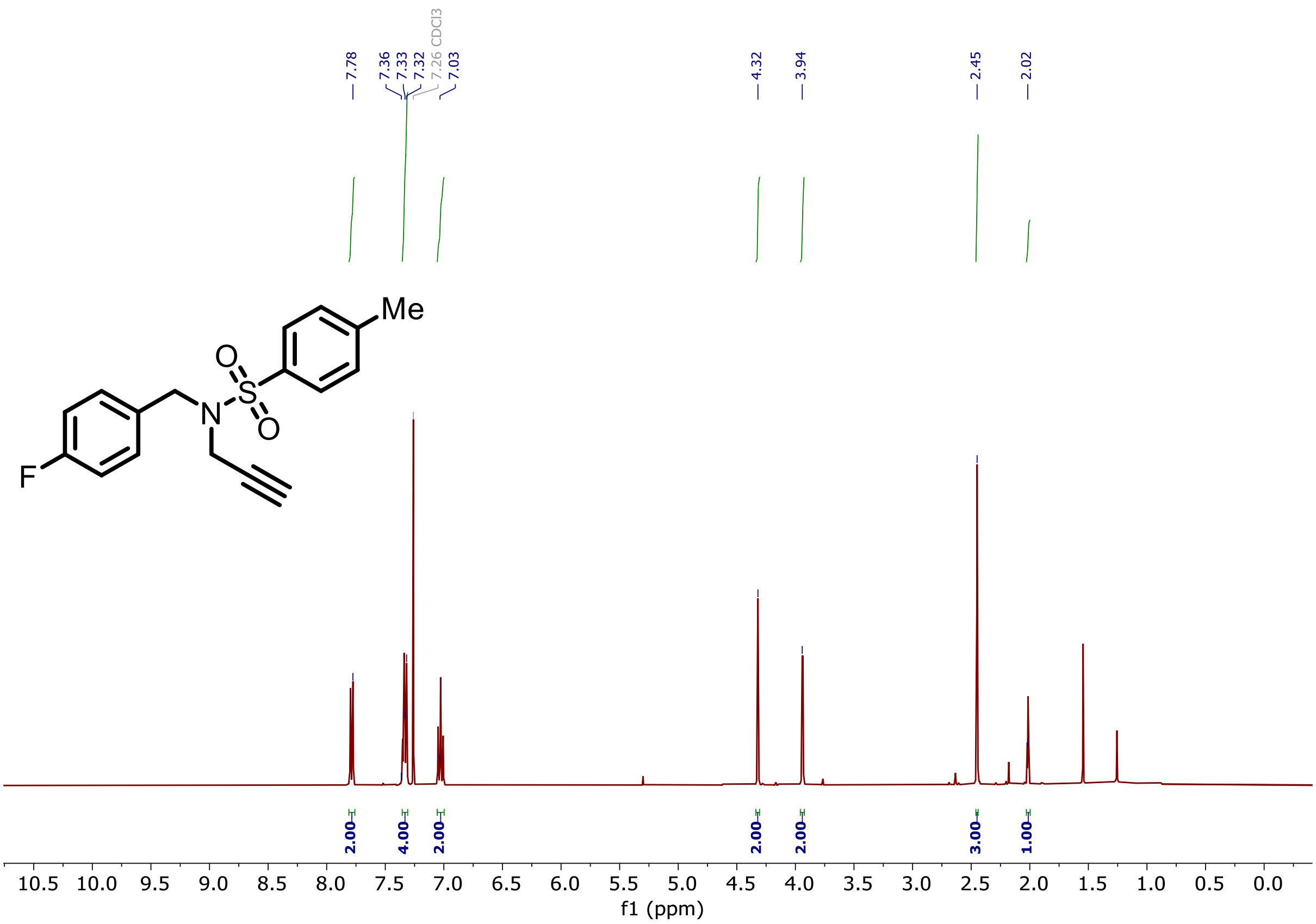

^{13}C NMR (151MHz, $CDCl_3$)

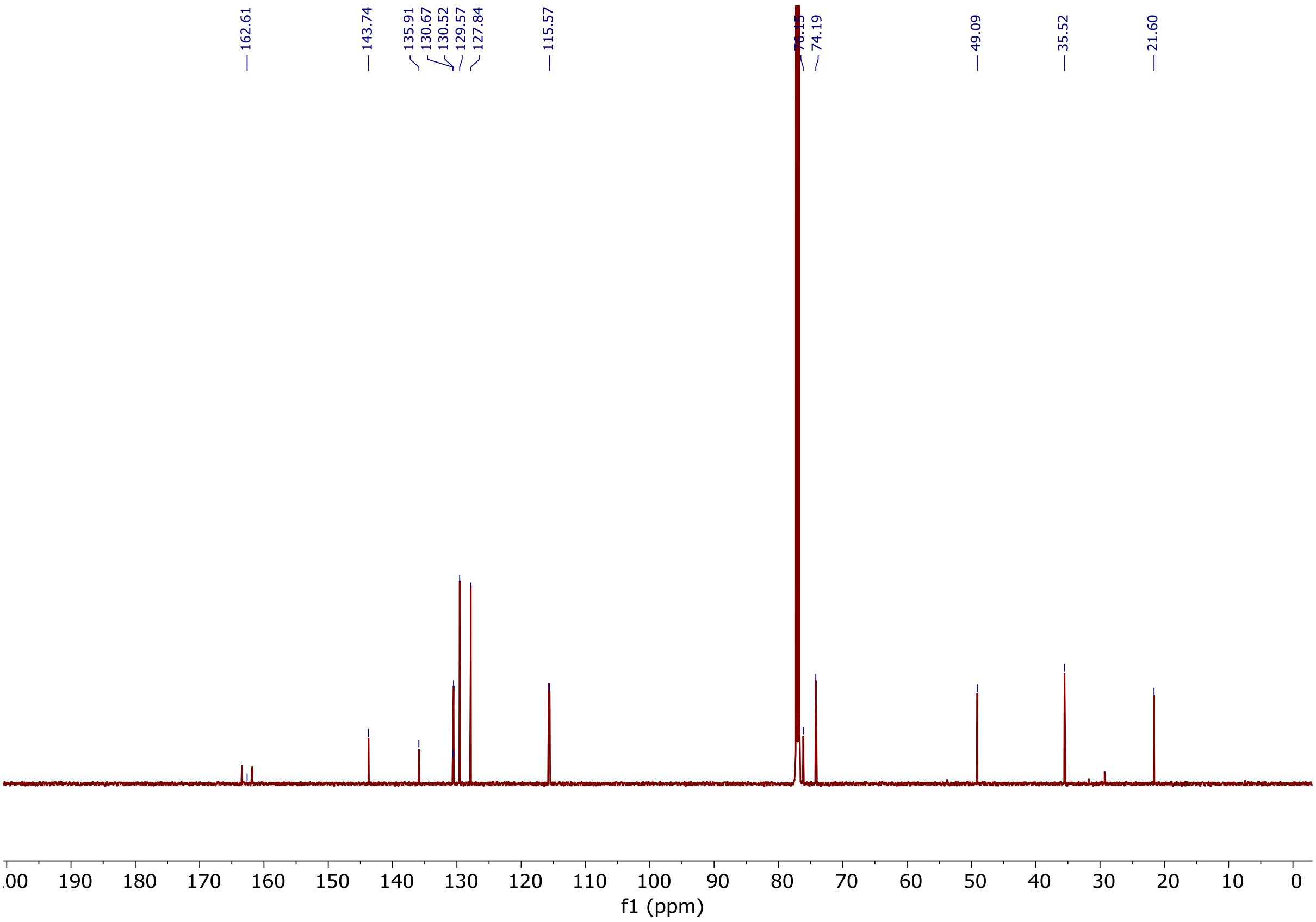

^{19}F NMR (376 MHz, $CDCl_3$)

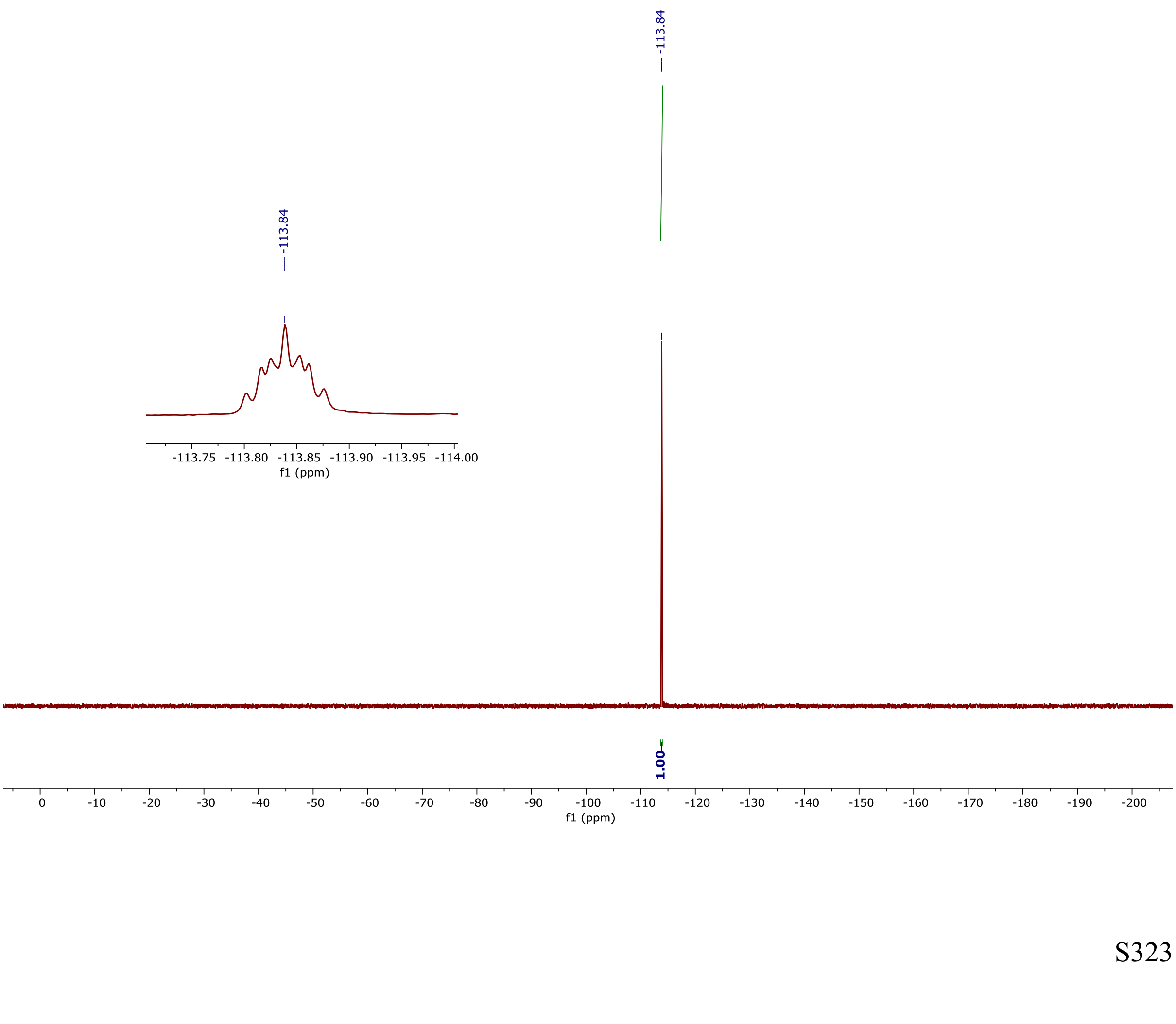

N-((4-fluorophenyl)methyl-d)-4-methyl-N-(prop-2-yn-1-yl)benzenesulfonamide ([²H]-1e)

¹H NMR (500 MHz, CDCl₃)

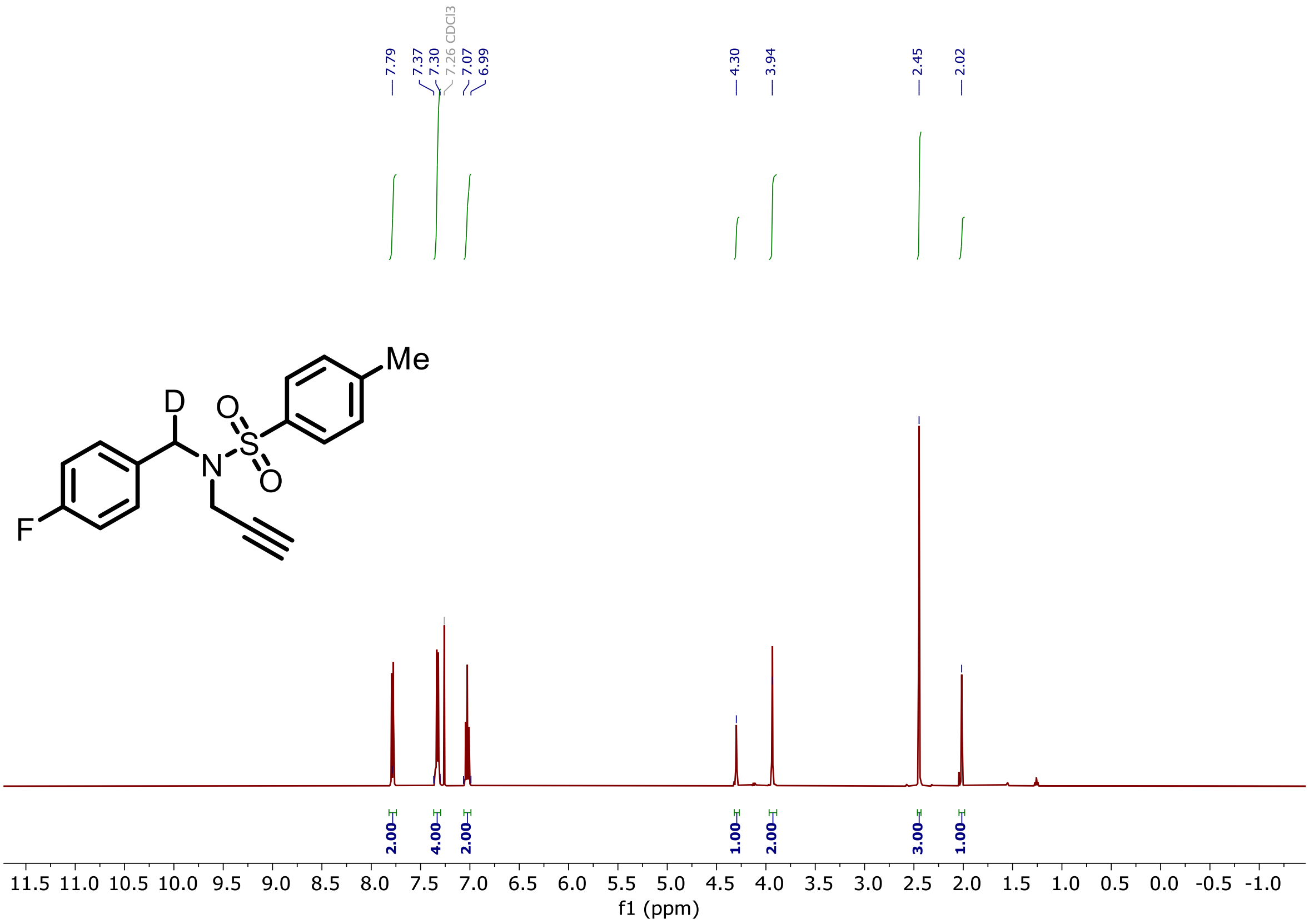

¹³C NMR (126 MHz, CDCl₃)

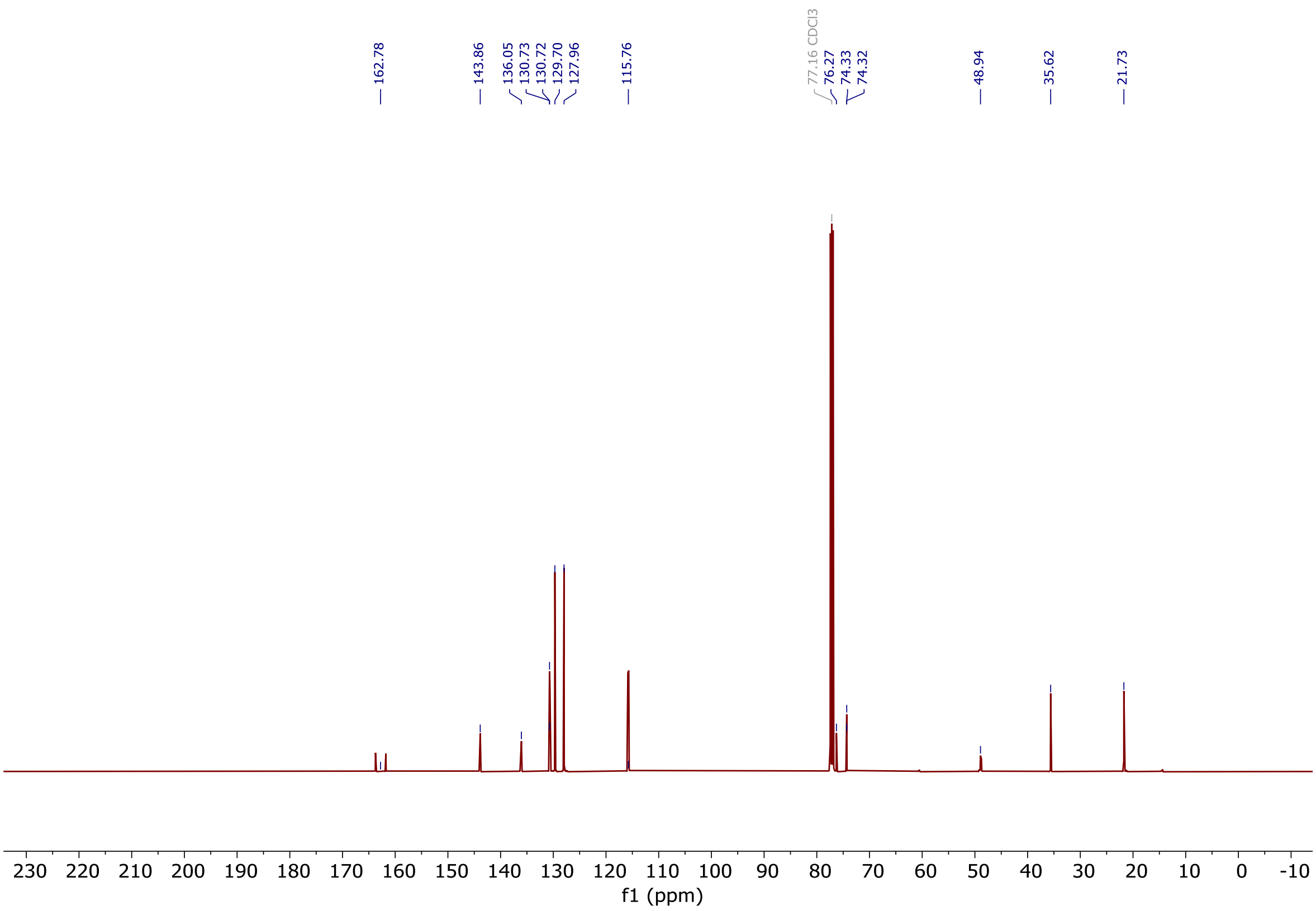

¹⁹F NMR (376 MHz, CDCl₃)

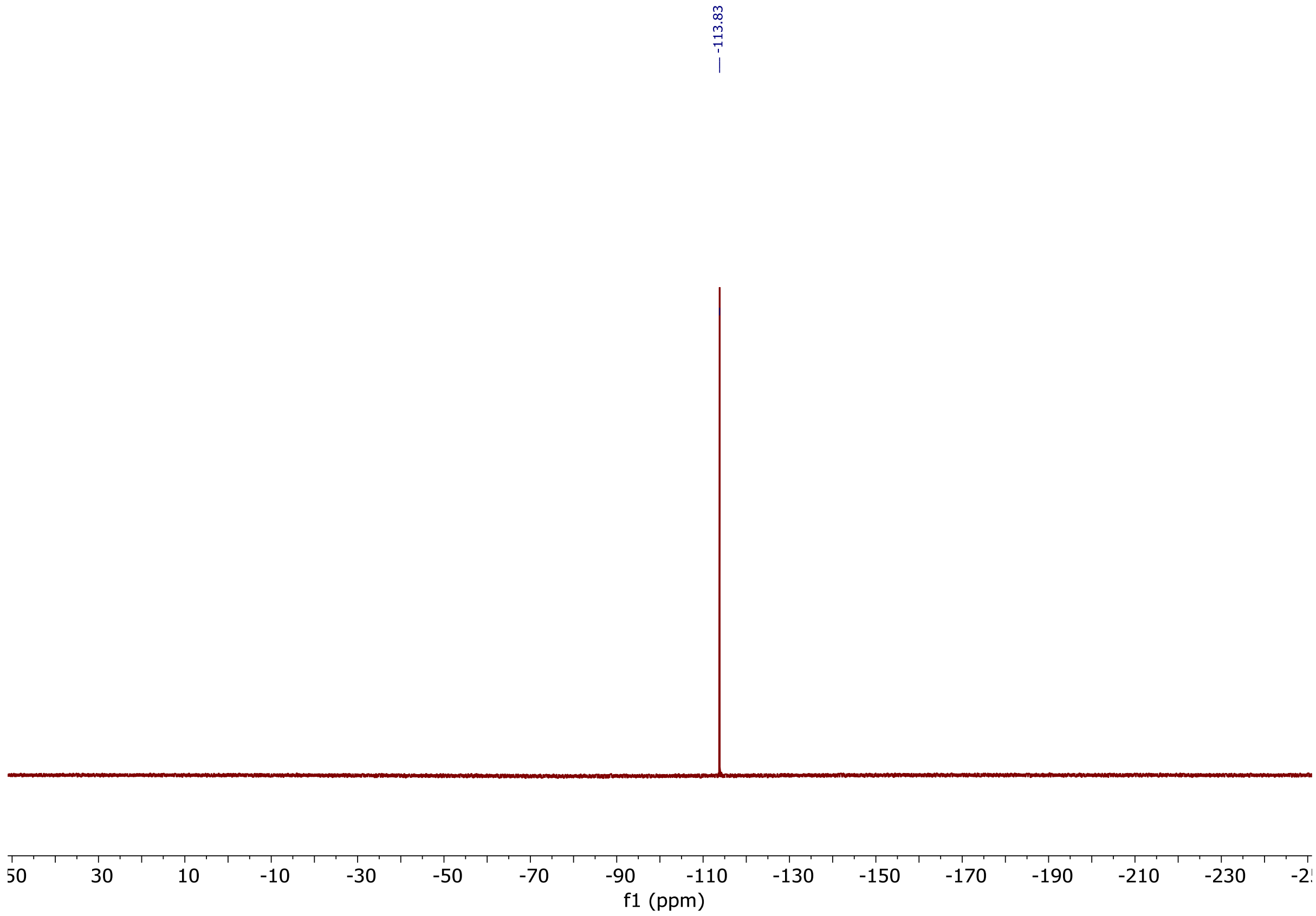
-113.83
50
30
10
-10
-30
-50
-70
-90
-110
-130
-150
-170
-190
-210
-230
f1 (ppm)

***4-methyl-N-(prop-2-yn-1-yl)-N-(4-(trifluoromethyl)benzyl)benzenesulfonamide* (1f)**

^{1}H NMR (400 MHz, CDCl$_3$)

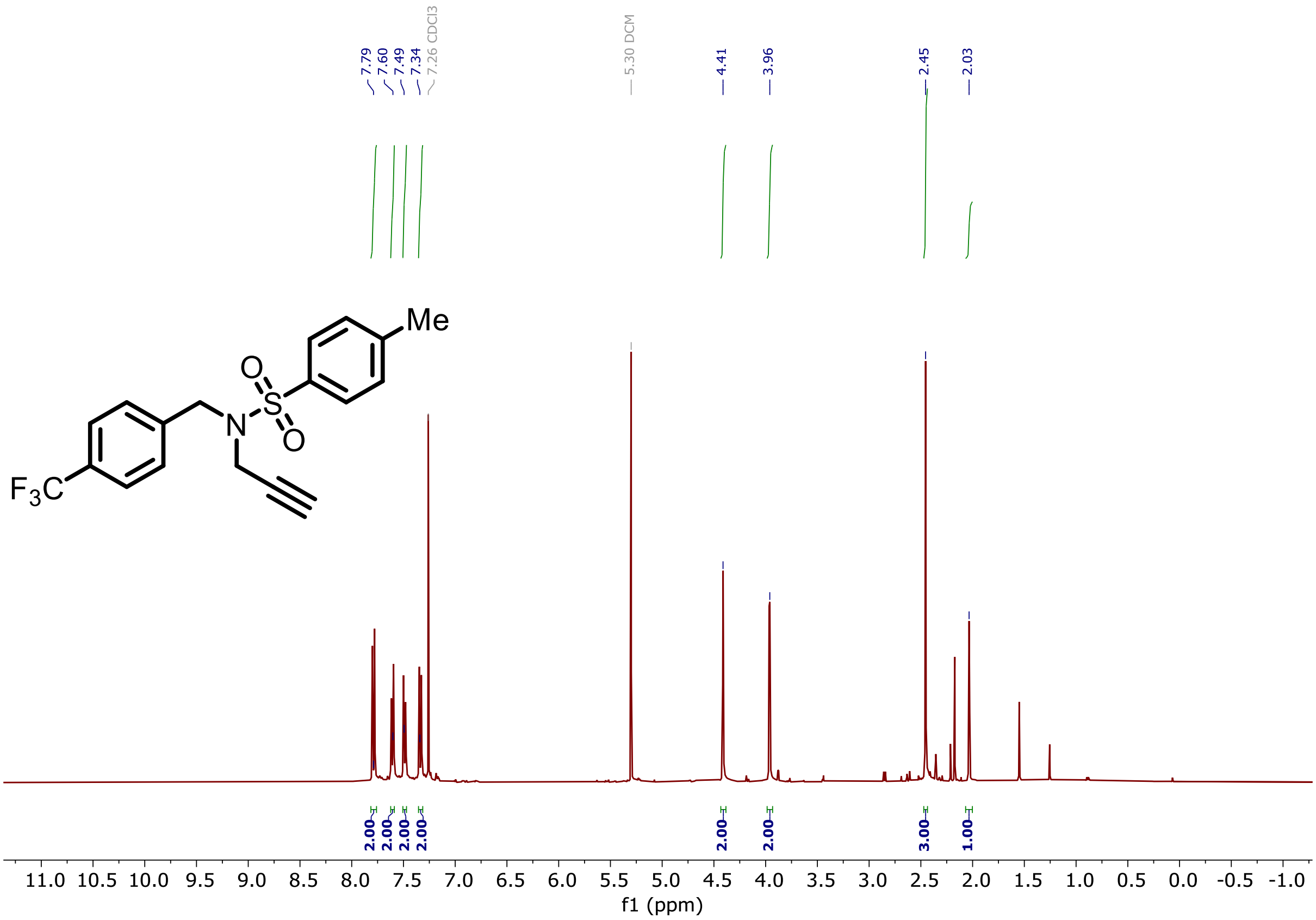

^{13}C NMR (151 MHz, CDCl$_3$)

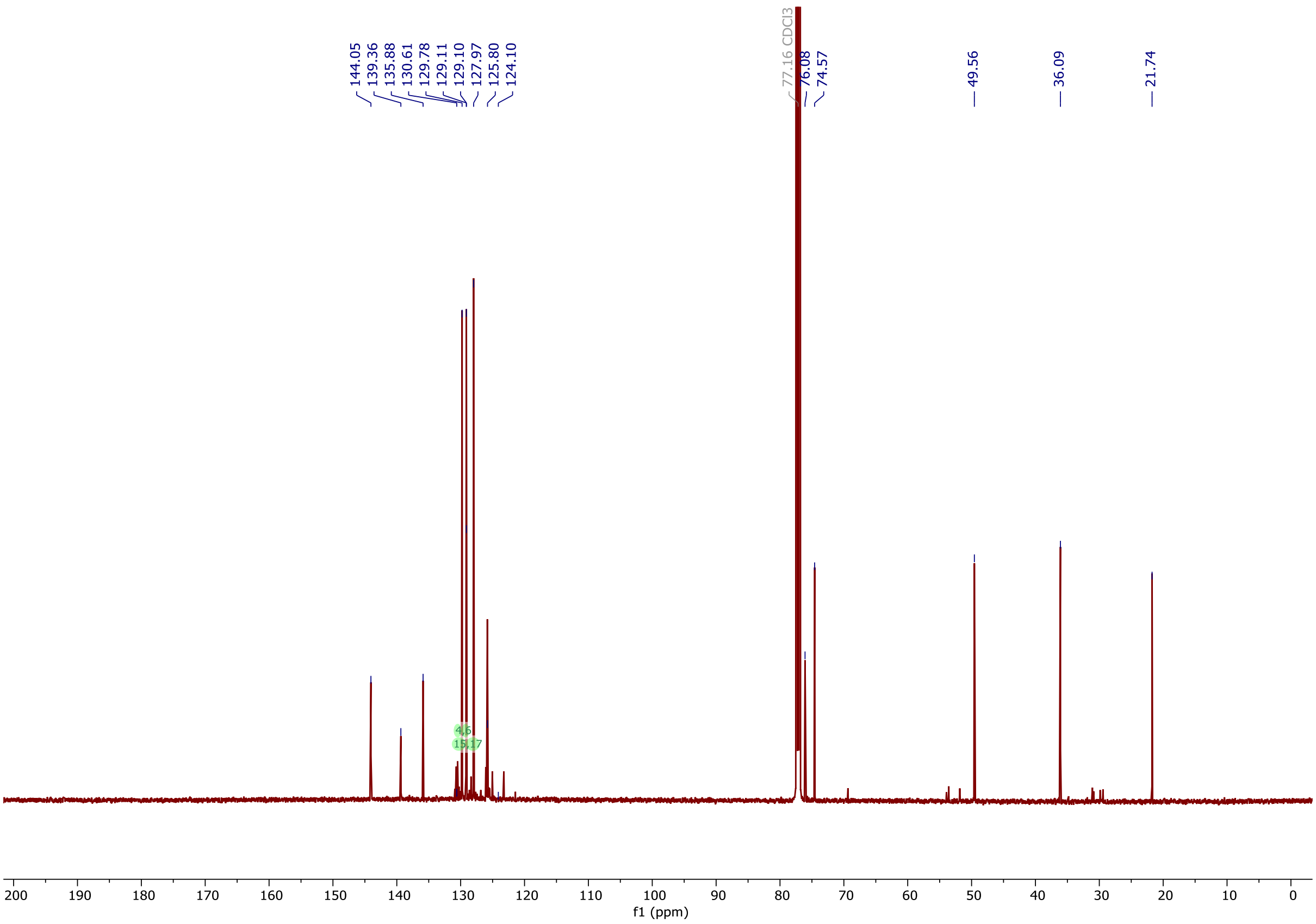

^{19}F NMR (376 MHz, $CDCl_3$)

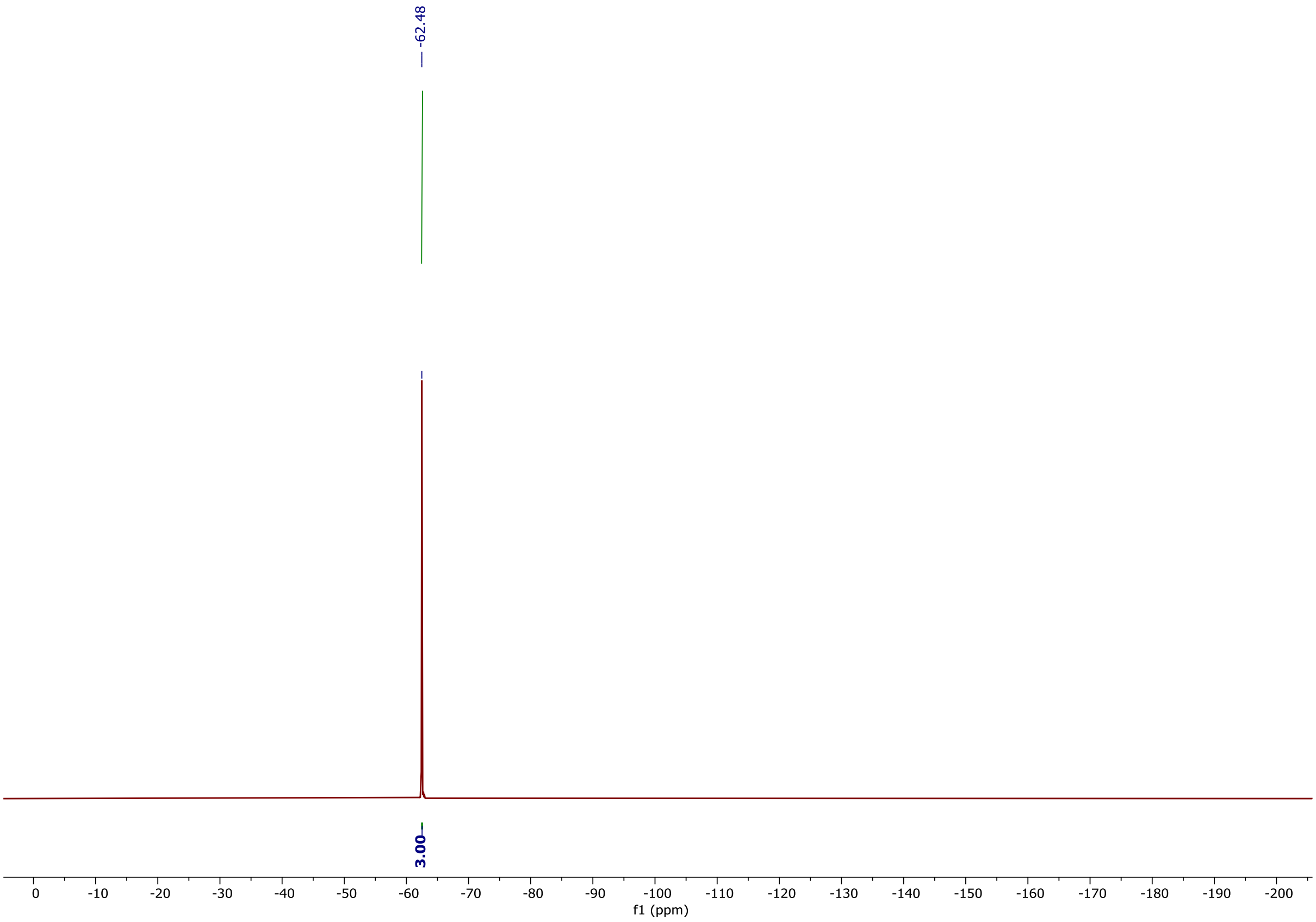

4-methyl-N-(4-nitrobenzyl)-N-(prop-2-yn-1-yl)benzenesulfonamide **(1g)**

^{1}H NMR (400 MHz, CDCl$_3$)

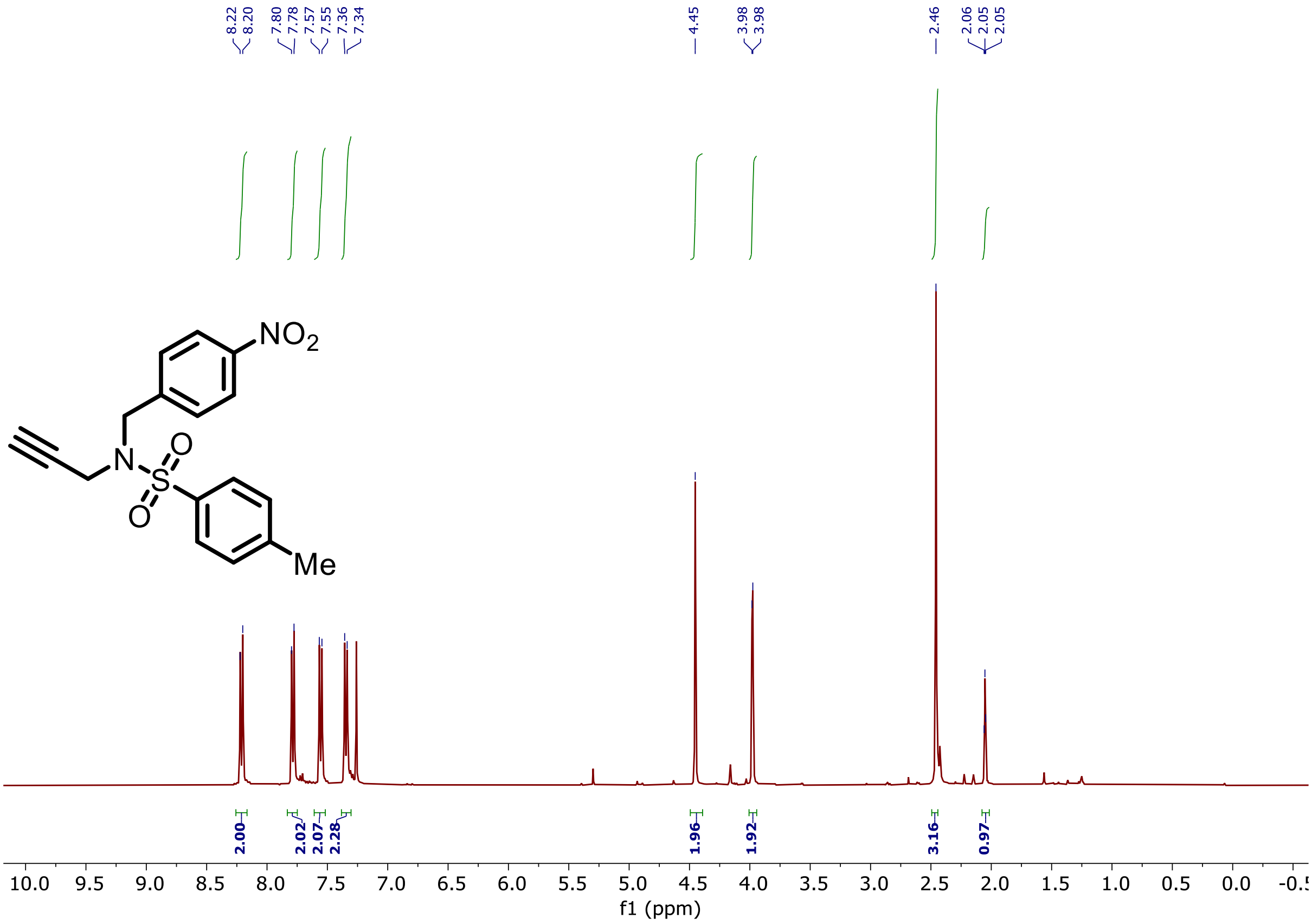

^{13}C NMR (101 MHz, CDCl$_3$)

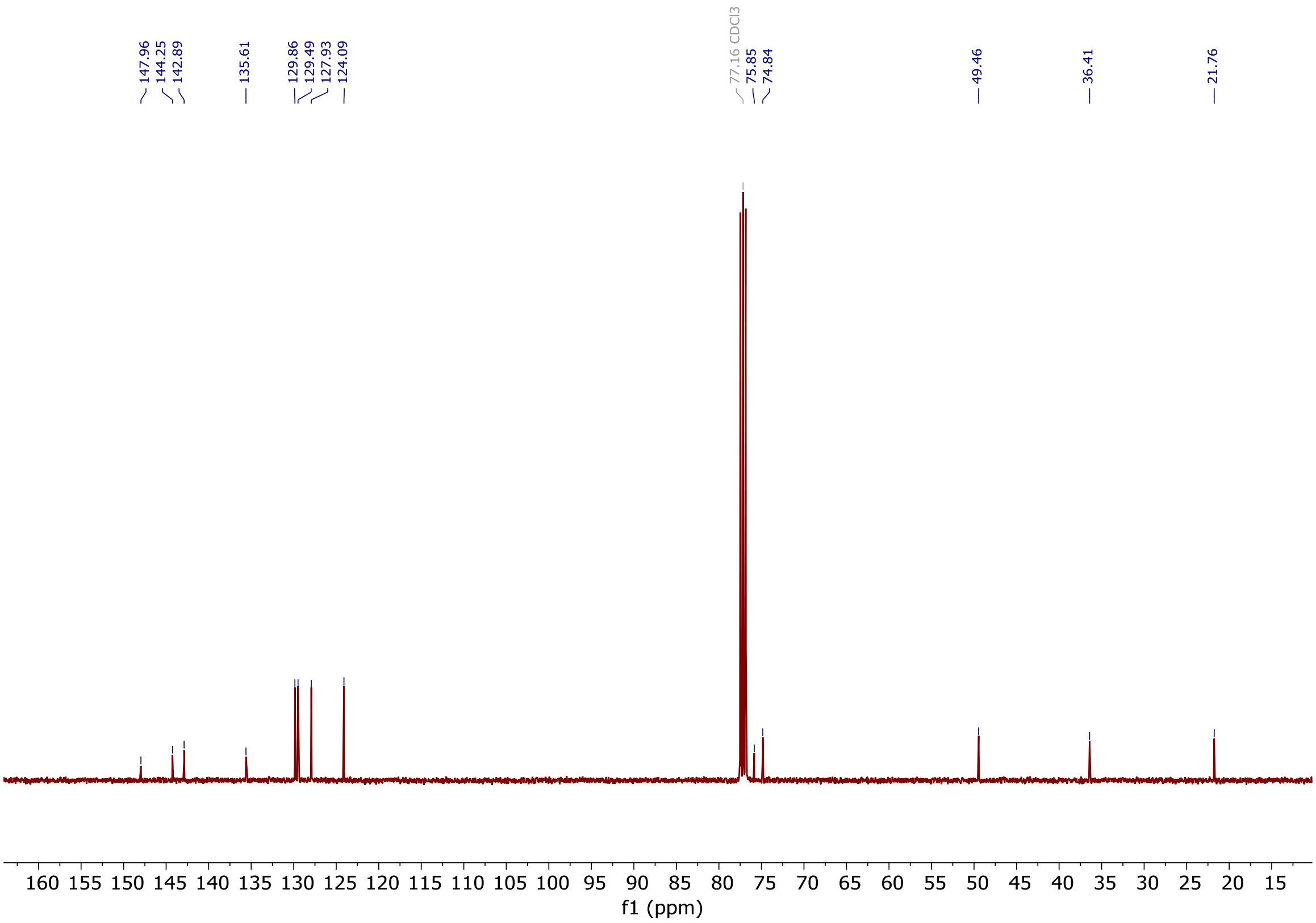

N-benzyl-4-methyl-N-(2-methylbut-3-yn-2-yl)benzenesulfonamide **(1h)**

^{1}H NMR (500 MHz, $CDCl_3$)

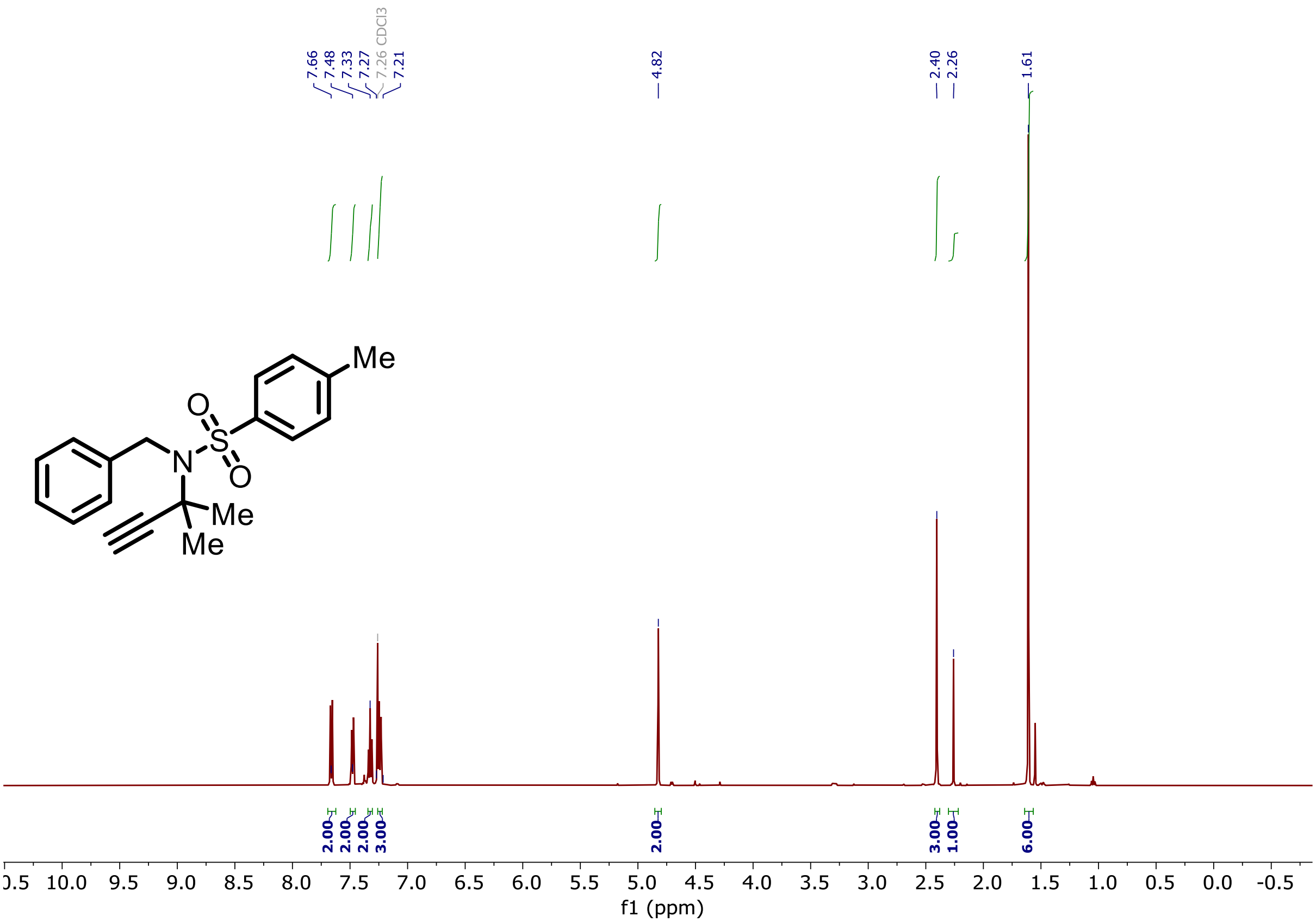

^{13}C NMR (126 MHz, $CDCl_3$)

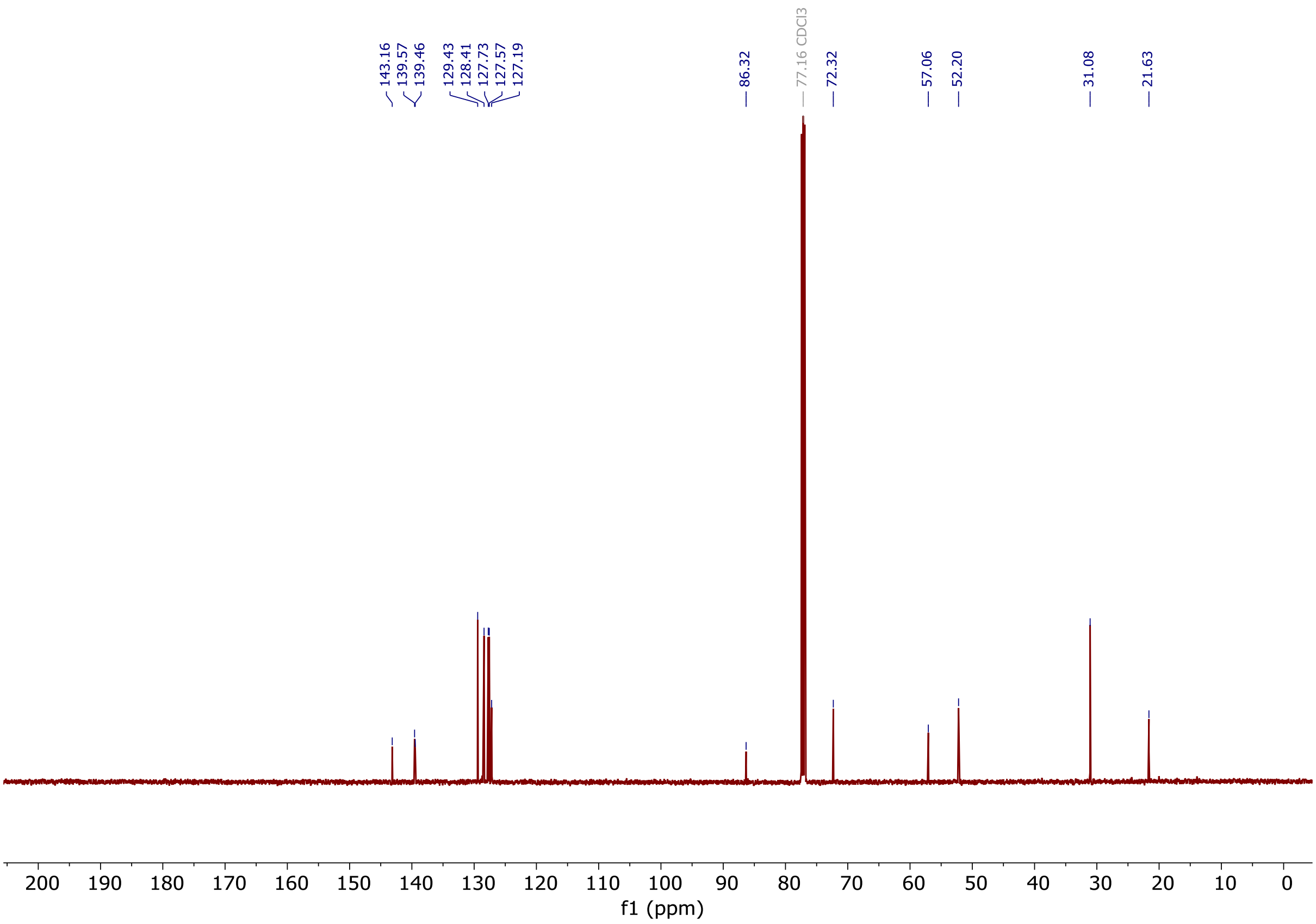

***N-butyl-4-nitro-N-(prop-2-yn-1-yl)benzenesulfonamide* (1i)**

^{1}H NMR (500 MHz, $CDCl_3$)

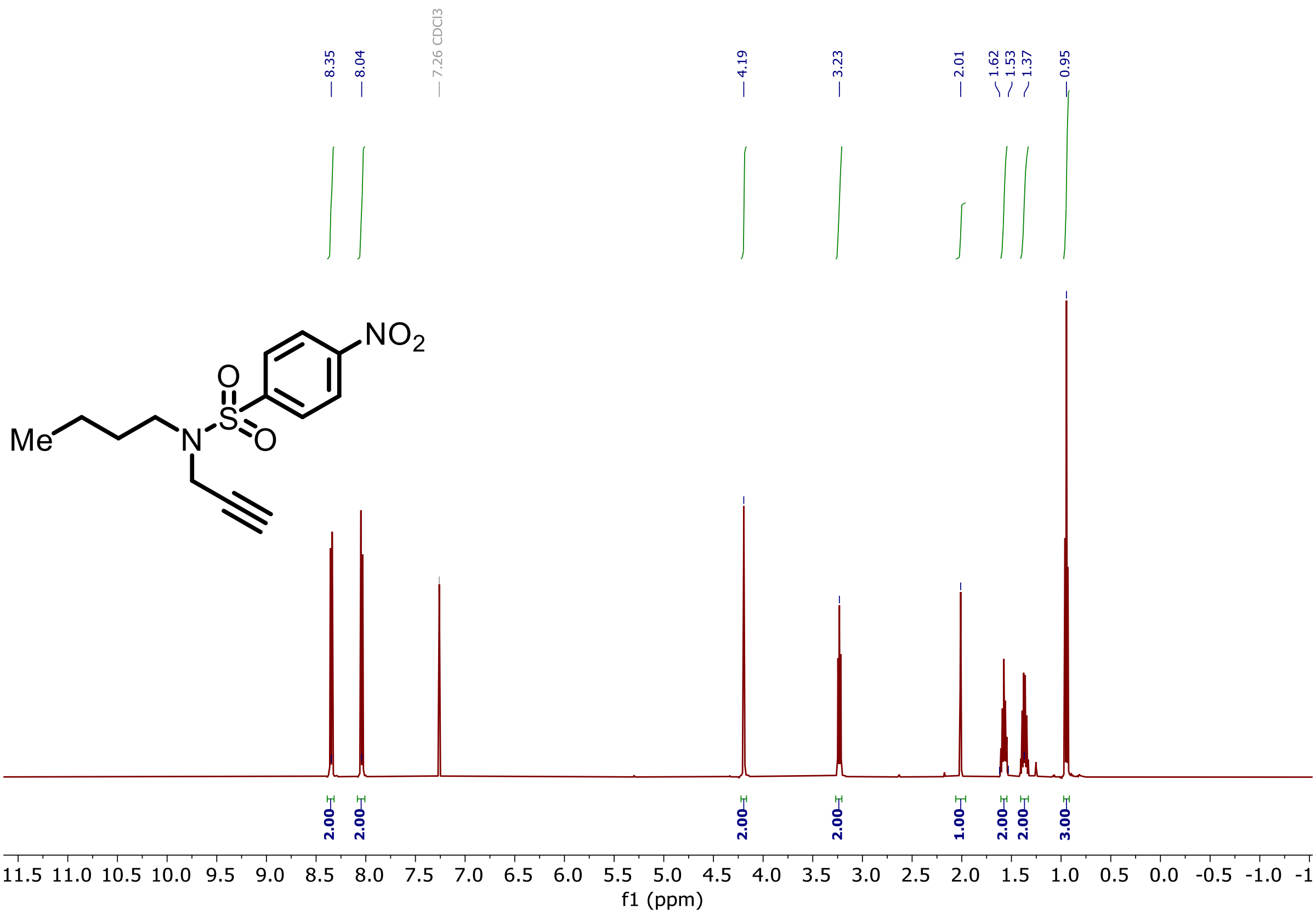

^{13}C NMR (126 MHz, $CDCl_3$)

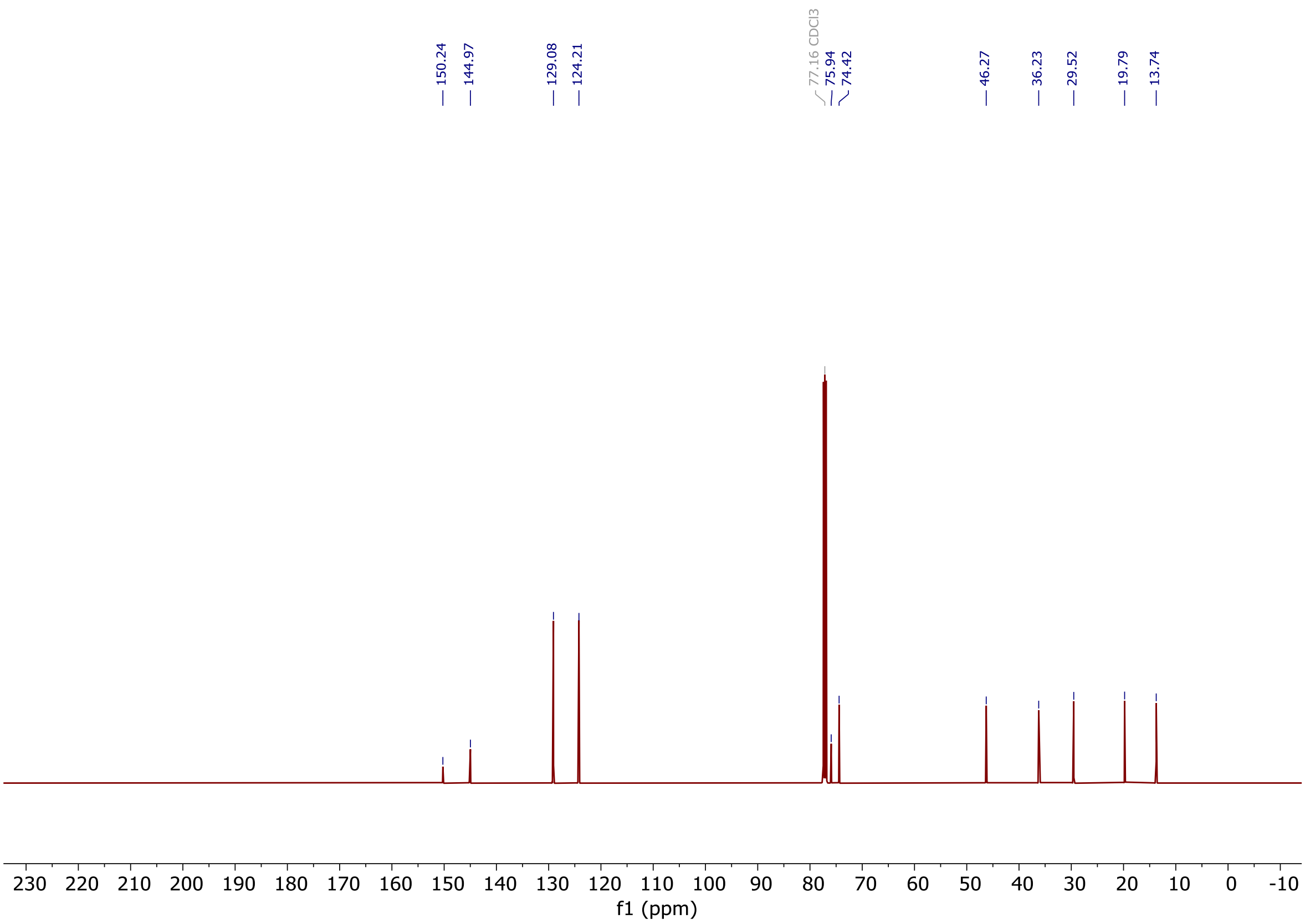

N-(but-3-yn-2-yl)-N-(4-fluorobenzyl)-4-methylbenzenesulfonamide **(1j)**

^{1}H NMR (400 MHz, CDCl$_3$)

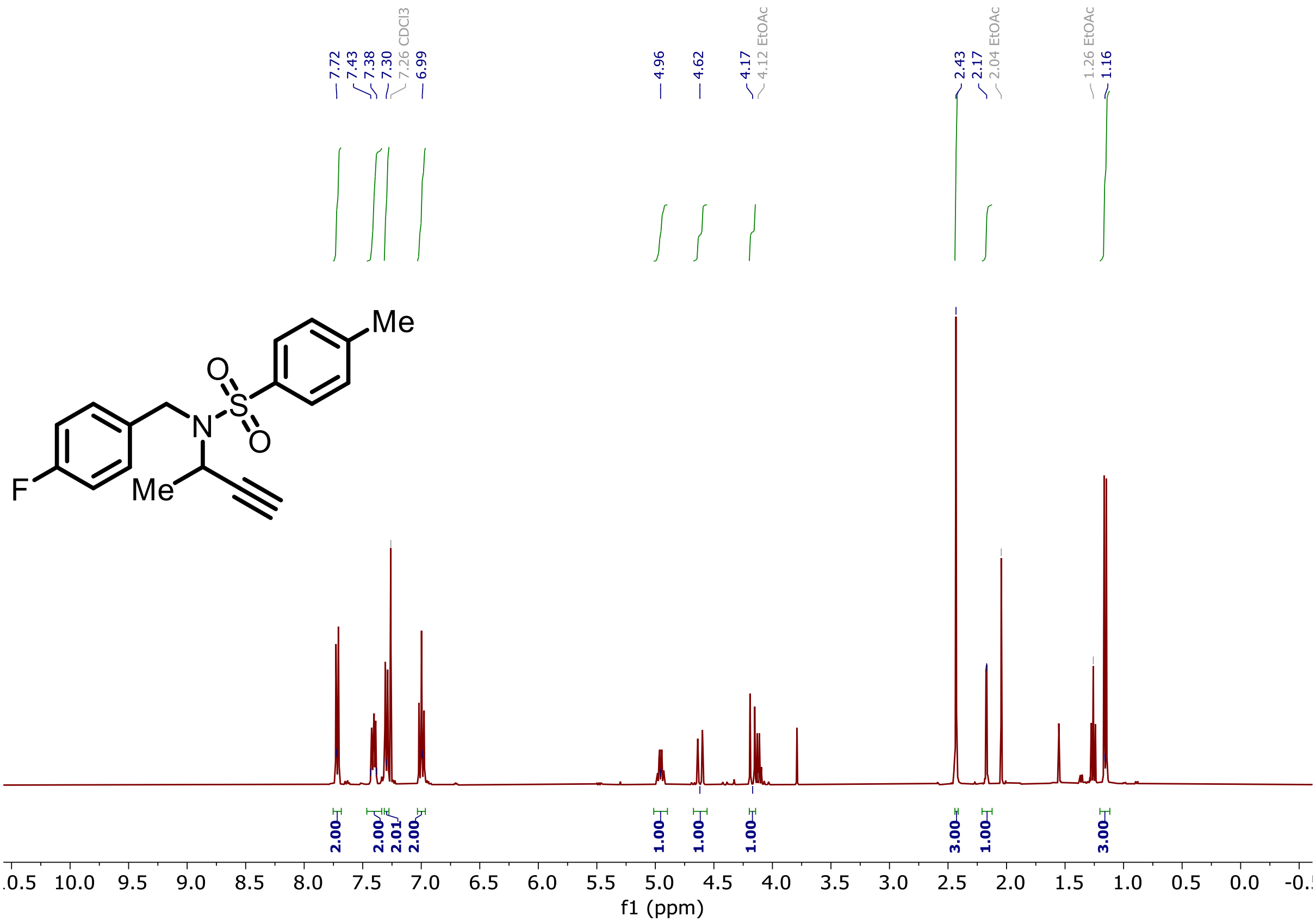

^{13}C NMR (126 MHz, CDCl$_3$)

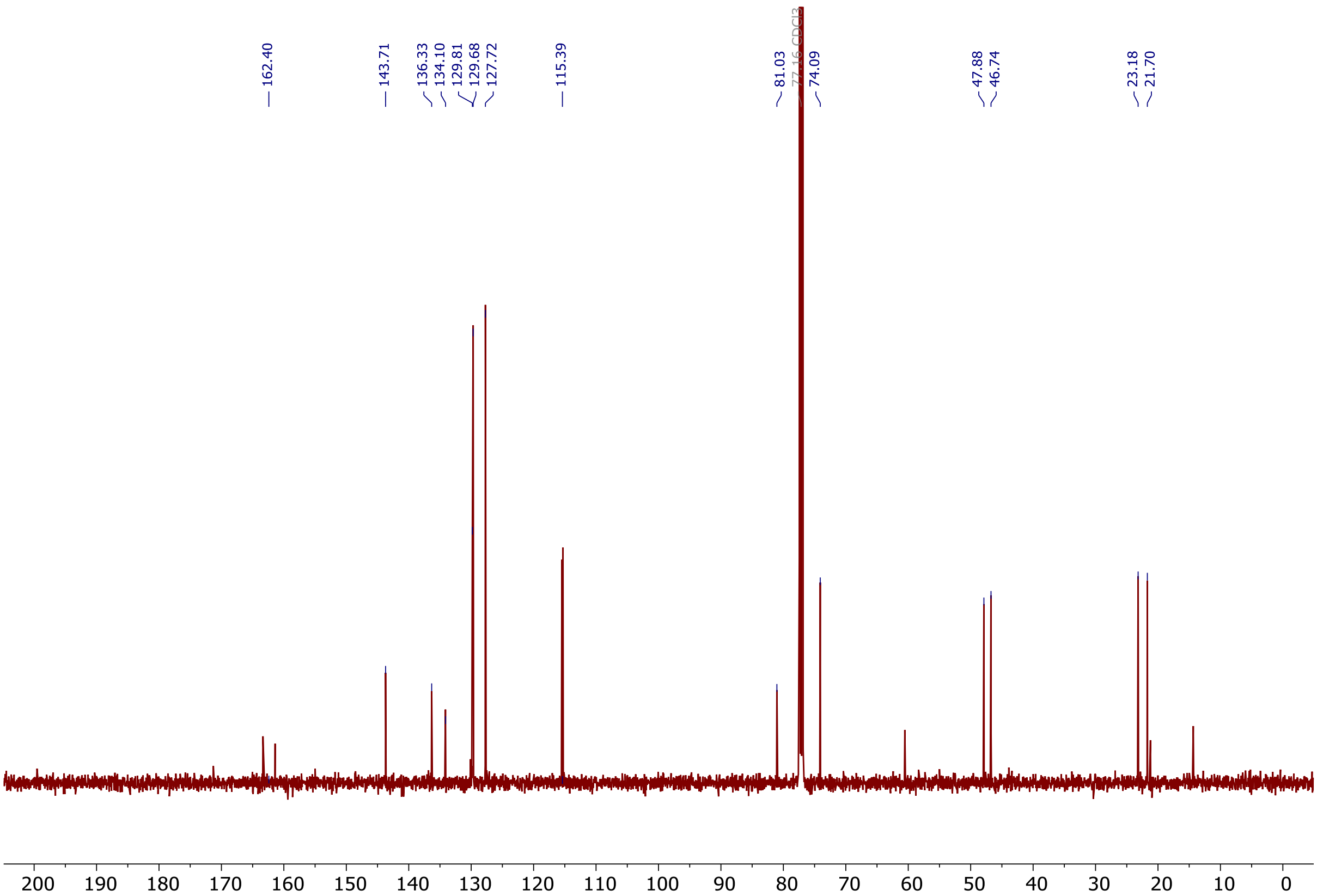

^{19}F NMR (471 MHz, CDCl$_3$)

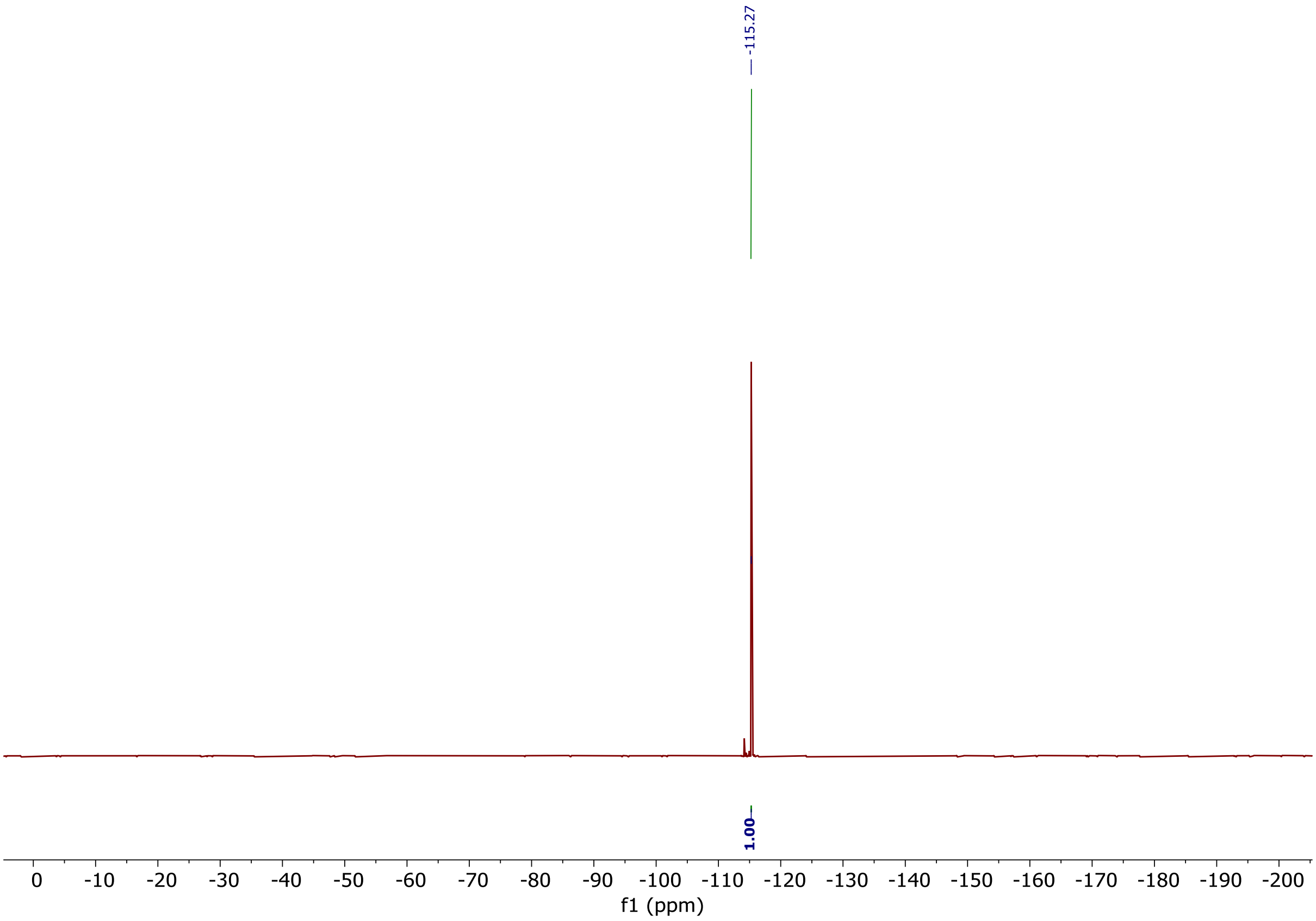

***N-(but-3-yn-2-yl)-N-(4-fluorobenzyl)methanesulfonamide* (1k)**

^{1}H NMR (500 MHz, $CDCl_3$)

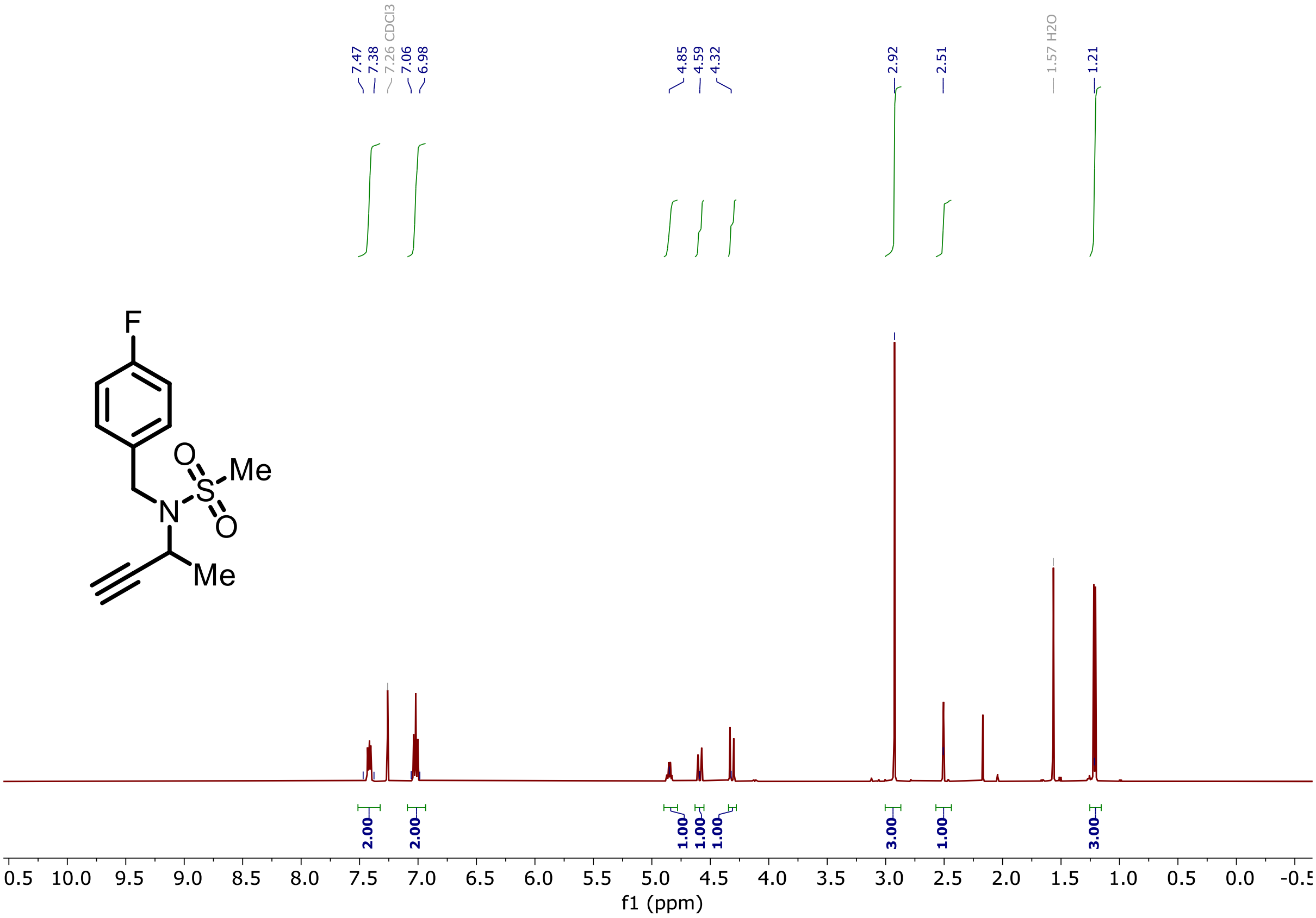

^{13}C NMR (126 MHz, $CDCl_3$)

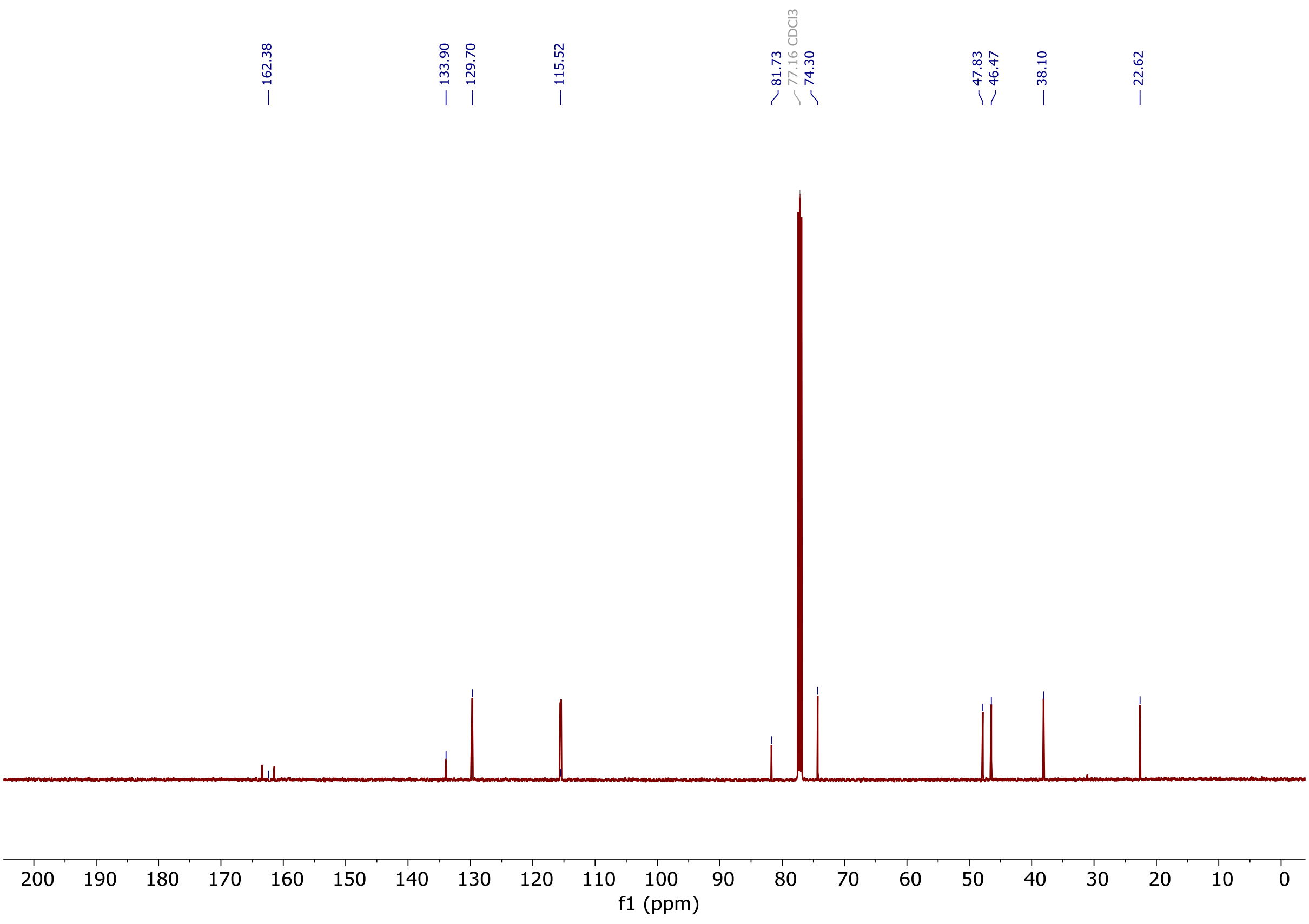

^{19}F NMR (471 MHz, $CDCl_3$)

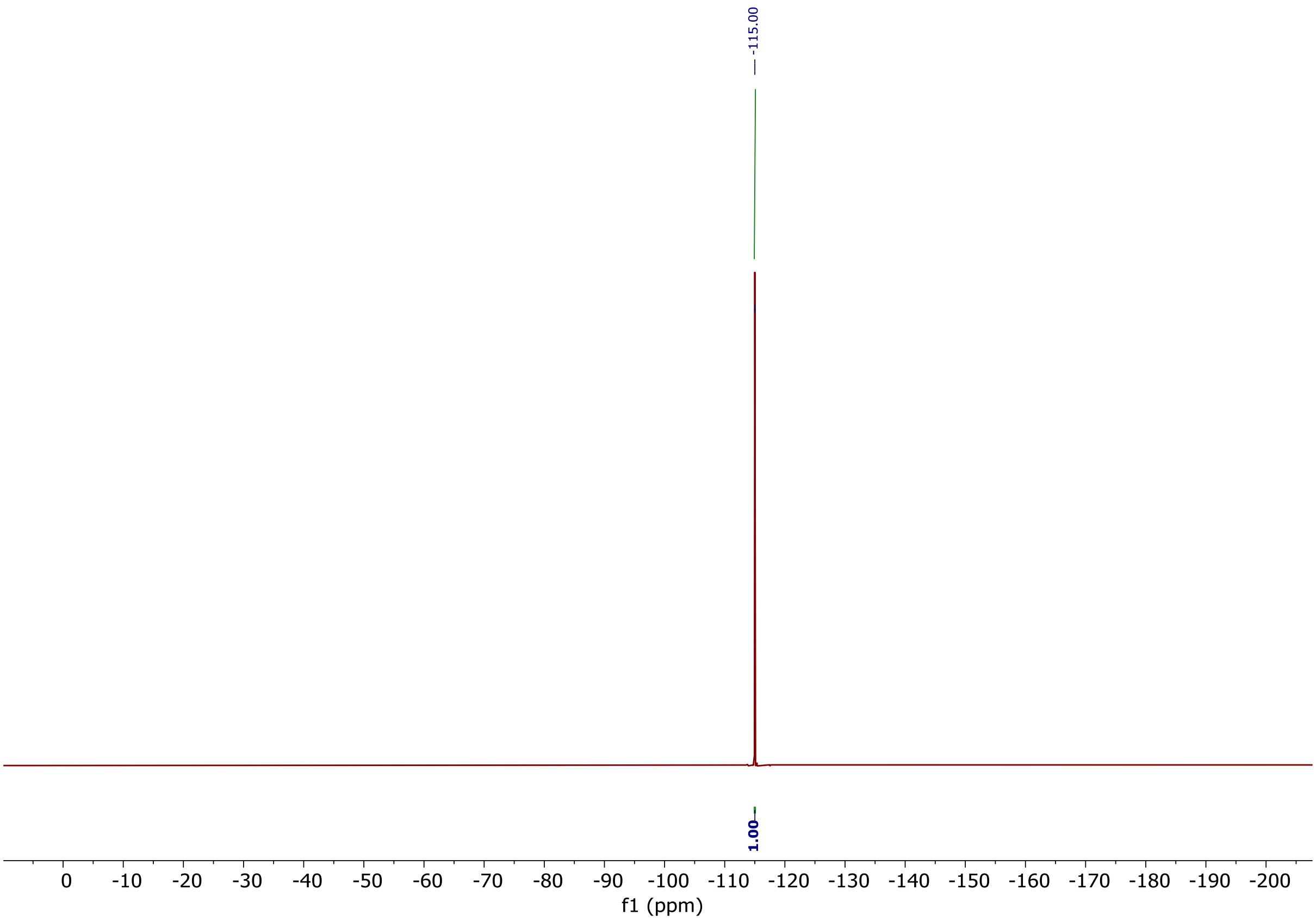

N-(4-bromobenzyl)-N-(but-3-yn-2-yl)-4-methylbenzenesulfonamide **(1l)**

^{1}H NMR (400 MHz, $CDCl_3$)

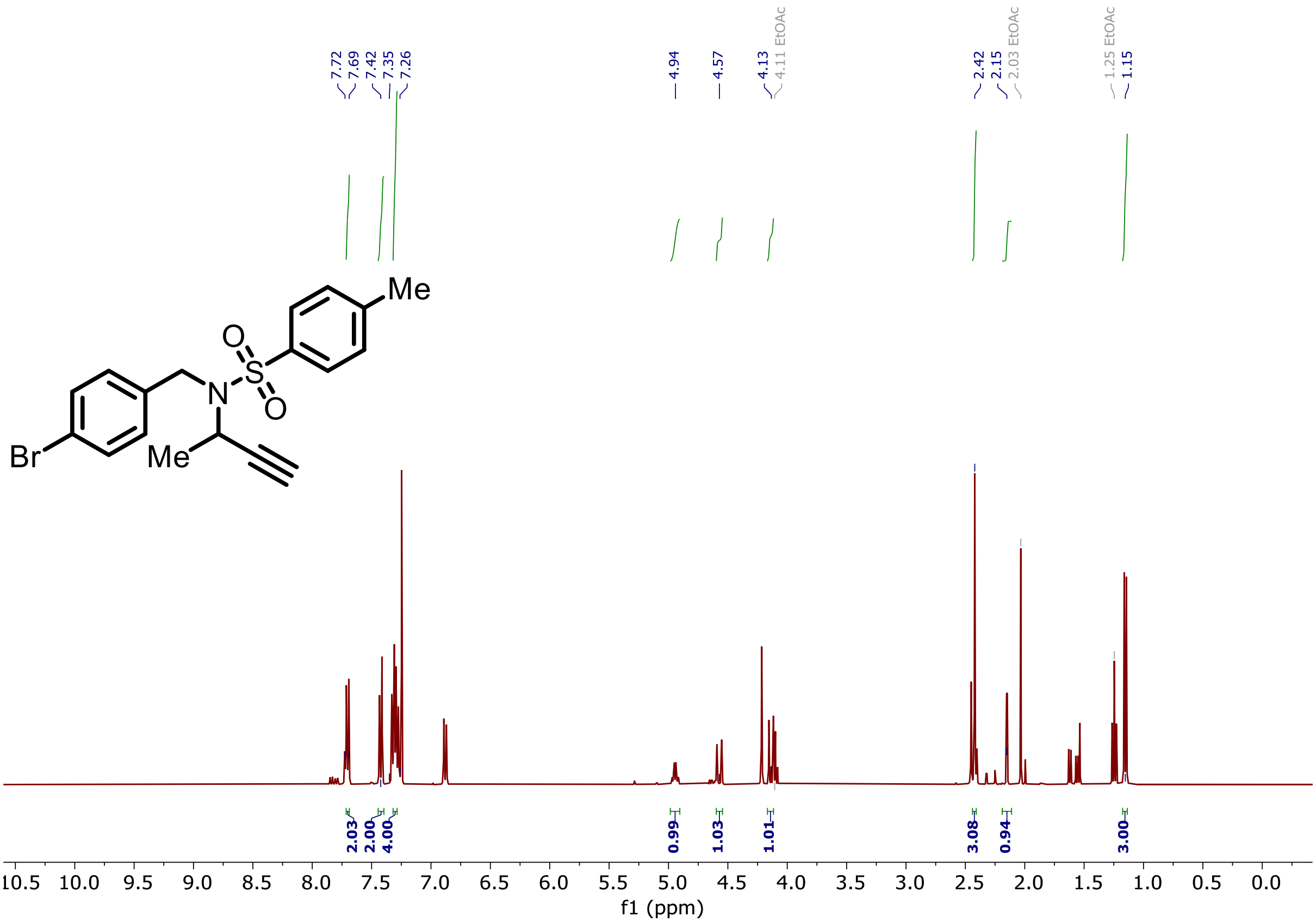

^{13}C NMR (126MHz, $CDCl_3$)

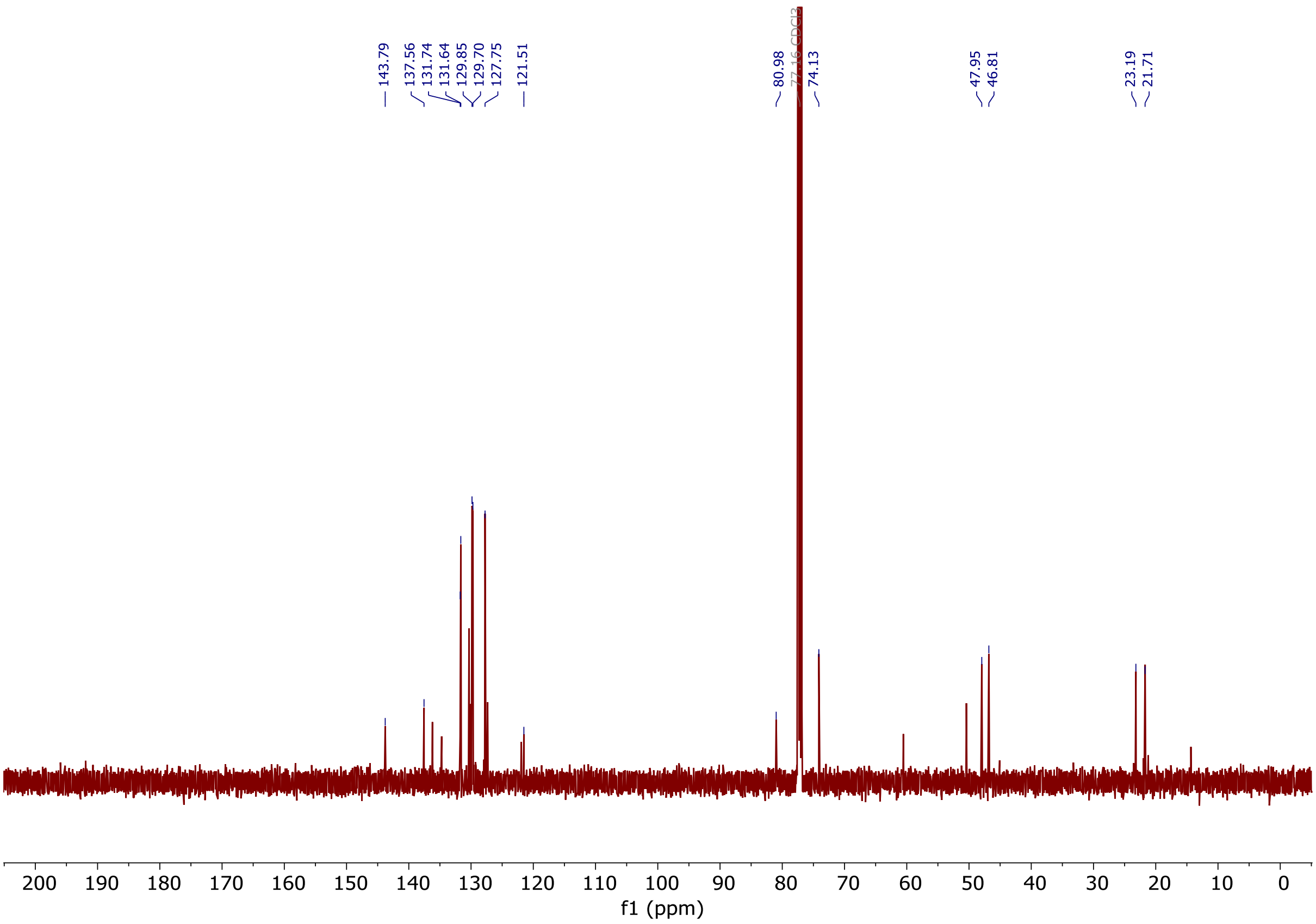

***N*-(but-3-yn-2-yl)-4-methyl-*N*-(naphthalen-2-ylmethyl)benzenesulfonamide* (1m)**

^{1}H NMR (500 MHz, CDCl$_3$)

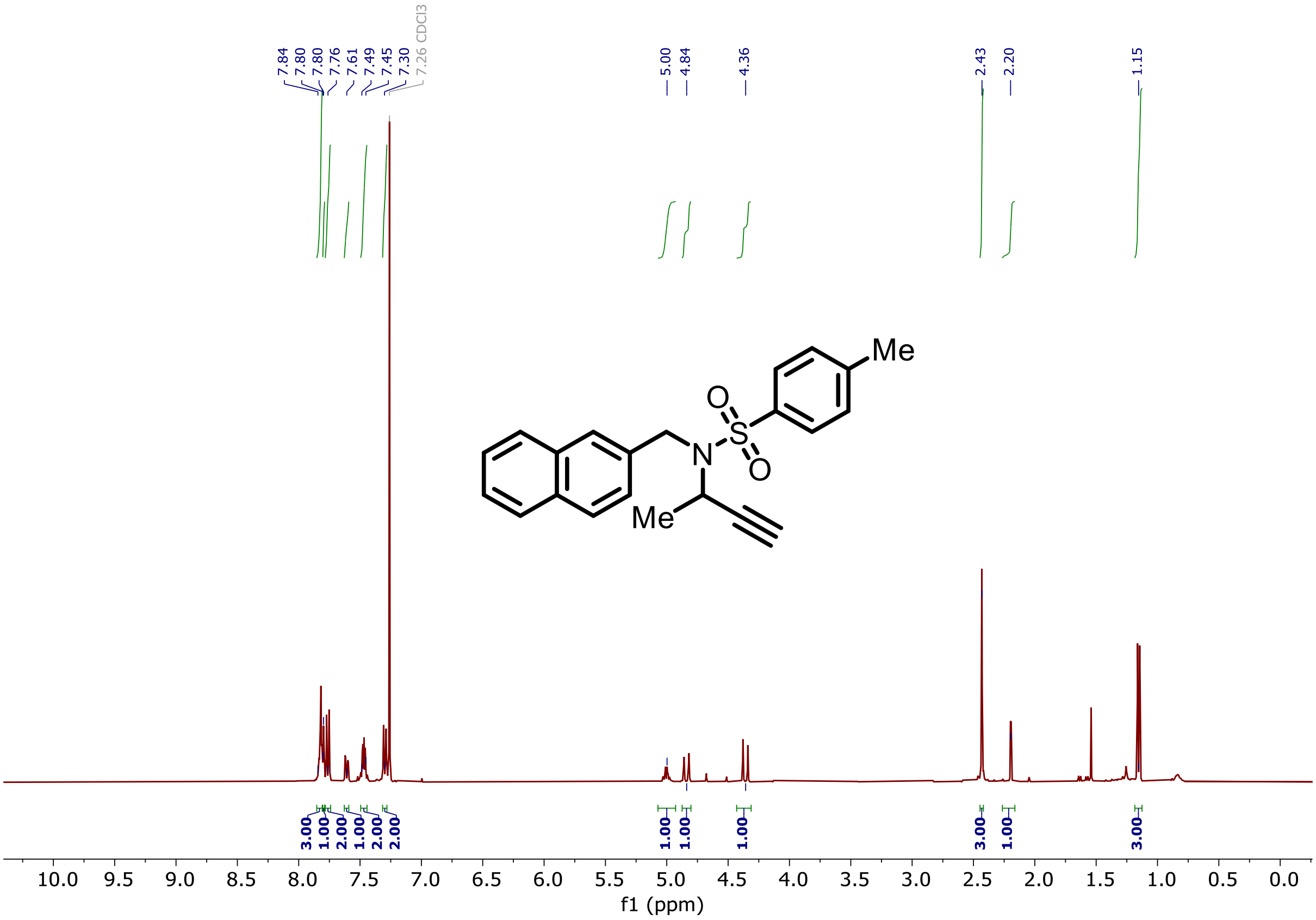

^{13}C NMR (126 MHz, CDCl$_3$)

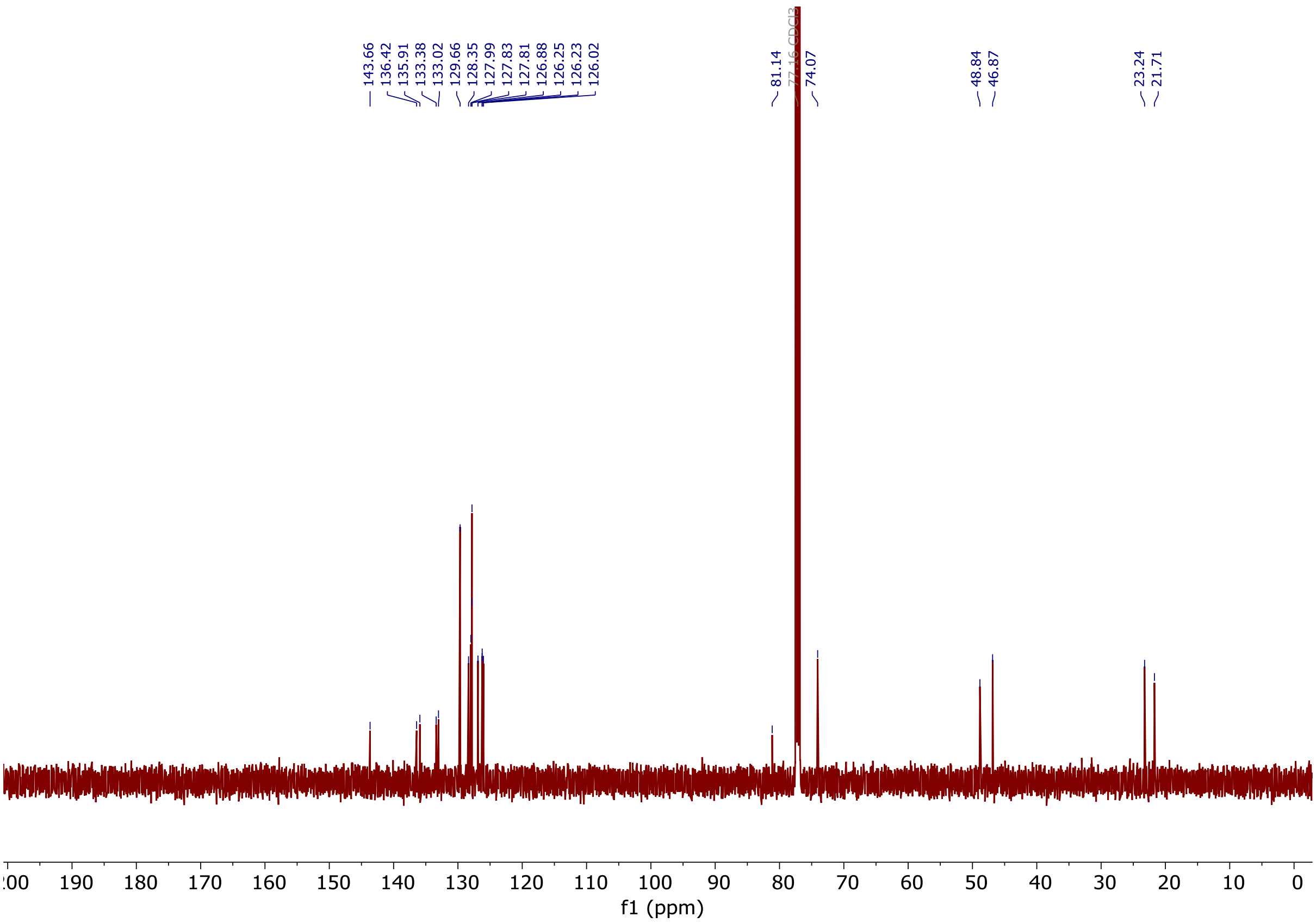

***N*-(4-fluorobenzyl)-4-methyl-*N*-(1-phenylprop-2-yn-1-yl)benzenesulfonamide** **(1n)**

^{1}H NMR (500 MHz, CDCl$_3$)

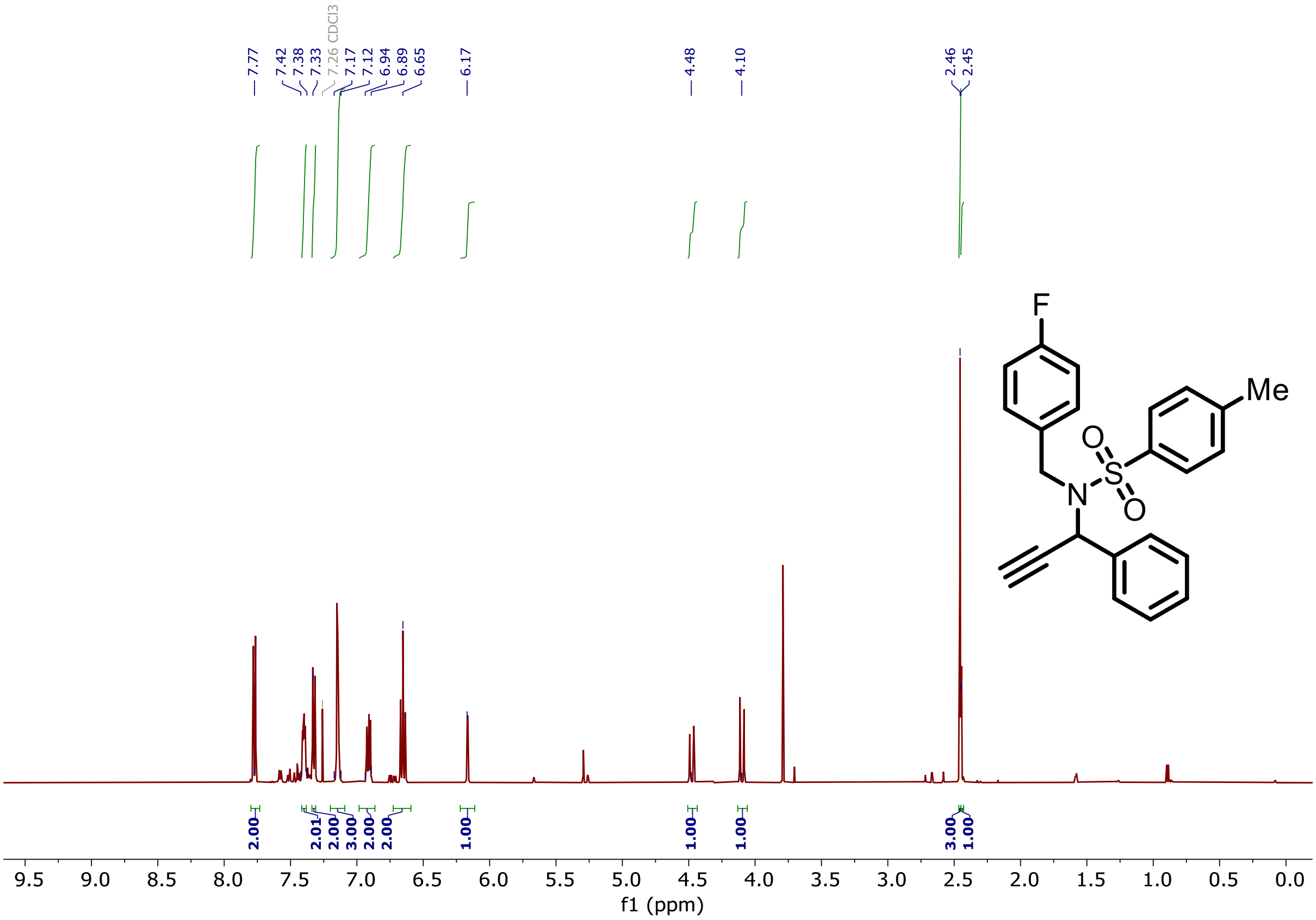

^{13}C NMR (126 MHz, CDCl$_3$)

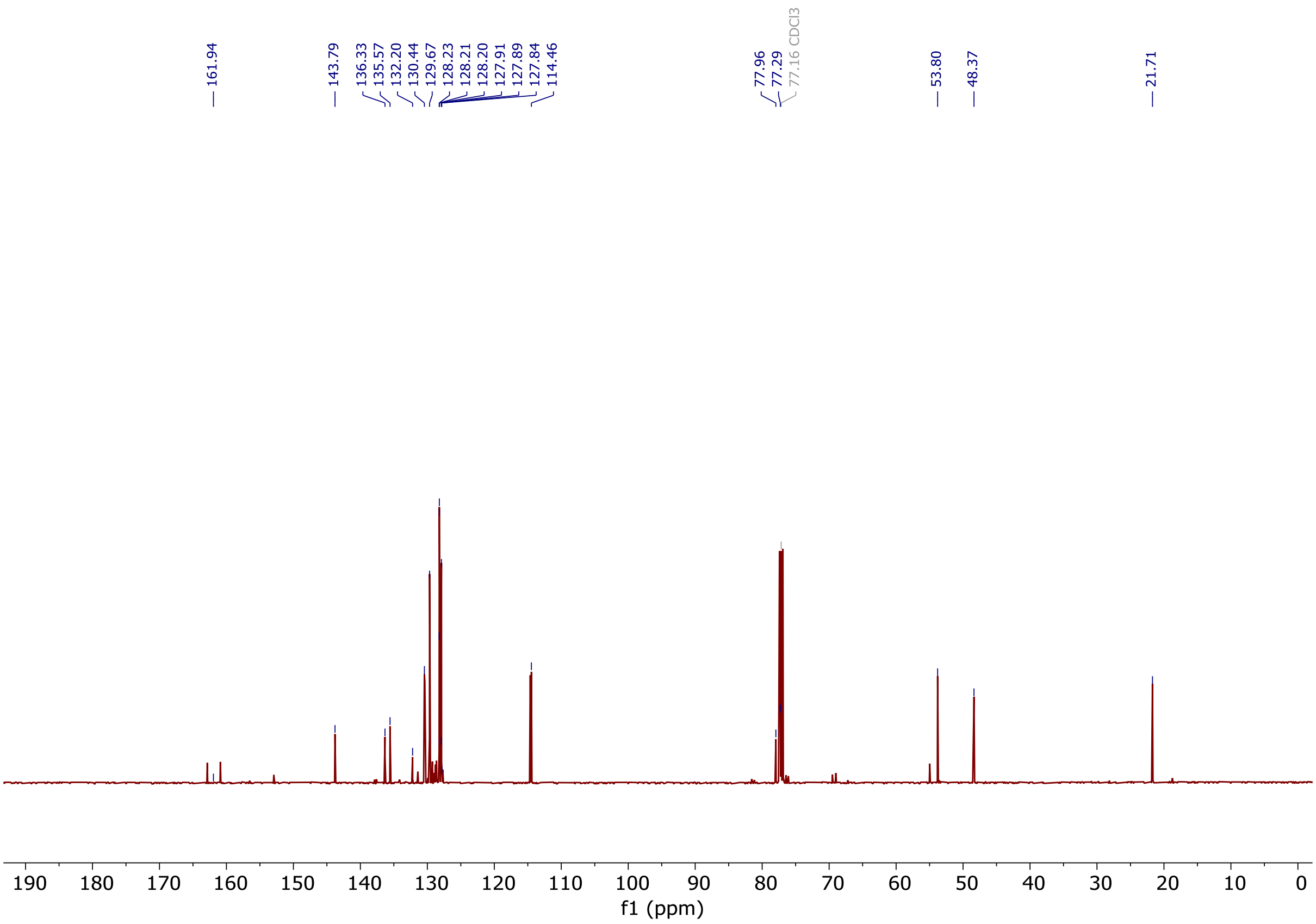

^{19}F NMR (471 MHz, $CDCl_3$)

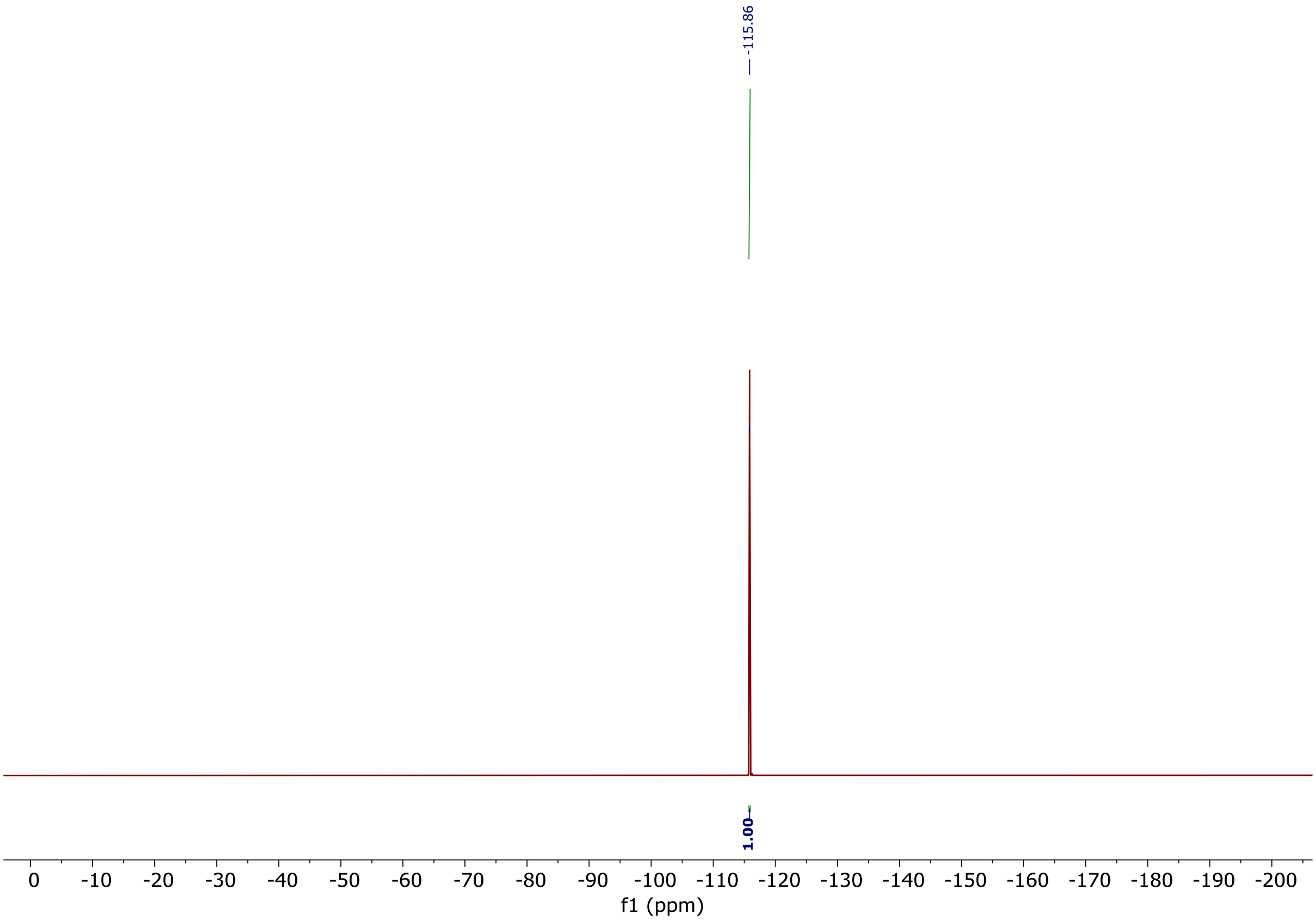

***rac*-4-methyl-N-(1-phenylethyl)-N-(prop-2-yn-1-yl)benzenesulfonamide** (1p)

^{1}H NMR (400MHz, $CDCl_3$)

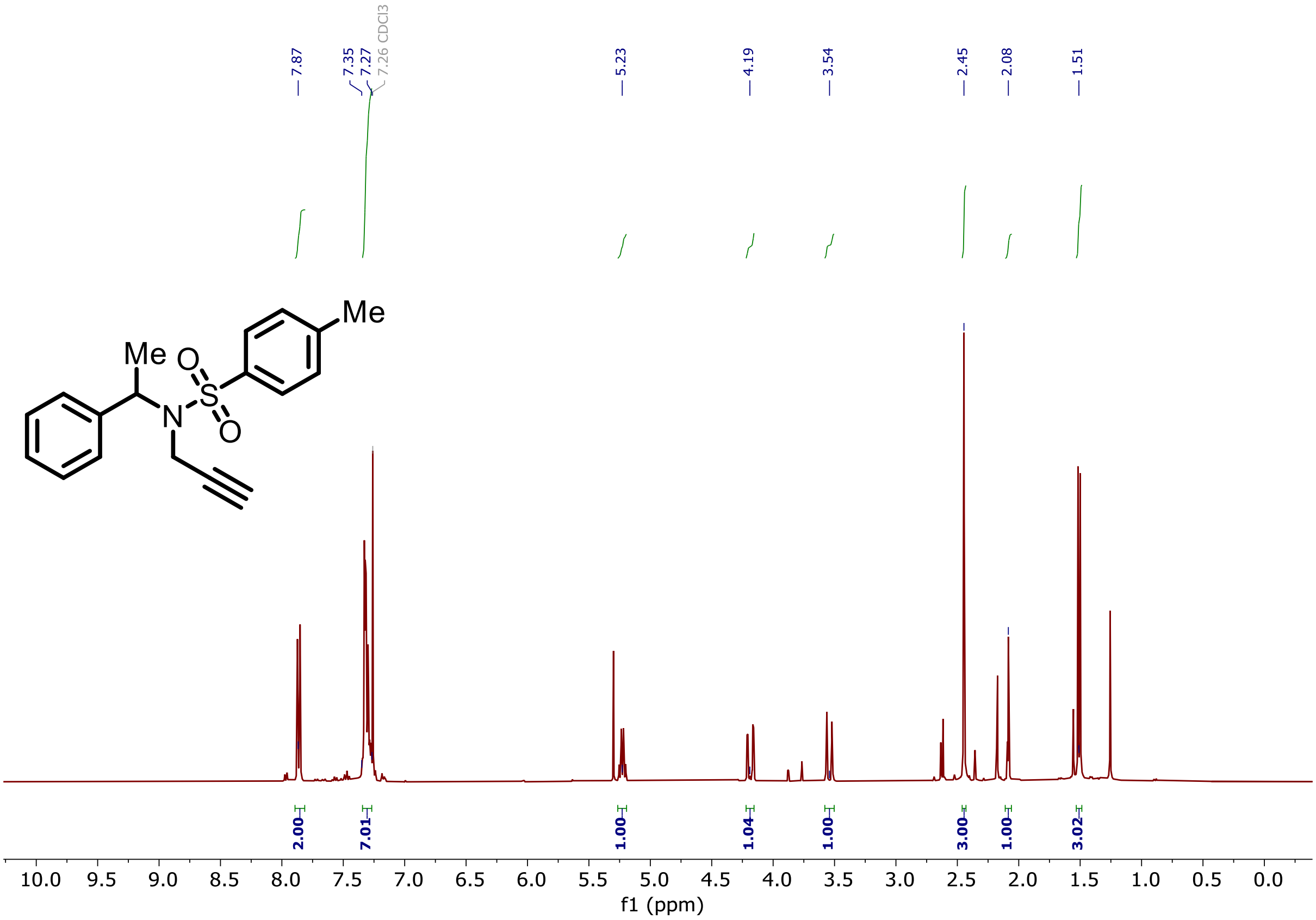

^{13}C NMR (126 MHz, $CDCl_3$)

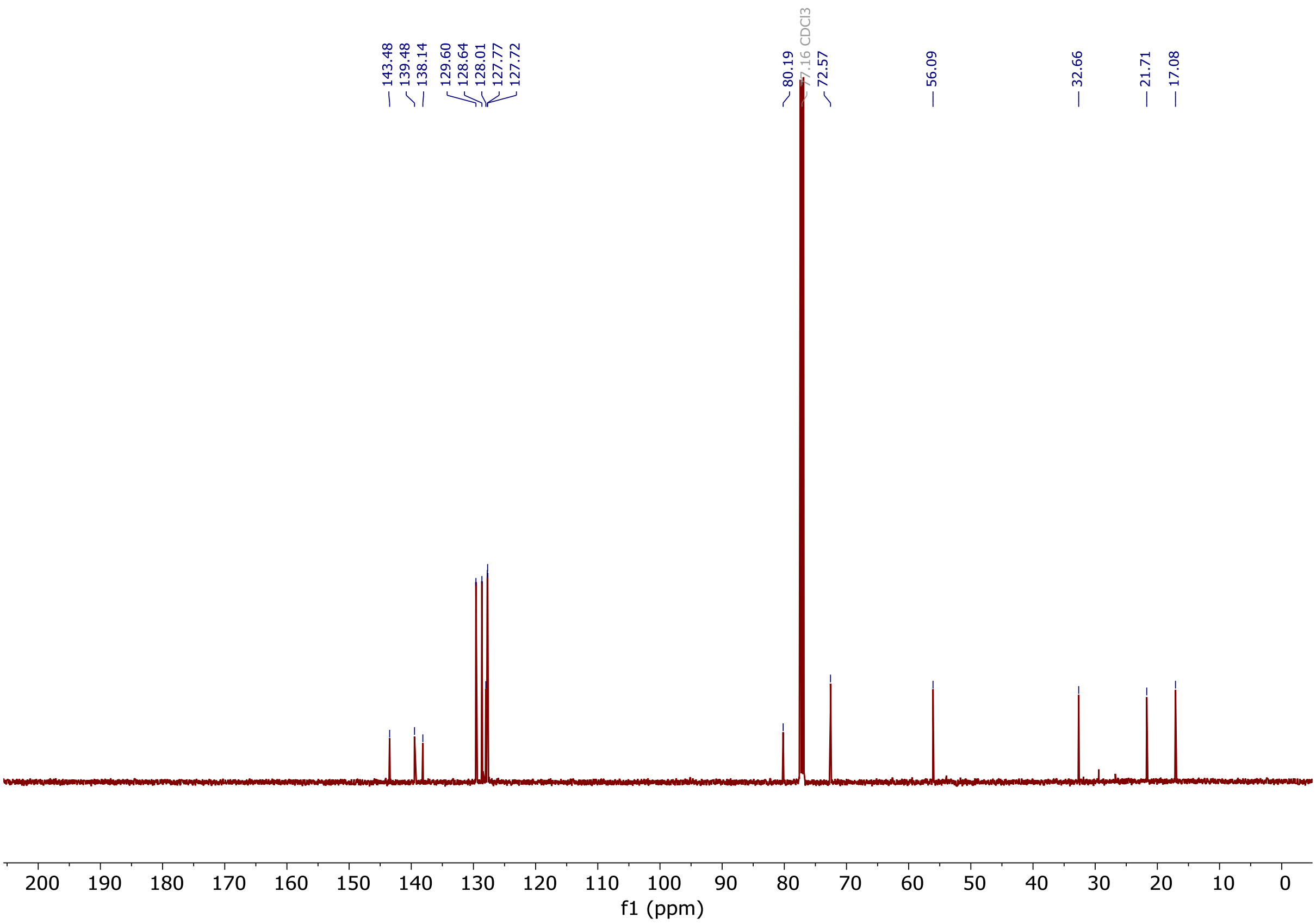

(S)-4-methyl-N-(1-phenylethyl)-N-(prop-2-yn-1-yl)benzenesulfonamide ((*S*)-1p)

^{1}H NMR (400MHz, CDCl$_3$)

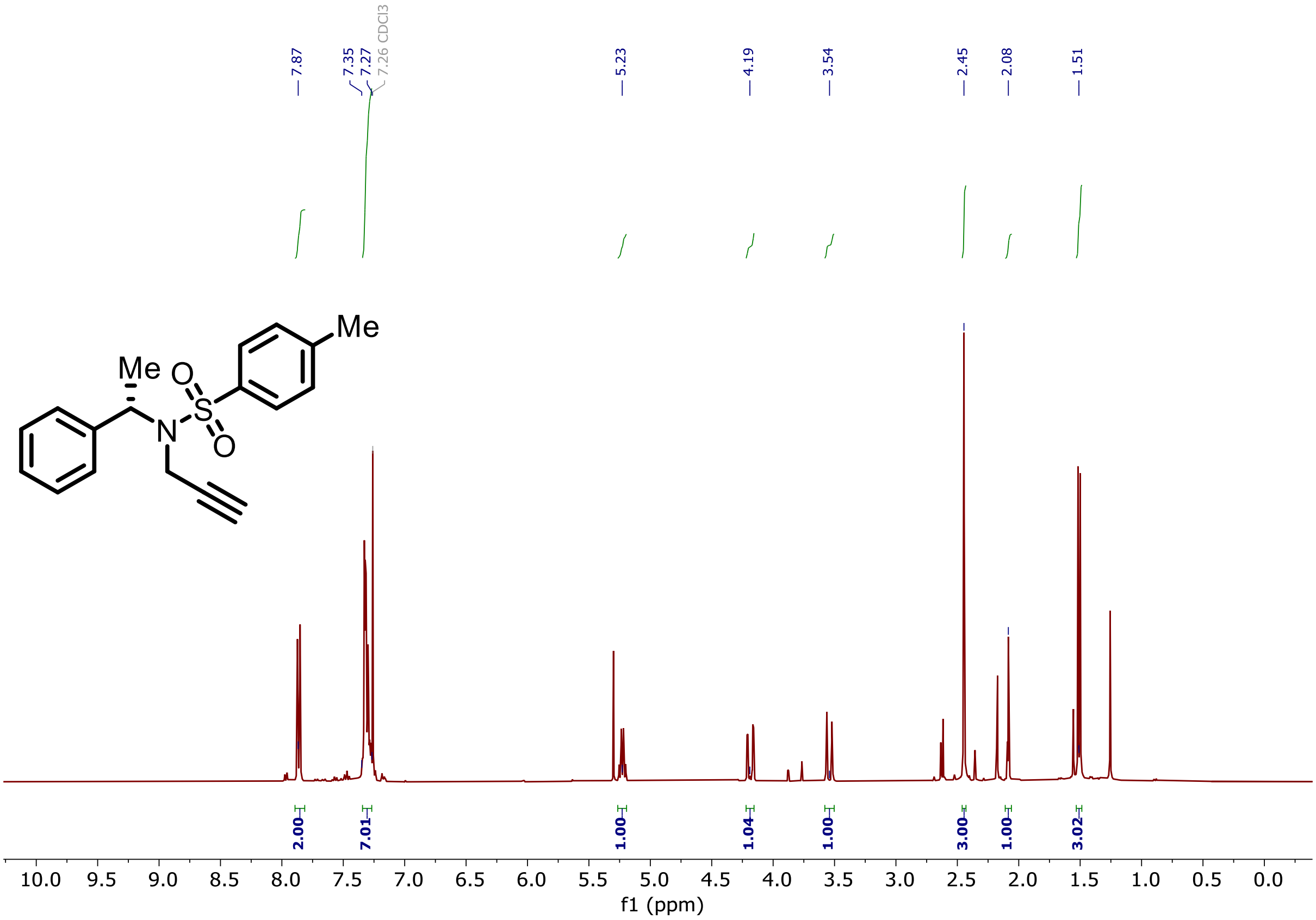

^{13}C NMR (126 MHz, CDCl$_3$)

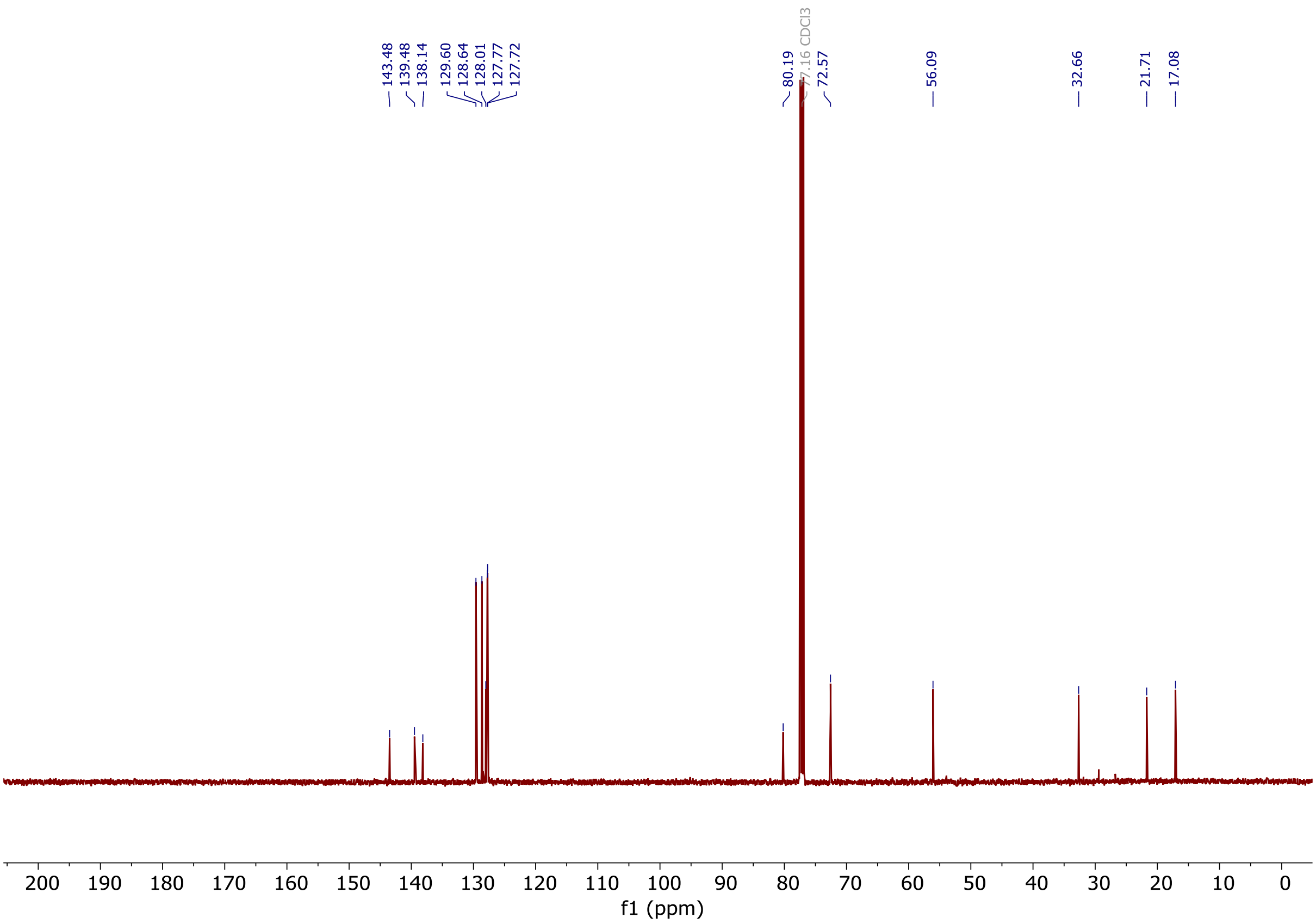

***4-methyl-N-(prop-2-yn-1-yl)-N-(1-tosylpiperidin-4-yl)benzenesulfonamide* (1r)**

^{1}H NMR (400 MHz, CDCl₃)

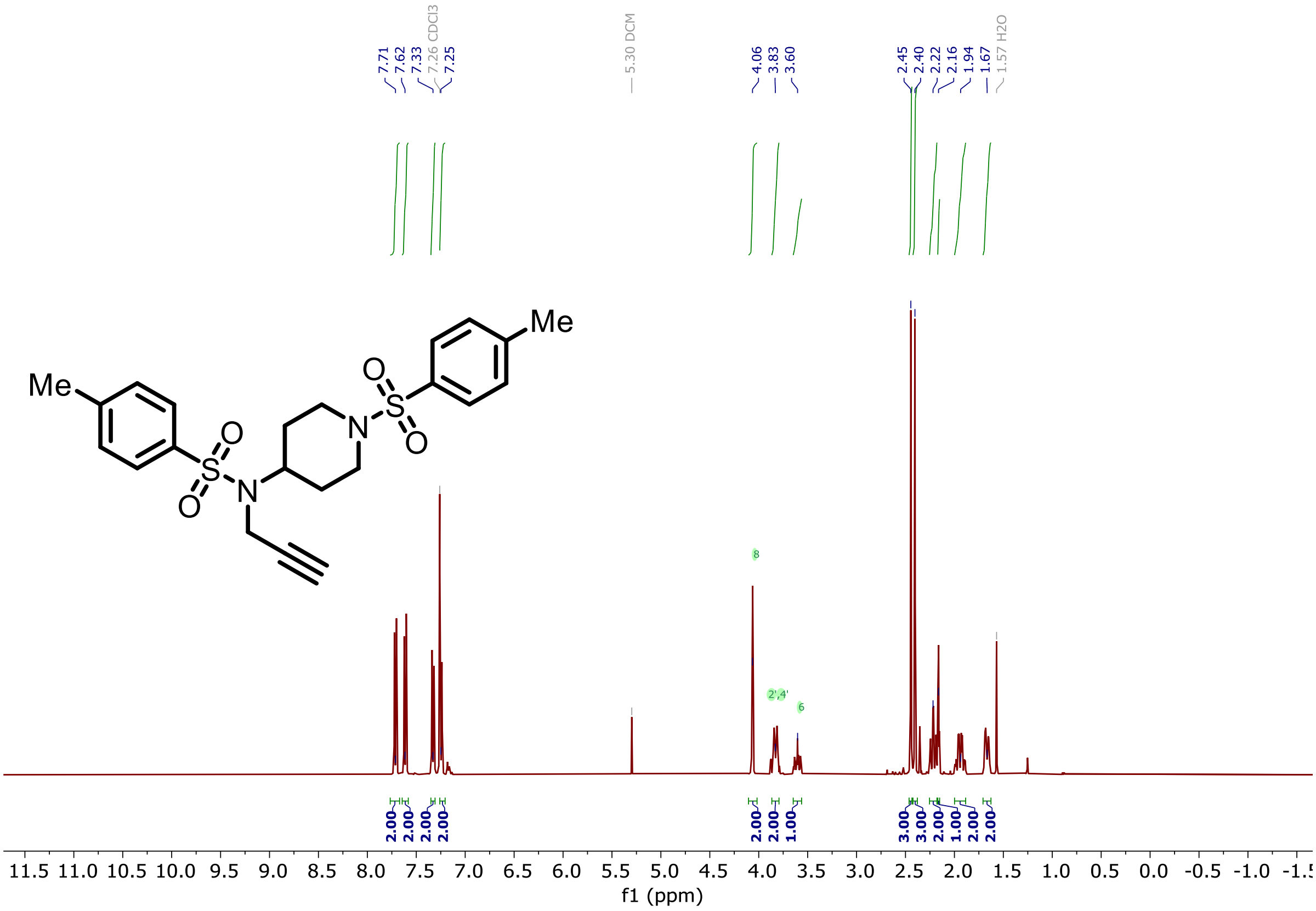

^{13}C NMR (101 MHz, CDCl₃)

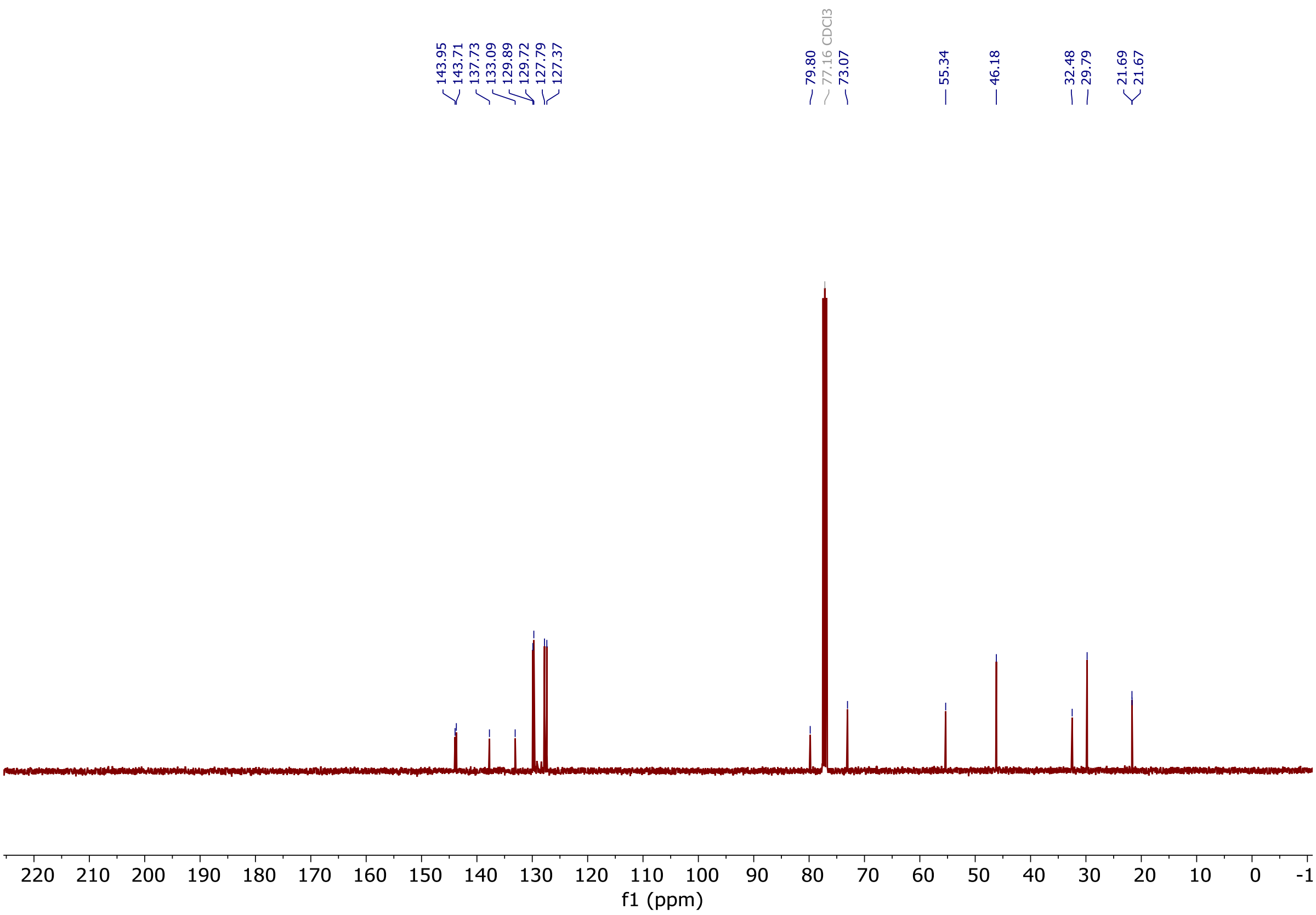

***N-butyl-4-methyl-N-(prop-2-yn-1-yl)benzenesulfonamide* (1v)**

^{1}H NMR (500 MHz, CDCl$_3$)

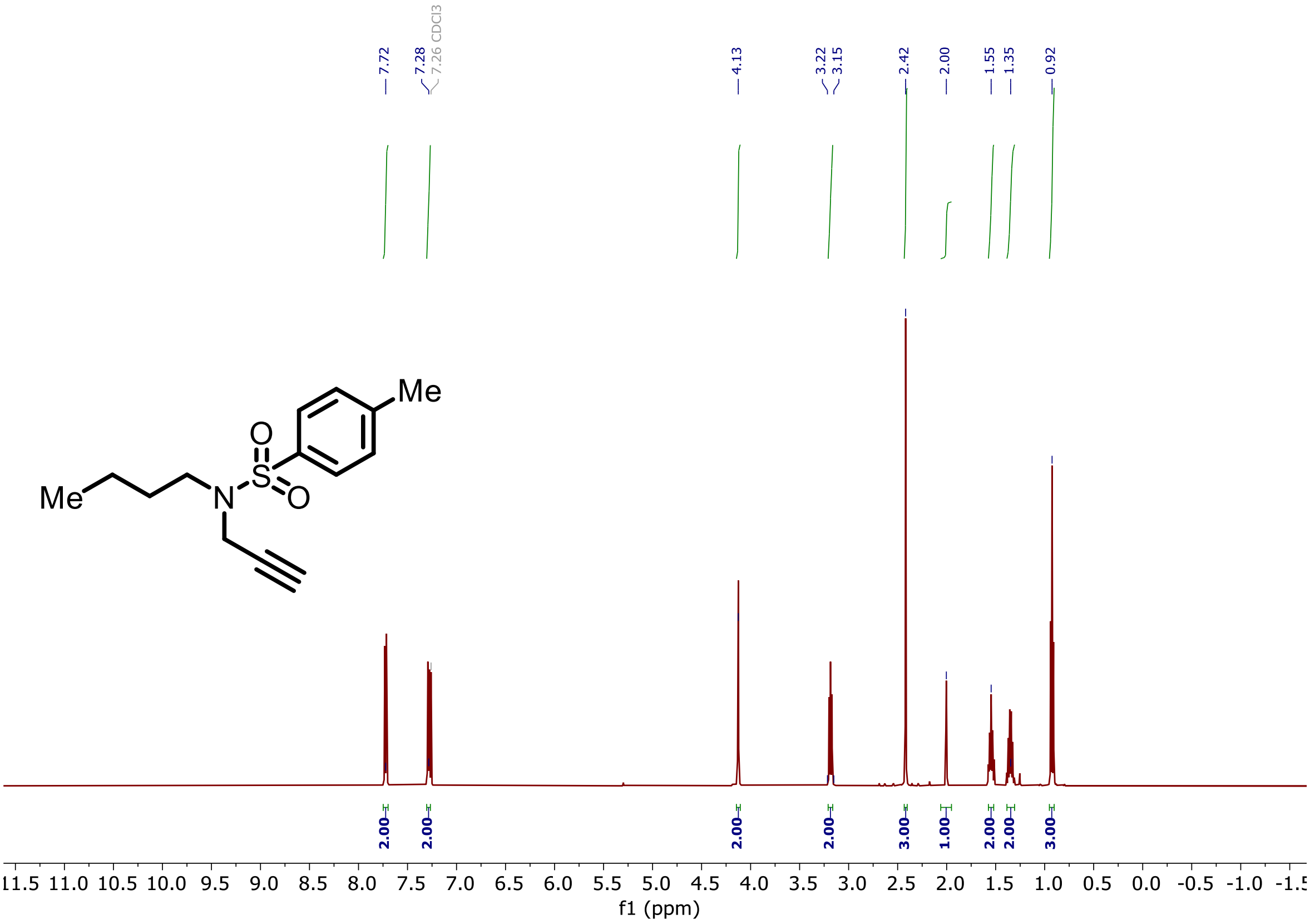

^{13}C NMR (126 MHz, CDCl$_3$)

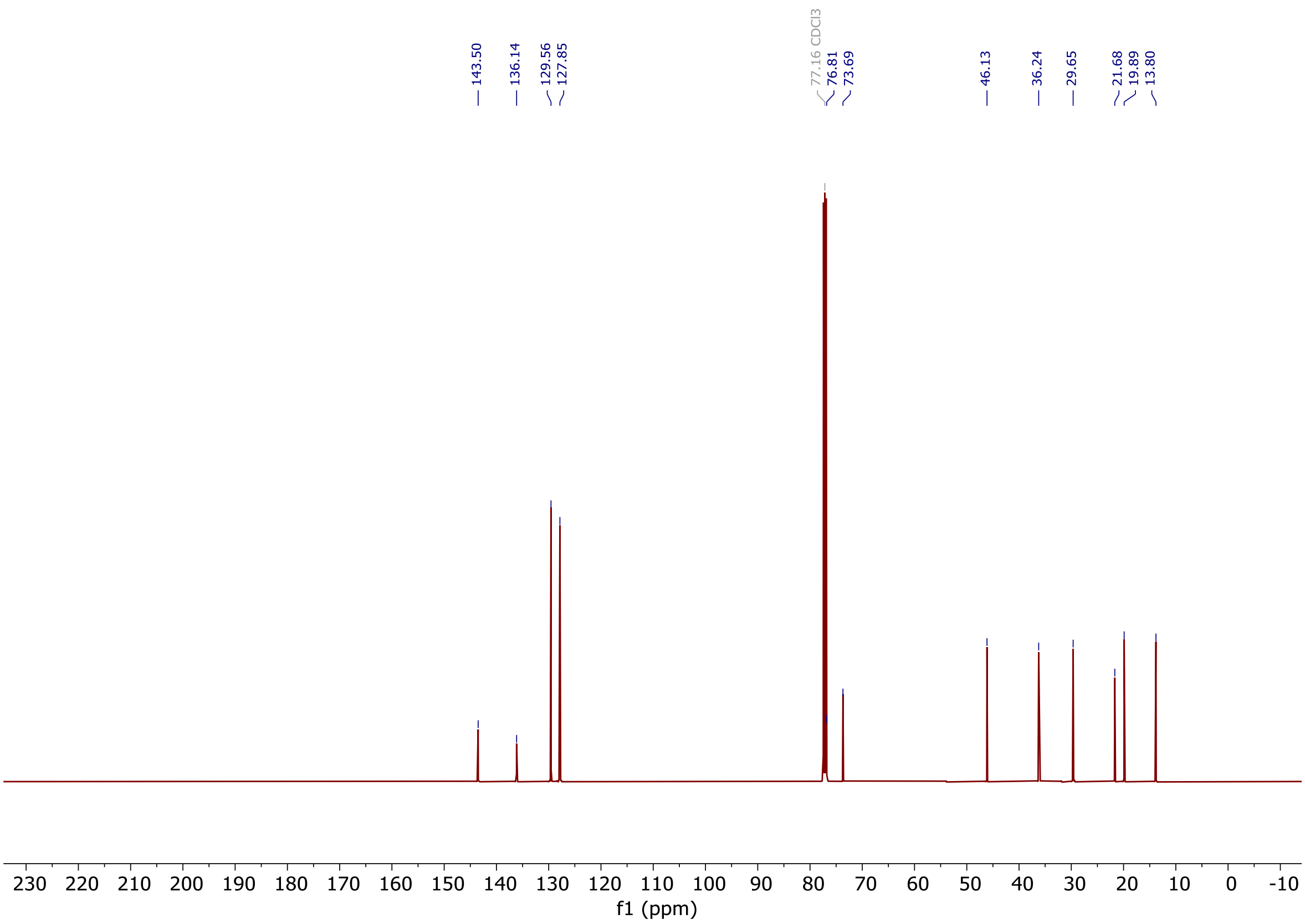

***4-methyl-N-(prop-2-yn-1-yl)-N-(2-(thiophen-2-yl)ethyl)benzenesulfonamide* (1w)**

^{1}H NMR (500 MHz, $CDCl_3$)

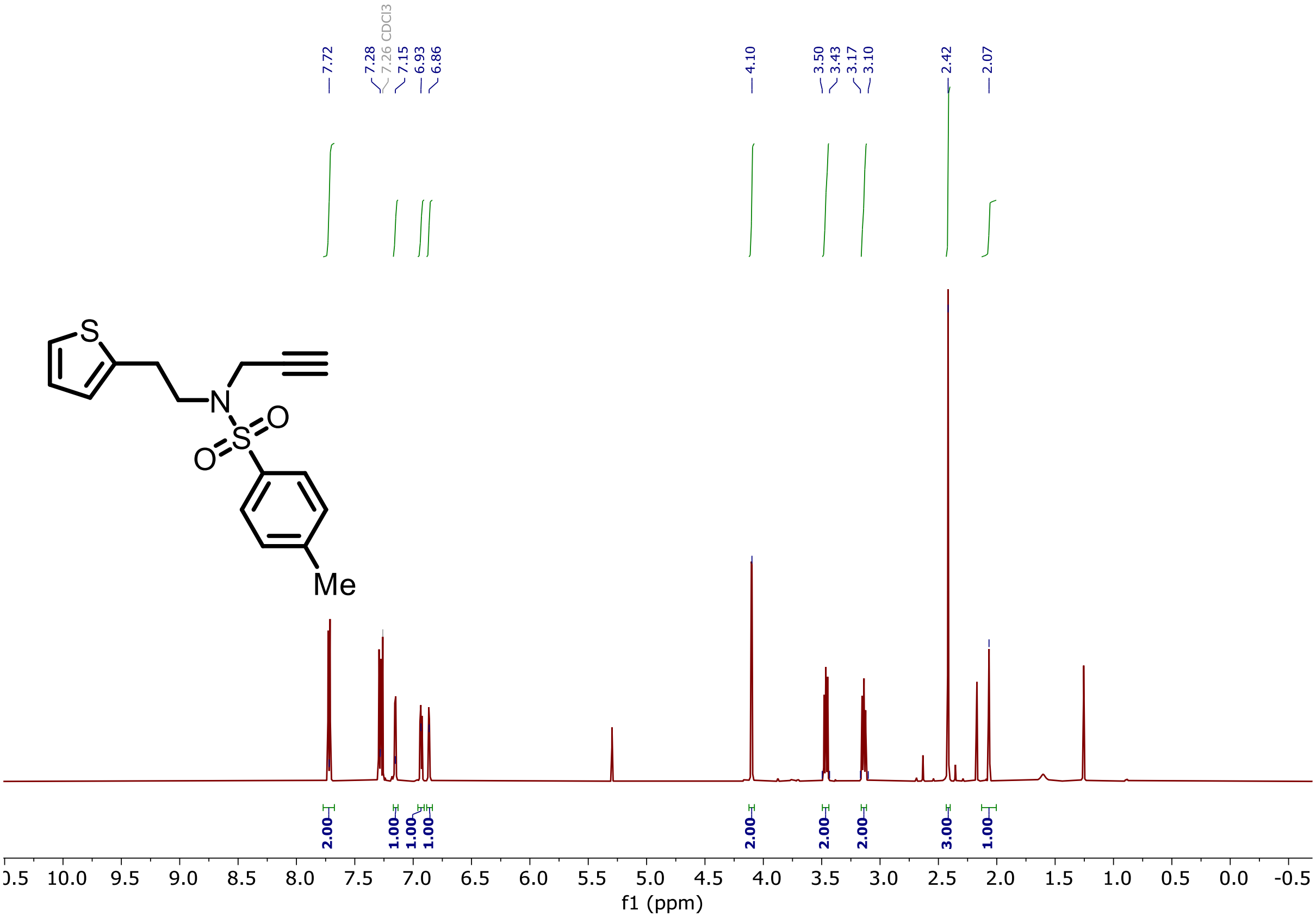

^{13}C NMR (126 MHz, $CDCl_3$)

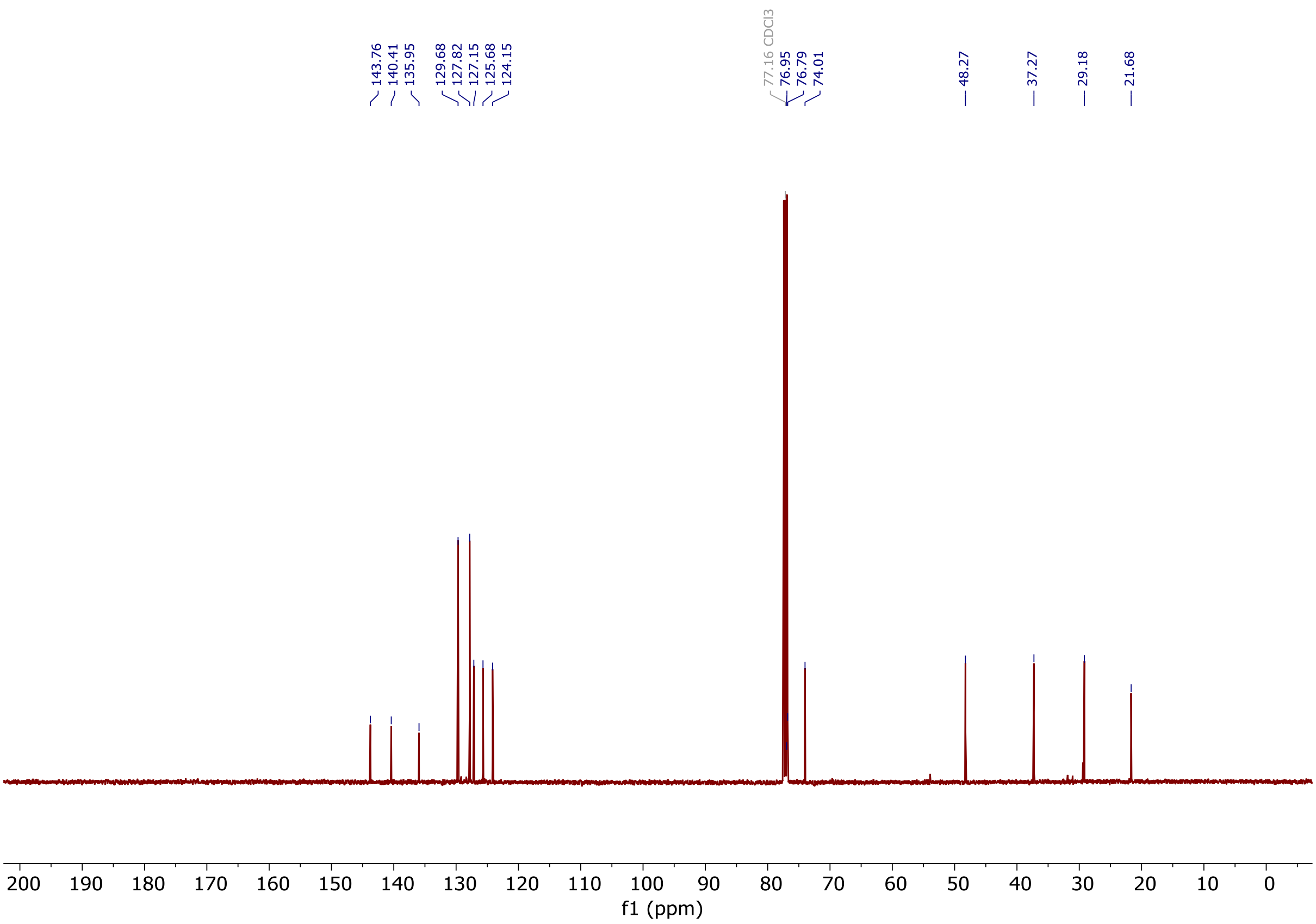

4-methyl-N-(prop-2-yn-1-yl)-N-((tetrahydrofuran-2-yl)methyl)benzenesulfonamide (1x)

^{1}H NMR (500 MHz, CDCl$_3$)

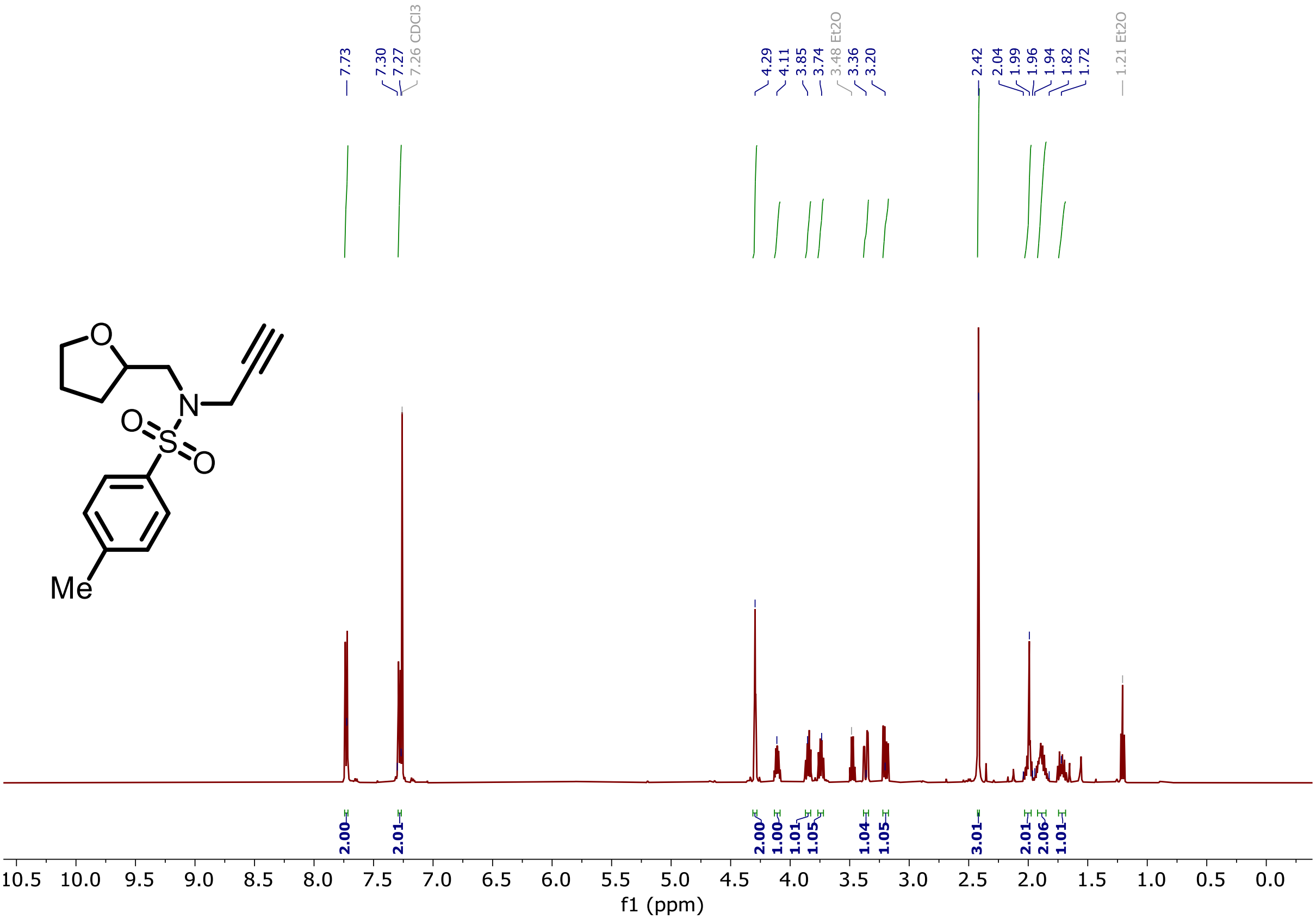

^{13}C NMR (126 MHz, CDCl$_3$)

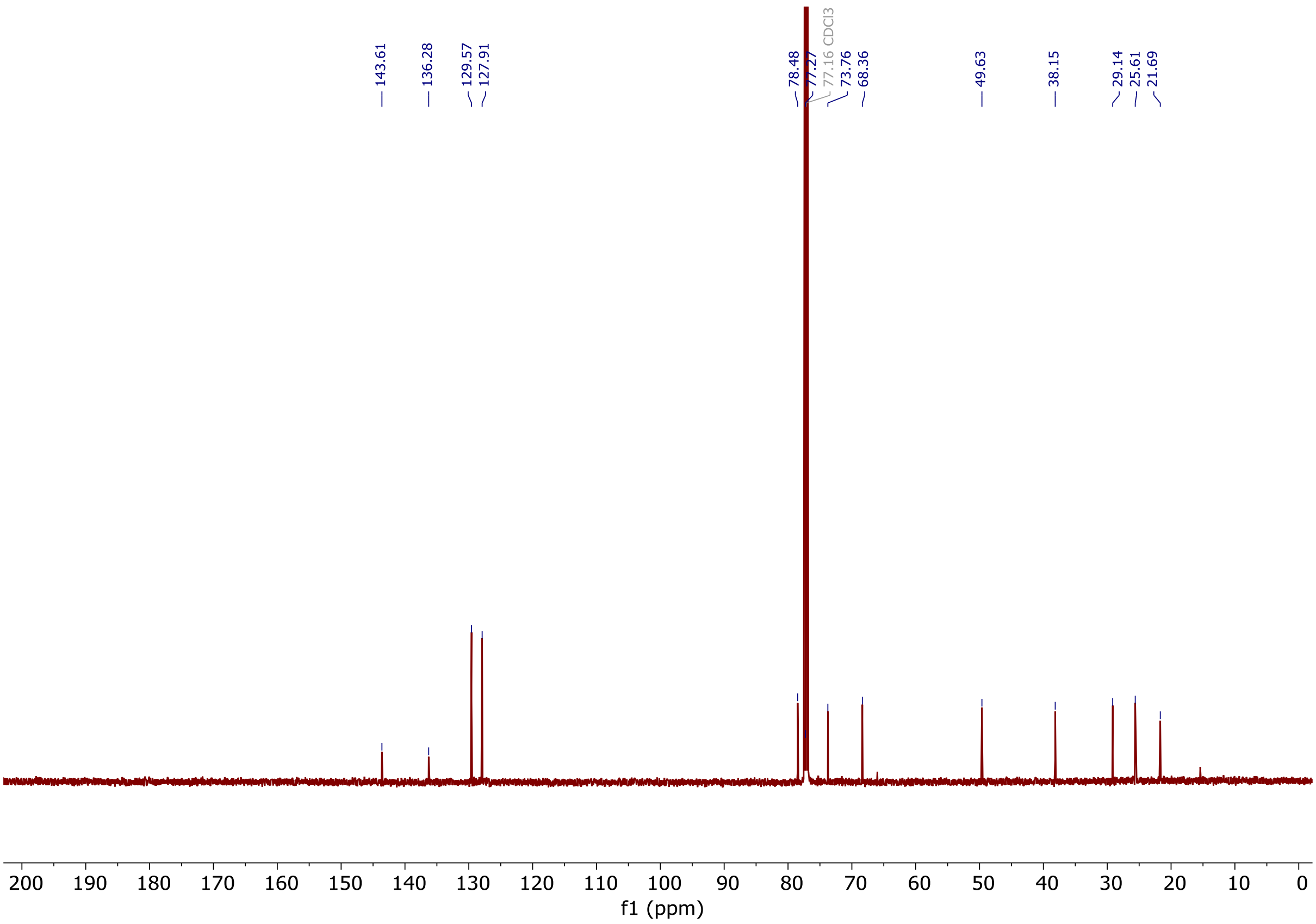

2-(prop-2-yn-1-yl)isothiazolidine 1,1-dioxide **(1y)**

^{1}H NMR (500 MHz, $CDCl_3$)

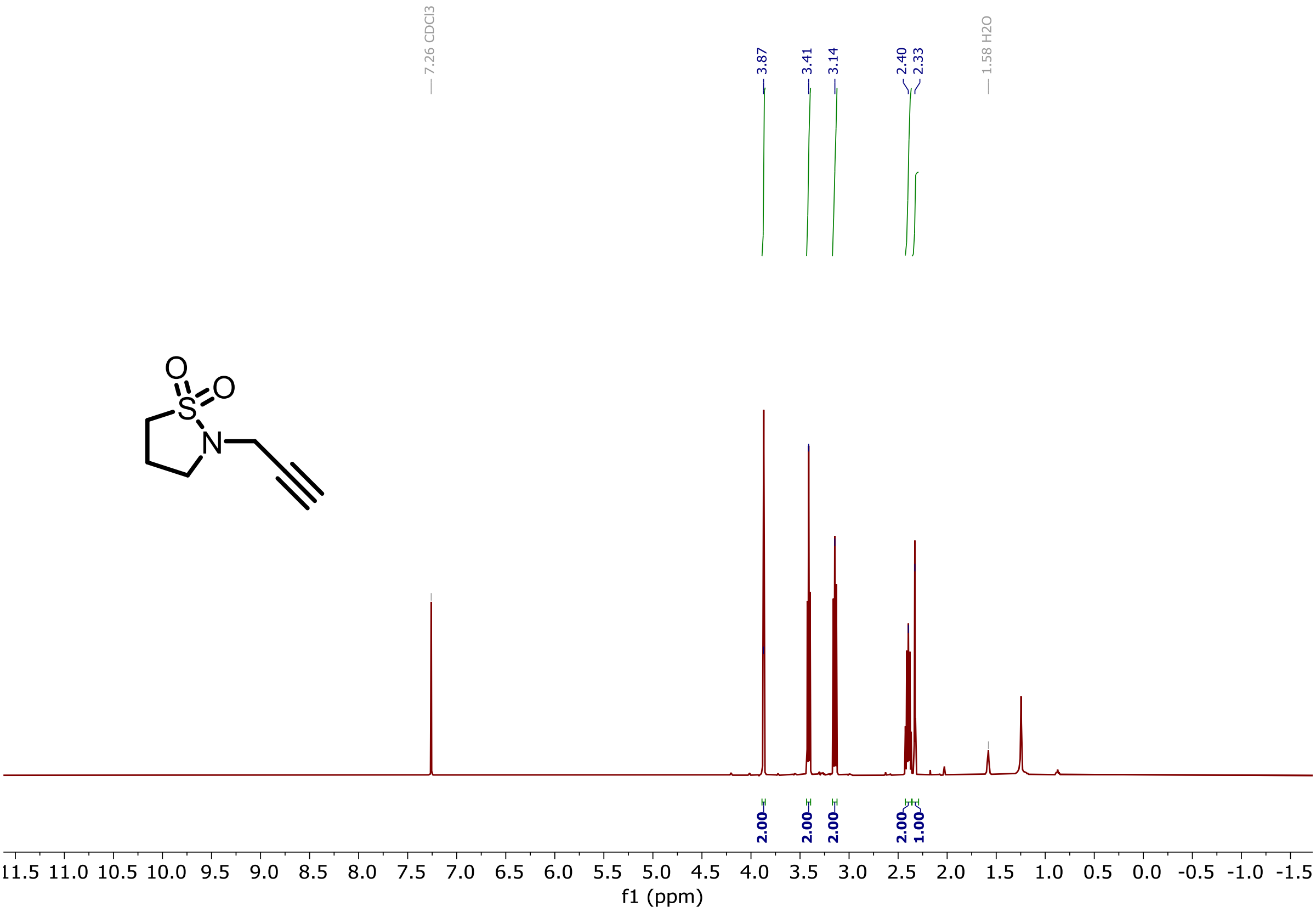

^{13}C NMR (126 MHz, $CDCl_3$)

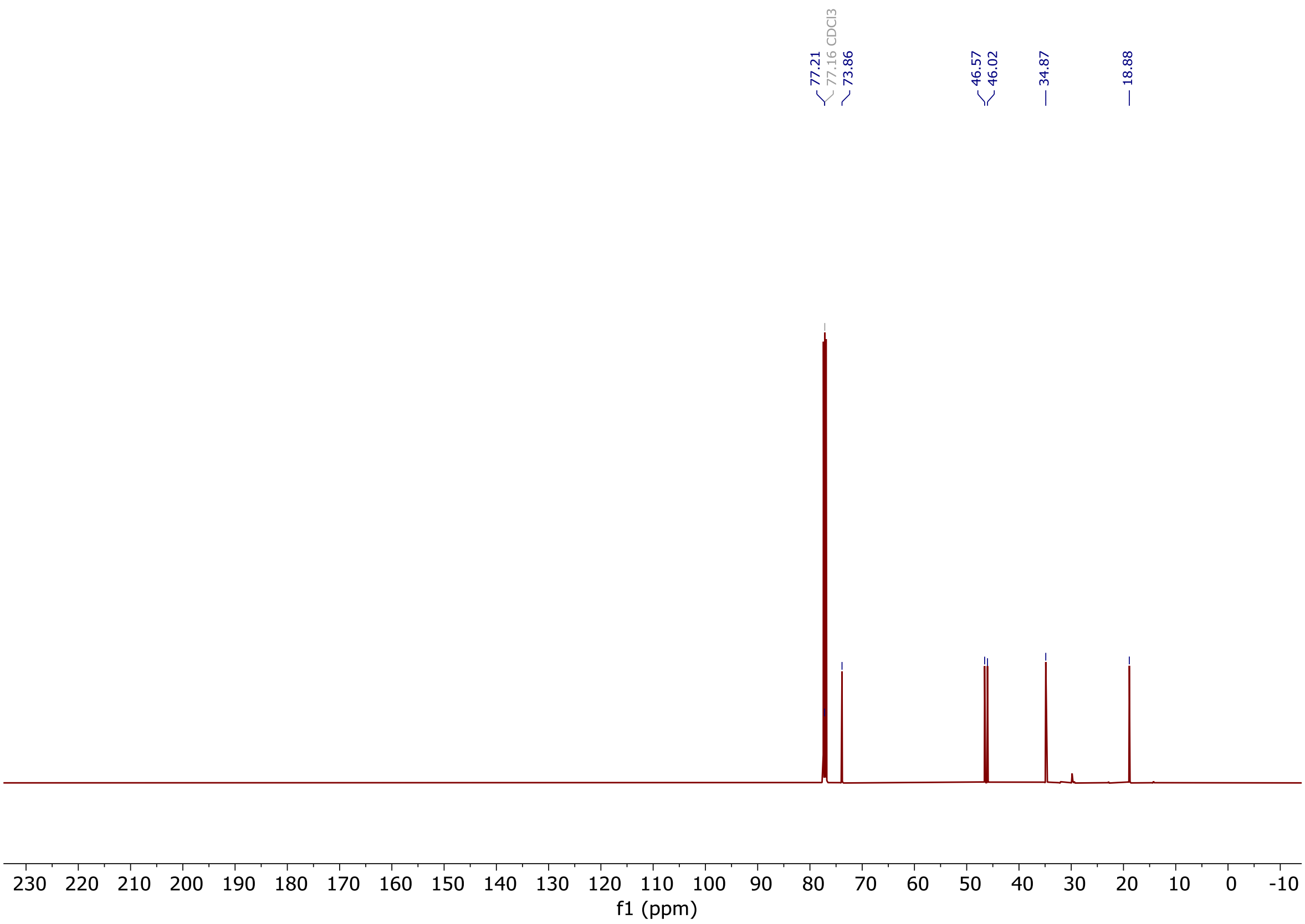

N-(1-ethynylcyclohexyl)-N-(3-fluorobenzyl)-4-methylbenzenesulfonamide **(1aa)**

^{1}H NMR (500 MHz, CDCl$_3$)

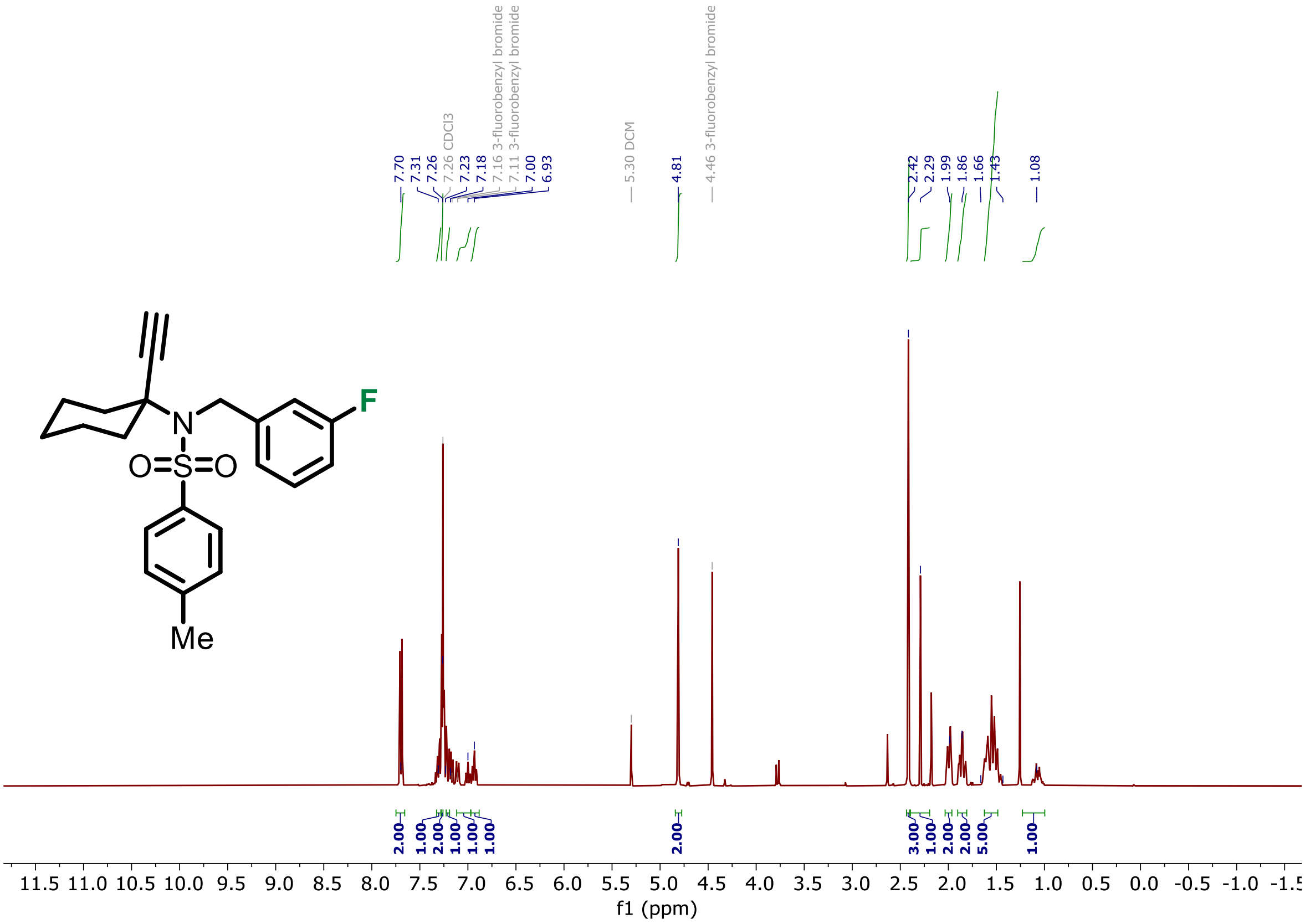

^{13}C NMR (126 MHz, CDCl$_3$)

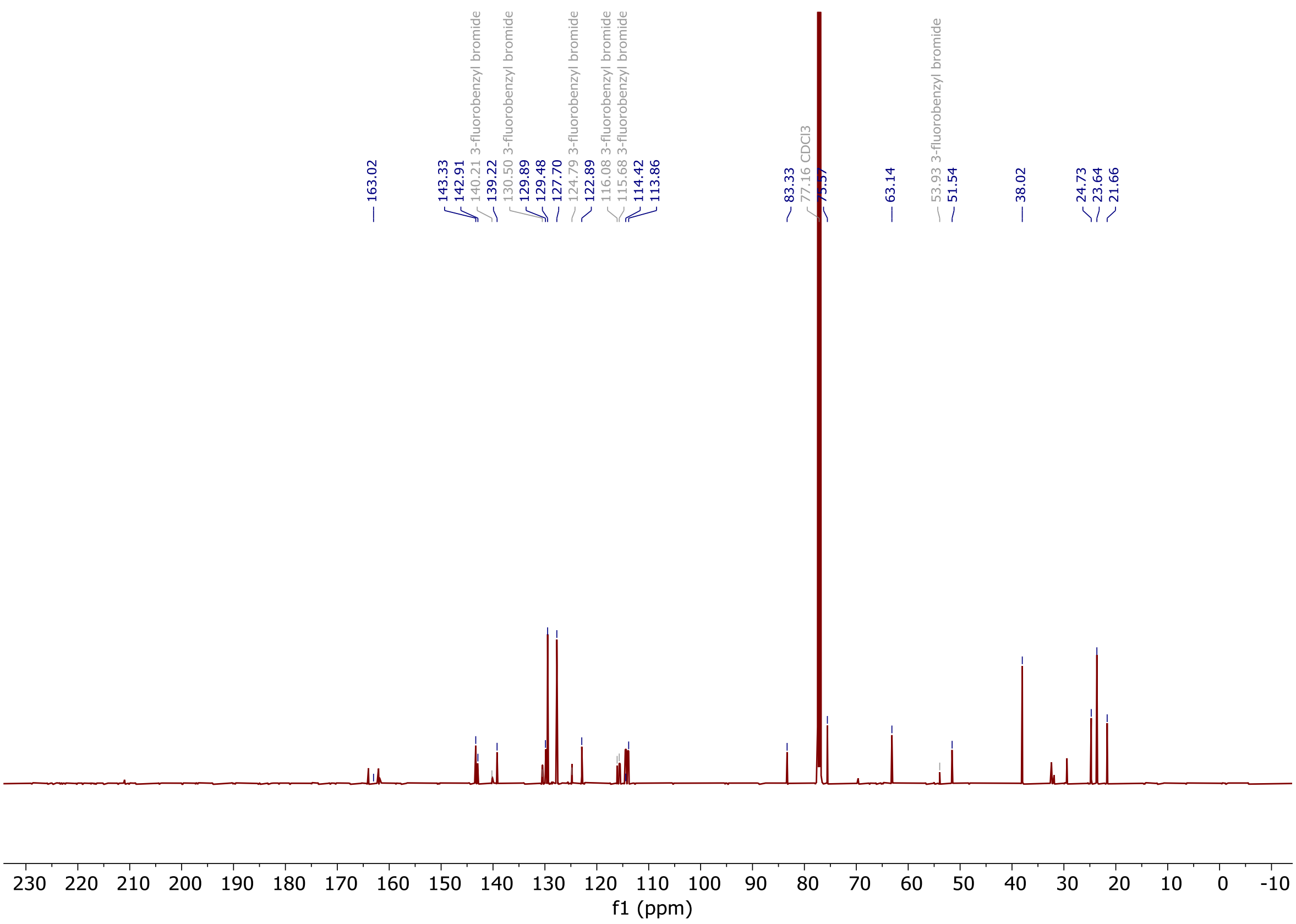

^{19}F NMR (376 MHz, CDCl$_3$)

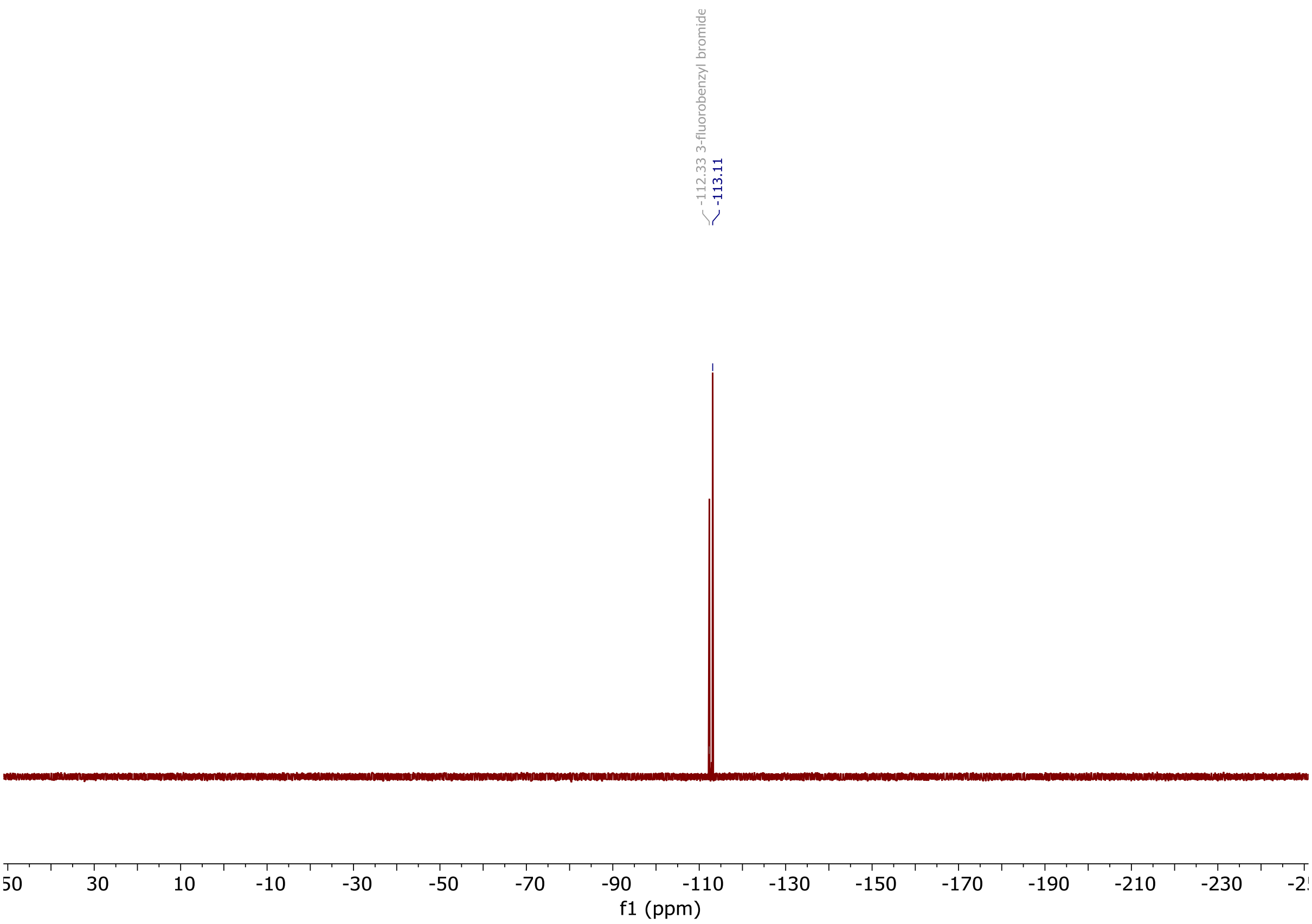

N-(1-ethynylcyclohexyl)-N,4-dimethylbenzenesulfonamide **(1ab)**

^{1}H NMR (500 MHz, CDCl$_3$)

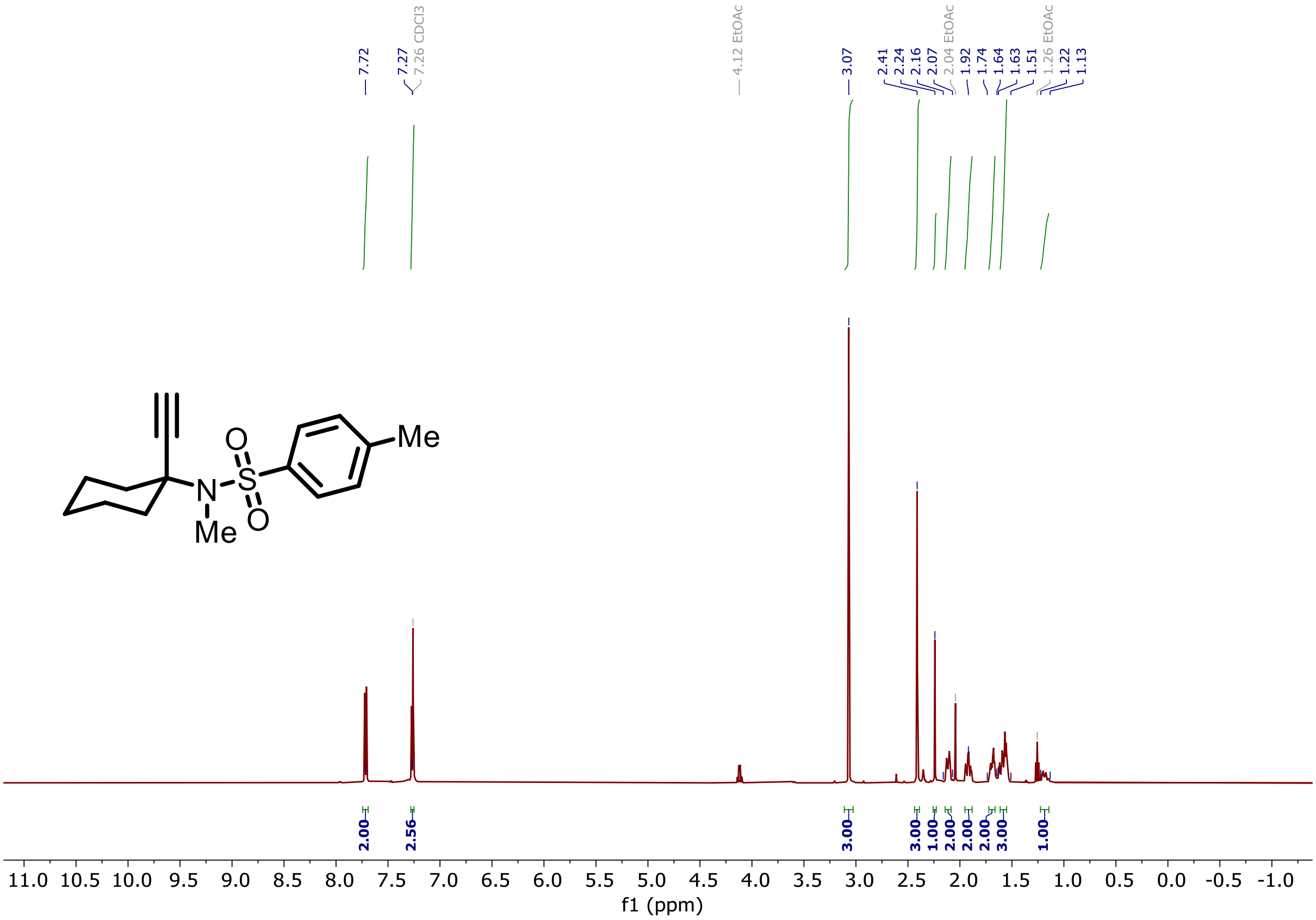

^{13}C NMR (126 MHz, CDCl$_3$)

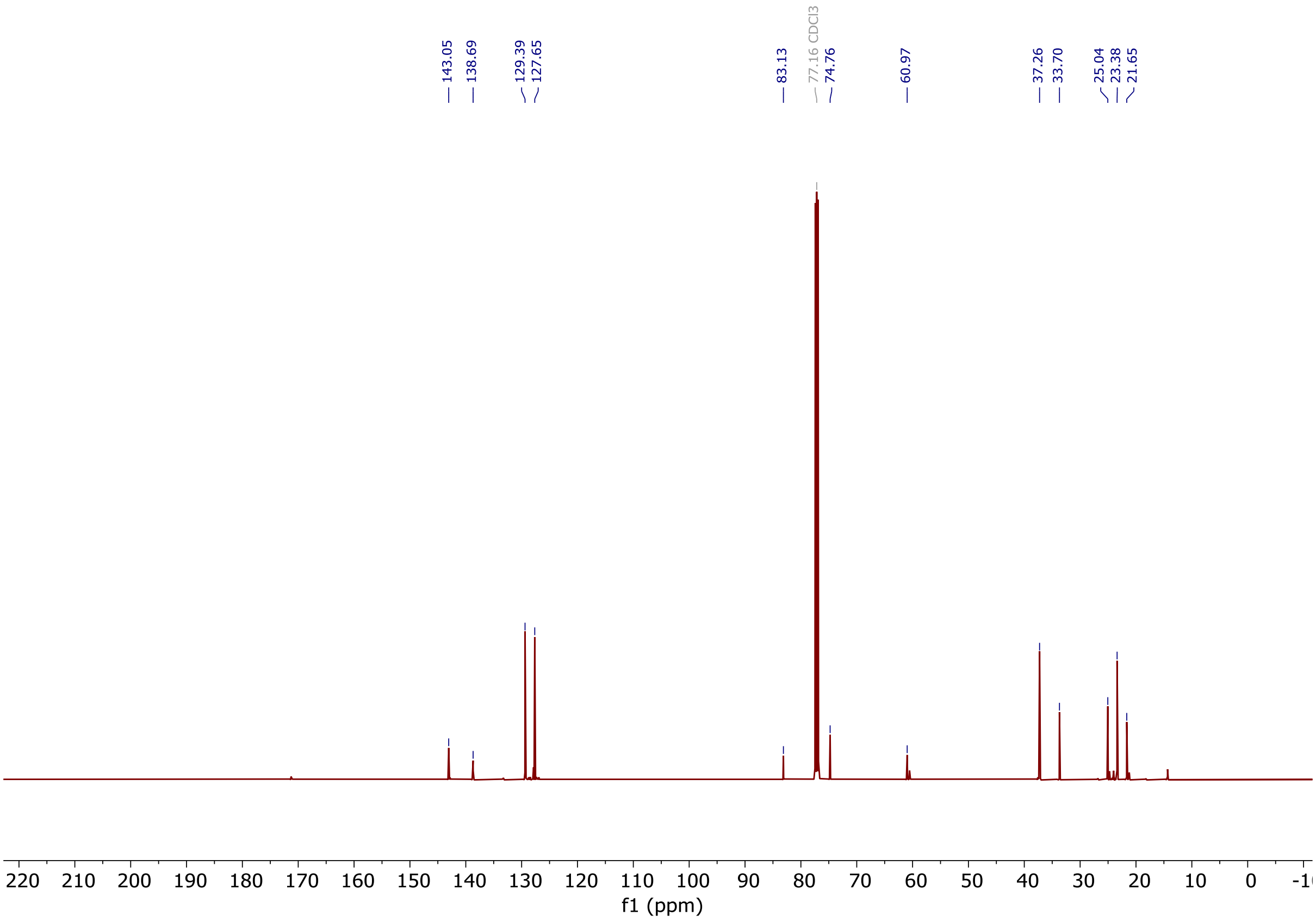

***1-bromo-3-(((2-methylbut-3-yn-2-yl)oxy)methyl)benzene* (1ae)**

^{1}H NMR (500 MHz, $CDCl_3$)

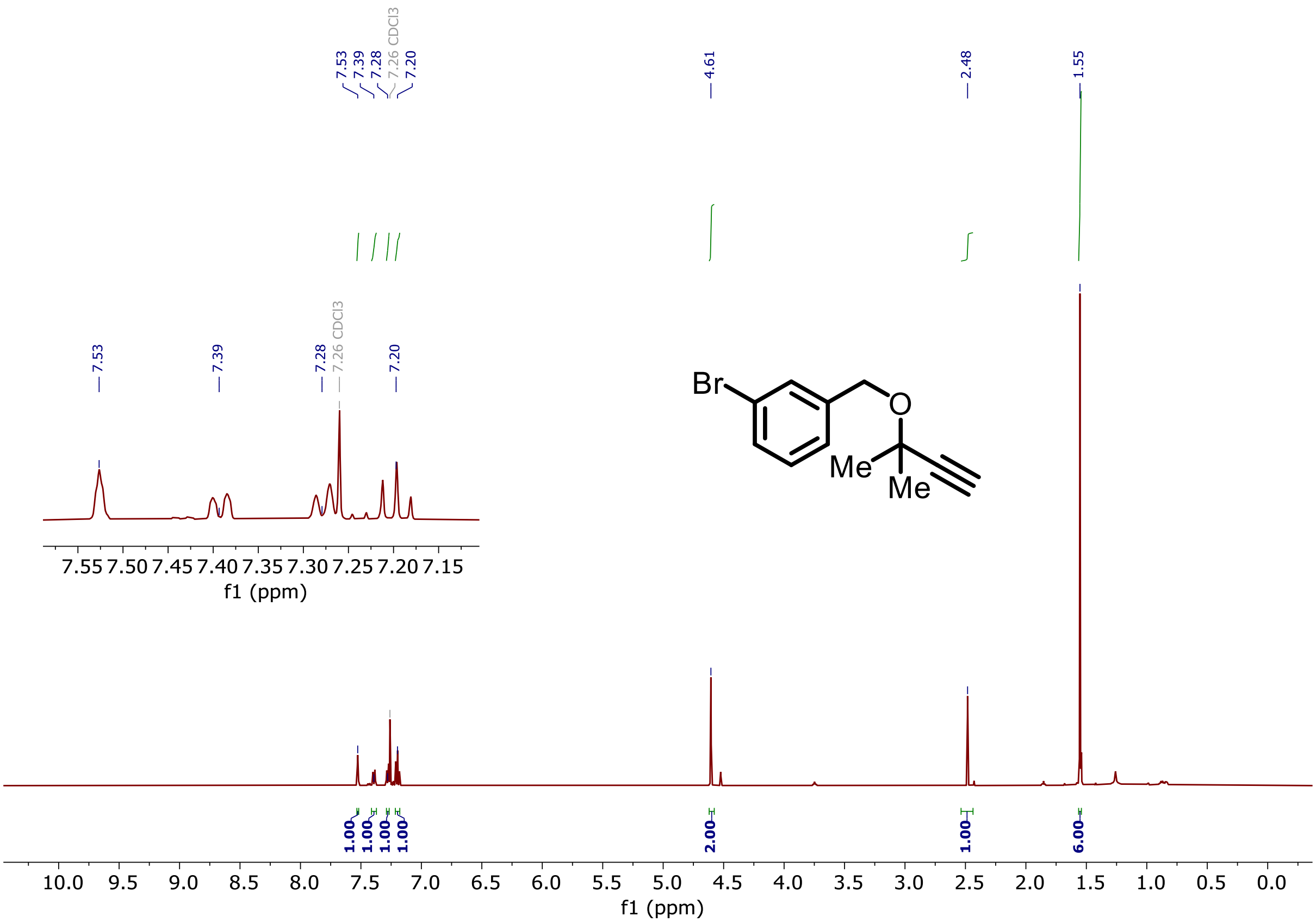

^{13}C NMR (126 MHz, $CDCl_3$)

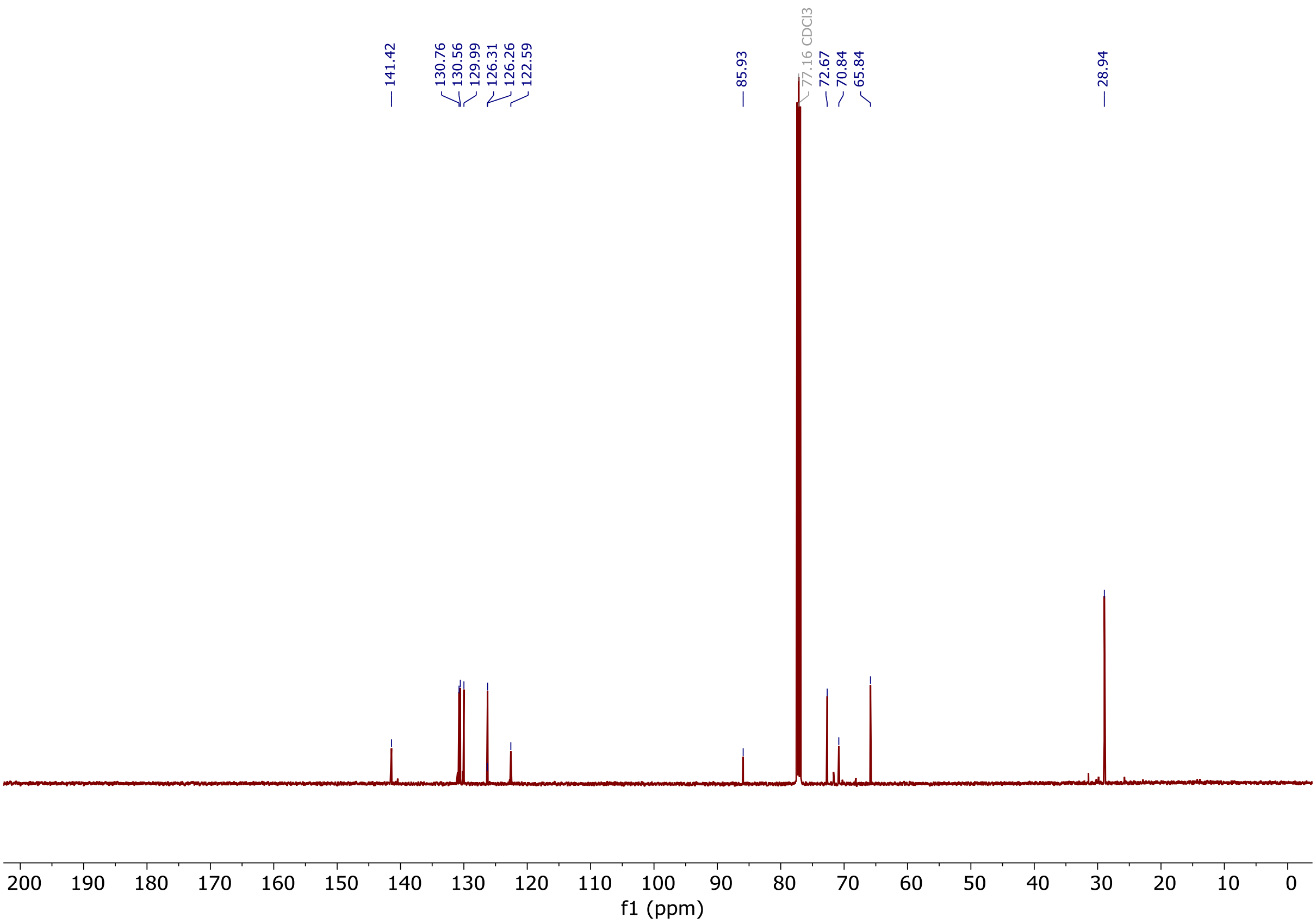

(1-((3-nitrobenzyl)oxy)prop-2-yne-1,1-diyl)dibenzene (**1af**)

^{1}H NMR (500 MHz, $CDCl_3$)

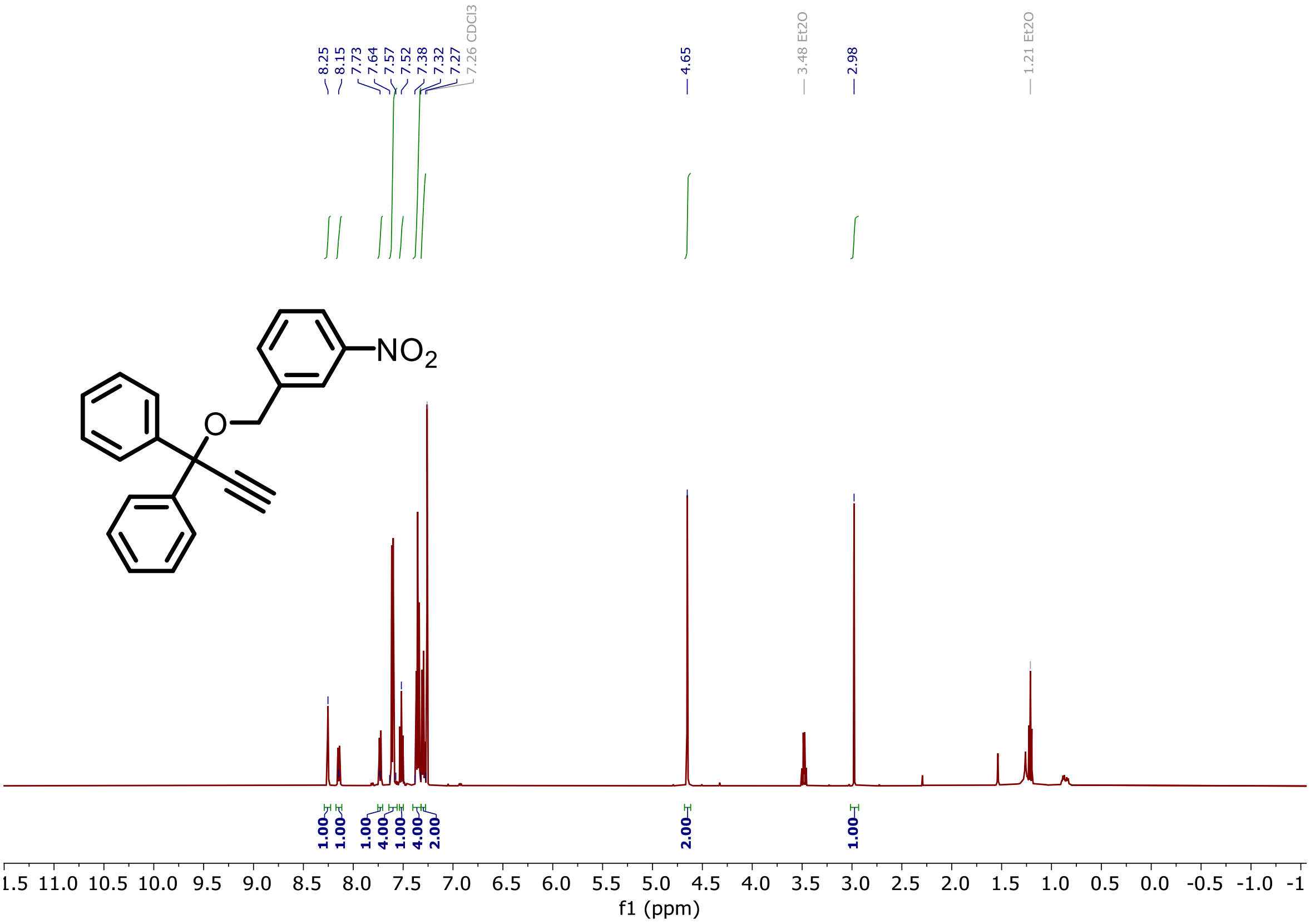

^{13}C NMR (126 MHz, $CDCl_3$)

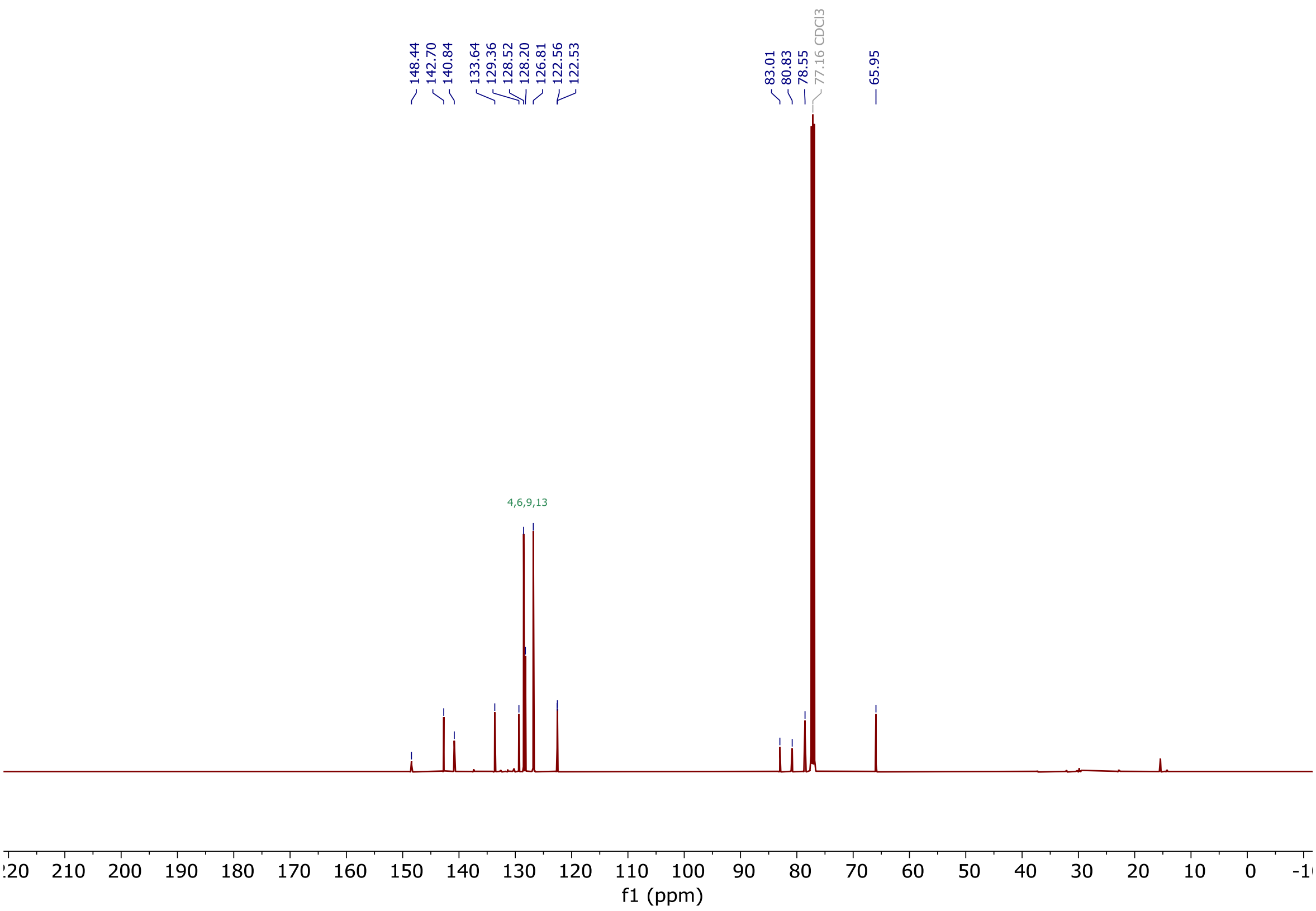

(4-(((1,1-diphenylprop-2-yn-1-yl)oxy)methyl)phenyl)(phenyl)methanone (1ag)

^{1}H NMR (400 MHz, CDCl$_3$)

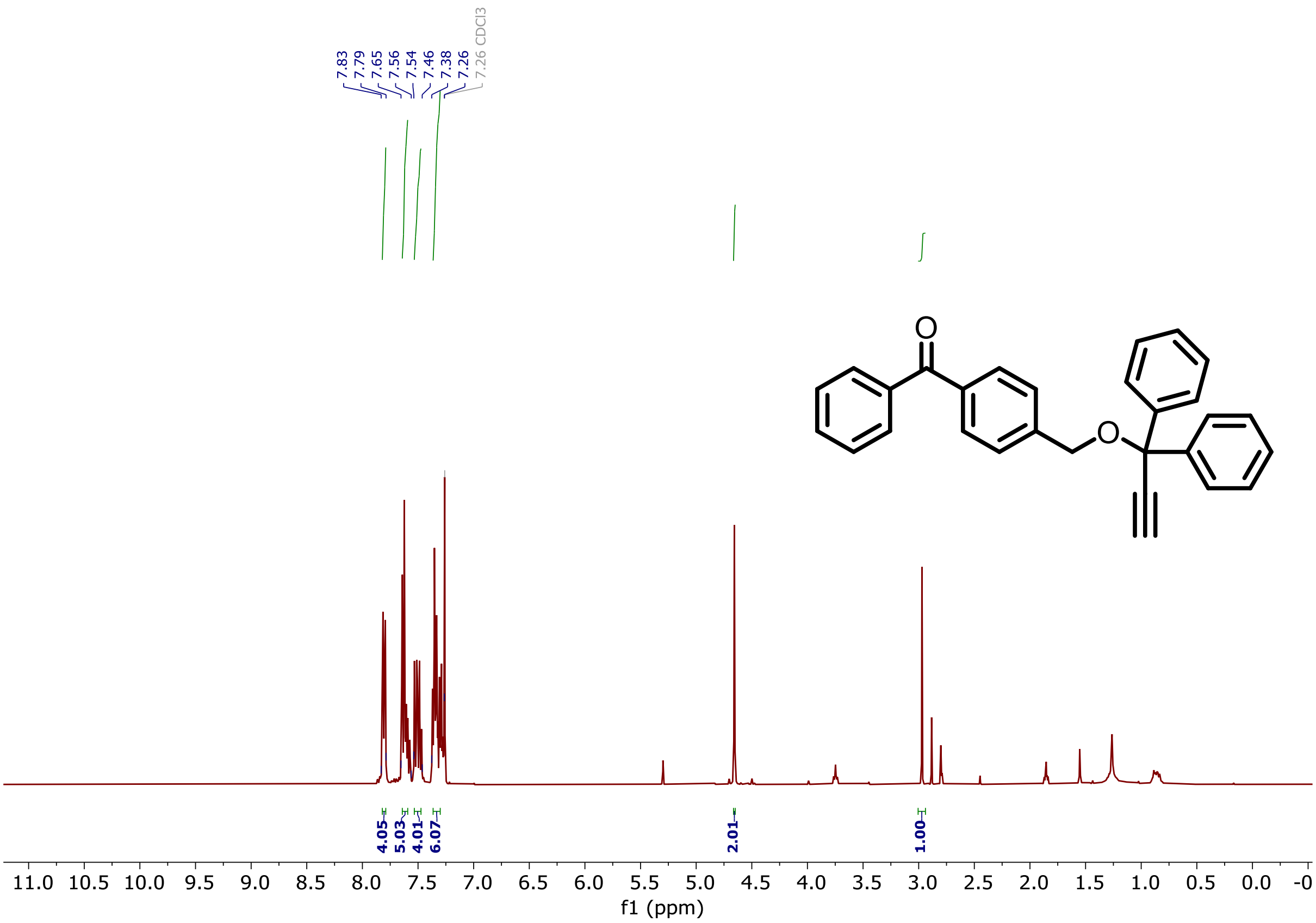

^{13}C NMR (126 MHz, CDCl$_3$)

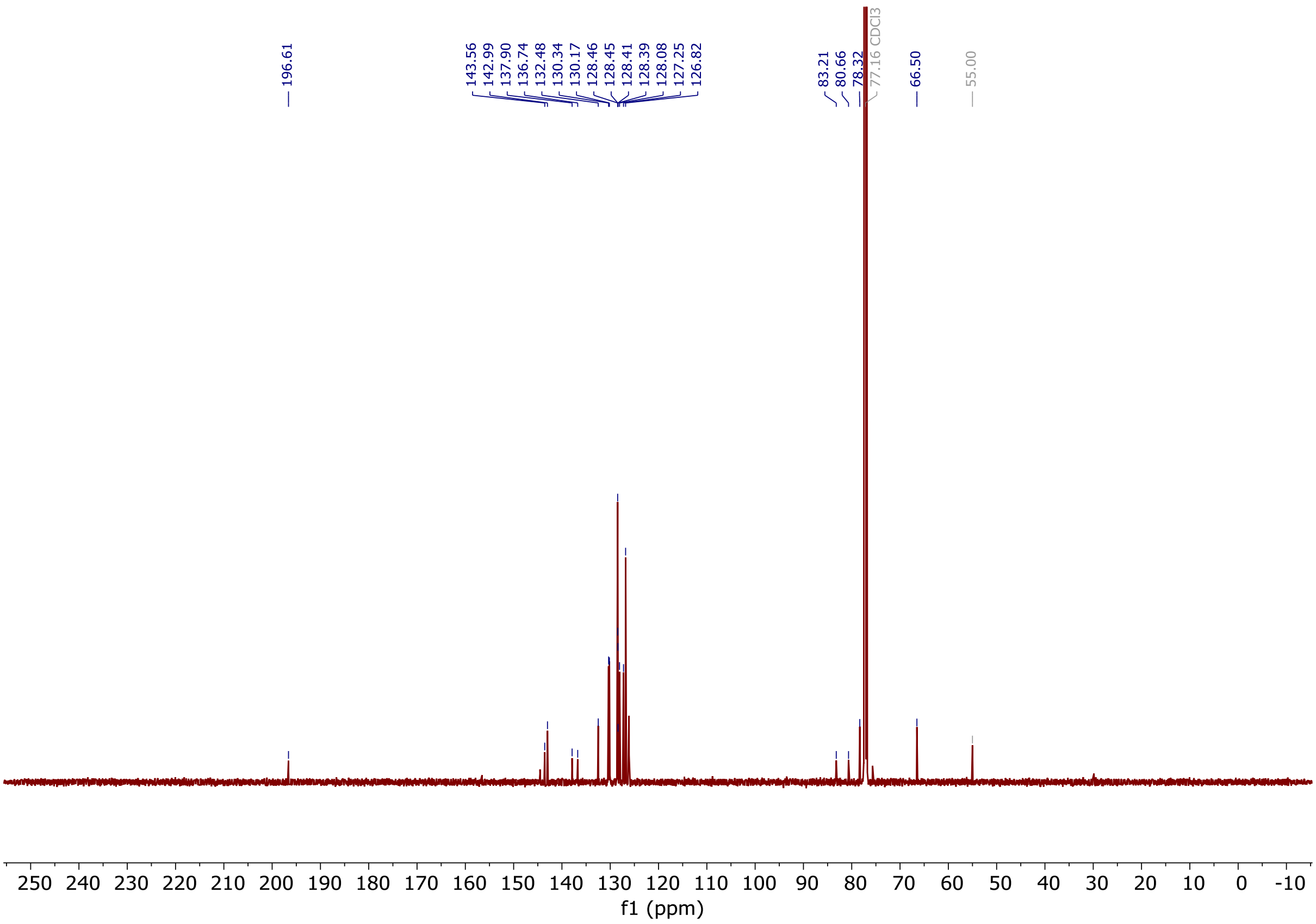

***2-(((1-ethynylcyclohexyl)oxy)methyl)benzonitrile* (1ai)**

^{1}H NMR (400 MHz, $CDCl_3$)

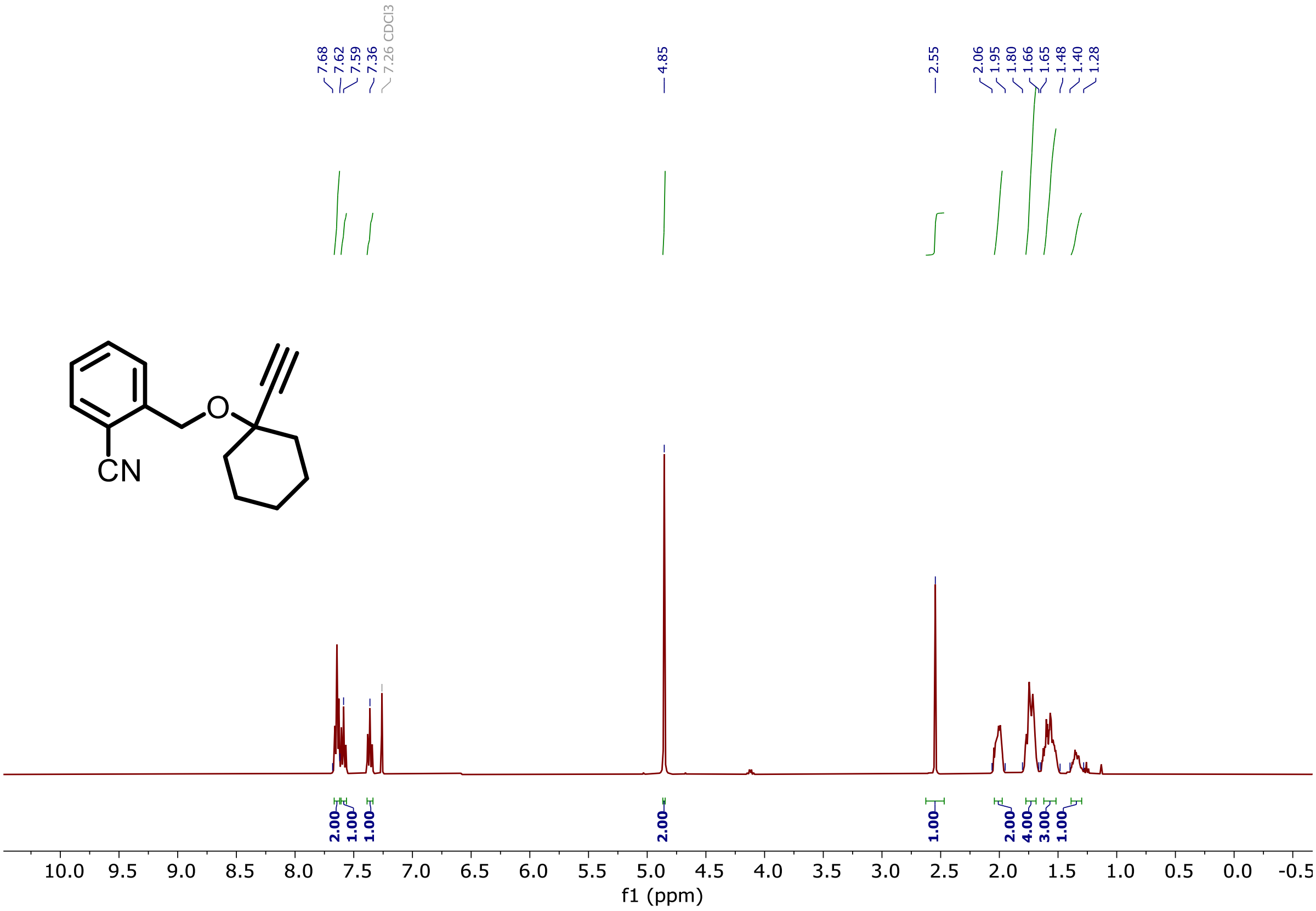

^{13}C NMR (101 MHz, $CDCl_3$)

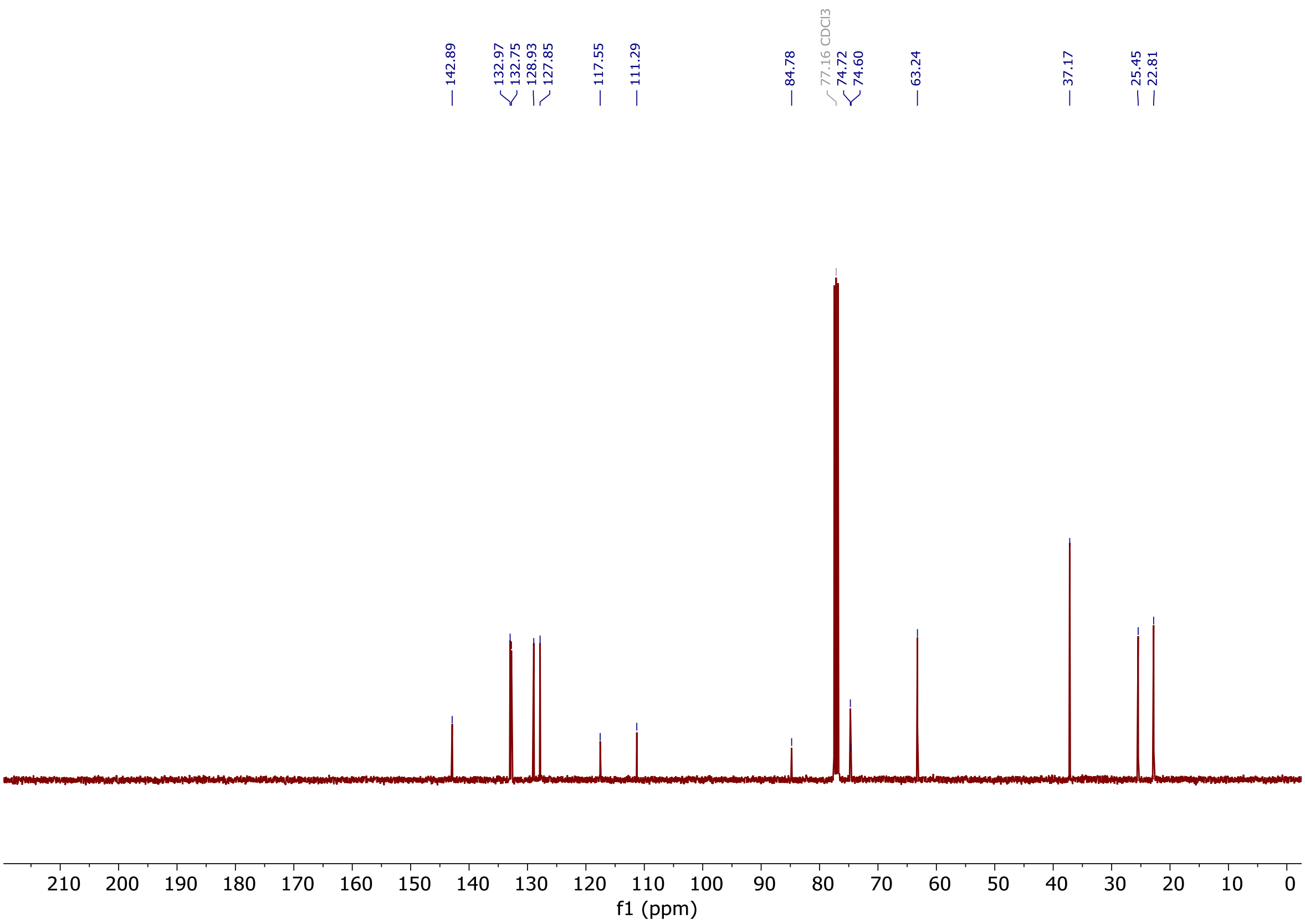

***(1-(but-3-yn-2-yloxy)ethyl)benzene* (1am)**

^{1}H NMR (400 MHz, CDCl$_3$)

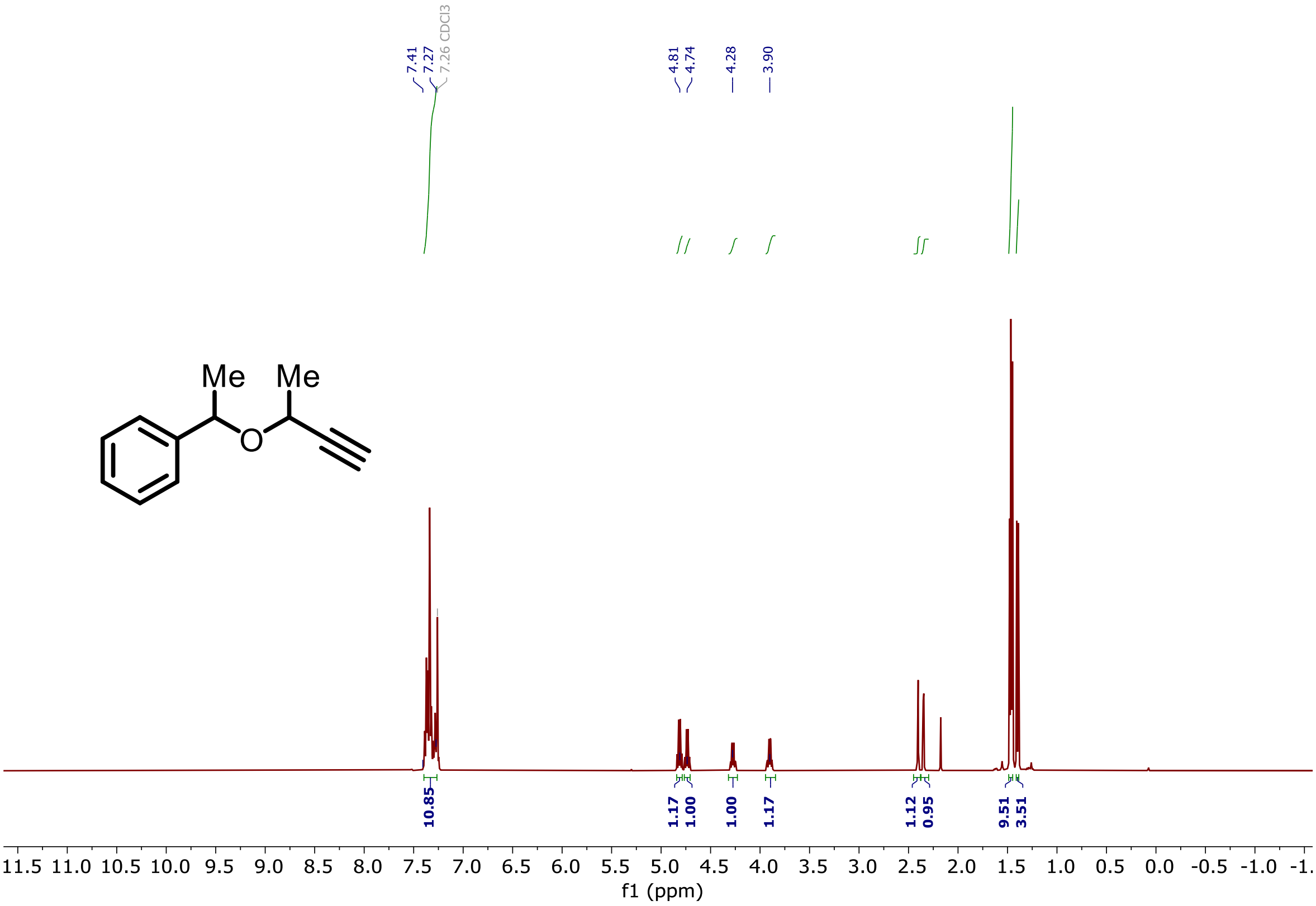

^{13}C NMR (126 MHz, CDCl$_3$)

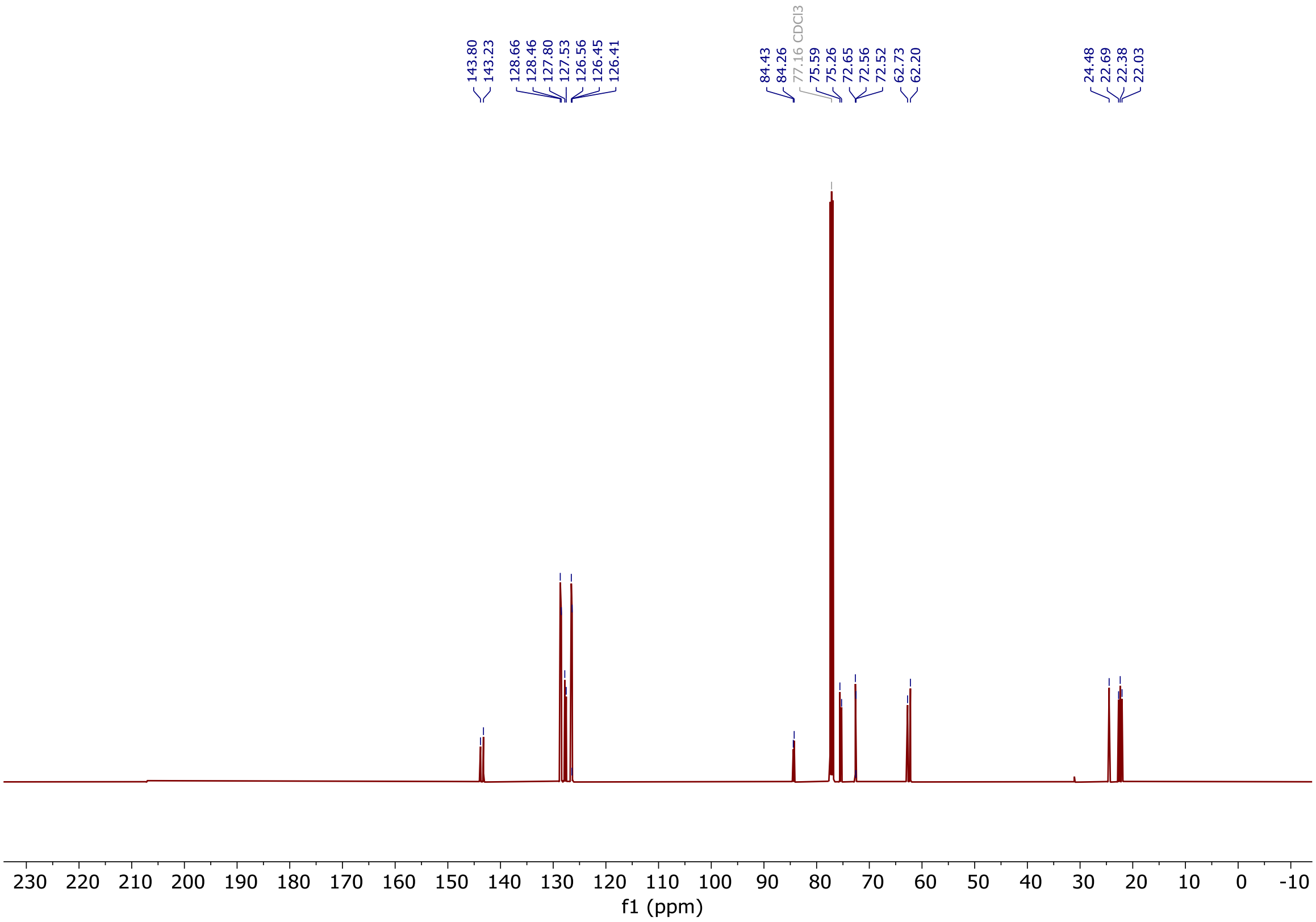

1-chloro-4-(2-(prop-2-yn-1-yloxy)ethoxy)benzene (1ao)

^{1}H NMR (500 MHz, $CDCl_3$)

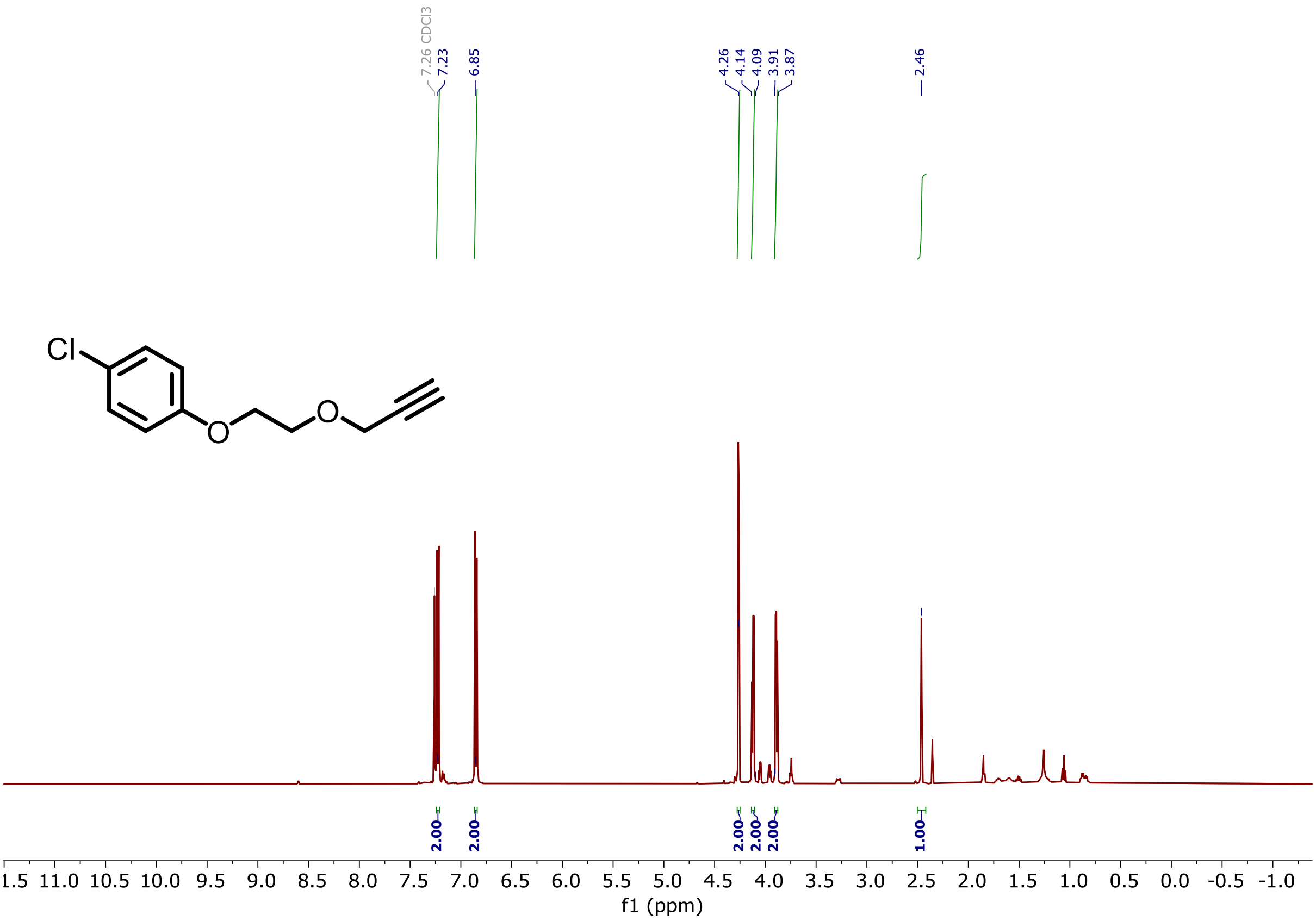

^{13}C NMR (126 MHz, $CDCl_3$)

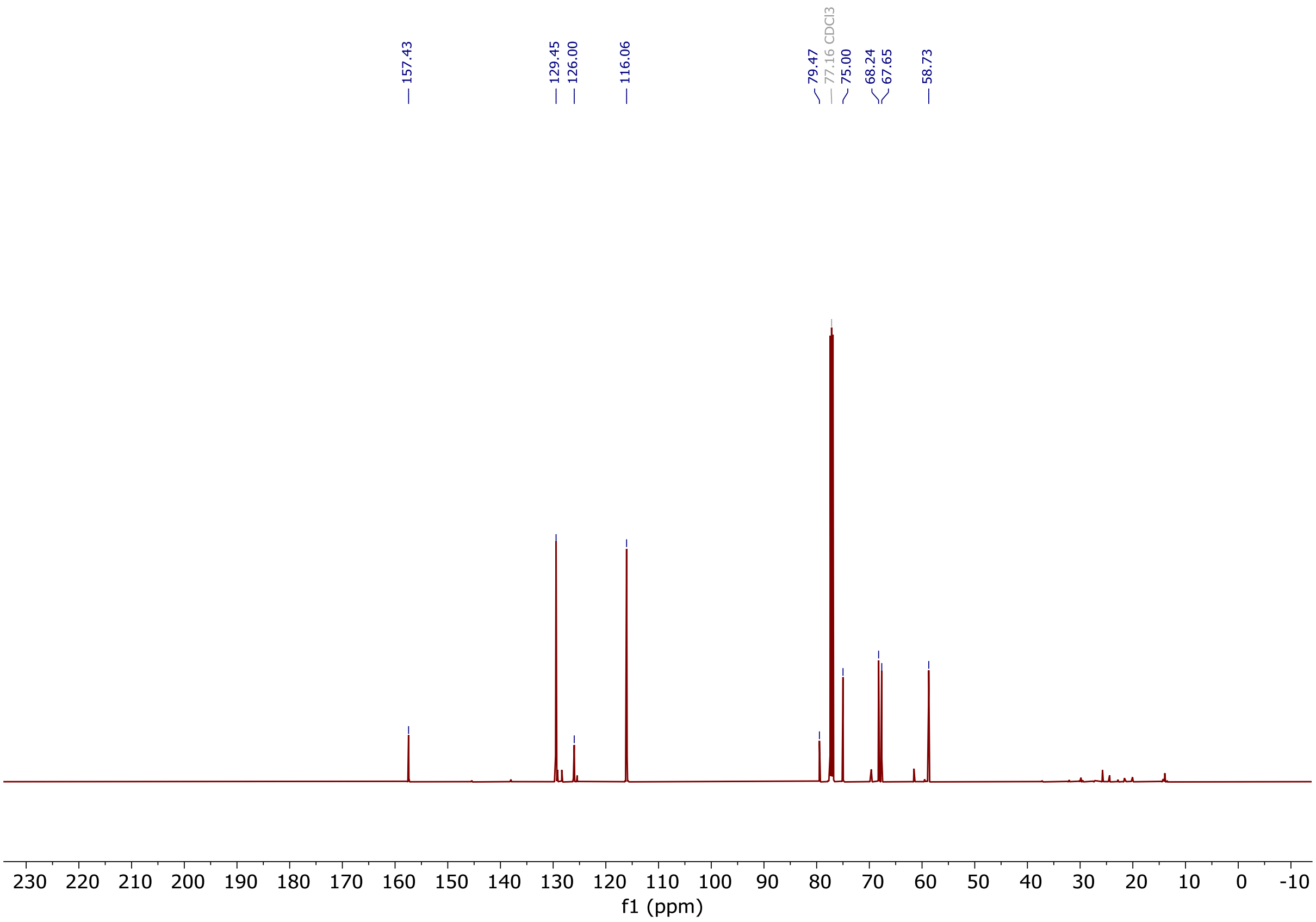

***4-(prop-2-yn-1-yloxy)-1-tosylpiperidine* (1ap)**

¹H NMR (500 MHz, CDCl₃)

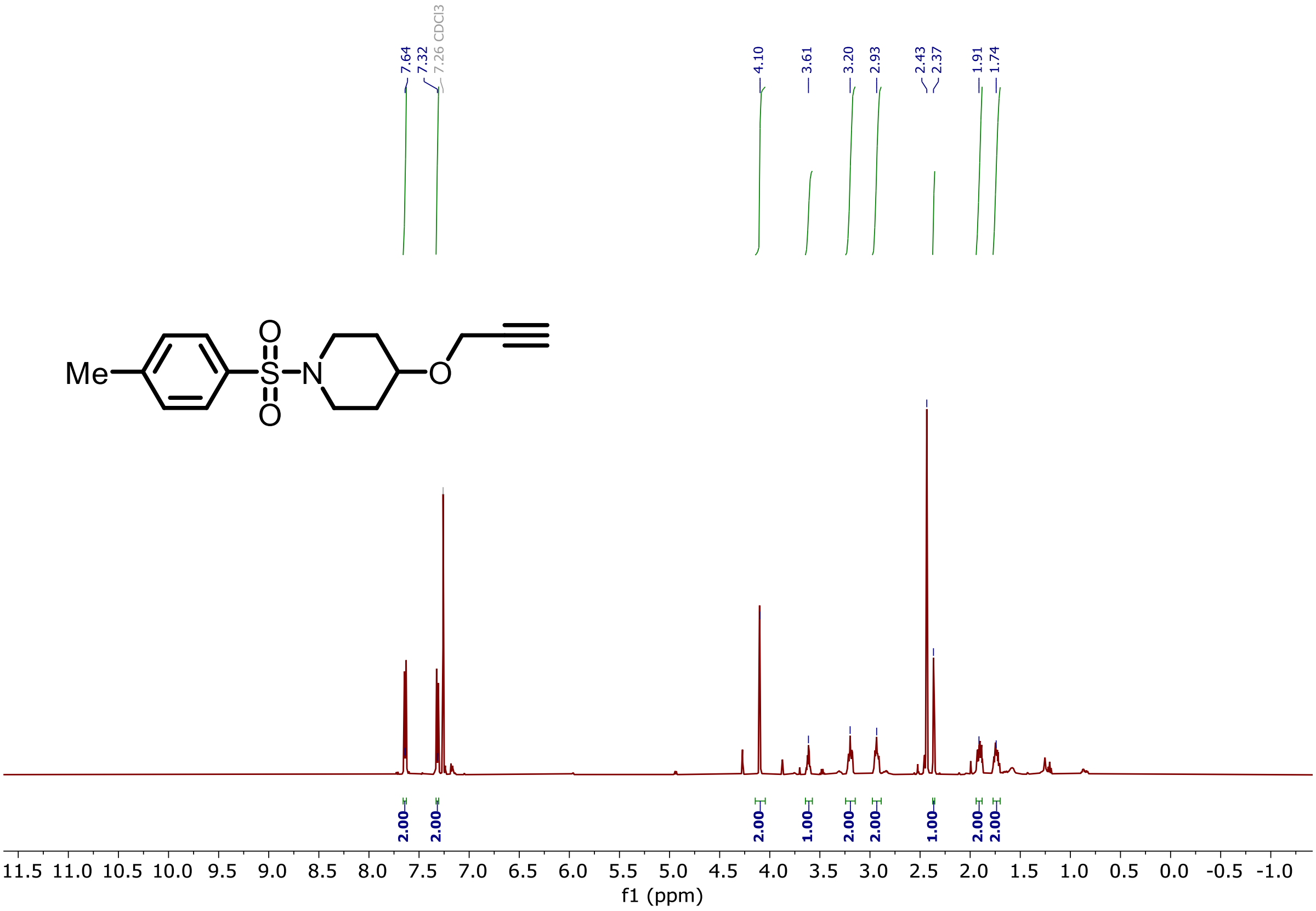

¹³C NMR (126 MHz, CDCl₃)

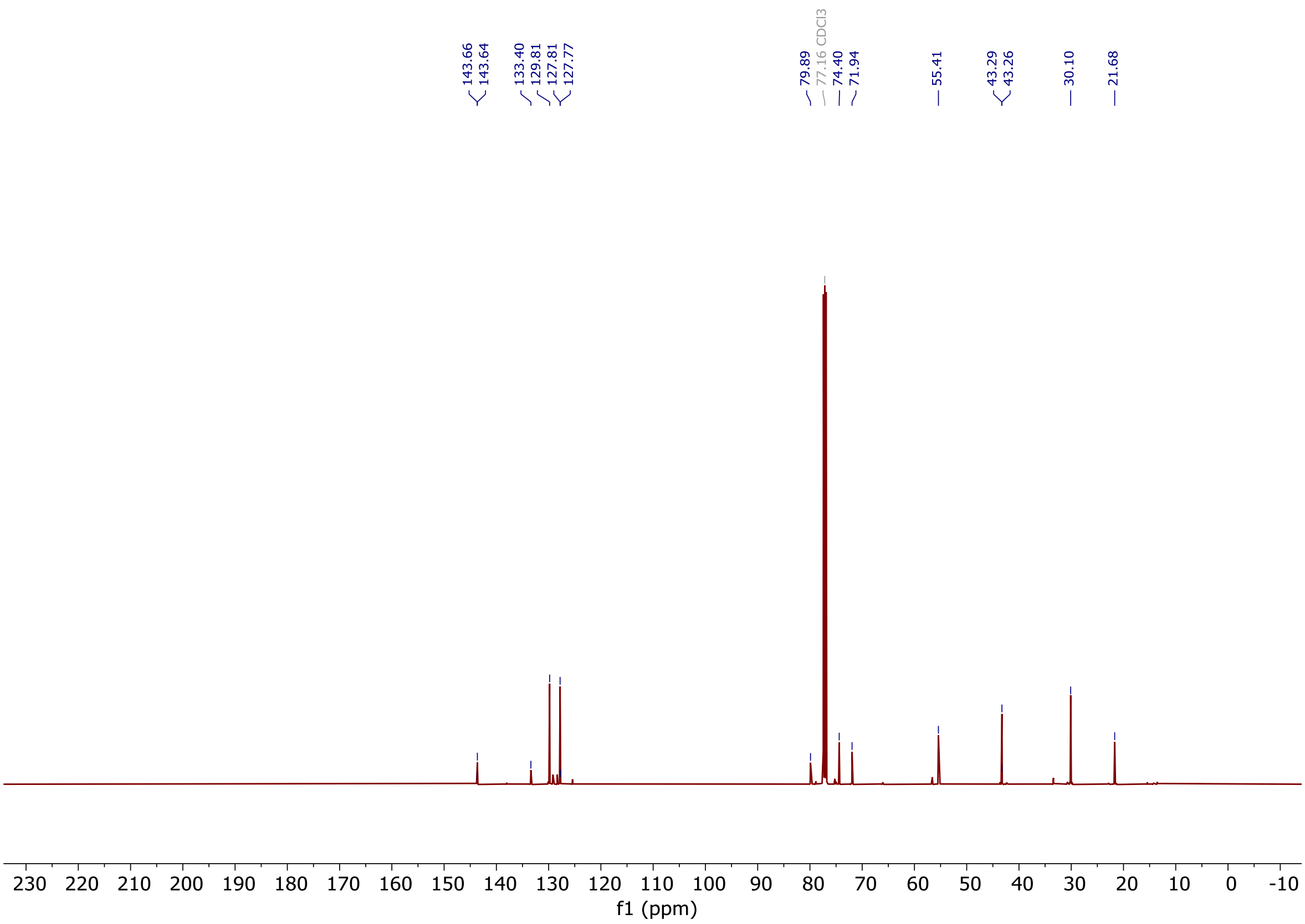

***N-(1-(4-fluorophenyl)-3-(trimethylsilyl)prop-2-yn-1-yl)-4-methylbenzenesulfonamide* (1ax)**

^{1}H NMR (500 MHz, CDCl$_3$)

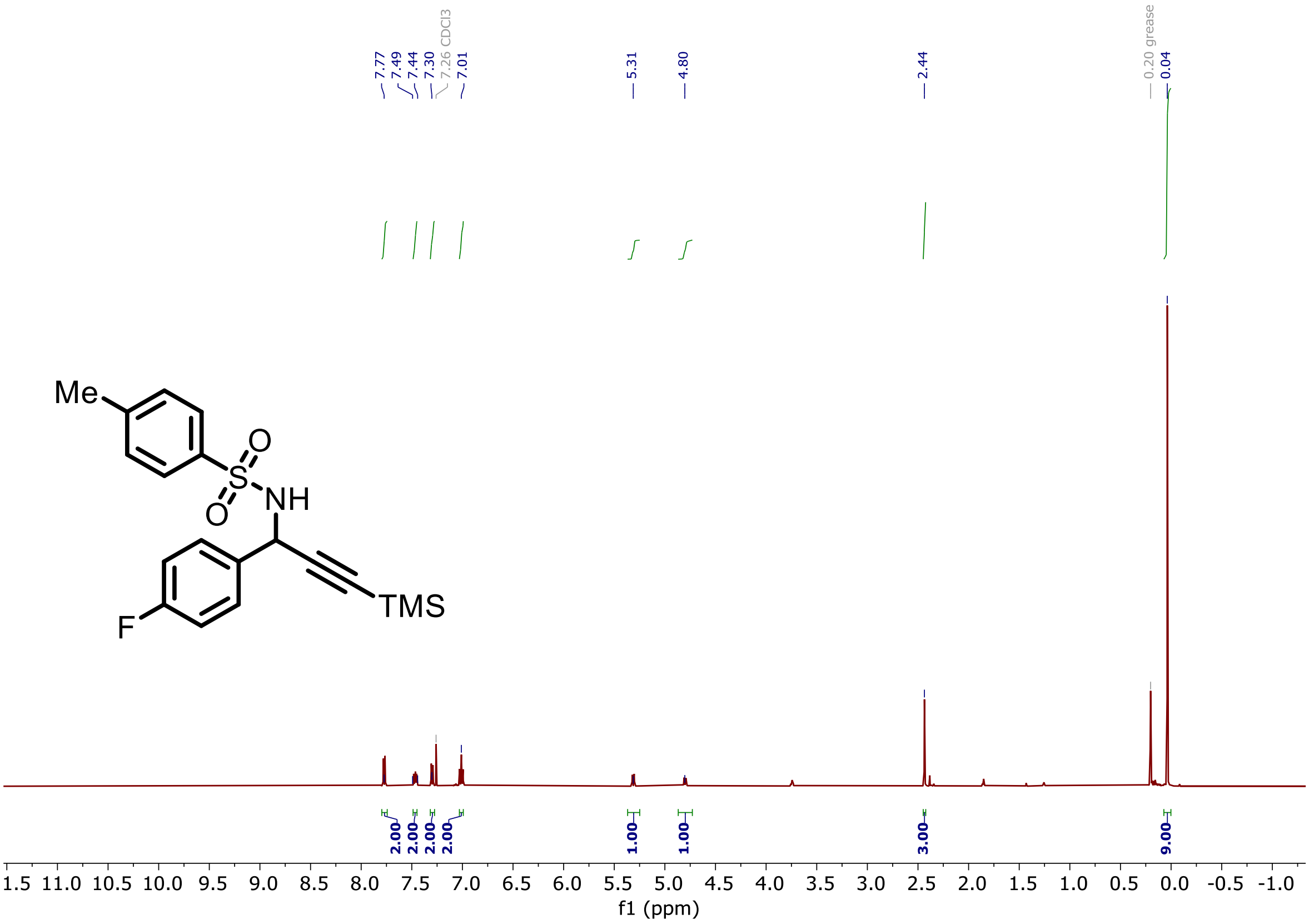

^{13}C NMR (126 MHz, CDCl$_3$)

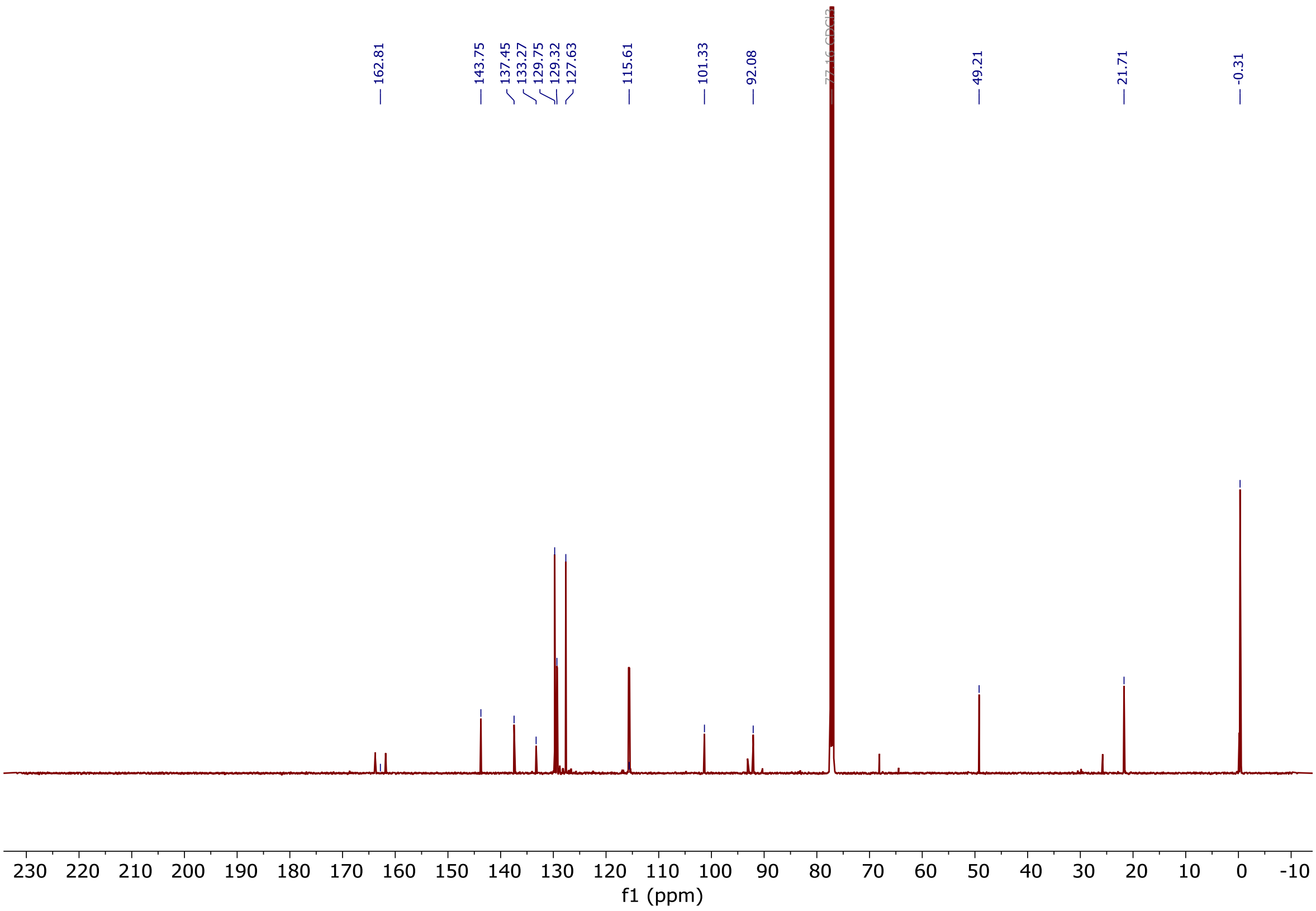

^{19}F NMR (376 MHz, $CDCl_3$)

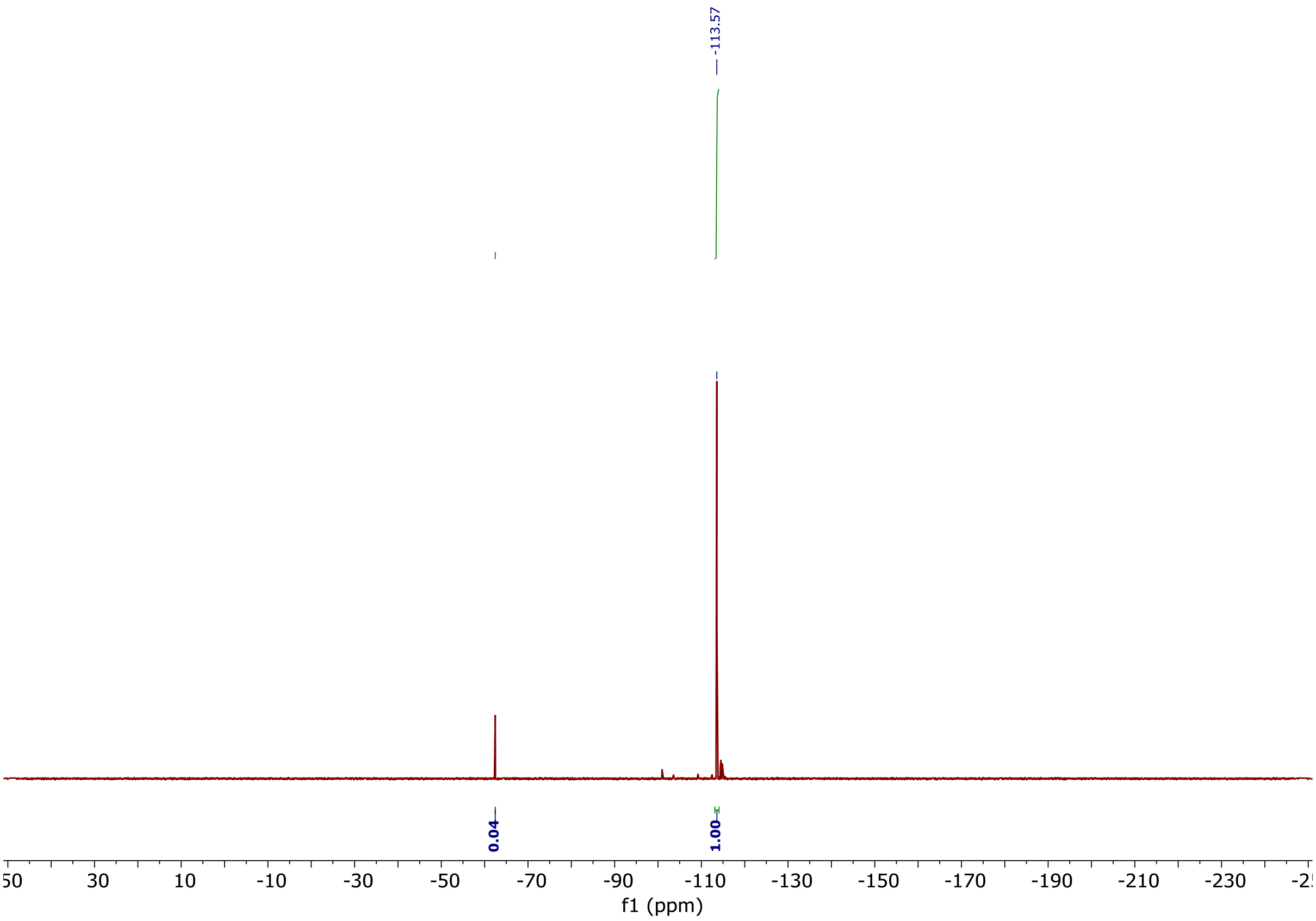

N-(1-([1,1'-biphenyl]-4-yl)prop-2-yn-1-yl)-4-methylbenzenesulfonamide **(1ay)**

^{1}H NMR (500 MHz, CDCl$_3$)

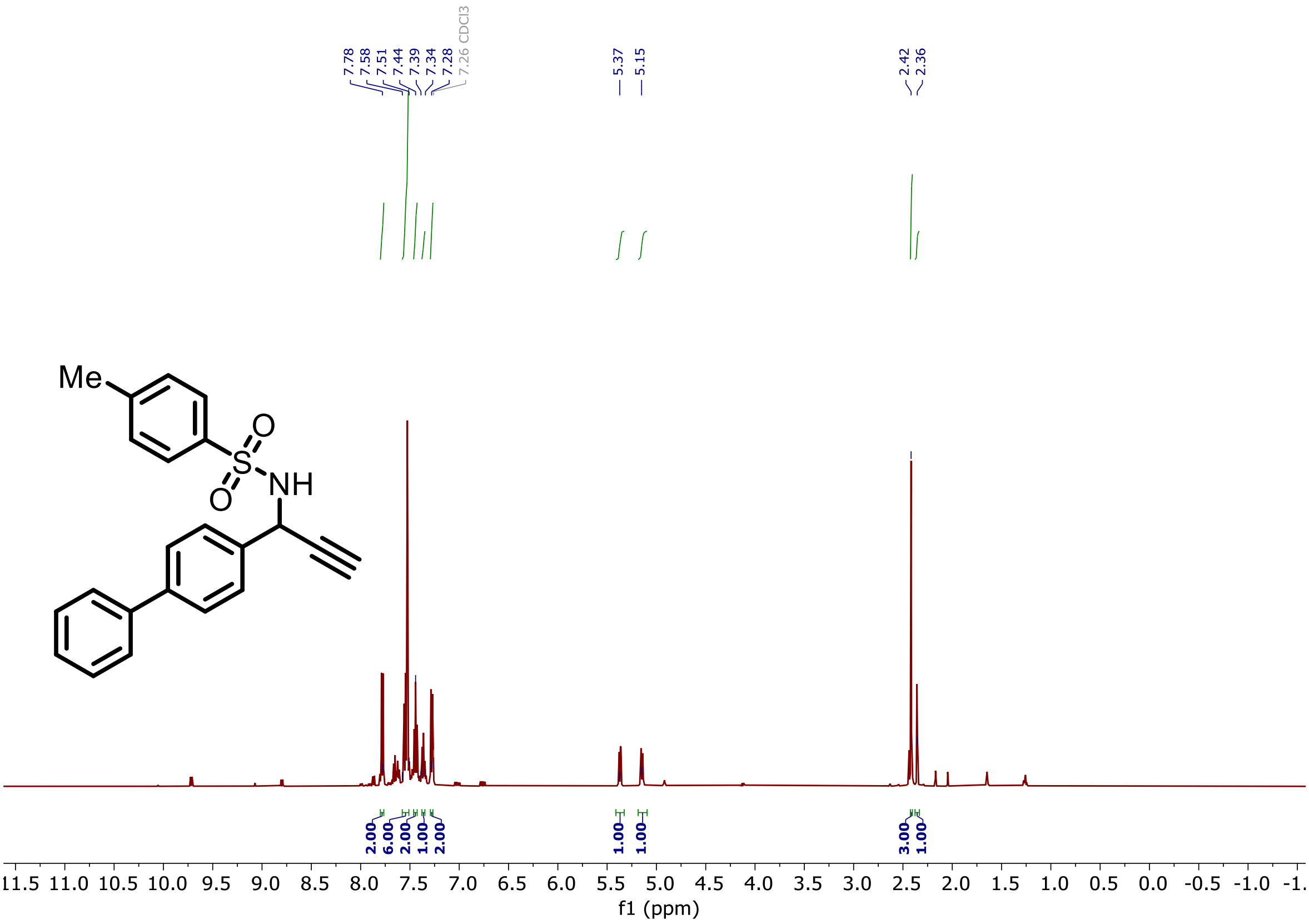

^{13}C NMR (126 MHz, CDCl$_3$)

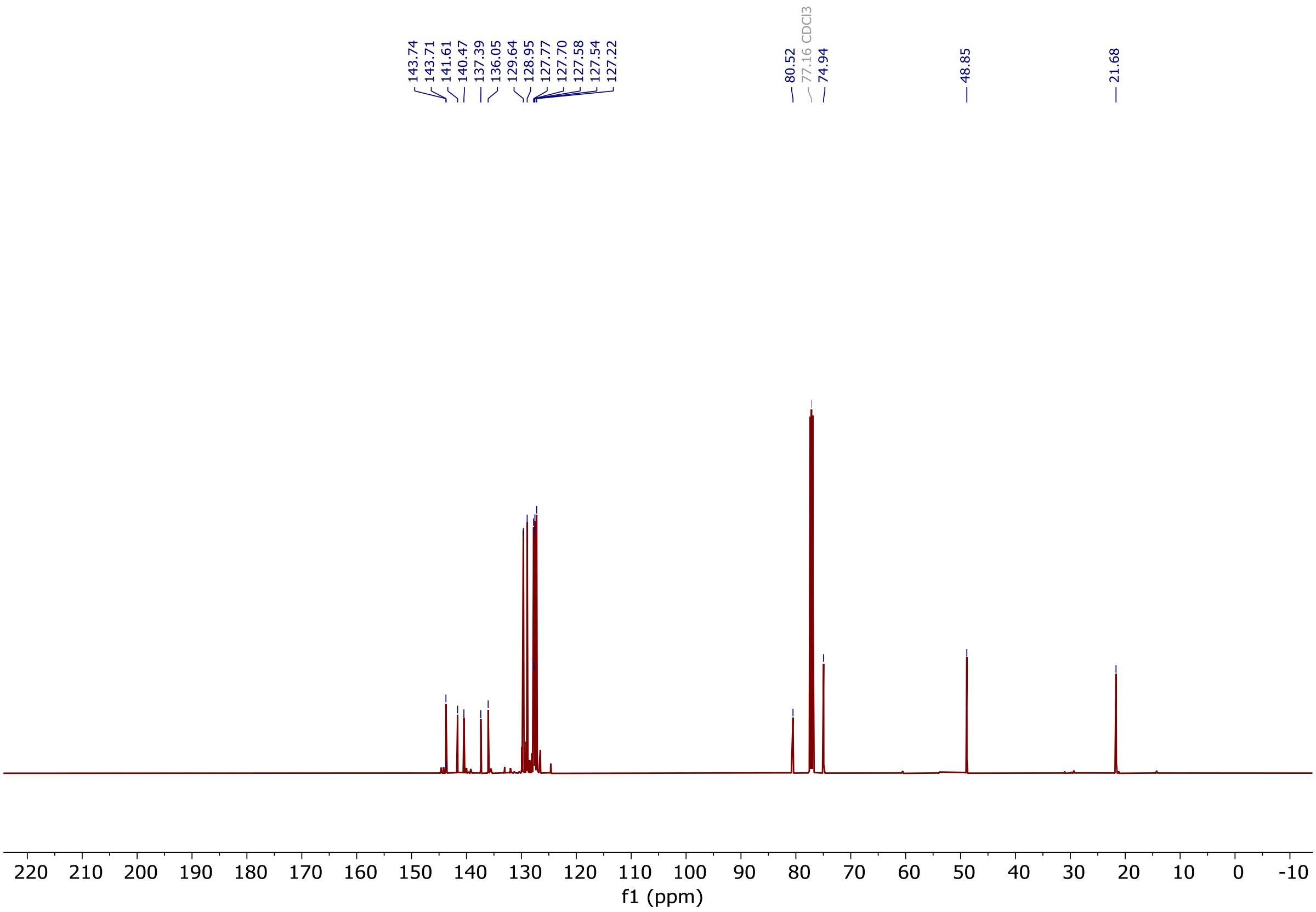

***4-methyl-N-(1-(4-(trifluoromethyl)phenyl)-3-(trimethylsilyl)prop-2-yn-1-yl)benzenesulfonamide* (1az)**

^{1}H NMR (500 MHz, $CDCl_3$)

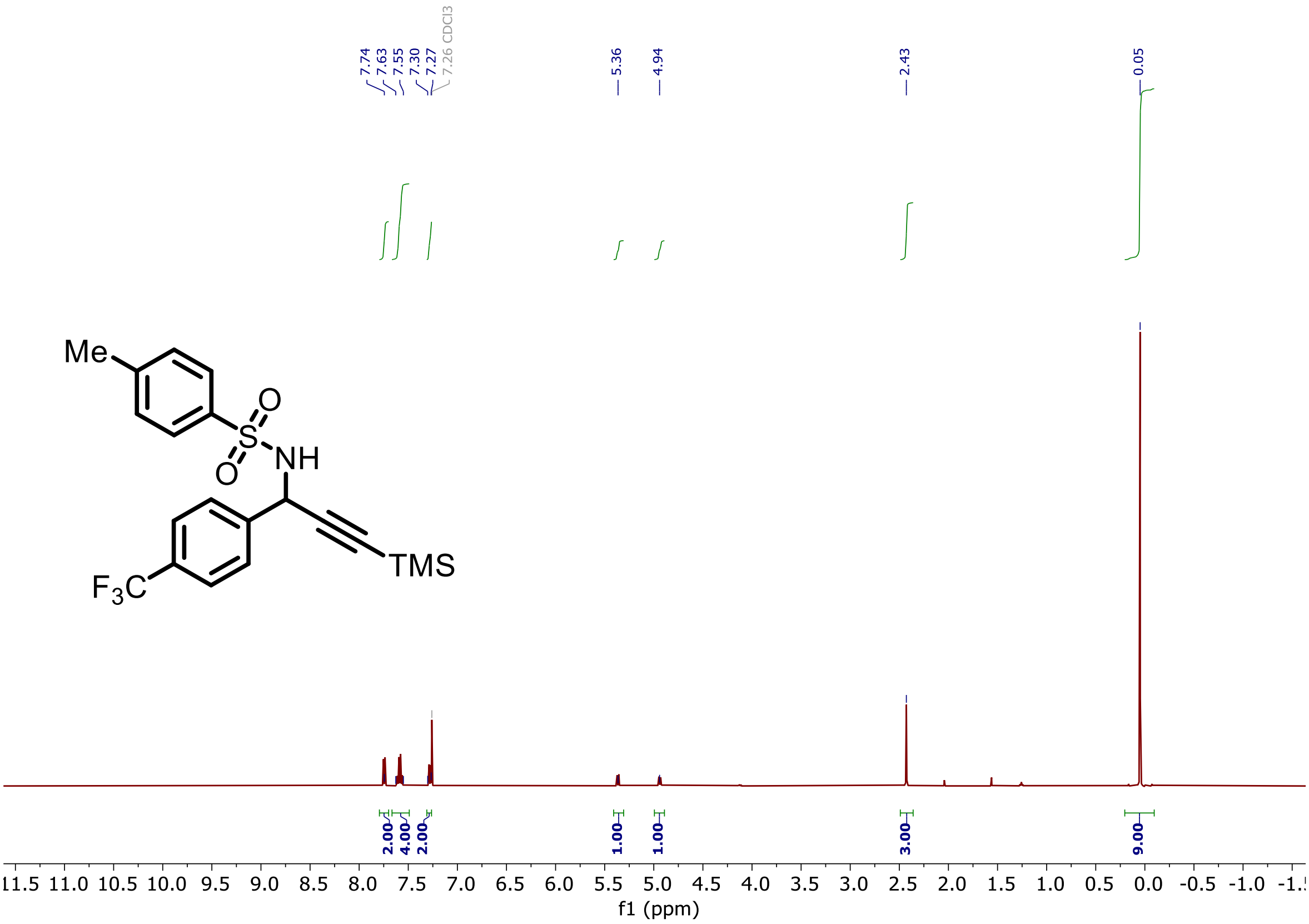

^{13}C NMR (126 MHz, $CDCl_3$)

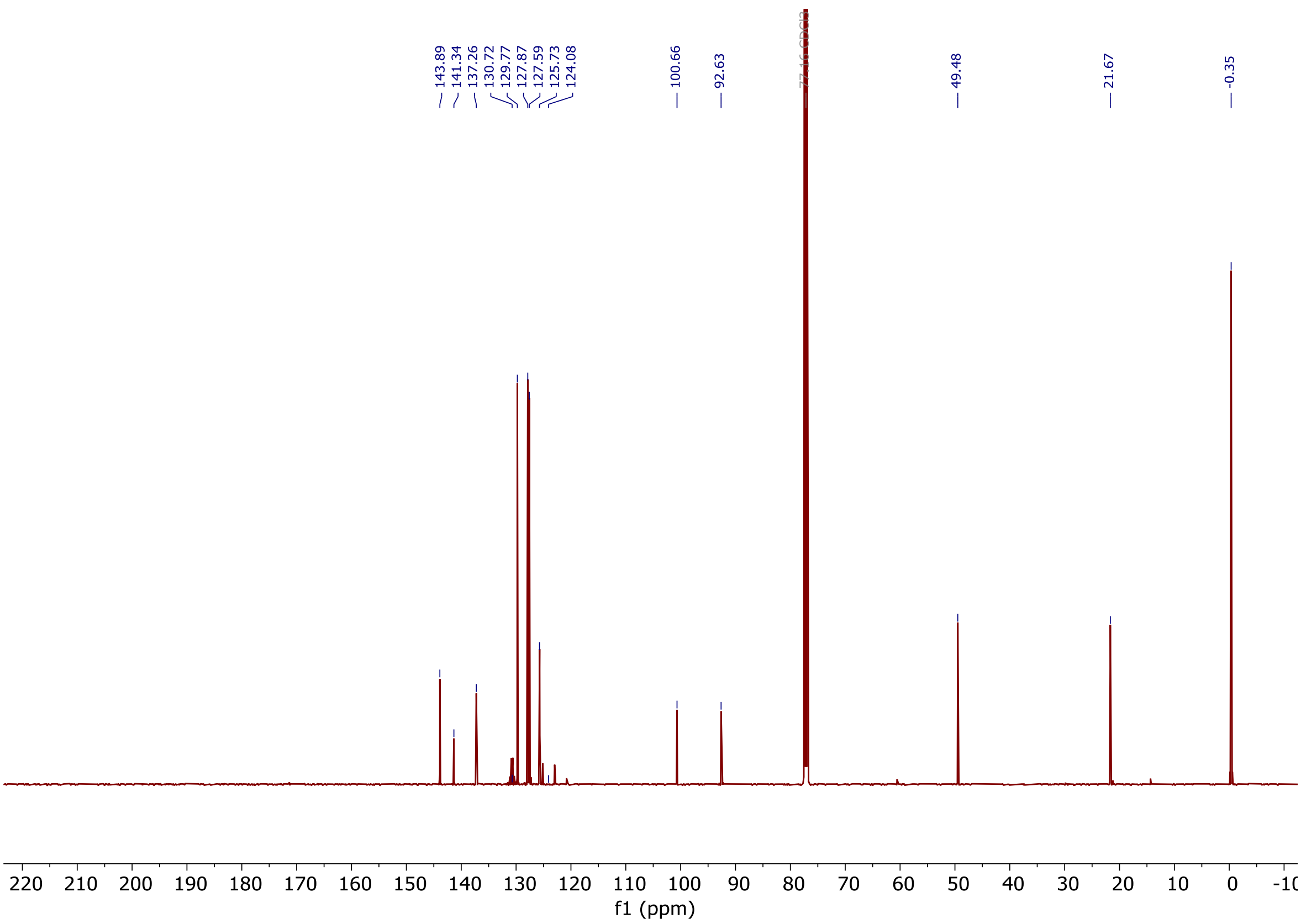

^{19}F NMR (376 MHz, $CDCl_3$)

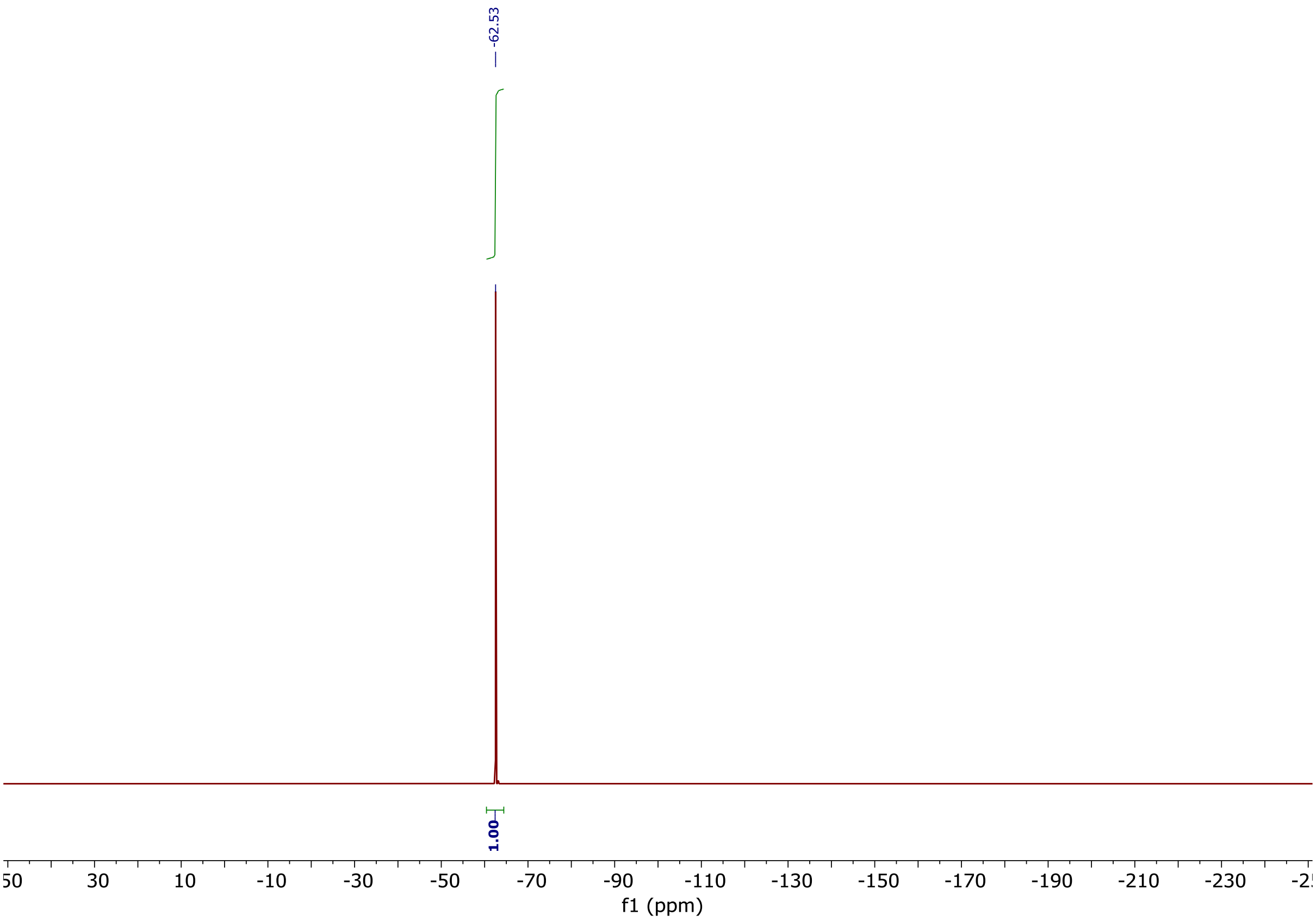

***N-(1-(4-bromophenyl)-3-(trimethylsilyl)prop-2-yn-1-yl)-4-methylbenzenesulfonamide* (1ba)**

^{1}H NMR (500 MHz, $CDCl_3$):

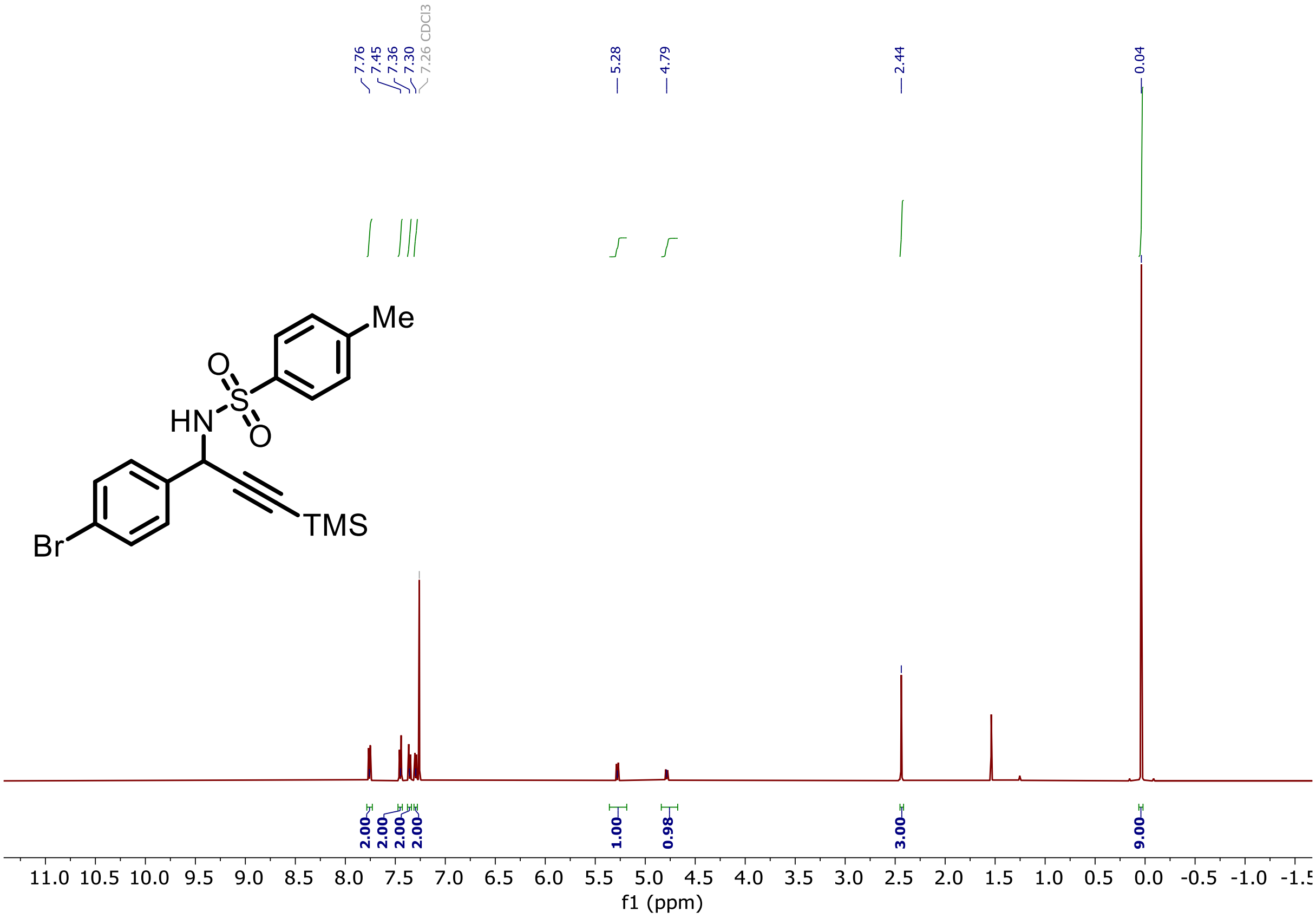

^{13}C NMR (126 MHz, $CDCl_3$):

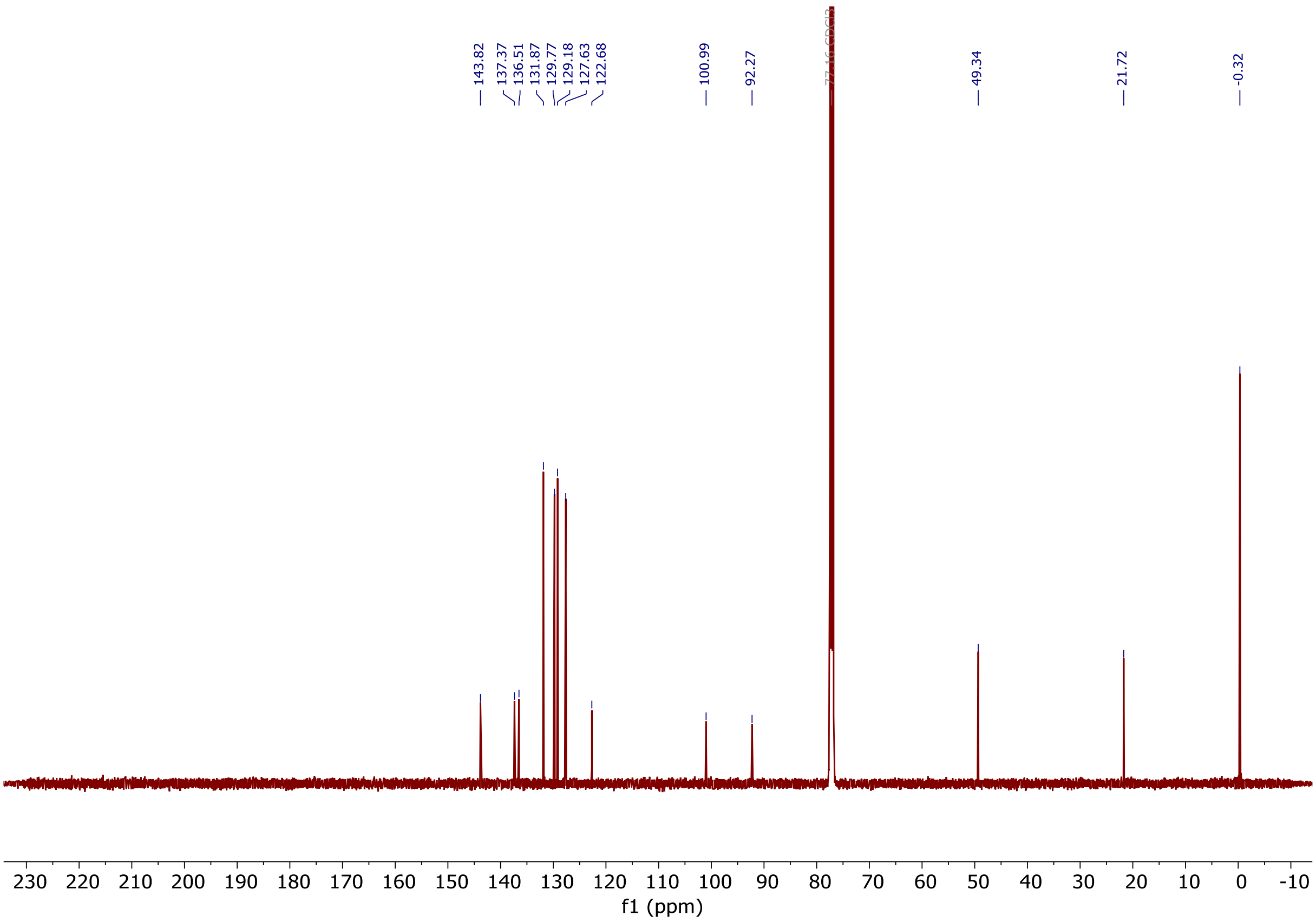

N-(1-(4-chlorophenyl)-3-(trimethylsilyl)prop-2-yn-1-yl)-4-methylbenzenesulfonamide **(1bb)**

^{1}H NMR (500 MHz, CDCl$_3$)

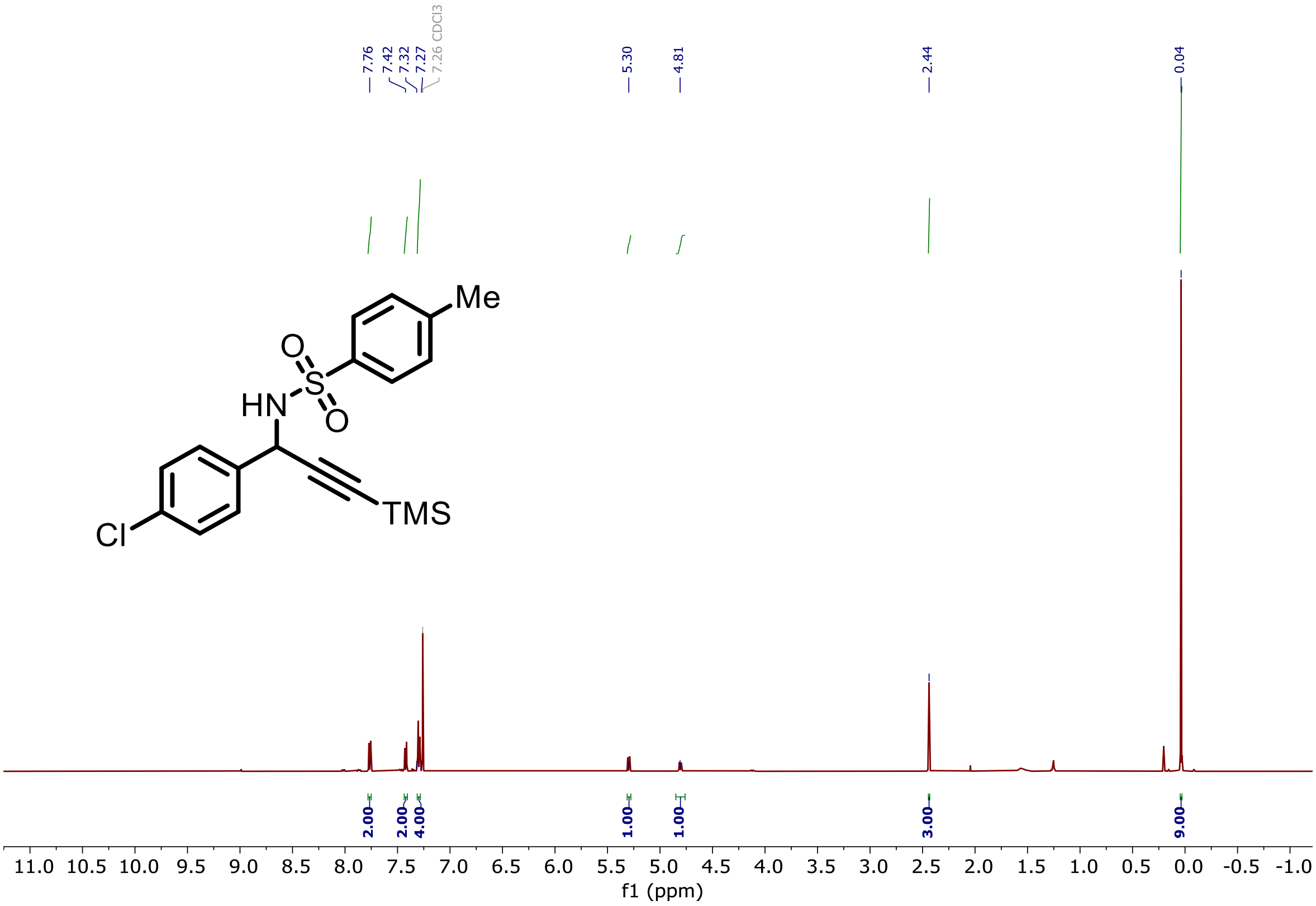

^{13}C NMR (126 MHz, CDCl$_3$)

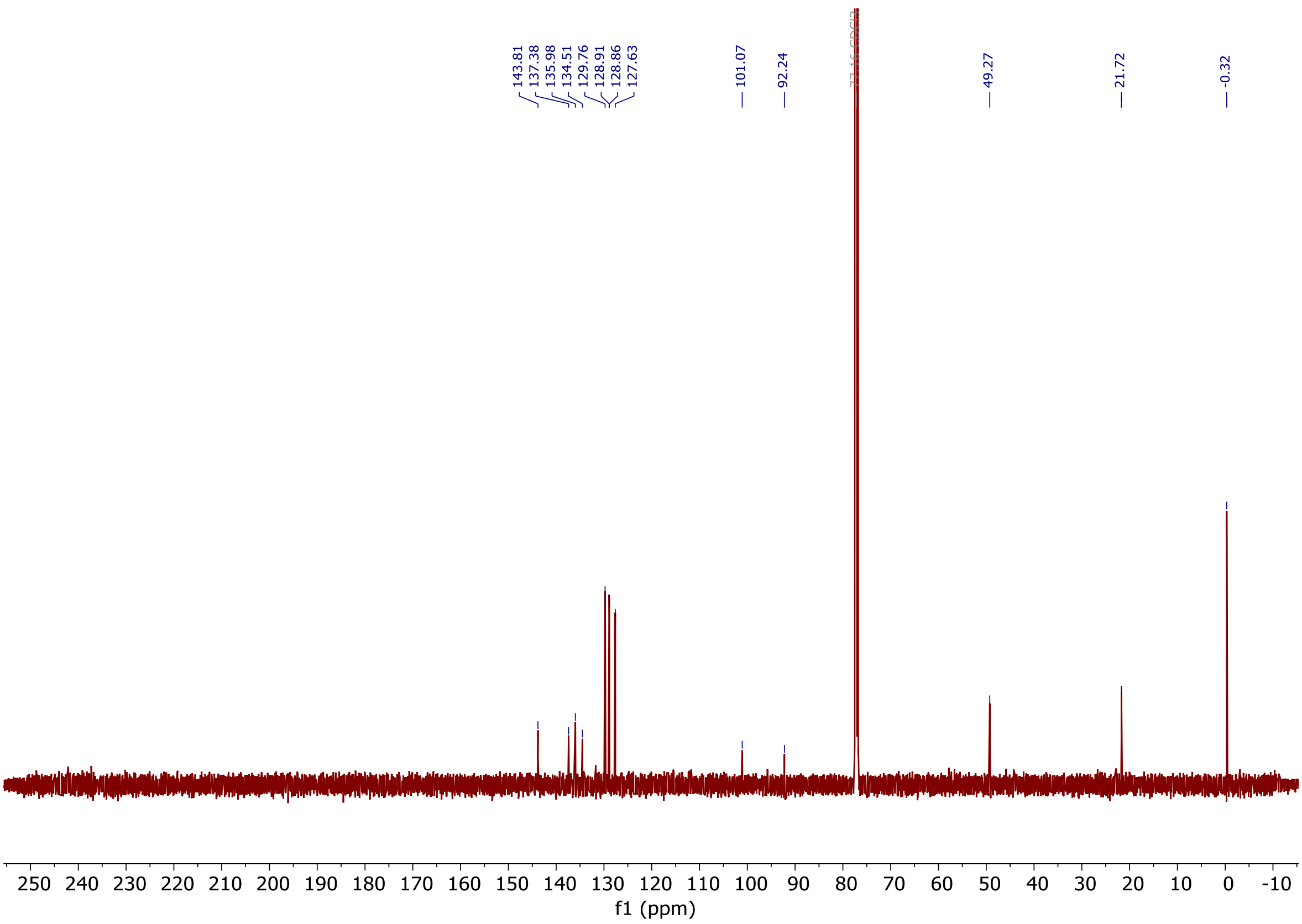

***4-methyl-N-(1-(1-tosyl-1H-indol-5-yl)-3-(trimethylsilyl)prop-2-yn-1-yl)benzenesulfonamide* (1bc)**
(contains 25 mol% 1-tosyl-1*H*-indole-5-carbaldehyde impurity.)
^{1}H NMR (500 MHz, CDCl$_3$)

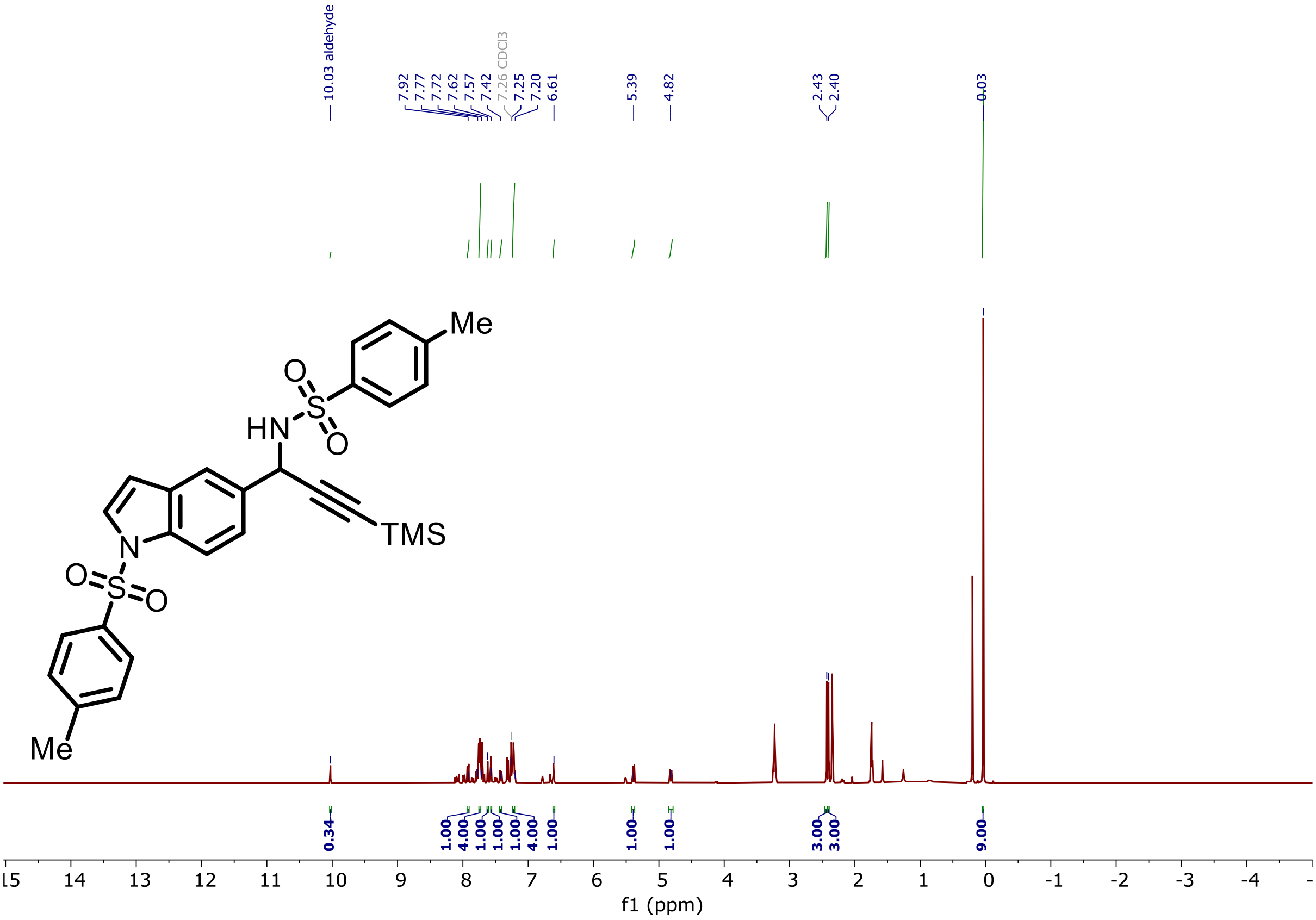

^{13}C NMR (126 MHz, CDCl$_3$)

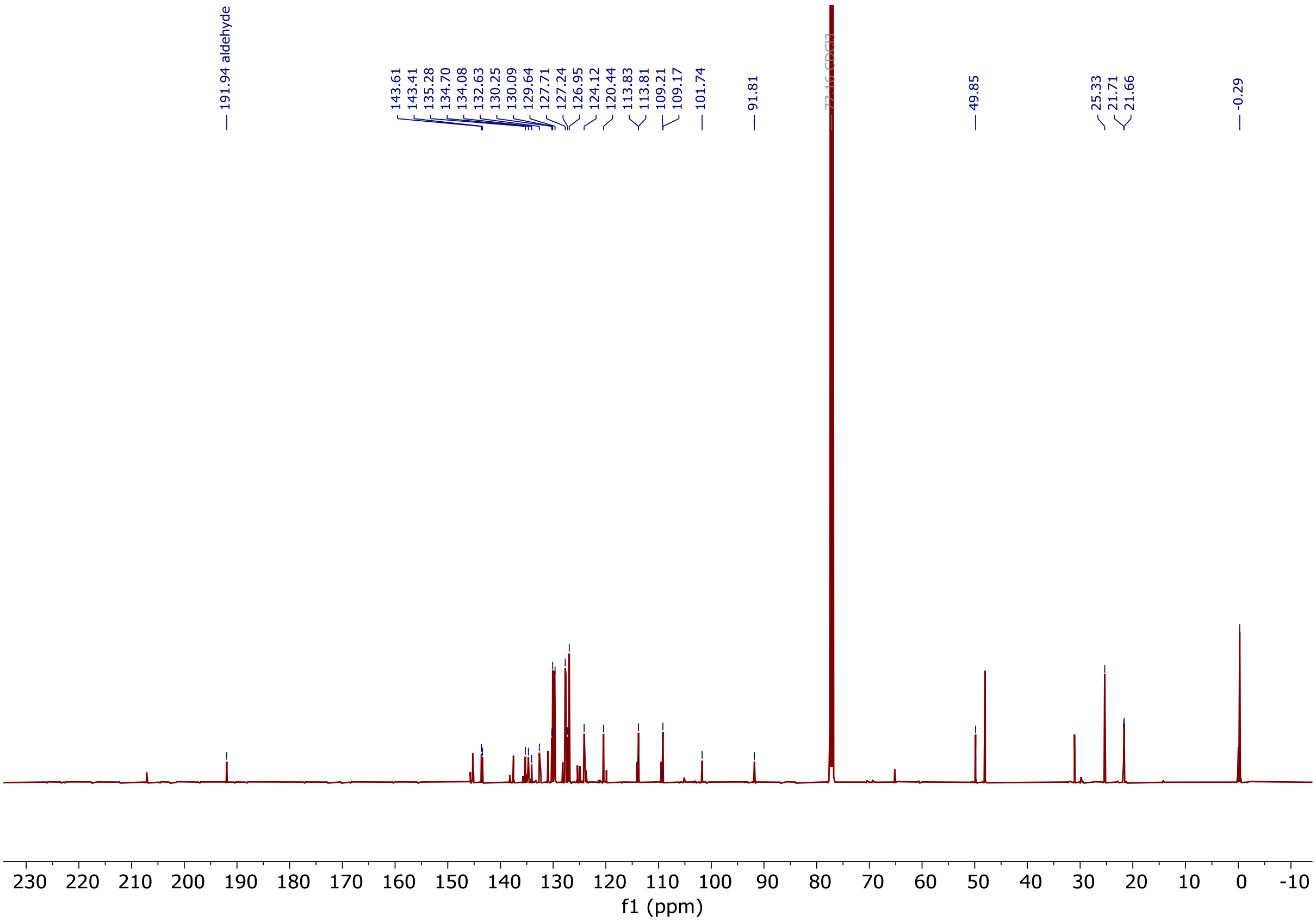

N-(cyanomethyl)-4-nitro-N-(prop-2-yn-1-yl)benzenesulfonamide (**1bi**)

^{1}H NMR (500 MHz, CDCl$_3$)

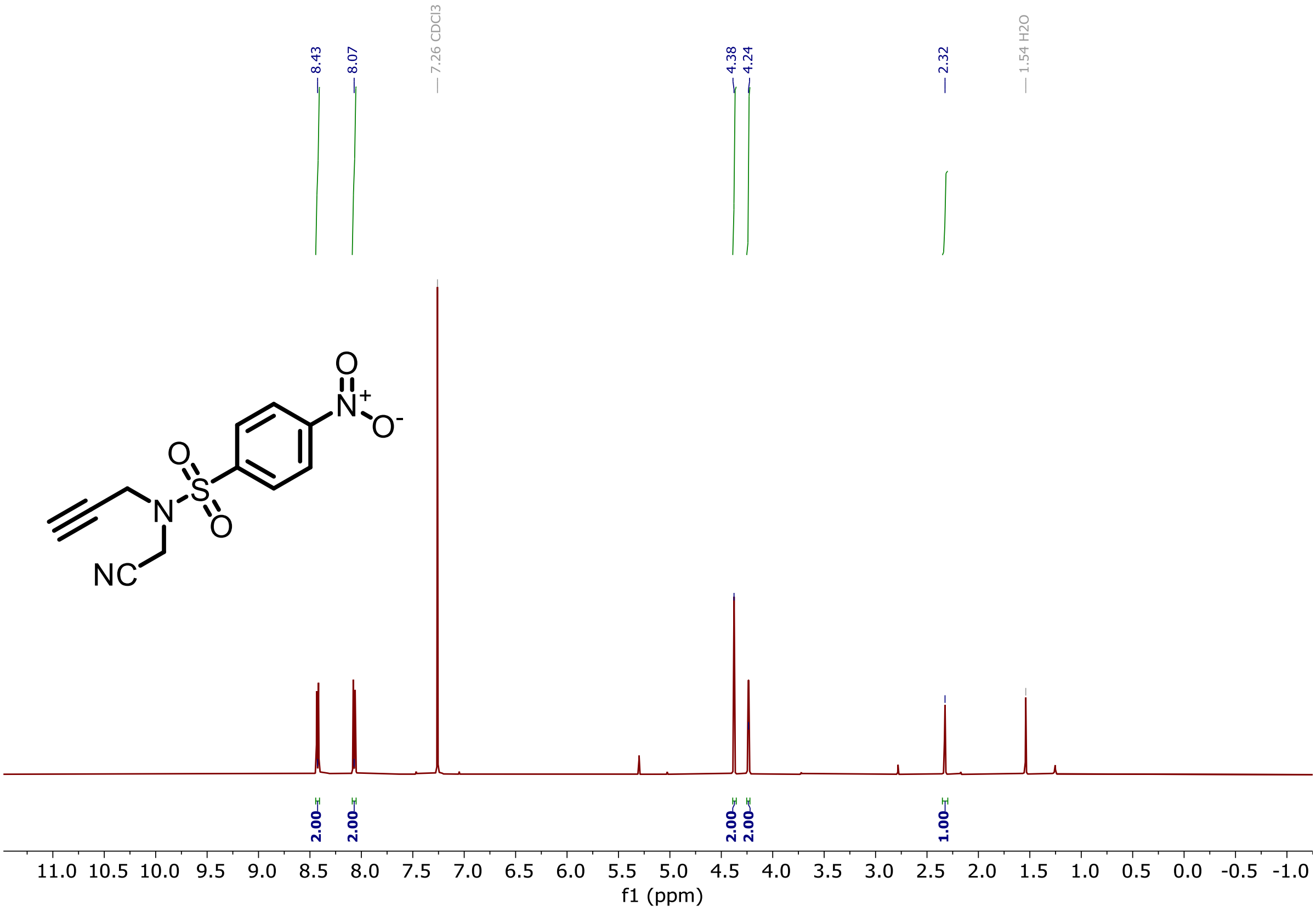

^{13}C NMR (126 MHz, CDCl$_3$)

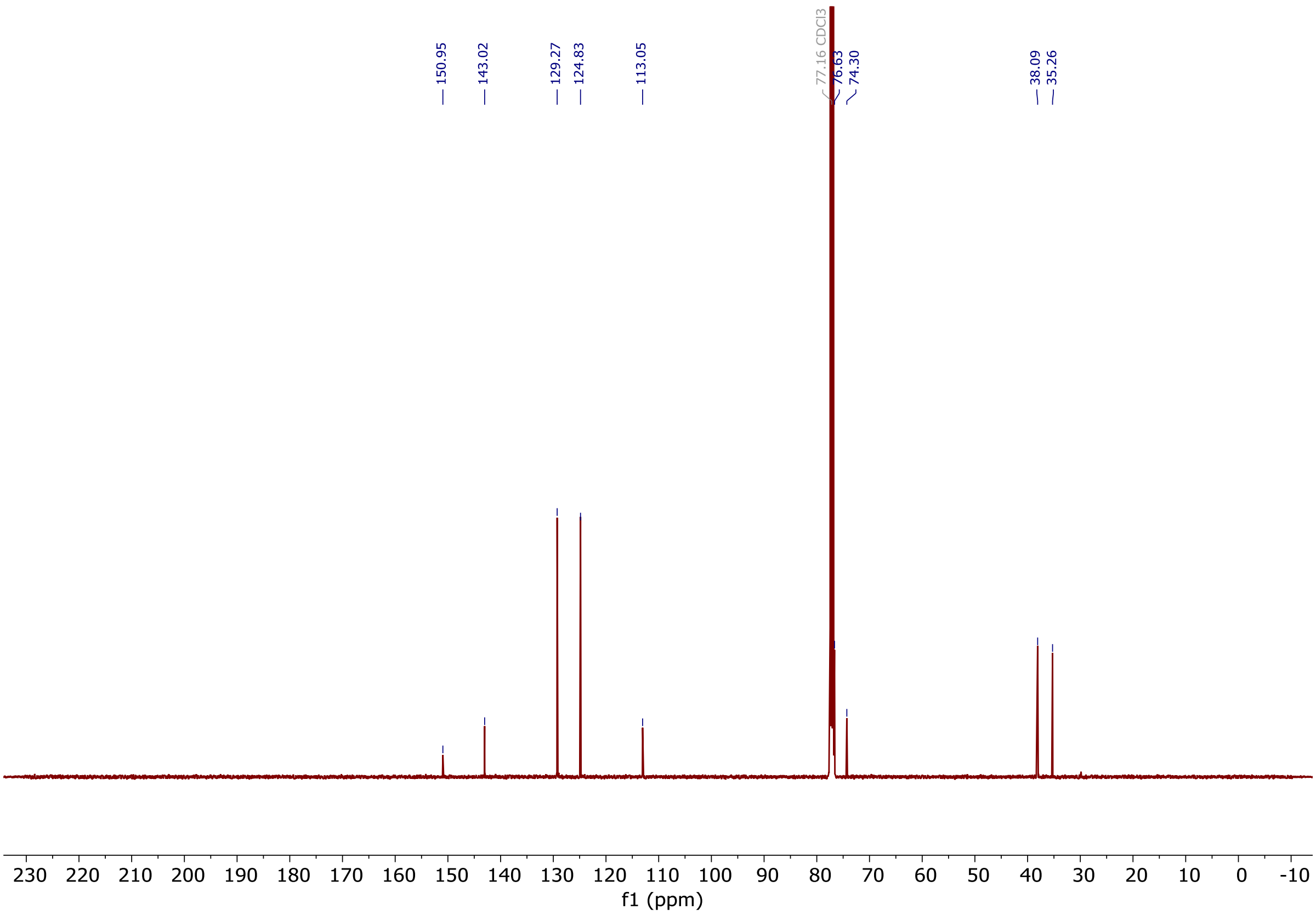

***(R)-4,4-dimethyl-3-(prop-2-yn-1-yloxy)dihydrofuran-2(3H)-one* (1bj)**

^{1}H NMR (500 MHz, $CDCl_3$)

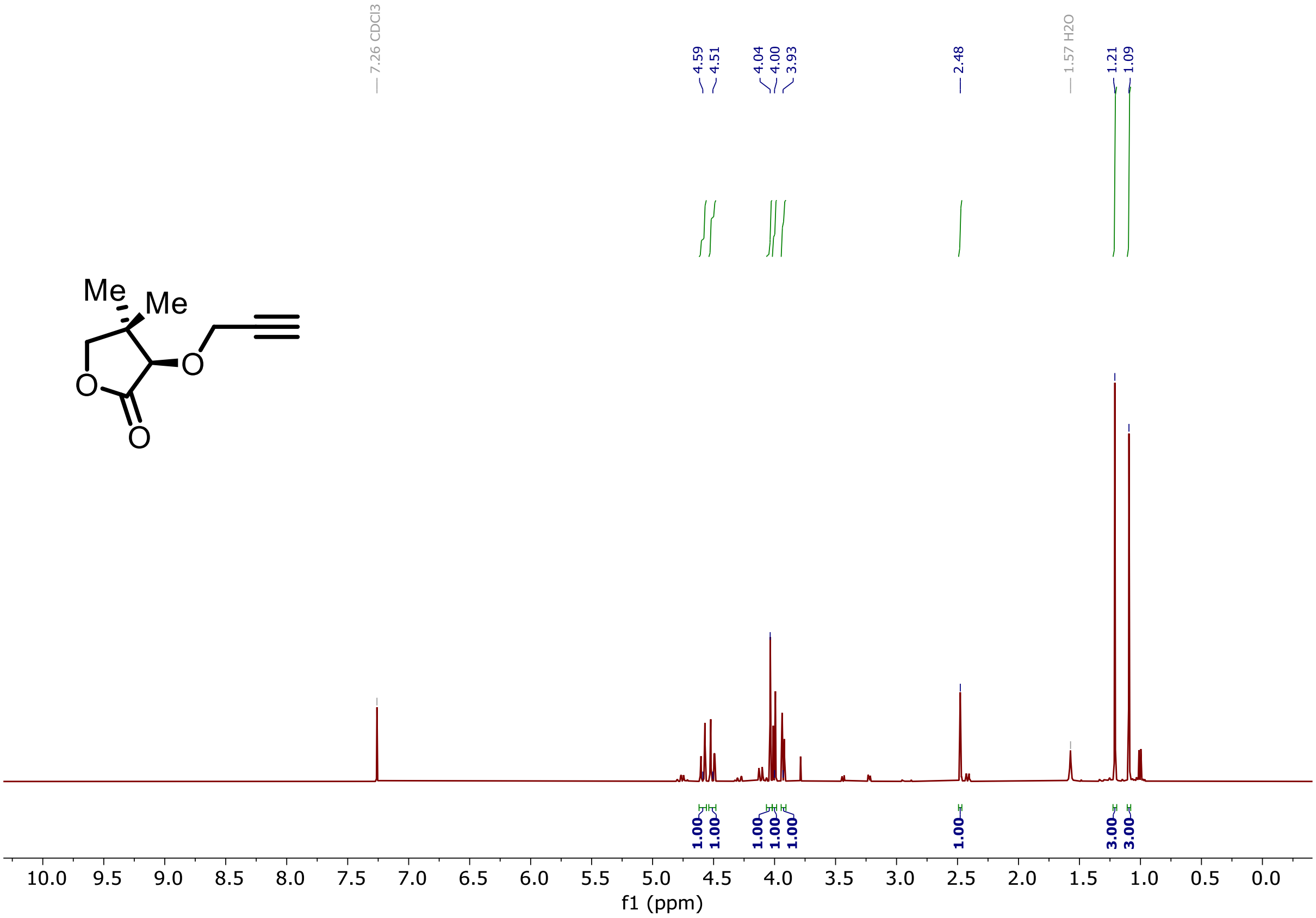

^{13}C NMR (126 MHz, $CDCl_3$)

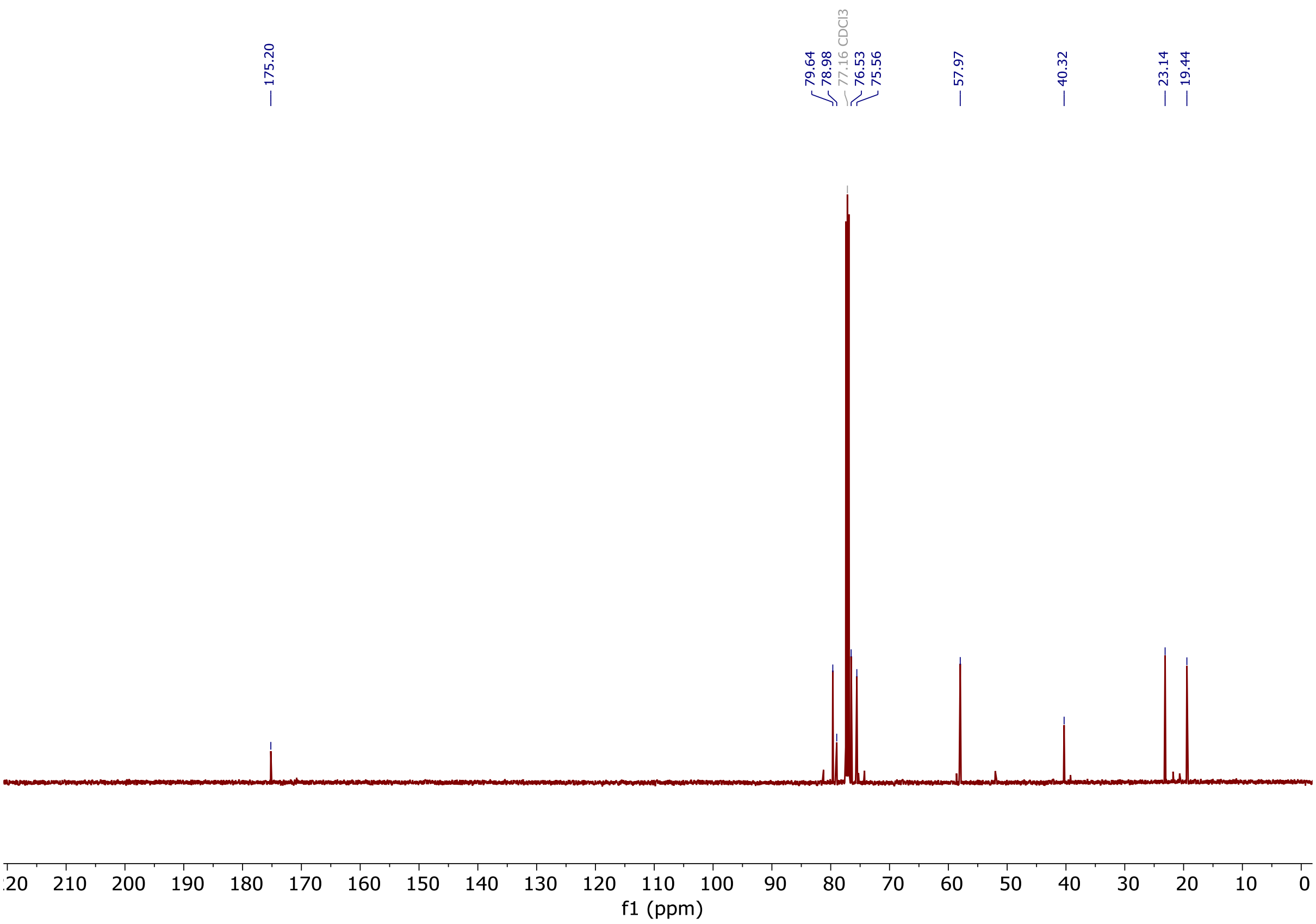

N-butyl-2-nitro-N-(prop-2-yn-1-yl)benzenesulfonamide **(1bp)**

^{1}H NMR (500 MHz, $CDCl_3$)

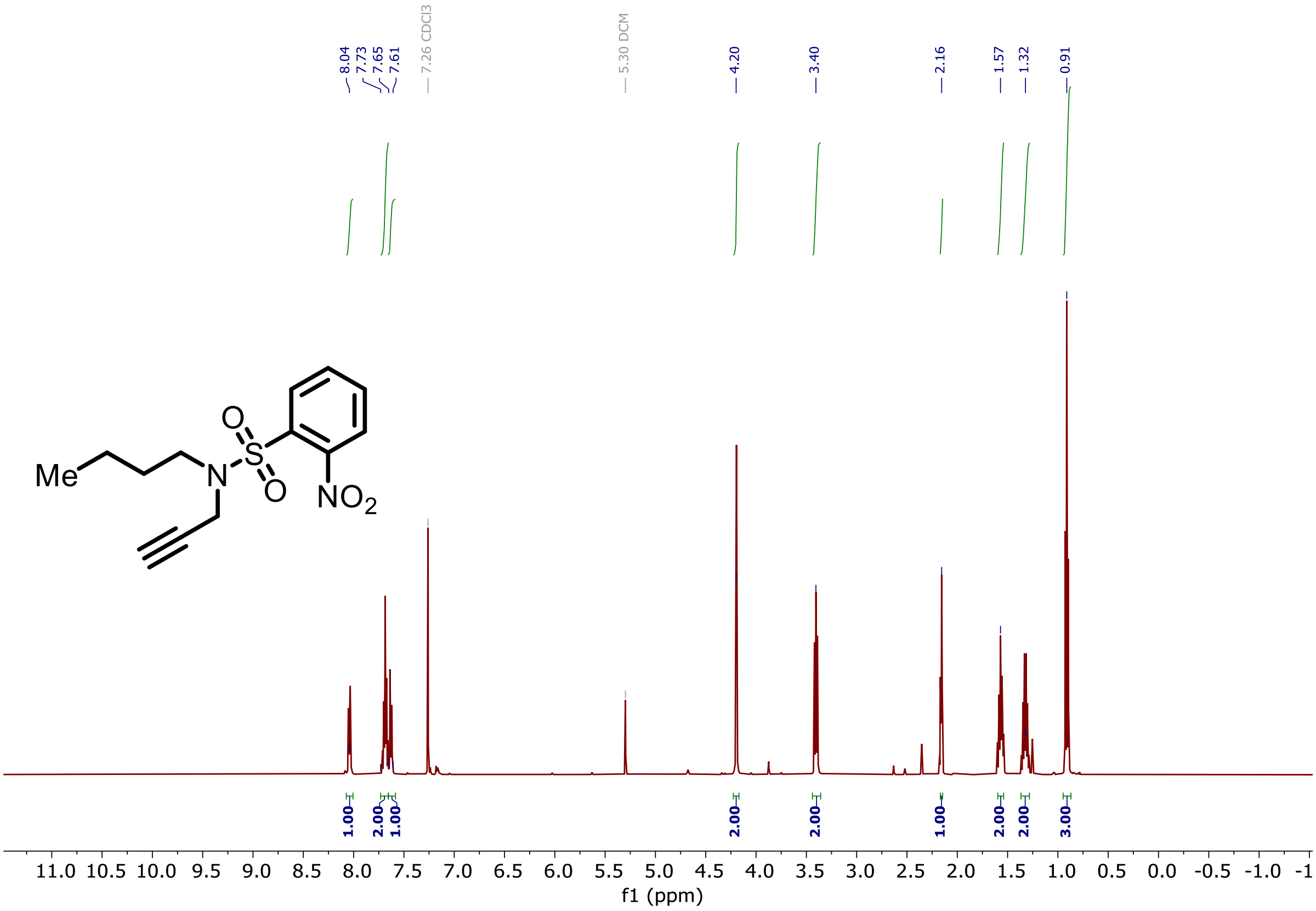

^{13}C NMR (126 MHz, $CDCl_3$)

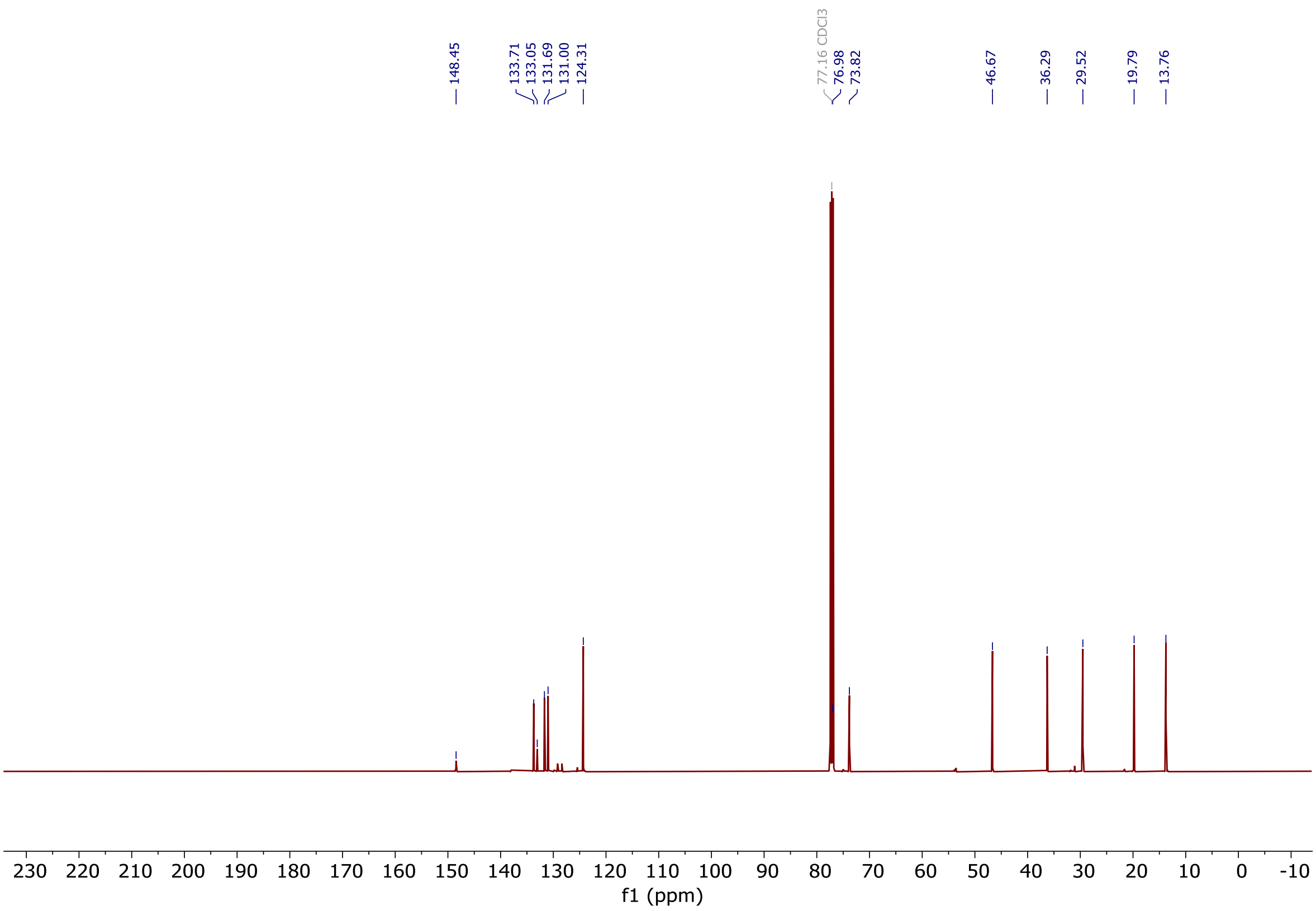

***N-(4-fluorobenzyl)-2,4-dinitro-N-(prop-2-yn-1-yl)benzenesulfonamide* (1bq)**

^{1}H NMR (500 MHz, $CDCl_3$)

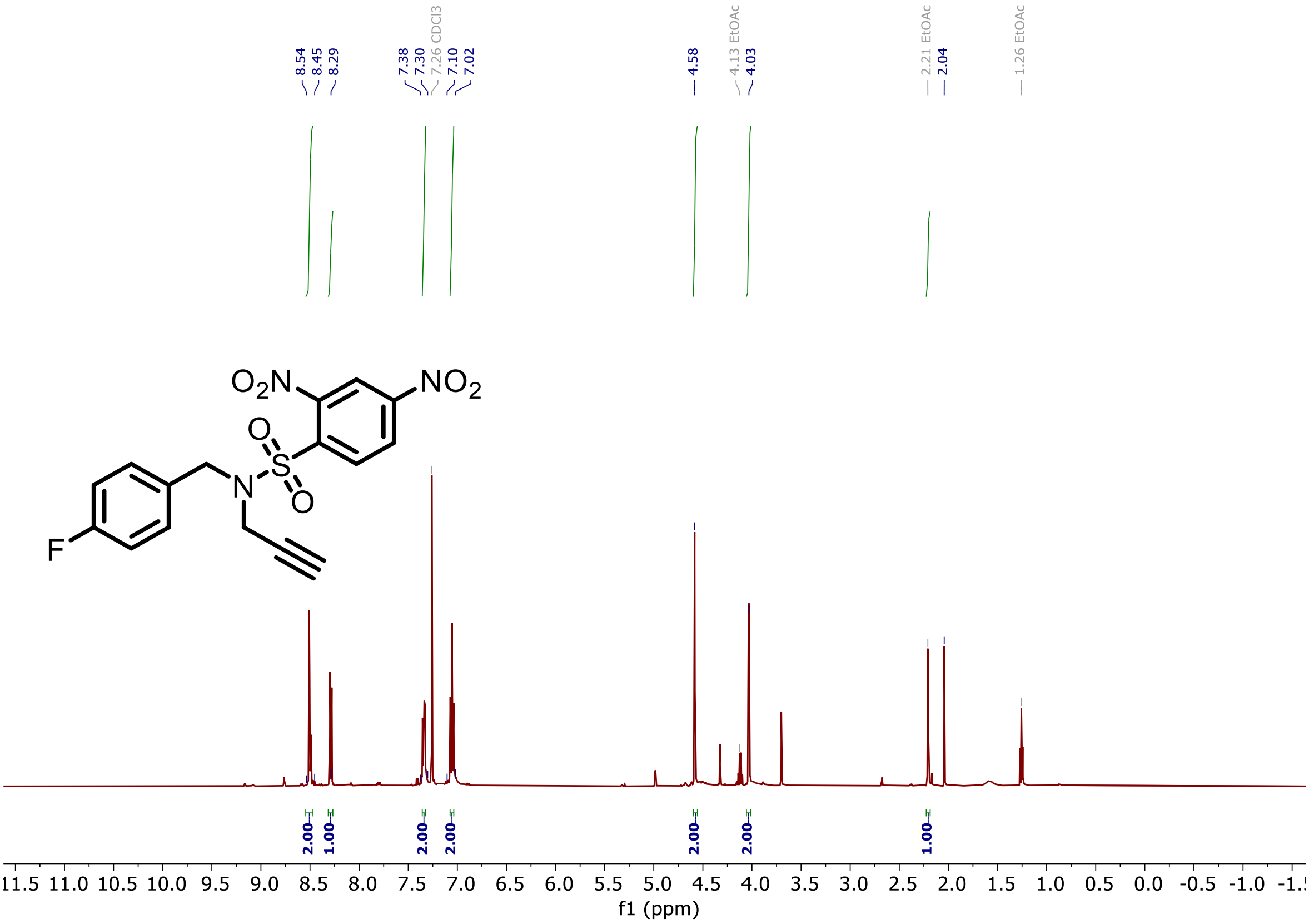

^{13}C NMR (126 MHz, $CDCl_3$)

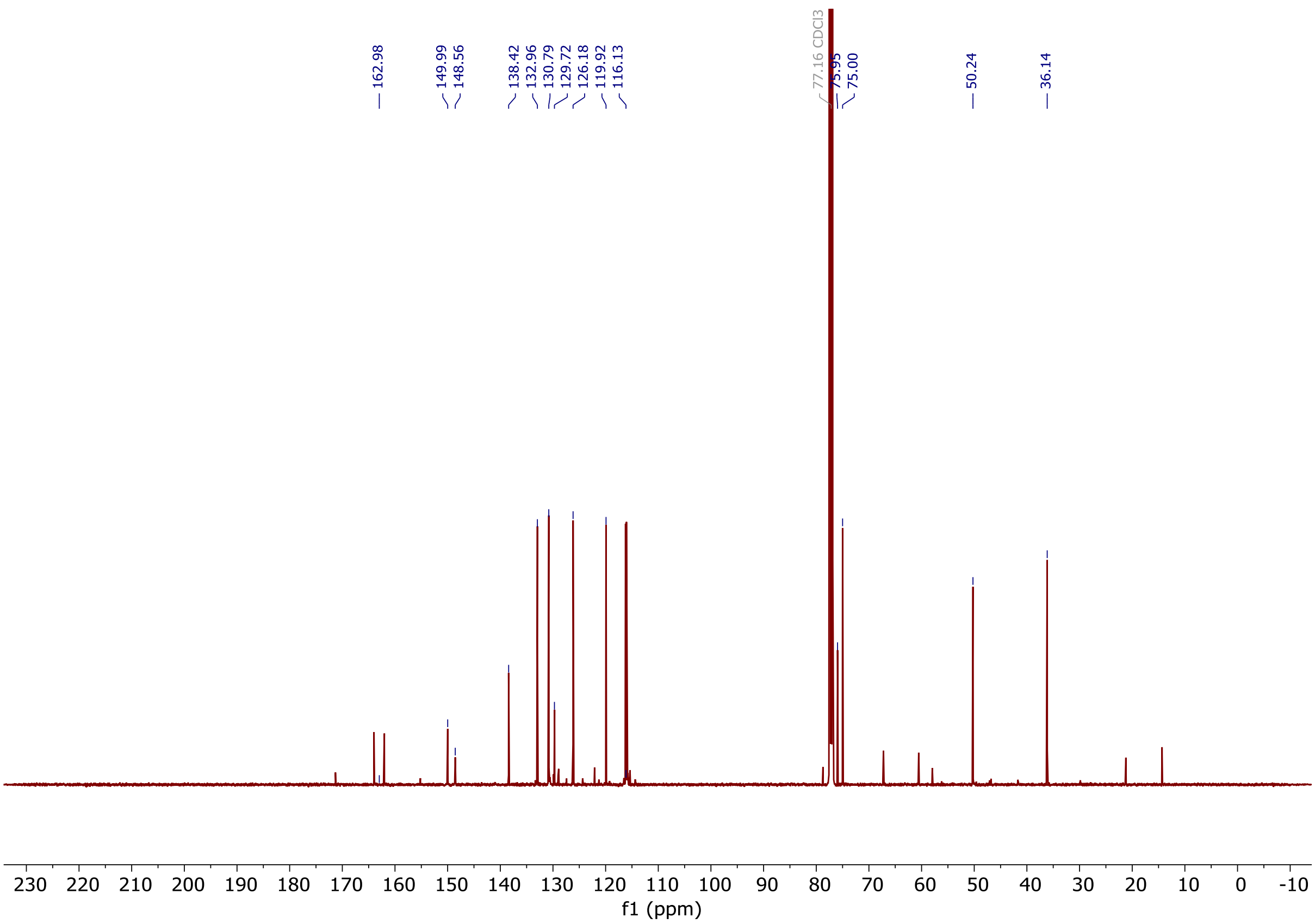

^{19}F NMR (376 MHz, $CDCl_3$)

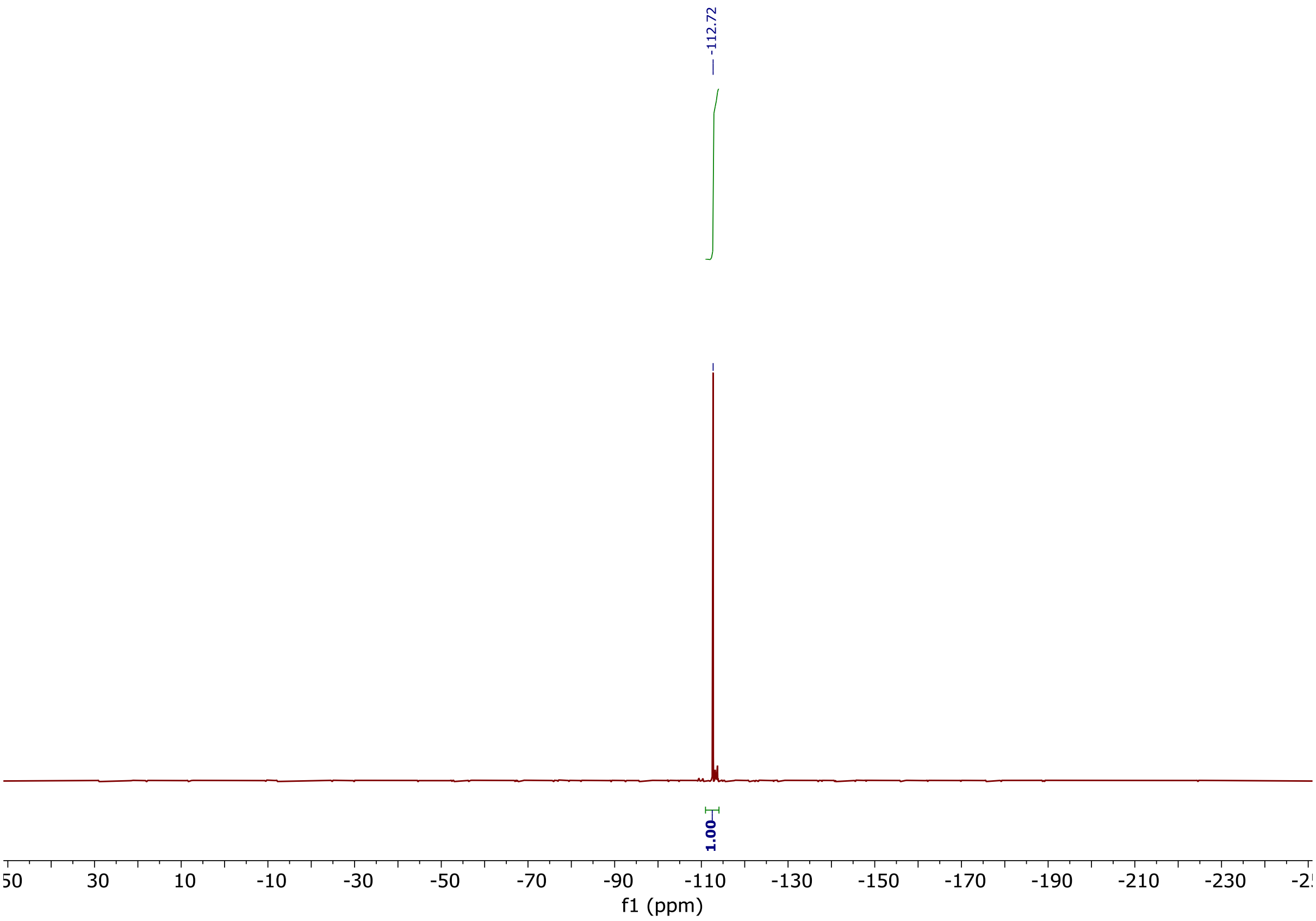

N-(but-3-yn-1-yl)-4-methyl-N-(4-methylbenzyl)benzenesulfonamide (**1br**)

^{1}H NMR (400 MHz, CDCl$_3$)

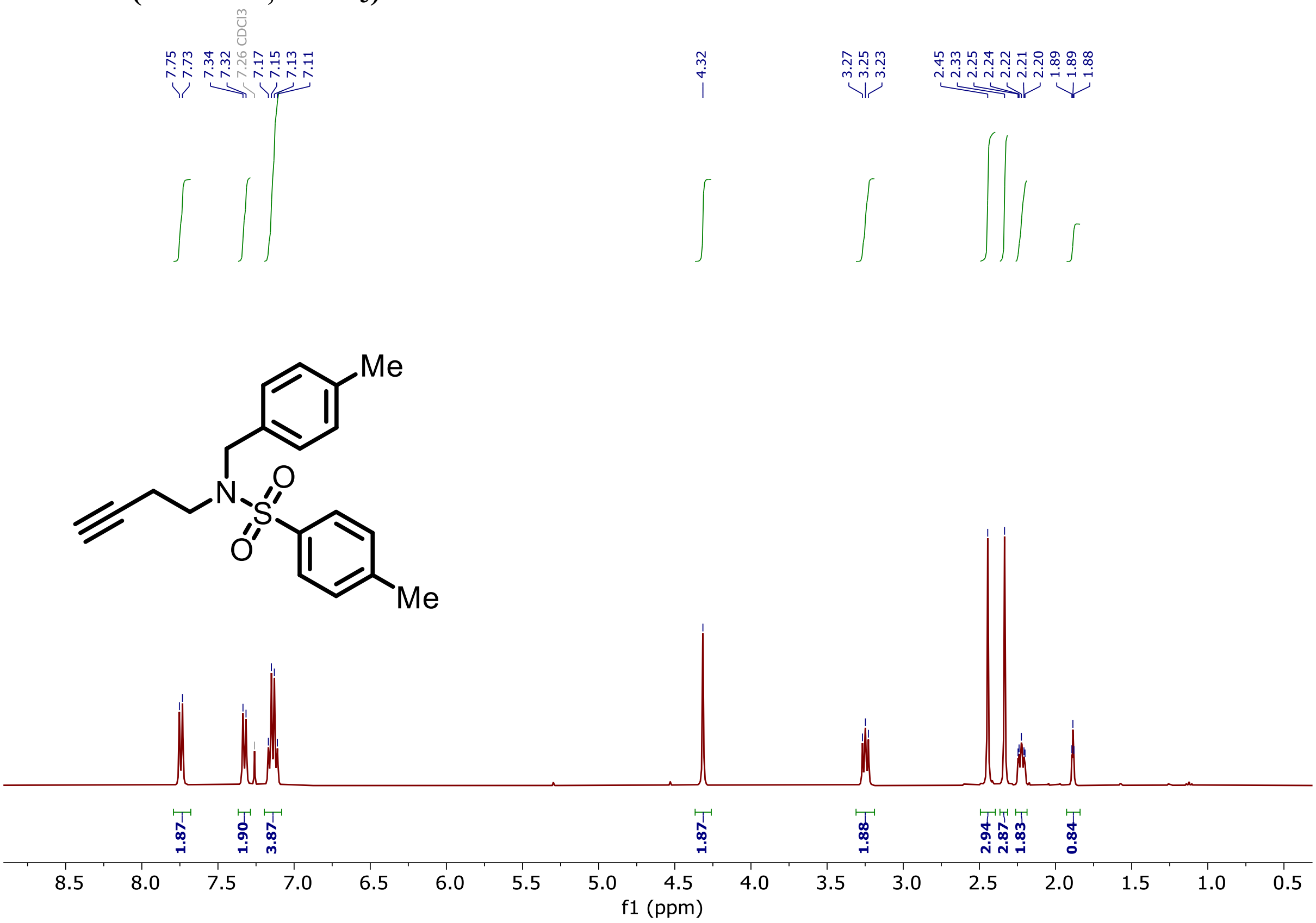

^{13}C NMR (101 MHz, CDCl$_3$)

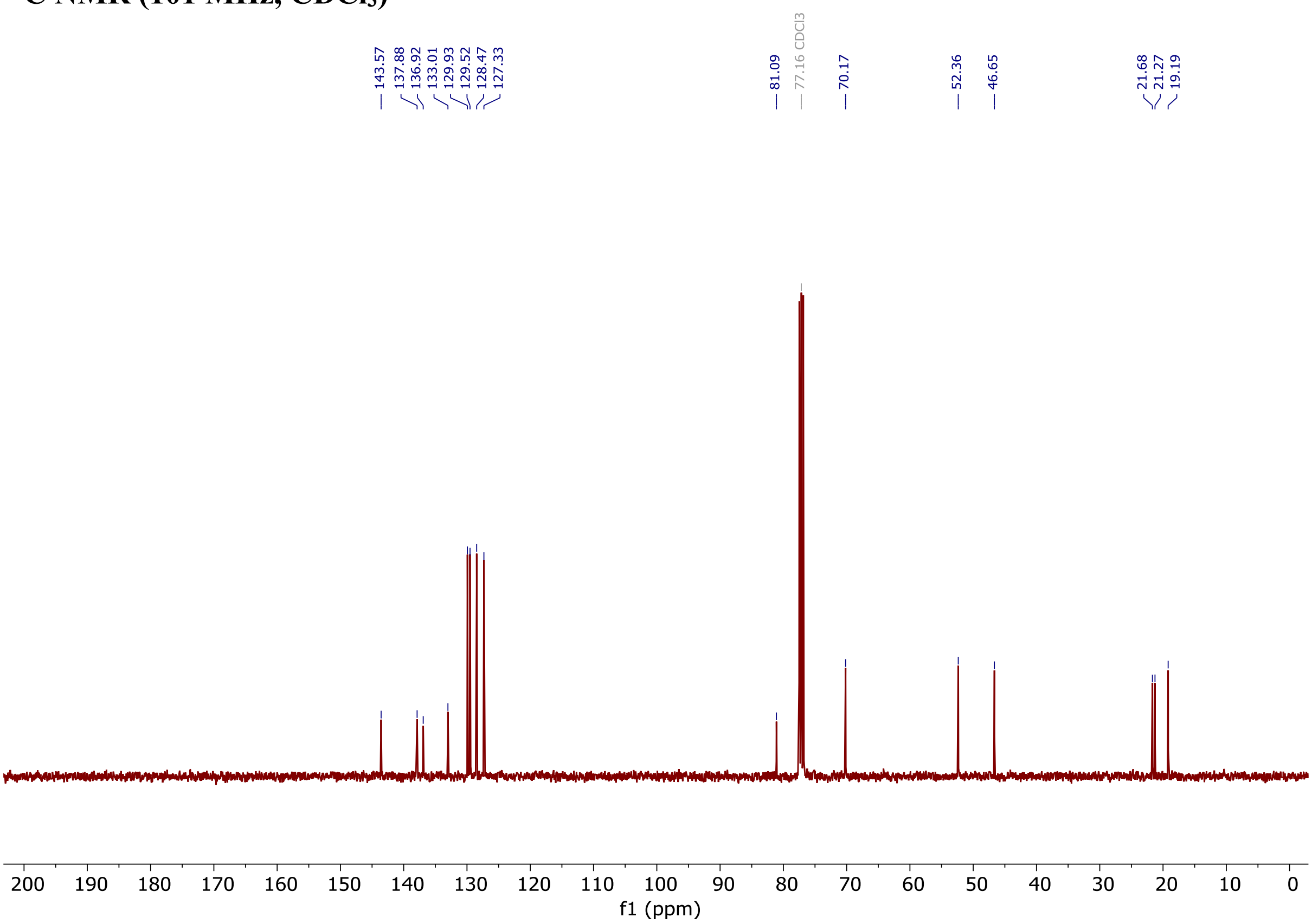

N-(but-3-yn-1-yl)-4-methyl-N-(4-fluorobenzyl)benzenesulfonamide **(1bs)**

^{1}H NMR (400 MHz, CDCl$_3$)

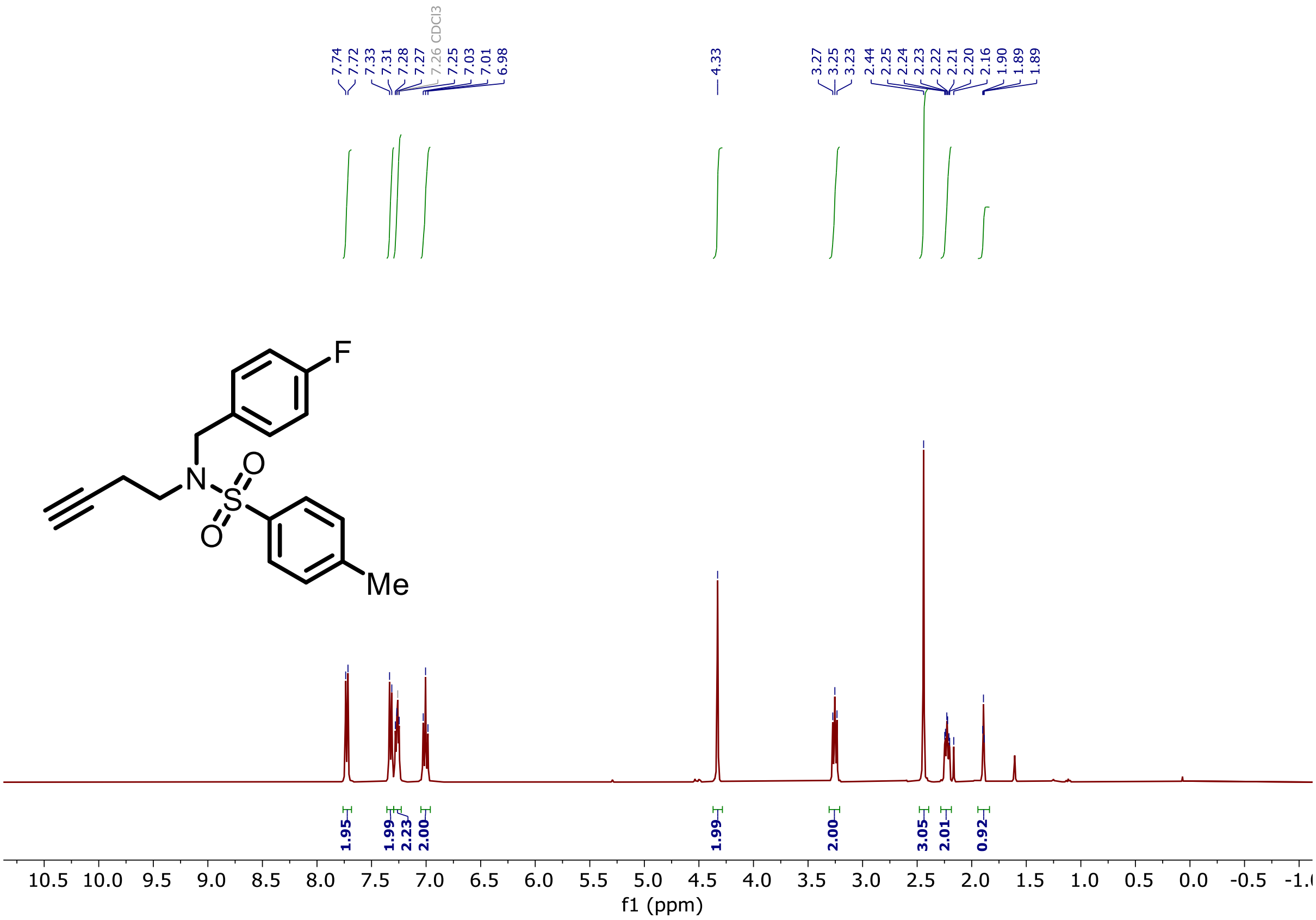

^{13}C NMR (101 MHz, CDCl$_3$)

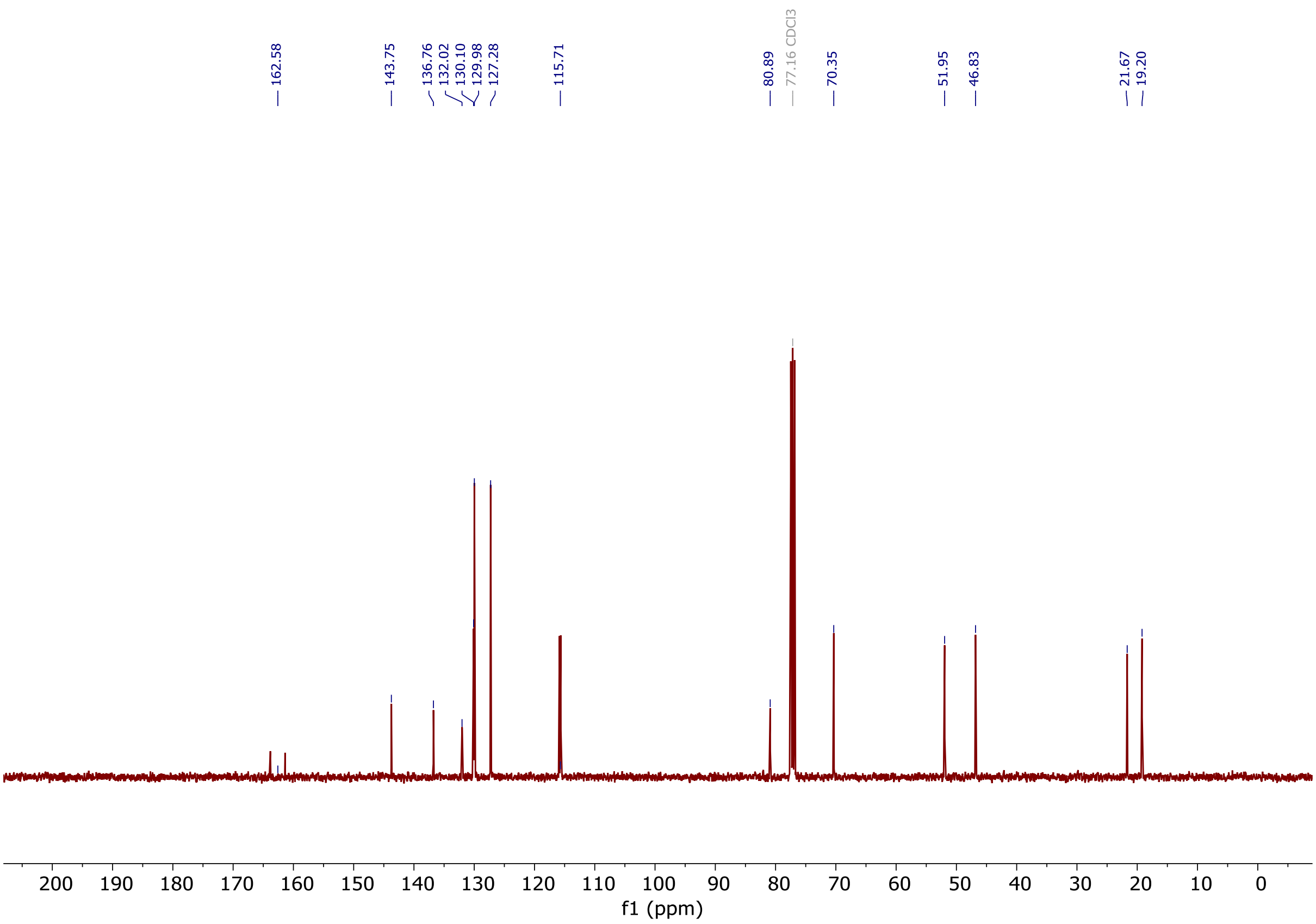

^{19}F NMR (376 MHz,CDCl₃)

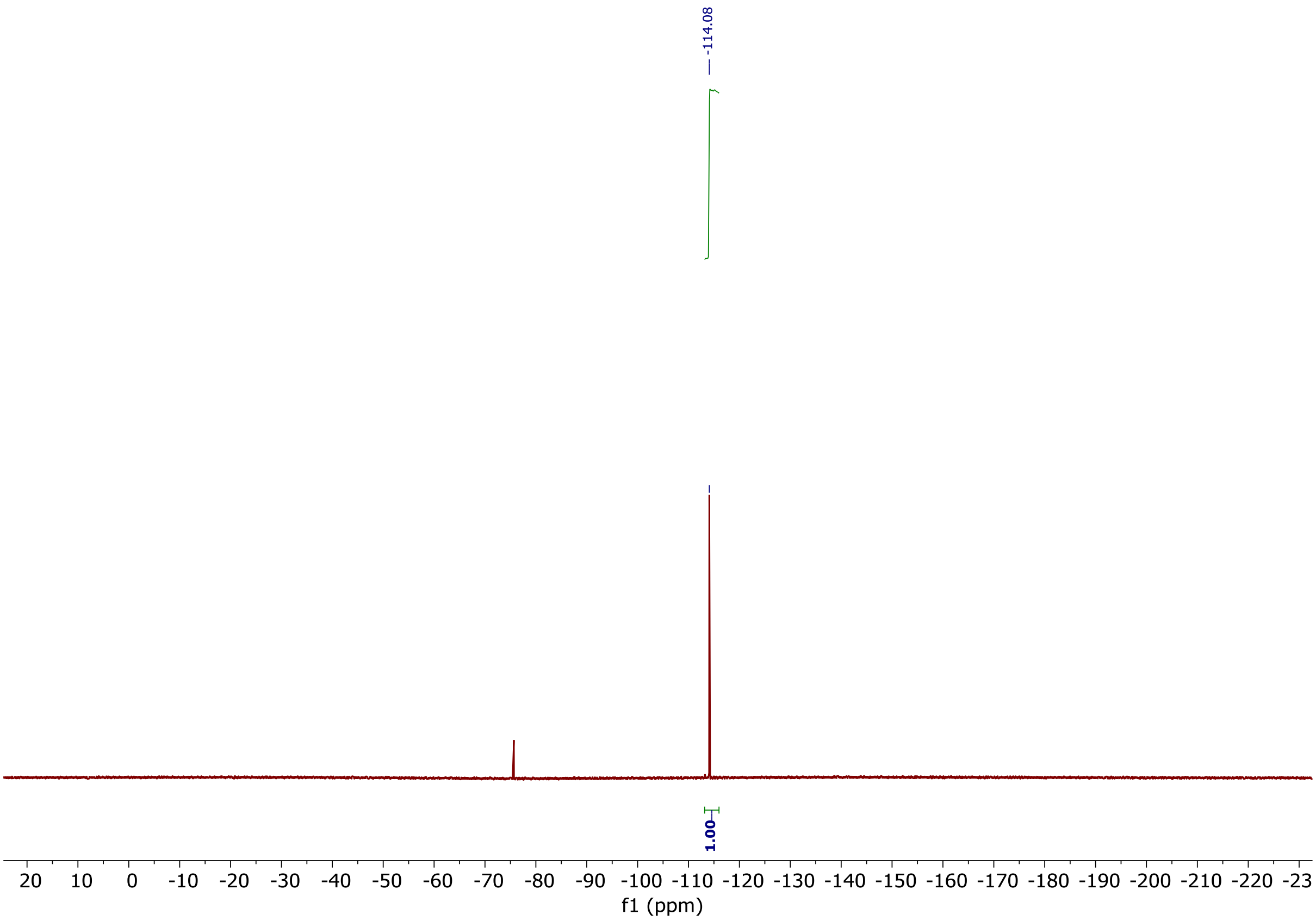

9.2. FVIs (3)

***(Z)-(2-fluoro-3-((N-(4-methoxybenzyl)-4-methylphenyl)sulfonamido)prop-1-en-1-yl)(p-tolyl)iodonium tetrafluoroborate* (3a)**

^{1}H NMR (500 MHz, Acetone-D_6)

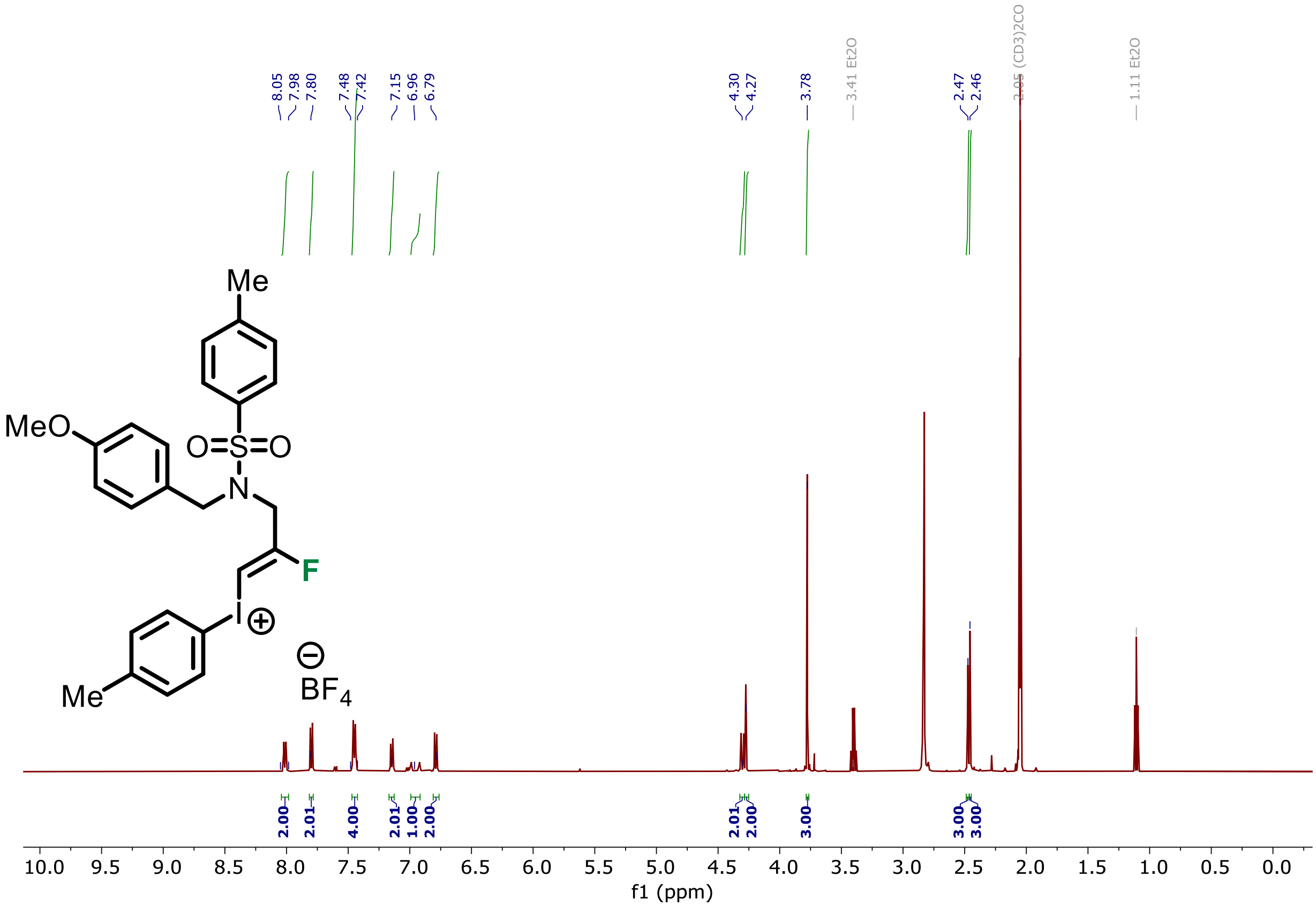

^{13}C NMR (126 MHz, Acetone-D_6)

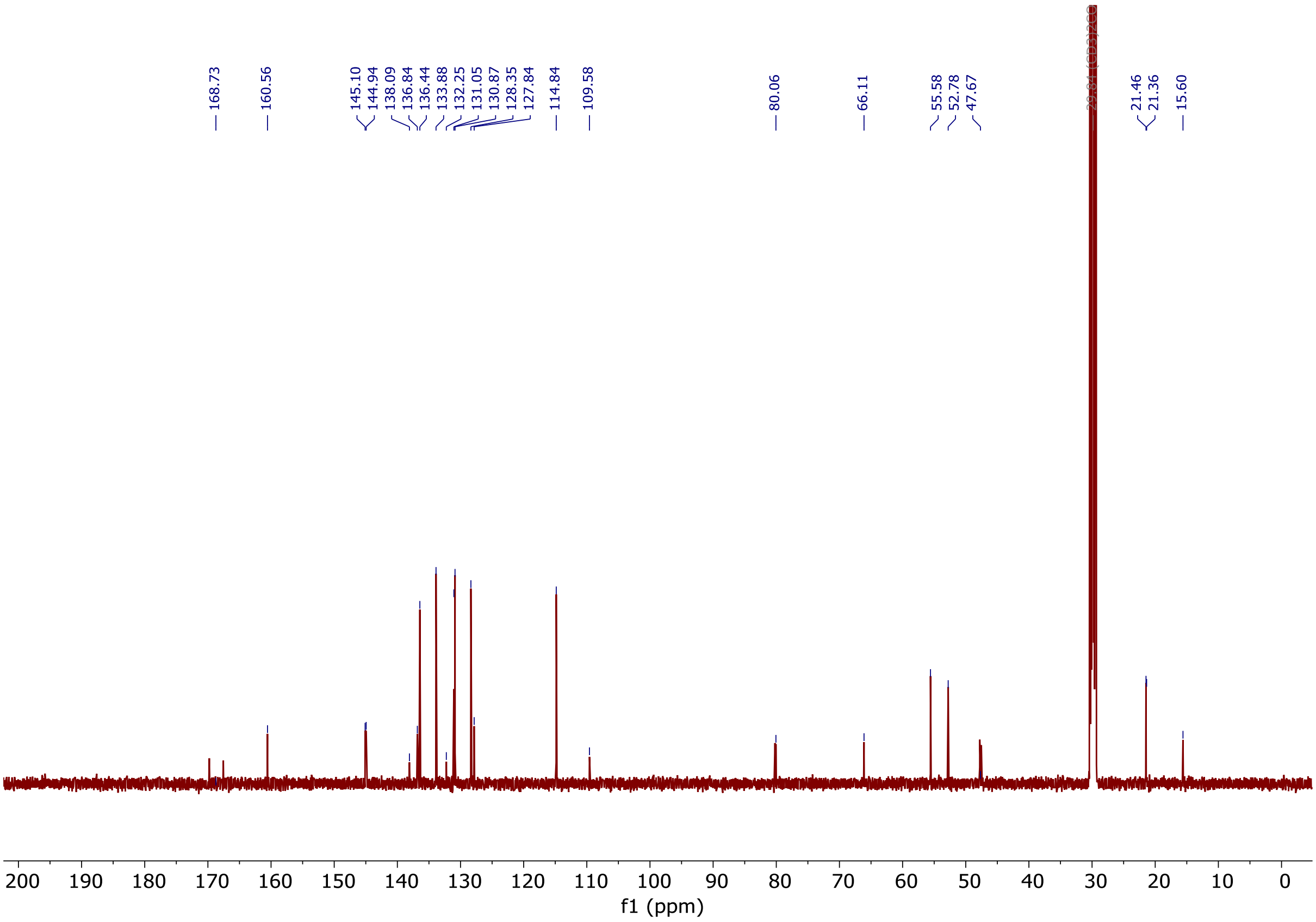

^{19}F NMR (376 MHz, Acetone-D_6)

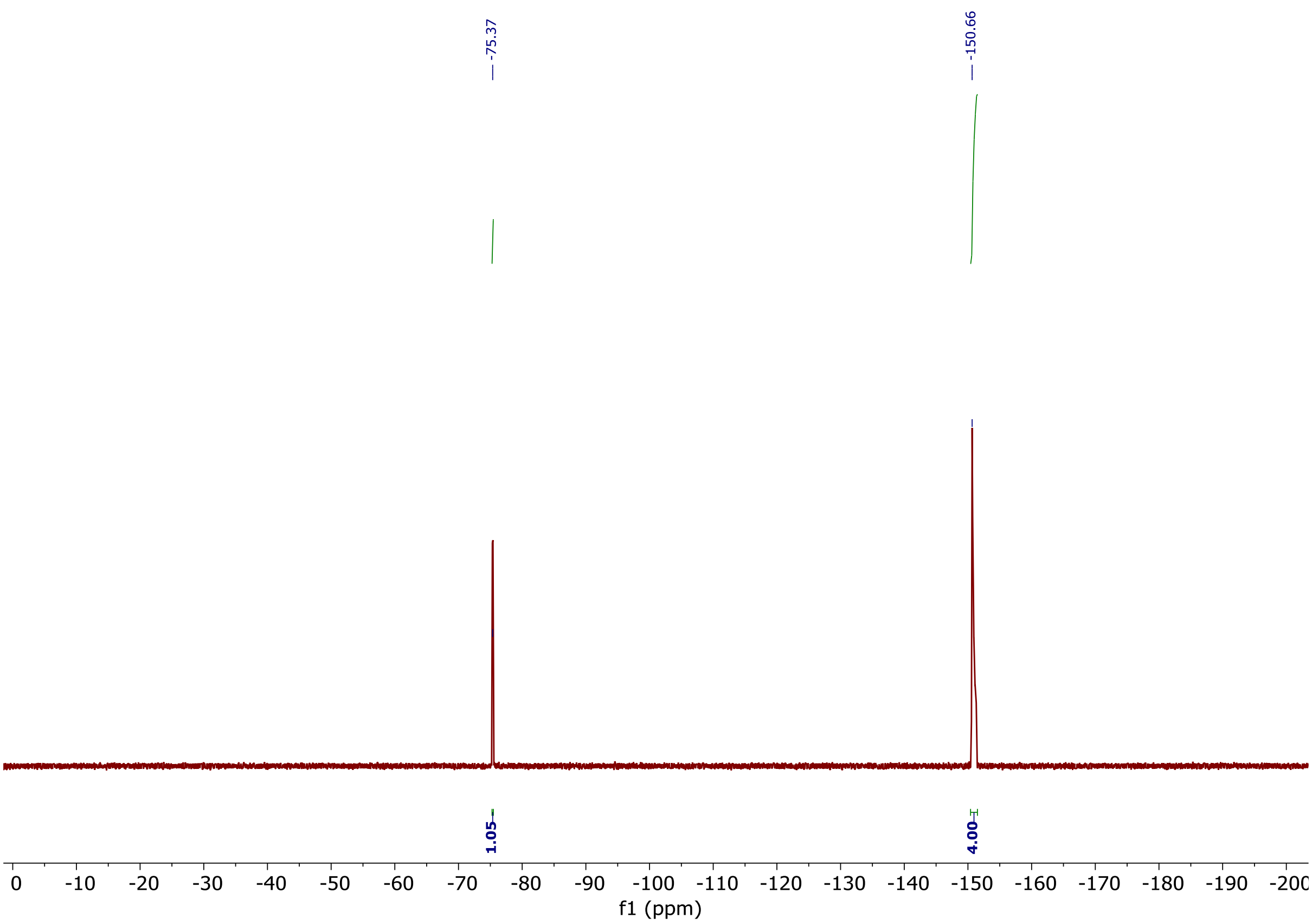

***(Z)*-(2-fluoro-3-((4-methyl-N-(4-methylbenzyl)phenyl)sulfonamido)prop-1-en-1-yl)(p-tolyl)iodonium tetrafluoroborate* (3b)**

^{1}H NMR (400 MHz, Acetone-D_6)

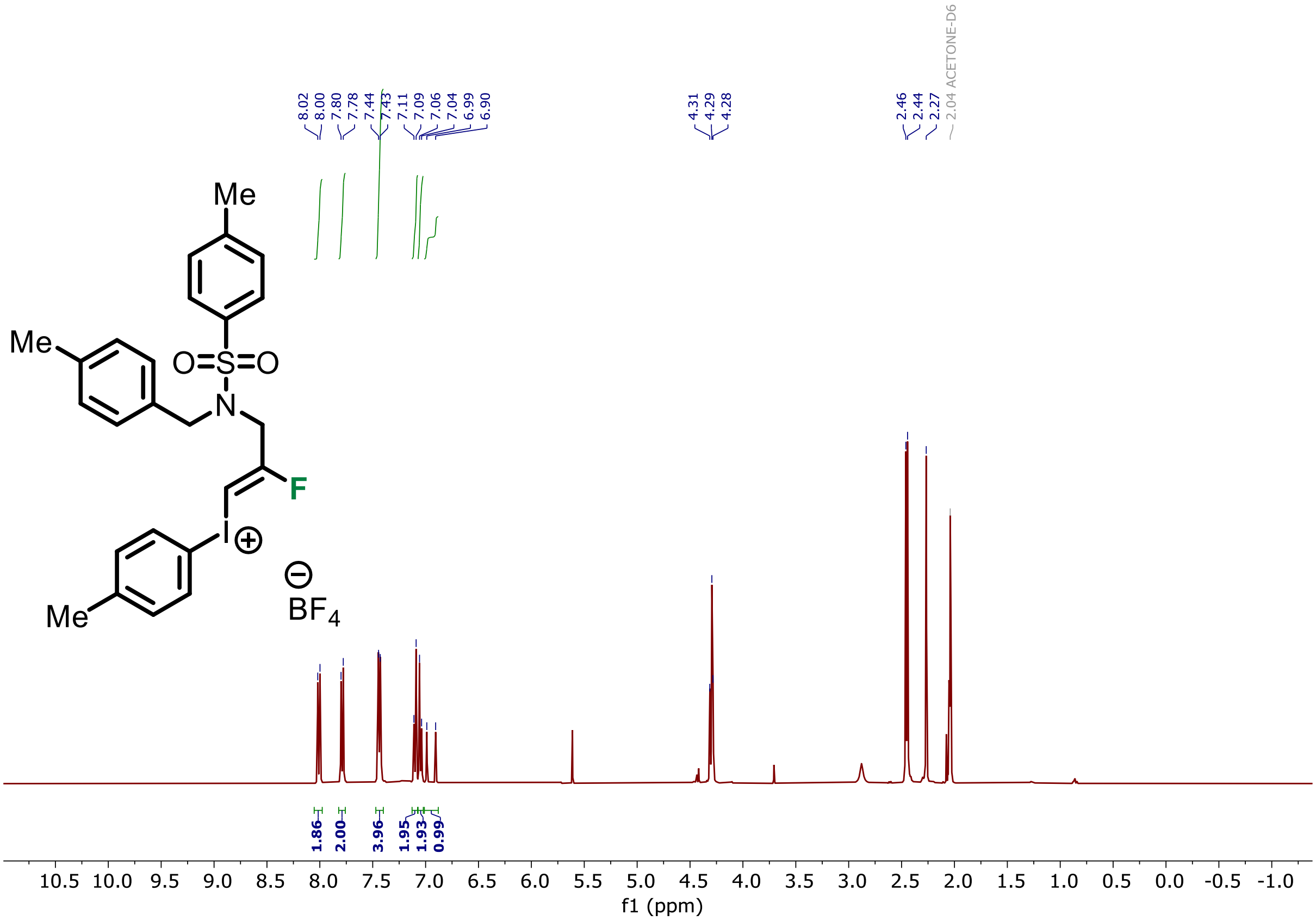

^{13}C NMR (126 MHz, Acetone-D_6)

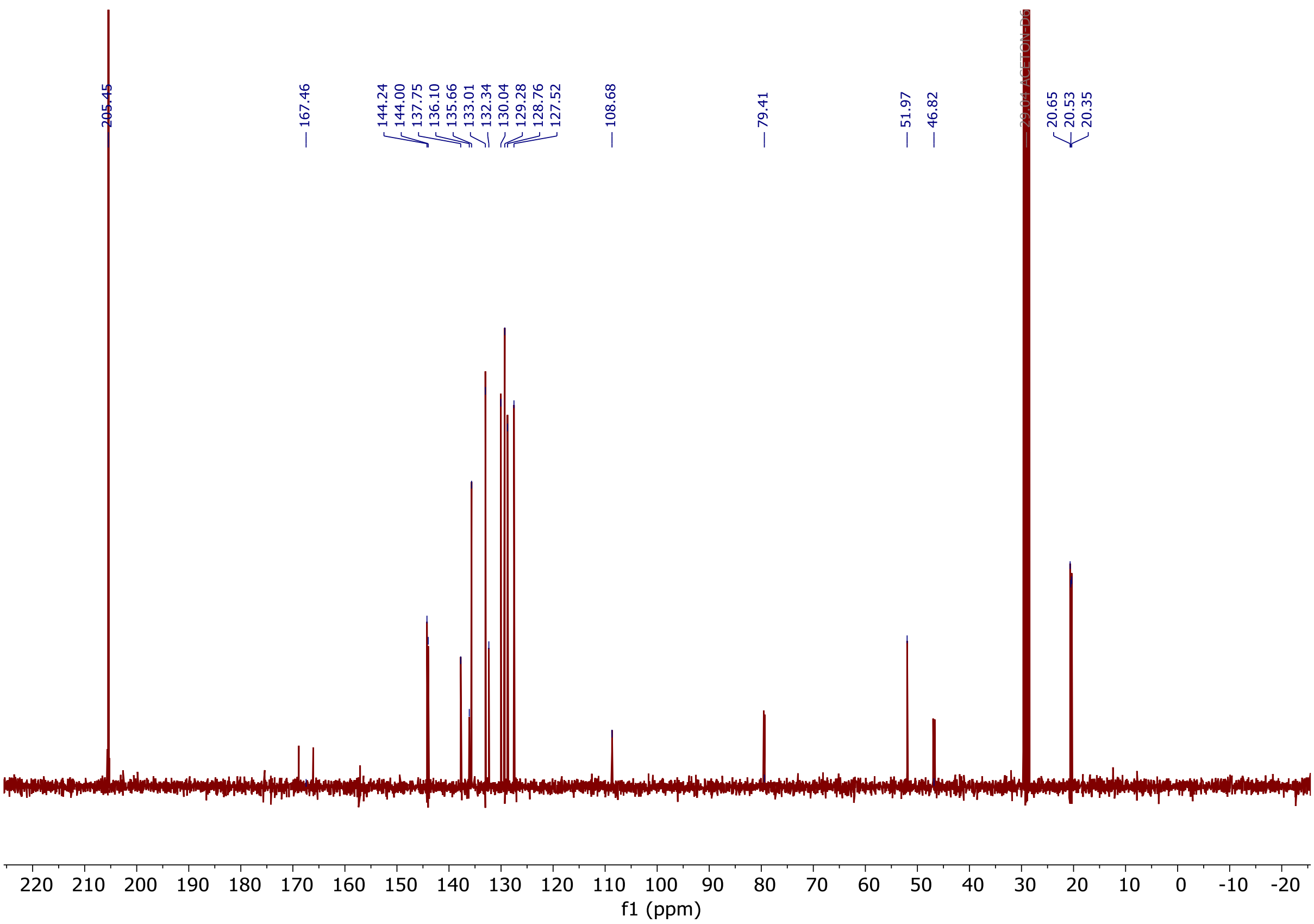

^{19}F NMR (371 MHz, Acetone-D_6)

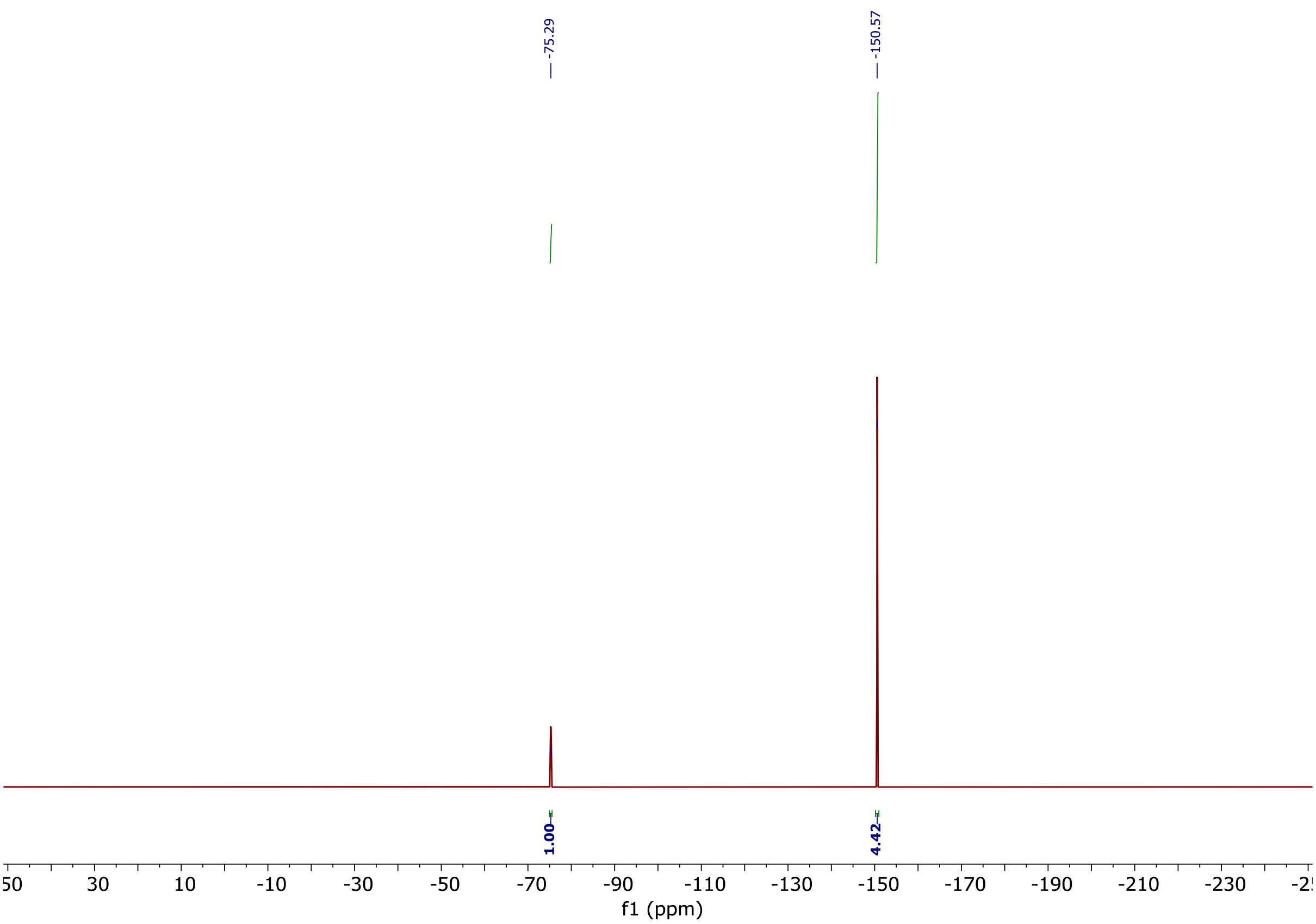

(Z)-(3-((N-(4-bromobenzyl)-4-methylphenyl)sulfonamido)-2-fluoroprop-1-en-1-yl)(p-tolyl)iodonium tetrafluoroborate **(3c)**

^{1}H NMR (500 MHz, Acetone-*D_6*)

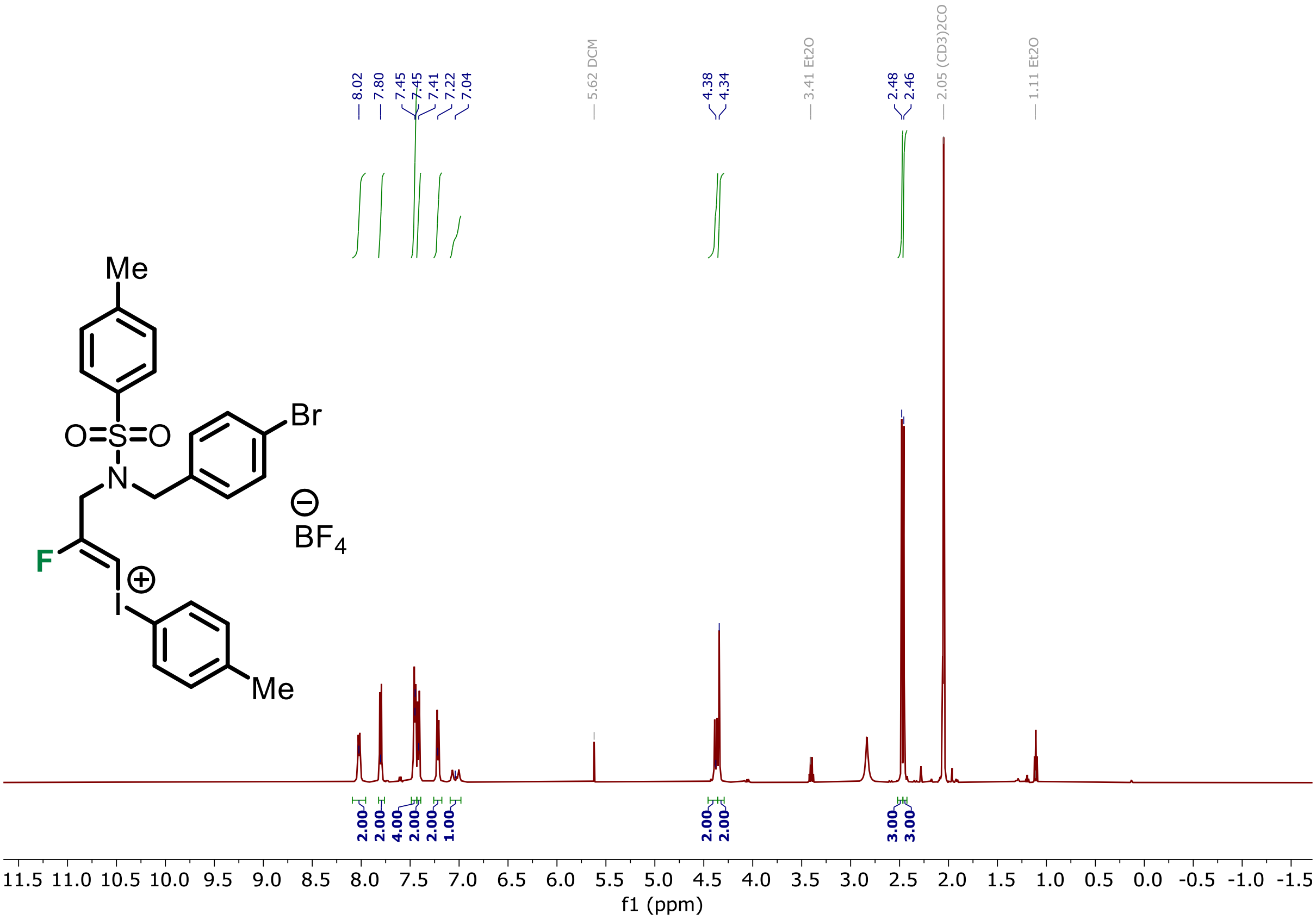

^{13}C NMR (126 MHz, Acetone-D_6)

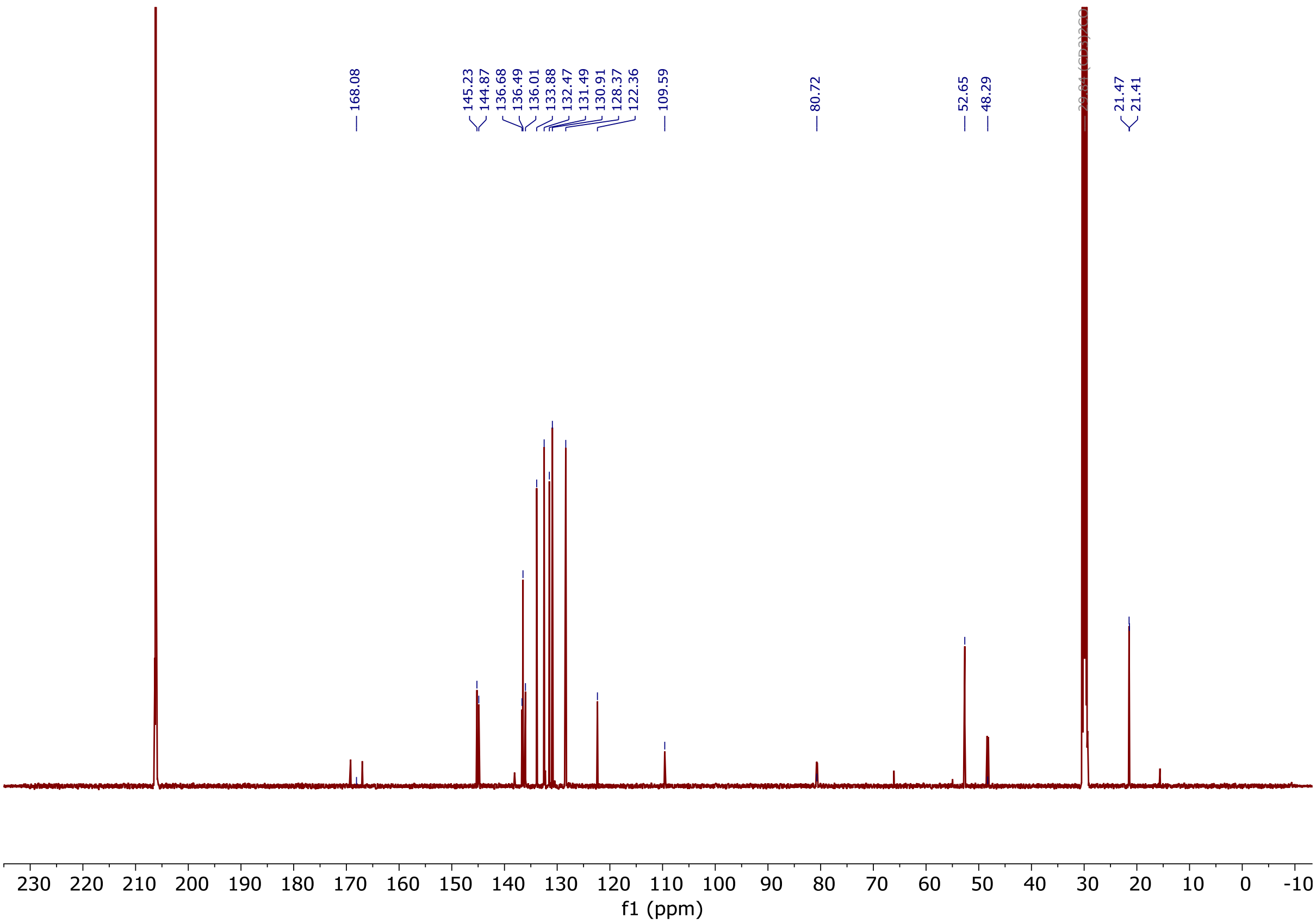

^{19}F NMR (377 MHz, Acetone-D_6)

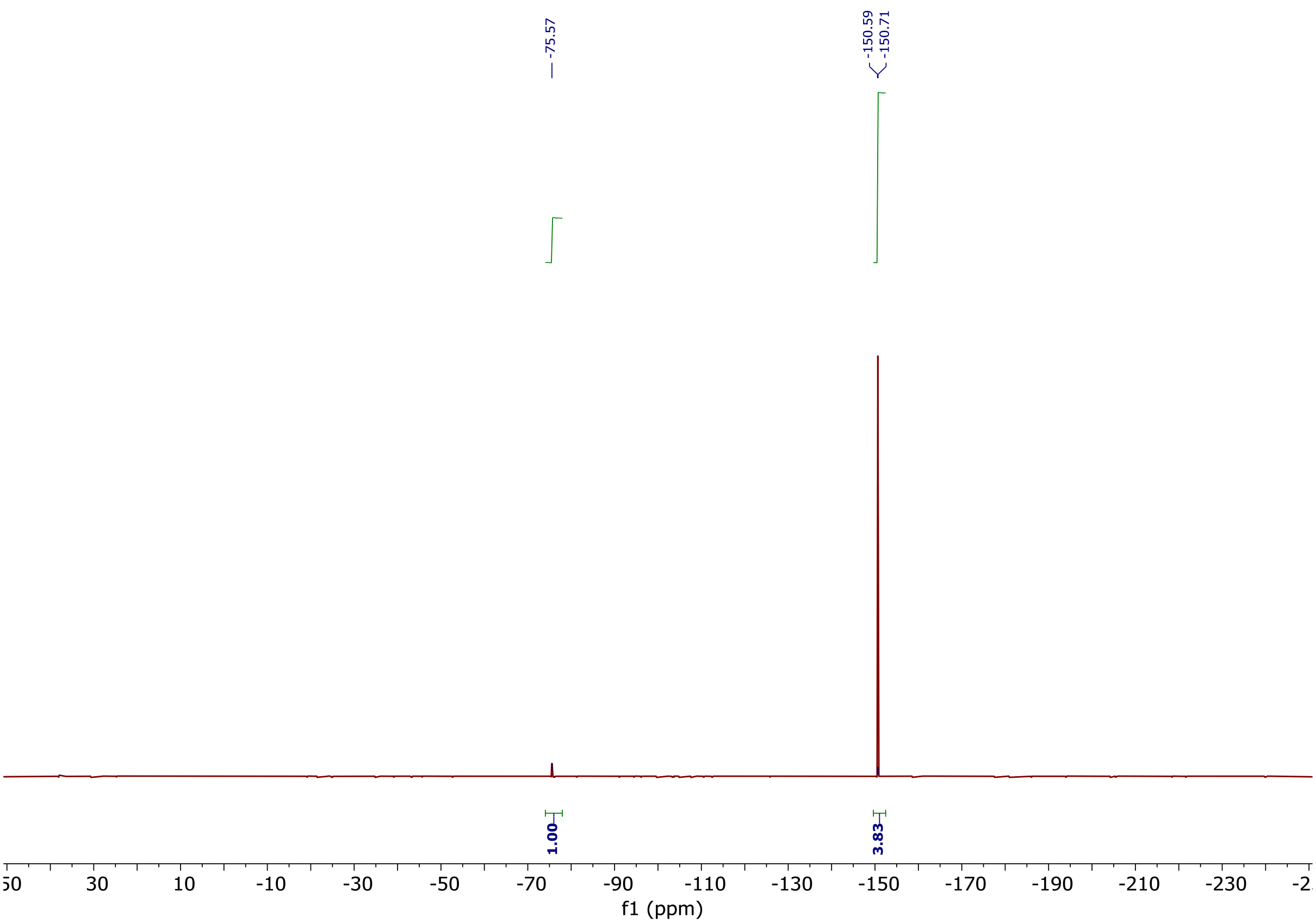

(Z)-(3-((N-(4-chlorobenzyl)-4-methylphenyl)sulfonamido)-2-fluoroprop-1-en-1-yl)(p-tolyl)iodonium tetrafluoroborate (3d)
^{1}H NMR (400 MHz, Acetone-D_6)

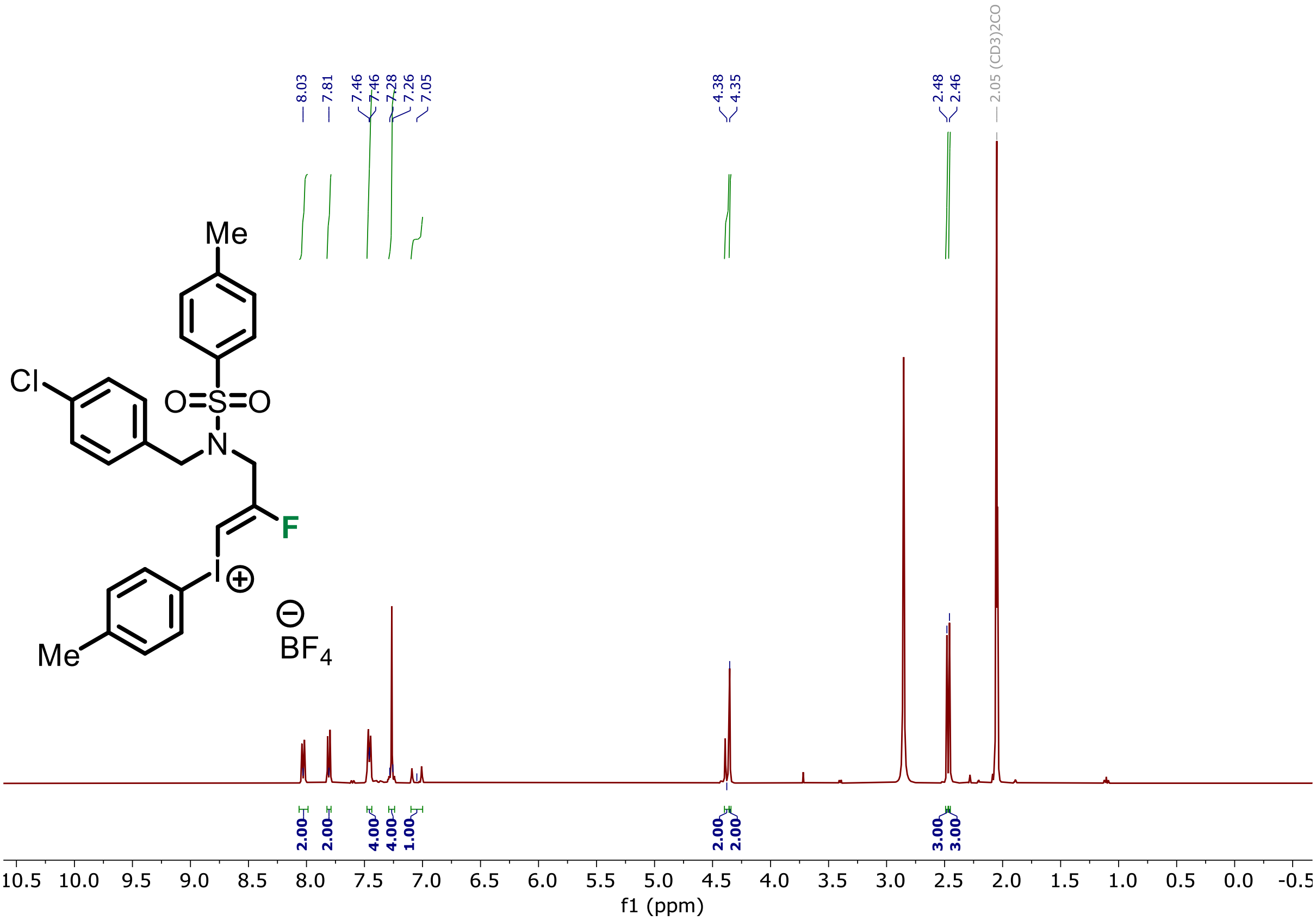

^{13}C NMR (126 MHz, Acetone-D_6)

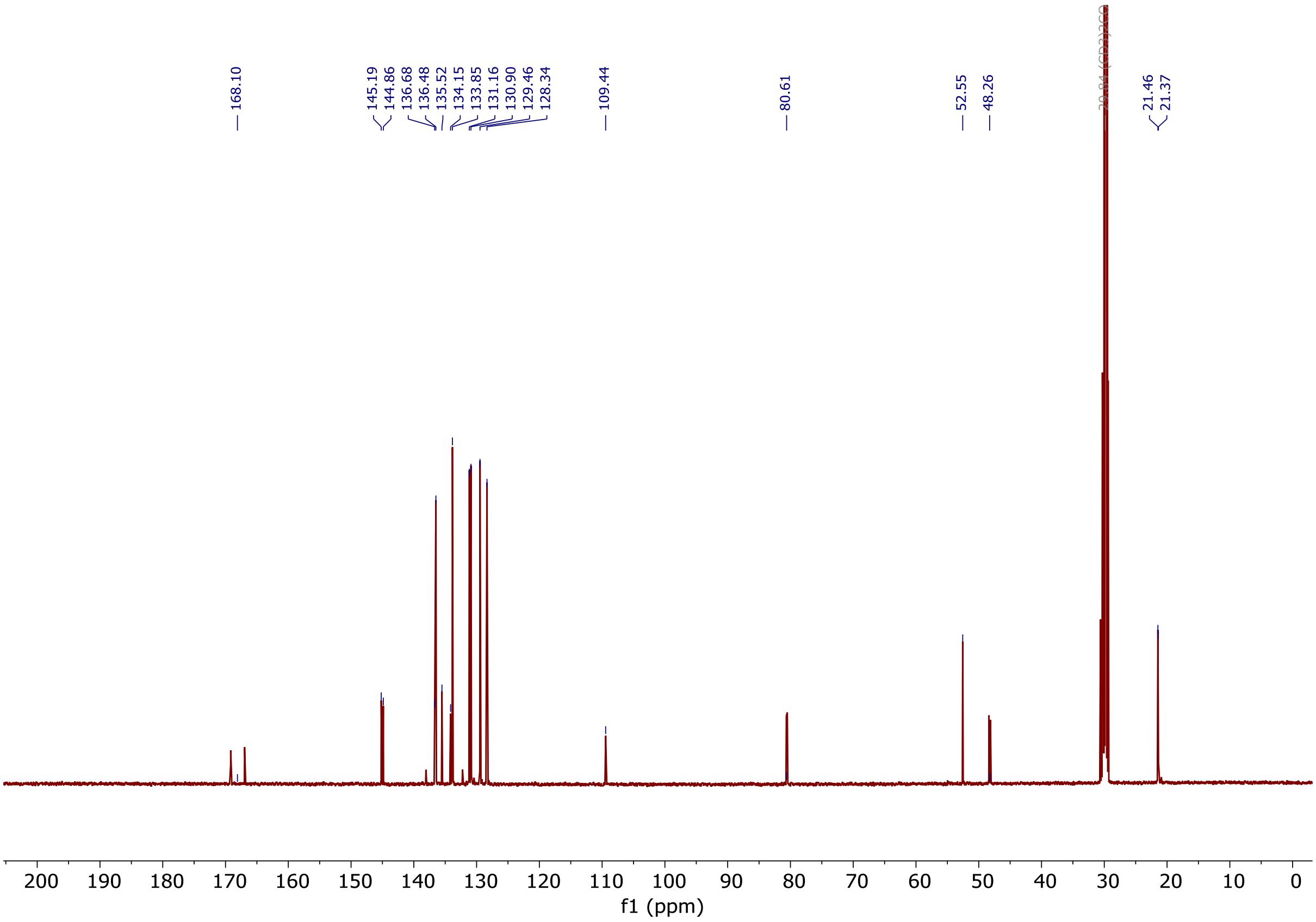

^{19}F NMR (471 MHz, Acetone-D_6)

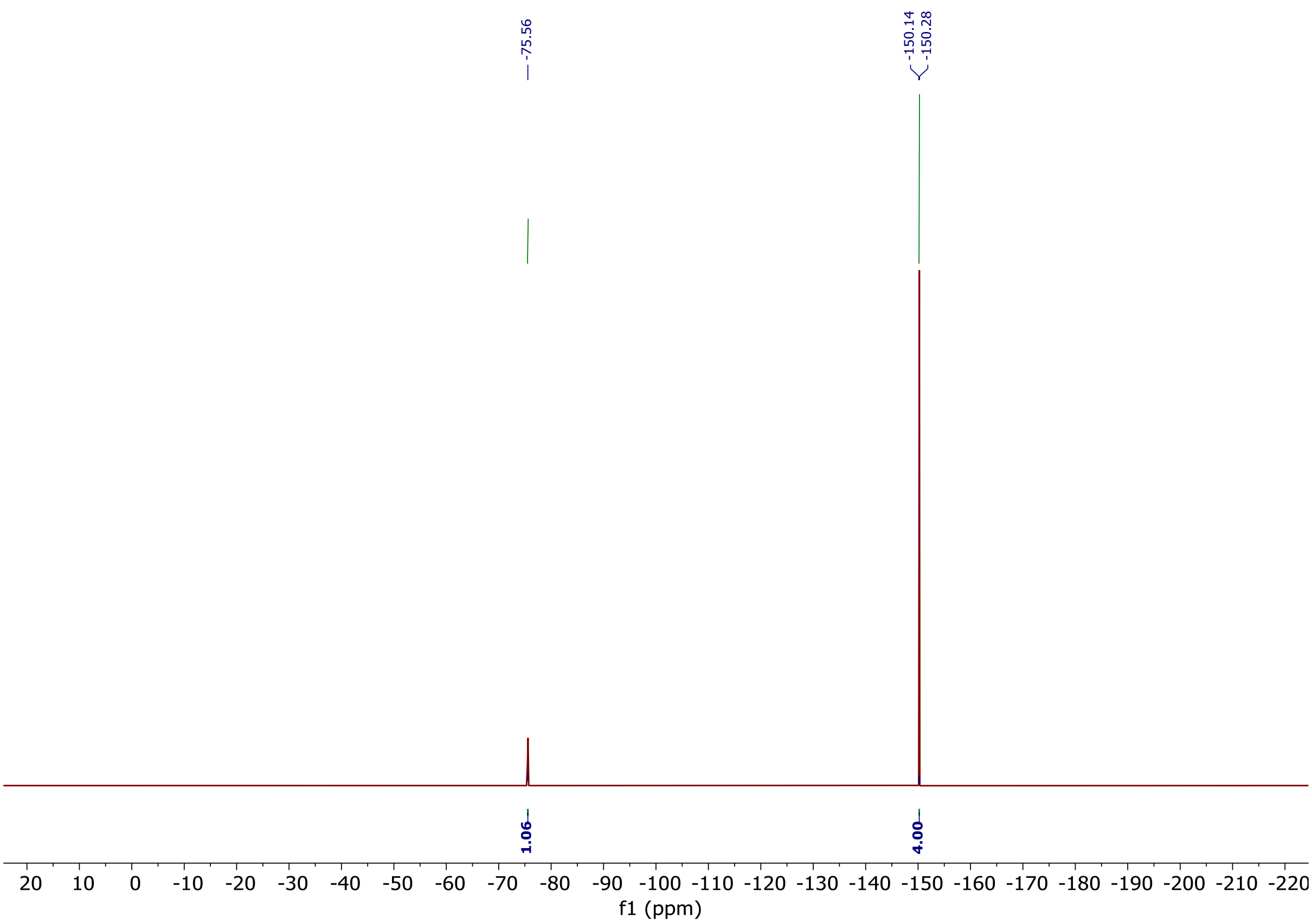

(Z)-(2-fluoro-3-((N-(4-fluorobenzyl)-4-methylphenyl)sulfonamido)prop-1-en-1-yl)(p-tolyl)iodonium tetrafluoroborate (3e)

^{1}H NMR (400 MHz, Acetone-D_6)

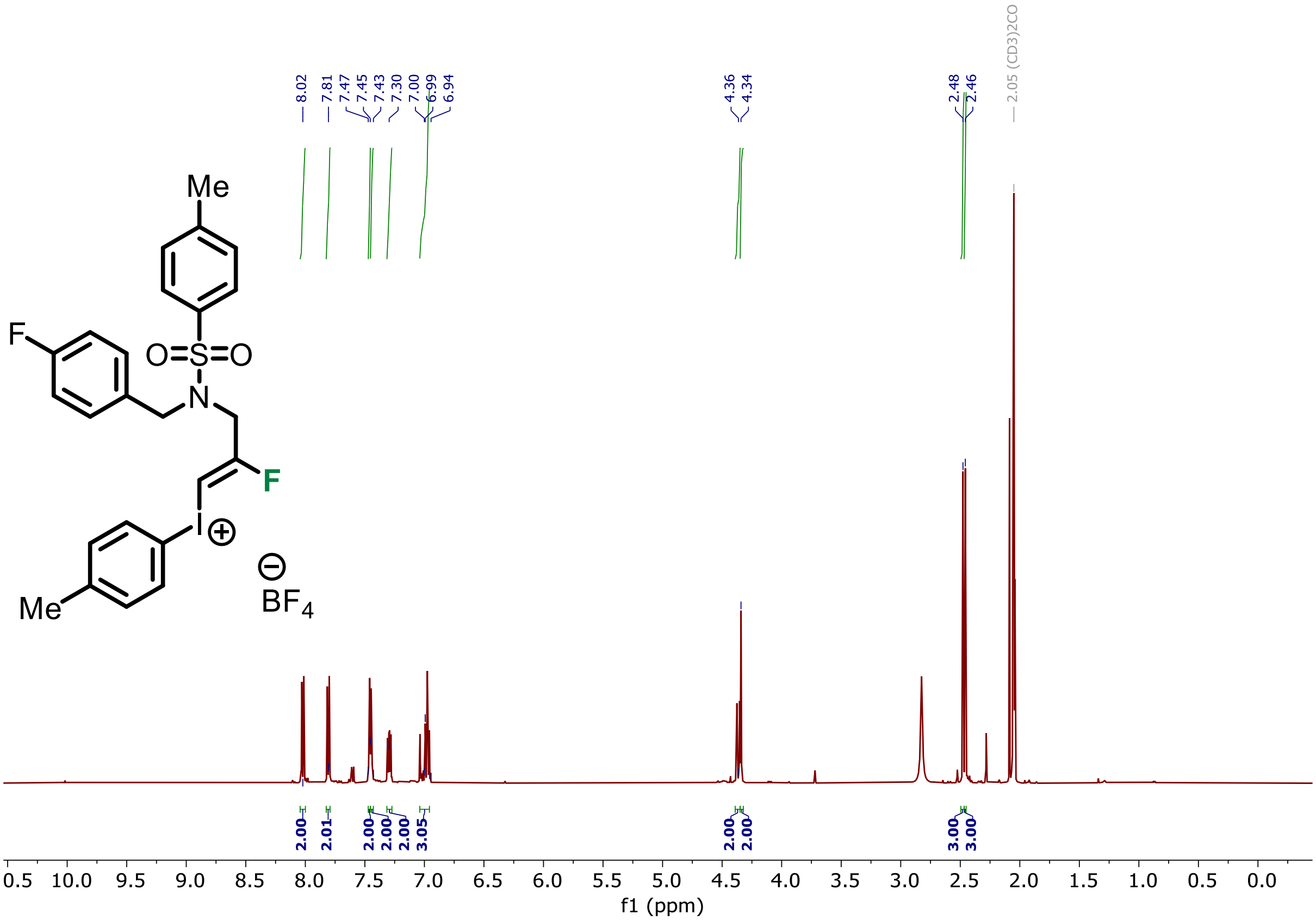

^{13}C NMR (126 MHz, Acetone-D_6)

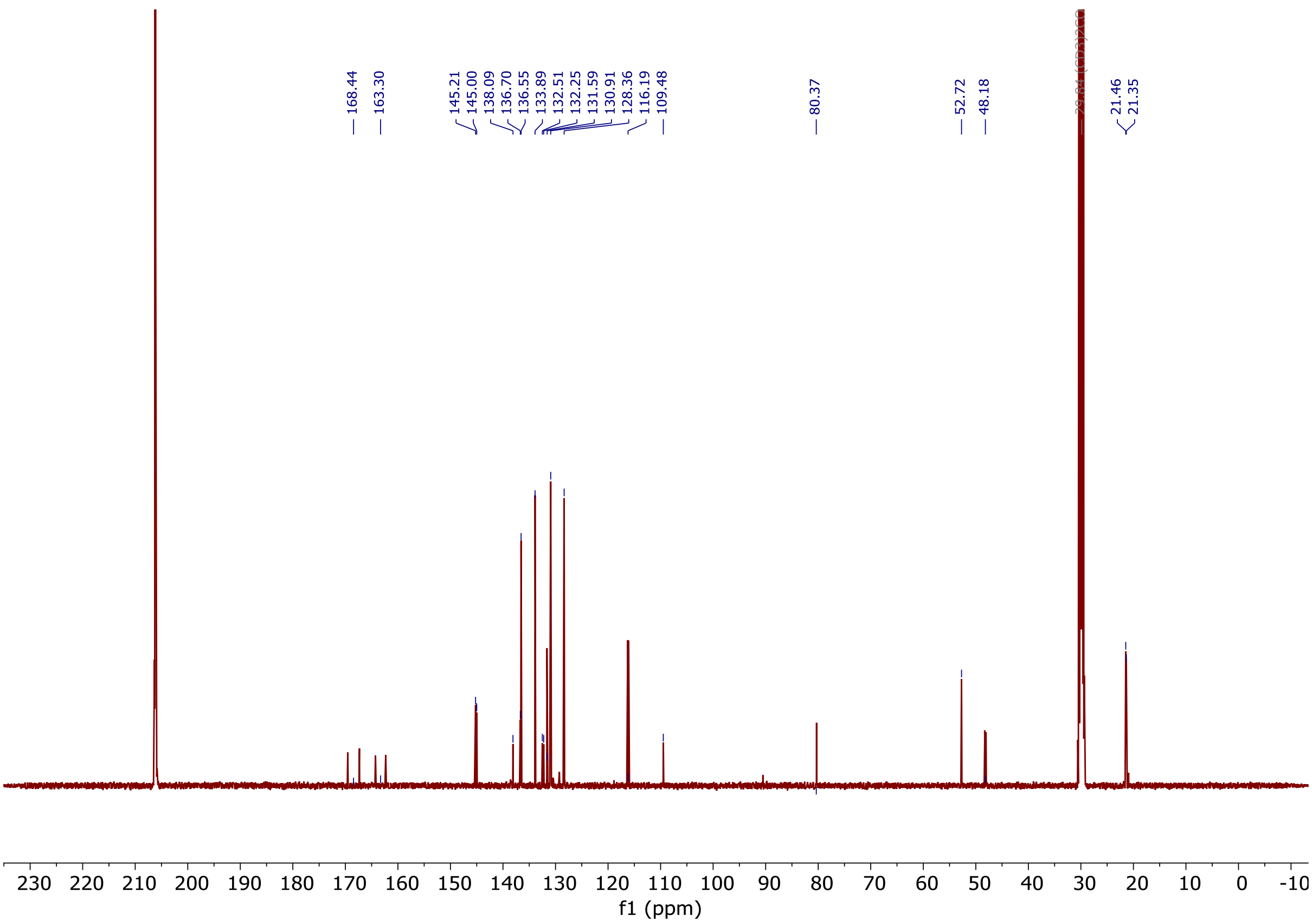

^{19}F NMR (371 MHz, Acetone-D_6)

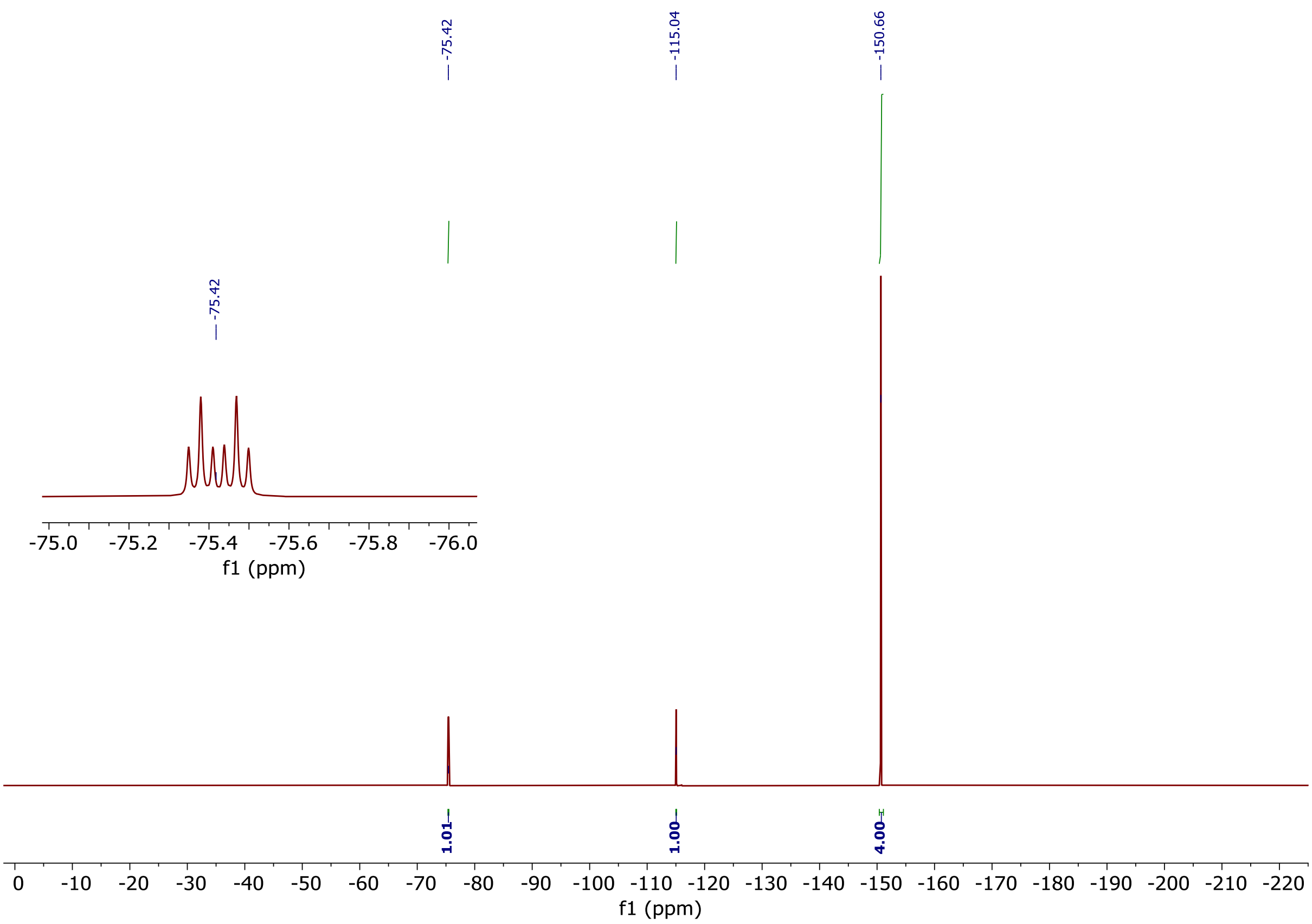

(Z)-(2-fluoro-3-((4-methyl-N-(4-(trifluoromethyl)benzyl)phenyl)sulfonamido)prop-1-en-1-yl)(p-tolyl)iodonium tetrafluoroborate **(3f)**

^{1}H NMR (600 MHz, Acetone-*D*$_6$)

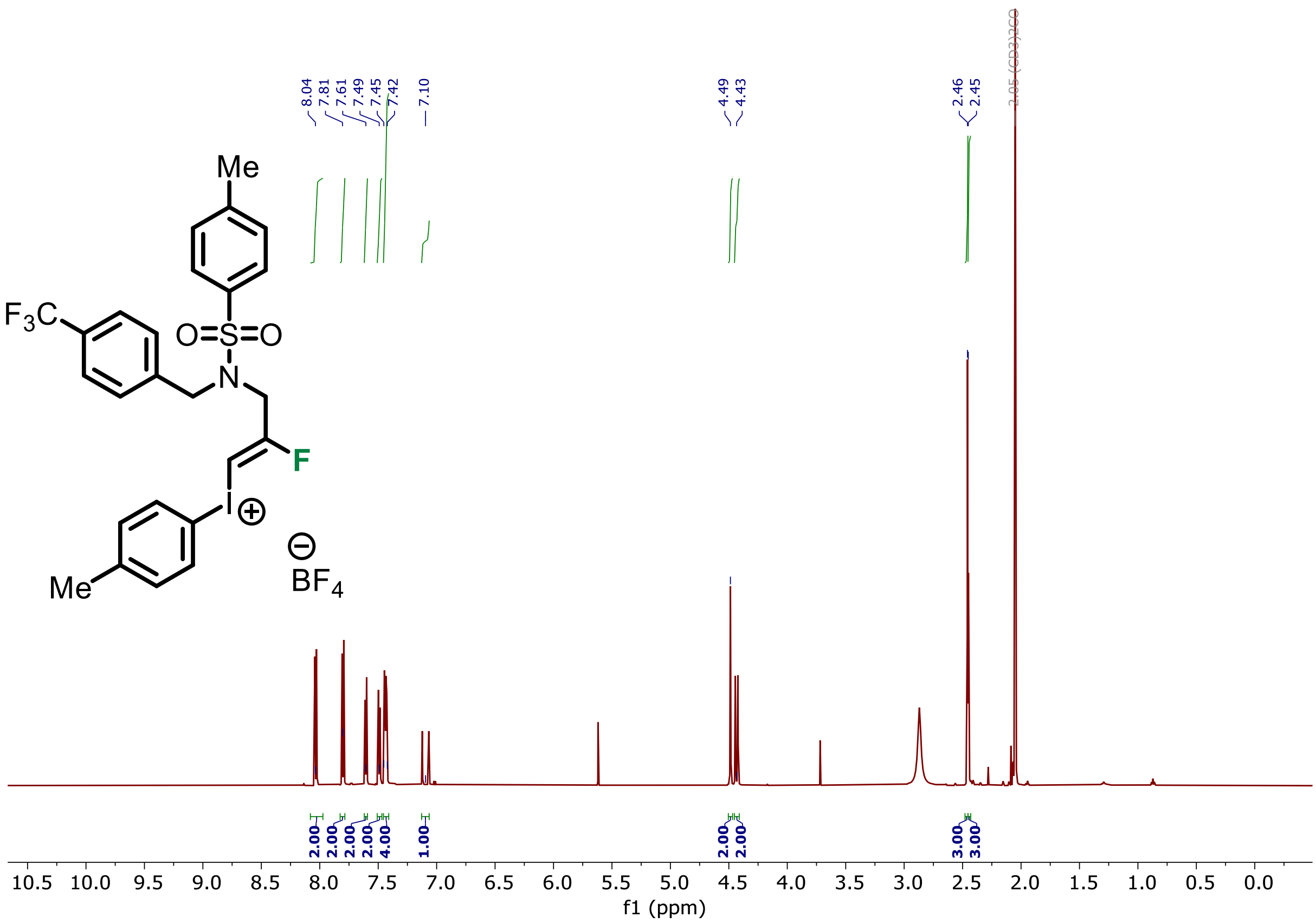

^{13}C NMR (151 MHz, Acetone-D_6)

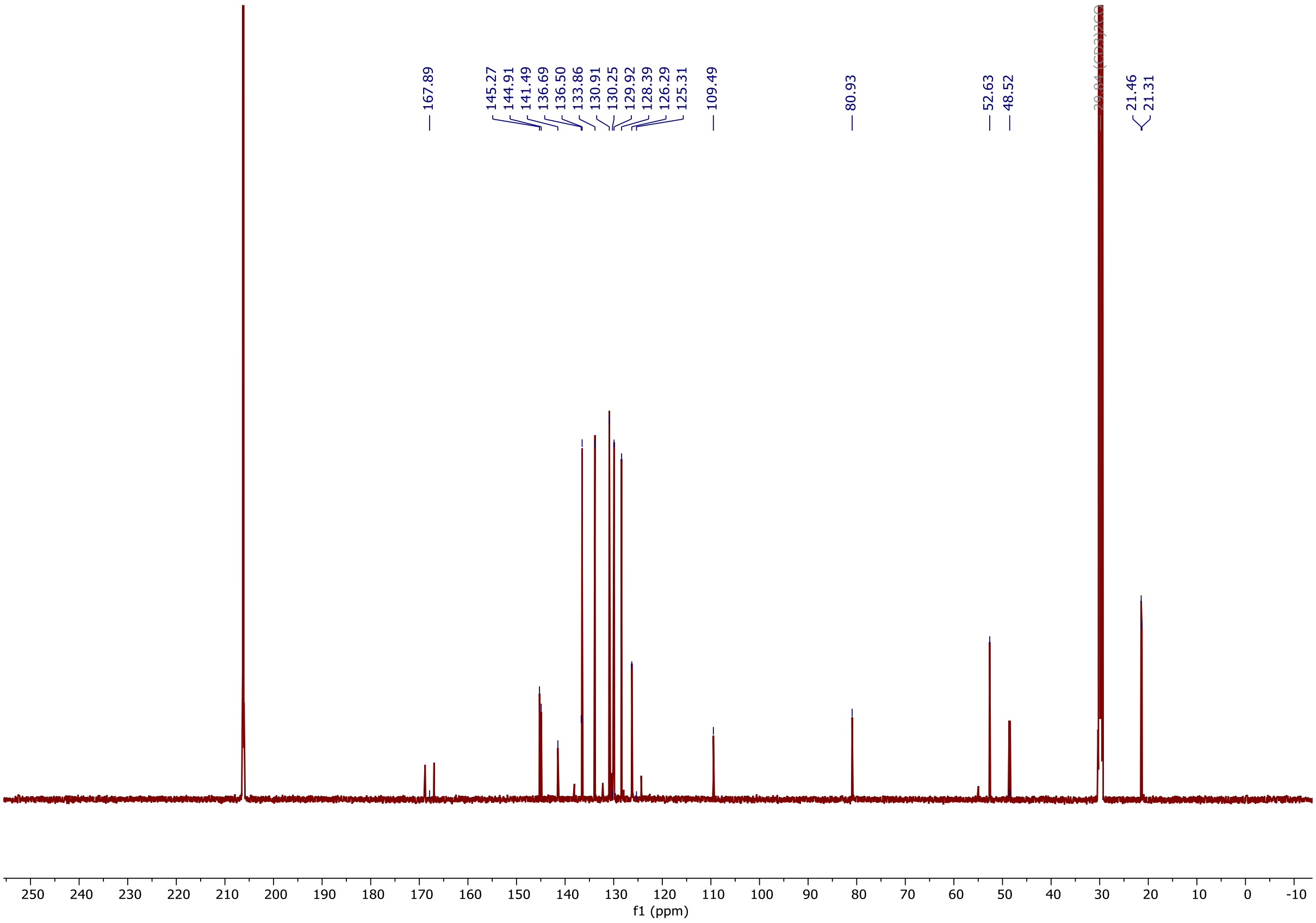

^{19}F NMR (376 MHz, Acetone-D_6)

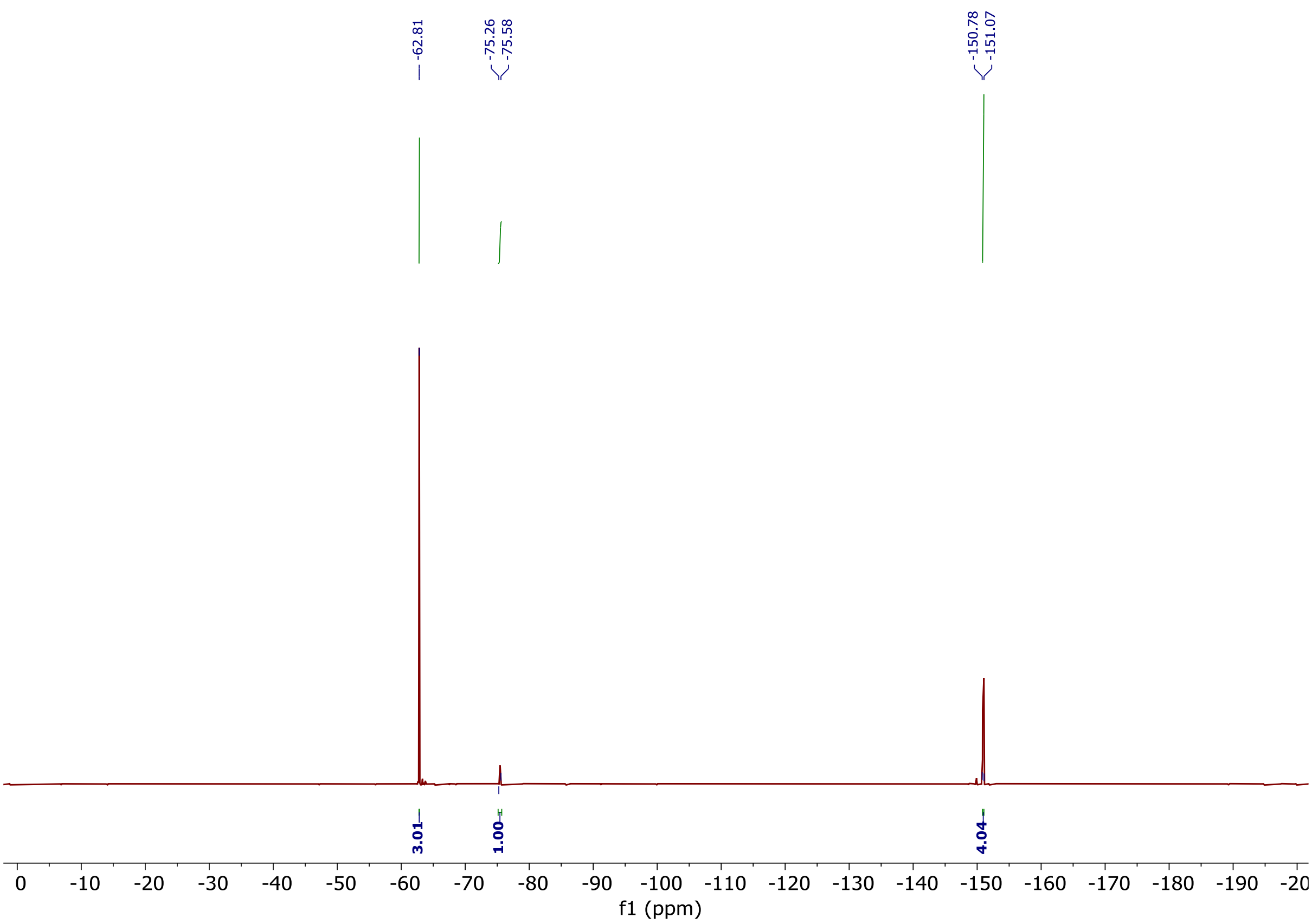

(Z)-(2-fluoro-3-((4-methyl-N-(4-nitrobenzyl)phenyl)sulfonamido)prop-1-en-1-yl)(p-tolyl)iodonium tetrafluoroborate **(3g)**
^{1}H NMR (400 MHz, Acetone-D_6)

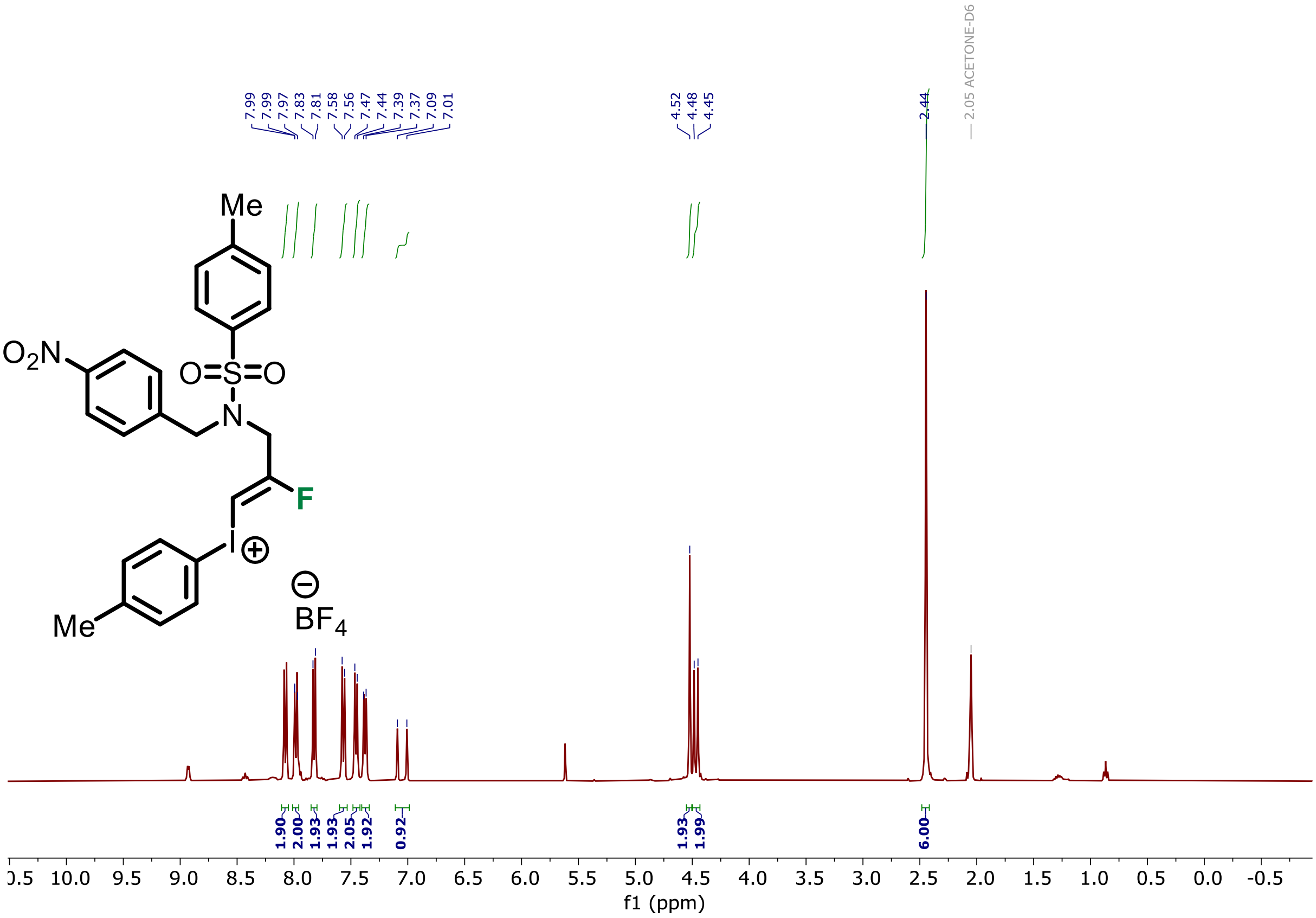

^{13}C NMR (126 MHz, Acetone-D_6)

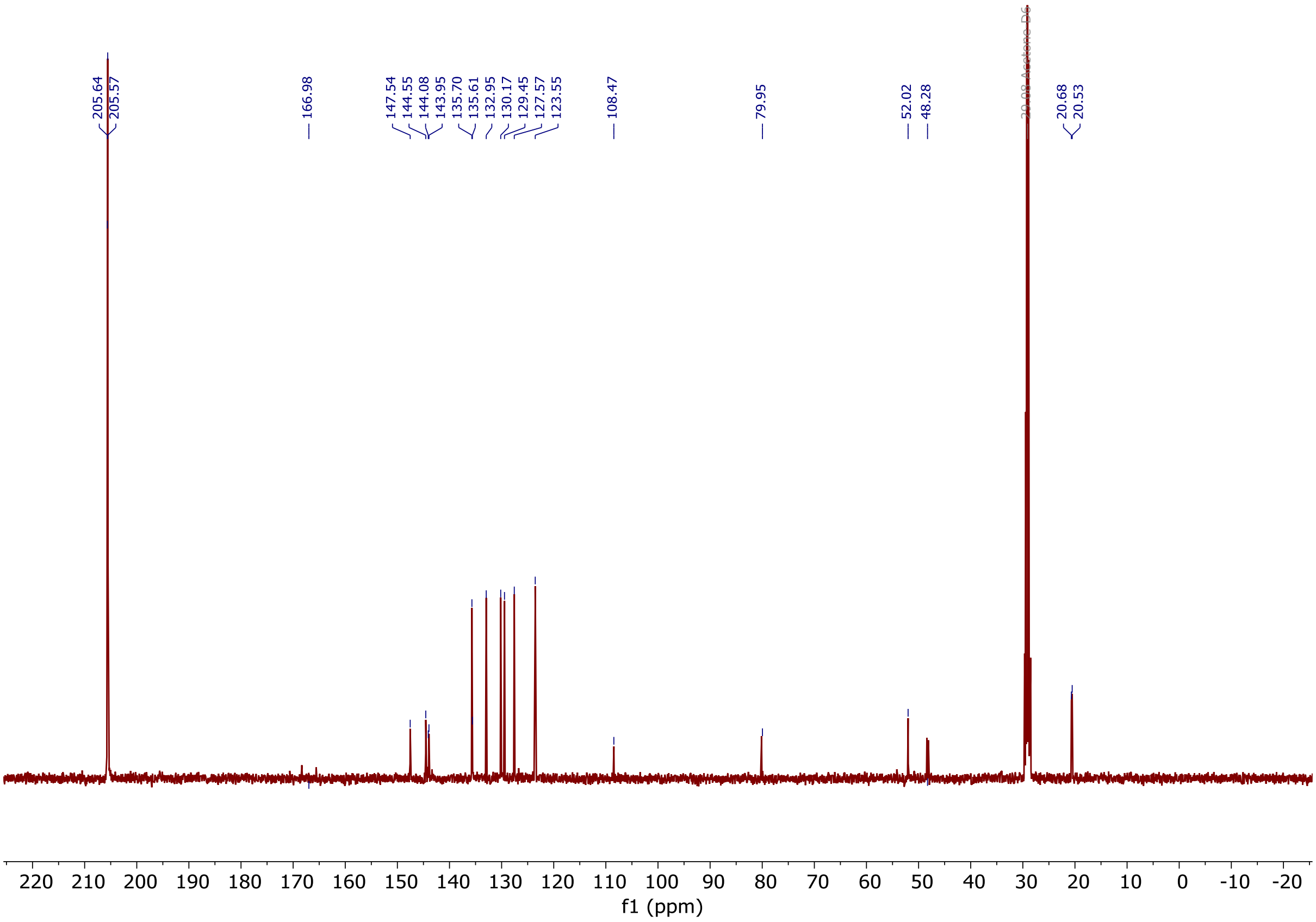

^{19}F NMR (371 MHz, Acetone-D_6)

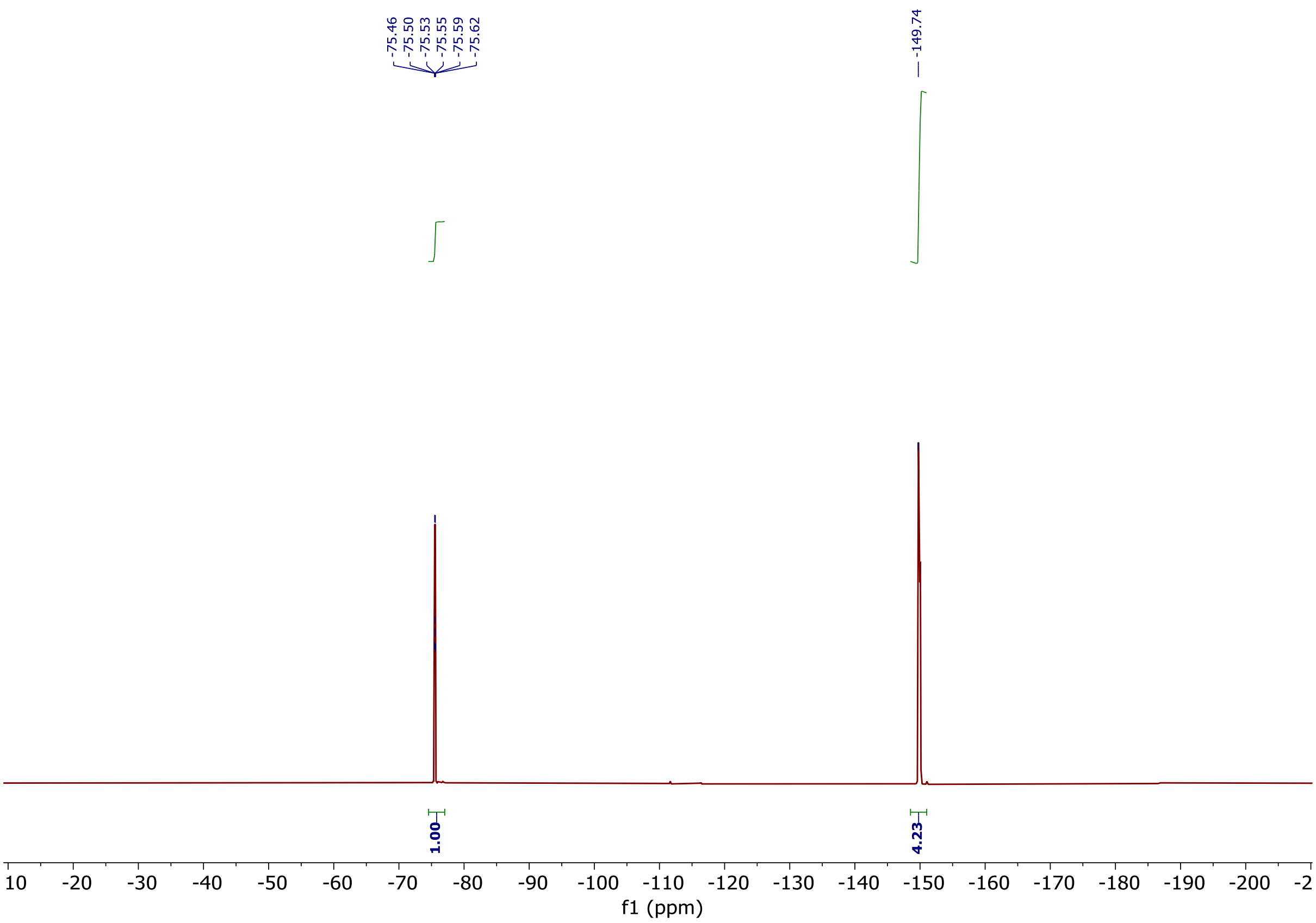

***(Z)-(3-((N-benzyl-4-methylphenyl)sulfonamido)-2-fluoro-3-methylbut-1-en-1-yl)(p-tolyl)iodonium tetrafluoroborate* (3h)**

^{1}H NMR (500 MHz, Acetone-*D*$_6$)

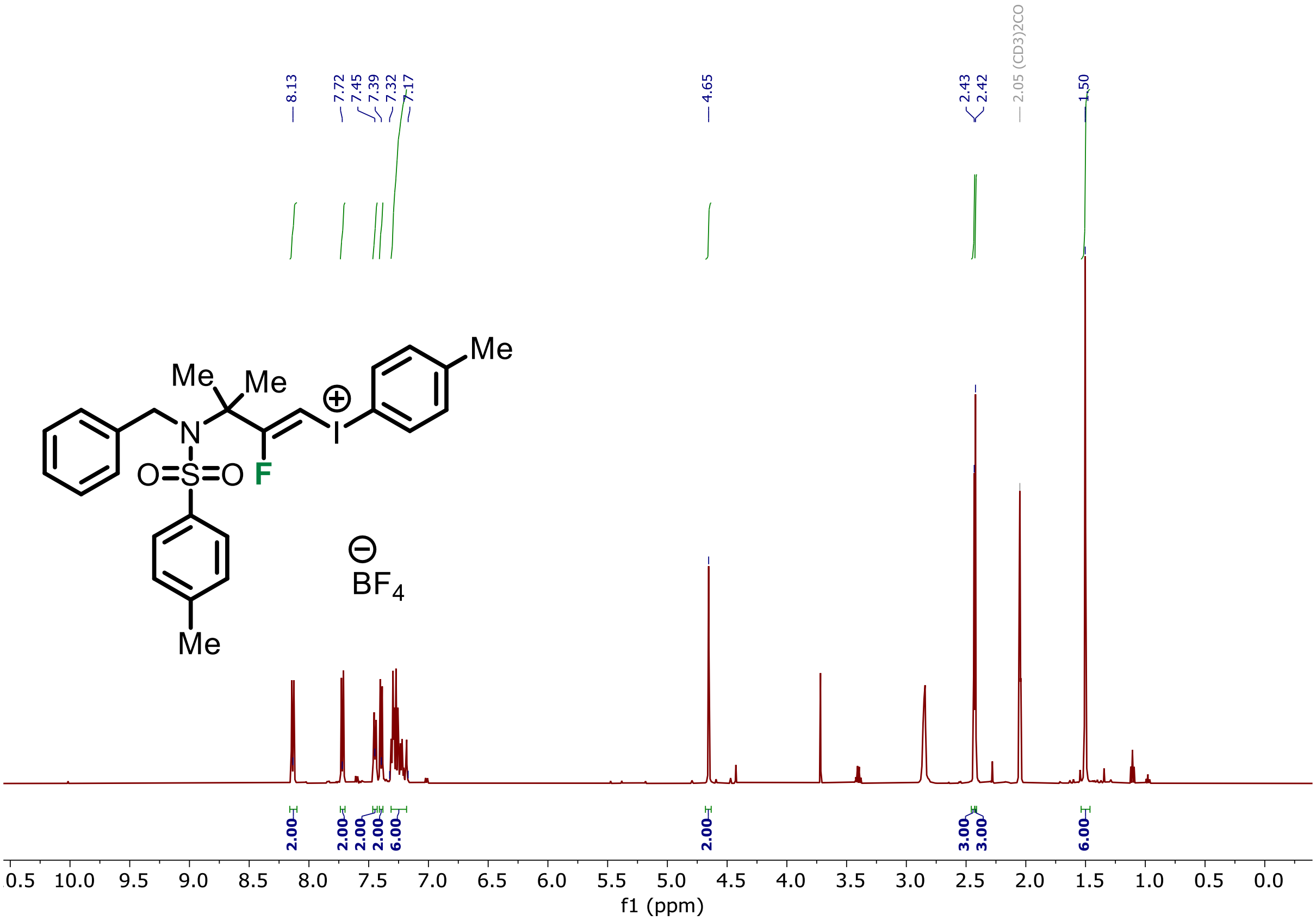

^{13}C NMR (126 MHz, Acetone-D_6)

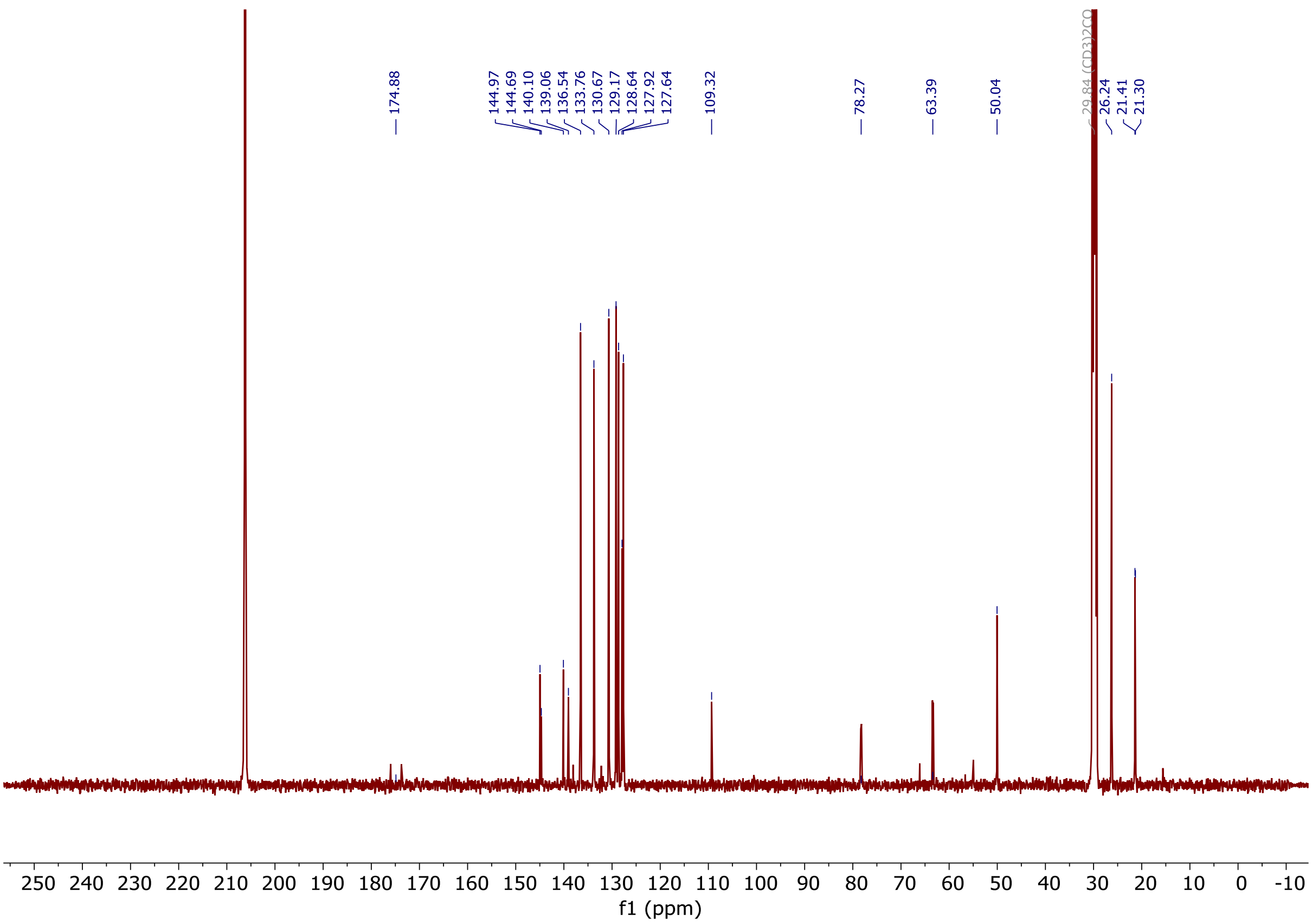

^{19}F NMR (471 MHz, Acetone)

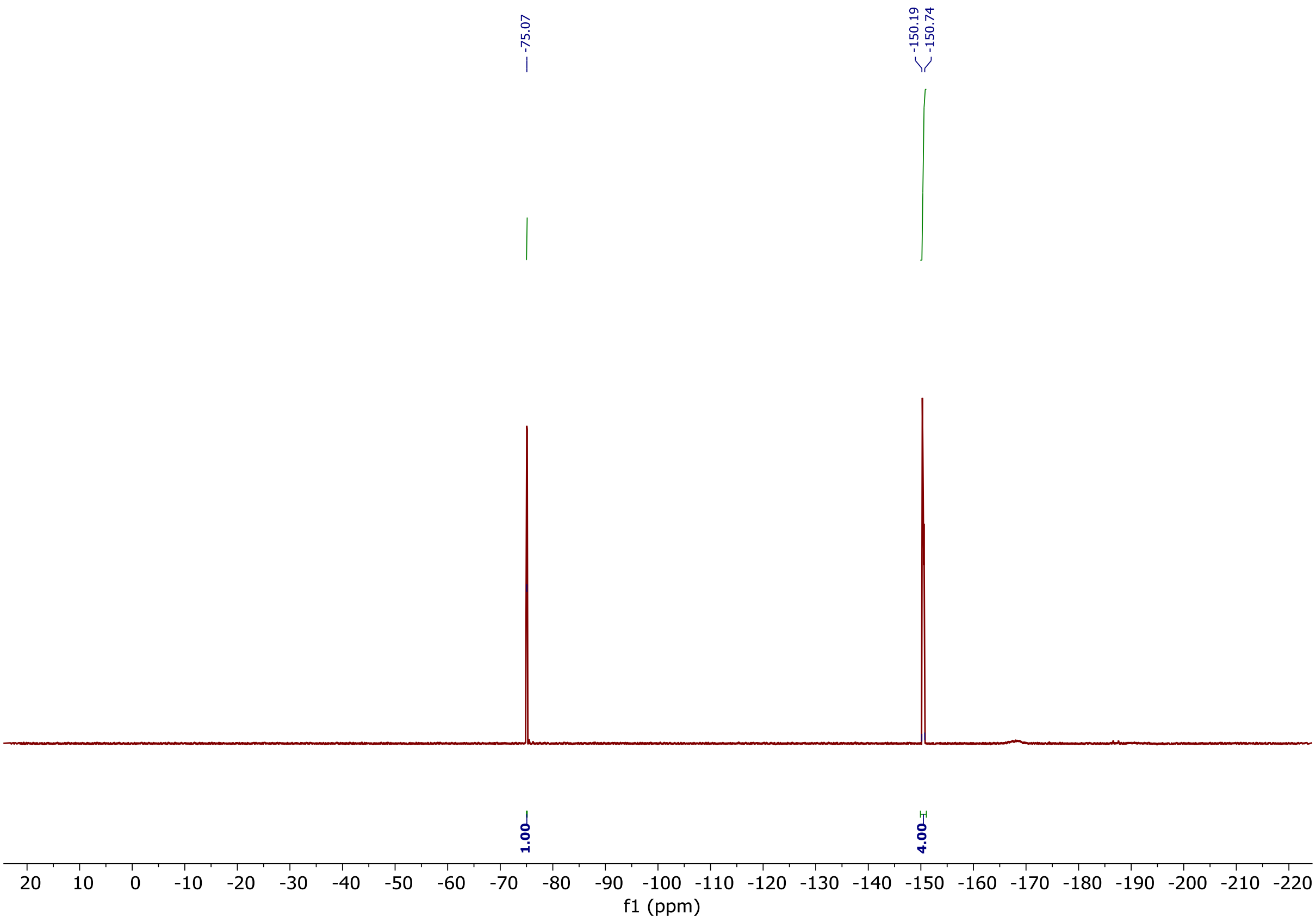

(Z)-(2-fluoro-3-((N-(4-fluorobenzyl)-4-nitrophenyl)sulfonamido)prop-1-en-1-yl)(p-tolyl)iodonium tetrafluoroborate **(3i)**

^{1}H NMR (500 MHz, Acetone-D_6)

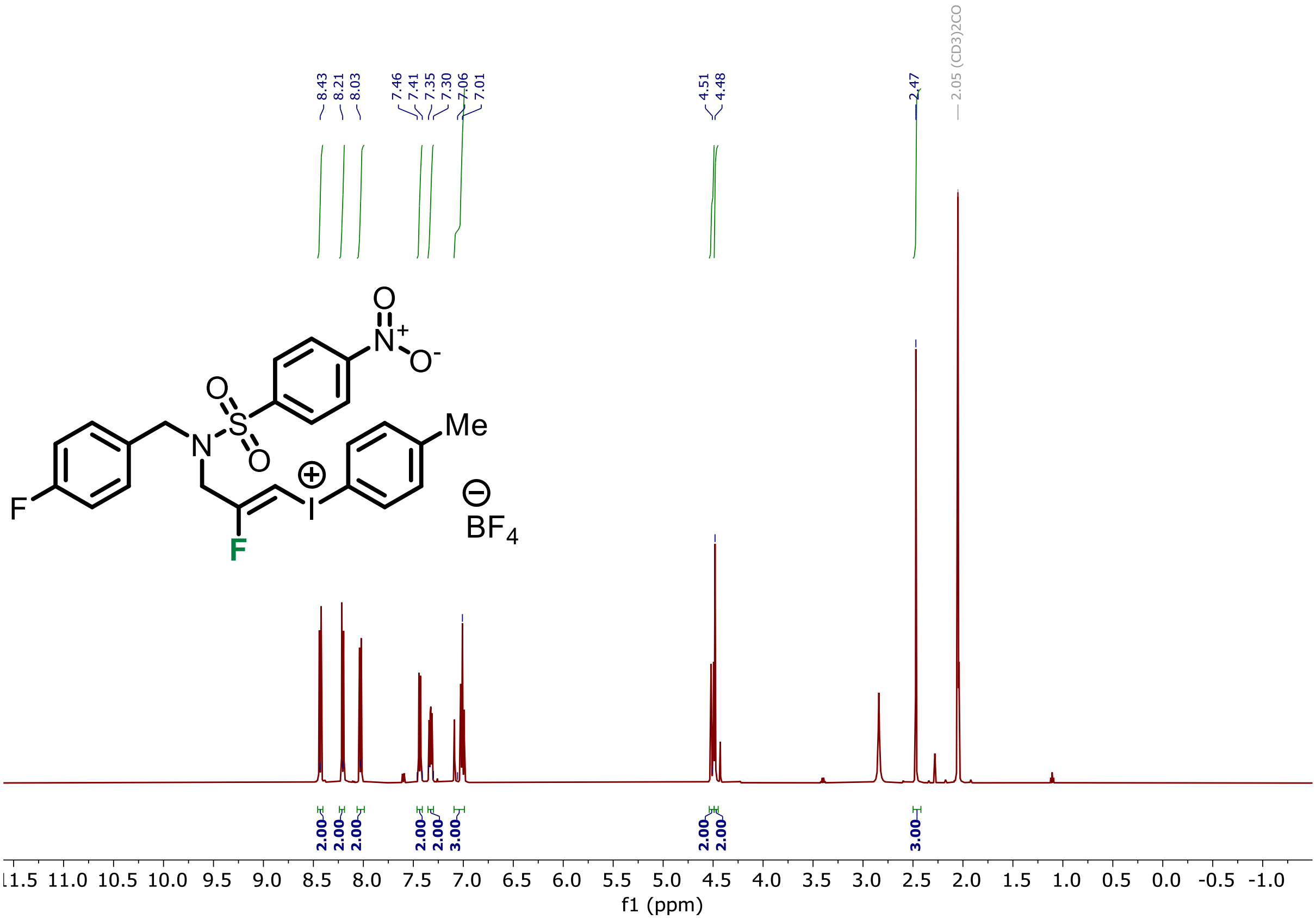

^{13}C NMR (126 MHz, Acetone-D_6)

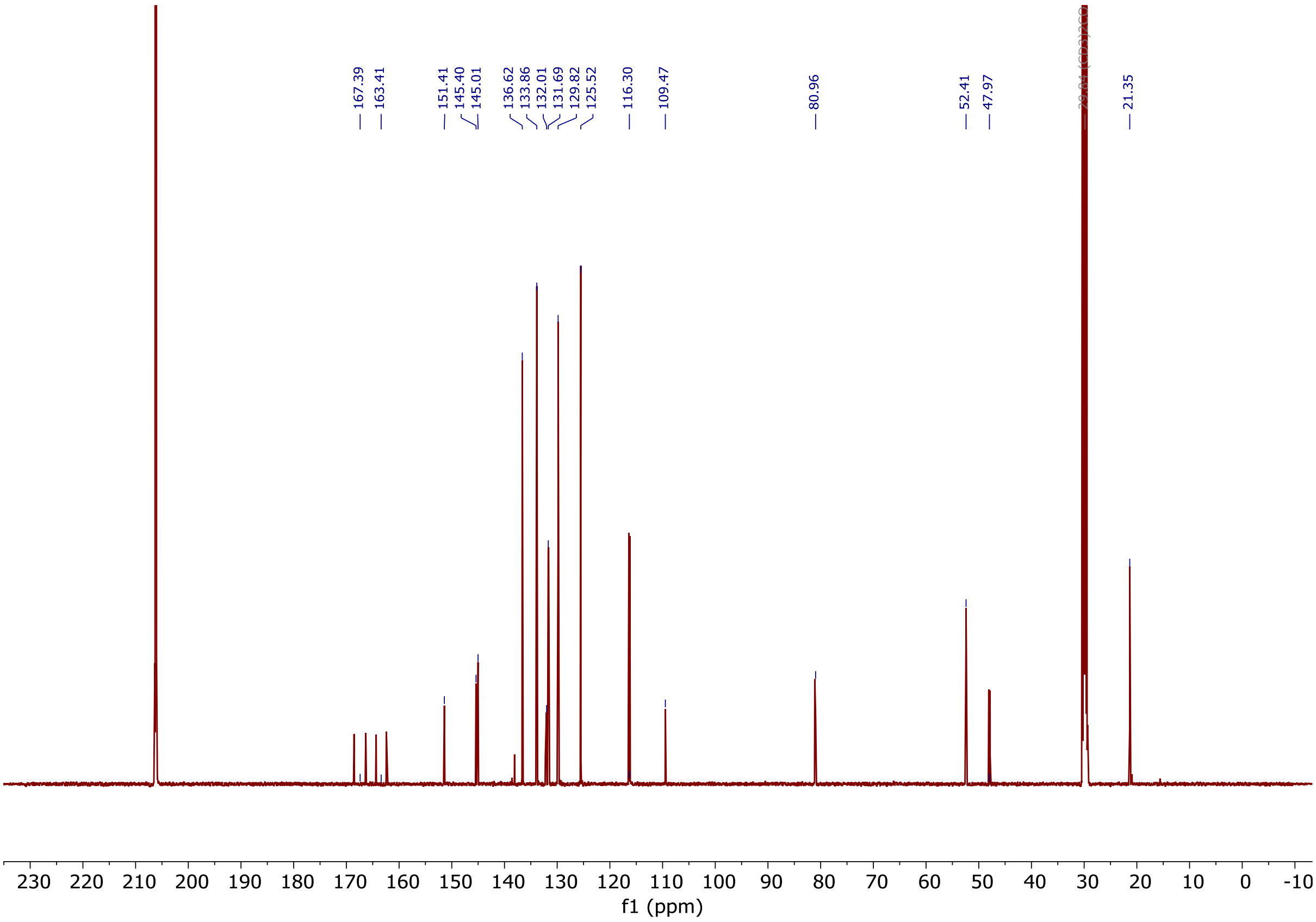

^{19}F NMR (376 MHz, Acetone-D_6)

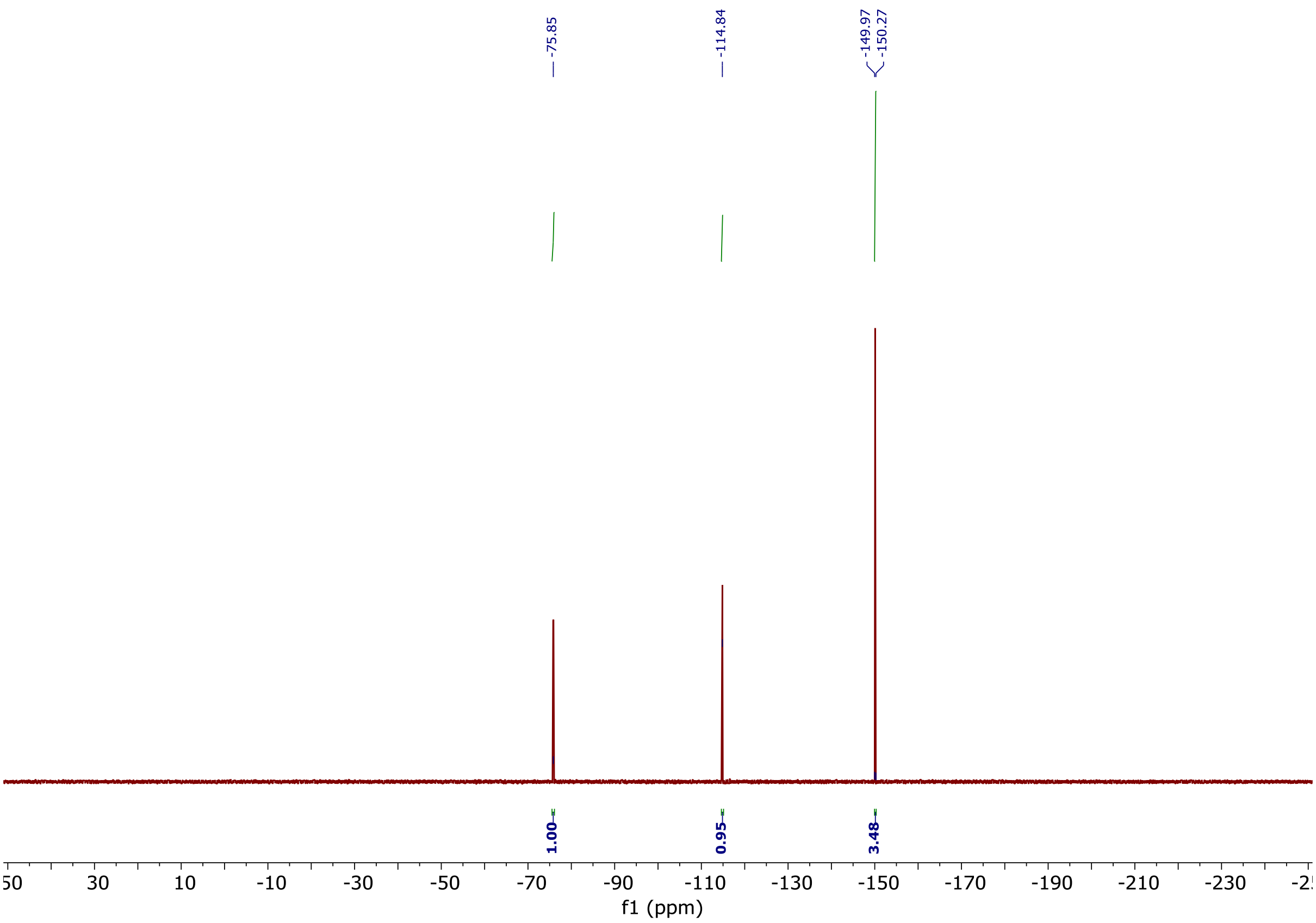

(Z)-(2-fluoro-3-((N-(4-fluorobenzyl)-4-methylphenyl)sulfonamido)but-1-en-1-yl)(p-tolyl)iodonium tetrafluoroborate **(3j)**

^{1}H NMR (400 MHz, Acetone-D_6)

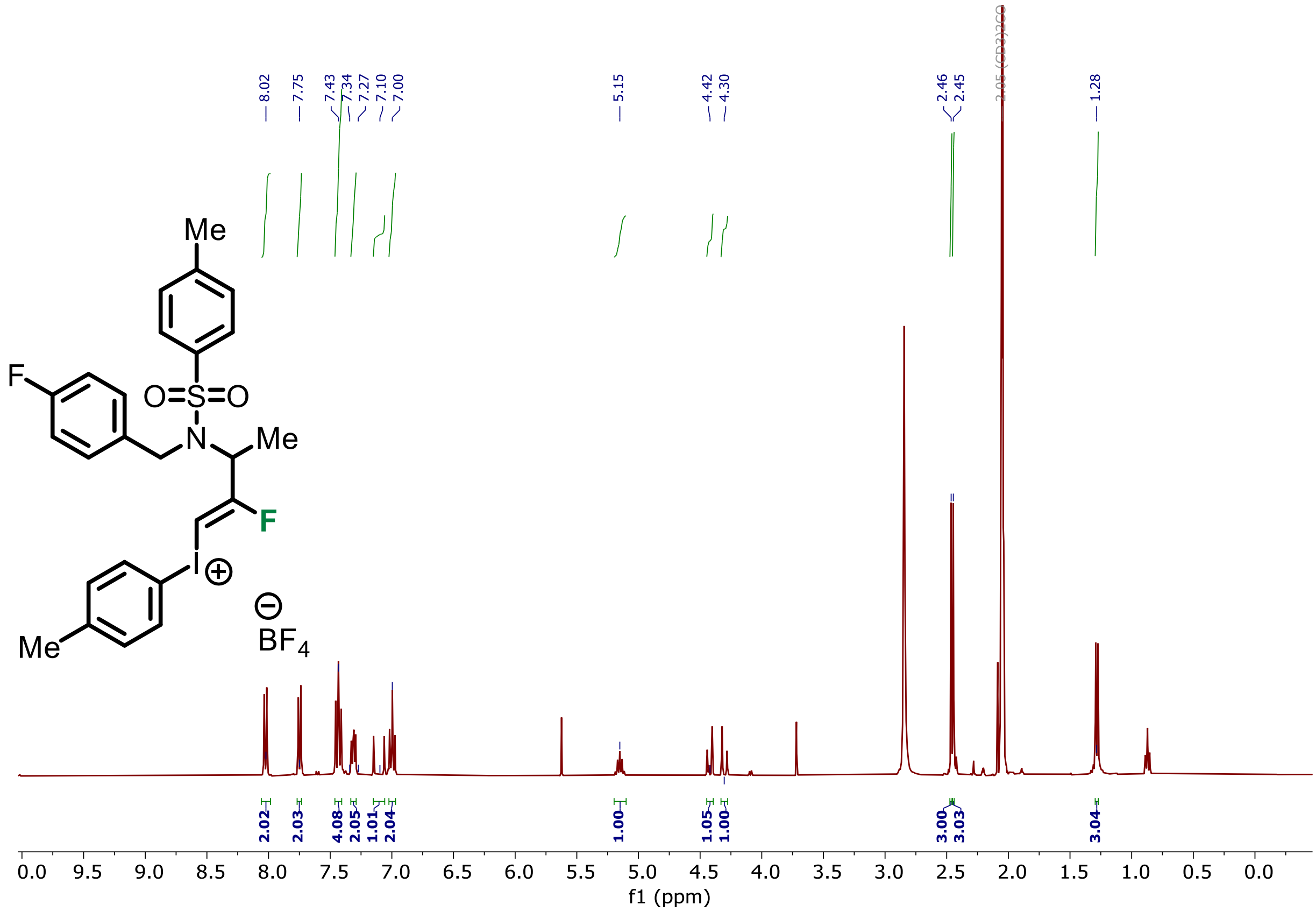

^{13}C NMR (126 MHz, Acetone-D_6)

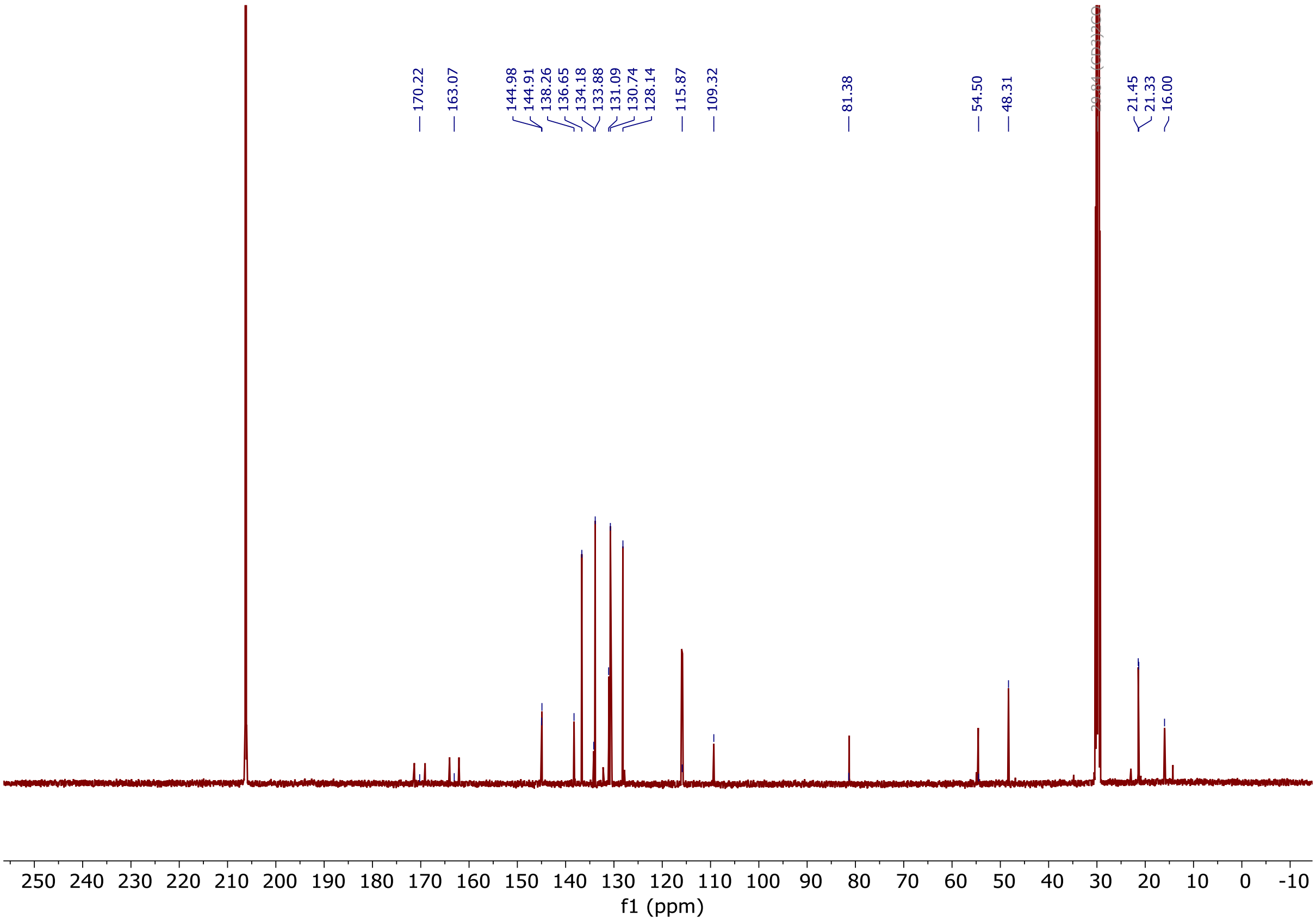

^{19}F NMR (376 MHz, Acetone-D_6)

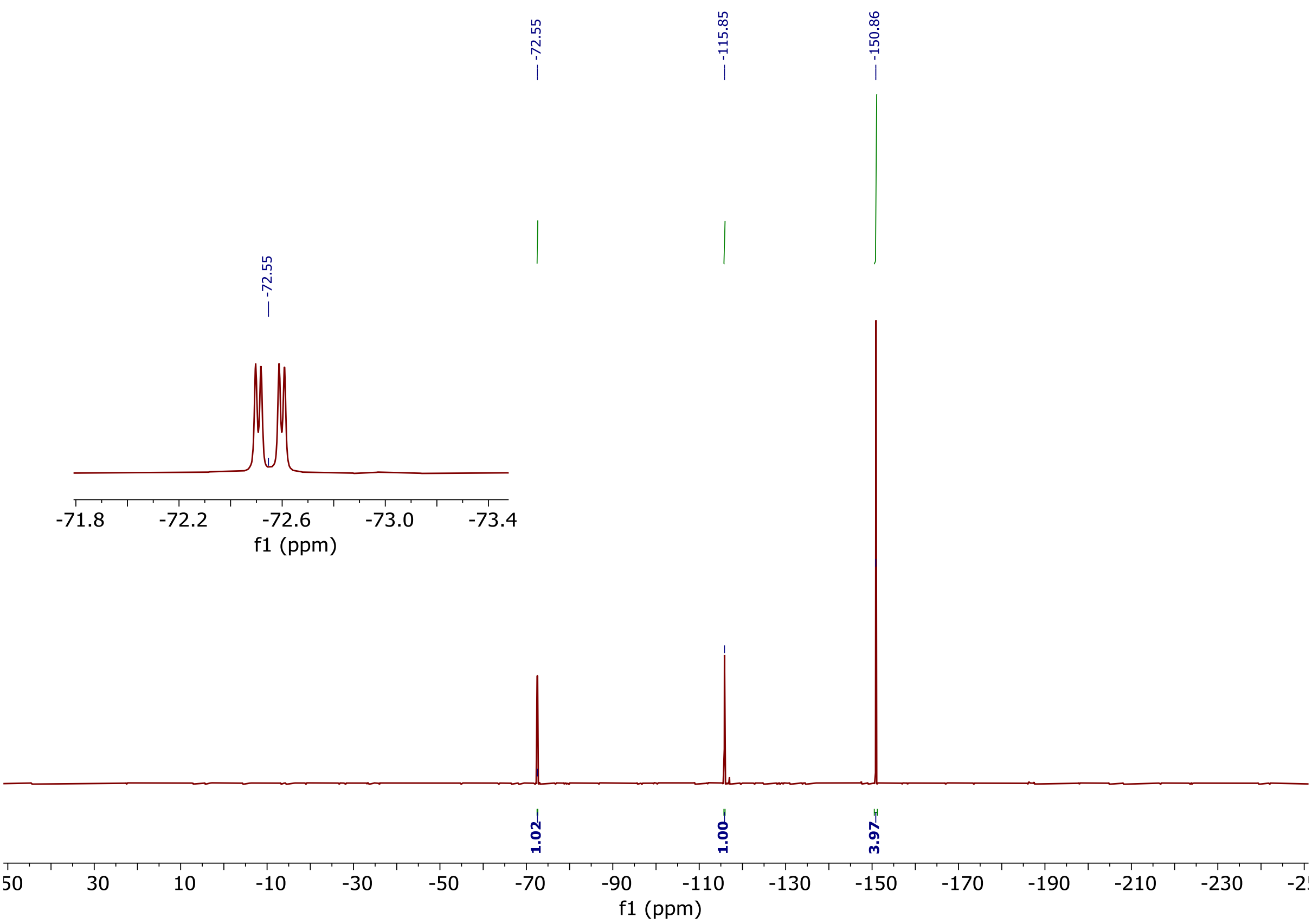

***(Z)-(2-fluoro-3-(N-(4-fluorobenzyl)methylsulfonamido)but-1-en-1-yl)(p-tolyl)iodonium tetrafluoroborate* (3k)**

^{1}H NMR (500 MHz, Acetone-D_6)

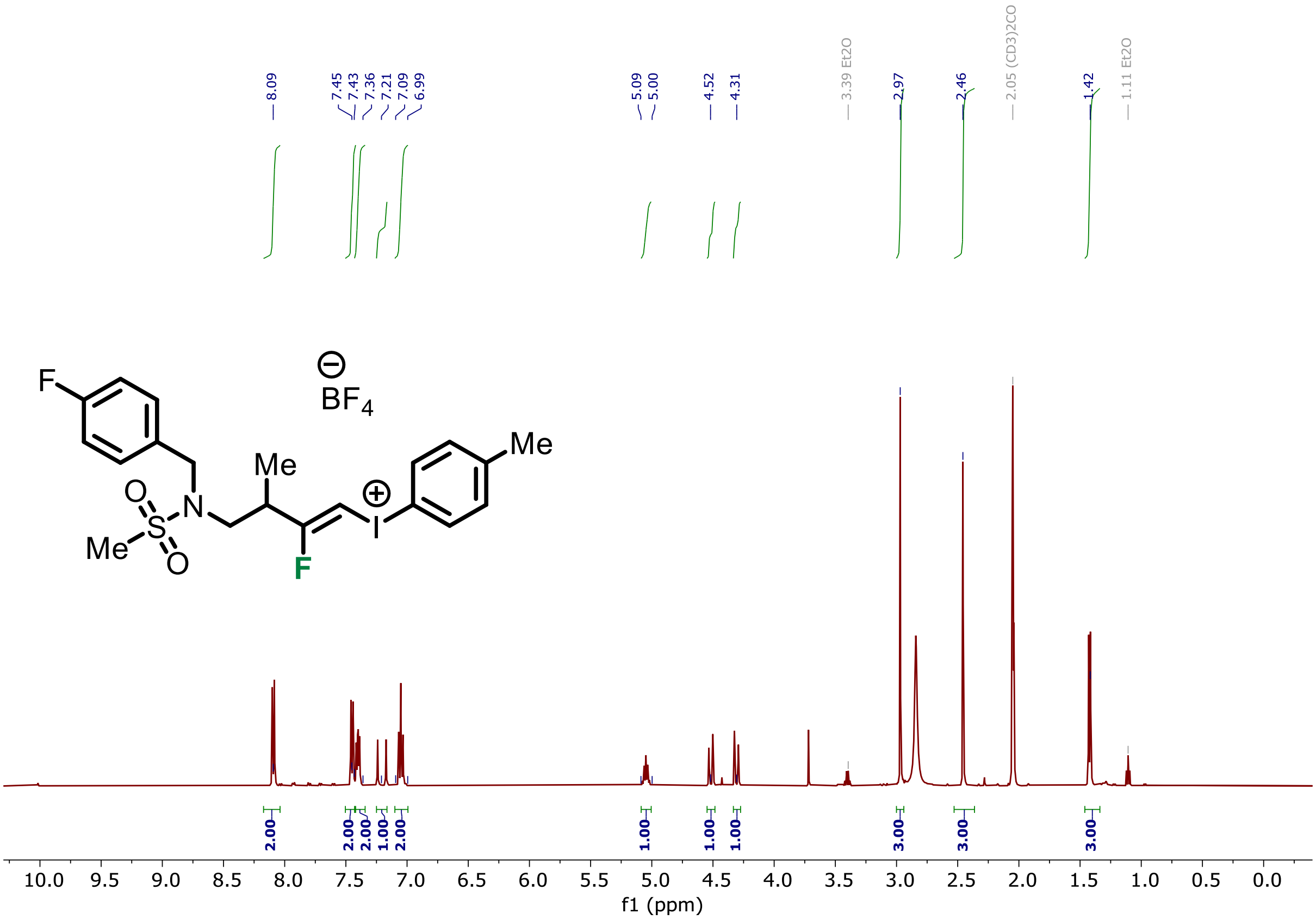

^{13}C NMR (126 MHz, Acetone-D_6)

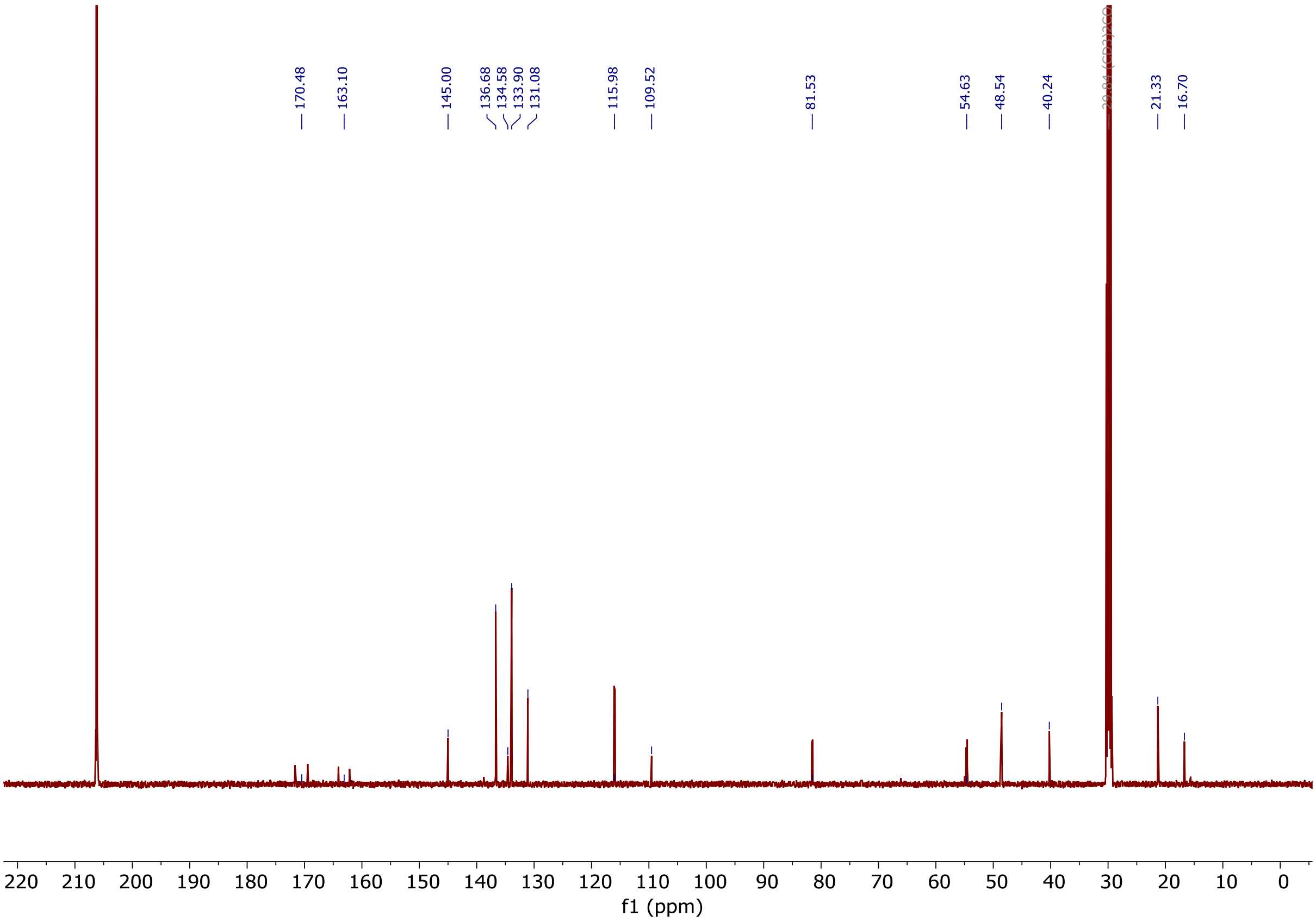

^{19}F NMR (471 MHz, Acetone-D_6)

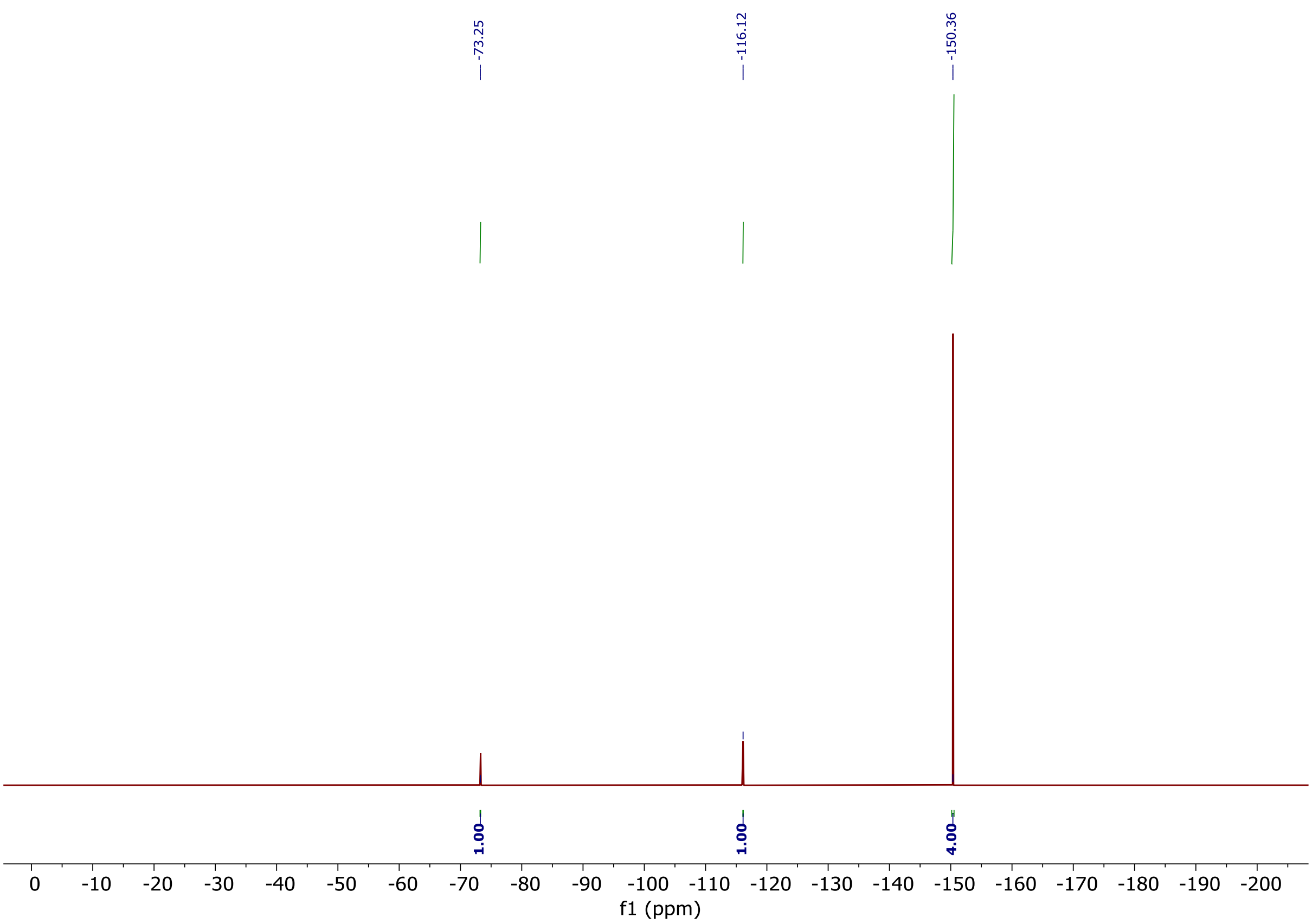

***(Z)-(3-((N-(4-bromobenzyl)-4-methylphenyl)sulfonamido)-2-fluorobut-1-en-1-yl)(p-tolyl)iodonium tetrafluoroborate* (3l)**

^{1}H NMR (500 MHz, Acetone-D_6)

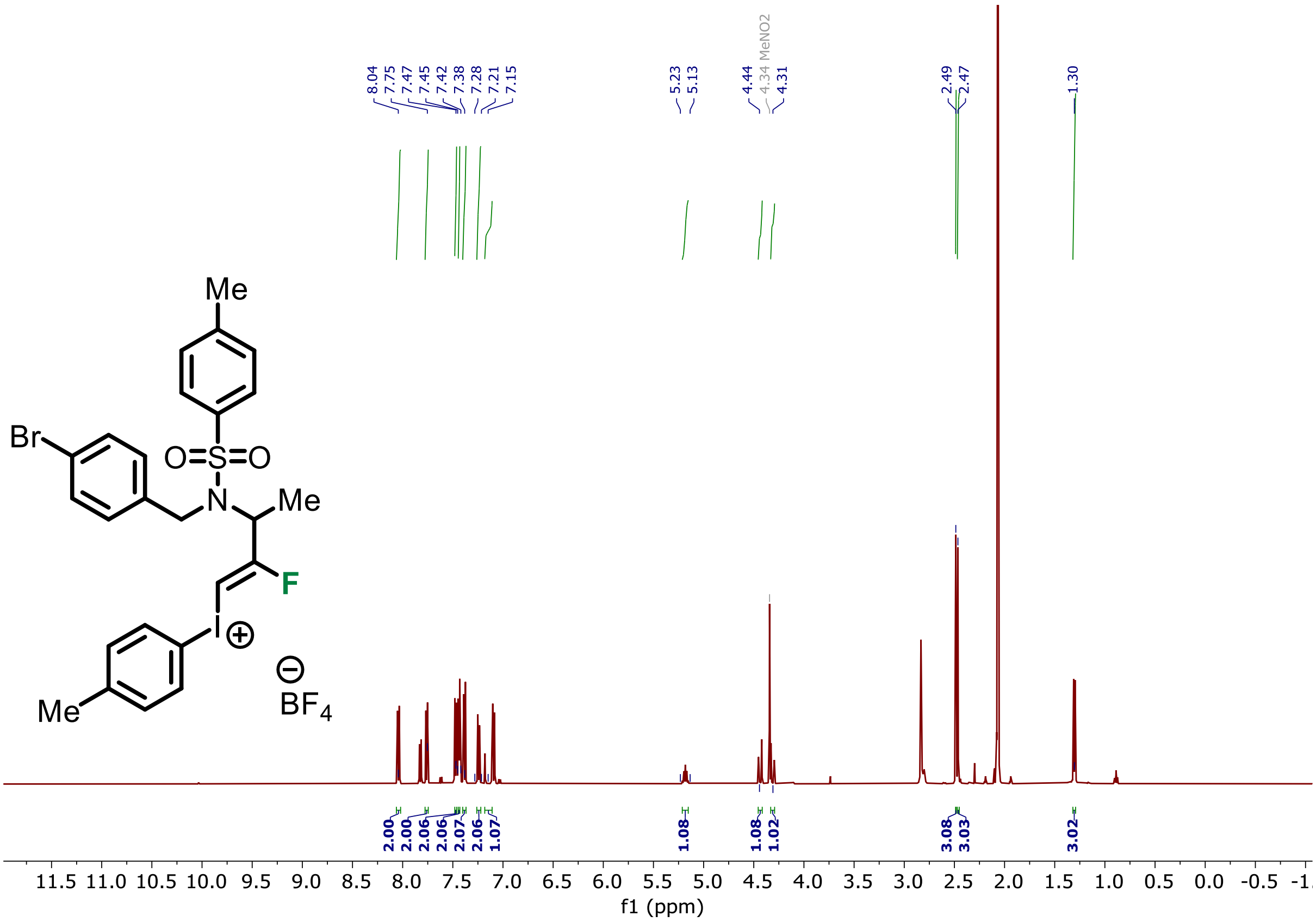

^{13}C NMR (126 MHz, Acetone-D_6)

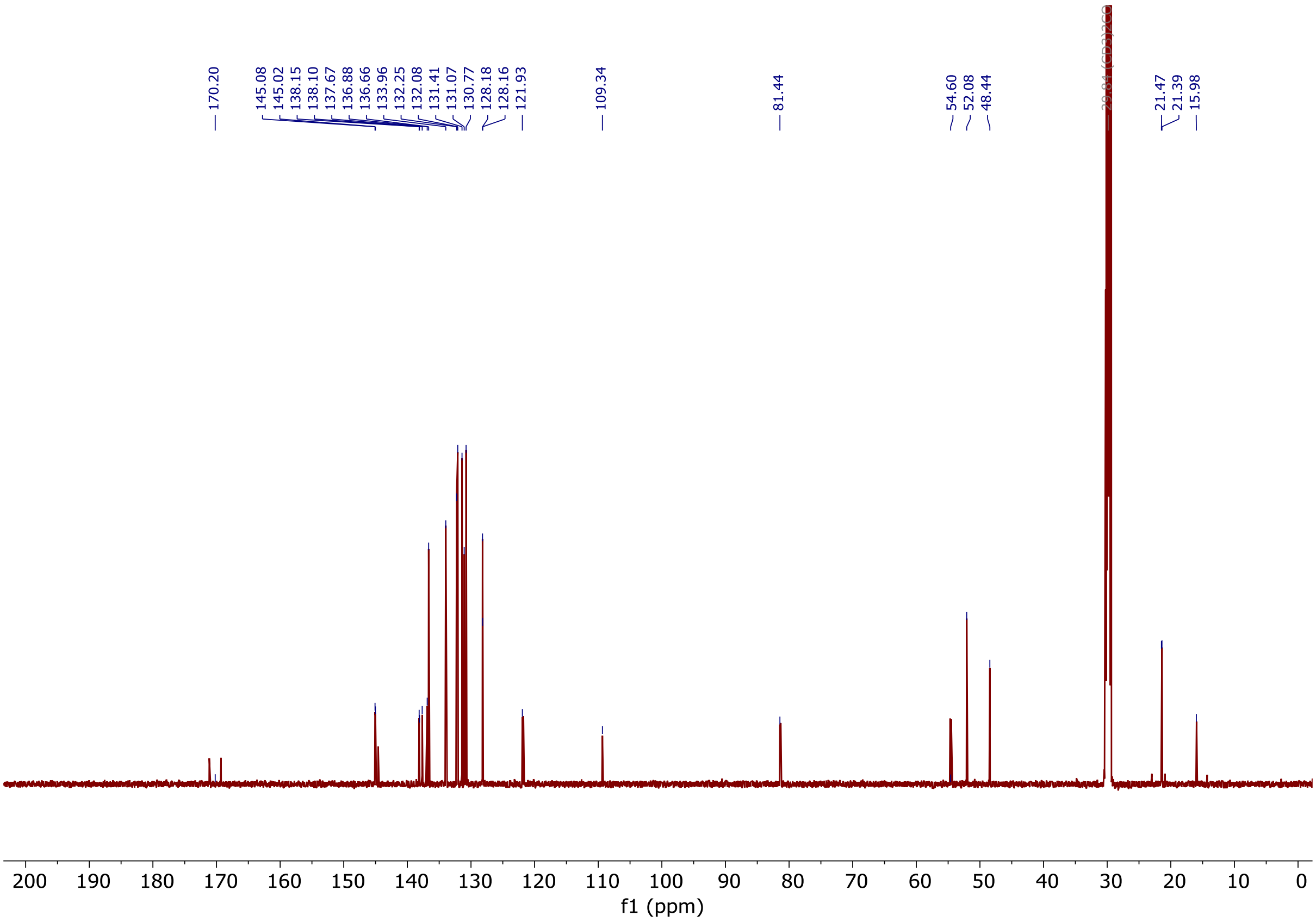

^{19}F NMR (471 MHz, Acetone-D_6)

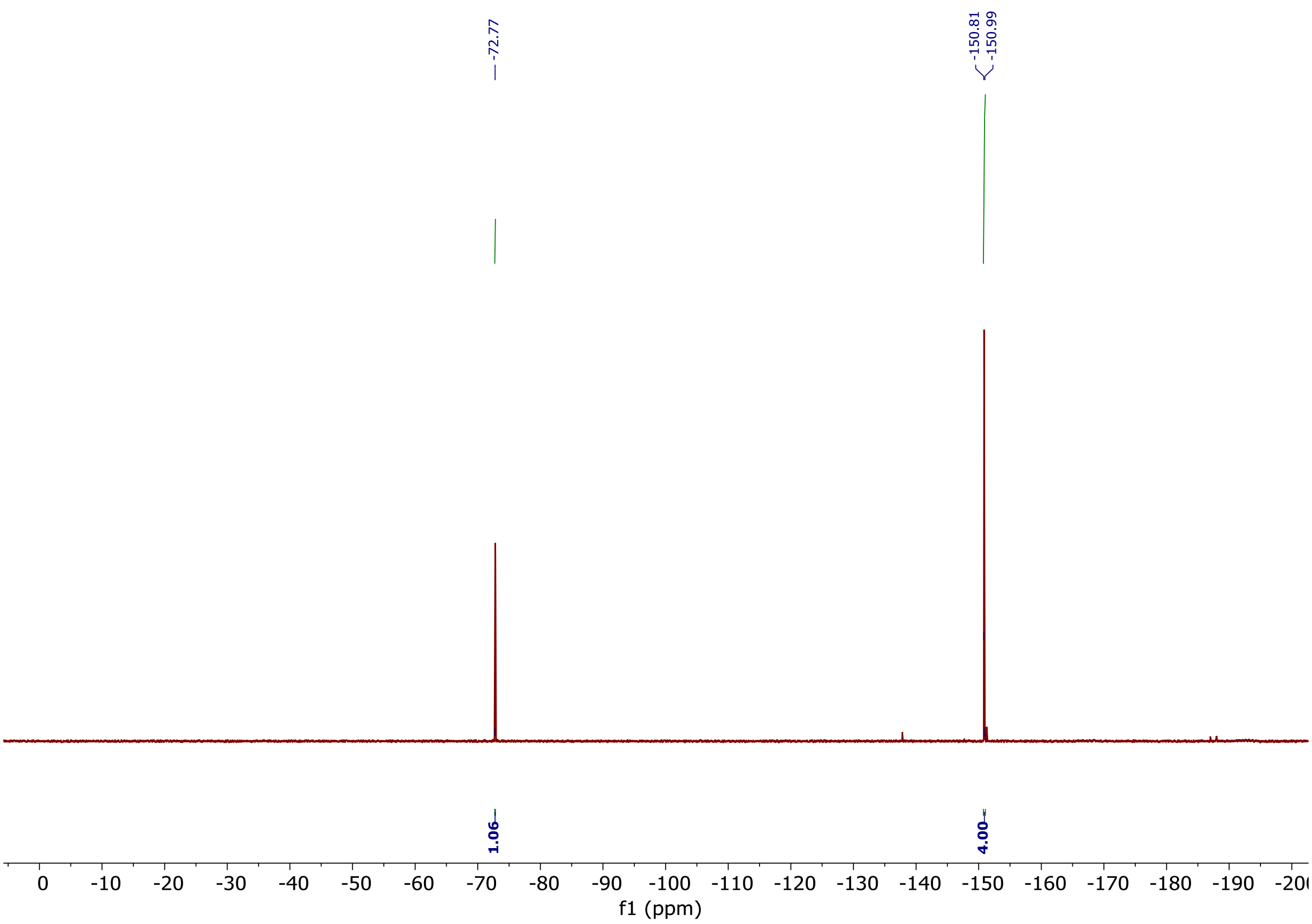

***(Z)-(2-fluoro-3-((N-(4-fluorobenzyl)-4-methylphenyl)sulfonamido)-3-phenylprop-1-en-1-yl)(p-tolyl)iodonium tetrafluoroborate* (3n)**

^{1}H NMR (500 MHz, Acetone-D_6)

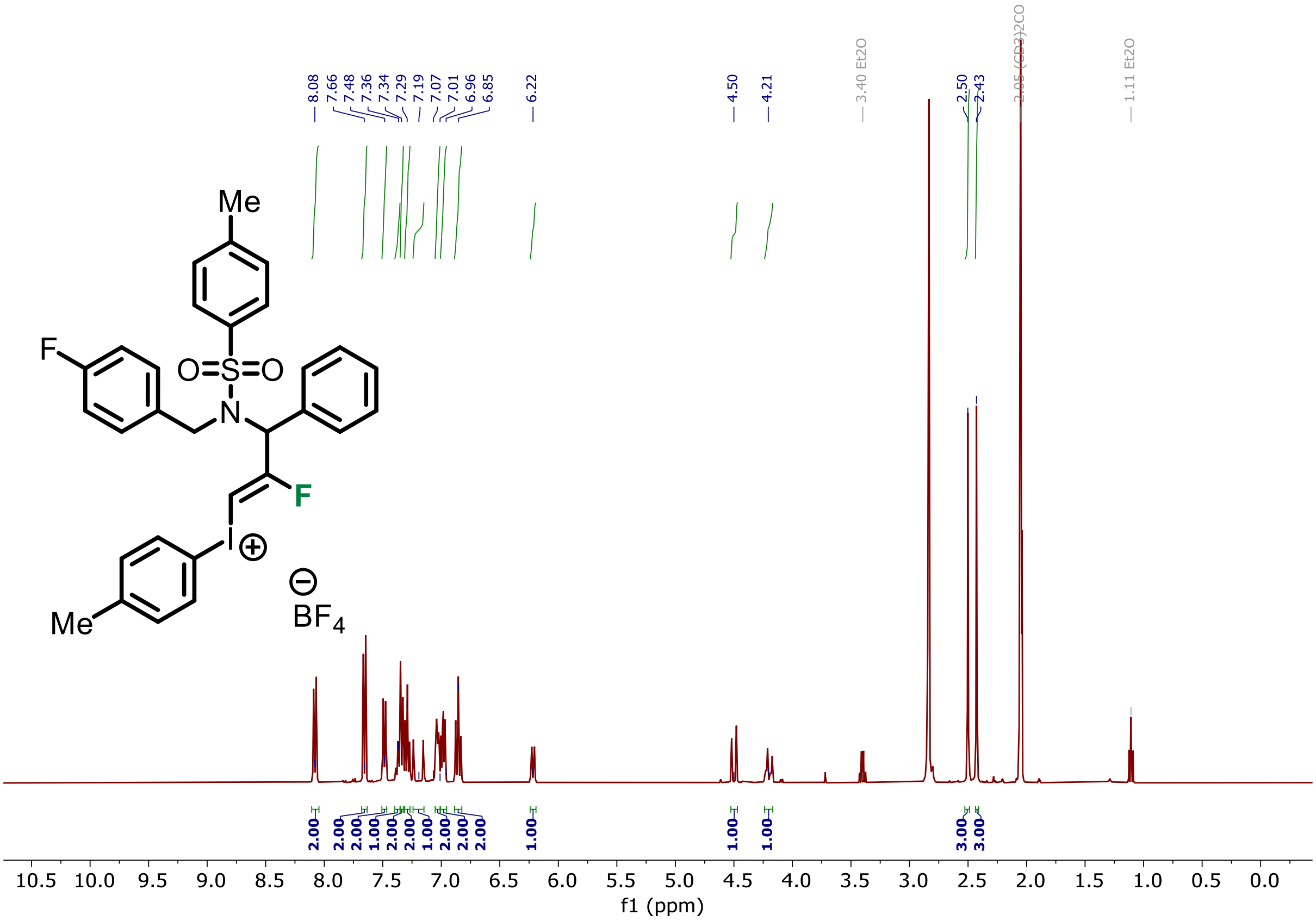

^{13}C NMR (126 MHz, Acetone-D_6)

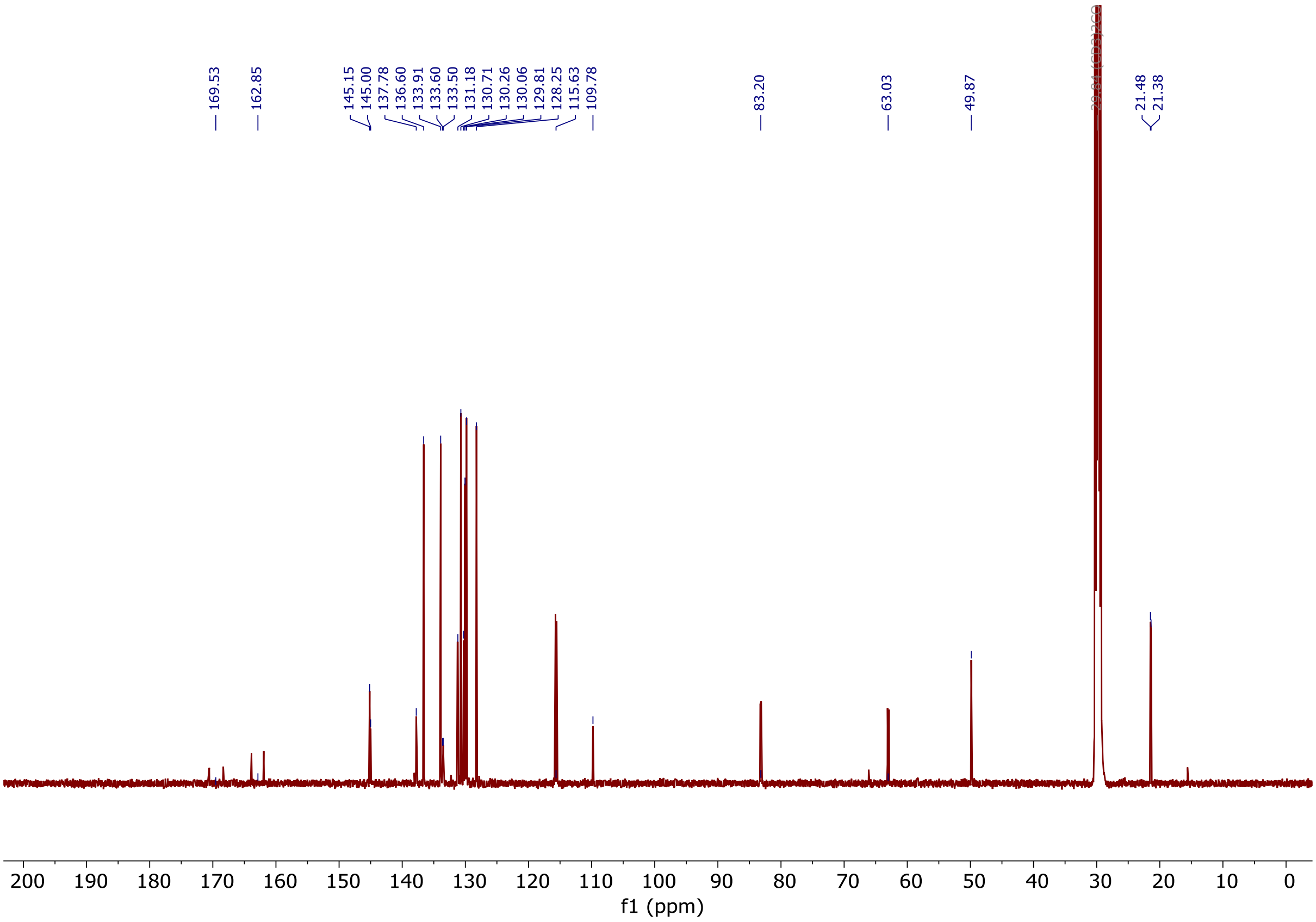

^{19}F NMR (471 MHz, Acetone-D_6)

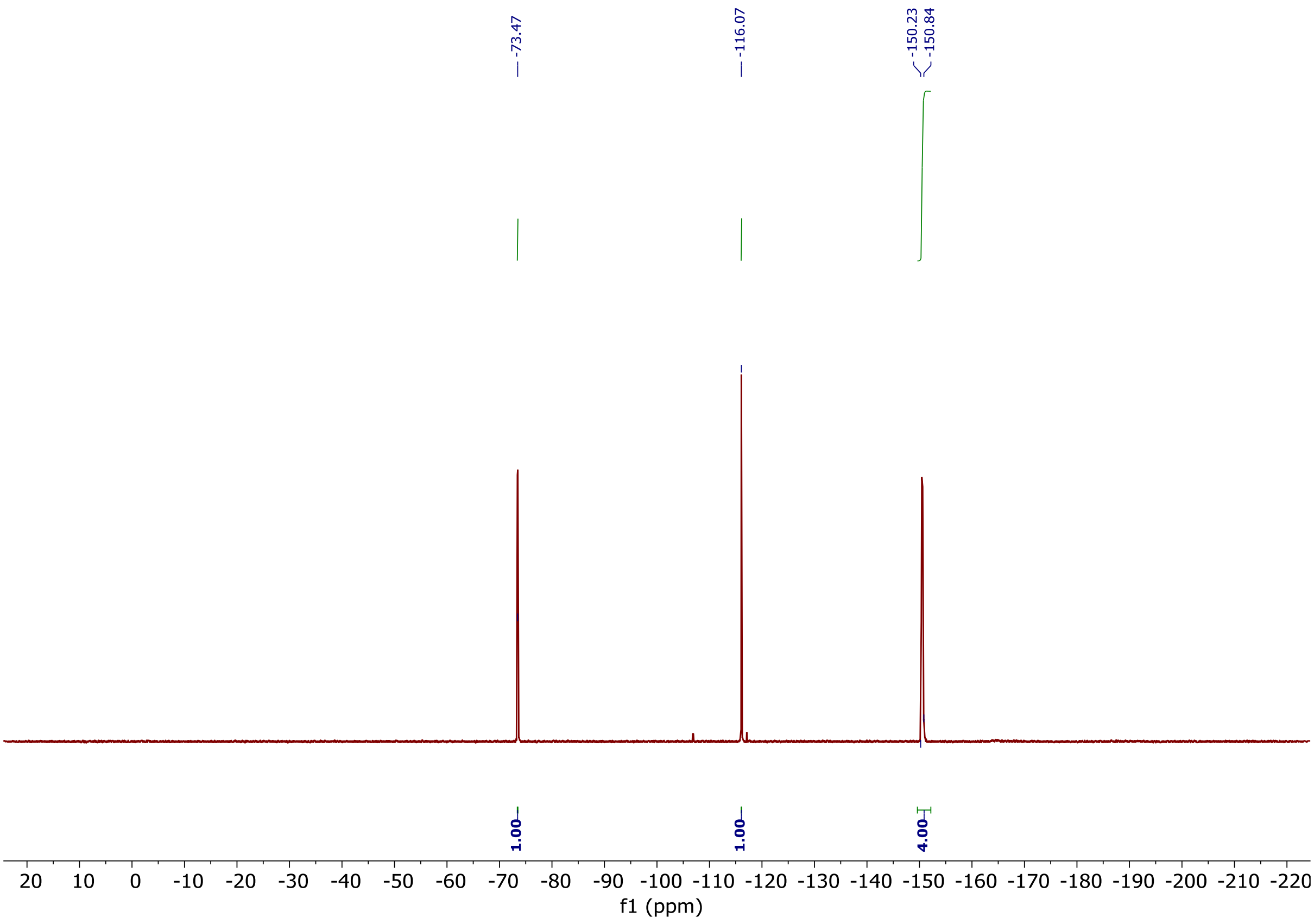

***(Z)-(3-((N-benzhydryl-4-methylphenyl)sulfonamido)-2-fluoroprop-1-en-1-yl)(p-tolyl)iodonium tetrafluoroborate* (3o)**

^{1}H NMR (500 MHz, Acetone-D_6)

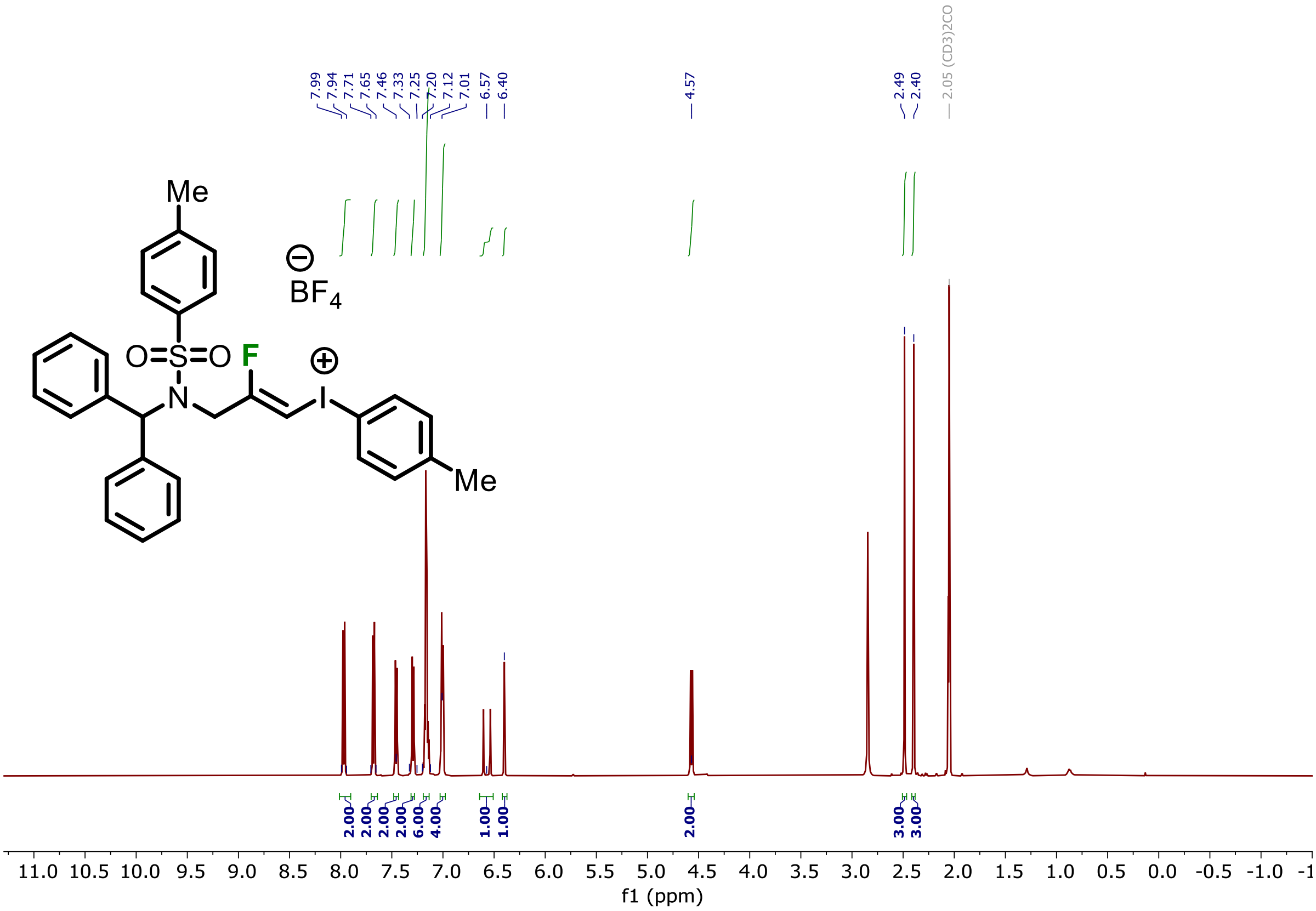

^{13}C NMR (126 MHz, Acetone-D_6)

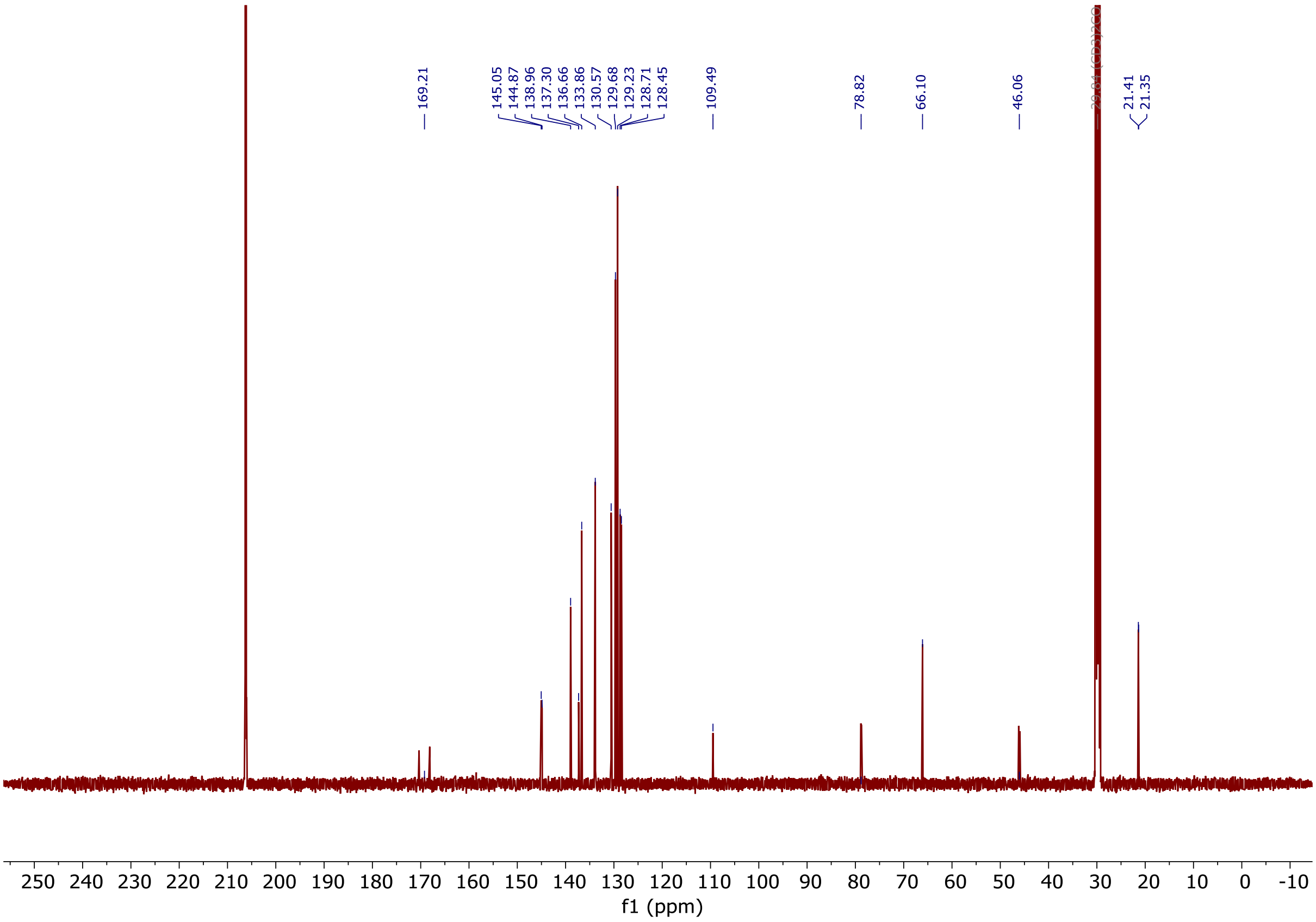

^{19}F NMR (376 MHz, Acetone-D_6)

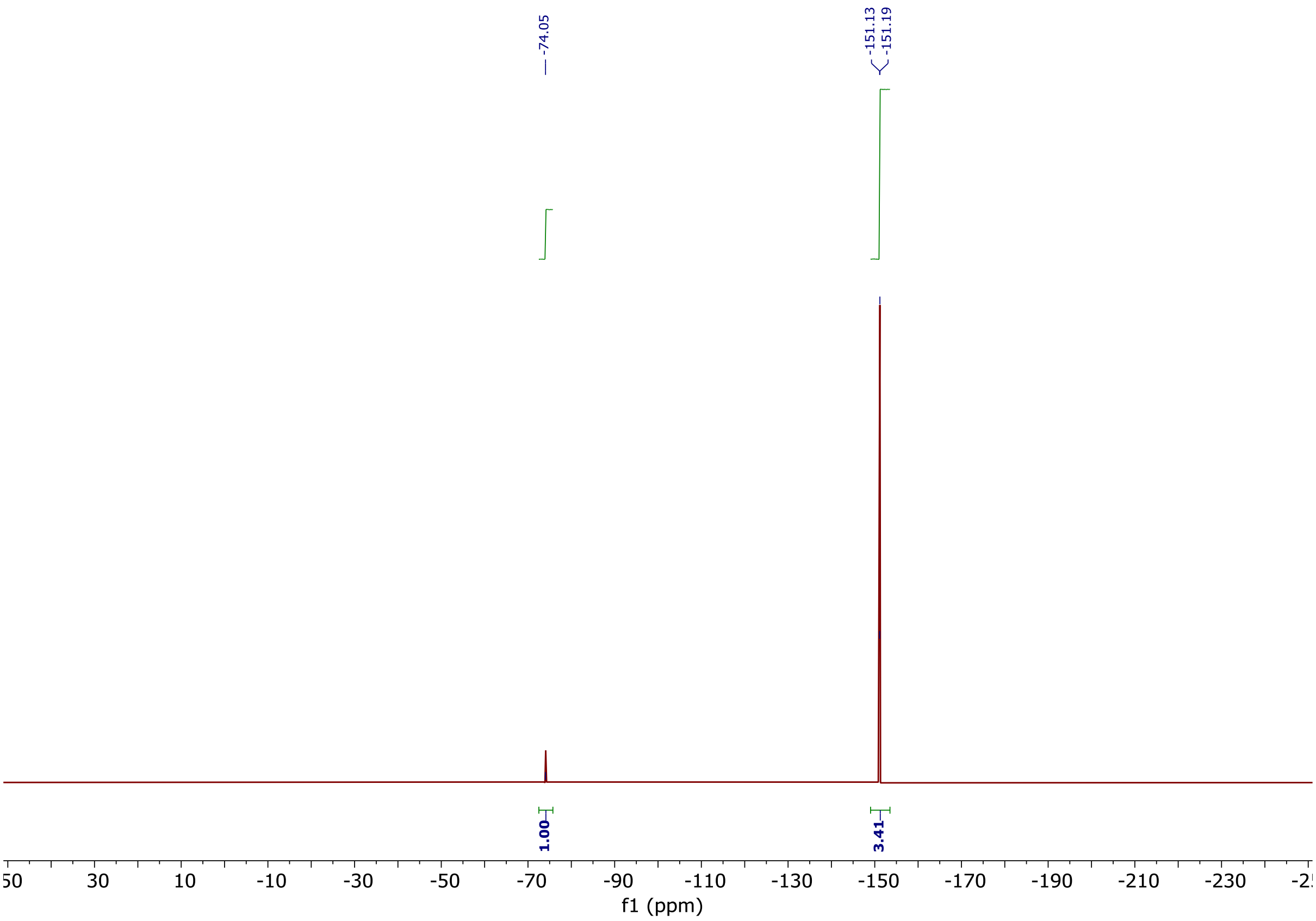

***(Z)-(2-fluoro-3-((4-methyl-N-(1-phenylethyl)phenyl)sulfonamido)prop-1-en-1-yl)(p-tolyl)iodonium tetrafluoroborate* (3p)**

^{1}H NMR (400 MHz, Acetone-D_6)

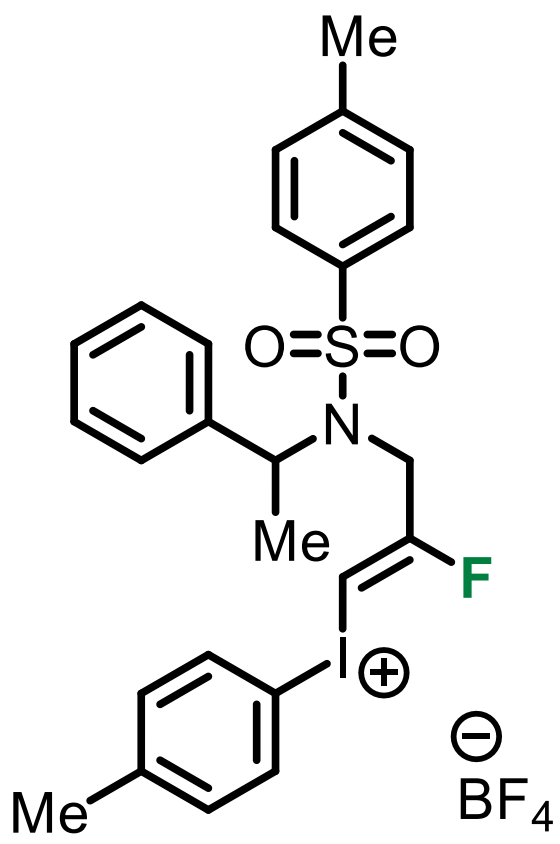

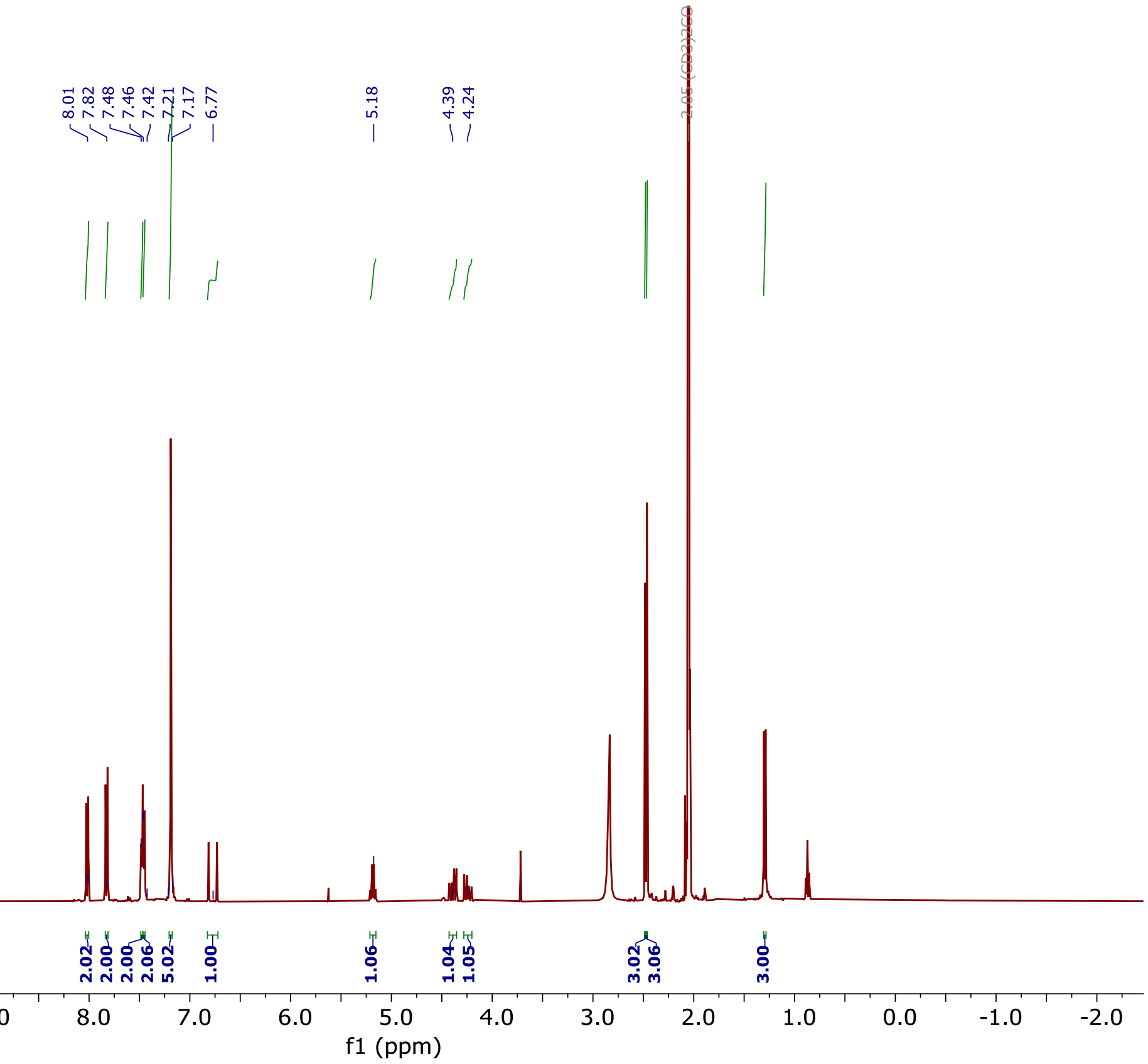

^{13}C NMR (101 MHz, Acetone-D_6)

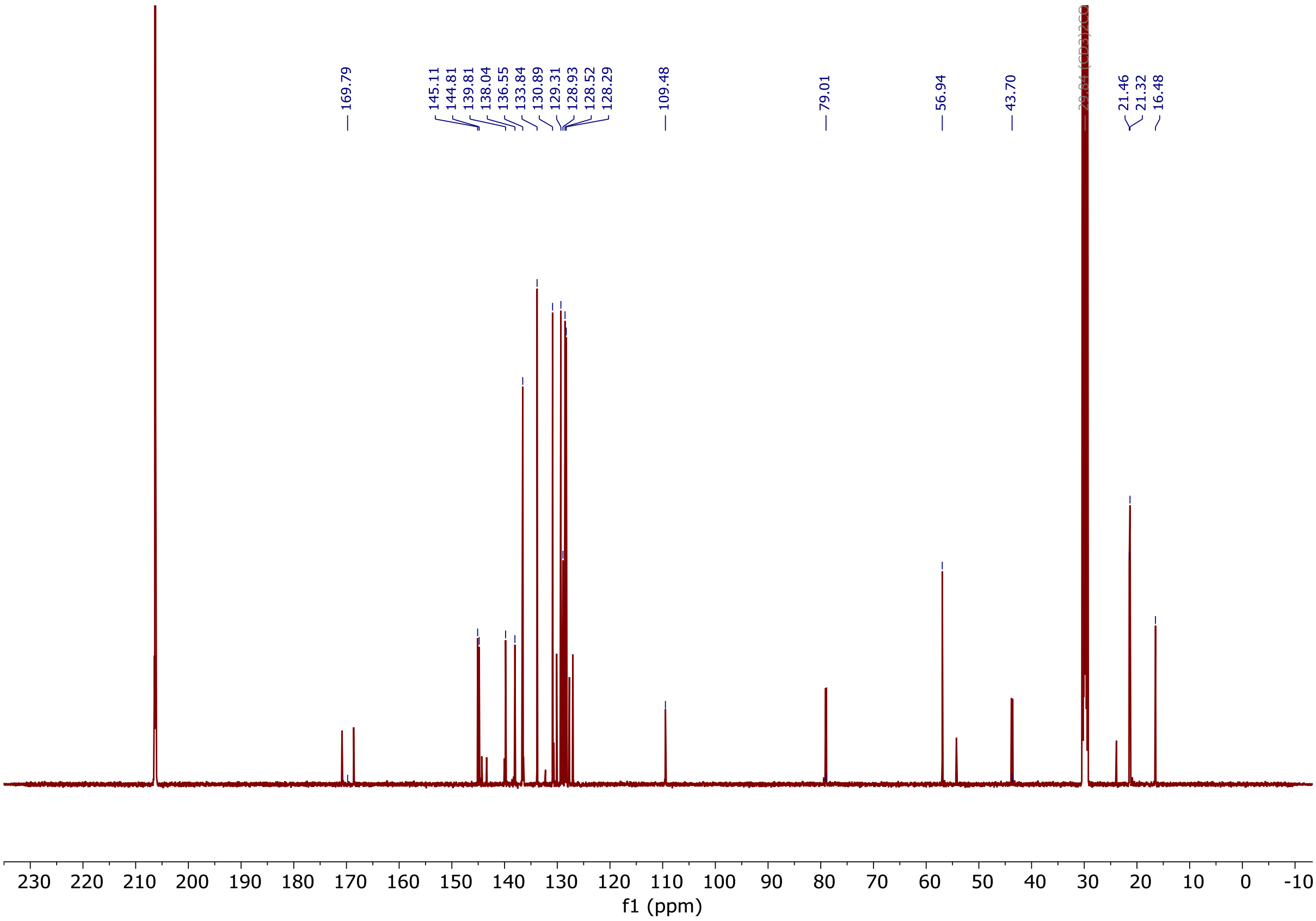

^{19}F NMR (377 MHz, Acetone-D_6)

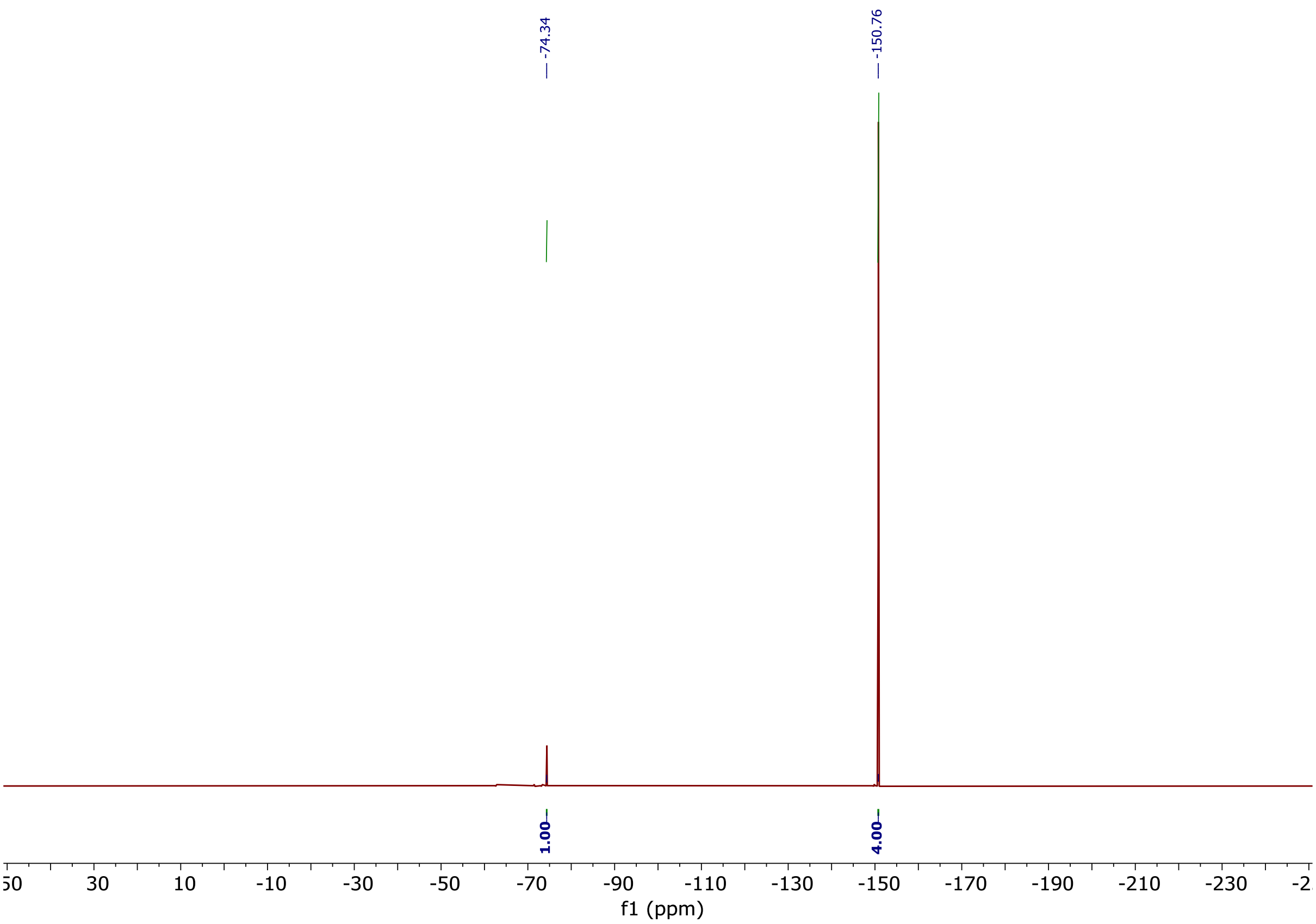

(S,Z)-(2-fluoro-3-((4-methyl-N-(1-phenylethyl)phenyl)sulfonamido)prop-1-en-1-yl)(p-tolyl)iodonium tetrafluoroborate **((*S*)-3p)**

^{1}H NMR (400 MHz, Acetone-*D*$_6$)

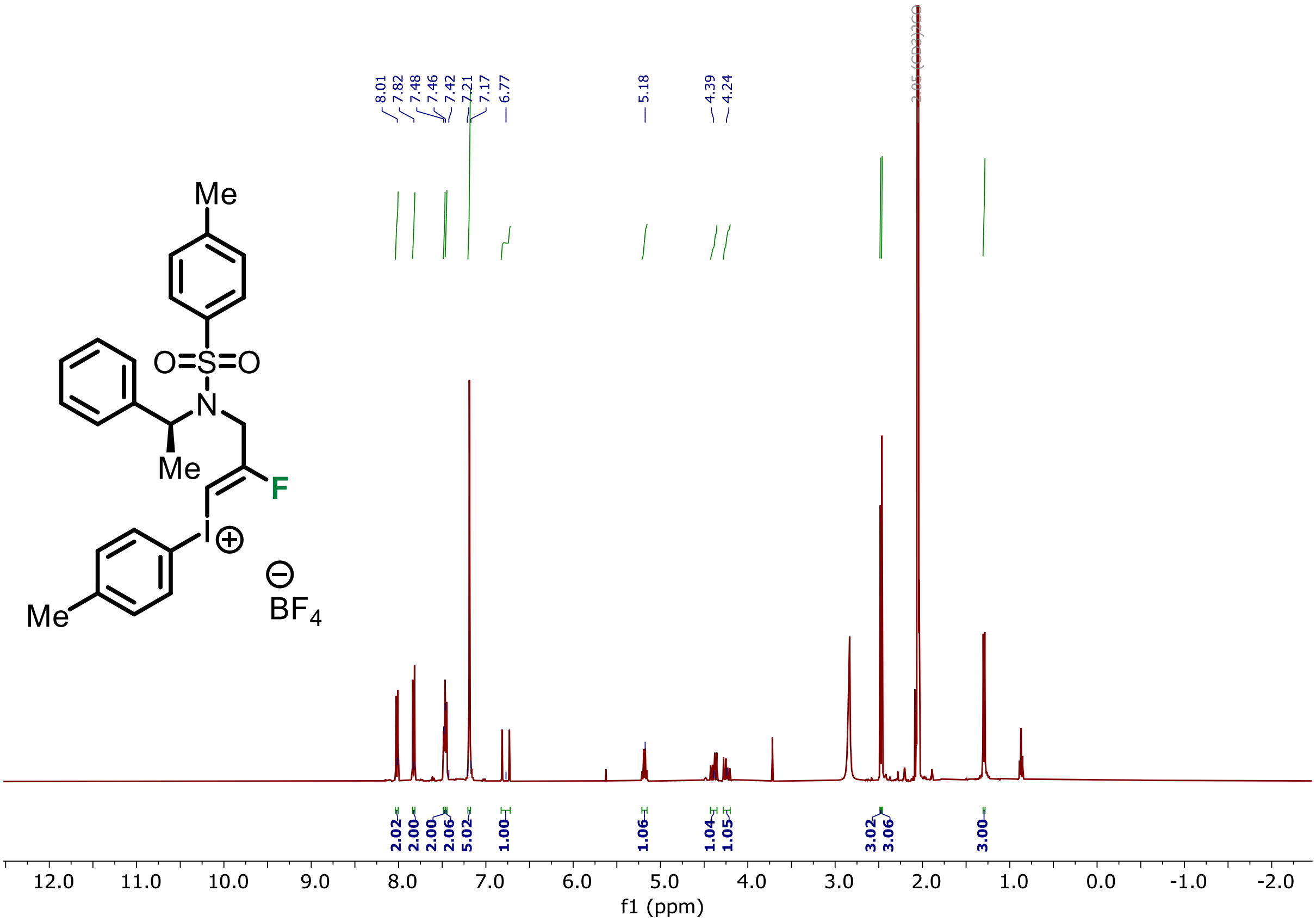

^{13}C NMR (101 MHz, Acetone-D_6)

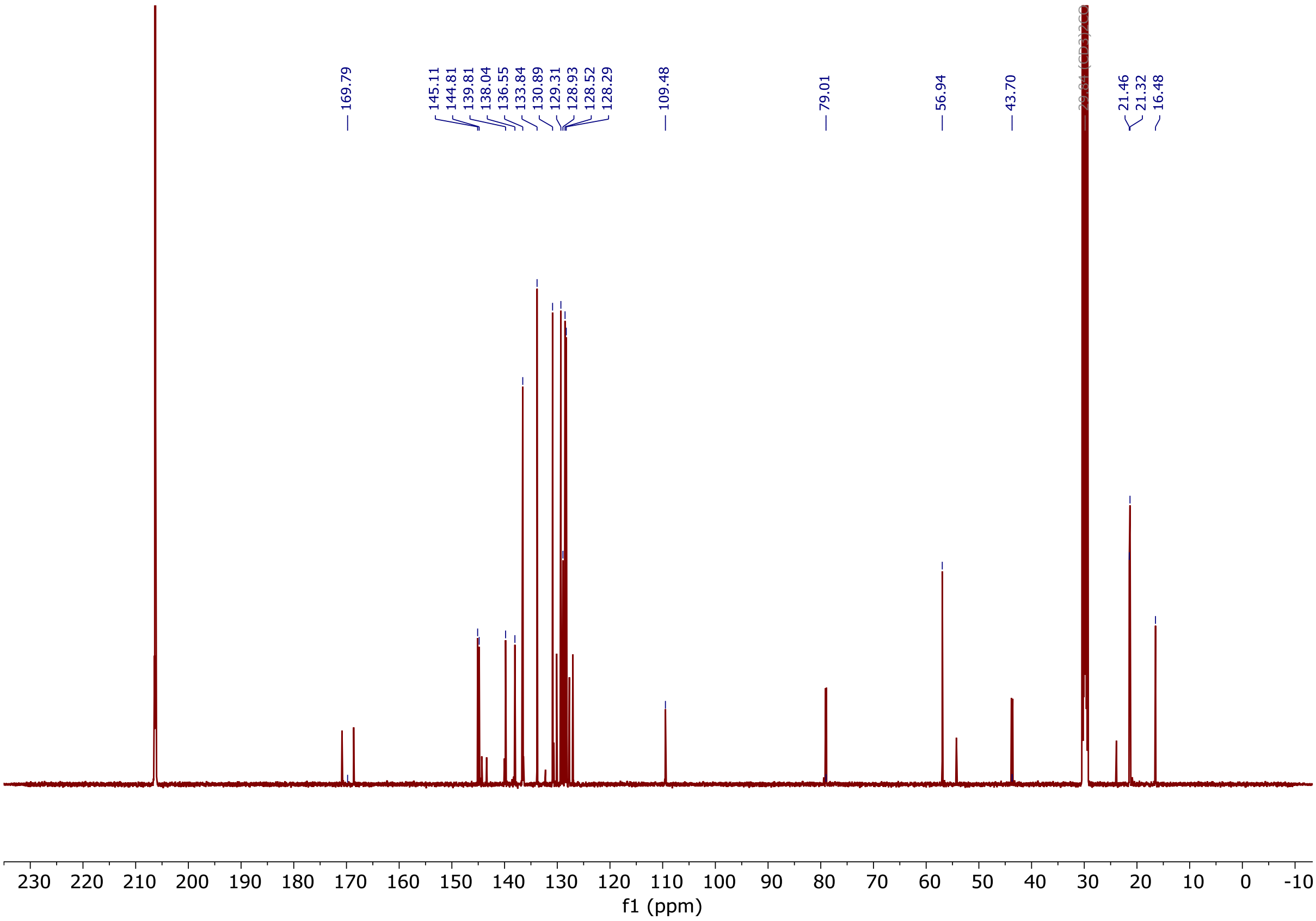

^{19}F NMR (377 MHz, Acetone-D_6)

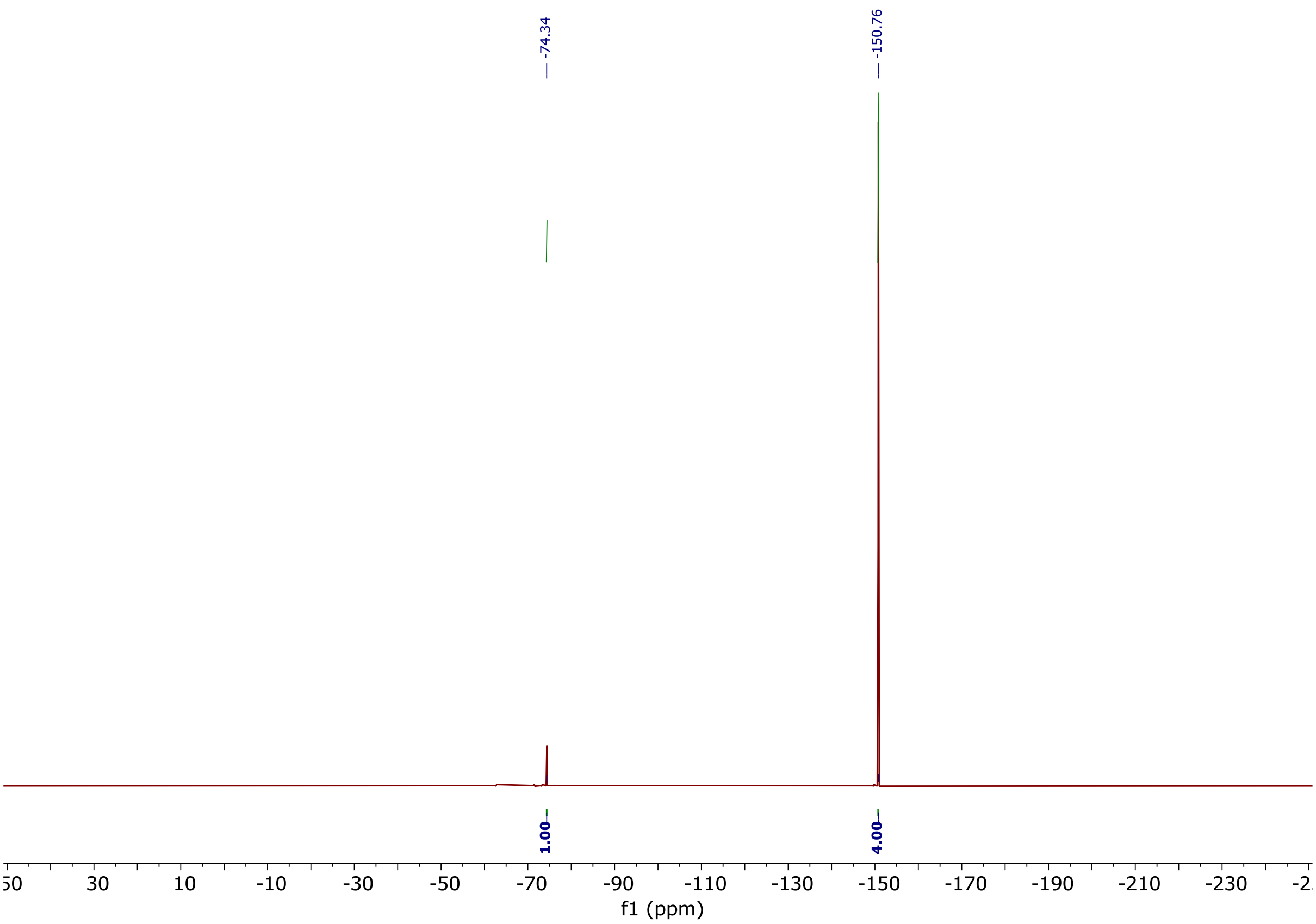

(Z)-(2-fluoro-3-((4-methyl-N-(1-tosylpiperidin-4-yl)phenyl)sulfonamido)prop-1-en-1-yl)(p-tolyl)iodonium tetrafluoroborate (3r)
^{1}H NMR (400 MHz, Acetone-D_6)

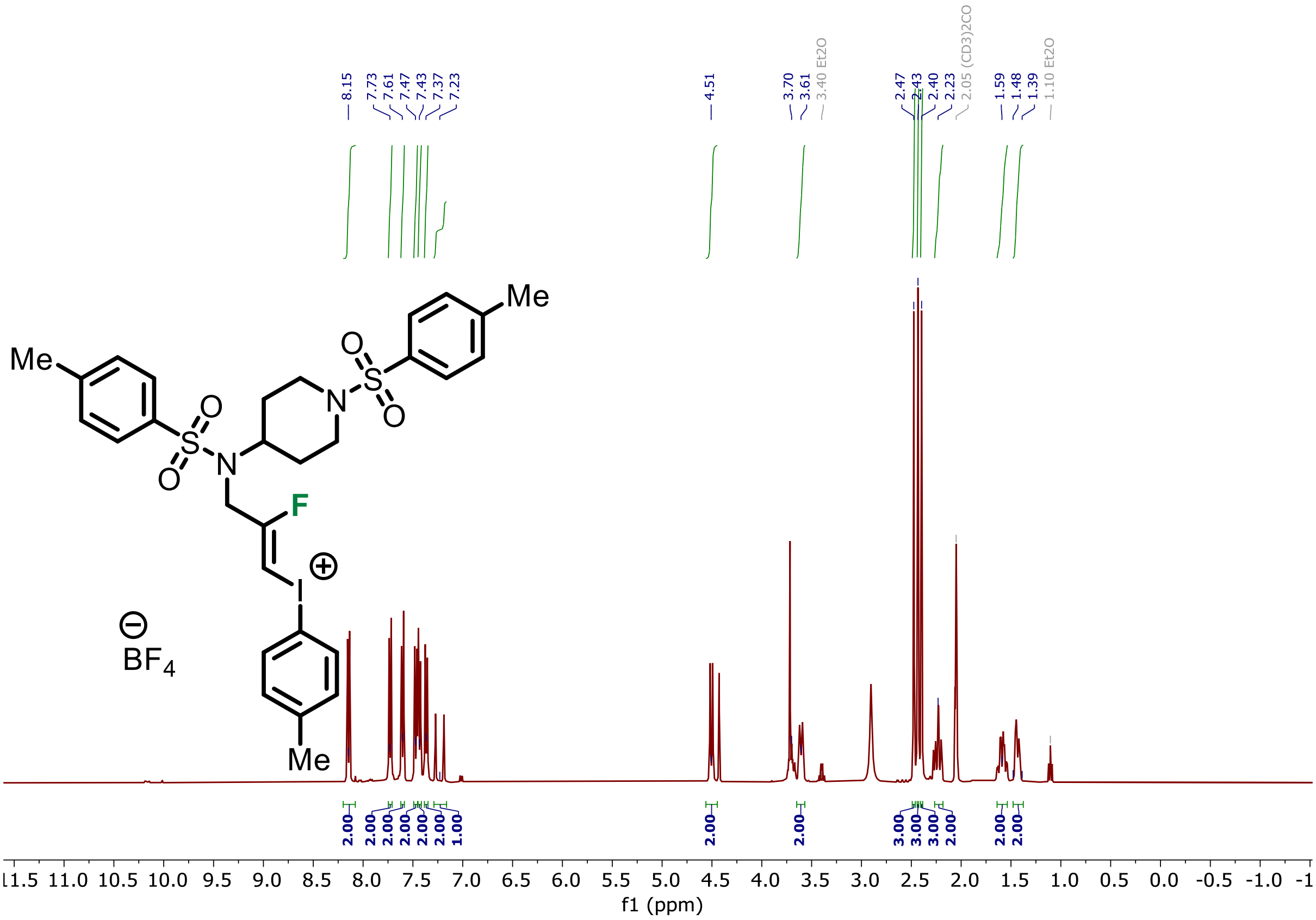

^{13}C NMR (126 MHz, Acetone-D_6)

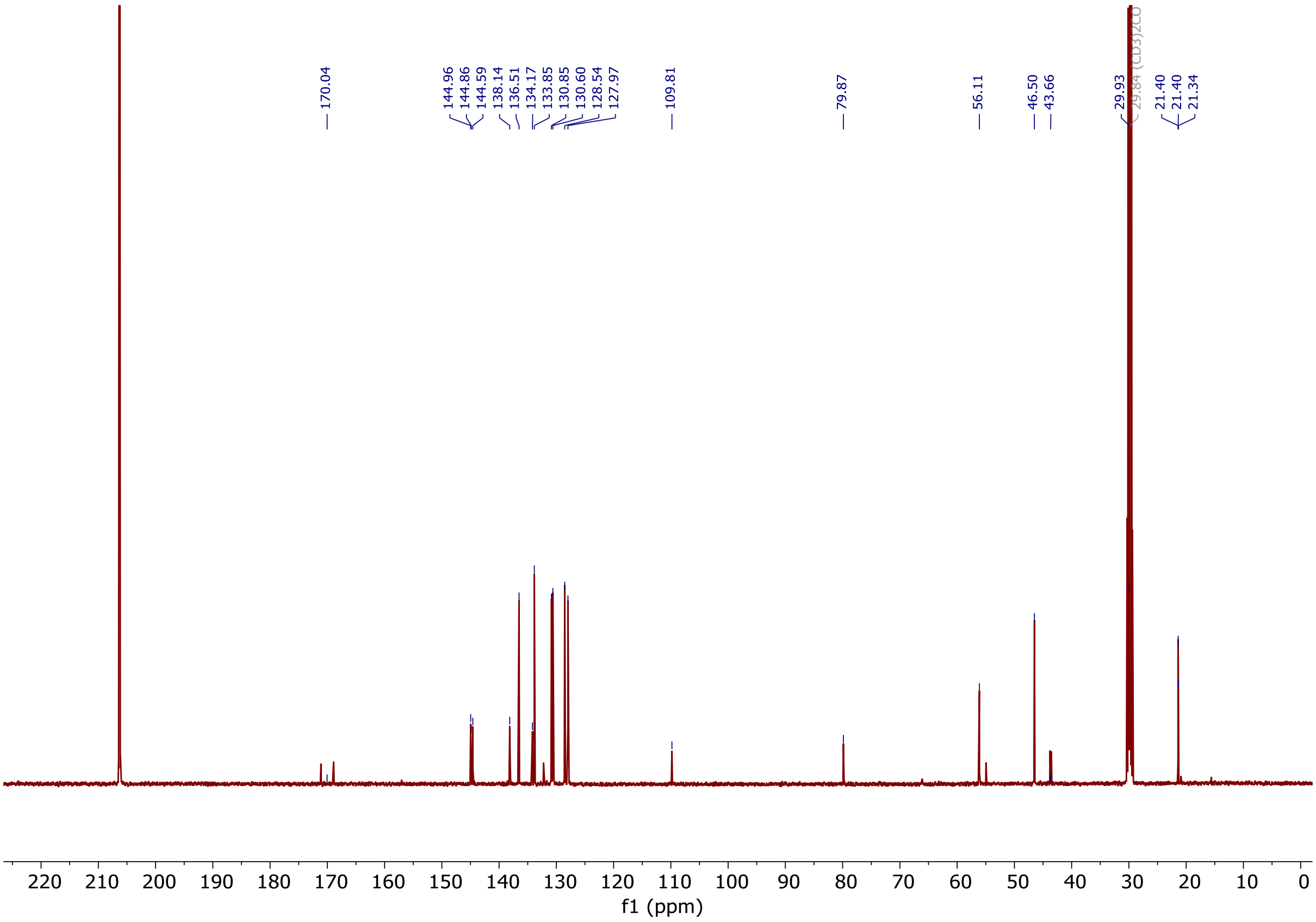

^{19}F NMR (376 MHz, Acetone-D_6)

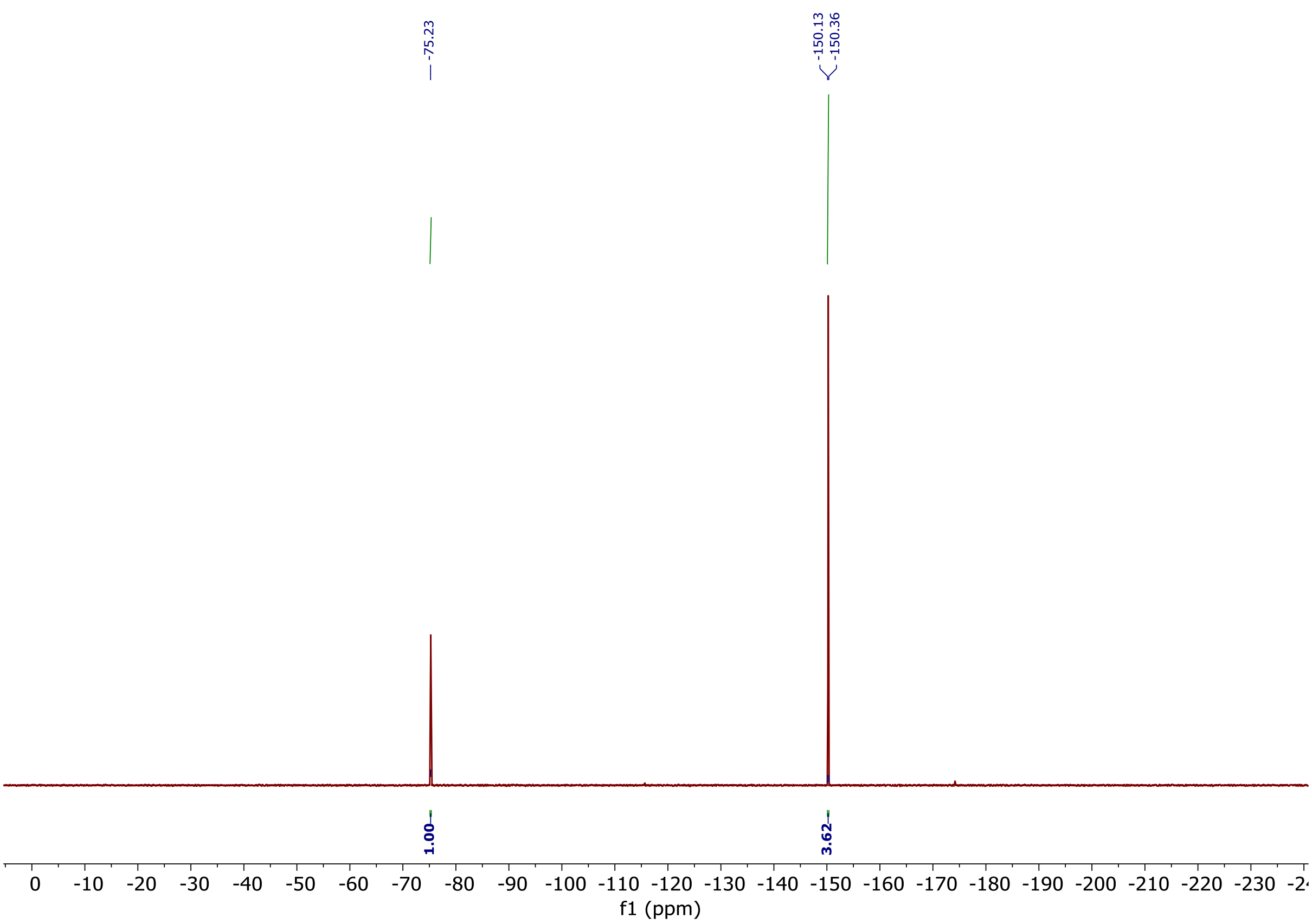

***(Z)-(3-((N-cyclohexyl-4-methylphenyl)sulfonamido)-2-fluoroprop-1-en-1-yl)(p-tolyl)iodonium tetrafluoroborate* (3s)**

^{1}H NMR (500 MHz, Acetone-D_6)

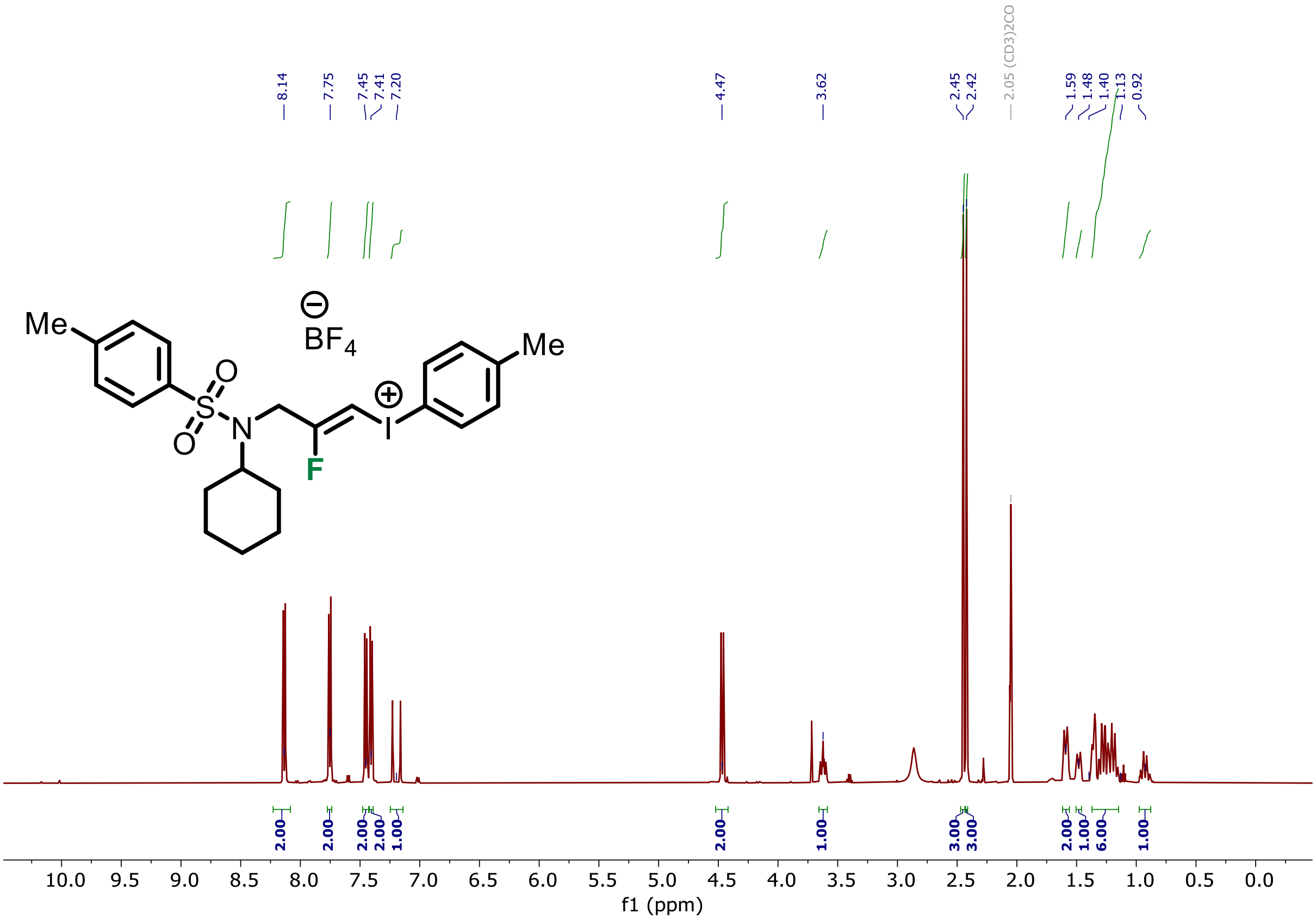

^{13}C NMR (126 MHz, Acetone-D_6)

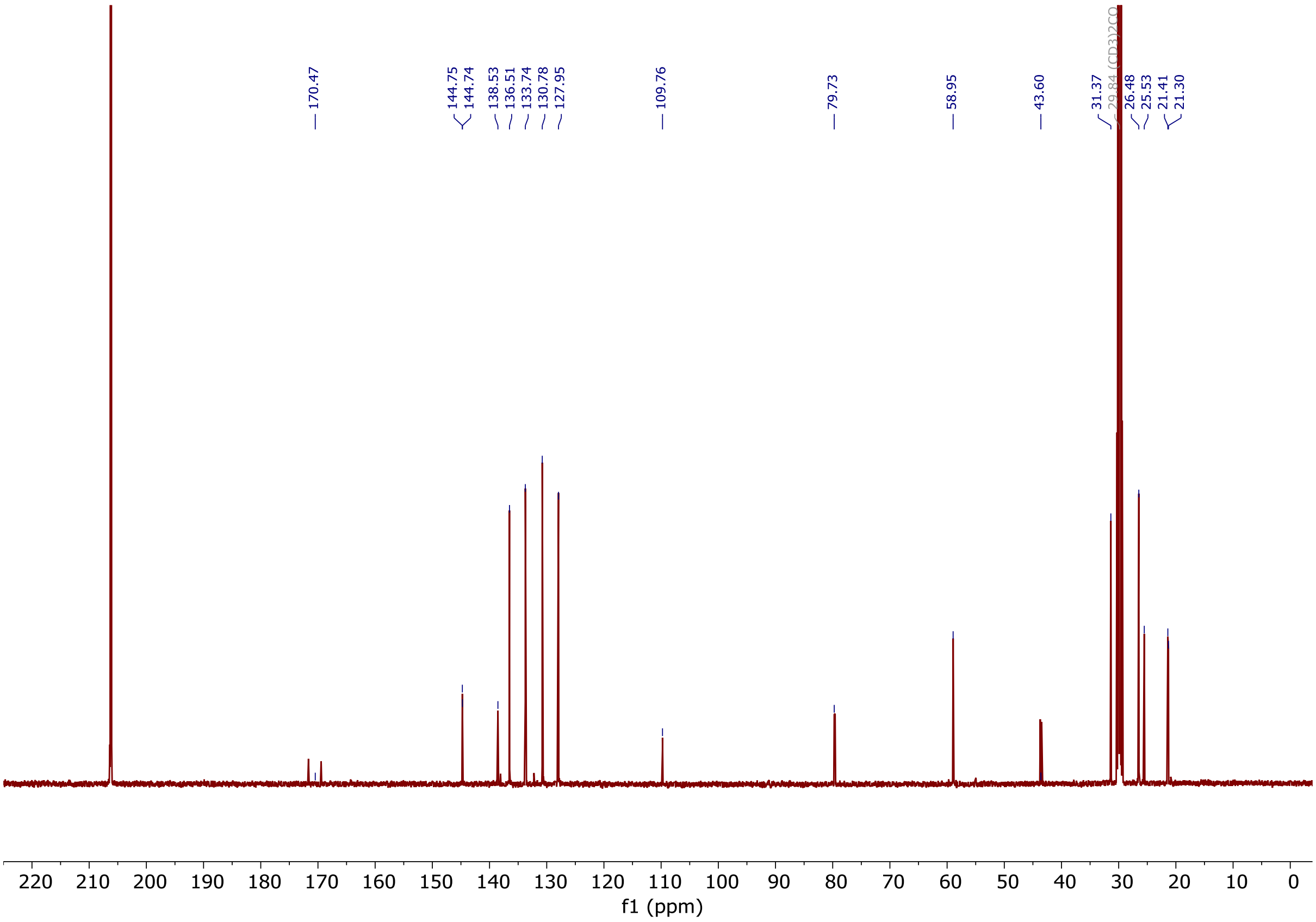

^{19}F NMR (376 MHz, Acetone-D_6)

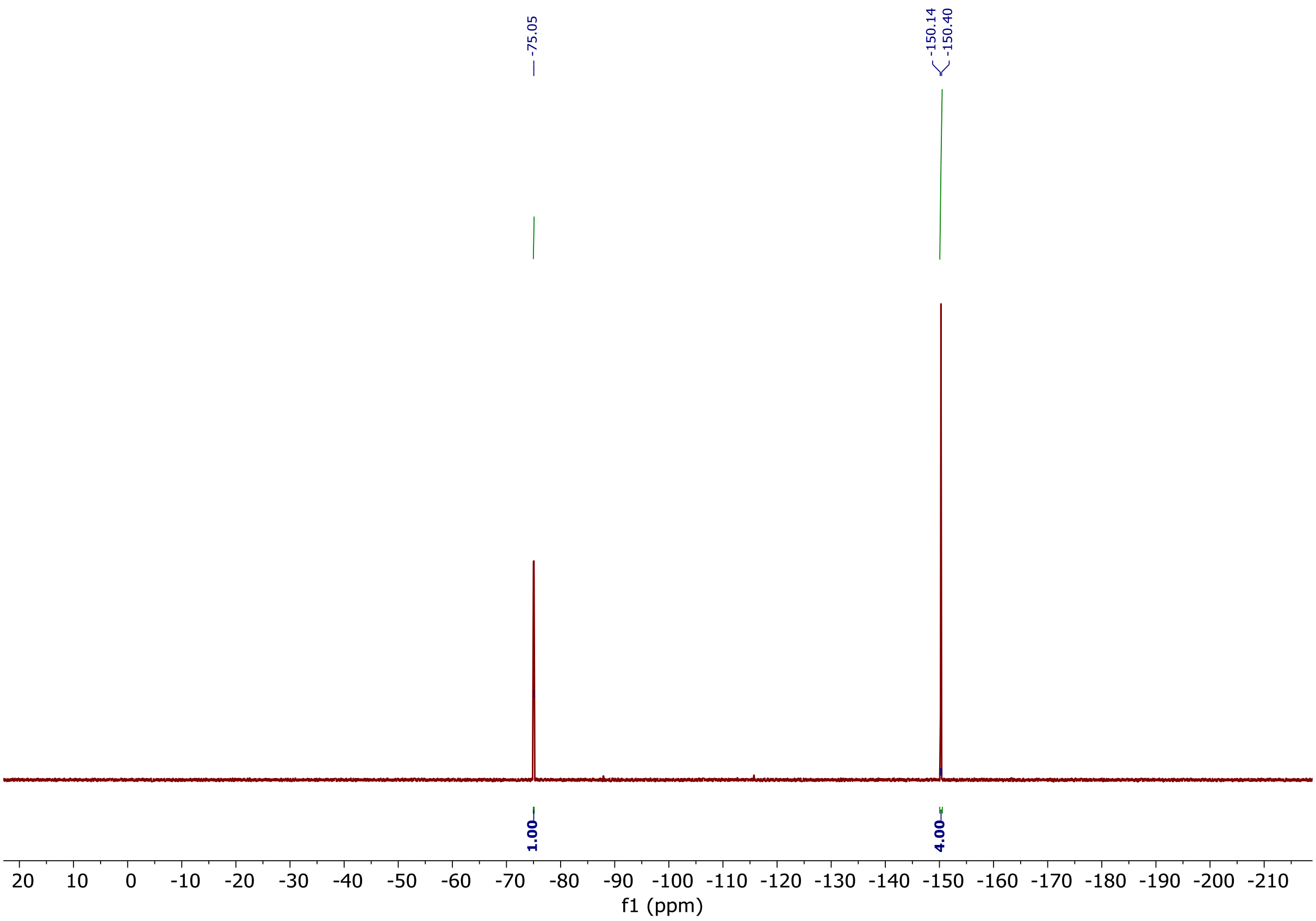

***(Z)-(2-fluoro-3-((4-methyl-N-phenethylphenyl)sulfonamido)prop-1-en-1-yl)(p-tolyl)iodonium tetrafluoroborate* (3t)**

^{1}H NMR (400 MHz, Acetone-D_6)

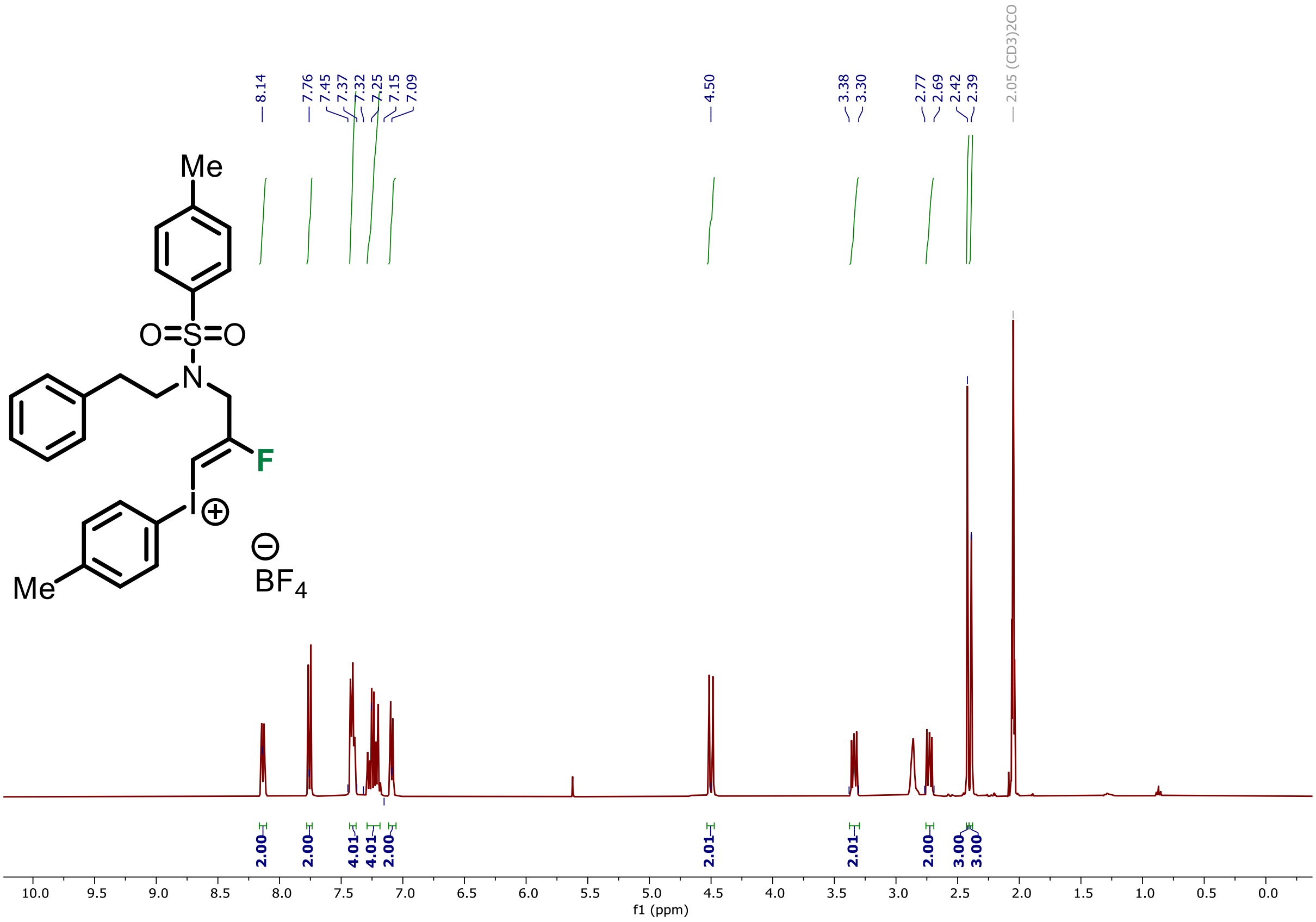

^{13}C NMR (151 MHz, Acetone-D_6)

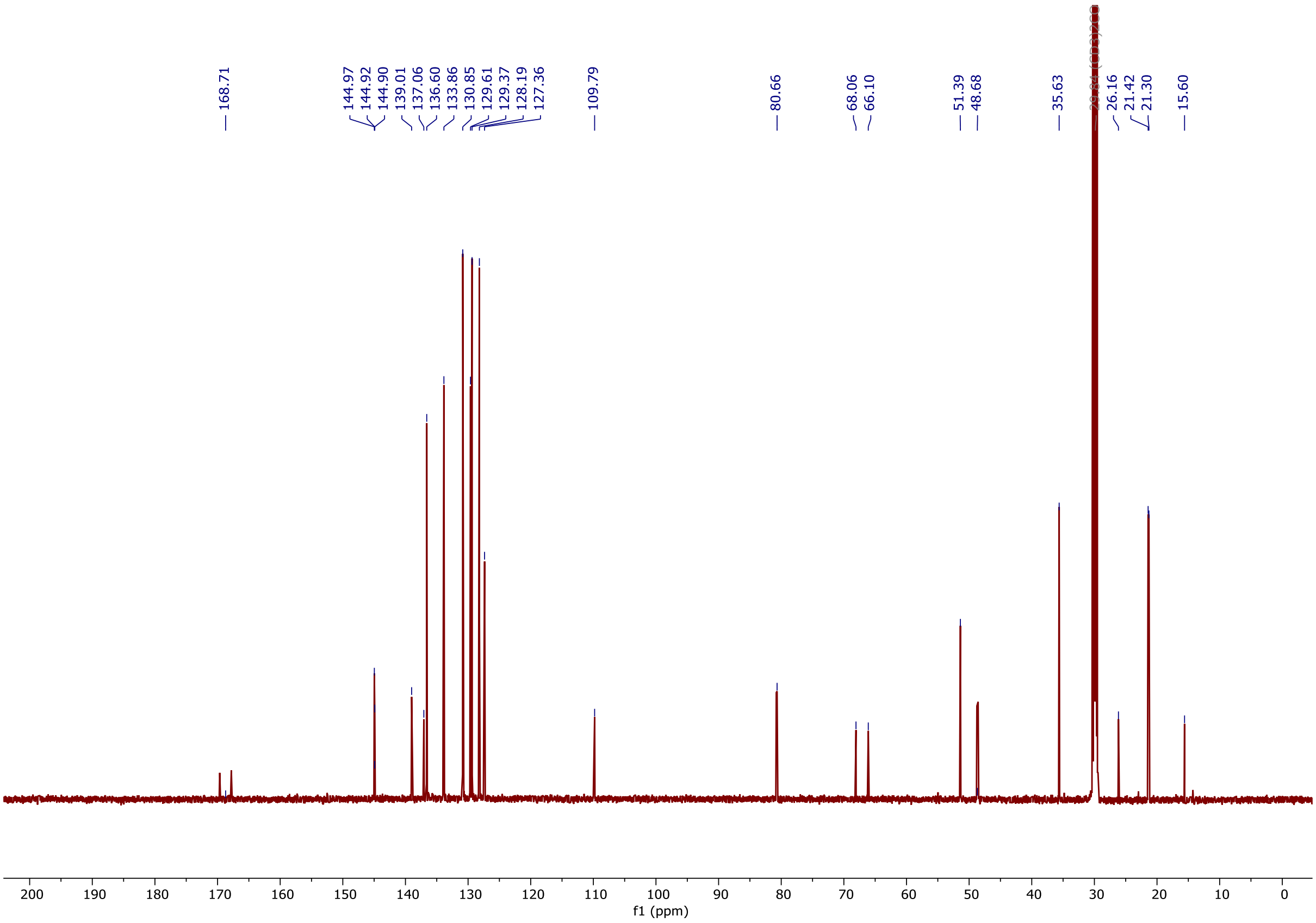

^{19}F NMR (376 MHz, Acetone-D_6)

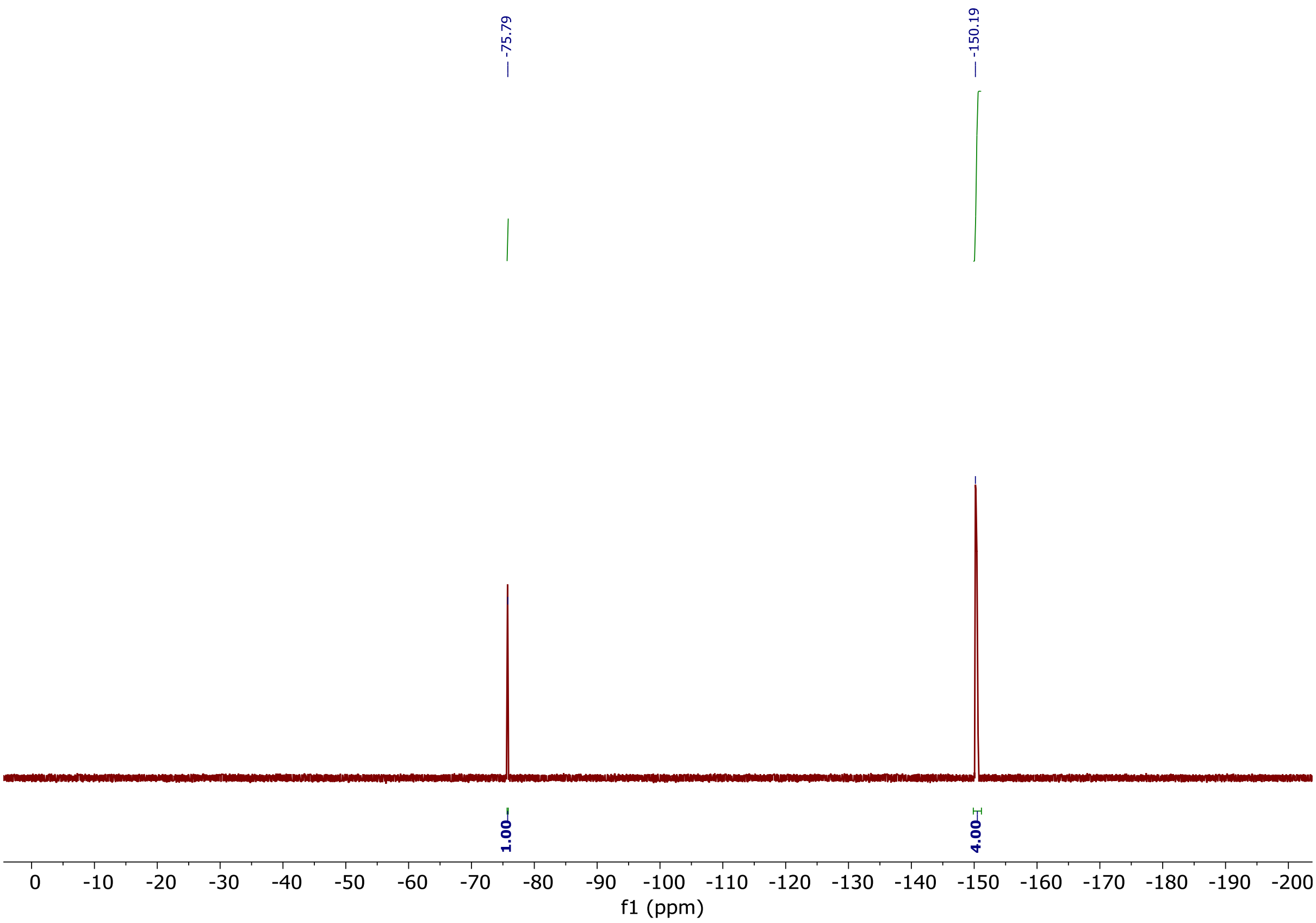

(Z)-(2-fluoro-3-((4-methyl-N-(naphthalen-2-ylmethyl)phenyl)sulfonamido)but-1-en-1-yl)(p-tolyl)iodonium tetrafluoroborate **(3m)**

^{1}H NMR (500 MHz, Acetone-*D*$_6$)

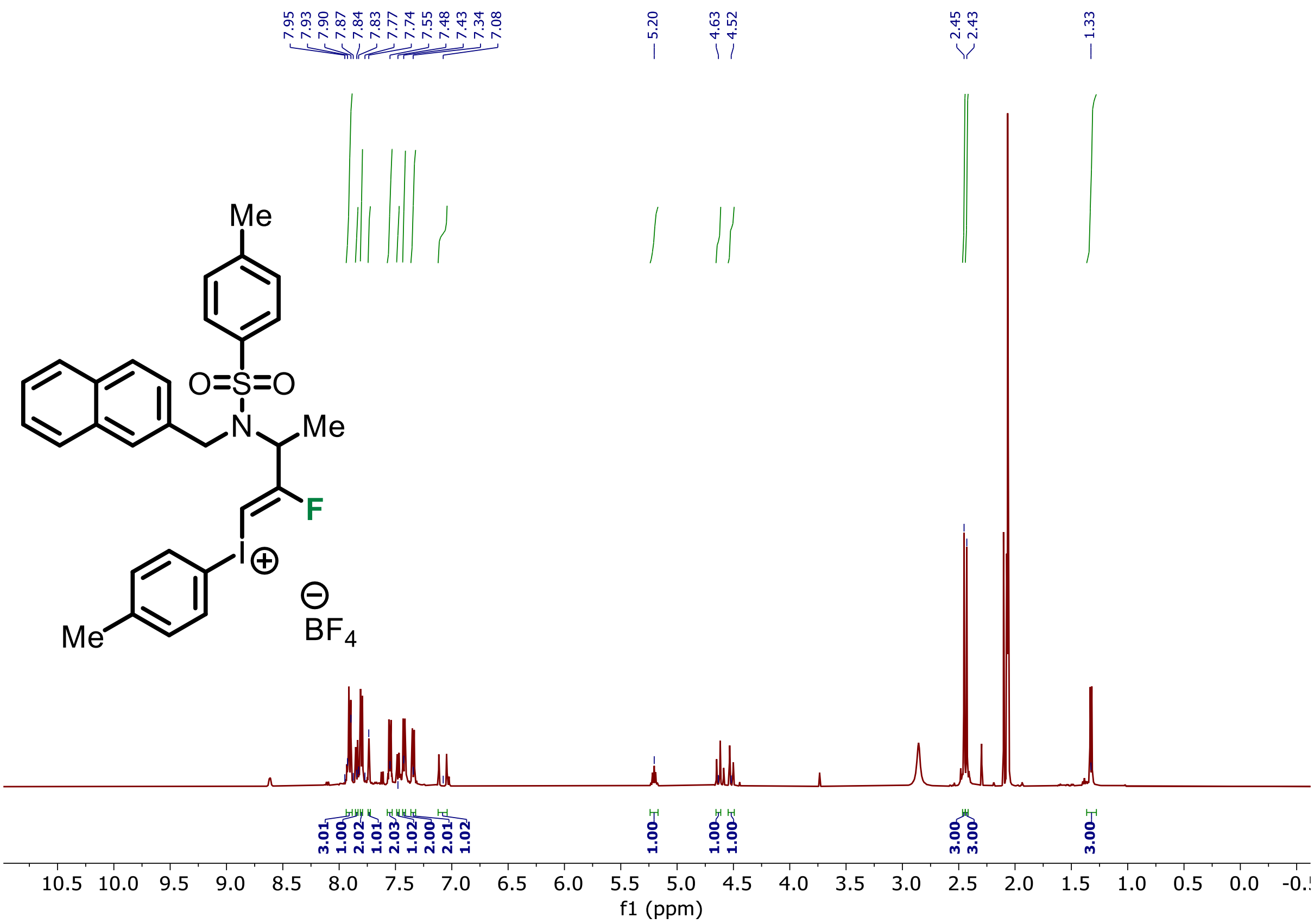

^{13}C NMR (126 MHz, Acetone-D_6)

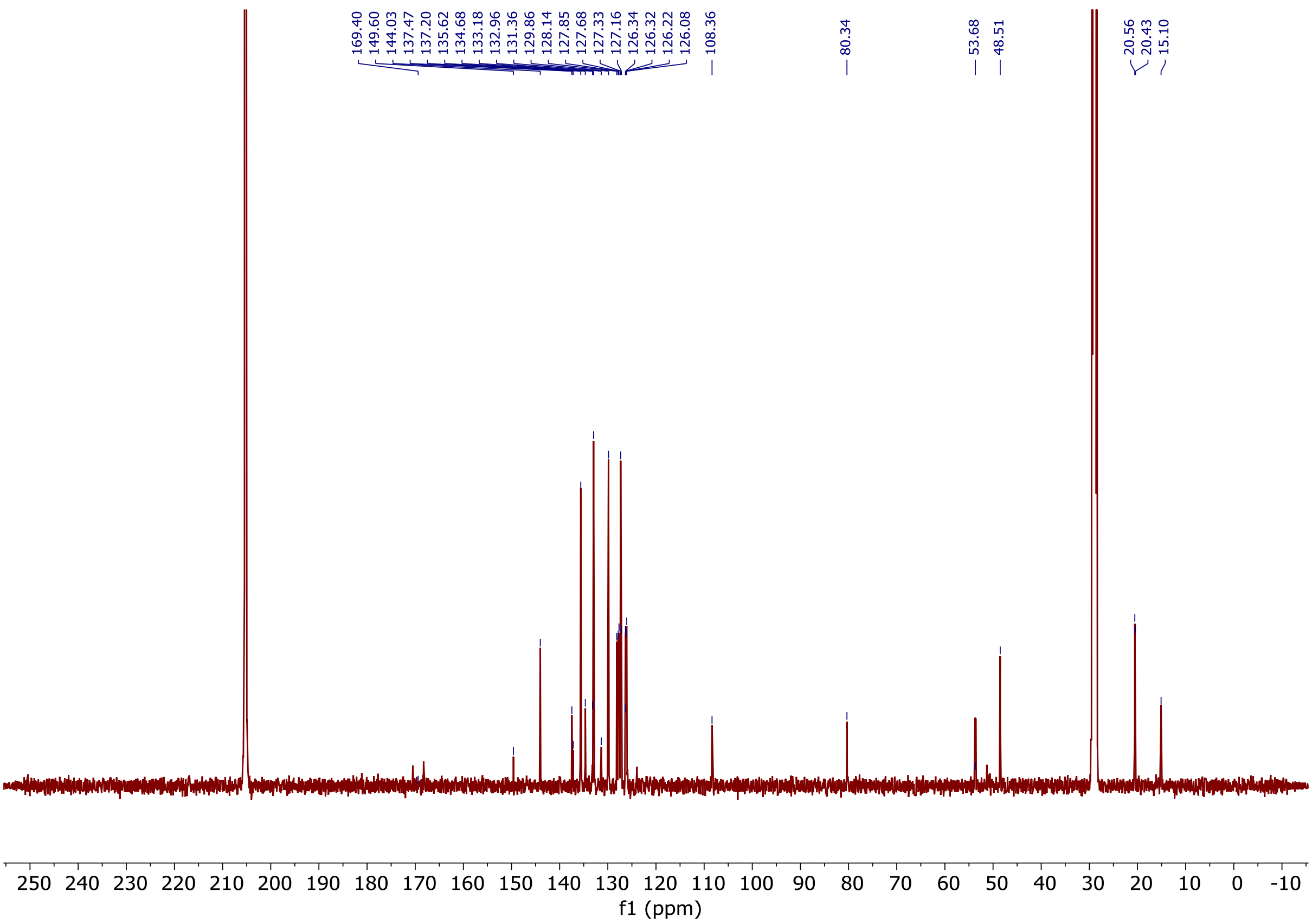

^{19}F NMR (471 MHz, Acetone-D_6)

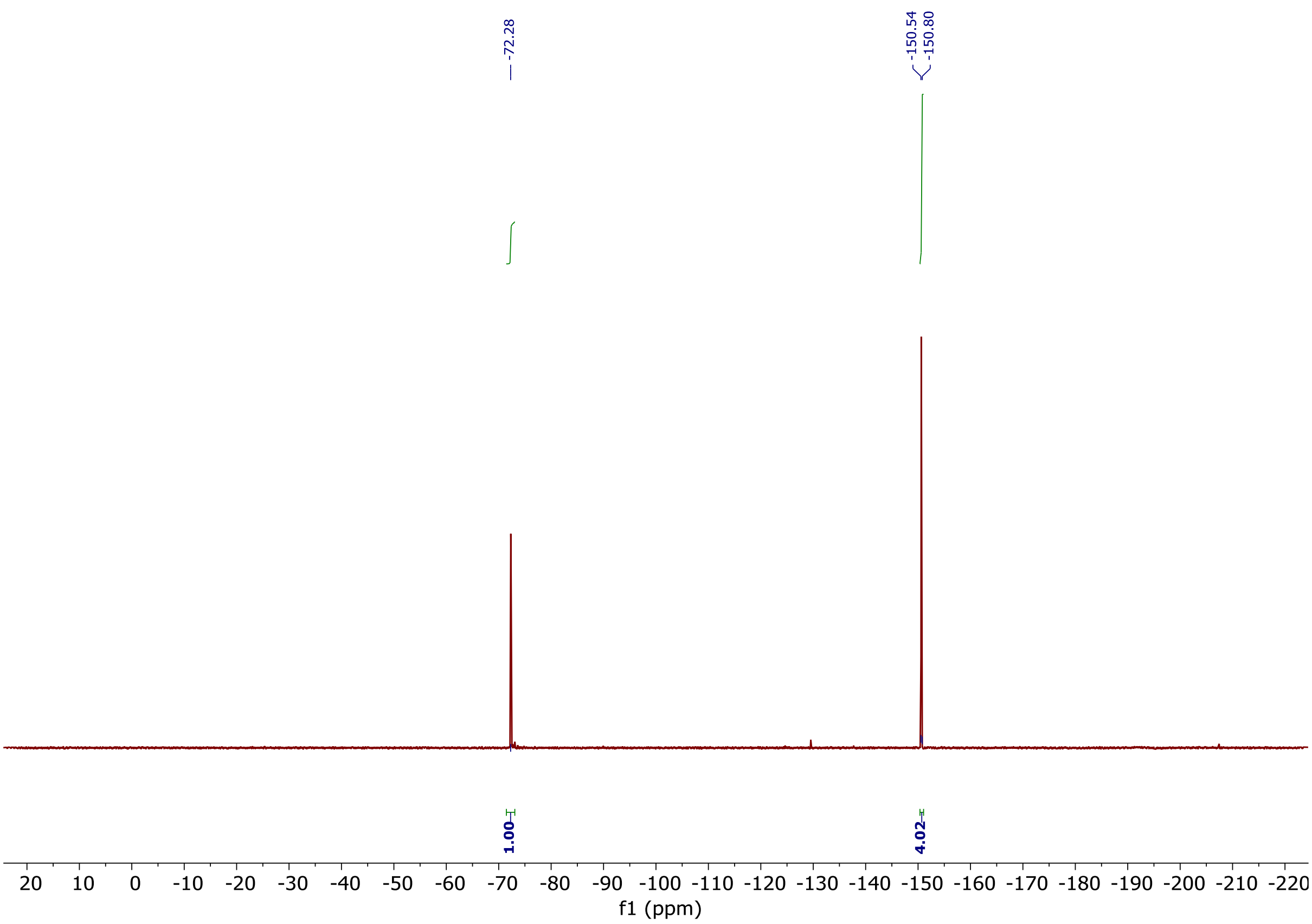

***(Z)-(3-((N-butyl-4-nitrophenyl)sulfonamido)-2-fluoroprop-1-en-1-yl)(p-tolyl)iodonium tetrafluoroborate* (3u)**

^{1}H NMR (500 MHz, Acetone-*D_6*)

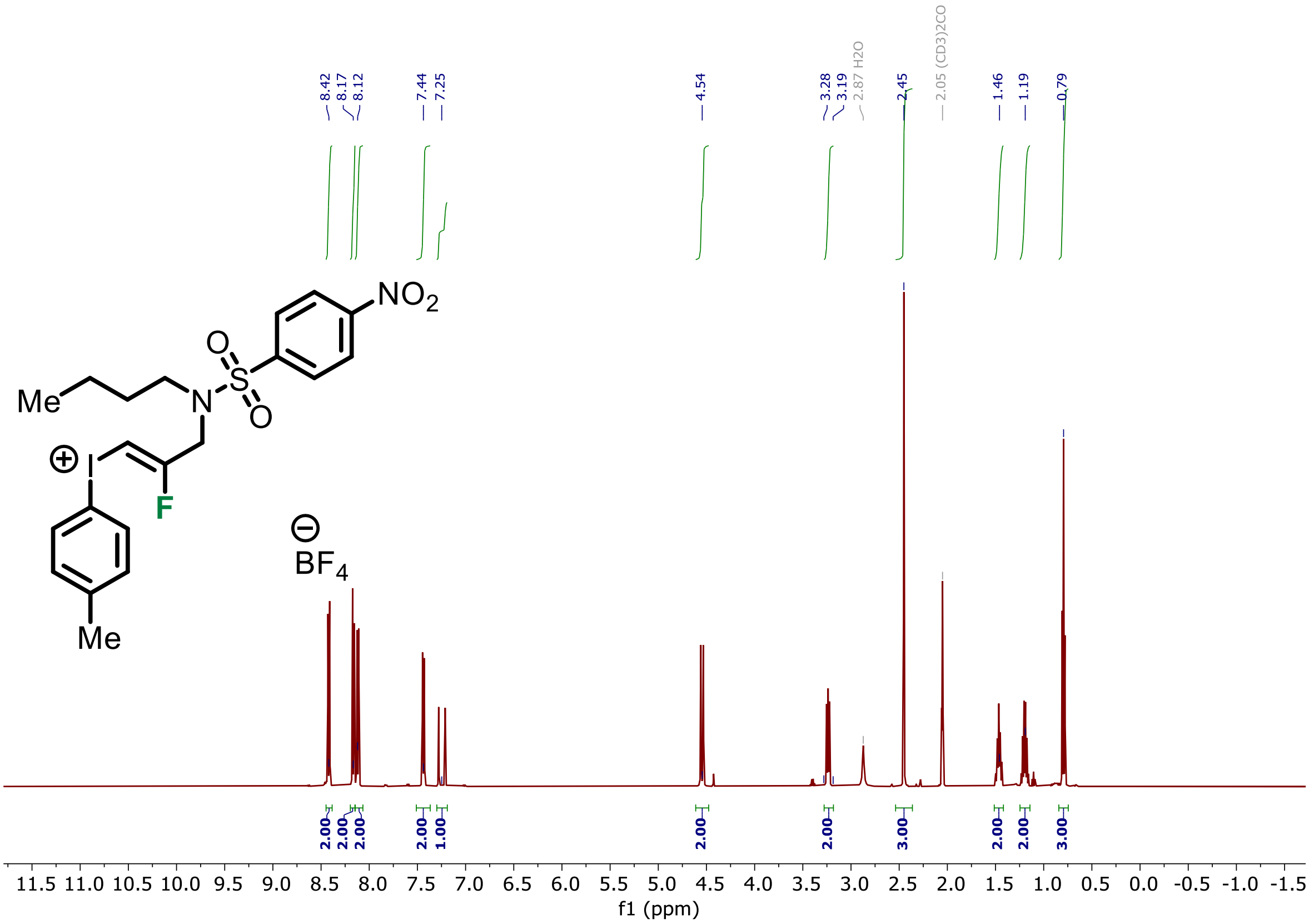

^{13}C NMR (126 MHz, Acetone-*D_6*)

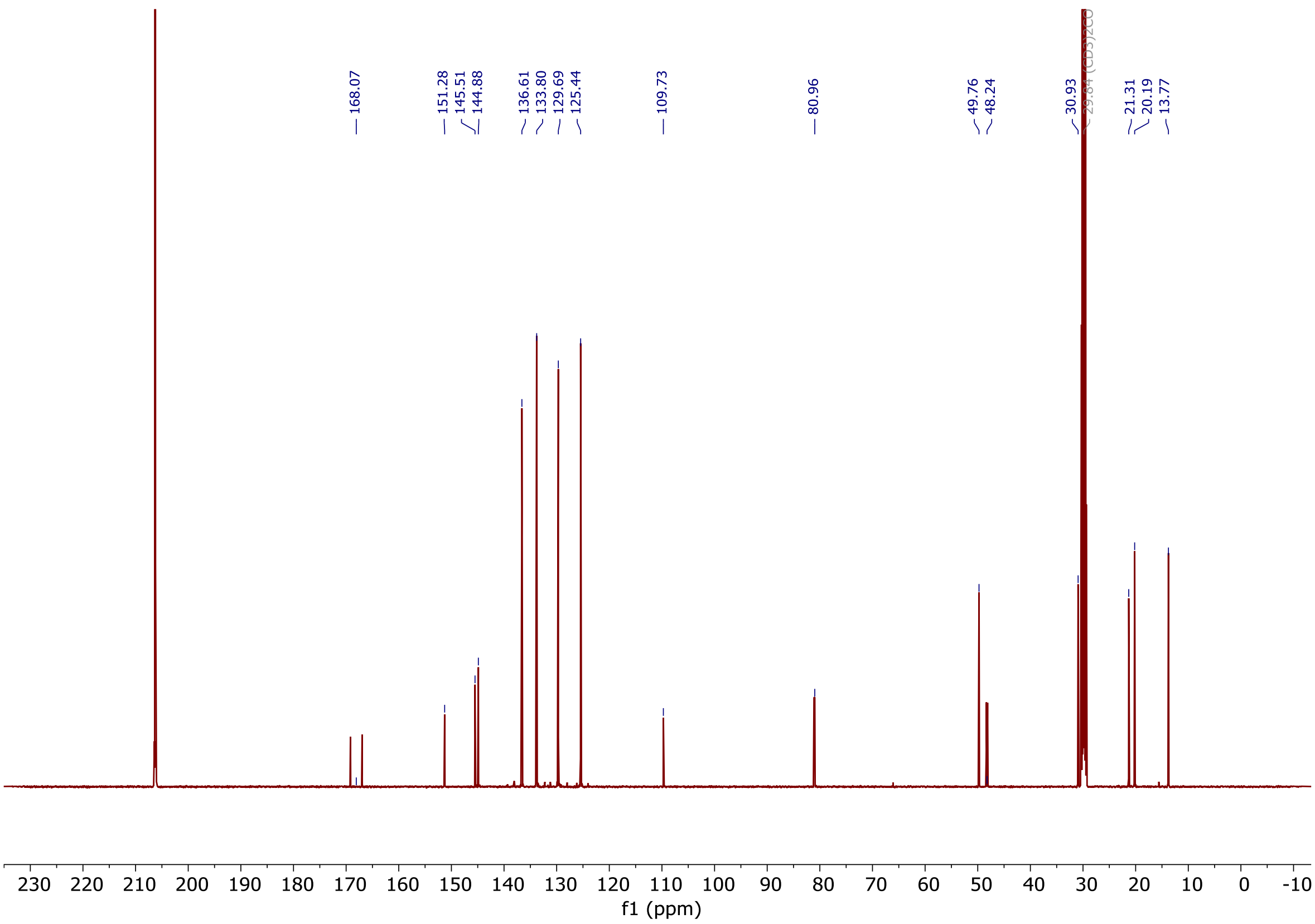

^{19}F NMR (376 MHz, Acetone-D_6)

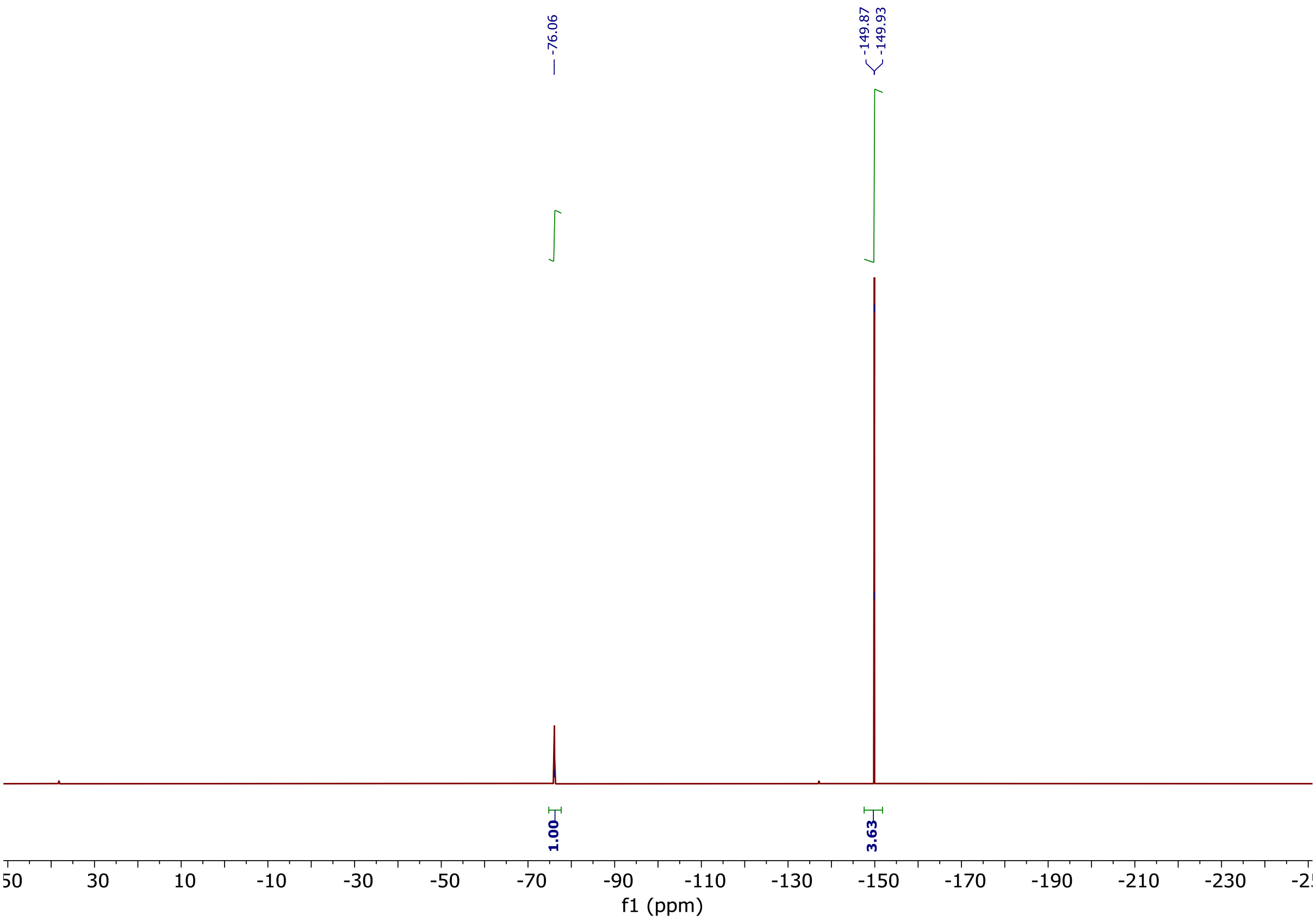

(Z)-(3-((N-butyl-4-methylphenyl)sulfonamido)-2-fluoroprop-1-en-1-yl)(p-tolyl)iodonium tetrafluoroborate (3v)

^{1}H NMR (500 MHz, Acetone-D_6)

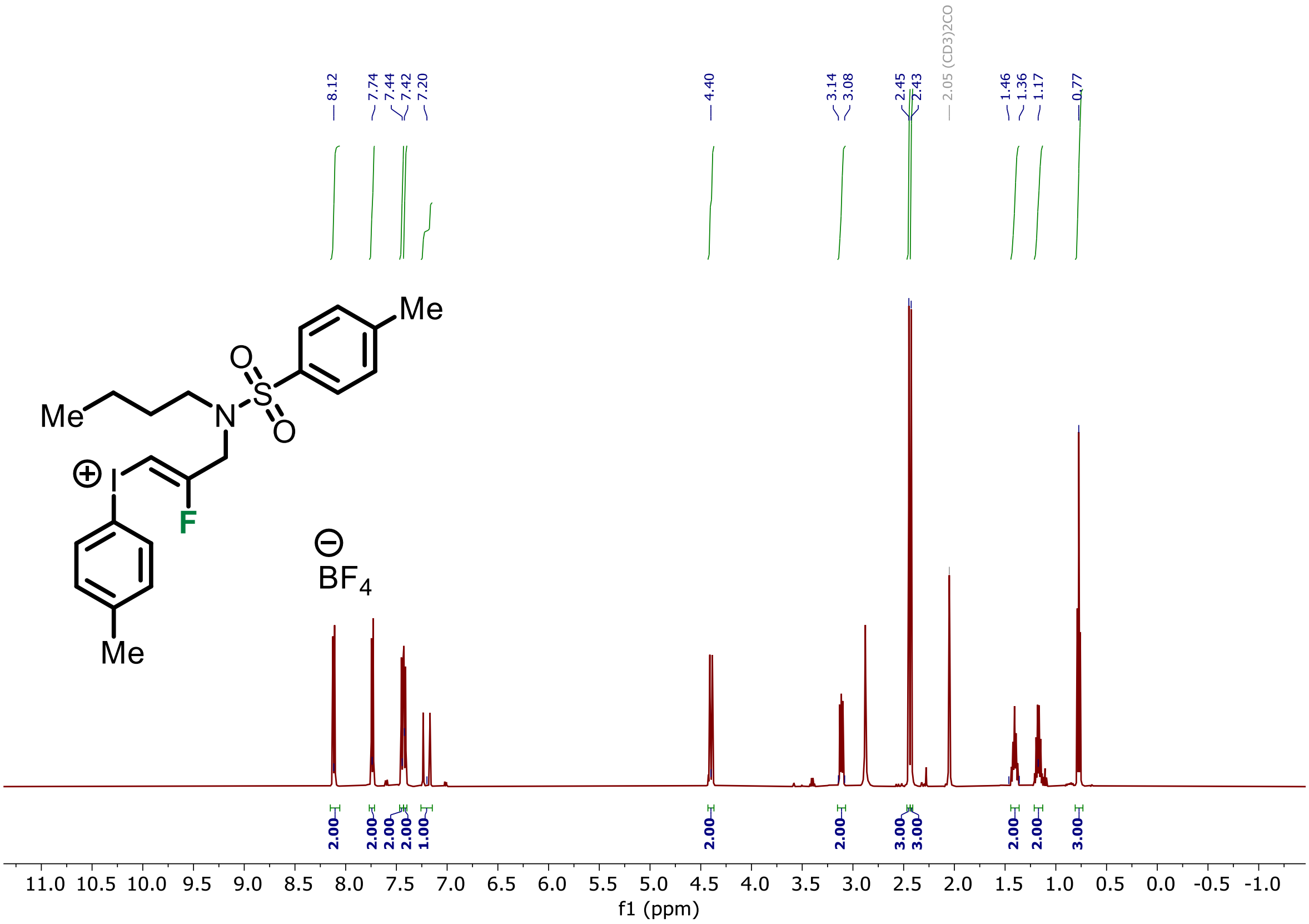

^{13}C NMR (126 MHz, Acetone-D_6)

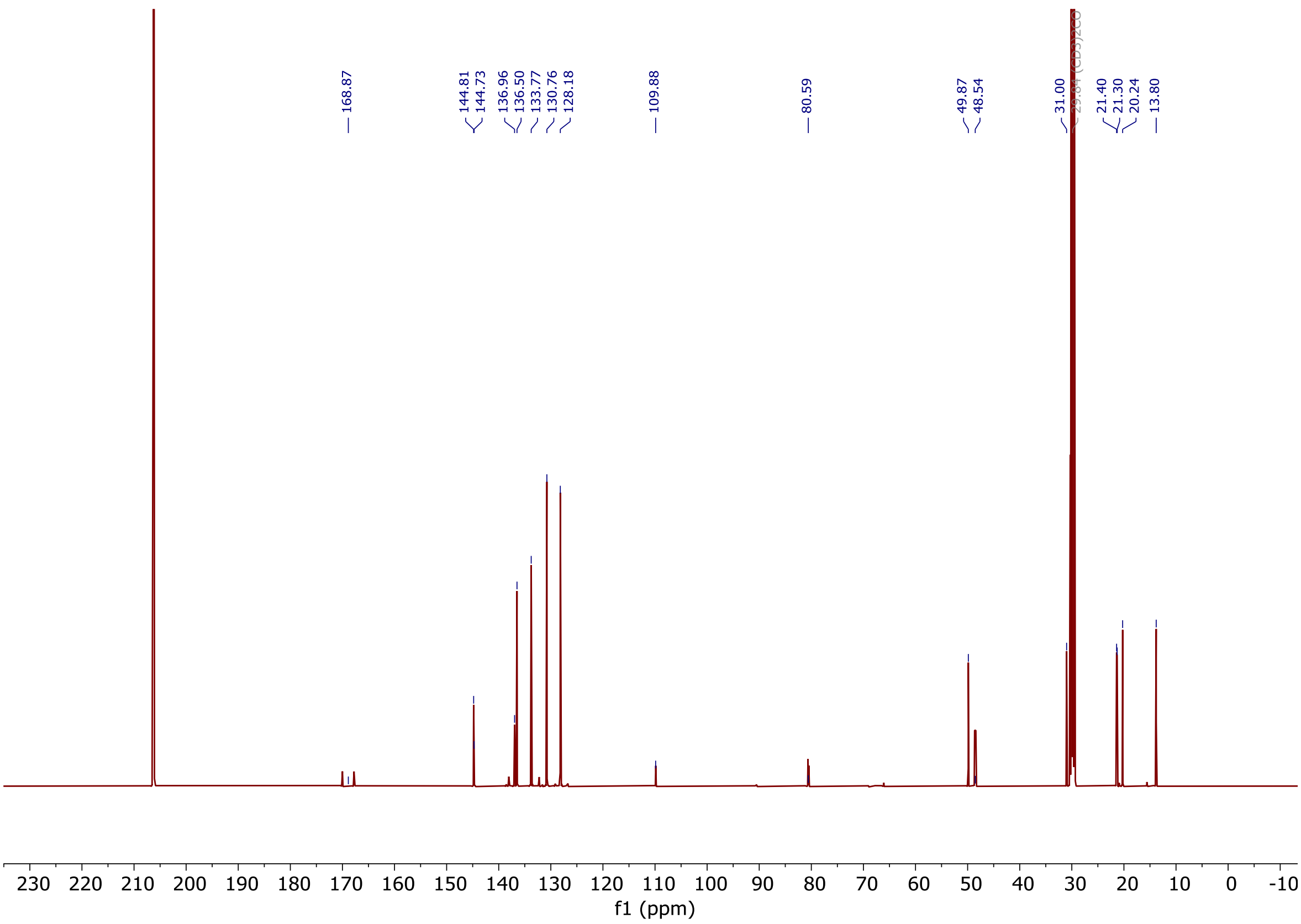

^{19}F NMR (376 MHz, Acetone-D_6)

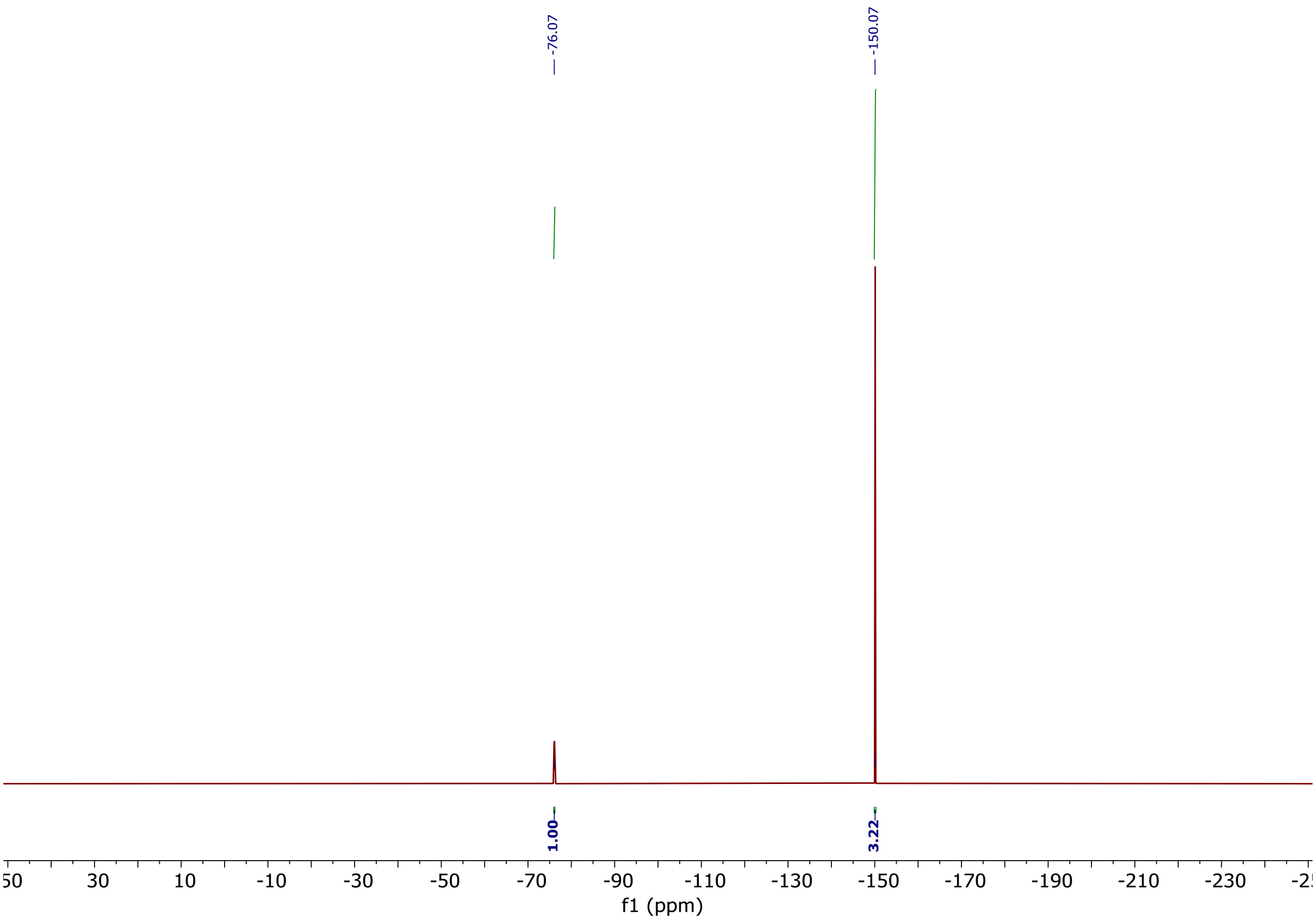

(Z)-(2-fluoro-3-((4-methyl-N-(2-(thiophen-2-yl)ethyl)phenyl)sulfonamido)prop-1-en-1-yl)(p-tolyl)iodonium tetrafluoroborate **(3w)**

^{1}H NMR (500 MHz, Acetone-D_6)

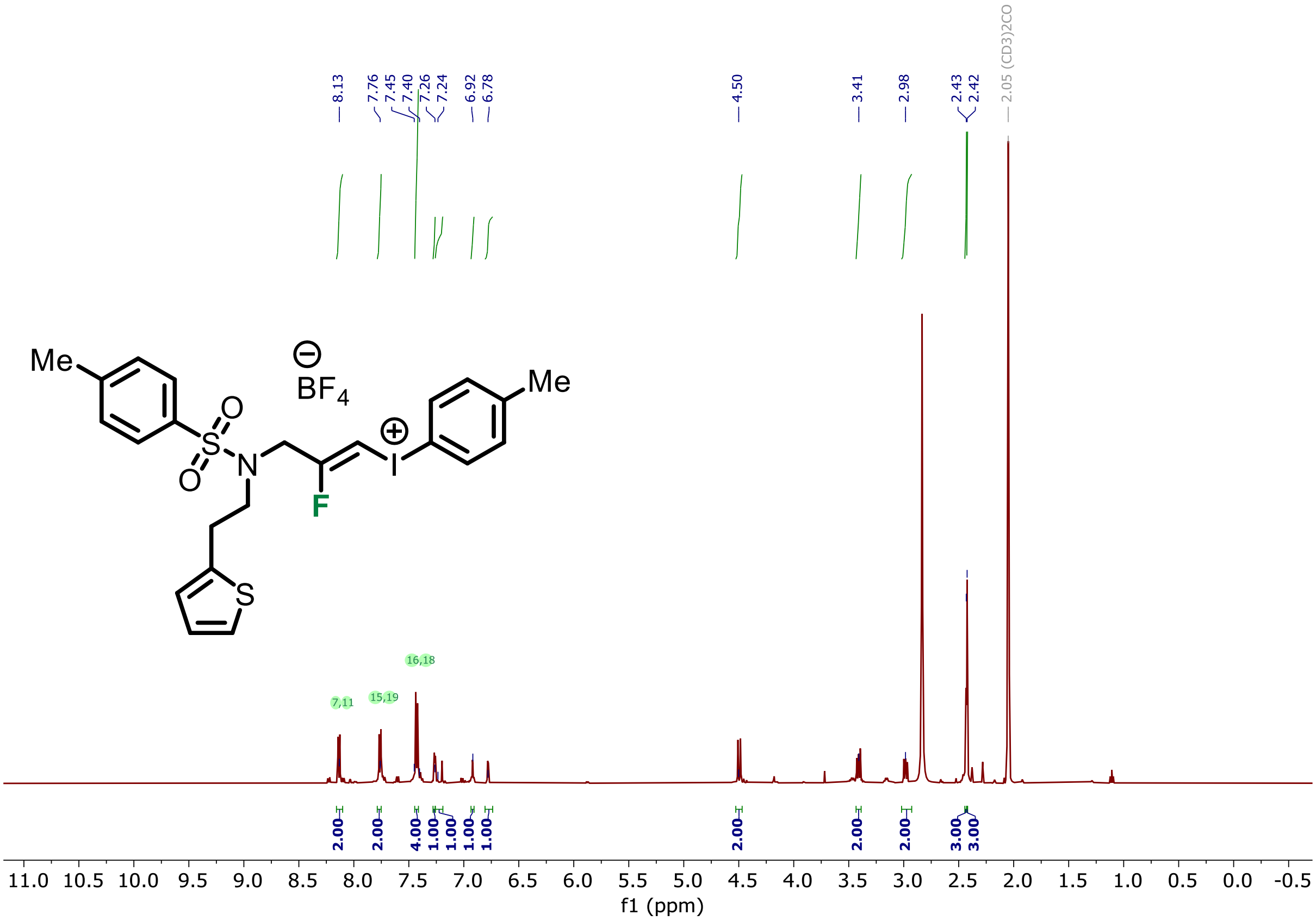

^{13}C NMR (126 MHz, Acetone-D_6)

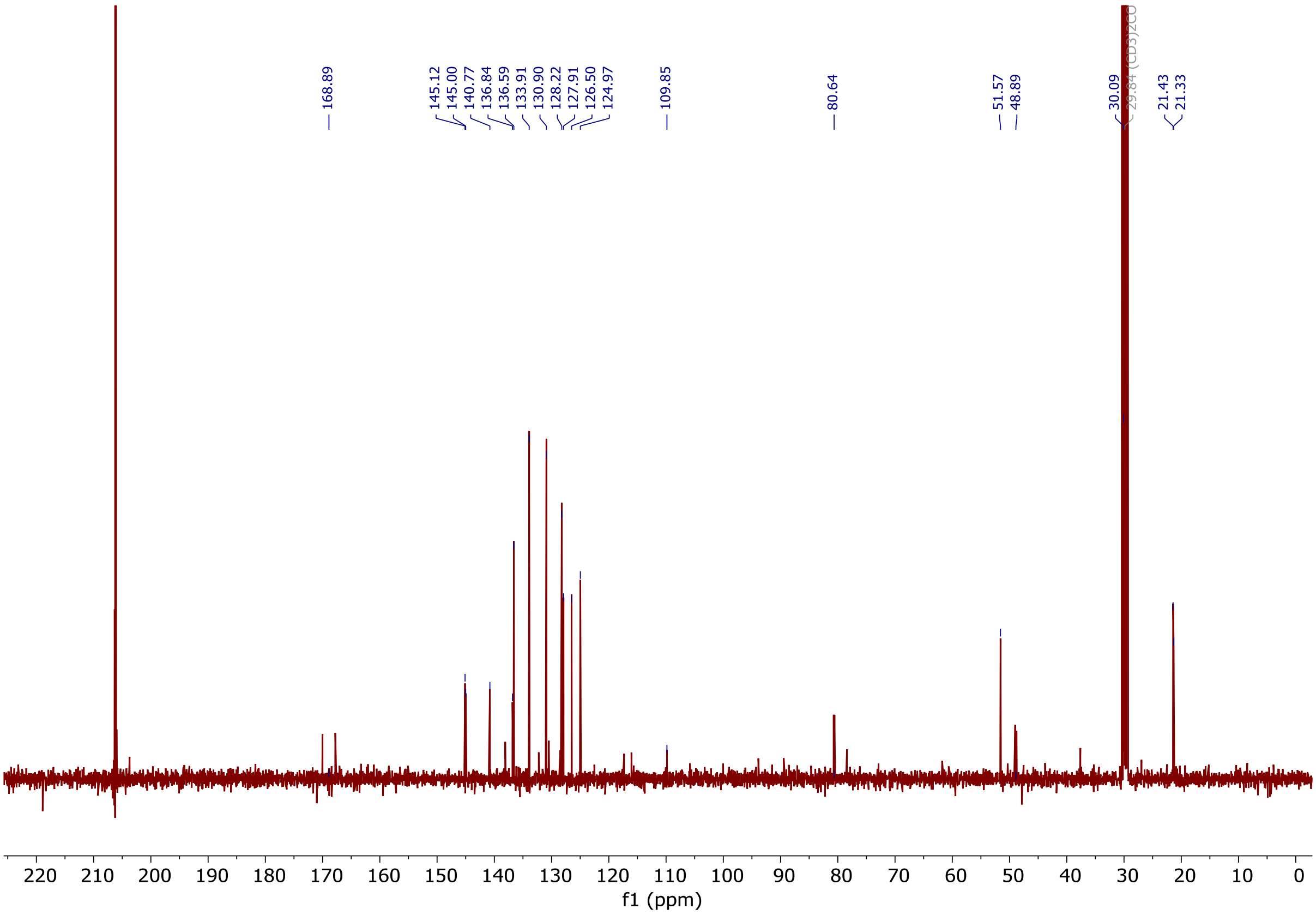

^{19}F NMR (471 MHz, Acetone-D_6)

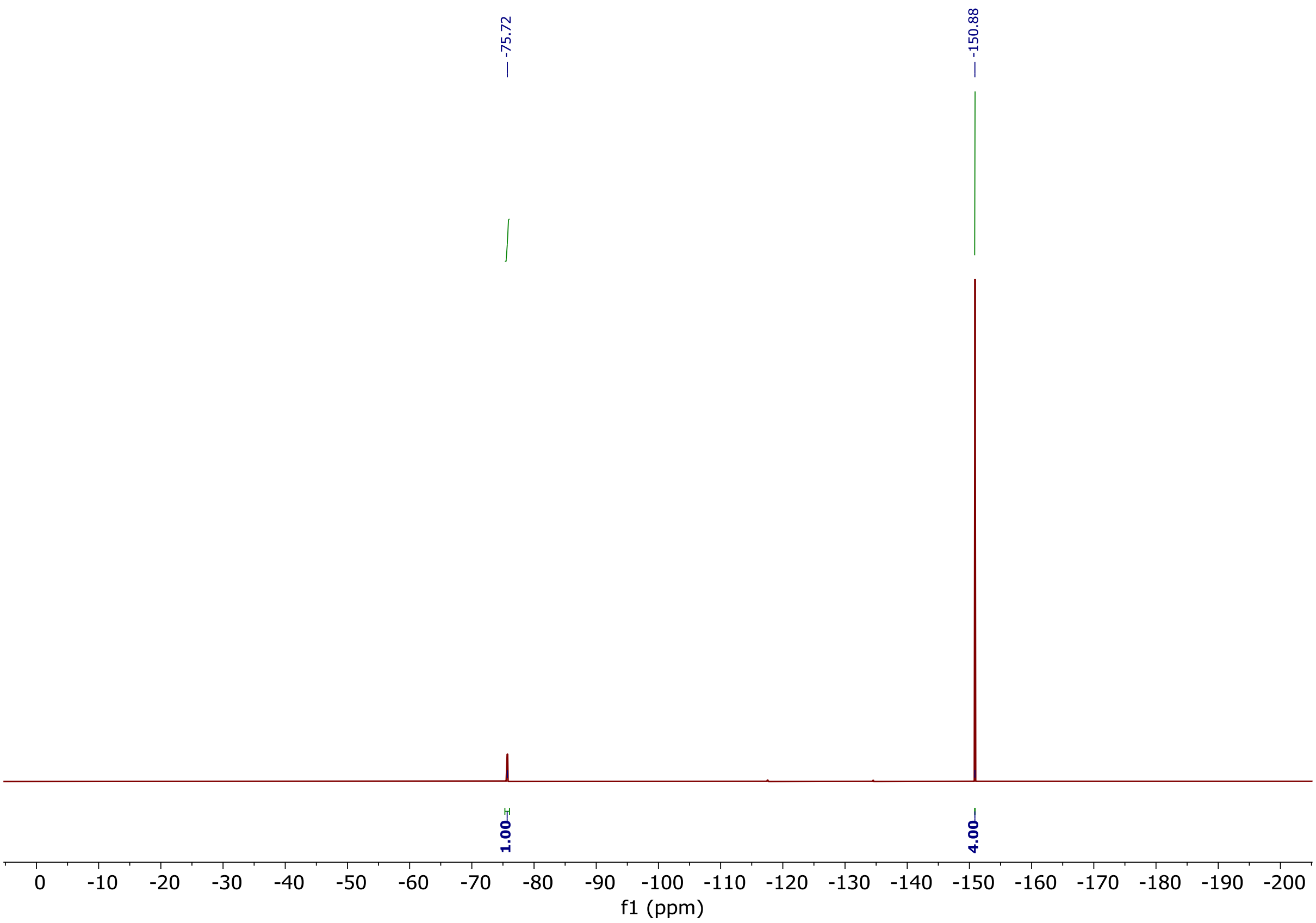

(Z)-(2-fluoro-3-((4-methyl-N-((tetrahydrofuran-2-yl)methyl)phenyl)sulfonamido)prop-1-en-1-yl)(p-tolyl)iodonium tetrafluoroborate (3x)
^{1}H NMR (400 MHz, Acetone-D_6)

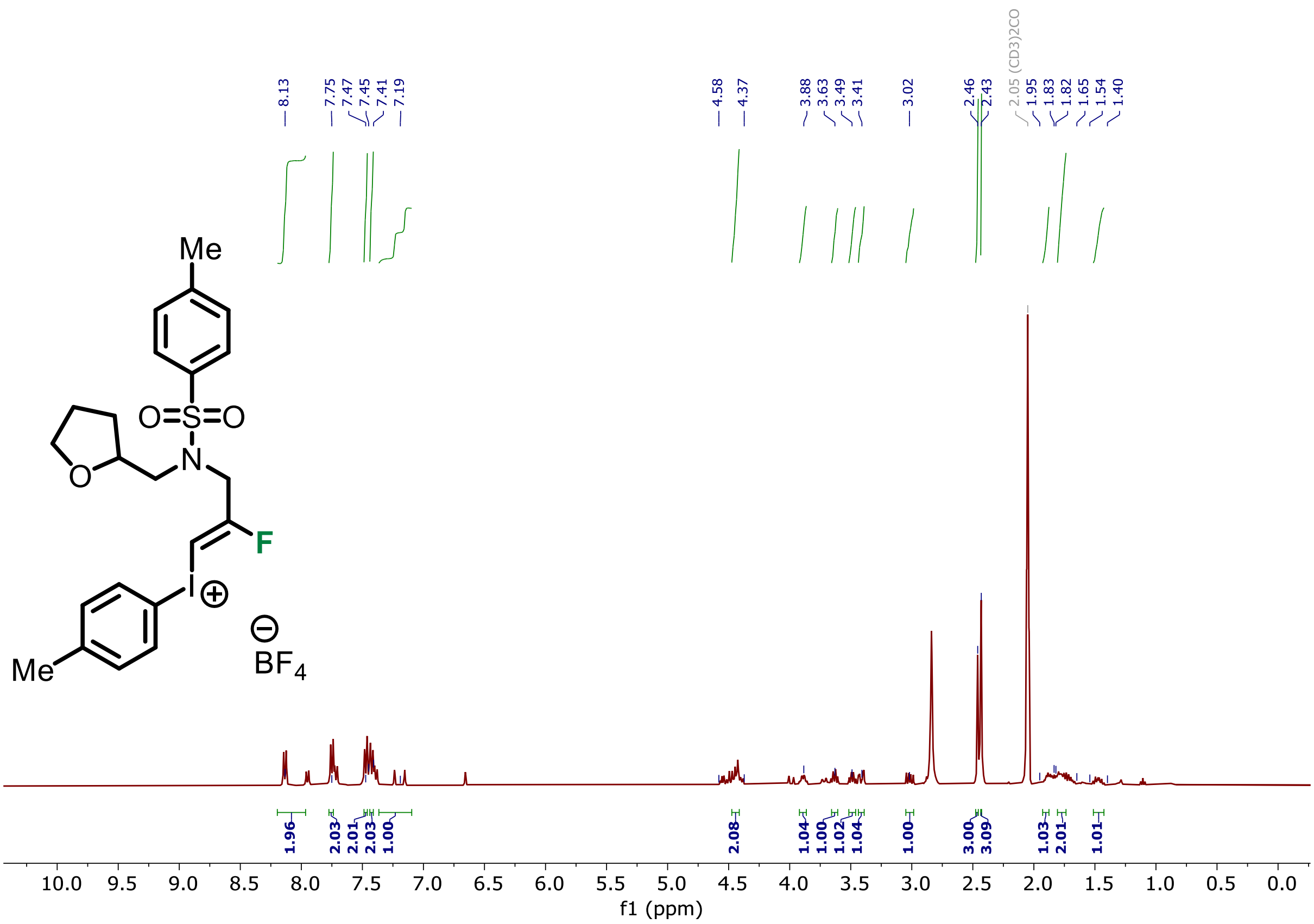

^{13}C NMR (126 MHz, Acetone-D_6)

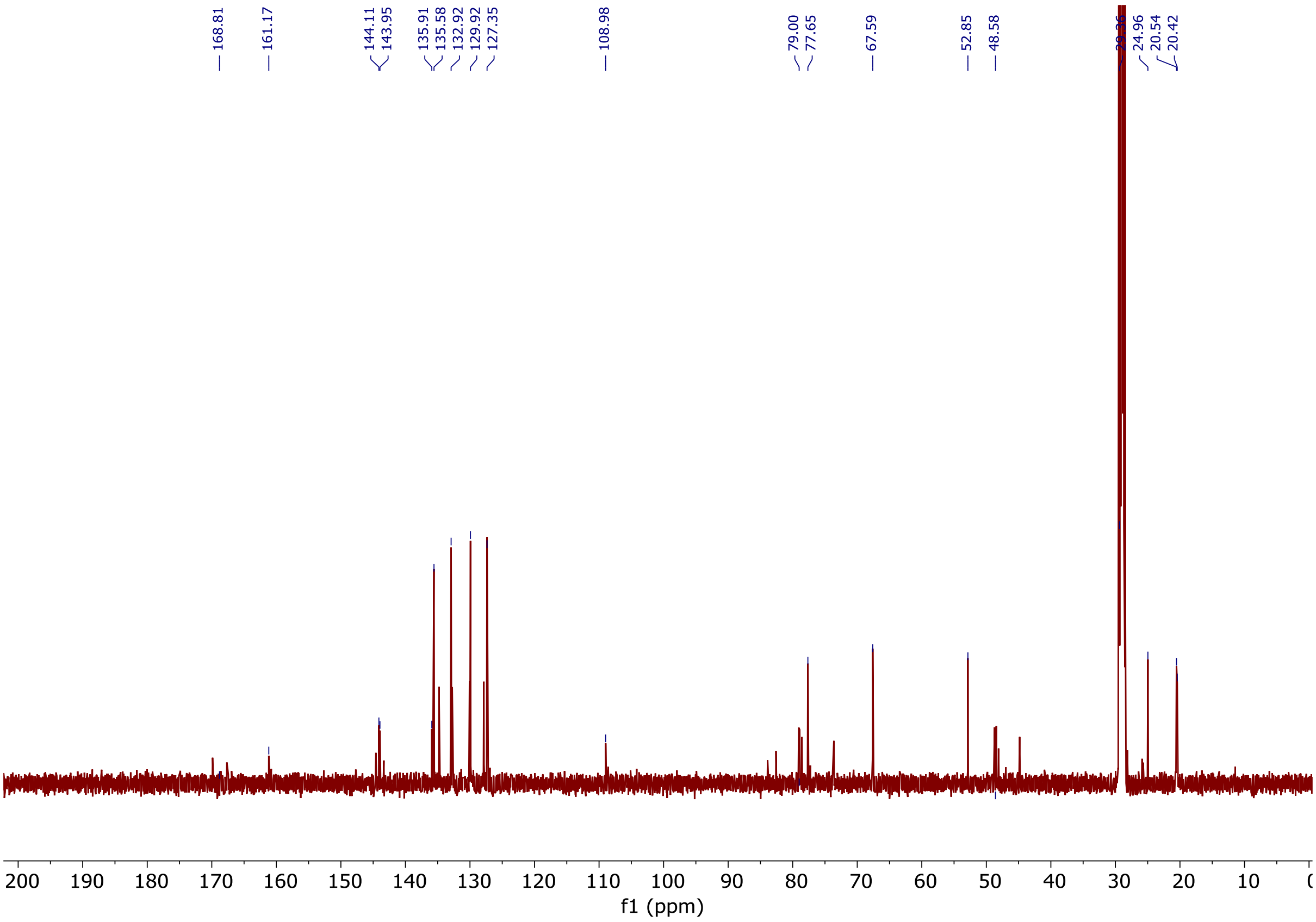

^{19}F NMR (471 MHz, Acetone-D_6)

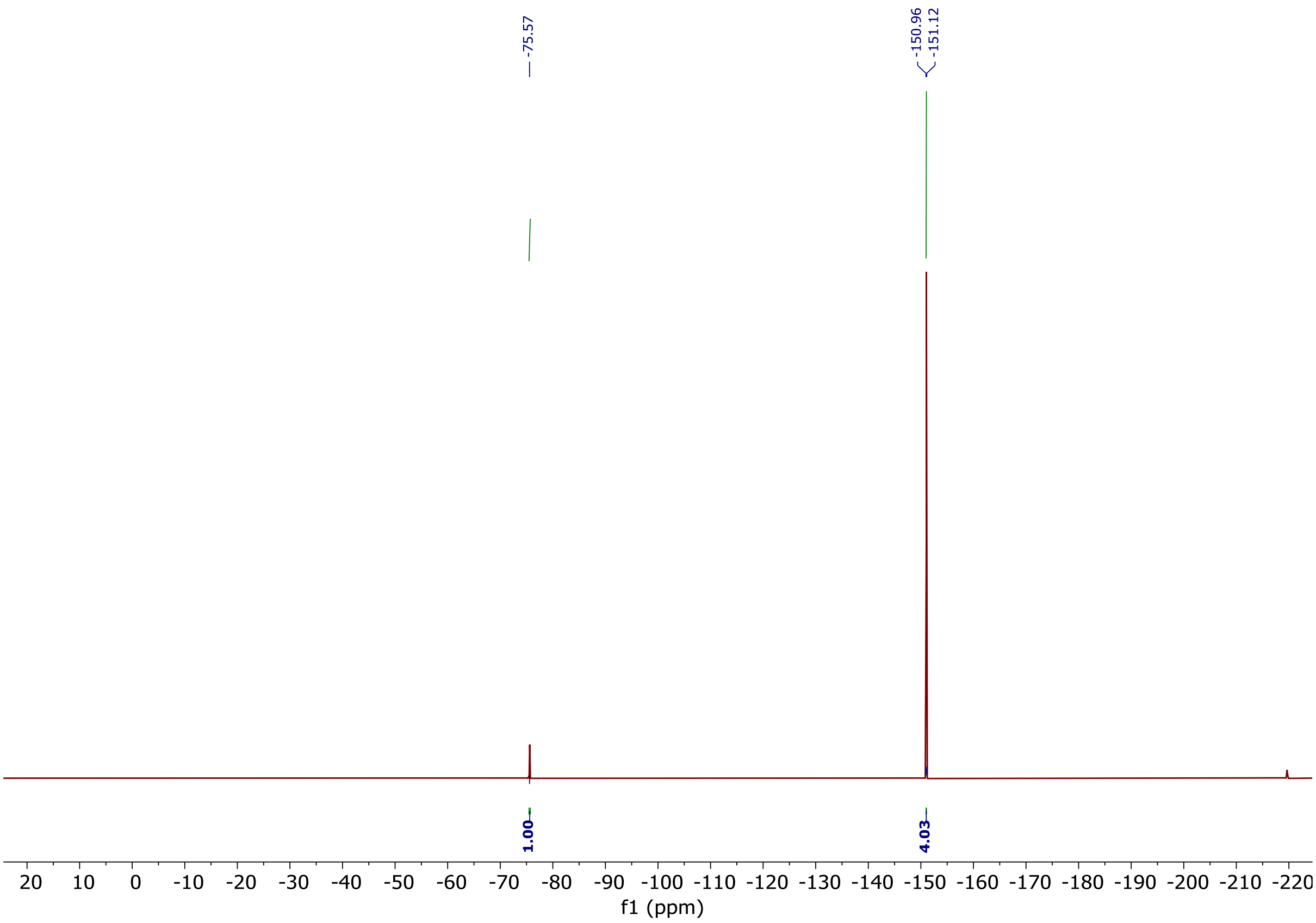

***(Z)-(3-(1,1-dioxidoisothiazolidin-2-yl)-2-fluoroprop-1-en-1-yl)(p-tolyl)iodonium tetrafluoroborate* (3y)**

^{1}H NMR (500 MHz, Acetone-D_6)

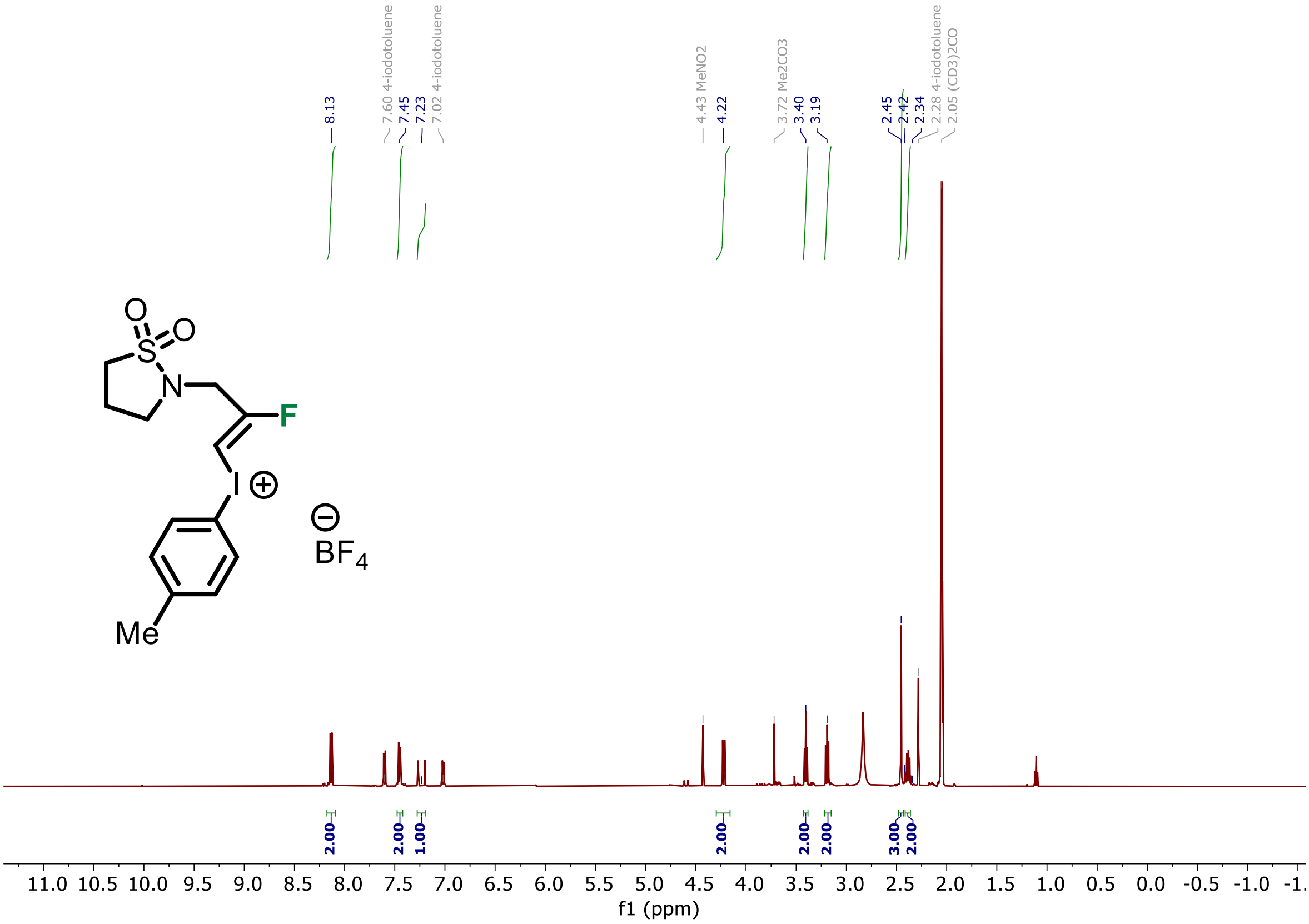

^{13}C NMR (126 MHz, Acetone-D_6)

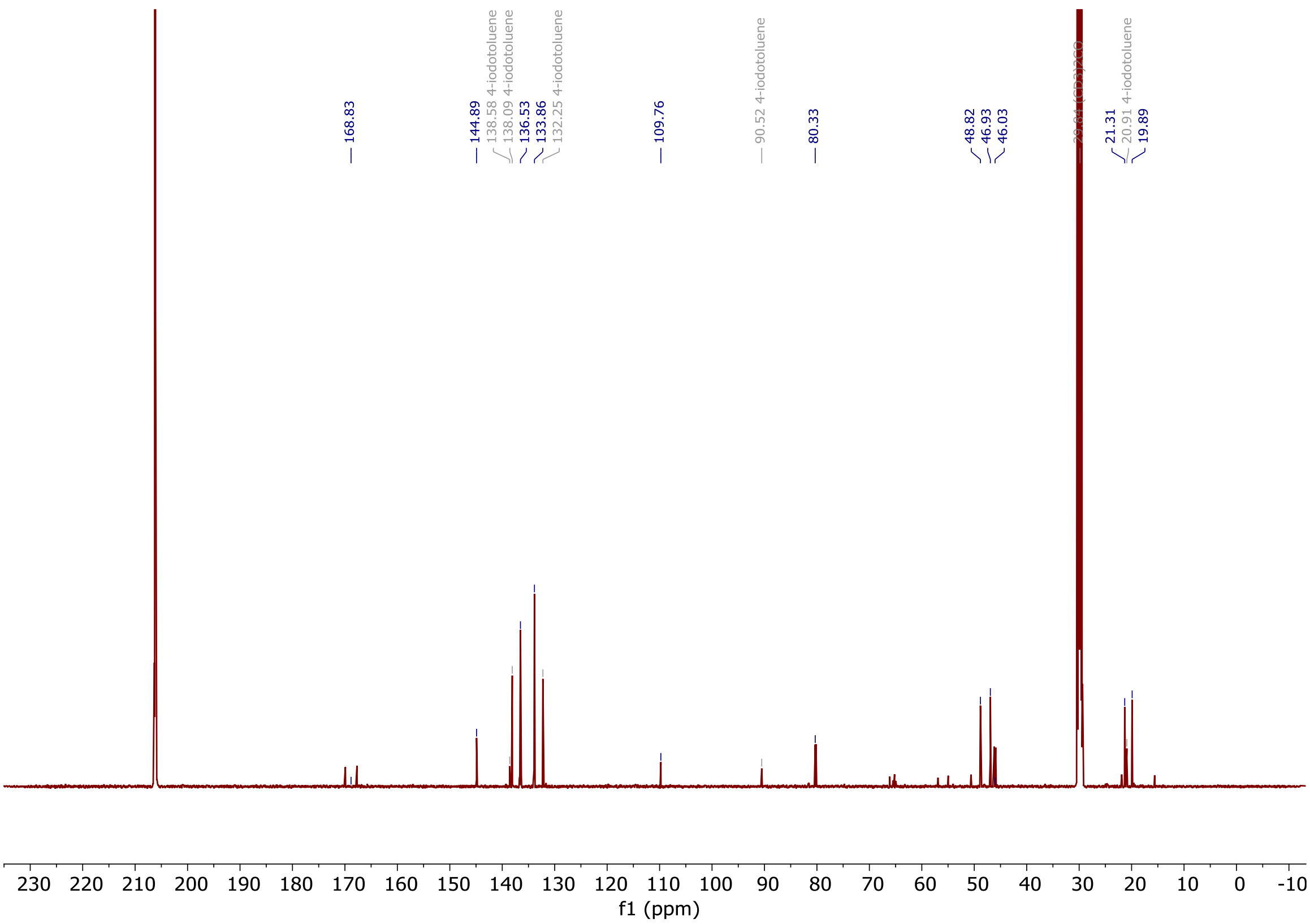

^{19}F NMR (376 MHz, Acetone-D_6)

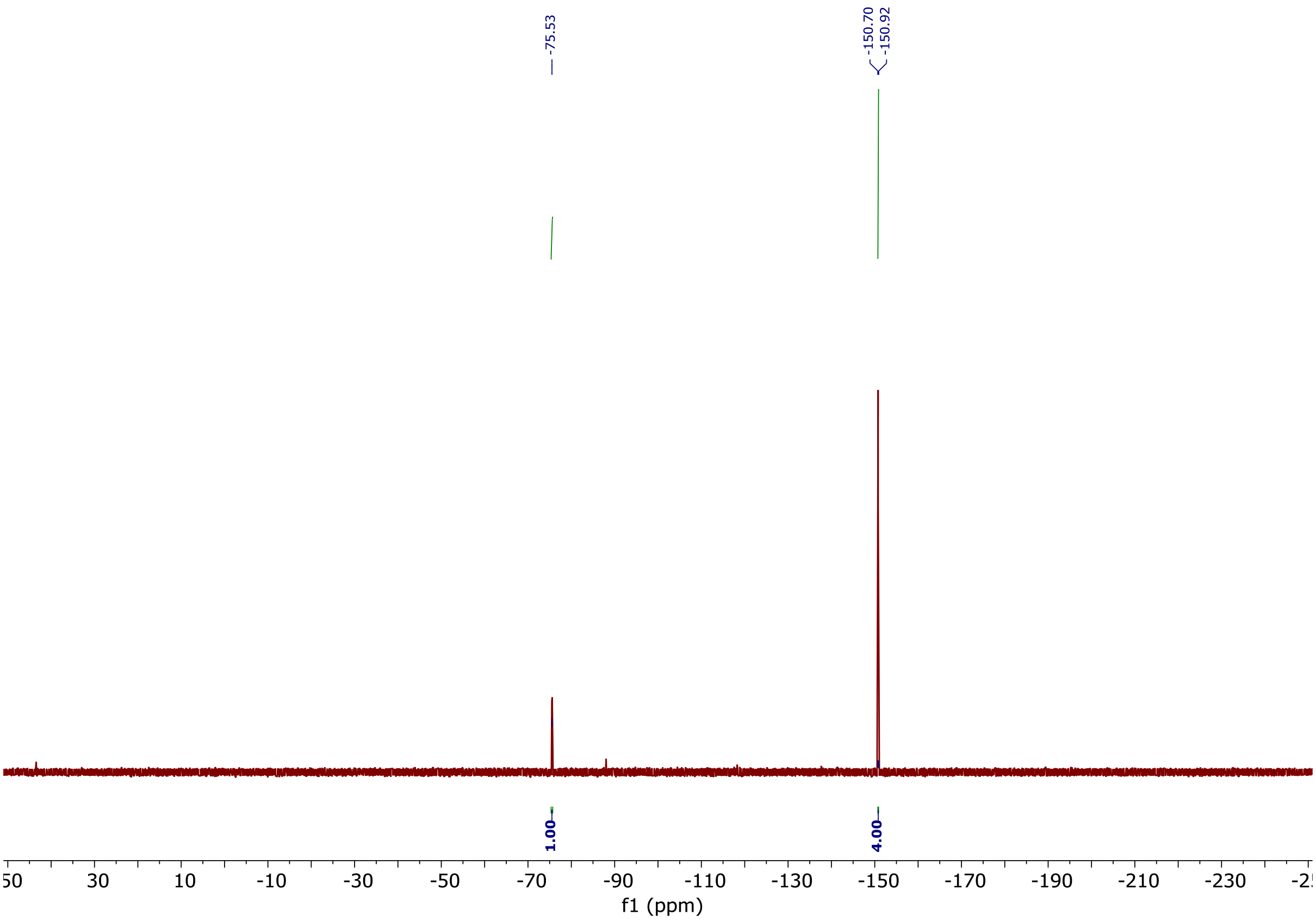

(Z)-(2-fluoro-2-(1-tosylpiperidin-2-yl)vinyl)(p-tolyl)iodonium tetrafluoroborate **(3z)**

^{1}H NMR (400 MHz, Acetone-D_6)

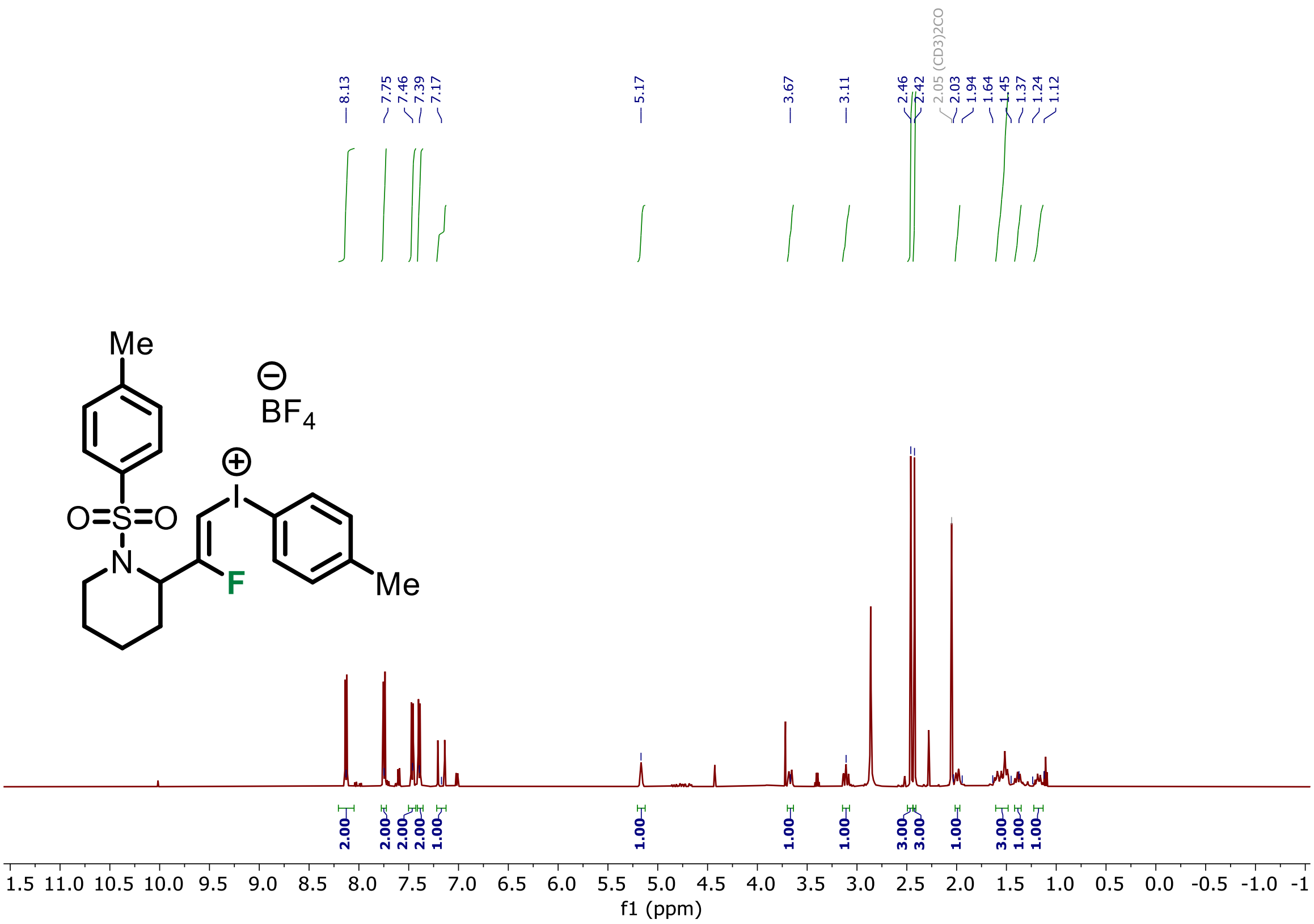

^{13}C NMR (126 MHz, Acetone-D_6)

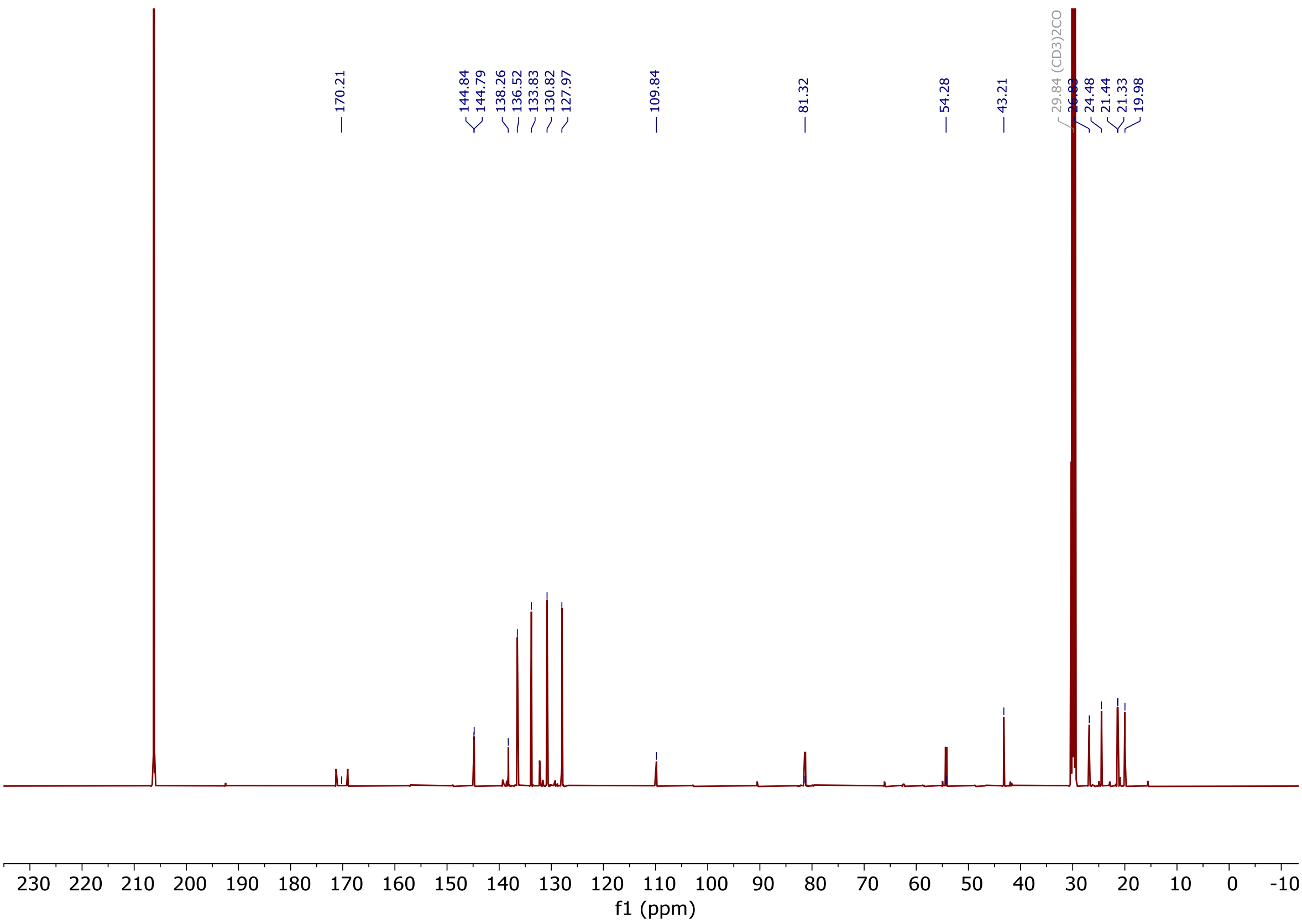

^{19}F NMR (376 MHz, Acetone-D_6)

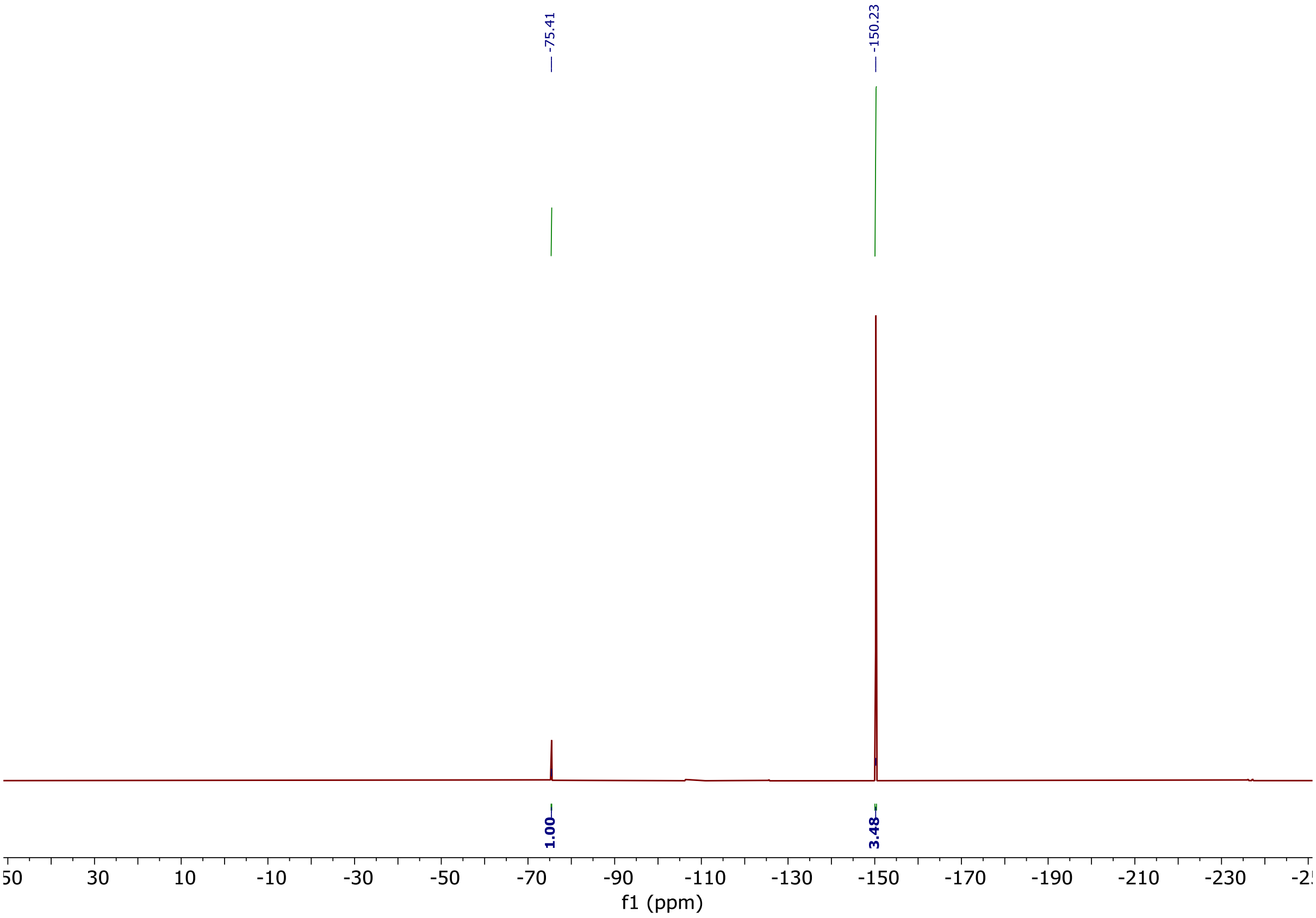

(Z)-(2-fluoro-2-(1-((N-(3-fluorobenzyl)-4-methylphenyl)sulfonamido)cyclohexyl)vinyl)(p-tolyl)iodonium tetrafluoroborate (3aa)

^{1}H NMR (500 MHz, Acetone-*D$_6$*)

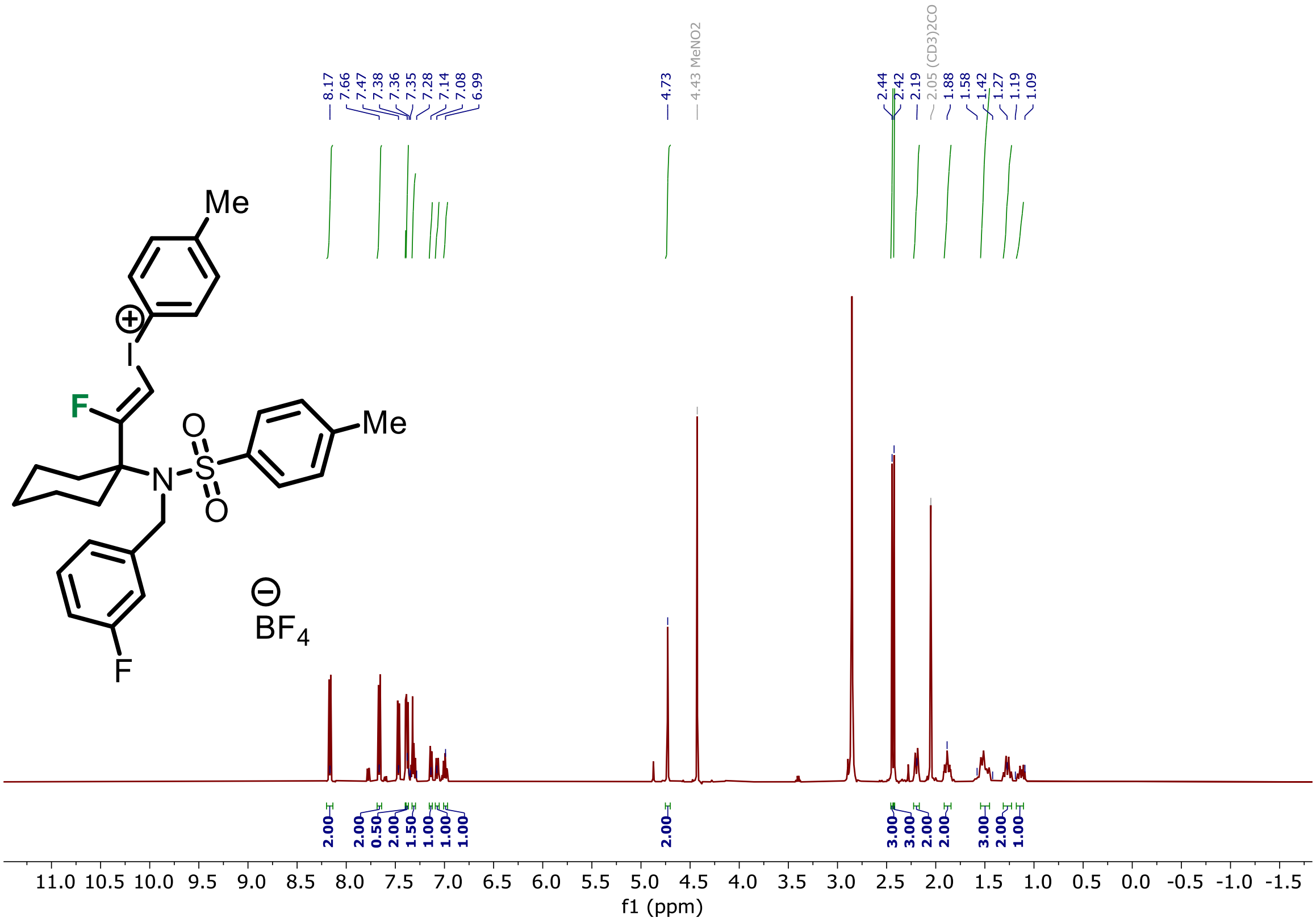

^{13}C NMR (126 MHz, Acetone-*D$_6$*)

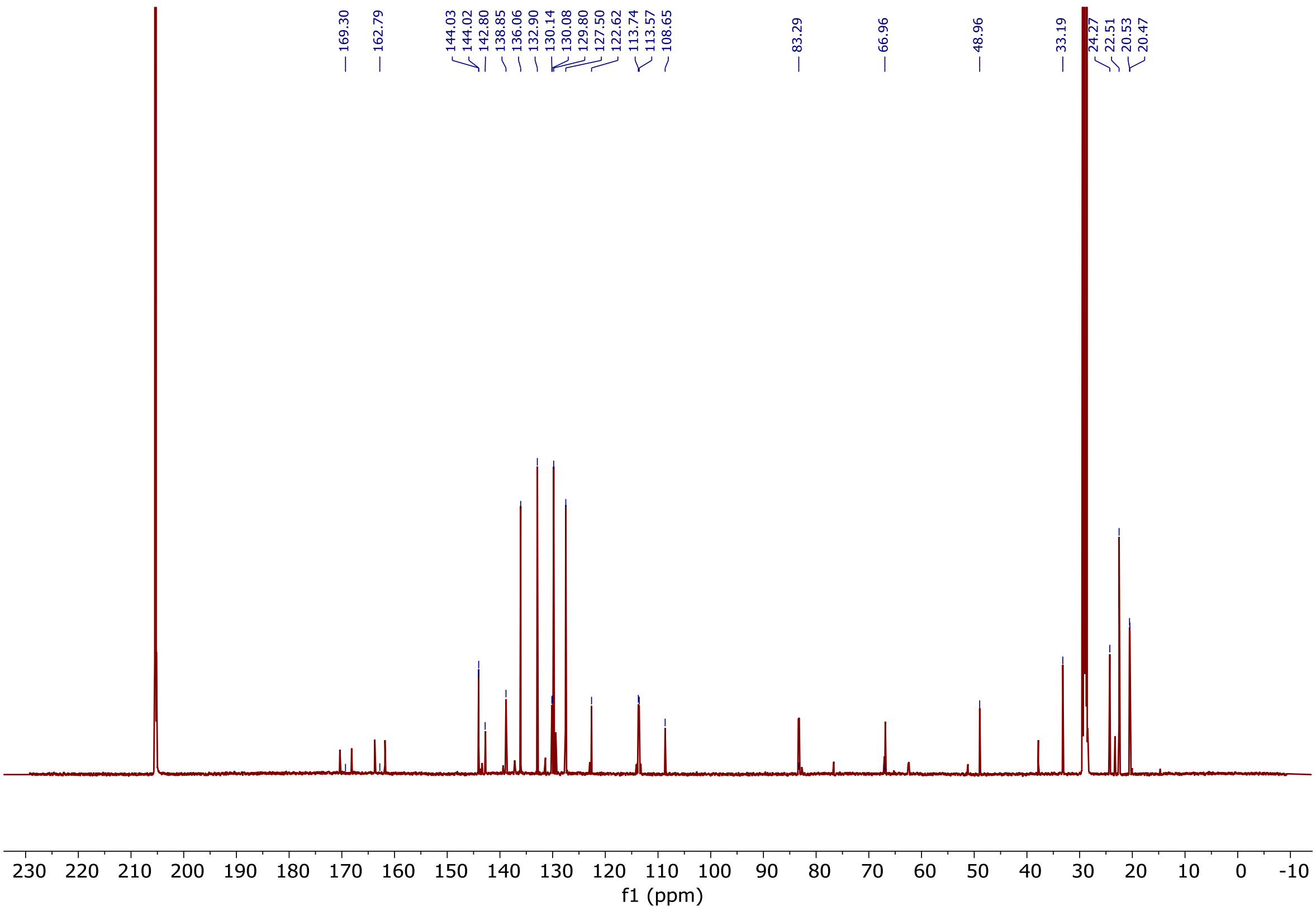

^{19}F NMR (376 MHz, Acetone-D_6)

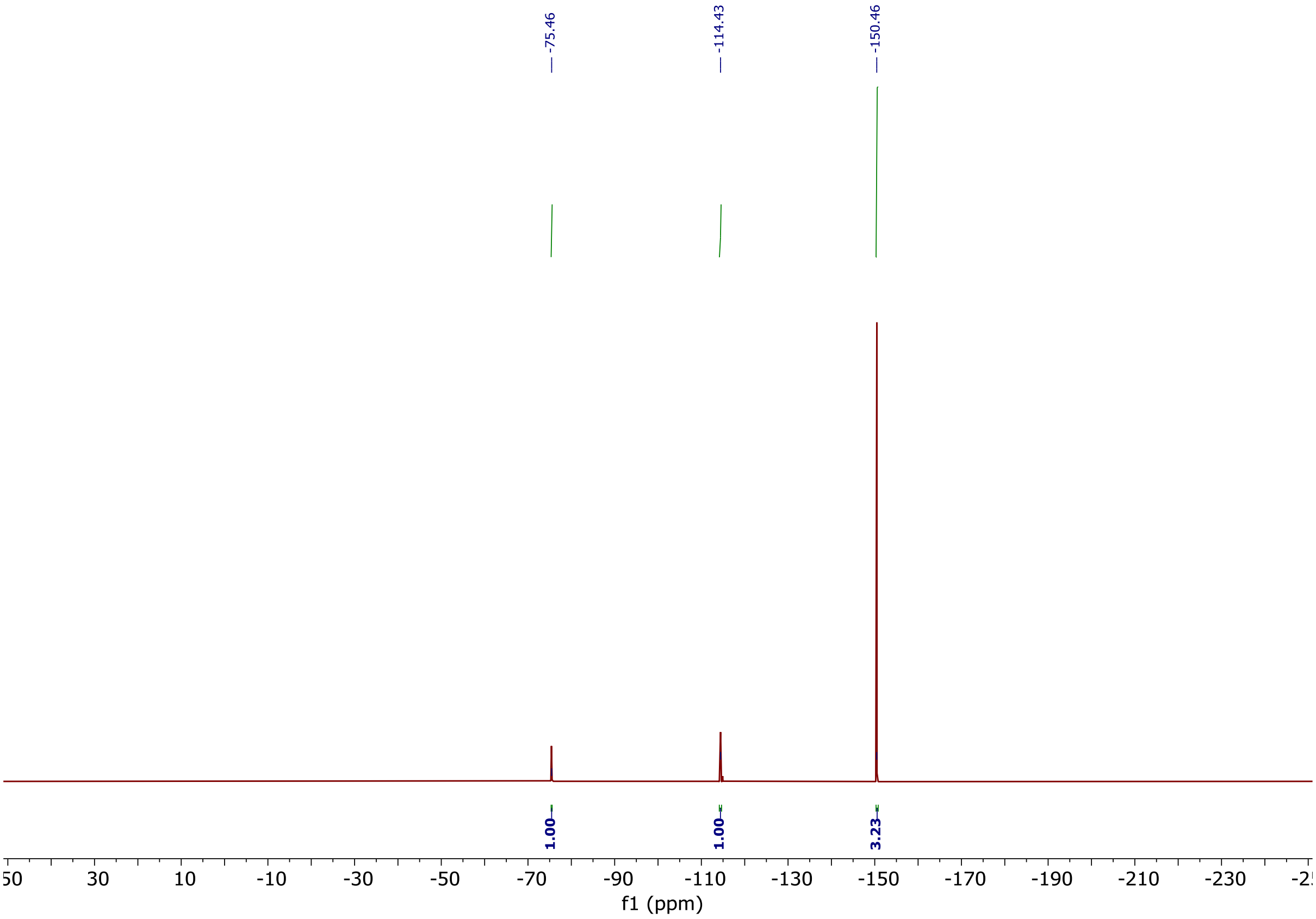

***(Z)-(2-(1-((N,4-dimethylphenyl)sulfonamido)cyclohexyl)-2-fluorovinyl)(p-tolyl)iodonium tetrafluoroborate* (3ab)**

^{1}H NMR (500 MHz, Acetone-D_6)

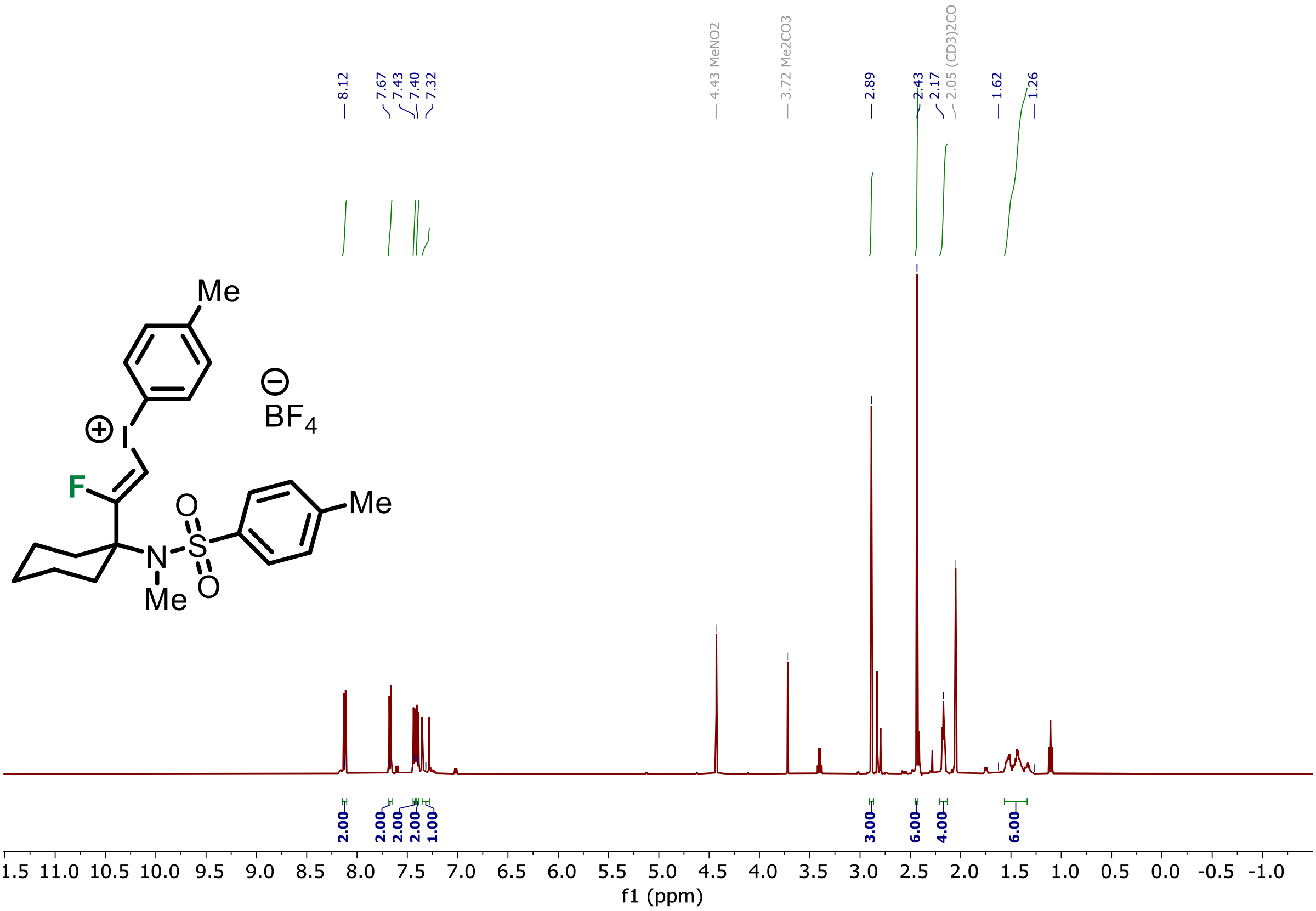

^{13}C NMR (126 MHz, Acetone-D_6)

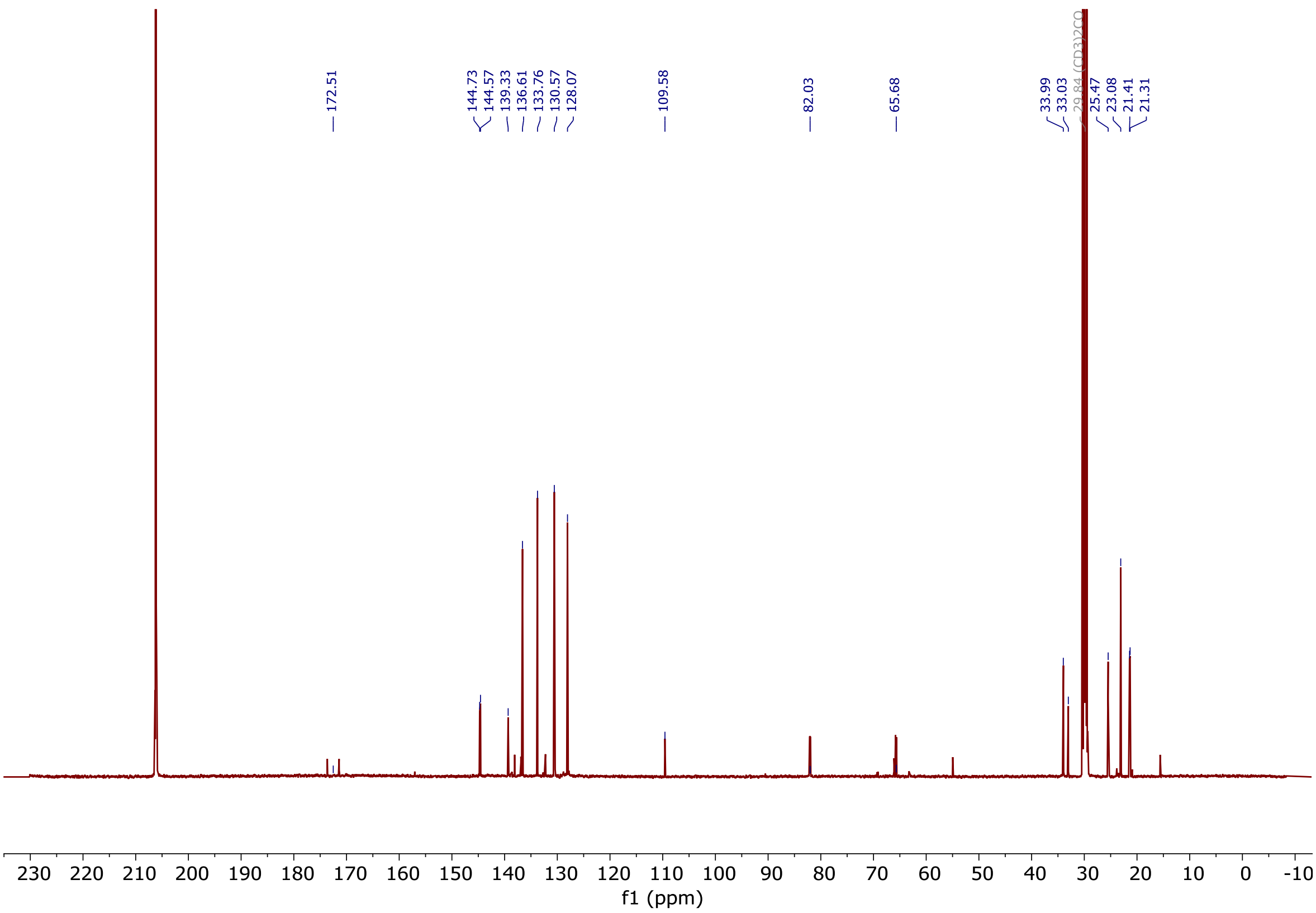

^{19}F NMR (376 MHz, Acetone-D_6)

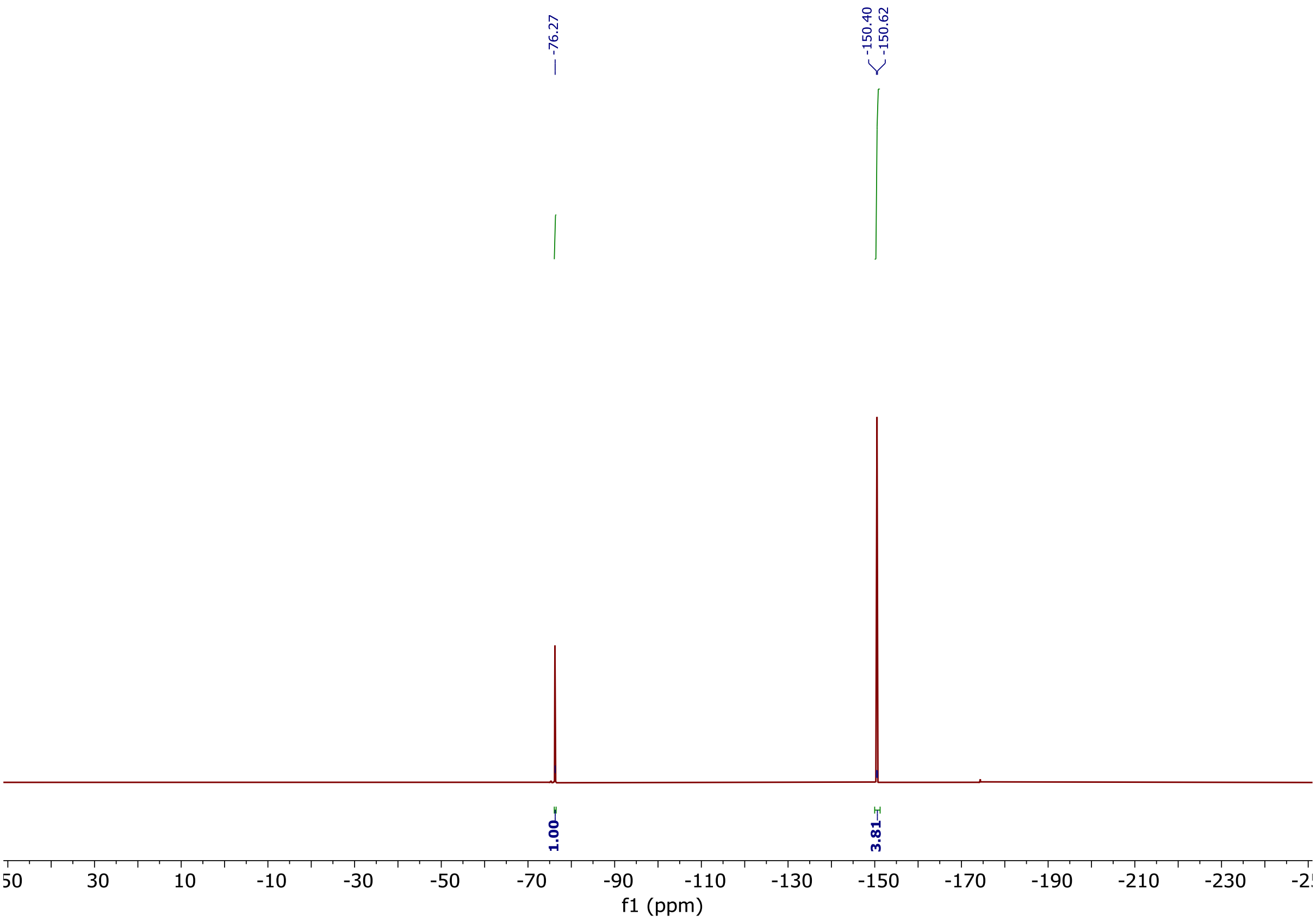

***(Z)-(3-((N,4-dimethylphenyl)sulfonamido)-2-fluoroprop-1-en-1-yl)(p-tolyl)iodonium tetrafluoroborate* (3ac)**

^{1}H NMR (500 MHz, Acetone-D_6)

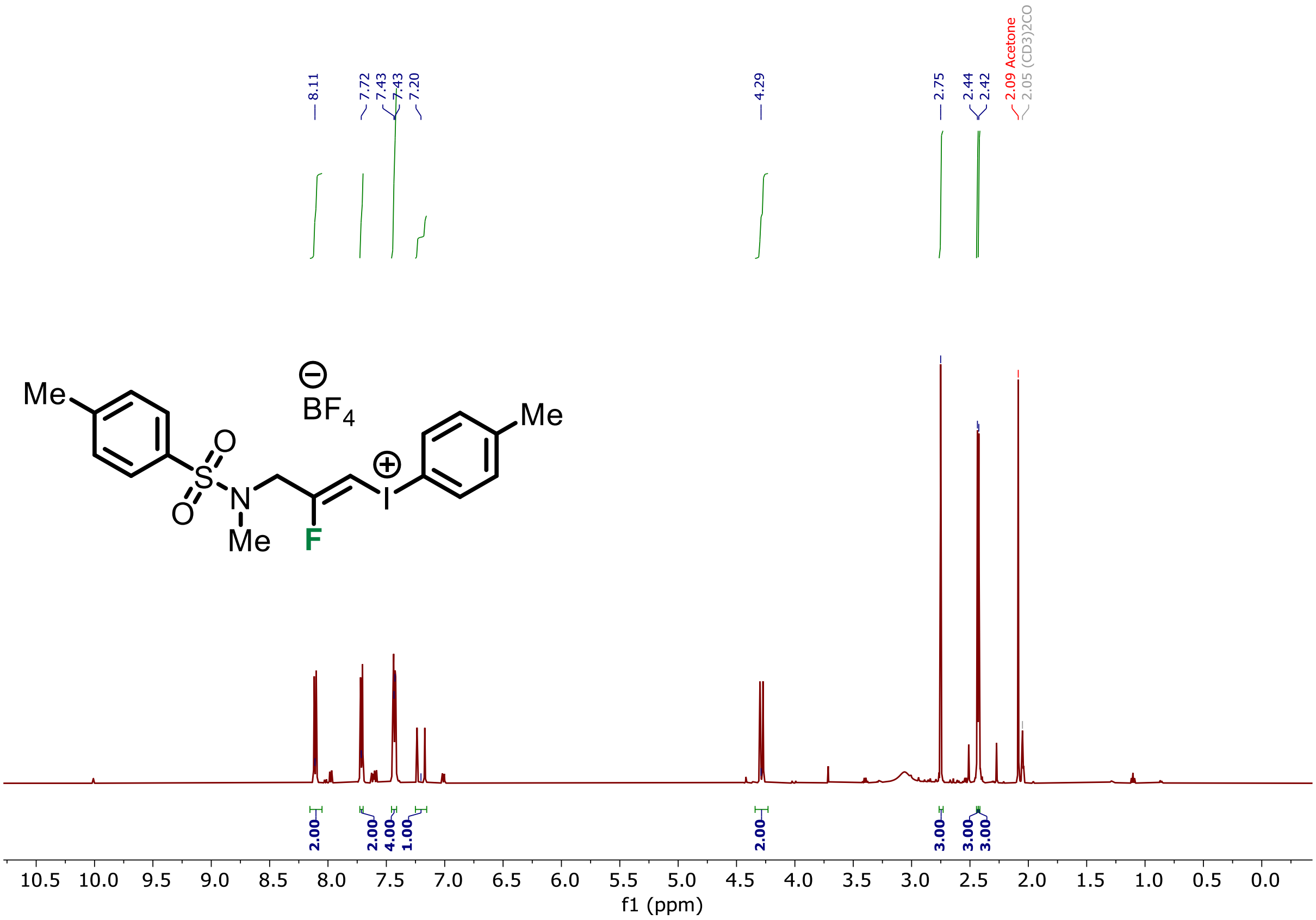

^{13}C NMR (126 MHz, Acetone-*D₆*)

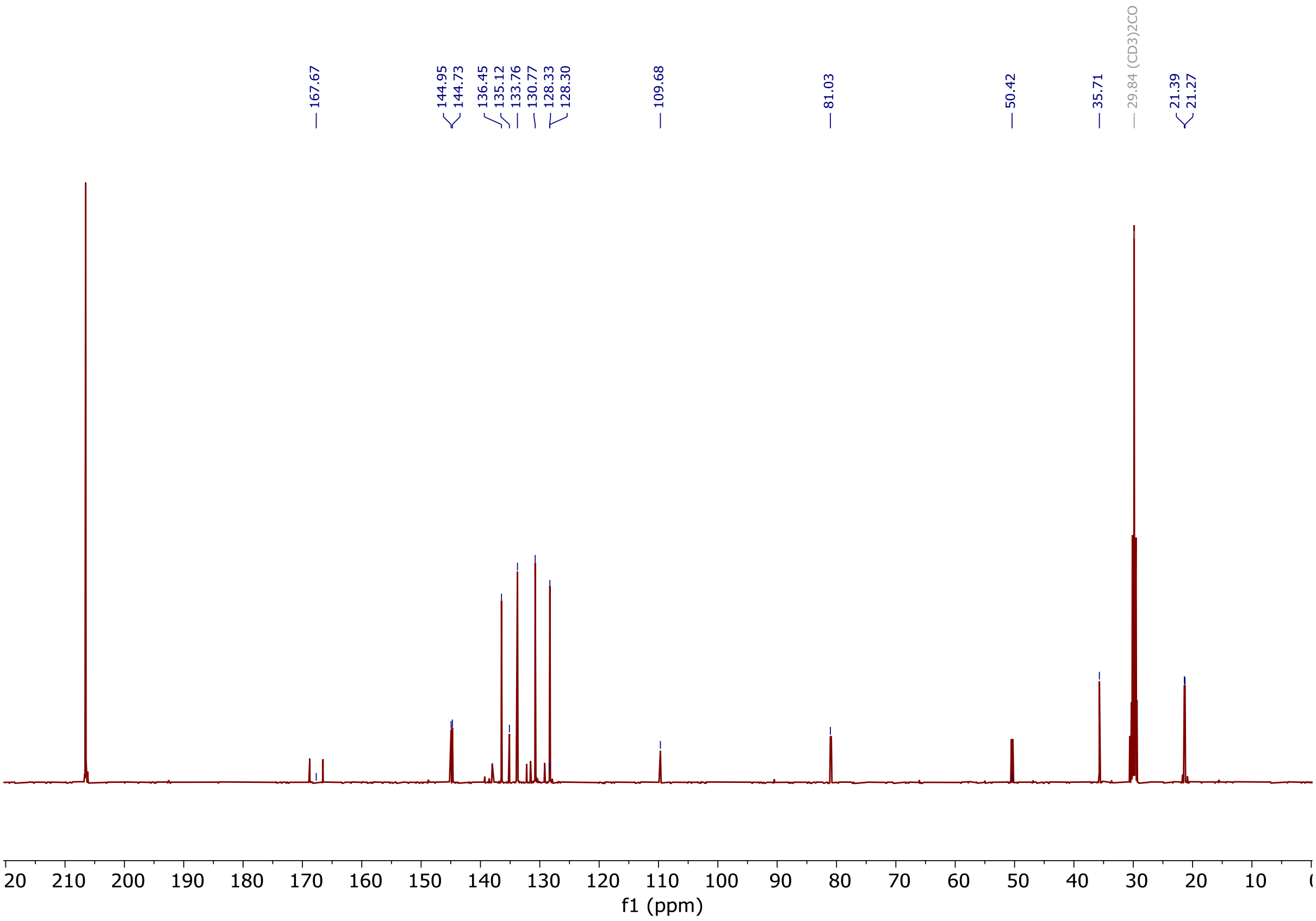

^{19}F NMR (471 MHz, Acetone-*D₆*)

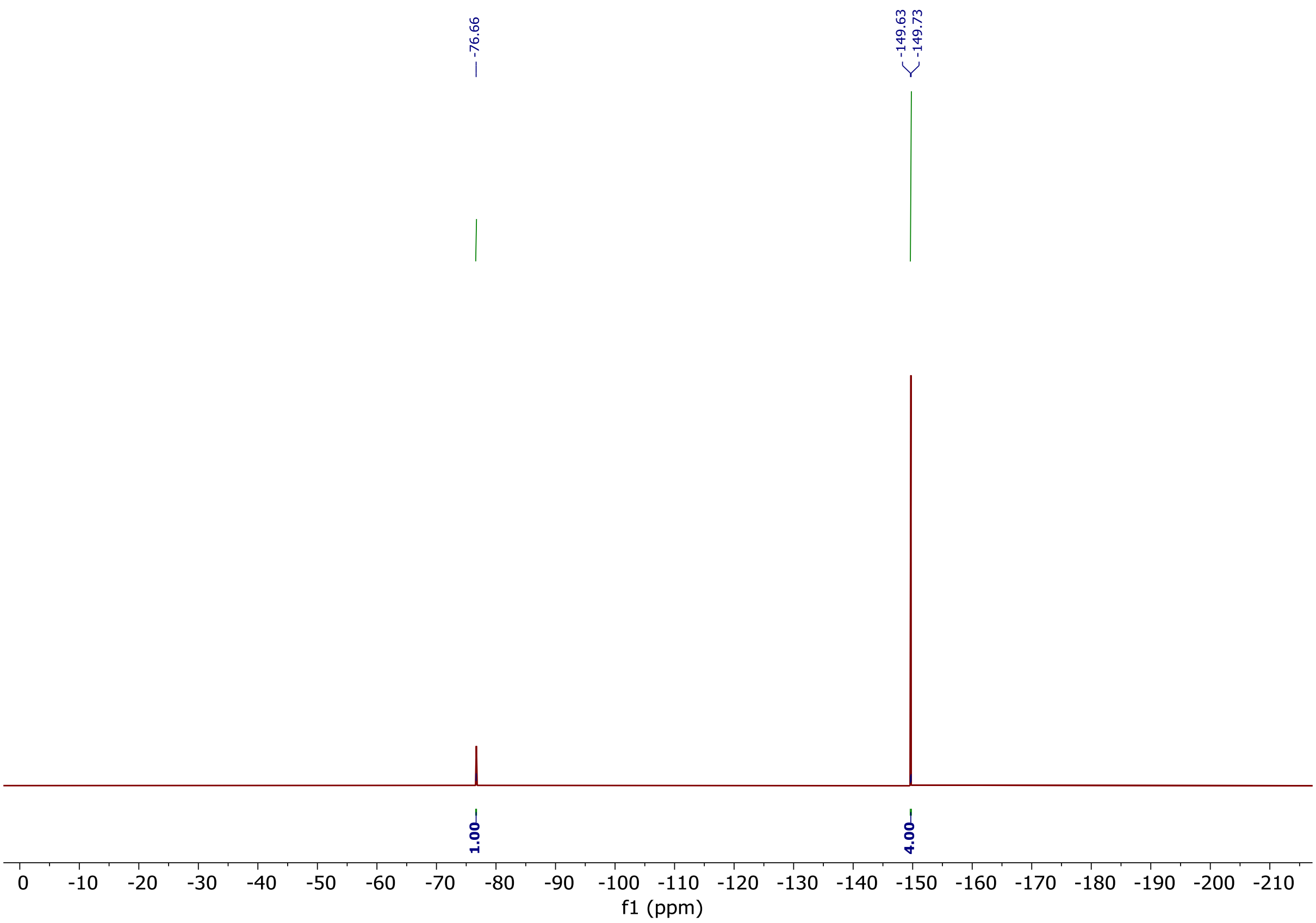

***(Z)-(3-(benzyloxy)-2-fluorobut-1-en-1-yl)(p-tolyl)iodonium tetrafluoroborate* (3ad)**

^{1}H NMR (600 MHz, Acetone-D_6)

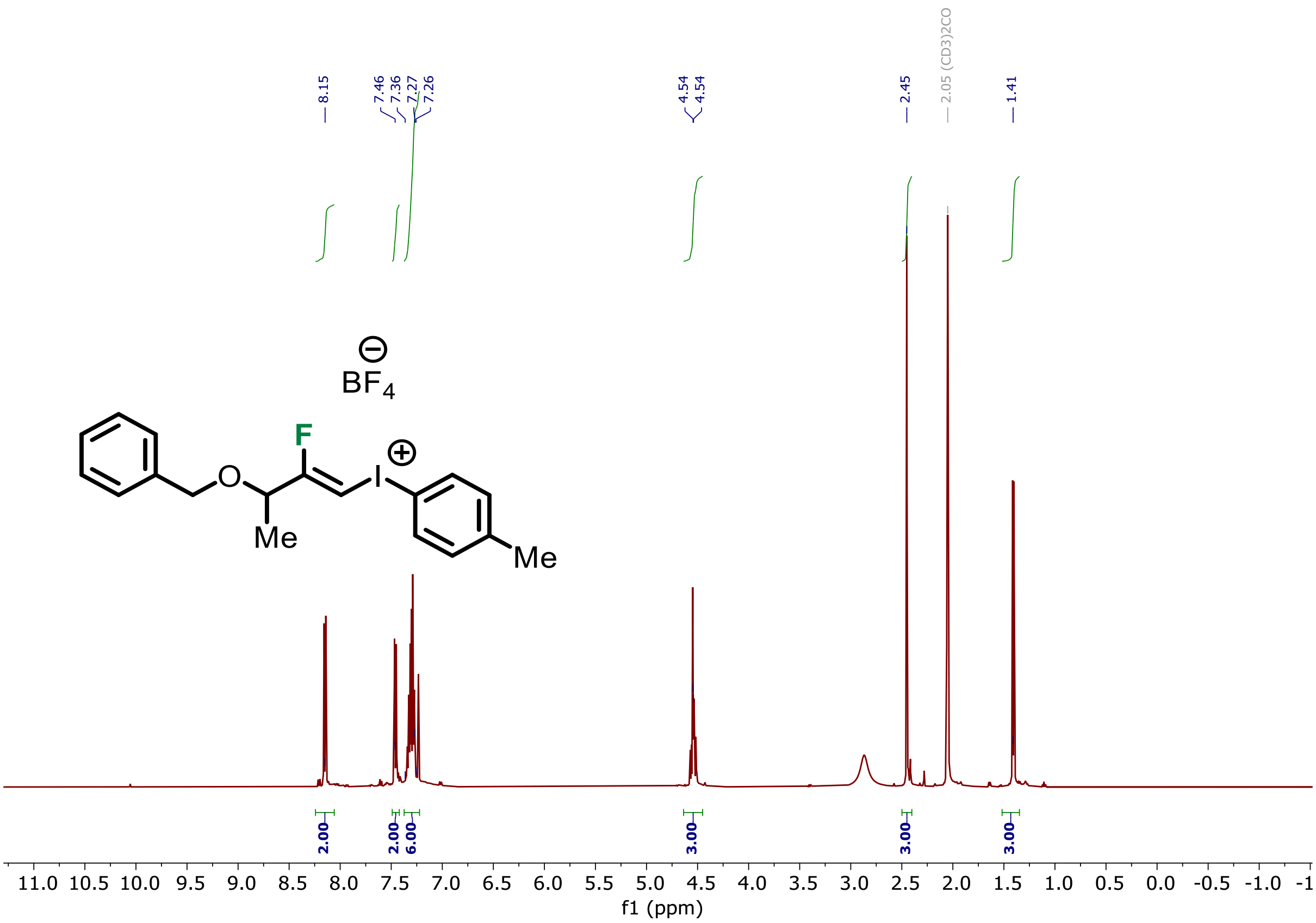

^{13}C NMR (151 MHz, Acetone-D_6)

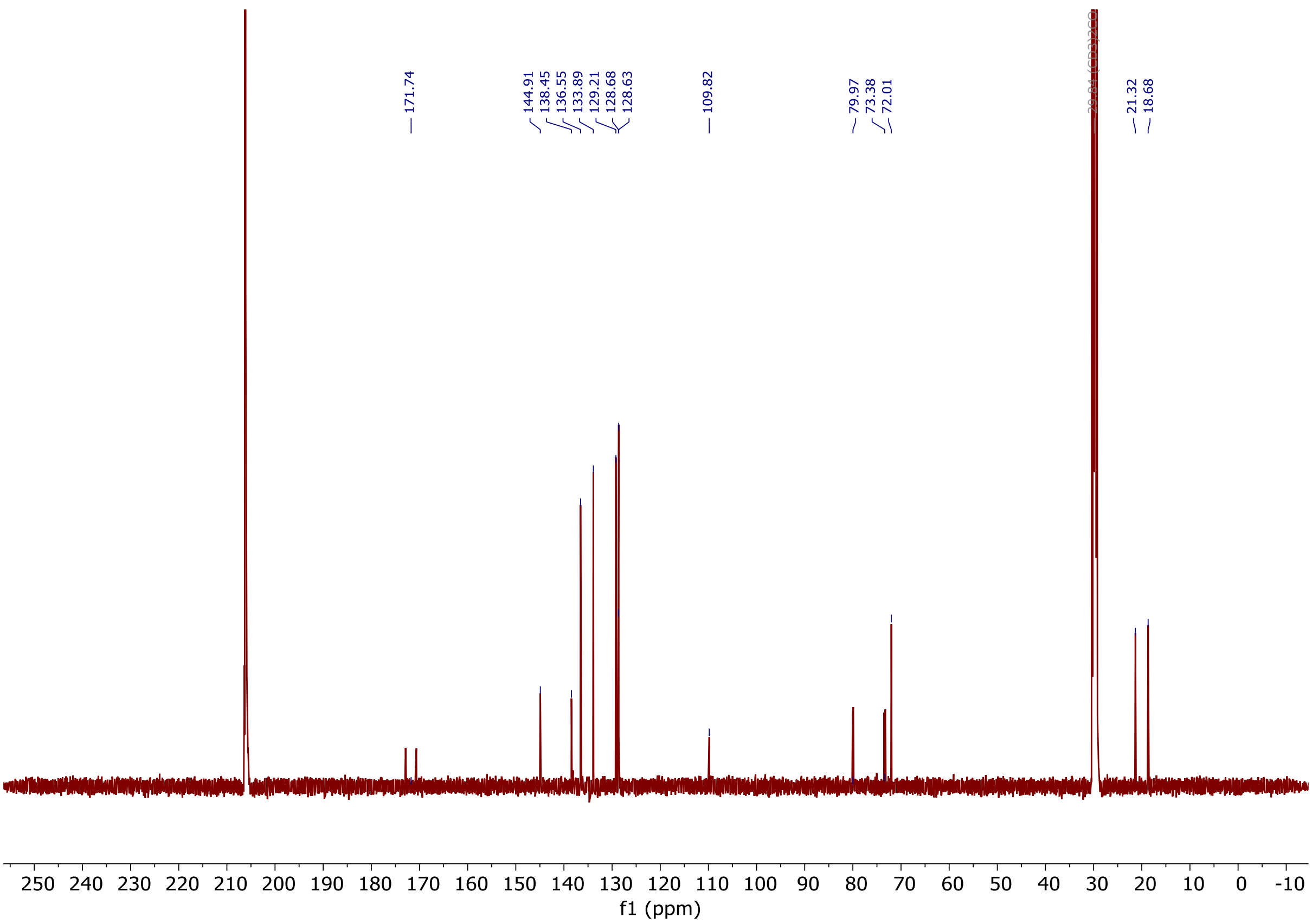

^{19}F NMR (471 MHz, Acetone-D_6)

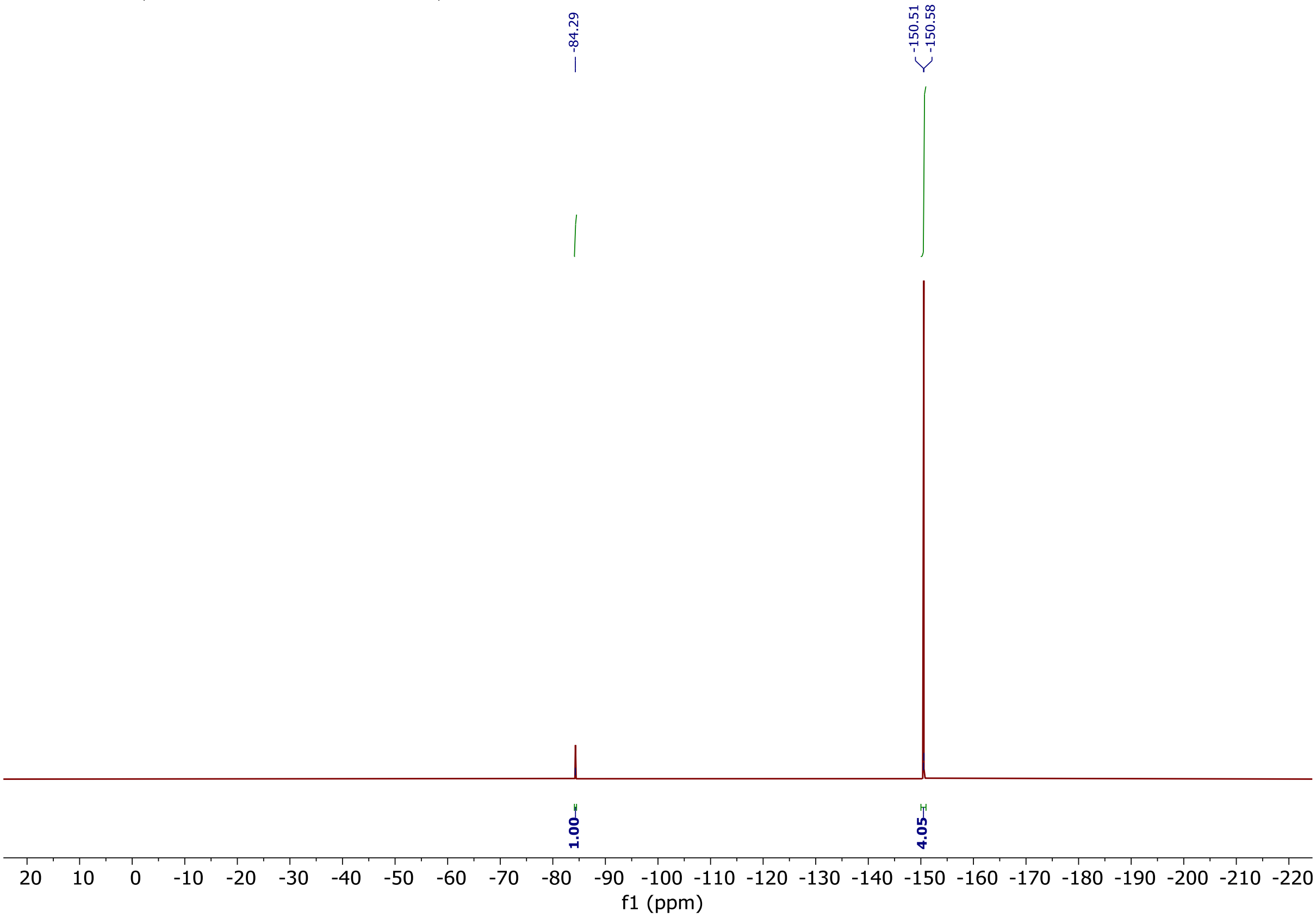

***(Z)-(3-((3-bromobenzyl)oxy)-2-fluoro-3-methylbut-1-en-1-yl)(p-tolyl)iodonium tetrafluoroborate* (3ae)**

^{1}H NMR (400 MHz, Acetone-*D*$_6$)

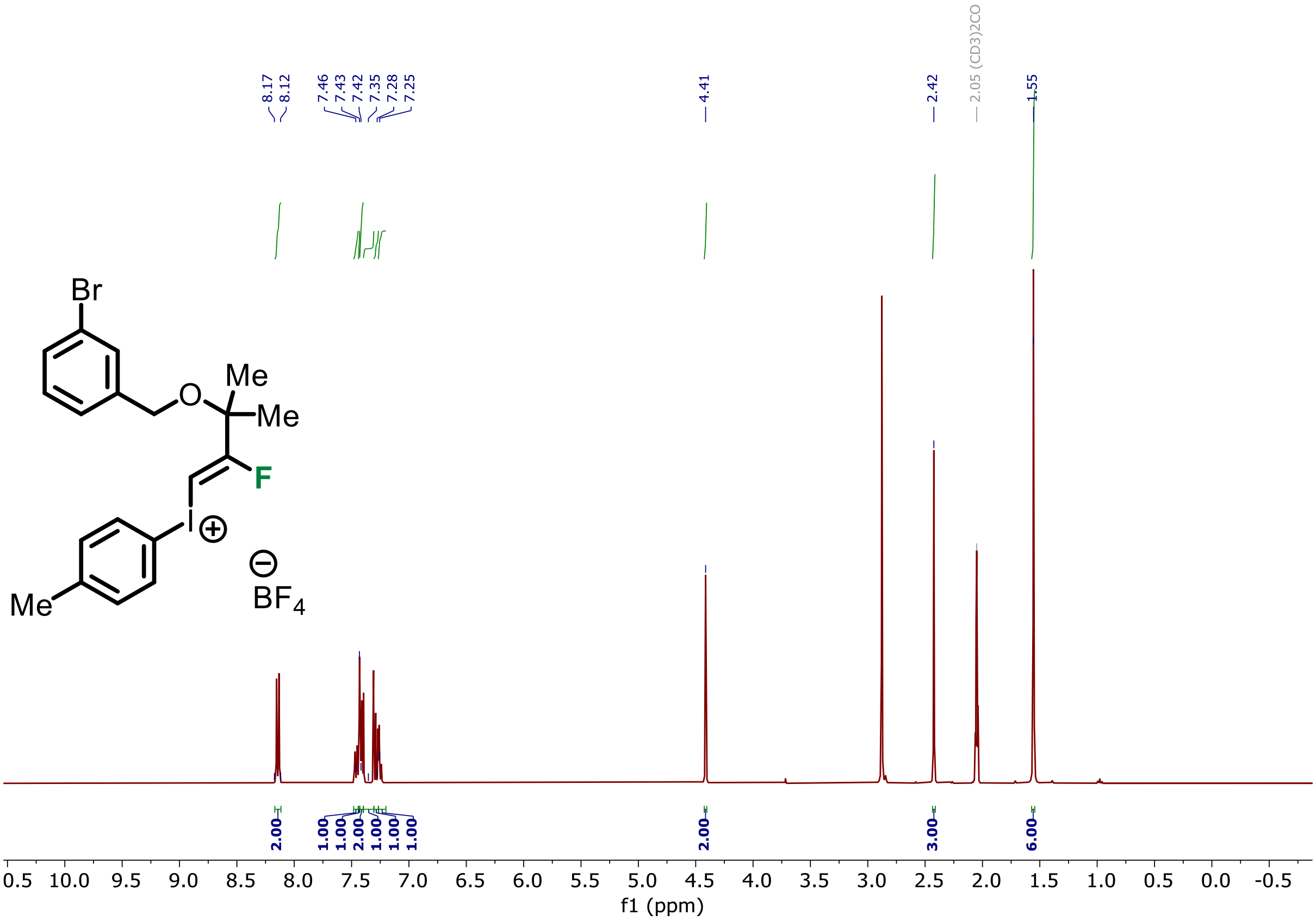

^{13}C NMR (126 MHz, Acetone-D_6)

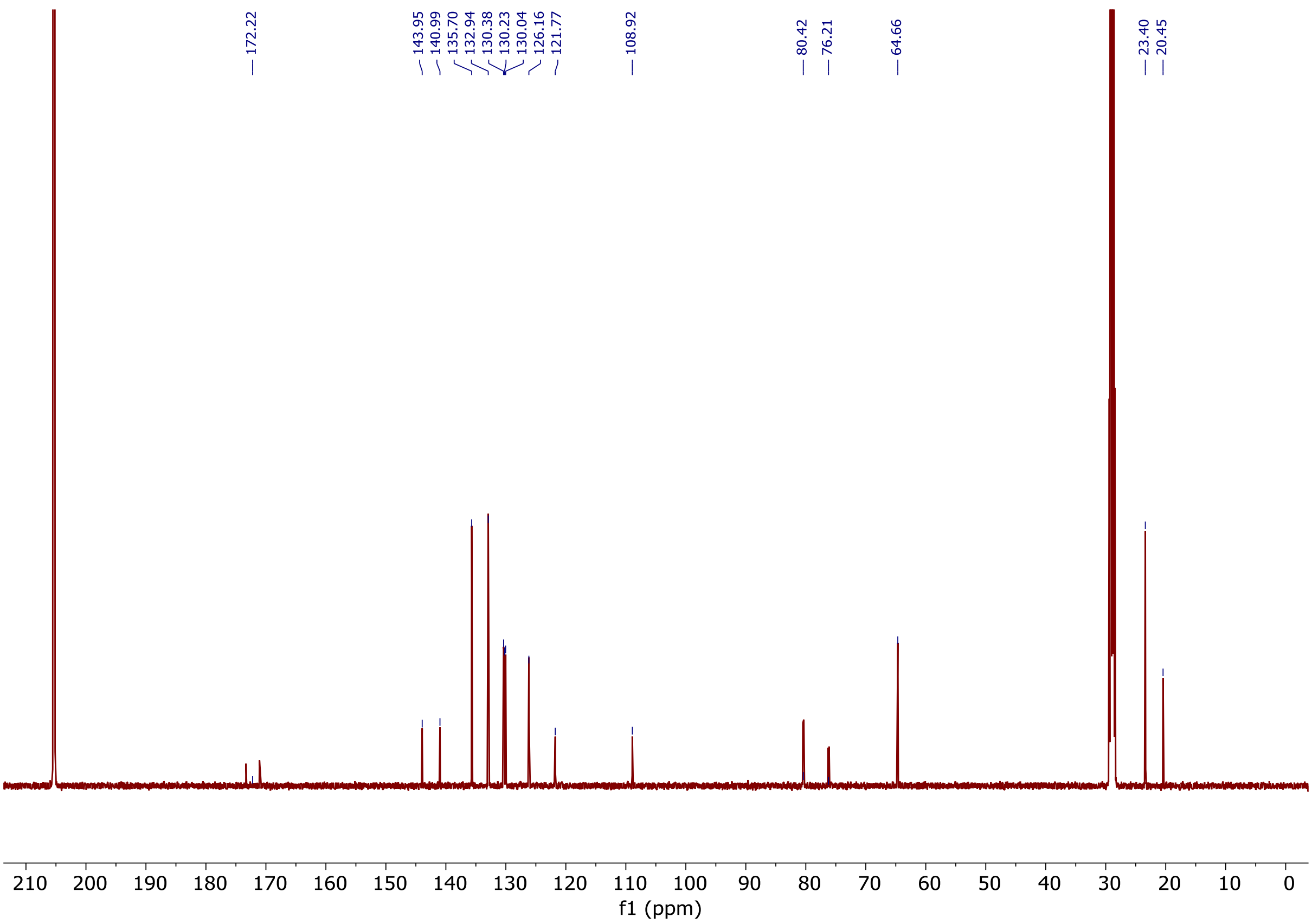

^{19}F NMR (471 MHz, Acetone-D_6)

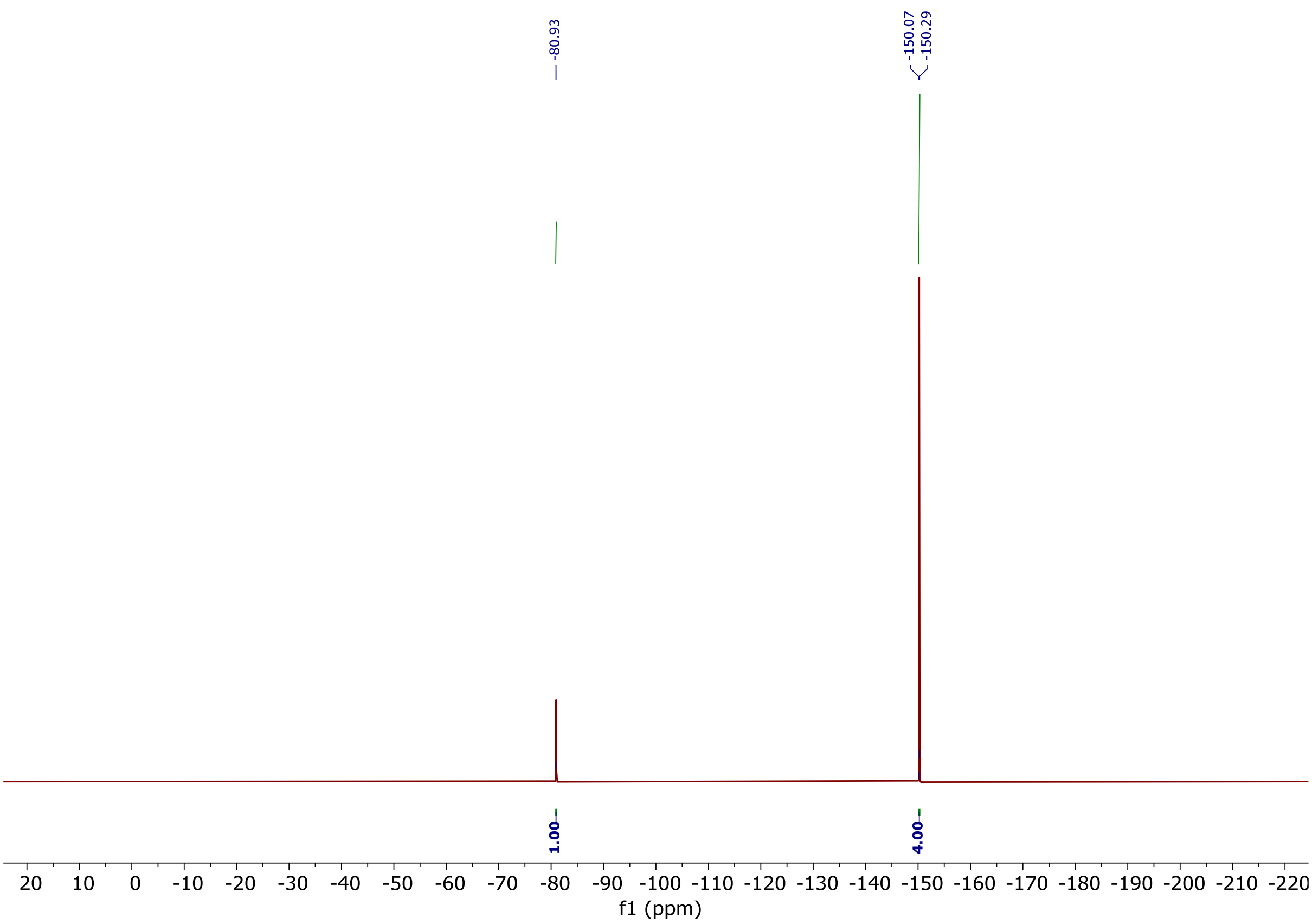

***(Z)-(2-fluoro-3-((3-nitrobenzyl)oxy)-3,3-diphenylprop-1-en-1-yl)(p-tolyl)iodonium tetrafluoroborate* (3af)**

^{1}H NMR (400 MHz, Acetone-D_6)

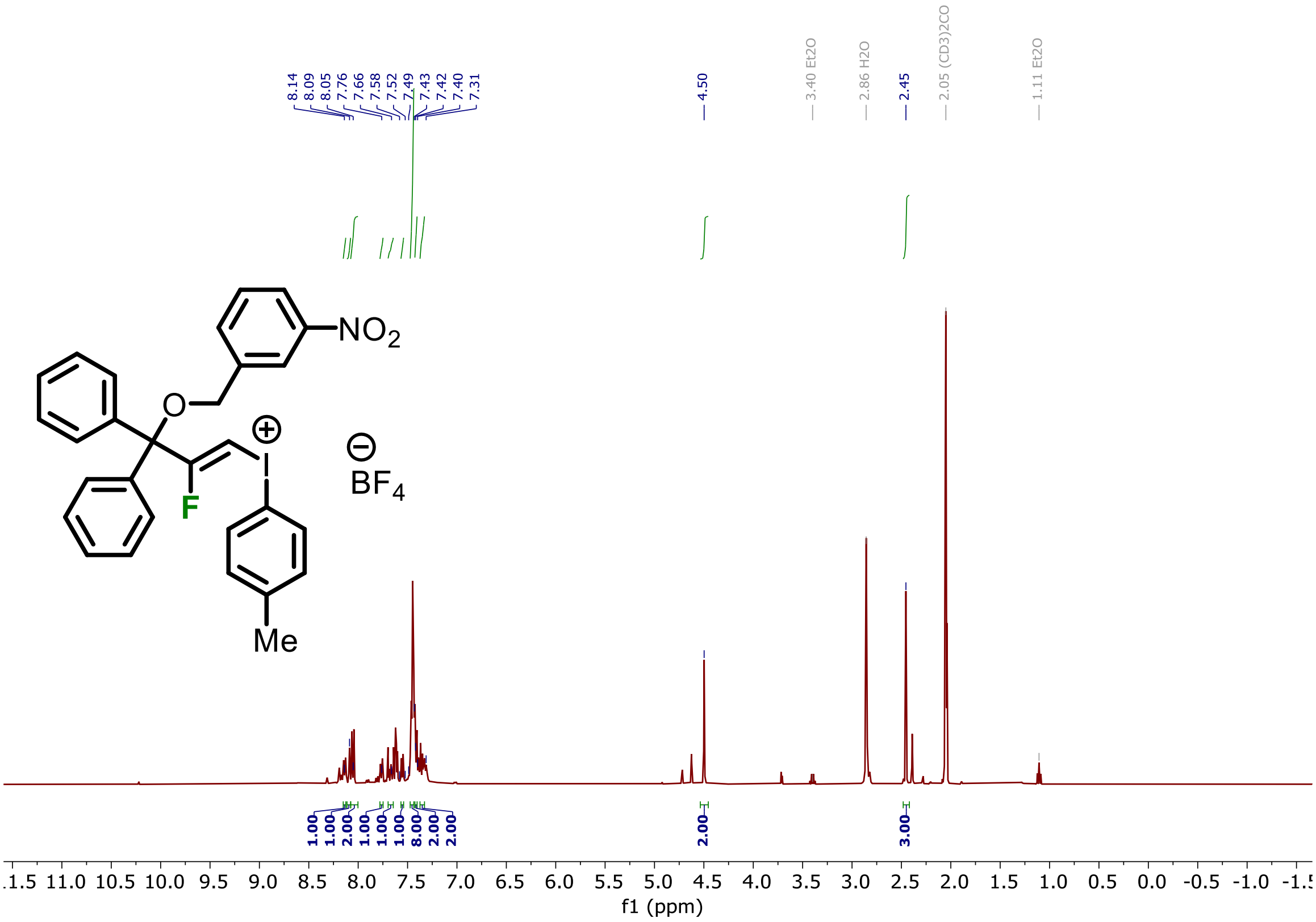

^{13}C NMR (126 MHz, Acetone-D_6)

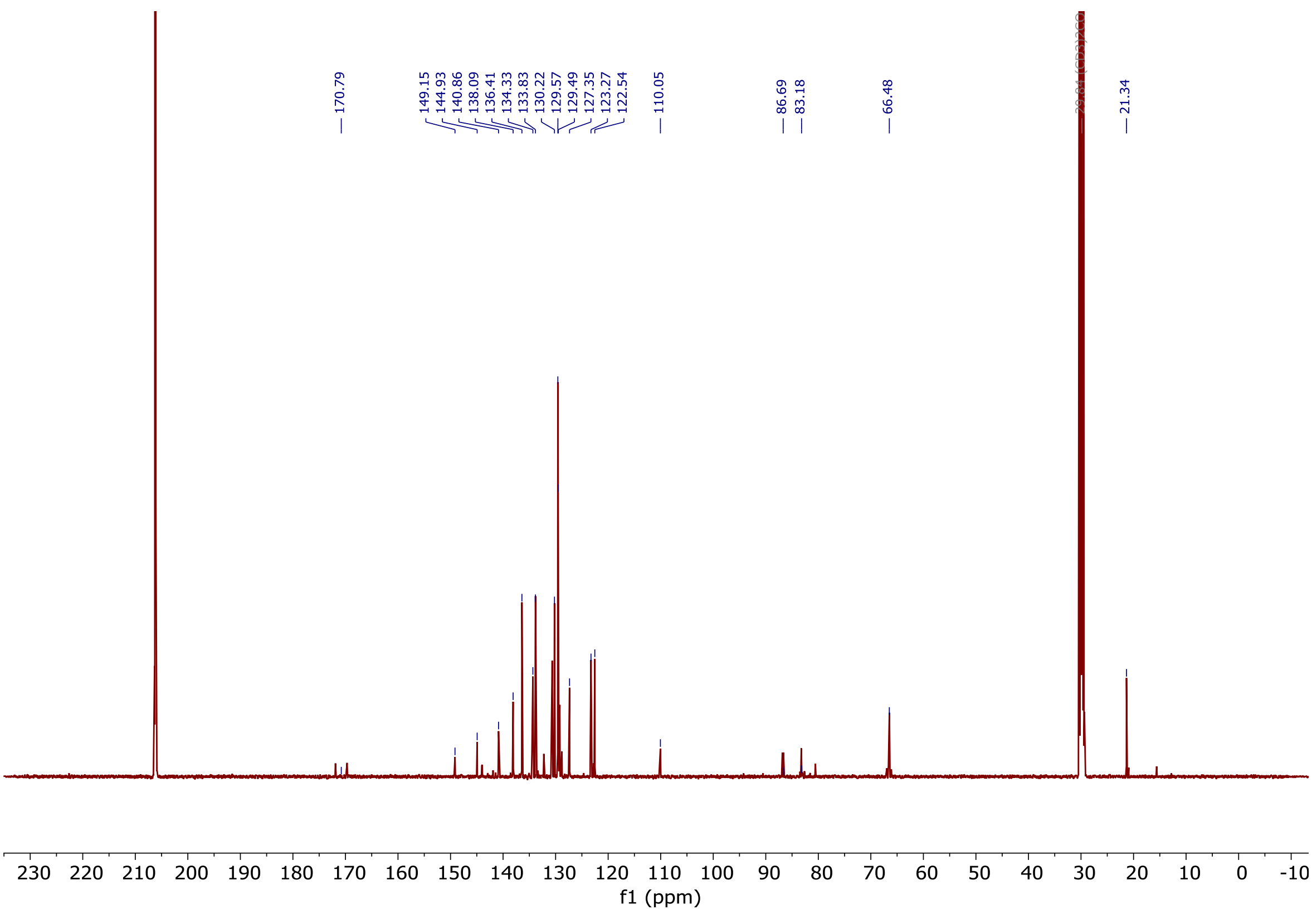

^{19}F NMR (376 MHz, Acetone-*D_6*)

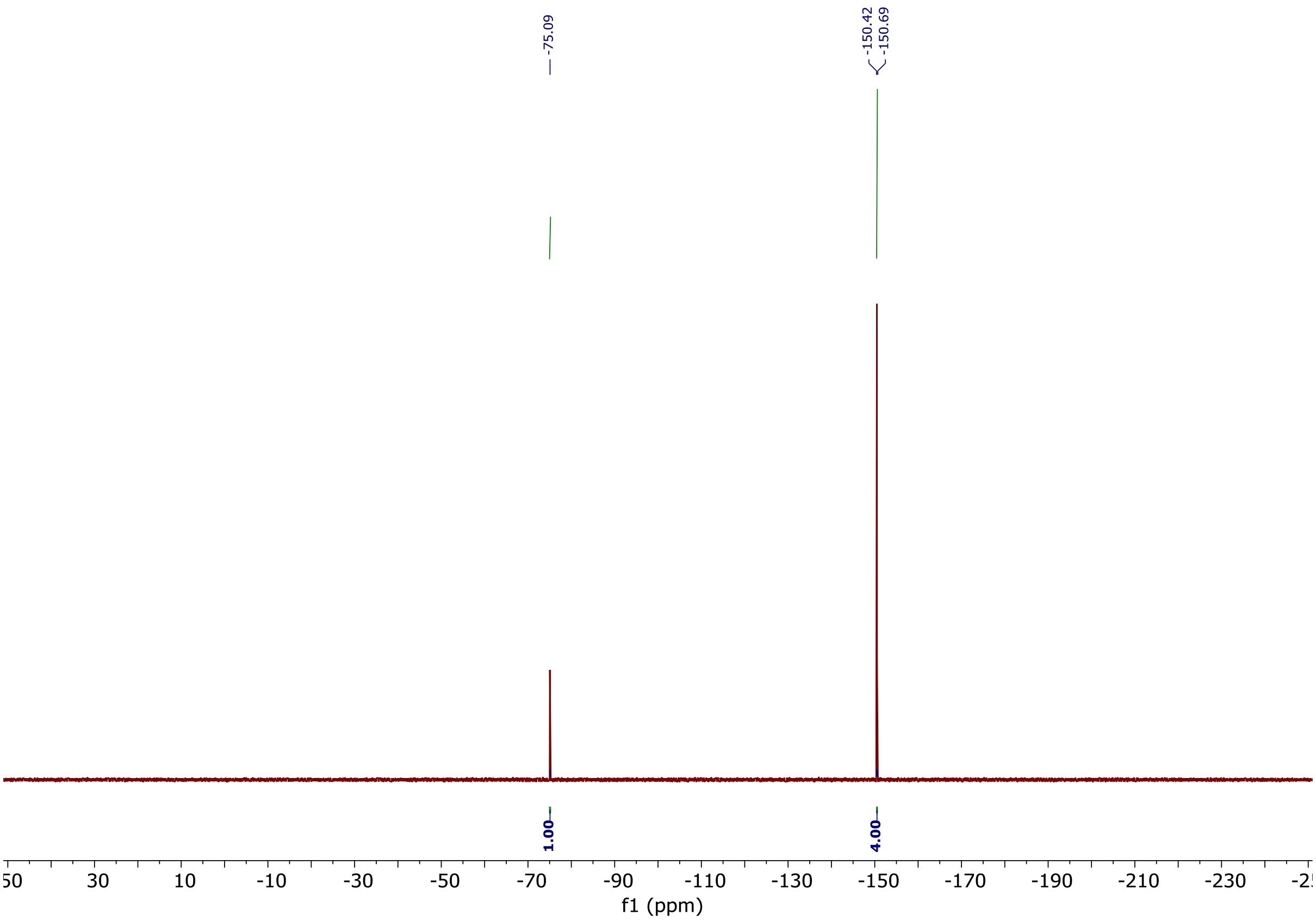

***(Z)-(3-((4-benzoylbenzyl)oxy)-2-fluoro-3,3-diphenylprop-1-en-1-yl)(p-tolyl)iodonium tetrafluoroborate* (3ag)**

^{1}H NMR (500 MHz, Acetone-D_6)

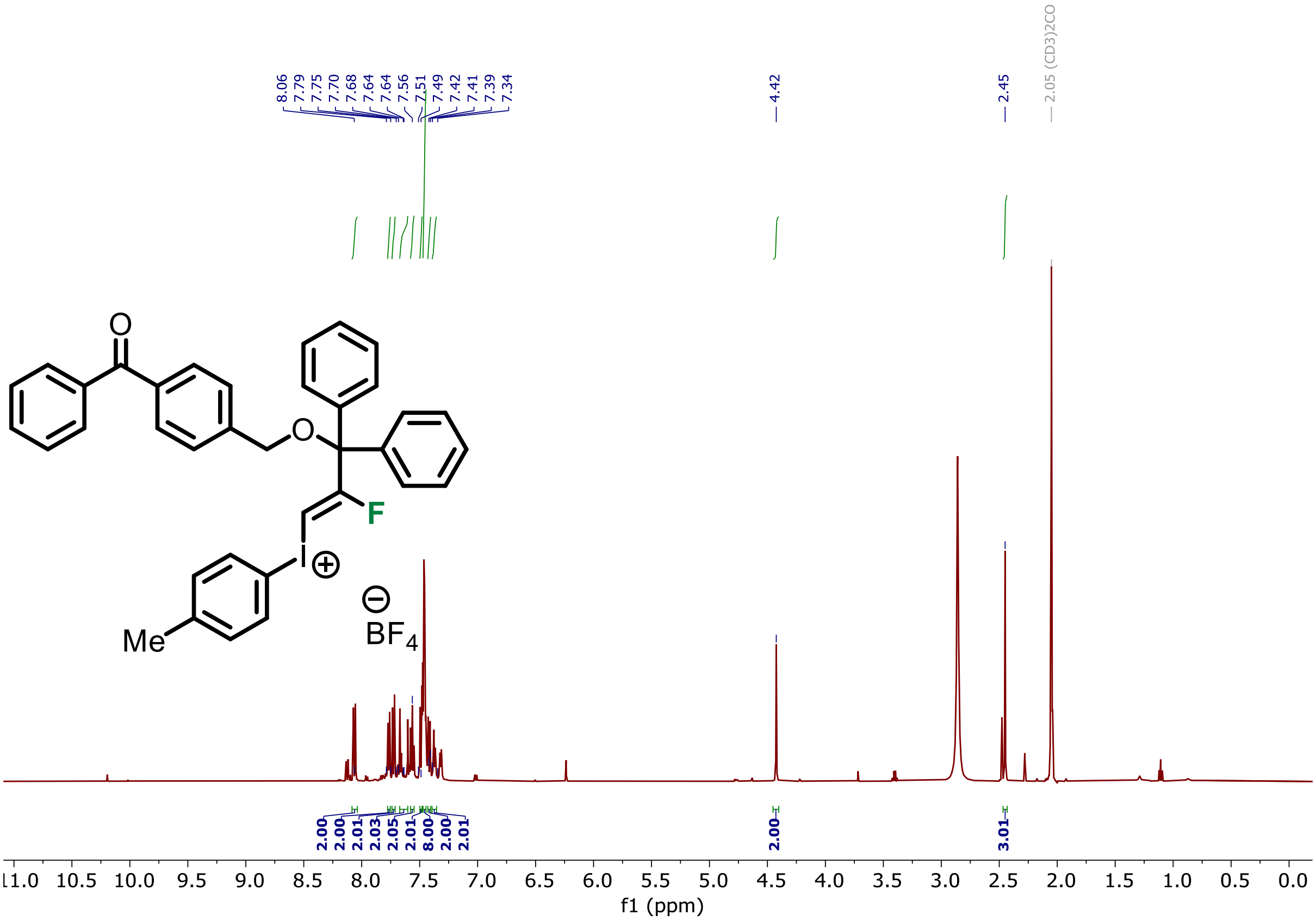

^{13}C NMR (126 MHz, Acetone-D_6)

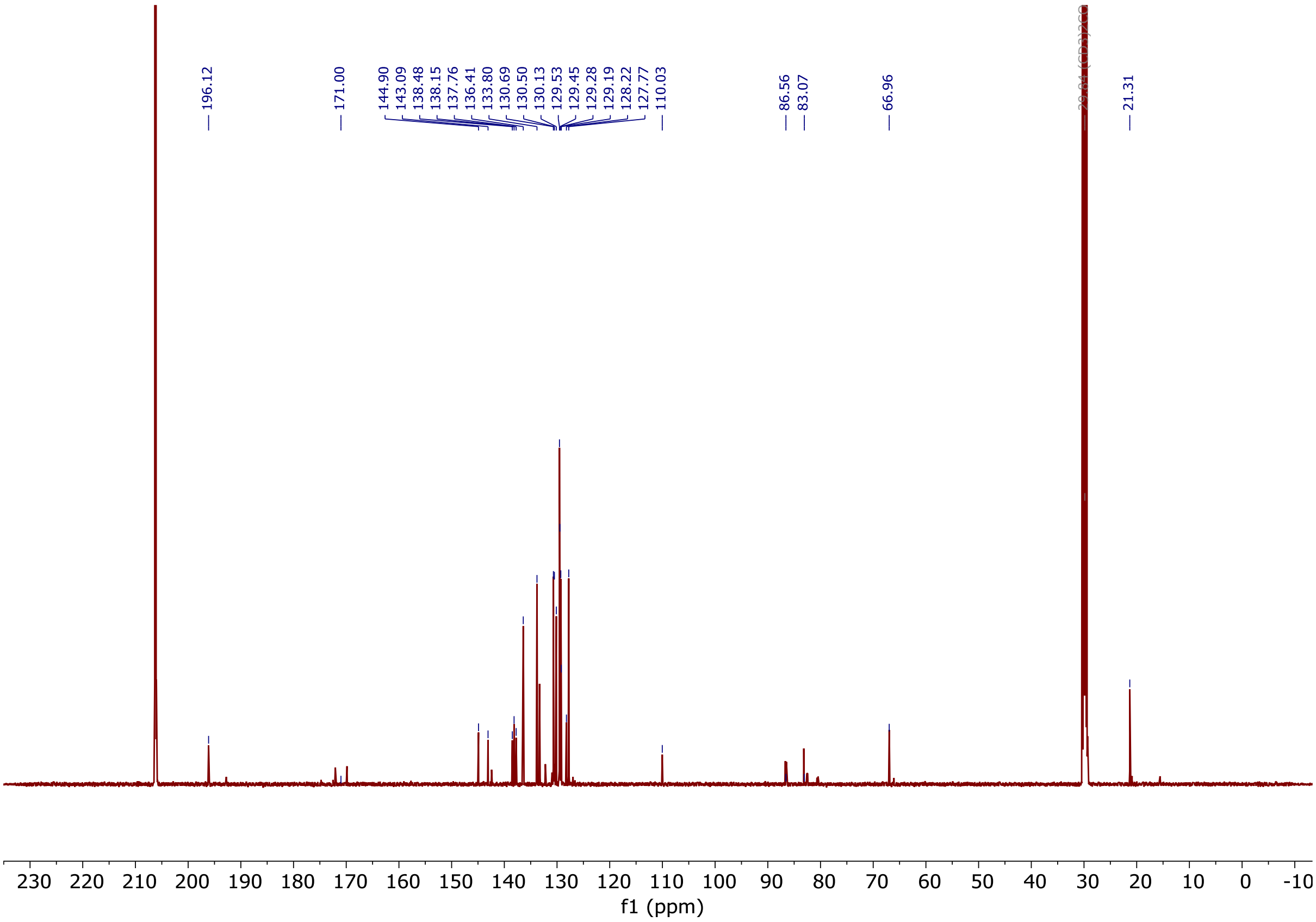

^{19}F NMR (376 MHz, Acetone-D_6)

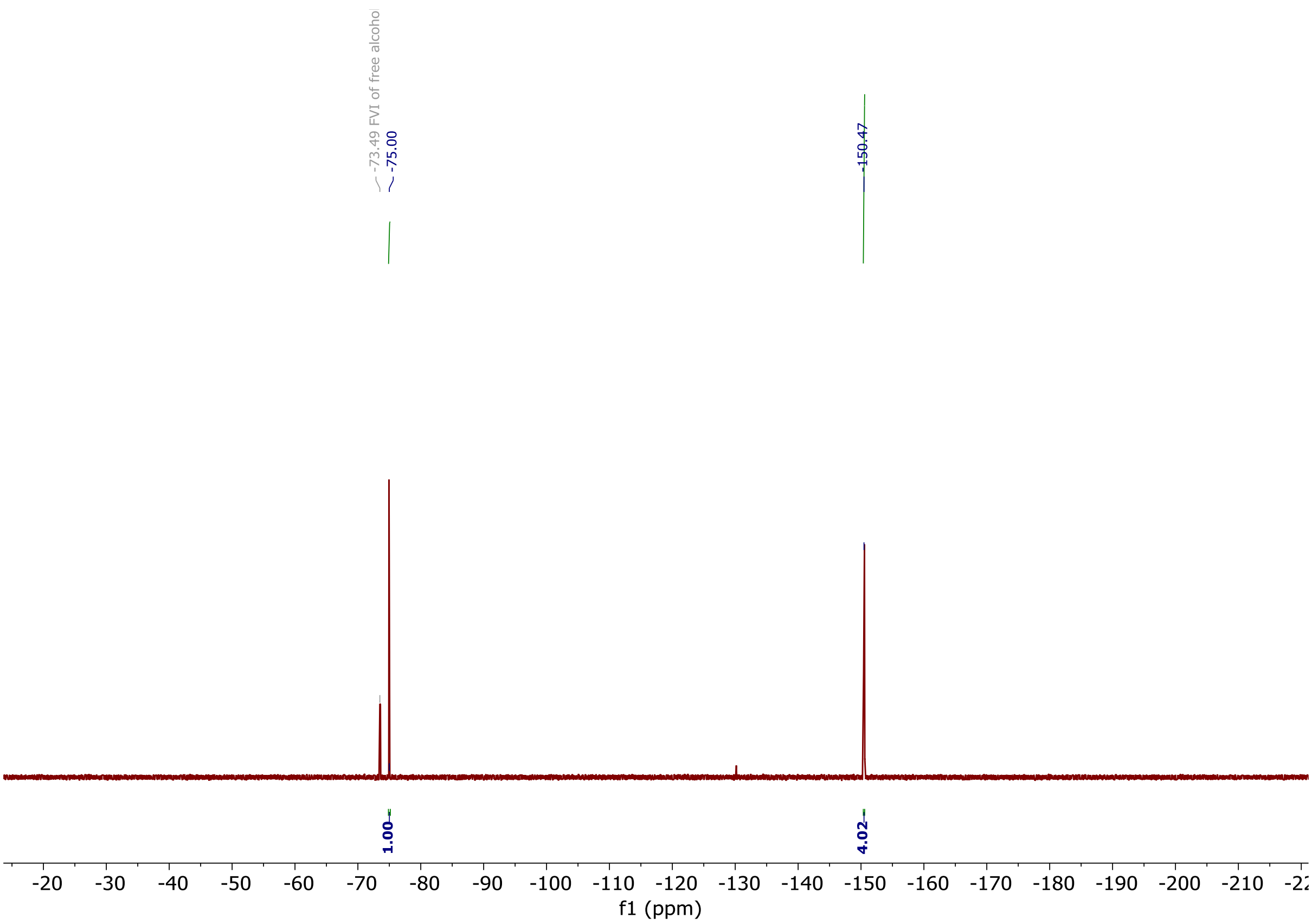

***(Z)*-(2-(1-((2-cyanobenzyl)oxy)cyclohexyl)-2-fluorovinyl)(p-tolyl)iodonium tetrafluoroborate** (3ai)*

^{1}H NMR (400 MHz, Acetone-*D$_6$*)

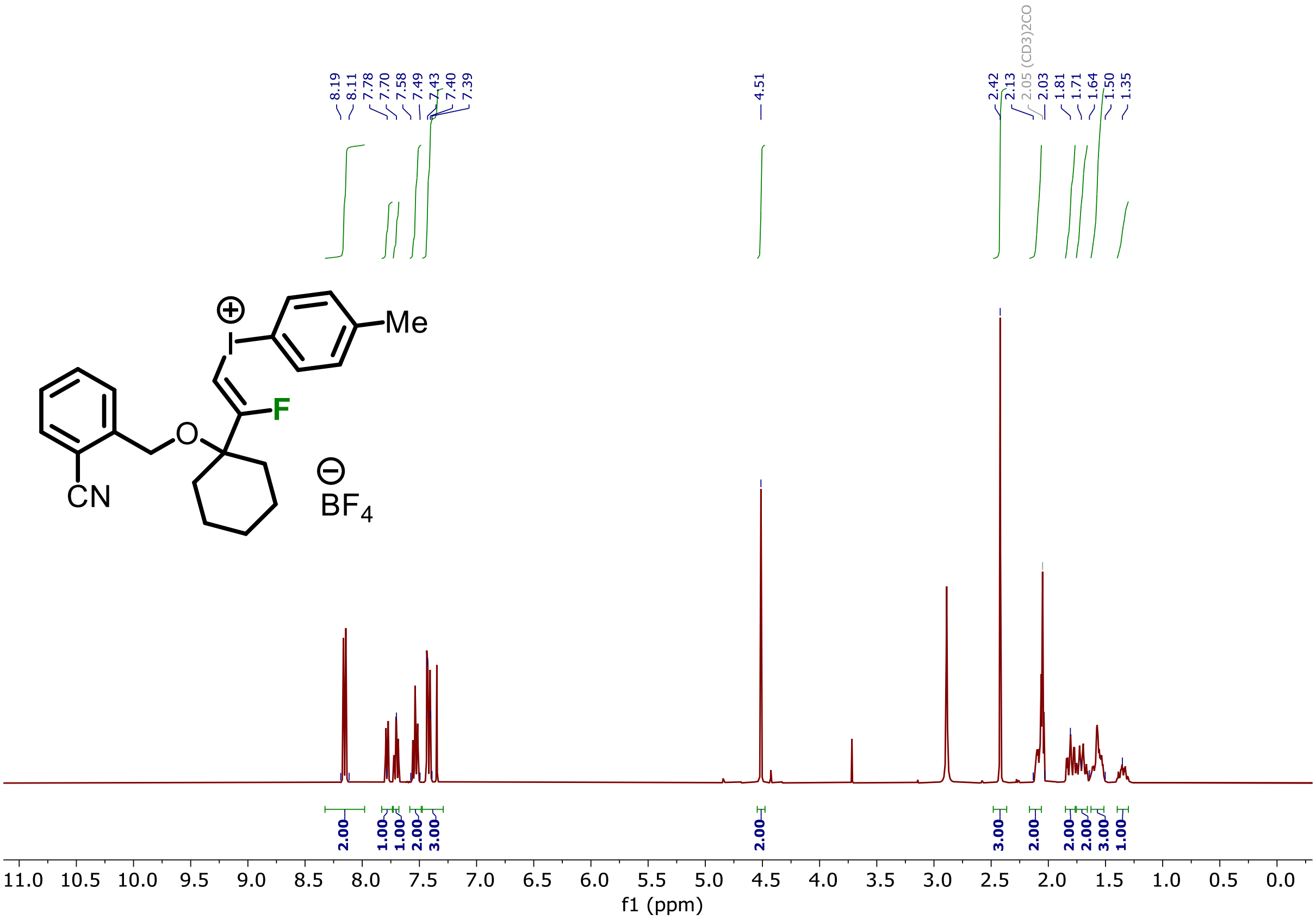

^{13}C NMR (101 MHz, Acetone-*D$_6$*)

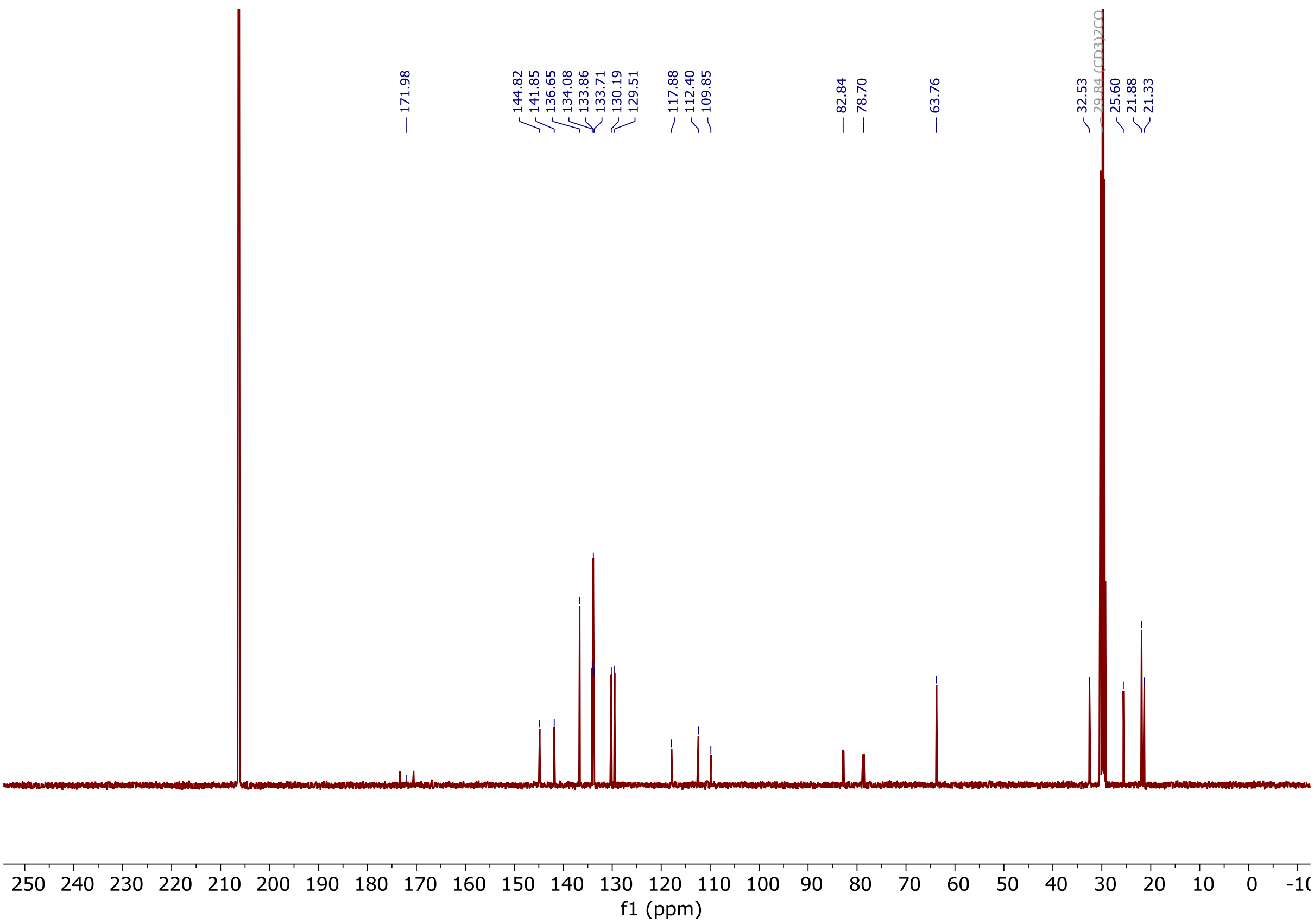

^{19}F NMR (376 MHz, Acetone-D_6)

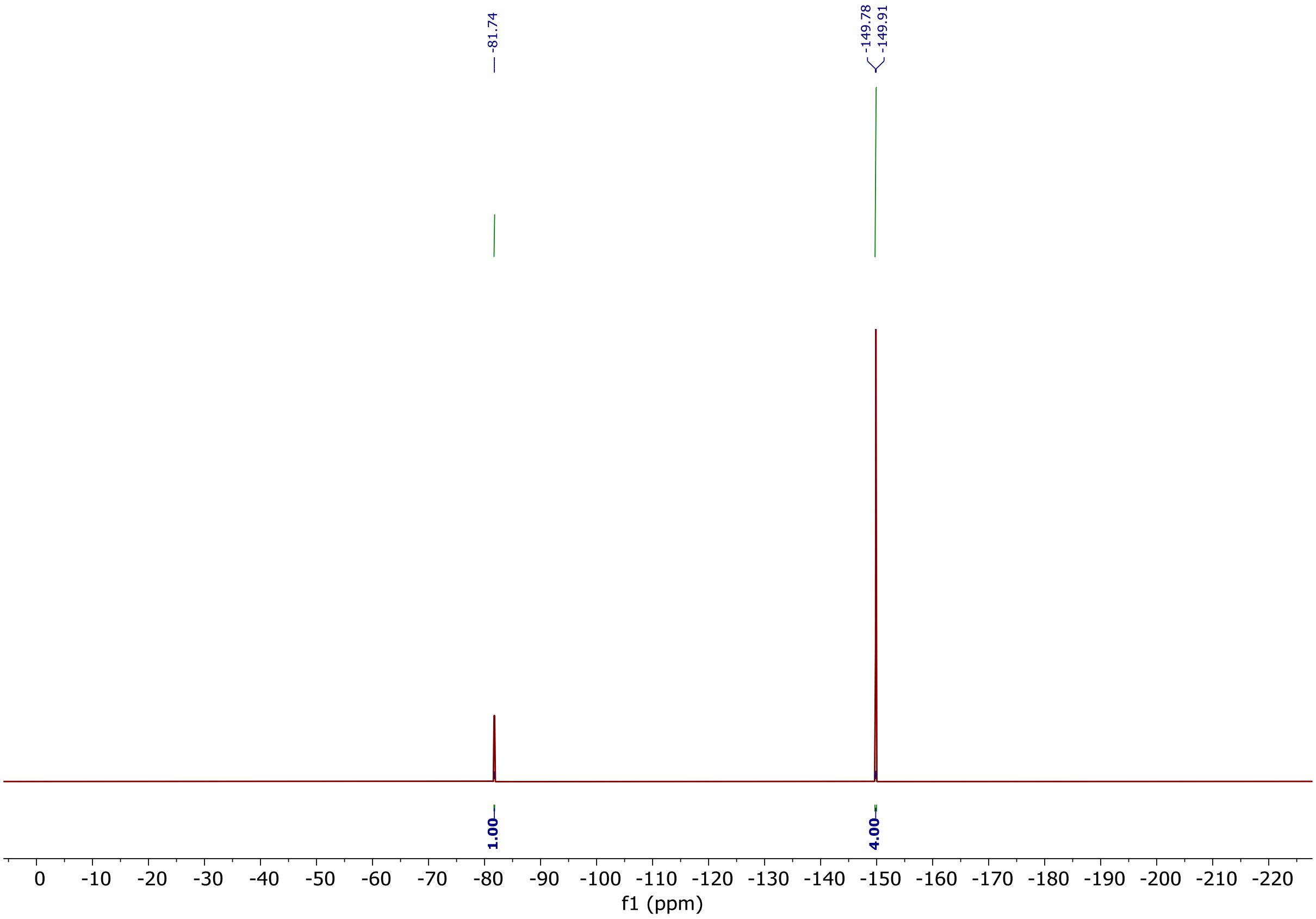

***(Z)-(2-fluoro-3-(1-phenylethoxy)prop-1-en-1-yl)(p-tolyl)iodonium tetrafluoroborate* (3aj)**

^{1}H NMR (600 MHz, Acetone-D_6)

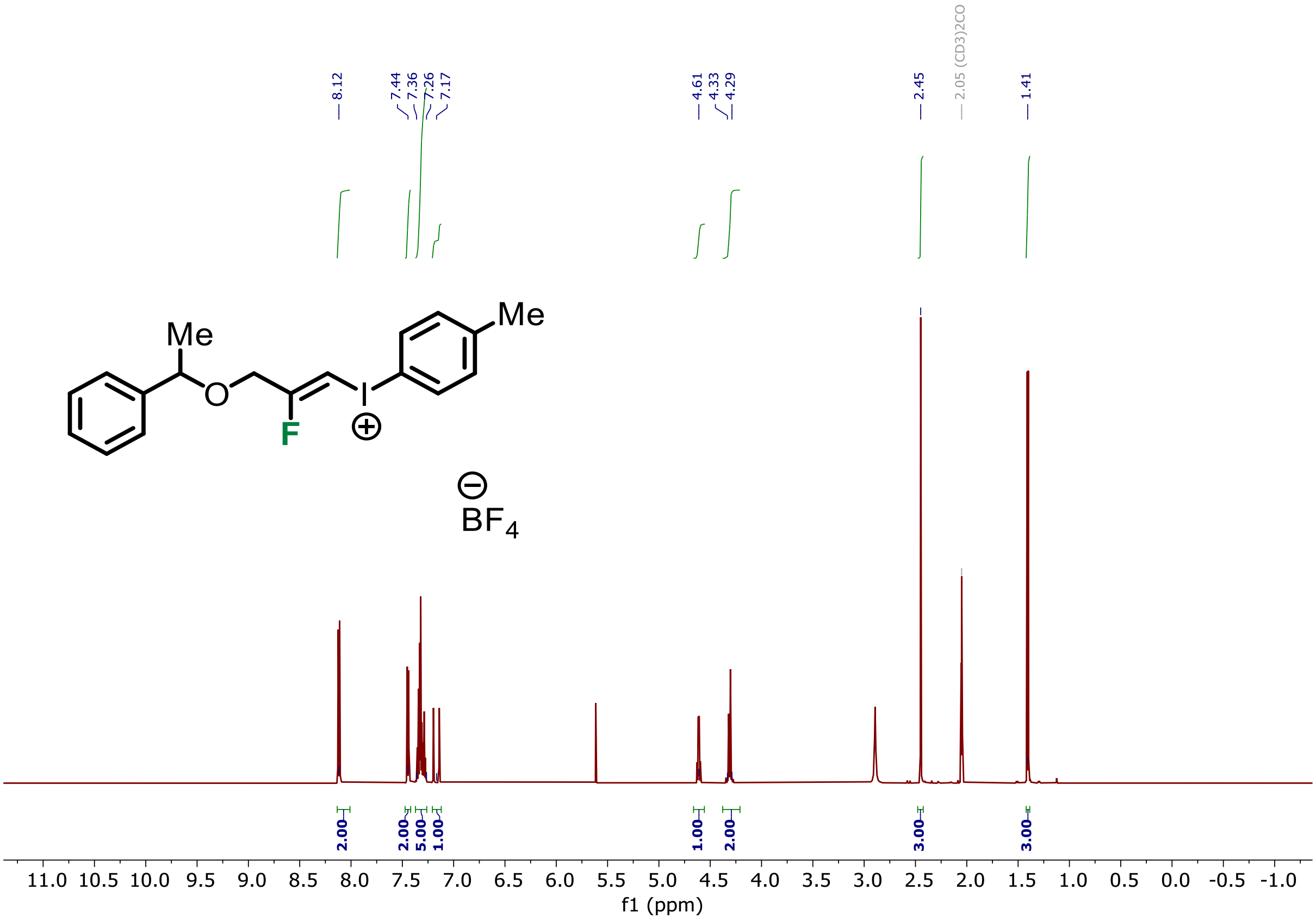

^{13}C NMR (151 MHz, Acetone-D_6)

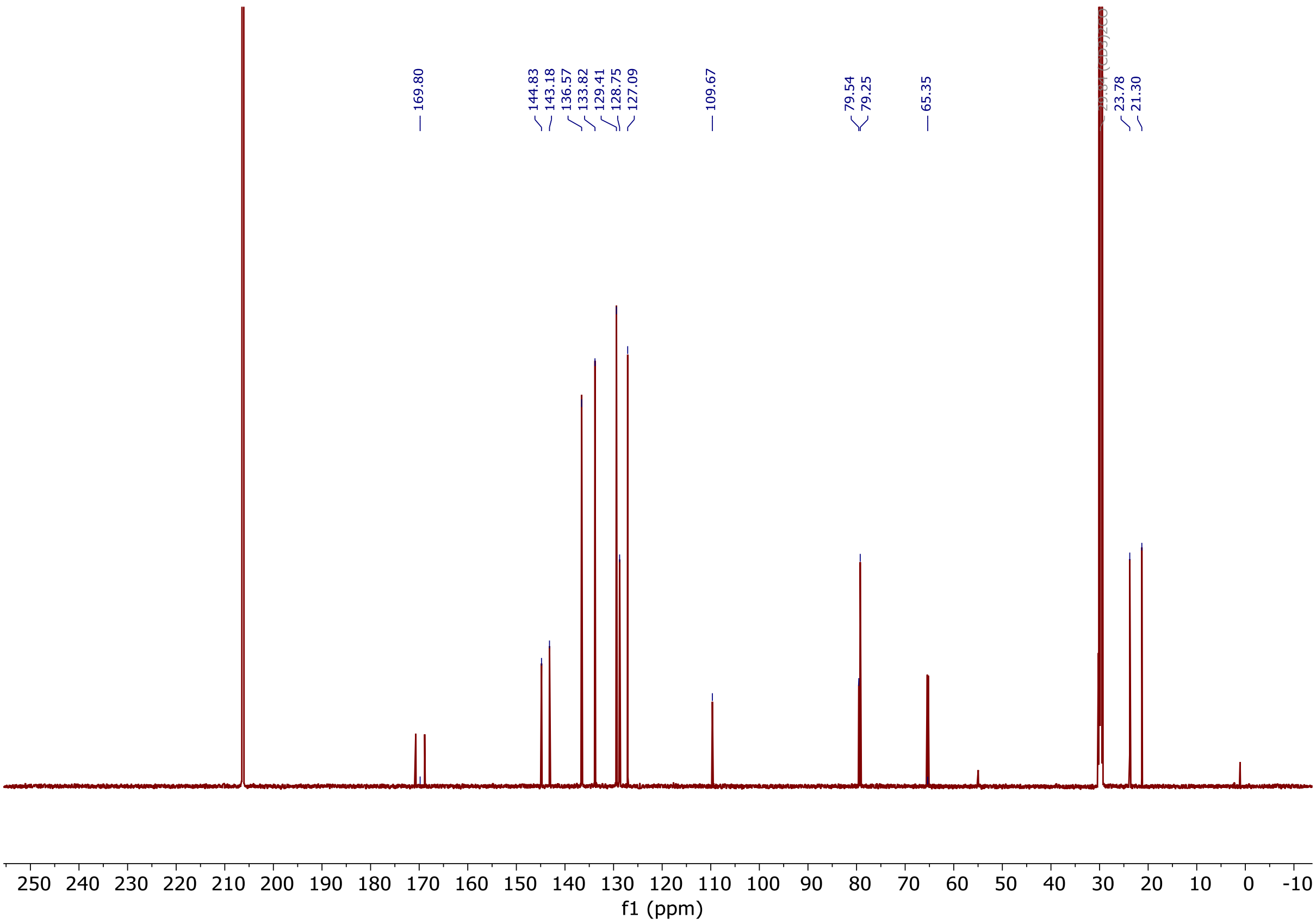

^{19}F NMR (471 MHz, Acetone-D_6)

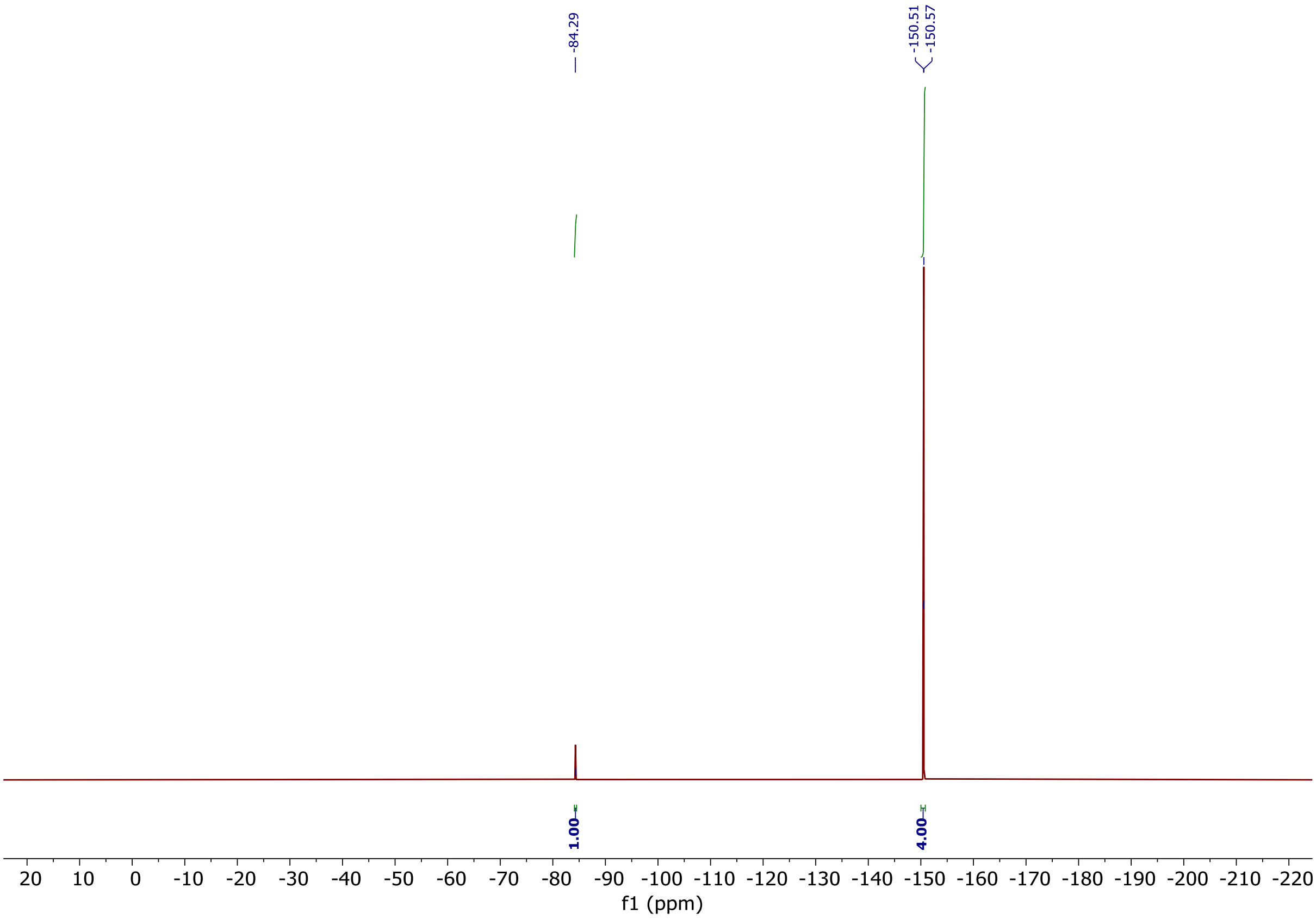

(Z)-(3-(benzhydryloxy)-2-fluoroprop-1-en-1-yl)(p-tolyl)iodonium tetrafluoroborate **(3ak)**
^{1}H NMR (500 MHz, CDCl$_3$)

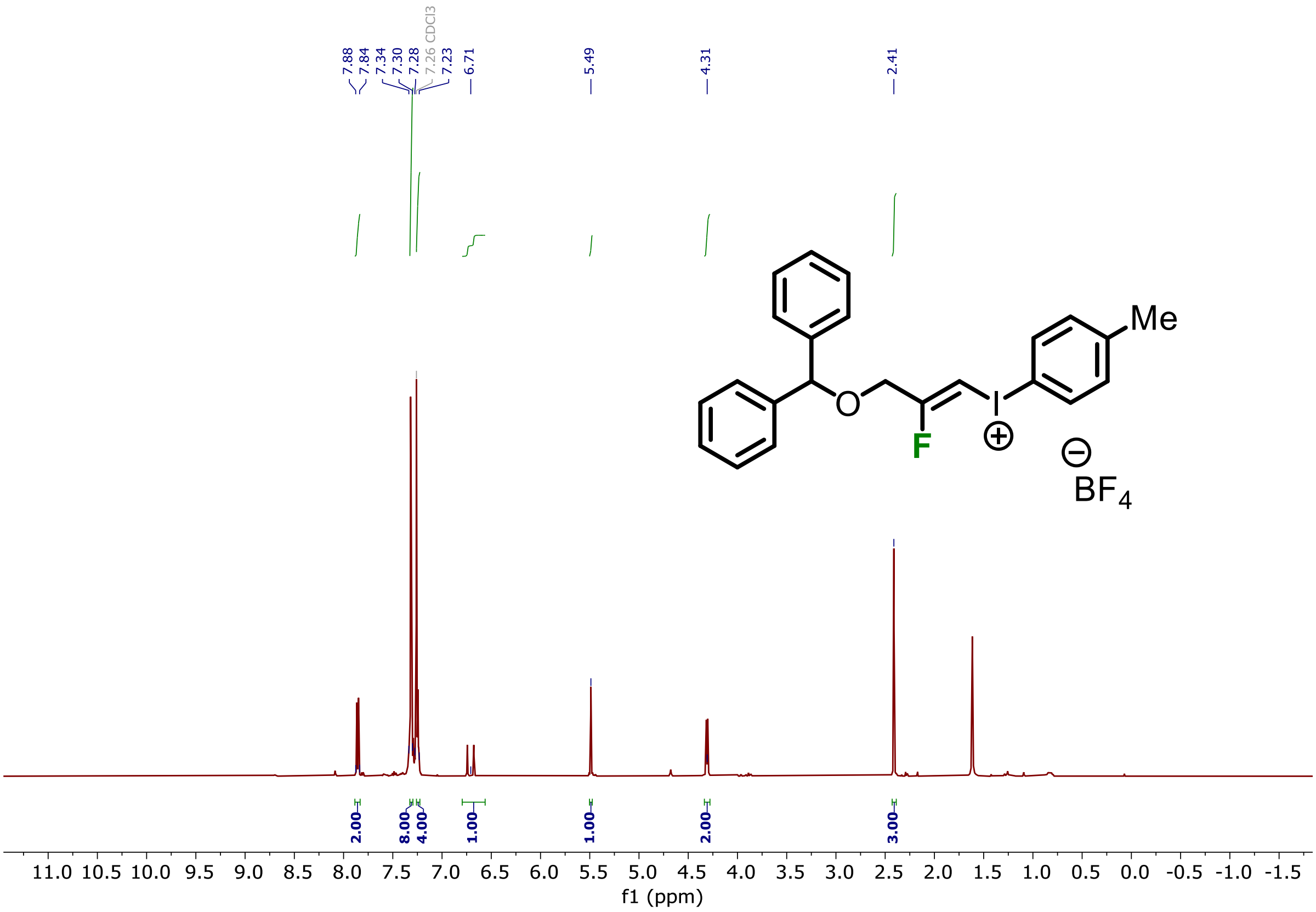

^{13}C NMR (126 MHz, CDCl$_3$)

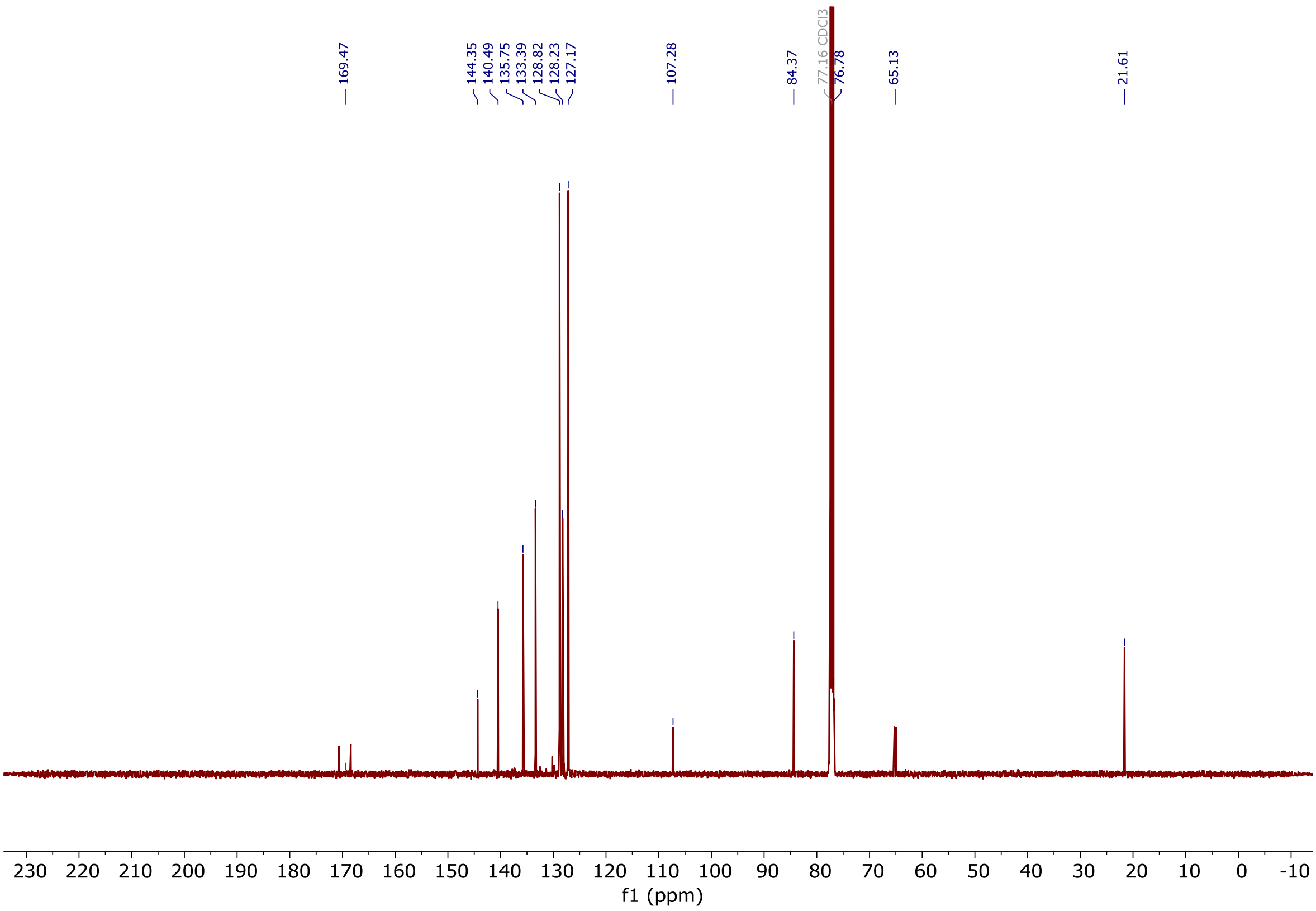

^{19}F NMR (376 MHz, $CDCl_3$)

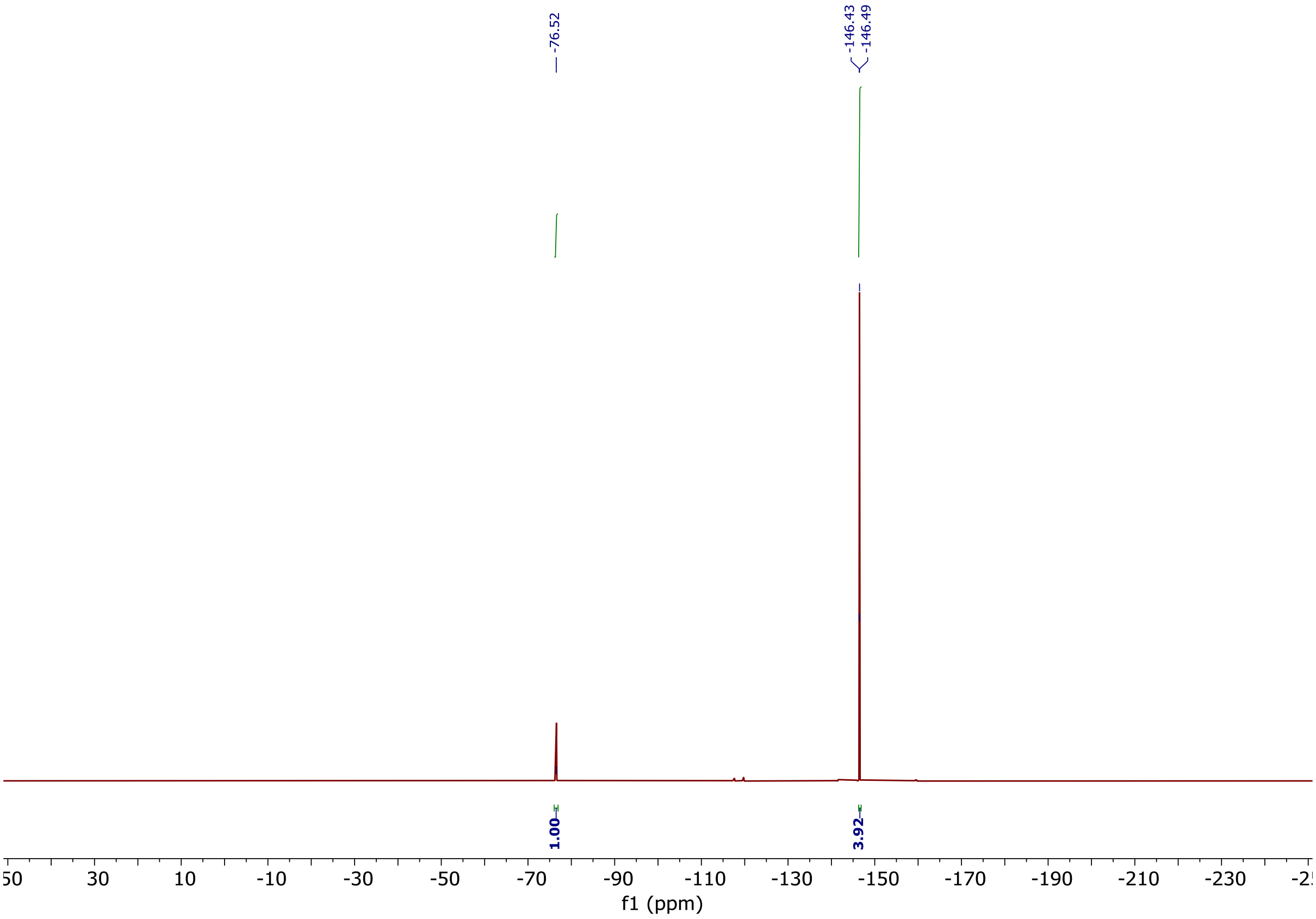

(Z)-(3-(chroman-4-yloxy)-2-fluoroprop-1-en-1-yl)(p-tolyl)iodonium tetrafluoroborate **(3al)**
^{19}F NMR (376 MHz, CDCl$_3$):

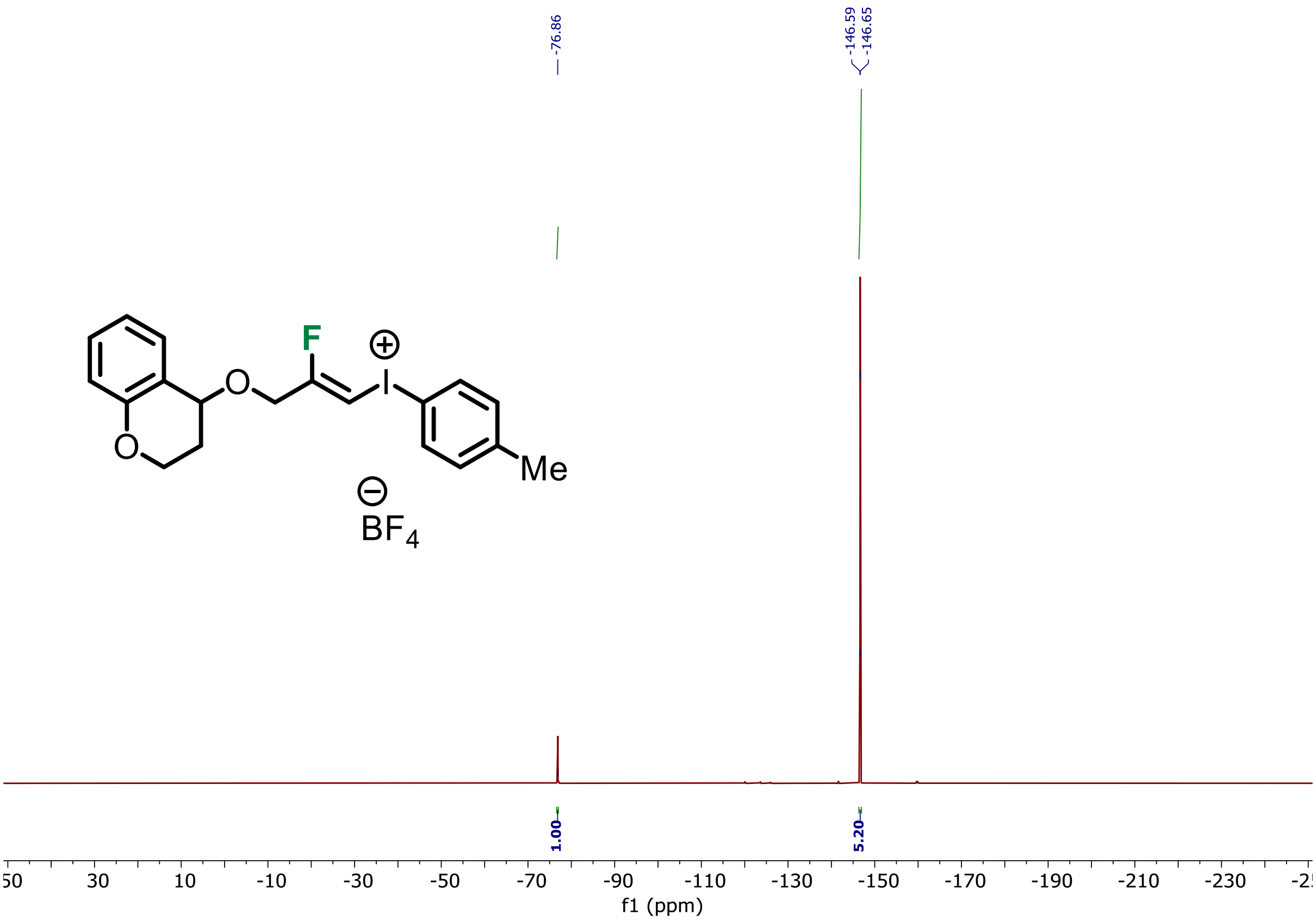

***(Z)-(2-fluoro-3-(1-phenylethoxy)but-1-en-1-yl)(p-tolyl)iodonium tetrafluoroborate* (3am)**

^{1}H NMR (500 MHz, Acetone-D_6)

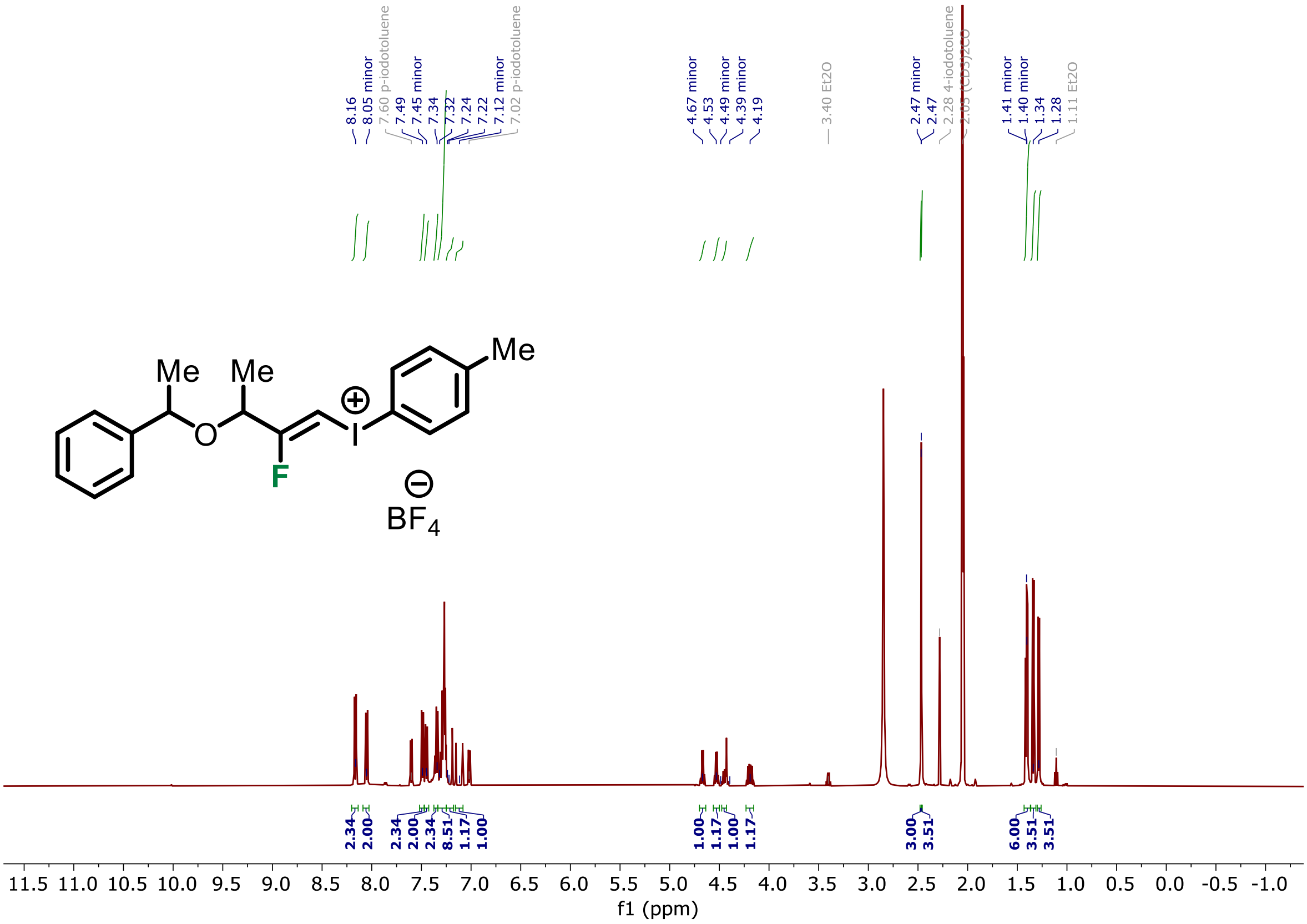

^{13}C NMR (126 MHz, Acetone-D_6)

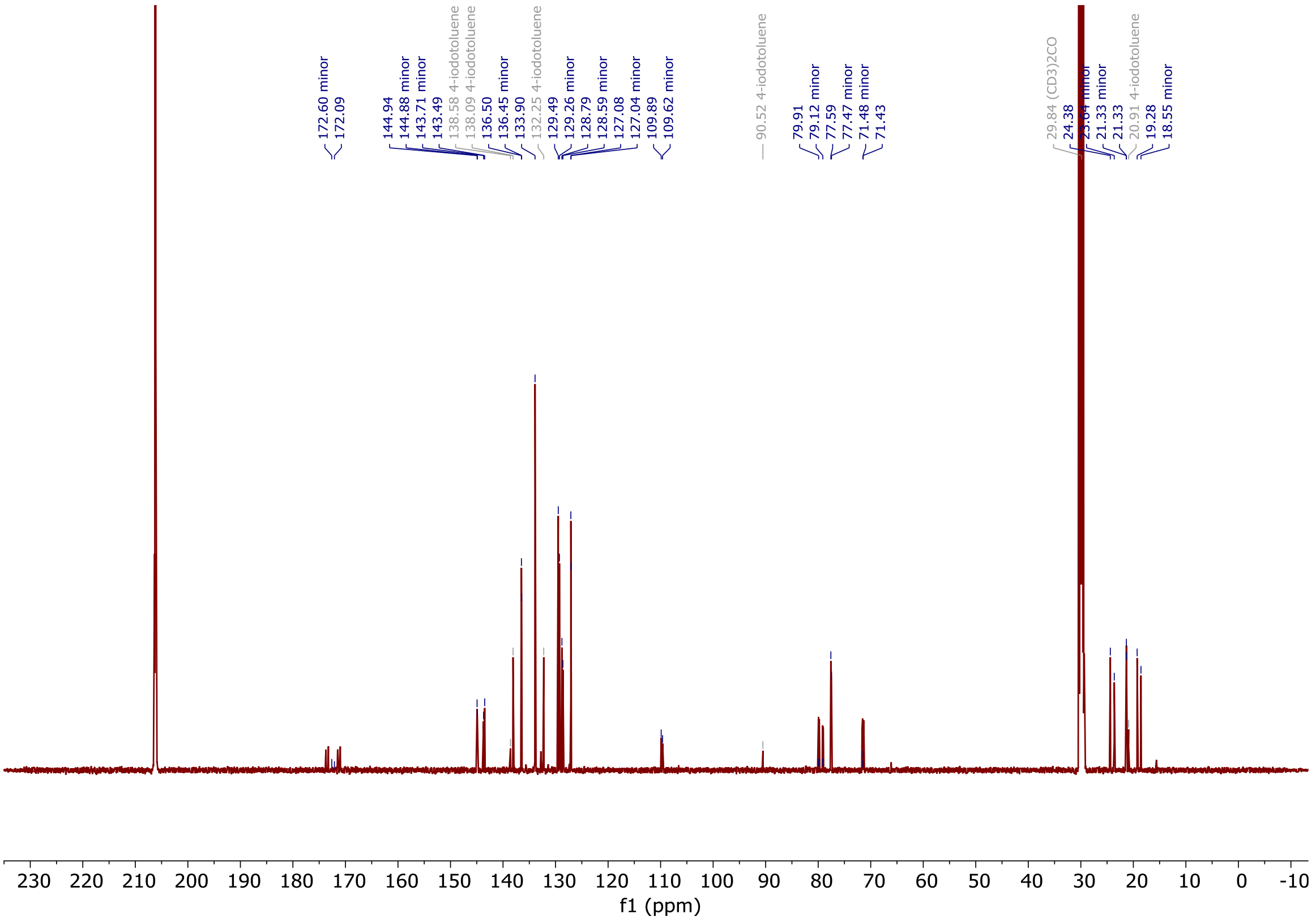

^{19}F NMR (376 MHz, Acetone-D_6)

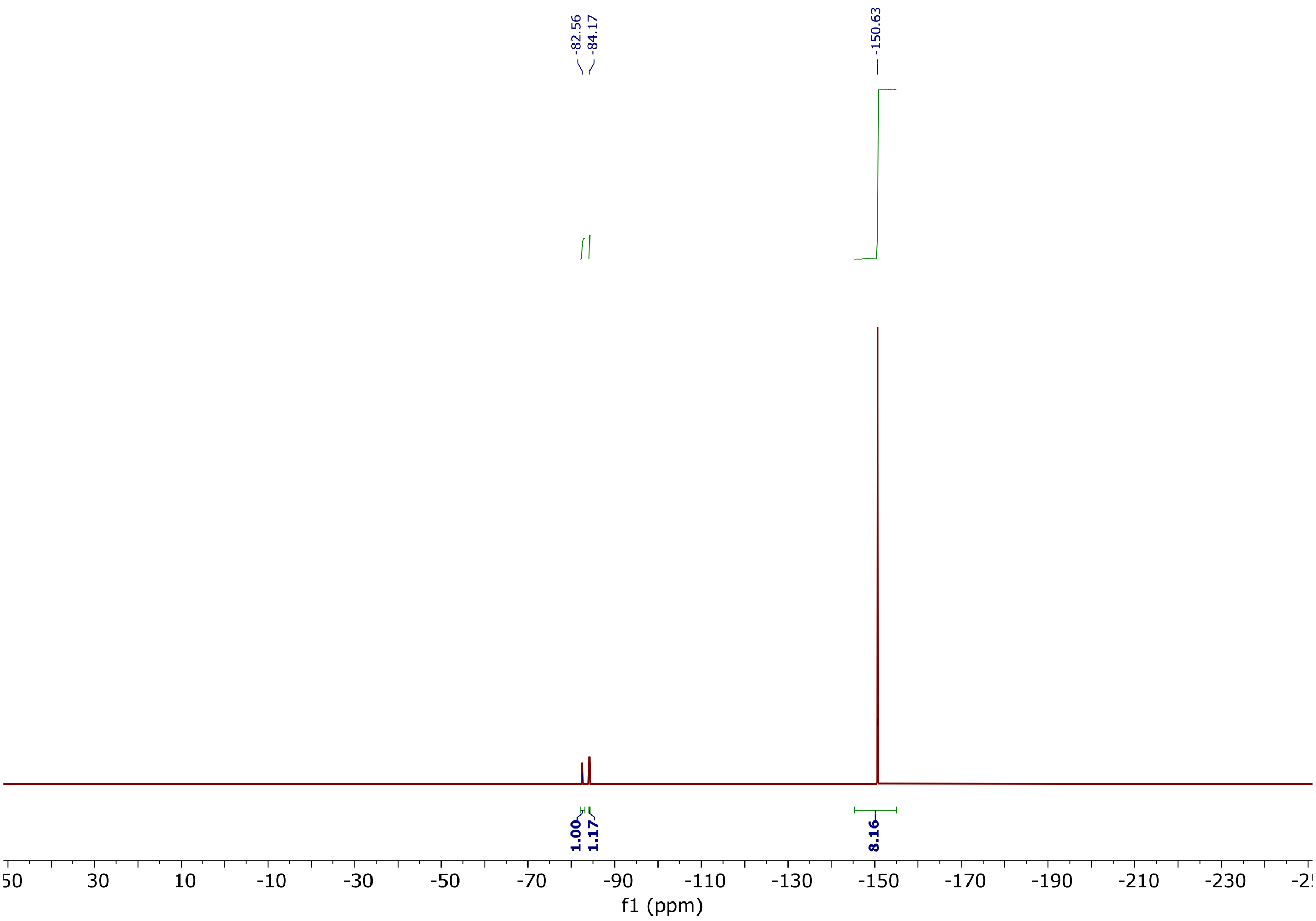

(Z)-(2-fluoro-3-hydroxy-3-phenylprop-1-en-1-yl)(p-tolyl)iodonium tetrafluoroborate (3bn) **^{1}H NMR (400 MHz, Acetone-D_6)**

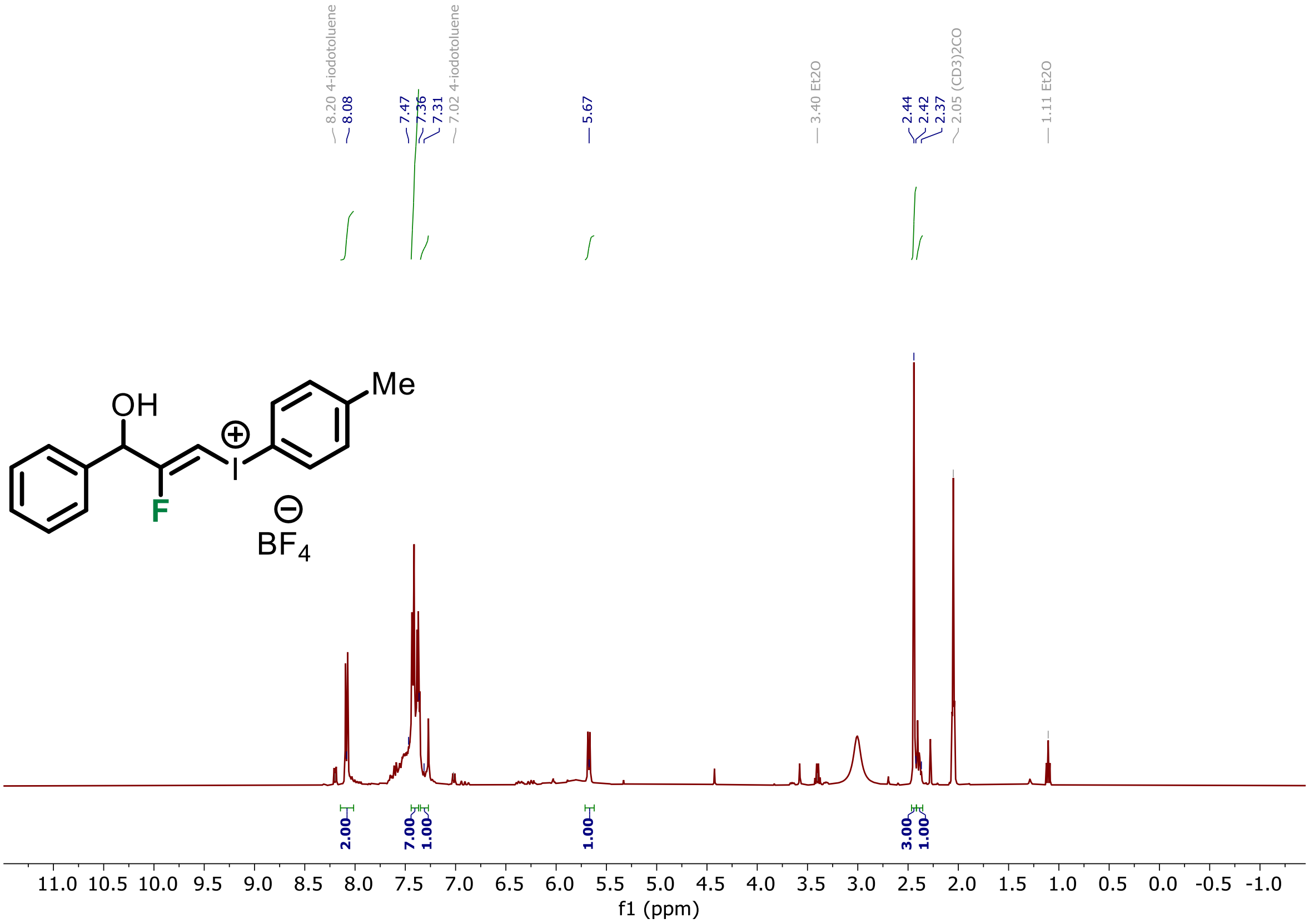

^{13}C NMR (126 MHz, Acetone-D_6)

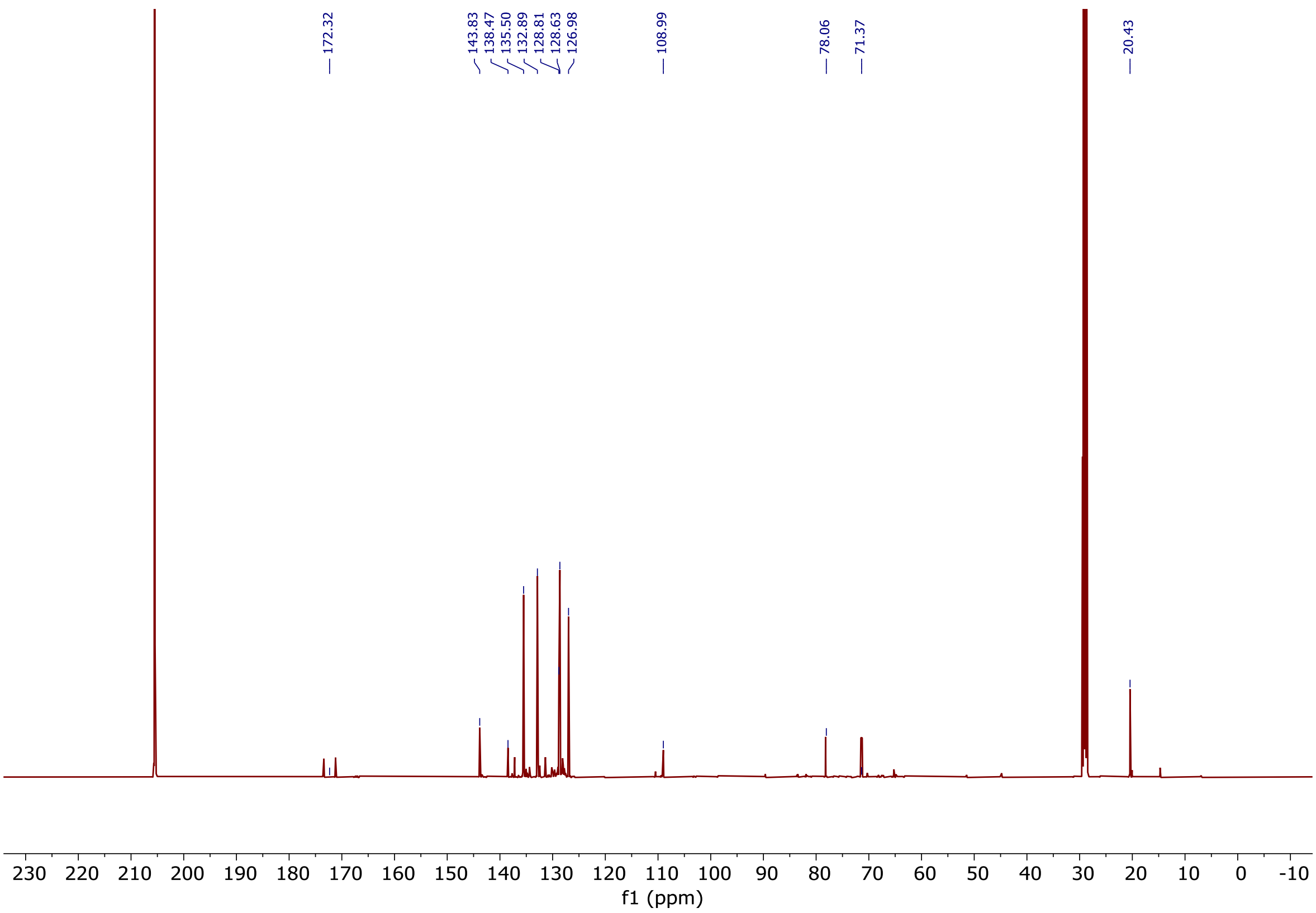

^{19}F NMR (376 MHz, Acetone-D_6)

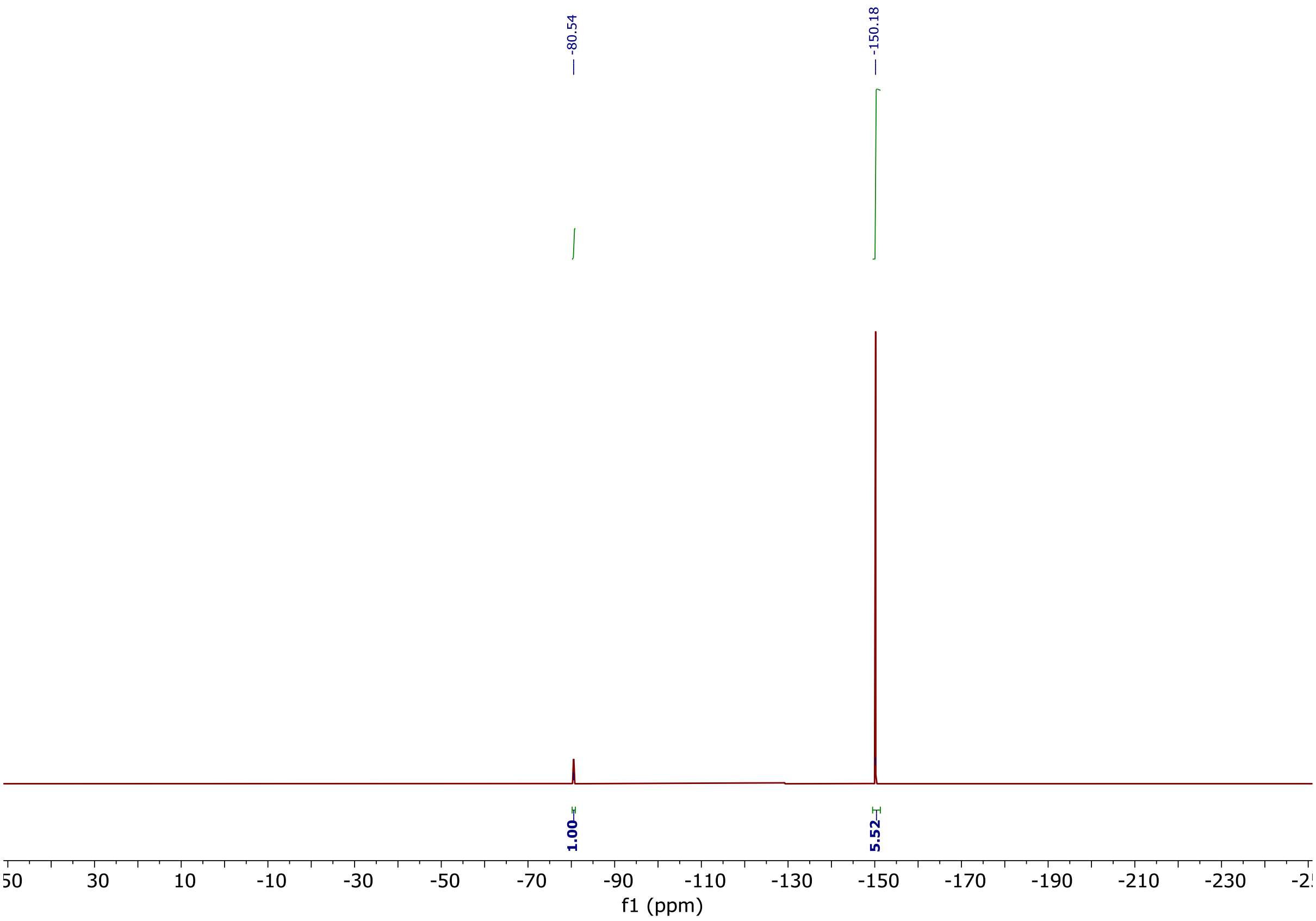

(Z)-(2-fluoro-2-(1-methoxycyclohexyl)vinyl)(p-tolyl)iodonium tetrafluoroborate **(3an)**

^{1}H NMR (400 MHz, Acetone-*D*$_6$)

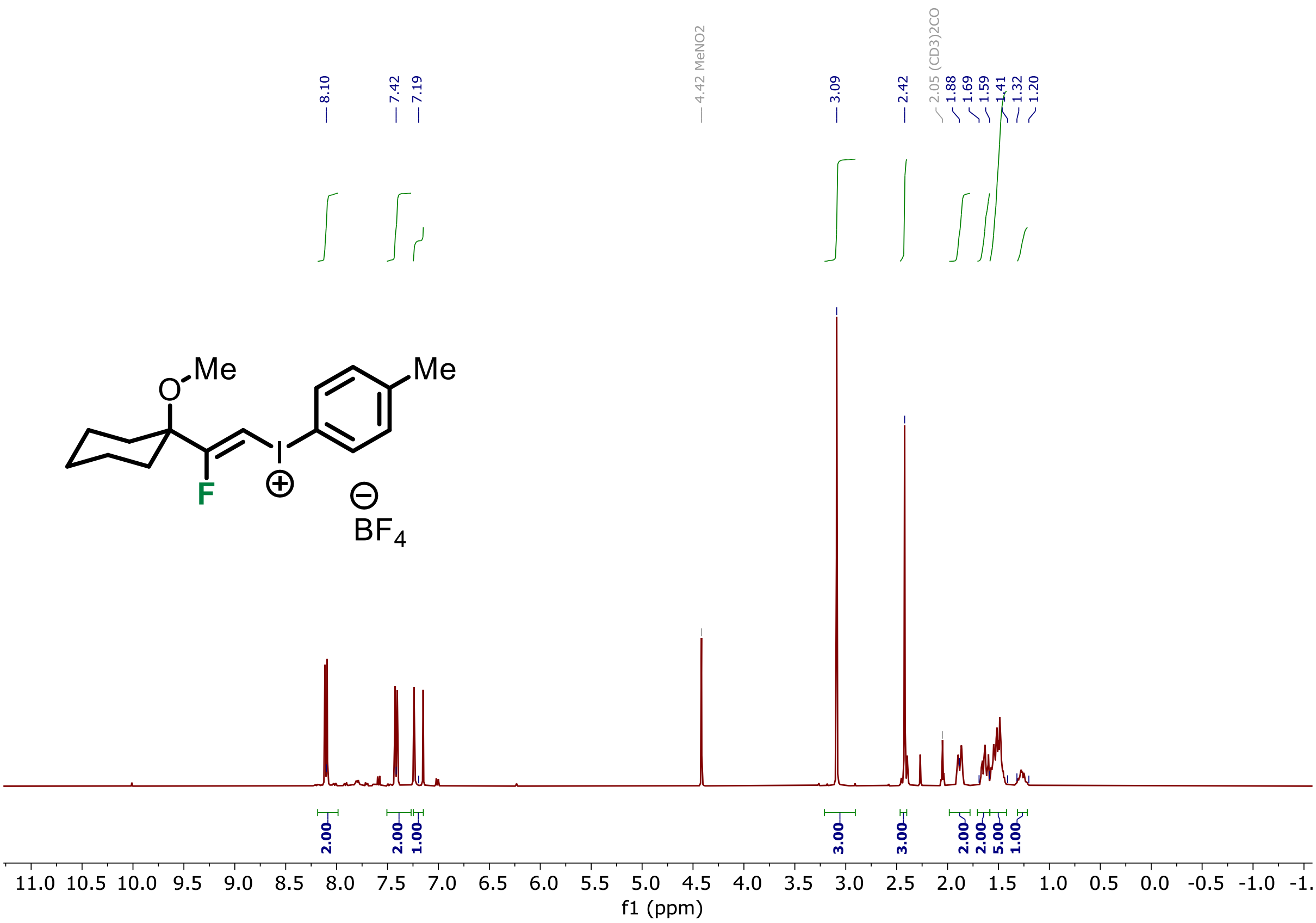

^{13}C NMR (126 MHz, Acetone-*D*$_6$)

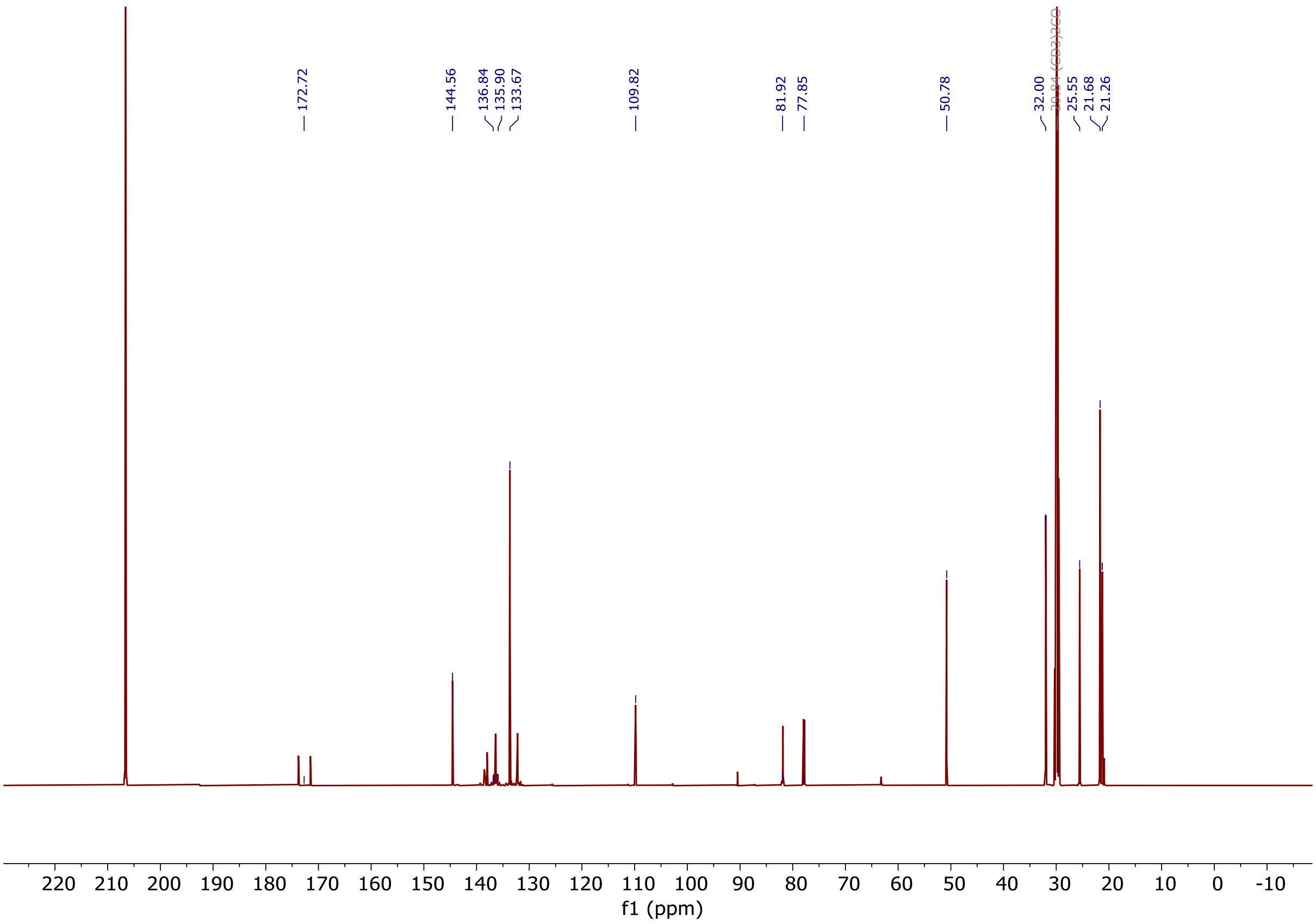

^{19}F NMR (376 MHz, Acetone-D_6)

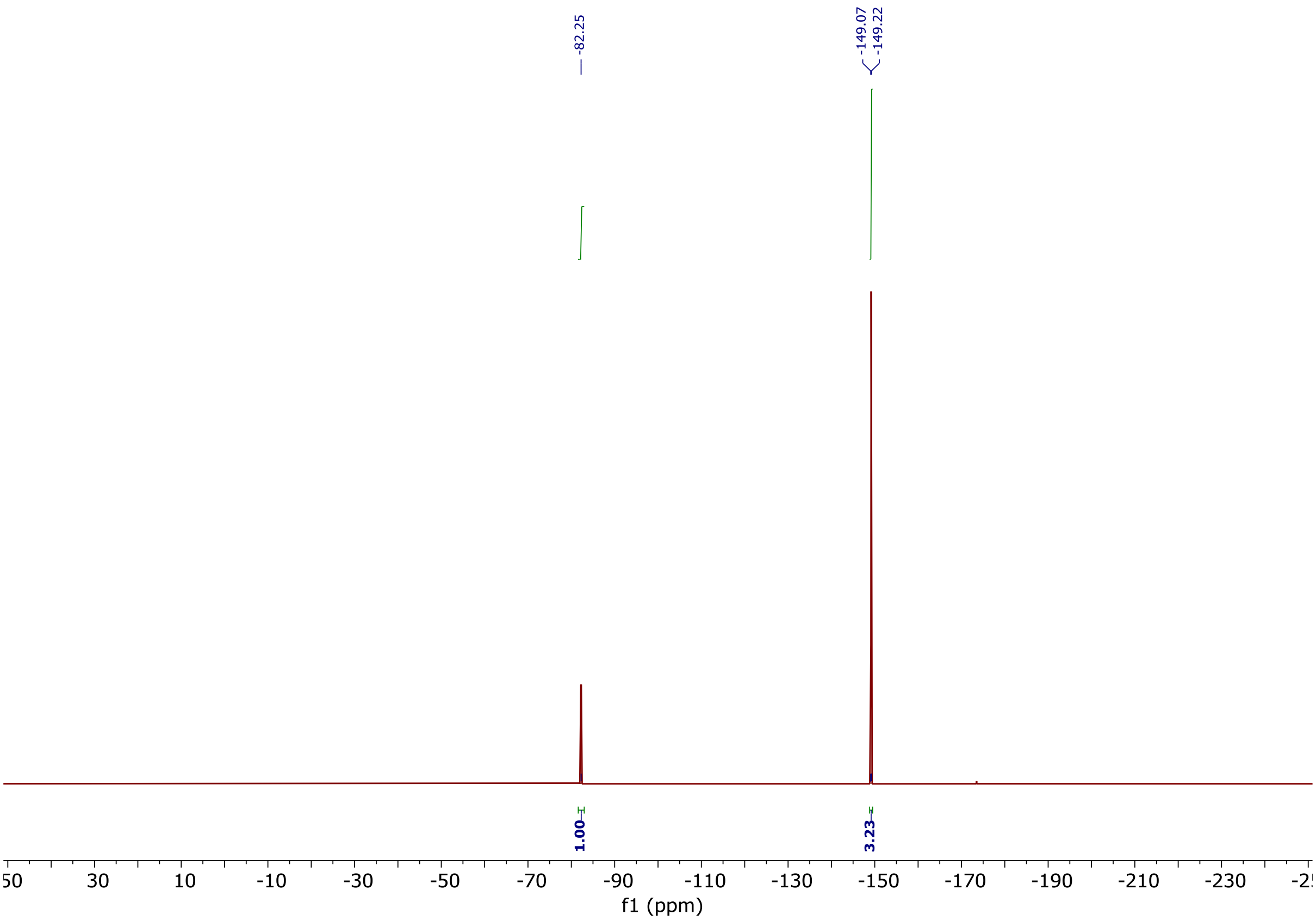

***(Z)-(3-(2-(4-chlorophenoxy)ethoxy)-2-fluoroprop-1-en-1-yl)(p-tolyl)iodonium tetrafluoroborate* (3ao)**

^{1}H NMR (500 MHz, Acetone-D_6)

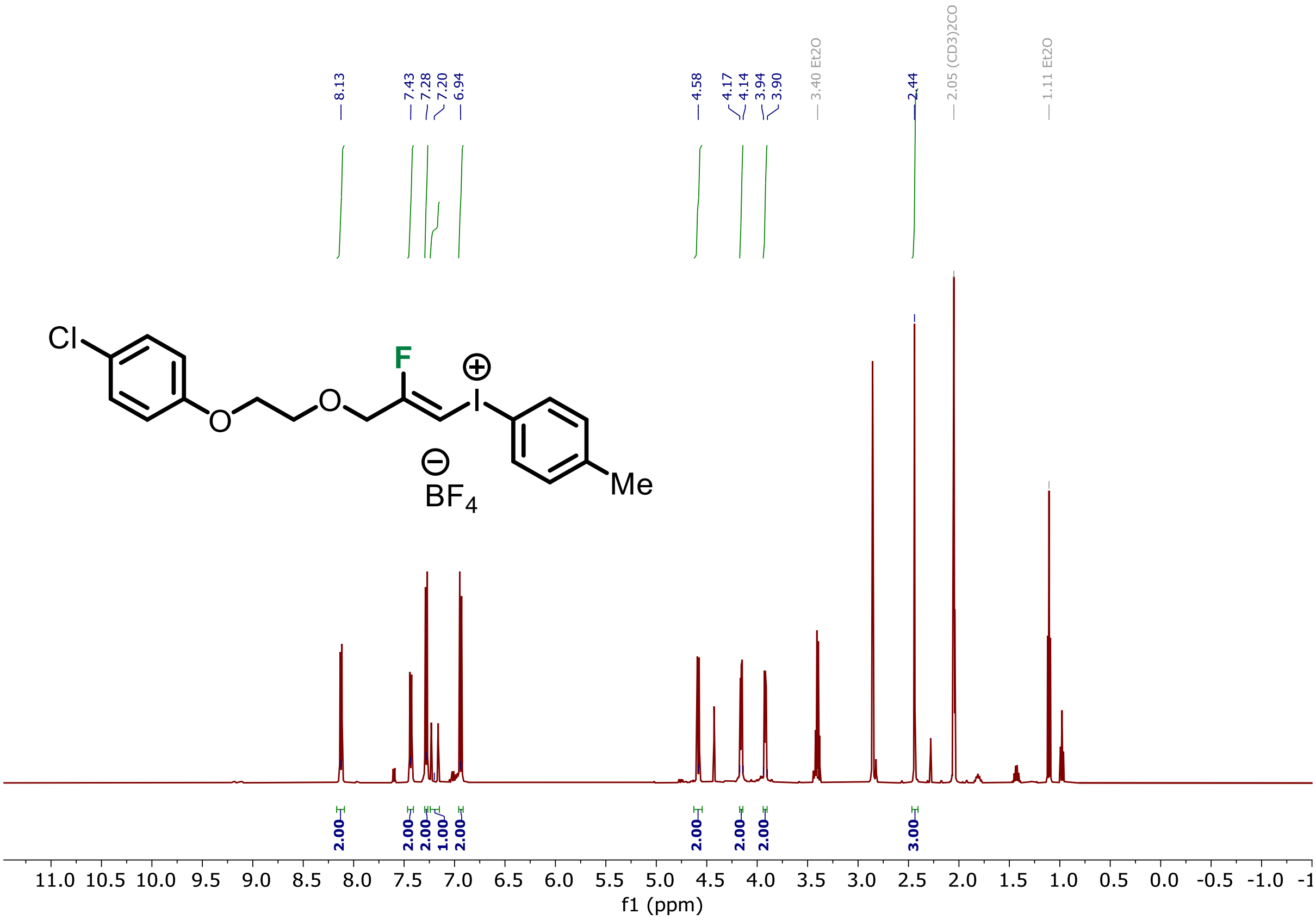

^{13}C NMR (126 MHz, Acetone-D_6)

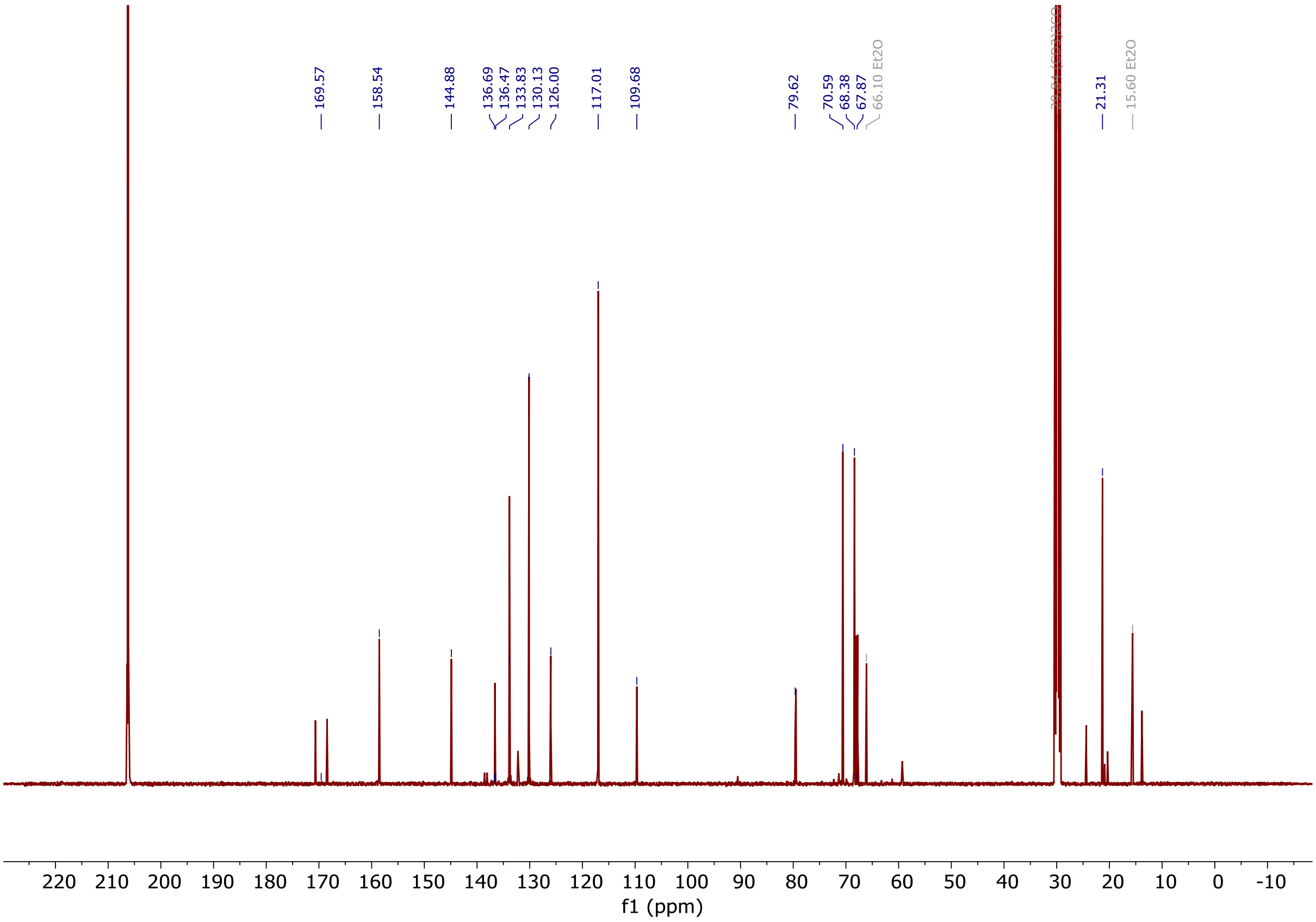

^{19}F NMR (376 MHz, Acetone-D_6)

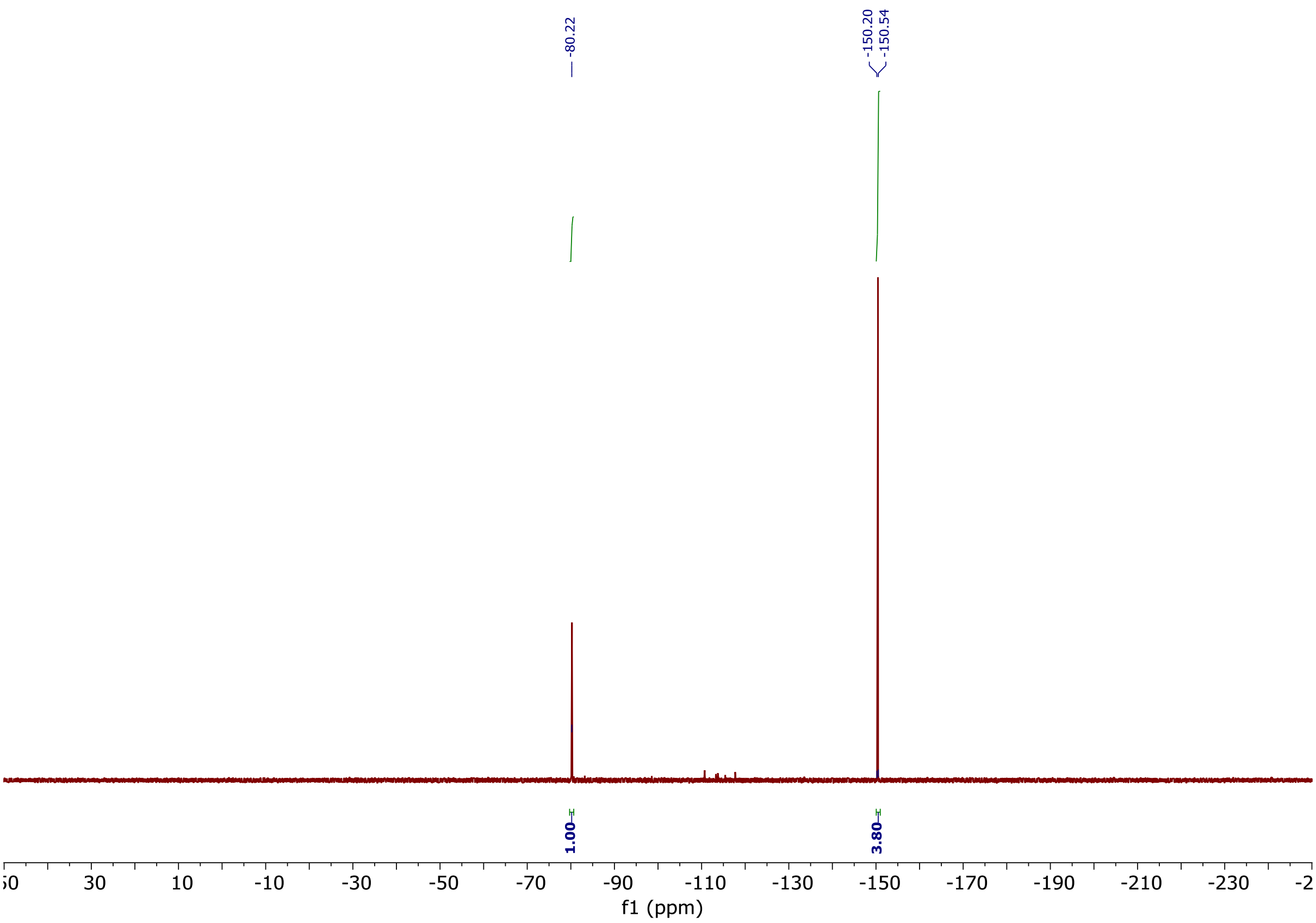

(Z)-(2-fluoro-3-((1-tosylpiperidin-4-yl)oxy)prop-1-en-1-yl)(p-tolyl)iodonium tetrafluoroborate **(3ap)**

^{1}H NMR (400 MHz, Acetone-D_6)

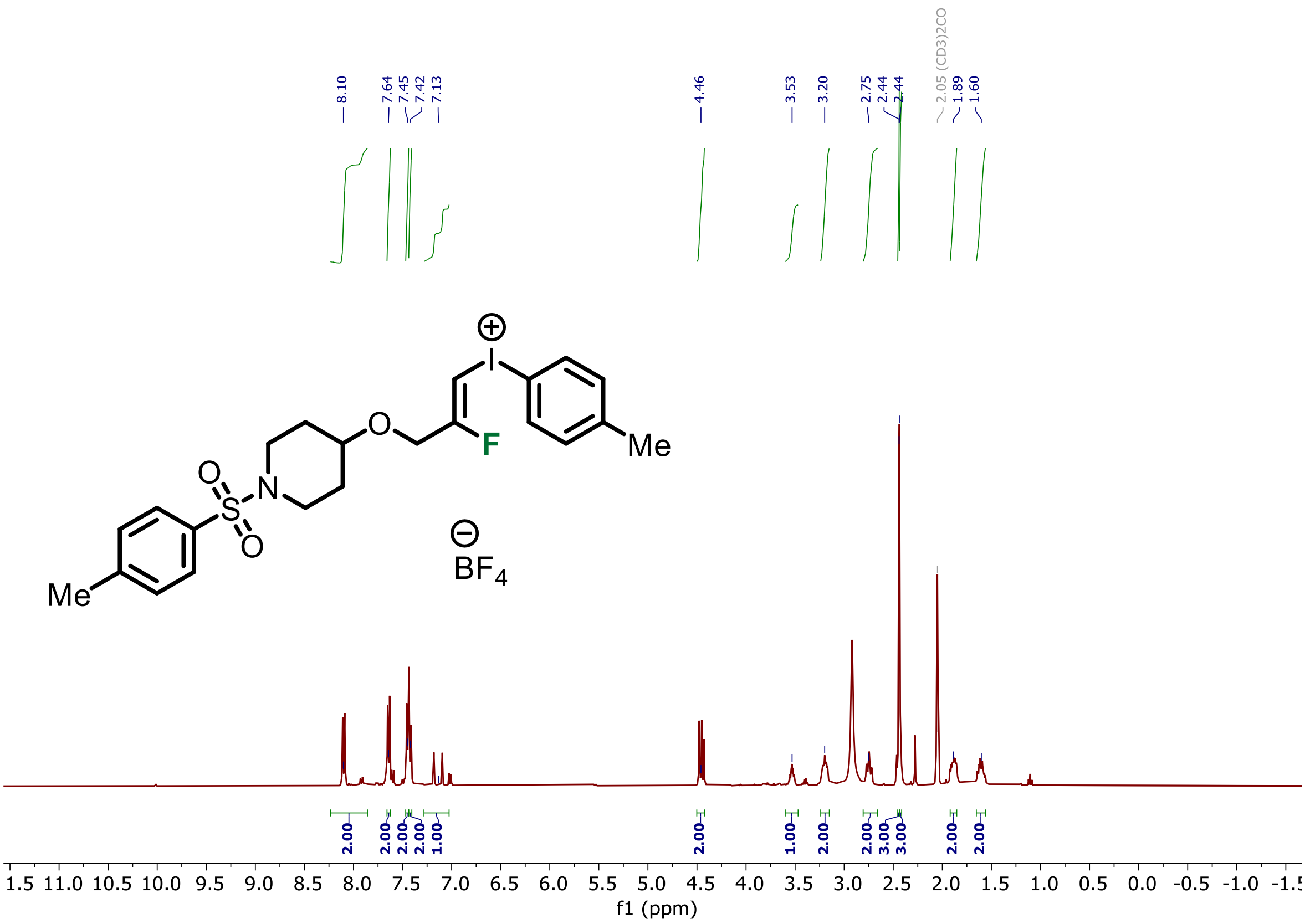

^{13}C NMR (126 MHz, Acetone-D_6)

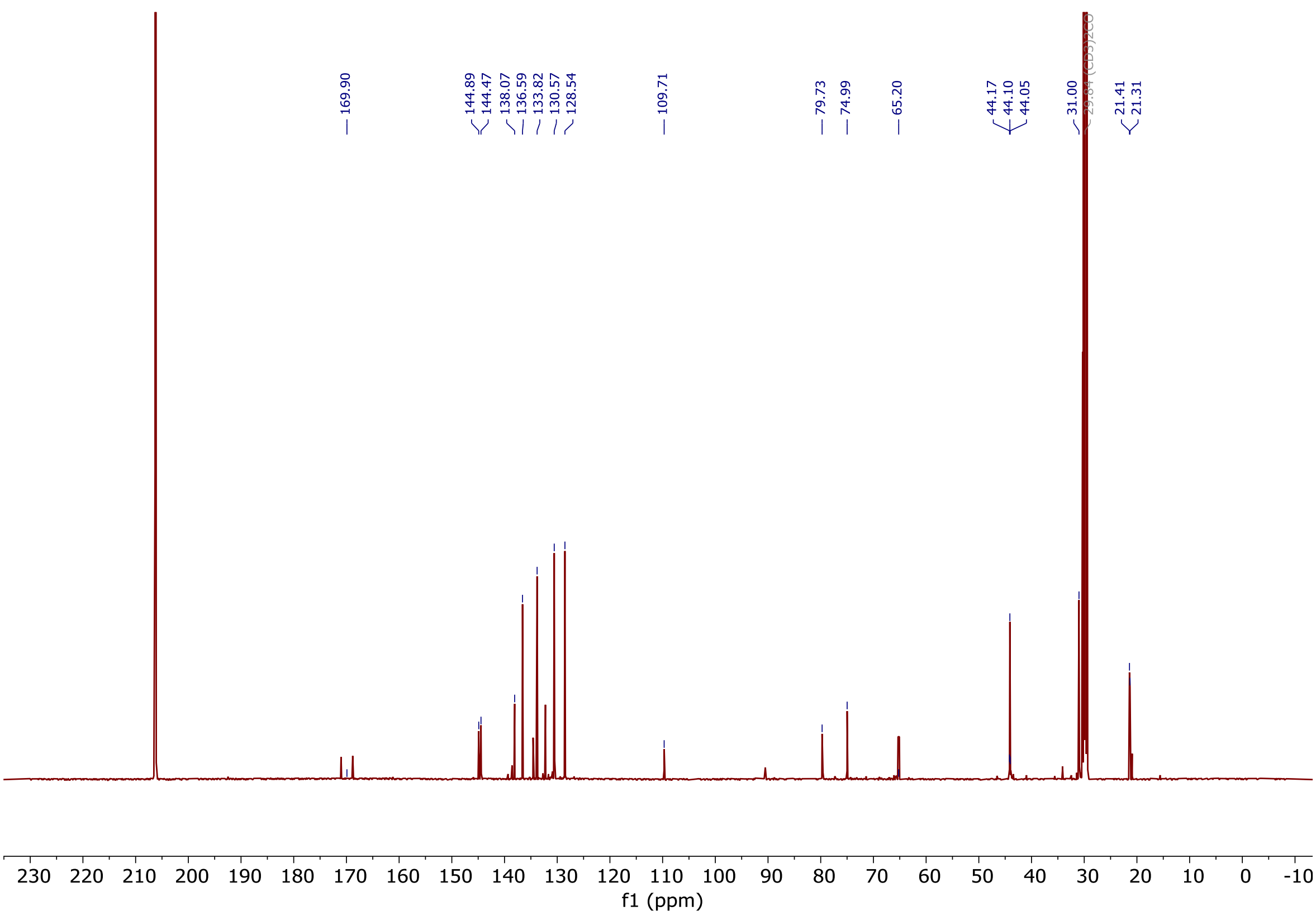

^{19}F NMR (376 MHz, Acetone-D_6)

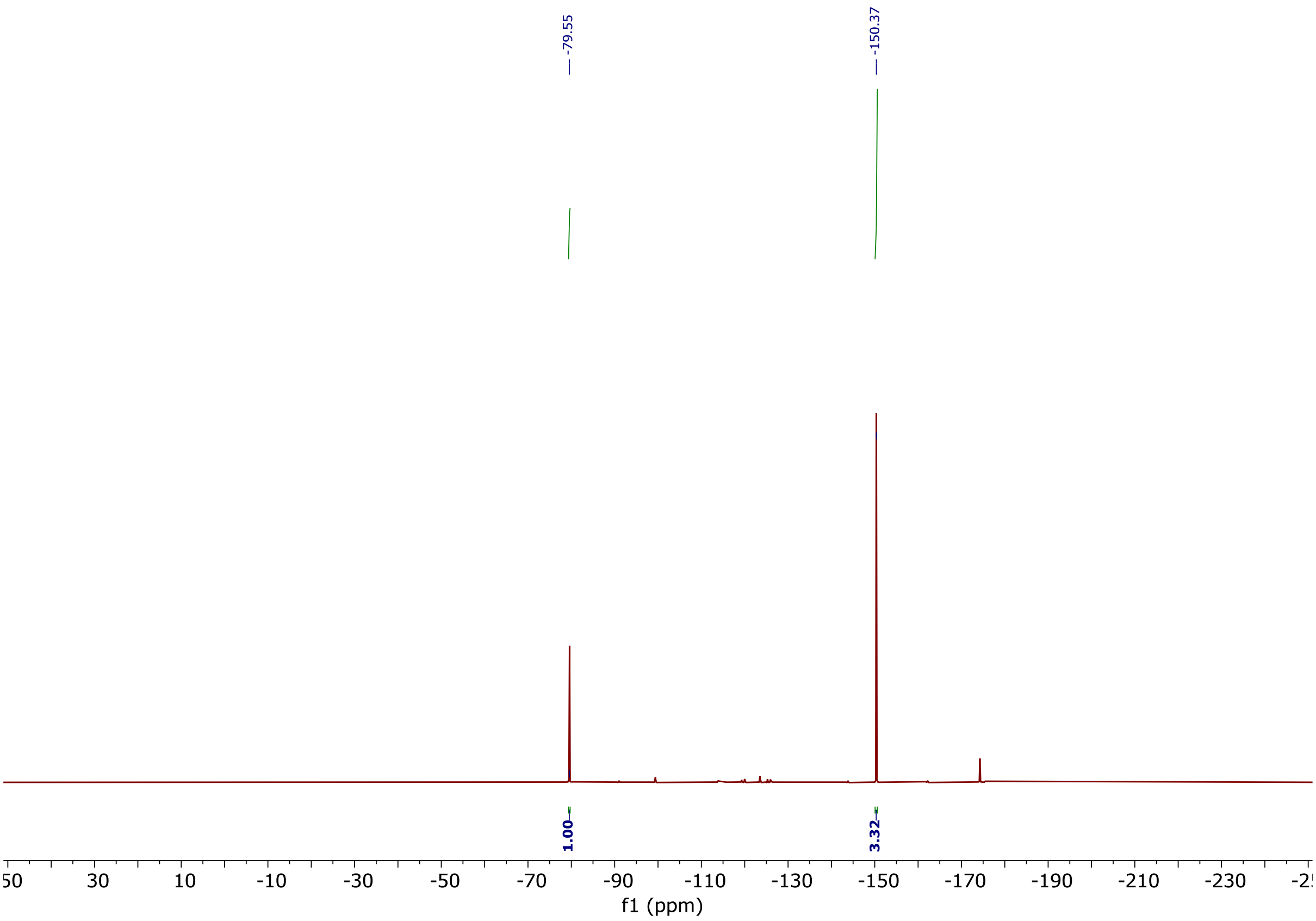

***(Z)-(2-fluoro-3-((4-methylphenyl)sulfonamido)prop-1-en-1-yl)(p-tolyl)iodonium tetrafluoroborate* (3ar)**

^{1}H NMR (500 MHz, Acetone-D_6)

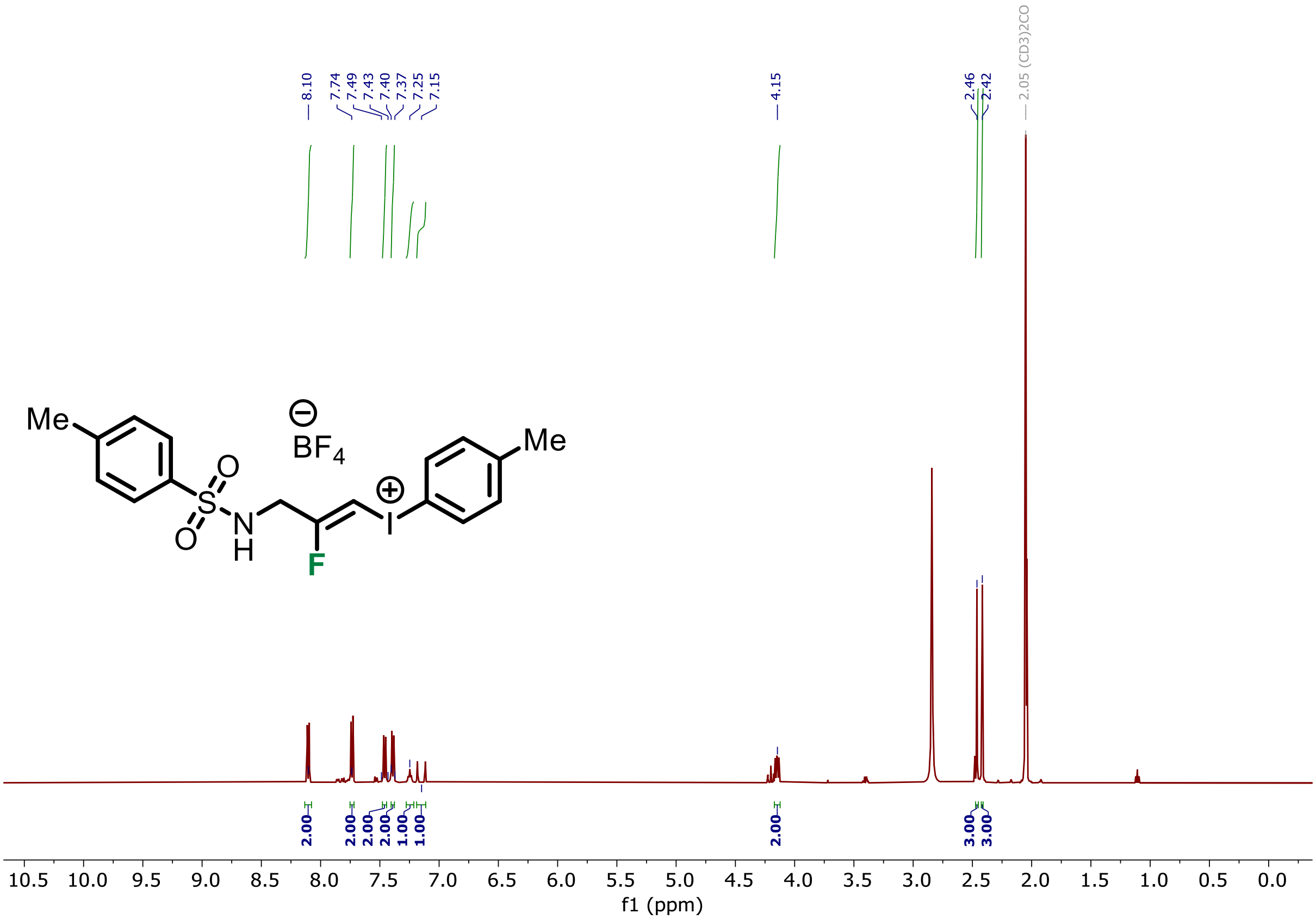

^{13}C NMR (126 MHz, Acetone-D_6)

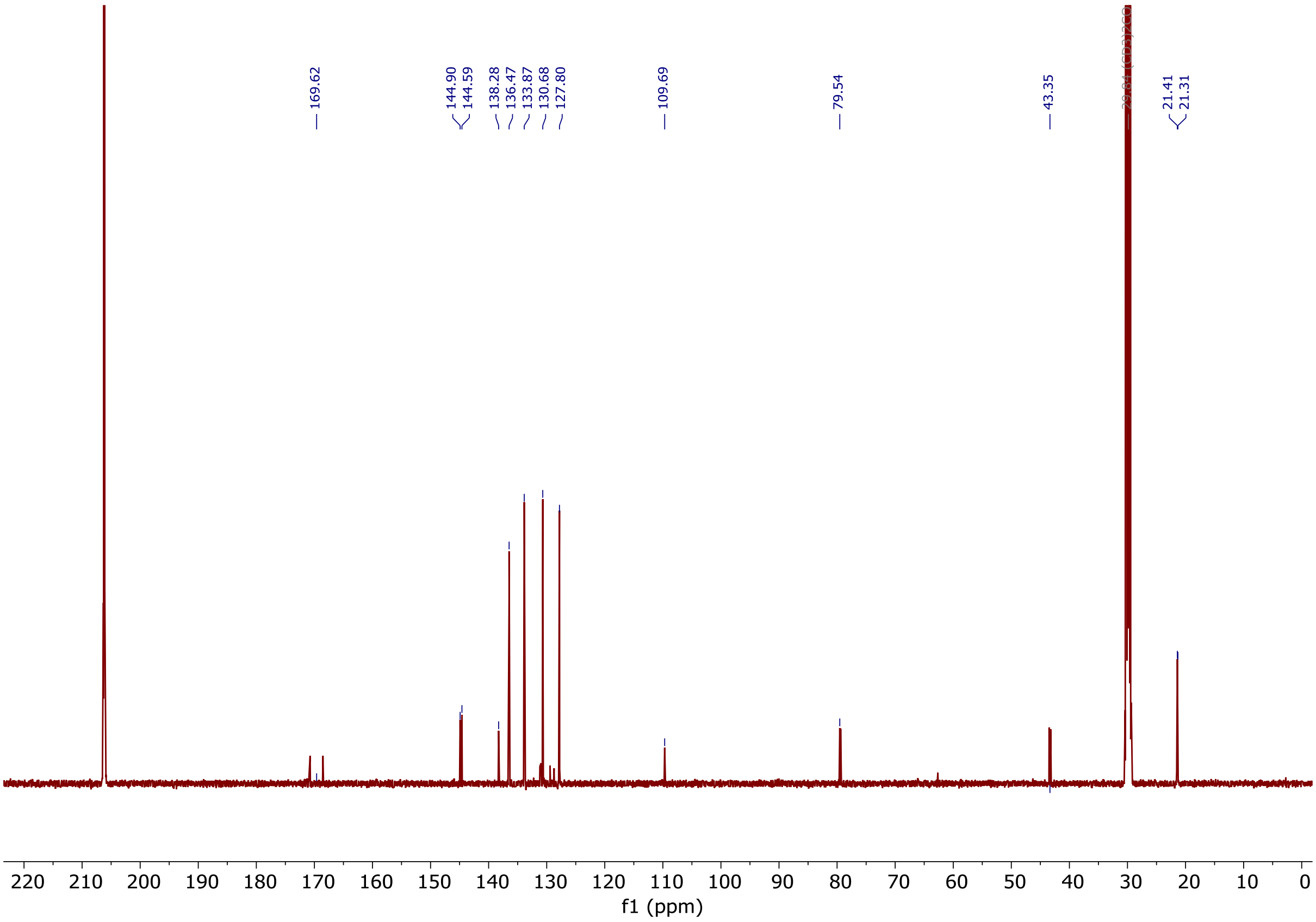

^{19}F NMR (376 MHz, Acetone-D_6)

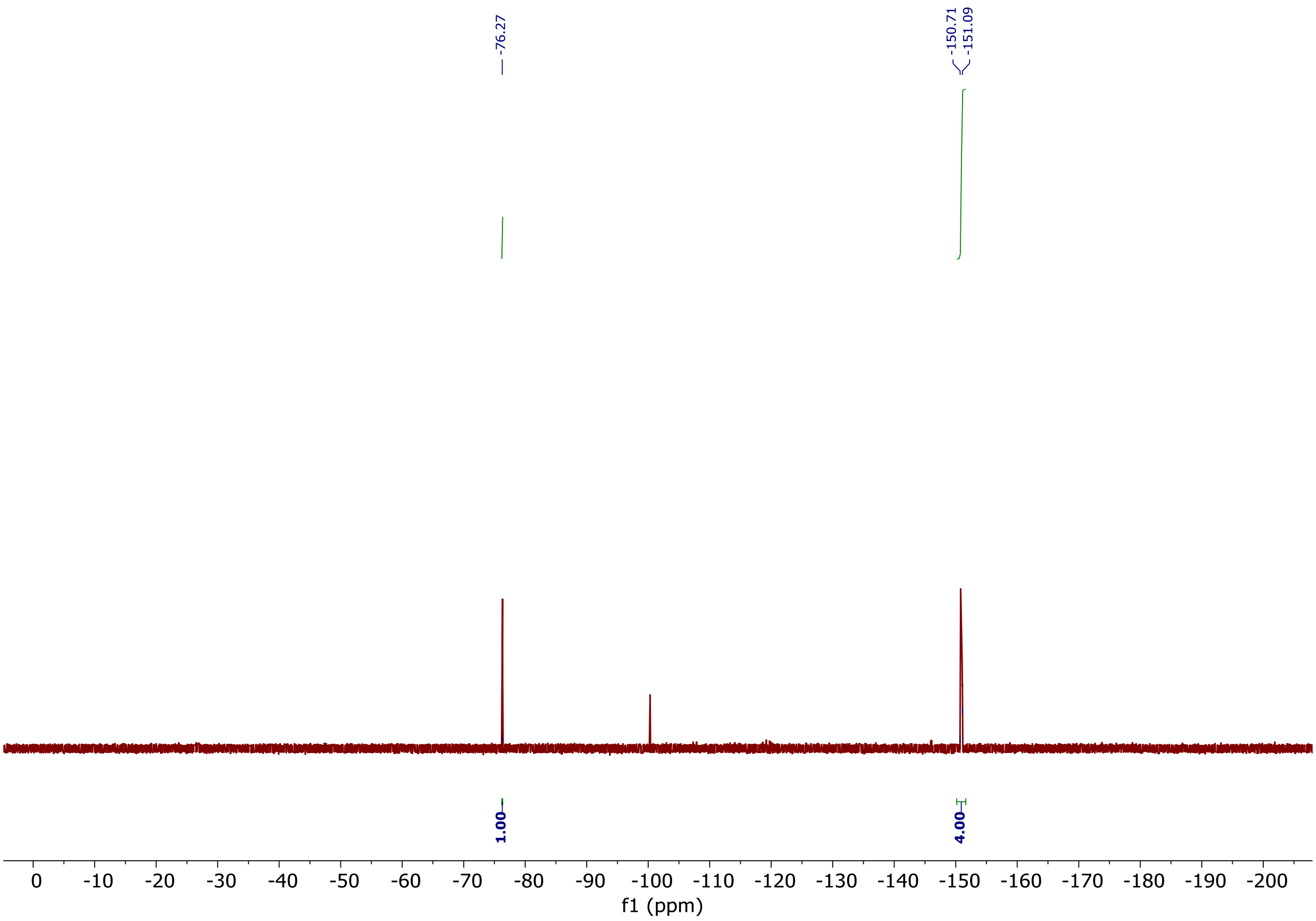

***(Z)-(2-fluoro-3-((4-methylphenyl)sulfonamido)prop-1-en-1-yl)(phenyl)iodonium tetrafluoroborate* (3ar')**

^{1}H NMR (500 MHz, Acetone-D_6)

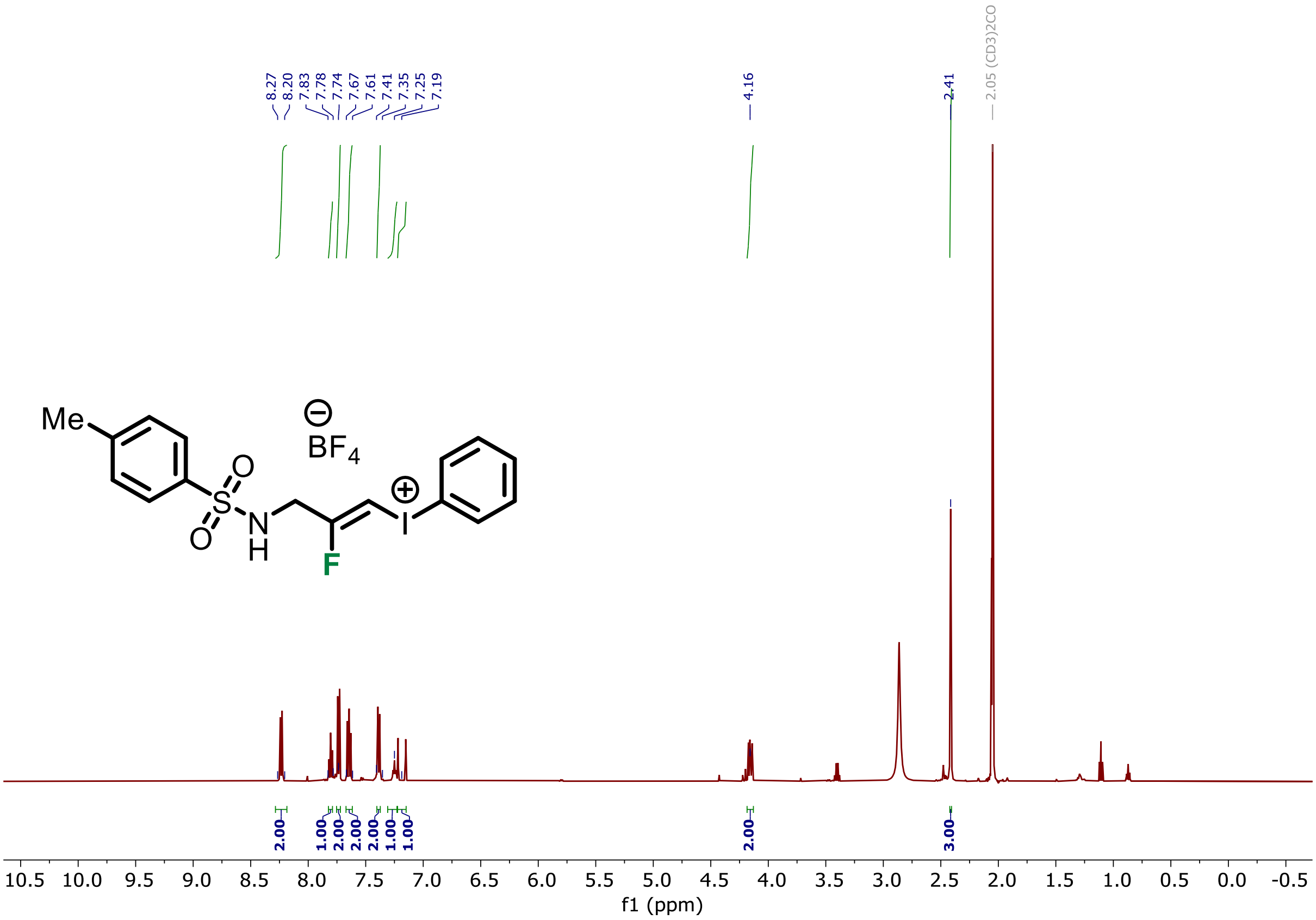

^{13}C NMR (126 MHz, Acetone-D_6)

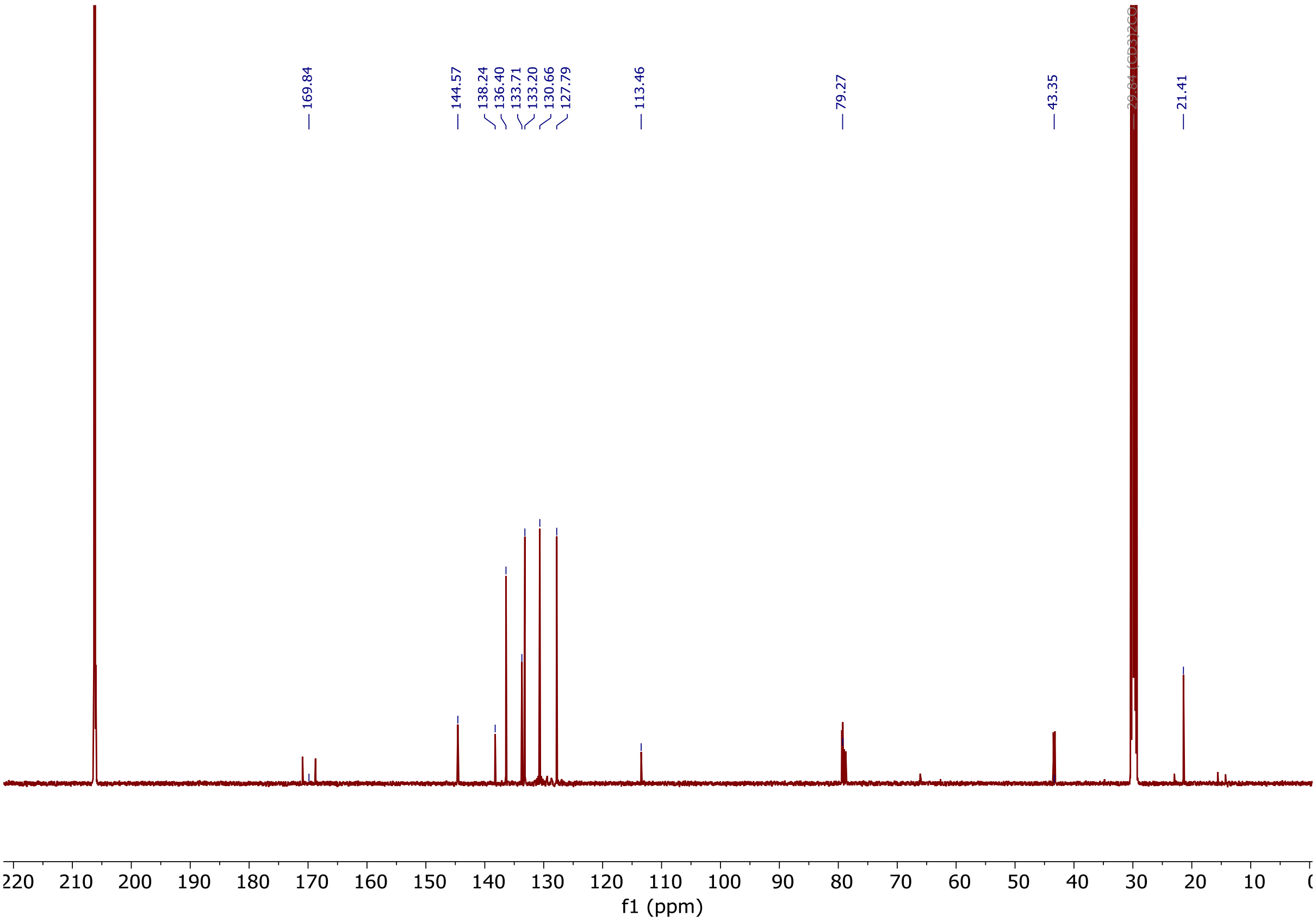

^{19}F NMR (376 MHz, Acetone-D_6)

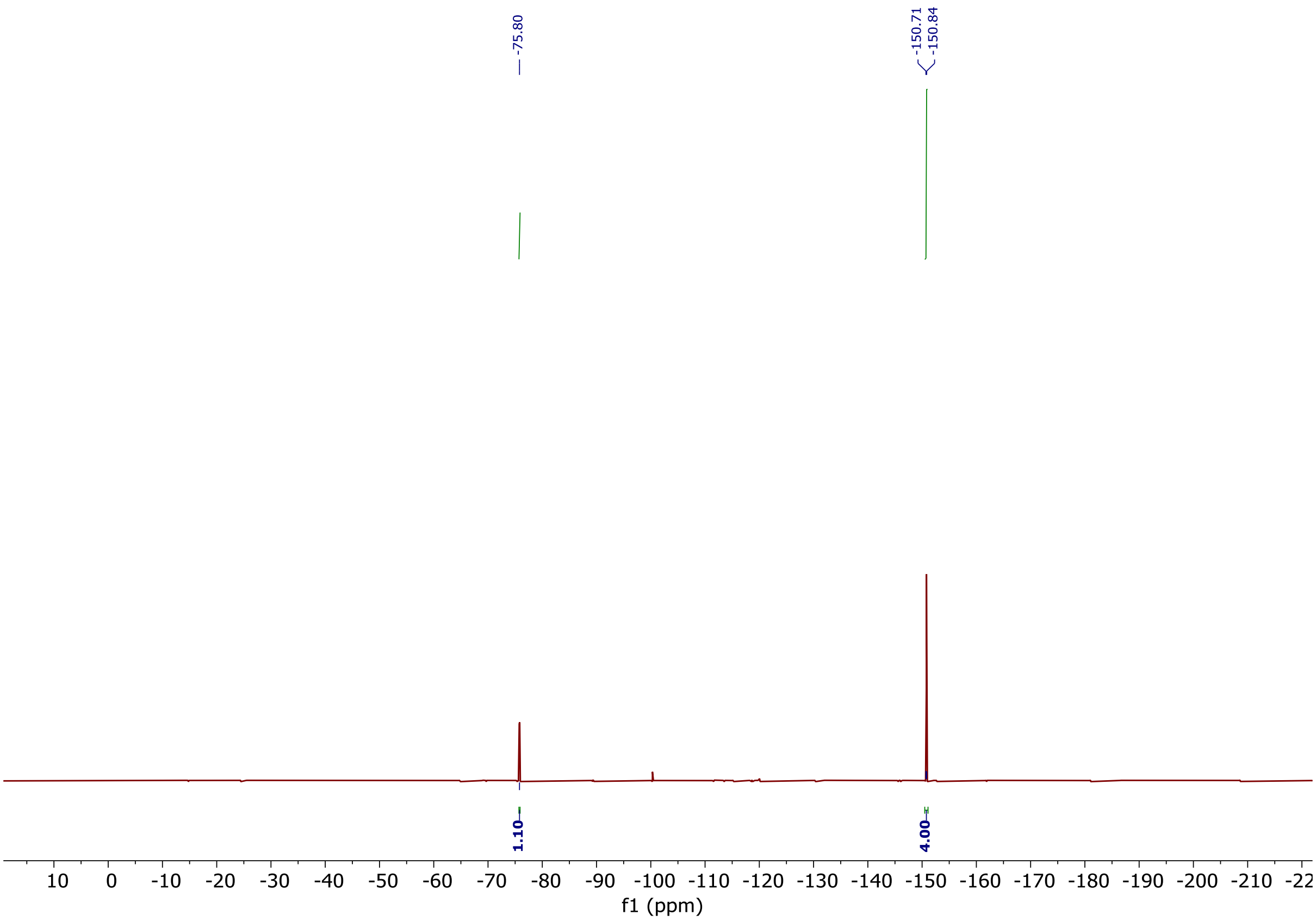

***(Z)-(2-fluoro-3-((4-nitrophenyl)sulfonamido)prop-1-en-1-yl)(p-tolyl)iodonium tetrafluoroborate* (3as)**

^{1}H NMR (500 MHz, Acetone-D_6)

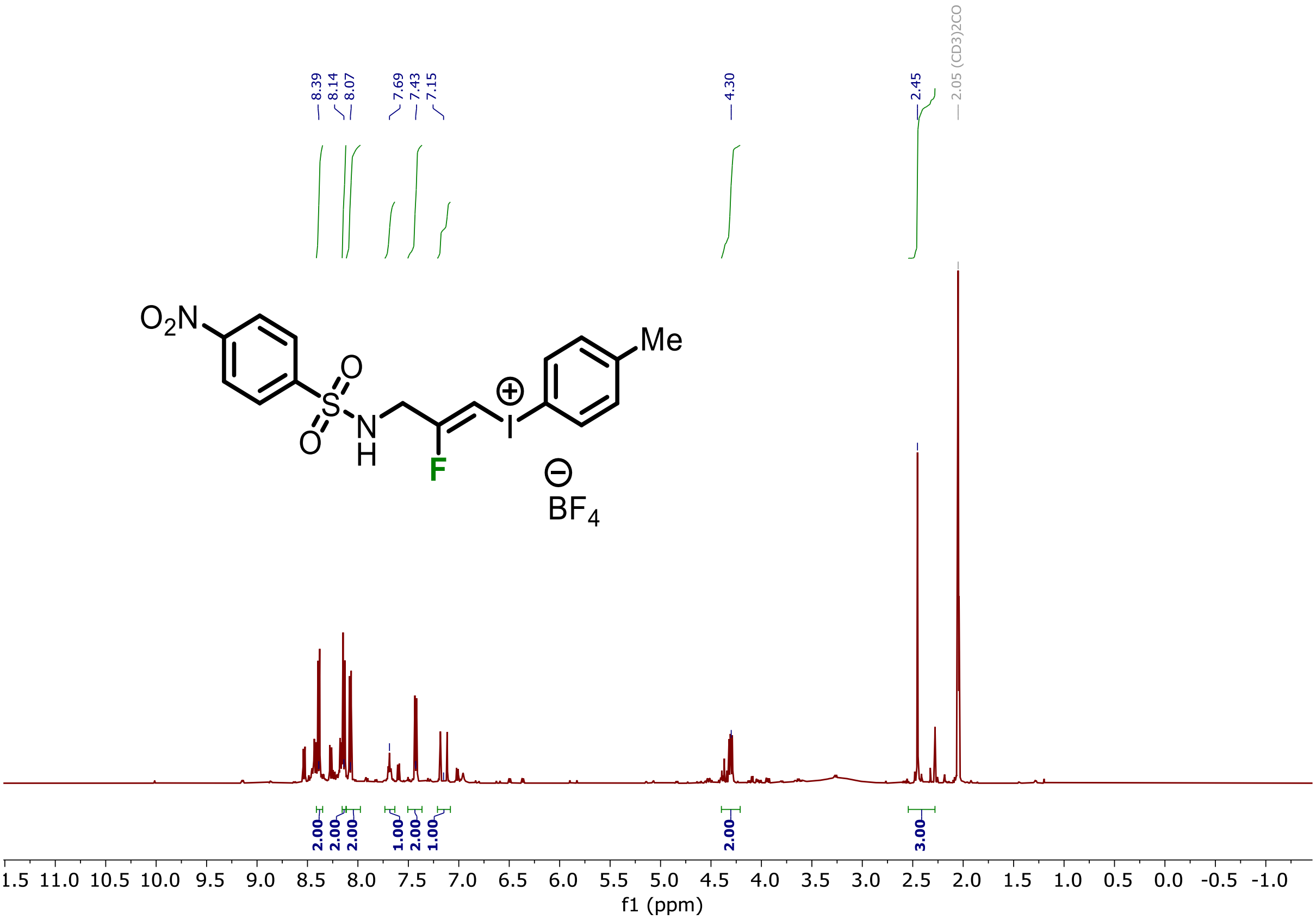

^{13}C NMR (126 MHz, Acetone-D_6)

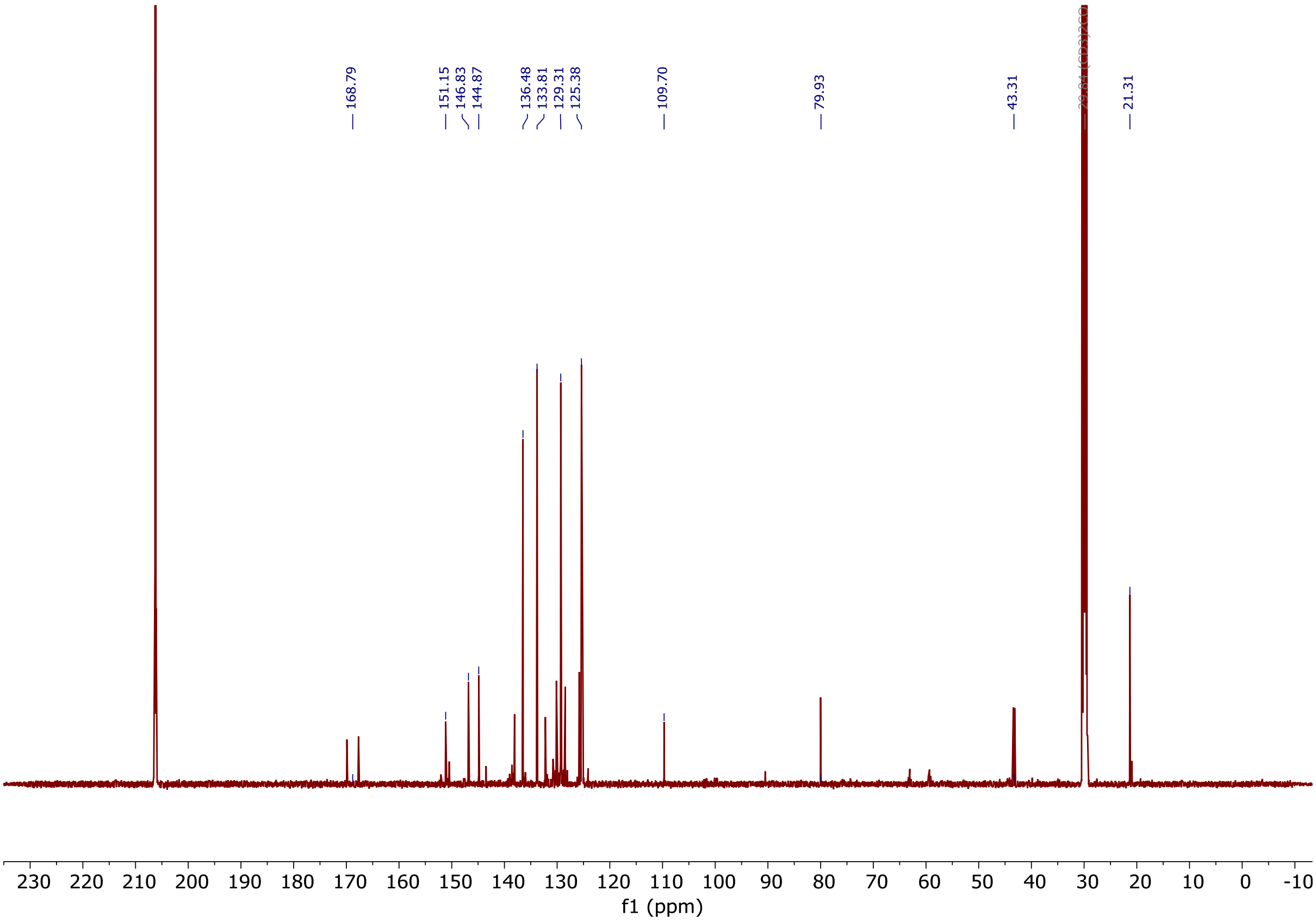

^{19}F NMR (376 MHz, Acetone-D_6)

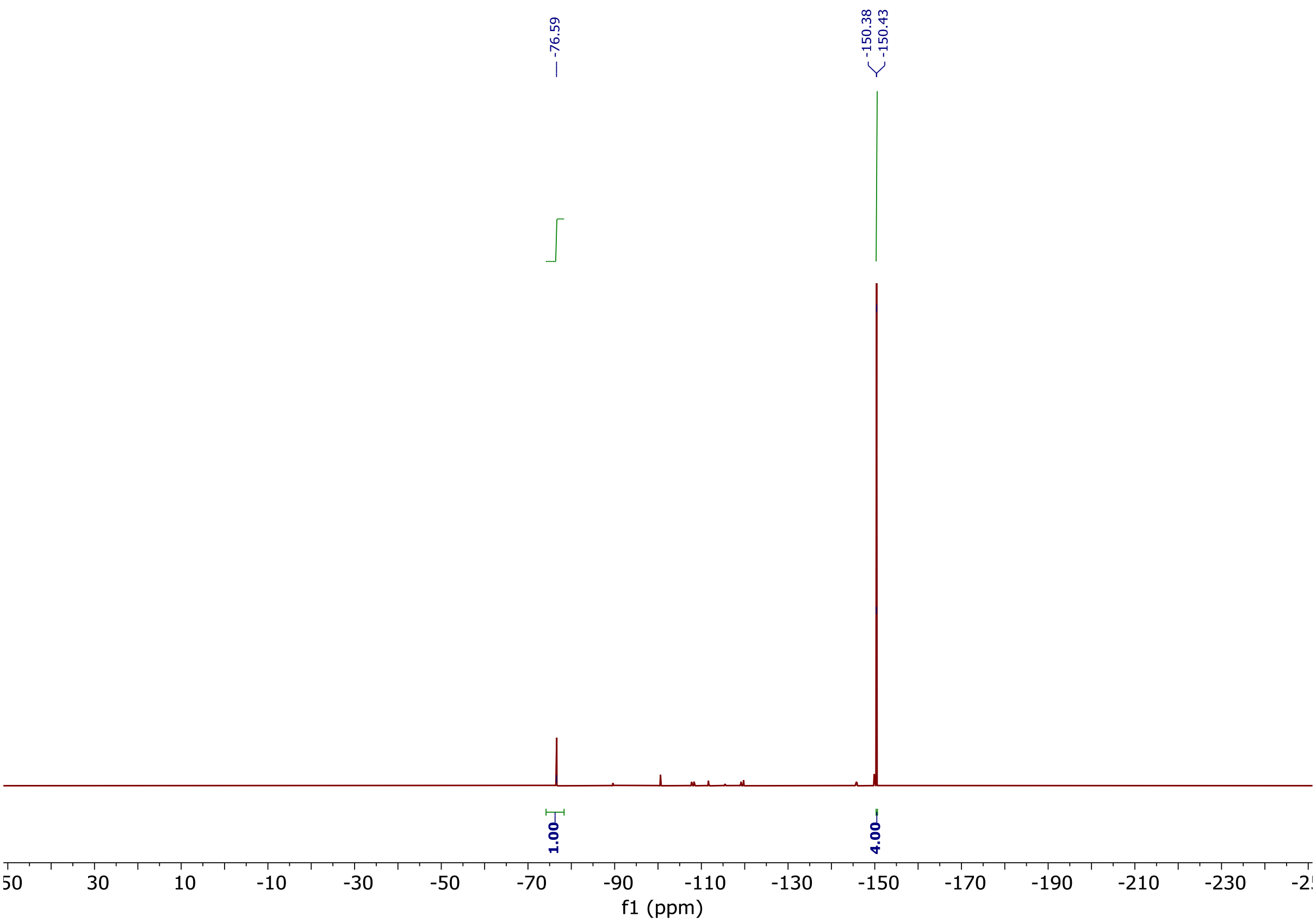

***(Z)-(2-fluoro-3-((4-methylphenyl)sulfonamido)but-1-en-1-yl)(p-tolyl)iodonium tetrafluoroborate* (3at)**

^{1}H NMR (400 MHz, Acetone-D_6)

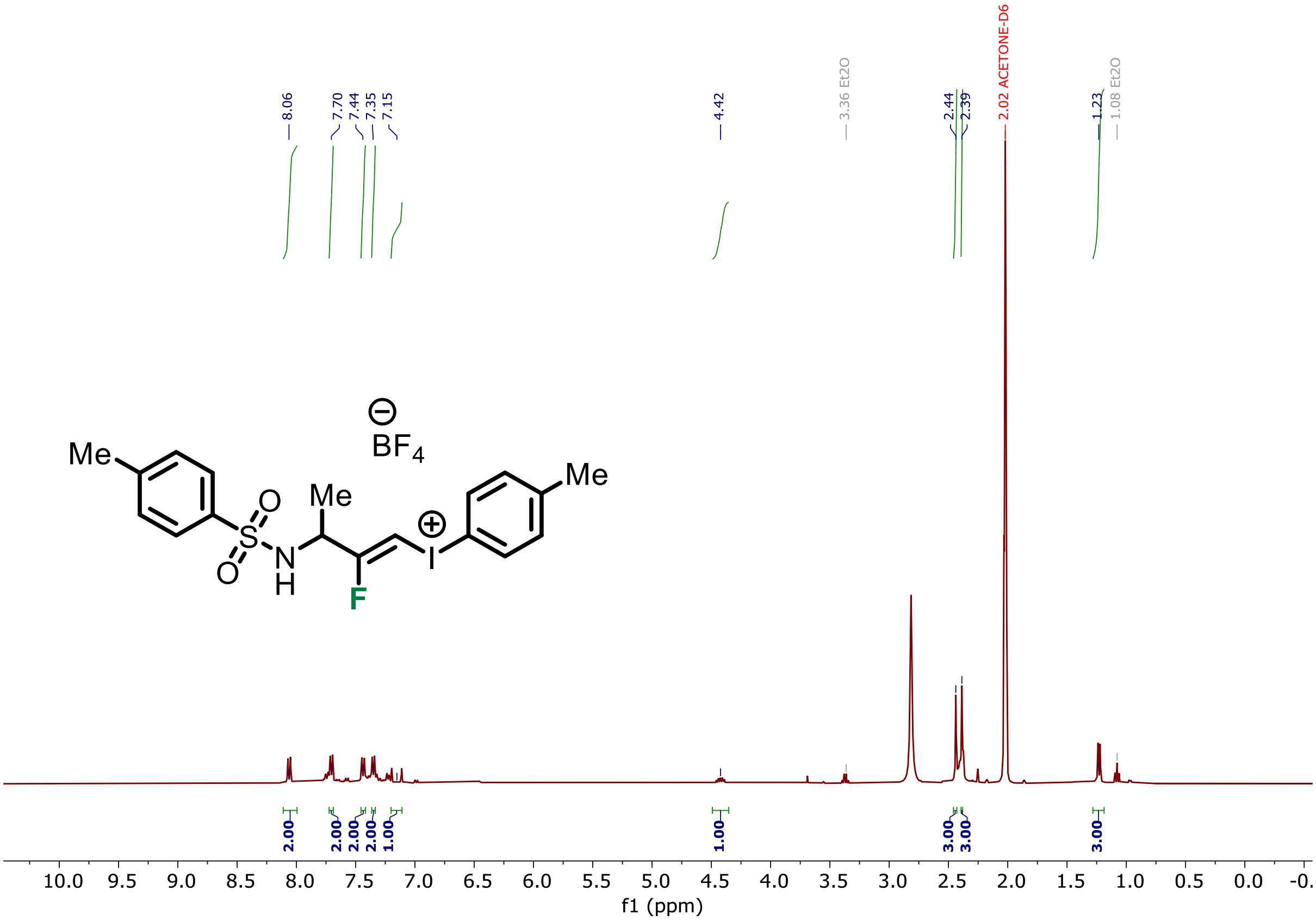

^{13}C NMR (126 MHz, Acetone-D_6)

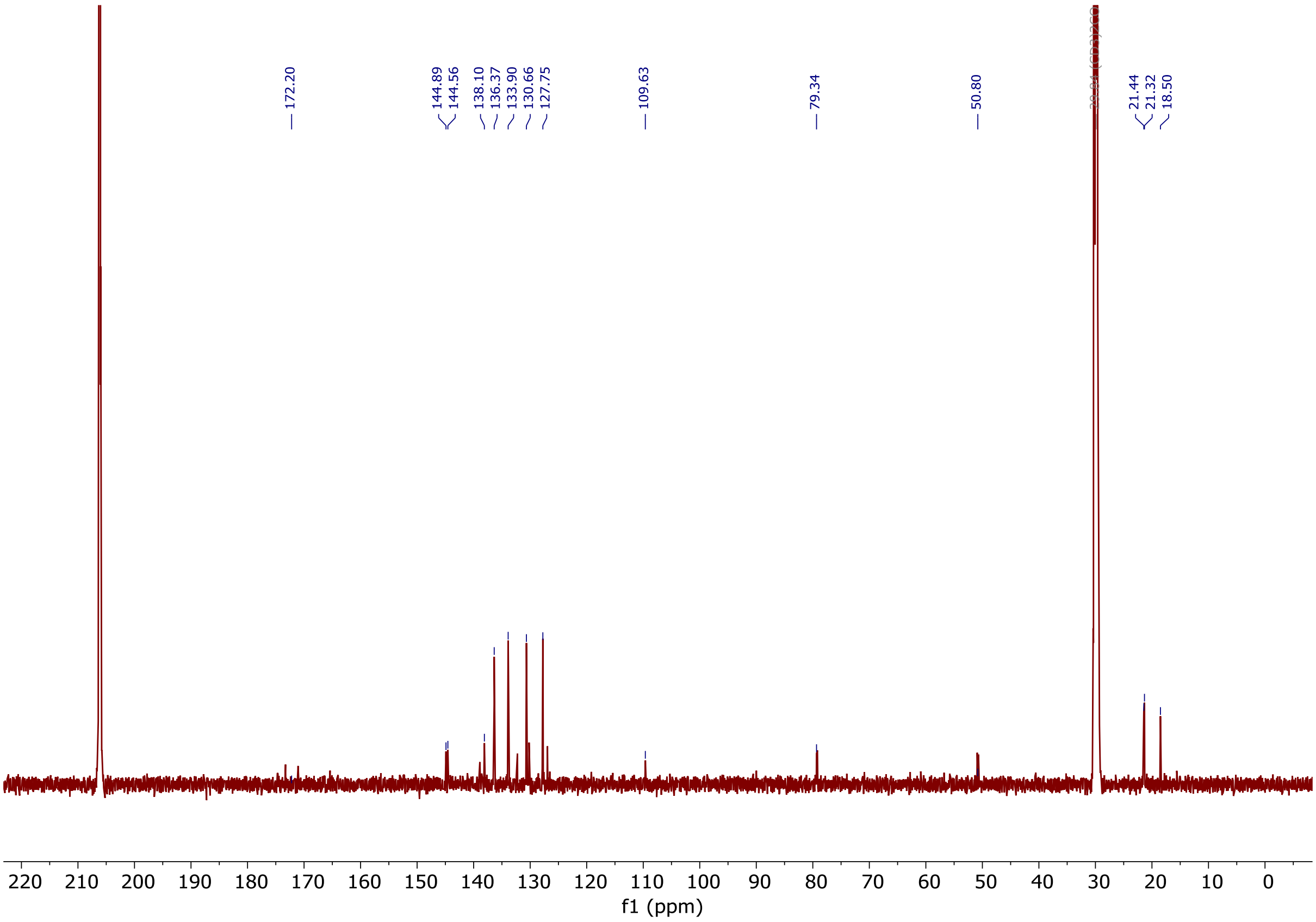

^{19}F NMR (376 MHz, Acetone-D_6)

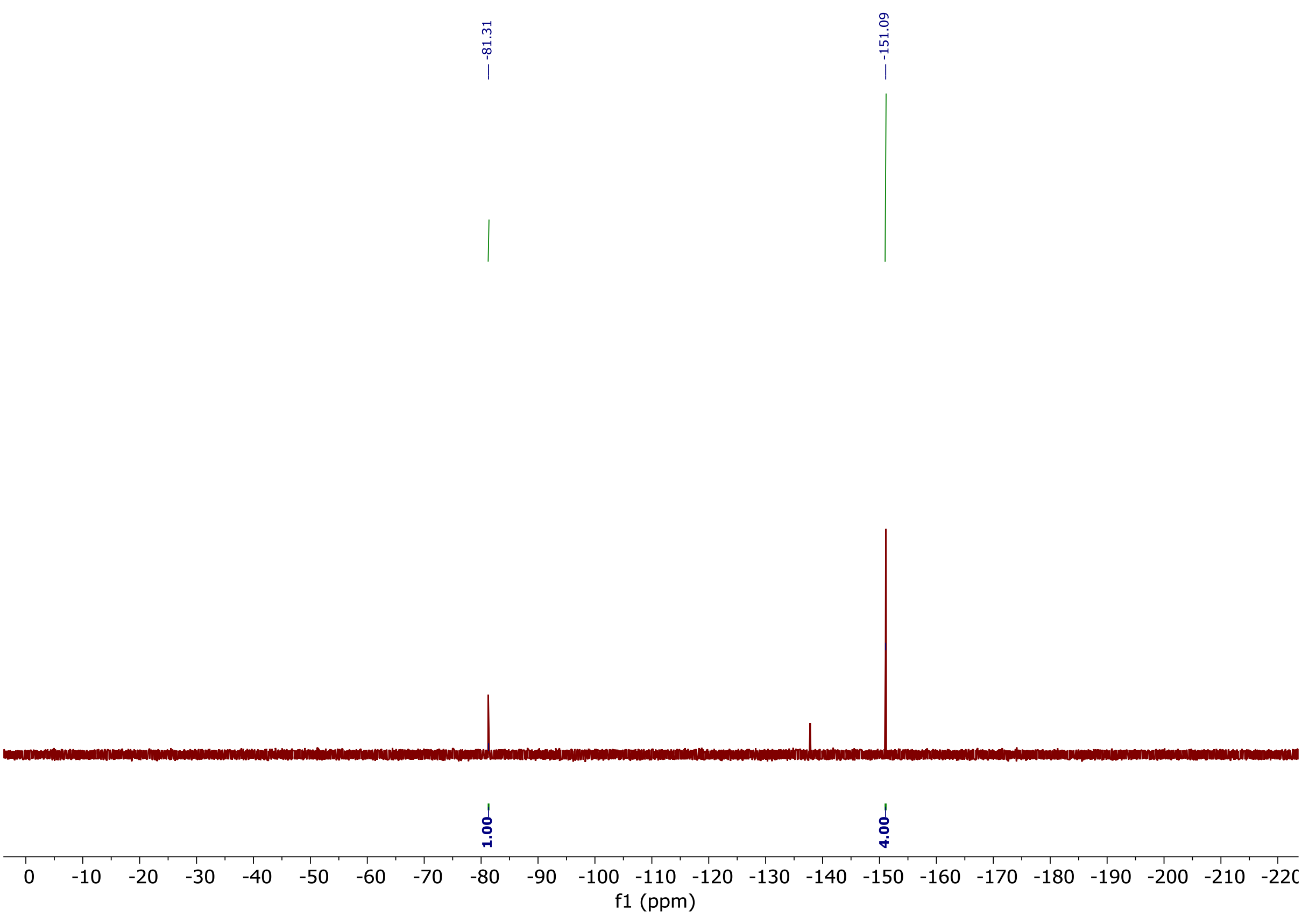

(Z)-(2-fluoro-2-(1-((4-methylphenyl)sulfonamido)cyclohexyl)vinyl)(p-tolyl)iodonium tetrafluoroborate **(3av)**

^{1}H NMR (500 MHz, Acetone-D_6)

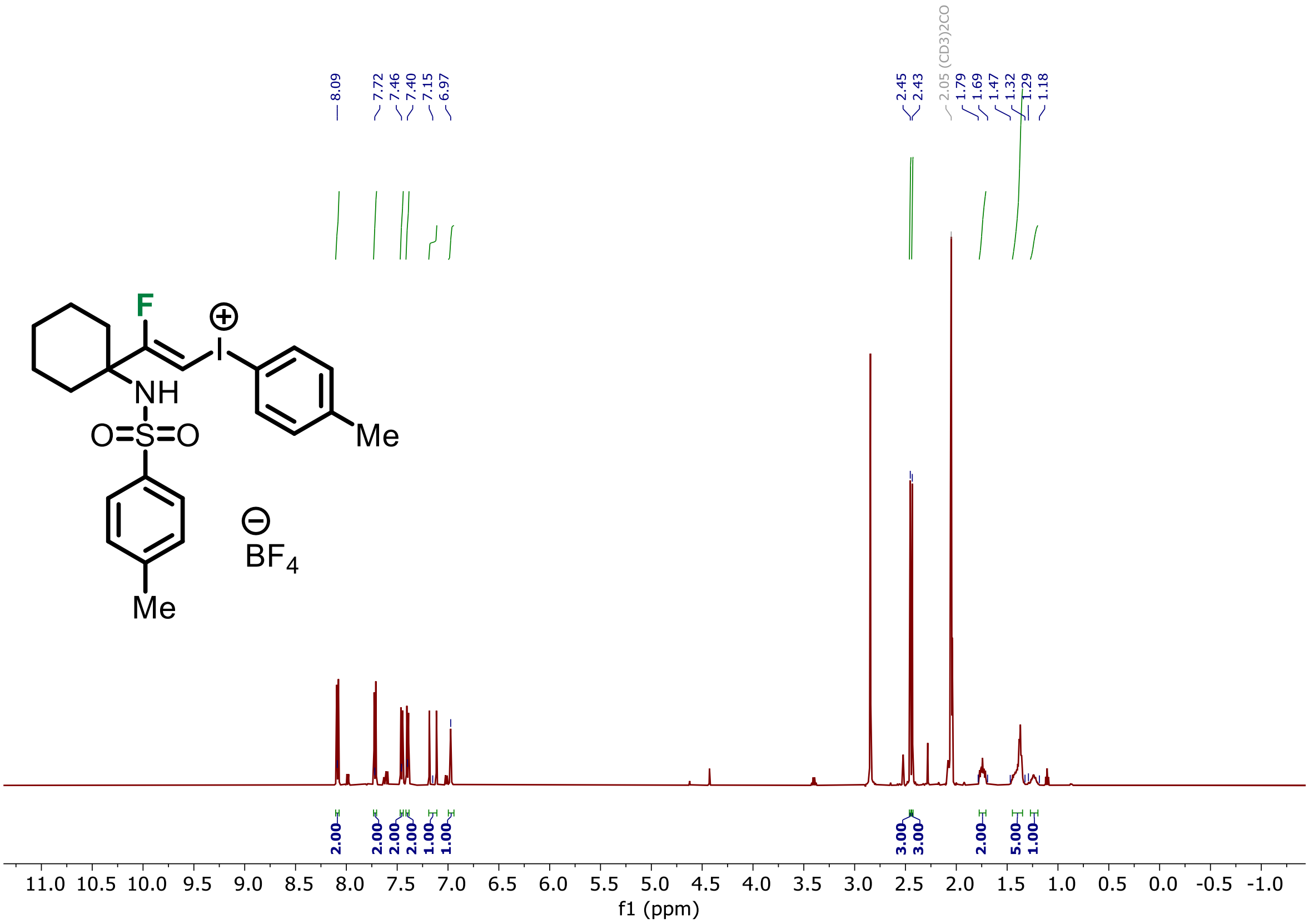

^{13}C NMR (126 MHz, Acetone-D_6)

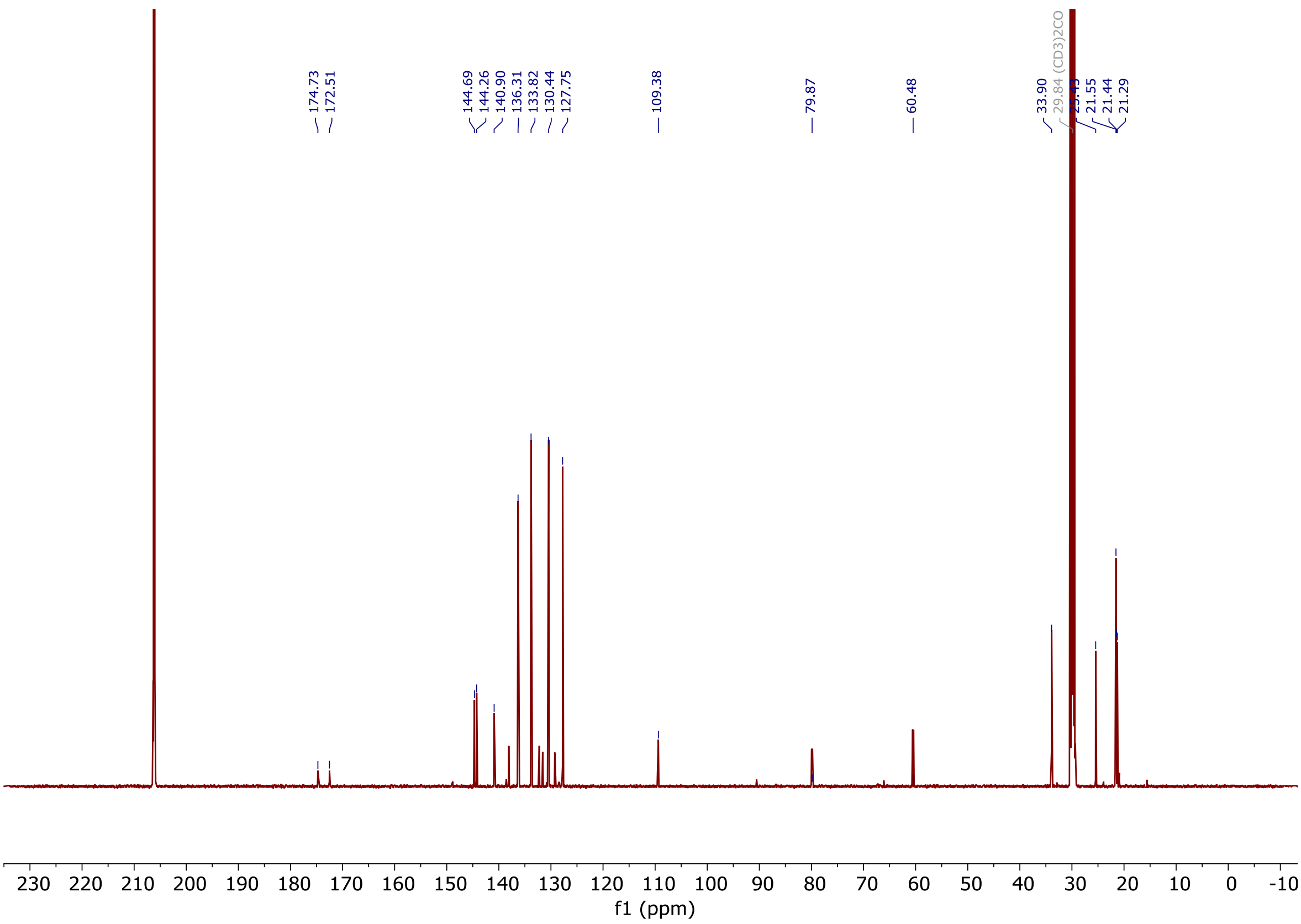

^{19}F NMR (377 MHz, Acetone-D_6)

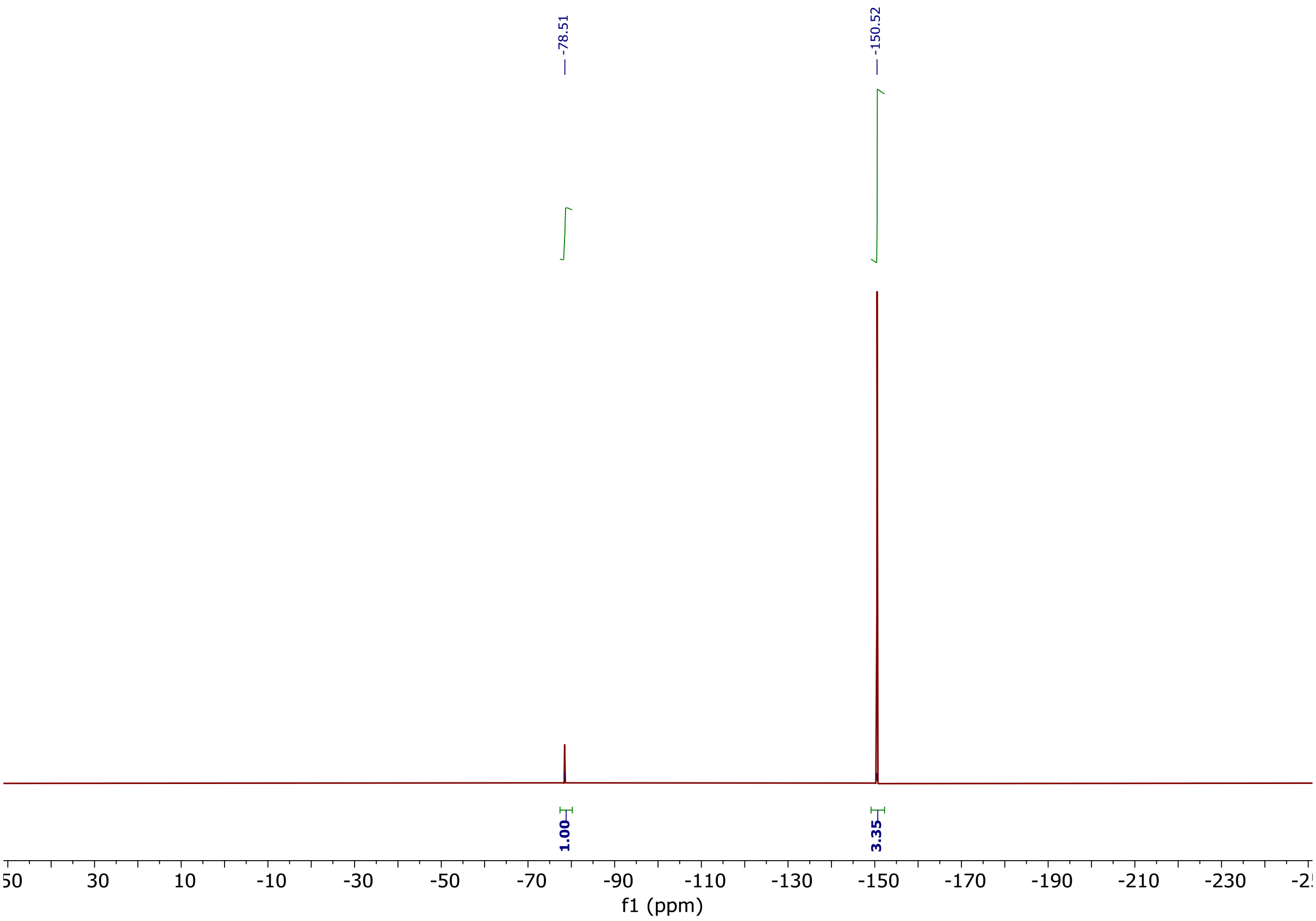

***(Z)-(2-fluoro-3-methyl-3-((4-methylphenyl)sulfonamido)but-1-en-1-yl)(p-tolyl)iodonium tetrafluoroborate* (3au)**

^{1}H NMR (400 MHz, Acetone-D_6)

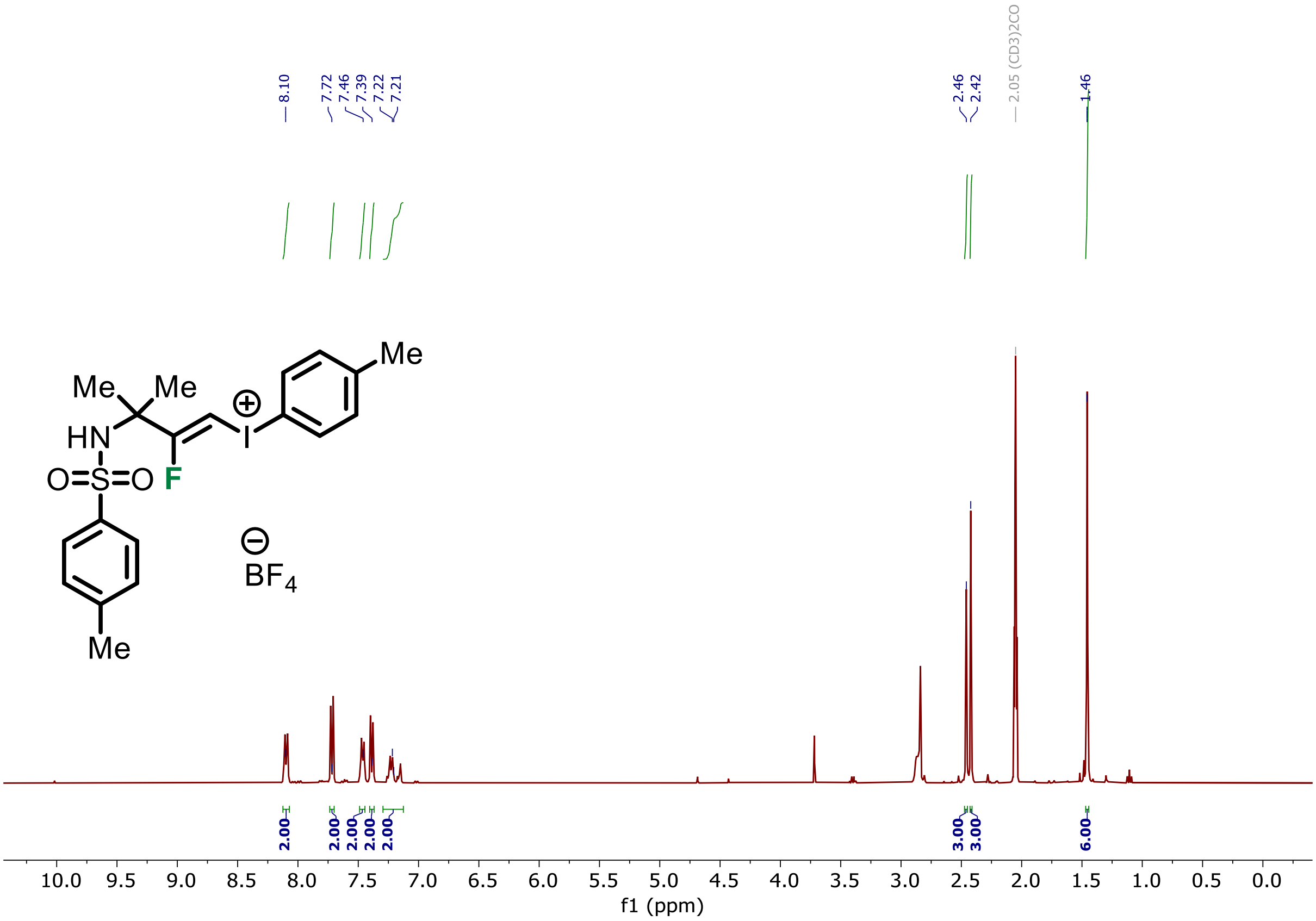

^{13}C NMR (126 MHz, Acetone-D_6)

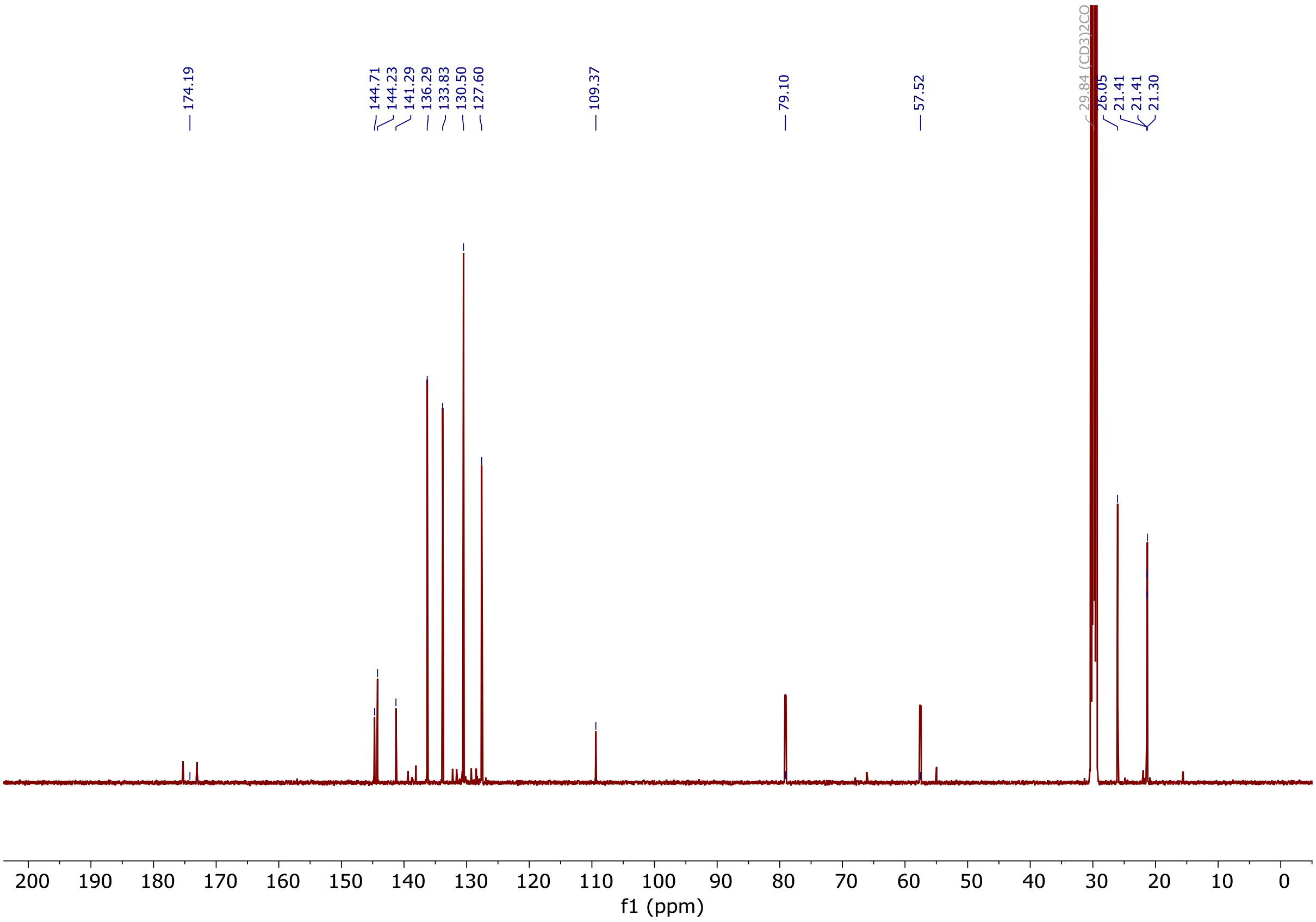

^{19}F NMR (376 MHz, Acetone-D_6)

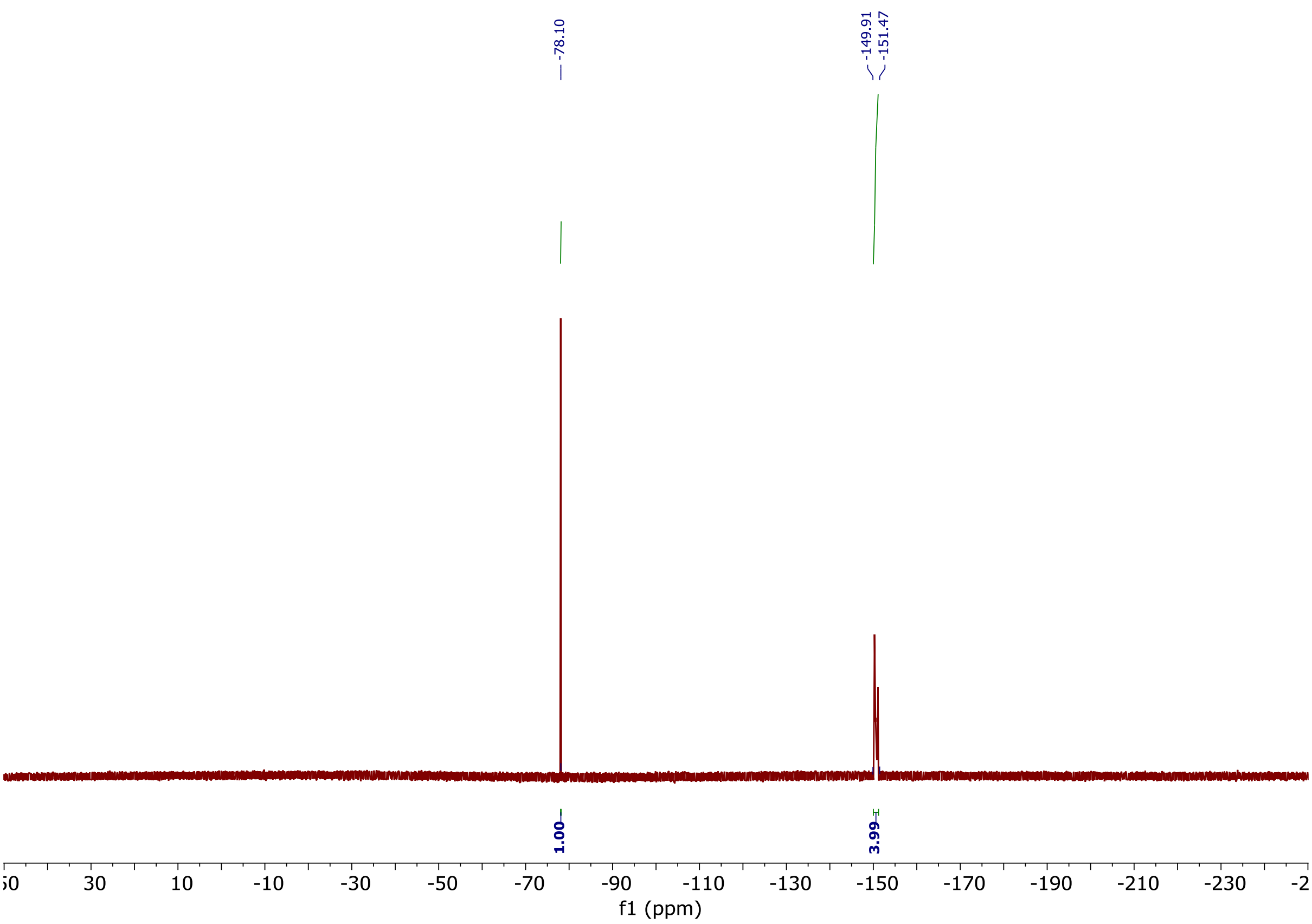

***(Z)-(2-fluoro-3-((4-methylphenyl)sulfonamido)-3-phenylprop-1-en-1-yl)(p-tolyl)iodonium tetrafluoroborate* (3aw)**

^{1}H NMR (500 MHz, Acetone-D_6)

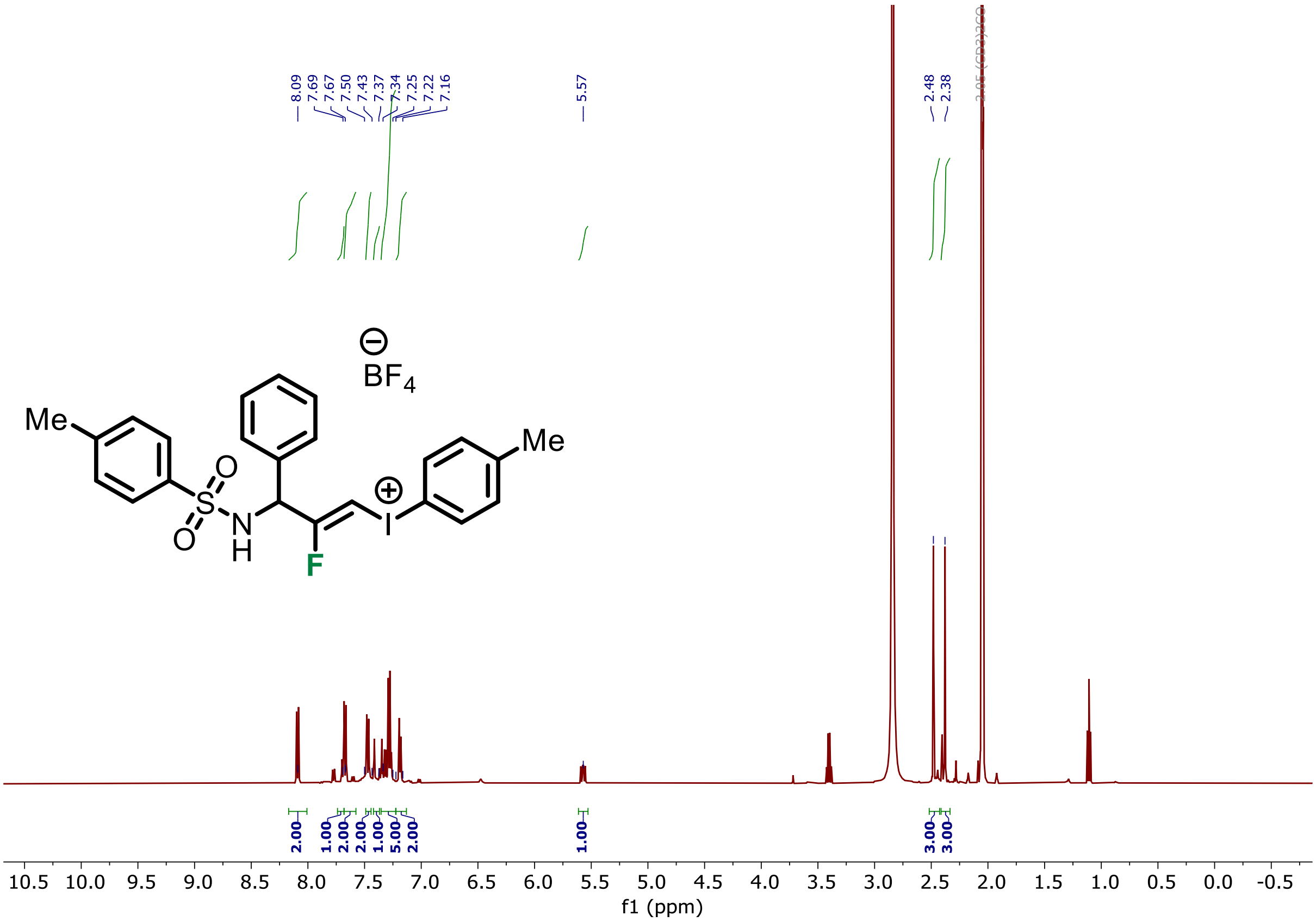

^{13}C NMR (126 MHz, Acetone-D_6)

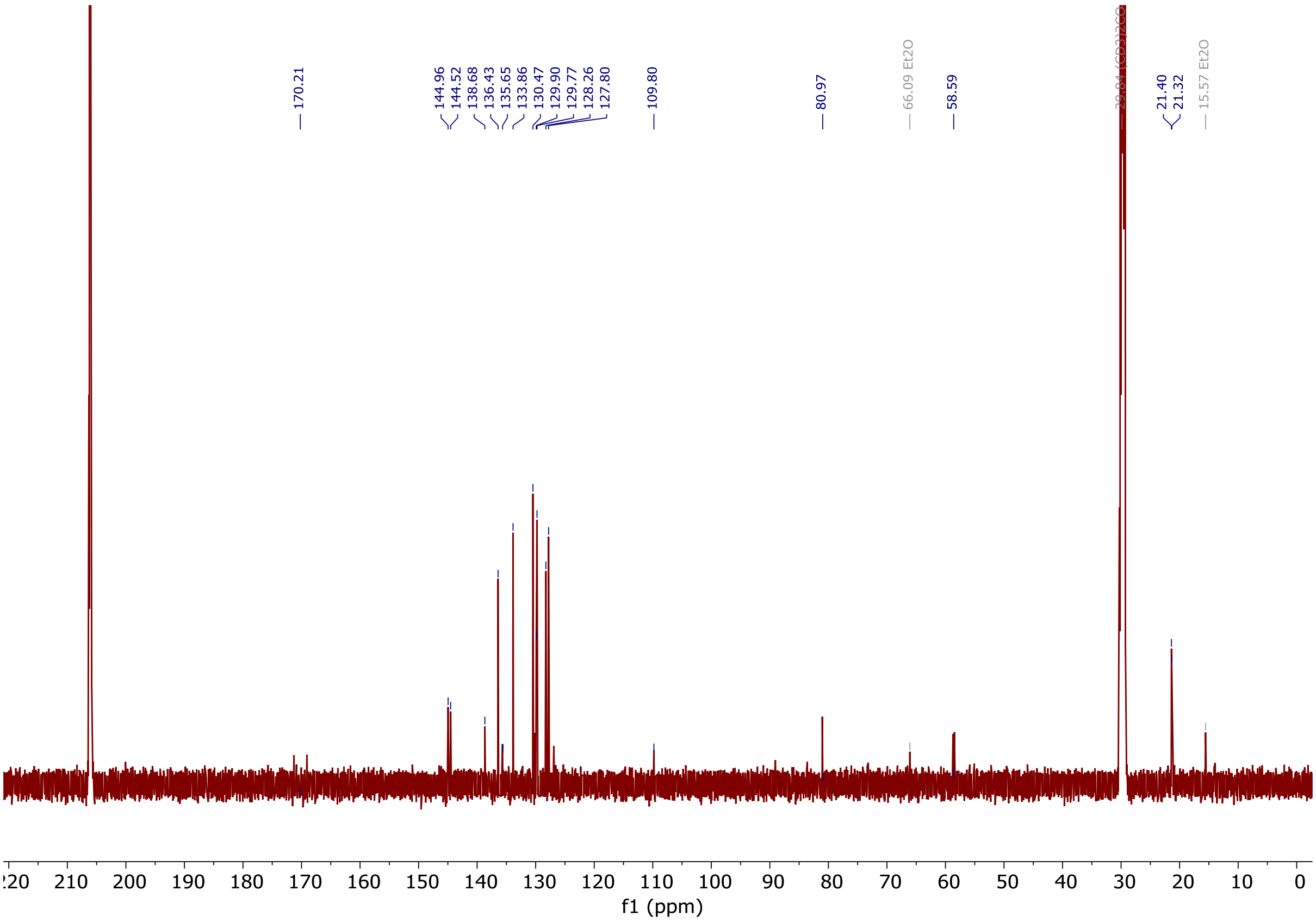

^{19}F NMR (471 MHz, Acetone-D_6)

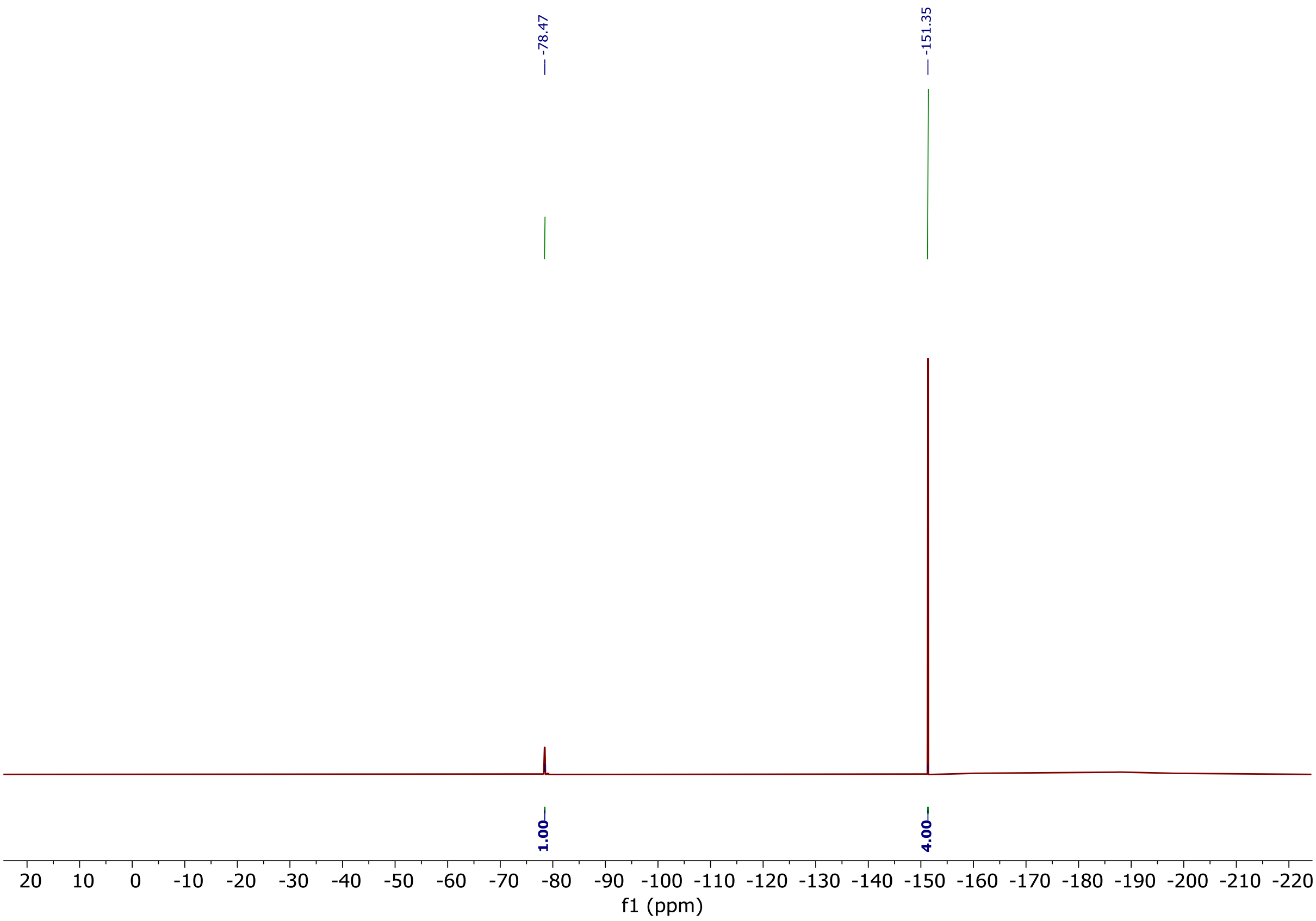

(Z)-(2-fluoro-3-(4-fluorophenyl)-3-((4-methylphenyl)sulfonamido)prop-1-en-1-yl)(p-tolyl)iodonium tetrafluoroborate **(3ax)**

^{1}H NMR (500 MHz, Acetone-*D_6*)

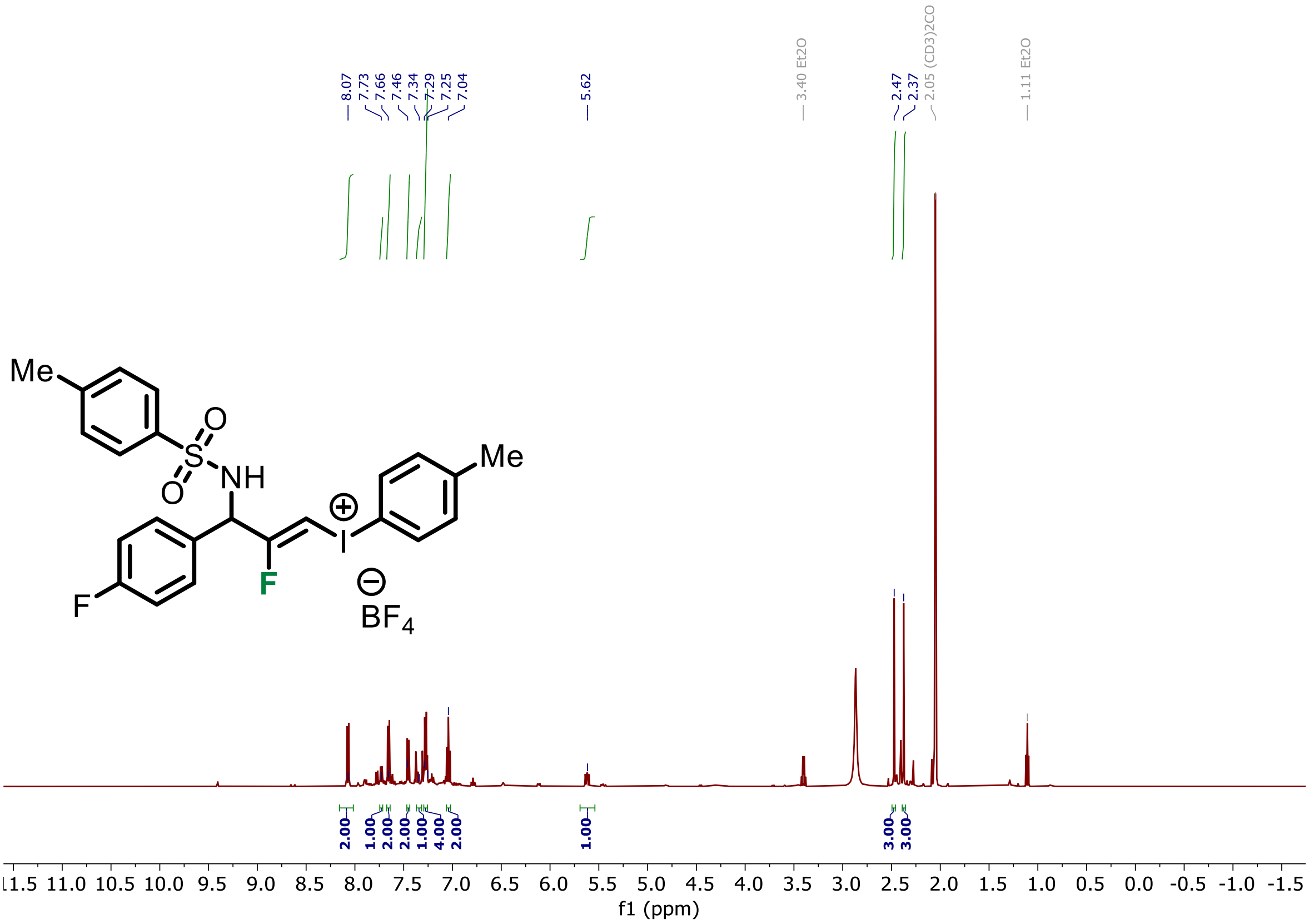

^{13}C NMR (126 MHz, Acetone-*D_6*)

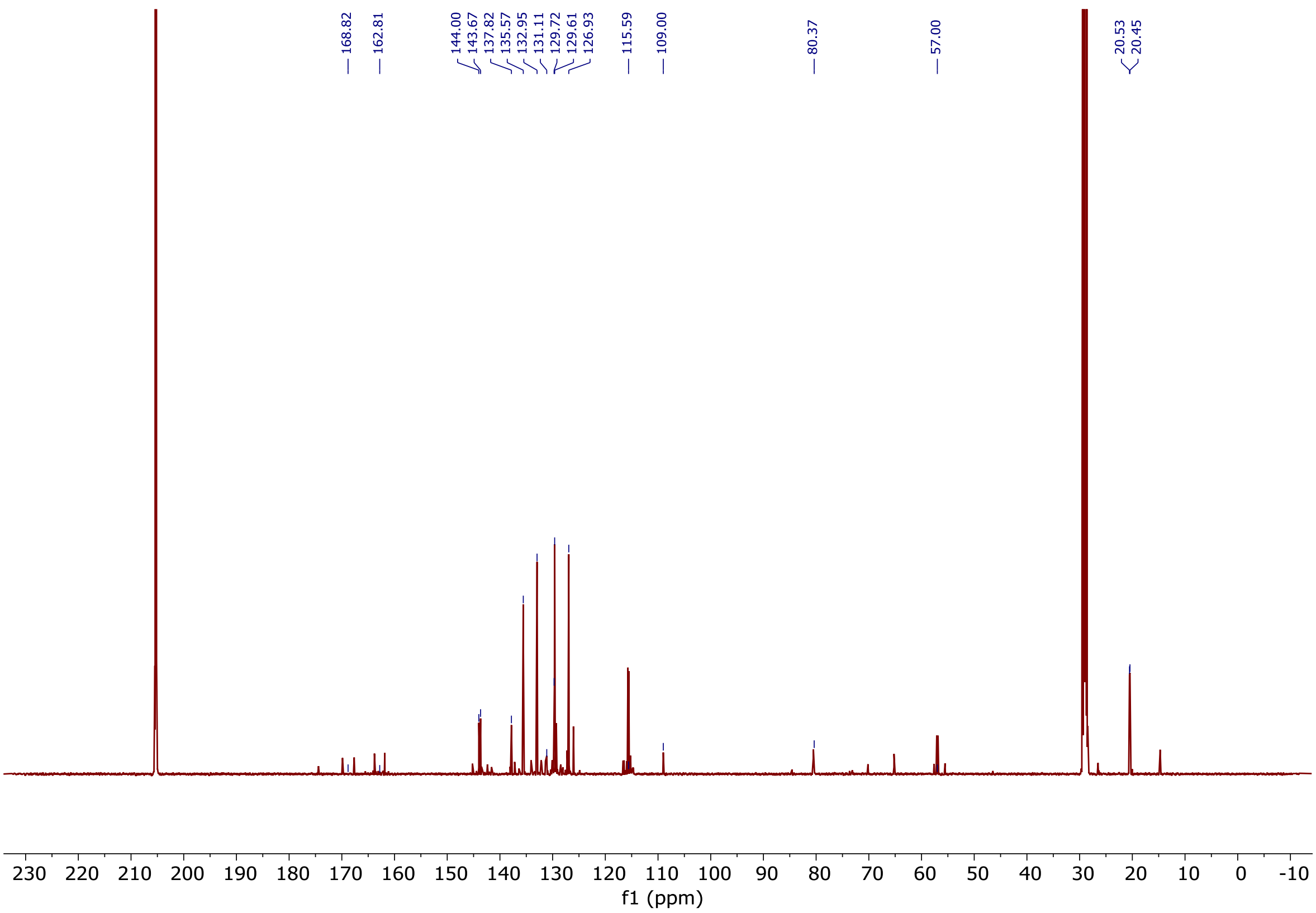

^{19}F NMR (376 MHz, Acetone-D_6)

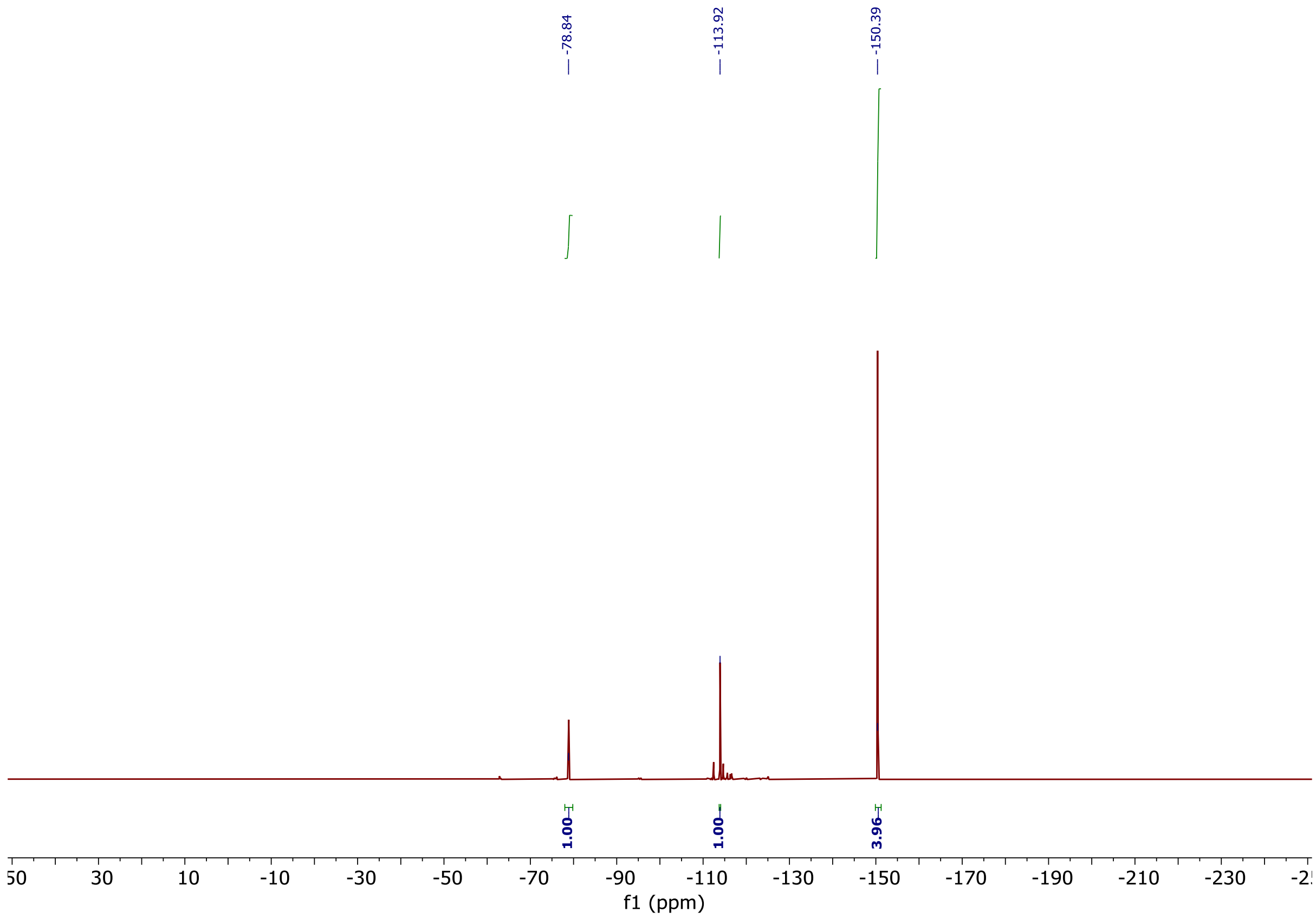

***(Z)-(2-fluoro-3-((4-methylphenyl)sulfonamido)-3-(4-(trifluoromethyl)phenyl)prop-1-en-1-yl)(p-tolyl)iodonium tetrafluoroborate* (3az)**
^{1}H NMR (400 MHz, Acetone-*D*$_6$)

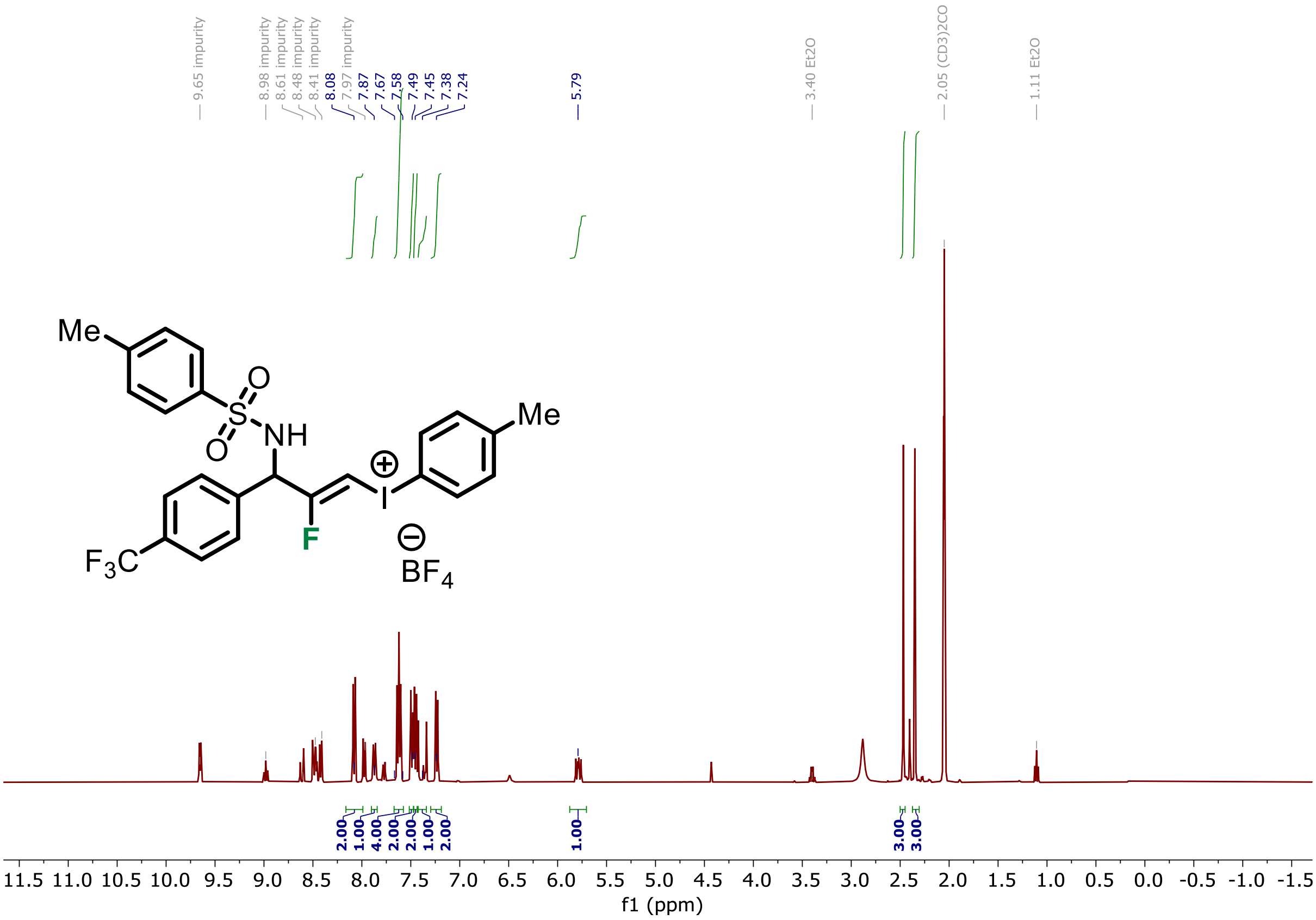

^{13}C NMR (126 MHz, Acetone-D_6)

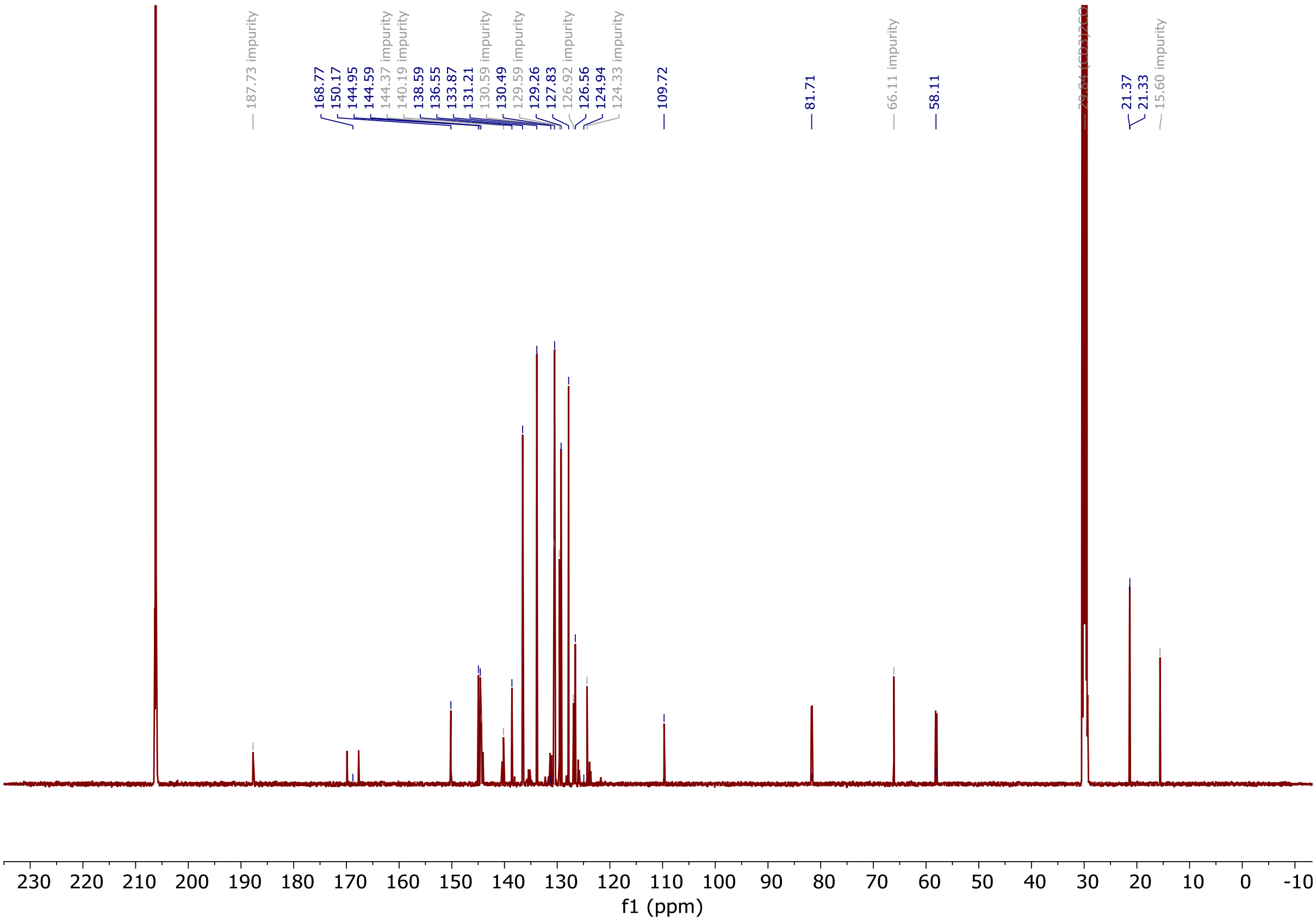

^{19}F NMR (376 MHz, Acetone-D_6)

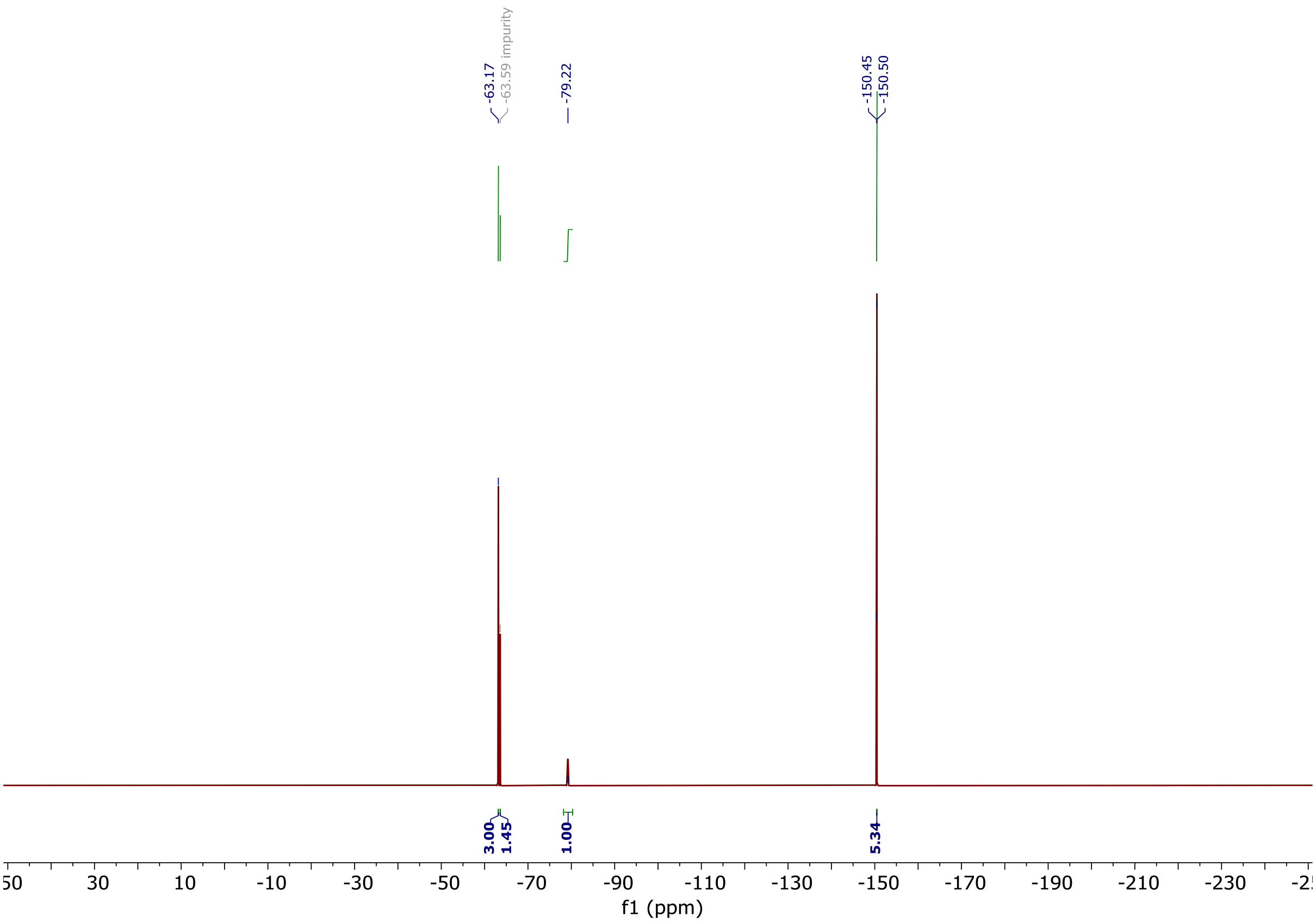

(Z)-(3-(4-bromophenyl)-2-fluoro-3-((4-methylphenyl)sulfonamido)prop-1-en-1-yl)(p-tolyl)iodonium tetrafluoroborate (3ba)

^{19}F NMR (376 MHz, Acetone-D_6):

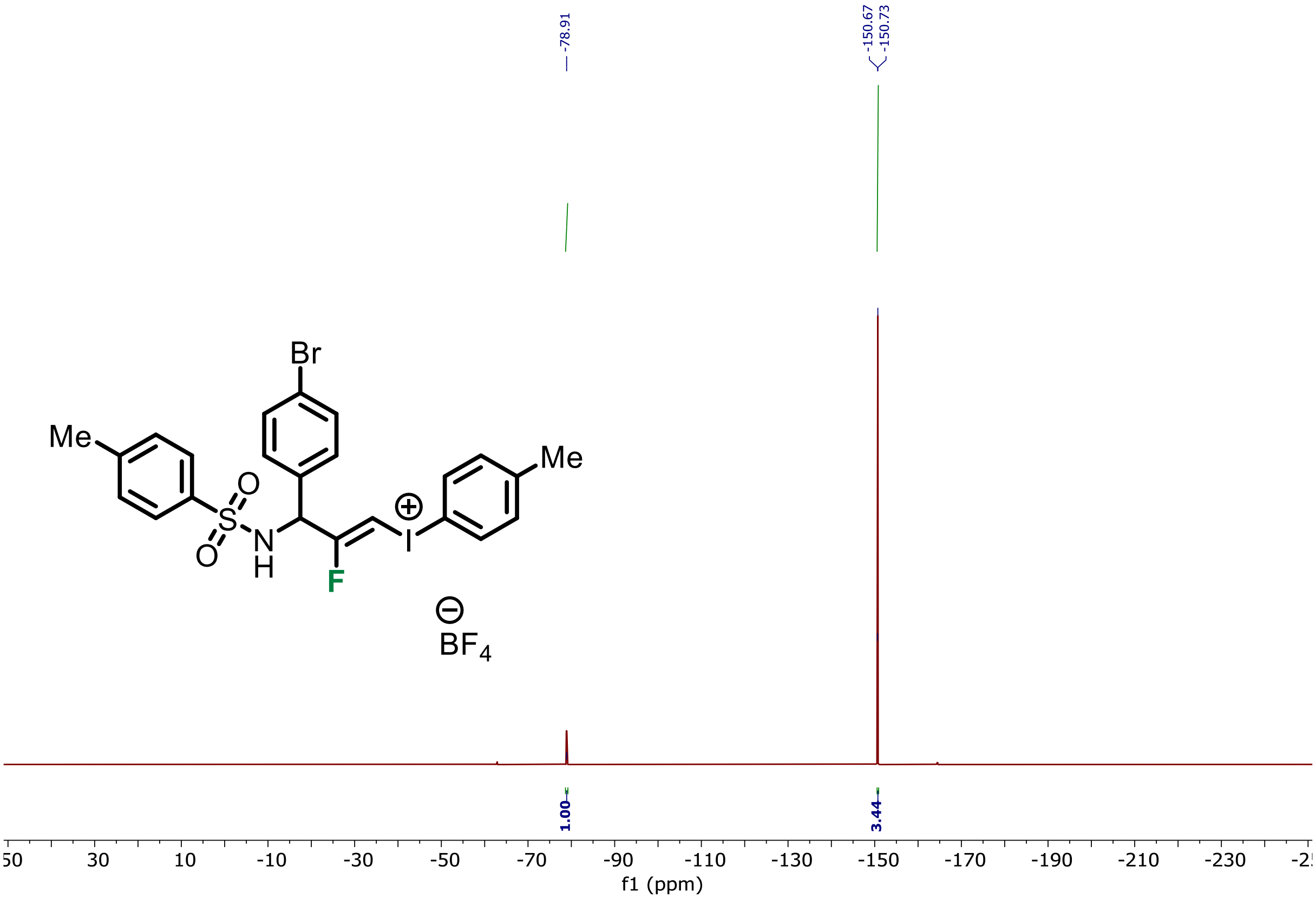

***(Z)*-(3-(4-chlorophenyl)-2-fluoro-3-((4-methylphenyl)sulfonamido)prop-1-en-1-yl)(p-tolyl)iodonium tetrafluoroborate* (3bb)**

^{1}H NMR (500 MHz, Acetone-D_6)

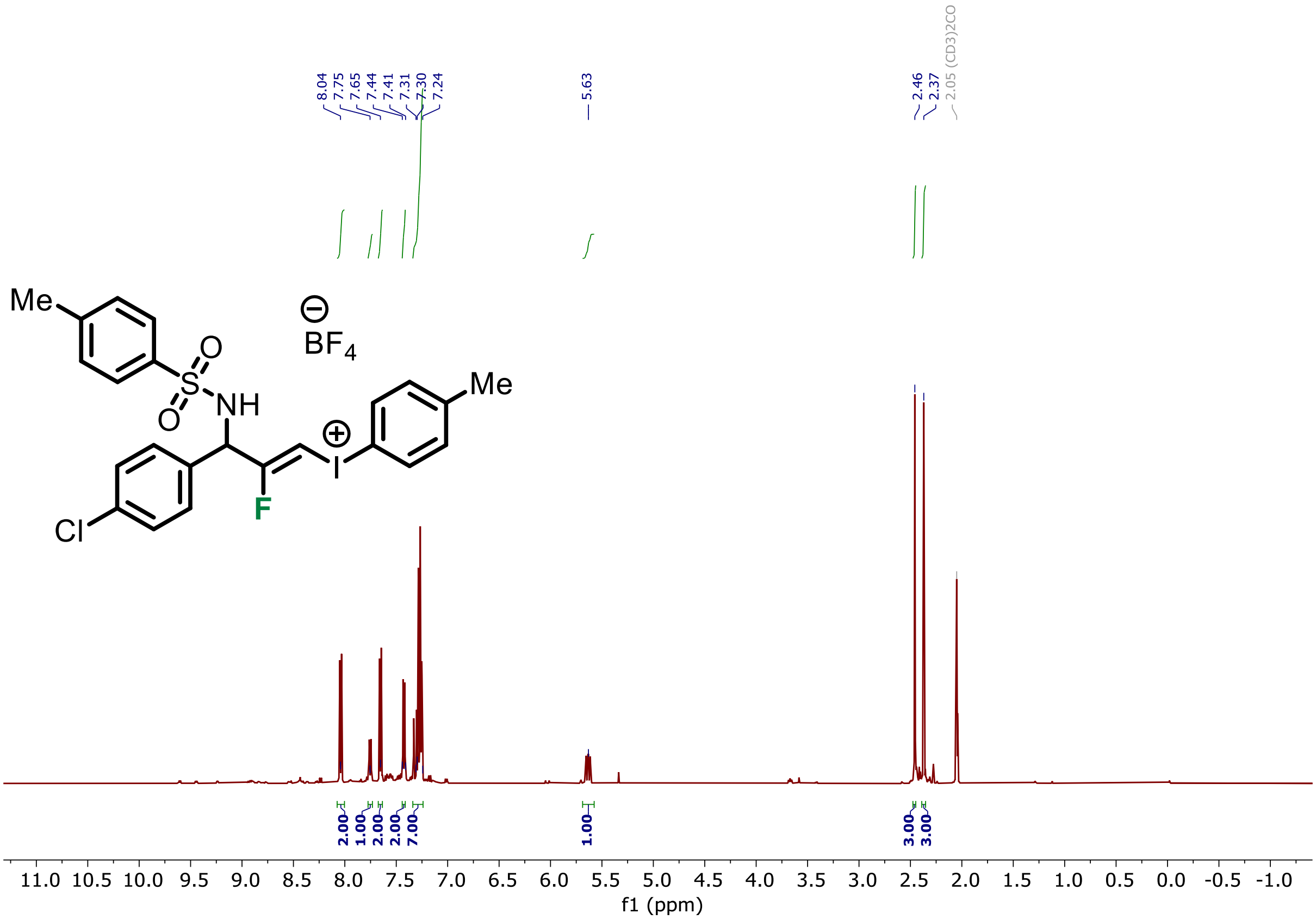

^{13}C NMR (126 MHz, Acetone-D_6)

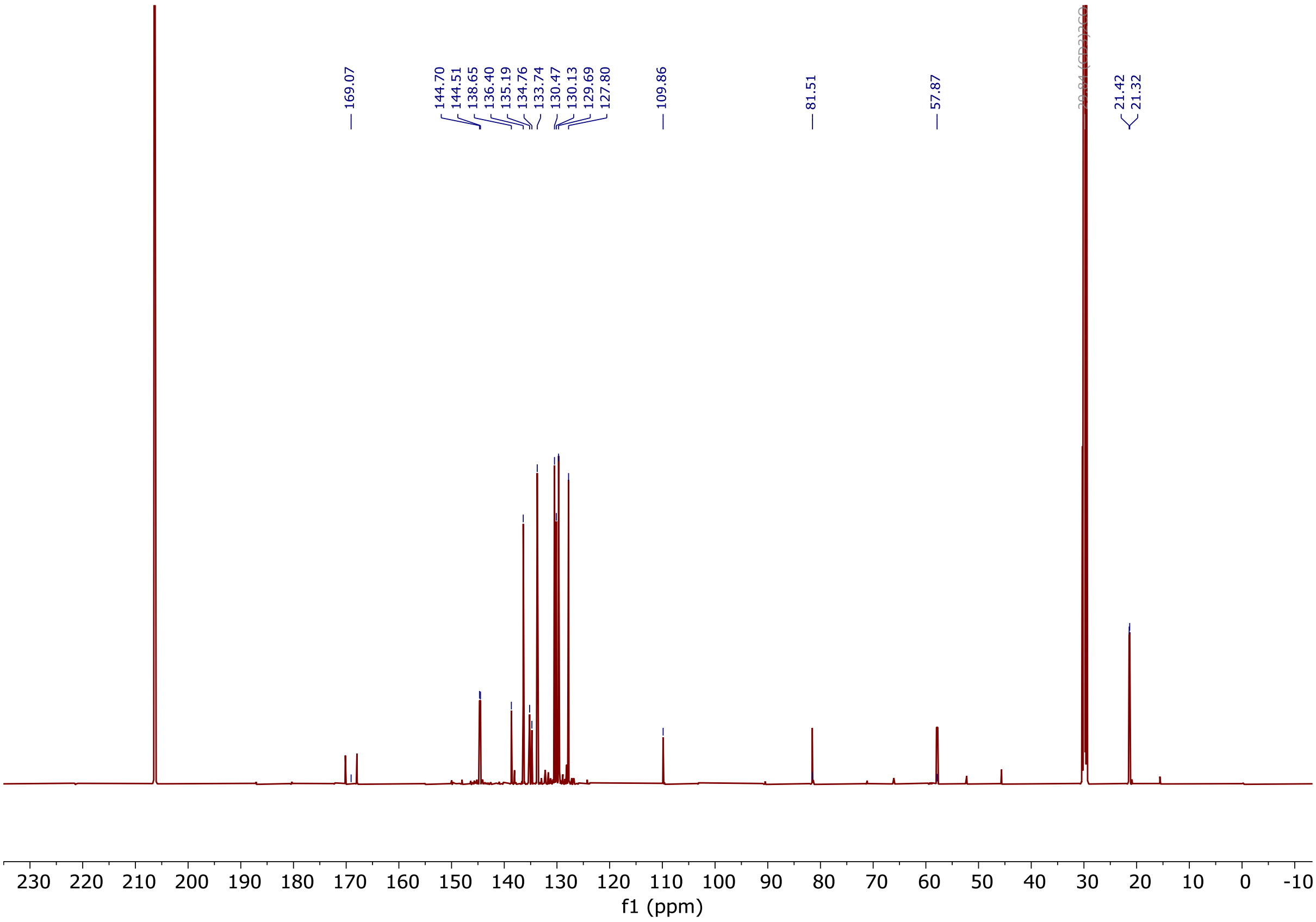

^{19}F NMR (376 MHz, Acetone-D_6)

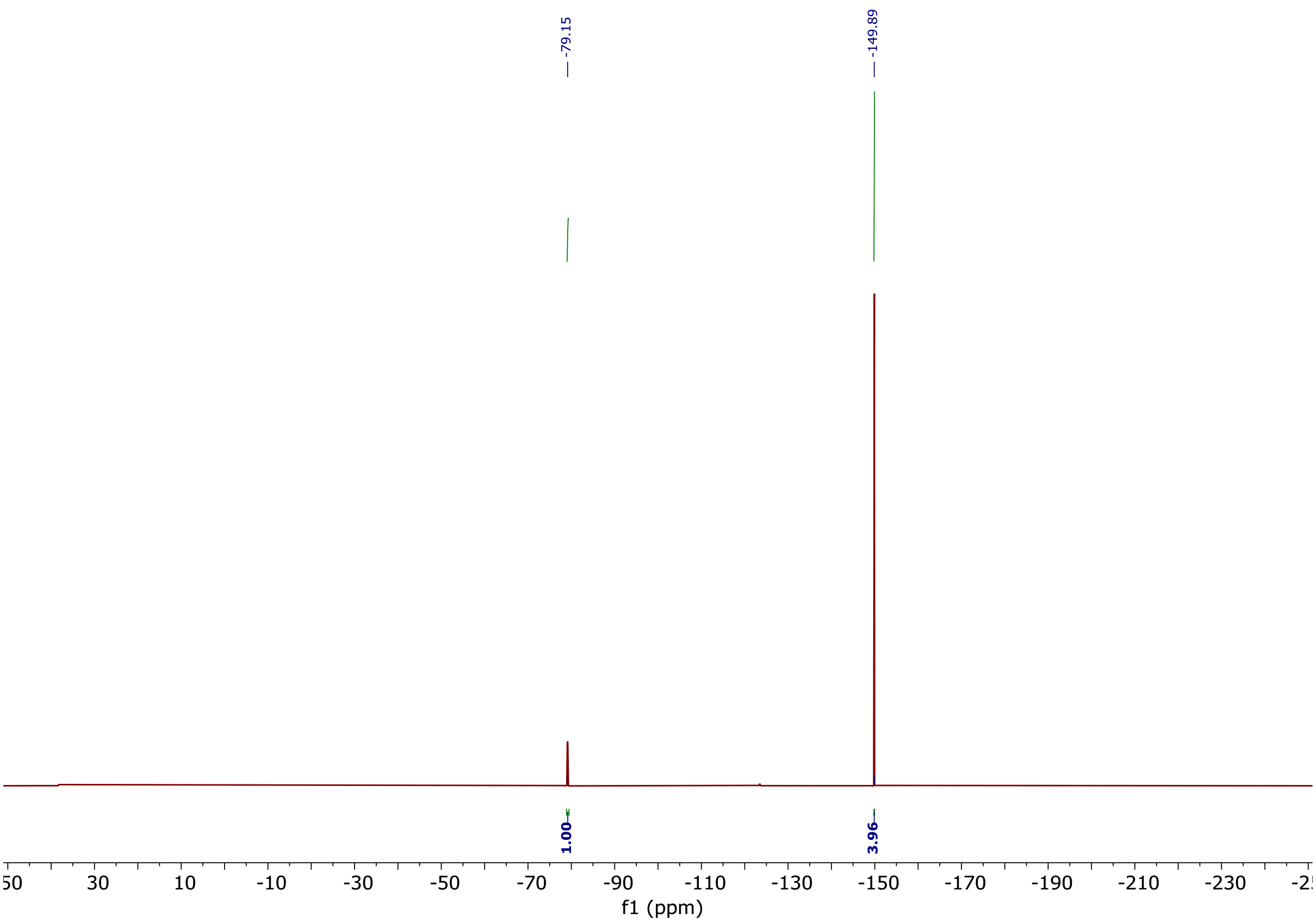

***(Z)-(2-fluoro-4-((4-methylphenyl)sulfonamido)but-1-en-1-yl)(p-tolyl)iodonium tetrafluoroborate* (3be)**

^{1}H NMR (600 MHz, Acetone-*D₆*)

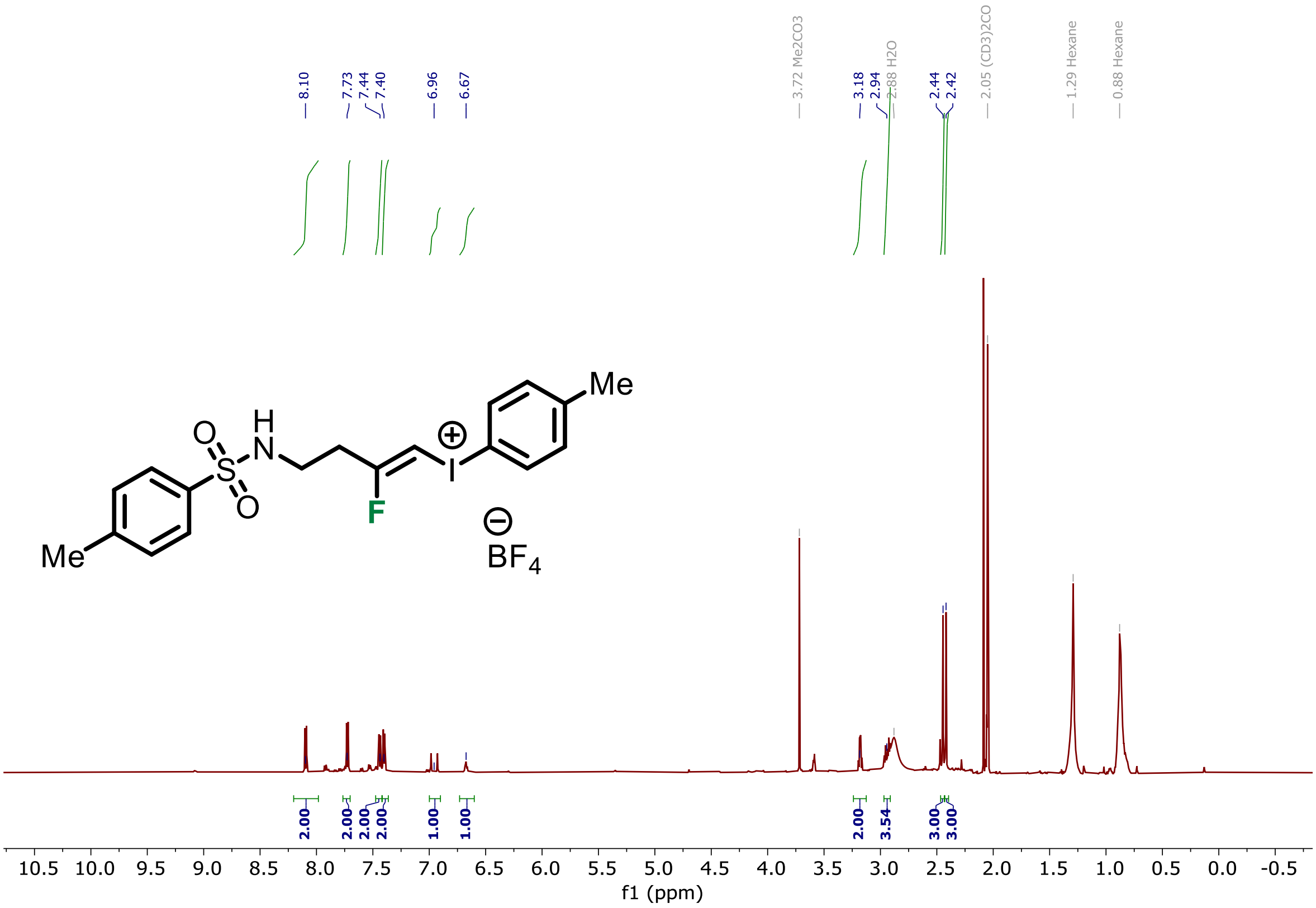

^{13}C NMR (151 MHz, Acetone-*D₆*)

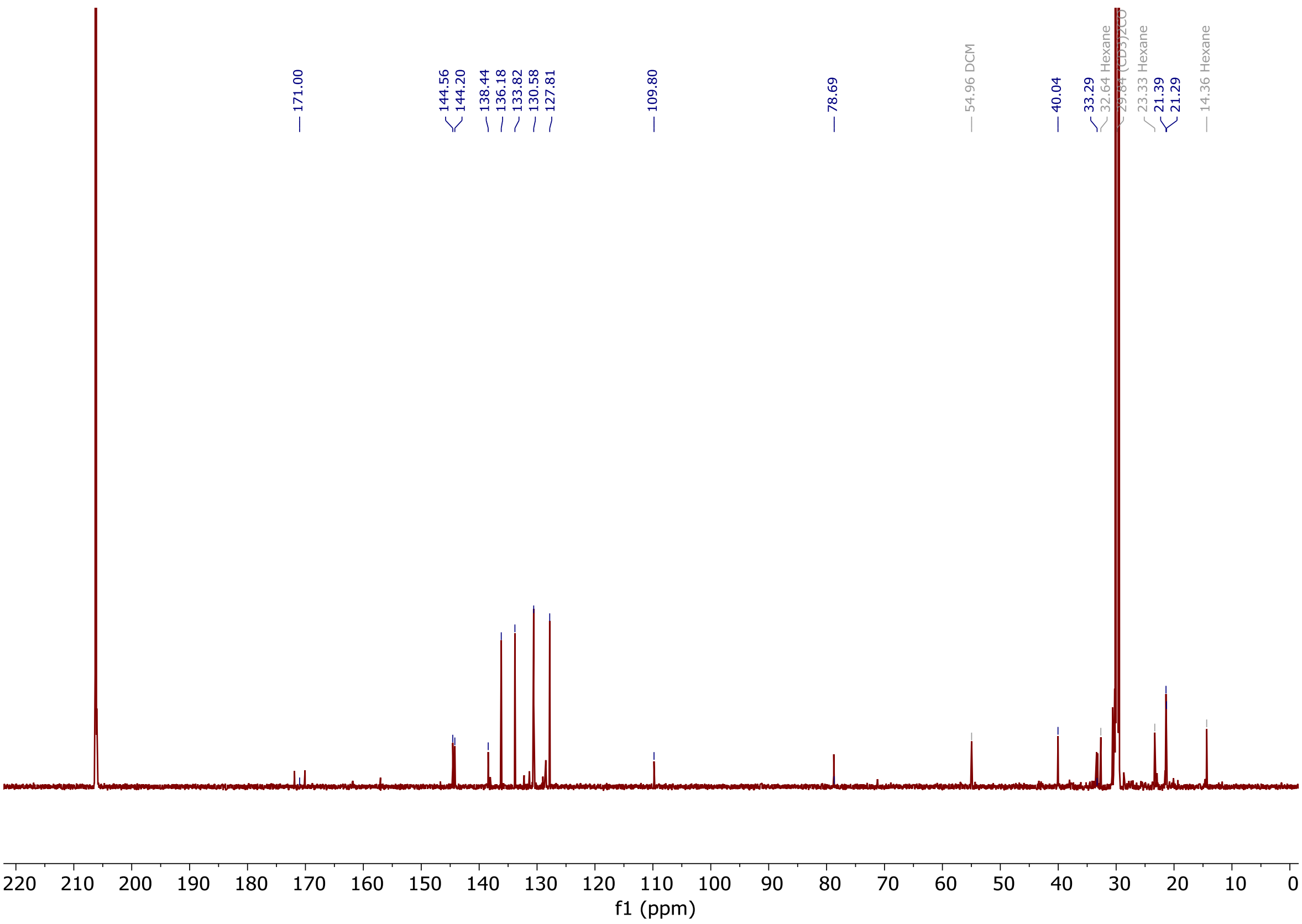

^{19}F NMR (376 MHz, Acetone-D_6)

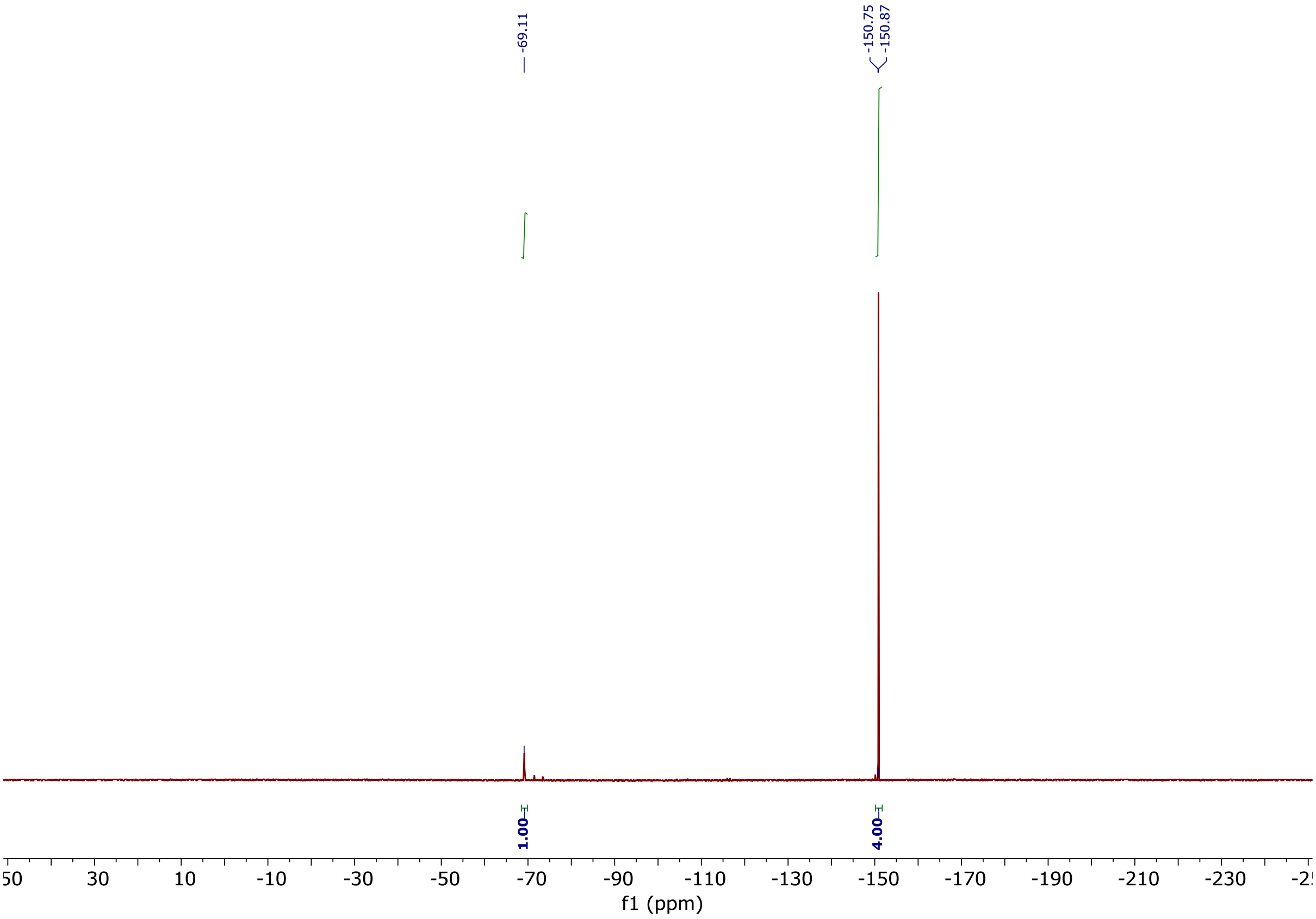

***(Z)-(3-((N-(cyanomethyl)-4-nitrophenyl)sulfonamido)-2-fluoroprop-1-en-1-yl)(p-tolyl)iodonium tetrafluoroborate* (3bi)**

^{1}H NMR (400 MHz, Acetone-D_6)

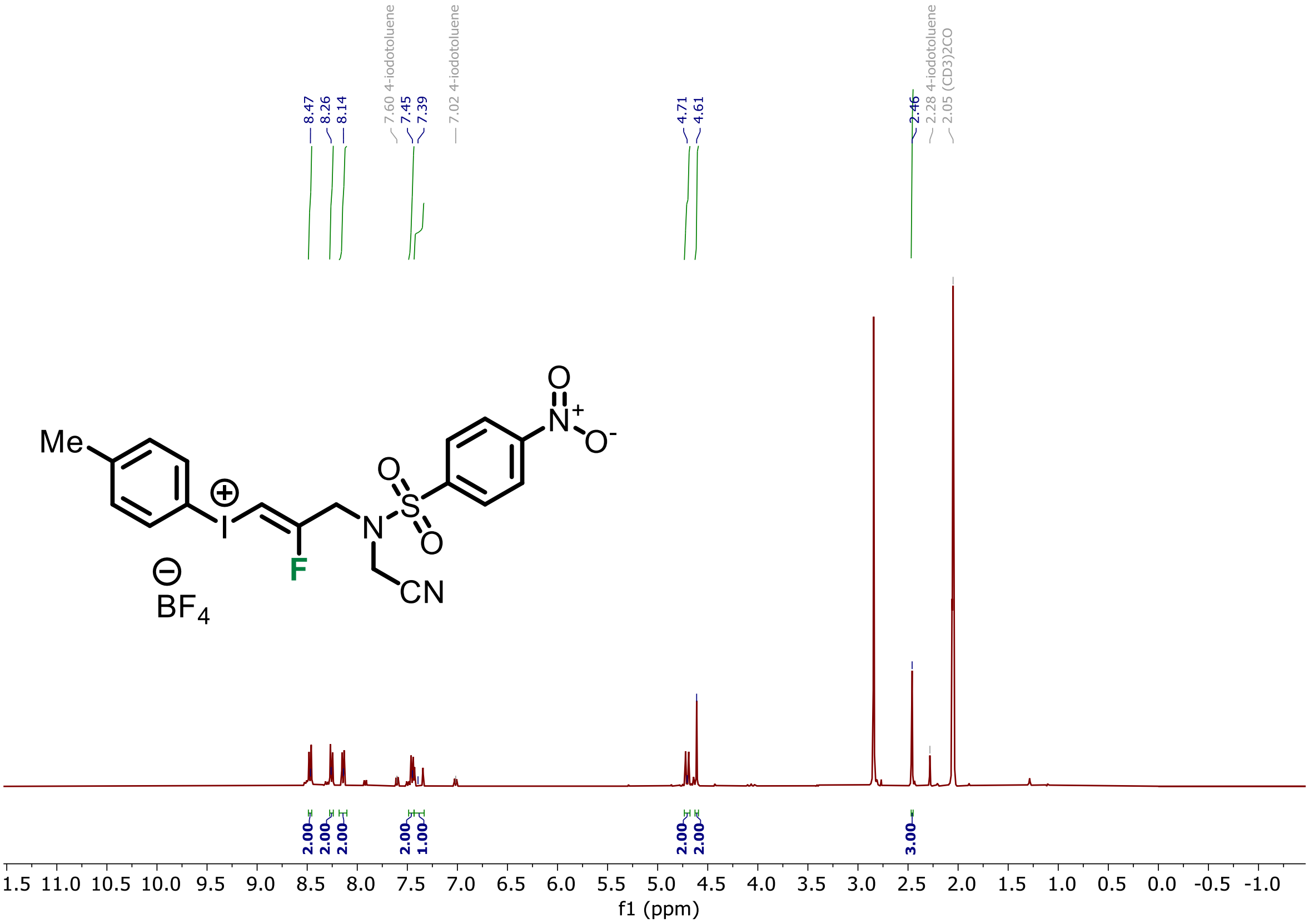

^{13}C NMR (126 MHz, Acetone-D_6)

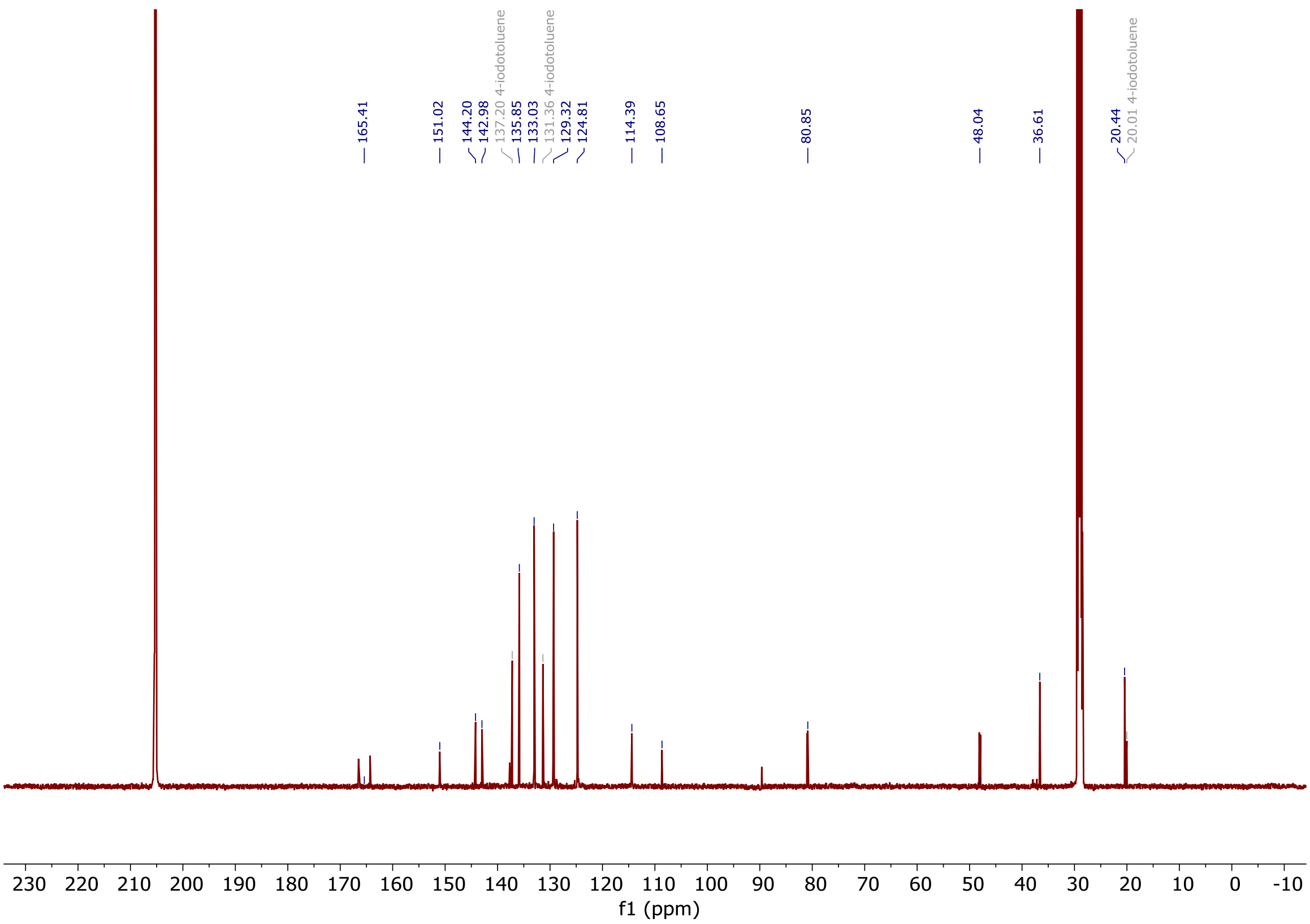

^{19}F NMR (376 MHz, Acetone-D_6)

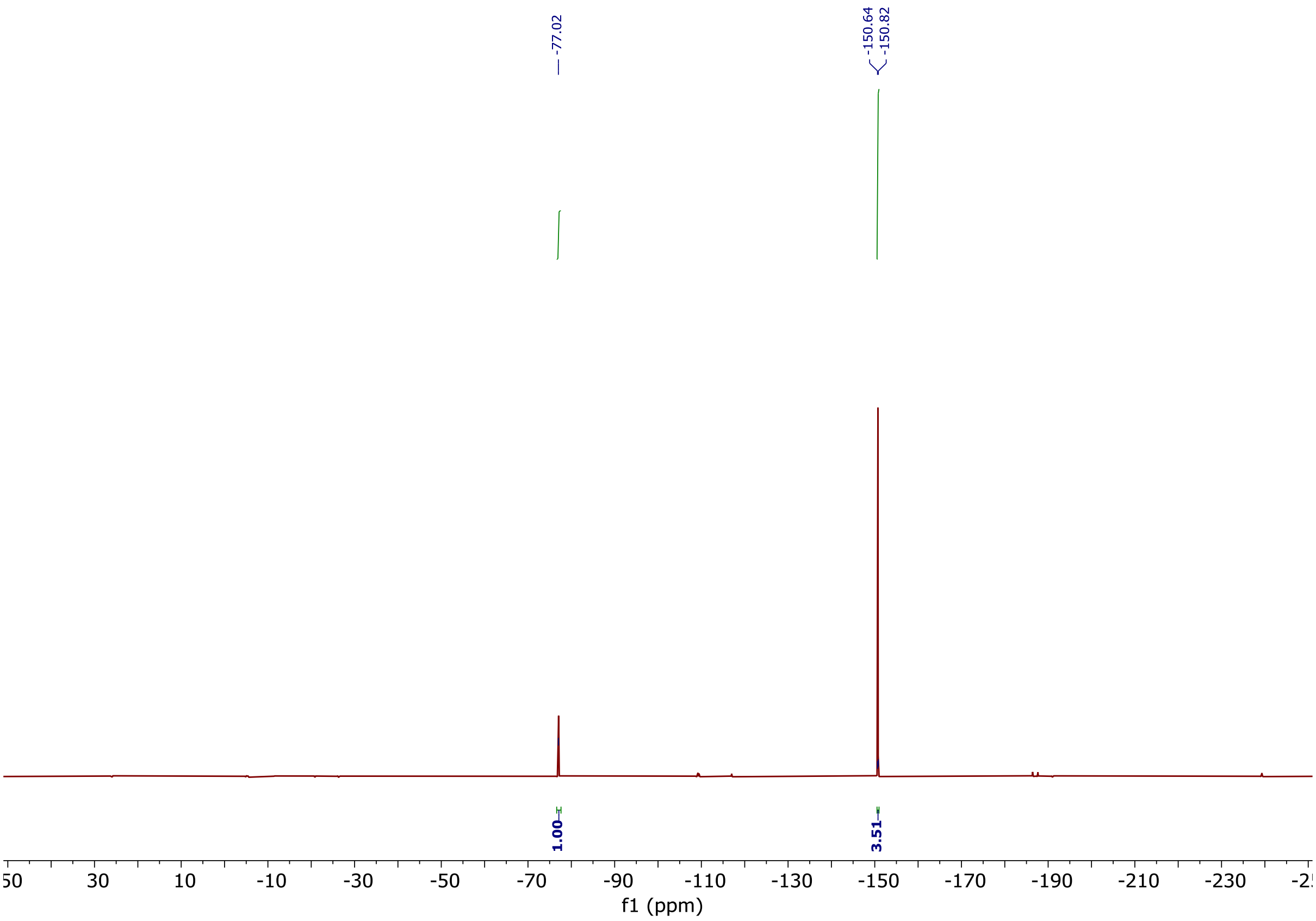

(R,Z)-(3-((4,4-dimethyl-2-oxotetrahydrofuran-3-yl)oxy)-2-fluoroprop-1-en-1-yl)(p-tolyl)iodonium tetrafluoroborate **(3bj)**

^{1}H NMR (500 MHz, Acetone-*D_6*)

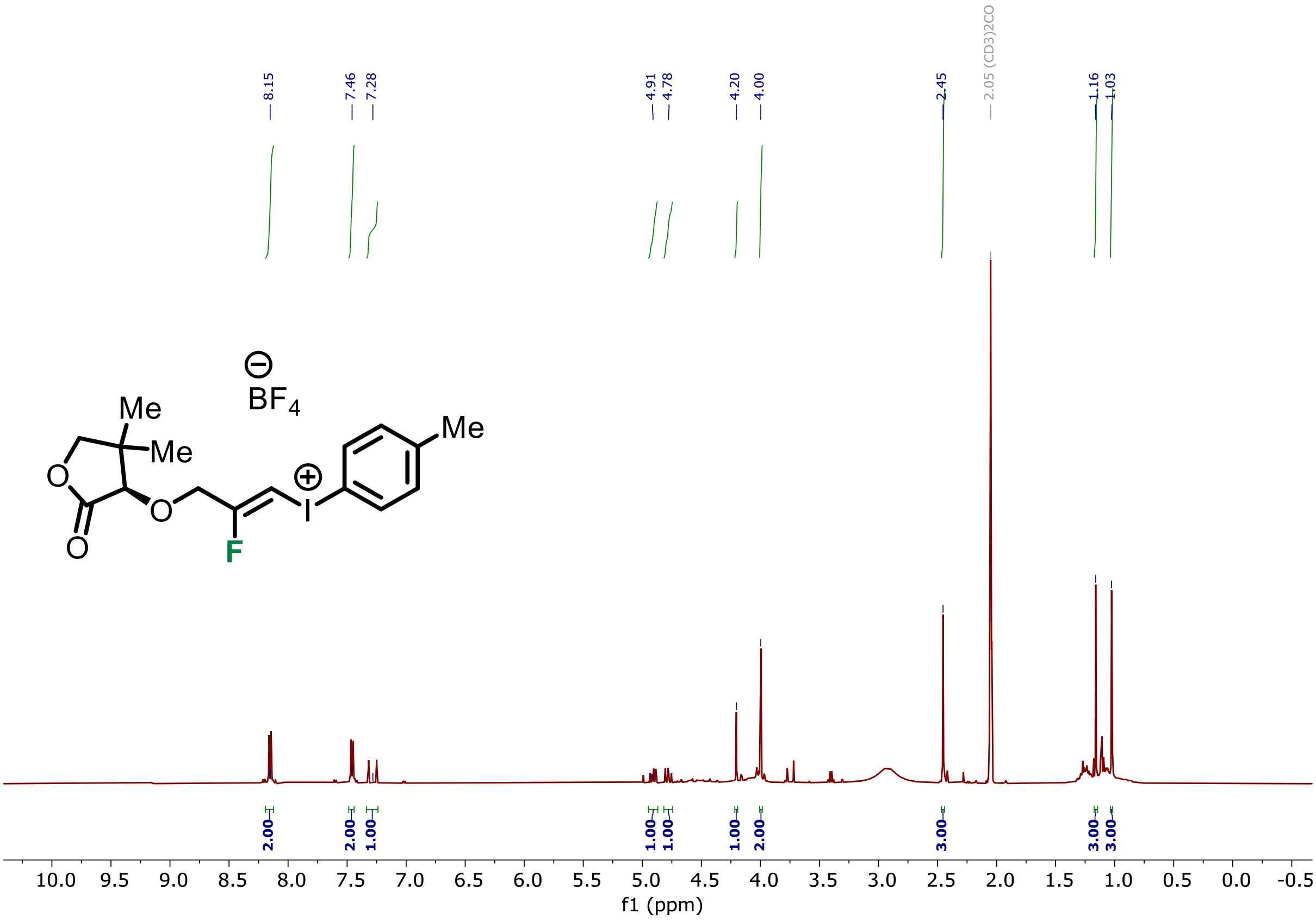

^{13}C NMR (126 MHz, Acetone-D_6)

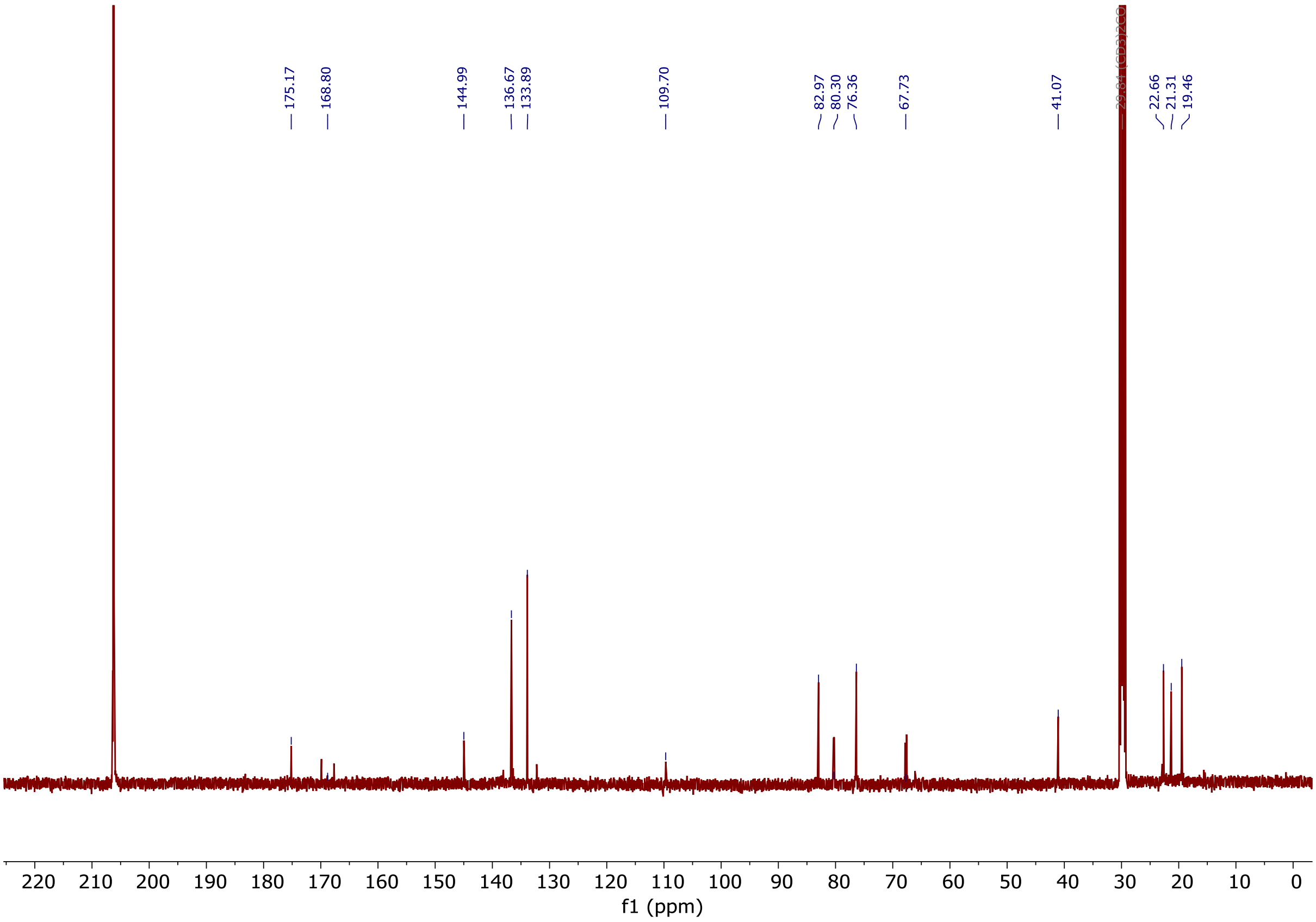

^{19}F NMR (471 MHz, Acetone-D_6)

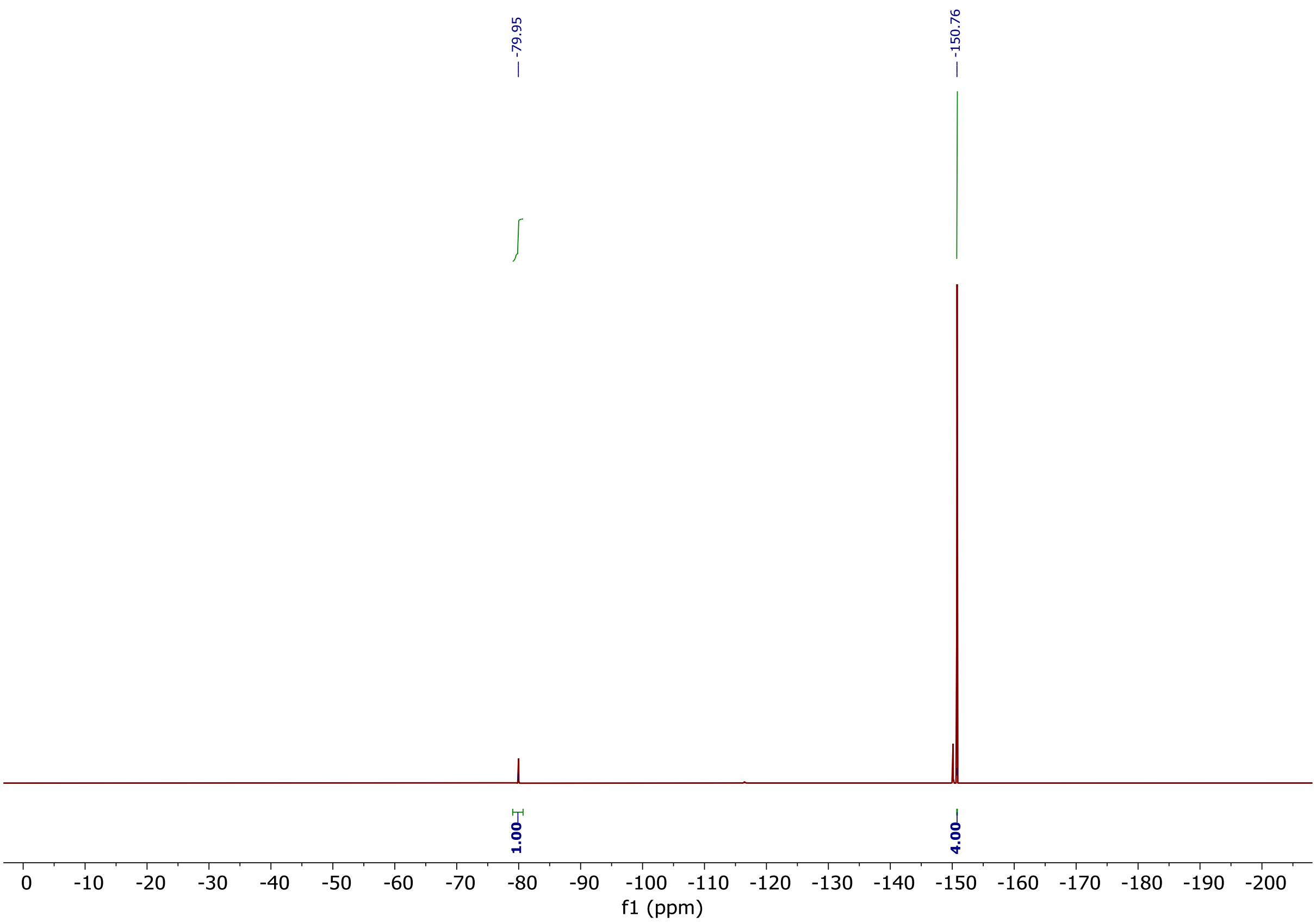

***(Z)-(2-fluoro-6-((4-methylphenyl)sulfonamido)hex-1-en-1-yl)(p-tolyl)iodonium tetrafluoroborate* (3bk)**

^{1}H NMR (500 MHz, Acetone-D_6)

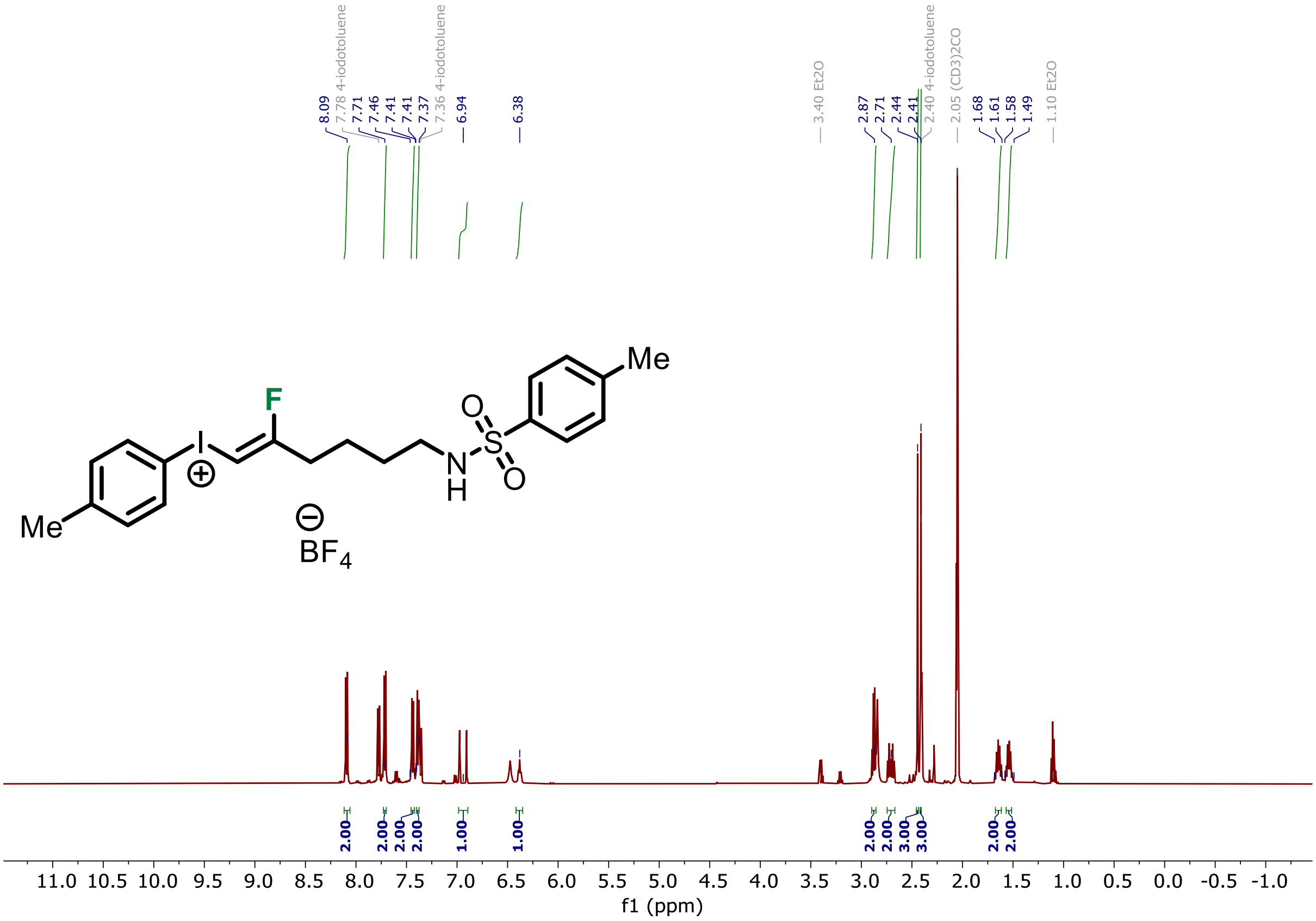

^{13}C NMR (126 MHz, Acetone- *D_6*)

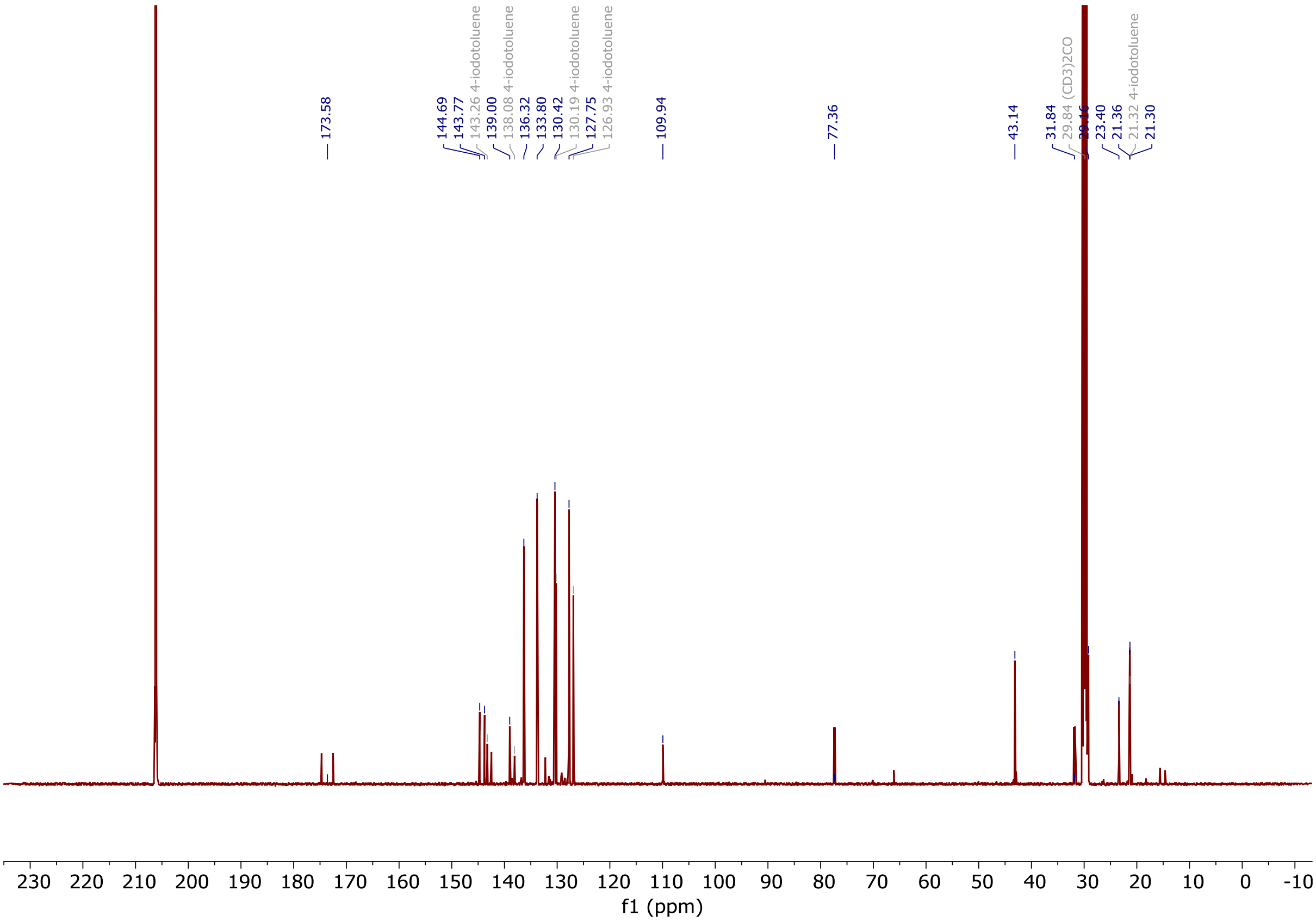

^{19}F NMR (377 MHz, Acetone-*D_6*)

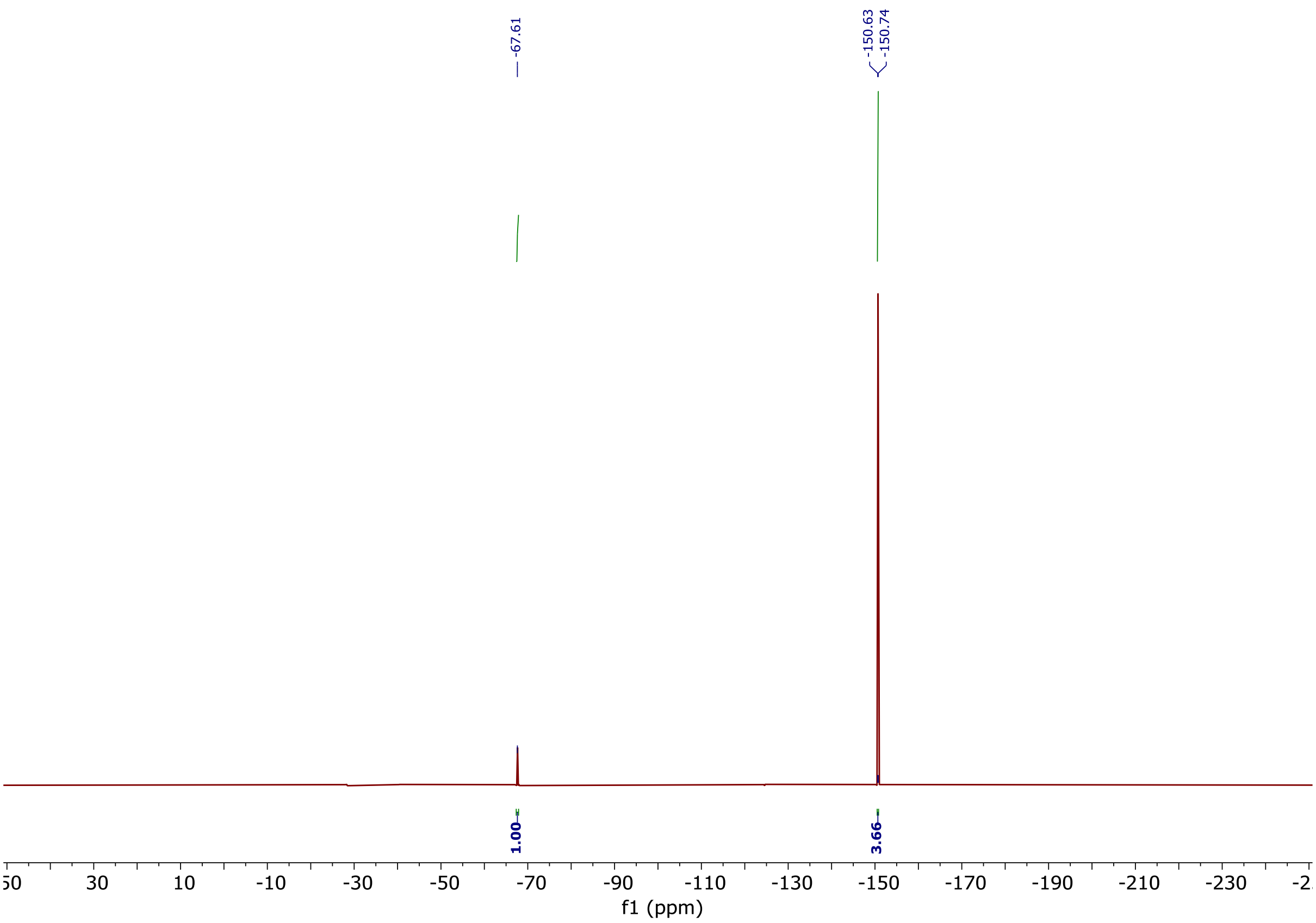

(Z)-(2-fluoro-3-hydroxy-3-(4-nitrophenyl)prop-1-en-1-yl)(p-tolyl)iodonium tetrafluoroborate **(3bm)**

^{1}H NMR (500 MHz, Acetone-D_6)

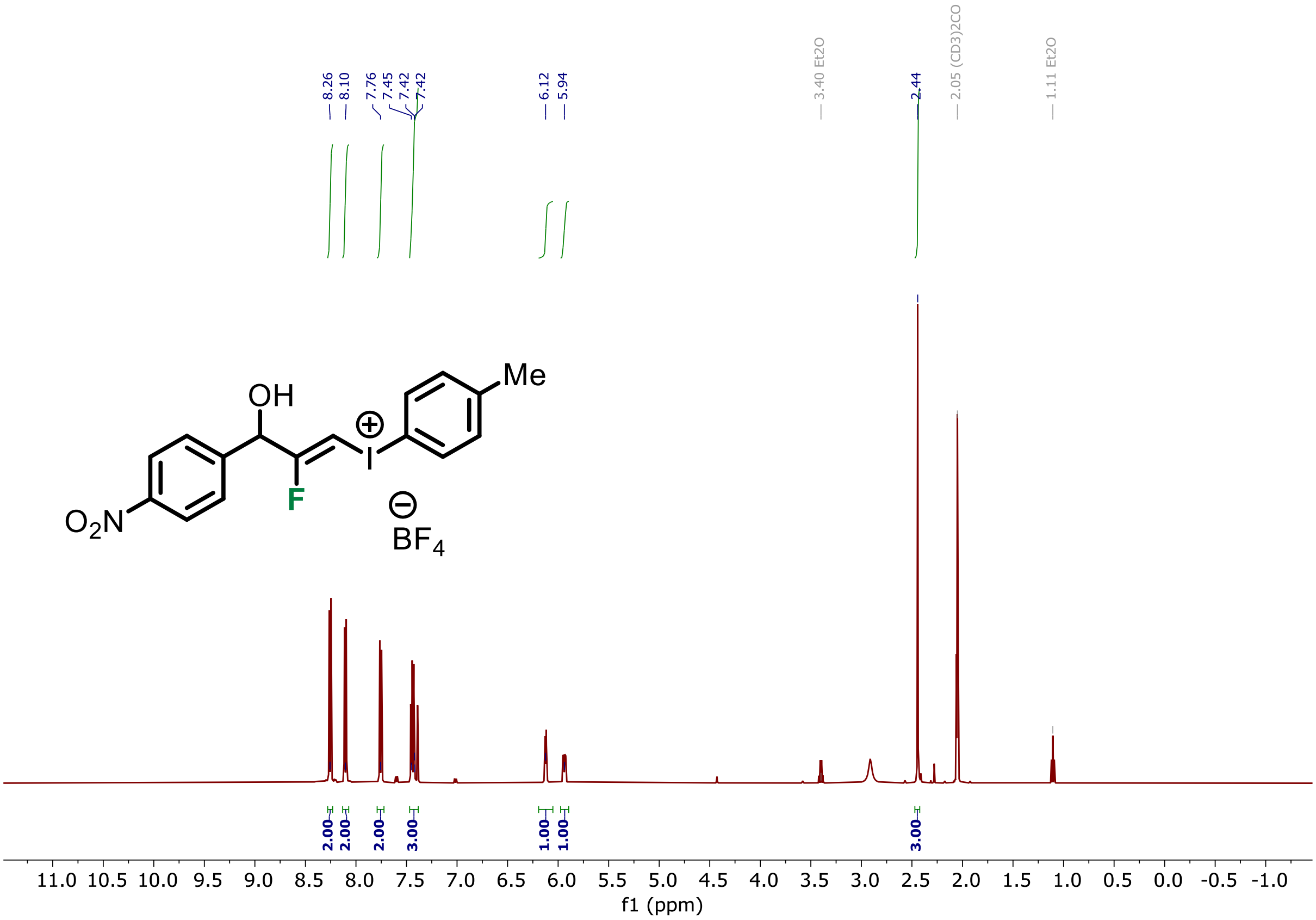

^{13}C NMR (126 MHz, Acetone-D_6)

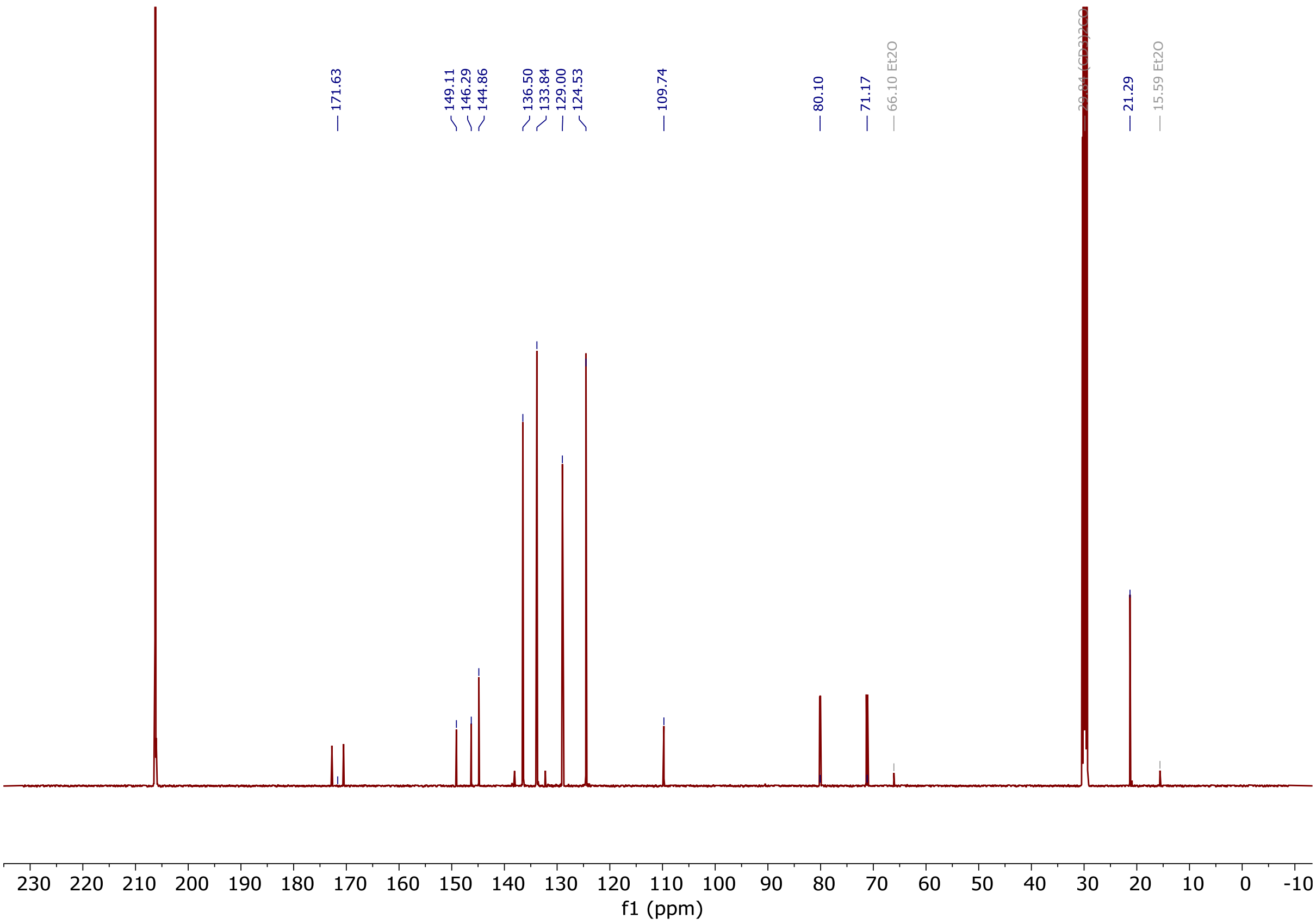

^{19}F NMR (376 MHz, Acetone-D_6)

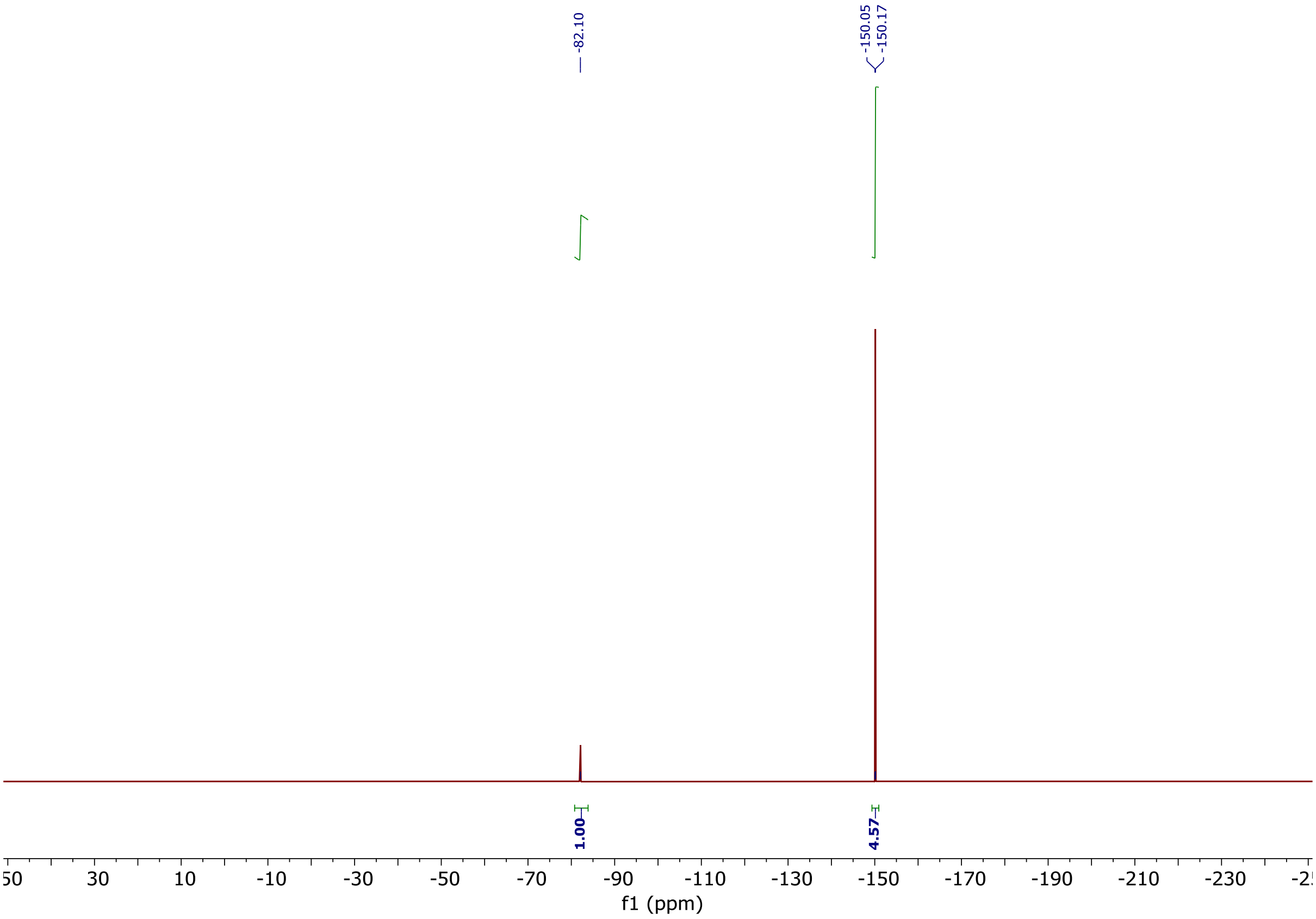

***(Z)-(3-((N-butyl-2-nitrophenyl)sulfonamido)-2-fluoroprop-1-en-1-yl)(p-tolyl)iodonium tetrafluoroborate* (3bp)**

^{1}H NMR (500 MHz, Acetone-*D_6*)

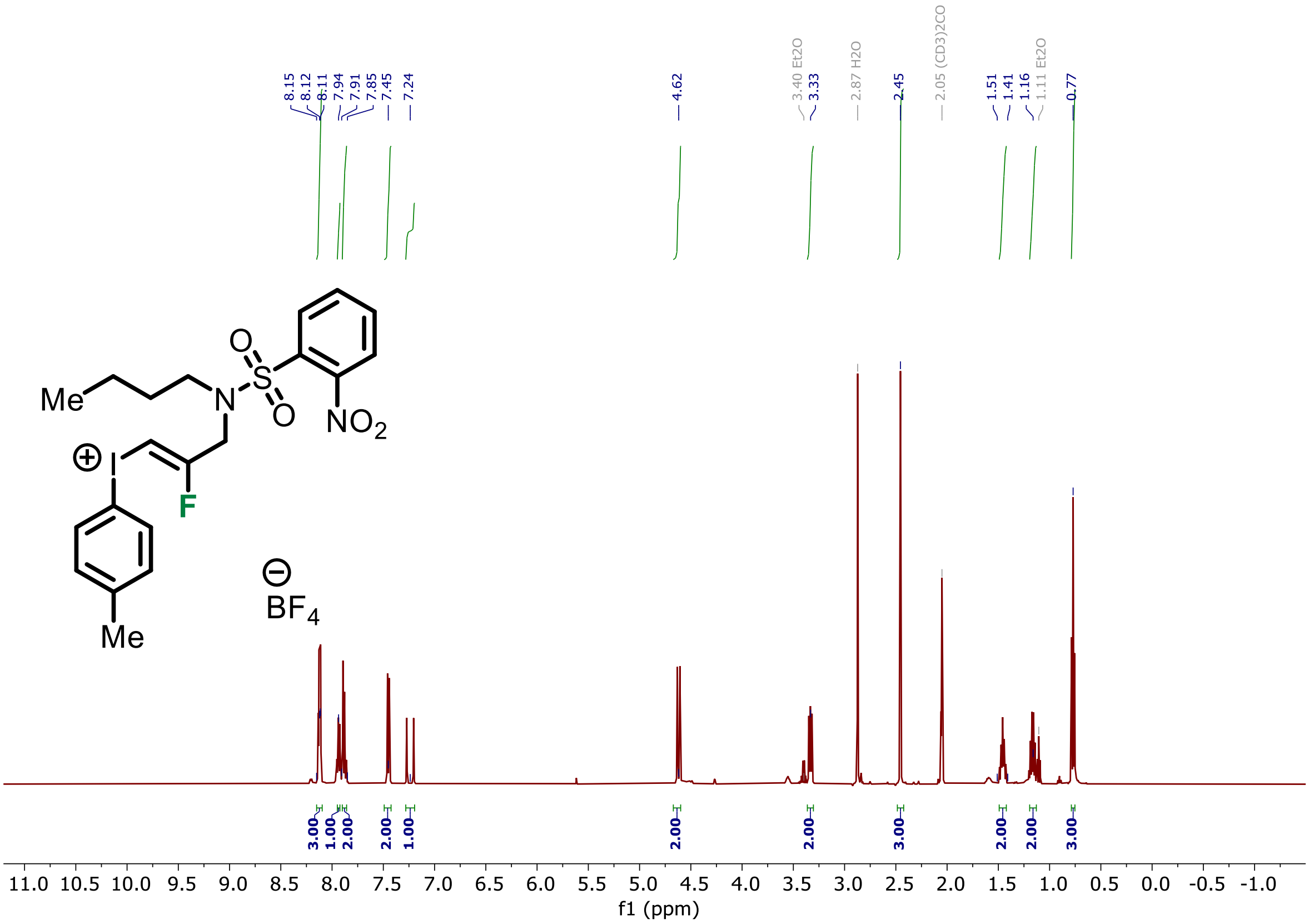

^{13}C NMR (126 MHz, Acetone-*D_6*)

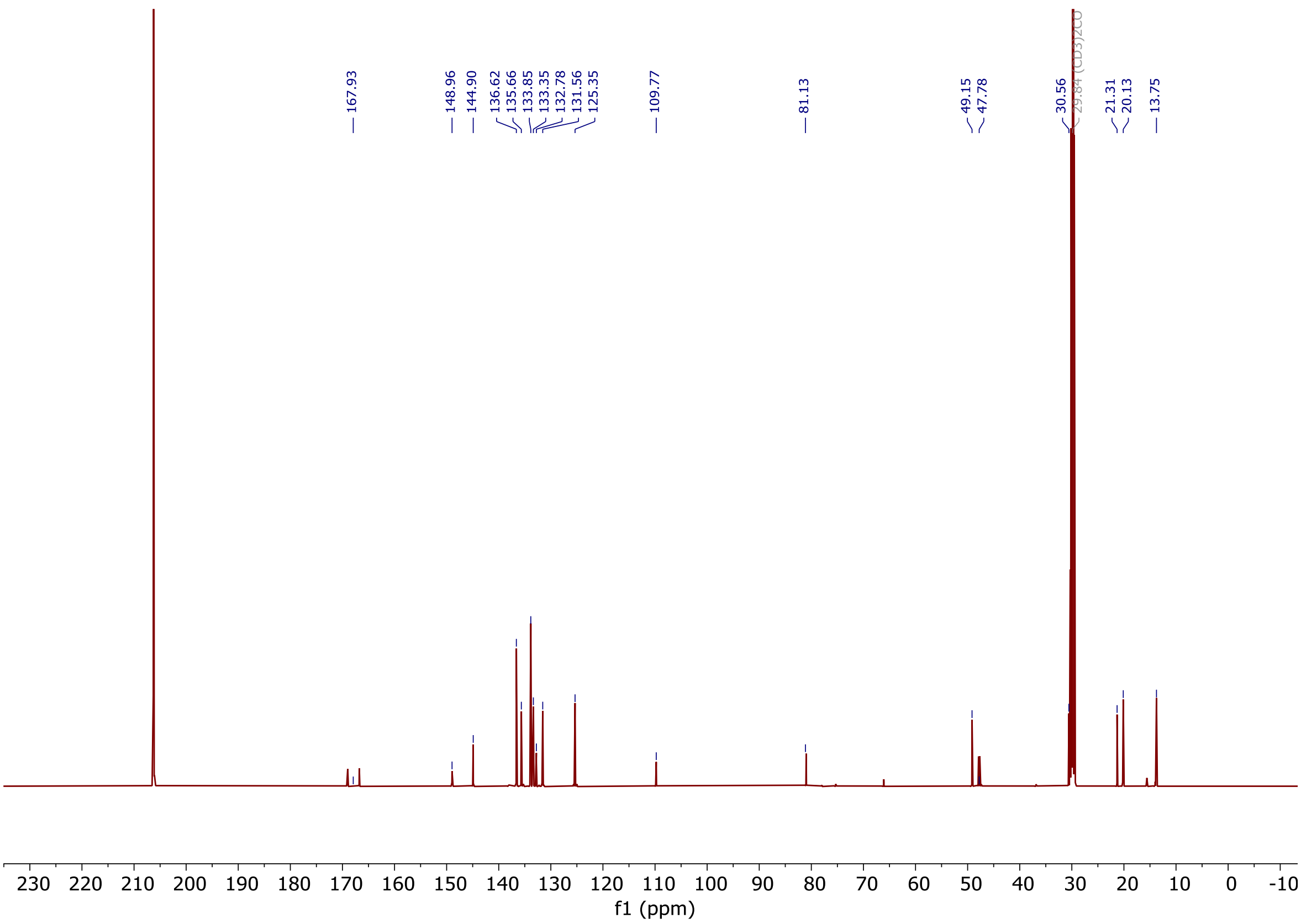

^{19}F NMR (376 MHz, Acetone-D_6)

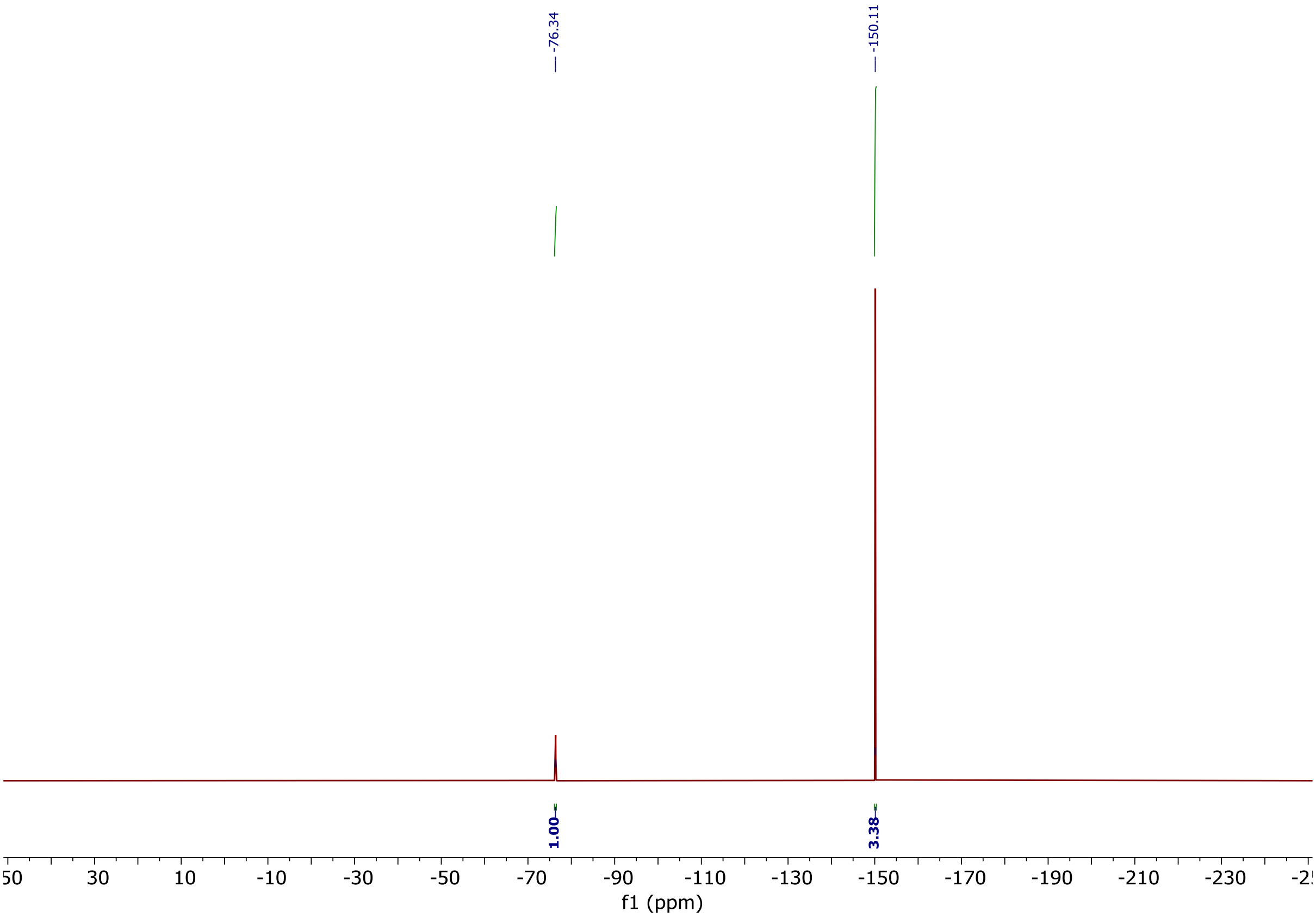

(Z)-(2-fluoro-3-((N-(4-fluorobenzyl)-2,4-dinitrophenyl)sulfonamido)prop-1-en-1-yl)(p-tolyl)iodonium tetrafluoroborate **(3bq)**

^{1}H NMR (500 MHz, Acetone-*D6*)

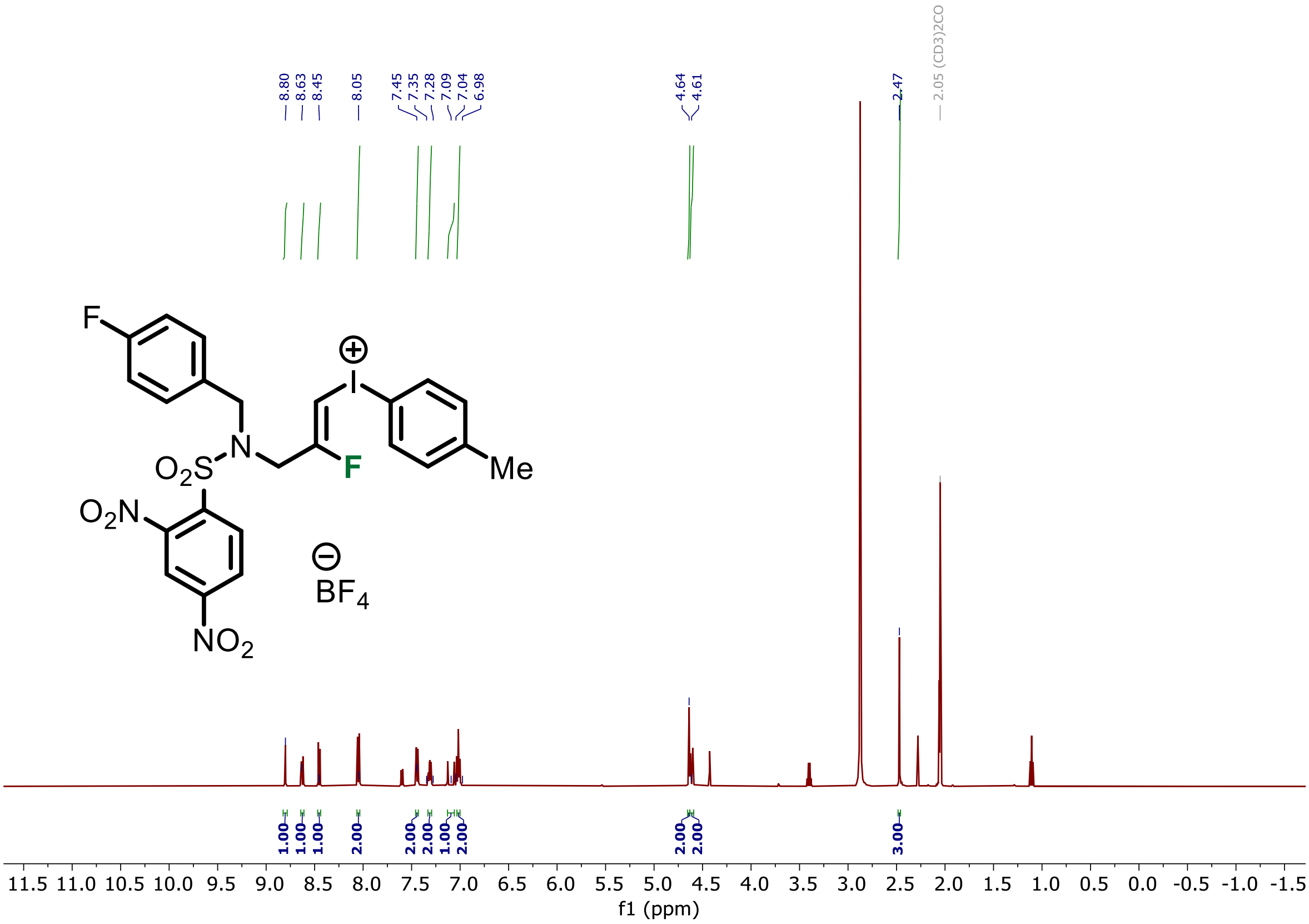

^{13}C NMR (126 MHz, Acetone-*D6*)

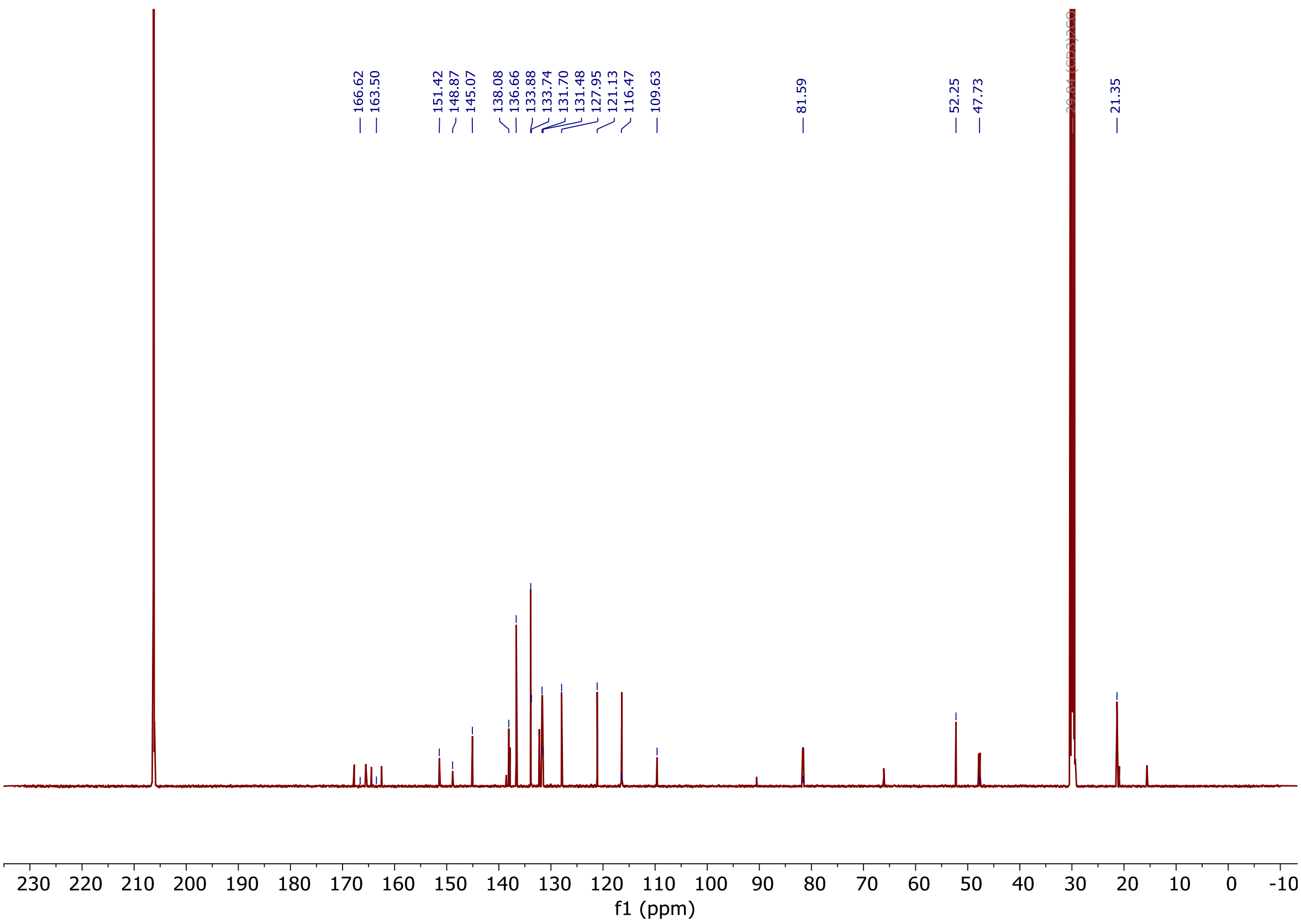

^{19}F NMR (376 MHz, Acetone-*D*$_6$)

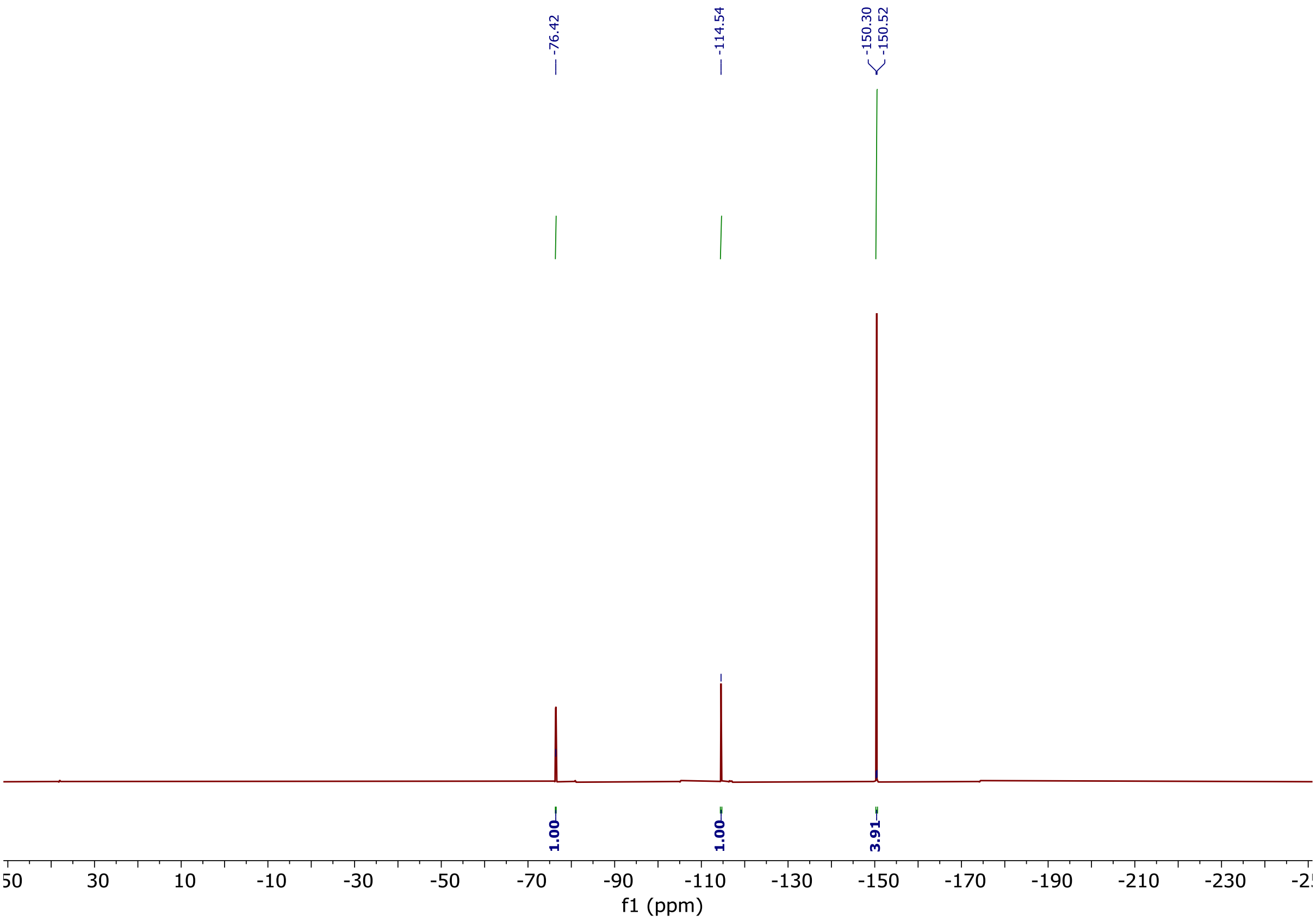

***(Z)-(2-fluoro-4-((4-methyl-N-(4-methylbenzyl)phenyl)sulfonamido)but-1-en-1-yl)(p-tolyl)iodonium tetrafluoroborate* (3br)**

^{1}H NMR (400 MHz, Acetone-D_6)

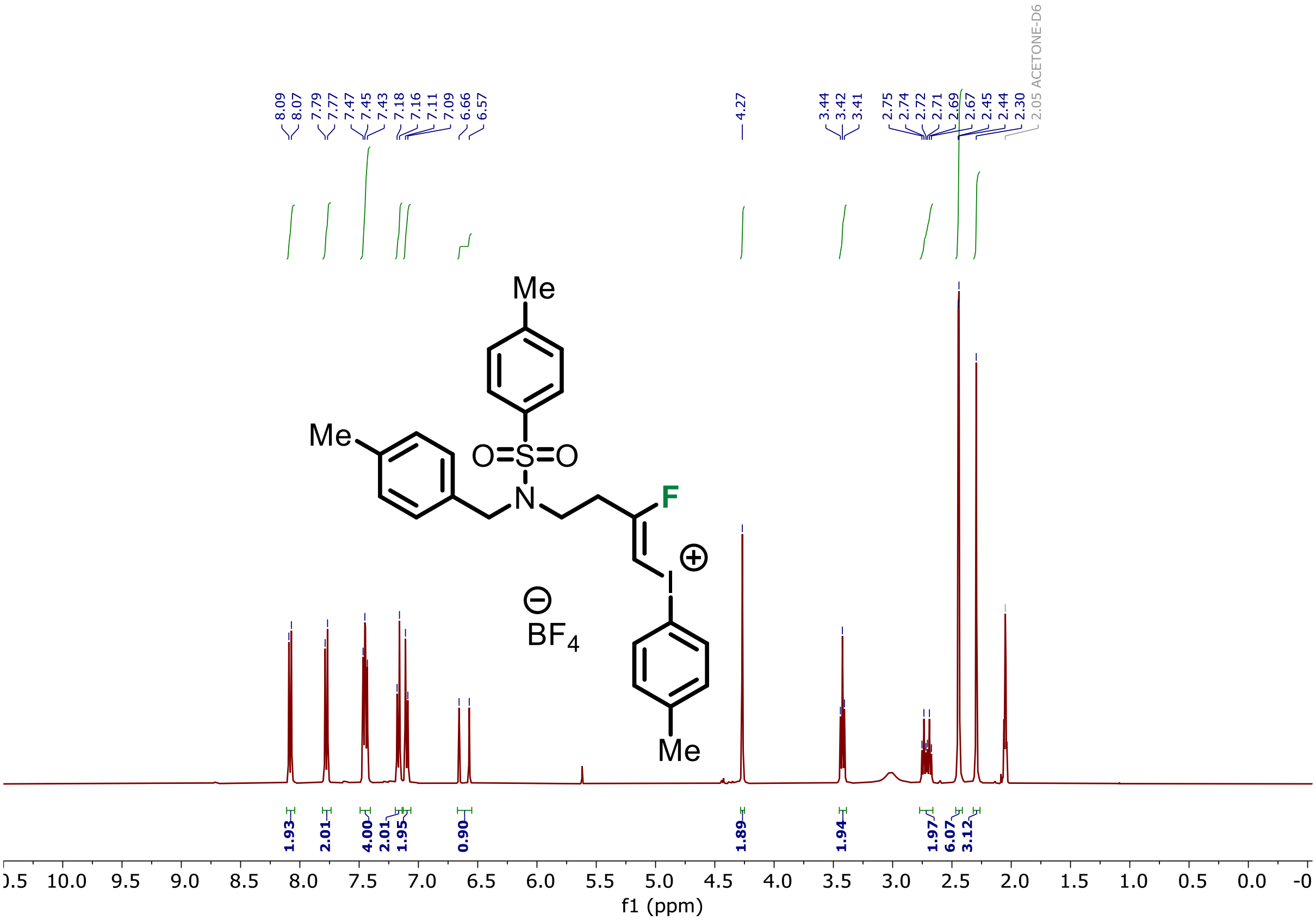

^{13}C NMR (126 MHz, Acetone-D_6)

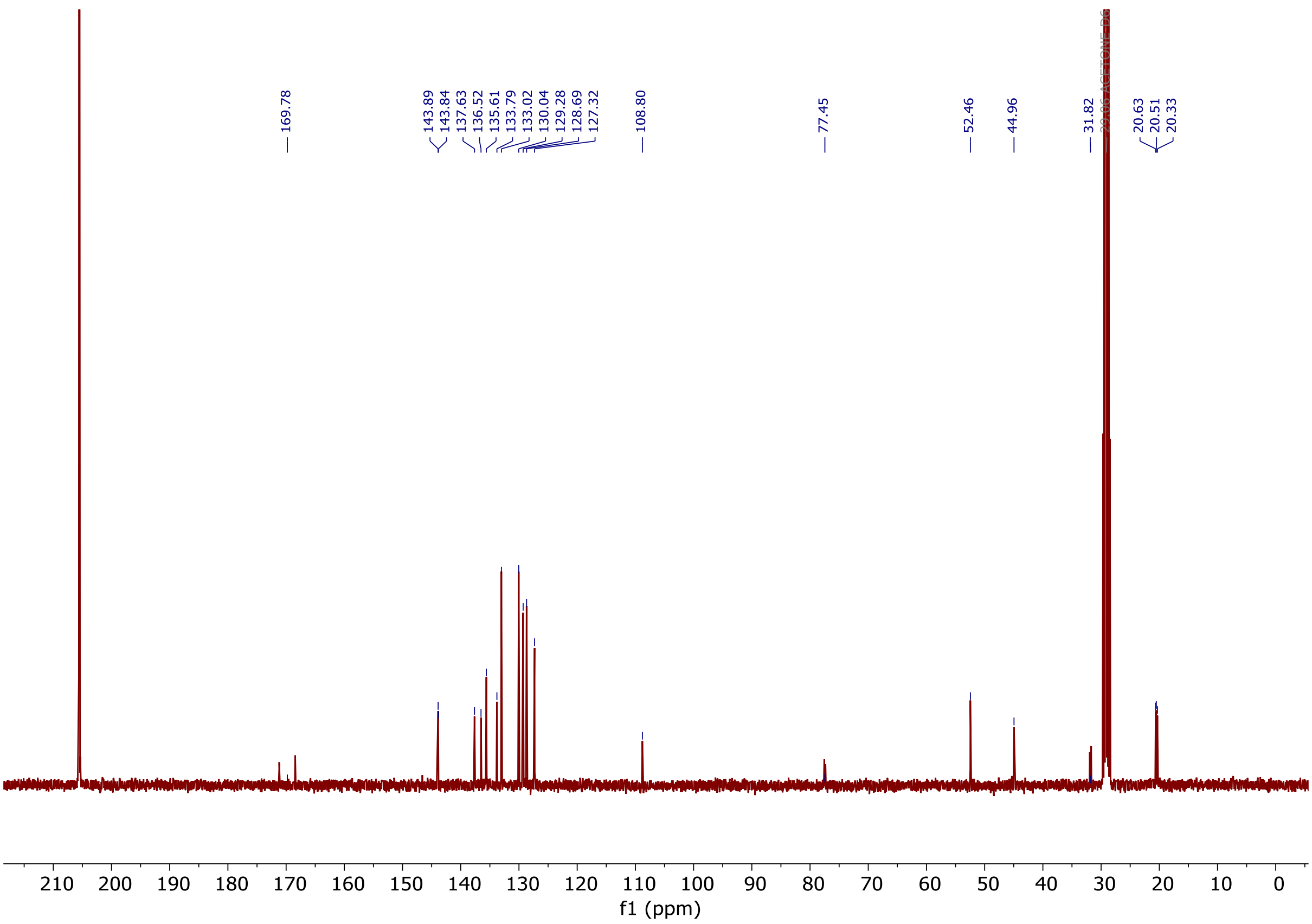

^{19}F NMR (371 MHz, Acetone-D_6)

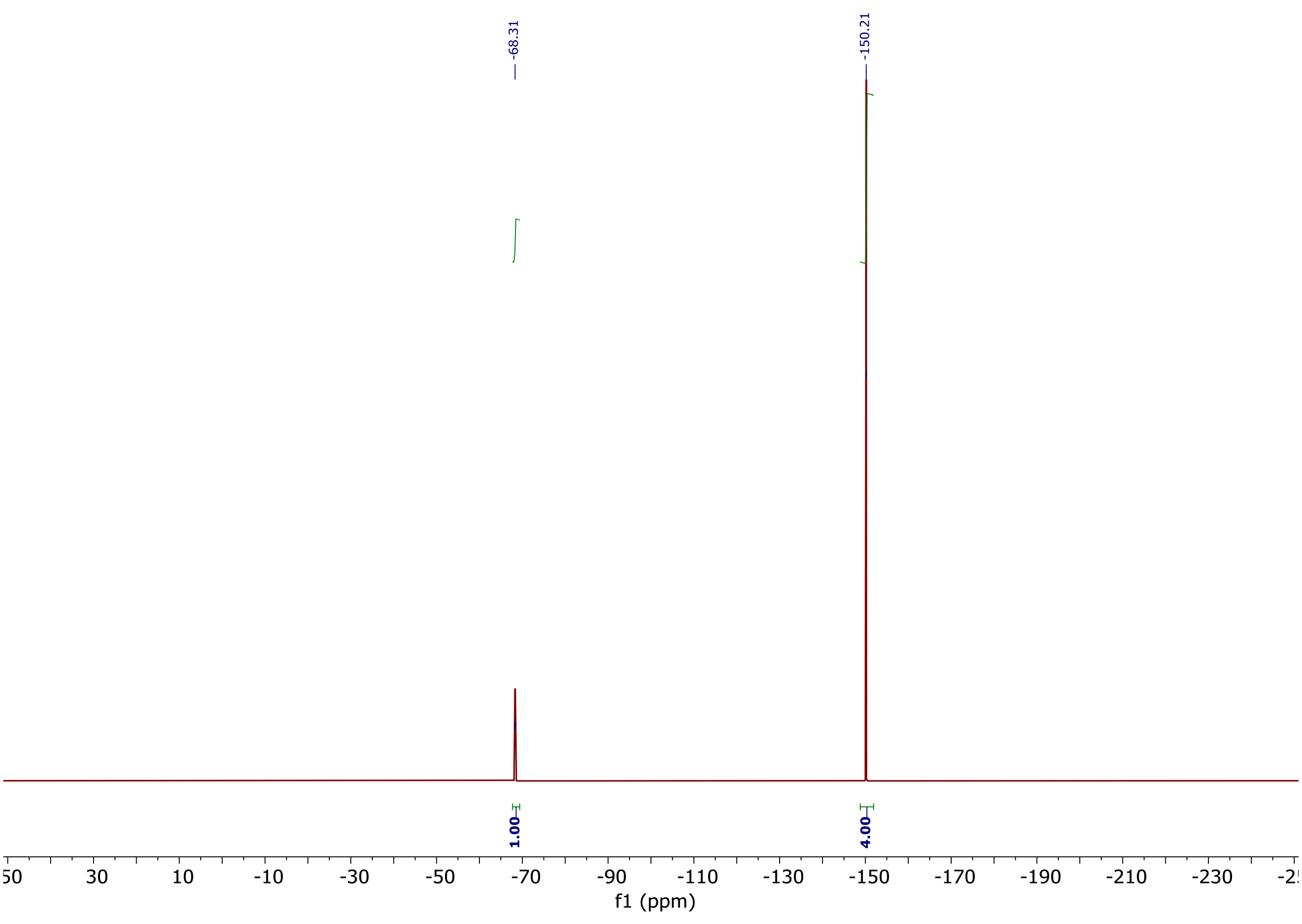

(Z)-(2-fluoro-4-((N-(4-fluorobenzyl)-4-methylphenyl)sulfonamido)but-1-en-1-yl)(p-tolyl)iodonium tetrafluoroborate **(3bs)**
^{1}H NMR (400 MHz, Acetone-*D_6*)

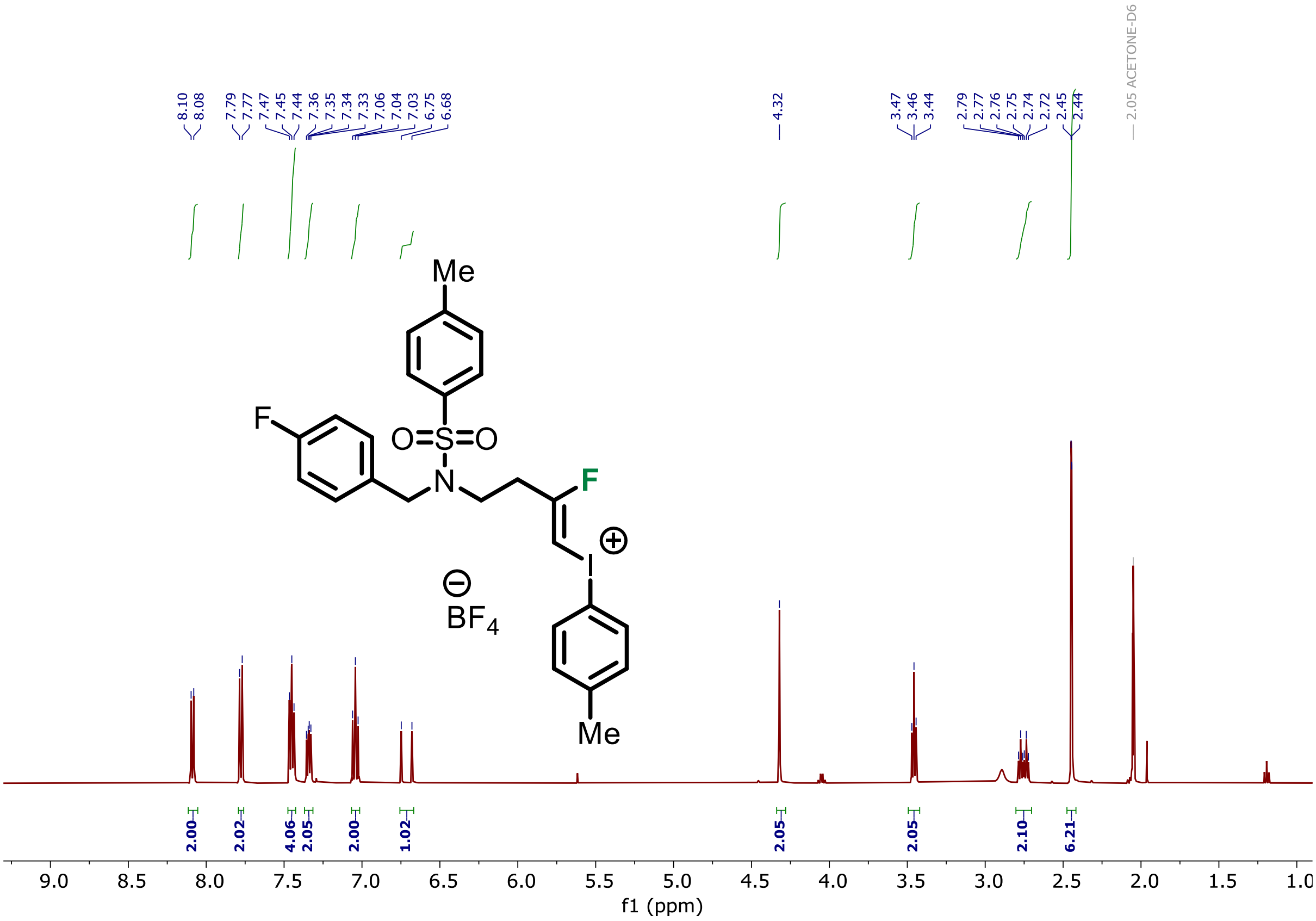

^{13}C NMR (126 MHz, Acetone-*D*$_6$)

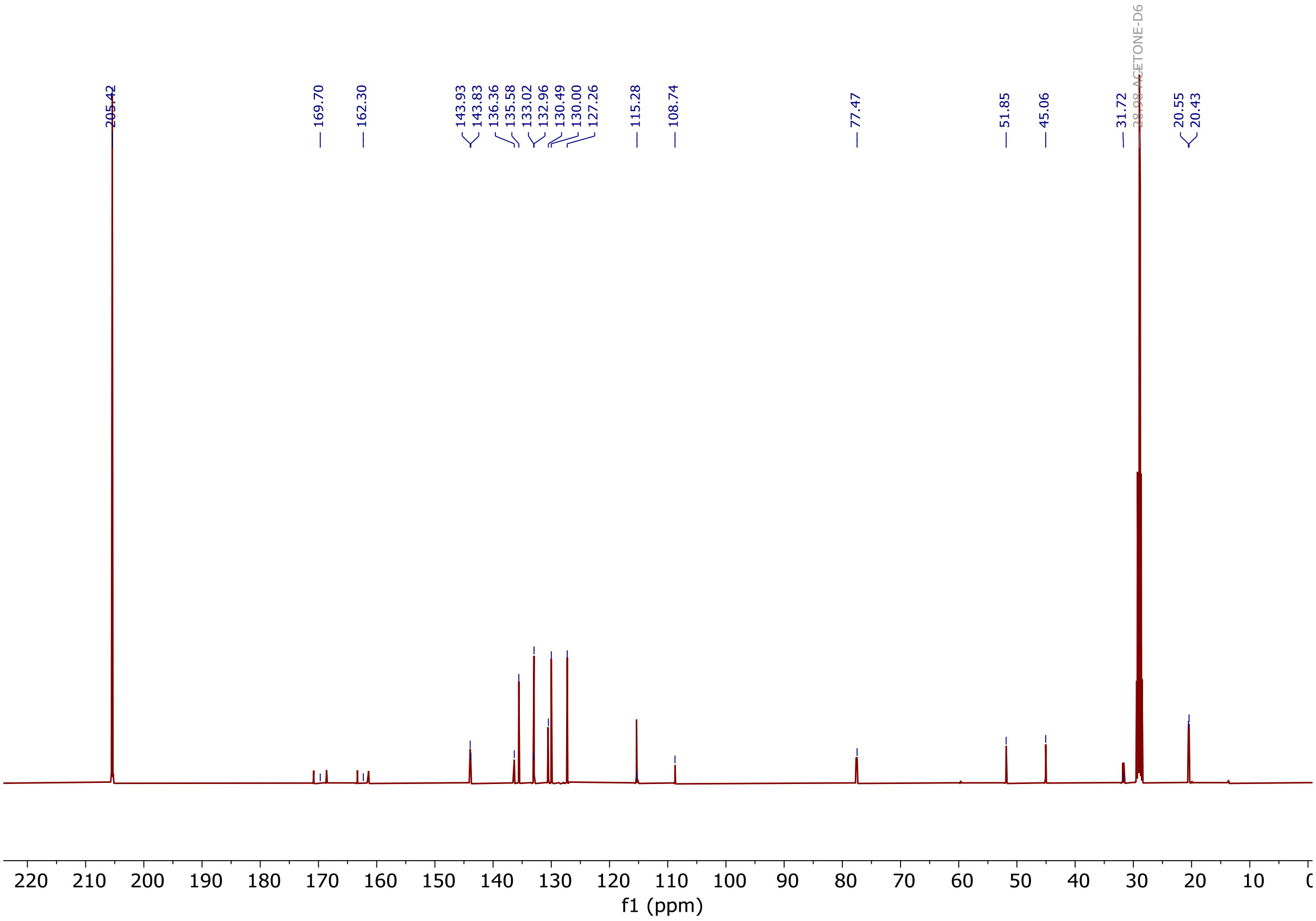

^{19}F NMR (371 MHz, Acetone-*D*$_6$)

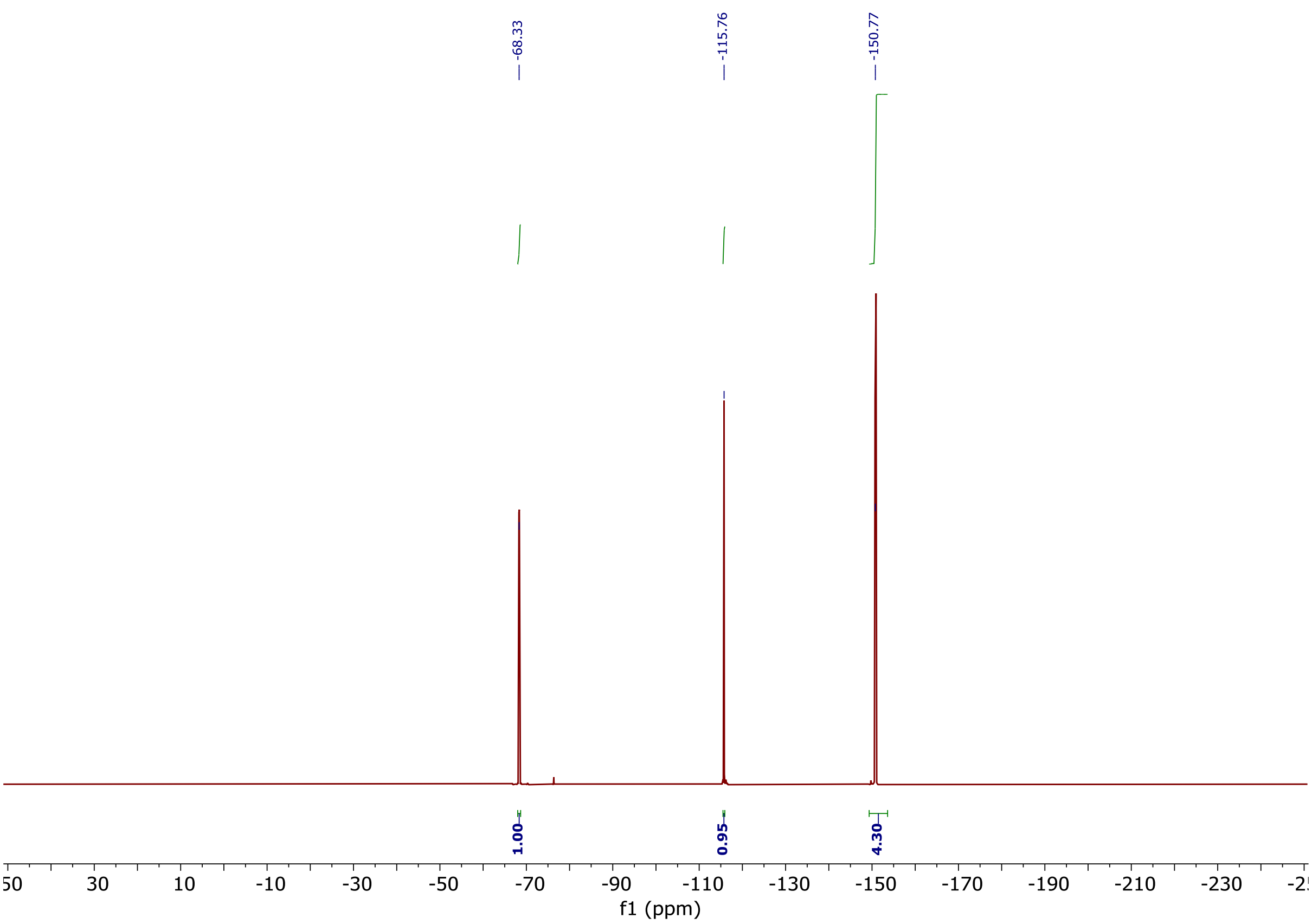

9.3. 3-Pyrrolines (4)

4-fluoro-2-(4-methoxyphenyl)-1-tosyl-2,5-dihydro-1H-pyrrole (4a)

^{1}H NMR (500 MHz, CDCl$_3$)

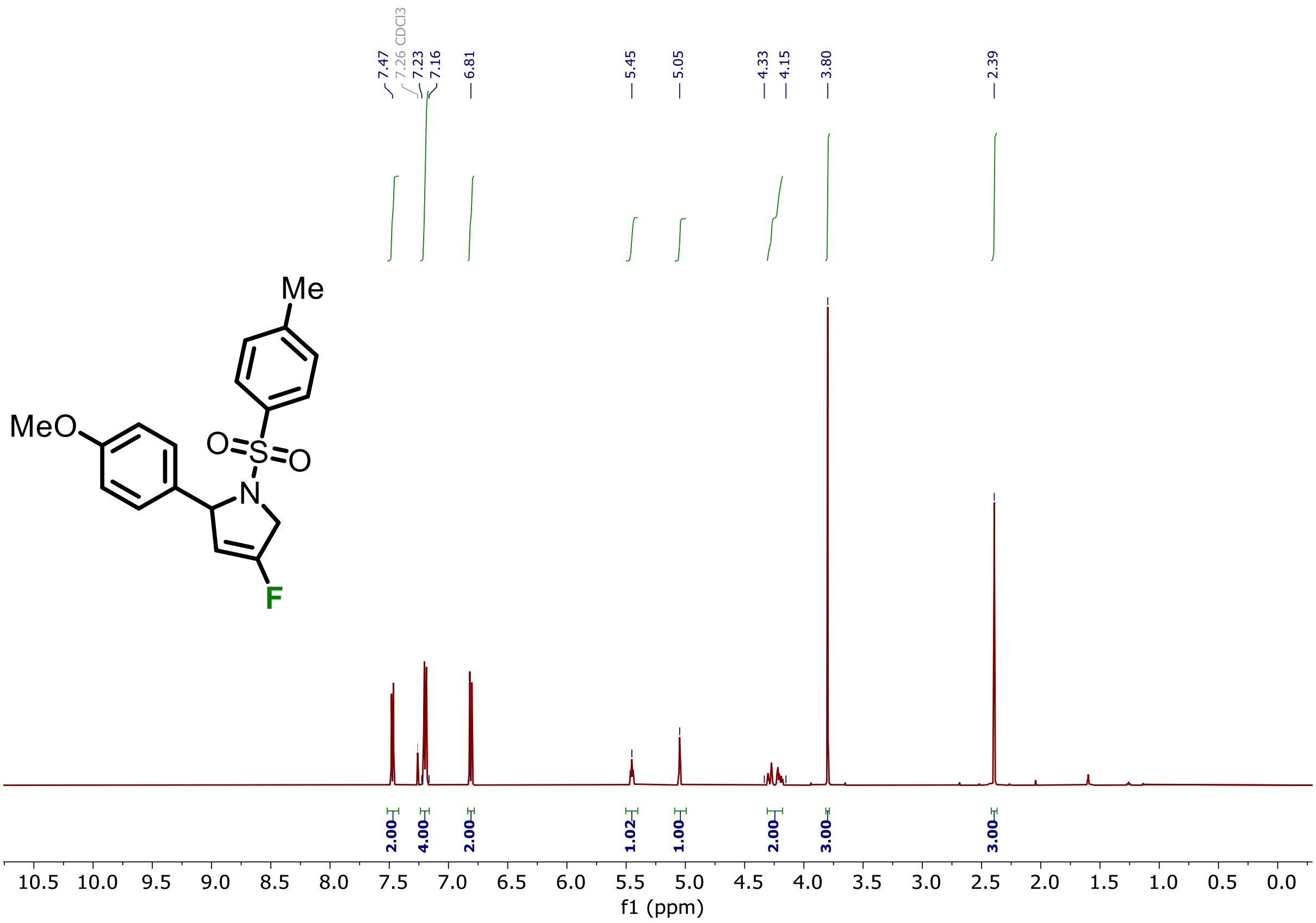

^{13}C NMR (126 MHz, CDCl$_3$)

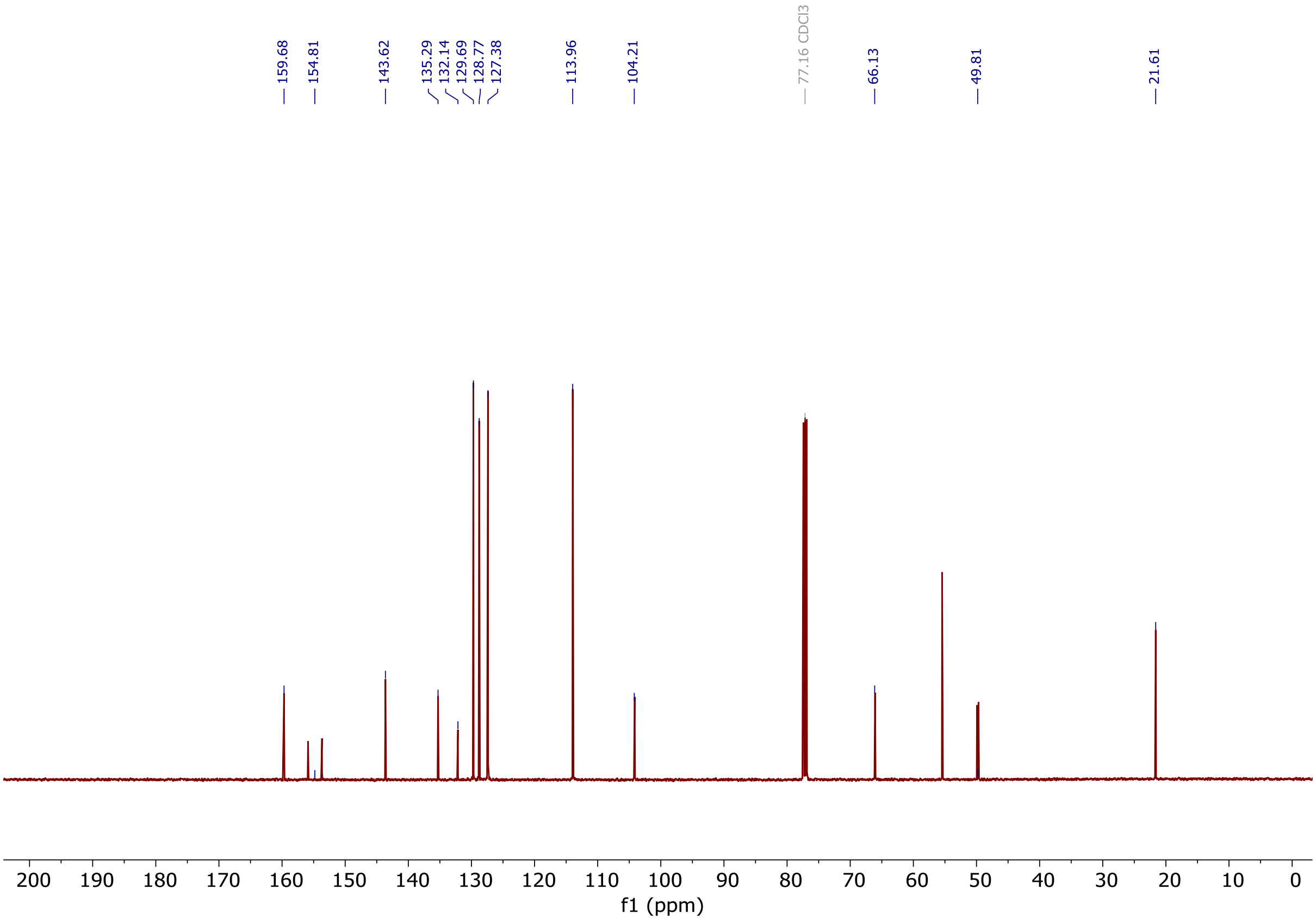

^{19}F NMR (471 MHz, CDCl$_3$)

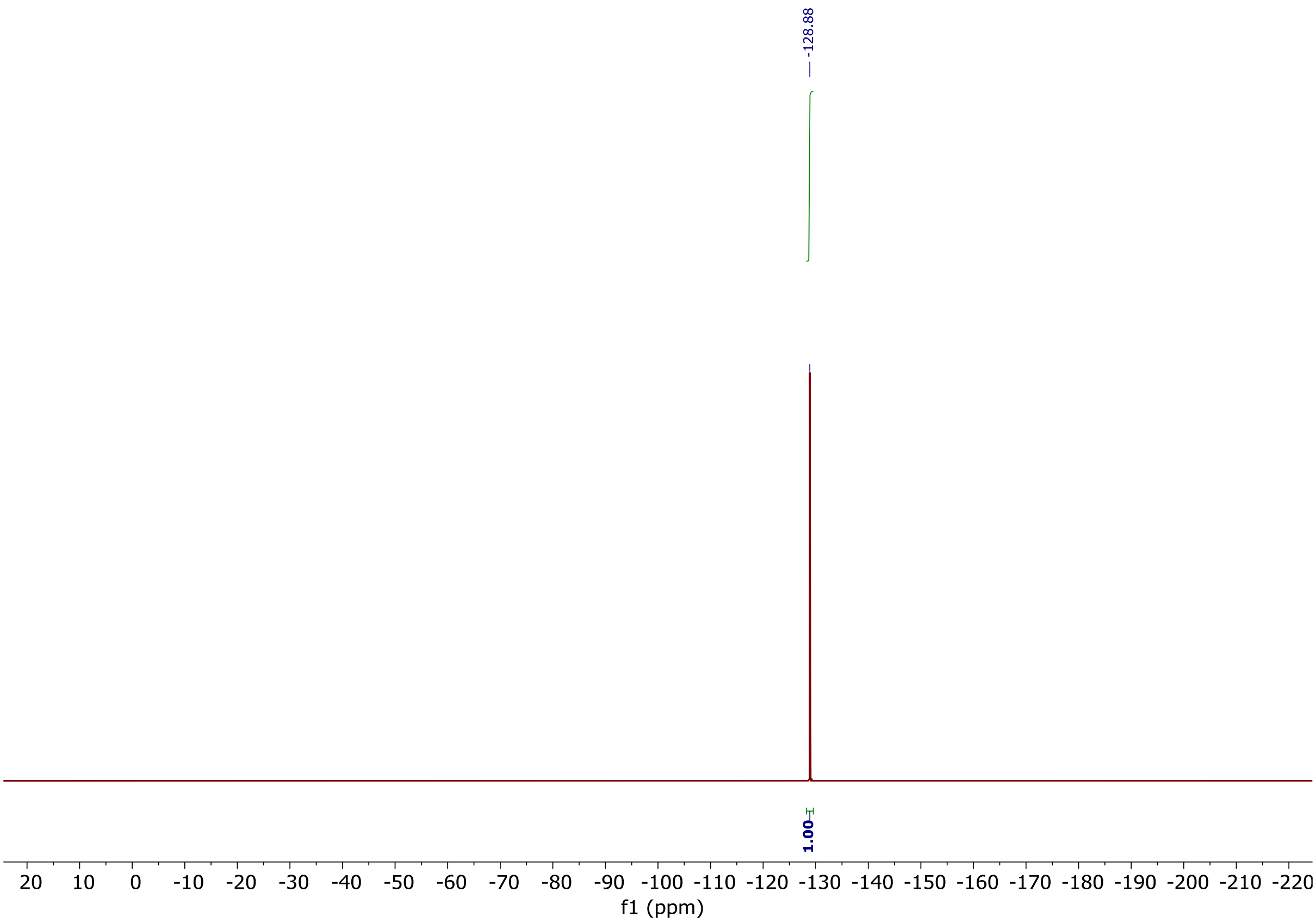

4-fluoro-2-(p-tolyl)-1-tosyl-2,5-dihydro-1H-pyrrole **(4b)**

^{1}H NMR (400 MHz, CDCl$_3$)

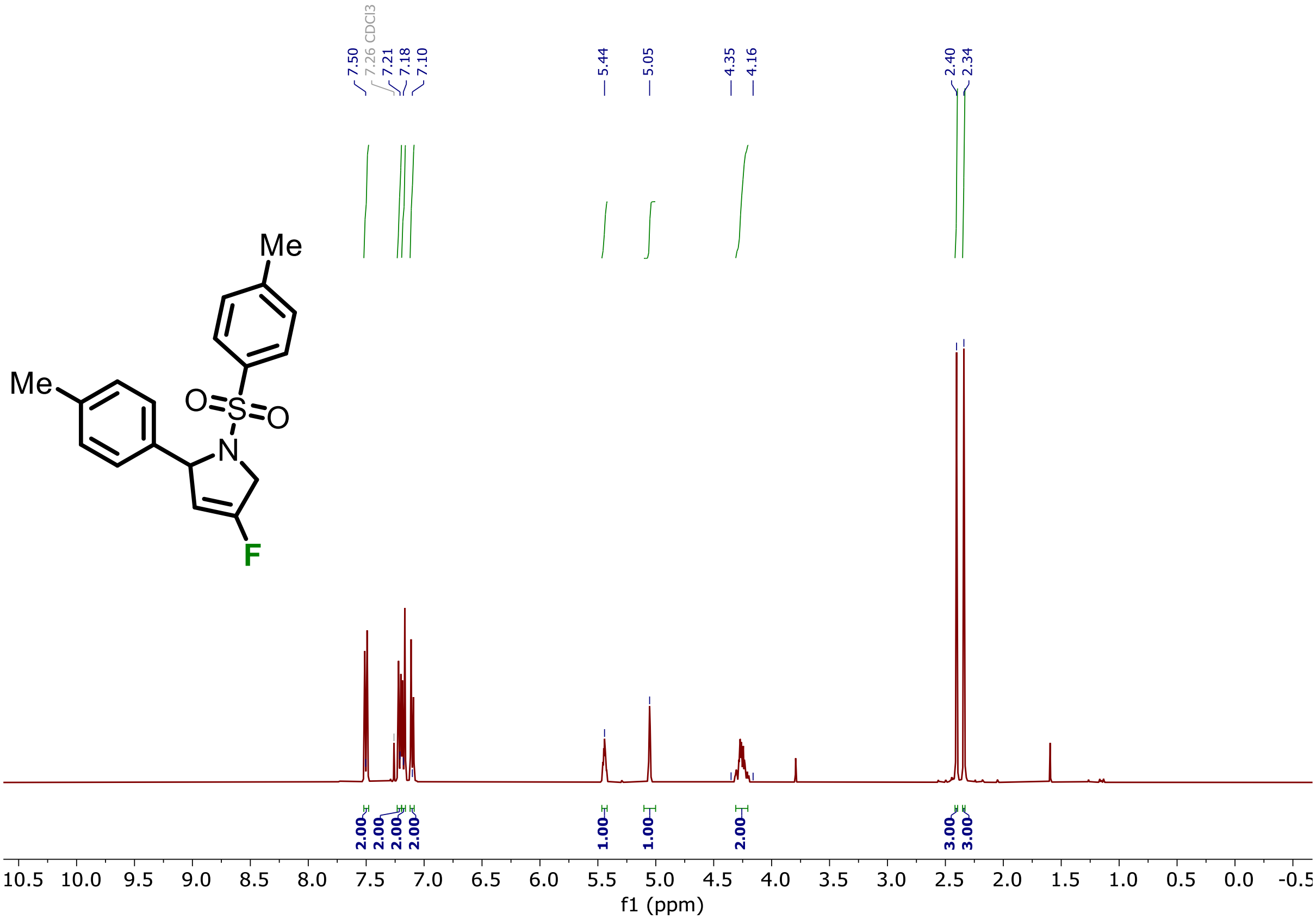

^{13}C NMR (126 MHz, CDCl$_3$)

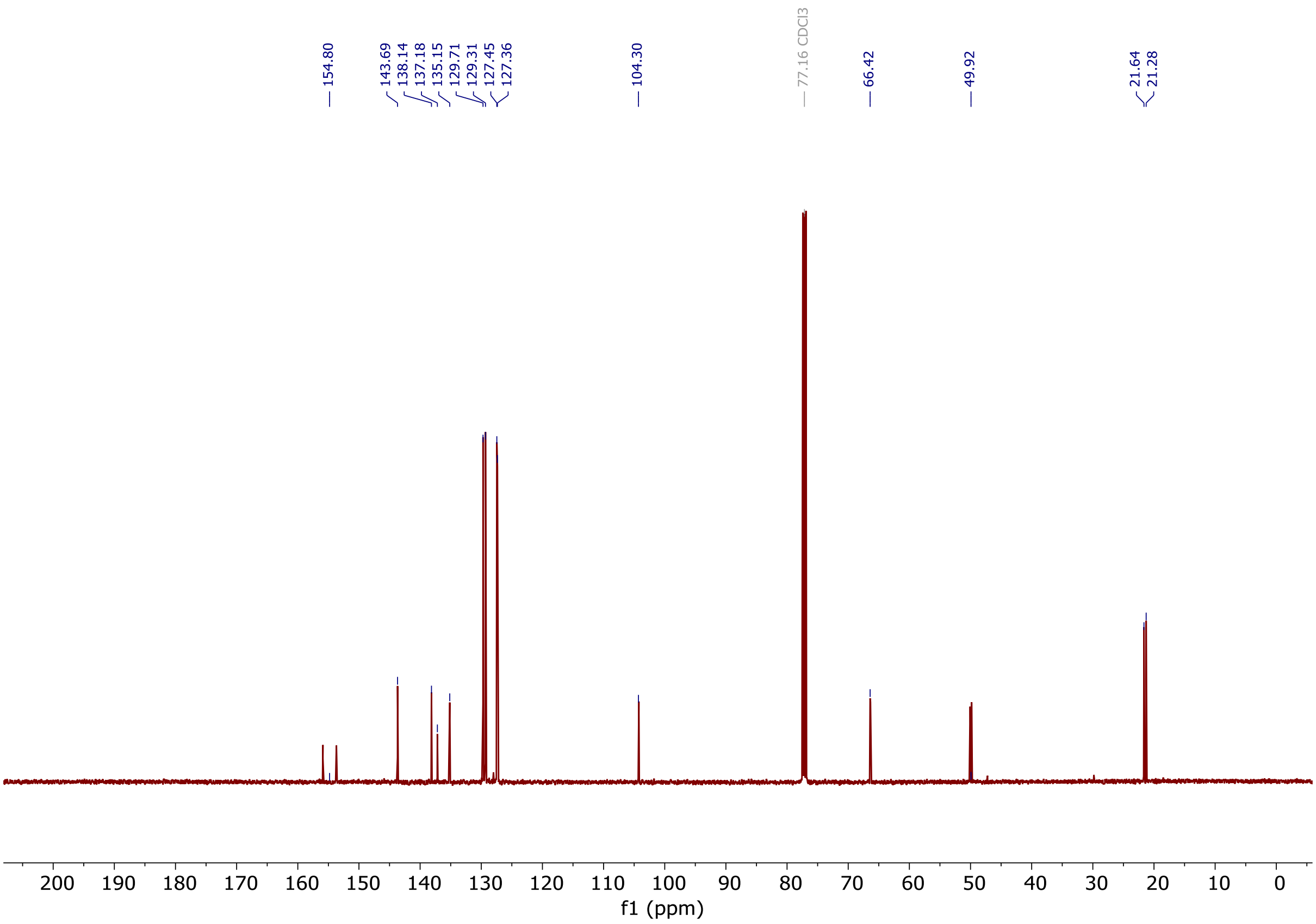

^{19}F NMR (376 MHz, $CDCl_3$)

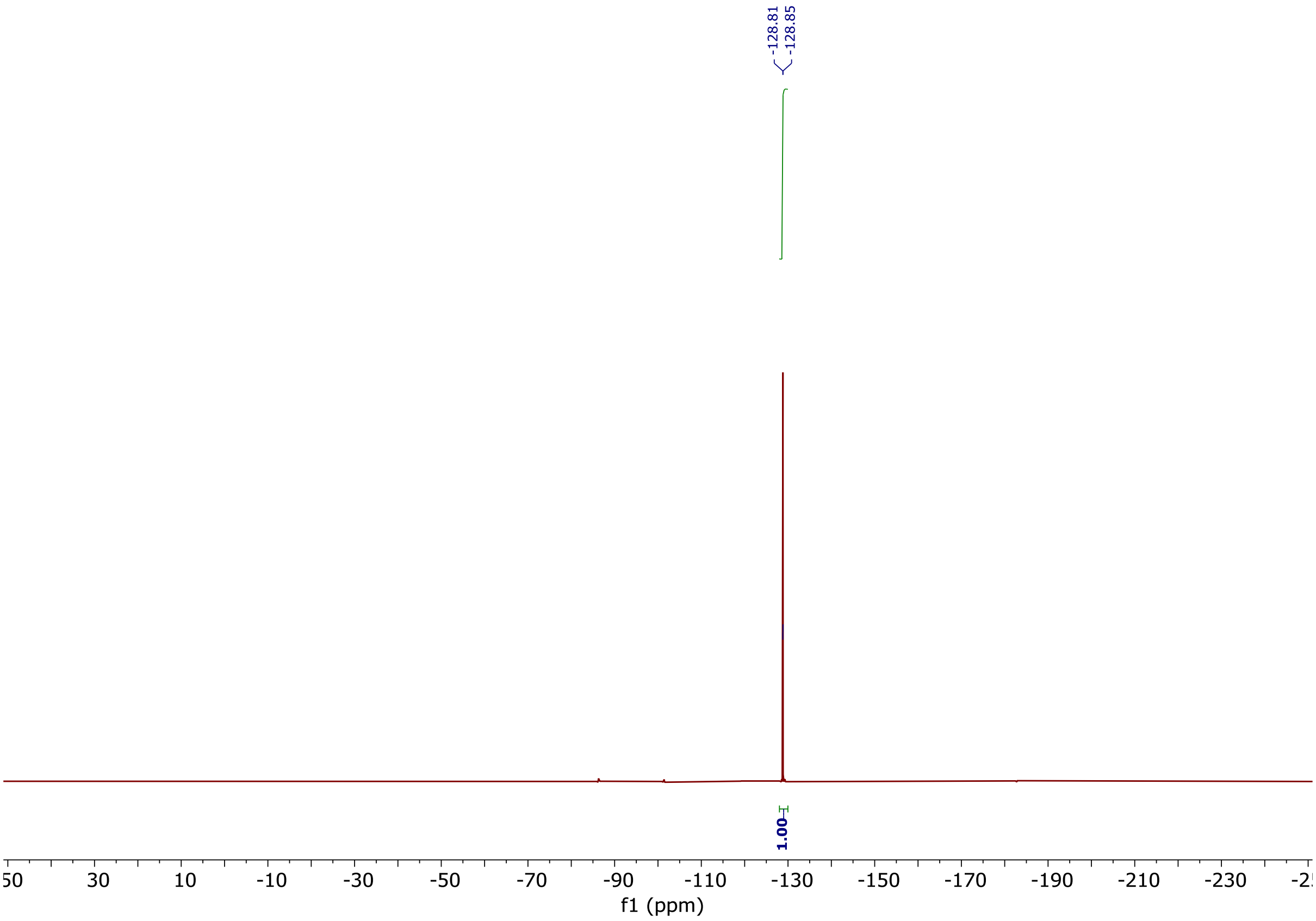

2-(4-bromophenyl)-4-fluoro-1-tosyl-2,5-dihydro-1H-pyrrole **(4c)**

^{1}H NMR (500 MHz, CDCl$_3$)

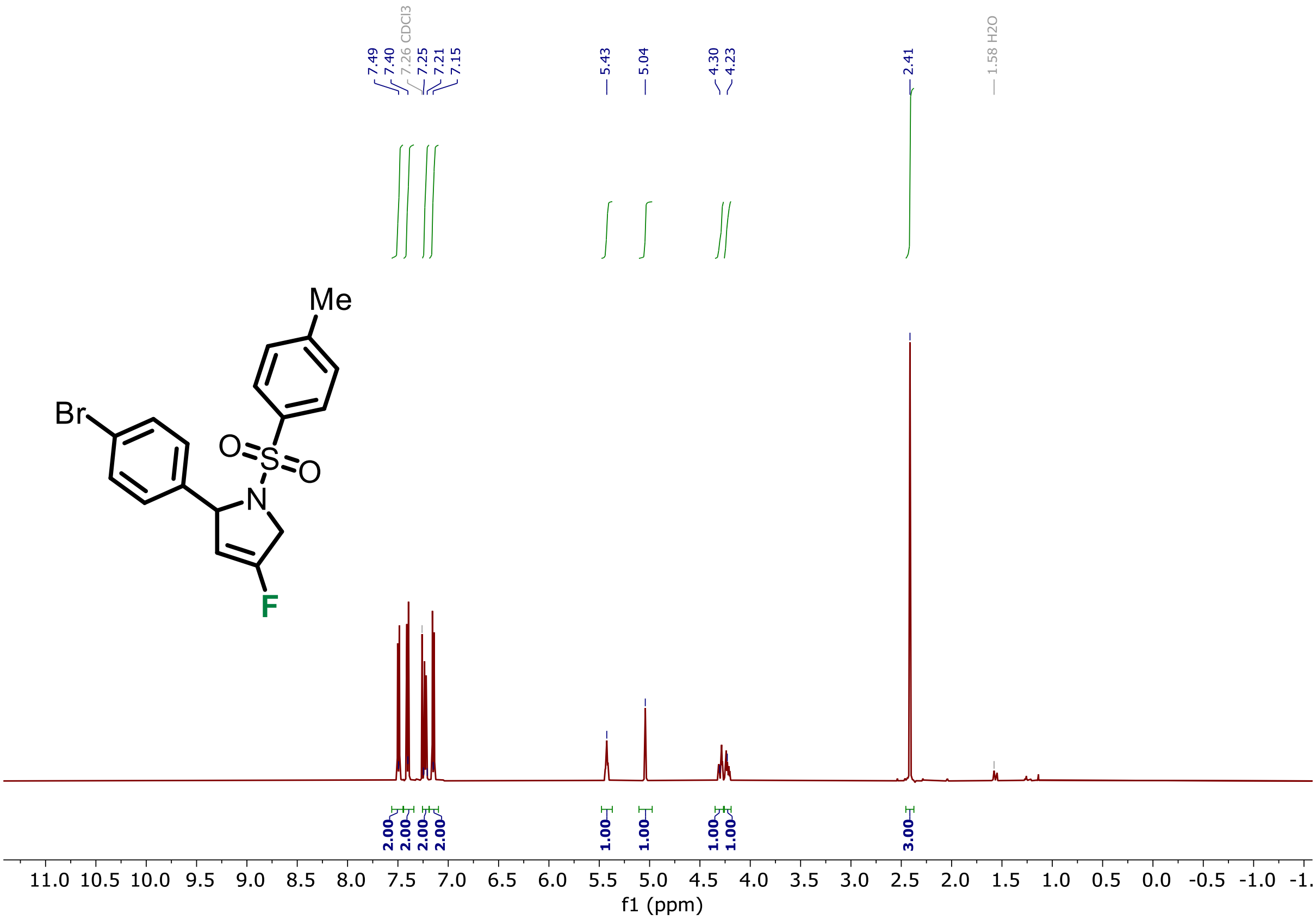

^{13}C NMR (126 MHz, CDCl$_3$)

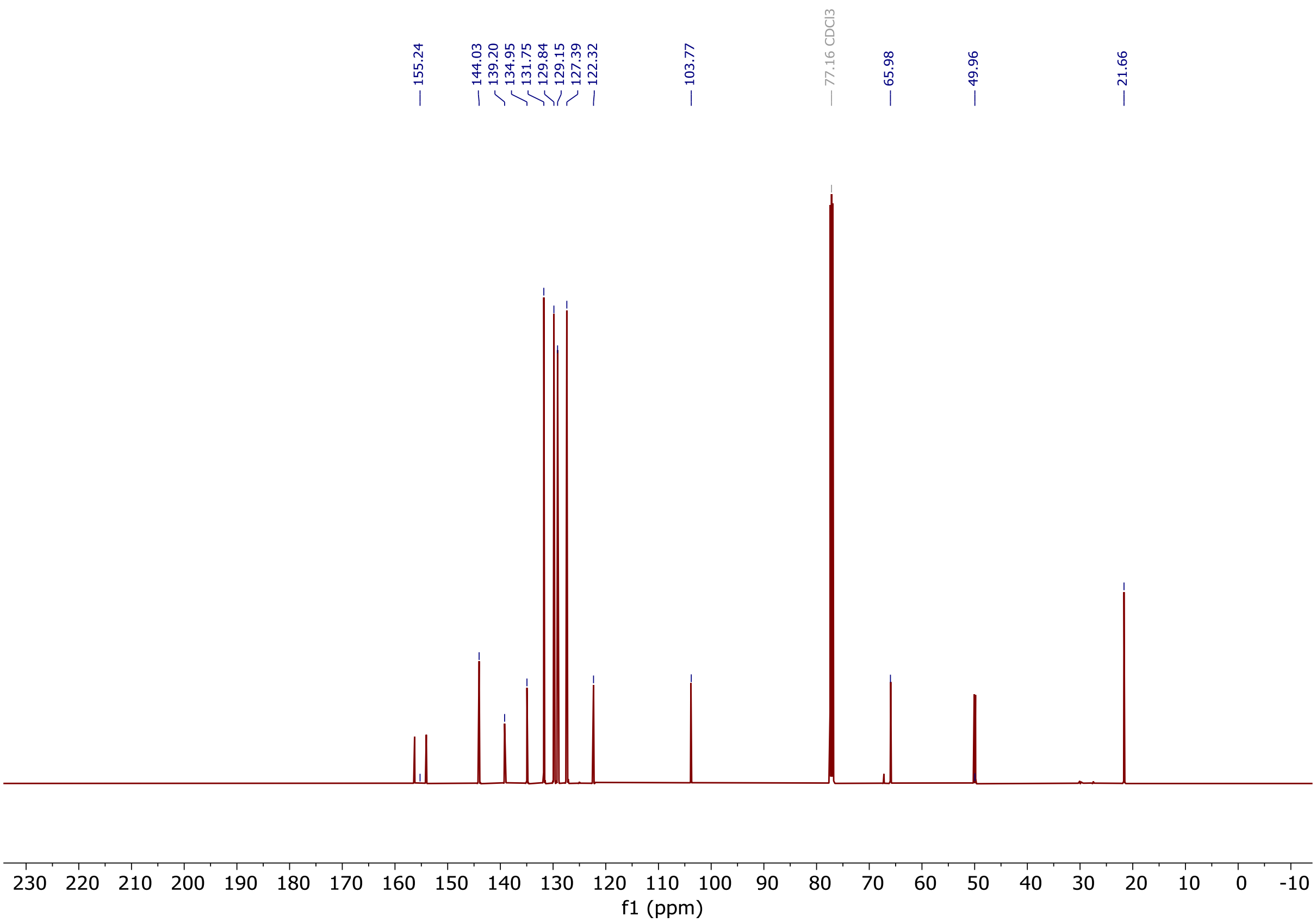

^{19}F NMR (376 MHz, $CDCl_3$)

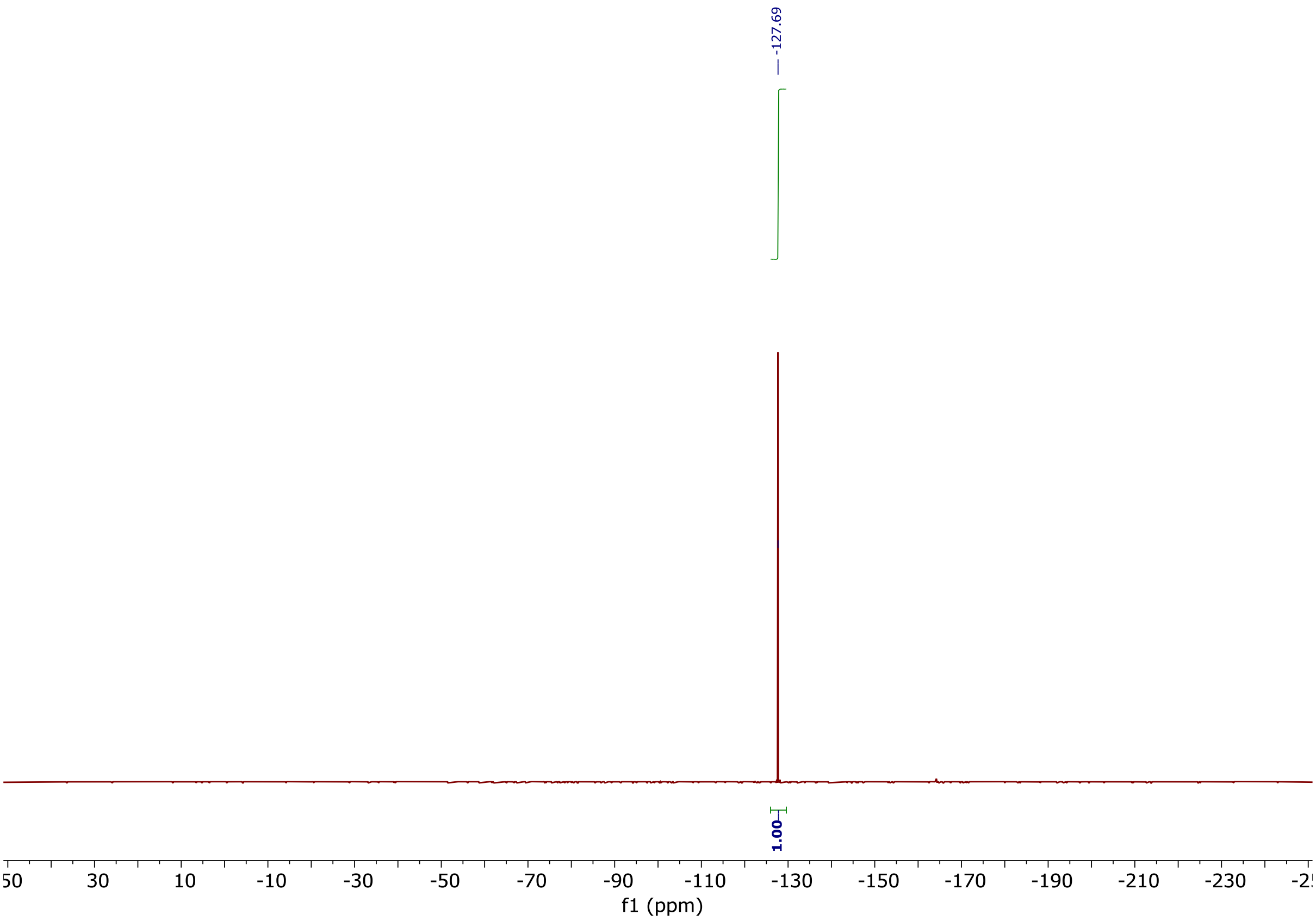

2-(4-chlorophenyl)-4-fluoro-1-tosyl-2,5-dihydro-1H-pyrrole **(4d)**

^{1}H NMR (500 MHz, CDCl$_3$)

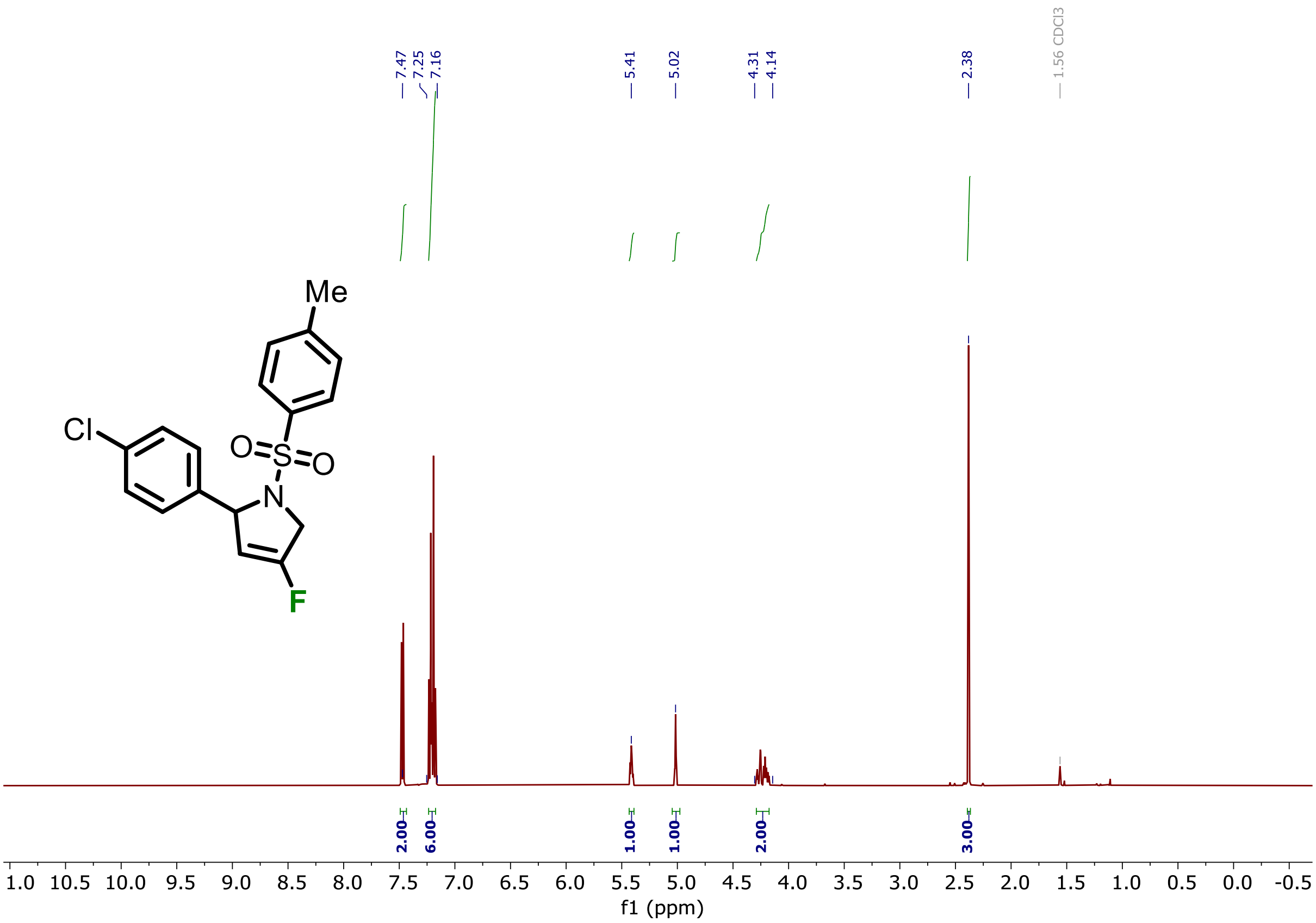

^{13}C NMR (126 MHz, CDCl$_3$)

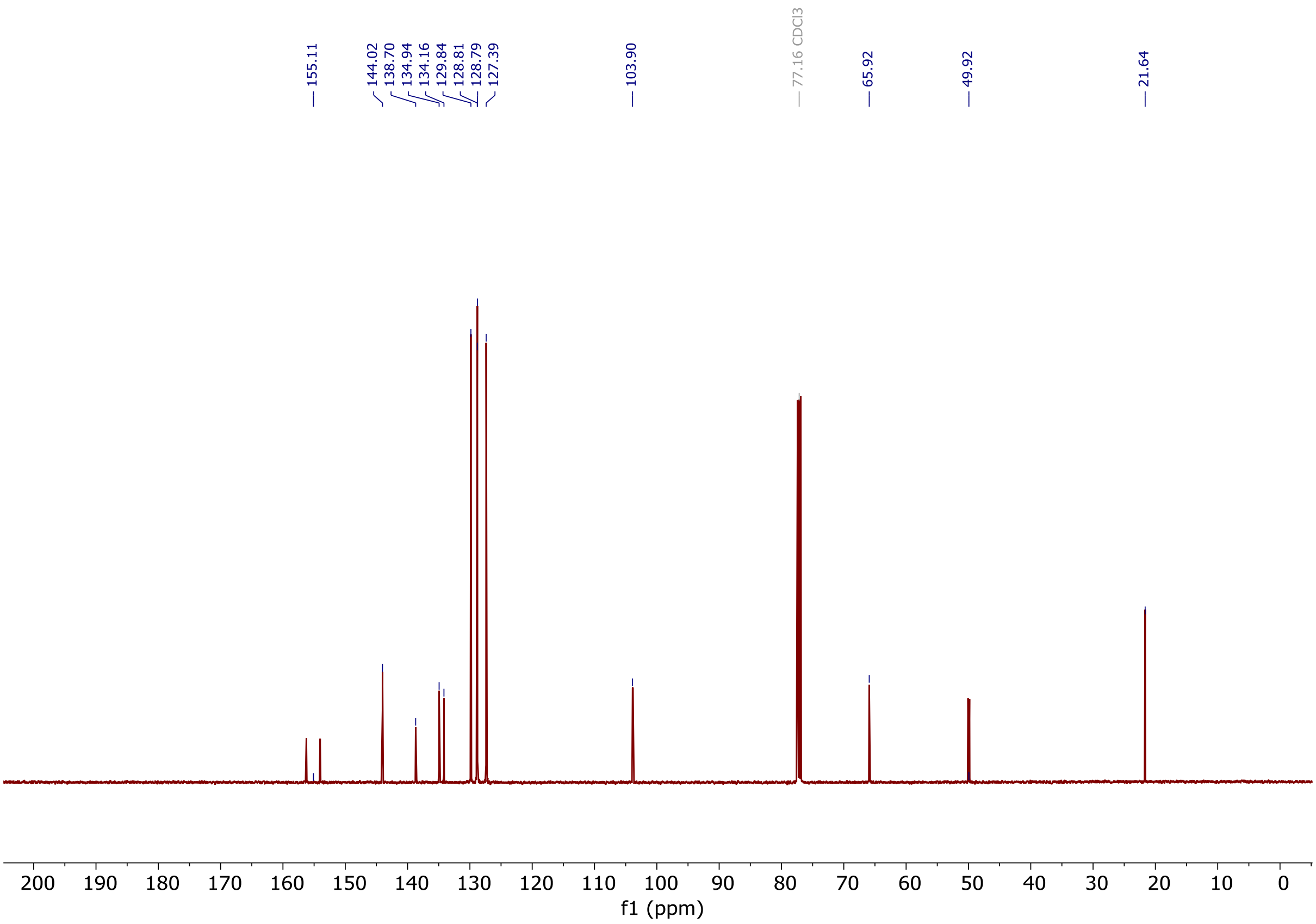

^{19}F NMR (471 MHz, $CDCl_3$)

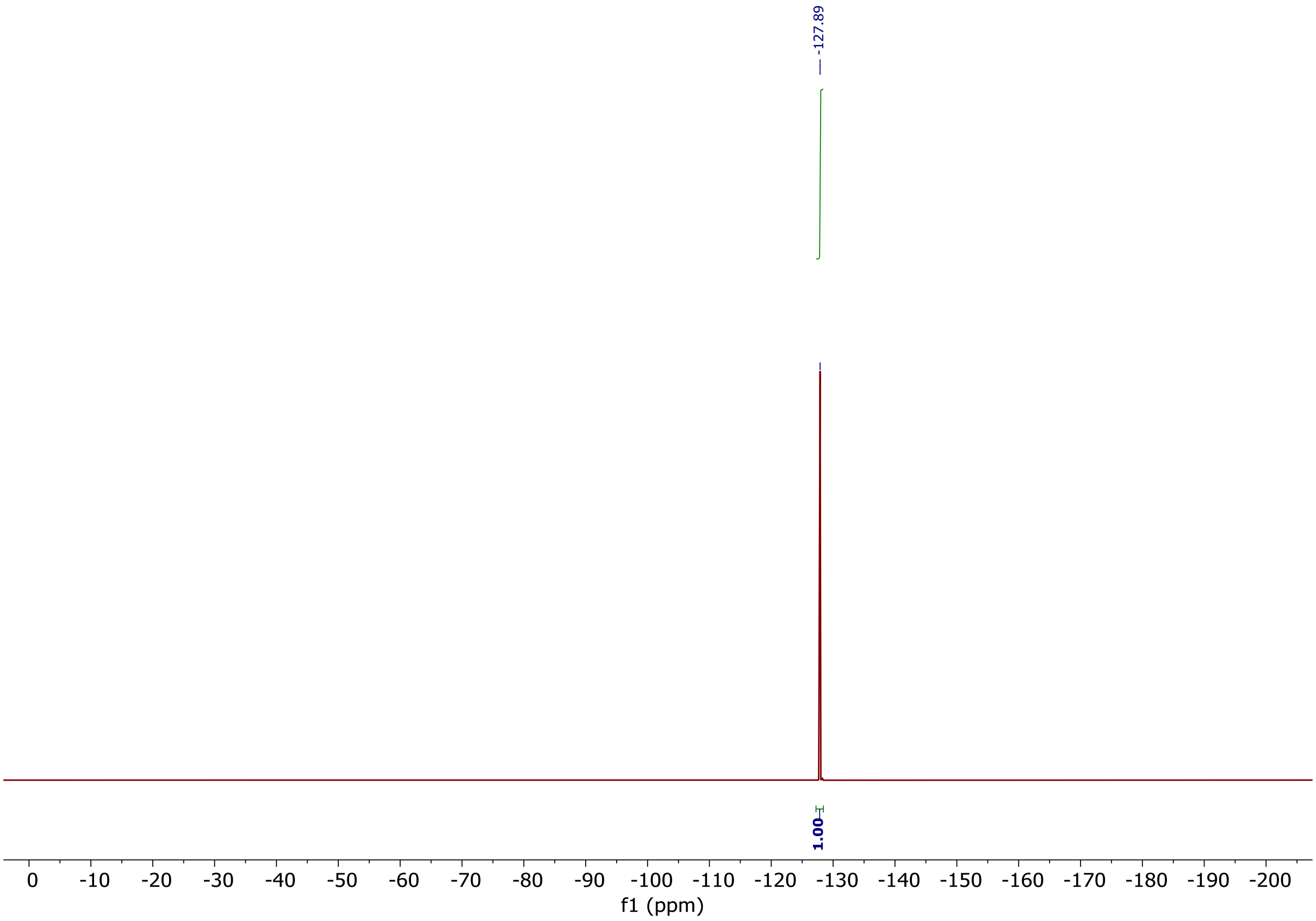

4-fluoro-2-(4-fluorophenyl)-1-tosyl-2,5-dihydro-1H-pyrrole (4e)

^{1}H NMR (500 MHz, CDCl$_3$)

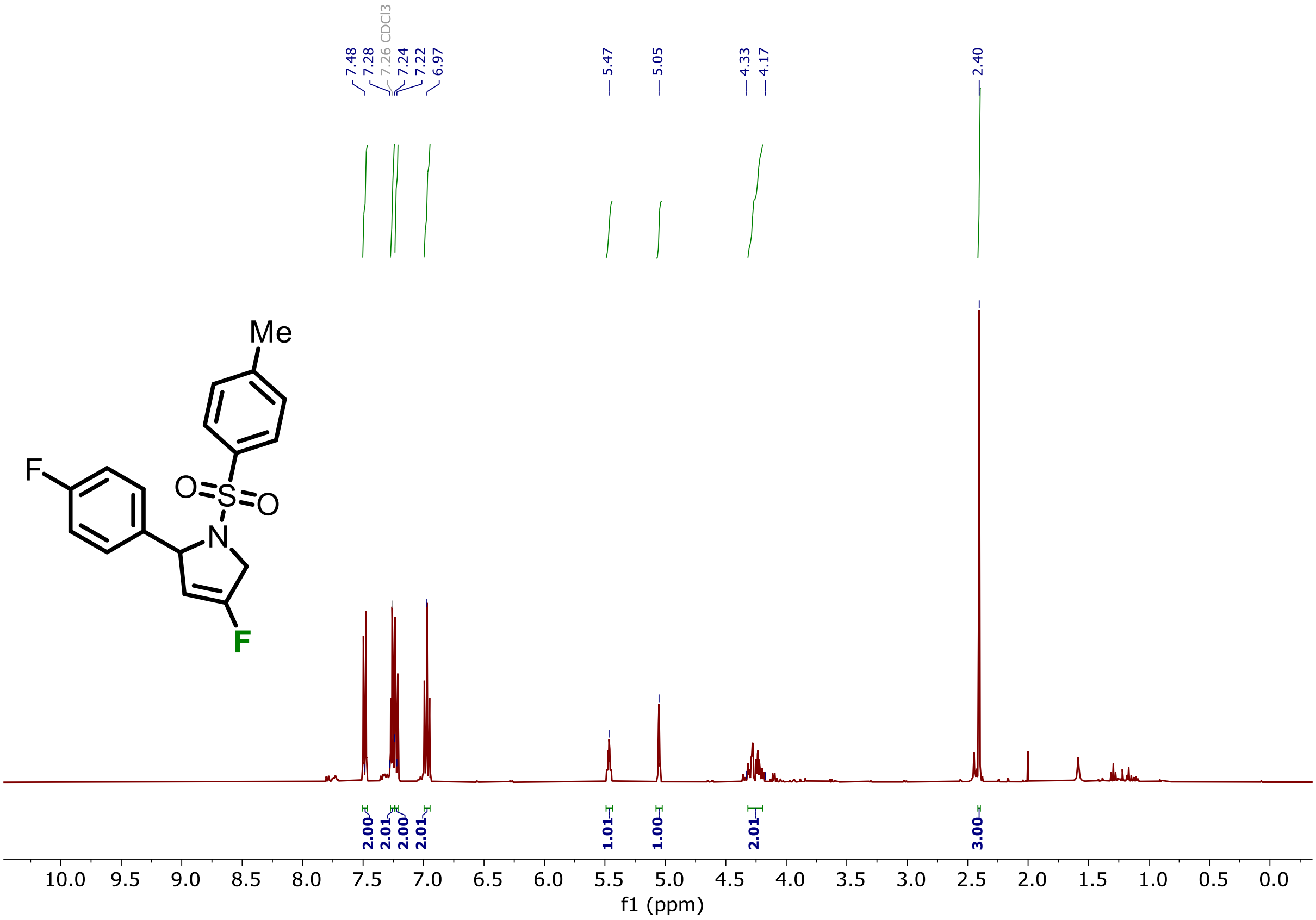

^{13}C NMR (126 MHz, CDCl$_3$)

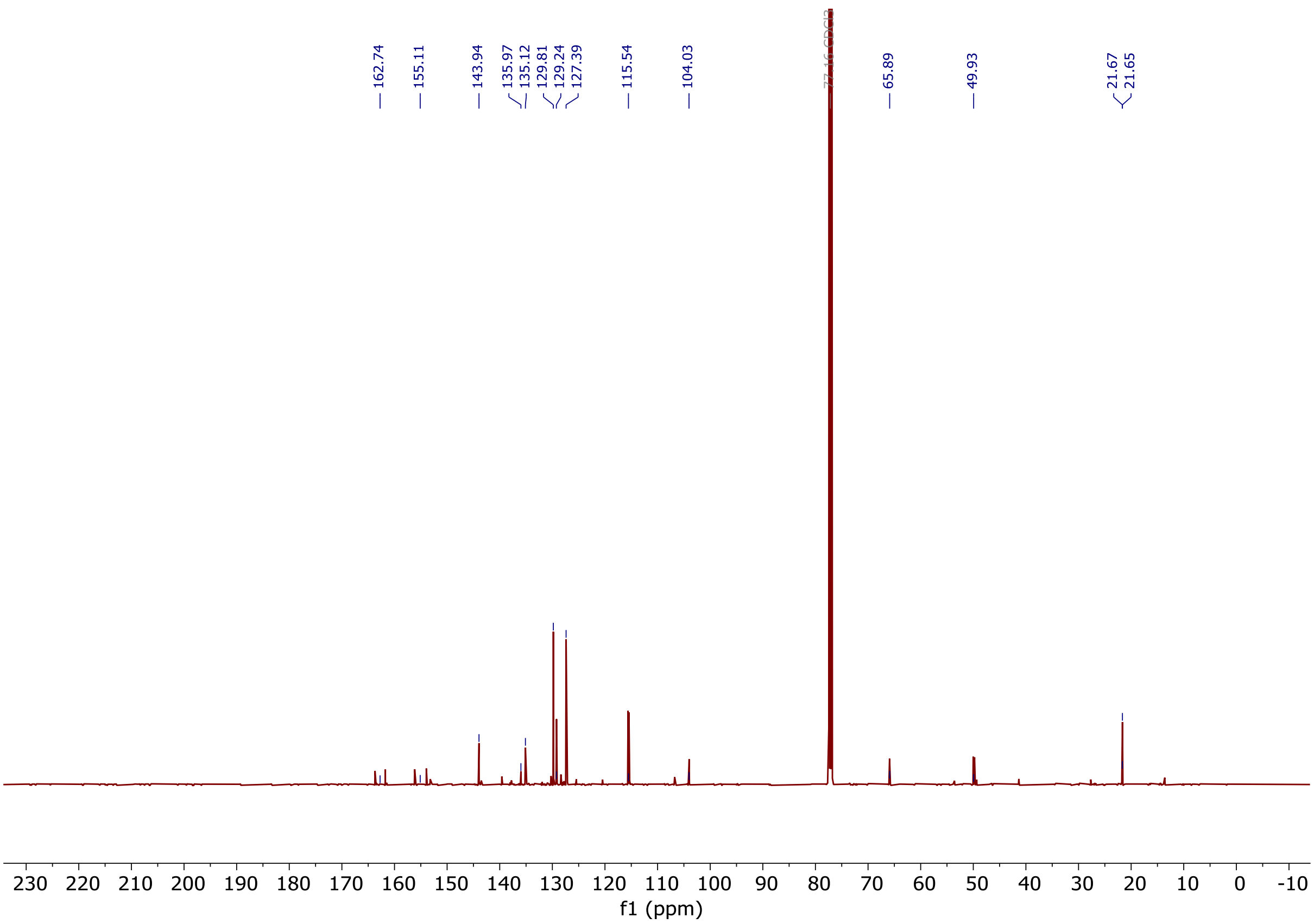

^{19}F NMR (376 MHz, $CDCl_3$)

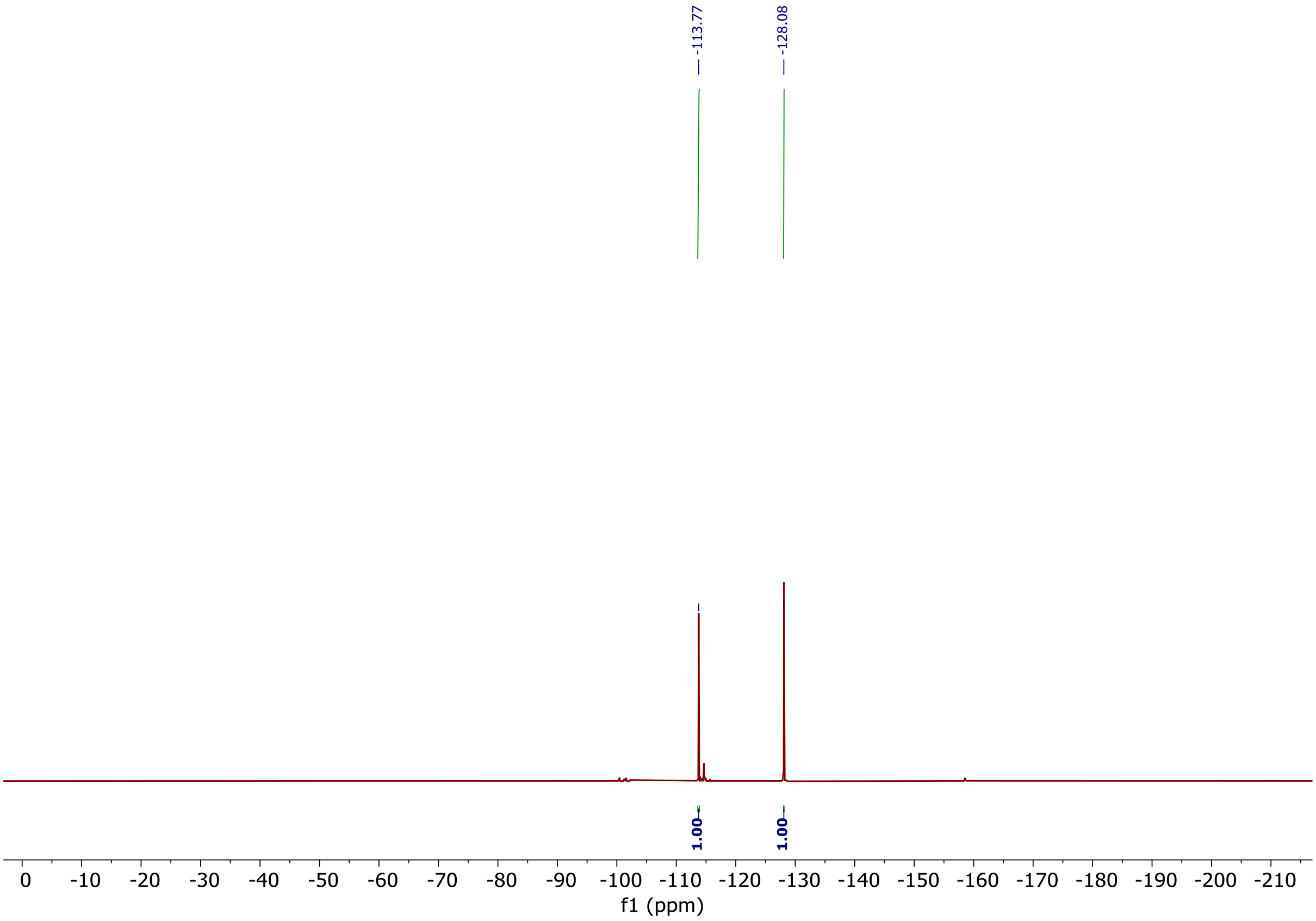

***4-fluoro-1-tosyl-2-(4-(trifluoromethyl)phenyl)-2,5-dihydro-1H-pyrrole* (4f)**

^{1}H NMR (600 MHz, CDCl$_3$)

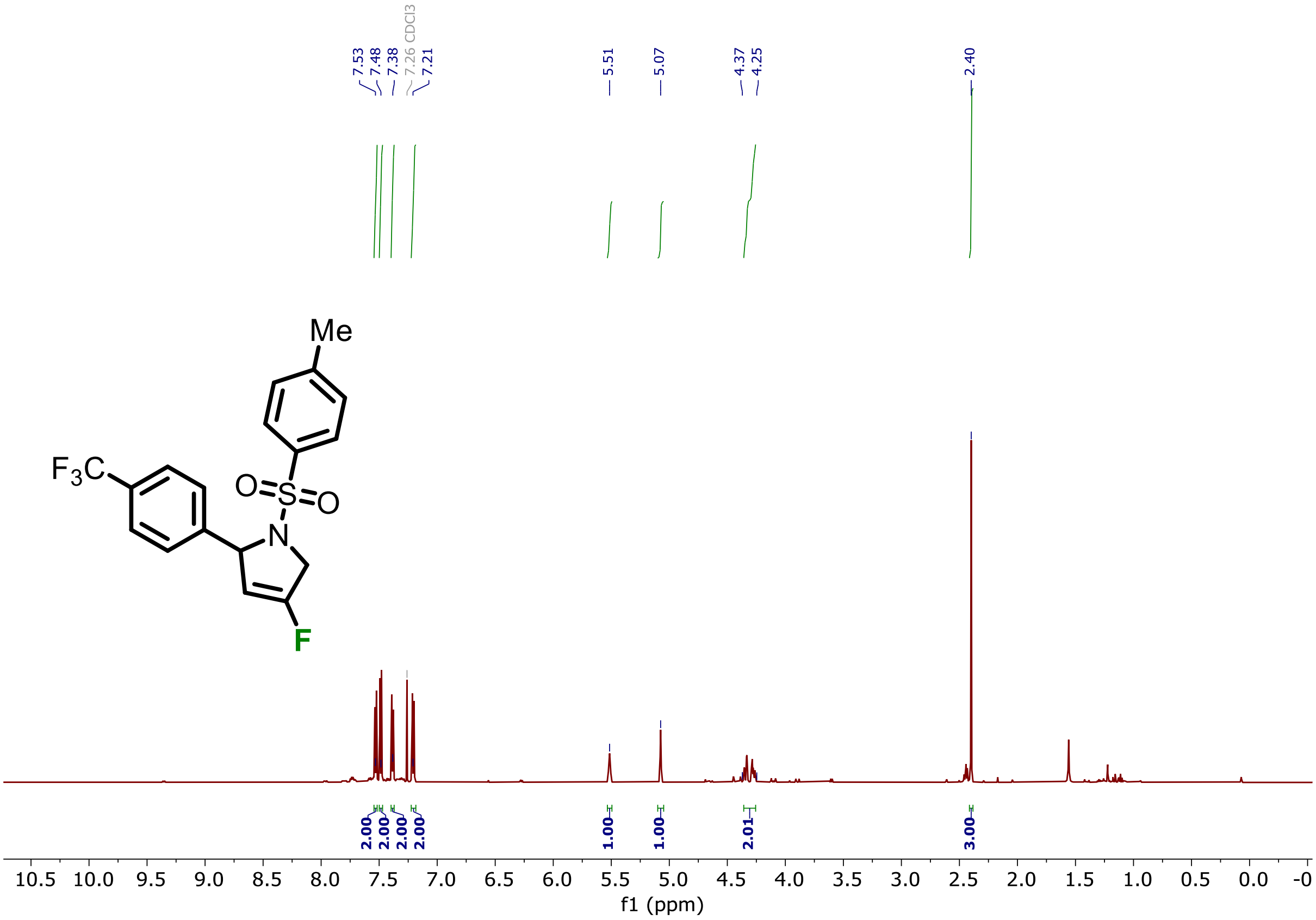

^{13}C NMR (151 MHz, CDCl$_3$)

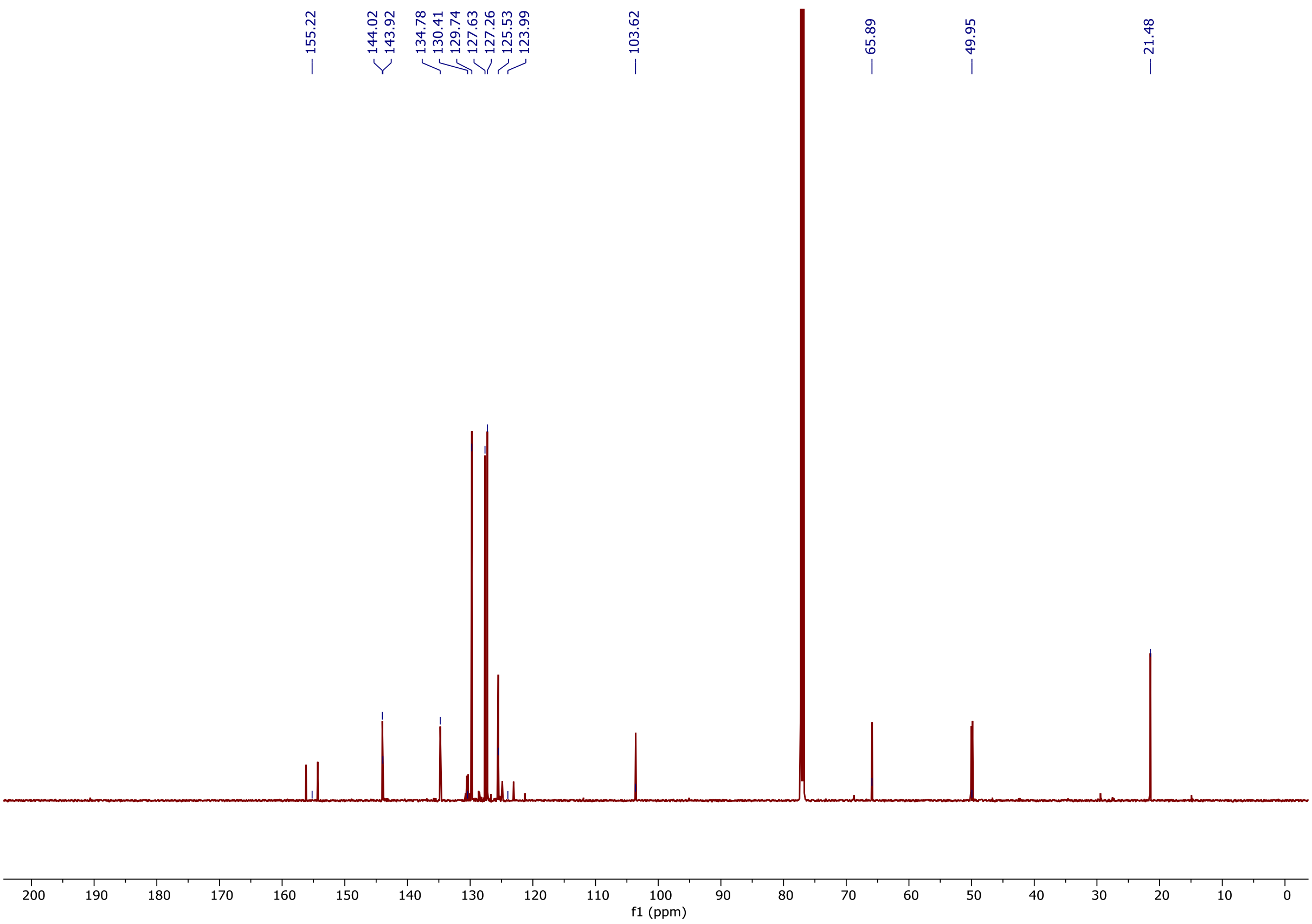

^{19}F NMR (376 MHz, CDCl$_3$)

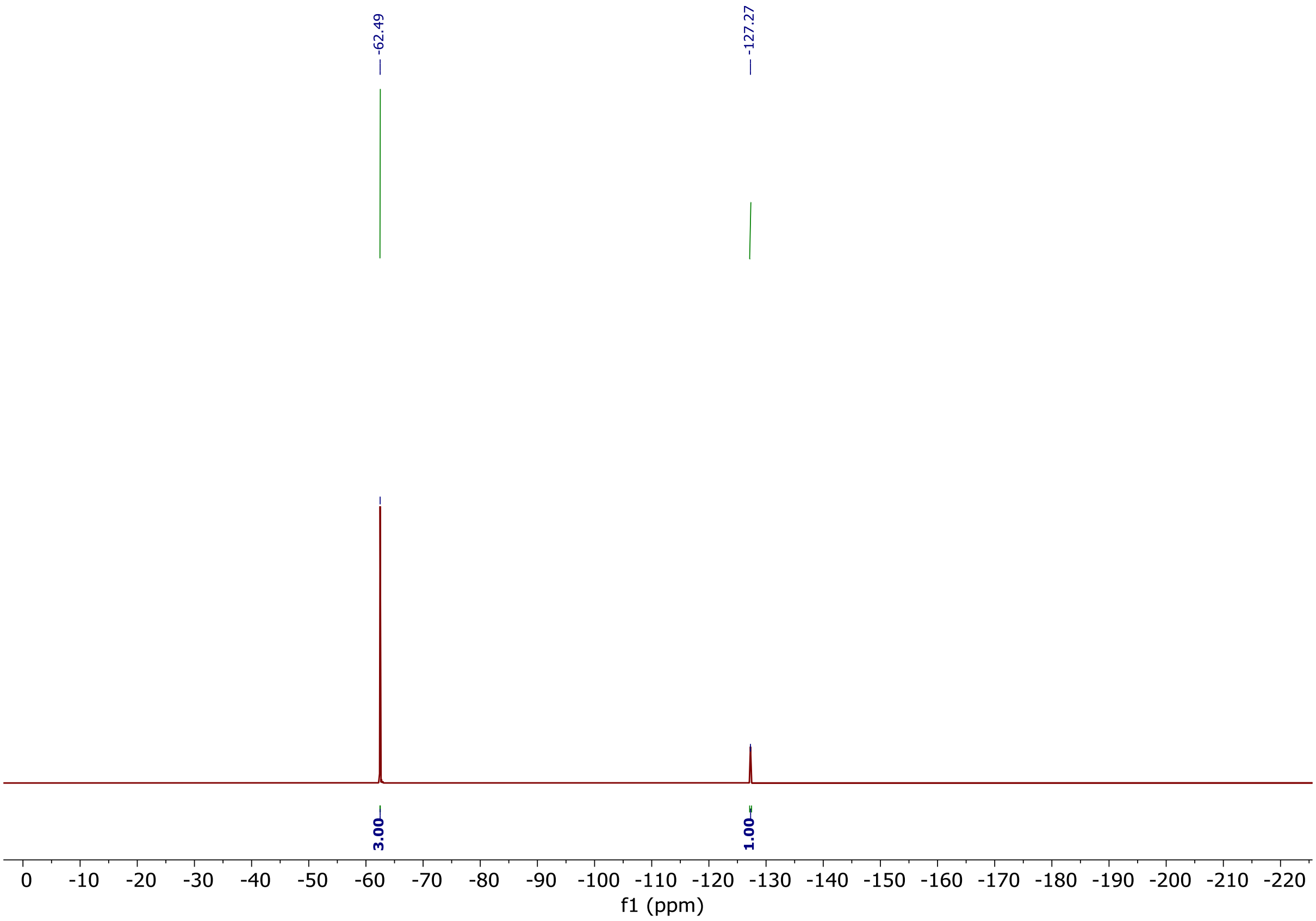

***3-fluoro-2,2-dimethyl-5-phenyl-1-tosyl-2,5-dihydro-1H-pyrrole* (4h)**

^{1}H NMR (400 MHz, CDCl$_3$)

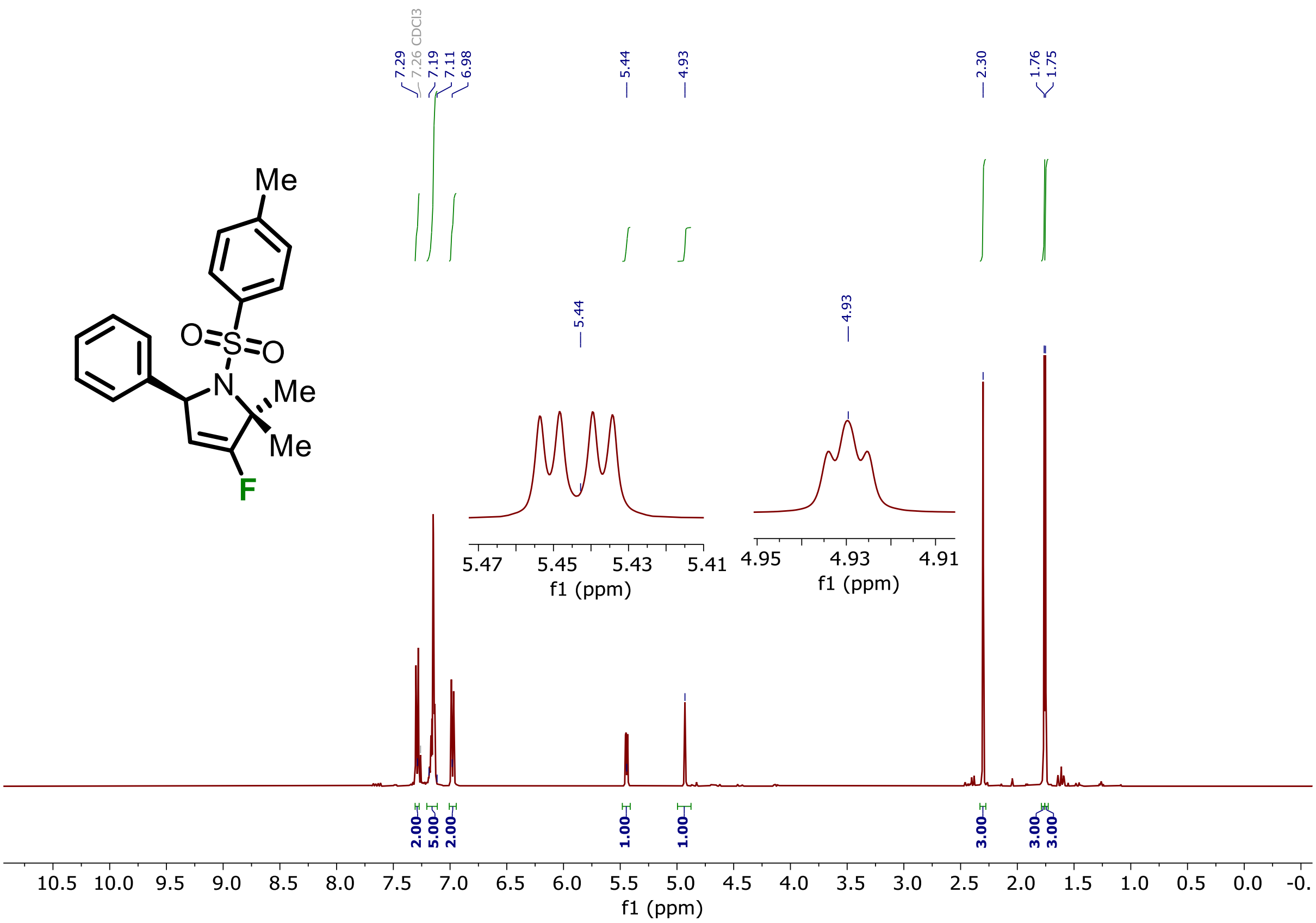

^{13}C NMR (101 MHz, CDCl$_3$)

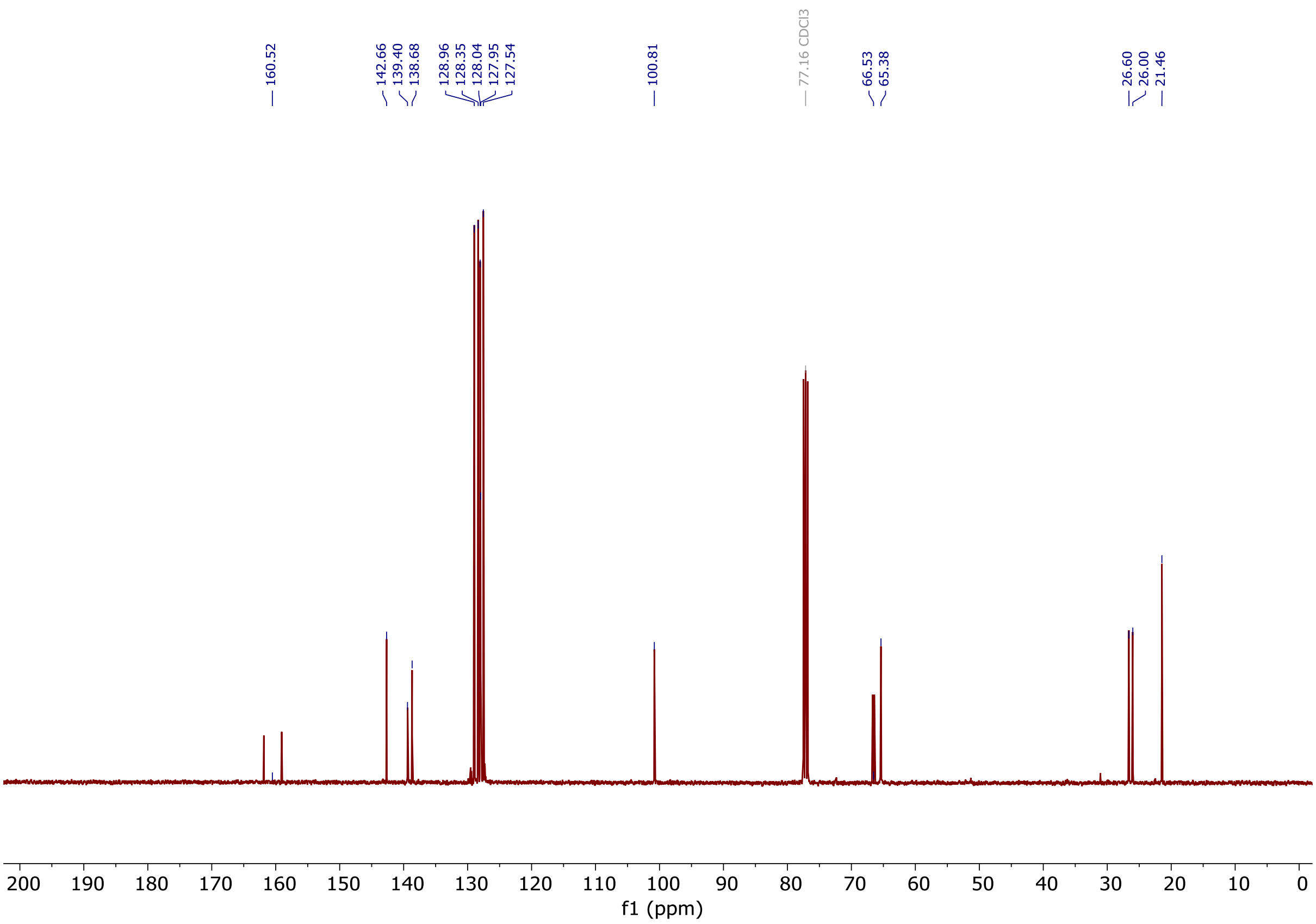

^{19}F NMR (376 MHz, $CDCl_3$)

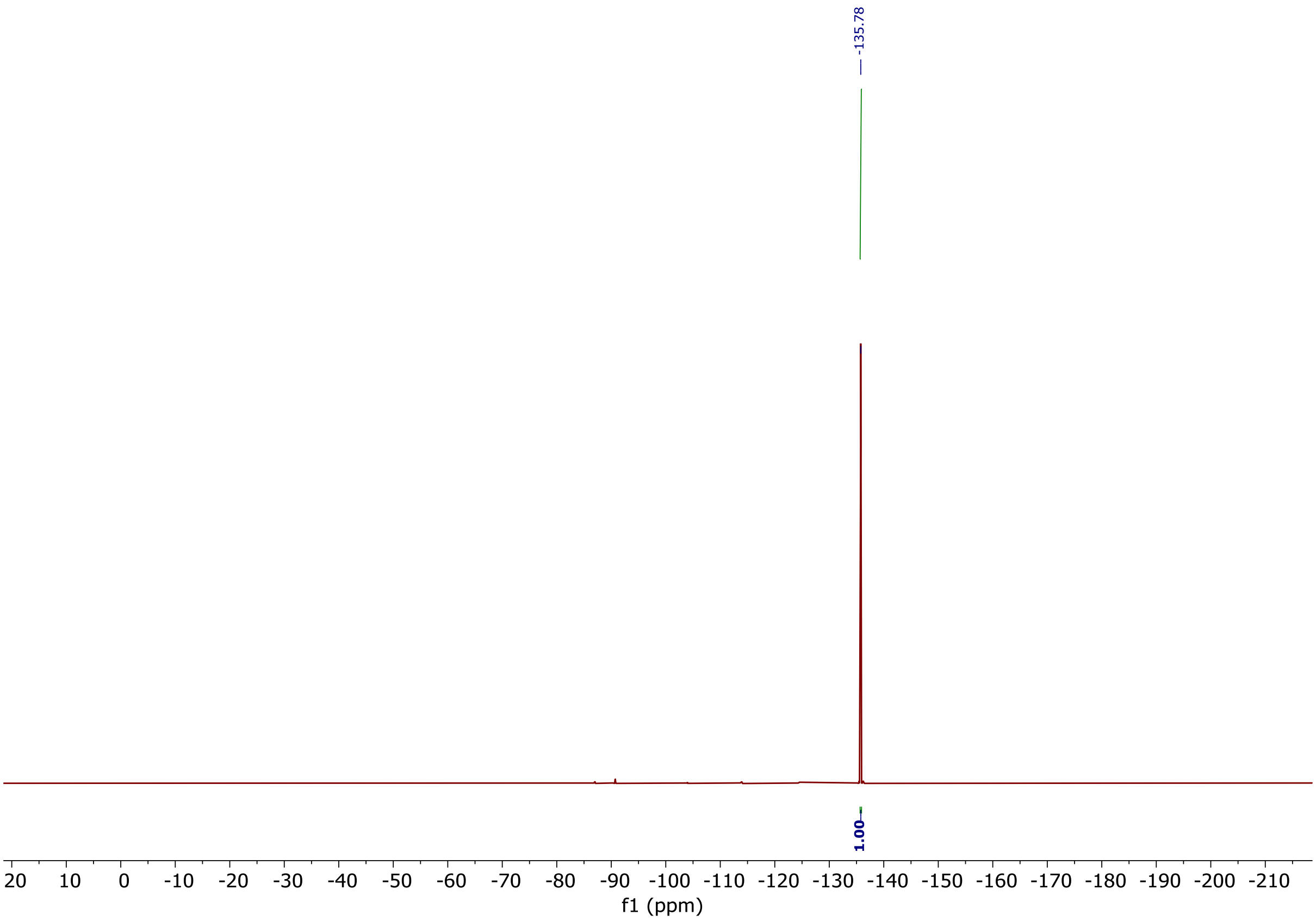

***4-fluoro-2-(4-fluorophenyl)-1-((4-nitrophenyl)sulfonyl)-2,5-dihydro-1H-pyrrole* (4i)**

^{1}H NMR (500 MHz, $CDCl_3$)

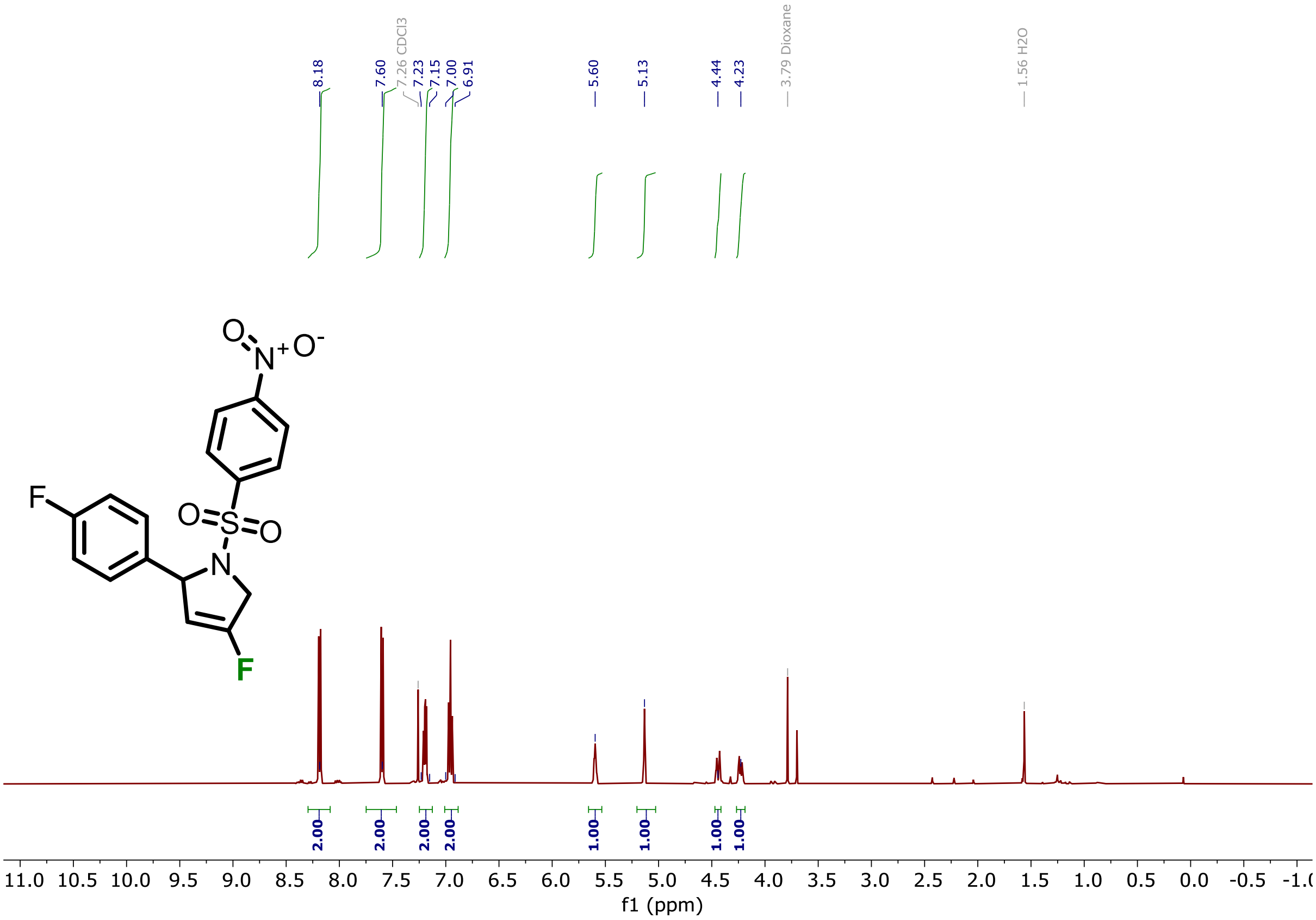

^{13}C NMR (126 MHz, $CDCl_3$)

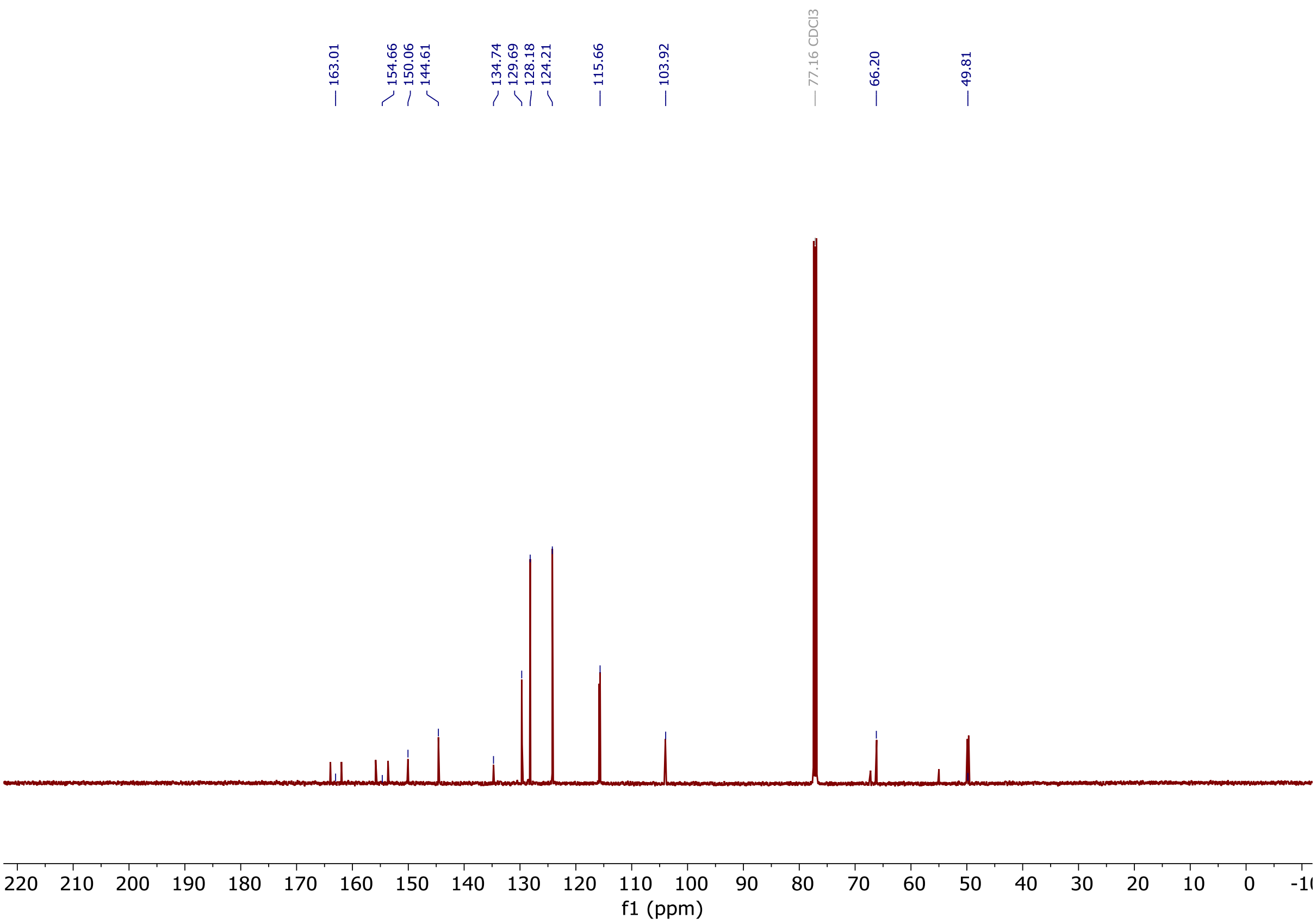

^{19}F NMR (376 MHz, CDCl$_3$)

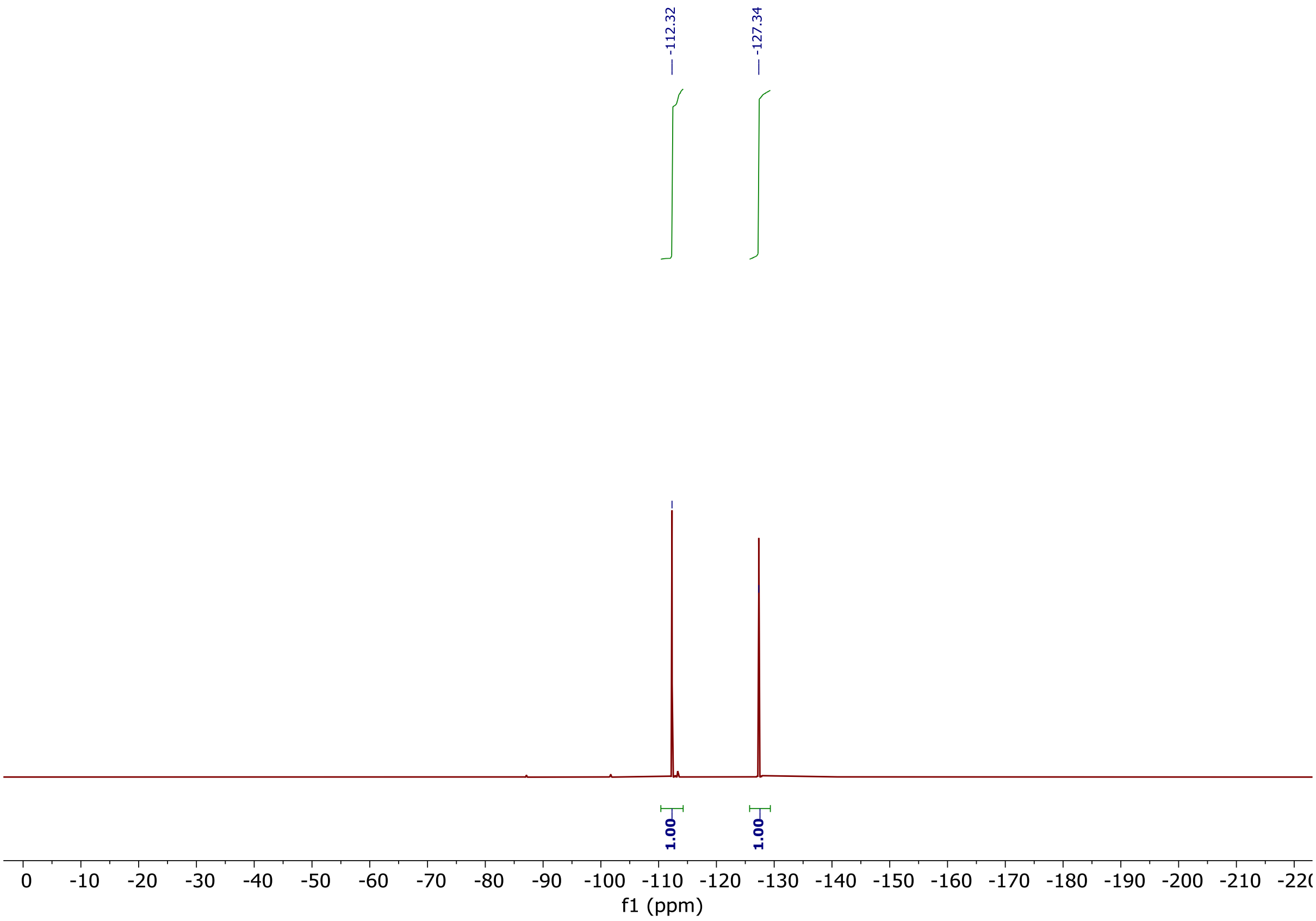

***cis*-3-fluoro-5-(4-fluorophenyl)-2-methyl-1-tosyl-2,5-dihydro-1*H*-pyrrole (4j)**

^{1}H NMR (400 MHz, $CDCl_3$)

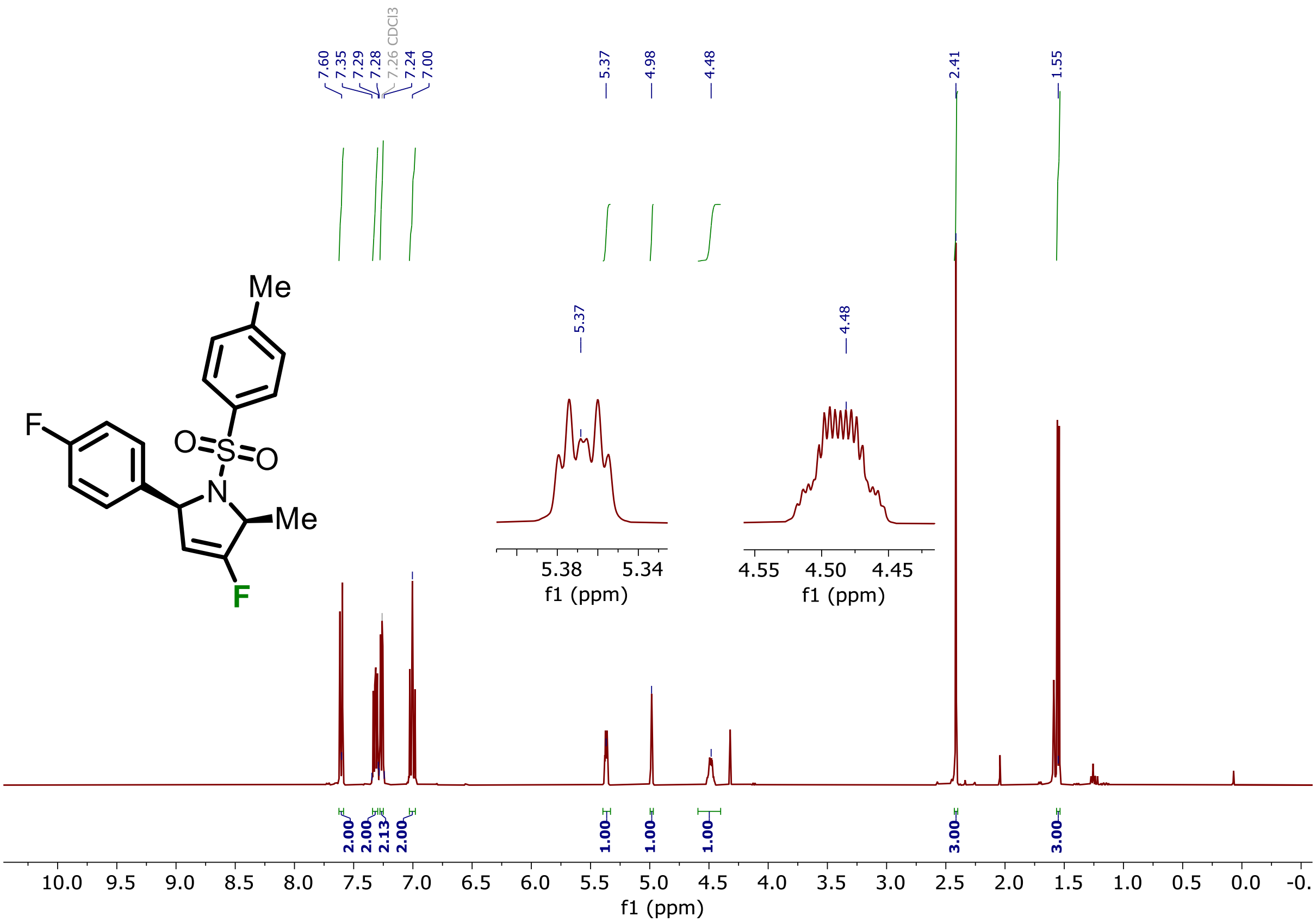

^{13}C NMR (126 MHz, $CDCl_3$)

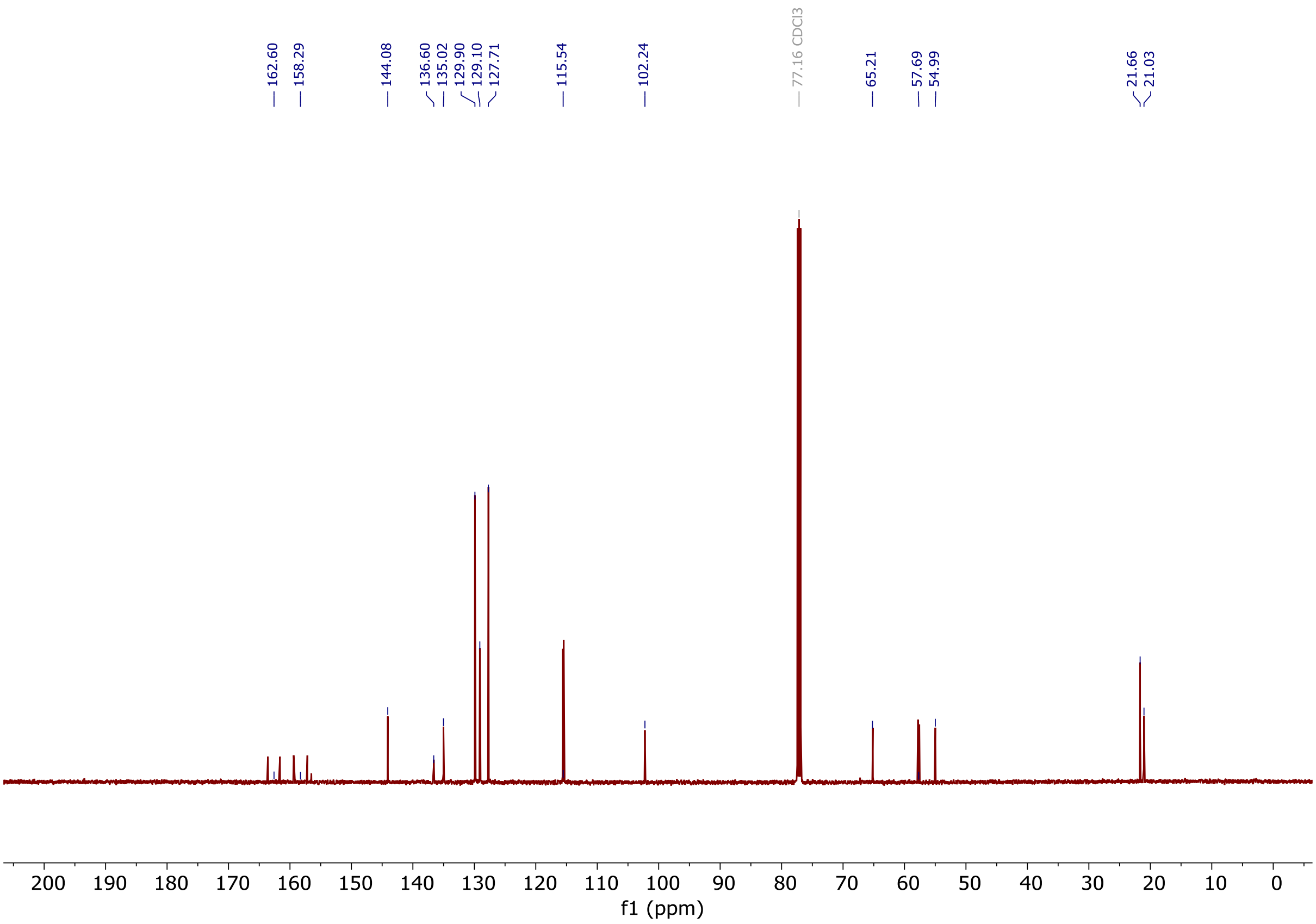

^{19}F NMR (376 MHz, $CDCl_3$)

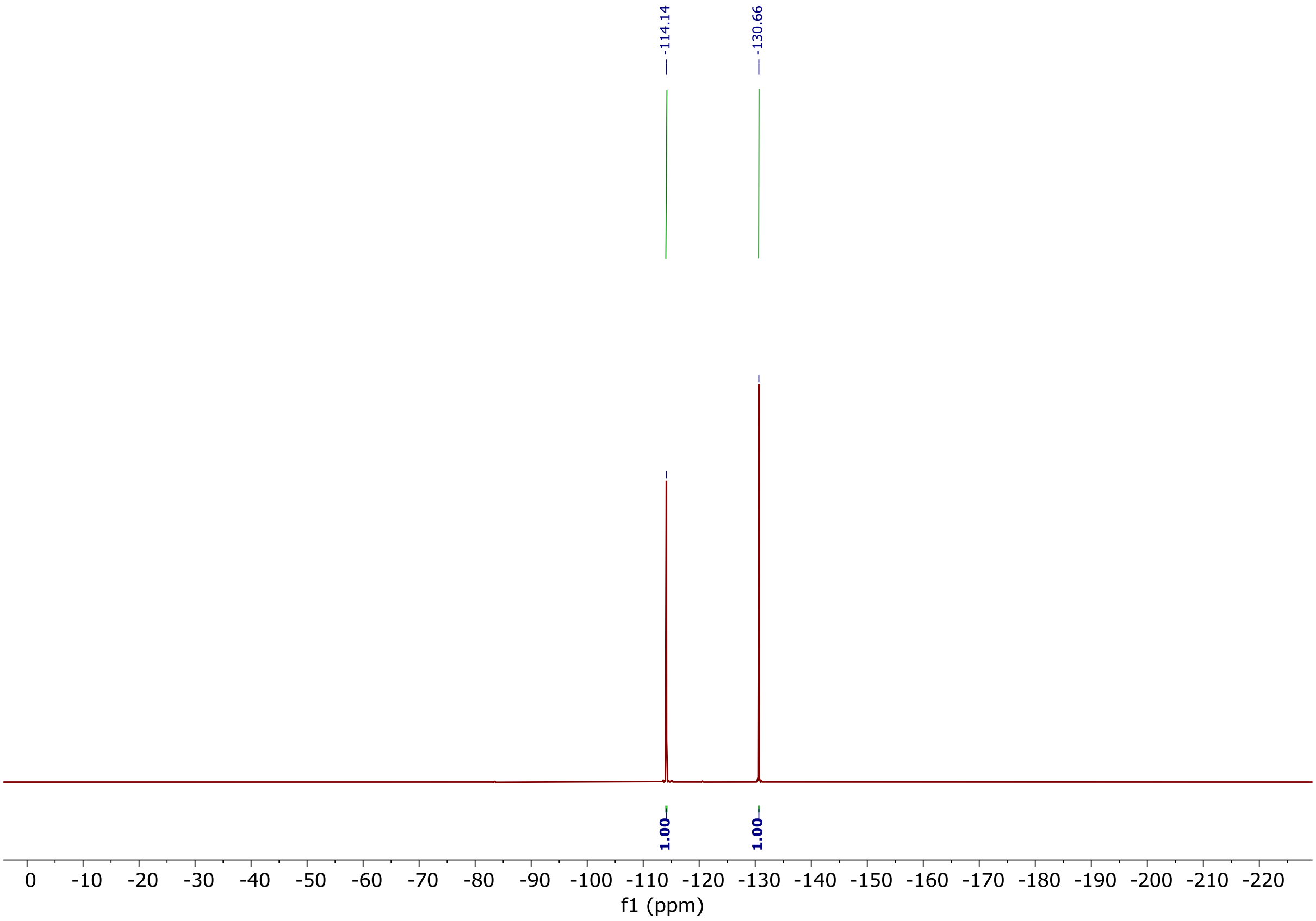

***cis*-3-fluoro-5-(4-fluorophenyl)-2-methyl-1-(methylsulfonyl)-2,5-dihydro-1*H*-pyrrole** **(4k)**

^{1}H NMR (500 MHz, CDCl$_3$)

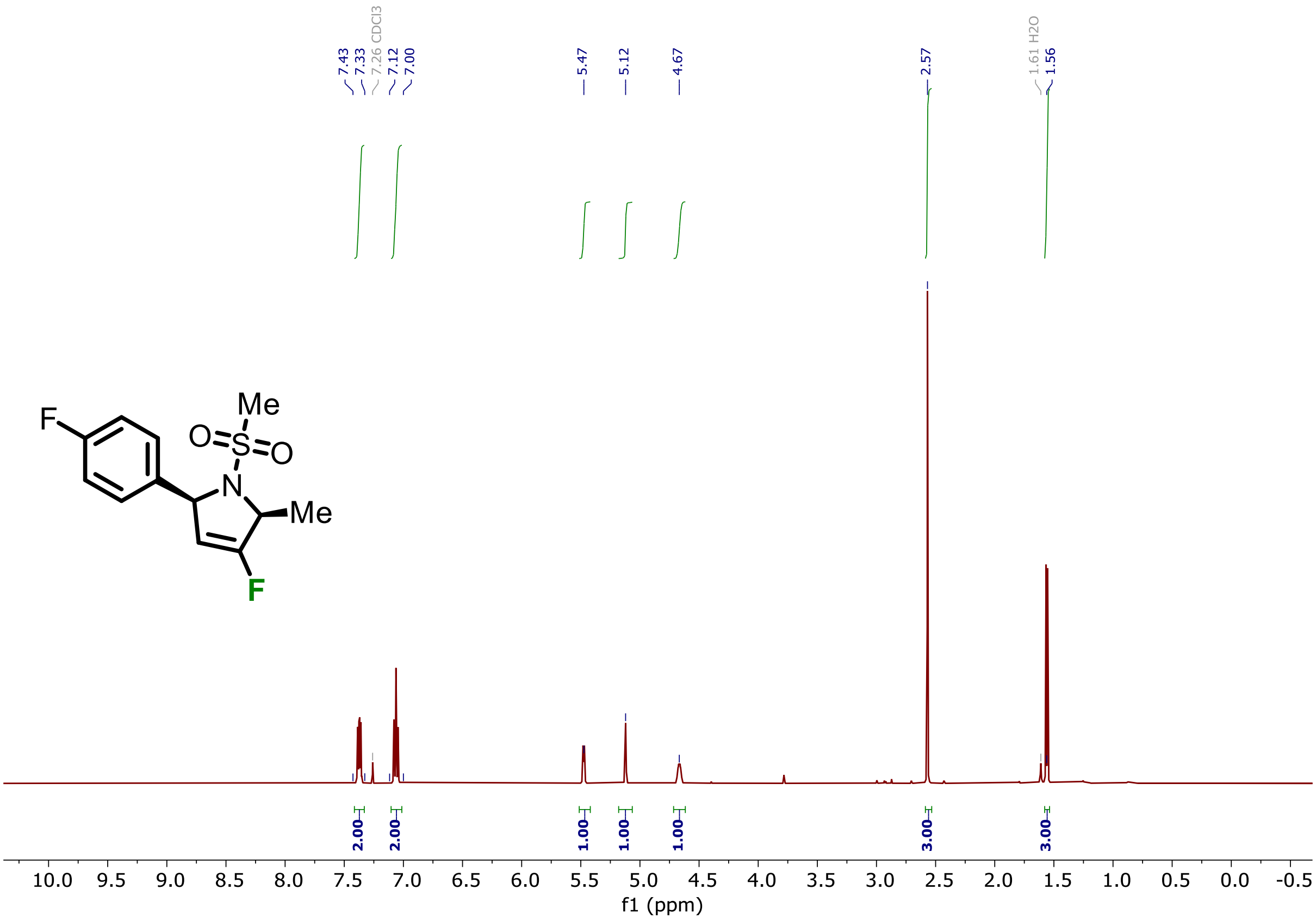

^{13}C NMR (126 MHz, CDCl$_3$)

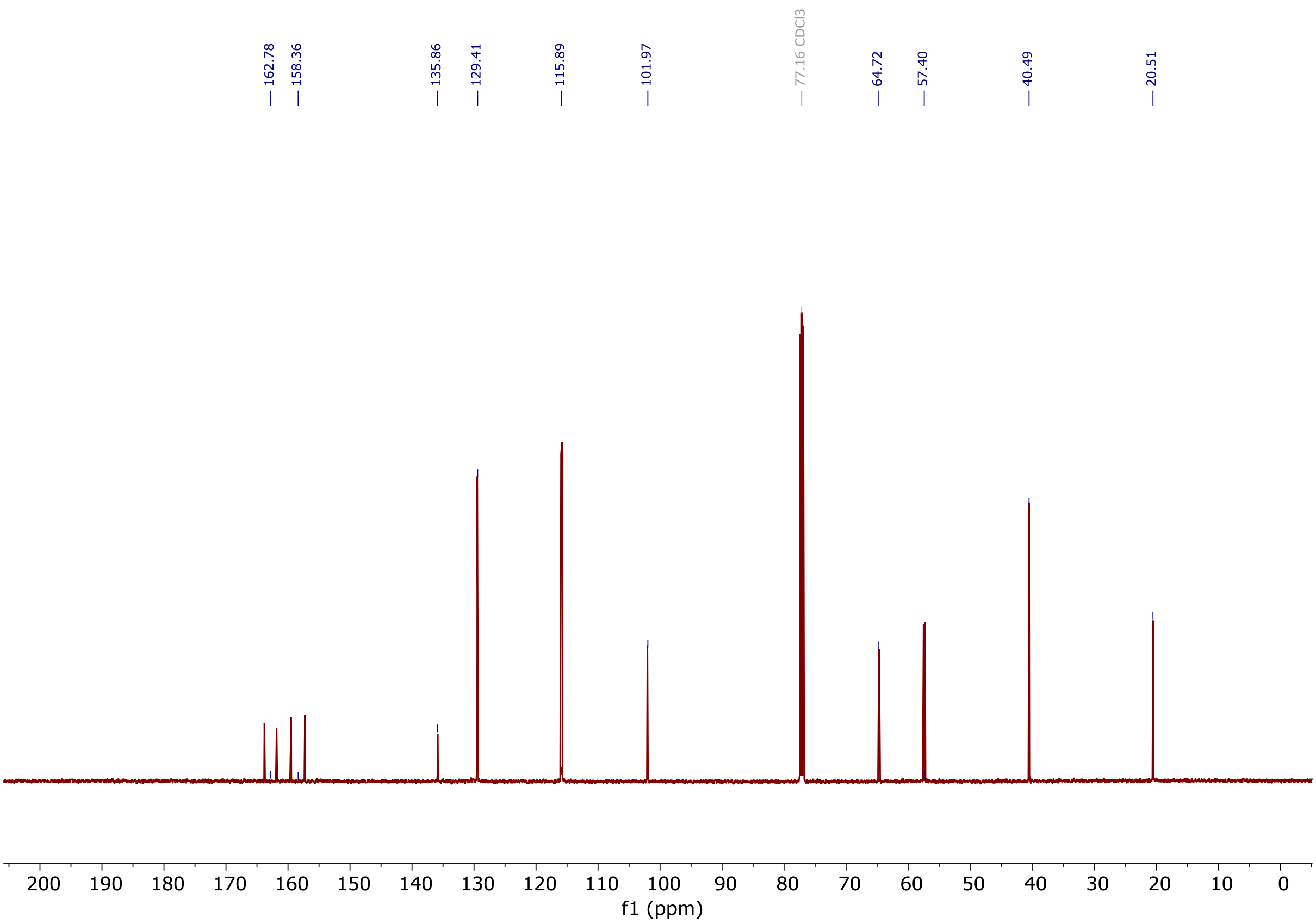

^{19}F NMR (471 MHz, $CDCl_3$)

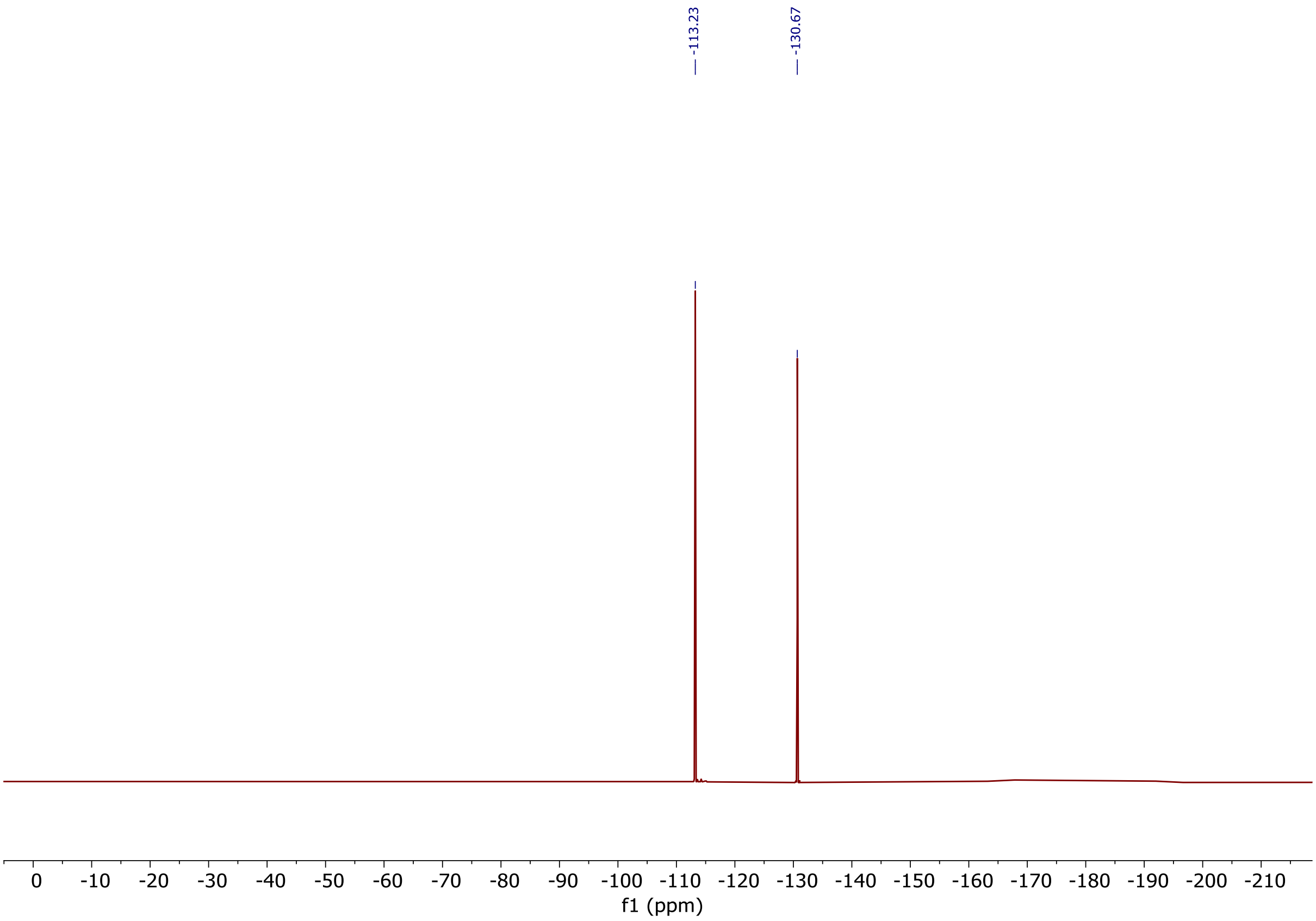

***cis*-5-(4-bromophenyl)-3-fluoro-2-methyl-1-tosyl-2,5-dihydro-1H-pyrrole** **(4l)**

^{1}H NMR (500 MHz, CDCl$_3$)

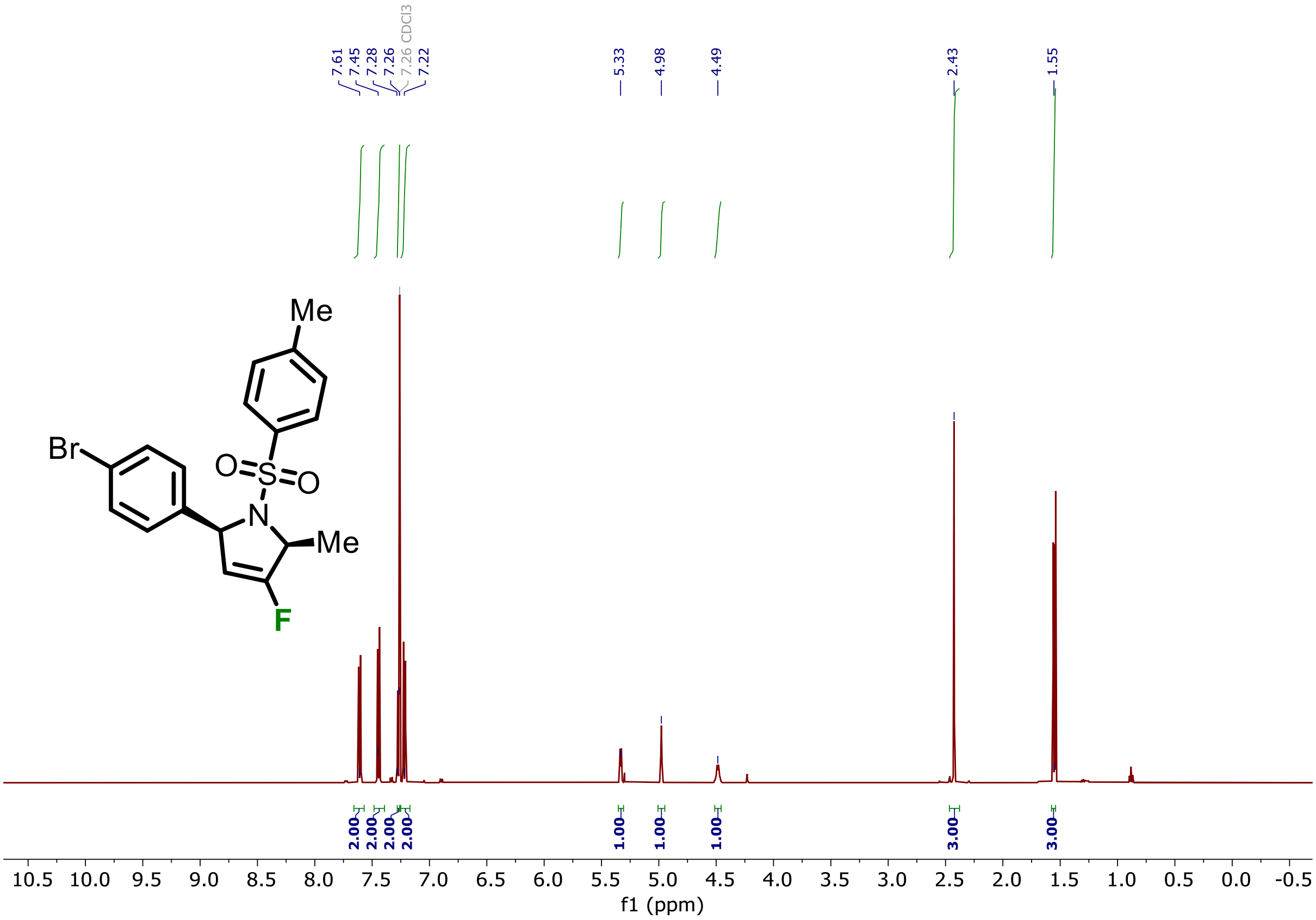

^{13}C NMR (126 MHz, CDCl$_3$)

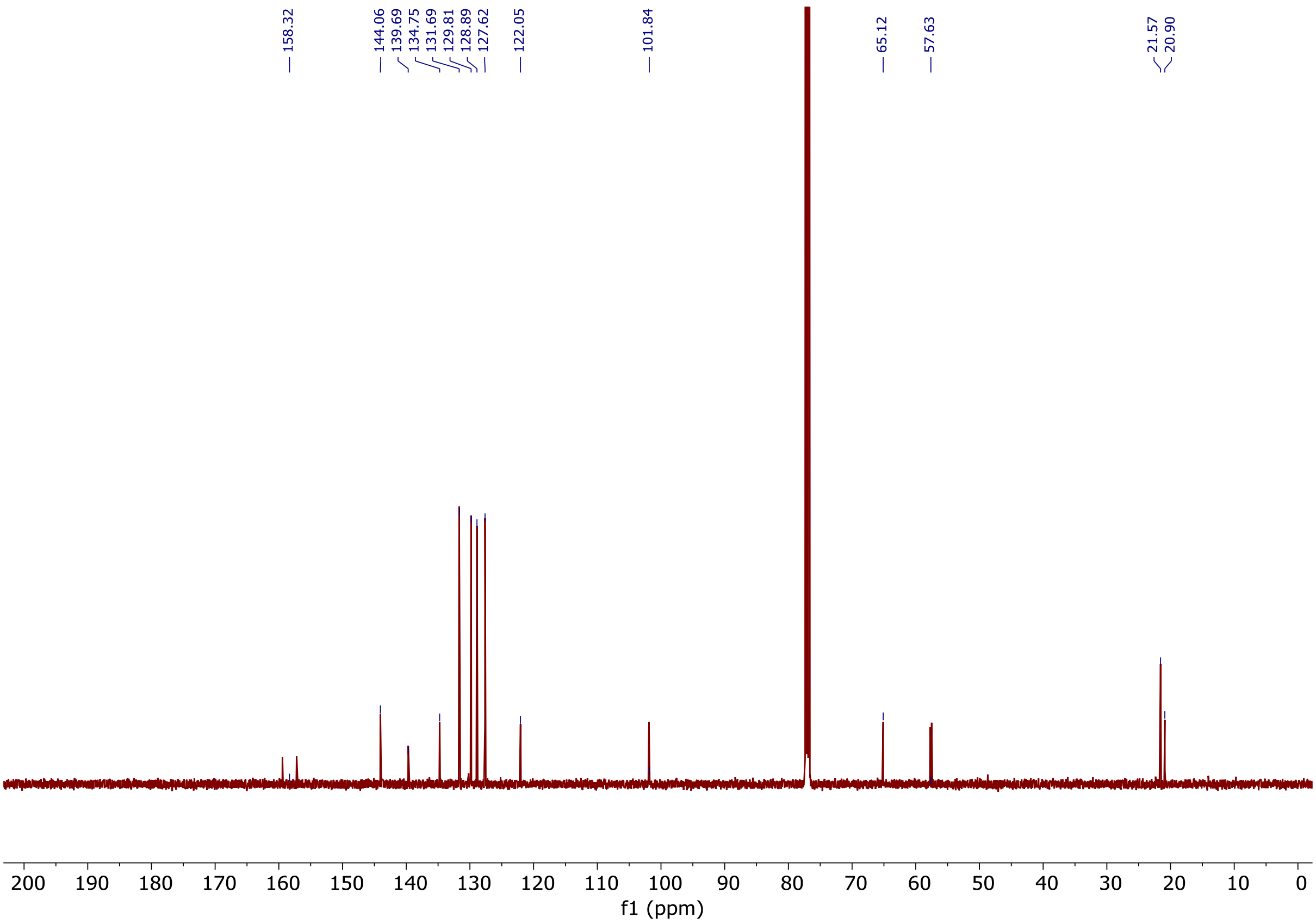

^{19}F NMR (471 MHz, $CDCl_3$)

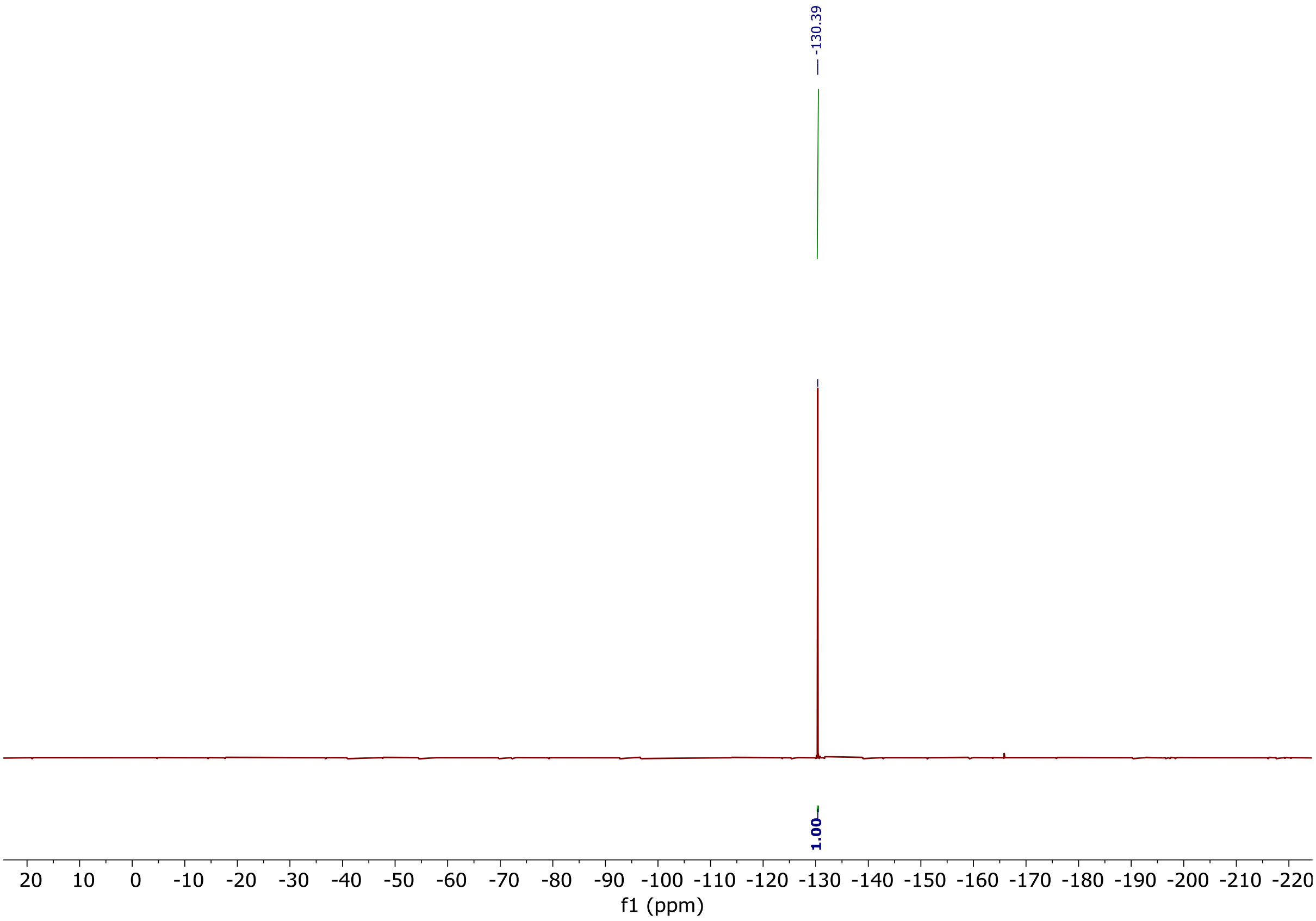

***cis-3-fluoro-2-methyl-5-(naphthalen-2-yl)-1-tosyl-2,5-dihydro-1H-pyrrole* (4m)**
^{1}H NMR (500 MHz, $CDCl_3$)

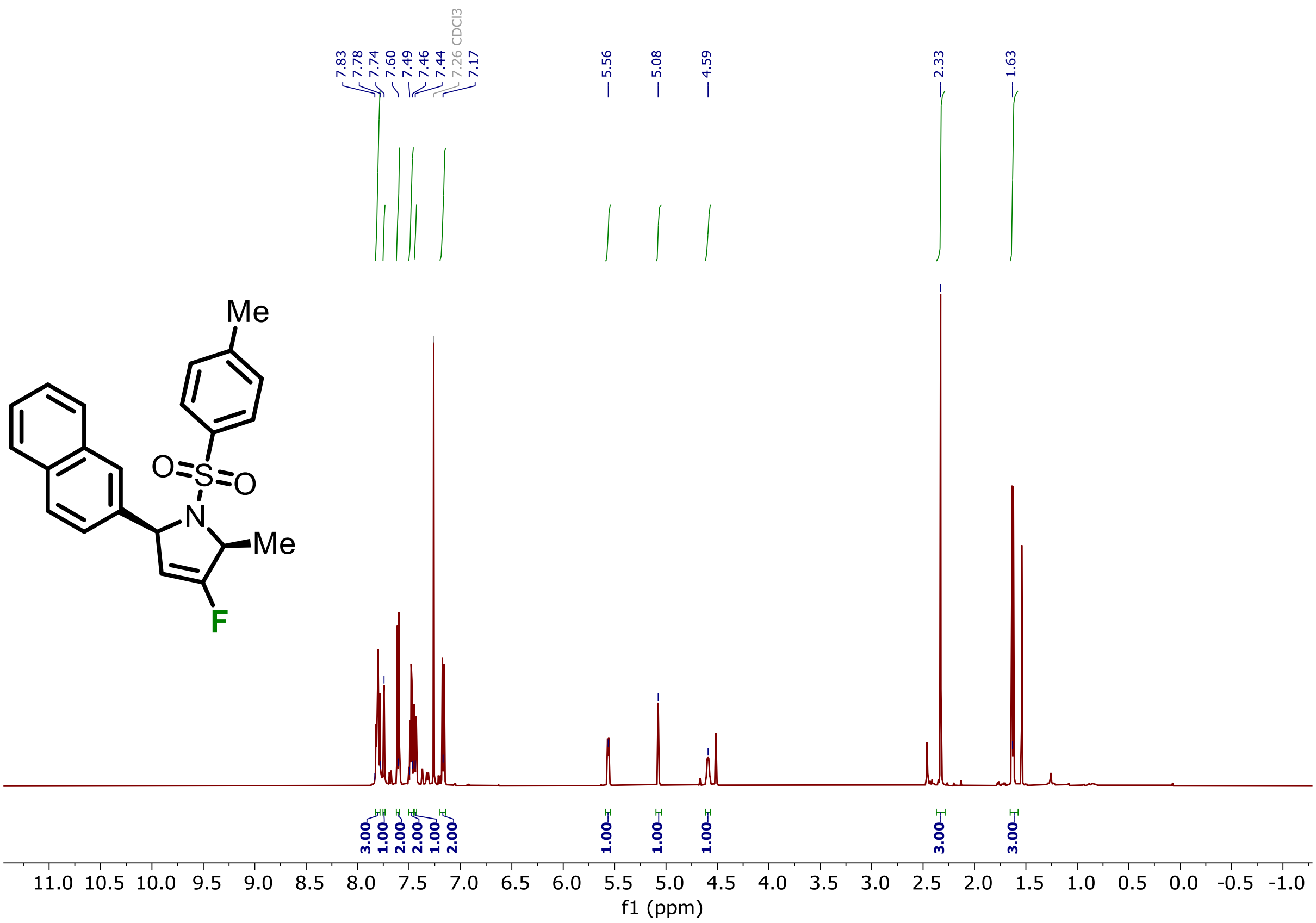

^{13}C NMR (126 MHz, CDCl$_3$)

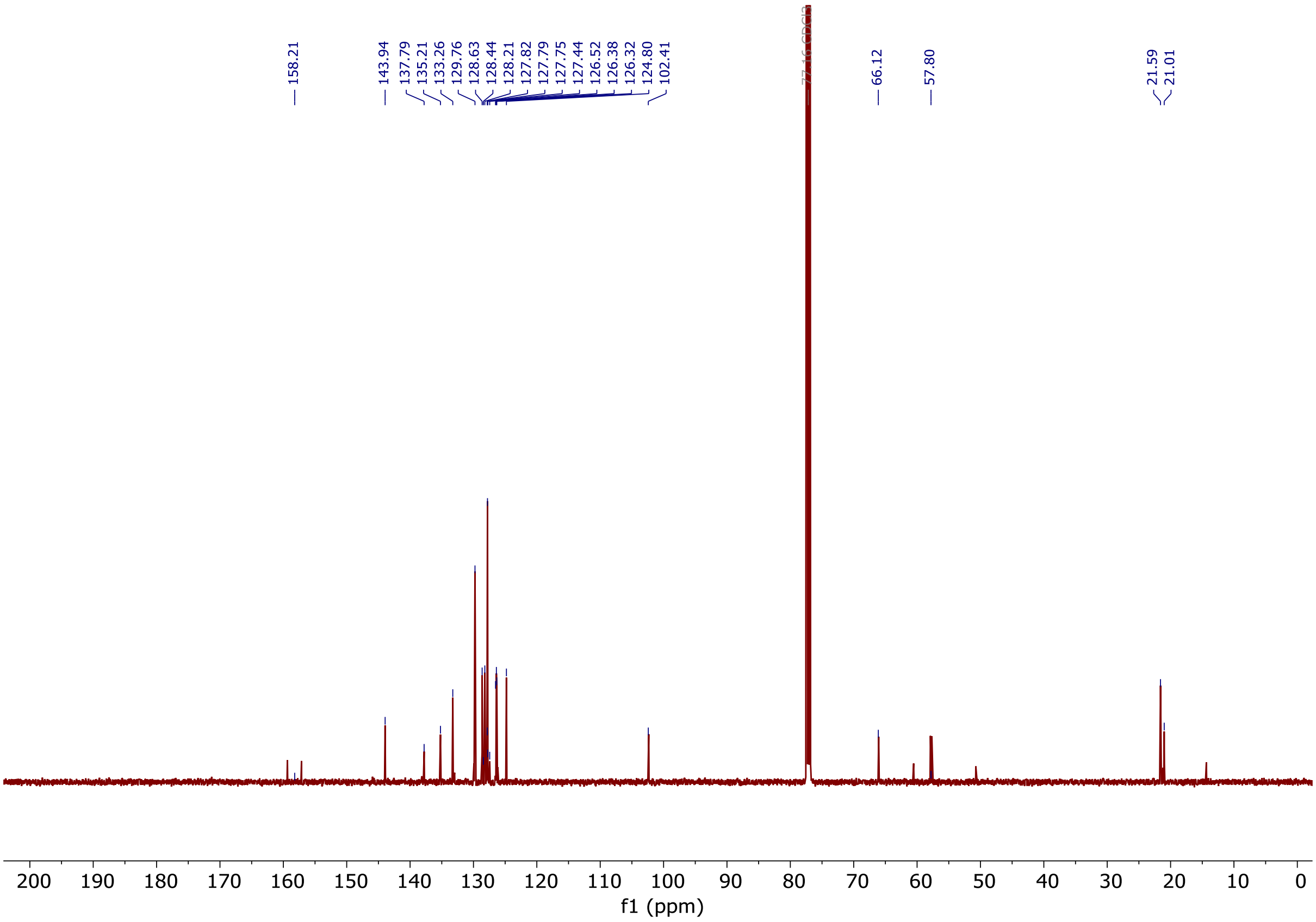

^{19}F NMR (471 MHz, CDCl$_3$)

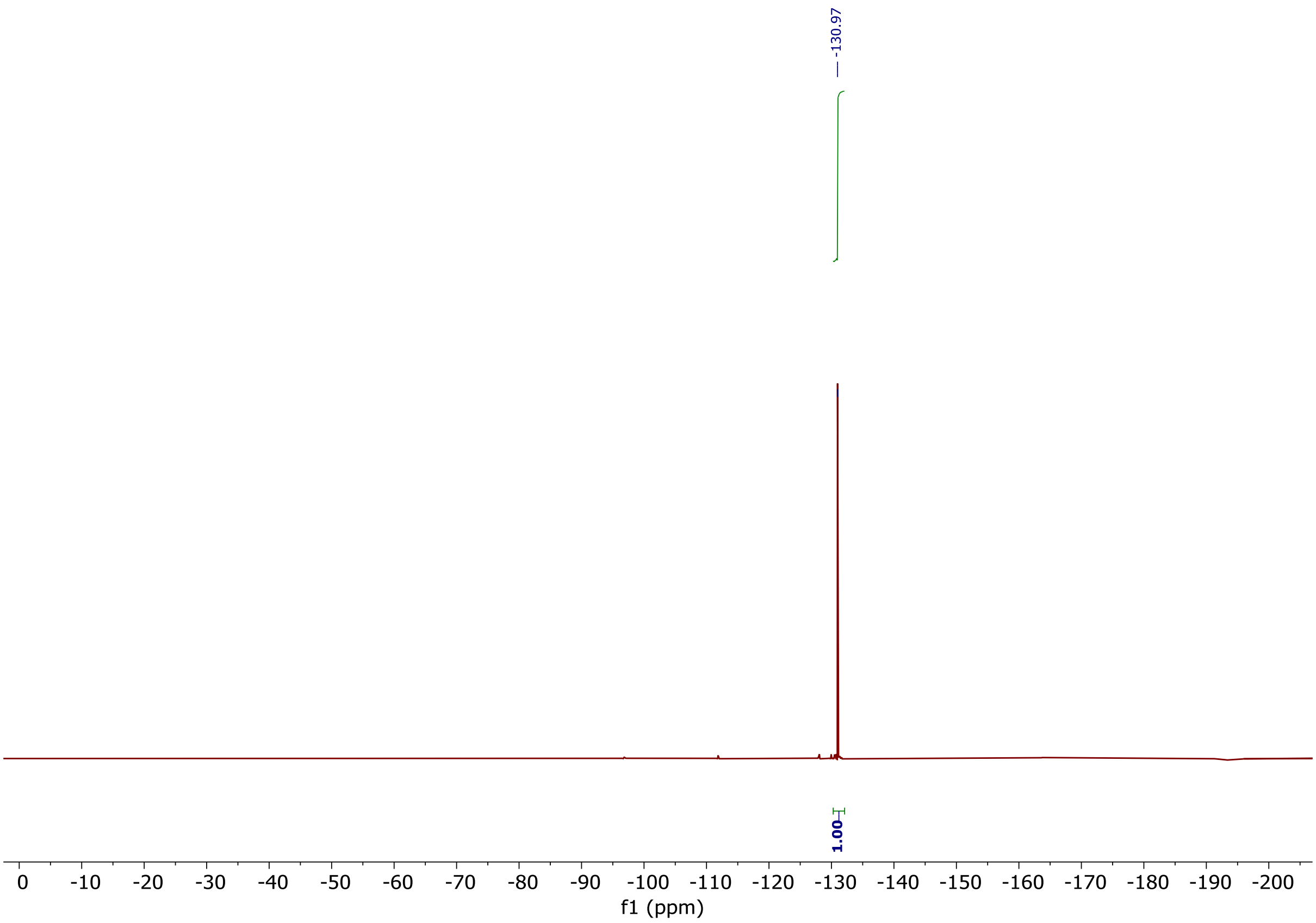

***cis-3-fluoro-5-(4-fluorophenyl)-2-phenyl-1-tosyl-2,5-dihydro-1H-pyrrole* (4n)**

^{1}H NMR (500 MHz, $CDCl_3$)

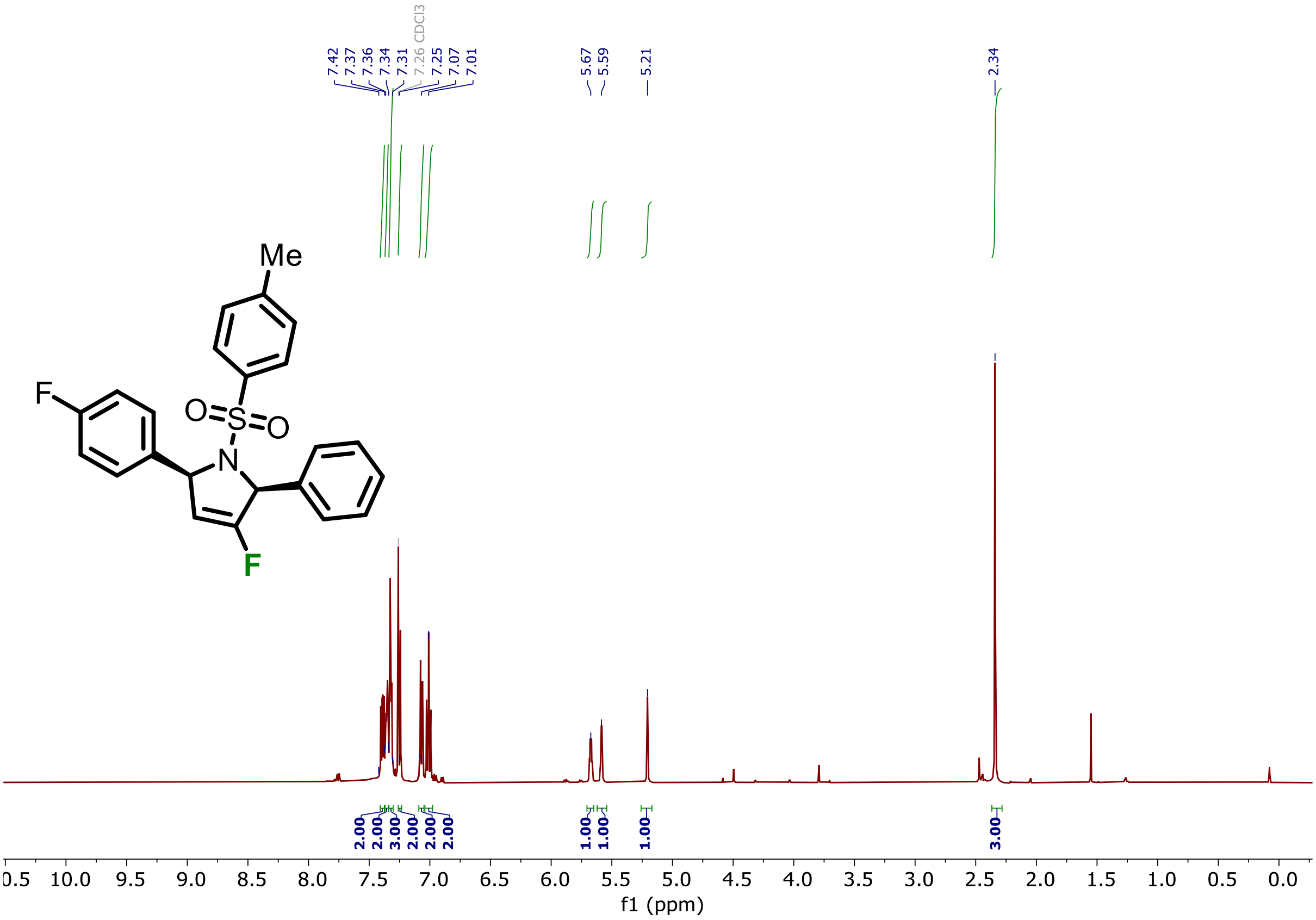

^{13}C NMR (126 MHz, $CDCl_3$)

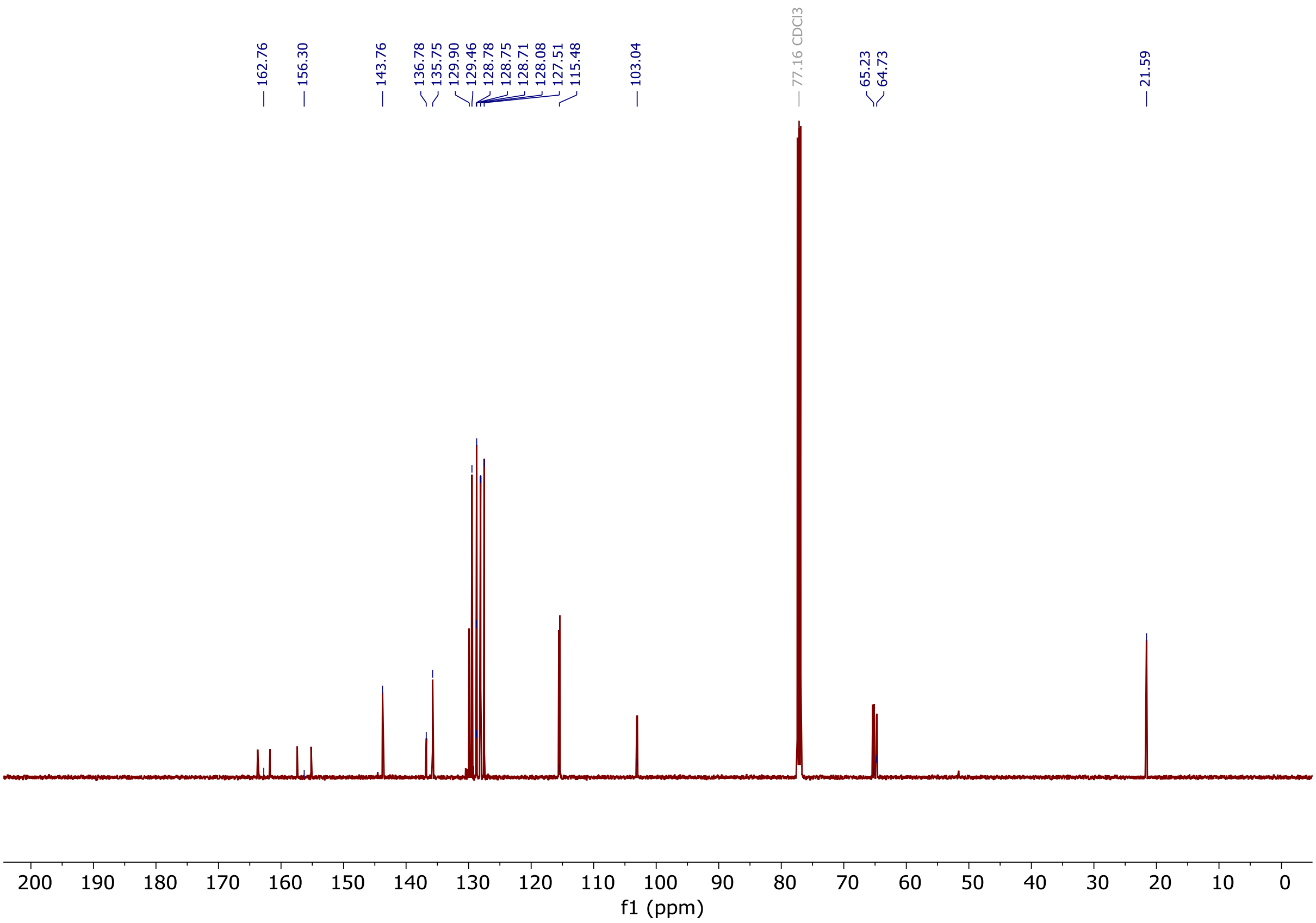

^{19}F NMR (471 MHz, CDCl$_3$)

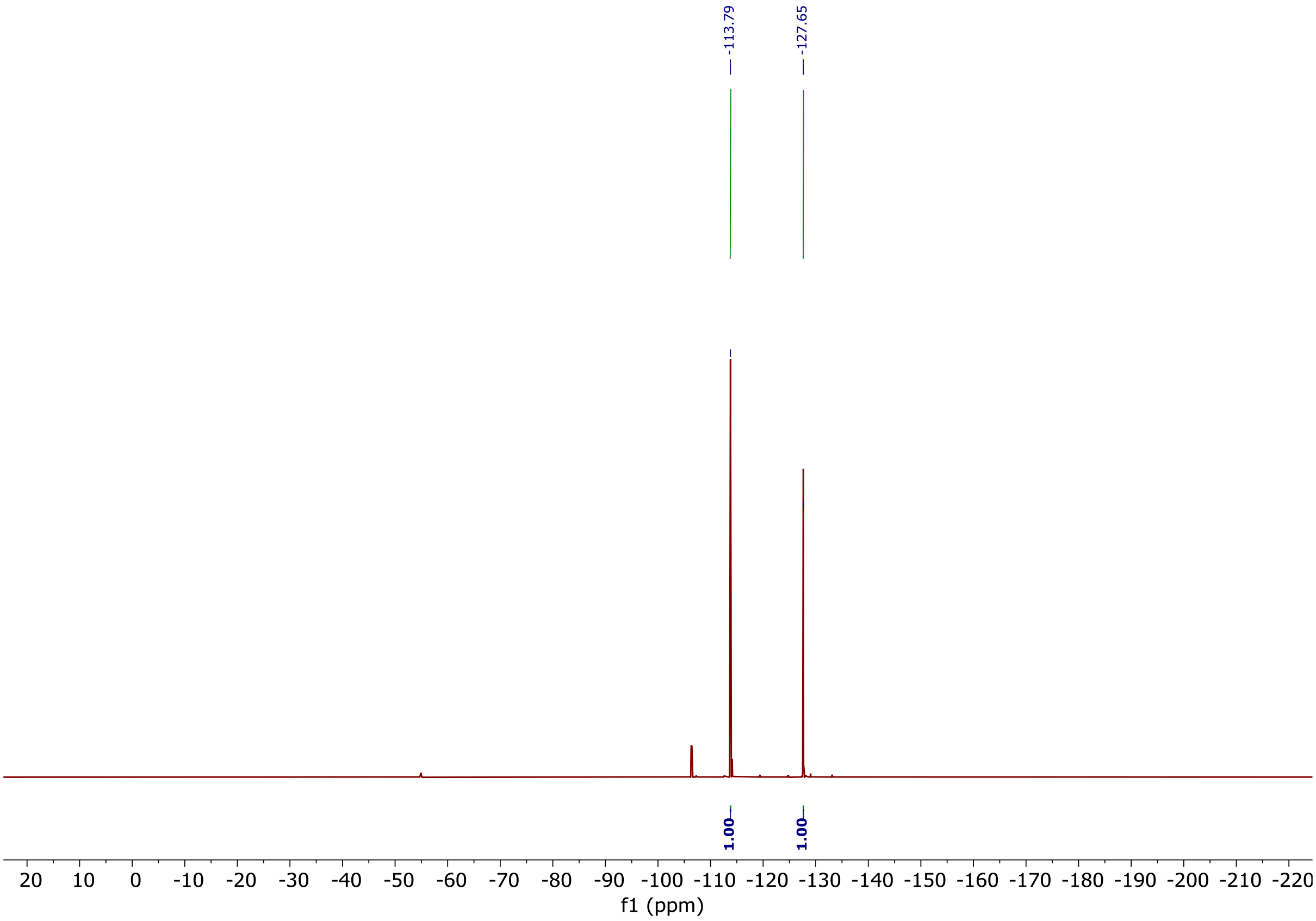

***4-fluoro-2,2-diphenyl-1-tosyl-2,5-dihydro-1H-pyrrole* (4o)**

^{1}H NMR (500 MHz, $CDCl_3$)

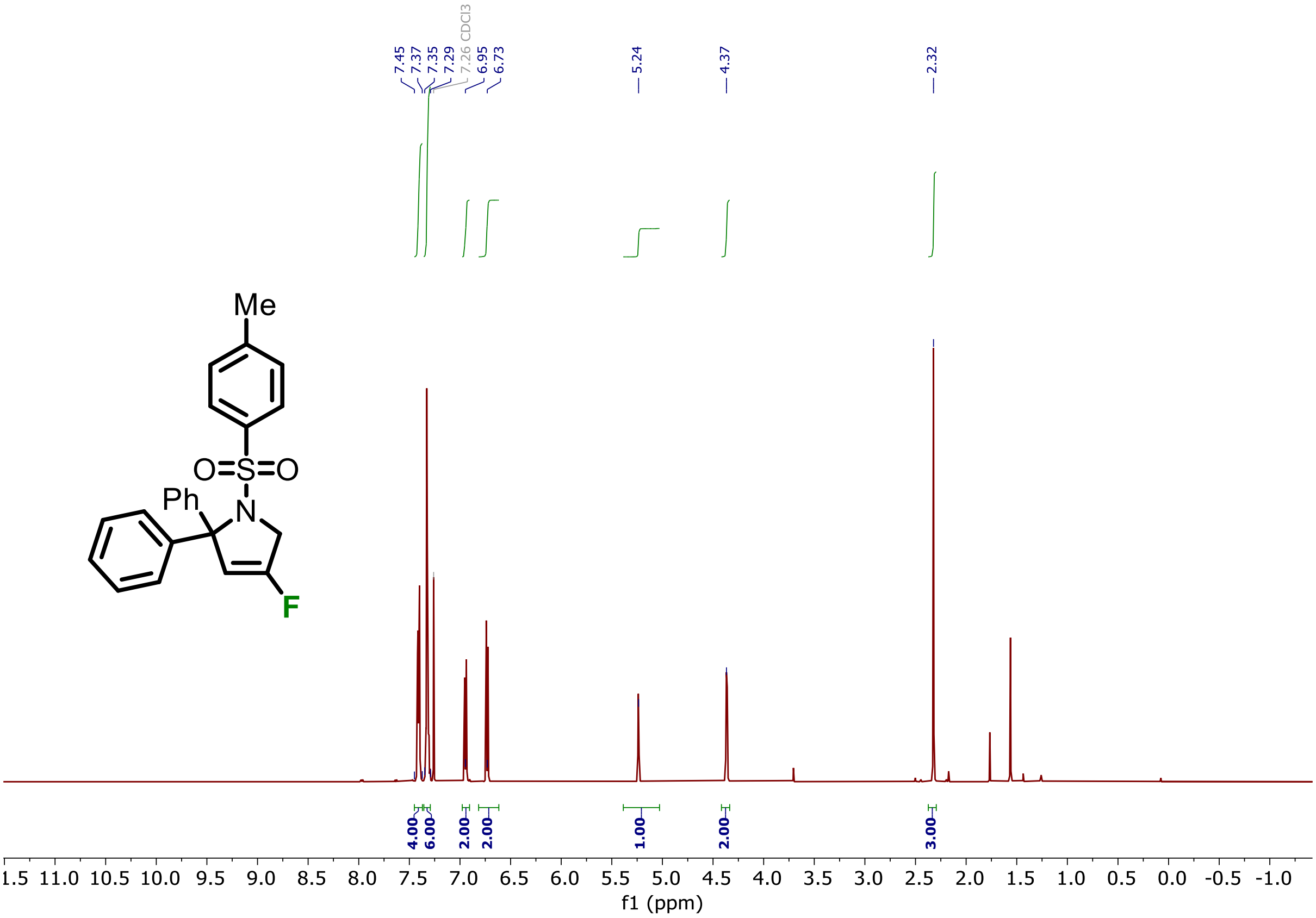

^{13}C NMR (126 MHz, $CDCl_3$)

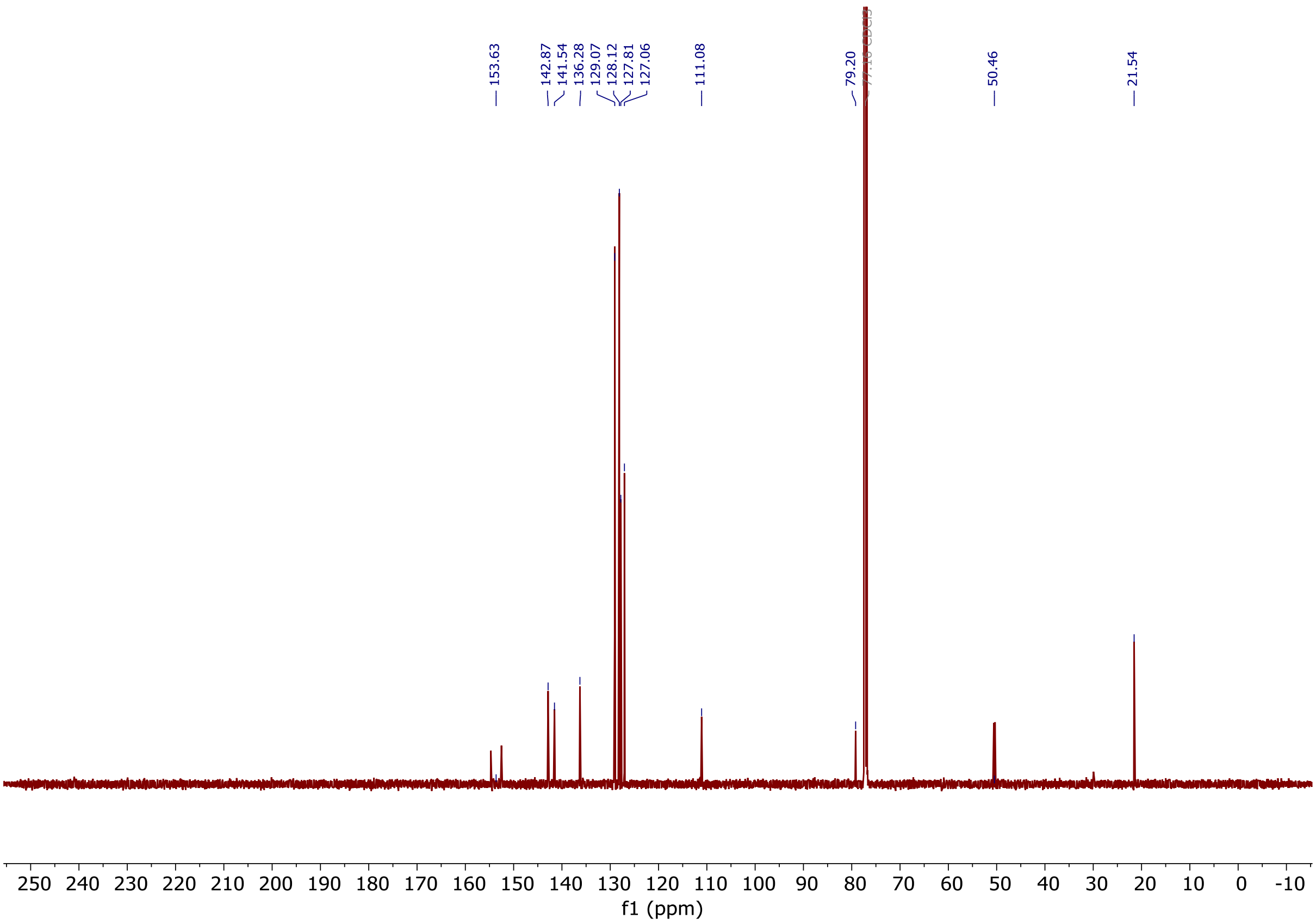

^{19}F NMR (471 MHz, CDCl$_3$)

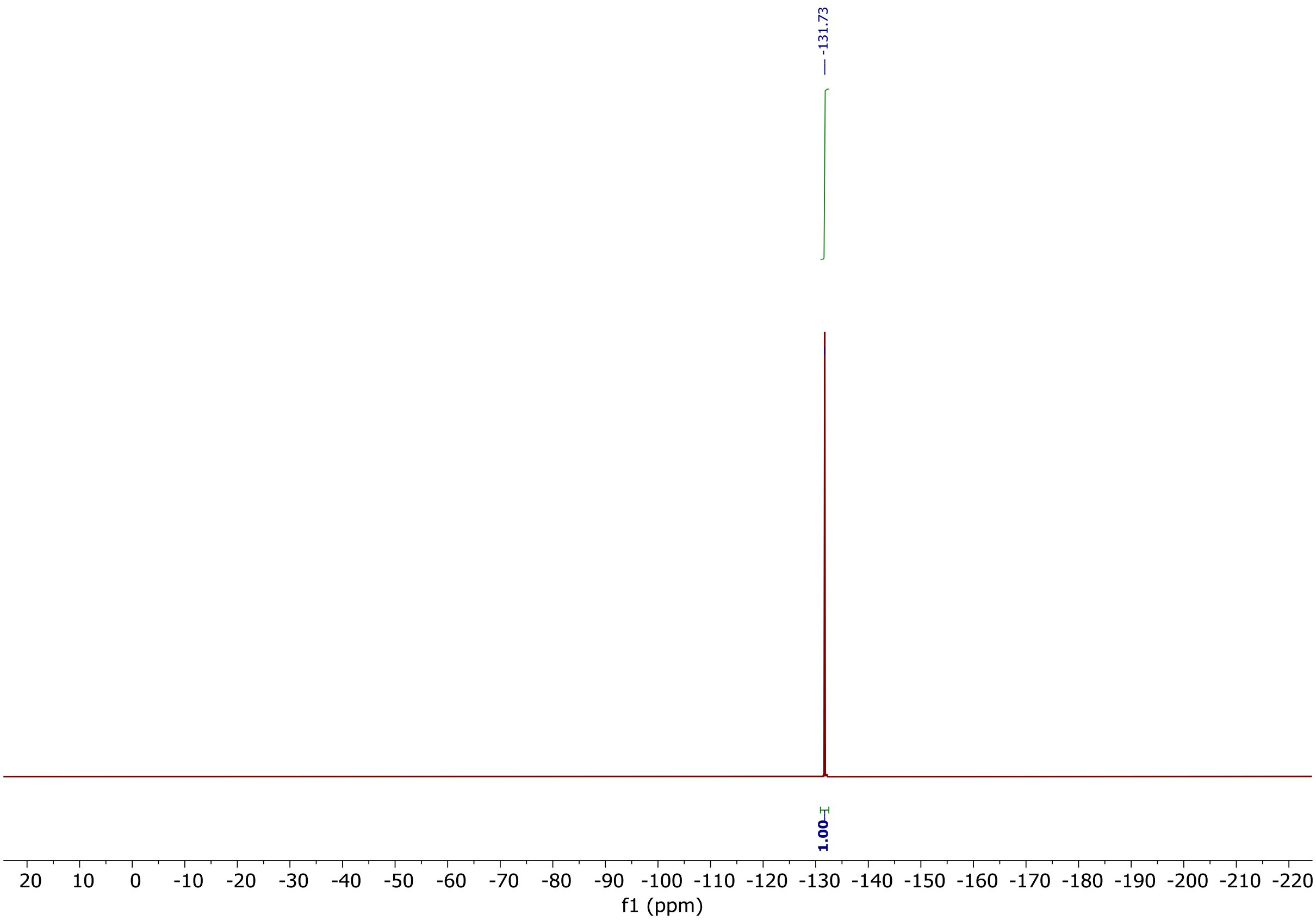

***4-fluoro-2-methyl-2-phenyl-1-tosyl-2,5-dihydro-1H-pyrrole* (4p)**

^{1}H NMR (500 MHz, CDCl$_3$)

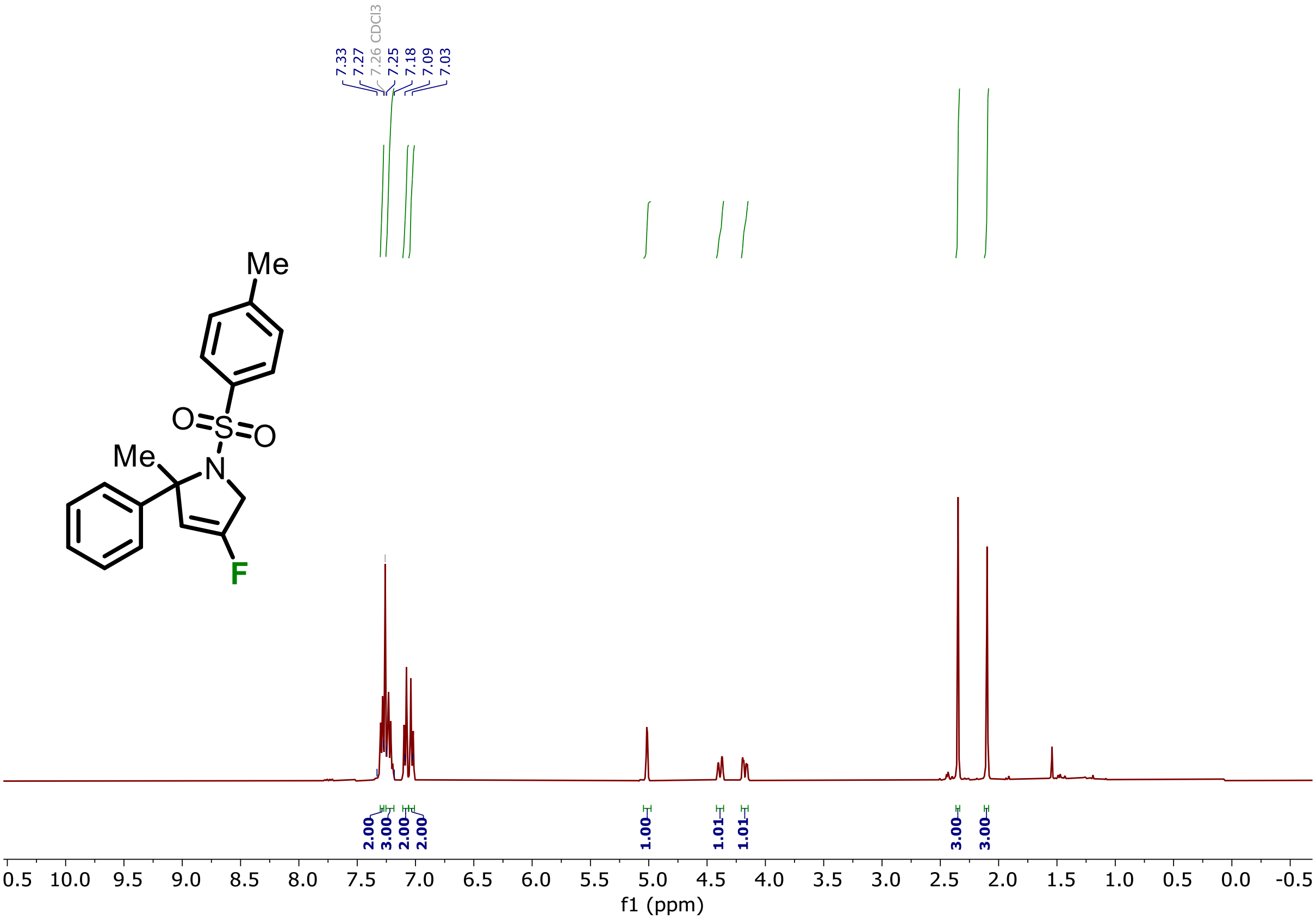

^{13}C NMR (151 MHz, CDCl$_3$)

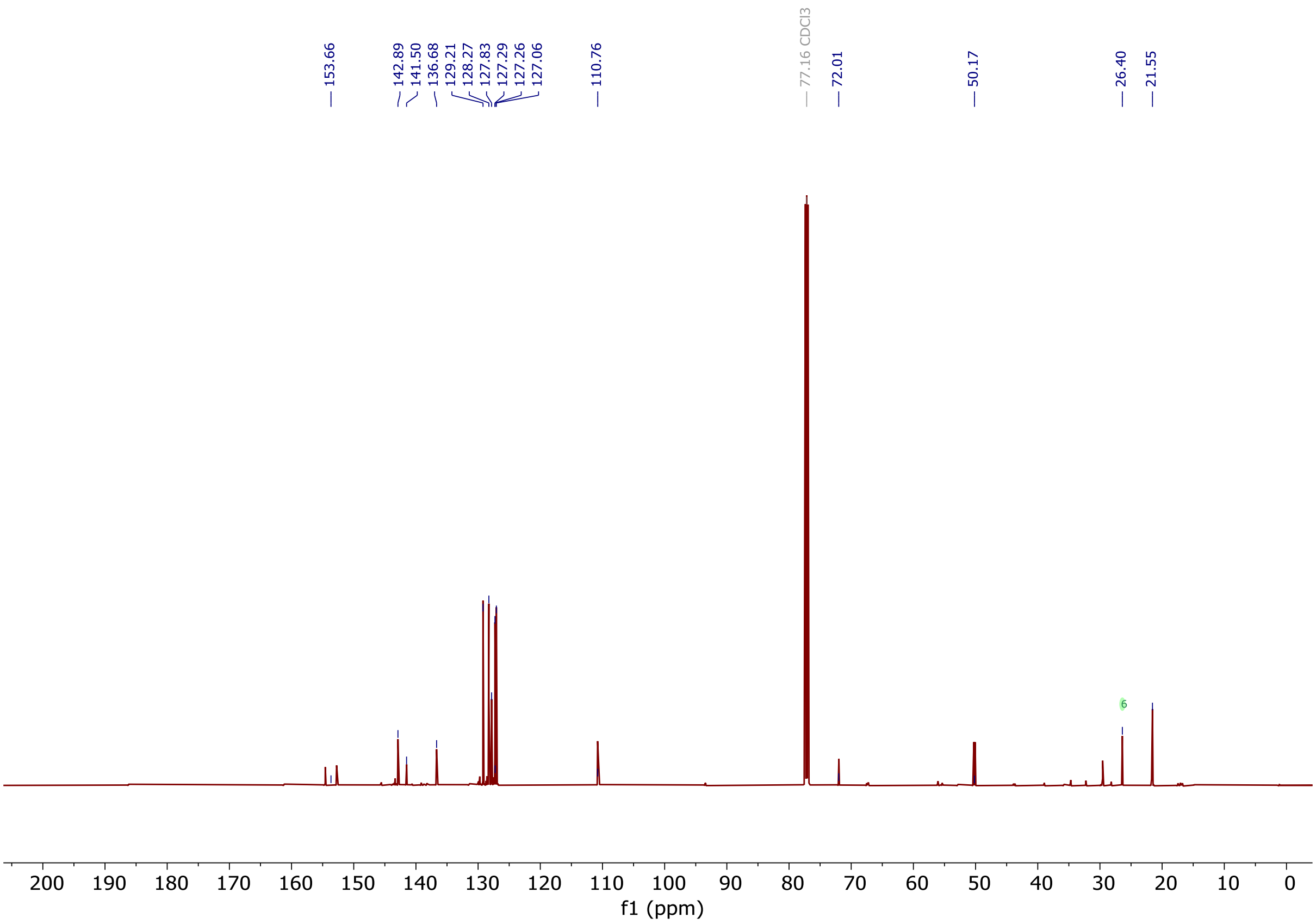

^{19}F NMR (376 MHz, $CDCl_3$)

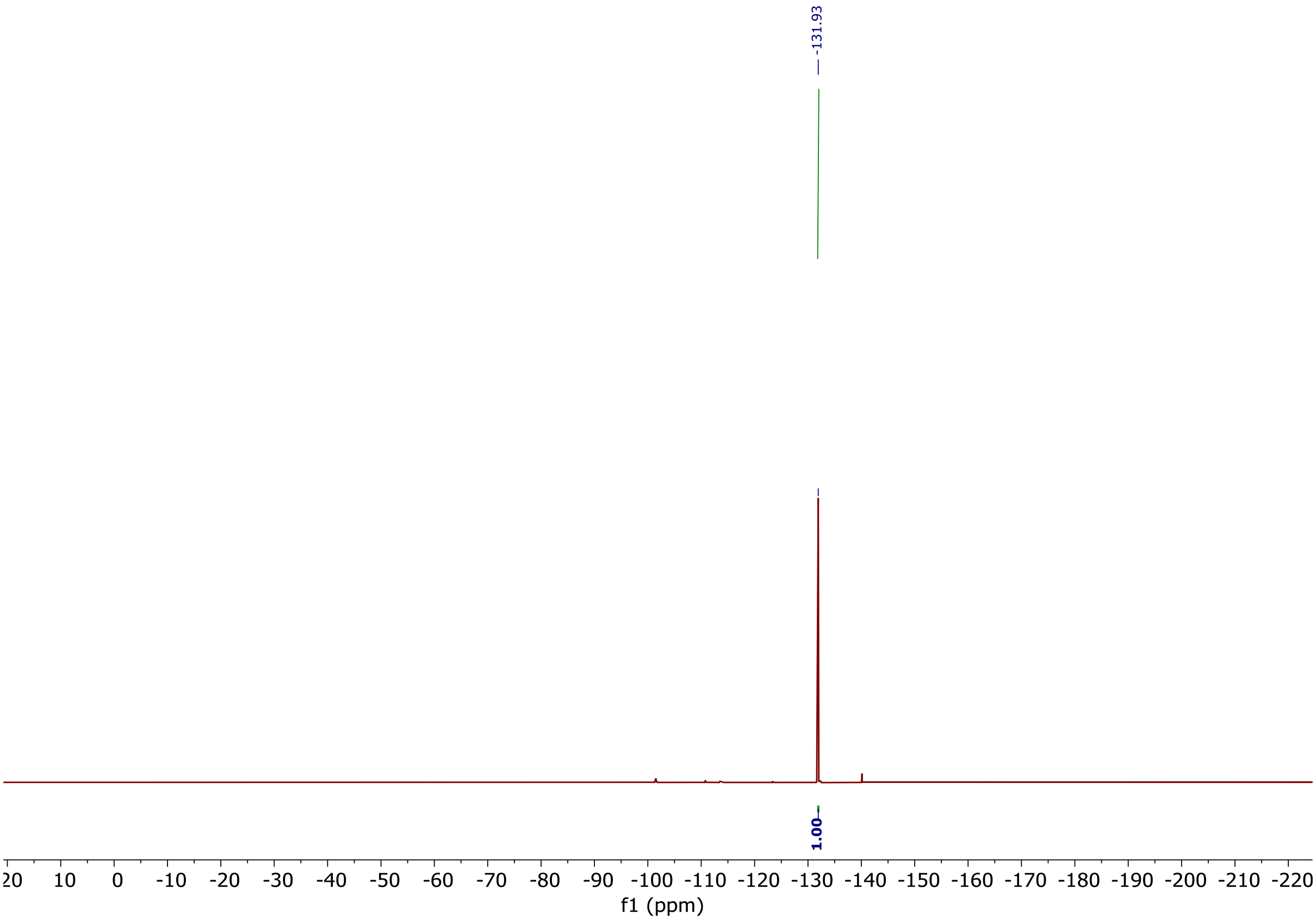

***6-fluoro-4-tosyl-4-azaspiro[2.4]hept-6-ene* (4q)**

^{1}H NMR (500 MHz, CDCl$_3$)

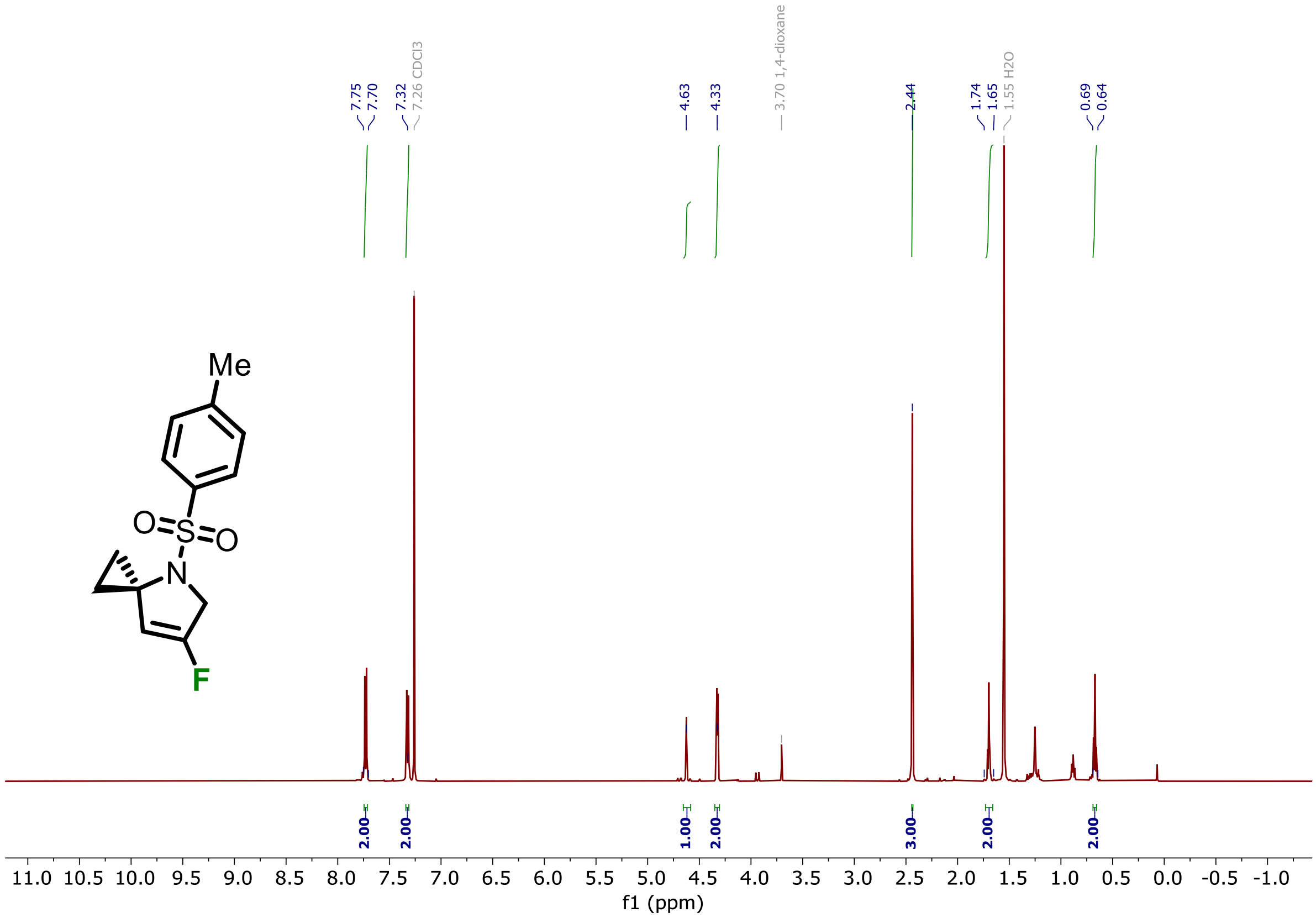

^{13}C NMR (126 MHz, CDCl$_3$)

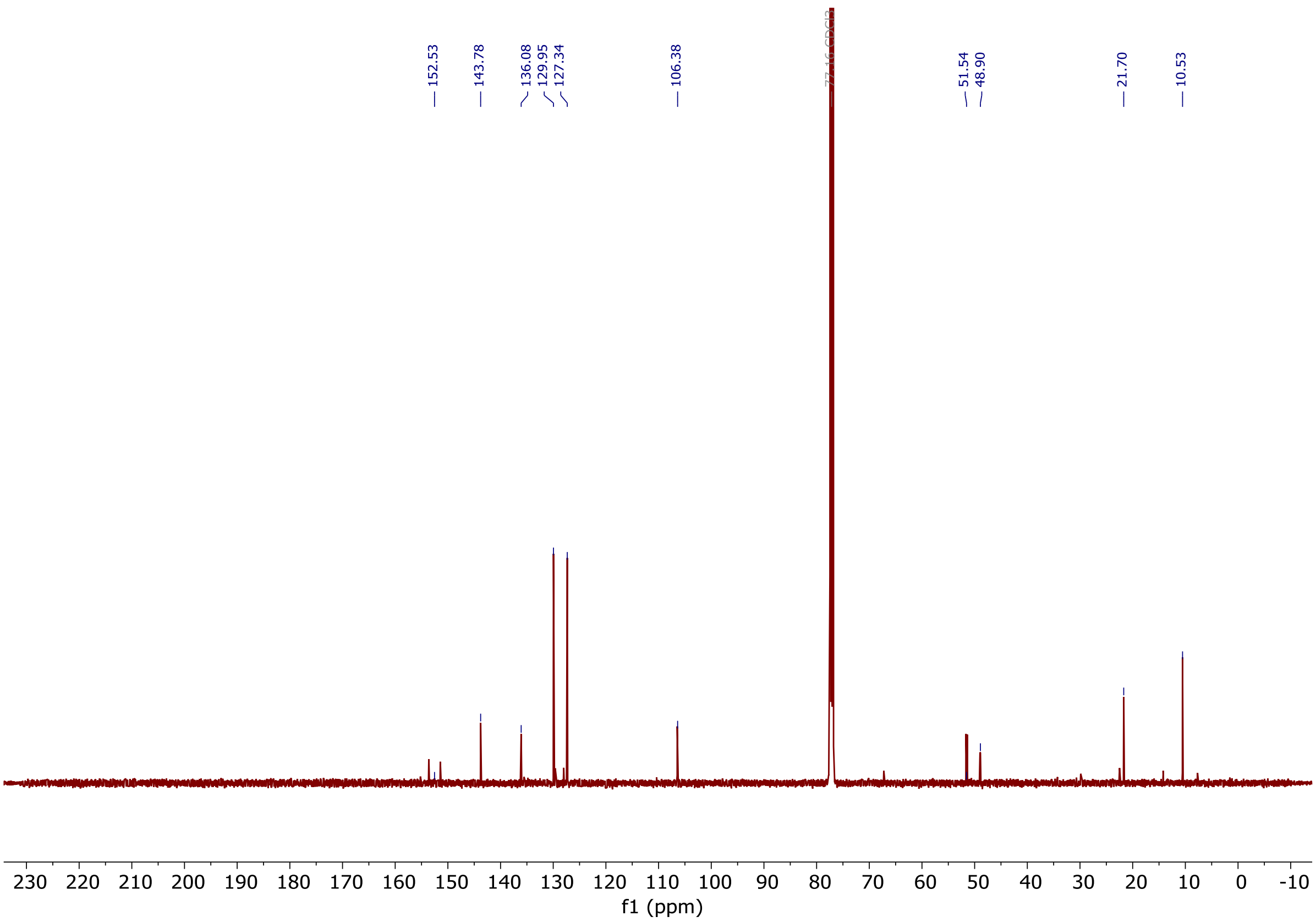

^{19}F NMR (471 MHz, $CDCl_3$)

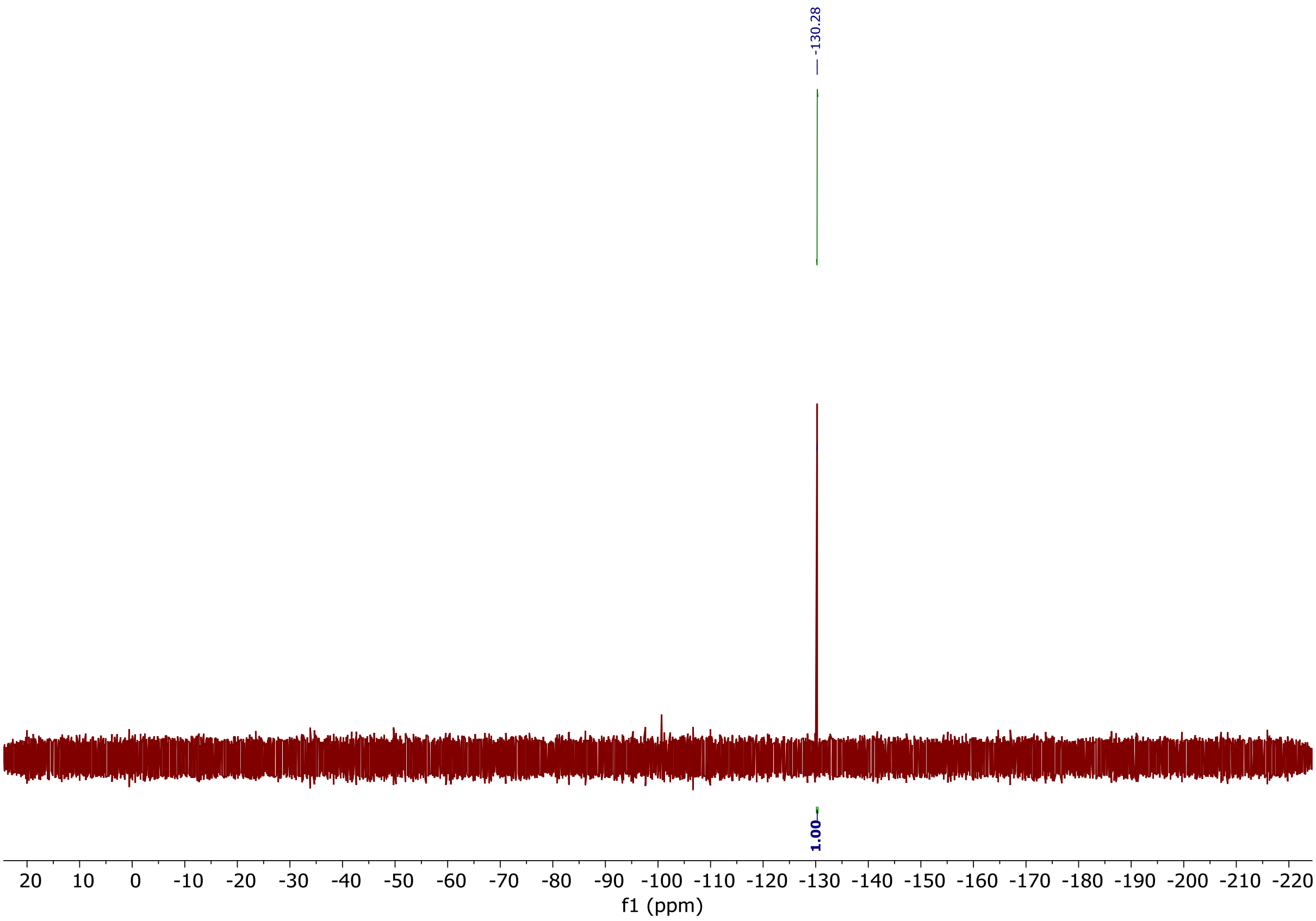

3-fluoro-1,8-ditosyl-1,8-diazaspiro[4.5]dec-3-ene **(4r)**

^{1}H NMR (500 MHz, CDCl$_3$)

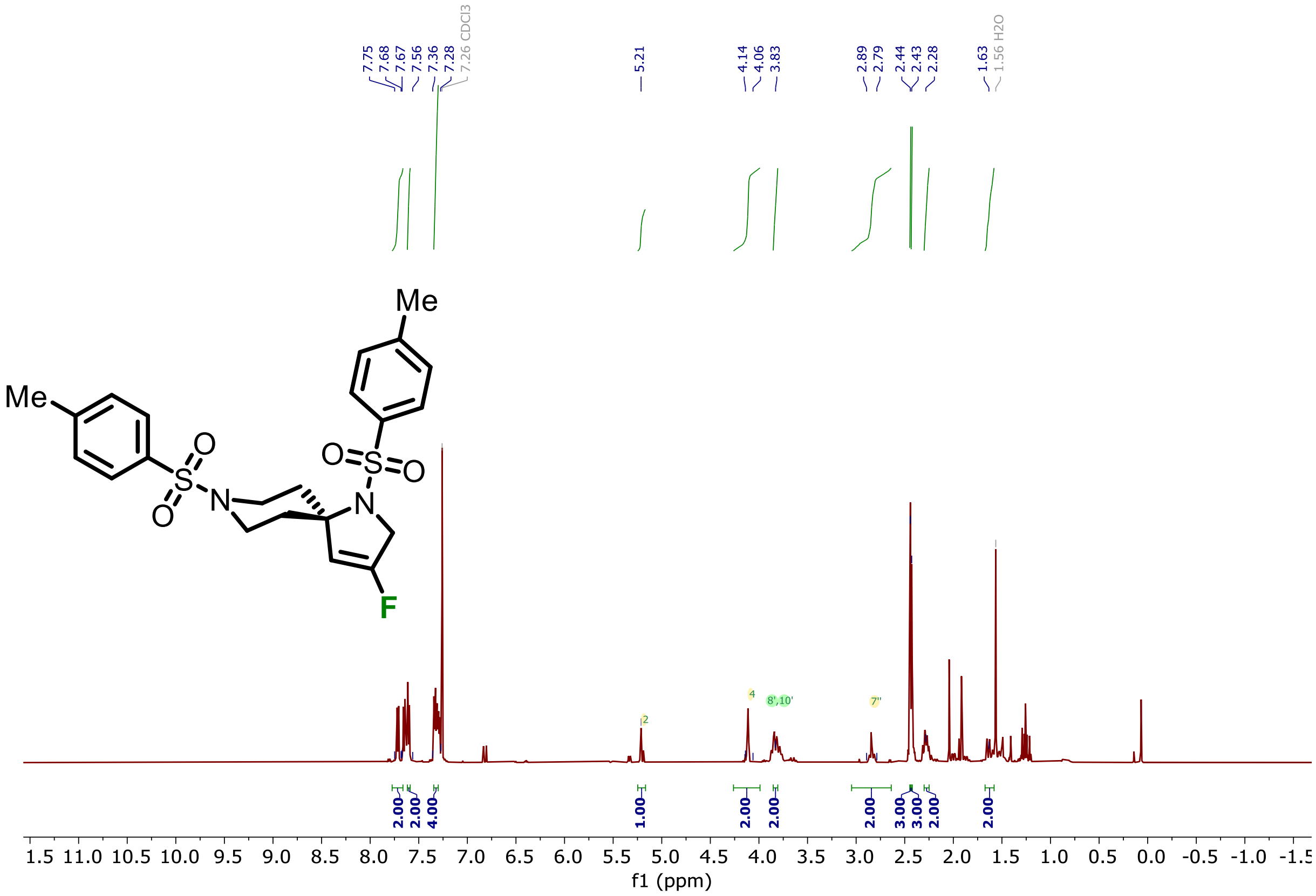

^{13}C NMR (126 MHz, CDCl$_3$)

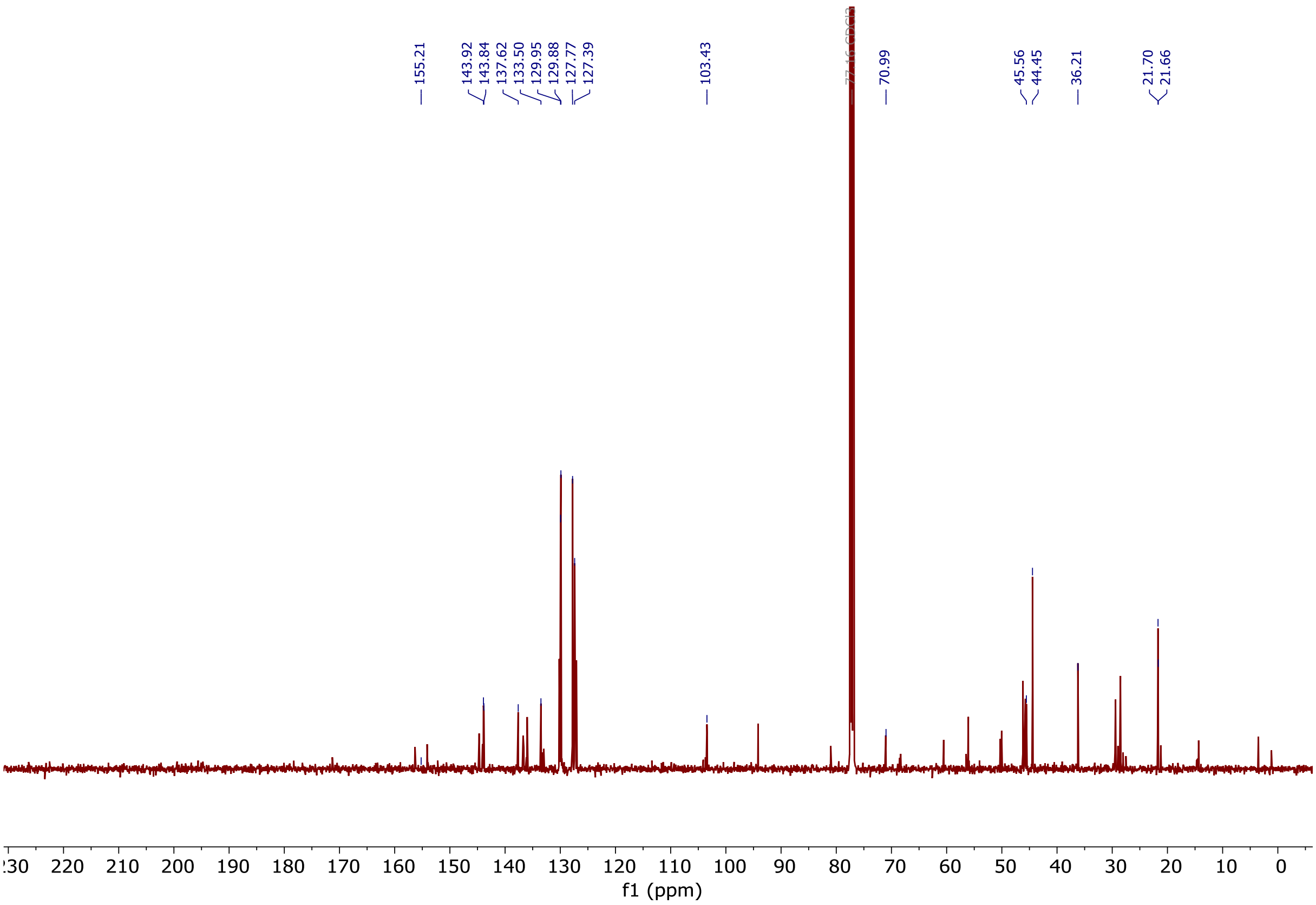

^{19}F NMR (376 MHz, $CDCl_3$)

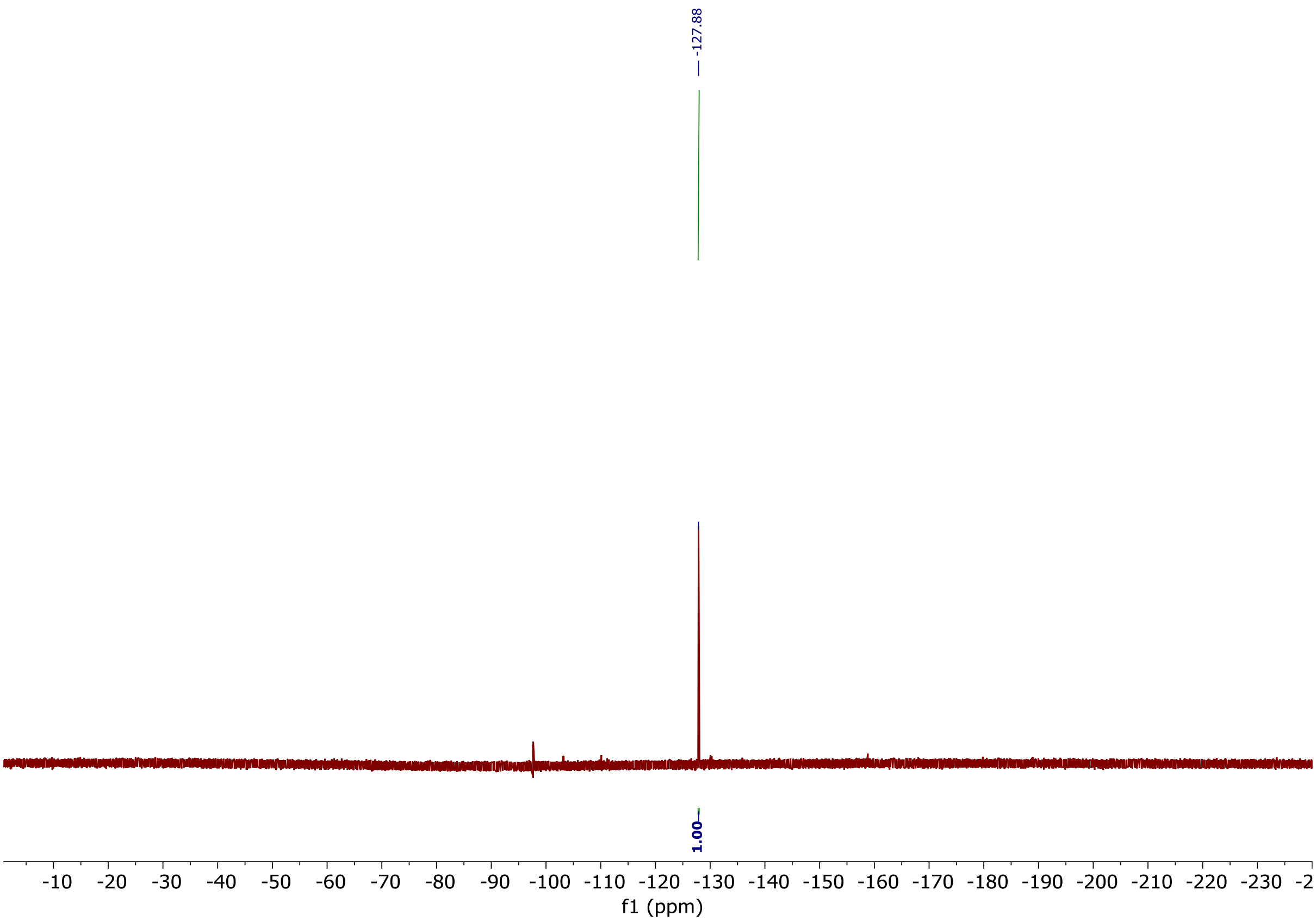

***3-fluoro-1-tosyl-1-azaspiro[4.5]dec-3-ene* (4s)**

^{1}H NMR (500 MHz, $CDCl_3$)

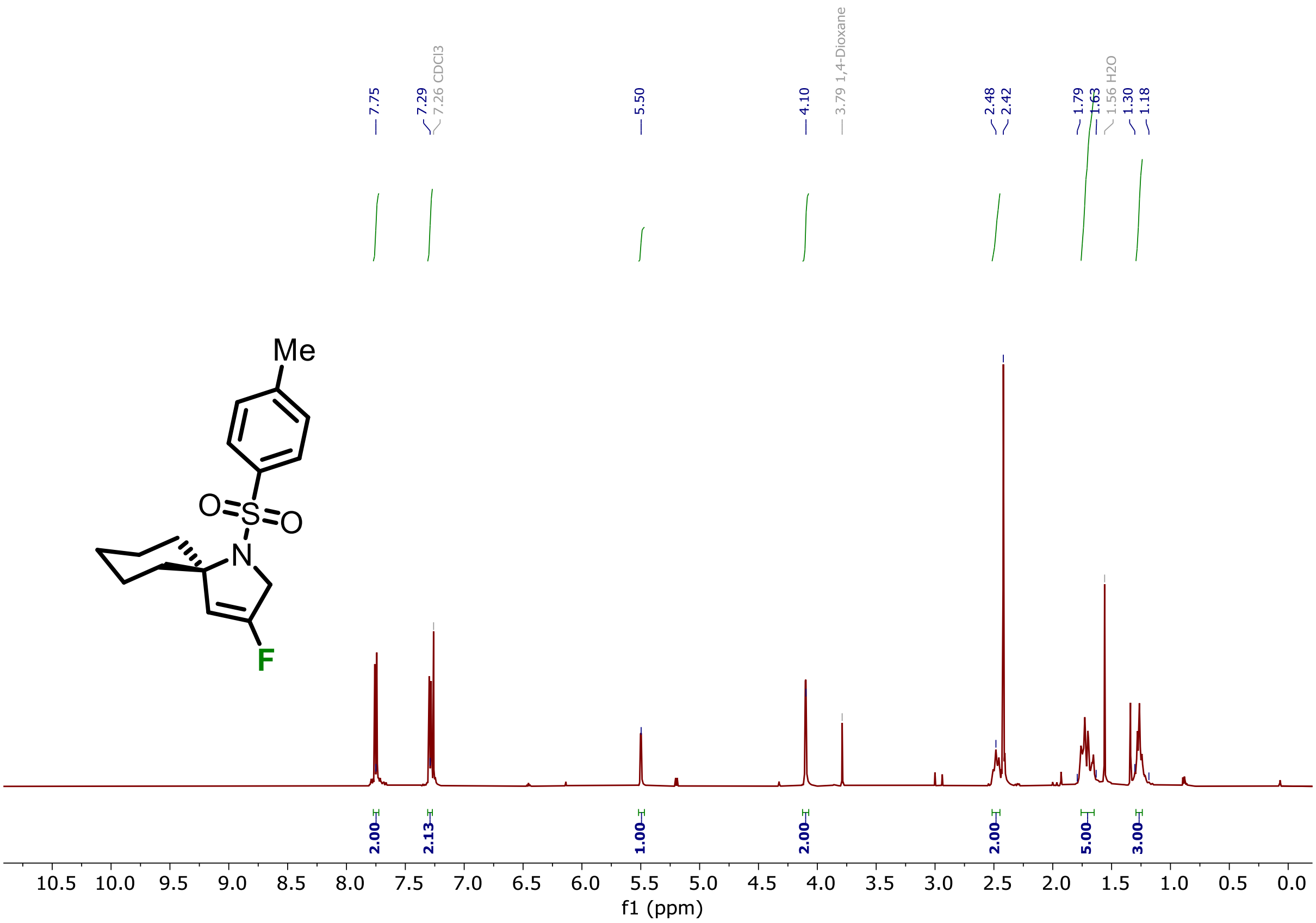

^{13}C NMR (126 MHz, $CDCl_3$)

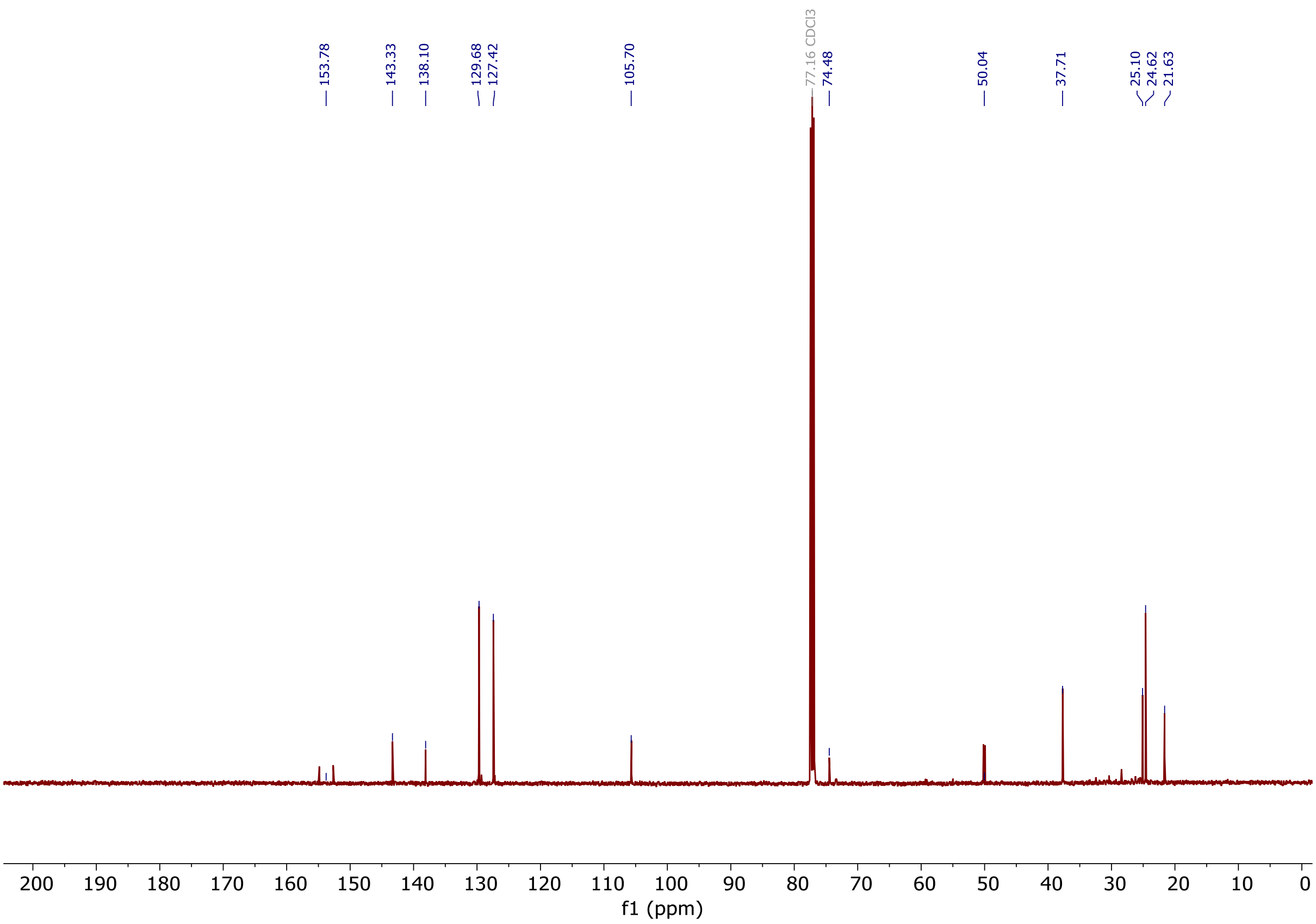

^{19}F NMR (376 MHz, CDCl$_3$)

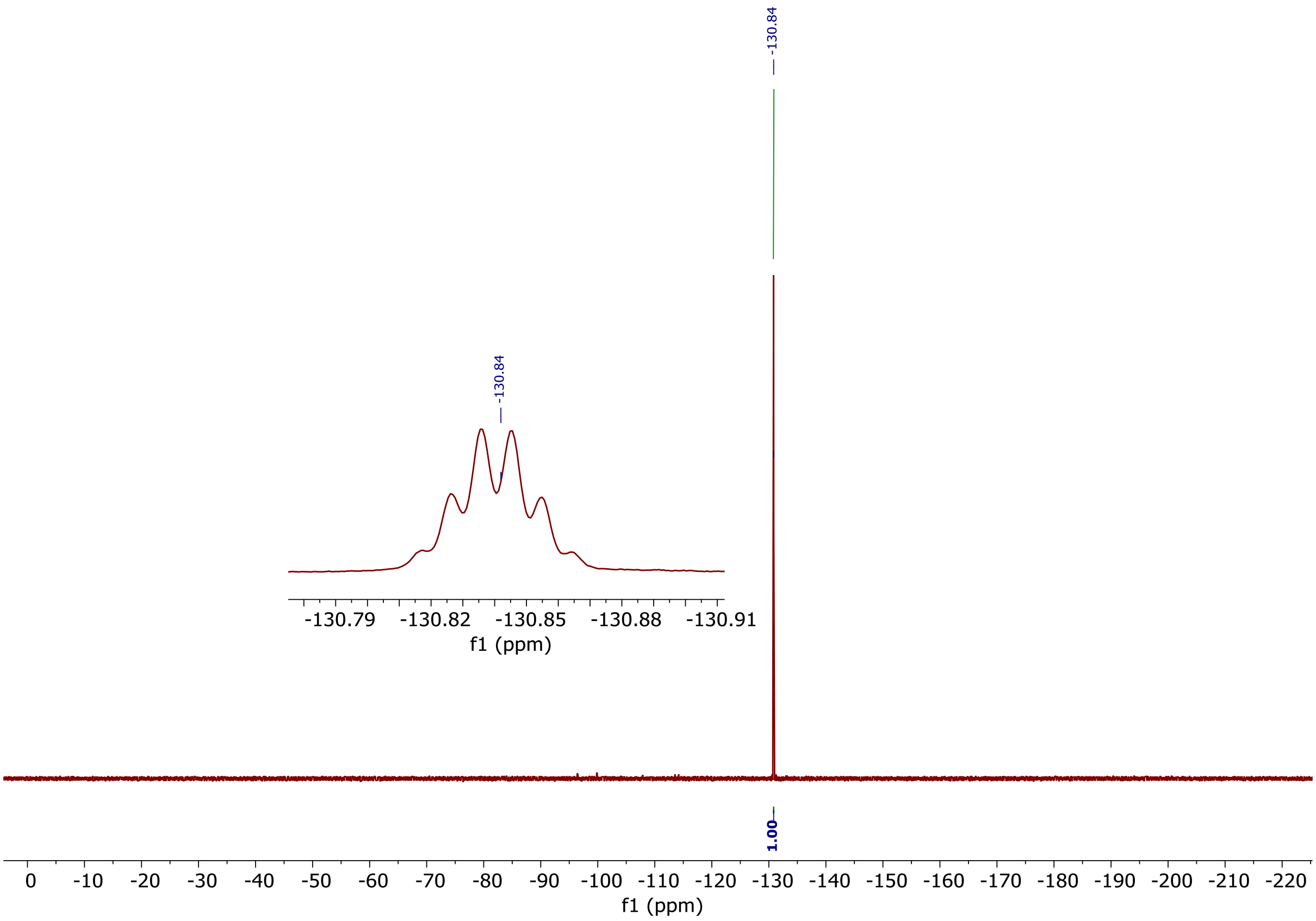

2-benzyl-4-fluoro-1-tosyl-2,5-dihydro-1H-pyrrole (**4t**)

^{1}H NMR (400 MHz, CDCl$_3$)

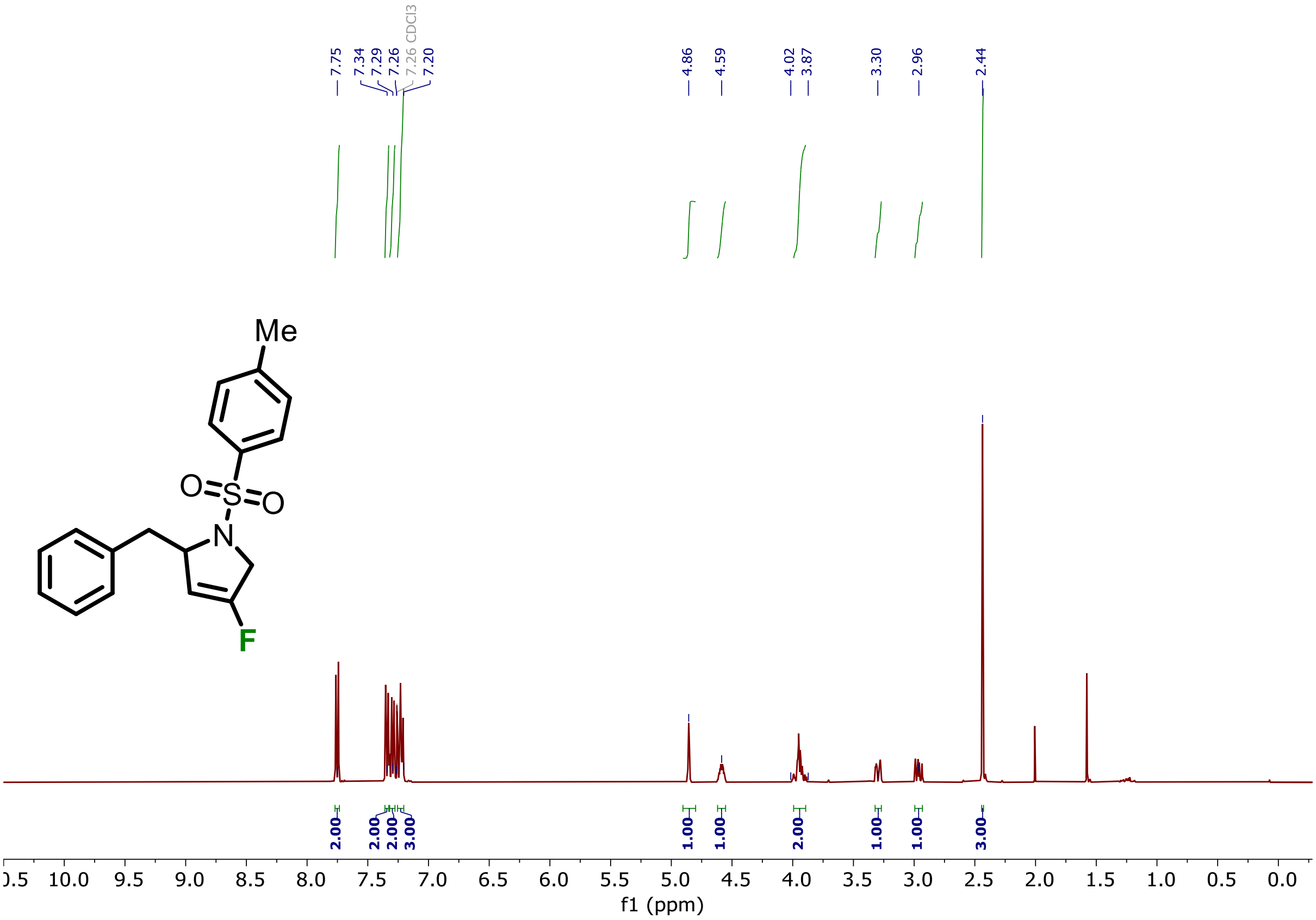

^{13}C NMR (126 MHz, CDCl$_3$)

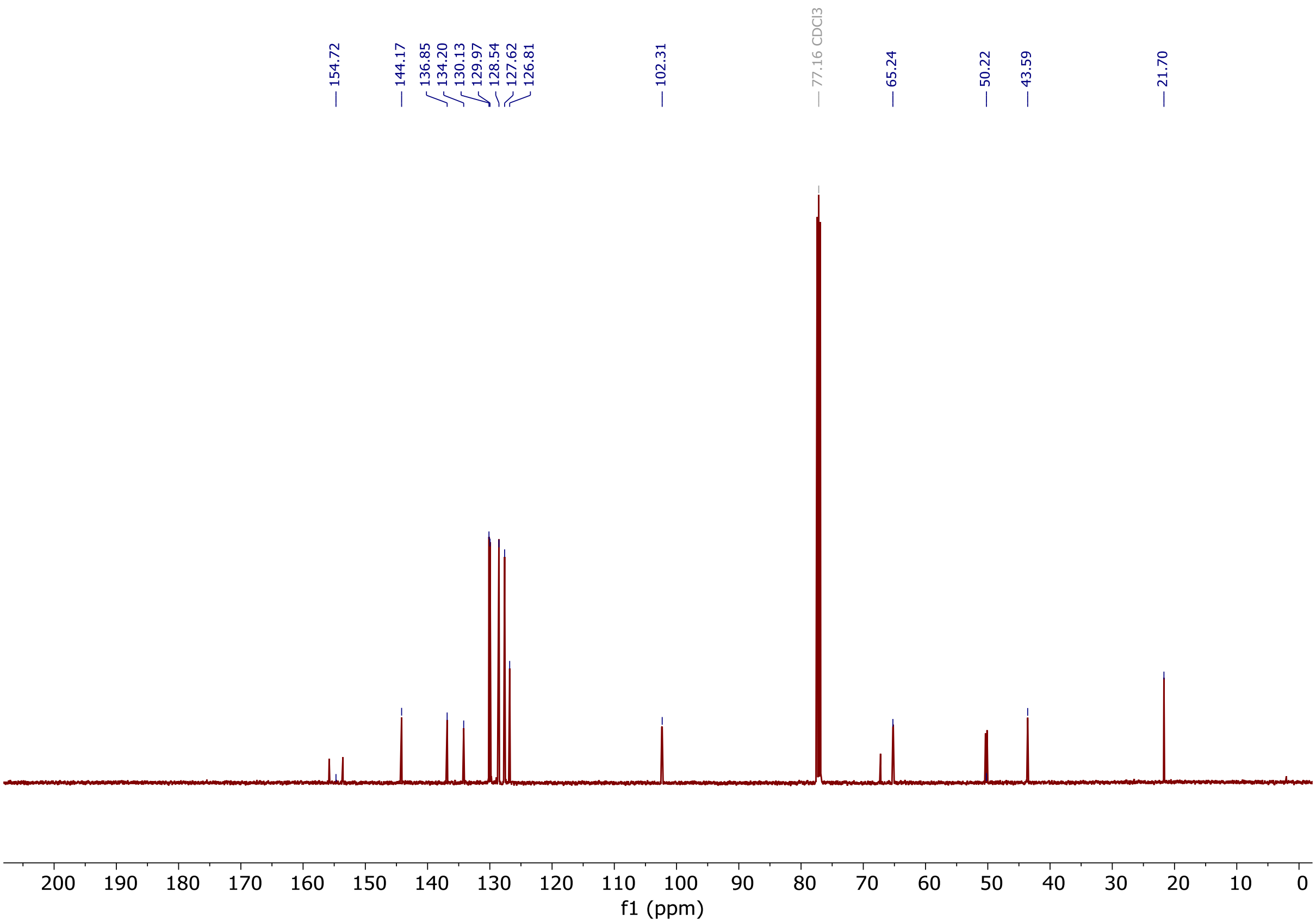

^{19}F NMR (376 MHz, CDCl$_3$)

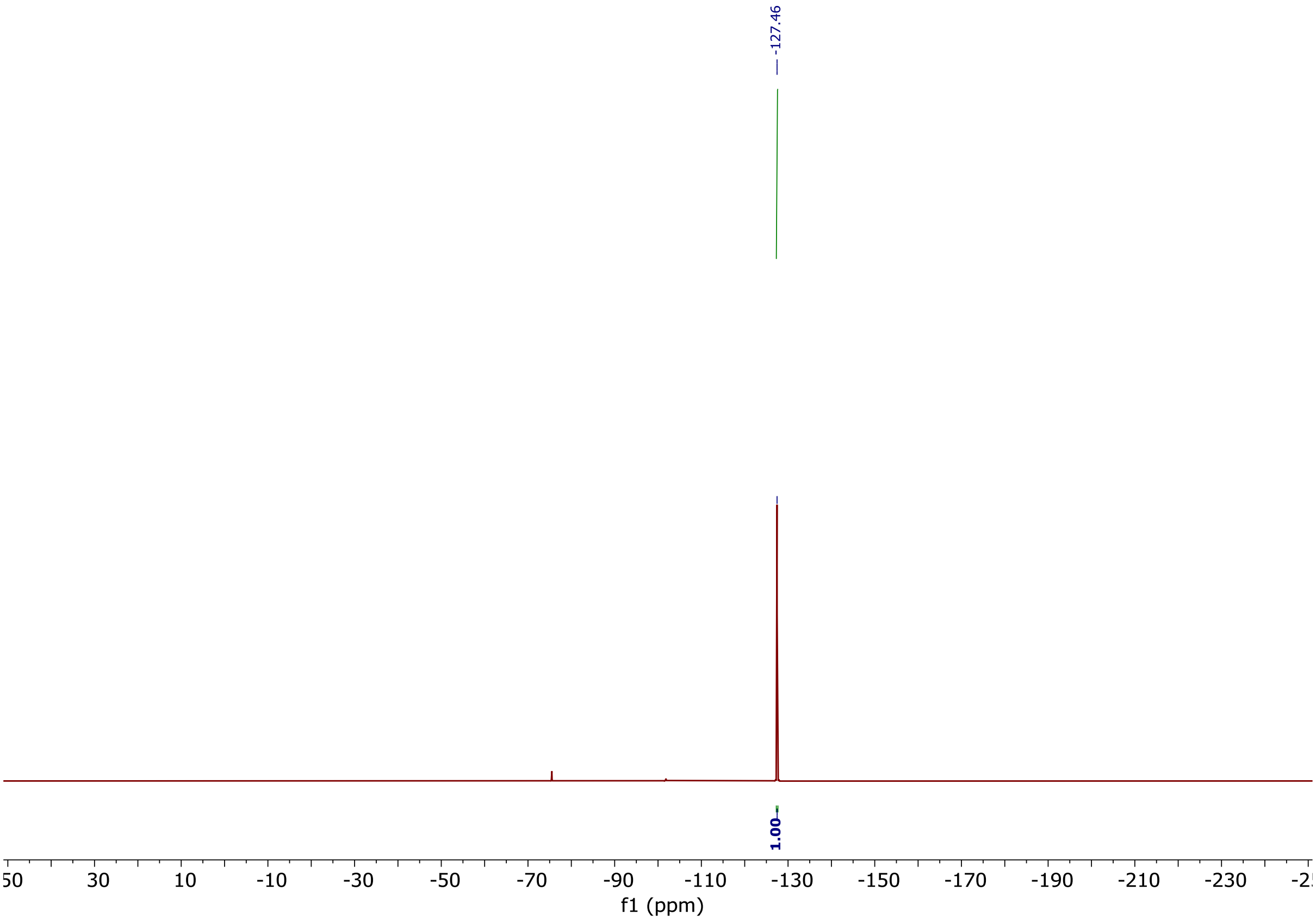

***4-fluoro-1-((4-nitrophenyl)sulfonyl)-2-propyl-2,5-dihydro-1H-pyrrole* (4u)**

^{1}H NMR (500 MHz, CDCl$_3$)

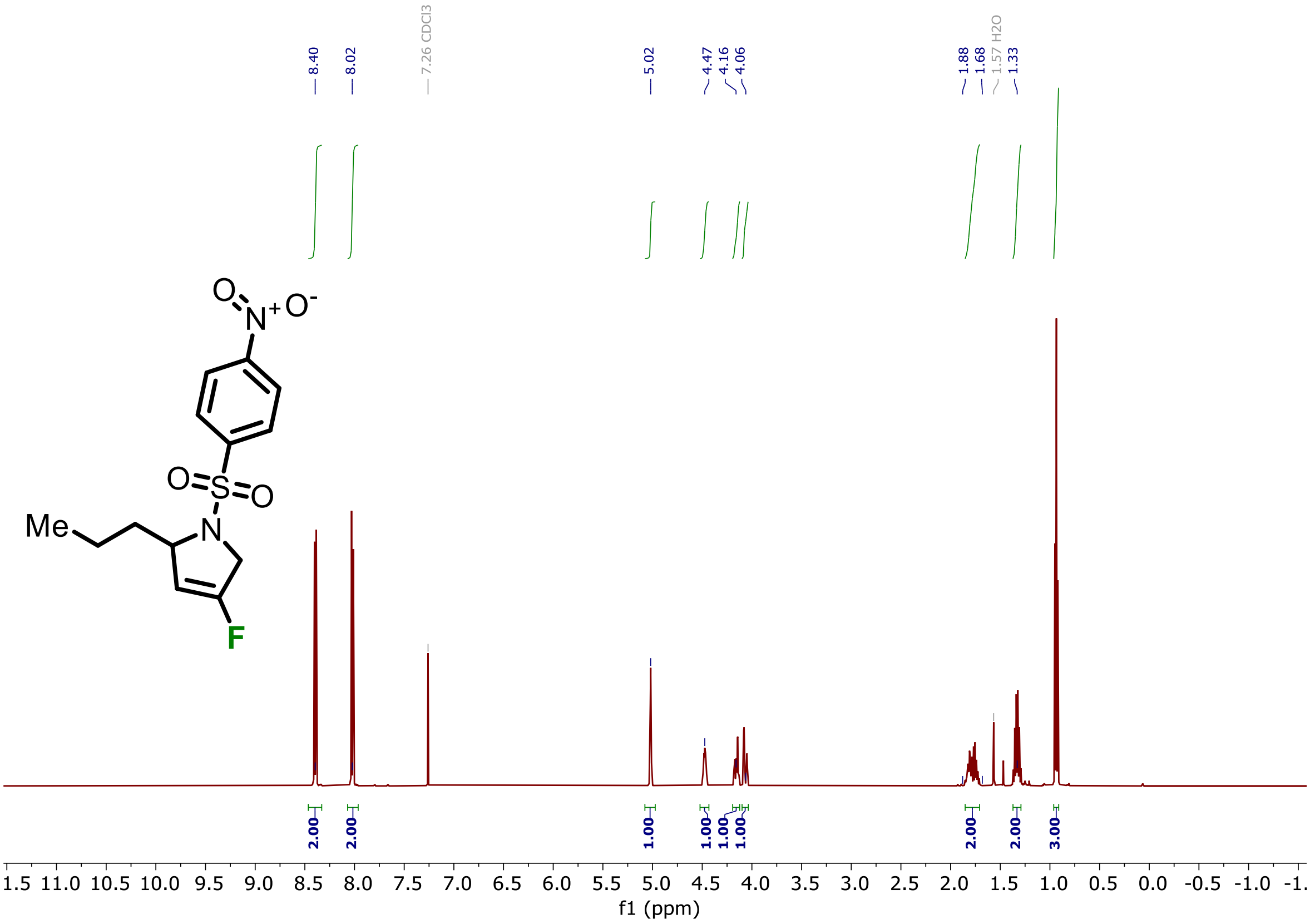

^{13}C NMR (126 MHz, CDCl$_3$)

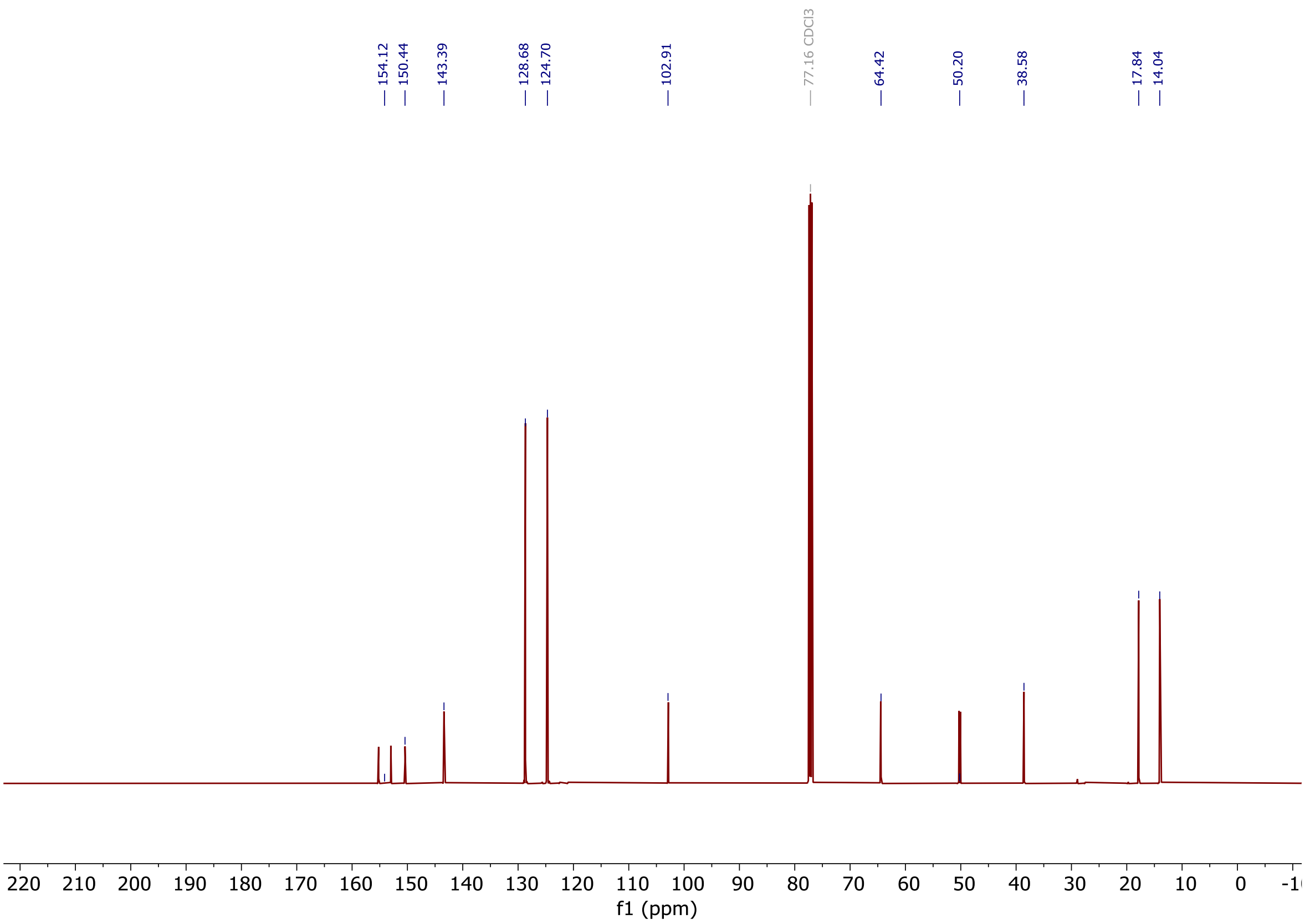

^{19}F NMR (376 MHz, $CDCl_3$)

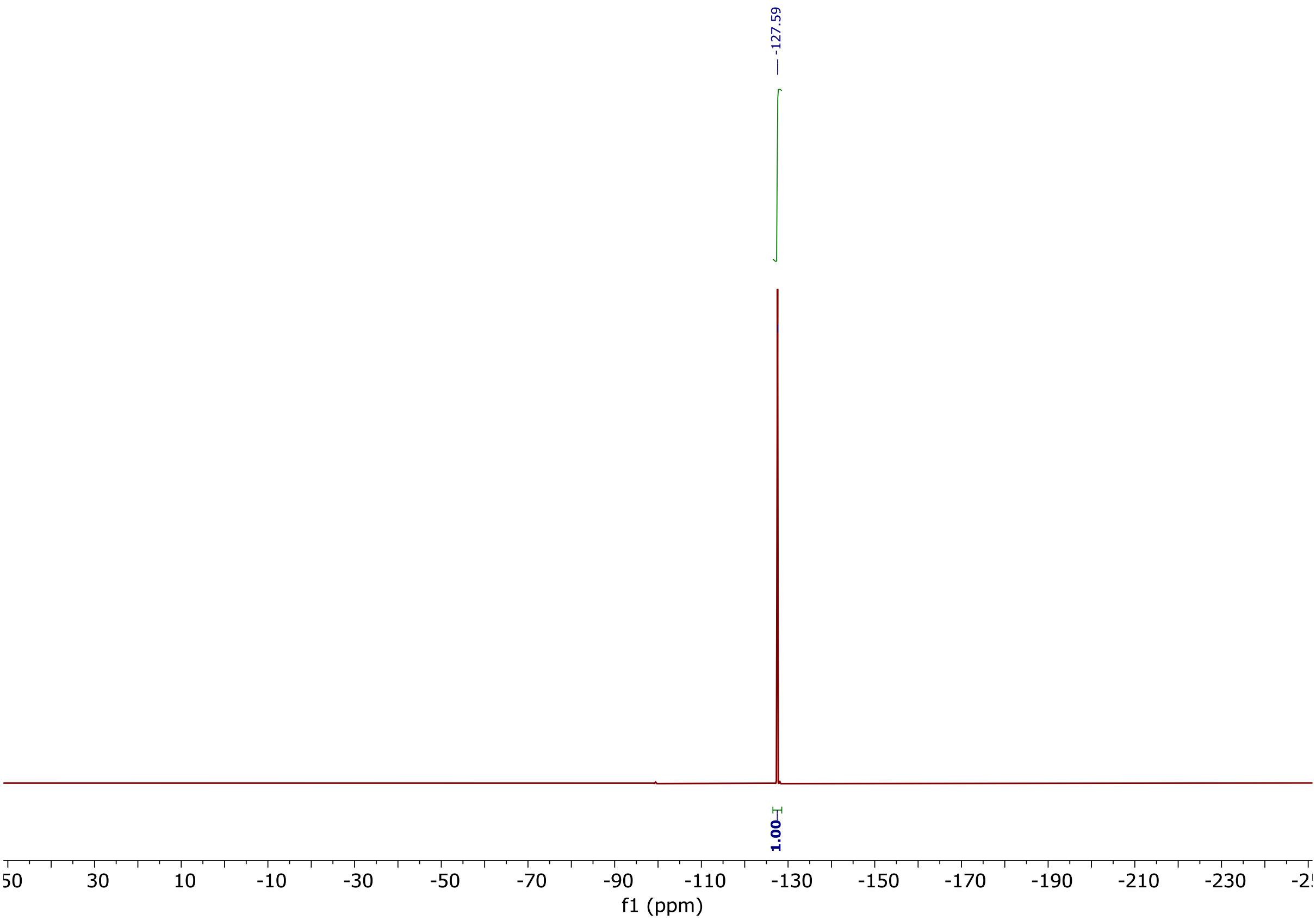

4-fluoro-2-propyl-1-tosyl-2,5-dihydro-1H-pyrrole (**4v**)

¹H NMR (400 MHz, $CDCl_3$)

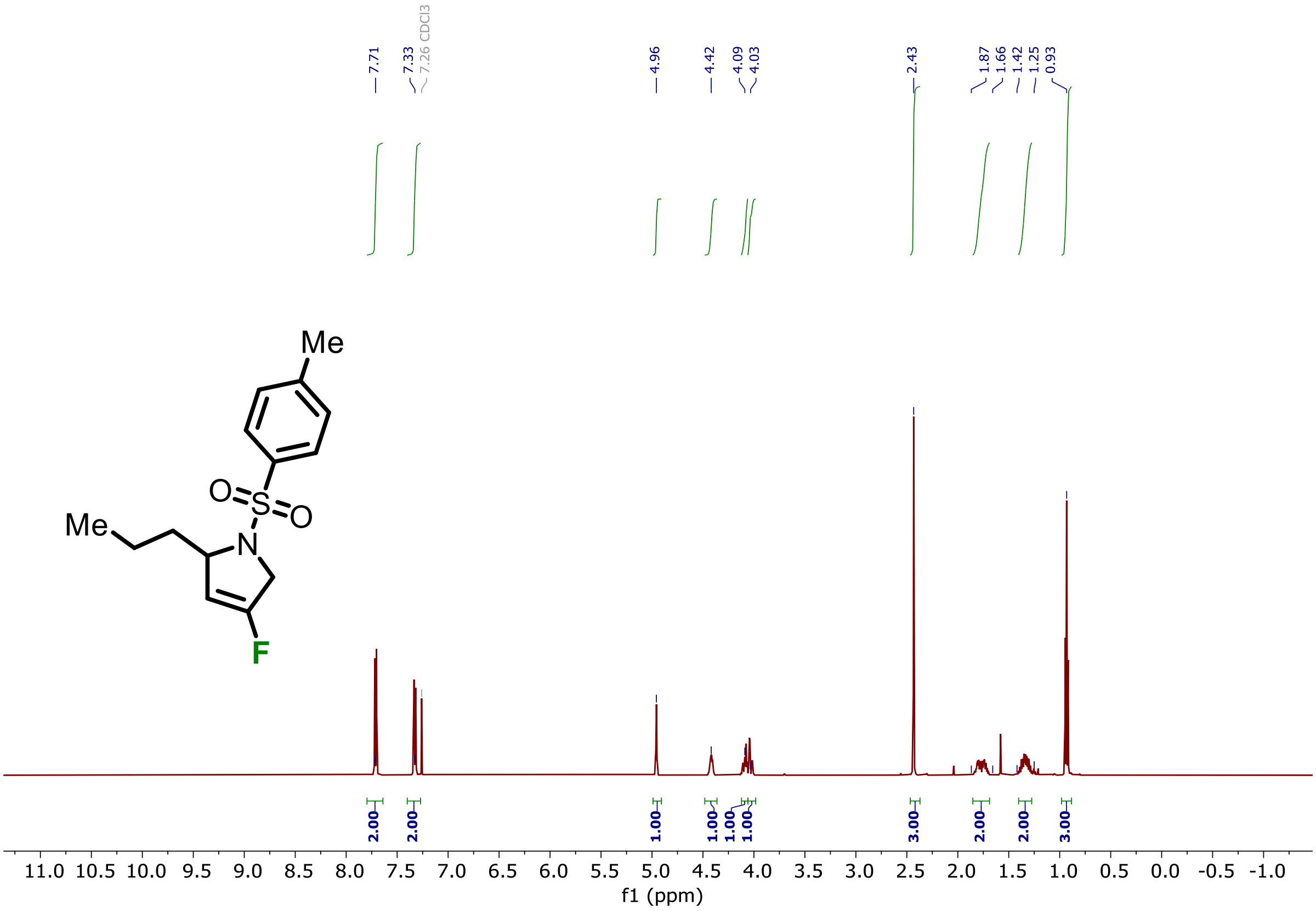

¹³C NMR (126 MHz, $CDCl_3$)

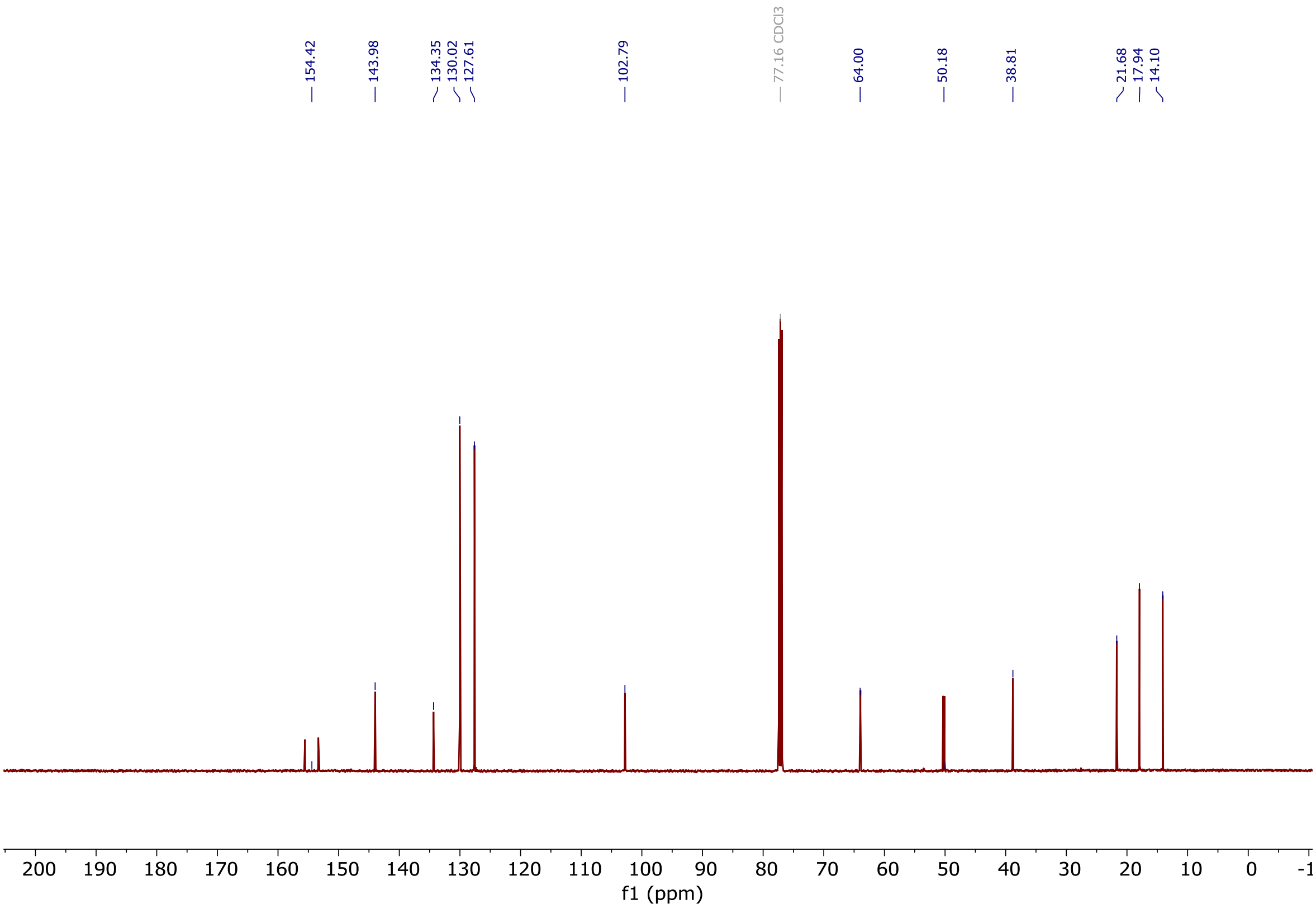

^{19}F NMR (471 MHz, CDCl$_3$)

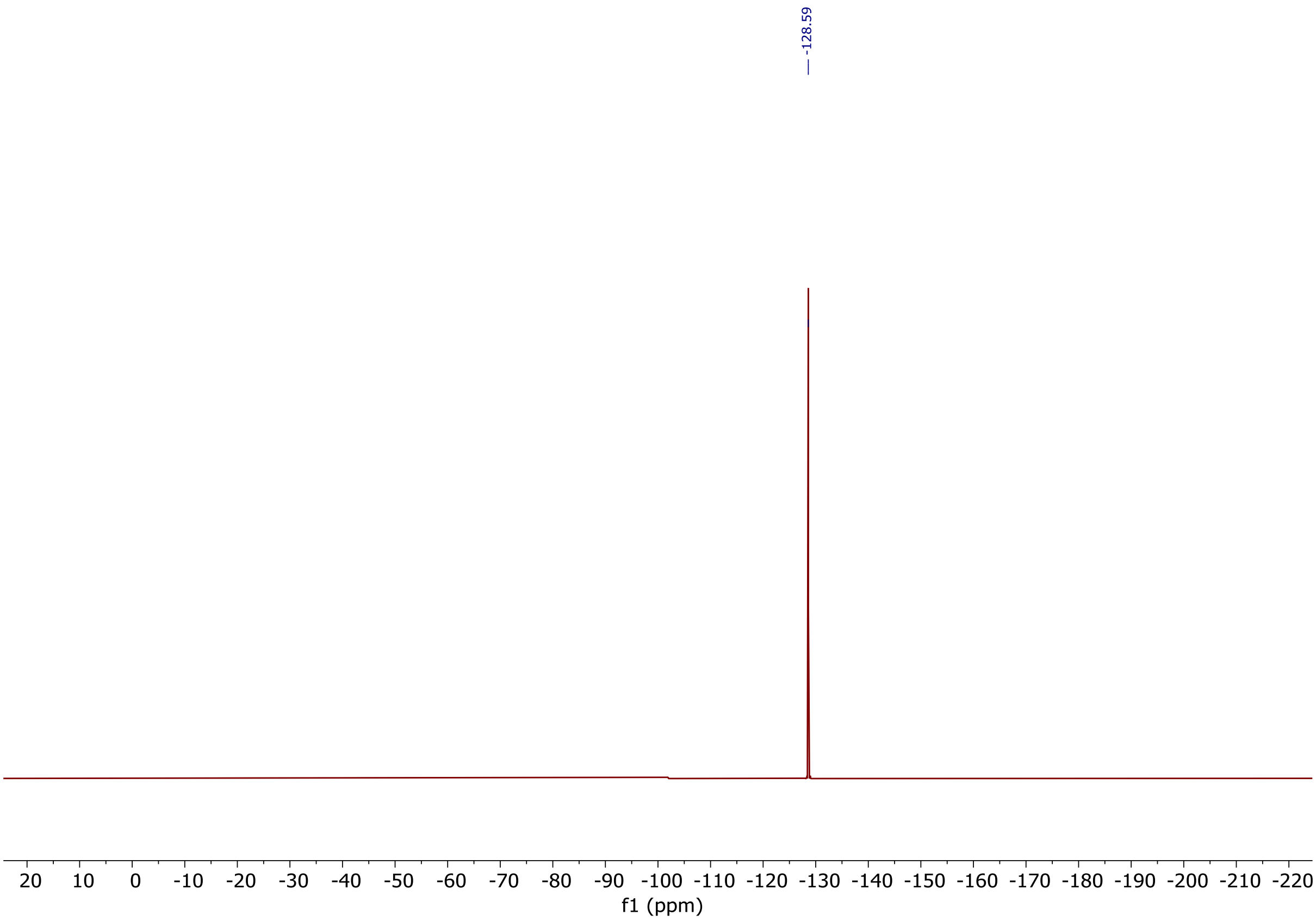

***4-fluoro-2-(thiophen-2-ylmethyl)-1-tosyl-2,5-dihydro-1H-pyrrole* (4w)**

^{1}H NMR (500 MHz, $CDCl_3$)

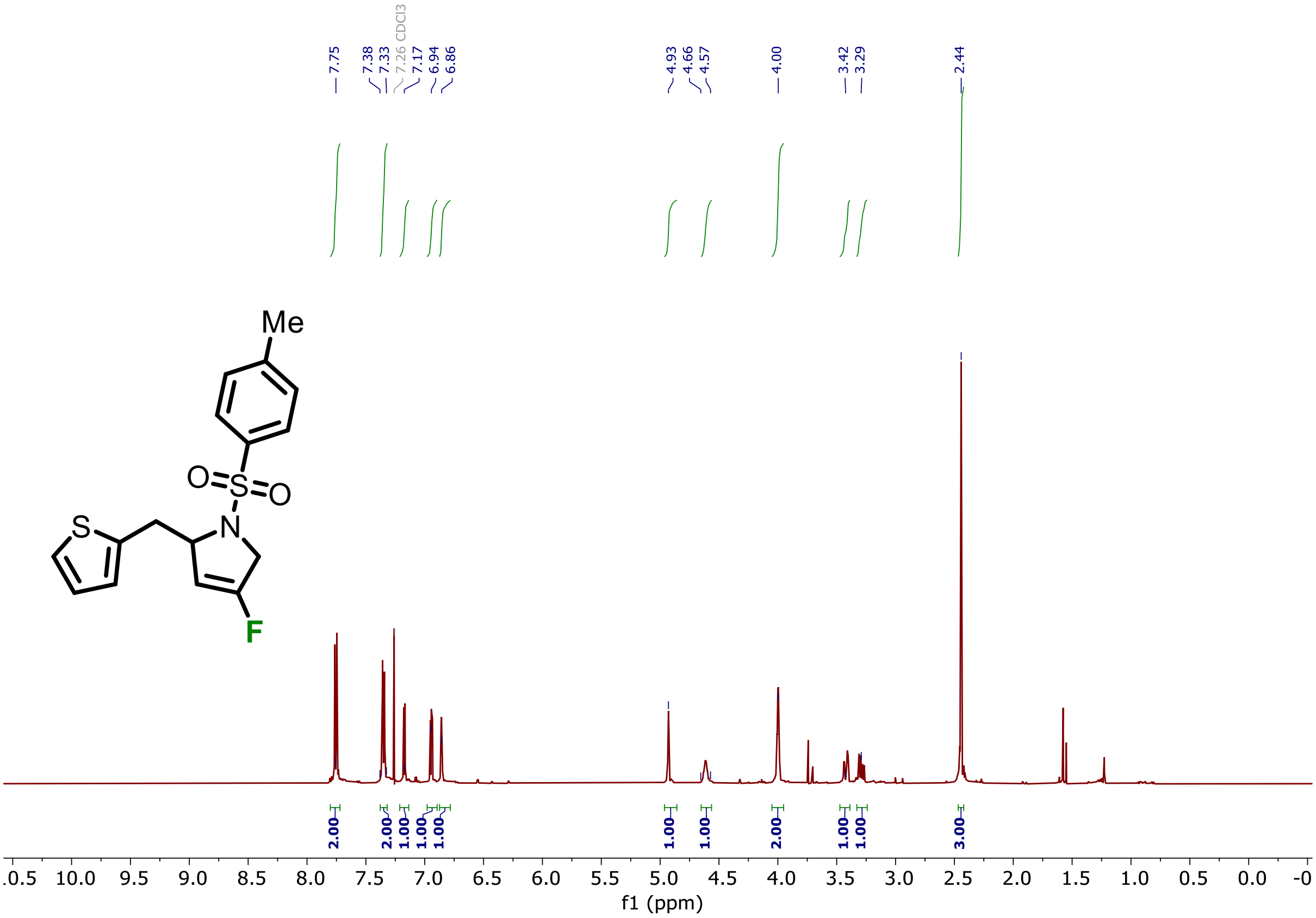

^{13}C NMR (126 MHz, $CDCl_3$)

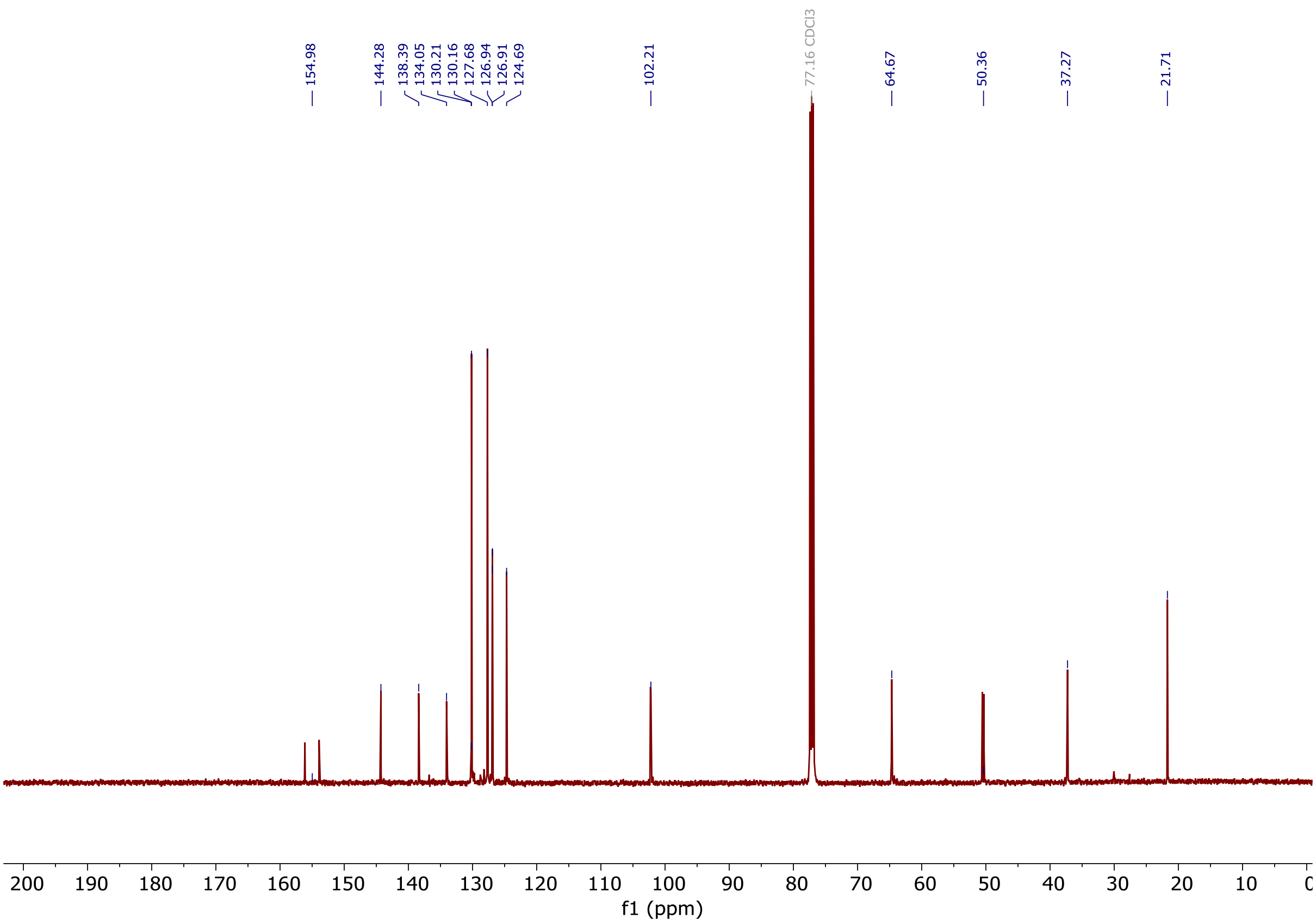

^{19}F NMR (376 MHz, $CDCl_3$)

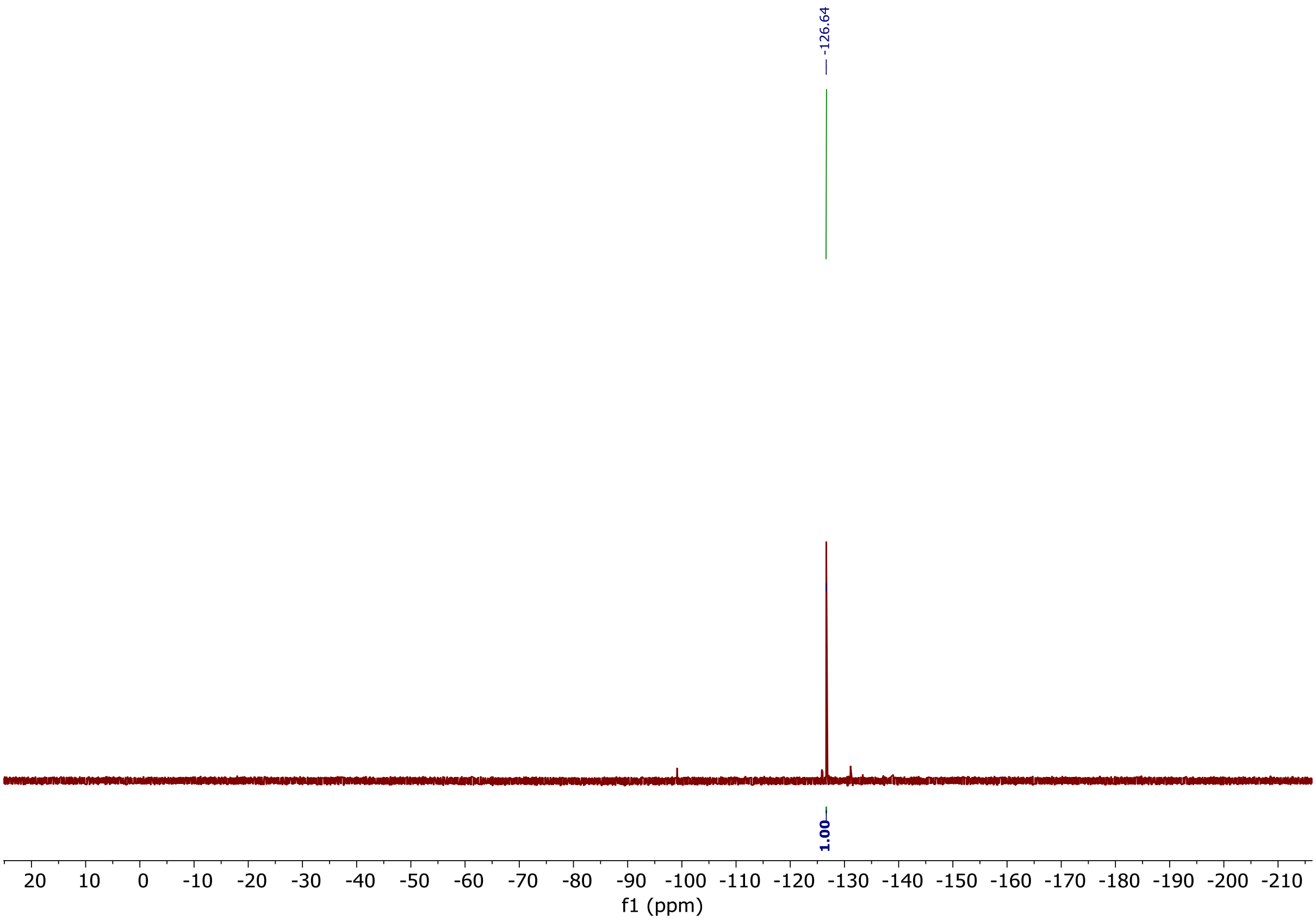

***4-fluoro-2-(tetrahydrofuran-2-yl)-1-tosyl-2,5-dihydro-1H-pyrrole* (4x)**

^{1}H NMR (500 MHz, $CDCl_3$) Mixture of diastereomers (1:0.71 *dr*)

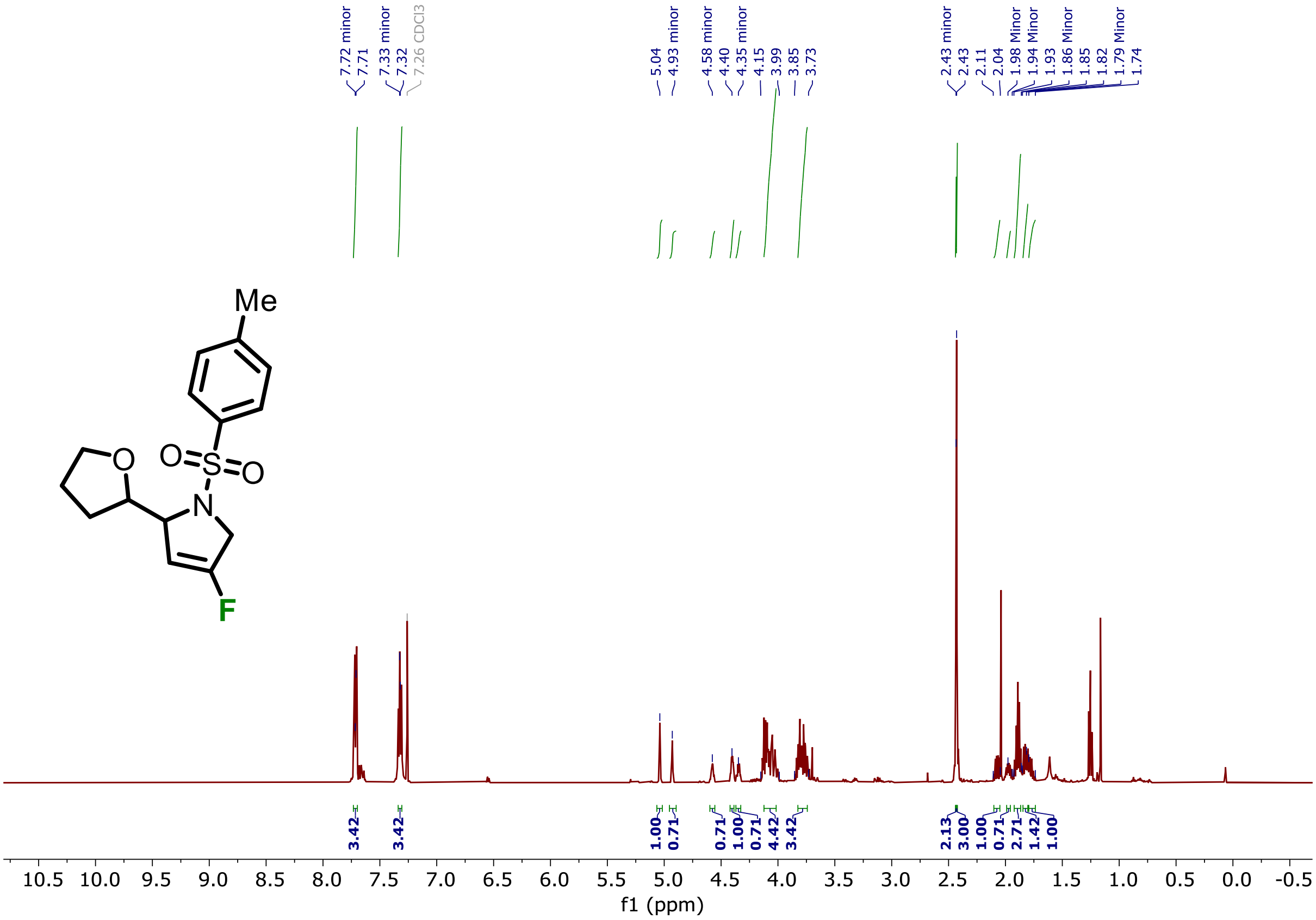

^{13}C NMR (126 MHz, $CDCl_3$)

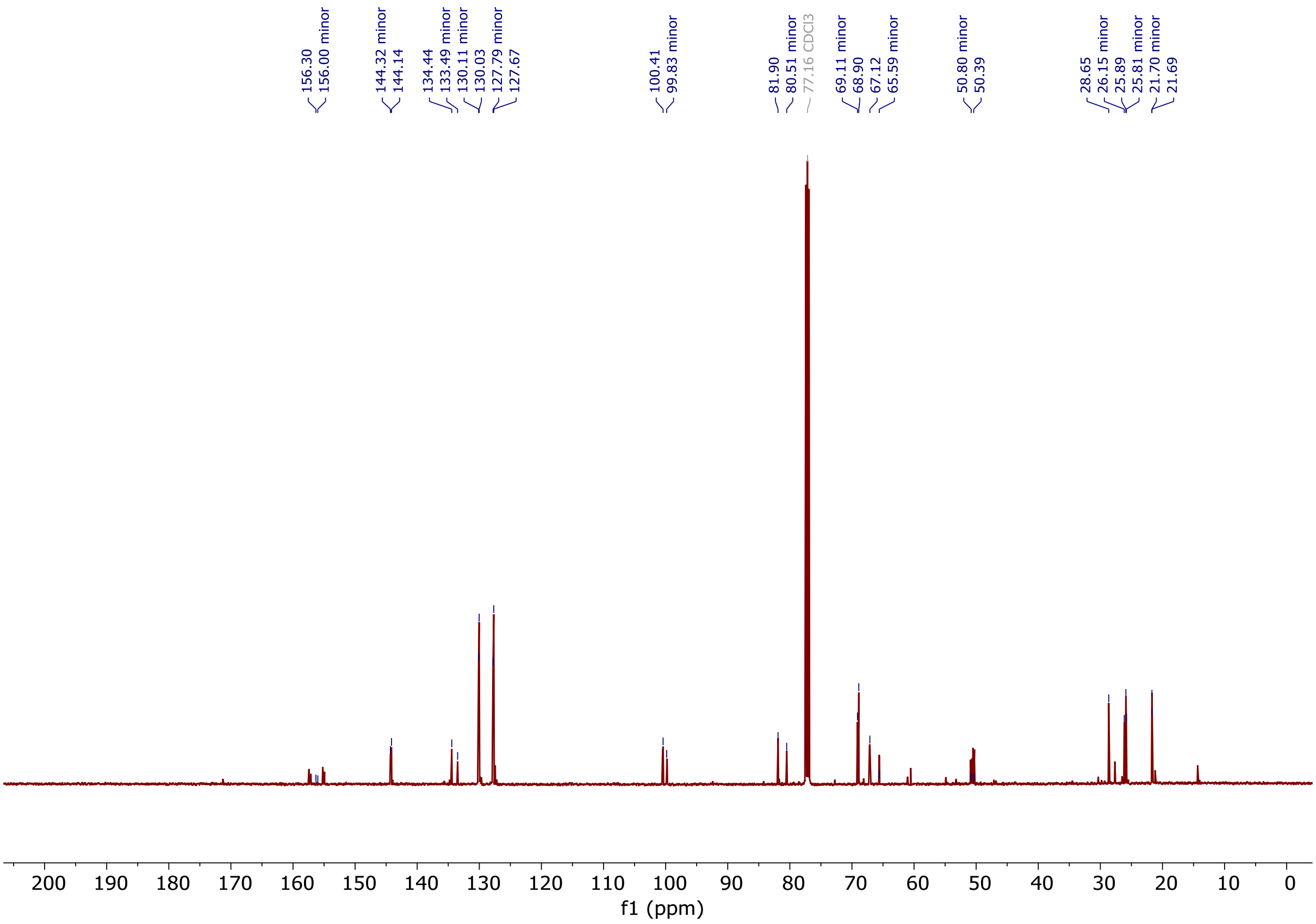

^{19}F NMR (471 MHz, $CDCl_3$)

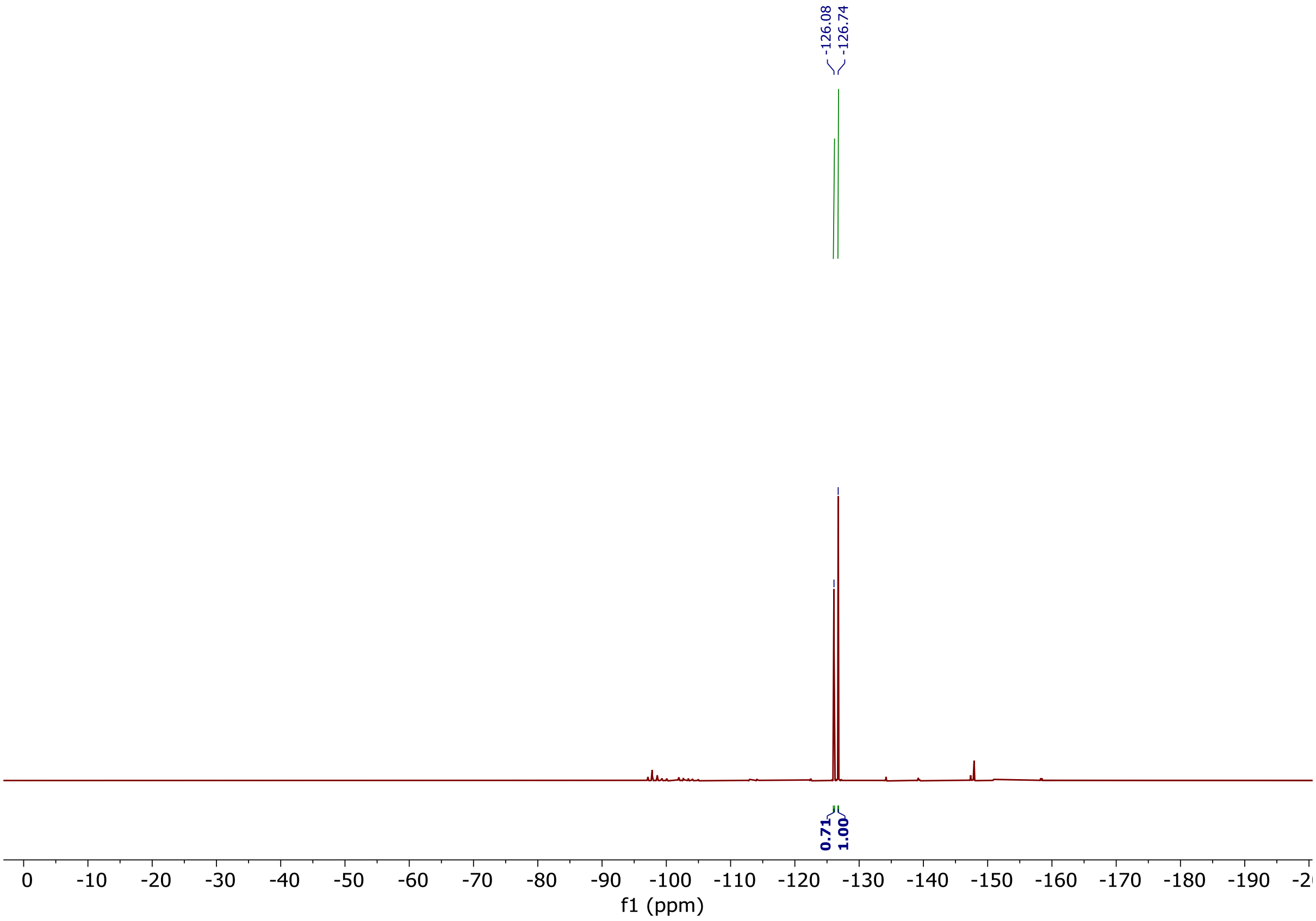

***5-fluoro-2,3,3a,6-tetrahydropyrrolo[1,2-b]isothiazole 1,1-dioxide* (4y)**

^{1}H NMR (400 MHz, CDCl$_3$)

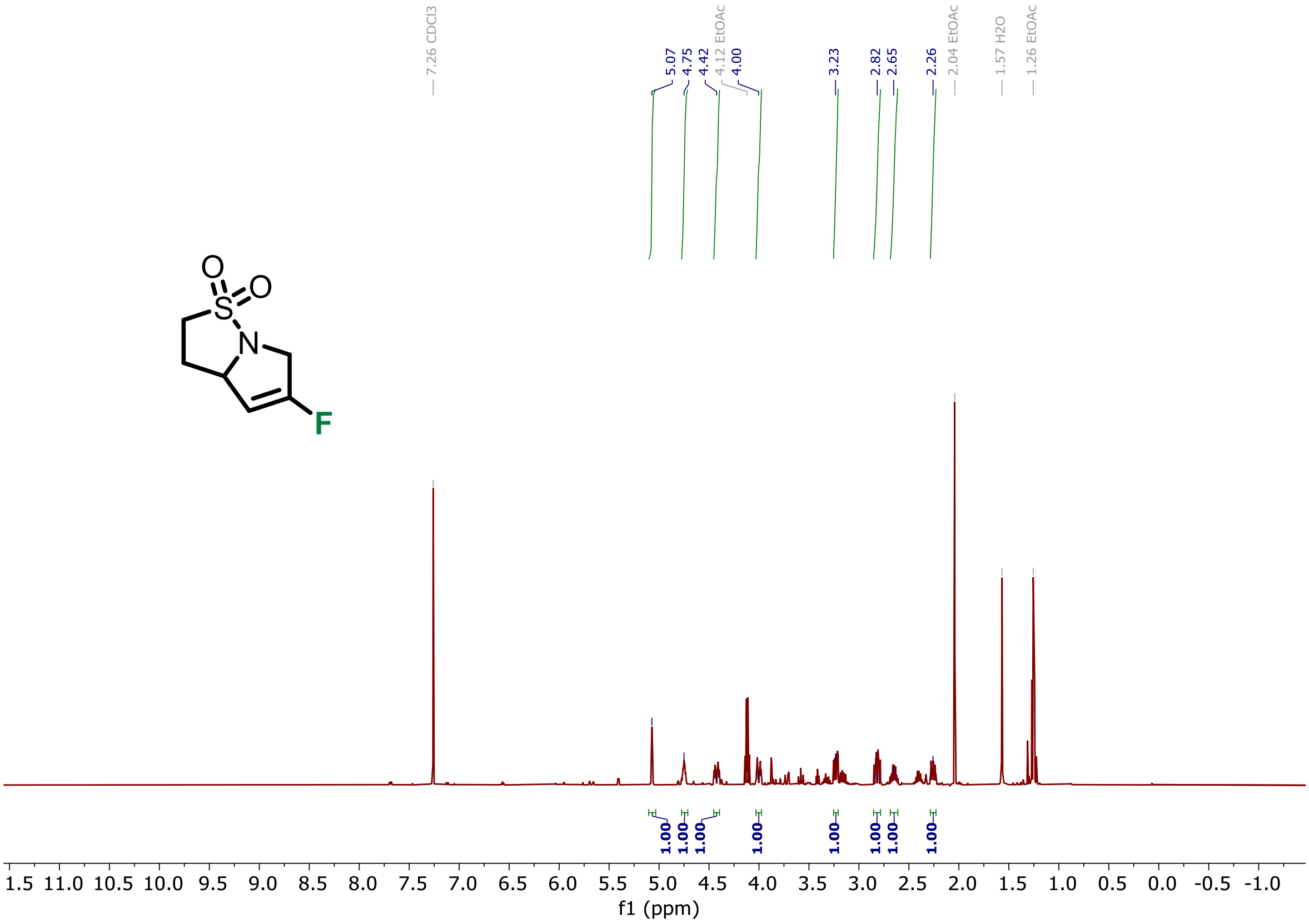

^{13}C NMR (126 MHz, CDCl$_3$)

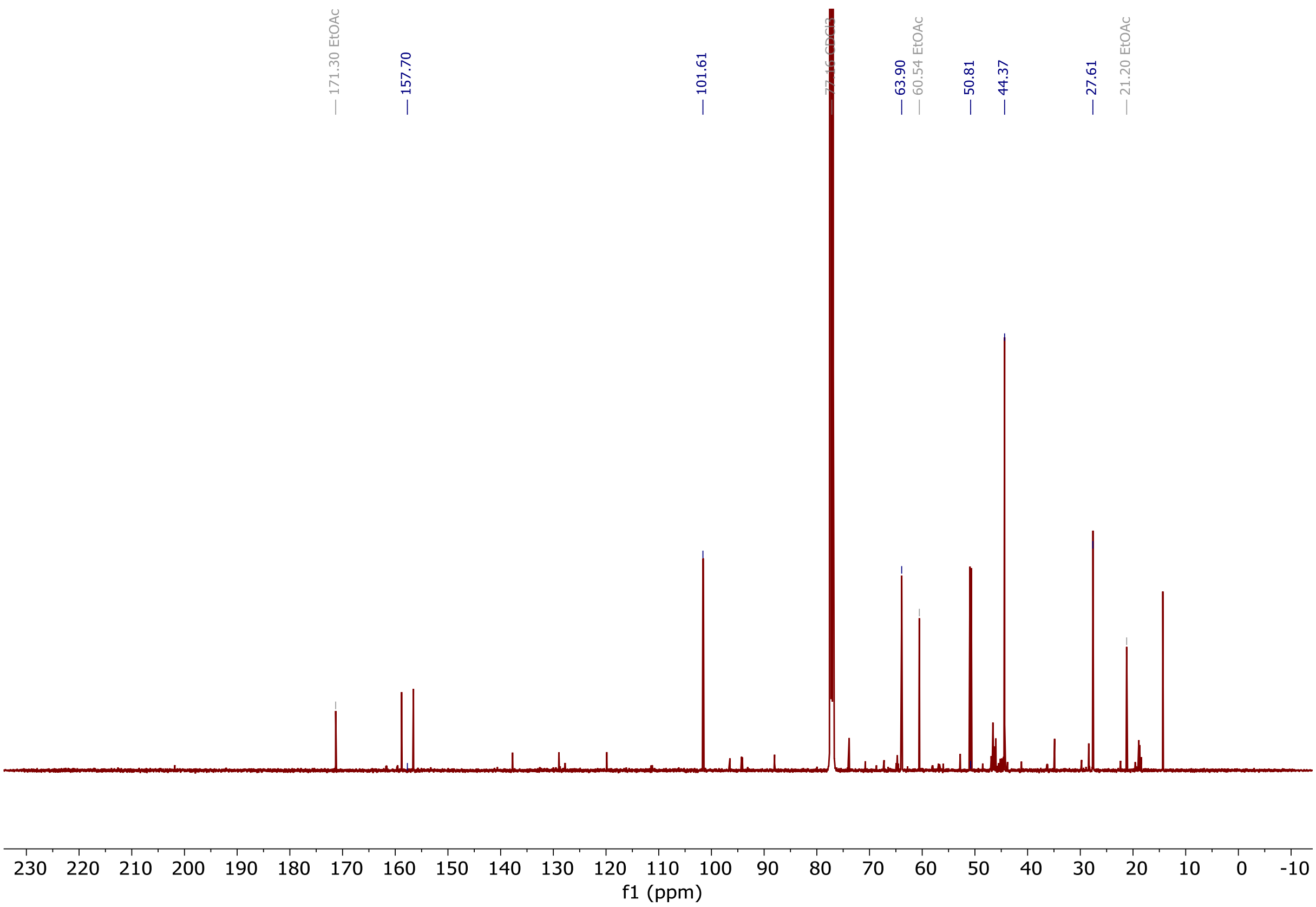

^{19}F NMR (376 MHz, $CDCl_3$)

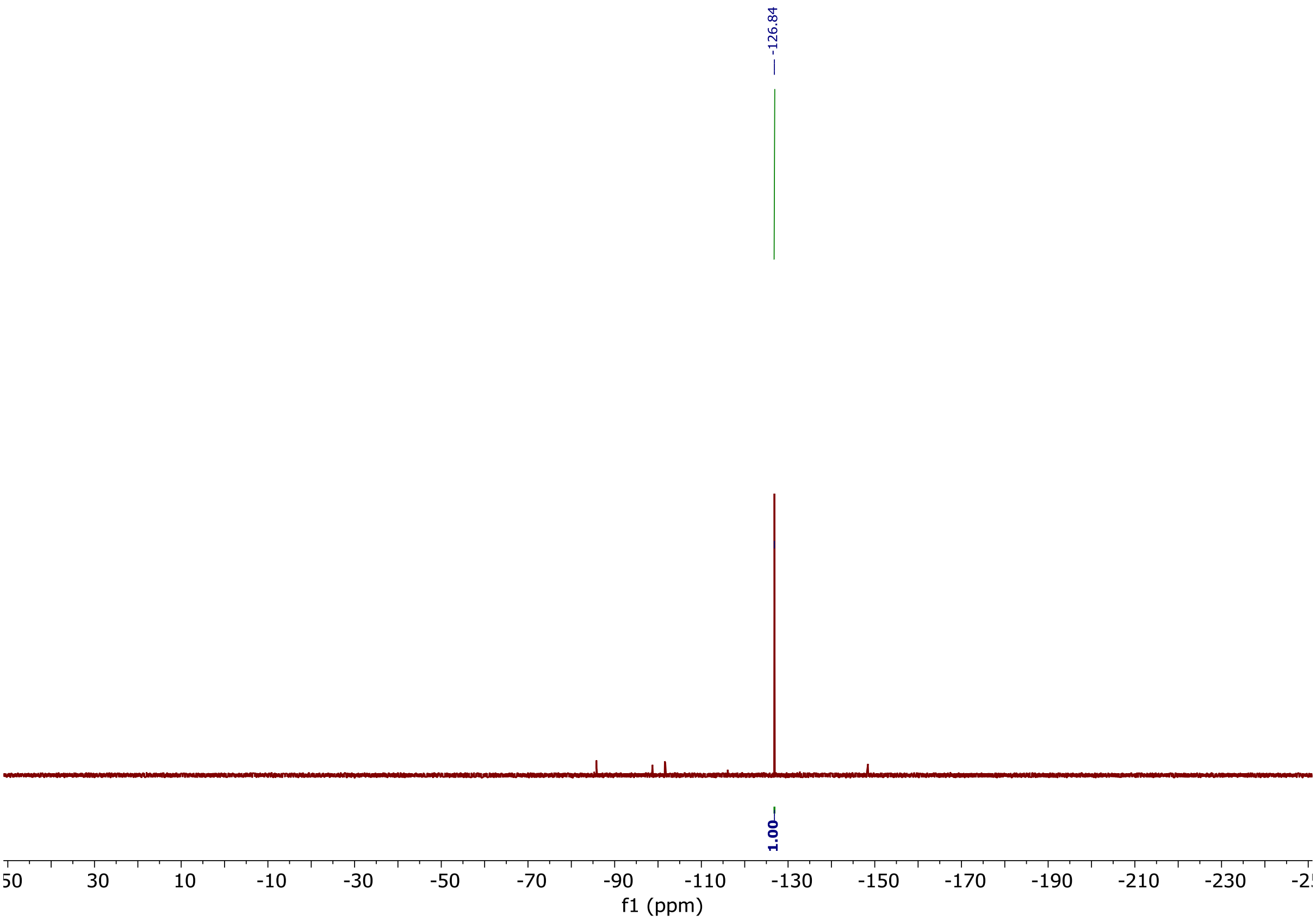

(syn)-6-fluoro-8-tosyl-8-azabicyclo[3.2.1]oct-6-ene (4z)

^{1}H NMR (500 MHz,CDCl$_3$)

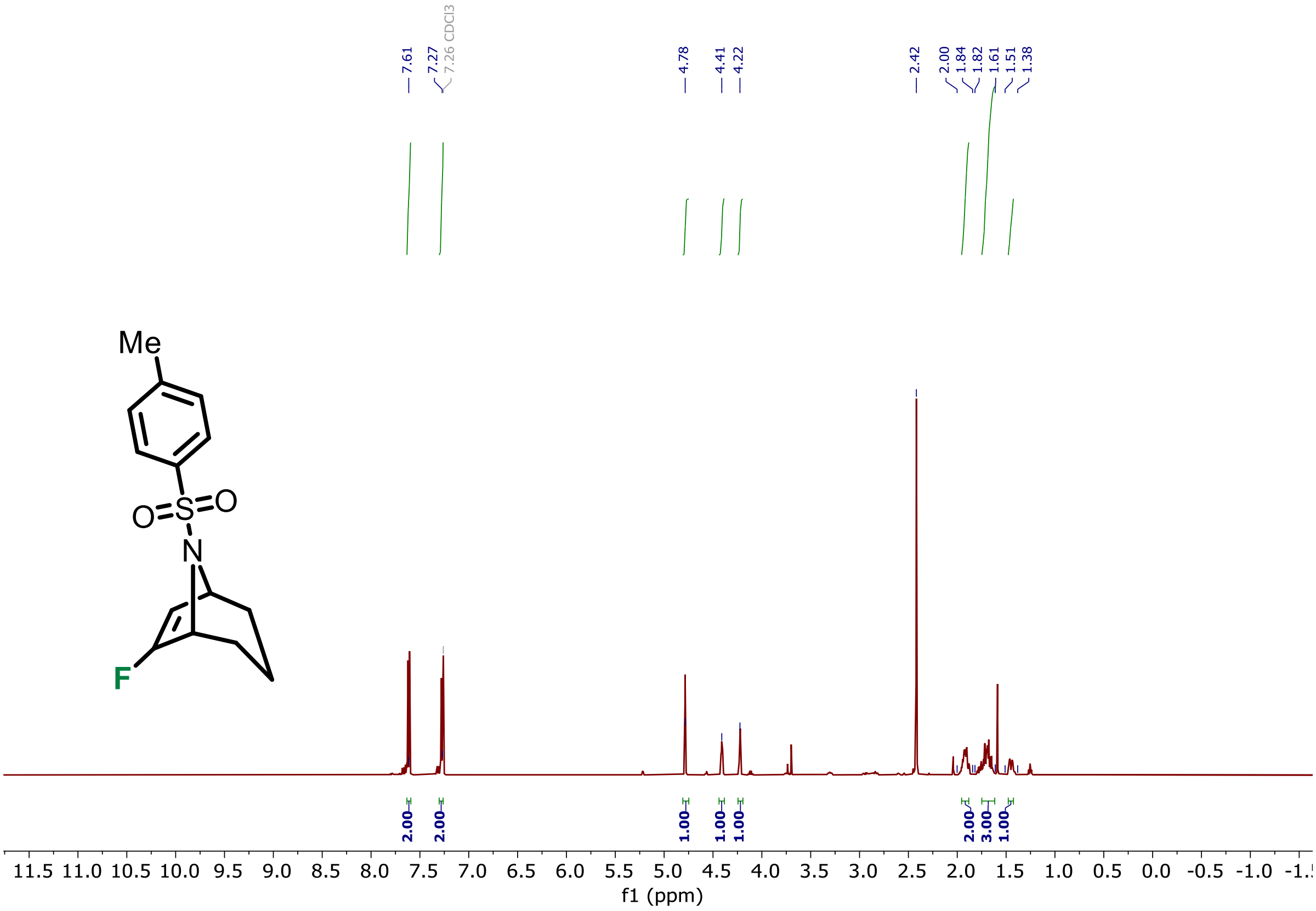

^{13}C NMR (126 MHz, CDCl$_3$)

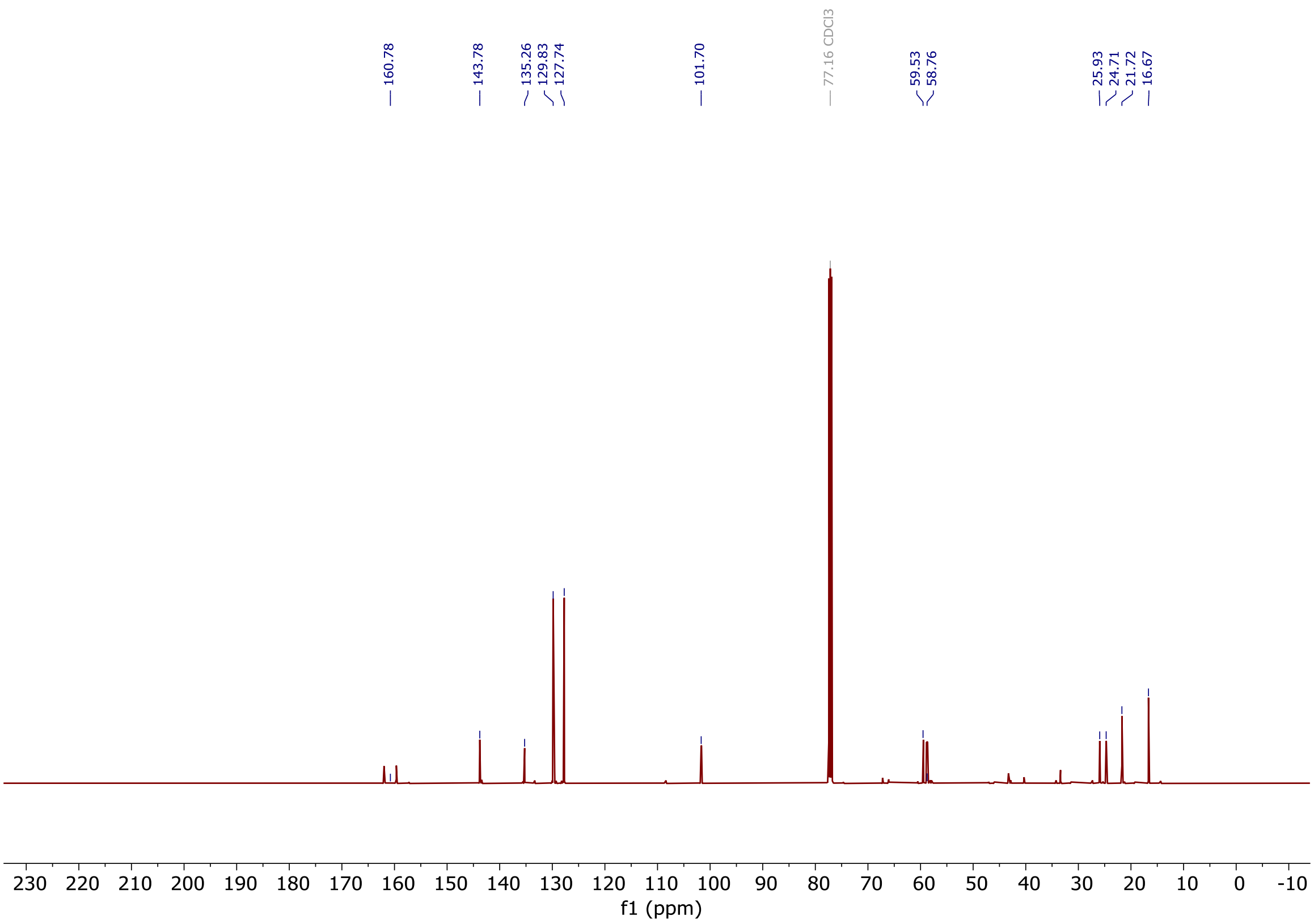

^{19}F NMR (376 MHz, CDCl$_3$)

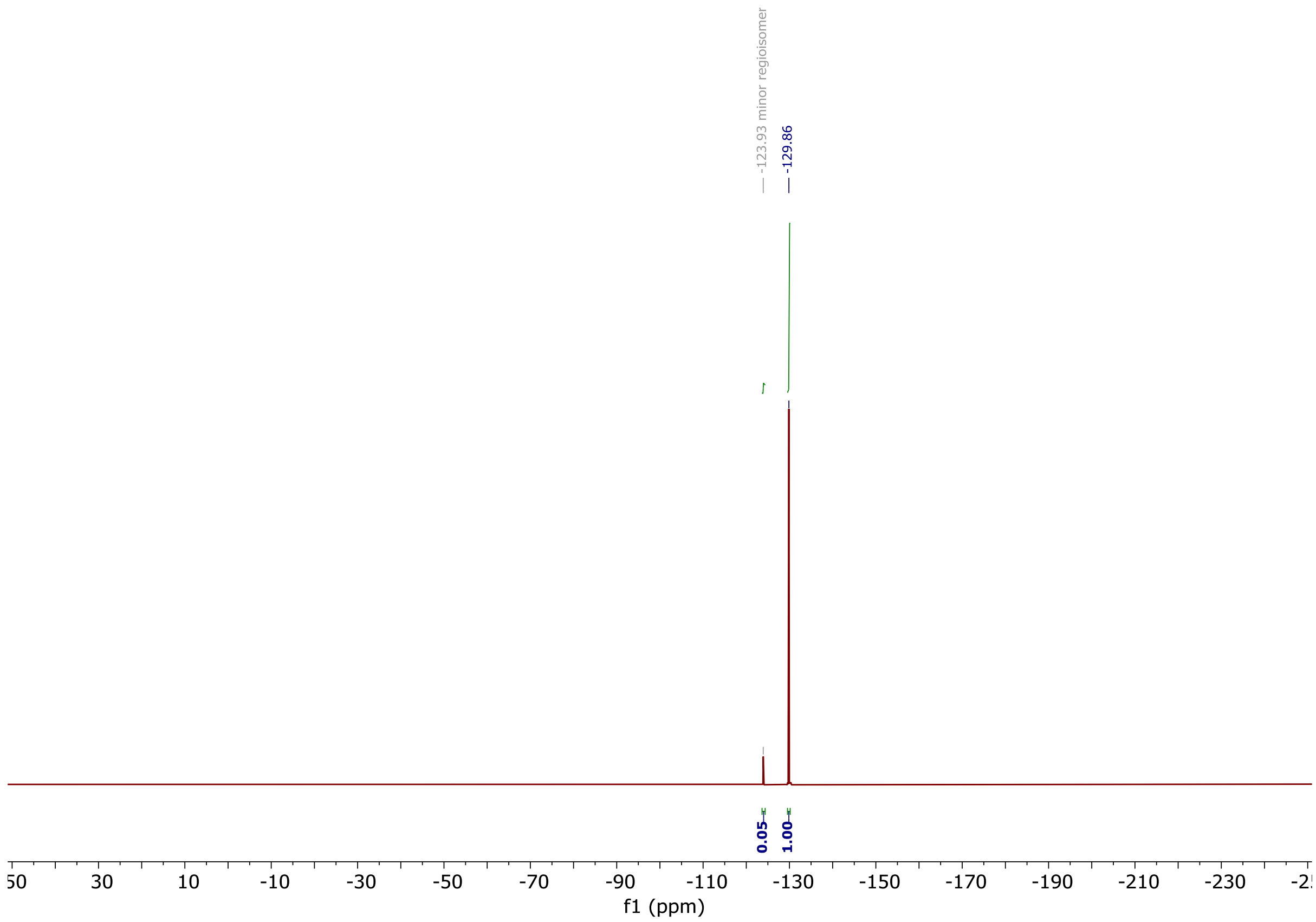

-123.93 minor regioisomer
-129.86
0.05
1.00
50
30
10
-10
-30
-50
-70
-90
-110
-130
-150
-170
-190
-210
-230
f1 (ppm)

3-fluoro-1-tosyl-2,5-dihydro-1H-pyrrole **(4ac)**

^{1}H NMR (500 MHz, $CDCl_3$)

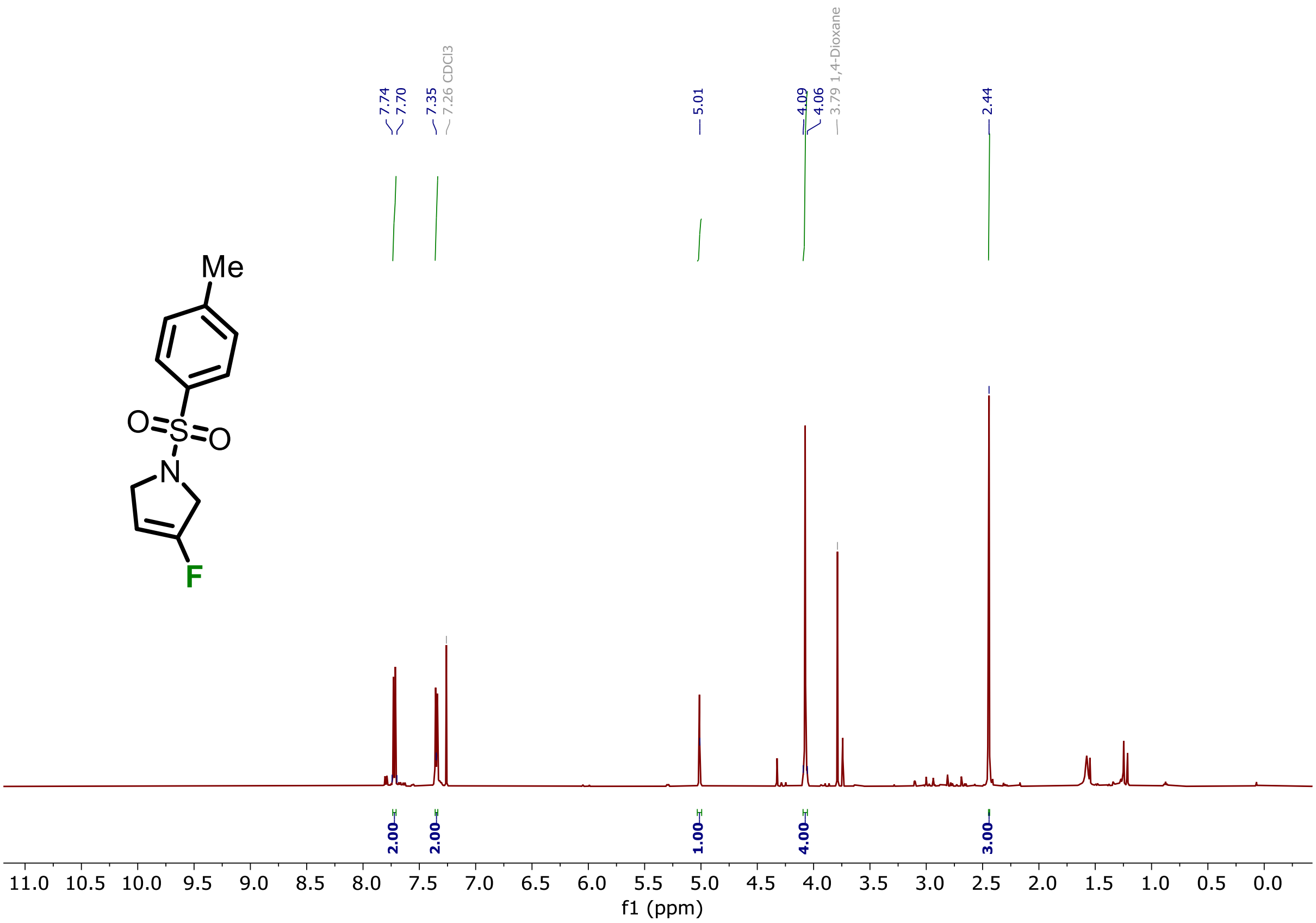

^{13}C NMR (126 MHz, $CDCl_3$)

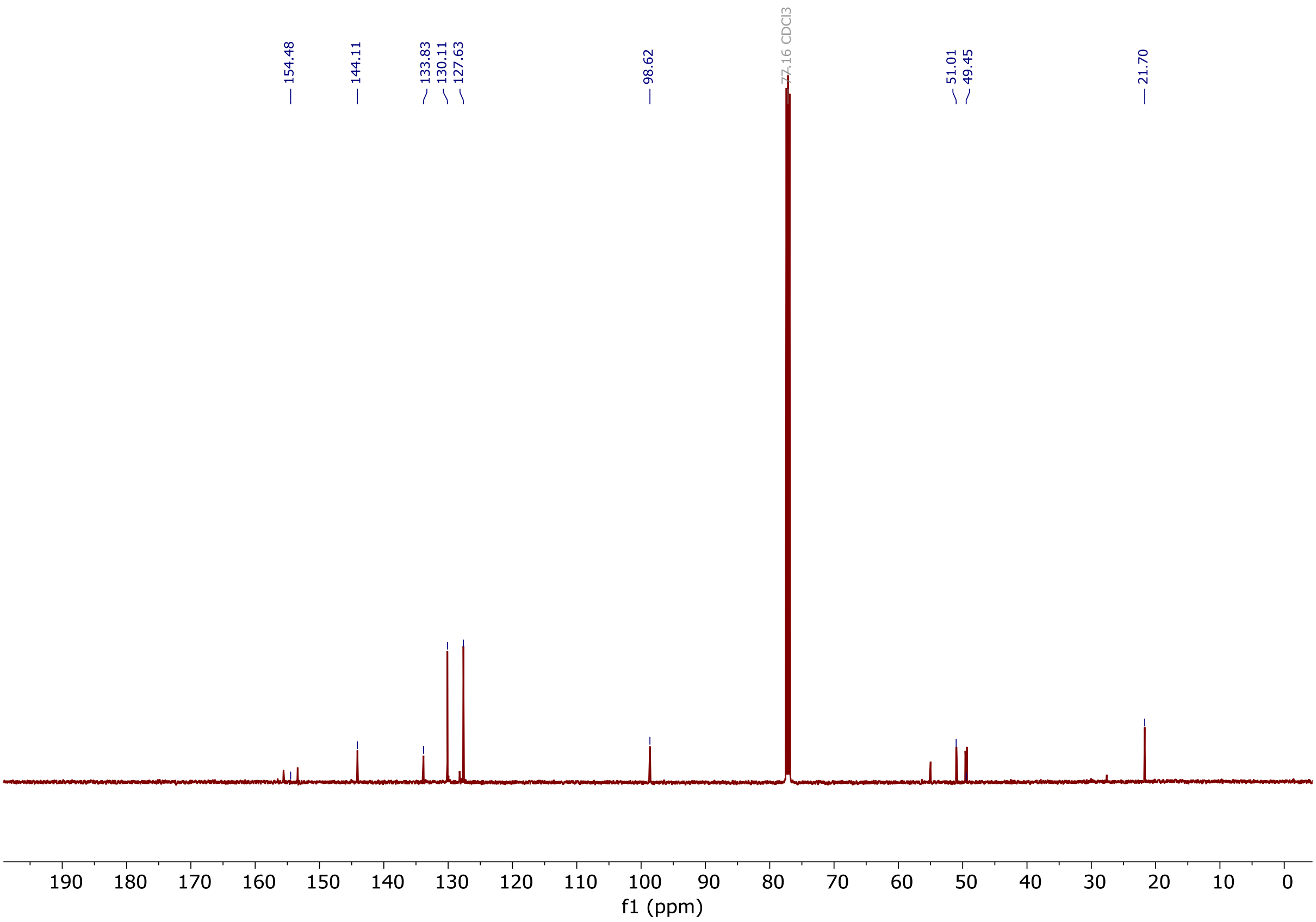

^{19}F NMR (377 MHz, CDCl$_3$)

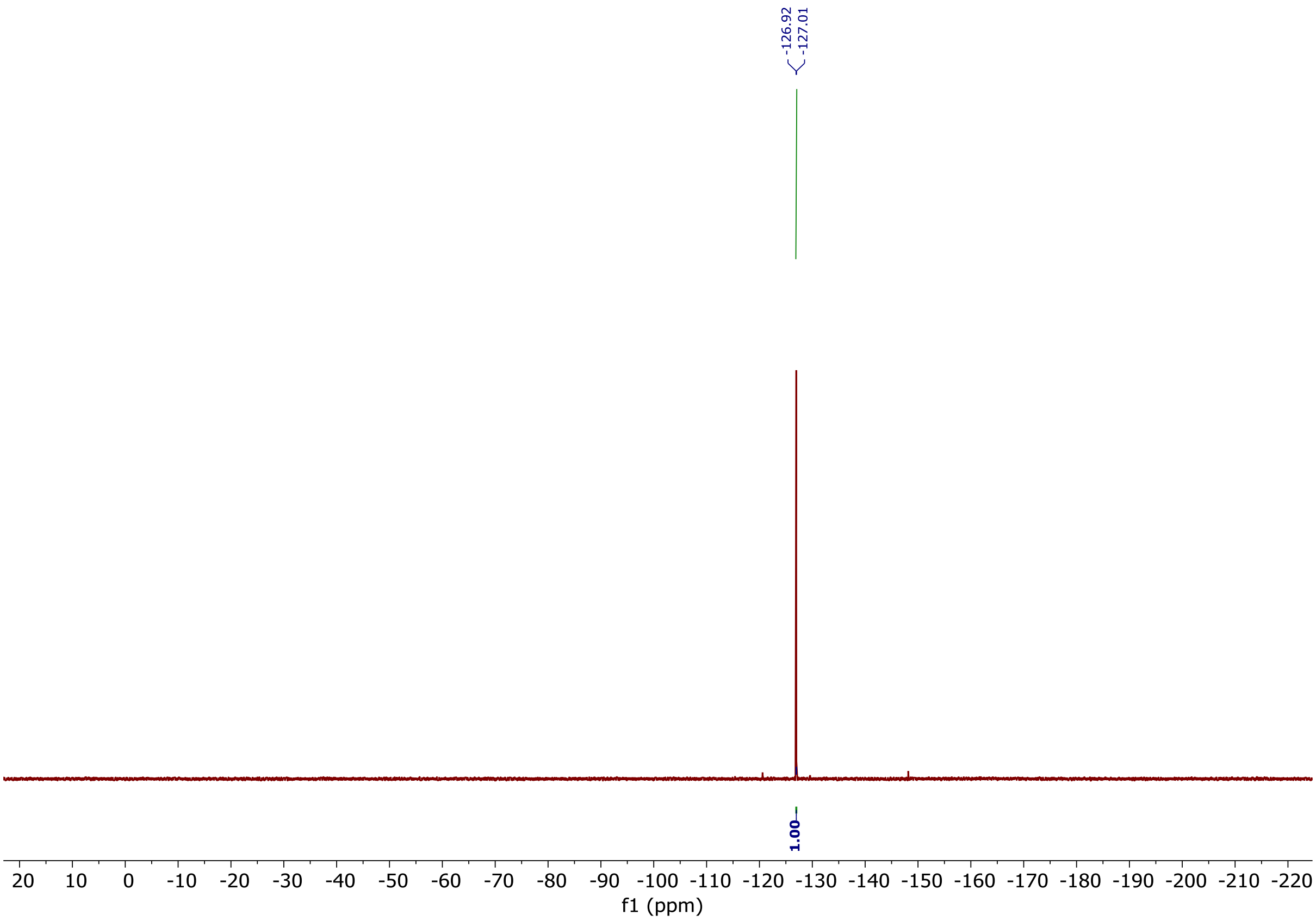

9.4. Other Cycles (5)

***(syn)-7-fluoro-2-tosyl-2-azabicyclo[3.2.1]oct-6-ene* (5z)**

^{1}H NMR (500 MHz, $CDCl_3$)

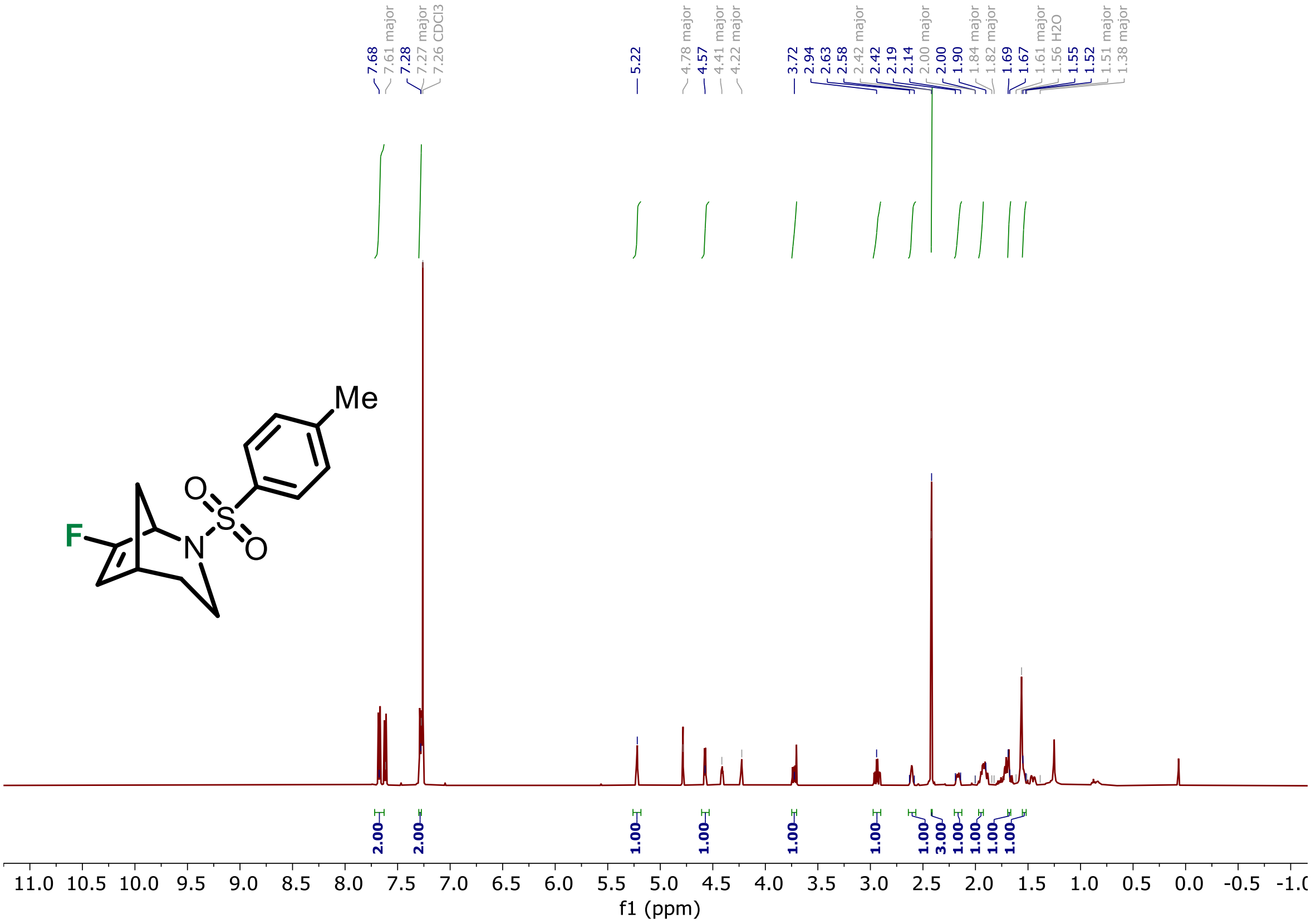

^{13}C NMR (126 MHz, $CDCl_3$)

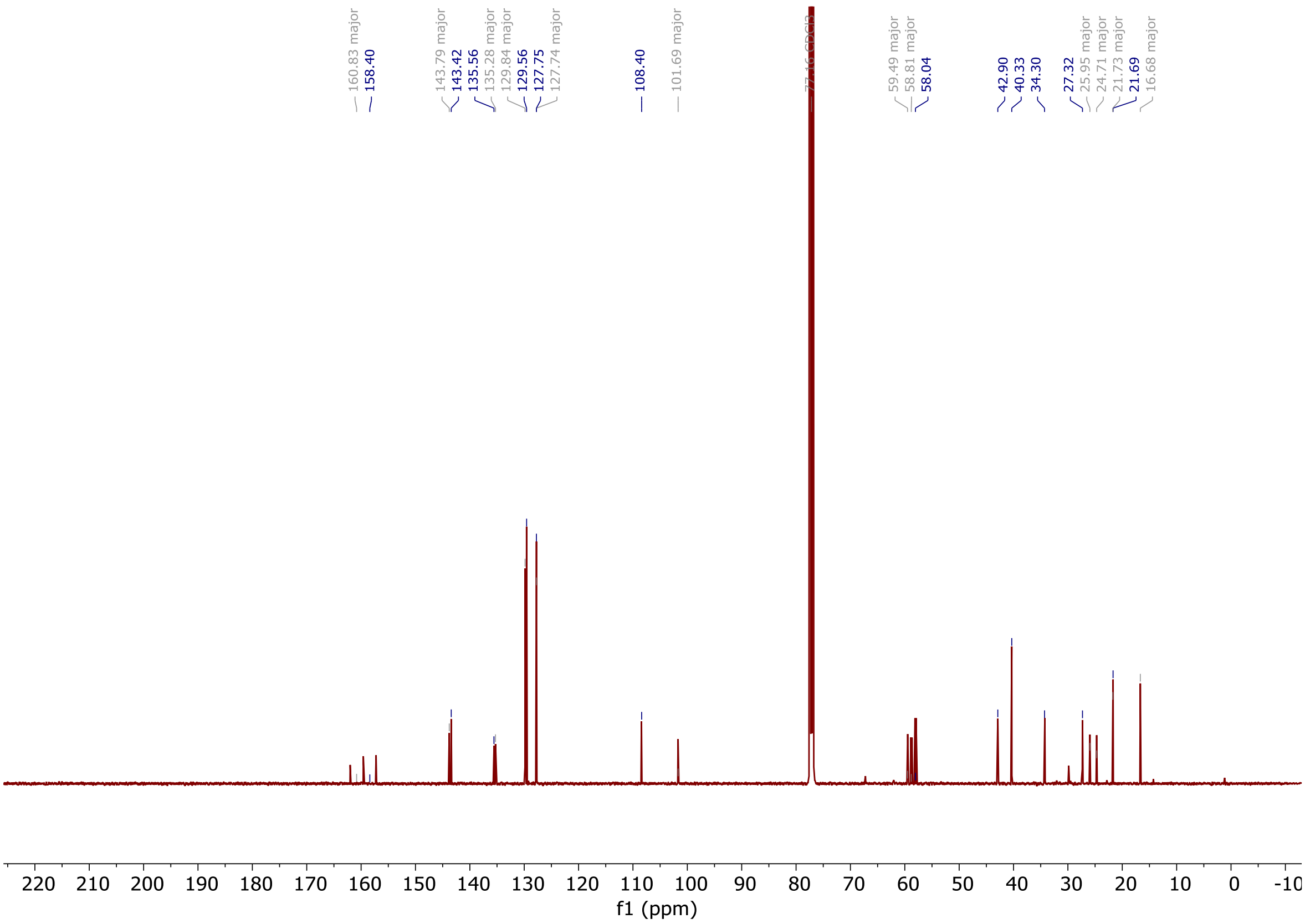

^{19}F NMR (377 MHz, CDCl$_3$)

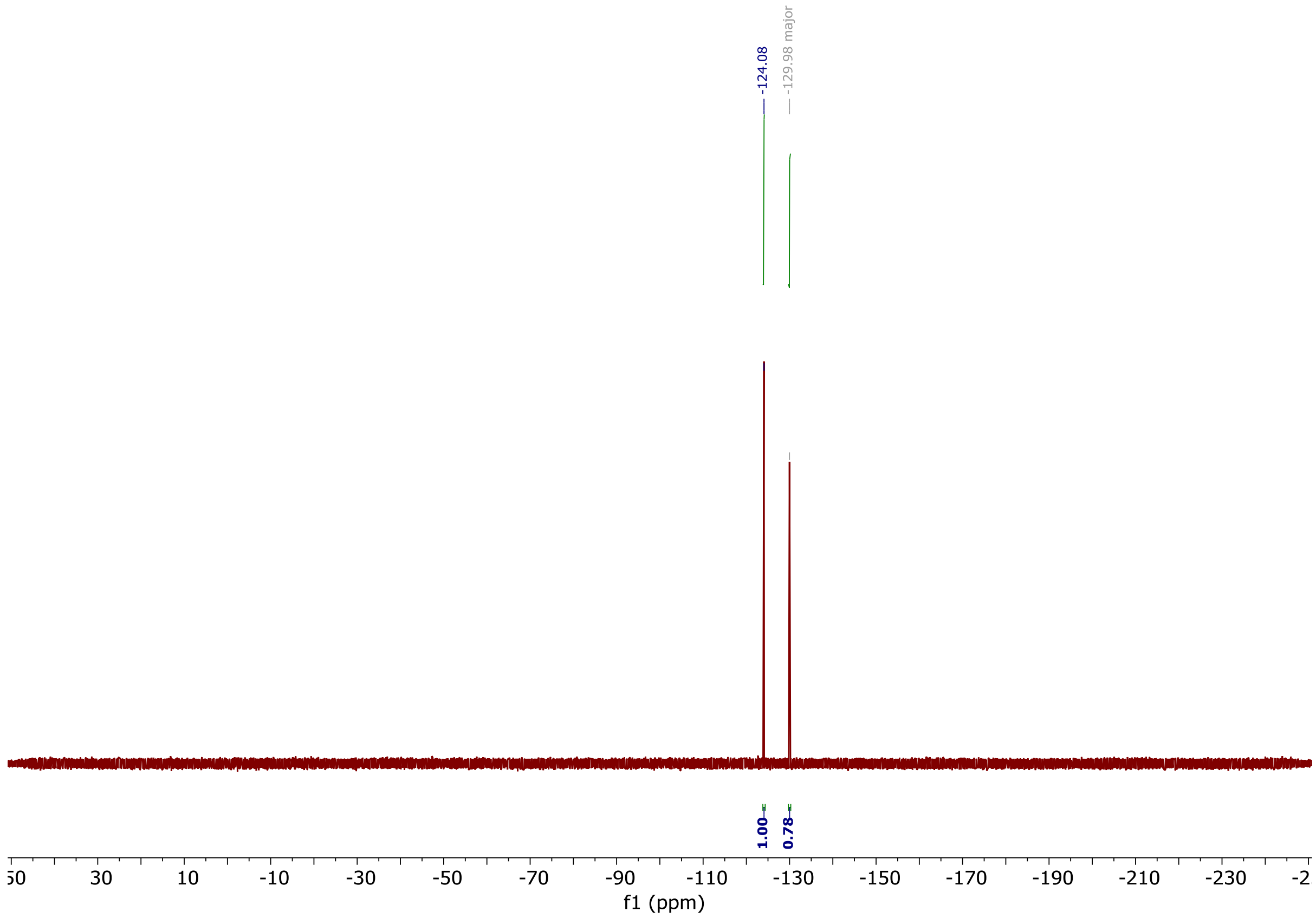

N-(3-fluorobenzyl)-N-((syn)-7-fluorobicyclo[3.2.1]oct-6-en-1-yl)-4-methylbenzenesulfonamide **(5aa)**

^{1}H NMR (500 MHz, $CDCl_3$)

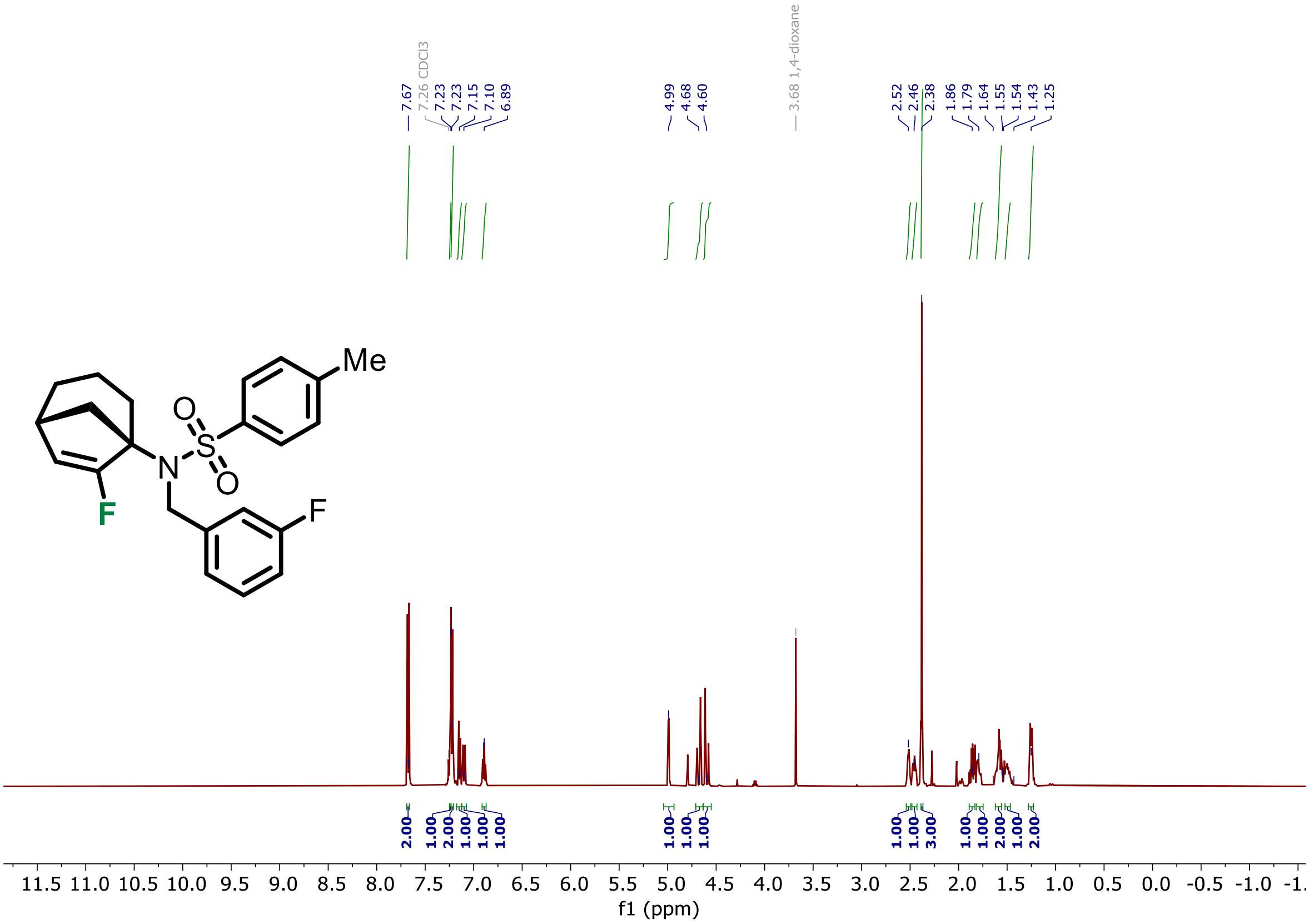

^{13}C NMR (126 MHz, $CDCl_3$)

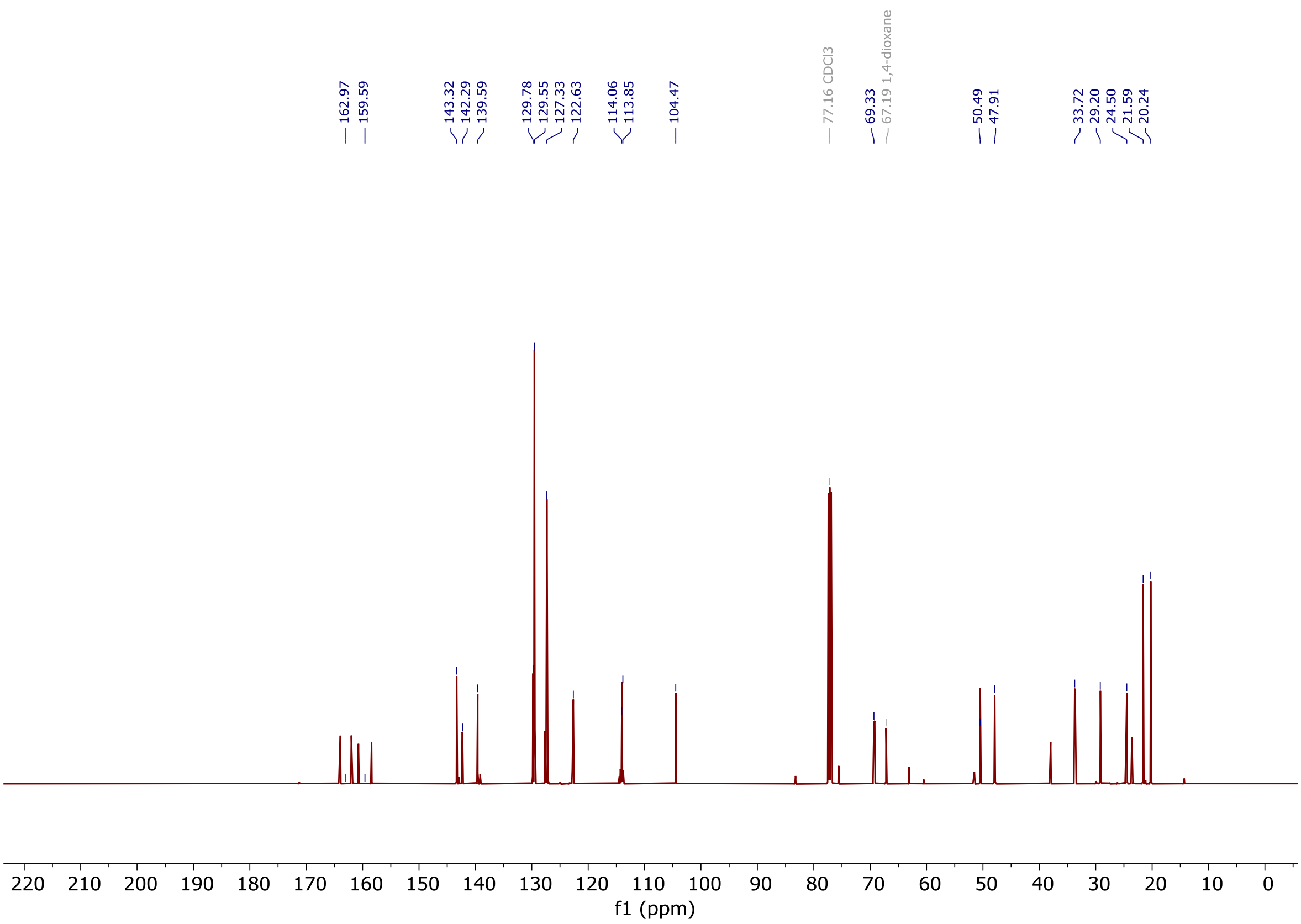

^{19}F NMR (376 MHz, CDCl$_3$)

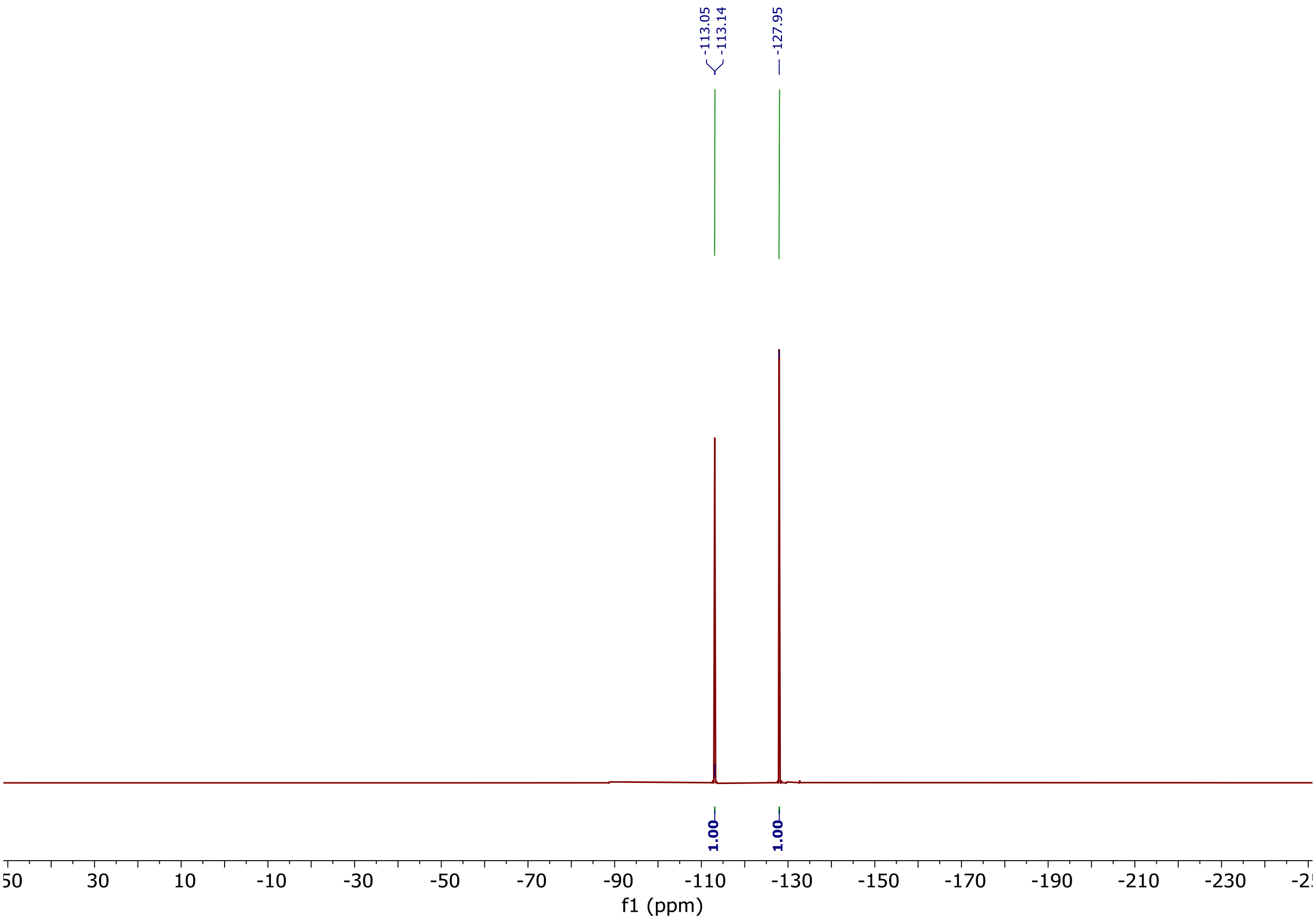

N-((syn)-7-fluorobicyclo[3.2.1]oct-6-en-1-yl)-N,4-dimethylbenzenesulfonamide **(5ab)**

^{1}H NMR (500 MHz, CDCl$_3$)

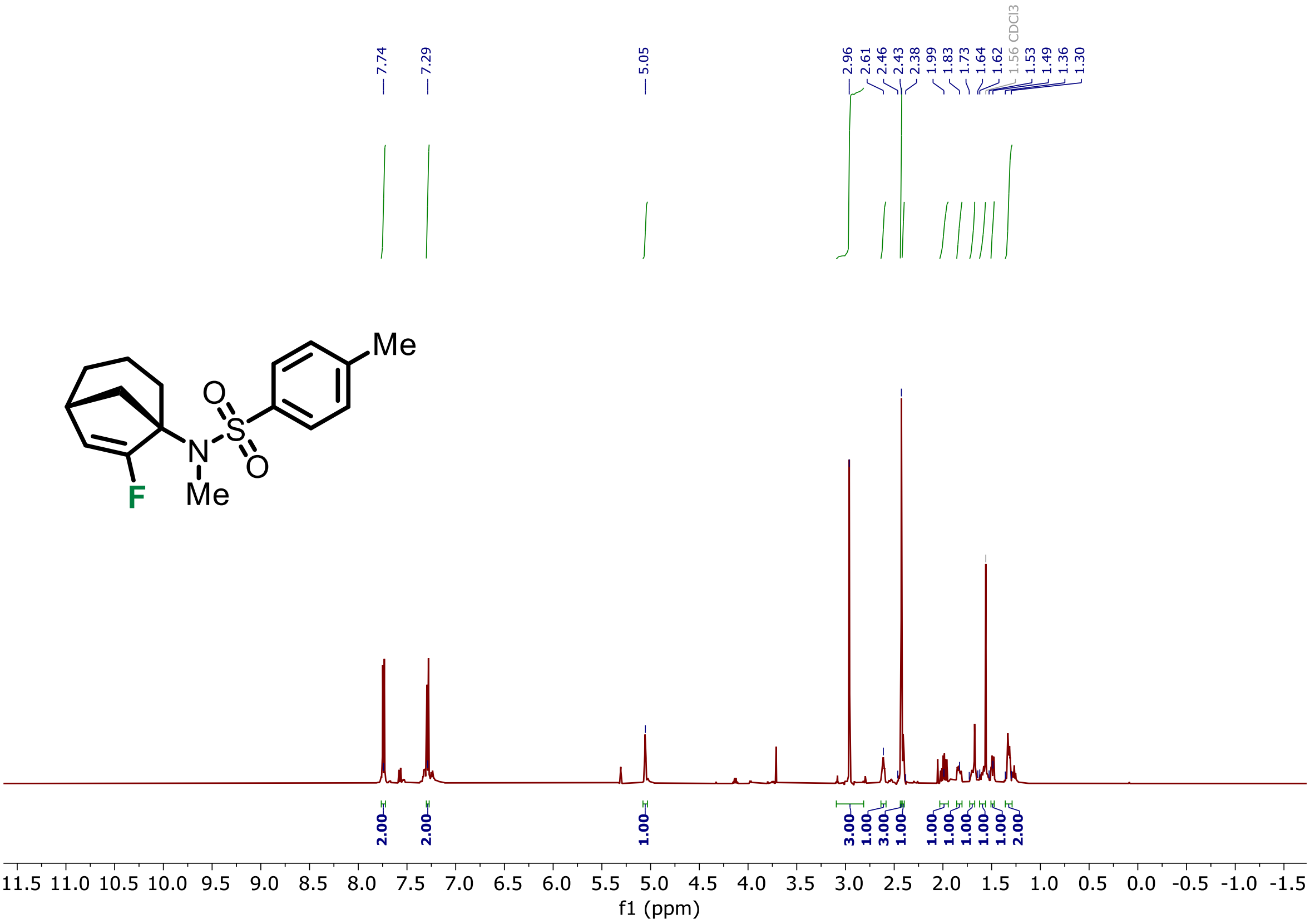

^{13}C NMR (126 MHz, CDCl$_3$)

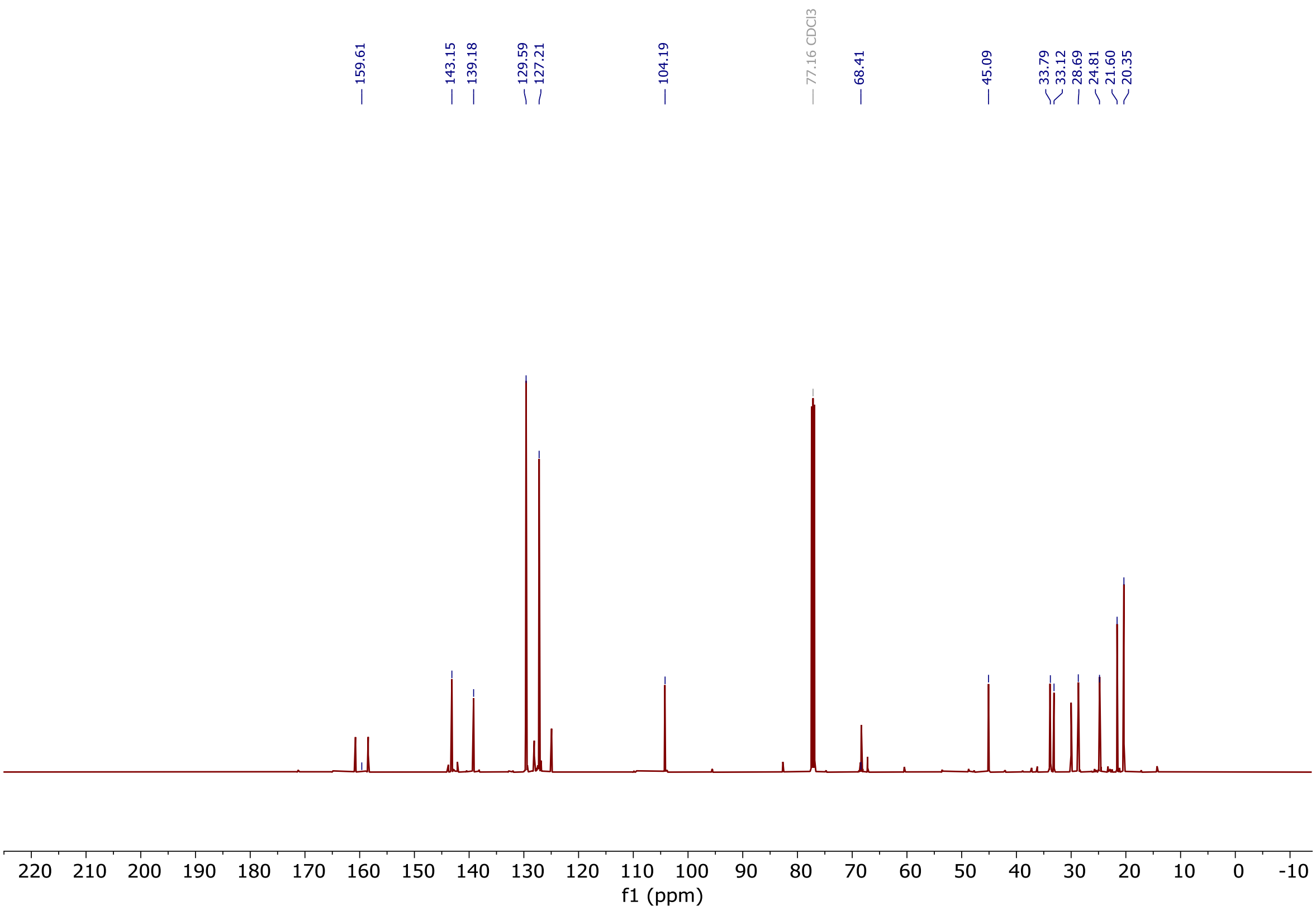

^{19}F NMR (376 MHz, $CDCl_3$)

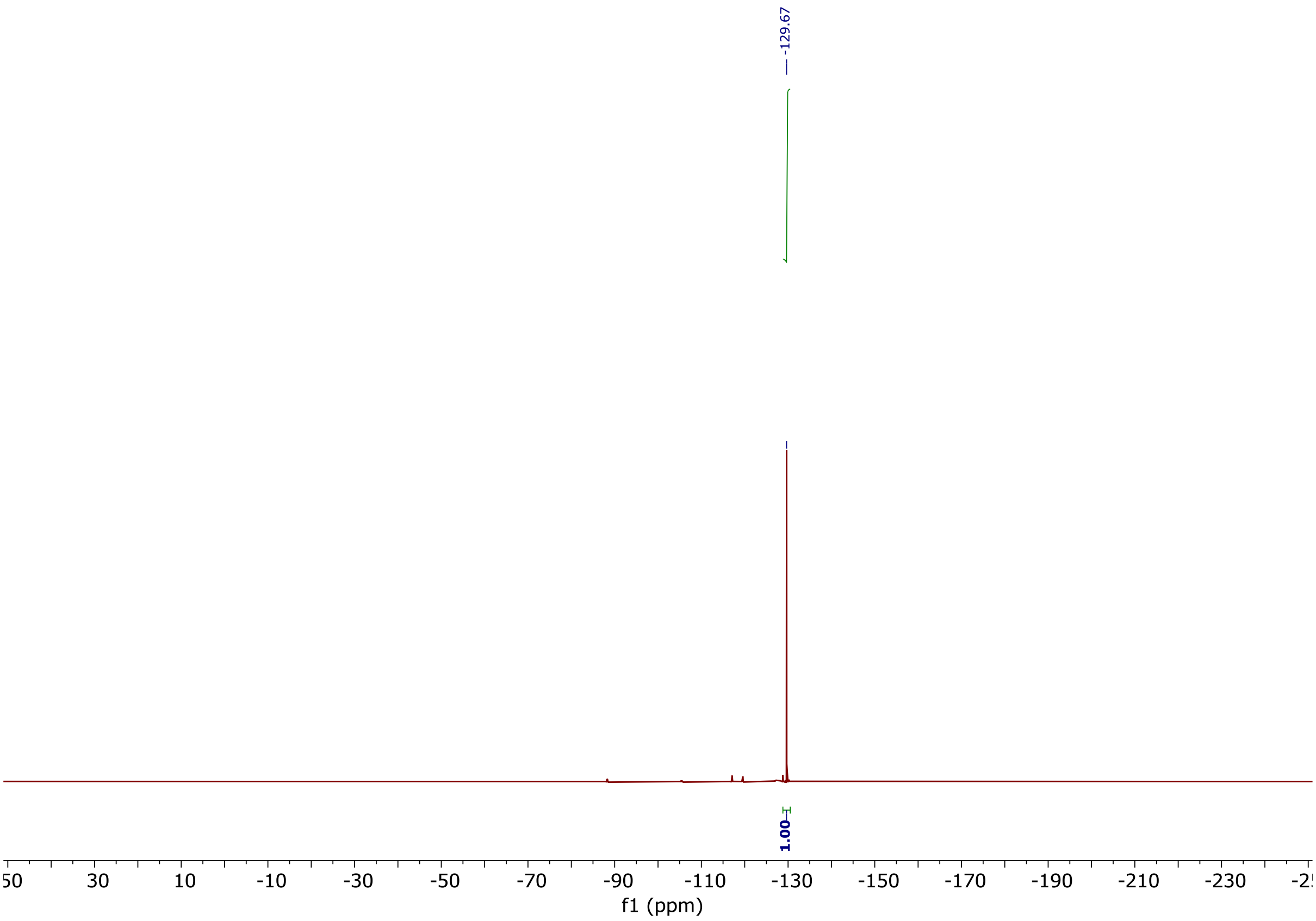

2-(((cis-7-fluorobicyclo[3.2.1]oct-6-en-1-yl)oxy)methyl)benzonitrile **(5ai)**

^{1}H NMR (400 MHz, $CDCl_3$)

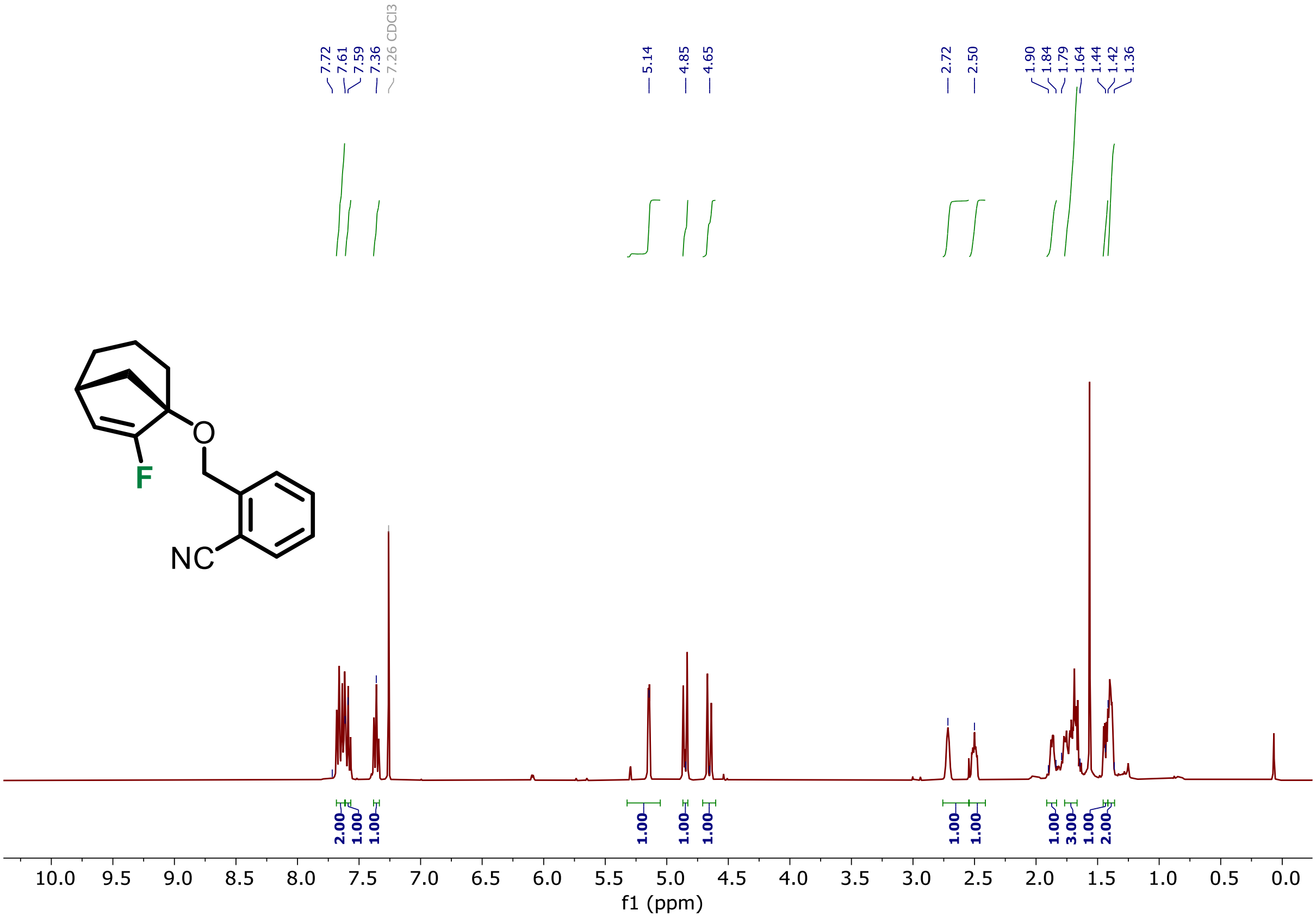

^{13}C NMR (101 MHz, $CDCl_3$)

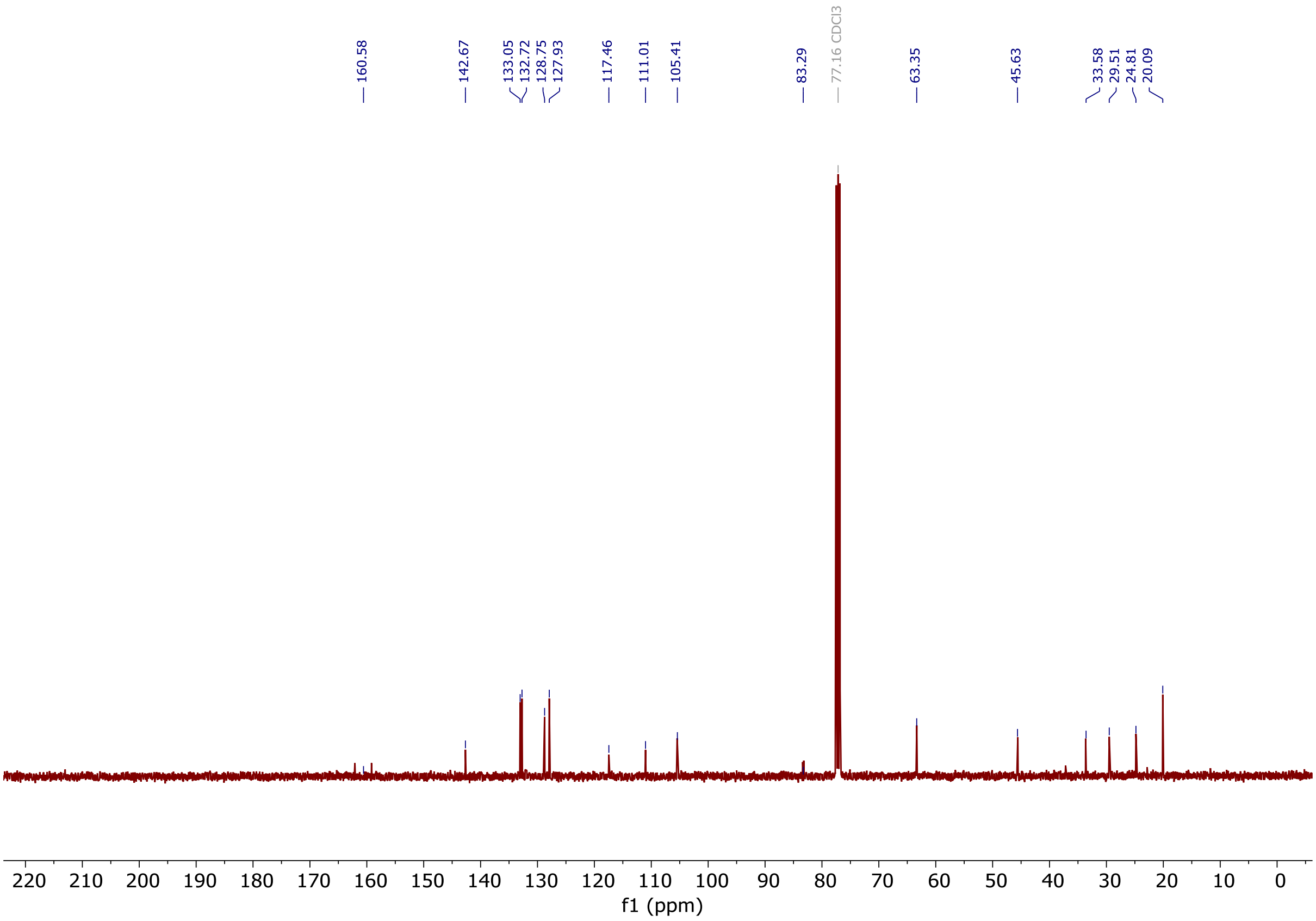

^{19}F NMR (376 MHz, CDCl$_3$

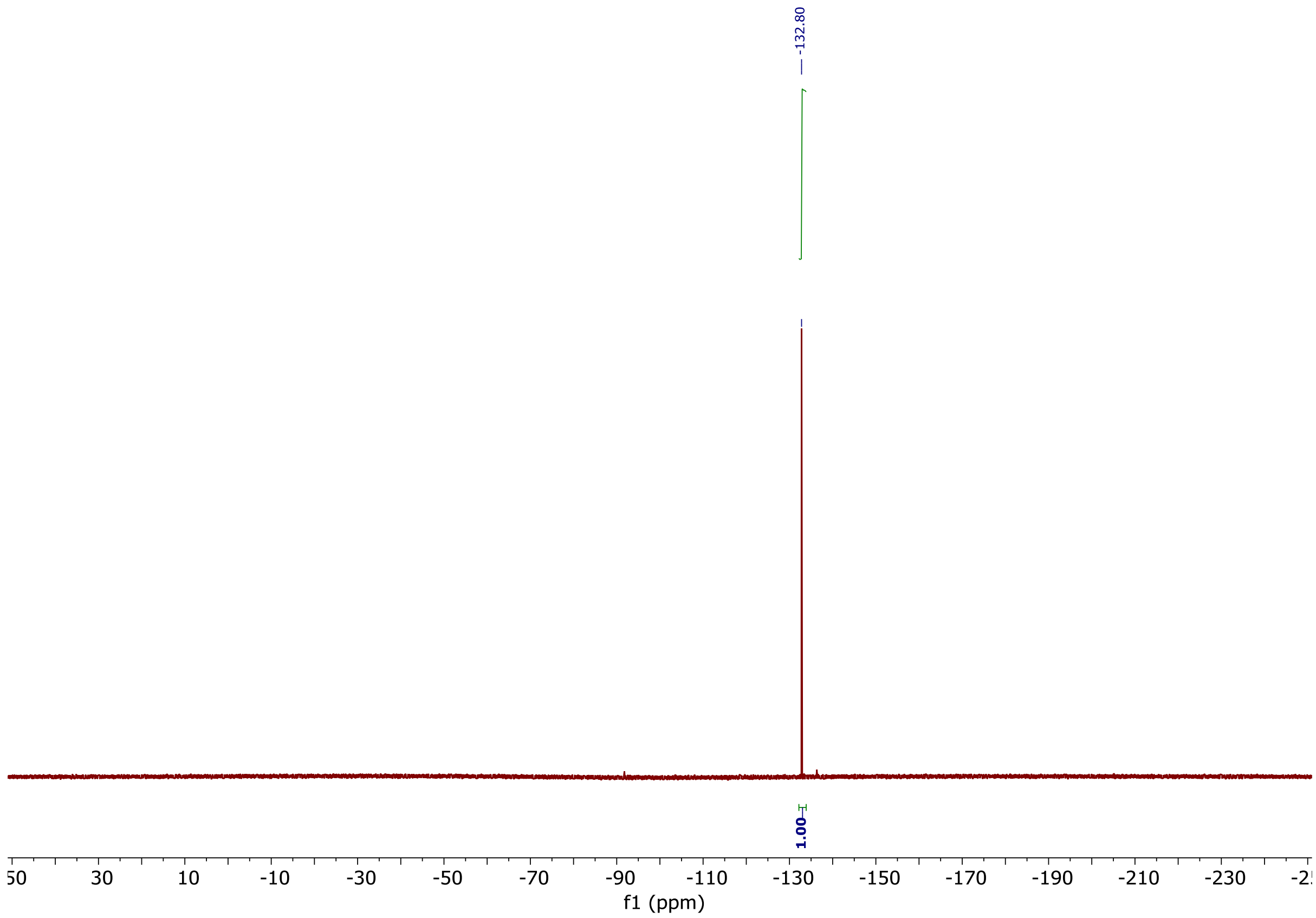

9.5. Oxolenes (6)

***3-fluoro-2-methyl-5-phenyl-2,5-dihydrofuran* (6ad)**

^{1}H NMR (500 MHz, $CDCl_3$)

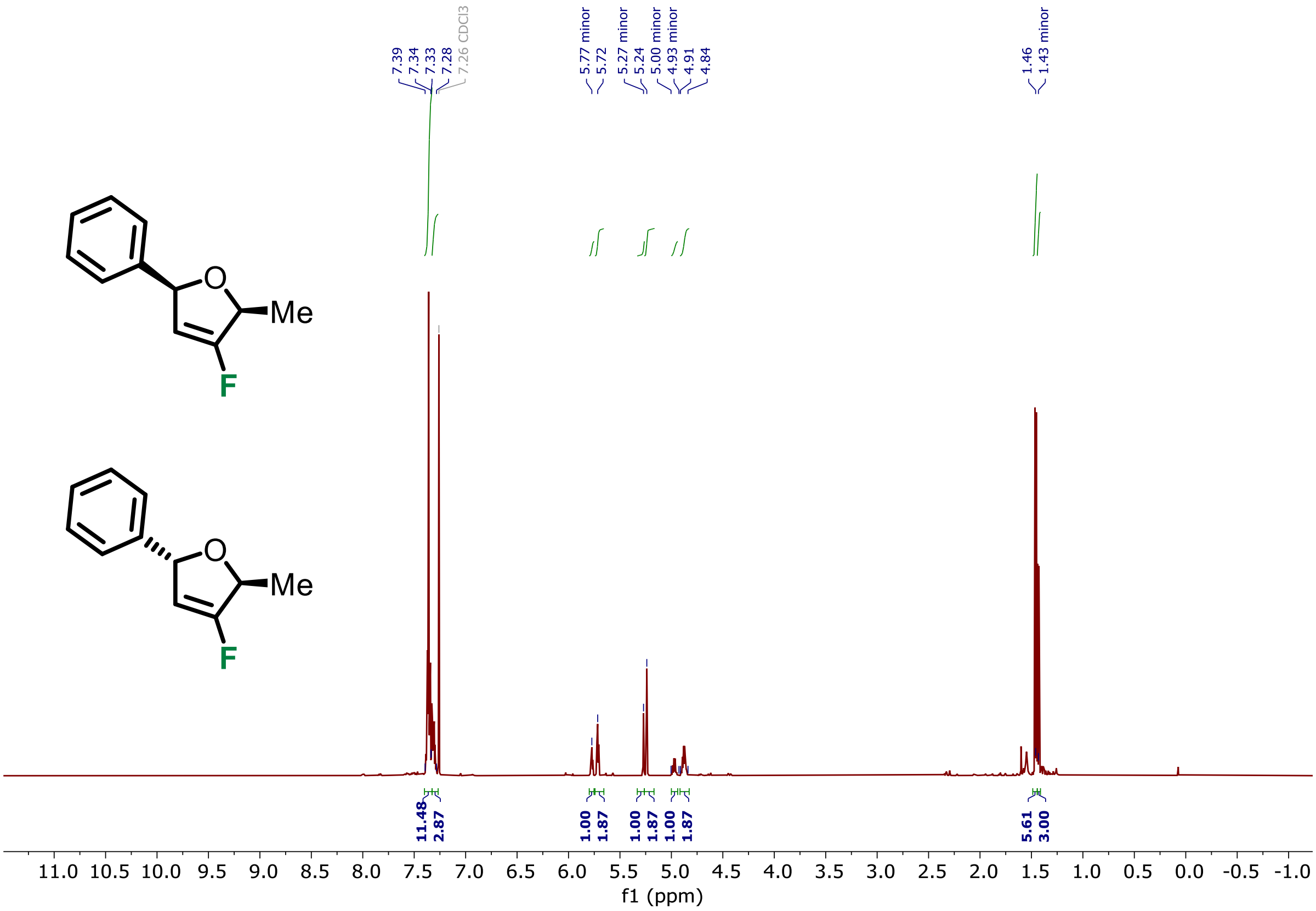

^{13}C NMR (126 MHz, $CDCl_3$)

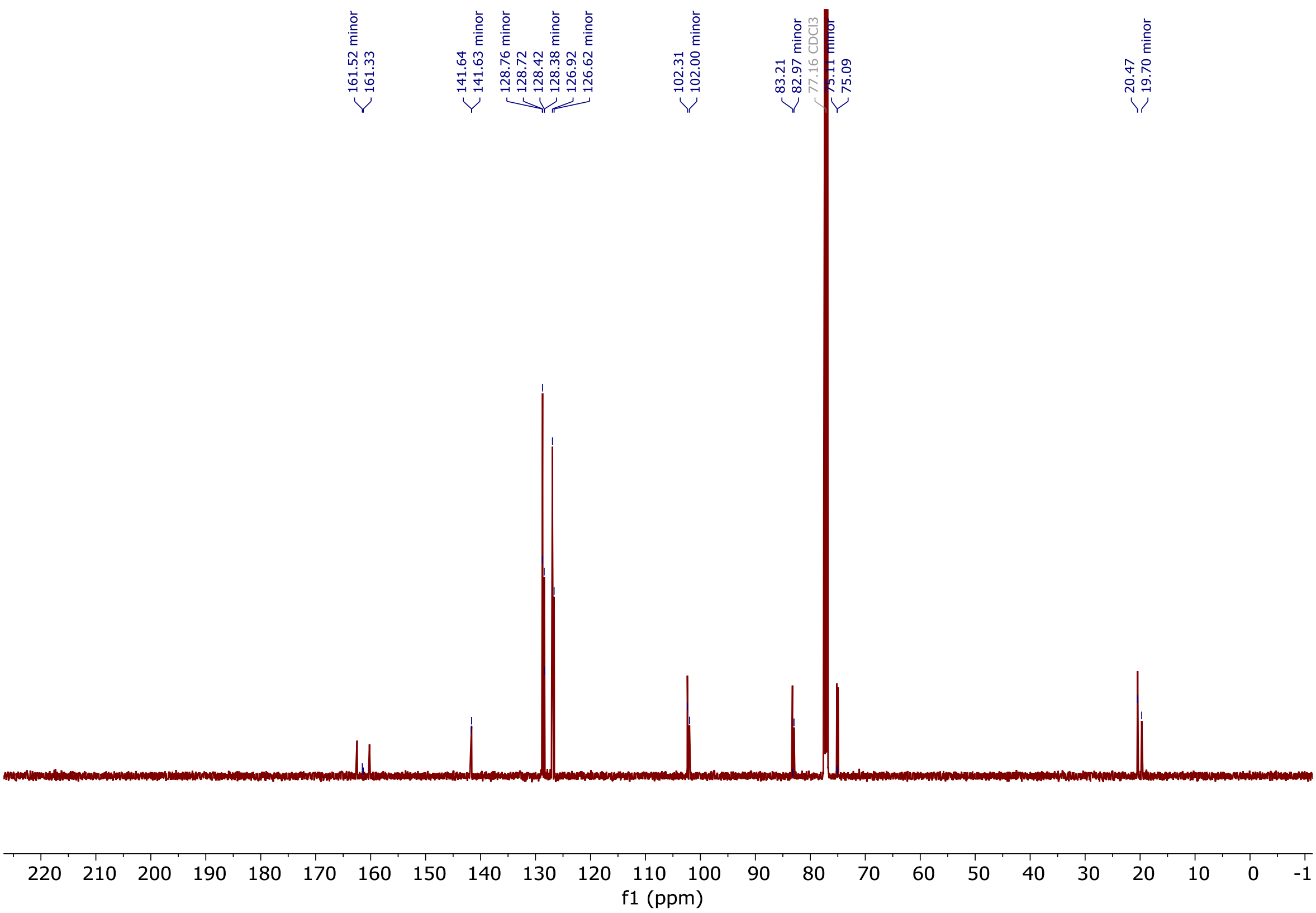

^{19}F NMR (376 MHz, CDCl$_3$)

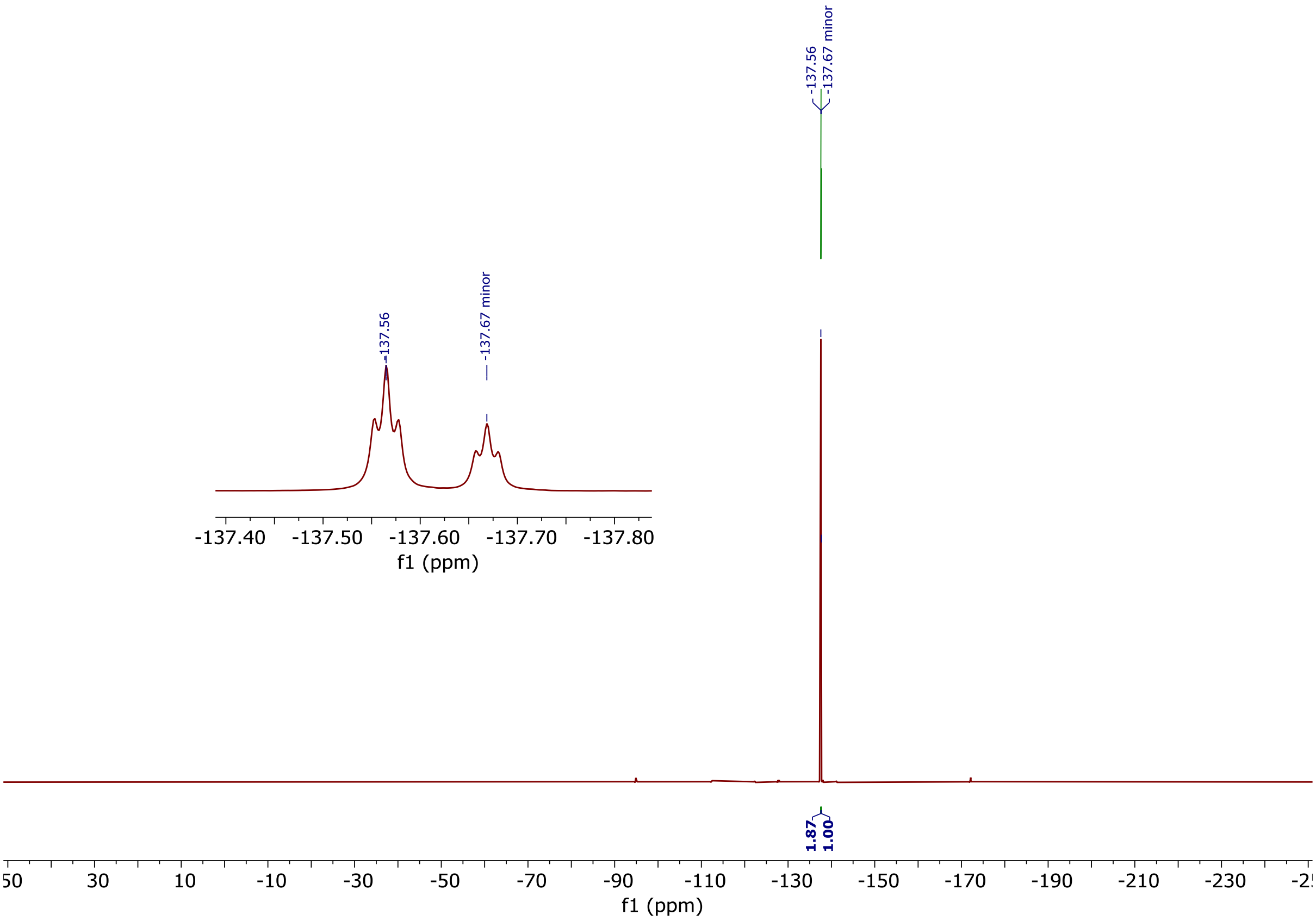

5-(3-bromophenyl)-3-fluoro-2,2-dimethyl-2,5-dihydrofuran (**6ae**)

^{1}H NMR (400 MHz, $CDCl_3$)

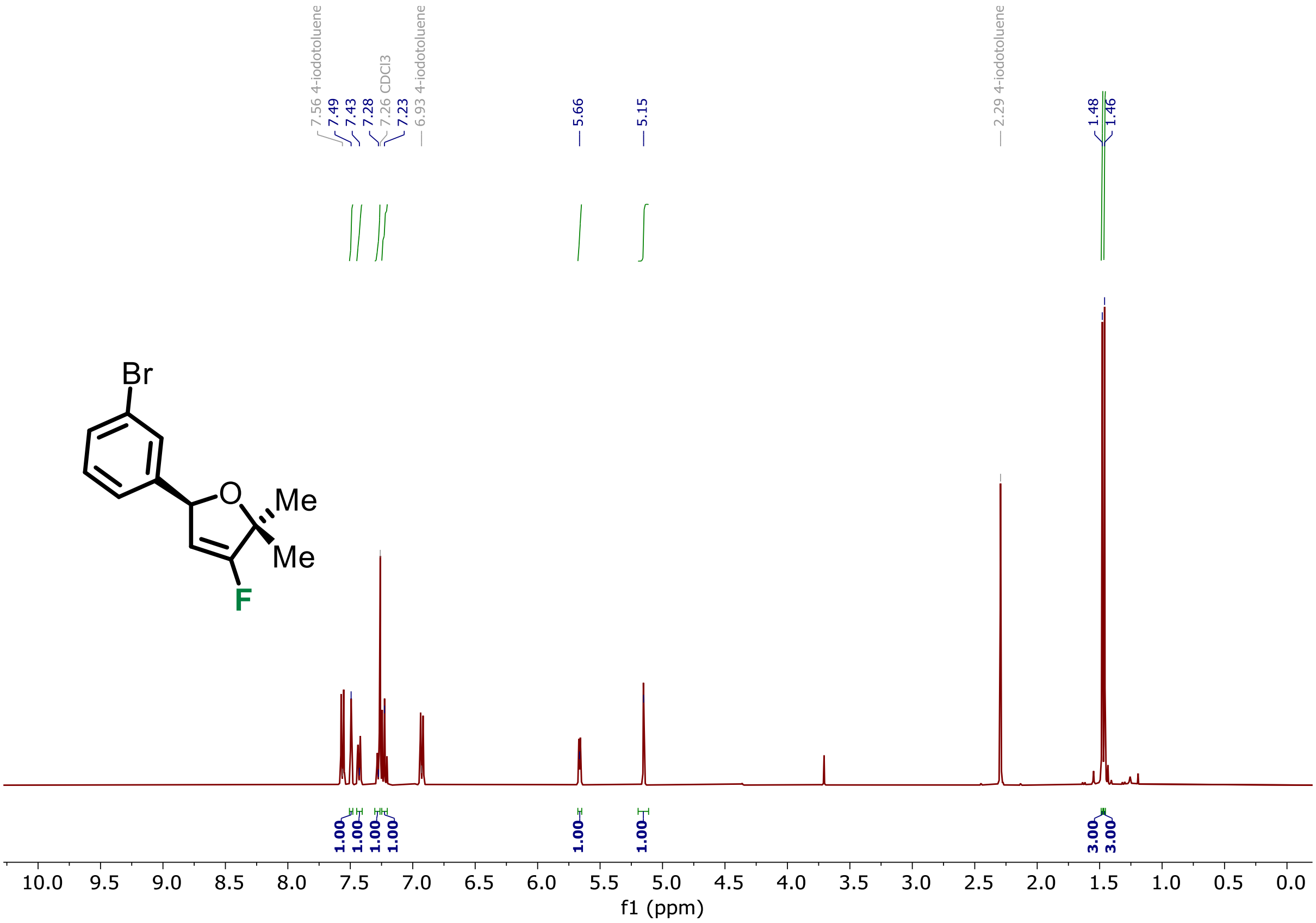

^{13}C NMR (126 MHz, CDCl$_3$)

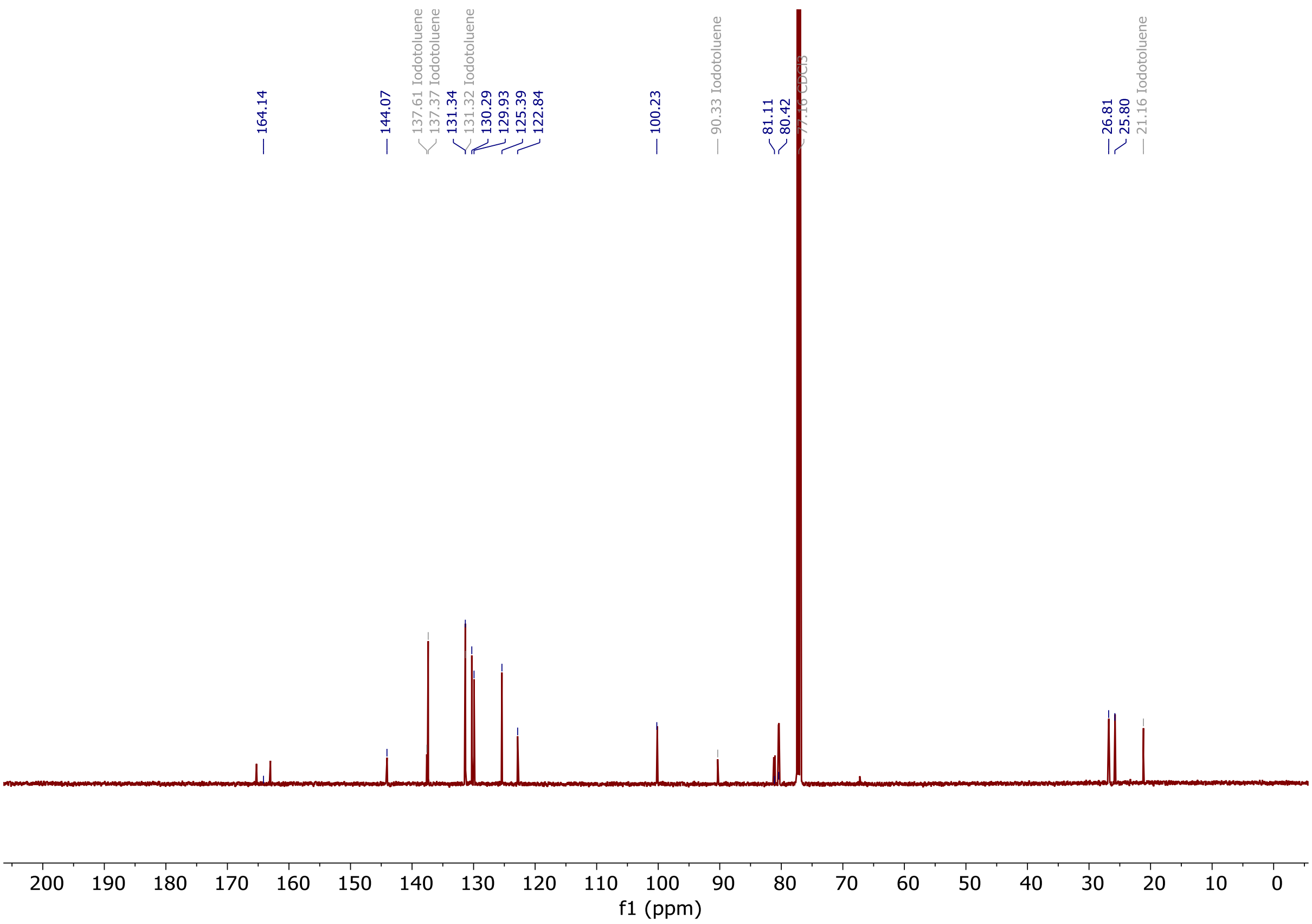

^{19}F NMR (376 MHz, CDCl$_3$)

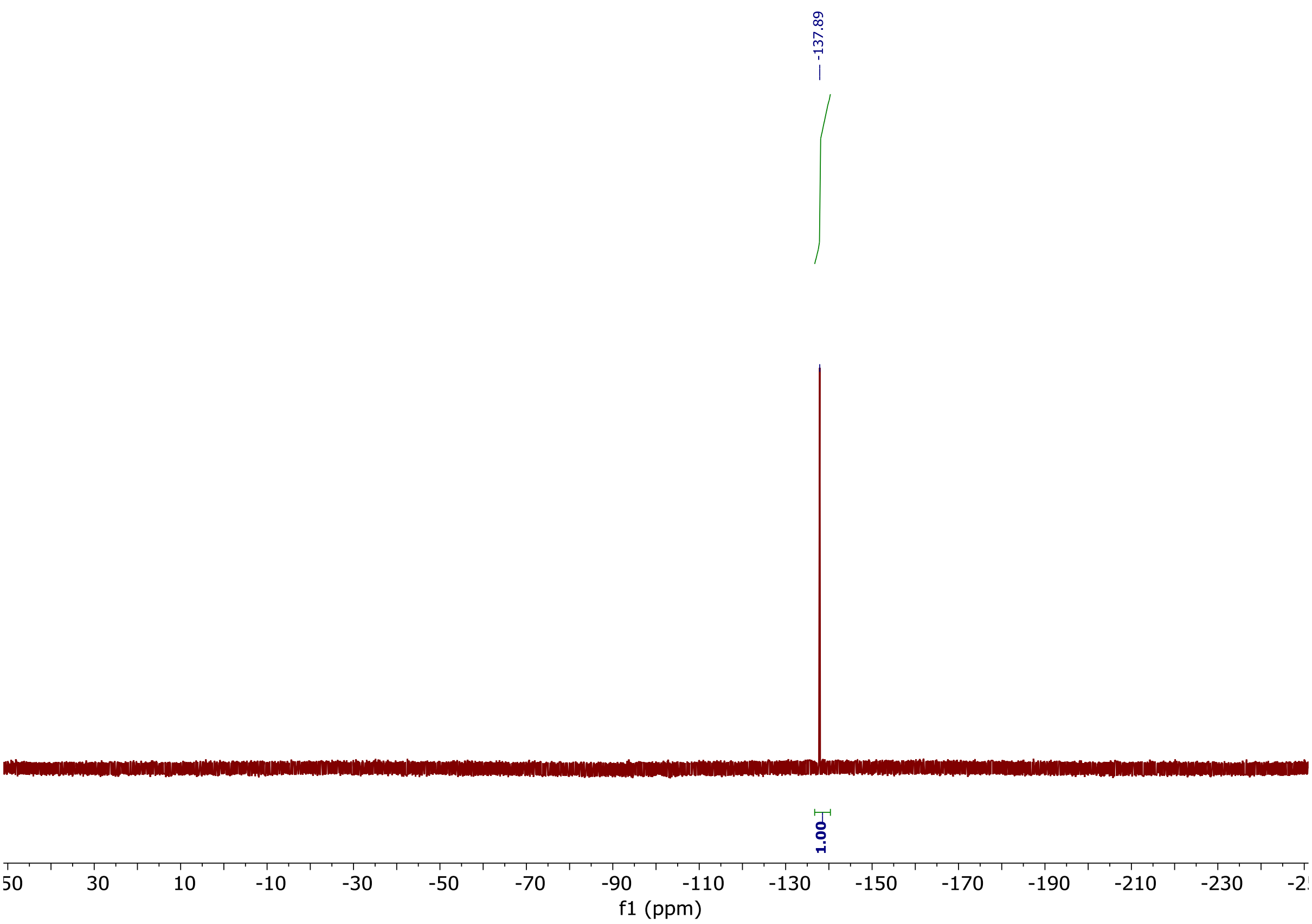

***3-fluoro-5-(3-nitrophenyl)-2,2-diphenyl-2,5-dihydrofuran* (6af)**

^{1}H NMR (500 MHz, CDCl$_3$)

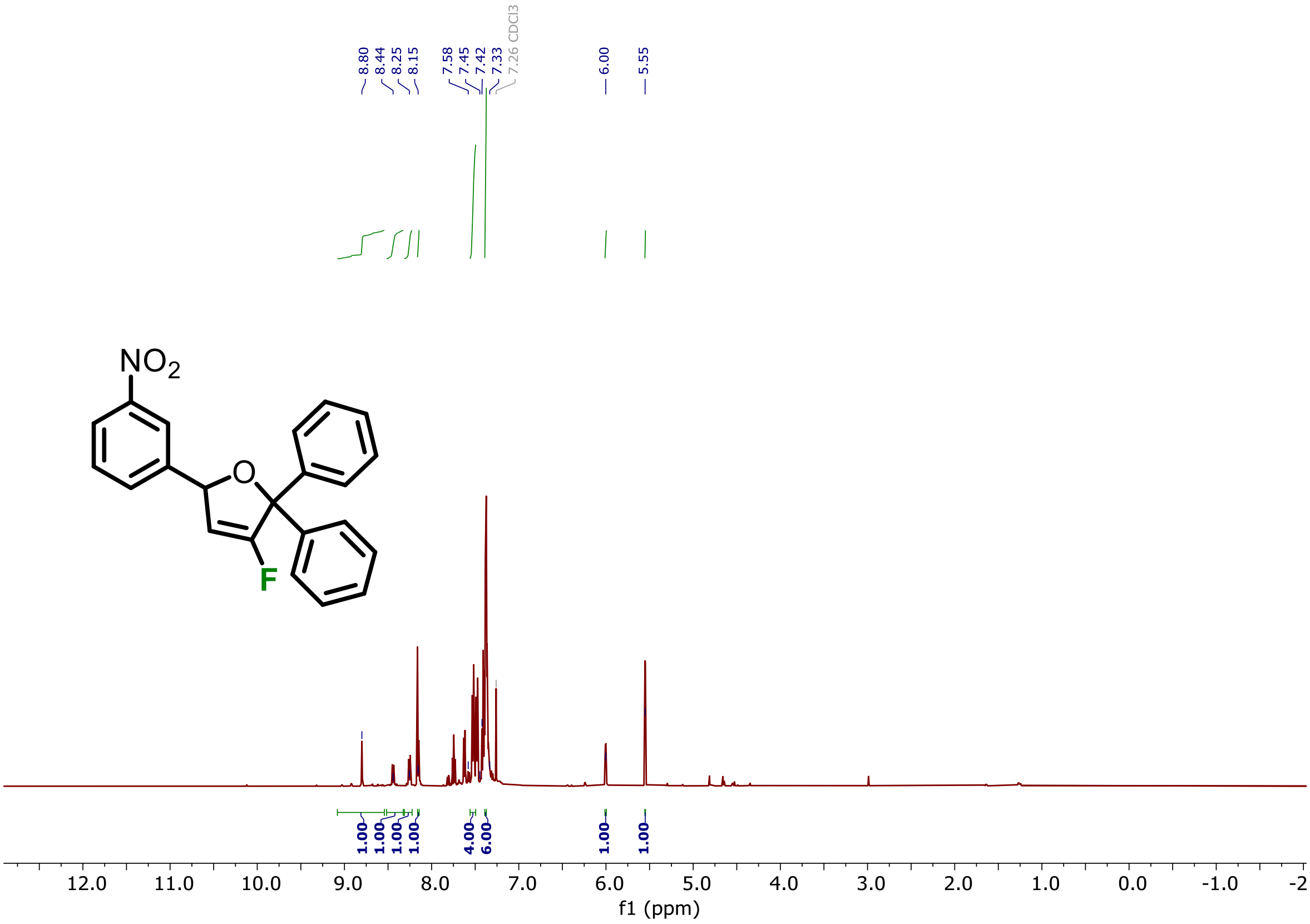

^{13}C NMR (126 MHz, CDCl$_3$)

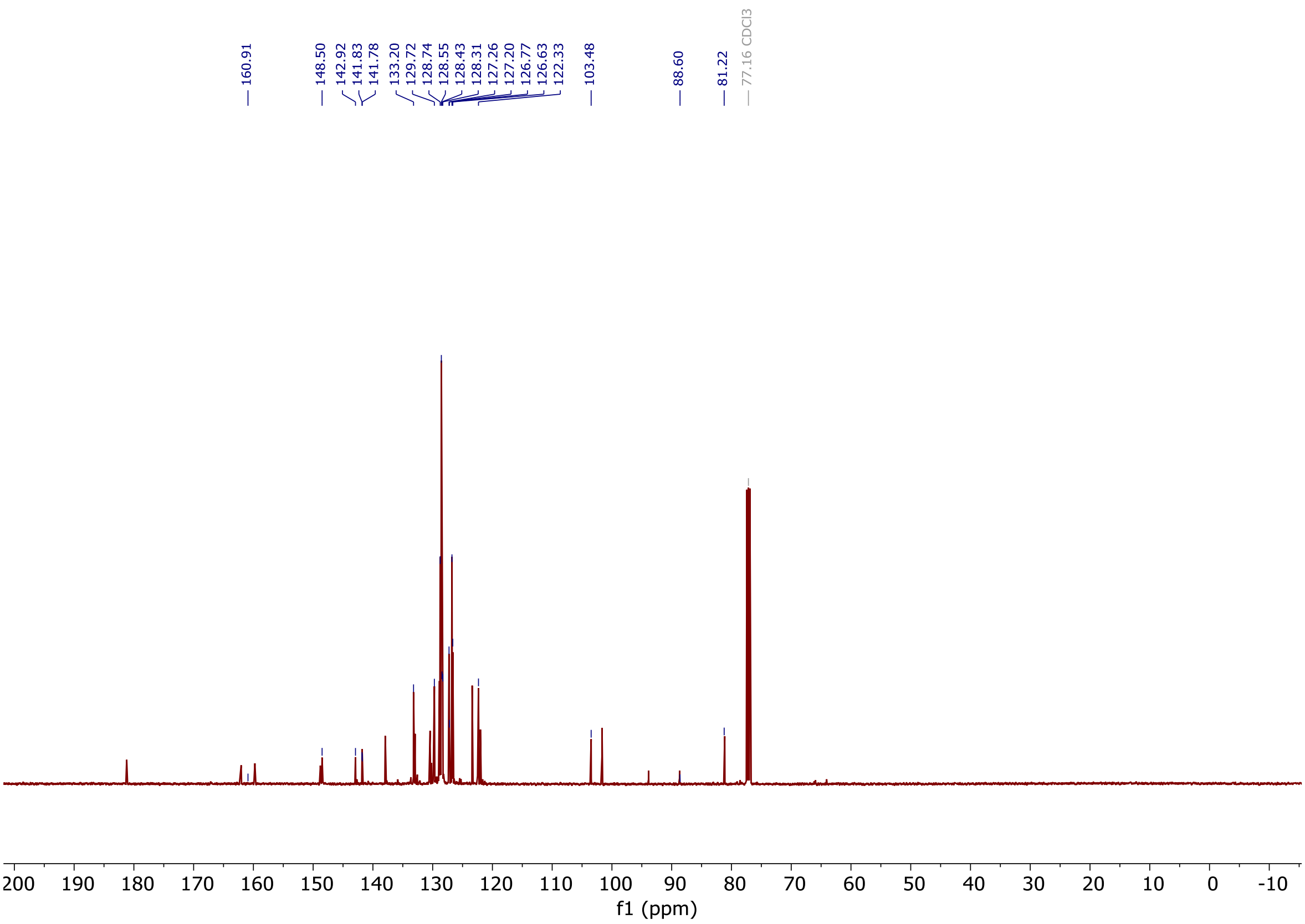

^{19}F NMR (471 MHz, $CDCl_3$)

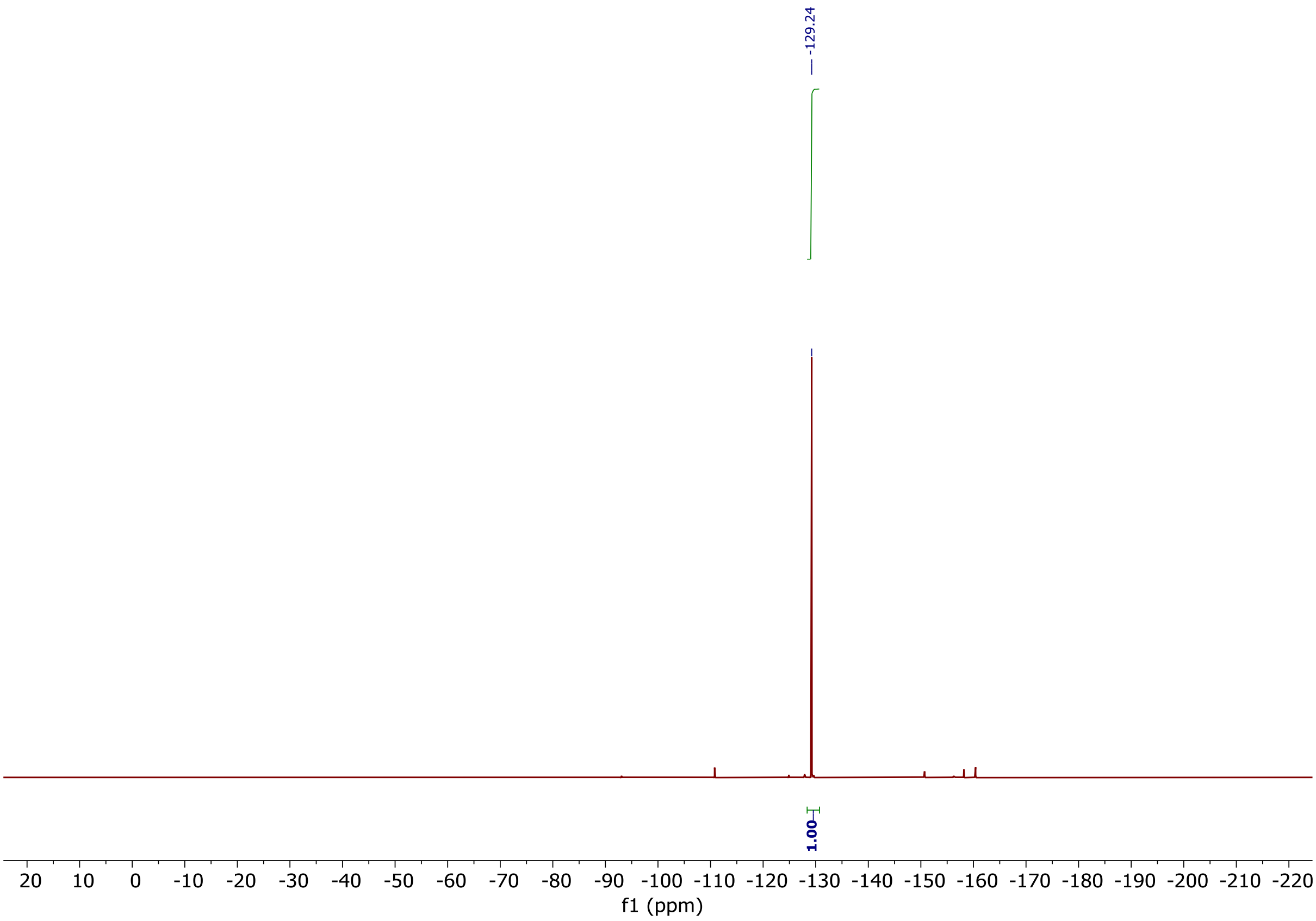

***(4-(4-fluoro-5,5-diphenyl-2,5-dihydrofuran-2-yl)phenyl)(phenyl)methanone* (6ag)**

^{1}H NMR (500 MHz, CDCl$_3$)

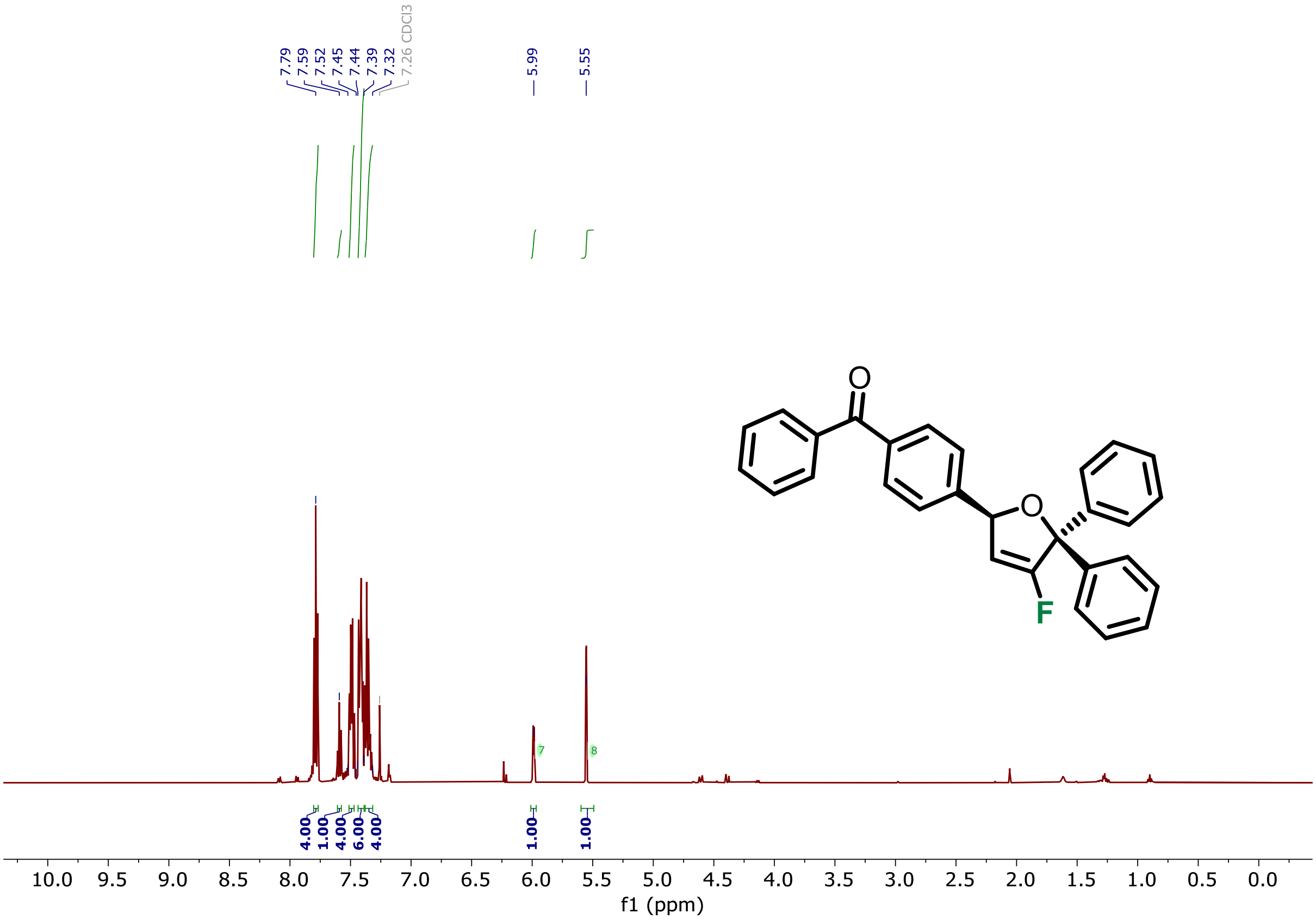

^{13}C NMR (126 MHz, CDCl$_3$)

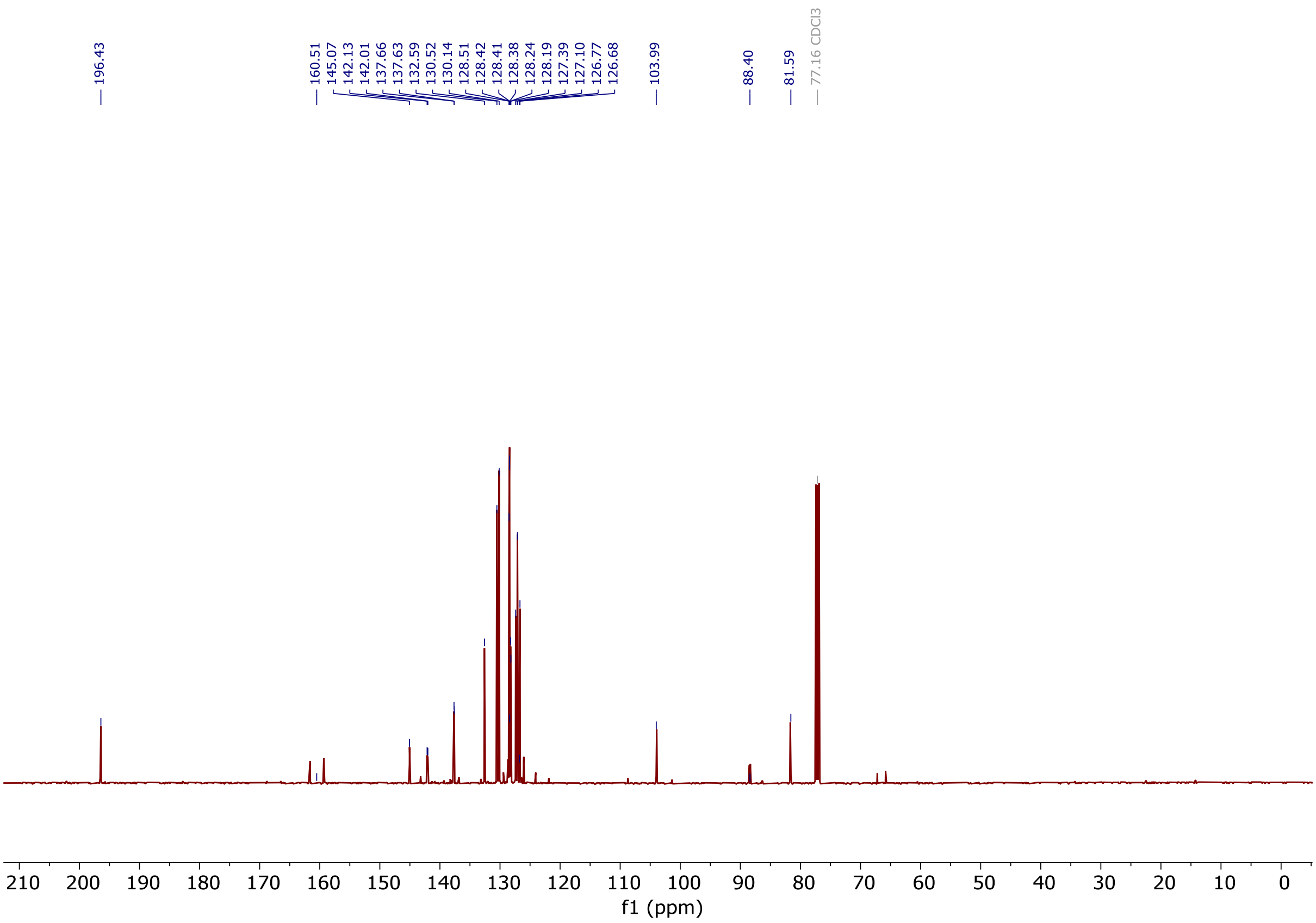

^{19}F NMR (471 MHz, $CDCl_3$)

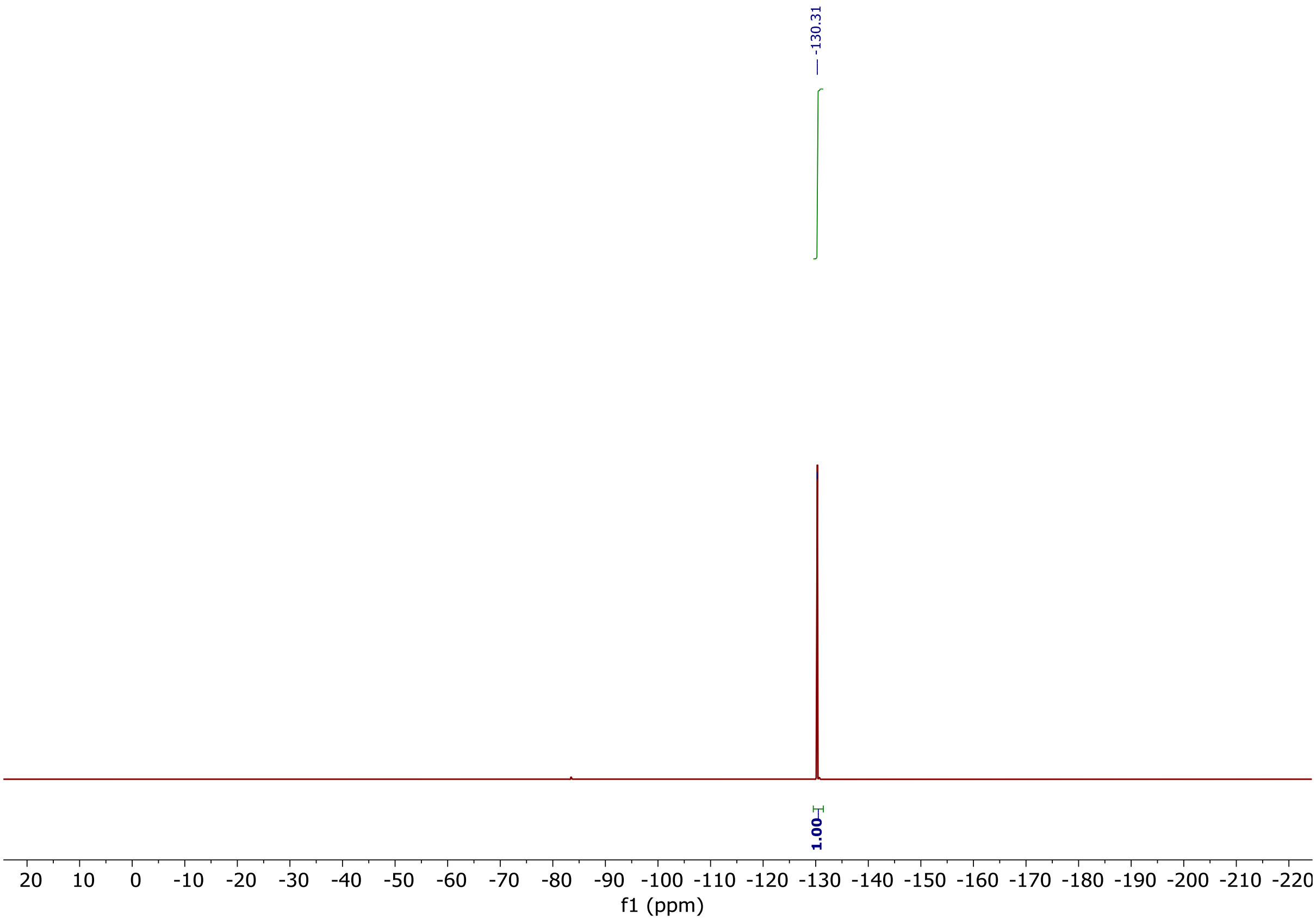

***4-fluoro-2-(4-(trifluoromethyl)phenyl)-2,5-dihydrofuran* (6ah)**

Compound not isolated, but reported as NMR yield. Spectra are provided to show diagnostic peaks.

^{1}H NMR (400 MHz, CDCl$_3$)

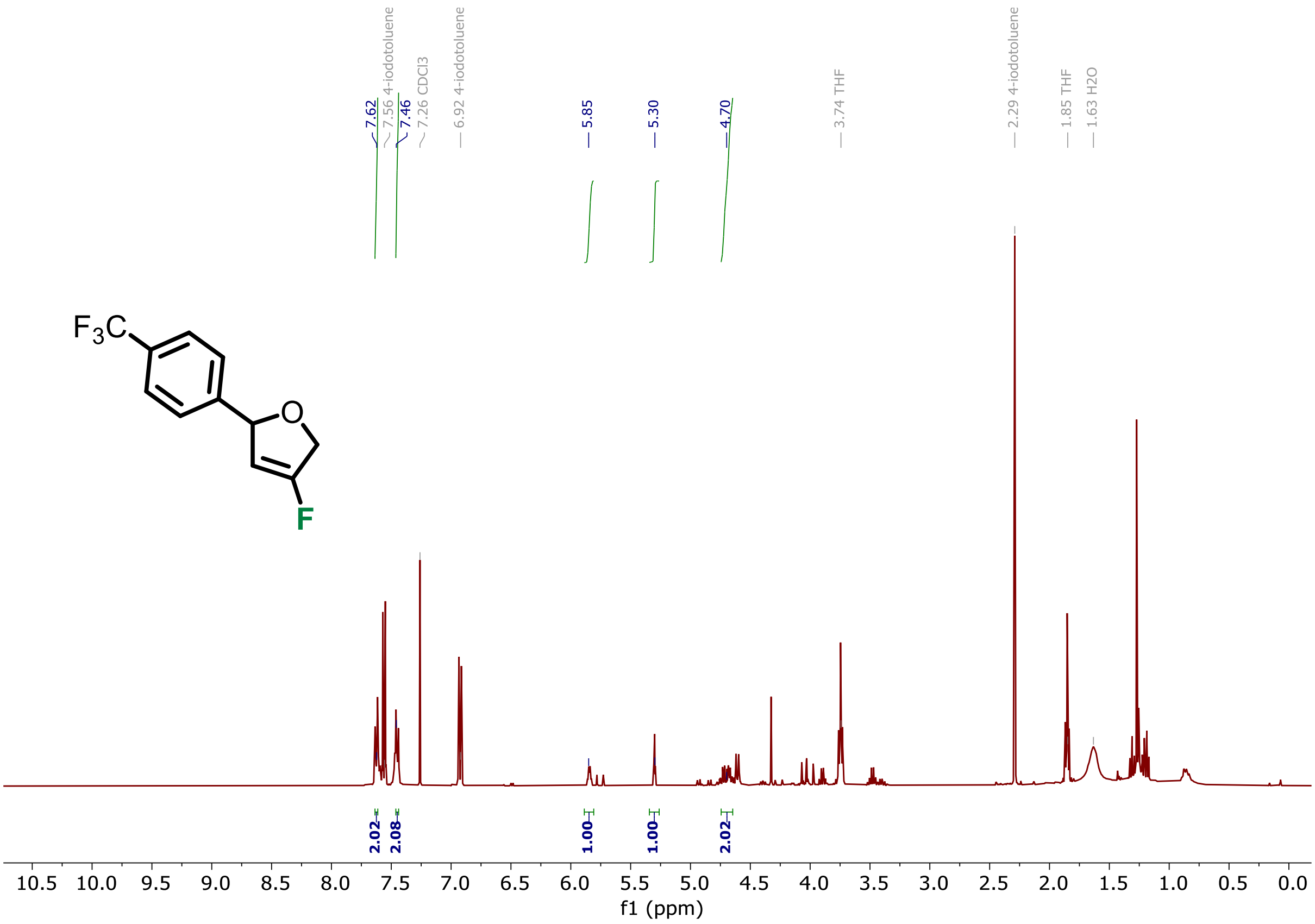

^{13}C NMR (126 MHz, CDCl$_3$)

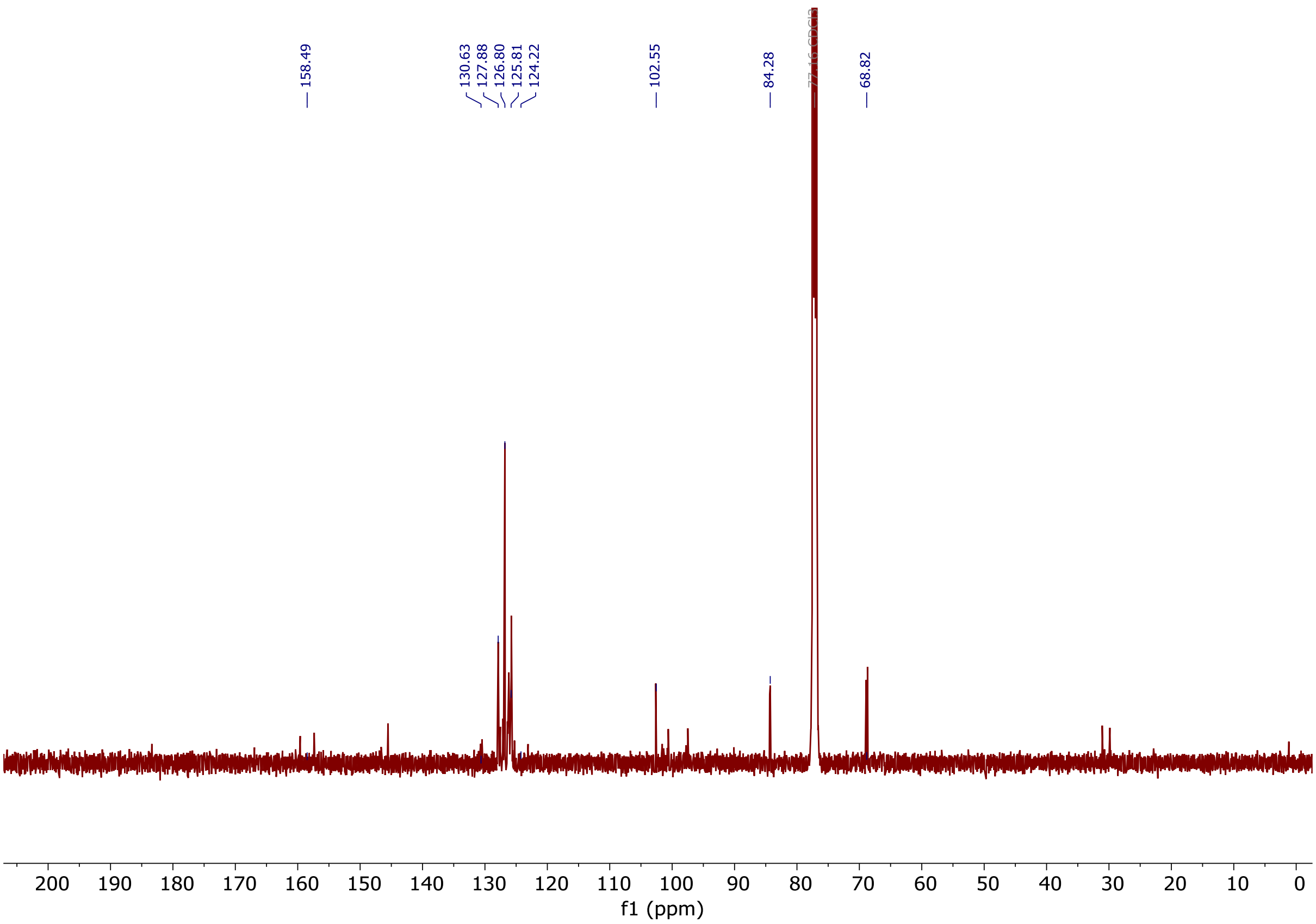

^{19}F NMR (376 MHz, $CDCl_3$)

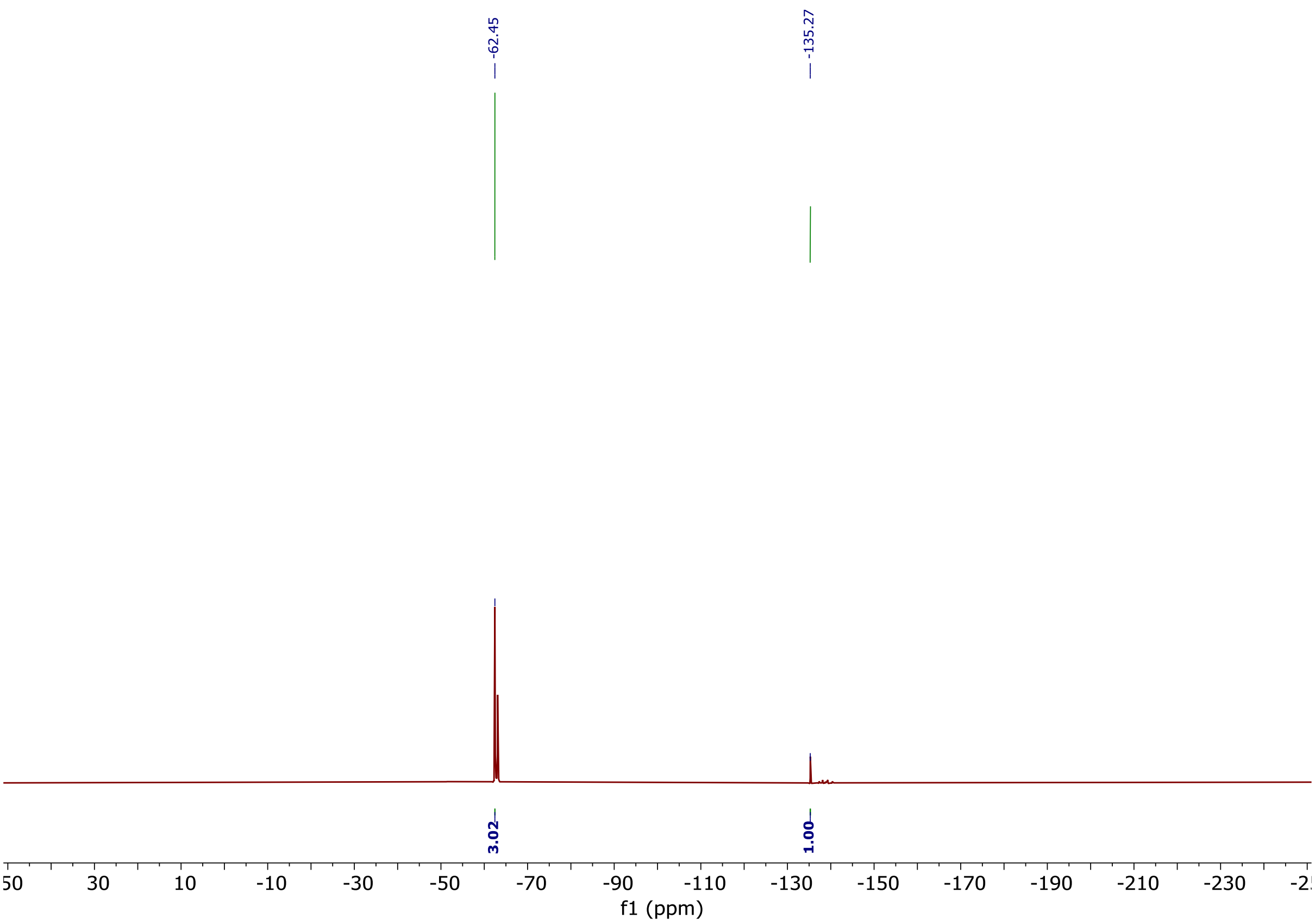

***2-(4-fluoro-1-oxaspiro[4.5]dec-3-en-2-yl)benzonitrile* (6ai)**

^{1}H NMR (400 MHz, $CDCl_3$)

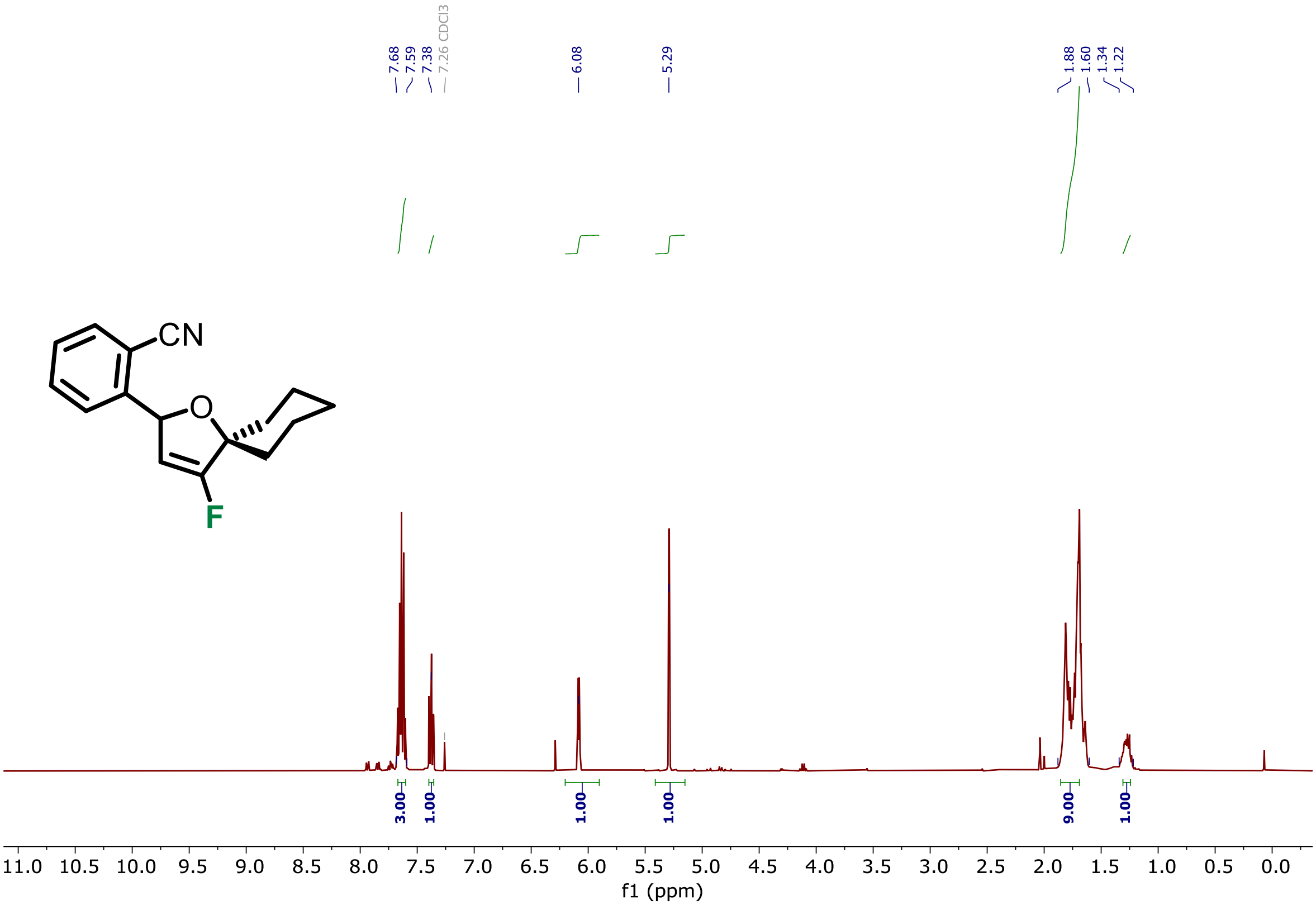

^{13}C NMR (101 MHz, $CDCl_3$)

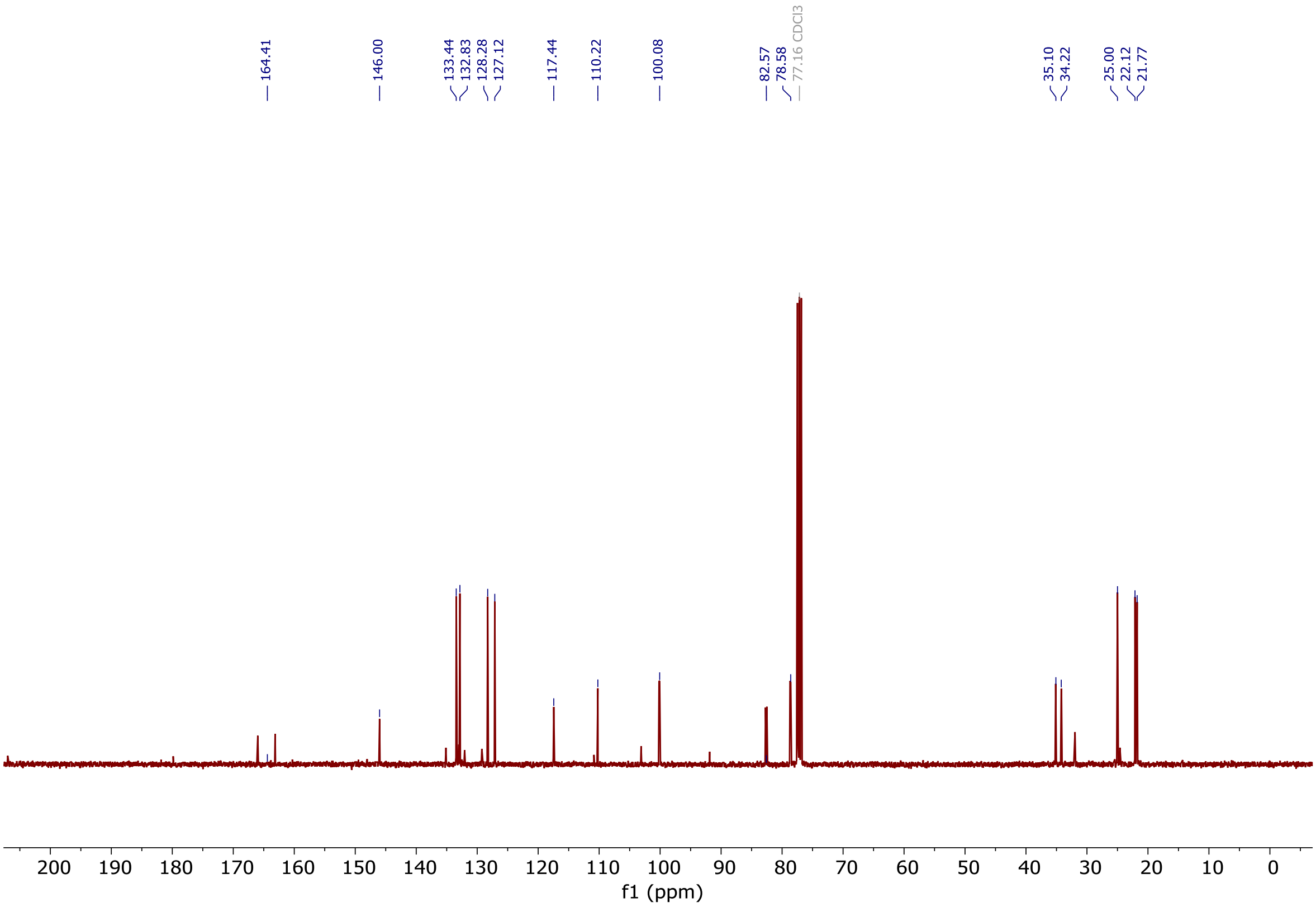

^{19}F NMR (377 MHz, $CDCl_3$)

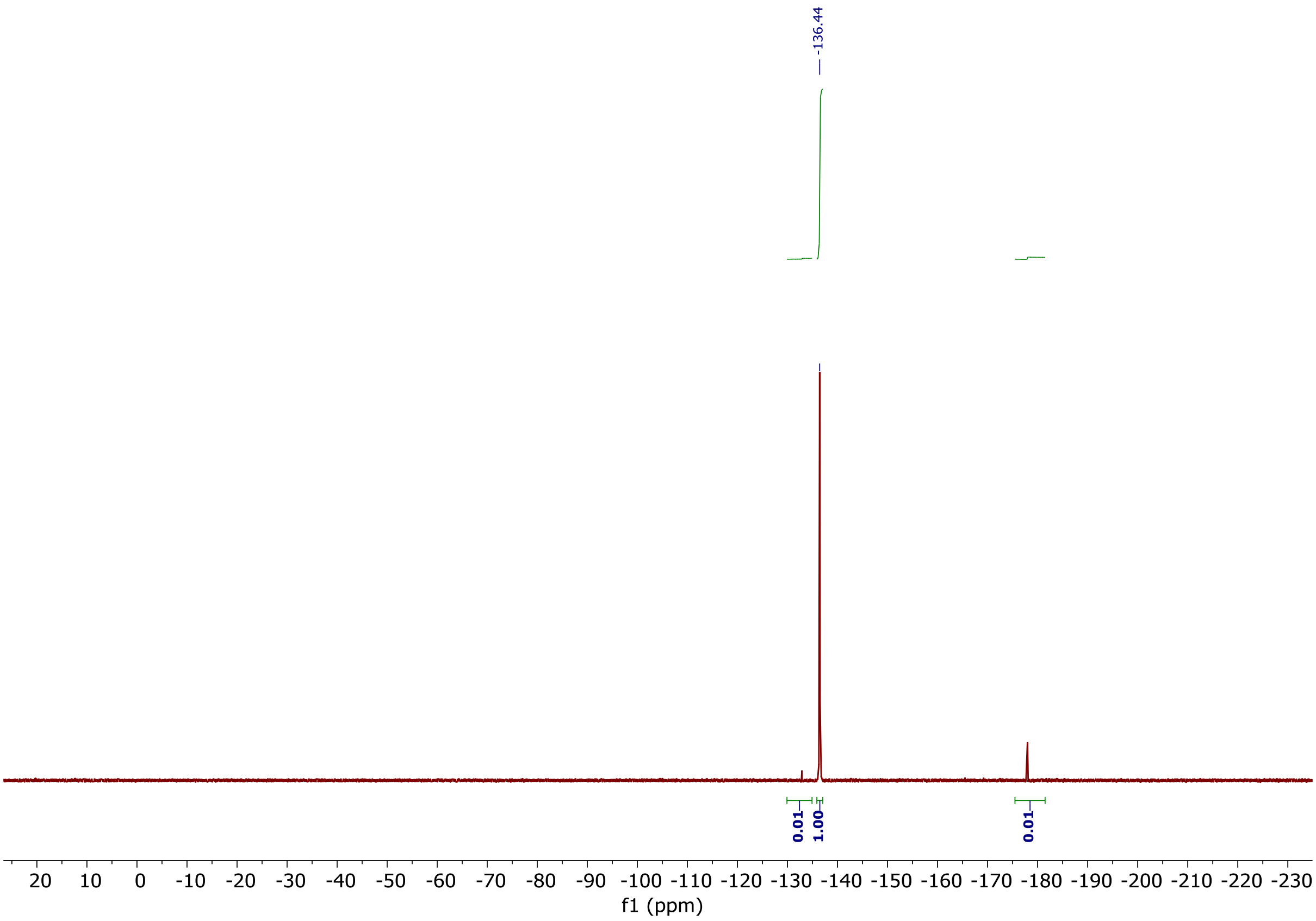

4-fluoro-2-methyl-2-phenyl-2,5-dihydrofuran **(6aj)**

^{1}H NMR (400 MHz, $CDCl_3$)

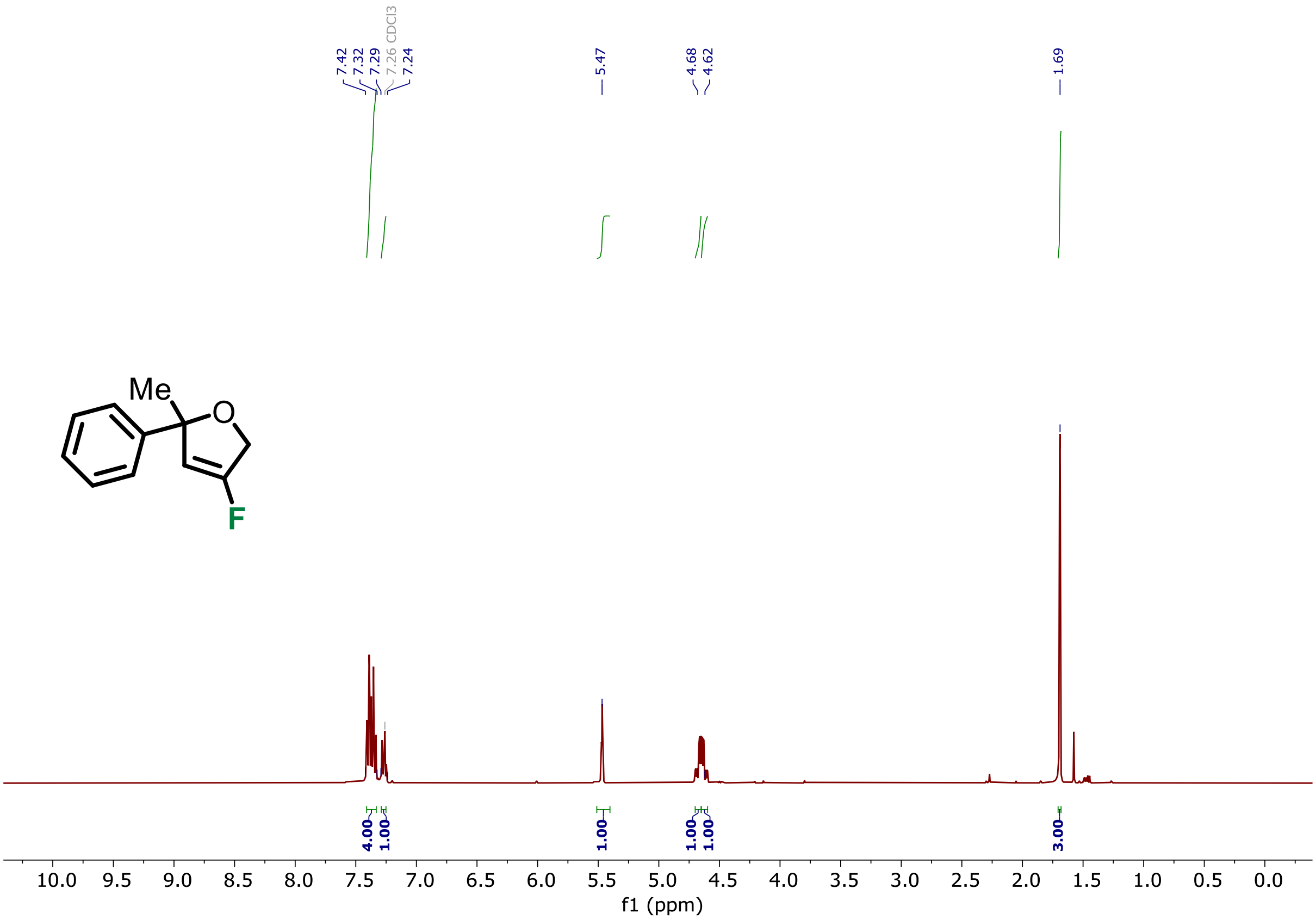

^{13}C NMR (101 MHz, $CDCl_3$)

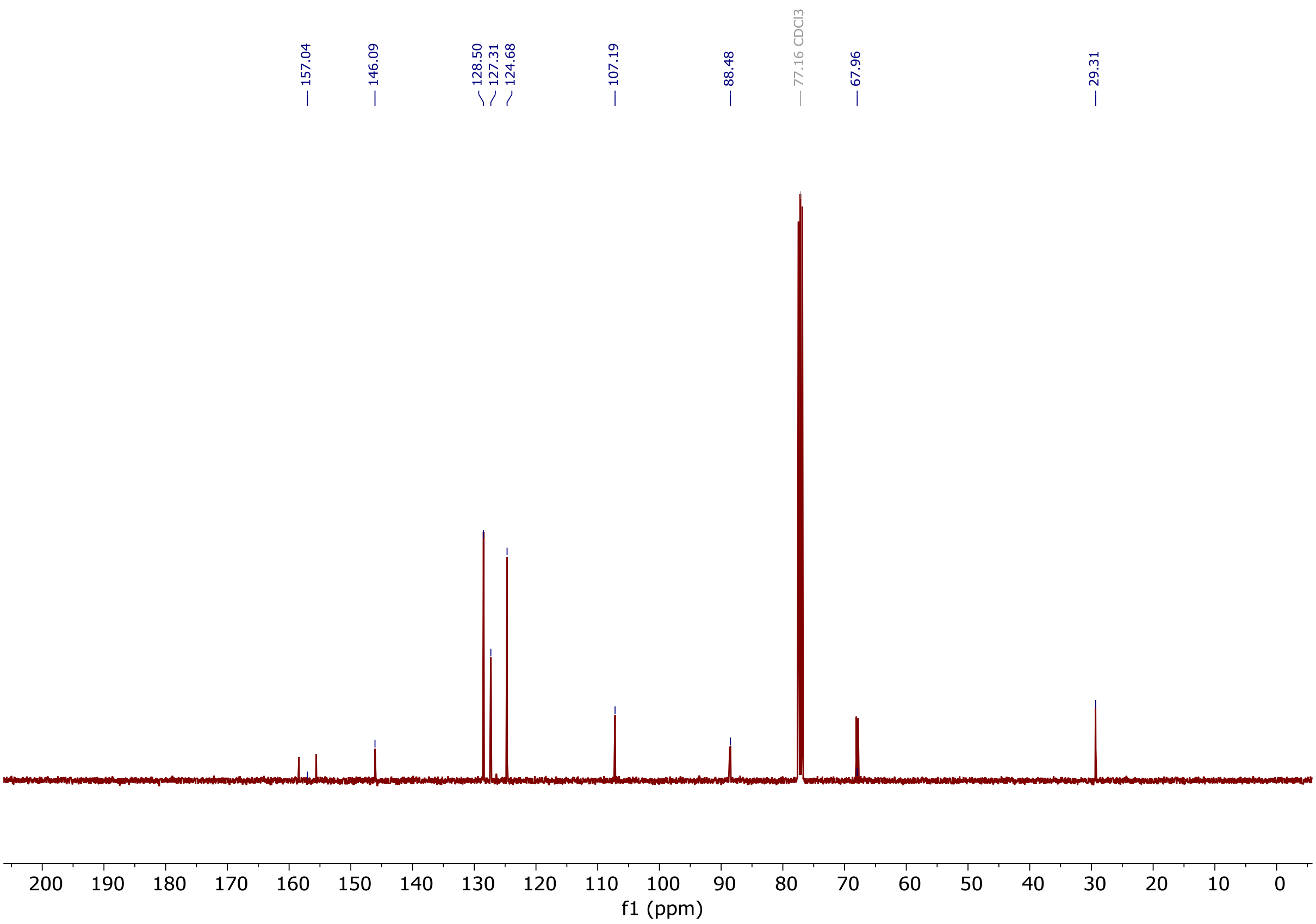

^{19}F NMR (376 MHz, $CDCl_3$)

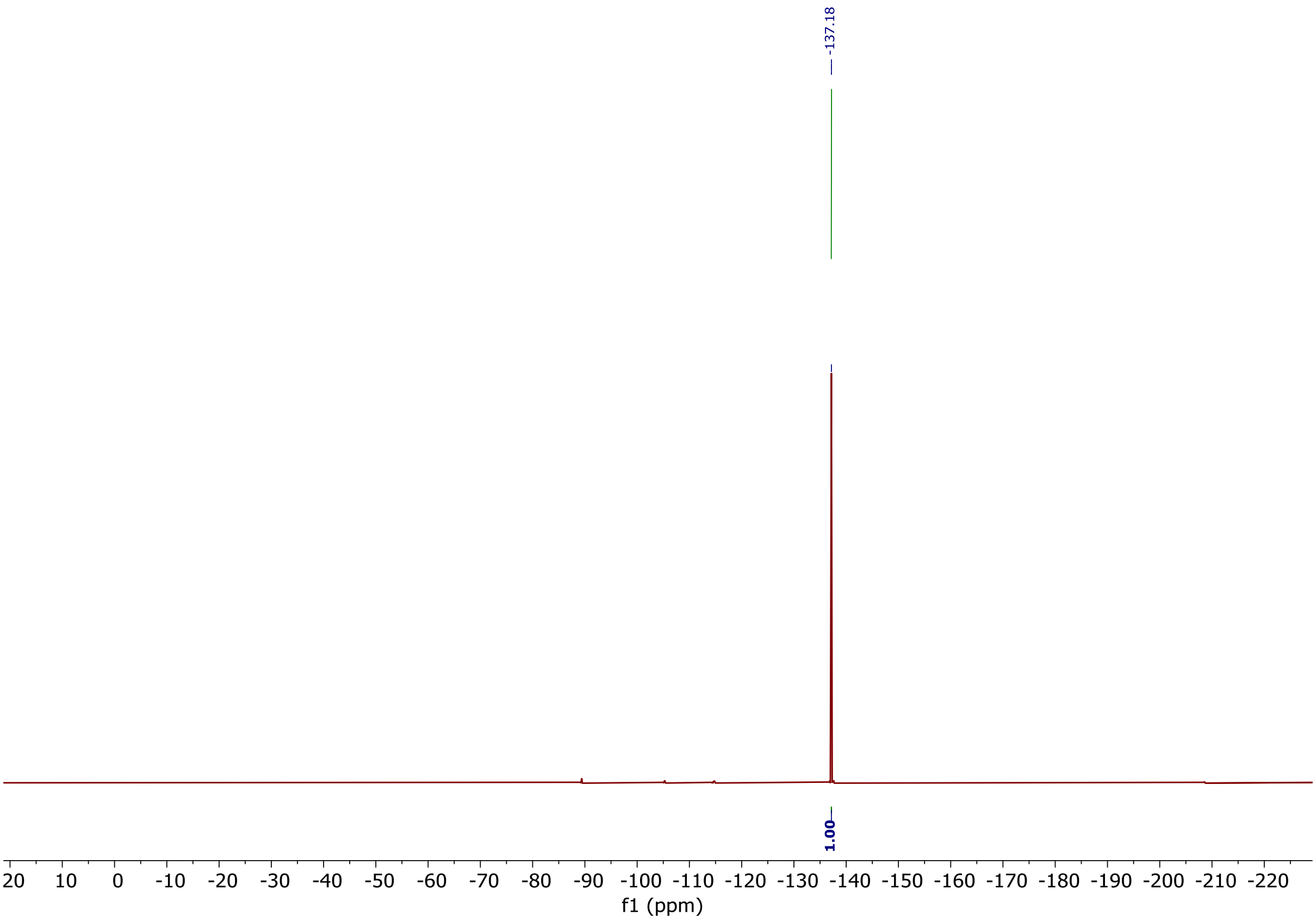

***4-fluoro-2,2-diphenyl-2,5-dihydrofuran* (6ak)**

^{1}H NMR (400 MHz, $CDCl_3$)

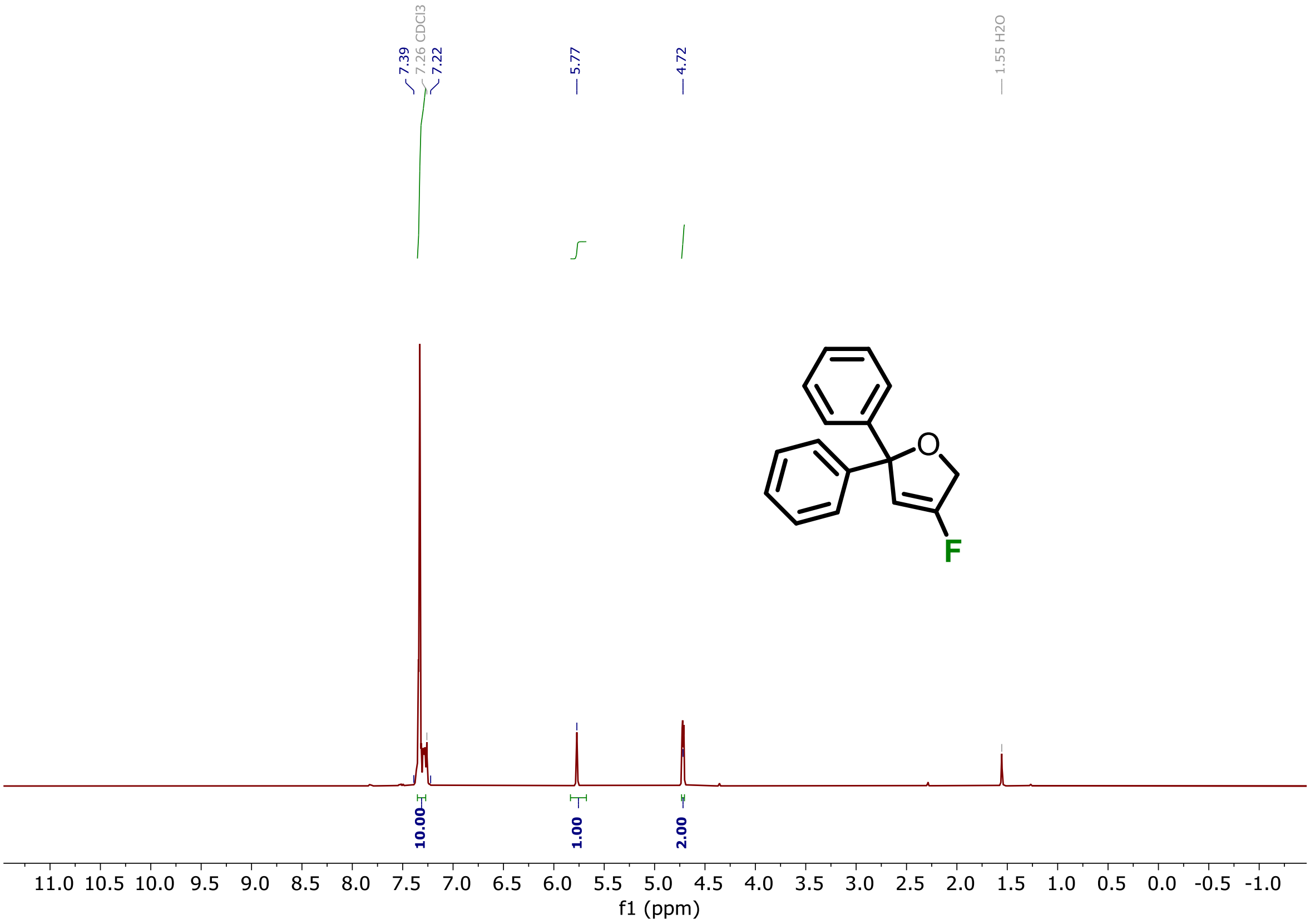

^{13}C NMR (101 MHz, $CDCl_3$)

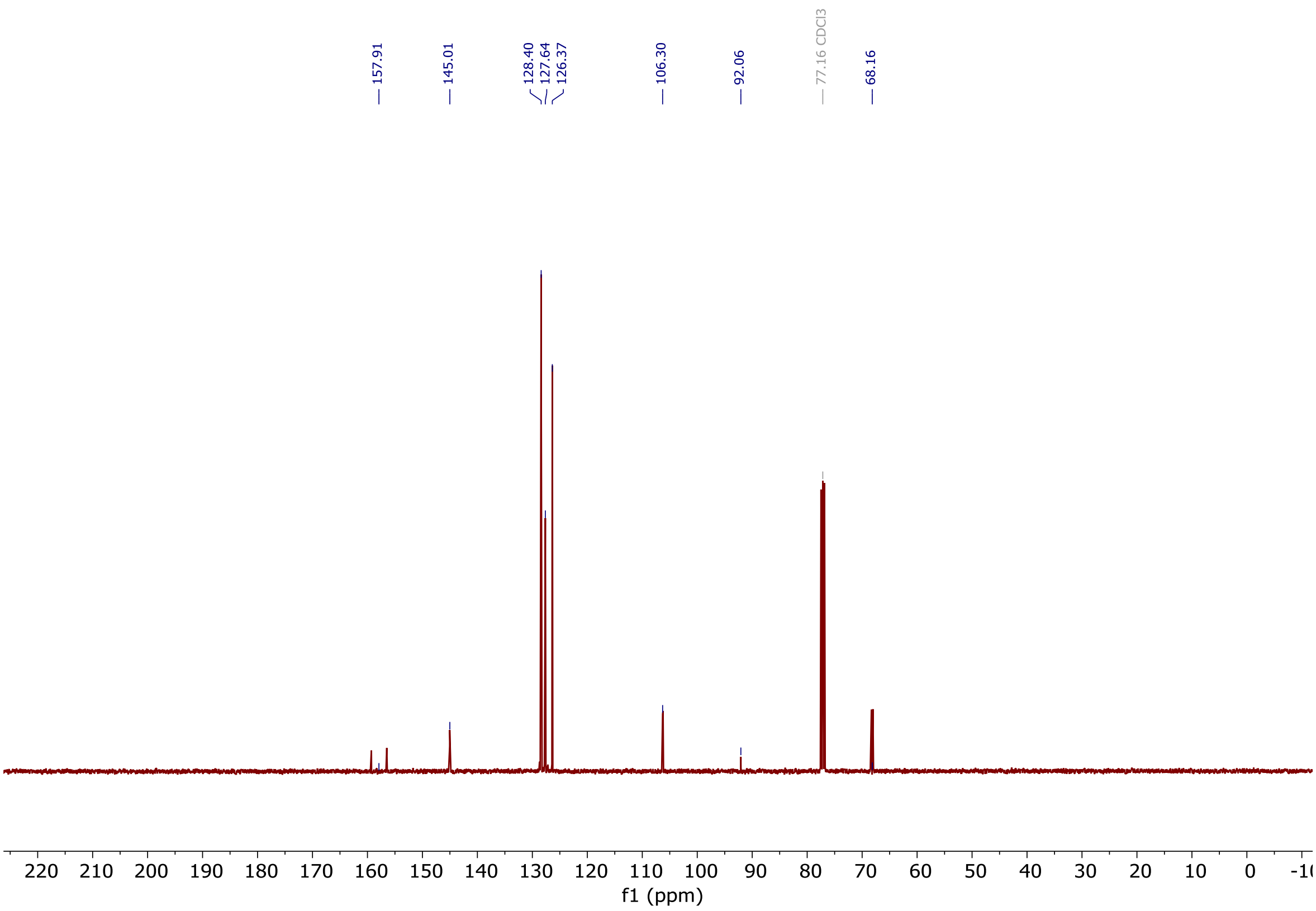

^{19}F NMR (377 MHz, $CDCl_3$)

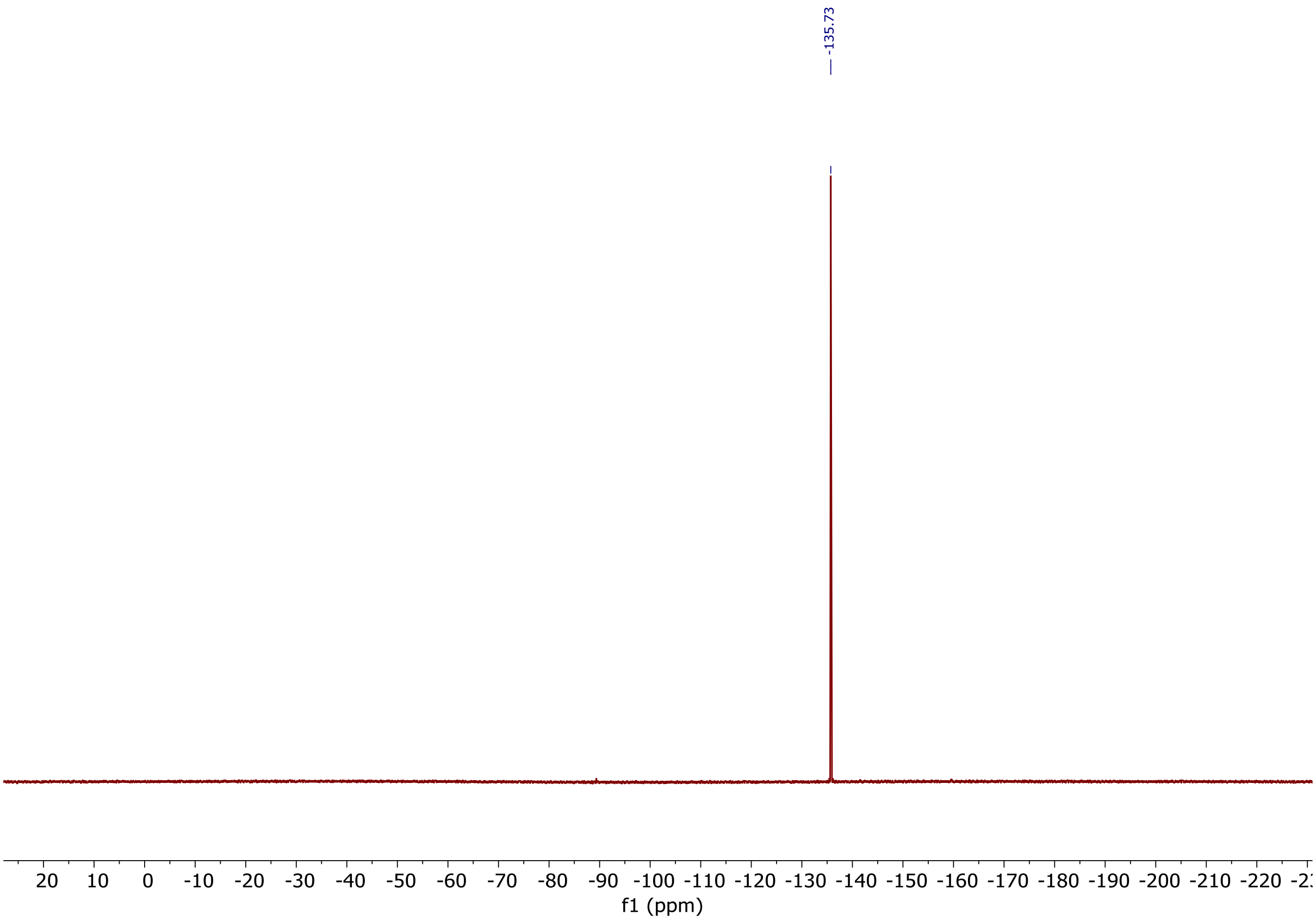

***4'-fluoro-5'H-spiro[chromane-4,2'-furan]* (6al)**

^{1}H NMR (500 MHz, CDCl$_3$)

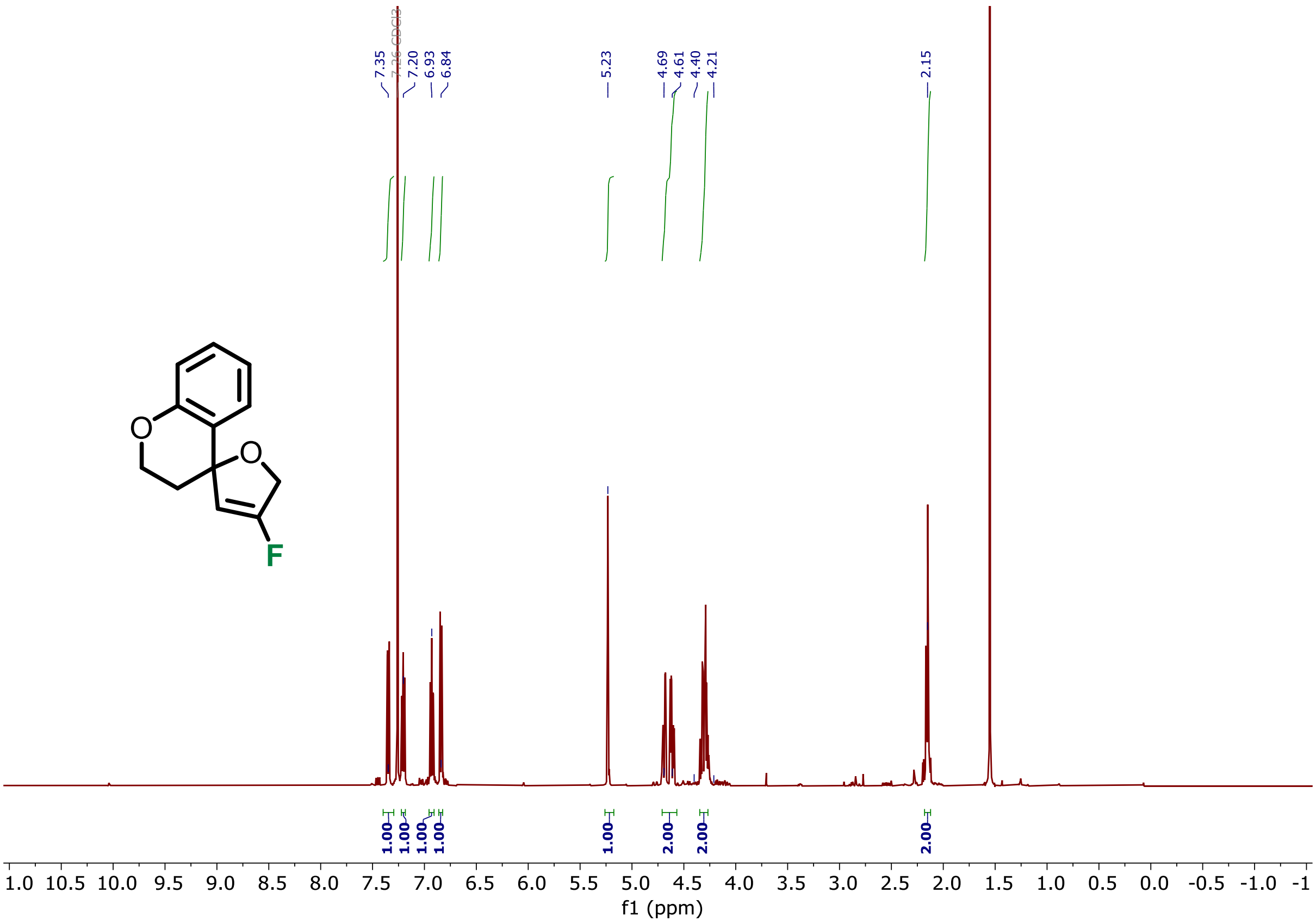

^{13}C NMR (126 MHz, CDCl$_3$)

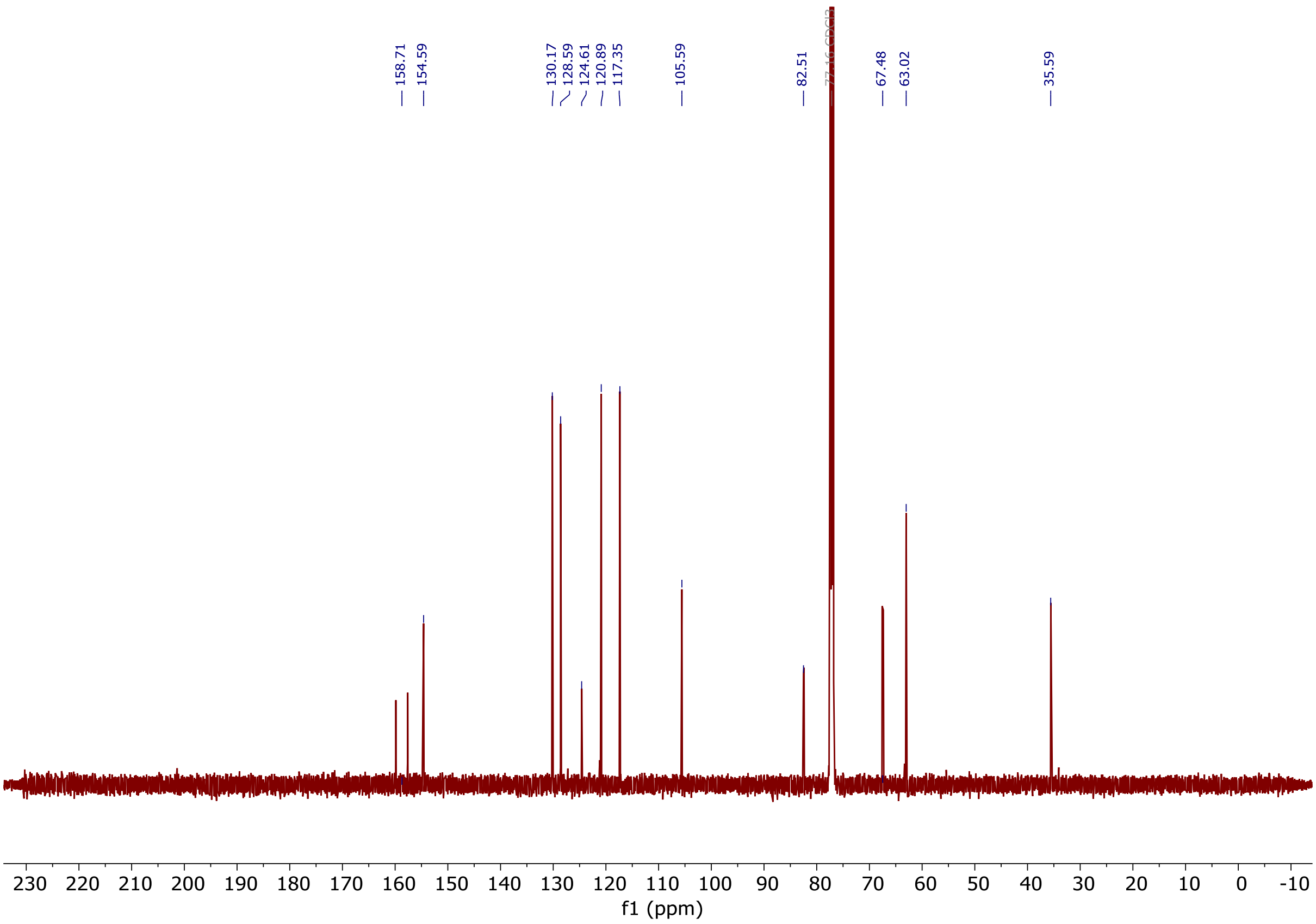

^{19}F NMR (376 MHz, $CDCl_3$)

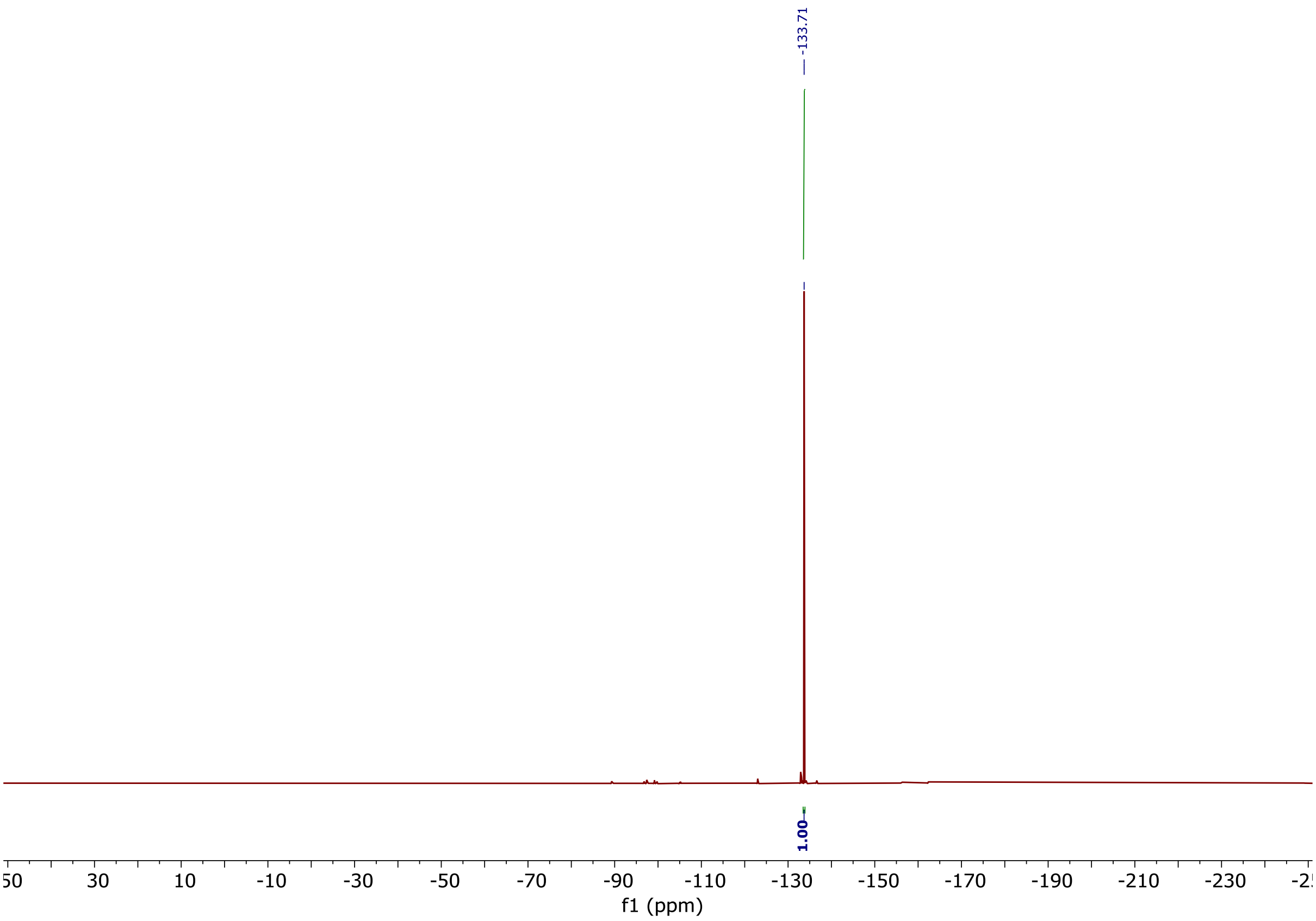

4-fluoro-1-oxaspiro[4.5]dec-3-ene (Major regioisomer) **(6an)**
(syn)-7-fluoro-1-methoxybicyclo[3.2.1]oct-6-ene (Minor regioisomer) **(5an)**

^{1}H NMR (500 MHz, CDCl$_3$)

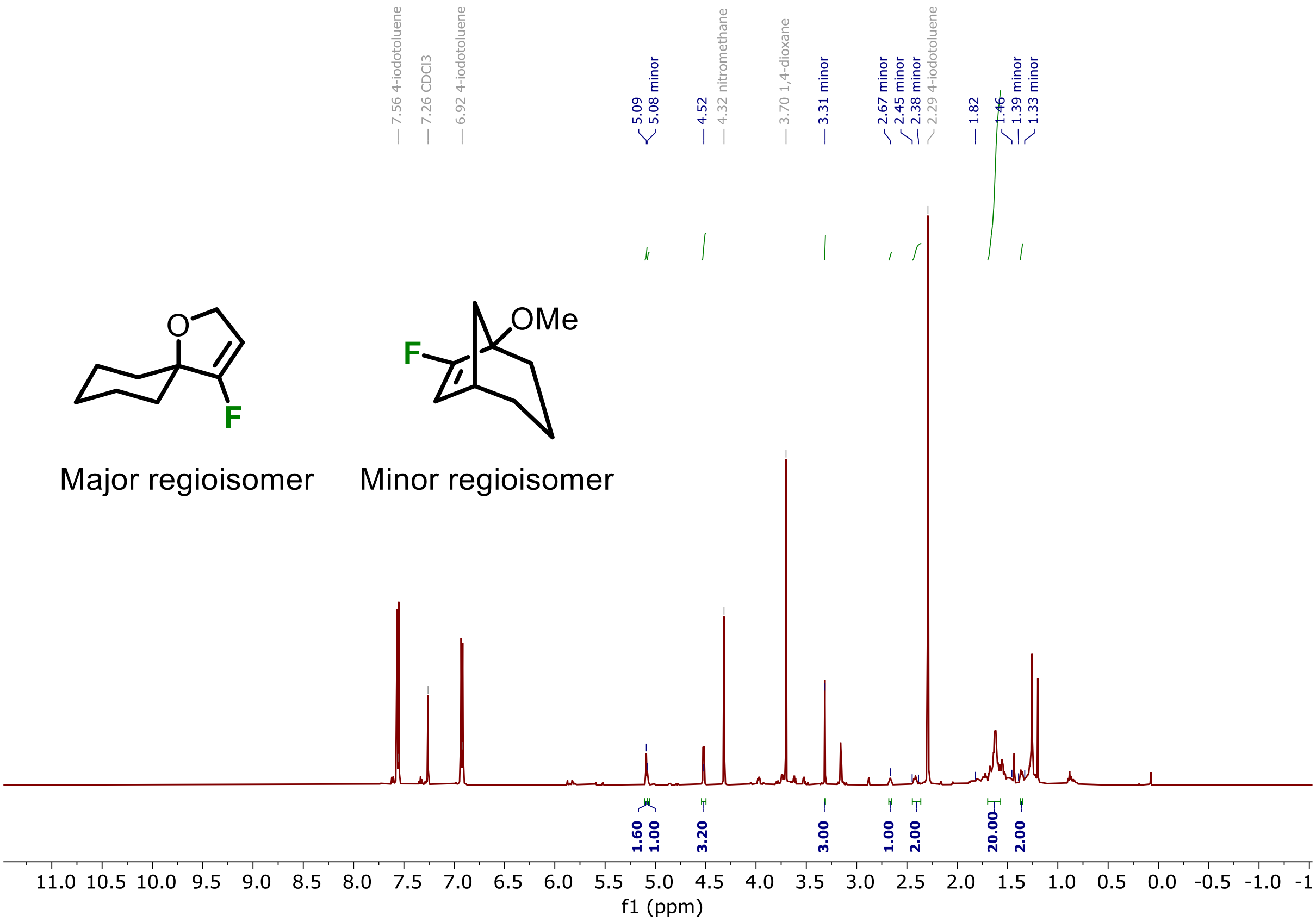

^{13}C NMR (126 MHz, $CDCl_3$)

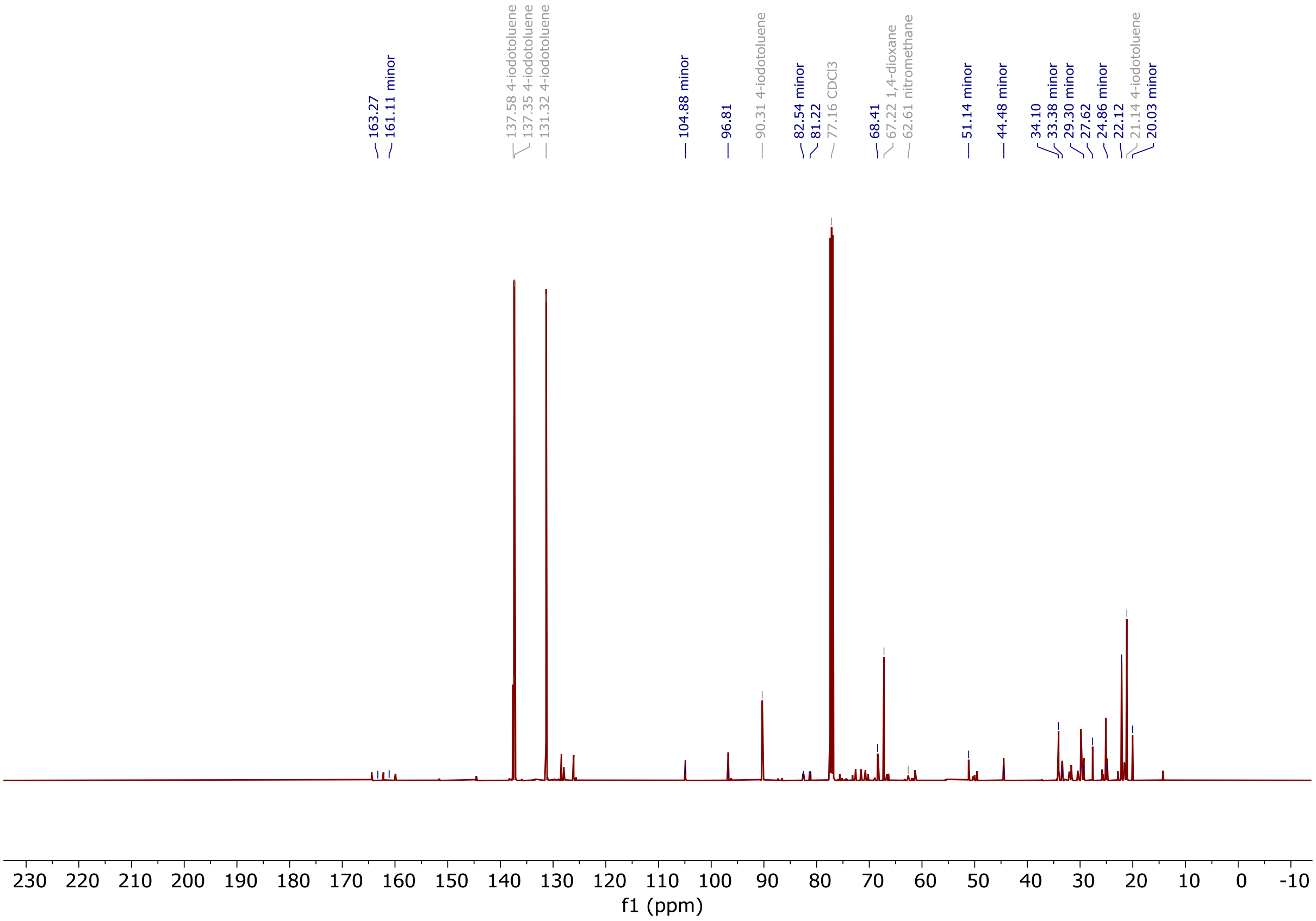

^{19}F NMR (376 MHz, $CDCl_3$)

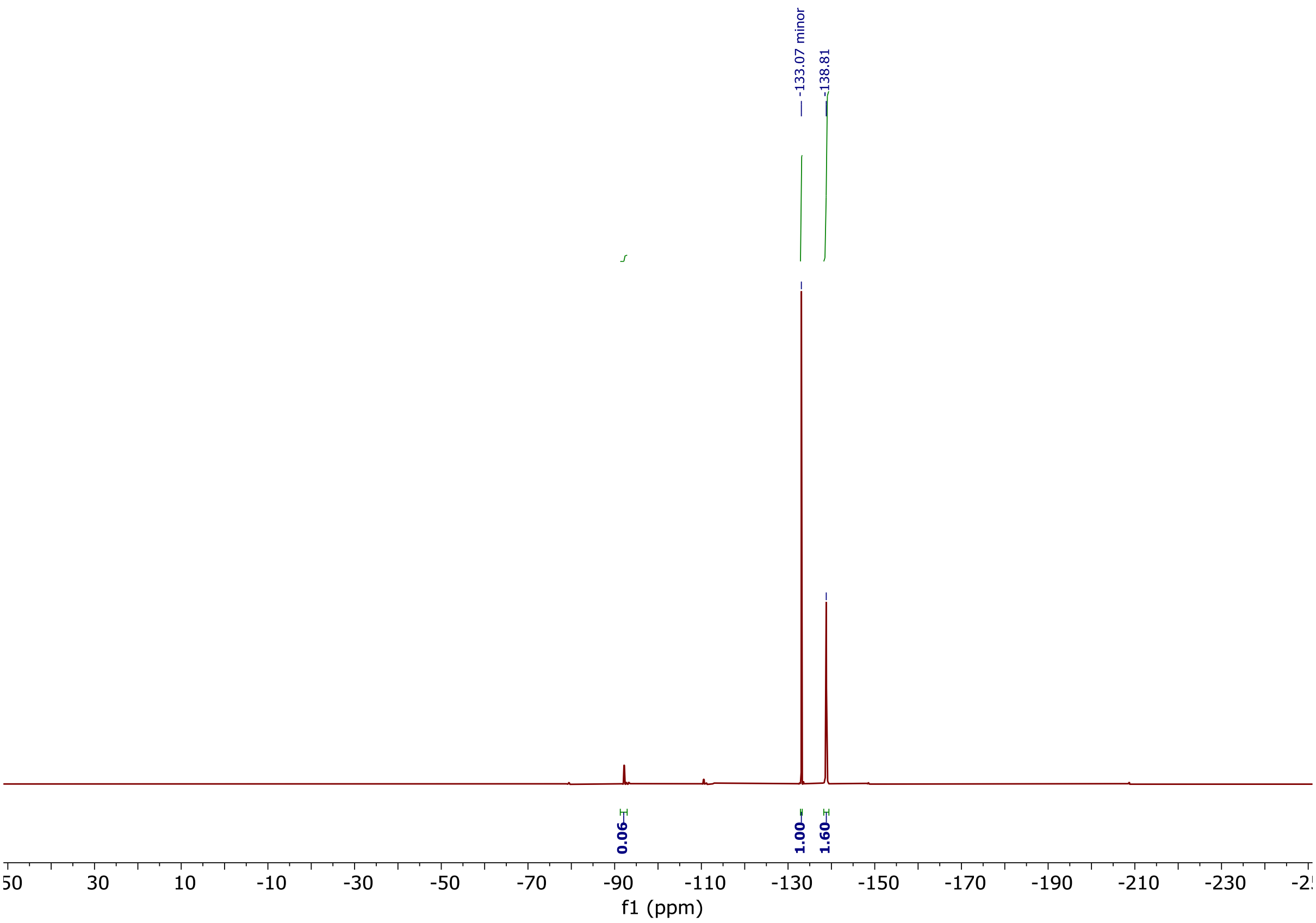

***2-((4-chlorophenoxy)methyl)-4-fluoro-2,5-dihydrofuran* (6ao)**

^{1}H NMR (500 MHz, $CDCl_3$)

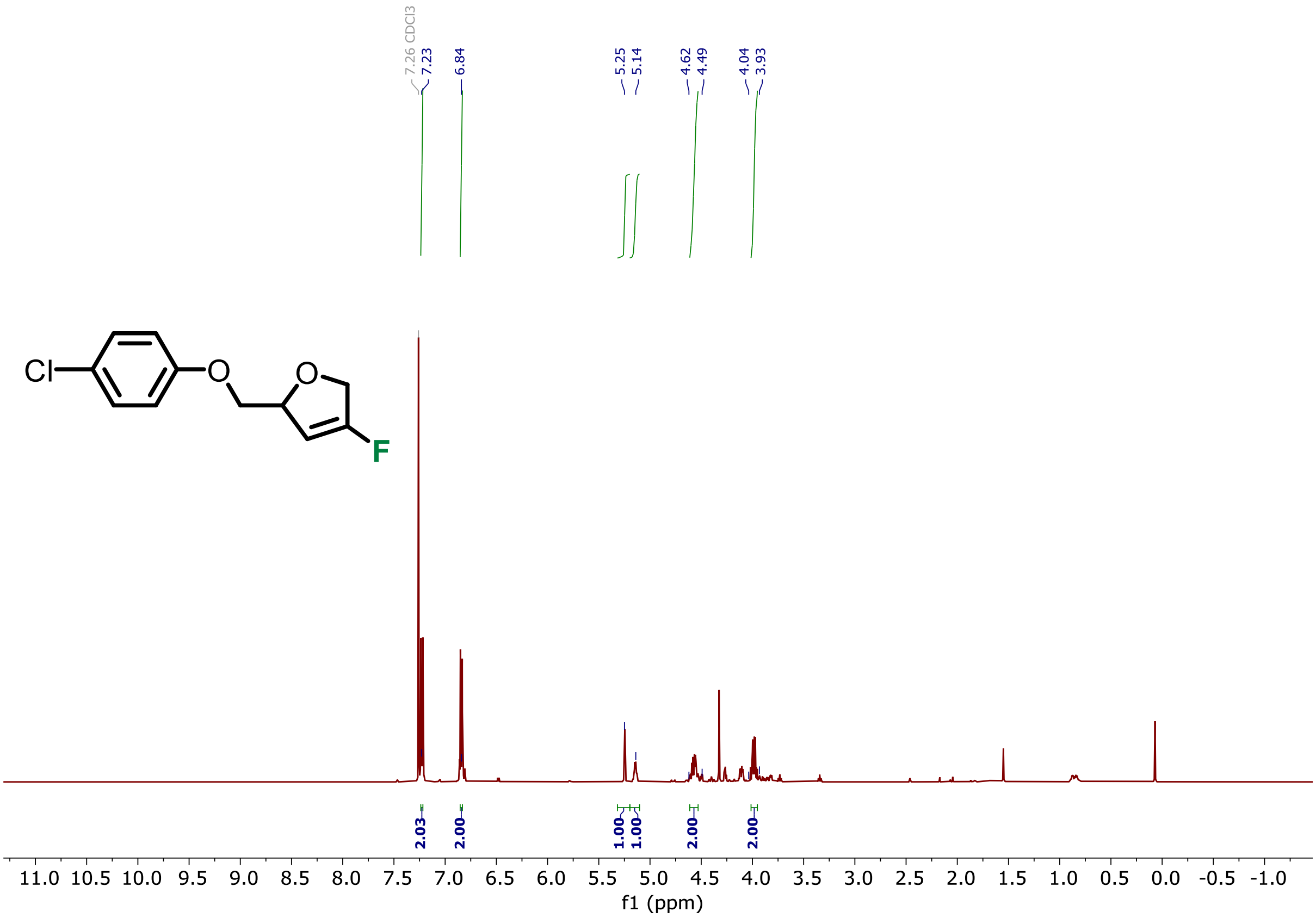

^{13}C NMR (126 MHz, $CDCl_3$)

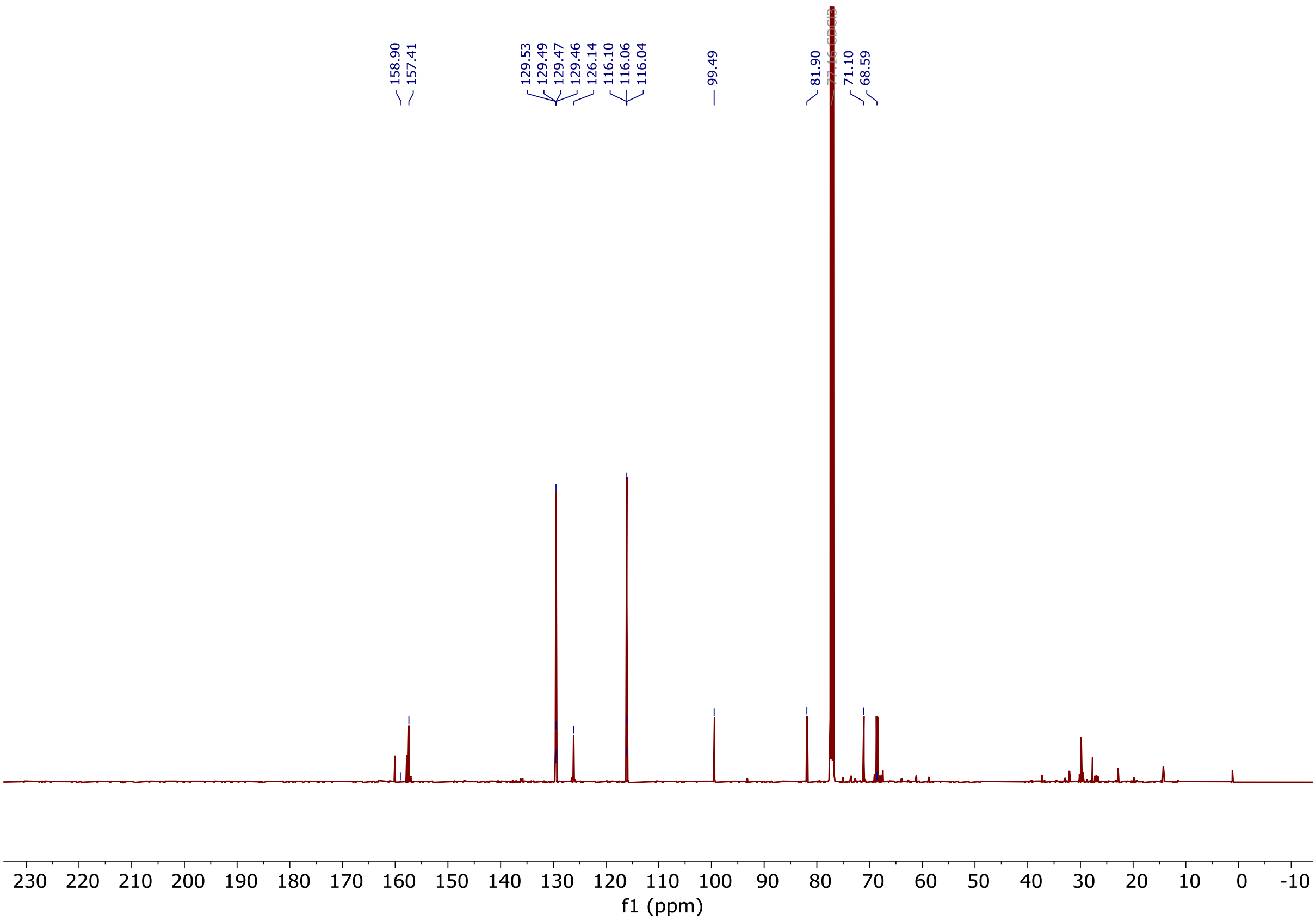

^{19}F NMR (471 MHz, $CDCl_3$)

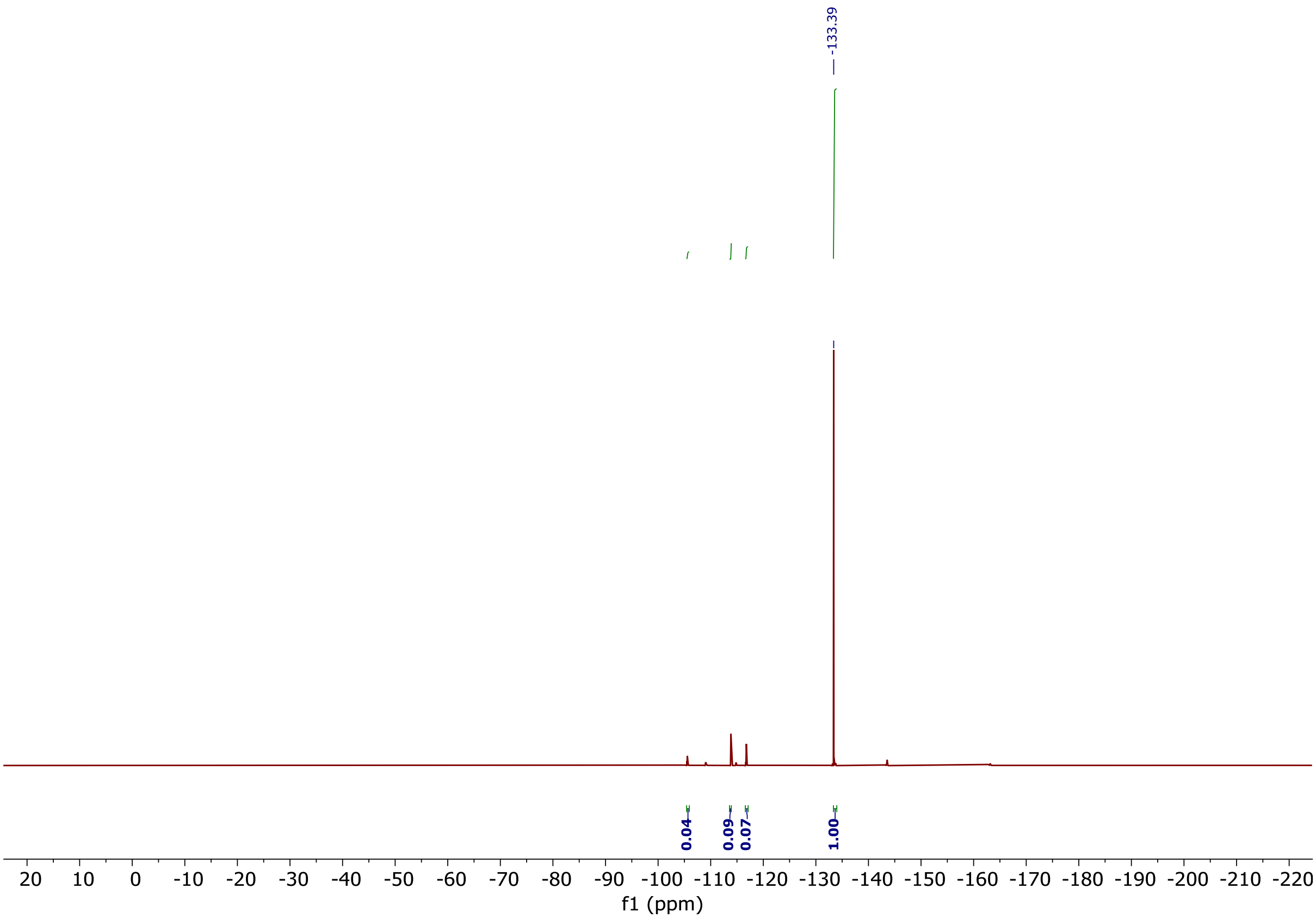

***3-fluoro-8-tosyl-1-oxa-8-azaspiro[4.5]dec-3-ene* (6ap)**

^{1}H NMR (500 MHz, $CDCl_3$)

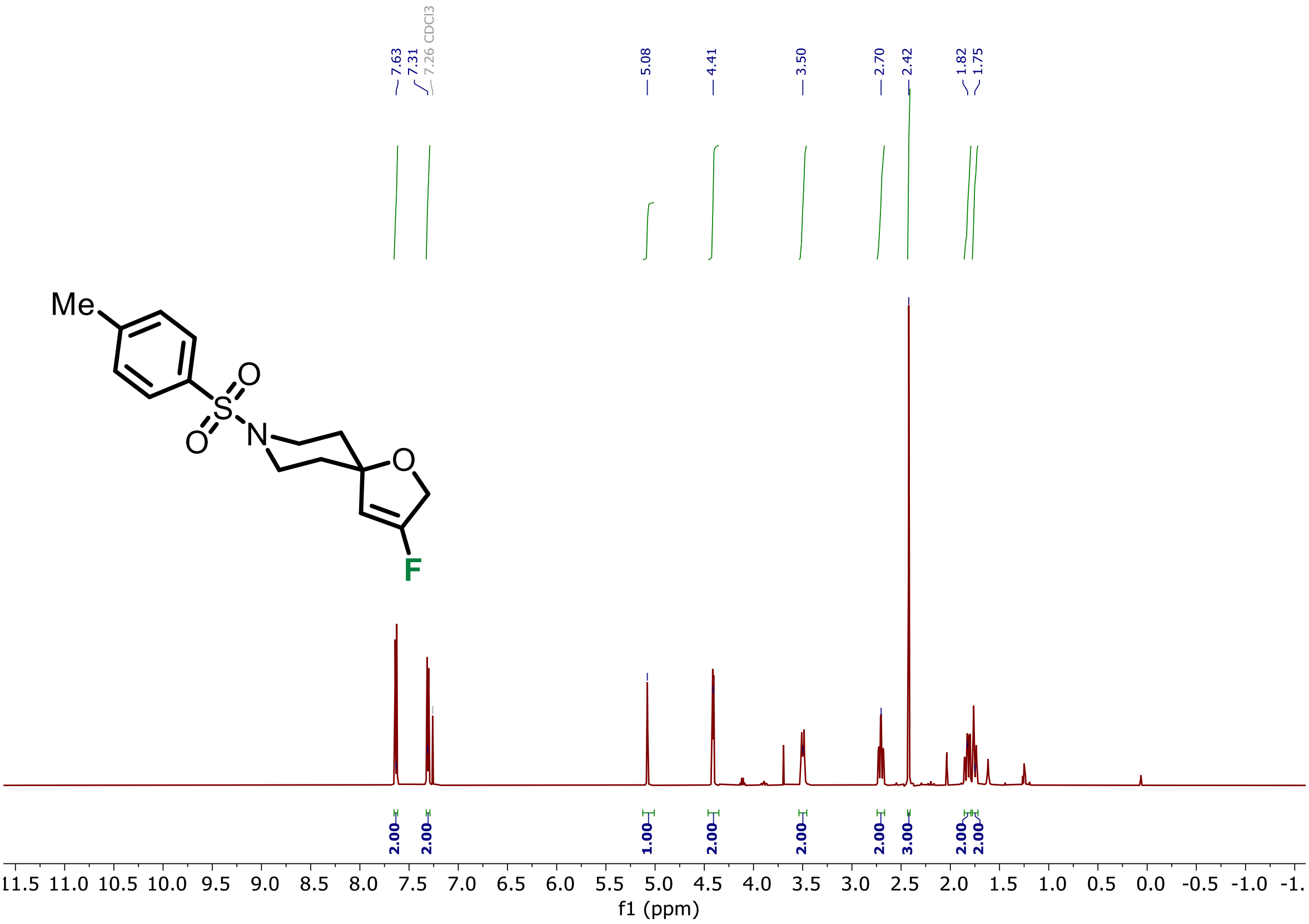

^{13}C NMR (126 MHz, $CDCl_3$)

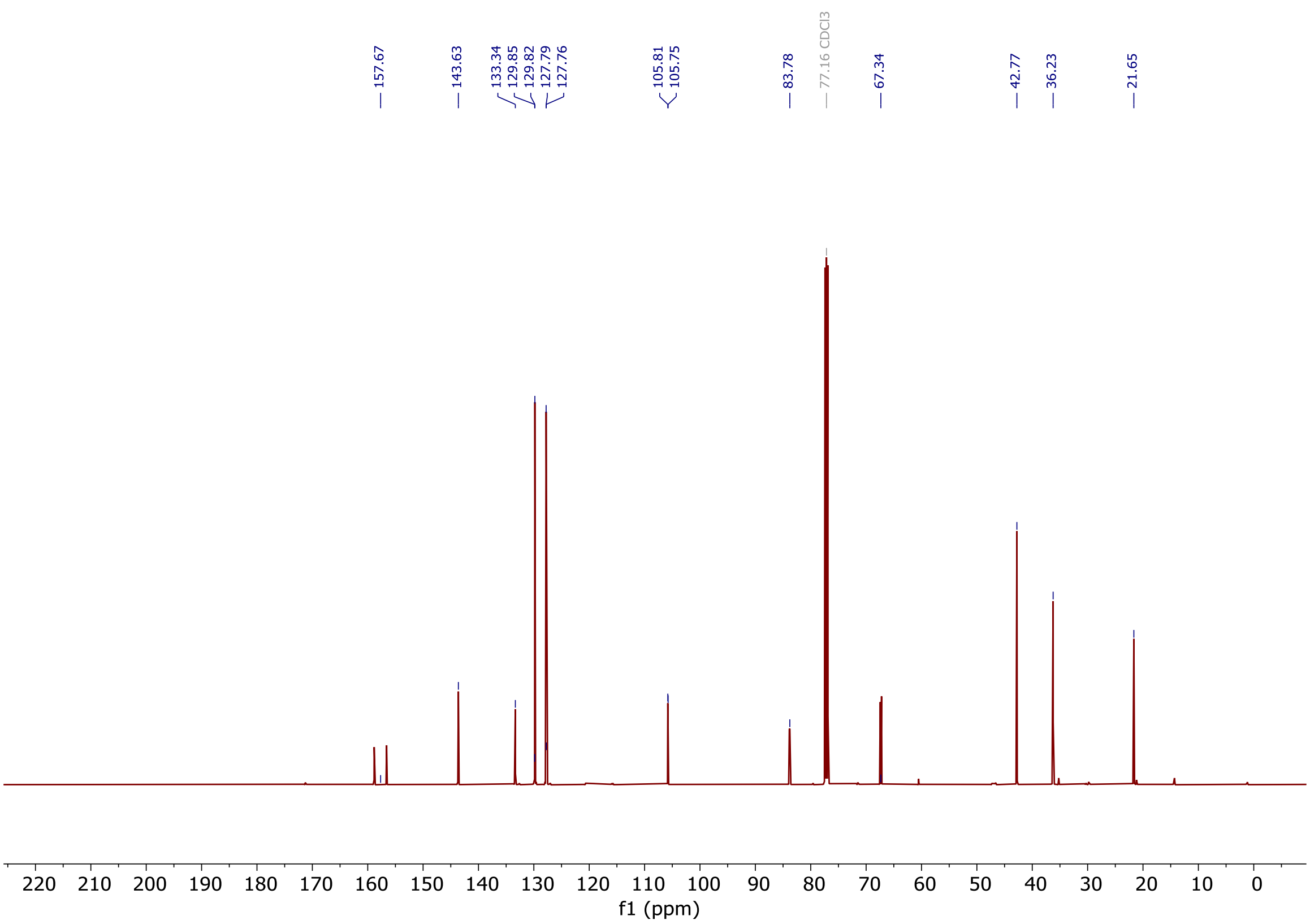

^{19}F NMR (376 MHz, $CDCl_3$)

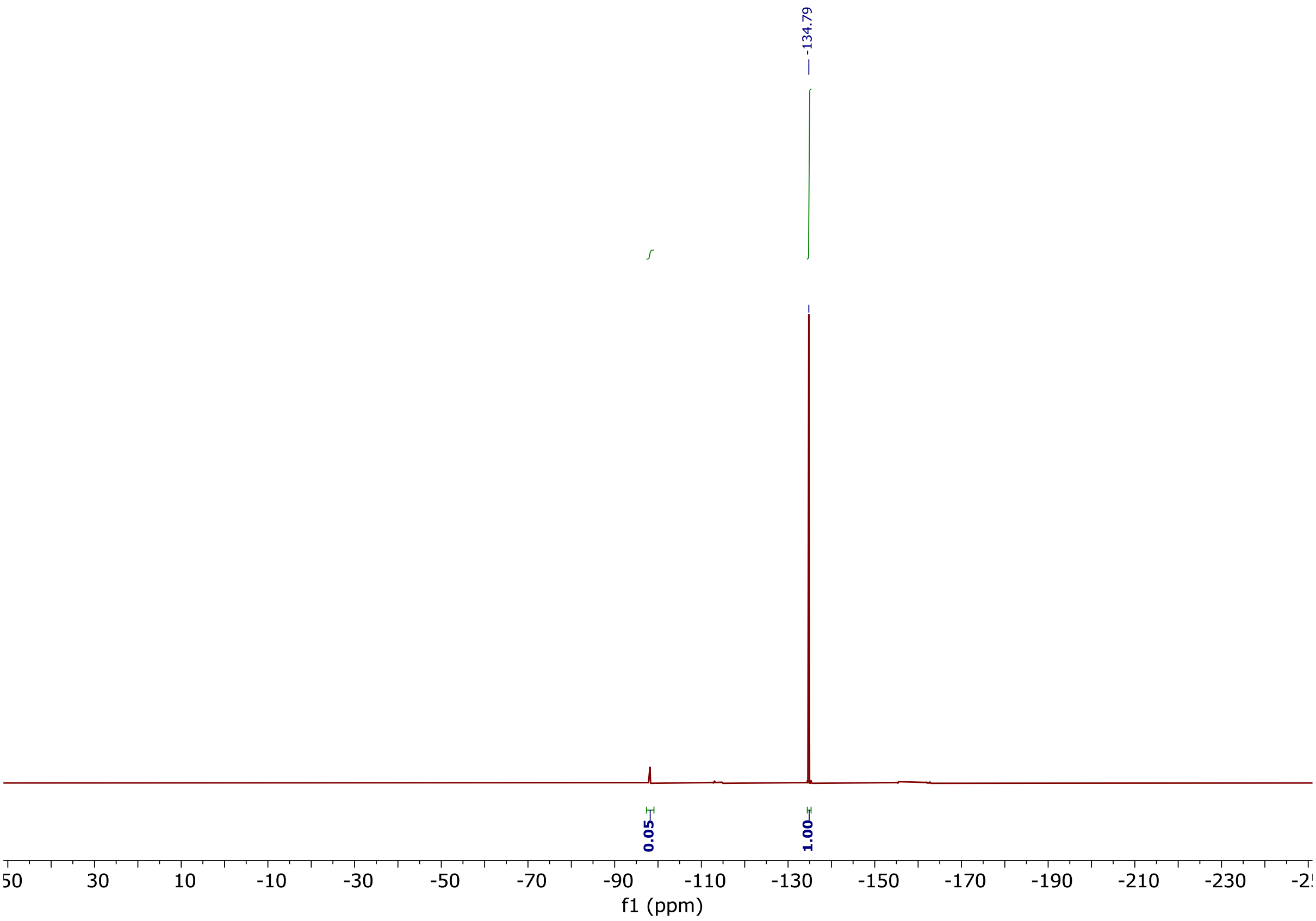

(5R,6S,9R)-3-fluoro-6-isopropyl-9-methyl-1-oxaspiro[4.5]dec-3-ene (menthol derivative) **(6aq)**

^{1}H NMR (500 MHz, $CDCl_3$)

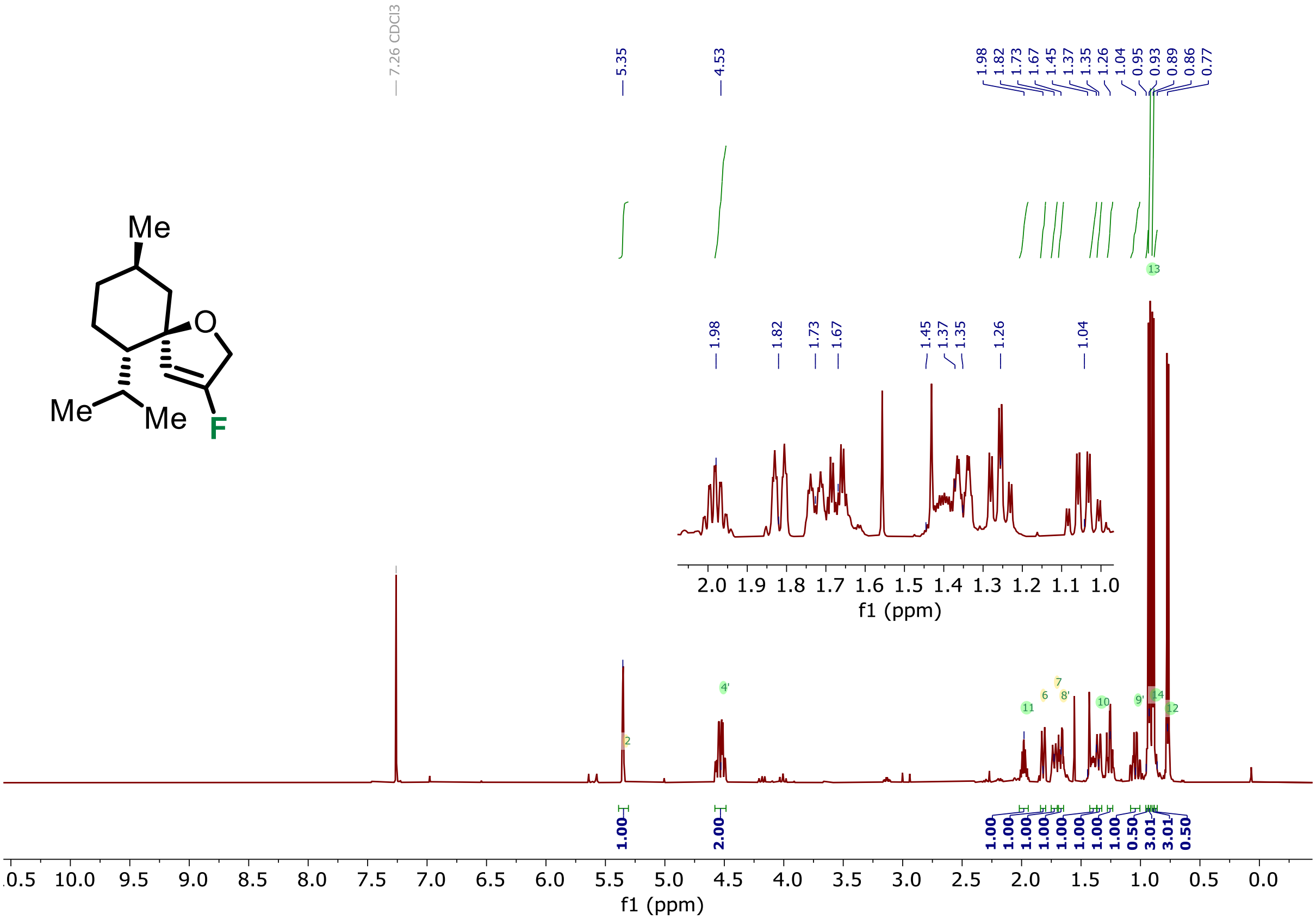

^{13}C NMR (126 MHz, $CDCl_3$)

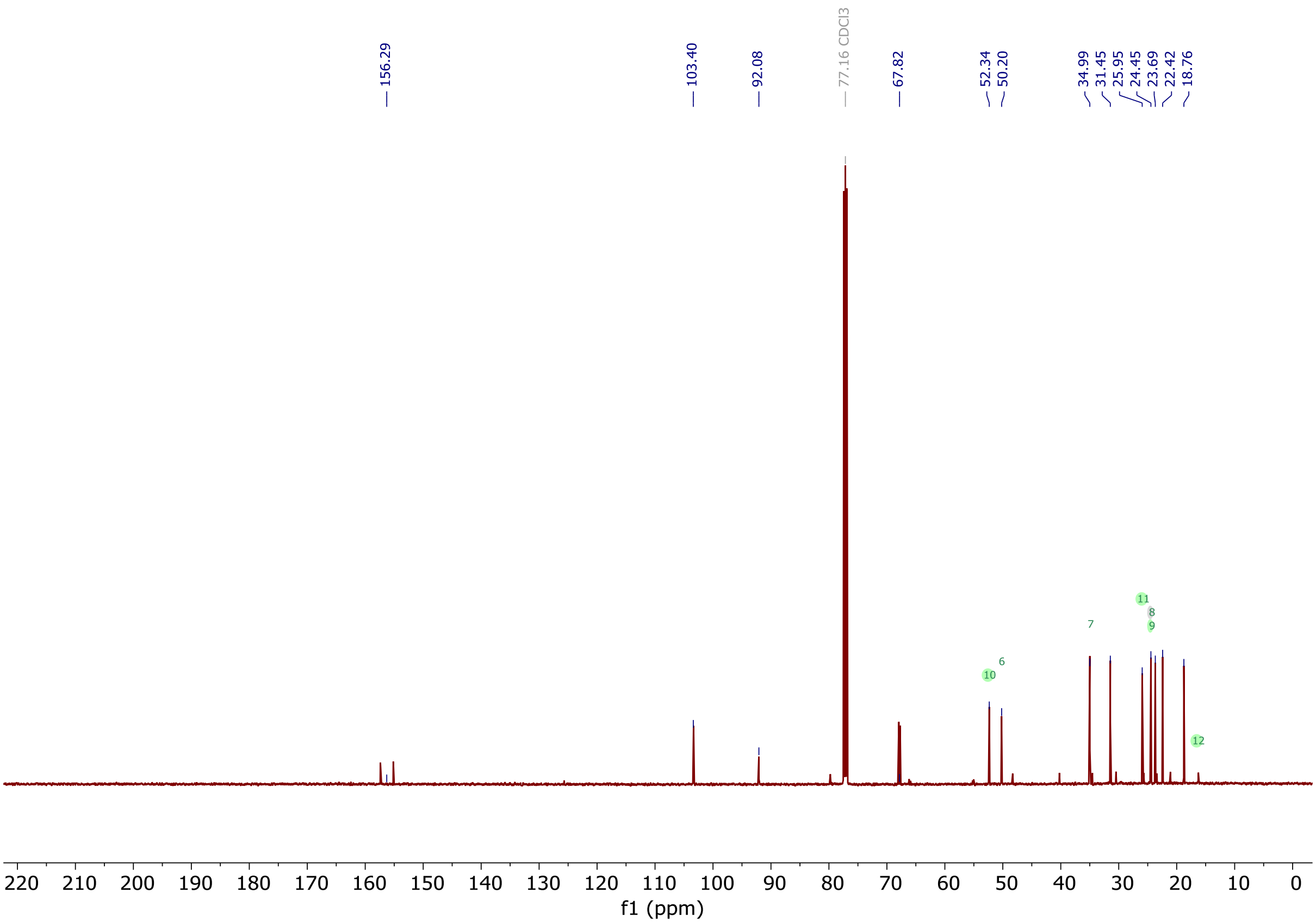

^{19}F NMR (471 MHz, $CDCl_3$)

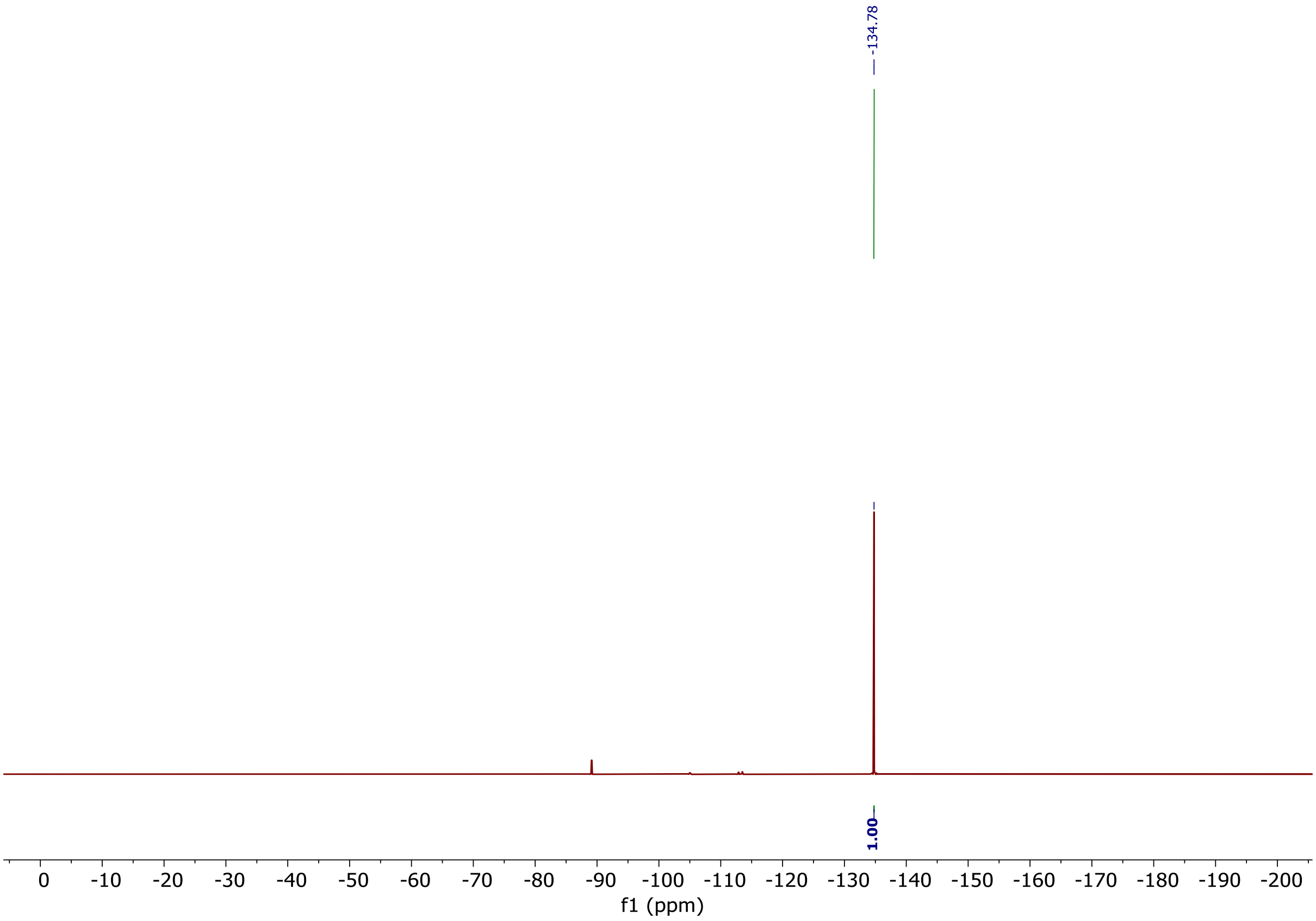

9.6. Azetines (7)

***3-fluoro-1-tosyl-1,2-dihydroazete* (7ar)**

^{1}H NMR (500 MHz, $CDCl_3$)

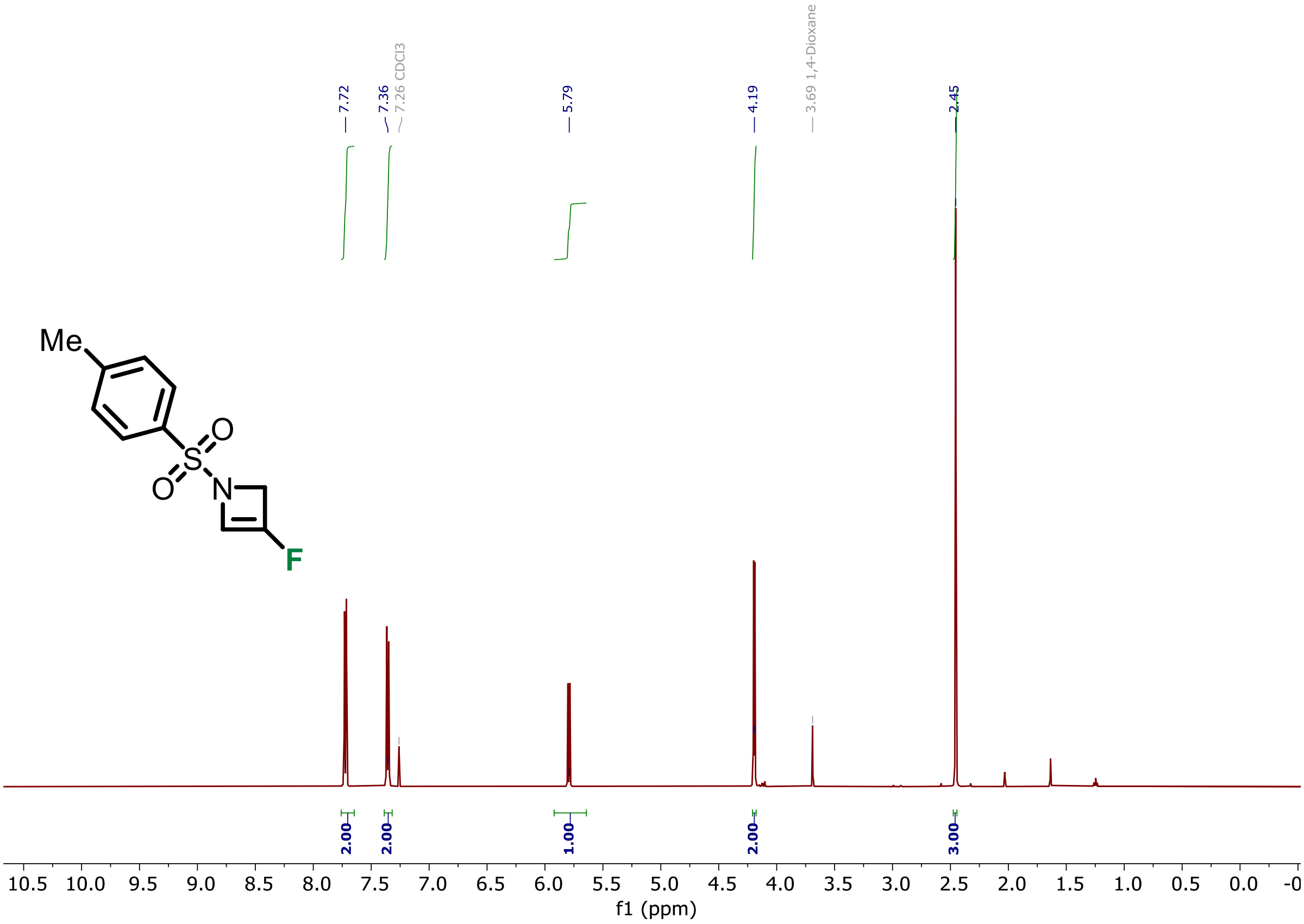

^{13}C NMR (126 MHz, CDCl$_3$)

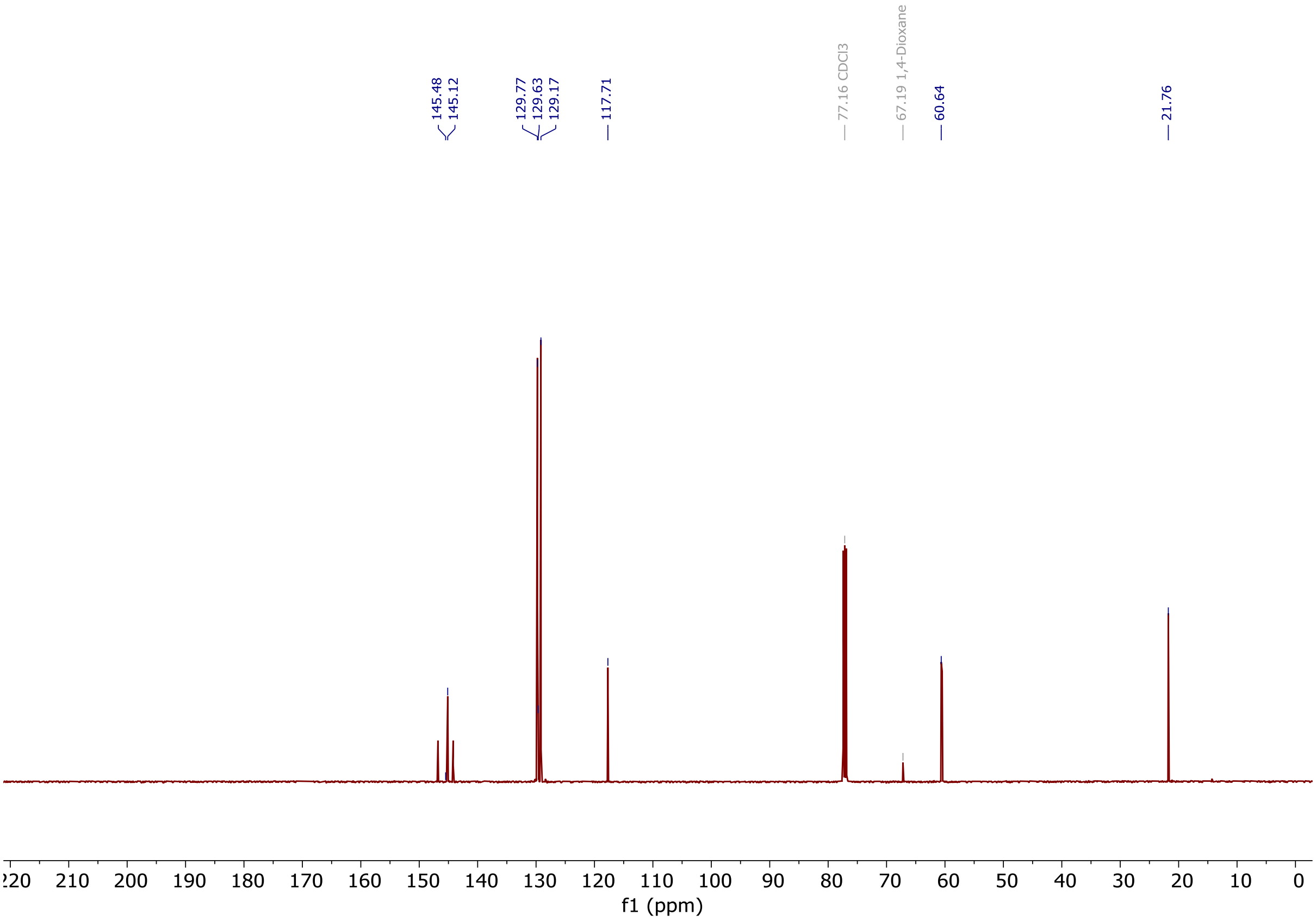

^{19}F NMR (471 MHz, CDCl$_3$)

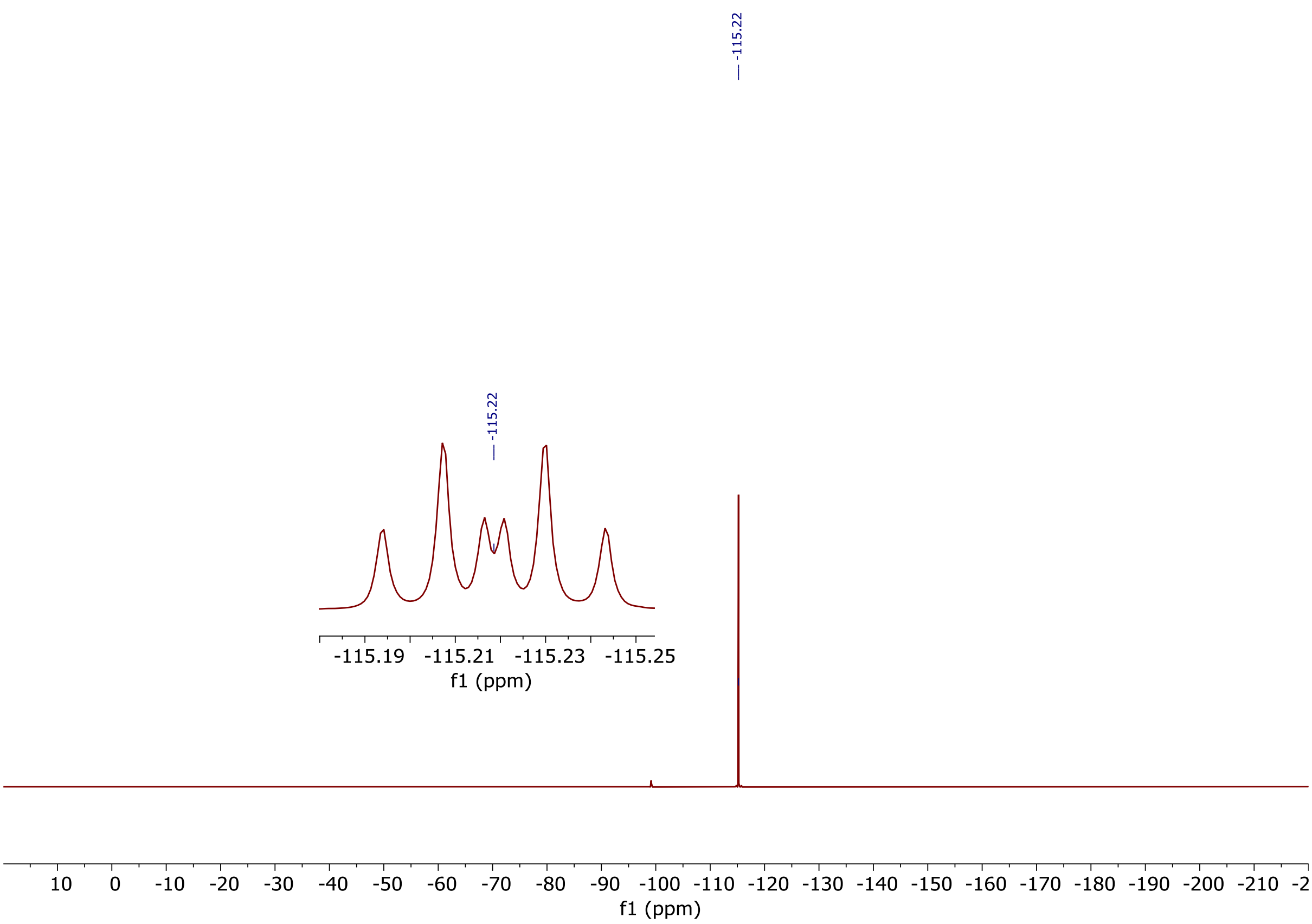

3-fluoro-1-((4-nitrophenyl)sulfonyl)-1,2-dihydroazete (**7as**)

^{1}H NMR (500 MHz, $CDCl_3$)

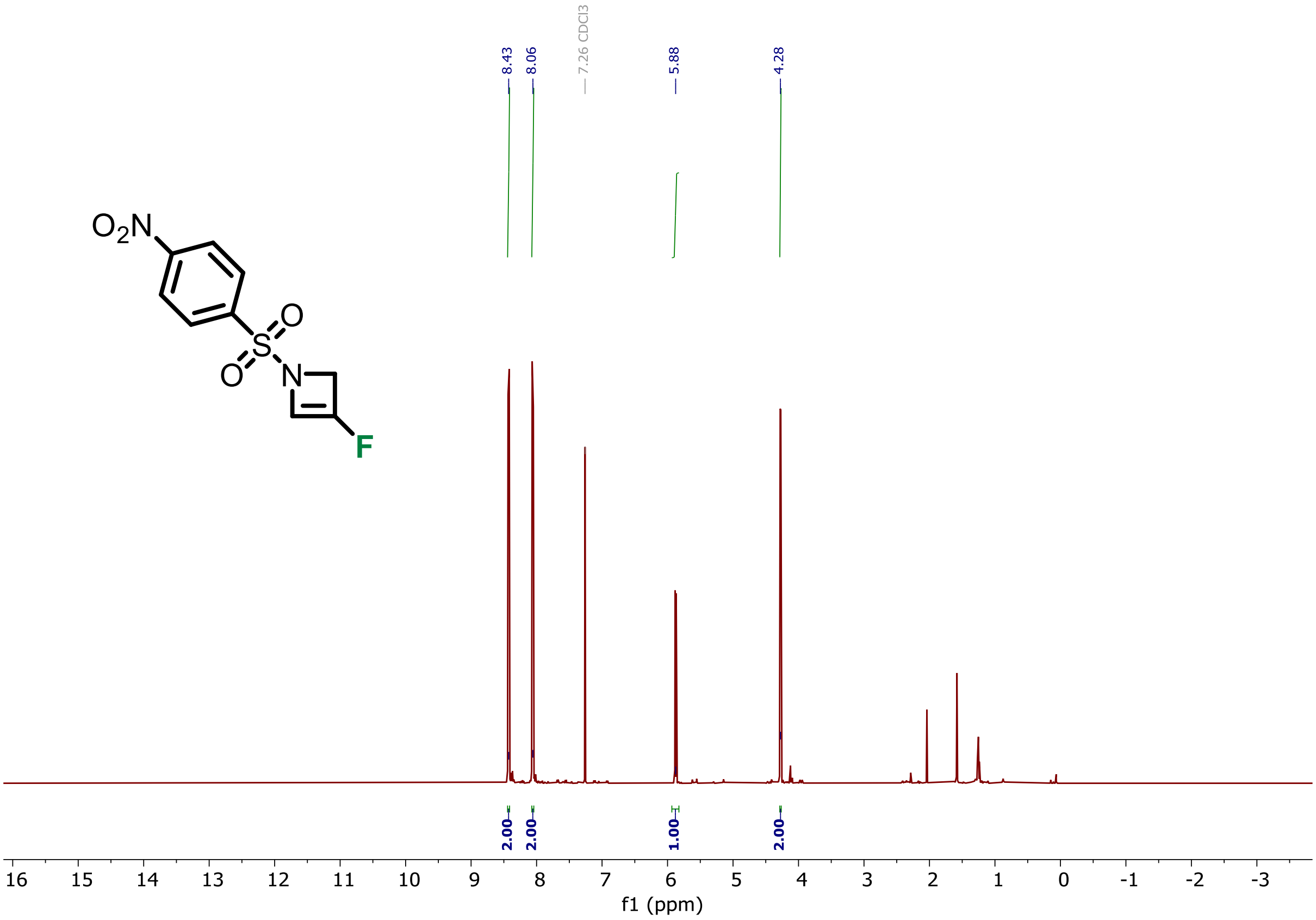

^{13}C NMR (126 MHz, $CDCl_3$)

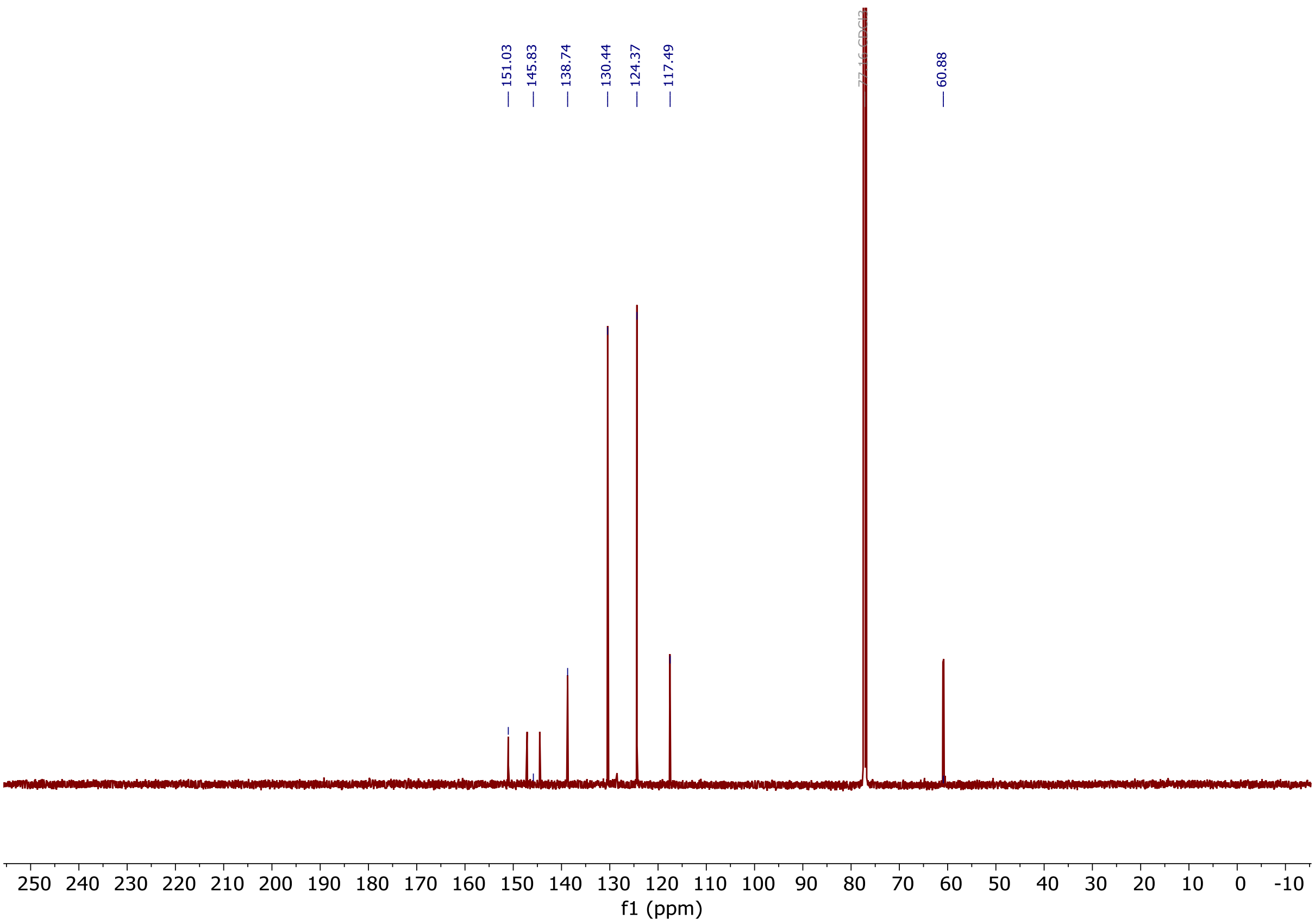

^{19}F NMR (471 MHz, $CDCl_3$)

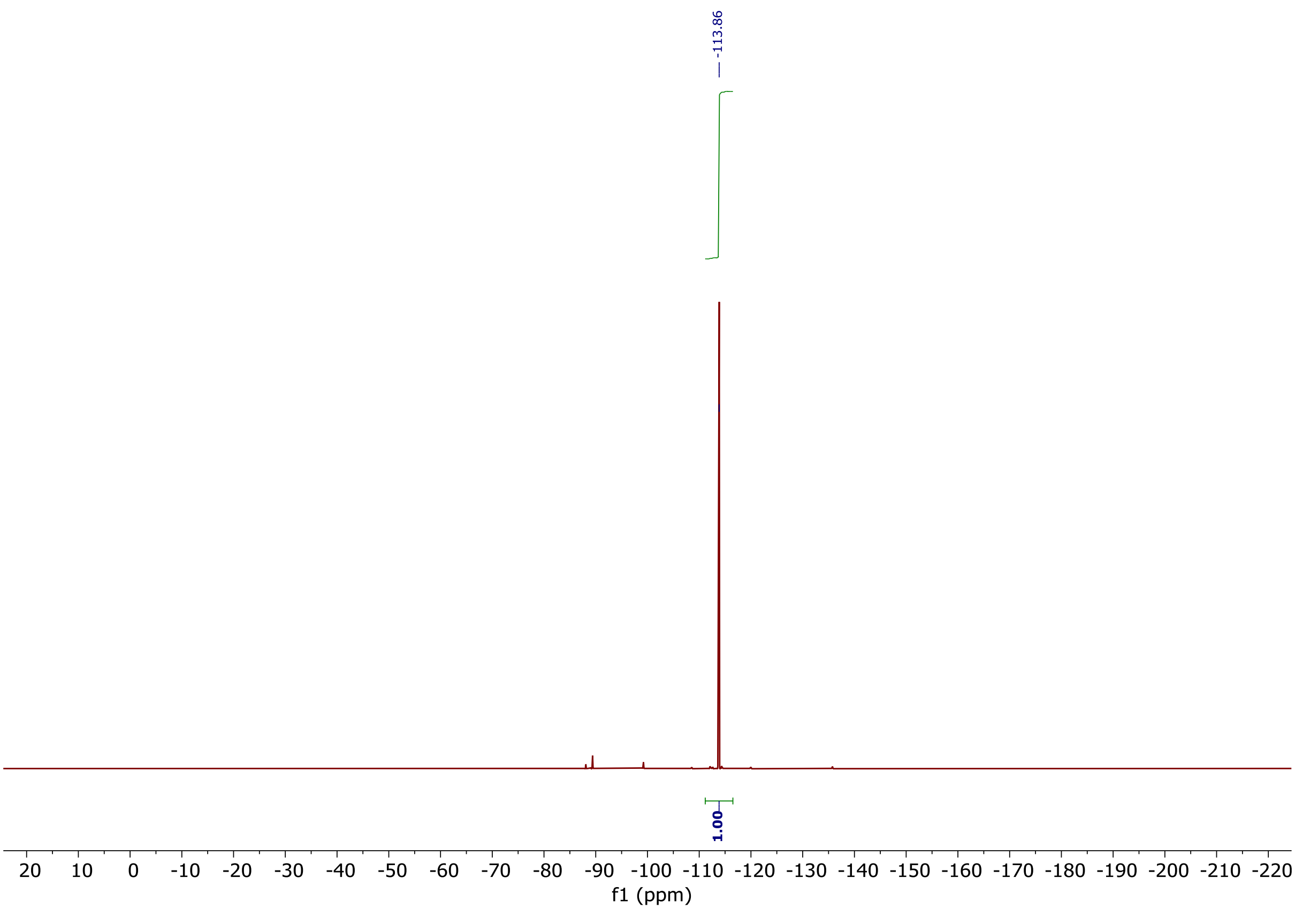

***3-fluoro-2-methyl-1-tosyl-1,2-dihydroazete* (7at)**

^{1}H NMR (400 MHz, CDCl₃)

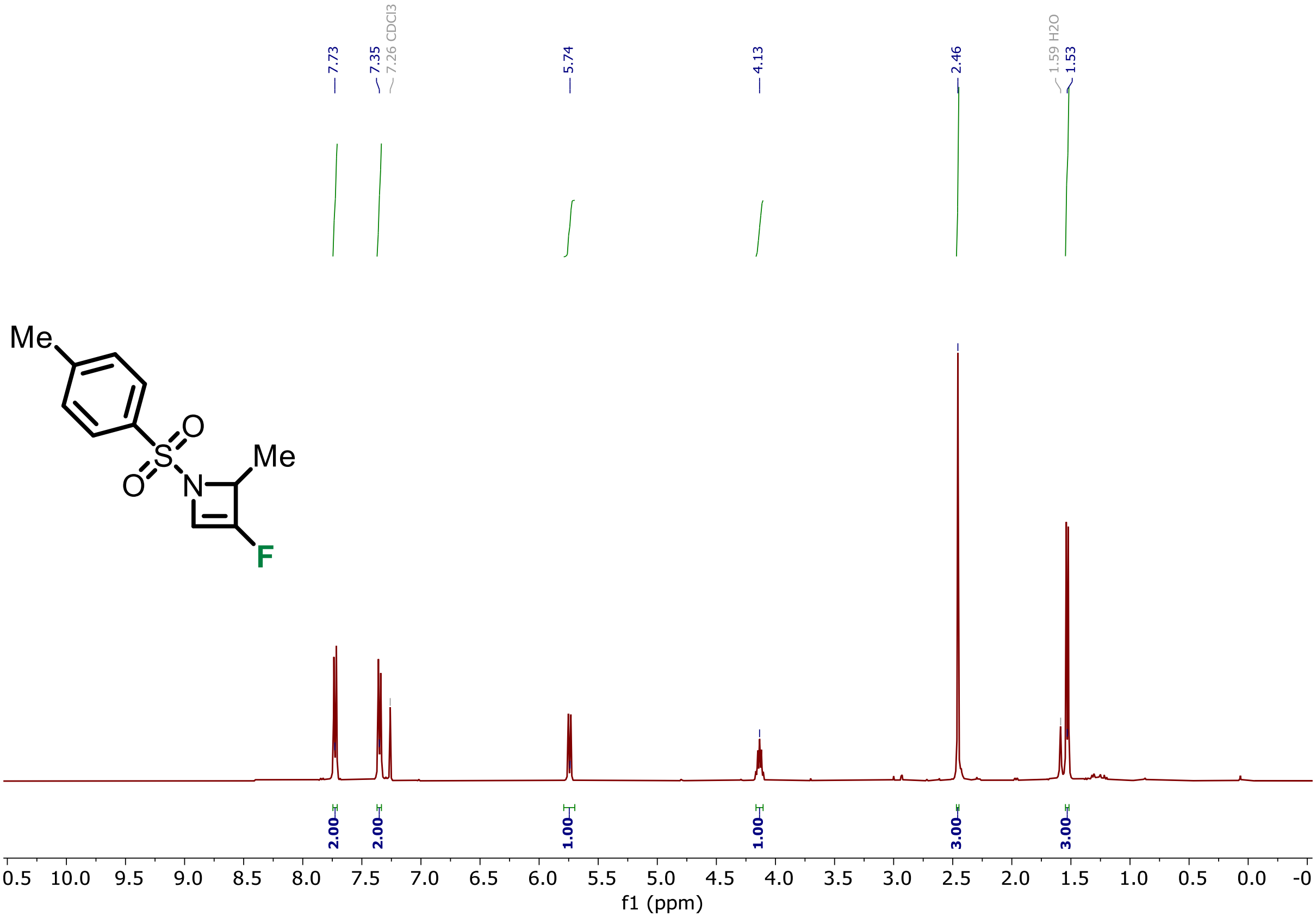

^{13}C NMR (126 MHz, CDCl₃)

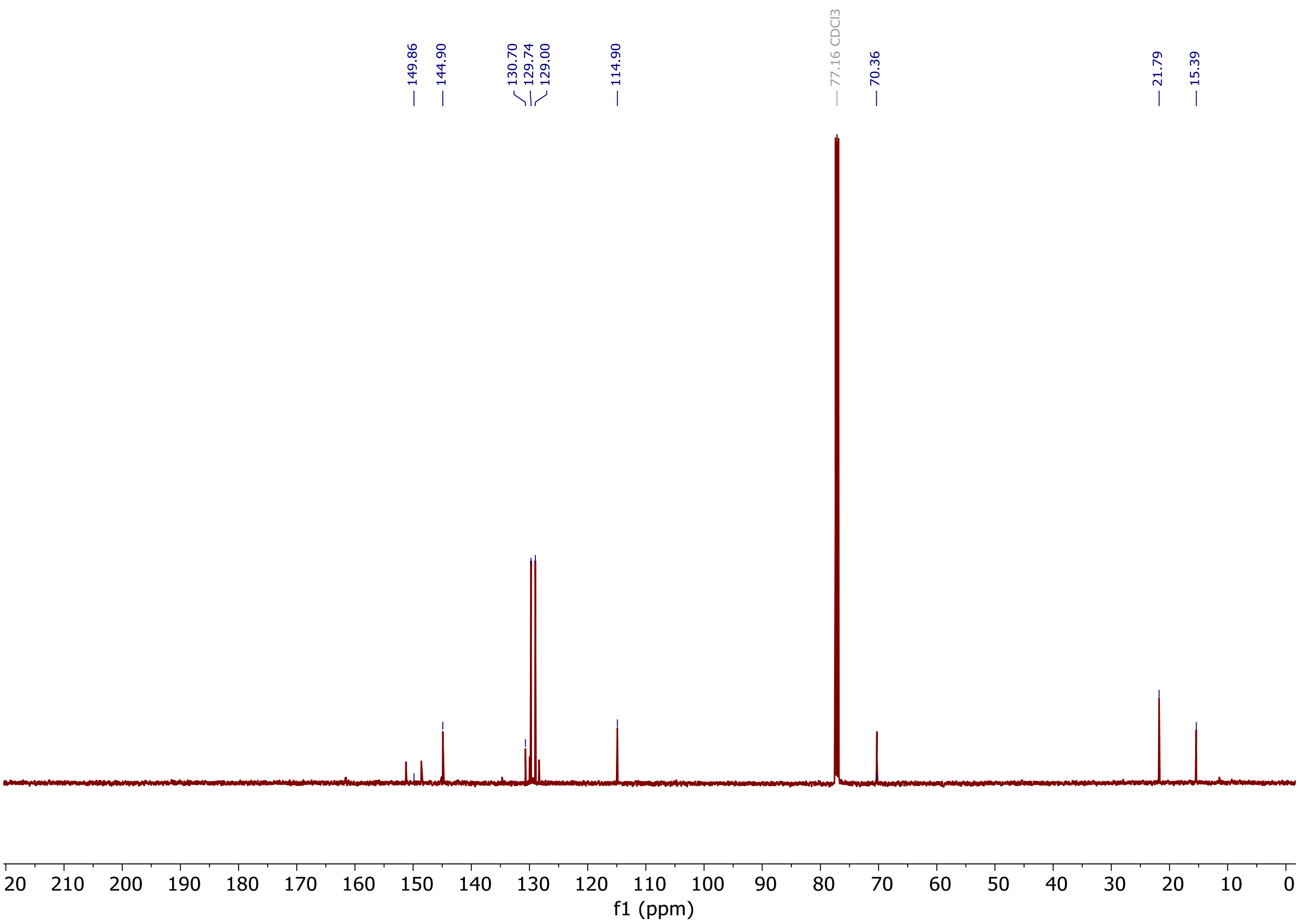

^{19}F NMR (376 MHz, $CDCl_3$)

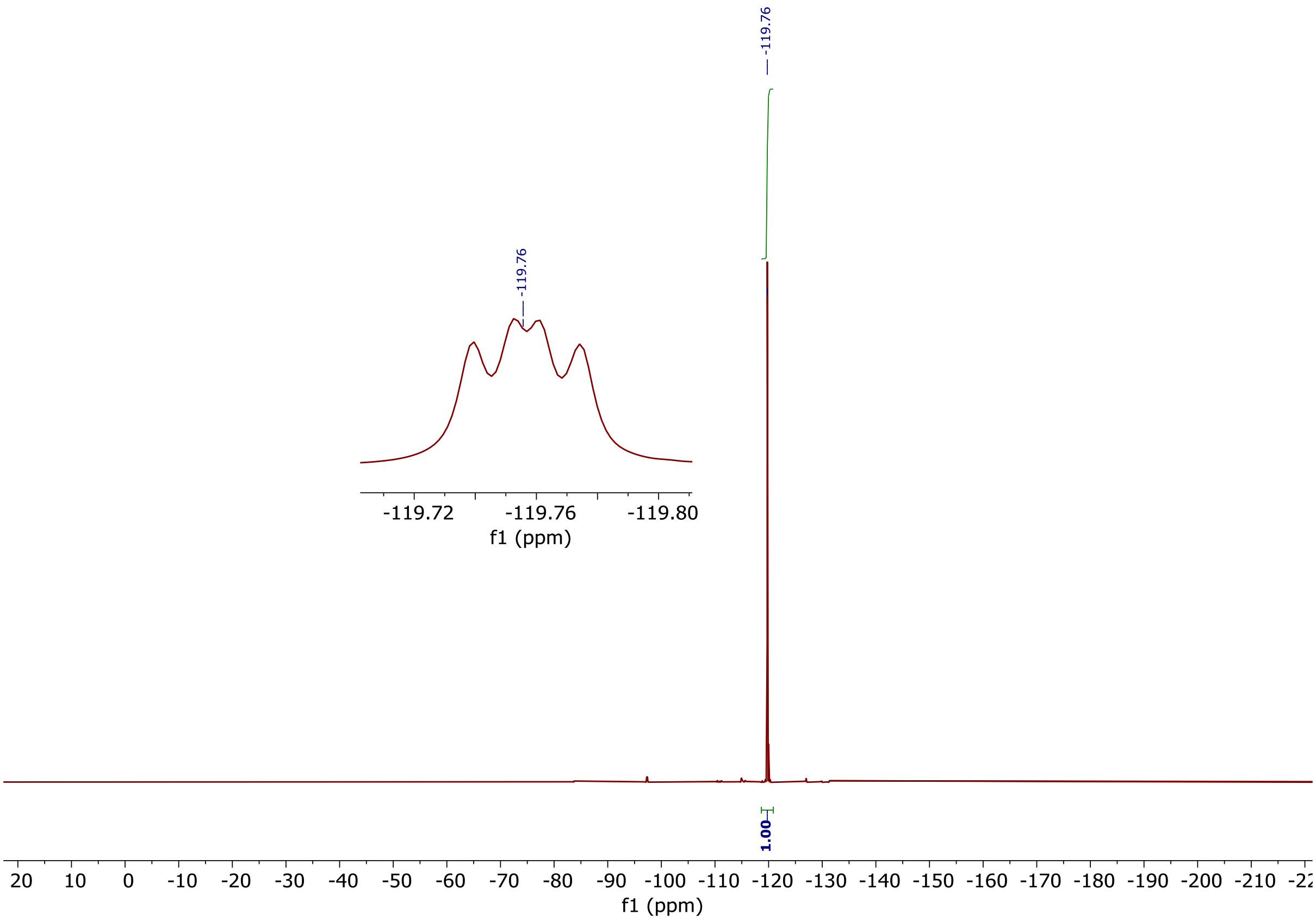

***3-fluoro-1-tosyl-1-azaspiro[3.5]non-2-ene* (7av)**

^{1}H NMR (500 MHz, $CDCl_3$)

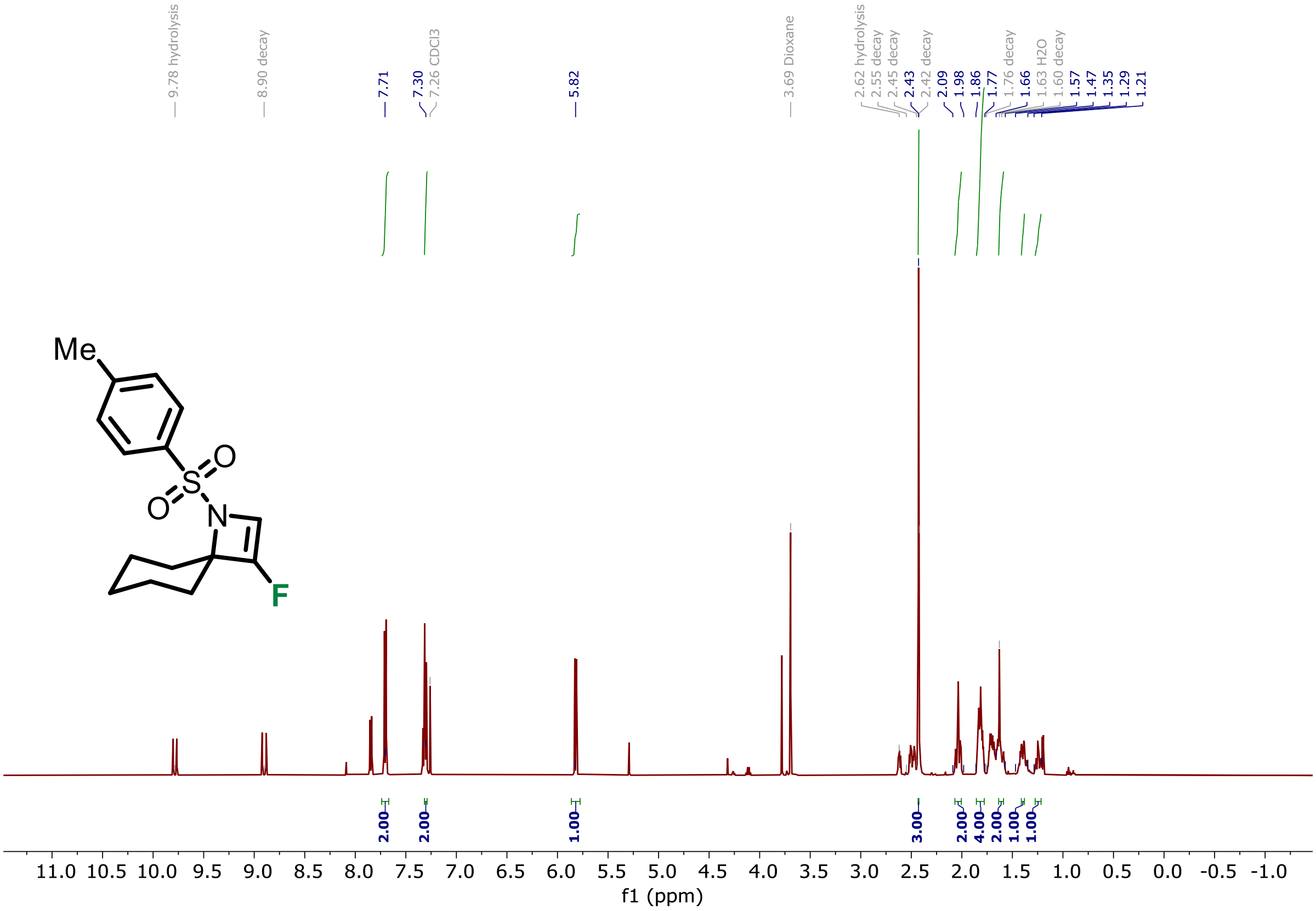

^{13}C NMR (126 MHz, $CDCl_3$)

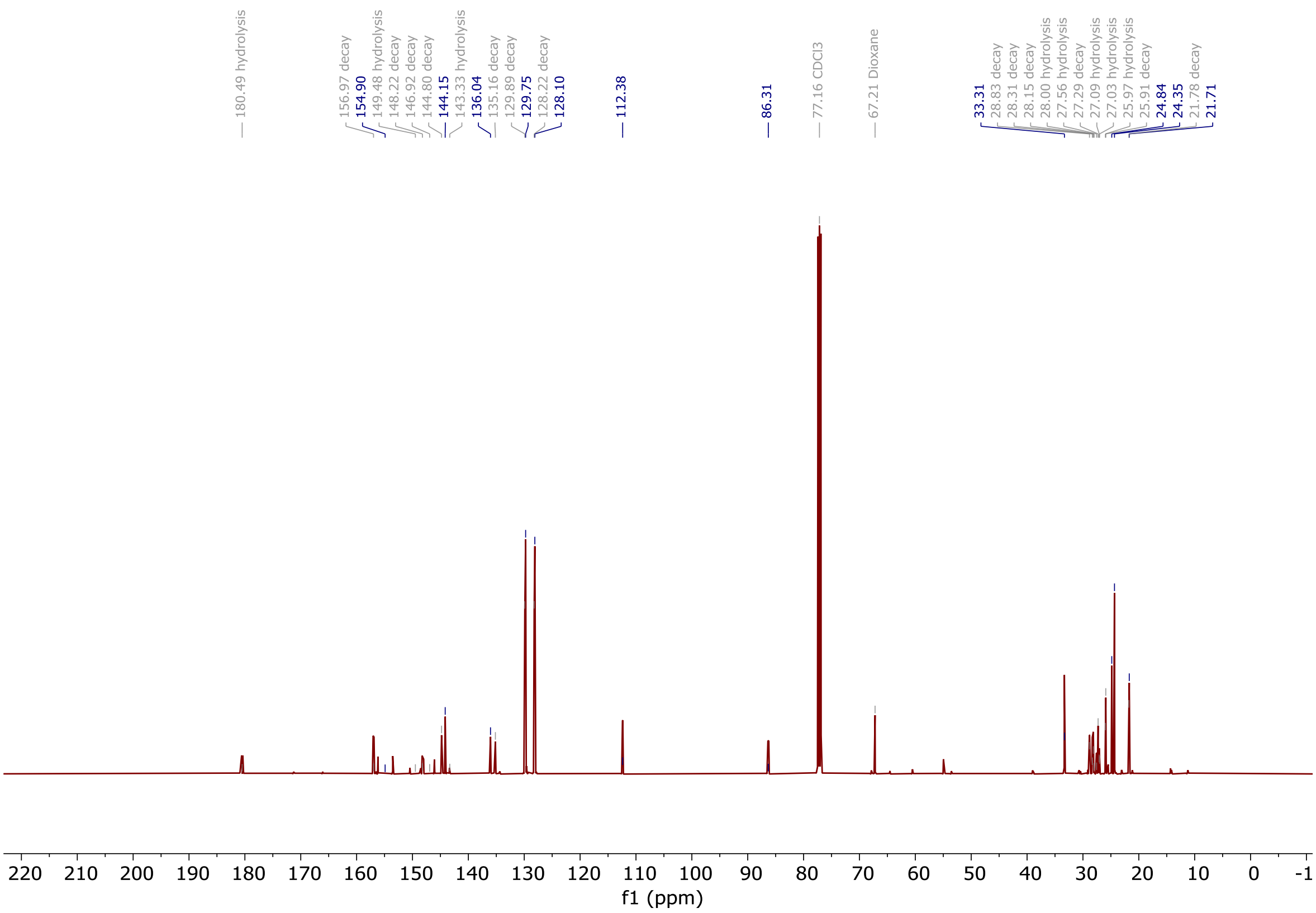

^{19}F NMR (376 MHz, $CDCl_3$)

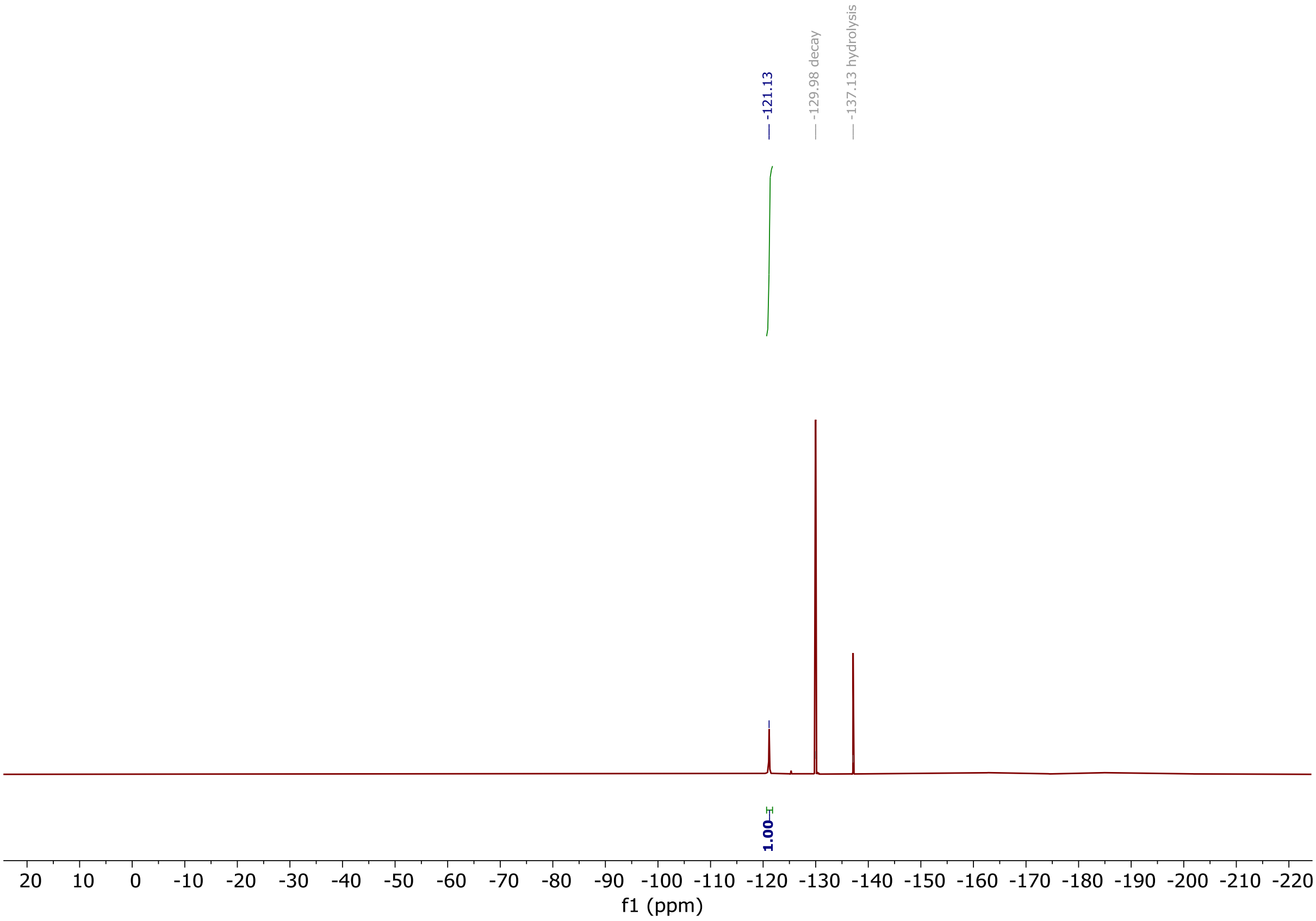

***(E)-N-(2-cyclohexylidene-2-fluoroethylidene)-4-methylbenzenesulfonamide* (7av')**

^{1}H NMR (500 MHz, CDCl$_3$)

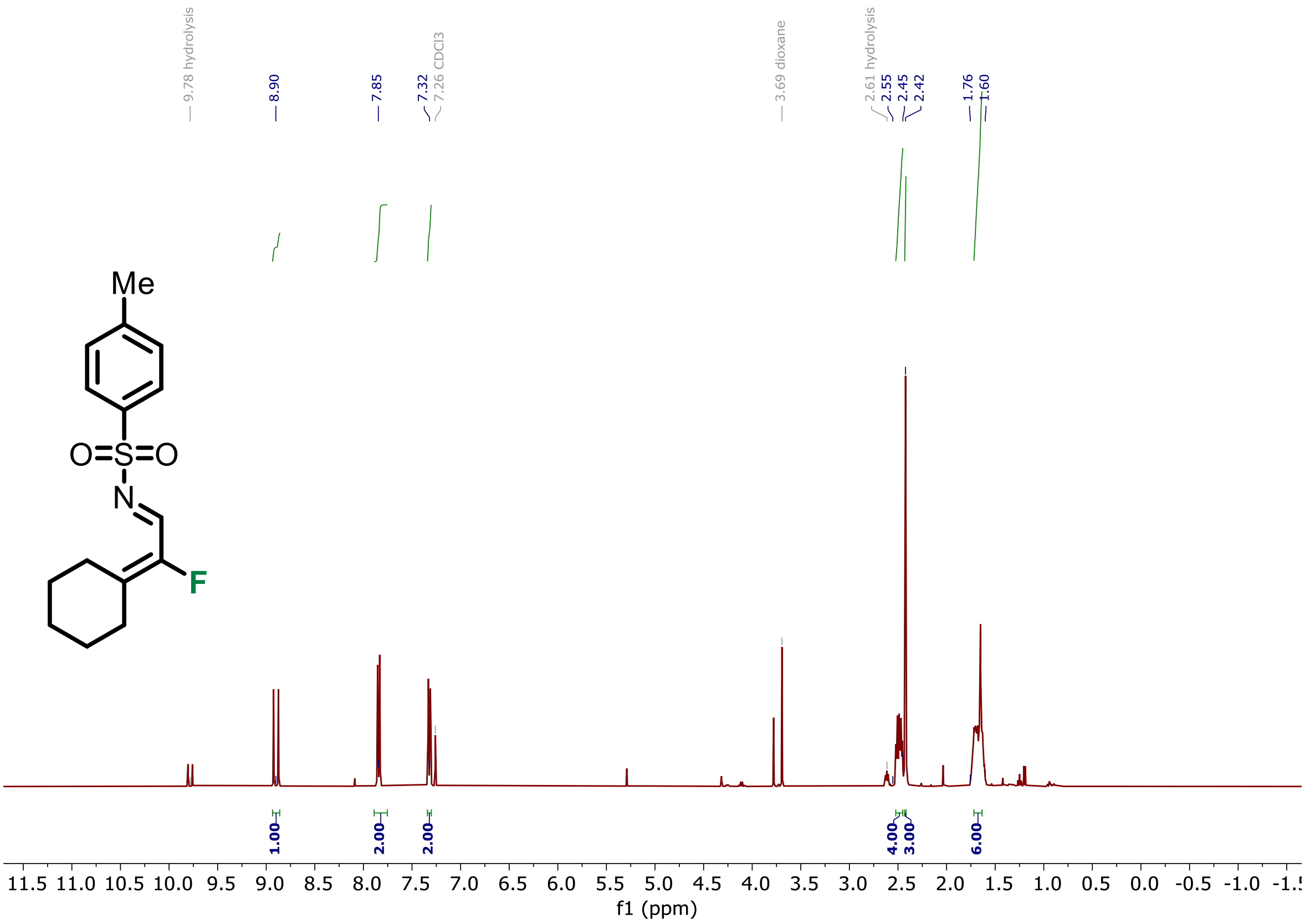

^{13}C NMR (126 MHz, CDCl$_3$)

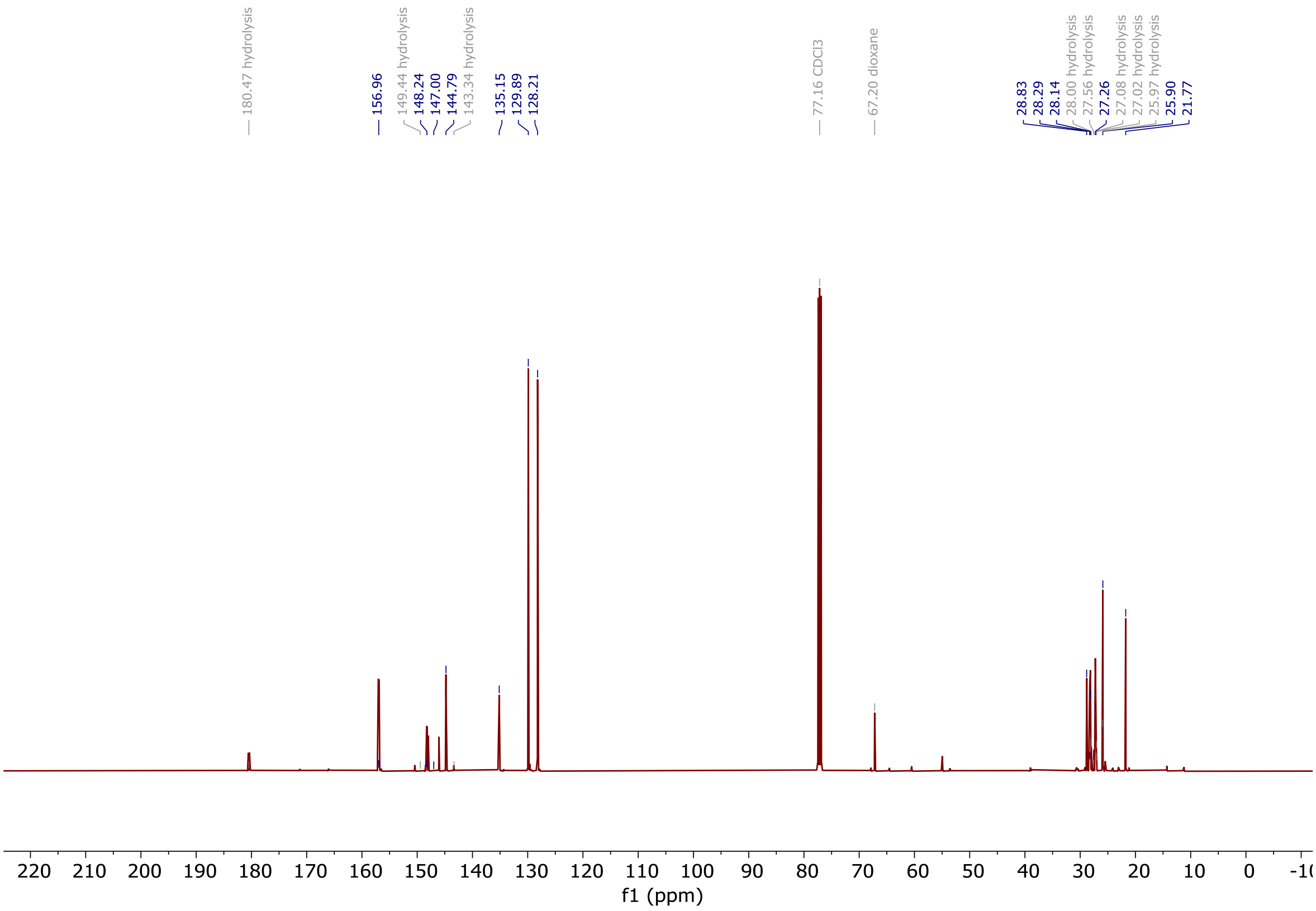

^{19}F NMR (376 MHz, CDCl$_3$)

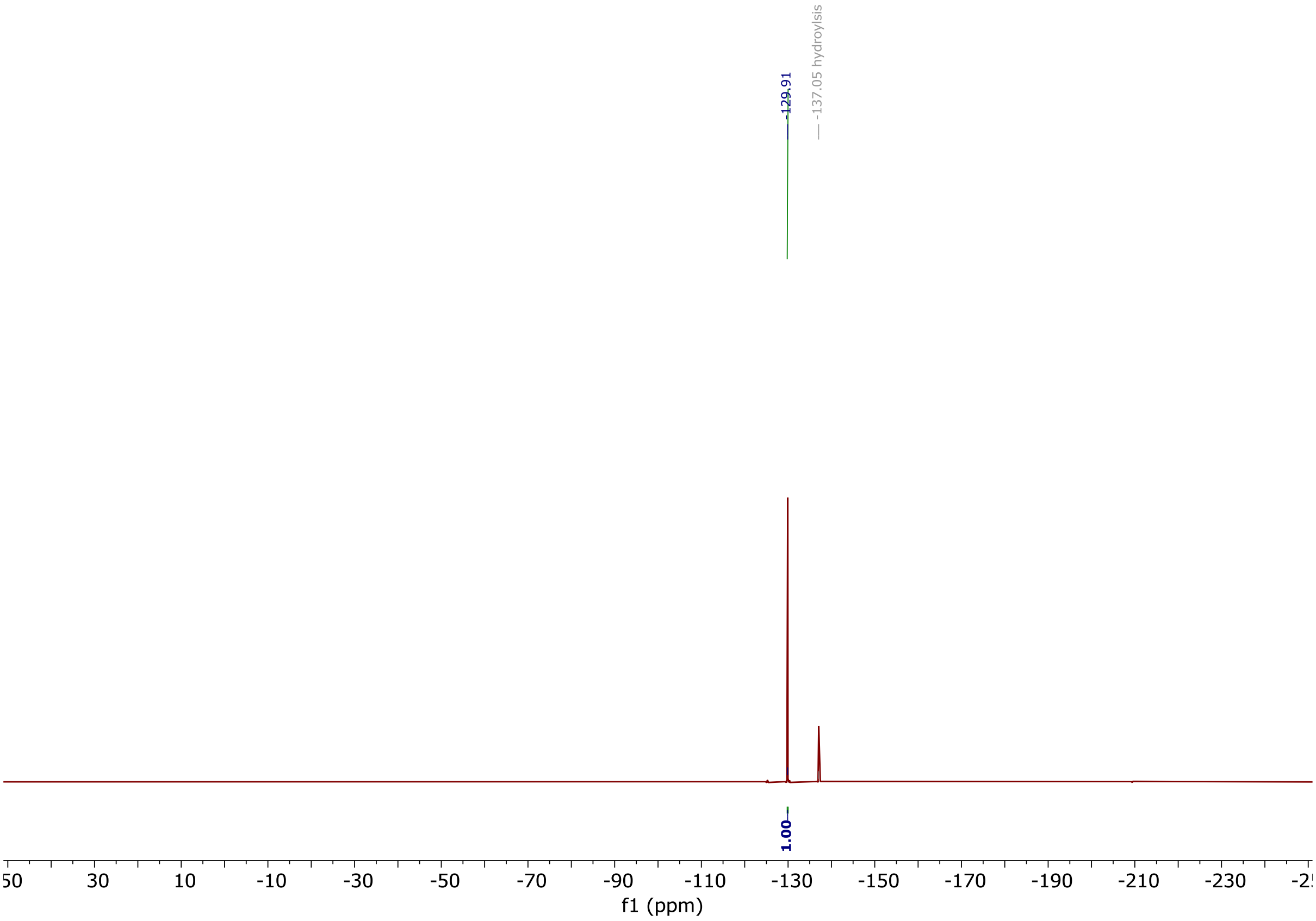

3-fluoro-2,2-dimethyl-1-tosyl-1,2-dihydroazete **(7au)**
^{1}H NMR (400 MHz, $CDCl_3$)

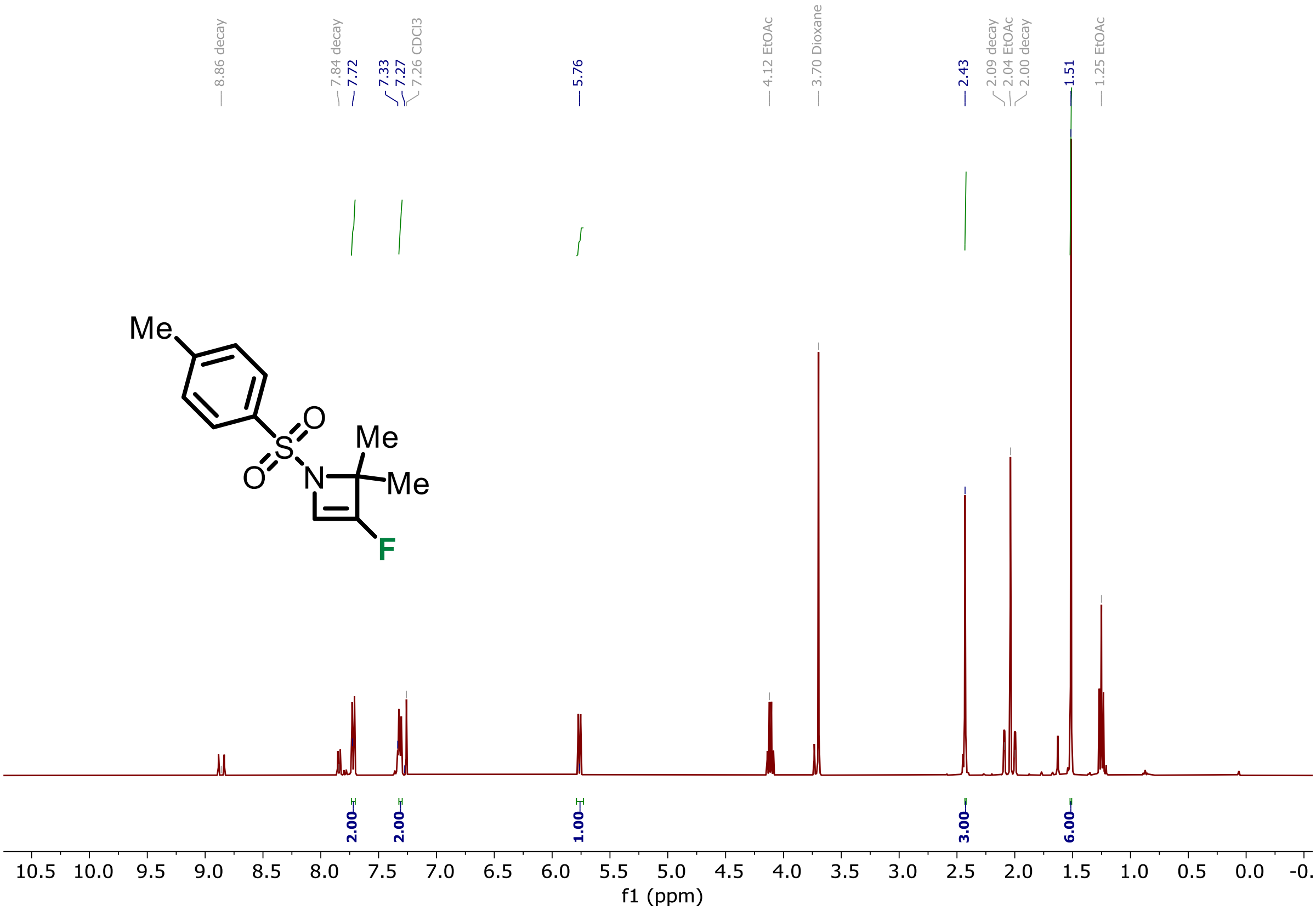

^{13}C NMR (126 MHz, CDCl$_3$)

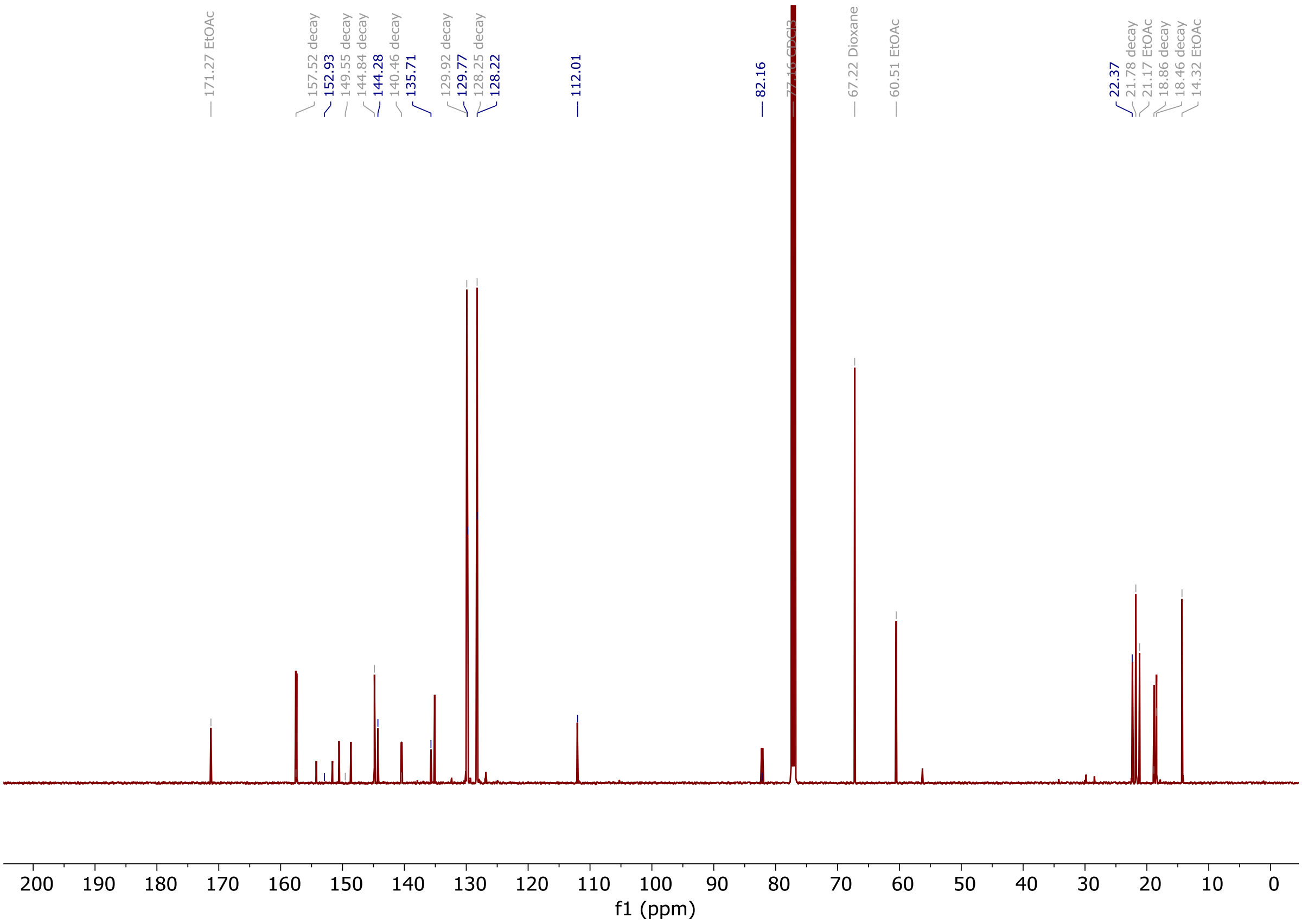

^{19}F NMR (376 MHz, CDCl$_3$)

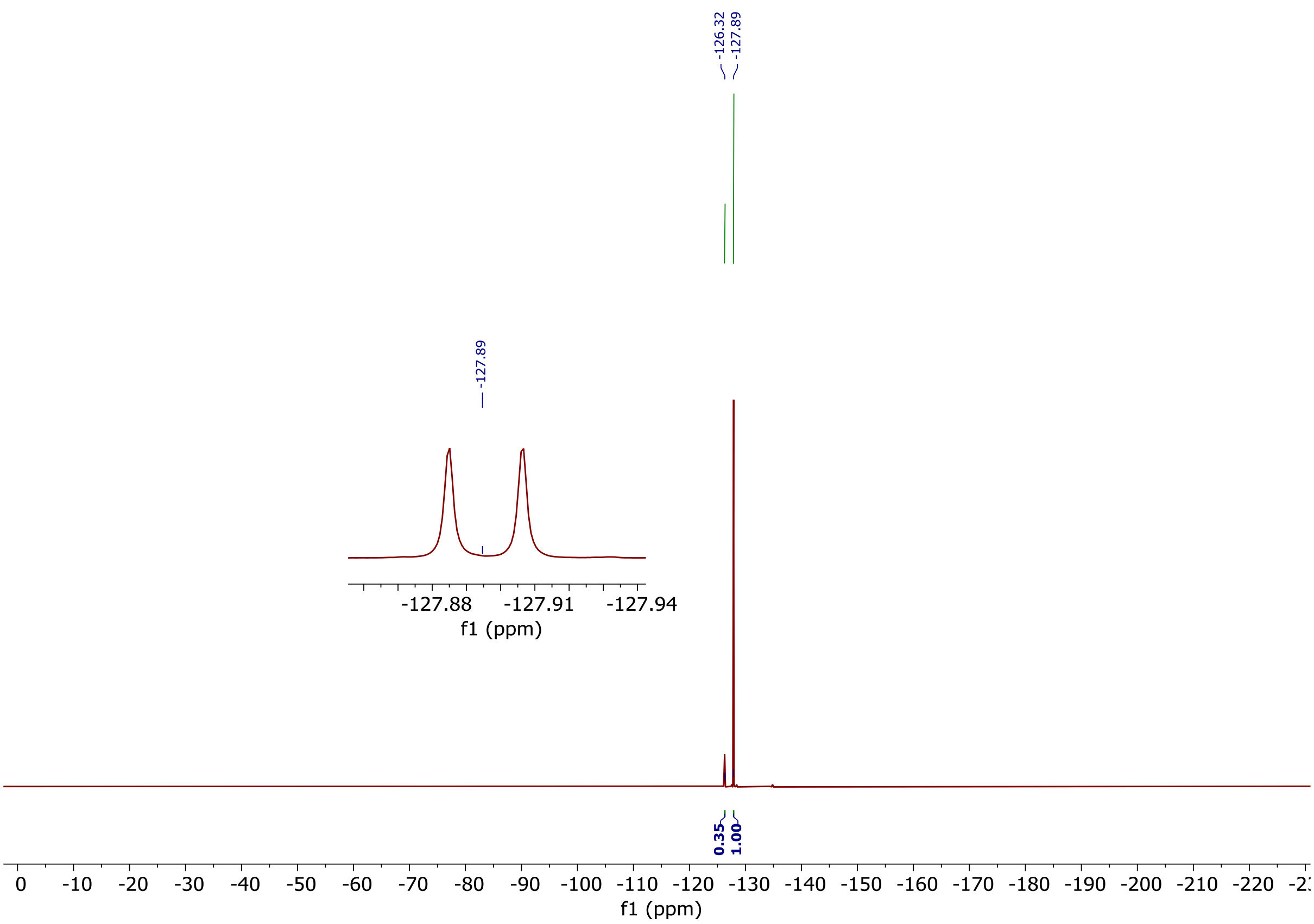

(E)-N-(2-fluoro-3-methylbut-2-en-1-ylidene)-4-methylbenzenesulfonamide **(7au')**

^{1}H NMR (500 MHz, $CDCl_3$)

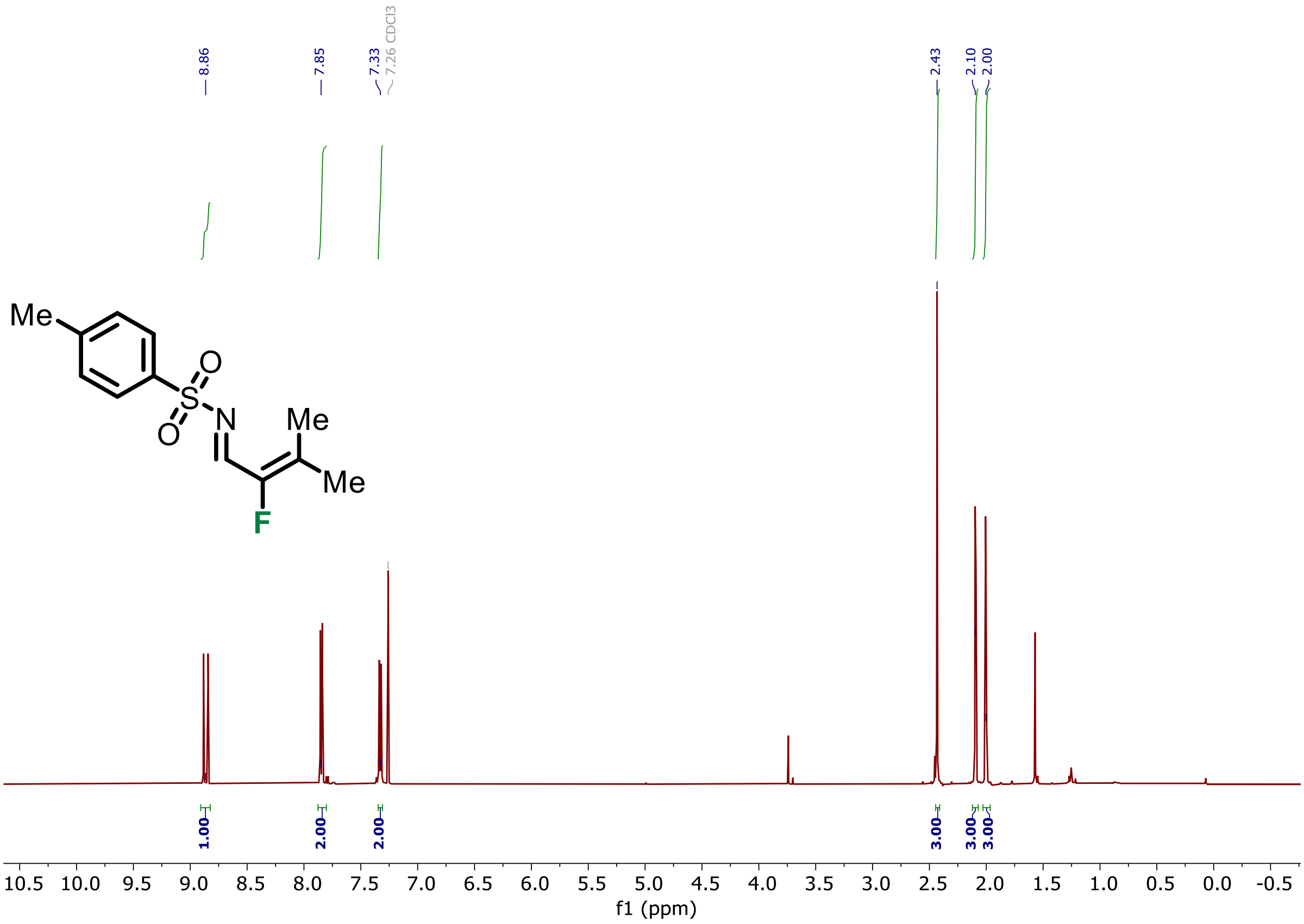

^{13}C NMR (126 MHz, $CDCl_3$)

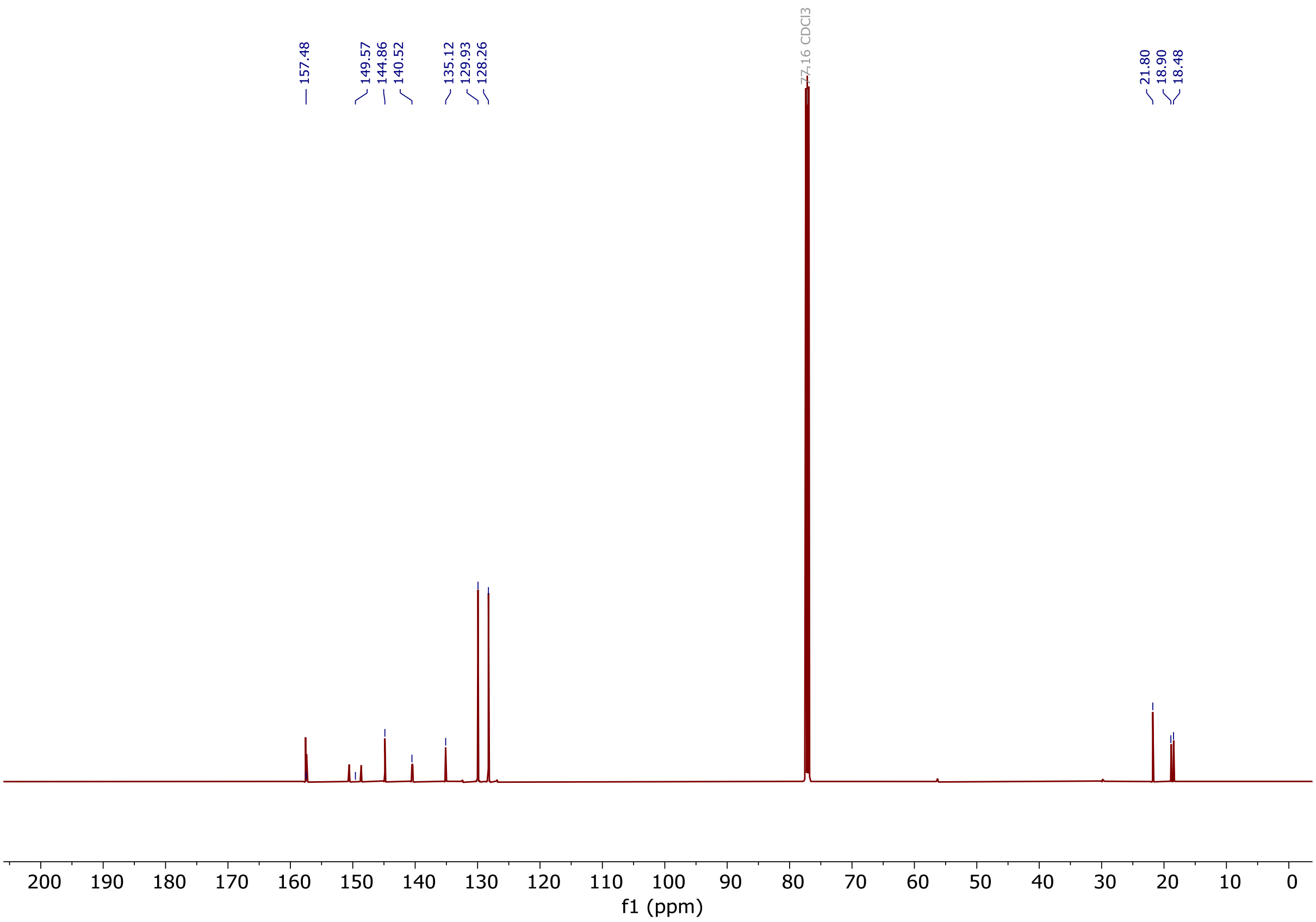

^{19}F NMR (376 MHz, $CDCl_3$)

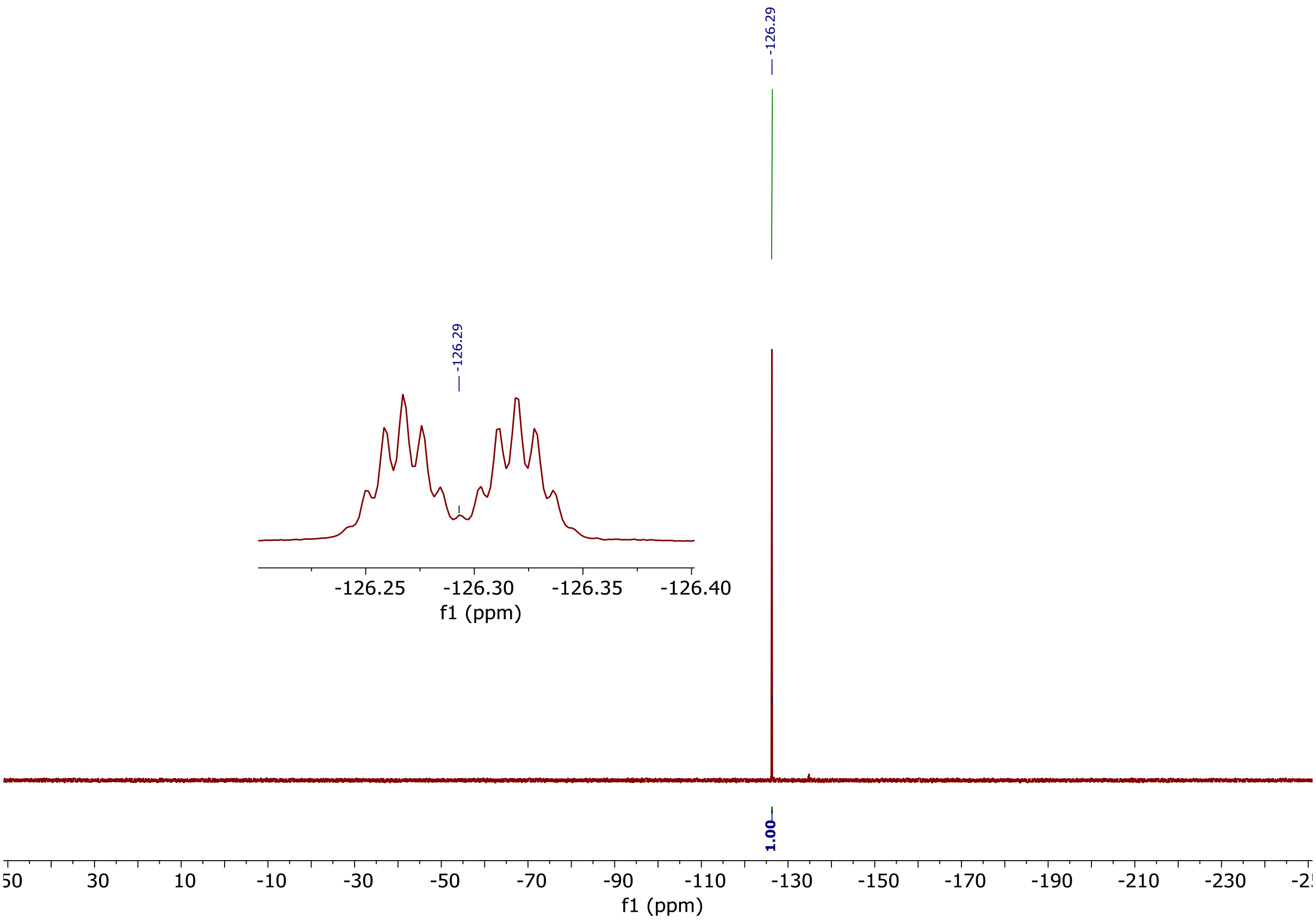

3-fluoro-4-phenyl-1-tosyl-1,2-dihydroazete (**7aw**)

^{1}H NMR (500 MHz, CDCl$_3$)

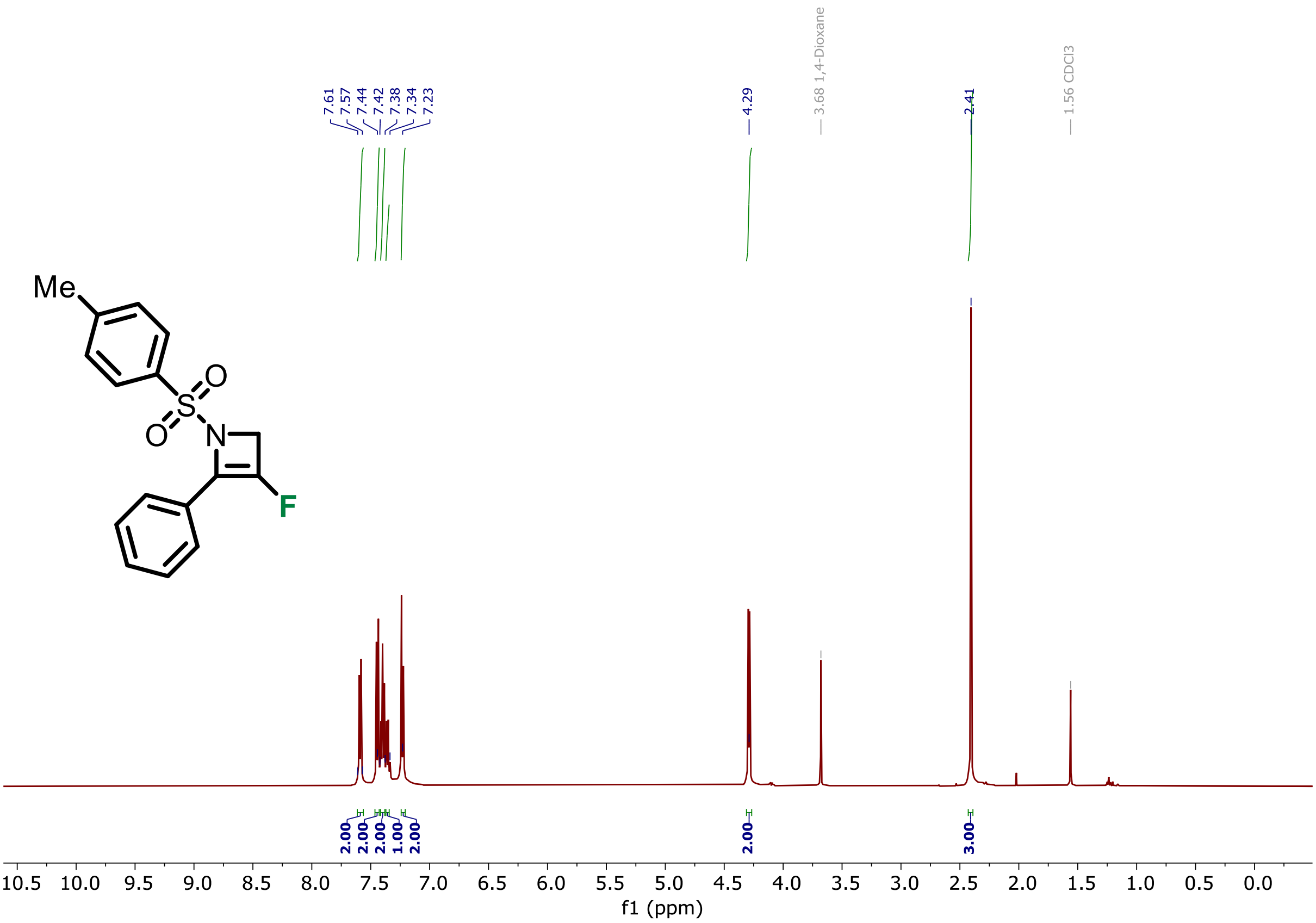

^{13}C NMR (126 MHz, CDCl$_3$)

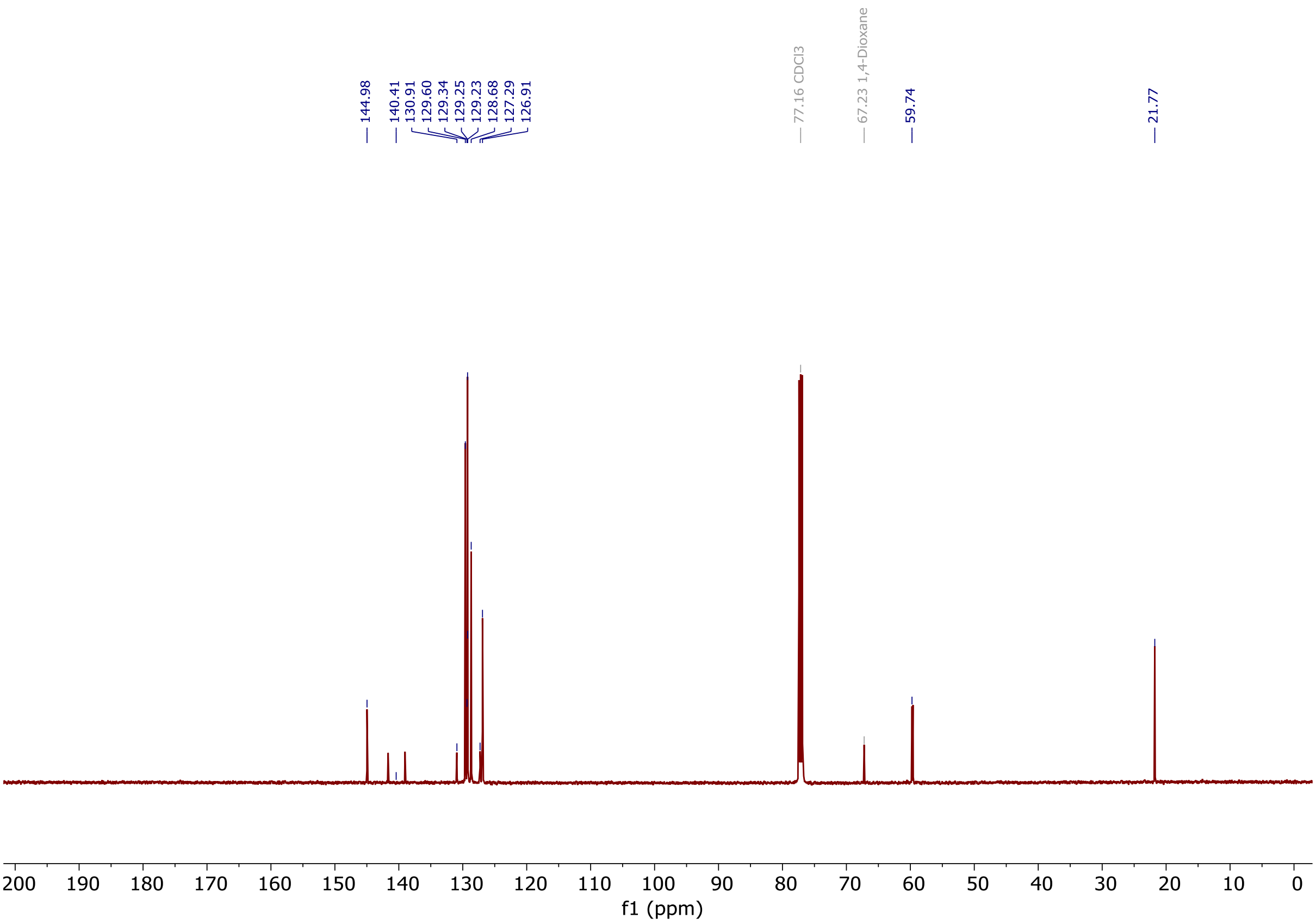

^{19}F NMR (471 MHz, $CDCl_3$)

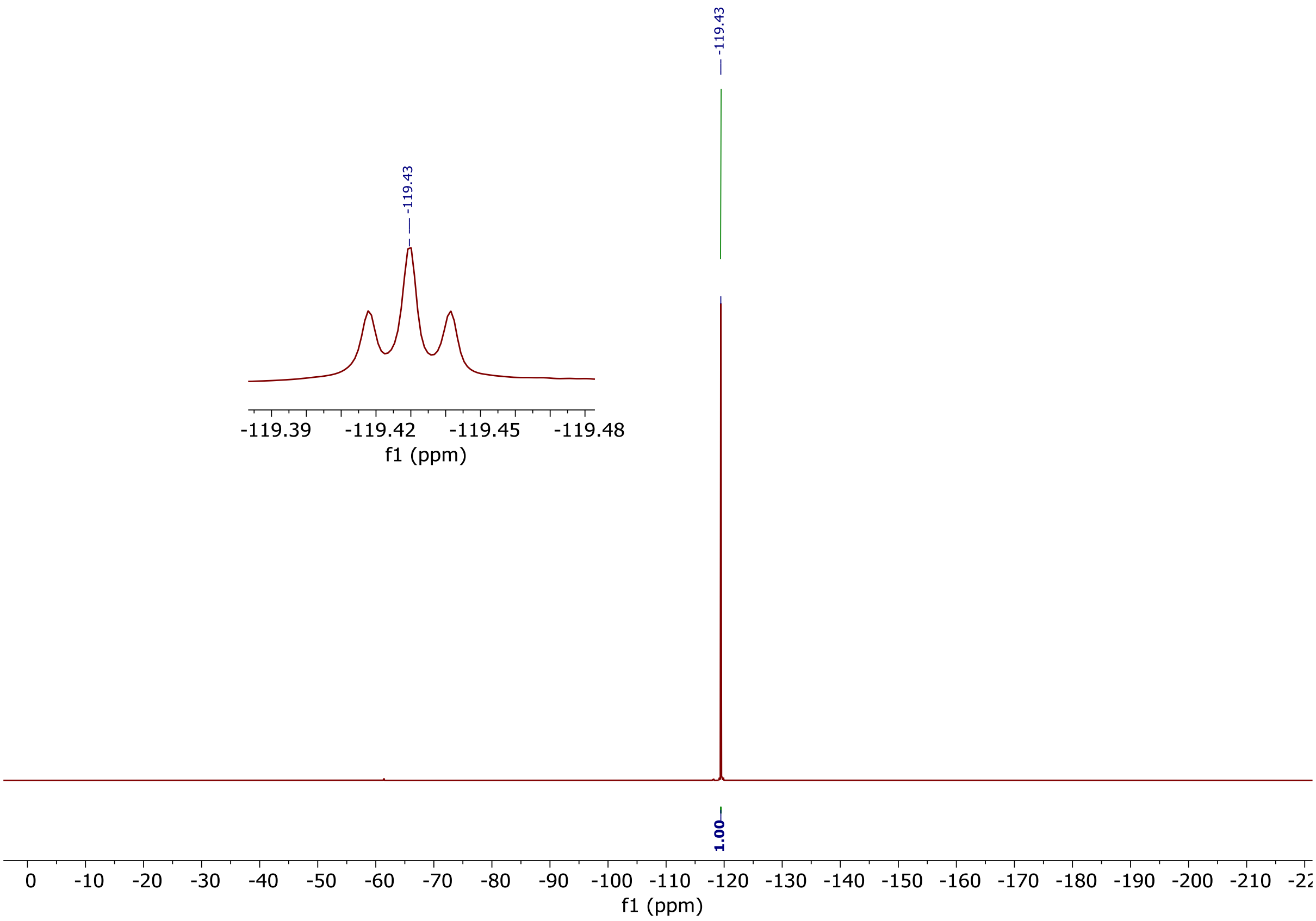

***3-fluoro-4-(4-fluorophenyl)-1-tosyl-1,2-dihydroazete* (7ax)**

^{1}H NMR (500 MHz, CDCl$_3$)

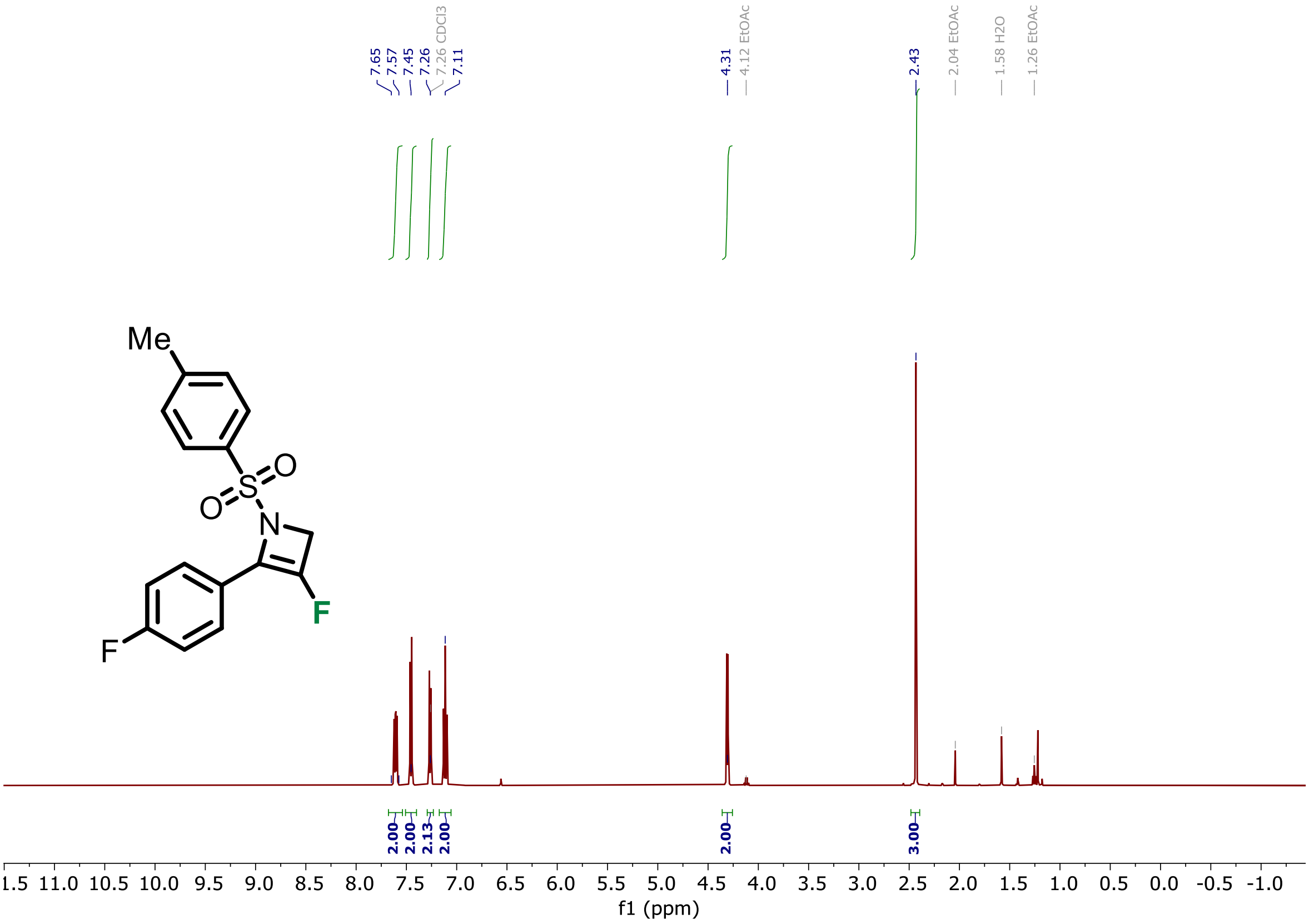

^{13}C NMR (126 MHz, CDCl$_3$)

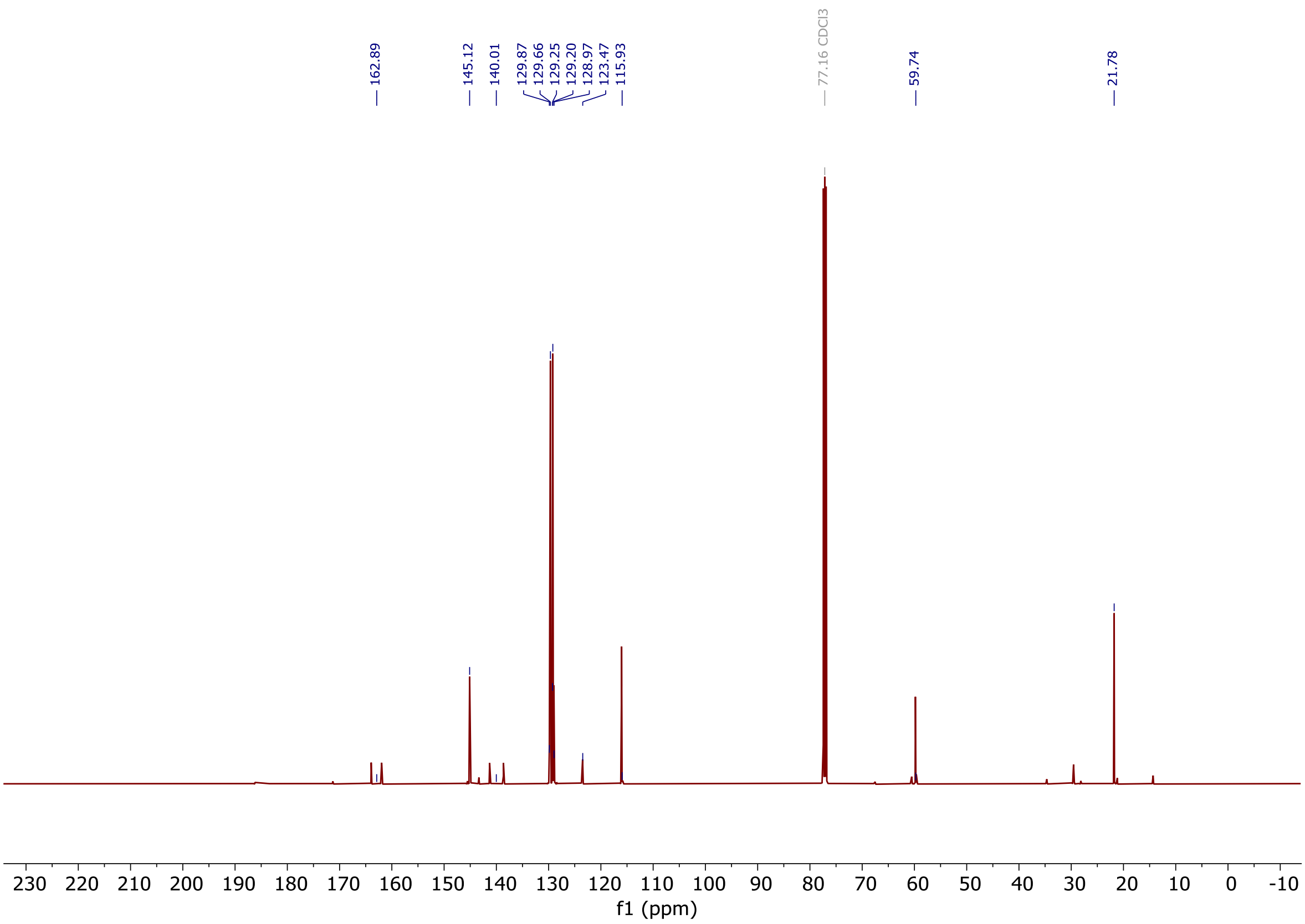

^{19}F NMR (376 MHz, CDCl$_3$)

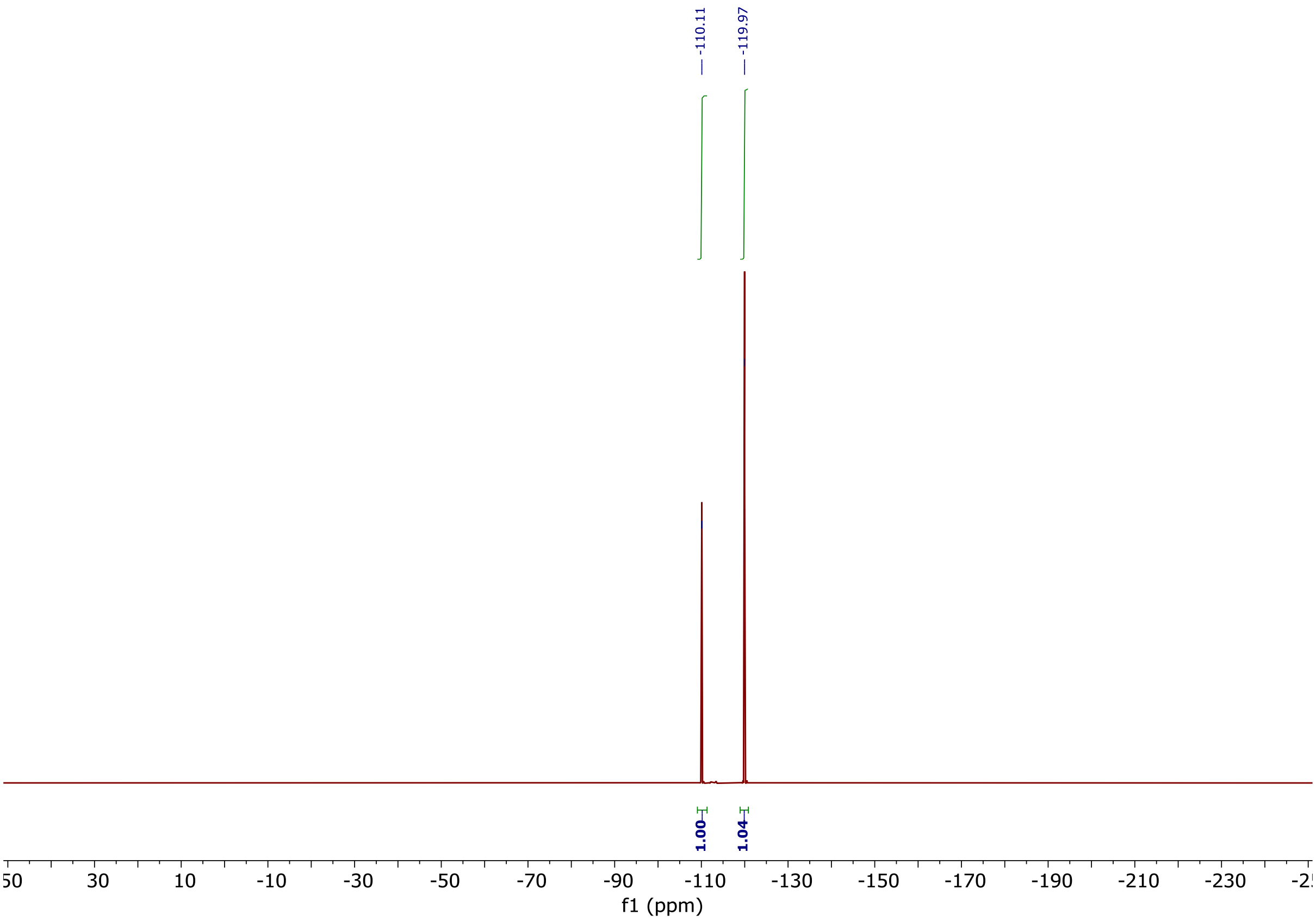

4-([1,1'-biphenyl]-4-yl)-3-fluoro-1-tosyl-1,2-dihydroazete **(7ay)**

^{1}H NMR (500 MHz, CDCl$_3$)

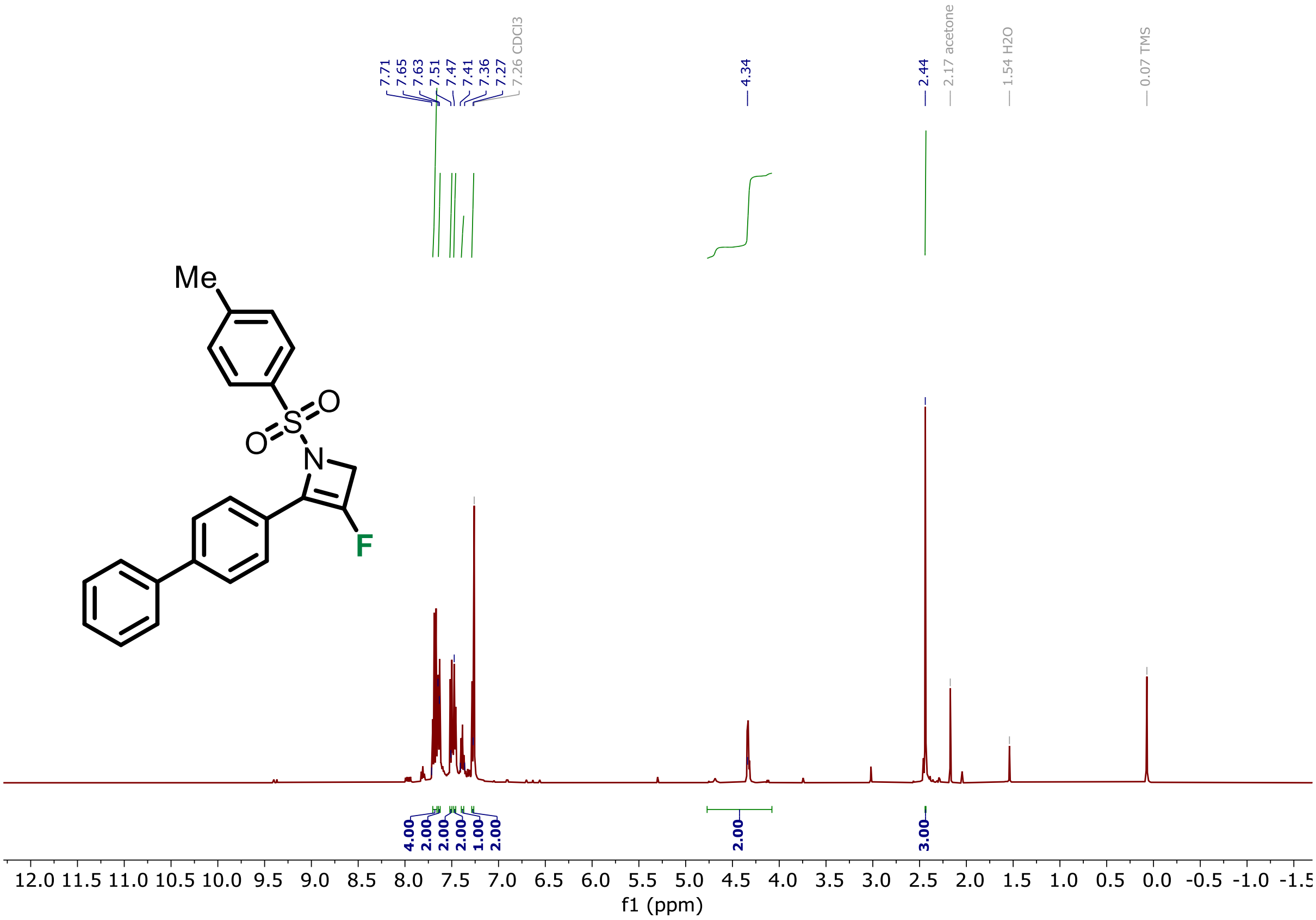

^{13}C NMR (126 MHz, CDCl$_3$)

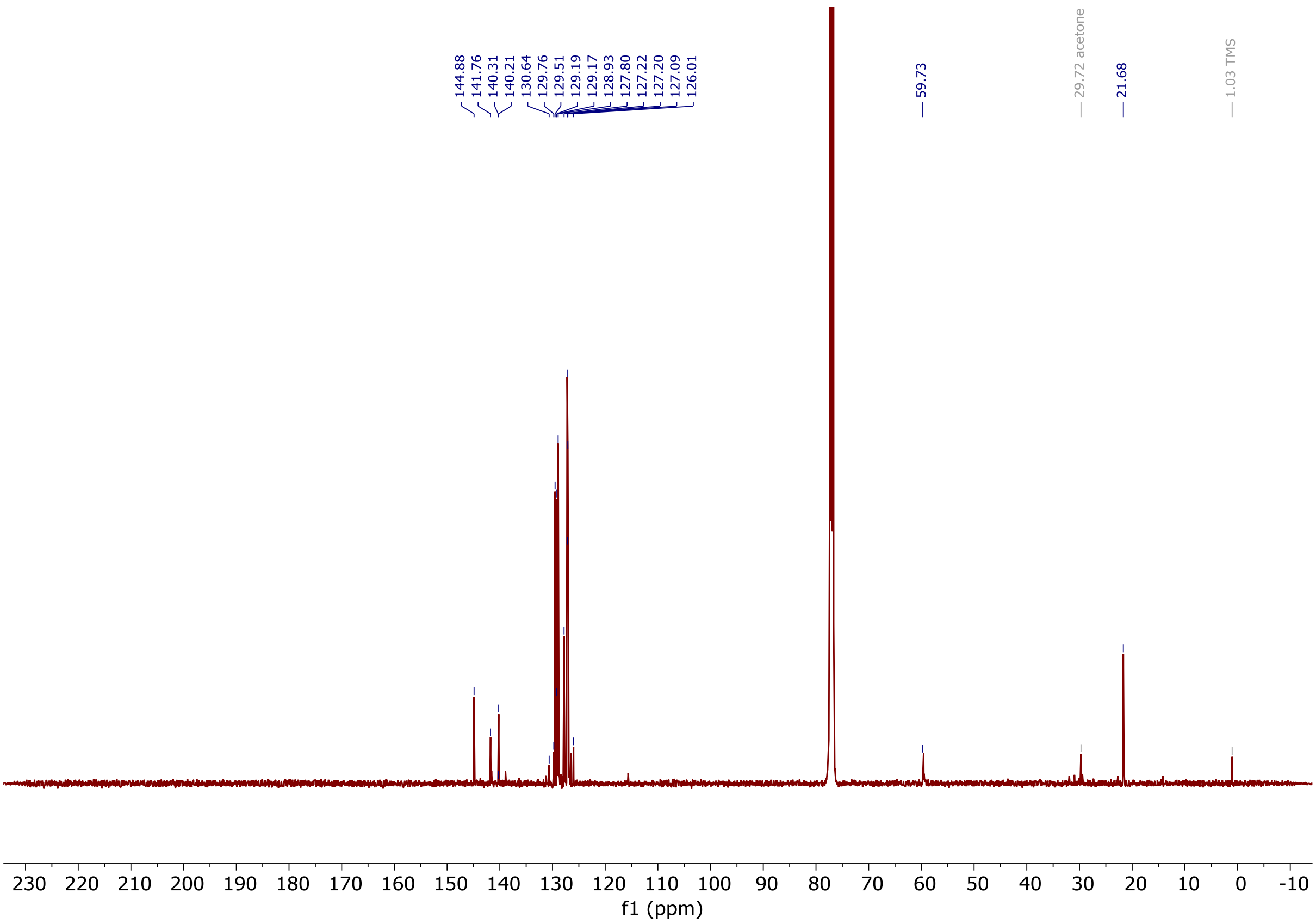

^{19}F NMR (376 MHz, $CDCl_3$)

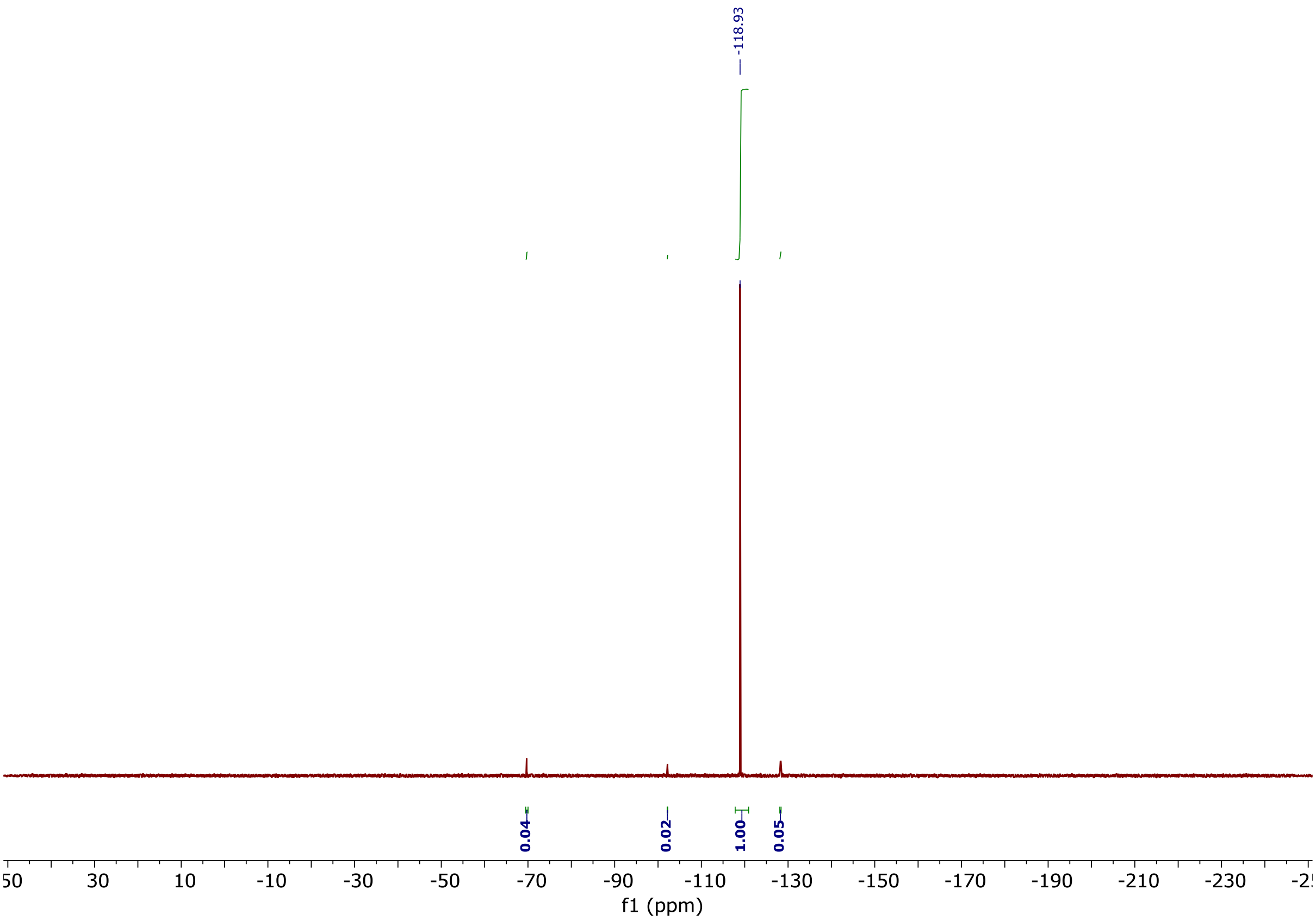

***3-fluoro-1-tosyl-4-(4-(trifluoromethyl)phenyl)-1,2-dihydroazete* (7az)**

^{1}H NMR (500 MHz, CDCl₃)

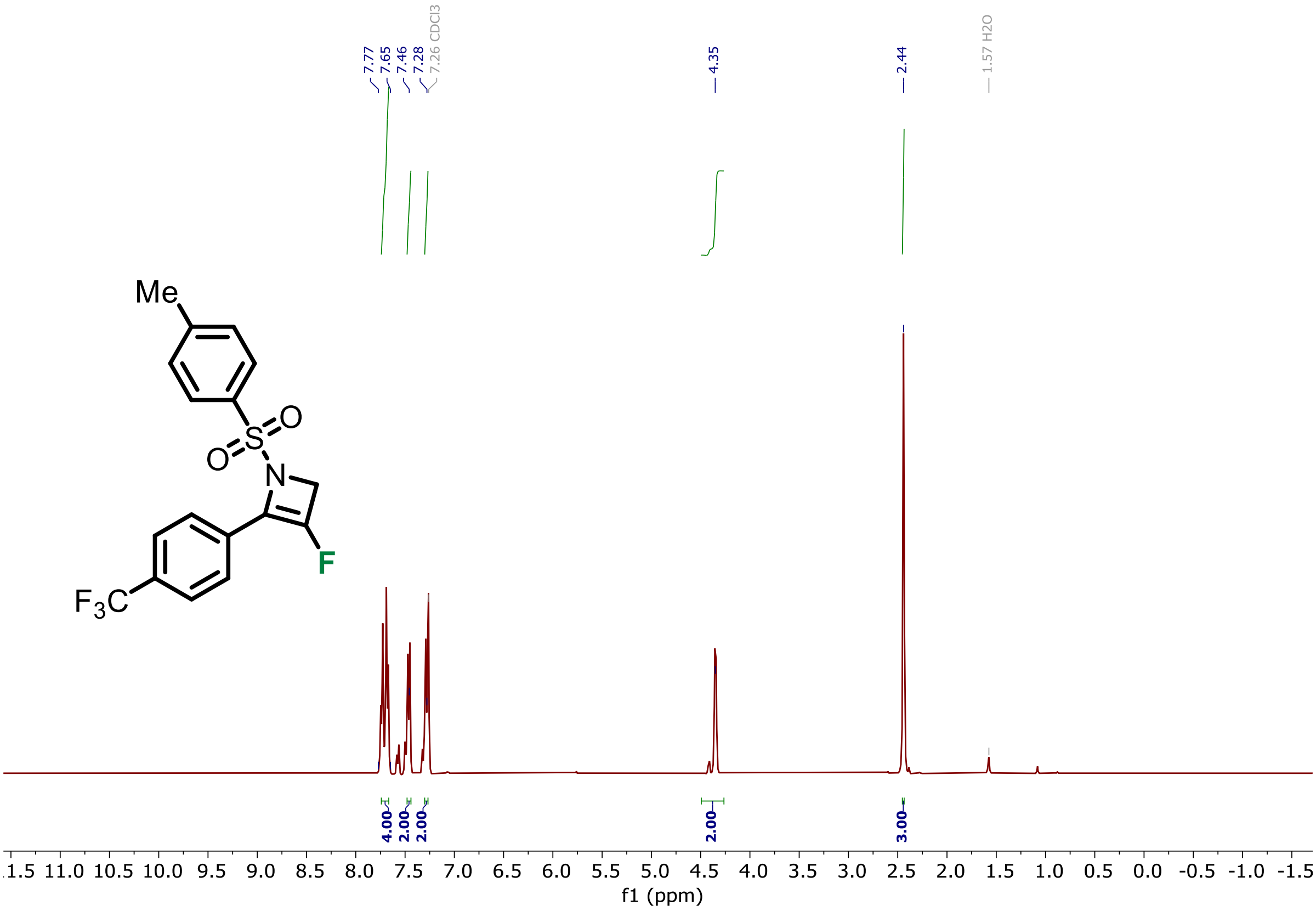

^{13}C NMR (126 MHz, CDCl₃)

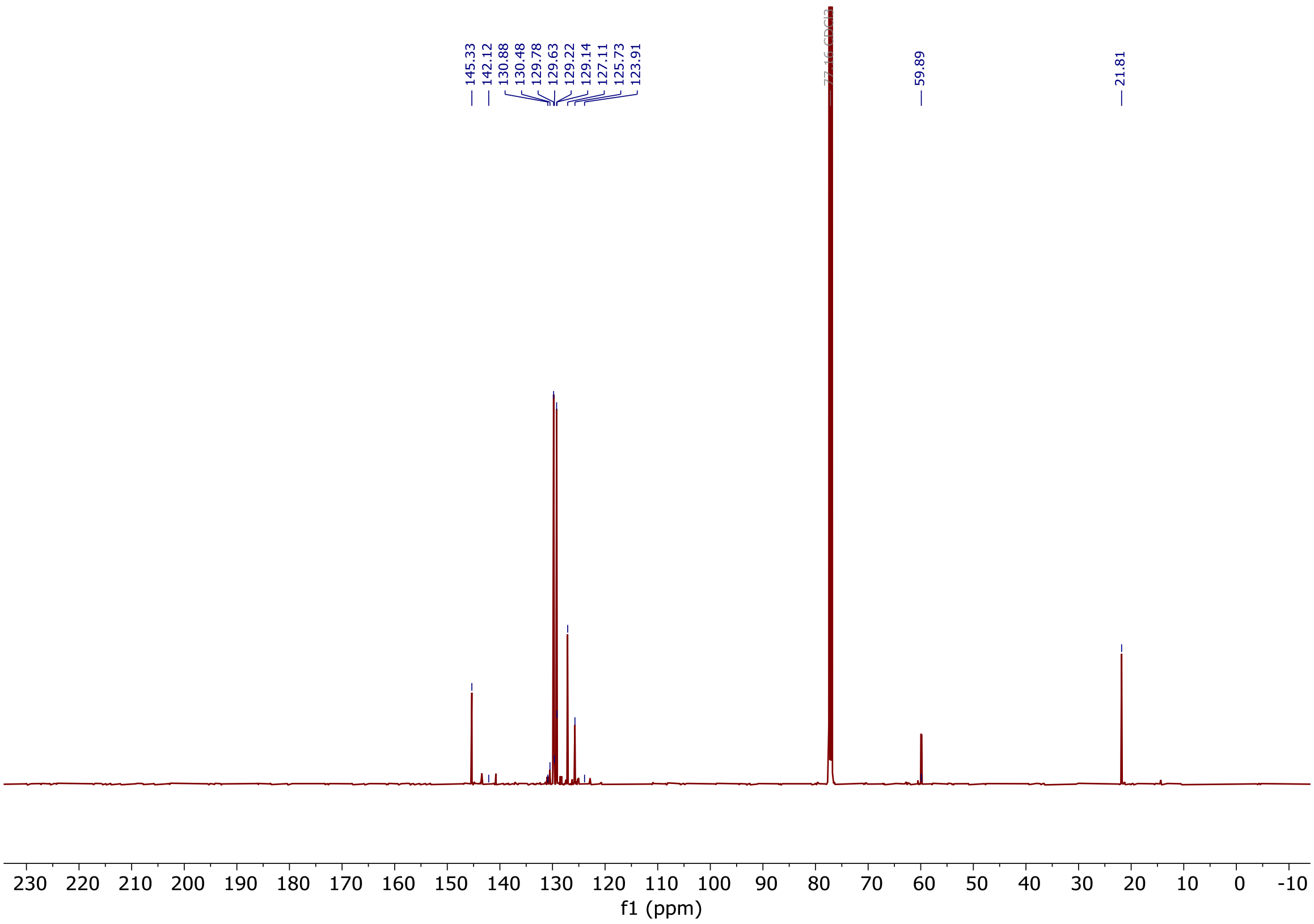

^{19}F NMR (376 MHz, $CDCl_3$)

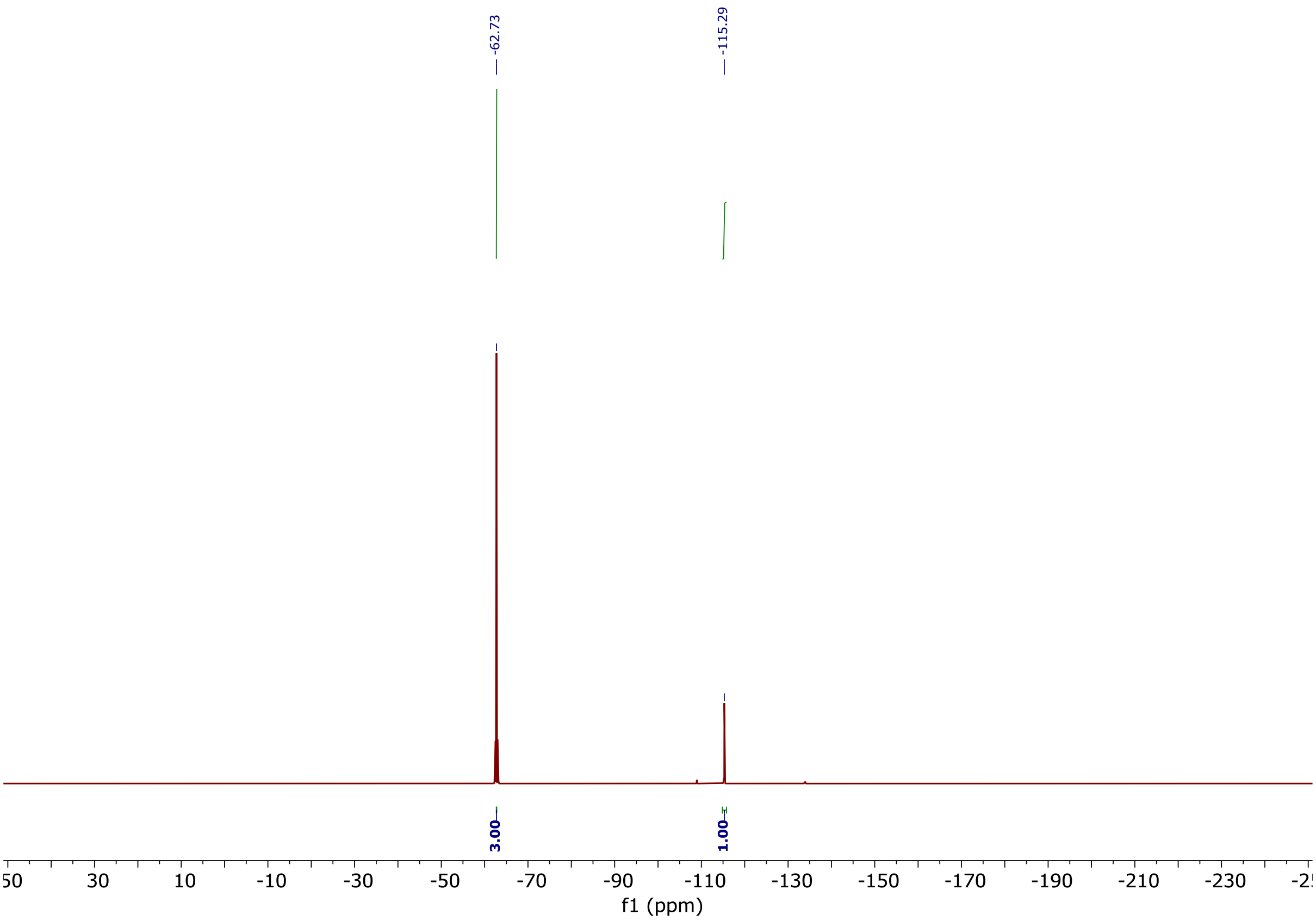

4-(4-bromophenyl)-3-fluoro-1-tosyl-1,2-dihydroazete (**7ba**)

^{1}H NMR (400 MHz, $CDCl_3$)

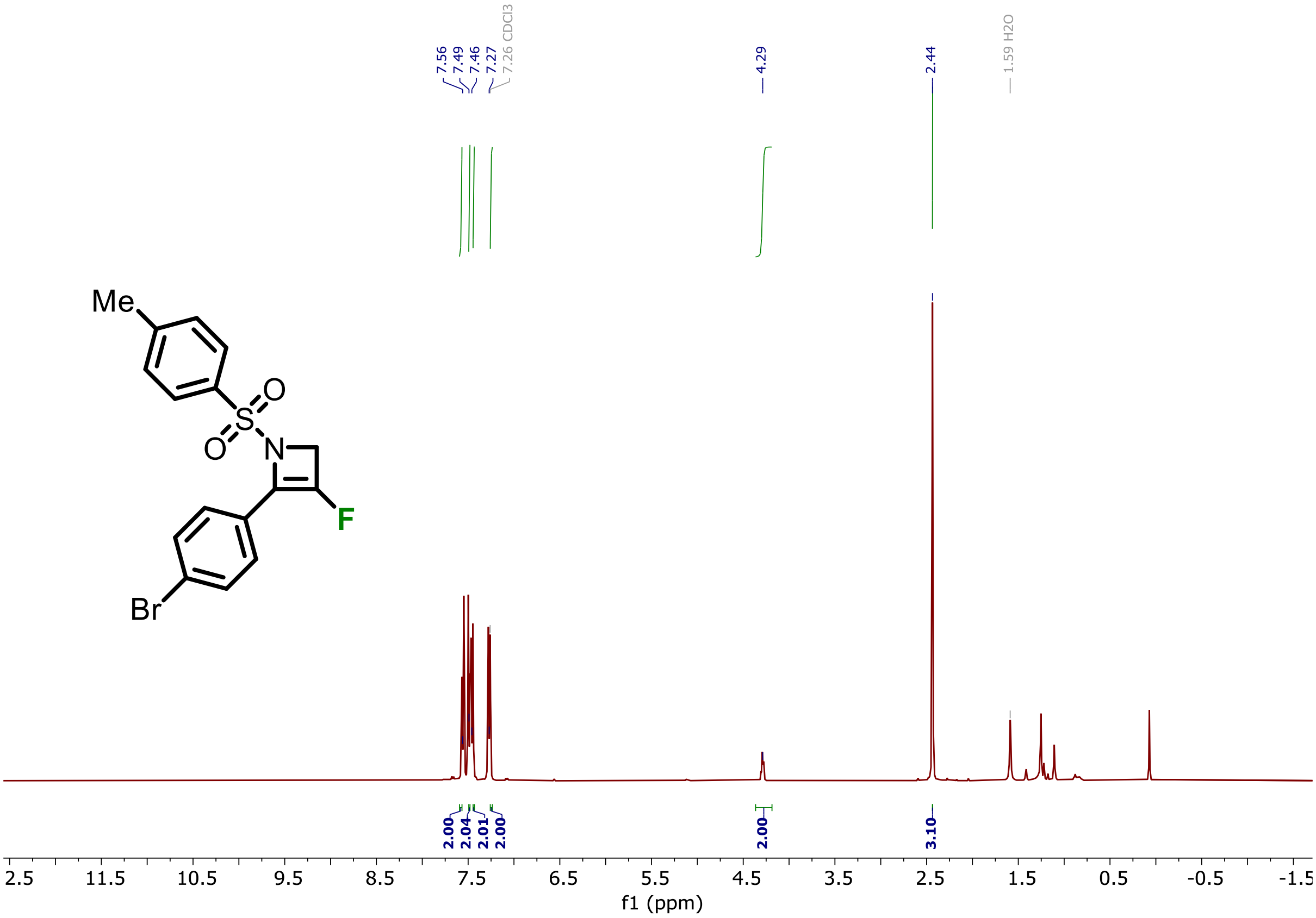

^{13}C NMR (126 MHz, $CDCl_3$)

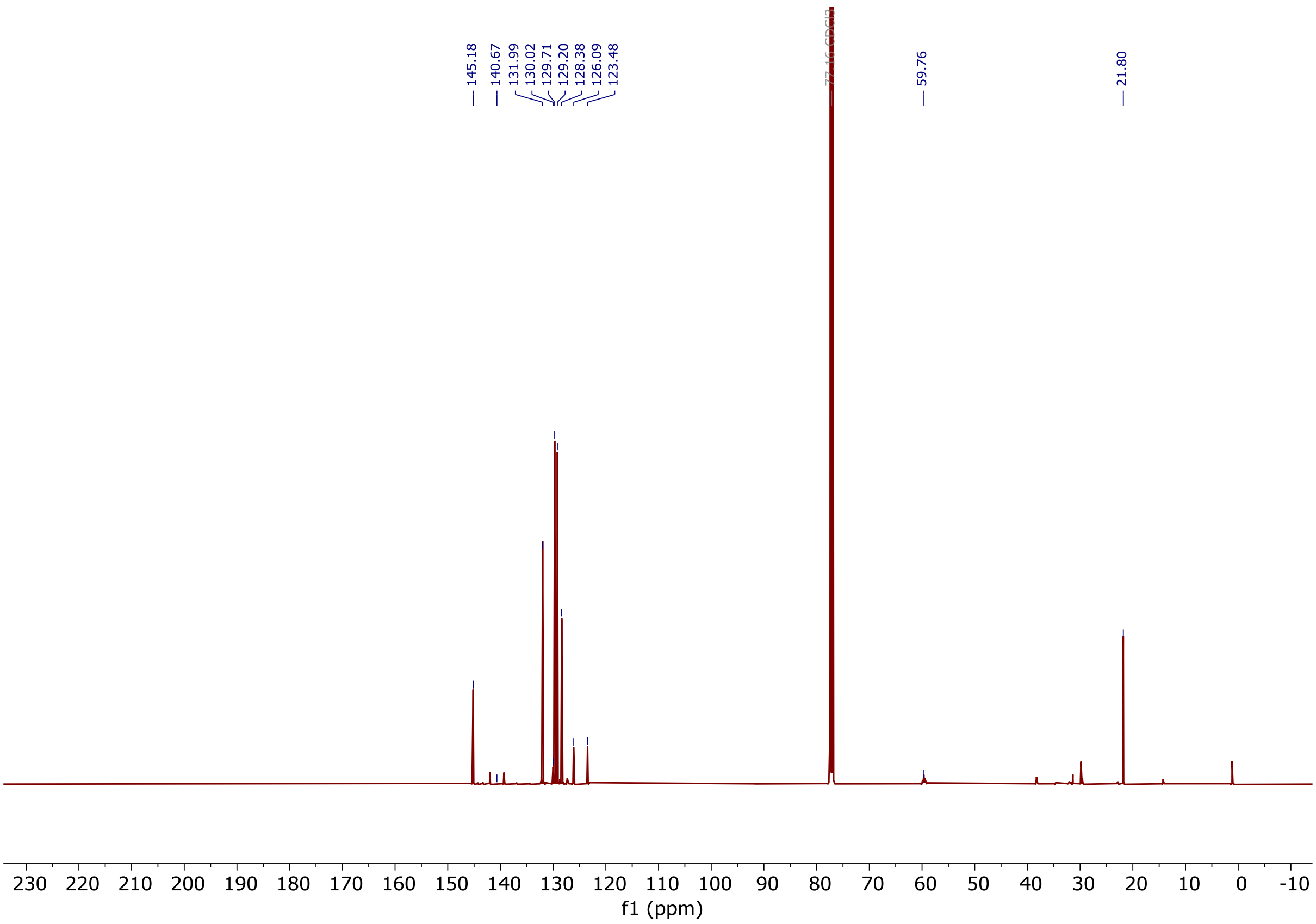

^{19}F NMR (376 MHz, $CDCl_3$)

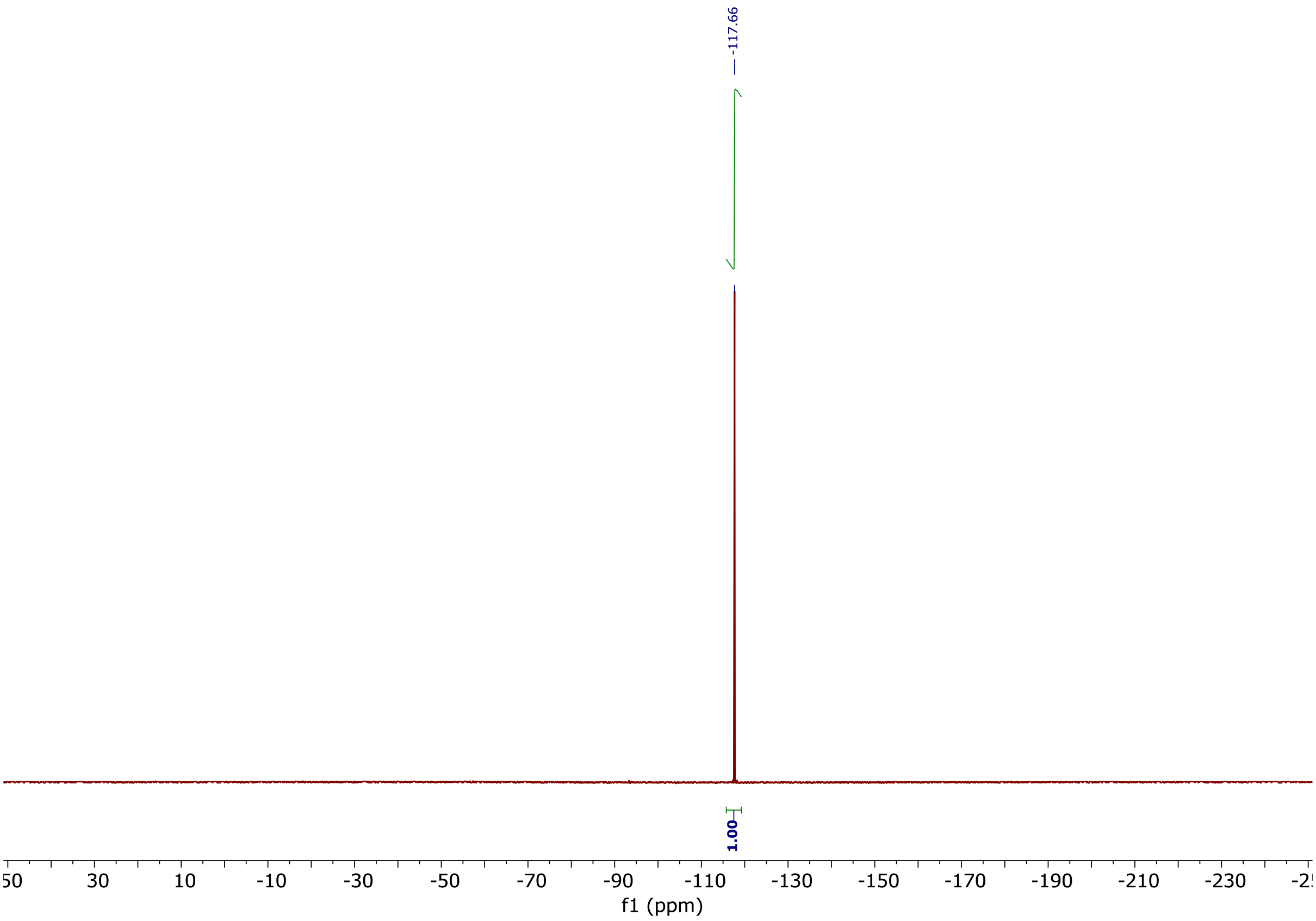

***4-(4-chlorophenyl)-3-fluoro-1-tosyl-1,2-dihydroazete* (7bb)**

^{1}H NMR (500 MHz, $CDCl_3$)

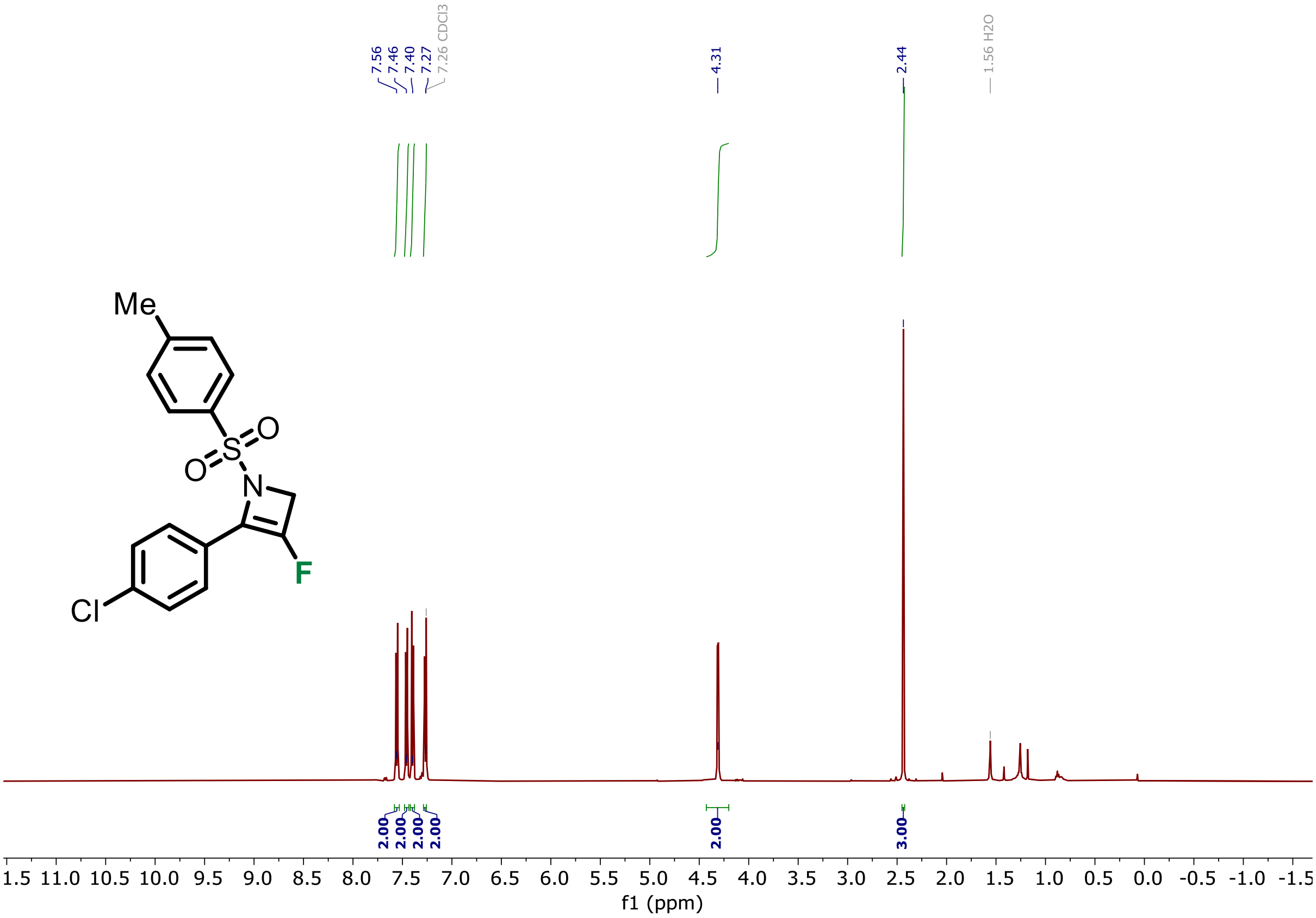

^{13}C NMR (126 MHz, $CDCl_3$)

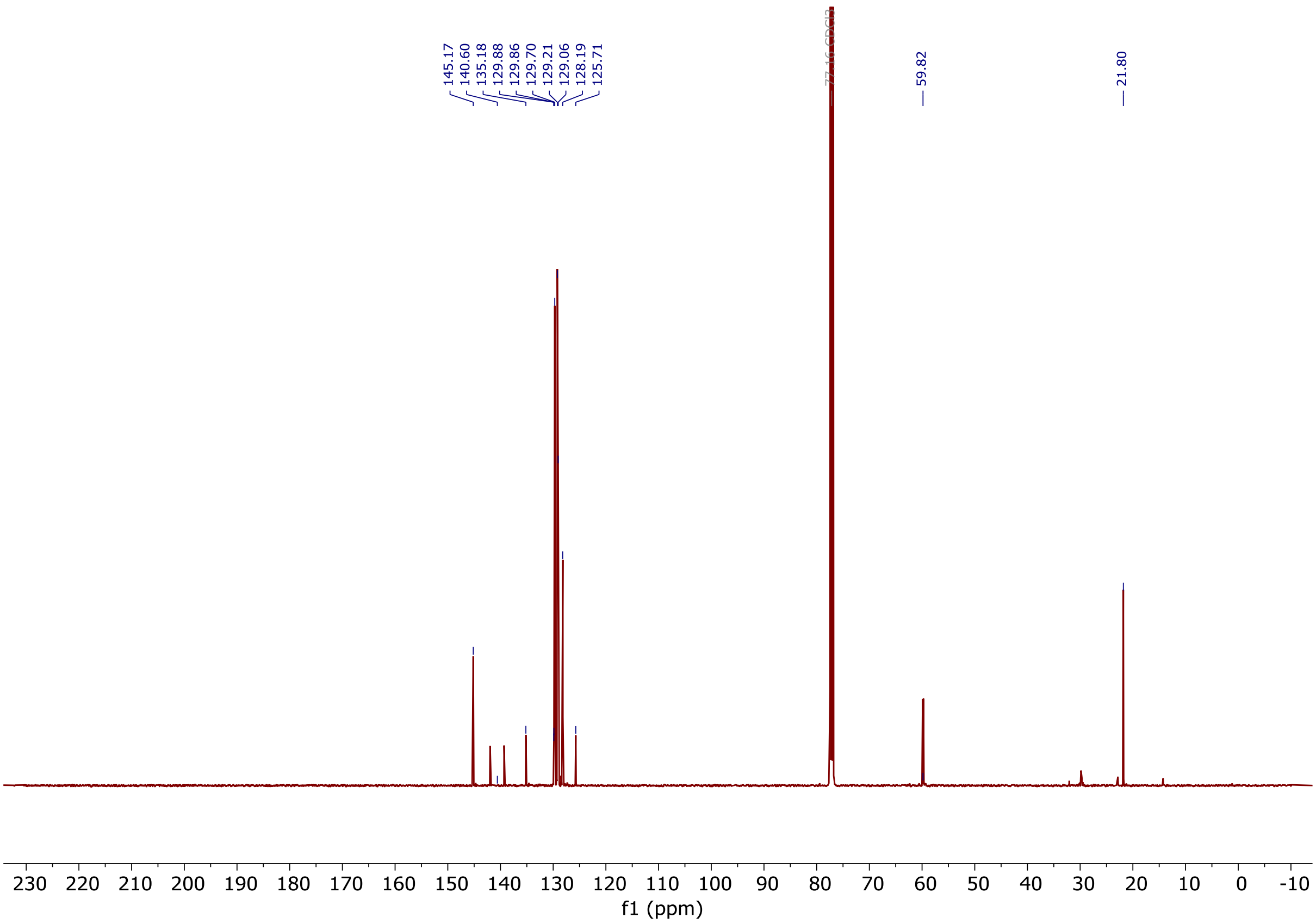

^{19}F NMR (376 MHz, $CDCl_3$)

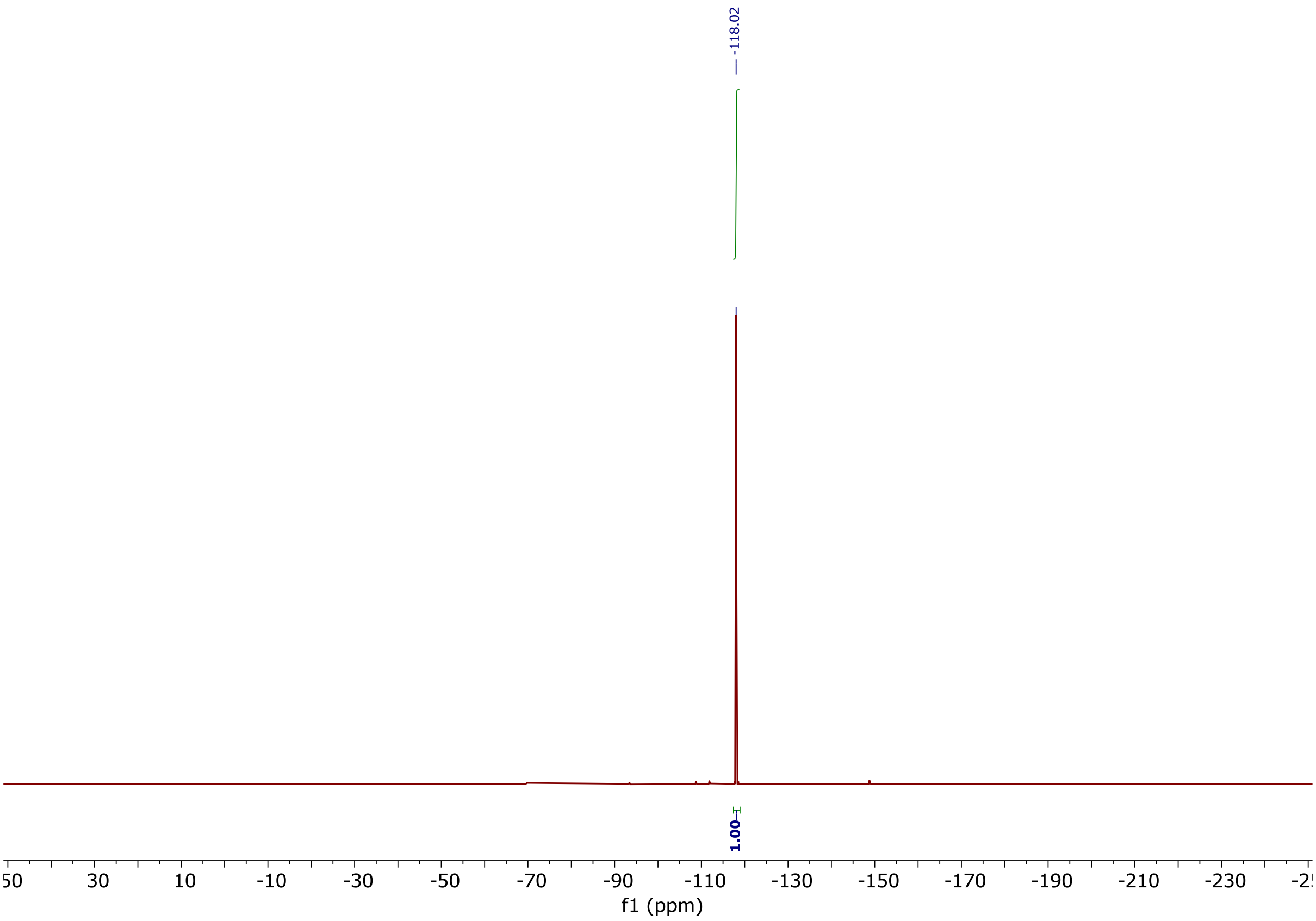

***5-(3-fluoro-1-tosyl-1,4-dihydroazet-2-yl)-1-tosyl-1H-indole* (7bc)**

^{1}H NMR (500 MHz, $CDCl_3$)

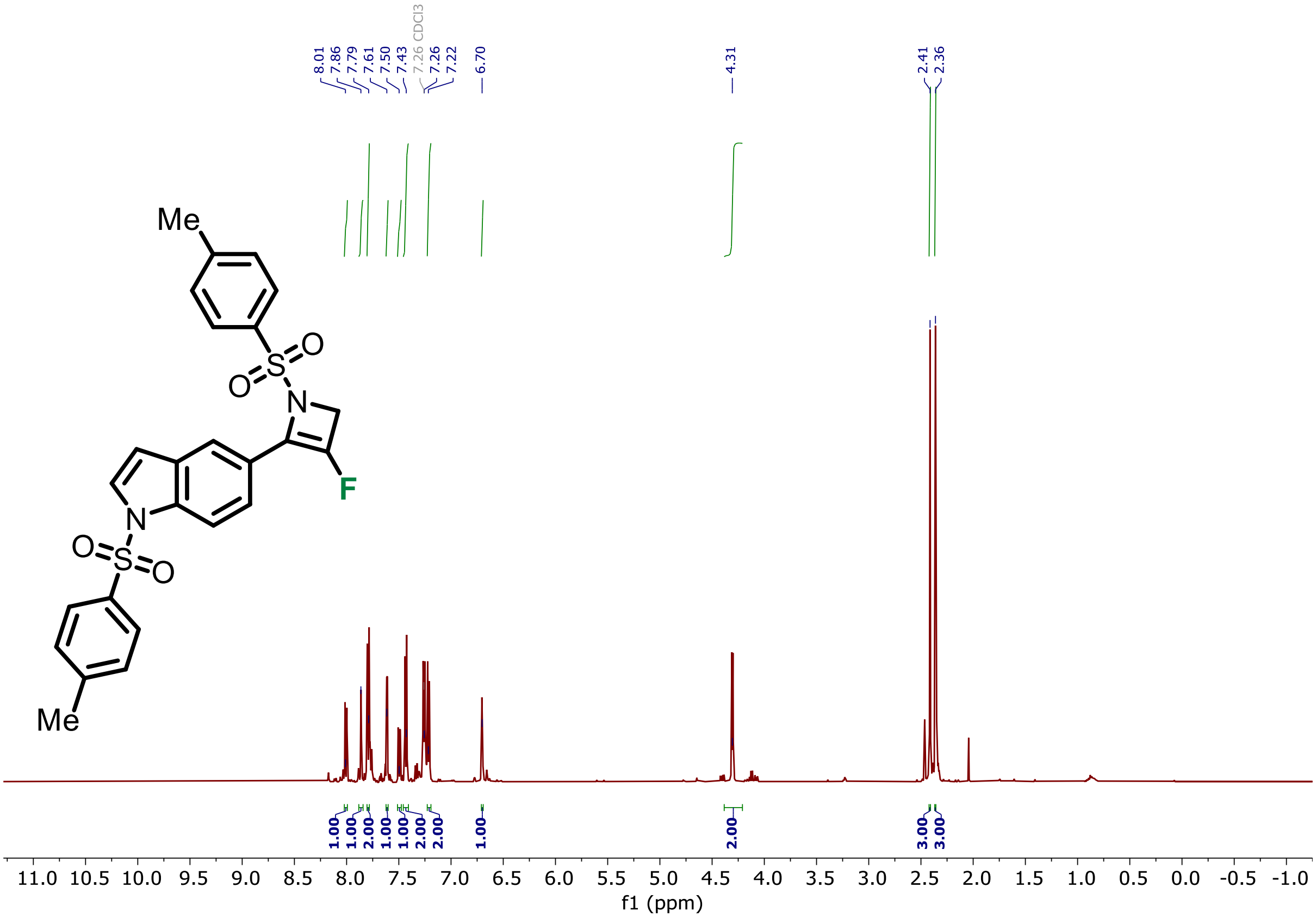

^{13}C NMR (126 MHz, $CDCl_3$)

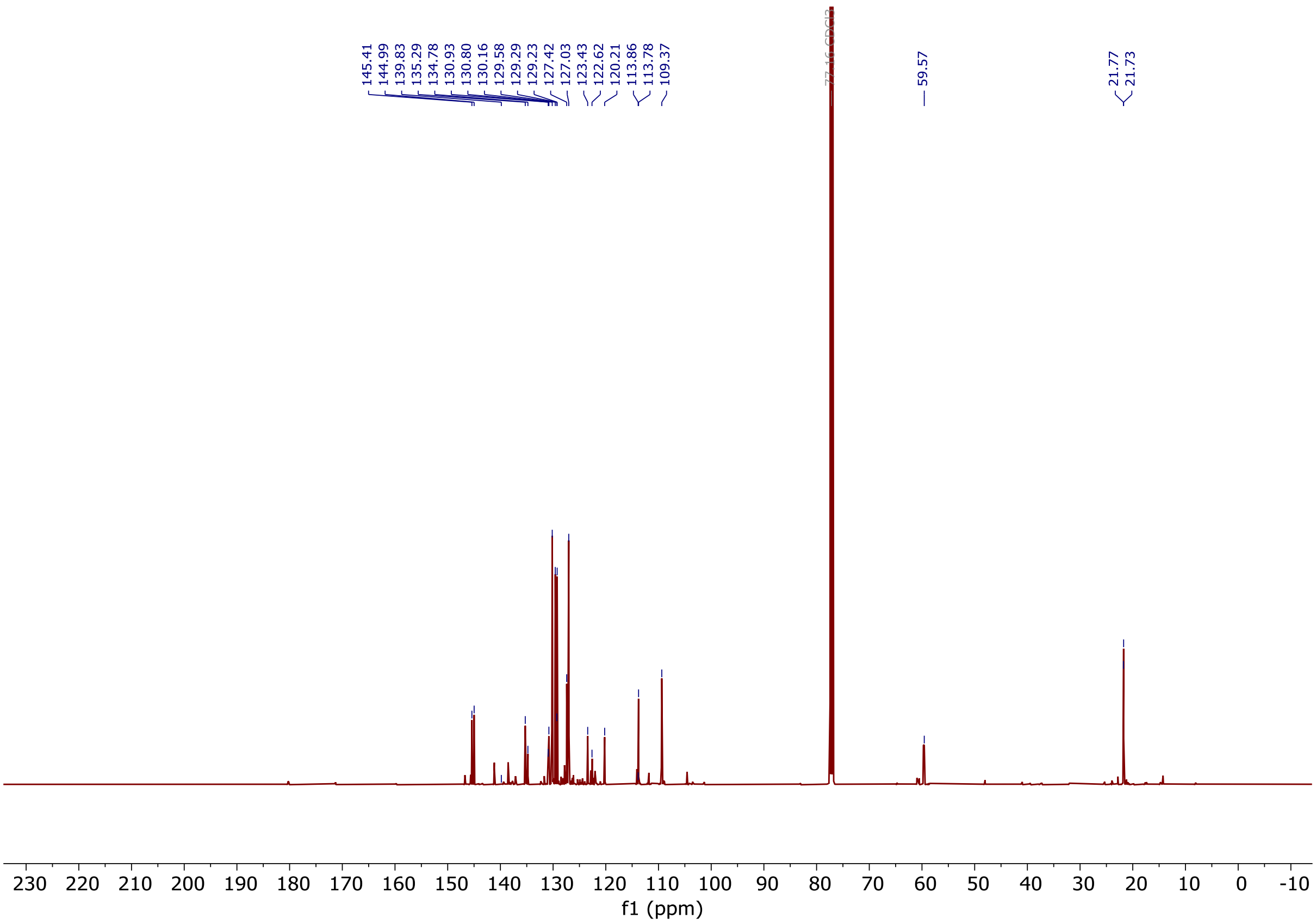

^{19}F NMR (377 MHz, $CDCl_3$)

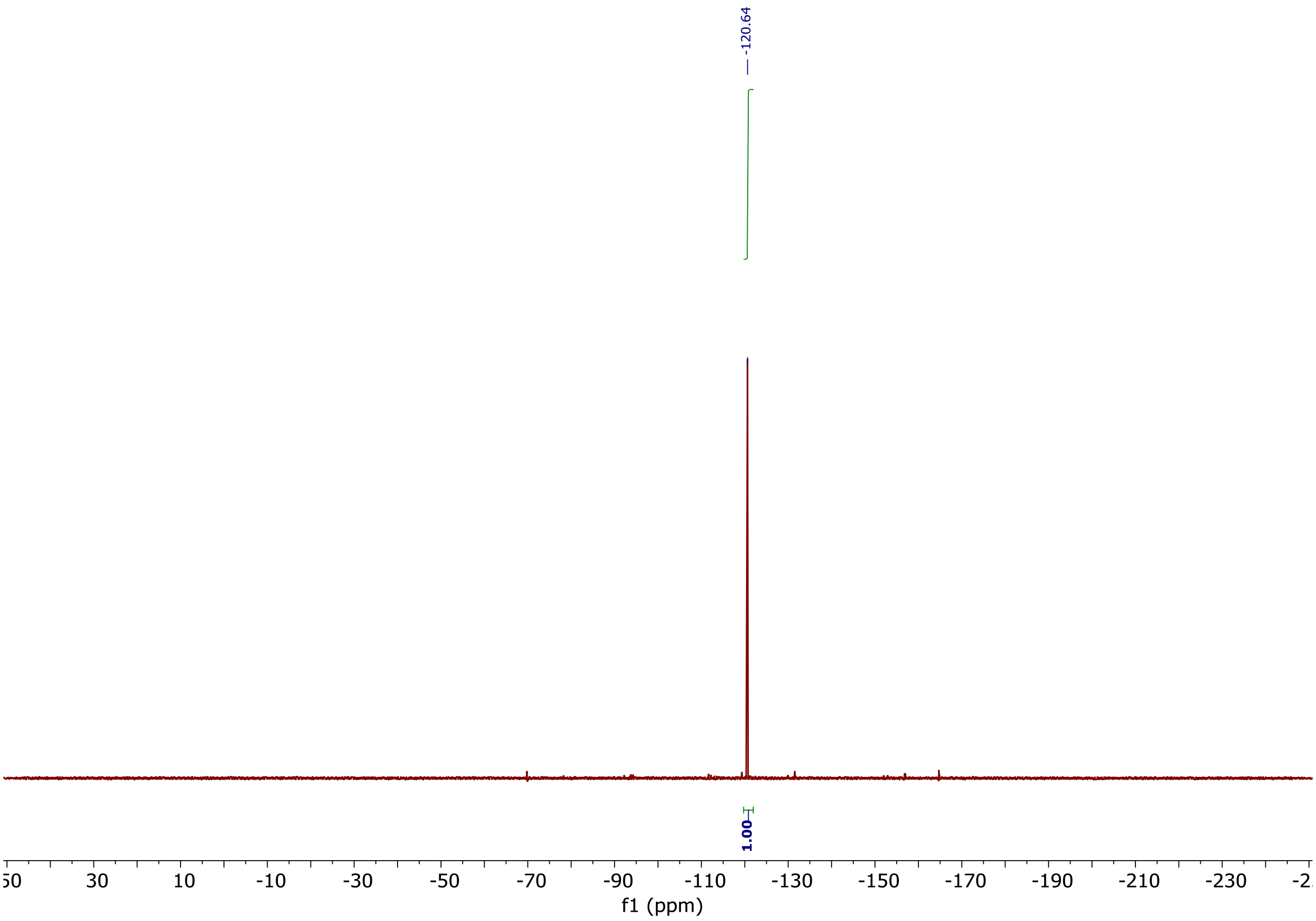

9.7. 2-Pyrrolines (8)

4-fluoro-1-tosyl-2,3-dihydro-1H-pyrrole (**8be**)

^{1}H NMR (400 MHz, CDCl$_3$)

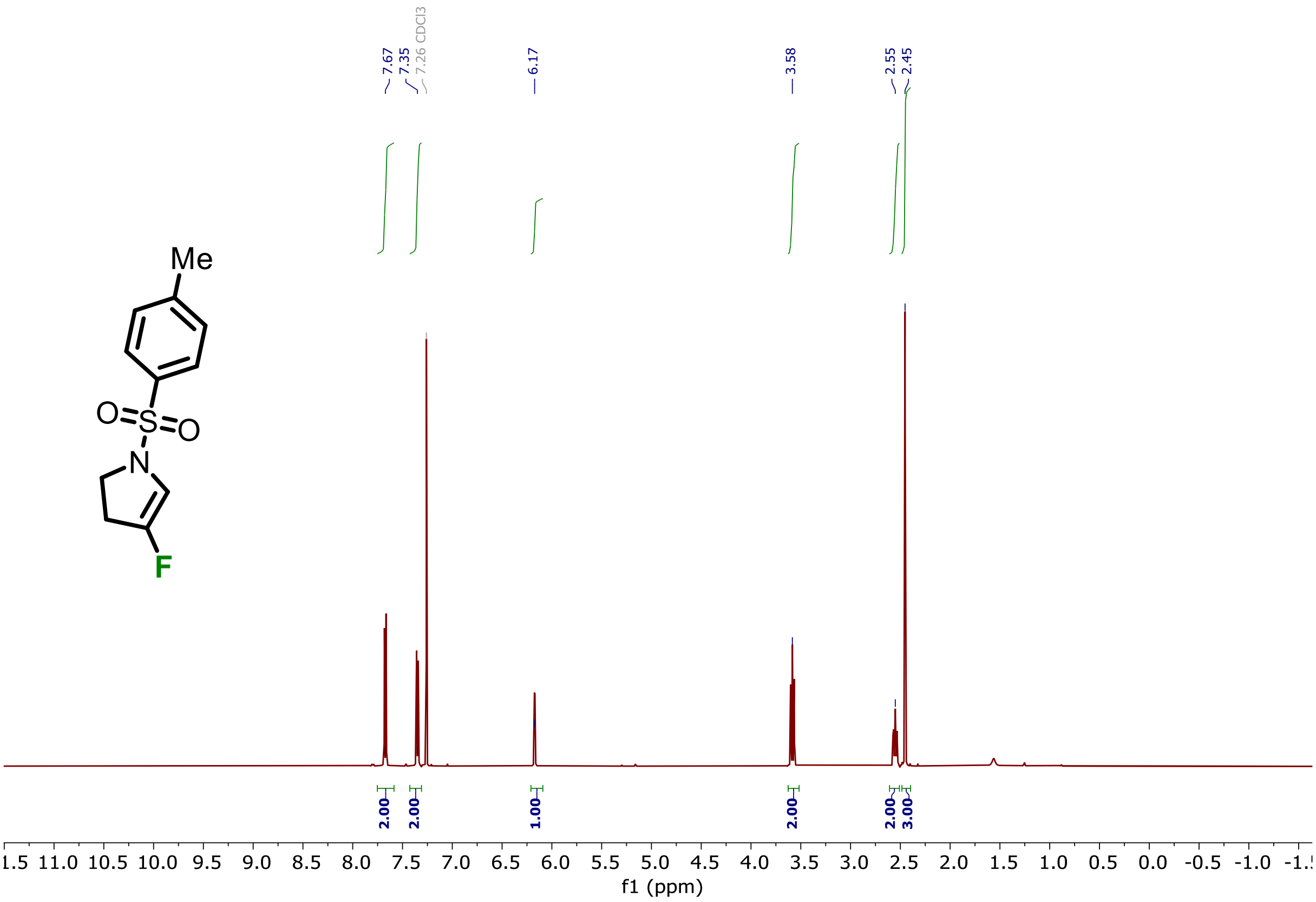

^{13}C NMR (151 MHz, CDCl$_3$)

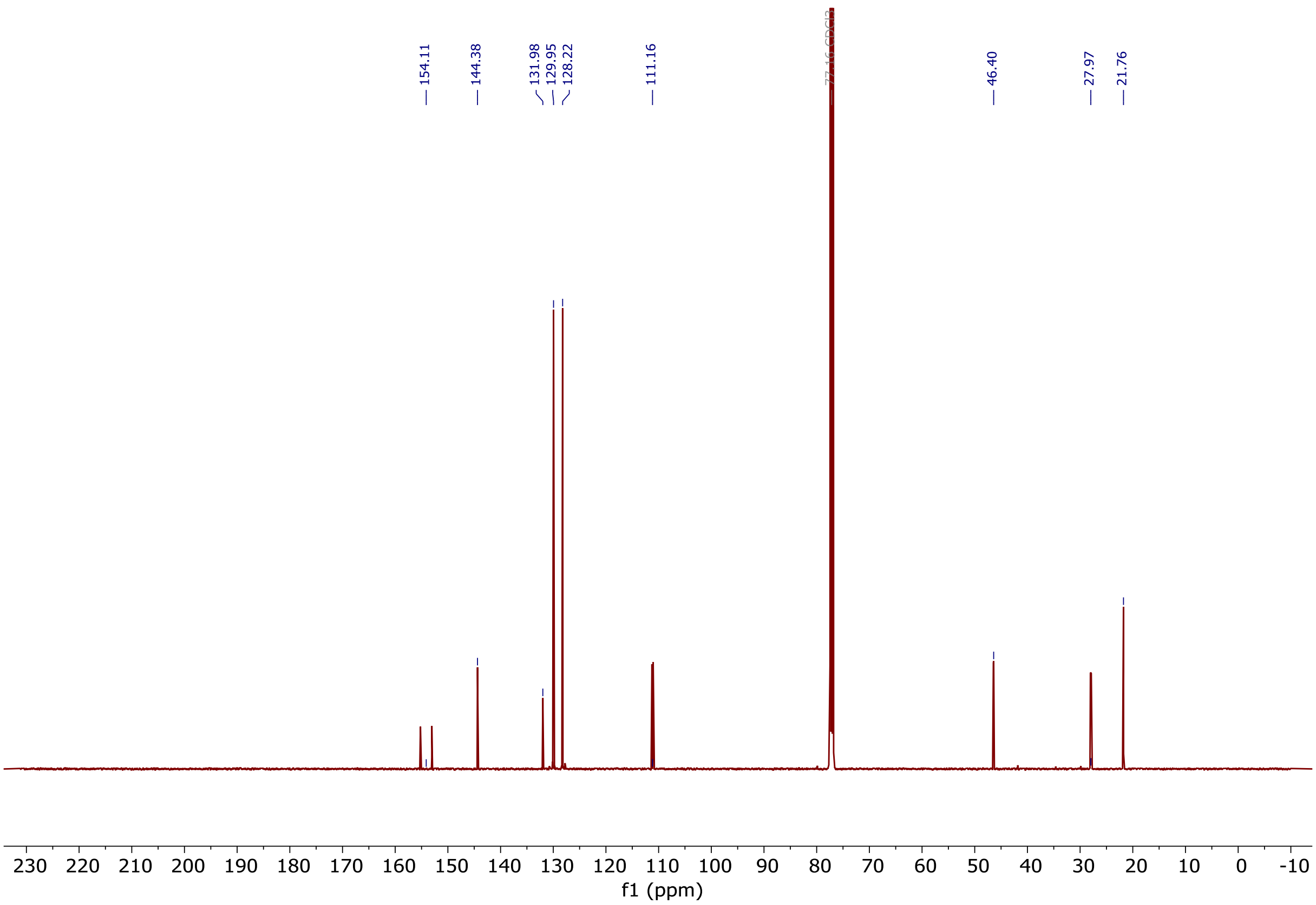

^{19}F NMR (376 MHz, $CDCl_3$)

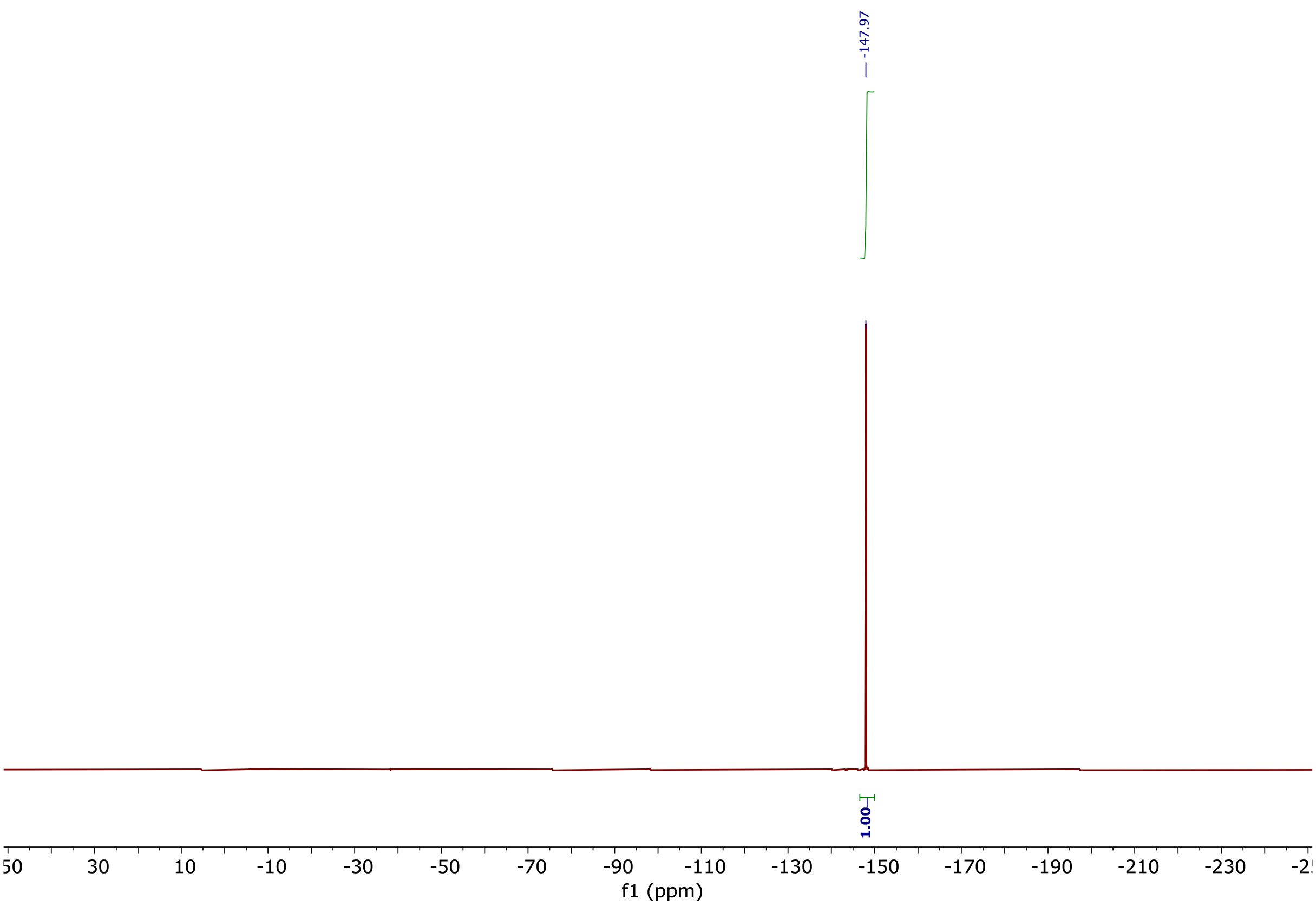

9.8. Pyrroles (9)

***4-fluoro-2-(4-fluorophenyl)-1-tosyl-1H-pyrrole* (9e)**

^{1}H NMR (600 MHz, CDCl$_3$)

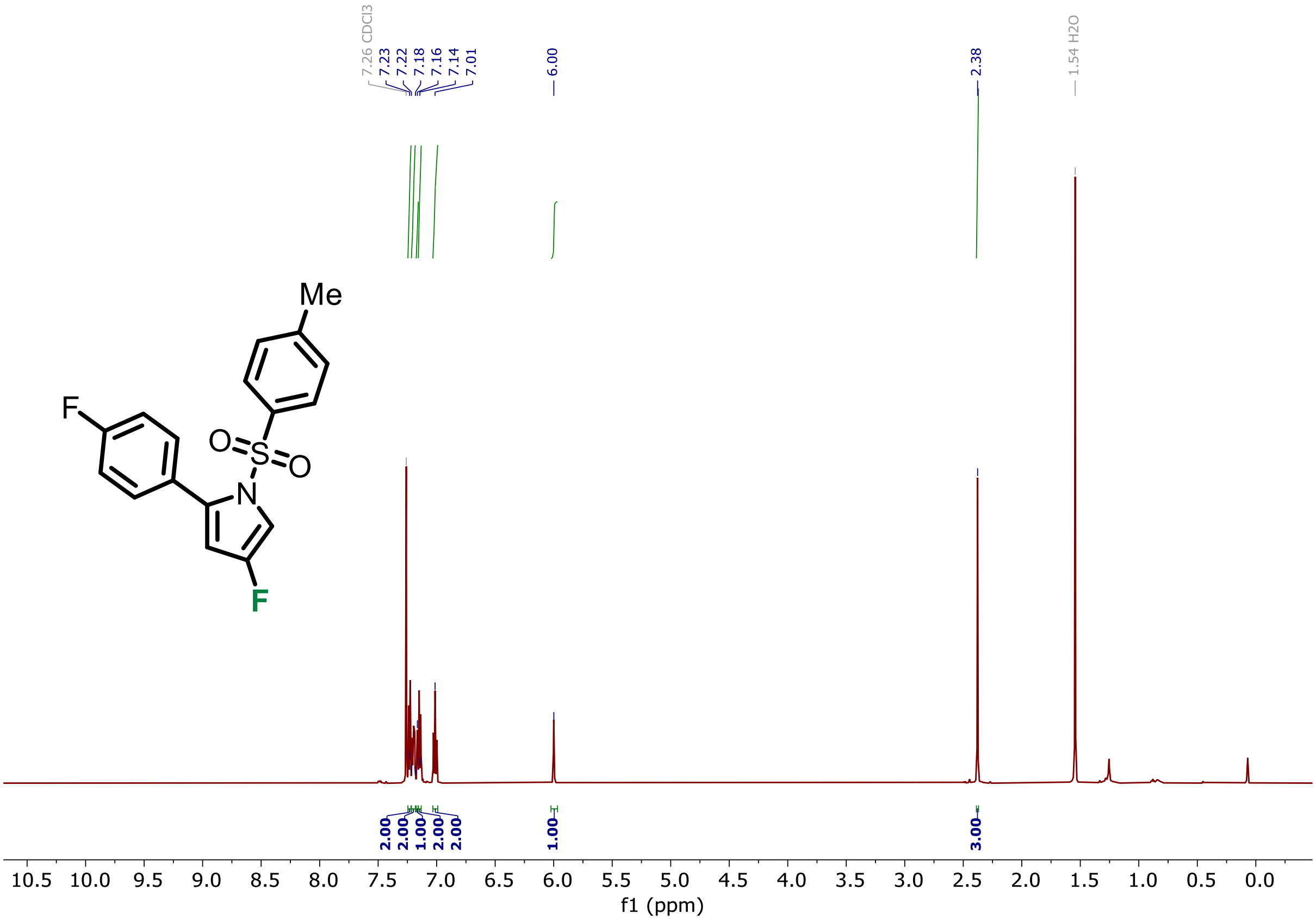

^{13}C NMR (151 MHz, $CDCl_3$)

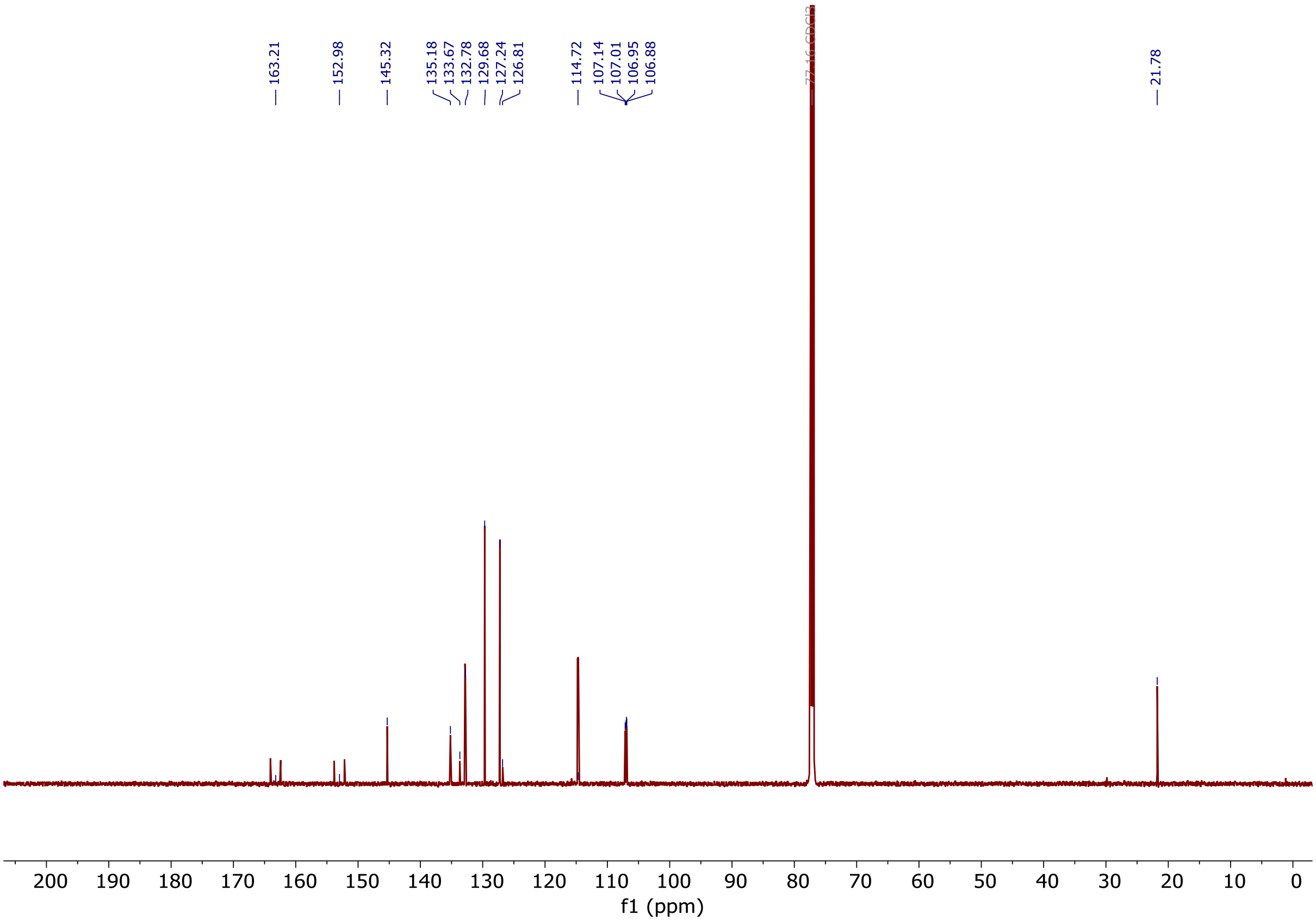

^{19}F NMR (376 MHz, $CDCl_3$)

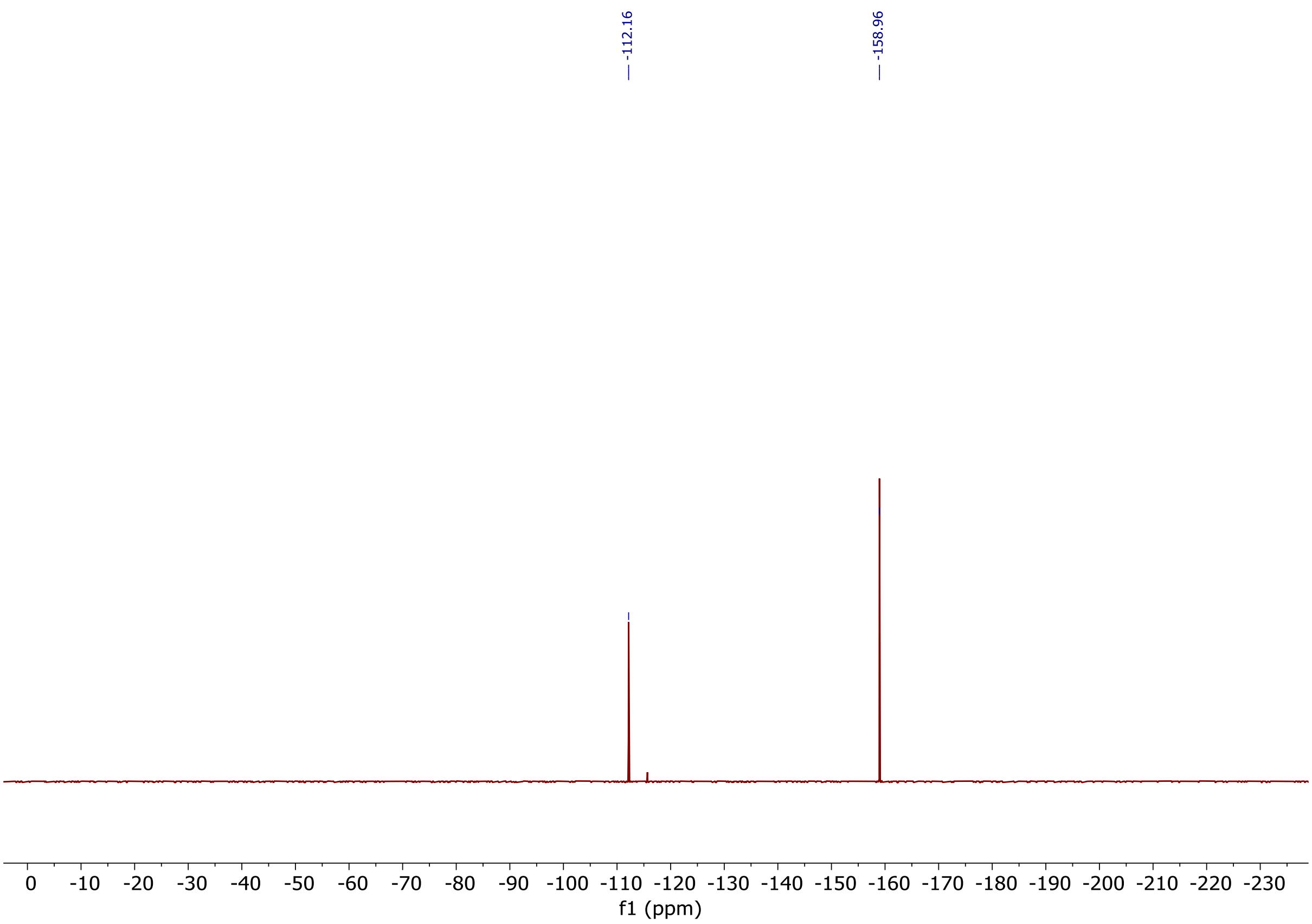

***4-fluoro-1-tosyl-2-(4-(trifluoromethyl)phenyl)-1H-pyrrole* (9f)**

^{1}H NMR (500 MHz, CDCl$_3$)

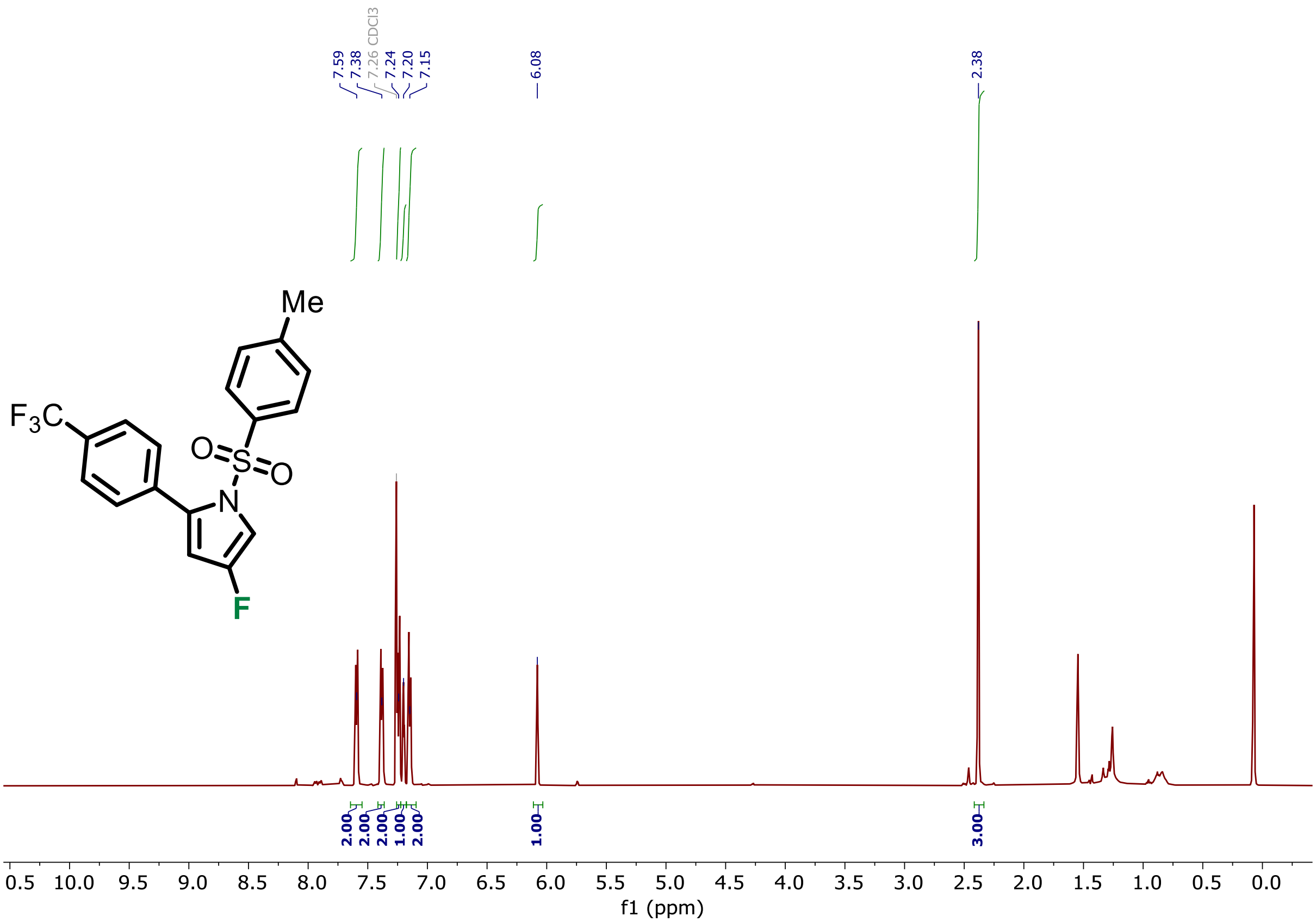

^{13}C NMR (126 MHz, CDCl$_3$)

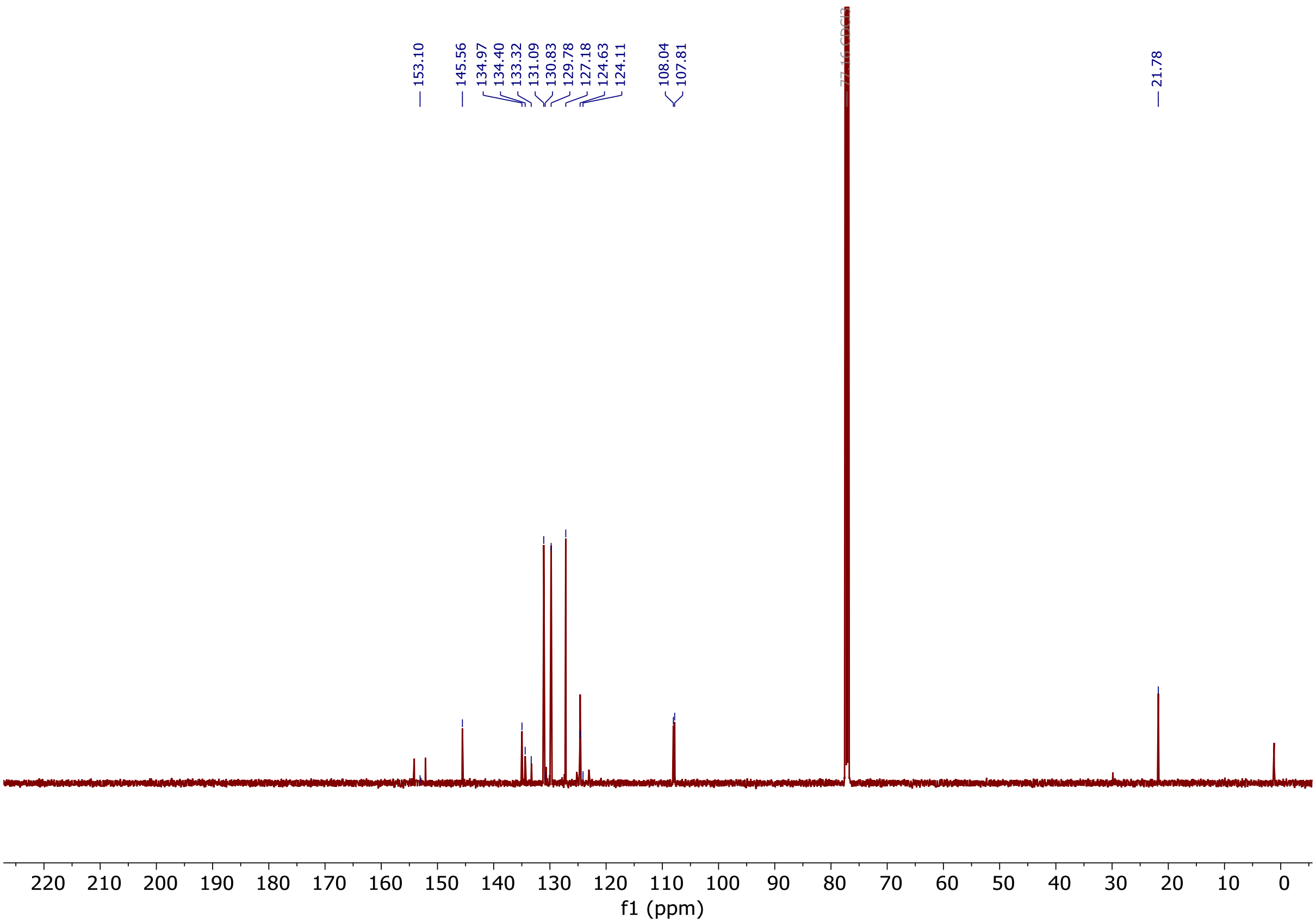

^{19}F NMR (471 MHz, $CDCl_3$)

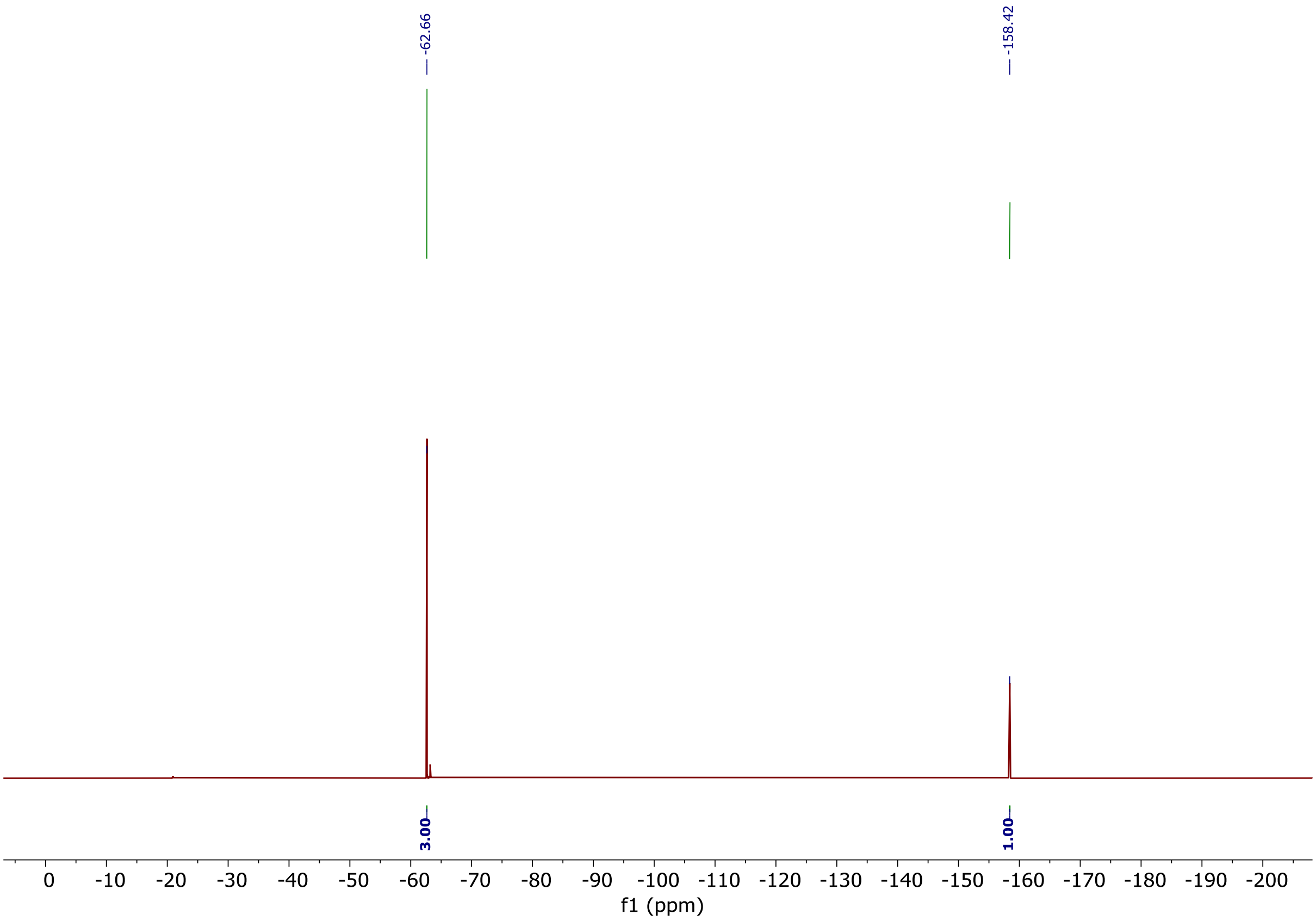

***3-fluoro-5-(4-fluorophenyl)-2-phenyl-1H-pyrrole* (9n)**

^{1}H NMR (600 MHz, $CDCl_3$)

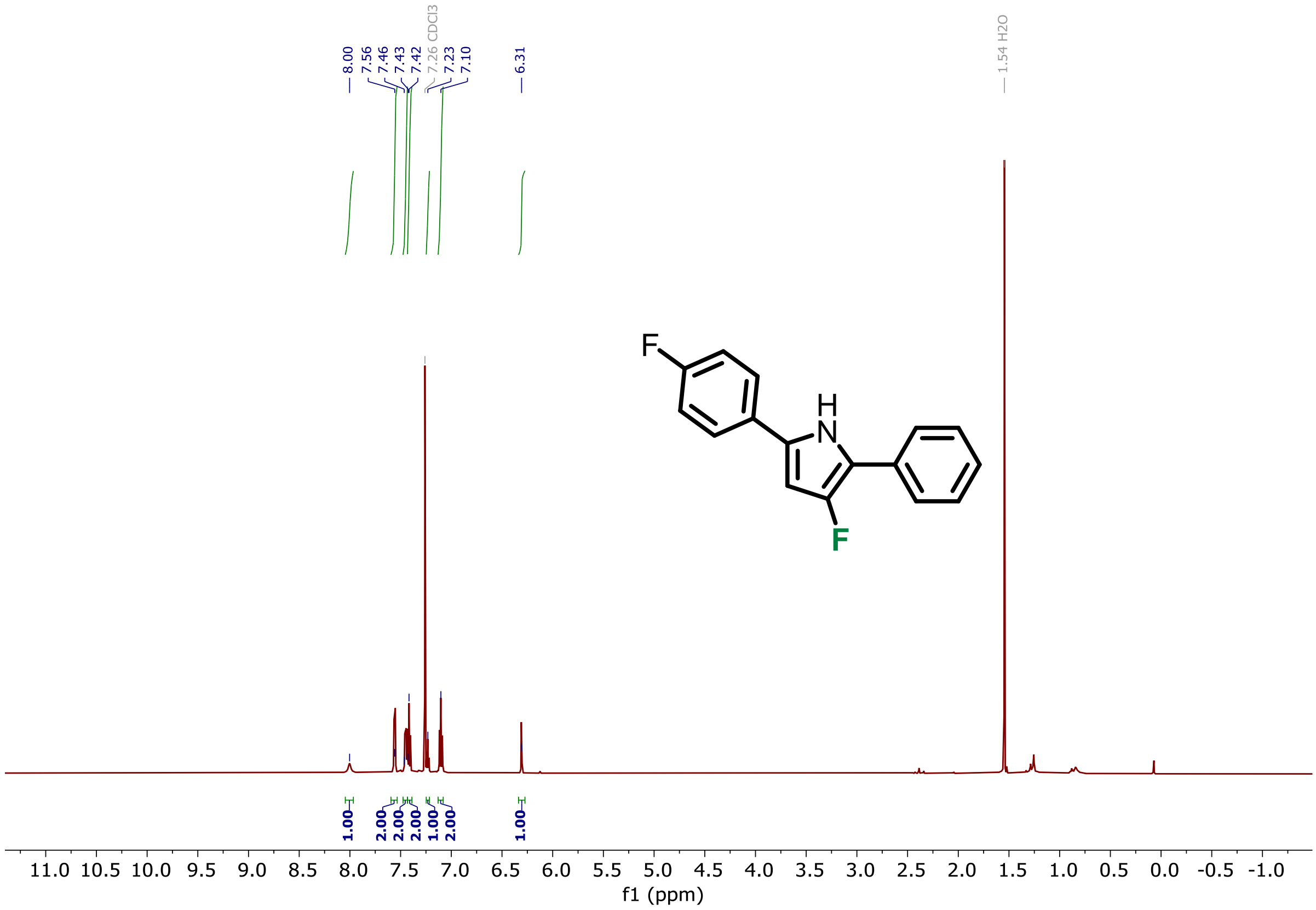

^{13}C NMR (151 MHz, $CDCl_3$)

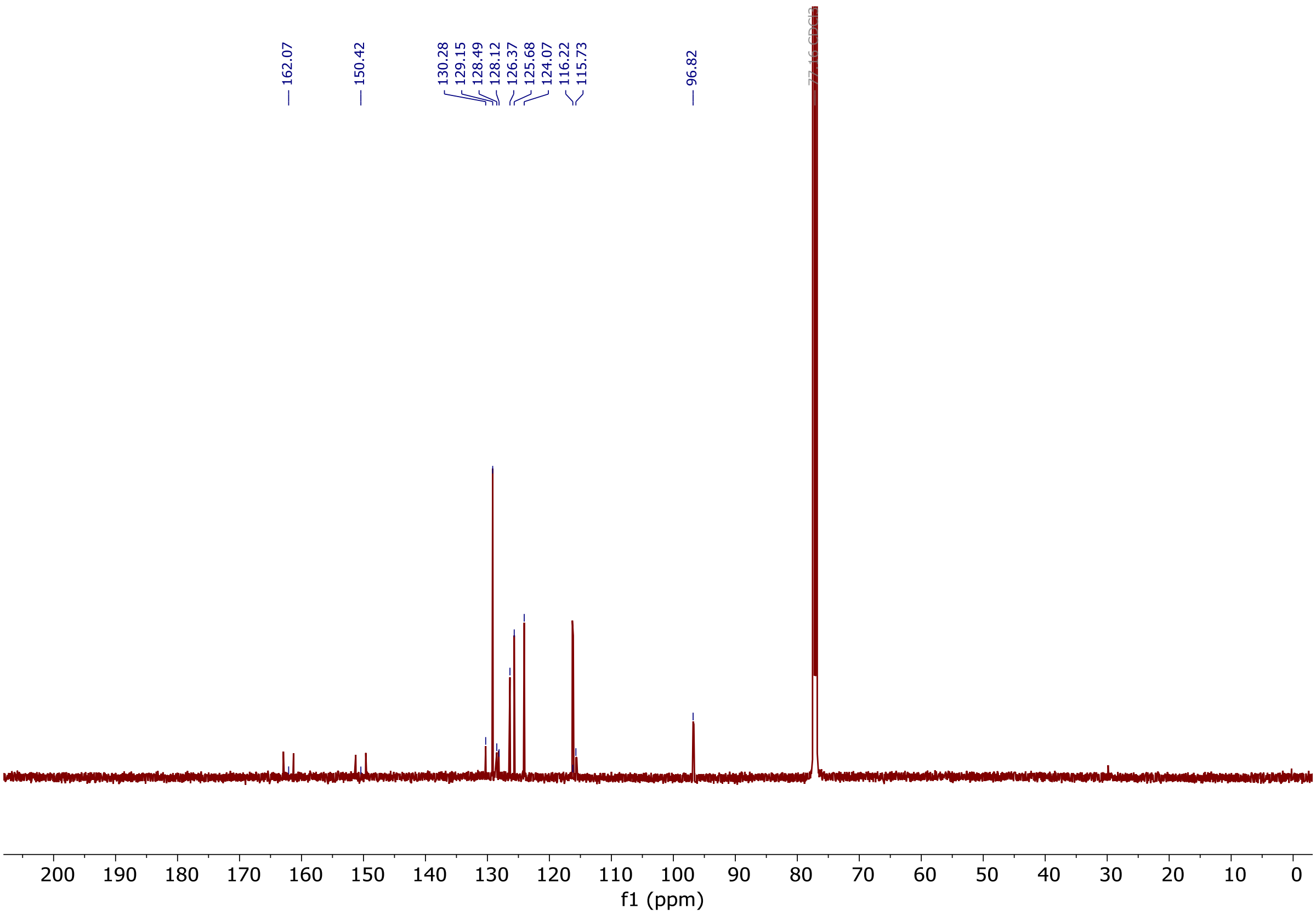

^{19}F NMR (471 MHz, $CDCl_3$)

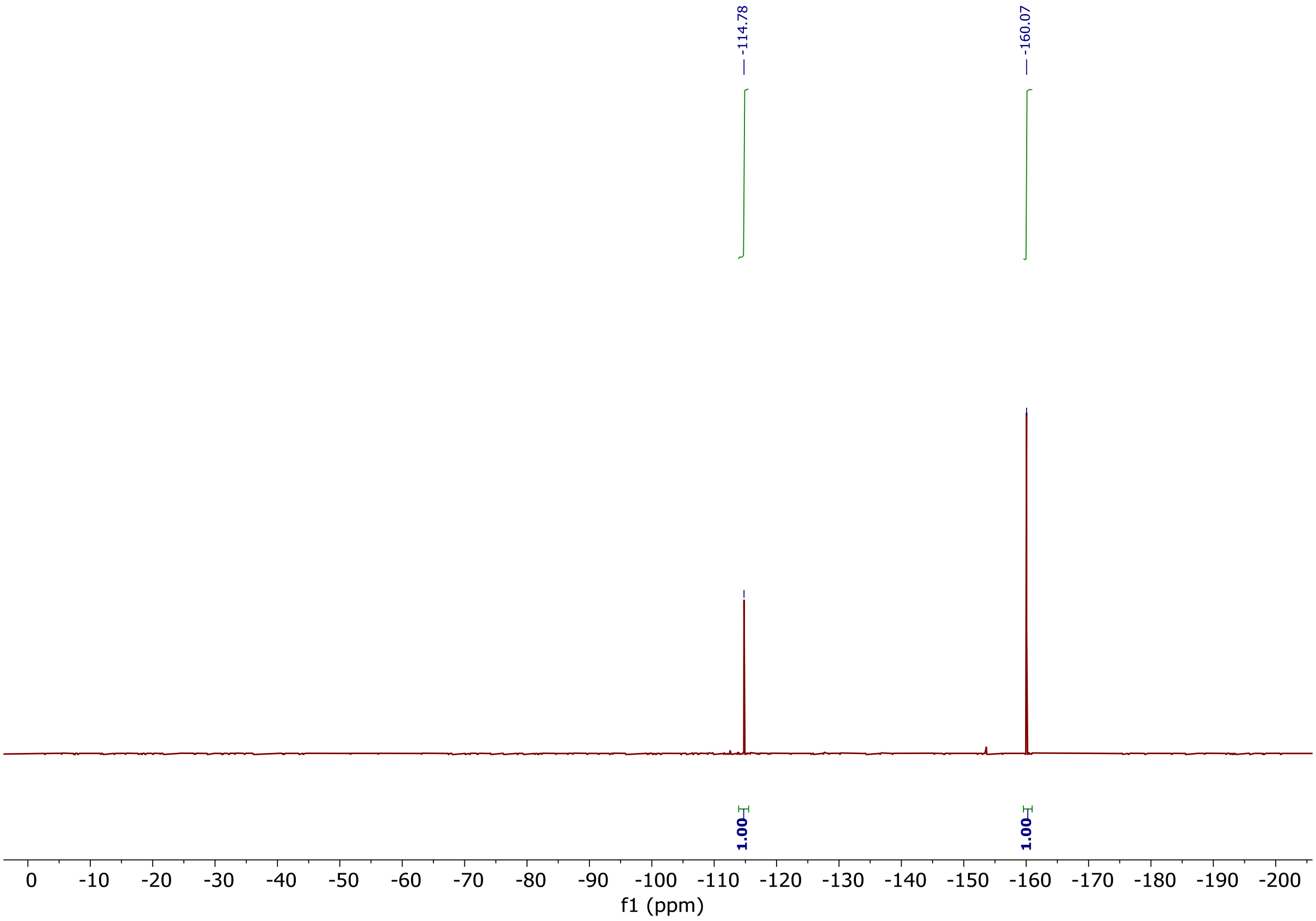

3-fluoro-5-(4-fluorophenyl)-2-phenyl-4-tosyl-1H-pyrrole **(9n')**

^{1}H NMR (500 MHz, CDCl$_3$)

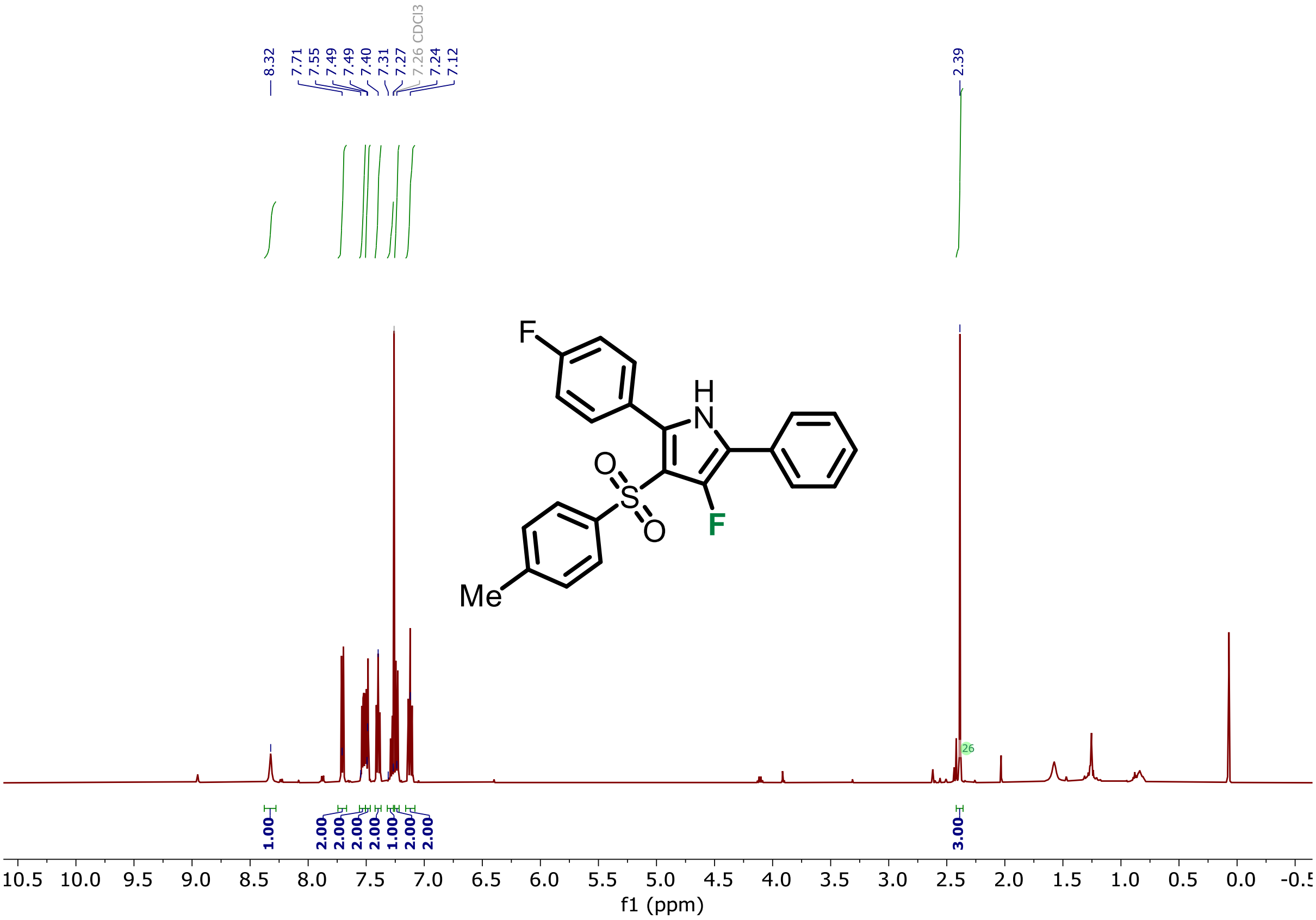

^{13}C NMR (126 MHz, CDCl$_3$)

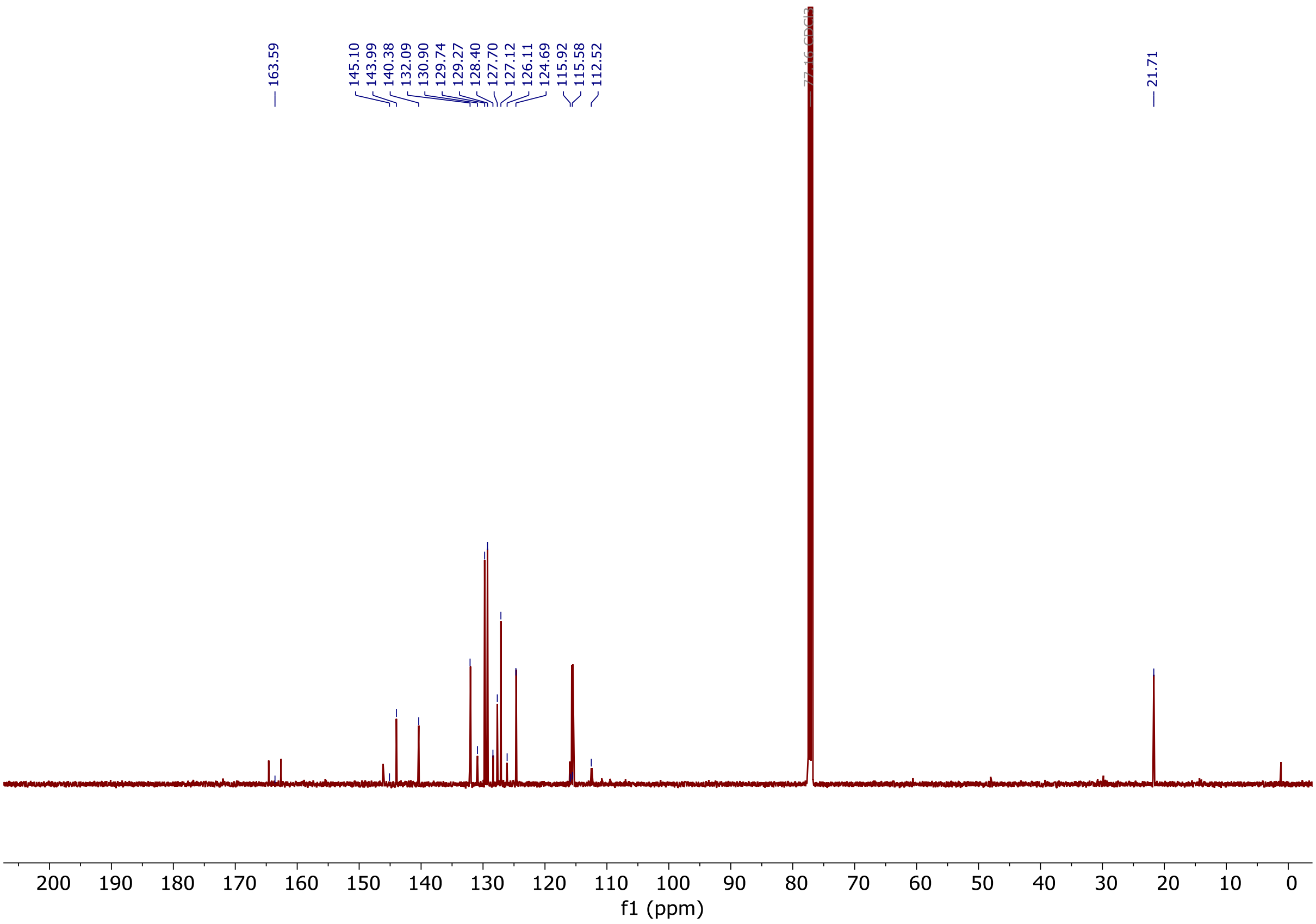

^{19}F NMR (471 MHz, $CDCl_3$)

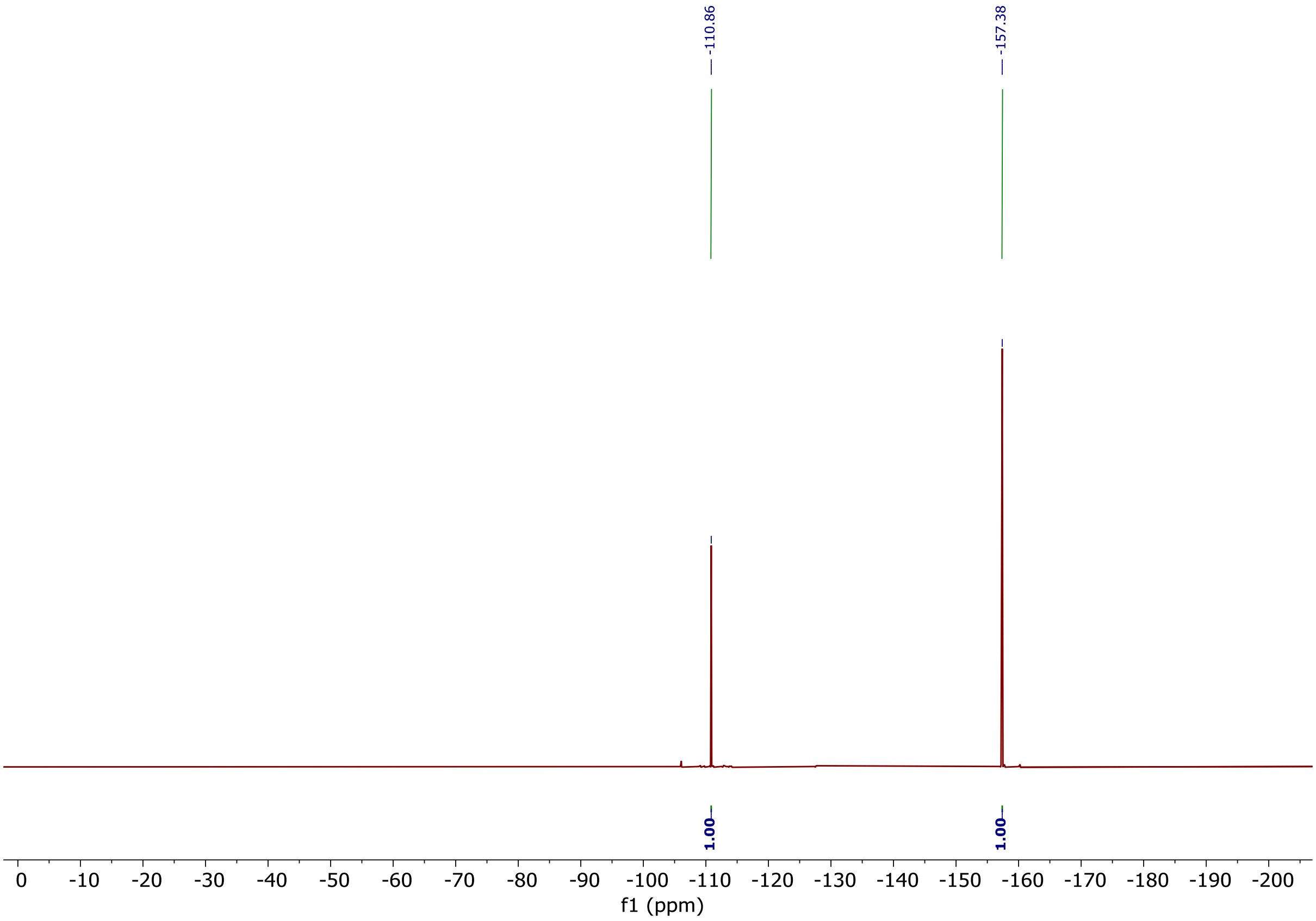

4-fluoro-2-propyl-1-tosyl-1H-pyrrole **(9v)**
(contains 16 mol% 4-methylbenzophenone)

^{1}H NMR (500 MHz, CDCl$_3$)

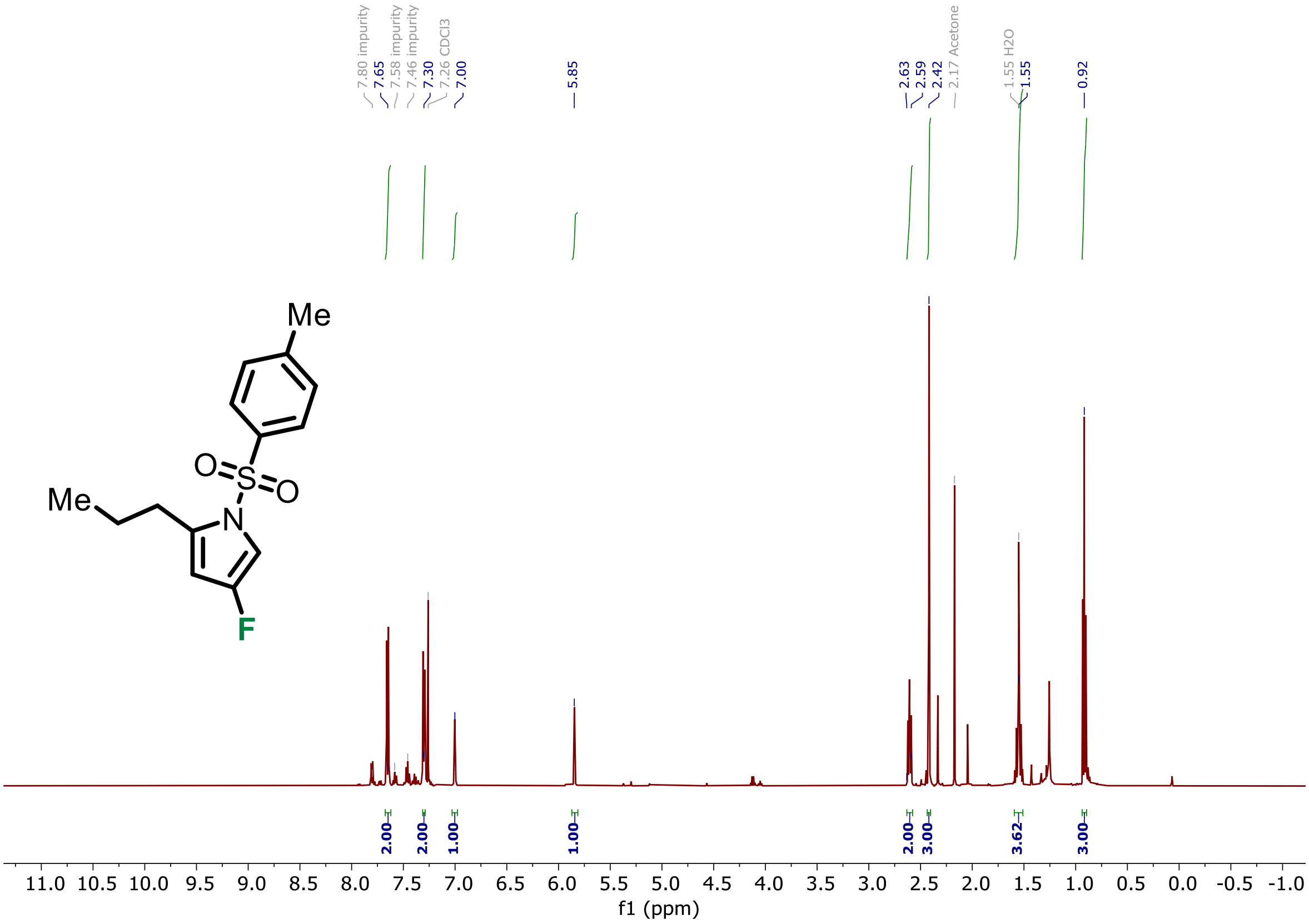

^{13}C NMR (126 MHz, $CDCl_3$)

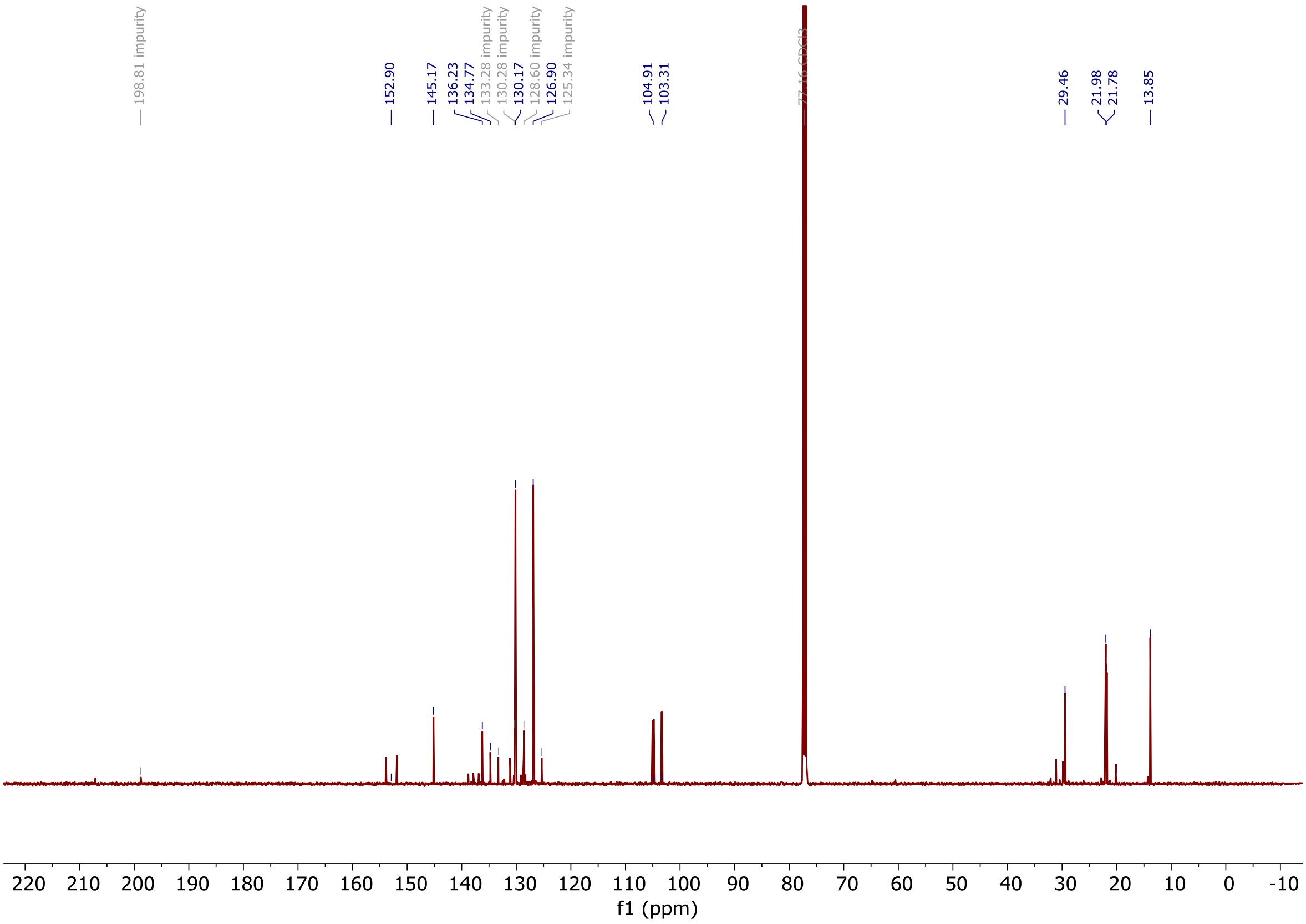

^{19}F NMR (376 MHz, $CDCl_3$)

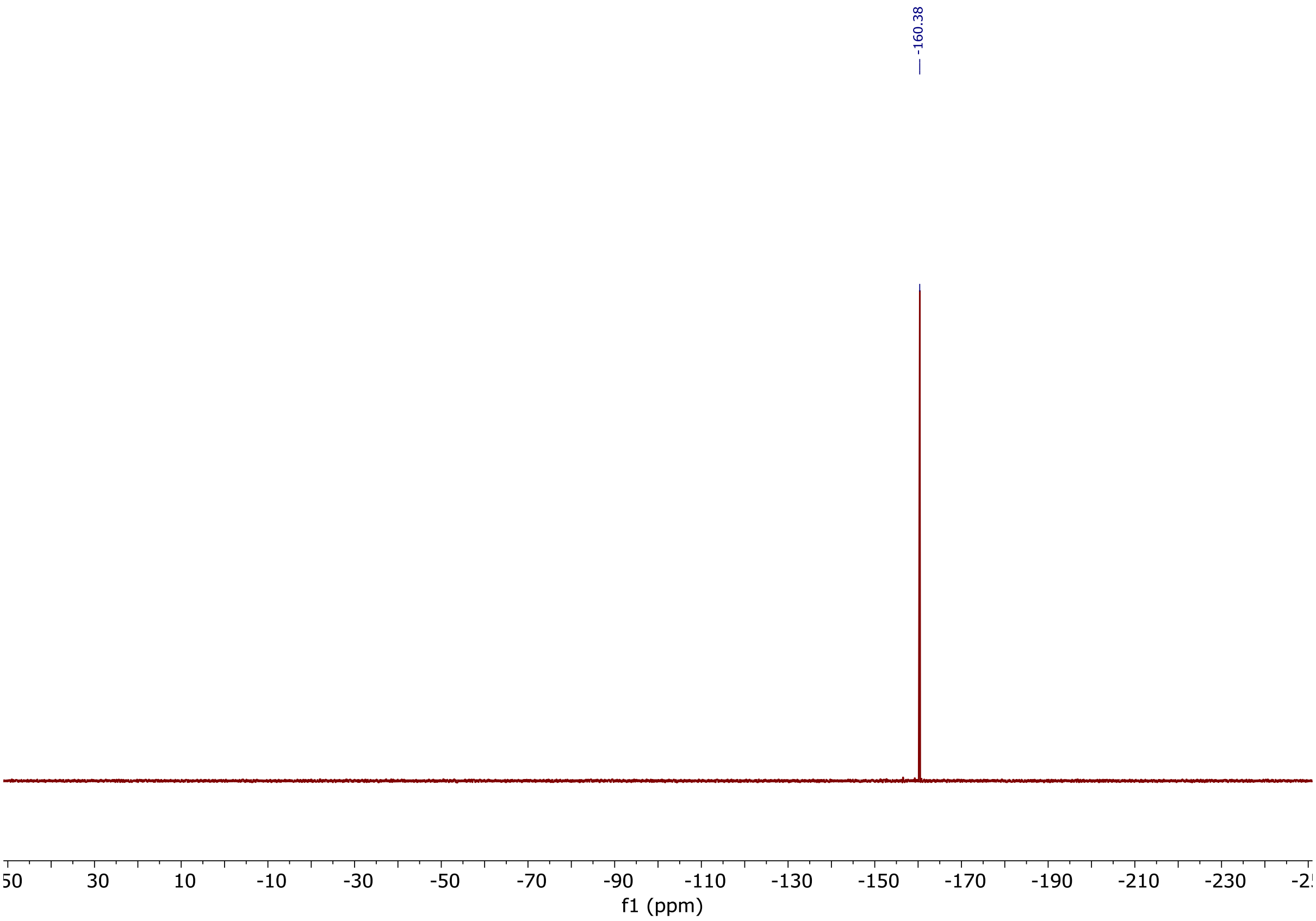

9.9. Pyrrolidines (10)

***4-fluoro-2-(p-tolyl)-1-tosylpyrrolidine* (10b)**

^{1}H NMR (500 MHz, $CDCl_3$)

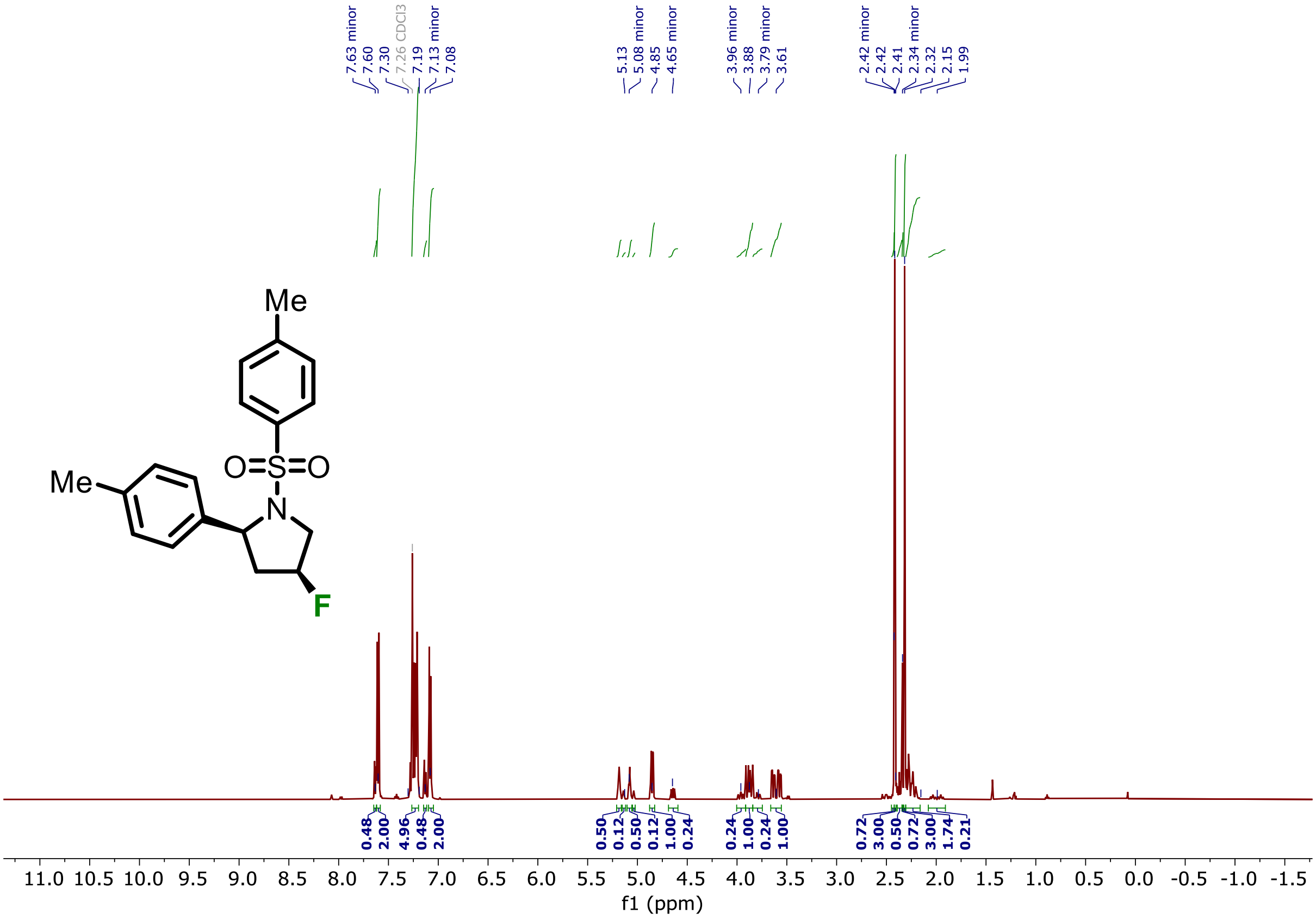

^{13}C NMR (126 MHz, $CDCl_3$)

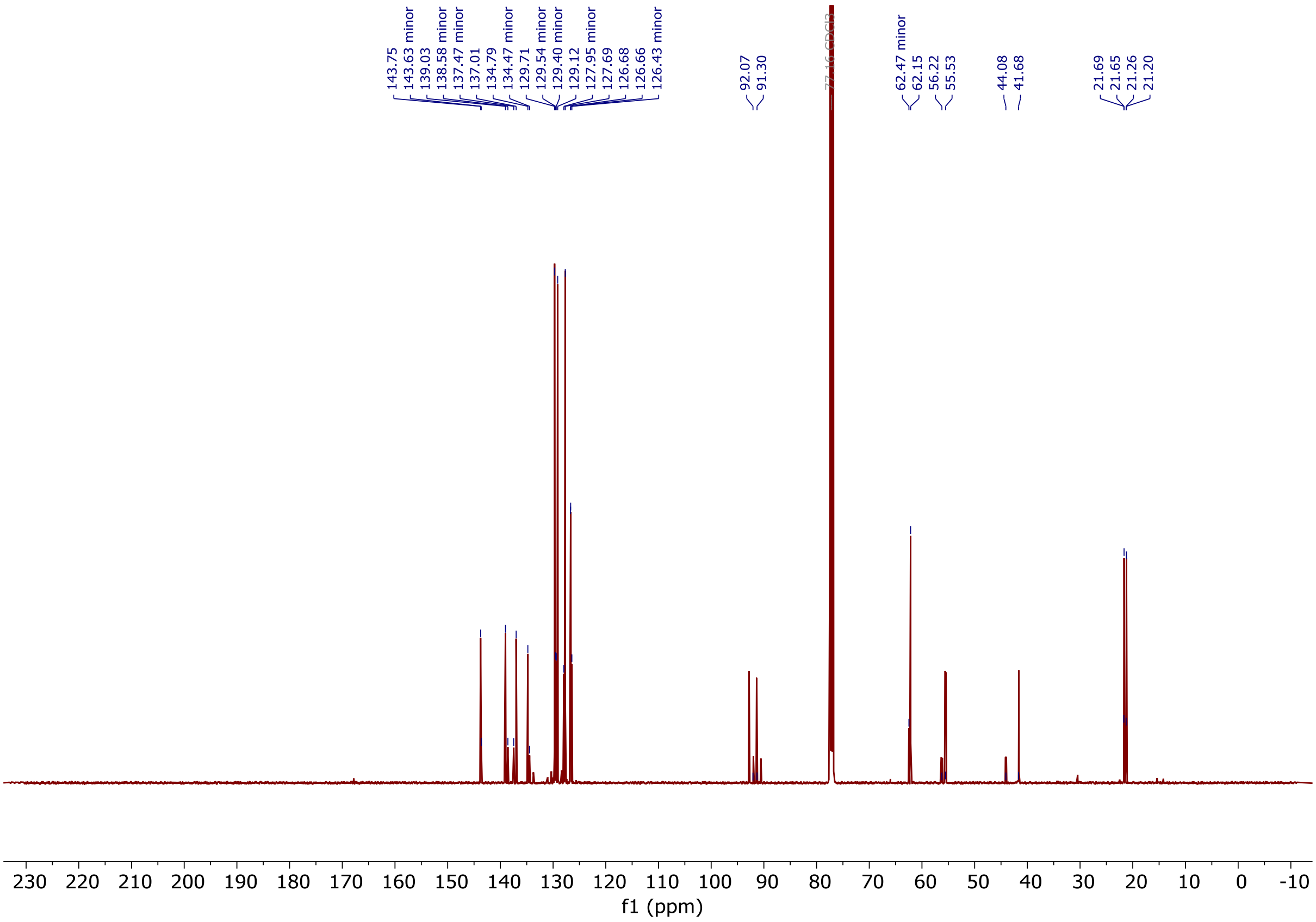

^{19}F NMR (471 MHz, $CDCl_3$)

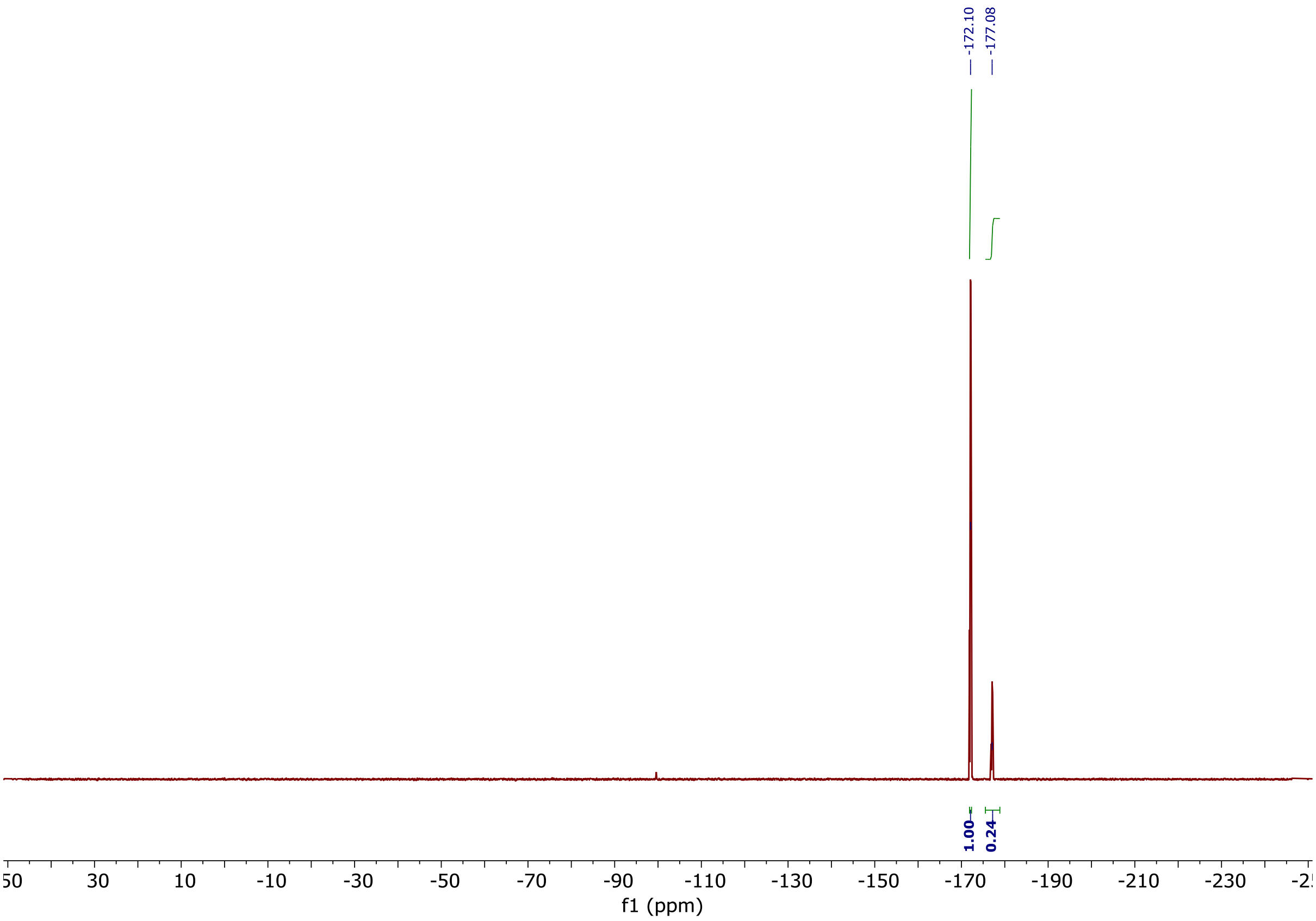

2-(4-chlorophenyl)-4-fluoro-1-tosylpyrrolidine **(10d)**

^{1}H NMR (500 MHz, $CDCl_3$)

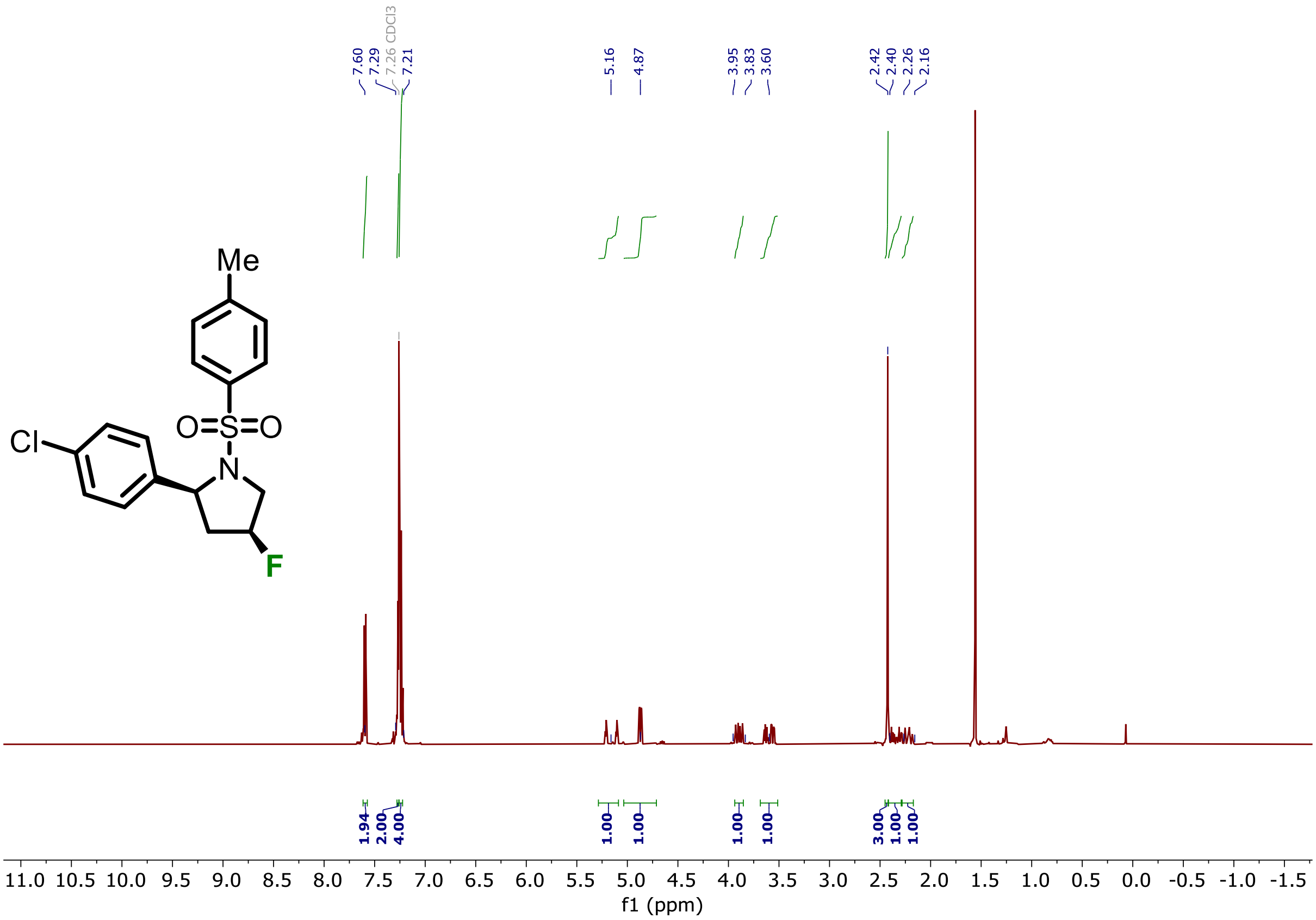

^{13}C NMR (126 MHz, $CDCl_3$)

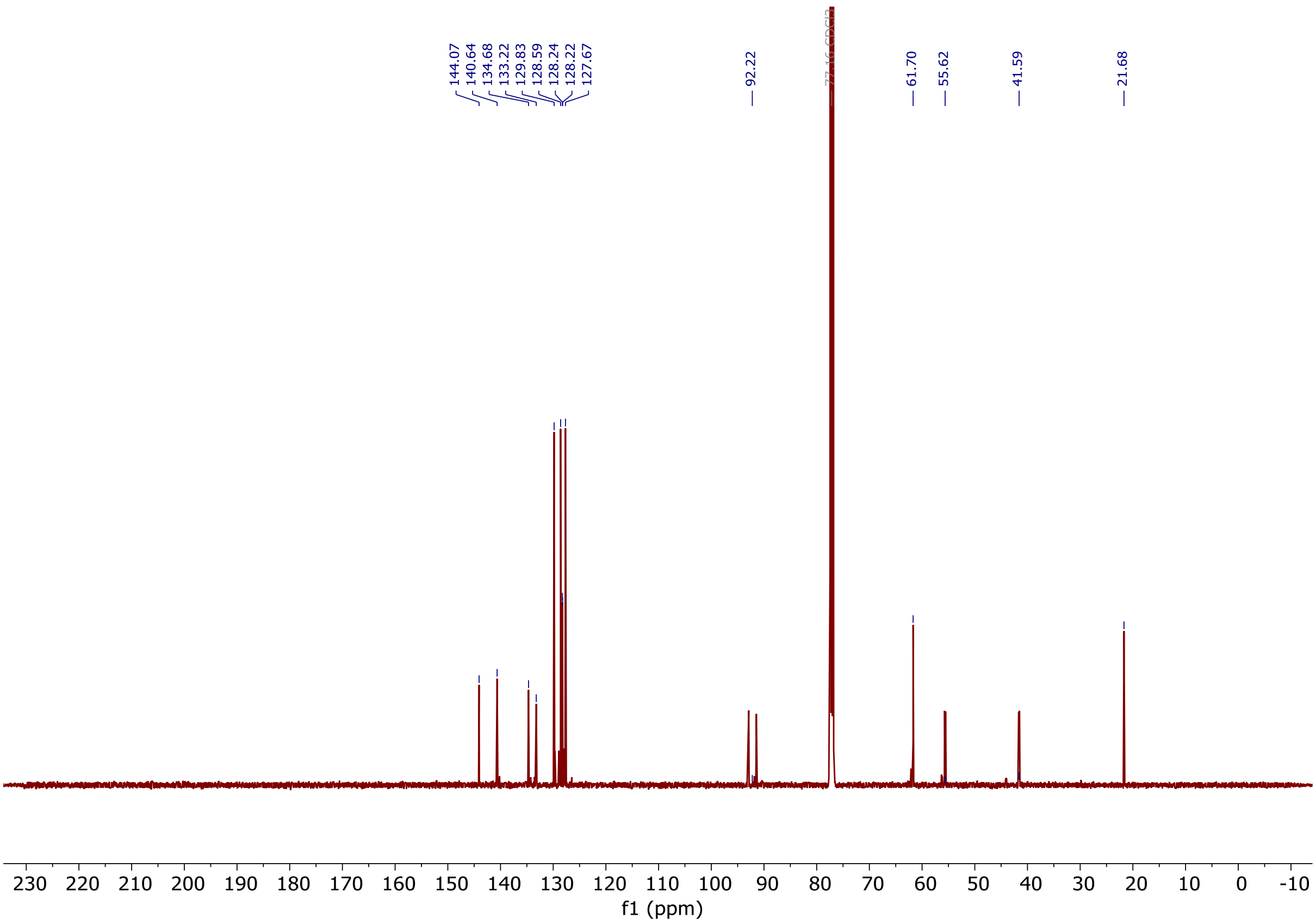

^{19}F NMR (471 MHz, CDCl$_3$)

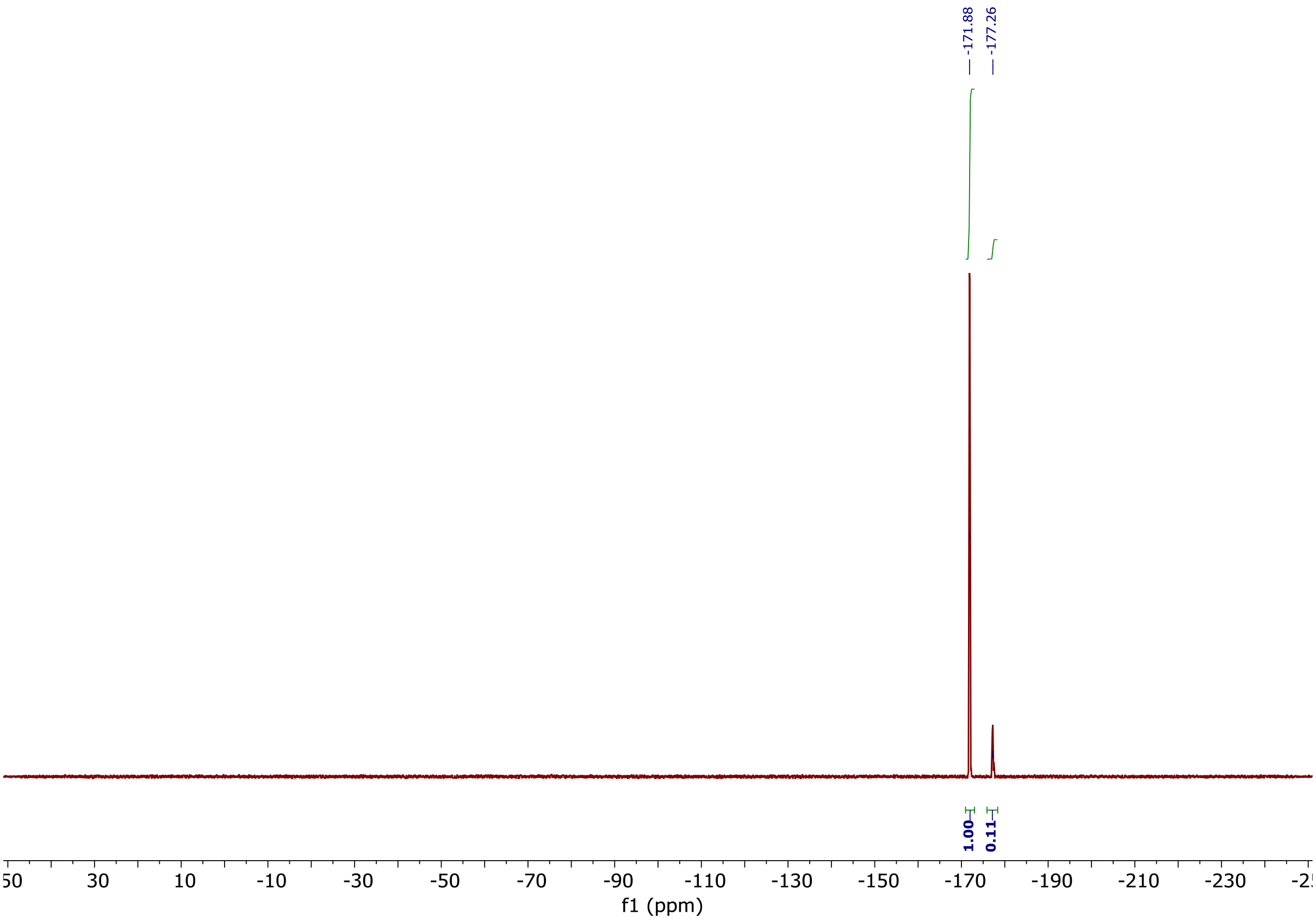

***cis-4-fluoro-2-(4-fluorophenyl)-1-tosylpyrrolidine* (10e)**

^{1}H NMR (500 MHz, $CDCl_3$)

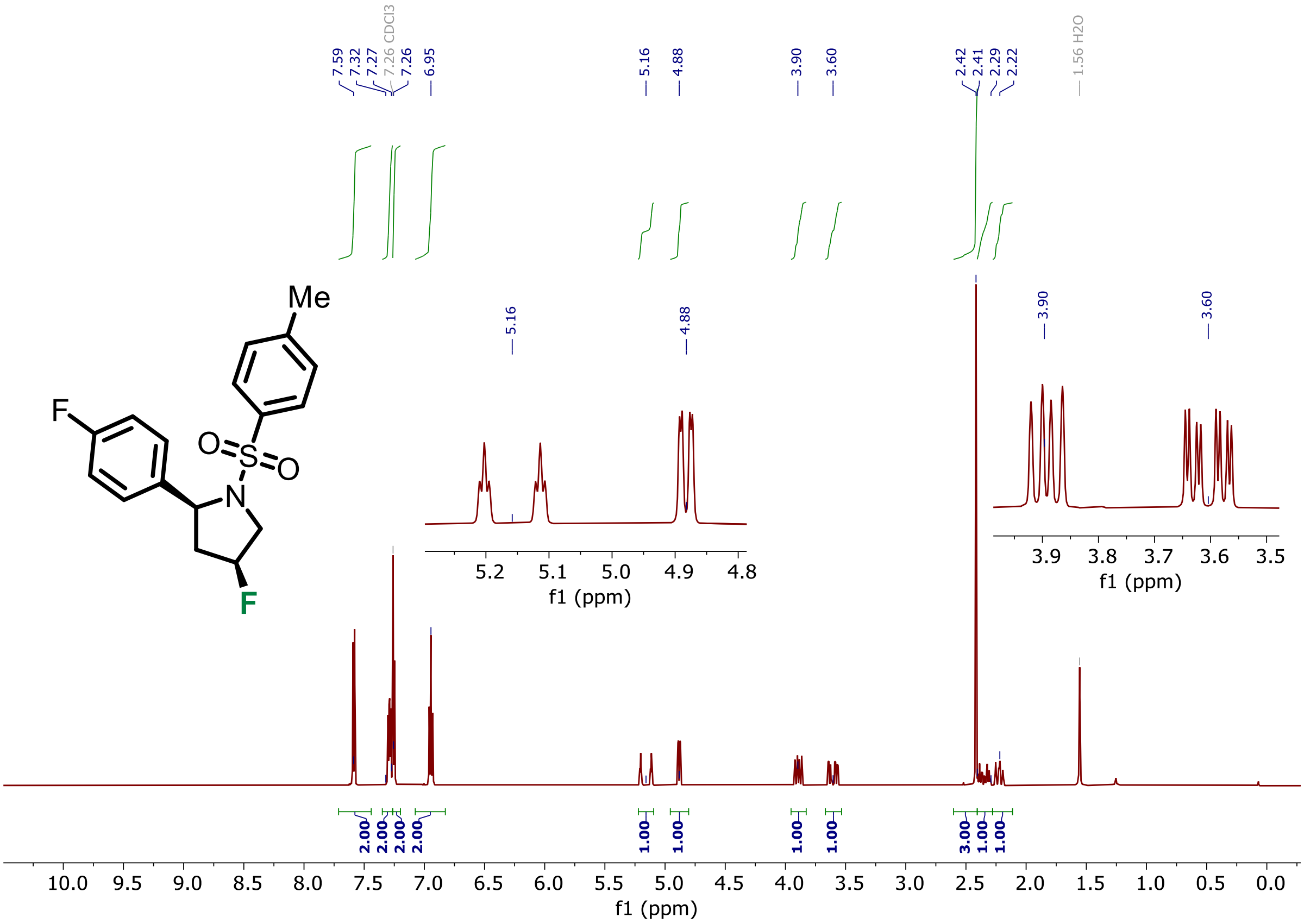

^{13}C NMR (151 MHz, $CDCl_3$)

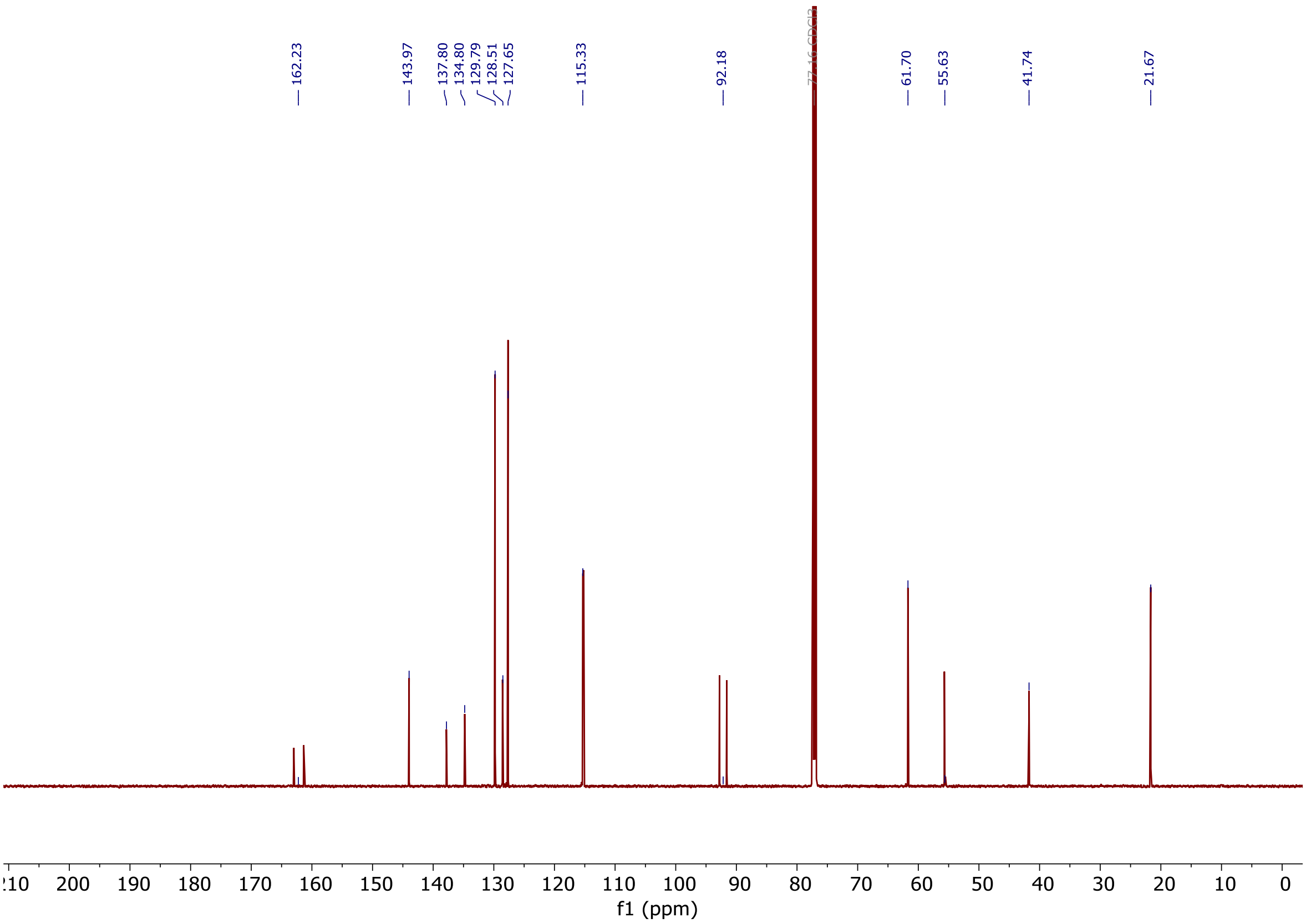

^{19}F NMR (376 MHz, $CDCl_3$)

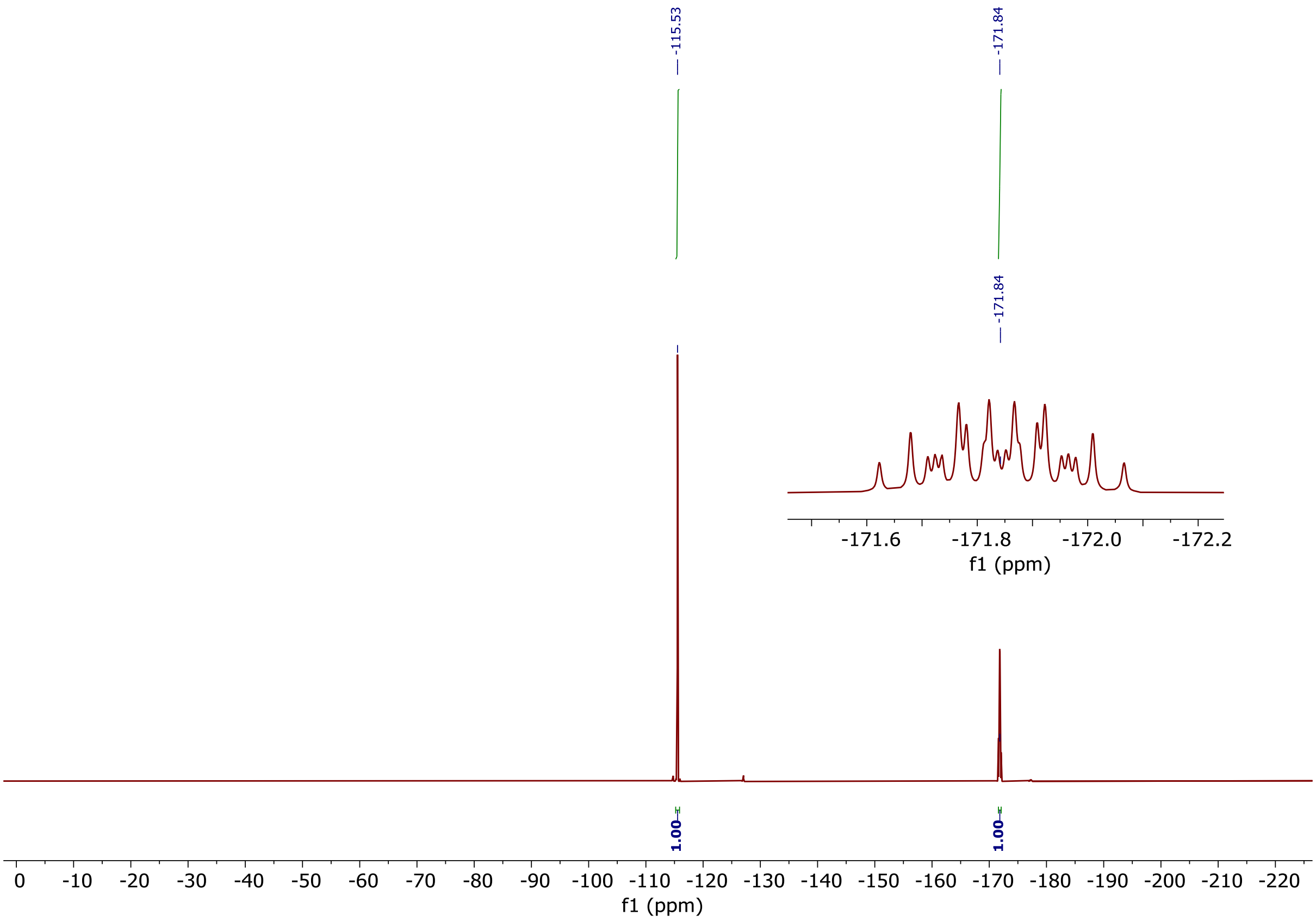

4-fluoro-1-tosyl-2-(4-(trifluoromethyl)phenyl)pyrrolidine **(10f)**

^{1}H NMR (500 MHz, CDCl$_3$)

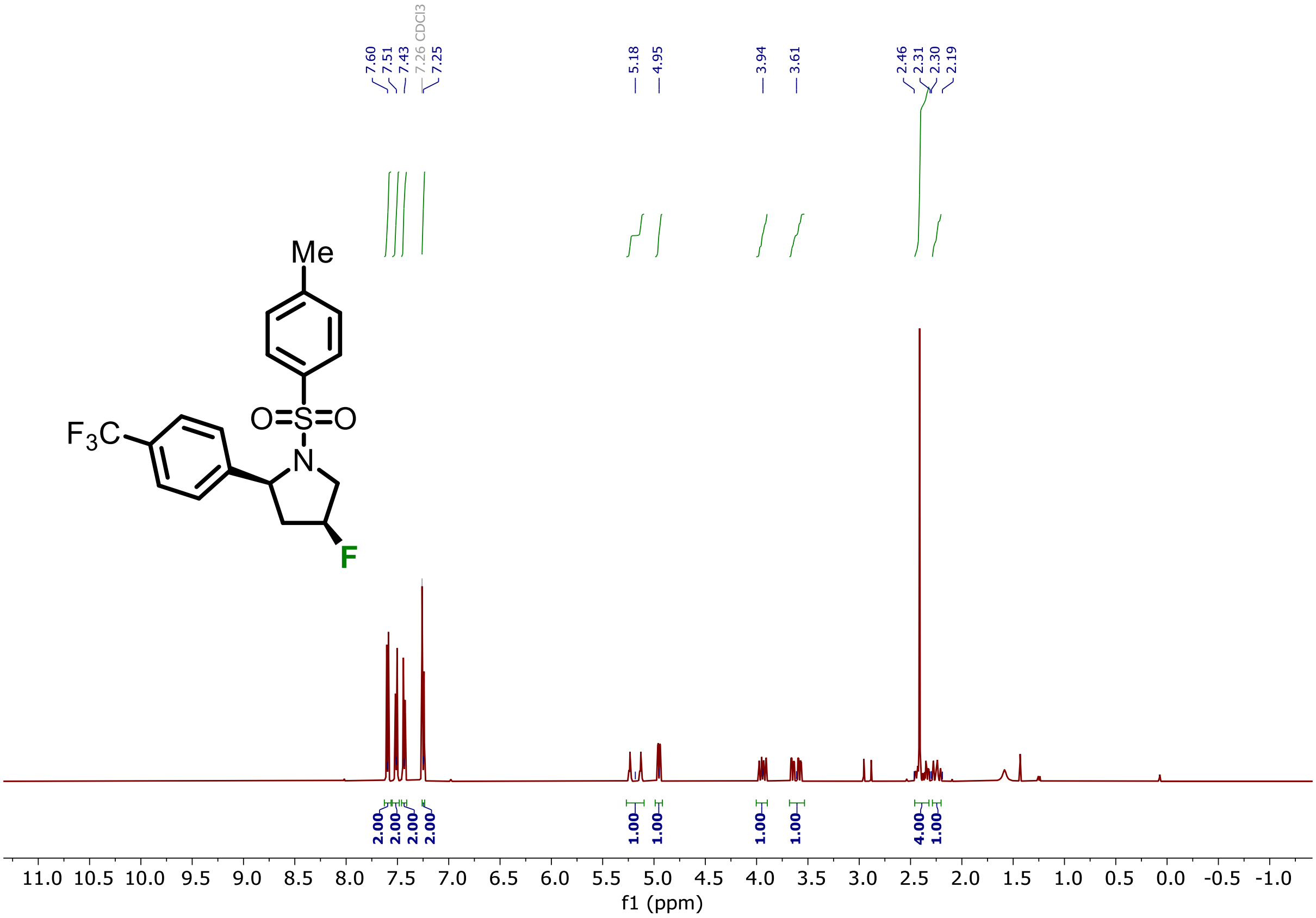

^{13}C NMR (126 MHz, CDCl$_3$)

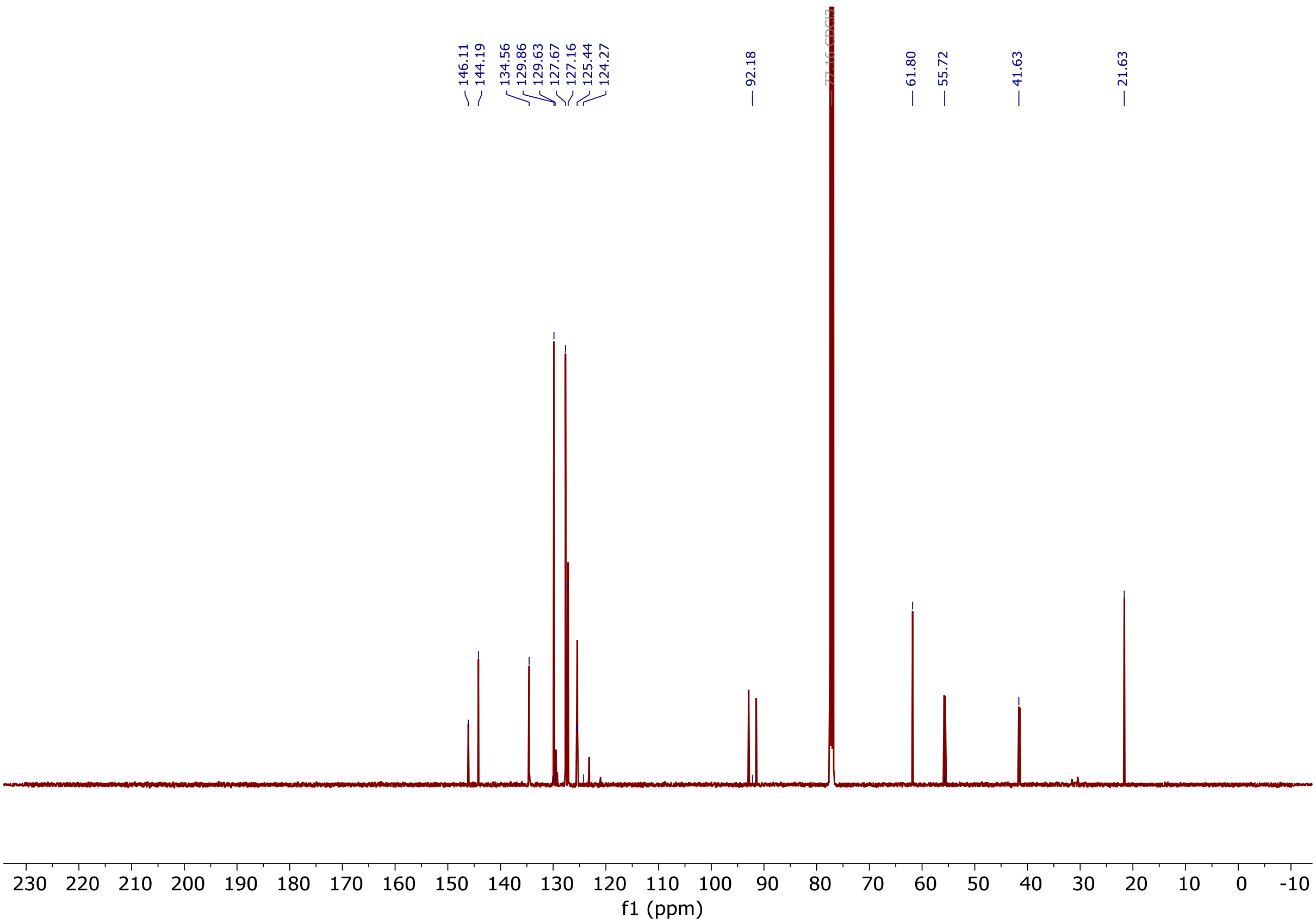

^{19}F NMR (376 MHz, $CDCl_3$)

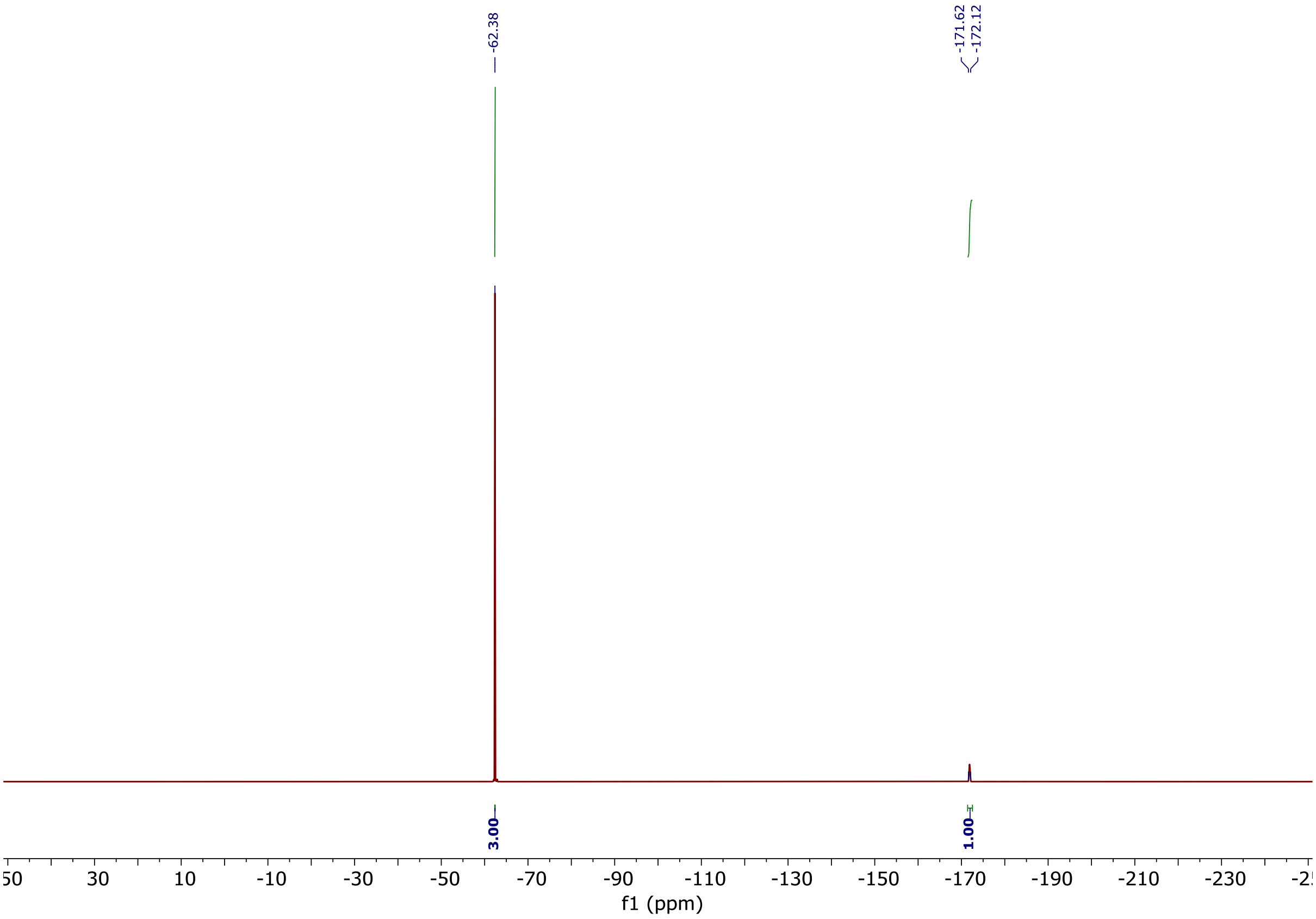

***(2R,3R,5R)-3-fluoro-5-(4-fluorophenyl)-2-methyl-1-tosylpyrrolidine* ((*R,R,R*)-10j)**

^{1}H NMR (500 MHz, CDCl$_3$)

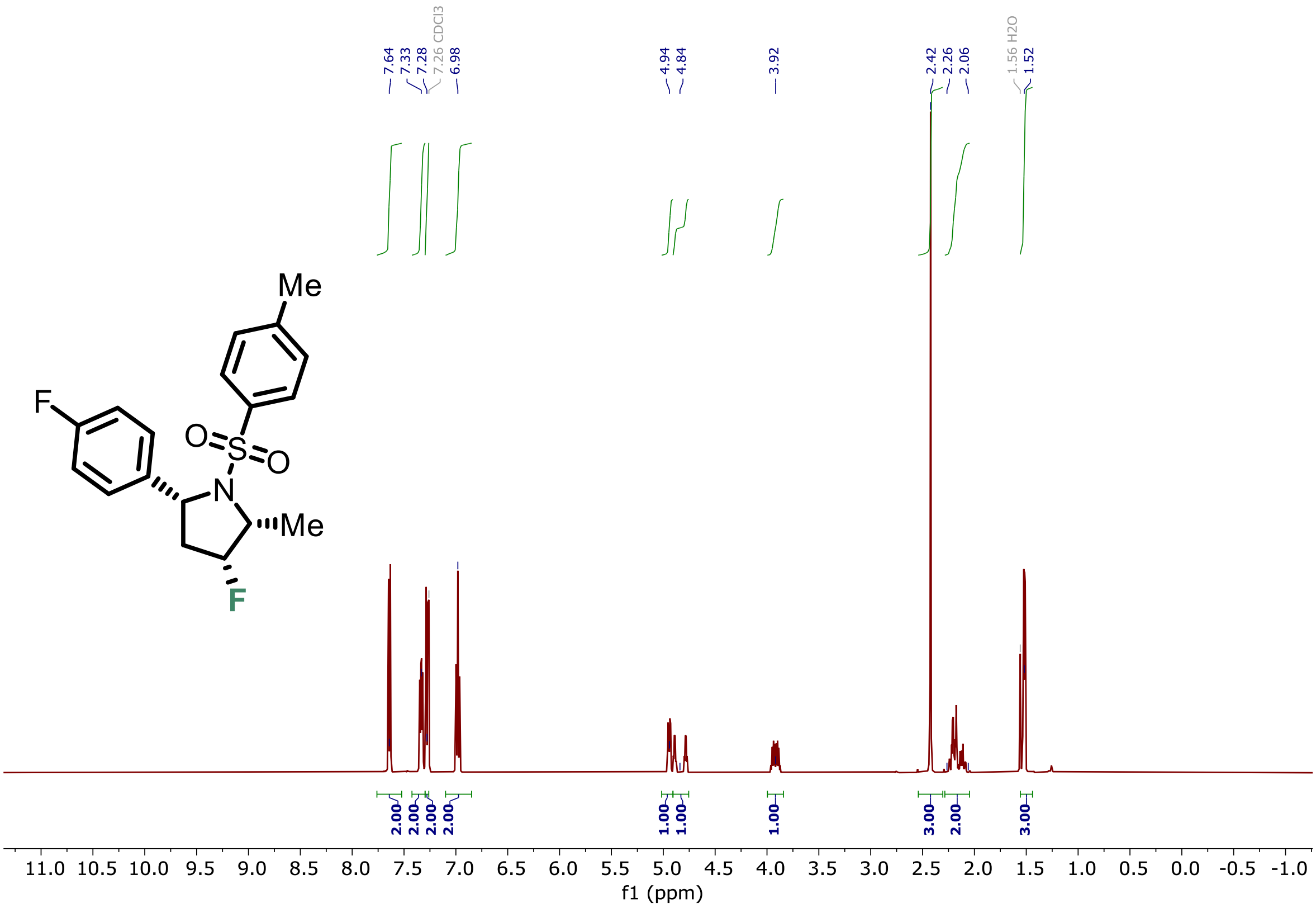

^{13}C NMR (126 MHz, CDCl$_3$)

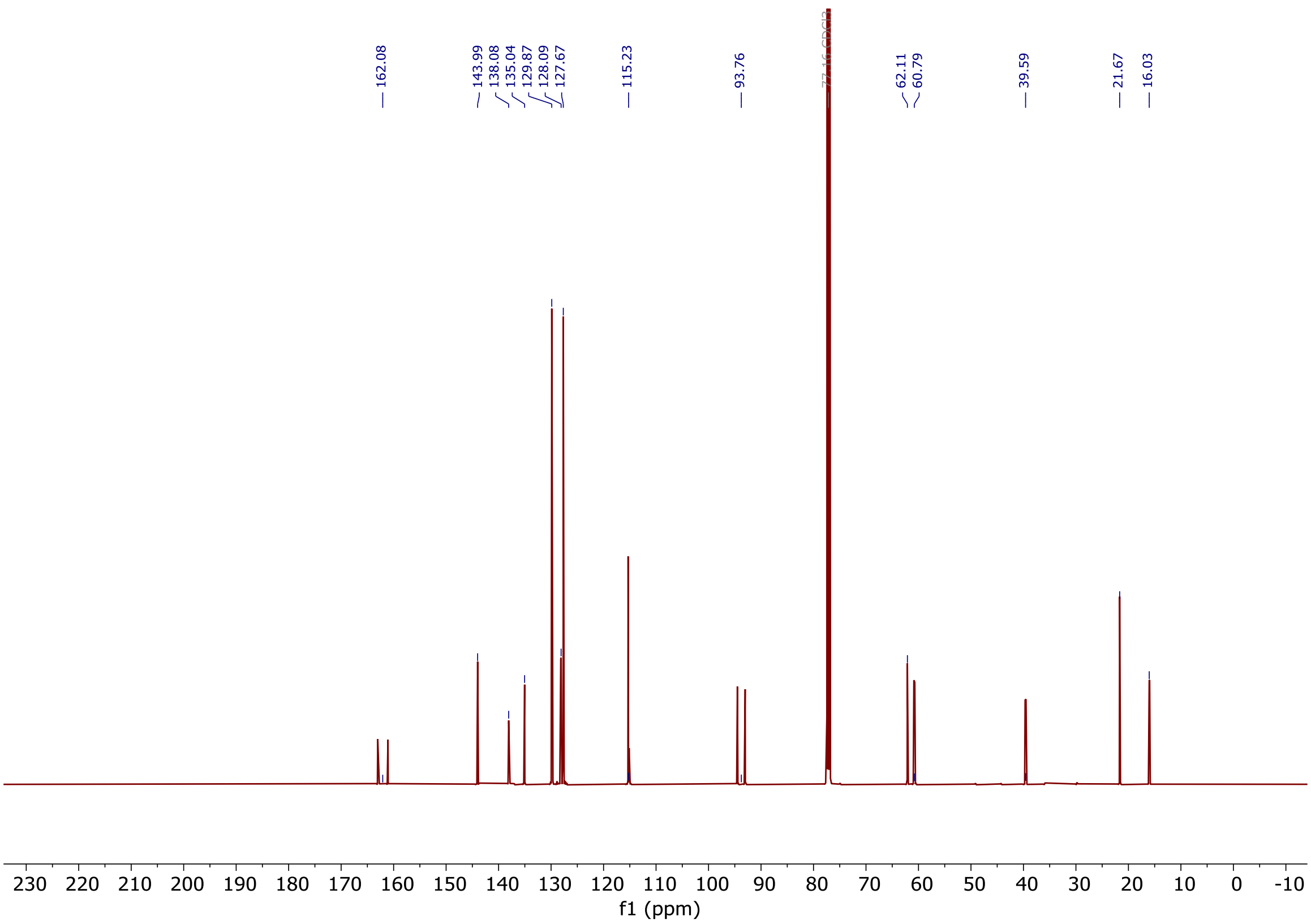

^{19}F NMR (376 MHz, CDCl$_3$)

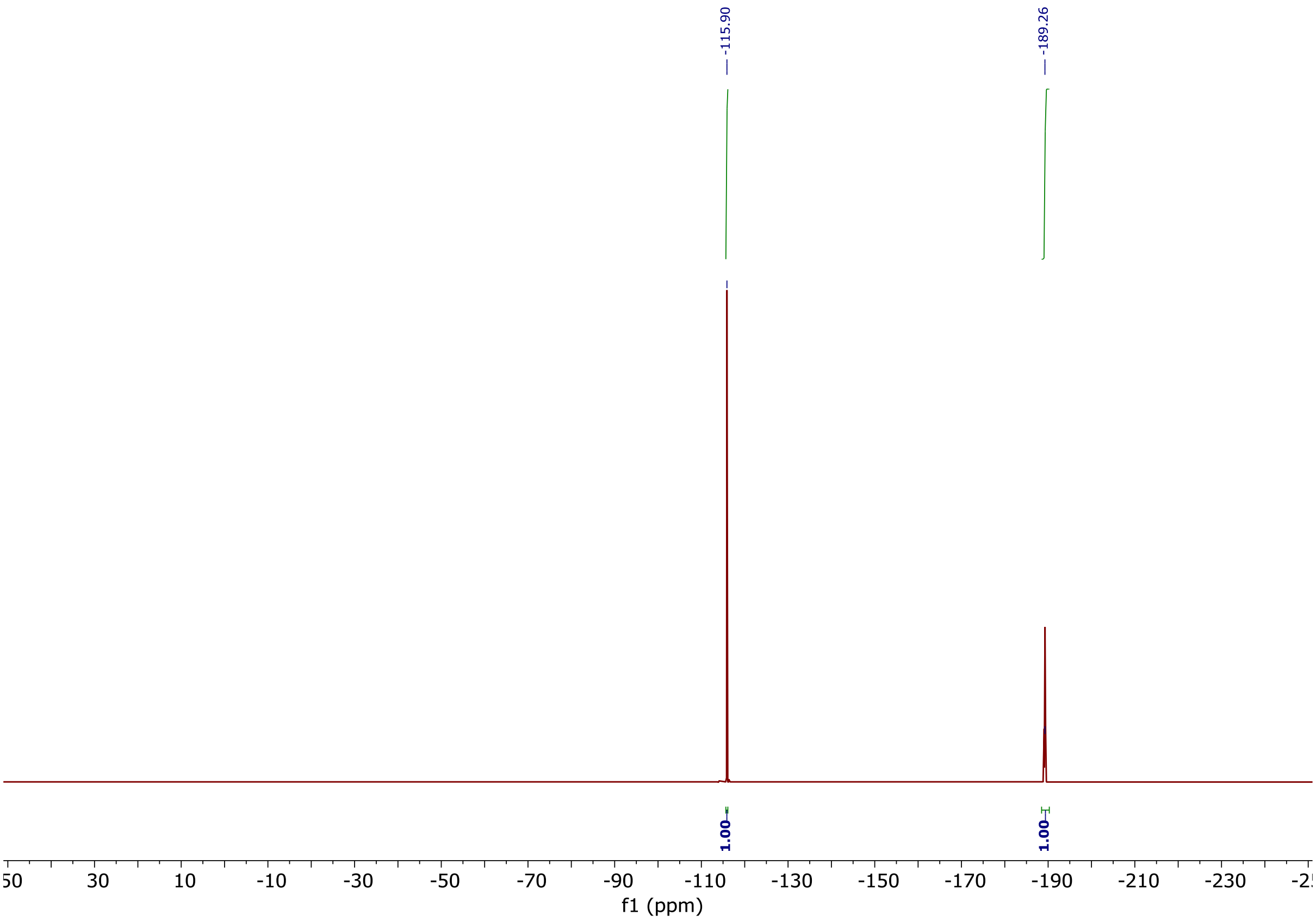

cis-3-fluoro-2-methyl-5-(naphthalen-2-yl)-1-tosylpyrrolidine **(10m)**

^{1}H NMR (500 MHz, CDCl$_3$)

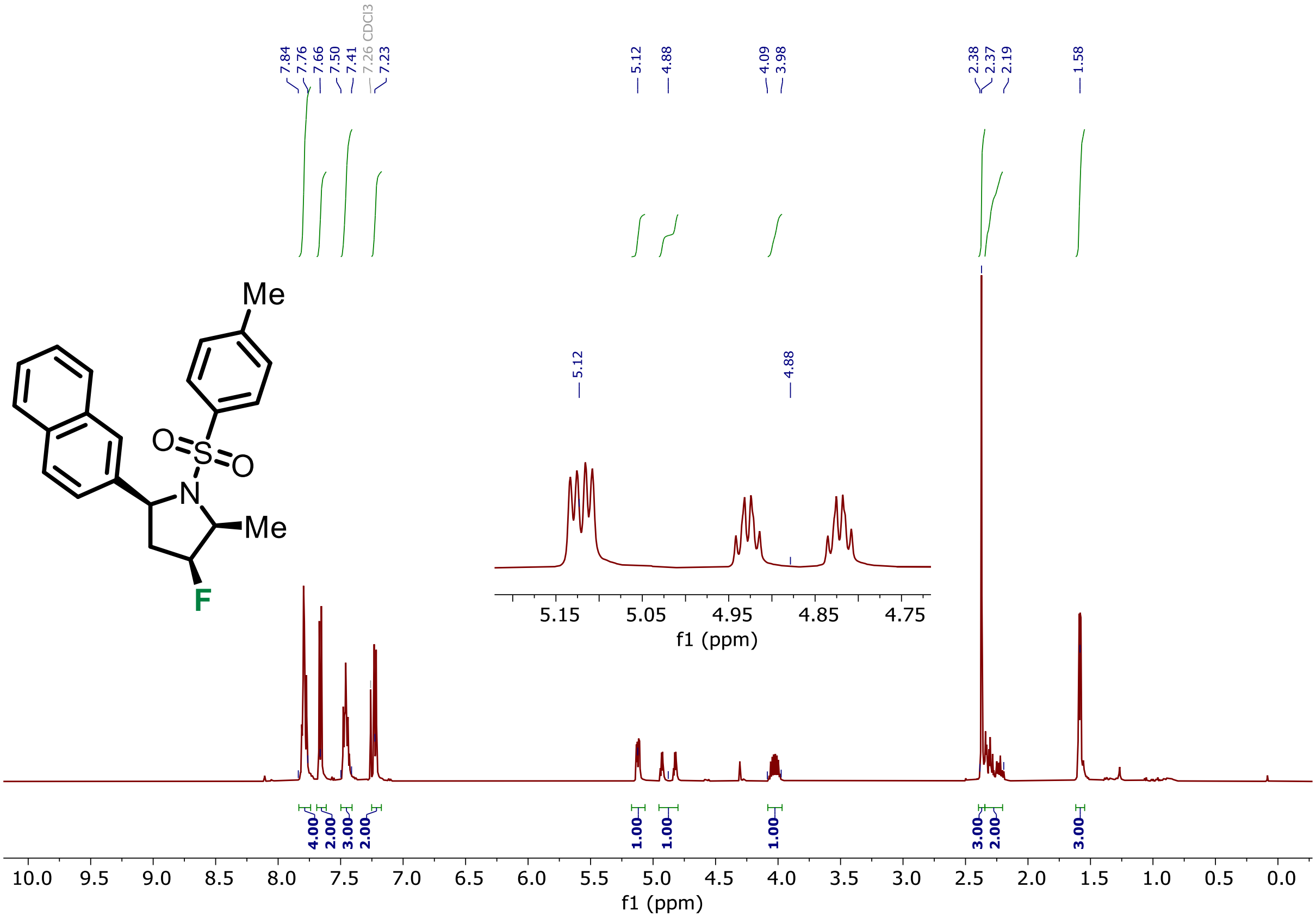

^{13}C NMR (126 MHz, CDCl$_3$)

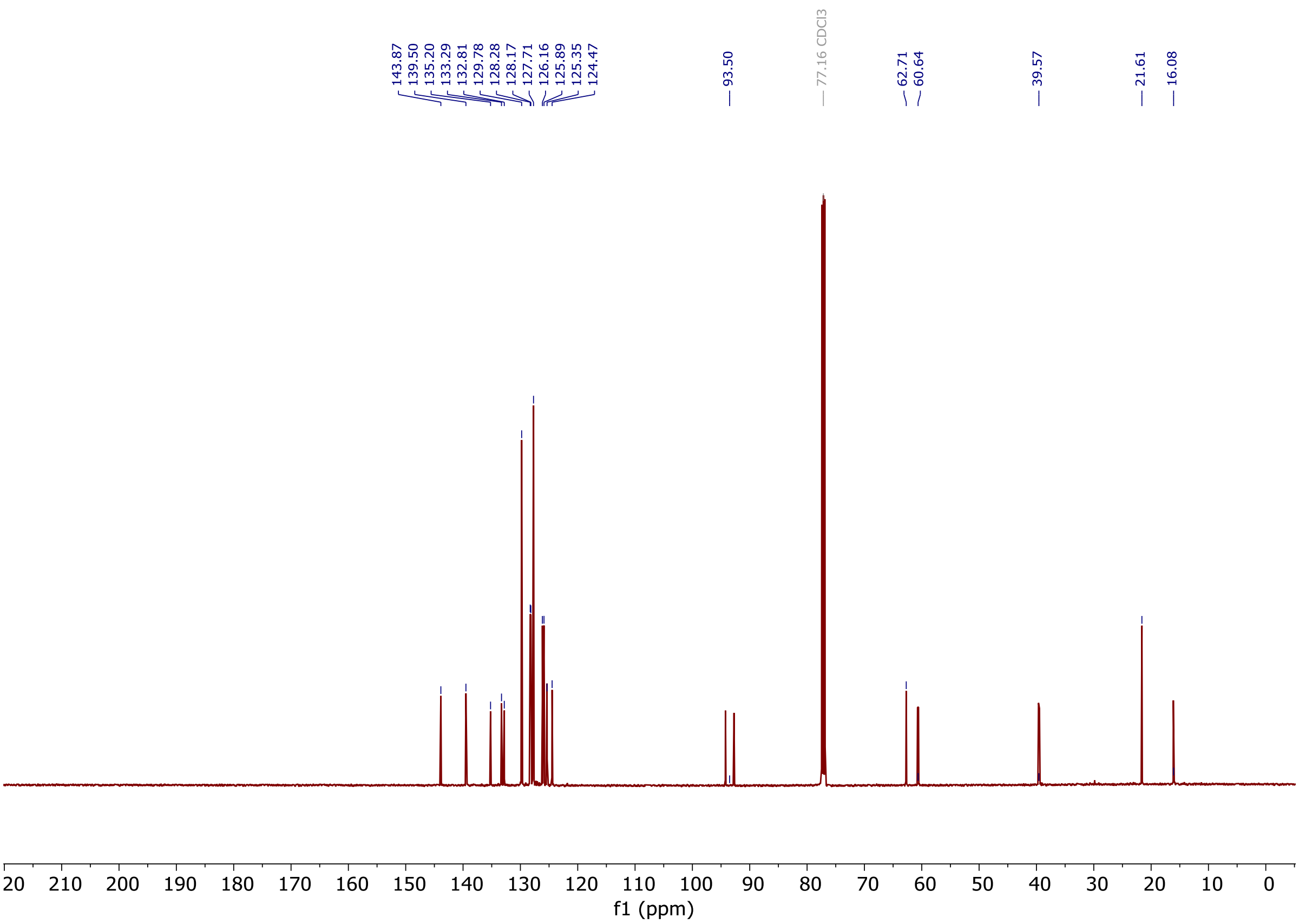

^{19}F NMR (471 MHz, CDCl$_3$)

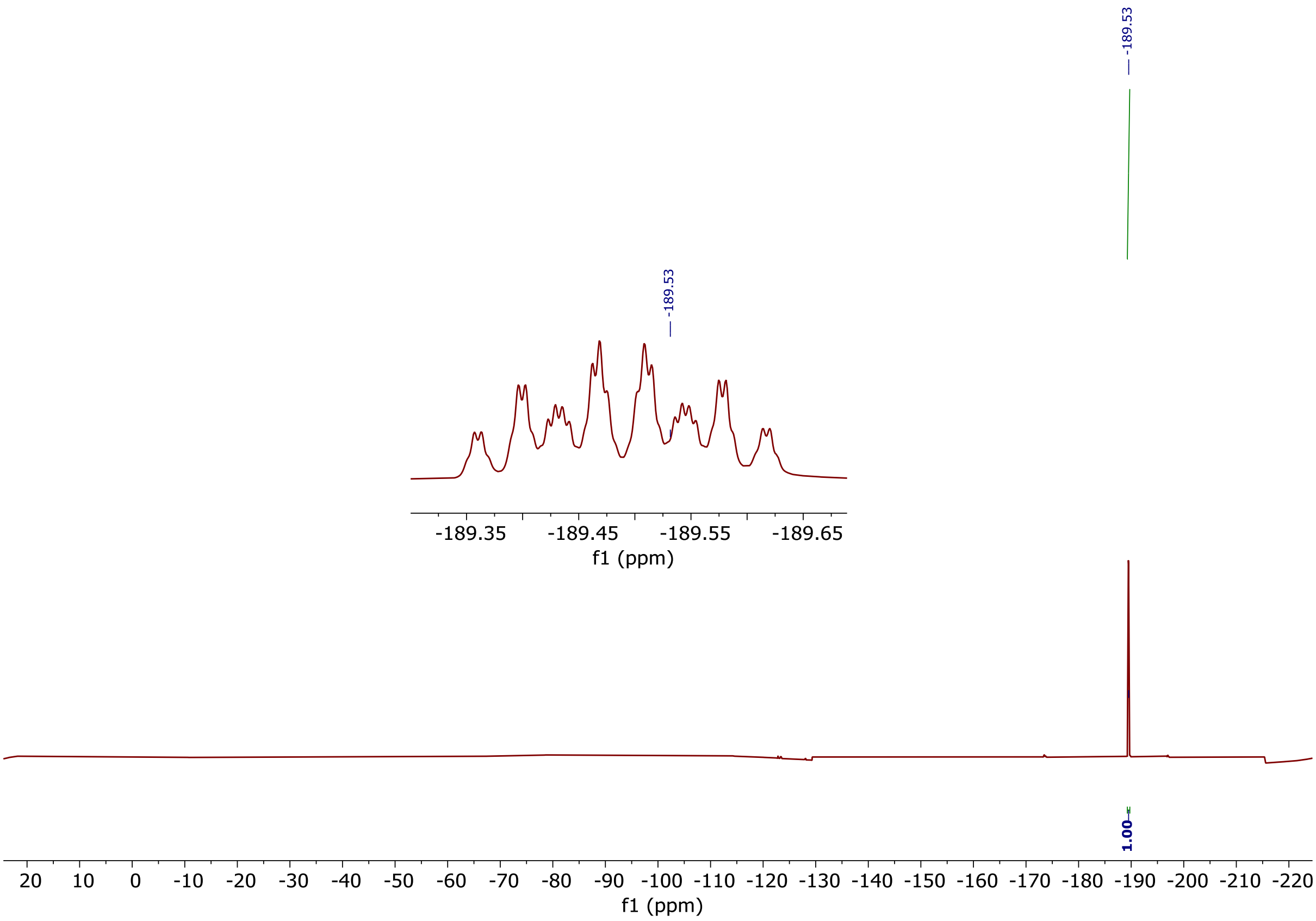

3-fluoro-2-methyl-5-(naphthalen-2-yl)-1-tosylpyrrolidine* (10m)*(mixture of diastereomers)

^{1}H NMR (500 MHz, $CDCl_3$)

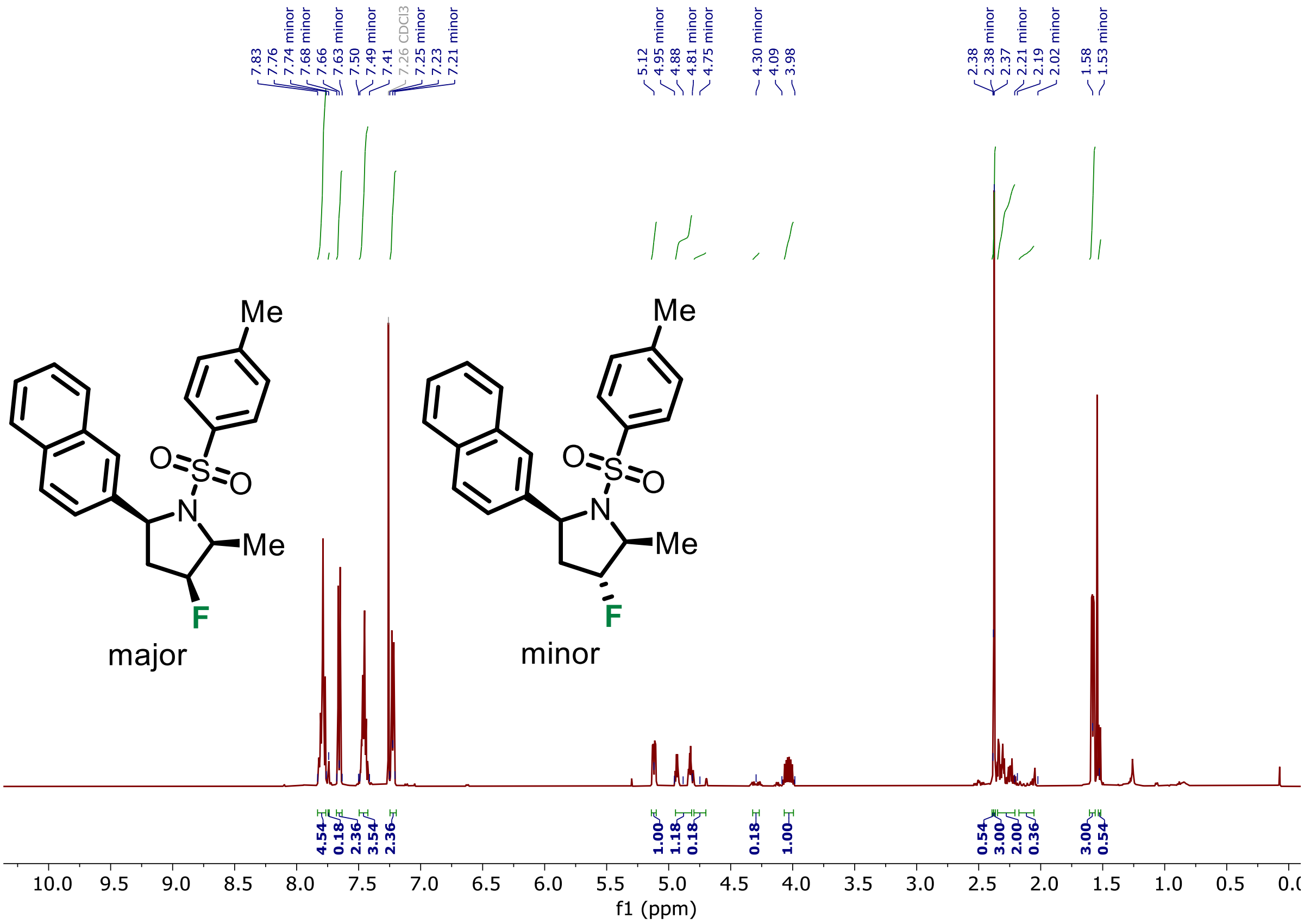

^{13}C NMR (126 MHz, $CDCl_3$)

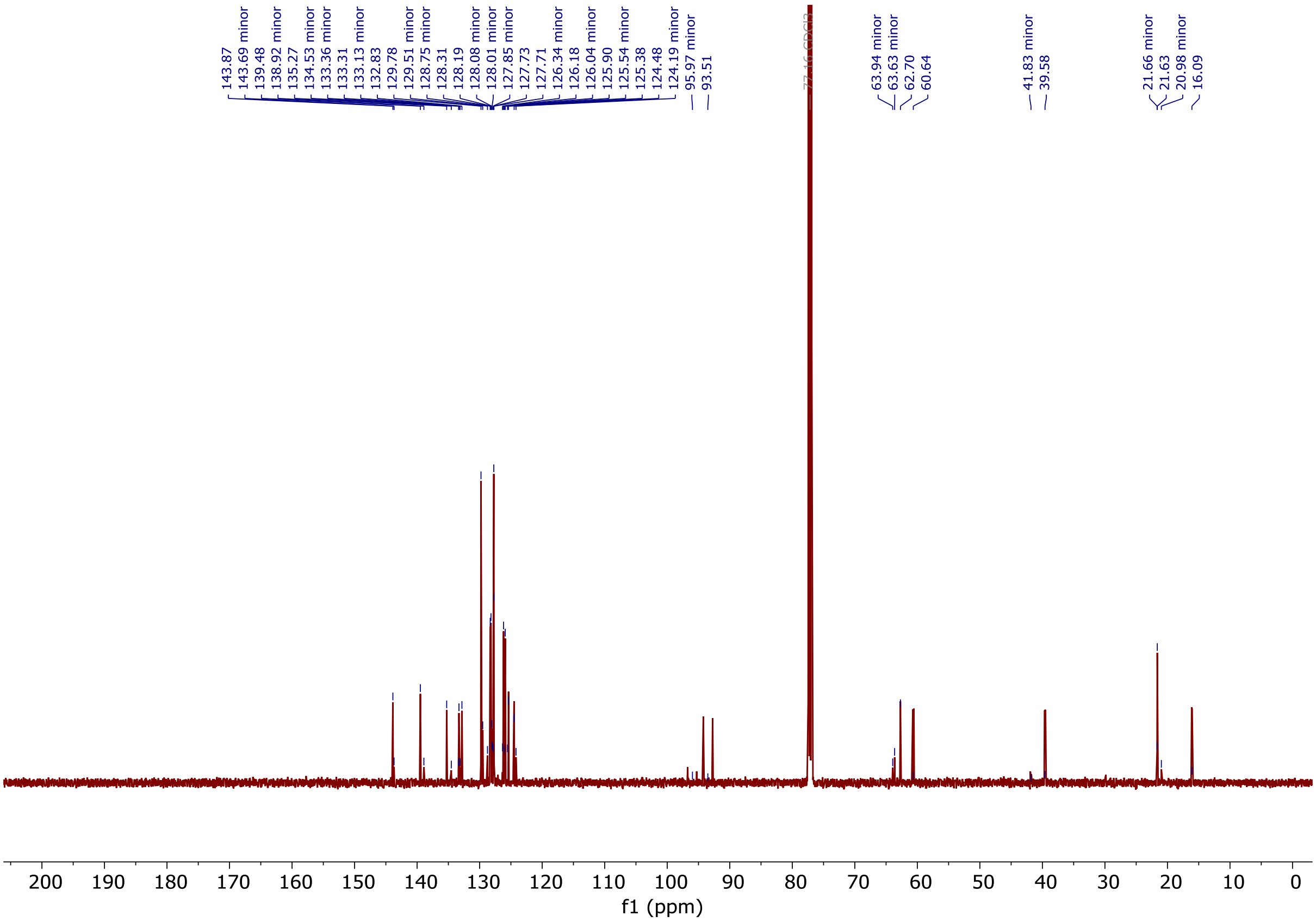

^{19}F NMR (471 MHz, CDCl$_3$)

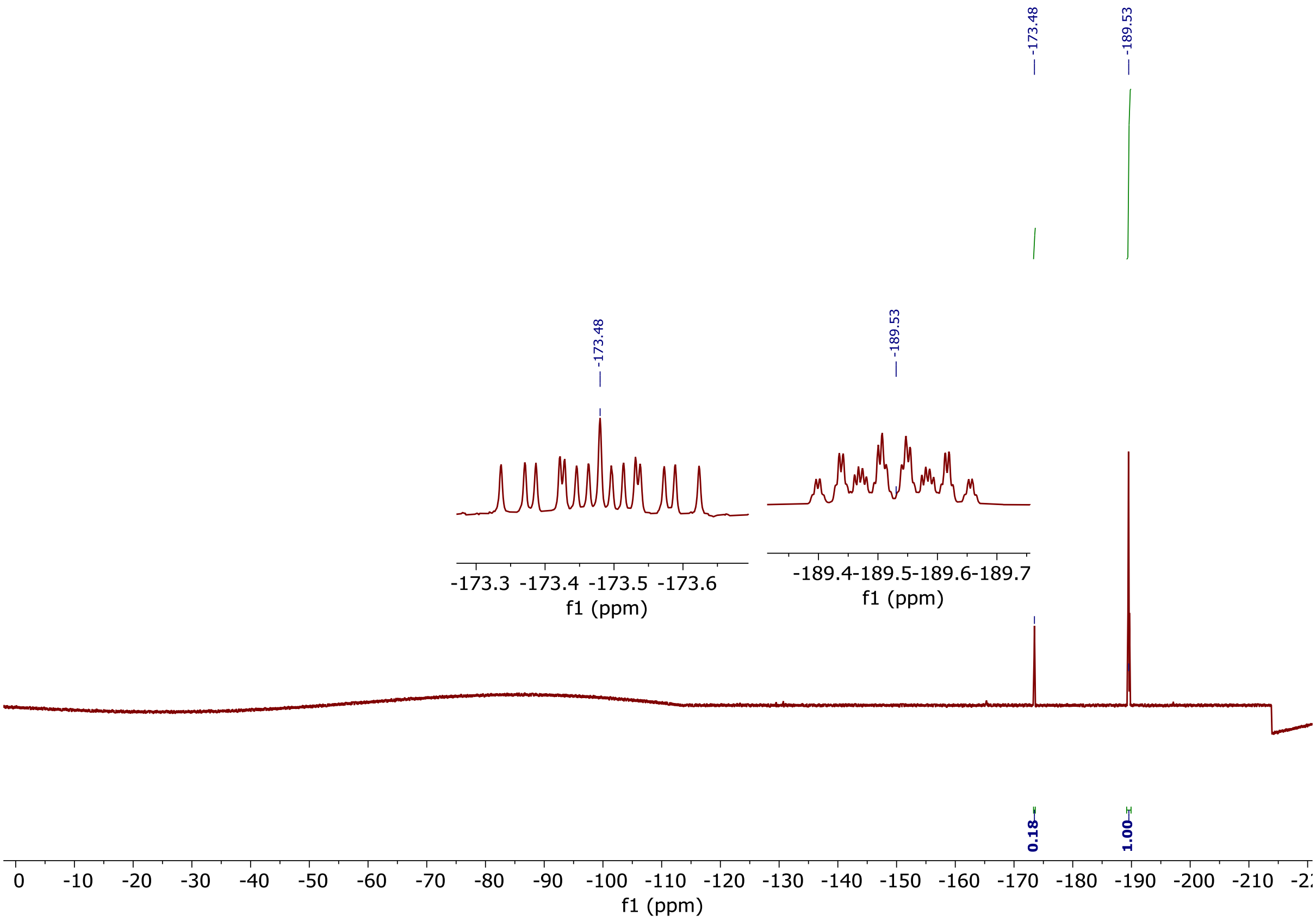

2-benzyl-4-fluoro-1-tosylpyrrolidine **(10t)**

^{1}H NMR (500 MHz, $CDCl_3$)

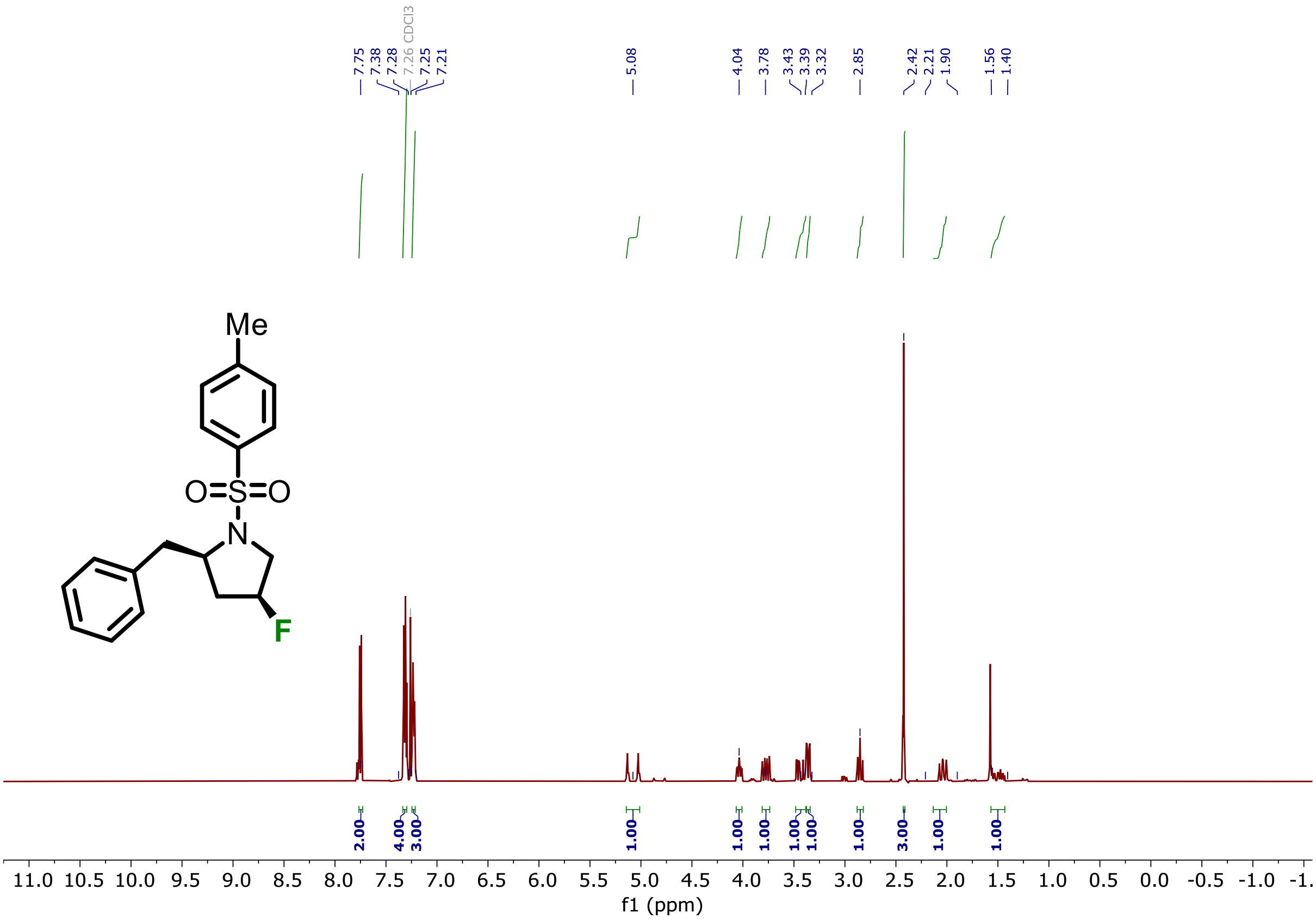

^{13}C NMR (126 MHz, $CDCl_3$)

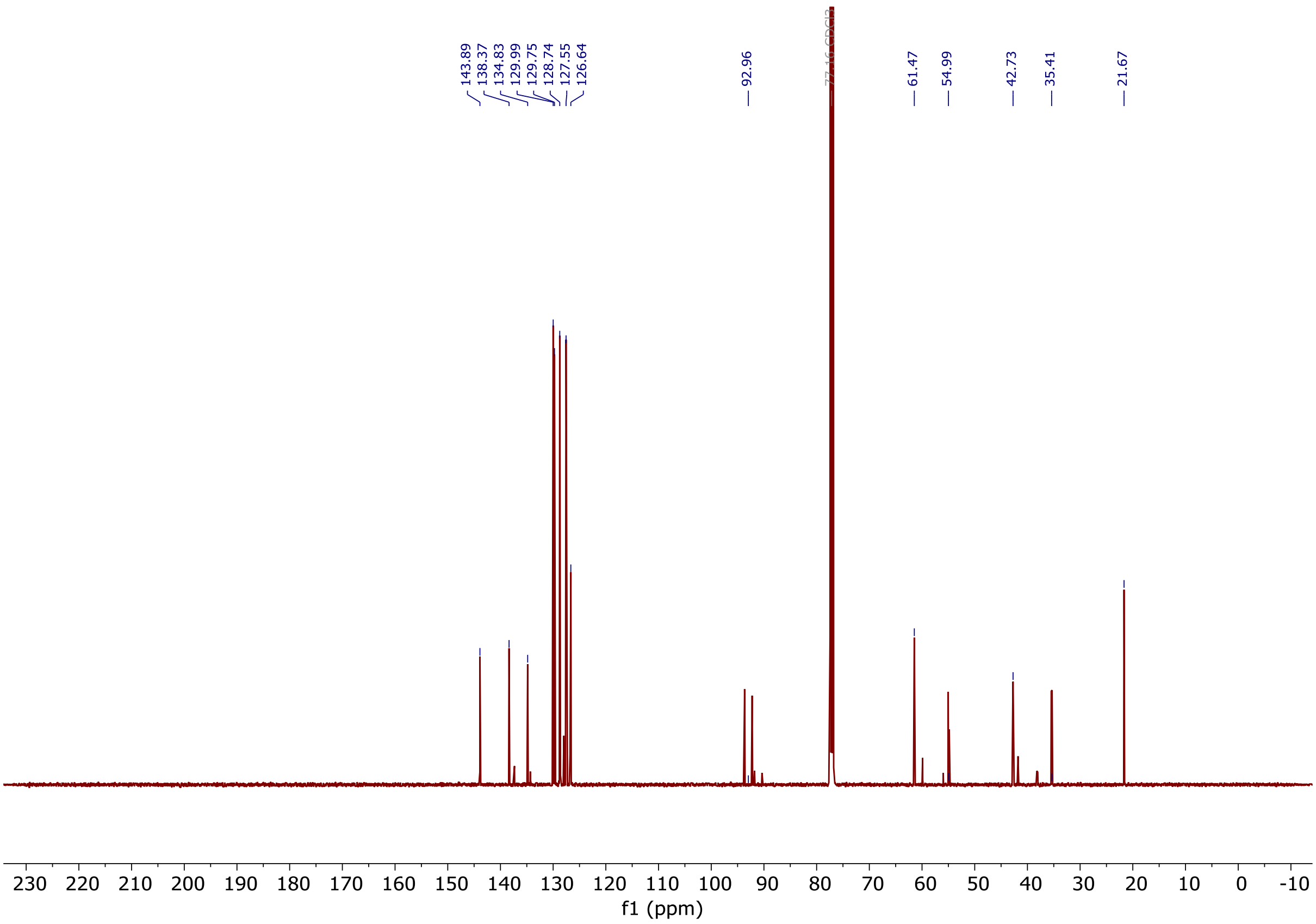

^{19}F NMR (376 MHz, CDCl$_3$)

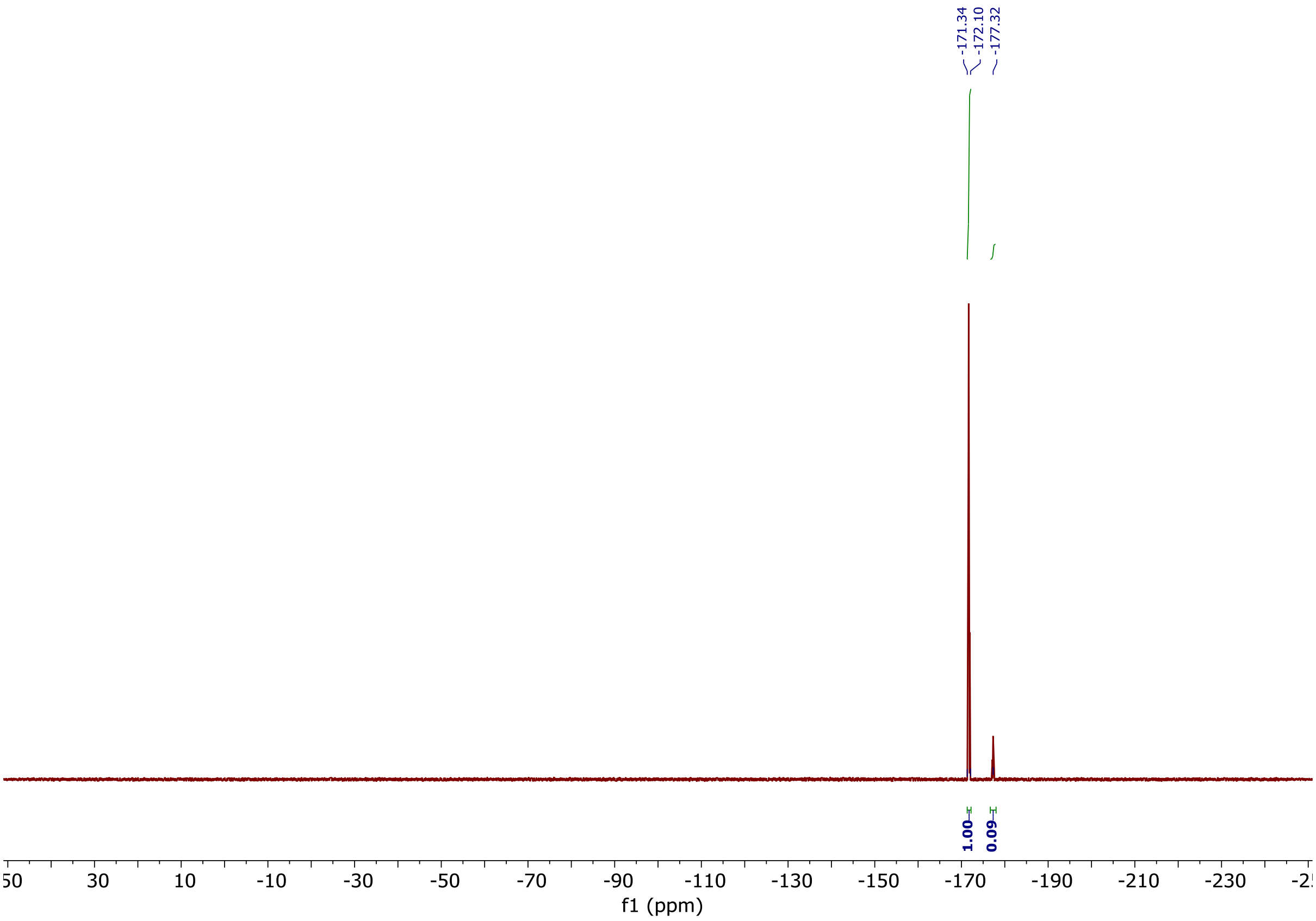

***cis*-4-((4-fluoro-2-propylpyrrolidin-1-yl)sulfonyl)aniline** (**10u**)

^{1}H NMR (500 MHz, $CDCl_3$)

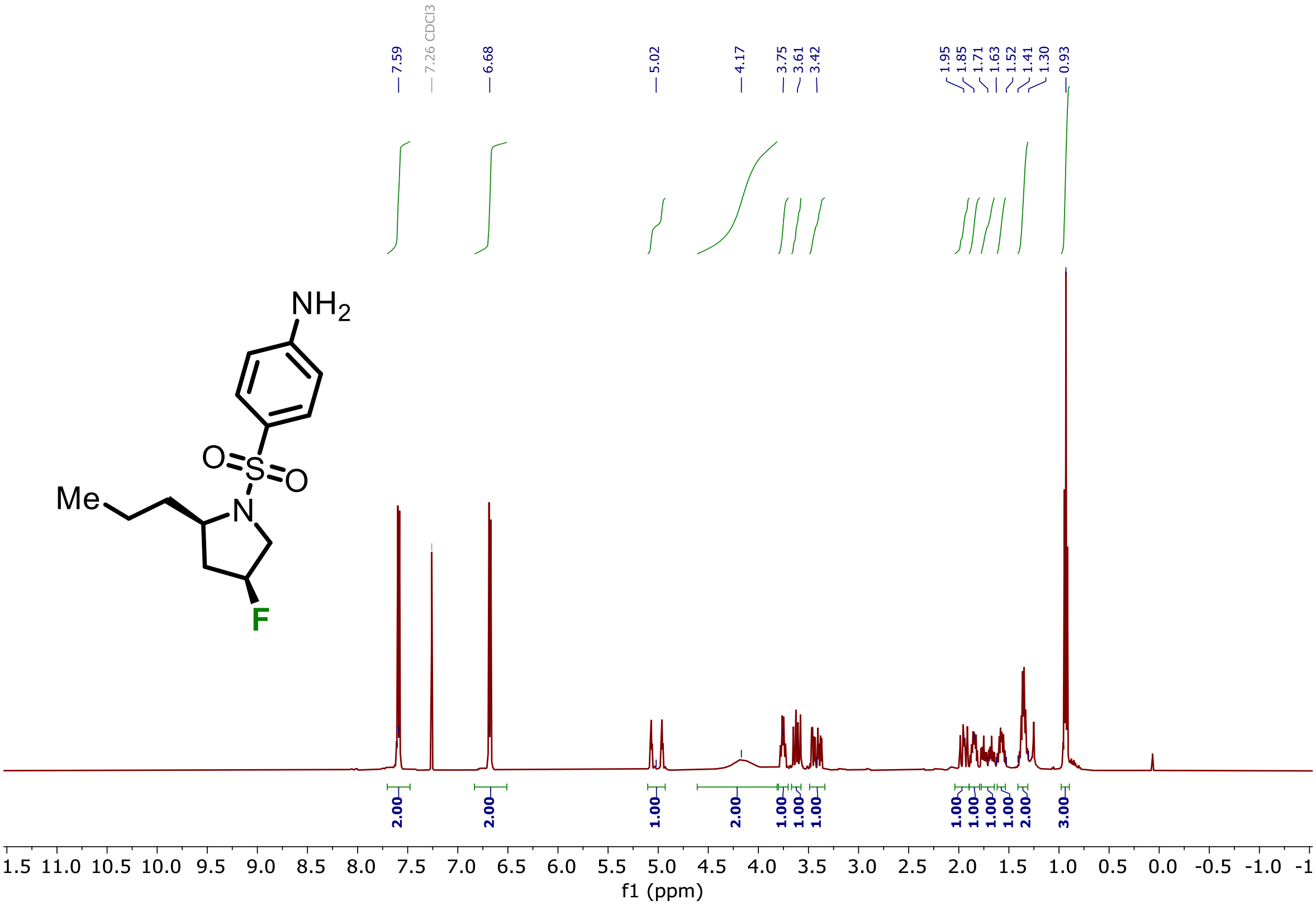

^{13}C NMR (126 MHz, $CDCl_3$)

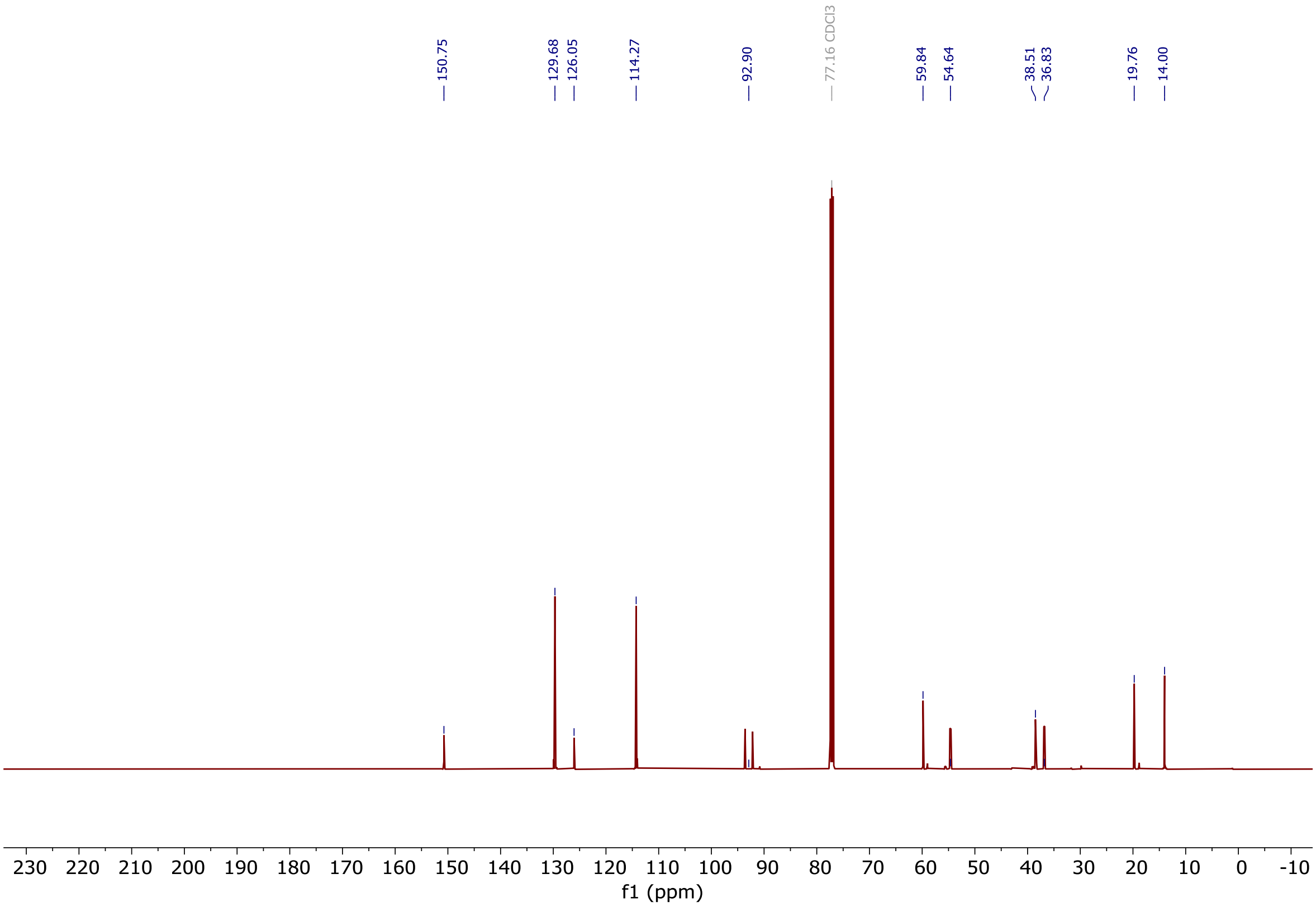

^{19}F NMR (376 MHz, $CDCl_3$)

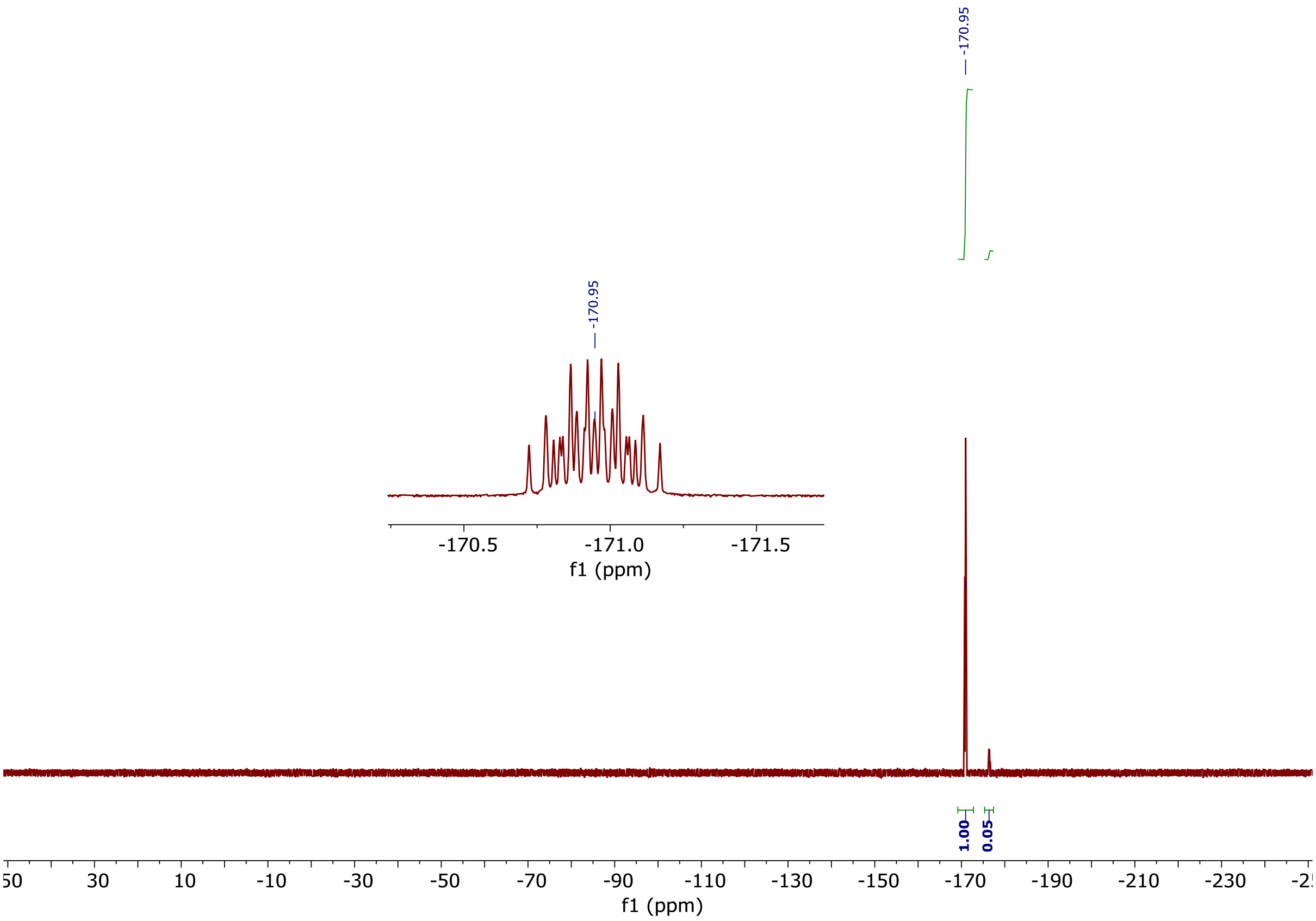

***cis-4-fluoro-2-propyl-1-tosylpyrrolidine* (10v)**

^{1}H NMR (500 MHz, CDCl$_3$)

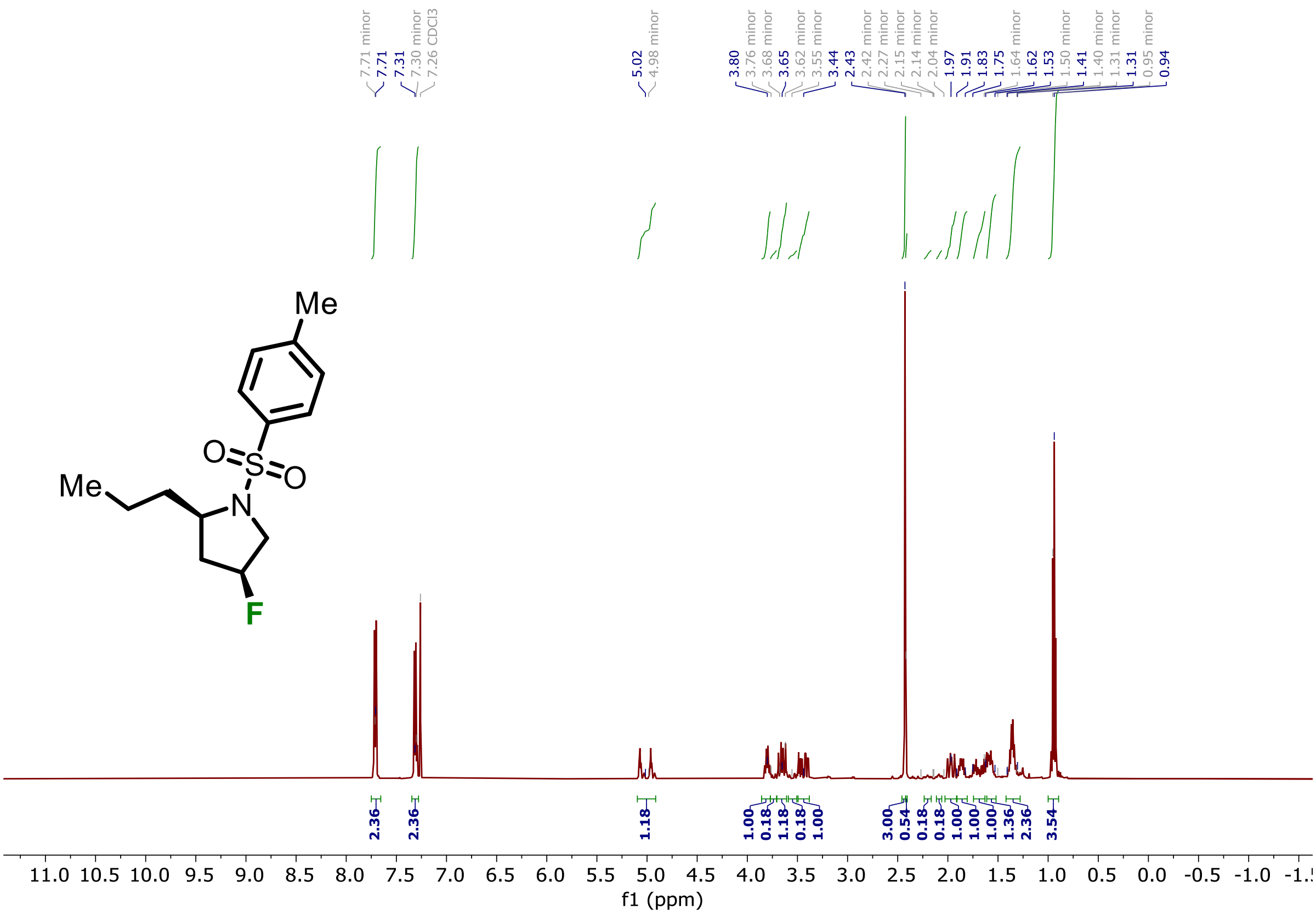

^{13}C NMR (126 MHz, CDCl$_3$)

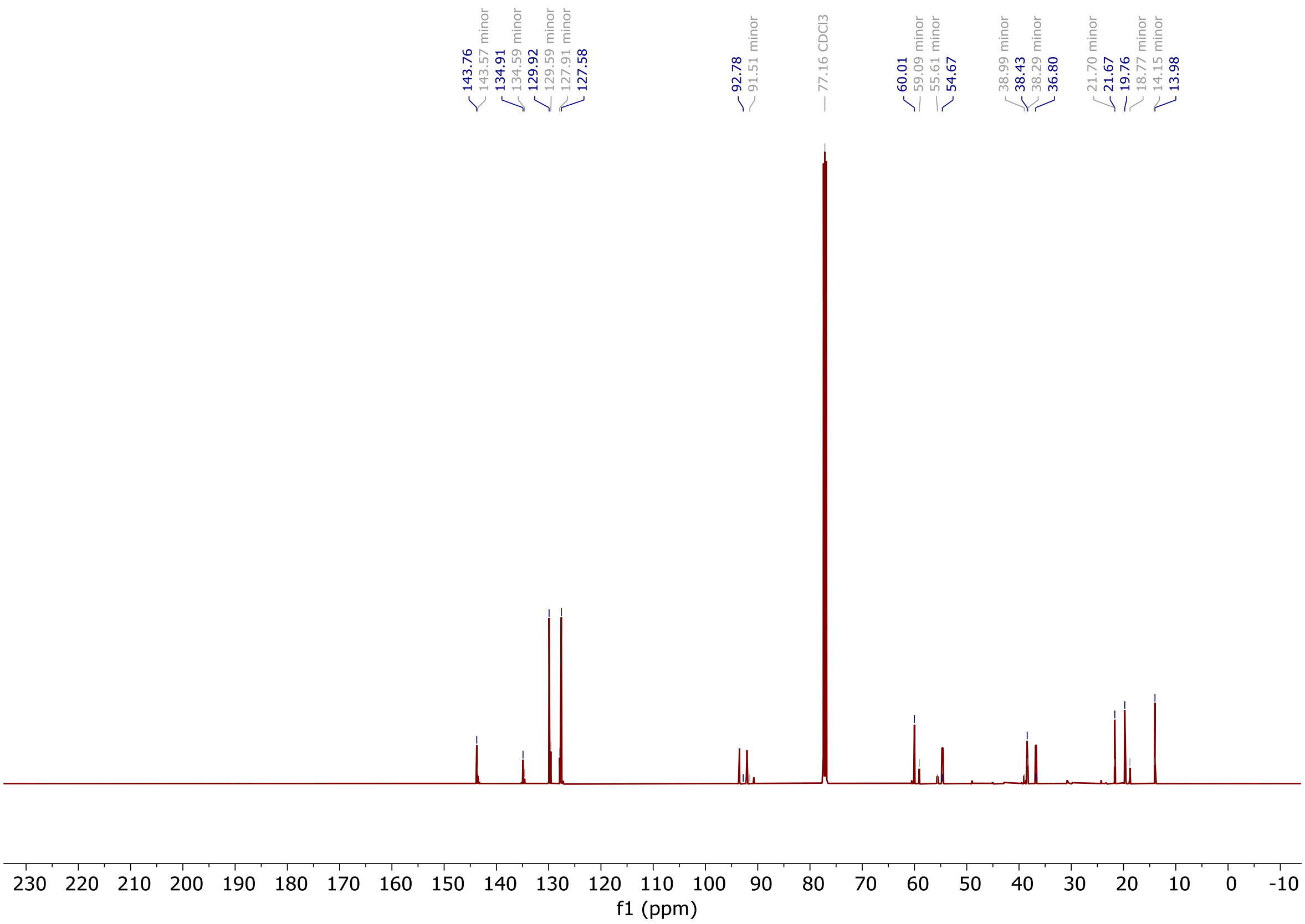

^{19}F NMR (376 MHz, $CDCl_3$)

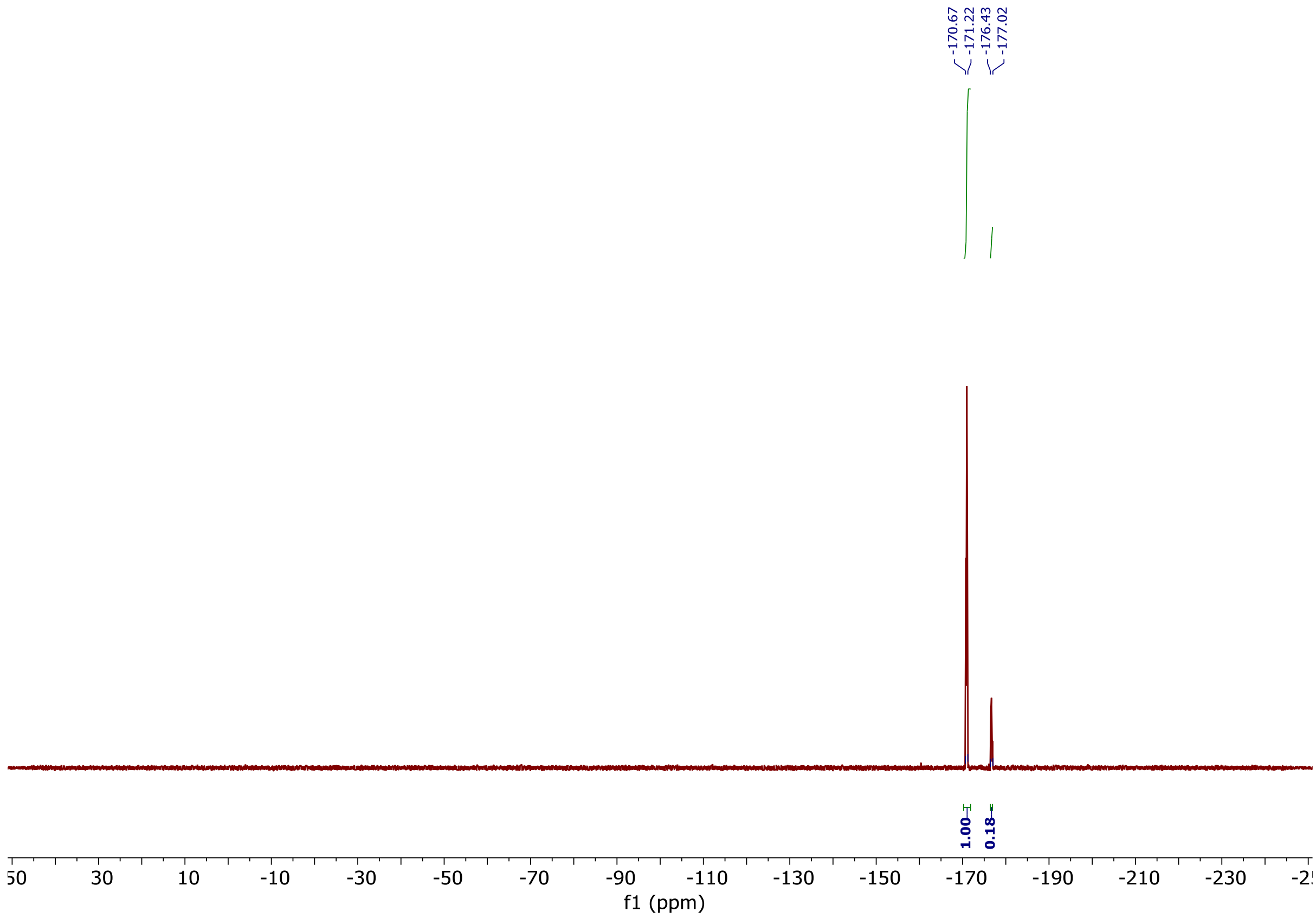

cis-4-fluoro-2-propylpyrrolidin-1-yl)(phenyl)methanone **(10w)**

^{1}H NMR (500 MHz, CDCl$_3$)

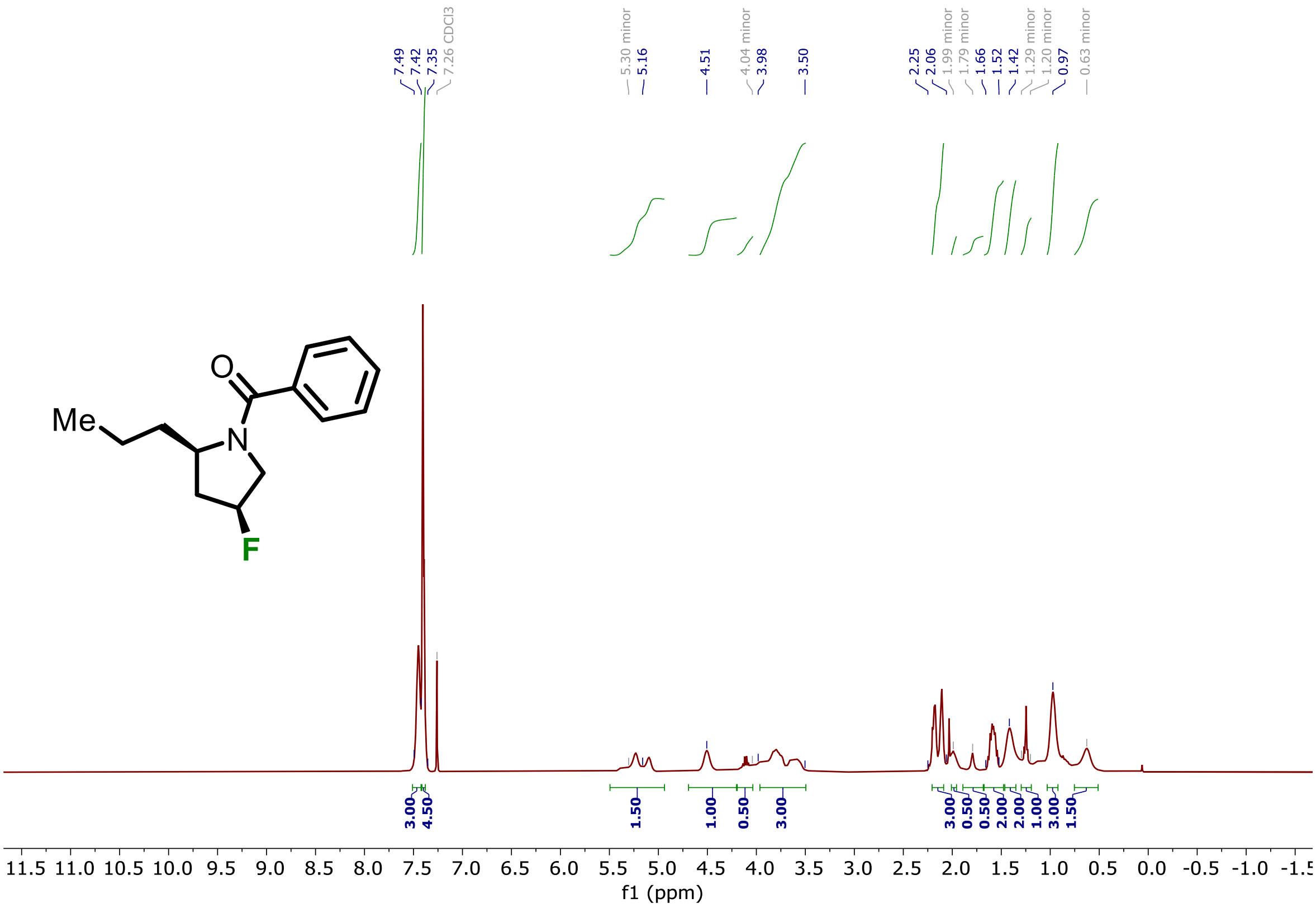

^{13}C NMR (126 MHz, CDCl$_3$)

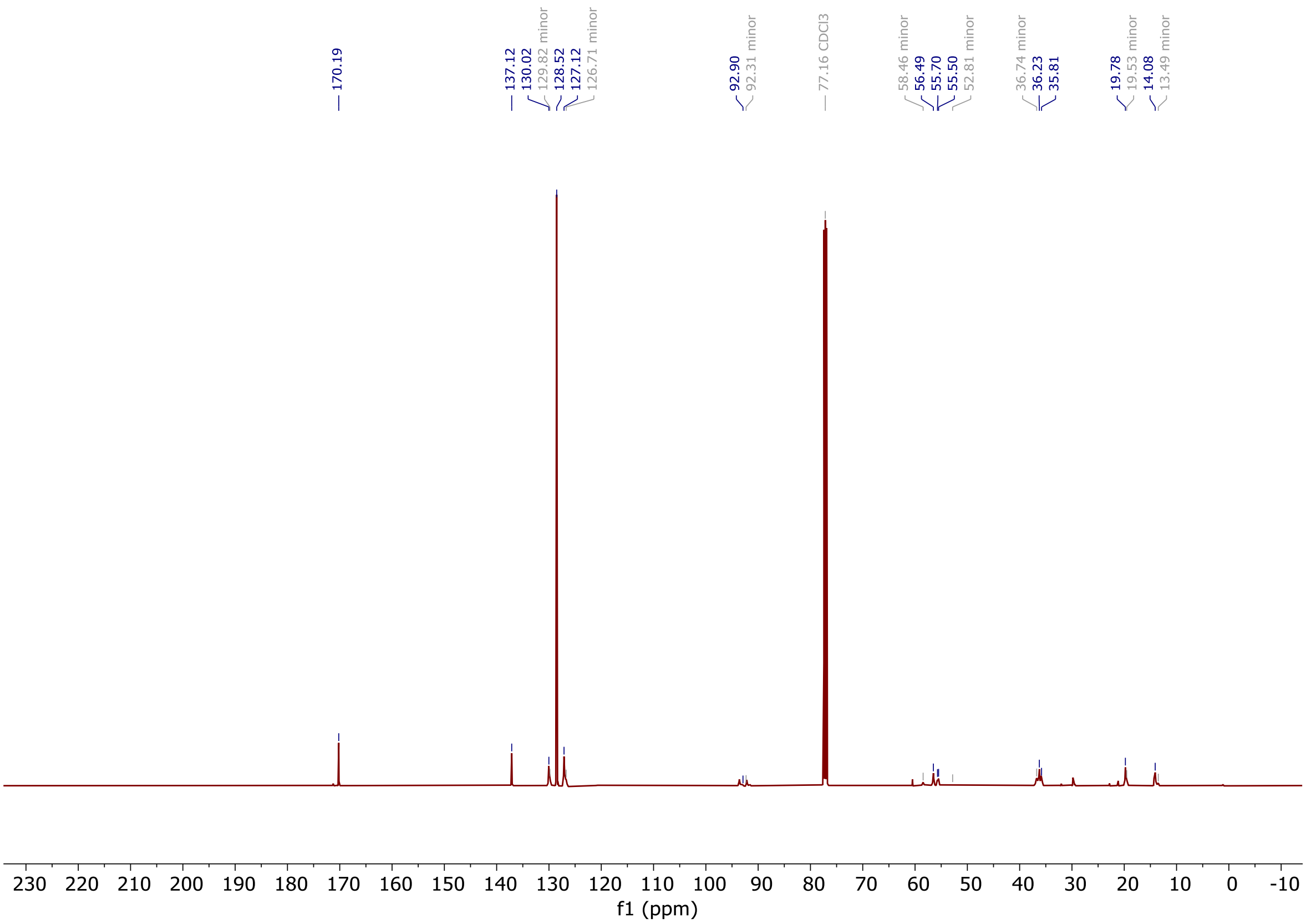

^{19}F NMR (376 MHz, CDCl$_3$)

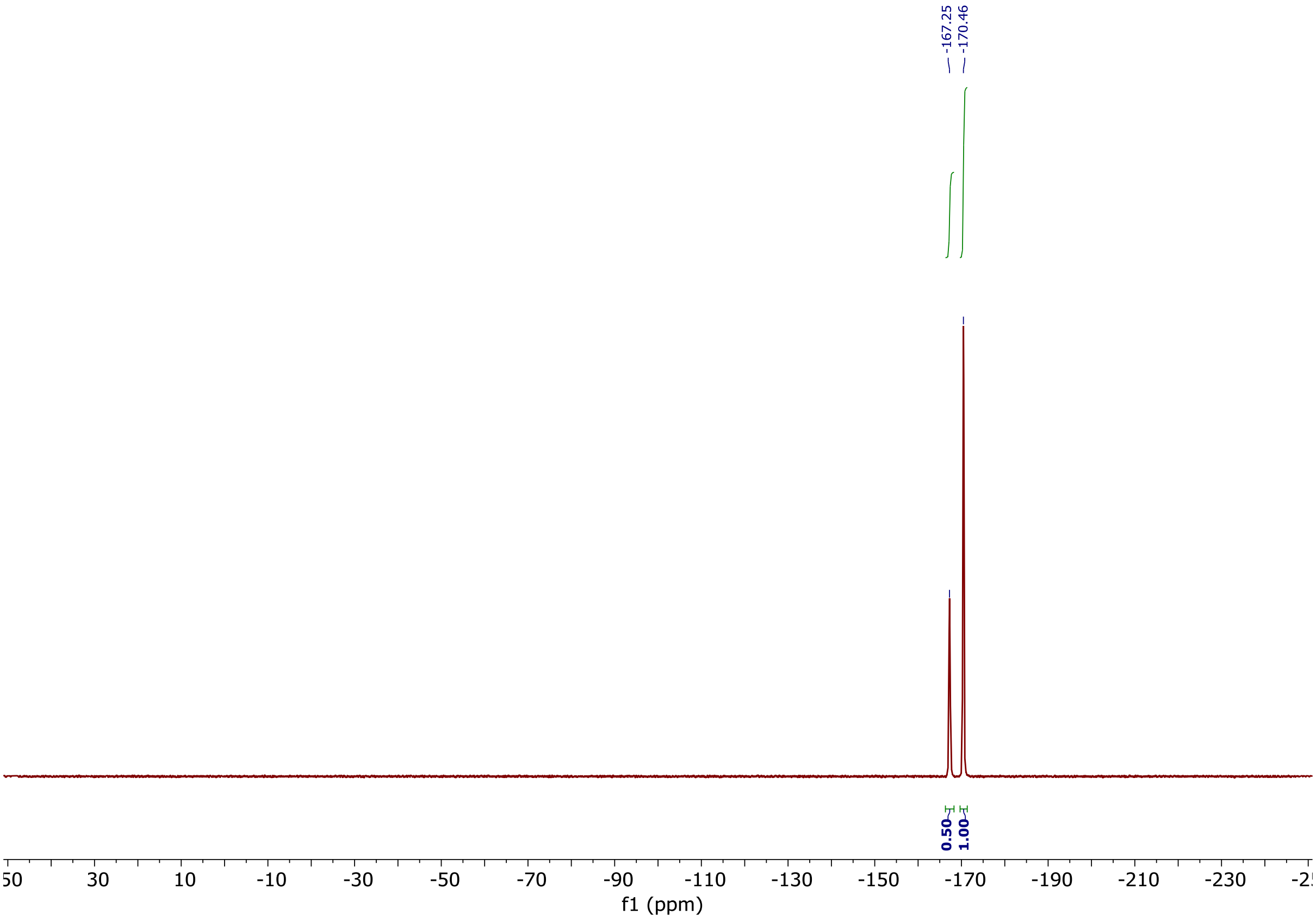

[100°C] ^{1}H NMR (500 MHz, DMSO-D_6)

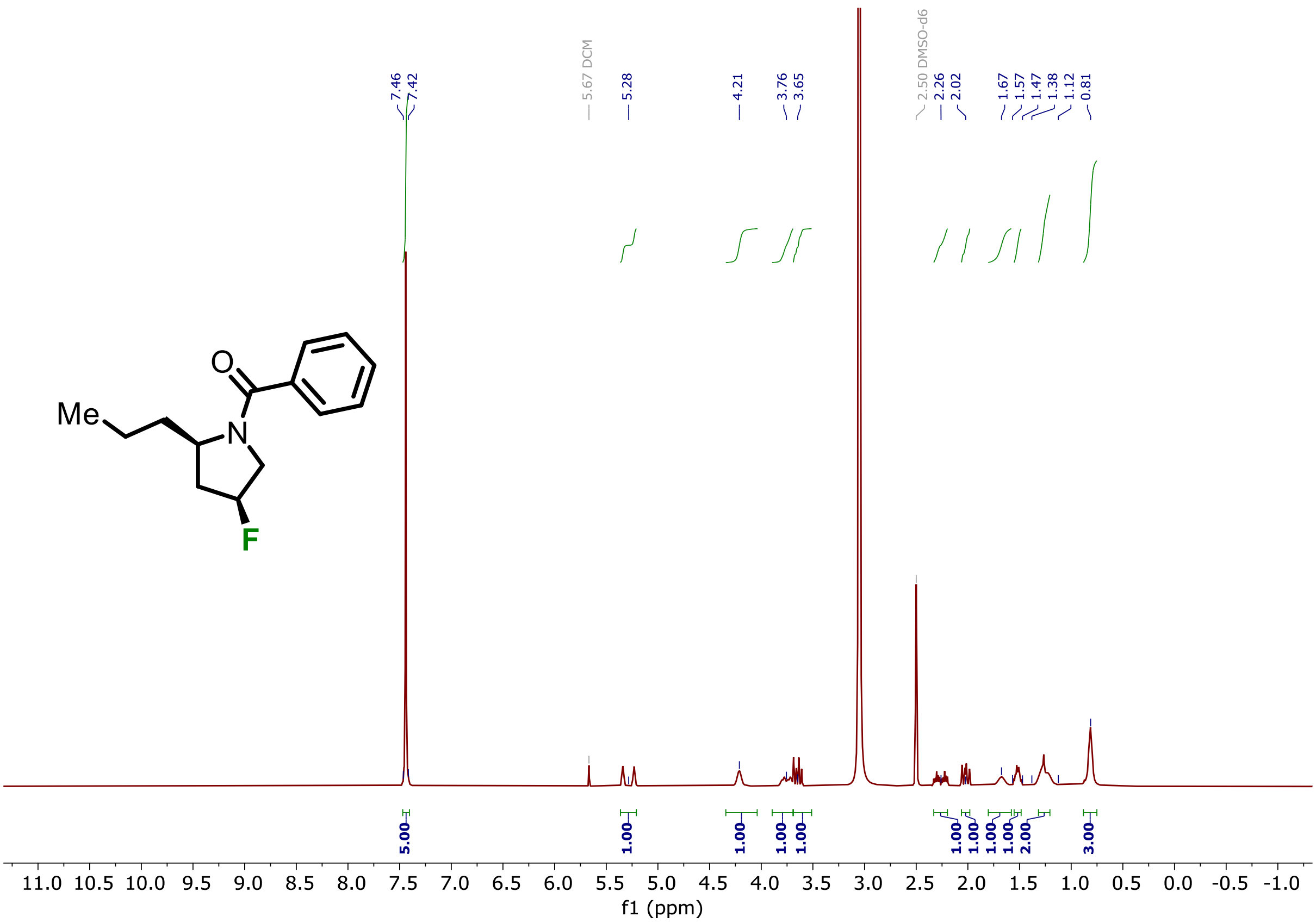

[100°C] ^{19}F NMR (470 MHz, DMSO-D_6)

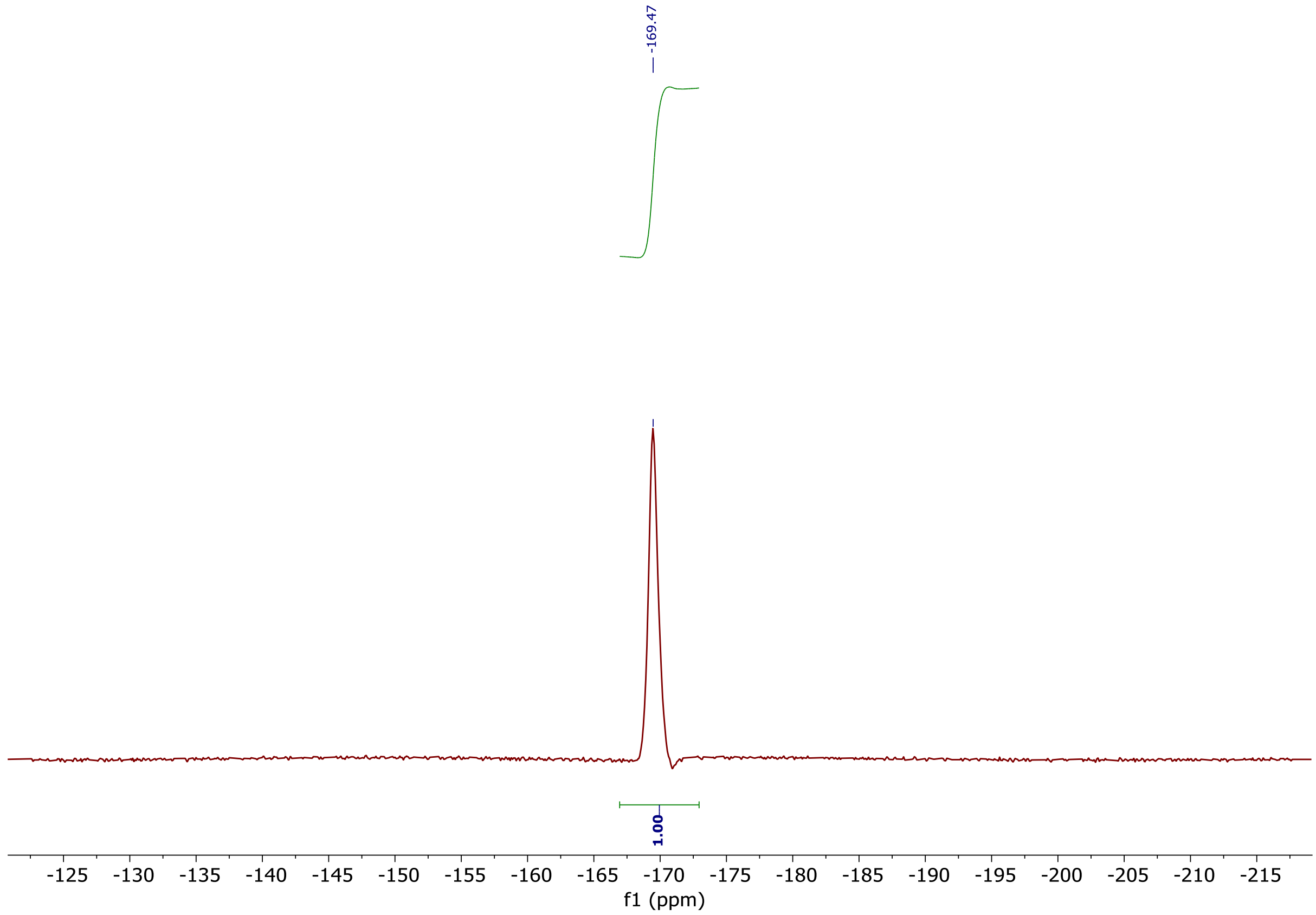

-169.47
1.00
-125 -130 -135 -140 -145 -150 -155 -160 -165 -170 -175 -180 -185 -190 -195 -200 -205 -210 -215
f1 (ppm)

9.10. Oxolanes (11)

***3-fluoro-8-tosyl-1-oxa-8-azaspiro[4.5]decane* (11ap)**

^{1}H NMR (500 MHz, CDCl$_3$)

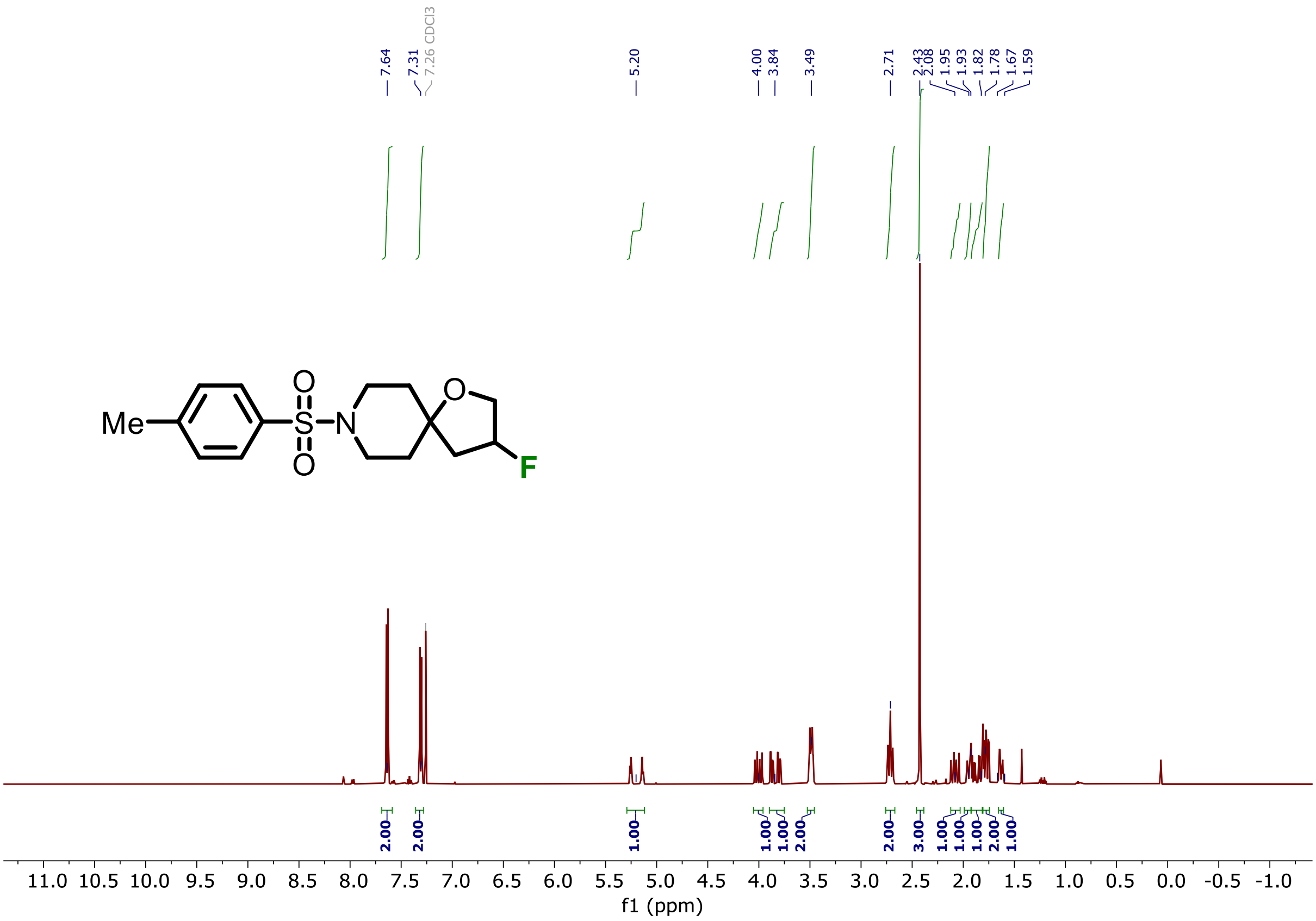

^{13}C NMR (126 MHz, $CDCl_3$)

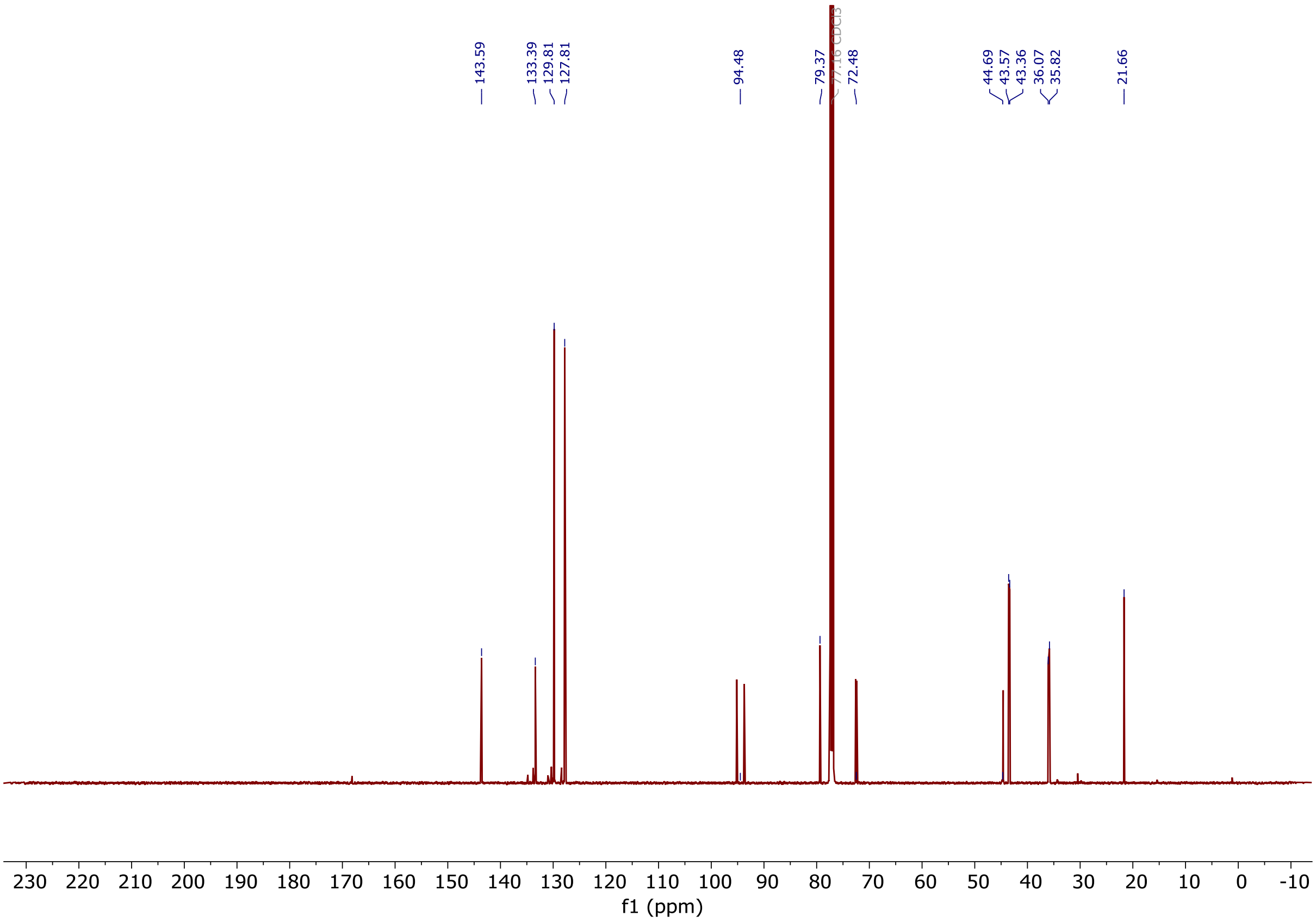

^{19}F NMR (376 MHz, $CDCl_3$)

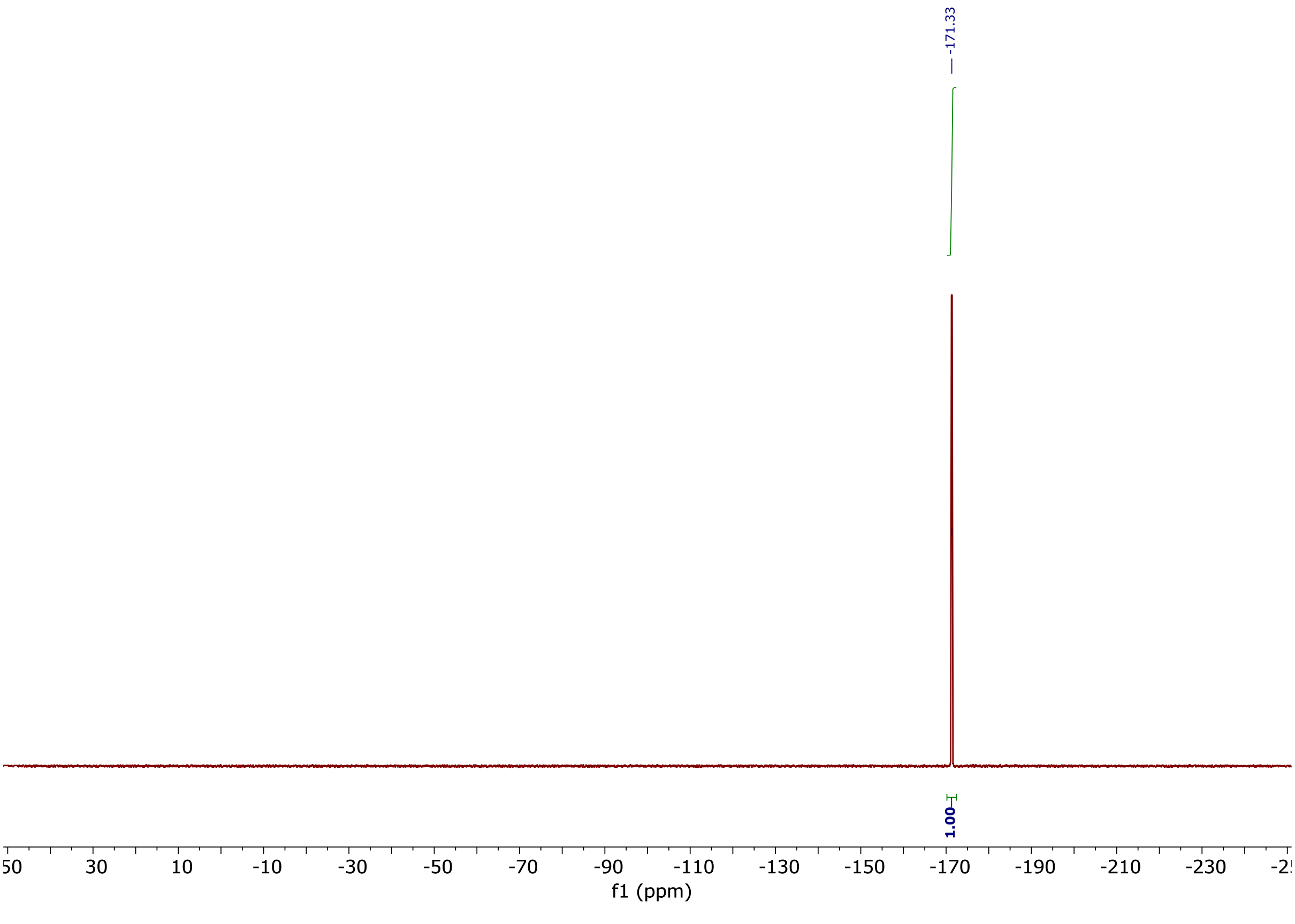

9.11. Azetidines (12)

***3-fluoro-1-tosylazetidine* (12ar)**

^{1}H NMR (500 MHz, $CDCl_3$)

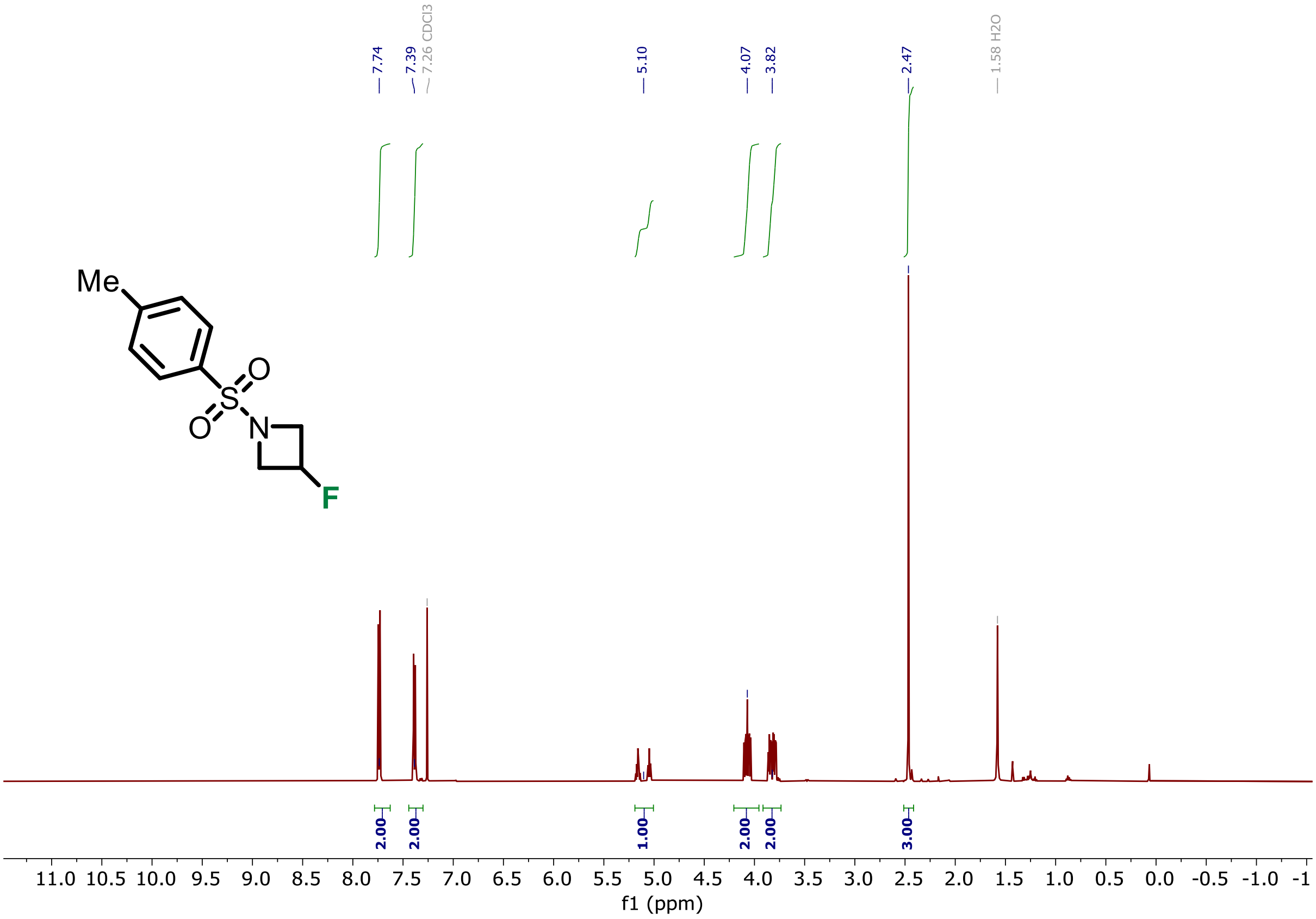

^{13}C NMR (126 MHz, CDCl$_3$)

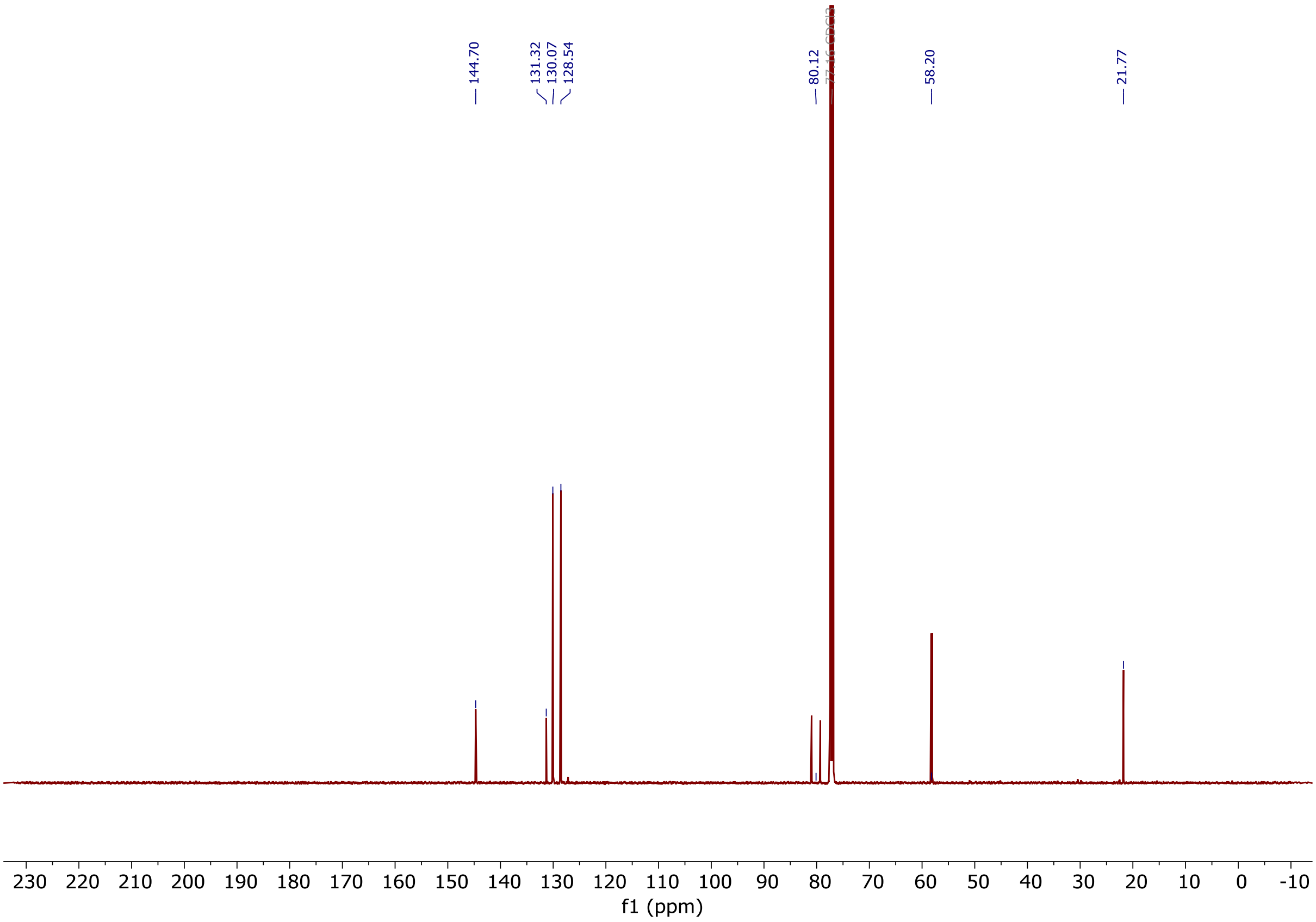

^{19}F NMR (376 MHz, CDCl$_3$)

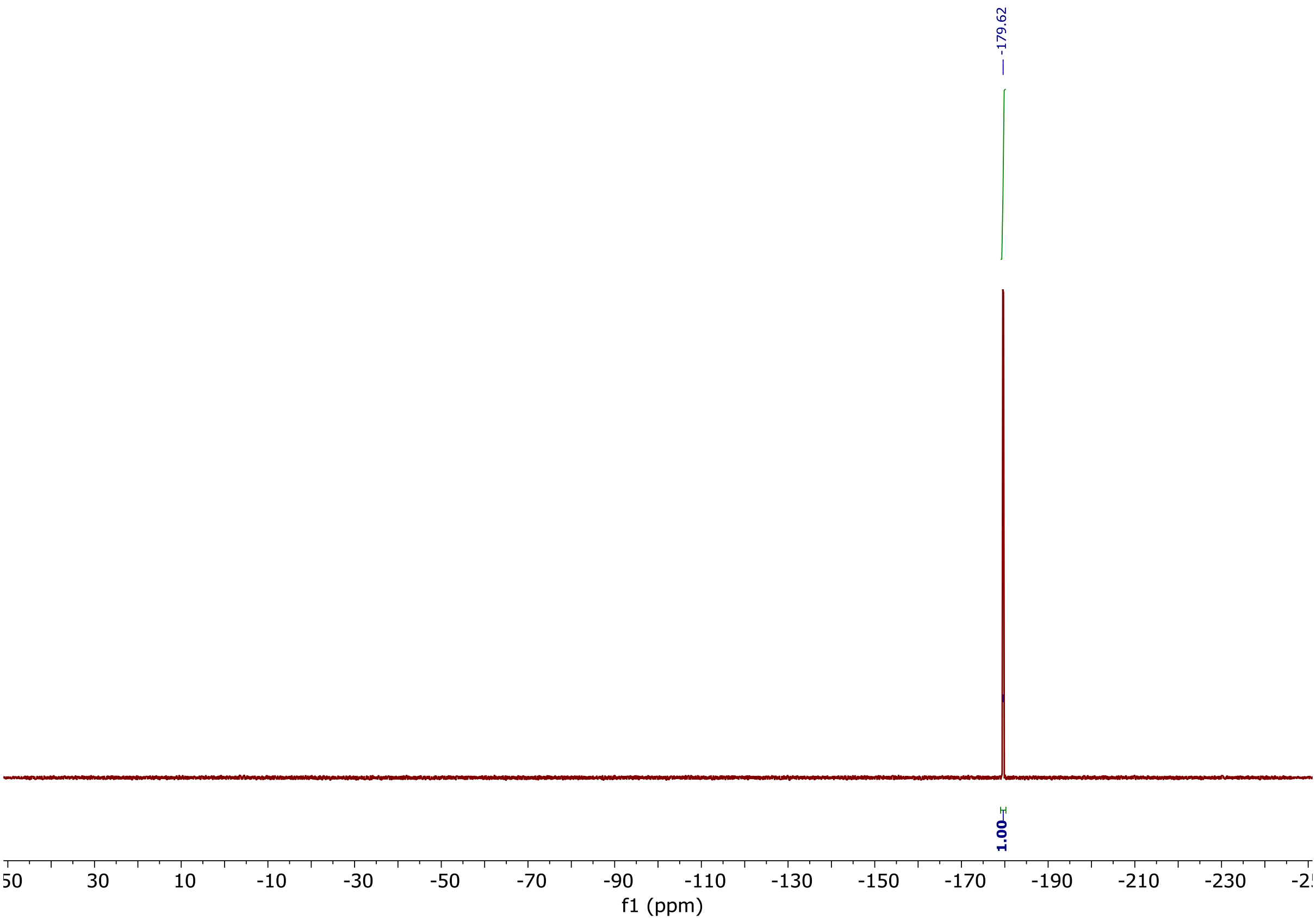

***3-fluoro-1-tosyl-1-azaspiro[3.5]nonane* (12av)**

^{1}H NMR (500 MHz, $CDCl_3$) (30 mol% 4-methylanisole)

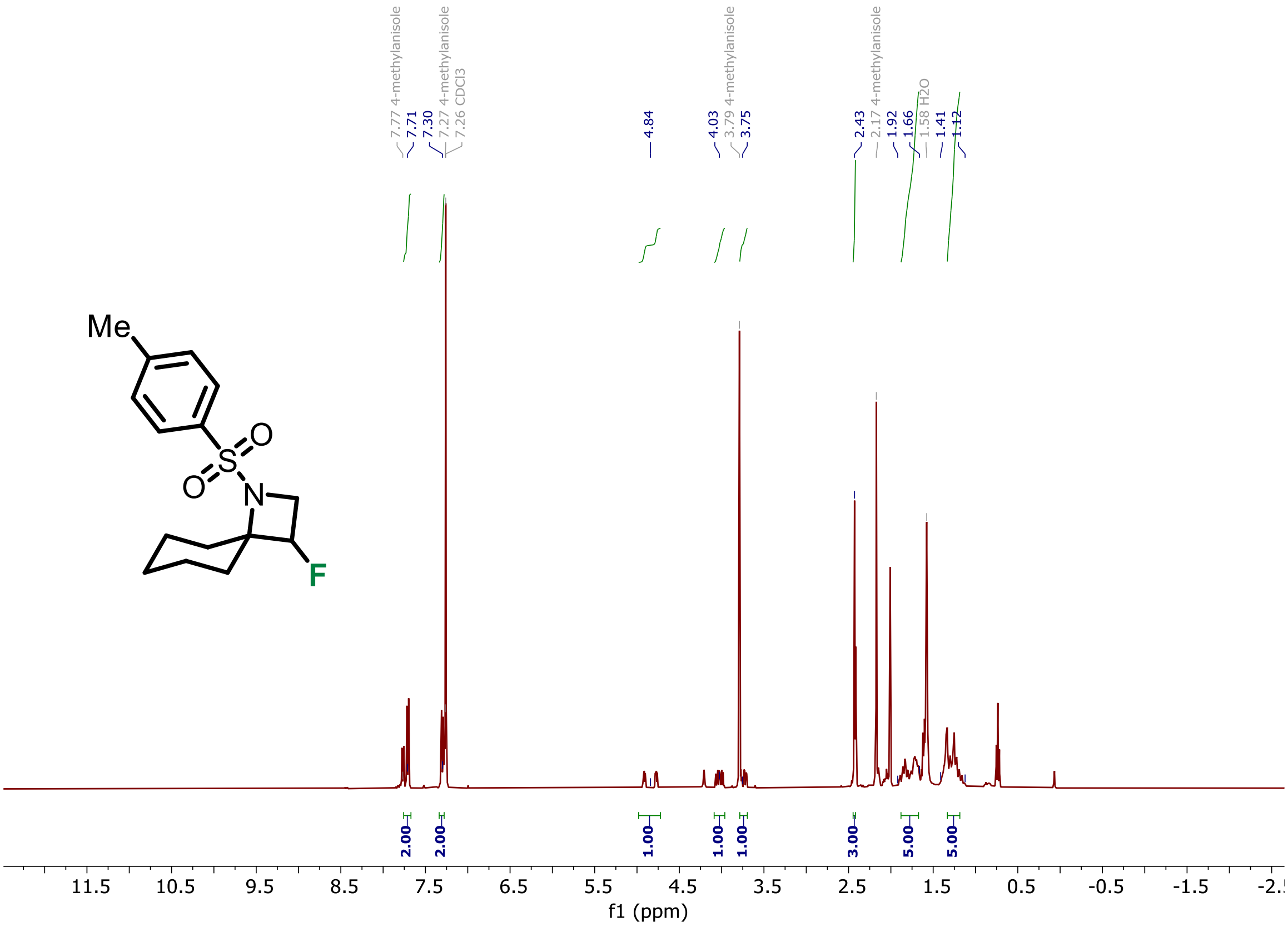

^{1}H NMR (500 MHz, $CDCl_3$) (54 mol% ring-opened product **12au'**)

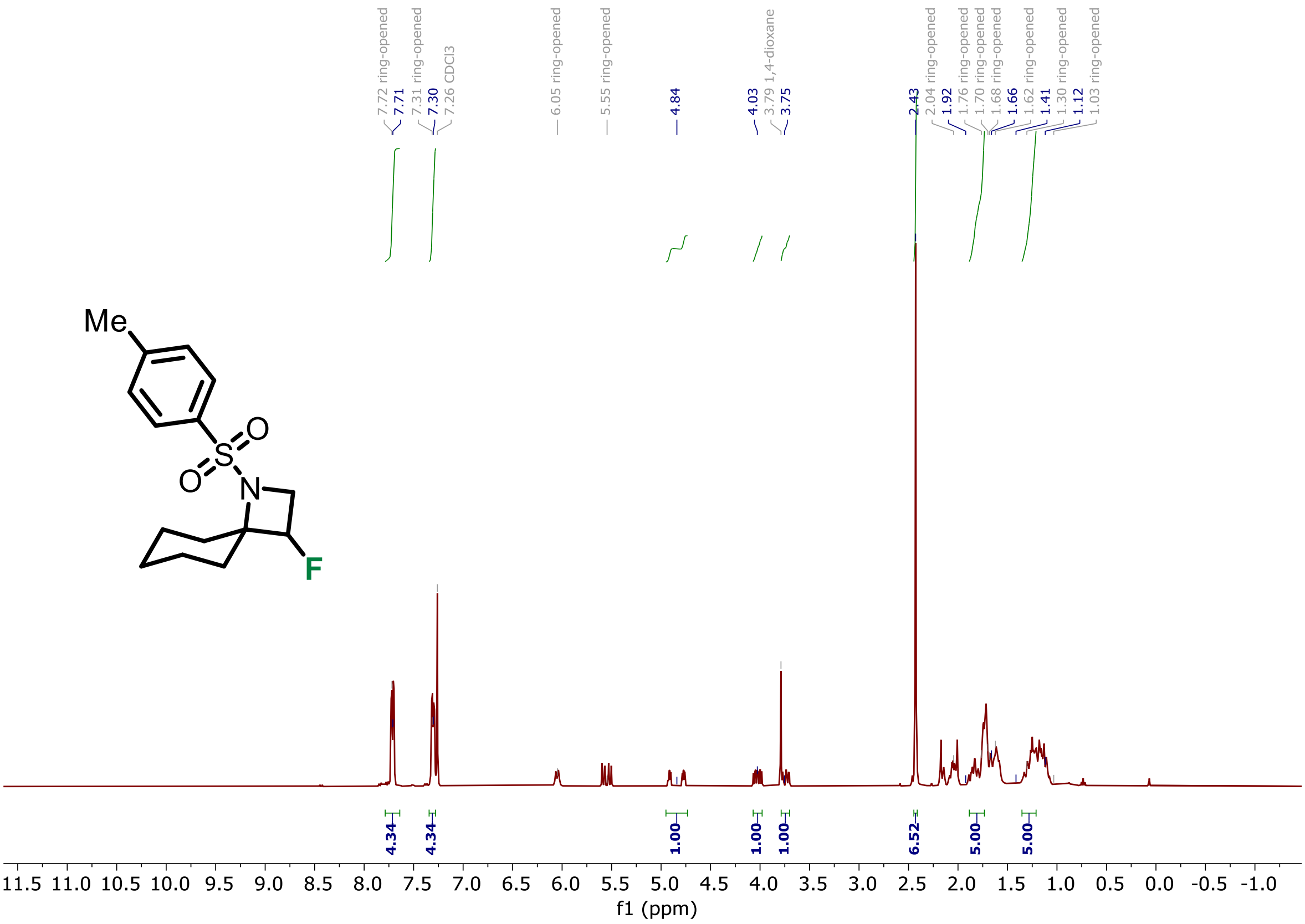

^{13}C NMR (126 MHz, $CDCl_3$)

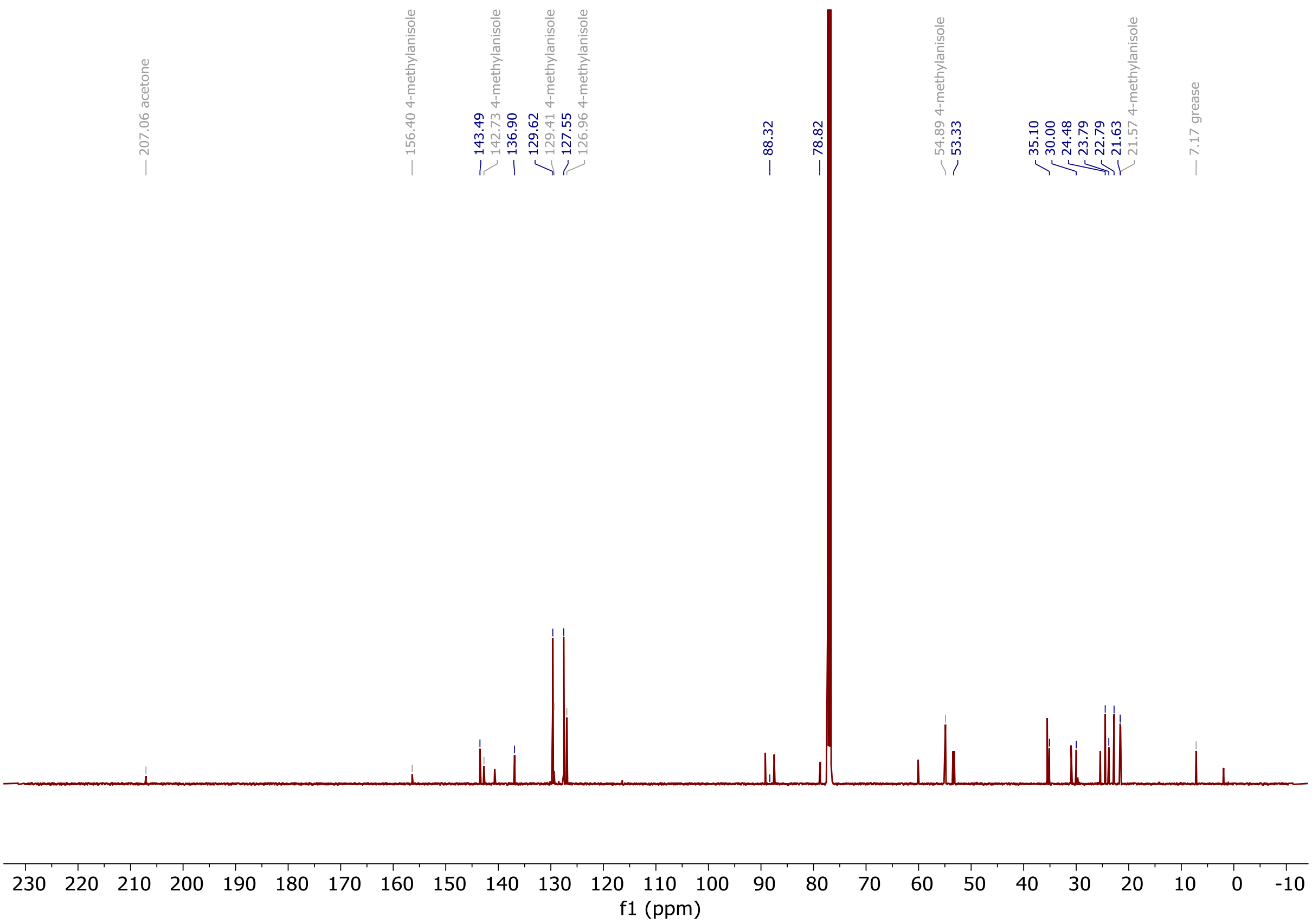

^{19}F NMR (376 MHz, $CDCl_3$)

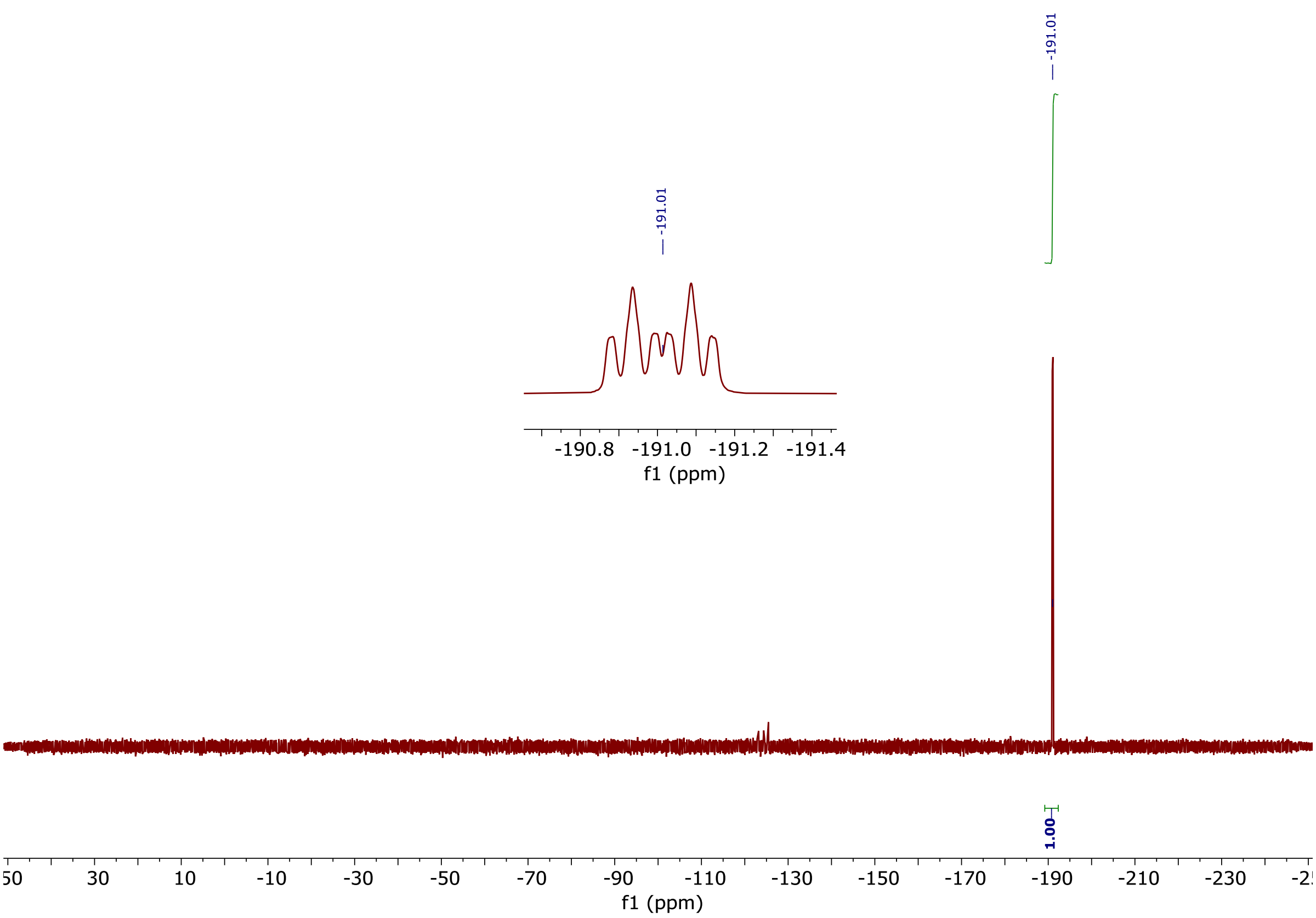

***(ring-opened) (E)-N-(2-cyclohexyl-2-fluorovinyl)-4-methylbenzenesulfonamide* (12av')**

^{1}H NMR (500 MHz, CDCl$_3$)

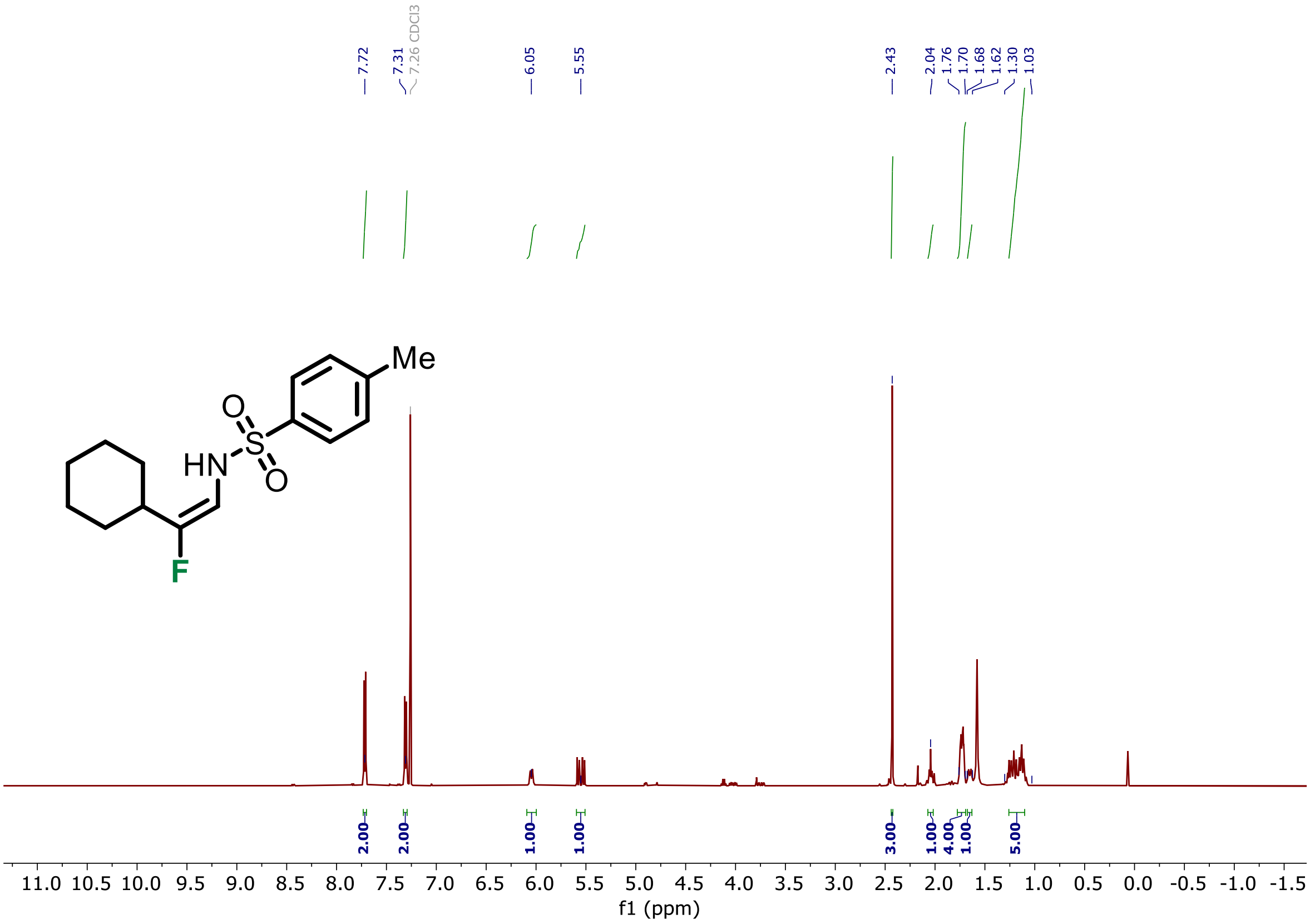

^{13}C NMR (126 MHz, CDCl$_3$)

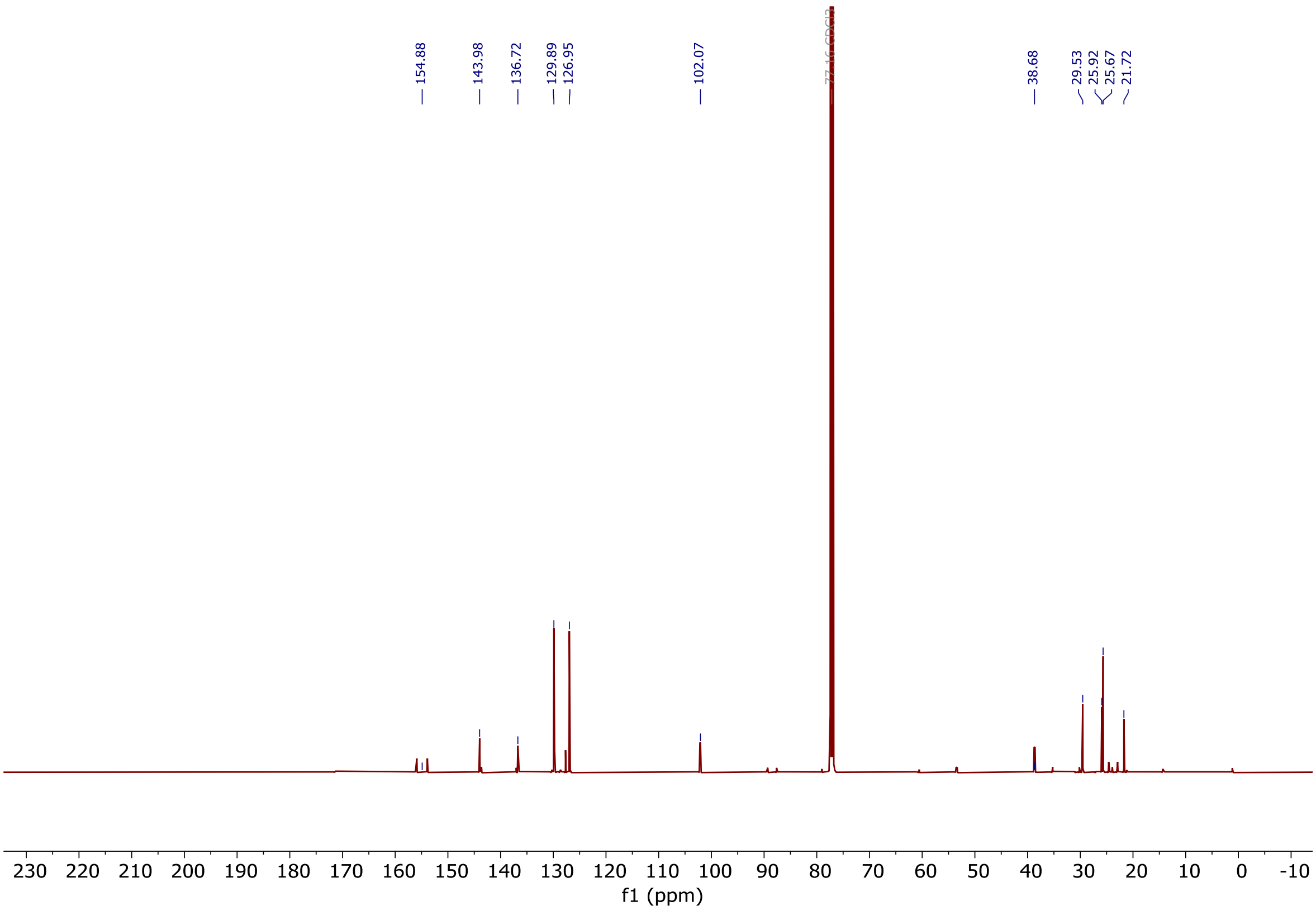

^{19}F NMR (376 MHz, $CDCl_3$)

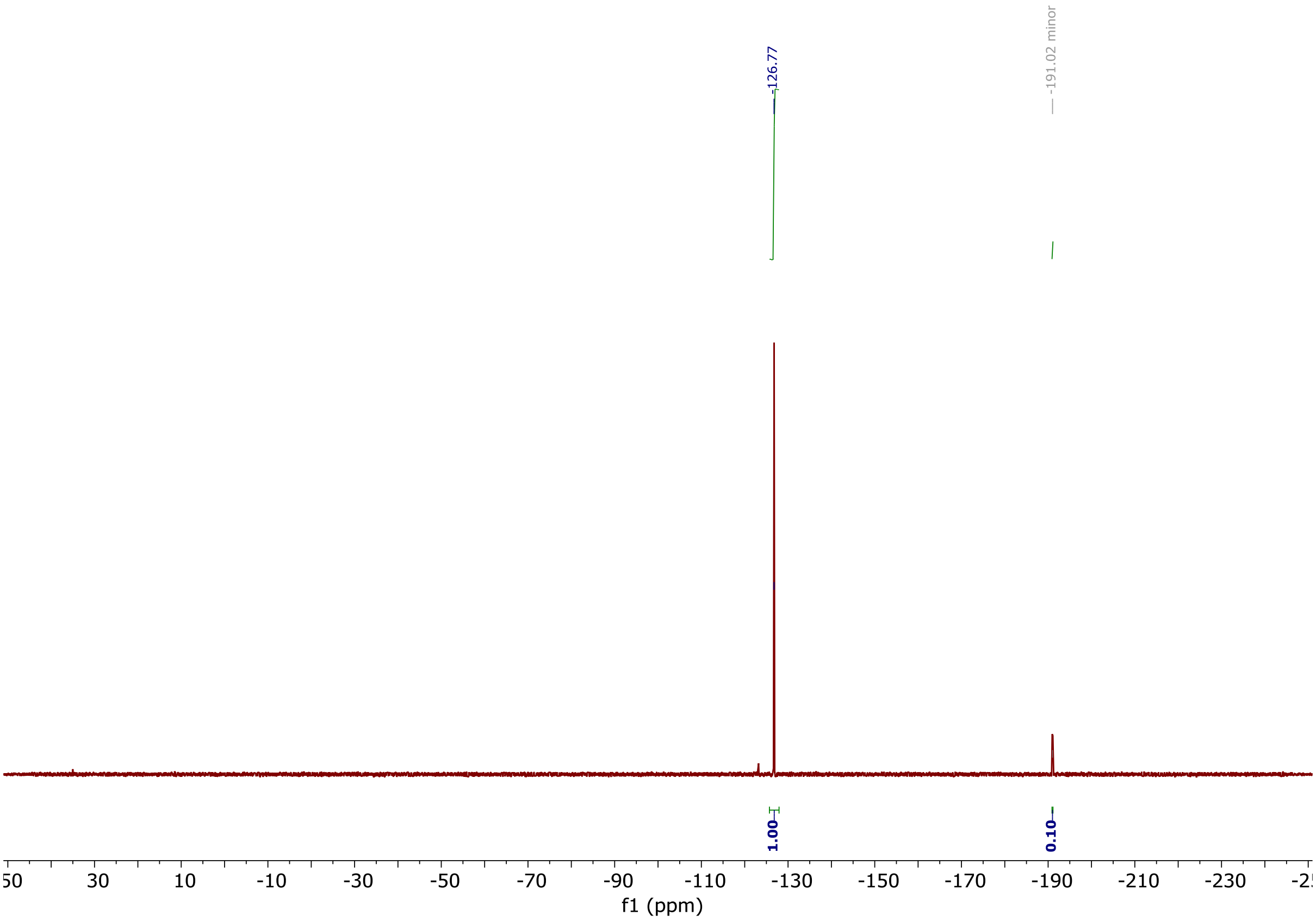

***3-fluoro-2,2-dimethyl-1-tosylazetidine* (12au)**

^{1}H NMR (500 MHz, $CDCl_3$)

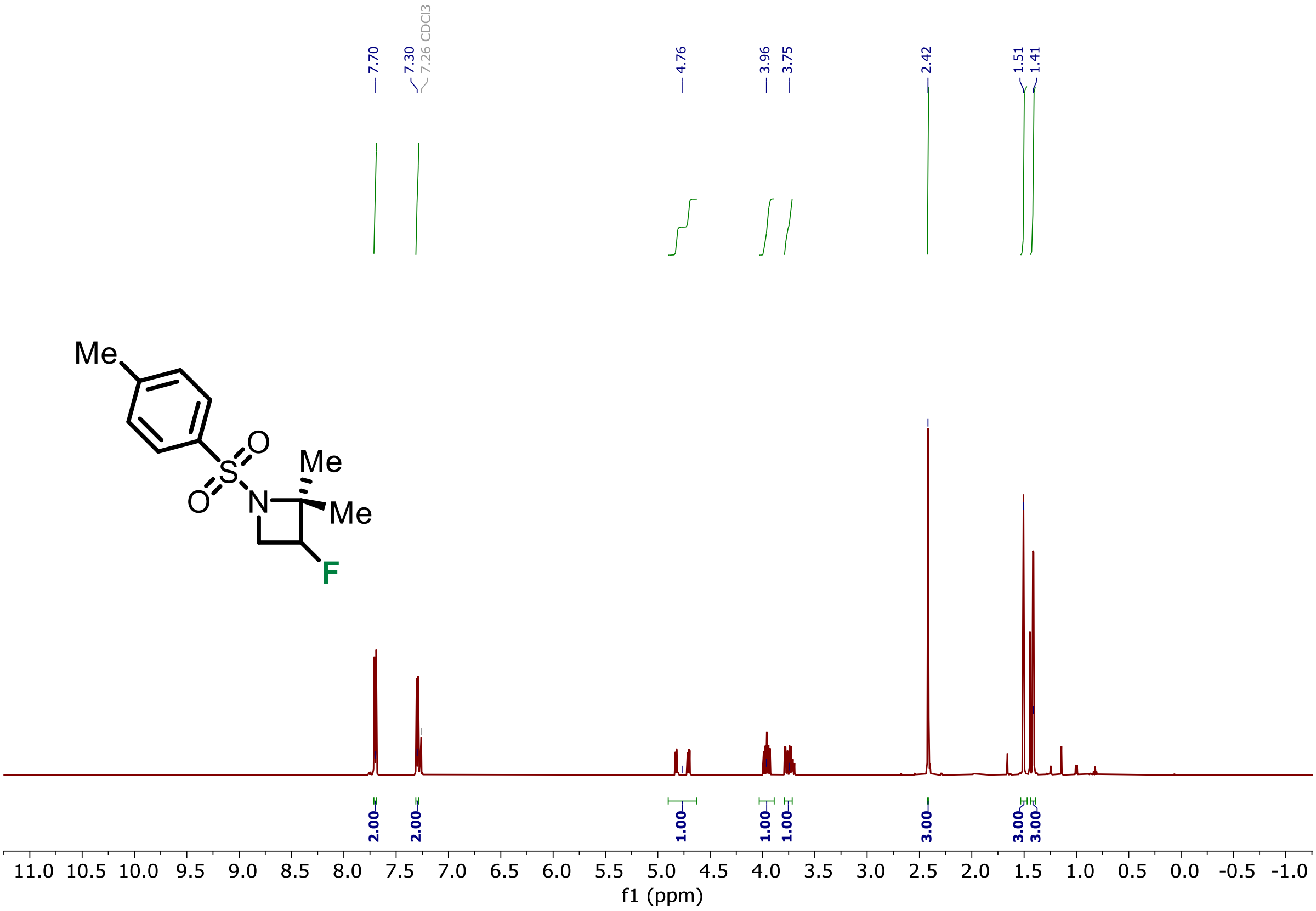

^{13}C NMR (126 MHz, $CDCl_3$)

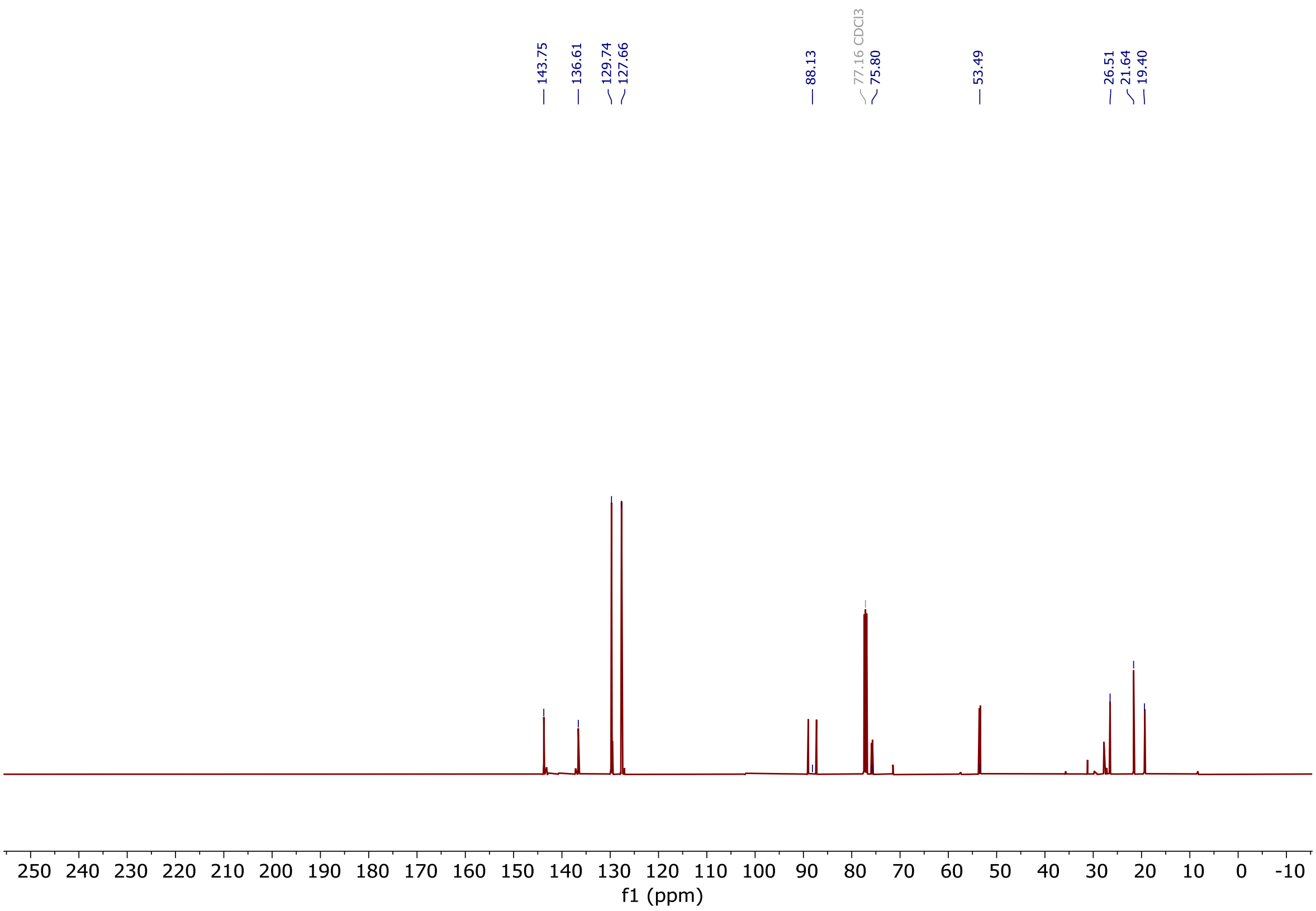

^{19}F NMR (376 MHz, $CDCl_3$)

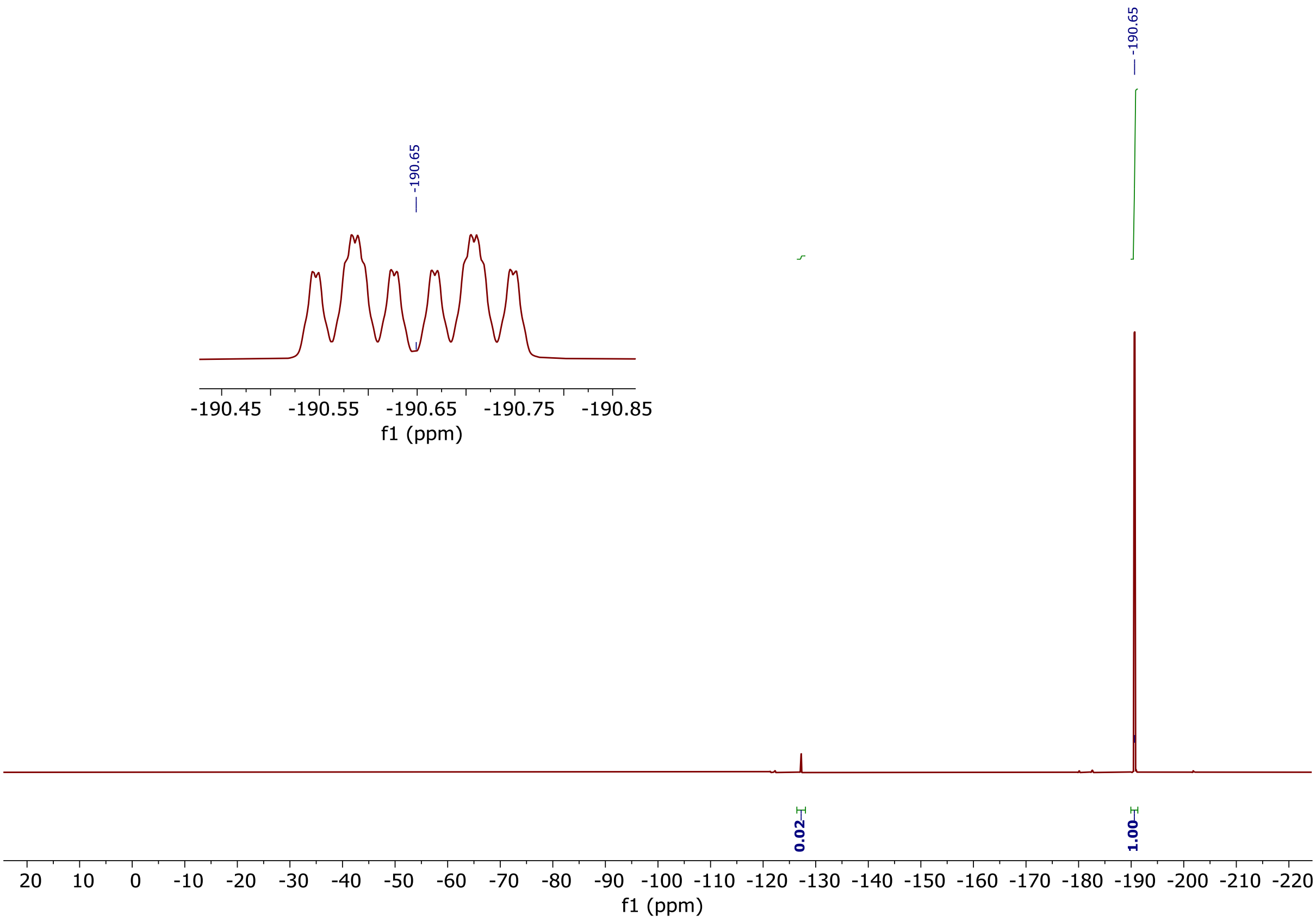

cis-3-fluoro-2-phenyl-1-tosylazetidine **(12aw)**

^{1}H NMR (500 MHz, $CDCl_3$)

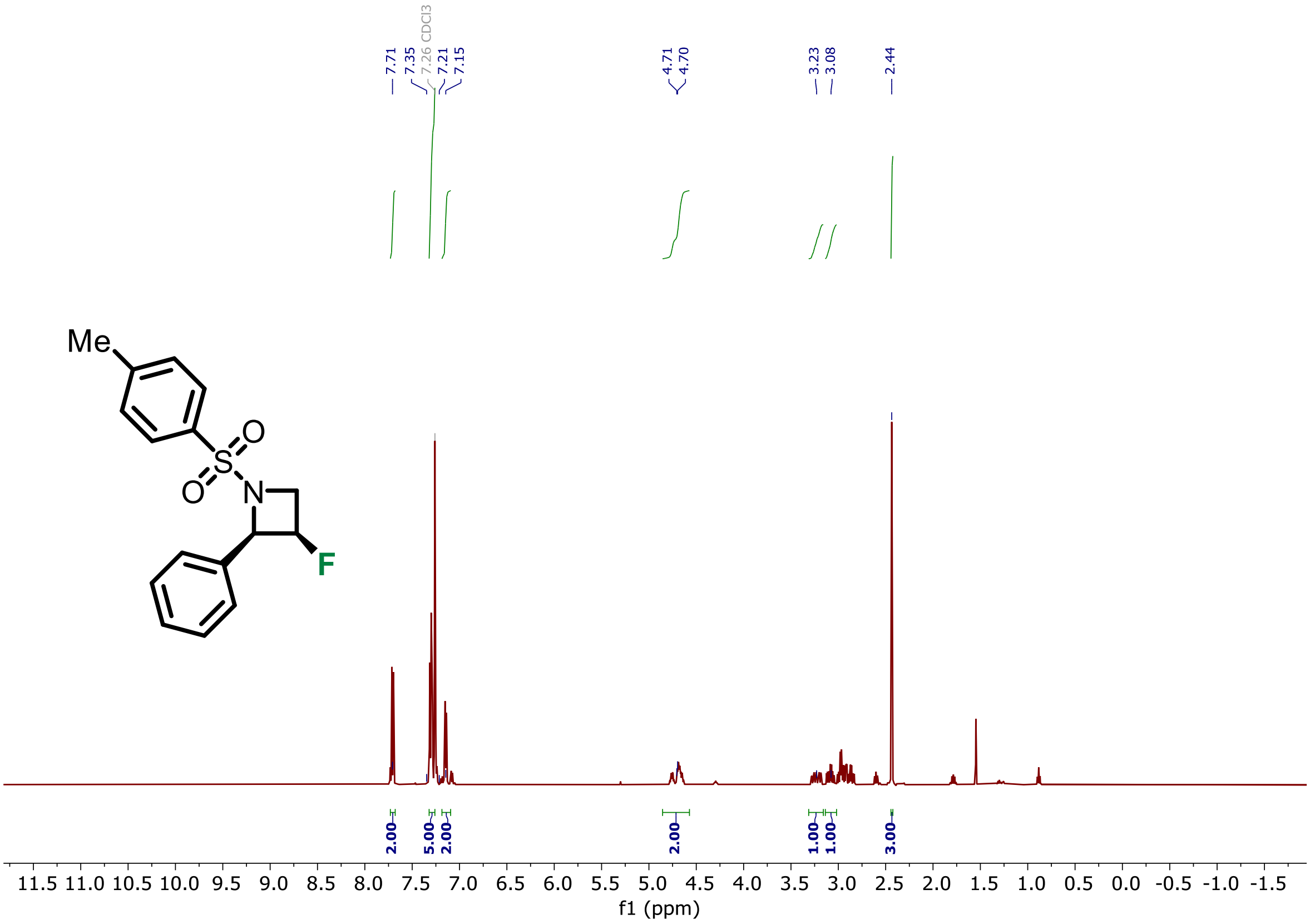

^{13}C NMR (126 MHz, $CDCl_3$)

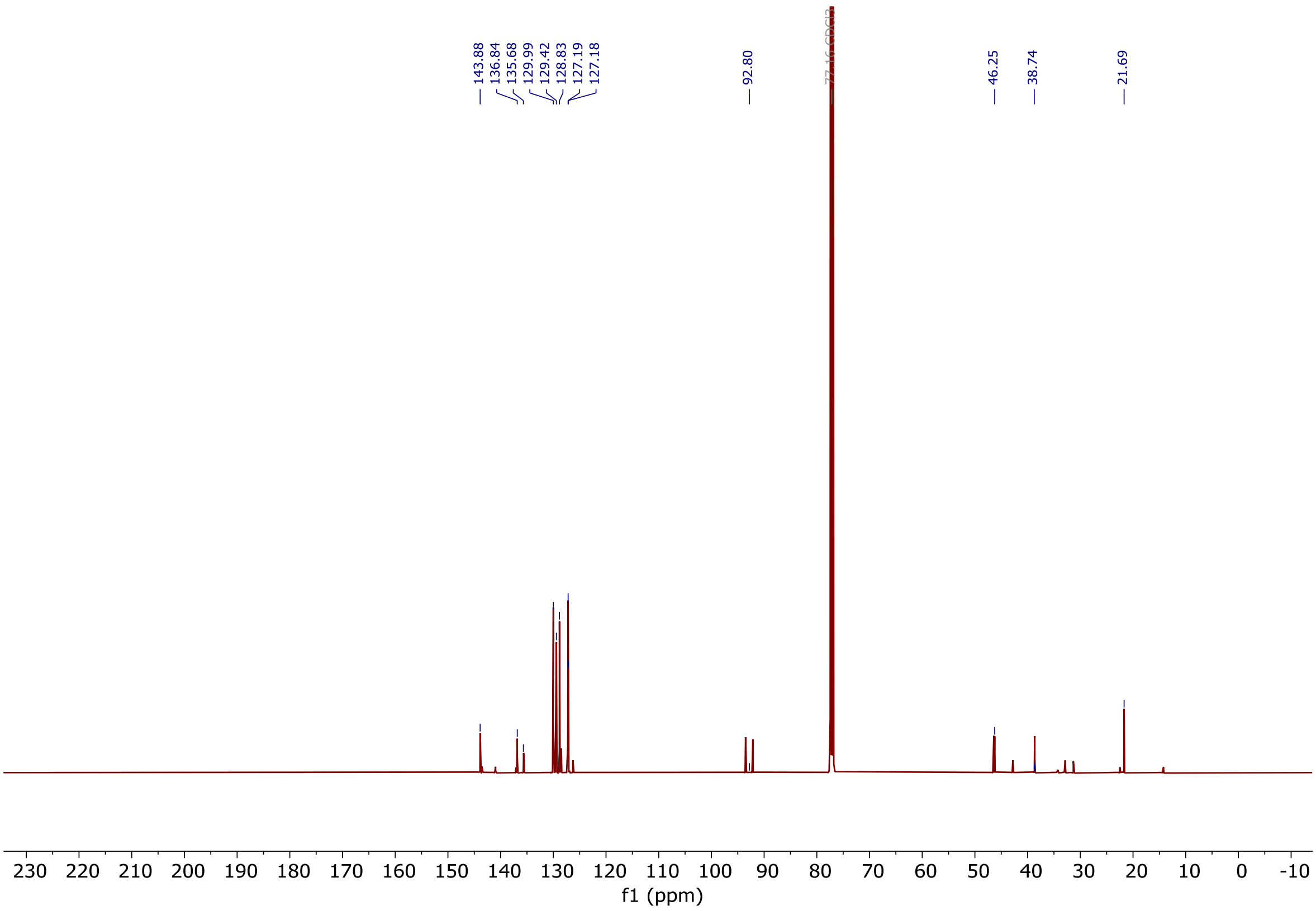

^{19}F NMR (376 MHz, $CDCl_3$)

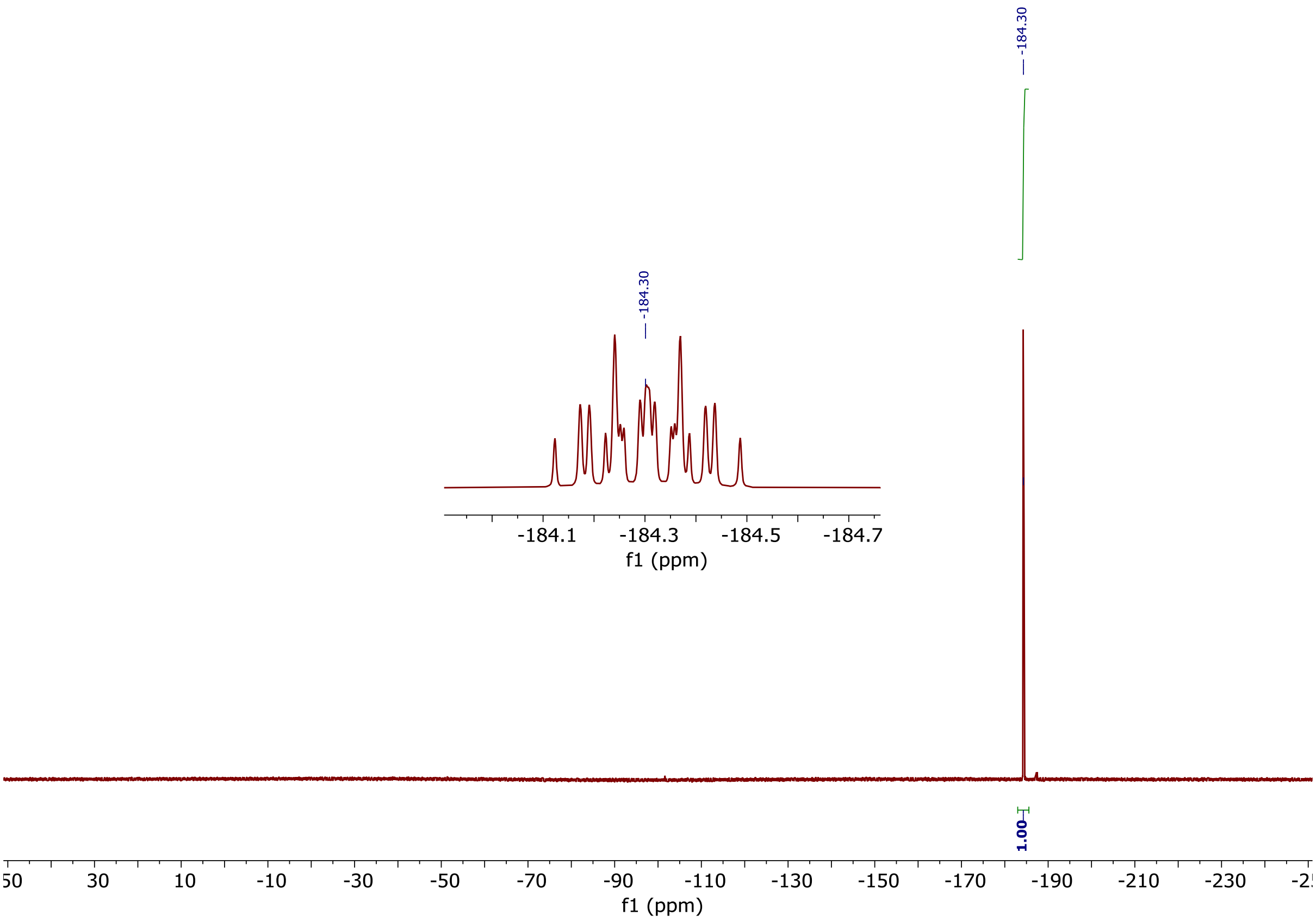

9.12. Dibromides (13-14)

***2-benzyl-3,4-dibromo-4-fluoro-1-tosylpyrrolidine* (13t)**

^{1}H NMR (500 MHz, CDCl$_3$)

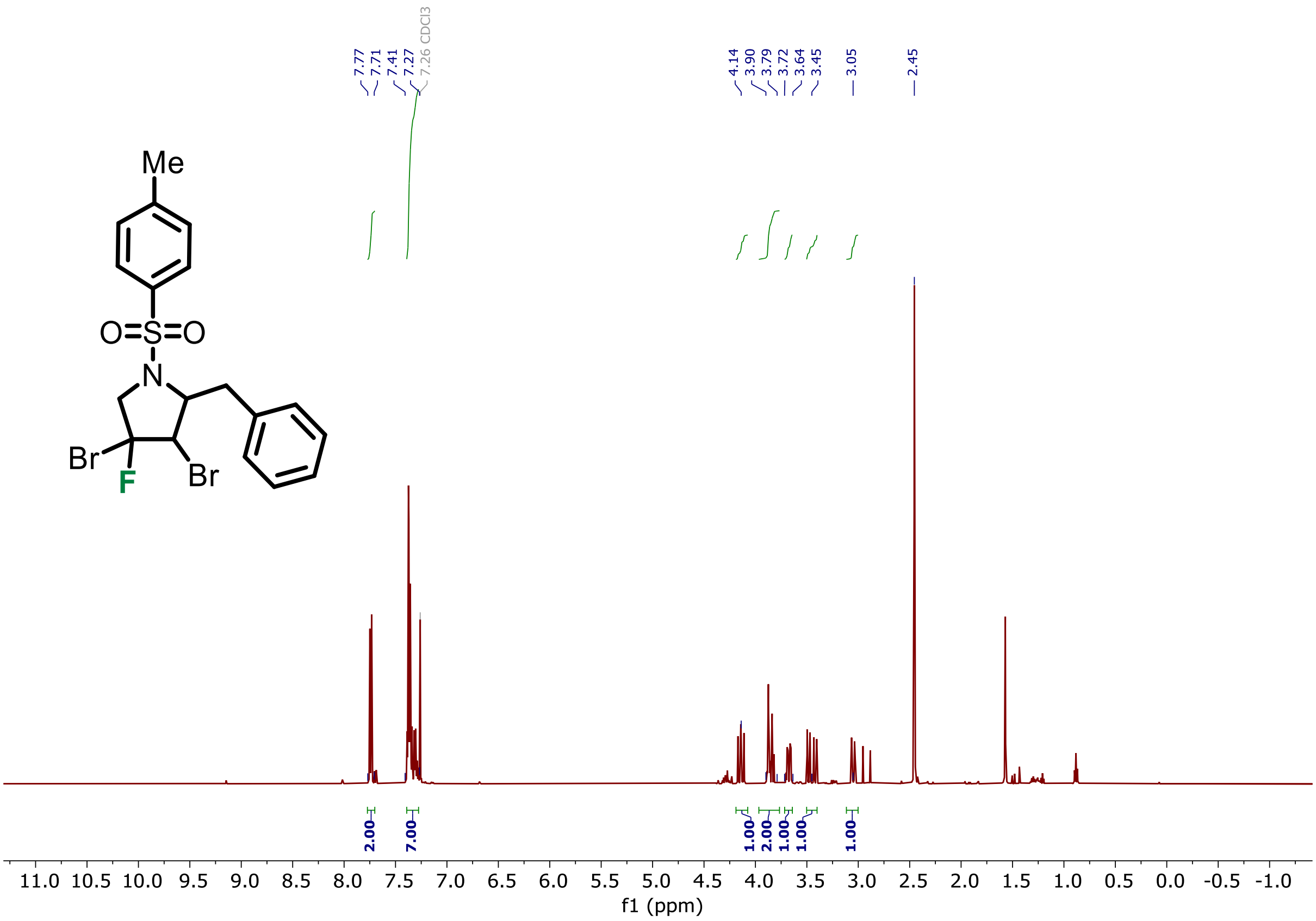

^{13}C NMR (126 MHz, $CDCl_3$)

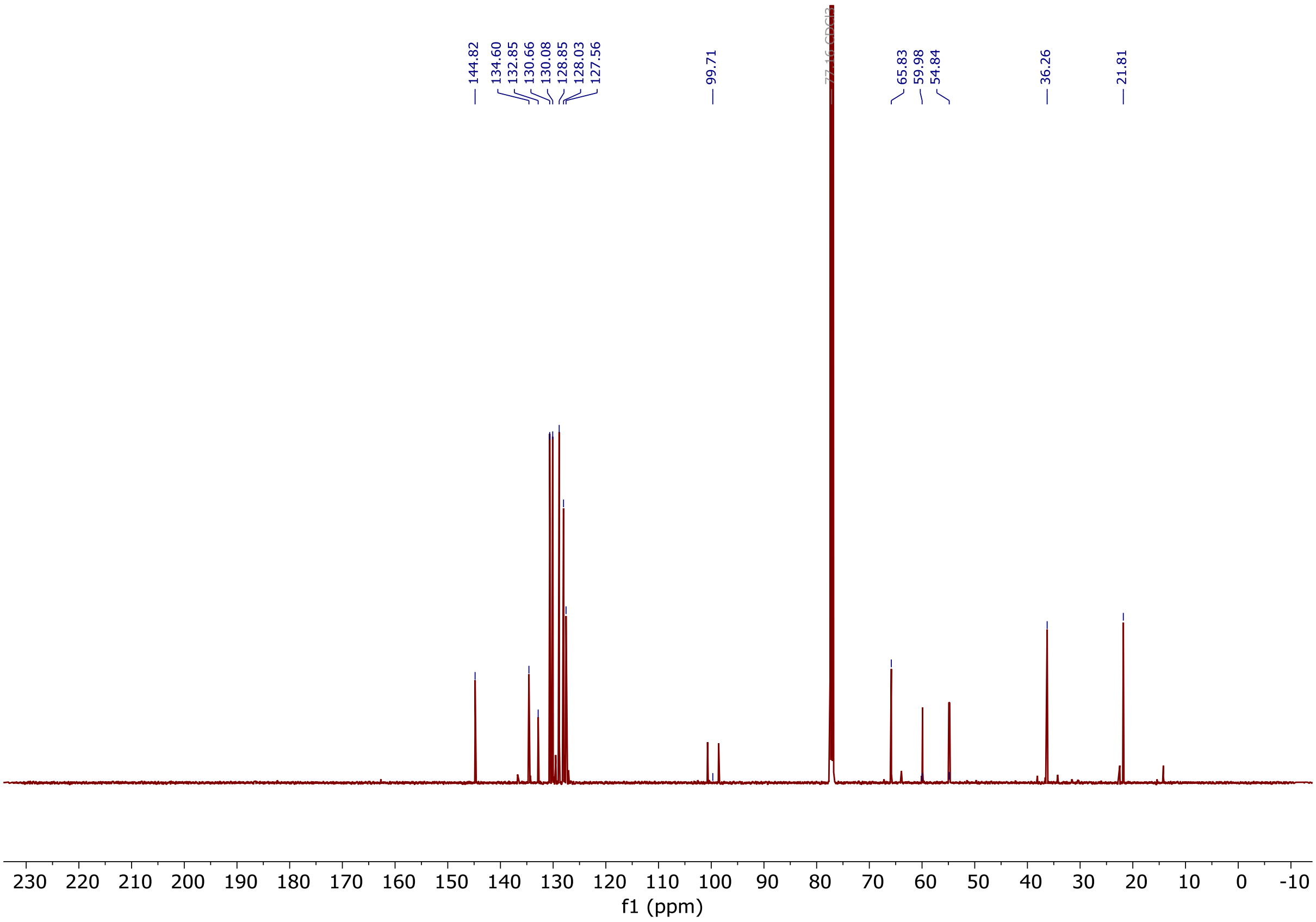

^{19}F NMR (377 MHz, $CDCl_3$)

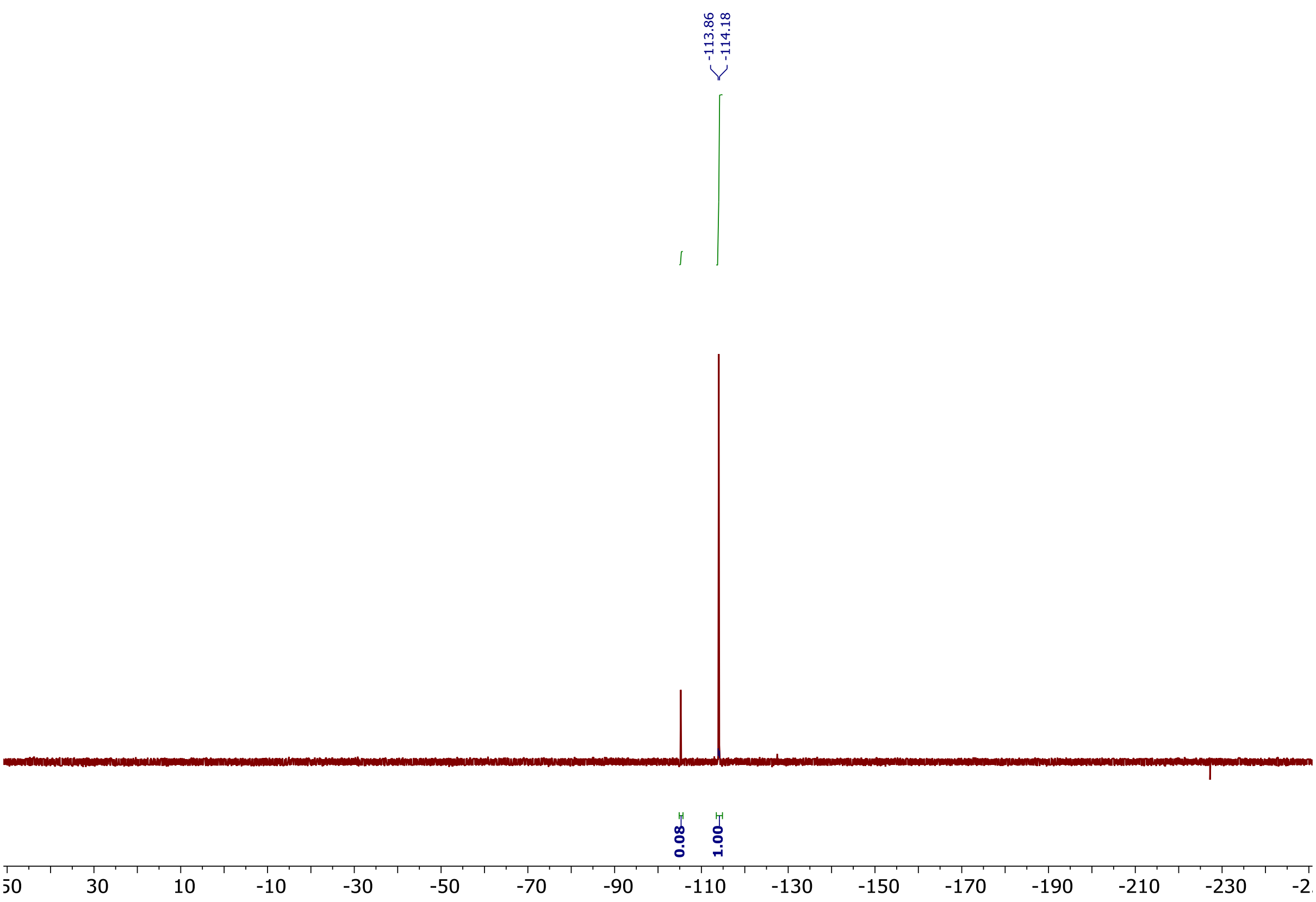

2,3-dibromo-3-fluoro-1-tosylazetidine **(14ar)**

^{1}H NMR (500 MHz, CDCl$_3$)

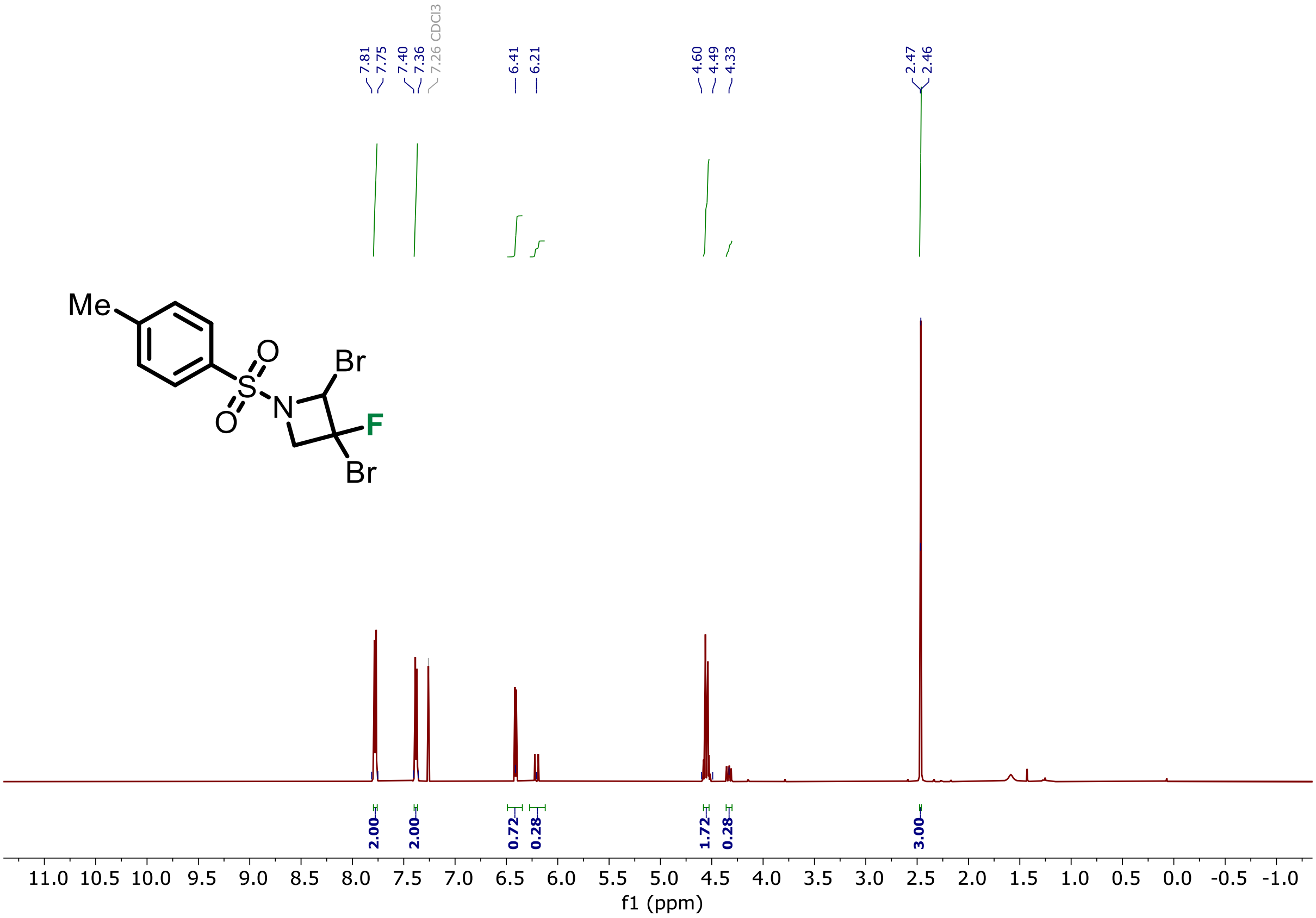

^{13}C NMR (126 MHz, CDCl$_3$)

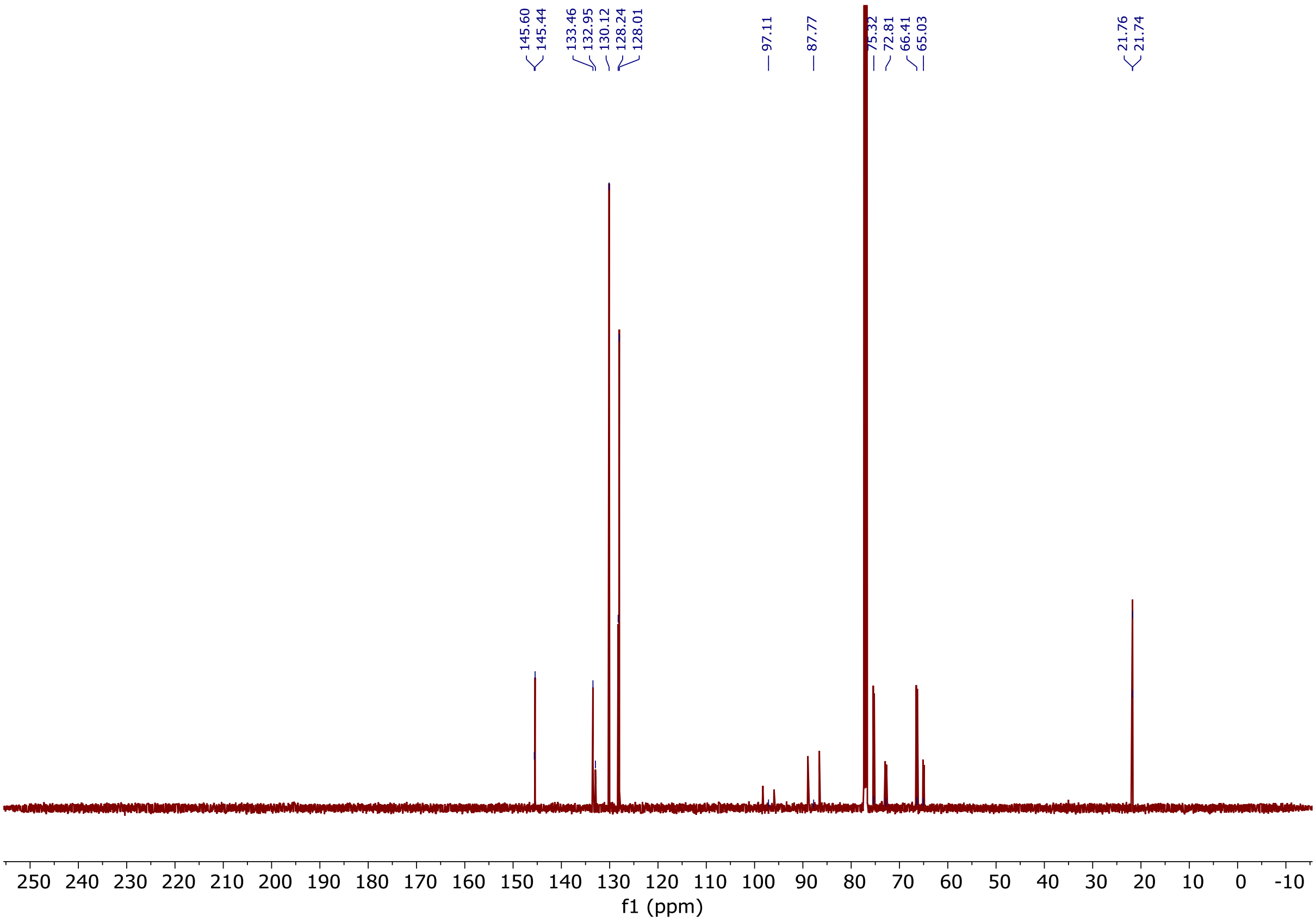

^{19}F NMR (471 MHz, CDCl$_3$)

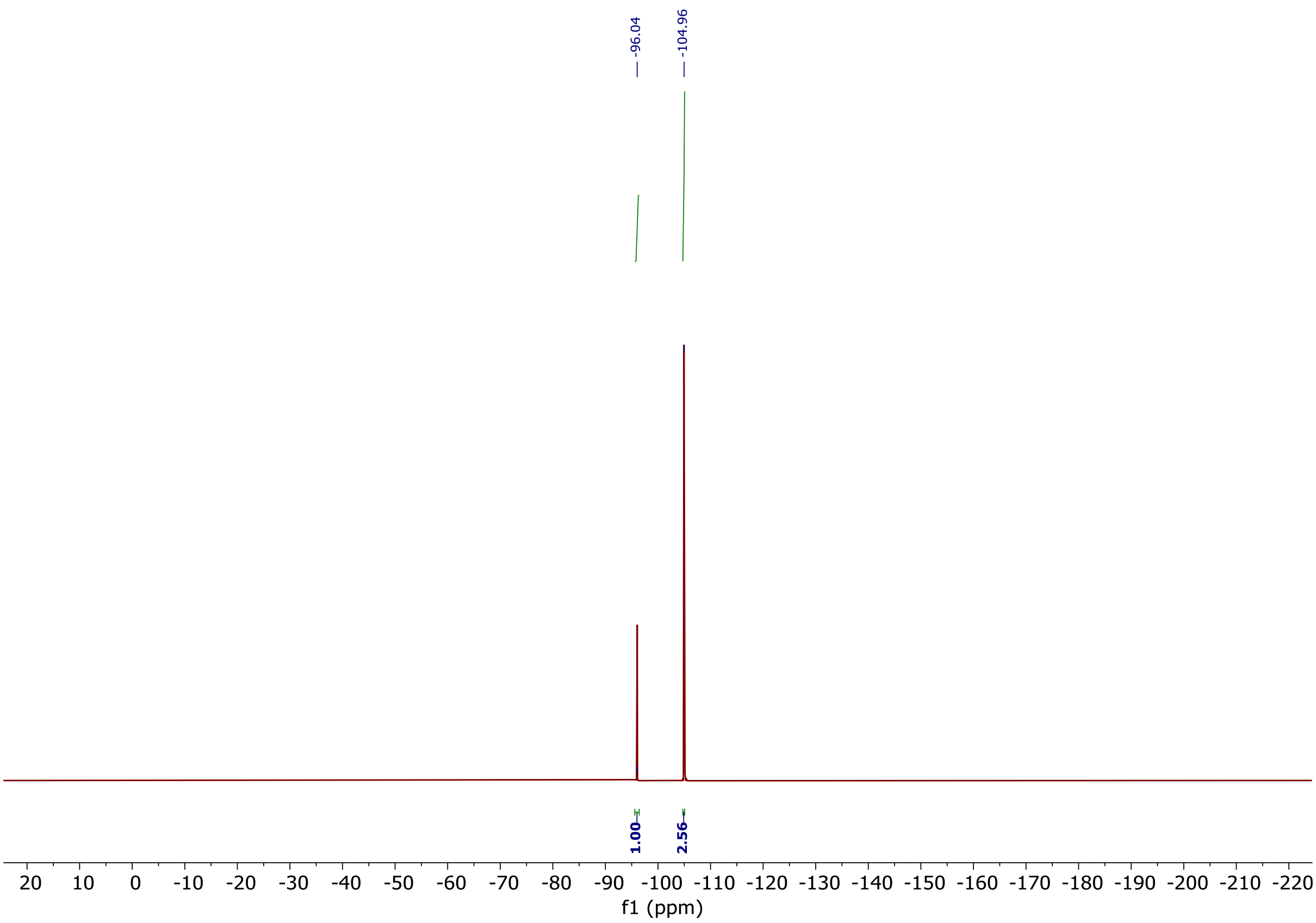

9.13. Cycloadditions (15)

***5-fluoro-4-phenyl-7-tosyl-2-oxa-3,7-diazabicyclo[3.2.0]hept-3-ene* (15ar)**

^{1}H NMR (500 MHz, $CDCl_3$)

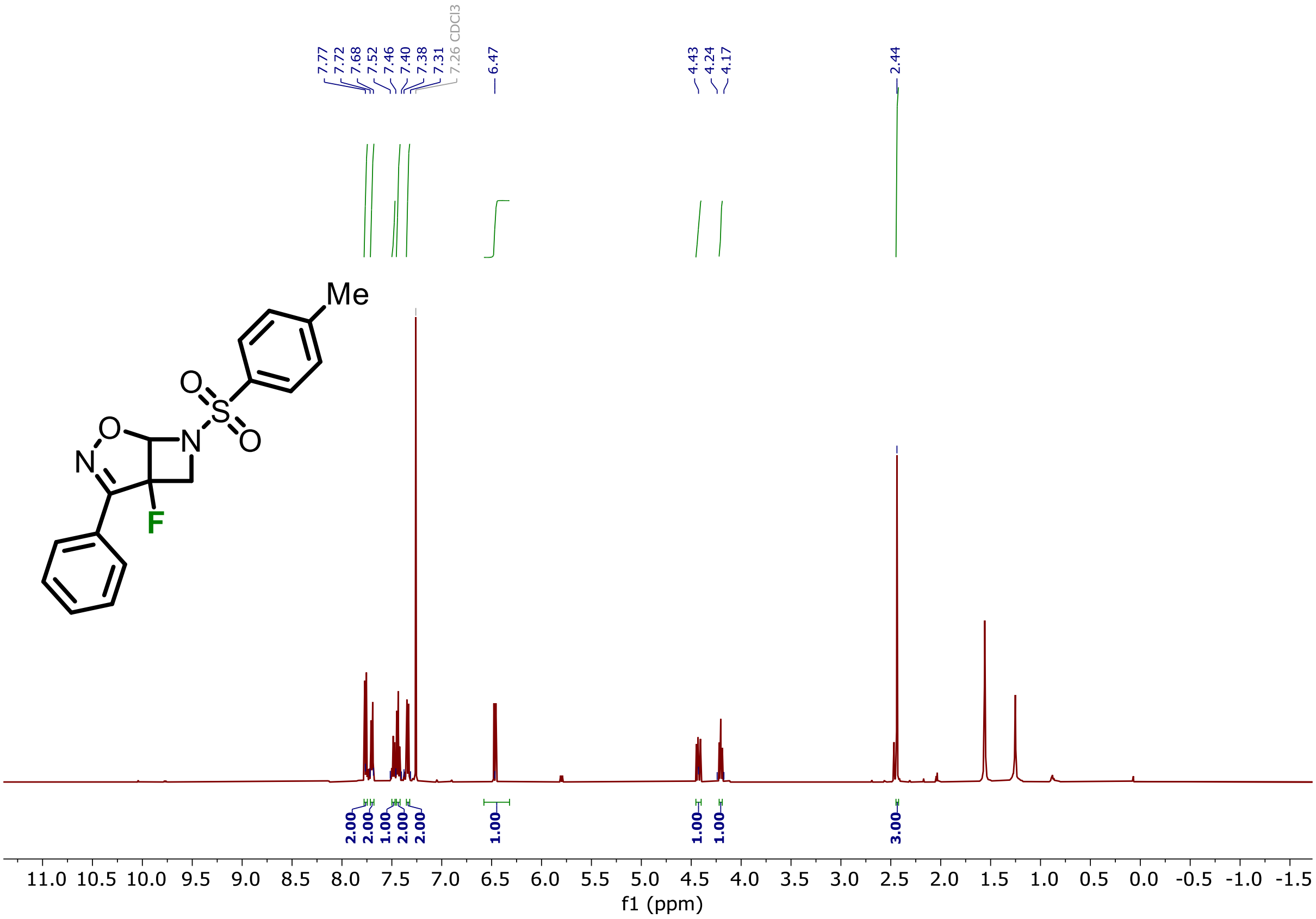

^{13}C NMR (126 MHz, CDCl$_3$)

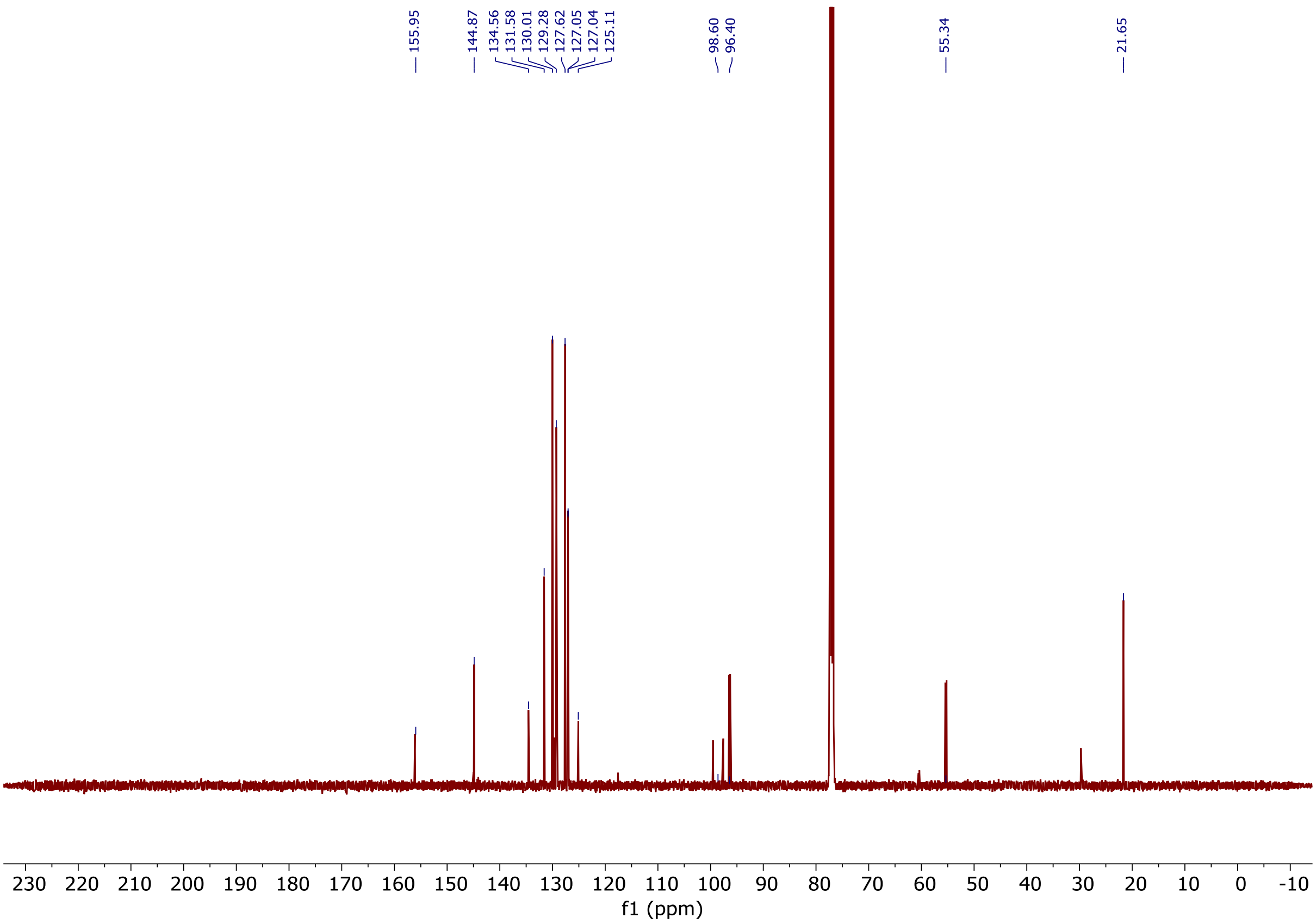

^{19}F NMR (376 MHz, CDCl$_3$)

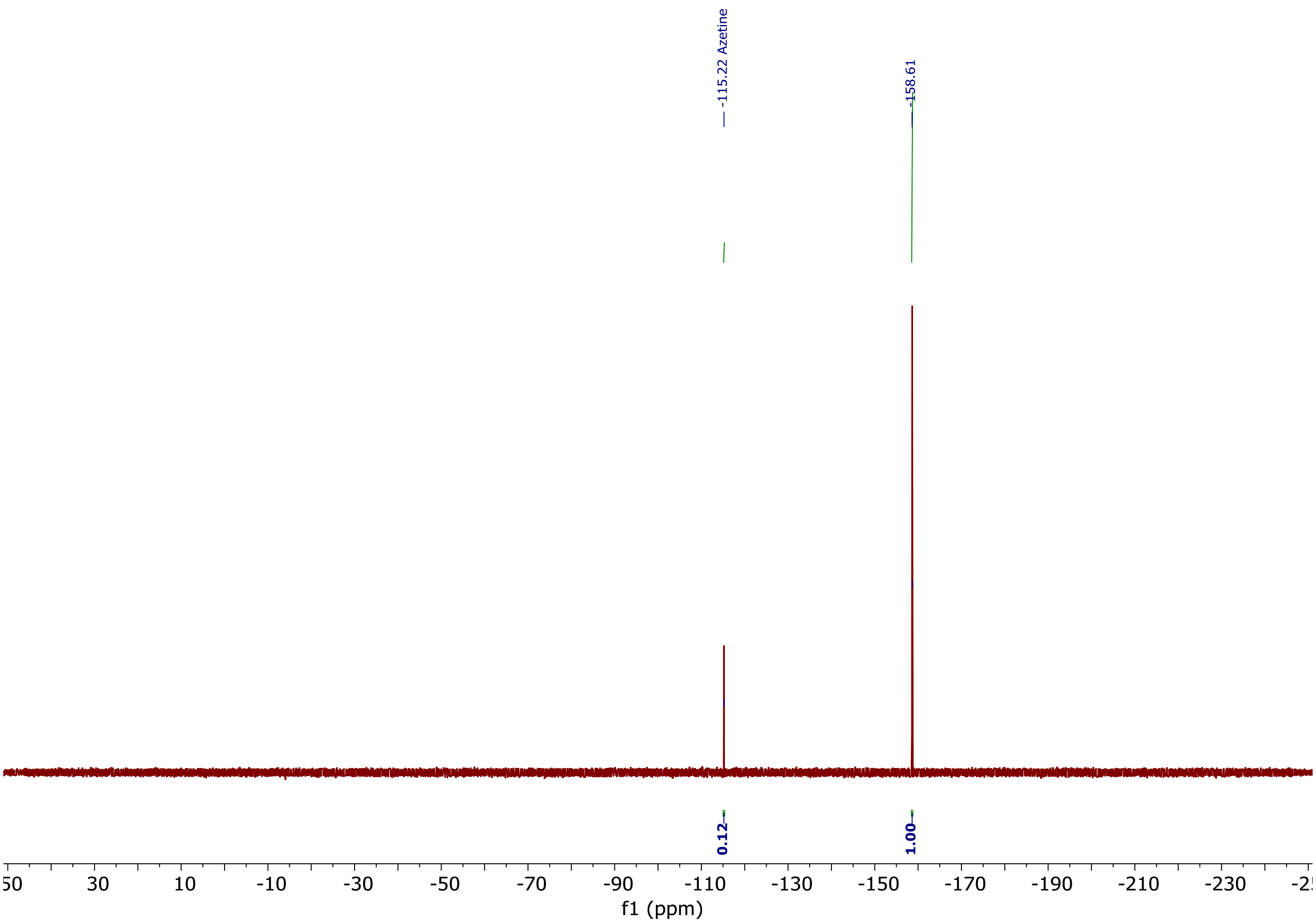

9.14. Hydrations (16)

***N-(2-fluoro-3-oxo-3-phenylpropyl)-4-methylbenzenesulfonamide* (16aw)**

^{1}H NMR (500 MHz, $CDCl_3$)

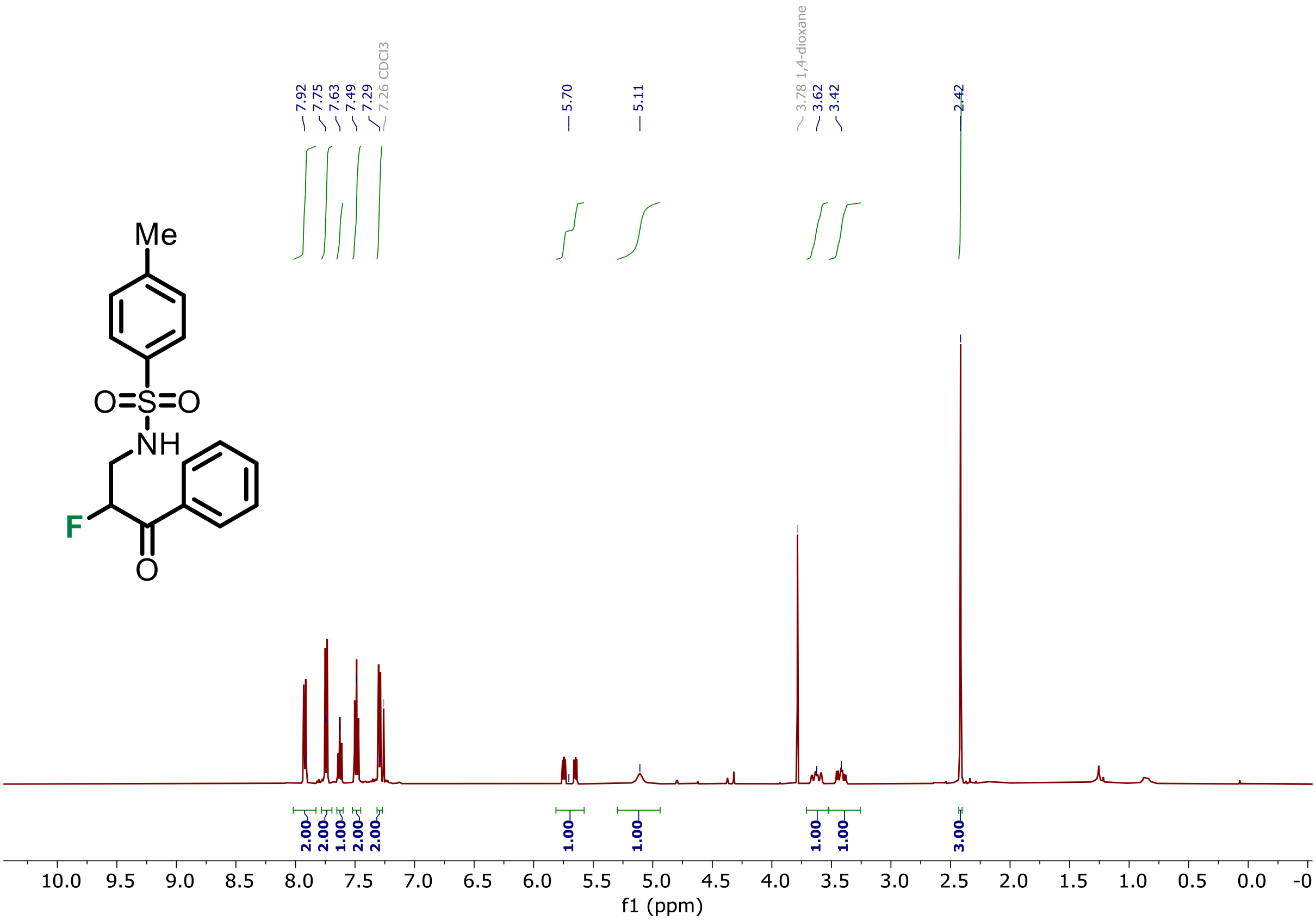

^{13}C NMR (126 MHz, $CDCl_3$)

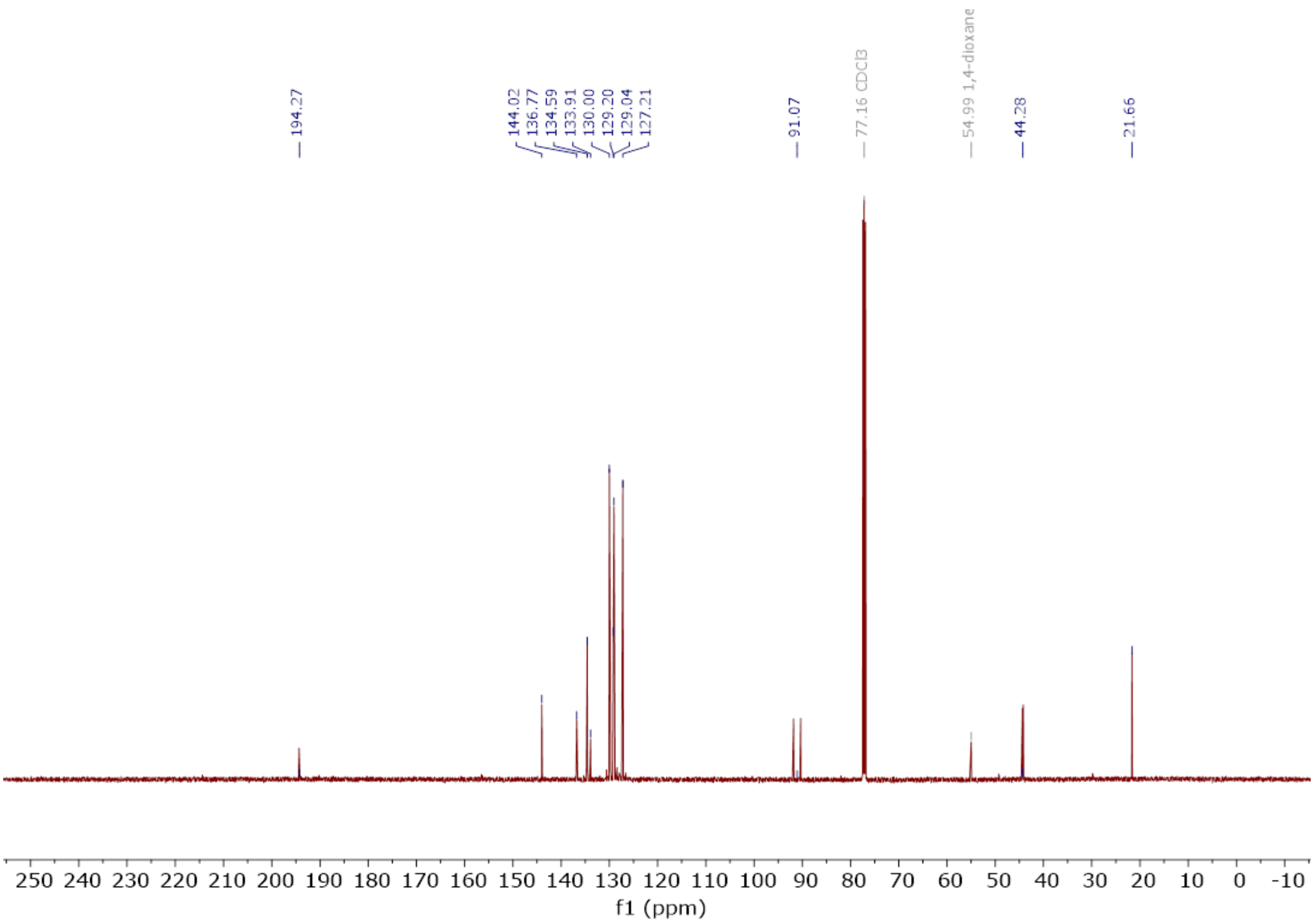

^{19}F NMR (471 MHz, $CDCl_3$)

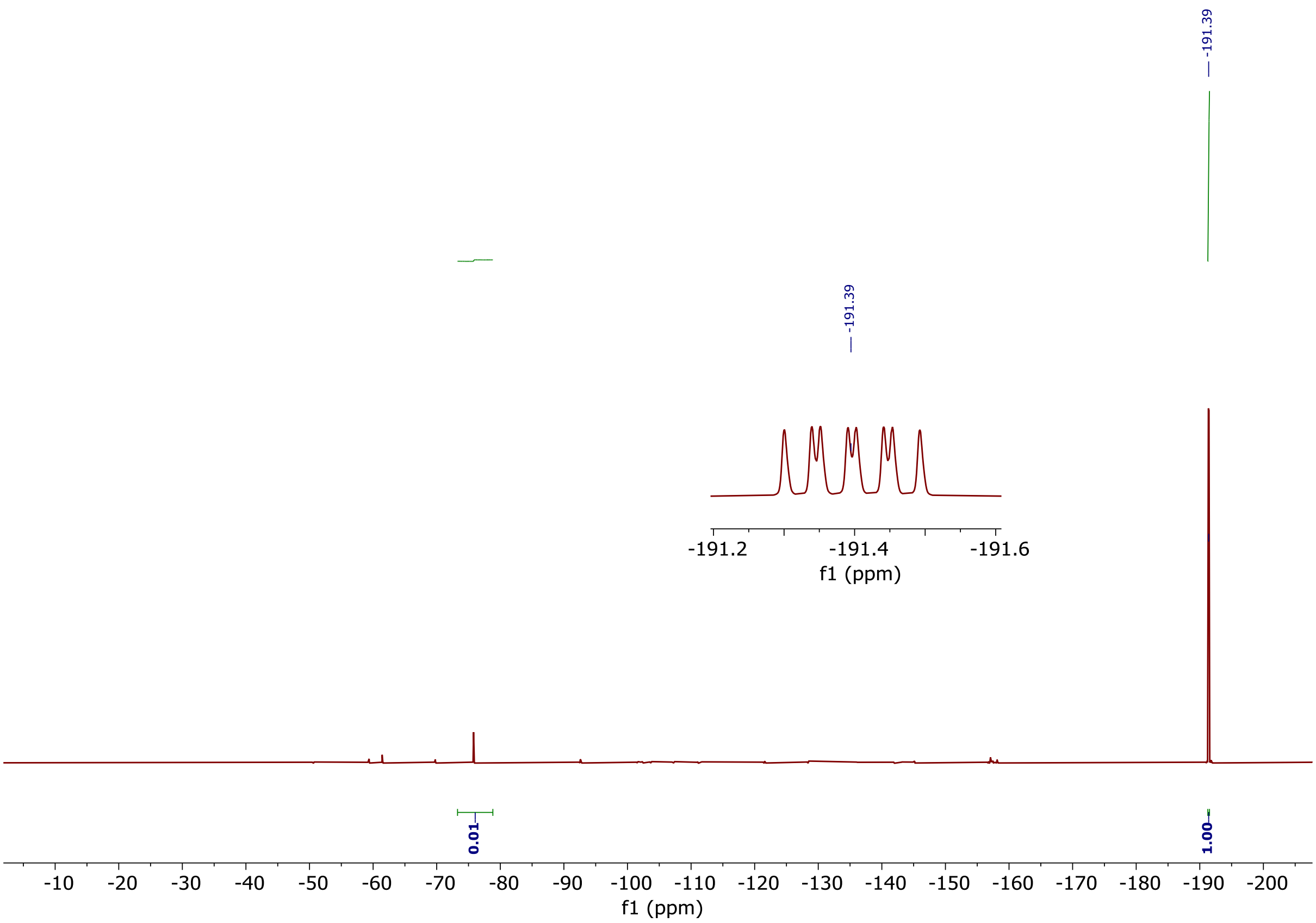

9.15. Epoxides (17)

(1S,2R,4S,5S)-1-fluoro-4-(4-fluorophenyl)-2-methyl-3-tosyl-6-oxa-3-azabicyclo[3.1.0]hexane
((*S*,*R*,*S*,*S*)-17j)
^{1}H NMR (400 MHz, $CDCl_3$)

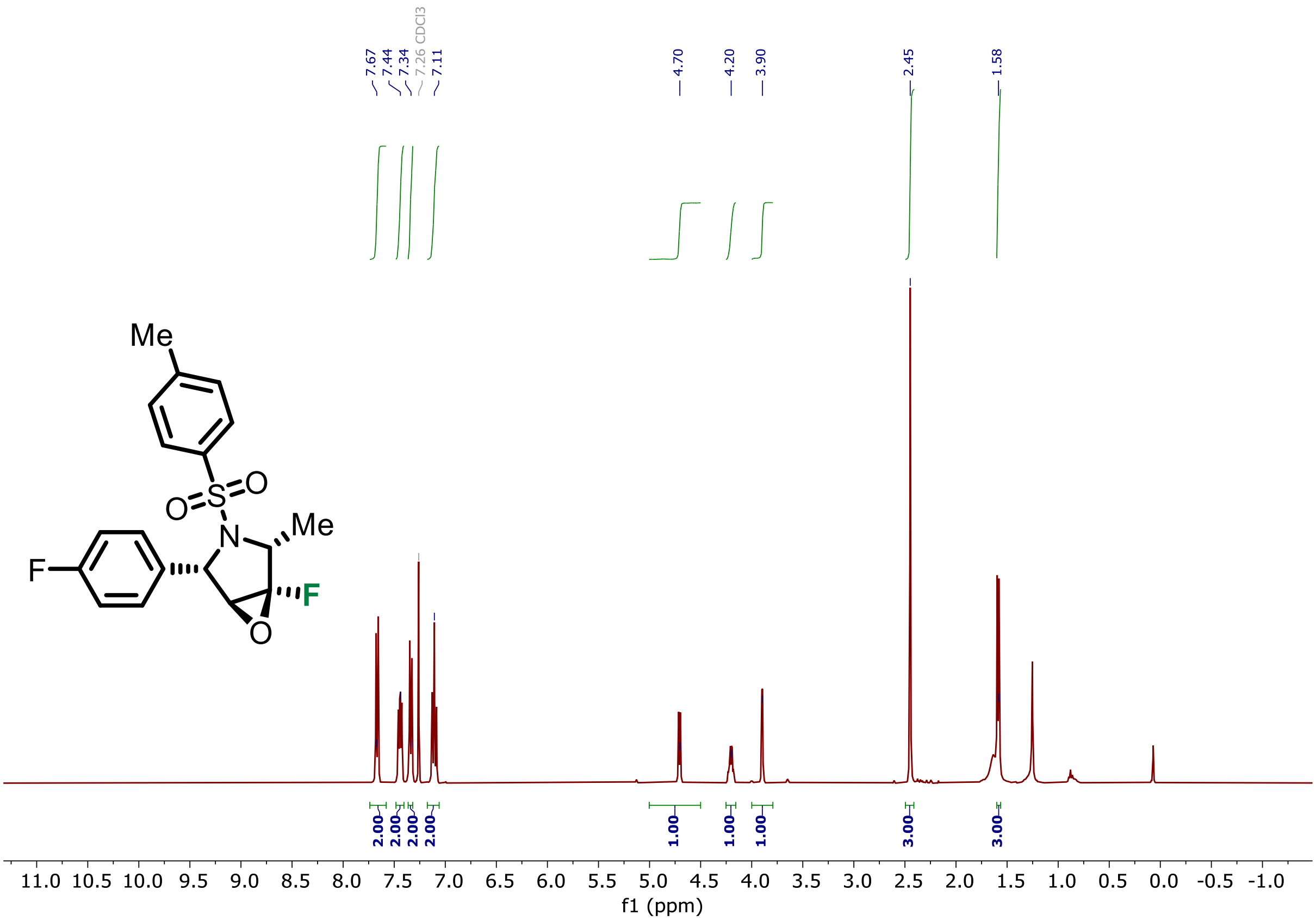

^{13}C NMR (101 MHz, $CDCl_3$)

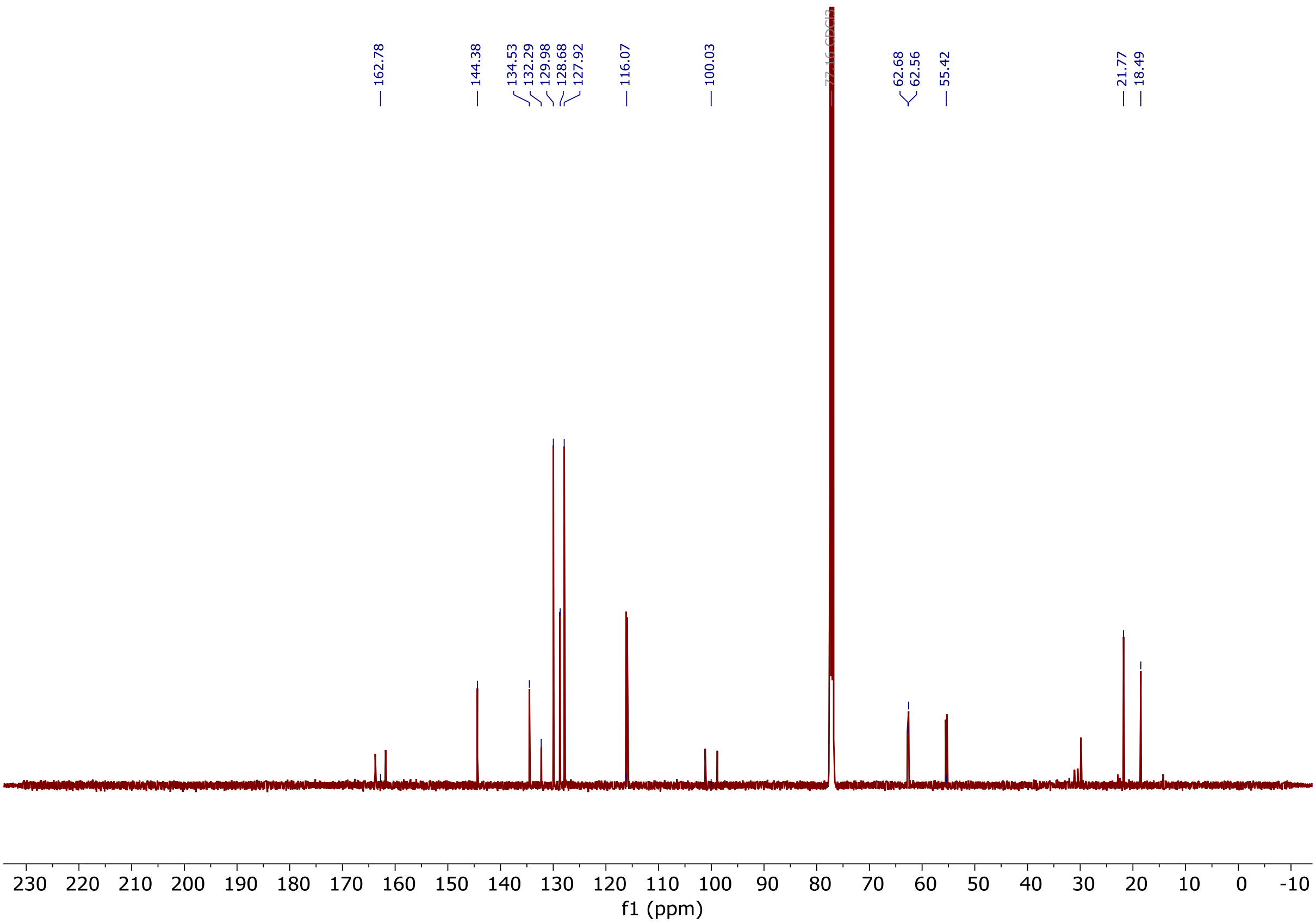

^{19}F NMR (376 MHz, $CDCl_3$)

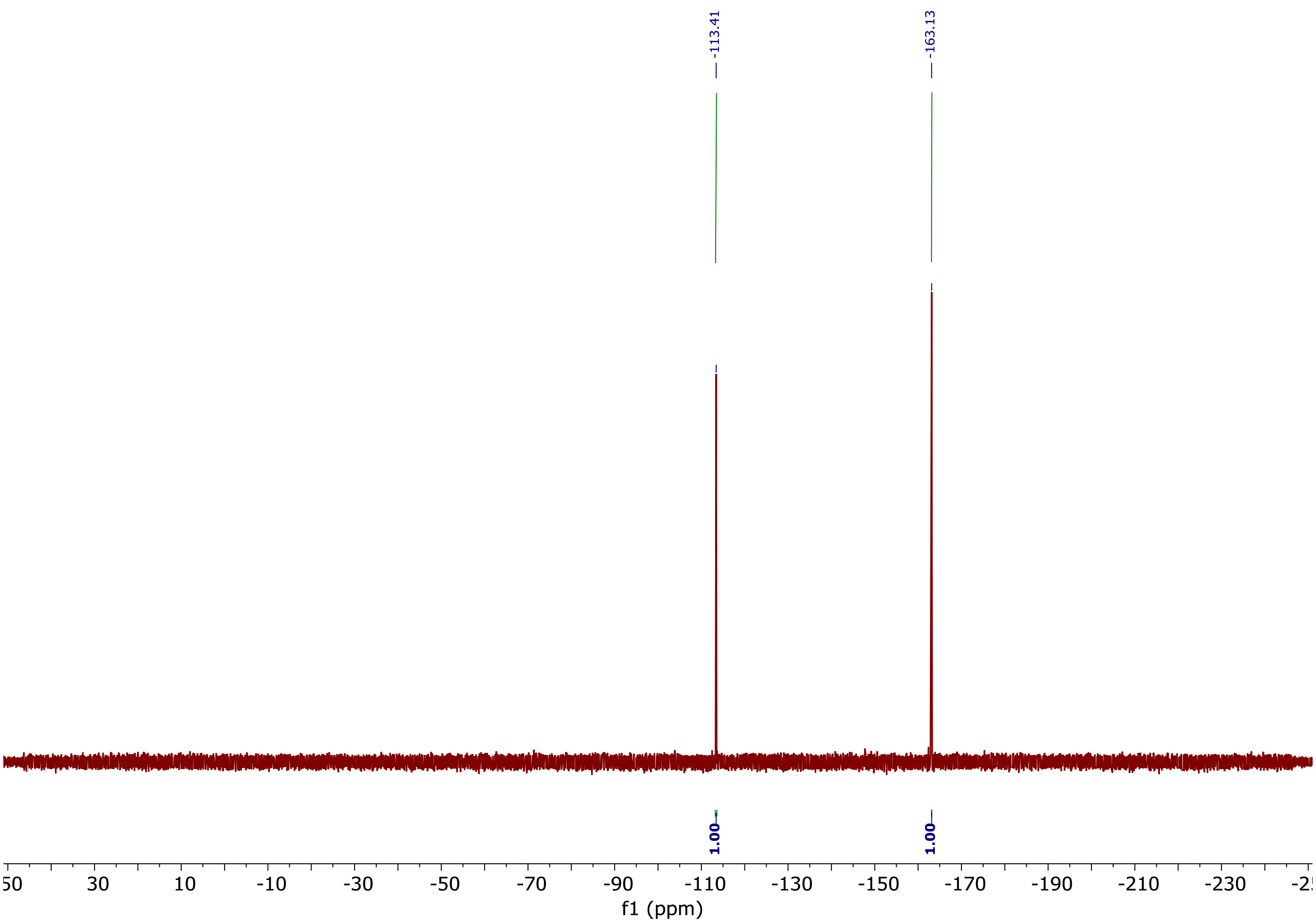

9.16. Pyrrolinones (18)

3-fluoro-5-methyl-5-phenyl-1-tosyl-1,5-dihydro-2H-pyrrol-2-one **((*R*)-18p)**

^{1}H NMR (500 MHz, CDCl$_3$)

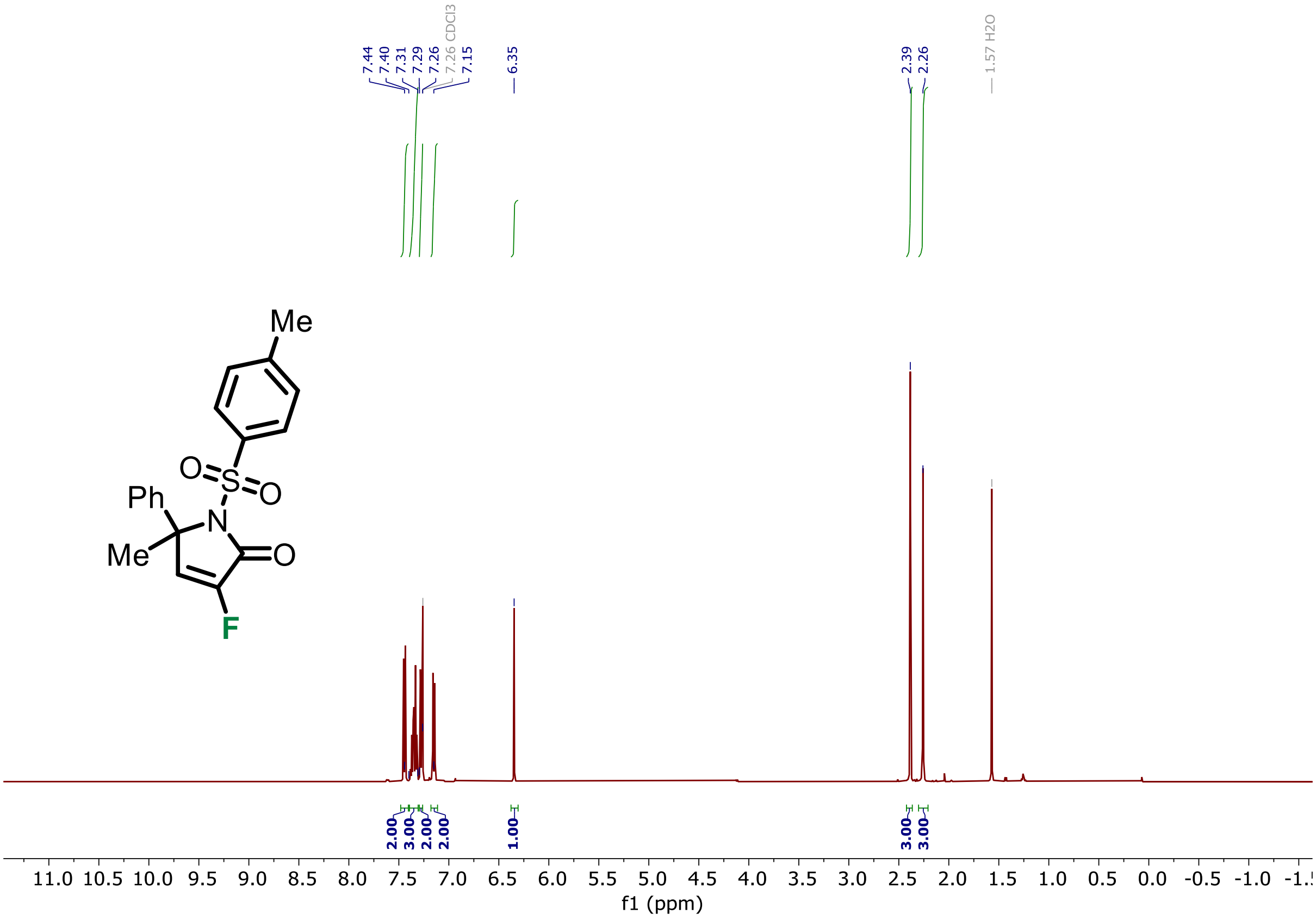

^{13}C NMR (126 MHz, $CDCl_3$)

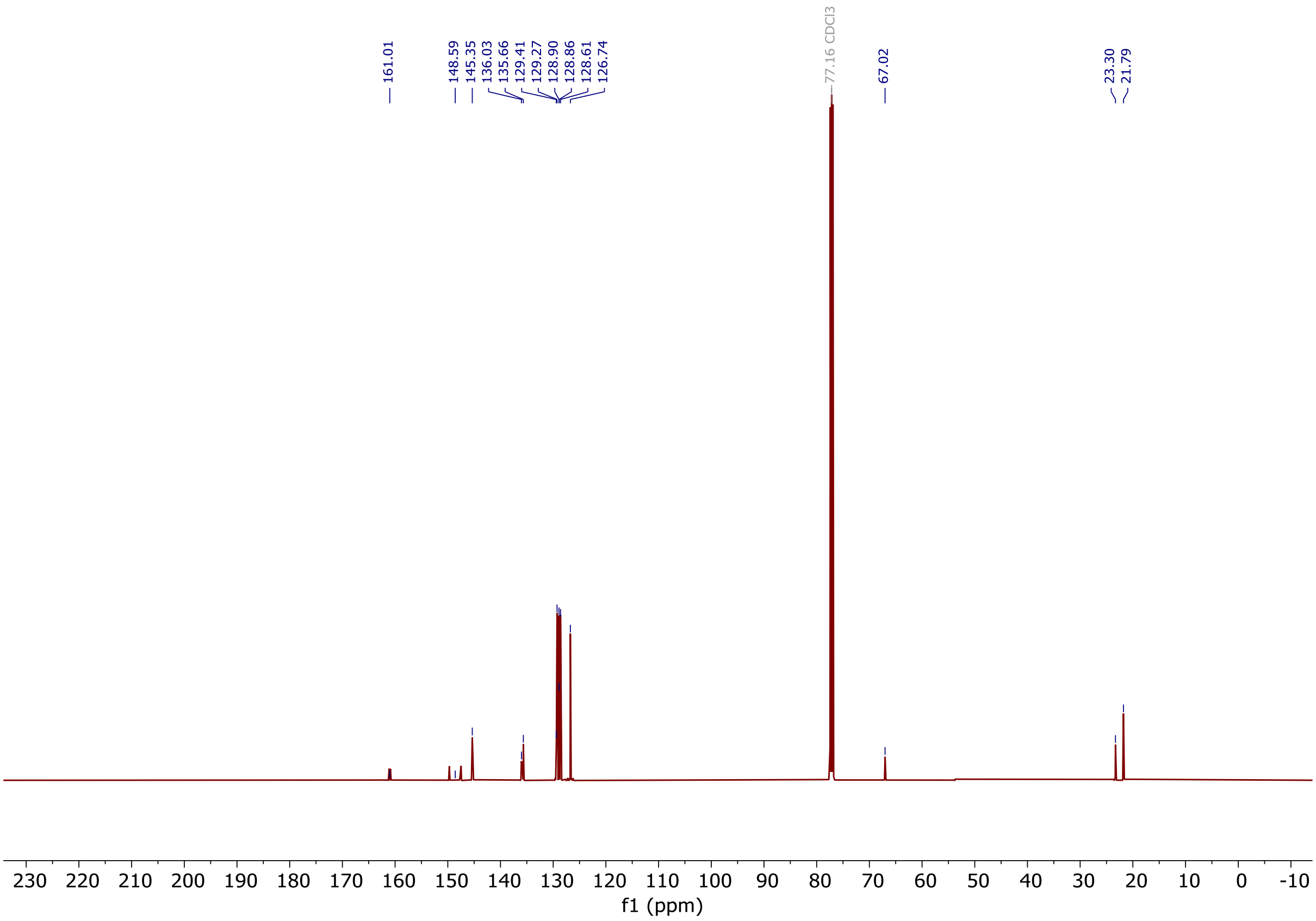

^{19}F NMR (376 MHz, $CDCl_3$)

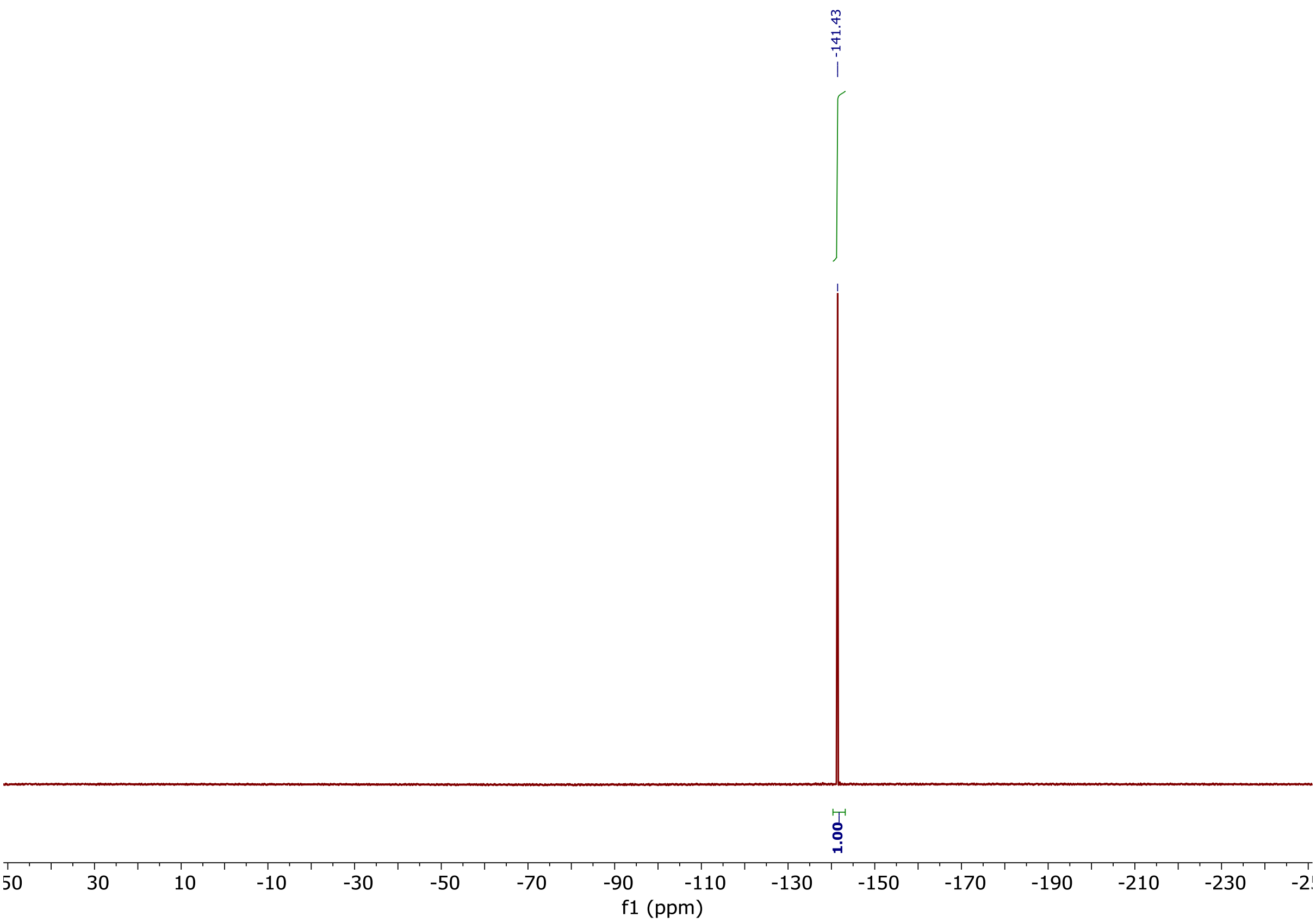

9.17. Deprotected Pyrrolines (19-25)

***2-benzyl-4-fluoro-2,5-dihydro-1H-pyrrole* (19t)**

^{1}H NMR (500 MHz, CDCl$_3$)

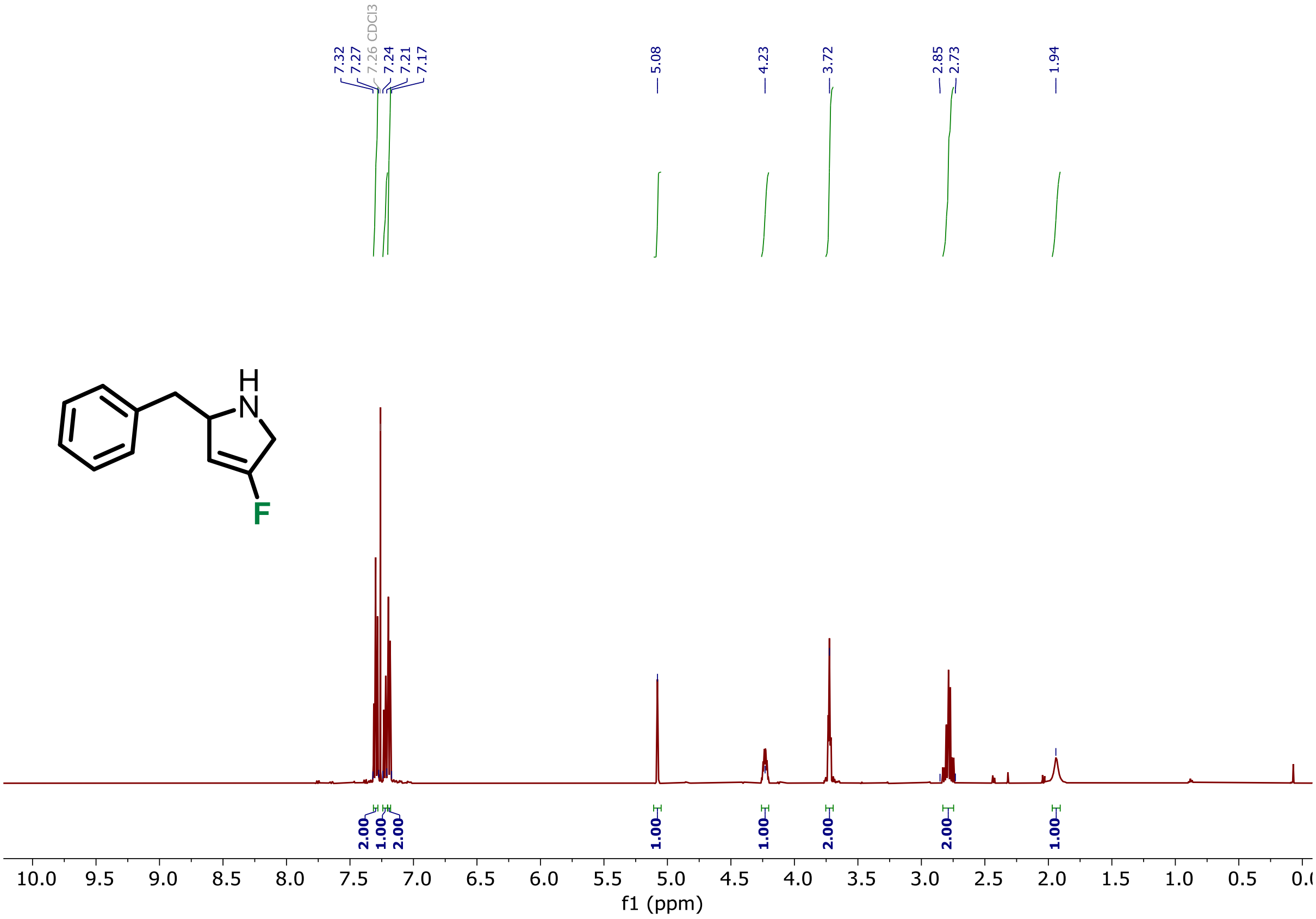

^{13}C NMR (126 MHz, CDCl3)

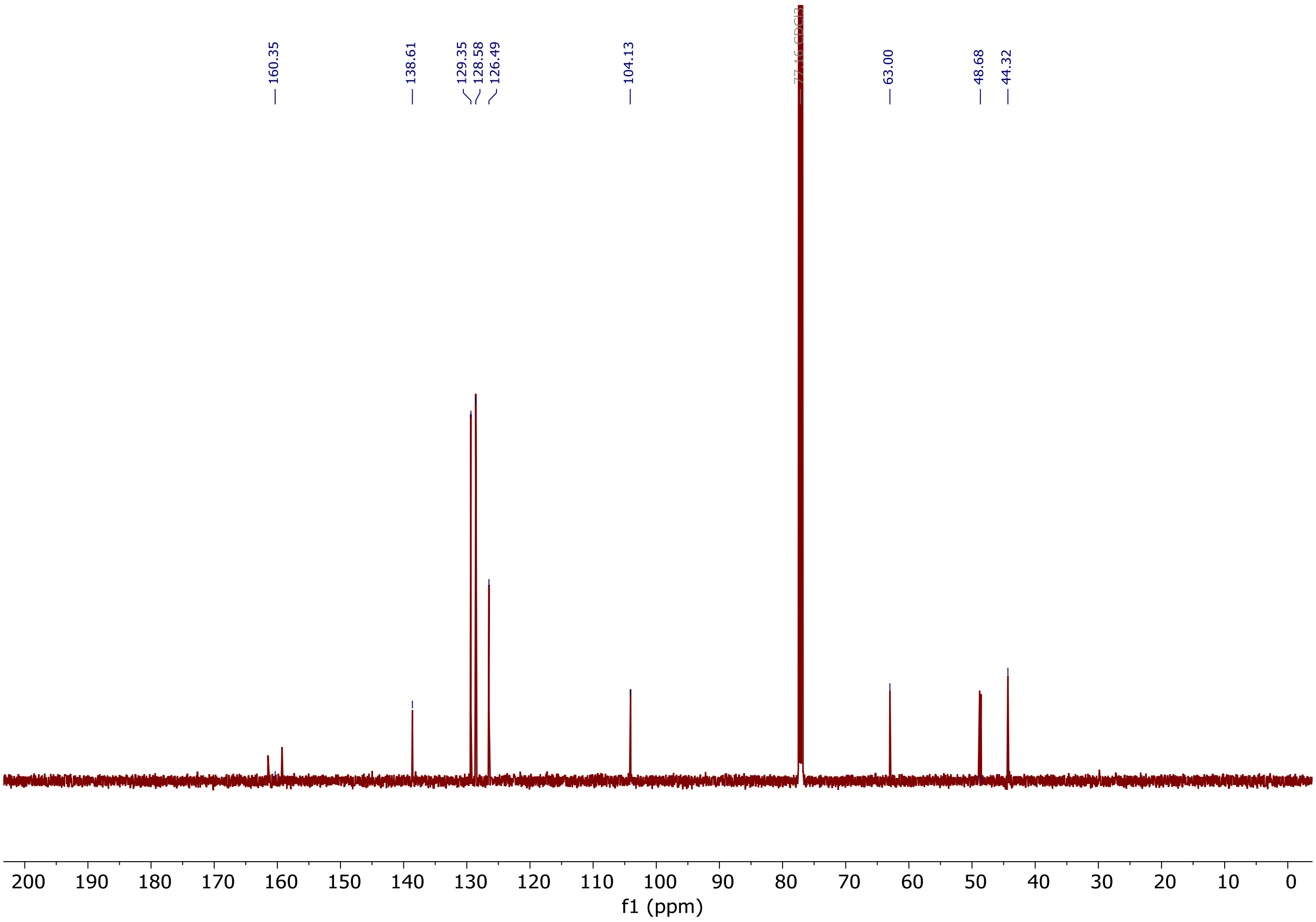

^{19}F NMR (376 MHz, CDCl3)

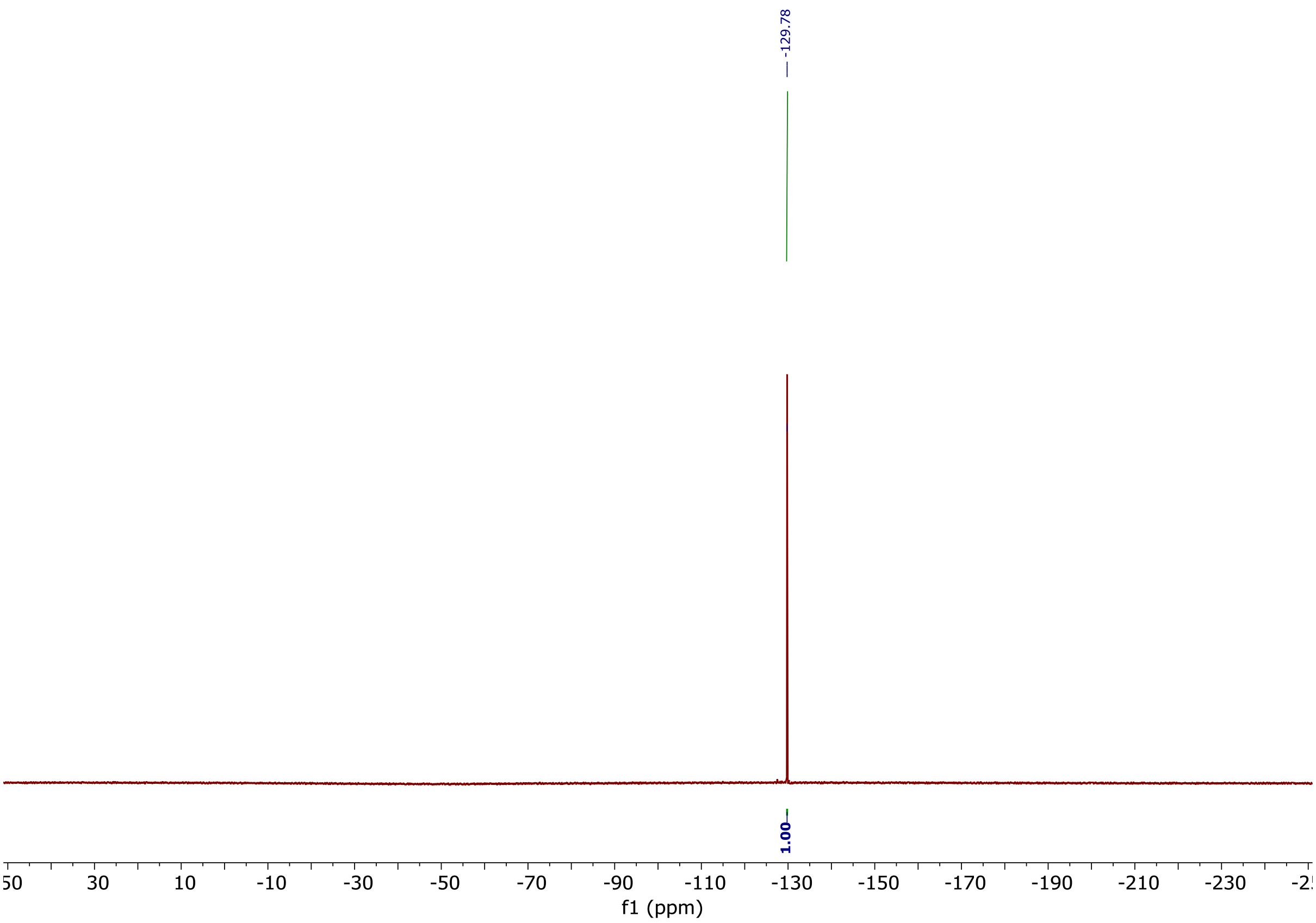

cis-3-fluoro-5-(4-fluorophenyl)-2-methyl-2,5-dihydro-1H-pyrrole **(19j)**

^{1}H NMR (500 MHz, CDCl$_3$)

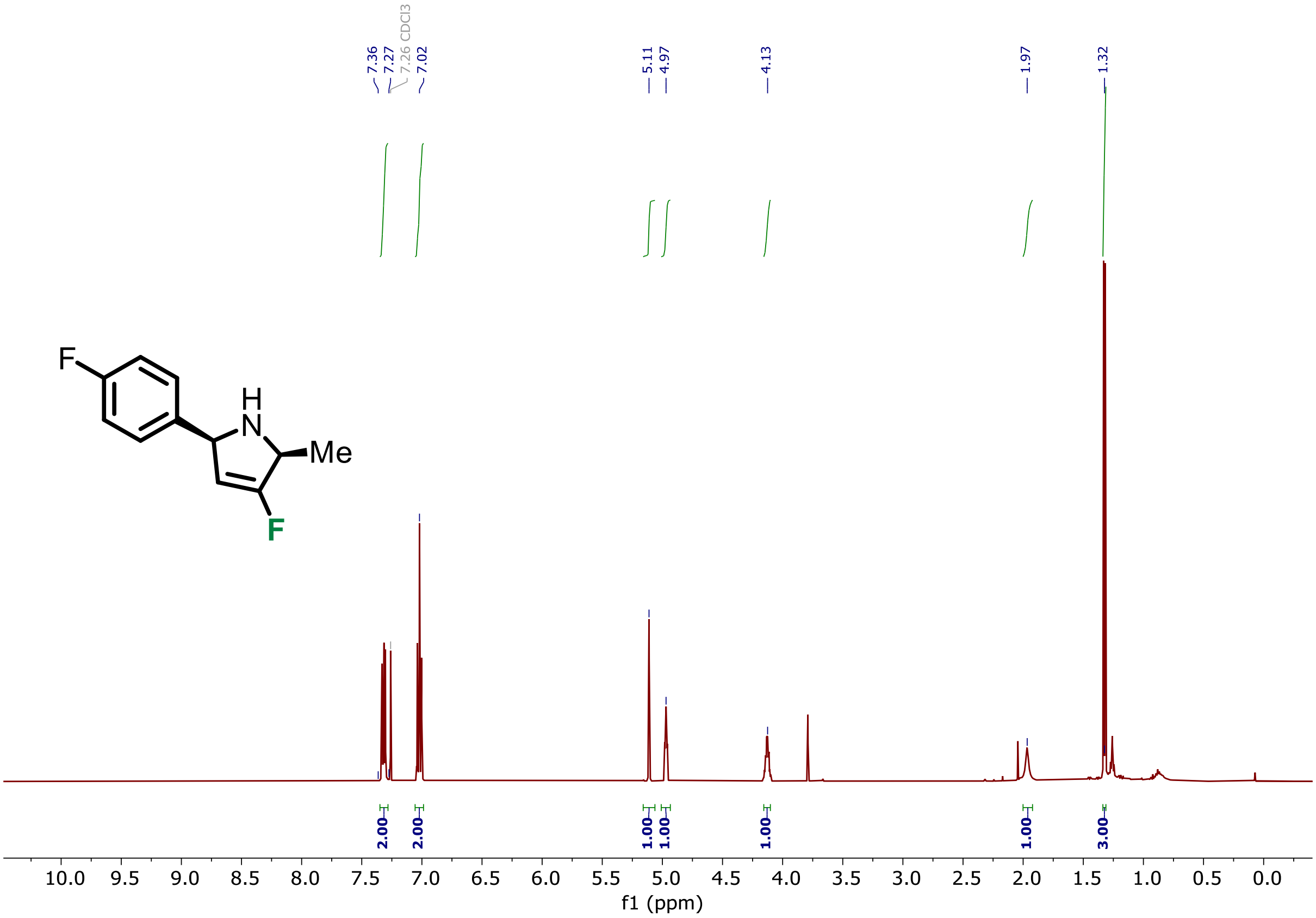

^{13}C NMR (126 MHz, CDCl$_3$)

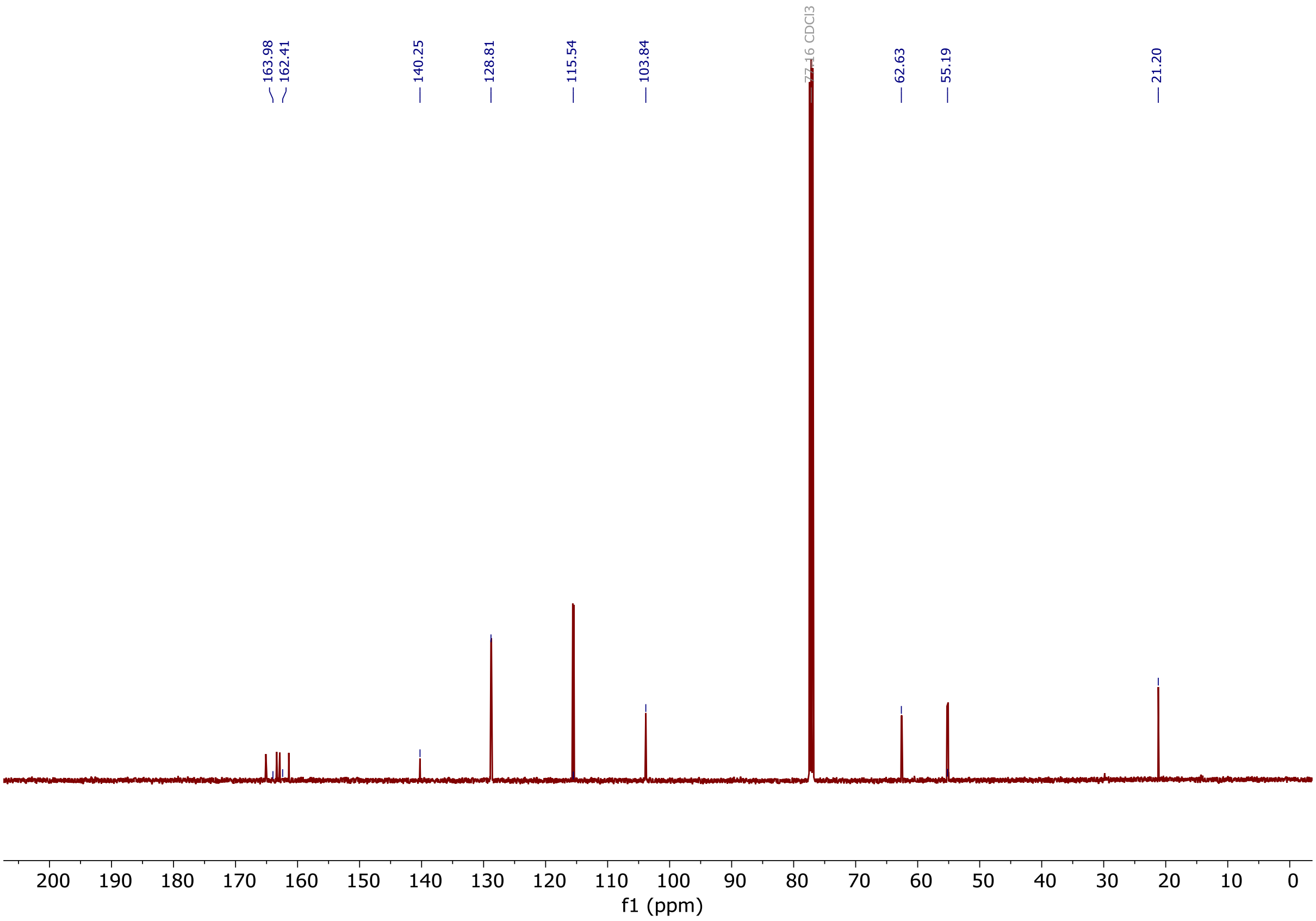

^{19}F NMR (376 MHz, $CDCl_3$)

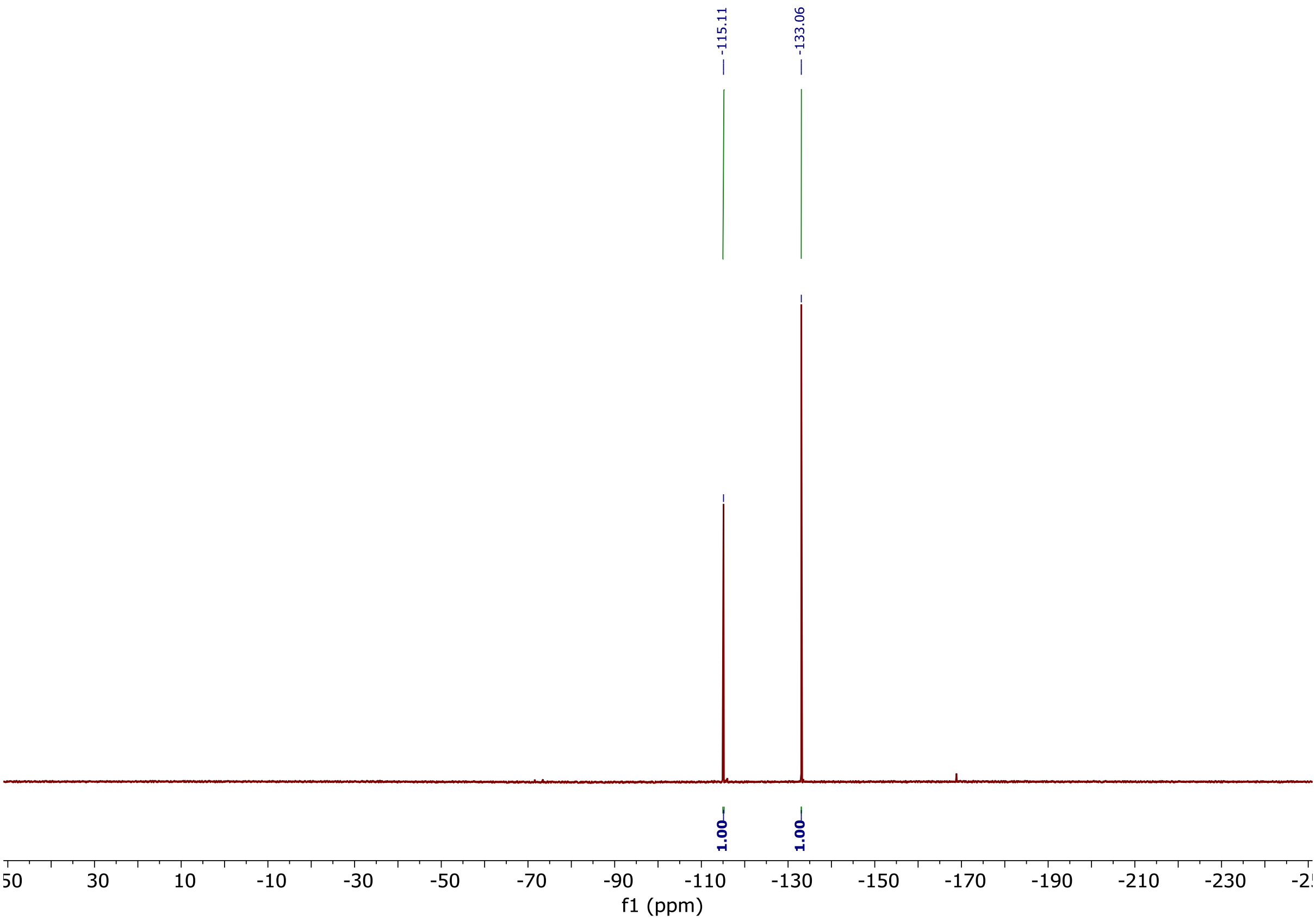

(2R,5R)-3-fluoro-5-(4-fluorophenyl)-2-methyl-2,5-dihydro-1H-pyrrole **((*R*,*R*)-19j)**

^{1}H NMR (400 MHz, CDCl$_3$)

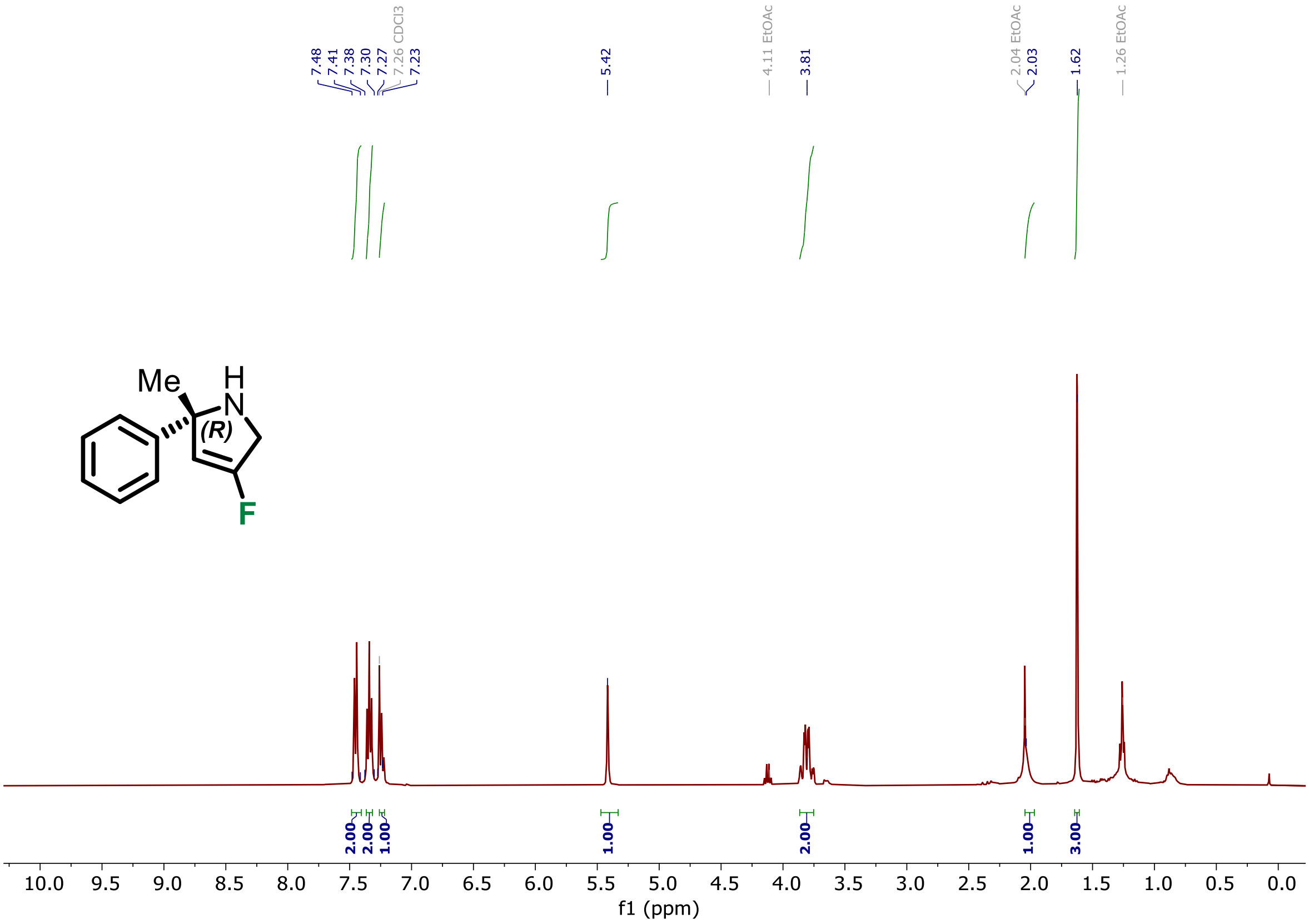

^{13}C NMR (101 MHz, CDCl$_3$)

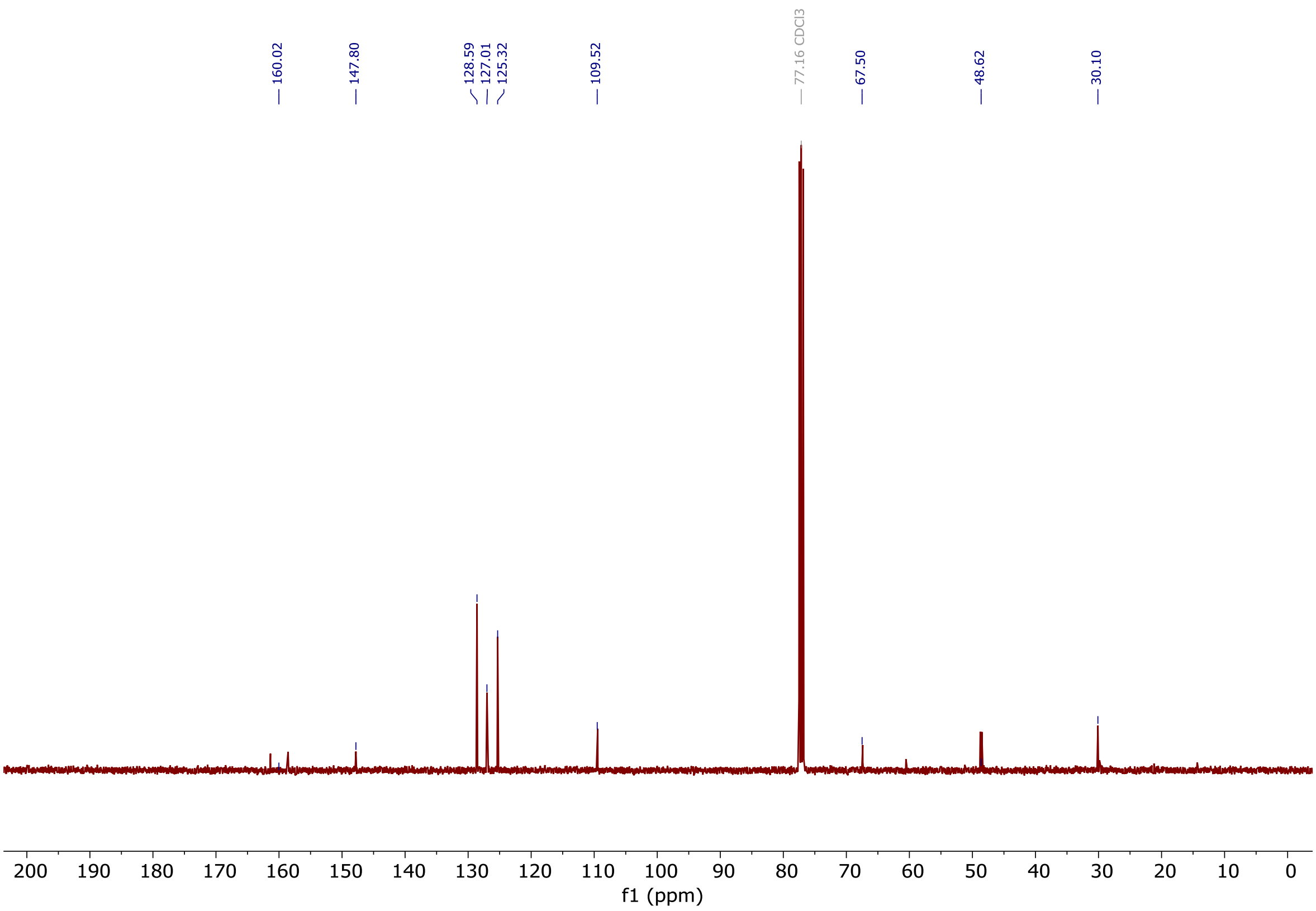

^{19}F NMR (376 MHz, CDCl$_3$)

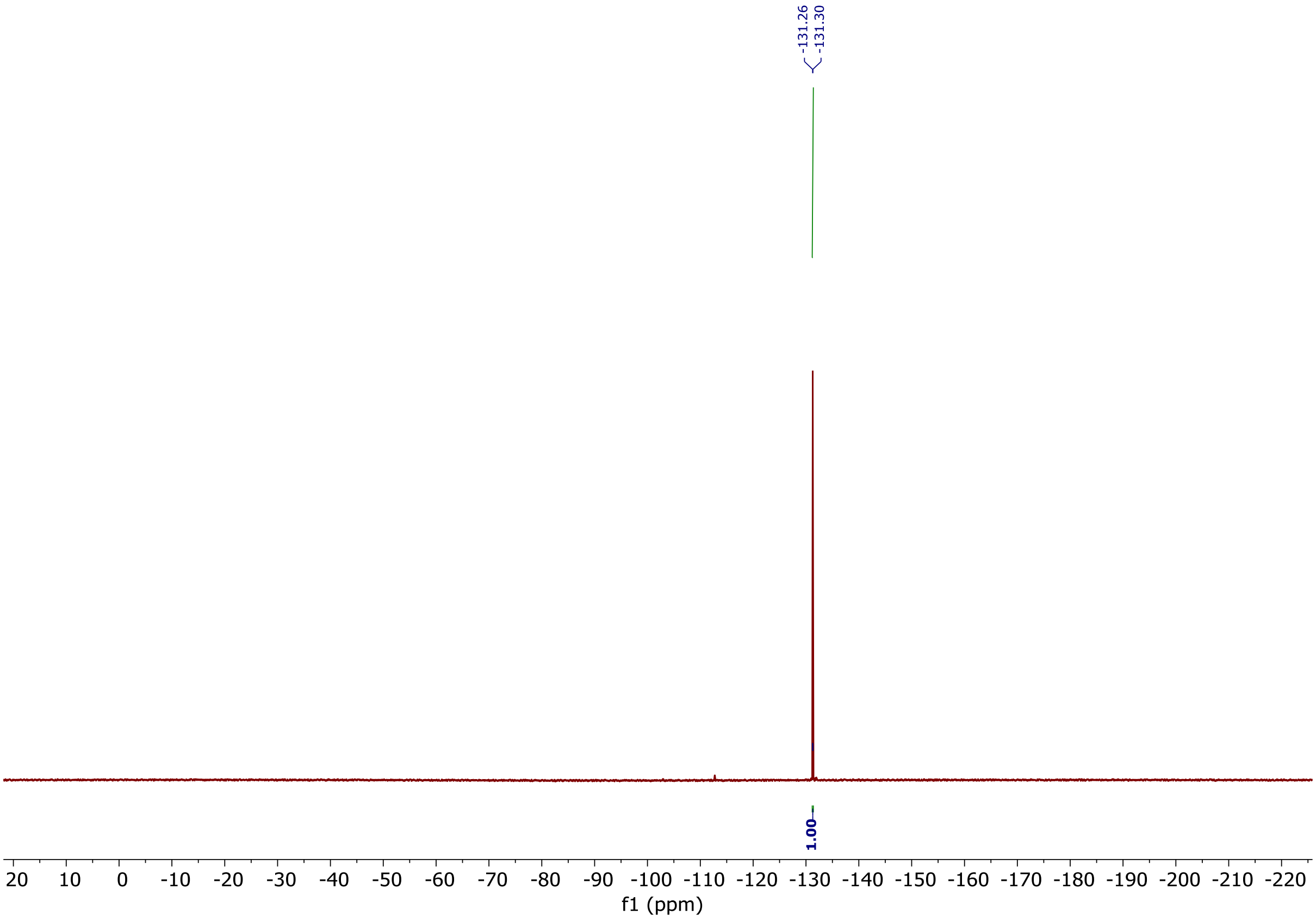

-131.26
-131.30
1.00
20 10 0 -10 -20 -30 -40 -50 -60 -70 -80 -90 -100 -110 -120 -130 -140 -150 -160 -170 -180 -190 -200 -210 -220
f1 (ppm)

(R)-4-fluoro-2-methyl-2-phenyl-2,5-dihydro-1H-pyrrole **((*R*)-19p)**

^{1}H NMR (500 MHz, CDCl$_3$)

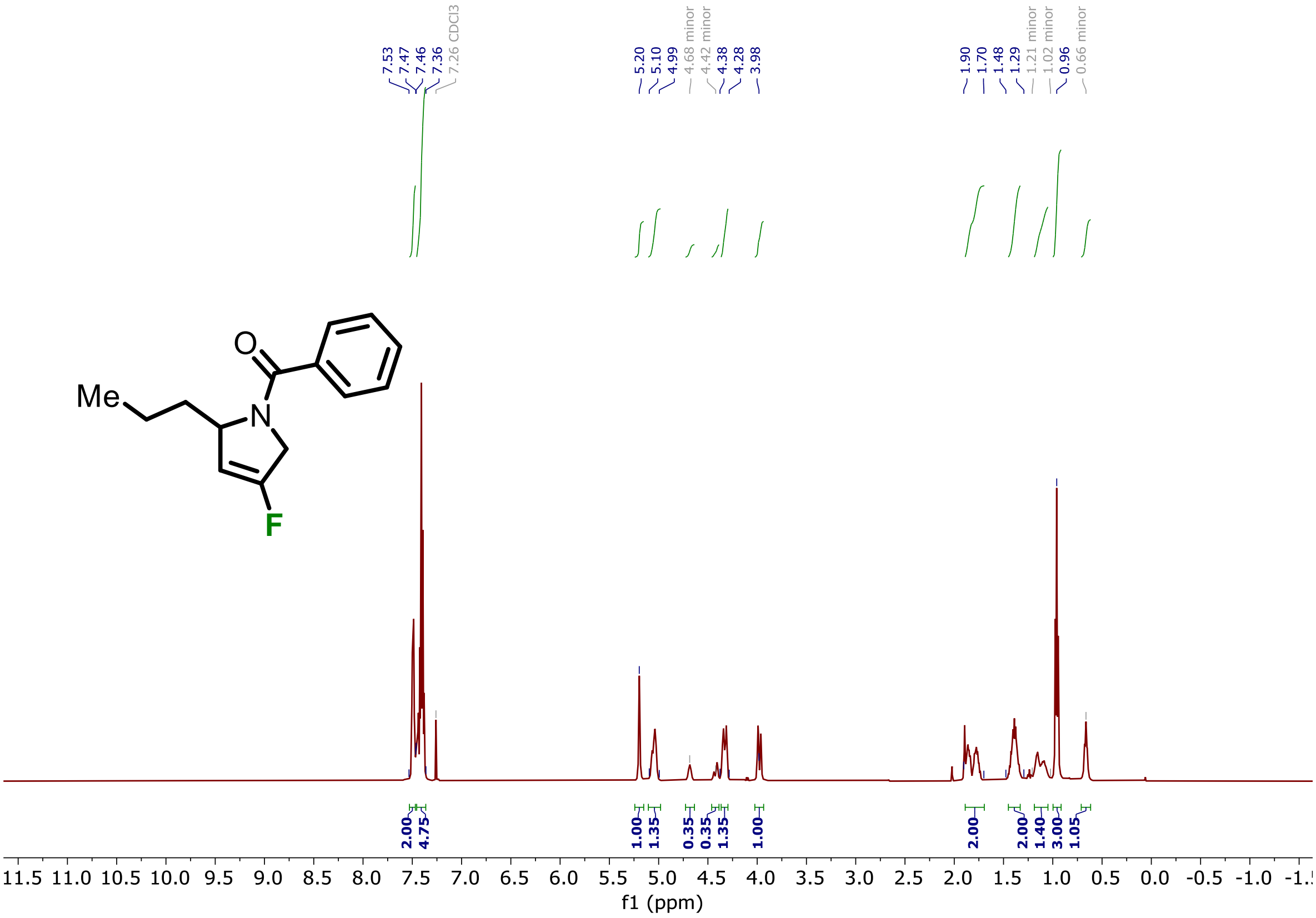

^{13}C NMR (126 MHz, CDCl$_3$)

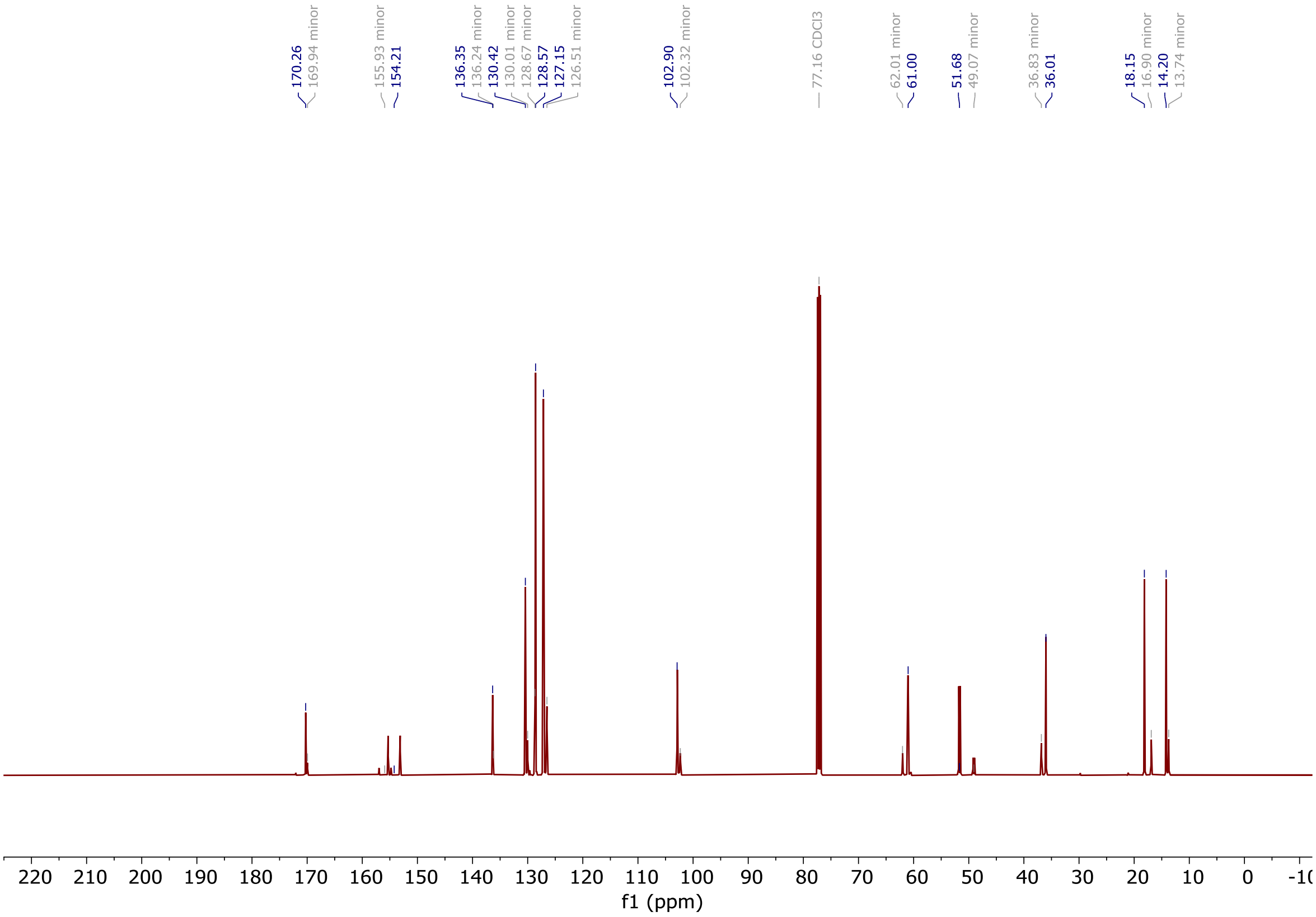

^{19}F NMR (376 MHz, $CDCl_3$)

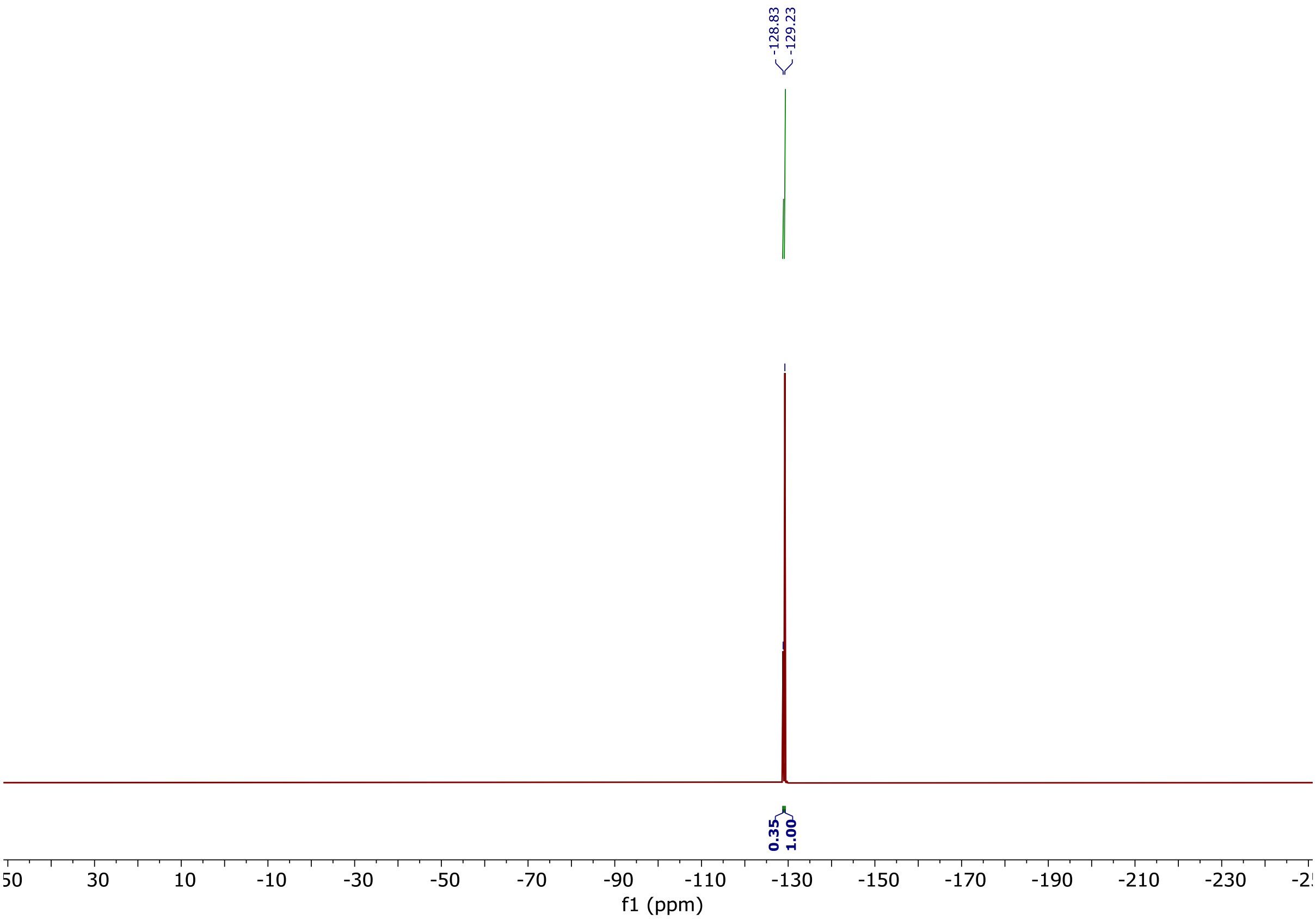

4-fluoro-2-propyl-2,5-dihydro-1H-pyrrole **(19v)**

^{1}H NMR (500 MHz, CDCl$_3$)

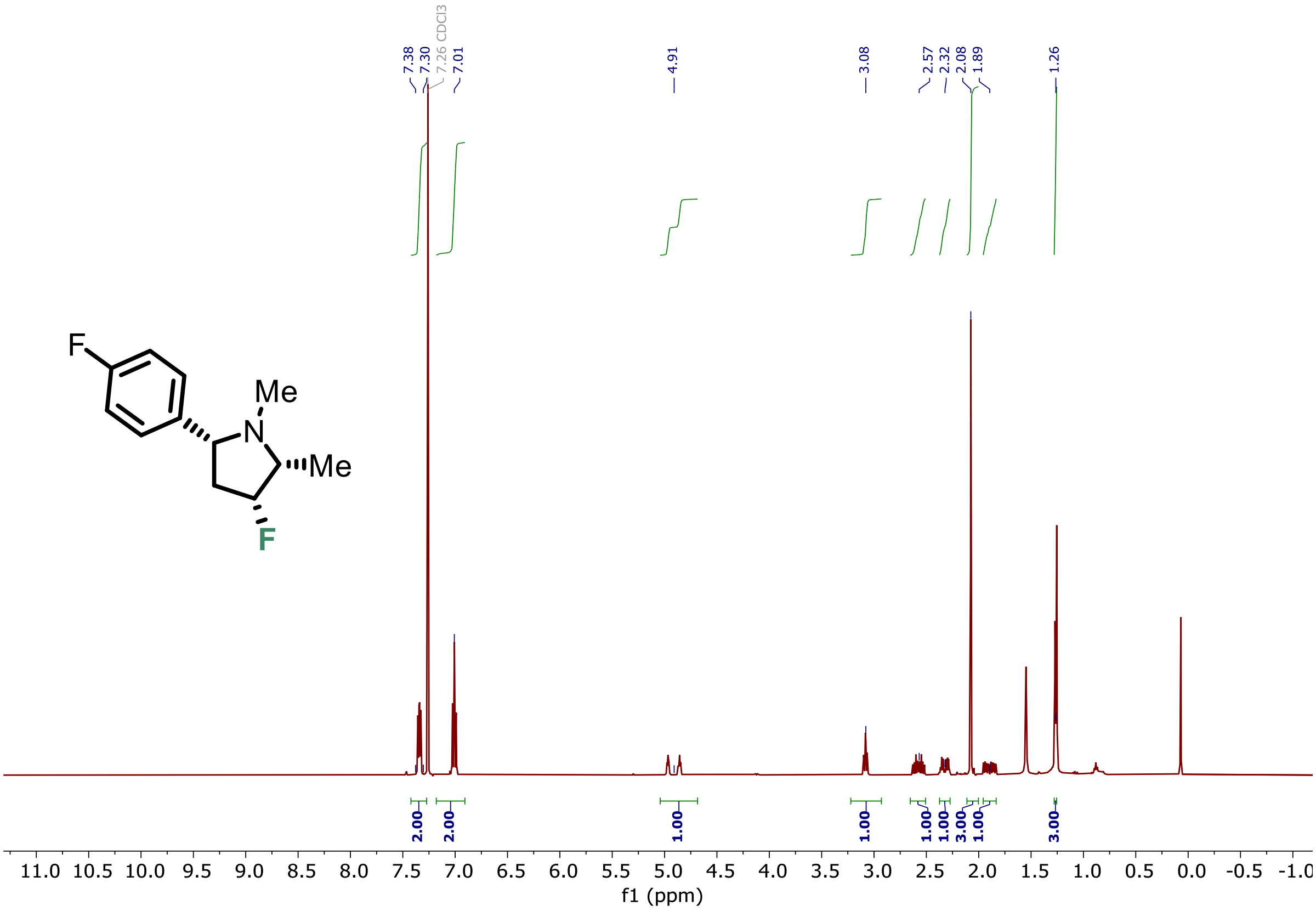

^{13}C NMR (126 MHz, CDCl$_3$)

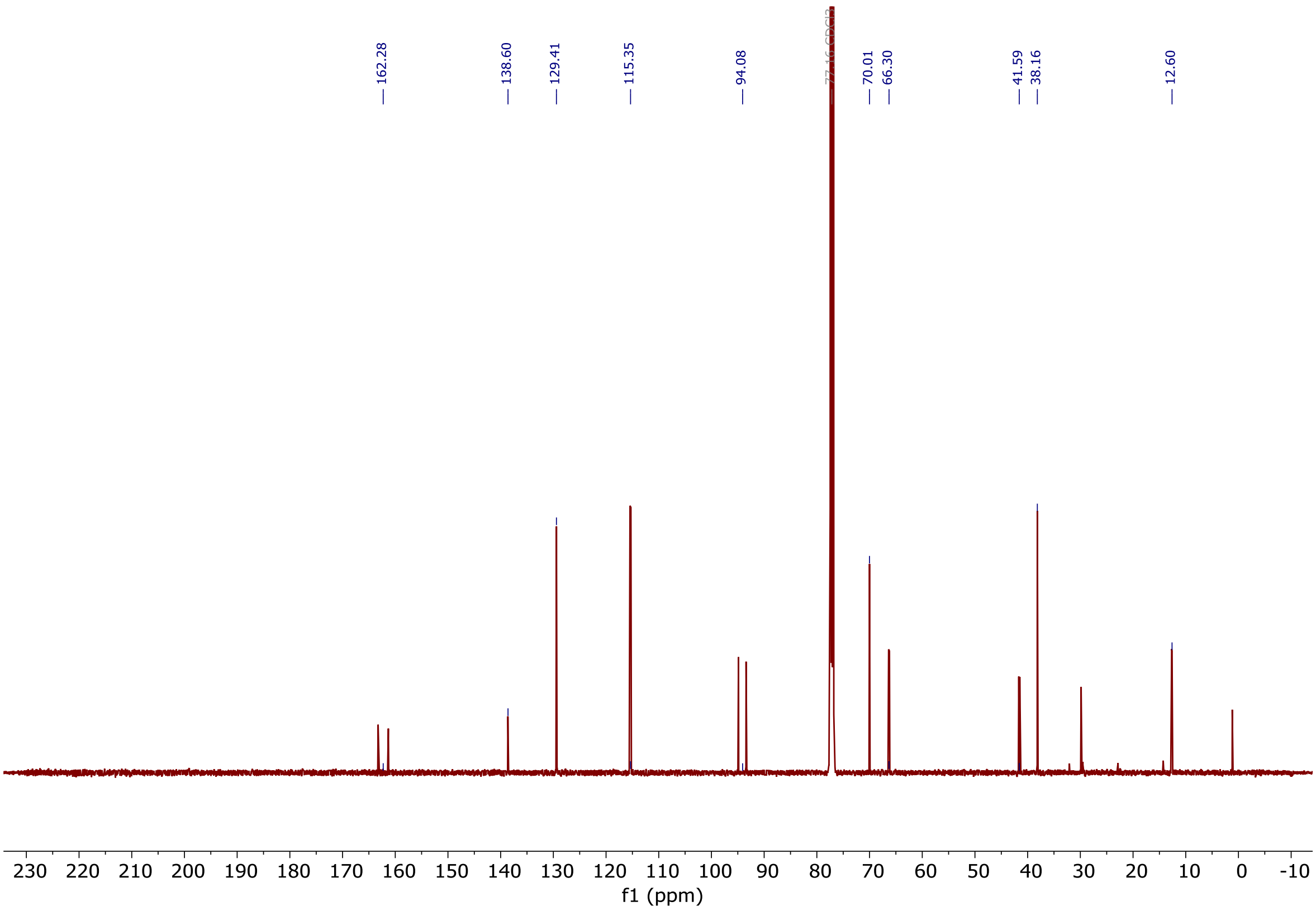

^{19}F NMR (376 MHz, CDCl$_3$)

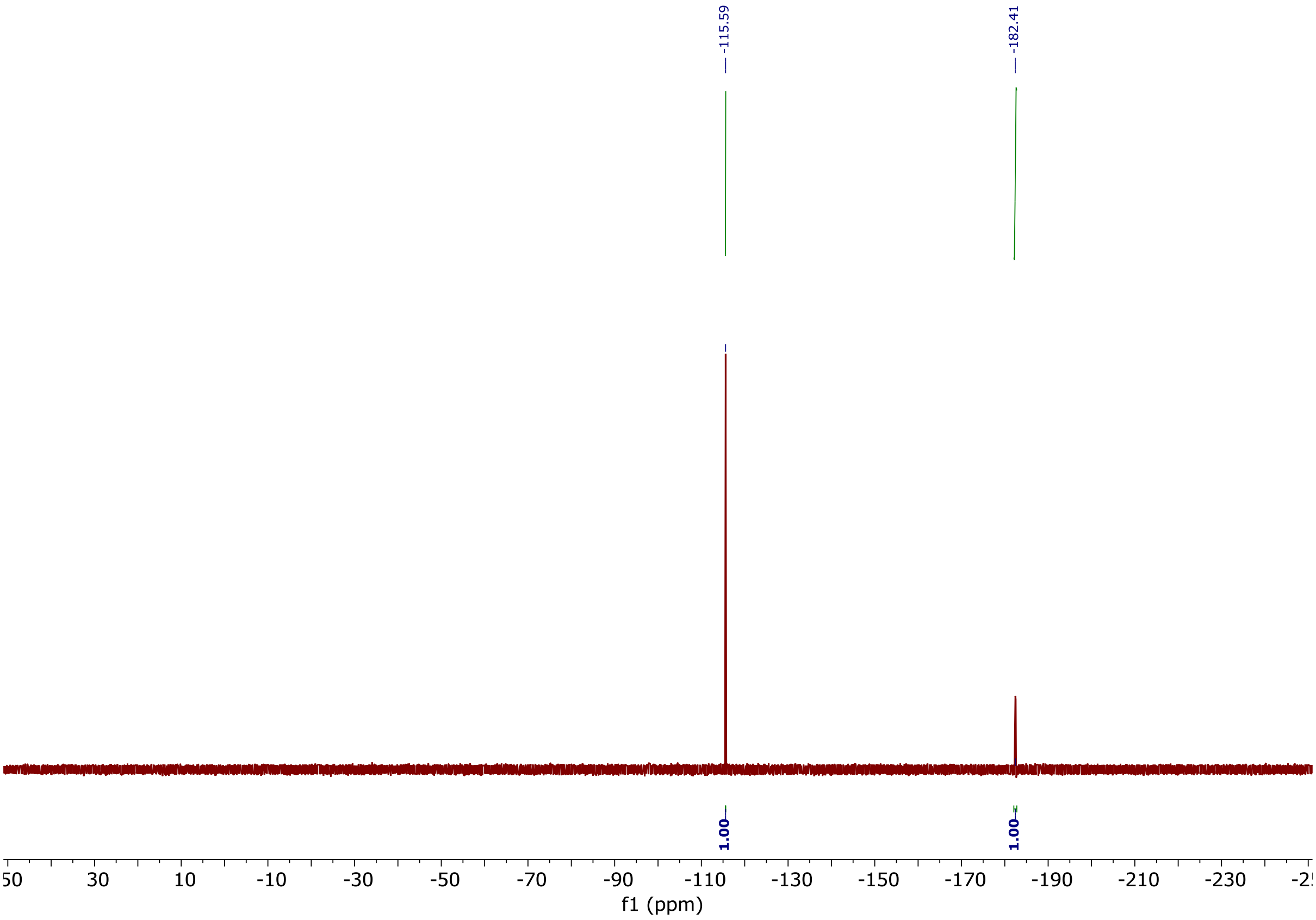
-115.59
-182.41
1.00
1.00
50
30
10
-10
-30
-50
-70
-90
-110
-130
-150
-170
-190
-210
-230
f1 (ppm)

(4-fluoro-2-propyl-2,5-dihydro-1H-pyrrol-1-yl)(phenyl)methanone **(21u)**

^{1}H NMR (500 MHz, CDCl$_3$)

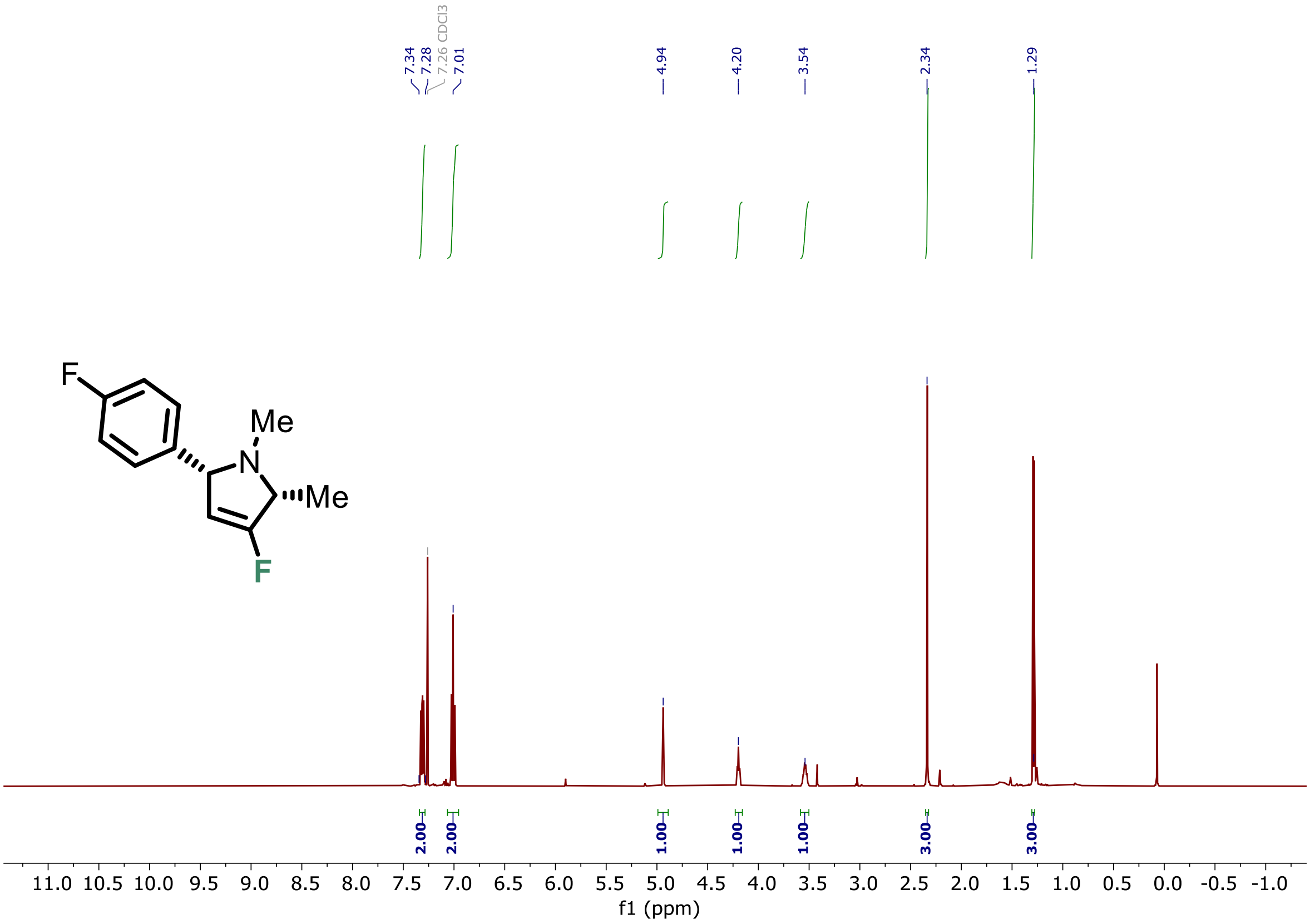

^{13}C NMR (126 MHz, CDCl$_3$)

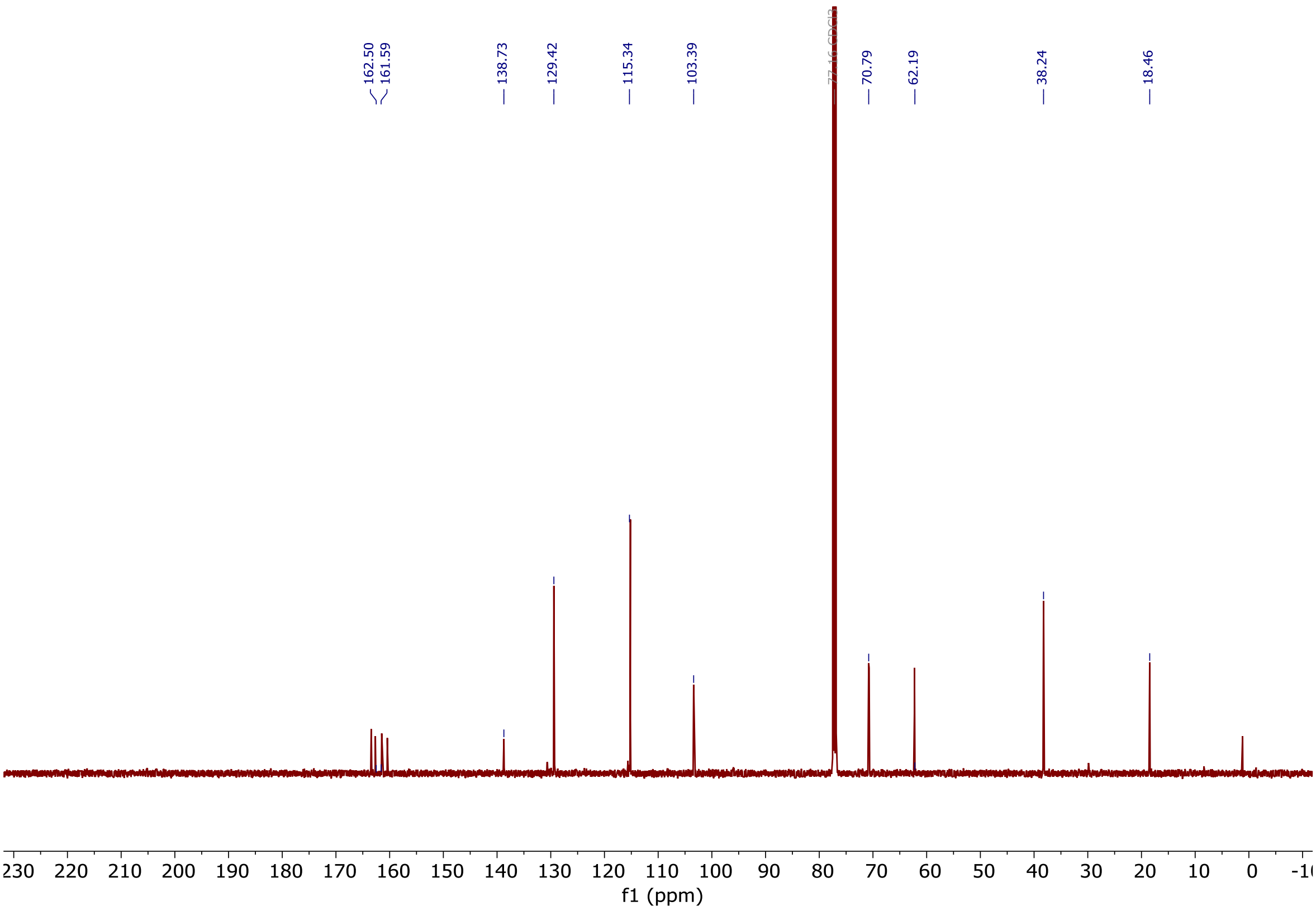

^{19}F NMR (471 MHz, CDCl$_3$)

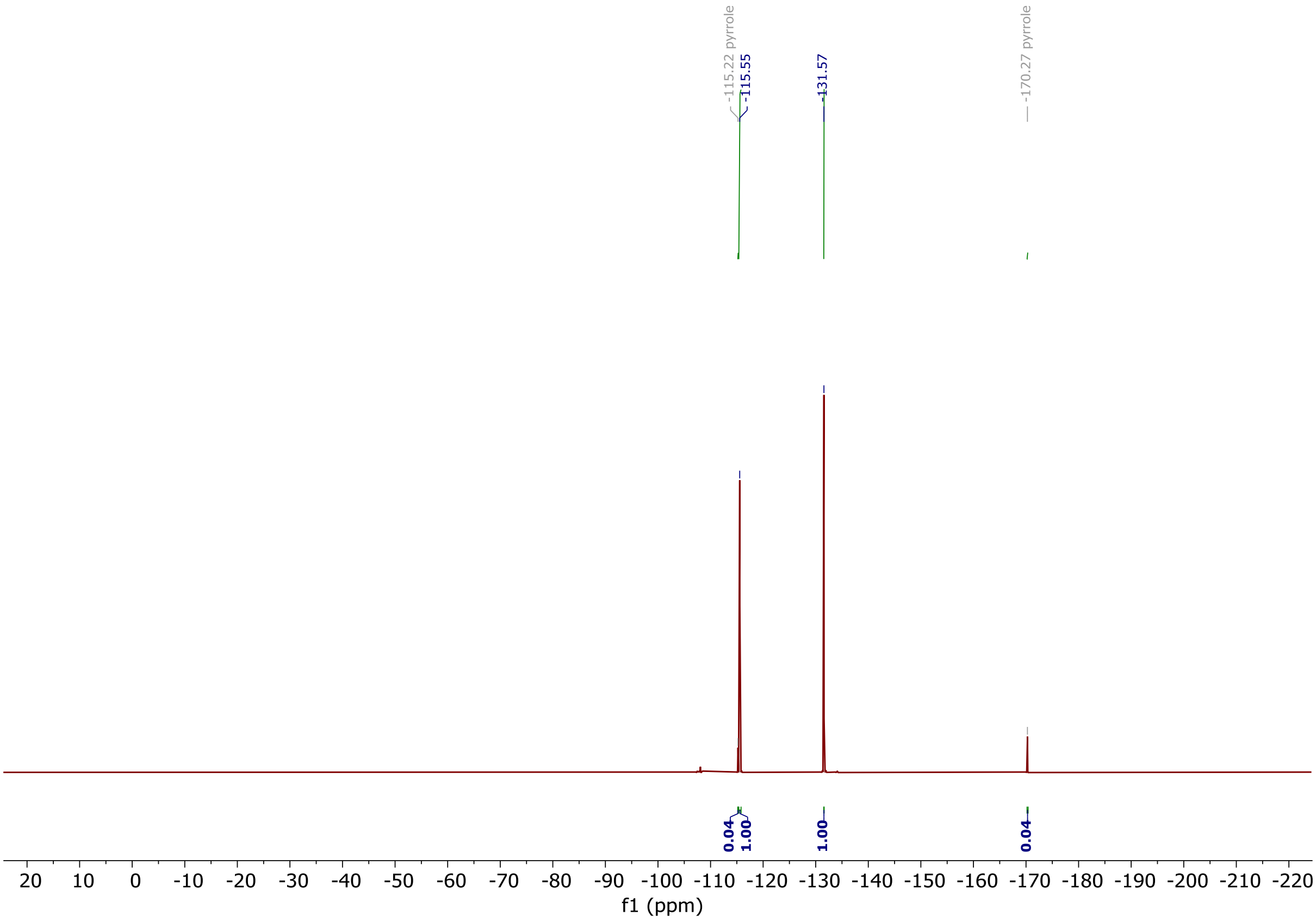

phenyl(2-propyl-2,5-dihydro-1H-pyrrol-1-yl)methanone **(21bf)**

^{1}H NMR (500 MHz, $CDCl_3$)

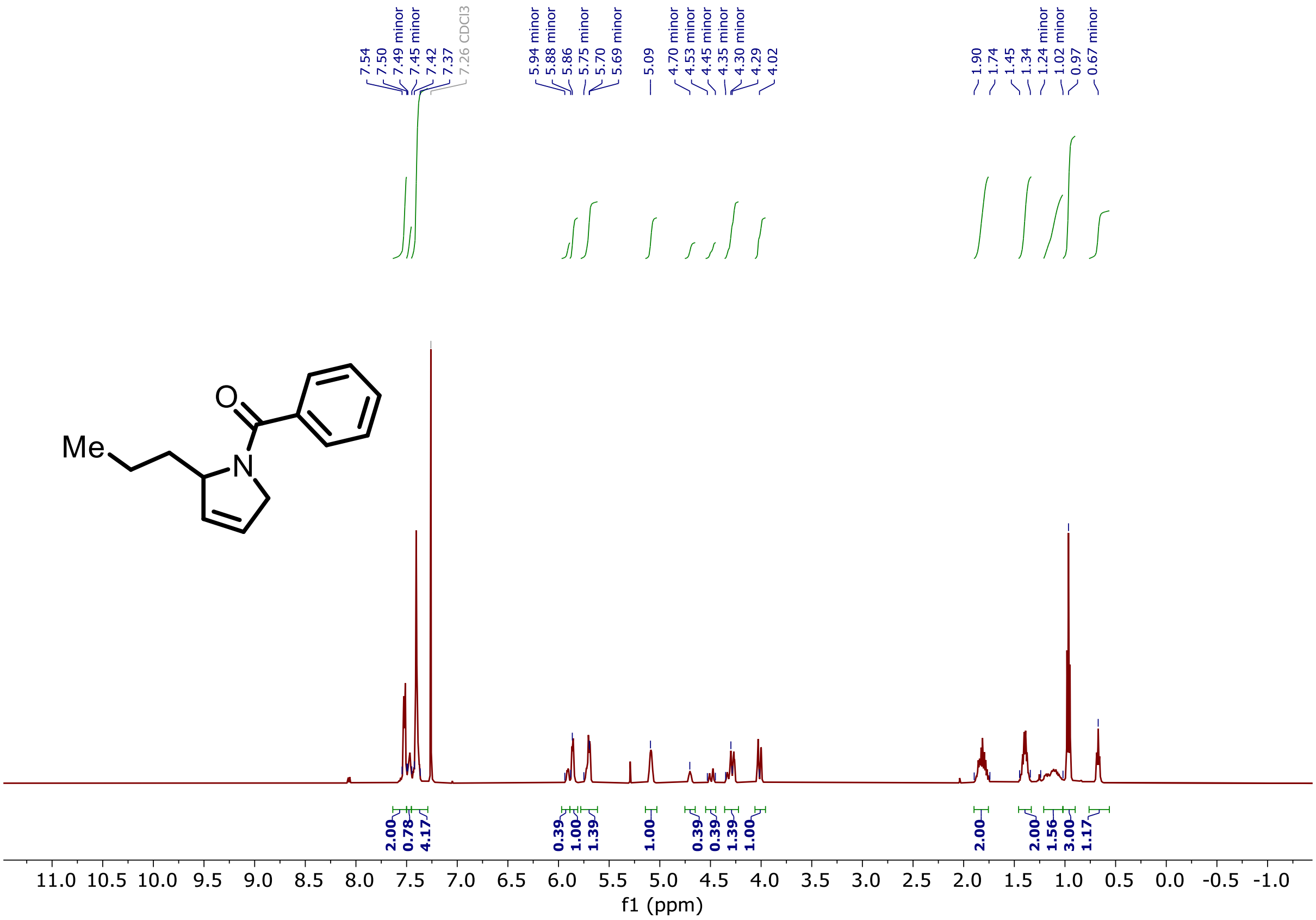

^{13}C NMR (126 MHz, $CDCl_3$)

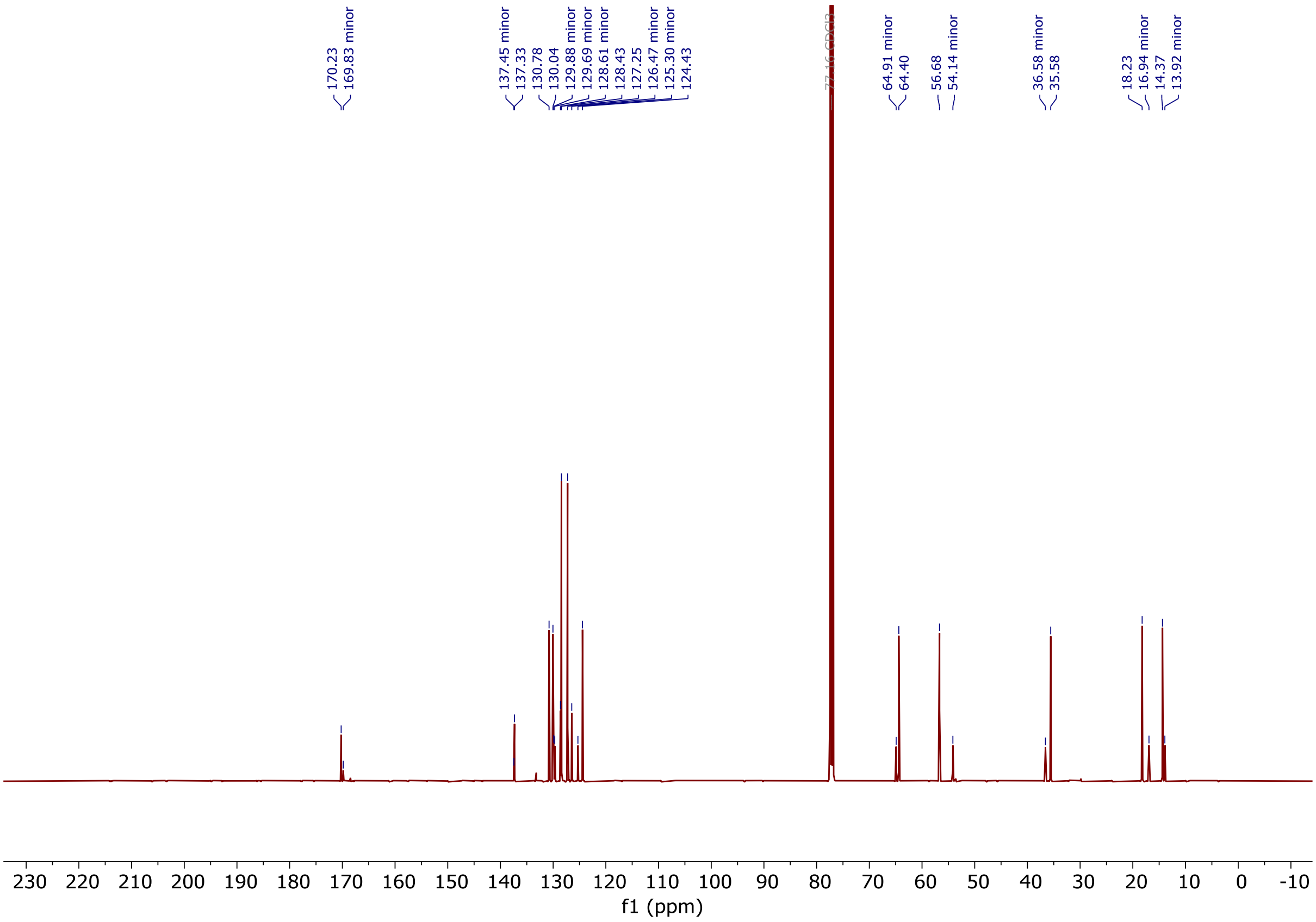

***(4-fluoro-2-propyl-2,5-dihydro-1H-pyrrol-1-yl)(trans-2-phenylcyclopropyl)methanone* (22v)**

^{1}H NMR (500 MHz, CDCl$_3$)

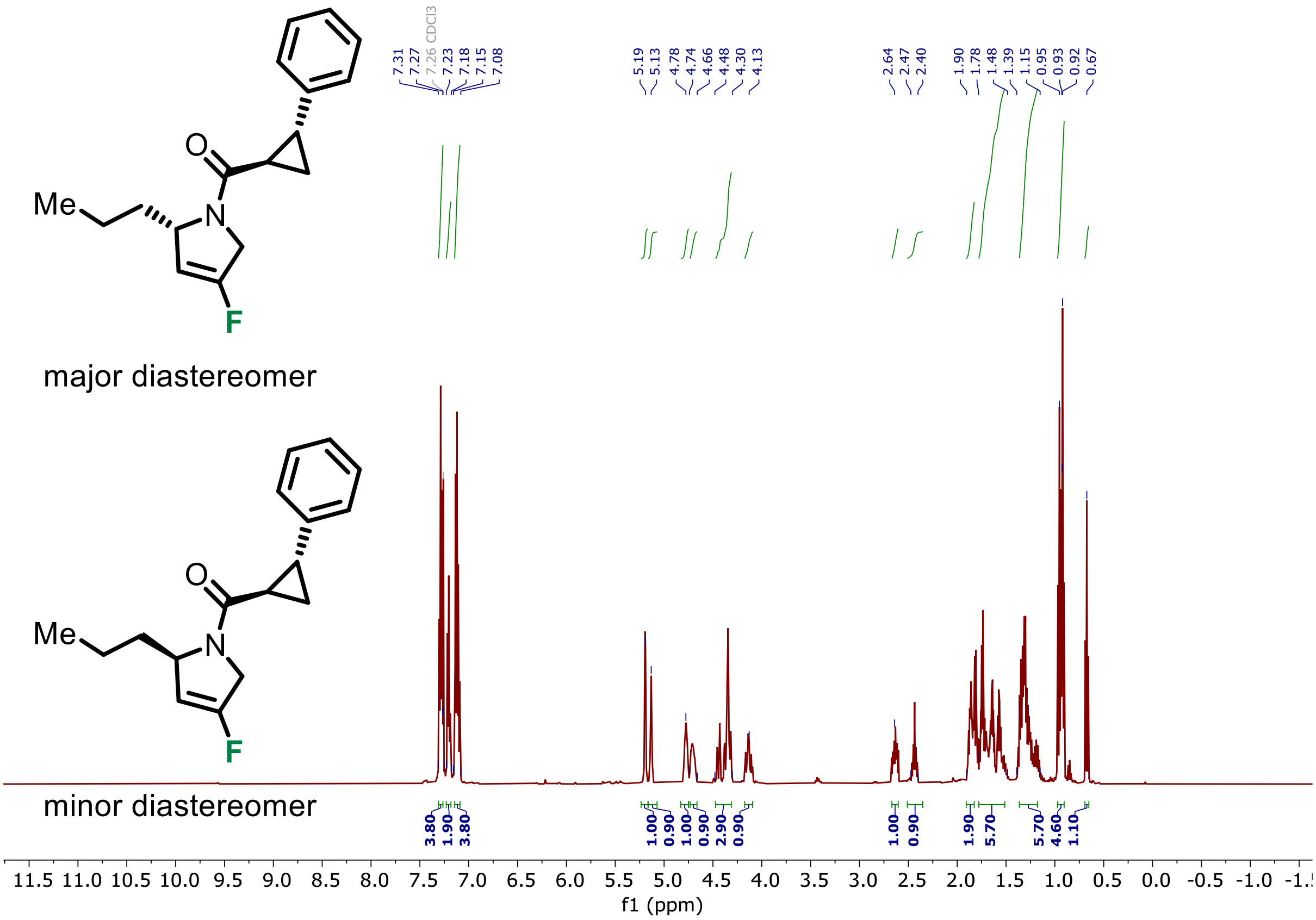

^{13}C NMR (126 MHz, CDCl$_3$)

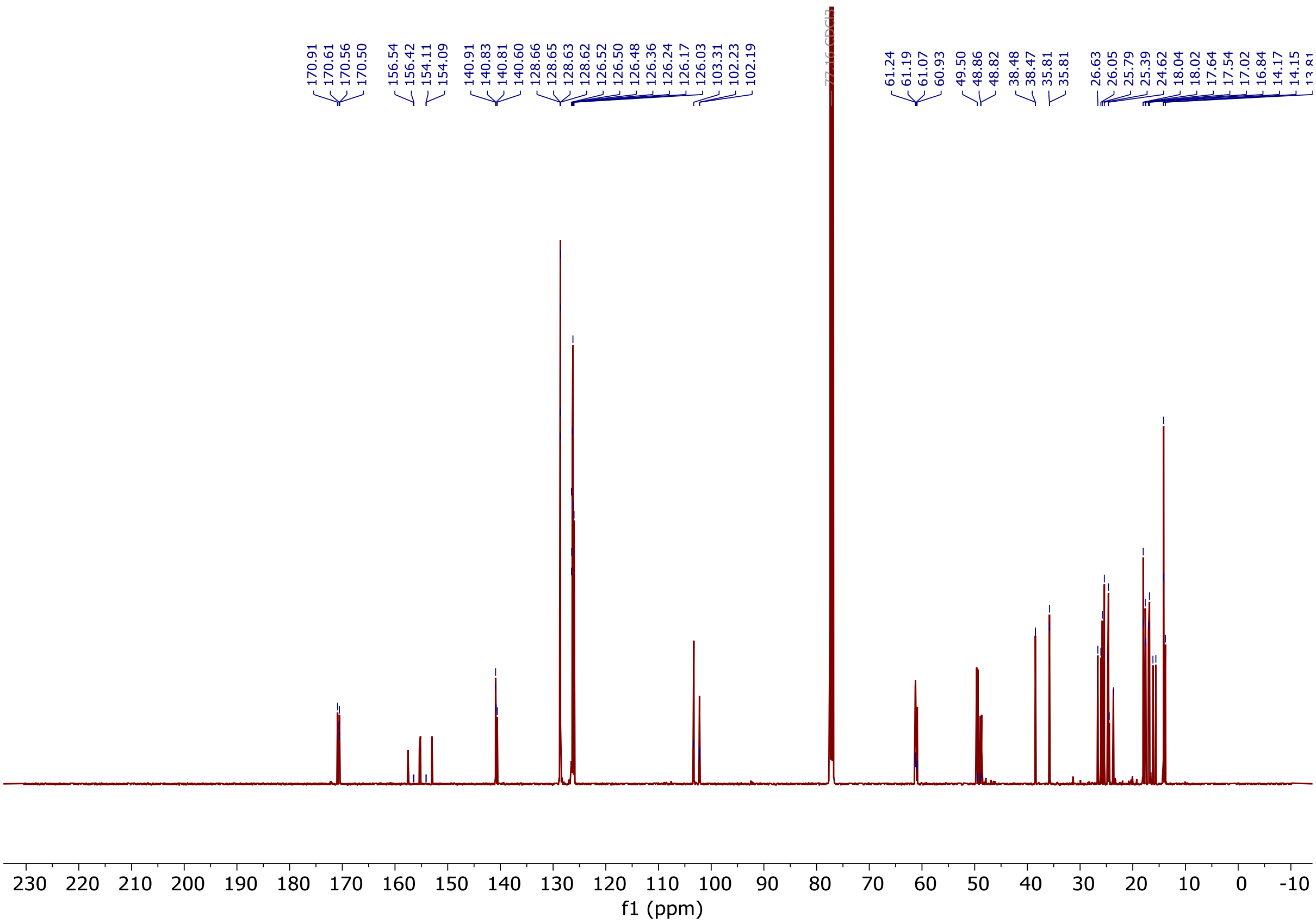

^{19}F NMR (376 MHz, CDCl$_3$)

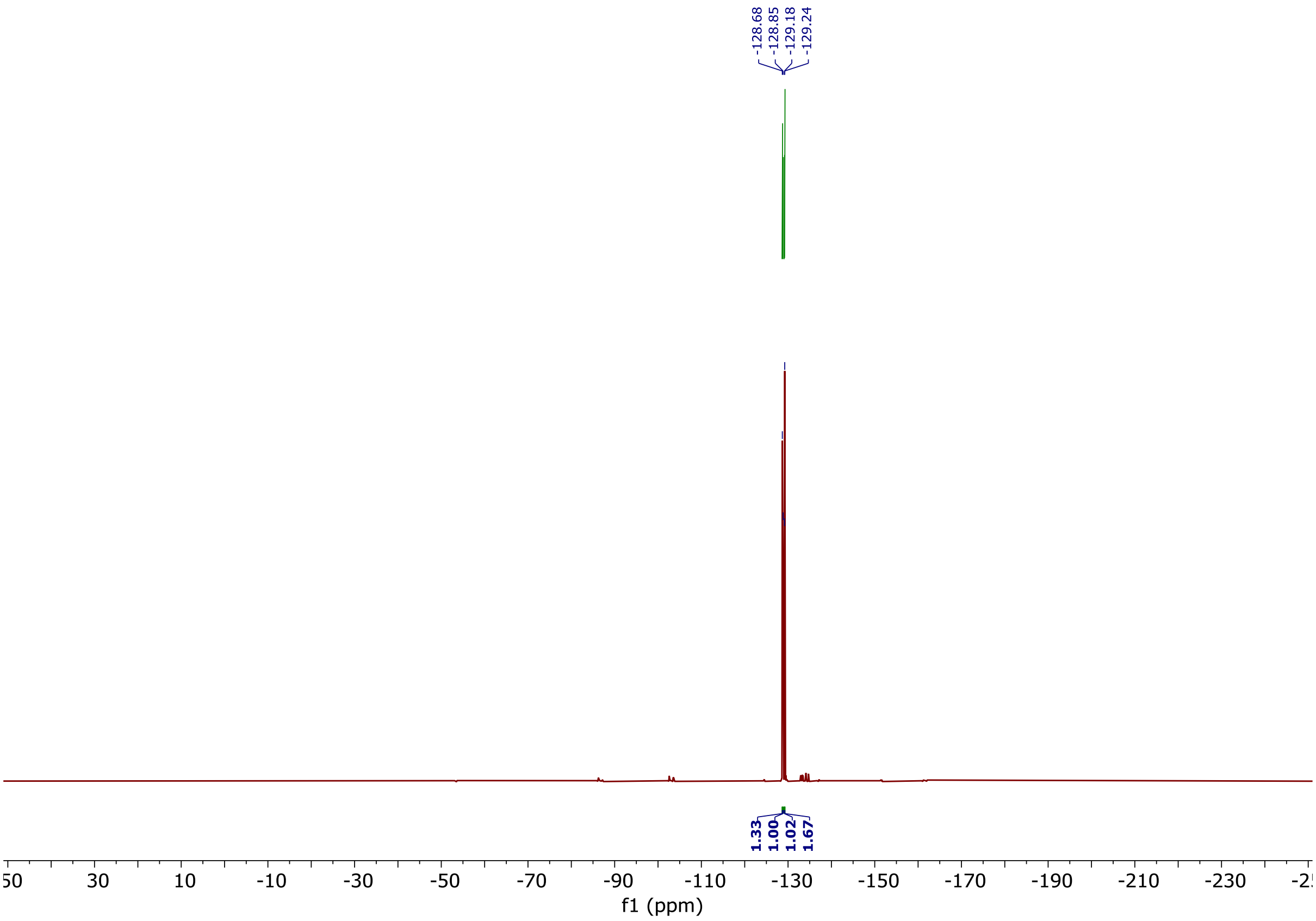

(3aS,7R,7aS)-2-benzoyl-7-(4-fluoro-2-(4-fluorophenyl)-2,5-dihydro-1H-pyrrole-1-carbonyl)-5-(3-methoxyphenyl)-2,3,3a,6,7,7a-hexahydro-1H-isoindol-1-one (**23e**)
(2 diastereomers, 4 rotamers each)

^{1}H NMR (500 MHz, CDCl3)

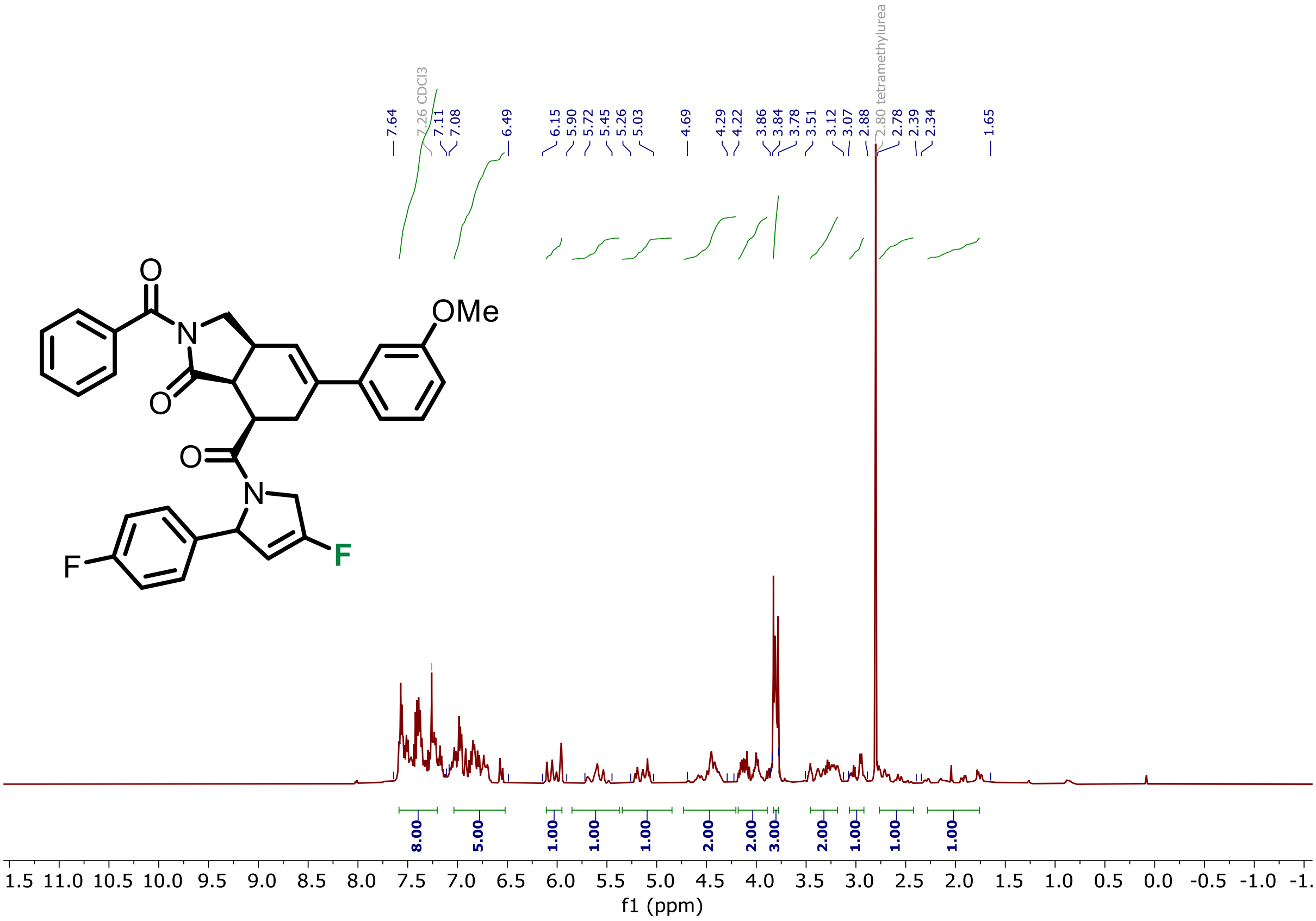

^{13}C NMR (126 MHz, $CDCl_3$)

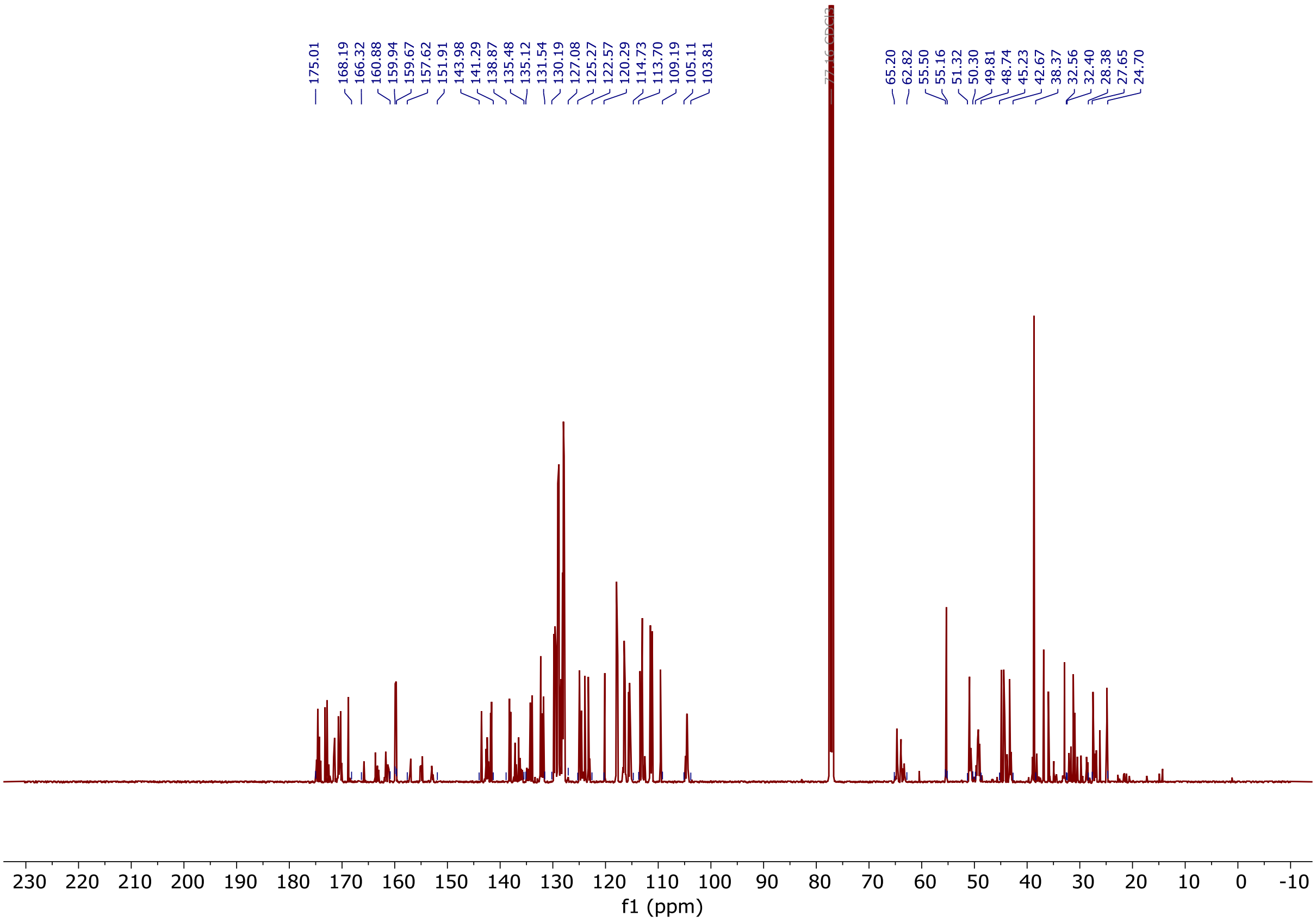

^{19}F NMR (376 MHz, $CDCl_3$)

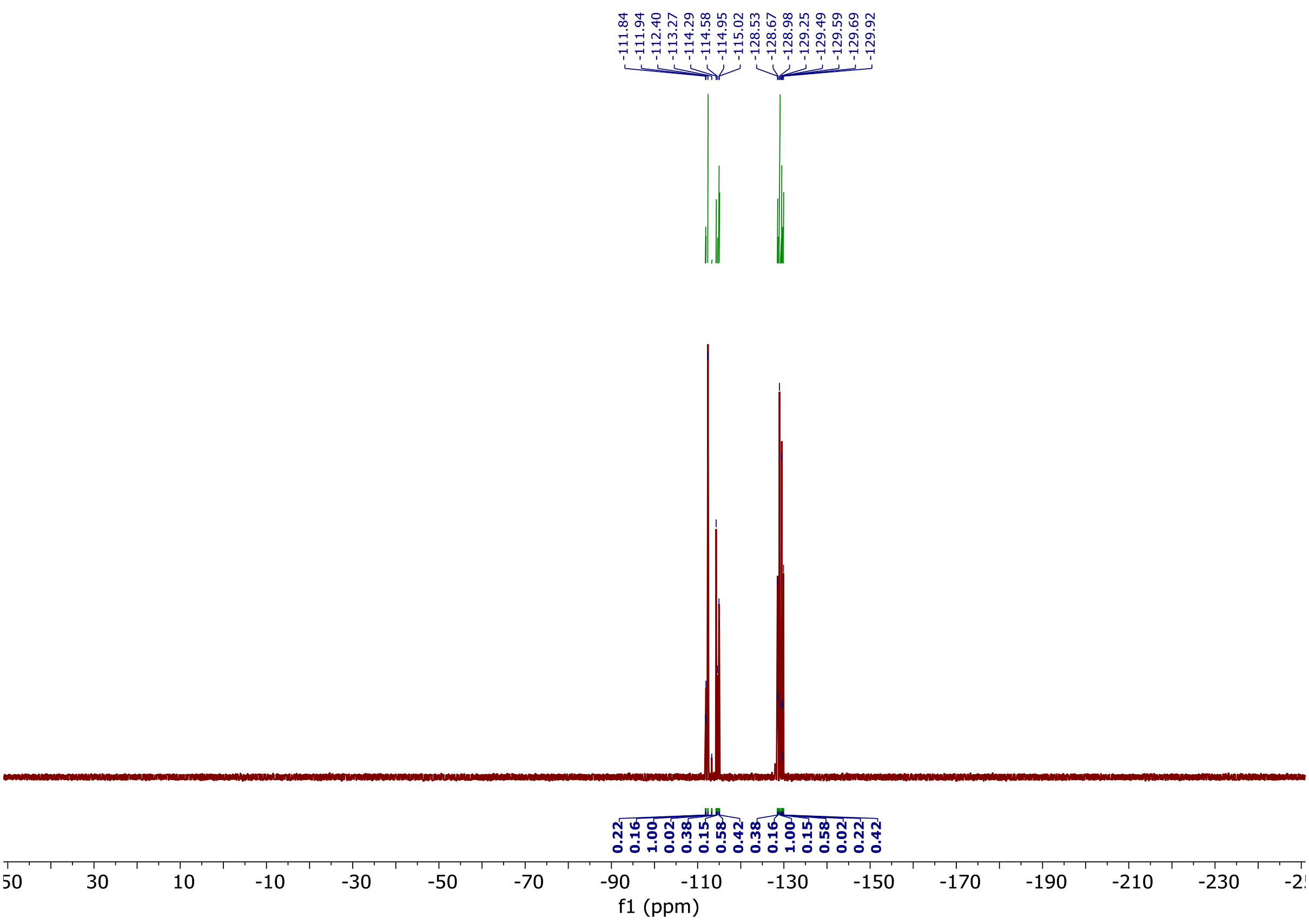

***(1R,4S)-1-((R)-4-fluoro-2-propyl-2,5-dihydro-1H-pyrrole-1-carbonyl)-4,7,7-trimethyl-2-oxabicyclo[2.2.1]heptan-3-one* (24v)**

^{1}H NMR (500 MHz, $CDCl_3$)

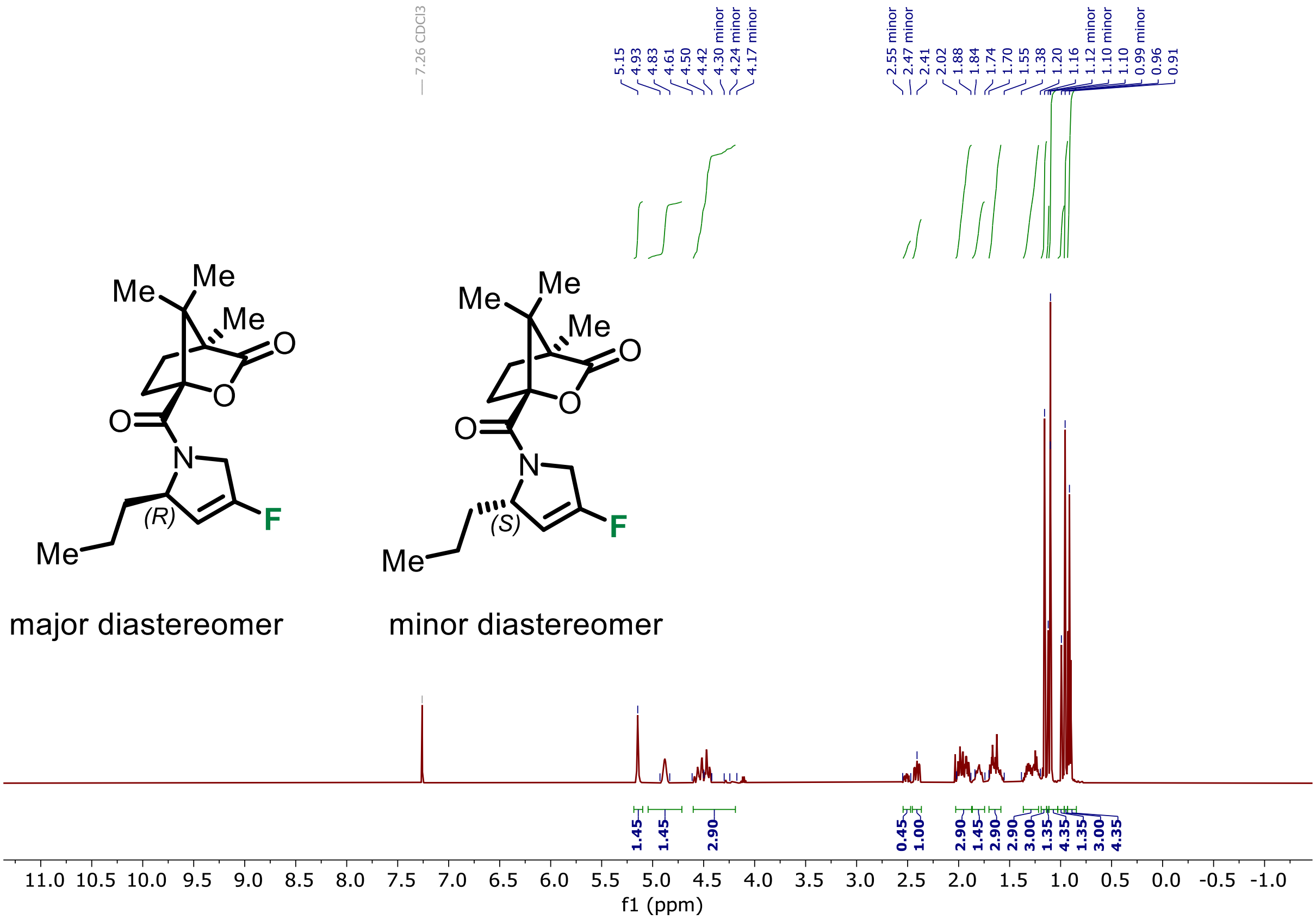

^{13}C NMR (126 MHz, $CDCl_3$)

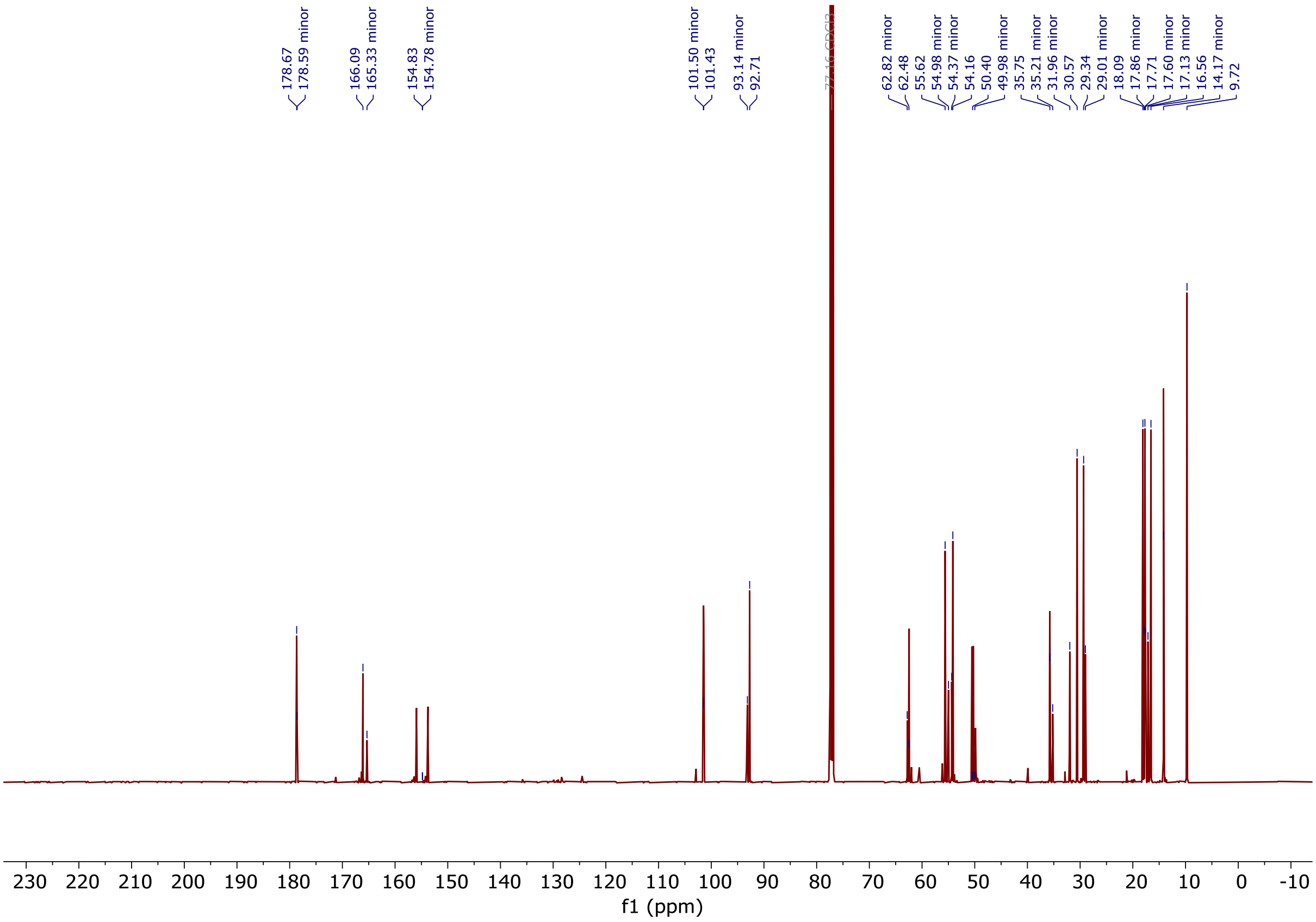

^{19}F NMR (376 MHz, $CDCl_3$)

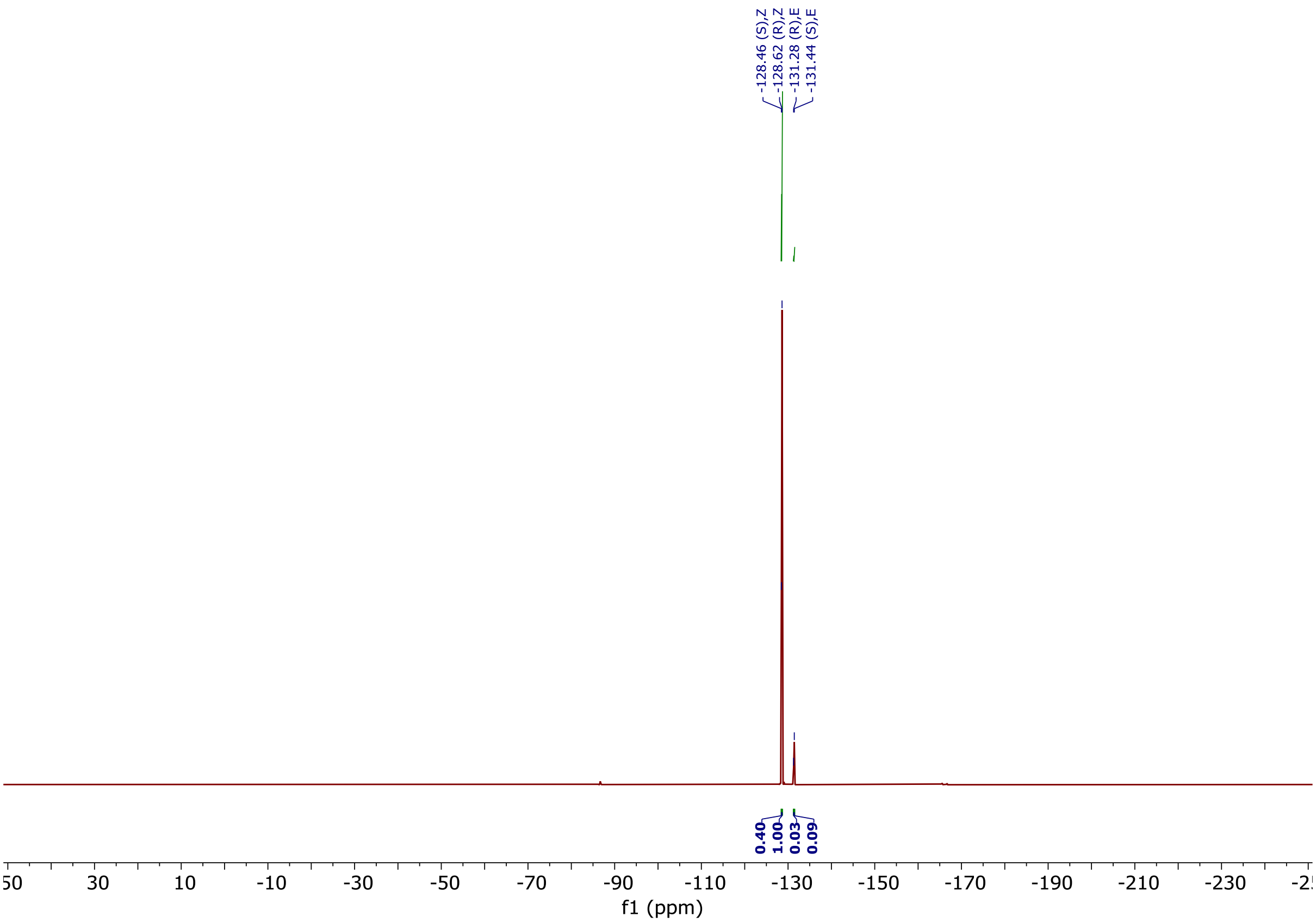

(3aS,7R,7aS)-2-benzoyl-5-(3-bromophenyl)-7-((2R,5R)-3-fluoro-5-(4-fluorophenyl)-2-methyl-2,5-dihydro-1H-pyrrole-1-carbonyl)-2,3,3a,6,7,7a-hexahydro-1H-isoindol-1-one **((*R*,*R*)-25j)**
(4 rotamers)

^{1}H NMR (500 MHz, $CDCl_3$)

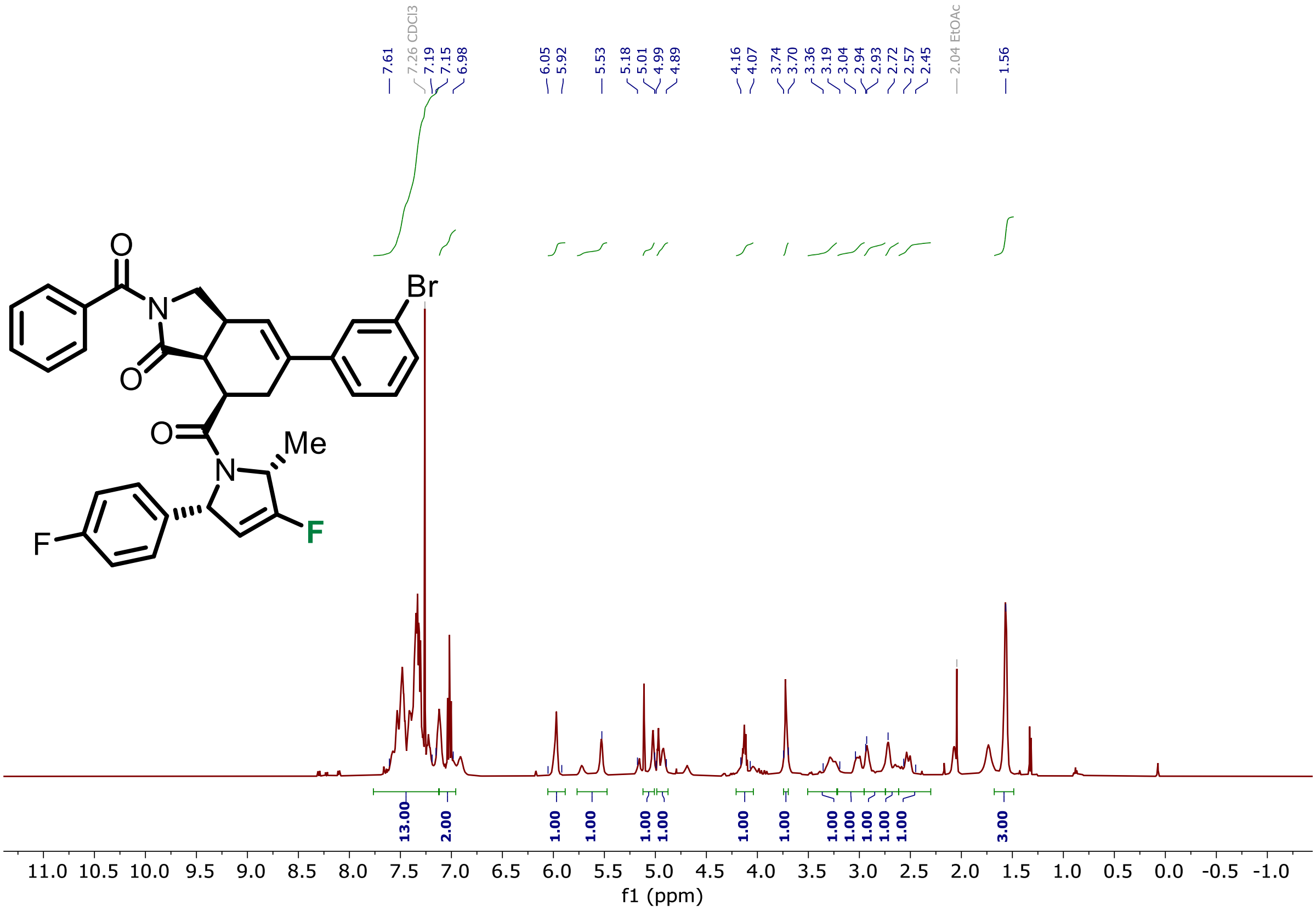

^{13}C NMR (126 MHz, $CDCl_3$)

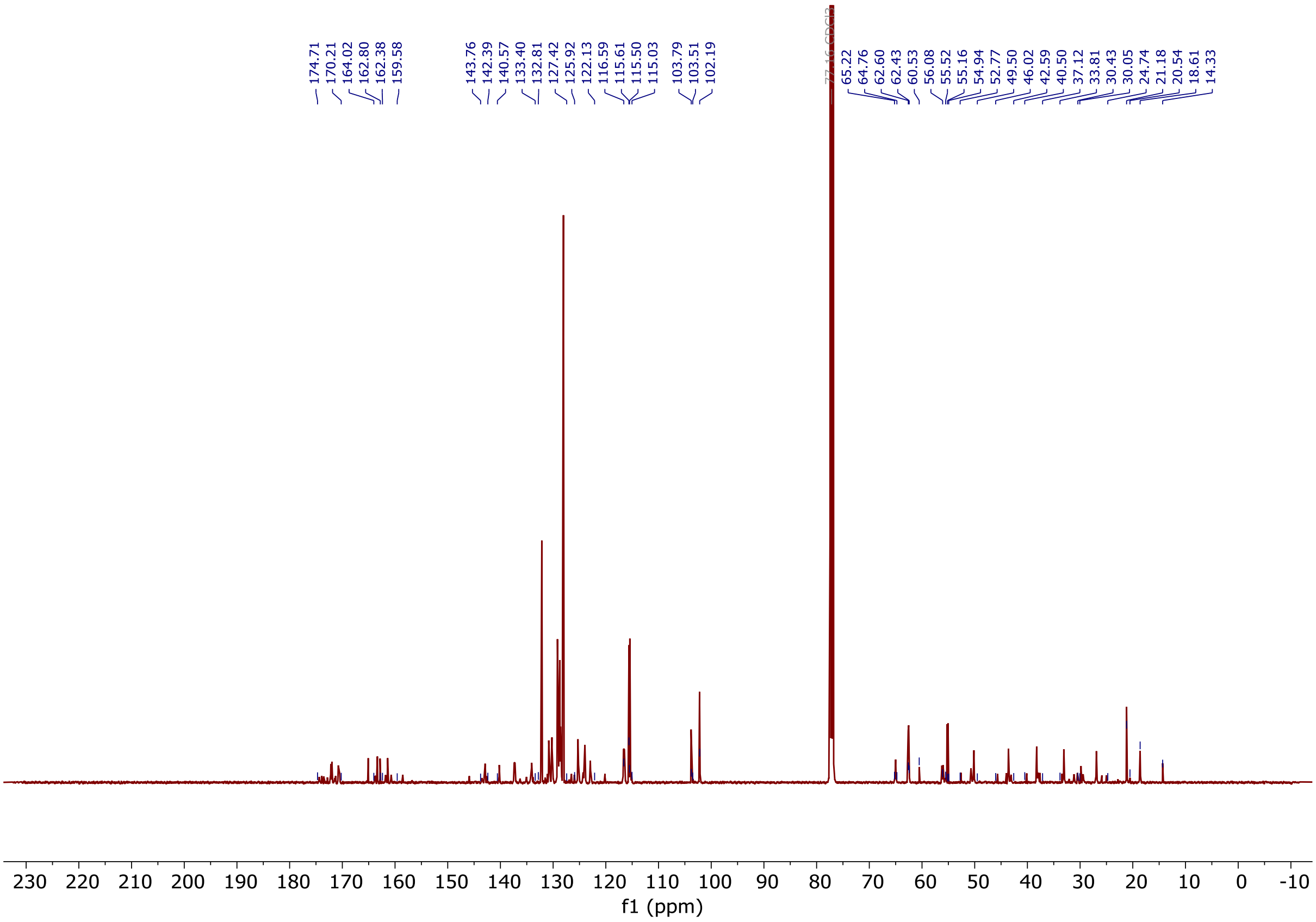

^{19}F NMR (471 MHz, $CDCl_3$)

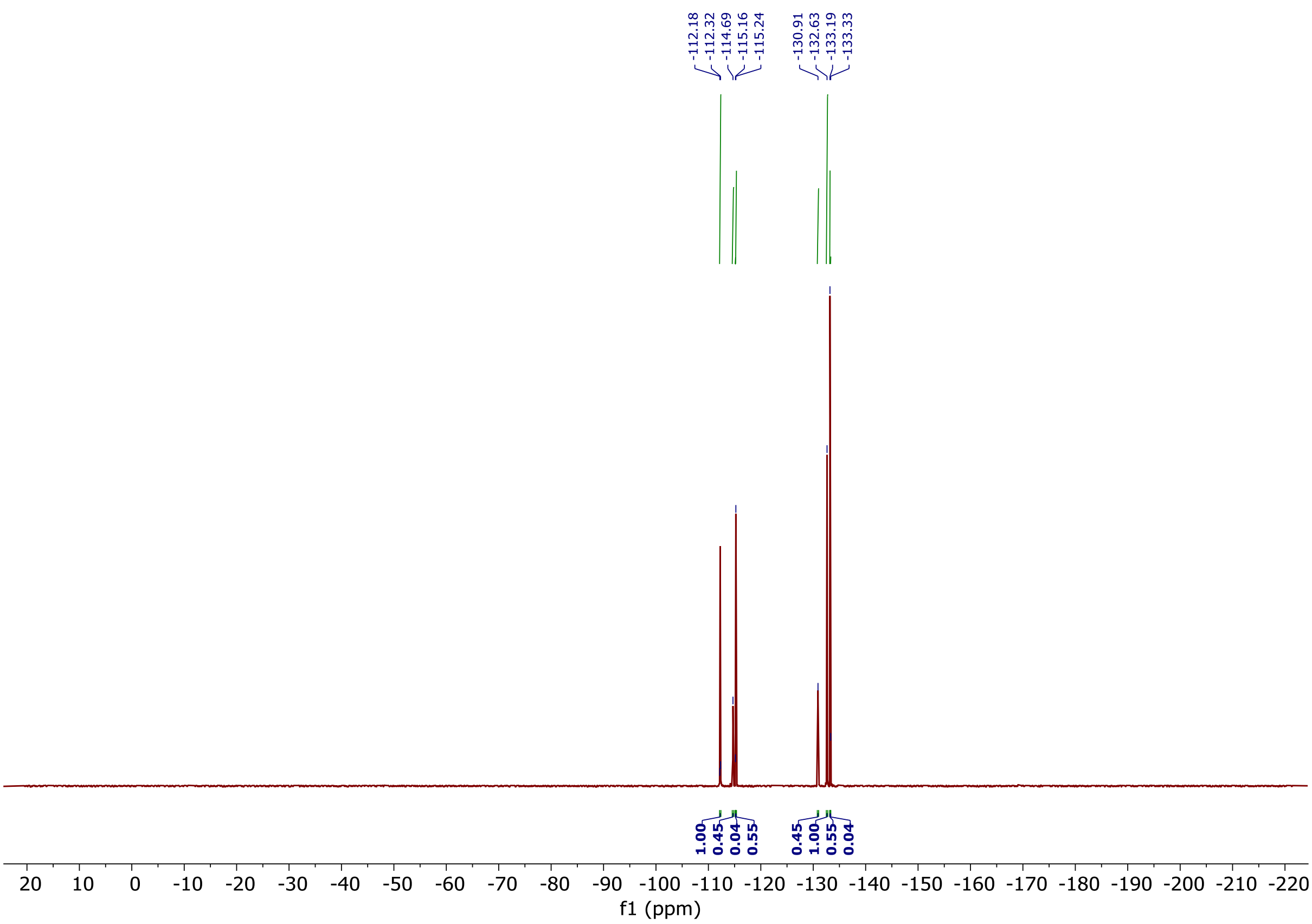

9.18. Pre-alkyne intermediates (26)

N-((4-fluorophenyl)methyl-d)prop-2-yn-1-amine **([²H]-26e)**

¹H NMR (500 MHz, CDCl₃)

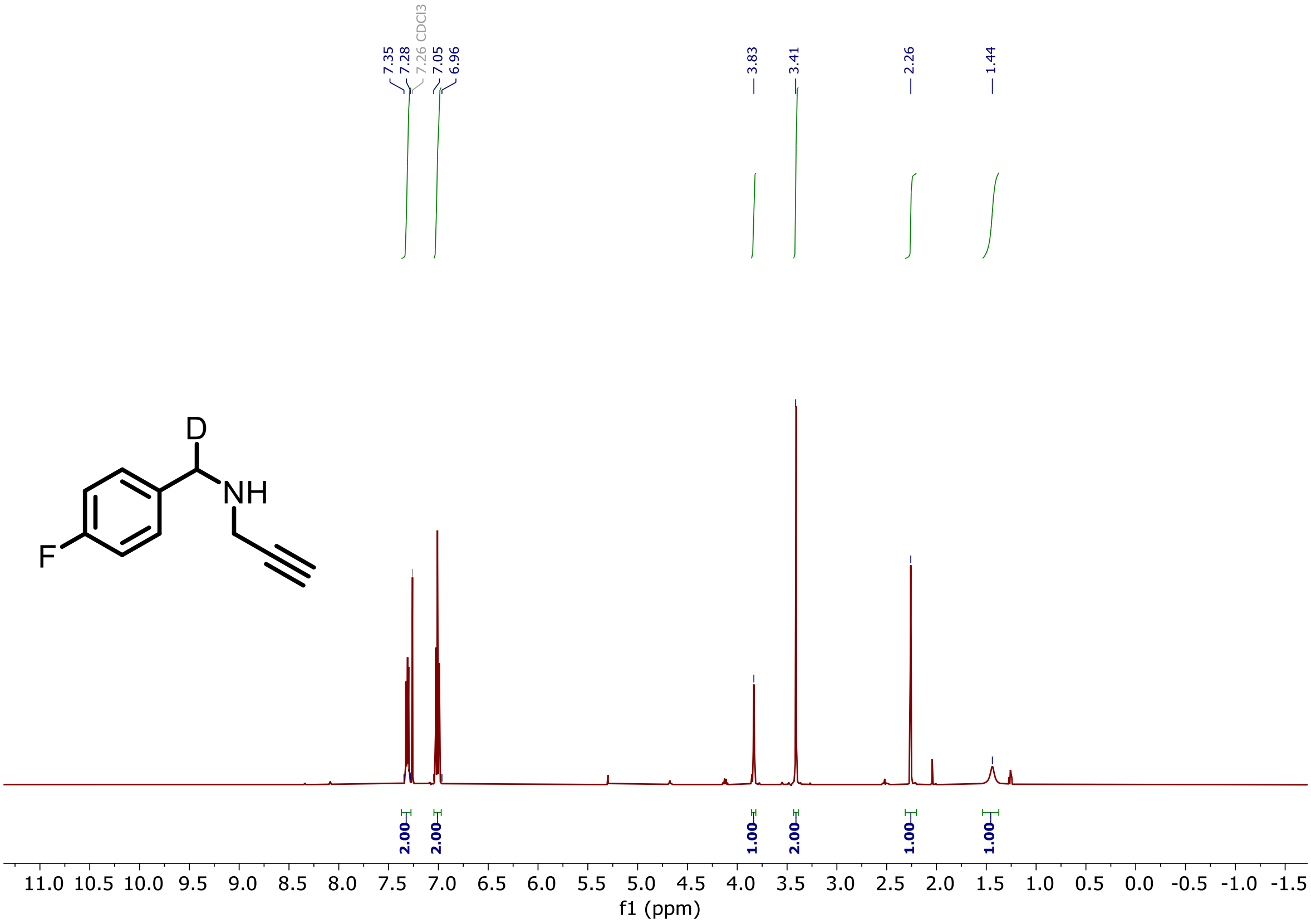

¹³C NMR (126 MHz, CDCl₃)

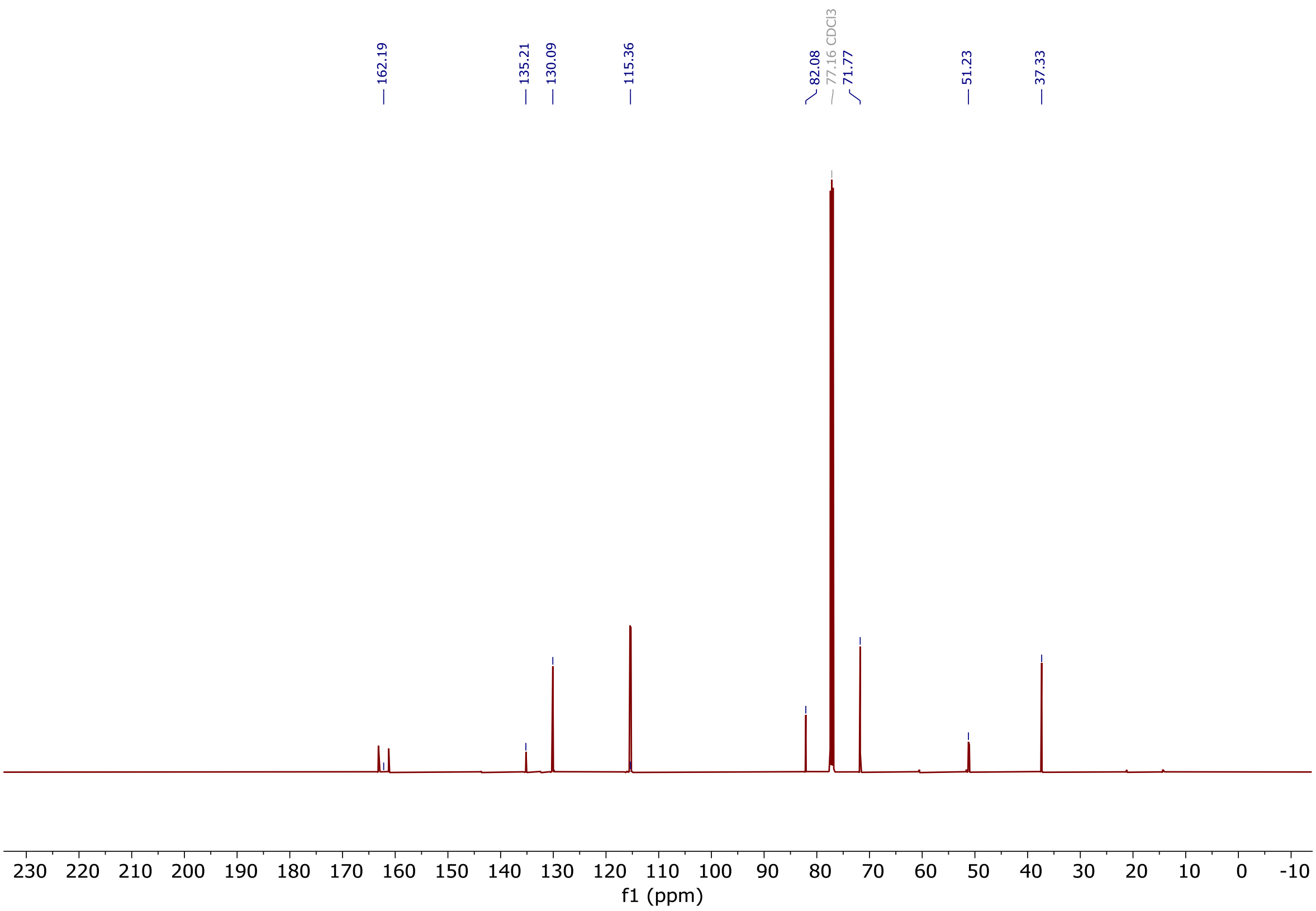

^{19}F NMR (376 MHz, CDCl$_3$)

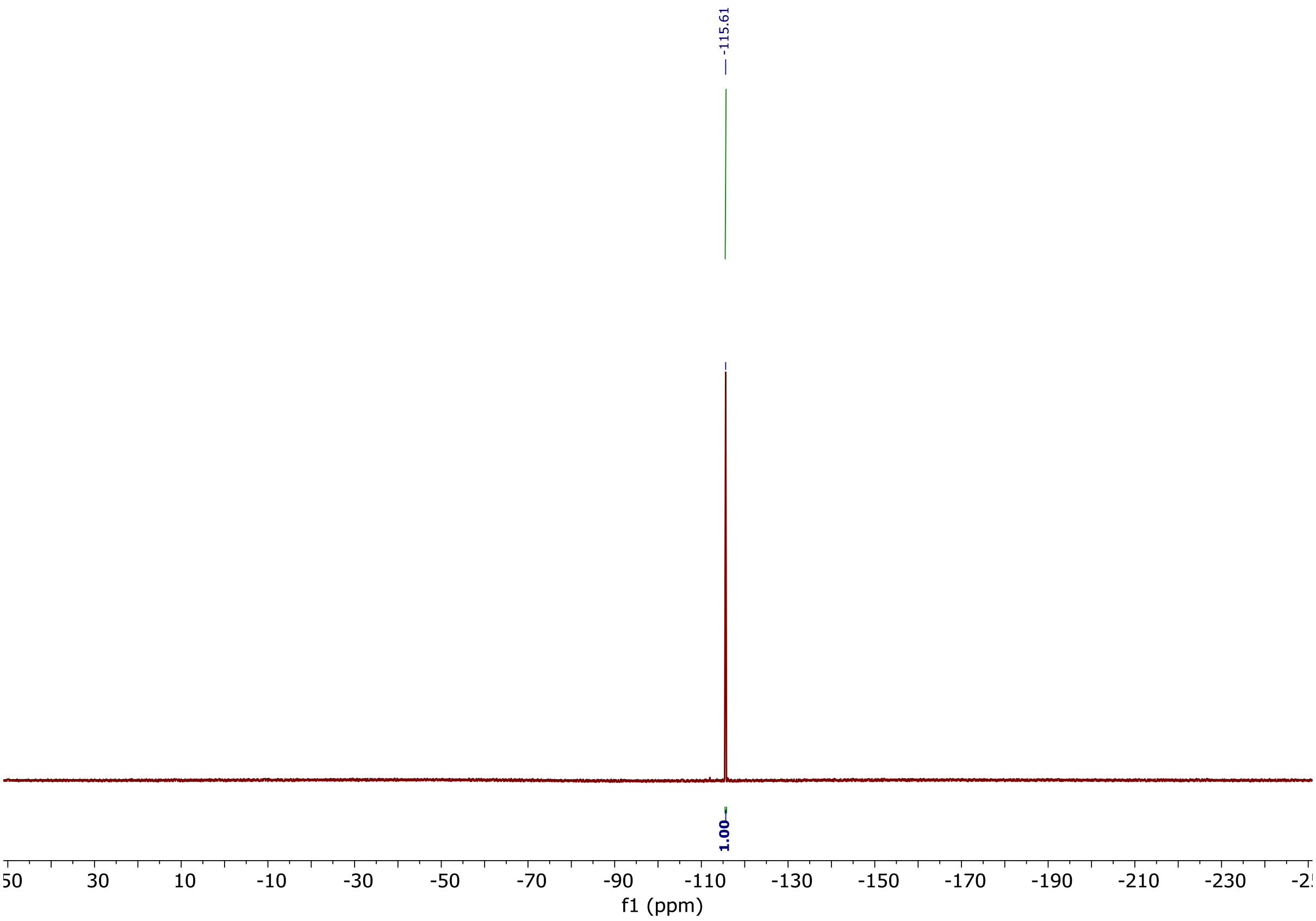

N-(4-fluorobenzyl)-2,4-dinitrobenzenesulfonamide **(26bq)**

^{1}H NMR (500 MHz, Acetone-D_6)

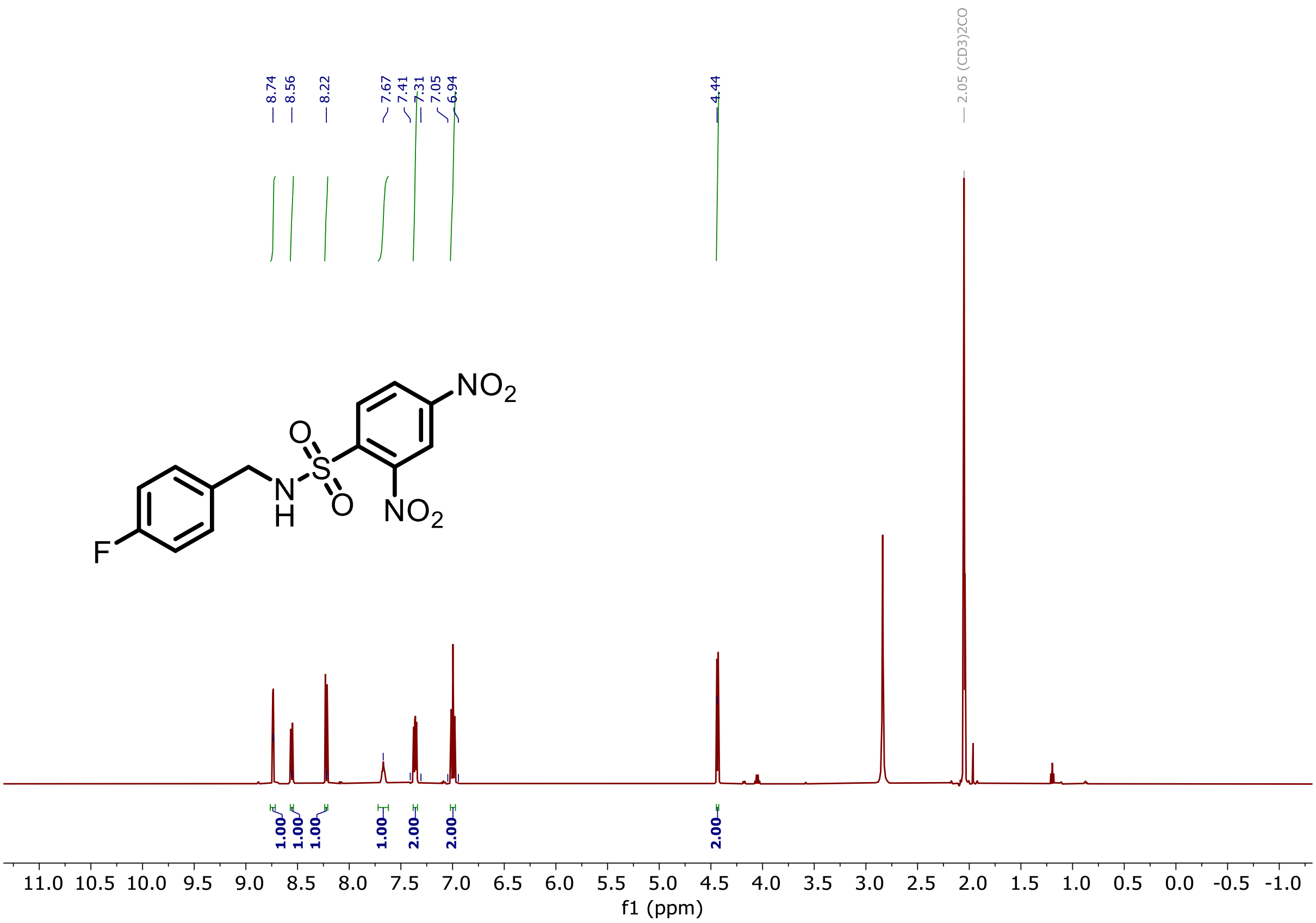

^{13}C NMR (126 MHz, Acetone-D_6)

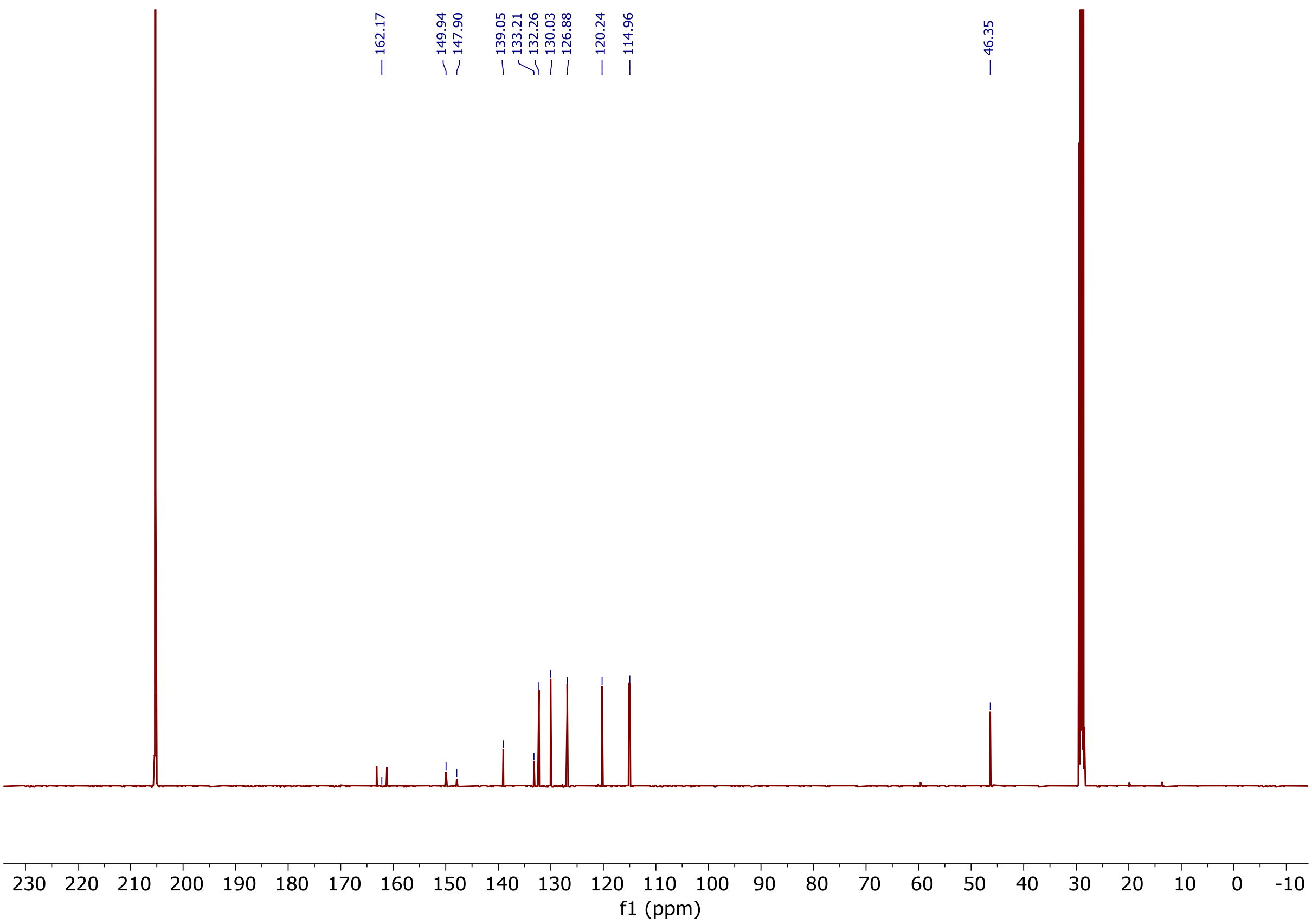

^{19}F NMR (376 MHz, Acetone-D_6)

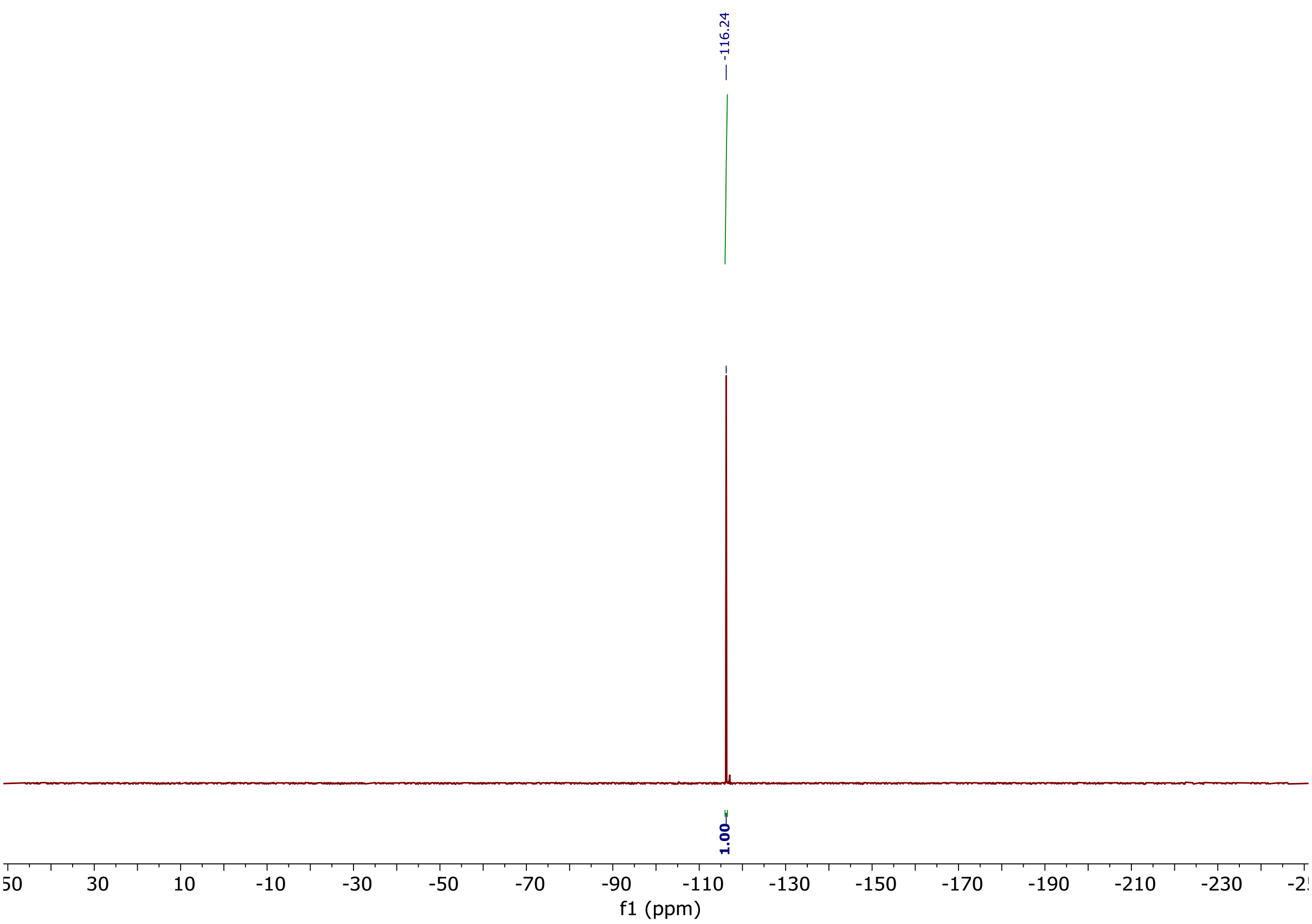

9.19. Miscellaneous (27-28)

***(Z)-N-(4-bromobenzyl)-N-(2-fluoro-3-iodoallyl)-4-methylbenzenesulfonamide* (27c)**

^{1}H NMR (500 MHz, CDCl$_3$)

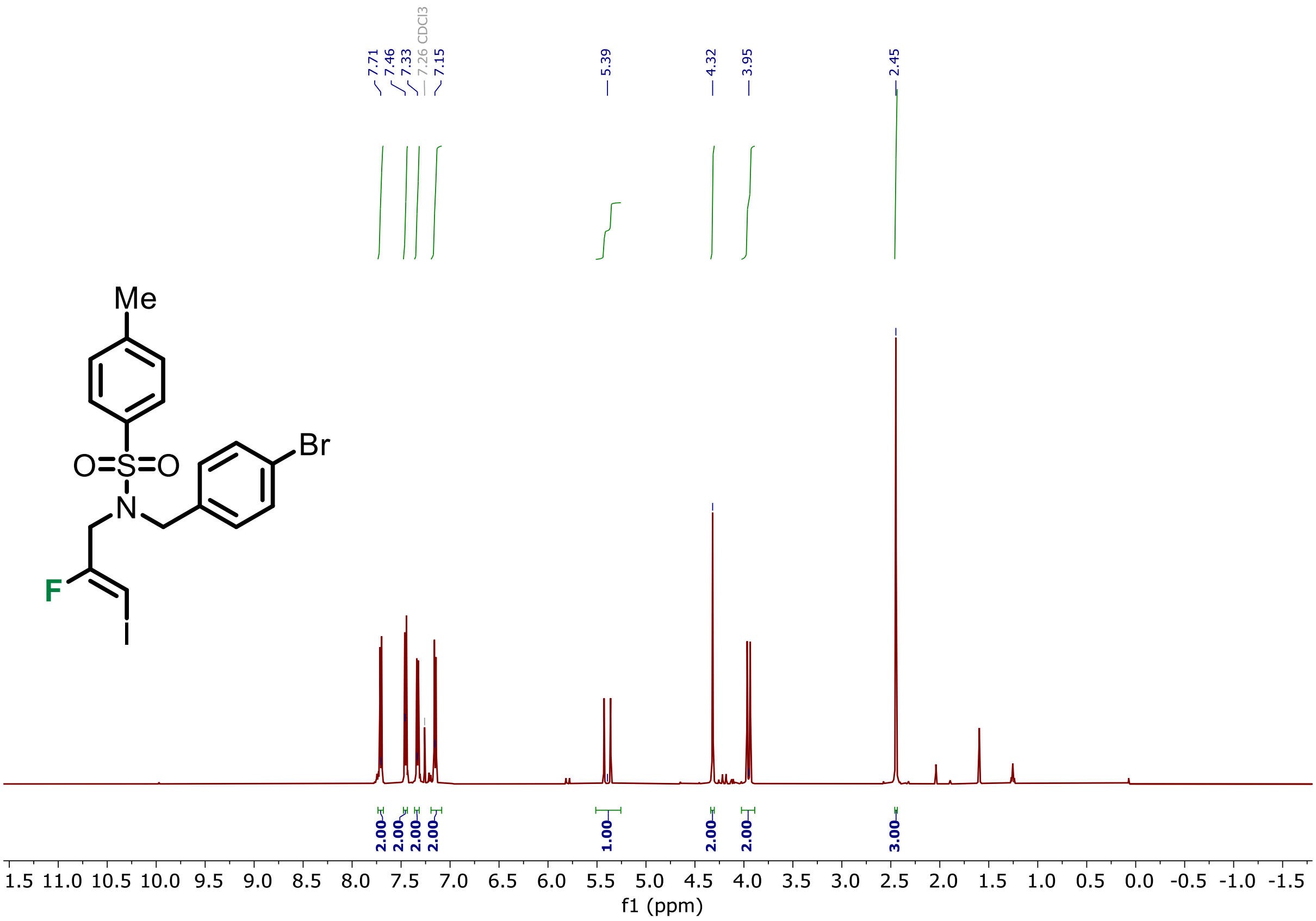

^{13}C NMR (126 MHz, CDCl$_3$)

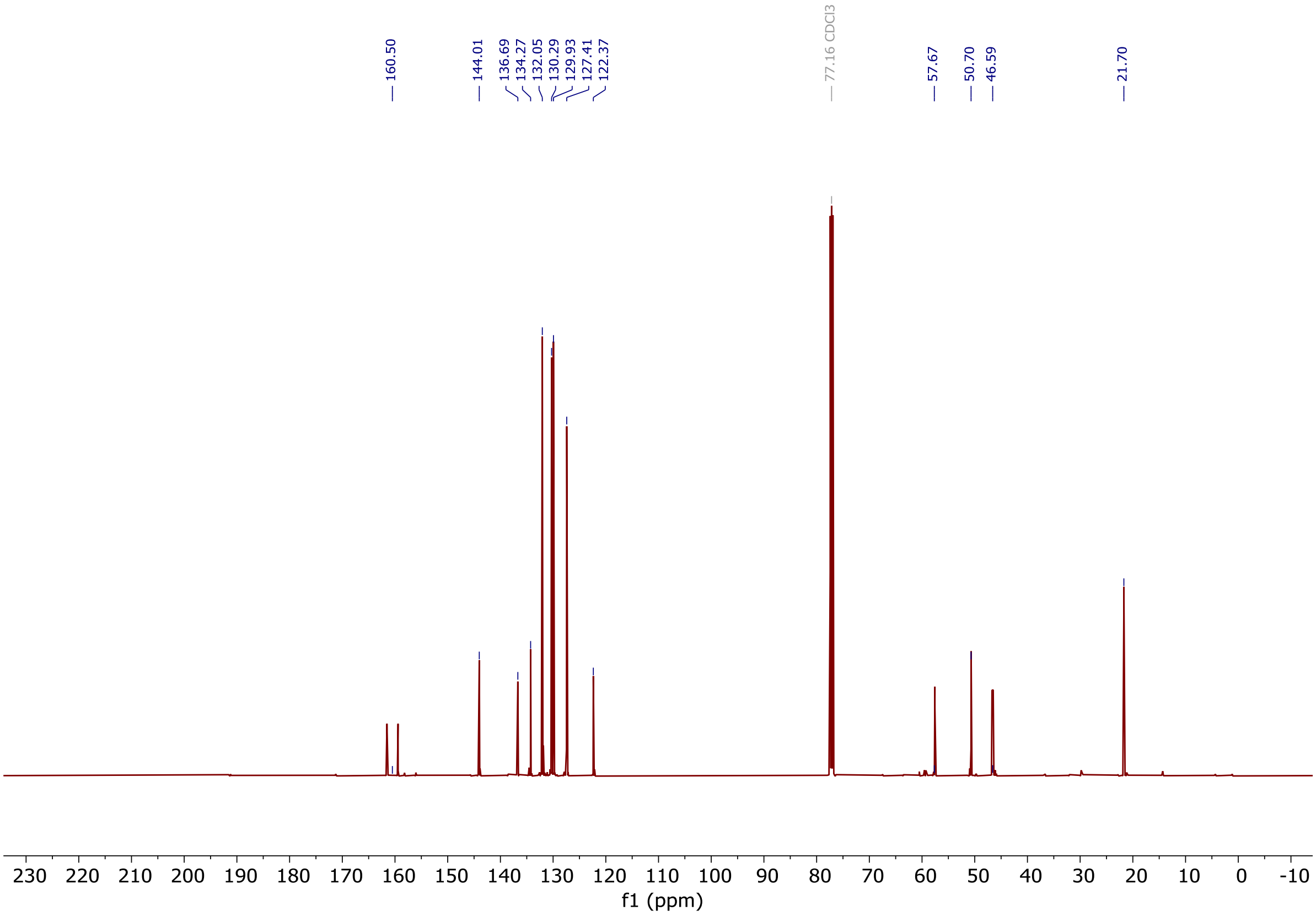

^{19}F NMR (376 MHz, CDCl$_3$)

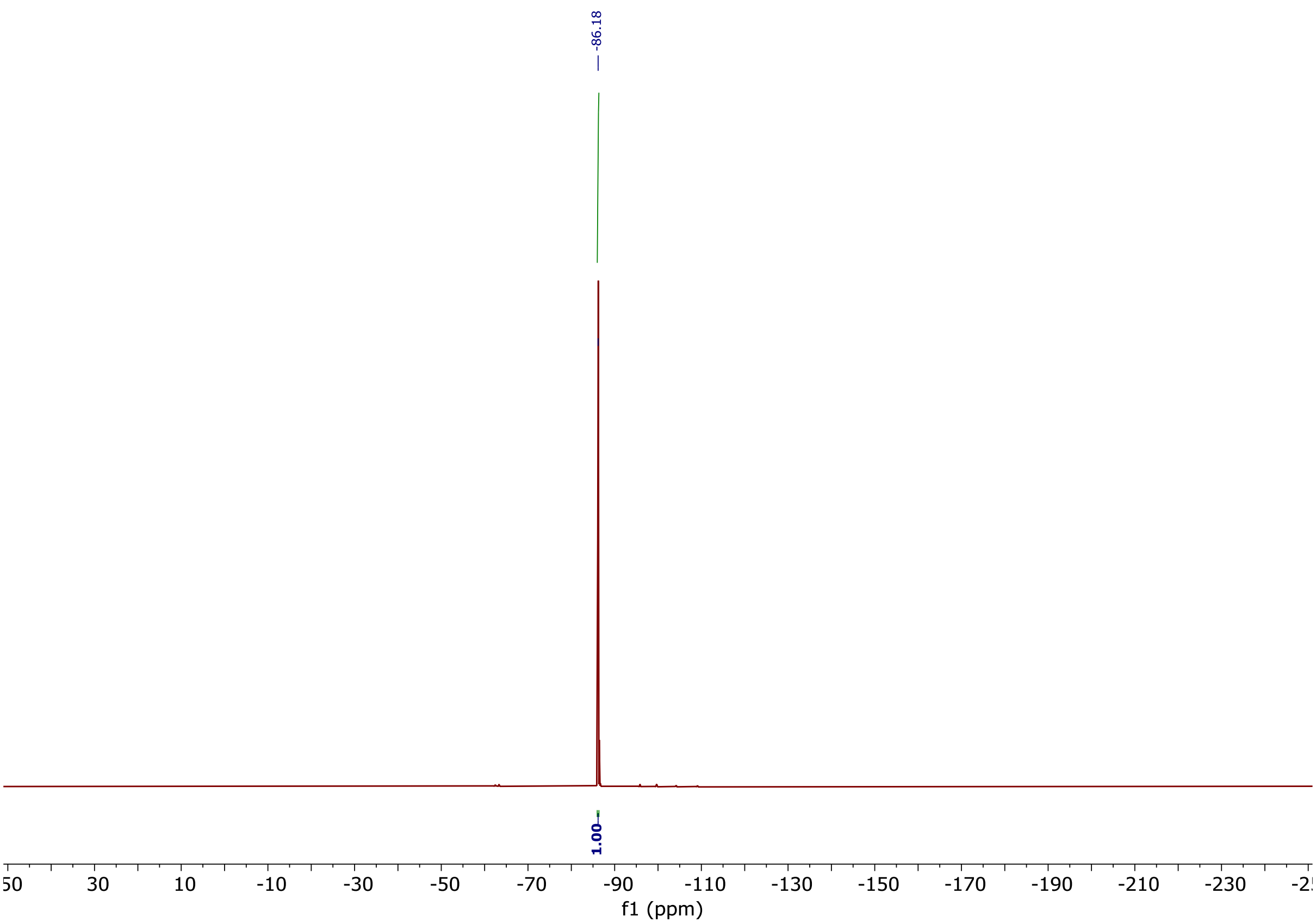

***N*-(4-bromobenzyl)-*N*-(3-iodoprop-2-yn-1-yl)-4-methylbenzenesulfonamide** **(28c)**

^{1}H NMR (500 MHz, CDCl$_3$)

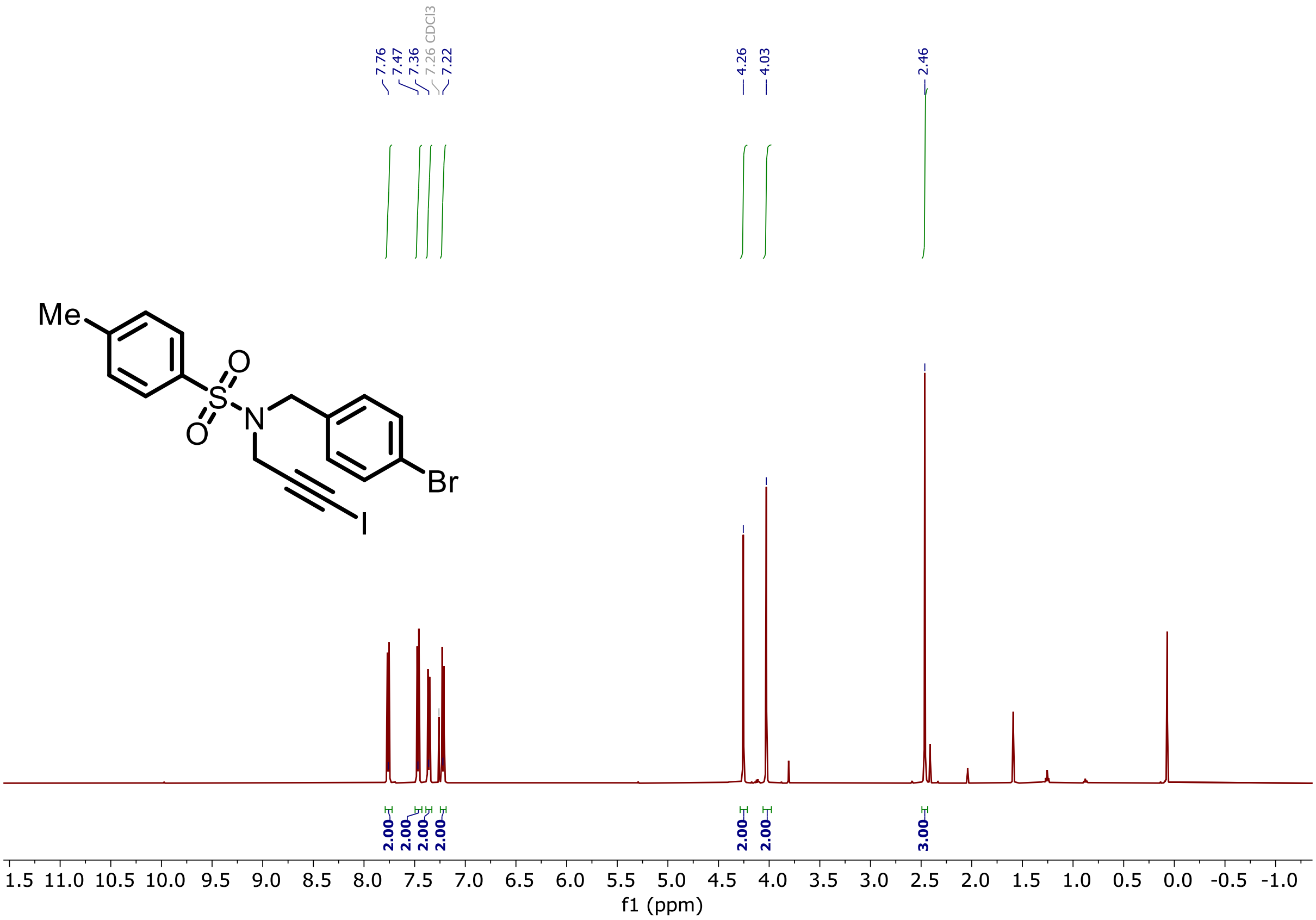

^{13}C NMR (126 MHz, CDCl$_3$)

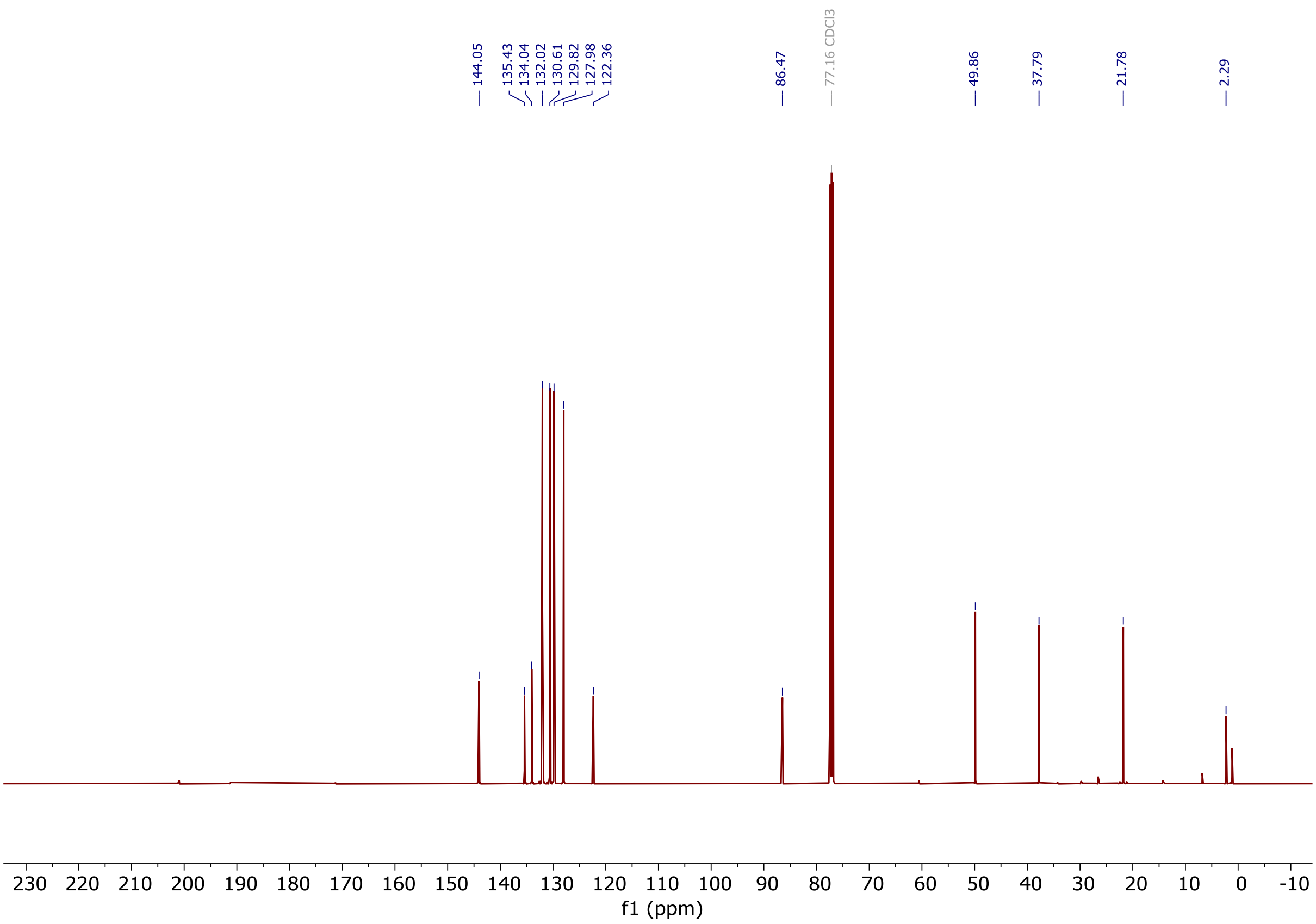

10. Crystal Data

10.1. Pyrroline 4l (CCDC Deposition Number 2485038)

Structure Report for 4l

Crystallographic data are presented in the Tables below. A single crystal of **4l** was coated in perfluoropolyether oil and mounted on a glass fibre. X-ray measurements were made using a Bruker Apex II kappa CCD area detector diffractometer with Mo K_{α} radiation (λ = 0.71073 Å).[61] Intensities were integrated[61] from several series of exposures, each exposure covering a fractional angle in ω or ϕ. Absorption corrections were applied, based on multiple and symmetry-equivalent measurements.[62] The structure was solved by direct methods and refined by least squares on weighted F^2 values for all reflections (see **Supplementary Table 12**).[63]

All non-hydrogen atoms were assigned anisotropic displacement parameters and refined without positional constraints. The positions of the methyl hydrogen atoms were assigned by a rotating group refinement with fixed, idealised C-H distances. All other hydrogen atoms were constrained to ideal geometries. All hydrogen atoms were assigned isotropic displacement parameters equal to 1.5 times (methyl hydrogen atoms) or 1.2 times (all other hydrogen atoms) that of their parent atom.

Refinement proceeded smoothly to give the residuals shown in **Supplementary Table 12**. Complex neutral-atom scattering factors were used.[64]

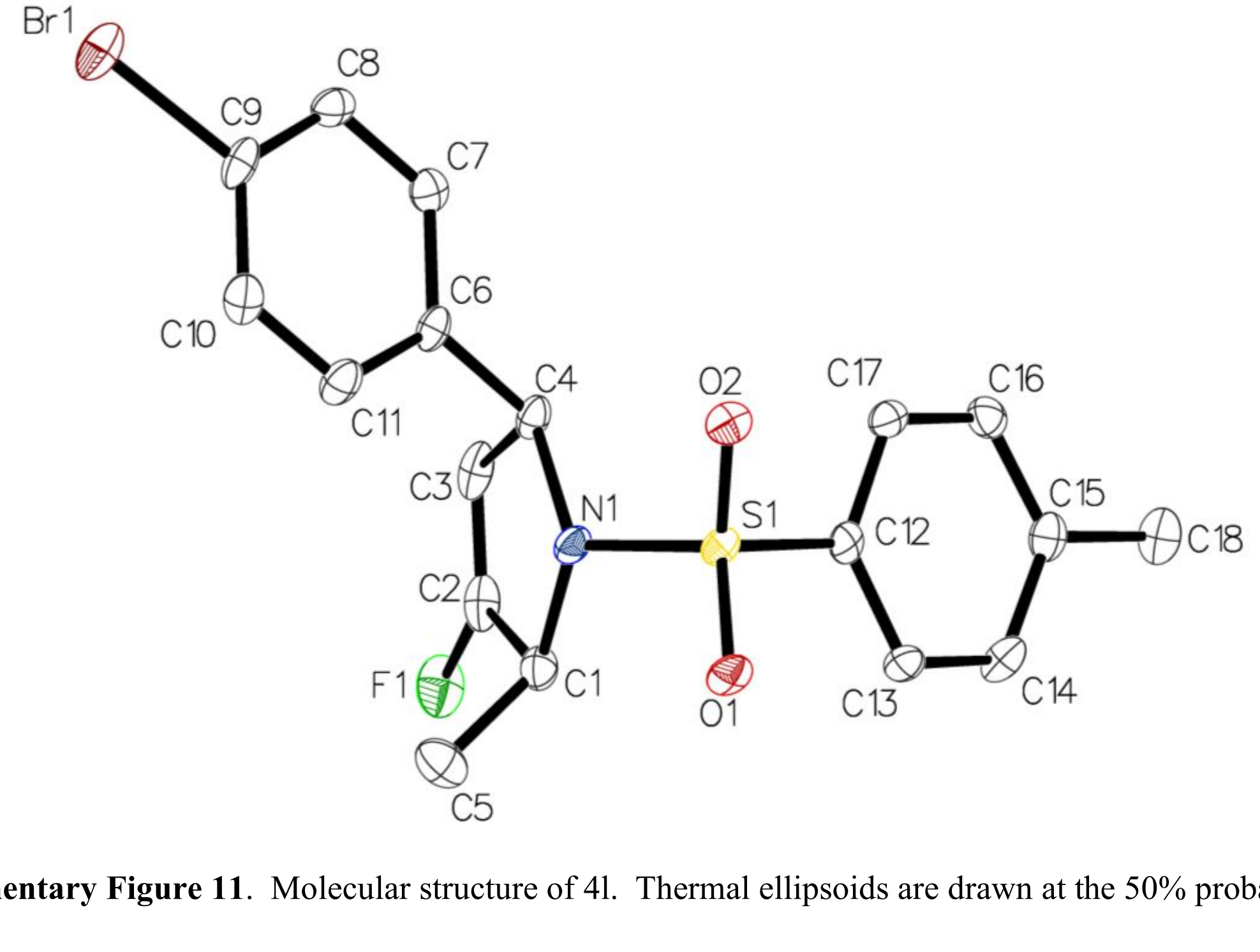

Supplementary Figure 11. Molecular structure of 4l. Thermal ellipsoids are drawn at the 50% probability level.

Supplementary Table 12. Crystal data and structure refinement for **4l**.

Identification code	HR077	
Empirical formula	$C_{18}H_{17}BrFNO_2S$	
Formula weight	410.29	
Temperature	100(2) K	
Wavelength	0.71073 Å	
Crystal system	Monoclinic	
Space group	$P2_1/n$	
Unit cell dimensions	a = 10.3090(5)	α = 90º
	b = 15.8353(8)	β = 104.715(4)º
	c = 10.9383(6)	γ = 90º
Volume	1727.07(16) ^{3}	
Z	4	
Density (calculated)	1.578 Mg/m^3	
Absorption coefficient	2.521 mm^{-1}	
F(000)	832	
Crystal size	0.342 x 0.336 x 0.245 mm	
θ range for data collection	2.572 to 27.948°	
Index ranges	$-13<=h<=13$, $-20<=k<=20$, $-14<=l<=14$	
Reflections collected	23111	
Independent reflections	4134 [R_{int} = 0.0779]	
Completeness to θ = 25.242°	99.8 %	
Absorption correction	Semi-empirical from equivalents	
Max. and min. transmission	0.7456 and 0.5259	
Refinement method	Full-matrix least-squares on F^2	
Data / restraints / parameters	4134 / 0 / 219	
Goodness-of-fit on F^2	S = 1.024	
R indices [for 3047reflections with $I>2\sigma(I)$]	R_1 = 0.0386, wR_2 = 0.0818	
R indices (for all 4134 data)	R_1 = 0.0657, wR_2 = 0.0922	
Weighting scheme	$w^{-1} = \sigma^2(F_o^2) + (aP)^2 + (bP)$,	
	where $P = [\max(F_o^2, 0) + 2F_c^2]/3$	
	a = 0.0375, b = 1.2580	
Largest diff. peak and hole	0.574 and −0.575 eÅ^{-3}	

Supplementary Table 13. Bond lengths [Å] and angles [°] for **4l**.

Br(1)-C(9)	1.905(3)
S(1)-O(2)	1.432(2)
S(1)-O(1)	1.4333(19)
S(1)-N(1)	1.640(2)
S(1)-C(12)	1.755(3)
F(1)-C(2)	1.352(3)
N(1)-C(1)	1.493(3)
N(1)-C(4)	1.505(3)
C(13)-H(13)	0.9500
C(13)-C(12)	1.389(4)
C(13)-C(14)	1.380(4)
C(6)-C(11)	1.385(4)
C(6)-C(4)	1.522(4)
C(6)-C(7)	1.392(4)
C(9)-C(8)	1.380(4)
C(9)-C(10)	1.376(4)
C(17)-H(17)	0.9500
C(17)-C(12)	1.397(4)
C(17)-C(16)	1.378(4)
C(8)-H(8)	0.9500
C(8)-C(7)	1.385(4)
C(16)-H(16)	0.9500
C(16)-C(15)	1.393(4)
C(14)-H(14)	0.9500
C(14)-C(15)	1.396(4)
C(1)-H(1)	1.0000
C(1)-C(2)	1.488(4)
C(1)-C(5)	1.520(4)
C(11)-H(11)	0.9500
C(11)-C(10)	1.392(4)
C(2)-C(3)	1.308(4)
C(10)-H(10)	0.9500
C(4)-H(4)	1.0000
C(4)-C(3)	1.505(4)
C(7)-H(7)	0.9500
C(3)-H(3)	0.9500

C(18)-H(18A)	0.9800
C(18)-H(18B)	0.9800
C(18)-H(18C)	0.9800
C(18)-C(15)	1.503(4)
C(5)-H(5A)	0.9800
C(5)-H(5B)	0.9800
C(5)-H(5C)	0.9800

O(2)-S(1)-O(1)	120.16(12)
O(2)-S(1)-N(1)	106.42(12)
O(2)-S(1)-C(12)	108.44(12)
O(1)-S(1)-N(1)	105.89(12)
O(1)-S(1)-C(12)	107.56(12)
N(1)-S(1)-C(12)	107.81(12)
C(1)-N(1)-S(1)	115.83(18)
C(1)-N(1)-C(4)	111.4(2)
C(4)-N(1)-S(1)	117.09(18)
C(12)-C(13)-H(13)	120.4
C(14)-C(13)-H(13)	120.4
C(14)-C(13)-C(12)	119.1(3)
C(11)-C(6)-C(4)	122.6(3)
C(11)-C(6)-C(7)	118.9(3)
C(7)-C(6)-C(4)	118.3(2)
C(8)-C(9)-Br(1)	118.9(2)
C(10)-C(9)-Br(1)	119.7(2)
C(10)-C(9)-C(8)	121.4(3)
C(12)-C(17)-H(17)	120.3
C(16)-C(17)-H(17)	120.3
C(16)-C(17)-C(12)	119.4(3)
C(13)-C(12)-S(1)	119.6(2)
C(13)-C(12)-C(17)	120.4(3)
C(17)-C(12)-S(1)	119.9(2)
C(9)-C(8)-H(8)	120.5
C(9)-C(8)-C(7)	119.0(3)
C(7)-C(8)-H(8)	120.5
C(17)-C(16)-H(16)	119.4
C(17)-C(16)-C(15)	121.2(3)
C(15)-C(16)-H(16)	119.4

C(13)-C(14)-H(14)	119.2
C(13)-C(14)-C(15)	121.5(3)
C(15)-C(14)-H(14)	119.2
N(1)-C(1)-H(1)	109.8
N(1)-C(1)-C(5)	112.8(2)
C(2)-C(1)-N(1)	100.0(2)
C(2)-C(1)-H(1)	109.8
C(2)-C(1)-C(5)	114.3(2)
C(5)-C(1)-H(1)	109.8
C(6)-C(11)-H(11)	119.7
C(6)-C(11)-C(10)	120.6(3)
C(10)-C(11)-H(11)	119.7
F(1)-C(2)-C(1)	117.1(3)
C(3)-C(2)-F(1)	126.5(3)
C(3)-C(2)-C(1)	116.4(3)
C(9)-C(10)-C(11)	119.2(3)
C(9)-C(10)-H(10)	120.4
C(11)-C(10)-H(10)	120.4
N(1)-C(4)-C(6)	113.6(2)
N(1)-C(4)-H(4)	109.2
N(1)-C(4)-C(3)	102.6(2)
C(6)-C(4)-H(4)	109.2
C(3)-C(4)-C(6)	112.8(2)
C(3)-C(4)-H(4)	109.2
C(6)-C(7)-H(7)	119.5
C(8)-C(7)-C(6)	120.9(3)
C(8)-C(7)-H(7)	119.5
C(2)-C(3)-C(4)	109.6(3)
C(2)-C(3)-H(3)	125.2
C(4)-C(3)-H(3)	125.2
H(18A)-C(18)-H(18B)	109.5
H(18A)-C(18)-H(18C)	109.5
H(18B)-C(18)-H(18C)	109.5
C(15)-C(18)-H(18A)	109.5
C(15)-C(18)-H(18B)	109.5
C(15)-C(18)-H(18C)	109.5
C(16)-C(15)-C(14)	118.2(3)
C(16)-C(15)-C(18)	121.8(3)

C(14)-C(15)-C(18)	120.0(3)
C(1)-C(5)-H(5A)	109.5
C(1)-C(5)-H(5B)	109.5
C(1)-C(5)-H(5C)	109.5
H(5A)-C(5)-H(5B)	109.5
H(5A)-C(5)-H(5C)	109.5
H(5B)-C(5)-H(5C)	109.5

10.2. Pyrrolidine 10m (CCDC Deposition Number 2485039)

Structure Report for 10m

Crystallographic data are presented in the Tables below. A single crystal of **10m** was coated in perfluoropolyether oil and mounted on a glass fibre. X-ray measurements were made using a Bruker D8 Venture dual source kappa CPAD diffractometer with Mo K_{α} radiation (λ = 0.71073 Å).[61] Intensities were integrated[61] from several series of exposures, each exposure covering 0.5° in ω or ϕ. Absorption corrections were applied, based on multiple and symmetry-equivalent measurements.[62] The structure was solved by direct methods and refined by least squares on weighted F^2 values for all reflections (see **Supplementary Table 14**).[63] All non-hydrogen atoms were assigned anisotropic displacement parameters and refined without positional constraints. The positions of the methyl hydrogen atoms were assigned by a rotating group refinement with fixed, idealised C-H distances. All other hydrogen atoms were constrained to ideal geometries. All hydrogen atoms were assigned isotropic displacement parameters equal to 1.5 times (methyl hydrogen atoms) or 1.2 times (all other hydrogen atoms) that of their parent atom.

Refinement proceeded smoothly to give the residuals shown in **Supplementary Table 14**. Complex neutral-atom scattering factors were used.[64]

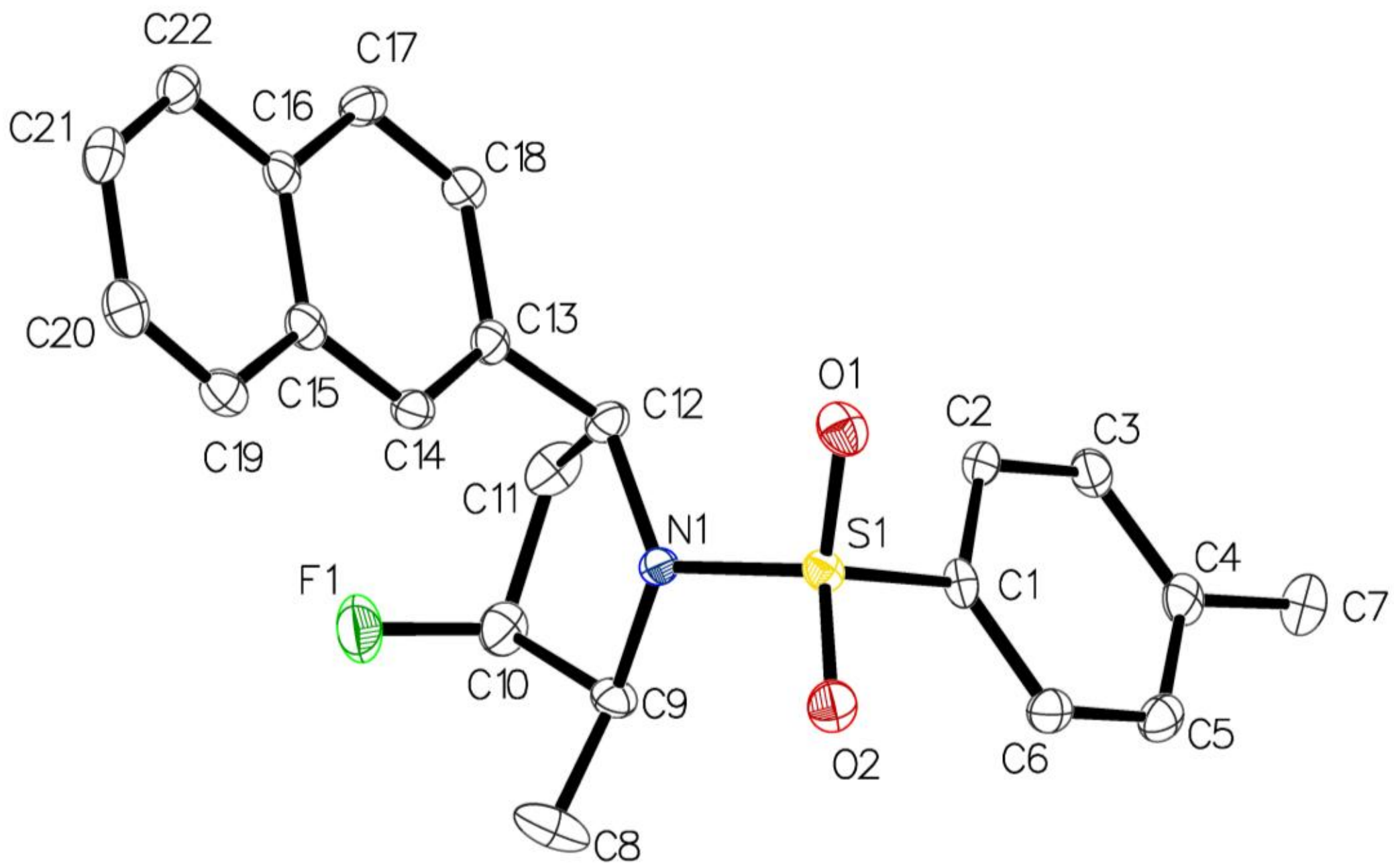

Supplementary Figure 12. Molecular structure of 10m. Thermal ellipsoids are drawn at the 50% probability level.

Supplementary Table 14. Crystal data and structure refinement for **10m**.

Identification code	HR106	
Empirical formula	$C_{22}H_{22}FNO_2S$	
Formula weight	383.46	
Temperature	100(2) K	
Wavelength	0.71073 Å	
Crystal system	Monoclinic	
Space group	Cc	
Unit cell dimensions	a = 20.4974(7) Å	α = 90°
	b = 11.8807(4) Å	β = 93.6010(10)°
	c = 7.6051(3) Å	γ = 90°
Volume	1848.36(11) Å^3	
Z	4	
Density (calculated)	1.378 Mg/m^3	
Absorption coefficient	0.202 mm^{-1}	
F(000)	808	
Crystal size	0.401 x 0.397 x 0.204 mm	
θ range for data collection	1.982 to 27.994°	
Index ranges	−26<=h<=27, −15<=k<=15, −10<=l<=10	
Reflections collected	25150	
Independent reflections	4398 [R_{int} = 0.0420]	
Completeness to θ = 25.242°	100.0 %	
Absorption correction	Semi-empirical from equivalents	
Max. and min. transmission	0.7456 and 0.5902	
Refinement method	Full-matrix least-squares on F^2	
Data / restraints / parameters	4398 / 2 / 246	
Goodness-of-fit on F^2	S = 1.040	
R indices [for 4157reflections with I>2σ(I)]	R_1 = 0.0333, wR_2 = 0.0752	
R indices (for all 4398 data)	R_1 = 0.0362, wR_2 = 0.0764	
Weighting scheme	$w^{\circ 1} = \sigma^2(F_o^{\ 2}) + (aP)^2 + (bP)$,	
	where $P = [\max(F_o^{\ 2}, 0) + 2F_c^{\ 2}]/3$	
	a = 0.0382, b = 1.0079	
Absolute structure (Flack) parameter	0.01(2)	
Extinction coefficient	n/a	
Largest diff. peak and hole	0.412 and 0.291 eÅ^{-3}	

Supplementary Table 15.Bond lengths [Å] and angles [°] for **10m**.

S(1)-O(1)	1.4356(19)
S(1)-N(1)	1.633(2)
S(1)-C(1)	1.778(2)
S(1)-O(2)	1.4356(19)
F(1)-C(10)	1.410(3)
N(1)-C(9)	1.496(3)
N(1)-C(12)	1.492(3)
C(1)-C(2)	1.392(3)
C(1)-C(6)	1.388(4)
C(2)-H(2)	0.9500
C(2)-C(3)	1.393(4)
C(3)-H(3)	0.9500
C(3)-C(4)	1.396(4)
C(4)-C(5)	1.395(4)
C(4)-C(7)	1.503(4)
C(5)-H(5)	0.9500
C(5)-C(6)	1.395(4)
C(6)-H(6)	0.9500
C(7)-H(7A)	0.9800
C(7)-H(7B)	0.9800
C(7)-H(7C)	0.9800
C(8)-H(8A)	0.9800
C(8)-H(8B)	0.9800
C(8)-H(8C)	0.9800
C(8)-C(9)	1.520(4)
C(9)-H(9)	1.0000
C(9)-C(10)	1.524(4)
C(10)-H(10)	1.0000
C(10)-C(11)	1.506(4)
C(11)-H(11A)	0.9900
C(11)-H(11B)	0.9900
C(11)-C(12)	1.535(3)
C(12)-H(12)	1.0000
C(12)-C(13)	1.515(3)
C(13)-C(14)	1.364(3)
C(13)-C(18)	1.421(4)

C(14)-H(14)	0.9500
C(14)-C(15)	1.424(3)
C(15)-C(16)	1.418(3)
C(15)-C(19)	1.421(3)
C(16)-C(17)	1.419(4)
C(16)-C(22)	1.422(3)
C(17)-H(17)	0.9500
C(17)-C(18)	1.371(3)
C(18)-H(18)	0.9500
C(19)-H(19)	0.9500
C(19)-C(20)	1.369(4)
C(20)-H(20)	0.9500
C(20)-C(21)	1.413(4)
C(21)-H(21)	0.9500
C(21)-C(22)	1.363(4)
C(22)-H(22)	0.9500
O(1)-S(1)-N(1)	106.52(11)
O(1)-S(1)-C(1)	107.83(11)
O(1)-S(1)-O(2)	119.40(11)
N(1)-S(1)-C(1)	108.06(11)
O(2)-S(1)-N(1)	107.44(10)
O(2)-S(1)-C(1)	107.15(12)
C(9)-N(1)-S(1)	117.75(16)
C(12)-N(1)-S(1)	118.05(16)
C(12)-N(1)-C(9)	111.80(19)
C(2)-C(1)-S(1)	119.64(18)
C(6)-C(1)-S(1)	119.80(19)
C(6)-C(1)-C(2)	120.5(2)
C(1)-C(2)-H(2)	120.3
C(1)-C(2)-C(3)	119.4(2)
C(3)-C(2)-H(2)	120.3
C(2)-C(3)-H(3)	119.5
C(2)-C(3)-C(4)	121.0(2)
C(4)-C(3)-H(3)	119.5
C(3)-C(4)-C(7)	119.9(2)
C(5)-C(4)-C(3)	118.8(2)
C(5)-C(4)-C(7)	121.3(2)

C(4)-C(5)-H(5)	119.6
C(6)-C(5)-C(4)	120.8(2)
C(6)-C(5)-H(5)	119.6
C(1)-C(6)-C(5)	119.6(2)
C(1)-C(6)-H(6)	120.2
C(5)-C(6)-H(6)	120.2
C(4)-C(7)-H(7A)	109.5
C(4)-C(7)-H(7B)	109.5
C(4)-C(7)-H(7C)	109.5
H(7A)-C(7)-H(7B)	109.5
H(7A)-C(7)-H(7C)	109.5
H(7B)-C(7)-H(7C)	109.5
H(8A)-C(8)-H(8B)	109.5
H(8A)-C(8)-H(8C)	109.5
H(8B)-C(8)-H(8C)	109.5
C(9)-C(8)-H(8A)	109.5
C(9)-C(8)-H(8B)	109.5
C(9)-C(8)-H(8C)	109.5
N(1)-C(9)-C(8)	113.1(2)
N(1)-C(9)-H(9)	108.7
N(1)-C(9)-C(10)	102.70(19)
C(8)-C(9)-H(9)	108.7
C(8)-C(9)-C(10)	114.6(2)
C(10)-C(9)-H(9)	108.7
F(1)-C(10)-C(9)	108.8(2)
F(1)-C(10)-H(10)	111.4
F(1)-C(10)-C(11)	108.3(2)
C(9)-C(10)-H(10)	111.4
C(11)-C(10)-C(9)	105.1(2)
C(11)-C(10)-H(10)	111.4
C(10)-C(11)-H(11A)	110.8
C(10)-C(11)-H(11B)	110.8
C(10)-C(11)-C(12)	104.6(2)
H(11A)-C(11)-H(11B)	108.9
C(12)-C(11)-H(11A)	110.8
C(12)-C(11)-H(11B)	110.8
N(1)-C(12)-C(11)	102.89(19)
N(1)-C(12)-H(12)	108.6

N(1)-C(12)-C(13)	113.3(2)
C(11)-C(12)-H(12)	108.6
C(13)-C(12)-C(11)	114.6(2)
C(13)-C(12)-H(12)	108.6
C(14)-C(13)-C(12)	123.9(2)
C(14)-C(13)-C(18)	119.7(2)
C(18)-C(13)-C(12)	116.4(2)
C(13)-C(14)-H(14)	119.6
C(13)-C(14)-C(15)	120.9(2)
C(15)-C(14)-H(14)	119.6
C(16)-C(15)-C(14)	119.1(2)
C(16)-C(15)-C(19)	118.6(2)
C(19)-C(15)-C(14)	122.3(2)
C(15)-C(16)-C(17)	119.2(2)
C(15)-C(16)-C(22)	119.4(2)
C(17)-C(16)-C(22)	121.4(2)
C(16)-C(17)-H(17)	119.9
C(18)-C(17)-C(16)	120.1(2)
C(18)-C(17)-H(17)	119.9
C(13)-C(18)-H(18)	119.5
C(17)-C(18)-C(13)	121.0(2)
C(17)-C(18)-H(18)	119.5
C(15)-C(19)-H(19)	119.5
C(20)-C(19)-C(15)	120.9(2)
C(20)-C(19)-H(19)	119.5
C(19)-C(20)-H(20)	119.9
C(19)-C(20)-C(21)	120.1(2)
C(21)-C(20)-H(20)	119.9
C(20)-C(21)-H(21)	119.7
C(22)-C(21)-C(20)	120.6(2)
C(22)-C(21)-H(21)	119.7
C(16)-C(22)-H(22)	119.8
C(21)-C(22)-C(16)	120.4(2)
C(21)-C(22)-H(22)	119.8

10.3. Azetine 7ar (CCDC Deposition Number 2485040)

Structure Report for 7ar

Crystallographic data are presented in the Tables below. A single crystal of **7ar** was coated in perfluoropolyether oil and mounted on a glass fibre. X-ray measurements were made using a Bruker D8 Venture dual source kappa CPAD diffractometer with Mo K_{α} radiation (λ = 0.71073 Å).[61] Intensities were integrated[61] from several series of exposures, each exposure covering 0.5° in ω or ϕ. Absorption corrections were applied, based on multiple and symmetry-equivalent measurements.[62] The structure was solved by direct methods and refined by least squares on weighted F^2 values for all reflections (see **Supplementary Table 16**).[63] All non-hydrogen atoms were assigned anisotropic displacement parameters and refined without positional constraints. The positions of the methyl hydrogen atoms were assigned by a rotating group refinement with fixed, idealised C-H distances. All other hydrogen atoms were constrained to ideal geometries. All hydrogen atoms were assigned isotropic displacement parameters equal to 1.5 times (methyl hydrogen atoms) or 1.2 times (all other hydrogen atoms) that of their parent atom.

Refinement proceeded smoothly to give the residuals shown in **Supplementary Table 16**. Complex neutral-atom scattering factors were used.[64]

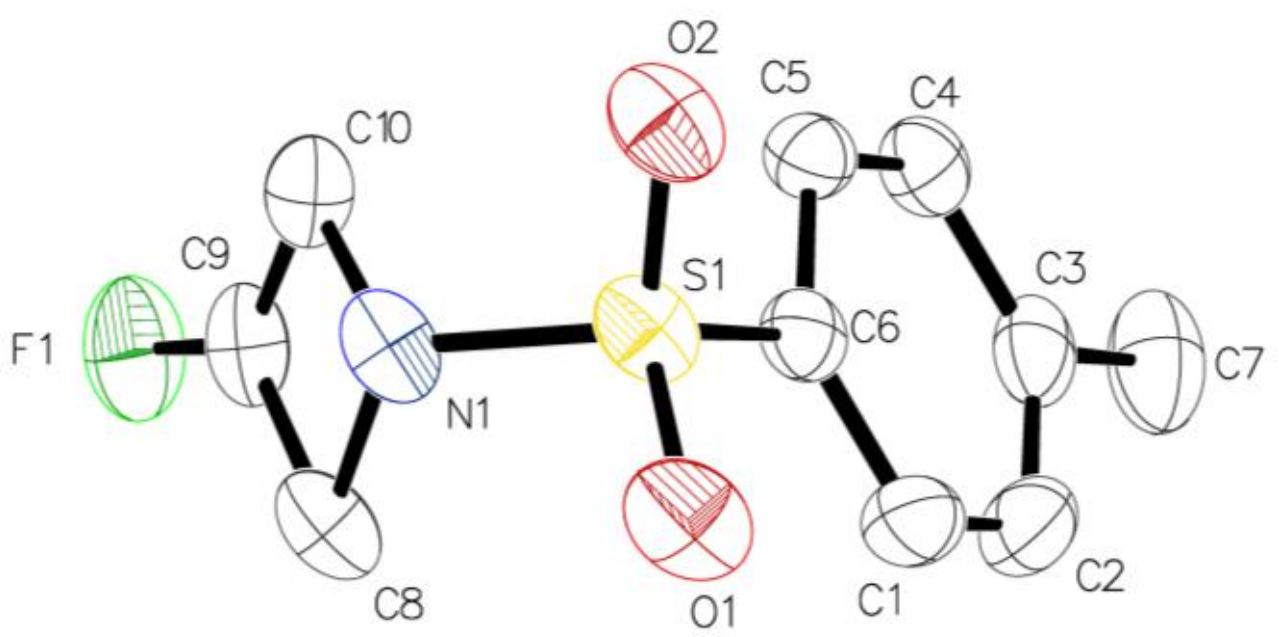

Supplementary Figure 13. Molecular structure of 7ar. Thermal ellipsoids are drawn at the 50% probability level.

Supplementary Table 16. Crystal data and structure refinement for **7ar**.

Identification code	HR157	
Empirical formula	$C_{10}H_{10}FNO_2S$	
Formula weight	227.25	
Temperature	200(2) K	
Wavelength	0.71073 Å	
Crystal system	Monoclinic	
Space group	$P2_1/n$	
Unit cell dimensions	a = 7.8161(2) Å	α = 90°
	b = 16.7322(5) Å	β = 110.8160(10)°
	c = 8.3486(2) Å	γ = 90°
Volume	1020.57(5) $Å^3$	
Z	4	
Density (calculated)	1.479 Mg/m^3	
Absorption coefficient	0.309 mm^{-1}	
F(000)	472	
Crystal size	0.44 x 0.436 x 0.416 mm	
θ range for data collection	2.434 to 27.913°	
Index ranges	$-10<=h<=10$, $-22<=k<=22$, $-10<=l<=10$	
Reflections collected	32284	
Independent reflections	2437 [R_{int} = 0.0501]	
Completeness to θ = 25.242°	100.0 %	
Absorption correction	Semi-empirical from equivalents	
Max. and min. transmission	0.7456 and 0.6146	
Refinement method	Full-matrix least-squares on F^2	
Data / restraints / parameters	2437 / 0 / 137	
Goodness-of-fit on F^2	S = 1.084	
R indices [for 2035reflections with $I>2\sigma(I)$]	R_1 = 0.0401, wR_2 = 0.0998	
R indices (for all 2437 data)	R_1 = 0.0511, wR_2 = 0.1084	
Weighting scheme	$w^{-1} = \sigma^2(F_o^2) + (aP)^2 + (bP)$,	
	where $P = [\max(F_o^2, 0) + 2F_c^2]/3$	
	a = 0.0453, b = 0.4615	
Largest diff. peak and hole	0.226 and −0.468 $eÅ^{-3}$	

Supplementary Table 17.Bond lengths [Å] and angles [°] for **7ar**.

S(1)-O(1)	1.4319(14)
S(1)-N(1)	1.6605(15)
S(1)-O(2)	1.4267(14)
S(1)-C(6)	1.7592(17)
F(1)-C(9)	1.323(2)
N(1)-C(8)	1.521(2)
N(1)-C(10)	1.457(2)
C(1)-H(1)	0.9500
C(1)-C(2)	1.379(3)
C(1)-C(6)	1.388(3)
C(2)-H(2)	0.9500
C(2)-C(3)	1.386(3)
C(3)-C(4)	1.387(3)
C(3)-C(7)	1.506(3)
C(4)-H(4)	0.9500
C(4)-C(5)	1.385(2)
C(5)-H(5)	0.9500
C(5)-C(6)	1.388(2)
C(7)-H(7A)	0.9800
C(7)-H(7B)	0.9800
C(7)-H(7C)	0.9800
C(8)-H(8A)	0.9900
C(8)-H(8B)	0.9900
C(8)-C(9)	1.486(3)
C(9)-C(10)	1.320(3)
C(10)-H(10)	0.9500
O(1)-S(1)-N(1)	105.31(8)
O(1)-S(1)-C(6)	108.75(10)
N(1)-S(1)-C(6)	107.90(7)
O(2)-S(1)-O(1)	120.02(9)
O(2)-S(1)-N(1)	104.95(9)
O(2)-S(1)-C(6)	109.24(8)
C(8)-N(1)-S(1)	117.60(13)
C(10)-N(1)-S(1)	118.07(11)
C(10)-N(1)-C(8)	88.37(13)

C(2)-C(1)-H(1)	120.3
C(2)-C(1)-C(6)	119.44(17)
C(6)-C(1)-H(1)	120.3
C(1)-C(2)-H(2)	119.2
C(1)-C(2)-C(3)	121.57(17)
C(3)-C(2)-H(2)	119.2
C(2)-C(3)-C(4)	118.03(17)
C(2)-C(3)-C(7)	120.71(18)
C(4)-C(3)-C(7)	121.26(19)
C(3)-C(4)-H(4)	119.2
C(5)-C(4)-C(3)	121.57(17)
C(5)-C(4)-H(4)	119.2
C(4)-C(5)-H(5)	120.4
C(4)-C(5)-C(6)	119.15(16)
C(6)-C(5)-H(5)	120.4
C(1)-C(6)-S(1)	120.22(14)
C(1)-C(6)-C(5)	120.22(16)
C(5)-C(6)-S(1)	119.51(13)
C(3)-C(7)-H(7A)	109.5
C(3)-C(7)-H(7B)	109.5
C(3)-C(7)-H(7C)	109.5
H(7A)-C(7)-H(7B)	109.5
H(7A)-C(7)-H(7C)	109.5
H(7B)-C(7)-H(7C)	109.5
N(1)-C(8)-H(8A)	114.7
N(1)-C(8)-H(8B)	114.7
H(8A)-C(8)-H(8B)	111.8
C(9)-C(8)-N(1)	83.82(14)
C(9)-C(8)-H(8A)	114.7
C(9)-C(8)-H(8B)	114.7
F(1)-C(9)-C(8)	129.86(17)
C(10)-C(9)-F(1)	134.53(19)
C(10)-C(9)-C(8)	95.27(15)
N(1)-C(10)-H(10)	133.8
C(9)-C(10)-N(1)	92.48(16)
C(9)-C(10)-H(10)	133.8

10.4. Azetine 7aw (CCDC Deposition Number 2486256)

Central Analytical Services		
**EF-SM-LH9 Solid State Analytics, Crystallography**		**Date: 23.04.2025**
Carolina von Essen	☎ ++49 151 1454 4924 Carolina.von-Essen@merckgroup.com	Page 1 of 3

Single Crystal Structure Report CE25-01

Client: Julien Lefranc, HC-RD-XC3A

Order date: 11.04.2025

Objective: Solution of the single-crystal structure of AL/HR N409 HR174

Document history:

Version	Date	Changes
first version	15.04.2025	-
Second version	23.04.2025	Molecular structure corrected

Sample: Crystals of AL/HR N409 HR174 in pentane.

The crystal used for the experiment was block shaped. The colour of the crystal was clear and colourless.

Summary: **Molecule Structure**

As proposed:

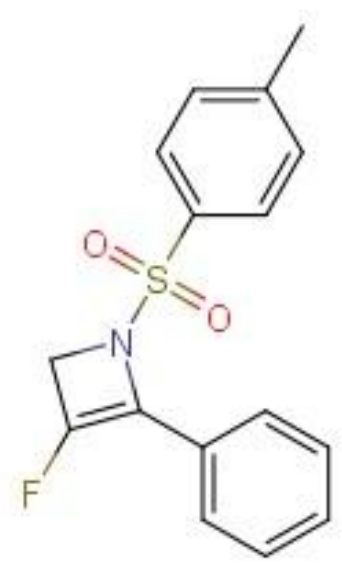

Comment

The delivered sample contained different crystals. The crystallographic report of the second crystal structure is CE25-02.

absolute structure: -

(Pseudo-) Polymorph: -

Dataset Quality Level[1]: A

Inhouse CSD Code: 001614

This report has been generated electronically and thus is not signed.

The report and the data have been critically reviewed and checked for plausibility.

[1] A: high quality, element assignment and hydrogen atom positions unequivocal
B: (absolute) structure unequivocally determined, not sufficient to determine chemical element of all atom positions
C: molecule connectivity established, absolute structure cannot be derived

Left: Thermal ellipsoids are drawn at the 50% probability level

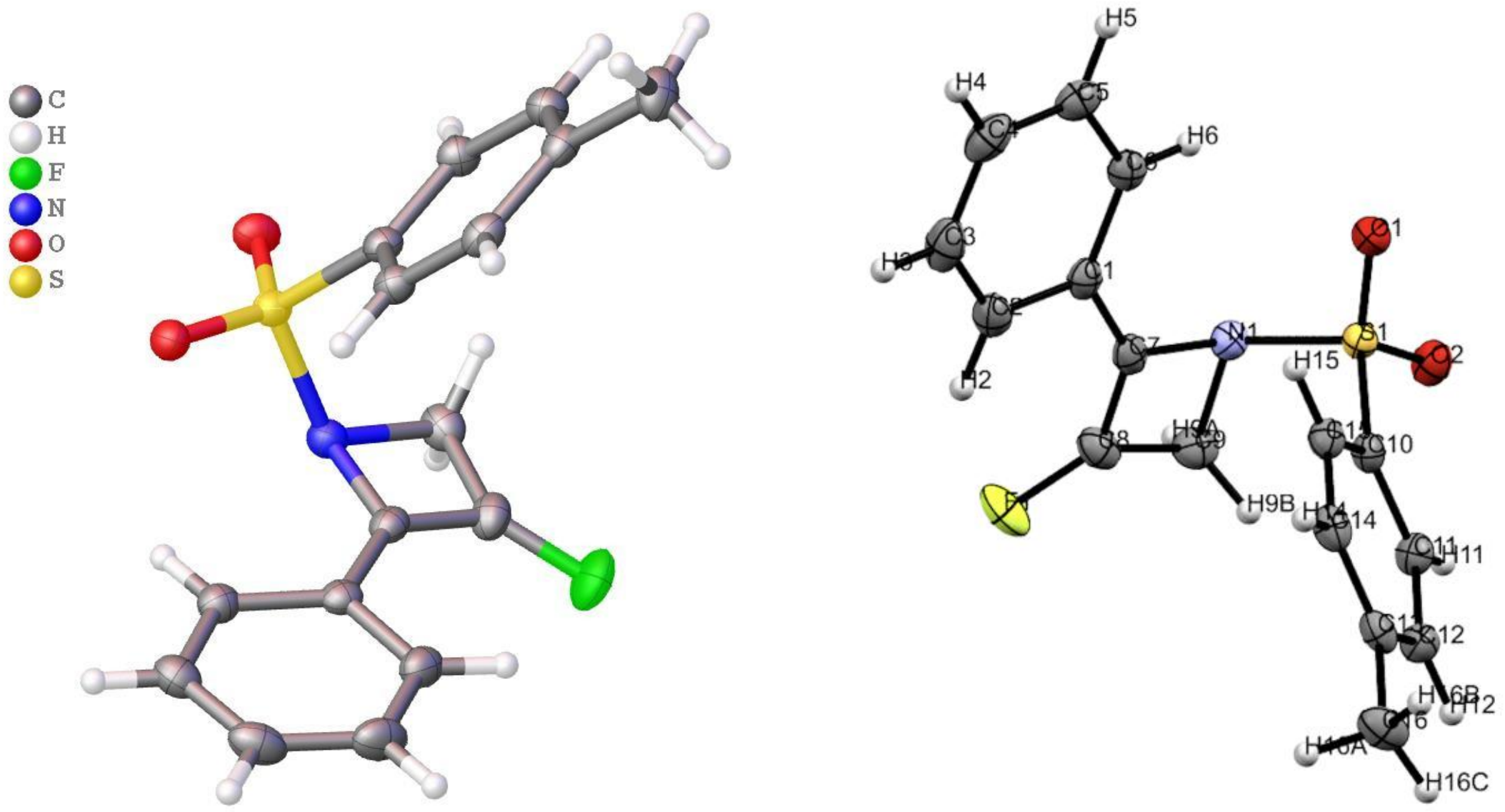

Plot of AL/HR N409 HR174 according to the structure solution.

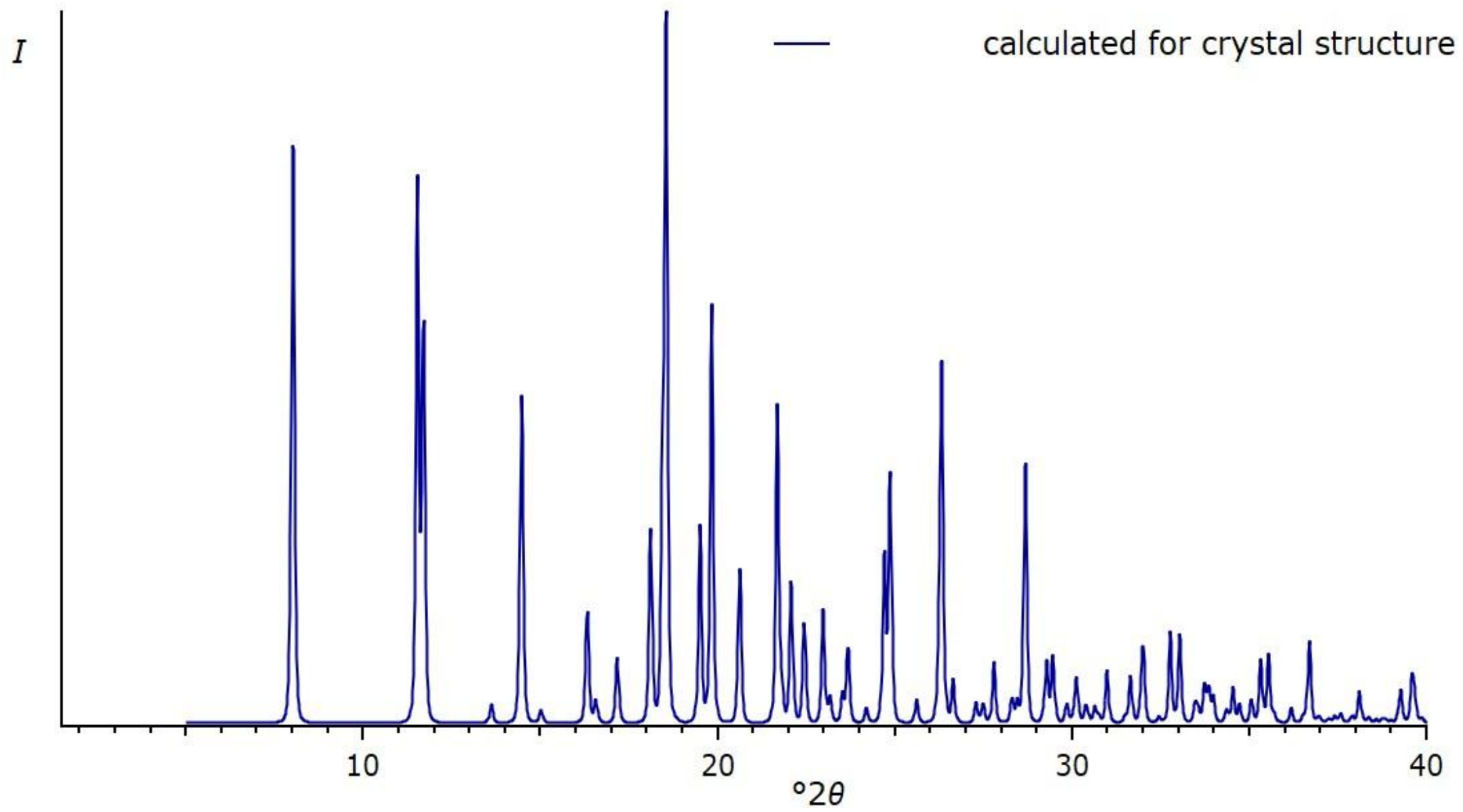

Calculated Powder X-ray diffraction pattern.

Comment: The amount of powder delivered is not sufficient to perform a Powder-Xray experiment of the sample.

Supplementary Table 18: Structure solution and refinement parameters

Empirical formula	$C_{16}H_{14}NO_2FS$
Molecular weight	303.34 g/mol
Diffractometer/Detector	Synergy-R (Rigaku), HyPix 6000 Detector
Radiation	Cu K□ (1.5418 Å), X-ray mirrors
Crystal size	(0.15 × 0.12 × 0.04) mm³
Temperature	99.98(16) K
Diffraction Experiment ID	exp_5487
Crystal system	orthorhombic
Space group	*Pbca*
Lattice parameters:	
a, *b*, *c*	10.6844(2) Å, 12.2416(2) Å, 22.0590(3) Å
□, □, □	90°, 90°, 90°
Unit cell volume	2885.19(8) Å³
Formula units per unit cell	8
F(000)	1264
Calculated density	1.397 g/cm3
Linear absorption coeff.	2.134 mm^{-1}
Scan	□-scans
Measured reflections	16609
Index limits	-11 ≤ h ≤ 13; -14 ≤ k ≤ 15; -27 ≤ l ≤ 22
R_{int} (internal consistency of the dataset)	0.0373
Reflections used	2924
Refined parameters / restraints	191 / 0
*R*1 (I > 2□)	0.0311
wR^2 (all data)	0.0799
GooF *S*	1.029
Max. shift in final cycle	≤ 0
Max./min. differential electron density	max: 0.24 / min: -0.38 e^-/Å³
Structure Solution / Refinement	Olex2.1.5 (see reference)[63]
Automated Report	SHELX-2018/1 (see reference)[65]

11. Supplementary References

1. Sedikides, A. T. & Lennox, A. J. J. Silver-Catalyzed (*Z*)-β-Fluoro-vinyl Iodonium Salts from Alkynes: Efficient and Selective Syntheses of *Z* -Monofluoroalkenes. *J. Am. Chem. Soc.* **146**, 15672–15680 (2024).

2. Taber, D. F. & Neubert, T. D. Enantioselective Construction of Cyclic Quaternary Centers: (−)-Mesembrine. *J. Org. Chem.* **66**, 143–147 (2001).

3. Taber, D. F. & Meagley, R. P. Diastereoselectivity in uncatalyzed intramolecular C-H insertion by an alkylidene carbene. *Tetrahedron Lett.* **35**, 7909–7910 (1994).

4. Taber, D. F., Sahli, A., Yu, H. & Meagley, R. P. Efficient Intramolecular C-H Insertion by an Alkylidene Carbene Generated from a Vinyl Chloride. *J. Org. Chem.* **60**, 6571–6573 (1995).

5. Green, M. P., Prodger, J. C., Sherlock, A. E. & Hayes, C. J. A Convenient Method for 3-Pyrroline Synthesis. *Org. Lett.* **3**, 3377–3379 (2001).

6. Sparr, C., Schweizer, W. B., Senn, H. M. & Gilmour, R. The Fluorine-Iminium Ion *Gauche* Effect: Proof of Principle and Application to Asymmetric Organocatalysis. *Angew. Chem. Int. Ed.* **48**, 3065–3068 (2009).

7. Bürger, H. & Sommer, S. Isolation, characterization and some properties of free difluoroethyne, FC□CF. *J. Chem. Soc., Chem. Commun.* 456–458 (1991).

8. Klapötke, T. M. *et al.* Halogenotrinitromethanes: A Combined Study in the Crystalline and Gaseous Phase and Using Quantum Chemical Methods. *Chem. – Eur. J.* **20**, 12962–12973 (2014).

9. Chang, C. H., Porter, R. F. & Bauer, S. H. The molecular structures of perfluorocyclobutane and perfluorocyclobutene, determined by electron diffraction. *J. Mol. Struct.* **7**, 89–99 (1971).

10. Zhang, W., Lu, F., Zhang, C., Guo, Q. & Quan, H. Synthesis of hydrohalocyclobutenes through dechlorination of hydrohalocyclobutanes in amide solvents. *J. Fluor. Chem.* **233**, 109506 (2020).

11. Maurer, C. K., Fang, Z., Harlfinger, S. & Petersson, C. Evaluation of the disconnect between hepatocyte and microsomal intrinsic clearance at intracellular and extracellular pH. *Drug Metabolism and Disposition* **54**, 100288 (2026).

12. De Matteis, V. *et al.* An Improved Ring-Closing Metathesis Approach to Fluorinated and Trifluoromethylated Nitrogen Heterocycles. *European J. Org. Chem.* **2007**, 2667–2675 (2007).

13. Marhold, M., Buer, A., Hiemstra, H., van Maarseveen, J. H. & Haufe, G. Synthesis of vinyl fluorides by ring-closing metathesis. *Tetrahedron Lett.* **45**, 57–60 (2004).

14. Ueji, T., Tadano, R., Inagi, S. & Inoue, M. Ring-Closing Metathesis for Fluoroolefins Using Ruthenium Catalysts with Six-Membered NHC Ligands. *Org. Lett.* **27**, 1470–1474 (2025).

15. Salim, S. S., Bellingham, R. K., Satcharoen, V. & Brown, R. C. D. Synthesis of Heterocyclic and Carbocyclic Fluoro-olefins by Ring-Closing Metathesis. *Org. Lett.* **5**, 3403–3406 (2003).

16. Xu, T., Mu, X., Peng, H. & Liu, G. Silver-Catalyzed Intramolecular Aminofluorination of Activated Allenes. *Angew. Chem. Int. Ed.* **50**, 8176–8179 (2011).

17. Choi, Y., Lee, K., Hong, J. H., Schinazi, R. F. & Chu, C. K. Synthesis and anti-HIV activity of l-2′-fluoro-2′,3′-unsaturated purine nucleosides. *Tetrahedron Lett.* **39**, 4437–4440 (1998).

18. Lee, K. *et al.* Structure−Activity Relationships of 2‘-Fluoro-2‘,3‘-unsaturated <scp>d</scp> -Nucleosides as Anti-HIV-1 Agents. *J. Med. Chem.* **45**, 1313–1320 (2002).

19. Chen, X., Zhou, W., Schinazi, R. F. & Chu, C. K. Synthesis of 3‘-Fluoro-2‘,3‘-dideoxy-2‘,3‘-didehydro-4‘-ethynyl-(D)- and -(L)-furanosyl Nucleosides. *J. Org. Chem.* **69**, 6034–6041 (2004).

20. Fontenelle, C. Q., Thierry, T., Laporte, R., Pfund, E. & Lequeux, T. Selective preparation of tetrasubstituted fluoroalkenes by fluorine-directed oxetane ring-opening reactions. *Beilstein J. Org. Chem.* **16**, 1936–1946 (2020).

21. Ma, J. *et al.* Dual Catalysis: Proton/Metal-Catalyzed Tandem Benzofuran Annulation/Carbene Transfer Reaction. *Org. Lett.* **18**, 1322–1325 (2016).

22. Zheng, W., Sun, G., Chen, L. & Kang, Q. Enantioselective Synthesis of *trans* -Vicinal Diamines *via* Rhodium-Catalyzed [2+2] Cycloaddition of Allenamides. *Adv. Synth. Catal.* **360**, 1790–1794 (2018).

23. Huang, W., Shen, Q., Wang, J. & Zhou, X. One-Step Synthesis of Substituted Dihydro- and Tetrahydroisoquinolines by $FeCl_3\cdot 6H_2O$ Catalyzed Intramolecular Friedel−Crafts Reaction of Benzylamino-Substituted Propargylic Alcohols. *J. Org. Chem.* **73**, 1586–1589 (2008).

24. Marcyk, P. T. & Cook, S. P. Iron-Catalyzed Hydroamination and Hydroetherification of Unactivated Alkenes. *Org. Lett.* **21**, 1547–1550 (2019).

25. Greiner, L. C. *et al.* Access to Indole-Fused Benzannulated Medium-Sized Rings through a Gold(I)-Catalyzed Cascade Cyclization of Azido-Alkynes. *Chem. – Eur. J.* **27**, 12992–12997 (2021).

26. Blieck, R. *et al.* Copper-Catalysed Hydroamination of N-Allenylsulfonamides: The Key Role of Ancillary Coordinating Groups. *Synthesis (Stuttg).* **51**, 1225–1234 (2019).

27. Shade, R. E., Hyde, A. M., Olsen, J.-C. & Merlic, C. A. Copper-Promoted Coupling of Vinyl Boronates and Alcohols: A Mild Synthesis of Allyl Vinyl Ethers. *J. Am. Chem. Soc.* **132**, 1202–1203 (2010).

28. Nangunuri, B. G., Shirke, R. P. & Kim, M. Metal-free synthesis of dihydrofuran derivatives as anti-vicinal amino alcohol isosteres. *Org. Biomol. Chem.* **21**, 960–965 (2023).

29. Xu, L. *et al.* Visible-light-mediated O–H functionalization reactions of acetylenic alcohols with 3-diazooxindoles: Facile access to spirooxindole-fused oxacycles. *Tetrahedron* **159**, 134014 (2024).

30. Wagner, C. E. & Shea, K. J. Synthesis and First Molecular Structure of a Bis-2-spiro-1-boraadamantane Derivative. *Org. Lett.* **6**, 313–316 (2004).

31. Nieto-Oberhuber, C. *et al.* Gold(I)-Catalyzed Cyclizations of 1,6-Enynes: Alkoxycyclizations and *exo*/*endo* Skeletal Rearrangements. *Chem. – Eur. J.* **12**, 1677–1693 (2006).

32. James, T., Simpson, I., Grant, J. A., Sridharan, V. & Nelson, A. Modular, Gold-Catalyzed Approach to the Synthesis of Lead-like Piperazine Scaffolds. *Org. Lett.* **15**, 6094–6097 (2013).

33. Grishina, A. A., Polyakova, S. M., Kunetskiy, R. A., Císařová, I. & Lyapkalo, I. M. 4,5-Disubstituted *N* , *N′* -Di- *tert* -alkyl Imidazolium Salts: New Synthesis and Structural Features. *Chem. – Eur. J.* **17**, 96–100 (2011).

34. Kim, S. & Chung, Y. K. Rhodium-Catalyzed Carbonylative [3+2+1] Cycloaddition of Alkyne-Tethered Alkylidenecyclopropanes to Phenols in the Presence of Carbon Monoxide. *Org. Lett.* **16**, 4352–4355 (2014).

35. Liu, R., Ni, Z., Giordano, L. & Tenaglia, A. Ruthenium-Catalyzed Hydroalkynylative Cyclization of 1,6-Enynes Induced by Substituent Effects. *Org. Lett.* **18**, 4040–4043 (2016).

36. Zhuang, X. *et al.* Alkynyl Borrowing: Silver-Catalyzed Amination of Secondary Propargylic Alcohols via C(sp ^{3})–C(sp) Bond Cleavage. *J. Org. Chem.* **87**, 5395–5403 (2022).

37. Rong, Z., Hu, W., Dai, N. & Qian, G. A $Hg(OTf)_2$ -Catalyzed Enolate Umpolung Reaction Enables the Synthesis of Coumaran-3-ones and Indolin-3-ones. *Org. Lett.* **22**, 3286–3290 (2020).

38. Zhang, Q. *et al.* Formal Synthesis of (−)-Cephalotaxine Based on a Tandem Hydroamination/Semipinacol Rearrangement Reaction. *Chem. Asian J.* **7**, 894–898 (2012).

39. Zhang, Y.-Y., Wei, Y., Tang, X.-Y. & Shi, M. Dual-role of $PtCl_2$ catalysis in the intramolecular cyclization of (hetero)aryl-allenes for the facile construction of substituted 2,3-dihydropyrroles and polyheterocyclic skeletons. *Chem. Commun.* **53**, 5966–5969 (2017).

40. Altman, R. A. *et al.* Total Synthesis of (+)-Nankakurines A and B and (±)-5- *epi* -Nankakurine A. *J. Org. Chem.* **75**, 7519–7534 (2010).

41. Andrade, F. C. D. *et al.* A Straightforward Sequential Approach for the Enantioselective Synthesis of Optically Active α-Arylmethanol-1,2,3-Triazoles. *European J. Org. Chem.* **2018**, 5467–5476 (2018).

42. Szafrański, P. W., Trybula, M. E., Kasza, P. & Cegła, M. T. Following the oxidation state of organosulfur compounds with NMR: Experimental data versus DFT calculations and database-powered NMR prediction. *J. Mol. Struct.* **1202**, 127346 (2020).

43. Bondzić, B. P. & Eilbracht, P. A Novel Access to Tetrahydro-β-Carbolines via One-Pot Hydroformylation/Fischer Indole Synthesis: Rearrangement of 3,3-Spiroindoleninium Cations. *Org. Lett.* **10**, 3433–3436 (2008).

44. Torres, JoséC., Garden, S. J., Pinto, A. C., da Silva, F. S. Q. & Boechat, N. A synthesis of 3-fluoroindoles and 3,3-difluoroindolines by reduction of 3,3-difluoro-2-oxindoles using a borane tetrahydrofuran complex. *Tetrahedron* **55**, 1881–1892 (1999).

45. Shafi, S., Kędziorek, M. & Grela, K. Cross Metathesis of N-Allylamines and α,β-Unsaturated Carbonyl Compounds: A One-Pot Synthesis of Substituted Pyrroles. *Synlett* **2011**, 124–128 (2011).

46. Brom, J., Maruani, A., Micouin, L. & Benedetti, E. Light-Driven Reductive Cleavage of Sulfonamides Promoted by Thiourea Organophotosensitizers. *J. Org. Chem.* **88**, 5923–5935 (2023).

47. Chatterjee, I. & Oestreich, M. $B(C_6F_5)_3$ -Catalyzed Transfer Hydrogenation of Imines and Related Heteroarenes Using Cyclohexa-1,4-dienes as a Dihydrogen Source. *Angew. Chem. Int. Ed.* **54**, 1965–1968 (2015).

48. Reed-Berendt, B. G. & Morrill, L. C. Manganese-Catalyzed *N* -Alkylation of Sulfonamides Using Alcohols. *J. Org. Chem.* **84**, 3715–3724 (2019).

49. Grishina, A. A., Polyakova, S. M., Kunetskiy, R. A., Císařová, I. & Lyapkalo, I. M. 4,5-Disubstituted *N* , *N′* -Di- *tert* -alkyl Imidazolium Salts: New Synthesis and Structural Features. *Chem. – Eur. J.* **17**, 96–100 (2011).

50. Ban, Y. *et al.* Indium Promotes Direct Sulfonamidation of Unactivated Alcohols. *J. Org. Chem.* **89**, 6345–6352 (2024).

51. Hu, N. *et al.* Synthesis of Chiral α-Amino Tertiary Boronic Esters by Enantioselective Hydroboration of α-Arylenamides. *J. Am. Chem. Soc.* **137**, 6746–6749 (2015).

52. Lv, X., Abrams, R. & Martin, R. Copper-Catalyzed C(sp^3)-Amination of Ketone-Derived Dihydroquinazolinones by Aromatization-Driven C−C Bond Scission. *Angew. Chem. Int. Ed.* **62**, (2023).

53. Fu, Y. *et al.* NaI-Catalyzed Oxidative Amination of Aromatic Sodium Sulfinates: Synergetic Effect of Ethylene Dibromide and Air as Oxidants. *European J. Org. Chem.* **2018**, 6966–6970 (2018).

54. Tang, X. *et al.* Copper-catalyzed sulfonamides formation from sodium sulfinates and amines. *Chem. Commun.* **49**, 6102 (2013).

55. Patil, R. D. & Pratihar, S. Ruthenium(II)-Catalyzed Hydrogenation and Tandem (De)Hydrogenation *via* Metal–Ligand Cooperation: Base- and Solvent-Assisted Switchable Selectivity. *J. Org. Chem.* **89**, 1361–1378 (2024).

56. Sarkar, D., Ghosh, M. K. & Rout, N. PTAB mediated open air synthesis of sulfonamides, thiosulfonates and symmetrical disulfanes. *Tetrahedron Lett.* **59**, 2360–2364 (2018).

57. Borys, A. M., Kunzmann, T., Gil-Negrete, J. M. & Hevia, E. Atom-efficient arylation of *N* -tosylimines mediated by cooperative $ZnAr_2/Zn(C_6F_5)_2$ combinations. *Chem. Commun.* **59**, 7583–7586 (2023).

58. Ito, K., Tamashima, H., Iwasawa, N. & Kusama, H. Photochemically Promoted Transition Metal-Free Cross-Coupling of Acylsilanes with Organoboronic Esters. *J. Am. Chem. Soc.* **133**, 3716–3719 (2011).

59. Li, Q.-Q., Xiao, Z.-F., Yao, C.-Z., Zheng, H.-X. & Kang, Y.-B. Direct Alkylation of Amines with Alcohols Catalyzed by Base. *Org. Lett.* **17**, 5328–5331 (2015).

60. Yang, Y., Hammond, G. B. & Umemoto, T. Self-Sustaining Fluorination of Active Methylene Compounds and High-Yielding Fluorination of Highly Basic Aryl and Alkenyl Lithium Species with a Sterically Hindered *N* -Fluorosulfonamide Reagent. *Angew. Chem. Int. Ed.* **61**, (2022).

61. *SAINT integration software version 8.38A*, Bruker AXS Inc., Madison,WI, 2018.

62. *SADABS version 2016/2*, Bruker AXS Inc., 2016.

63. *Olex2 1.5*, O. V. Dolomanov, L. J. Bourhis, R. J. Gildea, J. A. K. Howard and H. Puschmann, *J. Appl. Cryst.*, 2009, **42**, 339.

64. *International Tables for Crystallography*, Wiley, Oxford, 2016, vol. C.

65. SHELX-2018/1; G.M. Sheldrick, *Acta Cryst. A*, **71** (2015) G.M. Sheldrick, *Acta Cryst. C*, **71** (2015).
