# Supplementary material for: Synthesis of 4- and 5-membered fluorine-stabilised semi-saturated heterocycles via alkylidene carbenes
Source: Nat Commun. 2026 Sep 28;17:11200. doi: 10.1038/s41467-026-77771-y (PMC13714270; doi:10.1038/s41467-026-77771-y)
Supplement: Supplementary file 2 [file 41467_2026_77771_MOESM2_ESM.pdf]

nature portfolio

Peer Review File

A General Strategy for 4- and 5-Membered Fluorine-Stabilised Semi-Saturated Heterocycles via Alkylidene Carbenes

Corresponding Author: Dr Alastair Lennox

This file contains all reviewer reports in order by version, followed by all author rebuttals in order by version.

Version 0:

Reviewer comments:

Reviewer #1

(Remarks to the Author)
This is an excellent manuscript. The case for devoting resource for the development of small fluorinated semi-saturated rings is well made, and this work will be of great interest.

The authors provide a convincing case of this transformation for a novel entry into semi-saturated heterocycles. Good to excellent yields are obtained, and a broad substrate scope demonstrated. Noteworthy results include the azetine formation, as well as the demonstration that the reactivity of the formed cyclic enamines is substantially attenuated by fluorination. Interesting observations are included, for example when two different C–H bonds can react, with the selectivity of azetidine over cyclopentene formation particulatly remarkable, as well as the occurrence of a spontaneous allylic proton shift to arrive at a cross-conjugated structure. Hence, this methodology development is sound and carried out in great detail, with a choice of substrates that allow to describe different cases.

The Hara precedent, the demonstration that iodonium leaving groups for carbene generation are compatible with alkene fluorine substitution is quite substantial though, and should be depicted in Figure 2A, mentioning their optimal conditions. The synthetic methodology to access the FVI salts was developed by the authors.

The addition of the description of physicochemical properties and of experiments to gauge the increased stability against oxidation, both chemical and through metabolism, significantly adds to the quality of the work, as it shows the potential of this type of compounds in the pharmaceutical industry. I am not sure about the dipole argument. Surely N-inversion of the saturated structures leads to diastereomeric structures with different dipole directions and relative energies (so not a 1:1 population ratio), for which the resulting averaged dipole moment cannot be in the plane? In contrast, N-inversion of the azetine structure leads to an enantiomeric structure, hence this averaged dipole moment should also be in the plane? Could the authors clarify?

The mechanistic investigation is also of interest. Have the authors investigated whether there is a correlation with Hammett parameters? I am not sure why the KIE observed for the reaction of 3e would suggest that the reaction is initiated with a hydride transfer, as opposed to being a manifestation of the C-D bond being stronger than the C-H bond, and being broken in the rate determining step. Could the authors elaborate?

I am not sure about the explanation for the diastereoselectivity (Figure 2C). The dihedral angle between the Ar and sulfonyl groups is the same in the anti- and syn- transition states. It is the 1,3-diaxial interaction with a C–H group that is different. Could the authors give more information or precedent from comparable cyclisation reactions?

Finally, the extensive demonstration of the synthetic utility of the semi-unsaturated heterocycles is very useful.

The SI is excellent. Congratulations to the authors for providing fully assigned NMR characterisation data. There is enough detail for reproduction of the work, except for the data listed under Sections 6.1 and 6.2. If these are Merck-specific protocols, then it should be provided or at least explained how logD, Human CL etc were determined. If these are literature procedures, then citations should be given.

It is good that a list of cycles that could not be synthesised is included in the SI. However, the main text should be more explicit in referring to this list. For example, with Figure 4 it should be mentioned that oxetenes could not be obtained in this way (and evidence of their formation, as explained in SI section 4.4 should also be mentioned in the text).

Small comments:
In Figures 2-4 it is good that the reaction sequence is shown from the alkyne starting material. It is not clear though whether the yields reported refer to the whole sequence, or just for the cyclisation starting from the FVI salts. From the SI I understand it is the latter. Please indicate the range of yields obtained for the conversion of the alkyne to the FVI salt under the reaction conditions.

In figure 3, the structure of 5an should be shown together with 6an.

In Figure 4, some of the NMR yields are marked with a '*' (7ay, 7bc, 8bd), but it is not explained what this refers to. Please include.

In the SI:
- To have known and novel alkynes in different sections makes it more difficult to find the data for a specific alkyne (as one doesn't generally know which are known and novel). Please list the characterisation data of substrates etc according to compound numbering.
- Page S22: the 't-butyl alcohol byproduct' should be 'the tBuOD byproduct'? There should also eb an explicit reference to Scheme S4.6b

In summary, this is an excellent and well-constructed manuscript which will be of great interest to the synthetic and medicinal chemistry community. It provides convenient access to rare types of heterocyclic ring structures and is bound to find much use. The quality of the research is excellent. I recommend publication subject to a satisfactory addressing of the minor comments made.

Reviewer #2

(Remarks to the Author)
Lennox and co-workers report a general and versatile strategy to access fluorine-stabilized semi-saturated heterocycles (N, O). The authors demonstrate a unified approach to these exotic compounds and justify their synthesis by potential applications in medicinal chemistry as underexplored scaffolds, thus paving the way to new chemical space for drug-discovery programs. The authors conceptualize the idea of fluorine-stabilized semi-saturated rings and provide experimental proof. It is also good to see the high syn-diastereoselectivity observed; the stereochemical model appears reasonable. (Regarding terminology: would it be more appropriate to use cis/trans for cyclic products and syn/anti for open-chain products?)

Remarkably, oxidation of these species is achieved under rather harsh conditions, supporting the proposed idea that fluorine is retained; this also provides a new approach to access monofluorinated unsaturated heterocycles, some of which would be difficult to obtain by other means. One of the most intriguing parts of the work is the synthesis of previously unreported monofluorinated azetines — compounds that appear unstable but, due to the fluorine effect, were isolated and characterized in this study. I find this a very important contribution.

From a curiosity standpoint, the very high 1J(C–F) coupling (≈330 Hz) in the NMR is interesting. Although this is a spectroscopic curiosity, some readers may find it useful for understanding molecular properties of these small rings. In addition, the mechanism is supported by DFT calculations and by deuteration experiments. Oxidative stability was compared both experimentally and by cyclic voltammetry using a non-fluorinated analogue as reference. Microsomal stability was also evaluated, showing improved properties and potential medicinal-chemistry application. Overall, I recommend publication of this work in Nature Communications after minor revisions.

Manuscript — required edits and suggestions

-Please format FVI in bold throughout.
-From the main text it is not clear which FVI was used for optimization. Please mention the model substrate(s) in the main text.
-Comment on the usefulness of the "full-factorial Design of Experiments (DoE)". Was it helpful; did it make optimization more efficient in this study? If not in the main text, include a brief description and conclusions in the SI, and add the corresponding reference.
-N- and O-heterocycles were synthesized. Any particular reason S-heterocycles were not explored? There is one unsuccessful example (3bo) containing a sulfone moiety, but what about simple thioethers?
-Strictly speaking, please cite the Tamura work: J. Chem. Soc., Perkin Trans. 1, 1990, 975–981.
-Add compound numbers in the text. For example: "Using this methodology, a second semi-saturated heterocycle could be obtained by replacing the nitrogen atom with oxygen to produce fluorinated 3-oxolenes (Figure 3)." Add the corresponding compound labels.

SI — required edits and suggestions

-Add compound numbering to schemes and procedures in the SI (e.g., General Procedure 2, 2A, 3, 3A and other instances).
-In General Procedure 3, the abbreviation FVI is not explained in the SI. Define FVI independently in the experimental section (even if defined in the main article). If it is a compound label, reflect it in the scheme and format it in bold.
-I recommend avoiding Arabic numbering for General Procedures (1, 2, etc.) as this may be confused with compound numbers. Use Roman numerals or letters instead.
-To improve reproducibility, indicate how reaction progress was monitored in General Procedures (TLC, LC–MS, crude NMR, etc.). For example, General Procedure 4 lists an exact time (2 h). Is that time consistent for every analogue? Please state how conversion was assessed before work-up.
-Section numbering "3:, 3.1.:" looks awkward in titles. Use standard numbering like "3." and "3.1." followed by the title.
-Figure S4.2: Please add information on how these graphs were created (HPLC control, NMR sampling?) and clarify what the graphs indicate.
-Figure S4.3: How was deuteration determined — NMR or MS? Please state the method.
-Scheme S4.1: The note "Yield shown is relative to the alcohol FVI impurity in 4ag." is unclear. Please elaborate what this means and how yields were determined.

Reviewer #3

(Remarks to the Author)
This manuscript describes a high-impact and general synthetic strategy for constructing fluorinated semi-saturated four- and five-membered N- and O-heterocycles via alkylidene carbene intermediates. The chemistry is highly novel: fluorinated azetines are previously unknown, and the diastereoselective synthesis of fluorinated 3-pyrrolines and 3-oxolenes fills a long-standing gap in heterocyclic chemistry. Fluorine-mediated stabilization of these inherently unstable scaffolds is convincingly supported by controlled oxidation experiments, cyclic voltammetry, and human liver microsomal metabolism assays. The methodology is operationally simple, broad in scope, and directly relevant to drug discovery and pharmaceutical development. This work provides a general, practical, and conceptually novel method to access valuable fluorinated semi-saturated heterocycles that are highly attractive for drug development. The chemistry is well-designed, carefully executed, and broadly significant. This work meets the scope and impact criteria for Nature Communications. Publication is recommended after minor-to-moderate revision to address the scientific issues detailed below.
1.Mechanistic Evidence for Carbene Formation and N–H Insertion
The deuterium-labeling experiment supports the proposed N–H insertion pathway but does not fully exclude alternative anionic substitution mechanisms. Direct mechanistic evidence is required. Perform intermolecular carbene-trapping experiments (e.g., with external alkenes, methanol, or TEMPO) to verify the formation of free β-fluoro alkylidene carbene intermediates. Provide DFT-calculated energy profiles for the key steps: α elimination to generate the carbene, 1,5-C–H insertion for five-membered rings, and 1,4-N–H insertion for four-membered azetines, to rationalize the chemo- and diastereoselectivity.
2.Structural Characterization of Novel Heterocyclic Scaffolds
Several unprecedented fluorinated heterocycles are characterized only by NMR and MS. Unambiguous structural confirmation is necessary. Obtain and present X ray single-crystal structures for at least three representative compounds, including one fluorinated azetine, one diastereoselective pyrroline, and one spirocyclic oxolene. Report full analytical data (HRMS, $^{1}H/^{13}C/^{19}F$ NMR, and optical rotation for chiral products) consistently for all new compounds in the Supporting Information.
3.Substrate Scope and Methodological Limitations
The manuscript only presents successful substrates and does not discuss failed cases or functional group compatibility. Add a table in the Supporting Information summarizing unsuccessful substrates (e.g., those containing –OH, $–CO_2H$, –CN, aldehyde, or unprotected basic amine groups) and provide mechanistic explanations for failed examples such as the p-NO_2-substituted substrate 4g. Clearly state the functional group tolerance and sensitivity of the reaction to air, moisture, and temperature.
4.Stability and Physicochemical Characterization
Fluorine-induced stability is the central design principle of this work and requires deeper validation. Extend stability studies to include long-term storage stability (at least 7–30 days at room temperature) and stability across a pH range (pH 2, 7, and 12), monitored by NMR or HPLC. Include metabolic stability data for a broader set of analogs to better support the claim of improved drug-like properties.
5.Comparison with Previous Methods
The advances of this methodology should be quantitatively benchmarked against existing synthetic routes. Add a comparative table that contrasts this work with previous methods in terms of step count, isolated yield, diastereoselectivity, substrate scope, fluorination pattern, and synthetic scalability.

Reviewer #4

(Remarks to the Author)
This manuscript by Lennox and co-workers describes a general protocol for the synthesis of four- and five-membered heterocycles (oxolenes, pyrrolines, and azetines) bearing a fluorinated unsaturation.
The authors' strategy relies on the generation of alkylidene carbenes from hypervalent iodine intermediates upon treatment with a base (NaOtBu). The corresponding fluorovinyl iodonium species are readily accessed through a silver-catalyzed

fluoroiodination reaction. This two-step sequence provides efficient access to highly valuable partially saturated fluorinated heterocycles. The originality of the work lies in the elegant exploitation of the unique reactivity of alkylidene carbenes and their facile generation from vinyl iodonium precursors. Building upon the Lennox group's recognized expertise in fluorinated hypervalent iodine chemistry, this study constitutes a well-executed and insightful contribution to the field.
The target compounds are highly valuable motifs with considerable potential for application in medicinal chemistry. Furthermore, the work aligns well with current efforts to access non-planar heterocyclic architectures and fluorinated analogues, both of which are of significant contemporary interest. The potential utility of these scaffolds is further highlighted by the determination of LogD values. In addition, the authors support their mechanistic proposal through a thorough and carefully designed mechanistic investigation and further demonstrate the synthetic utility of the products as versatile building blocks.
Overall, the originality of this work is high, and its scientific relevance is substantial. The study addresses important contemporary challenges in synthetic methodology and is likely to inspire further developments in the field. I therefore recommend publication of this manuscript in Nature Communications after minor revision.
1/ Have the authors explored whether other chalcogen-containing heterocycles, such as sulfur-, sulfone-, or selenium-based analogues, can be accessed under the standard reaction conditions?
2/ The authors may wish to cite a relevant example demonstrating the synthetic value of fluorinated pyrrolines. In particular, the application of fluorinated pyrrolines in the synthesis of a strategic API building block has been reported (OPRD, 2020, 24, 792) and would further illustrate the practical significance of this scaffold.

Version 1:

Reviewer comments:

Reviewer #1

(Remarks to the Author)
The authors have provided satisfactory replies and corrections to the referee comments.
I only have a small comment, in that in the Hammett plot provided, the Hammett parameter is correlated with yield, not with reaction rate. I am not sure this is of much value, certainly not with the information provided. At the very least it should be specified whether the reaction conditions, especially reaction time, was the same in all cases. If not, then this should be removed.
Apart from this comment, which needs to be addressed, I am happy to recommend acceptance.

Reviewer #2

(Remarks to the Author)
All comments/suggestions more or less were addressed. I recommend publication of this manuscript as it is.

Reviewer #3

(Remarks to the Author)
The authors have addressed my previous comments satisfactorily and have responded to the concerns raised during the initial review. The revisions have improved the clarity and quality of the manuscript, and I believe the work is now suitable for publication.

Reviewer #4

(Remarks to the Author)
I carefully checked the revised manuscript and I am fully happy with the revised version.
Hence, I warmly recommend this work for publication in nature Communications.

Open Access This Peer Review File is licensed under a Creative Commons Attribution 4.0 International License, which permits use, sharing, adaptation, distribution and reproduction in any medium or format, as long as you give appropriate credit to the original author(s) and the source, provide a link to the Creative Commons license, and indicate if changes were made.
In cases where reviewers are anonymous, credit should be given to 'Anonymous Referee' and the source.
The images or other third party material in this Peer Review File are included in the article's Creative Commons license, unless indicated otherwise in a credit line to the material. If material is not included in the article's Creative Commons license and your intended use is not permitted by statutory regulation or exceeds the permitted use, you will need to obtain permission directly from the copyright holder.
To view a copy of this license, visit https://creativecommons.org/licenses/by/4.0/

RESPONSE TO REVIEWER COMMENTS

We would also like to thank each of the reviewers for their very careful reading of our manuscript. The comments provided have been extremely constructive and have subsequently substantially increased the quality of our review, for which we are very grateful.

Please find a revised manuscript (with and without highlights of changes), in which we have addressed all the issues noted by the reviewers. Quotations from the reviewers are copied in their entirety and provided in *blue italicized font.*

Reviewer #1 (Remarks to the Author):

This is an excellent manuscript. The case for devoting resource for the development of small fluorinated semi-saturated rings is well made, and this work will be of great interest.

The authors provide a convincing case of this transformation for a novel entry into semi-saturated heterocycles. Good to excellent yields are obtained, and a broad substrate scope demonstrated. Noteworthy results include the azetine formation, as well as the demonstration that the reactivity of the formed cyclic enamines is substantially attenuated by fluorination. Interesting observations are included, for example when two different C–H bonds can react, with the selectivity of azetidine over cyclopentene formation particulatly remarkable, as well as the occurrence of a spontaneous allylic proton shift to arrive at a cross-conjugated structure. Hence, this methodology development is sound and carried out in great detail, with a choice of substrates that allow to describe different cases.

We would like to thank the reviewer very much for their careful assessment of our manuscript. These comments and suggestions have been incredibly useful, especially for a couple points, detailed below, in which we made minor errors in our analysis. These comments have been extremely useful to us, so *thank you very much*.

The Hara precedent, the demonstration that iodonium leaving groups for carbene generation are compatible with alkene fluorine substitution is quite substantial though, and should be depicted in Figure 2A, mentioning their optimal conditions. The synthetic methodology to access the FVI salts was developed by the authors.

We would like to thank the reviewer for highlighting point. While the precedent from Hara is clearly described in the text, we agree his name should be featured in the figure, as we have done with "*Taber, Hayes and Komenda*". We have now done this now. We have not

included his optimal conditions in the figure, because we felt this level of detail was not necessary or relevant for the Figure at this stage in the manuscript.

The addition of the description of physicochemical properties and of experiments to gauge the increased stability against oxidation, both chemical and through metabolism, significantly adds to the quality of the work, as it shows the potential of this type of compounds in the pharmaceutical industry.

We are grateful that this reviewer agrees with us on the importance of these studies.

I am not sure about the dipole argument. Surely N-inversion of the saturated structures leads to diastereomeric structures with different dipole directions and relative energies (so not a 1:1 population ratio), for which the resulting averaged dipole moment cannot be in the plane? In contrast, N-inversion of the azetine structure leads to an enantiomeric structure, hence this averaged dipole moment should also be in the plane? Could the authors clarify?

We would like to thank the reviewer for pointing this out. Indeed, considerations of other N-inverted conformers was, unfortunately, inadvertently missed. We now appreciate for the unsubstituted pyrrolines, which we were comparing the pyrroles and pyrrolidines to, N-inversion gives an enantiomeric conformer (population ratio of 1:1), which therefore eliminates the out-of-plane dipole that we had assumed from our calculations. This is also the case for the azetines.

We have since calculated the relative energies and population ratios of various substituted derivatives of the saturated, unsaturated and our semi saturated heterocycles, in which the population ratios will significantly differ. However, these studies do not paint a uniform and consistent picture, as the dipole is, as expected, highly reliant on the substituent and its positioning. We have therefore decided to remove this analysis from the paper in order to avoid any potential confusion.

Thank you to the reviewer again for highlighting this to us.

The mechanistic investigation is also of interest. Have the authors investigated whether there is a correlation with Hammett parameters?

We had already investigated this, but had inadvertently missed it out of the supporting information, so we are very grateful for the prompt. We have now added it to the SI S4.10, p S41, and have pasted it here:

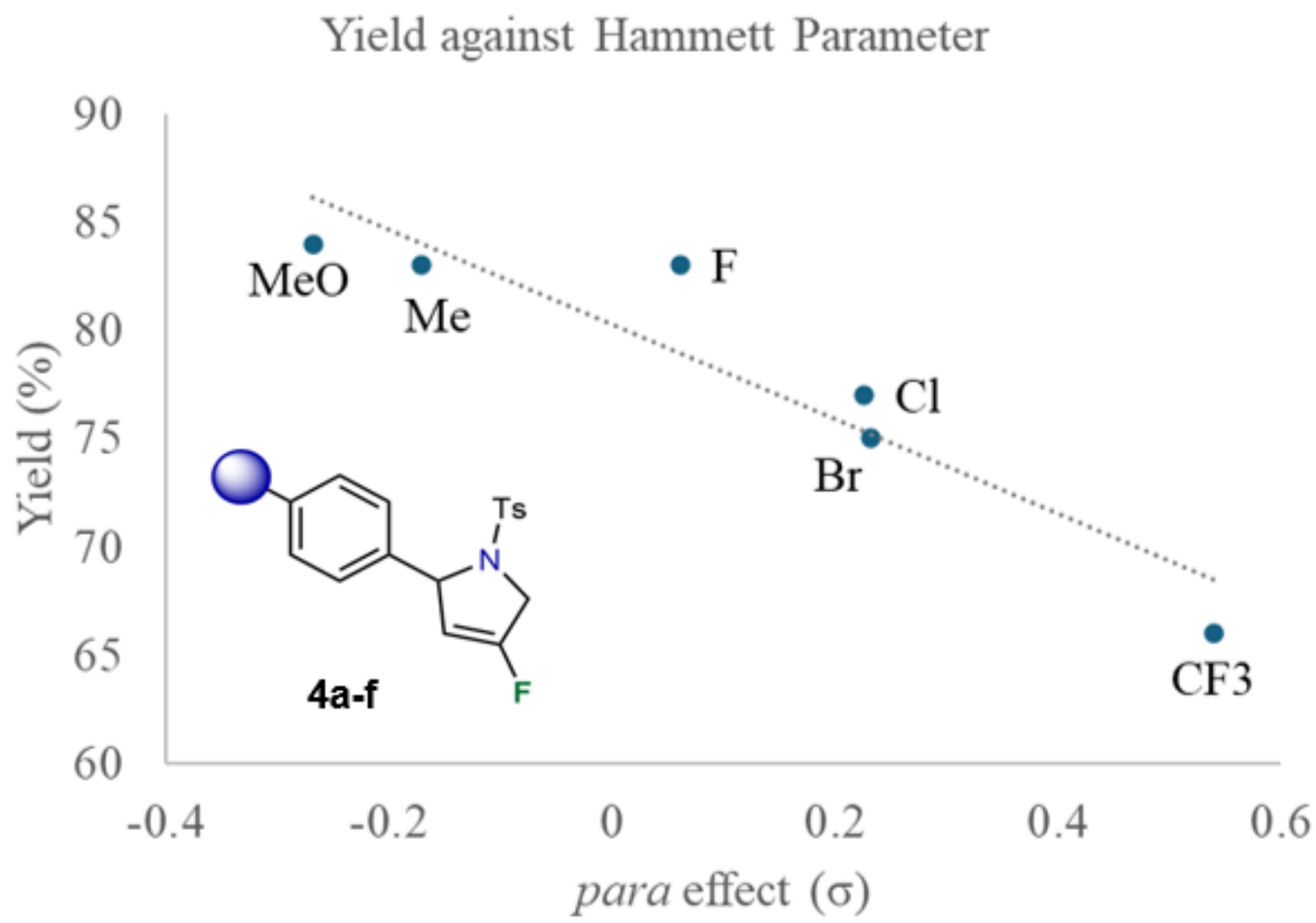

Figure S4.8: A graph of yield against Hammett parameters of *para*-substituents on an aromatic ring adjacent to the insertion site (**4a-f**). Results show lower yields for more electron-withdrawing substituents. *p*-NO_2 did not cyclise at all.

The plot shows a significant negative correlation between yield and Hammett parameter, with more electron-withdrawing substituents affording lower yields. Literature shows alkylidene carbenes to already be electrophilic, and fluorination of this carbene will cause it to be even more so. This is consistent with these data, as a more electrophilic carbene will react faster with electron-rich C-H bonds to give higher yields.

I am not sure why the KIE observed for the reaction of 3e would suggest that the reaction is initiated with a hydride transfer, as opposed to being a manifestation of the C-D bond being stronger than the C-H bond, and being broken in the rate determining step. Could the authors elaborate?

We would like to thank the reviewer for this critique of our analysis. Indeed, the KIE demonstrates C-D/C-H cleavage occurs in the product-determining step, which is not necessarily the rate-determining step, and does not unequivocally indicate the process is initiated by a hydride transfer. We have edited the text in the manuscript to reflect this point:

"An intramolecular KIE of 2.0 was measured from the reaction of monodeuterated **3e** (Figure 5C), in which insertion into the weaker C–H bond was preferred. This KIE reflects an expected energy barrier increase of 1.7 kcal/mol for insertion into a C–D bond over a C–H bond."

I am not sure about the explanation for the diastereoselectivity (Figure 2C). The dihedral angle between the Ar and sulfonyl groups is the same in the anti- and syn- transition

states. It is the 1,3-diaxial interaction with a C–H group that is different. Could the authors give more information or precedent from comparable cyclisation reactions?

The reviewer highlights an important need for clarification here; while the dihedral angle is indeed the same, due to rotation of the N-S bond, the relative positions of the aryl and tolyl groups are different. The steric clash is with the tolyl group rather than the SO2, as the SO2 is angled close to 90 degrees, pointing the tolyl group's C-S bond axially perpendicular to the plane of the transition state. This causes it to block one face of the transition state, and force *both* substituents onto the same side. We have edited the text and figure caption to clarify this explanation.

"Formation of the *trans*-product should be attenuated due to steric clash between the tosyl group and the substituents on the ring in the 6-membered transition state; the tosyl group occupies one side of the ring, forcing both substituents onto the opposite side, which leads to the excellent selectivity observed (inset Figure 2).[36]"

We have also edited the inset figure to add further explanation.

Finally, the extensive demonstration of the synthetic utility of the semi-unsaturated heterocycles is very useful.

We are happy the reviewer appreciates the extensive efforts we have made to show the utility of these heterocycles.

The SI is excellent. Congratulations to the authors for providing fully assigned NMR characterisation data. There is enough detail for reproduction of the work, except for the data listed under Sections 6.1 and 6.2. If these are Merck-specific protocols, then it should be provided or at least explained how logD, Human CL etc were determined. If these are literature procedures, then citations should be given.

We would like to thank the reviewer for pointing out these inadvertent omissions. The experimental protocols used by Merck have been added to the SI at the end of sections 6.1 and 6.2 respectively, with the appropriate reference added for the HLM (Human Liver Microsomal) assay procedure. The protocols added after each set of results read as follows:

6.1. Metabolic stability

LogD:

LogD (distribution coefficient) is a shake flask method measuring a compound's distribution between two immiscible solvents typically octanol and water. The compound's varying solubility in each phase, dictated by its polarity, leads to different concentration levels, which are quantified through HPLC analysis.

For the sample 8µL of the 10mM compound solution in DMSO are pipetted into one safe-lock tube. 200µL of Octanol saturated Phosphate Buffer 20mM pH 7.4 and 200µL of Phosphate Buffer pH 7.4 saturated Octanol is added to this. Afterwards the tube is placed on the overhead rotator to invert for 60 minutes. After centrifugation for 2 min at 1000 rpm, both the upper phase and the buffer-phase (lower phase) taken with an insulin jab through the bottom of the safe-lock are analysed. Dilution Octanol-phase can be diluted with Methanol (dilution factor = 10) if required.

Chromatographic conditions: Column Waters XBridge Column C8 3.5µm (Waters Cat No 186003053); Column temperature 30°C; Autosampler Room temperature (approx. 25°C); Injection volume 10 µL (HPLC) / 3µL (UPLC); Wavelength 220 nm, 254 nm, 290 nm using DAD detector. Eluant A: Place 630mg Ammonium formiate in a 2000mL volumetric flask, add 4mL Formic acid and bring up to volume with Ultrapure Water; Eluant B: 50 mL Eluant A + 950 mL Acetonitrile.

HPLC Gradient:

Time	Eluent A (%)	Eluent B (%)	Flow (ml/min)
0.0	95	5	2.0
4.0	0	100	2.0
4.1	95	2	2.0
6.0	95	5	2.0

HLM: See literature procedure.[11]

Microsomal stability was assessed on a liquid handling platform (Tecan Freedom EVO-2 200 MCA384) using pooled mixed genders HLM (HLM UltraPool 150 Corning catalog no. 452117, lot no. 38291). Compounds (1 μM final) were preincubated with microsomes (final 0.5 mg/mL) for 5 minutes at 37 °C in 50 mM phosphate buffer at pH 7.4 or 7.0, followed by the start of the incubation by the addition of cofactor NADPH (final 1.5 mM). The final DMSO concentration did not exceed 0.2% (v/v). Incubations were carried out in singlicate. Aliquots were taken at time points 0, 5, 10, 20, and 30 minutes. Additionally, 2 control

samples (0 and 30 minutes) lacking the addition of NADPH were included to flag compounds with nonmicrosomal metabolism. After incubation, samples were quenched by adding sample aliquots from 4 different compound incubations to the 2-fold volume of acetonitrile for bioanalytical cocktailing designed to avoid analytical interference. After centrifugation, supernatants were analyzed by a ultra-high performance liquid chromatography coupled to tandem mass spectrometry (UHPLC-MS/MS).

The data were subjected to postacquisition analysis by the software Analyst (version 1.7.2), and the percentage of compound remaining (defined as the ratio of peak area at a given time point and that at 0-minute time point) was plotted against incubation time. The first-order elimination rate constant (k_{el}) was derived by nonlinear regression using the software Biorails version 6.0 (The Edge Software Consultancy) and CL_{int} values (expressed in μL/min/mg) obtained by normalizing k_{el} with the microsomal concentration.

6.2. Blood plasma stability

Measurement methods:

**Procedure:**	The incubation was done on a Hamilton ML STAR pipetting robot.
	Compounds were available in a 10 mM DMSO solution.
	Compounds were mixed in a cocktail with up to 9 compounds and a verification standard.
	5 different matrices were used for the process: Simulated Gastric fluid, phosphate buffer, human plasma, mouse plasma and rat plasma.
	The 3 plasma types were set to a pH of 7.4 using 5% 1M phosphate buffer.
	The incubation period lased for 4 hours at 37°C while shaking. Samples were generated for 4h, 2h, 1h 0h and 4h total workup.
	Precipitation was achieved using acetonitrile. An internal standard was added during the precipitation step. The final dilution was done using water.
	Calibration and blank samples were also processed by the robot.
**Measurement:**	The quantitative determination was performed using an LC-MS/MS assay.
	One calibration and one blank sample per matrix were prepared.
	Measurements were performed on a UPLC.
	Tuning data for all compounds was generated beforehand.

	Detection was performed on a Tandem MS using multiple reaction monitoring (MRM) for MS/MS detection of the compounds.
	The concentrations of the compounds in all timepoints and calibration standards were calculated by interpolation of the peak area ratio of the analyte/internal standard versus the ratio of their nominal concentration into the regression line obtained from the calibration standards through zero.
**Equipment:**	Waters H-Class UPLC,<br>Sciex Triple QTrap API 5500 Mass Spectrometer<br>Hamilton ML STAR
**Chemicals Used:**	Water Milli-Q<br>Formic Acid (FAC) Art. 1.00264.0100<br>Acetonitrile (ACN) Art. 34998<br>Dimethyl sulfoxide (DMSO) Art. 1.01900.0500<br>di-Potassium hydrogen phosphate-trihydrate Art. 1.05099<br>Sodium chloride Art.1.06404<br>Hydrochloric acid 25% Art.1.00316<br>Pepsin Art. P7000<br>Human plasma was sourced from internal donors<br>CD1 mouse plasma and wistar rat plasma bought from CliniScience

It is good that a list of cycles that could not be synthesised is included in the SI. However, the main text should be more explicit in referring to this list. For example, with Figure 4 it should be mentioned that oxetenes could not be obtained in this way (and evidence of their formation, as explained in SI section 4.4 should also be mentioned in the text).

We'd like to thank the reviewer for this comment. The failed synthesis of oxetenes is mentioned in the text, alongside a short explanation for the evidence of their formation. We have now added an explicit figure reference. The text now reads:

"When alcohols were employed for O–H insertion, products were observed from the electrocyclic ring opening of the, evidently unstable, 4-membered oxetenes (see Scheme S4.1 for mechanism and Scheme S5.1 for failed oxetene substrates)."

We have also now highlighted limitations for other heterocycles in the text and referred to the SI accordingly:

“Carbene insertion into secondary benzylic C–H bonds gave moderate to excellent yields of pyrrolines **4a**-**f** containing differentially substituted rings (Figure 2Ca), except *p*-NO_2 (**4g**) that was unsuccessful. Insertion into more acidic C-H sites such as this proved difficult, as is shown by a comparison of yield against Hammett parameter (Figure S4.9) and our attempted C-H insertions α to sulfur, nitrile or carbonyl groups (Scheme S5.1).”

Small comments:

In Figures 2-4 it is good that the reaction sequence is shown from the alkyne starting material. It is not clear though whether the yields reported refer to the whole sequence, or just for the cyclisation starting from the FVI salts. From the SI I understand it is the latter. Please indicate the range of yields obtained for the conversion of the alkyne to the FVI salt under the reaction conditions.

Indeed, the yields are from the FVI salt. We have clarified this in each of the figure captions to read:

“Isolated yields from **3**, with NMR yields in parentheses”

We have also indicated the yield range of making the FVIs in each reaction scheme.

In figure 3, the structure of 5an should be shown together with 6an.

We have now included both structures in the figure.

In Figure 4, some of the NMR yields are marked with a ‘’ (7ay, 7bc, 8bd), but it is not explained what this refers to. Please include.*

We thank the reviewer for highlighting this omission. The * refers to a footnote that was mistakenly omitted from the figure caption. It has now been edited, and the end of the caption now reads:

“*Yield over 2 steps from alkyne.”

In the SI:

- To have known and novel alkynes in different sections makes it more difficult to find the data for a specific alkyne (as one doesn’t generally know which are known and novel). Please list the characterisation data of substrates etc according to compound numbering.

Thank you for pointing this out. We completely agree that the compounds should not be ordered in this way, and so we have reordered them according to their compound number.

- Page S22: the 't-butyl alcohol byproduct' should be 'the tBuOD byproduct'?

There should also be an explicit reference to Scheme S4.6b

Thank you for highlighting these points. We have now corrected and edited the SI to reflect them.

"Since it has been shown that the pK_a of the α-iodo proton (10-13) is much lower than that of a sulfonamide (16-17) through the base optimisation (**Table S3.2**), this exchange will only go in this direction. The results of these tests are shown in **scheme S4.6b**. Since the cyclisation results in a ~86% reduction in D incorporation, we conclude that the major mechanism is carbene formation, and the remaining 14% proceeds via either N-H deprotonation followed by D exchange with either the FVI or *tert*-butyl alcohol byproduct and then a carbene insertion, or via S_NV. To investigate this, the reaction was spiked with 1 equivalent of either *t*-BuOH or *t*-BuOD. The result was a 5% increase in product deuterium incorporation from the deuterated alcohol, and a 5% decrease from the non-deuterated alcohol. We conclude from this that deuterium incorporation in the product is primarily a result of exchange between the sulfonamide and the *t*-BuOD byproduct."

In summary, this is an excellent and well-constructed manuscript which will be of great interest to the synthetic and medicinal chemistry community. It provides convenient access to rare types of heterocyclic ring structures and is bound to find much use. The quality of the research is excellent. I recommend publication subject to a satisfactory addressing of the minor comments made.

Reviewer #2 (Remarks to the Author):

Lennox and co-workers report a general and versatile strategy to access fluorine-stabilized semi-saturated heterocycles (N, O). The authors demonstrate a unified approach to these exotic compounds and justify their synthesis by potential applications in medicinal chemistry as underexplored scaffolds, thus paving the way to new chemical space for drug-discovery programs. The authors conceptualize the idea of fluorine-stabilized semi-saturated rings and provide experimental proof. It is also good to see the high syn-diastereoselectivity observed; the stereochemical model appears reasonable.

We would like to thank this reviewer for their very constructive remarks, which have substantially helped to improve the manuscript.

(Regarding terminology: would it be more appropriate to use cis/trans for cyclic products and syn/anti for open-chain products?)

Thank you for highlighting this to us. We have checked literature data and IUPAC recommendations, which indeed recommend *cis/trans* for aliphatic rings. Therefore, we have switched all instances of *syn* and *anti* in the manuscript and SI to *cis* and *trans*.

Remarkably, oxidation of these species is achieved under rather harsh conditions, supporting the proposed idea that fluorine is retained; this also provides a new approach to access monofluorinated unsaturated heterocycles, some of which would be difficult to obtain by other means. One of the most intriguing parts of the work is the synthesis of previously unreported monofluorinated azetines — compounds that appear unstable but, due to the fluorine effect, were isolated and characterized in this study. I find this a very important contribution.

From a curiosity standpoint, the very high 1J(C–F) coupling (≈330 Hz) in the NMR is interesting. Although this is a spectroscopic curiosity, some readers may find it useful for understanding molecular properties of these small rings. In addition, the mechanism is supported by DFT calculations and by deuteration experiments. Oxidative stability was compared both experimentally and by cyclic voltammetry using a non-fluorinated analogue as reference. Microsomal stability was also evaluated, showing improved properties and potential medicinal-chemistry application. Overall, I recommend publication of this work in Nature Communications after minor revisions.

Manuscript — required edits and suggestions

-Please format FVI in bold throughout.

'FVI' has now been formatted in bold throughout the manuscript.

-From the main text it is not clear which FVI was used for optimization. Please mention the model substrate(s) in the main text.
-Comment on the usefulness of the "full-factorial Design of Experiments (DoE)". Was it helpful; did it make optimization more efficient in this study? If not in the main text, include a brief description and conclusions in the SI, and add the corresponding reference.

The model substrates have now been explicitly mentioned in the text and Figure 2B, and a brief description of the usefulness of the DoE optimisation has been added to the manuscript, which now reads:

"The synthesis of 3-fluoro-3-pyrrolines (**4**) was studied first, using β-fluorovinyl iodonium (**FVI**) salts that were formed from unactivated alkynes in a single, silver-catalysed step.[49] Optimisation of solvent and base with **3e** as the model substrate revealed 1,4-dioxane and sodium *tert*-butoxide as a suitable combination (see SI, Tables S3.1, S3.2, S3.3). A wide range of conditions were then surveyed with **3j** as the model substrate for the remaining variables, where a full-factorial Design of Experiments (DoE) approach was used to scan the multi-dimensional chemical space for both yield and diastereoselectivity (Figure 2B and SI, Table S3.4).[50] This enabled reaction optimisation to be carried out in relatively few experiments and determined which variables were significant. While complete diastereoselectivity was observed under all conditions tested, optimal yields of product and minimal yields of side-products were achieved with the slow addition of **FVI** to the basic solution. Through the initial screening, the reaction was shown to be unaffected by air and moisture, and the DoE optimisation showed the reaction to not be temperature sensitive."

-N- and O-heterocycles were synthesized. Any particular reason S-heterocycles were not explored? There is one unsuccessful example (3bo) containing a sulfone moiety, but what about simple thioethers?

Thioethers and sulfoxides unfortunately are too easily oxidised in the synthesis of the FVI starting materials. Due to the oxidising difluoro(aryl)-λ^3-iodane reagents used, several easily oxidised functional groups cannot be used. For a comprehensive study of this functional group tolerance, see previous work from our group:

J. Am. Chem. Soc. **146**, 15672–15680 (2024).

-Strictly speaking, please cite the Tamura work: J. Chem. Soc., Perkin Trans. 1, 1990, 975–981.

This reference has been added where azetines are introduced in the manuscript:

Results and discussion:

"However, insertion into the corresponding N–H bond of a sulfonamide gave novel 3-fluoro-2-azetine heterocycles **7** (Figure 4A).[60]"

References:

"60. Dimerised perfluorinated azetes have previously been formed, see: Chambers, R. D., Shepherd, T. & Tamura, M. Photochemistry of halogenocarbon compounds. Part 5. Photolysis of fluorinated 1,2,3-triazine derivatives. *J. Chem. Soc. Perkin 1* 975 (1990). "

-Add compound numbers in the text. For example: "Using this methodology, a second semi-saturated heterocycle could be obtained by replacing the nitrogen atom with oxygen to produce fluorinated 3-oxolenes (Figure 3)." Add the corresponding compound labels.

Thank you for highlighting this omission. We have now added compounds numbers after each mention of a family of compounds, e.g.

"Using this methodology, a second semi-saturated heterocycle could be obtained by replacing the nitrogen atom with oxygen to produce fluorinated 3-oxolenes **(6)**"

SI — required edits and suggestions

-Add compound numbering to schemes and procedures in the SI (e.g., General Procedure 2, 2A, 3, 3A and other instances).

Compound numbering has now been added to the schemes and general procedures in the supporting information. An example is shown below:

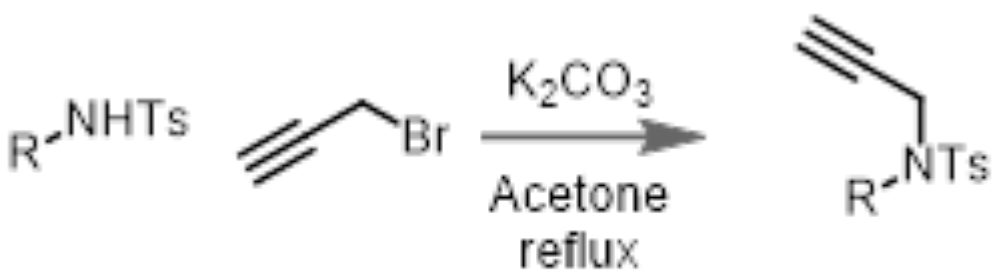

26 **1**

-In General Procedure 3, the abbreviation FVI is not explained in the SI. Define FVI independently in the experimental section (even if defined in the main article). If it is a compound label, reflect it in the scheme and format it in bold.

We have defined the abbreviation 'FVI' in the figure title and now in the body of the text as well.

-I recommend avoiding Arabic numbering for General Procedures (1, 2, etc.) as this may be confused with compound numbers. Use Roman numerals or letters instead.

All Arabic numbering in general procedures has been replaced with roman numerals to avoid confusion, example below:

"General procedure III (Fluorovinyl iodonium salt (FVI) synthesis - from original FVI paper).[1]

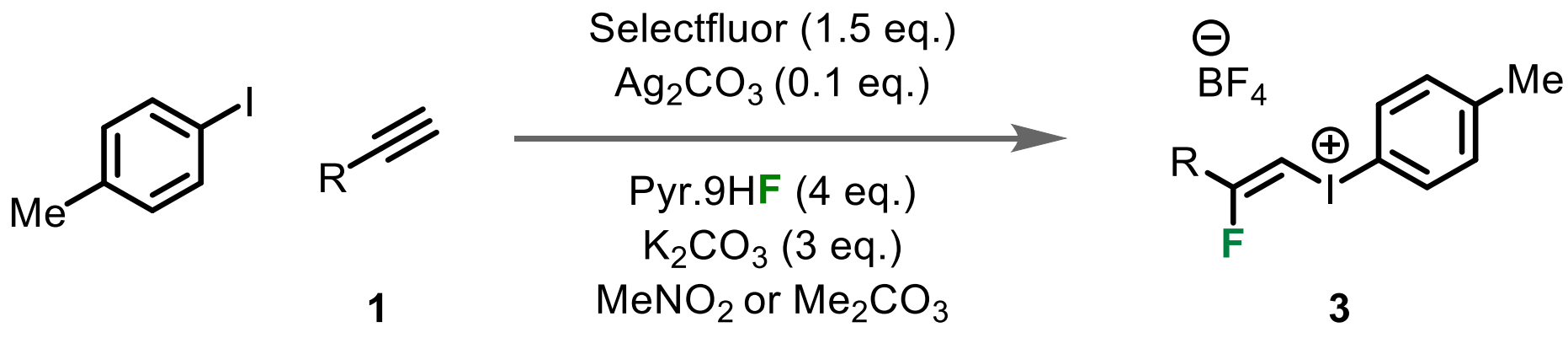

An HDPE vial was charged with *p*-iodotoluene (2.2 mmol, 1.1 equiv.), Selectfluor (3 mmol, 1.5 equiv.), Ag_2CO_3 (0.2 mmol, 10 mol%), K_2CO_3 (6 mmol, 3 equiv.), and $MeNO_2$ (12 mL). The mixture was cooled to 0 °C, 9HF.Pyridine was added (1.88 mL, 72 mmol, 36 HF equiv.), and the reaction mixture was stirred vigorously at 0 °C for 10 minutes. The relevant alkyne (2 mmol, 1 equiv.) was then dissolved in 2 mL $MeNO_2$ and added dropwise to the HDPE vial. The vial was warmed to RT and stirred for 18 h. Upon completion, the reaction was diluted with H_2O (100 mL) and dimethyl carbonate (50 mL). The organic layer was washed with H_2O (4 × 100 mL) and a 2 M aqueous solution of $NaBF_4$ (100 mL) and the aqueous washes were quenched with excess sat. aq. $NaHCO_3$. The organic layer was then filtered and concentrated *in vacuo* to afford the crude fluorovinyl iodonium salt (**FVI**), which was triturated using Et_2O with sonication to give the pure FVI."

-To improve reproducibility, indicate how reaction progress was monitored in General Procedures (TLC, LC–MS, crude NMR, etc.). For example, General Procedure 4 lists an exact time (2 h). Is that time consistent for every analogue? Please state how conversion was assessed before work-up.

We would like to thank the reviewer for highlighting this important omission.

For most of the general procedures, we have now added how the reaction was monitored. For example:

"A Schlenk tube was charged with tosyl chloride (6 mmol, 1.2 equiv.) and purged with N_2. Pyridine (10 mL) was then added, and the tube was cooled to 0 °C. The relevant amine (5 mmol, 1 equiv.) was then added dropwise to the tube via needle (added as a solution in 1 mL pyridine if solid) and stirred at 0 °C for 2 h. The tube was then warmed to RT and stirred for a

further 18 h, monitoring reaction progress by TLC. Upon completion, the reaction mixture was diluted with EtOAc (30 mL) and washed with aqueous 1 M $HCl_{(aq)}$ (2 × 30 mL). The organic layer was dried over $MgSO_4$, filtered, and concentrated *in vacuo* to afford the crude product."

However, for the synthesis of the FVI, we did not monitor the reaction since quenching aliquots with base would degrade the FVI, and unquenched HF would react with a TLC plate, causing unreliable results. Taking NMRs of unquenched aliquots would be possible, but inconvenient and unnecessary. No issues with incomplete FVI formation were encountered, see previous work within the group for a study of the time this reaction takes.

J. Am. Chem. Soc. **146**, 15672–15680 (2024).

-Section numbering "3:, 3.1.:" looks awkward in titles. Use standard numbering like "3." and "3.1." followed by the title.

Colons in all section numbers in the SI document have now been replaced with full stops.

-Figure S4.2: Please add information on how these graphs were created (HPLC control, NMR sampling?) and clarify what the graphs indicate.

Section 4.2 has now been edited to clarify these points, and now reads as:

"**4.2. Oxidation Kinetics**

The graph below shows the relative rates of oxidation by DDQ of a non-fluorinated pyrroline **4bf** and a fluorinated pyrroline **4v** to exemplify the stabilising effect of fluorine on oxidisable alkenes.

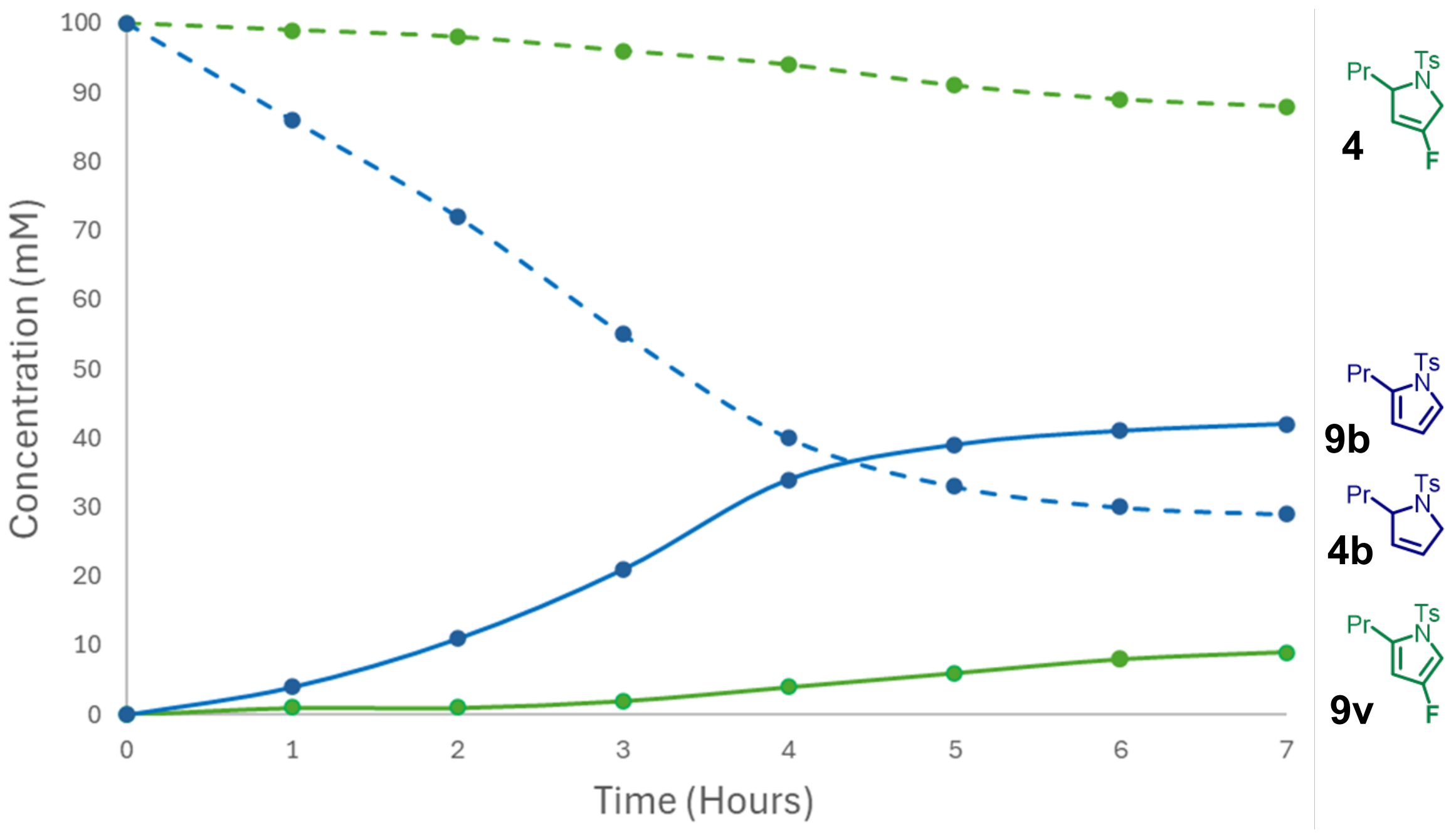

Figure S4.2: Relative rates of dehydrogenative oxidation of the fluorinated *n*-propyl pyrroline substrate **4v** to the pyrrole **9v**, and the non-fluorinated pyrroline **4bf** to pyrrole **9bf**. Concentration of each compound at each time point is shown on the *y*-axis, and time points are shown on the *x*-axis. Conditions: 5 equiv. DDQ, 110°C, 0.1 M, Toluene. Concentrations were monitored via NMR sampling, using 1,3,5-trimethoxybenzene (^{1}H) and 4,4'-difluorobiphenyl (^{19}F) as NMR standards."

-Figure S4.3: How was deuteration determined — NMR or MS? Please state the method.

The deuteration level was determined by NMR, and the figure caption has now been changed to clarify this, and appears as below. Deuterium incorporation of the starting material has also now been included.

"4.3. KIE study

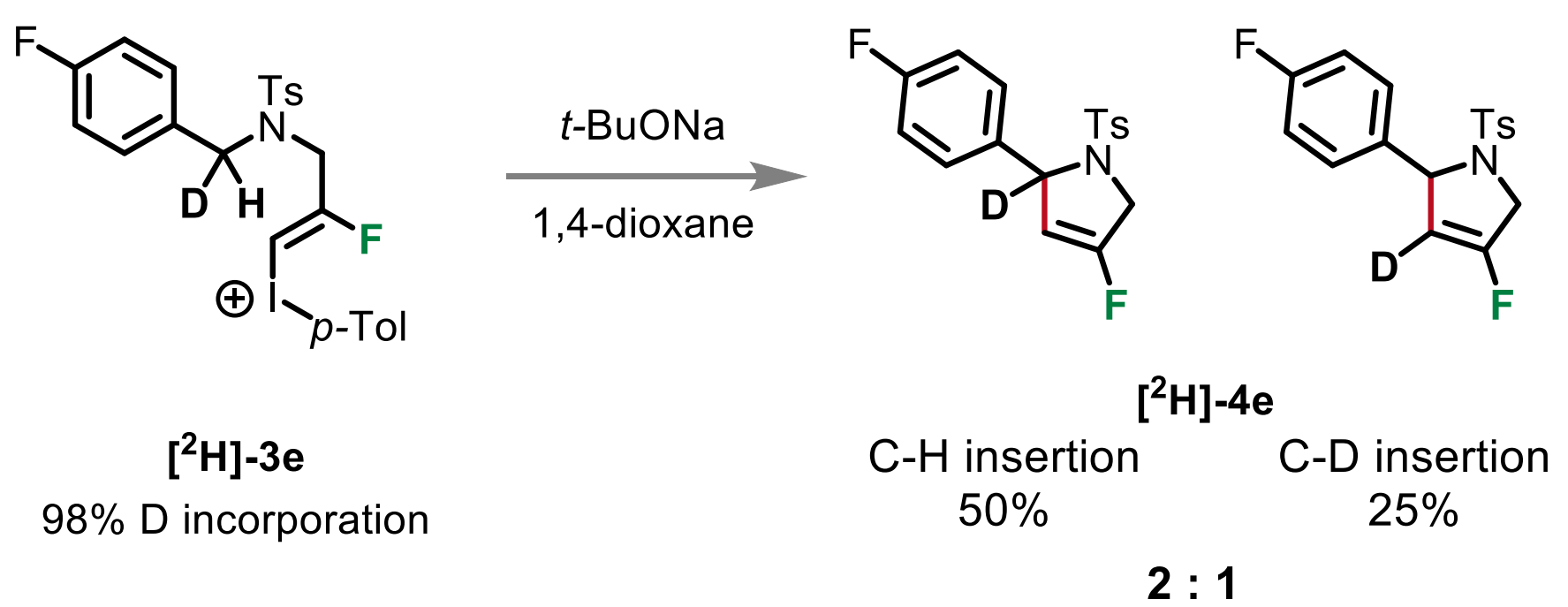

Figure S4.3: Cyclisation of monodeuterated fluorovinyl iodonium salt (FVI) **[^{2}H]-3e** to pyrroline **[^{2}H]-4e** according to **General procedure IV**. NMR yields were measured from the crude reaction mixture, showing a kinetic isotope effect of 2.0. Products were isolated as an inseparable mixture, and deuterium incorporation was measured via NMR. See next pages for characterisation data of deuterated FVI and pyrrolines."

For convenience to the reader, characterisation data of the deuterated FVI starting material and pyrroline products have now been moved to immediately after this section, and the same is done for the azetine deuteration experiment.

-Scheme S4.1: The note "Yield shown is relative to the alcohol FVI impurity in 4ag." is unclear. Please elaborate what this means and how yields were determined.

We recognise that this was very unclear, and therefore we have clarified this with edits to the figure and caption, and given an explicit compound number to the compound in question. This section now appears as shown:

"4.4. Oxetene Intermediate

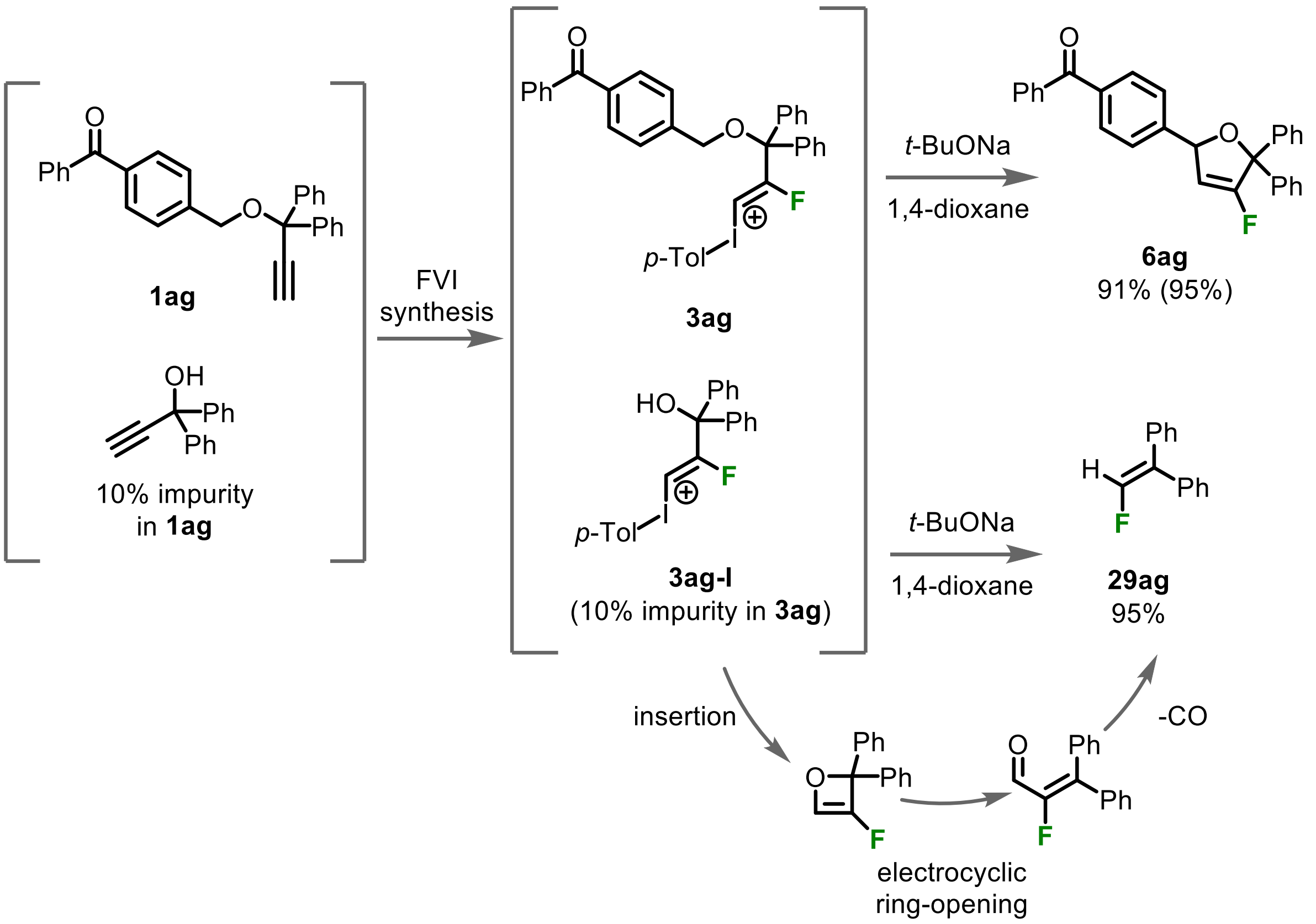

Scheme S4.1: Formation of alkene **29ag** during the synthesis of oxolene **6ag**.

10% of 1,1-diphenylprop-2-yn-1-ol was present in alkyne **1ag**. These were both converted into FVIs without separation, and the final post-cyclisation products **6ag** and **29ag** were separated by flash column chromatography. The formation of **29ag** in 95% yield provides evidence for an oxetene intermediate formed via O-H insertion (**Scheme S4.1**). Yields were determined by ^{19}F NMR using 4,4'-difluorobiphenyl as an internal standard. Yield of **29ag** was calculated relative to the quantity of **3ag-I** present in the starting material."

Reviewer #3 (Remarks to the Author):

This manuscript describes a high-impact and general synthetic strategy for constructing fluorinated semi-saturated four- and five-membered N- and O-heterocycles via alkylidene carbene intermediates. The chemistry is highly novel: fluorinated azetines are previously unknown, and the diastereoselective synthesis of fluorinated 3-pyrrolines and 3-oxolenes fills a long-standing gap in heterocyclic chemistry. Fluorine-mediated stabilization of these inherently unstable scaffolds is convincingly supported by controlled oxidation experiments, cyclic voltammetry, and human liver microsomal metabolism assays. The methodology is operationally simple, broad in scope, and directly relevant to drug discovery and pharmaceutical development. This work provides a general, practical, and conceptually novel method to access valuable fluorinated semi-saturated heterocycles that are highly attractive for drug development. The chemistry is well-designed, carefully executed, and broadly significant. This work meets the scope and impact criteria for Nature Communications. Publication is recommended after minor-to-moderate revision to address the scientific issues detailed below.

We are happy the reviewer agrees with us on the importance and significance of the work, and we would like to thank them for their constructive comments.

1.Mechanistic Evidence for Carbene Formation and N–H Insertion
The deuterium-labeling experiment supports the proposed N–H insertion pathway but does not fully exclude alternative anionic substitution mechanisms. Direct mechanistic evidence is required. Perform intermolecular carbene-trapping experiments (e.g., with external alkenes, methanol, or TEMPO) to verify the formation of free β-fluoro alkylidene carbene intermediates.

We would like to thank the reviewer for this valid suggestion. The alkylidene carbene is extremely short lived because the fluorine destabilises it through inductive withdrawal of electron density. Hence, they possess only very short lifetimes and, despite our efforts, we have not been able to observe any intermolecular reactivity. When we have added alkenes or alcohols, we do not observe any of the expected intermolecular trapping products.

1,5-C-H insertion is a characteristic reaction of an alkylidene carbene and not feasible by a different mechanism in this instance. For additional evidence of alkylidene carbene formation by similar methods, see Hara *Tetrahedron Lett.* **49**, 76–79 (2008). For studies on 1,5-C-H insertion of fluorovinyl carbenes and carbene rearrangement to alkynes for non-fluorinated analogues, see Ochiai *J. Am. Chem. Soc.* **115**, 2528–2529 (1993), *J. Am. Chem. Soc.* **110**, 6565–6566 (1988). Finally, see Stang *et al*. *J. Org. Chem.* **42**, 1802–1804 (1977) for intermolecular alkene trapping of similar alkylidene carbenes generated by α-elimination.

Provide DFT-calculated energy profiles for the key steps: α elimination to generate the carbene, 1,5-C–H insertion for five-membered rings, and 1,4-N–H insertion for four-membered azetines, to rationalize the chemo- and diastereoselectivity.

We would like to thank the reviewer for this good suggestion. We have conducted DFT studies, however, they are currently inconclusive. In the case of 1,5-and 1,4-N-H insertion, a transition state with a thermodynamically feasible energy barrier for the insertion mechanism could not be obtained (27.6 kcal/mol is too high for a reaction complete in minutes at RT). We have added this DFT data into an appendix of this document. This is the case despite experimental data showing the insertion mechanism to occur over the alternative vinylic substitution pathway. A more extensive study is, unfortunately, beyond the scope of this study.

Nevertheless, computational studies have been conducted by Taber and co-workers into 1,5-C-H insertions of alkylidene carbenes generated through α-elimination. (Taber, D. F., Meagley, R. P. & Doren, D. J. Intramolecular Alkylidene C-H insertion: https://www1.udel.edu/chem/valhalla/alkylidene.html.). Grainger (*Tetrahedron* **71**, 7795–7835 (2015)) wrote an extensive review on the chemistry of alkylidene carbenes including 1,5-C-H insertion and formation via base-induced α-elimination.

2.Structural Characterization of Novel Heterocyclic Scaffolds
Several unprecedented fluorinated heterocycles are characterized only by NMR and MS. Unambiguous structural confirmation is necessary. Obtain and present X ray single-crystal structures for at least three representative compounds, including one fluorinated azetine, one diastereoselective pyrroline, and one spirocyclic oxolene.

Thank you for this suggestion. Indeed, we have several X-ray structures present in the manuscript that confirm the structures of the products.

Diastereoselective pyrroline **4l** crystal structure and CCDC deposition number is shown in Figure 2 (CCSD (**4l**) = 2485038.). Azetines **7ar** and **7aw** have crystal structures and CCDC deposition numbers are shown in Figure 4, (CCSD (**7ar**) = 2485040; (**7aw**) = 2486256). A crystal structure for pyrrolidine derivative **10m** is also shown in figure 6 (CCSD (**10m**) = 2485039.).

Report full analytical data (HRMS, $^{1}H/^{13}C/^{19}F$ NMR, and optical rotation for chiral products) consistently for all new compounds in the Supporting Information.

Thank you for this suggestion. The suggested data has been collected and reported for all substrates that could be isolated. The only compounds missing full characterisation data

were those not stable enough to isolate, such as oxolenes **6ah** or **6am**, which are identified by characteristic NMR peaks present in similar products that could be isolated.

3.Substrate Scope and Methodological Limitations
The manuscript only presents successful substrates and does not discuss failed cases or functional group compatibility. Add a table in the Supporting Information summarizing unsuccessful substrates (e.g., those containing –OH, –CO_2H, –CN, aldehyde, or unprotected basic amine groups) and provide mechanistic explanations for failed examples such as the p-NO_2-substituted substrate 4g.

Thank you for this suggestion. A section detailing failed substrates is shown in the SI (Scheme S5.1). For more detail on functional groups that are not tolerated in the FVI synthesis step, see the FVI synthesis paper previously published by our group:

J. Am. Chem. Soc. **146**, 15672–15680 (2024).

A mechanistic explanation for the *p*-NO_2 substrate and other similar failures has now been added in the manuscript, which now reads as:

"With an optimised set of conditions for the synthesis of semi-saturated fluorinated pyrrolines **(4)**, the generality of the substrate scope was explored (Figure 2C). Carbene insertion into secondary benzylic C–H bonds gave moderate to excellent yields of pyrrolines **4a**-**f** containing differentially substituted rings (Figure 2Ca), except *p*-NO_2 (**4g**) that was unsuccessful. Insertion into more acidic C-H sites such as this proved difficult, as is shown by a comparison of yield against Hammett parameter (see SI, figure S4.9) and our attempted C-H insertions α to sulfur, nitrile, or carbonyl groups (see SI, scheme S5.1)."

Clearly state the functional group tolerance and sensitivity of the reaction to air, moisture, and temperature.

The functional group tolerance is shown by failed substrates section (S5, figure 5.1). Sensitivity to air, moisture and temperature is detailed in the SI at the top of table S3.1. and has now also been detailed in the second paragraph of the results and discussion, which now reads:

"The reaction outcomes were found to be unaffected by air and moisture, and the DoE optimisation showed the reaction to not be temperature sensitive."

4.Stability and Physicochemical Characterization
Fluorine-induced stability is the central design principle of this work and requires deeper validation. Extend stability studies to include long-term storage stability (at least

7–30 days at room temperature) and stability across a pH range (pH 2, 7, and 12), monitored by NMR or HPLC.

Thank you for this good suggestion. NMR spectra were taken of representative examples from four families of products, which had been in storage for extended periods of time under air *at room temperature*. A section has been added to the SI which details the results of this, including the date of synthesis and the date the NMR spectrum was taken for the long-term stability test, the result, and the calculated half-life ($t_{1/2}$). The table is shown below:

"4.10. Long-term storage stability at room temperature

Structure	Compound no.	Synthesised	Tested	Result	$t_{1/2}$
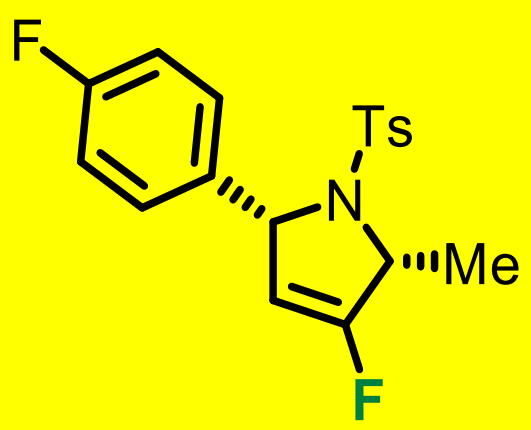	**(*R*,*R*)-4j**	21/06/2024	16/06/2026 (2 years)	Unchanged	>200 years*
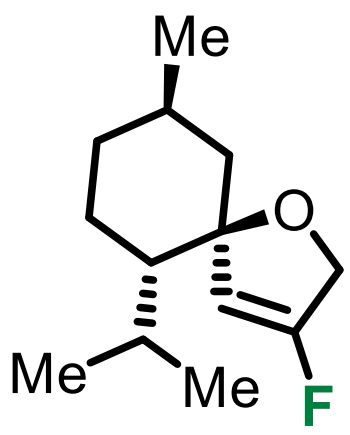	**6aq**	26/04/2024	16/06/2026 (2 years 2 months)	58% **6aq**<br>42% degraded	2.8 years
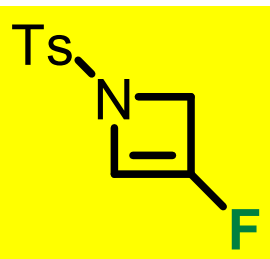	**7ar**	14/05/2025	16/06/2026 (13 months)	48% **7ar**<br>23% ring-opened<br>29% degraded	12.3 months
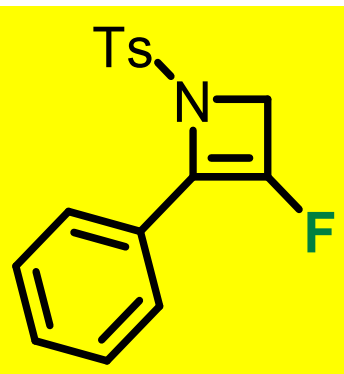	**7aw**	13/12/2024	16/06/2026 (1 year 7 months)	96% **16aw**<br>4% degraded	n/a

Table S4.2: Effects of long-term storage of various substrate classes under air at RT. Representative examples from 4 substrate classes (pyrroline, oxolene, alkyl azetine, aryl azetine) were chosen based on sample availability. Results were quantified by NMR analysis. *Based on 1% margin of error.

Pyrroline **(*R*,*R*)-4j** showed no signs of degradation after 2 years of storage under air at RT. Oxolene **6aq** showed 58% remaining material after 2 years and 2 months under air at RT (half-life 2.8 years). Azetine **7ar** showed 48% remaining material after 13 months under air at RT (half-life 12.3 months). To prevent thermal electrocyclic ring-opening, azetines should be stored in a freezer. Aryl azetine **7aw** showed complete conversion to hydrated open chain product **16aw** after 1 year 7 months under air at RT. To prevent this hydration, this type of azetine should be stored in a sealed container under dry conditions. See below for NMR spectra

of samples after the specified amount of time in storage. See section S8 for NMR spectra of the newly synthesised compounds."

The NMR spectra of these compounds after the specified time in storage are shown in the SI, just below this section.

A second section has also been added to show the stability in acidic, basic, and neutral aqueous media. A sample of each compound was combined with an NMR standard, measured, and then exposed to each set of conditions, before re-measuring by NMR to determine sample integrity. This section is shown below:

"4.11. Acid and base stability

A pyrroline, an oxolene, and an azetine were subjected to acidic, basic, and neutral conditions in aqueous media for extended periods of time, and the remaining quantity of material recorded. Solutions were neutralised with either sat. aq. Na_2CO_3 or 2M aq. HCl as required.

Structure	Compound no.	pH 0 (24 h)<br>% remaining	pH 14 (24 h)<br>% remaining	pH 7 (48 h)<br>% remaining
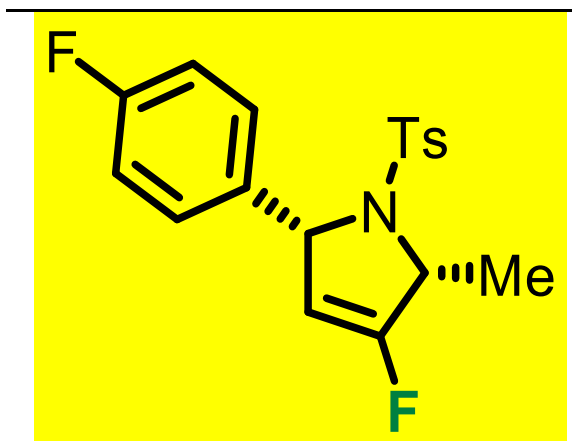	**(*R*,*R*)-4j**	100	100	100
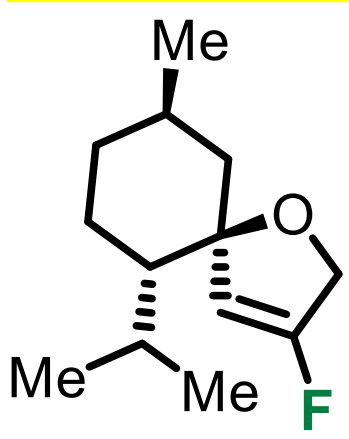	**6aq**	44	51	86
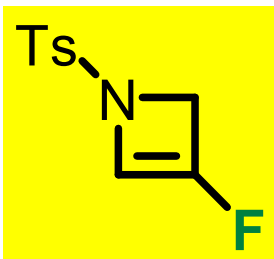	**7ar**	91	66	95

Table S4.3: Remaining quantity of a selected example from each product family after stirring in either acid or base for 24 h, and stability in aqueous media over 48 h. Solutions were made up in 2:1 MeCN/H_2O, and made up to the required pH using HCl (37% w/w aqueous) for the acidic solutions, and DBU for the basic solutions. Substrate concentration was 0.1M. After neutralising, results were measured by ^{19}F NMR using 4,4'-difluorobiphenyl as an internal standard."

Include metabolic stability data for a broader set of analogs to better support the claim of improved drug-like properties.

Thank you for this good suggestion. We have included metabolism data for several representative examples of these heterocycles. Further expansion of these tests would would be more appropriate for a separate, more medicinally focussed, article, and

therefore re beyond the scope of this study. We wish to communicate our results rapidly, so that these types of extended studies can be undertaken also by others.

5.Comparison with Previous Methods

The advances of this methodology should be quantitatively benchmarked against existing synthetic routes. Add a comparative table that contrasts this work with previous methods in terms of step count, isolated yield, diastereoselectivity, substrate scope, fluorination pattern, and synthetic scalability.

Thank you for this great suggestion. We have now added tables in the SI benchmarking this work against previous methods for pyrrolines and oxolenes, which are shown below. Azetines are not included as there are no previous methods for their synthesis. Fluorination pattern is not benchmarked as fluorine is always in the 3-position.

"7. Methodology Benchmarking

Existing methods of synthesis for 3-fluoro-3-pyrrolines and 3-fluoro-3-oxolenes are benchmarked against this work in the tables below. No table has been included for fluorinated azetines are there are currently no methods for their synthesis other than this work. Ring-closing metathesis (RCM) work on pyrrolines is by Rutjes,[12] Haufe,[13] Inoue,[14] and Brown,[15] and in the case of oxolenes is only by Inoue. Brown's attempt to synthesise a 3-fluoro-3-pyrroline was unsuccessful, and Haufe's products were pyrrolinones rather than strictly pyrrolines. Stated step count is given from simple non-fluorinated or commercially available starting materials. For more details on limitations of this work's functional group compatibility, see section S5 (failed cyclisation substrates) and previous work from our group for limitations in the FVI synthesis step.[1]

7.1. 3-Pyrrolines

**Metric**	**Liu *et al.***	**RCM**	**This work**
**Step count**	2	2-4	2
**Isolated yield**	28-92%	19-86%	10-79% (see manuscript)
**Diastereoselectivity**	1:1 (none)	n/a	>100:1 *cis*
**Substitution Patterns**	Broad substitution patterns, but 4-position must be ester or aryl	Limited to 1 methyl group in 2- or 4-position	Broad (see manuscript), but no substitution at 4-position
**Functional Group Tolerance**	Limited to esters, sulfonates, and aryl halides	Simple alkyl only	Broad (see manuscript) but no groups highly sensitive to oxidants
**Synthetic Scalability**	Only tested on 0.1 mmol scale (<100 mg)	20.2 mg scale	Tested on multi-gram scale

Table 7.1: Benchmarking of existing methods of synthesis for 3-fluoro-3-pyrrolines against this work. Methods shown are ring-closing metathesis (RCM), and a general method from allenes by Liu *et al.*[16]

7.2. 3-Oxolenes

Metric	Chu *et al.*	Lequeux *et al.*	RCM	This work
**Step count**	6	4 steps from 3-fluorooxetane	2	2
**Isolated yield**	75-80%	75%	74%	15-95% (see manuscript)
**Diastereoselectivity**	1.3:1 *trans*	n/a	n/a	2:1 *cis*
**Substitution Patterns**	Single substitution pattern, small variations at 1 position	1 example, 1 position	1 example, 1 position	Broad (see manuscript), but no substitution at 4-position
**Functional Group Tolerance**	Only alcohol and ether groups shown	Only alcohol shown	Alkyl/aryl only	Broad (see manuscript) but no groups highly sensitive to oxidants
**Synthetic Scalability**	Up to 280 mg scale	30 mg scale	14.2 mg scale	Tested on multi-gram scale

Table 7.2: Benchmarking of existing methods of synthesis for 3-fluoro-3-oxolenes against this work. Methods shown are a multi-step synthesis used by Chu *et al.* in the synthesis of nucleotide bioisosteres,[17–19] a single example by Lequeux *et al.*,[20] and ring-closing metathesis (RCM)."

Reviewer #4 (Remarks to the Author):

This manuscript by Lennox and co-workers describes a general protocol for the synthesis of four- and five-membered heterocycles (oxolenes, pyrrolines, and azetines) bearing a fluorinated unsaturation.
The authors' strategy relies on the generation of alkylidene carbenes from hypervalent iodine intermediates upon treatment with a base (NaOtBu). The corresponding fluorovinyl iodonium species are readily accessed through a silver-catalyzed fluoroiodination reaction. This two-step sequence provides efficient access to highly valuable partially saturated fluorinated heterocycles. The originality of the work lies in the elegant exploitation of the unique reactivity of alkylidene carbenes and their facile generation from vinyl iodonium precursors. Building upon the Lennox group's recognized expertise in fluorinated hypervalent iodine chemistry, this study constitutes a well-executed and insightful contribution to the field.
The target compounds are highly valuable motifs with considerable potential for application in medicinal chemistry. Furthermore, the work aligns well with current efforts to access non-planar heterocyclic architectures and fluorinated analogues, both of which are of significant contemporary interest. The potential utility of these scaffolds is further highlighted by the determination of LogD values. In addition, the authors support their mechanistic proposal through a thorough and carefully designed mechanistic investigation and further demonstrate the synthetic utility of the products as versatile building blocks.

Overall, the originality of this work is high, and its scientific relevance is substantial. The study addresses important contemporary challenges in synthetic methodology and is likely to inspire further developments in the field. I therefore recommend publication of this manuscript in Nature Communications after minor revision.

We would like to thank this reviewer for their careful reading of the manuscript and we are happy they share our enthusiasm for the work.

1/ Have the authors explored whether other chalcogen-containing heterocycles, such as sulfur-, sulfone-, or selenium-based analogues, can be accessed under the standard reaction conditions?

A sulfone has been tested, but the cyclisation failed, see the failed substrates section, scheme S5.1 in the SI. Thioethers and sulfoxides are too easily oxidised in the FVI synthesis due to the highly oxidising nature of the difluoro(aryl)-λ^3-iodane reagents required for their formation. For greater detail on functionalities not tolerated by this reaction, see previous work from the group (*J. Am. Chem. Soc.* **146**, 15672–15680 (2024)). Selenium has not been attempted, as it is even more easily oxidised than sulfur.

2/ The authors may wish to cite a relevant example demonstrating the synthetic value of fluorinated pyrrolines. In particular, the application of fluorinated pyrrolines in the synthesis of a strategic API building block has been reported (OPRD, 2020, 24, 792) and would further illustrate the practical significance of this scaffold.

This reference has been added to the end of the introduction, which now reads:

"Hence, the development of a general and accessible method to semi-saturated fluorinated oxygen and nitrogen heterocycles would provide new building blocks for drug development.[31]"

31. René, A. *et al.* Practical Synthesis of Ethyl 3-Fluoro-1-pyrrole-2-carboxylate: A Key Fragment of a Potent Drug Candidate against Hepatitis B Virus. *Org. Process Res. Dev.* **24**, 792–801 (2020).

Appendix (DFT data)

The following pages contain the preliminary DFT data for the N-H insertion.

N-H insertion:

2 possible mechanisms are considered: either direct carbene insertion, or deprotonation of the sulfonamide followed by vinylic substitution.

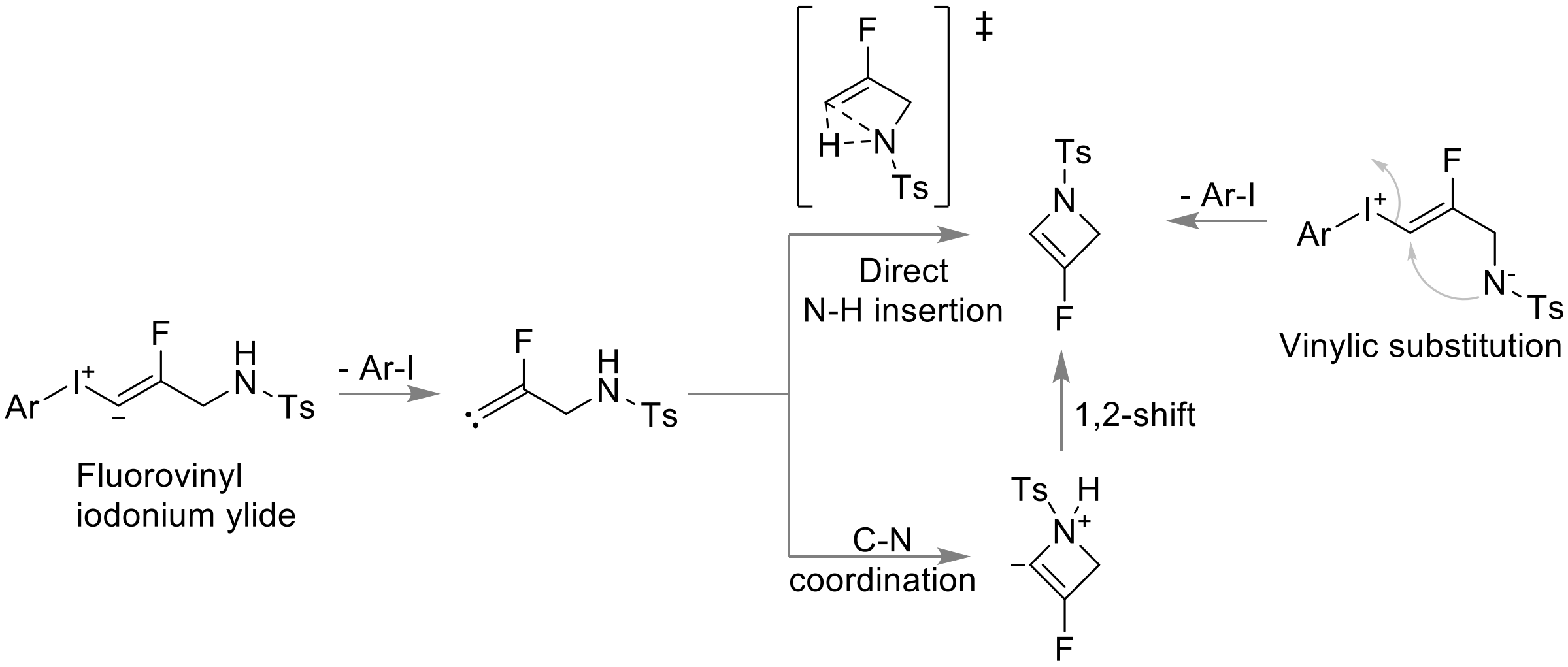

N-H Insertion Pathway: 4-Membered Ring (Azetine) – No Protecting Group

M06-2X/Def2-TZVP, IEFPCM(1,4-Dioxane)
Energies in kcal/mol

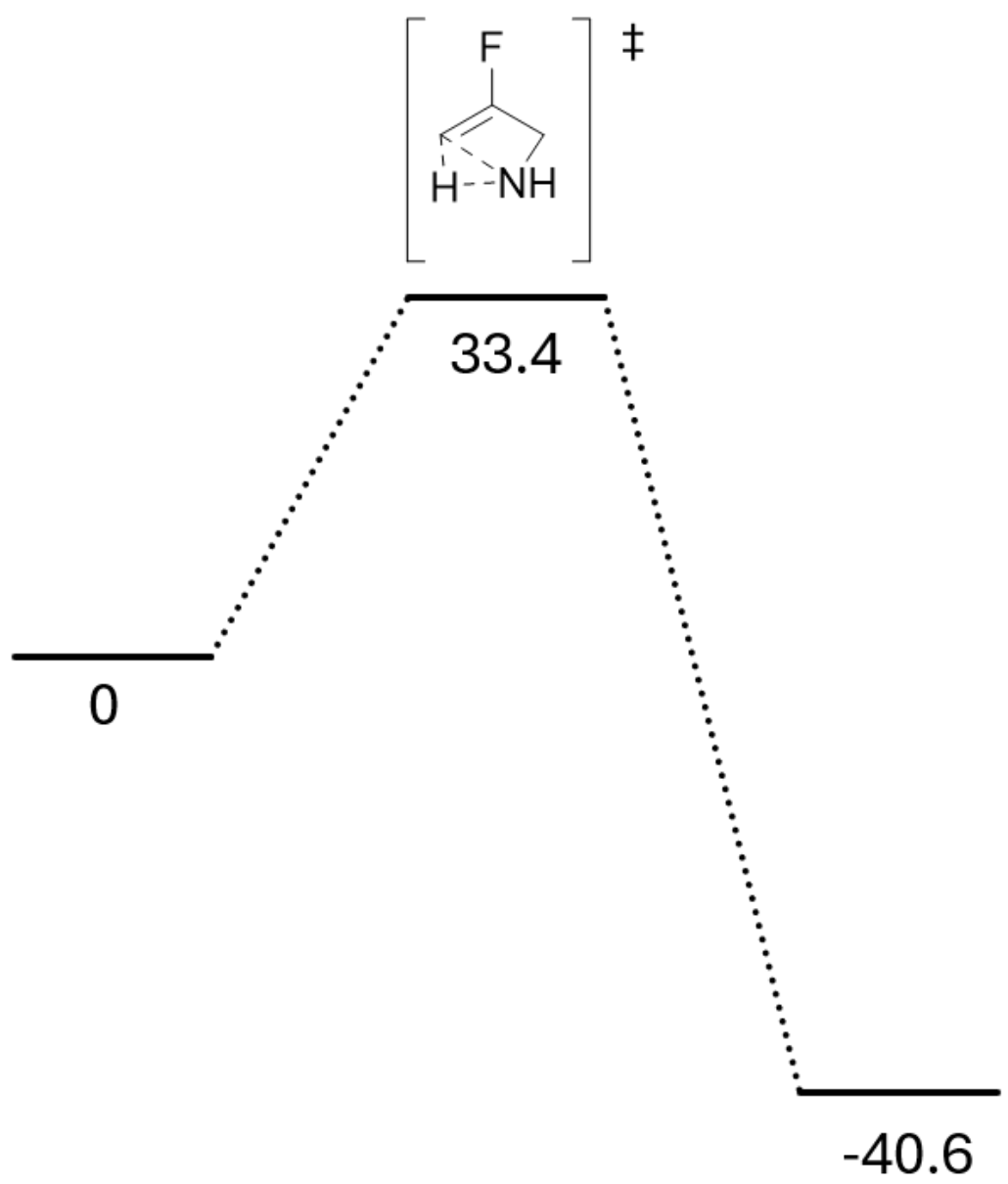

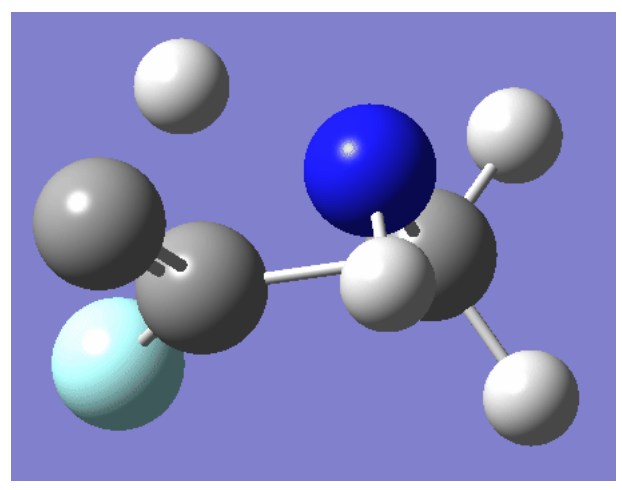

$\Delta G^{\ddagger}$ = 33.4 kcal / mol

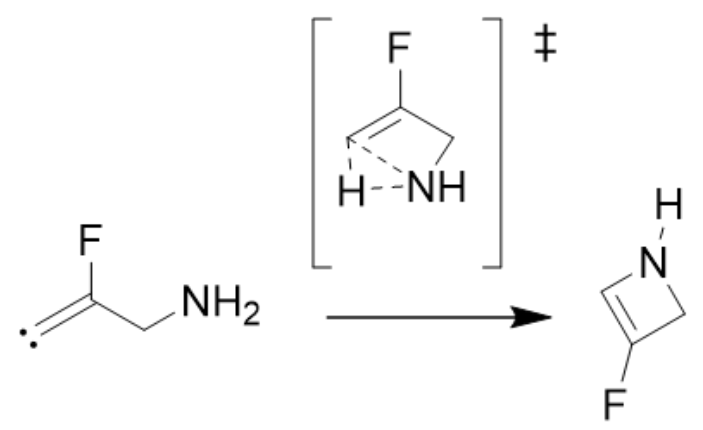

N-H Insertion Pathway: 4-Membered Ring (Azetine, tosyl protected)

ΔG‡ = 27.6 kcal / mol

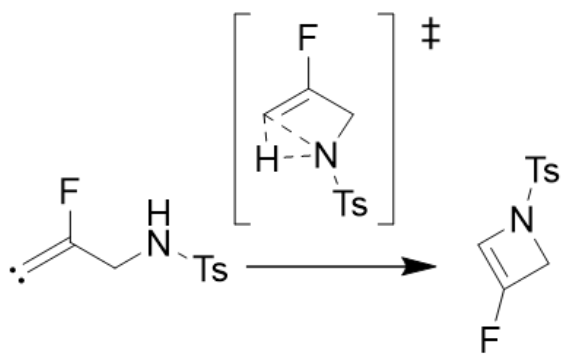

Ts Protecting group on nitrogen

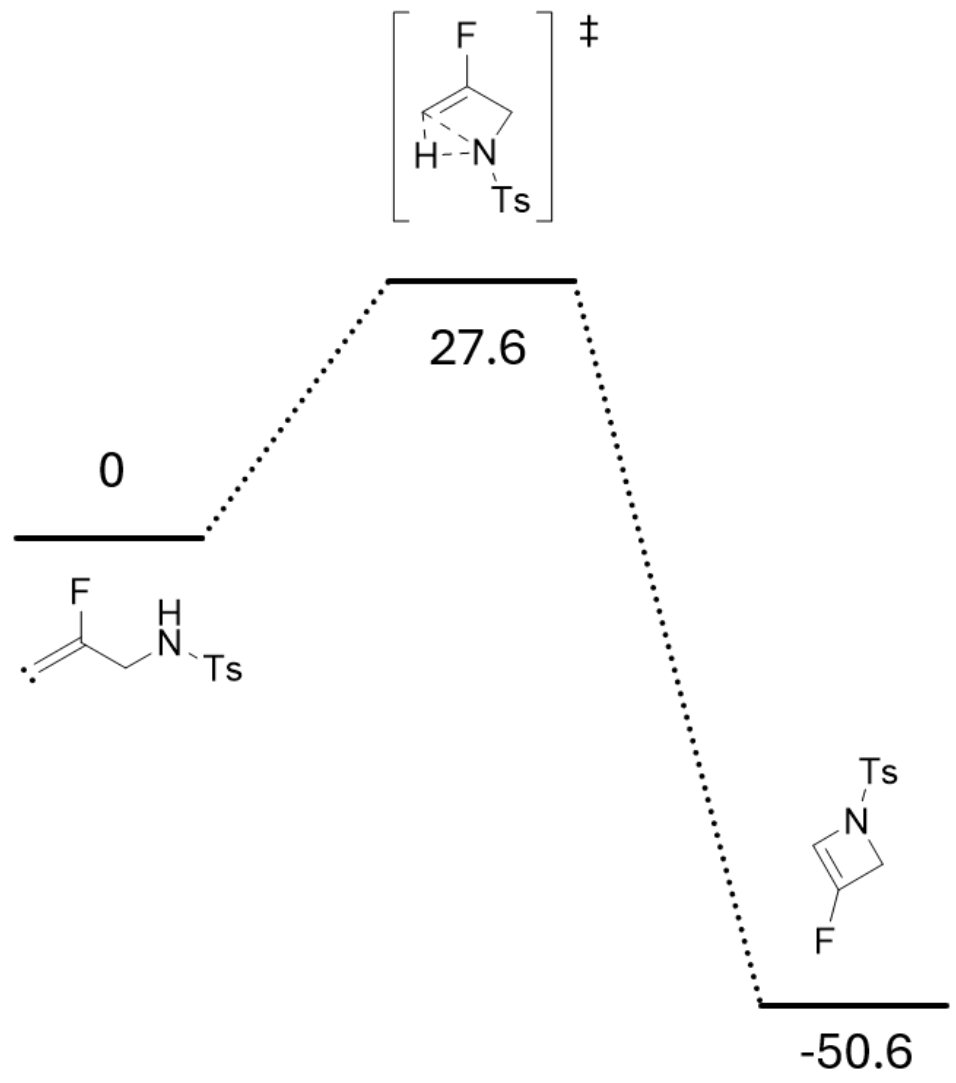

4-Membered Ring (Azetine, tosyl protected): Vinylic Substitution

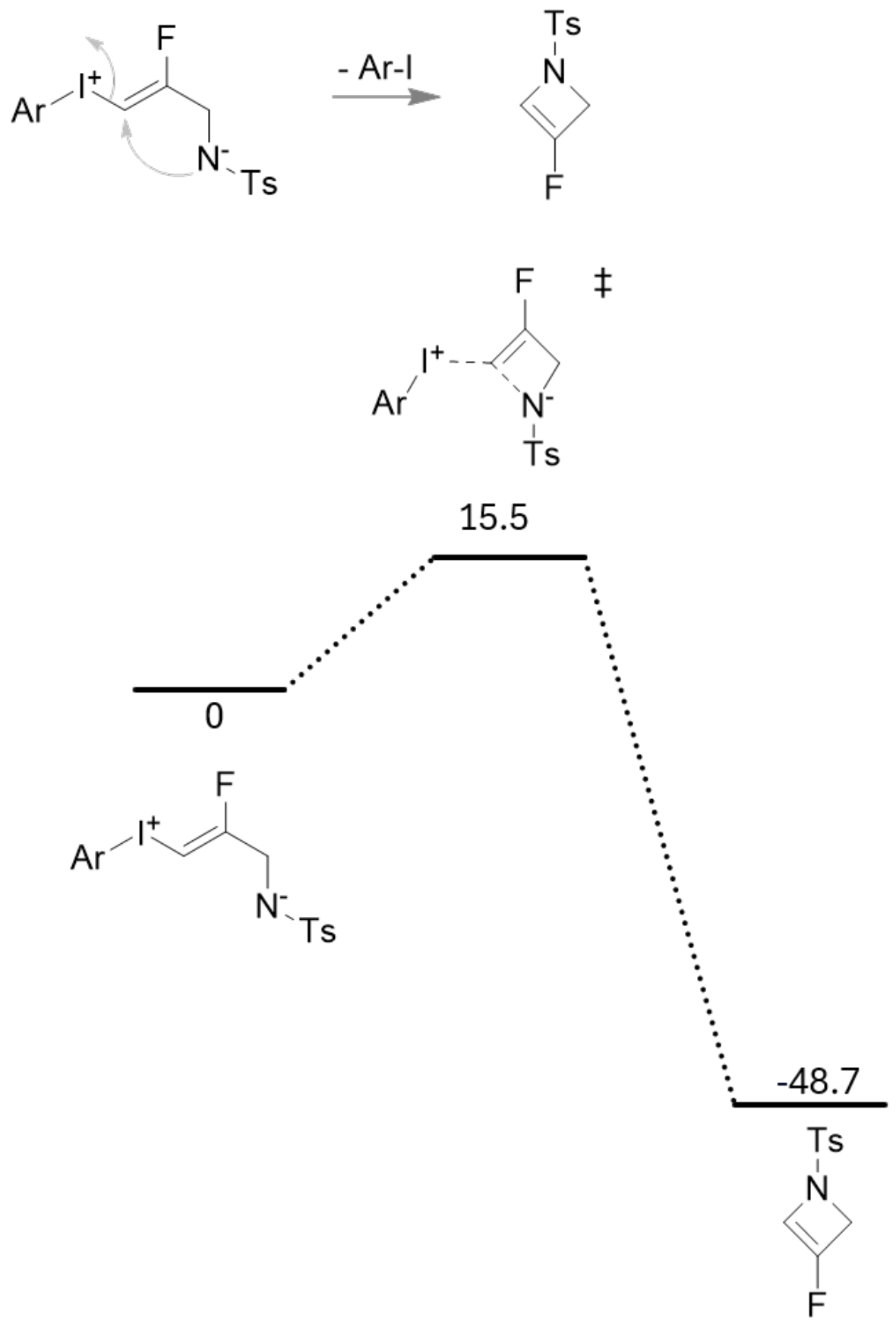

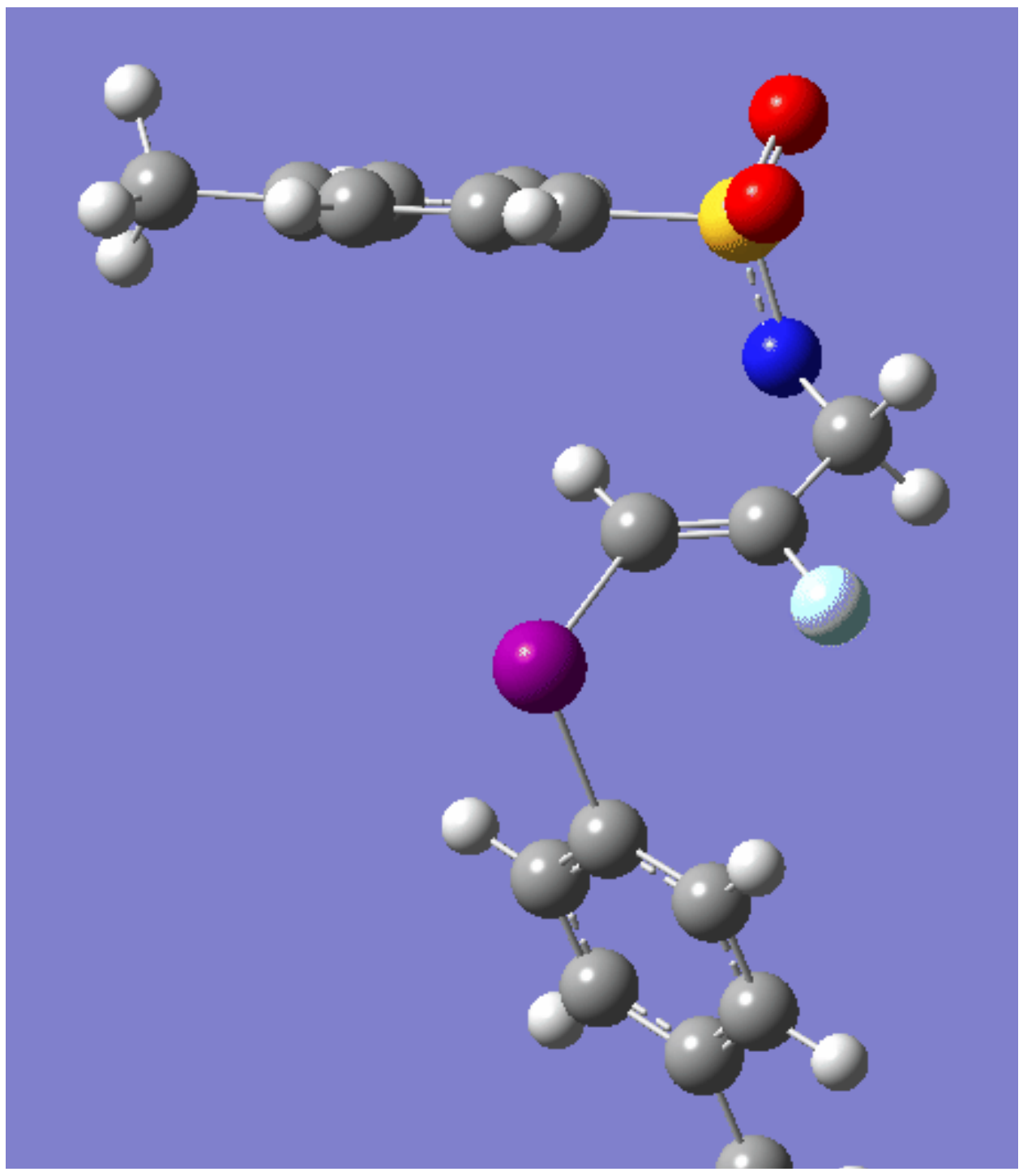

Energies in kcal / mol

N-H Insertion Pathway: 5-Membered Ring, concerted N-H insertion

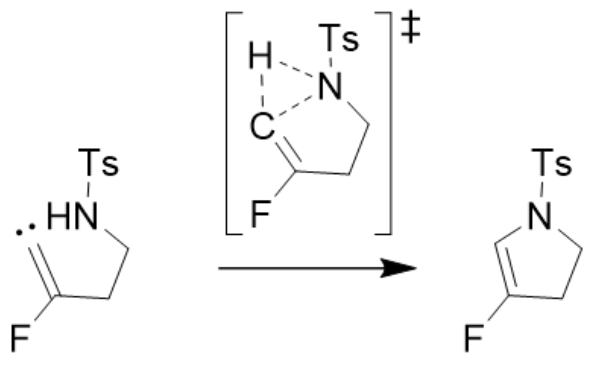

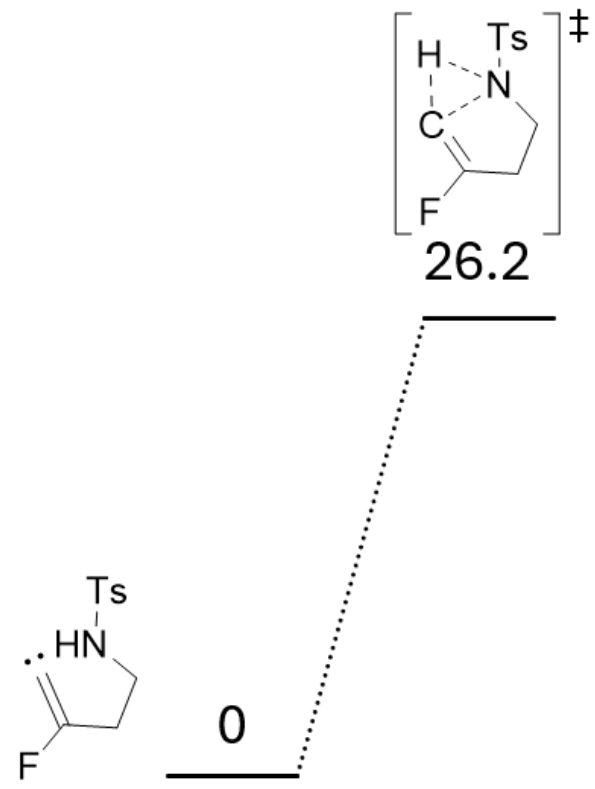

N-H Insertion Pathway: 5-Membered Ring, 1,2-Proton shift

$\Delta G^{\ddagger}$ = 25.9 kcal / mol

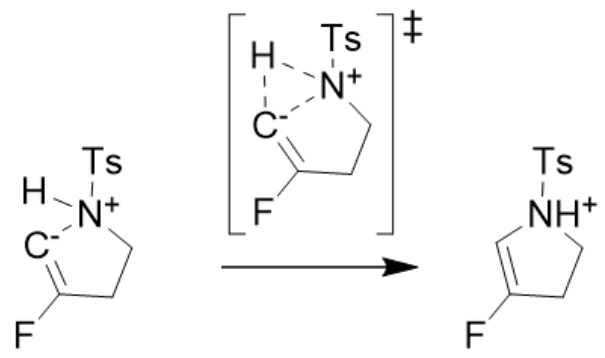

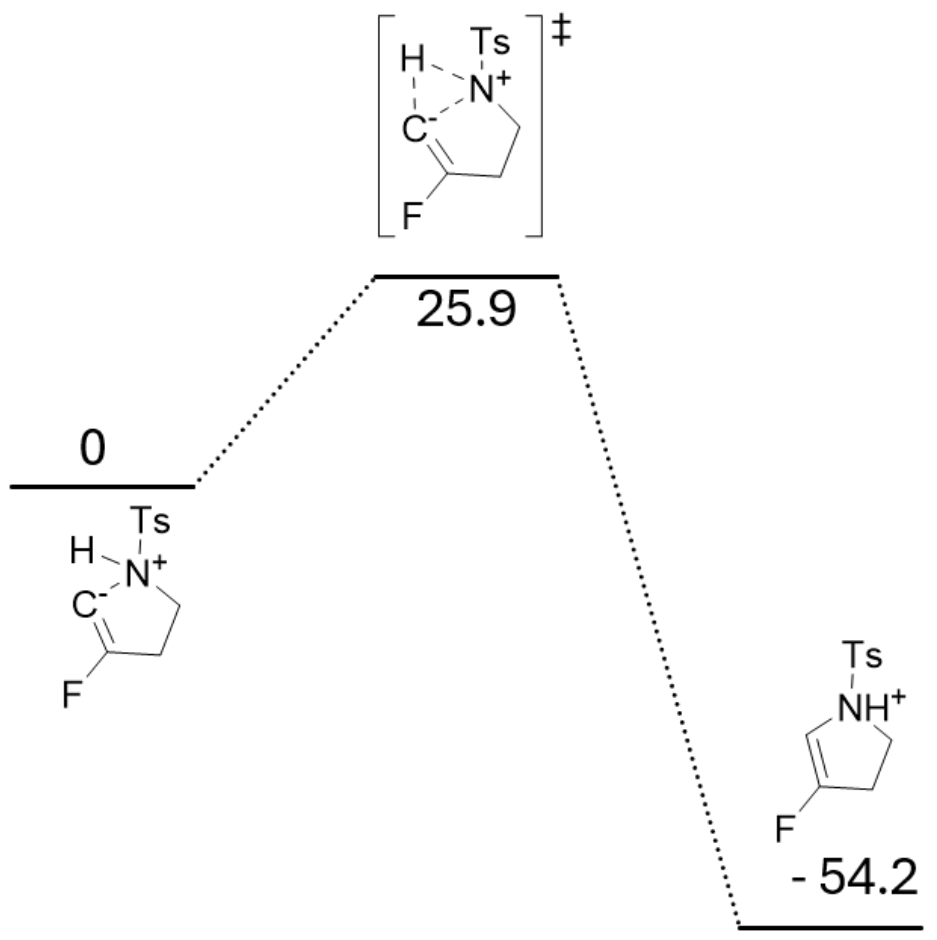

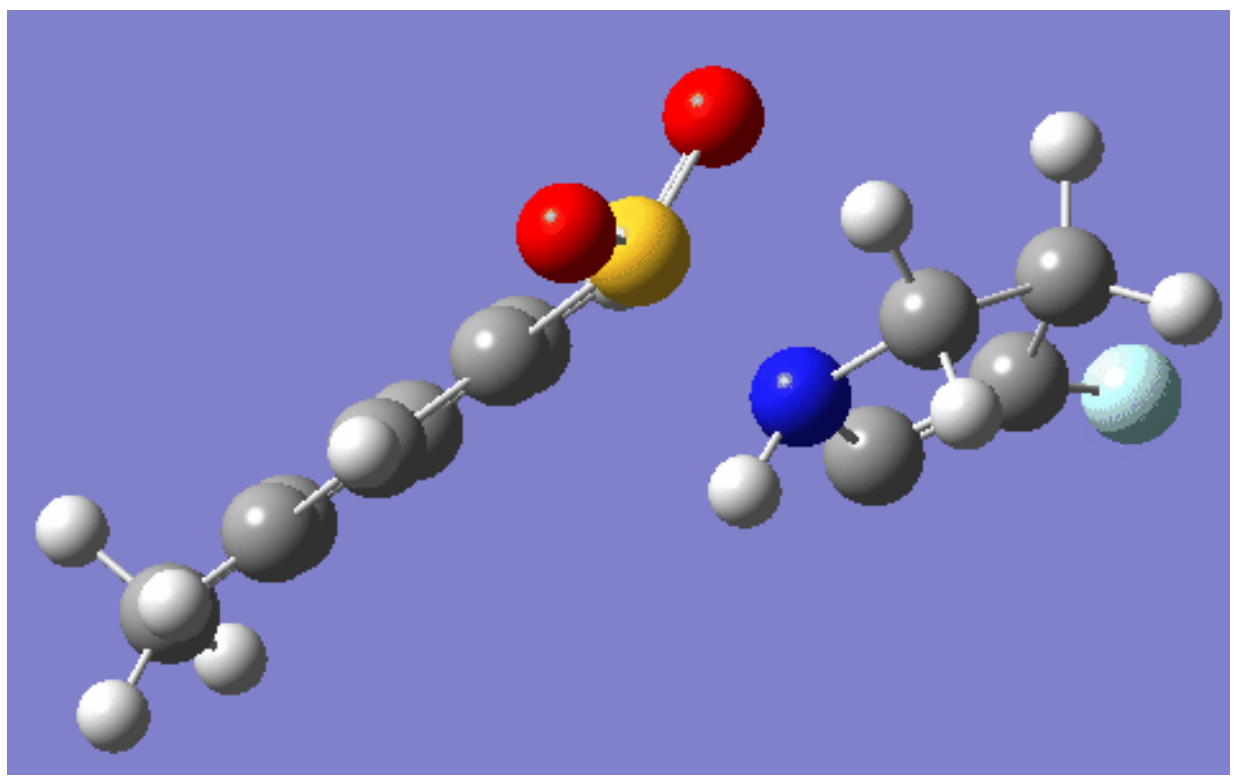

5-Membered Ring: Vinylic Substitution

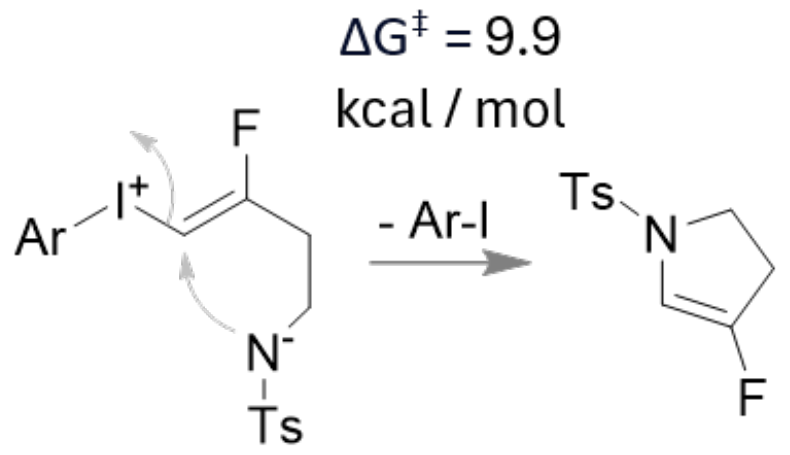

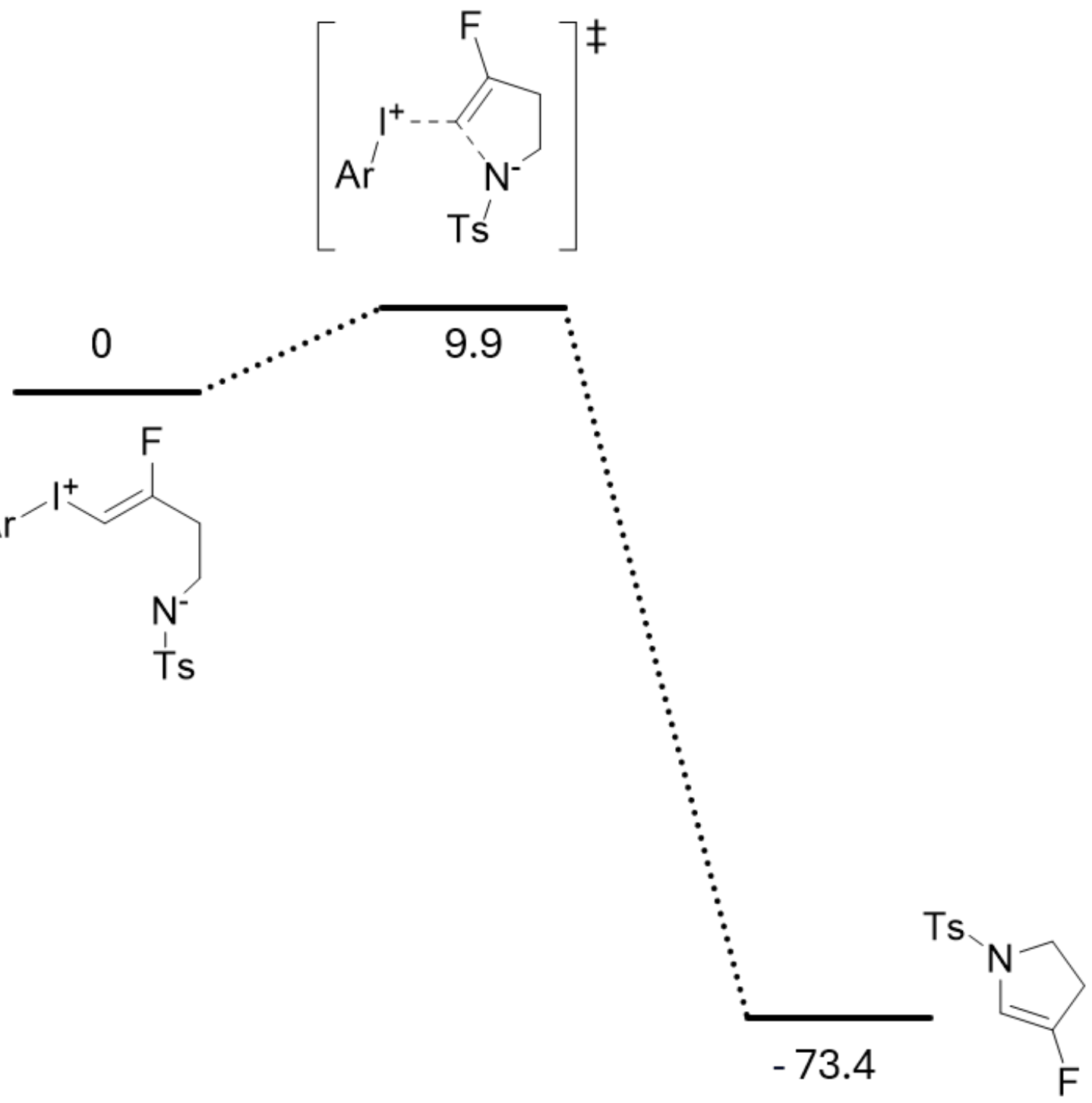

Computational Data

The following computational work was carried out using the computational facilities of the Advanced Computing Research Centre, University of Bristol - http://www.bristol.ac.uk/acrc/. Density functional theory (DFT) calculations were undertaken using Gaussian 16. Geometry optimisations and frequency calculations were performed using the M062x functional, using 6-31+G(d,p) for H, C, B, and F atoms, and the SDD basis set along with its associated pseudopotential for I. frequency calculations were conducted after geometry optimisations, both to verify stationary points as minima (no imaginary frequencies) or transition states (exactly one imaginary frequency), and to calculate thermodynamic data. Single-point energy calculations were conducted on the geometry-optimised structures using Def2TZVP on all atoms. Intrinsic reaction coordinate (IRC) calculations were carried out on each transition state (TS) to verify the reaction pathway, and to find/confirm the intermediates either side of the transition state. All calculations included implicit solvation (1,4-Dioxane) using the integral equation formalism variant (IEFPCM) of the Polarisable Continuum Model (PCM). CYLview20 was used for the visualisation of the optimised structures in this text.

Pyrroline formation, SNV, deprotonated Nitrogen

Reactant

C	-1.14256	3.339199	-0.88697
H	-1.75875	3.142175	-1.77176
H	-0.70403	4.33907	-1.01874
C	-2.07014	3.424948	0.343111
H	-1.51463	3.785189	1.219028
H	-2.88901	4.131313	0.159386
C	-2.67155	2.124363	0.752045
C	-2.37704	0.93148	0.249702
N	-0.13023	2.295886	-0.84142
H	-1.60469	0.75213	-0.49358
S	0.925606	2.469601	0.312356
C	2.172355	1.260456	-0.1261
C	2.032052	0.396733	-1.2049
C	3.287328	1.175637	0.709595
C	3.011503	-0.57165	-1.43748
H	1.159826	0.490113	-1.84233
C	4.258195	0.211138	0.463103
H	3.383169	1.859415	1.548024
C	4.134009	-0.67911	-0.6136
H	2.900188	-1.25169	-2.27875
H	5.128756	0.14724	1.111569
O	1.581901	3.797825	0.371995
O	0.448779	2.032472	1.663633
F	-3.57248	2.222445	1.746709
C	5.208062	-1.70238	-0.88298
H	6.130252	-1.21658	-1.21772
H	5.448508	-2.27235	0.020077
H	4.899347	-2.40612	-1.66016
I	-3.17497	-0.81843	1.134357
C	-1.25838	-1.80694	1.070154
C	-0.14505	-1.04894	1.385966
C	-1.21848	-3.13569	0.675022
C	1.093725	-1.68357	1.278756
H	-0.18949	0.005494	1.663207
C	0.037374	-3.7358	0.595152
H	-2.11426	-3.69258	0.423349
C	1.204648	-3.01873	0.88323
H	1.989171	-1.10512	1.493563
H	0.103037	-4.77485	0.284663
C	2.559476	-3.65499	0.722916
H	2.887629	-3.58098	-0.32013
H	3.305417	-3.14534	1.337542
H	2.539817	-4.713	0.995776

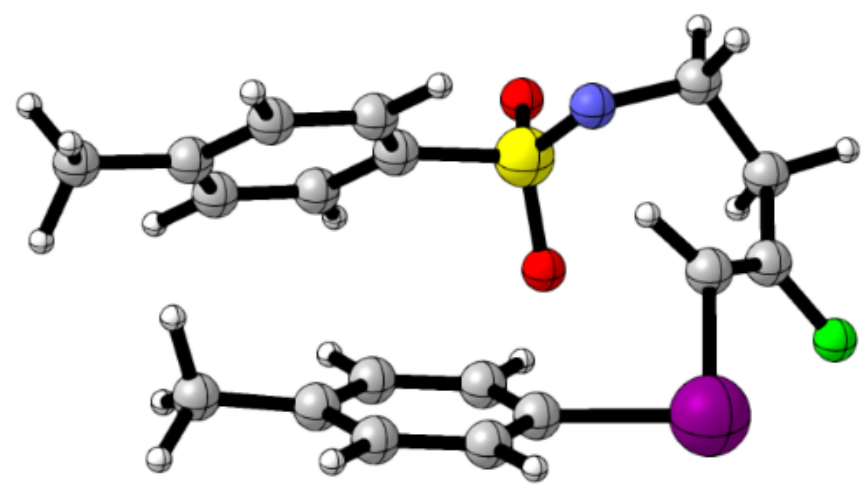

Thermal correction to G: 0.279752
Electronic energies = -1698.03129212
Charge = 0 Multiplicity = 1

Transition State

C	1.9629127	3.336768676	-1.26773
H	2.5436865	3.285558278	-2.19436
H	2.502493	3.993587645	-0.57243
C	0.5827156	3.912949689	-1.56833
H	0.0831786	4.251920678	-0.65143
H	0.601327	4.749659365	-2.27502
C	-0.286365	2.829451421	-2.13876
C	-0.120362	1.515534311	-2.04575
N	1.8391344	1.97612632	-0.76841
H	0.4772282	0.669093747	-1.77511
S	1.4147245	1.865232609	0.778885
C	1.7083324	0.127361654	1.066522
C	0.6449204	-0.76518806	1.035951
C	3.0098981	-0.315956129	1.296011
C	0.8902669	-2.1254553	1.234179
H	-0.361324	-0.391494338	0.87519
C	3.2386331	-1.671850259	1.495459
H	3.8238684	0.401884549	1.32098
C	2.1839808	-2.59643458	1.466329
H	0.0583008	-2.825541428	1.213491
H	4.2504923	-2.024310645	1.679329
O	2.3164077	2.636447347	1.658886
O	-0.031307	2.085963404	1.025786
F	-1.380654	3.328785911	-2.74572
C	2.454184	-4.06187439	1.693815
H	3.2091824	-4.43360935	0.994654
H	2.8320992	-4.235018165	2.706293
H	1.5469857	-4.656515934	1.564752
I	-2.072089	0.379919802	-2.93983
C	-2.478942	-0.299106511	-0.95711
C	-2.646045	0.652898654	0.039467
C	-2.527973	-1.664056996	-0.70515
C	-2.869499	0.207550617	1.340454
H	-2.564685	1.71466647	-0.16523
C	-2.758612	-2.080141519	0.605584
H	-2.383172	-2.391539811	-1.49617
C	-2.923043	-1.156929393	1.643801
H	-2.970227	0.942530914	2.1335
H	-2.796753	-3.144648013	0.821061
C	-3.093186	-1.619485918	3.066397
H	-2.119432	-1.652746132	3.567176
H	-3.732	-0.93629	3.630824
H	-3.529	-2.62032	3.109755

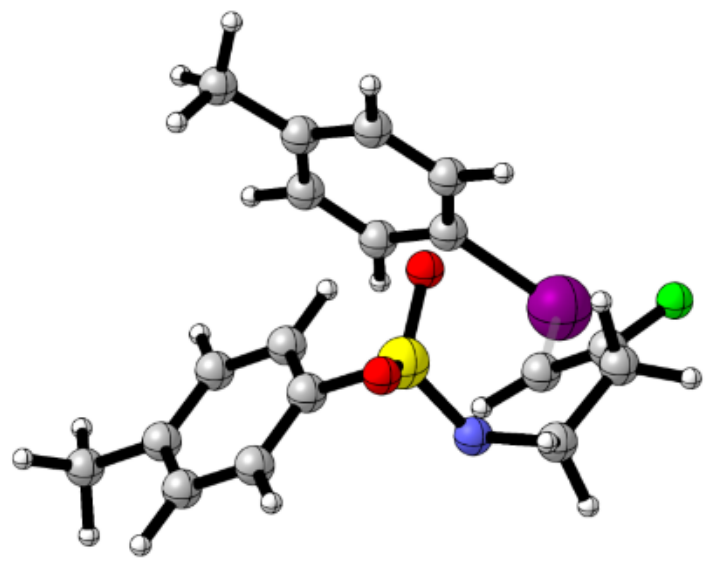

Thermal correction to G: 0.275171
Electronic energies = -1698.01101631
Charge = 0 Multiplicity = 1

Product

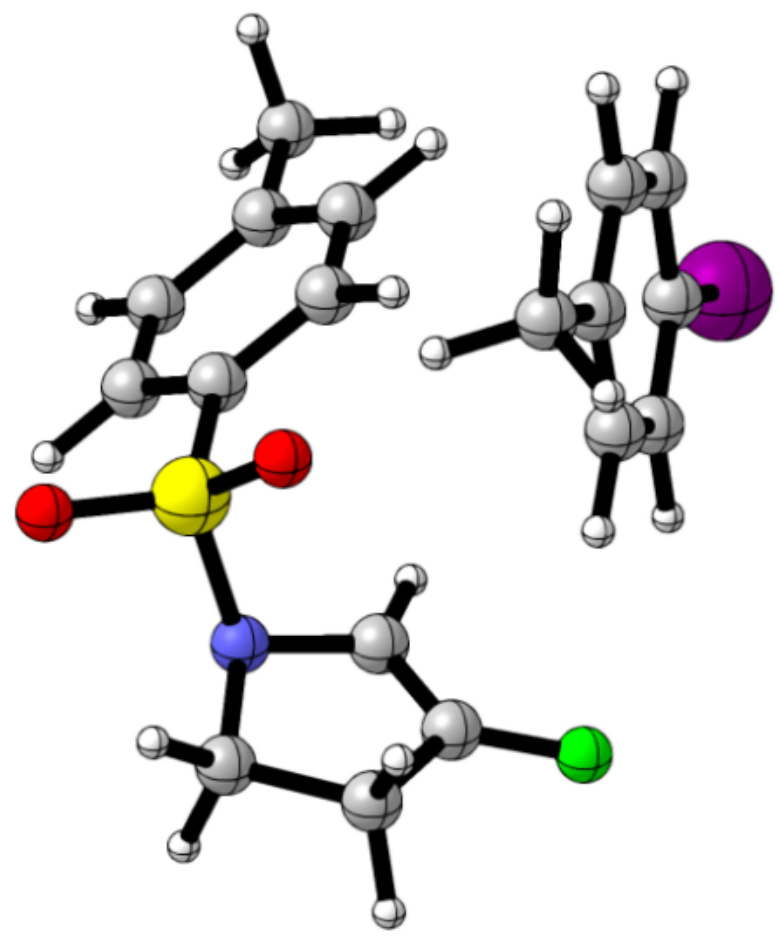

C	0.220397028	0.861034	1.332155
H	0.9988725	1.334544	0.730318
H	0.288116017	1.24198	2.351605
C	-1.160337247	1.047036	0.67473
H	-1.979387168	1.142942	1.397196
H	-1.178636748	1.913942	0.010307
C	-1.261613618	-0.24706	-0.07266
C	-0.360746154	-1.15217	0.295829
N	0.505198977	-0.61219	1.304858
H	-0.228513985	-2.16571	-0.06001
S	0.290126413	-1.33095	2.836067
C	0.724222664	-3.00598	2.472997
C	-0.284024146	-3.9627	2.398505
C	2.058735096	-3.31901	2.223741
C	0.063718428	-5.26772	2.063488
H	-1.314108596	-3.68187	2.597501
C	2.382702498	-4.62902	1.887763
H	2.821446723	-2.5495	2.290598
C	1.393088542	-5.6173	1.800926
H	-0.713400471	-6.02469	1.995781
H	3.417771475	-4.88926	1.68648
O	1.292767683	-0.7124	3.696252
O	-1.115169752	-1.28696	3.243309
F	-2.202319512	-0.42911	-1.0082
C	1.745528903	-7.03832	1.449218
H	2.731630372	-7.10014	0.984215
H	1.757049596	-7.66281	2.348503
H	1.008595376	-7.46198	0.761533
I	-2.40876874	-5.98605	-0.97834
C	-3.107889809	-4.70105	0.565877
C	-3.11853048	-3.32357	0.35882
C	-3.520864215	-5.24403	1.777334
C	-3.5326992	-2.48702	1.392239
H	-2.805930364	-2.8997	-0.59
C	-3.93282	-4.38644	2.8001
H	-3.52093	-6.31782	1.934016
C	-3.93698	-2.99945	2.629945
H	-3.52302	-1.41091	1.237465
H	-4.24757	-4.81114	3.749622
C	-4.32832	-2.07069	3.748132
H	-3.44017	-1.5569	4.129157
H	-5.0311	-1.30938	3.397771
H	-4.79446	-2.61596	4.57186

Thermal correction to G: 0.279712
Electronic energies = -1698.14774569
Charge = 0 Multiplicity = 1

Azetine Formation, SNV

Reactant

C	-1.10807	2.742447	0.246636
H	-1.51849	2.825169	1.267778
N	-2.06873	2.923511	-0.81272
S	-3.55536	2.555964	-0.40337
C	-3.59747	0.756071	-0.25779
C	-3.67866	-0.01711	-1.41496
C	-3.32383	0.155011	0.96838
C	-3.45002	-1.38905	-1.34608
H	-3.90027	0.468708	-2.3612
C	-3.09488	-1.22016	1.029614
H	-3.28256	0.769844	1.862964
C	-3.14478	-2.01105	-0.12471
H	-3.51479	-1.99277	-2.24873
H	-2.87776	-1.68736	1.987544
O	-3.93738	3.02616	0.946163
O	-4.45282	2.883878	-1.51817
C	-0.42876	1.397062	0.179496
C	-0.5018	0.574071	-0.86375
F	0.265291	1.049163	1.27202
H	-0.31707	3.502313	0.173024
C	-2.93466	-3.50333	-0.05073
H	-3.88503	-4.01734	0.126746
H	-2.52203	-3.89543	-0.98503
H	-2.26046	-3.77126	0.768047
H	-1.06469	0.795932	-1.75803
I	0.226998	-1.38581	-0.71767
C	2.306287	-1.03942	-1.07744
C	2.762228	-1.01304	-2.38908
C	3.138773	-0.83946	0.015343
C	4.118166	-0.77717	-2.60217
H	2.09156	-1.16227	-3.22822
C	4.49092	-0.6069	-0.22977
H	2.75737	-0.85209	1.030471
C	4.998476	-0.57547	-1.53276
H	4.493594	-0.74661	-3.62092
H	5.157203	-0.44249	0.611926
C	6.466274	-0.35412	-1.78286
H	6.982357	-1.31459	-1.88229
H	6.62767	0.206956	-2.70588
H	6.930256	0.191229	-0.95871

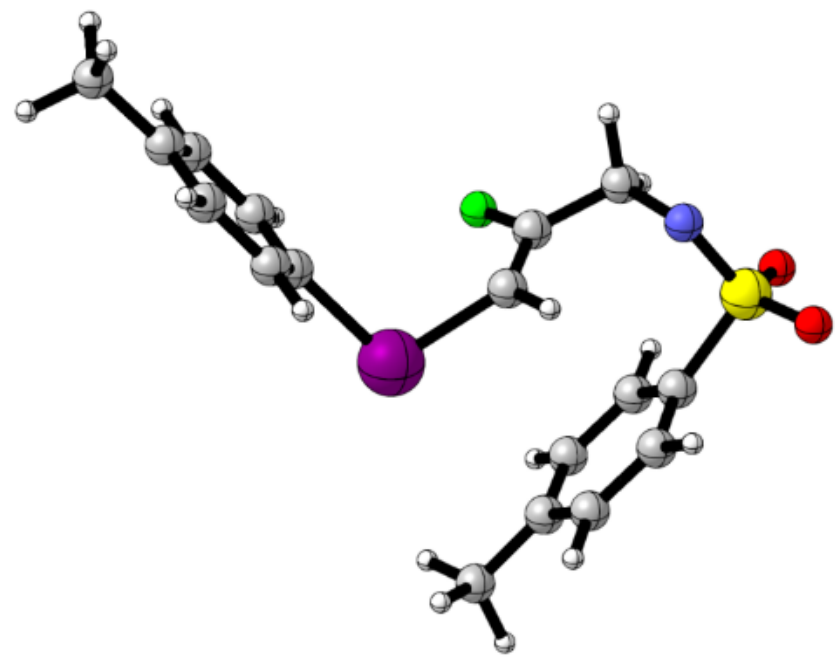

Thermal correction to G: 0.249842
Electronic energies = -1658.72150095
Charge = 0 Multiplicity = 1

Transition state

C	-0.51649	2.947016	0.579015
H	-0.90827	3.542918	1.413243
N	-1.5127	2.168537	-0.16787
S	-2.99717	2.224407	0.466752
C	-3.60914	0.618547	-0.01442
C	-3.97654	0.390326	-1.33991
C	-3.68936	-0.39444	0.934939
C	-4.41858	-0.87428	-1.71197
H	-3.9173	1.1988	-2.06176
C	-4.13976	-1.65733	0.546638
H	-3.41718	-0.18091	1.964368
C	-4.5087	-1.91449	-0.77626
H	-4.7075	-1.05922	-2.74351
H	-4.21476	-2.45027	1.286158
O	-2.98166	2.265119	1.945016
O	-3.85229	3.227941	-0.18809
C	0.264685	1.802593	1.086969
C	-0.24013	0.607125	0.780299
F	1.399109	1.973729	1.77744
H	0.076853	3.600327	-0.0708
C	-5.02059	-3.26949	-1.19324
H	-6.0971	-3.23133	-1.38856
H	-4.53266	-3.60893	-2.11129
H	-4.84688	-4.01579	-0.4148
H	-0.96008	-0.03931	0.318828
I	1.229358	-1.07573	1.737118
C	2.971486	-0.57968	0.614551
C	3.085962	-1.04719	-0.68892
C	3.941854	0.216894	1.207273
C	4.222758	-0.69923	-1.41531
H	2.313471	-1.66159	-1.13806
C	5.069841	0.547469	0.45773
H	3.826348	0.585116	2.220473
C	5.228401	0.096632	-0.85617
H	4.322852	-1.05265	-2.43773
H	5.83462	1.174425	0.907132
C	6.464216	0.437884	-1.6465
H	7.201071	-0.36829	-1.5696
H	6.229779	0.573327	-2.70486
H	6.930293	1.353077	-1.27546

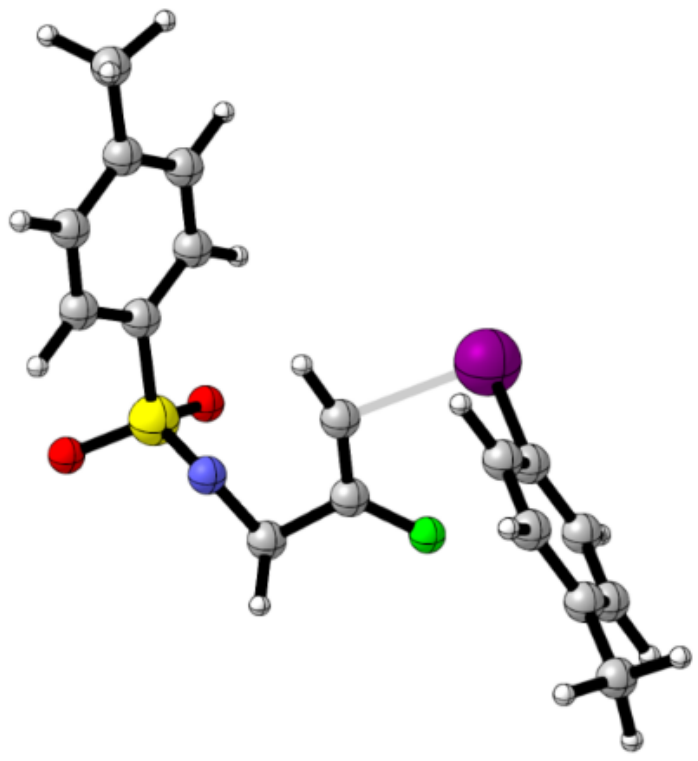

Thermal correction to G: 0.243821
Electronic energies = -1658.69084863
Charge = 0 Multiplicity = 1

Product

C	0.86764	1.856608	0.021624
H	0.532062	2.878216	-0.17387
N	-0.19773	0.810908	-0.24369
S	-1.62461	1.451233	-0.91225
C	-2.65235	0.01083	-0.89228
C	-2.44113	-0.98185	-1.84908
C	-3.6352	-0.10923	0.083968
C	-3.23732	-2.11884	-1.81518
H	-1.67075	-0.85428	-2.6031
C	-4.42359	-1.25859	0.100138
H	-3.77975	0.687735	0.806312
C	-4.23771	-2.27266	-0.84367
H	-3.0871	-2.90047	-2.55471
H	-5.19672	-1.36493	0.855418
O	-2.218	2.460474	-0.03736
O	-1.27395	1.808045	-2.28227
C	0.765375	1.380812	1.437773
C	-0.21047	0.51297	1.179351
F	1.53851	1.631997	2.482622
H	1.791219	1.623146	-0.5112
C	-5.10529	-3.50359	-0.84084
H	-5.80711	-3.47807	-1.68059
H	-4.50062	-4.40853	-0.94475
H	-5.68473	-3.57856	0.081405
H	-0.79136	-0.25093	1.676213
I	2.444446	-2.00862	2.28351
C	3.071015	-0.88159	0.592936
C	2.512342	-1.14717	-0.65516
C	4.047582	0.097592	0.742709
C	2.956192	-0.43004	-1.76421
H	1.737965	-1.89844	-0.76687
C	4.479151	0.80501	-0.38132
H	4.474218	0.309964	1.717151
C	3.946783	0.552345	-1.64843
H	2.514546	-0.63415	-2.73633
H	5.245434	1.566394	-0.26285
C	4.397836	1.329877	-2.85819
H	4.633135	0.659439	-3.68931
H	3.609505	2.009598	-3.19788
H	5.285585	1.926033	-2.63653

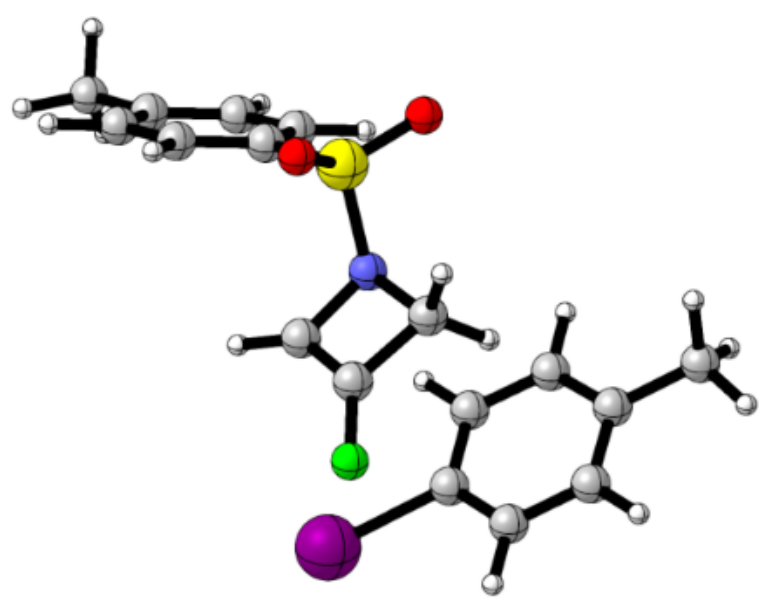

Thermal correction to G: 0.246983
Electronic energies = -1658.79588955
Charge = 0 Multiplicity = 1

Azetine formation, concerted N-H insertion

Reactant

C	3.157231	0.226148	-0.55797
H	3.418106	0.851068	0.30051
N	1.654192	0.135503	-0.74808
H	1.340965	0.374116	-1.69469
S	0.709089	1.135037	0.380619
C	-0.90122	0.464276	0.193511
C	-1.80119	1.109849	-0.65075
C	-1.23044	-0.69919	0.891151
C	-3.07367	0.566458	-0.79687
H	-1.51348	2.020782	-1.16541
C	-2.50576	-1.21977	0.726316
H	-0.49821	-1.17948	1.530285
C	-3.44065	-0.59867	-0.11478
H	-3.79213	1.057863	-1.44577
H	-2.78284	-2.12686	1.255359
O	1.259346	0.829397	1.683586
O	0.775378	2.471152	-0.18842
C	3.009913	-1.23983	-0.34053
C	1.683479	-1.44231	-0.4673
F	4.001514	-2.10493	-0.10555
H	3.654189	0.582598	-1.46353
C	-4.82008	-1.1818	-0.26495
H	-5.37194	-1.10447	0.676948
H	-5.38975	-0.66272	-1.03779
H	-4.76475	-2.24178	-0.52759

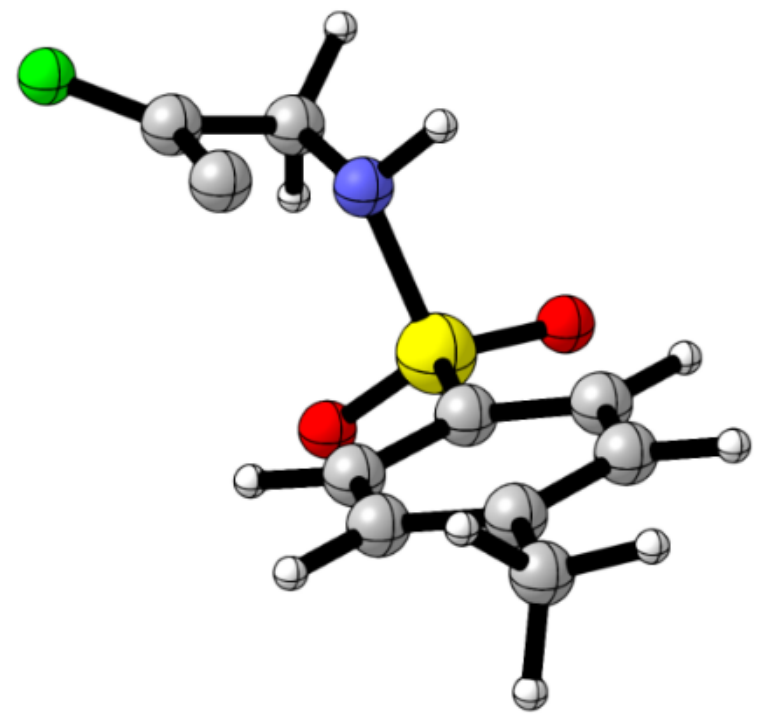

Thermal correction to G: 0.145624

Electronic energies = -1090.13935731

Charge = 0 Multiplicity = 1

Transition State

C	3.07599	0.274663	-0.49984
H	3.323038	1.026318	0.256744
N	1.592334	0.184751	-0.6805
H	1.113794	-0.9376	-1.00613
S	0.660215	1.213935	0.326971
C	-0.93374	0.481503	0.158261
C	-1.75402	0.892852	-0.89016
C	-1.32962	-0.49361	1.07136
C	-3.00499	0.300997	-1.02288
H	-1.41647	1.66347	-1.5757
C	-2.58542	-1.07002	0.918597
H	-0.66308	-0.78824	1.874973
C	-3.43711	-0.68162	-0.1231
H	-3.65874	0.609624	-1.83329
H	-2.91043	-1.83366	1.619075
O	1.128614	1.089459	1.699781
O	0.696763	2.511375	-0.33295
C	3.039083	-1.12624	-0.03452
C	1.736513	-1.45651	-0.04843
F	4.072019	-1.89205	0.290538
H	3.607497	0.478903	-1.43114
C	-4.80769	-1.29084	-0.25458
H	-5.52813	-0.73605	0.355596
H	-5.15573	-1.2629	-1.28939
H	-4.81225	-2.32822	0.087405

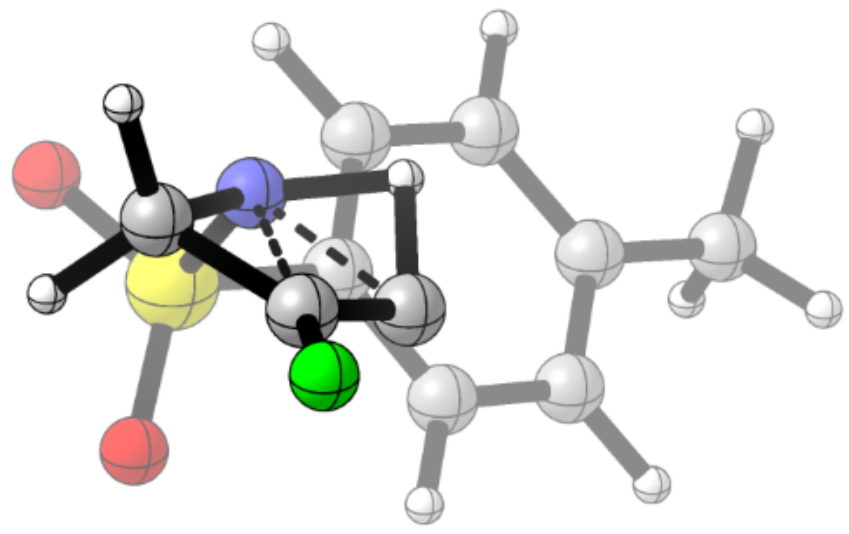

Thermal correction to G: 0.138458
Electronic energies = -1090.08831914
Charge = 0 Multiplicity = 1

Product

C	0.082553	0.779512	-0.05345
H	0.366005	1.491985	0.724936
N	-1.41155	0.524848	-0.12451
H	-1.89651	-1.62587	0.42704
S	-2.30495	1.506302	0.946925
C	-3.92103	0.818027	0.73528
C	-4.64383	1.136195	-0.41453
C	-4.42959	-0.03376	1.709714
C	-5.90401	0.578748	-0.58264
H	-4.22208	1.81116	-1.15253
C	-5.69693	-0.5822	1.521011
H	-3.84571	-0.24863	2.599034
C	-6.44733	-0.28543	0.379831
H	-6.48145	0.818572	-1.47123
H	-6.10866	-1.24626	2.275297
O	-1.8823	1.293084	2.329423
O	-2.24047	2.844625	0.372899
C	0.157573	-0.67096	0.314542
C	-1.16166	-0.84395	0.299682
F	1.204107	-1.47498	0.42457
H	0.481219	1.069704	-1.02597
C	-7.82478	-0.86208	0.184405
H	-8.58295	-0.07653	0.26182
H	-7.92082	-1.31513	-0.80617
H	-8.04686	-1.62281	0.93521

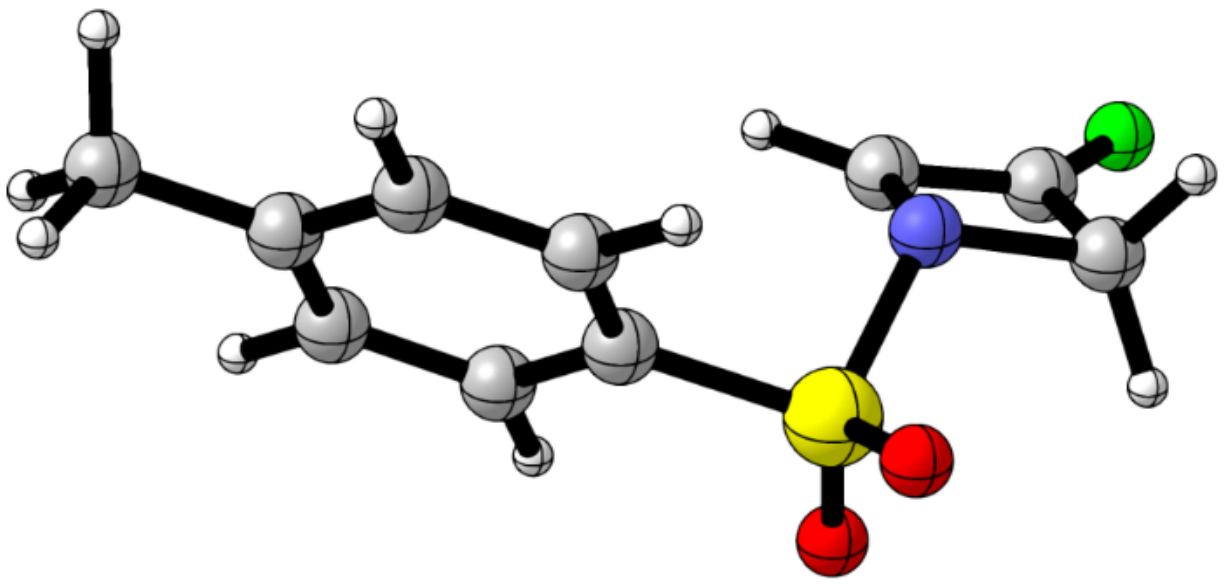

Thermal correction to G: 0.145325
Electronic energies = -1090.21933873
Charge = 0 Multiplicity = 1

Pyrroline NH insertion – 1,2-proton shift

Reactant

C	-1.49896	1.039708	-0.04173
H	-1.10366	1.163495	0.966924
H	-1.62315	2.020091	-0.50428
C	-0.70451	0.019465	-0.8452
H	-0.64683	0.275193	-1.90786
H	0.311616	-0.09386	-0.45779
C	-1.5396	-1.21325	-0.61577
C	-2.75518	-1.13938	-0.06162
N	-2.86177	0.359833	0.128054
H	-3.25731	0.573357	1.048697
S	-4.03968	1.119372	-1.03476
C	-5.52041	0.206794	-0.80388
C	-5.6932	-0.99184	-1.49794
C	-6.48627	0.716271	0.059941
C	-6.8762	-1.69024	-1.30911
H	-4.91213	-1.36149	-2.15224
C	-7.66317	-0.00603	0.232466
H	-6.32447	1.660372	0.569534
C	-7.87268	-1.21123	-0.44547
H	-7.0306	-2.62804	-1.83456
H	-8.43076	0.376573	0.898063
O	-4.19542	2.470316	-0.5162
O	-3.4565	0.864182	-2.33543
F	-0.9838	-2.38081	-1.01127
C	-9.151	-1.98606	-0.27007
H	-9.76017	-1.5724	0.535678
H	-9.74104	-1.96157	-1.19159
H	-8.93959	-3.03436	-0.04261

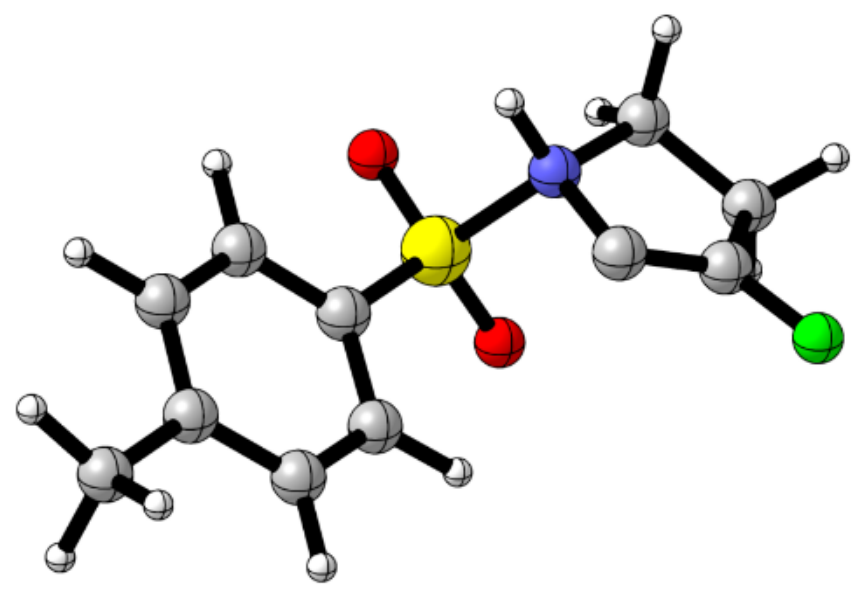

Thermal correction to G: 0.173724
Electronic energies = -1129.48043378
Charge = 0 Multiplicity = 1

Transition State

C	0.64872	-0.98833	0.004478
H	0.938011	-0.91757	1.054703
H	0.581021	0.01611	-0.4163
C	1.532988	-1.97308	-0.75986
H	1.620371	-1.7356	-1.8251
H	2.535924	-2.03397	-0.33099
C	0.767559	-3.25459	-0.53519
C	-0.51142	-3.17863	-0.16381
N	-0.70587	-1.63945	-0.02066
H	-1.18829	-2.44425	0.729677
S	-1.79469	-0.94102	-1.19007
C	-3.28421	-1.83624	-0.9006
C	-3.53113	-2.99621	-1.63186
C	-4.17582	-1.35744	0.056447
C	-4.70873	-3.69128	-1.38691
H	-2.81018	-3.34003	-2.36564
C	-5.34623	-2.07266	0.286246
H	-3.95752	-0.44208	0.59703
C	-5.62821	-3.24225	-0.4298
H	-4.91724	-4.60001	-1.9438
H	-6.05389	-1.71455	1.028043
O	-1.95197	0.441981	-0.76411
O	-1.26675	-1.24763	-2.51024
F	1.42706	-4.41249	-0.70017
C	-6.91241	-3.9932	-0.1985
H	-7.31319	-3.79561	0.7979
H	-7.66692	-3.68682	-0.93056
H	-6.76294	-5.0699	-0.30725

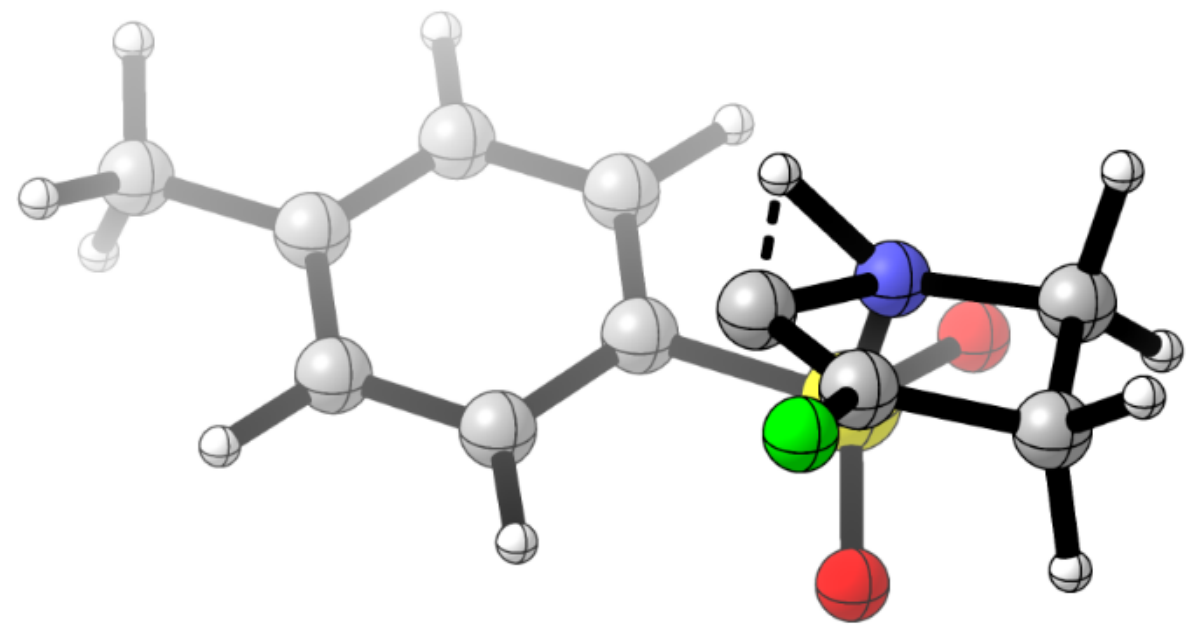

Thermal correction to G: 0.168109
Electronic energies = -1129.43377483
Charge = 0 Multiplicity = 1

Pyrroline formation, concerted NH insertion, benzenesulfonyl protecting group

Reactant

C	2.504259	1.070753	0.709454
H	2.880144	1.213751	1.722923
H	2.388958	2.042219	0.226112
C	3.313704	0.035244	-0.05845
H	3.390358	0.269034	-1.12496
H	4.322561	-0.06916	0.349718
C	2.475416	-1.19344	0.180642
C	1.248664	-1.10999	0.708563
N	1.138368	0.393622	0.866223
H	0.724511	0.625998	1.774356
S	-0.01391	1.125082	-0.33752
C	-1.49928	0.210401	-0.10716
C	-1.64762	-1.00302	-0.77728
C	-2.47735	0.74054	0.732031
C	-2.83151	-1.70808	-0.59198
H	-0.8517	-1.37748	-1.41053
C	-3.65397	0.016767	0.905324
H	-2.32332	1.698306	1.217703
C	-3.82586	-1.20065	0.246444
H	-2.97509	-2.65649	-1.09744
H	-4.43566	0.406896	1.547693
H	-4.74569	-1.75956	0.385246
O	-0.18373	2.488808	0.140266
O	0.591146	0.836017	-1.62065
F	3.039764	-2.36767	-0.18025

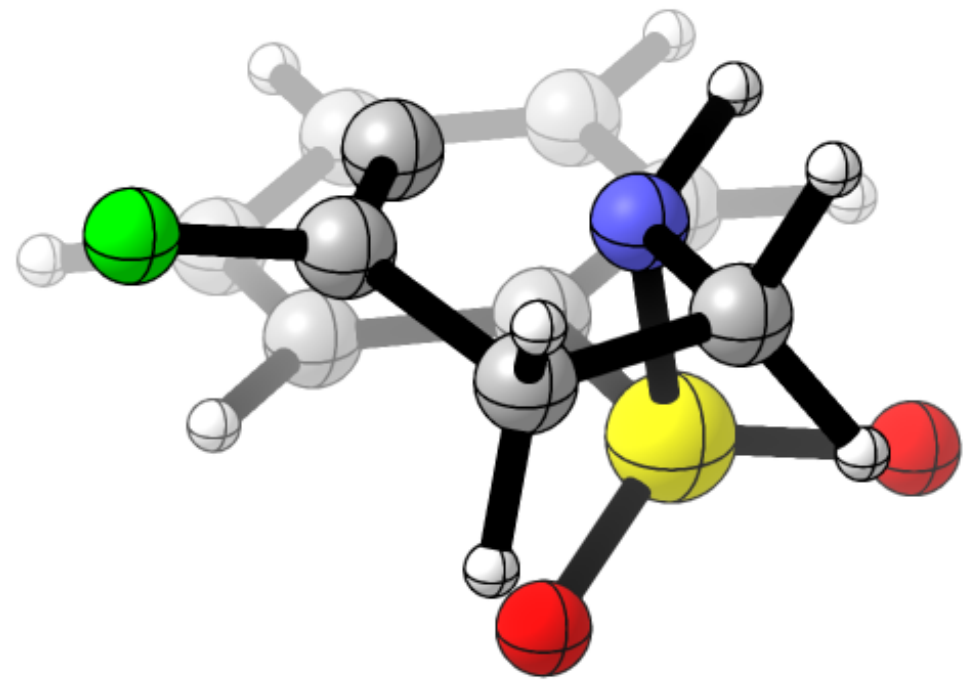

Thermal correction to G: 0.150225
Electronic energy: -1090.16831742
Charge = 0 Multiplicity = 1

Pyrroline formation, concerted NH insertion, benzenesulfonyl protecting group

Transition state

C	2.517941	-0.87805	-0.71904
H	2.790595	-0.97905	-1.7712
H	2.519221	-1.86473	-0.25313
C	3.355532	0.191031	-0.01799
H	3.479537	0.006436	1.054051
H	4.343742	0.293323	-0.47236
C	2.509216	1.413286	-0.27916
C	1.23	1.245638	-0.61868
N	1.126917	-0.30814	-0.69296
H	0.584507	0.432173	-1.46708
S	0.105454	-1.01902	0.527036
C	-1.44143	-0.2191	0.230707
C	-1.73209	0.955924	0.919729
C	-2.32065	-0.79592	-0.68342
C	-2.95446	1.574668	0.675655
H	-1.0137	1.366643	1.620653
C	-3.53738	-0.16081	-0.91691
H	-2.05764	-1.72253	-1.18308
C	-3.84918	1.018742	-0.23967
H	-3.20623	2.491298	1.197535
H	-4.24253	-0.59001	-1.62033
H	-4.80004	1.507926	-0.4249
O	0.016831	-2.42506	0.162015
O	0.63755	-0.62747	1.822457
F	3.099947	2.614426	-0.17723

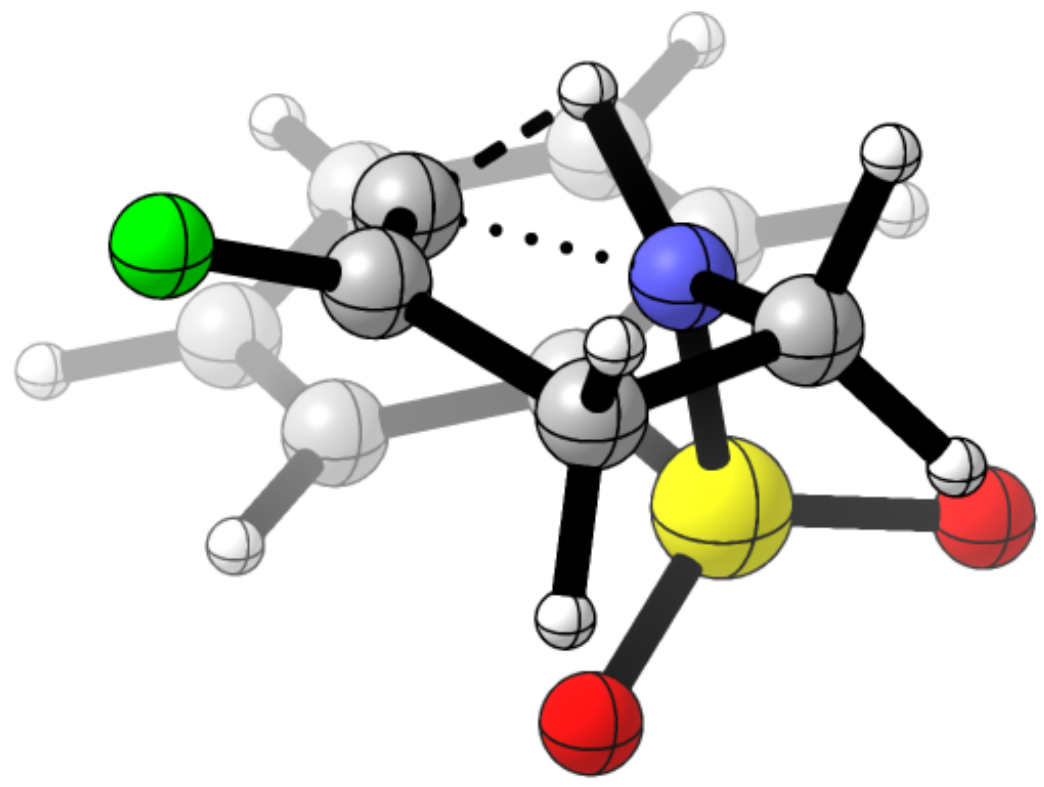

Thermal correction to G: 0.143688
Electronic energy: -1090.12180756
Charge = 0 Multiplicity = 1

Pyrroline formation, concerted NH insertion, benzenesulfonyl protecting group

Product

H	2.552191	1.395995	1.762319
H	2.326433	2.065481	0.136879
C	3.389055	0.113352	0.211063
H	3.657504	0.243252	-0.84327
H	4.304051	0.129526	0.807621
C	2.607751	-1.14996	0.406312
C	1.314763	-0.95003	0.640957
N	1.026923	0.455855	0.687562
H	0.532772	-1.67942	0.802919
S	0.040154	1.00088	-0.5871
C	-1.47651	0.156294	-0.2154
C	-1.86539	-0.91587	-1.01254
C	-2.23541	0.594298	0.868487
C	-3.05696	-1.57272	-0.70759
H	-1.24689	-1.21568	-1.85222
C	-3.42094	-0.07216	1.161331
H	-1.90082	1.44078	1.459627
C	-3.82813	-1.15238	0.375007
H	-3.38139	-2.40902	-1.31752
H	-4.02929	0.252846	1.998532
H	-4.75526	-1.66659	0.607125
O	-0.11836	2.433843	-0.36563
O	0.526043	0.528107	-1.8825
F	3.208835	-2.34218	0.347313

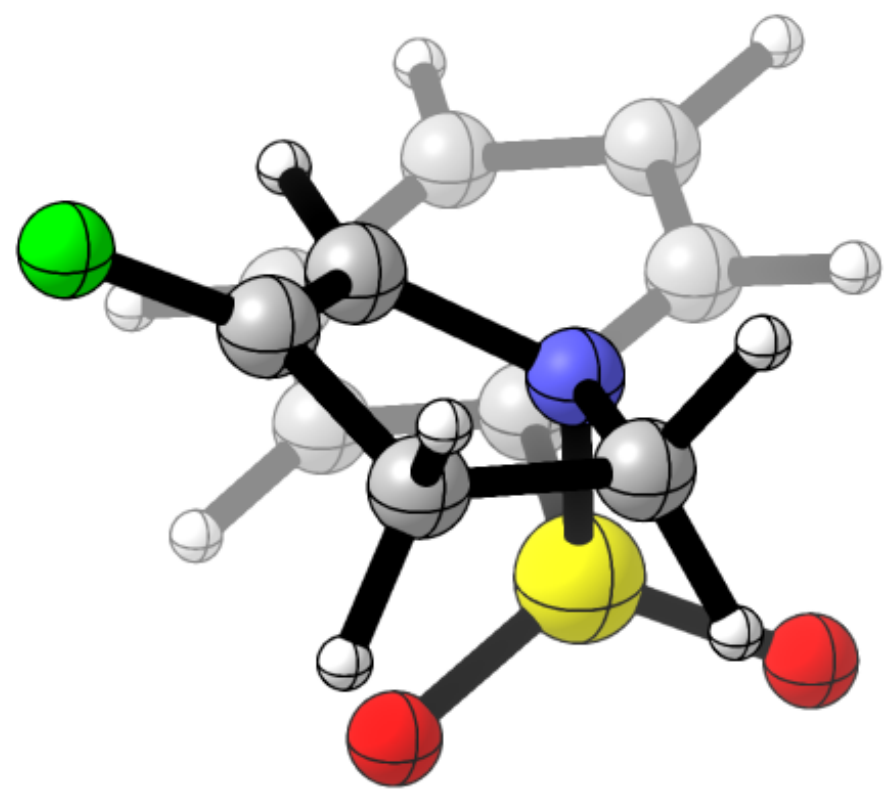

Thermal correction to G: 0.149521
Electronic energy: -1090.25644896
Charge = 0 Multiplicity = 1

Product

C	1.043918	0.8661	-0.01318
H	1.239422	1.092458	1.037018
H	1.005011	1.802571	-0.56982
C	2.069821	-0.14944	-0.55044
H	2.33356	0.008237	-1.60215
H	2.987329	-0.1479	0.042518
C	1.290317	-1.41797	-0.38453
C	-0.00147	-1.22523	-0.13768
N	-0.29077	0.178449	-0.05362
H	-0.78256	-1.95904	0.007635
S	-1.28143	0.75307	-1.31362
C	-2.78953	-0.11129	-0.97383
C	-3.17101	-1.16451	-1.79778
C	-3.55885	0.281691	0.120486
C	-4.35512	-1.84091	-1.51153
H	-2.55065	-1.43949	-2.64472
C	-4.73488	-0.40655	0.389138
H	-3.23694	1.111275	0.742083
C	-5.1498	-1.47302	-0.42156
H	-4.66555	-2.6654	-2.14684
H	-5.34505	-0.11264	1.238599
O	-1.44844	2.178245	-1.05024
O	-0.78541	0.323094	-2.62042
F	1.89187	-2.60811	-0.47785
C	-6.44469	-2.18645	-0.13432
H	-6.60054	-2.30156	0.941159
H	-7.2896	-1.61452	-0.53174
H	-6.463	-3.1758	-0.59611

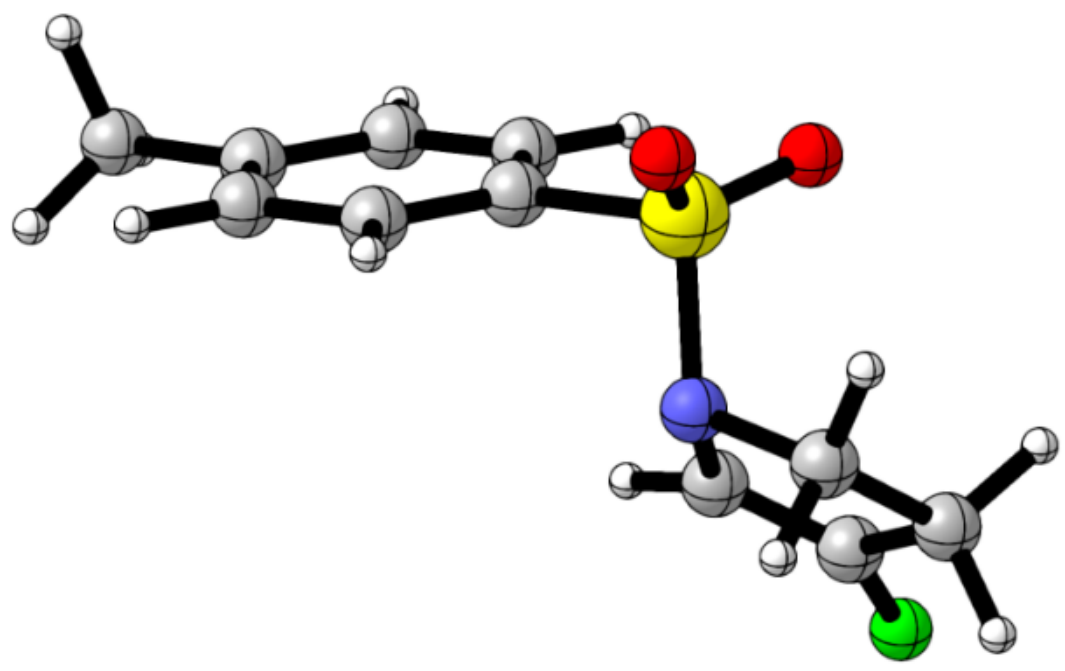

Thermal correction to G: 0.17529
Electronic energies = -1129.56805977
Charge = 0 Multiplicity = 1

Azetine formation, concerted N-H insertion, free amine

Reactant

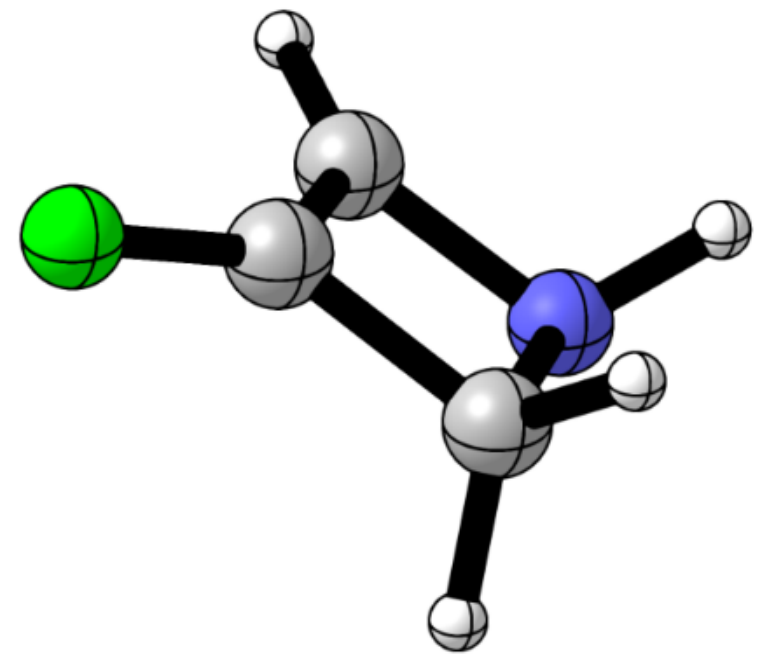

C	-0.09395	0.056281	-0.00124
C	-1.11552	-1.02731	-0.0734
C	-0.36268	-2.14313	-0.10696
F	-2.44725	-0.86339	-0.09603
H	0.030977	0.714188	-0.86537
H	-0.00941	0.633131	0.923565
N	0.862909	-1.10671	-0.03234
H	1.470856	-1.13868	-0.8497
H	1.433506	-1.21368	0.805323

Thermal correction to G: 0.042441
Electronic energies = -271.174402989
Charge = 0 Multiplicity = 1

Transition state

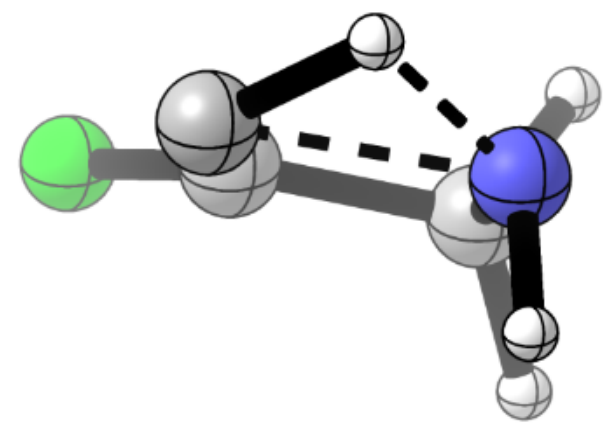

C	-0.35759	0.19448	0
C	-1.37959	-0.86953	-0.04479
C	-0.6752	-2.00414	-0.05781
F	-2.70067	-0.72149	-0.06503
H	-0.26646	0.83992	-0.88018
H	-0.3452	0.81518	0.90007
N	0.7119	-0.84521	0.01076
H	1.11616	-0.97331	-0.9165
H	0.41281	-2.01531	0.4771

Thermal correction to G: 0.035602
Electronic energies = -271.114571119
Charge = 0 Multiplicity = 1

Product

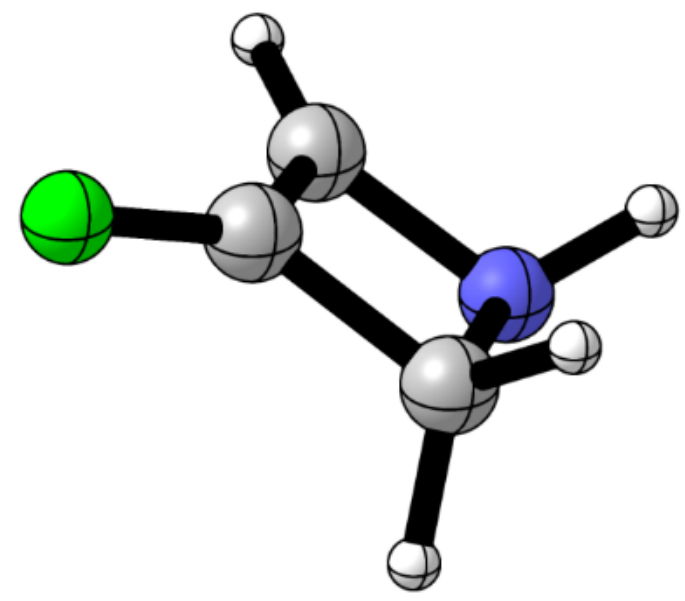

C	0.489874	1.06664	-0.02829
C	-0.47181	-0.08181	0.028333
C	0.506527	-0.98245	0.112282
F	-1.80029	-0.10918	0.158817
H	0.545507	1.609623	-0.975
H	0.467536	1.760663	0.814514
N	1.55513	0.014427	0.127916
H	2.167195	-0.03414	-0.68612
H	0.618285	-2.05201	0.224794

Thermal correction to G: 0.041762
Electronic energies = -271.238171429
Charge = 0 Multiplicity = 1

REVIEWERS' COMMENTS

Reviewer #1 (Remarks to the Author):

The authors have provided satisfactory replies and corrections to the referee comments.
I only have a small comment, in that in the Hammett plot provided, the Hammett parameter is correlated with yield, not with reaction rate. I am not sure this is of much value, certainly not with the information provided. At the very least it should be specified whether the reaction conditions, especially reaction time, was the same in all cases. If not, then this should be removed.
Apart from this comment, which needs to be addressed, I am happy to recommend acceptance.

Thank you for this comment. We do not claim this to be a formal "Hammett plot" and have removed this title in the Supplementary Information. Hence, from "Hammett plot", we have renamed it to: "A plot of yield against Hammett parameter".

We are simply correlating yield with Hammett parameter and we are not trying to extract the type of mechanistic information with regards to the build of charge in the transition state.

Reviewer #2 (Remarks to the Author):

All comments/suggestions more or less were addressed. I recommend publication of this manuscript as it is.
thank you

Reviewer #3 (Remarks to the Author):

The authors have addressed my previous comments satisfactorily and have responded to the concerns raised during the initial review. The revisions have improved the clarity and quality of the manuscript, and I believe the work is now suitable for publication.
thank you

Reviewer #4 (Remarks to the Author):

I carefully checked the revised manuscript and I am fully happy with the revised version.
Hence, I warmly recommend this work for publication in nature Communications.
thank you
